# 96 sample parallel acoustic fragmentation for high throughput next generation sequencing library preparation

**DOI:** 10.1371/journal.pone.0341139

**Published:** 2026-02-17

**Authors:** Marjan Mehrab-Mohseni, Kathlyne Jayne B. Bautista, Yusha Liu, Erika E. Maldonado-Rosado, Haofan Zheng, Matthew J. Niederhuber, Jonathan D. Rosen, Michell D. Carroll, Brian Velasco, James K. Tsuruta, Jinwook Kim, James S. Marron, Perry D. Haaland, Sandeep K. Kasoji, Piotr A. Mieczkowski, Austin J. Hepperla, Paul A. Dayton, Samantha G. Pattenden

**Affiliations:** 1 Division of Chemical Biology and Medicinal Chemistry, Eshelman School of Pharmacy, The University of North Carolina, Chapel Hill, North Carolina, United States of America; 2 Lampe Joint Department of Biomedical Engineering, The University of North Carolina, Chapel Hill, North Carolina, United States of America; 3 UNC Lineberger Comprehensive Cancer Center, Department of Biostatistics, University of North Carolina at Chapel Hill, Chapel Hill, North Carolina, United States of America; 4 Bioinformatics and Analytics Research Collaborative, University of North Carolina at Chapel Hill, Chapel Hill, North Carolina, United States of America; 5 Statistics & Operations Research, College of Arts and Sciences, University of North Carolina at Chapel Hill, Chapel Hill, North Carolina, United States of America; 6 Department of Genetics, UNC School of Medicine, University of North Carolina at Chapel Hill, Chapel Hill, North Carolina, United States of America; 7 Department of Biostatistics, Gillings School of Global Public Health, University of North Carolina at Chapel Hill, Chapel Hill, North Carolina, United States of America; 8 Triangle Biotechnology, Inc., Durham, N.C., United States; 9 Neuroscience Center, UNC School of Medicine, University of North Carolina at Chapel Hill, Chapel Hill, North Carolina, United States of America; 10 Lineberger Comprehensive Cancer Center, UNC School of Medicine, University of North Carolina at Chapel Hill, Chapel Hill, North Carolina, United States of America; University of Waterloo, CANADA

## Abstract

Random, unbiased fragmentation of genomic DNA is necessary for next generation sequencing (NGS), yet the process of DNA fragmentation is still a bottleneck in NGS pipelines. A technology that increases the efficiency and consistency of this step will be highly desirable for both research laboratories and in clinical diagnostics. Previously, we reported the development of a novel cavitation enhancement reagent that dramatically decreases the time and acoustic energy required for genomic DNA fragmentation. The inclusion of this reagent in standard protocols facilitates highly efficient sonication enabling the use of widely available and inexpensive equipment, including water bath-based sonicators. Here, we report that cavitation enhancement facilitates parallel fragmentation of up to 96 samples of genomic DNA in a modified sonication device. The parallel processing of multiple samples significantly reduces processing time, while maintaining fragment range reproducibility and preserving DNA quality for NGS. Consequently, this system removes a key bottleneck in standard NGS pipelines and facilitates efforts toward research and personalized genomics.

## Introduction

Over the past two decades, next generation sequencing (NGS) has become routine for a variety of research and clinical applications, including whole genome sequencing to identify disease-related variations in the DNA sequence, and transcriptomics to monitor changes in gene expression during treatment or disease progression [1]. While NGS applications have become streamlined and more cost effective, bottlenecks in the process still exist. For instance, short read sequencers require input DNA fragments from about 200–500 base pairs (bp) in size (depending on application) for the preparation of libraries with sample-identifying adaptors for sequencing. Thus, random, unbiased, and size-specific fragmentation of DNA is a key step in the preparation of NGS libraries, however, this process remains problematic [1–6].

DNA fragmentation is commonly performed through mechanical shearing or enzymatic digestion [1,7,8]. The most widely used method for mechanical shearing of DNA is acoustic sonication. Sonication instruments generally consist of an ultrasound probe that is submerged in a sample tube, or for higher throughput, an ultrasonic transducer inverted in a water bath, with samples suspended in tubes above the transducer [7–9]. Historically, sonication is considered the “gold standard” method for DNA fragmentation since the process is random and largely unbiased [4,6–8].

In recent years, however, mechanical sonication has waned in popularity due to low throughput and reliance on specialized instruments that often exceed the budget of the average laboratory. Instead, enzymatic DNA fragmentation has become more widespread since it is relatively inexpensive, can be performed in a high throughput format (such as 384-well plates), and protocols for fragmentation are already optimized for many sequencing library preparation kits [1,4,6]. Unlike sonication, however, enzymatic digestion produces inherently biased DNA fragments that require correction using bioinformatic methods following sequencing [3–5].

Since mechanical fragmentation by sonication is a method that produces random DNA fragments with limited bias compared to enzyme digestion, innovative technologies that improve the efficiency and throughput of sonication would have a significant impact on the NGS sample preparation pipeline. Previously, we demonstrated that cavitation enhancing nanodroplets could mediate efficient DNA fragmentation in a bench top ultrasonic water bath [9]. Cavitation, which is the rapid growth and collapse of gas cavities in a varying fluid pressure field, causes powerful liquid jets and shock waves on a micro-scale. The addition of ‘cavitation nuclei’ such as nanodroplets to a sample prior to sonication serves to lower the amount of energy required to generate the cavitation effect [10].

The nanodroplet reagent consists of a solution of 150–200 nanometer droplets with a lipid monolayer surrounding a perfluorocarbon liquid core [11,12]. Nanodroplets are added directly to a sample prior to sonication, where they provide cavitation enhancement to dramatically decrease the time and acoustic energy required for DNA fragmentation [9]. Here, we show that nanodroplets can facilitate consistent parallel fragmentation of up to 96 samples in an inexpensive thin-walled polymerase chain reaction (PCR) plate. This system is presented as a high throughput alternative for current methods of DNA fragmentation for NGS applications.

## Materials and methods

### Human Genomic DNA (gDNA) preparation

Human embryonic kidney (HEK) 293T cells were cultured as previously described [13]. Cell gDNA was extracted using the Quick-gDNA Midi Prep Kit (Zymo Research, Cat. #11–317MD, USA) according to the manufacturer’s instructions. DNA concentrations were determined using a Nanodrop spectrophotometer (Thermo Scientific, MA, USA).

### Nanodroplet preparation

Nanodroplets were prepared as described by Kasoji *et al*. [9] and Durham *et al*. [14]. Briefly, microbubbles were generated by mechanically agitating a lipid solution in the presence of decafluorobutane (DFB) gas. The lipid mixture consisted of DSPC and DSPE-PEG2000 (9:1 molar ratio, Avanti Polar Lipids, Alabaster, AL, USA) in phosphate-buffered saline containing 15% (v/v) propylene glycol and 5% (v/v) glycerol, at a final lipid concentration of 1 mg/mL. Aliquots (1.5 mL) were dispensed into 3 mL vials, sealed with butyl rubber septa, and the headspace was replaced with DFB gas after degassing under vacuum for 30 min. Vials were shaken for 45 s using a Vialmix shaker (Lantheus Medical Imaging, North Billerica, MA, USA), producing microbubbles (~10¹⁰ bubbles/mL; mean diameter 1.07 ± 0.9 μm, measured with an Accusizer 780). Nanodroplets were obtained by cooling and pressurizing the microbubble suspension to induce DFB condensation. Vials were submerged in −13°C ethanol bath while pressure was applied to the vial with nitrogen gas via a needle connected to the headspace, condensing microbubbles to liquid nano-scale droplets. The resulting nanodroplet suspension was stored at −20°C.

### Human gDNA fragmentation using the Covaris LE220 sonicator

Fragmentation of 100 ng human gDNA was performed in either Covaris microTUBEs or borosilicate glass vials (Wheaton, 9360000392) with a final volume of 100 μL in TE (10 mM Tris-HCl, pH 8.0; 1 mM EDTA) buffer. For samples in glass vials, the reaction mix consisted of either 100 μL TE (no nanodroplets) or 90 μL TE with 10 μL nanodroplets. Glass vials were crimp sealed with caps (Fisher C4008-2A).

All samples were sonicated using the Covaris LE220 instrument using the 96 microTUBE rack (500282) at water level 6 according to manufacturer instructions. Samples sonicated with nanodroplets or in microTUBEs for 80, 120, and 240 seconds, while samples without nanodroplets were sonicated for 720, 840, 1080, and 1296 seconds. Sonication parameters were: Peak Incident Power (PIP) 450, Duty Factor 20%, and Cycles per Burst 200. DNA fragment size and quality were analyzed using the Agilent D5000 or D1000 ScreenTape systems.

### Human gDNA fragmentation using the adapted Qsonica Q700MPX instrument with translator

For fragmentation of DNA in the Qsonica Q700MPX instrument (Qsonica, LLC., USA), 100 ng of human gDNA in 100 μL of either TE buffer alone or in 90 μL TE with 10 μL nanodroplets was loaded into wells of a 96-well PCR plate (Olympus, Cat. #24–300). Nanodroplet-containing samples were kept on ice during the procedure. Plates were sealed with an adhesive sealing sheet (Thermo Scientific, AB-0558).

A custom 3 dimensionally (3D) printed 96-well plate holder was mounted on a linear translator, maintaining a 0.5-centimeter (cm) gap between the sonication horn and the bottom of the wells. The water bath level was aligned with the sample volume, and the temperature was controlled at 4–7°C using a refrigerated chiller (Brinkman Chiller RK 20). Sonication was performed at 20% amplitude for a total of 21 minutes and 12 seconds (8 cycles of 2 minutes 39 seconds ON, 30 seconds OFF). During each cycle while sonication is ON, the plate was linearly translated 83.75 mm from one side of the sonication horn to another using a stepper motor with a linear stage actuator (SainSmart Nema17). A quadratic speed profile was programmed for the motor using a microcontroller (Arduino UNO R3) such that the plate moves approximately two times faster near the edges of the sonication horn compared to the center. The plate was kept stationary while sonication is OFF.

For samples without linear translation, DNA was visualized via electrophoresis on a 1.5% agarose gel, and gel images were acquired using the Typhoon FLA 9500 scanner. Densitometry was conducted using custom analysis software (see companion manuscript, Bautista *et al*., GelInsight: open-source software for large-sample DNA fragmentation quality control in gel electrophoresis images). Samples with linear translation were analyzed using the Agilent TapeStation with D1000 ScreenTapes.

### Yeast gDNA preparation

Genomic DNA was extracted from a 10 mL saturated culture of Saccharomyces cerevisiae wild-type haploid strain BY4741 (MAT**a** *his3*Δ*1 leu2*Δ*0 met15*Δ*0 ura3*Δ*0*) using the YeaStar Genomic DNA Kit (Zymo Research, Cat. #D2002, USA) according to the manufacturer’s instructions. DNA concentrations were measured with a Qubit 2.0 Fluorometer (Life Technologies, Grand Island, NY, USA).

### Yeast gDNA fragmentation using the adapted Qsonica instrument

A total of 800 ng of yeast gDNA in 100 μL TE buffer containing 10 μL nanodroplets was loaded into a 96-well plate (Olympus, Cat. #24–300). Samples were randomly distributed, and all empty wells were filled with water to ensure thermal uniformity. The plate was sealed (Thermo Scientific, AB-0558) and mounted on a linear translator plate holder, and the water level was adjusted to align with the sample volume.

Sonication was performed using the Qsonica Q700MPX instrument under the same settings described above (20% amplitude; total duration: 21 minutes and 12 seconds; 8 cycles of 2 minutes 39 seconds ON, 30 seconds OFF). Fragment size and quality were assessed using the Agilent D1000 ScreenTape system.

### Yeast gDNA fragmentation using the Covaris LE220

Fragmentation of 800 ng of yeast gDNA was performed in either Covaris microTUBEs or borosilicate glass vials (Wheaton, 9360000392) with a final volume of 100 μL in TE (10 mM Tris-HCl, pH 8.0; 1 mM EDTA) buffer. For samples in glass vials, the reaction mix consisted of either 100 μL TE (no nanodroplets) or 90 μL TE with 10 μL nanodroplets. Glass vials were crimp sealed with caps (Fisher C4008-2A). Samples were sonicated for 80 seconds at peak incidence time 450, duty factor 20%, and cycles per burst 200.

### NGS library preparation

Fragmented yeast gDNA (250 ng per reaction) was used for NGS library preparation using the xGen ssDNA & Low-Input DNA Library Prep Kit (Integrated DNA Technologies, 1009859). Final library concentrations were quantified using a Qubit 2.0 Fluorometer, and fragment size distributions were analyzed using the Agilent D1000 ScreenTape system.

### Yeast whole genome DNA sequencing analyses

Whole genome sequencing of yeast gDNA libraries was performed on the Illumina NextSeq 2000 instrument with paired end 2x100 cycles. Whole Genome data was processed using the Nextflow (v23.04.2) nf-core/atacseq pipeline (v2.1.2) [1,15]. Briefly, the nf-core/atacseq pipeline performed the following default steps. The pipeline was used only for the purpose of quality filtering, trimming and alignment, and all post bigwig generation pipeline steps were skipped. Sequencing adapters were removed using Trim Galore (v0.6.7) (https://github.com/FelixKrueger/TrimGalore) and Cutdadapt (v3.4) (https://github.com/marcelm/cutadapt/). Reads were aligned to the NCBI *Saccharomyces cerevisiae* genome reference (sacCer3) using bowtie2 (v2.5.4) [16]. Duplicates were marked with Picard (v3.0.0) (https://github.com/broadinstitute/picard) but not removed and downstream replicates were merged. Normalized bigwig files were created using BEDTools (v2.31.1) [17] and bedGraphToBigWig (https://github.com/ucscGenomeBrowser/kent/tree/master/src/utils/bedGraphToBigWig). A comprehensive list of pipeline steps, software, and citations for the nf-core/atacseq pipeline can be found on the nf-core/atacseq website.

Python (v3.10.9) (https://www.python.org/) was used to analyze data and generate graphs. For data manipulation and stats using numpy (v1.25.0) [18] and pandas (v2.1.4) [19].

Insert size line graphs were generated using the output for picards insert size. Figures were generated using matplotlib (v3.8.4) [20].

Coverage line graphs were generated using multiBigwigSummary a function on deeptools (v3.5.4) on the merged replicated bigwig files with a bin of 1000. PlotCorrelation function in deeptools was used to generate the Pearson correlation coefficent from the output npz file from multiBigwigSummary. The line graph represents the score of those samples across the genome. Mean quality score across the read was calculated and plotted using the adapter-trimmed fastq files, the same files that were used as input for alignment. After the first 10 bp, quality scores were binned into 2 bp groups for plot readability. The data were plotted using Python package matplotlib.

To calculate sequencing error, variants were identified across chrI from the *sacCer3* aligned bam files for each replicate (n = 3) per fragmentation method. Per base error was calculated in a similar manner as previously described (Method #1) [7]. In brief, SAMtools (v1.21)/BCFtools mpileup [21] was run with default settings aside from the following options: -h 5, -r chrI, and -O v. Default settings include minimum base quality filter (-Q) of 13 and read filtering to exclude UNMAP, SECONDARY, QCFAIL, and DUP flags. To calculate the per base error percentage, the total alternate allele counts were divided by the sum of total alternate and total reference counts $\frac{total_{alt}}{total_{alt}+total_{ref}}\times \text{1}\text{0}\text{0}$. Error rates were found not to be significantly different between fragmentation methods (p = 0.4597, Welch Two Sample t-test). The statistical test and bar plot depicting replicate means ±standard deviation were generated with ggplot2 [22] (v3.5.2) in R (v4.4.2).

Sequencing data have been deposited to the BioProject database (National Center for Biotechnology Information), BioProject accession PRJNA1370072.

### Statistical analyses

The observed DNA fragment sizes at a given sonication time were modeled using a normal distribution to calculate the average DNA fragment size and its standard deviation, separately for each fragmentation method. A logistic regression was fitted to estimate the probability of falling into the fragmentation target range, as a function of fragmentation method (LE220 DFB + , Covaris microTUBE) and sonication time.

For plate-based analyses, the observed DNA fragment sizes were collapsed over wells and replicates and modeled using a normal distribution to calculate the average DNA fragment size and its standard deviation, separately for each fragmentation method (with or without translator, with or without nanodroplets).

For each fragmentation method (with or without translator, with or without nanodroplets), the average DNA fragment size and its variability were summarized with heat maps using the median and the median absolute variation (MAD) across 3 replicates, separately for each of the 96 well locations. The median and MAD provided a more robust measure of the average size and the variability of fragment size against outliers, compared to using the mean and standard deviation. The probability of reaching 150–300 bp range (across 3 replicates) was calculated as a function of well location, for each fragmentation method, and visualized using heatmaps.

All statistical analyses were performed in R (versions 4.3.1 or 4.2.2 [see **Yeast whole genome DNA sequencing analyses**]).

## Results and discussion

### Genomic DNA fragmentation with nanodroplets is comparable to a commercially available method

**Fig 1:**
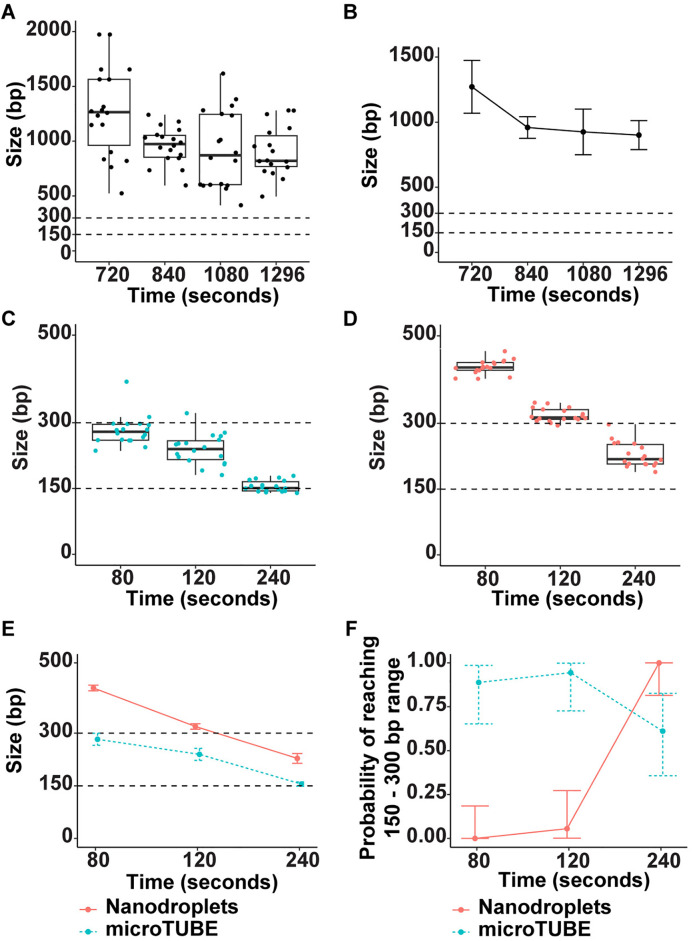
Nanodroplets Facilitate Efficient Genomic DNA Fragmentation in a Commercial Sonication Device. **(A)** Box plot showing fragment size distribution of 18 samples of human gDNA fragmented without cavitation enhancement in a Covaris LE220 sonicator. X-axis indicates sonication time (seconds), and Y-axis indicates peak DNA size (base pairs, bp) as determined by Agilent TapeStation. **(B)** Average DNA fragment size and standard deviation of gDNA fragment size without cavitation enhancement. Box plot showing fragment size distribution of 18 samples of human gDNA each sonicated in a microTUBE (C) or in a borosilicate glass tube with nanodroplets **(D)**. Fragmentation target range of 150–300 bp is indicated with dotted lines. **(E)** Comparison of average fragment size with standard deviation for samples sonicated in microTUBEs (green line) versus with nanodroplets (pink line). **(F)** Probability (Y-axis) that gDNA sample will reach target range of 150–300 bp in the time indicated (seconds, X-axis) when sonicated in a microTUBE (green line) or with nanodroplets (pink line).

Initially, we investigated the efficiency of gDNA fragmentation using the nanodroplet cavitation enhancement reagent in a commercially available sonicator. Fragmentation of gDNA was performed in a Covaris LE220 instrument using either the Covaris microTUBE or in a borosilicate glass tube with nanodroplet cavitation enhancement. We used this comparison as a benchmark, confirming data that we previously published using a Covaris E110 sonicator, which has a spherically focused instead of cylindrically focused ultrasonic transducer [9]. Purified human gDNA (100 ng) was added to an empty borosilicate glass tube, a Covaris microTUBE containing a plastic rod, or a borosilicate glass tube with nanodroplets. Each condition had 18 replicates with a total volume of 100 µL per sample. Sonication was performed for the duration of time indicated (Fig 1). We set a fragment target range of 150–300 base pairs (bp) since the recommended gDNA fragment size range for whole genome or whole exome Illumina short read sequencing applications is ~ 200–250 bp [2,8]. In the absence of cavitation enhancement, we were unable to fragment gDNA below an average size of approximately 900 bp after over 20 minutes sonication time (Figs 1A and 1B). Sonication of gDNA in the microTUBE yielded an average DNA fragment size in the target range within 80 seconds, with all samples averaging 150 bp by 240 seconds (Fig 1C). In the presence of nanodroplets, all gDNA samples reached the target fragment range at 240 seconds (Fig 1D). Average gDNA fragment size using both methods are compared (Fig 1E), and the peak probability of reaching the target range was 120 seconds for the microTUBE and 240 seconds for the nanodroplets (Fig 1F). Even though the microTUBE was able to fragment the majority of DNA samples two-fold faster than the nanodroplets in the Covaris LE220 instrument (120 seconds versus 240 seconds), both methods produced consistent and predictable DNA fragmentation patterns over time. These data from the Covaris LE220 instrument are comparable to previous results demonstrating that nanodroplets mediate efficient fragmentation of gDNA in the Covaris E110 instrument [9].

### Design and implementation of a sonication system for parallel fragmentation of 96 samples of gDNA

**Fig 2:**
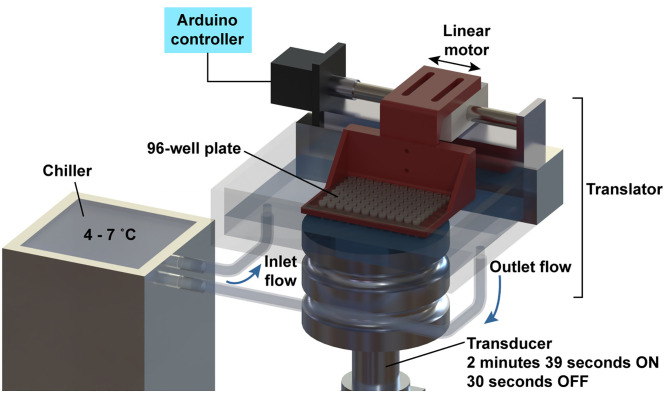
Schematic of QSonica Q700MPX Instrument With Linear Translator. A rectangular collar was fitted around the transducer to create a water bath. Ports were inserted in the bath to circulate water from a chiller set at 4-7˚C. A 3D printed plate holder was fitted to the linear transducer. A sealed 96-well plate was fitted in the holder and moved back and forth across the transducer during sonication.

Previously, we had demonstrated that nanodroplets facilitated sonication of up to 14 gDNA samples in a standard bench top ultrasonic water bath [9]. Since fragmentation of gDNA is a bottleneck in high throughput sequencing pipelines, we sought to expand sample throughput to a 96-well format. The advantage of our original ultrasonic water bath setup was that it utilized a low-powered, inexpensive transducer, while the disadvantage was that 14 samples was the maximum throughput for this device. To significantly expand our sample throughput, we modified a Qsonica Q700MPX instrument (Fig 2). This device utilizes a 20 kilohertz (kHz) 133-millimeter diameter transducer, which is significantly lower-powered than the 1-MegaHertz (MHz) transducer in the Covaris LE220 instrument. Initially, we designed a rectangular collar water bath from plexiglass surrounding the transducer. Water circulation ports were added to the bath for sample cooling to 4˚C, which is standard for current DNA fragmentation methods. We previously found that the thin-walled plastic used for polymerase chain reaction (PCR) tubes permitted efficient transmission of ultrasound waves [9]. Therefore, we placed a thin-walled 96-well PCR plate over the transducer using a 3D-printed, stationary plate holder.

**Fig 3:**
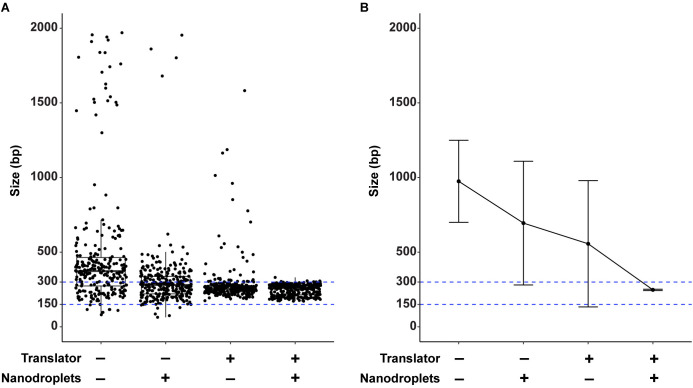
A Combination of Nanodroplets and Linear Translation Permits Efficient and Reproducible Parallel Fragmentation of 96 Samples of Genomic DNA. **(A)** Box plots showing the DNA fragment size distribution (base pairs, bp) of gDNA in a 96-well plate format with (+) or without (–) nanodroplets or the translator. Data represents 3 technical replicate 96-well plates (288 samples total) for each condition. **(B)** Average gDNA fragment size and standard deviation with outlier points removed. Target DNA size range of 150–300 bp is indicated with blue dotted lines.

**Fig 4:**
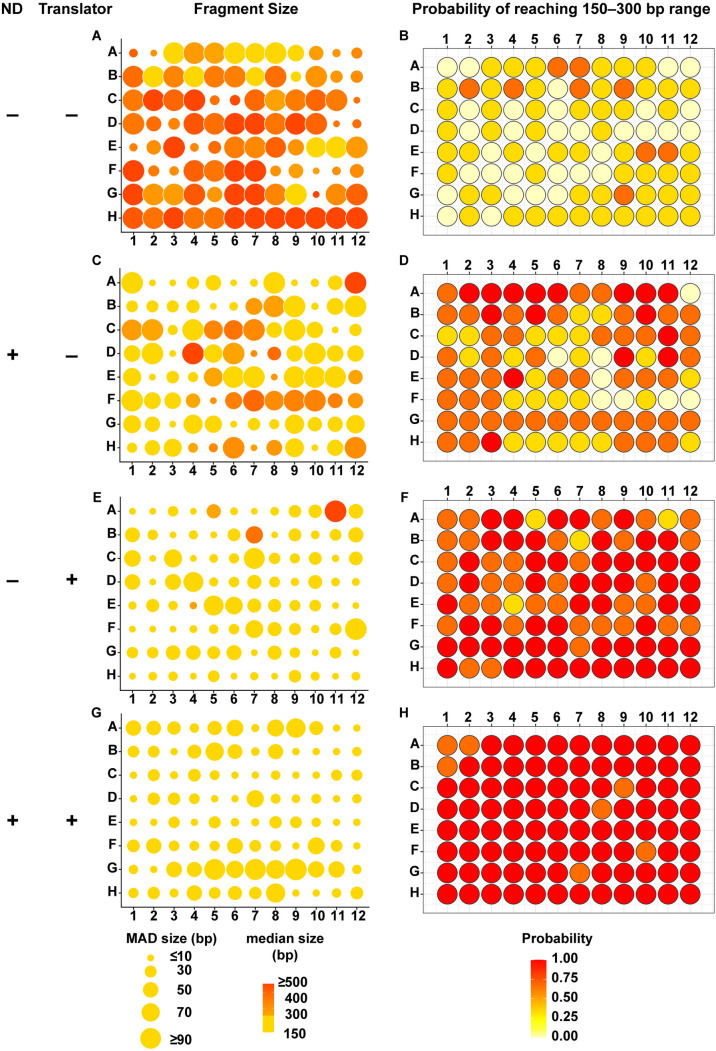
DNA Fragment Size Distribution is Consistent and Reproducible With a Combination of Nanodroplets and Linear Translation. The median distribution of gDNA fragment sizes across a 96-well plate and probability of reaching the target fragment size range of 150–300 base pairs (bp) is shown without nanodroplets or translation **(A, B)**, with nanodroplets and without translation **(C, D)**, without nanodroplets and with translation **(E, F)**, and with both nanodroplets and translation **(G, H)**. Data represents a total of 3 replicate 96-well plates (total of 288 samples) per condition.

To assess gDNA fragmentation efficiency in this device, each well of the 96-well plate was loaded with 100 ng of human gDNA in 100 μL of TE buffer with or without nanodroplets and plates were sealed with a plastic adhesive plate seal. Sonication was performed with triplicate plates at 20% amplitude for a total of 21 minutes and 12 seconds (8 cycles of 2 minutes 39 seconds ON, 30 seconds OFF). This sonication method resulted in a large fragment size distribution, with sizes that were consistently outside the range of the D1000 ScreenTape recommended for our target range of 150–300 bp. Therefore, average DNA fragment size for these samples was determined by gel electrophoresis followed by DNA size analysis using custom software that we designed for this purpose that produces DNA size distribution data comparable to Agilent TapeStation (Figs 3 and 4, and companion manuscript, Bautista *et al*., GelInsight: open-source software for large-sample DNA fragmentation quality control in gel electrophoresis images).

Even in the presence of nanodroplets, the stationary plate holder did not consistently fragment gDNA in the target range (Fig 3A and 3B). When we analyzed the gDNA fragment distribution within each well, we noted that there was significant well-to-well variation in median DNA fragment size (Fig 4A) and a low probability of reaching the target fragment range (Fig 4B) that were improved but not eliminated with the addition of nanodroplets (Figs 4C and 4D).

We hypothesized that the fragmentation inconsistency was due to variability in the ultrasonic field across the 96-well plate. Specifically, the ultrasound energy was lower near the center of the transducer relative to the outer edge (S1 Fig). To mitigate this variation, we designed a plate holder that could be linearly translated left to right (and right to left) across the transducer during sonication (Fig 2). The speed at which the plate was translated was specifically programmed to be lower while moving near the center of the transducer to functionally increase the relative sonication time at the center position compared to the outer edge. The linear translation effectively normalized the amount of ultrasound energy received by each well. Fragmentation of human gDNA in the 96-well plate format was repeated in technical triplicate as described above with or without nanodroplets and with linear translation. Since this sonication method produced DNA fragment sizes in the recommended range for a D1000 ScreenTape, DNA size measurements were performed on the Agilent TapeStation. Translation alone slightly improved the overall fragment distribution (Fig 3A and 3B) and well-to-well fragment variation (Figs 4E and 4F), but only nanodroplets combined with linear translation produced an average fragment size within 150–300 bp (bp) (Fig 3A and 3B), a consistent median DNA base pair size across the plate (Fig 4G) and a high probability of fragmentation within the target range (Fig 4H). These data demonstrate that it is possible to consistently fragment 96 samples of gDNA in parallel in a low-powered ultrasonic water bath sonicator.

### High throughput sequencing demonstrates that the quality of gDNA fragmented with nanodroplets using linear translation is comparable to gDNA fragmented with a commercial sonicator

To evaluate the quality of gDNA fragmented in the adapted 96-well plate sonicator, we performed high throughput sequencing. The gDNA used for these experiments was extracted from a strain of BY4741 *Saccharomyces cerevisiae* (yeast), because the small yeast genome allows for high density sequencing coverage [9]. Three randomly selected wells of a 96-well plate were loaded with 800 ng yeast gDNA in a final volume of 100 µL TE buffer with nanodroplets. The remaining wells were filled with 100 µL water to ensure even sonication across the plate. Sonication was performed as described for human gDNA.

Sequencing libraries were prepared for Illumina short read sequencing. Since the library preparation kit recommended the use of a Covaris ultrasonicator to fragment DNA, we also performed sonication of yeast gDNA in the Covaris LE220 instrument. A total of 800 ng of yeast gDNA in a final volume of 100 µL TE was loaded into microTUBEs in triplicate and sonication was performed in the Covaris LE220 instrument for 80 seconds as previously described.

**Fig 5:**
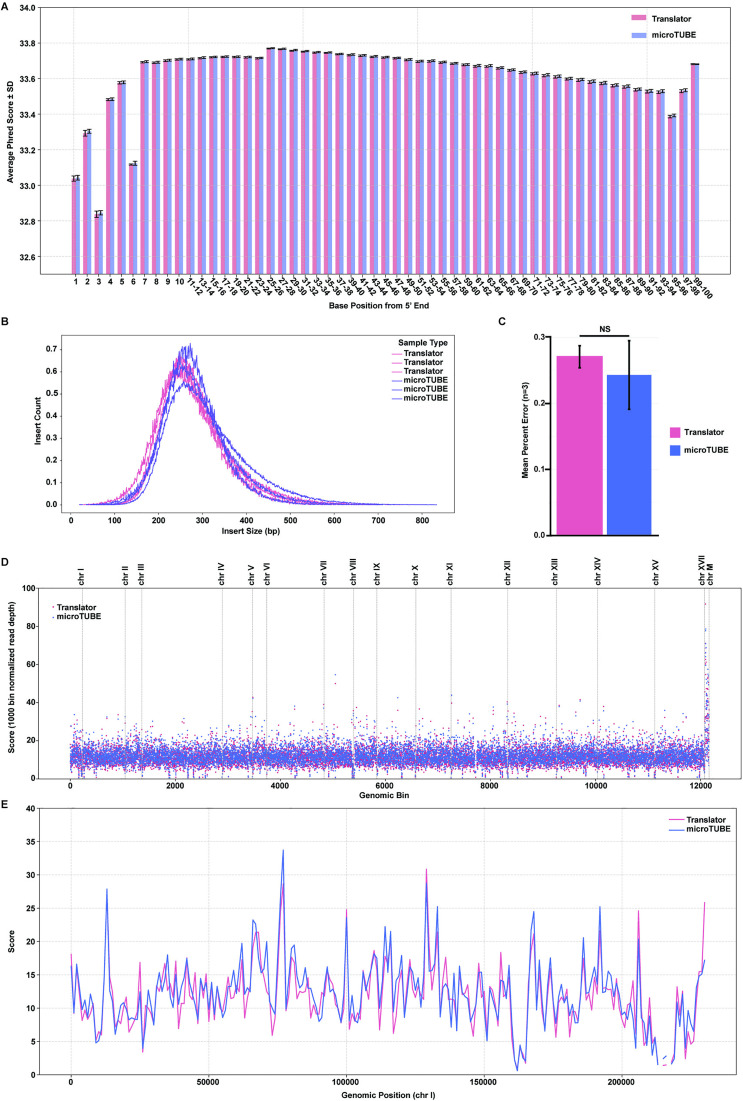
Quality of Genomic DNA Fragmented With Nanodroplets and Linear Translation is Comparable to Genomic DNA Fragmented in a microTUBE, as Shown By Sequencing Fragment Size, Mean Percent Error, Phred Score, and Genome Coverage. Yeast genomic DNA was fragmented in triplicate in either the QSonica Q700MPX instrument with nanodroplets and linear translator (pink) or in a Covaris LE220 sonicator with microTUBE (purple). **(A)** Average sequencing quality scores (Phred) across the sequencing reads. **(B)** DNA insert size distribution for each replicate. **(C)** Average, per base percent sequencing error including missense, deletion and insertion errors. **(D)** Sequence coverage across the entire yeast genome in 1,000 bp genomic bins. **(E)** Sequence coverage across chromosome I in 1,000 bp genomic bins.

Sequencing of triplicate samples from the 96-well plate format (translator) or Covaris microTUBE was performed on the Illumina NEXTseq 2000 instrument with paired end 2x100 cycles to provide accuracy and appropriate coverage for the relatively small yeast genome by reading 100 bp from both ends of the DNA fragments. DNA quality was assessed by calculating the average Phred score, which is a measure of base calling accuracy, where a score of >30 is equivalent to a 99.9% probability that a base is called accurately [23,24]. The average Phred scores for both methods were remarkably similar with all base positions from the 5’ end having an average score greater than 30 (Fig 5A). The average insert size was 254 ± 0.19 bp for the microTUBE and 241 ± 0.19 bp for the translator samples (Fig 5B), with a KS test p-value of 0.139 indicating no significant difference in fragment size distribution. The mean per base error was calculated as previously described in Knierim *et al*. (Method #1) [7] and includes missense mutations, deletions, and insertions (Fig 5C). Both methods produced a mean percent error rate less than 0.3%, with no statistically significant difference between the translator and microTUBE gDNA.

Next, we compared the sequence coverage across the entire yeast genome in 1,000 bp genomic bins (Fig 5D). For each chromosome, coverage for both methods overlapped significantly, with a Pearson correlation coefficient of 0.9926. We assessed coverage on chromosome I (Fig 5E) and found that both methods provided complete and overlapping coverage across the entire genomic region.

Overall, fragmentation of gDNA with a combination of nanodroplets and linear plate translation produces consistent DNA size distributions and high-quality whole genome sequencing data comparable to the Illumina recommended Covaris instrument. Unlike the LE220 instrument, which processes only 8 samples in parallel, our modified platform can accommodate up to 96 samples in parallel.

## Conclusions

We have demonstrated that cavitation-enhancing nanodroplets facilitate efficient gDNA fragmentation in a sonication device with a low-powered (20 kHz) transducer. The quality of gDNA sonicated in our platform was confirmed by whole genome sequencing to be comparable to a lower-throughput commercial sonication system. Low-powered transducers have the advantage of producing less heat during sonication compared to high-powered transducers (e.g., 1 MHz Covaris transducer), which minimizes sample degradation including single strand breaks, and mitigates DNA oxidation [25,26]. Further, we showed that the addition of a simple translation platform significantly improves the consistency of DNA fragmentation, allowing for parallel processing of 96 samples of gDNA in less than 22 minutes. To facilitate throughput, the open top water bath and standardized plate holder have the potential to accommodate retrieval of plates using an automation arm. We previously demonstrated that nanodroplets were useful for biospecimen processing [13,27], so future applications for this type of system could include disruption of bacteria, yeast or human tissue culture cells for genomic or epigenomic applications.

## Supporting information

S1 Fig Acoustic field characterization of the QSonica Q700MPX transducer. The acoustic pressure across the transducer was measured with a hydrophone as indicated (left box) at room temperature with no water circulation (middle panel) or at −8˚C with water circulation (right panel). Pressure level in decibels (dB) is indicated to the right of the middle and right panels. The ultrasound energy was lower near the center of the transducer relative to the outer edge.
(DOCX)

S2 Fig Raw data. (ZIP)

S3 File QSonica nanodroplets no translator gel analysis raw data. (ZIP)

S4 File QSonica no cav enhance no translator gel analysis raw data. (ZIP)

S5 File QSonica translator TapeStation raw data. (ZIP)

S6 File Summary of data for Figures 3 and 4. (XLSX)

S7 File Statistical analysis R code. (ZIP)
