# Supplementary figures and images for: 96 sample parallel acoustic fragmentation for high throughput next generation sequencing library preparation
Source: PLoS One. 2026 Feb 17;21(2):e0341139. doi: 10.1371/journal.pone.0341139 (PMC12912608; doi:10.1371/journal.pone.0341139)

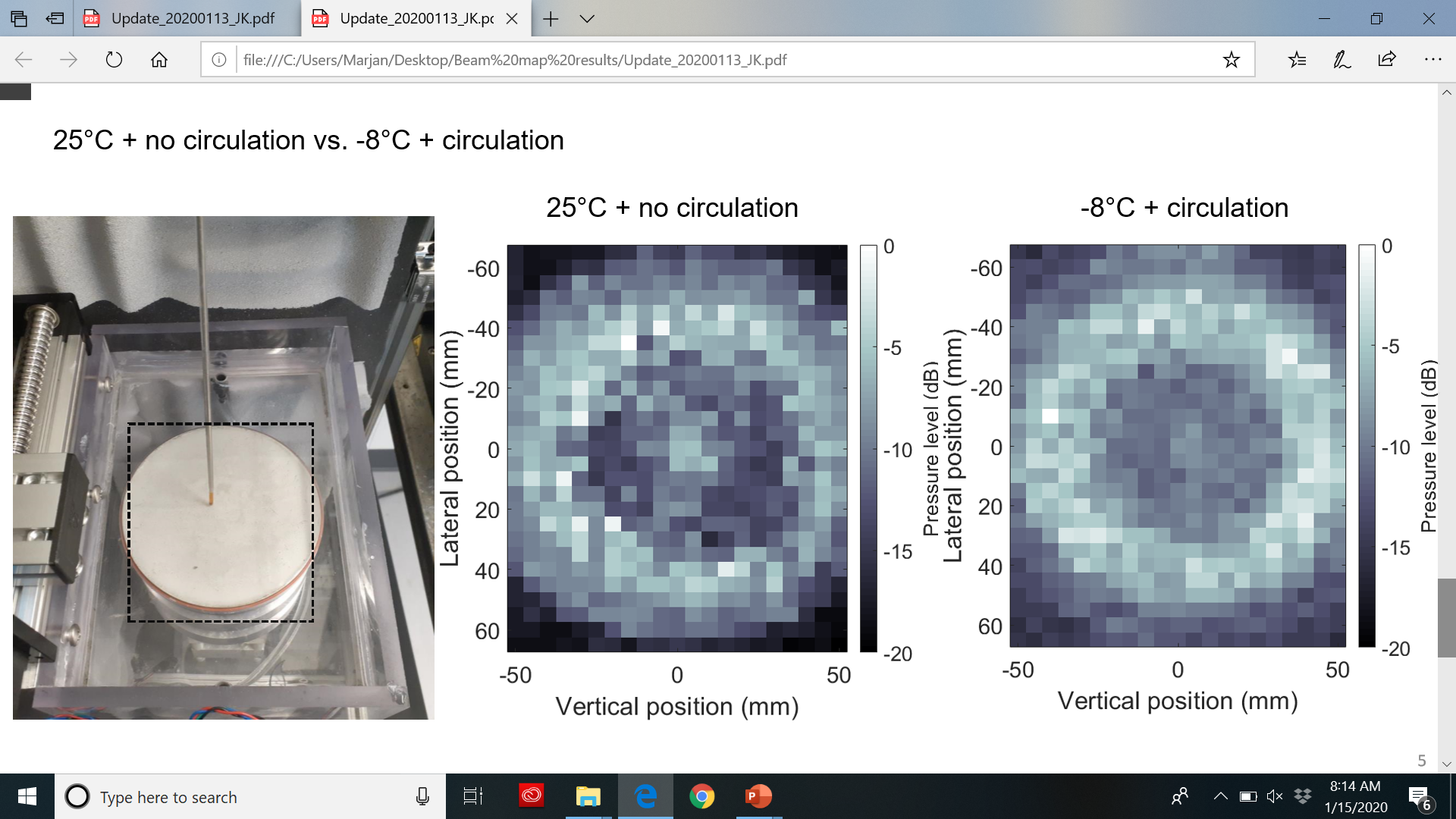

Supplement: S1 Fig — The acoustic pressure across the transducer was measured with a hydrophone as indicated (left box) at room temperature with no water circulation (middle panel) or at −8˚C with water circulation (right panel). Pressure level in decibels (dB) is indicated to the right of the middle and right panels. The ultrasound energy was lower near the center of the transducer relative to the outer edge. (DOCX) [file pone.0341139.s001.docx]

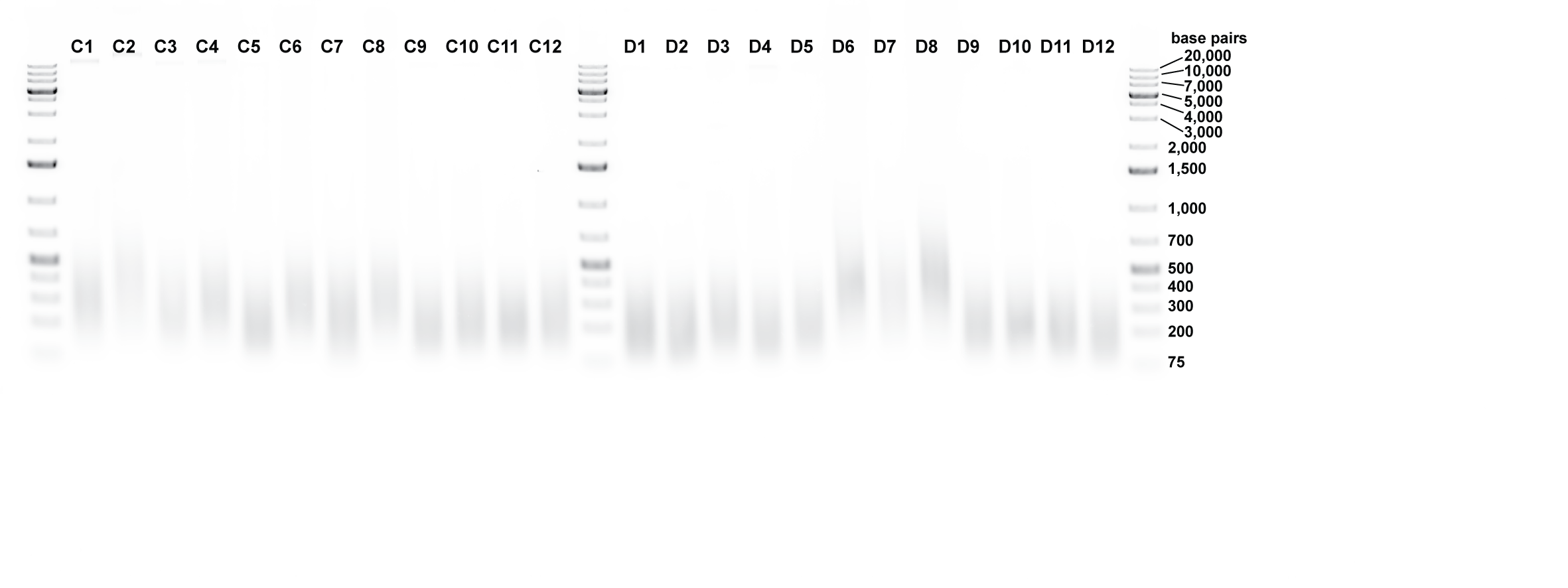

Supplement: S3 File — (ZIP) [file pone.0341139.s003.zip › QSonica nanodroplets no translator gel analysis/Gel Pictures/R1 QSonica nanodroplets no translator C,D 1-12.tif]

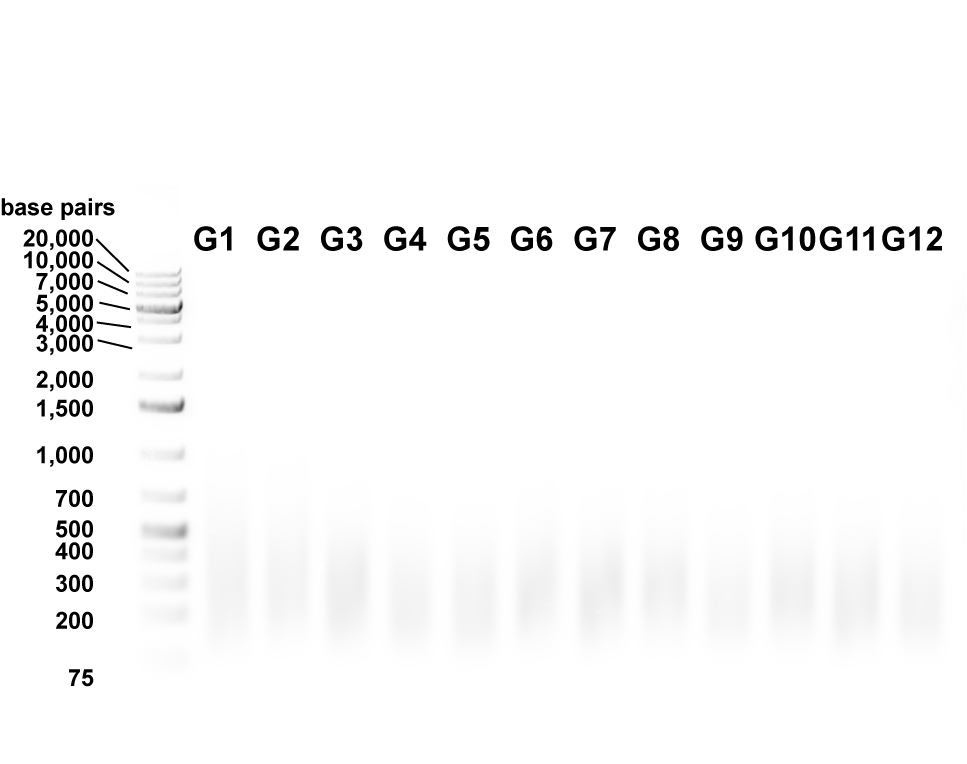

Supplement: S3 File — (ZIP) [file pone.0341139.s003.zip › QSonica nanodroplets no translator gel analysis/Gel Pictures/R1 QSonica nanodroplets no translator G 1-12.tif]

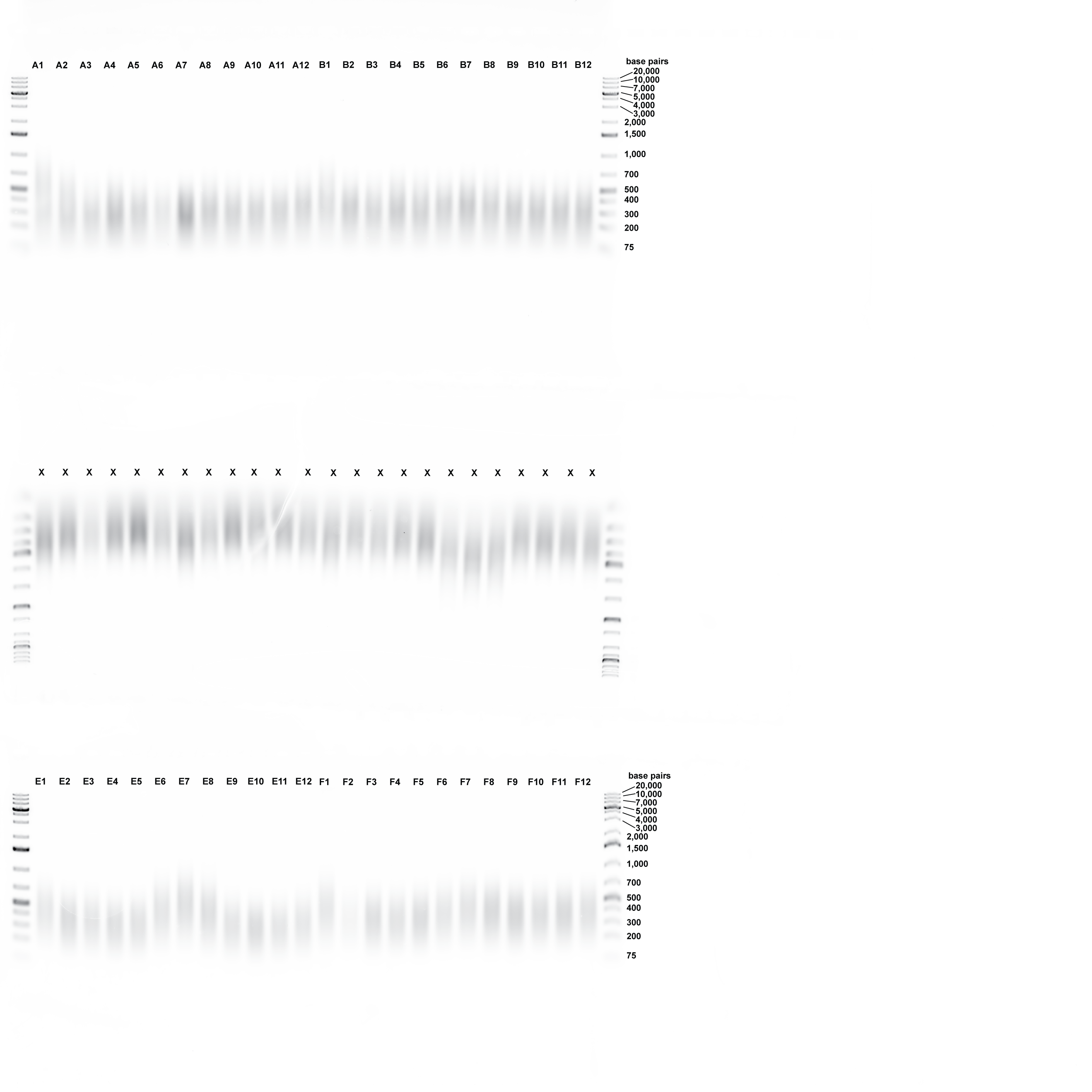

Supplement: S3 File — (ZIP) [file pone.0341139.s003.zip › QSonica nanodroplets no translator gel analysis/Gel Pictures/R1 QSonica nanodroplets no translator A,B,E,F 1-12.tif]

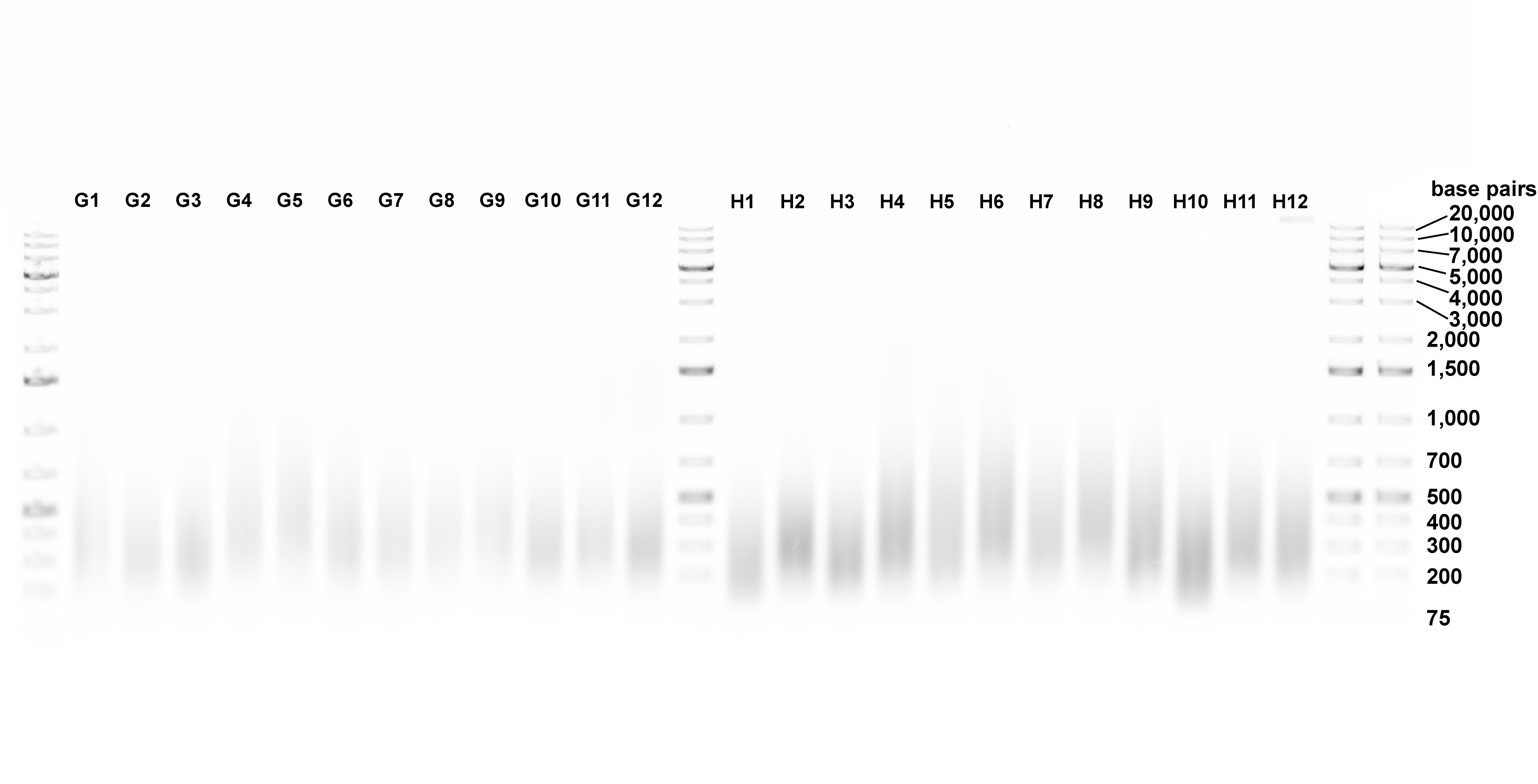

Supplement: S3 File — (ZIP) [file pone.0341139.s003.zip › QSonica nanodroplets no translator gel analysis/Gel Pictures/R2 QSonica nanodroplets no translator G,H 1-12.tif]

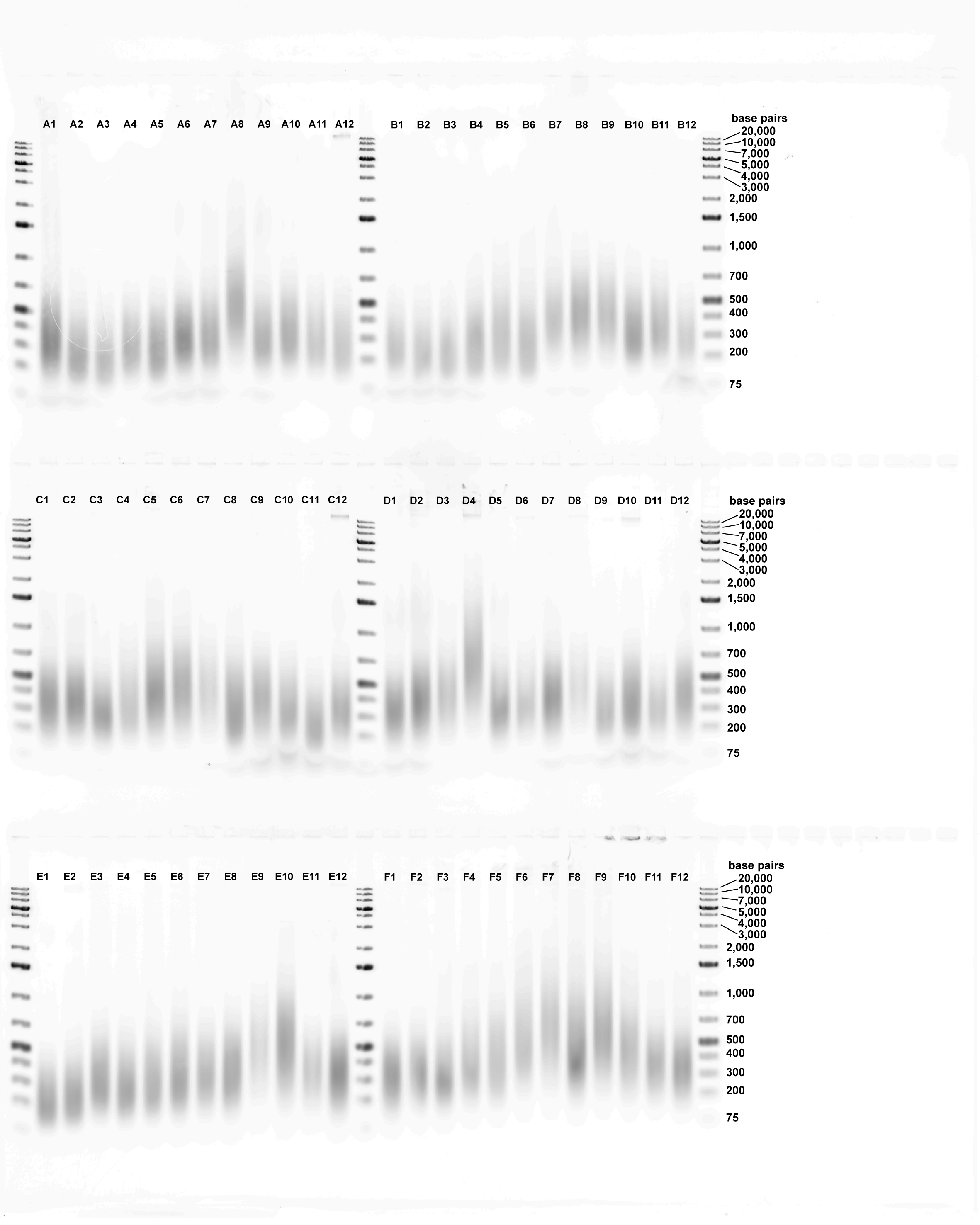

Supplement: S3 File — (ZIP) [file pone.0341139.s003.zip › QSonica nanodroplets no translator gel analysis/Gel Pictures/R2 QSonica nanodroplets no translator A,B,C,D,E,F 1-12.tif]

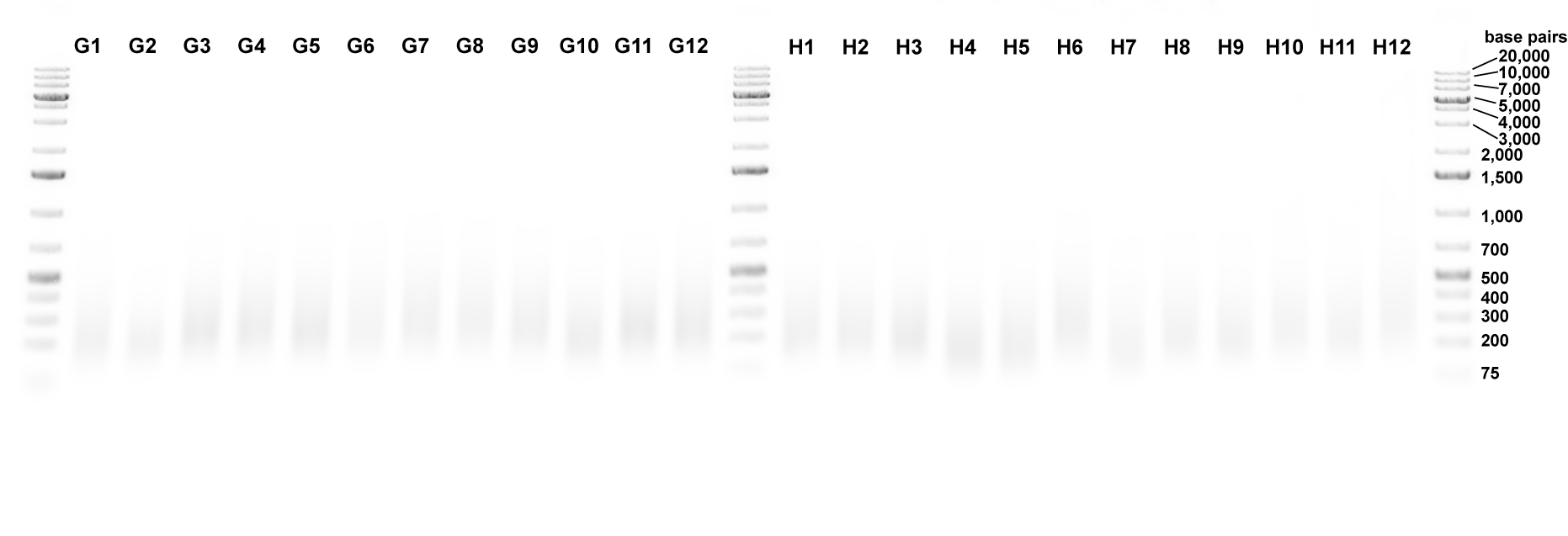

Supplement: S3 File — (ZIP) [file pone.0341139.s003.zip › QSonica nanodroplets no translator gel analysis/Gel Pictures/R3 QSonica nanodroplets no translator G,H 1-12.tif]

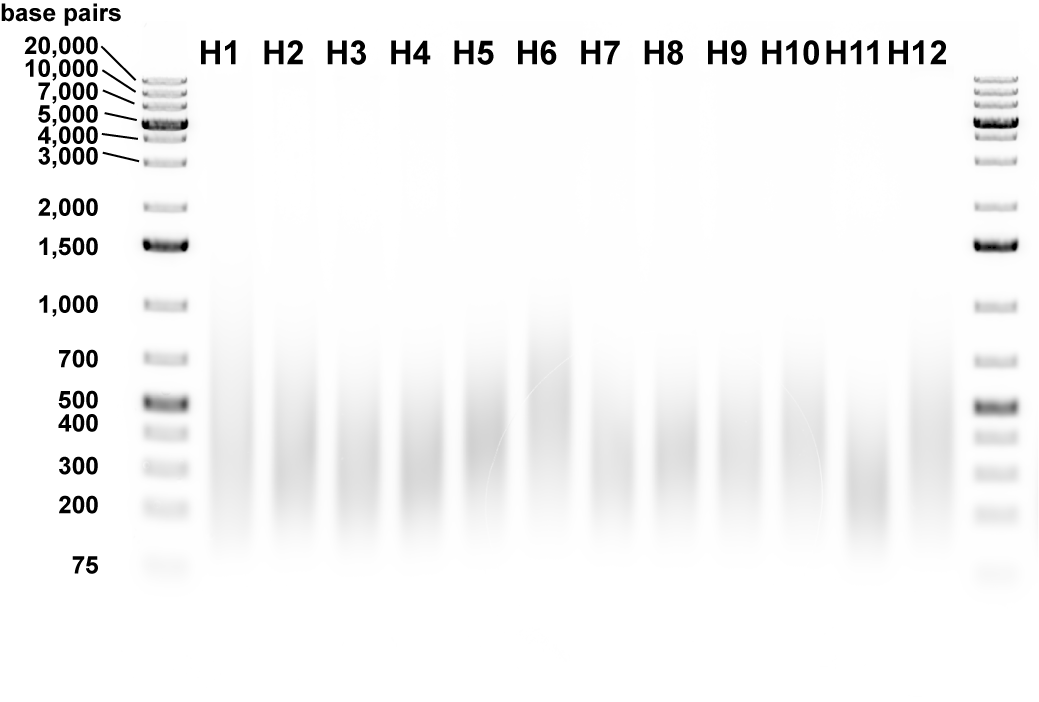

Supplement: S3 File — (ZIP) [file pone.0341139.s003.zip › QSonica nanodroplets no translator gel analysis/Gel Pictures/R1 QSonica nanodroplets no translator H 1-12.tif]

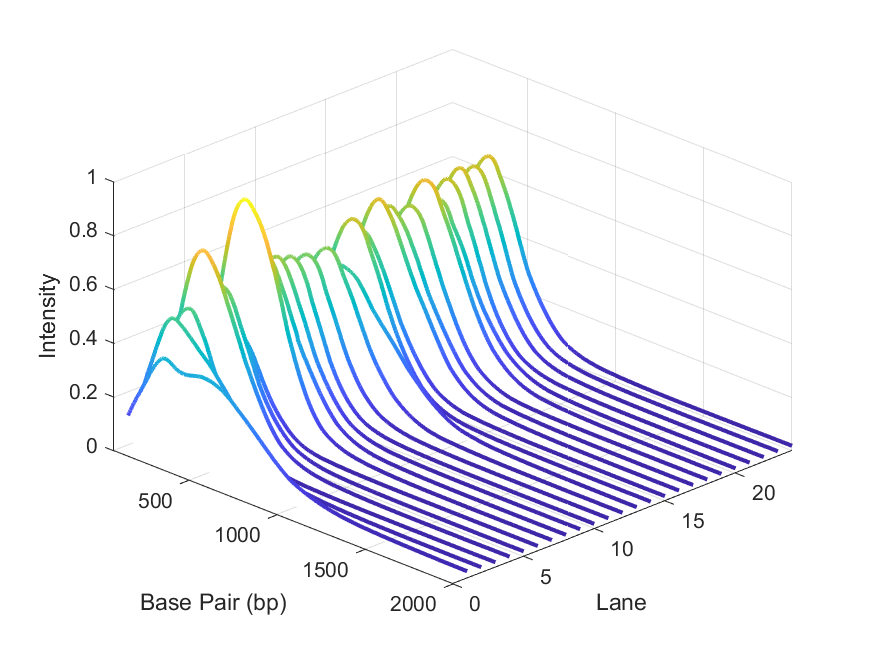

Supplement: S3 File — (ZIP) [file pone.0341139.s003.zip › QSonica nanodroplets no translator gel analysis/Densitometry Analysis/R1 QSonica nanodroplets no translator densitometry/QS,PND,R1,A1-12, B1-12-wf.png]

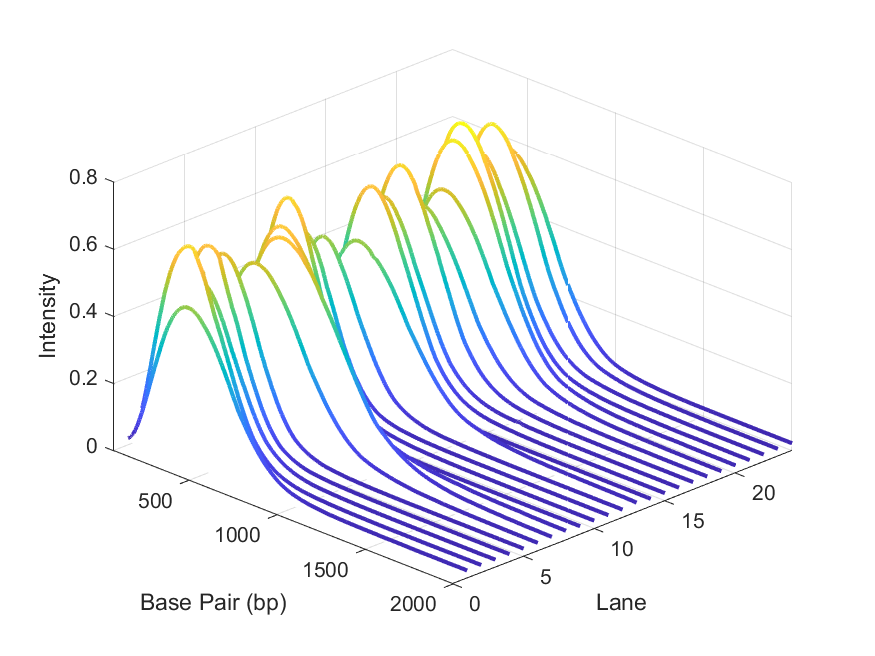

Supplement: S3 File — (ZIP) [file pone.0341139.s003.zip › QSonica nanodroplets no translator gel analysis/Densitometry Analysis/R1 QSonica nanodroplets no translator densitometry/QS,PND,R1,E1-12, F1-12.-wf.png]

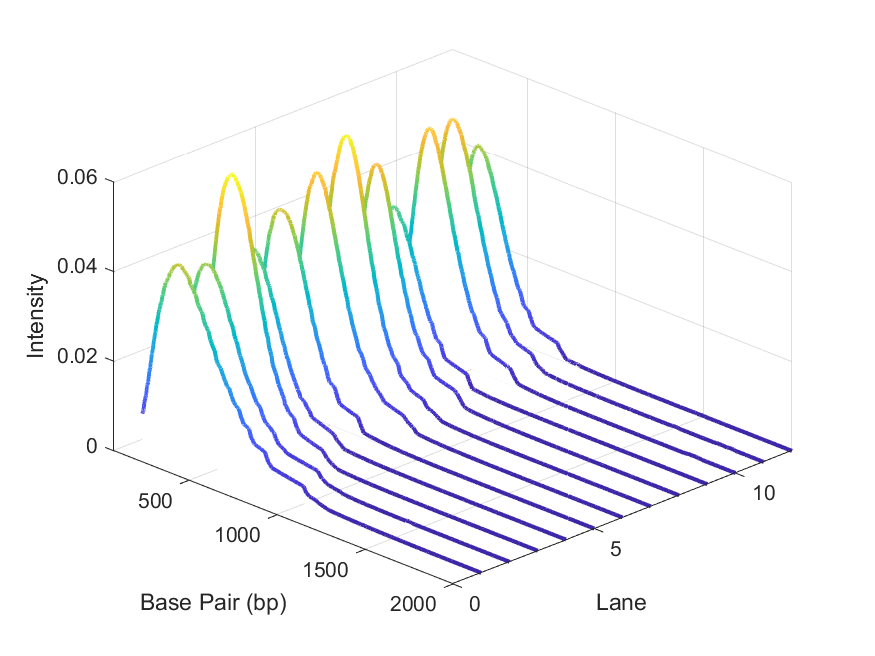

Supplement: S3 File — (ZIP) [file pone.0341139.s003.zip › QSonica nanodroplets no translator gel analysis/Densitometry Analysis/R1 QSonica nanodroplets no translator densitometry/QS,PND,R1,G1-12,-wf.png]

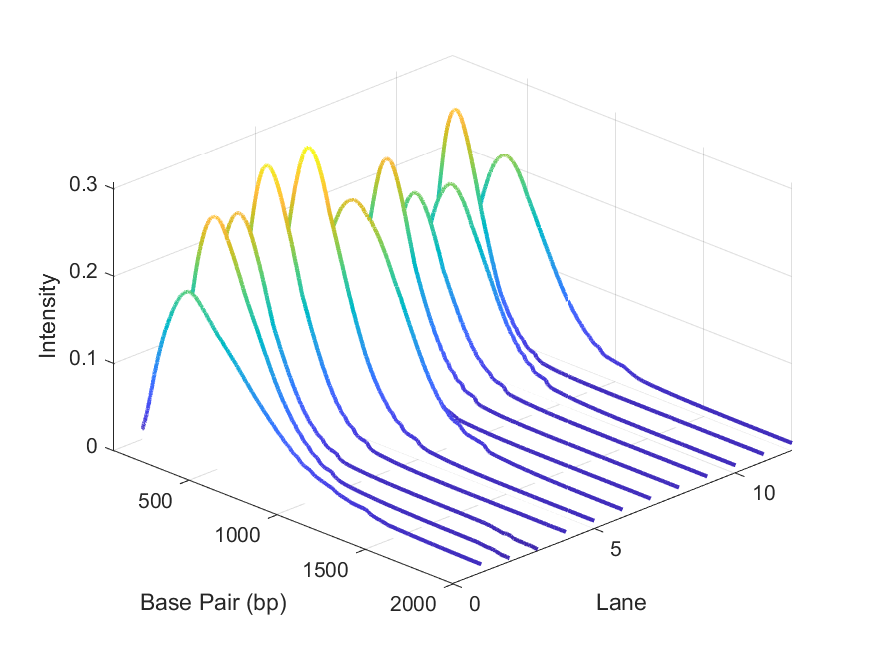

Supplement: S3 File — (ZIP) [file pone.0341139.s003.zip › QSonica nanodroplets no translator gel analysis/Densitometry Analysis/R1 QSonica nanodroplets no translator densitometry/QS,PND,R1,H1-12.adj-wf.png]

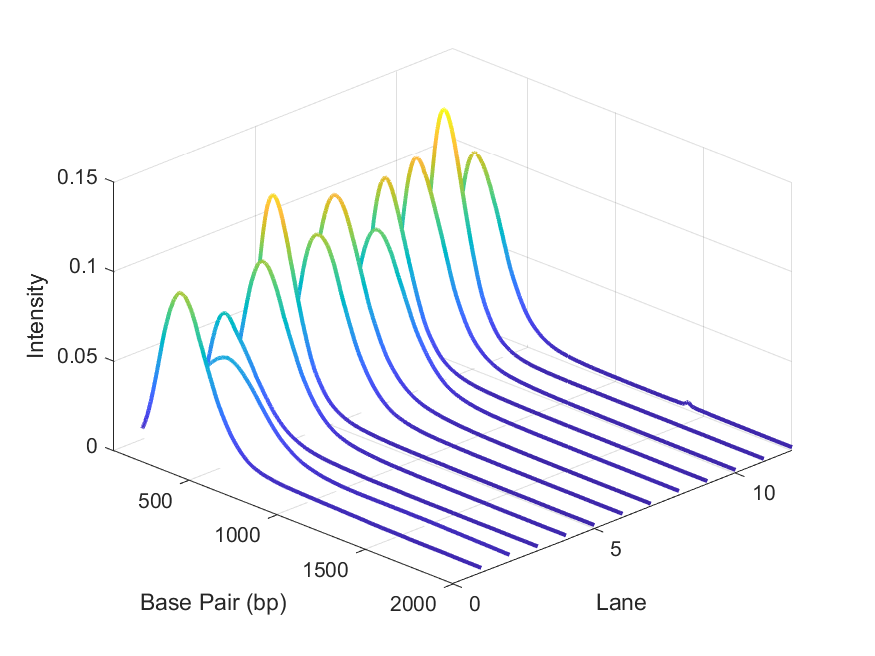

Supplement: S3 File — (ZIP) [file pone.0341139.s003.zip › QSonica nanodroplets no translator gel analysis/Densitometry Analysis/R1 QSonica nanodroplets no translator densitometry/QS,PND,R1,C1-12-wf.png]

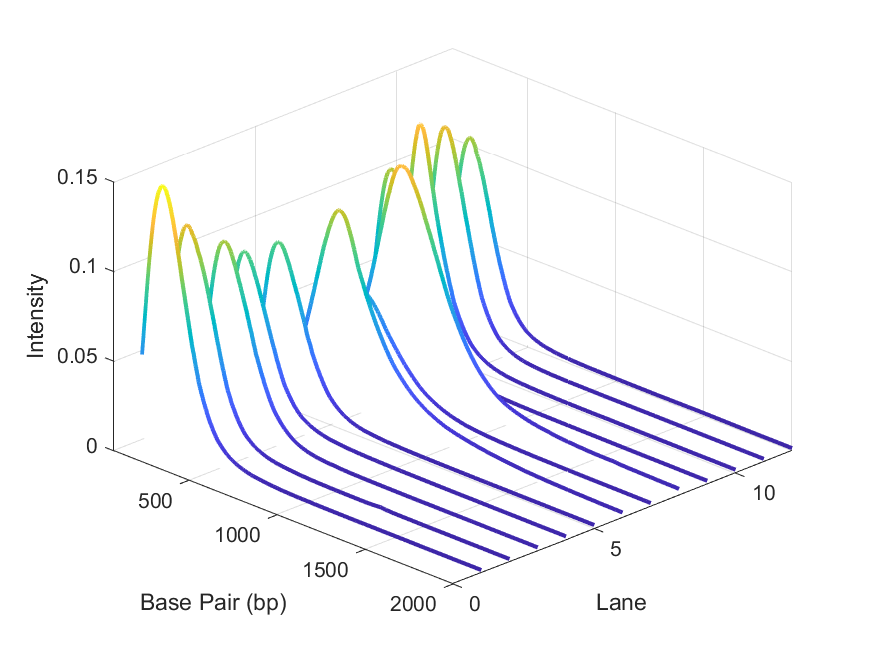

Supplement: S3 File — (ZIP) [file pone.0341139.s003.zip › QSonica nanodroplets no translator gel analysis/Densitometry Analysis/R1 QSonica nanodroplets no translator densitometry/QS,PND,R1,D1-12-wf.png]

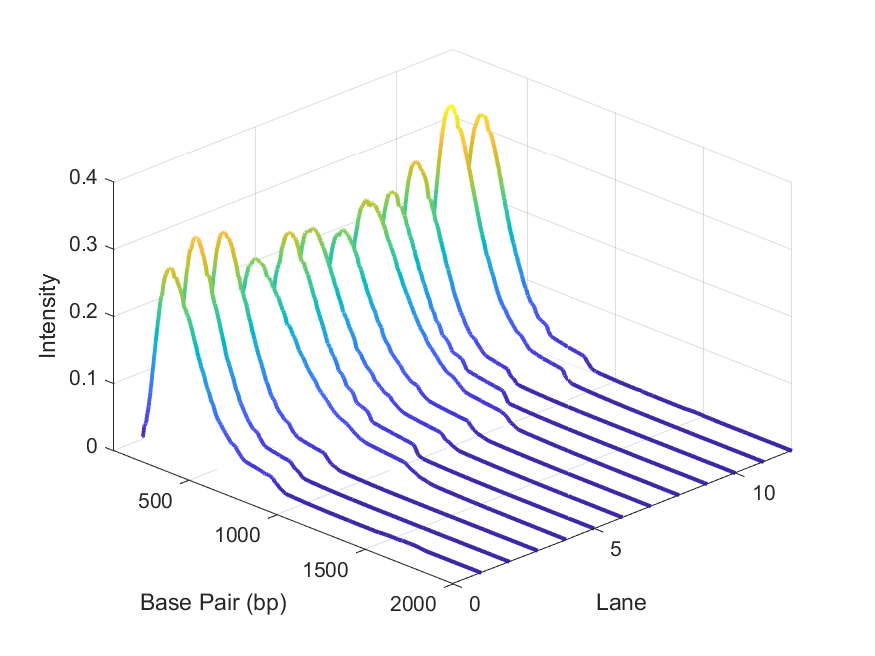

Supplement: S3 File — (ZIP) [file pone.0341139.s003.zip › QSonica nanodroplets no translator gel analysis/Densitometry Analysis/R3 QSonica nanodroplets no translator densitometry/QS PND,R3,B1-12-wf.png]

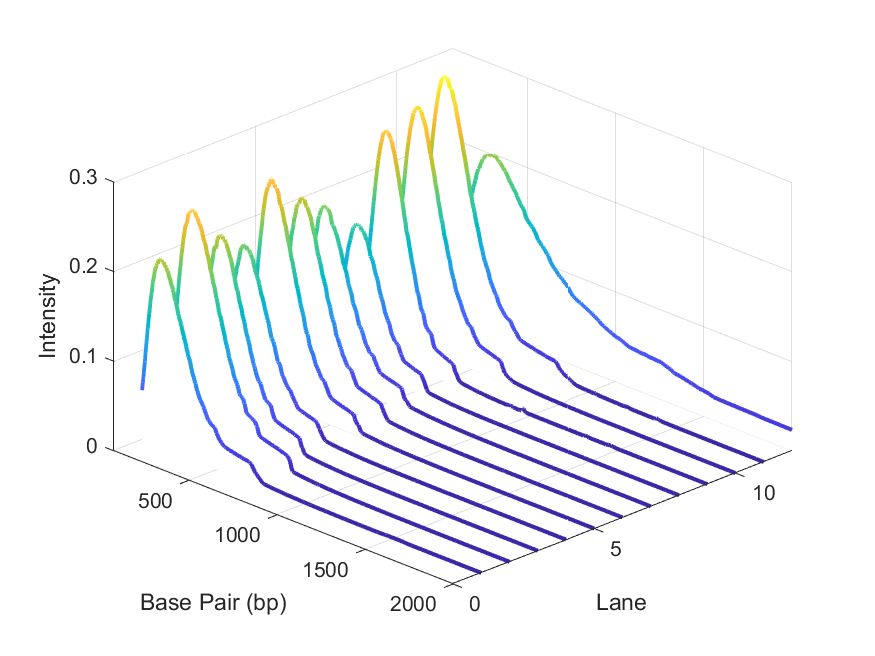

Supplement: S3 File — (ZIP) [file pone.0341139.s003.zip › QSonica nanodroplets no translator gel analysis/Densitometry Analysis/R3 QSonica nanodroplets no translator densitometry/QS PND,R3,A-12-wf.png]

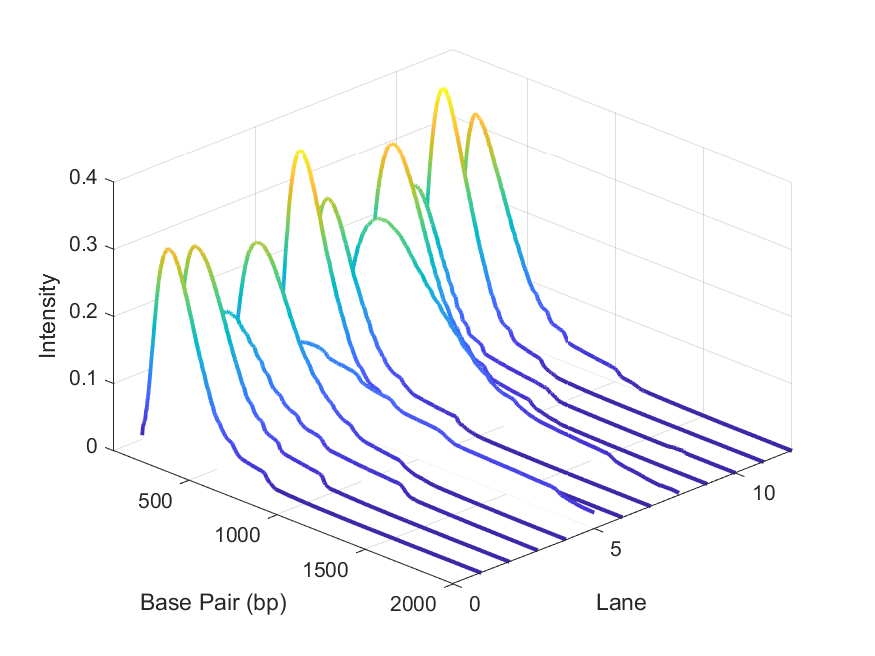

Supplement: S3 File — (ZIP) [file pone.0341139.s003.zip › QSonica nanodroplets no translator gel analysis/Densitometry Analysis/R3 QSonica nanodroplets no translator densitometry/QS PND,R3,E1-12-wf.png]

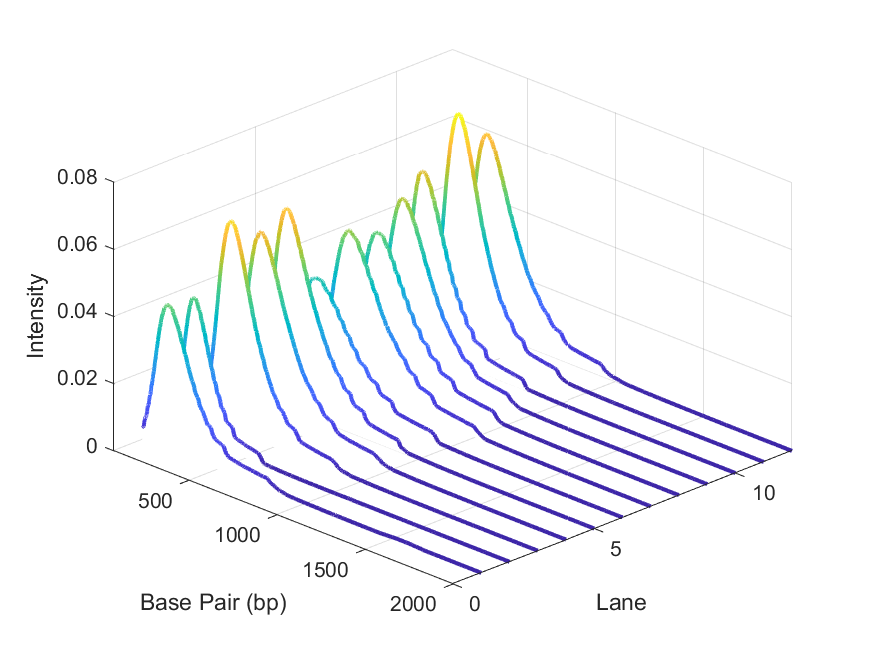

Supplement: S3 File — (ZIP) [file pone.0341139.s003.zip › QSonica nanodroplets no translator gel analysis/Densitometry Analysis/R3 QSonica nanodroplets no translator densitometry/QS,PND, R3, G1-12-wf.png]

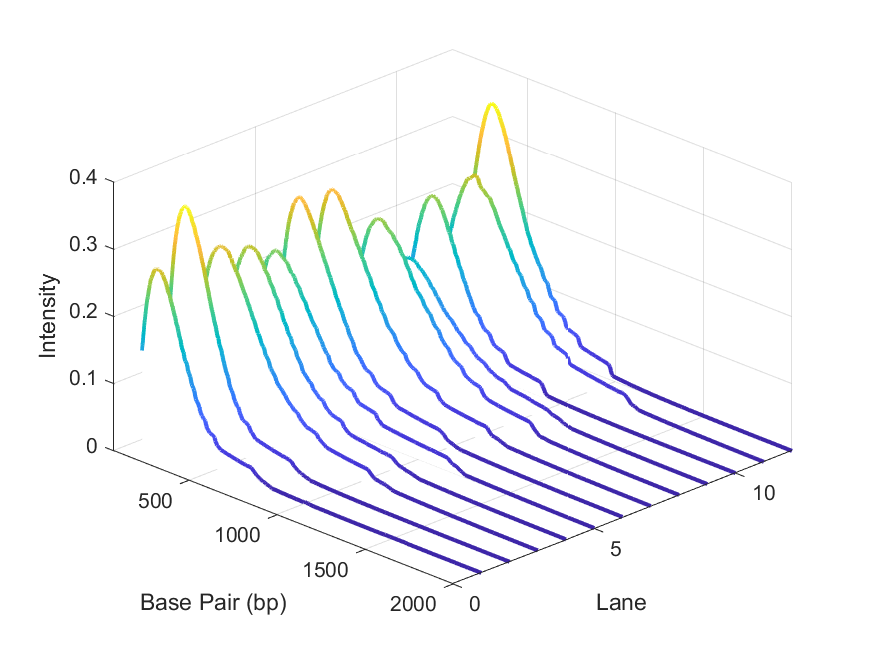

Supplement: S3 File — (ZIP) [file pone.0341139.s003.zip › QSonica nanodroplets no translator gel analysis/Densitometry Analysis/R3 QSonica nanodroplets no translator densitometry/QS PND,R3,F1-12-wf.png]

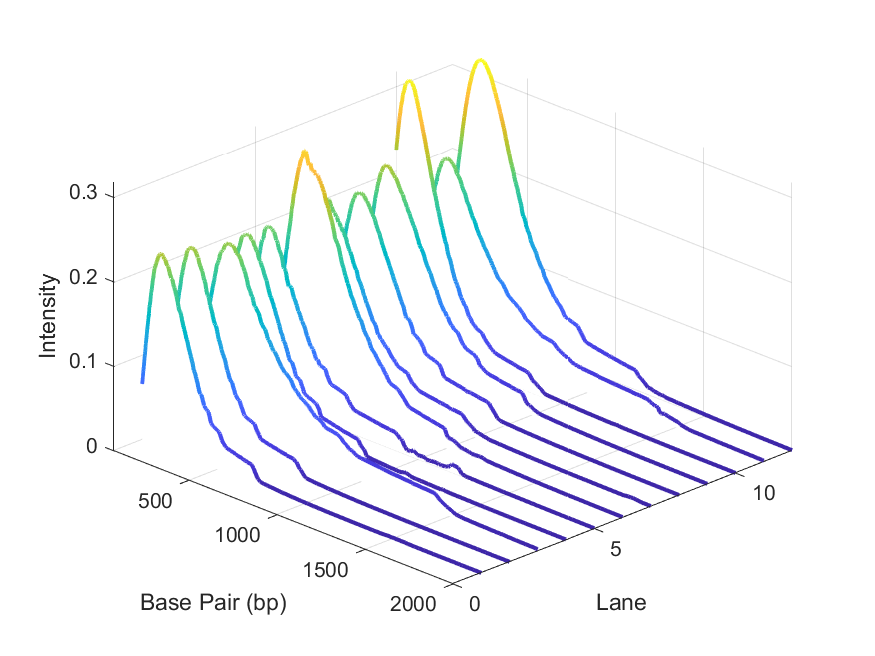

Supplement: S3 File — (ZIP) [file pone.0341139.s003.zip › QSonica nanodroplets no translator gel analysis/Densitometry Analysis/R3 QSonica nanodroplets no translator densitometry/QS PND,R3,C1-12-wf.png]

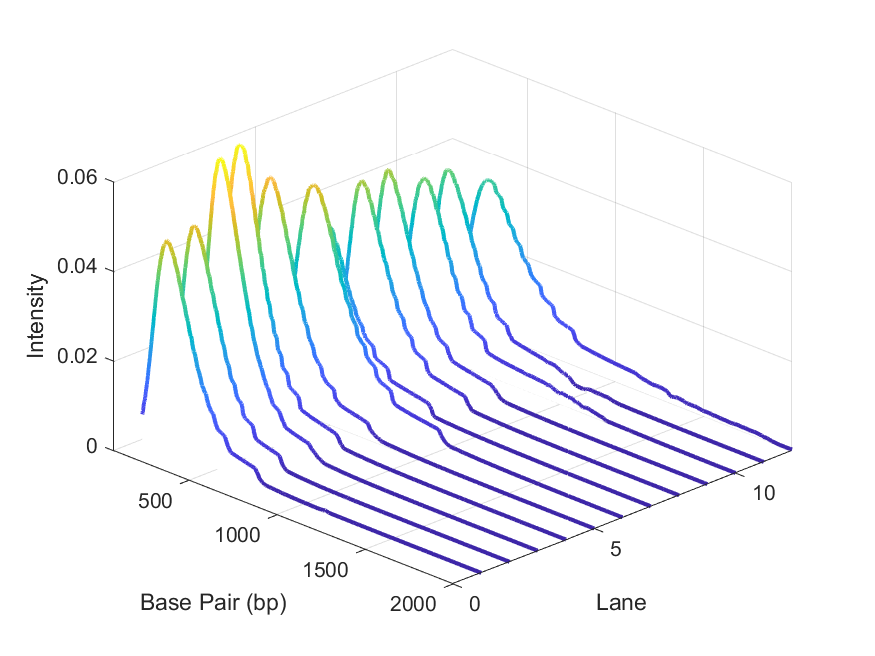

Supplement: S3 File — (ZIP) [file pone.0341139.s003.zip › QSonica nanodroplets no translator gel analysis/Densitometry Analysis/R3 QSonica nanodroplets no translator densitometry/QS,PND, R3, H1-12-wf.png]

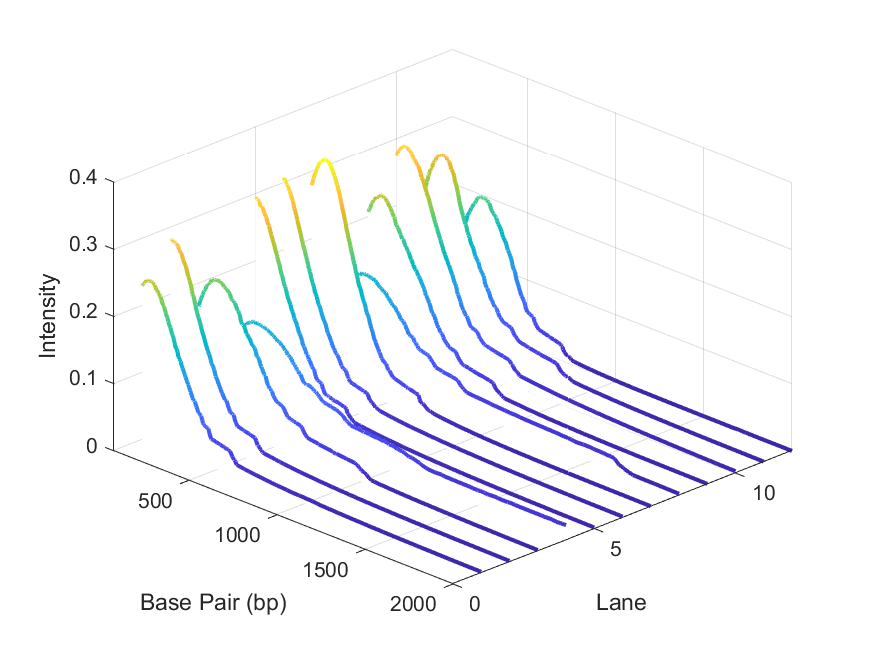

Supplement: S3 File — (ZIP) [file pone.0341139.s003.zip › QSonica nanodroplets no translator gel analysis/Densitometry Analysis/R3 QSonica nanodroplets no translator densitometry/QS PND,R3,D1-12-wf.png]

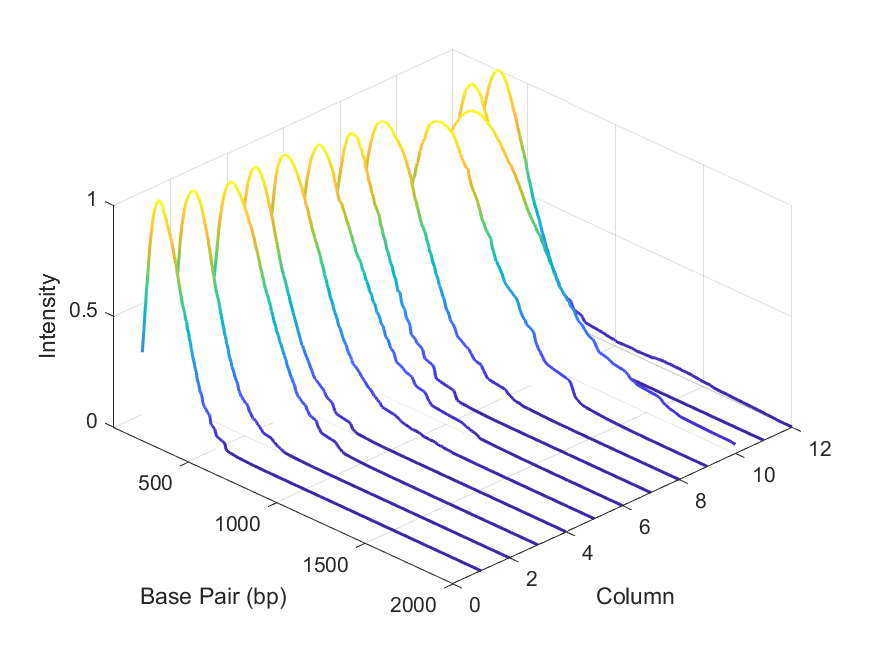

Supplement: S3 File — (ZIP) [file pone.0341139.s003.zip › QSonica nanodroplets no translator gel analysis/Densitometry Analysis/R2 QSonica nanodroplets no translator densitometry/Q-S,PNDR2,E1-E12-wf-norm.png]

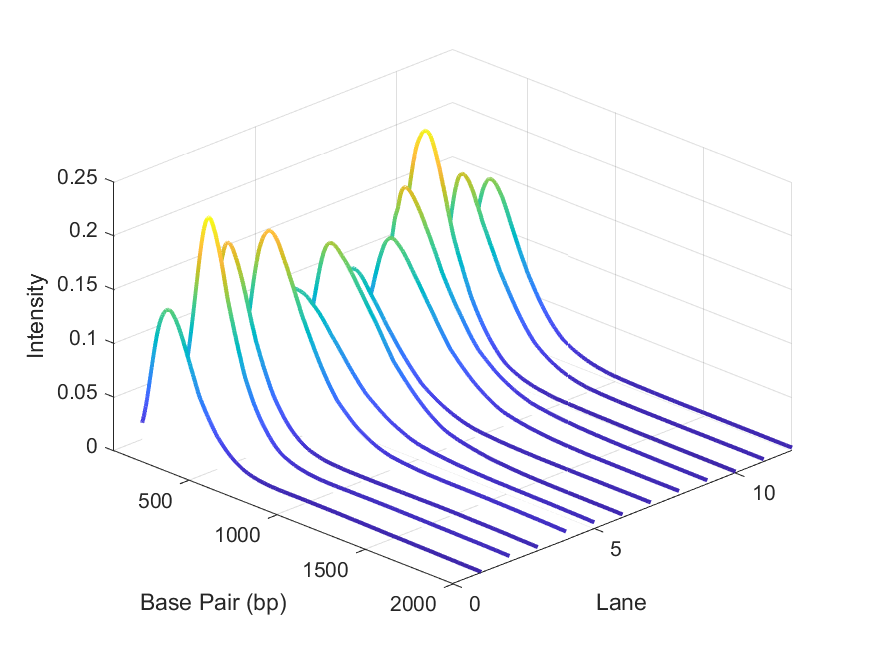

Supplement: S3 File — (ZIP) [file pone.0341139.s003.zip › QSonica nanodroplets no translator gel analysis/Densitometry Analysis/R2 QSonica nanodroplets no translator densitometry/Q-S,PND,R2,H1-12-wf.png]

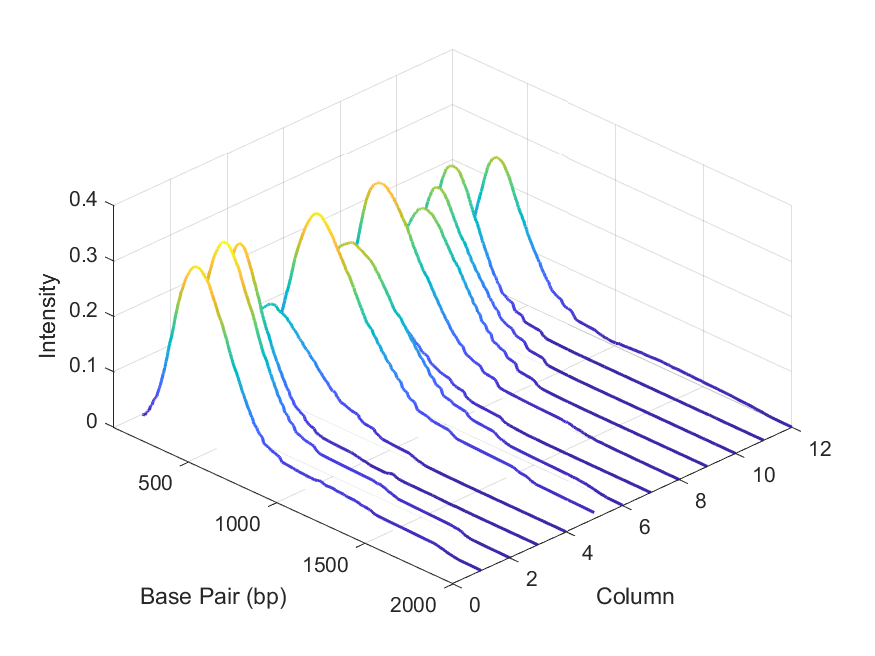

Supplement: S3 File — (ZIP) [file pone.0341139.s003.zip › QSonica nanodroplets no translator gel analysis/Densitometry Analysis/R2 QSonica nanodroplets no translator densitometry/Q-S.PND,R2 C1-C12-wf.png]

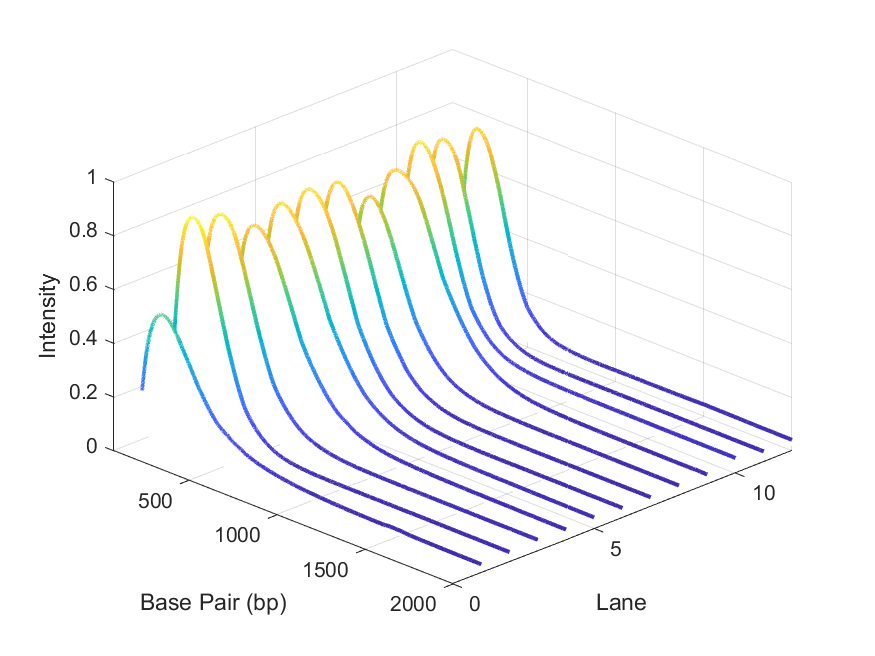

Supplement: S3 File — (ZIP) [file pone.0341139.s003.zip › QSonica nanodroplets no translator gel analysis/Densitometry Analysis/R2 QSonica nanodroplets no translator densitometry/QS,R2,PND,B1-12-wf.png]

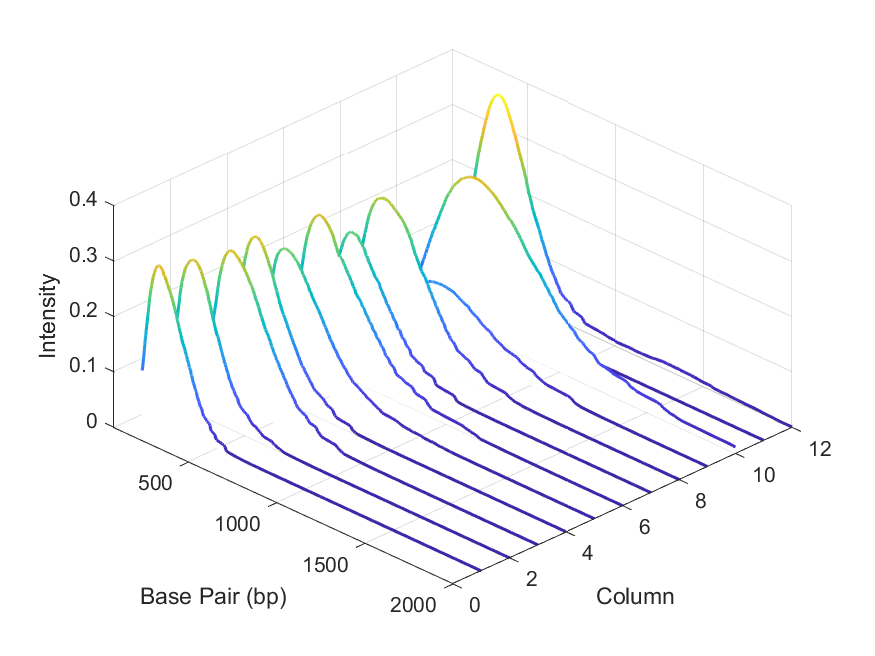

Supplement: S3 File — (ZIP) [file pone.0341139.s003.zip › QSonica nanodroplets no translator gel analysis/Densitometry Analysis/R2 QSonica nanodroplets no translator densitometry/Q-S,PND,R2,E1-E12-wf.png]

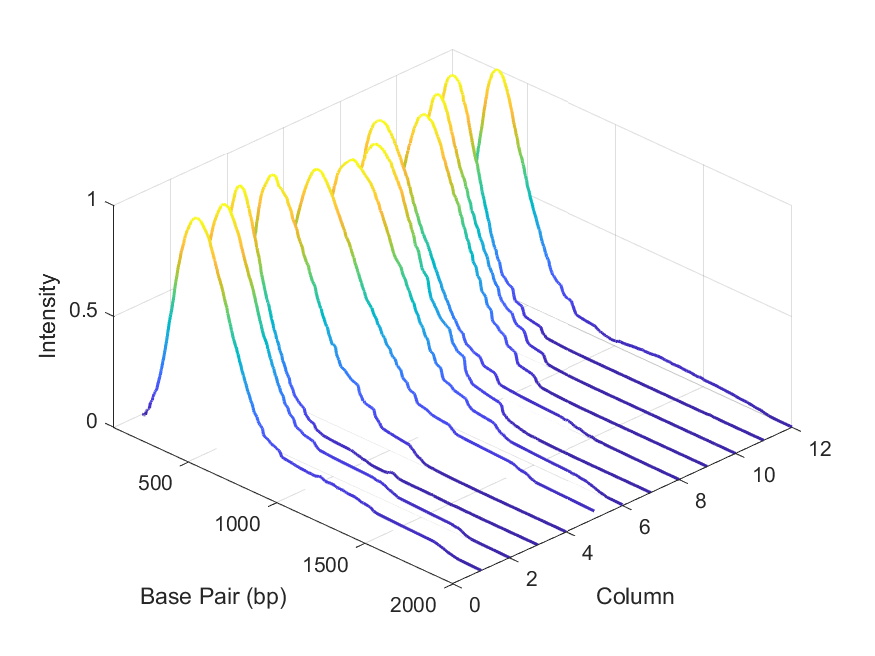

Supplement: S3 File — (ZIP) [file pone.0341139.s003.zip › QSonica nanodroplets no translator gel analysis/Densitometry Analysis/R2 QSonica nanodroplets no translator densitometry/Q-S,PND,R2,C1-C12-wf-norm.png]

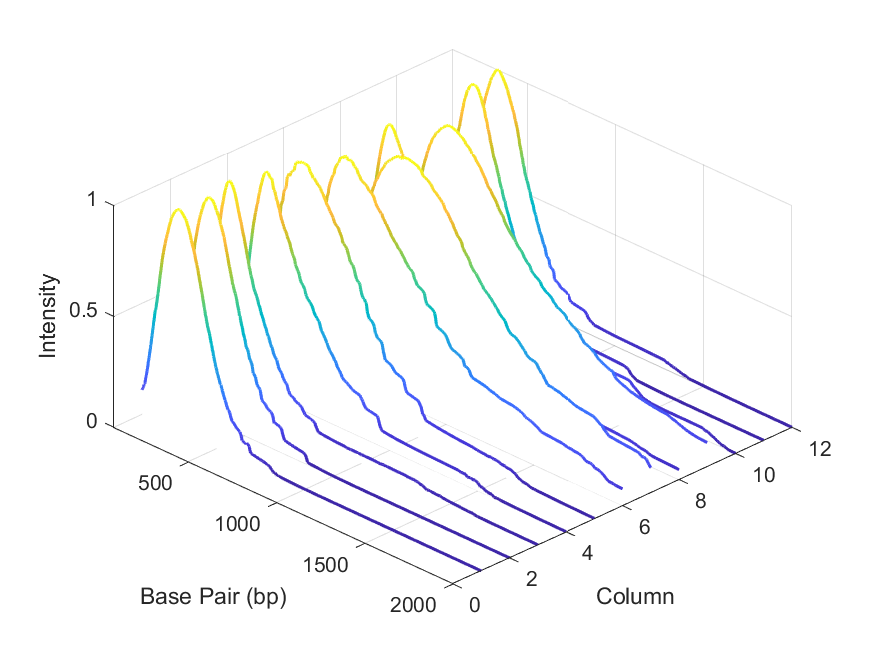

Supplement: S3 File — (ZIP) [file pone.0341139.s003.zip › QSonica nanodroplets no translator gel analysis/Densitometry Analysis/R2 QSonica nanodroplets no translator densitometry/Q-S,PND,R2, F1-F12-wf-norm.png]

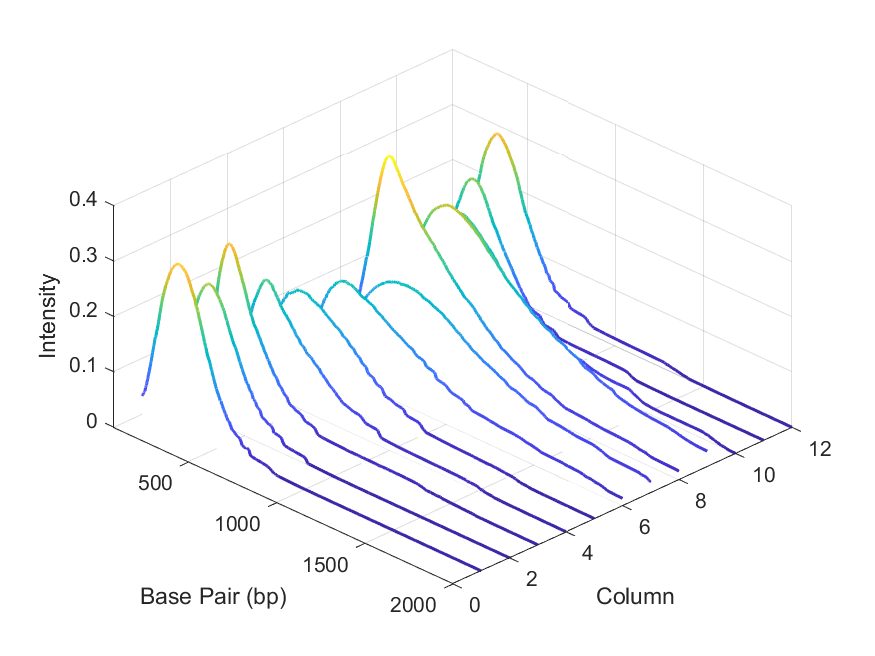

Supplement: S3 File — (ZIP) [file pone.0341139.s003.zip › QSonica nanodroplets no translator gel analysis/Densitometry Analysis/R2 QSonica nanodroplets no translator densitometry/Q-S,PND,R2, F1-F12-wf.png]

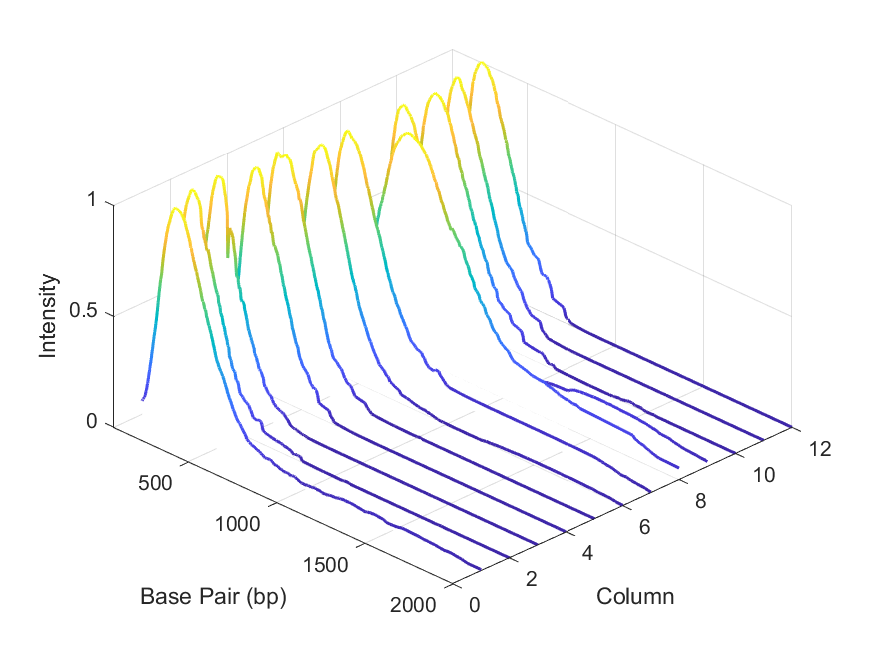

Supplement: S3 File — (ZIP) [file pone.0341139.s003.zip › QSonica nanodroplets no translator gel analysis/Densitometry Analysis/R2 QSonica nanodroplets no translator densitometry/Q-S,PND,R2,A1-12-wf-norm.png]

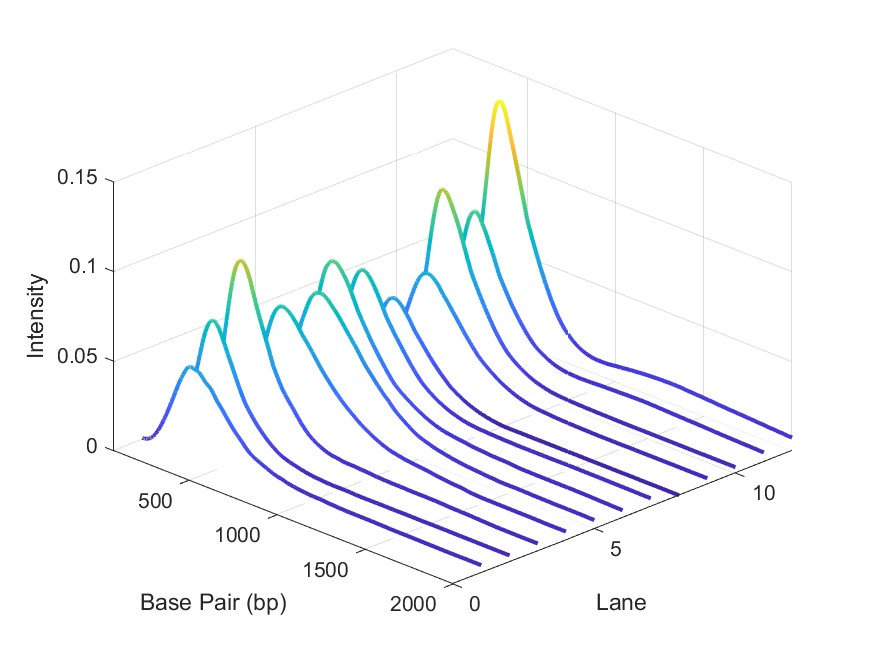

Supplement: S3 File — (ZIP) [file pone.0341139.s003.zip › QSonica nanodroplets no translator gel analysis/Densitometry Analysis/R2 QSonica nanodroplets no translator densitometry/5.24.23 Q-S,R2,PND,G1-12-wf.png]

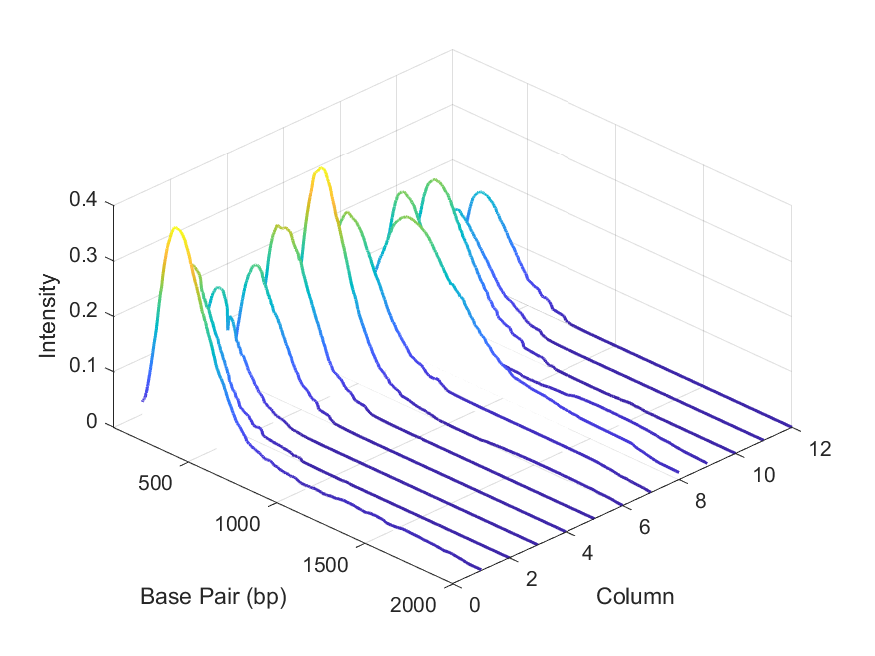

Supplement: S3 File — (ZIP) [file pone.0341139.s003.zip › QSonica nanodroplets no translator gel analysis/Densitometry Analysis/R2 QSonica nanodroplets no translator densitometry/Q-S,PND, R2,A1-12-wf.png]

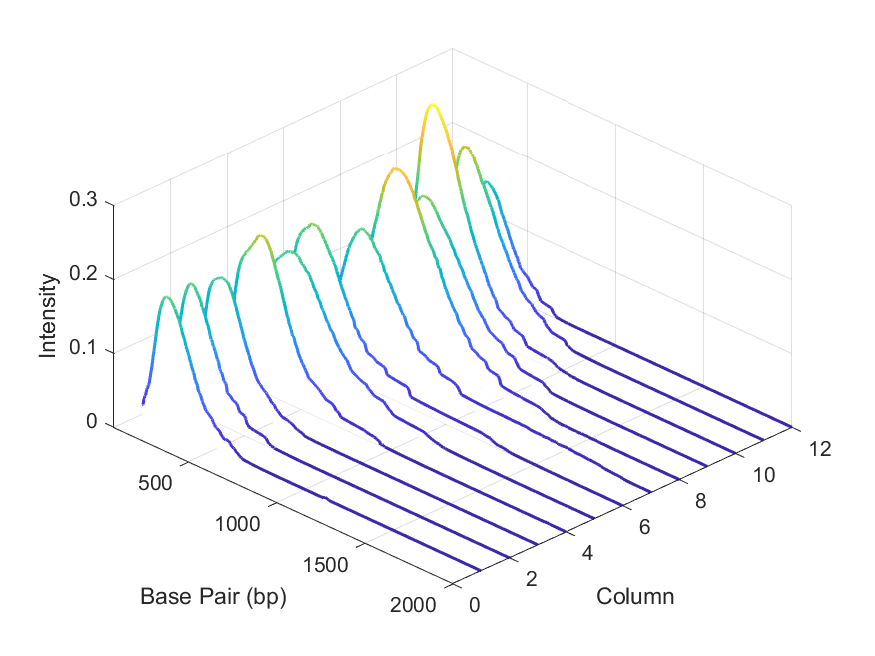

Supplement: S3 File — (ZIP) [file pone.0341139.s003.zip › QSonica nanodroplets no translator gel analysis/Densitometry Analysis/R2 QSonica nanodroplets no translator densitometry/Q-S,PND,R2 ,B1-B12-wf.png]

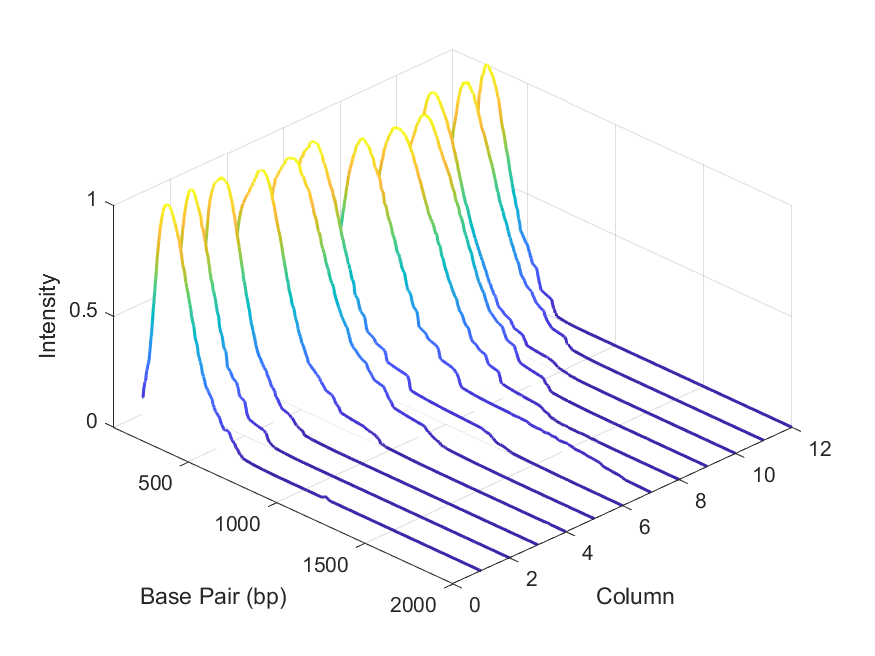

Supplement: S3 File — (ZIP) [file pone.0341139.s003.zip › QSonica nanodroplets no translator gel analysis/Densitometry Analysis/R2 QSonica nanodroplets no translator densitometry/Q-S,PND,R2,B12-wf-norm.png]

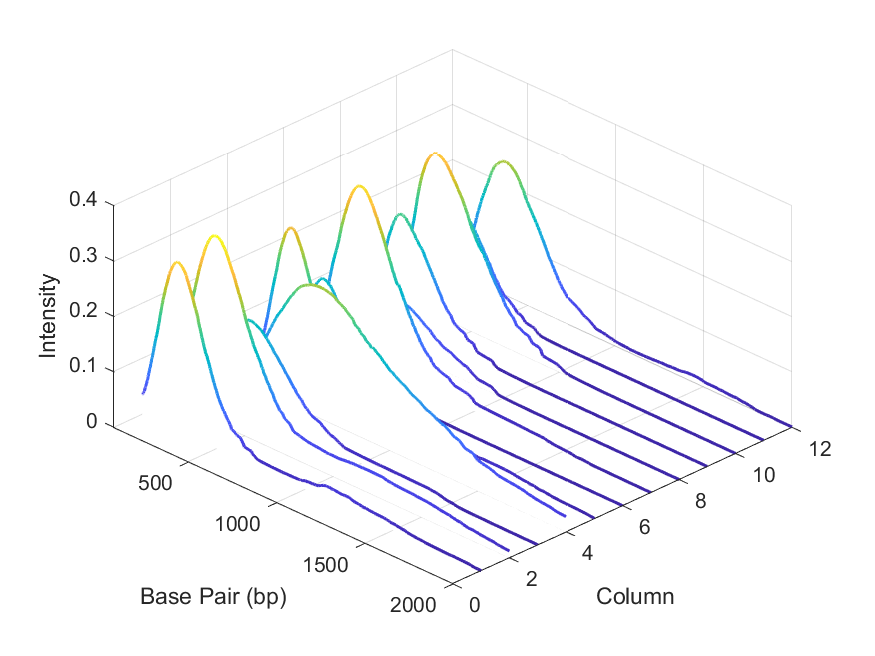

Supplement: S3 File — (ZIP) [file pone.0341139.s003.zip › QSonica nanodroplets no translator gel analysis/Densitometry Analysis/R2 QSonica nanodroplets no translator densitometry/Q-S,PND,R2,D1-D12-wf.png]

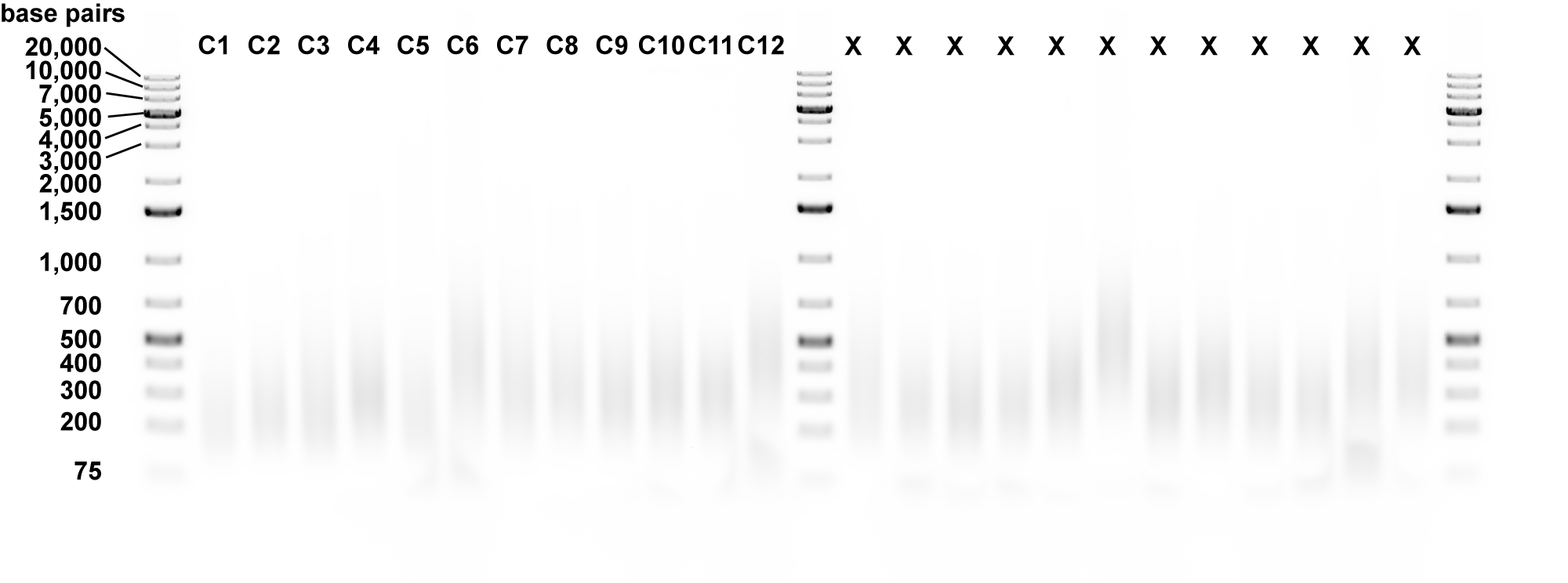

Supplement: S4 File — (ZIP) [file pone.0341139.s004.zip › QSonica no cavitation enhancement no translator gel analysis/Gel Pictures/R1 QSonica no cavitation enhancement no translator C 1-12.tif]

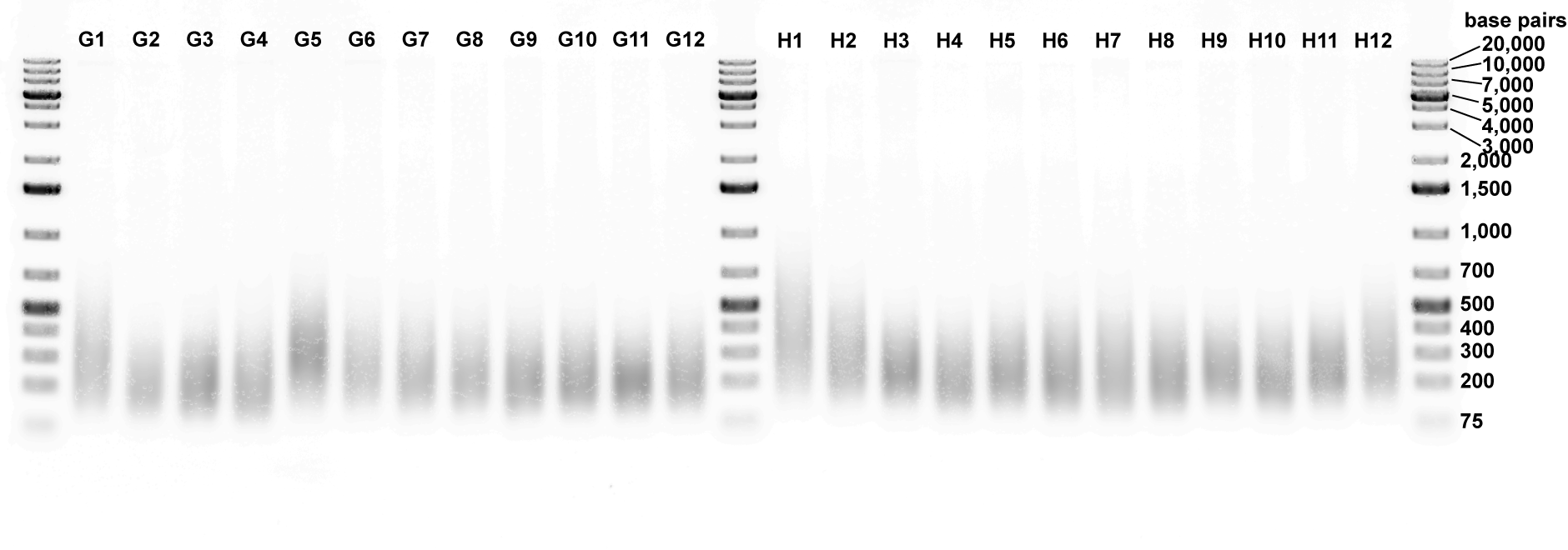

Supplement: S4 File — (ZIP) [file pone.0341139.s004.zip › QSonica no cavitation enhancement no translator gel analysis/Gel Pictures/R2 QSonica no cavitation enhancement no translator G,H 1-12.tif]

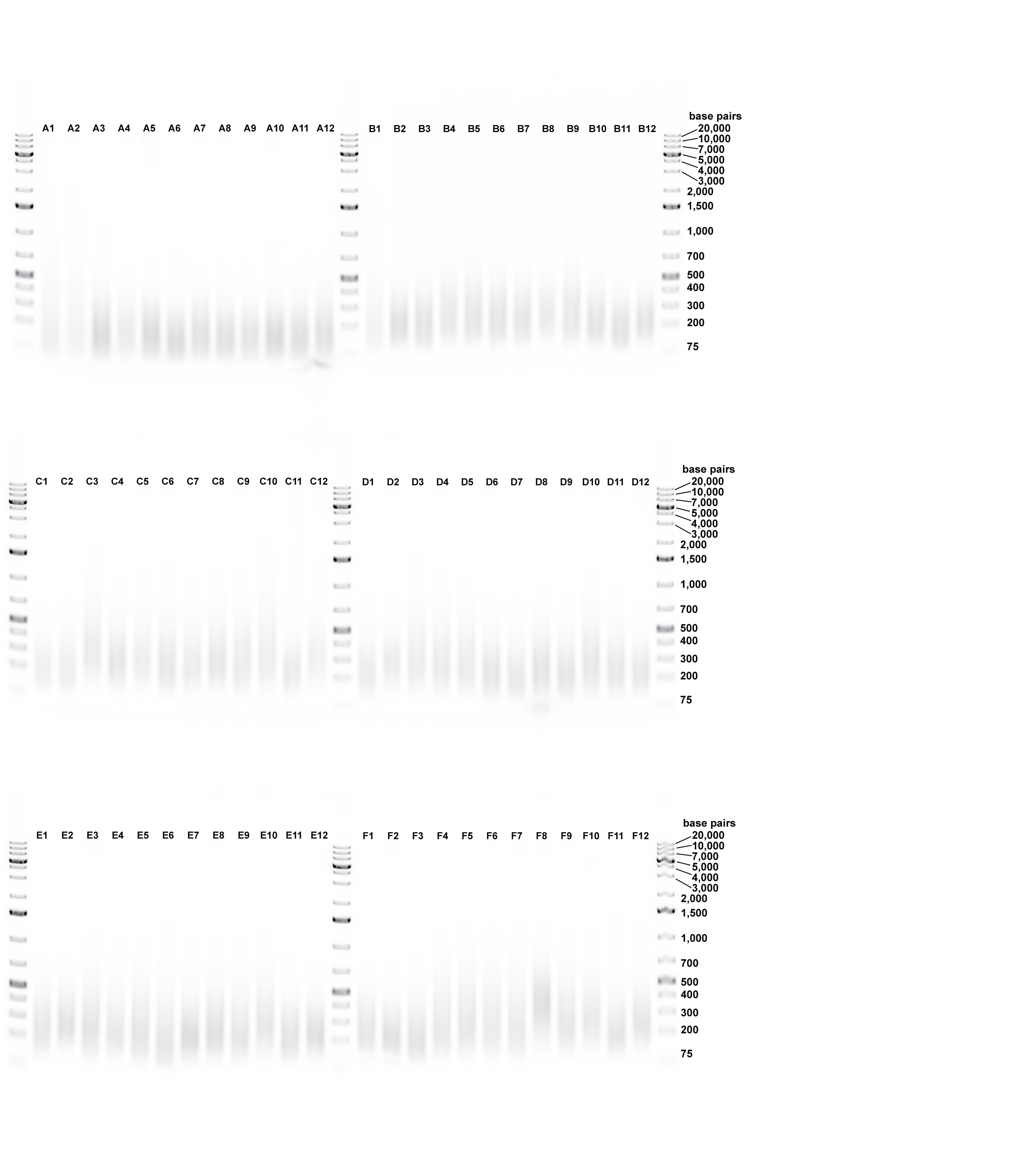

Supplement: S4 File — (ZIP) [file pone.0341139.s004.zip › QSonica no cavitation enhancement no translator gel analysis/Gel Pictures/R2 QSonica no cavitation enhancement no translator A,B,C,D,E,F 1-12.tif]

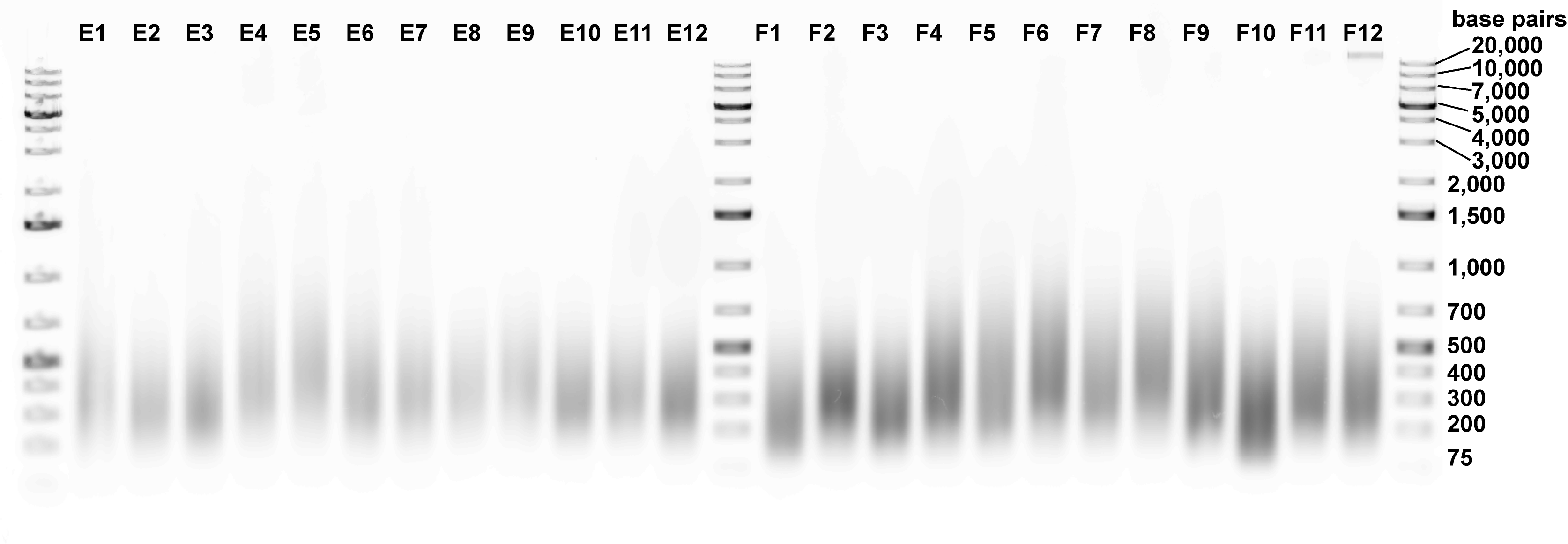

Supplement: S4 File — (ZIP) [file pone.0341139.s004.zip › QSonica no cavitation enhancement no translator gel analysis/Gel Pictures/R1 QSonica no cavitation enhancement no translator E,F,1-12.tif]

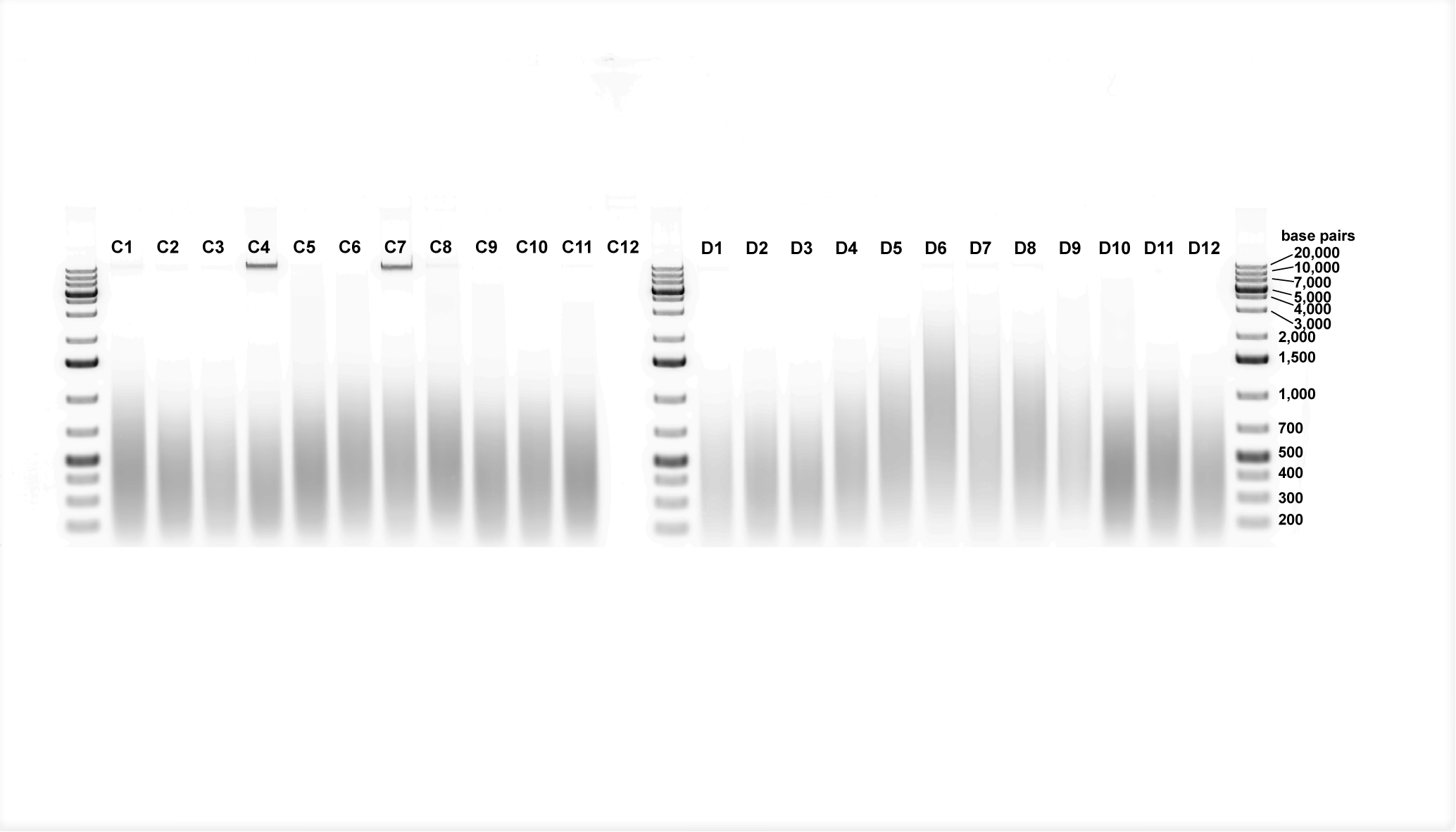

Supplement: S4 File — (ZIP) [file pone.0341139.s004.zip › QSonica no cavitation enhancement no translator gel analysis/Gel Pictures/R3 QSonica no cavitation enhancement no translator C,D 1-12.tif]

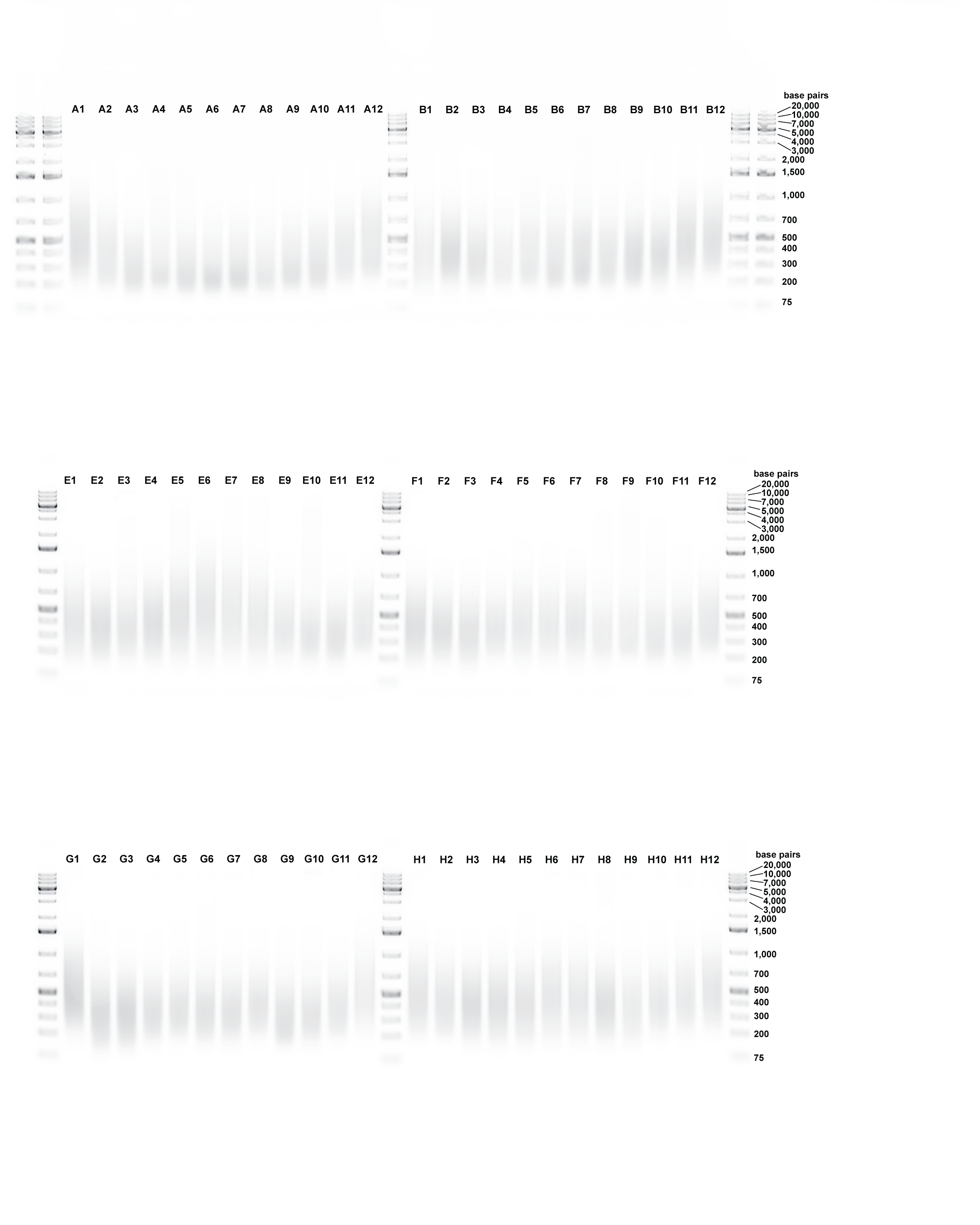

Supplement: S4 File — (ZIP) [file pone.0341139.s004.zip › QSonica no cavitation enhancement no translator gel analysis/Gel Pictures/R3 QSonica no cavitation enhancement no translator A,B,E,F,G,H 1-12.tif]

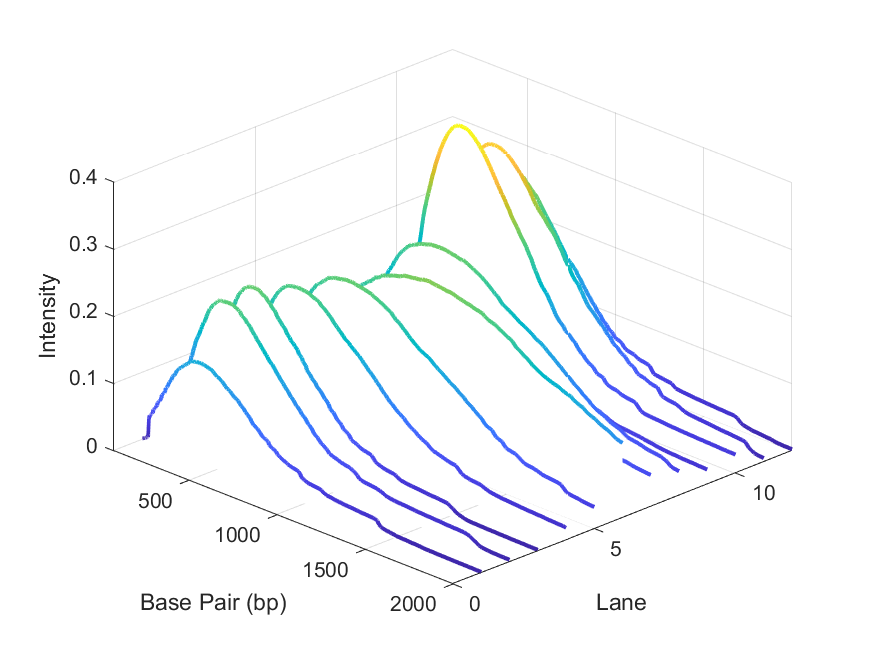

Supplement: S4 File — (ZIP) [file pone.0341139.s004.zip › QSonica no cavitation enhancement no translator gel analysis/Densitometry analysis/R3 QSonica no cavitation enhancement no translator densitometry/11.15.22 Q-S Minus ND, R3. D1-`12-wf.png]

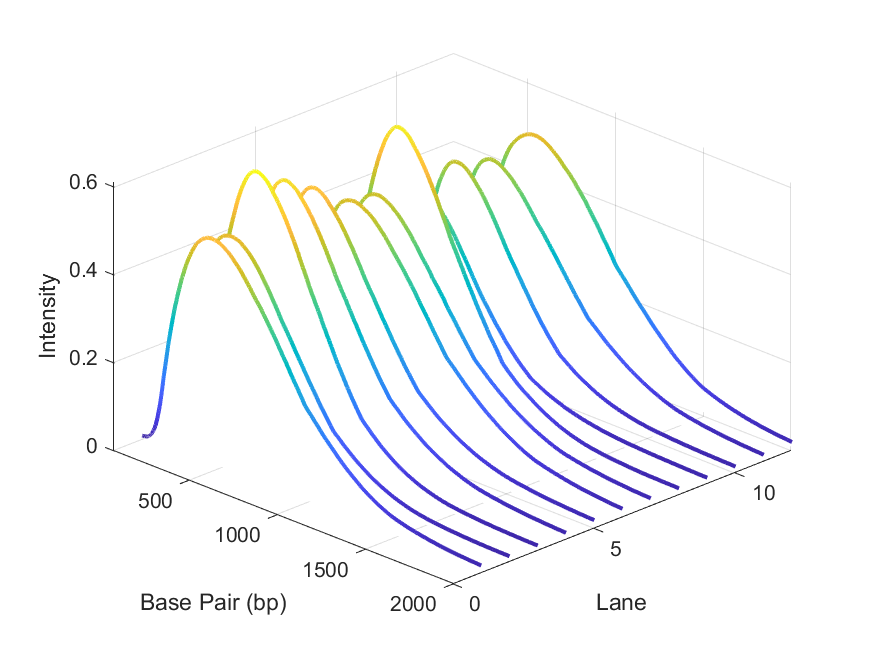

Supplement: S4 File — (ZIP) [file pone.0341139.s004.zip › QSonica no cavitation enhancement no translator gel analysis/Densitometry analysis/R3 QSonica no cavitation enhancement no translator densitometry/10.11.23 QS,MND, R3, H,1-12,adj-wf.png]

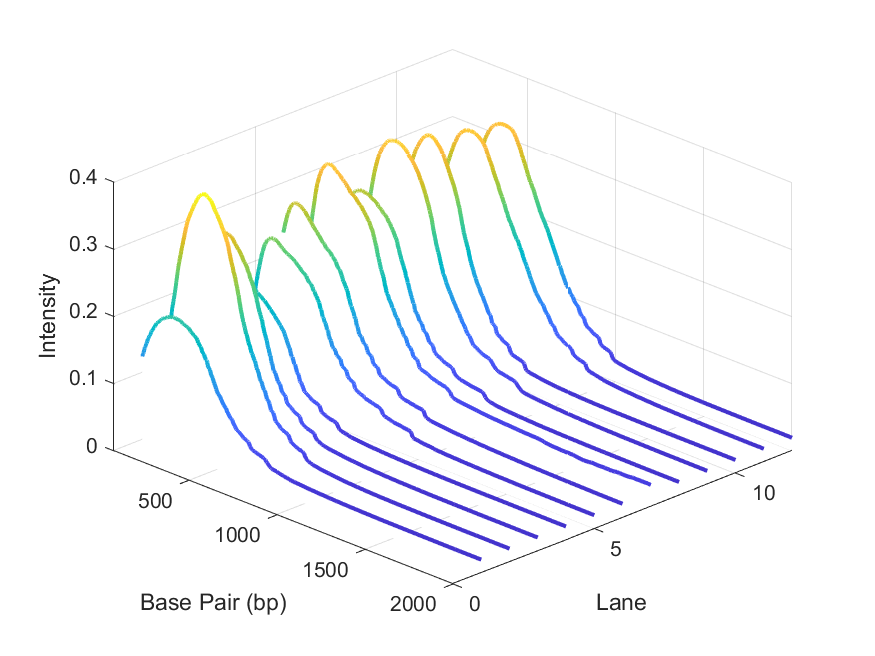

Supplement: S4 File — (ZIP) [file pone.0341139.s004.zip › QSonica no cavitation enhancement no translator gel analysis/Densitometry analysis/R3 QSonica no cavitation enhancement no translator densitometry/11.21.22 Q-S Minus ND. R3,B1-12-wf.png]

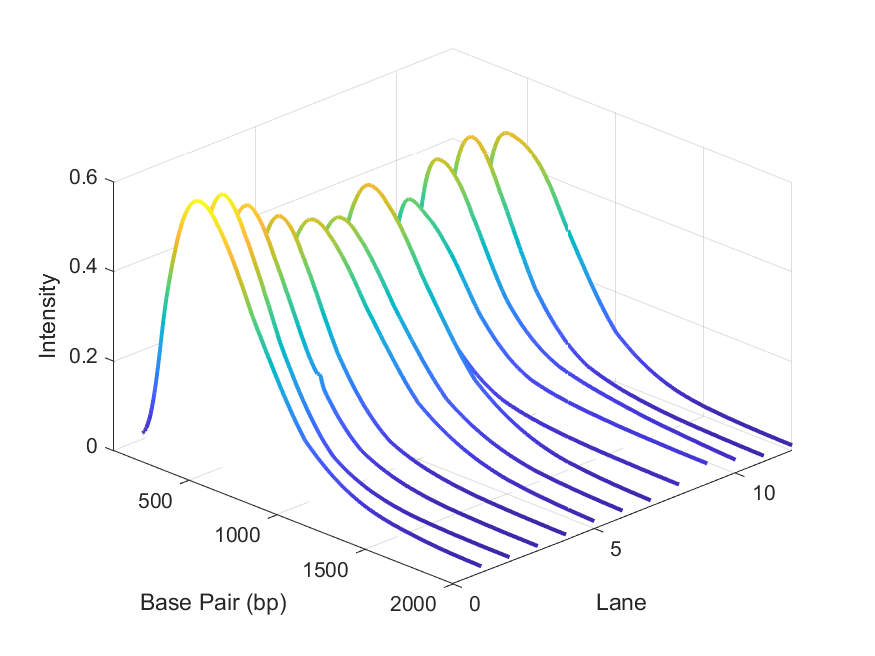

Supplement: S4 File — (ZIP) [file pone.0341139.s004.zip › QSonica no cavitation enhancement no translator gel analysis/Densitometry analysis/R3 QSonica no cavitation enhancement no translator densitometry/QS,MND, R3, F1-12-wf.png]

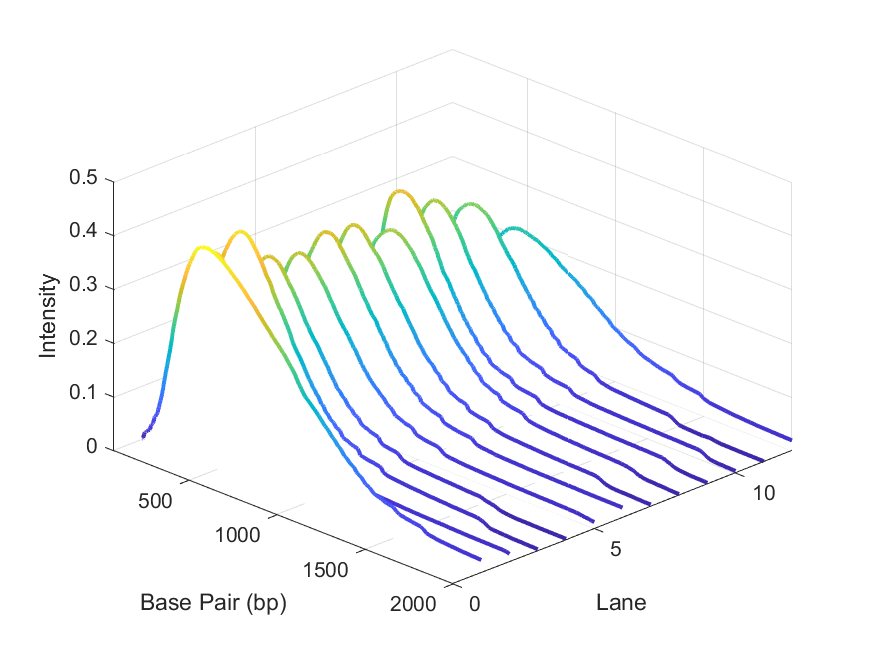

Supplement: S4 File — (ZIP) [file pone.0341139.s004.zip › QSonica no cavitation enhancement no translator gel analysis/Densitometry analysis/R3 QSonica no cavitation enhancement no translator densitometry/11.21.22 Q-S Minus ND, R3, G1-12-wf.png]

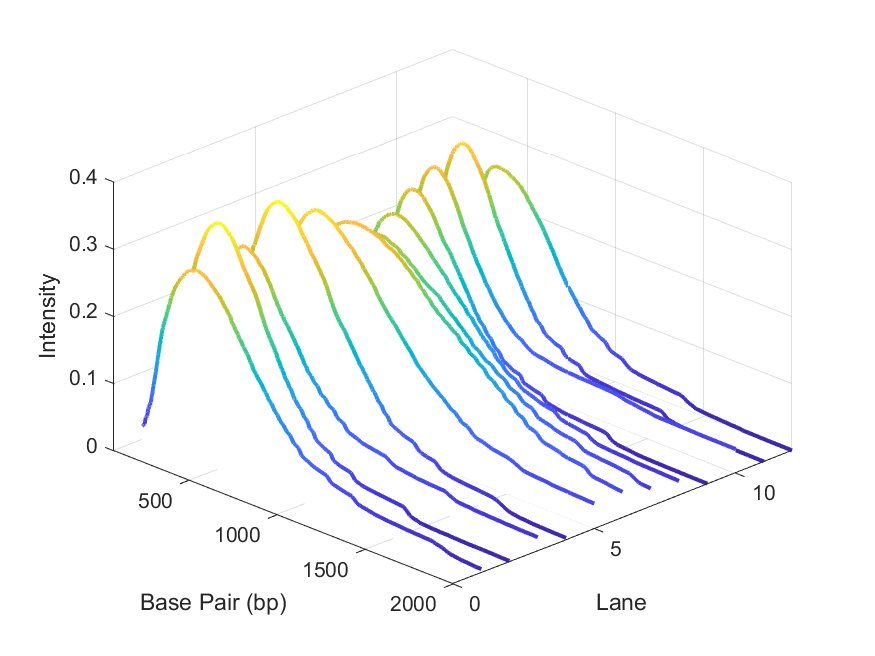

Supplement: S4 File — (ZIP) [file pone.0341139.s004.zip › QSonica no cavitation enhancement no translator gel analysis/Densitometry analysis/R3 QSonica no cavitation enhancement no translator densitometry/11.21.22 Q_S Minus ND, R3, E1-12-wf.png]

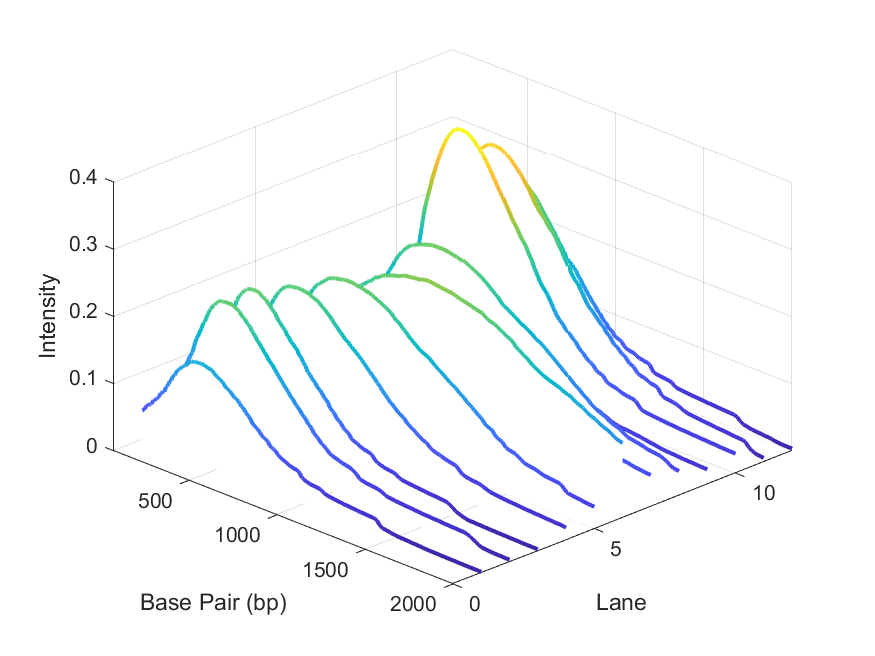

Supplement: S4 File — (ZIP) [file pone.0341139.s004.zip › QSonica no cavitation enhancement no translator gel analysis/Densitometry analysis/R3 QSonica no cavitation enhancement no translator densitometry/11.15.22 Q-S Minus ND, R3. D1-12-wf.png]

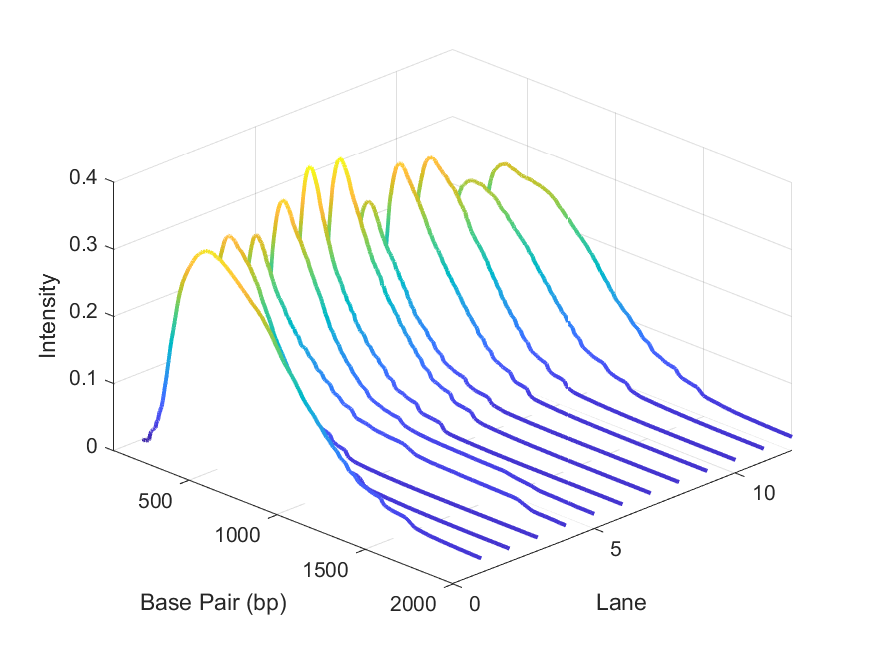

Supplement: S4 File — (ZIP) [file pone.0341139.s004.zip › QSonica no cavitation enhancement no translator gel analysis/Densitometry analysis/R3 QSonica no cavitation enhancement no translator densitometry/11.21.22 Q-S Minus ND, R3, A1-12-wf.png]

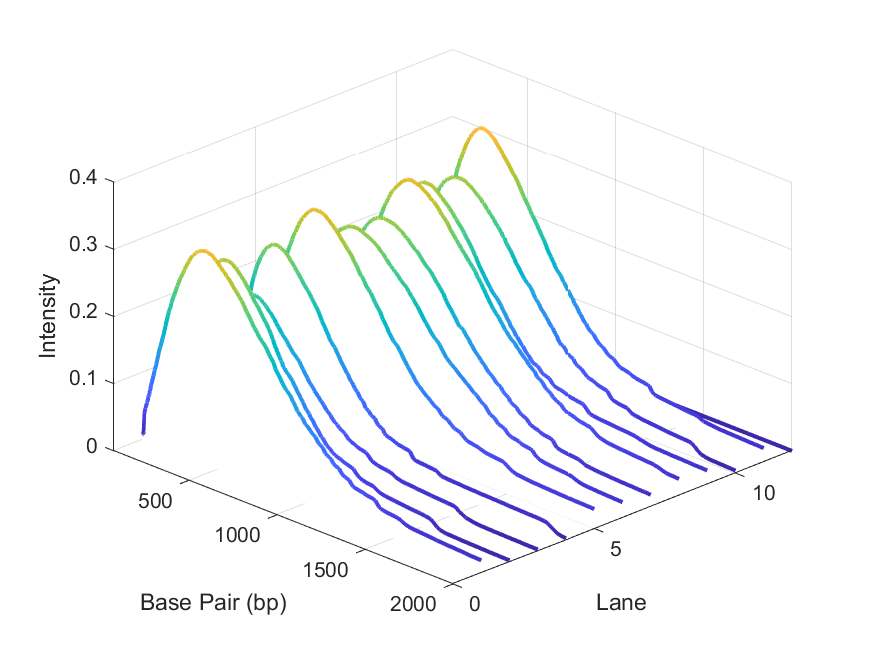

Supplement: S4 File — (ZIP) [file pone.0341139.s004.zip › QSonica no cavitation enhancement no translator gel analysis/Densitometry analysis/R3 QSonica no cavitation enhancement no translator densitometry/11.15.22 Q-S Minus ND, R3. C1-12-wf.png]

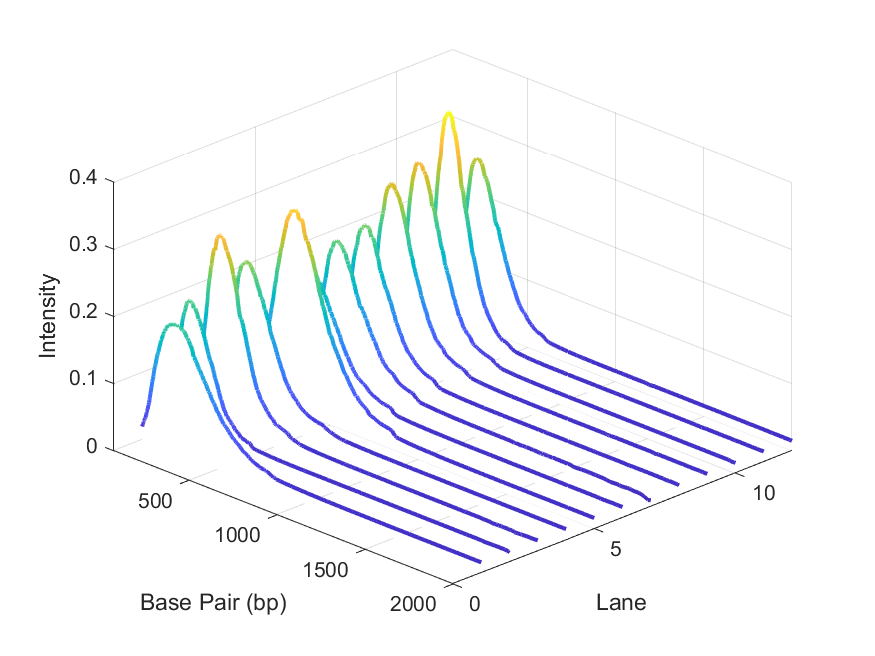

Supplement: S4 File — (ZIP) [file pone.0341139.s004.zip › QSonica no cavitation enhancement no translator gel analysis/Densitometry analysis/R2 QSonica no cavitation enhancement no translator densitometry/QS,MND, R2, G1-12-wf.png]

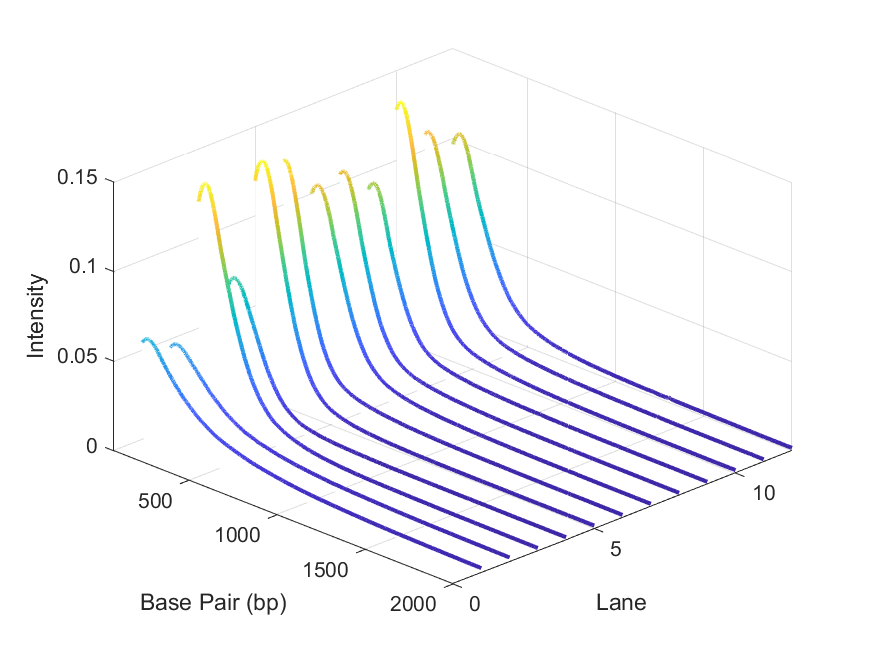

Supplement: S4 File — (ZIP) [file pone.0341139.s004.zip › QSonica no cavitation enhancement no translator gel analysis/Densitometry analysis/R2 QSonica no cavitation enhancement no translator densitometry/QS,R2,MND,A1-12-wf.png]

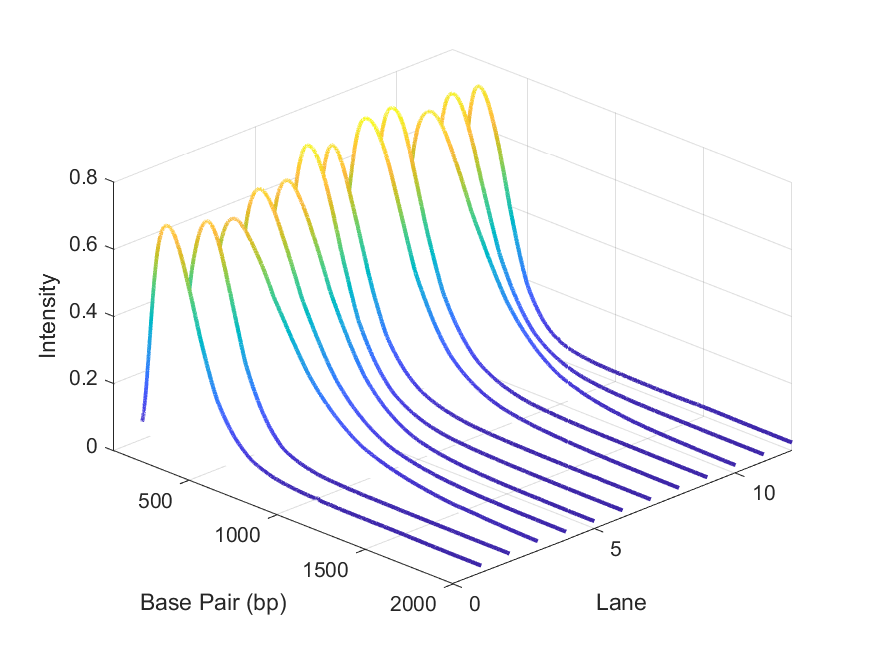

Supplement: S4 File — (ZIP) [file pone.0341139.s004.zip › QSonica no cavitation enhancement no translator gel analysis/Densitometry analysis/R2 QSonica no cavitation enhancement no translator densitometry/QS,R2,MND,D1-12-wf.png]

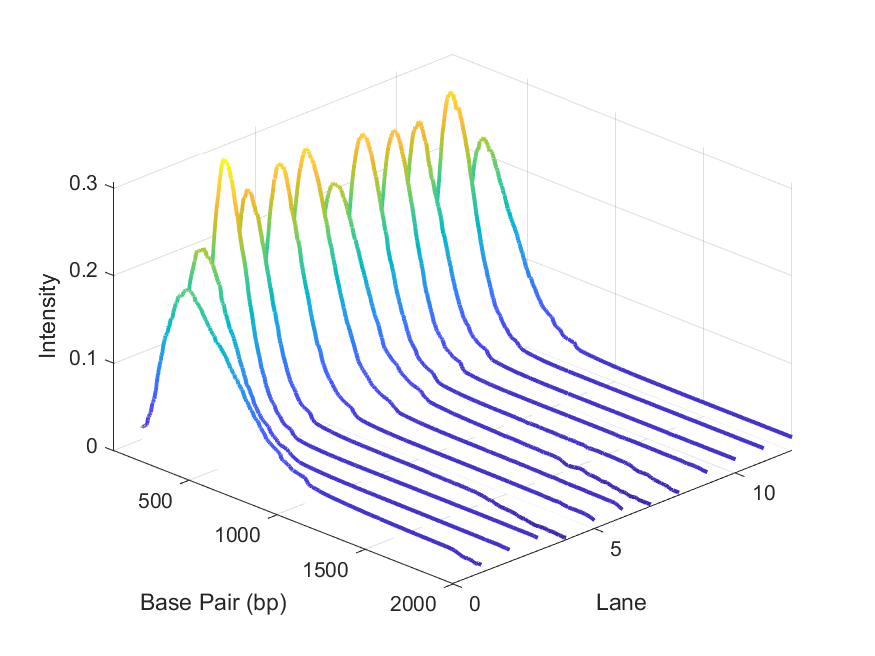

Supplement: S4 File — (ZIP) [file pone.0341139.s004.zip › QSonica no cavitation enhancement no translator gel analysis/Densitometry analysis/R2 QSonica no cavitation enhancement no translator densitometry/QS,MND, R2, H1-12-wf.png]

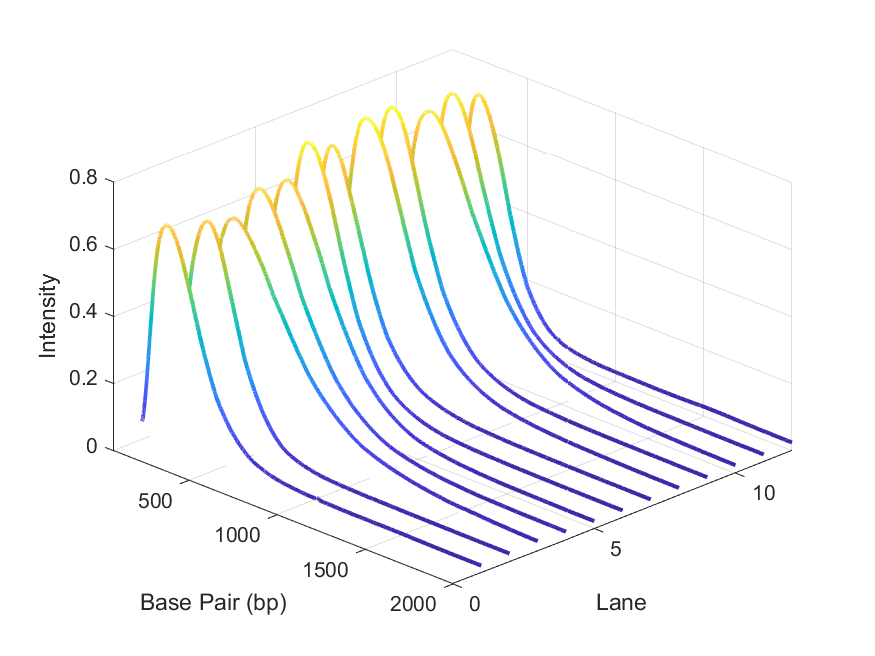

Supplement: S4 File — (ZIP) [file pone.0341139.s004.zip › QSonica no cavitation enhancement no translator gel analysis/Densitometry analysis/R2 QSonica no cavitation enhancement no translator densitometry/QS,R2,MND,C1-12-wf.png]

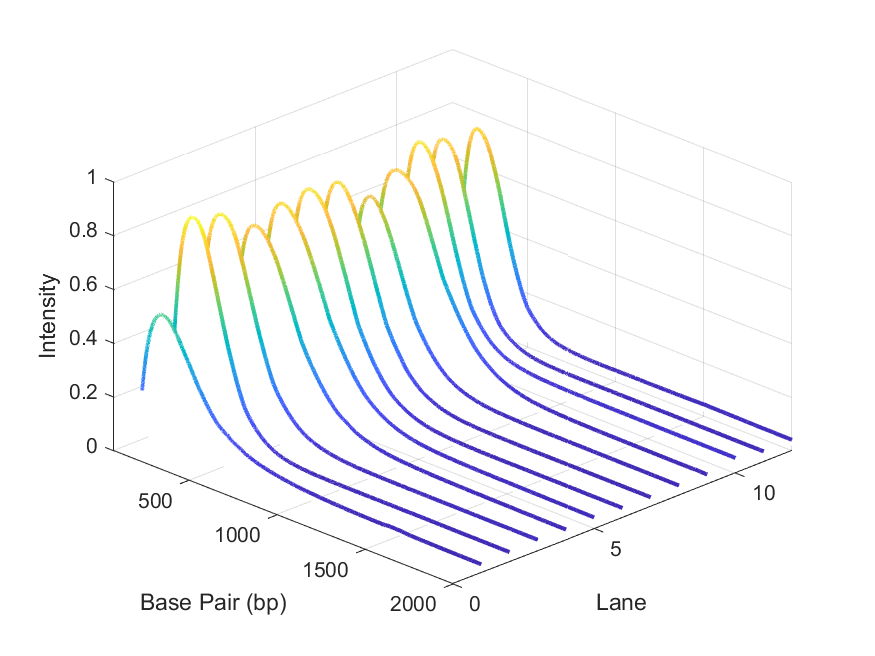

Supplement: S4 File — (ZIP) [file pone.0341139.s004.zip › QSonica no cavitation enhancement no translator gel analysis/Densitometry analysis/R2 QSonica no cavitation enhancement no translator densitometry/QS,MND, R2,B1-12-wf.png]

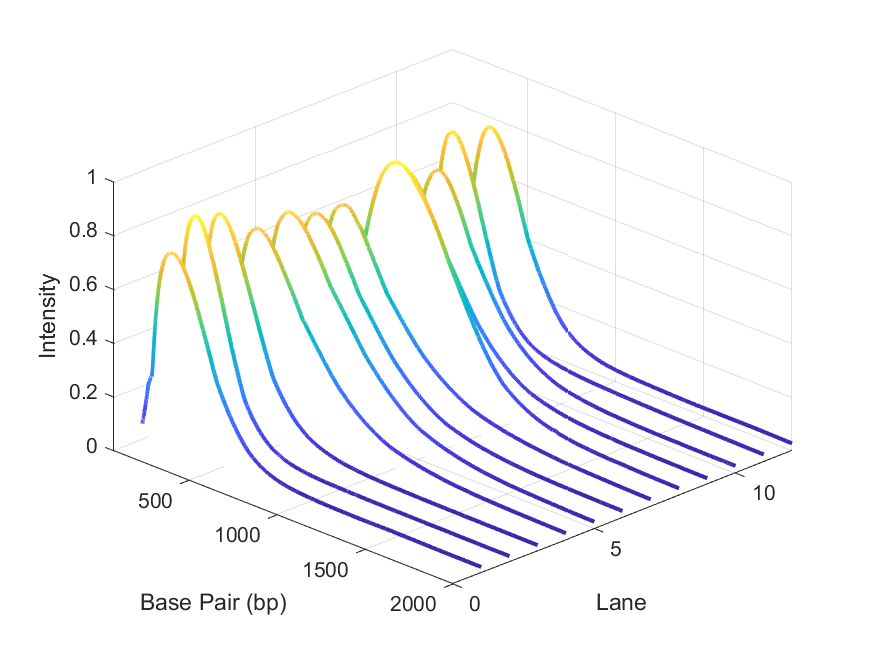

Supplement: S4 File — (ZIP) [file pone.0341139.s004.zip › QSonica no cavitation enhancement no translator gel analysis/Densitometry analysis/R2 QSonica no cavitation enhancement no translator densitometry/QS,R2,MND,F1-12-wf.png]

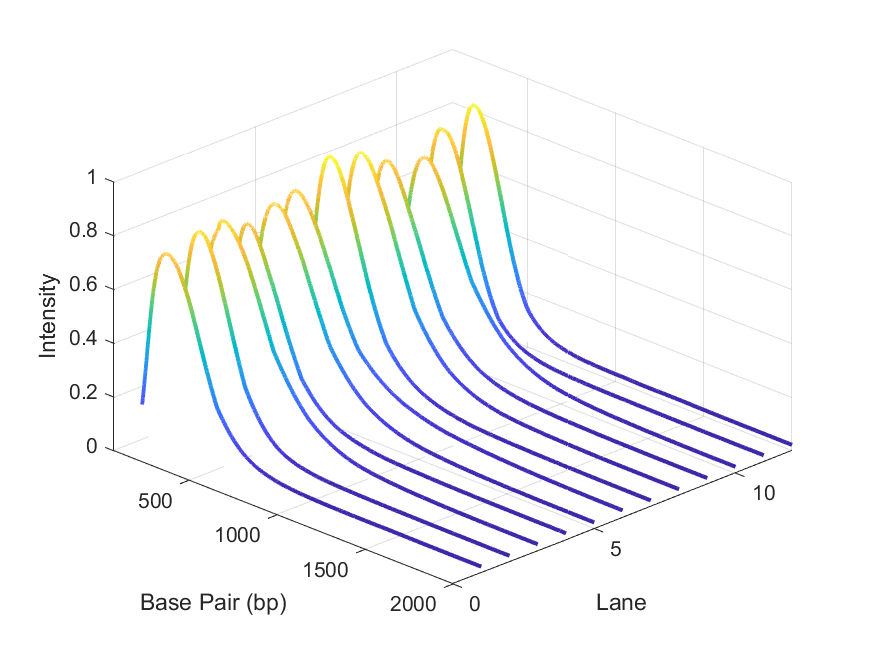

Supplement: S4 File — (ZIP) [file pone.0341139.s004.zip › QSonica no cavitation enhancement no translator gel analysis/Densitometry analysis/R2 QSonica no cavitation enhancement no translator densitometry/QS,R2,MND,E1-12-wf.png]
