# Supplementary material for: 96 sample parallel acoustic fragmentation for high throughput next generation sequencing library preparation
Source: PLoS One. 2026 Feb 17;21(2):e0341139. doi: 10.1371/journal.pone.0341139 (PMC12912608; doi:10.1371/journal.pone.0341139)
Supplement: S2 Fig — (ZIP) [file pone.0341139.s002.zip › Figure 1 Raw Data/glass tube with nanodroplets 240 seconds.pdf]

Filename: 2019-03-26-02- LE220 plus 240 sec 120 (last 8 line).D5000

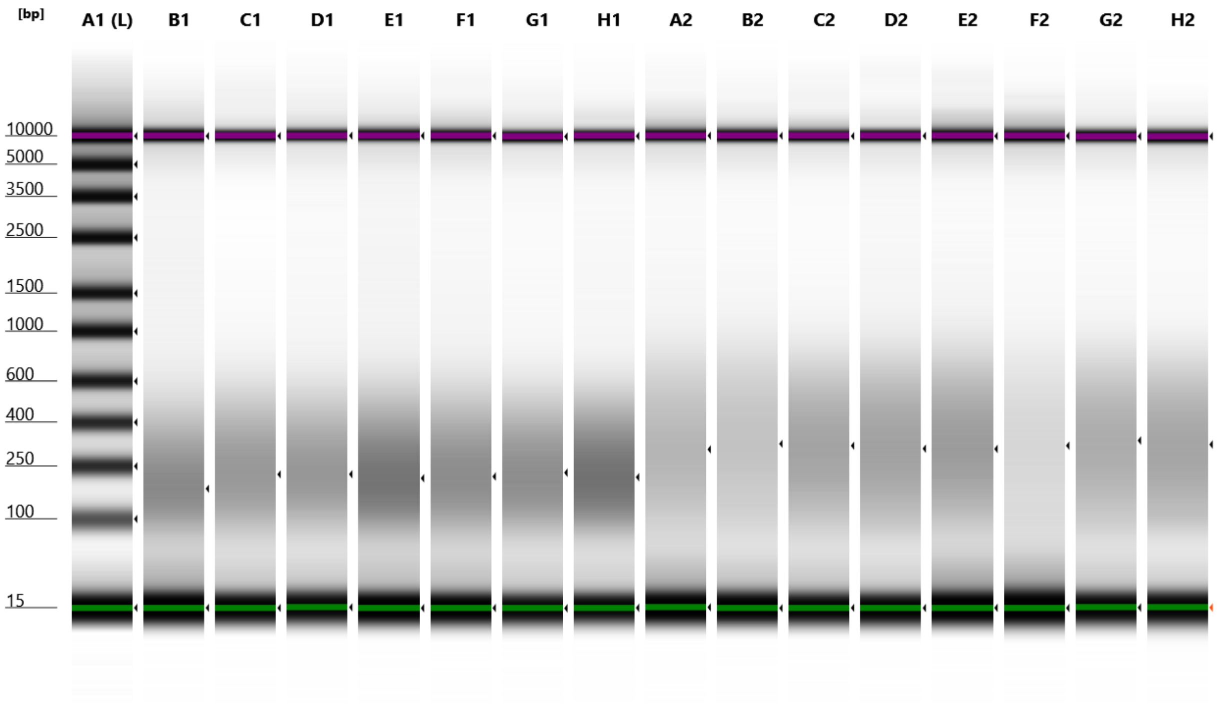

Default image (Contrast 100%)

Sample Info

Well	Conc. [ng/µl]	Sample Description	Alert	Observations
A1	27.8	Ladder		Ladder
B1	2.72			
C1	3.15			
D1	6.23			
E1	4.36			
F1	3.50	DFB plus 240 sec		
G1	4.72	DFB plus 240 sec		
H1	5.55	DFB plus 240 sec		
A2	0.389	DFB plus 240 sec		
B2	0.235			
C2	0.808			
D2	0.577			
E2	0.569	DFB plus 120 sec		
F2	0.217	DFB plus 120 sec		
G2	0.488	DFB plus 120 sec		
H2	0.458	DFB plus 120 sec		

 © Agilent Technologies, Inc. 2017

A1: Ladder

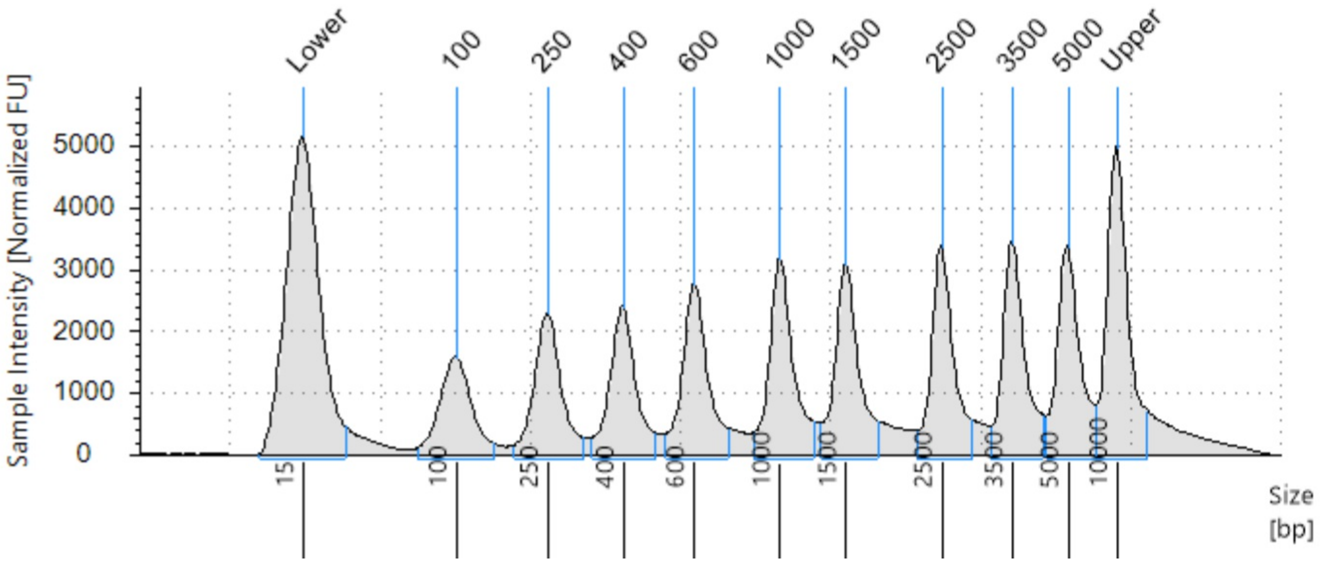

Sample Table

Well	Conc. [ng/µl]	Sample Description	Alert	Observations
A1	27.8	Ladder		Ladder

Peak Table

Size [bp]	Calibrated Conc. [ng/µl]	Assigned Conc. [ng/µl]	Peak Molarity [nmol/l]	% Integrated Area	Peak Comment	Observations
15	6.88	-	706	-		Lower Marker
100	2.27	-	34.9	8.15		
250	2.75	-	17.0	9.90		
400	2.77	-	10.7	9.97		
600	3.12	-	8.01	11.23		
1000	3.41	-	5.25	12.25		
1500	3.28	-	3.36	11.78		
2500	3.35	-	2.06	12.03		
3500	3.44	-	1.51	12.35		
5000	3.43	-	1.06	12.34		
10000	3.25	3.25	0.500	-		Upper Marker

© Agilent Technologies, Inc. 2017

F1: DFB plus 240 sec

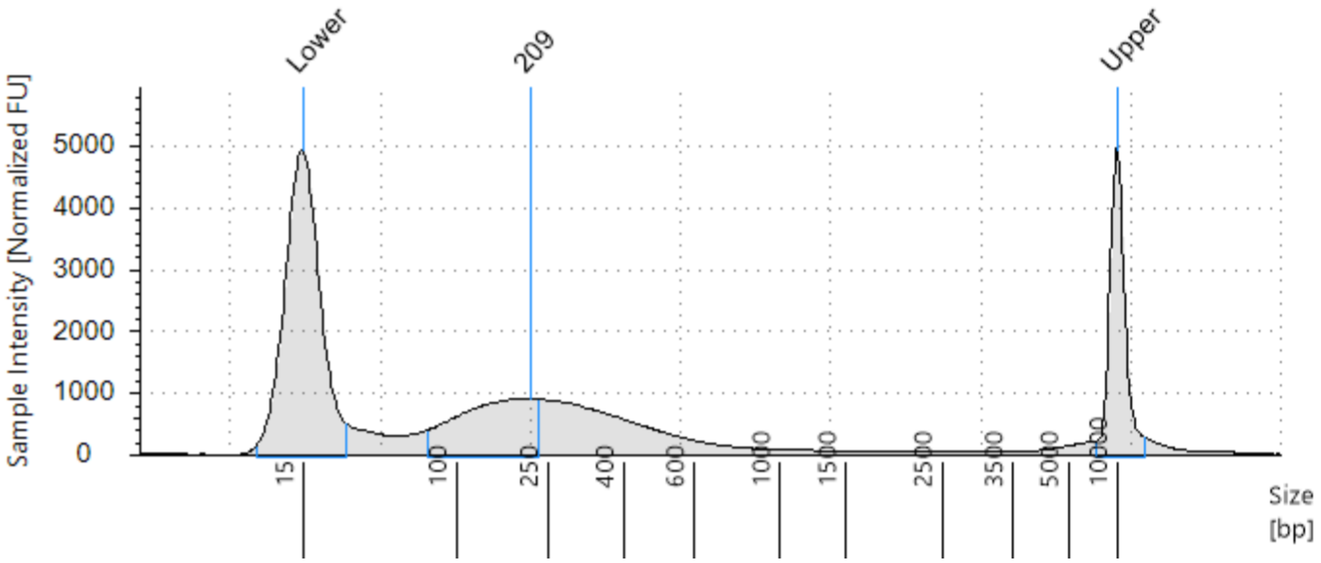

Sample Table

Well	Conc. [ng/µl]	Sample Description	Alert	Observations
F1	3.50	DFB plus 240 sec		

Peak Table

Size [bp]	Calibrated Conc. [ng/µl]	Assigned Conc. [ng/µl]	Peak Molarity [nmol/l]	% Integrated Area	Peak Comment	Observations
15	7.37	-	756	-		Lower Marker
209	3.50	-	25.7	100.00		
10000	3.25	3.25	0.500	-		Upper Marker

© Agilent Technologies, Inc. 2017

G1: DFB plus 240 sec

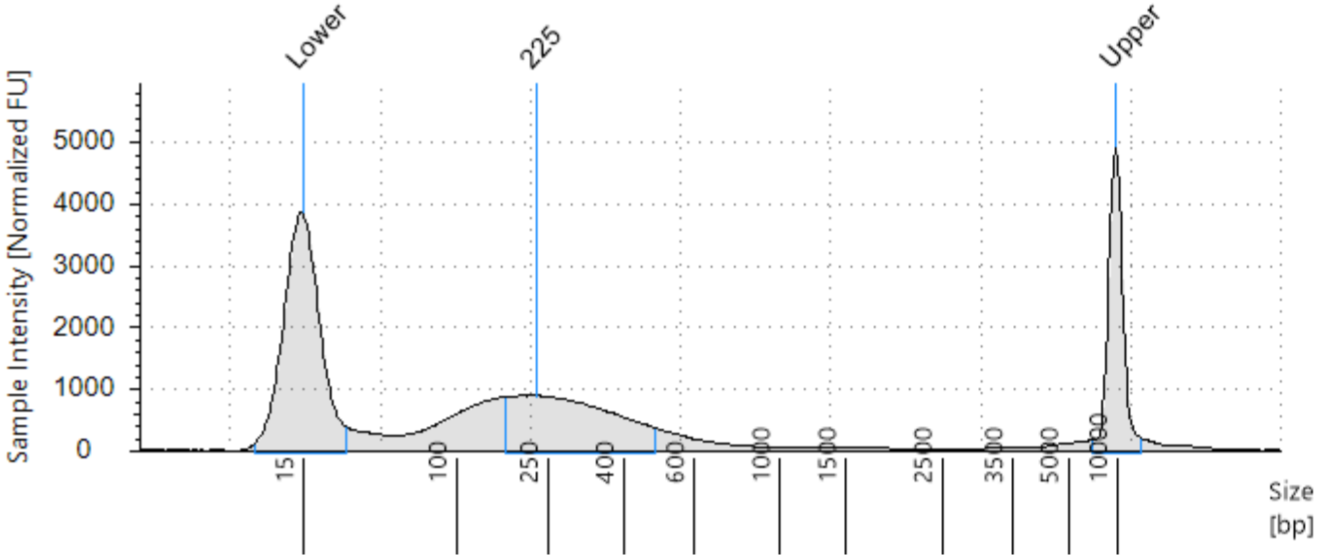

Sample Table

Well	Conc. [ng/µl]	Sample Description	Alert	Observations
G1	4.72	DFB plus 240 sec		

Peak Table

Size [bp]	Calibrated Conc. [ng/µl]	Assigned Conc. [ng/µl]	Peak Molarity [nmol/l]	% Integrated Area	Peak Comment	Observations
15	6.12	-	628	-		Lower Marker
225	4.72	-	32.2	100.00		
10000	3.25	3.25	0.500	-		Upper Marker

© Agilent Technologies, Inc. 2017

H1: DFB plus 240 sec

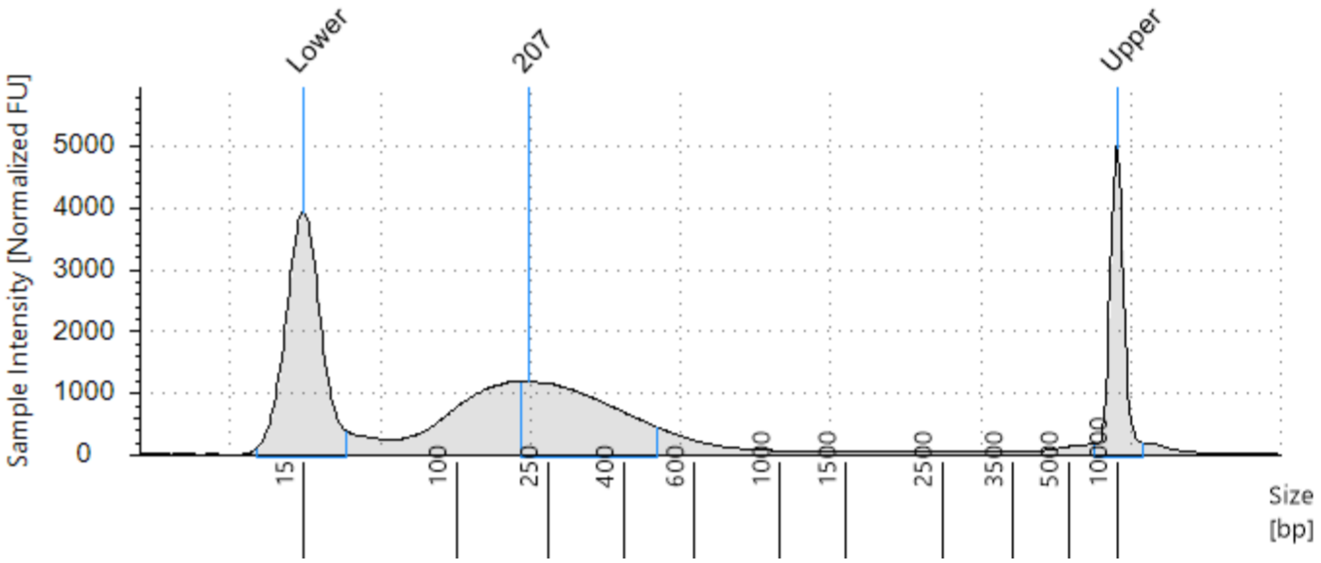

Sample Table

Well	Conc. [ng/µl]	Sample Description	Alert	Observations
H1	5.55	DFB plus 240 sec		

Peak Table

Size [bp]	Calibrated Conc. [ng/µl]	Assigned Conc. [ng/µl]	Peak Molarity [nmol/l]	% Integrated Area	Peak Comment	Observations
15	6.19	-	635	-		Lower Marker
207	5.55	-	41.3	100.00		
10000	3.25	3.25	0.500	-		Upper Marker

© Agilent Technologies, Inc. 2017

A2: DFB plus 240 sec

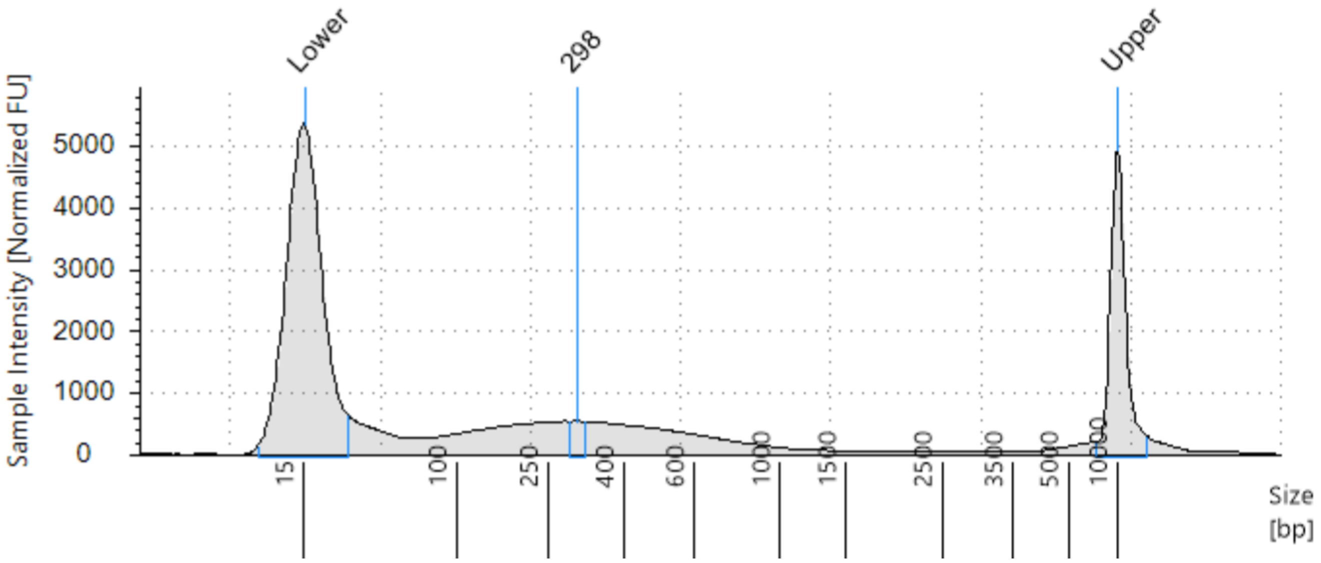

Sample Table

Well	Conc. [ng/µl]	Sample Description	Alert	Observations
A2	0.389	DFB plus 240 sec		

Peak Table

Size [bp]	Calibrated Conc. [ng/µl]	Assigned Conc. [ng/µl]	Peak Molarity [nmol/l]	% Integrated Area	Peak Comment	Observations
15	8.12	-	833	-		Lower Marker
298	0.389	-	2.01	100.00		
10000	3.25	3.25	0.500	-		Upper Marker

© Agilent Technologies, Inc. 2017

Filename: 2019-05-17- LE220 DFB PLUS FIRST 8 240 SEC LAST 8 120 SEC.D5000

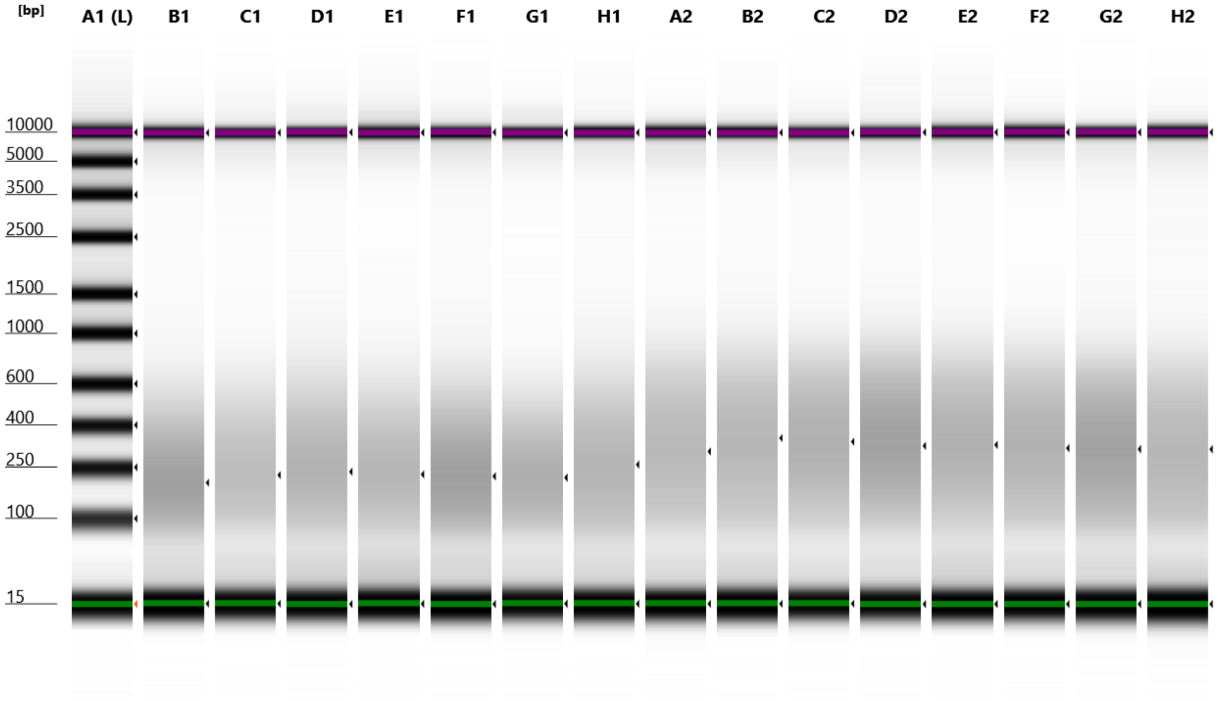

Default image (Contrast 100%)

Sample Info

Well	Conc. [ng/µl]	Sample Description	Alert	Observations
A1	31.7	Ladder		Ladder
B1	1.07	R2 DFB 1 PLUS 240 SEC		
C1	0.544	R2DFB 2 PLUS 240 SEC		
D1	2.72	R2 DFB 3 PLUS 240 SEC		
E1	0.349	R2 DFB 4 PLUS 240 SEC		
F1	0.613	R2 DFB 5 PLUS 240 SEC		
G1	3.00	R2 DFB 6 PLUS 240 SEC		
H1	0.333	R2 DFB 7 PLUS 240 SEC		
A2	0.427	R2 DFB 1 PLUS 240 SEC		
B2	0.366	R2 DFB 2 PLUS 120 SEC		
C2	0.422	R2 DFB 3 PLUS 120 SEC		
D2	0.798	R2 DFB4 PLUS 120 SEC		
E2	0.487	R2 DFB 5PLUS 120 SEC		
F2	0.417	R2 DFB6 PLUS 120 SEC		
G2	0.610	R2 DFB7 PLUS 120 SEC		
H2	0.441	R2 DFB8 PLUS 120 SEC		

© Agilent Technologies, Inc. 2017

A1: Ladder

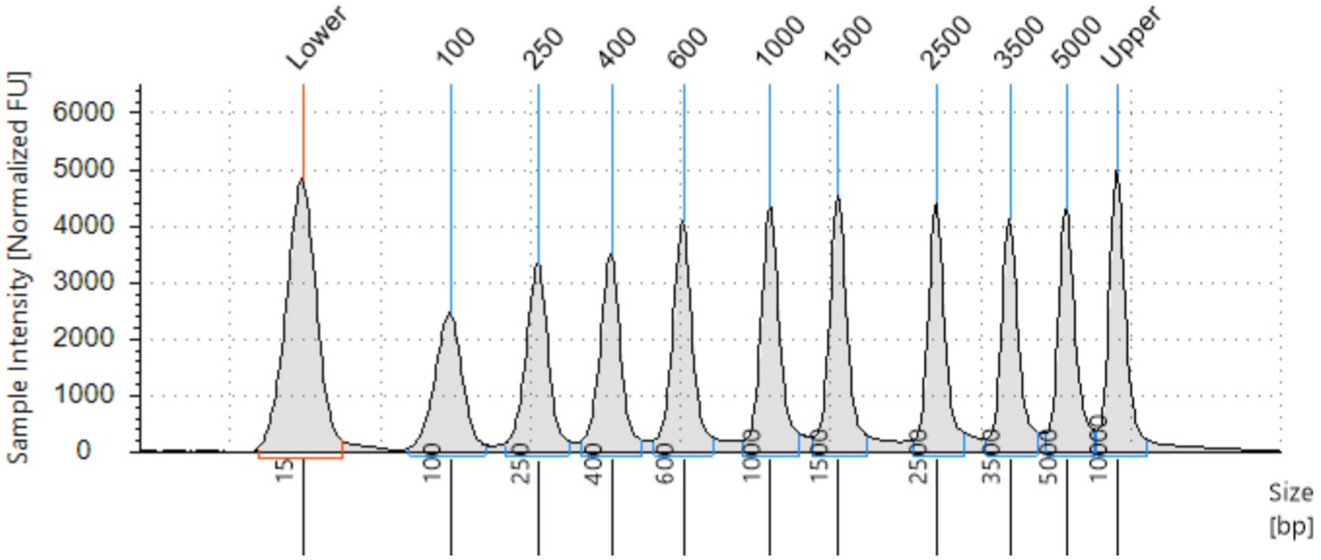

Sample Table

Well	Conc. [ng/µl]	Sample Description	Alert	Observations
A1	31.7	Ladder		Ladder

Peak Table

Size [bp]	Calibrated Conc. [ng/µl]	Assigned Conc. [ng/µl]	Peak Molarity [nmol/l]	% Integrated Area	Peak Comment	Observations
15	6.37	-	653	-		Lower Marker
100	3.15	-	48.4	9.92		
250	3.47	-	21.3	10.92		
400	3.40	-	13.1	10.71		
600	3.76	-	9.64	11.85		
1000	3.80	-	5.85	11.99		
1500	3.80	-	3.90	11.99		
2500	3.52	-	2.16	11.09		
3500	3.35	-	1.47	10.55		
5000	3.49	-	1.07	10.99		
10000	3.25	3.25	0.500	-		Upper Marker

© Agilent Technologies, Inc. 2017

B1: R2 DFB 1 PLUS 240 SEC

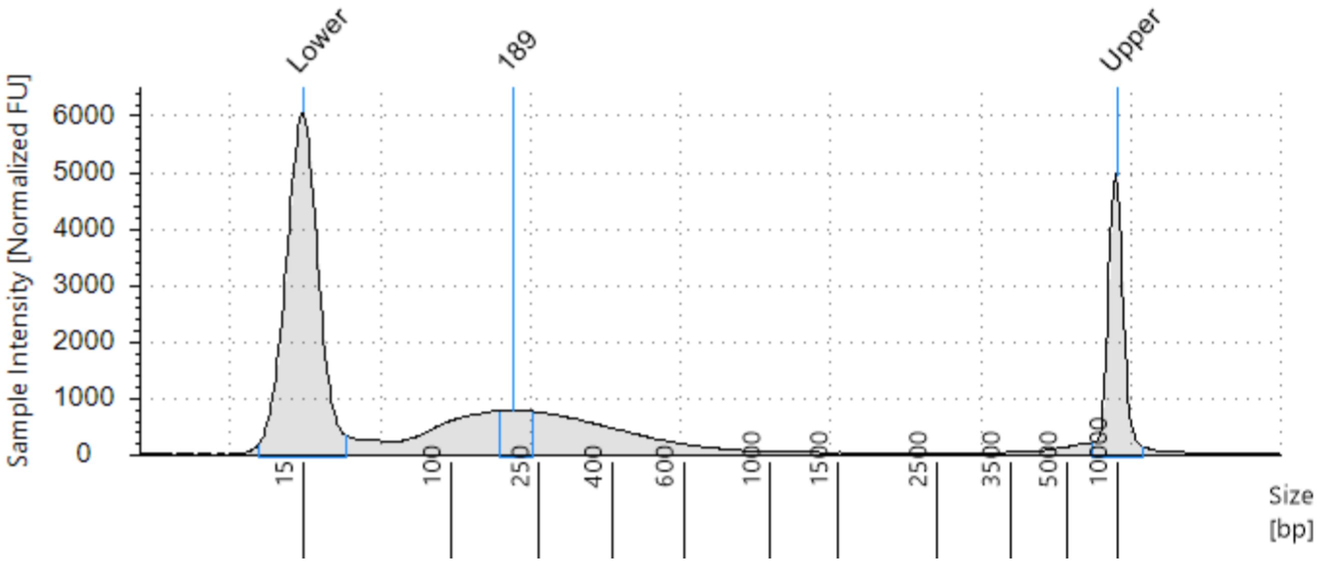

Sample Table

Well	Conc. [ng/µl]	Sample Description	Alert	Observations
B1	1.07	R2 DFB 1 PLUS 240 SEC		

Peak Table

Size [bp]	Calibrated Conc. [ng/µl]	Assigned Conc. [ng/µl]	Peak Molarity [nmol/l]	% Integrated Area	Peak Comment	Observations
15	8.26	-	847	-		Lower Marker
189	1.07	-	8.73	100.00		
10000	3.25	3.25	0.500	-		Upper Marker

© Agilent Technologies, Inc. 2017

C1: R2DFB 2 PLUS 240 SEC

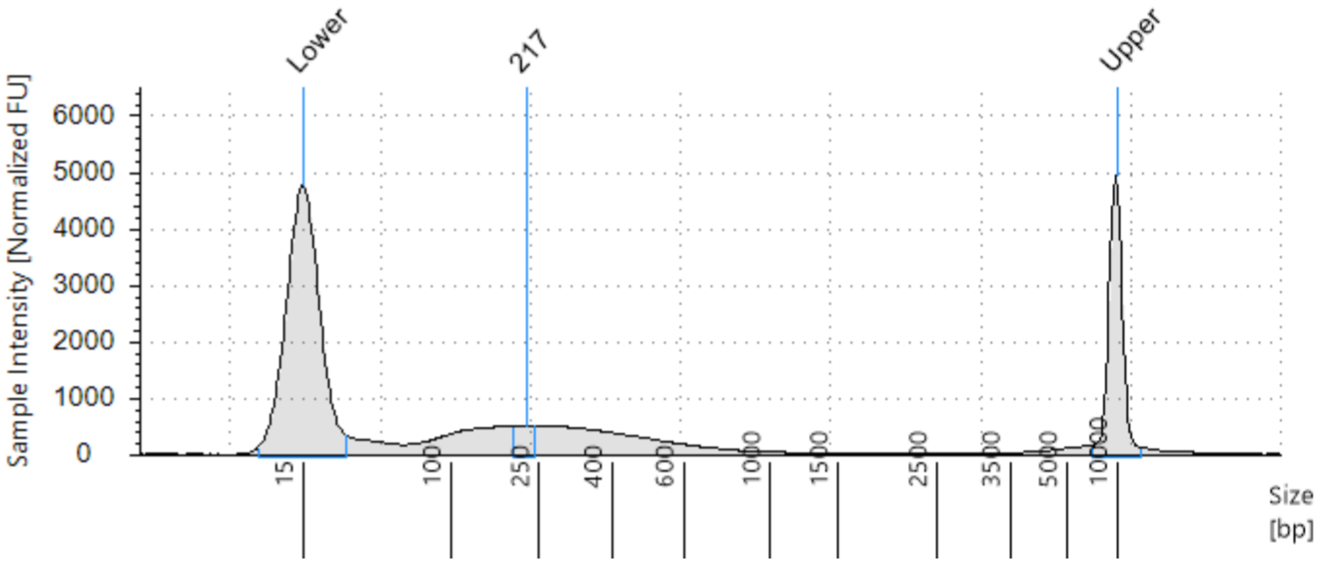

Sample Table

Well	Conc. [ng/µl]	Sample Description	Alert	Observations
C1	0.544	R2DFB 2 PLUS 240 SEC		

Peak Table

Size [bp]	Calibrated Conc. [ng/µl]	Assigned Conc. [ng/µl]	Peak Molarity [nmol/l]	% Integrated Area	Peak Comment	Observations
15	7.48	-	767	-		Lower Marker
217	0.544	-	3.86	100.00		
10000	3.25	3.25	0.500	-		Upper Marker

 © Agilent Technologies, Inc. 2017

D1: R2 DFB 3 PLUS 240 SEC

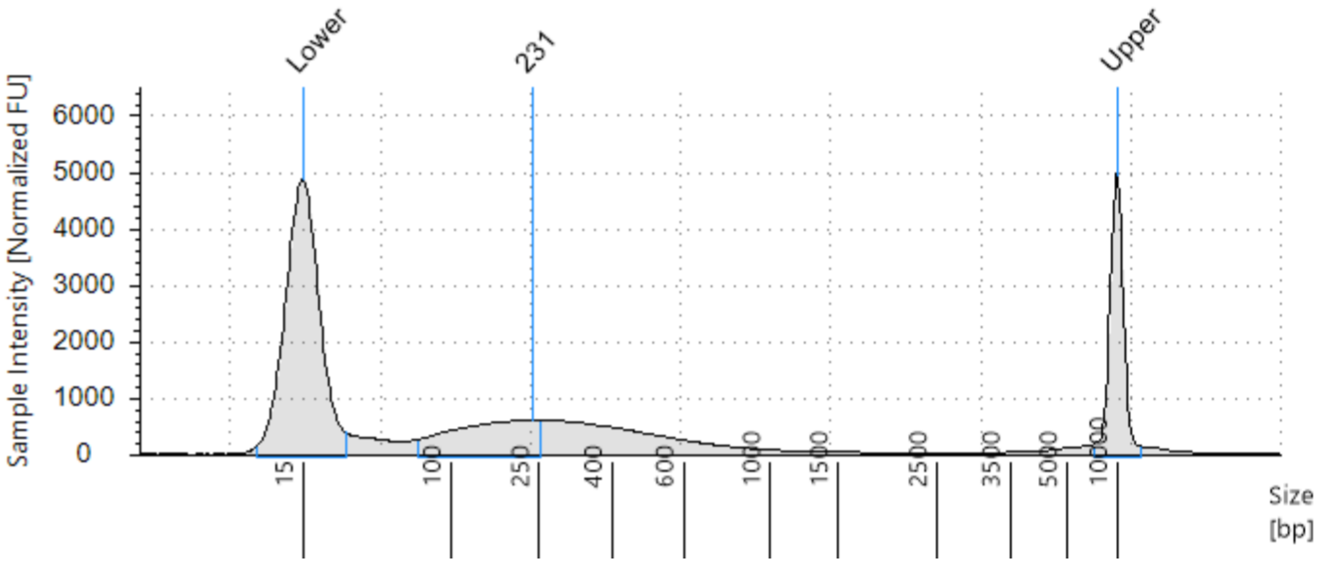

Sample Table

Well	Conc. [ng/µl]	Sample Description	Alert	Observations
D1	2.72	R2 DFB 3 PLUS 240 SEC		

Peak Table

Size [bp]	Calibrated Conc. [ng/µl]	Assigned Conc. [ng/µl]	Peak Molarity [nmol/l]	% Integrated Area	Peak Comment	Observations
15	7.71	-	791	-		Lower Marker
231	2.72	-	18.1	100.00		
10000	3.25	3.25	0.500	-		Upper Marker

© Agilent Technologies, Inc. 2017

E1: R2 DFB 4 PLUS 240 SEC

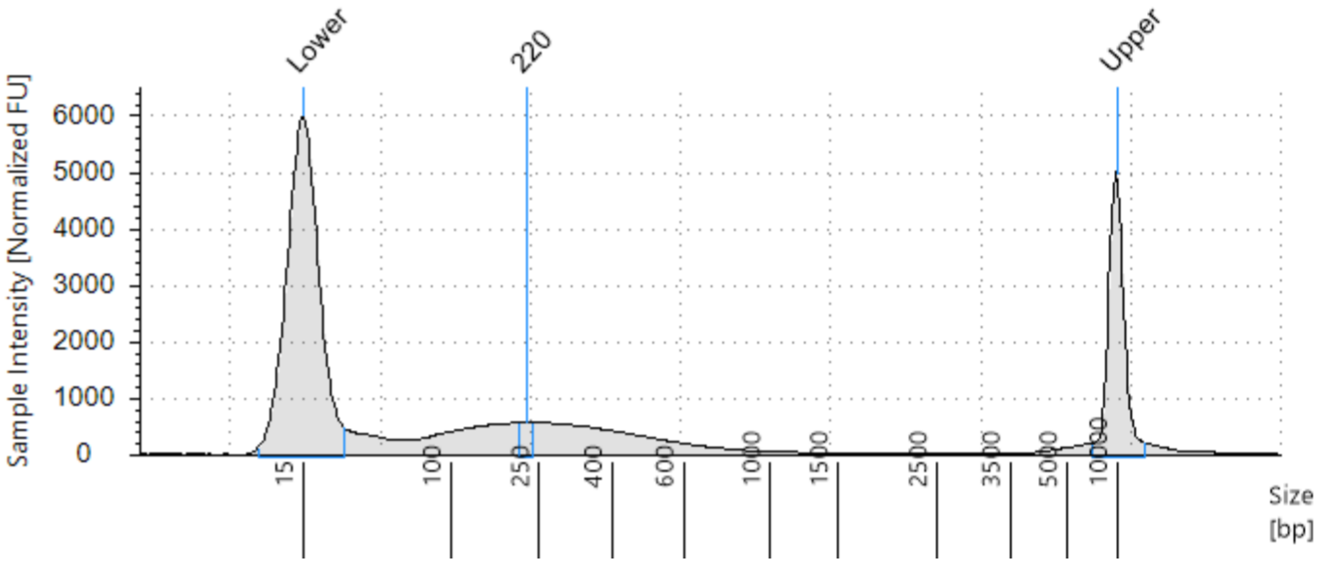

Sample Table

Well	Conc. [ng/µl]	Sample Description	Alert	Observations
E1	0.349	R2 DFB 4 PLUS 240 SEC		

Peak Table

Size [bp]	Calibrated Conc. [ng/µl]	Assigned Conc. [ng/µl]	Peak Molarity [nmol/l]	% Integrated Area	Peak Comment	Observations
15	8.04	-	825	-		Lower Marker
220	0.349	-	2.45	100.00		
10000	3.25	3.25	0.500	-		Upper Marker

 © Agilent Technologies, Inc. 2017

F1: R2 DFB 5 PLUS 240 SEC

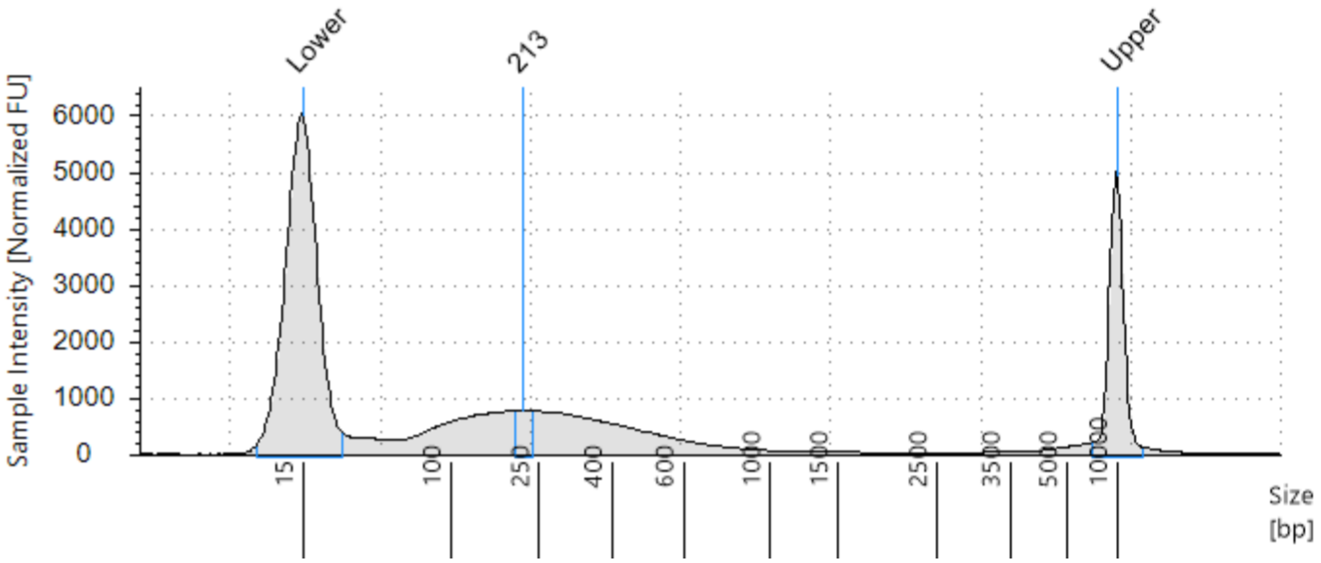

Sample Table

Well	Conc. [ng/µl]	Sample Description	Alert	Observations
F1	0.613	R2 DFB 5 PLUS 240 SEC		

Peak Table

Size [bp]	Calibrated Conc. [ng/µl]	Assigned Conc. [ng/µl]	Peak Molarity [nmol/l]	% Integrated Area	Peak Comment	Observations
15	8.48	-	869	-		Lower Marker
213	0.613	-	4.44	100.00		
10000	3.25	3.25	0.500	-		Upper Marker

 © Agilent Technologies, Inc. 2017

G1: R2 DFB 6 PLUS 240 SEC

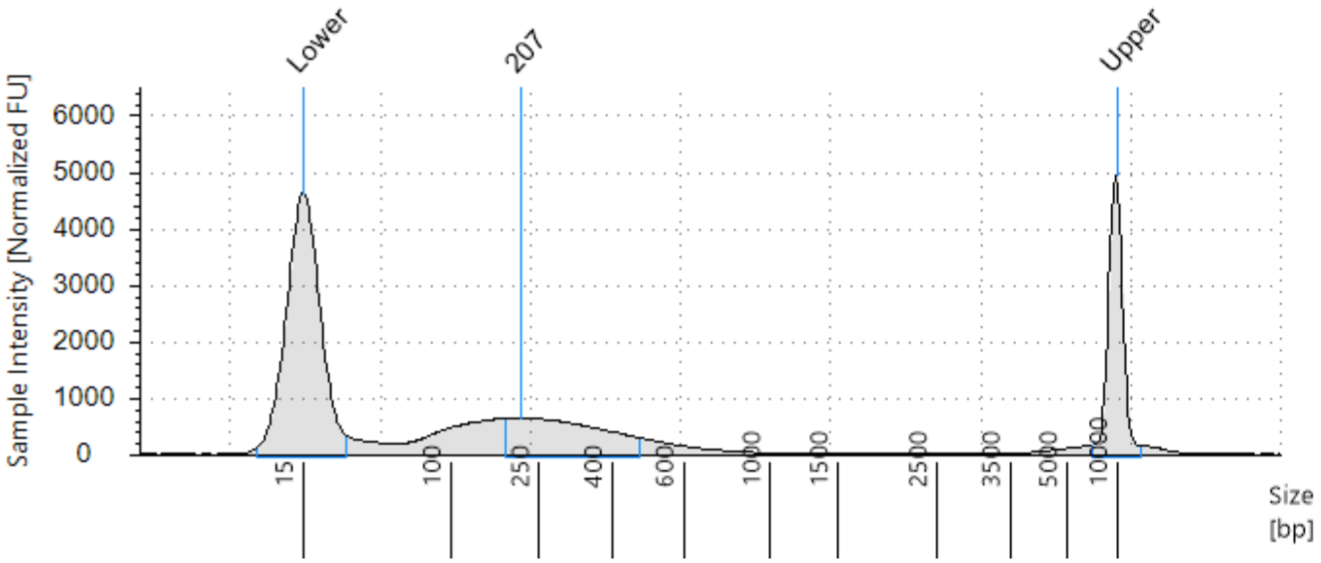

Sample Table

Well	Conc. [ng/µl]	Sample Description	Alert	Observations
G1	3.00	R2 DFB 6 PLUS 240 SEC		

Peak Table

Size [bp]	Calibrated Conc. [ng/µl]	Assigned Conc. [ng/µl]	Peak Molarity [nmol/l]	% Integrated Area	Peak Comment	Observations
15	7.00	-	718	-		Lower Marker
207	3.00	-	22.3	100.00		
10000	3.25	3.25	0.500	-		Upper Marker

© Agilent Technologies, Inc. 2017

H1: R2 DFB 7 PLUS 240 SEC

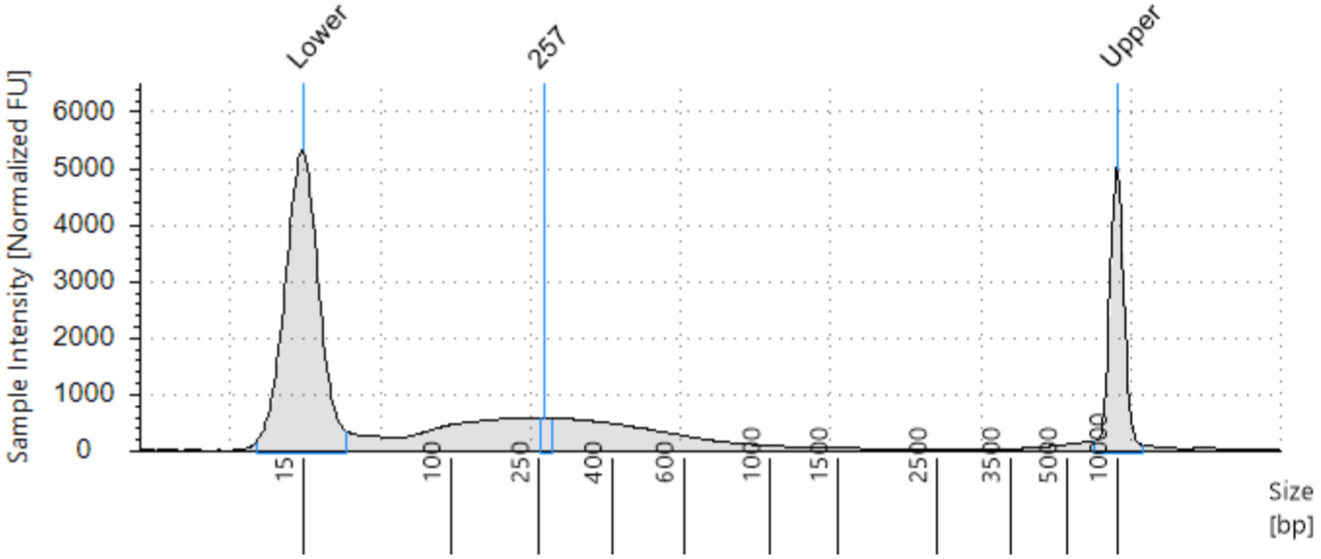

Sample Table

Well	Conc. [ng/µl]	Sample Description	Alert	Observations
H1	0.333	R2 DFB 7 PLUS 240 SEC		

Peak Table

Size [bp]	Calibrated Conc. [ng/µl]	Assigned Conc. [ng/µl]	Peak Molarity [nmol/l]	% Integrated Area	Peak Comment	Observations
15	7.71	-	791	-		Lower Marker
257	0.333	-	1.99	100.00		
10000	3.25	3.25	0.500	-		Upper Marker

© Agilent Technologies, Inc. 2017

Filename: 2019-05-21-01 DFB plus, first 7, 240 sec last 8 120 sec.D5000

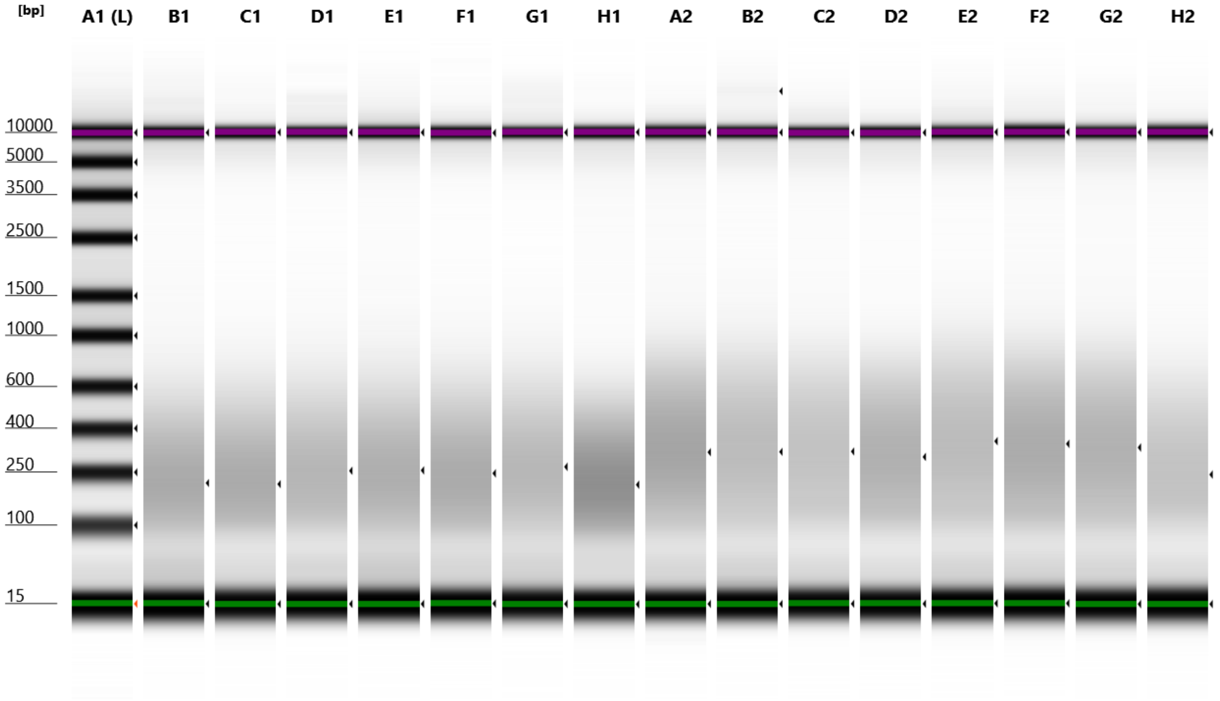

Default image (Contrast 100%)

Sample Info

Well	Conc. [ng/µl]	Sample Description	Alert	Observations
A1	28.3	Ladder		Ladder
B1	2.35	DFB 1 plus 240 sec R3		
C1	2.31	DFB2 plus 240 sec R3		
D1	0.683	DFB 3plus 240 sec R3		
E1	2.71	DFB4 plus 240 sec R3		
F1	0.491	DFB5 plus 240 sec R3		
G1	2.21	DFB6 plus 240 sec R3		
H1	3.52	DFB7 plus 240 sec R3		
A2	0.980	DFB1 plus 120 sec R3		
B2	0.961	DFB2 plus 120 sec R3		
C2	0.381	DFB3 plus 120 sec R3		
D2	0.490	DFB4 plus 120 sec R3		
E2	0.359	DFB5 plus 120 sec R3		
F2	0.428	DFB6 plus 120 sec R3		
G2	0.595	DFB7 plus 120 sec R3		
H2	0.326	DFB8 plus 120 sec R3		

© Agilent Technologies, Inc. 2017

A1: Ladder

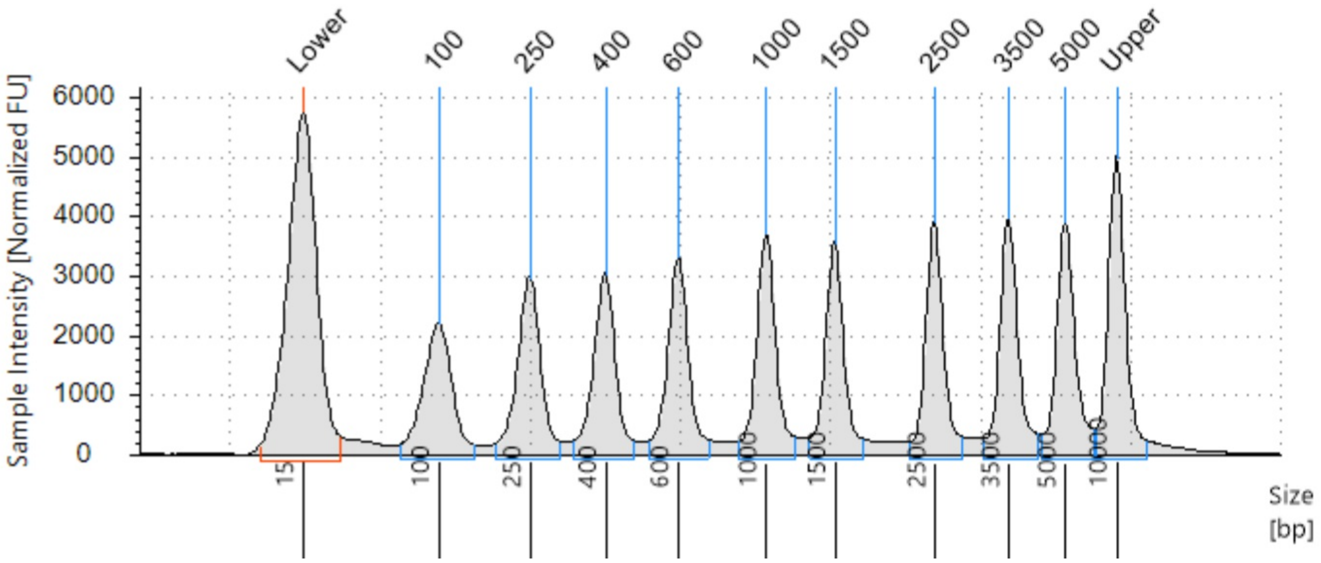

Sample Table

Well	Conc. [ng/µl]	Sample Description	Alert	Observations
A1	28.3	Ladder		Ladder

Peak Table

Size [bp]	Calibrated Conc. [ng/µl]	Assigned Conc. [ng/µl]	Peak Molarity [nmol/l]	% Integrated Area	Peak Comment	Observations
15	6.84	-	701	-		Lower Marker
100	2.88	-	44.4	10.21		
250	3.15	-	19.4	11.14		
400	3.02	-	11.6	10.71		
600	3.14	-	8.04	11.10		
1000	3.26	-	5.01	11.52		
1500	3.02	-	3.10	10.70		
2500	3.17	-	1.95	11.21		
3500	3.32	-	1.46	11.75		
5000	3.30	-	1.01	11.67		
10000	3.25	3.25	0.500	-		Upper Marker

 © Agilent Technologies, Inc. 2017

B1: DFB 1 plus 240 sec R3

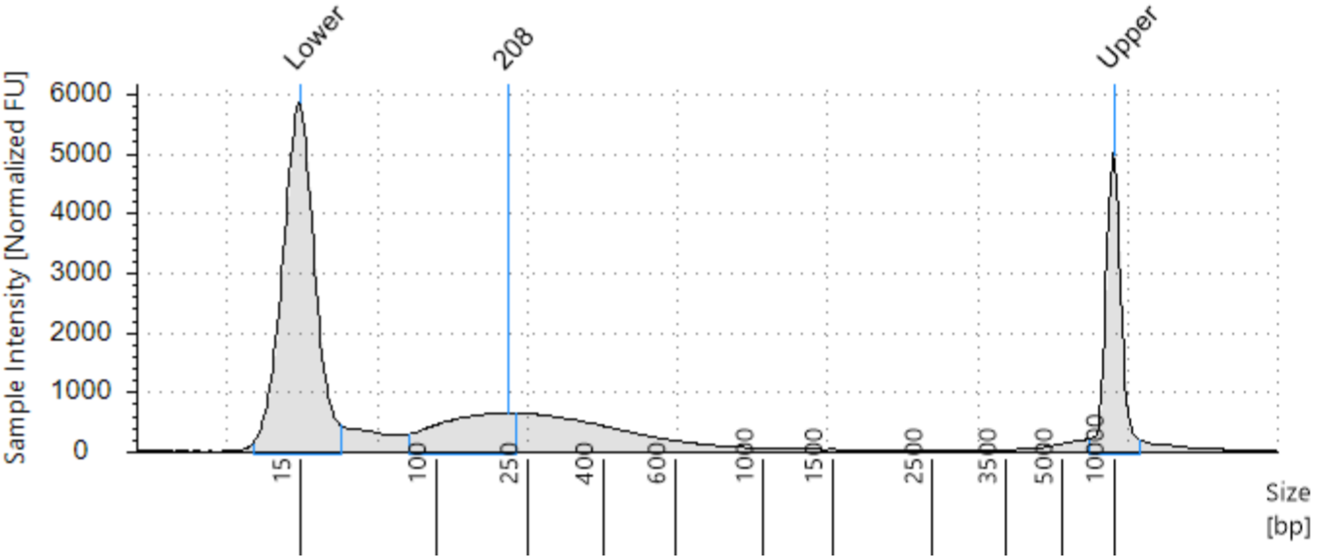

Sample Table

Well	Conc. [ng/µl]	Sample Description	Alert	Observations
B1	2.35	DFB 1 plus 240 sec R3		

Peak Table

Size [bp]	Calibrated Conc. [ng/µl]	Assigned Conc. [ng/µl]	Peak Molarity [nmol/l]	% Integrated Area	Peak Comment	Observations
15	7.92	-	813	-		Lower Marker
208	2.35	-	17.4	100.00		
10000	3.25	3.25	0.500	-		Upper Marker

 © Agilent Technologies, Inc. 2017

C1: DFB2 plus 240 sec R3

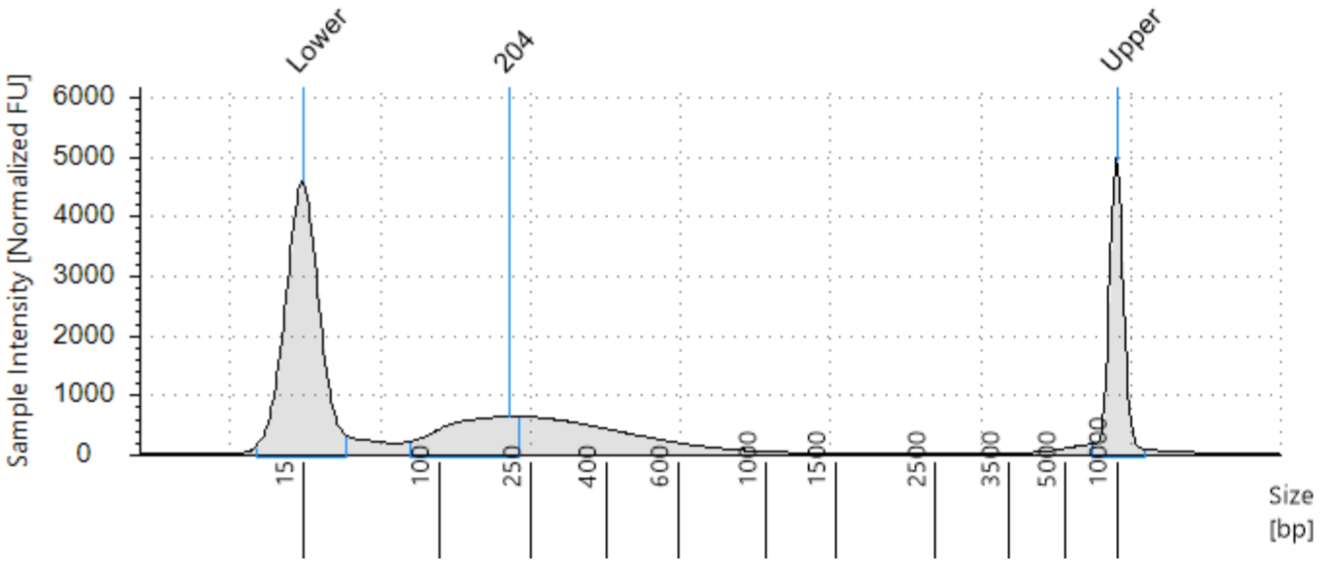

Sample Table

Well	Conc. [ng/µl]	Sample Description	Alert	Observations
C1	2.31	DFB2 plus 240 sec R3		

Peak Table

Size [bp]	Calibrated Conc. [ng/µl]	Assigned Conc. [ng/µl]	Peak Molarity [nmol/l]	% Integrated Area	Peak Comment	Observations
15	6.78	-	695	-		Lower Marker
204	2.31	-	17.4	100.00		
10000	3.25	3.25	0.500	-		Upper Marker

© Agilent Technologies, Inc. 2017

D1: DFB 3plus 240 sec R3

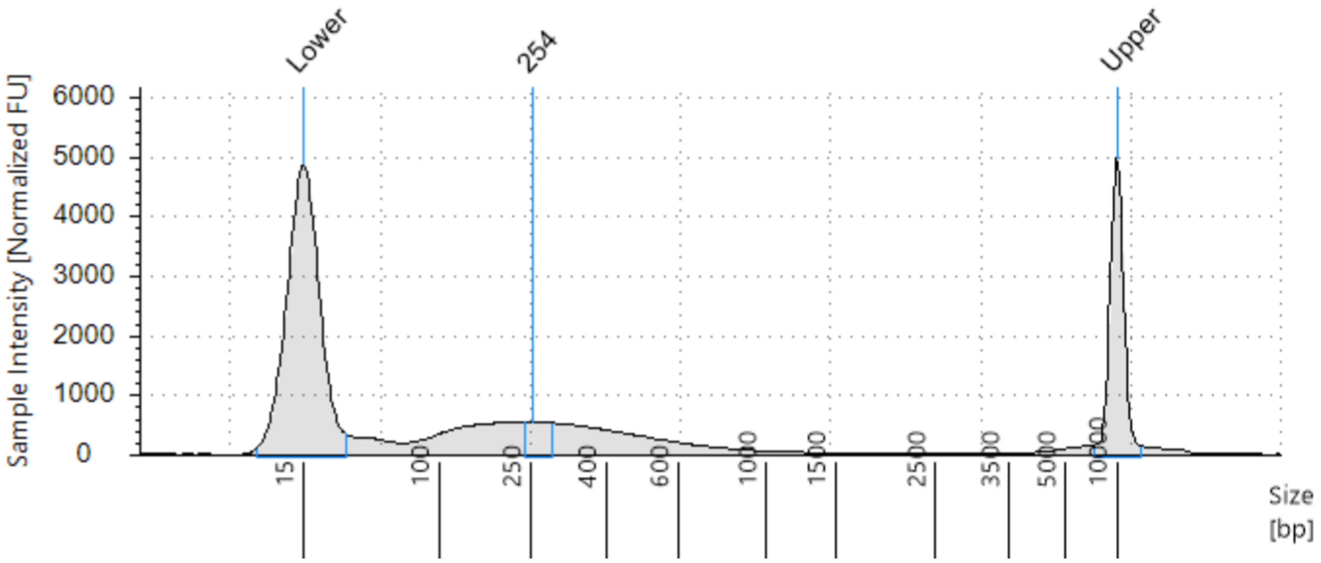

Sample Table

Well	Conc. [ng/µl]	Sample Description	Alert	Observations
D1	0.683	DFB 3plus 240 sec R3		

Peak Table

Size [bp]	Calibrated Conc. [ng/µl]	Assigned Conc. [ng/µl]	Peak Molarity [nmol/l]	% Integrated Area	Peak Comment	Observations
15	7.34	-	753	-		Lower Marker
254	0.683	-	4.13	100.00		
10000	3.25	3.25	0.500	-		Upper Marker

© Agilent Technologies, Inc. 2017

E1: DFB4 plus 240 sec R3

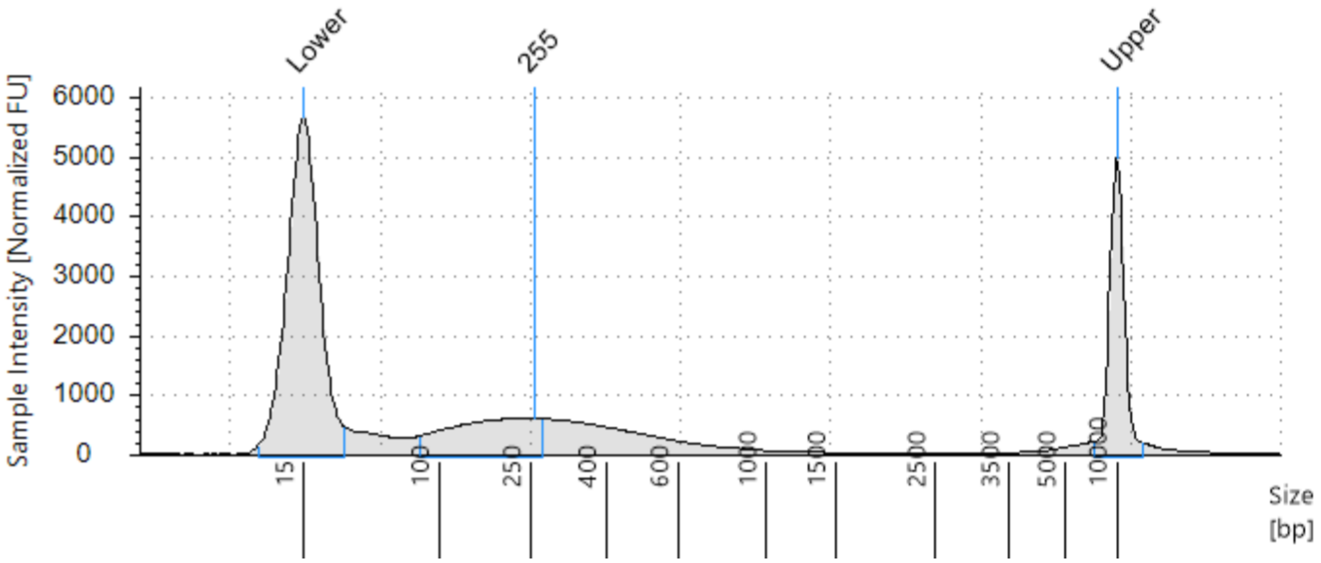

Sample Table

Well	Conc. [ng/µl]	Sample Description	Alert	Observations
E1	2.71	DFB4 plus 240 sec R3		

Peak Table

Size [bp]	Calibrated Conc. [ng/µl]	Assigned Conc. [ng/µl]	Peak Molarity [nmol/l]	% Integrated Area	Peak Comment	Observations
15	7.91	-	812	-		Lower Marker
255	2.71	-	16.3	100.00		
10000	3.25	3.25	0.500	-		Upper Marker

© Agilent Technologies, Inc. 2017

F1: DFB5 plus 240 sec R3

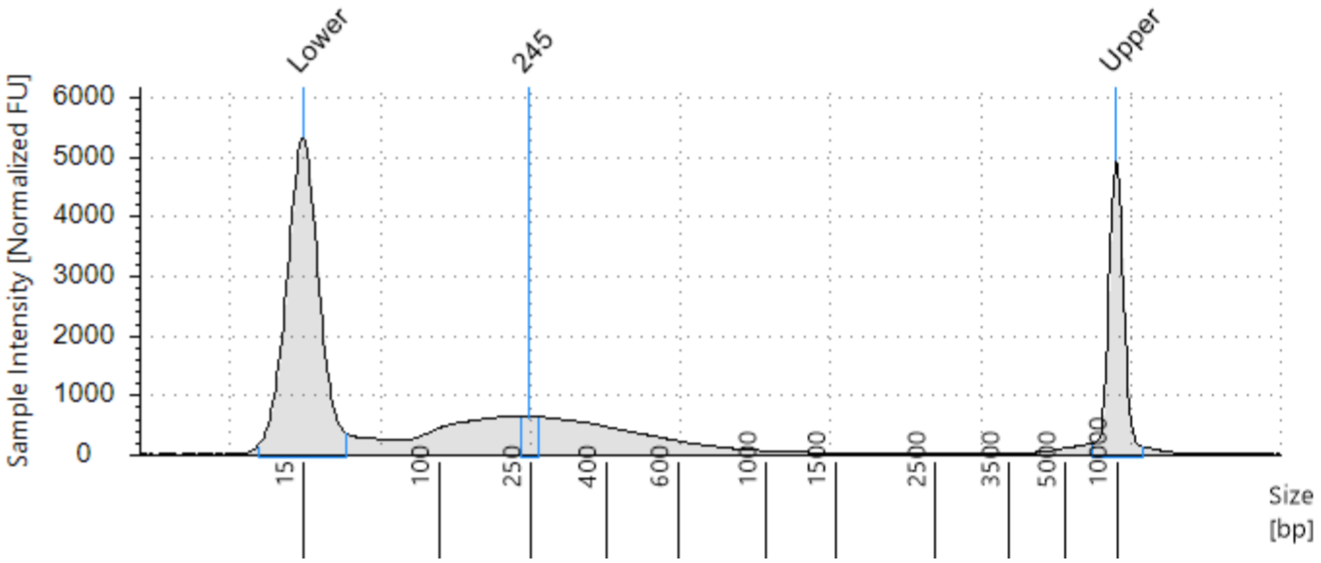

Sample Table

Well	Conc. [ng/µl]	Sample Description	Alert	Observations
F1	0.491	DFB5 plus 240 sec R3		

Peak Table

Size [bp]	Calibrated Conc. [ng/µl]	Assigned Conc. [ng/µl]	Peak Molarity [nmol/l]	% Integrated Area	Peak Comment	Observations
15	7.27	-	746	-		Lower Marker
245	0.491	-	3.09	100.00		
10000	3.25	3.25	0.500	-		Upper Marker

 © Agilent Technologies, Inc. 2017

G1: DFB6 plus 240 sec R3

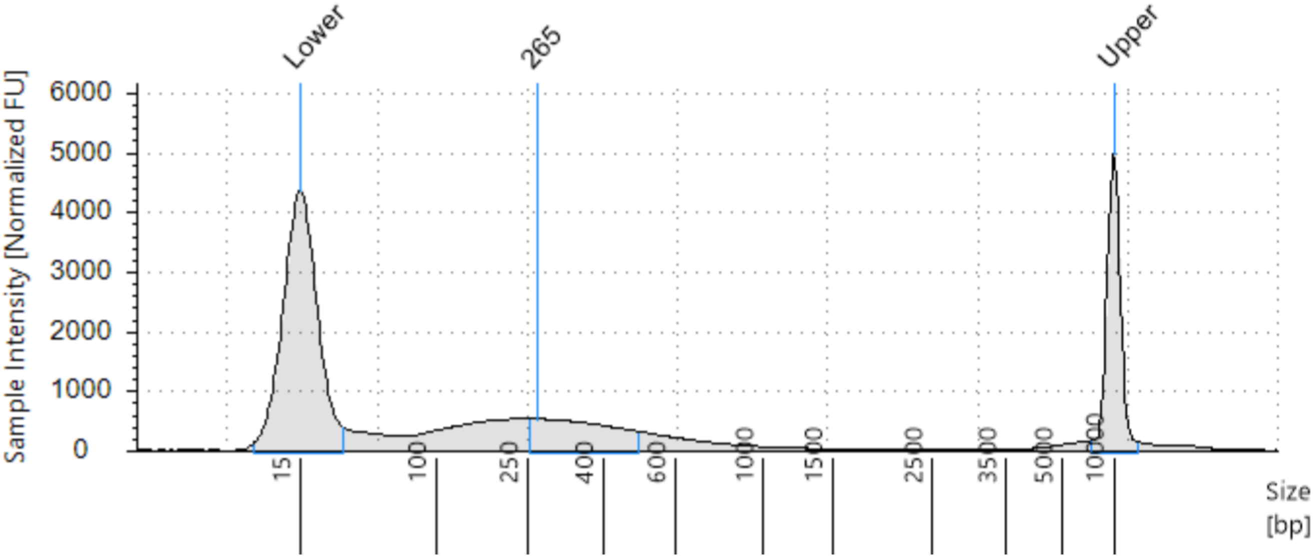

Sample Table

Well	Conc. [ng/µl]	Sample Description	Alert	Observations
G1	2.21	DFB6 plus 240 sec R3		

Peak Table

Size [bp]	Calibrated Conc. [ng/µl]	Assigned Conc. [ng/µl]	Peak Molarity [nmol/l]	% Integrated Area	Peak Comment	Observations
15	6.77	-	695	-		Lower Marker
265	2.21	-	12.8	100.00		
10000	3.25	3.25	0.500	-		Upper Marker

© Agilent Technologies, Inc. 2017

H1: DFB7 plus 240 sec R3

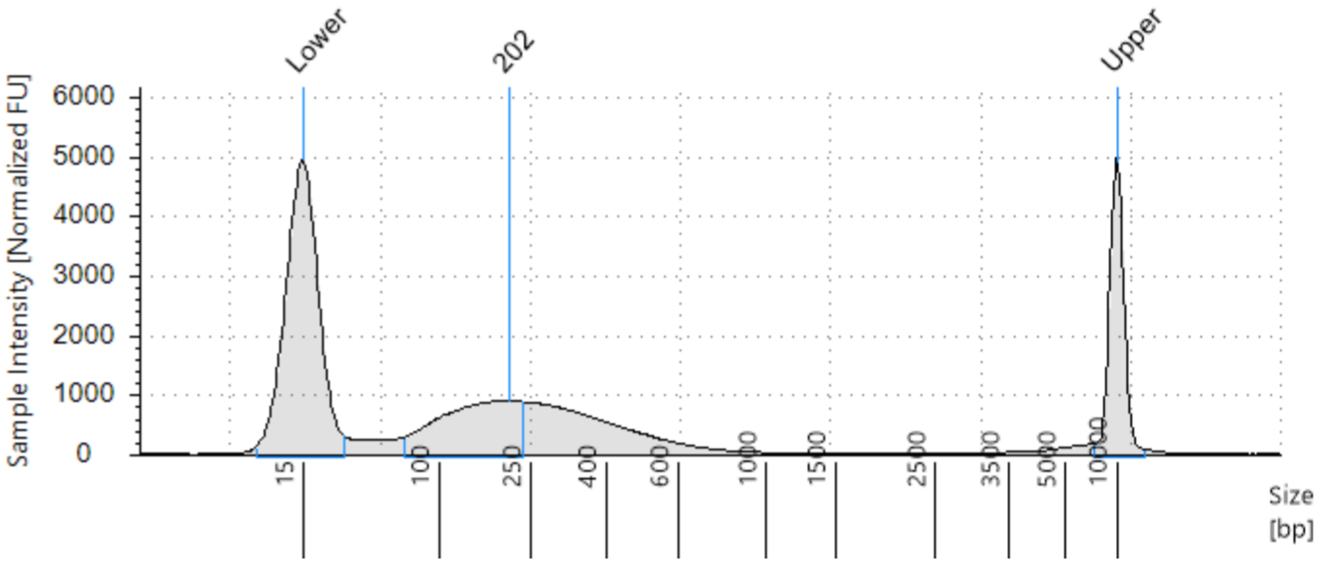

Sample Table

Well	Conc. [ng/µl]	Sample Description	Alert	Observations
H1	3.52	DFB7 plus 240 sec R3		

Peak Table

Size [bp]	Calibrated Conc. [ng/µl]	Assigned Conc. [ng/µl]	Peak Molarity [nmol/l]	% Integrated Area	Peak Comment	Observations
15	7.11	-	730	-		Lower Marker
202	3.52	-	26.8	100.00		
10000	3.25	3.25	0.500	-		Upper Marker

 © Agilent Technologies, Inc. 2017
