# Supplementary material for: 96 sample parallel acoustic fragmentation for high throughput next generation sequencing library preparation
Source: PLoS One. 2026 Feb 17;21(2):e0341139. doi: 10.1371/journal.pone.0341139 (PMC12912608; doi:10.1371/journal.pone.0341139)
Supplement: S2 Fig — (ZIP) [file pone.0341139.s002.zip › Figure 1 Raw Data/No cavitation enhancement 1296 seconds.pdf]

Filename: 2019-05-29-02- 1-8 1296 sec for 6.28, 8-15 1296 sec for 6.28.D5000

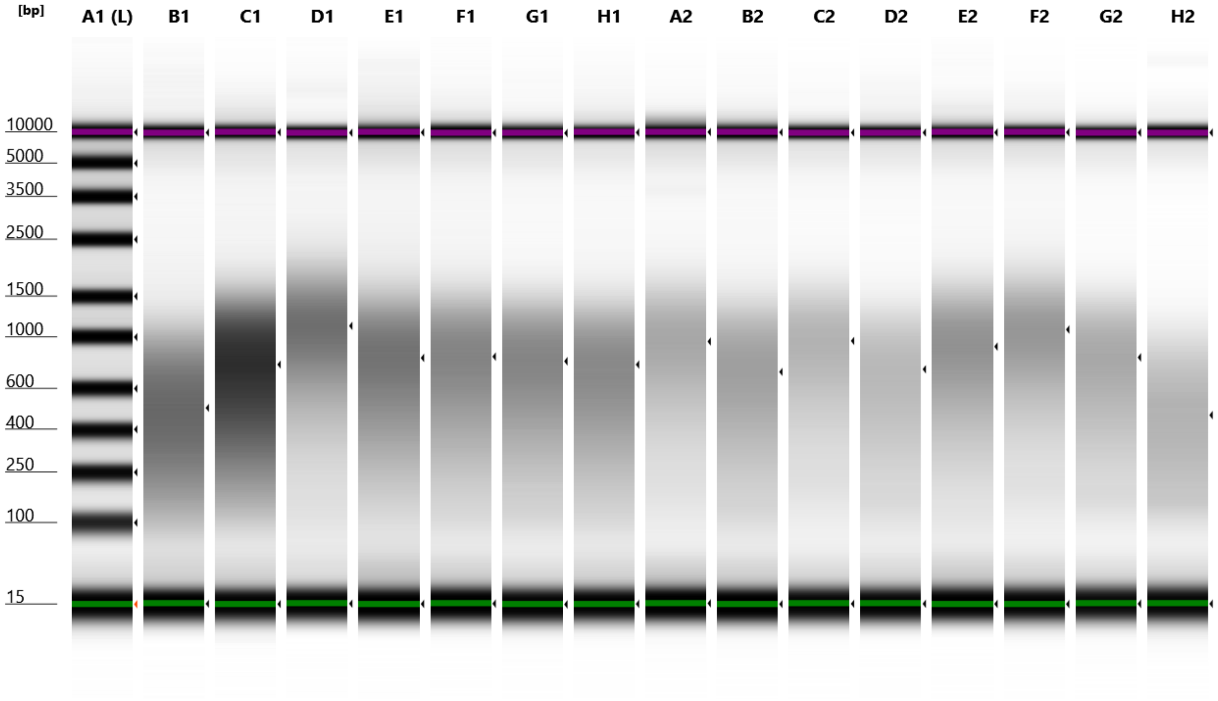

Default image (Contrast 100%)

Sample Info

Well	Conc. [ng/µl]	Sample Description	Alert	Observations
A1	28.9	Ladder		Ladder
B1	0.934	DFB1 MINUS LE220 1296 sec from 6.28 first row		
C1	16.3	DFB2 MINUS LE220 1296 sec from 6.28 first row		
D1	7.35	DFB3 MINUS LE220 1296 sec from 6.28 first row		
E1	4.10	DFB4 MINUS LE220 1296 sec from 6.28 first row		
F1	3.31	DFB5 MINUS LE220 1296 sec from 6.28 first row		
G1	6.93	DFB6 MINUS LE220 1296 sec from 6.28 first row		
H1	0.778	DFB7 MINUS LE220 1296 sec from 6.28 first row		
A2	1.87	DFB 1 MINUS LE220 1296 sec from 6.28 second row		
B2	0.743	DFB2 MINUS LE220 1296 sec from 6.28 second row		
C2	1.72	DFB3 MINUS LE220 1296 sec from 6.28 second row		
D2	0.399	DFB3 MINUS LE220 1296 sec from 6.28 second row		
E2	3.00	DFB4 MINUS LE220 1296 sec from 6.28 second row		
F2	2.49	DFB5 MINUS LE220 1296 sec from 6.28 second row		
G2	4.40	DFB6 MINUS LE220 1296 sec from 6.28 second row		
H2	2.31	DFB7 MINUS LE220 1296 sec from 6.28 second row		

 © Agilent Technologies, Inc. 2017

A1: Ladder

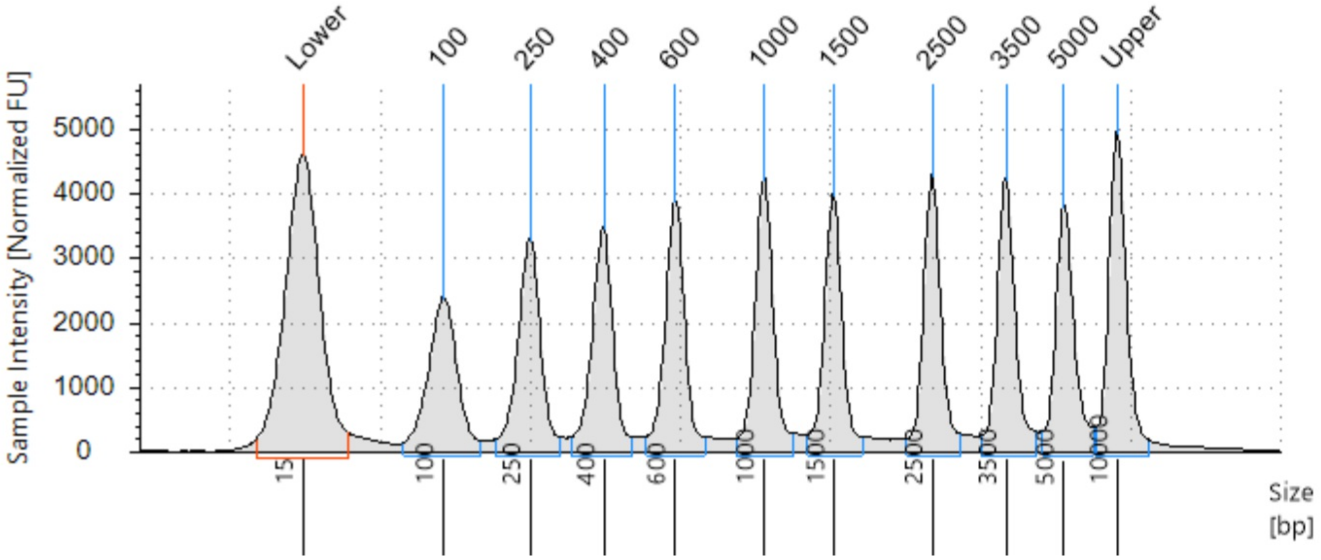

Sample Table

Well	Conc. [ng/µl]	Sample Description	Alert	Observations
A1	28.9	Ladder		Ladder

Peak Table

Size [bp]	Calibrated Conc. [ng/µl]	Assigned Conc. [ng/µl]	Peak Molarity [nmol/l]	% Integrated Area	Peak Comment	Observations
15	5.98	-	614	-		Lower Marker
100	3.00	-	46.1	10.36		
250	3.24	-	19.9	11.20		
400	3.16	-	12.1	10.91		
600	3.31	-	8.48	11.43		
1000	3.40	-	5.24	11.76		
1500	3.12	-	3.20	10.78		
2500	3.26	-	2.01	11.28		
3500	3.35	-	1.47	11.60		
5000	3.09	-	0.951	10.68		
10000	3.25	3.25	0.500	-		Upper Marker

© Agilent Technologies, Inc. 2017

B1: DFB1 MINUS LE220 1296 sec from 6.28 first row

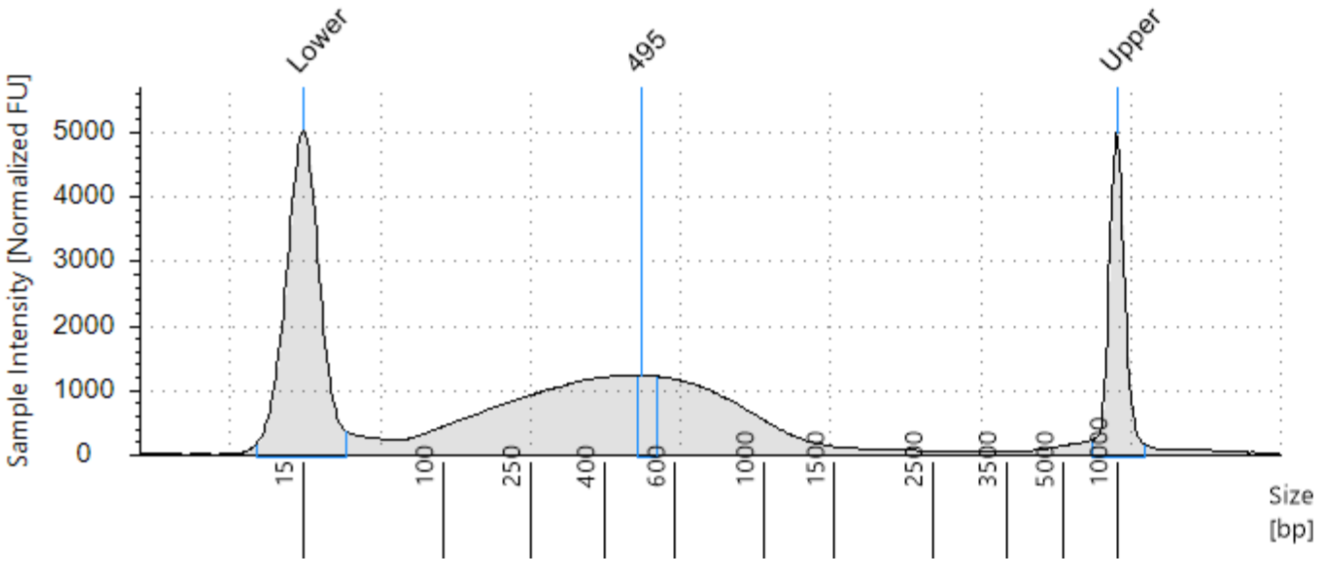

Sample Table

Well	Conc. [ng/µl]	Sample Description	Alert	Observations
B1	0.934	DFB1 MINUS LE220 1296 sec from 6.28 first row		

Peak Table

Size [bp]	Calibrated Conc. [ng/µl]	Assigned Conc. [ng/µl]	Peak Molarity [nmol/l]	% Integrated Area	Peak Comment	Observations
15	6.77	-	695	-		Lower Marker
495	0.934	-	2.90	100.00		
10000	3.25	3.25	0.500	-		Upper Marker

 © Agilent Technologies, Inc. 2017

C1: DFB2 MINUS LE220 1296 sec from 6.28 first row

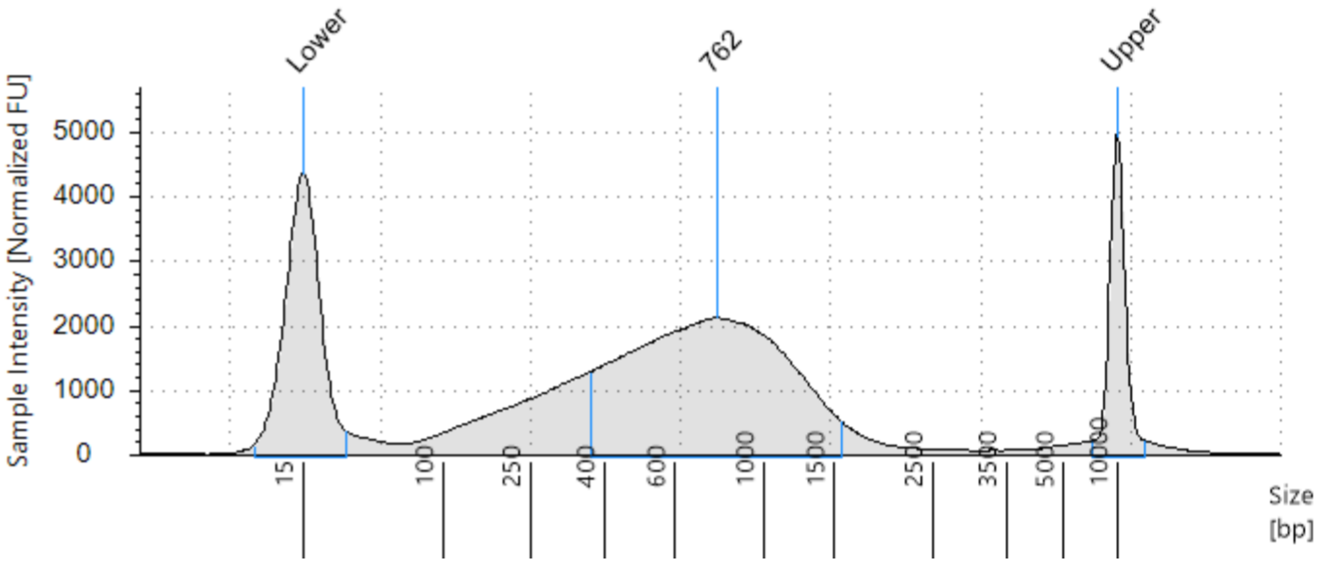

Sample Table

Well	Conc. [ng/µl]	Sample Description	Alert	Observations
C1	16.3	DFB2 MINUS LE220 1296 sec from 6.28 first row		

Peak Table

Size [bp]	Calibrated Conc. [ng/µl]	Assigned Conc. [ng/µl]	Peak Molarity [nmol/l]	% Integrated Area	Peak Comment	Observations
15	6.25	-	641	-		Lower Marker
762	16.3	-	33.0	100.00		
10000	3.25	3.25	0.500	-		Upper Marker

© Agilent Technologies, Inc. 2017

D1: DFB3 MINUS LE220 1296 sec from 6.28 first row

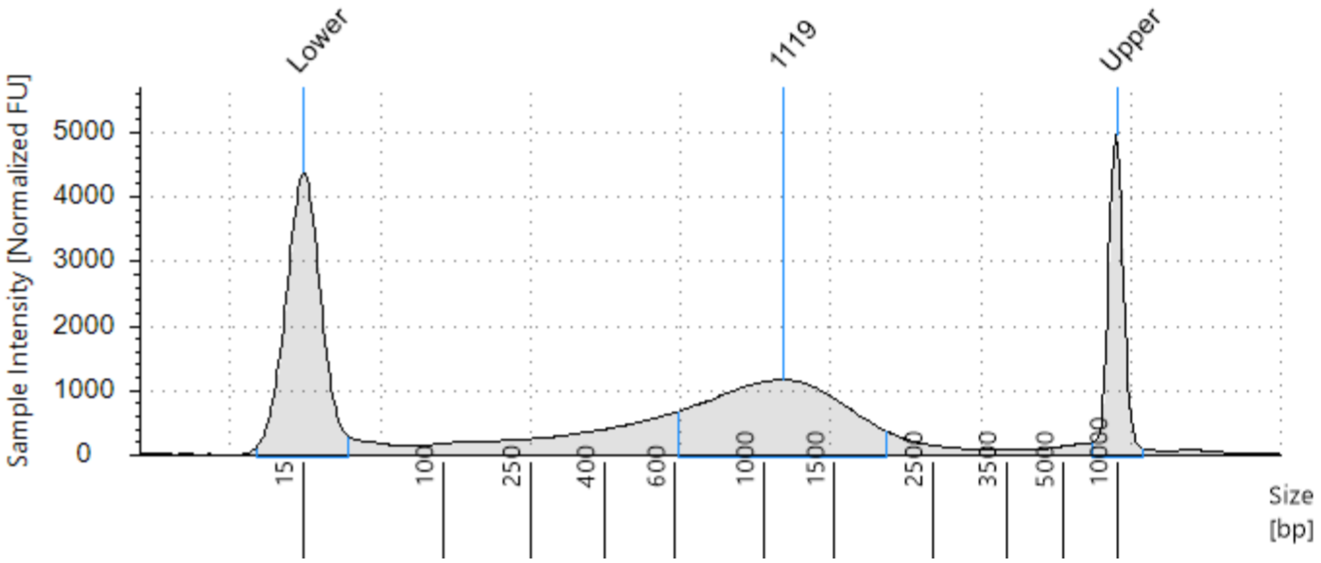

Sample Table

Well	Conc. [ng/µl]	Sample Description	Alert	Observations
D1	7.35	DFB3 MINUS LE220 1296 sec from 6.28 first row		

Peak Table

Size [bp]	Calibrated Conc. [ng/µl]	Assigned Conc. [ng/µl]	Peak Molarity [nmol/l]	% Integrated Area	Peak Comment	Observations
15	6.38	-	655	-		Lower Marker
1119	7.35	-	10.1	100.00		
10000	3.25	3.25	0.500	-		Upper Marker

© Agilent Technologies, Inc. 2017

E1: DFB4 MINUS LE220 1296 sec from 6.28 first row

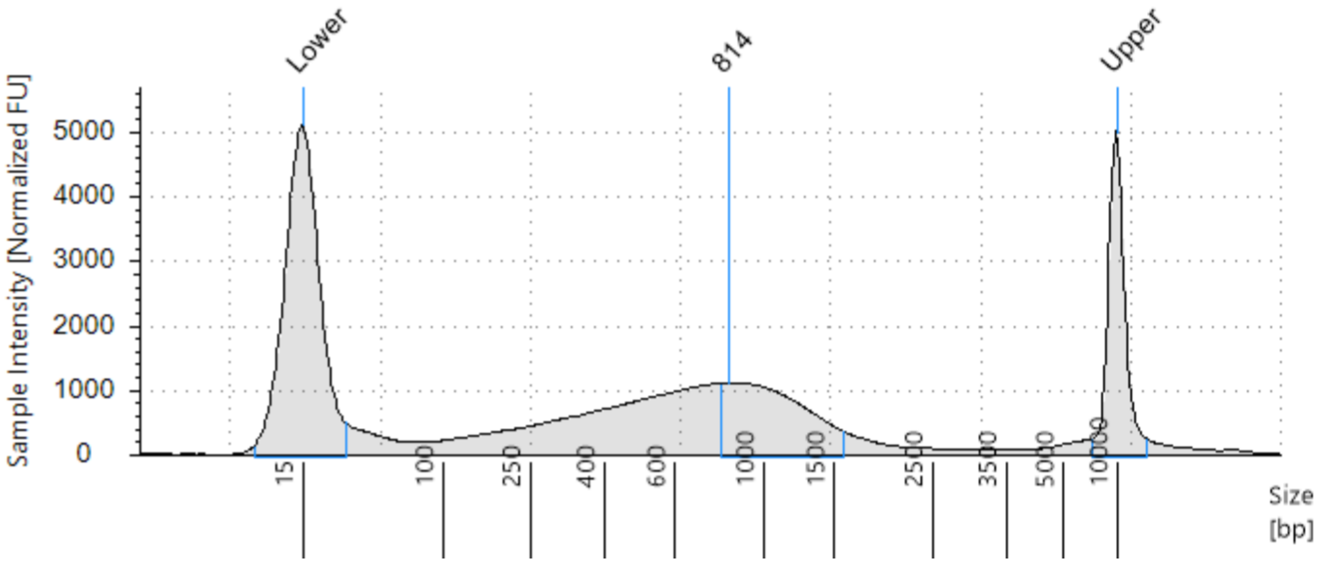

Sample Table

Well	Conc. [ng/µl]	Sample Description	Alert	Observations
E1	4.10	DFB4 MINUS LE220 1296 sec from 6.28 first row		

Peak Table

Size [bp]	Calibrated Conc. [ng/µl]	Assigned Conc. [ng/µl]	Peak Molarity [nmol/l]	% Integrated Area	Peak Comment	Observations
15	6.95	-	713	-		Lower Marker
814	4.10	-	7.76	100.00		
10000	3.25	3.25	0.500	-		Upper Marker

© Agilent Technologies, Inc. 2017

F1: DFB5 MINUS LE220 1296 sec from 6.28 first row

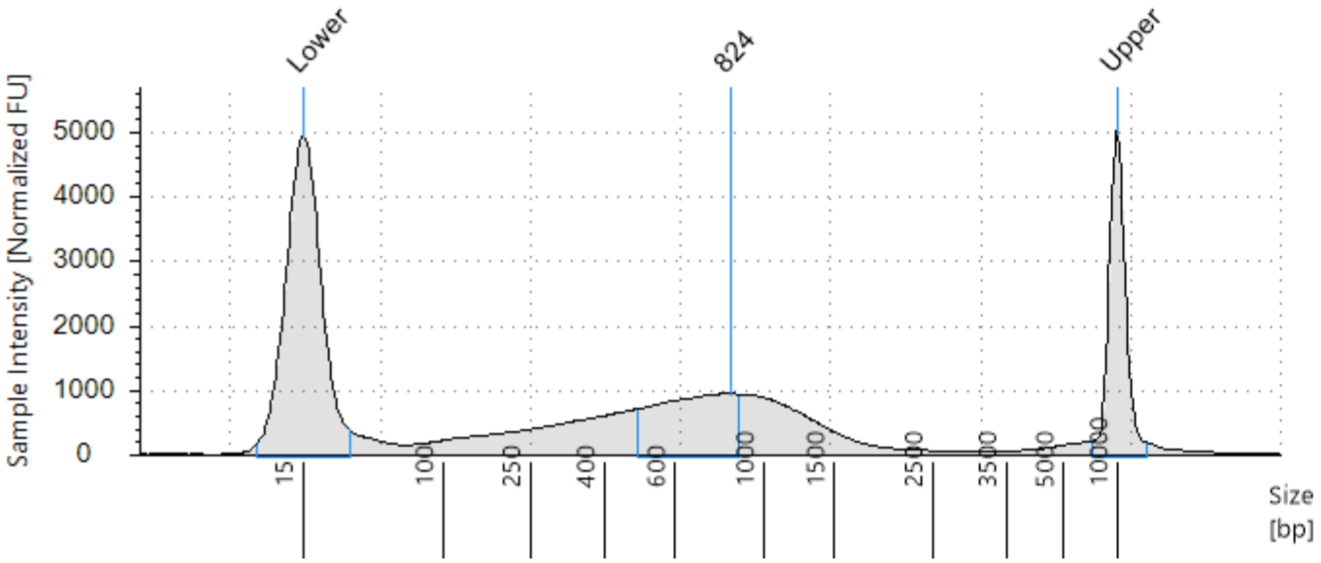

Sample Table

Well	Conc. [ng/µl]	Sample Description	Alert	Observations
F1	3.31	DFB5 MINUS LE220 1296 sec from 6.28 first row		

Peak Table

Size [bp]	Calibrated Conc. [ng/µl]	Assigned Conc. [ng/µl]	Peak Molarity [nmol/l]	% Integrated Area	Peak Comment	Observations
15	6.79	-	697	-		Lower Marker
824	3.31	-	6.18	100.00		
10000	3.25	3.25	0.500	-		Upper Marker

© Agilent Technologies, Inc. 2017

G1: DFB6 MINUS LE220 1296 sec from 6.28 first row

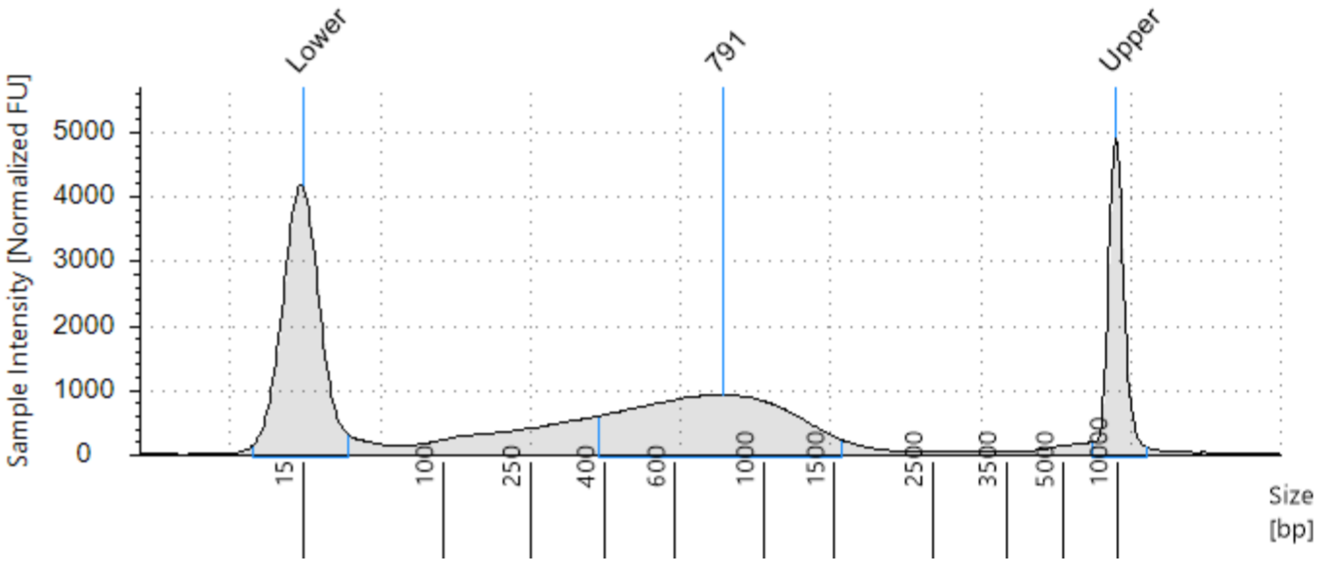

Sample Table

Well	Conc. [ng/µl]	Sample Description	Alert	Observations
G1	6.93	DFB6 MINUS LE220 1296 sec from 6.28 first row		

Peak Table

Size [bp]	Calibrated Conc. [ng/µl]	Assigned Conc. [ng/µl]	Peak Molarity [nmol/l]	% Integrated Area	Peak Comment	Observations
15	6.31	-	648	-		Lower Marker
791	6.93	-	13.5	100.00		
10000	3.25	3.25	0.500	-		Upper Marker

© Agilent Technologies, Inc. 2017

H1: DFB7 MINUS LE220 1296 sec from 6.28 first row

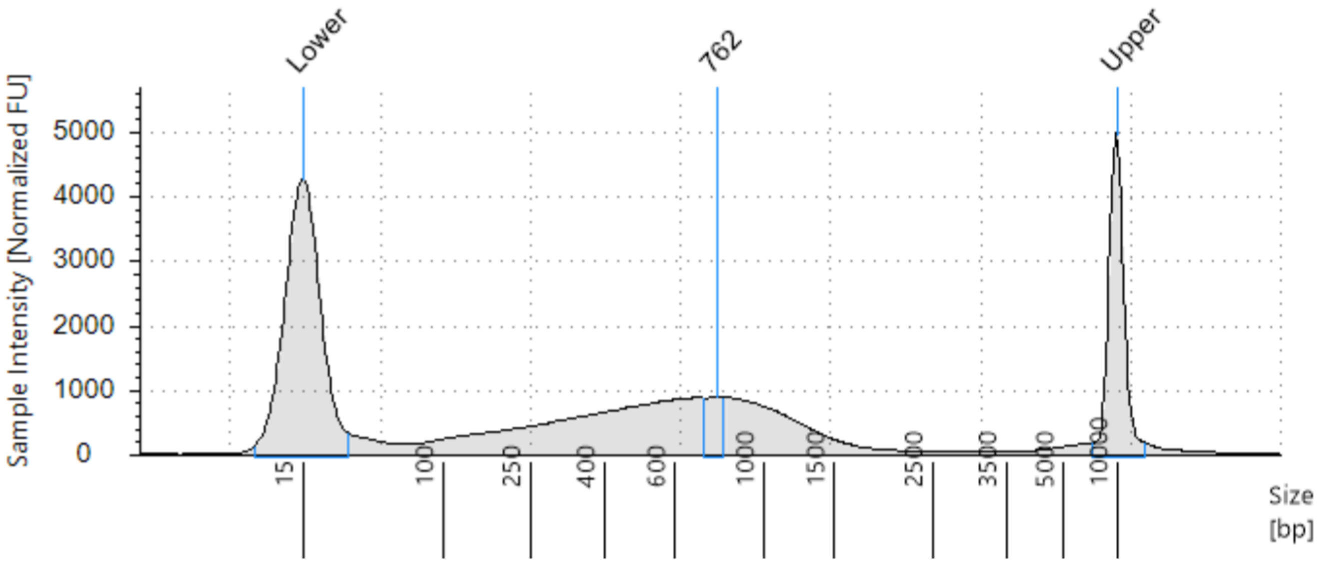

Sample Table

Well	Conc. [ng/µl]	Sample Description	Alert	Observations
H1	0.778	DFB7 MINUS LE220 1296 sec from 6.28 first row		

Peak Table

Size [bp]	Calibrated Conc. [ng/µl]	Assigned Conc. [ng/µl]	Peak Molarity [nmol/l]	% Integrated Area	Peak Comment	Observations
15	6.47	-	664	-		Lower Marker
762	0.778	-	1.57	100.00		
10000	3.25	3.25	0.500	-		Upper Marker

 © Agilent Technologies, Inc. 2017

A2: DFB 1 MINUS LE220 1296 sec from 6.28 second row

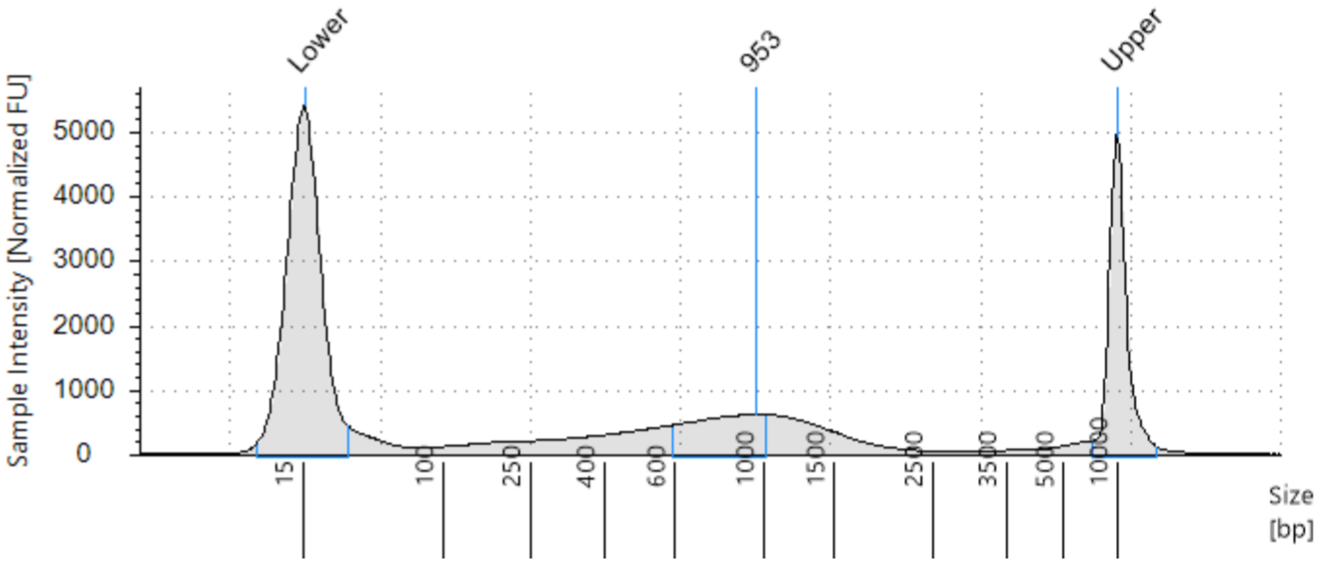

Sample Table

Well	Conc. [ng/µl]	Sample Description	Alert	Observations
A2	1.87	DFB 1 MINUS LE220 1296 sec from 6.28 second row		

Peak Table

Size [bp]	Calibrated Conc. [ng/µl]	Assigned Conc. [ng/µl]	Peak Molarity [nmol/l]	% Integrated Area	Peak Comment	Observations
15	6.77	-	694	-		Lower Marker
953	1.87	-	3.01	100.00		
10000	3.25	3.25	0.500	-		Upper Marker

© Agilent Technologies, Inc. 2017

B2: DFB2 MINUS LE220 1296 sec from 6.28 second row

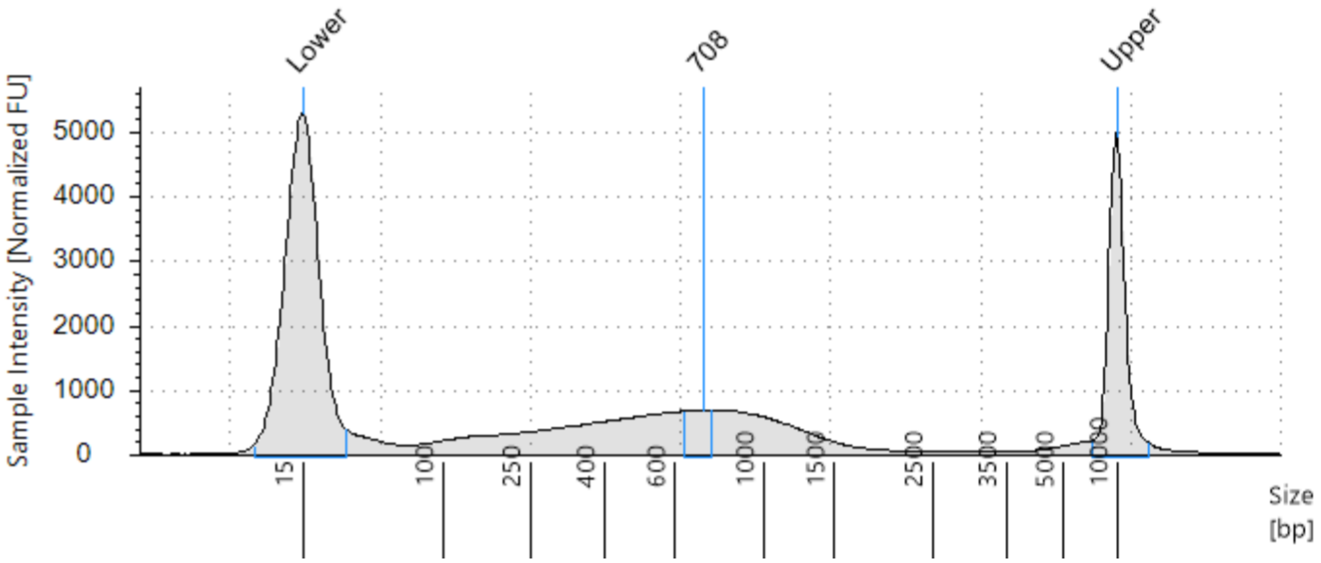

Sample Table

Well	Conc. [ng/µl]	Sample Description	Alert	Observations
B2	0.743	DFB2 MINUS LE220 1296 sec from 6.28 second row		

Peak Table

Size [bp]	Calibrated Conc. [ng/µl]	Assigned Conc. [ng/µl]	Peak Molarity [nmol/l]	% Integrated Area	Peak Comment	Observations
15	7.18	-	736	-		Lower Marker
708	0.743	-	1.61	100.00		
10000	3.25	3.25	0.500	-		Upper Marker

© Agilent Technologies, Inc. 2017

C2: DFB3 MINUS LE220 1296 sec from 6.28 second row

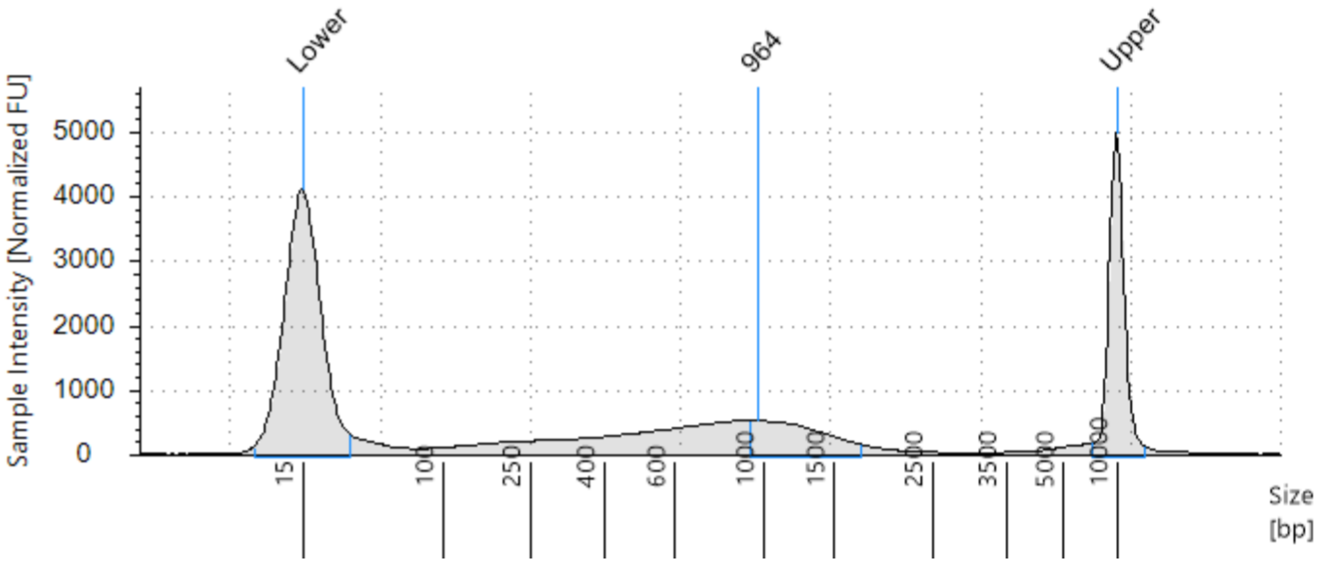

Sample Table

Well	Conc. [ng/µl]	Sample Description	Alert	Observations
C2	1.72	DFB3 MINUS LE220 1296 sec from 6.28 second row		

Peak Table

Size [bp]	Calibrated Conc. [ng/µl]	Assigned Conc. [ng/µl]	Peak Molarity [nmol/l]	% Integrated Area	Peak Comment	Observations
15	6.24	-	640	-		Lower Marker
964	1.72	-	2.75	100.00		
10000	3.25	3.25	0.500	-		Upper Marker

© Agilent Technologies, Inc. 2017

D2: DFB3 MINUS LE220 1296 sec from 6.28 second row

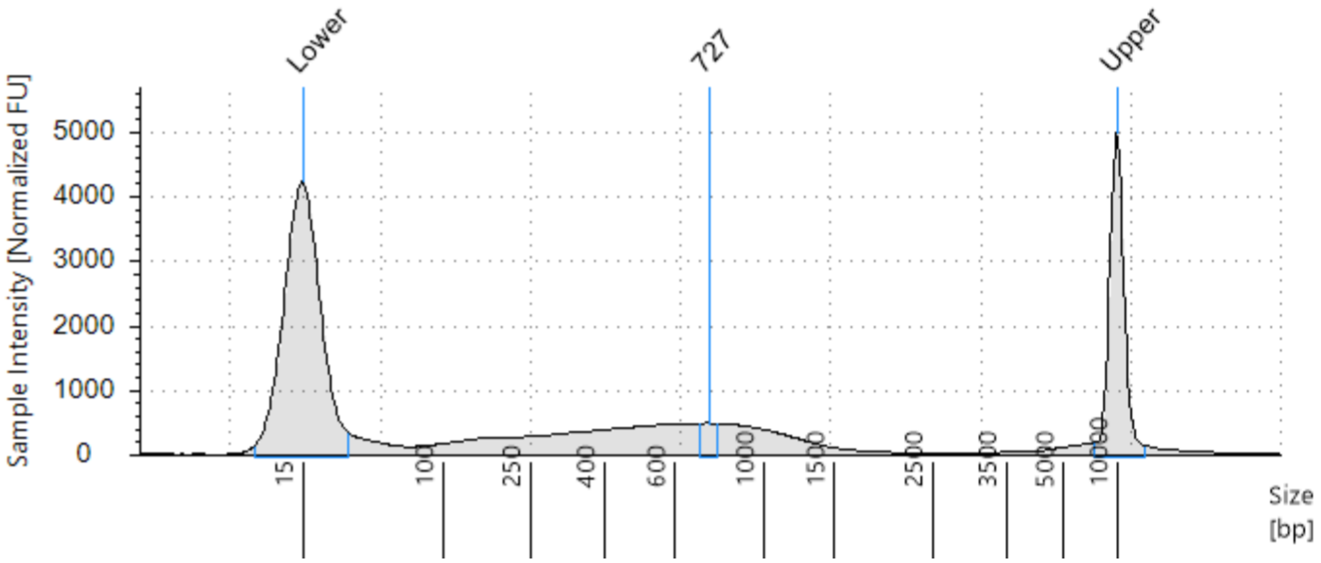

Sample Table

Well	Conc. [ng/µl]	Sample Description	Alert	Observations
D2	0.399	DFB3 MINUS LE220 1296 sec from 6.28 second row		

Peak Table

Size [bp]	Calibrated Conc. [ng/µl]	Assigned Conc. [ng/µl]	Peak Molarity [nmol/l]	% Integrated Area	Peak Comment	Observations
15	6.66	-	683	-		Lower Marker
727	0.399	-	0.843	100.00		
10000	3.25	3.25	0.500	-		Upper Marker

© Agilent Technologies, Inc. 2017

E2: DFB4 MINUS LE220 1296 sec from 6.28 second row

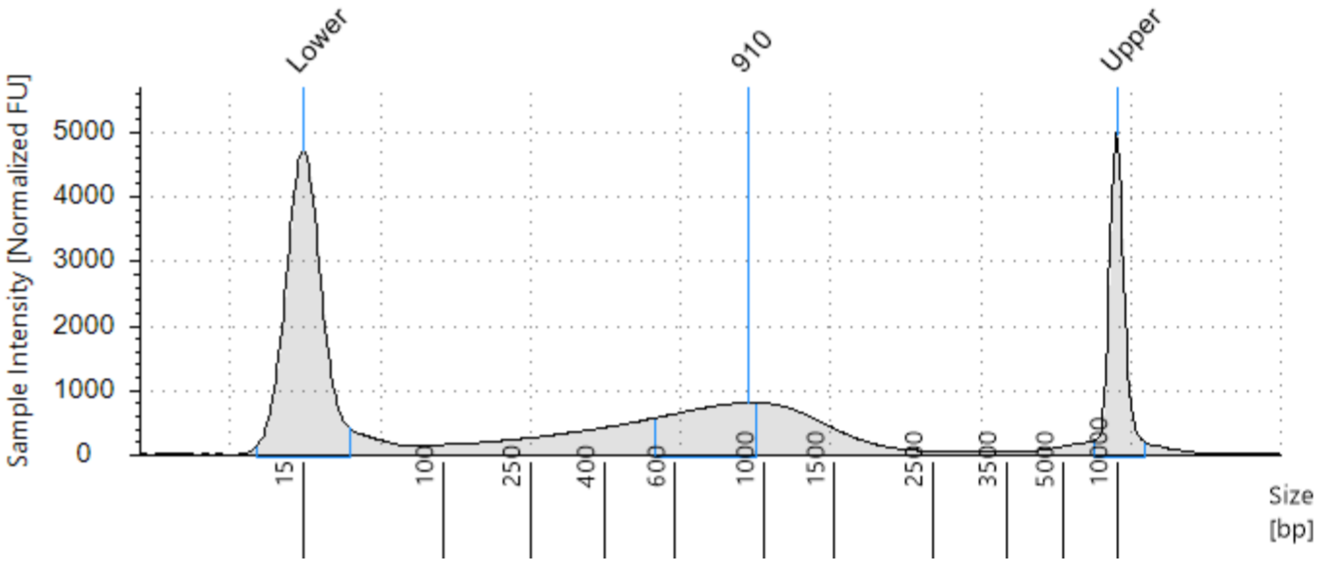

Sample Table

Well	Conc. [ng/µl]	Sample Description	Alert	Observations
E2	3.00	DFB4 MINUS LE220 1296 sec from 6.28 second row		

Peak Table

Size [bp]	Calibrated Conc. [ng/µl]	Assigned Conc. [ng/µl]	Peak Molarity [nmol/l]	% Integrated Area	Peak Comment	Observations
15	7.10	-	729	-		Lower Marker
910	3.00	-	5.07	100.00		
10000	3.25	3.25	0.500	-		Upper Marker

© Agilent Technologies, Inc. 2017

F2: DFB5 MINUS LE220 1296 sec from 6.28 second row

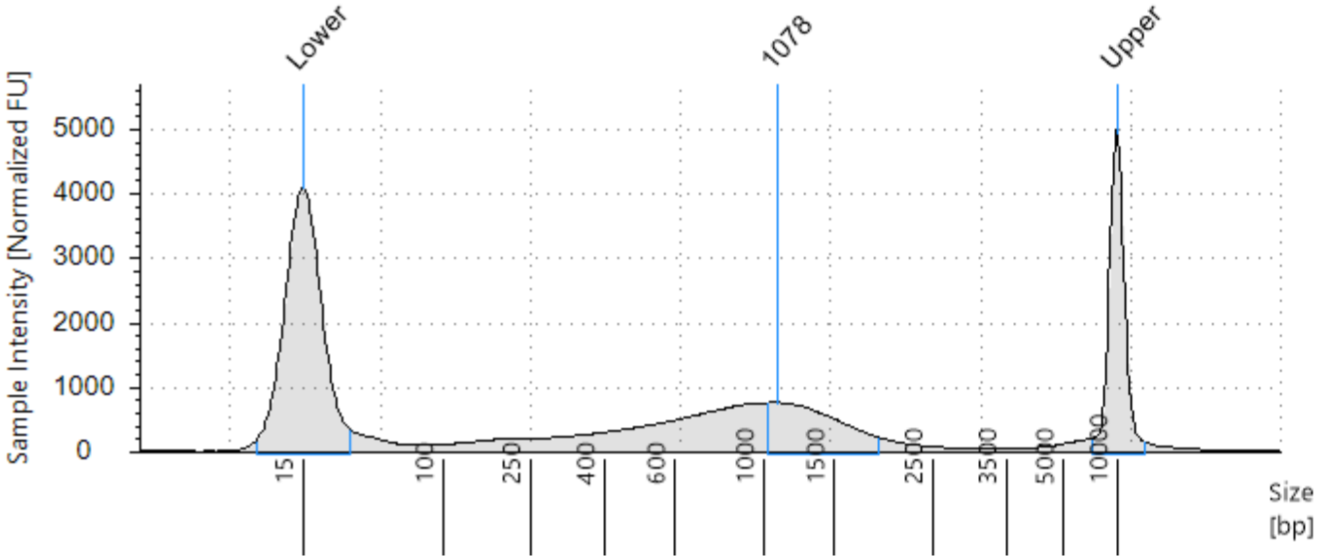

Sample Table

Well	Conc. [ng/µl]	Sample Description	Alert	Observations
F2	2.49	DFB5 MINUS LE220 1296 sec from 6.28 second row		

Peak Table

Size [bp]	Calibrated Conc. [ng/µl]	Assigned Conc. [ng/µl]	Peak Molarity [nmol/l]	% Integrated Area	Peak Comment	Observations
15	6.30	-	646	-		Lower Marker
1078	2.49	-	3.56	100.00		
10000	3.25	3.25	0.500	-		Upper Marker

© Agilent Technologies, Inc. 2017

G2: DFB6 MINUS LE220 1296 sec from 6.28 second row

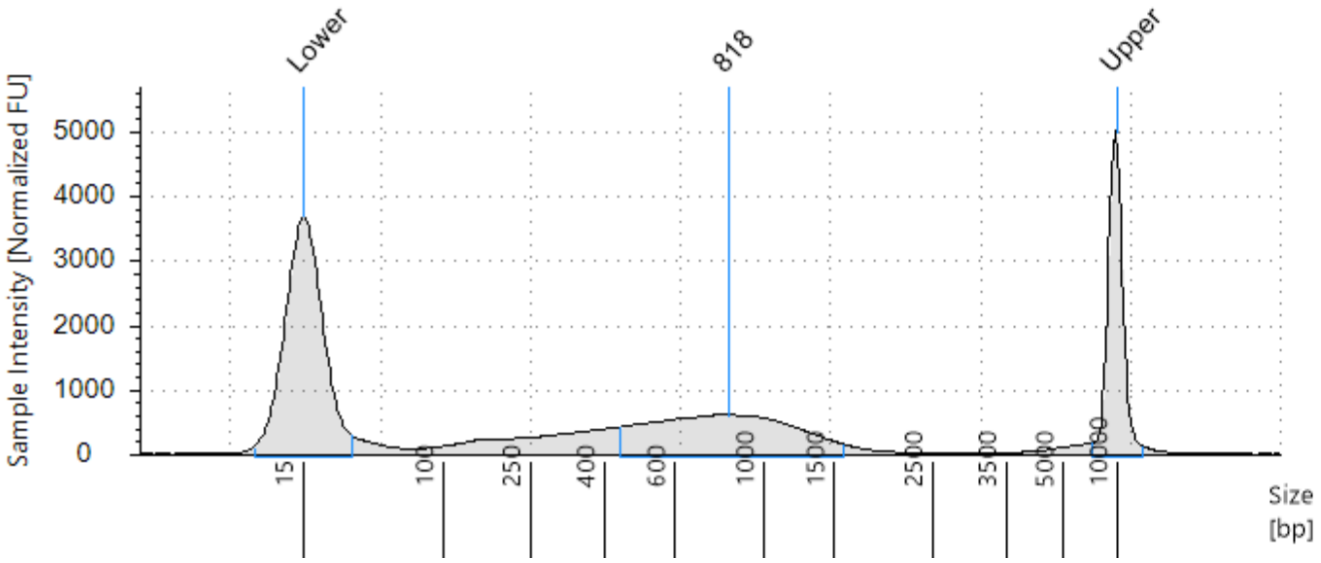

Sample Table

Well	Conc. [ng/µl]	Sample Description	Alert	Observations
G2	4.40	DFB6 MINUS LE220 1296 sec from 6.28 second row		

Peak Table

Size [bp]	Calibrated Conc. [ng/µl]	Assigned Conc. [ng/µl]	Peak Molarity [nmol/l]	% Integrated Area	Peak Comment	Observations
15	5.91	-	606	-		Lower Marker
818	4.40	-	8.27	100.00		
10000	3.25	3.25	0.500	-		Upper Marker

© Agilent Technologies, Inc. 2017

Filename: 2019-05-29-01- 1-8 1008 sec for 6.22, 8-15 1296 sec for 6.22.D5000

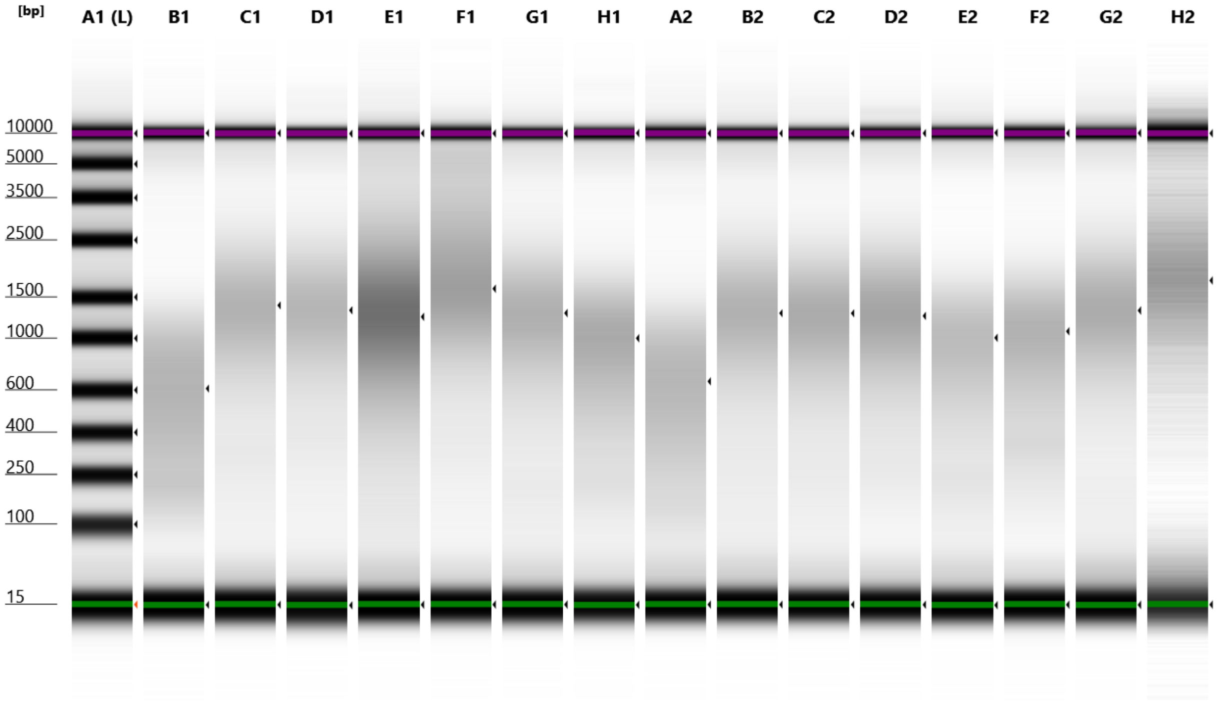

Default image (Contrast 100%)

Sample Info

Well	Conc. [ng/µl]	Sample Description	Alert	Observations
A1	32.1	Ladder		Ladder
B1	0.412	1 MINUS1E220 1008sec from 6.22		
C1	1.82	2 MINUS1E220 1008sec from 6.22		
D1	1.98	3 MINUS1E220 1008sec from 6.22		
E1	4.15	4 MINUS1E220 1008sec from 6.22		
F1	2.71	5 MINUS1E220 1008sec from 6.22		
G1	3.86	6MINUS1E220 1008sec from 6.22		
H1	2.22	7 MINUS1E220 1008sec from 6.22		
A2	0.398	DFB1 MINUS1E220 1296 sec from 6.22		
B2	3.40	DFB2 MINUS1E220 1296 sec from 6.22		
C2	2.10	DFB3 MINUS1E220 1296 sec from 6.22		
D2	2.25	DFB4 MINUS1E220 1296 sec from 6.22		
E2	1.65	DFB5 MINUS1E220 1296 sec from 6.22		
F2	0.412	DFB6 MINUS1E220 1296 sec from 6.22		
G2	0.473	DFB7 MINUS1E220 1296 sec from 6.22		
H2	0.442	DFB8 MINUS1E220 1296 sec from 6.22		

© Agilent Technologies, Inc. 2017

A1: Ladder

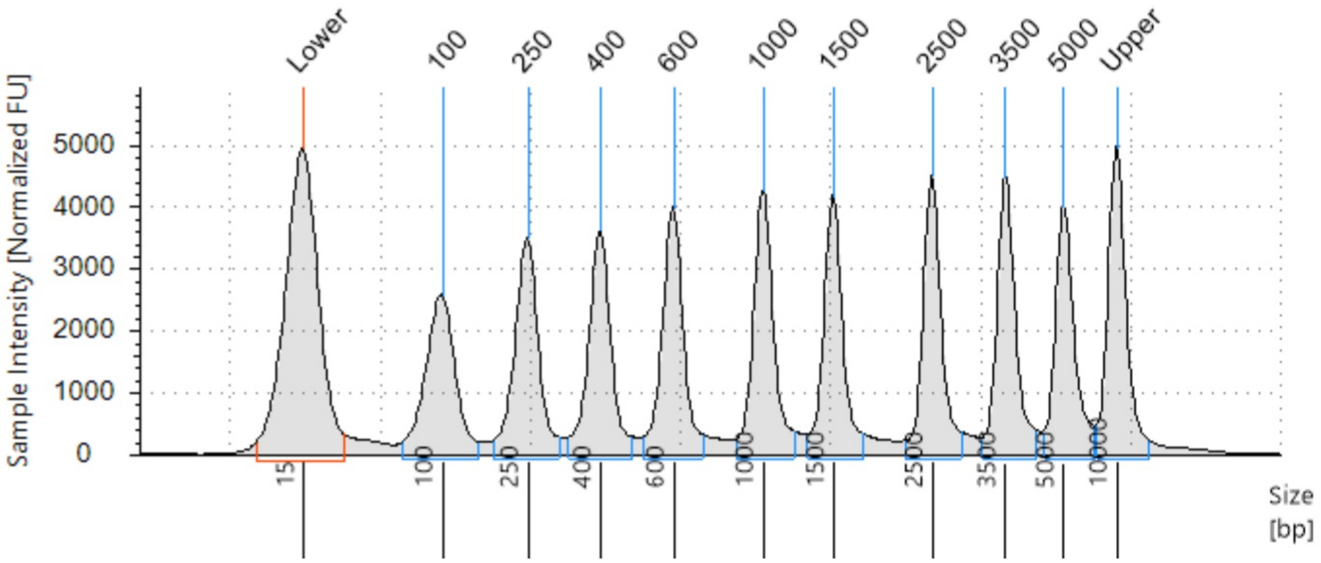

Sample Table

Well	Conc. [ng/µl]	Sample Description	Alert	Observations
A1	32.1	Ladder		Ladder

Peak Table

Size [bp]	Calibrated Conc. [ng/µl]	Assigned Conc. [ng/µl]	Peak Molarity [nmol/l]	% Integrated Area	Peak Comment	Observations
15	6.25	-	641	-		Lower Marker
100	3.36	-	51.7	10.45		
250	3.62	-	22.3	11.26		
400	3.51	-	13.5	10.92		
600	3.68	-	9.42	11.44		
1000	3.75	-	5.77	11.66		
1500	3.51	-	3.60	10.92		
2500	3.63	-	2.23	11.29		
3500	3.69	-	1.62	11.49		
5000	3.39	-	1.04	10.56		
10000	3.25	3.25	0.500	-		Upper Marker

© Agilent Technologies, Inc. 2017

A2: DFB1 MINUS IE220 1296 sec from 6.22

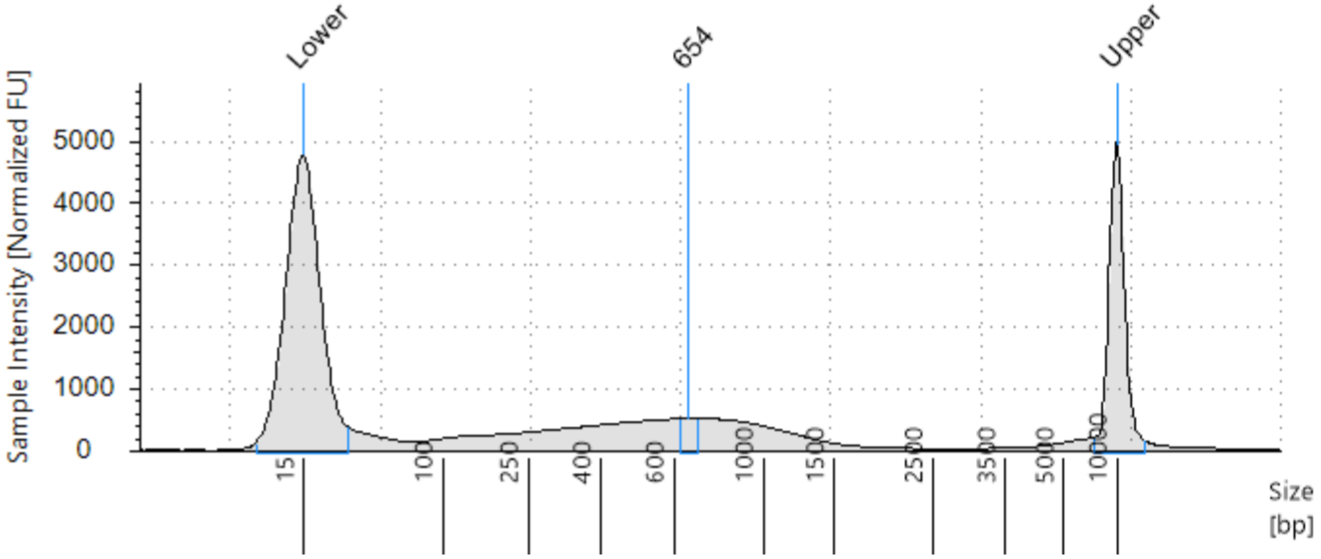

Sample Table

Well	Conc. [ng/µl]	Sample Description	Alert	Observations
A2	0.398	DFB1 MINUS IE220 1296 sec from 6.22		

Peak Table

Size [bp]	Calibrated Conc. [ng/µl]	Assigned Conc. [ng/µl]	Peak Molarity [nmol/l]	% Integrated Area	Peak Comment	Observations
15	6.82	-	699	-		Lower Marker
654	0.398	-	0.937	100.00		
10000	3.25	3.25	0.500	-		Upper Marker

© Agilent Technologies, Inc. 2017

B2: DFB2 MINUS IE220 1296 sec from 6.22

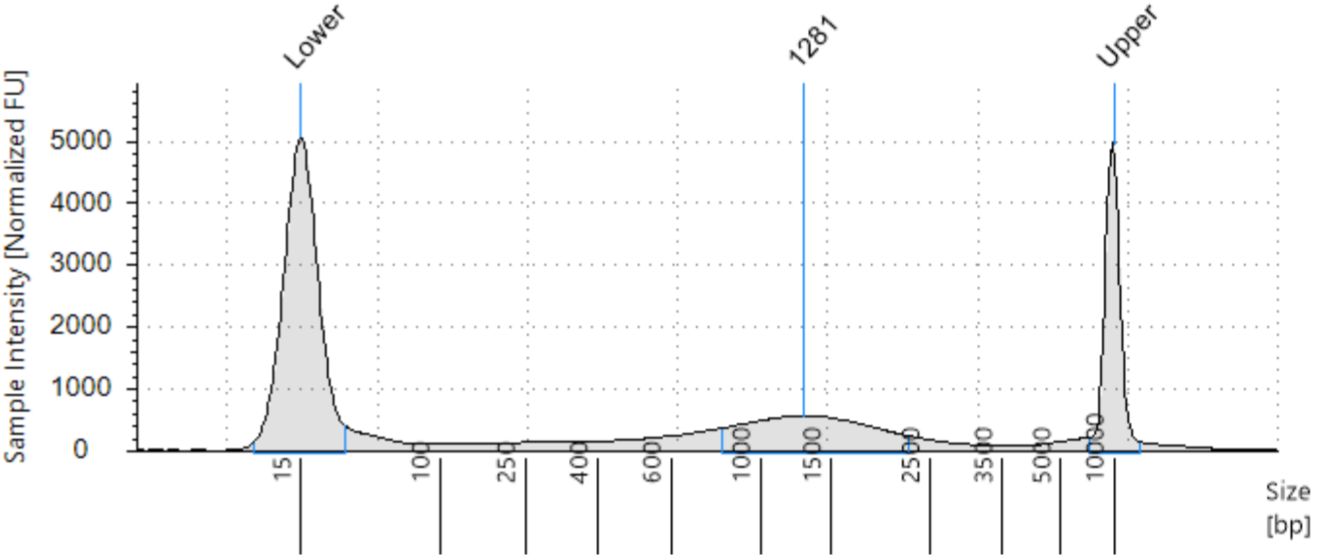

Sample Table

Well	Conc. [ng/µl]	Sample Description	Alert	Observations
B2	3.40	DFB2 MINUS IE220 1296 sec from 6.22		

Peak Table

Size [bp]	Calibrated Conc. [ng/µl]	Assigned Conc. [ng/µl]	Peak Molarity [nmol/l]	% Integrated Area	Peak Comment	Observations
15	7.30	-	749	-		Lower Marker
1281	3.40	-	4.09	100.00		
10000	3.25	3.25	0.500	-		Upper Marker

© Agilent Technologies, Inc. 2017

C2: DFB3 MINUS IE220 1296 sec from 6.22

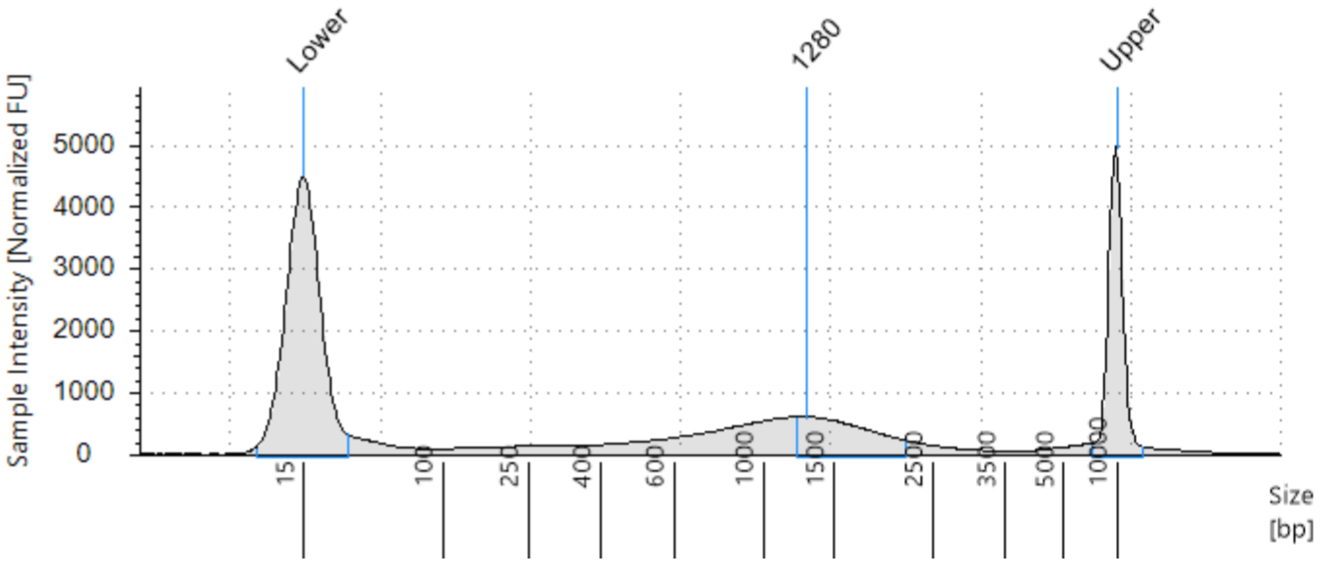

Sample Table

Well	Conc. [ng/µl]	Sample Description	Alert	Observations
C2	2.10	DFB3 MINUS IE220 1296 sec from 6.22		

Peak Table

Size [bp]	Calibrated Conc. [ng/µl]	Assigned Conc. [ng/µl]	Peak Molarity [nmol/l]	% Integrated Area	Peak Comment	Observations
15	6.81	-	698	-		Lower Marker
1280	2.10	-	2.52	100.00		
10000	3.25	3.25	0.500	-		Upper Marker

© Agilent Technologies, Inc. 2017

D2: DFB4 MINUS IE220 1296 sec from 6.22

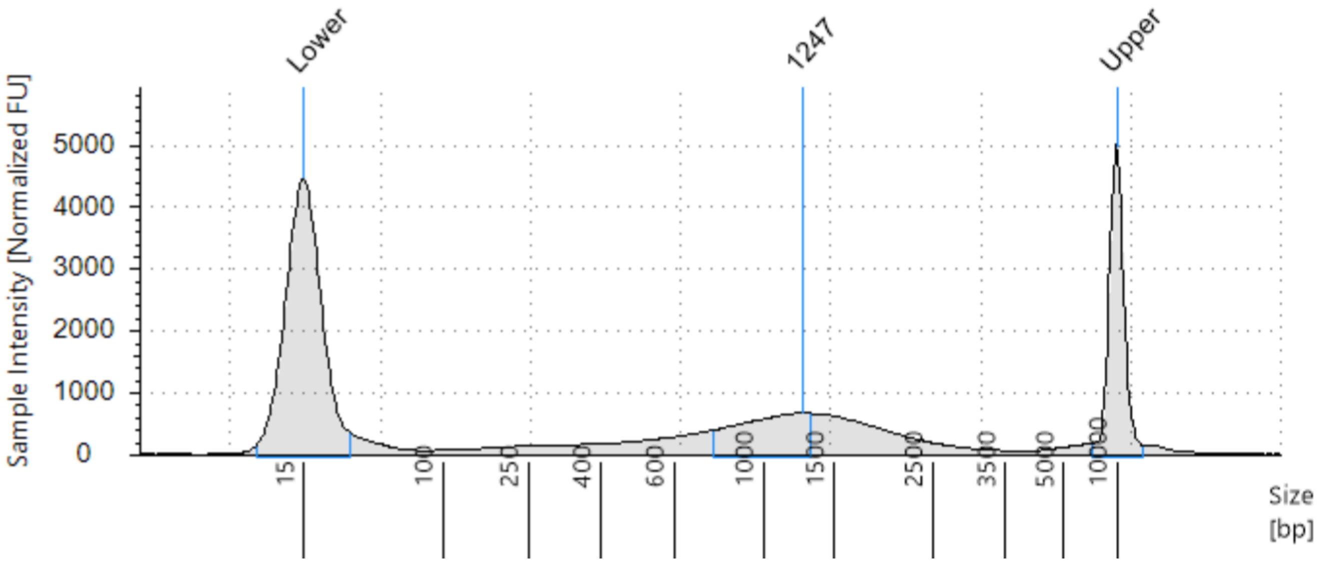

Sample Table

Well	Conc. [ng/µl]	Sample Description	Alert	Observations
D2	2.25	DFB4 MINUS IE220 1296 sec from 6.22		

Peak Table

Size [bp]	Calibrated Conc. [ng/µl]	Assigned Conc. [ng/µl]	Peak Molarity [nmol/l]	% Integrated Area	Peak Comment	Observations
15	6.80	-	698	-		Lower Marker
1247	2.25	-	2.78	100.00		
10000	3.25	3.25	0.500	-		Upper Marker

© Agilent Technologies, Inc. 2017
