# Supplementary material for: 96 sample parallel acoustic fragmentation for high throughput next generation sequencing library preparation
Source: PLoS One. 2026 Feb 17;21(2):e0341139. doi: 10.1371/journal.pone.0341139 (PMC12912608; doi:10.1371/journal.pone.0341139)
Supplement: S2 Fig — (ZIP) [file pone.0341139.s002.zip › Figure 1 Raw Data/glass tube with nanodroplets 80 seconds.pdf]

Filename: 2019-03-26-01- q-s 12 mint plus- LE220 80 sec (last 8 line).D5000

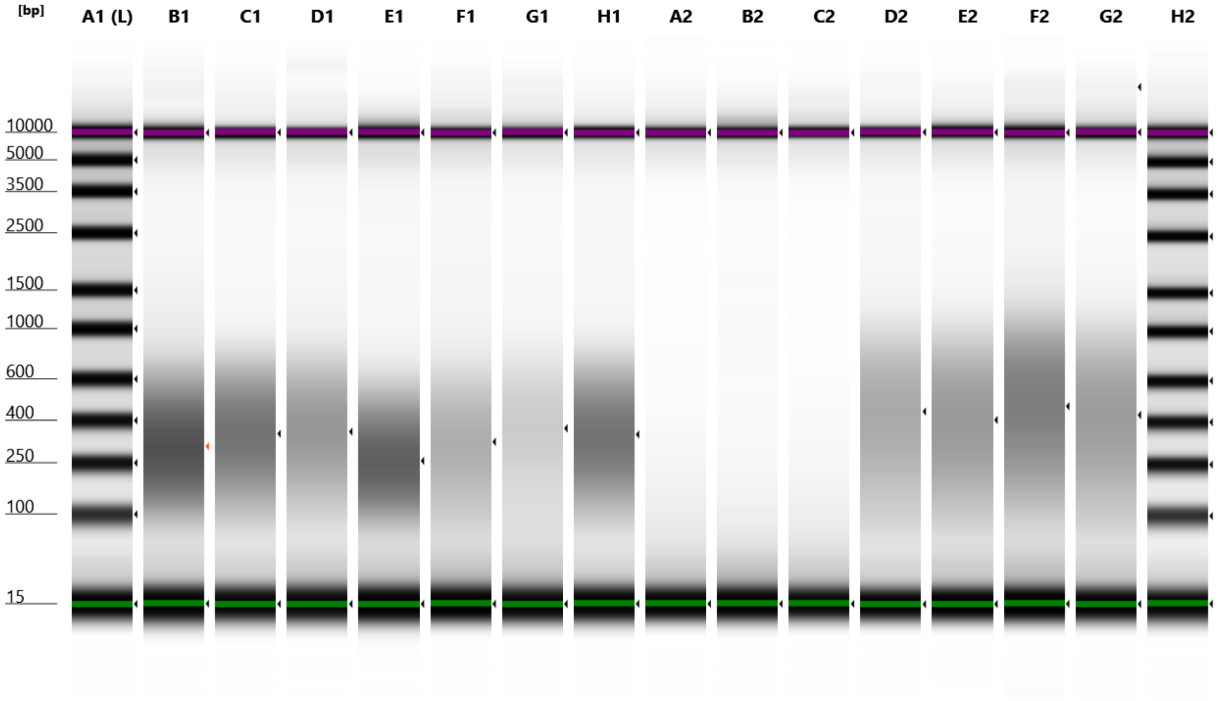

Default image (Contrast 100%)

Sample Info

Well	Conc. [ng/µl]	Sample Description	Alert	Observations
A1	27.5	Ladder		Ladder
B1	10.6	DFB Plus 80 sec		
C1	4.81	DFB plus 80 sec		
D1	3.52	DFB plus 80 sec		
E1	8.71	DFB plus 80sec		
F1	0.471	DFB plus 80 sec		
G1	0.227	SFb plus 80 sec		
H1	4.91	DFB plus 80 sec		
A2				
B2				
C2				
D2	0.489			
E2	0.554	DFB plus 80 sec		
F2	0.732	DFB plus 80 sec		
G2	0.498	DFB plus 80 sec		
H2	26.2	DFB plus 80 sec		

© Agilent Technologies, Inc. 2017

A1: Ladder

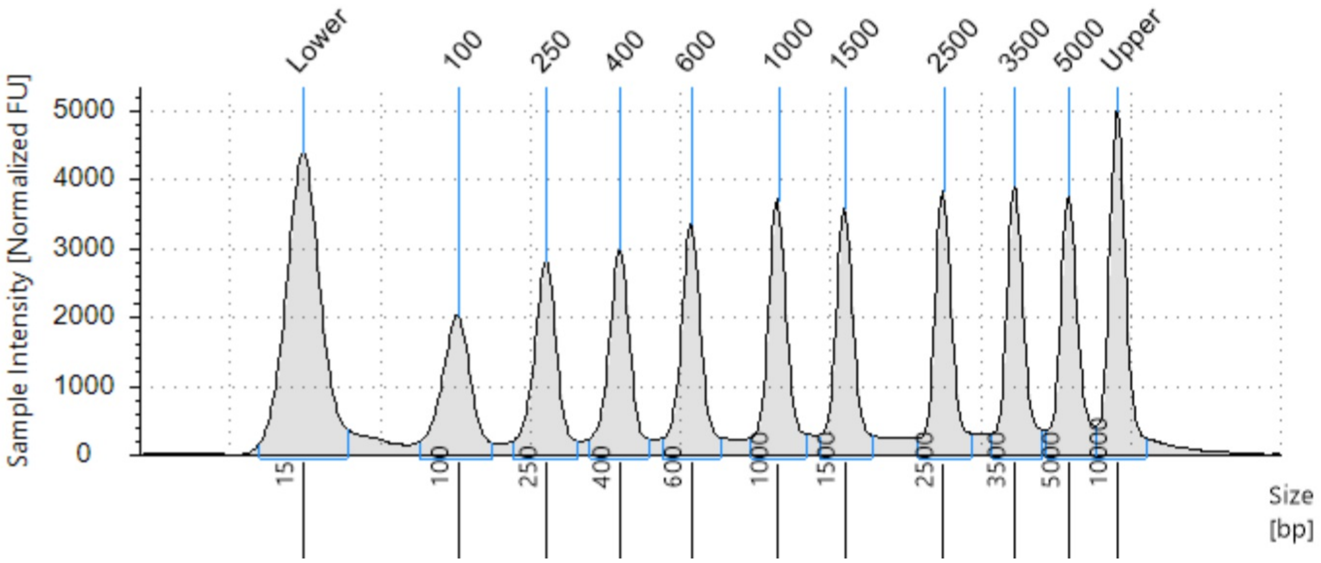

Sample Table

Well	Conc. [ng/µl]	Sample Description	Alert	Observations
A1	27.5	Ladder		Ladder

Peak Table

Size [bp]	Calibrated Conc. [ng/µl]	Assigned Conc. [ng/µl]	Peak Molarity [nmol/l]	% Integrated Area	Peak Comment	Observations
15	6.01	-	616	-		Lower Marker
100	2.70	-	41.6	9.83		
250	2.97	-	18.3	10.80		
400	2.95	-	11.3	10.73		
600	3.13	-	8.03	11.40		
1000	3.25	-	5.00	11.83		
1500	3.03	-	3.11	11.03		
2500	3.12	-	1.92	11.35		
3500	3.19	-	1.40	11.62		
5000	3.14	-	0.966	11.42		
10000	3.25	3.25	0.500	-		Upper Marker

© Agilent Technologies, Inc. 2017

G2: DFB plus 80 sec

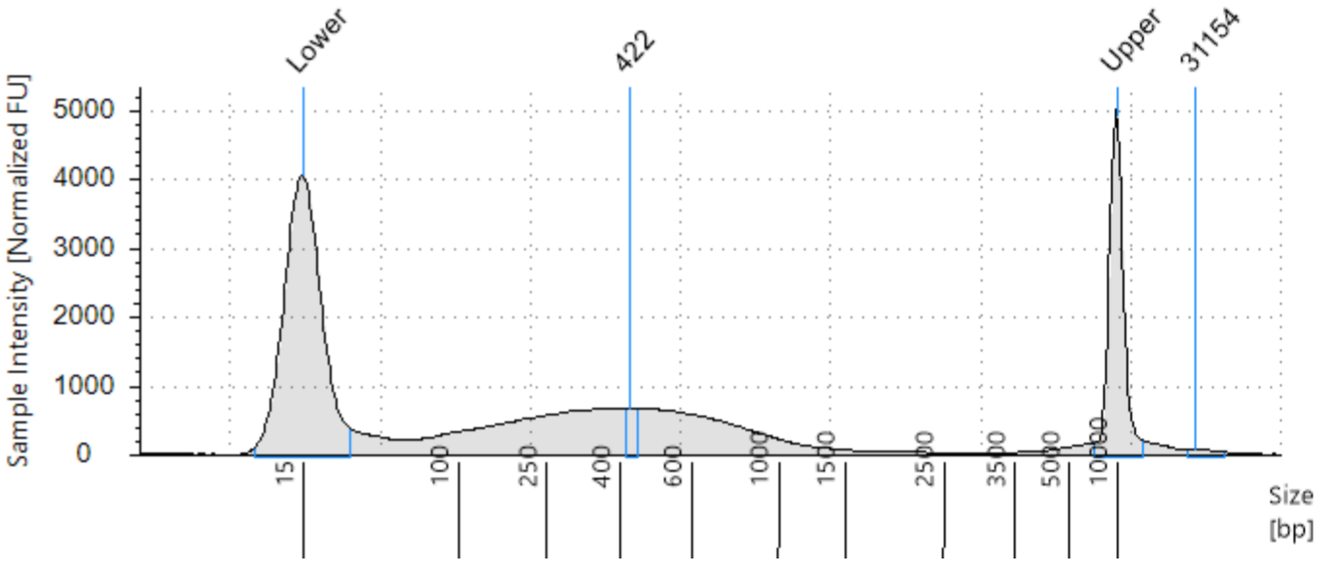

Sample Table

Well	Conc. [ng/µl]	Sample Description	Alert	Observations
G2	0.498	DFB plus 80 sec		

Peak Table

Size [bp]	Calibrated Conc. [ng/µl]	Assigned Conc. [ng/µl]	Peak Molarity [nmol/l]	% Integrated Area	Peak Comment	Observations
15	6.71	-	689	-		Lower Marker
422	0.397	-	1.45	79.72		
10000	3.25	3.25	0.500	-		Upper Marker
31154	0.101	-	0.00499	20.28		

© Agilent Technologies, Inc. 2017

Filename: 2019-05-17- LE220, DFB PLUS FIRST 8 80 SEC, LAST 8 DFB MINUS 240 SEC.D5000

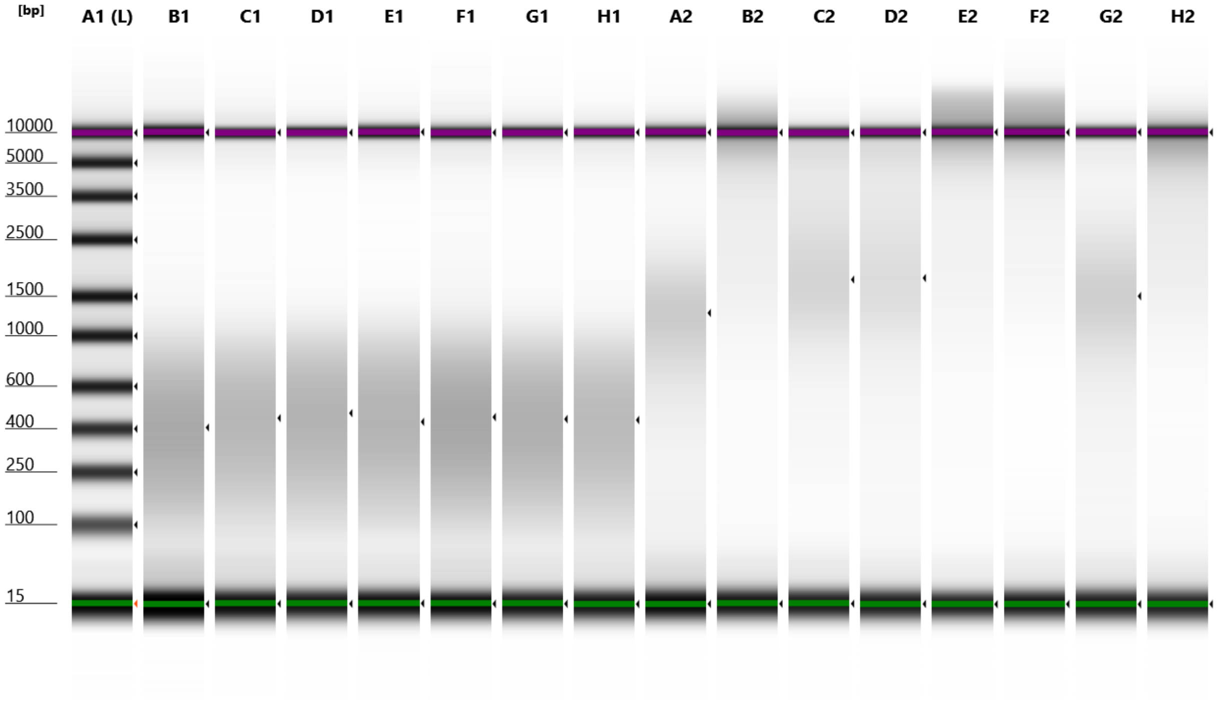

Default image (Contrast 100%)

Sample Info

Well	Conc. [ng/µl]	Sample Description	Alert	Observations
A1	30.7	Ladder		Ladder
B1	0.605	LE220 R2 DFB1 PLUS 80 SEC		
C1	0.918	LE220 R2 DFB2 PLUS 80 SEC		
D1	0.672	LE220 R2 DFB3 PLUS 80 SEC		
E1	3.04	LE220 R2 DFB4 PLUS 80 SEC		
F1	1.18	LE220 R2 DFB5 PLUS 80 SEC		
G1	1.13	LE220 R2 DFB6 PLUS 80 SEC		
H1	0.768	LE220 R2 DFB7 PLUS 80 SEC		
A2	1.82	LE220 R2 DFB1 MINUS 240 SEC		
B2		LE220 R2 DFB2 MINUS 240 SEC		
C2	1.50	LE220 R2 DFB3 MINUS 240 SEC		
D2	0.266	LE220 R2 DFB4 MINUS 240 SEC		
E2		LE220 R2 DFB5 MINUS 240 SEC		
F2		LE220 R2 DFB6 MINUS 240 SEC		
G2	0.391	LE220 R2 DFB7 MINUS 240 SEC		
H2		LE220 R2 DFB8 MINUS 240 SEC		

© Agilent Technologies, Inc. 2017

A1: Ladder

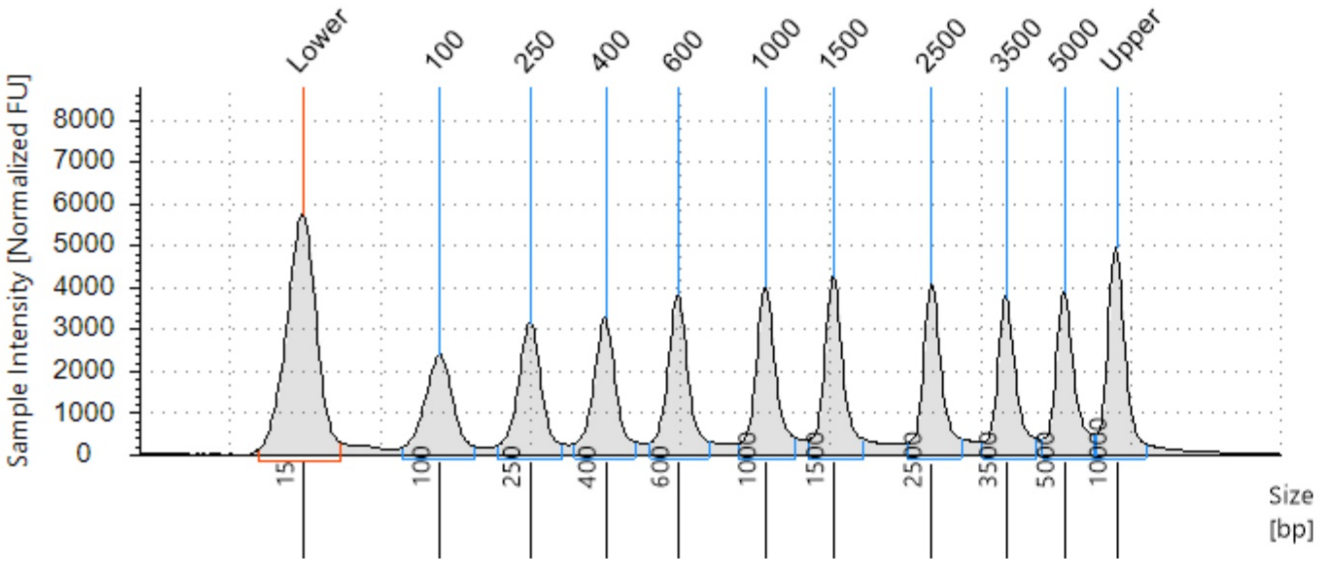

Sample Table

Well	Conc. [ng/µl]	Sample Description	Alert	Observations
A1	30.7	Ladder		Ladder

Peak Table

Size [bp]	Calibrated Conc. [ng/µl]	Assigned Conc. [ng/µl]	Peak Molarity [nmol/l]	% Integrated Area	Peak Comment	Observations
15	7.08	-	726	-		Lower Marker
100	3.09	-	47.6	10.09		
250	3.37	-	20.7	10.99		
400	3.30	-	12.7	10.77		
600	3.61	-	9.26	11.78		
1000	3.64	-	5.61	11.89		
1500	3.68	-	3.77	12.00		
2500	3.41	-	2.10	11.14		
3500	3.21	-	1.41	10.48		
5000	3.33	-	1.03	10.87		
10000	3.25	3.25	0.500	-		Upper Marker

© Agilent Technologies, Inc. 2017

B1: LE220 R2 DFB1 PLUS 80 SEC

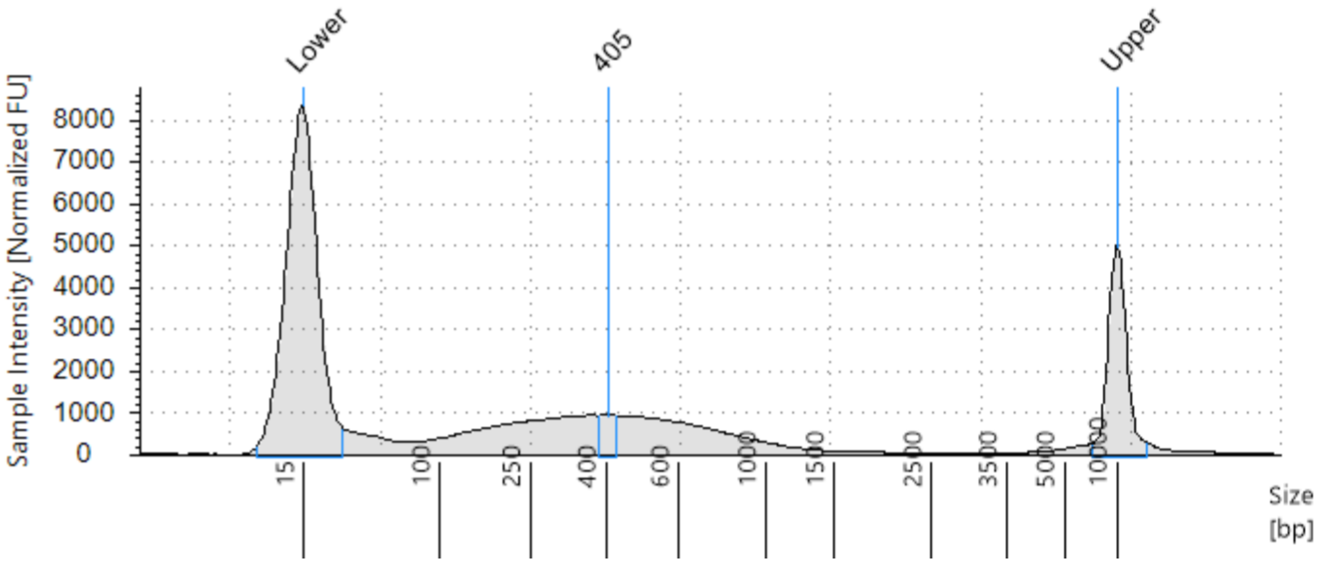

Sample Table

Well	Conc. [ng/µl]	Sample Description	Alert	Observations
B1	0.605	LE220 R2 DFB1 PLUS 80 SEC		

Peak Table

Size [bp]	Calibrated Conc. [ng/µl]	Assigned Conc. [ng/µl]	Peak Molarity [nmol/l]	% Integrated Area	Peak Comment	Observations
15	9.45	-	969	-		Lower Marker
405	0.605	-	2.29	100.00		
10000	3.25	3.25	0.500	-		Upper Marker

© Agilent Technologies, Inc. 2017

C1: LE220 R2 DFB2 PLUS 80 SEC

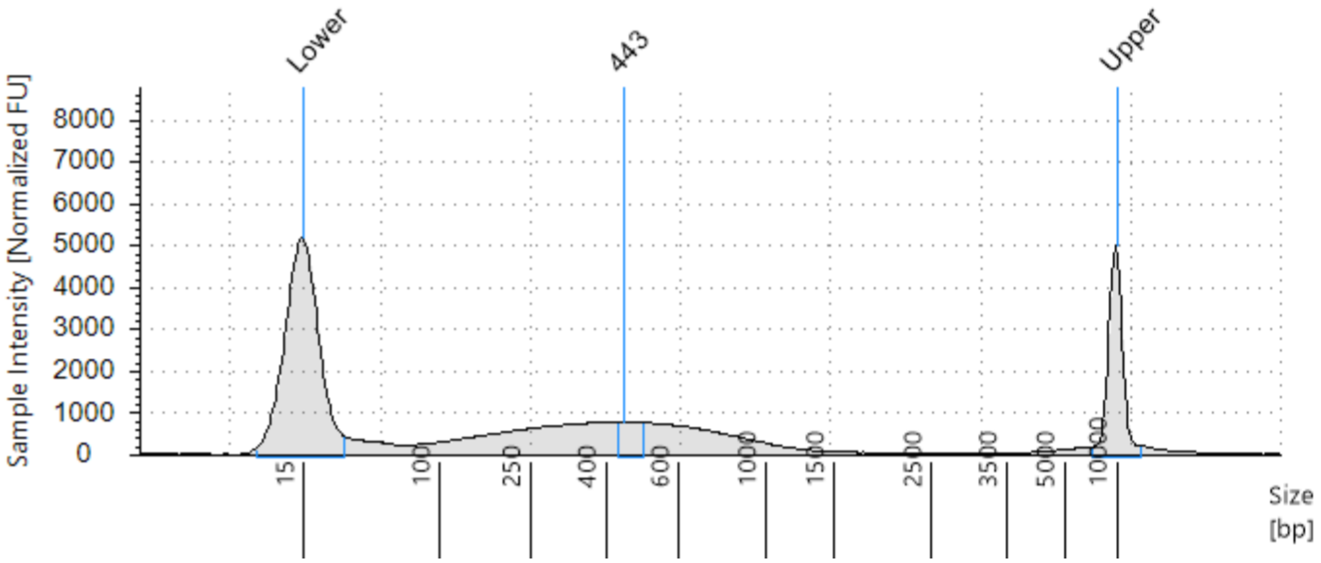

Sample Table

Well	Conc. [ng/µl]	Sample Description	Alert	Observations
C1	0.918	LE220 R2 DFB2 PLUS 80 SEC		

Peak Table

Size [bp]	Calibrated Conc. [ng/µl]	Assigned Conc. [ng/µl]	Peak Molarity [nmol/l]	% Integrated Area	Peak Comment	Observations
15	7.95	-	815	-		Lower Marker
443	0.918	-	3.19	100.00		
10000	3.25	3.25	0.500	-		Upper Marker

© Agilent Technologies, Inc. 2017

D1: LE220 R2 DFB3 PLUS 80 SEC

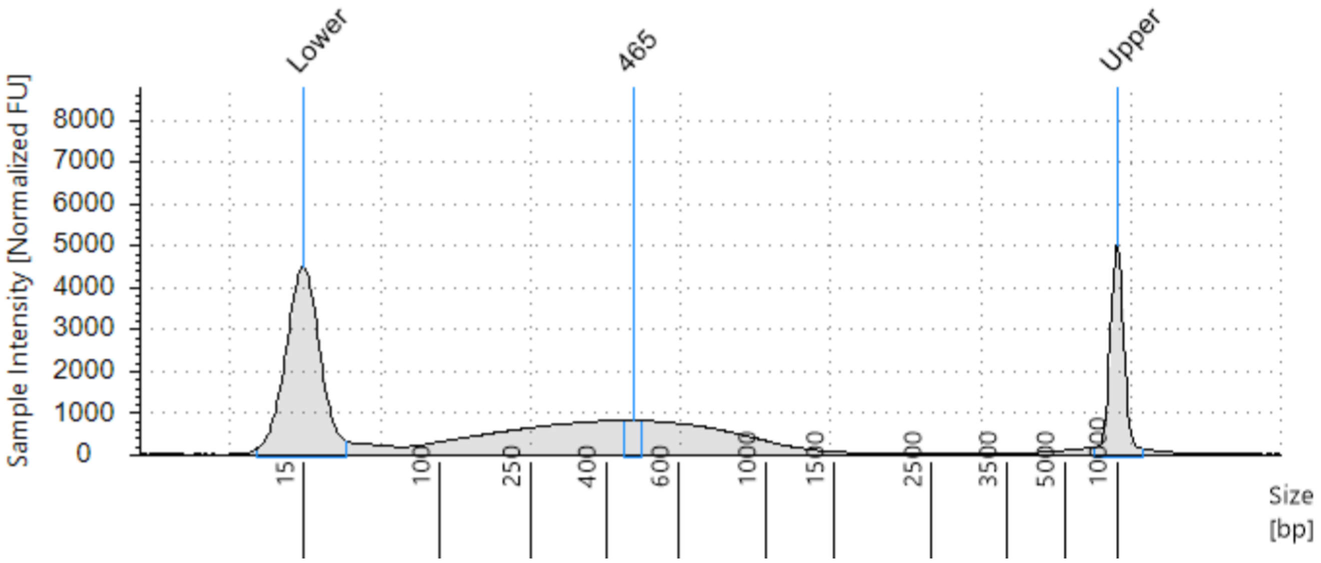

Sample Table

Well	Conc. [ng/µl]	Sample Description	Alert	Observations
D1	0.672	LE220 R2 DFB3 PLUS 80 SEC		

Peak Table

Size [bp]	Calibrated Conc. [ng/µl]	Assigned Conc. [ng/µl]	Peak Molarity [nmol/l]	% Integrated Area	Peak Comment	Observations
15	6.95	-	713	-		Lower Marker
465	0.672	-	2.22	100.00		
10000	3.25	3.25	0.500	-		Upper Marker

© Agilent Technologies, Inc. 2017

E1: LE220 R2 DFB4 PLUS 80 SEC

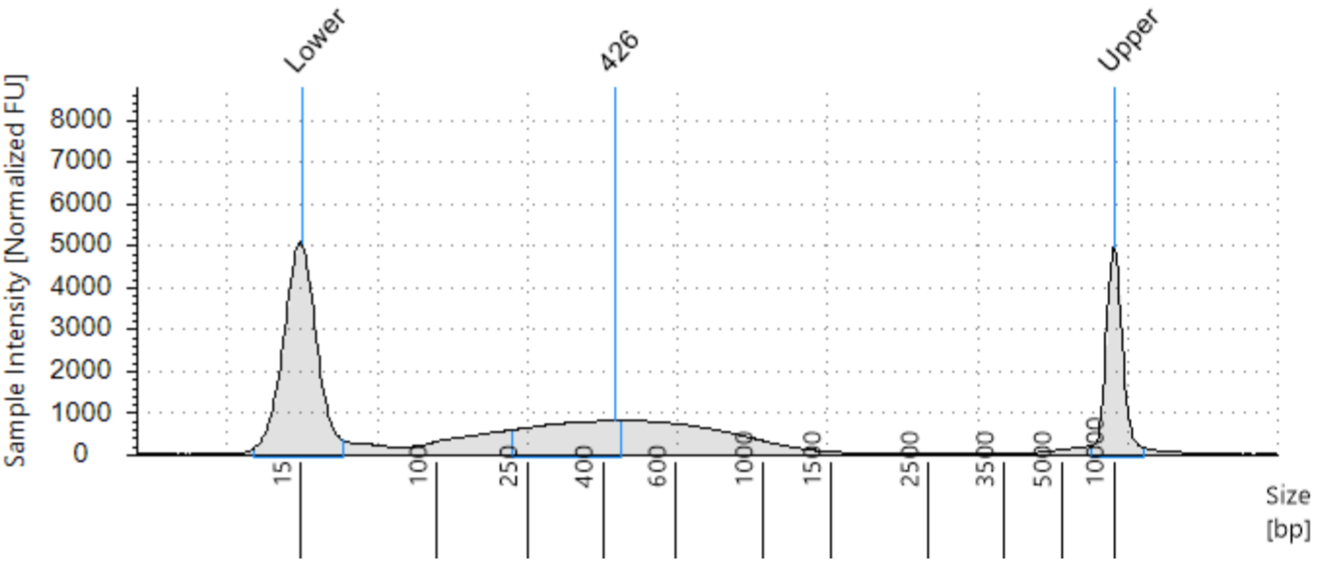

Sample Table

Well	Conc. [ng/µl]	Sample Description	Alert	Observations
E1	3.04	LE220 R2 DFB4 PLUS 80 SEC		

Peak Table

Size [bp]	Calibrated Conc. [ng/µl]	Assigned Conc. [ng/µl]	Peak Molarity [nmol/l]	% Integrated Area	Peak Comment	Observations
15	6.54	-	671	-		Lower Marker
426	3.04	-	11.0	100.00		
10000	3.25	3.25	0.500	-		Upper Marker

© Agilent Technologies, Inc. 2017

F1: LE220 R2 DFB5 PLUS 80 SEC

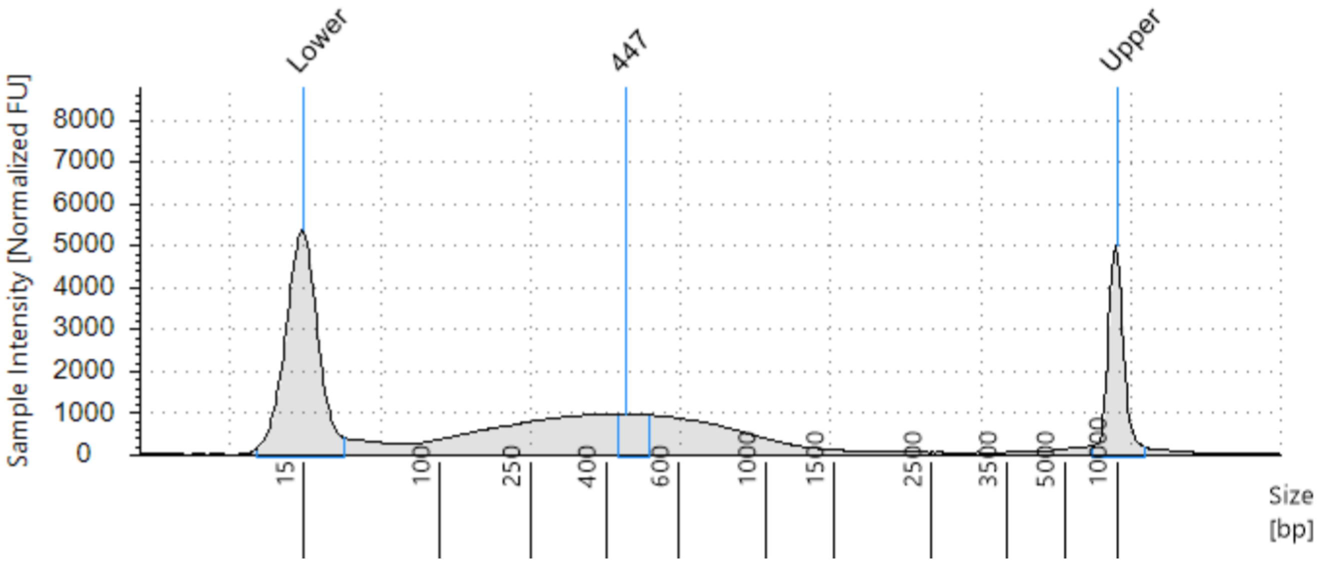

Sample Table

Well	Conc. [ng/µl]	Sample Description	Alert	Observations
F1	1.18	LE220 R2 DFB5 PLUS 80 SEC		

Peak Table

Size [bp]	Calibrated Conc. [ng/µl]	Assigned Conc. [ng/µl]	Peak Molarity [nmol/l]	% Integrated Area	Peak Comment	Observations
15	7.07	-	725	-		Lower Marker
447	1.18	-	4.07	100.00		
10000	3.25	3.25	0.500	-		Upper Marker

© Agilent Technologies, Inc. 2017

G1: LE220 R2 DFB6 PLUS 80 SEC

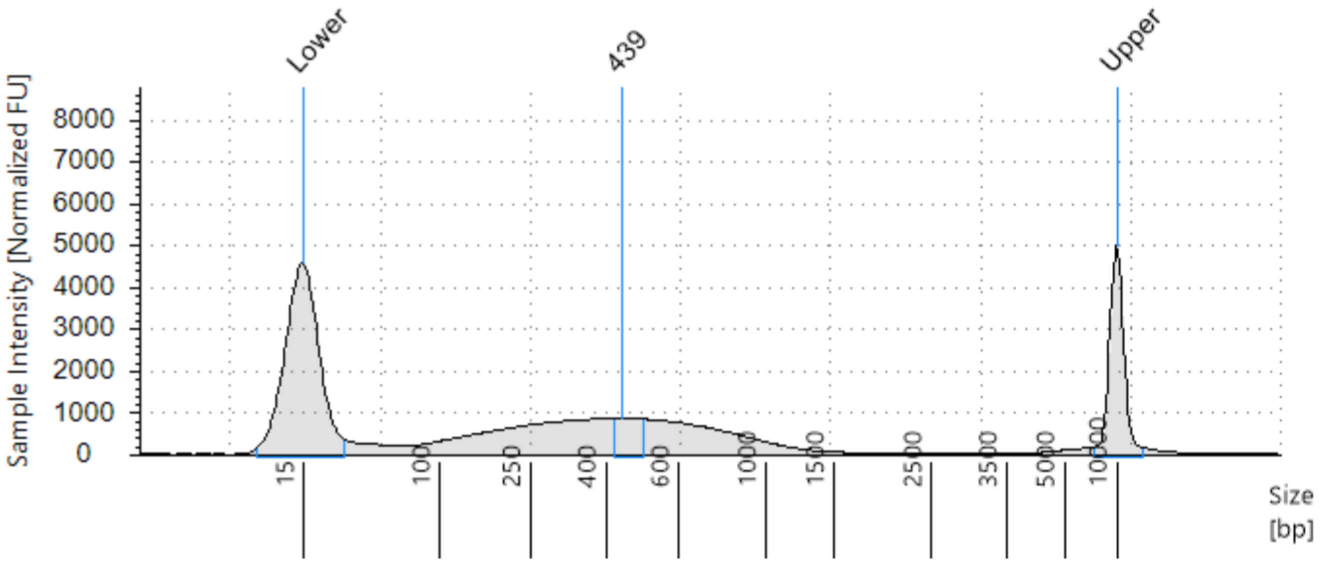

Sample Table

Well	Conc. [ng/µl]	Sample Description	Alert	Observations
G1	1.13	LE220 R2 DFB6 PLUS 80 SEC		

Peak Table

Size [bp]	Calibrated Conc. [ng/µl]	Assigned Conc. [ng/µl]	Peak Molarity [nmol/l]	% Integrated Area	Peak Comment	Observations
15	6.84	-	702	-		Lower Marker
439	1.13	-	3.96	100.00		
10000	3.25	3.25	0.500	-		Upper Marker

© Agilent Technologies, Inc. 2017

H1: LE220 R2 DFB7 PLUS 80 SEC

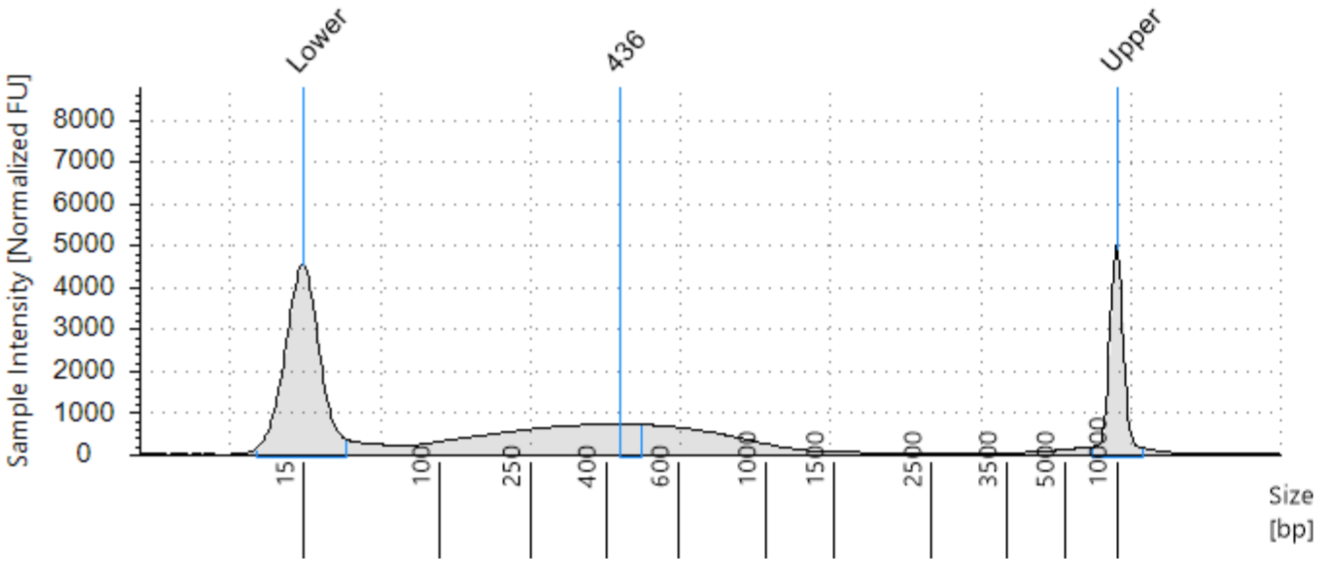

Sample Table

Well	Conc. [ng/µl]	Sample Description	Alert	Observations
H1	0.768	LE220 R2 DFB7 PLUS 80 SEC		

Peak Table

Size [bp]	Calibrated Conc. [ng/µl]	Assigned Conc. [ng/µl]	Peak Molarity [nmol/l]	% Integrated Area	Peak Comment	Observations
15	7.07	-	725	-		Lower Marker
436	0.768	-	2.71	100.00		
10000	3.25	3.25	0.500	-		Upper Marker

© Agilent Technologies, Inc. 2017

Filename: 2019-03-26-01- q-s 12 mint plus- LE220 80 sec (last 8 line).D5000

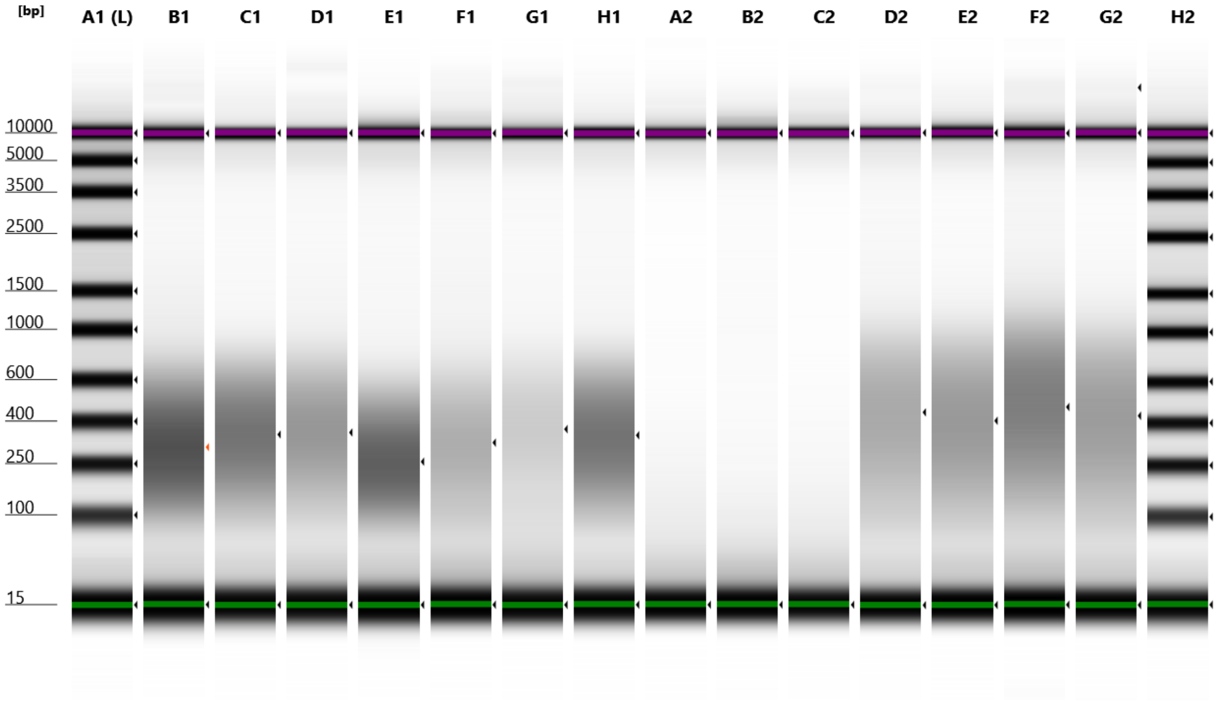

Default image (Contrast 100%)

Sample Info

Well	Conc. [ng/µl]	Sample Description	Alert	Observations
A1	27.5	Ladder		Ladder
B1	10.6	DFB Plus 80 sec		
C1	4.81	DFB plus 80 sec		
D1	3.52	DFB plus 80 sec		
E1	8.71	DFB plus 80sec		
F1	0.471	DFB plus 80 sec		
G1	0.227	SFb plus 80 sec		
H1	4.91	DFB plus 80 sec		
A2				
B2				
C2				
D2	0.489			
E2	0.554	DFB plus 80 sec		
F2	0.732	DFB plus 80 sec		
G2	0.498	DFB plus 80 sec		
H2	26.2	DFB plus 80 sec		

© Agilent Technologies, Inc. 2017

A1: Ladder

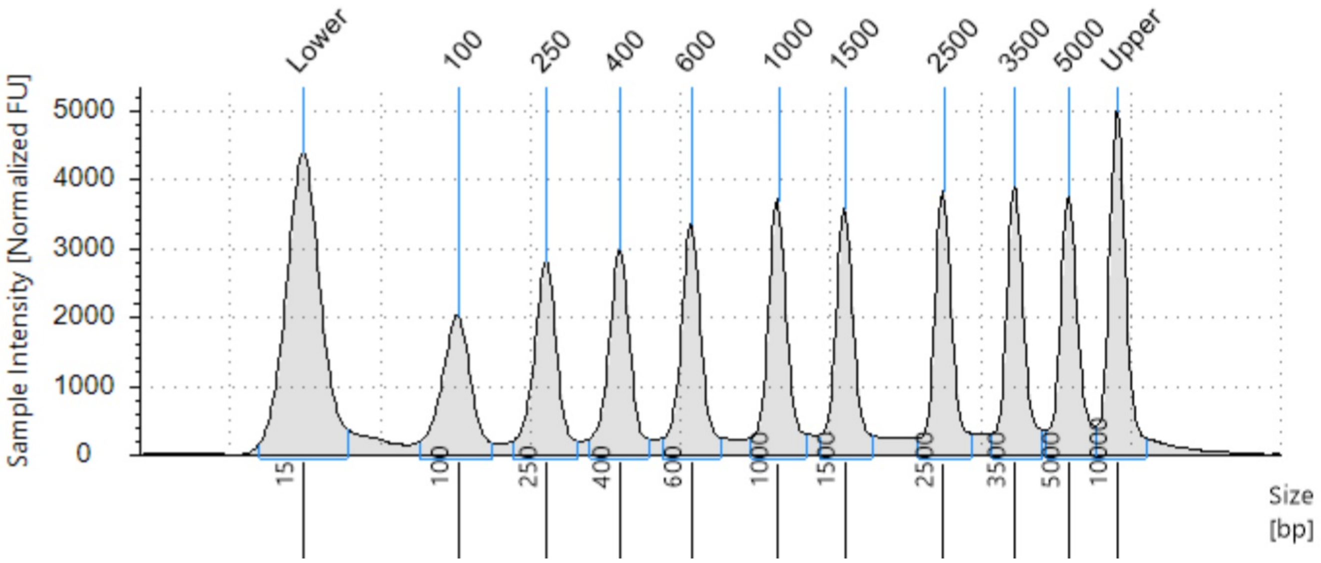

Sample Table

Well	Conc. [ng/µl]	Sample Description	Alert	Observations
A1	27.5	Ladder		Ladder

Peak Table

Size [bp]	Calibrated Conc. [ng/µl]	Assigned Conc. [ng/µl]	Peak Molarity [nmol/l]	% Integrated Area	Peak Comment	Observations
15	6.01	-	616	-		Lower Marker
100	2.70	-	41.6	9.83		
250	2.97	-	18.3	10.80		
400	2.95	-	11.3	10.73		
600	3.13	-	8.03	11.40		
1000	3.25	-	5.00	11.83		
1500	3.03	-	3.11	11.03		
2500	3.12	-	1.92	11.35		
3500	3.19	-	1.40	11.62		
5000	3.14	-	0.966	11.42		
10000	3.25	3.25	0.500	-		Upper Marker

© Agilent Technologies, Inc. 2017

E2: DFB plus 80 sec

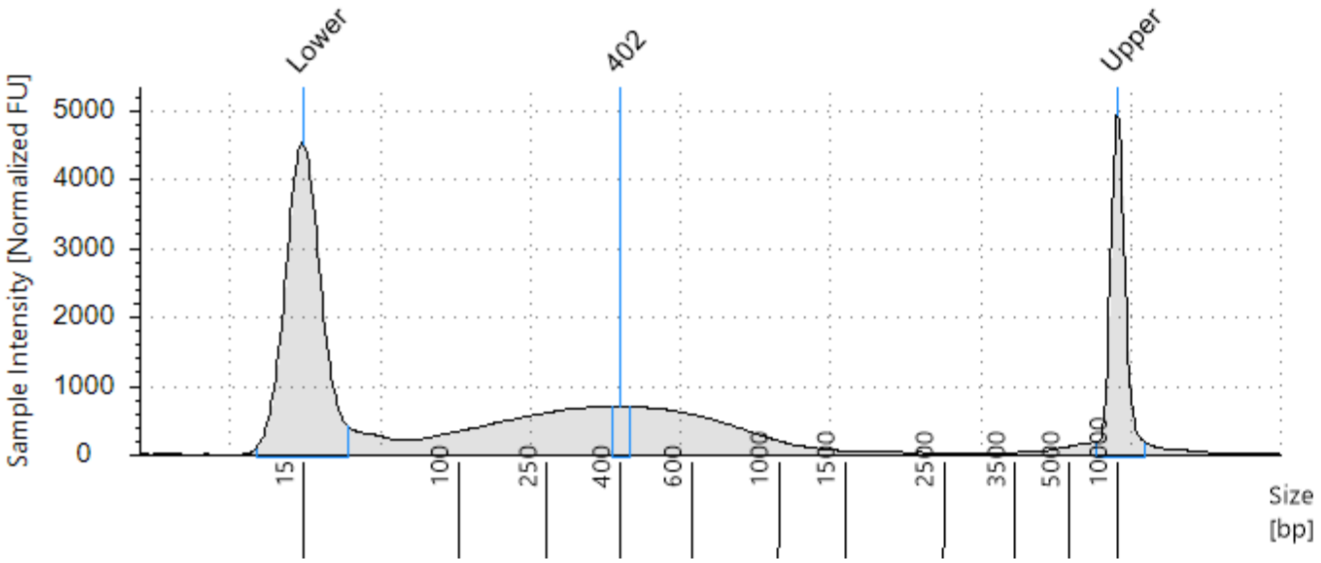

Sample Table

Well	Conc. [ng/µl]	Sample Description	Alert	Observations
E2	0.554	DFB plus 80 sec		

Peak Table

Size [bp]	Calibrated Conc. [ng/µl]	Assigned Conc. [ng/µl]	Peak Molarity [nmol/l]	% Integrated Area	Peak Comment	Observations
15	6.95	-	713	-		Lower Marker
402	0.554	-	2.12	100.00		
10000	3.25	3.25	0.500	-		Upper Marker

© Agilent Technologies, Inc. 2017

D2

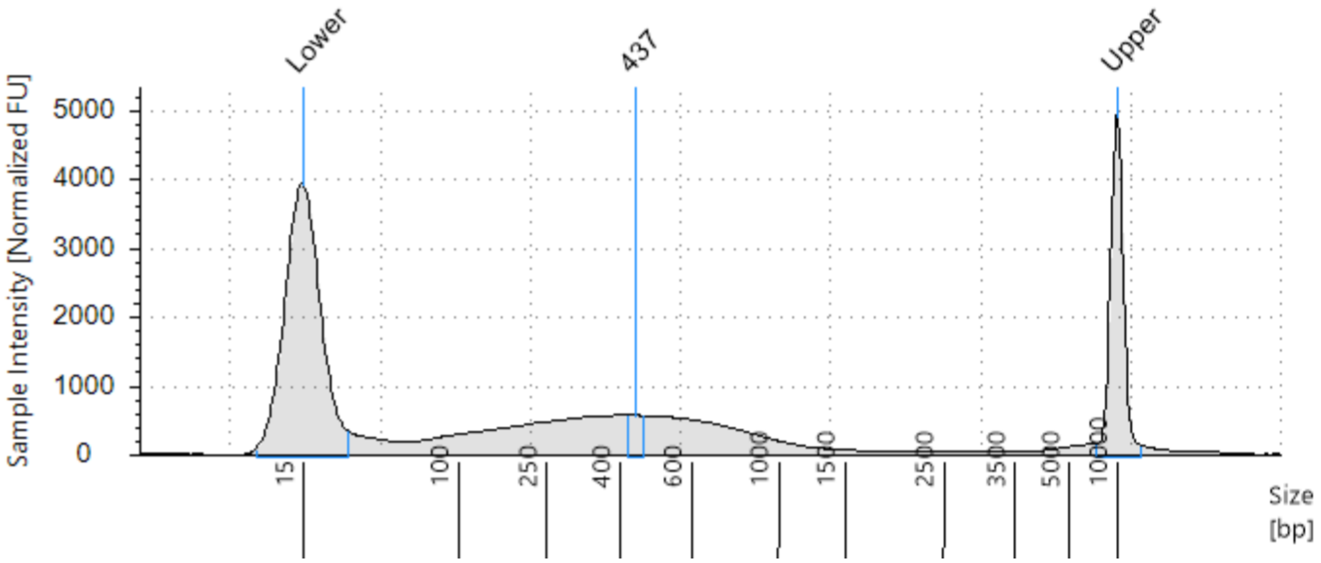

Sample Table

Well	Conc. [ng/µl]	Sample Description	Alert	Observations
D2	0.489			

Peak Table

Size [bp]	Calibrated Conc. [ng/µl]	Assigned Conc. [ng/µl]	Peak Molarity [nmol/l]	% Integrated Area	Peak Comment	Observations
15	6.81	-	699	-		Lower Marker
437	0.489	-	1.72	100.00		
10000	3.25	3.25	0.500	-		Upper Marker

 © Agilent Technologies, Inc. 2017

F2: DFB plus 80 sec

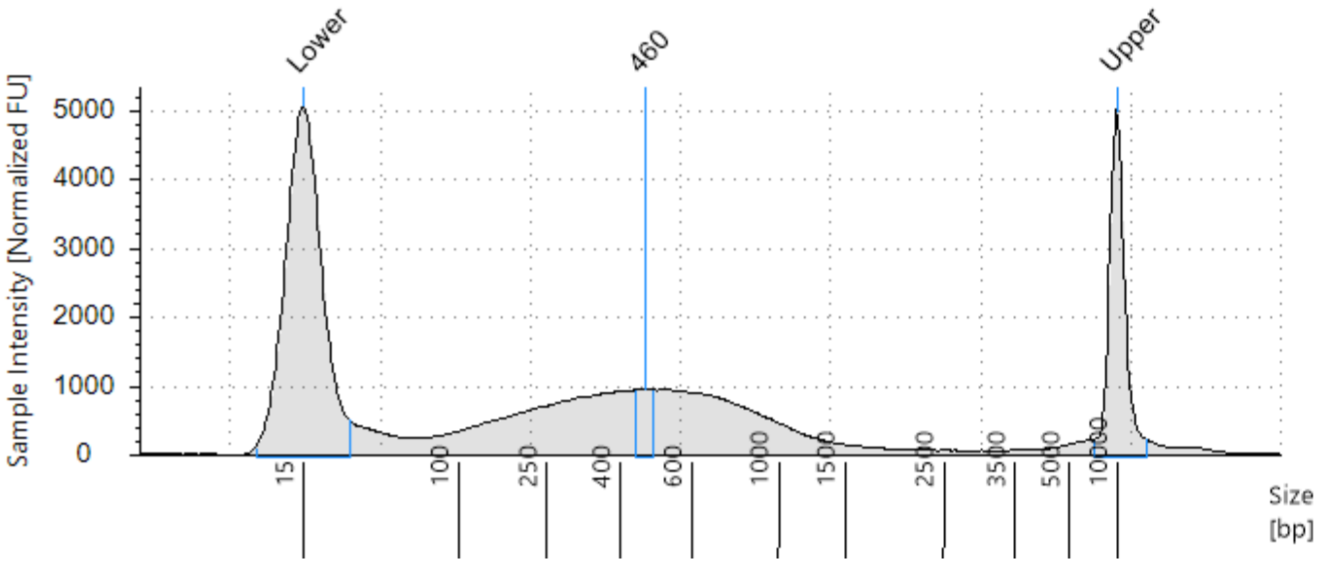

Sample Table

Well	Conc. [ng/µl]	Sample Description	Alert	Observations
F2	0.732	DFB plus 80 sec		

Peak Table

Size [bp]	Calibrated Conc. [ng/µl]	Assigned Conc. [ng/µl]	Peak Molarity [nmol/l]	% Integrated Area	Peak Comment	Observations
15	7.76	-	796	-		Lower Marker
460	0.732	-	2.45	100.00		
10000	3.25	3.25	0.500	-		Upper Marker

 © Agilent Technologies, Inc. 2017

Filename: 2019-05-24-02 DFB plus, E110, 240 sec, LE220 DFB2, 120 DFB 1-7 80 SEC.D5000

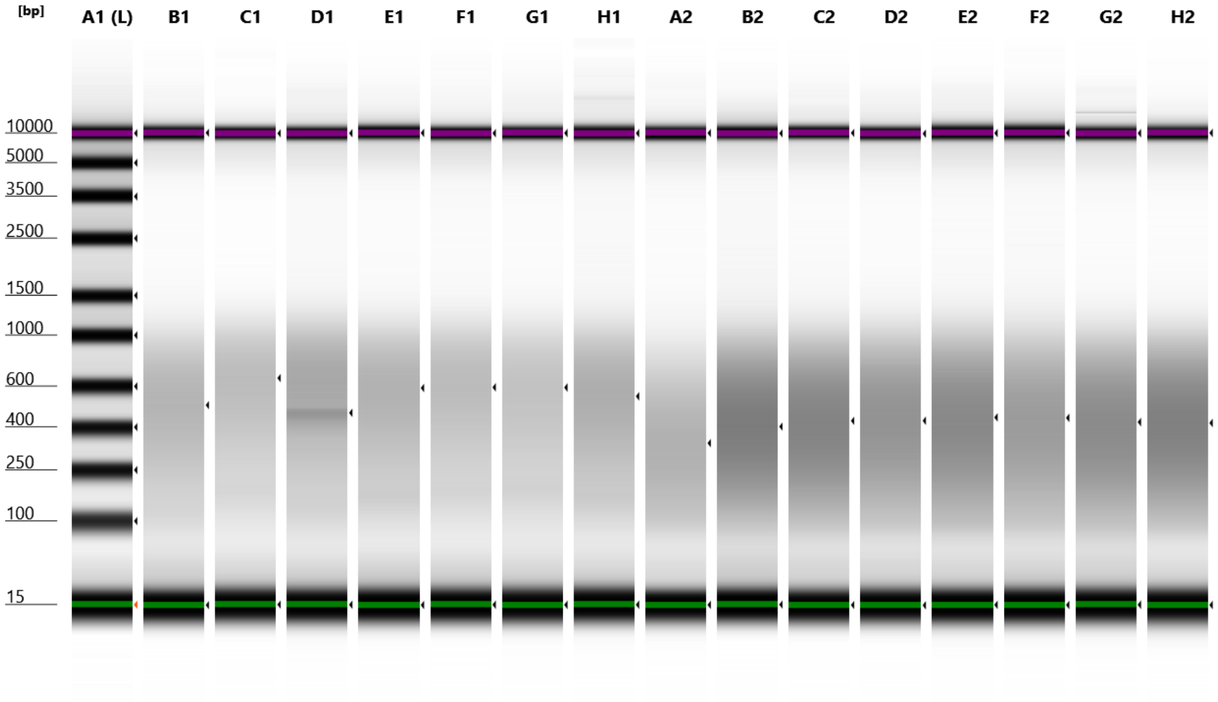

Default image (Contrast 100%)

Sample Info

Well	Conc. [ng/µl]	Sample Description	Alert	Observations
A1	28.3	Ladder		Ladder
B1	0.566	DFB1 PLUS 90 sec R2 E110		
C1	0.354	DFB2 PLUS 90 sec R2 E110		
D1	1.52	DFB3 PLUS 90 sec R2 E110		
E1	0.321	DFB4 PLUS 90 sec R2 E110		
F1	0.349	DFB5 PLUS 90 sec R2 E110		
G1	1.60	DFB6 PLUS 90 sec R2 E110		
H1	0.303	DFB6 PLUS 90 sec R2 E110		
A2	0.360	DFB2 plus120 sec R3 LE220		
B2	0.760	DFB1 plus 80 sec R3 LE220		
C2	4.36	DFB2 plus 80 sec R3 LE220		
D2	0.606	DFB3 plus 80 sec R3 LE220		
E2	5.29	DFB4 plus 80 sec R3 LE220		
F2	0.513	DFB5 plus 80 sec R3 LE220		
G2	0.657	DFB6 plus 80 sec R3 LE220		
H2	0.725	DFB7 plus 80 sec R3 LE220		

© Agilent Technologies, Inc. 2017

A1: Ladder

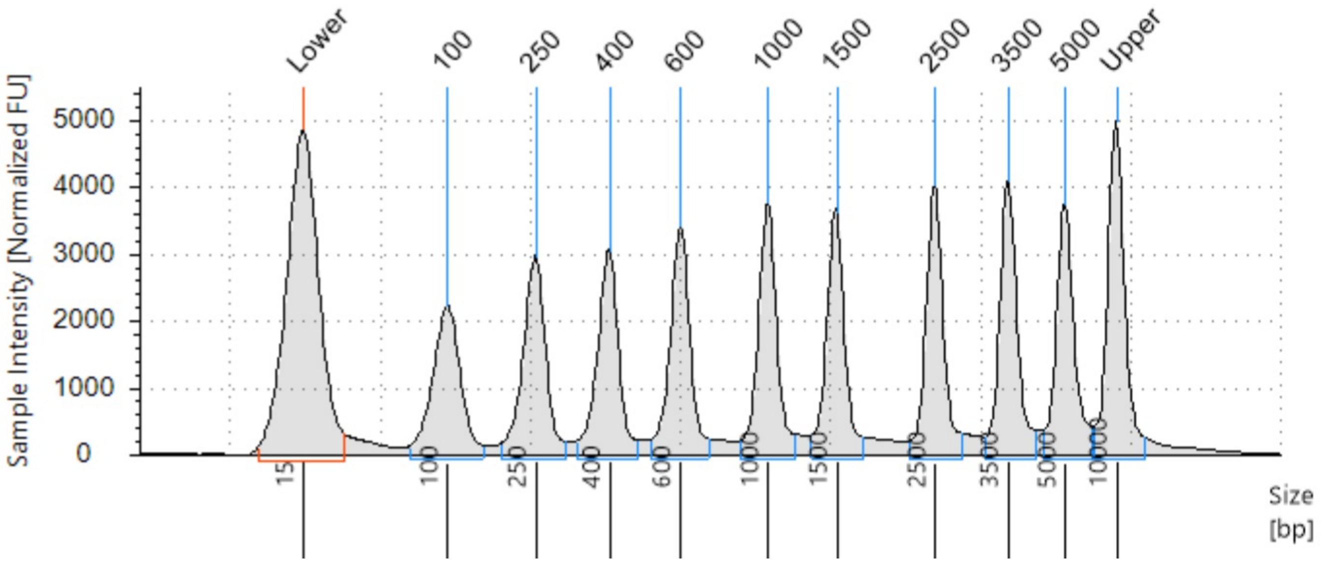

Sample Table

Well	Conc. [ng/µl]	Sample Description	Alert	Observations
A1	28.3	Ladder		Ladder

Peak Table

Size [bp]	Calibrated Conc. [ng/µl]	Assigned Conc. [ng/µl]	Peak Molarity [nmol/l]	% Integrated Area	Peak Comment	Observations
15	6.50	-	666	-		Lower Marker
100	2.88	-	44.3	10.18		
250	3.12	-	19.2	11.03		
400	3.00	-	11.5	10.62		
600	3.17	-	8.13	11.22		
1000	3.25	-	4.99	11.49		
1500	3.07	-	3.15	10.87		
2500	3.26	-	2.01	11.55		
3500	3.37	-	1.48	11.92		
5000	3.15	-	0.969	11.14		
10000	3.25	3.25	0.500	-		Upper Marker

© Agilent Technologies, Inc. 2017

B2: DFB1 plus 80 sec R3 LE220

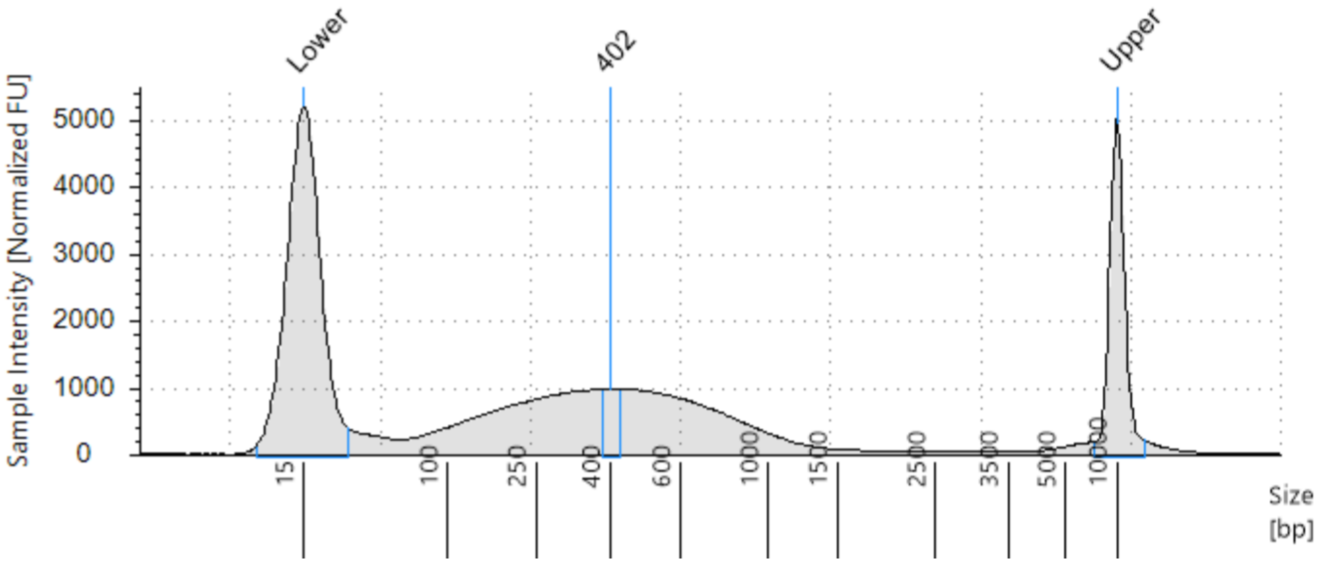

Sample Table

Well	Conc. [ng/µl]	Sample Description	Alert	Observations
B2	0.760	DFB1 plus 80 sec R3 LE220		

Peak Table

Size [bp]	Calibrated Conc. [ng/µl]	Assigned Conc. [ng/µl]	Peak Molarity [nmol/l]	% Integrated Area	Peak Comment	Observations
15	7.42	-	761	-		Lower Marker
402	0.760	-	2.91	100.00		
10000	3.25	3.25	0.500	-		Upper Marker

© Agilent Technologies, Inc. 2017

C2: DFB2 plus 80 sec R3 LE220

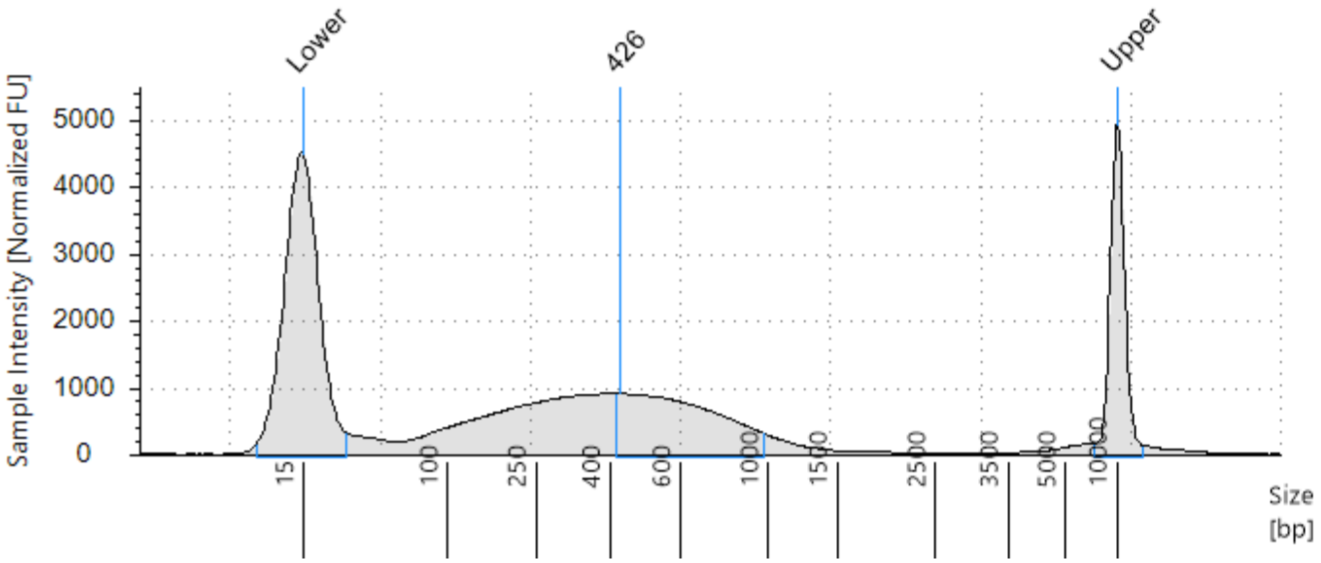

Sample Table

Well	Conc. [ng/µl]	Sample Description	Alert	Observations
C2	4.36	DFB2 plus 80 sec R3 LE220		

Peak Table

Size [bp]	Calibrated Conc. [ng/µl]	Assigned Conc. [ng/µl]	Peak Molarity [nmol/l]	% Integrated Area	Peak Comment	Observations
15	6.72	-	689	-		Lower Marker
426	4.36	-	15.8	100.00		
10000	3.25	3.25	0.500	-		Upper Marker

© Agilent Technologies, Inc. 2017

D2: DFB3 plus 80 sec R3 LE220

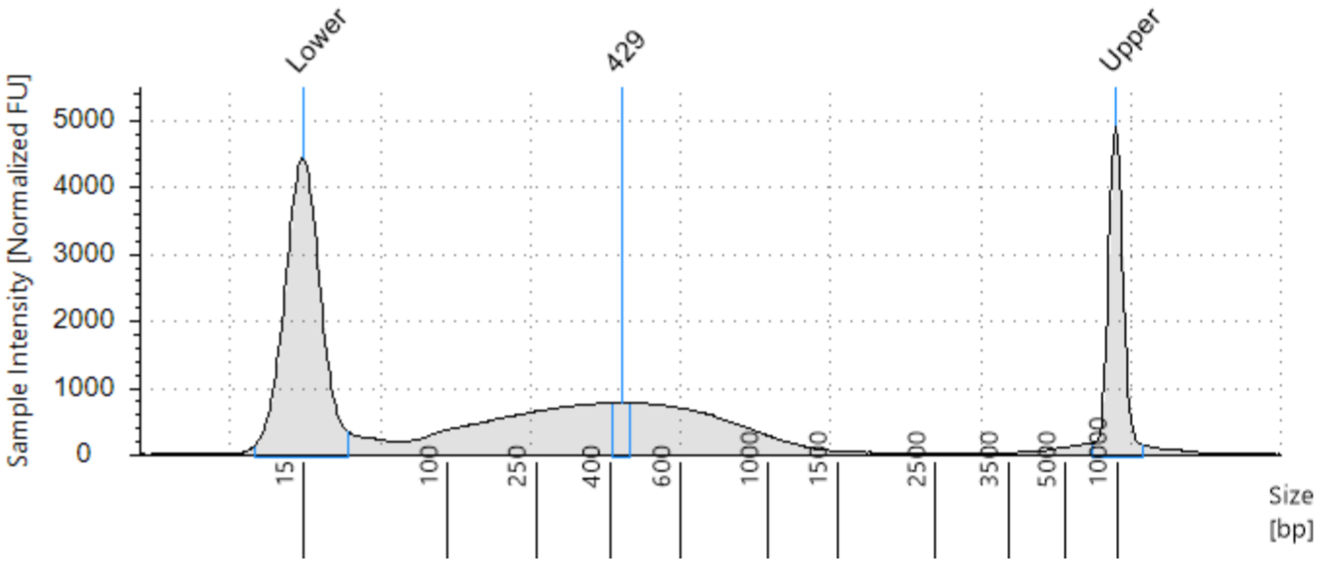

Sample Table

Well	Conc. [ng/µl]	Sample Description	Alert	Observations
D2	0.606	DFB3 plus 80 sec R3 LE220		

Peak Table

Size [bp]	Calibrated Conc. [ng/µl]	Assigned Conc. [ng/µl]	Peak Molarity [nmol/l]	% Integrated Area	Peak Comment	Observations
15	6.71	-	688	-		Lower Marker
429	0.606	-	2.18	100.00		
10000	3.25	3.25	0.500	-		Upper Marker

 © Agilent Technologies, Inc. 2017

E2: DFB4 plus 80 sec R3 LE220

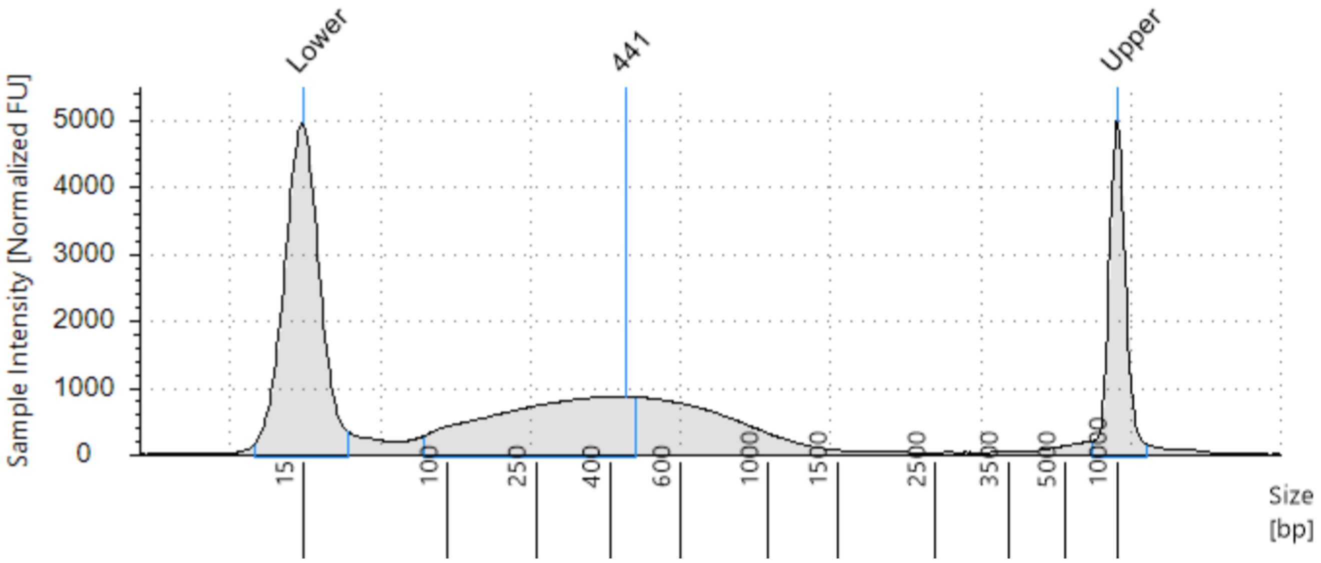

Sample Table

Well	Conc. [ng/µl]	Sample Description	Alert	Observations
E2	5.29	DFB4 plus 80 sec R3 LE220		

Peak Table

Size [bp]	Calibrated Conc. [ng/µl]	Assigned Conc. [ng/µl]	Peak Molarity [nmol/l]	% Integrated Area	Peak Comment	Observations
15	6.76	-	693	-		Lower Marker
441	5.29	-	18.5	100.00		
10000	3.25	3.25	0.500	-		Upper Marker

 © Agilent Technologies, Inc. 2017

F2: DFB5 plus 80 sec R3 LE220

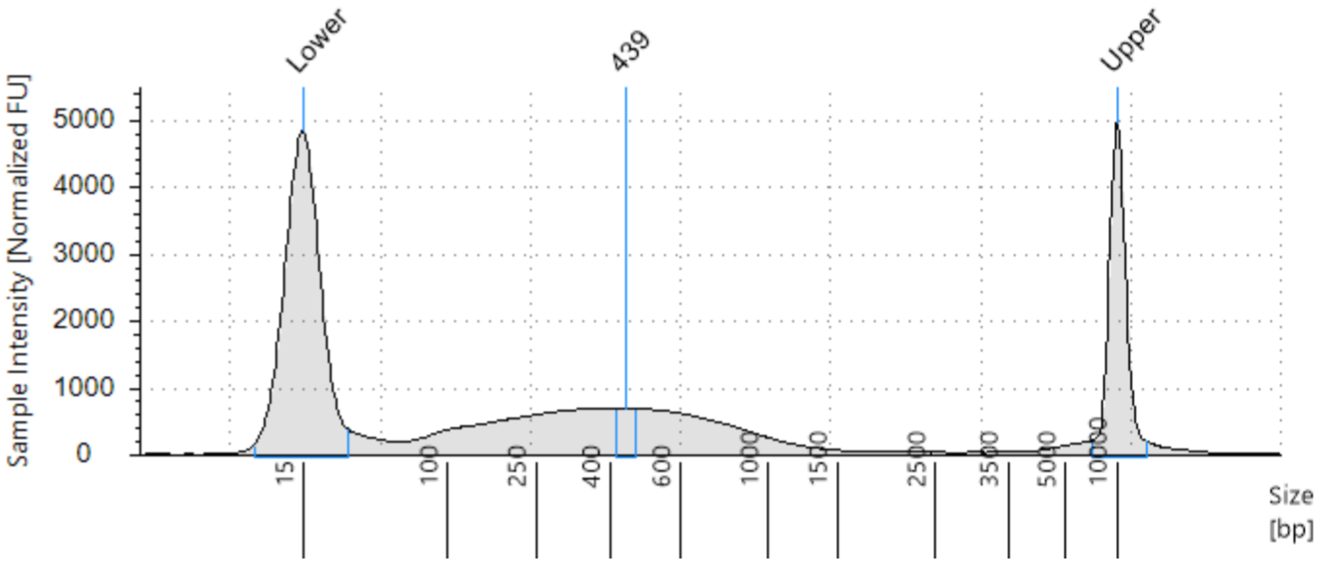

Sample Table

Well	Conc. [ng/µl]	Sample Description	Alert	Observations
F2	0.513	DFB5 plus 80 sec R3 LE220		

Peak Table

Size [bp]	Calibrated Conc. [ng/µl]	Assigned Conc. [ng/µl]	Peak Molarity [nmol/l]	% Integrated Area	Peak Comment	Observations
15	6.69	-	687	-		Lower Marker
439	0.513	-	1.80	100.00		
10000	3.25	3.25	0.500	-		Upper Marker

© Agilent Technologies, Inc. 2017

G2: DFB6 plus 80 sec R3 LE220

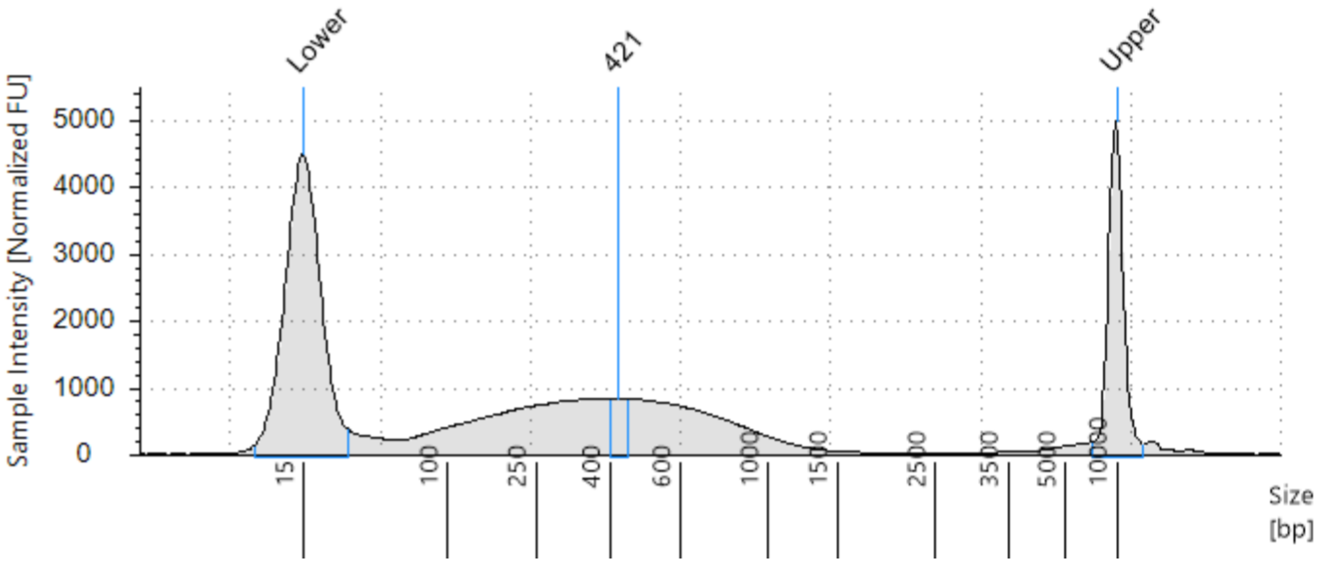

Sample Table

Well	Conc. [ng/µl]	Sample Description	Alert	Observations
G2	0.657	DFB6 plus 80 sec R3 LE220		

Peak Table

Size [bp]	Calibrated Conc. [ng/µl]	Assigned Conc. [ng/µl]	Peak Molarity [nmol/l]	% Integrated Area	Peak Comment	Observations
15	6.62	-	679	-		Lower Marker
421	0.657	-	2.41	100.00		
10000	3.25	3.25	0.500	-		Upper Marker

 © Agilent Technologies, Inc. 2017

H2: DFB7 plus 80 sec R3 LE220

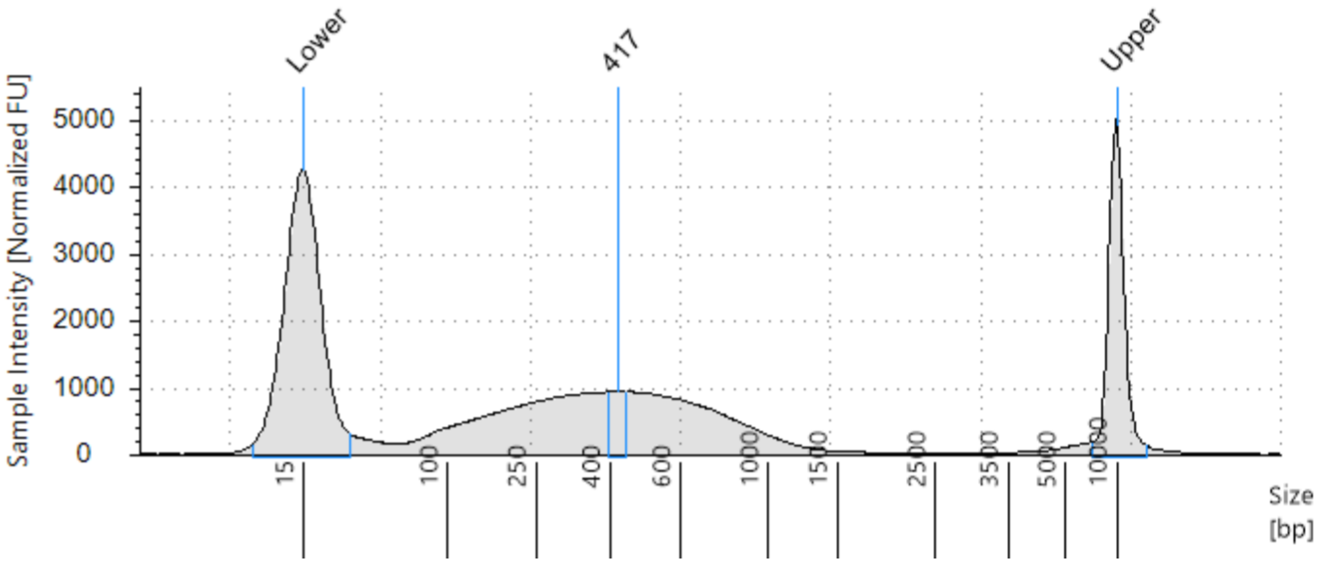

Sample Table

Well	Conc. [ng/µl]	Sample Description	Alert	Observations
H2	0.725	DFB7 plus 80 sec R3 LE220		

Peak Table

Size [bp]	Calibrated Conc. [ng/µl]	Assigned Conc. [ng/µl]	Peak Molarity [nmol/l]	% Integrated Area	Peak Comment	Observations
15	6.39	-	656	-		Lower Marker
417	0.725	-	2.67	100.00		
10000	3.25	3.25	0.500	-		Upper Marker

© Agilent Technologies, Inc. 2017
