# Supplementary material for: 96 sample parallel acoustic fragmentation for high throughput next generation sequencing library preparation
Source: PLoS One. 2026 Feb 17;21(2):e0341139. doi: 10.1371/journal.pone.0341139 (PMC12912608; doi:10.1371/journal.pone.0341139)
Supplement: S2 Fig — (ZIP) [file pone.0341139.s002.zip › Figure 1 Raw Data/Covaris microTUBE 120 seconds.pdf]

Filename: 4.22.19 Covaris LE220 first 8, 80 sec last 80 120 sec.D5000

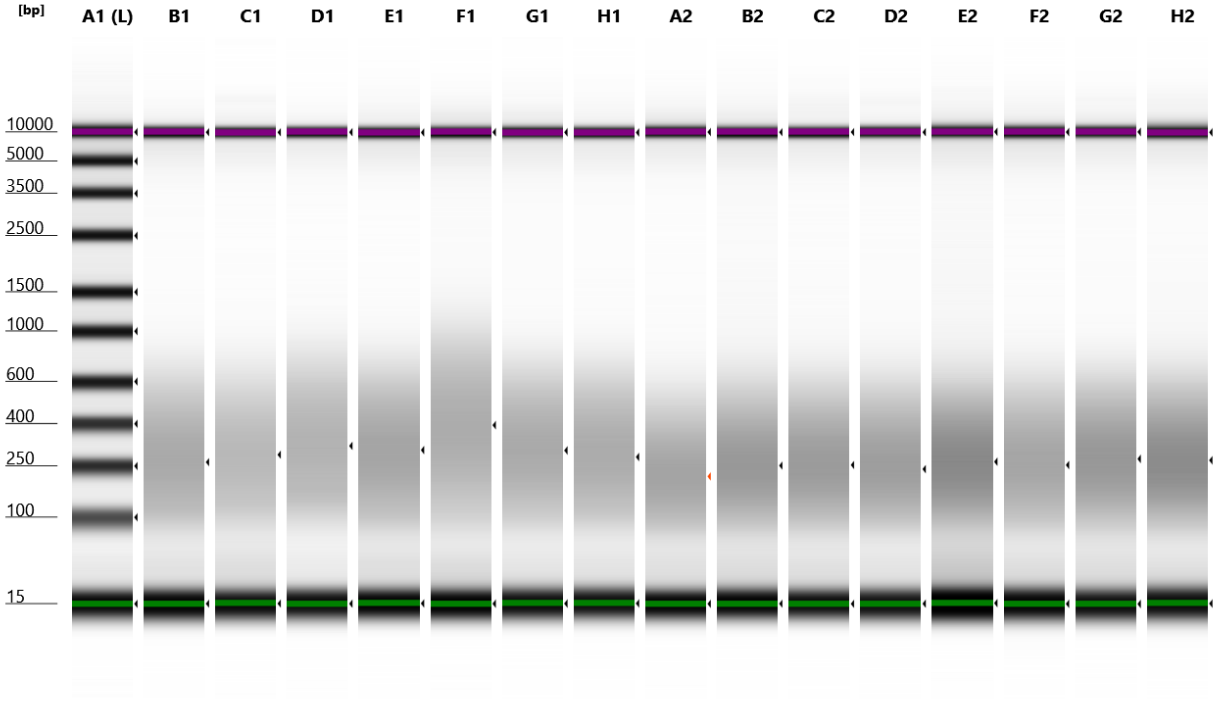

Default image (Contrast 100%)

Sample Info

Well	Conc. [ng/µl]	Sample Description	Alert	Observations
A1	27.0	Ladder		Ladder
B1	0.749	rod-LE220 80 sec		
C1	0.487	rod-LE220 80 sec		
D1	0.473	rod-LE220 80 sec		
E1	0.688	rod-LE220 80 sec		
F1	0.798	rod-LE220 80 sec		
G1	4.18	rod-LE220 80 sec		
H1	1.91	rod LE220 80 sec		
A2	3.44	rod LE220 120 sec		
B2	0.798	rod LE220 120 sec		
C2	0.930	rod LE220 120 sec		
D2	7.91	rod LE220 120 sec		
E2	0.946	rod LE220 120 sec		
F2	3.88	rod LE220 120 sec		
G2	0.674	rod LE220 120 sec		
H2	4.77			

 © Agilent Technologies, Inc. 2017

A1: Ladder

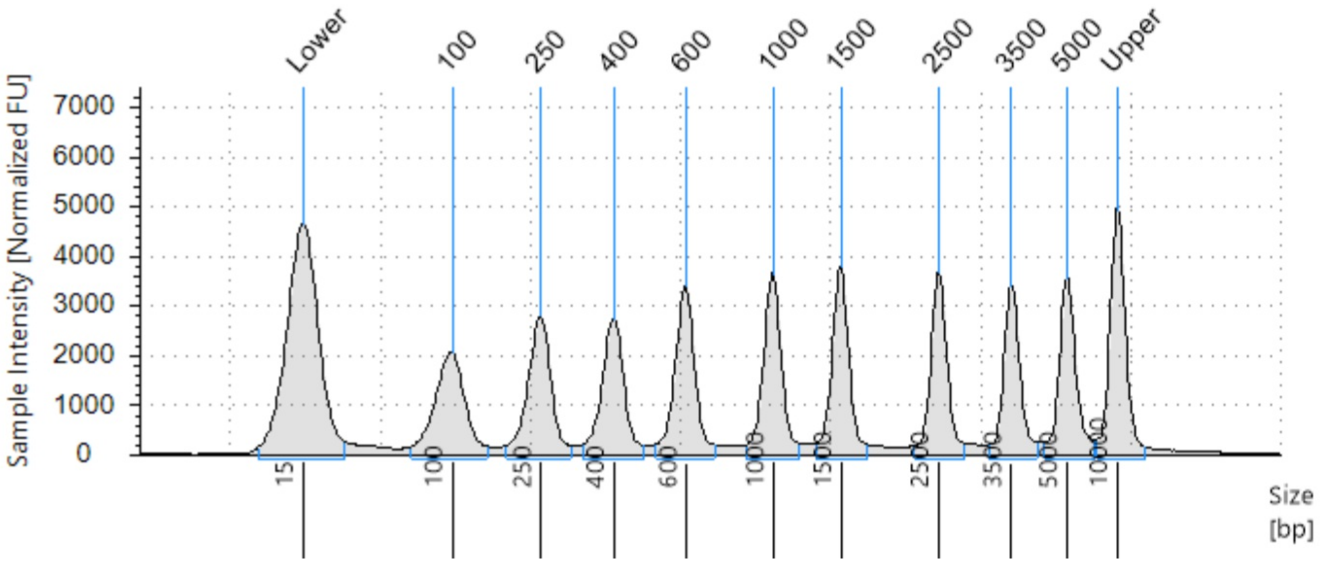

Sample Table

Well	Conc. [ng/µl]	Sample Description	Alert	Observations
A1	27.0	Ladder		Ladder

Peak Table

Size [bp]	Calibrated Conc. [ng/µl]	Assigned Conc. [ng/µl]	Peak Molarity [nmol/l]	% Integrated Area	Peak Comment	Observations
15	6.38	-	655	-		Lower Marker
100	2.98	-	45.8	11.03		
250	3.08	-	19.0	11.42		
400	2.92	-	11.2	10.82		
600	3.25	-	8.33	12.04		
1000	3.19	-	4.90	11.81		
1500	3.12	-	3.20	11.58		
2500	2.90	-	1.78	10.75		
3500	2.68	-	1.18	9.94		
5000	2.86	-	0.881	10.61		
10000	3.25	3.25	0.500	-		Upper Marker

 © Agilent Technologies, Inc. 2017

A2: rod LE220 120 sec

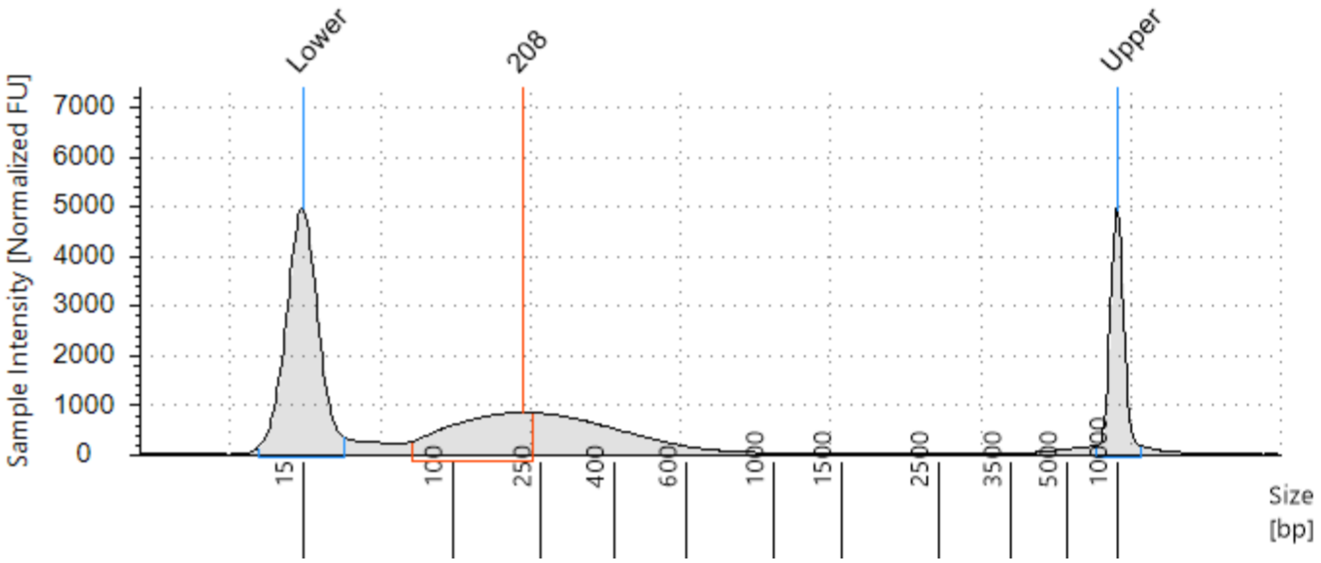

Sample Table

Well	Conc. [ng/µl]	Sample Description	Alert	Observations
A2	3.44	rod LE220 120 sec		

Peak Table

Size [bp]	Calibrated Conc. [ng/µl]	Assigned Conc. [ng/µl]	Peak Molarity [nmol/l]	% Integrated Area	Peak Comment	Observations
15	7.32	-	751	-		Lower Marker
208	3.44	-	25.5	100.00		
10000	3.25	3.25	0.500	-		Upper Marker

© Agilent Technologies, Inc. 2017

B2: rod LE220 120 sec

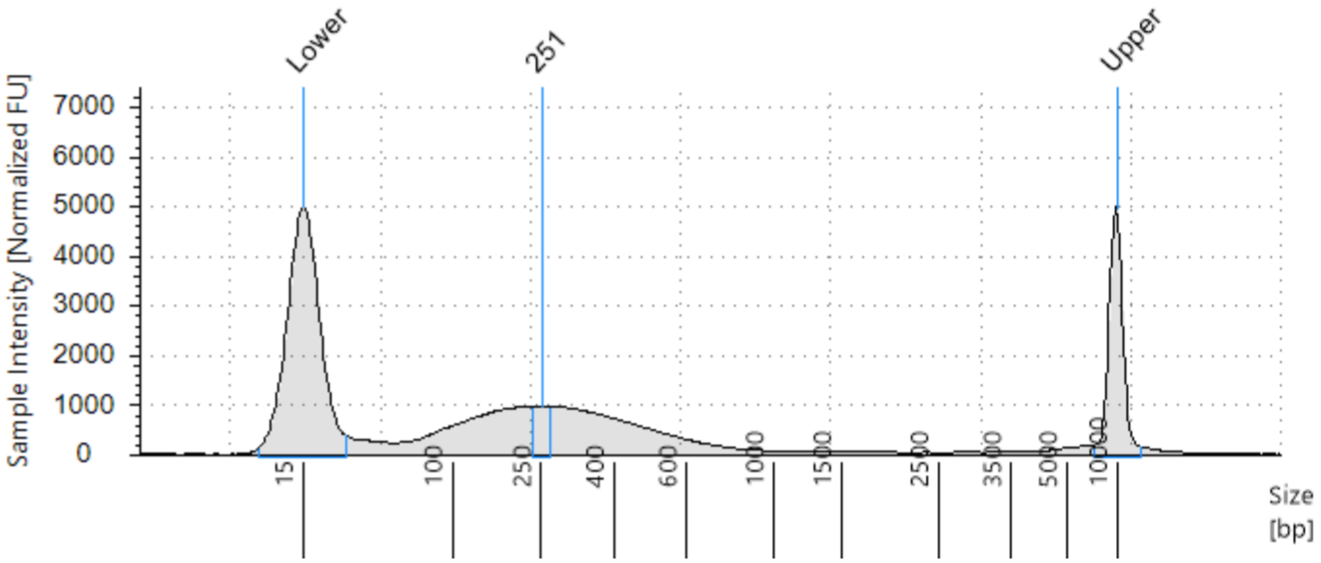

Sample Table

Well	Conc. [ng/µl]	Sample Description	Alert	Observations
B2	0.798	rod LE220 120 sec		

Peak Table

Size [bp]	Calibrated Conc. [ng/µl]	Assigned Conc. [ng/µl]	Peak Molarity [nmol/l]	% Integrated Area	Peak Comment	Observations
15	7.42	-	761	-		Lower Marker
251	0.798	-	4.89	100.00		
10000	3.25	3.25	0.500	-		Upper Marker

© Agilent Technologies, Inc. 2017

C2: rod LE220 120 sec

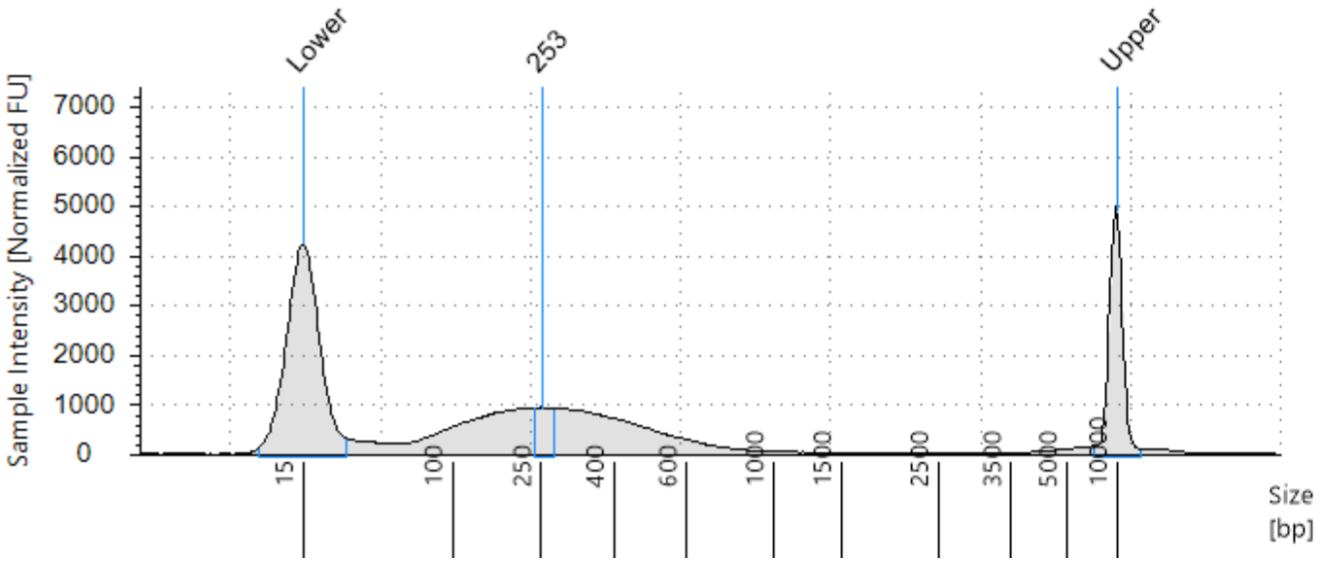

Sample Table

Well	Conc. [ng/µl]	Sample Description	Alert	Observations
C2	0.930	rod LE220 120 sec		

Peak Table

Size [bp]	Calibrated Conc. [ng/µl]	Assigned Conc. [ng/µl]	Peak Molarity [nmol/l]	% Integrated Area	Peak Comment	Observations
15	6.51	-	668	-		Lower Marker
253	0.930	-	5.66	100.00		
10000	3.25	3.25	0.500	-		Upper Marker

© Agilent Technologies, Inc. 2017

D2: rod LE220 120 sec

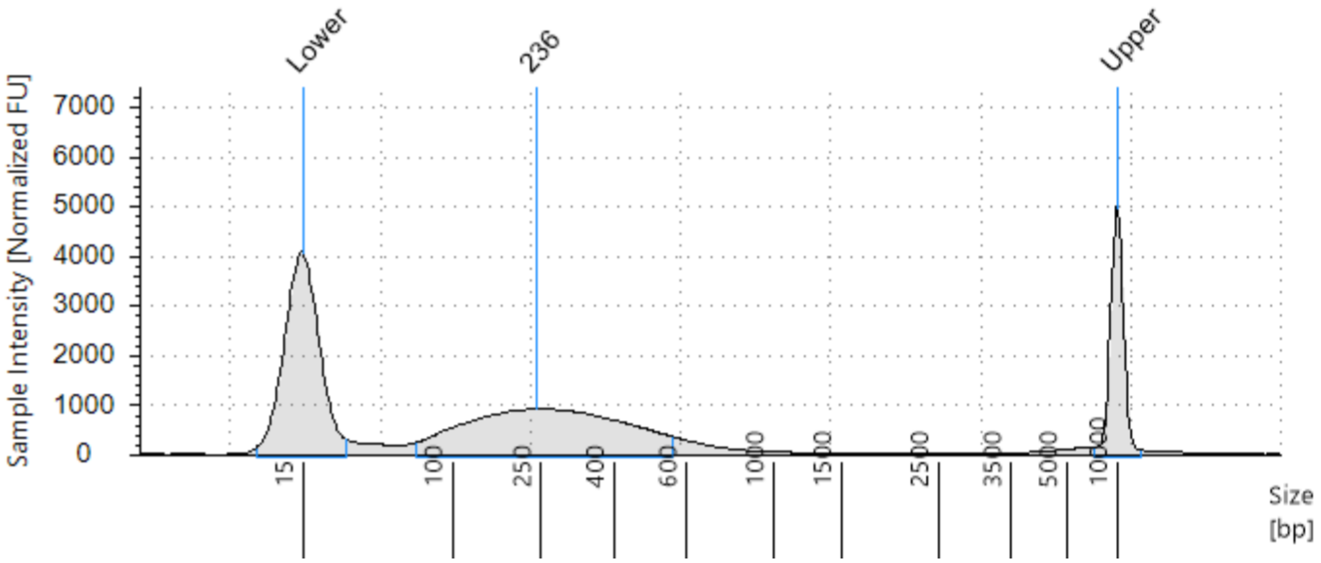

Sample Table

Well	Conc. [ng/µl]	Sample Description	Alert	Observations
D2	7.91	rod LE220 120 sec		

Peak Table

Size [bp]	Calibrated Conc. [ng/µl]	Assigned Conc. [ng/µl]	Peak Molarity [nmol/l]	% Integrated Area	Peak Comment	Observations
15	6.66	-	683	-		Lower Marker
236	7.91	-	51.5	100.00		
10000	3.25	3.25	0.500	-		Upper Marker

© Agilent Technologies, Inc. 2017

E2: rod LE220 120 sec

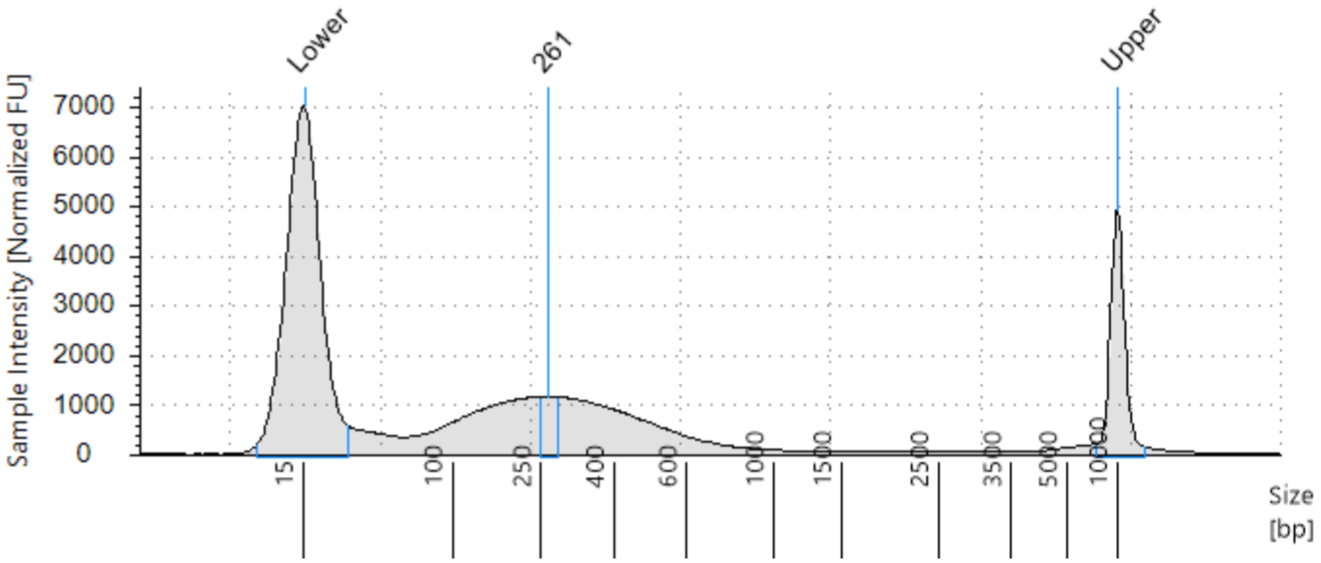

Sample Table

Well	Conc. [ng/µl]	Sample Description	Alert	Observations
E2	0.946	rod LE220 120 sec		

Peak Table

Size [bp]	Calibrated Conc. [ng/µl]	Assigned Conc. [ng/µl]	Peak Molarity [nmol/l]	% Integrated Area	Peak Comment	Observations
15	10.7	-	1090	-		Lower Marker
261	0.946	-	5.58	100.00		
10000	3.25	3.25	0.500	-		Upper Marker

© Agilent Technologies, Inc. 2017

F2: rod LE220 120 sec

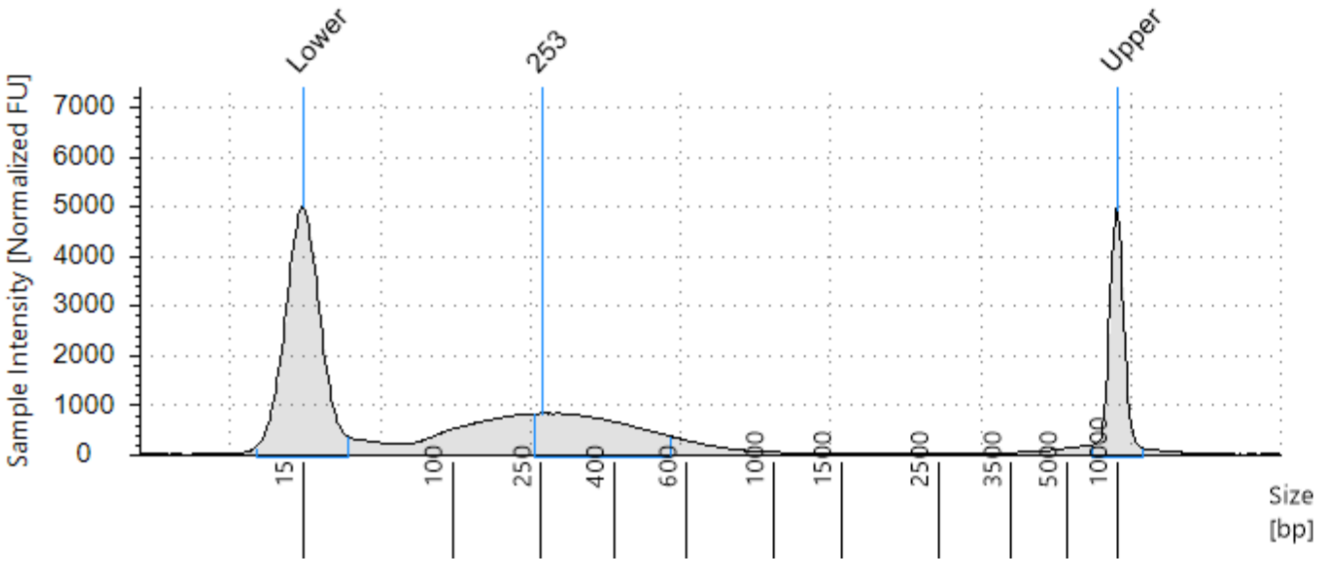

Sample Table

Well	Conc. [ng/µl]	Sample Description	Alert	Observations
F2	3.88	rod LE220 120 sec		

Peak Table

Size [bp]	Calibrated Conc. [ng/µl]	Assigned Conc. [ng/µl]	Peak Molarity [nmol/l]	% Integrated Area	Peak Comment	Observations
15	7.57	-	777	-		Lower Marker
253	3.88	-	23.6	100.00		
10000	3.25	3.25	0.500	-		Upper Marker

 © Agilent Technologies, Inc. 2017

G2: rod LE220 120 sec

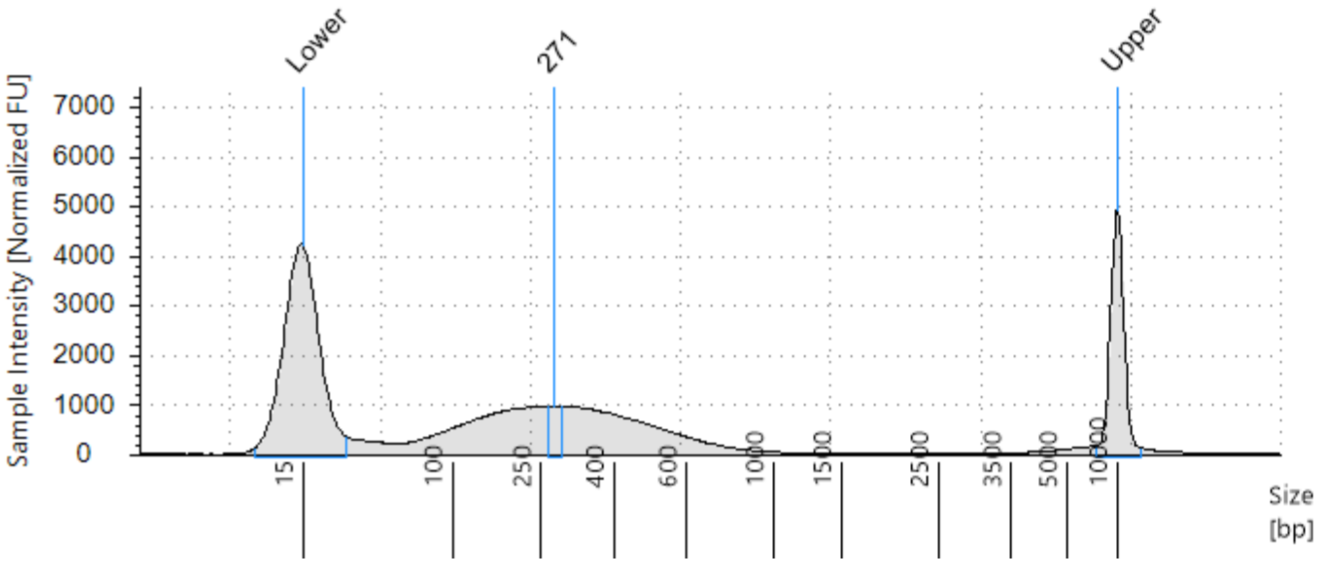

Sample Table

Well	Conc. [ng/µl]	Sample Description	Alert	Observations
G2	0.674	rod LE220 120 sec		

Peak Table

Size [bp]	Calibrated Conc. [ng/µl]	Assigned Conc. [ng/µl]	Peak Molarity [nmol/l]	% Integrated Area	Peak Comment	Observations
15	6.89	-	707	-		Lower Marker
271	0.674	-	3.83	100.00		
10000	3.25	3.25	0.500	-		Upper Marker

© Agilent Technologies, Inc. 2017

Filename: 2019-06-03-01LE220 covaris micro tube 80 sec 120 sec R2.D5000

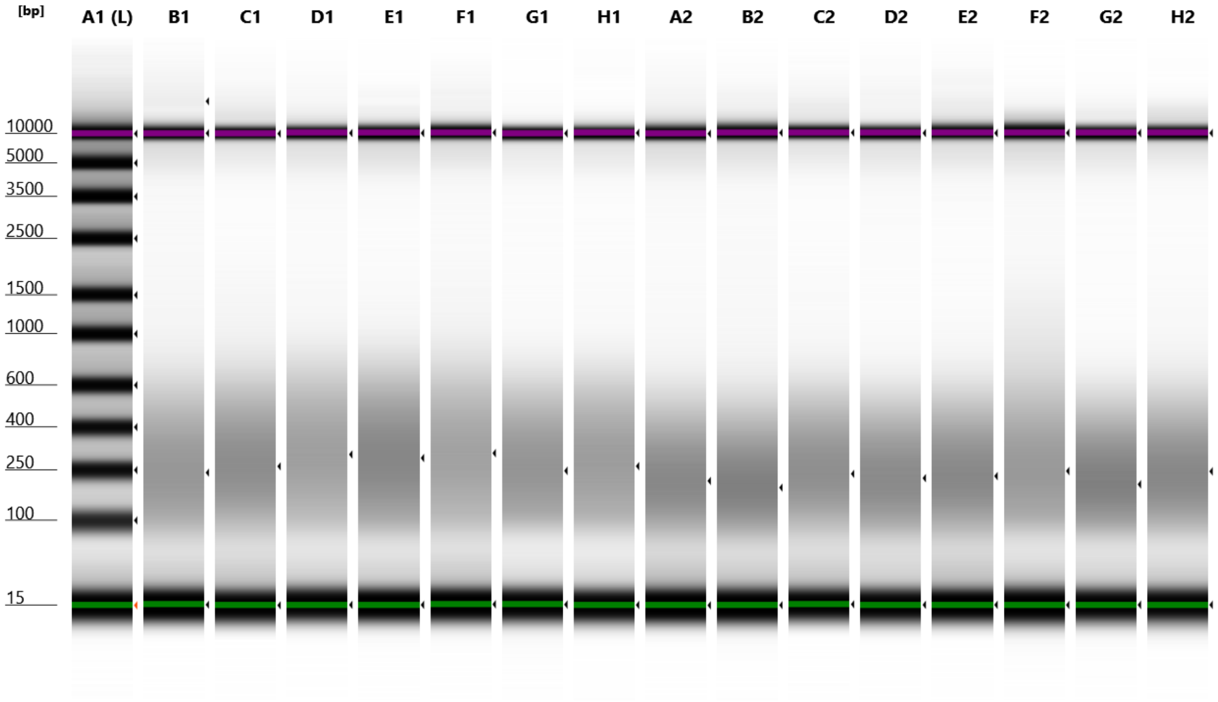

Default image (Contrast 100%)

Sample Info

Well	Conc. [ng/µl]	Sample Description	Alert	Observations
A1	35.9	Ladder		Ladder
B1	3.44	covaris micro tube 1 80 sec R2		
C1	0.704	covaris micro tube 2 80 sec R2		
D1	0.507	covaris micro tube 3 80 sec R2		
E1	0.668	covaris micro tube 4 80 sec R2		
F1	0.788	covaris micro tube 5 80 sec R2		
G1	3.65	covaris micro tube 6 80 sec R2		
H1	1.50	covaris micro tube 7 80 sec R2		
A2	3.07	covaris micro tube 1 120 sec R2		
B2	5.88	covaris micro tube 2 120 sec R2		
C2	3.16	covaris micro tube 3 120 sec R2		
D2	3.32	covaris micro tube 4 120 sec R2		
E2	3.47	covaris micro tube 5 120 sec R2		
F2	0.532	covaris micro tube 6 120 sec R2		
G2	3.37	covaris micro tube 7 120 sec R2		
H2	3.62	covaris micro tube 8 120 sec R2		

© Agilent Technologies, Inc. 2017

A1: Ladder

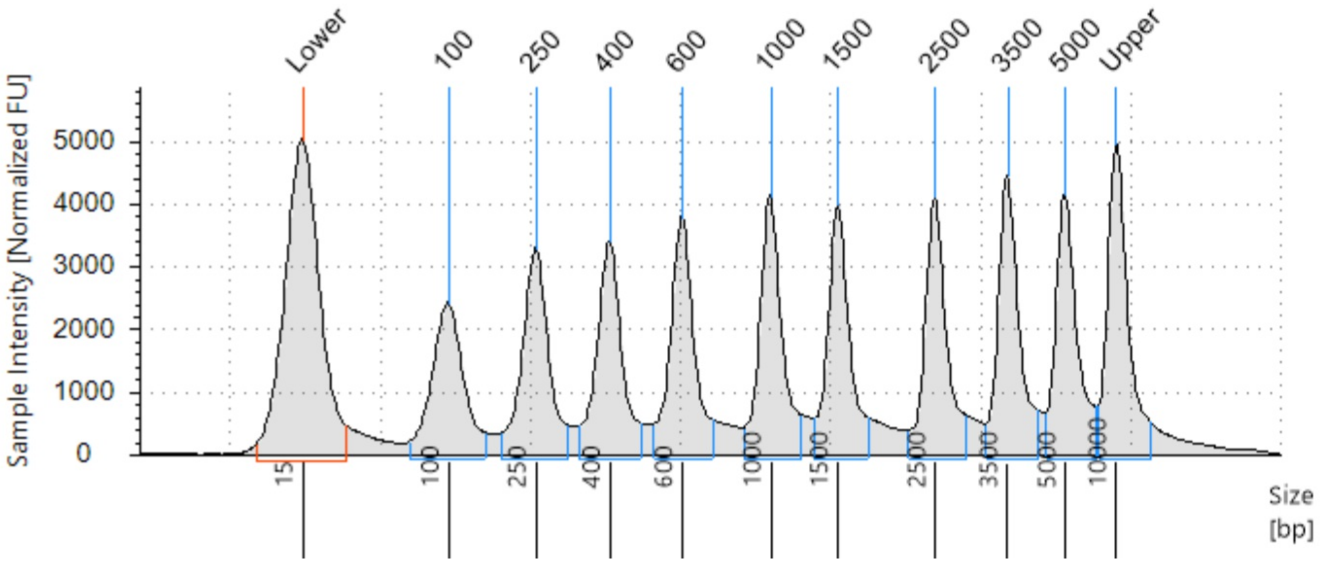

Sample Table

Well	Conc. [ng/µl]	Sample Description	Alert	Observations
A1	35.9	Ladder		Ladder

Peak Table

Size [bp]	Calibrated Conc. [ng/µl]	Assigned Conc. [ng/µl]	Peak Molarity [nmol/l]	% Integrated Area	Peak Comment	Observations
15	6.79	-	696	-		Lower Marker
100	3.49	-	53.7	9.72		
250	3.93	-	24.2	10.94		
400	3.85	-	14.8	10.72		
600	4.09	-	10.5	11.38		
1000	4.21	-	6.47	11.72		
1500	3.95	-	4.05	10.99		
2500	4.03	-	2.48	11.23		
3500	4.28	-	1.88	11.91		
5000	4.09	-	1.26	11.38		
10000	3.25	3.25	0.500	-		Upper Marker

© Agilent Technologies, Inc. 2017

A2: covaris micro tube 1 120 sec R2

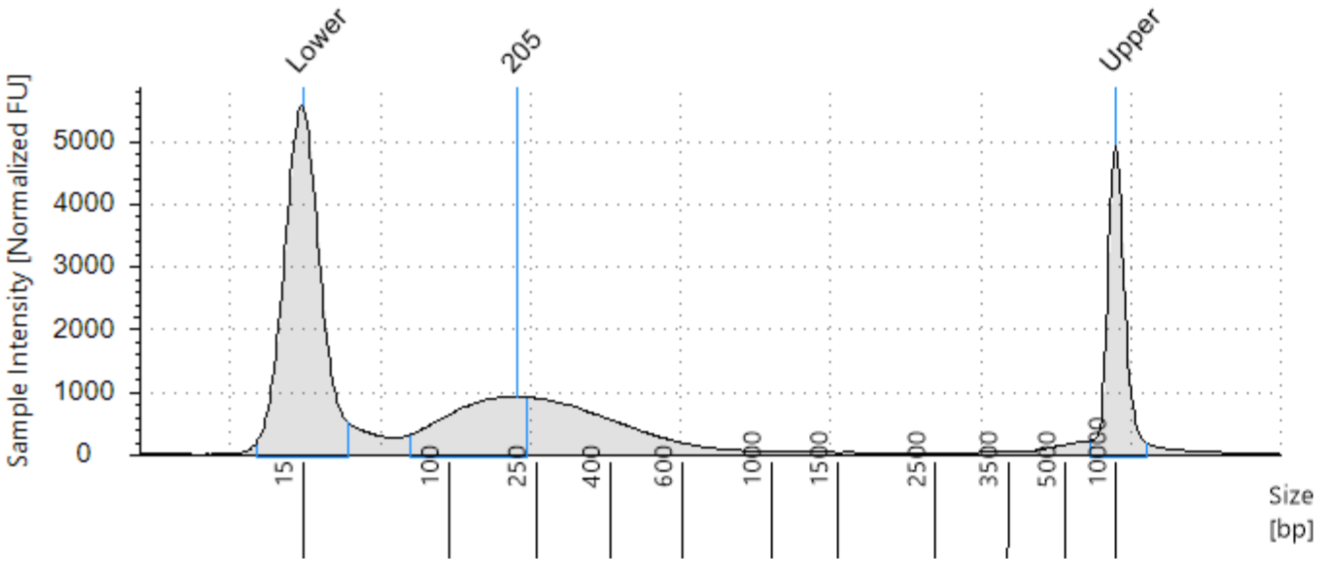

Sample Table

Well	Conc. [ng/µl]	Sample Description	Alert	Observations
A2	3.07	covaris micro tube 1 120 sec R2		

Peak Table

Size [bp]	Calibrated Conc. [ng/µl]	Assigned Conc. [ng/µl]	Peak Molarity [nmol/l]	% Integrated Area	Peak Comment	Observations
15	7.39	-	758	-		Lower Marker
205	3.07	-	23.0	100.00		
10000	3.25	3.25	0.500	-		Upper Marker

© Agilent Technologies, Inc. 2017

B2: covaris micro tube 2 120 sec R2

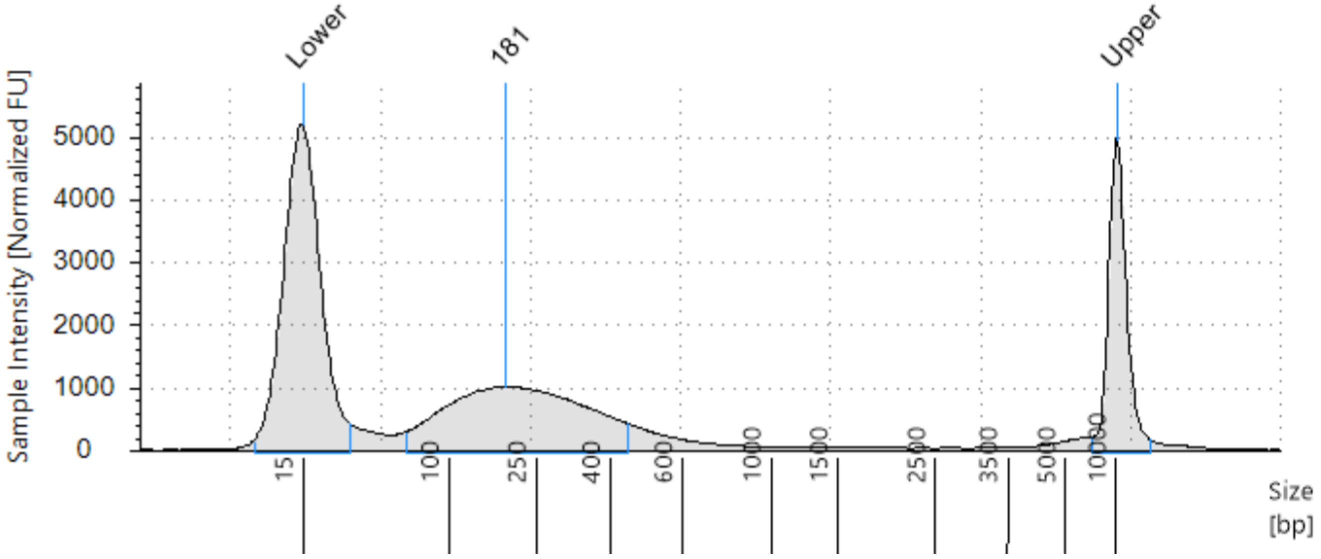

Sample Table

Well	Conc. [ng/µl]	Sample Description	Alert	Observations
B2	5.88	covaris micro tube 2 120 sec R2		

Peak Table

Size [bp]	Calibrated Conc. [ng/µl]	Assigned Conc. [ng/µl]	Peak Molarity [nmol/l]	% Integrated Area	Peak Comment	Observations
15	6.89	-	707	-		Lower Marker
181	5.88	-	50.1	100.00		
10000	3.25	3.25	0.500	-		Upper Marker

© Agilent Technologies, Inc. 2017

C2: covaris micro tube 3 120 sec R2

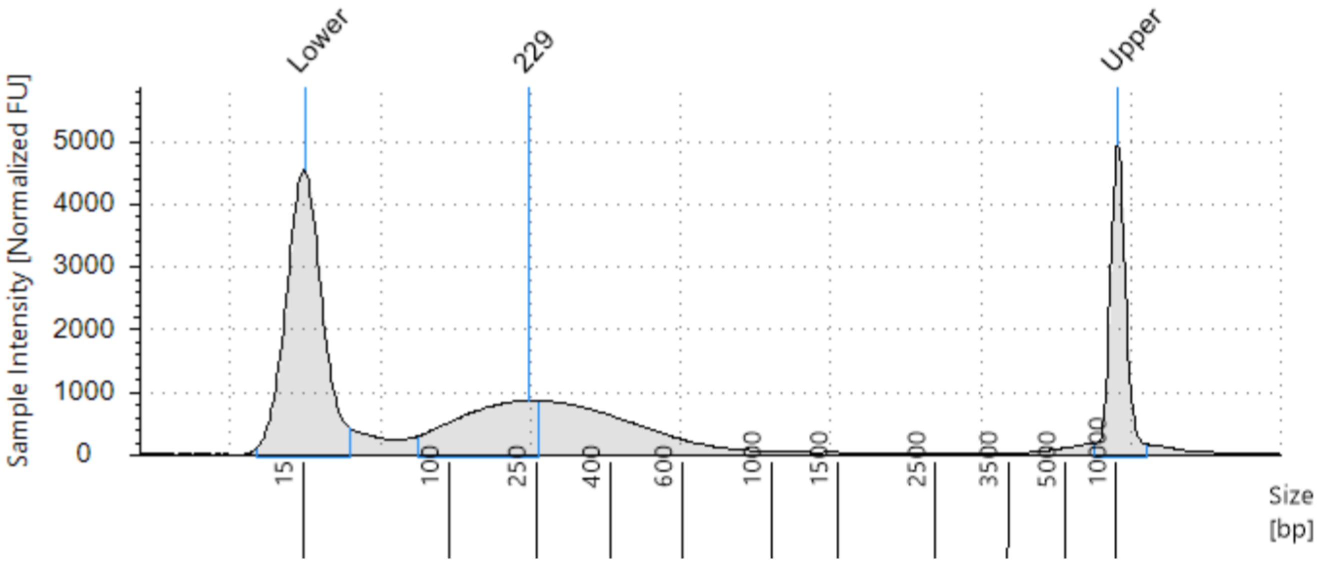

Sample Table

Well	Conc. [ng/µl]	Sample Description	Alert	Observations
C2	3.16	covaris micro tube 3 120 sec R2		

Peak Table

Size [bp]	Calibrated Conc. [ng/µl]	Assigned Conc. [ng/µl]	Peak Molarity [nmol/l]	% Integrated Area	Peak Comment	Observations
15	6.58	-	675	-		Lower Marker
229	3.16	-	21.2	100.00		
10000	3.25	3.25	0.500	-		Upper Marker

© Agilent Technologies, Inc. 2017

D2: covaris micro tube 4 120 sec R2

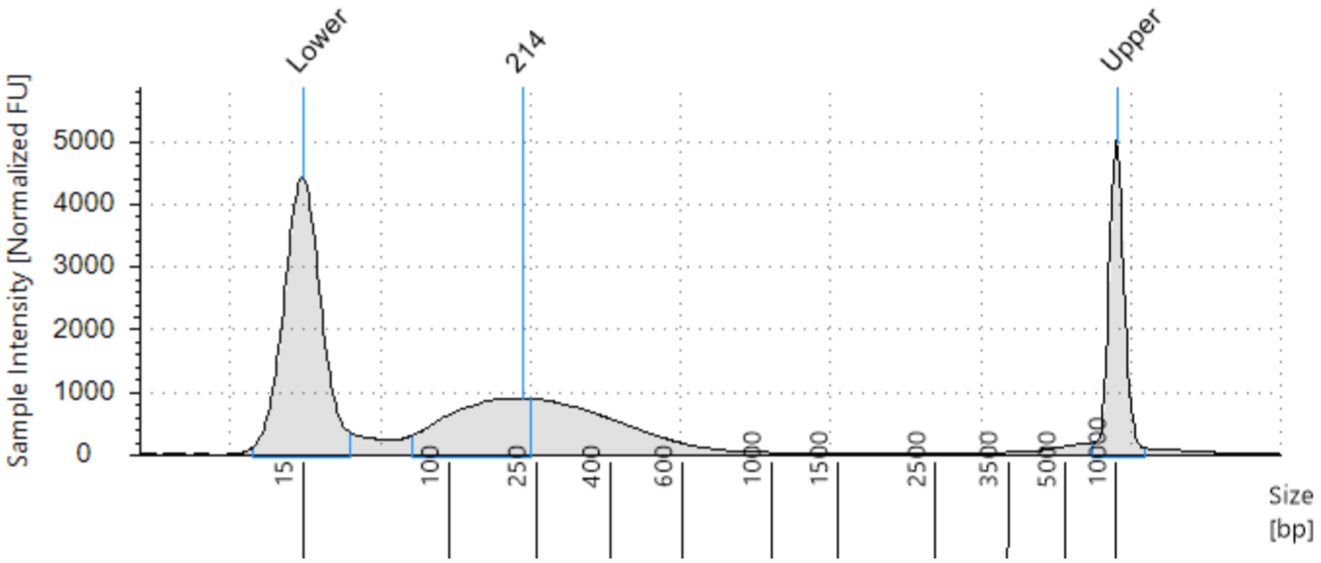

Sample Table

Well	Conc. [ng/µl]	Sample Description	Alert	Observations
D2	3.32	covaris micro tube 4 120 sec R2		

Peak Table

Size [bp]	Calibrated Conc. [ng/µl]	Assigned Conc. [ng/µl]	Peak Molarity [nmol/l]	% Integrated Area	Peak Comment	Observations
15	6.60	-	677	-		Lower Marker
214	3.32	-	23.8	100.00		
10000	3.25	3.25	0.500	-		Upper Marker

© Agilent Technologies, Inc. 2017

E2: covaris micro tube 5 120 sec R2

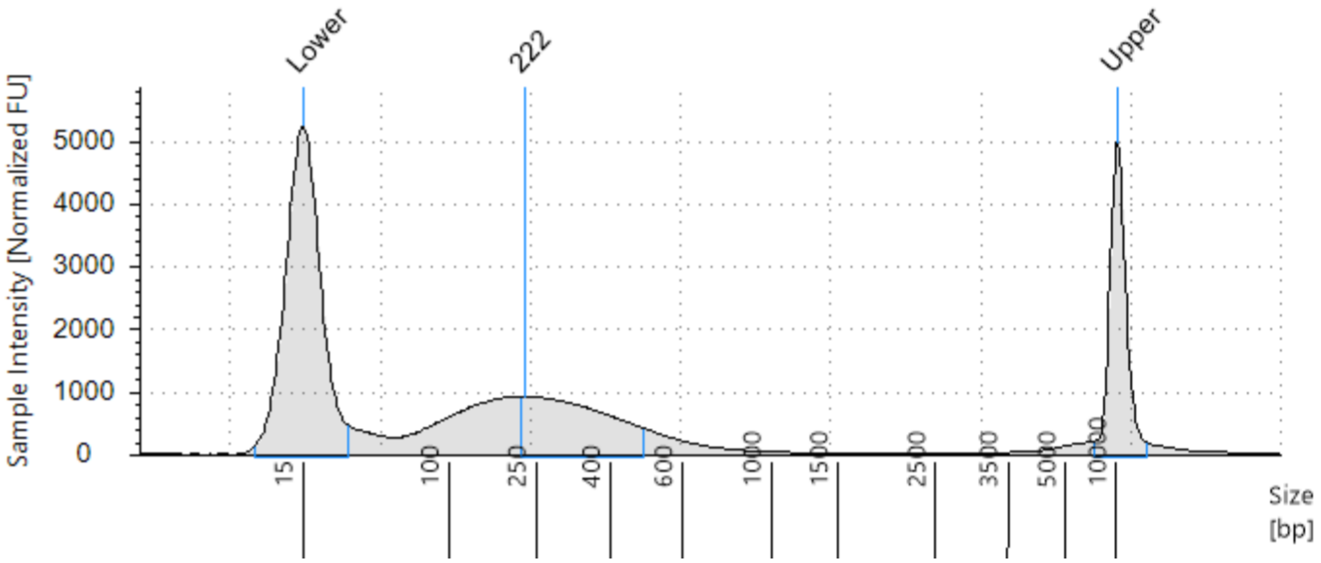

Sample Table

Well	Conc. [ng/µl]	Sample Description	Alert	Observations
E2	3.47	covaris micro tube 5 120 sec R2		

Peak Table

Size [bp]	Calibrated Conc. [ng/µl]	Assigned Conc. [ng/µl]	Peak Molarity [nmol/l]	% Integrated Area	Peak Comment	Observations
15	7.11	-	729	-		Lower Marker
222	3.47	-	24.1	100.00		
10000	3.25	3.25	0.500	-		Upper Marker

© Agilent Technologies, Inc. 2017

F2: covaris micro tube 6 120 sec R2

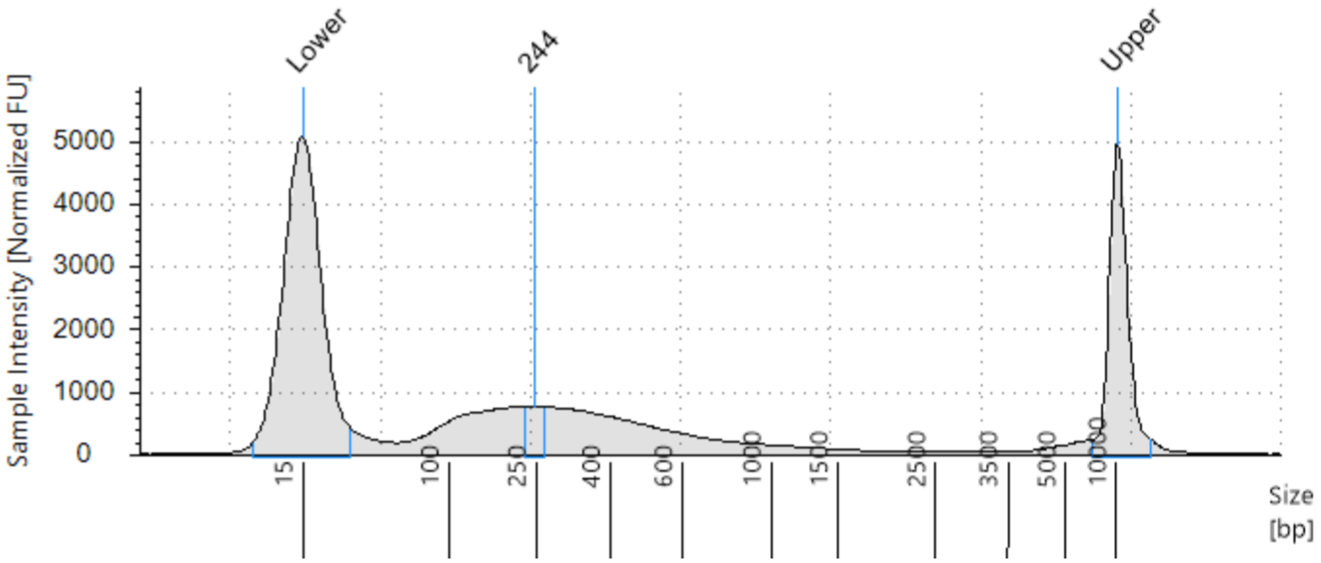

Sample Table

Well	Conc. [ng/µl]	Sample Description	Alert	Observations
F2	0.532	covaris micro tube 6 120 sec R2		

Peak Table

Size [bp]	Calibrated Conc. [ng/µl]	Assigned Conc. [ng/µl]	Peak Molarity [nmol/l]	% Integrated Area	Peak Comment	Observations
15	6.89	-	706	-		Lower Marker
244	0.532	-	3.35	100.00		
10000	3.25	3.25	0.500	-		Upper Marker

© Agilent Technologies, Inc. 2017

G2: covaris micro tube 7 120 sec R2

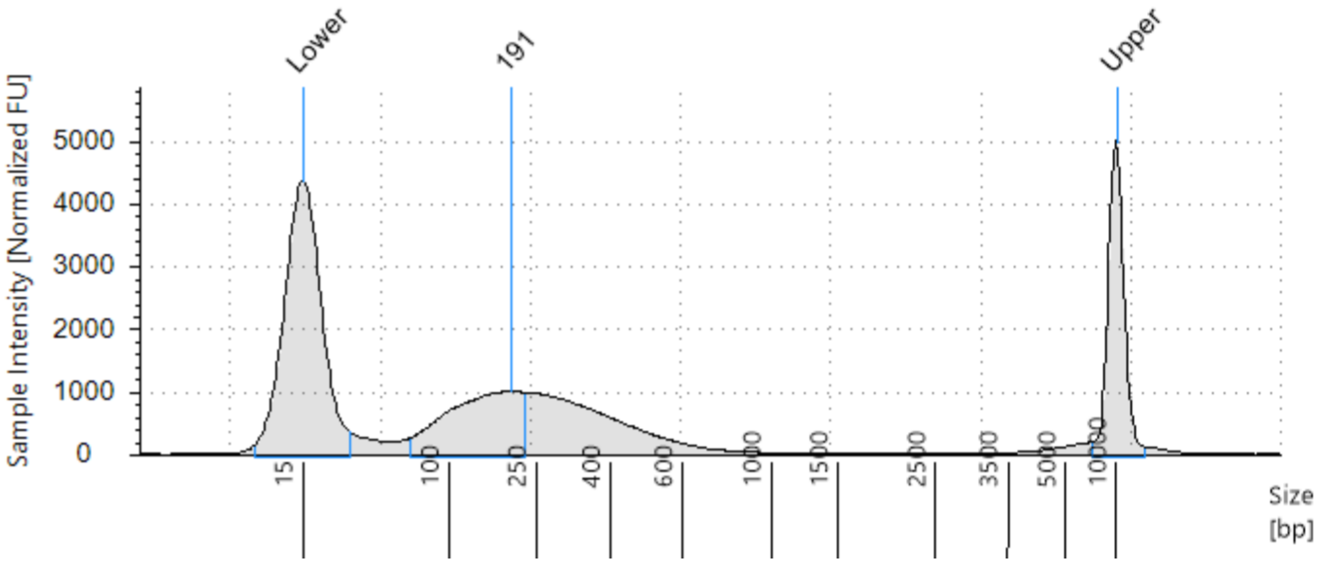

Sample Table

Well	Conc. [ng/µl]	Sample Description	Alert	Observations
G2	3.37	covaris micro tube 7 120 sec R2		

Peak Table

Size [bp]	Calibrated Conc. [ng/µl]	Assigned Conc. [ng/µl]	Peak Molarity [nmol/l]	% Integrated Area	Peak Comment	Observations
15	6.29	-	645	-		Lower Marker
191	3.37	-	27.1	100.00		
10000	3.25	3.25	0.500	-		Upper Marker

© Agilent Technologies, Inc. 2017

H2: covaris micro tube 8 120 sec R2

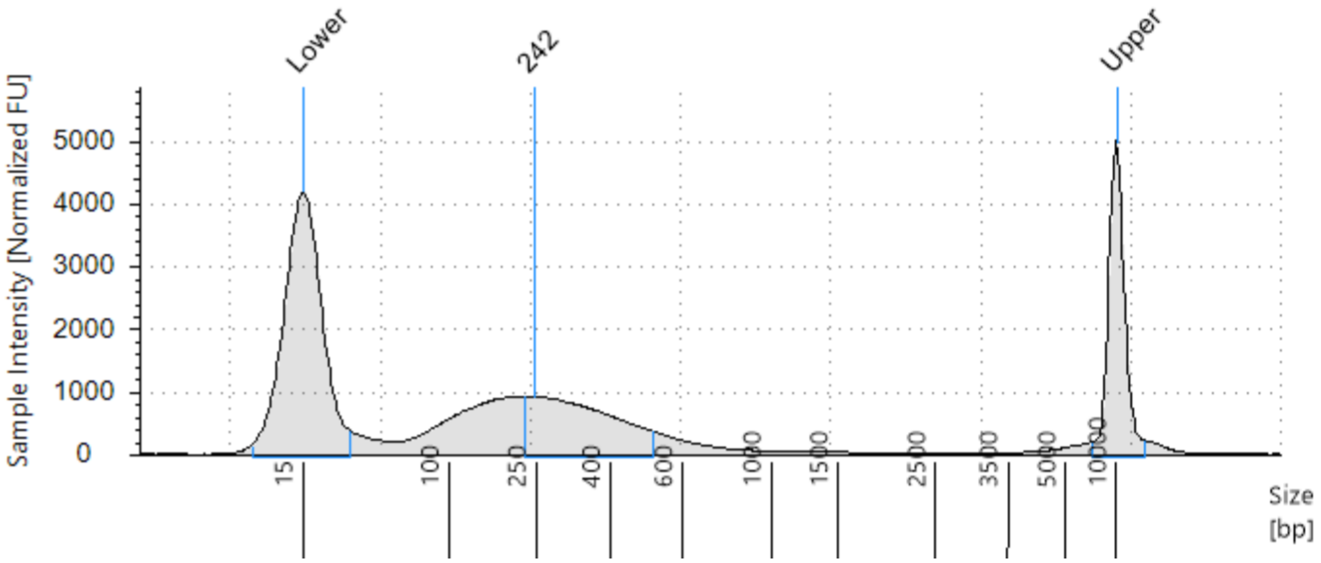

Sample Table

Well	Conc. [ng/µl]	Sample Description	Alert	Observations
H2	3.62	covaris micro tube 8 120 sec R2		

Peak Table

Size [bp]	Calibrated Conc. [ng/µl]	Assigned Conc. [ng/µl]	Peak Molarity [nmol/l]	% Integrated Area	Peak Comment	Observations
15	6.29	-	645	-		Lower Marker
242	3.62	-	23.0	100.00		
10000	3.25	3.25	0.500	-		Upper Marker

© Agilent Technologies, Inc. 2017

Filename: 2019-06-03-02LE220 covaris micro tube , 80 sec 120 sec R3.D5000

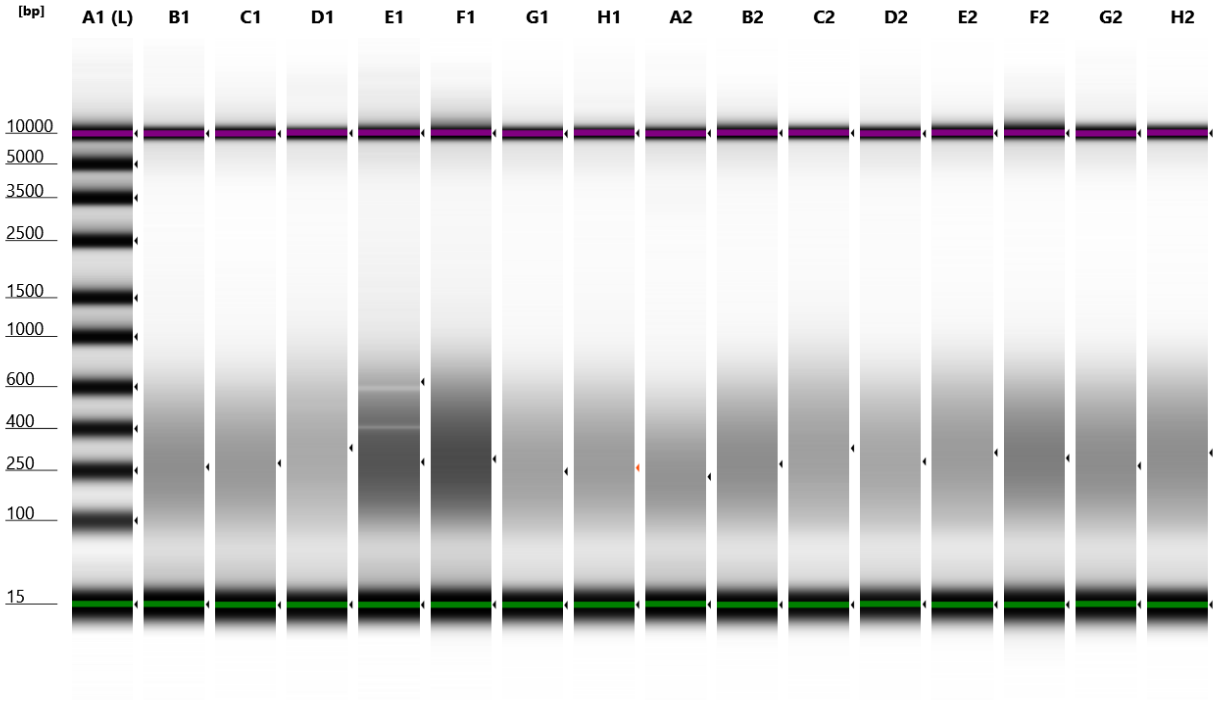

Default image (Contrast 100%)

Sample Info

Well	Conc. [ng/µl]	Sample Description	Alert	Observations
A1	30.6	Ladder		Ladder
B1	3.57	covaris micro tube 1 80 sec R3		
C1	3.28	covaris micro tube 2 80 sec R3		
D1	0.228	covaris micro tube 3 80 sec R3		
E1	6.98	covaris micro tube 4 80 sec R3		
F1	0.843	covaris micro tube 5 80 sec R3		
G1	0.700	covaris micro tube 6 80 sec R3		
H1	3.07	covaris micro tube 7 80 sec R3		
A2	3.35	covaris micro tube 1 120 sec R3		
B2	0.960	covaris micro tube 2 120 sec R3		
C2	0.348	covaris micro tube 3 120 sec R3		
D2	3.11	covaris micro tube 4 120 sec R3		
E2	0.683	covaris micro tube 5 120 sec R3		
F2	0.887	covaris micro tube 6 120 sec R3		
G2	3.21	covaris micro tube 7 120 sec R3		
H2	0.729	covaris micro tube 8 120 sec R3		

 © Agilent Technologies, Inc. 2017

A1: Ladder

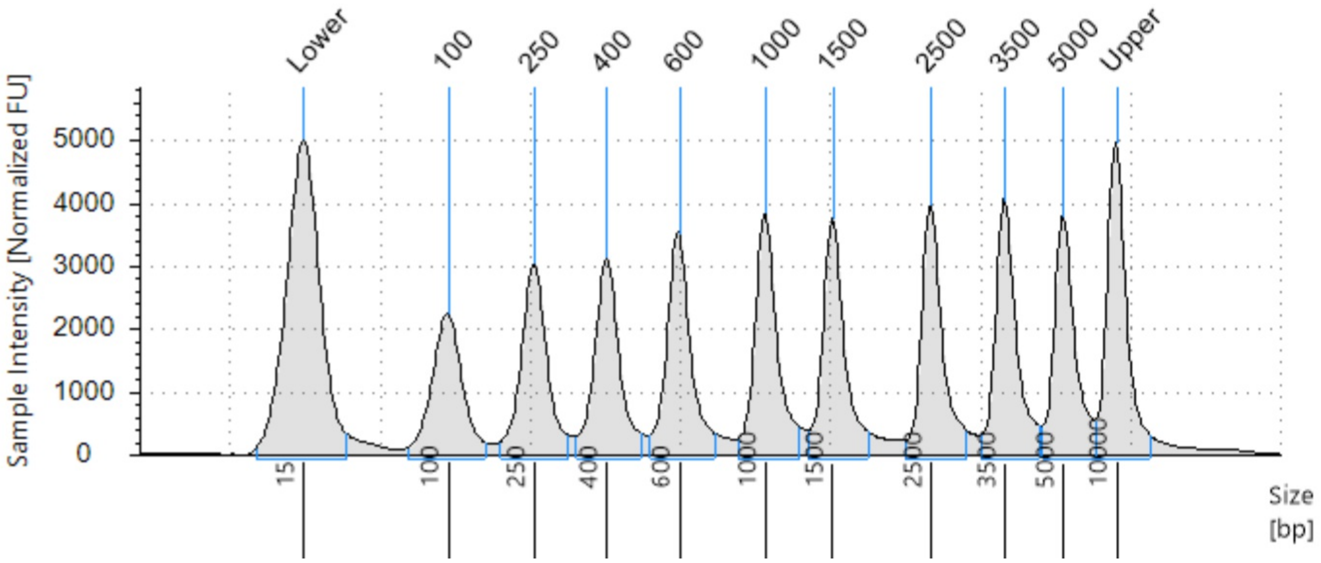

Sample Table

Well	Conc. [ng/µl]	Sample Description	Alert	Observations
A1	30.6	Ladder		Ladder

Peak Table

Size [bp]	Calibrated Conc. [ng/µl]	Assigned Conc. [ng/µl]	Peak Molarity [nmol/l]	% Integrated Area	Peak Comment	Observations
15	6.38	-	654	-		Lower Marker
100	2.87	-	44.1	9.36		
250	3.29	-	20.3	10.75		
400	3.27	-	12.6	10.66		
600	3.54	-	9.09	11.57		
1000	3.63	-	5.59	11.86		
1500	3.44	-	3.53	11.23		
2500	3.56	-	2.19	11.61		
3500	3.63	-	1.60	11.85		
5000	3.40	-	1.05	11.09		
10000	3.25	3.25	0.500	-		Upper Marker

 © Agilent Technologies, Inc. 2017

B2: covaris micro tube 2 120 sec R3

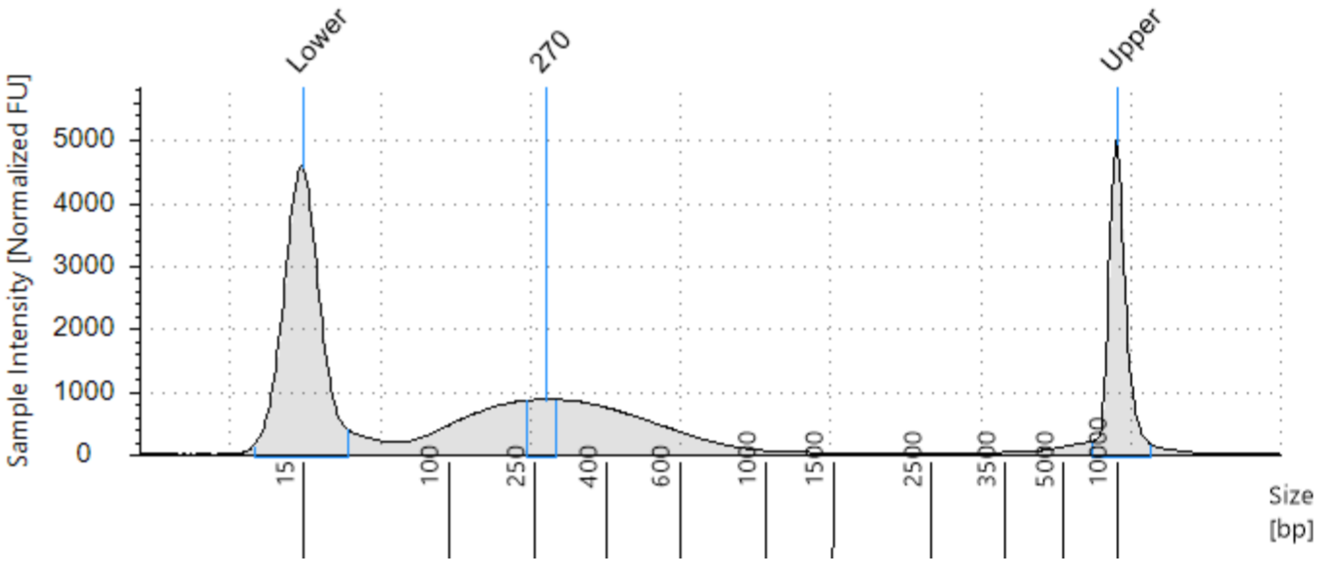

Sample Table

Well	Conc. [ng/µl]	Sample Description	Alert	Observations
B2	0.960	covaris micro tube 2 120 sec R3		

Peak Table

Size [bp]	Calibrated Conc. [ng/µl]	Assigned Conc. [ng/µl]	Peak Molarity [nmol/l]	% Integrated Area	Peak Comment	Observations
15	6.10	-	626	-		Lower Marker
270	0.960	-	5.48	100.00		
10000	3.25	3.25	0.500	-		Upper Marker

© Agilent Technologies, Inc. 2017

C2: covaris micro tube 3 120 sec R3

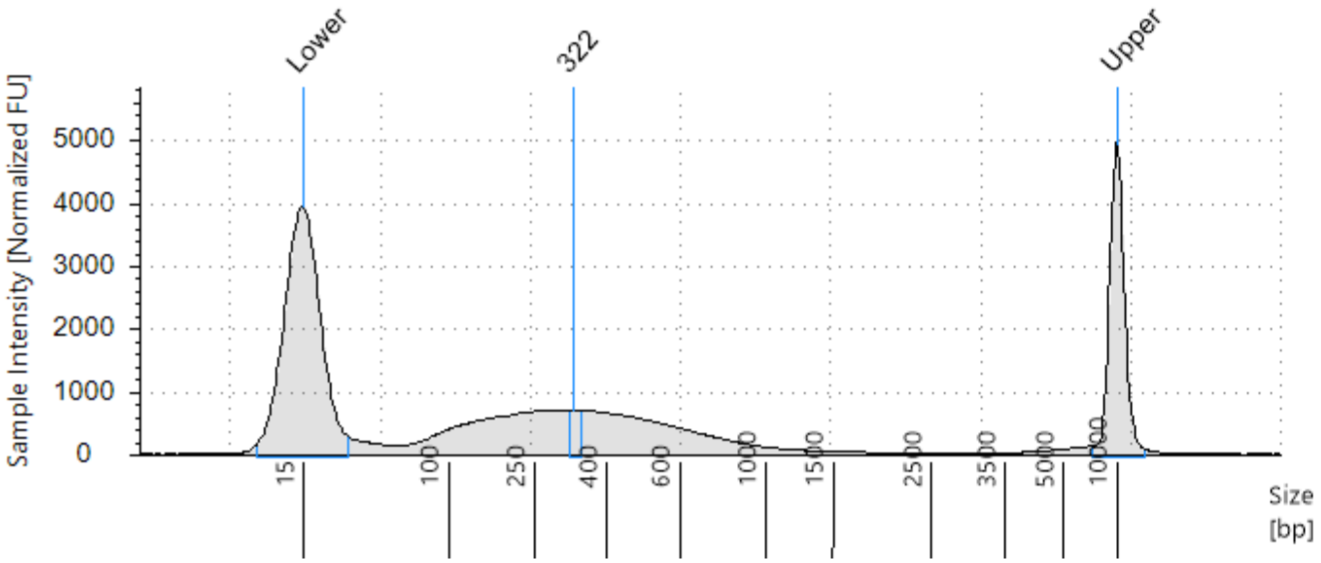

Sample Table

Well	Conc. [ng/µl]	Sample Description	Alert	Observations
C2	0.348	covaris micro tube 3 120 sec R3		

Peak Table

Size [bp]	Calibrated Conc. [ng/µl]	Assigned Conc. [ng/µl]	Peak Molarity [nmol/l]	% Integrated Area	Peak Comment	Observations
15	5.98	-	613	-		Lower Marker
322	0.348	-	1.67	100.00		
10000	3.25	3.25	0.500	-		Upper Marker

© Agilent Technologies, Inc. 2017

D2: covaris micro tube 4 120 sec R3

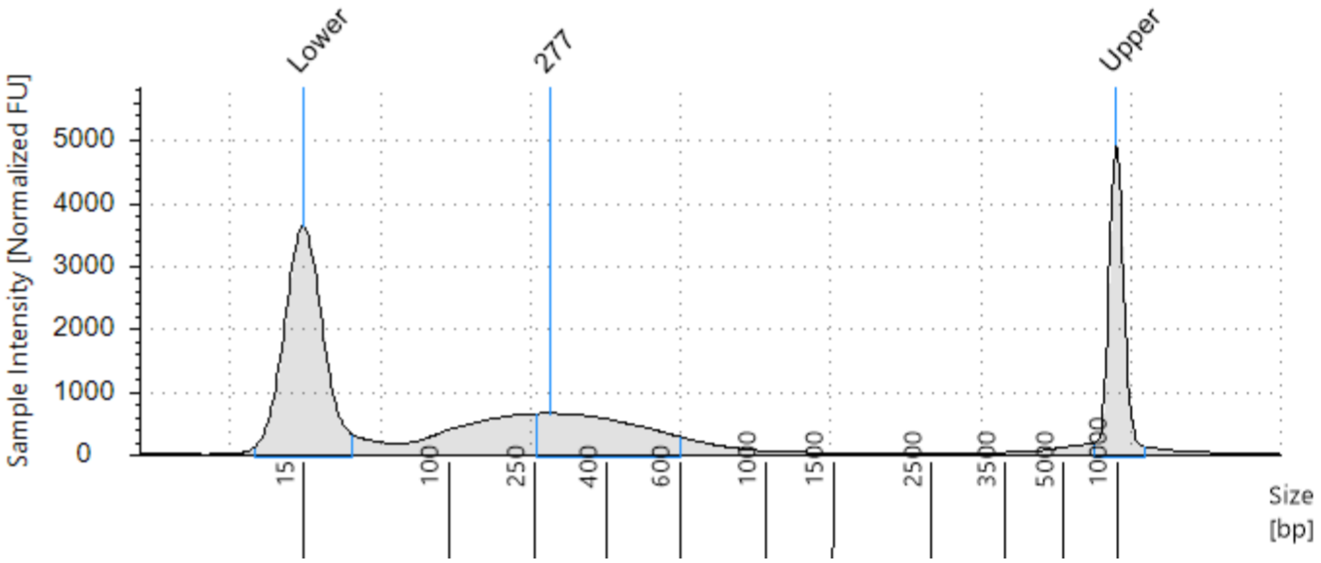

Sample Table

Well	Conc. [ng/µl]	Sample Description	Alert	Observations
D2	3.11	covaris micro tube 4 120 sec R3		

Peak Table

Size [bp]	Calibrated Conc. [ng/µl]	Assigned Conc. [ng/µl]	Peak Molarity [nmol/l]	% Integrated Area	Peak Comment	Observations
15	5.78	-	593	-		Lower Marker
277	3.11	-	17.3	100.00		
10000	3.25	3.25	0.500	-		Upper Marker

© Agilent Technologies, Inc. 2017
