# Supplementary material for: 96 sample parallel acoustic fragmentation for high throughput next generation sequencing library preparation
Source: PLoS One. 2026 Feb 17;21(2):e0341139. doi: 10.1371/journal.pone.0341139 (PMC12912608; doi:10.1371/journal.pone.0341139)
Supplement: S2 Fig — (ZIP) [file pone.0341139.s002.zip › Figure 1 Raw Data/Covaris microTUBE 240 seconds.pdf]

Filename: 2019-09-03-01- LE220,covaris microtube, 240 sec.D5000

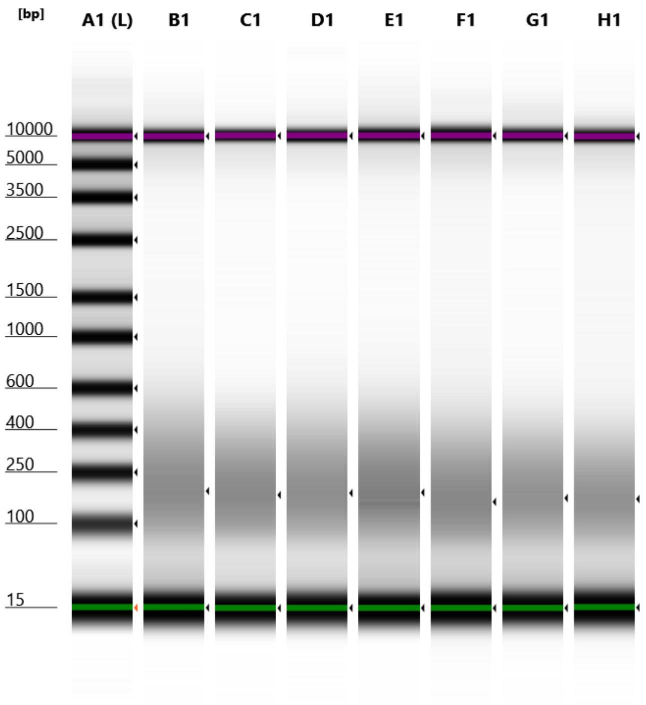

Default image (Contrast 100%)

Sample Info

Well	Conc. [ng/µl]	Sample Description	Alert	Observations
A1	29.8	Ladder		Ladder
B1	3.19	LE220 S1 covaris microtube 240sec R1		
C1	3.11	LE220 S2 covaris microtube 240sec R1		
D1	5.39	LE220 S3 covaris microtube 240sec R1		
E1	6.02	LE220 S4 covaris microtube 240sec R1		
F1	5.19	LE220 S5 covaris microtube 240sec R1		
G1	5.07	LE220 S6 covaris microtube 240sec R1		
H1	4.93	LE220 S7 covaris microtube 240sec R1		

© Agilent Technologies, Inc. 2017

A1: Ladder

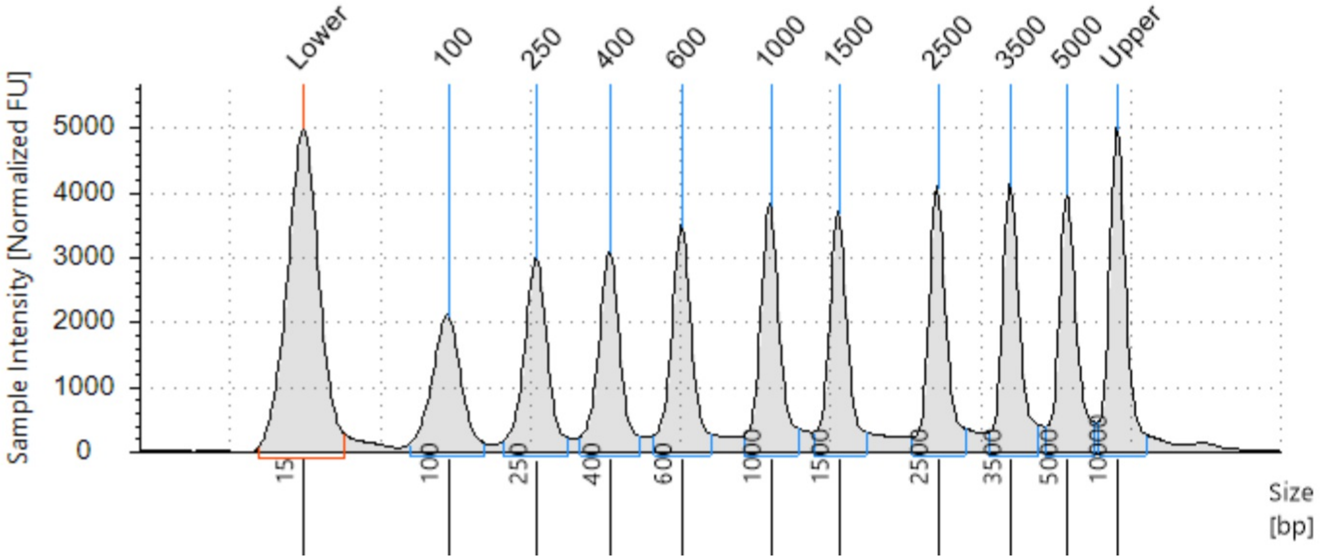

Sample Table

Well	Conc. [ng/µl]	Sample Description	Alert	Observations
A1	29.8	Ladder		Ladder

Peak Table

Size [bp]	Calibrated Conc. [ng/µl]	Assigned Conc. [ng/µl]	Peak Molarity [nmol/l]	% Integrated Area	Peak Comment	Observations
15	6.85	-	703	-		Lower Marker
100	2.91	-	44.7	9.77		
250	3.29	-	20.2	11.04		
400	3.17	-	12.2	10.65		
600	3.39	-	8.68	11.38		
1000	3.50	-	5.38	11.75		
1500	3.25	-	3.33	10.91		
2500	3.43	-	2.11	11.54		
3500	3.46	-	1.52	11.61		
5000	3.37	-	1.04	11.34		
10000	3.25	3.25	0.500	-		Upper Marker

© Agilent Technologies, Inc. 2017

B1: LE220 S1 covaris microtube 240sec R1

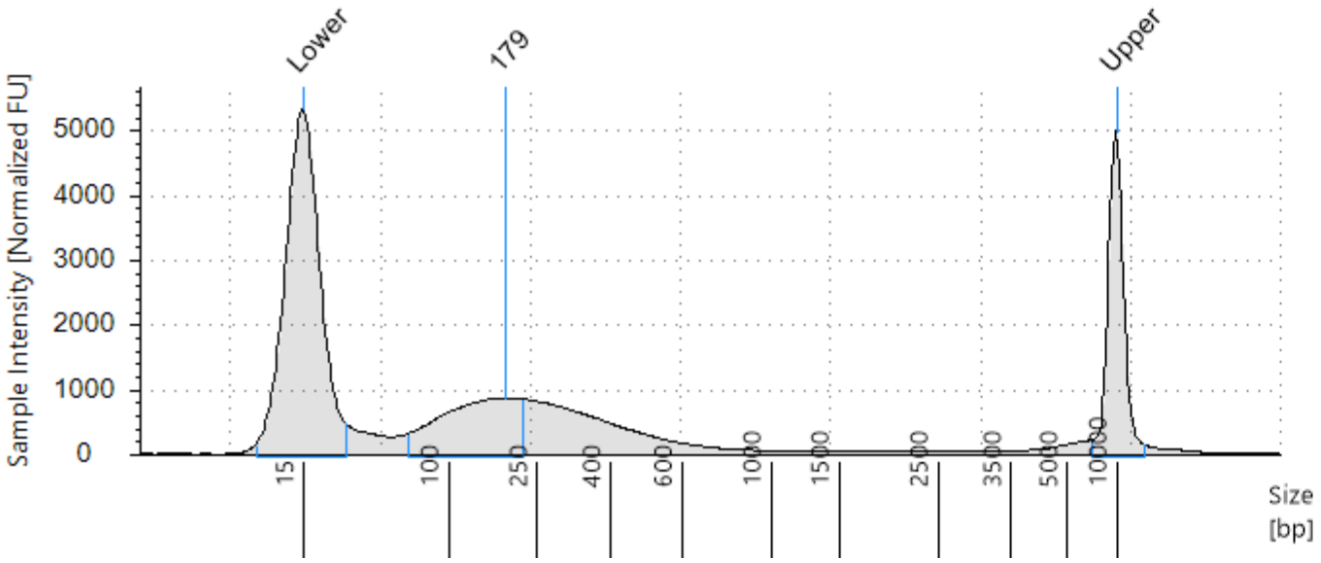

Sample Table

Well	Conc. [ng/µl]	Sample Description	Alert	Observations
B1	3.19	LE220 S1 covaris microtube 240sec R1		

Peak Table

Size [bp]	Calibrated Conc. [ng/µl]	Assigned Conc. [ng/µl]	Peak Molarity [nmol/l]	% Integrated Area	Peak Comment	Observations
15	7.48	-	767	-		Lower Marker
179	3.19	-	27.5	100.00		
10000	3.25	3.25	0.500	-		Upper Marker

© Agilent Technologies, Inc. 2017

C1: LE220 S2 covaris microtube 240sec R1

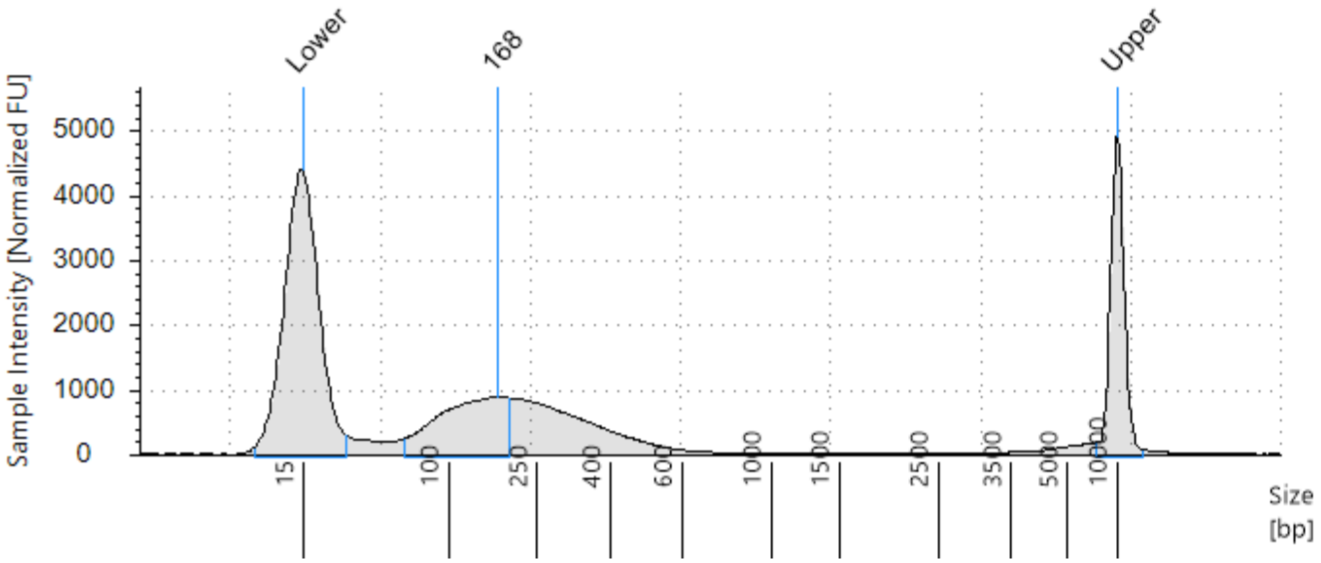

Sample Table

Well	Conc. [ng/µl]	Sample Description	Alert	Observations
C1	3.11	LE220 S2 covaris microtube 240sec R1		

Peak Table

Size [bp]	Calibrated Conc. [ng/µl]	Assigned Conc. [ng/µl]	Peak Molarity [nmol/l]	% Integrated Area	Peak Comment	Observations
15	6.95	-	713	-		Lower Marker
168	3.11	-	28.6	100.00		
10000	3.25	3.25	0.500	-		Upper Marker

© Agilent Technologies, Inc. 2017

D1: LE220 S3 covaris microtube 240sec R1

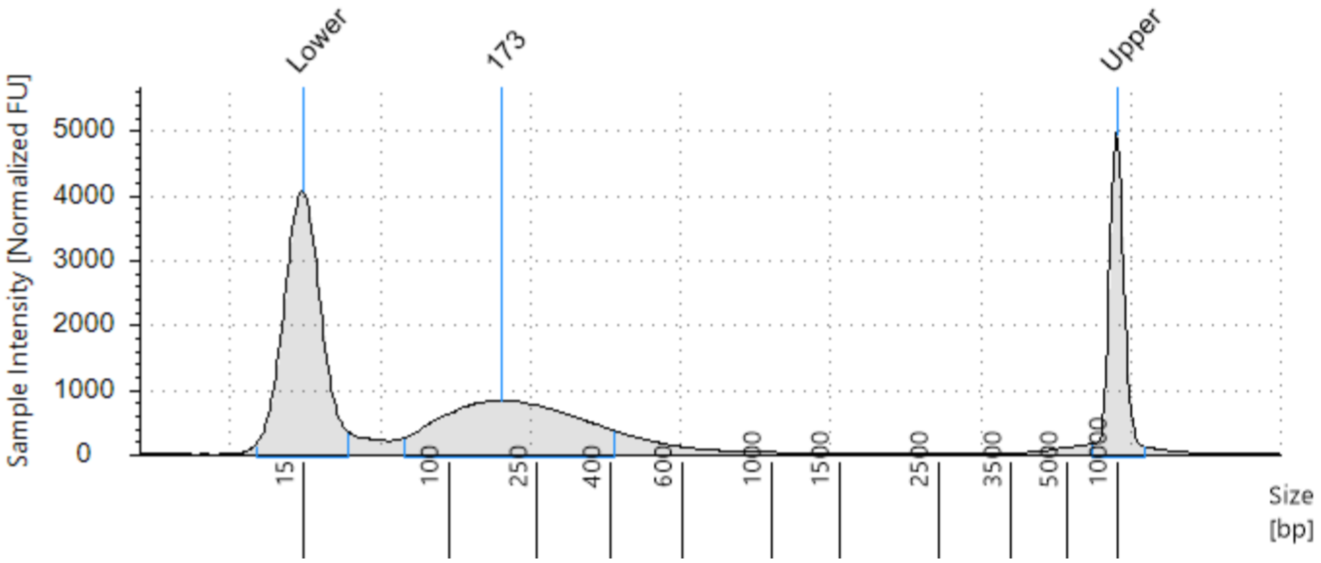

Sample Table

Well	Conc. [ng/µl]	Sample Description	Alert	Observations
D1	5.39	LE220 S3 covaris microtube 240sec R1		

Peak Table

Size [bp]	Calibrated Conc. [ng/µl]	Assigned Conc. [ng/µl]	Peak Molarity [nmol/l]	% Integrated Area	Peak Comment	Observations
15	6.28	-	644	-		Lower Marker
173	5.39	-	47.8	100.00		
10000	3.25	3.25	0.500	-		Upper Marker

 © Agilent Technologies, Inc. 2017

E1: LE220 S4 covaris microtube 240sec R1

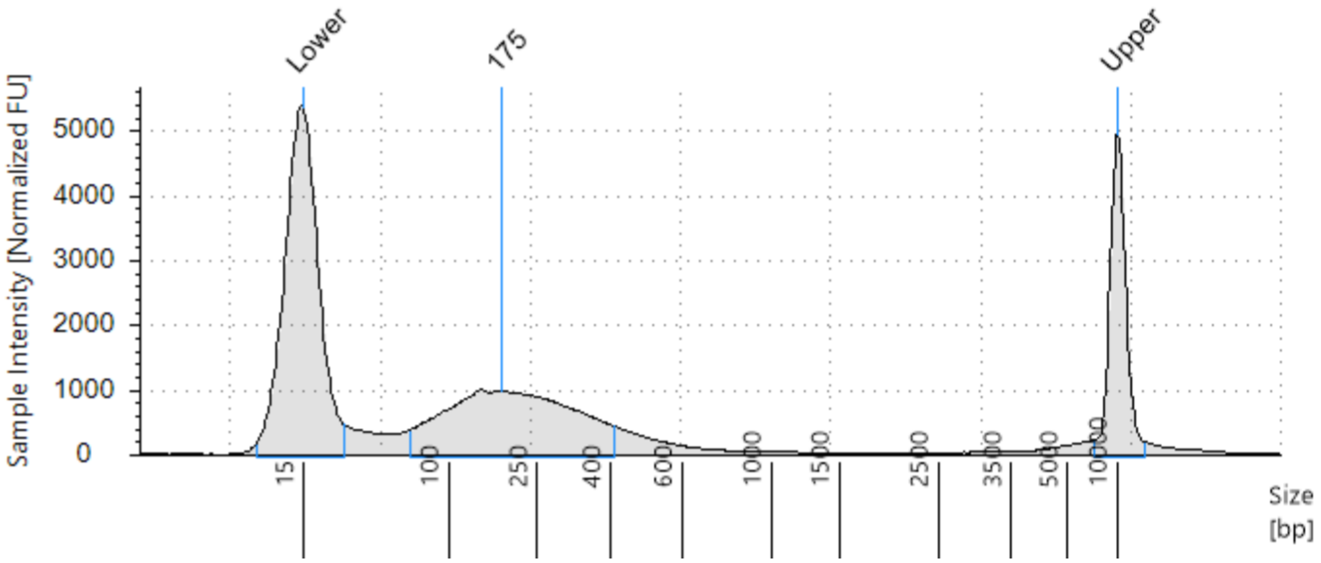

Sample Table

Well	Conc. [ng/µl]	Sample Description	Alert	Observations
E1	6.02	LE220 S4 covaris microtube 240sec R1		

Peak Table

Size [bp]	Calibrated Conc. [ng/µl]	Assigned Conc. [ng/µl]	Peak Molarity [nmol/l]	% Integrated Area	Peak Comment	Observations
15	7.22	-	740	-		Lower Marker
175	6.02	-	53.0	100.00		
10000	3.25	3.25	0.500	-		Upper Marker

 © Agilent Technologies, Inc. 2017

F1: LE220 S5 covaris microtube 240sec R1

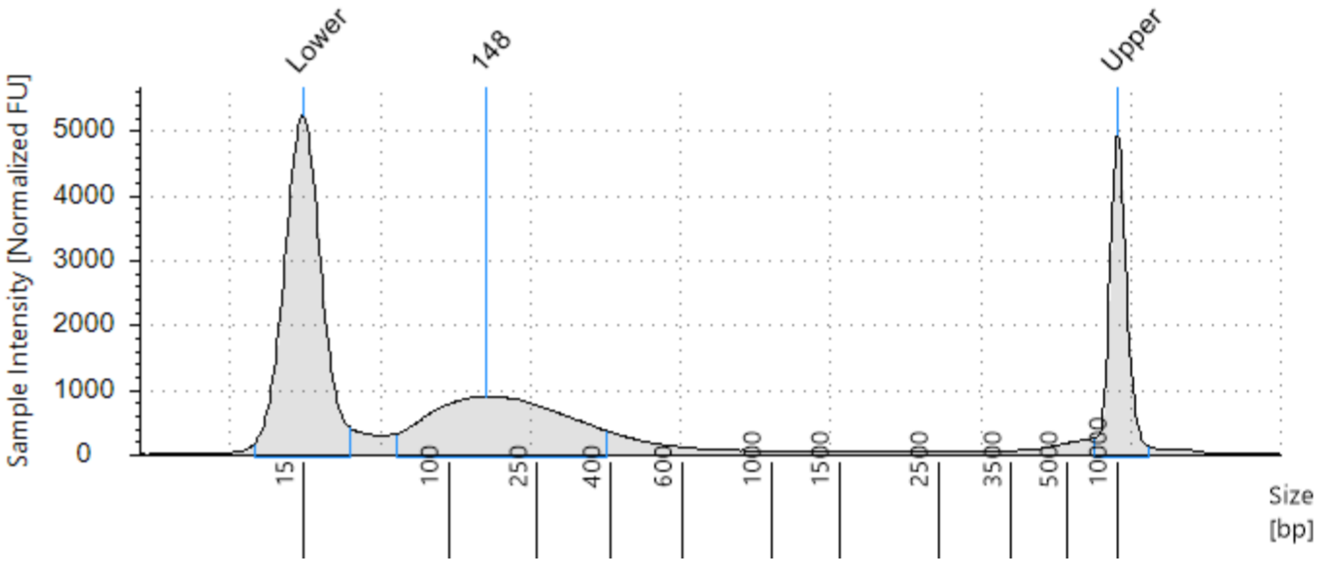

Sample Table

Well	Conc. [ng/µl]	Sample Description	Alert	Observations
F1	5.19	LE220 S5 covaris microtube 240sec R1		

Peak Table

Size [bp]	Calibrated Conc. [ng/µl]	Assigned Conc. [ng/µl]	Peak Molarity [nmol/l]	% Integrated Area	Peak Comment	Observations
15	7.13	-	732	-		Lower Marker
148	5.19	-	53.8	100.00		
10000	3.25	3.25	0.500	-		Upper Marker

© Agilent Technologies, Inc. 2017

G1: LE220 S6 covaris microtube 240sec R1

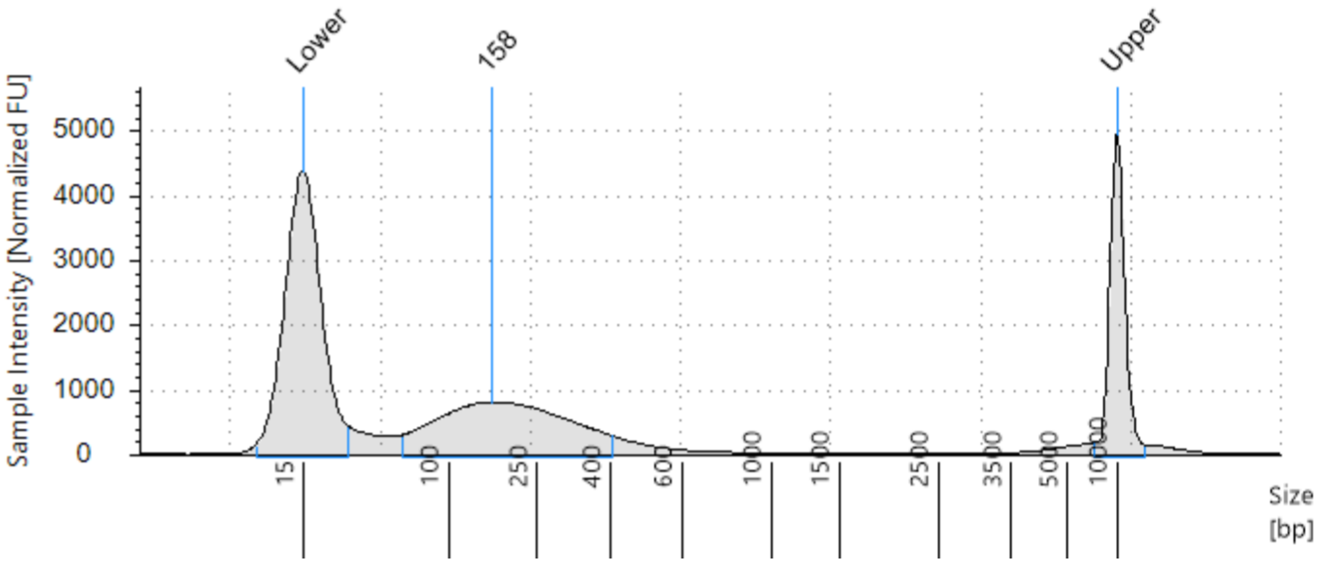

Sample Table

Well	Conc. [ng/µl]	Sample Description	Alert	Observations
G1	5.07	LE220 S6 covaris microtube 240sec R1		

Peak Table

Size [bp]	Calibrated Conc. [ng/µl]	Assigned Conc. [ng/µl]	Peak Molarity [nmol/l]	% Integrated Area	Peak Comment	Observations
15	6.54	-	671	-		Lower Marker
158	5.07	-	49.3	100.00		
10000	3.25	3.25	0.500	-		Upper Marker

© Agilent Technologies, Inc. 2017

H1: LE220 S7 covaris microtube 240sec R1

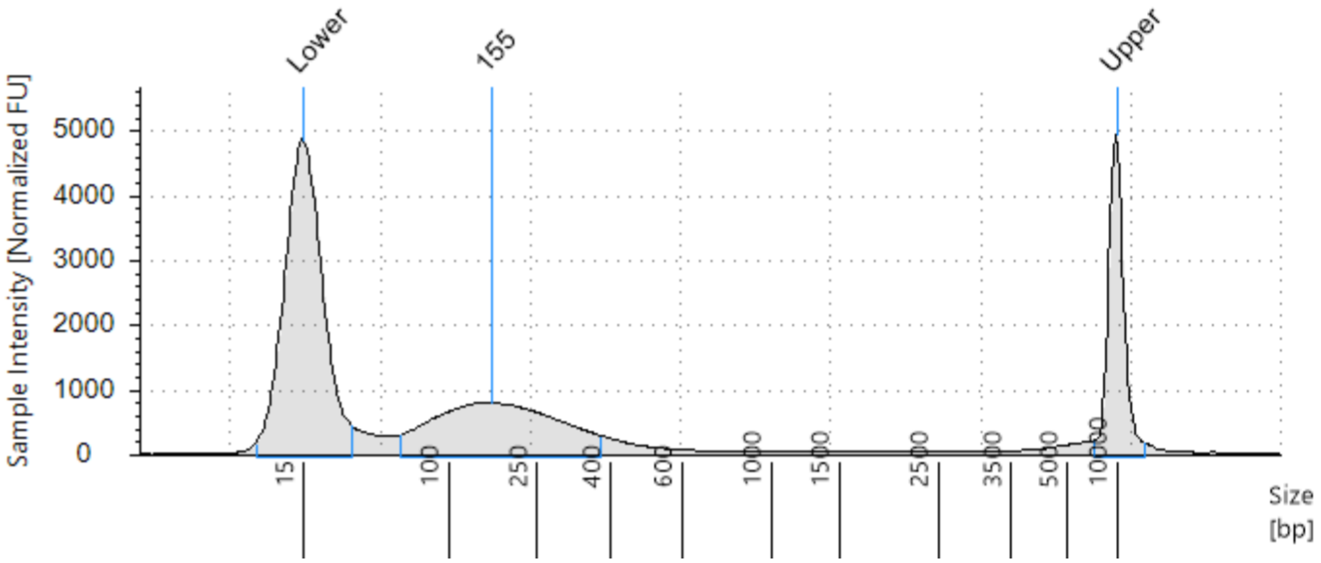

Sample Table

Well	Conc. [ng/µl]	Sample Description	Alert	Observations
H1	4.93	LE220 S7 covaris microtube 240sec R1		

Peak Table

Size [bp]	Calibrated Conc. [ng/µl]	Assigned Conc. [ng/µl]	Peak Molarity [nmol/l]	% Integrated Area	Peak Comment	Observations
15	7.67	-	787	-		Lower Marker
155	4.93	-	49.0	100.00		
10000	3.25	3.25	0.500	-		Upper Marker

© Agilent Technologies, Inc. 2017

Filename: 2019-06-06-01- covaris micro tube R2, R3 240 sec.D5000

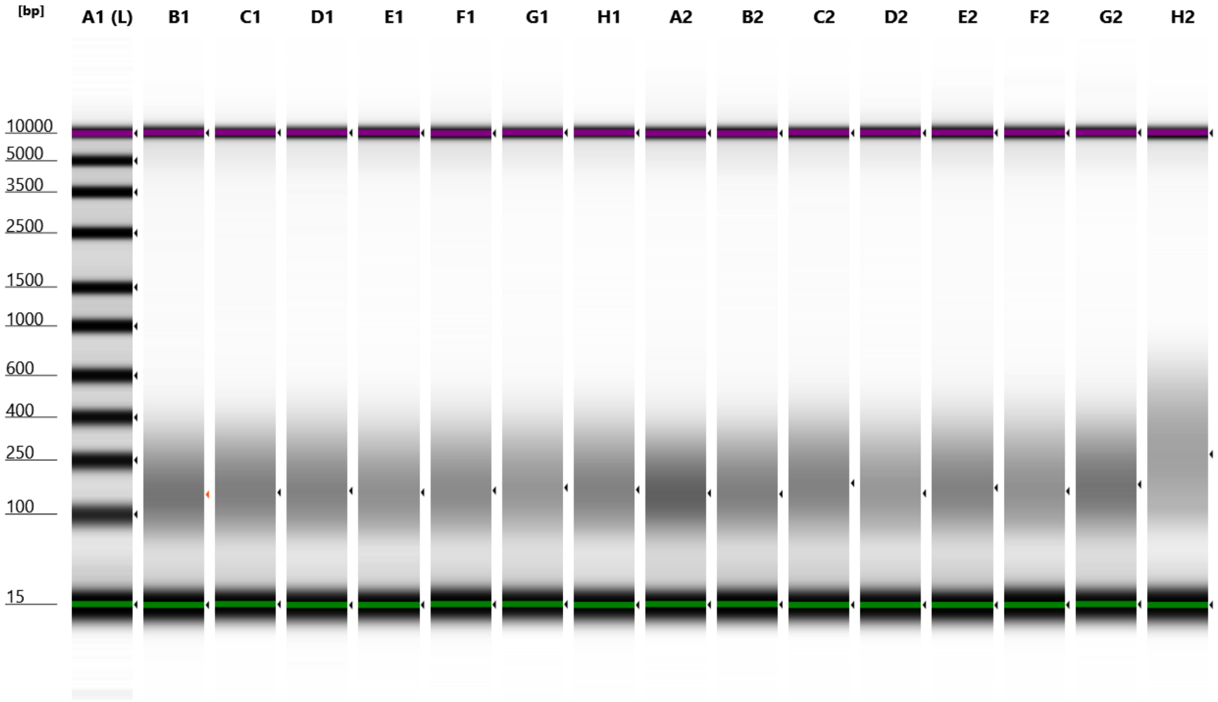

Default image (Contrast 100%)

Sample Info

Well	Conc. [ng/µl]	Sample Description	Alert	Observations
A1	39.1	Ladder		Ladder
B1	4.64	covaris micro tube1 R2 240 sec		
C1	7.58	covaris micro tube2 R2 240 sec		
D1	7.09	covaris micro tube3 R2 240 sec		
E1	5.86	covaris micro tube4 R2 240 sec		
F1	6.21	covaris micro tube5 R2 240 sec		
G1	6.12	covaris micro tube6 R2 240 sec		
H1	7.25	covaris micro tube7 R2 240 sec		
A2	8.94	covaris micro tube1 R3 240 sec		
B2	7.00	covaris micro tube2 R3 240 sec		
C2	4.35	covaris micro tube3 R3 240 sec		
D2	3.00	covaris micro tube4 R3 240 sec		
E2	6.71	covaris micro tube5 R3 240 sec		
F2	5.91	covaris micro tube6 R3 240 sec		
G2	8.36	covaris micro tube7 R3 240 sec		
H2	0.895	covaris micro tube8 R3 240 sec		

© Agilent Technologies, Inc. 2017

A1: Ladder

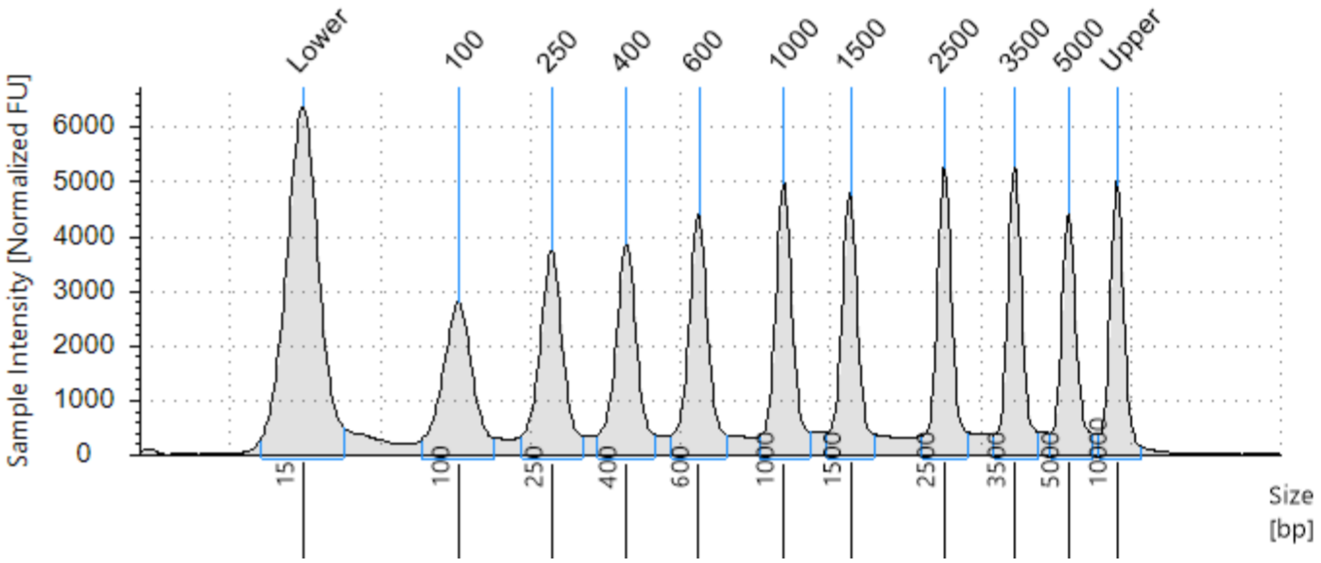

Sample Table

Well	Conc. [ng/µl]	Sample Description	Alert	Observations
A1	39.1	Ladder		Ladder

Peak Table

Size [bp]	Calibrated Conc. [ng/µl]	Assigned Conc. [ng/µl]	Peak Molarity [nmol/l]	% Integrated Area	Peak Comment	Observations
15	9.51	-	976	-		Lower Marker
100	4.43	-	68.2	11.34		
250	4.65	-	28.6	11.89		
400	4.43	-	17.1	11.34		
600	4.61	-	11.8	11.79		
1000	4.66	-	7.16	11.91		
1500	4.23	-	4.33	10.81		
2500	4.30	-	2.65	10.99		
3500	4.22	-	1.85	10.79		
5000	3.58	-	1.10	9.15		
10000	3.25	3.25	0.500	-		Upper Marker

© Agilent Technologies, Inc. 2017

B1: covaris micro tube1 R2 240 sec

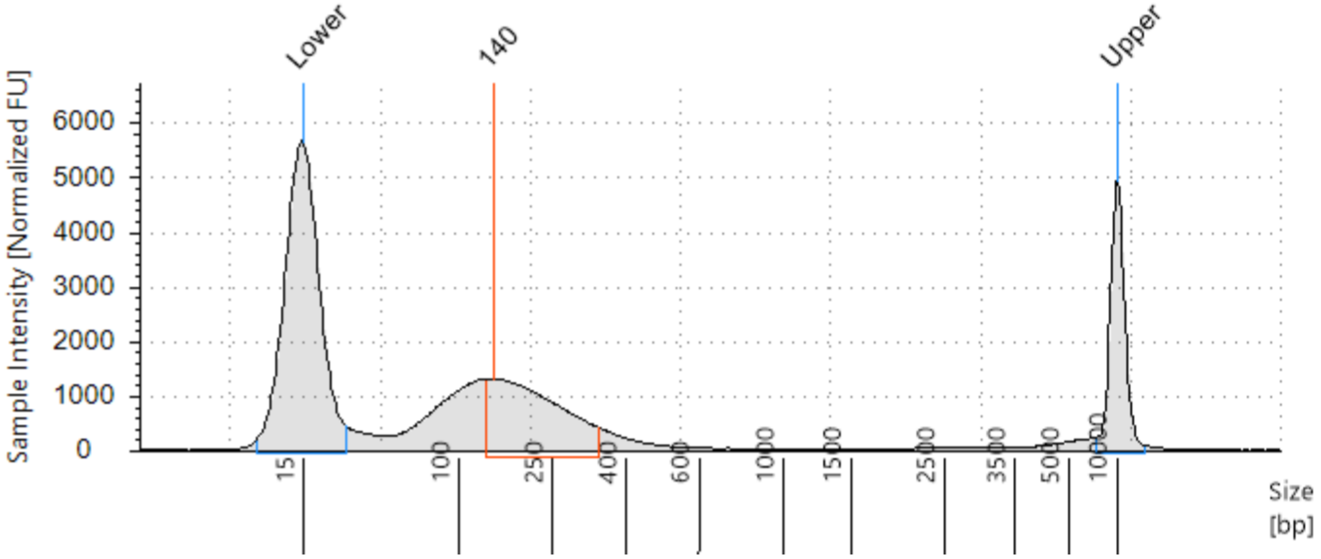

Sample Table

Well	Conc. [ng/µl]	Sample Description	Alert	Observations
B1	4.64	covaris micro tube1 R2 240 sec		

Peak Table

Size [bp]	Calibrated Conc. [ng/µl]	Assigned Conc. [ng/µl]	Peak Molarity [nmol/l]	% Integrated Area	Peak Comment	Observations
15	8.50	-	872	-		Lower Marker
140	4.64	-	50.9	100.00		
10000	3.25	3.25	0.500	-		Upper Marker

© Agilent Technologies, Inc. 2017

C1: covaris micro tube2 R2 240 sec

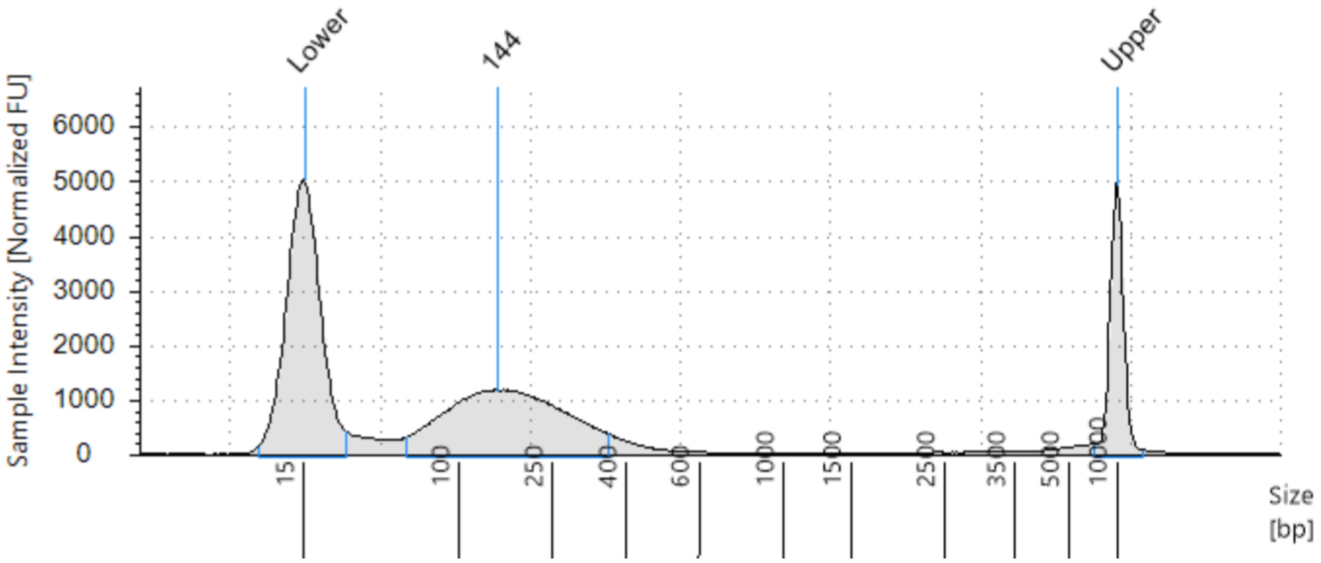

Sample Table

Well	Conc. [ng/µl]	Sample Description	Alert	Observations
C1	7.58	covaris micro tube2 R2 240 sec		

Peak Table

Size [bp]	Calibrated Conc. [ng/µl]	Assigned Conc. [ng/µl]	Peak Molarity [nmol/l]	% Integrated Area	Peak Comment	Observations
15	7.83	-	804	-		Lower Marker
144	7.58	-	81.0	100.00		
10000	3.25	3.25	0.500	-		Upper Marker

© Agilent Technologies, Inc. 2017

D1: covaris micro tube3 R2 240 sec

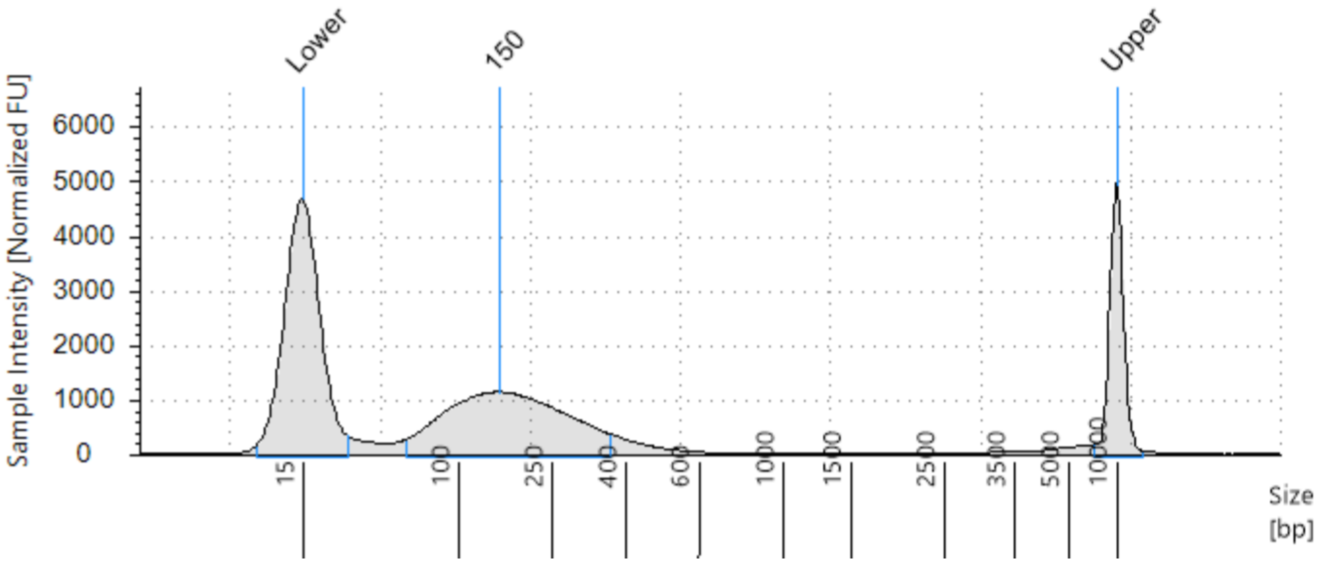

Sample Table

Well	Conc. [ng/µl]	Sample Description	Alert	Observations
D1	7.09	covaris micro tube3 R2 240 sec		

Peak Table

Size [bp]	Calibrated Conc. [ng/µl]	Assigned Conc. [ng/µl]	Peak Molarity [nmol/l]	% Integrated Area	Peak Comment	Observations
15	7.39	-	758	-		Lower Marker
150	7.09	-	72.8	100.00		
10000	3.25	3.25	0.500	-		Upper Marker

© Agilent Technologies, Inc. 2017

E1: covaris micro tube4 R2 240 sec

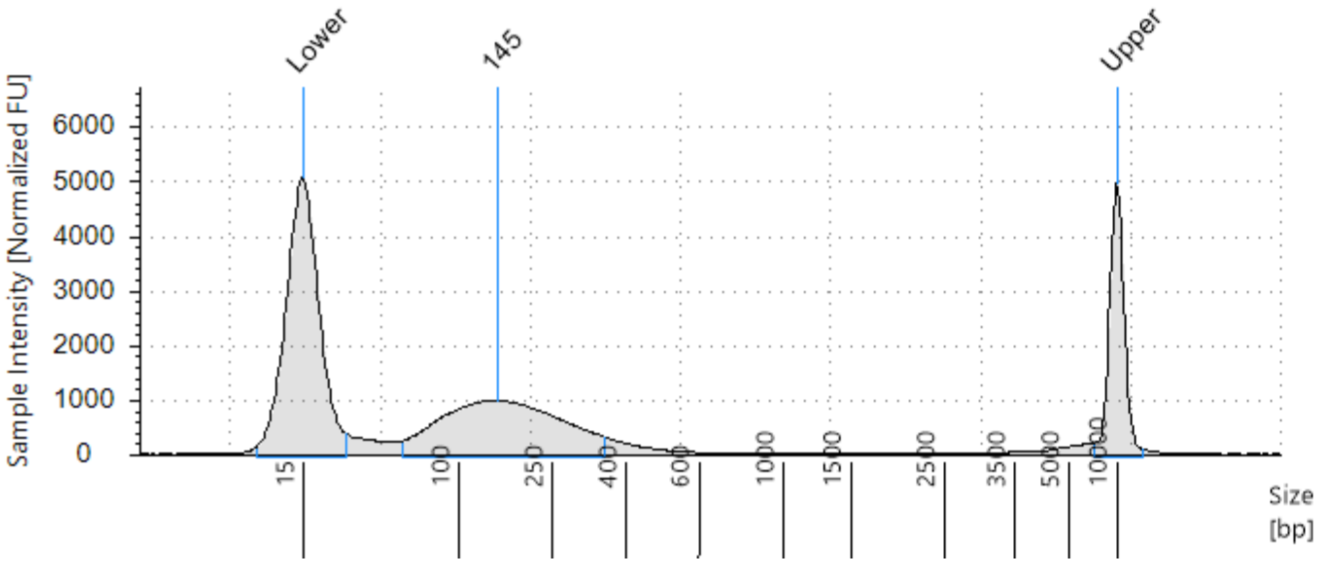

Sample Table

Well	Conc. [ng/µl]	Sample Description	Alert	Observations
E1	5.86	covaris micro tube4 R2 240 sec		

Peak Table

Size [bp]	Calibrated Conc. [ng/µl]	Assigned Conc. [ng/µl]	Peak Molarity [nmol/l]	% Integrated Area	Peak Comment	Observations
15	7.16	-	734	-		Lower Marker
145	5.86	-	62.0	100.00		
10000	3.25	3.25	0.500	-		Upper Marker

© Agilent Technologies, Inc. 2017

F1: covaris micro tube5 R2 240 sec

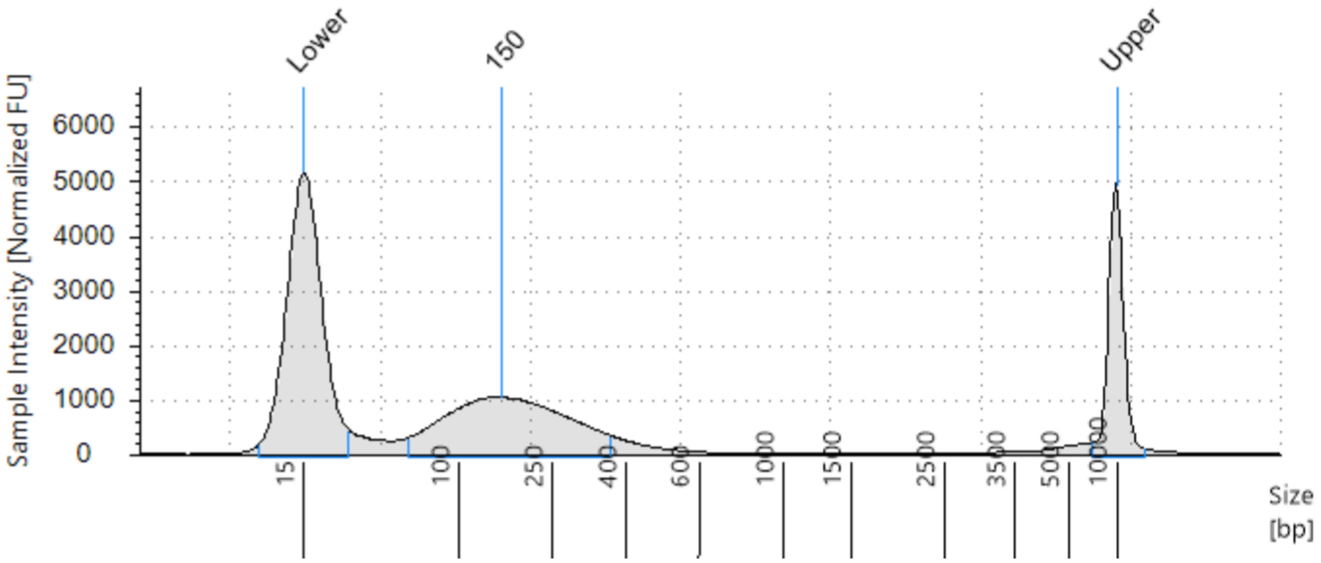

Sample Table

Well	Conc. [ng/µl]	Sample Description	Alert	Observations
F1	6.21	covaris micro tube5 R2 240 sec		

Peak Table

Size [bp]	Calibrated Conc. [ng/µl]	Assigned Conc. [ng/µl]	Peak Molarity [nmol/l]	% Integrated Area	Peak Comment	Observations
15	7.50	-	769	-		Lower Marker
150	6.21	-	63.8	100.00		
10000	3.25	3.25	0.500	-		Upper Marker

© Agilent Technologies, Inc. 2017

G1: covaris micro tube6 R2 240 sec

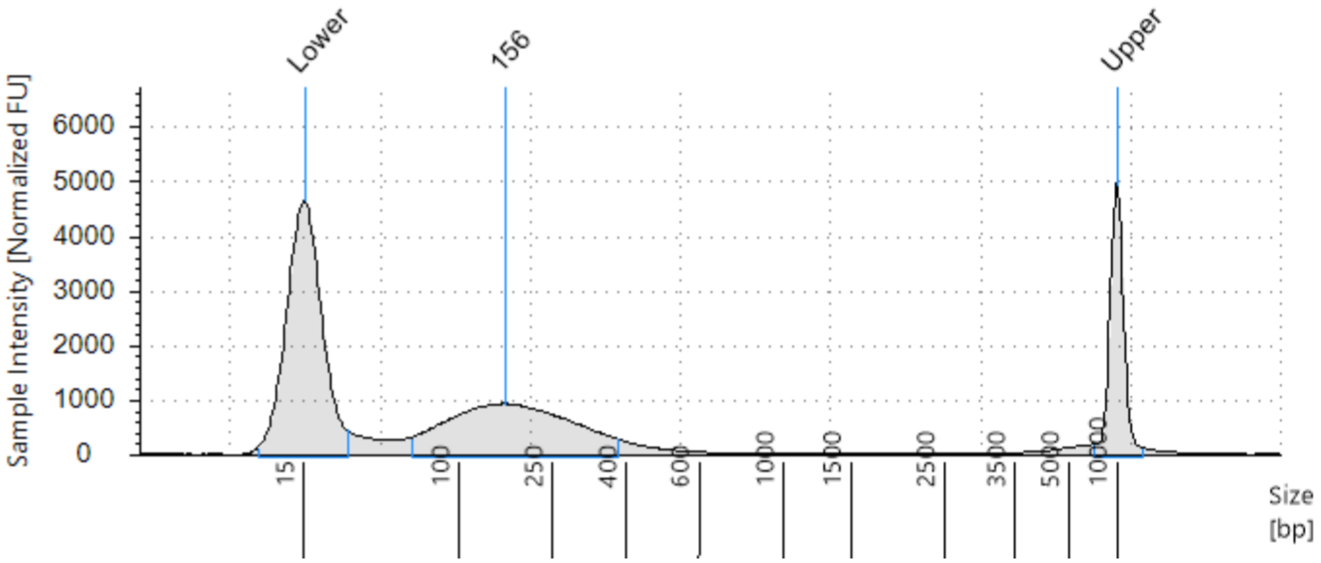

Sample Table

Well	Conc. [ng/µl]	Sample Description	Alert	Observations
G1	6.12	covaris micro tube6 R2 240 sec		

Peak Table

Size [bp]	Calibrated Conc. [ng/µl]	Assigned Conc. [ng/µl]	Peak Molarity [nmol/l]	% Integrated Area	Peak Comment	Observations
15	7.38	-	757	-		Lower Marker
156	6.12	-	60.3	100.00		
10000	3.25	3.25	0.500	-		Upper Marker

 © Agilent Technologies, Inc. 2017

H1: covaris micro tube7 R2 240 sec

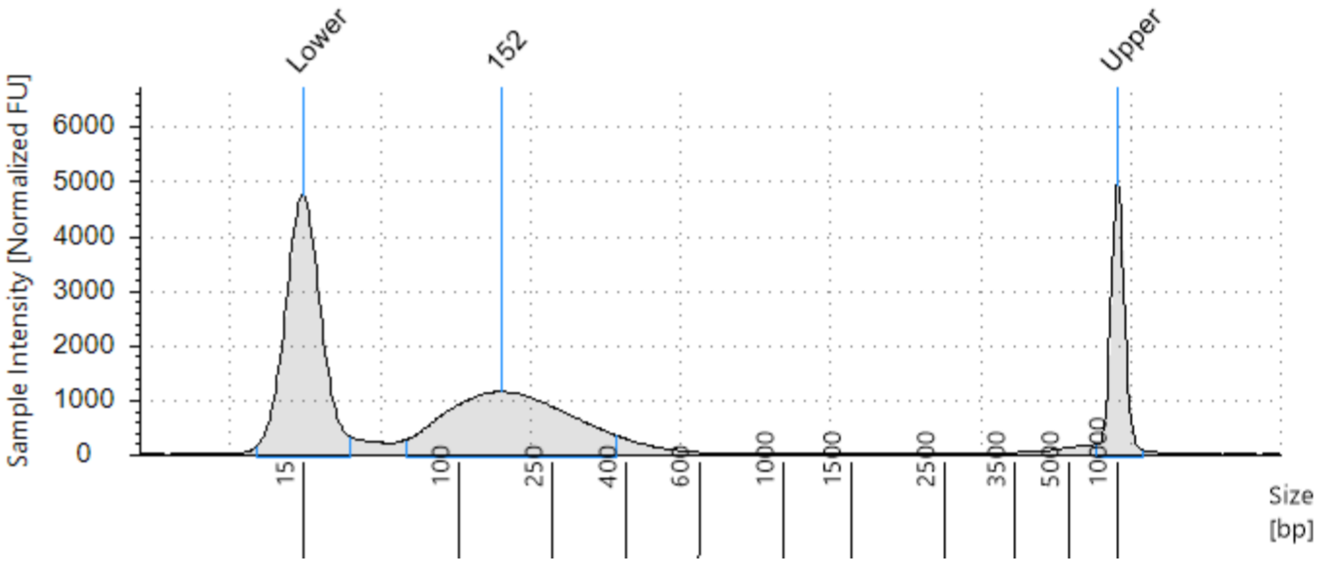

Sample Table

Well	Conc. [ng/µl]	Sample Description	Alert	Observations
H1	7.25	covaris micro tube7 R2 240 sec		

Peak Table

Size [bp]	Calibrated Conc. [ng/µl]	Assigned Conc. [ng/µl]	Peak Molarity [nmol/l]	% Integrated Area	Peak Comment	Observations
15	7.52	-	771	-		Lower Marker
152	7.25	-	73.3	100.00		
10000	3.25	3.25	0.500	-		Upper Marker

© Agilent Technologies, Inc. 2017

A2: covaris micro tube1 R3 240 sec

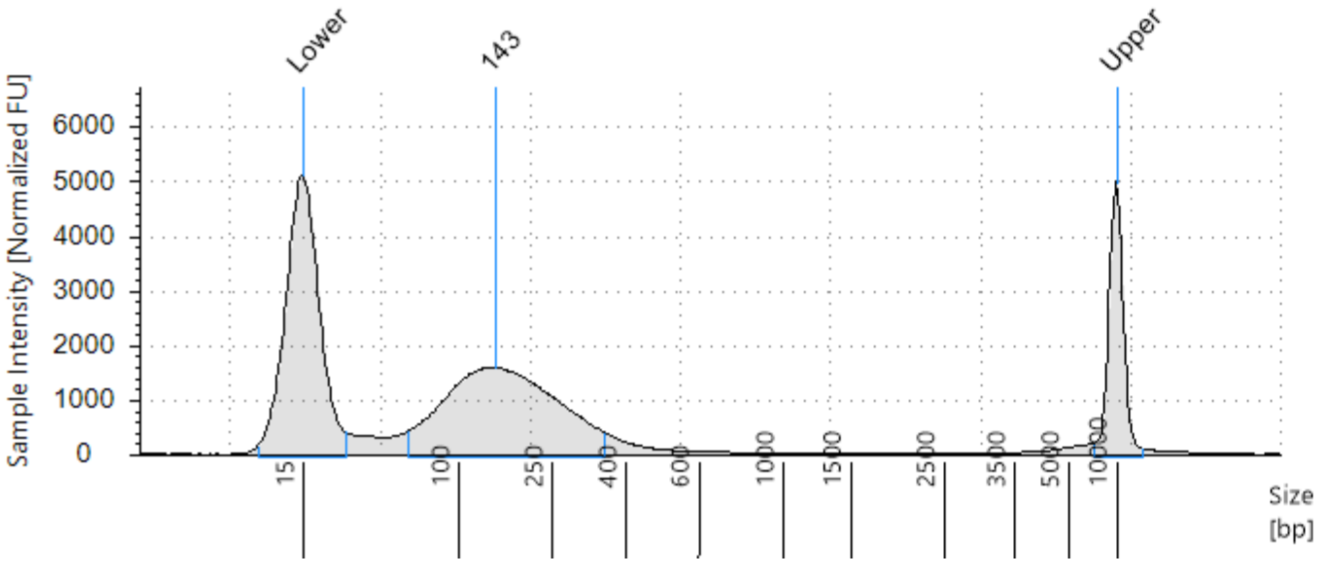

Sample Table

Well	Conc. [ng/µl]	Sample Description	Alert	Observations
A2	8.94	covaris micro tube1 R3 240 sec		

Peak Table

Size [bp]	Calibrated Conc. [ng/µl]	Assigned Conc. [ng/µl]	Peak Molarity [nmol/l]	% Integrated Area	Peak Comment	Observations
15	7.38	-	757	-		Lower Marker
143	8.94	-	96.0	100.00		
10000	3.25	3.25	0.500	-		Upper Marker

© Agilent Technologies, Inc. 2017

B2: covaris micro tube2 R3 240 sec

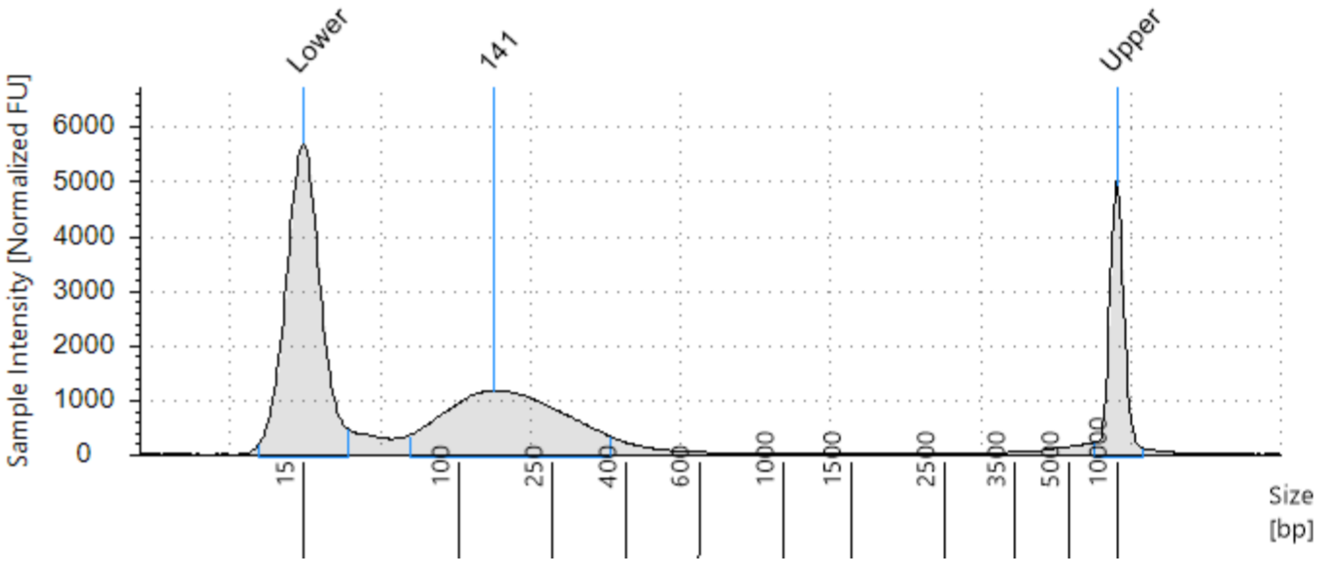

Sample Table

Well	Conc. [ng/µl]	Sample Description	Alert	Observations
B2	7.00	covaris micro tube2 R3 240 sec		

Peak Table

Size [bp]	Calibrated Conc. [ng/µl]	Assigned Conc. [ng/µl]	Peak Molarity [nmol/l]	% Integrated Area	Peak Comment	Observations
15	8.38	-	860	-		Lower Marker
141	7.00	-	76.4	100.00		
10000	3.25	3.25	0.500	-		Upper Marker

© Agilent Technologies, Inc. 2017

C2: covaris micro tube3 R3 240 sec

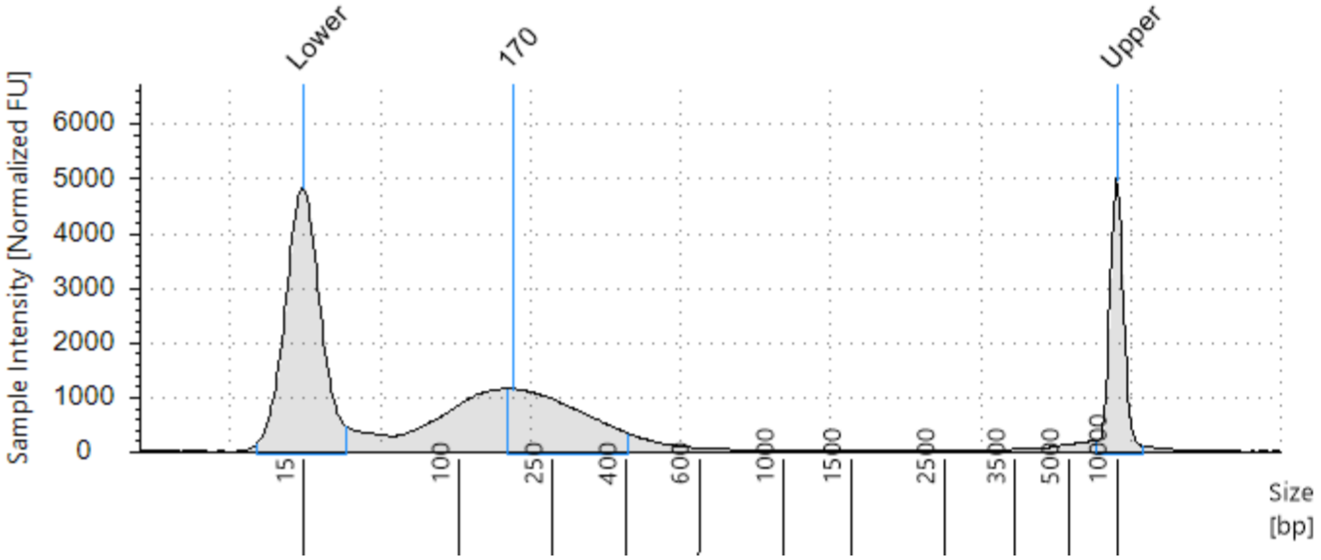

Sample Table

Well	Conc. [ng/µl]	Sample Description	Alert	Observations
C2	4.35	covaris micro tube3 R3 240 sec		

Peak Table

Size [bp]	Calibrated Conc. [ng/µl]	Assigned Conc. [ng/µl]	Peak Molarity [nmol/l]	% Integrated Area	Peak Comment	Observations
15	7.51	-	770	-		Lower Marker
170	4.35	-	39.3	100.00		
10000	3.25	3.25	0.500	-		Upper Marker

 © Agilent Technologies, Inc. 2017

D2: covaris micro tube4 R3 240 sec

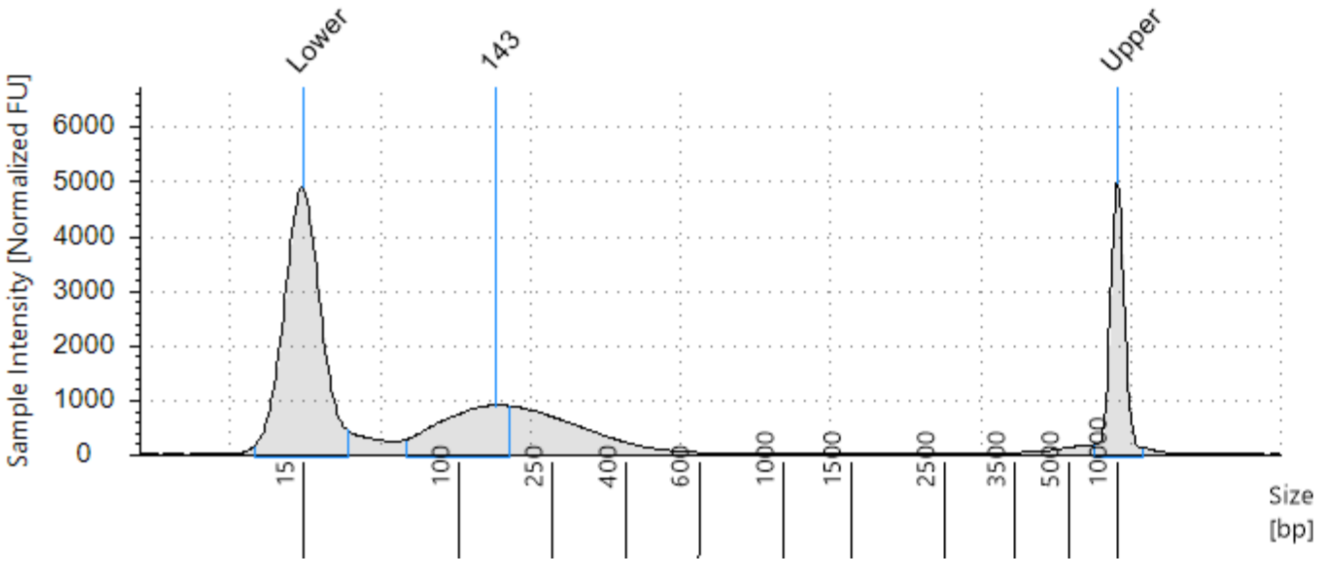

Sample Table

Well	Conc. [ng/µl]	Sample Description	Alert	Observations
D2	3.00	covaris micro tube4 R3 240 sec		

Peak Table

Size [bp]	Calibrated Conc. [ng/µl]	Assigned Conc. [ng/µl]	Peak Molarity [nmol/l]	% Integrated Area	Peak Comment	Observations
15	7.65	-	785	-		Lower Marker
143	3.00	-	32.3	100.00		
10000	3.25	3.25	0.500	-		Upper Marker

© Agilent Technologies, Inc. 2017
