# Supplementary material for: 96 sample parallel acoustic fragmentation for high throughput next generation sequencing library preparation
Source: PLoS One. 2026 Feb 17;21(2):e0341139. doi: 10.1371/journal.pone.0341139 (PMC12912608; doi:10.1371/journal.pone.0341139)
Supplement: S2 Fig — (ZIP) [file pone.0341139.s002.zip › Figure 1 Raw Data/No cavitation enhancement 840 seconds.pdf]

Filename: 2019-05-02-01- FFB minus firs 8 row 720 las 8 row 840 LE220 metal plat holder.D5000

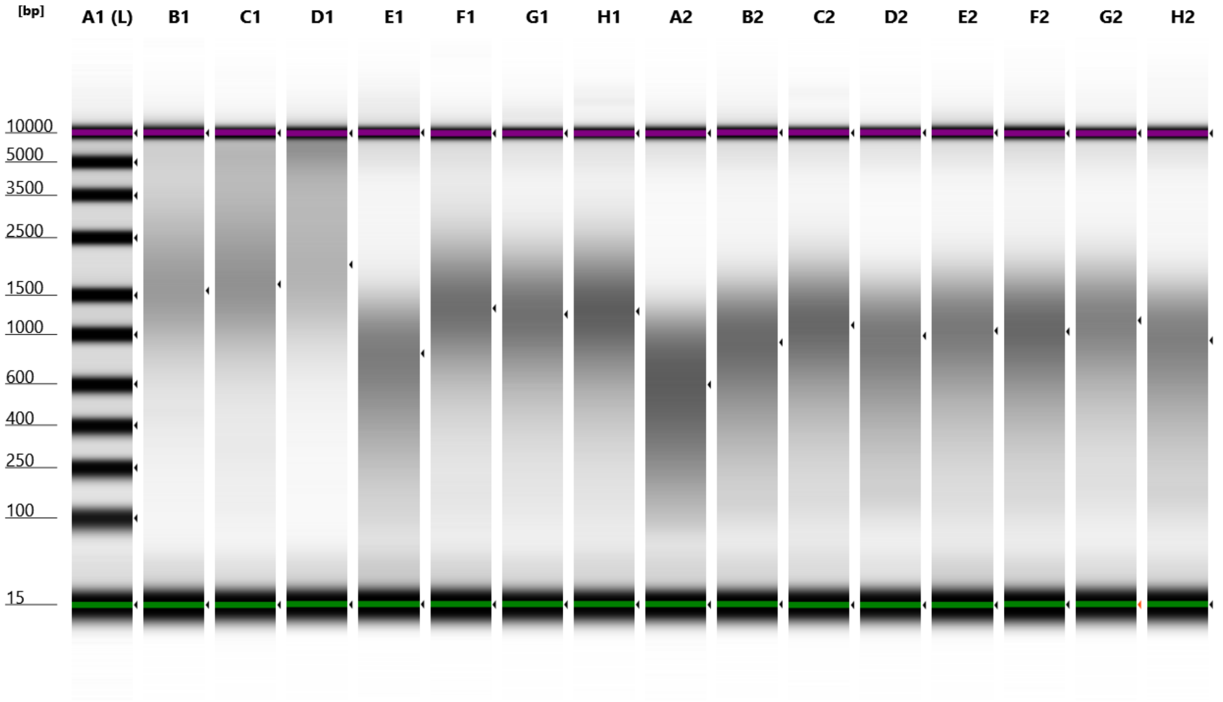

Default image (Contrast 100%)

Sample Info

Well	Conc. [ng/µl]	Sample Description	Alert	Observations
A1	33.7	Ladder		Ladder
B1	0.678	DFB minus 720 sec		
C1	0.649	DFB minus 720 sec		
D1	0.444	DFB minus 720 sec		
E1	3.98	DFB minus 720 sec		
F1	7.51	DFB minus 720 sec		
G1	7.93	DFB minus 720 sec		
H1	9.49	DFB minus 720 sec		
A2	5.83	DFB minus 840 sec		
B2	7.57	DFB minus 840 sec		
C2	7.92	DFB minus 840 sec		
D2	3.57	DFB minus 840 sec		
E2	3.65	DFB minus 840 sec		
F2	4.03	DFB minus 840 sec		
G2	6.39			
H2	6.35	DFB minus 840 sec		

© Agilent Technologies, Inc. 2017

A1: Ladder

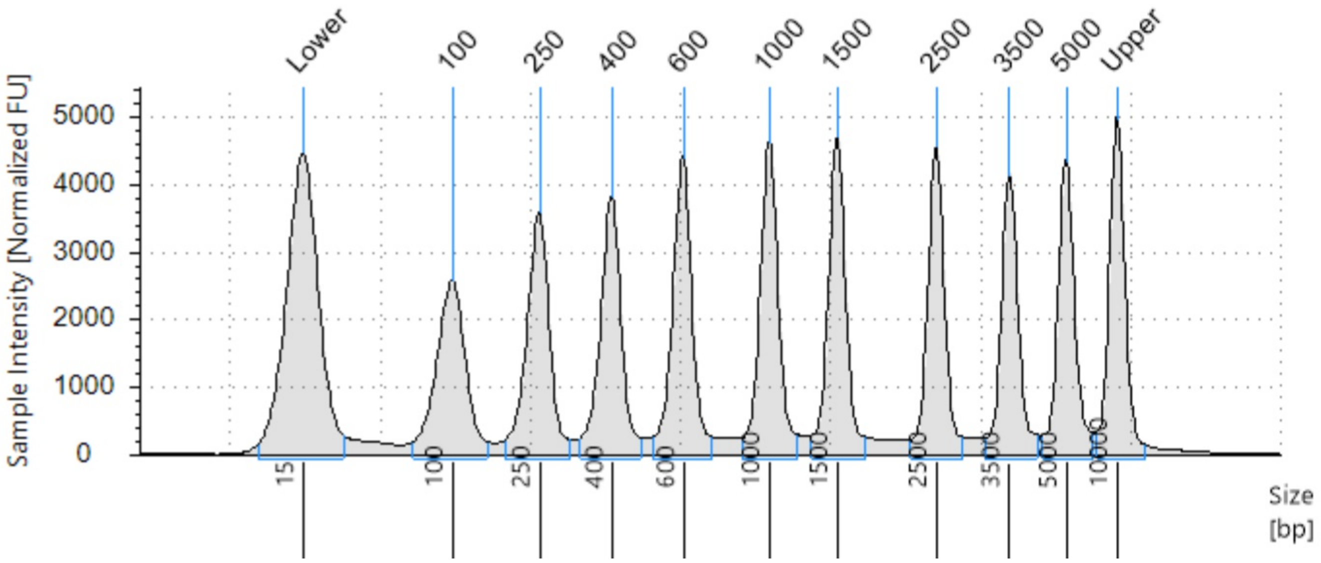

Sample Table

Well	Conc. [ng/µl]	Sample Description	Alert	Observations
A1	33.7	Ladder		Ladder

Peak Table

Size [bp]	Calibrated Conc. [ng/µl]	Assigned Conc. [ng/µl]	Peak Molarity [nmol/l]	% Integrated Area	Peak Comment	Observations
15	5.95	-	610	-		Lower Marker
100	3.54	-	54.5	10.53		
250	3.80	-	23.4	11.28		
400	3.77	-	14.5	11.21		
600	4.05	-	10.4	12.03		
1000	4.05	-	6.23	12.03		
1500	3.92	-	4.02	11.65		
2500	3.67	-	2.26	10.89		
3500	3.37	-	1.48	10.00		
5000	3.49	-	1.07	10.38		
10000	3.25	3.25	0.500	-		Upper Marker

© Agilent Technologies, Inc. 2017

A2: DFB minus 840 sec

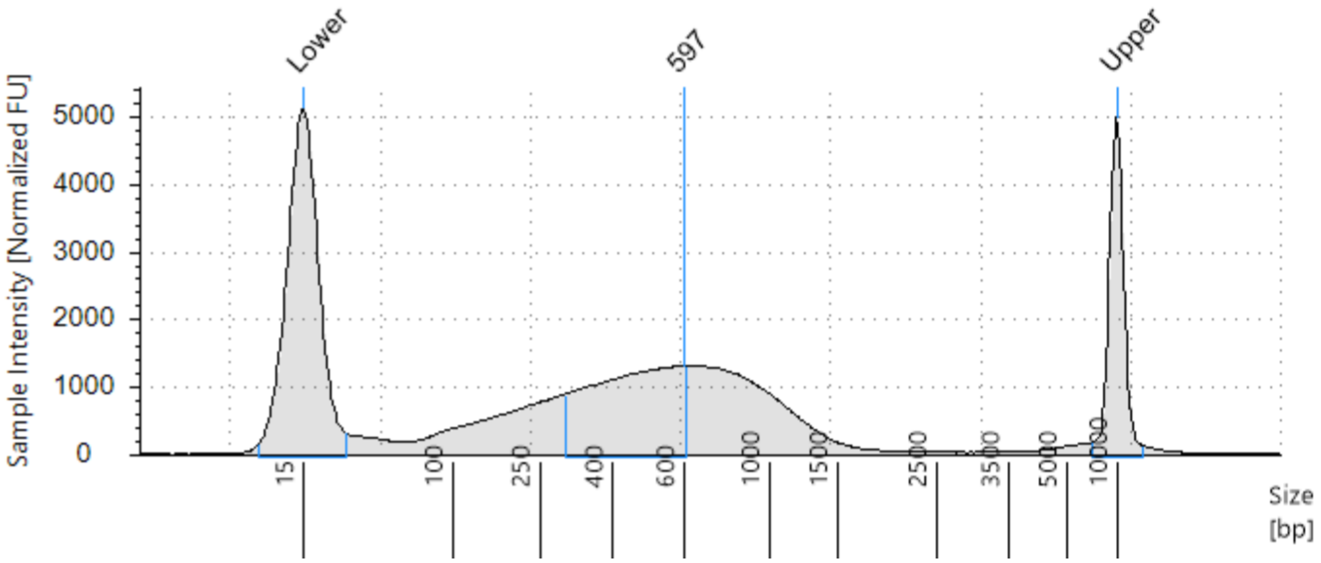

Sample Table

Well	Conc. [ng/µl]	Sample Description	Alert	Observations
A2	5.83	DFB minus 840 sec		

Peak Table

Size [bp]	Calibrated Conc. [ng/µl]	Assigned Conc. [ng/µl]	Peak Molarity [nmol/l]	% Integrated Area	Peak Comment	Observations
15	7.08	-	726	-		Lower Marker
597	5.83	-	15.0	100.00		
10000	3.25	3.25	0.500	-		Upper Marker

© Agilent Technologies, Inc. 2017

B2: DFB minus 840 sec

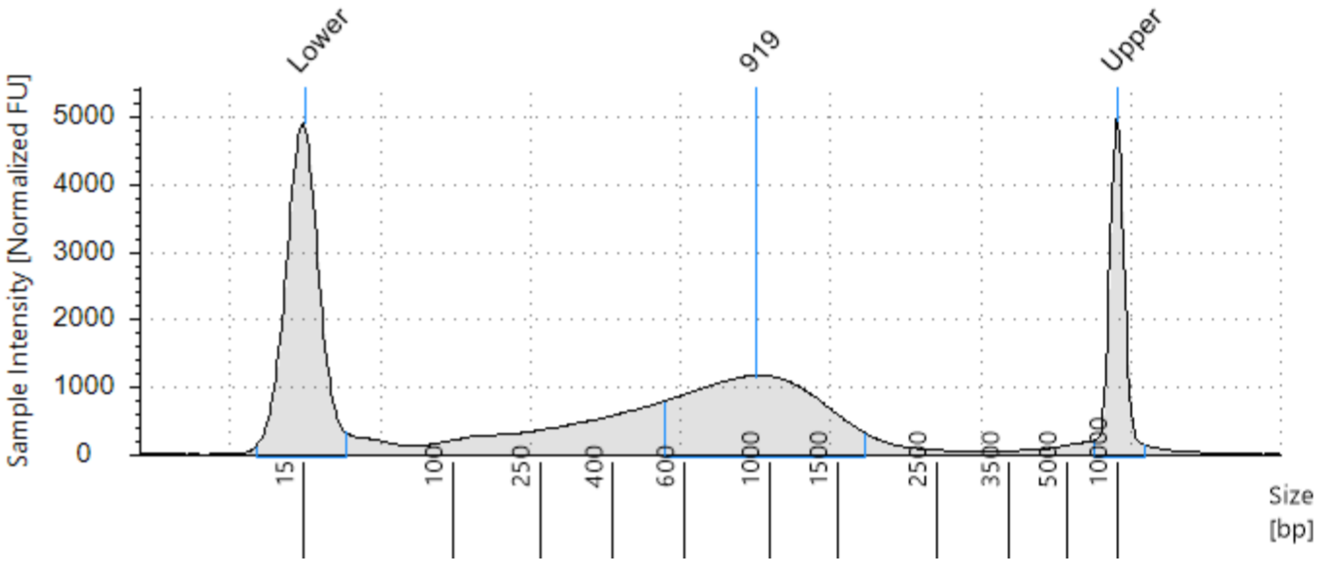

Sample Table

Well	Conc. [ng/µl]	Sample Description	Alert	Observations
B2	7.57	DFB minus 840 sec		

Peak Table

Size [bp]	Calibrated Conc. [ng/µl]	Assigned Conc. [ng/µl]	Peak Molarity [nmol/l]	% Integrated Area	Peak Comment	Observations
15	6.92	-	710	-		Lower Marker
919	7.57	-	12.7	100.00		
10000	3.25	3.25	0.500	-		Upper Marker

© Agilent Technologies, Inc. 2017

C2: DFB minus 840 sec

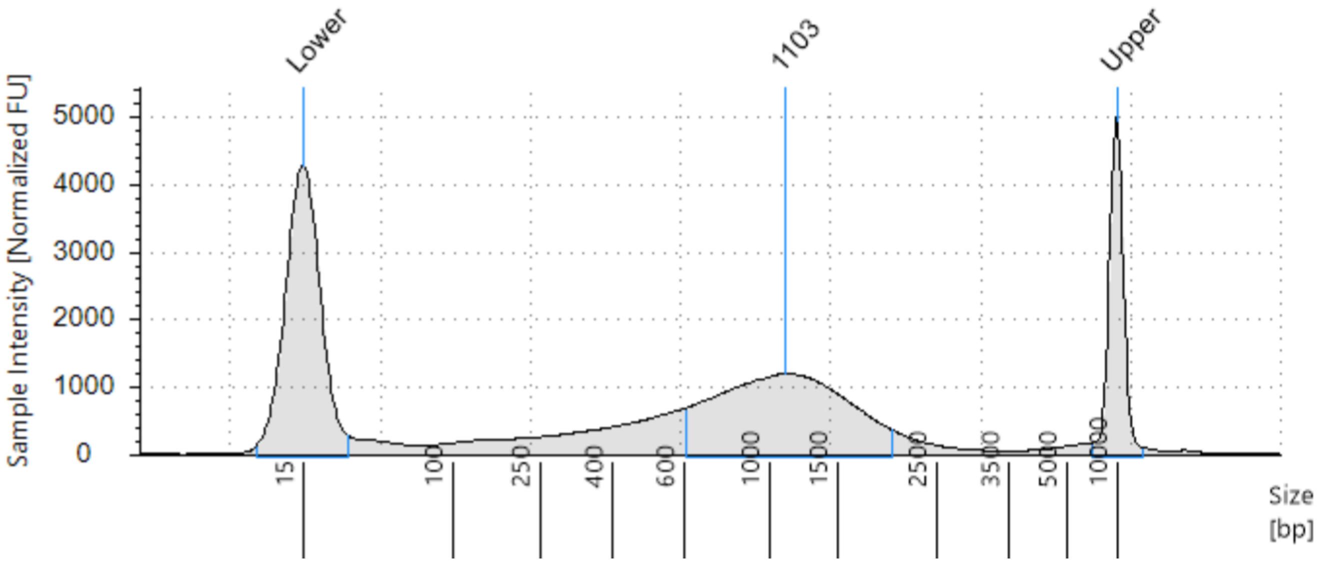

Sample Table

Well	Conc. [ng/µl]	Sample Description	Alert	Observations
C2	7.92	DFB minus 840 sec		

Peak Table

Size [bp]	Calibrated Conc. [ng/µl]	Assigned Conc. [ng/µl]	Peak Molarity [nmol/l]	% Integrated Area	Peak Comment	Observations
15	6.69	-	686	-		Lower Marker
1103	7.92	-	11.0	100.00		
10000	3.25	3.25	0.500	-		Upper Marker

© Agilent Technologies, Inc. 2017

D2: DFB minus 840 sec

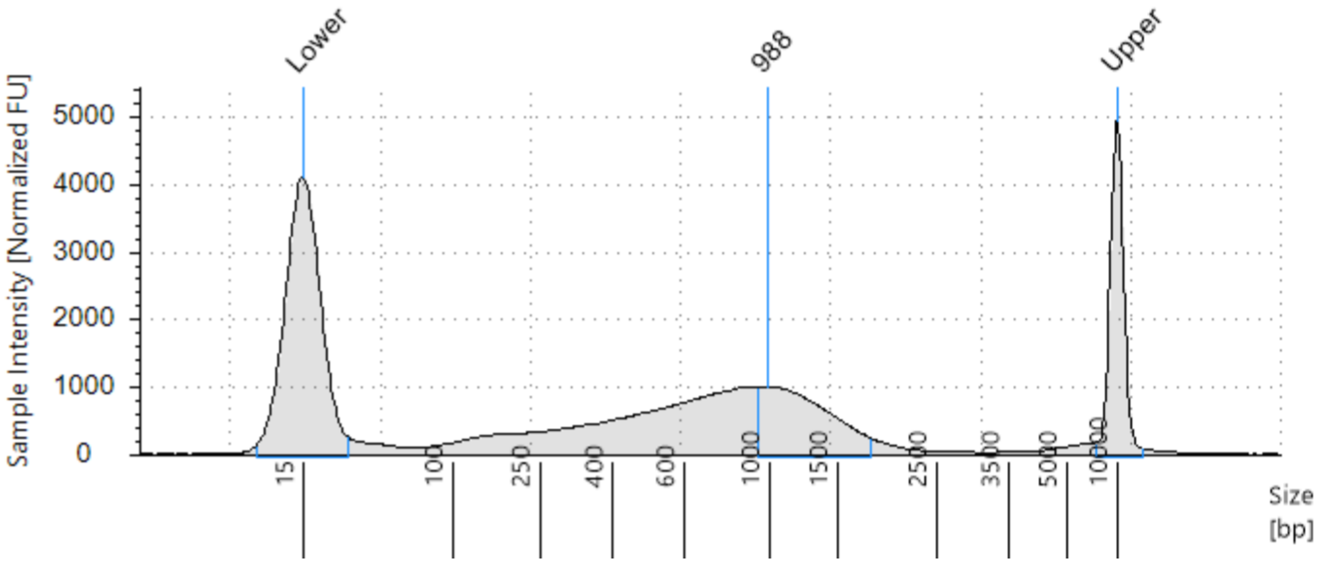

Sample Table

Well	Conc. [ng/µl]	Sample Description	Alert	Observations
D2	3.57	DFB minus 840 sec		

Peak Table

Size [bp]	Calibrated Conc. [ng/µl]	Assigned Conc. [ng/µl]	Peak Molarity [nmol/l]	% Integrated Area	Peak Comment	Observations
15	6.78	-	695	-		Lower Marker
988	3.57	-	5.55	100.00		
10000	3.25	3.25	0.500	-		Upper Marker

© Agilent Technologies, Inc. 2017

E2: DFB minus 840 sec

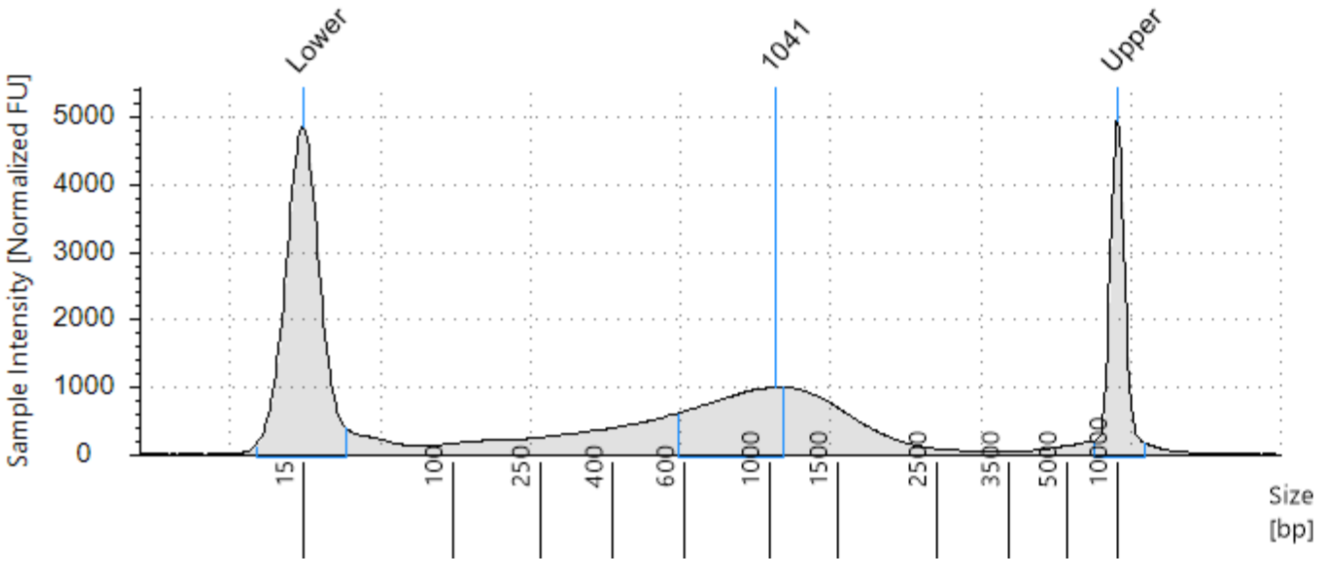

Sample Table

Well	Conc. [ng/µl]	Sample Description	Alert	Observations
E2	3.65	DFB minus 840 sec		

Peak Table

Size [bp]	Calibrated Conc. [ng/µl]	Assigned Conc. [ng/µl]	Peak Molarity [nmol/l]	% Integrated Area	Peak Comment	Observations
15	7.06	-	724	-		Lower Marker
1041	3.65	-	5.40	100.00		
10000	3.25	3.25	0.500	-		Upper Marker

© Agilent Technologies, Inc. 2017

F2: DFB minus 840 sec

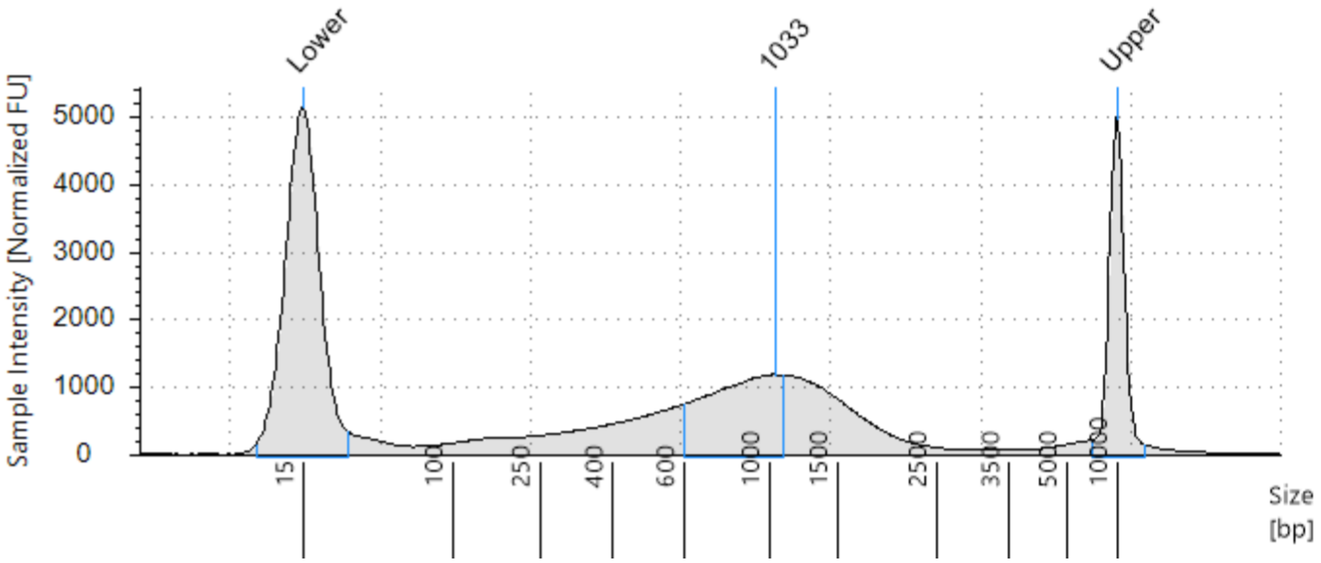

Sample Table

Well	Conc. [ng/µl]	Sample Description	Alert	Observations
F2	4.03	DFB minus 840 sec		

Peak Table

Size [bp]	Calibrated Conc. [ng/µl]	Assigned Conc. [ng/µl]	Peak Molarity [nmol/l]	% Integrated Area	Peak Comment	Observations
15	7.34	-	753	-		Lower Marker
1033	4.03	-	6.00	100.00		
10000	3.25	3.25	0.500	-		Upper Marker

© Agilent Technologies, Inc. 2017

H2: DFB minus 840 sec

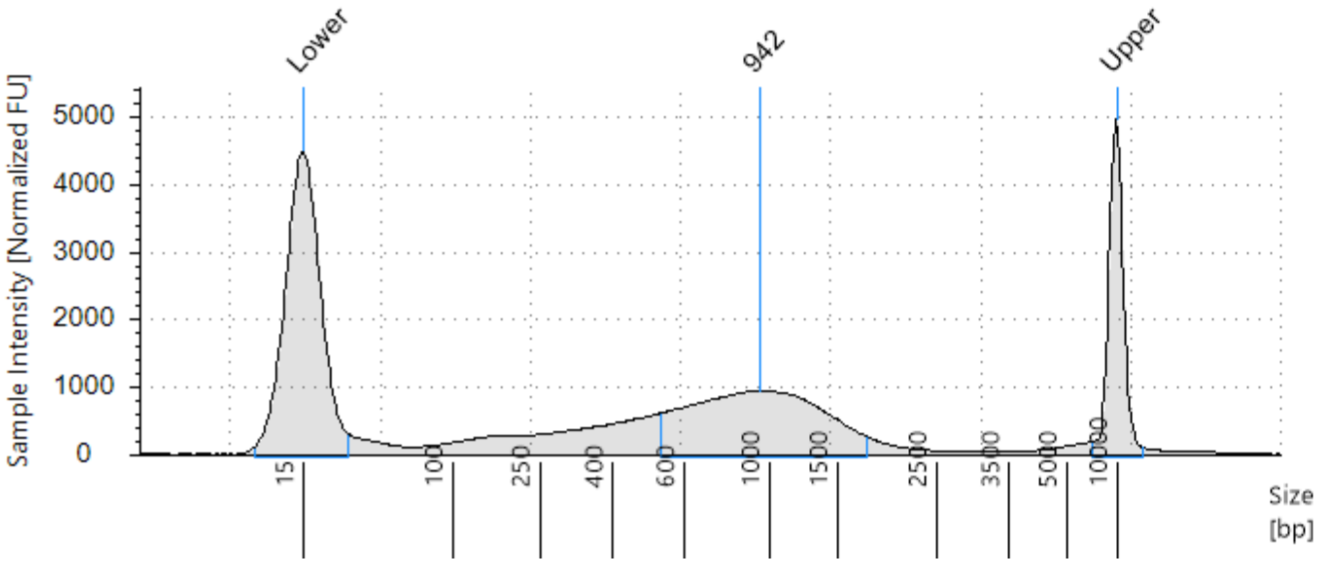

Sample Table

Well	Conc. [ng/µl]	Sample Description	Alert	Observations
H2	6.35	DFB minus 840 sec		

Peak Table

Size [bp]	Calibrated Conc. [ng/µl]	Assigned Conc. [ng/µl]	Peak Molarity [nmol/l]	% Integrated Area	Peak Comment	Observations
15	6.94	-	712	-		Lower Marker
942	6.35	-	10.4	100.00		
10000	3.25	3.25	0.500	-		Upper Marker

© Agilent Technologies, Inc. 2017

Filename: 2019-05-29-03- 1-8 1008 sec for 6.28 first row, 8-15 840 sec for 6.28 first row.D5000

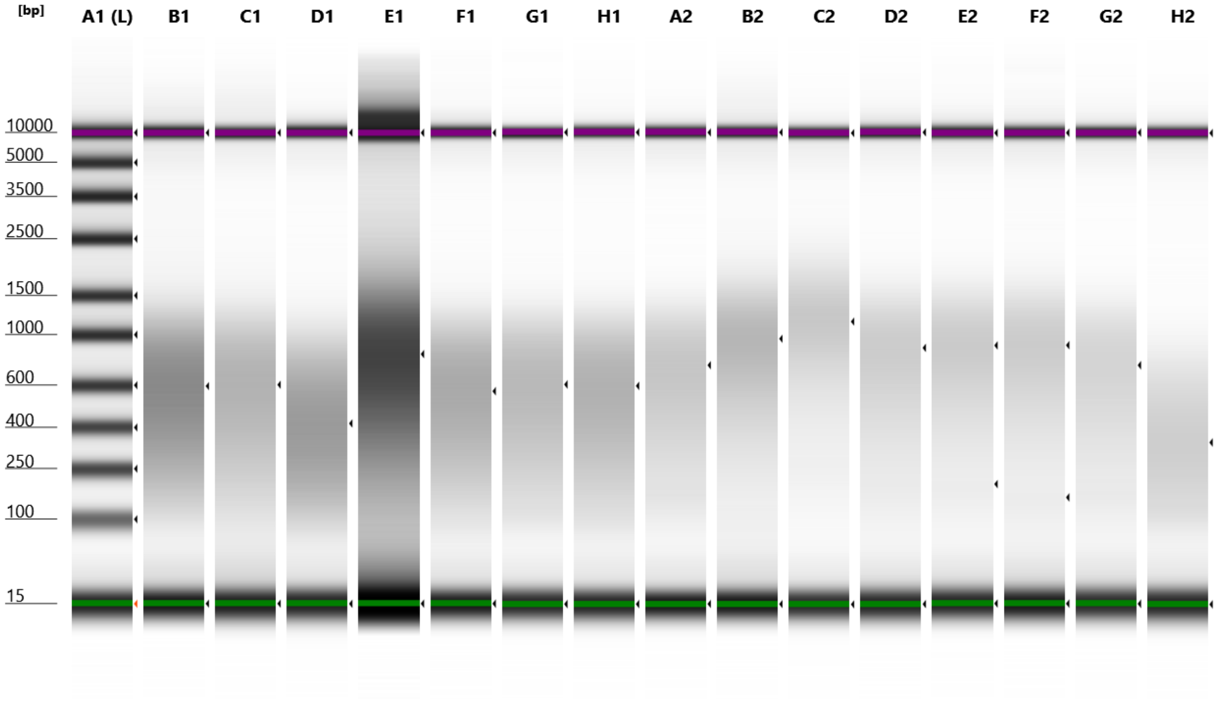

Default image (Contrast 100%)

Sample Info

Well	Conc. [ng/µl]	Sample Description	Alert	Observations
A1	29.6	Ladder		Ladder
B1	1.02	DFB 1 minus LE220 1008 sec from 6.28 first Row		
C1	0.717	DFB 2 minus LE220 1008 sec from 6.28 first Row		
D1	0.938	DFB 3 minus LE220 1008 sec from 6.28 first Row		
E1	10.5	DFB 4 minus LE220 1008 sec from 6.28 first Row		
F1	4.88	DFB 5 minus LE220 1008 sec from 6.28 first Row		
G1	0.610	DFB 6 minus LE220 1008 sec from 6.28 first Row		
H1	4.05	DFB 7 minus LE220 1008 sec from 6.28 first Row		
A2	0.520	DFB1 minus LE220 840 sec from 6.28 first Row		
B2	3.01	DFB2 minus LE220 840 sec from 6.28 first Row		
C2	2.28	DFB 3minus LE220 840 sec from 6.28 first Row		
D2	2.08	DFB4 minus LE220 840 sec from 6.28 first Row		
E2	2.20	DFB5 minus LE220 840 sec from 6.28 first Row		
F2	2.22	DFB6 minus LE220 840 sec from 6.28 first Row		
G2	1.93	DFB7 minus LE220 840 sec from 6.28 first Row		
H2	2.26	DFB8 minus LE220 840 sec from 6.28 first Row		

 © Agilent Technologies, Inc. 2017

A1: Ladder

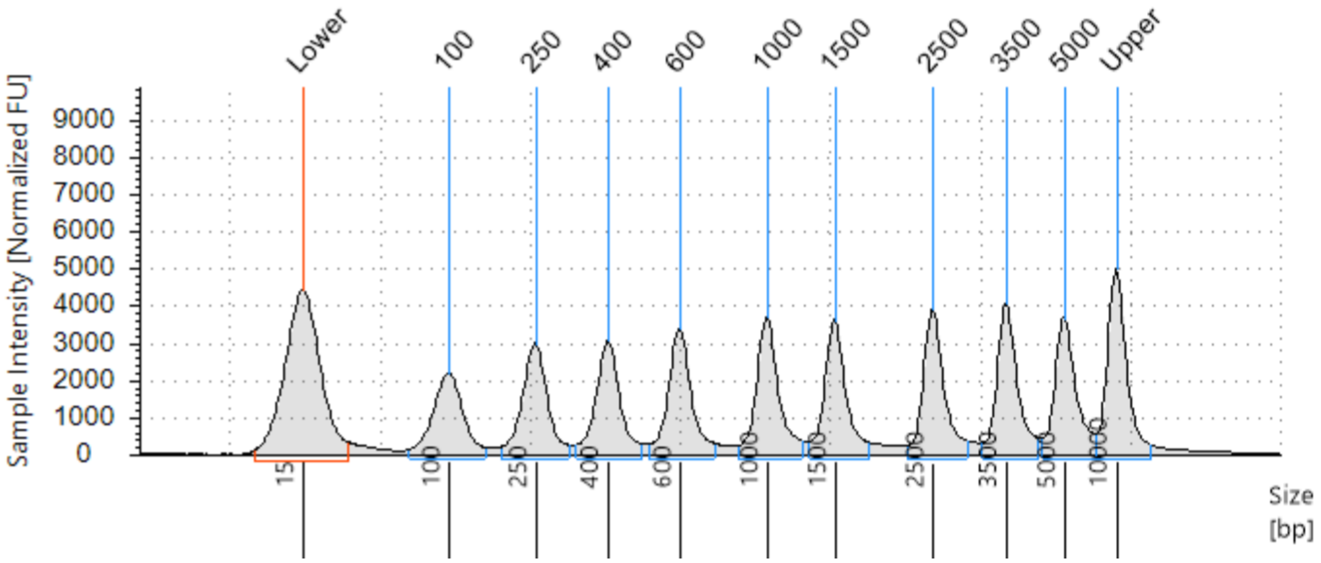

Sample Table

Well	Conc. [ng/µl]	Sample Description	Alert	Observations
A1	29.6	Ladder		Ladder

Peak Table

Size [bp]	Calibrated Conc. [ng/µl]	Assigned Conc. [ng/µl]	Peak Molarity [nmol/l]	% Integrated Area	Peak Comment	Observations
15	5.87	-	602	-		Lower Marker
100	2.80	-	43.1	9.46		
250	3.17	-	19.5	10.70		
400	3.12	-	12.0	10.52		
600	3.34	-	8.58	11.29		
1000	3.48	-	5.35	11.75		
1500	3.29	-	3.37	11.10		
2500	3.48	-	2.14	11.74		
3500	3.59	-	1.58	12.11		
5000	3.35	-	1.03	11.32		
10000	3.25	3.25	0.500	-		Upper Marker

© Agilent Technologies, Inc. 2017

A2: DFB1 minus LE220 840 sec from 6.28 first Row

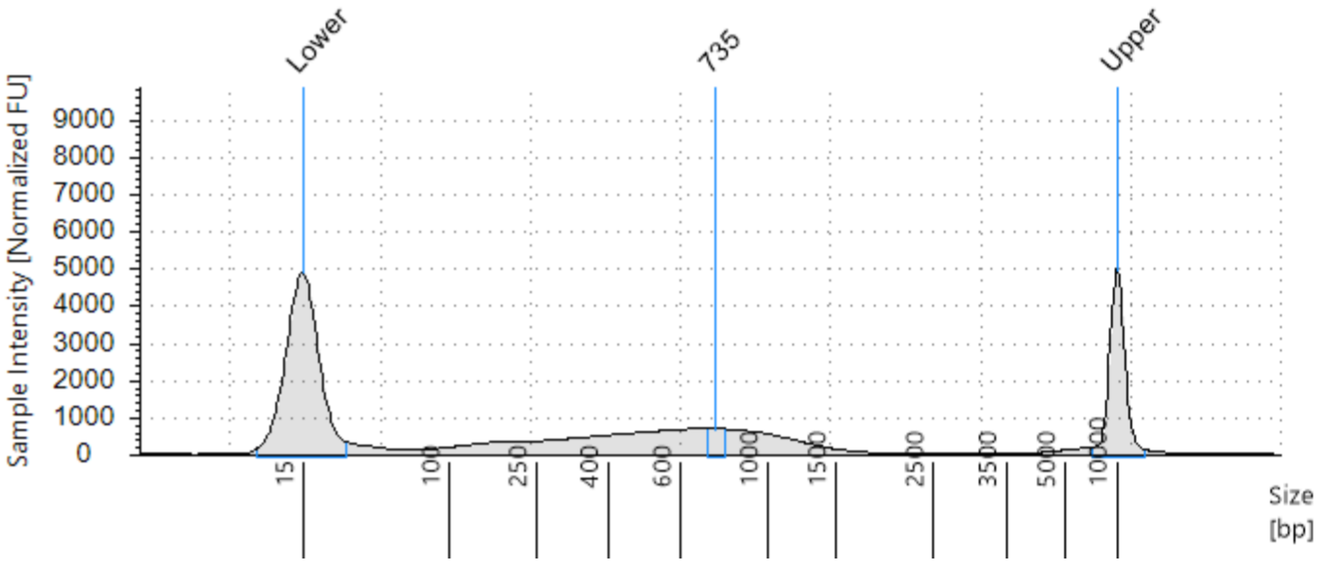

Sample Table

Well	Conc. [ng/µl]	Sample Description	Alert	Observations
A2	0.520	DFB1 minus LE220 840 sec from 6.28 first Row		

Peak Table

Size [bp]	Calibrated Conc. [ng/µl]	Assigned Conc. [ng/µl]	Peak Molarity [nmol/l]	% Integrated Area	Peak Comment	Observations
15	6.71	-	688	-		Lower Marker
735	0.520	-	1.09	100.00		
10000	3.25	3.25	0.500	-		Upper Marker

© Agilent Technologies, Inc. 2017

B2: DFB2 minus LE220 840 sec from 6.28 first Row

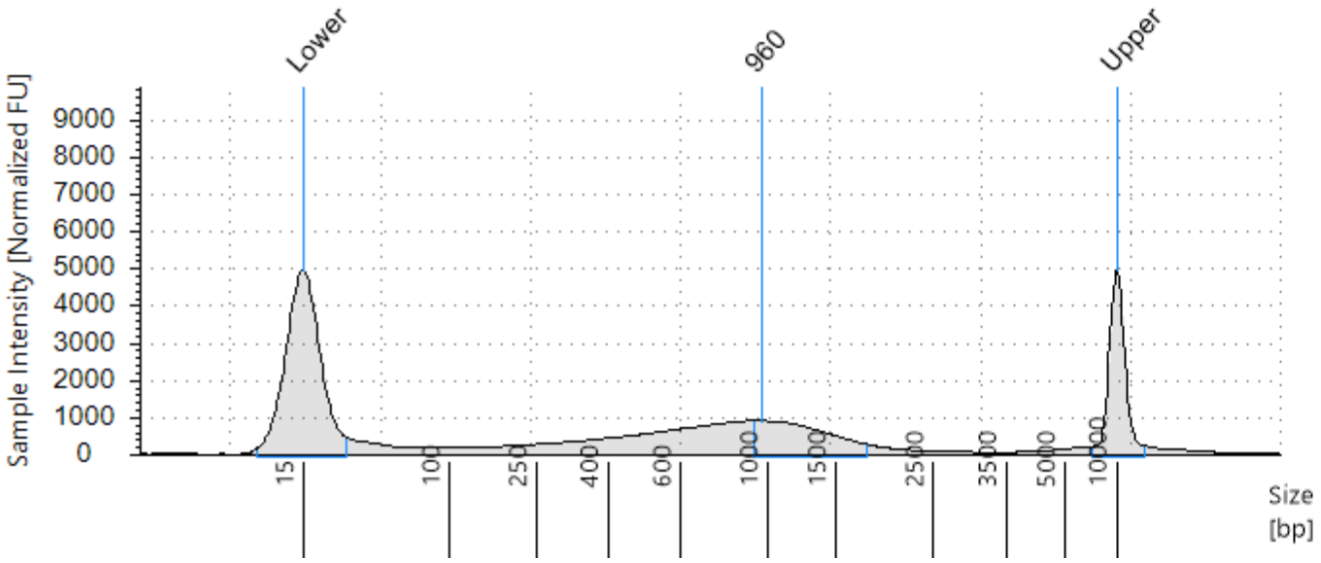

Sample Table

Well	Conc. [ng/µl]	Sample Description	Alert	Observations
B2	3.01	DFB2 minus LE220 840 sec from 6.28 first Row		

Peak Table

Size [bp]	Calibrated Conc. [ng/µl]	Assigned Conc. [ng/µl]	Peak Molarity [nmol/l]	% Integrated Area	Peak Comment	Observations
15	7.06	-	725	-		Lower Marker
960	3.01	-	4.83	100.00		
10000	3.25	3.25	0.500	-		Upper Marker

© Agilent Technologies, Inc. 2017

C2: DFB 3minus LE220 840 sec from 6.28 first Row

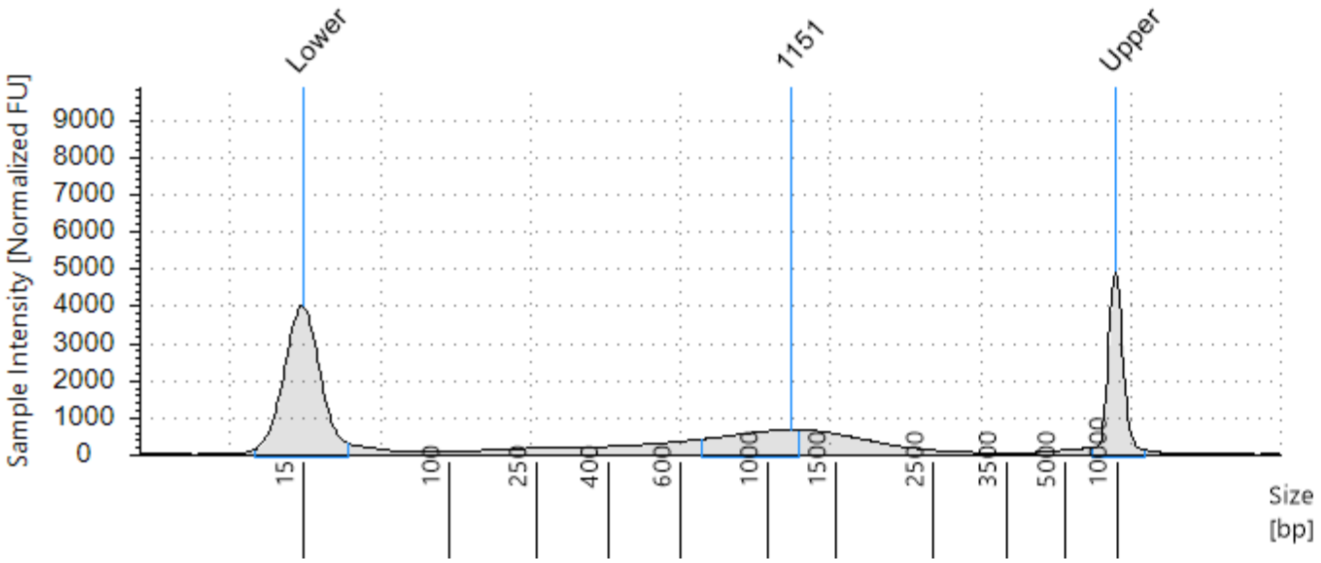

Sample Table

Well	Conc. [ng/µl]	Sample Description	Alert	Observations
C2	2.28	DFB 3minus LE220 840 sec from 6.28 first Row		

Peak Table

Size [bp]	Calibrated Conc. [ng/µl]	Assigned Conc. [ng/µl]	Peak Molarity [nmol/l]	% Integrated Area	Peak Comment	Observations
15	6.14	-	630	-		Lower Marker
1151	2.28	-	3.05	100.00		
10000	3.25	3.25	0.500	-		Upper Marker

© Agilent Technologies, Inc. 2017

D2: DFB4 minus LE220 840 sec from 6.28 first Row

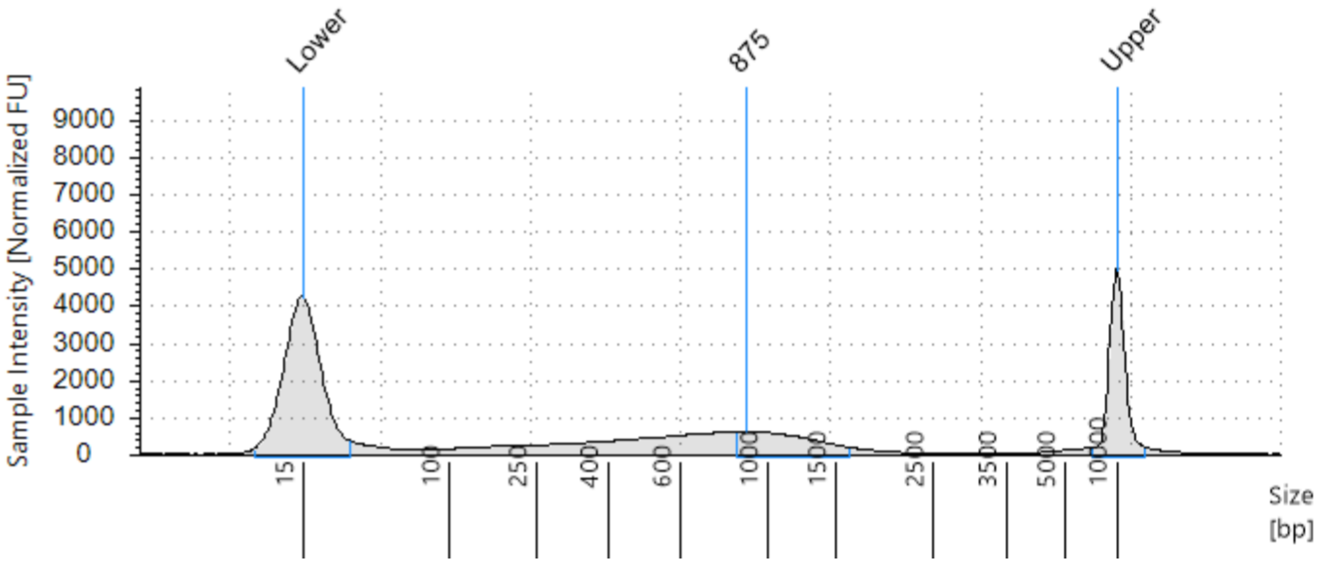

Sample Table

Well	Conc. [ng/µl]	Sample Description	Alert	Observations
D2	2.08	DFB4 minus LE220 840 sec from 6.28 first Row		

Peak Table

Size [bp]	Calibrated Conc. [ng/µl]	Assigned Conc. [ng/µl]	Peak Molarity [nmol/l]	% Integrated Area	Peak Comment	Observations
15	6.43	-	660	-		Lower Marker
875	2.08	-	3.65	100.00		
10000	3.25	3.25	0.500	-		Upper Marker

© Agilent Technologies, Inc. 2017

E2: DFB5 minus LE220 840 sec from 6.28 first Row

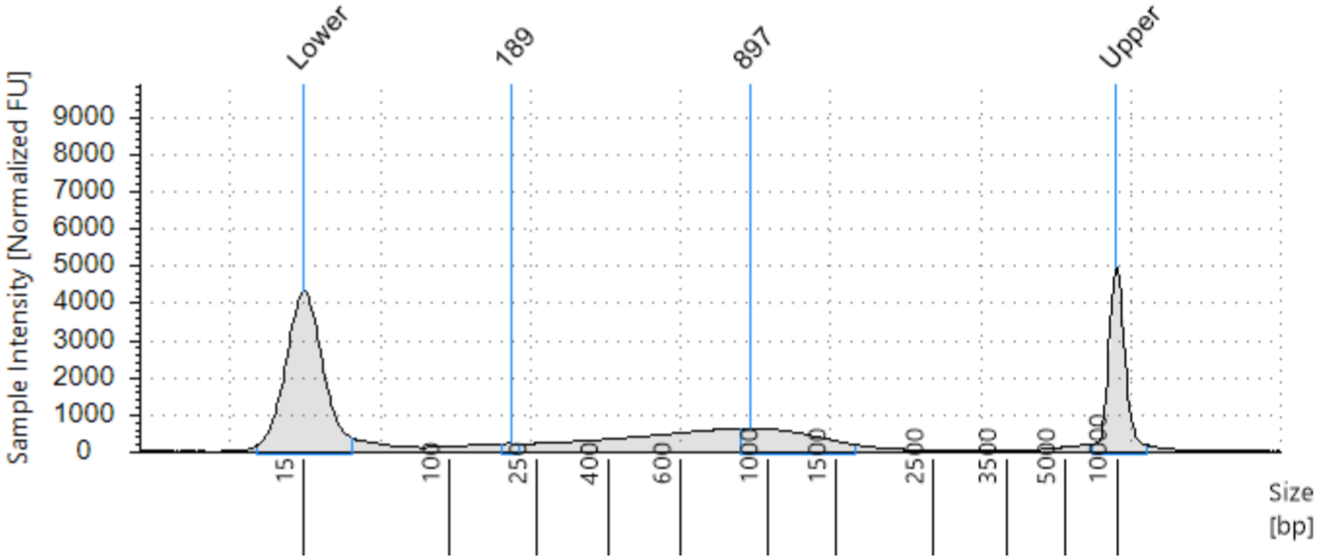

Sample Table

Well	Conc. [ng/µl]	Sample Description	Alert	Observations
E2	2.20	DFB5 minus LE220 840 sec from 6.28 first Row		

Peak Table

Size [bp]	Calibrated Conc. [ng/µl]	Assigned Conc. [ng/µl]	Peak Molarity [nmol/l]	% Integrated Area	Peak Comment	Observations
15	6.18	-	634	-		Lower Marker
189	0.152	-	1.24	6.88		
897	2.05	-	3.52	93.12		
10000	3.25	3.25	0.500	-		Upper Marker

 © Agilent Technologies, Inc. 2017

F2: DFB6 minus LE220 840 sec from 6.28 first Row

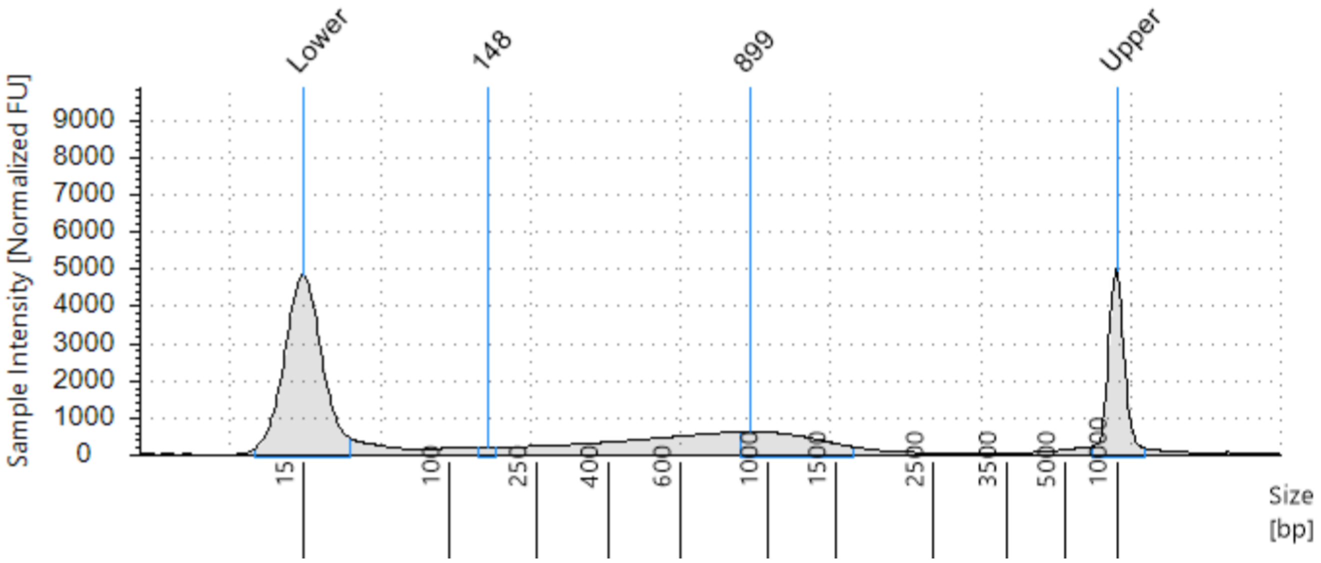

Sample Table

Well	Conc. [ng/µl]	Sample Description	Alert	Observations
F2	2.22	DFB6 minus LE220 840 sec from 6.28 first Row		

Peak Table

Size [bp]	Calibrated Conc. [ng/µl]	Assigned Conc. [ng/µl]	Peak Molarity [nmol/l]	% Integrated Area	Peak Comment	Observations
15	7.08	-	726	-		Lower Marker
148	0.153	-	1.59	6.91		
899	2.06	-	3.53	93.09		
10000	3.25	3.25	0.500	-		Upper Marker

© Agilent Technologies, Inc. 2017

G2: DFB7 minus LE220 840 sec from 6.28 first Row

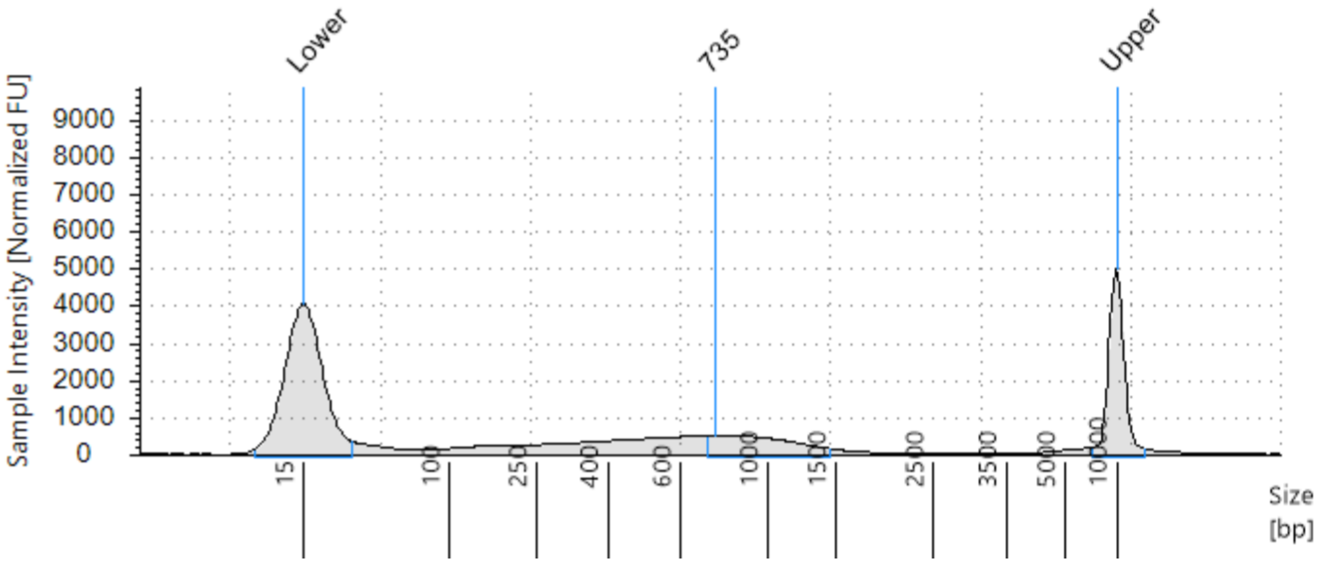

Sample Table

Well	Conc. [ng/µl]	Sample Description	Alert	Observations
G2	1.93	DFB7 minus LE220 840 sec from 6.28 first Row		

Peak Table

Size [bp]	Calibrated Conc. [ng/µl]	Assigned Conc. [ng/µl]	Peak Molarity [nmol/l]	% Integrated Area	Peak Comment	Observations
15	6.25	-	641	-		Lower Marker
735	1.93	-	4.04	100.00		
10000	3.25	3.25	0.500	-		Upper Marker

© Agilent Technologies, Inc. 2017

Filename: 2019-05-21-03 DFB minus , first 7, 840 sec last 8 DFB minus 720 sec.D5000

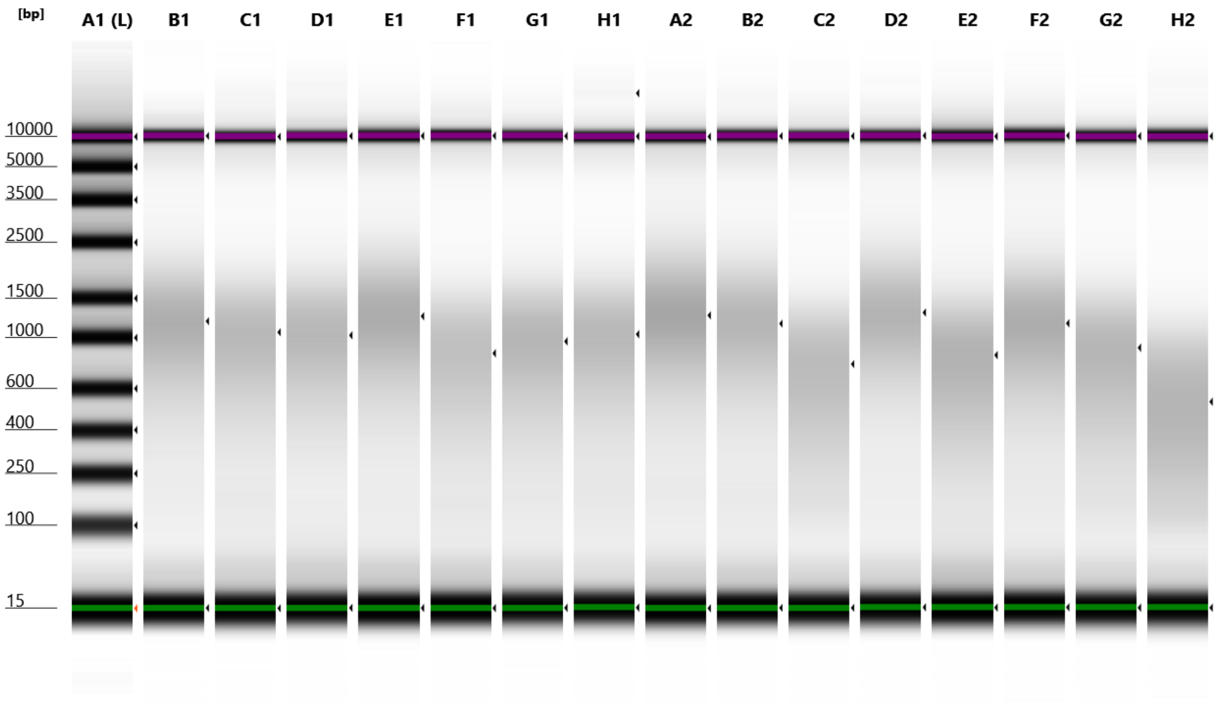

Default image (Contrast 100%)

Sample Info

Well	Conc. [ng/µl]	Sample Description	Alert	Observations
A1	33.1	Ladder		Ladder
B1	0.469	DFB1 minus 840 sec		
C1	1.74	DFB2 minus 840 sec		
D1	0.431	DFB3 minus 840 sec		
E1	3.74	DFB4 minus 840 sec		
F1	0.528	DFB5 minus 840 sec		
G1	2.01	DFB6 minus 840 sec		
H1	1.87	DFB7 minus 840 sec		
A2	4.24	DFB1 minus 720 sec		
B2	3.21	DFB2 minus 720 sec		
C2	1.82	DF3 minus 720 sec		
D2	3.47	DFB4 minus 720 sec		
E2	0.433	DFB5 minus 720 sec		
F2	2.17	DFB6 minus 720 sec		
G2	1.72	DFB7 minus 720 sec		
H2	0.270	DFB8 minus 720 sec		

© Agilent Technologies, Inc. 2017

A1: Ladder

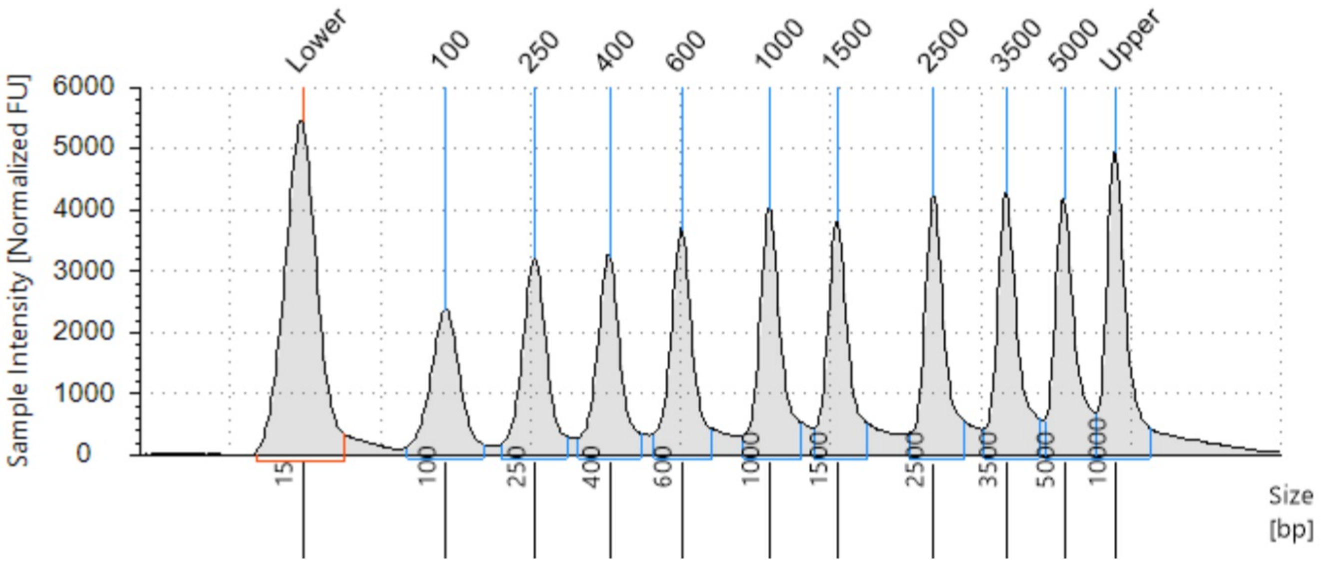

Sample Table

Well	Conc. [ng/µl]	Sample Description	Alert	Observations
A1	33.1	Ladder		Ladder

Peak Table

Size [bp]	Calibrated Conc. [ng/µl]	Assigned Conc. [ng/µl]	Peak Molarity [nmol/l]	% Integrated Area	Peak Comment	Observations
15	7.22	-	741	-		Lower Marker
100	3.15	-	48.5	9.51		
250	3.54	-	21.8	10.70		
400	3.43	-	13.2	10.34		
600	3.67	-	9.40	11.06		
1000	3.92	-	6.03	11.83		
1500	3.62	-	3.71	10.92		
2500	3.86	-	2.37	11.64		
3500	4.02	-	1.77	12.14		
5000	3.93	-	1.21	11.86		
10000	3.25	3.25	0.500	-		Upper Marker

© Agilent Technologies, Inc. 2017

B1: DFB1 minus 840 sec

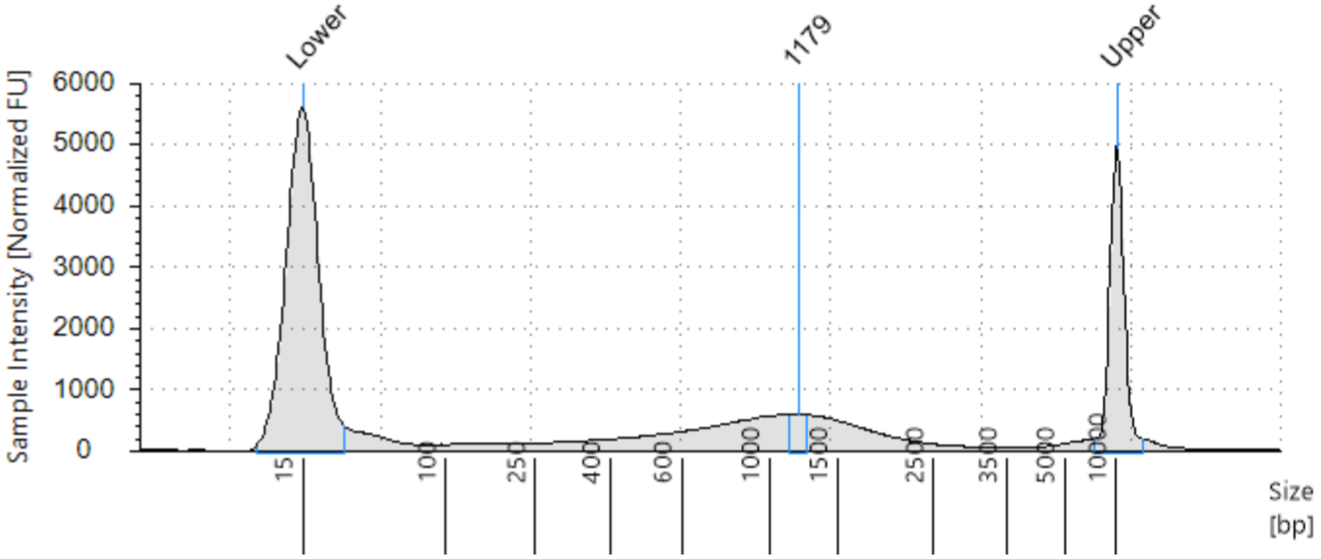

Sample Table

Well	Conc. [ng/µl]	Sample Description	Alert	Observations
B1	0.469	DFB1 minus 840 sec		

Peak Table

Size [bp]	Calibrated Conc. [ng/µl]	Assigned Conc. [ng/µl]	Peak Molarity [nmol/l]	% Integrated Area	Peak Comment	Observations
15	8.17	-	838	-		Lower Marker
1179	0.469	-	0.612	100.00		
10000	3.25	3.25	0.500	-		Upper Marker

© Agilent Technologies, Inc. 2017

C1: DFB2 minus 840 sec

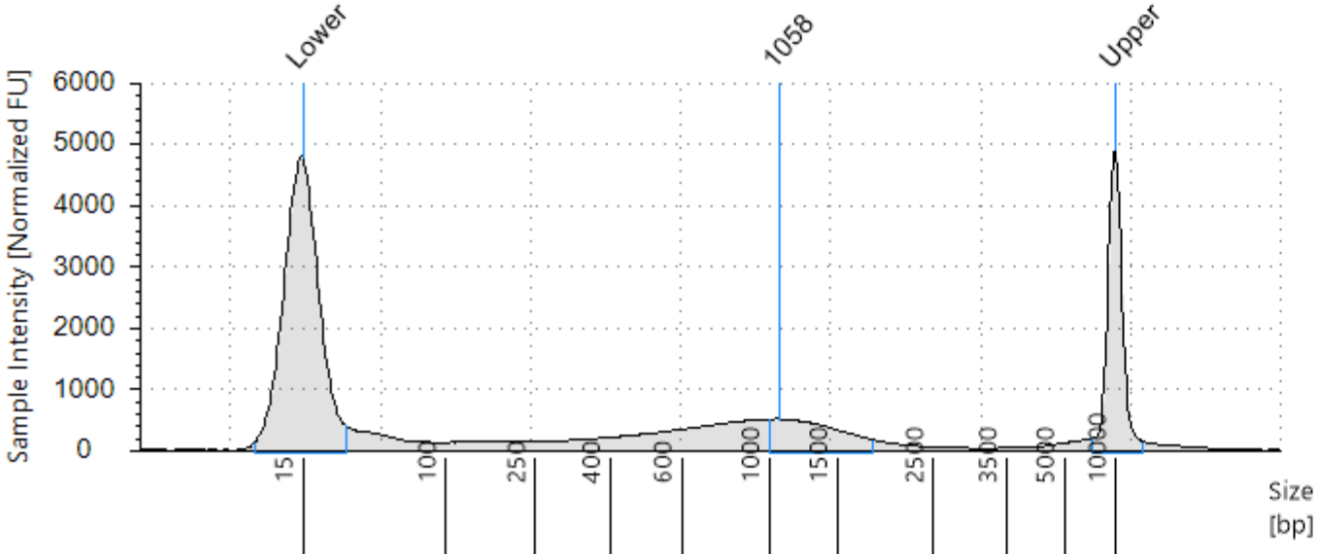

Sample Table

Well	Conc. [ng/µl]	Sample Description	Alert	Observations
C1	1.74	DFB2 minus 840 sec		

Peak Table

Size [bp]	Calibrated Conc. [ng/µl]	Assigned Conc. [ng/µl]	Peak Molarity [nmol/l]	% Integrated Area	Peak Comment	Observations
15	7.51	-	770	-		Lower Marker
1058	1.74	-	2.53	100.00		
10000	3.25	3.25	0.500	-		Upper Marker

© Agilent Technologies, Inc. 2017

D1: DFB3 minus 840 sec

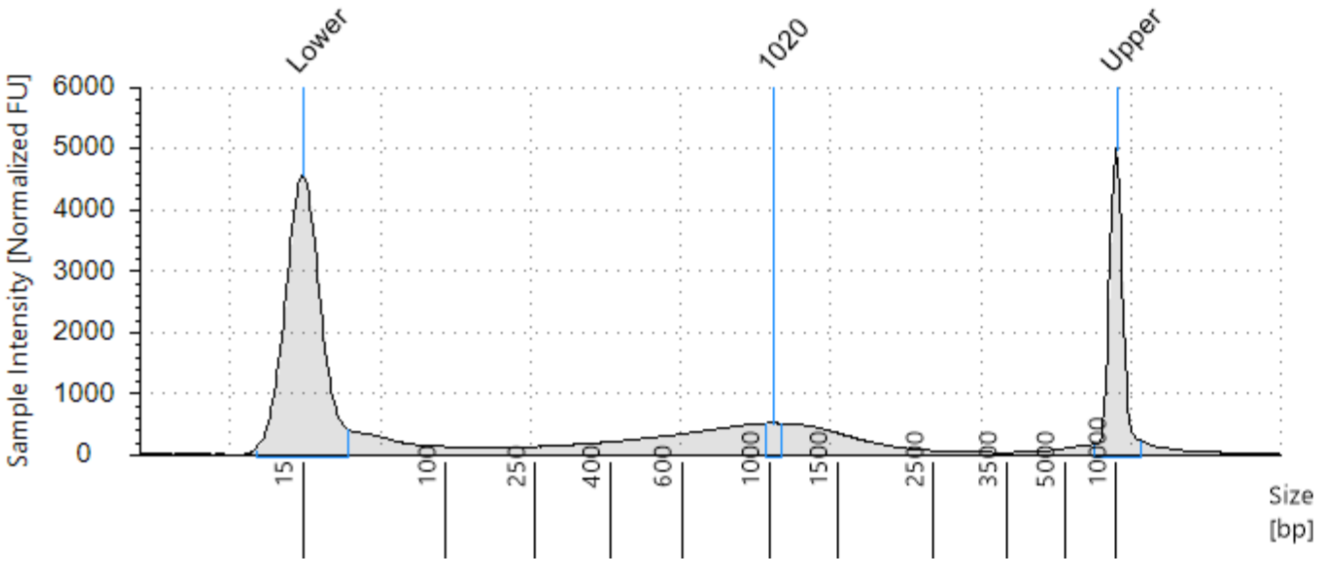

Sample Table

Well	Conc. [ng/µl]	Sample Description	Alert	Observations
D1	0.431	DFB3 minus 840 sec		

Peak Table

Size [bp]	Calibrated Conc. [ng/µl]	Assigned Conc. [ng/µl]	Peak Molarity [nmol/l]	% Integrated Area	Peak Comment	Observations
15	7.50	-	769	-		Lower Marker
1020	0.431	-	0.651	100.00		
10000	3.25	3.25	0.500	-		Upper Marker

© Agilent Technologies, Inc. 2017

E1: DFB4 minus 840 sec

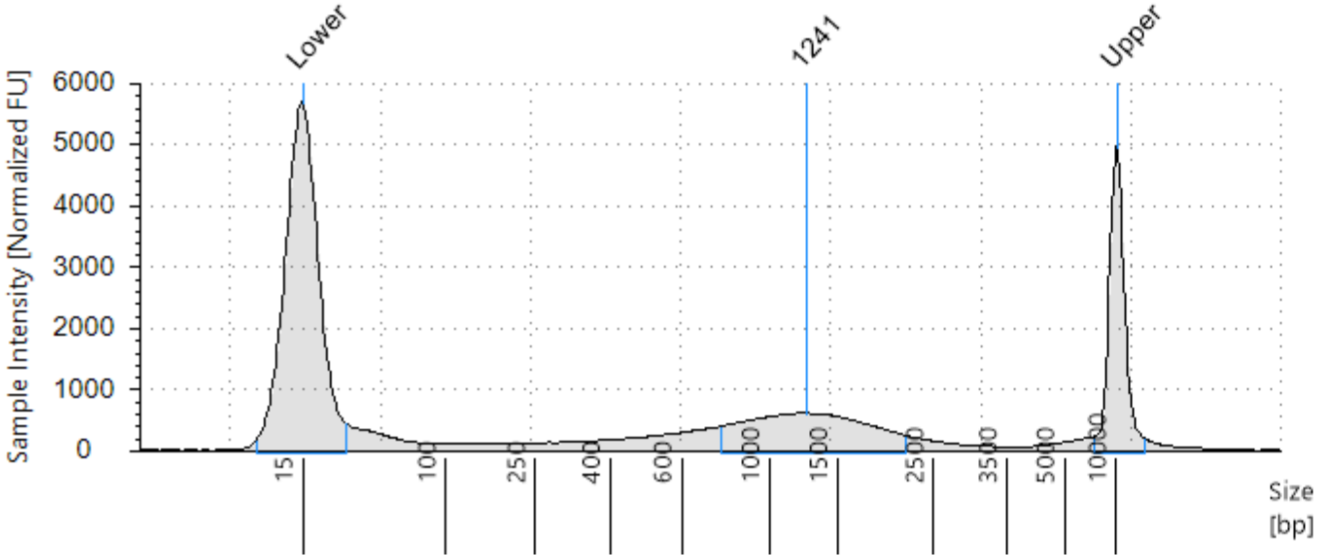

Sample Table

Well	Conc. [ng/µl]	Sample Description	Alert	Observations
E1	3.74	DFB4 minus 840 sec		

Peak Table

Size [bp]	Calibrated Conc. [ng/µl]	Assigned Conc. [ng/µl]	Peak Molarity [nmol/l]	% Integrated Area	Peak Comment	Observations
15	8.04	-	825	-		Lower Marker
1241	3.74	-	4.63	100.00		
10000	3.25	3.25	0.500	-		Upper Marker
