# Supplementary material for: 96 sample parallel acoustic fragmentation for high throughput next generation sequencing library preparation
Source: PLoS One. 2026 Feb 17;21(2):e0341139. doi: 10.1371/journal.pone.0341139 (PMC12912608; doi:10.1371/journal.pone.0341139)
Supplement: S2 Fig — (ZIP) [file pone.0341139.s002.zip › Figure 1 Raw Data/No cavitation enhancement 1080 seconds.pdf]

Filename: 2019-05-29-03- 1-8 1008 sec for 6.28 first row, 8-15 840 sec for 6.28 first row.D5000

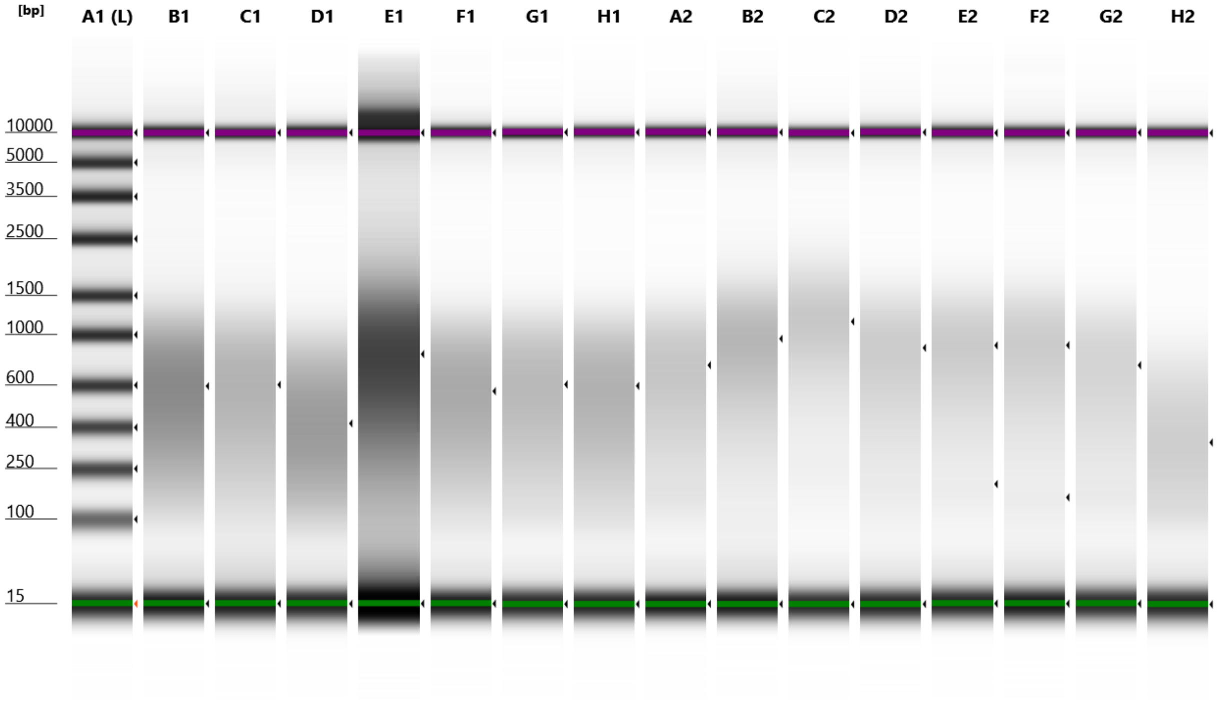

Default image (Contrast 100%)

Sample Info

Well	Conc. [ng/µl]	Sample Description	Alert	Observations
A1	29.6	Ladder		Ladder
B1	1.02	DFB 1 minus LE220 1008 sec from 6.28 first Row		
C1	0.717	DFB 2 minus LE220 1008 sec from 6.28 first Row		
D1	0.938	DFB 3 minus LE220 1008 sec from 6.28 first Row		
E1	10.5	DFB 4 minus LE220 1008 sec from 6.28 first Row		
F1	4.88	DFB 5 minus LE220 1008 sec from 6.28 first Row		
G1	0.610	DFB 6 minus LE220 1008 sec from 6.28 first Row		
H1	4.05	DFB 7 minus LE220 1008 sec from 6.28 first Row		
A2	0.520	DFB1 minus LE220 840 sec from 6.28 first Row		
B2	3.01	DFB2 minus LE220 840 sec from 6.28 first Row		
C2	2.28	DFB 3minus LE220 840 sec from 6.28 first Row		
D2	2.08	DFB4 minus LE220 840 sec from 6.28 first Row		
E2	2.20	DFB5 minus LE220 840 sec from 6.28 first Row		
F2	2.22	DFB6 minus LE220 840 sec from 6.28 first Row		
G2	1.93	DFB7 minus LE220 840 sec from 6.28 first Row		
H2	2.26	DFB8 minus LE220 840 sec from 6.28 first Row		

© Agilent Technologies, Inc. 2017

A1: Ladder

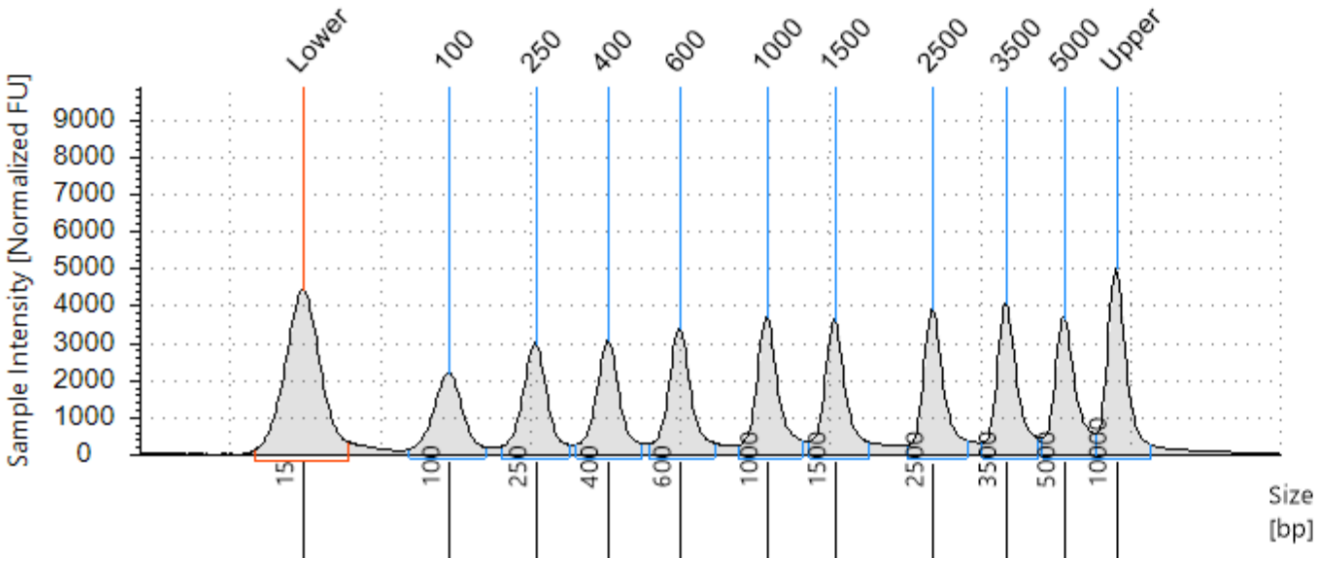

Sample Table

Well	Conc. [ng/µl]	Sample Description	Alert	Observations
A1	29.6	Ladder		Ladder

Peak Table

Size [bp]	Calibrated Conc. [ng/µl]	Assigned Conc. [ng/µl]	Peak Molarity [nmol/l]	% Integrated Area	Peak Comment	Observations
15	5.87	-	602	-		Lower Marker
100	2.80	-	43.1	9.46		
250	3.17	-	19.5	10.70		
400	3.12	-	12.0	10.52		
600	3.34	-	8.58	11.29		
1000	3.48	-	5.35	11.75		
1500	3.29	-	3.37	11.10		
2500	3.48	-	2.14	11.74		
3500	3.59	-	1.58	12.11		
5000	3.35	-	1.03	11.32		
10000	3.25	3.25	0.500	-		Upper Marker

© Agilent Technologies, Inc. 2017

B1: DFB 1 minus LE220 1008 sec from 6.28 first Row

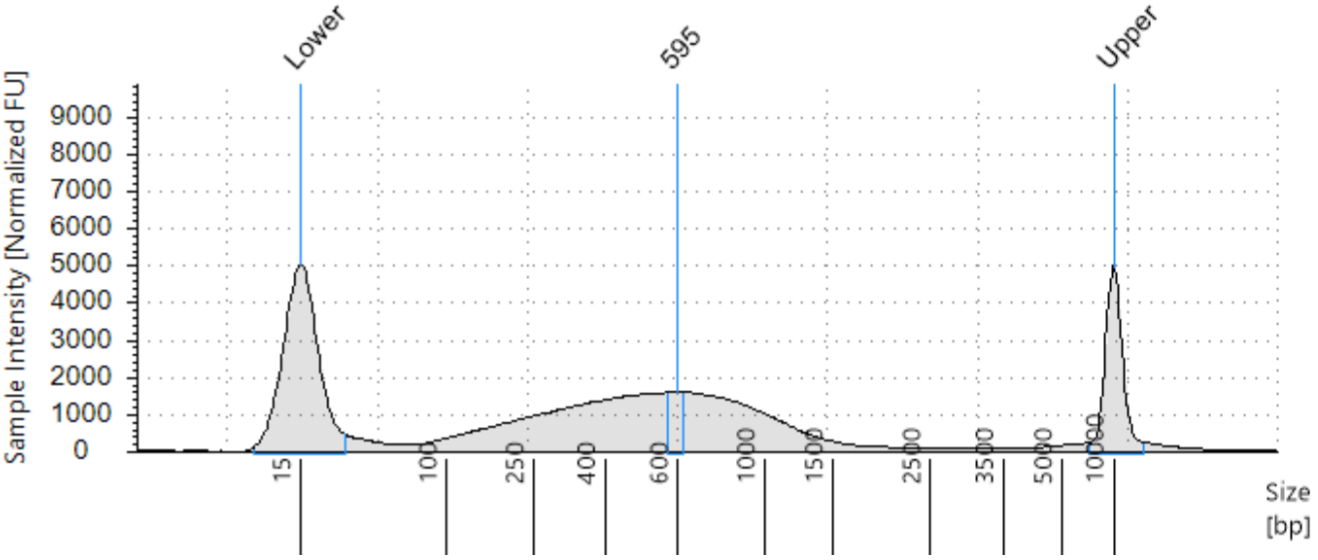

Sample Table

Well	Conc. [ng/µl]	Sample Description	Alert	Observations
B1	1.02	DFB 1 minus LE220 1008 sec from 6.28 first Row		

Peak Table

Size [bp]	Calibrated Conc. [ng/µl]	Assigned Conc. [ng/µl]	Peak Molarity [nmol/l]	% Integrated Area	Peak Comment	Observations
15	6.60	-	677	-		Lower Marker
595	1.02	-	2.63	100.00		
10000	3.25	3.25	0.500	-		Upper Marker

© Agilent Technologies, Inc. 2017

C1: DFB 2 minus LE220 1008 sec from 6.28 first Row

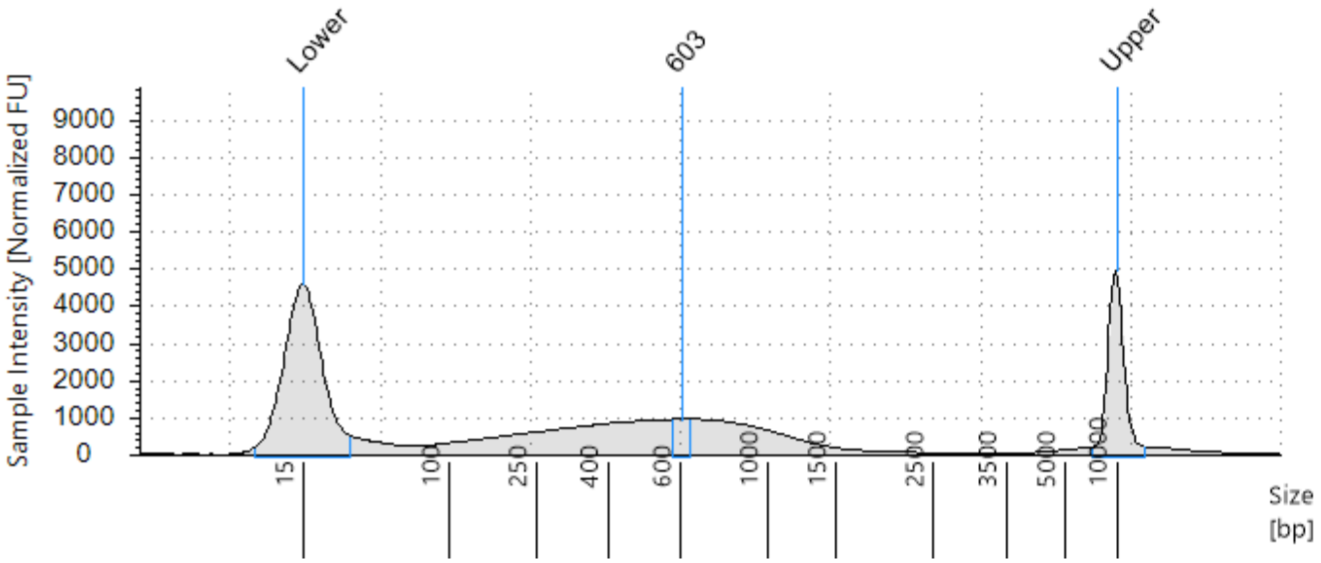

Sample Table

Well	Conc. [ng/µl]	Sample Description	Alert	Observations
C1	0.717	DFB 2 minus LE220 1008 sec from 6.28 first Row		

Peak Table

Size [bp]	Calibrated Conc. [ng/µl]	Assigned Conc. [ng/µl]	Peak Molarity [nmol/l]	% Integrated Area	Peak Comment	Observations
15	6.69	-	686	-		Lower Marker
603	0.717	-	1.83	100.00		
10000	3.25	3.25	0.500	-		Upper Marker

© Agilent Technologies, Inc. 2017

D1: DFB 3 minus LE220 1008 sec from 6.28 first Row

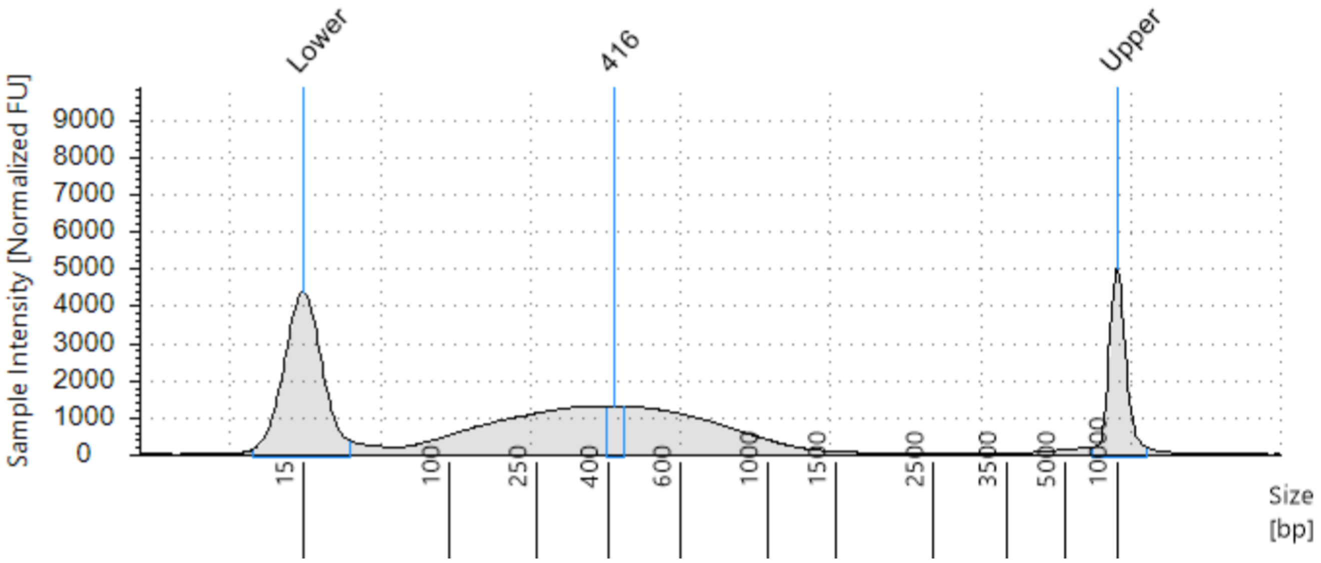

Sample Table

Well	Conc. [ng/µl]	Sample Description	Alert	Observations
D1	0.938	DFB 3 minus LE220 1008 sec from 6.28 first Row		

Peak Table

Size [bp]	Calibrated Conc. [ng/µl]	Assigned Conc. [ng/µl]	Peak Molarity [nmol/l]	% Integrated Area	Peak Comment	Observations
15	6.23	-	639	-		Lower Marker
416	0.938	-	3.47	100.00		
10000	3.25	3.25	0.500	-		Upper Marker

© Agilent Technologies, Inc. 2017

E1: DFB 4 minus LE220 1008 sec from 6.28 first Row

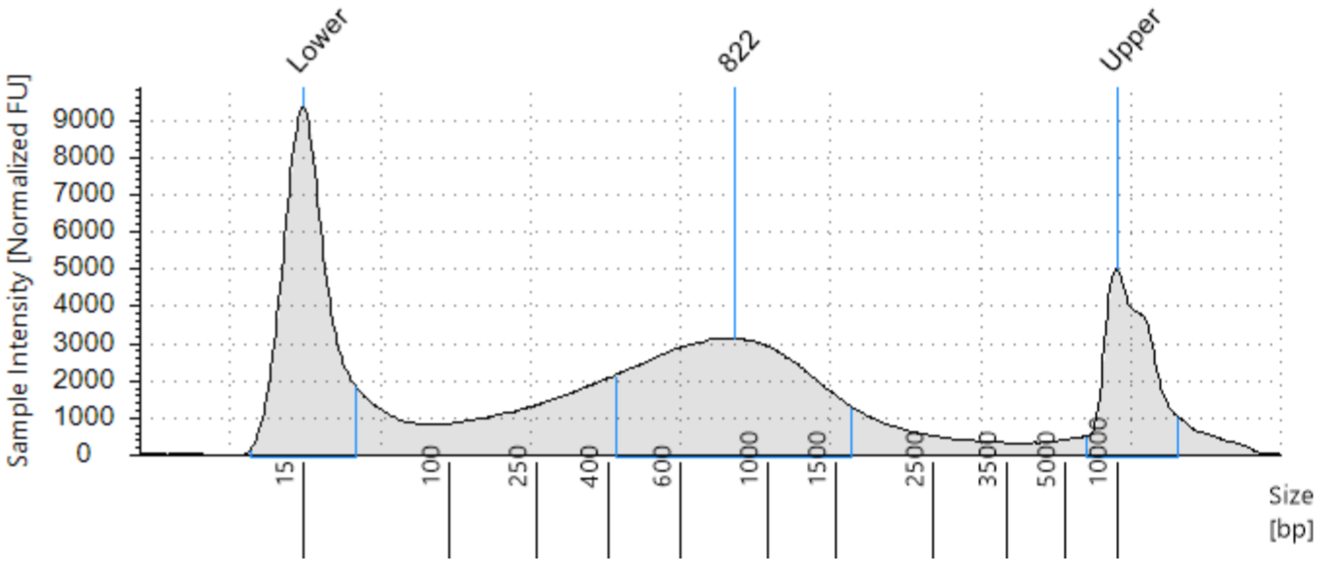

Sample Table

Well	Conc. [ng/µl]	Sample Description	Alert	Observations
E1	10.5	DFB 4 minus LE220 1008 sec from 6.28 first Row		

Peak Table

Size [bp]	Calibrated Conc. [ng/µl]	Assigned Conc. [ng/µl]	Peak Molarity [nmol/l]	% Integrated Area	Peak Comment	Observations
15	6.38	-	654	-		Lower Marker
822	10.5	-	19.6	100.00		
10000	3.25	3.25	0.500	-		Upper Marker

© Agilent Technologies, Inc. 2017

F1: DFB 5 minus LE220 1008 sec from 6.28 first Row

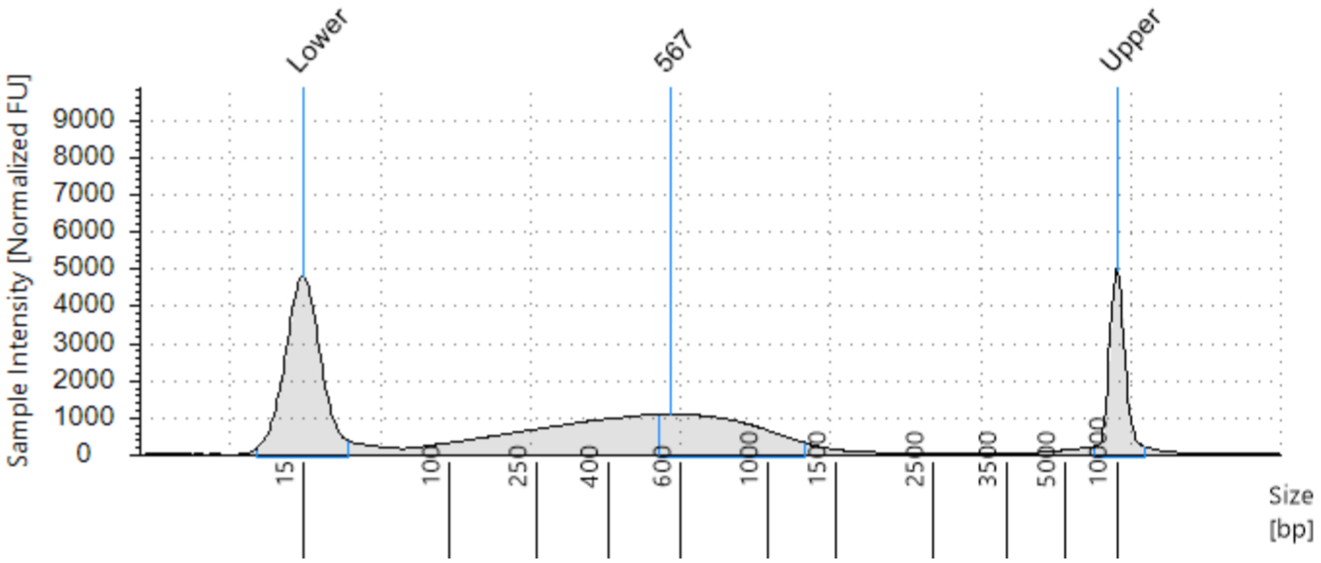

Sample Table

Well	Conc. [ng/µl]	Sample Description	Alert	Observations
F1	4.88	DFB 5 minus LE220 1008 sec from 6.28 first Row		

Peak Table

Size [bp]	Calibrated Conc. [ng/µl]	Assigned Conc. [ng/µl]	Peak Molarity [nmol/l]	% Integrated Area	Peak Comment	Observations
15	6.87	-	705	-		Lower Marker
567	4.88	-	13.3	100.00		
10000	3.25	3.25	0.500	-		Upper Marker

 © Agilent Technologies, Inc. 2017

G1: DFB 6 minus LE220 1008 sec from 6.28 first Row

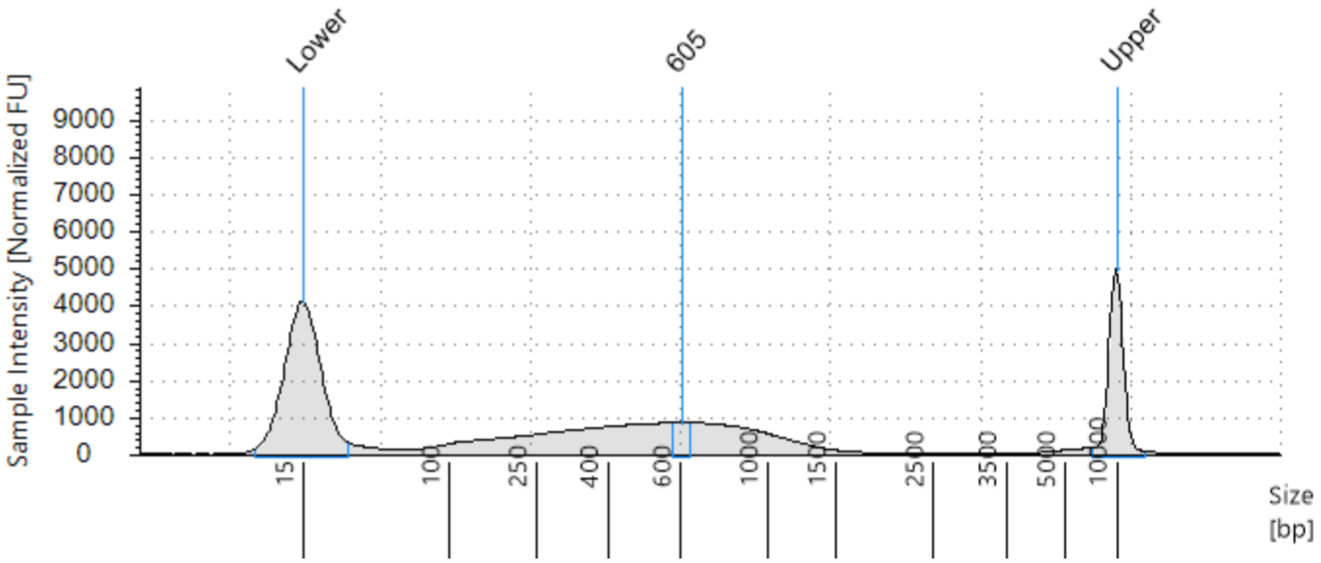

Sample Table

Well	Conc. [ng/µl]	Sample Description	Alert	Observations
G1	0.610	DFB 6 minus LE220 1008 sec from 6.28 first Row		

Peak Table

Size [bp]	Calibrated Conc. [ng/µl]	Assigned Conc. [ng/µl]	Peak Molarity [nmol/l]	% Integrated Area	Peak Comment	Observations
15	6.43	-	660	-		Lower Marker
605	0.610	-	1.55	100.00		
10000	3.25	3.25	0.500	-		Upper Marker

© Agilent Technologies, Inc. 2017

H1: DFB 7 minus LE220 1008 sec from 6.28 first Row

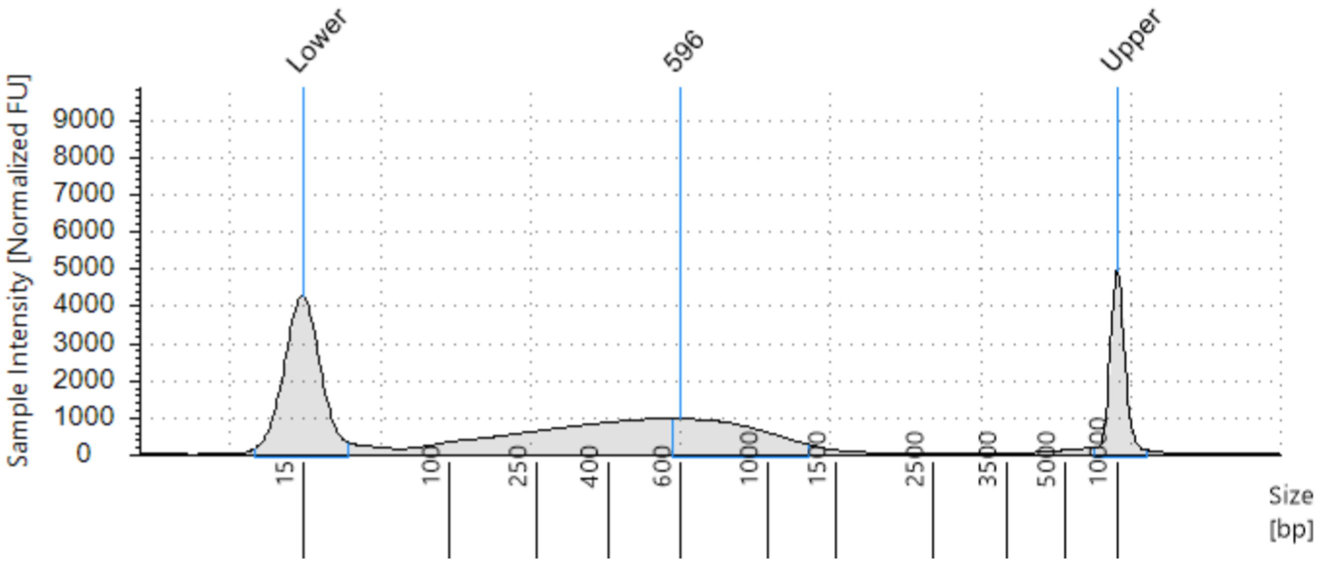

Sample Table

Well	Conc. [ng/µl]	Sample Description	Alert	Observations
H1	4.05	DFB 7 minus LE220 1008 sec from 6.28 first Row		

Peak Table

Size [bp]	Calibrated Conc. [ng/µl]	Assigned Conc. [ng/µl]	Peak Molarity [nmol/l]	% Integrated Area	Peak Comment	Observations
15	6.52	-	668	-		Lower Marker
596	4.05	-	10.5	100.00		
10000	3.25	3.25	0.500	-		Upper Marker

© Agilent Technologies, Inc. 2017

Filename: 2019-05-29-01- 1-8 1008 sec for 6.22, 8-15 1296 sec for 6.22.D5000

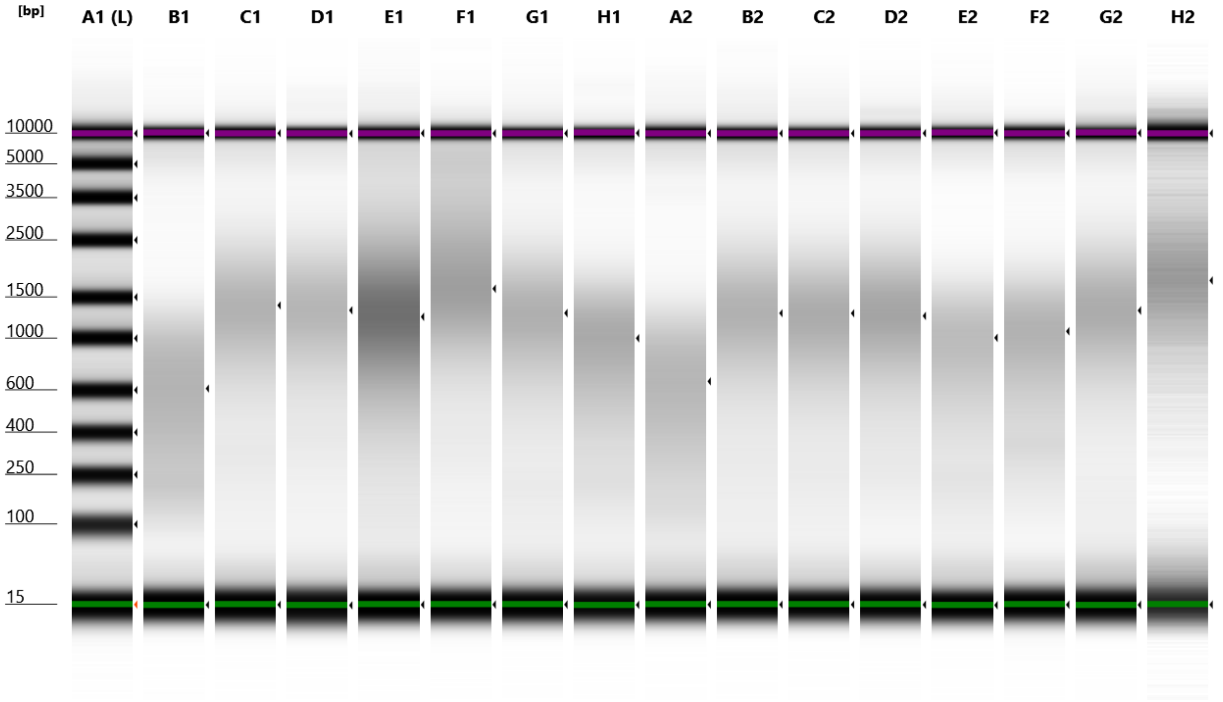

Default image (Contrast 100%)

Sample Info

Well	Conc. [ng/µl]	Sample Description	Alert	Observations
A1	32.1	Ladder		Ladder
B1	0.412	1 MINUS1E220 1008sec from 6.22		
C1	1.82	2 MINUS1E220 1008sec from 6.22		
D1	1.98	3 MINUS1E220 1008sec from 6.22		
E1	4.15	4 MINUS1E220 1008sec from 6.22		
F1	2.71	5 MINUS1E220 1008sec from 6.22		
G1	3.86	6MINUS1E220 1008sec from 6.22		
H1	2.22	7 MINUS1E220 1008sec from 6.22		
A2	0.398	DFB1 MINUS1E220 1296 sec from 6.22		
B2	3.40	DFB2 MINUS1E220 1296 sec from 6.22		
C2	2.10	DFB3 MINUS1E220 1296 sec from 6.22		
D2	2.25	DFB4 MINUS1E220 1296 sec from 6.22		
E2	1.65	DFB5 MINUS1E220 1296 sec from 6.22		
F2	0.412	DFB6 MINUS1E220 1296 sec from 6.22		
G2	0.473	DFB7 MINUS1E220 1296 sec from 6.22		
H2	0.442	DFB8 MINUS1E220 1296 sec from 6.22		

© Agilent Technologies, Inc. 2017

A1: Ladder

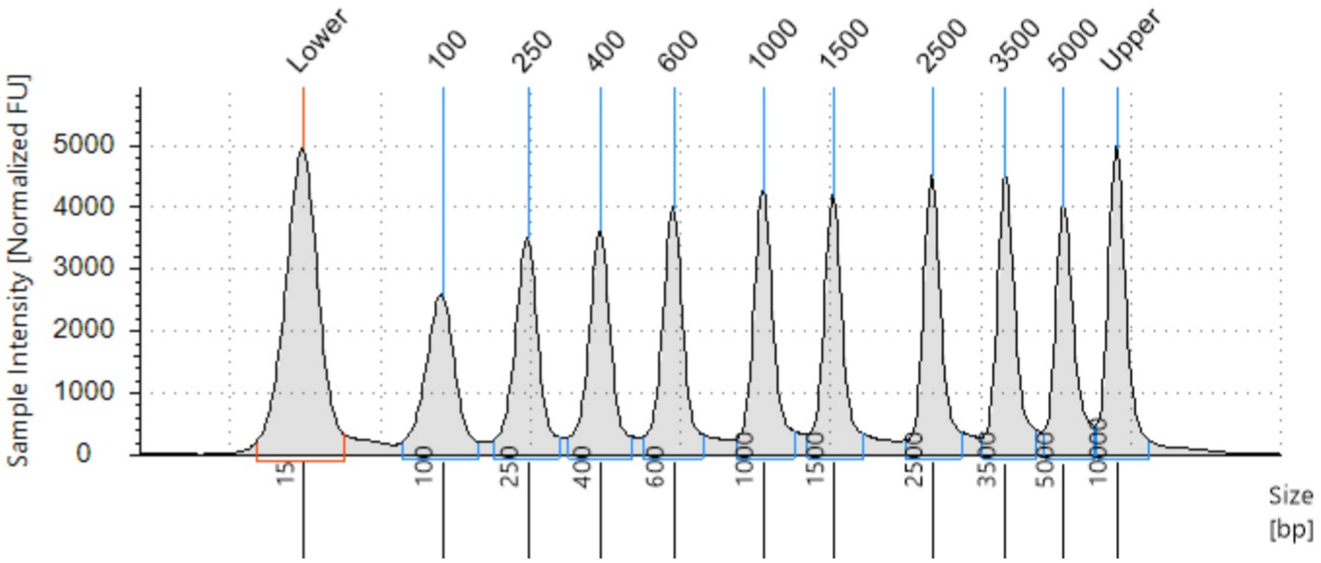

Sample Table

Well	Conc. [ng/µl]	Sample Description	Alert	Observations
A1	32.1	Ladder		Ladder

Peak Table

Size [bp]	Calibrated Conc. [ng/µl]	Assigned Conc. [ng/µl]	Peak Molarity [nmol/l]	% Integrated Area	Peak Comment	Observations
15	6.25	-	641	-		Lower Marker
100	3.36	-	51.7	10.45		
250	3.62	-	22.3	11.26		
400	3.51	-	13.5	10.92		
600	3.68	-	9.42	11.44		
1000	3.75	-	5.77	11.66		
1500	3.51	-	3.60	10.92		
2500	3.63	-	2.23	11.29		
3500	3.69	-	1.62	11.49		
5000	3.39	-	1.04	10.56		
10000	3.25	3.25	0.500	-		Upper Marker

© Agilent Technologies, Inc. 2017

B1: 1 MINUS IE220 1008sec from 6.22

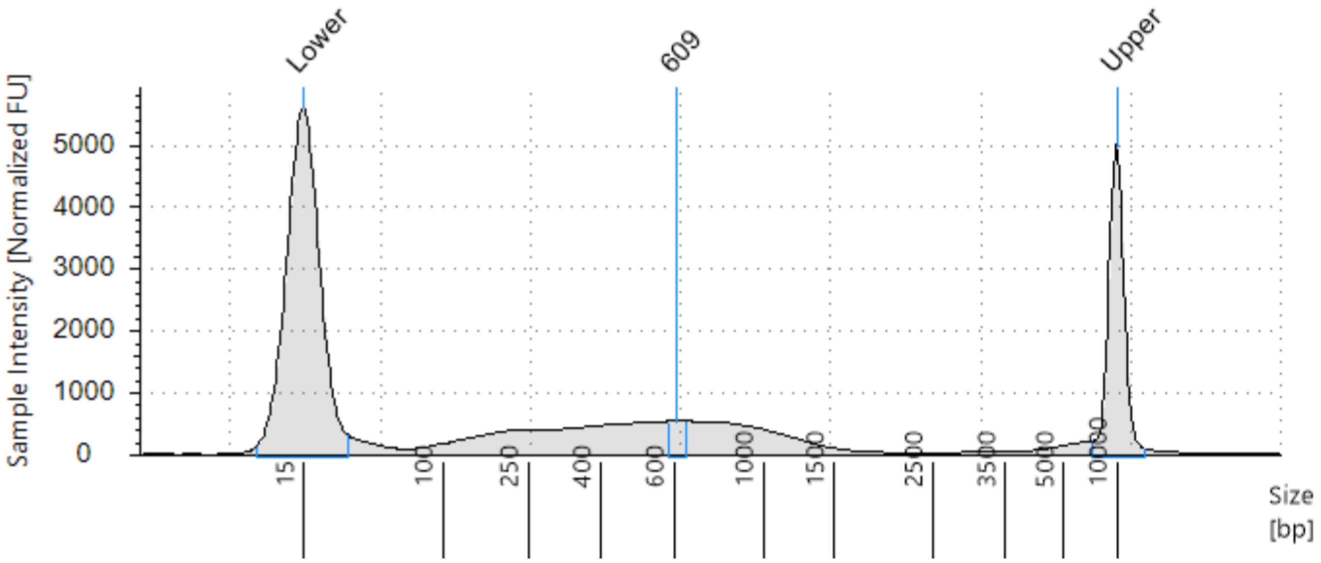

Sample Table

Well	Conc. [ng/µl]	Sample Description	Alert	Observations
B1	0.412	1 MINUS IE220 1008sec from 6.22		

Peak Table

Size [bp]	Calibrated Conc. [ng/µl]	Assigned Conc. [ng/µl]	Peak Molarity [nmol/l]	% Integrated Area	Peak Comment	Observations
15	7.81	-	801	-		Lower Marker
609	0.412	-	1.04	100.00		
10000	3.25	3.25	0.500	-		Upper Marker

© Agilent Technologies, Inc. 2017

C1: 2 MINUS IE220 1008sec from 6.22

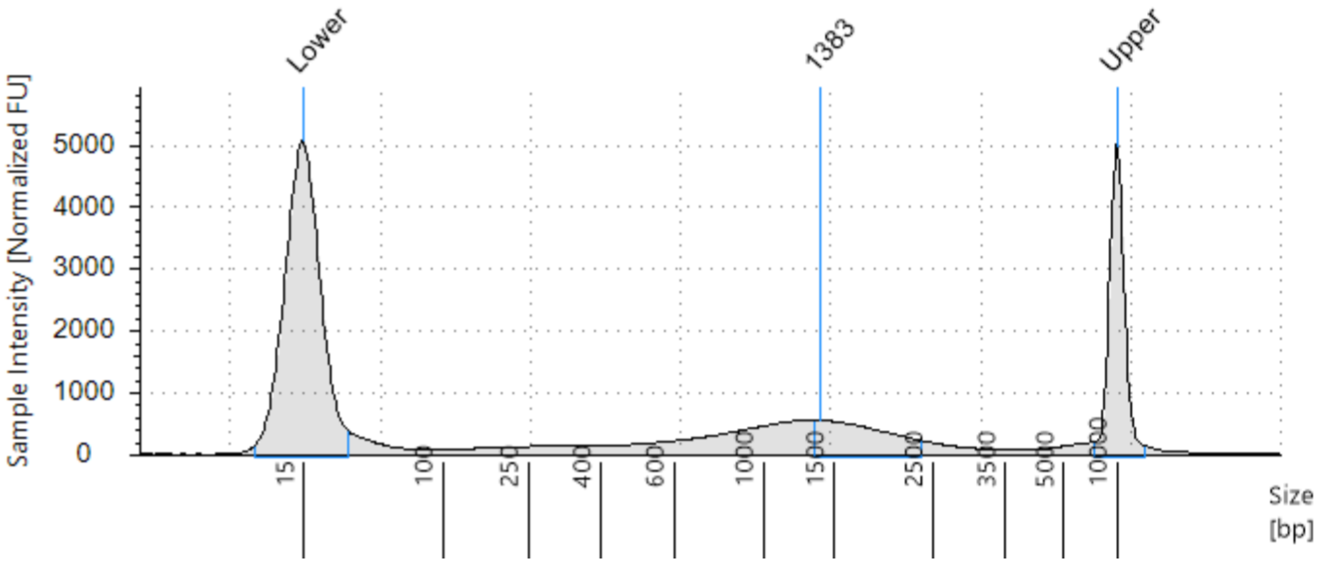

Sample Table

Well	Conc. [ng/µl]	Sample Description	Alert	Observations
C1	1.82	2 MINUSIE220 1008sec from 6.22		

Peak Table

Size [bp]	Calibrated Conc. [ng/µl]	Assigned Conc. [ng/µl]	Peak Molarity [nmol/l]	% Integrated Area	Peak Comment	Observations
15	7.59	-	779	-		Lower Marker
1383	1.82	-	2.02	100.00		
10000	3.25	3.25	0.500	-		Upper Marker

© Agilent Technologies, Inc. 2017

D1: 3 MINUS IE220 1008sec from 6.22

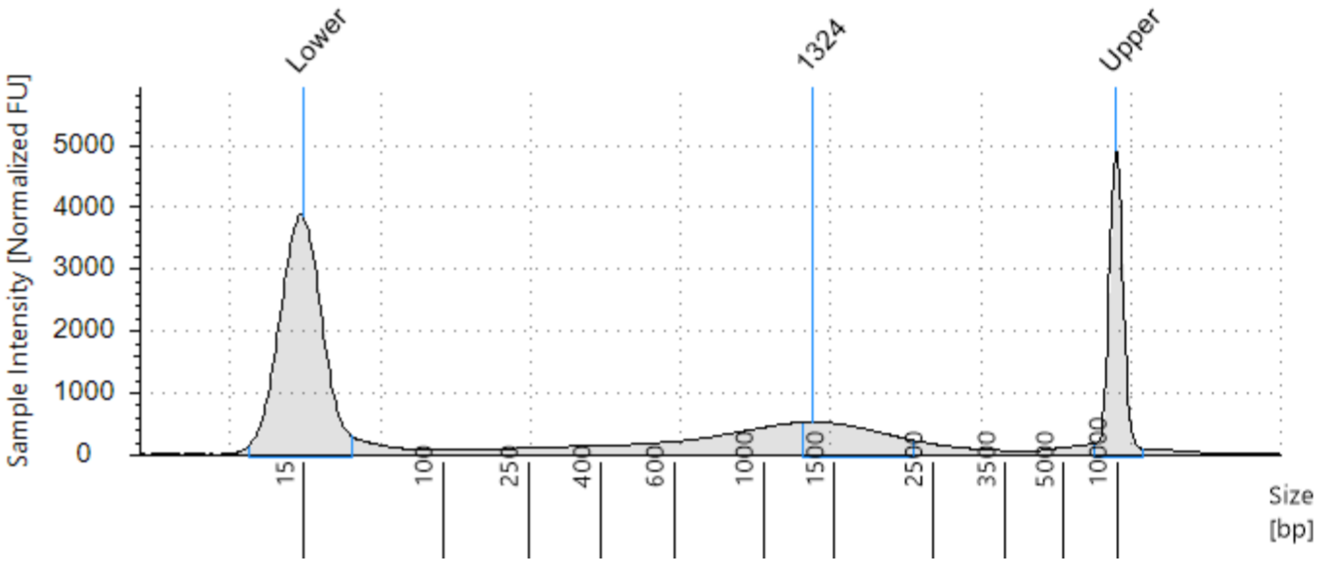

Sample Table

Well	Conc. [ng/µl]	Sample Description	Alert	Observations
D1	1.98	3 MINUS IE220 1008sec from 6.22		

Peak Table

Size [bp]	Calibrated Conc. [ng/µl]	Assigned Conc. [ng/µl]	Peak Molarity [nmol/l]	% Integrated Area	Peak Comment	Observations
15	7.13	-	731	-		Lower Marker
1324	1.98	-	2.30	100.00		
10000	3.25	3.25	0.500	-		Upper Marker

© Agilent Technologies, Inc. 2017

E1: 4 MINUS IE220 1008sec from 6.22

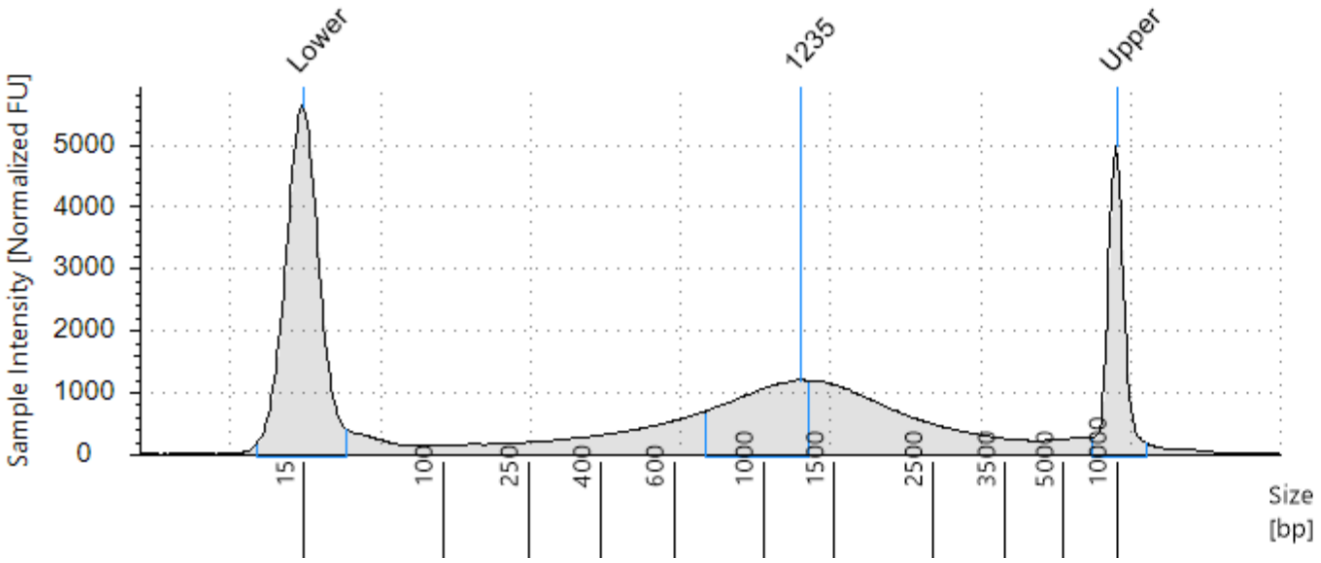

Sample Table

Well	Conc. [ng/µl]	Sample Description	Alert	Observations
E1	4.15	4 MINUS IE220 1008sec from 6.22		

Peak Table

Size [bp]	Calibrated Conc. [ng/µl]	Assigned Conc. [ng/µl]	Peak Molarity [nmol/l]	% Integrated Area	Peak Comment	Observations
15	7.75	-	795	-		Lower Marker
1235	4.15	-	5.17	100.00		
10000	3.25	3.25	0.500	-		Upper Marker

© Agilent Technologies, Inc. 2017

F1: 5 MINUS IE220 1008sec from 6.22

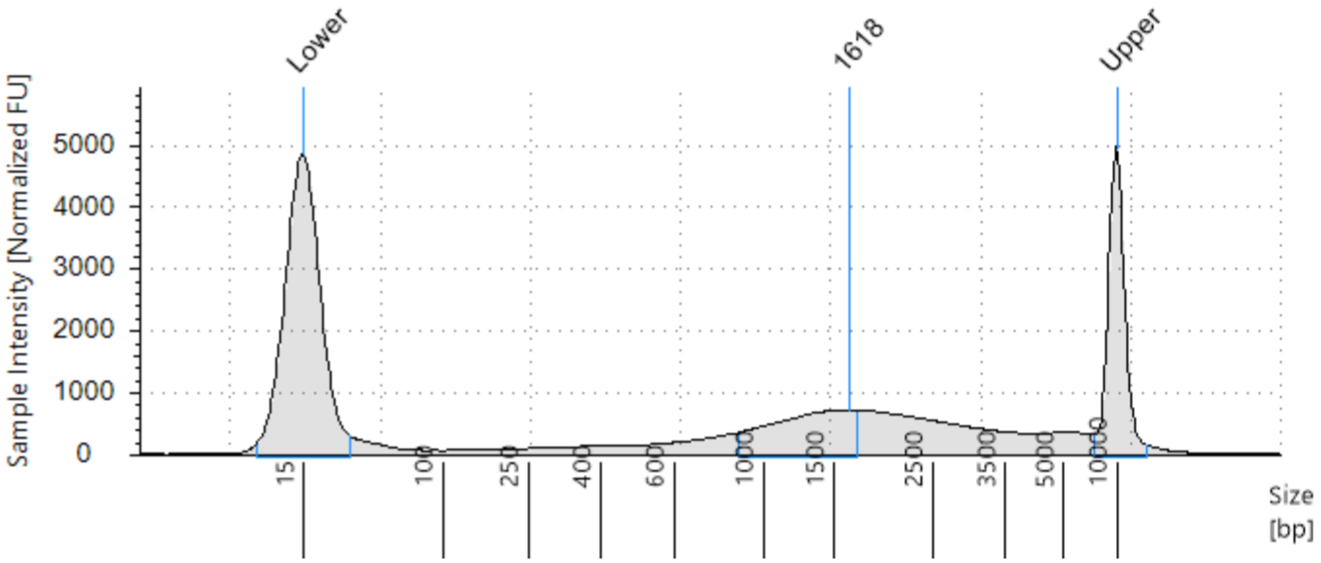

Sample Table

Well	Conc. [ng/µl]	Sample Description	Alert	Observations
F1	2.71	5 MINUSIE220 1008sec from 6.22		

Peak Table

Size [bp]	Calibrated Conc. [ng/µl]	Assigned Conc. [ng/µl]	Peak Molarity [nmol/l]	% Integrated Area	Peak Comment	Observations
15	6.96	-	714	-		Lower Marker
1618	2.71	-	2.58	100.00		
10000	3.25	3.25	0.500	-		Upper Marker

© Agilent Technologies, Inc. 2017

G1: 6MINUS IE220 1008sec from 6.22

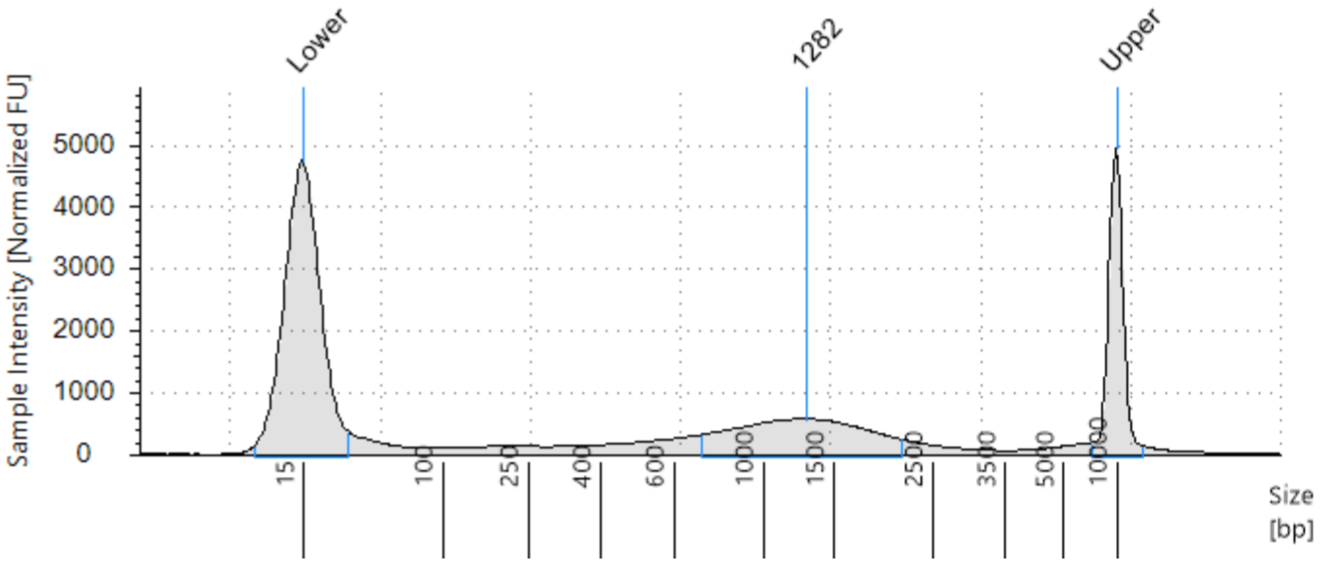

Sample Table

Well	Conc. [ng/µl]	Sample Description	Alert	Observations
G1	3.86	6MINUS IE220 1008sec from 6.22		

Peak Table

Size [bp]	Calibrated Conc. [ng/µl]	Assigned Conc. [ng/µl]	Peak Molarity [nmol/l]	% Integrated Area	Peak Comment	Observations
15	7.45	-	764	-		Lower Marker
1282	3.86	-	4.63	100.00		
10000	3.25	3.25	0.500	-		Upper Marker

© Agilent Technologies, Inc. 2017

H1: 7 MINUS IE220 1008sec from 6.22

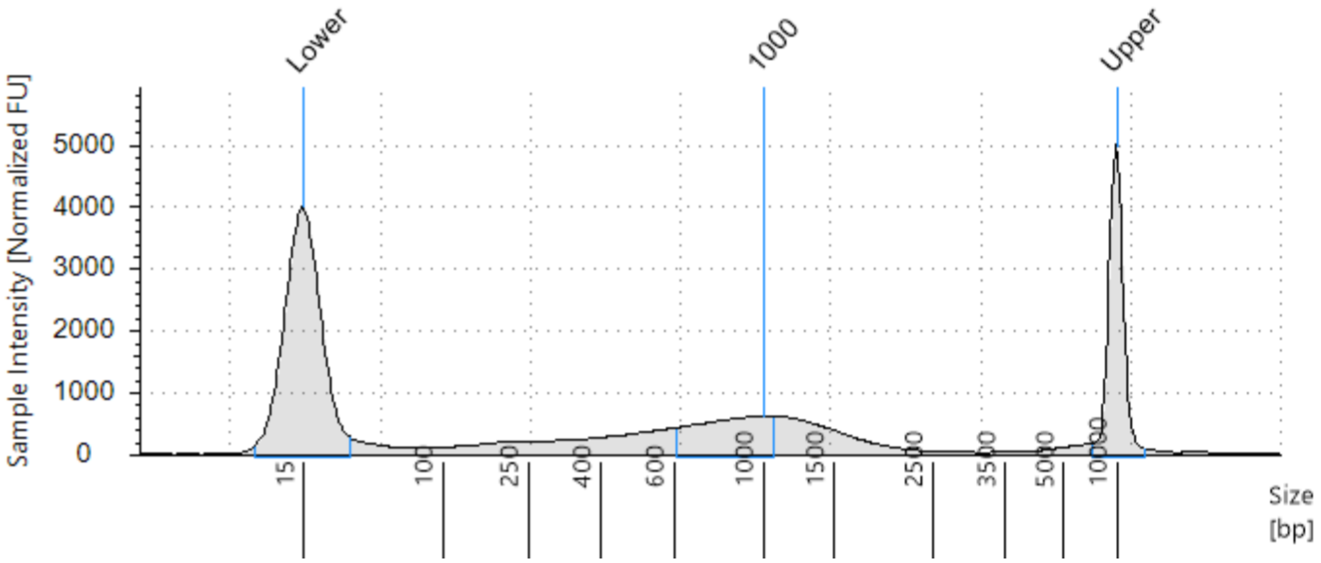

Sample Table

Well	Conc. [ng/µl]	Sample Description	Alert	Observations
H1	2.22	7 MINUSIE220 1008sec from 6.22		

Peak Table

Size [bp]	Calibrated Conc. [ng/µl]	Assigned Conc. [ng/µl]	Peak Molarity [nmol/l]	% Integrated Area	Peak Comment	Observations
15	6.31	-	647	-		Lower Marker
1000	2.22	-	3.41	100.00		
10000	3.25	3.25	0.500	-		Upper Marker

© Agilent Technologies, Inc. 2017

Filename: 2019-05-21-02 DFB plus, first 7, 80 sec last 8 minus 1080 sec.D5000

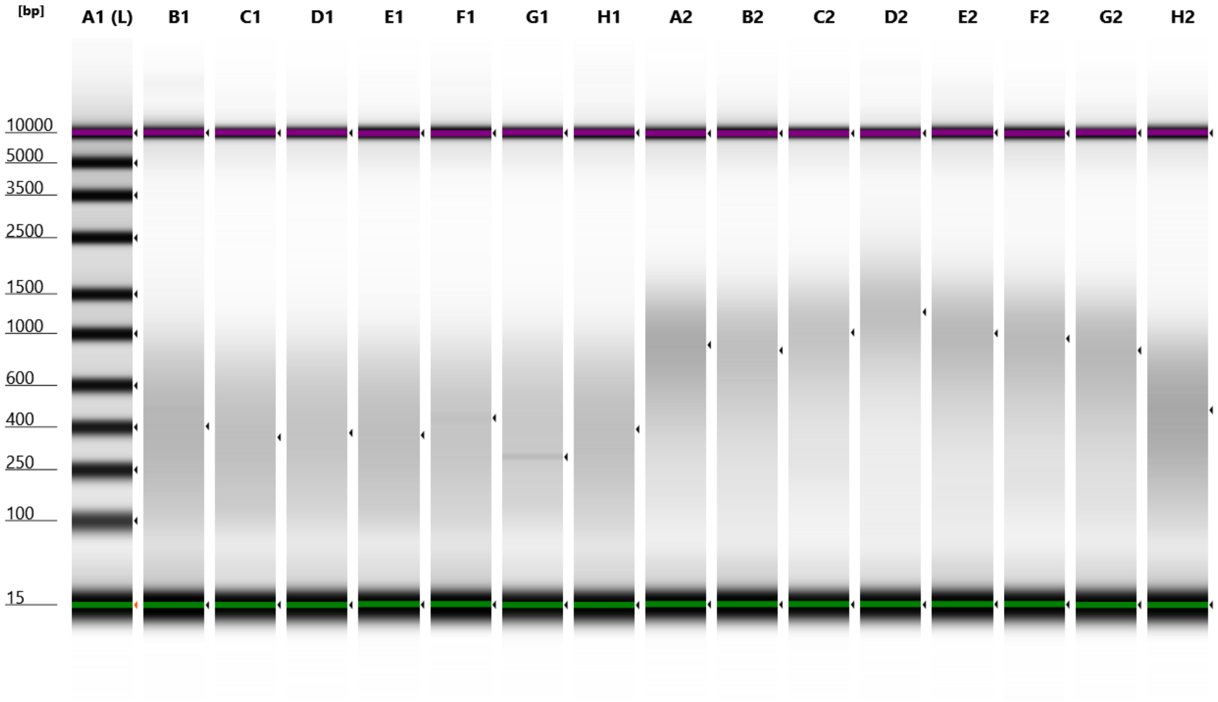

Default image (Contrast 100%)

Sample Info

Well	Conc. [ng/µl]	Sample Description	Alert	Observations
A1	30.9	Ladder		Ladder
B1	1.30	DFB 1 plus 80 sec R3		
C1	0.419	DFB2 plus 80 sec R3		
D1	0.386	DFB3 plus 80 sec R3		
E1	0.915	DFB4 plus 80 sec R3		
F1	0.815	DFB5 plus 80 sec R3		
G1	0.846	DFB6 plus 80 sec R3		
H1	0.360	DFB7 plus 80 sec R3		
A2	2.30	DFB1 minus 1080 sec R3		
B2	0.391	DFB2 minus 1080 sec R3		
C2	1.69	DFB3 minus 1080 sec R3		
D2	3.24	DFB4 minus 1080 sec R3		
E2	3.38	DFB5 minus 1080 sec R3		
F2	3.36	DFB6 minus 1080 sec R3		
G2	2.07	DFB7 minus 1080 sec R3		
H2	0.925	DFB8 minus 1080 sec R3		

© Agilent Technologies, Inc. 2017

A1: Ladder

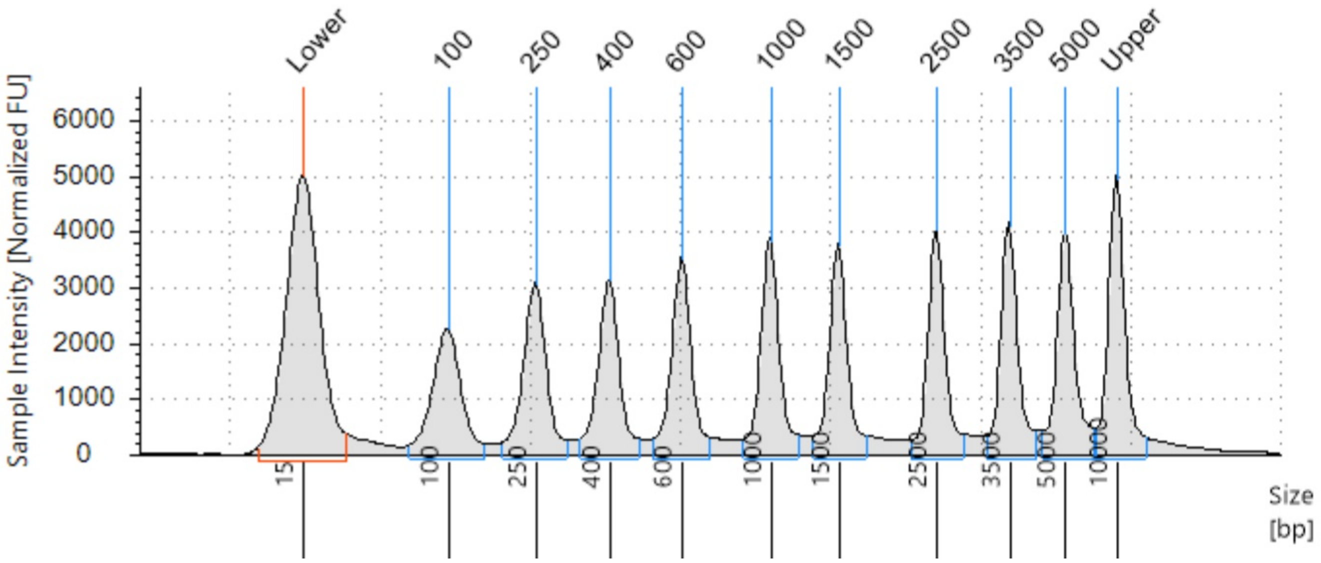

Sample Table

Well	Conc. [ng/µl]	Sample Description	Alert	Observations
A1	30.9	Ladder		Ladder

Peak Table

Size [bp]	Calibrated Conc. [ng/µl]	Assigned Conc. [ng/µl]	Peak Molarity [nmol/l]	% Integrated Area	Peak Comment	Observations
15	7.12	-	731	-		Lower Marker
100	3.20	-	49.2	10.36		
250	3.46	-	21.3	11.23		
400	3.28	-	12.6	10.62		
600	3.47	-	8.89	11.24		
1000	3.57	-	5.50	11.58		
1500	3.37	-	3.45	10.92		
2500	3.43	-	2.11	11.12		
3500	3.54	-	1.56	11.47		
5000	3.54	-	1.09	11.48		
10000	3.25	3.25	0.500	-		Upper Marker

© Agilent Technologies, Inc. 2017

A2: DFB1 minus 1080 sec R3

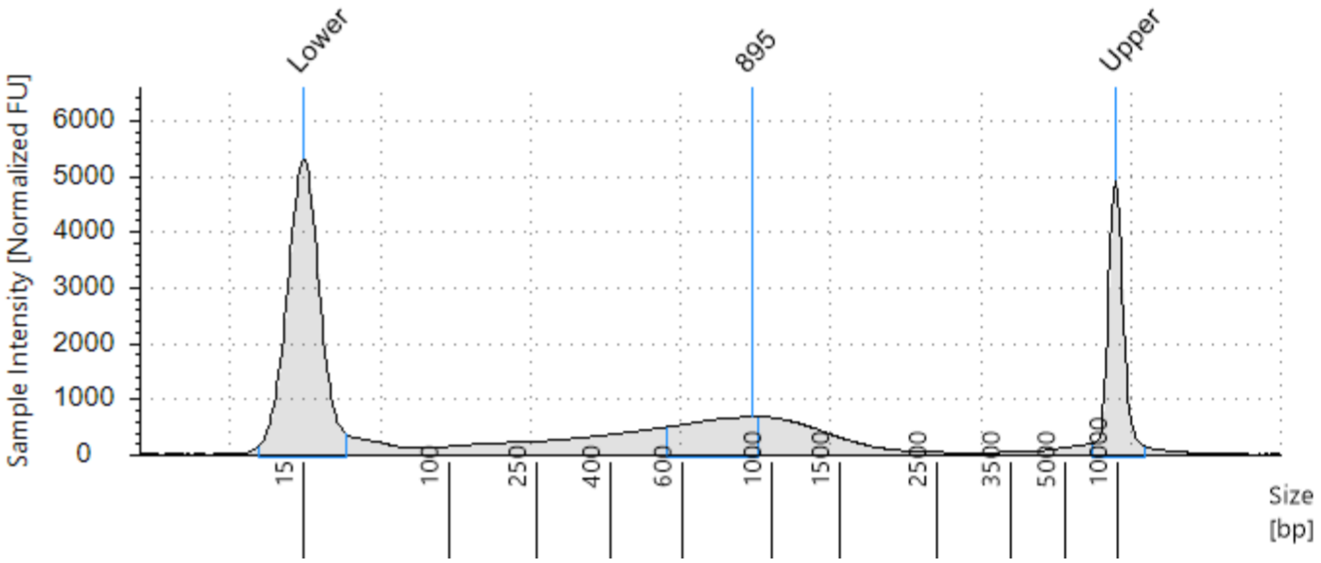

Sample Table

Well	Conc. [ng/µl]	Sample Description	Alert	Observations
A2	2.30	DFB1 minus 1080 sec R3		

Peak Table

Size [bp]	Calibrated Conc. [ng/µl]	Assigned Conc. [ng/µl]	Peak Molarity [nmol/l]	% Integrated Area	Peak Comment	Observations
15	7.33	-	752	-		Lower Marker
895	2.30	-	3.96	100.00		
10000	3.25	3.25	0.500	-		Upper Marker

© Agilent Technologies, Inc. 2017

B2: DFB2 minus 1080 sec R3

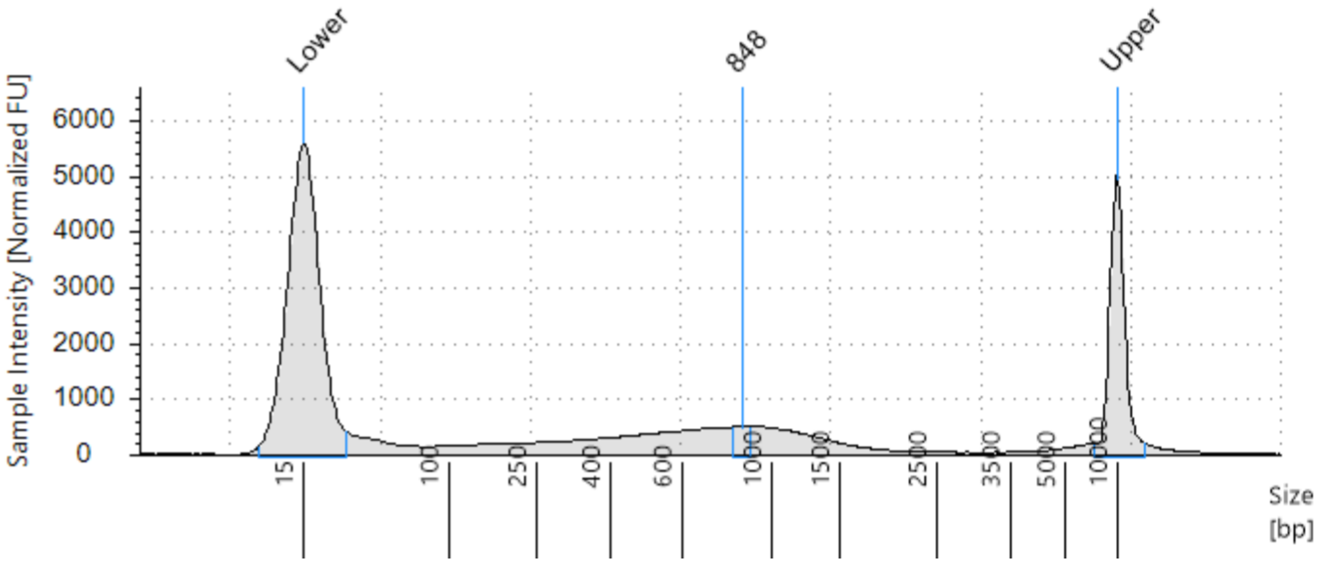

Sample Table

Well	Conc. [ng/µl]	Sample Description	Alert	Observations
B2	0.391	DFB2 minus 1080 sec R3		

Peak Table

Size [bp]	Calibrated Conc. [ng/µl]	Assigned Conc. [ng/µl]	Peak Molarity [nmol/l]	% Integrated Area	Peak Comment	Observations
15	7.84	-	804	-		Lower Marker
848	0.391	-	0.709	100.00		
10000	3.25	3.25	0.500	-		Upper Marker

© Agilent Technologies, Inc. 2017

C2: DFB3 minus 1080 sec R3

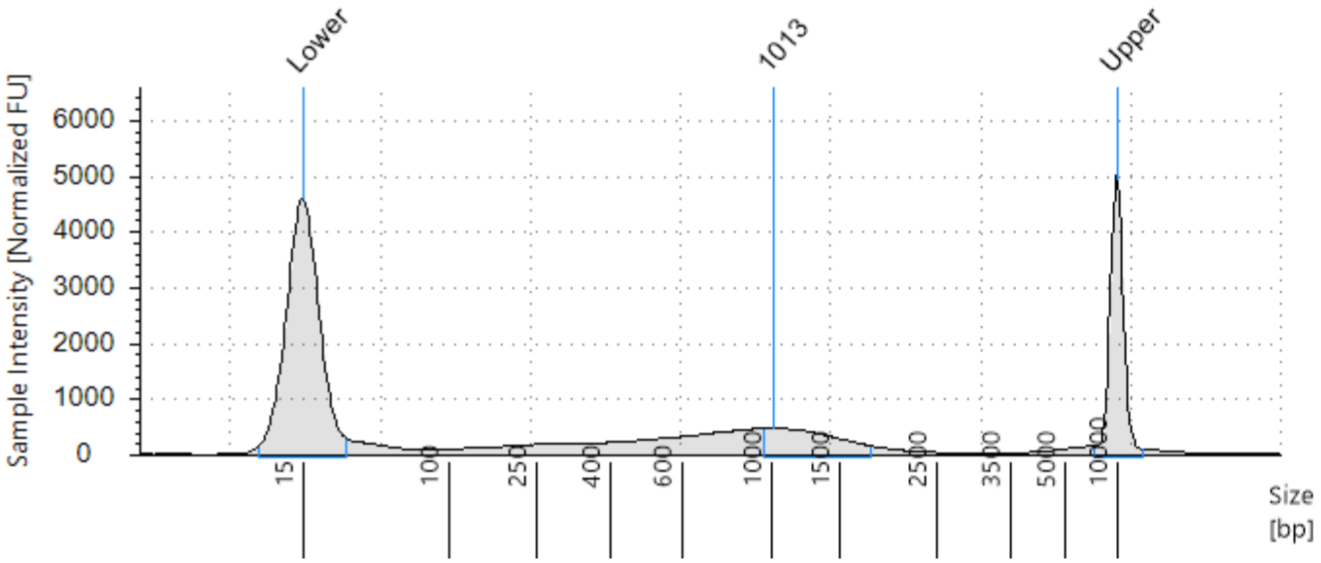

Sample Table

Well	Conc. [ng/µl]	Sample Description	Alert	Observations
C2	1.69	DFB3 minus 1080 sec R3		

Peak Table

Size [bp]	Calibrated Conc. [ng/µl]	Assigned Conc. [ng/µl]	Peak Molarity [nmol/l]	% Integrated Area	Peak Comment	Observations
15	7.09	-	728	-		Lower Marker
1013	1.69	-	2.57	100.00		
10000	3.25	3.25	0.500	-		Upper Marker

© Agilent Technologies, Inc. 2017

D2: DFB4 minus 1080 sec R3

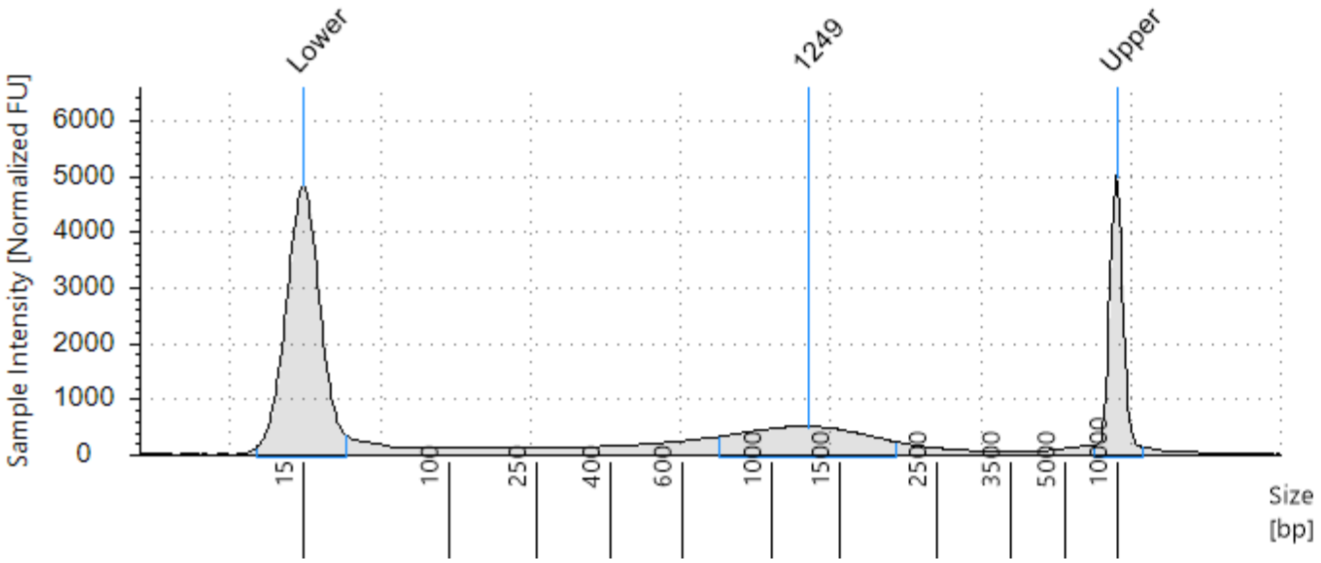

Sample Table

Well	Conc. [ng/µl]	Sample Description	Alert	Observations
D2	3.24	DFB4 minus 1080 sec R3		

Peak Table

Size [bp]	Calibrated Conc. [ng/µl]	Assigned Conc. [ng/µl]	Peak Molarity [nmol/l]	% Integrated Area	Peak Comment	Observations
15	7.58	-	777	-		Lower Marker
1249	3.24	-	3.99	100.00		
10000	3.25	3.25	0.500	-		Upper Marker

© Agilent Technologies, Inc. 2017
