# Supplementary material for: 96 sample parallel acoustic fragmentation for high throughput next generation sequencing library preparation
Source: PLoS One. 2026 Feb 17;21(2):e0341139. doi: 10.1371/journal.pone.0341139 (PMC12912608; doi:10.1371/journal.pone.0341139)
Supplement: S5 File — (ZIP) [file pone.0341139.s005.zip › QSonica translator TapeStation raw data/QSonica no cavitation enhancement translator 96-well plate replicate 3.pdf]

Filename: 2020-10-13 Q-S MINUS A1- H2 EXP H1 R3.D1000

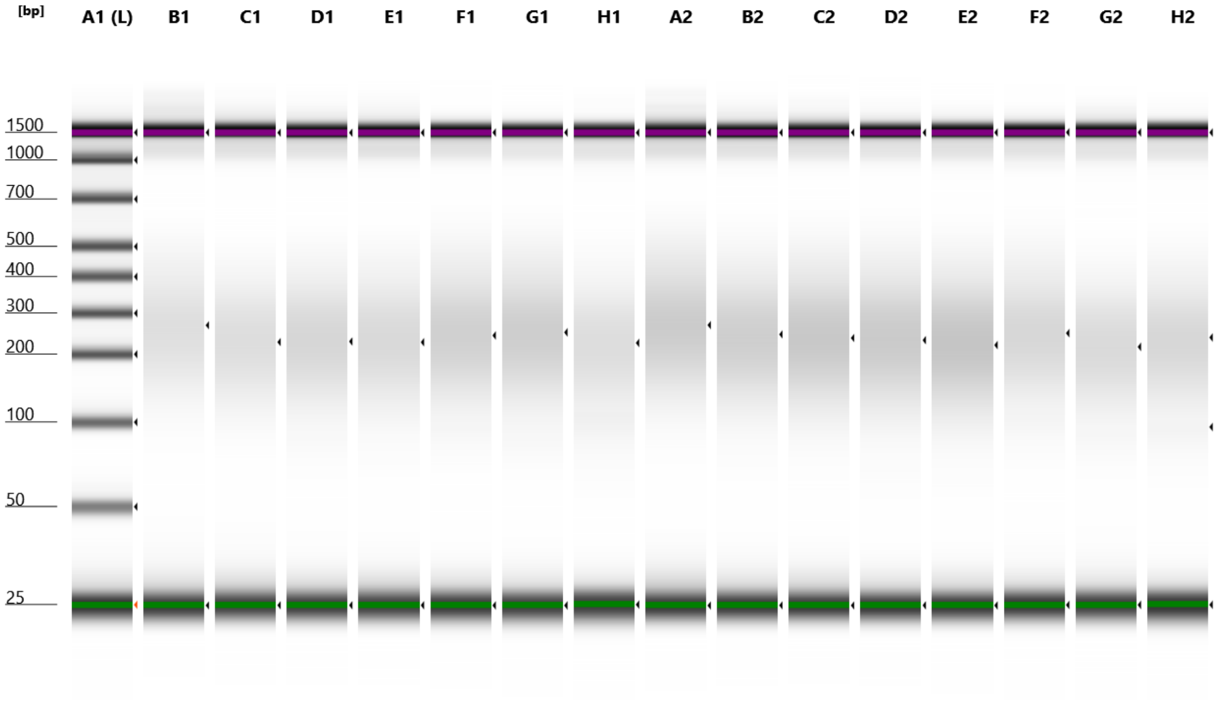

Default image (Contrast 100%)

Sample Info

Well	Conc. [ng/µl]	Sample Description	Alert	Observations
A1	15.8	Ladder		Ladder
B1	0.999	A1 M R3		
C1	0.231	B1 M R3		
D1	1.44	C1 M R3		
E1	0.207	D1 M R3		
F1	1.86	E1 M R3		
G1	3.60	F1 M R3		
H1	1.24	G1 M R3		
A2	2.05	A2 M R3		
B2	3.37	B2 M R3		
C2	0.625	C2 M R3		
D2	0.342	D2 M R3		
E2	0.665	E2 M R3		
F2	1.56	F2 M R3		
G2	1.41	G2 M R3		
H2	1.55	H2 M R3		

 © Agilent Technologies, Inc. 2017

A1: Ladder

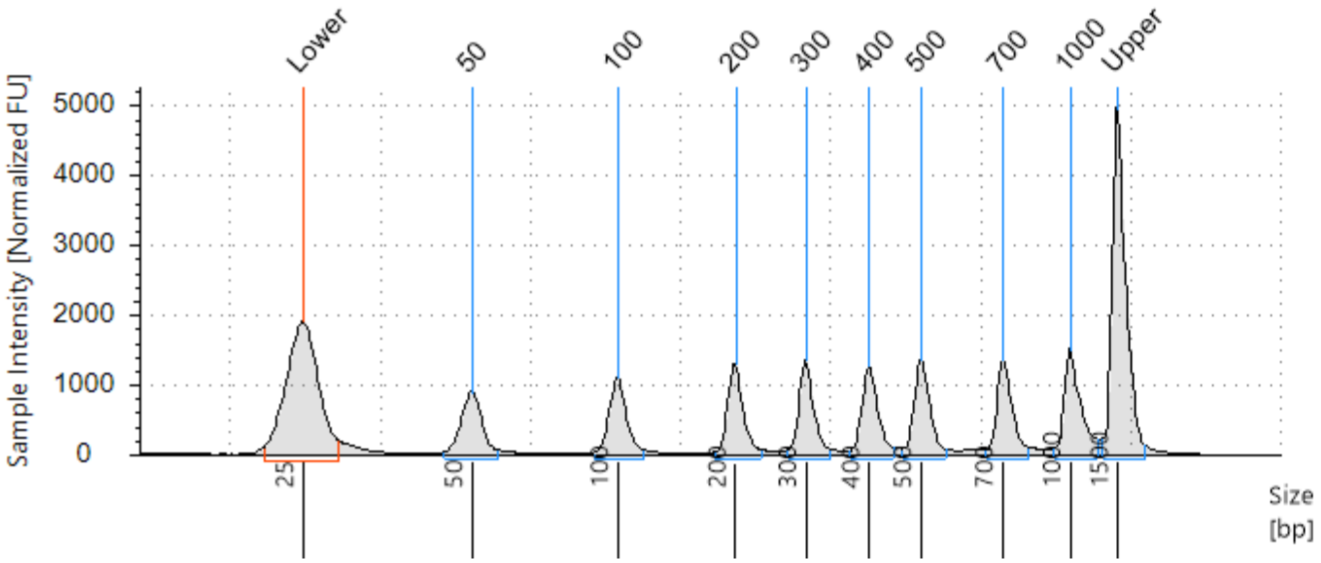

Sample Table

Well	Conc. [ng/µl]	Sample Description	Alert	Observations
A1	15.8	Ladder		Ladder

Peak Table

Size [bp]	Calibrated Conc. [ng/µl]	Assigned Conc. [ng/µl]	Peak Molarity [nmol/l]	% Integrated Area	Peak Comment	Observations
25	5.88	-	362	-		Lower Marker
50	1.78	-	54.8	11.25		
100	1.85	-	28.4	11.67		
200	1.95	-	15.0	12.30		
300	1.93	-	9.87	12.17		
400	1.95	-	7.49	12.30		
500	2.05	-	6.30	12.95		
700	2.01	-	4.43	12.73		
1000	2.32	-	3.56	14.63		
1500	6.50	6.50	6.67	-		Upper Marker

© Agilent Technologies, Inc. 2017

B1: A1 M R3

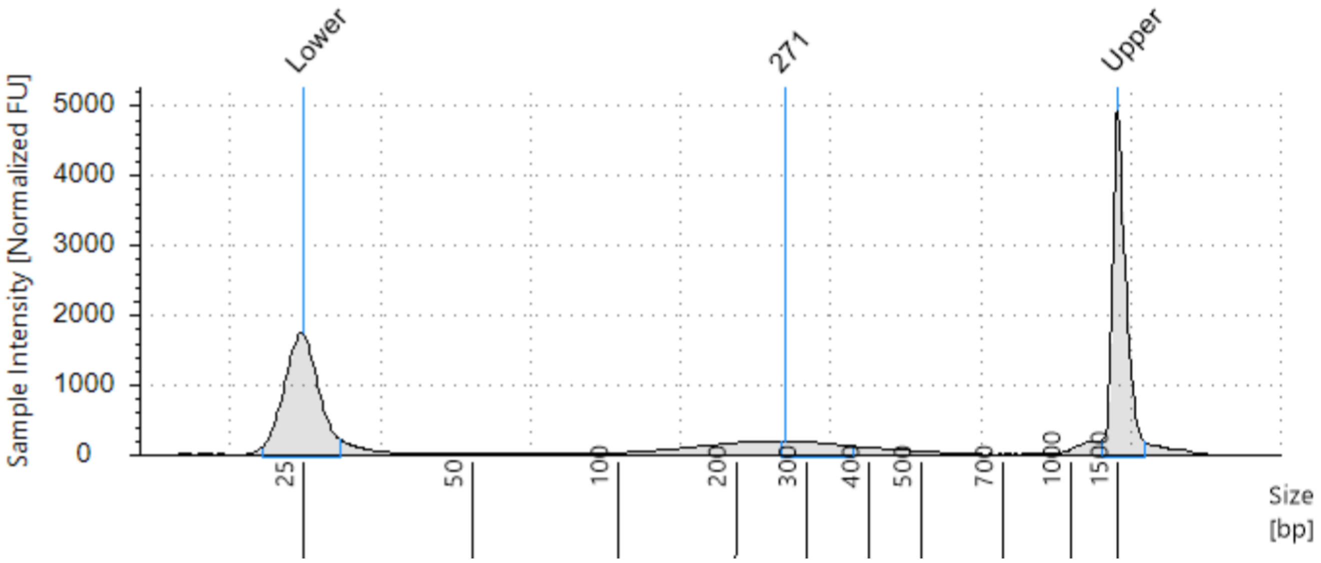

Sample Table

Well	Conc. [ng/µl]	Sample Description	Alert	Observations
B1	0.999	A1 M R3		

Peak Table

Size [bp]	Calibrated Conc. [ng/µl]	Assigned Conc. [ng/µl]	Peak Molarity [nmol/l]	% Integrated Area	Peak Comment	Observations
25	5.88	-	362	-		Lower Marker
271	0.999	-	5.67	100.00		
1500	6.50	6.50	6.67	-		Upper Marker

 © Agilent Technologies, Inc. 2017

C1: B1 M R3

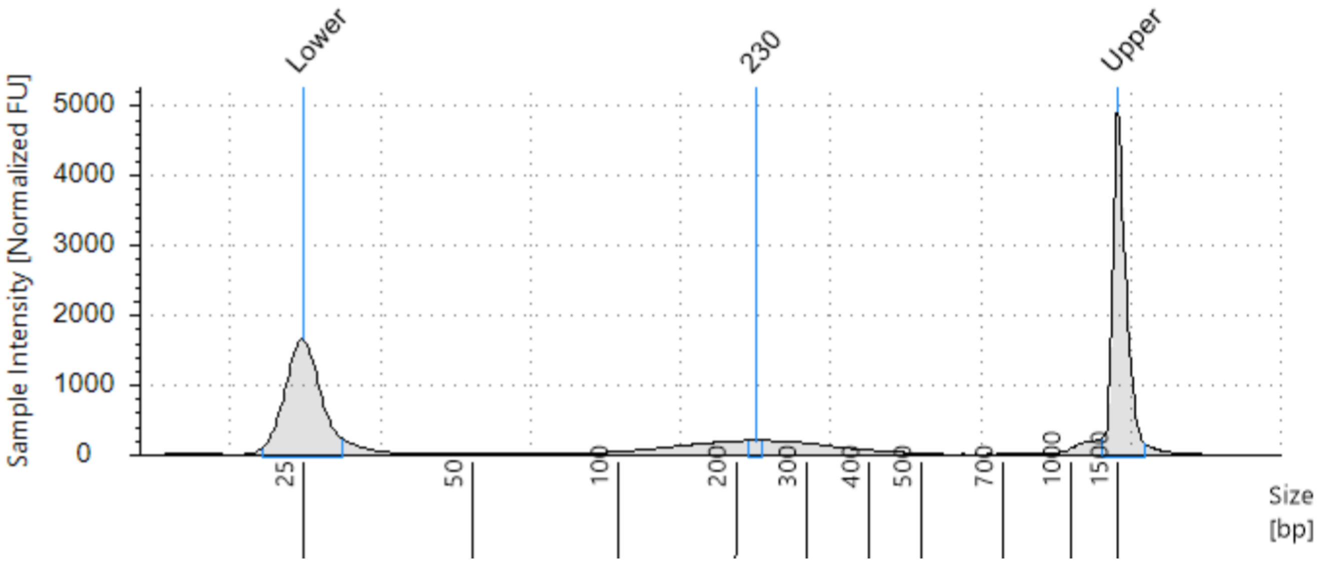

Sample Table

Well	Conc. [ng/µl]	Sample Description	Alert	Observations
C1	0.231	B1 M R3		

Peak Table

Size [bp]	Calibrated Conc. [ng/µl]	Assigned Conc. [ng/µl]	Peak Molarity [nmol/l]	% Integrated Area	Peak Comment	Observations
25	5.71	-	352	-		Lower Marker
230	0.231	-	1.54	100.00		
1500	6.50	6.50	6.67	-		Upper Marker

© Agilent Technologies, Inc. 2017

D1: C1 M R3

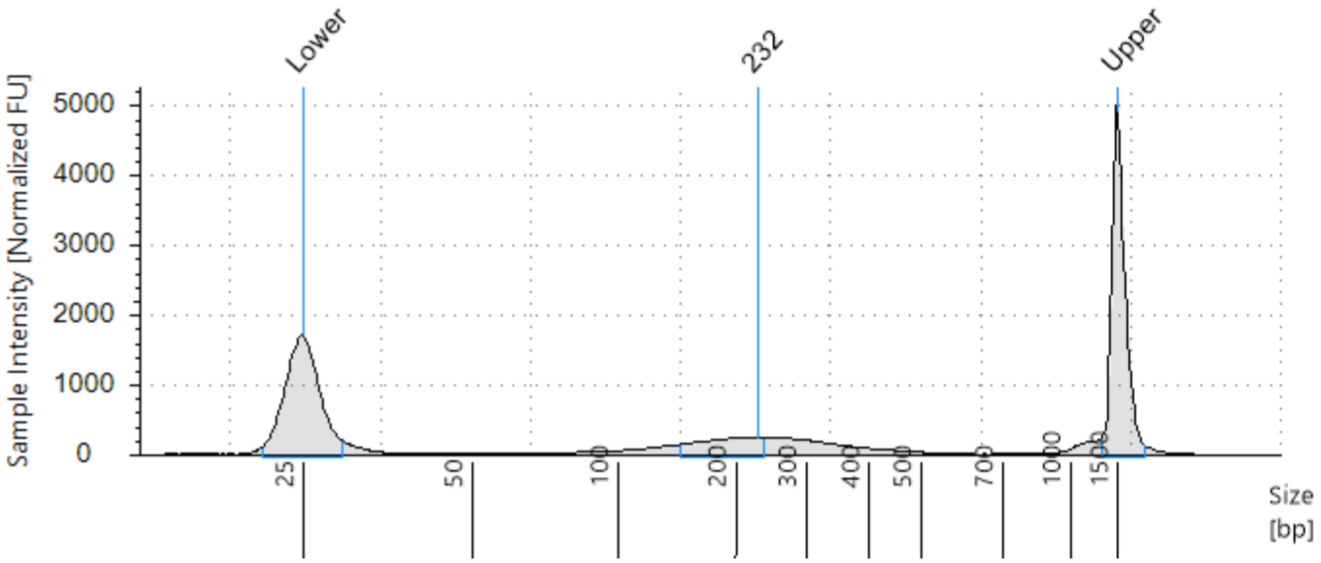

Sample Table

Well	Conc. [ng/µl]	Sample Description	Alert	Observations
D1	1.44	C1 M R3		

Peak Table

Size [bp]	Calibrated Conc. [ng/µl]	Assigned Conc. [ng/µl]	Peak Molarity [nmol/l]	% Integrated Area	Peak Comment	Observations
25	5.78	-	356	-		Lower Marker
232	1.44	-	9.58	100.00		
1500	6.50	6.50	6.67	-		Upper Marker

© Agilent Technologies, Inc. 2017

E1: D1 M R3

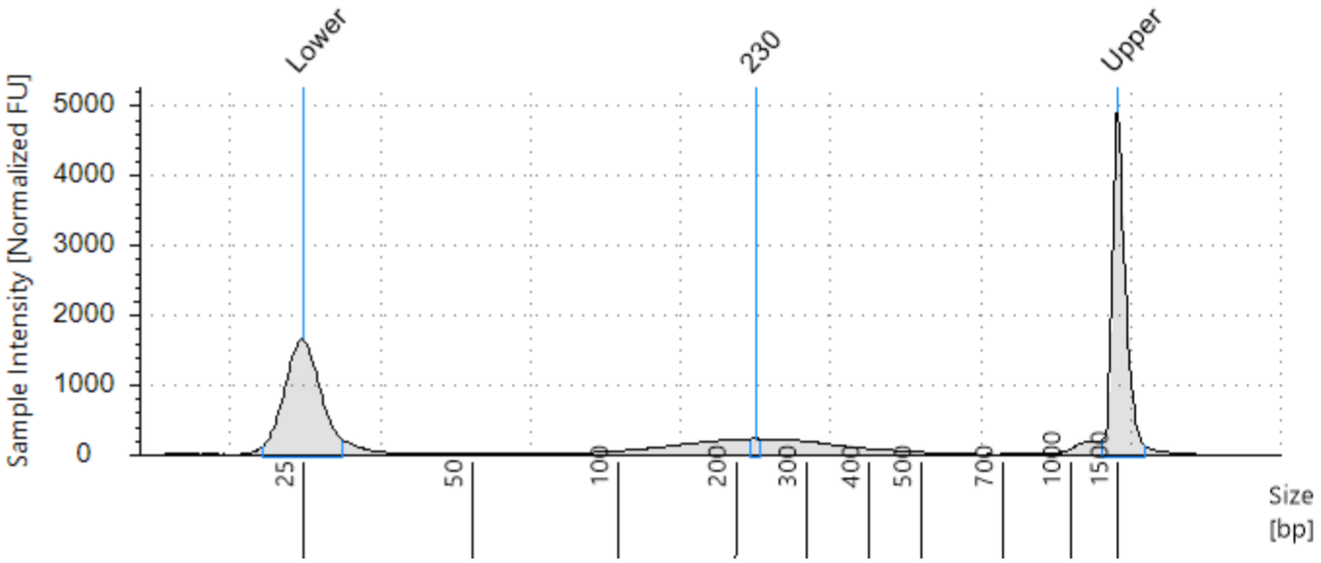

Sample Table

Well	Conc. [ng/µl]	Sample Description	Alert	Observations
E1	0.207	D1 M R3		

Peak Table

Size [bp]	Calibrated Conc. [ng/µl]	Assigned Conc. [ng/µl]	Peak Molarity [nmol/l]	% Integrated Area	Peak Comment	Observations
25	5.96	-	367	-		Lower Marker
230	0.207	-	1.39	100.00		
1500	6.50	6.50	6.67	-		Upper Marker

 © Agilent Technologies, Inc. 2017

F1: E1 M R3

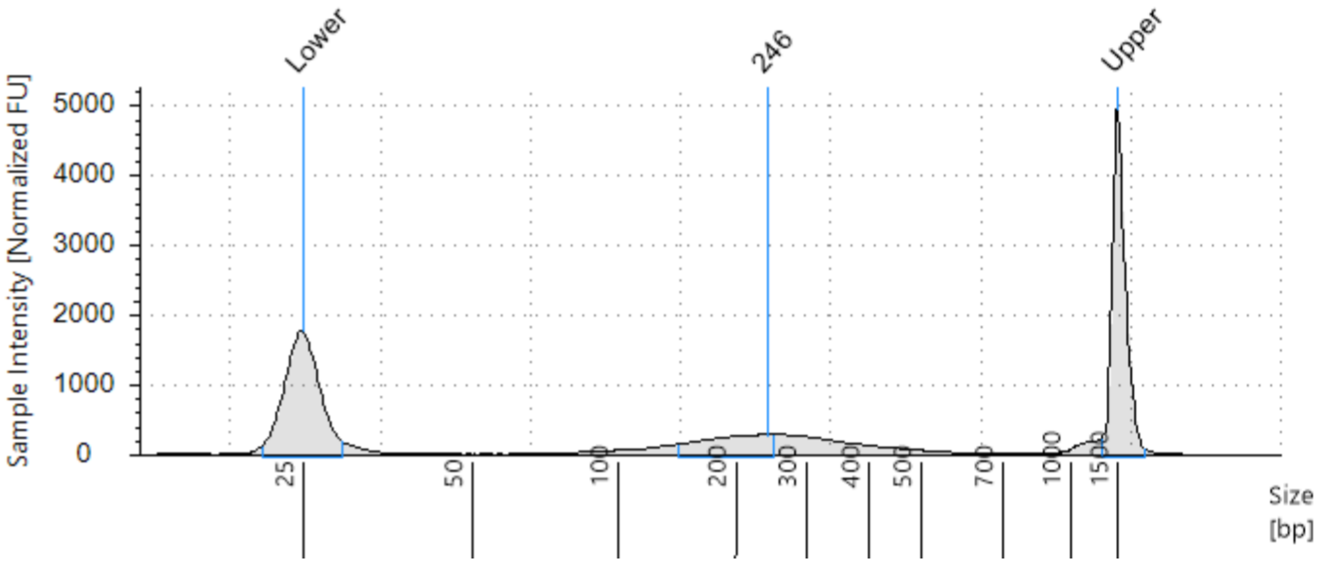

Sample Table

Well	Conc. [ng/µl]	Sample Description	Alert	Observations
F1	1.86	E1 M R3		

Peak Table

Size [bp]	Calibrated Conc. [ng/µl]	Assigned Conc. [ng/µl]	Peak Molarity [nmol/l]	% Integrated Area	Peak Comment	Observations
25	6.07	-	374	-		Lower Marker
246	1.86	-	11.6	100.00		
1500	6.50	6.50	6.67	-		Upper Marker

 © Agilent Technologies, Inc. 2017

G1: F1 M R3

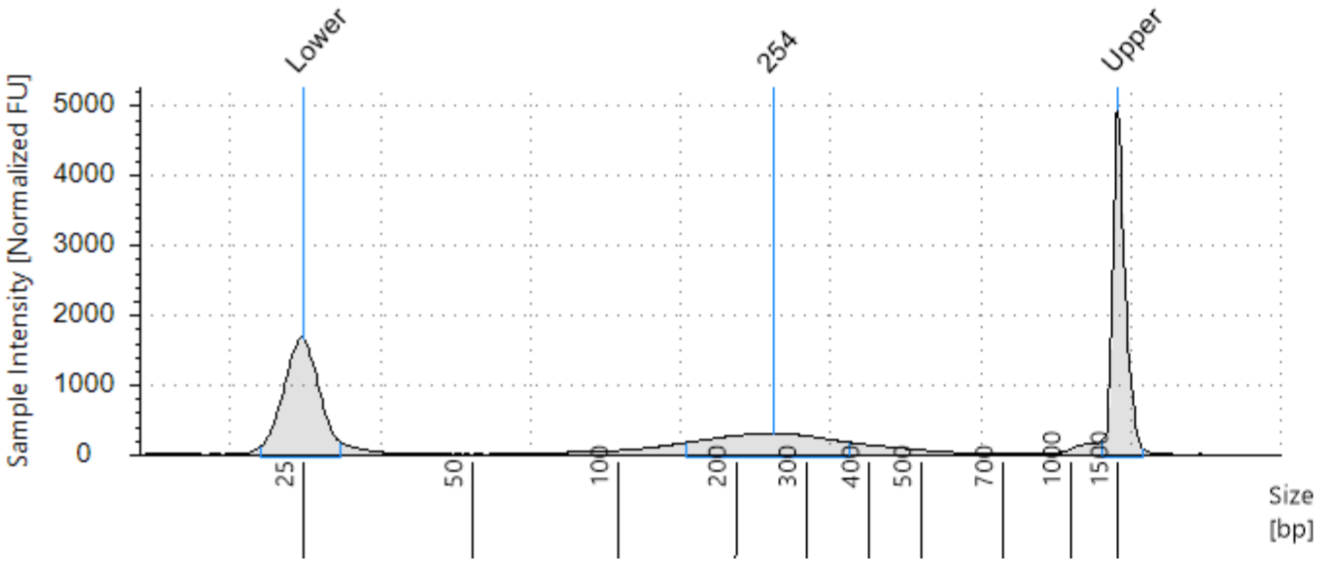

Sample Table

Well	Conc. [ng/µl]	Sample Description	Alert	Observations
G1	3.60	F1 M R3		

Peak Table

Size [bp]	Calibrated Conc. [ng/µl]	Assigned Conc. [ng/µl]	Peak Molarity [nmol/l]	% Integrated Area	Peak Comment	Observations
25	6.18	-	380	-		Lower Marker
254	3.60	-	21.8	100.00		
1500	6.50	6.50	6.67	-		Upper Marker

© Agilent Technologies, Inc. 2017

H1: G1 M R3

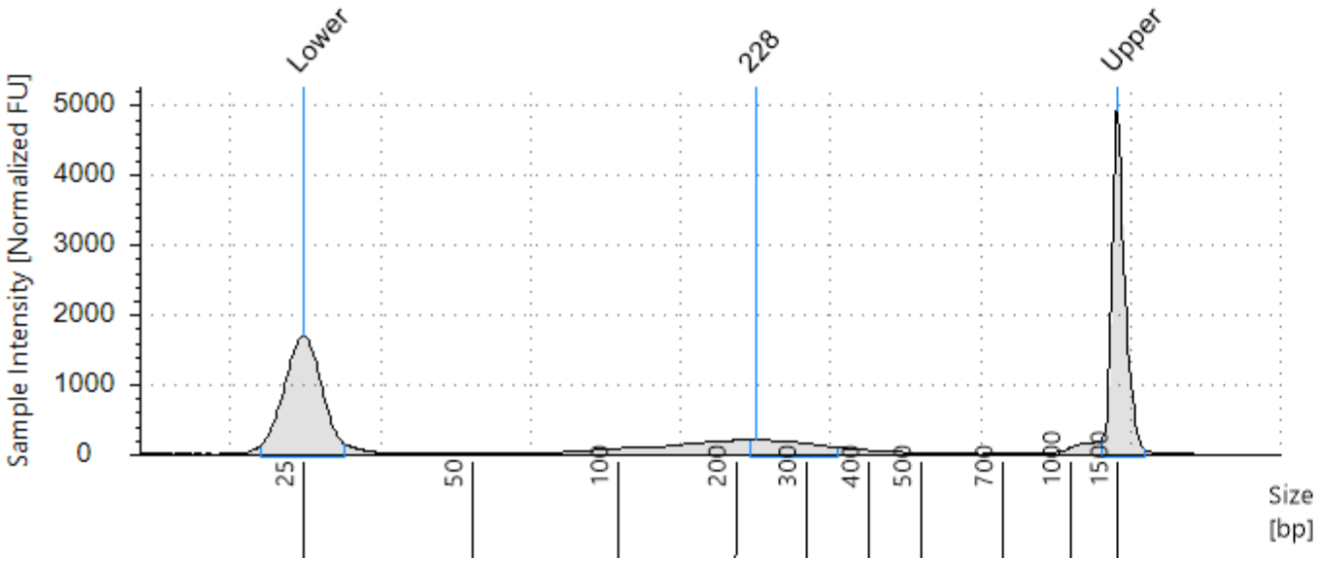

Sample Table

Well	Conc. [ng/µl]	Sample Description	Alert	Observations
H1	1.24	G1 M R3		

Peak Table

Size [bp]	Calibrated Conc. [ng/µl]	Assigned Conc. [ng/µl]	Peak Molarity [nmol/l]	% Integrated Area	Peak Comment	Observations
25	6.44	-	396	-		Lower Marker
228	1.24	-	8.40	100.00		
1500	6.50	6.50	6.67	-		Upper Marker

 © Agilent Technologies, Inc. 2017

A2: A2 M R3

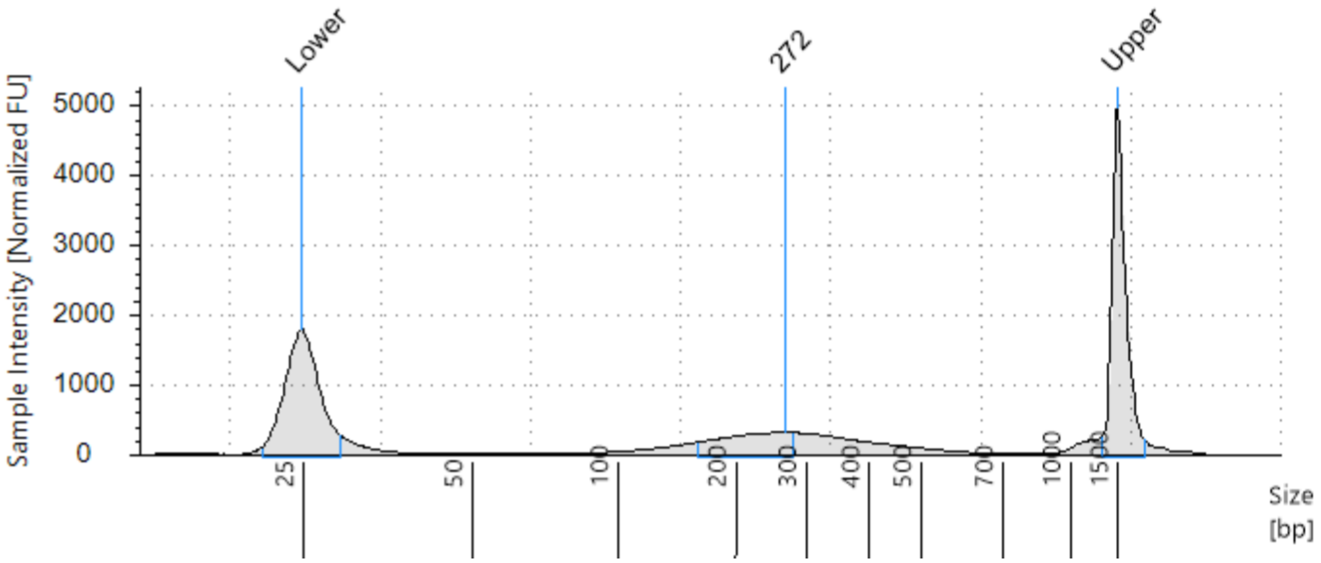

Sample Table

Well	Conc. [ng/µl]	Sample Description	Alert	Observations
A2	2.05	A2 M R3		

Peak Table

Size [bp]	Calibrated Conc. [ng/µl]	Assigned Conc. [ng/µl]	Peak Molarity [nmol/l]	% Integrated Area	Peak Comment	Observations
25	5.88	-	362	-		Lower Marker
272	2.05	-	11.6	100.00		
1500	6.50	6.50	6.67	-		Upper Marker

© Agilent Technologies, Inc. 2017

B2: B2 M R3

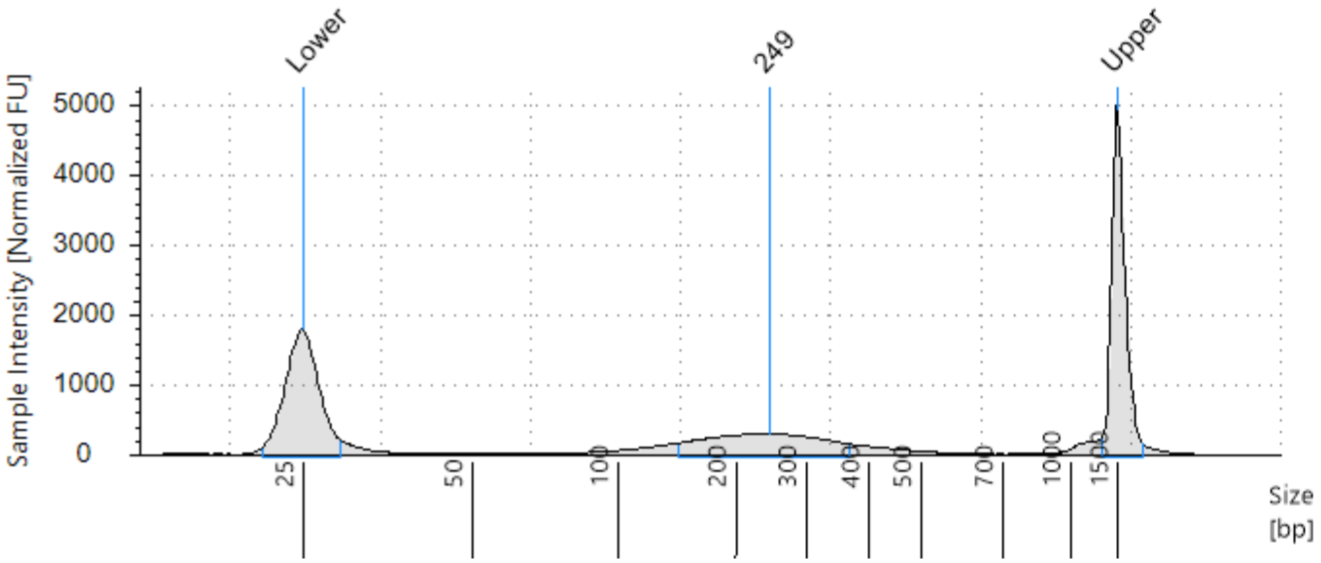

Sample Table

Well	Conc. [ng/µl]	Sample Description	Alert	Observations
B2	3.37	B2 M R3		

Peak Table

Size [bp]	Calibrated Conc. [ng/µl]	Assigned Conc. [ng/µl]	Peak Molarity [nmol/l]	% Integrated Area	Peak Comment	Observations
25	5.82	-	358	-		Lower Marker
249	3.37	-	20.8	100.00		
1500	6.50	6.50	6.67	-		Upper Marker

 © Agilent Technologies, Inc. 2017

C2: C2 M R3

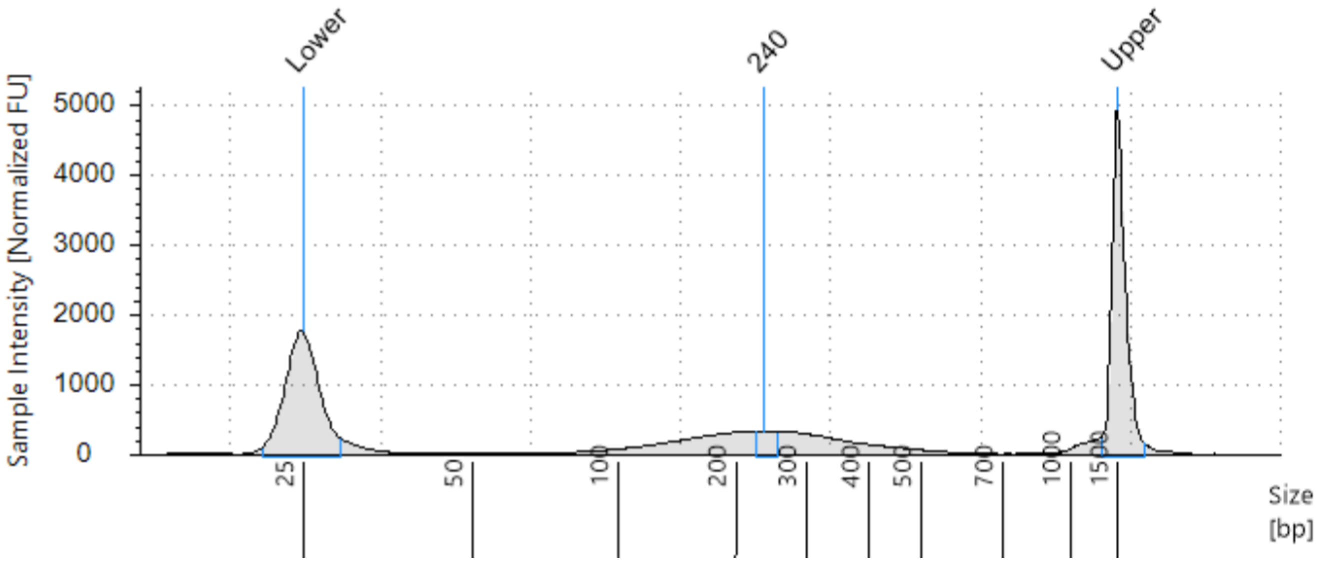

Sample Table

Well	Conc. [ng/µl]	Sample Description	Alert	Observations
C2	0.625	C2 M R3		

Peak Table

Size [bp]	Calibrated Conc. [ng/µl]	Assigned Conc. [ng/µl]	Peak Molarity [nmol/l]	% Integrated Area	Peak Comment	Observations
25	5.81	-	357	-		Lower Marker
240	0.625	-	4.00	100.00		
1500	6.50	6.50	6.67	-		Upper Marker

© Agilent Technologies, Inc. 2017

D2: D2 M R3

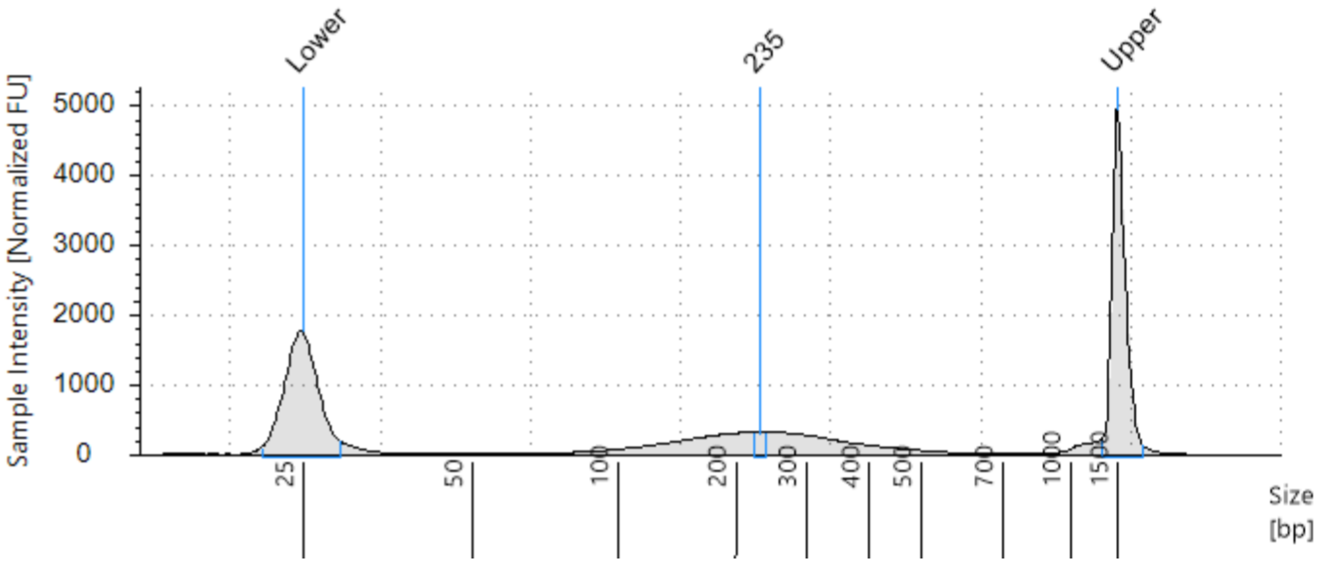

Sample Table

Well	Conc. [ng/µl]	Sample Description	Alert	Observations
D2	0.342	D2 M R3		

Peak Table

Size [bp]	Calibrated Conc. [ng/µl]	Assigned Conc. [ng/µl]	Peak Molarity [nmol/l]	% Integrated Area	Peak Comment	Observations
25	5.81	-	357	-		Lower Marker
235	0.342	-	2.24	100.00		
1500	6.50	6.50	6.67	-		Upper Marker

 © Agilent Technologies, Inc. 2017

E2: E2 M R3

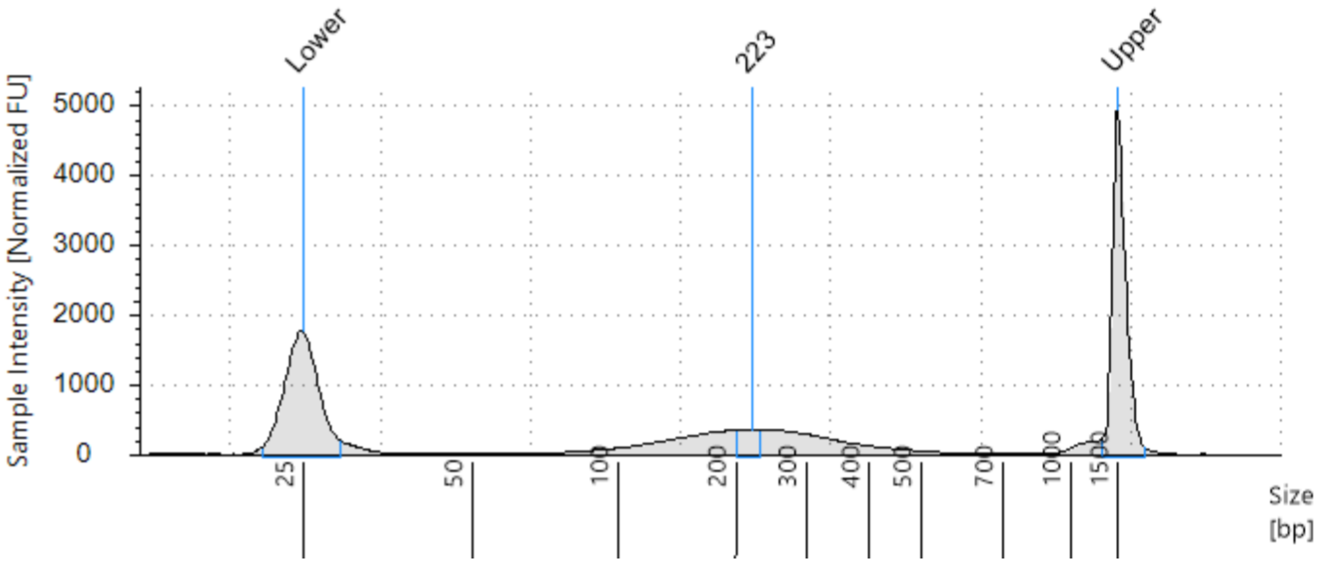

Sample Table

Well	Conc. [ng/µl]	Sample Description	Alert	Observations
E2	0.665	E2 M R3		

Peak Table

Size [bp]	Calibrated Conc. [ng/µl]	Assigned Conc. [ng/µl]	Peak Molarity [nmol/l]	% Integrated Area	Peak Comment	Observations
25	5.80	-	357	-		Lower Marker
223	0.665	-	4.59	100.00		
1500	6.50	6.50	6.67	-		Upper Marker

© Agilent Technologies, Inc. 2017

F2: F2 M R3

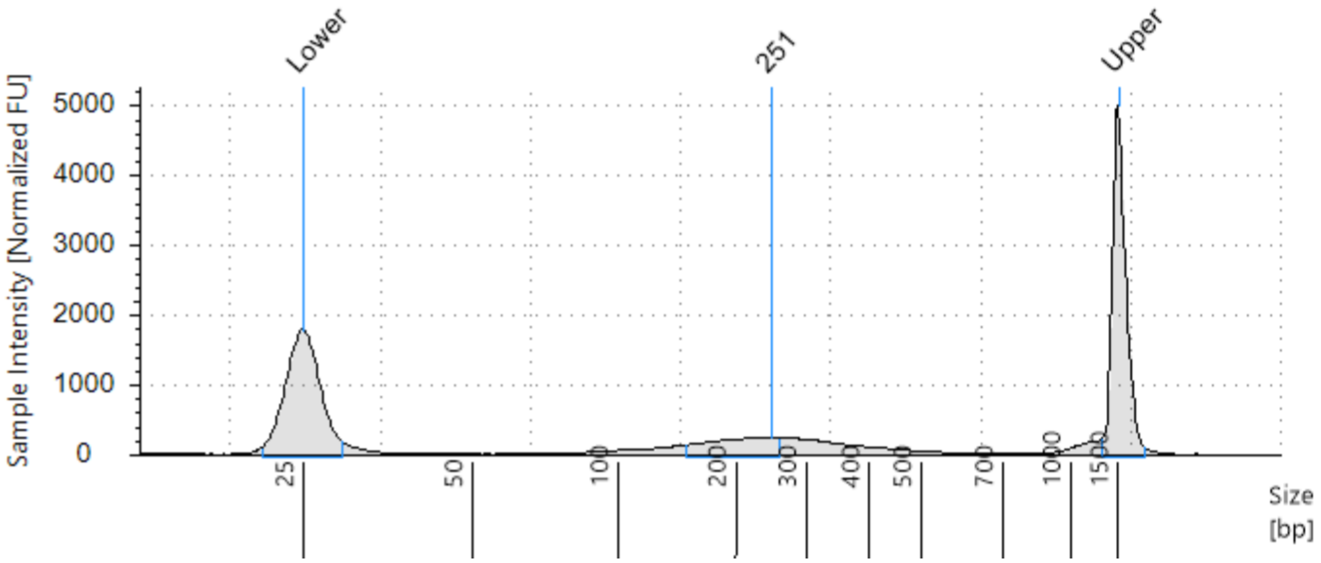

Sample Table

Well	Conc. [ng/µl]	Sample Description	Alert	Observations
F2	1.56	F2 M R3		

Peak Table

Size [bp]	Calibrated Conc. [ng/µl]	Assigned Conc. [ng/µl]	Peak Molarity [nmol/l]	% Integrated Area	Peak Comment	Observations
25	5.97	-	367	-		Lower Marker
251	1.56	-	9.54	100.00		
1500	6.50	6.50	6.67	-		Upper Marker

© Agilent Technologies, Inc. 2017

G2: G2 M R3

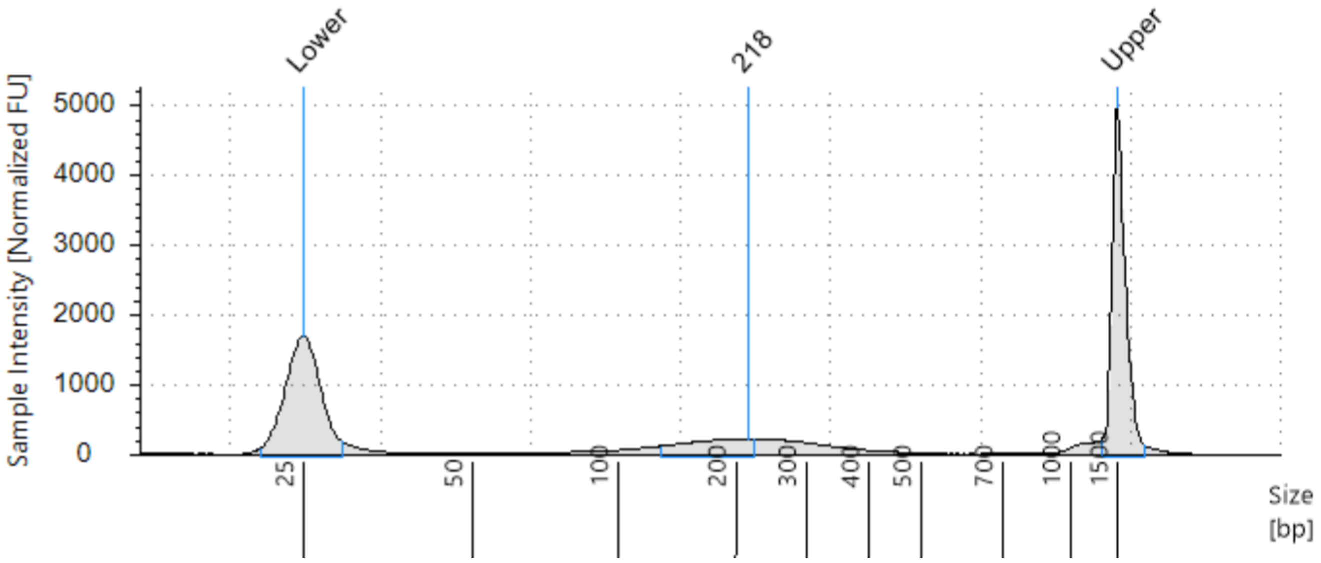

Sample Table

Well	Conc. [ng/µl]	Sample Description	Alert	Observations
G2	1.41	G2 M R3		

Peak Table

Size [bp]	Calibrated Conc. [ng/µl]	Assigned Conc. [ng/µl]	Peak Molarity [nmol/l]	% Integrated Area	Peak Comment	Observations
25	5.82	-	358	-		Lower Marker
218	1.41	-	9.96	100.00		
1500	6.50	6.50	6.67	-		Upper Marker

 © Agilent Technologies, Inc. 2017

H2: H2 M R3

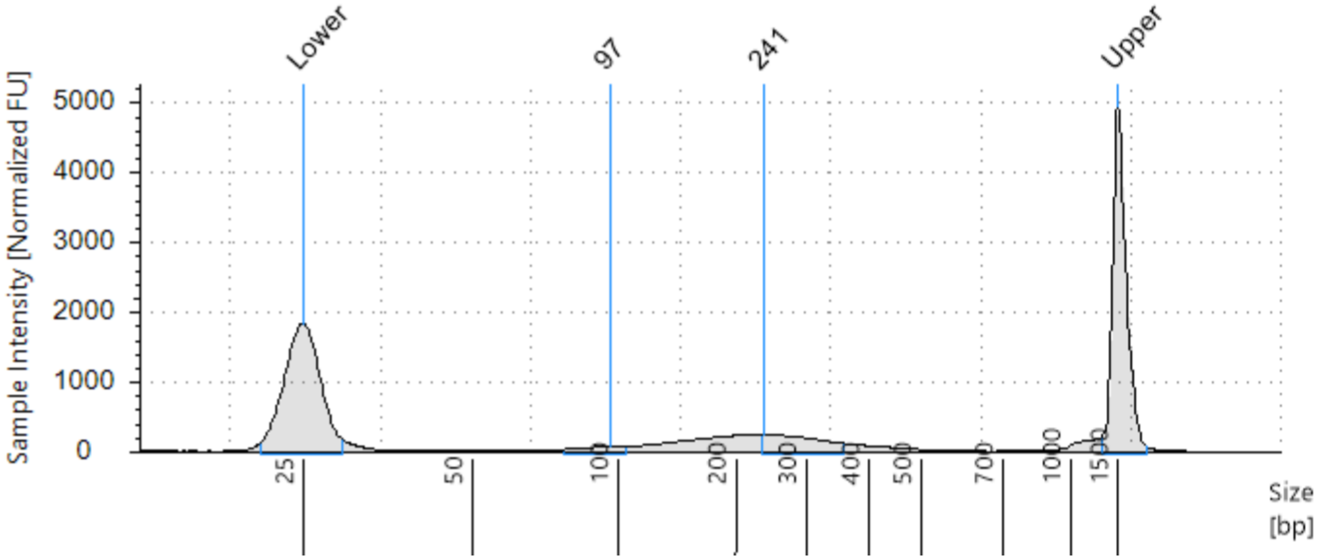

Sample Table

Well	Conc. [ng/µl]	Sample Description	Alert	Observations
H2	1.55	H2 M R3		

Peak Table

Size [bp]	Calibrated Conc. [ng/µl]	Assigned Conc. [ng/µl]	Peak Molarity [nmol/l]	% Integrated Area	Peak Comment	Observations
25	6.40	-	394	-		Lower Marker
97	0.260	-	4.12	16.78		
241	1.29	-	8.24	83.22		
1500	6.50	6.50	6.67	-		Upper Marker

 © Agilent Technologies, Inc. 2017

Filename: 2020-10-13 Q-S MINUS A3- H4 EXP H3 R3.D1000

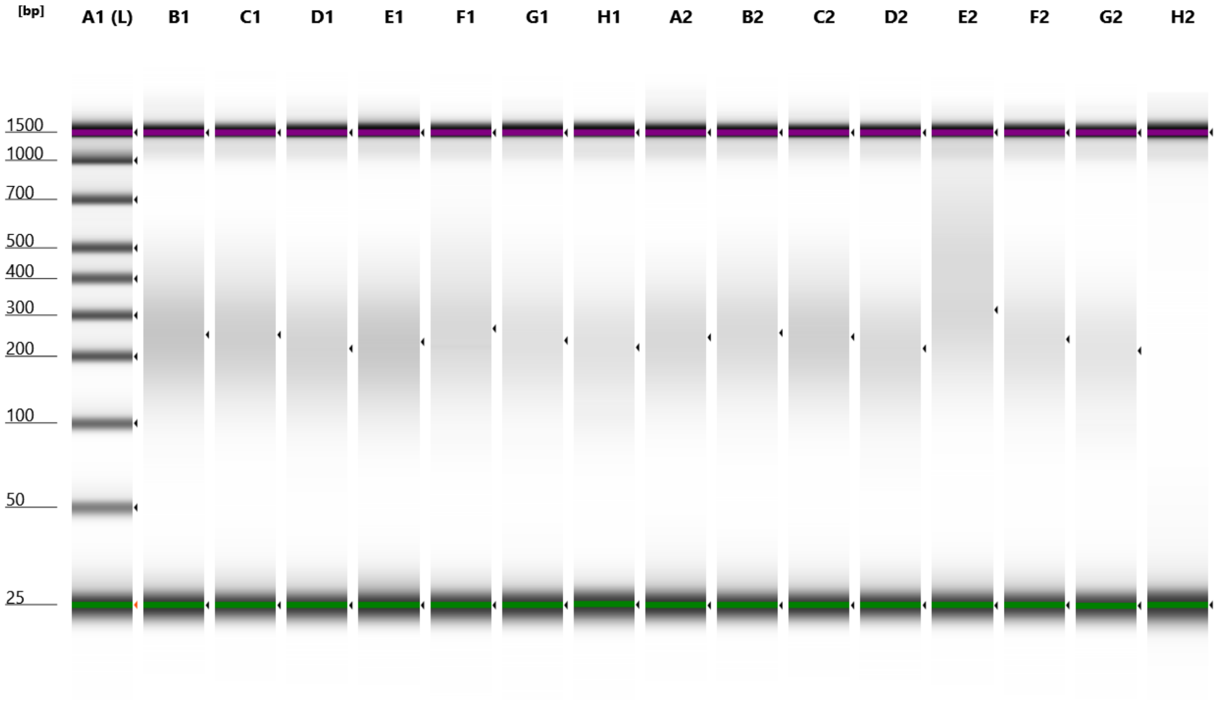

Default image (Contrast 100%)

Sample Info

Well	Conc. [ng/µl]	Sample Description	Alert	Observations
A1	15.7	Ladder		Ladder
B1	2.50	A3 M R3		
C1	0.434	B3 M R3		
D1	0.326	C3 M R3		
E1	4.02	D3 M R3		
F1	1.38	E3 M R3		
G1	0.294	F3 M R3		
H1	1.22	G3 M R3		
A2	1.29	A4 M R3		
B2	1.47	B4 M R3		
C2	2.00	C4 M R3		
D2	1.59	D4 M R3		
E2	1.52	E4 M R3		
F2	1.16	F4 M R3		
G2	1.91	G4 M R3		
H2		H4 M R3		

 © Agilent Technologies, Inc. 2017

A1: Ladder

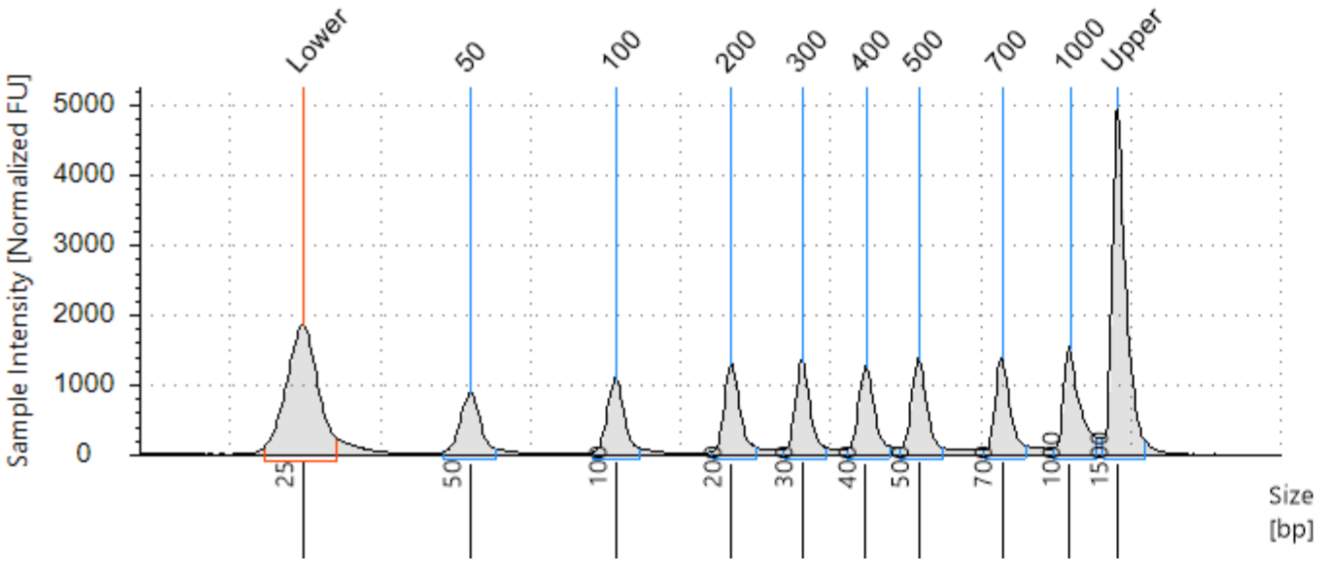

Sample Table

Well	Conc. [ng/µl]	Sample Description	Alert	Observations
A1	15.7	Ladder		Ladder

Peak Table

Size [bp]	Calibrated Conc. [ng/µl]	Assigned Conc. [ng/µl]	Peak Molarity [nmol/l]	% Integrated Area	Peak Comment	Observations
25	5.51	-	339	-		Lower Marker
50	1.69	-	51.9	10.75		
100	1.79	-	27.6	11.43		
200	1.96	-	15.1	12.48		
300	1.94	-	9.93	12.35		
400	1.92	-	7.38	12.24		
500	2.04	-	6.26	12.99		
700	1.99	-	4.38	12.72		
1000	2.36	-	3.62	15.03		
1500	6.50	6.50	6.67	-		Upper Marker

© Agilent Technologies, Inc. 2017

B1: A3 M R3

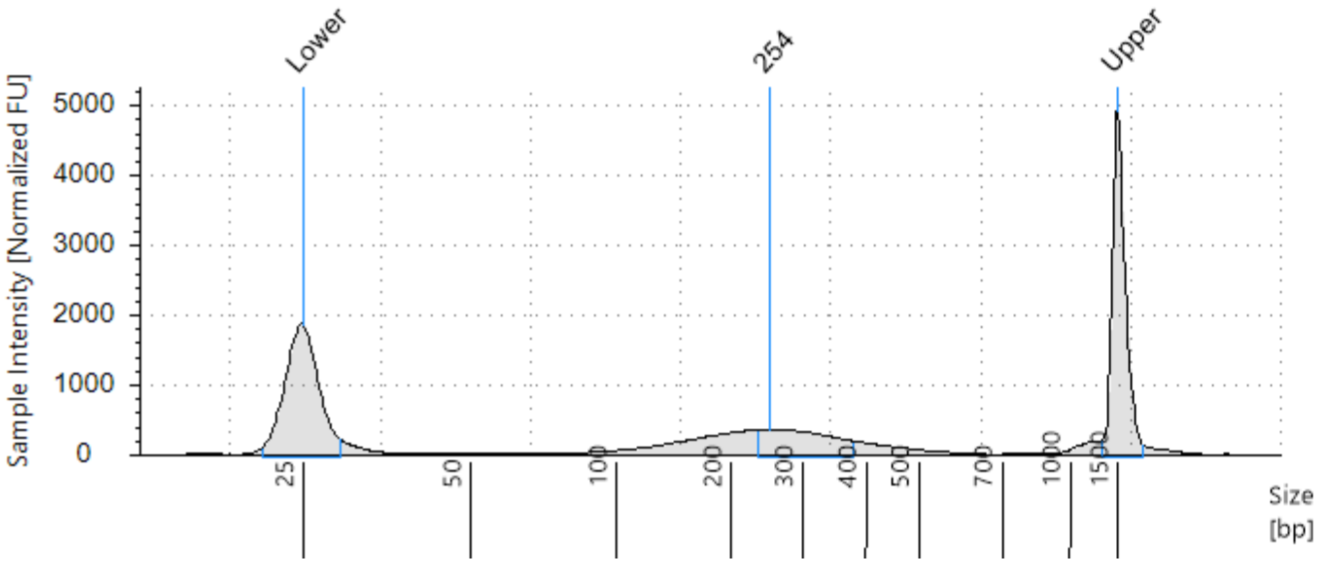

Sample Table

Well	Conc. [ng/µl]	Sample Description	Alert	Observations
B1	2.50	A3 M R3		

Peak Table

Size [bp]	Calibrated Conc. [ng/µl]	Assigned Conc. [ng/µl]	Peak Molarity [nmol/l]	% Integrated Area	Peak Comment	Observations
25	6.30	-	387	-		Lower Marker
254	2.50	-	15.2	100.00		
1500	6.50	6.50	6.67	-		Upper Marker

© Agilent Technologies, Inc. 2017

C1: B3 M R3

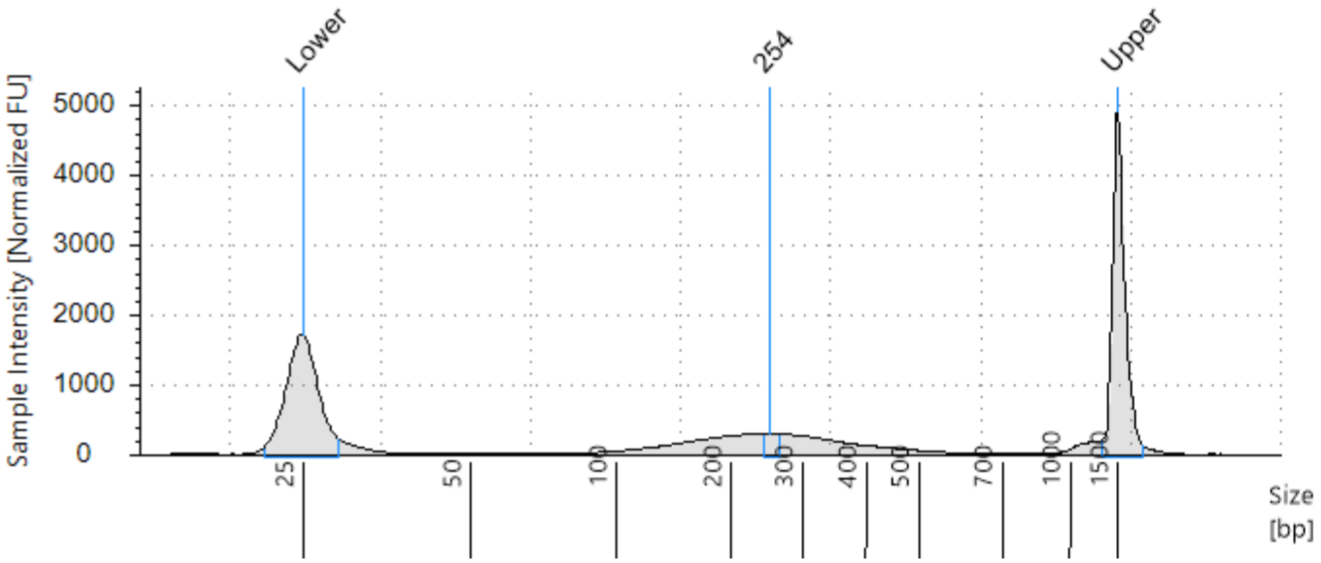

Sample Table

Well	Conc. [ng/µl]	Sample Description	Alert	Observations
C1	0.434	B3 M R3		

Peak Table

Size [bp]	Calibrated Conc. [ng/µl]	Assigned Conc. [ng/µl]	Peak Molarity [nmol/l]	% Integrated Area	Peak Comment	Observations
25	5.93	-	365	-		Lower Marker
254	0.434	-	2.63	100.00		
1500	6.50	6.50	6.67	-		Upper Marker

© Agilent Technologies, Inc. 2017

D1: C3 M R3

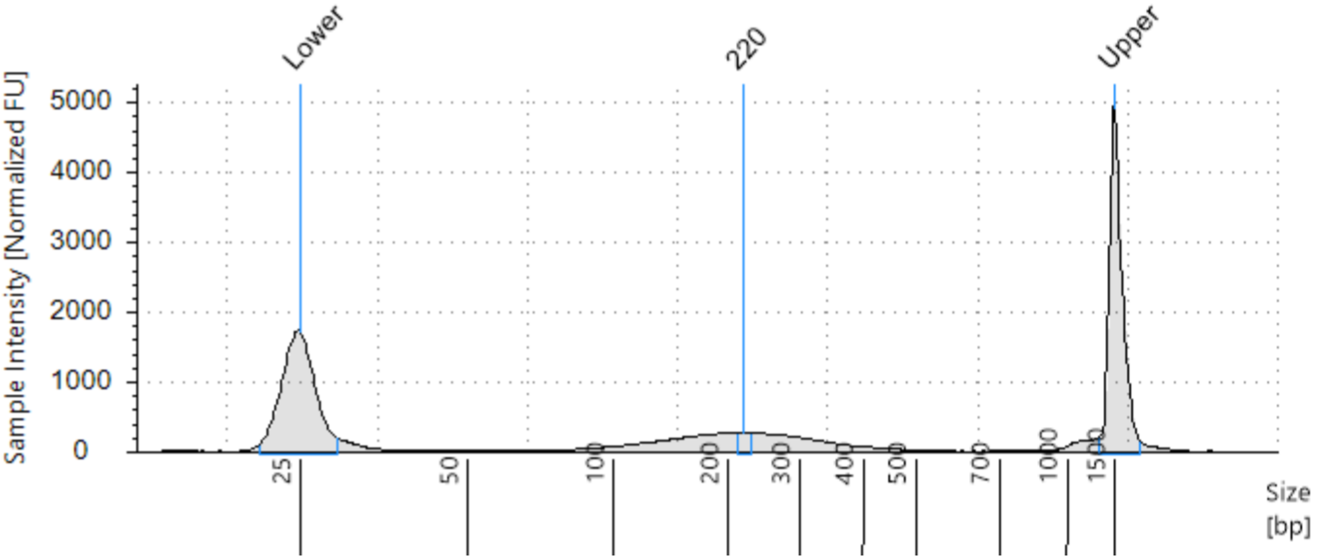

Sample Table

Well	Conc. [ng/µl]	Sample Description	Alert	Observations
D1	0.326	C3 M R3		

Peak Table

Size [bp]	Calibrated Conc. [ng/µl]	Assigned Conc. [ng/µl]	Peak Molarity [nmol/l]	% Integrated Area	Peak Comment	Observations
25	5.89	-	362	-		Lower Marker
220	0.326	-	2.28	100.00		
1500	6.50	6.50	6.67	-		Upper Marker

 © Agilent Technologies, Inc. 2017

E1: D3 M R3

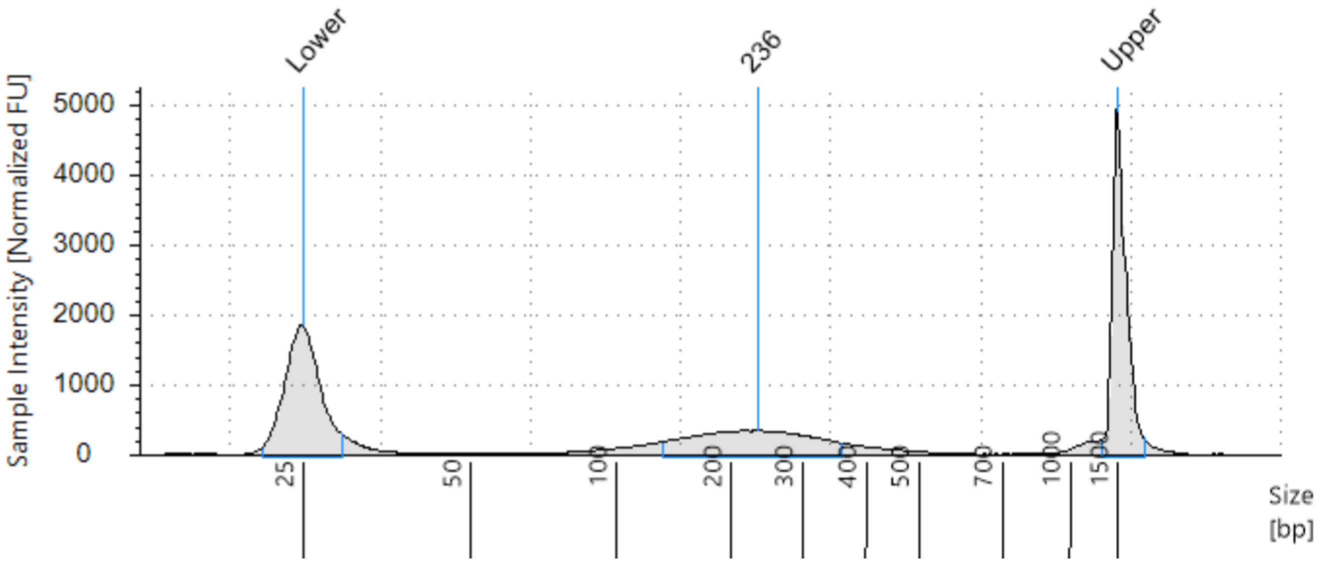

Sample Table

Well	Conc. [ng/µl]	Sample Description	Alert	Observations
E1	4.02	D3 M R3		

Peak Table

Size [bp]	Calibrated Conc. [ng/µl]	Assigned Conc. [ng/µl]	Peak Molarity [nmol/l]	% Integrated Area	Peak Comment	Observations
25	6.19	-	381	-		Lower Marker
236	4.02	-	26.2	100.00		
1500	6.50	6.50	6.67	-		Upper Marker

© Agilent Technologies, Inc. 2017

F1: E3 M R3

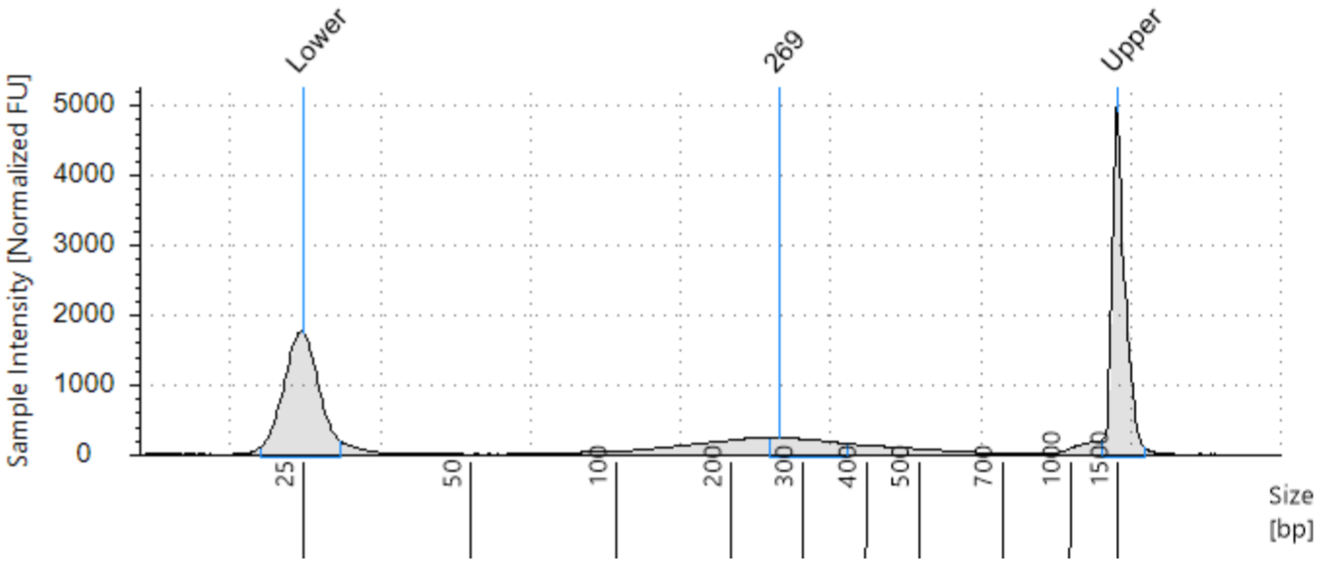

Sample Table

Well	Conc. [ng/µl]	Sample Description	Alert	Observations
F1	1.38	E3 M R3		

Peak Table

Size [bp]	Calibrated Conc. [ng/µl]	Assigned Conc. [ng/µl]	Peak Molarity [nmol/l]	% Integrated Area	Peak Comment	Observations
25	6.24	-	384	-		Lower Marker
269	1.38	-	7.89	100.00		
1500	6.50	6.50	6.67	-		Upper Marker

© Agilent Technologies, Inc. 2017

G1: F3 M R3

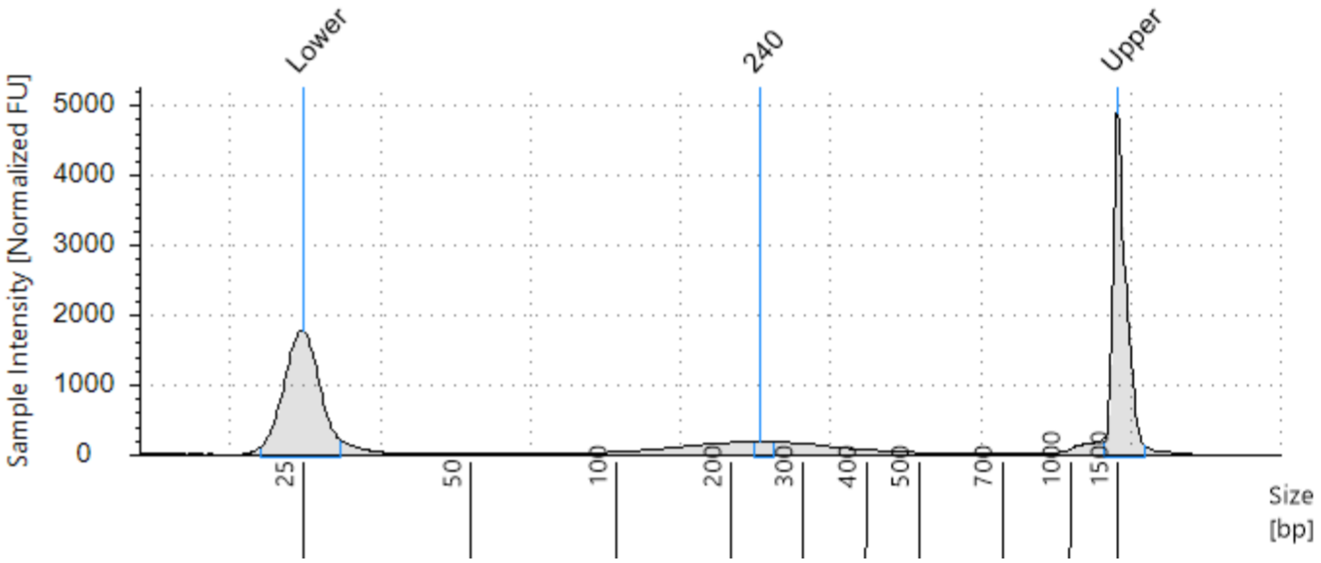

Sample Table

Well	Conc. [ng/µl]	Sample Description	Alert	Observations
G1	0.294	F3 M R3		

Peak Table

Size [bp]	Calibrated Conc. [ng/µl]	Assigned Conc. [ng/µl]	Peak Molarity [nmol/l]	% Integrated Area	Peak Comment	Observations
25	6.43	-	396	-		Lower Marker
240	0.294	-	1.88	100.00		
1500	6.50	6.50	6.67	-		Upper Marker

© Agilent Technologies, Inc. 2017

H1: G3 M R3

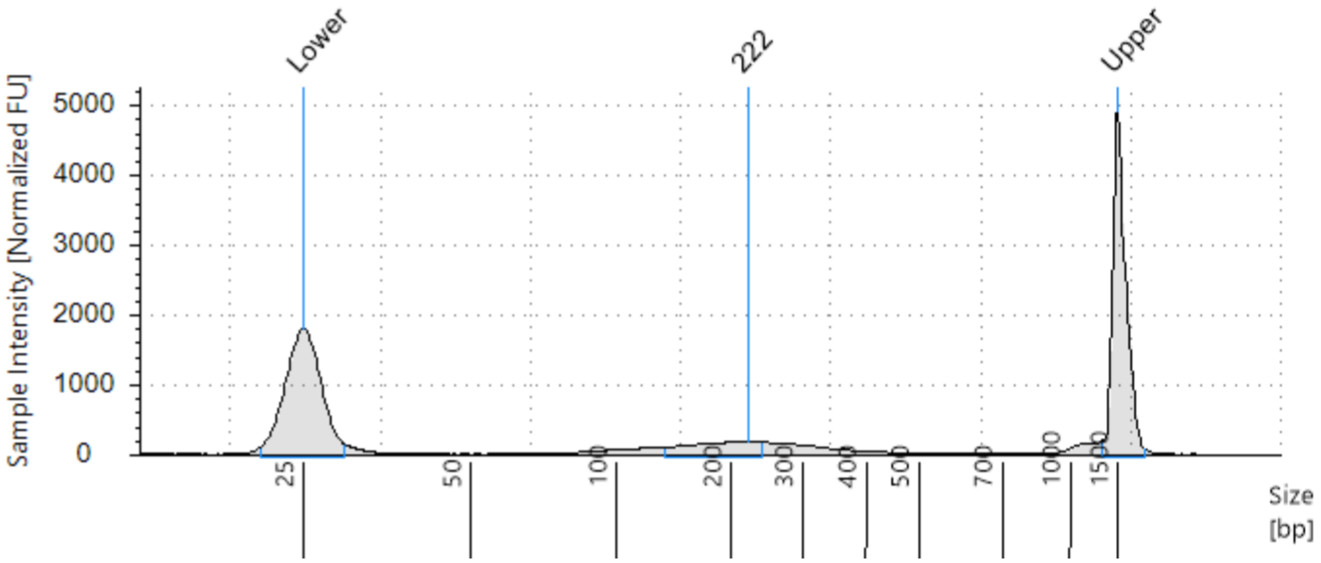

Sample Table

Well	Conc. [ng/µl]	Sample Description	Alert	Observations
H1	1.22	G3 M R3		

Peak Table

Size [bp]	Calibrated Conc. [ng/µl]	Assigned Conc. [ng/µl]	Peak Molarity [nmol/l]	% Integrated Area	Peak Comment	Observations
25	6.45	-	397	-		Lower Marker
222	1.22	-	8.47	100.00		
1500	6.50	6.50	6.67	-		Upper Marker

 © Agilent Technologies, Inc. 2017

A2: A4 M R3

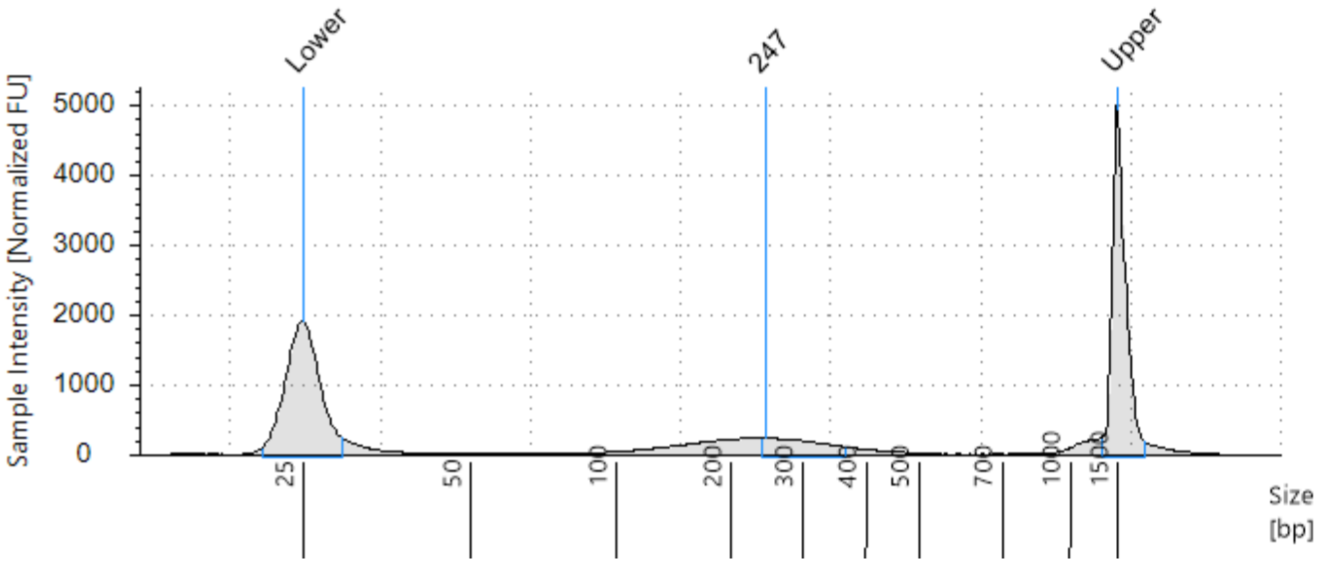

Sample Table

Well	Conc. [ng/µl]	Sample Description	Alert	Observations
A2	1.29	A4 M R3		

Peak Table

Size [bp]	Calibrated Conc. [ng/µl]	Assigned Conc. [ng/µl]	Peak Molarity [nmol/l]	% Integrated Area	Peak Comment	Observations
25	6.38	-	393	-		Lower Marker
247	1.29	-	8.00	100.00		
1500	6.50	6.50	6.67	-		Upper Marker

B2: B4 M R3

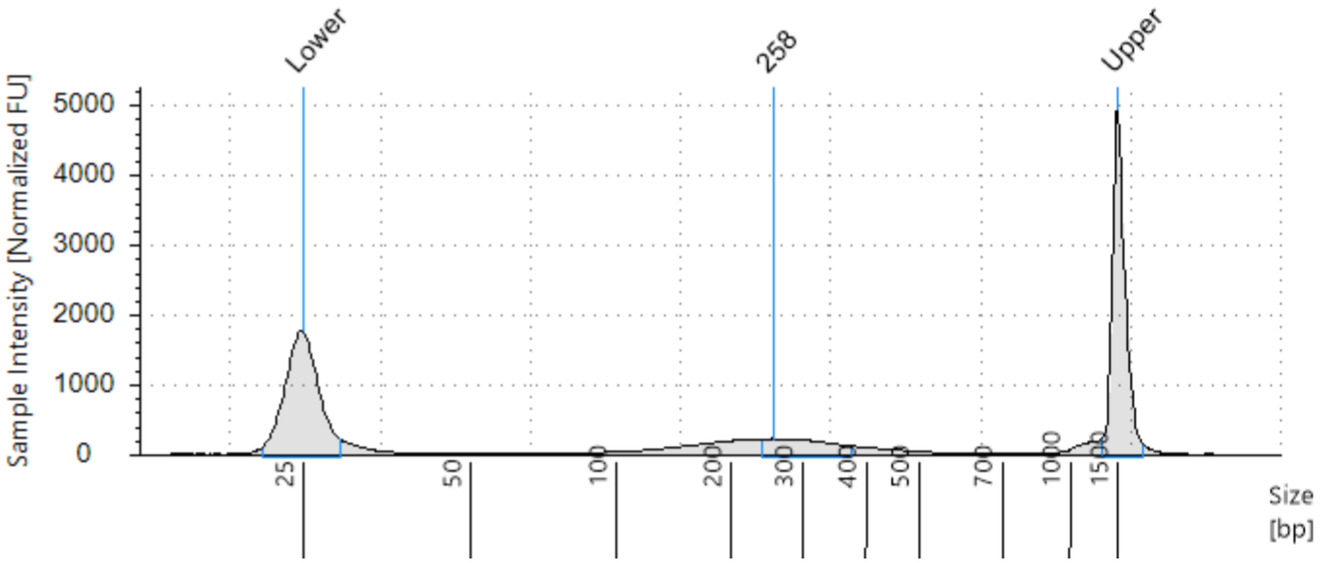

Sample Table

Well	Conc. [ng/µl]	Sample Description	Alert	Observations
B2	1.47	B4 M R3		

Peak Table

Size [bp]	Calibrated Conc. [ng/µl]	Assigned Conc. [ng/µl]	Peak Molarity [nmol/l]	% Integrated Area	Peak Comment	Observations
25	6.04	-	371	-		Lower Marker
258	1.47	-	8.76	100.00		
1500	6.50	6.50	6.67	-		Upper Marker

C2: C4 M R3

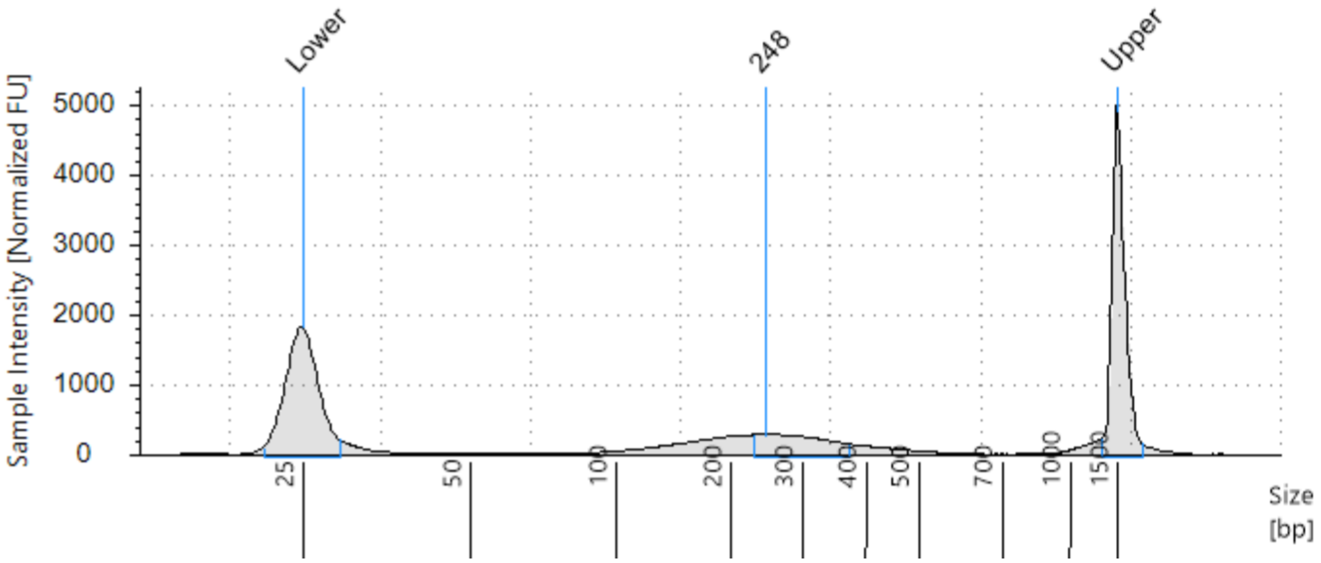

Sample Table

Well	Conc. [ng/µl]	Sample Description	Alert	Observations
C2	2.00	C4 M R3		

Peak Table

Size [bp]	Calibrated Conc. [ng/µl]	Assigned Conc. [ng/µl]	Peak Molarity [nmol/l]	% Integrated Area	Peak Comment	Observations
25	6.15	-	379	-		Lower Marker
248	2.00	-	12.4	100.00		
1500	6.50	6.50	6.67	-		Upper Marker

 © Agilent Technologies, Inc. 2017

D2: D4 M R3

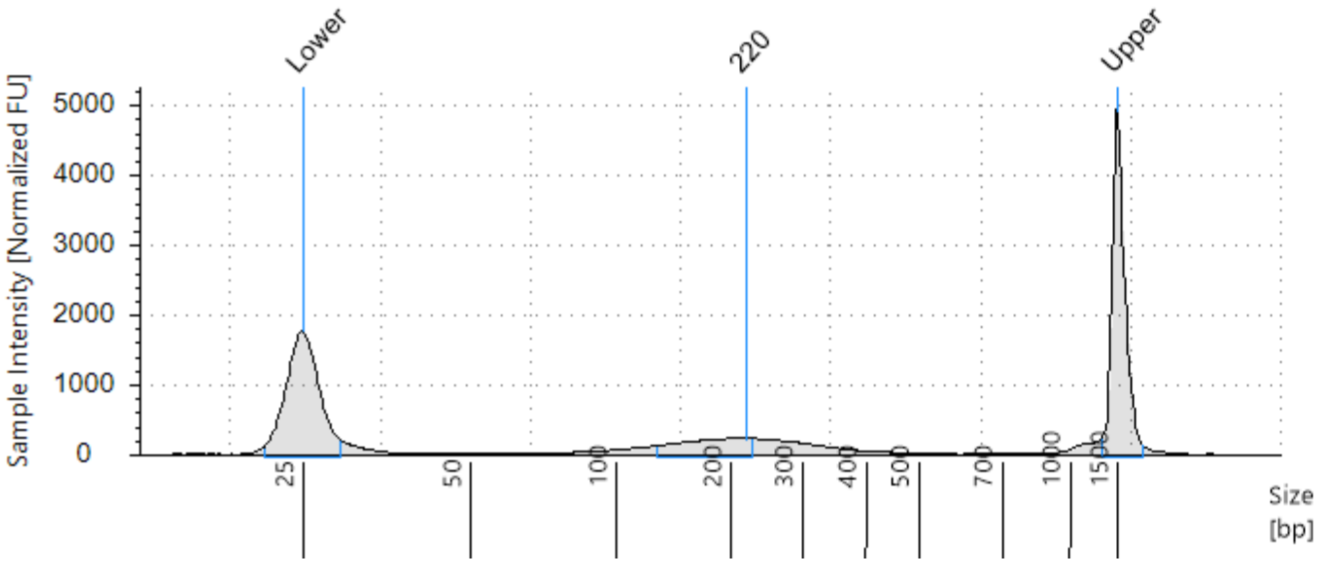

Sample Table

Well	Conc. [ng/µl]	Sample Description	Alert	Observations
D2	1.59	D4 M R3		

Peak Table

Size [bp]	Calibrated Conc. [ng/µl]	Assigned Conc. [ng/µl]	Peak Molarity [nmol/l]	% Integrated Area	Peak Comment	Observations
25	6.00	-	369	-		Lower Marker
220	1.59	-	11.1	100.00		
1500	6.50	6.50	6.67	-		Upper Marker

© Agilent Technologies, Inc. 2017

E2: E4 M R3

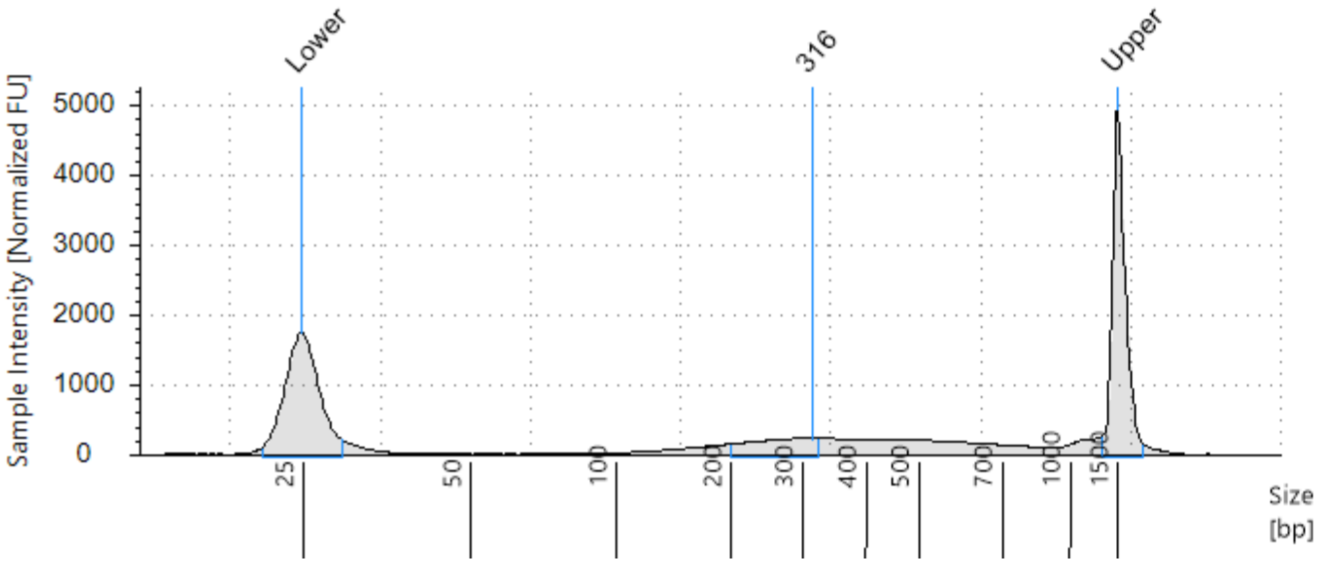

Sample Table

Well	Conc. [ng/µl]	Sample Description	Alert	Observations
E2	1.52	E4 M R3		

Peak Table

Size [bp]	Calibrated Conc. [ng/µl]	Assigned Conc. [ng/µl]	Peak Molarity [nmol/l]	% Integrated Area	Peak Comment	Observations
25	6.04	-	372	-		Lower Marker
316	1.52	-	7.41	100.00		
1500	6.50	6.50	6.67	-		Upper Marker

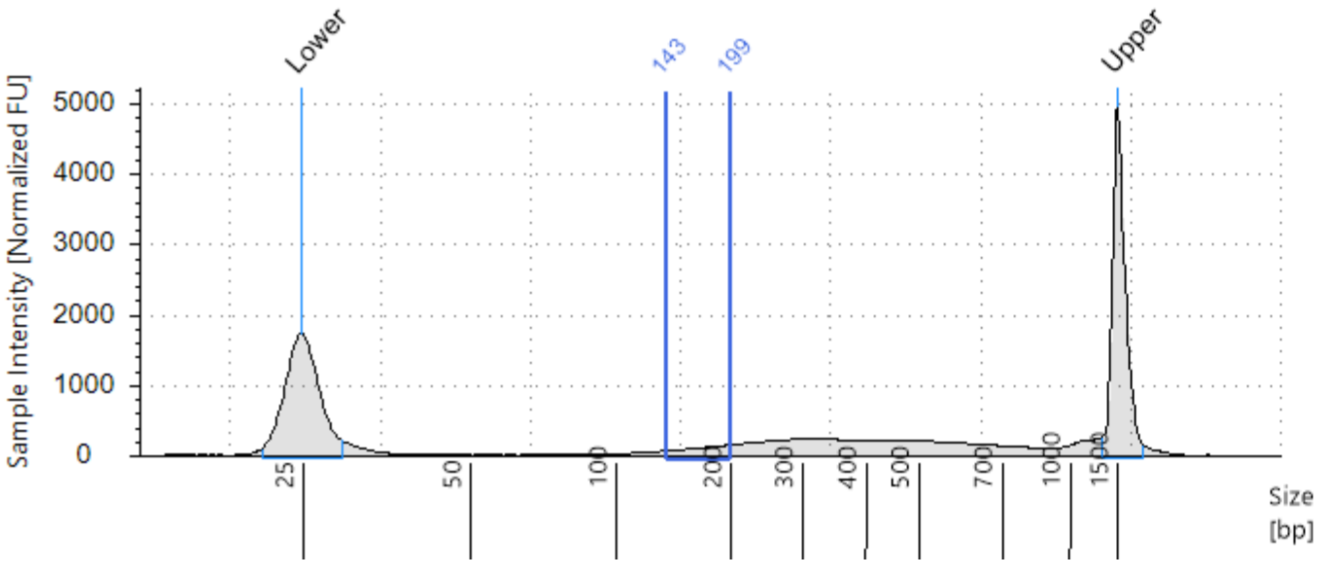

Region Table

From [bp]	To [bp]	Average Size [bp]	Conc. [ng/µl]	Region Molarity [nmol/l]	% of Total	Region Comment	Color
143	199	176	0.569	5.08	7.84		■

 © Agilent Technologies, Inc. 2017

F2: F4 M R3

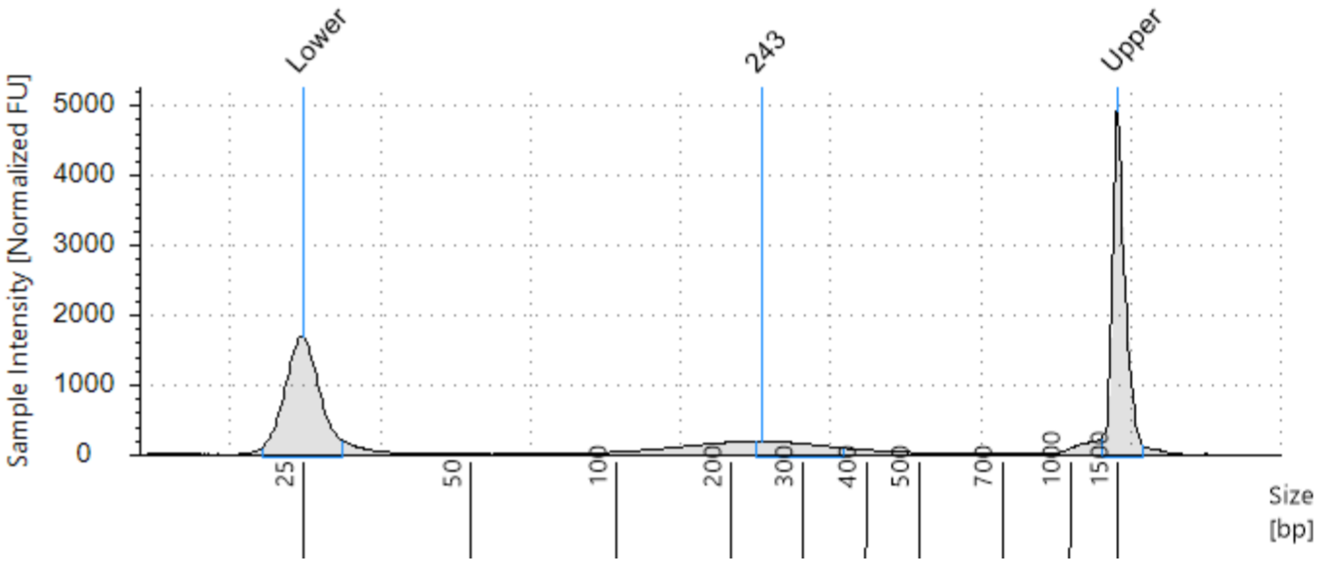

Sample Table

Well	Conc. [ng/µl]	Sample Description	Alert	Observations
F2	1.16	F4 M R3		

Peak Table

Size [bp]	Calibrated Conc. [ng/µl]	Assigned Conc. [ng/µl]	Peak Molarity [nmol/l]	% Integrated Area	Peak Comment	Observations
25	5.93	-	365	-		Lower Marker
243	1.16	-	7.38	100.00		
1500	6.50	6.50	6.67	-		Upper Marker

 © Agilent Technologies, Inc. 2017

G2: G4 M R3

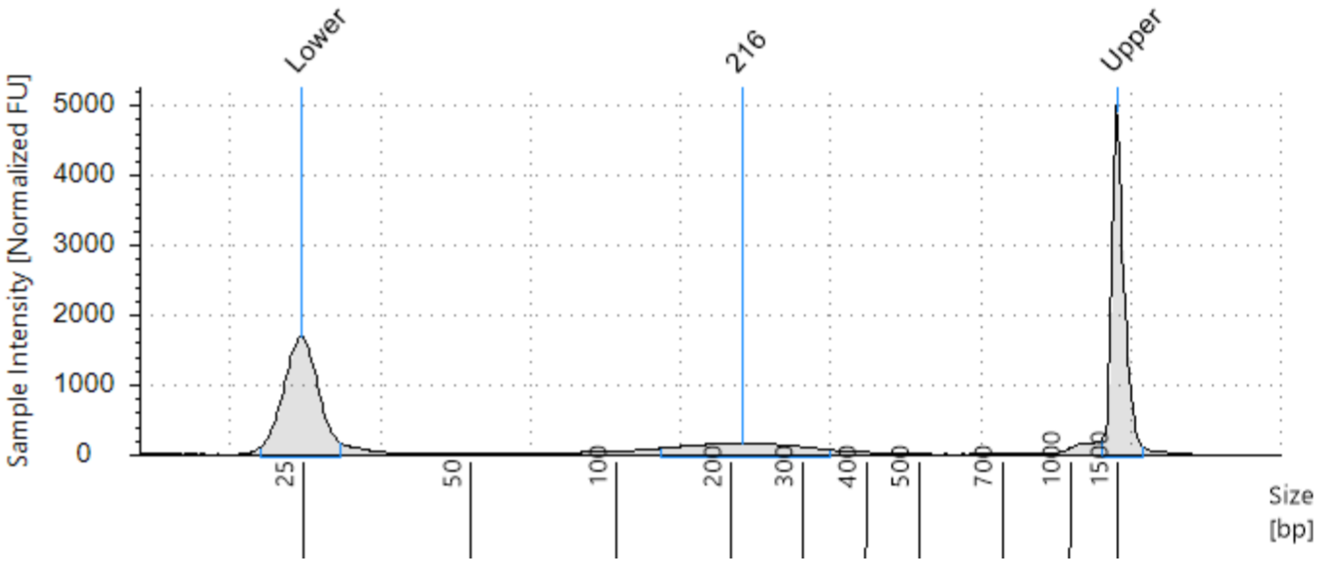

Sample Table

Well	Conc. [ng/µl]	Sample Description	Alert	Observations
G2	1.91	G4 M R3		

Peak Table

Size [bp]	Calibrated Conc. [ng/µl]	Assigned Conc. [ng/µl]	Peak Molarity [nmol/l]	% Integrated Area	Peak Comment	Observations
25	6.18	-	380	-		Lower Marker
216	1.91	-	13.6	100.00		
1500	6.50	6.50	6.67	-		Upper Marker

 © Agilent Technologies, Inc. 2017

H2: H4 M R3

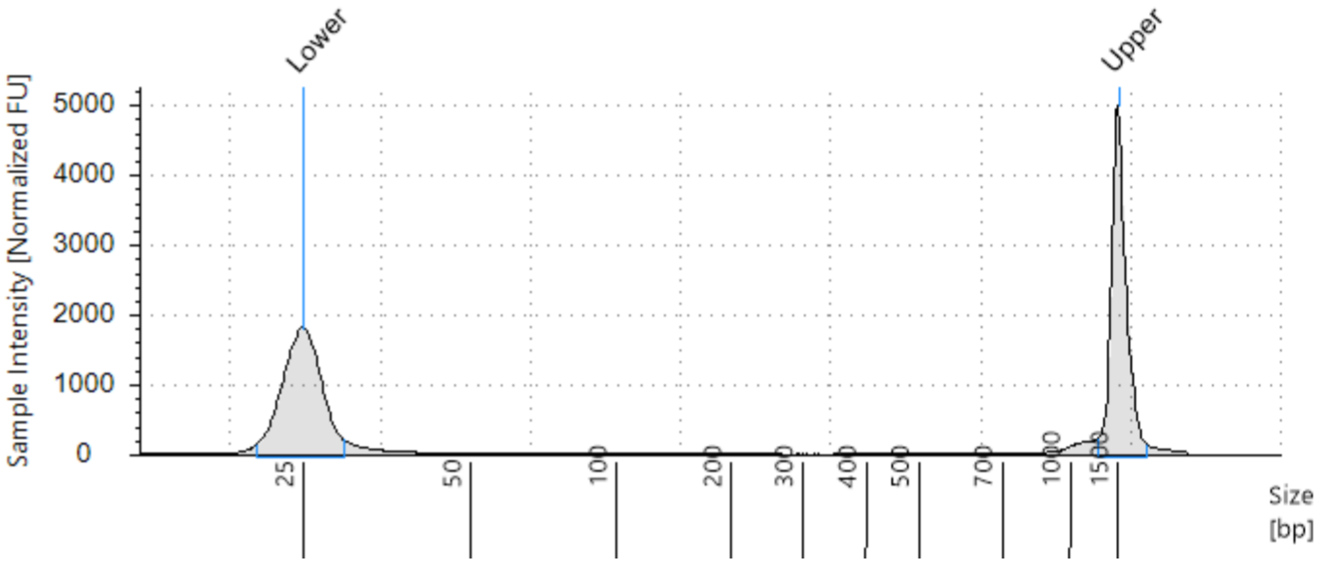

Sample Table

Well	Conc. [ng/µl]	Sample Description	Alert	Observations
H2		H4 M R3		

Peak Table

Size [bp]	Calibrated Conc. [ng/µl]	Assigned Conc. [ng/µl]	Peak Molarity [nmol/l]	% Integrated Area	Peak Comment	Observations
25	6.33	-	390	-		Lower Marker
1500	6.50	6.50	6.67	-		Upper Marker

© Agilent Technologies, Inc. 2017

Filename: 2020-10-13 Q-S MINUS A5- H6 EXP H5R3.D1000

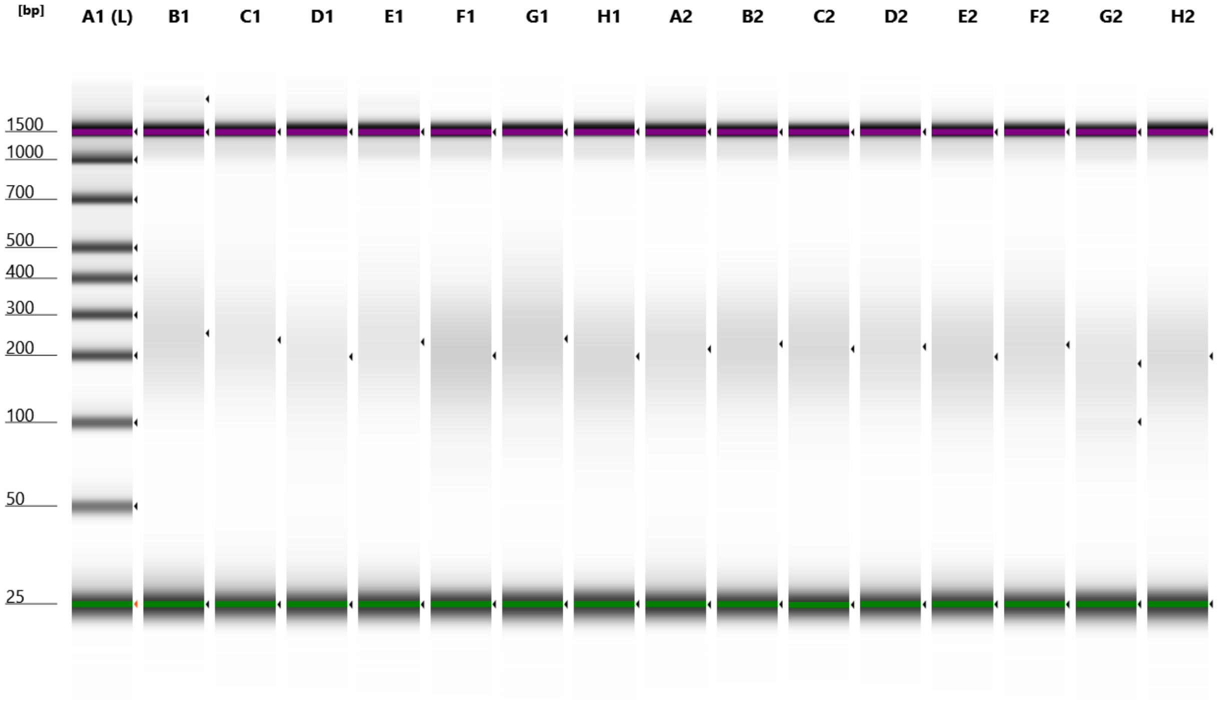

Default image (Contrast 100%)

Sample Info

Well	Conc. [ng/µl]	Sample Description	Alert	Observations
A1	17.3	Ladder		Ladder
B1	1.39	A5 M R3		
C1	0.238	B5 M R3		
D1	0.826	C5 M R3		
E1	0.948	D5 M R3		
F1	1.93	E5 M R3		
G1	0.245	F5 M R3		
H1	1.43	G5 M R3		
A2	1.10	A6 M R3		
B2	1.39	B6 M R3		
C2	1.30	C6 M R3		
D2	0.181	D6 M R3		
E2	1.44	E6 M R3		
F2	0.226	F6 M R3		
G2	2.35	G6 M R3		
H2	2.46	H6 M R3		

© Agilent Technologies, Inc. 2017

A1: Ladder

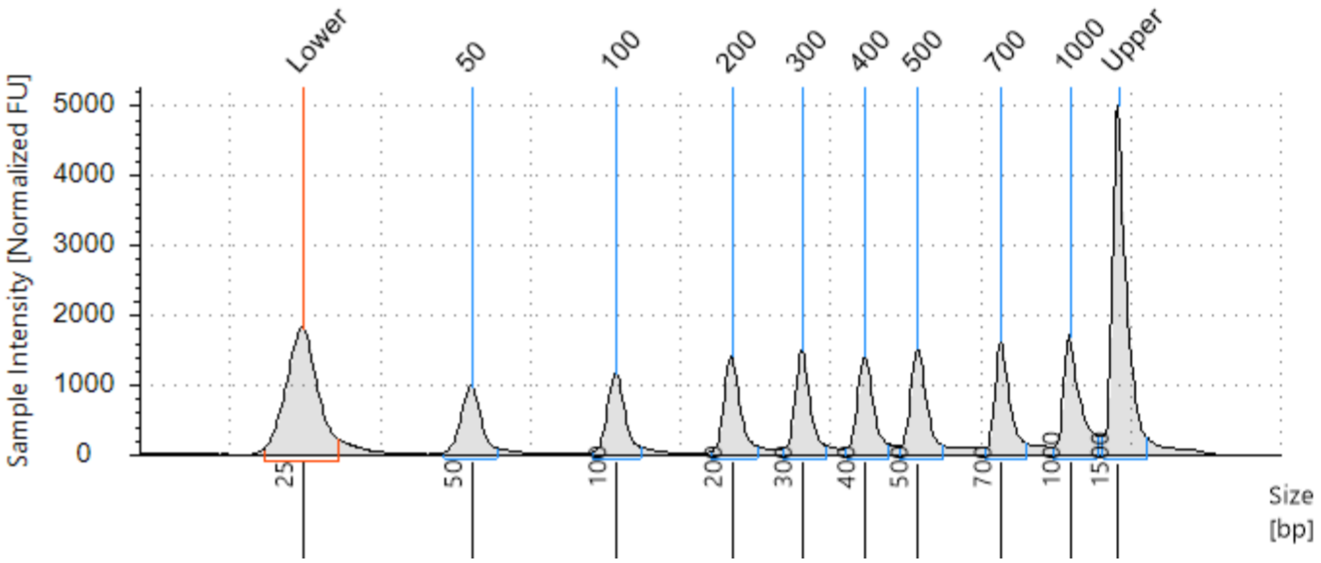

Sample Table

Well	Conc. [ng/µl]	Sample Description	Alert	Observations
A1	17.3	Ladder		Ladder

Peak Table

Size [bp]	Calibrated Conc. [ng/µl]	Assigned Conc. [ng/µl]	Peak Molarity [nmol/l]	% Integrated Area	Peak Comment	Observations
25	5.29	-	325	-		Lower Marker
50	1.82	-	56.0	10.52		
100	1.93	-	29.7	11.14		
200	2.12	-	16.3	12.27		
300	2.15	-	11.0	12.43		
400	2.15	-	8.26	12.40		
500	2.31	-	7.10	13.33		
700	2.27	-	4.98	13.09		
1000	2.56	-	3.94	14.81		
1500	6.50	6.50	6.67	-		Upper Marker

© Agilent Technologies, Inc. 2017

B1: A5 M R3

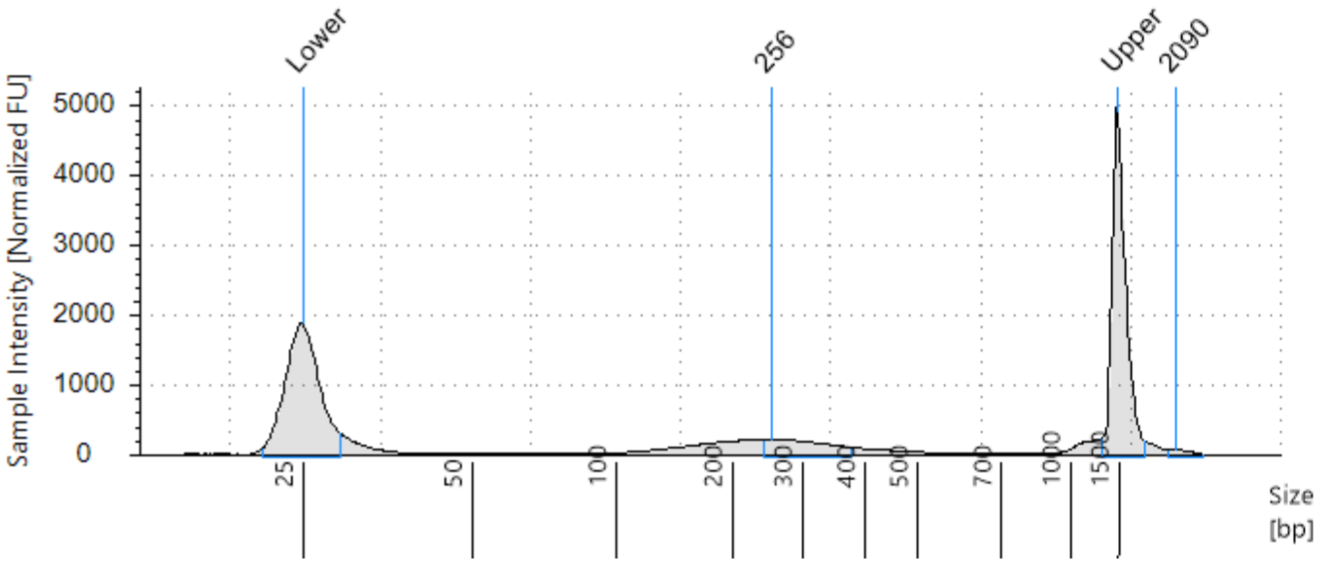

Sample Table

Well	Conc. [ng/µl]	Sample Description	Alert	Observations
B1	1.39	A5 M R3		

Peak Table

Size [bp]	Calibrated Conc. [ng/µl]	Assigned Conc. [ng/µl]	Peak Molarity [nmol/l]	% Integrated Area	Peak Comment	Observations
25	6.21	-	382	-		Lower Marker
256	1.25	-	7.51	89.60		
1500	6.50	6.50	6.67	-		Upper Marker
2090	0.145	-	0.107	10.40		

 © Agilent Technologies, Inc. 2017

C1: B5 M R3

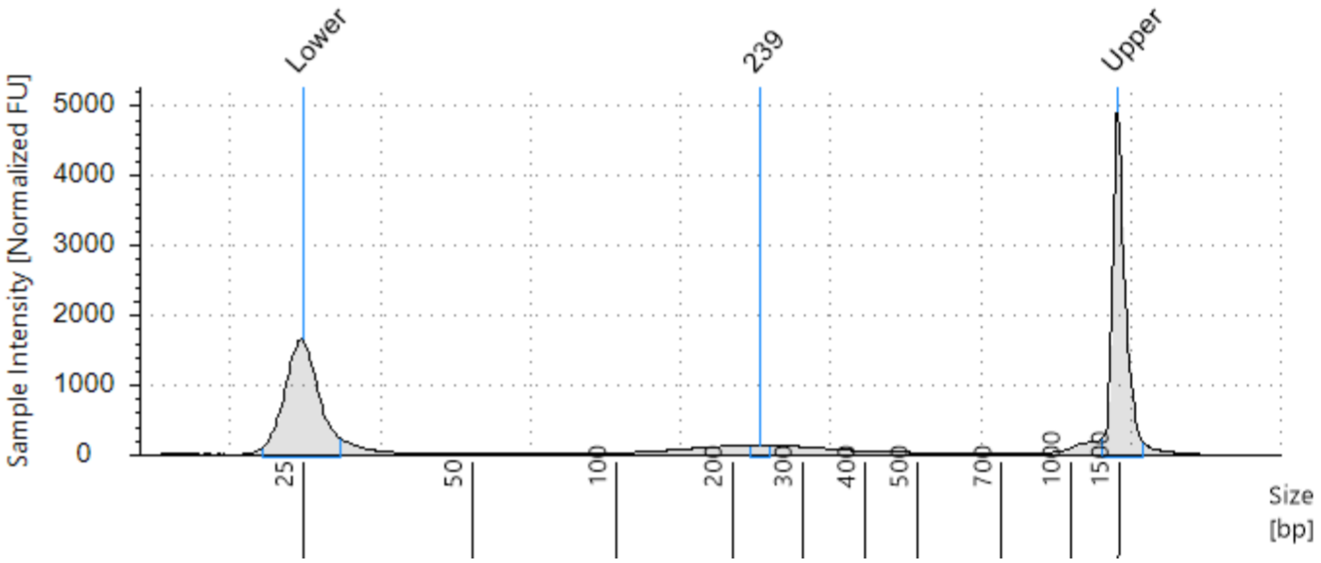

Sample Table

Well	Conc. [ng/µl]	Sample Description	Alert	Observations
C1	0.238	B5 M R3		

Peak Table

Size [bp]	Calibrated Conc. [ng/µl]	Assigned Conc. [ng/µl]	Peak Molarity [nmol/l]	% Integrated Area	Peak Comment	Observations
25	5.77	-	355	-		Lower Marker
239	0.238	-	1.53	100.00		
1500	6.50	6.50	6.67	-		Upper Marker

 © Agilent Technologies, Inc. 2017

D1: C5 M R3

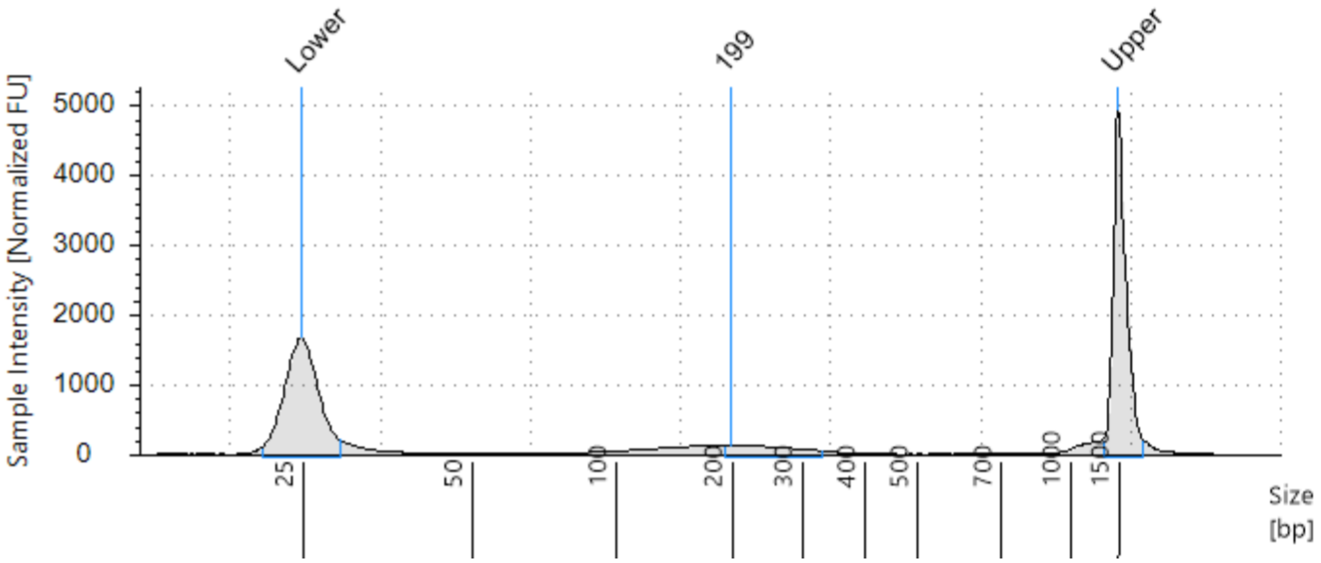

Sample Table

Well	Conc. [ng/µl]	Sample Description	Alert	Observations
D1	0.826	C5 M R3		

Peak Table

Size [bp]	Calibrated Conc. [ng/µl]	Assigned Conc. [ng/µl]	Peak Molarity [nmol/l]	% Integrated Area	Peak Comment	Observations
25	5.72	-	352	-		Lower Marker
199	0.826	-	6.40	100.00		
1500	6.50	6.50	6.67	-		Upper Marker

© Agilent Technologies, Inc. 2017

E1: D5 M R3

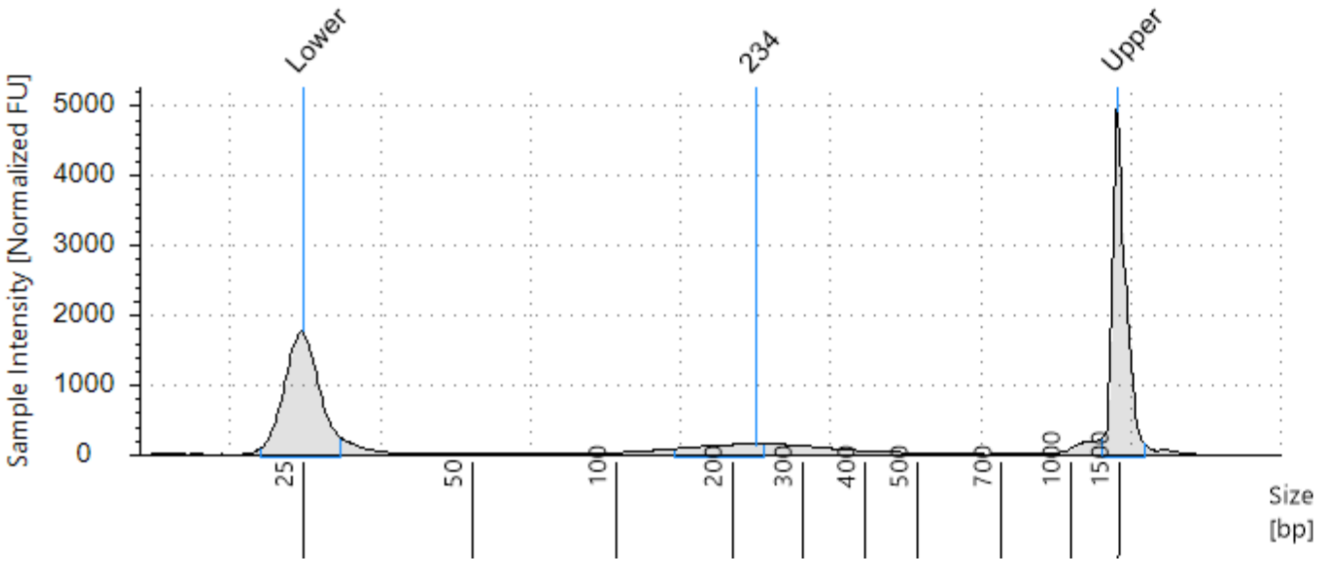

Sample Table

Well	Conc. [ng/µl]	Sample Description	Alert	Observations
E1	0.948	D5 M R3		

Peak Table

Size [bp]	Calibrated Conc. [ng/µl]	Assigned Conc. [ng/µl]	Peak Molarity [nmol/l]	% Integrated Area	Peak Comment	Observations
25	6.07	-	373	-		Lower Marker
234	0.948	-	6.23	100.00		
1500	6.50	6.50	6.67	-		Upper Marker

© Agilent Technologies, Inc. 2017

F1: E5 M R3

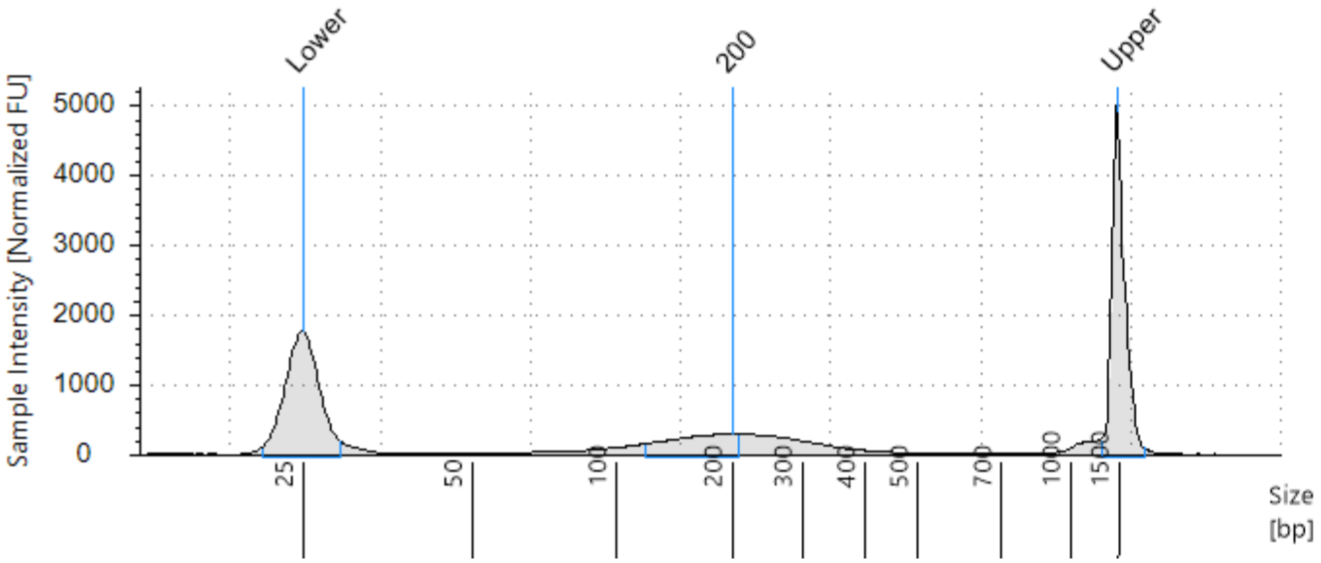

Sample Table

Well	Conc. [ng/µl]	Sample Description	Alert	Observations
F1	1.93	E5 M R3		

Peak Table

Size [bp]	Calibrated Conc. [ng/µl]	Assigned Conc. [ng/µl]	Peak Molarity [nmol/l]	% Integrated Area	Peak Comment	Observations
25	6.06	-	373	-		Lower Marker
200	1.93	-	14.8	100.00		
1500	6.50	6.50	6.67	-		Upper Marker

© Agilent Technologies, Inc. 2017

G1: F5 M R3

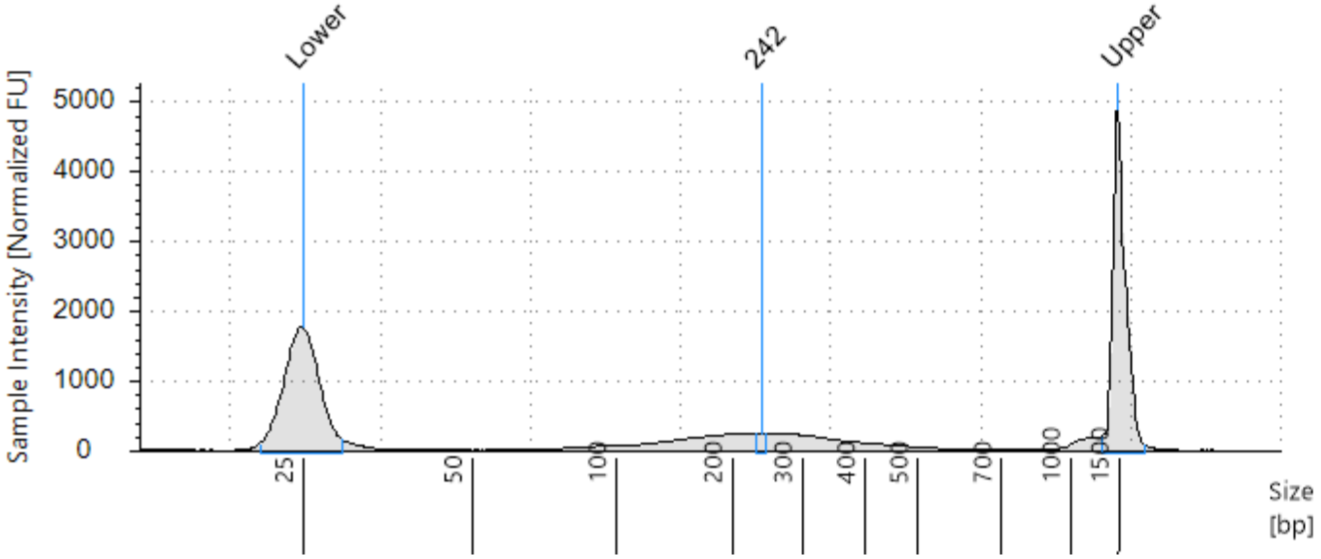

Sample Table

Well	Conc. [ng/µl]	Sample Description	Alert	Observations
G1	0.245	F5 M R3		

Peak Table

Size [bp]	Calibrated Conc. [ng/µl]	Assigned Conc. [ng/µl]	Peak Molarity [nmol/l]	% Integrated Area	Peak Comment	Observations
25	6.55	-	403	-		Lower Marker
242	0.245	-	1.55	100.00		
1500	6.50	6.50	6.67	-		Upper Marker

© Agilent Technologies, Inc. 2017

H1: G5 M R3

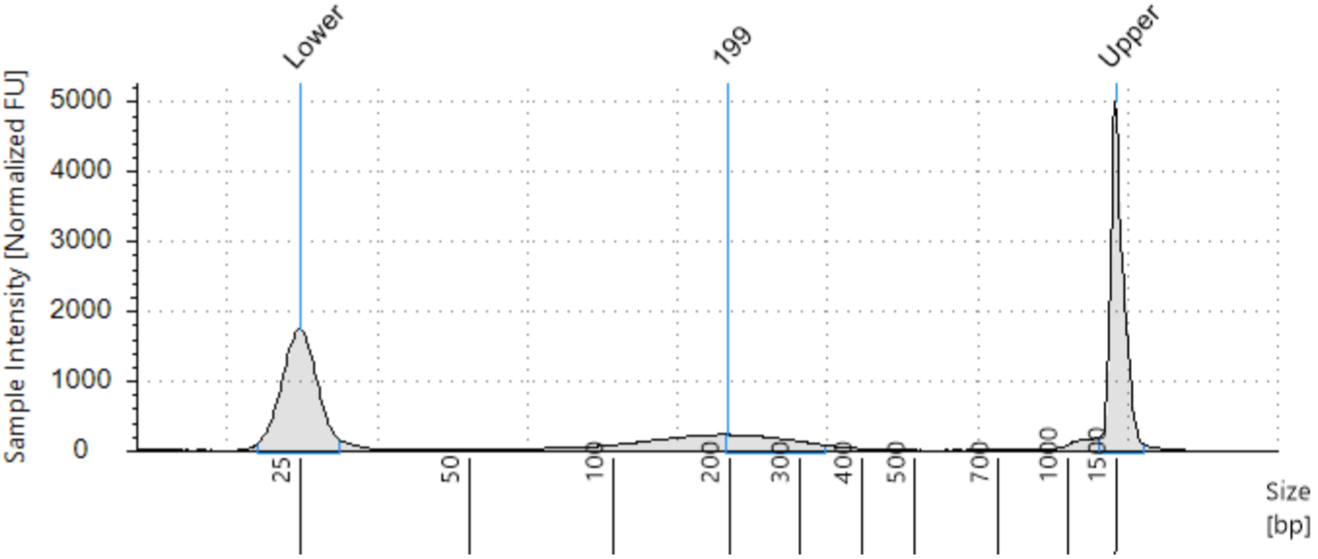

Sample Table

Well	Conc. [ng/µl]	Sample Description	Alert	Observations
H1	1.43	G5 M R3		

Peak Table

Size [bp]	Calibrated Conc. [ng/µl]	Assigned Conc. [ng/µl]	Peak Molarity [nmol/l]	% Integrated Area	Peak Comment	Observations
25	6.21	-	382	-		Lower Marker
199	1.43	-	11.1	100.00		
1500	6.50	6.50	6.67	-		Upper Marker

A2: A6 M R3

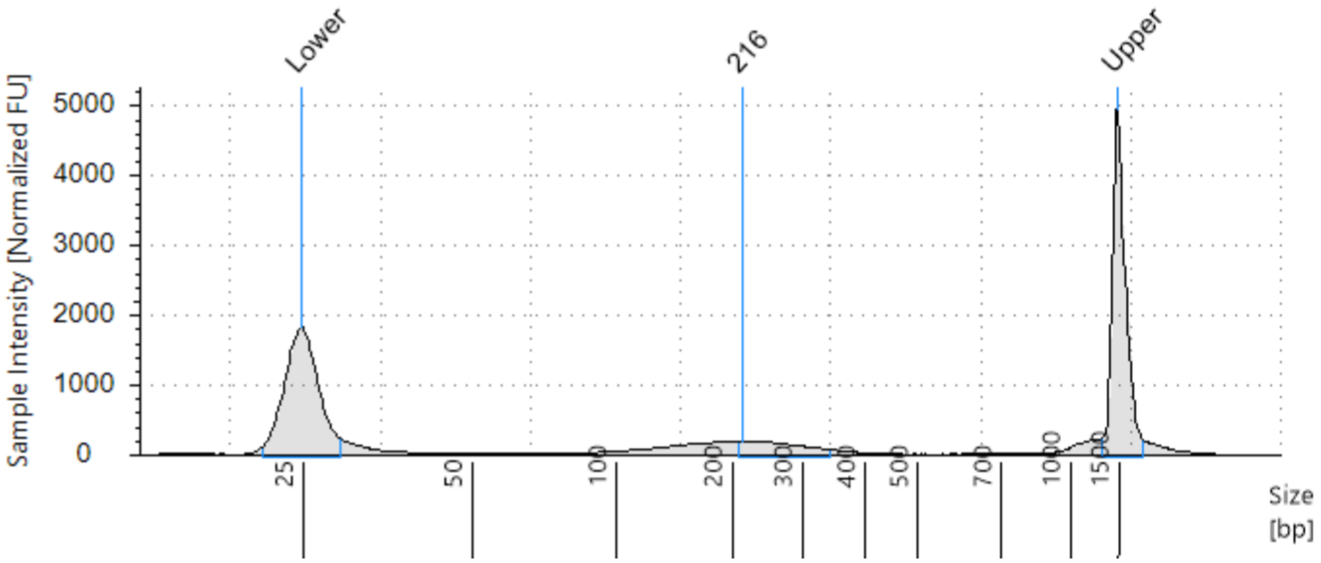

Sample Table

Well	Conc. [ng/µl]	Sample Description	Alert	Observations
A2	1.10	A6 M R3		

Peak Table

Size [bp]	Calibrated Conc. [ng/µl]	Assigned Conc. [ng/µl]	Peak Molarity [nmol/l]	% Integrated Area	Peak Comment	Observations
25	6.14	-	378	-		Lower Marker
216	1.10	-	7.84	100.00		
1500	6.50	6.50	6.67	-		Upper Marker

© Agilent Technologies, Inc. 2017

B2: B6 M R3

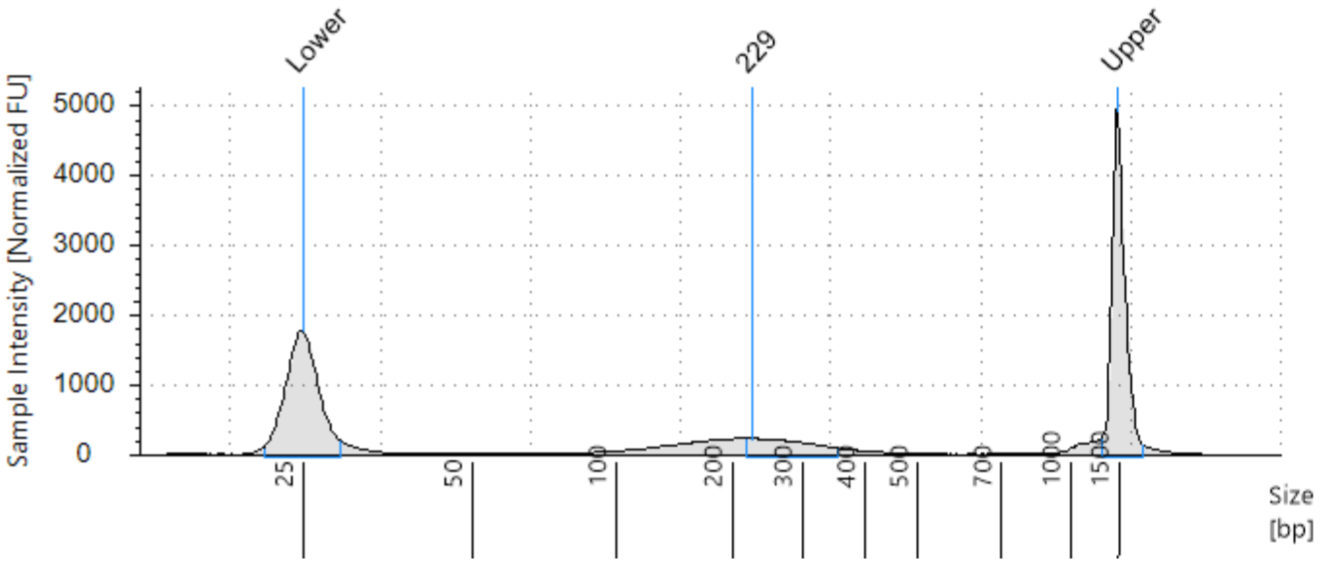

Sample Table

Well	Conc. [ng/µl]	Sample Description	Alert	Observations
B2	1.39	B6 M R3		

Peak Table

Size [bp]	Calibrated Conc. [ng/µl]	Assigned Conc. [ng/µl]	Peak Molarity [nmol/l]	% Integrated Area	Peak Comment	Observations
25	5.91	-	363	-		Lower Marker
229	1.39	-	9.34	100.00		
1500	6.50	6.50	6.67	-		Upper Marker

 © Agilent Technologies, Inc. 2017

C2: C6 M R3

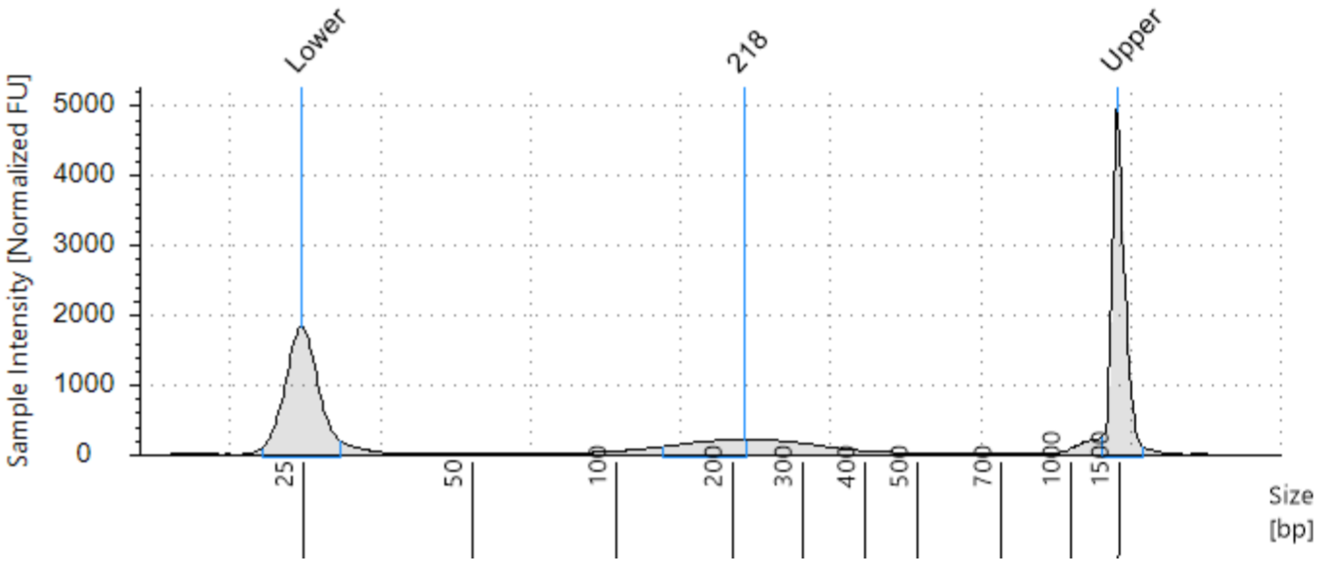

Sample Table

Well	Conc. [ng/µl]	Sample Description	Alert	Observations
C2	1.30	C6 M R3		

Peak Table

Size [bp]	Calibrated Conc. [ng/µl]	Assigned Conc. [ng/µl]	Peak Molarity [nmol/l]	% Integrated Area	Peak Comment	Observations
25	6.22	-	383	-		Lower Marker
218	1.30	-	9.21	100.00		
1500	6.50	6.50	6.67	-		Upper Marker

© Agilent Technologies, Inc. 2017

D2: D6 M R3

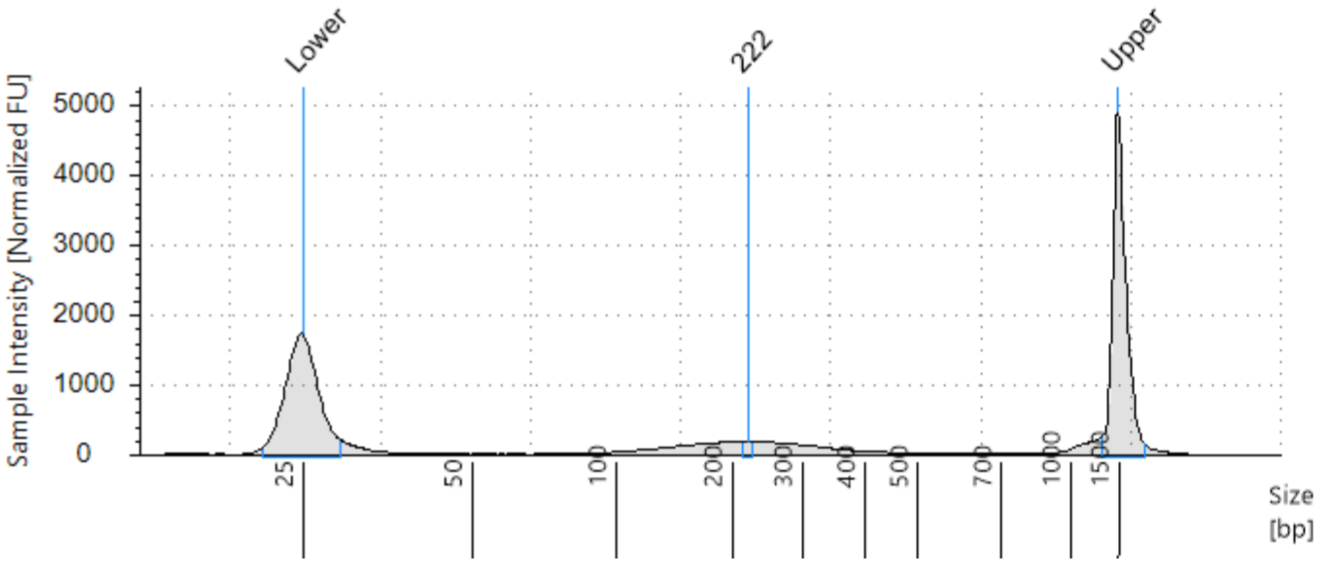

Sample Table

Well	Conc. [ng/µl]	Sample Description	Alert	Observations
D2	0.181	D6 M R3		

Peak Table

Size [bp]	Calibrated Conc. [ng/µl]	Assigned Conc. [ng/µl]	Peak Molarity [nmol/l]	% Integrated Area	Peak Comment	Observations
25	5.69	-	350	-		Lower Marker
222	0.181	-	1.25	100.00		
1500	6.50	6.50	6.67	-		Upper Marker

© Agilent Technologies, Inc. 2017

E2: E6 M R3

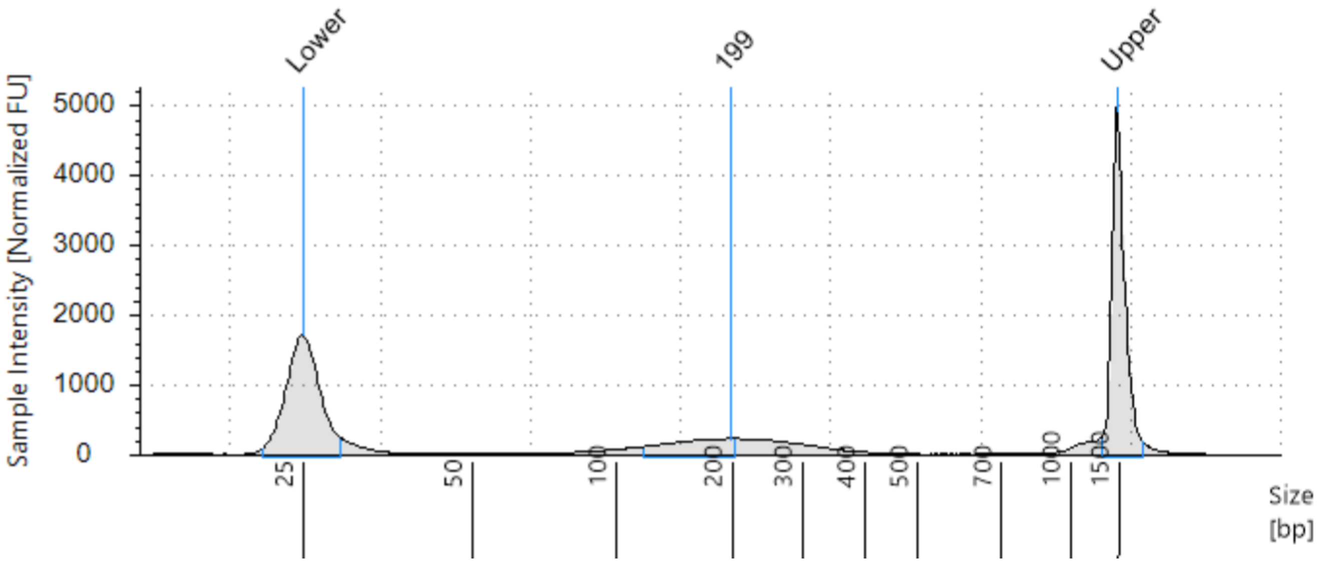

Sample Table

Well	Conc. [ng/µl]	Sample Description	Alert	Observations
E2	1.44	E6 M R3		

Peak Table

Size [bp]	Calibrated Conc. [ng/µl]	Assigned Conc. [ng/µl]	Peak Molarity [nmol/l]	% Integrated Area	Peak Comment	Observations
25	5.82	-	358	-		Lower Marker
199	1.44	-	11.2	100.00		
1500	6.50	6.50	6.67	-		Upper Marker

© Agilent Technologies, Inc. 2017

F2: F6 M R3

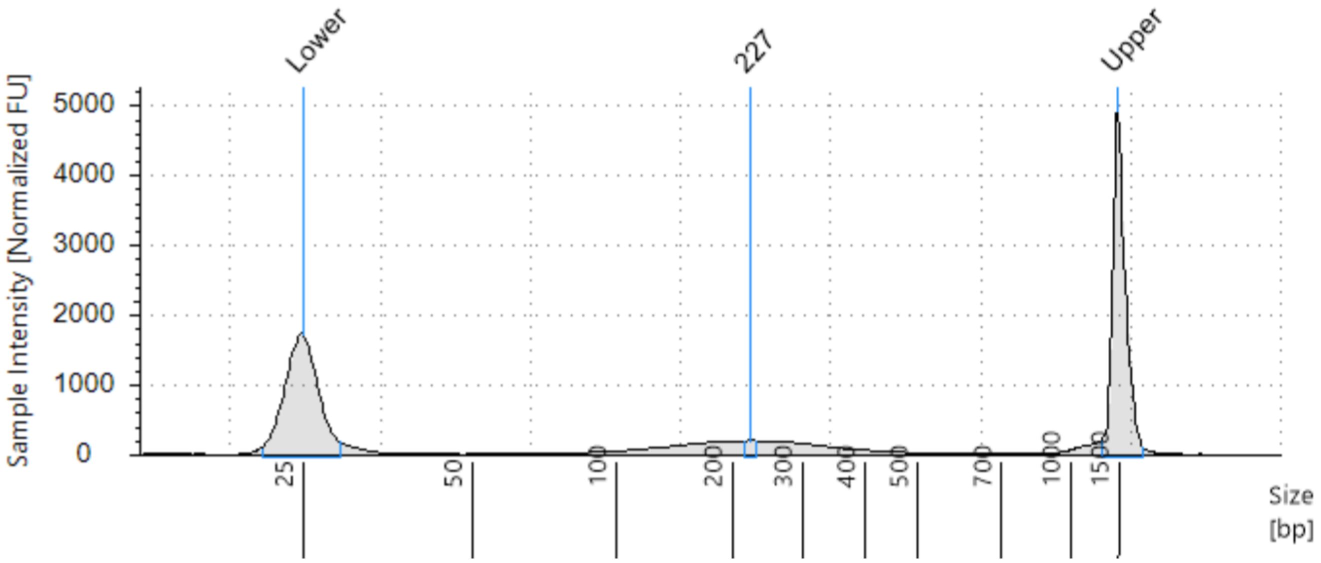

Sample Table

Well	Conc. [ng/µl]	Sample Description	Alert	Observations
F2	0.226	F6 M R3		

Peak Table

Size [bp]	Calibrated Conc. [ng/µl]	Assigned Conc. [ng/µl]	Peak Molarity [nmol/l]	% Integrated Area	Peak Comment	Observations
25	5.93	-	365	-		Lower Marker
227	0.226	-	1.53	100.00		
1500	6.50	6.50	6.67	-		Upper Marker

 © Agilent Technologies, Inc. 2017

G2: G6 M R3

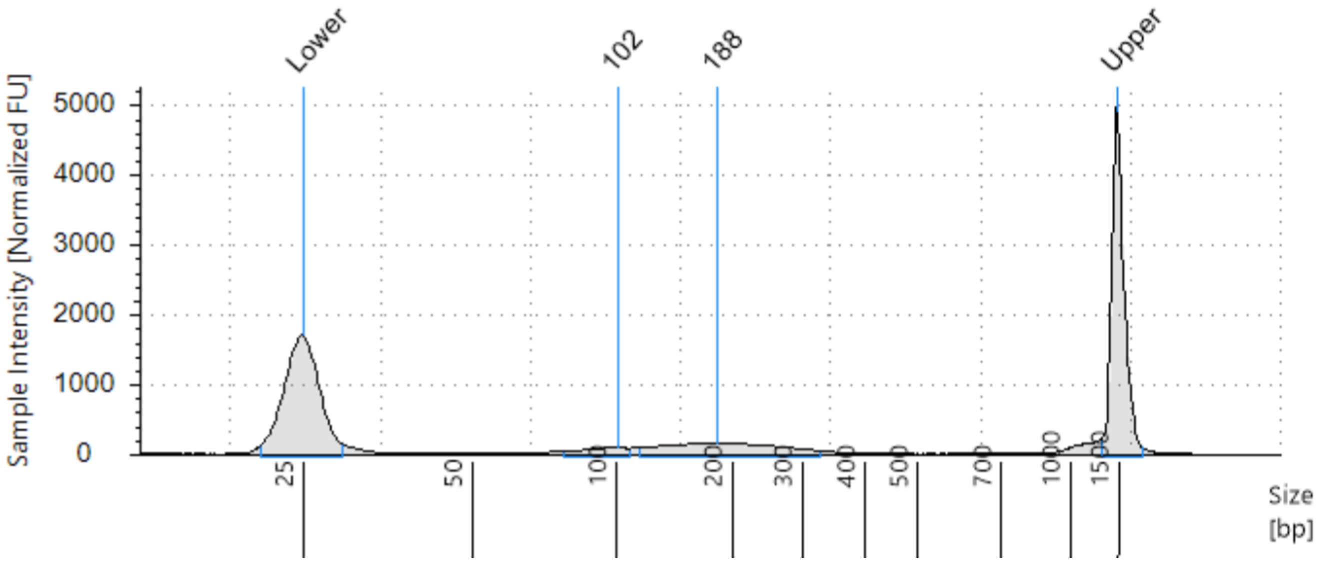

Sample Table

Well	Conc. [ng/µl]	Sample Description	Alert	Observations
G2	2.35	G6 M R3		

Peak Table

Size [bp]	Calibrated Conc. [ng/µl]	Assigned Conc. [ng/µl]	Peak Molarity [nmol/l]	% Integrated Area	Peak Comment	Observations
25	6.36	-	391	-		Lower Marker
102	0.414	-	6.27	17.60		
188	1.94	-	15.8	82.40		
1500	6.50	6.50	6.67	-		Upper Marker

H2: H6 M R3

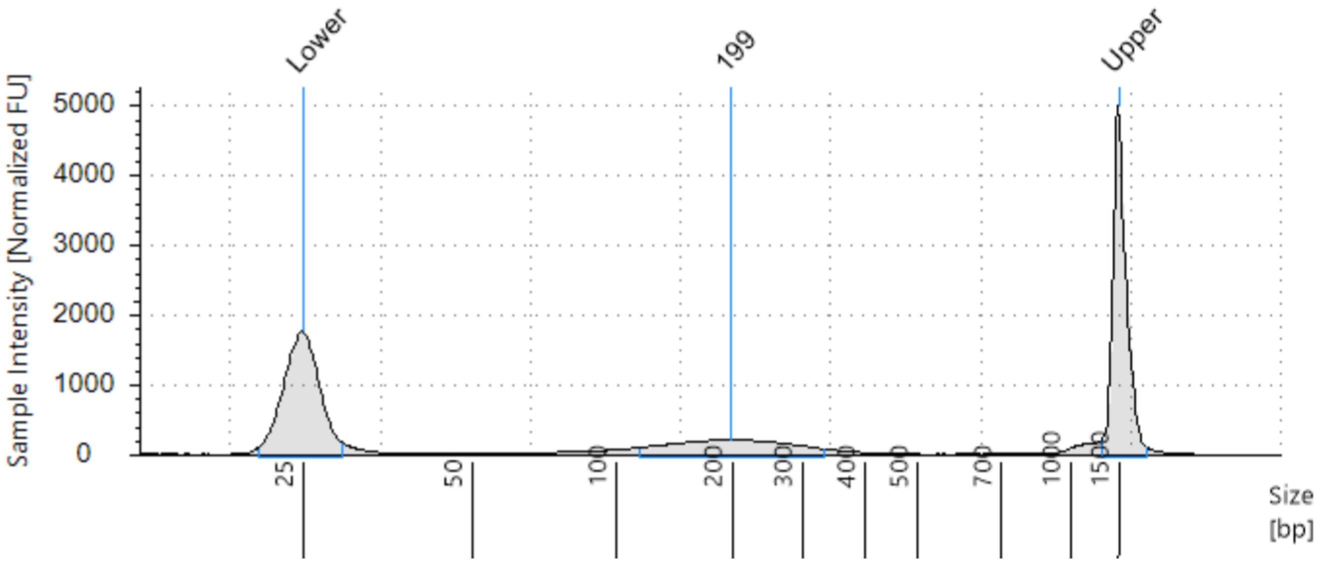

Sample Table

Well	Conc. [ng/µl]	Sample Description	Alert	Observations
H2	2.46	H6 M R3		

Peak Table

Size [bp]	Calibrated Conc. [ng/µl]	Assigned Conc. [ng/µl]	Peak Molarity [nmol/l]	% Integrated Area	Peak Comment	Observations
25	6.18	-	380	-		Lower Marker
199	2.46	-	19.0	100.00		
1500	6.50	6.50	6.67	-		Upper Marker

 © Agilent Technologies, Inc. 2017

Filename: 2020-10-20-01Q-S Minus A7-H8 EXP H7 R3.D1000

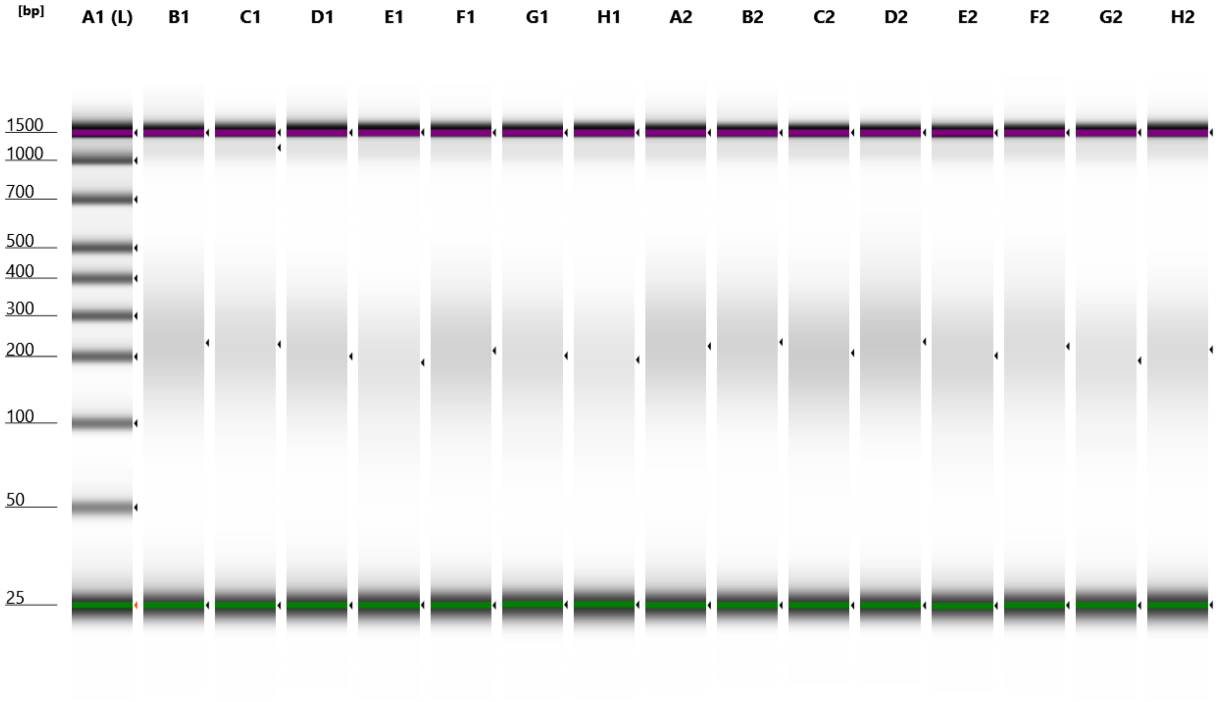

Default image (Contrast 100%)

Sample Info

Well	Conc. [ng/µl]	Sample Description	Alert	Observations
A1	14.0	Ladder		Ladder
B1	3.34	A7 M R3		
C1	1.65	B7 M R3		
D1	1.39	C7 M R3		
E1	0.977	D7 M R3		
F1	3.34	E7 M R3		
G1	0.299	F7 M R3		
H1	1.02	G7 M R3		
A2	1.63	A8 M R3		
B2	3.10	B8 M R3		
C2	1.98	C8 M R3		
D2	2.11	D8 M R3		
E2	0.223	E8 M R3		
F2	2.39	F8 M R3		
G2	0.170	G8 M R3		
H2	1.22	H8 M R3		

 © Agilent Technologies, Inc. 2017

A1: Ladder

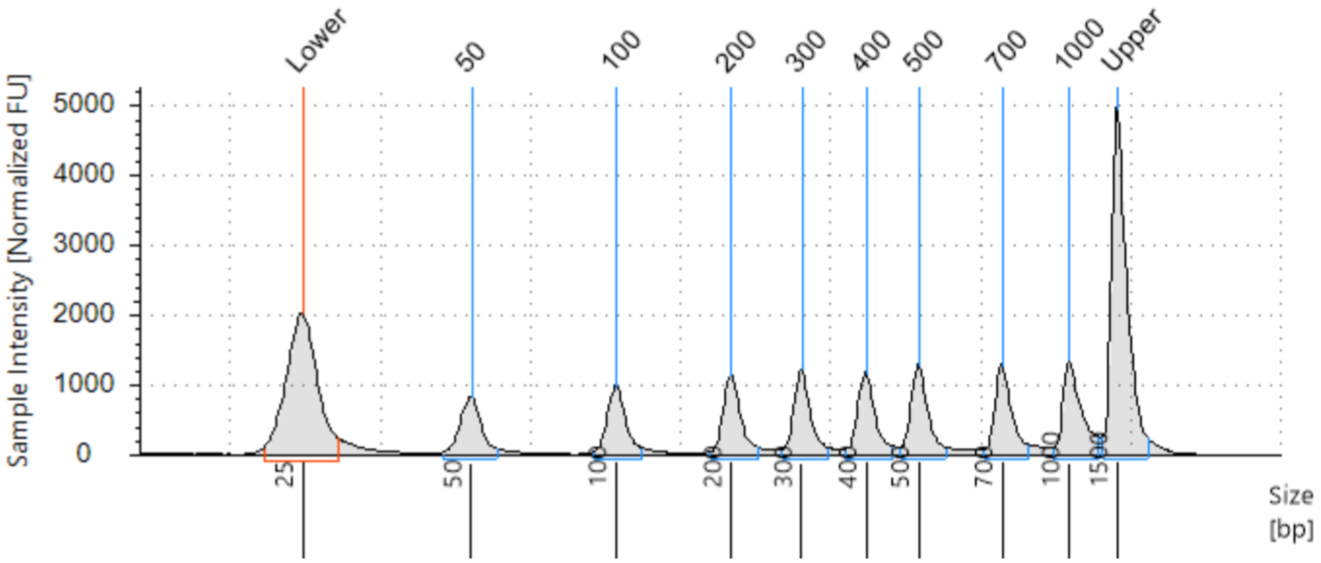

Sample Table

Well	Conc. [ng/µl]	Sample Description	Alert	Observations
A1	14.0	Ladder		Ladder

Peak Table

Size [bp]	Calibrated Conc. [ng/µl]	Assigned Conc. [ng/µl]	Peak Molarity [nmol/l]	% Integrated Area	Peak Comment	Observations
25	5.29	-	326	-		Lower Marker
50	1.47	-	45.3	10.54		
100	1.54	-	23.8	11.07		
200	1.70	-	13.1	12.19		
300	1.73	-	8.90	12.43		
400	1.75	-	6.72	12.53		
500	1.85	-	5.69	13.25		
700	1.83	-	4.02	13.12		
1000	2.08	-	3.19	14.87		
1500	6.50	6.50	6.67	-		Upper Marker

© Agilent Technologies, Inc. 2017

B1: A7 M R3

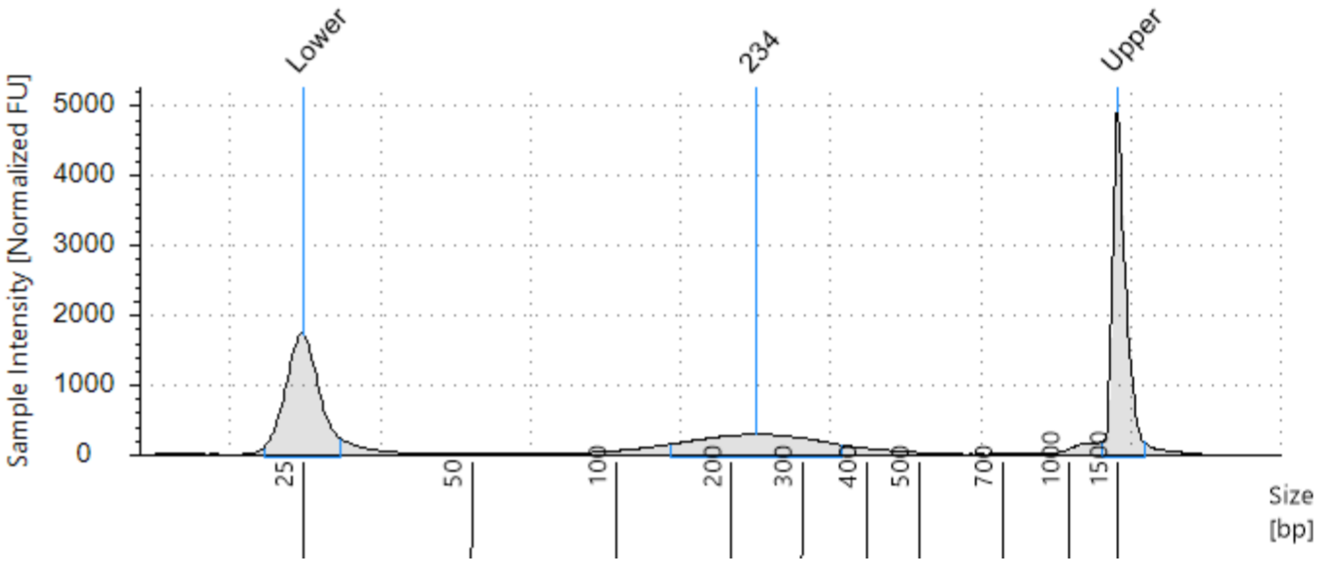

Sample Table

Well	Conc. [ng/µl]	Sample Description	Alert	Observations
B1	3.34	A7 M R3		

Peak Table

Size [bp]	Calibrated Conc. [ng/µl]	Assigned Conc. [ng/µl]	Peak Molarity [nmol/l]	% Integrated Area	Peak Comment	Observations
25	5.69	-	350	-		Lower Marker
234	3.34	-	21.9	100.00		
1500	6.50	6.50	6.67	-		Upper Marker

 © Agilent Technologies, Inc. 2017

C1: B7 M R3

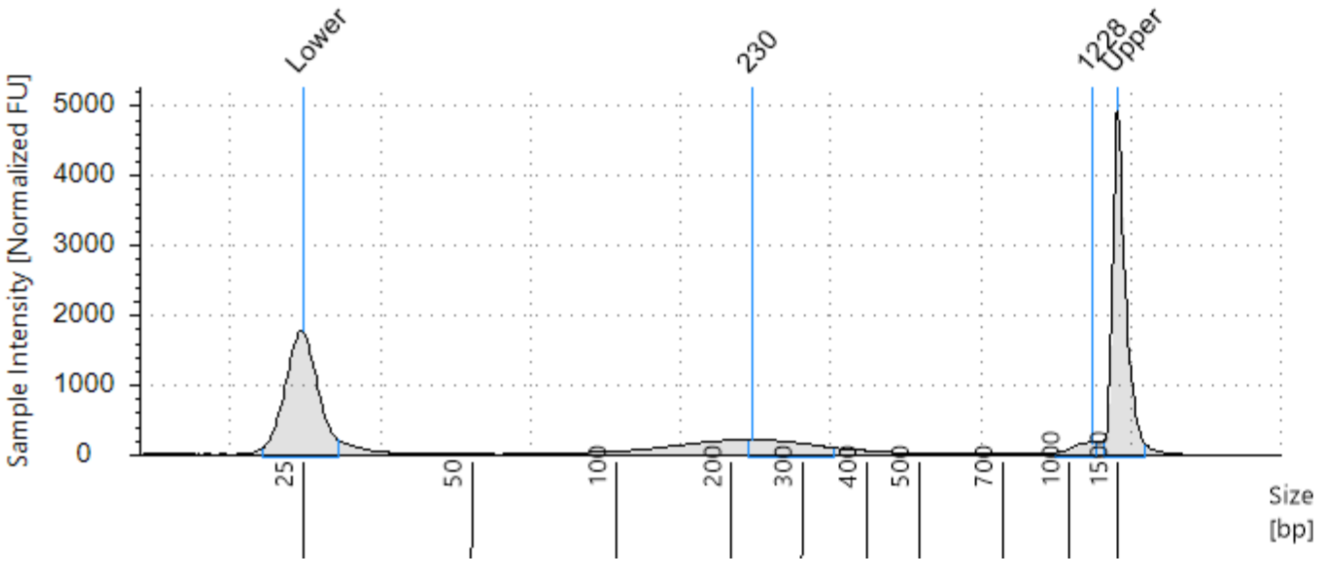

Sample Table

Well	Conc. [ng/µl]	Sample Description	Alert	Observations
C1	1.65	B7 M R3		

Peak Table

Size [bp]	Calibrated Conc. [ng/µl]	Assigned Conc. [ng/µl]	Peak Molarity [nmol/l]	% Integrated Area	Peak Comment	Observations
25	5.82	-	358	-		Lower Marker
230	1.26	-	8.43	76.40		
1228	0.390	-	0.488	23.60		
1500	6.50	6.50	6.67	-		Upper Marker

© Agilent Technologies, Inc. 2017

D1: C7 M R3

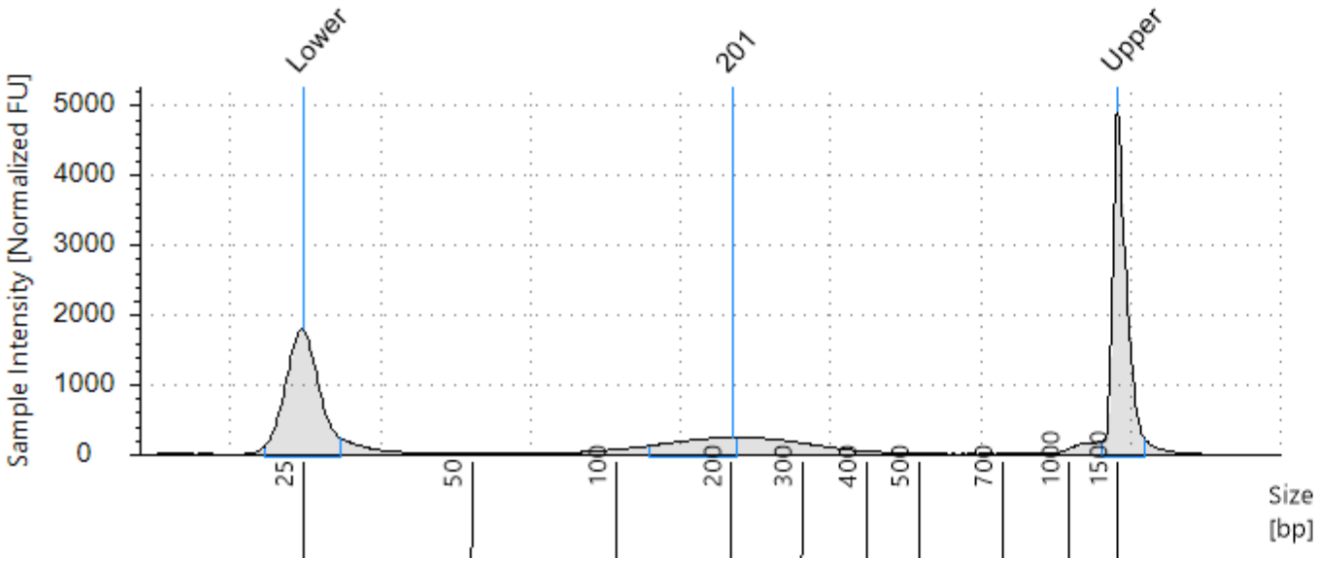

Sample Table

Well	Conc. [ng/µl]	Sample Description	Alert	Observations
D1	1.39	C7 M R3		

Peak Table

Size [bp]	Calibrated Conc. [ng/µl]	Assigned Conc. [ng/µl]	Peak Molarity [nmol/l]	% Integrated Area	Peak Comment	Observations
25	5.71	-	351	-		Lower Marker
201	1.39	-	10.7	100.00		
1500	6.50	6.50	6.67	-		Upper Marker

 © Agilent Technologies, Inc. 2017

E1: D7 M R3

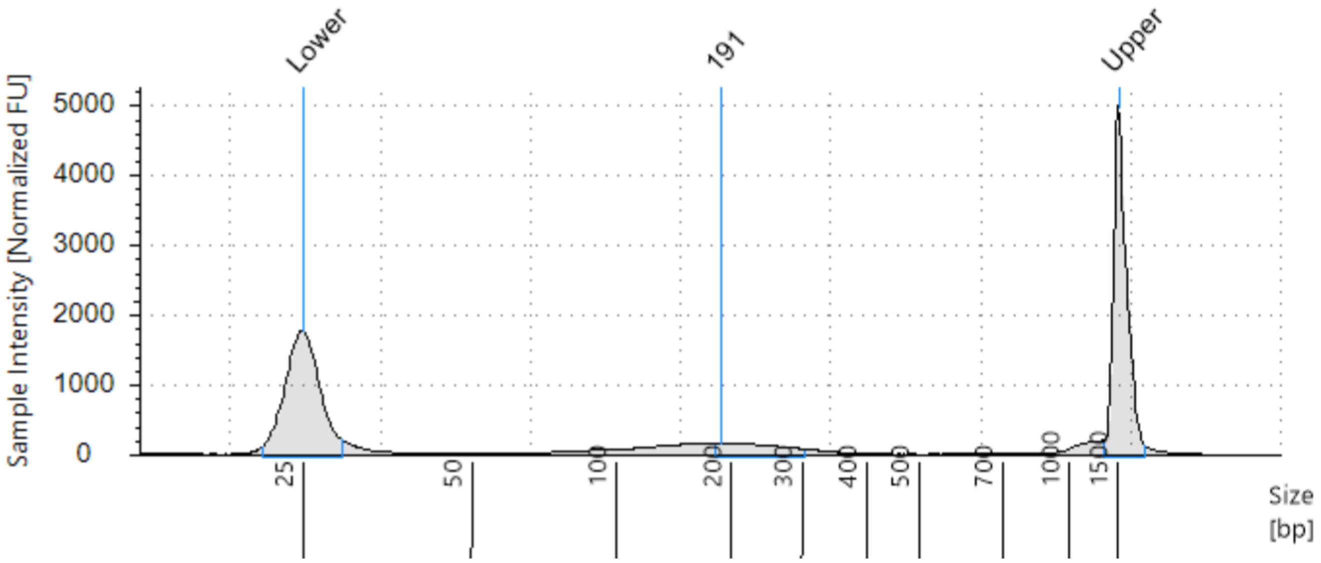

Sample Table

Well	Conc. [ng/µl]	Sample Description	Alert	Observations
E1	0.977	D7 M R3		

Peak Table

Size [bp]	Calibrated Conc. [ng/µl]	Assigned Conc. [ng/µl]	Peak Molarity [nmol/l]	% Integrated Area	Peak Comment	Observations
25	6.06	-	373	-		Lower Marker
191	0.977	-	7.89	100.00		
1500	6.50	6.50	6.67	-		Upper Marker

© Agilent Technologies, Inc. 2017

F1: E7 M R3

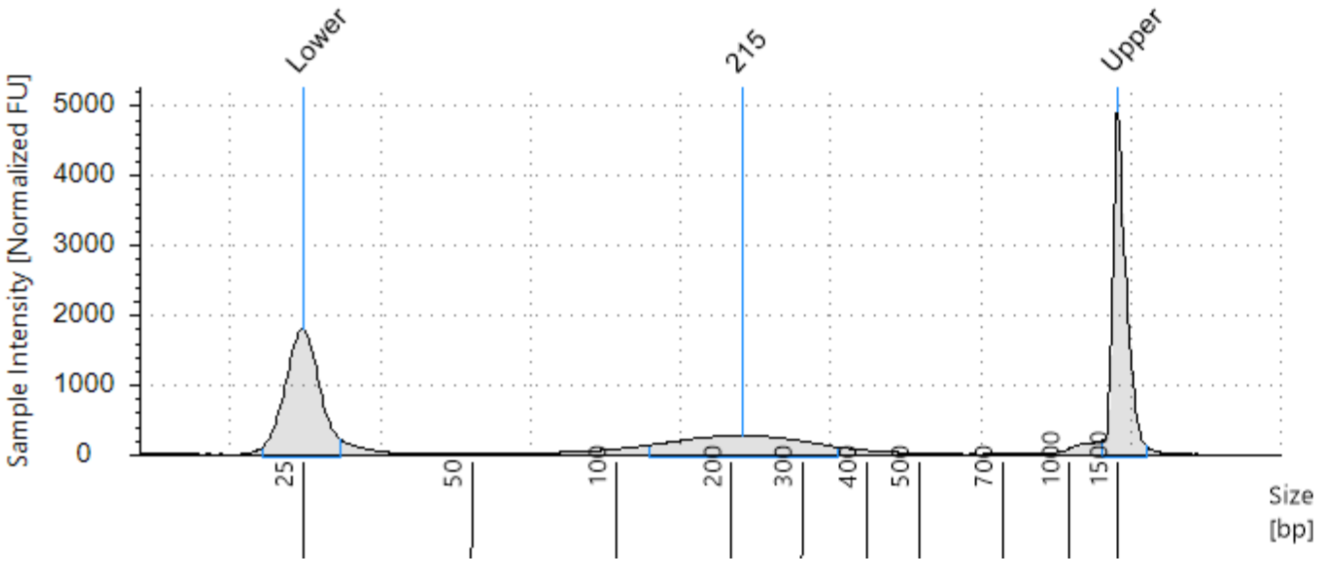

Sample Table

Well	Conc. [ng/µl]	Sample Description	Alert	Observations
F1	3.34	E7 M R3		

Peak Table

Size [bp]	Calibrated Conc. [ng/µl]	Assigned Conc. [ng/µl]	Peak Molarity [nmol/l]	% Integrated Area	Peak Comment	Observations
25	6.01	-	370	-		Lower Marker
215	3.34	-	24.0	100.00		
1500	6.50	6.50	6.67	-		Upper Marker

© Agilent Technologies, Inc. 2017

G1: F7 M R3

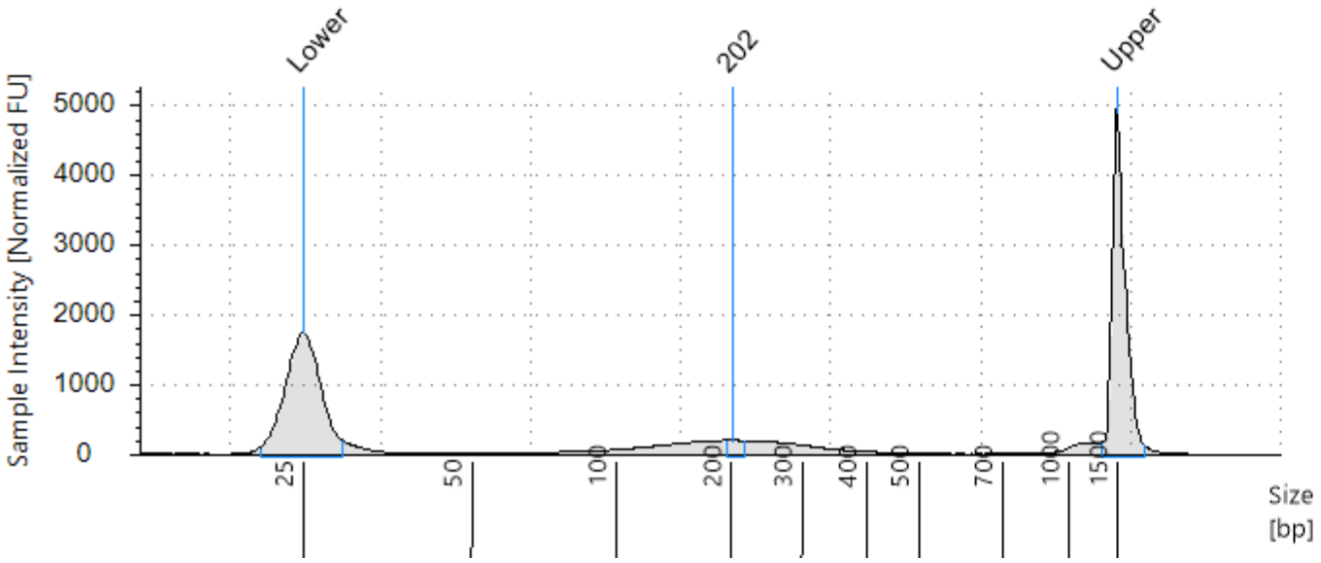

Sample Table

Well	Conc. [ng/µl]	Sample Description	Alert	Observations
G1	0.299	F7 M R3		

Peak Table

Size [bp]	Calibrated Conc. [ng/µl]	Assigned Conc. [ng/µl]	Peak Molarity [nmol/l]	% Integrated Area	Peak Comment	Observations
25	6.20	-	382	-		Lower Marker
202	0.299	-	2.28	100.00		
1500	6.50	6.50	6.67	-		Upper Marker

 © Agilent Technologies, Inc. 2017

H1: G7 M R3

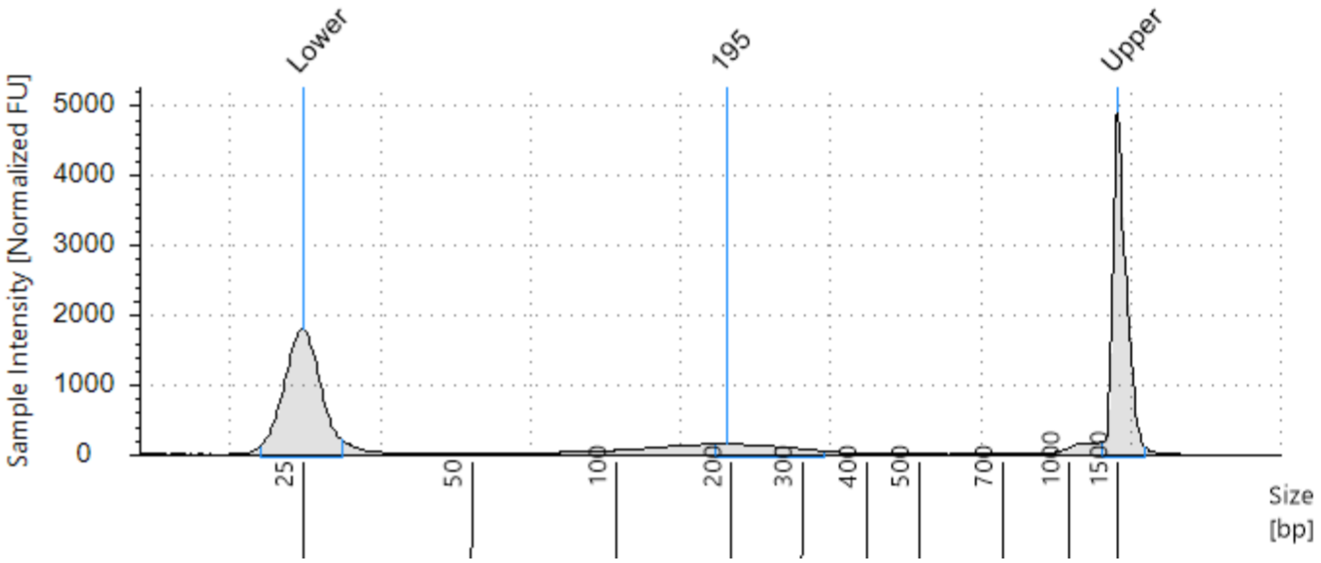

Sample Table

Well	Conc. [ng/µl]	Sample Description	Alert	Observations
H1	1.02	G7 M R3		

Peak Table

Size [bp]	Calibrated Conc. [ng/µl]	Assigned Conc. [ng/µl]	Peak Molarity [nmol/l]	% Integrated Area	Peak Comment	Observations
25	6.40	-	394	-		Lower Marker
195	1.02	-	8.07	100.00		
1500	6.50	6.50	6.67	-		Upper Marker

© Agilent Technologies, Inc. 2017

A2: A8 M R3

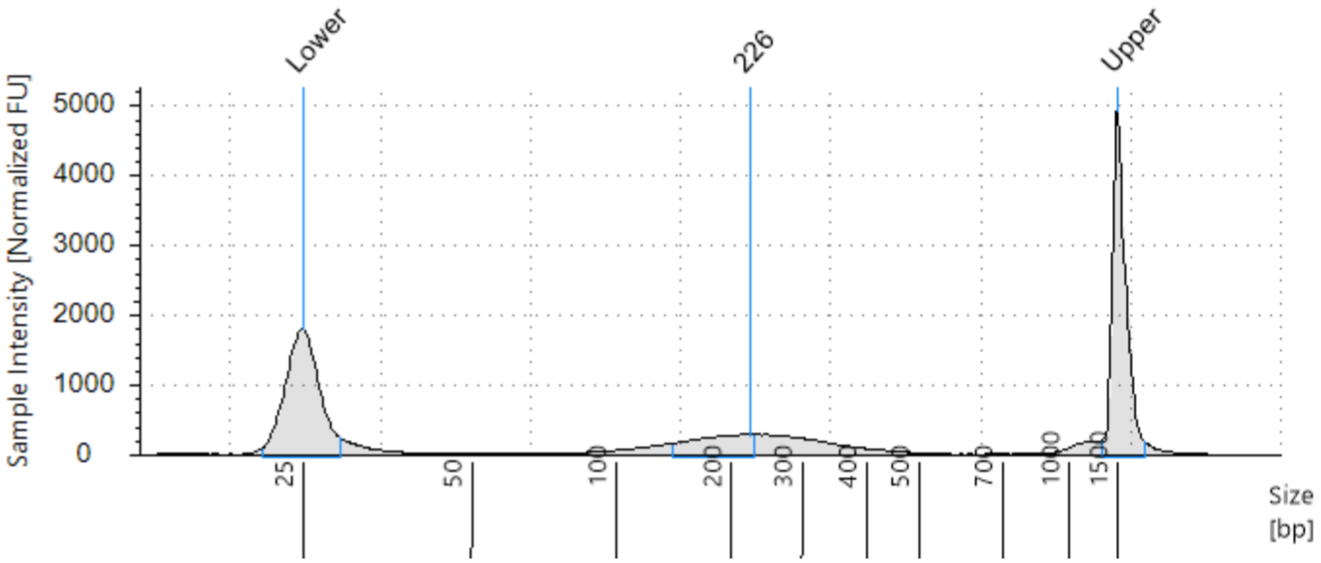

Sample Table

Well	Conc. [ng/µl]	Sample Description	Alert	Observations
A2	1.63	A8 M R3		

Peak Table

Size [bp]	Calibrated Conc. [ng/µl]	Assigned Conc. [ng/µl]	Peak Molarity [nmol/l]	% Integrated Area	Peak Comment	Observations
25	5.96	-	367	-		Lower Marker
226	1.63	-	11.1	100.00		
1500	6.50	6.50	6.67	-		Upper Marker

 © Agilent Technologies, Inc. 2017

B2: B8 M R3

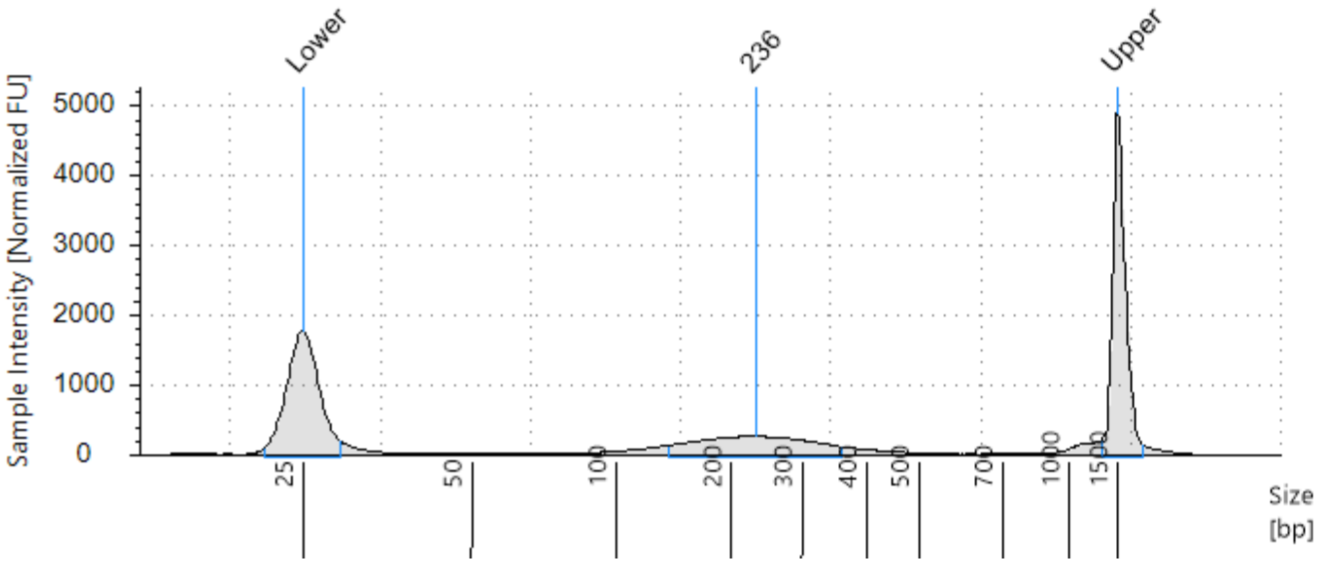

Sample Table

Well	Conc. [ng/µl]	Sample Description	Alert	Observations
B2	3.10	B8 M R3		

Peak Table

Size [bp]	Calibrated Conc. [ng/µl]	Assigned Conc. [ng/µl]	Peak Molarity [nmol/l]	% Integrated Area	Peak Comment	Observations
25	5.94	-	366	-		Lower Marker
236	3.10	-	20.2	100.00		
1500	6.50	6.50	6.67	-		Upper Marker

© Agilent Technologies, Inc. 2017

C2: C8 M R3

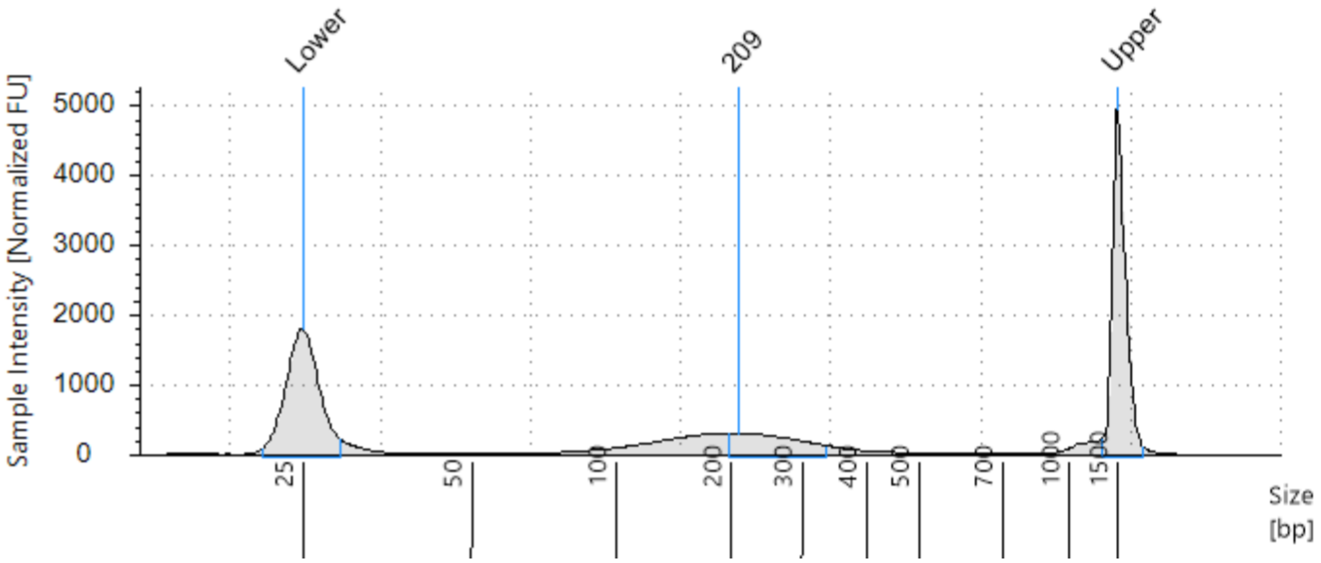

Sample Table

Well	Conc. [ng/µl]	Sample Description	Alert	Observations
C2	1.98	C8 M R3		

Peak Table

Size [bp]	Calibrated Conc. [ng/µl]	Assigned Conc. [ng/µl]	Peak Molarity [nmol/l]	% Integrated Area	Peak Comment	Observations
25	5.90	-	363	-		Lower Marker
209	1.98	-	14.6	100.00		
1500	6.50	6.50	6.67	-		Upper Marker

 © Agilent Technologies, Inc. 2017

D2: D8 M R3

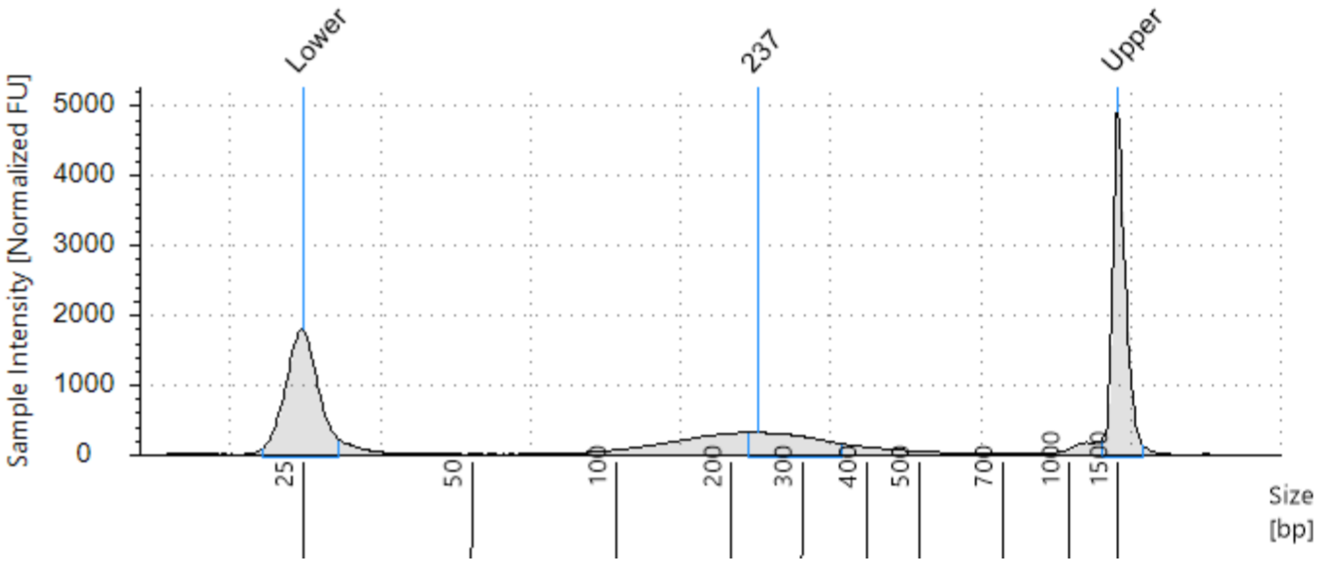

Sample Table

Well	Conc. [ng/µl]	Sample Description	Alert	Observations
D2	2.11	D8 M R3		

Peak Table

Size [bp]	Calibrated Conc. [ng/µl]	Assigned Conc. [ng/µl]	Peak Molarity [nmol/l]	% Integrated Area	Peak Comment	Observations
25	5.91	-	364	-		Lower Marker
237	2.11	-	13.7	100.00		
1500	6.50	6.50	6.67	-		Upper Marker

© Agilent Technologies, Inc. 2017

E2: E8 M R3

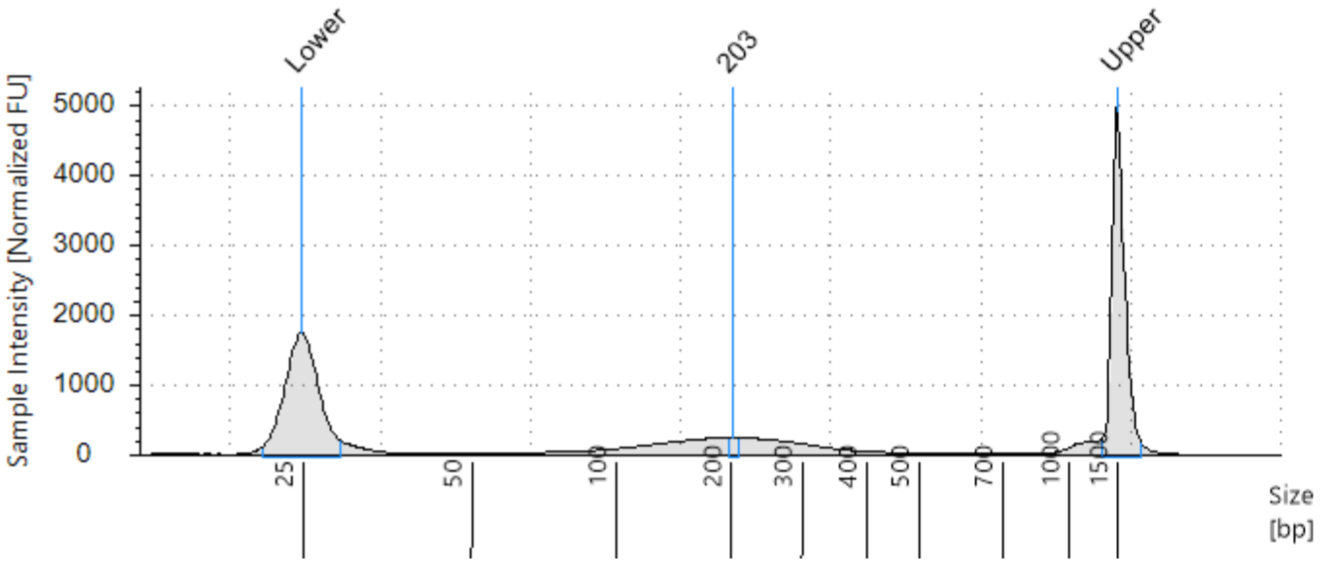

Sample Table

Well	Conc. [ng/µl]	Sample Description	Alert	Observations
E2	0.223	E8 M R3		

Peak Table

Size [bp]	Calibrated Conc. [ng/µl]	Assigned Conc. [ng/µl]	Peak Molarity [nmol/l]	% Integrated Area	Peak Comment	Observations
25	5.94	-	366	-		Lower Marker
203	0.223	-	1.69	100.00		
1500	6.50	6.50	6.67	-		Upper Marker

 © Agilent Technologies, Inc. 2017

F2: F8 M R3

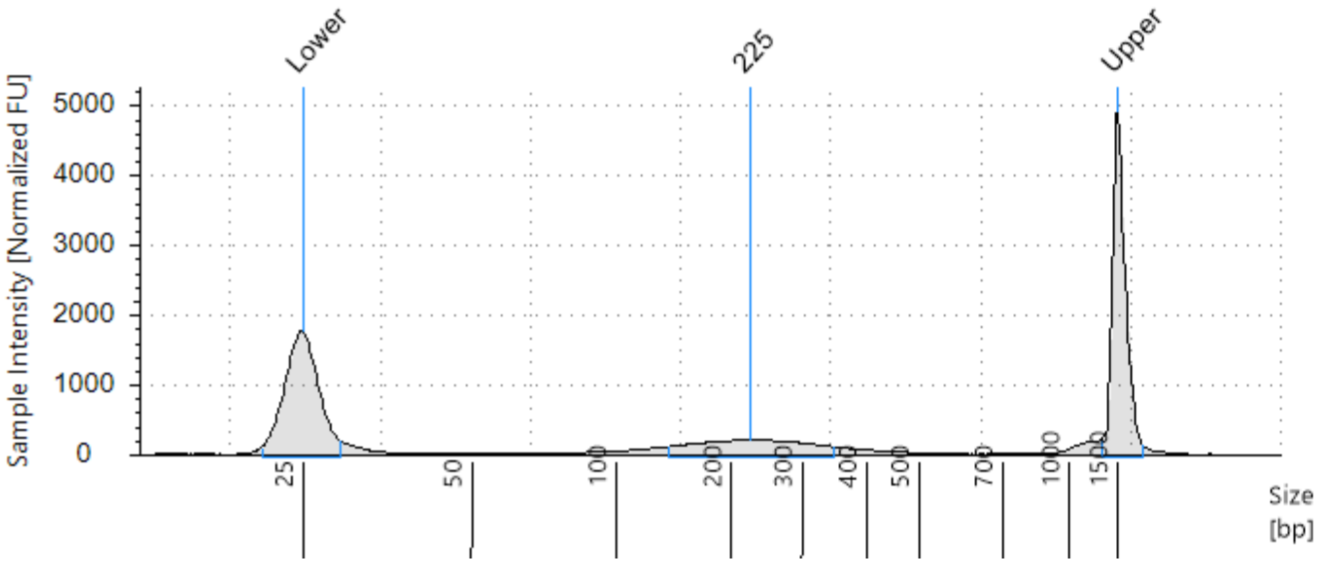

Sample Table

Well	Conc. [ng/µl]	Sample Description	Alert	Observations
F2	2.39	F8 M R3		

Peak Table

Size [bp]	Calibrated Conc. [ng/µl]	Assigned Conc. [ng/µl]	Peak Molarity [nmol/l]	% Integrated Area	Peak Comment	Observations
25	6.06	-	373	-		Lower Marker
225	2.39	-	16.3	100.00		
1500	6.50	6.50	6.67	-		Upper Marker

 © Agilent Technologies, Inc. 2017

G2: G8 M R3

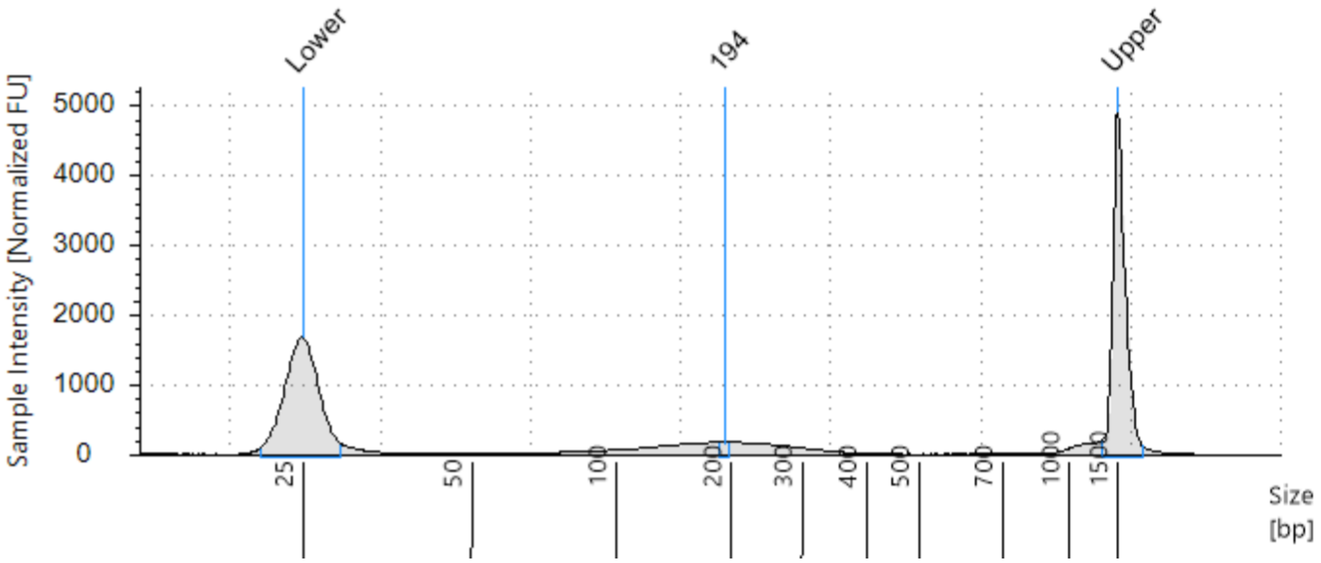

Sample Table

Well	Conc. [ng/µl]	Sample Description	Alert	Observations
G2	0.170	G8 M R3		

Peak Table

Size [bp]	Calibrated Conc. [ng/µl]	Assigned Conc. [ng/µl]	Peak Molarity [nmol/l]	% Integrated Area	Peak Comment	Observations
25	6.13	-	377	-		Lower Marker
194	0.170	-	1.35	100.00		
1500	6.50	6.50	6.67	-		Upper Marker

 © Agilent Technologies, Inc. 2017

H2: H8 M R3

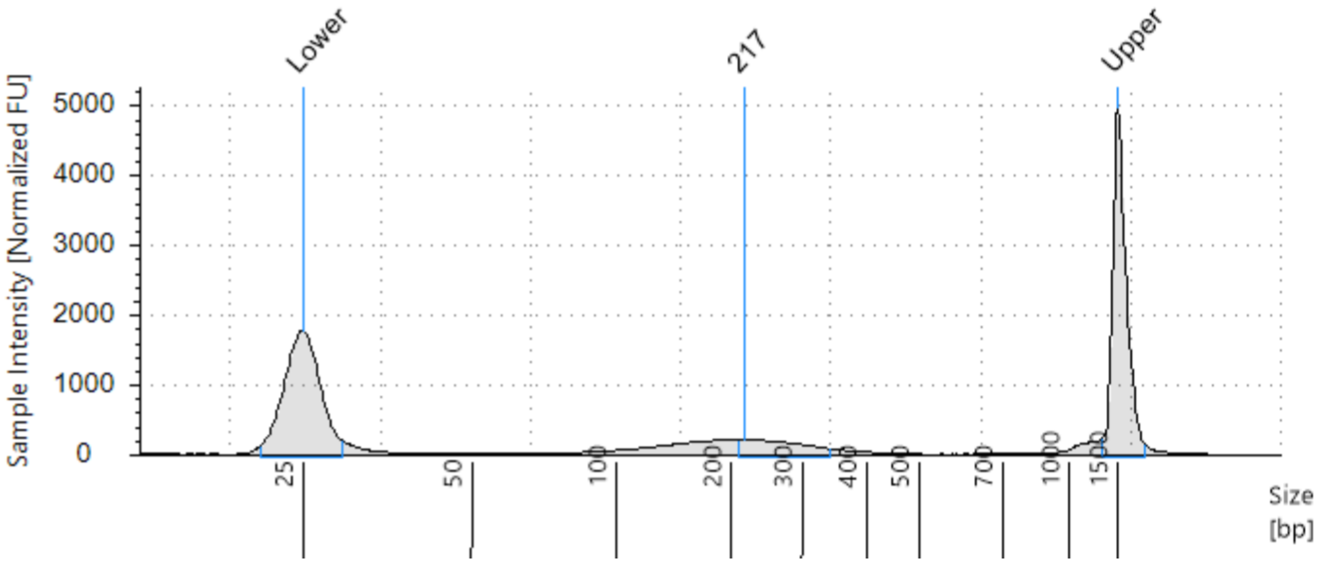

Sample Table

Well	Conc. [ng/µl]	Sample Description	Alert	Observations
H2	1.22	H8 M R3		

Peak Table

Size [bp]	Calibrated Conc. [ng/µl]	Assigned Conc. [ng/µl]	Peak Molarity [nmol/l]	% Integrated Area	Peak Comment	Observations
25	6.17	-	380	-		Lower Marker
217	1.22	-	8.69	100.00		
1500	6.50	6.50	6.67	-		Upper Marker

© Agilent Technologies, Inc. 2017

Filename: 2020-10-20-02Q-S Minus A9-H10 EXP H9 R3.D1000

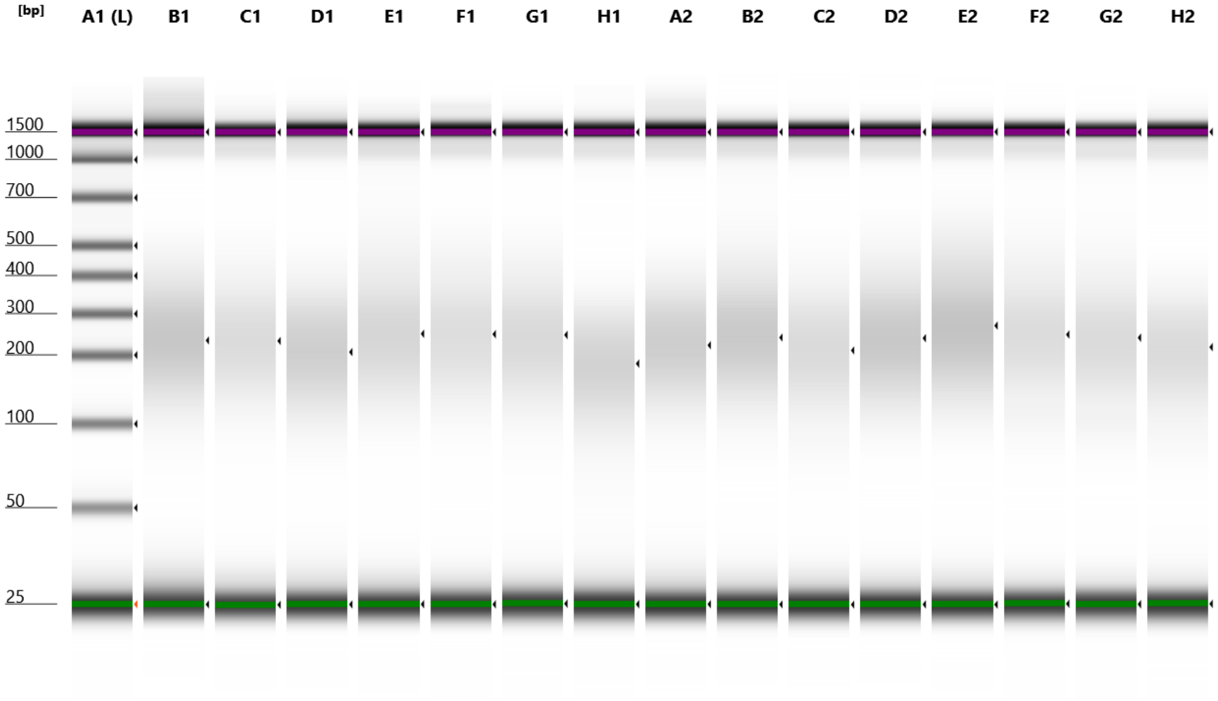

Default image (Contrast 100%)

Sample Info

Well	Conc. [ng/µl]	Sample Description	Alert	Observations
A1	12.1	Ladder		Ladder
B1	1.88	A9 M R3		
C1	0.227	B9 M R3		
D1	3.59	C9 M R3		
E1	1.60	D9 M R3		
F1	0.198	E9 M R3		
G1	1.26	F9 M R3		
H1	3.31	G9 M R3		
A2	0.490	A10 M R3		
B2	2.06	B10 M R3		
C2	0.273	C10 M R3		
D2	2.03	D10 M R3		
E2	2.79	E10 M R3		
F2	1.41	F10 M R3		
G2	1.33	G10 M R3		
H2	2.52	H10 M R3		

 © Agilent Technologies, Inc. 2017

A1: Ladder

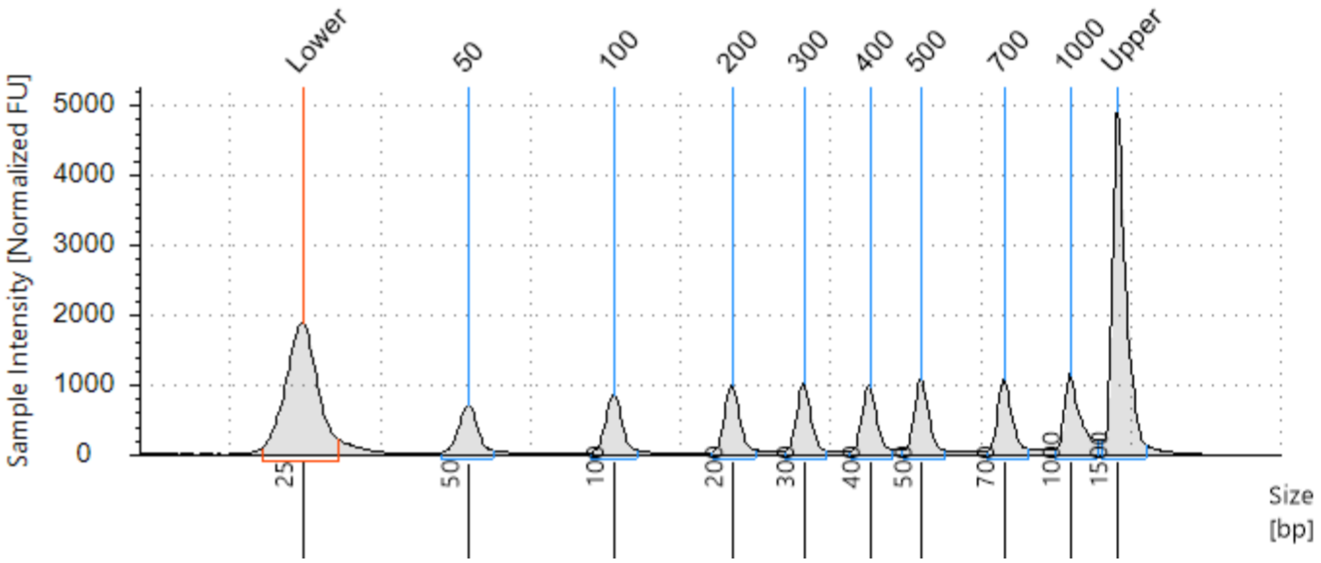

Sample Table

Well	Conc. [ng/µl]	Sample Description	Alert	Observations
A1	12.1	Ladder		Ladder

Peak Table

Size [bp]	Calibrated Conc. [ng/µl]	Assigned Conc. [ng/µl]	Peak Molarity [nmol/l]	% Integrated Area	Peak Comment	Observations
25	5.81	-	357	-		Lower Marker
50	1.33	-	41.0	11.02		
100	1.40	-	21.5	11.59		
200	1.50	-	11.6	12.46		
300	1.47	-	7.56	12.20		
400	1.47	-	5.67	12.20		
500	1.55	-	4.77	12.85		
700	1.56	-	3.42	12.88		
1000	1.79	-	2.75	14.80		
1500	6.50	6.50	6.67	-		Upper Marker

 © Agilent Technologies, Inc. 2017

B1: A9 M R3

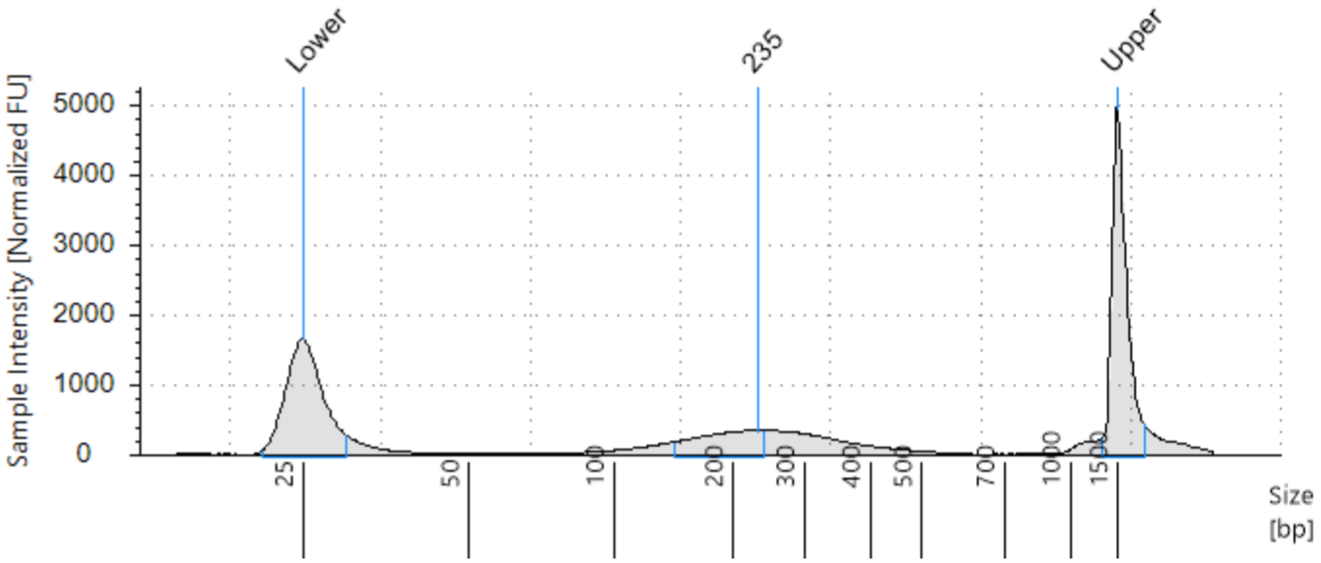

Sample Table

Well	Conc. [ng/µl]	Sample Description	Alert	Observations
B1	1.88	A9 M R3		

Peak Table

Size [bp]	Calibrated Conc. [ng/µl]	Assigned Conc. [ng/µl]	Peak Molarity [nmol/l]	% Integrated Area	Peak Comment	Observations
25	5.35	-	329	-		Lower Marker
235	1.88	-	12.3	100.00		
1500	6.50	6.50	6.67	-		Upper Marker

© Agilent Technologies, Inc. 2017

C1: B9 M R3

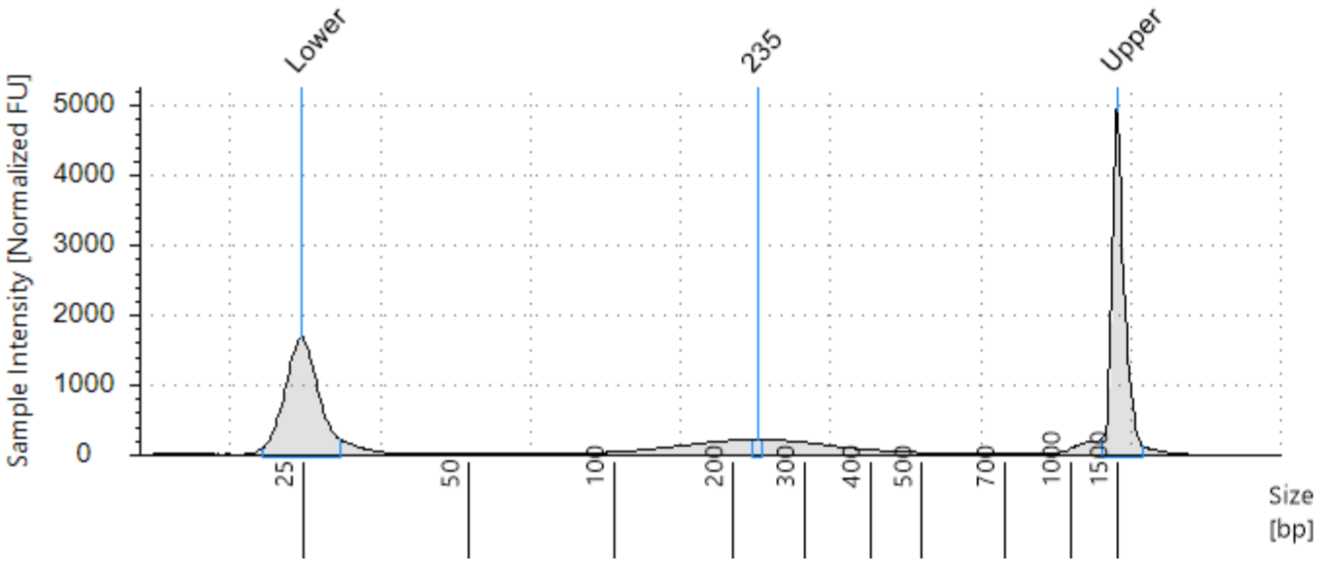

Sample Table

Well	Conc. [ng/µl]	Sample Description	Alert	Observations
C1	0.227	B9 M R3		

Peak Table

Size [bp]	Calibrated Conc. [ng/µl]	Assigned Conc. [ng/µl]	Peak Molarity [nmol/l]	% Integrated Area	Peak Comment	Observations
25	5.92	-	364	-		Lower Marker
235	0.227	-	1.48	100.00		
1500	6.50	6.50	6.67	-		Upper Marker

 © Agilent Technologies, Inc. 2017

D1: C9 M R3

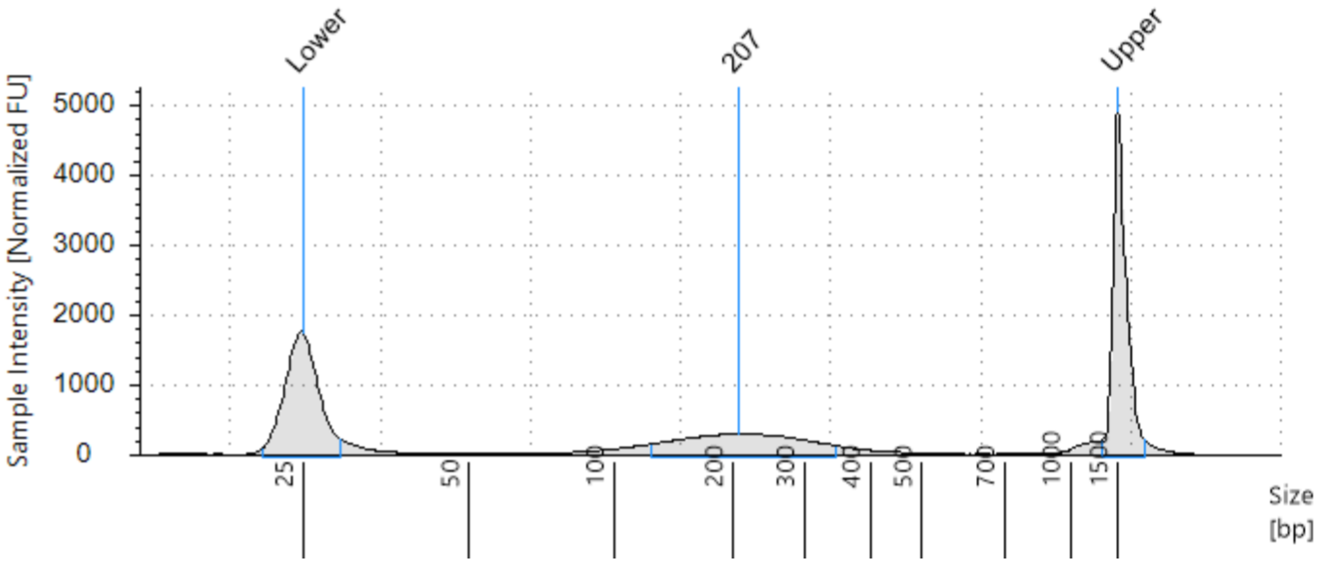

Sample Table

Well	Conc. [ng/µl]	Sample Description	Alert	Observations
D1	3.59	C9 M R3		

Peak Table

Size [bp]	Calibrated Conc. [ng/µl]	Assigned Conc. [ng/µl]	Peak Molarity [nmol/l]	% Integrated Area	Peak Comment	Observations
25	5.77	-	355	-		Lower Marker
207	3.59	-	26.6	100.00		
1500	6.50	6.50	6.67	-		Upper Marker

© Agilent Technologies, Inc. 2017

E1: D9 M R3

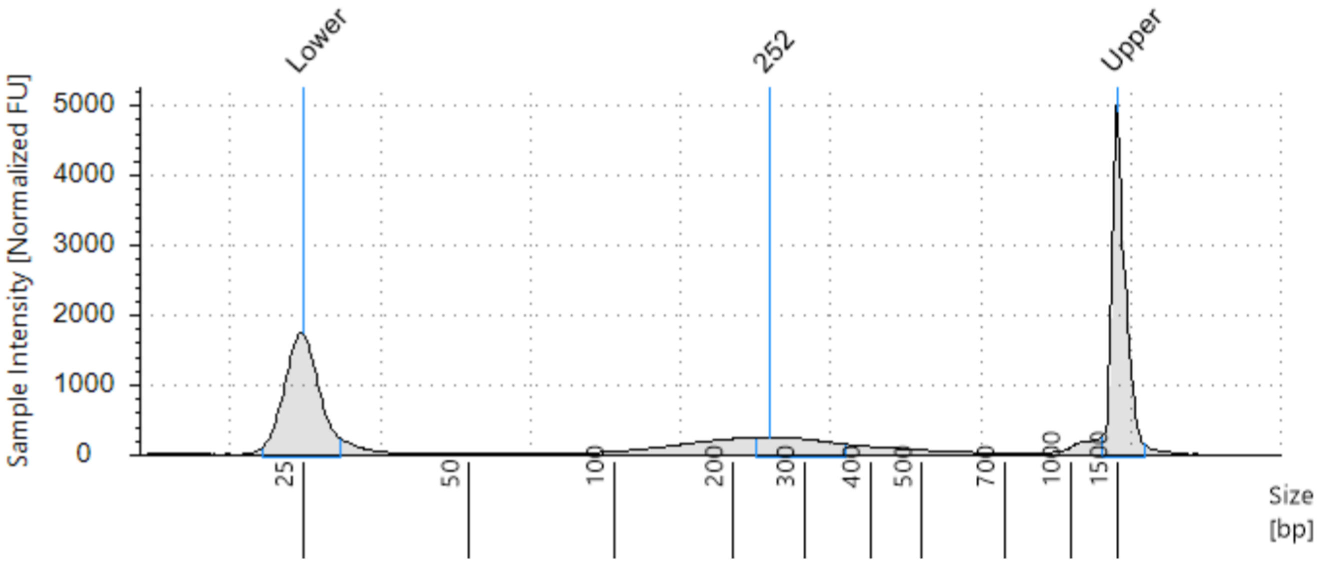

Sample Table

Well	Conc. [ng/µl]	Sample Description	Alert	Observations
E1	1.60	D9 M R3		

Peak Table

Size [bp]	Calibrated Conc. [ng/µl]	Assigned Conc. [ng/µl]	Peak Molarity [nmol/l]	% Integrated Area	Peak Comment	Observations
25	5.86	-	361	-		Lower Marker
252	1.60	-	9.76	100.00		
1500	6.50	6.50	6.67	-		Upper Marker

© Agilent Technologies, Inc. 2017

F1: E9 M R3

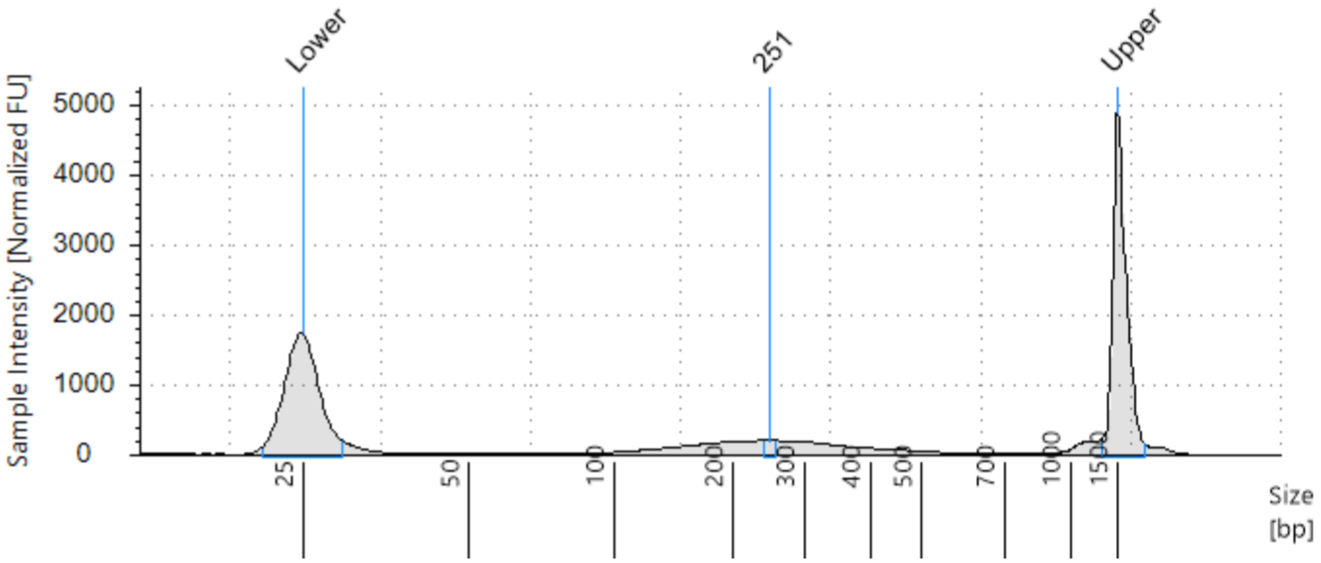

Sample Table

Well	Conc. [ng/µl]	Sample Description	Alert	Observations
F1	0.198	E9 M R3		

Peak Table

Size [bp]	Calibrated Conc. [ng/µl]	Assigned Conc. [ng/µl]	Peak Molarity [nmol/l]	% Integrated Area	Peak Comment	Observations
25	5.90	-	363	-		Lower Marker
251	0.198	-	1.22	100.00		
1500	6.50	6.50	6.67	-		Upper Marker

© Agilent Technologies, Inc. 2017

G1: F9 M R3

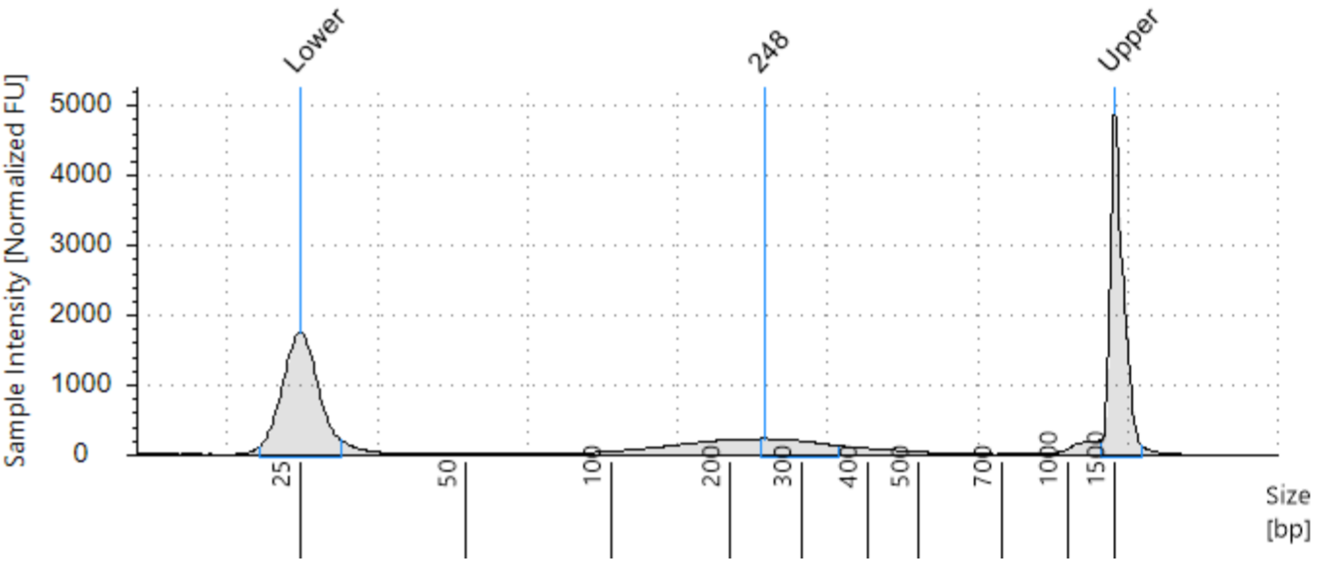

Sample Table

Well	Conc. [ng/µl]	Sample Description	Alert	Observations
G1	1.26	F9 M R3		

Peak Table

Size [bp]	Calibrated Conc. [ng/µl]	Assigned Conc. [ng/µl]	Peak Molarity [nmol/l]	% Integrated Area	Peak Comment	Observations
25	6.27	-	386	-		Lower Marker
248	1.26	-	7.84	100.00		
1500	6.50	6.50	6.67	-		Upper Marker

© Agilent Technologies, Inc. 2017

H1: G9 M R3

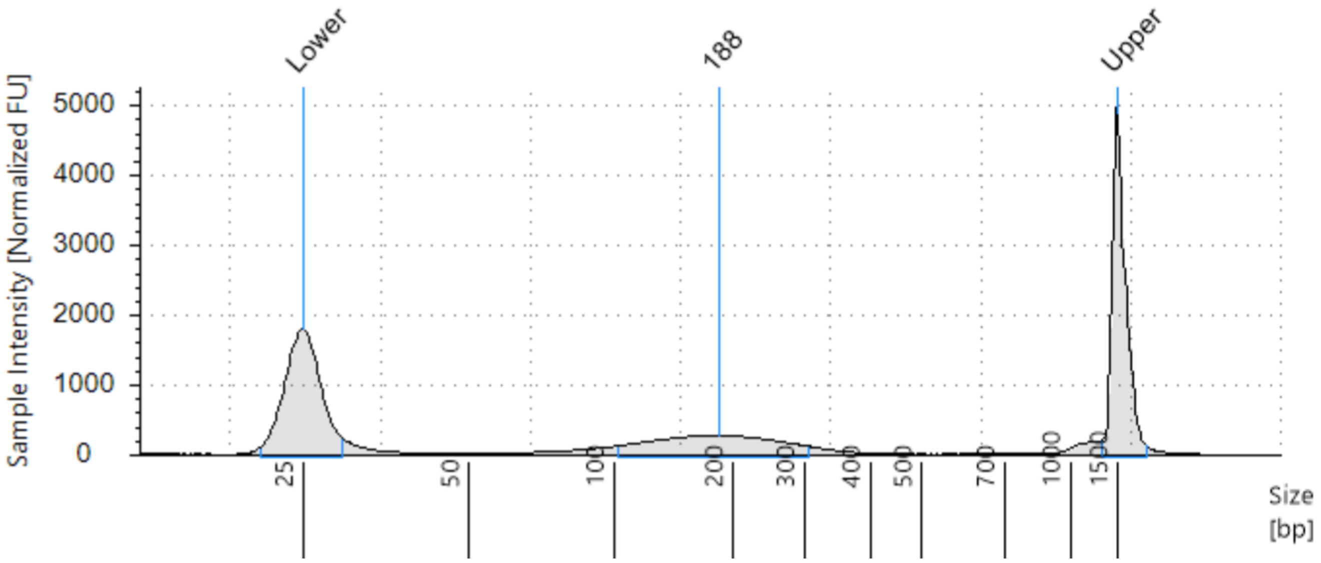

Sample Table

Well	Conc. [ng/µl]	Sample Description	Alert	Observations
H1	3.31	G9 M R3		

Peak Table

Size [bp]	Calibrated Conc. [ng/µl]	Assigned Conc. [ng/µl]	Peak Molarity [nmol/l]	% Integrated Area	Peak Comment	Observations
25	6.33	-	390	-		Lower Marker
188	3.31	-	27.2	100.00		
1500	6.50	6.50	6.67	-		Upper Marker

© Agilent Technologies, Inc. 2017

A2: A10 M R3

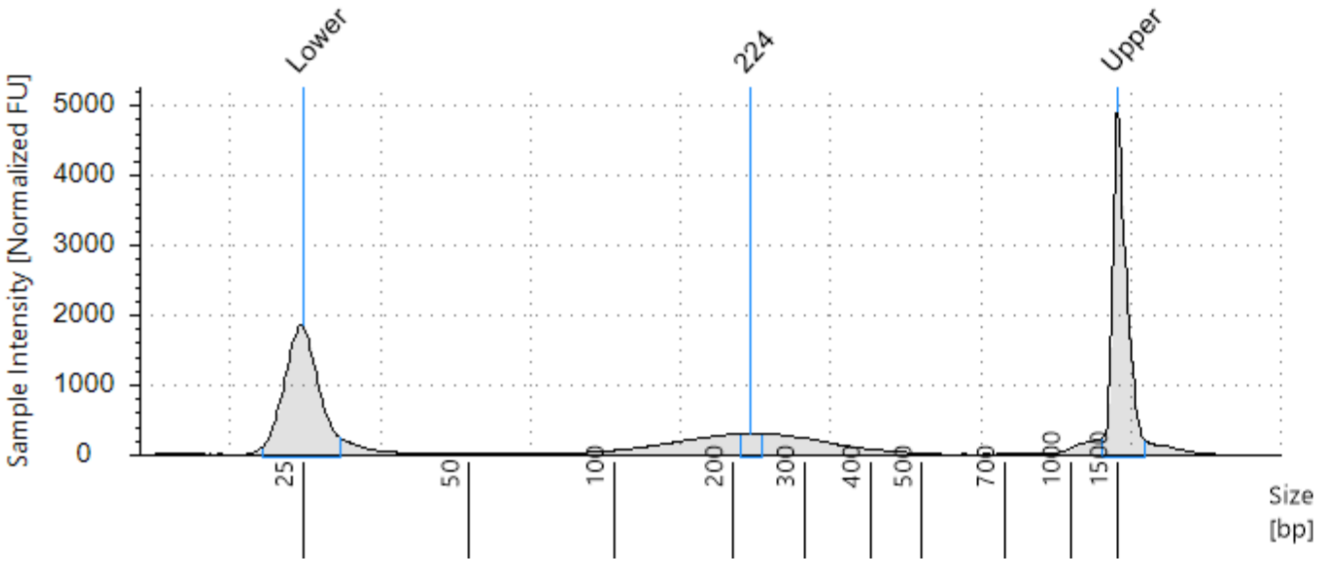

Sample Table

Well	Conc. [ng/µl]	Sample Description	Alert	Observations
A2	0.490	A10 M R3		

Peak Table

Size [bp]	Calibrated Conc. [ng/µl]	Assigned Conc. [ng/µl]	Peak Molarity [nmol/l]	% Integrated Area	Peak Comment	Observations
25	5.89	-	363	-		Lower Marker
224	0.490	-	3.37	100.00		
1500	6.50	6.50	6.67	-		Upper Marker

© Agilent Technologies, Inc. 2017

B2: B10 M R3

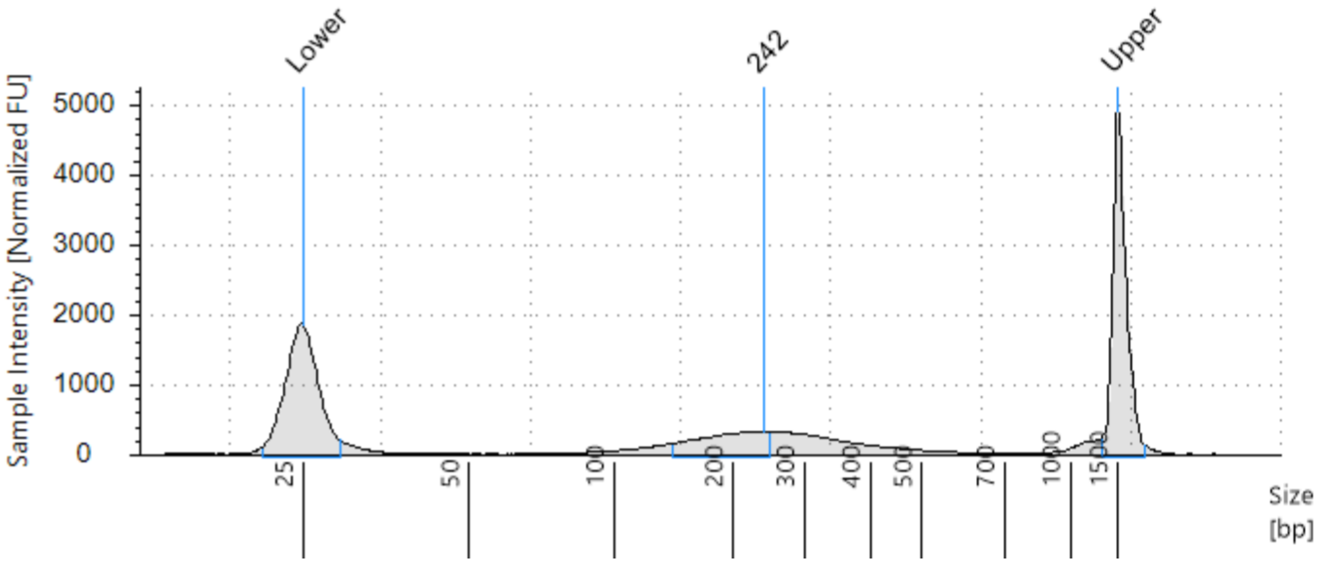

Sample Table

Well	Conc. [ng/µl]	Sample Description	Alert	Observations
B2	2.06	B10 M R3		

Peak Table

Size [bp]	Calibrated Conc. [ng/µl]	Assigned Conc. [ng/µl]	Peak Molarity [nmol/l]	% Integrated Area	Peak Comment	Observations
25	5.92	-	364	-		Lower Marker
242	2.06	-	13.1	100.00		
1500	6.50	6.50	6.67	-		Upper Marker

© Agilent Technologies, Inc. 2017

C2: C10 M R3

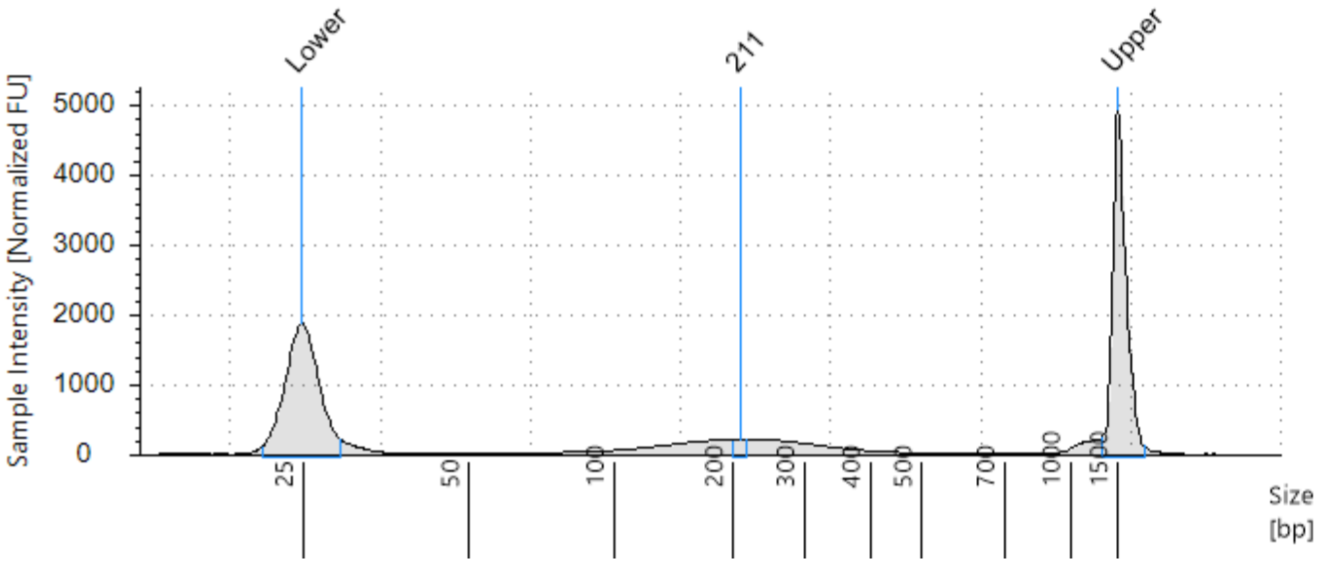

Sample Table

Well	Conc. [ng/µl]	Sample Description	Alert	Observations
C2	0.273	C10 M R3		

Peak Table

Size [bp]	Calibrated Conc. [ng/µl]	Assigned Conc. [ng/µl]	Peak Molarity [nmol/l]	% Integrated Area	Peak Comment	Observations
25	6.15	-	379	-		Lower Marker
211	0.273	-	1.99	100.00		
1500	6.50	6.50	6.67	-		Upper Marker

© Agilent Technologies, Inc. 2017

D2: D10 M R3

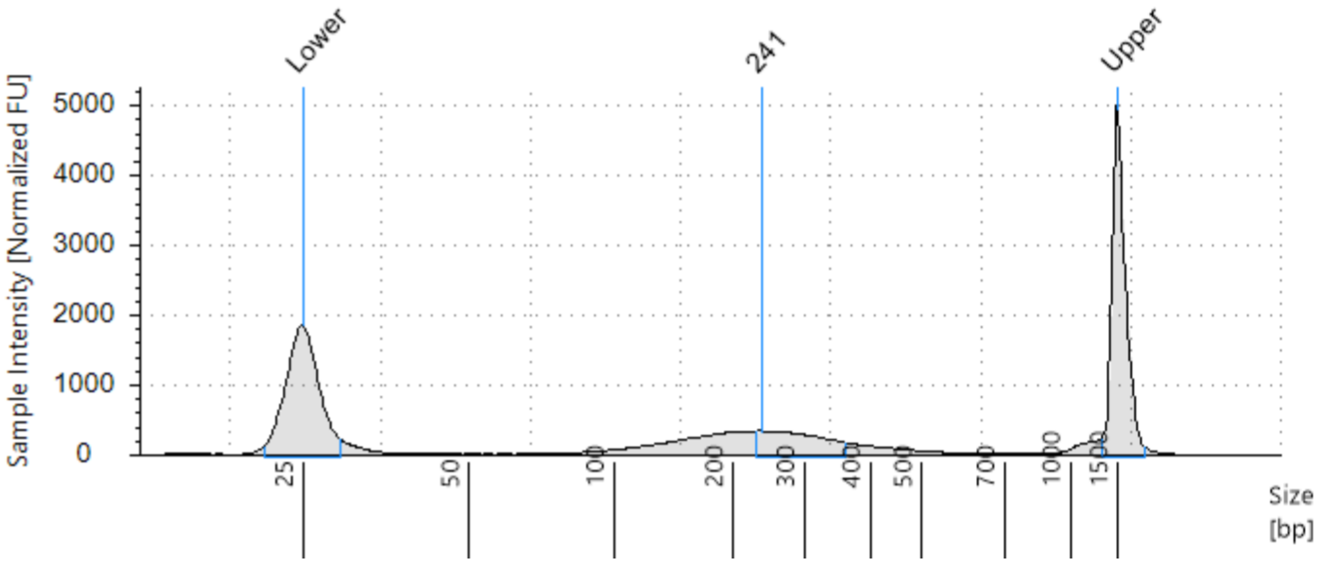

Sample Table

Well	Conc. [ng/µl]	Sample Description	Alert	Observations
D2	2.03	D10 M R3		

Peak Table

Size [bp]	Calibrated Conc. [ng/µl]	Assigned Conc. [ng/µl]	Peak Molarity [nmol/l]	% Integrated Area	Peak Comment	Observations
25	5.90	-	363	-		Lower Marker
241	2.03	-	12.9	100.00		
1500	6.50	6.50	6.67	-		Upper Marker

© Agilent Technologies, Inc. 2017

E2: E10 M R3

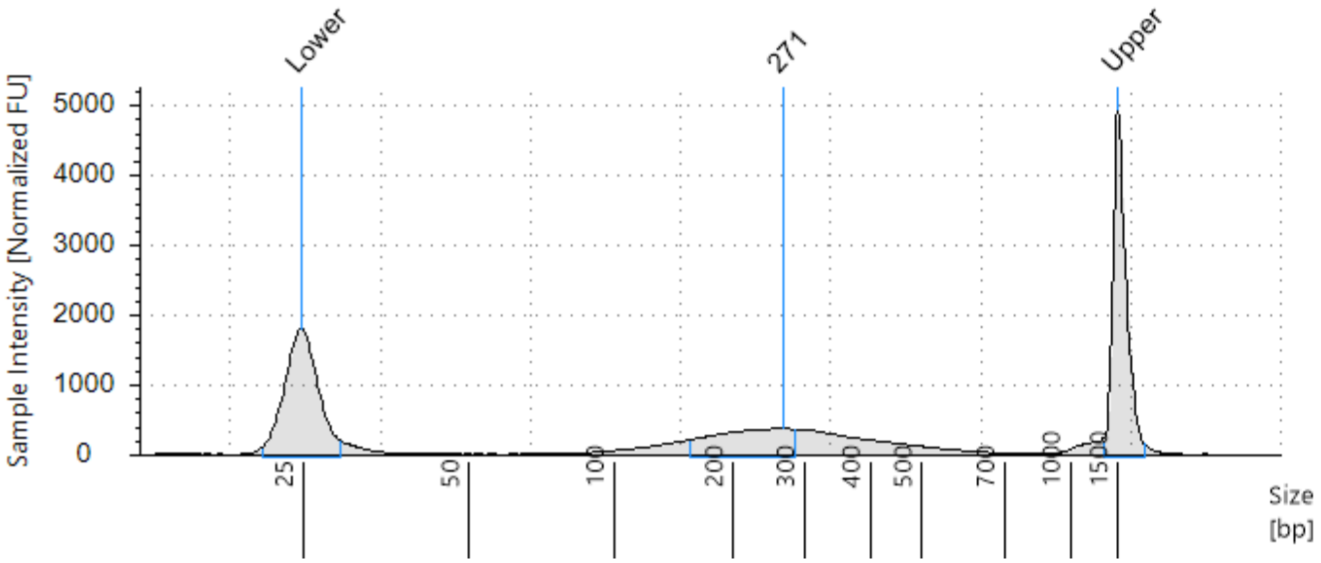

Sample Table

Well	Conc. [ng/µl]	Sample Description	Alert	Observations
E2	2.79	E10 M R3		

Peak Table

Size [bp]	Calibrated Conc. [ng/µl]	Assigned Conc. [ng/µl]	Peak Molarity [nmol/l]	% Integrated Area	Peak Comment	Observations
25	5.90	-	363	-		Lower Marker
271	2.79	-	15.8	100.00		
1500	6.50	6.50	6.67	-		Upper Marker

 © Agilent Technologies, Inc. 2017

F2: F10 M R3

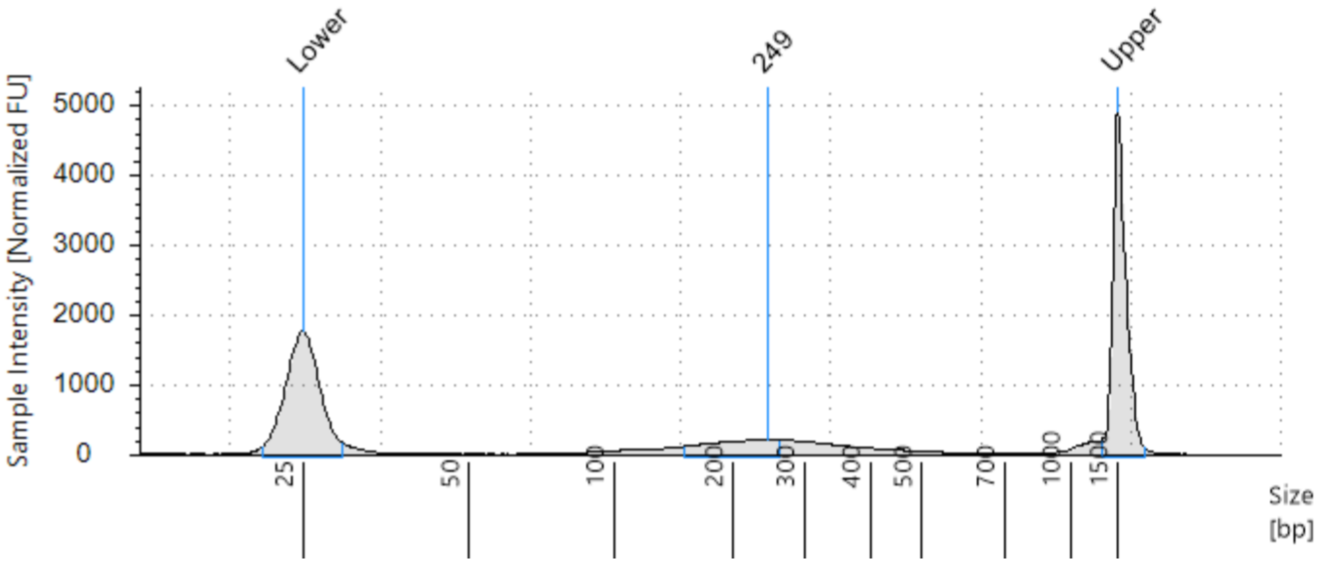

Sample Table

Well	Conc. [ng/µl]	Sample Description	Alert	Observations
F2	1.41	F10 M R3		

Peak Table

Size [bp]	Calibrated Conc. [ng/µl]	Assigned Conc. [ng/µl]	Peak Molarity [nmol/l]	% Integrated Area	Peak Comment	Observations
25	6.02	-	371	-		Lower Marker
249	1.41	-	8.71	100.00		
1500	6.50	6.50	6.67	-		Upper Marker

© Agilent Technologies, Inc. 2017

G2: G10 M R3

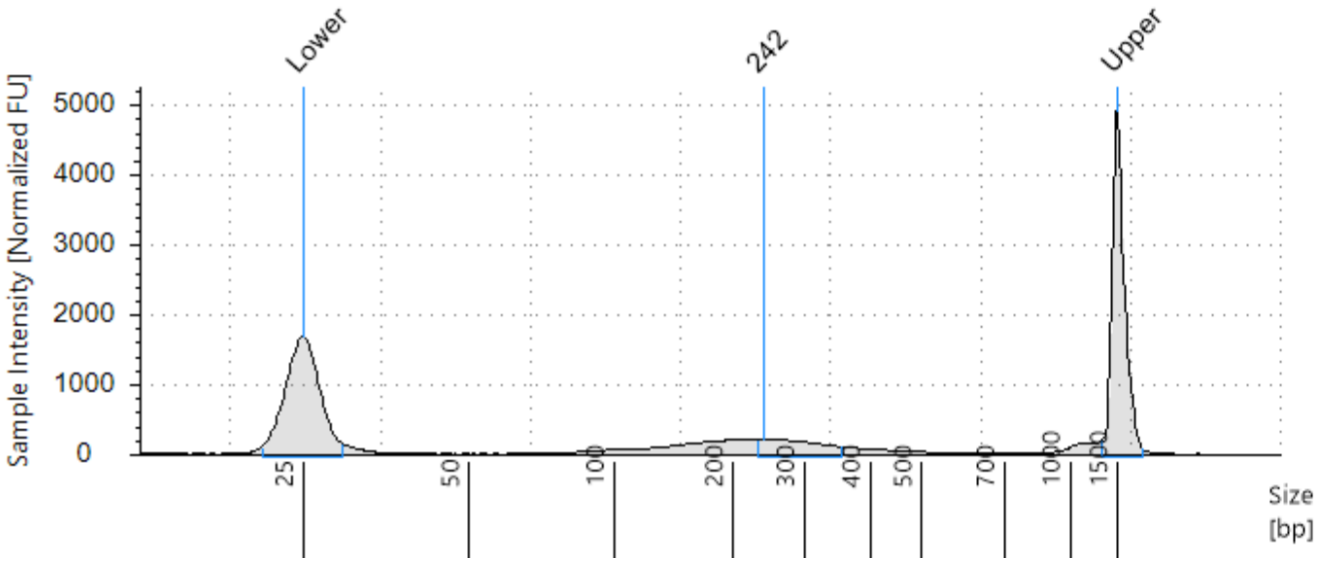

Sample Table

Well	Conc. [ng/µl]	Sample Description	Alert	Observations
G2	1.33	G10 M R3		

Peak Table

Size [bp]	Calibrated Conc. [ng/µl]	Assigned Conc. [ng/µl]	Peak Molarity [nmol/l]	% Integrated Area	Peak Comment	Observations
25	6.16	-	379	-		Lower Marker
242	1.33	-	8.45	100.00		
1500	6.50	6.50	6.67	-		Upper Marker

© Agilent Technologies, Inc. 2017

H2: H10 M R3

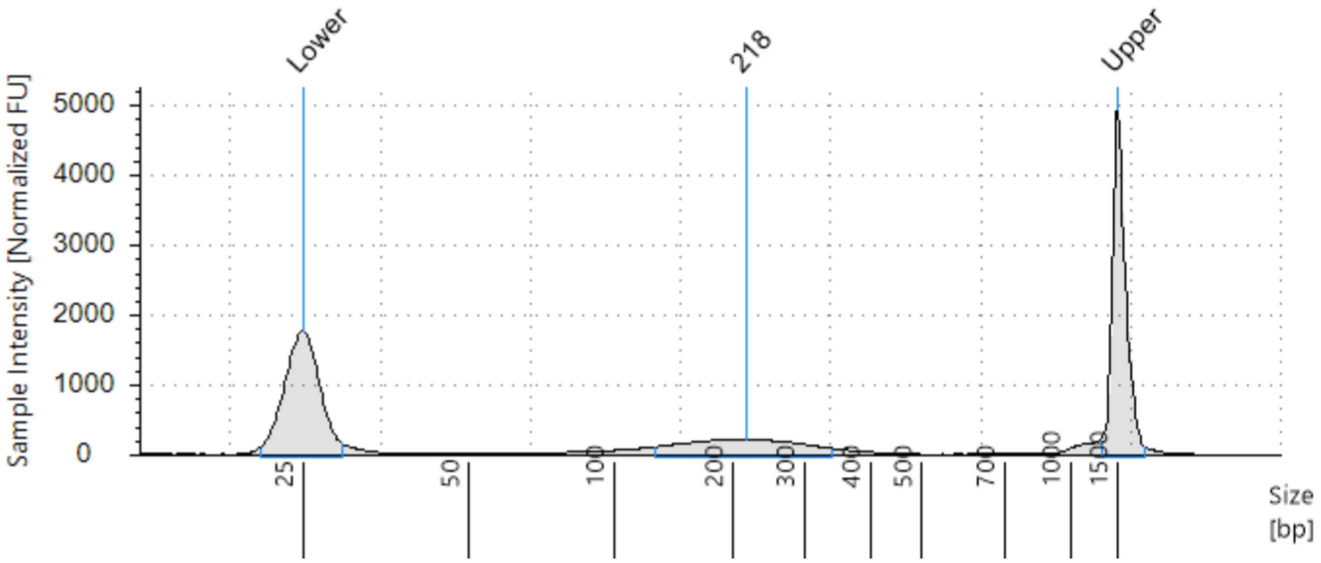

Sample Table

Well	Conc. [ng/µl]	Sample Description	Alert	Observations
H2	2.52	H10 M R3		

Peak Table

Size [bp]	Calibrated Conc. [ng/µl]	Assigned Conc. [ng/µl]	Peak Molarity [nmol/l]	% Integrated Area	Peak Comment	Observations
25	6.12	-	377	-		Lower Marker
218	2.52	-	17.7	100.00		
1500	6.50	6.50	6.67	-		Upper Marker

© Agilent Technologies, Inc. 2017

Filename: 2020-10-20-03Q-S Minus A11-H12 EXP H11 R3.D1000

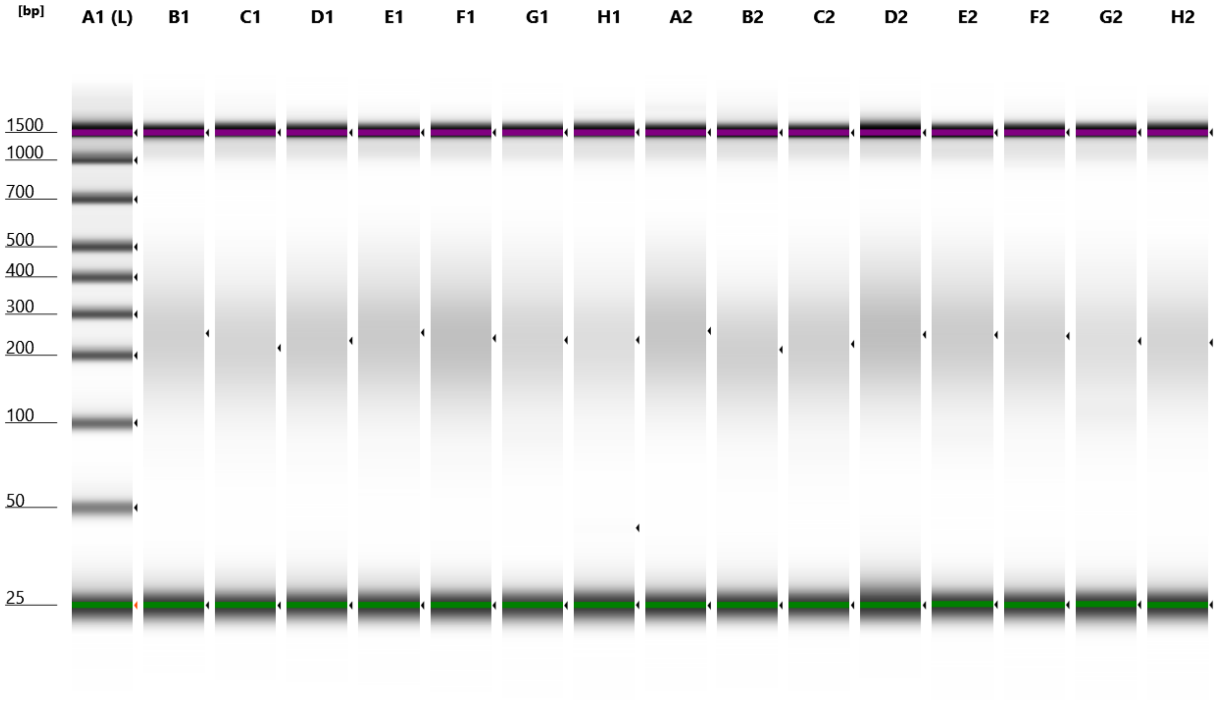

Default image (Contrast 100%)

Sample Info

Well	Conc. [ng/µl]	Sample Description	Alert	Observations
A1	16.0	Ladder		Ladder
B1	3.17	A11 M R3		
C1	1.60	B11 M R3		
D1	2.00	C11 M R3		
E1	1.97	D11 M R3		
F1	4.83	E11 M R3		
G1	1.74	F11 M R3		
H1	2.31	G11 M R3		
A2	0.811	A12 M R3		
B2	1.95	B12 M R3		
C2	1.85	C12 M R3		
D2	1.92	D12 M R3		
E2	3.60	E12 M R3		
F2	3.08	F12 M R3		
G2	2.18	G12 M R3		
H2	1.65	H12 M R3		

 © Agilent Technologies, Inc. 2017

A1: Ladder

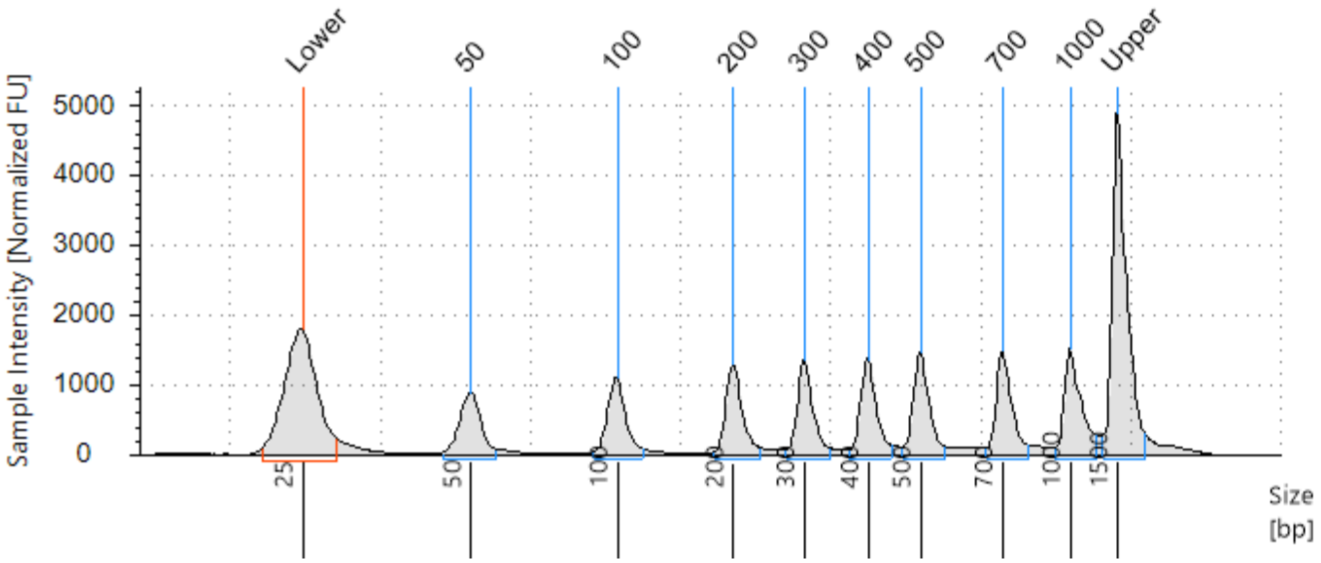

Sample Table

Well	Conc. [ng/µl]	Sample Description	Alert	Observations
A1	16.0	Ladder		Ladder

Peak Table

Size [bp]	Calibrated Conc. [ng/µl]	Assigned Conc. [ng/µl]	Peak Molarity [nmol/l]	% Integrated Area	Peak Comment	Observations
25	5.33	-	328	-		Lower Marker
50	1.72	-	52.9	10.76		
100	1.83	-	28.1	11.44		
200	1.94	-	15.0	12.17		
300	1.94	-	9.96	12.16		
400	2.00	-	7.70	12.54		
500	2.14	-	6.58	13.38		
700	2.11	-	4.64	13.23		
1000	2.29	-	3.52	14.33		
1500	6.50	6.50	6.67	-		Upper Marker

© Agilent Technologies, Inc. 2017

B1: A11 M R3

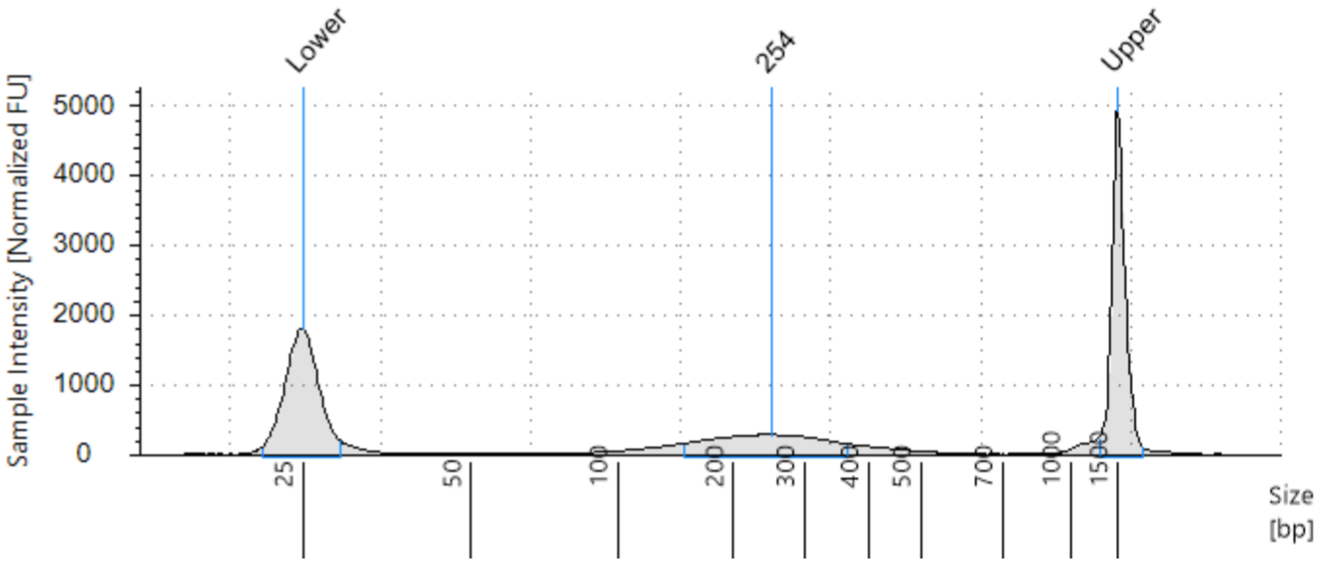

Sample Table

Well	Conc. [ng/µl]	Sample Description	Alert	Observations
B1	3.17	A11 M R3		

Peak Table

Size [bp]	Calibrated Conc. [ng/µl]	Assigned Conc. [ng/µl]	Peak Molarity [nmol/l]	% Integrated Area	Peak Comment	Observations
25	6.00	-	369	-		Lower Marker
254	3.17	-	19.2	100.00		
1500	6.50	6.50	6.67	-		Upper Marker

© Agilent Technologies, Inc. 2017

C1: B11 M R3

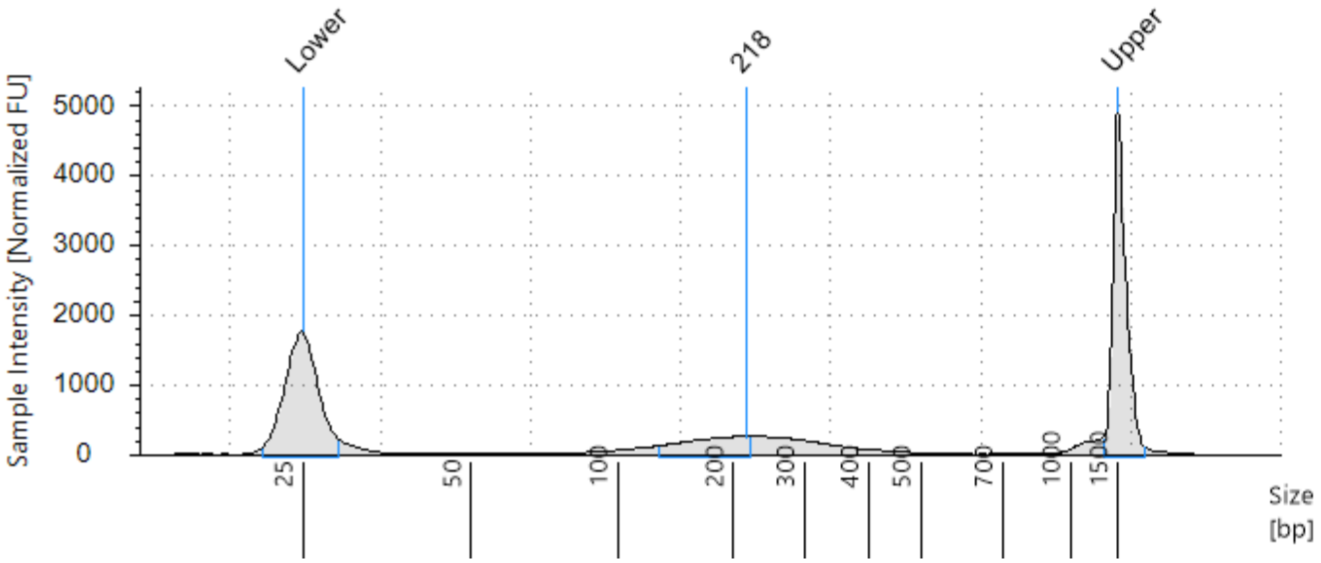

Sample Table

Well	Conc. [ng/µl]	Sample Description	Alert	Observations
C1	1.60	B11 M R3		

Peak Table

Size [bp]	Calibrated Conc. [ng/µl]	Assigned Conc. [ng/µl]	Peak Molarity [nmol/l]	% Integrated Area	Peak Comment	Observations
25	5.87	-	361	-		Lower Marker
218	1.60	-	11.3	100.00		
1500	6.50	6.50	6.67	-		Upper Marker

© Agilent Technologies, Inc. 2017

D1: C11 M R3

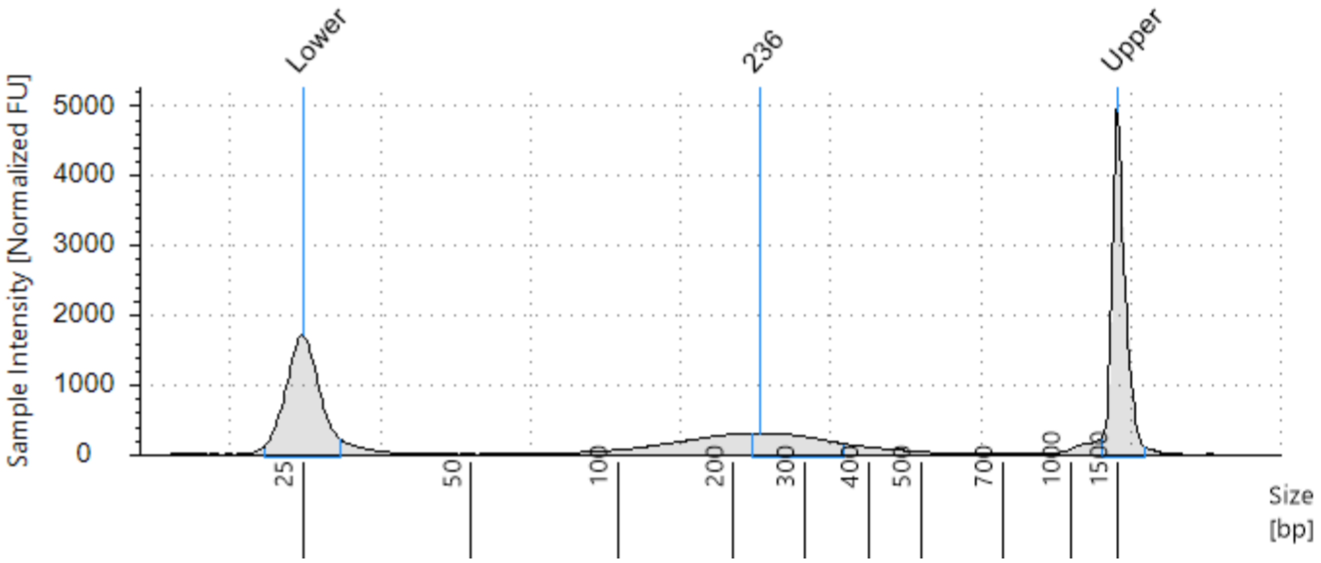

Sample Table

Well	Conc. [ng/µl]	Sample Description	Alert	Observations
D1	2.00	C11 M R3		

Peak Table

Size [bp]	Calibrated Conc. [ng/µl]	Assigned Conc. [ng/µl]	Peak Molarity [nmol/l]	% Integrated Area	Peak Comment	Observations
25	5.72	-	352	-		Lower Marker
236	2.00	-	13.1	100.00		
1500	6.50	6.50	6.67	-		Upper Marker

© Agilent Technologies, Inc. 2017

E1: D11 M R3

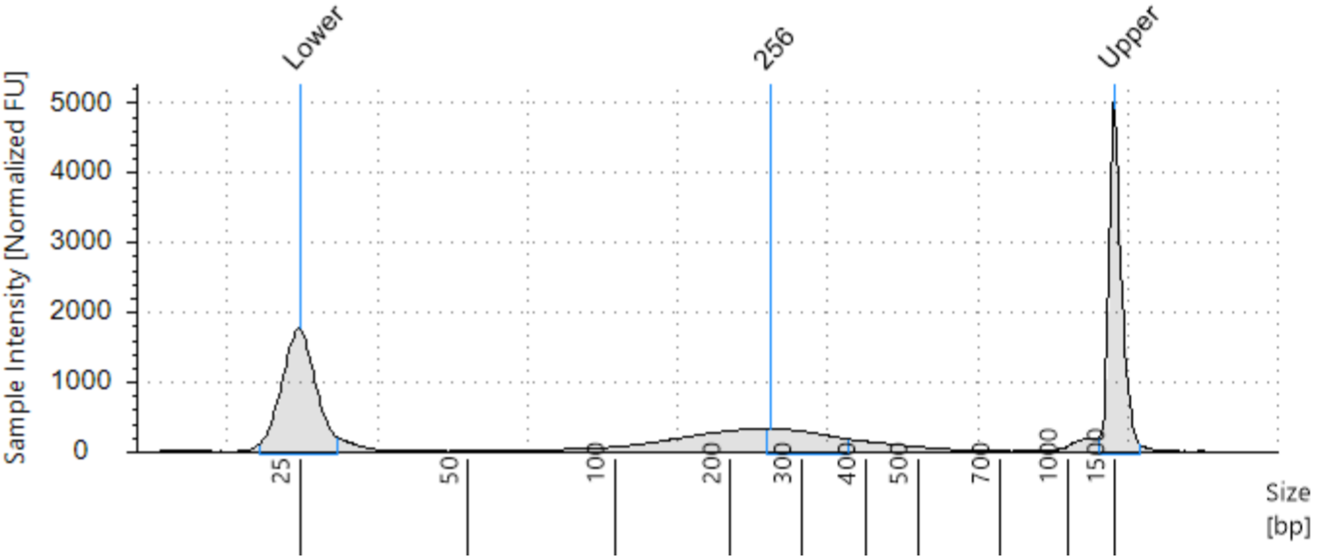

Sample Table

Well	Conc. [ng/µl]	Sample Description	Alert	Observations
E1	1.97	D11 M R3		

Peak Table

Size [bp]	Calibrated Conc. [ng/µl]	Assigned Conc. [ng/µl]	Peak Molarity [nmol/l]	% Integrated Area	Peak Comment	Observations
25	6.14	-	378	-		Lower Marker
256	1.97	-	11.8	100.00		
1500	6.50	6.50	6.67	-		Upper Marker

© Agilent Technologies, Inc. 2017

F1: E11 M R3

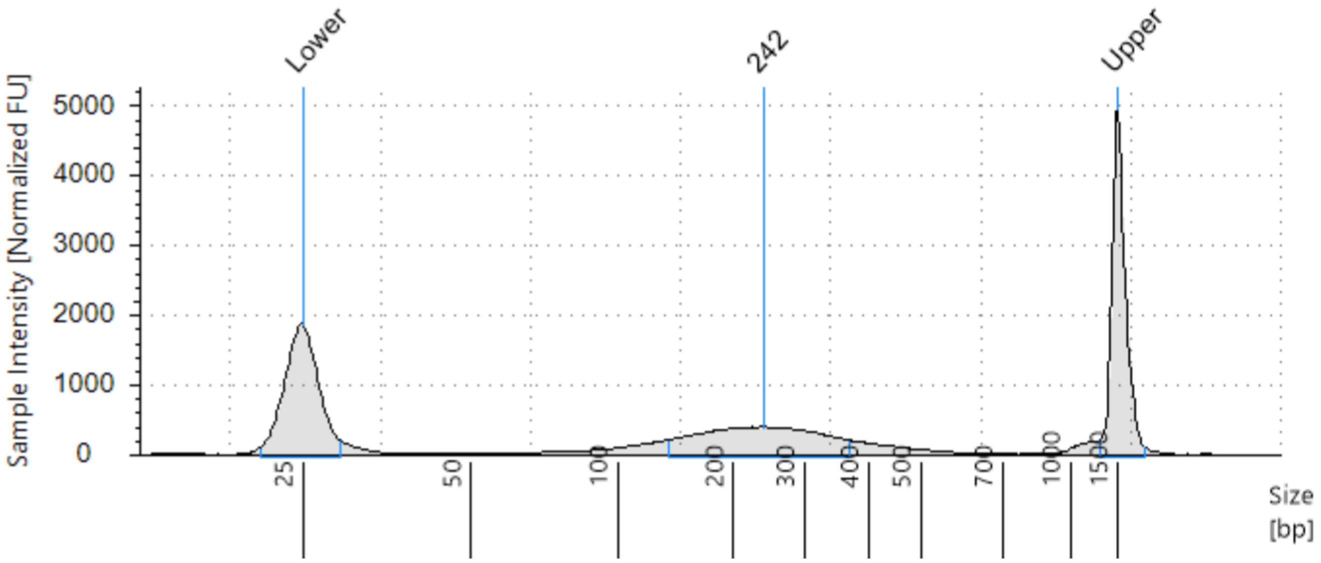

Sample Table

Well	Conc. [ng/µl]	Sample Description	Alert	Observations
F1	4.83	E11 M R3		

Peak Table

Size [bp]	Calibrated Conc. [ng/µl]	Assigned Conc. [ng/µl]	Peak Molarity [nmol/l]	% Integrated Area	Peak Comment	Observations
25	6.18	-	380	-		Lower Marker
242	4.83	-	30.7	100.00		
1500	6.50	6.50	6.67	-		Upper Marker

 © Agilent Technologies, Inc. 2017

G1: F11 M R3

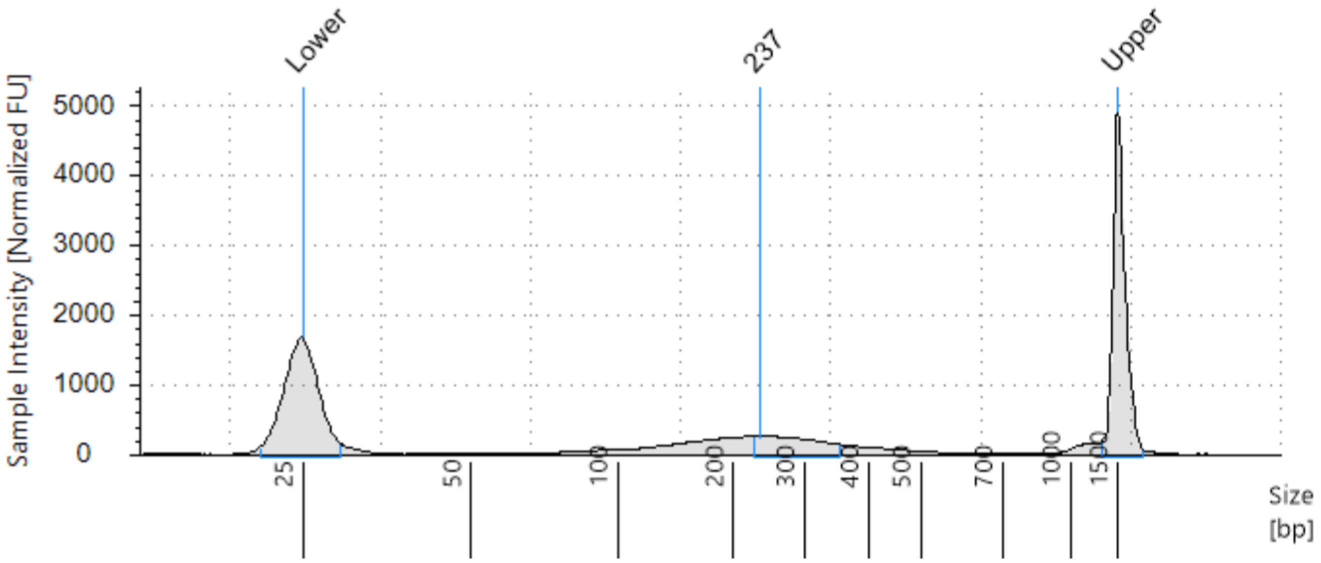

Sample Table

Well	Conc. [ng/µl]	Sample Description	Alert	Observations
G1	1.74	F11 M R3		

Peak Table

Size [bp]	Calibrated Conc. [ng/µl]	Assigned Conc. [ng/µl]	Peak Molarity [nmol/l]	% Integrated Area	Peak Comment	Observations
25	6.28	-	386	-		Lower Marker
237	1.74	-	11.3	100.00		
1500	6.50	6.50	6.67	-		Upper Marker

H1: G11 M R3

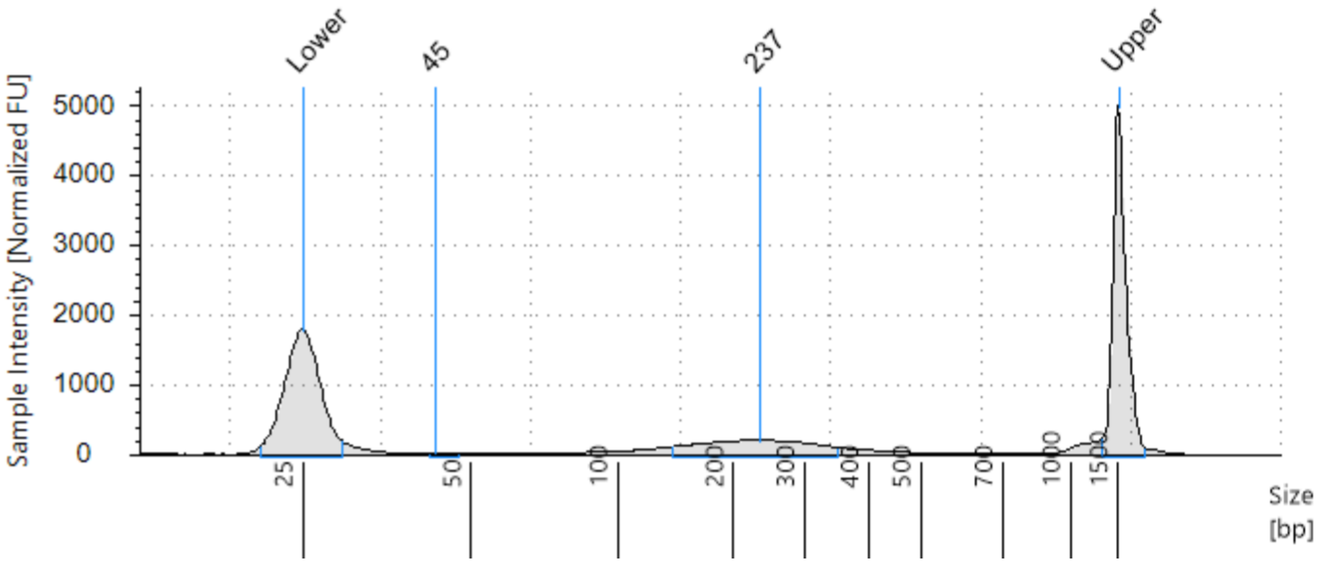

Sample Table

Well	Conc. [ng/µl]	Sample Description	Alert	Observations
H1	2.31	G11 M R3		

Peak Table

Size [bp]	Calibrated Conc. [ng/µl]	Assigned Conc. [ng/µl]	Peak Molarity [nmol/l]	% Integrated Area	Peak Comment	Observations
25	6.35	-	391	-		Lower Marker
45	0.0218	-	0.750	0.94		
237	2.29	-	14.9	99.06		
1500	6.50	6.50	6.67	-		Upper Marker

© Agilent Technologies, Inc. 2017

A2: A12 M R3

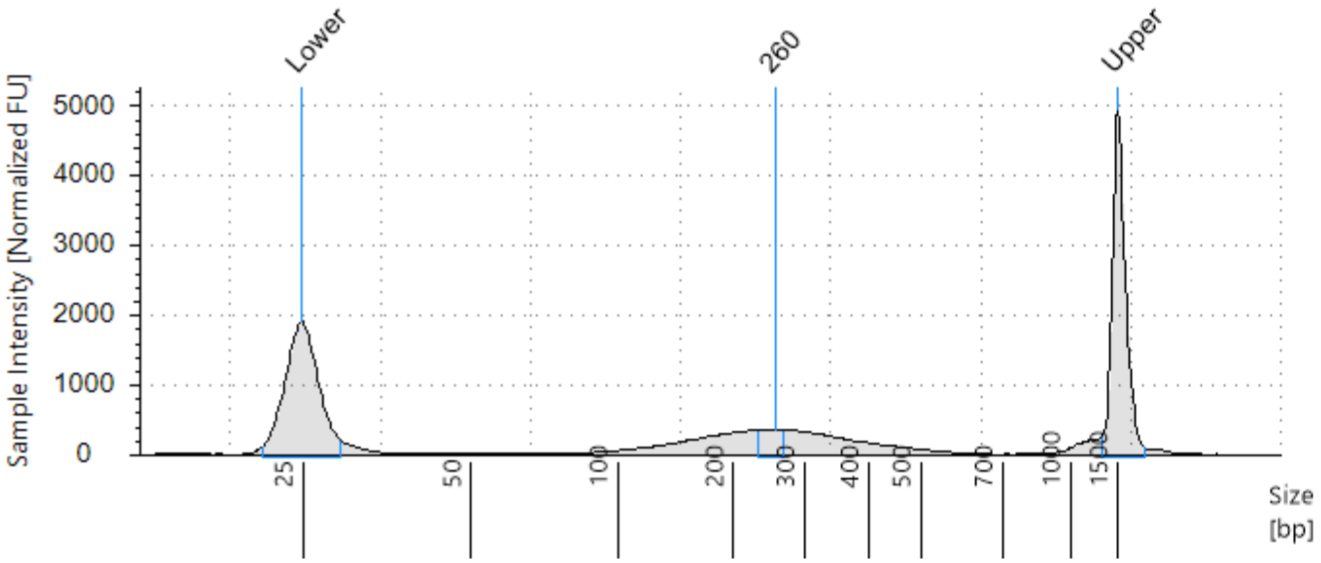

Sample Table

Well	Conc. [ng/µl]	Sample Description	Alert	Observations
A2	0.811	A12 M R3		

Peak Table

Size [bp]	Calibrated Conc. [ng/µl]	Assigned Conc. [ng/µl]	Peak Molarity [nmol/l]	% Integrated Area	Peak Comment	Observations
25	6.32	-	389	-		Lower Marker
260	0.811	-	4.81	100.00		
1500	6.50	6.50	6.67	-		Upper Marker

 © Agilent Technologies, Inc. 2017

B2: B12 M R3

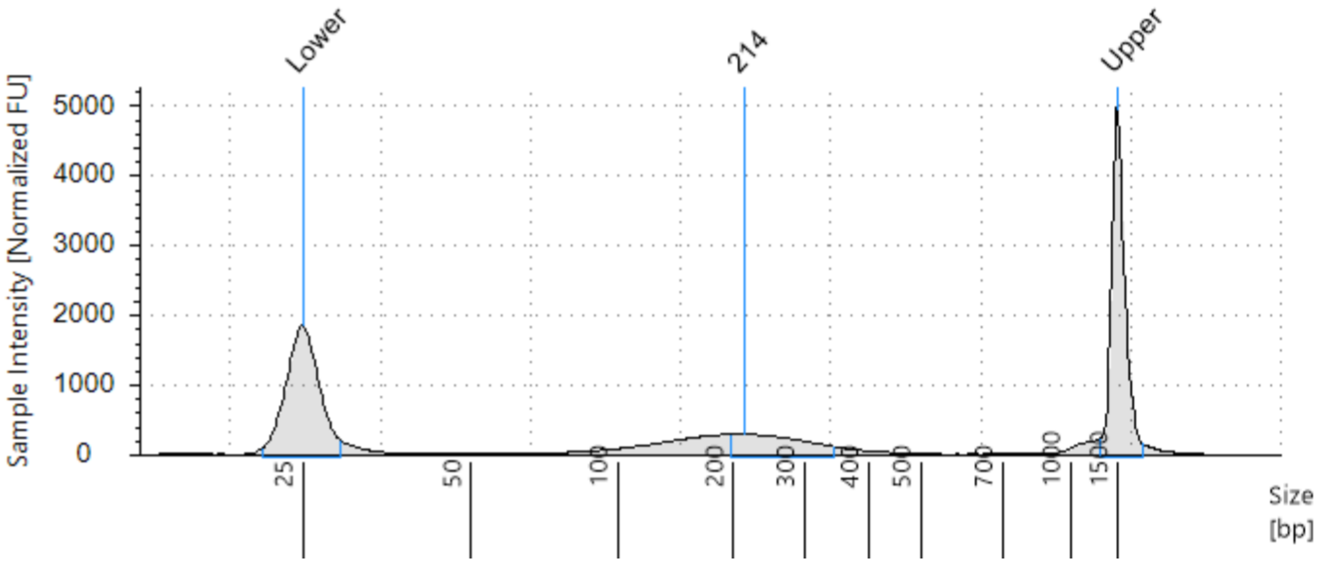

Sample Table

Well	Conc. [ng/µl]	Sample Description	Alert	Observations
B2	1.95	B12 M R3		

Peak Table

Size [bp]	Calibrated Conc. [ng/µl]	Assigned Conc. [ng/µl]	Peak Molarity [nmol/l]	% Integrated Area	Peak Comment	Observations
25	6.01	-	370	-		Lower Marker
214	1.95	-	14.0	100.00		
1500	6.50	6.50	6.67	-		Upper Marker

© Agilent Technologies, Inc. 2017

C2: C12 M R3

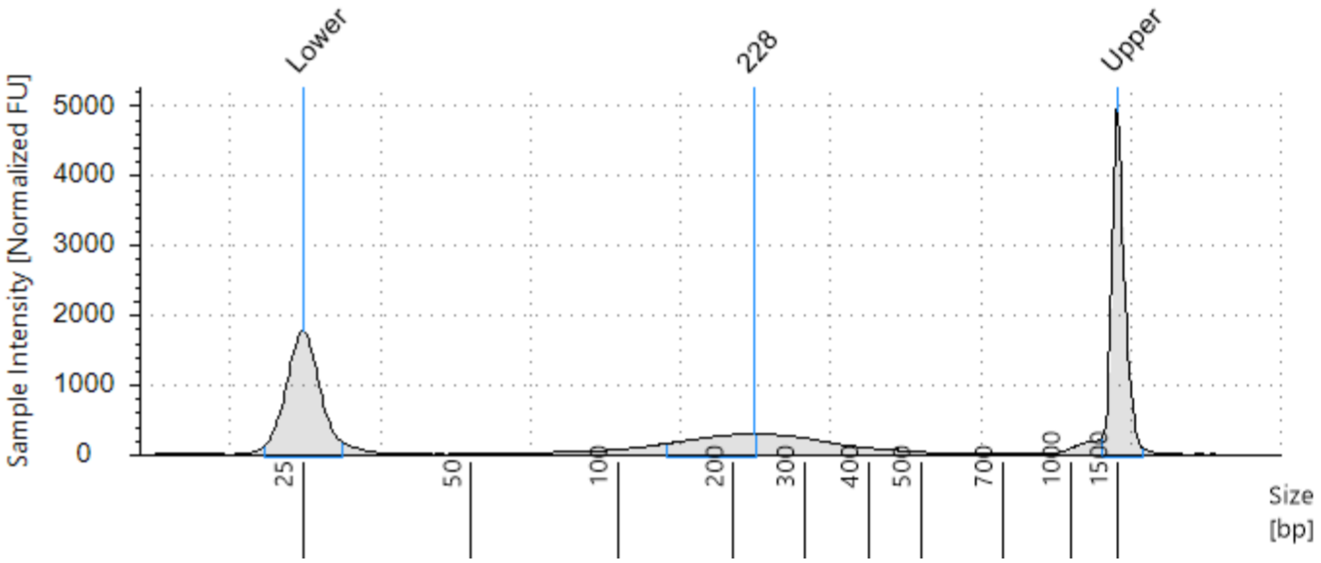

Sample Table

Well	Conc. [ng/µl]	Sample Description	Alert	Observations
C2	1.85	C12 M R3		

Peak Table

Size [bp]	Calibrated Conc. [ng/µl]	Assigned Conc. [ng/µl]	Peak Molarity [nmol/l]	% Integrated Area	Peak Comment	Observations
25	5.98	-	368	-		Lower Marker
228	1.85	-	12.5	100.00		
1500	6.50	6.50	6.67	-		Upper Marker

 © Agilent Technologies, Inc. 2017

D2: D12 M R3

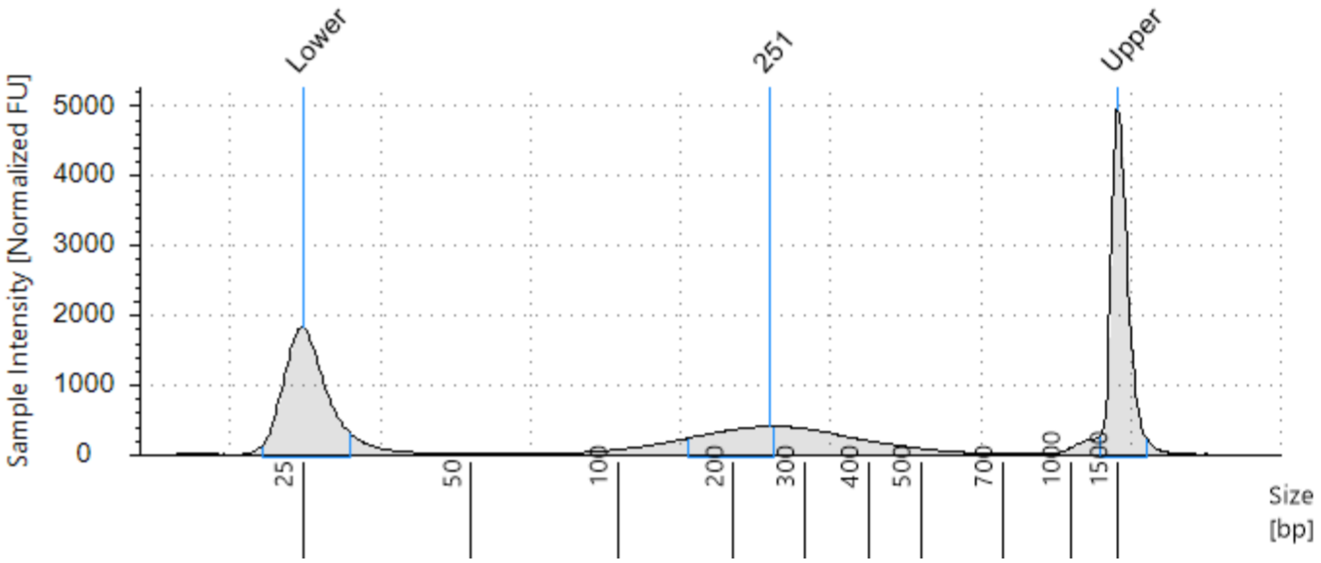

Sample Table

Well	Conc. [ng/µl]	Sample Description	Alert	Observations
D2	1.92	D12 M R3		

Peak Table

Size [bp]	Calibrated Conc. [ng/µl]	Assigned Conc. [ng/µl]	Peak Molarity [nmol/l]	% Integrated Area	Peak Comment	Observations
25	5.81	-	357	-		Lower Marker
251	1.92	-	11.8	100.00		
1500	6.50	6.50	6.67	-		Upper Marker

© Agilent Technologies, Inc. 2017

E2: E12 M R3

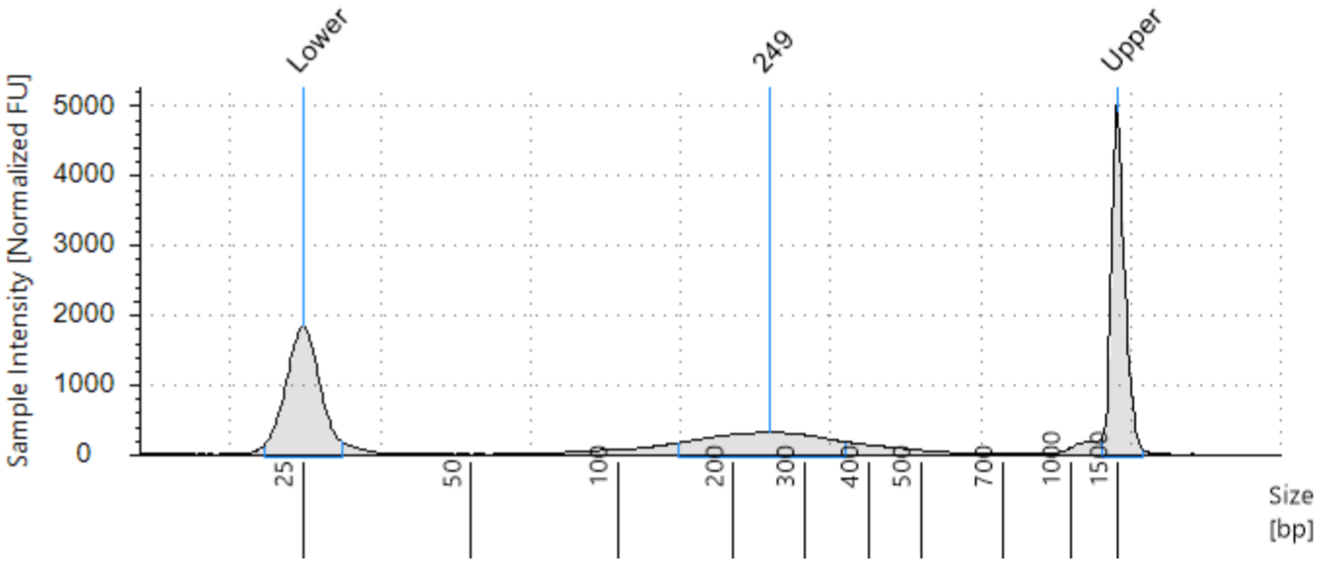

Sample Table

Well	Conc. [ng/µl]	Sample Description	Alert	Observations
E2	3.60	E12 M R3		

Peak Table

Size [bp]	Calibrated Conc. [ng/µl]	Assigned Conc. [ng/µl]	Peak Molarity [nmol/l]	% Integrated Area	Peak Comment	Observations
25	6.03	-	371	-		Lower Marker
249	3.60	-	22.2	100.00		
1500	6.50	6.50	6.67	-		Upper Marker

© Agilent Technologies, Inc. 2017

F2: F12 M R3

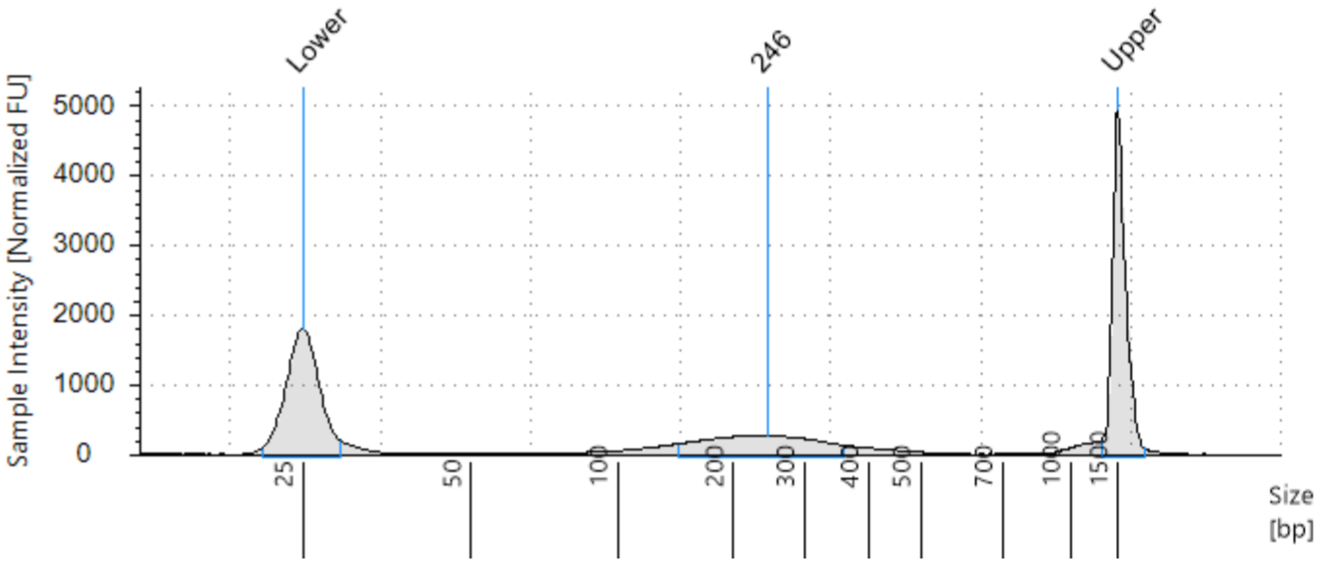

Sample Table

Well	Conc. [ng/µl]	Sample Description	Alert	Observations
F2	3.08	F12 M R3		

Peak Table

Size [bp]	Calibrated Conc. [ng/µl]	Assigned Conc. [ng/µl]	Peak Molarity [nmol/l]	% Integrated Area	Peak Comment	Observations
25	5.98	-	368	-		Lower Marker
246	3.08	-	19.2	100.00		
1500	6.50	6.50	6.67	-		Upper Marker

© Agilent Technologies, Inc. 2017

G2: G12 M R3

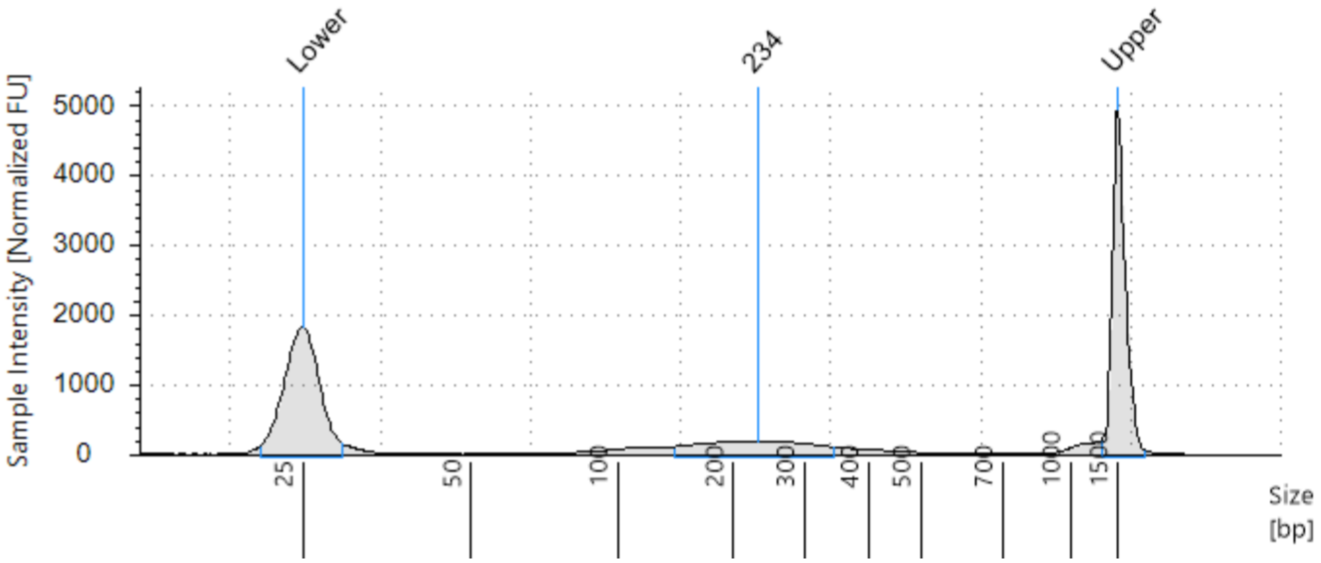

Sample Table

Well	Conc. [ng/µl]	Sample Description	Alert	Observations
G2	2.18	G12 M R3		

Peak Table

Size [bp]	Calibrated Conc. [ng/µl]	Assigned Conc. [ng/µl]	Peak Molarity [nmol/l]	% Integrated Area	Peak Comment	Observations
25	6.46	-	397	-		Lower Marker
234	2.18	-	14.3	100.00		
1500	6.50	6.50	6.67	-		Upper Marker

© Agilent Technologies, Inc. 2017

H2: H12 M R3

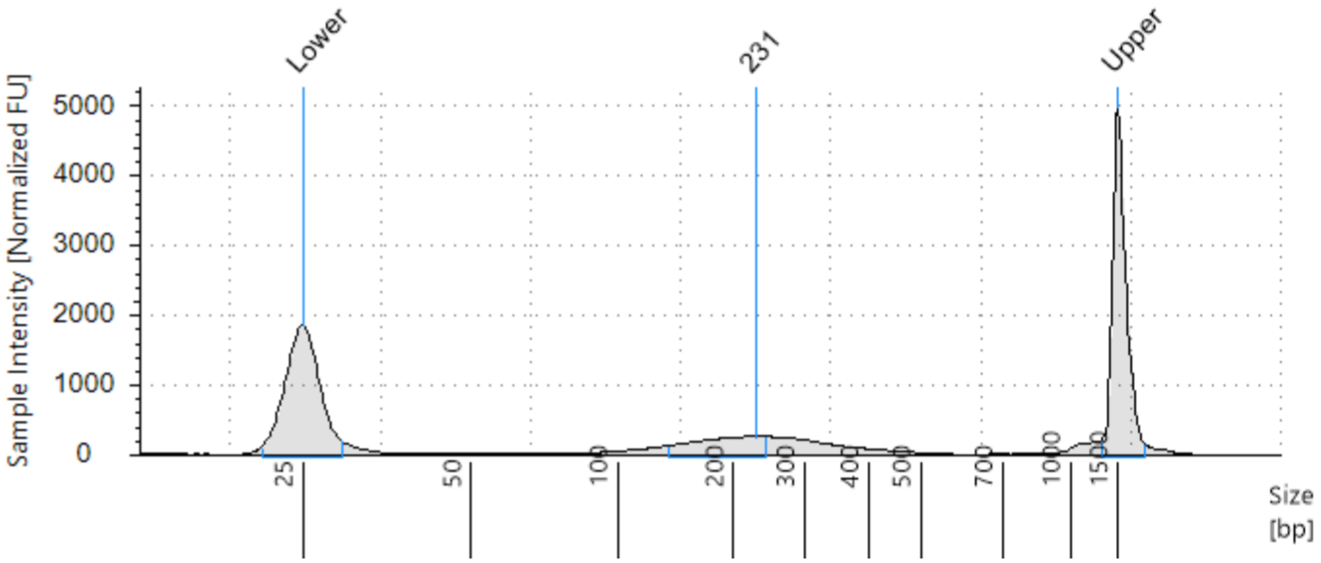

Sample Table

Well	Conc. [ng/µl]	Sample Description	Alert	Observations
H2	1.65	H12 M R3		

Peak Table

Size [bp]	Calibrated Conc. [ng/µl]	Assigned Conc. [ng/µl]	Peak Molarity [nmol/l]	% Integrated Area	Peak Comment	Observations
25	6.13	-	377	-		Lower Marker
231	1.65	-	11.0	100.00		
1500	6.50	6.50	6.67	-		Upper Marker

© Agilent Technologies, Inc. 2017

Filename: 2020-10-27-02 H1,3,5,5,9,11, F9 plus H1,2,3,5,9,11, H4 M R3.D1000

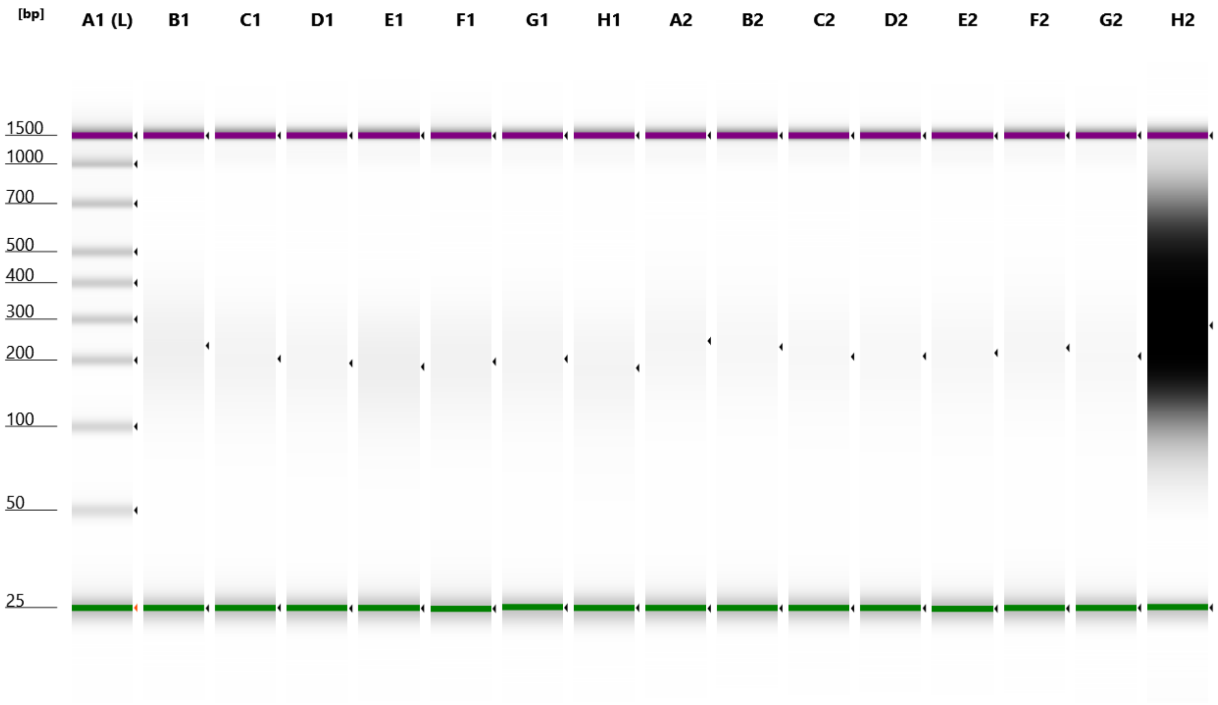

Default image (Contrast 100%)

Sample Info

Well	Conc. [ng/µl]	Sample Description	Alert	Observations
A1	15.4	Ladder		Ladder
B1	2.50	H1 P R3		
C1	3.32	H3 P R3		
D1	1.70	H5 P R3		
E1	5.13	H7 P R3		
F1	2.12	H9 P R3		
G1	3.50	H11 P R3		
H1	3.61	F9 P R3		
A2	0.304	H1 M R3		
B2	1.10	H3 M R3		
C2	2.11	H5 M R3		
D2	0.176	H7 M R3		
E2	1.28	H9 M R3		
F2	1.43	H11 M R3		
G2	1.18	H4 M R3		
H2	246	700 ng Plus undiluted		

 © Agilent Technologies, Inc. 2017

A1: Ladder

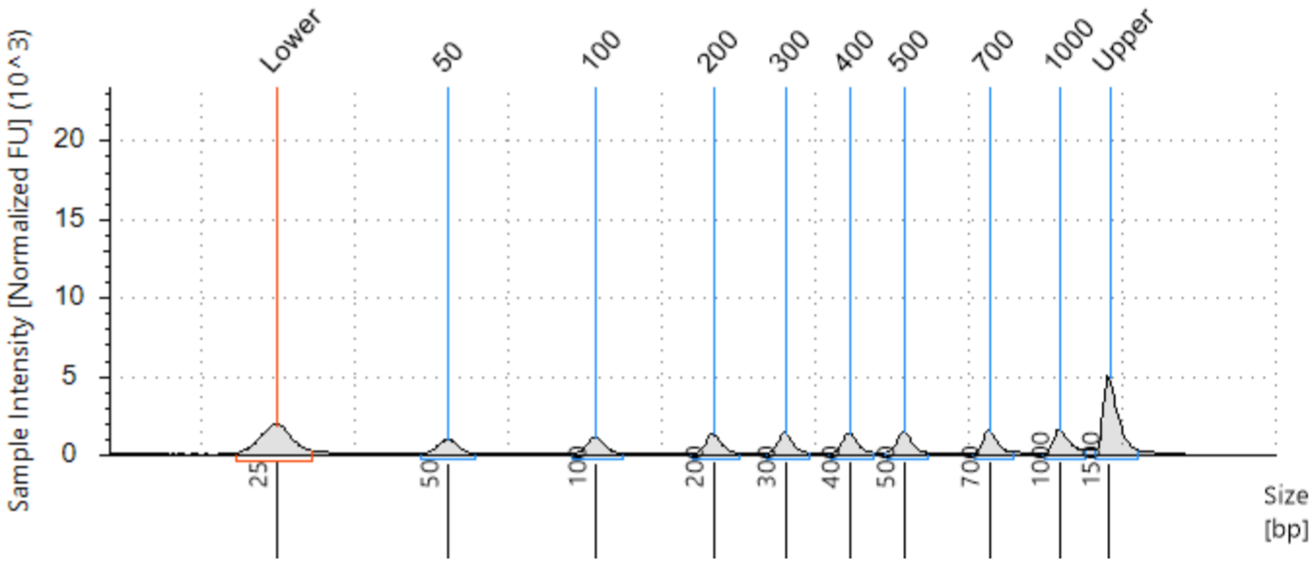

Sample Table

Well	Conc. [ng/µl]	Sample Description	Alert	Observations
A1	15.4	Ladder		Ladder

Peak Table

Size [bp]	Calibrated Conc. [ng/µl]	Assigned Conc. [ng/µl]	Peak Molarity [nmol/l]	% Integrated Area	Peak Comment	Observations
25	5.33	-	328	-		Lower Marker
50	1.67	-	51.5	10.88		
100	1.74	-	26.8	11.31		
200	1.87	-	14.4	12.18		
300	1.89	-	9.67	12.26		
400	1.92	-	7.38	12.47		
500	2.05	-	6.30	13.32		
700	1.98	-	4.36	12.90		
1000	2.26	-	3.47	14.68		
1500	6.50	6.50	6.67	-		Upper Marker

 © Agilent Technologies, Inc. 2017

B1: H1 P R3

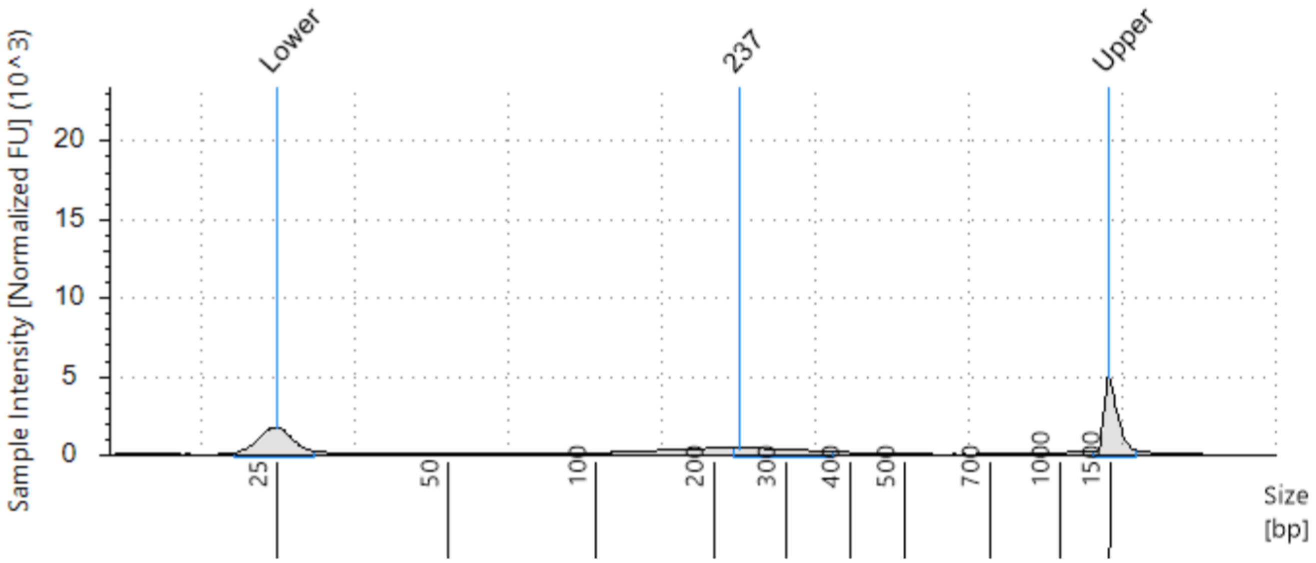

Sample Table

Well	Conc. [ng/µl]	Sample Description	Alert	Observations
B1	2.50	H1 P R3		

Peak Table

Size [bp]	Calibrated Conc. [ng/µl]	Assigned Conc. [ng/µl]	Peak Molarity [nmol/l]	% Integrated Area	Peak Comment	Observations
25	5.65	-	348	-		Lower Marker
237	2.50	-	16.3	100.00		
1500	6.50	6.50	6.67	-		Upper Marker

© Agilent Technologies, Inc. 2017

C1: H3 P R3

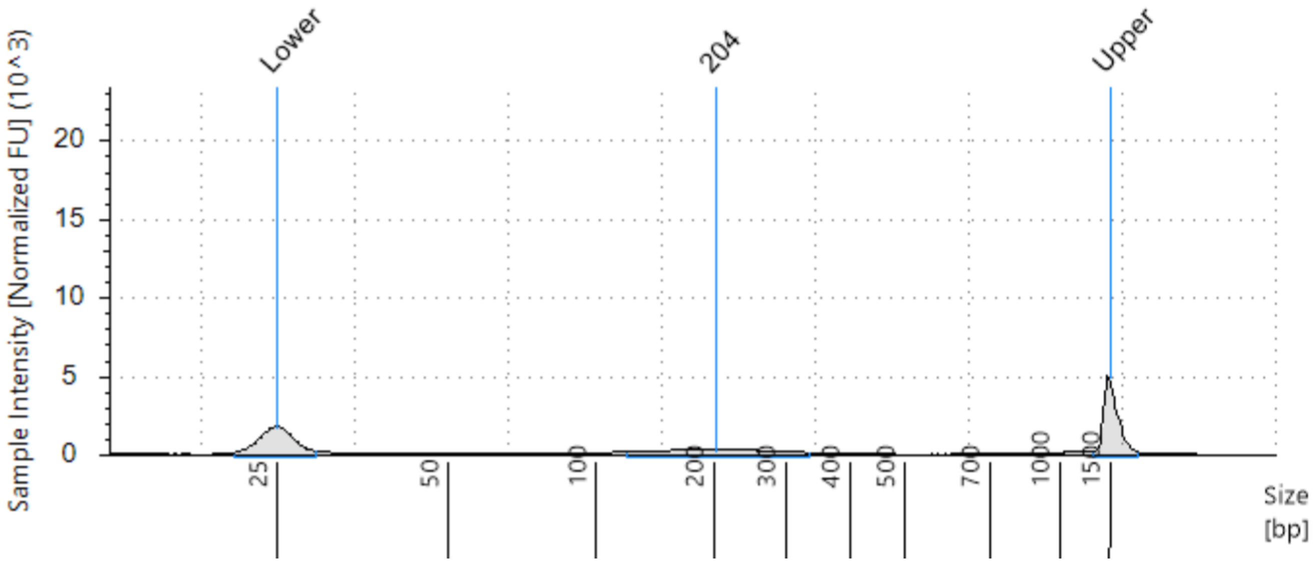

Sample Table

Well	Conc. [ng/µl]	Sample Description	Alert	Observations
C1	3.32	H3 P R3		

Peak Table

Size [bp]	Calibrated Conc. [ng/µl]	Assigned Conc. [ng/µl]	Peak Molarity [nmol/l]	% Integrated Area	Peak Comment	Observations
25	5.69	-	350	-		Lower Marker
204	3.32	-	25.0	100.00		
1500	6.50	6.50	6.67	-		Upper Marker

© Agilent Technologies, Inc. 2017

D1: H5 P R3

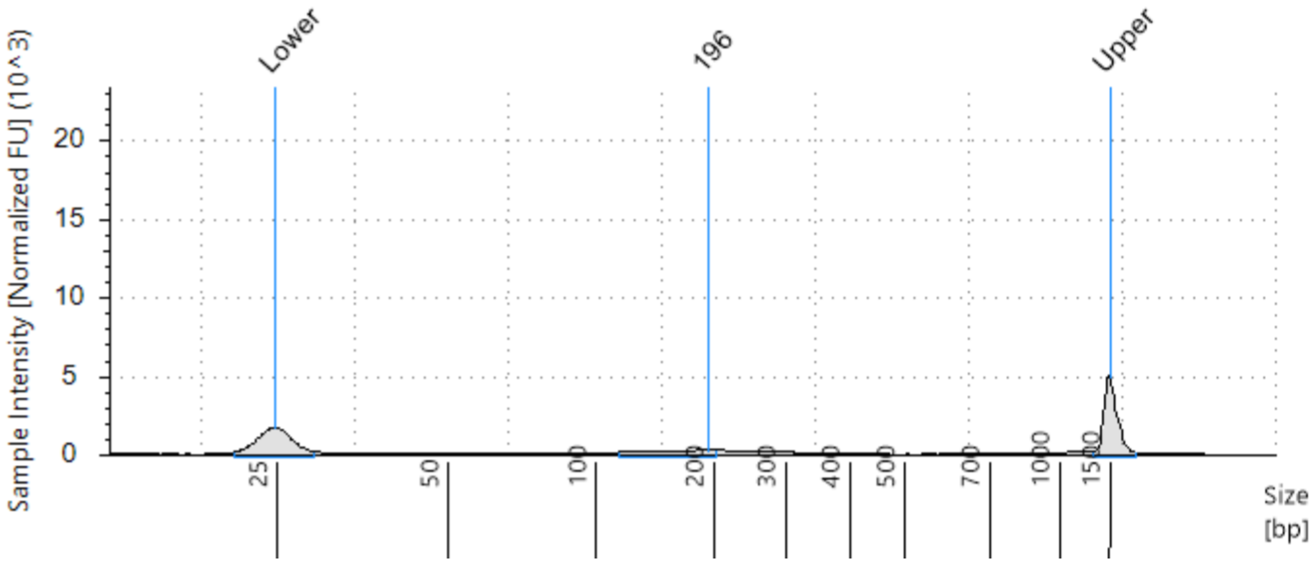

Sample Table

Well	Conc. [ng/µl]	Sample Description	Alert	Observations
D1	1.70	H5 P R3		

Peak Table

Size [bp]	Calibrated Conc. [ng/µl]	Assigned Conc. [ng/µl]	Peak Molarity [nmol/l]	% Integrated Area	Peak Comment	Observations
25	5.91	-	363	-		Lower Marker
196	1.70	-	13.3	100.00		
1500	6.50	6.50	6.67	-		Upper Marker

© Agilent Technologies, Inc. 2017

E1: H7 P R3

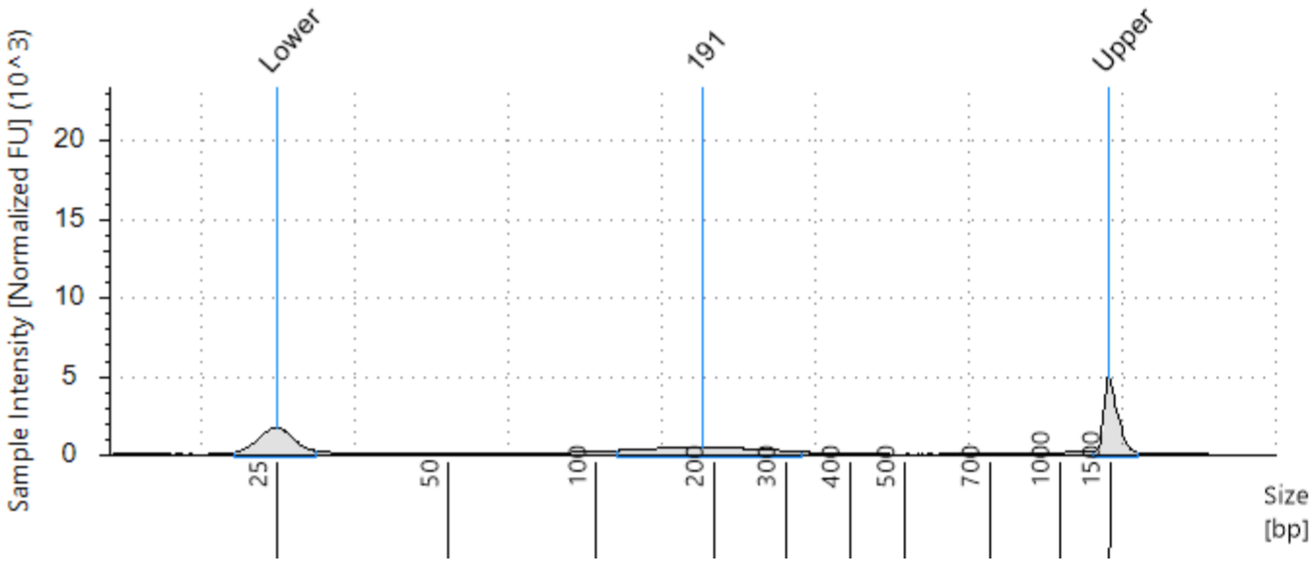

Sample Table

Well	Conc. [ng/µl]	Sample Description	Alert	Observations
E1	5.13	H7 P R3		

Peak Table

Size [bp]	Calibrated Conc. [ng/µl]	Assigned Conc. [ng/µl]	Peak Molarity [nmol/l]	% Integrated Area	Peak Comment	Observations
25	5.83	-	359	-		Lower Marker
191	5.13	-	41.4	100.00		
1500	6.50	6.50	6.67	-		Upper Marker

© Agilent Technologies, Inc. 2017

F1: H9 P R3

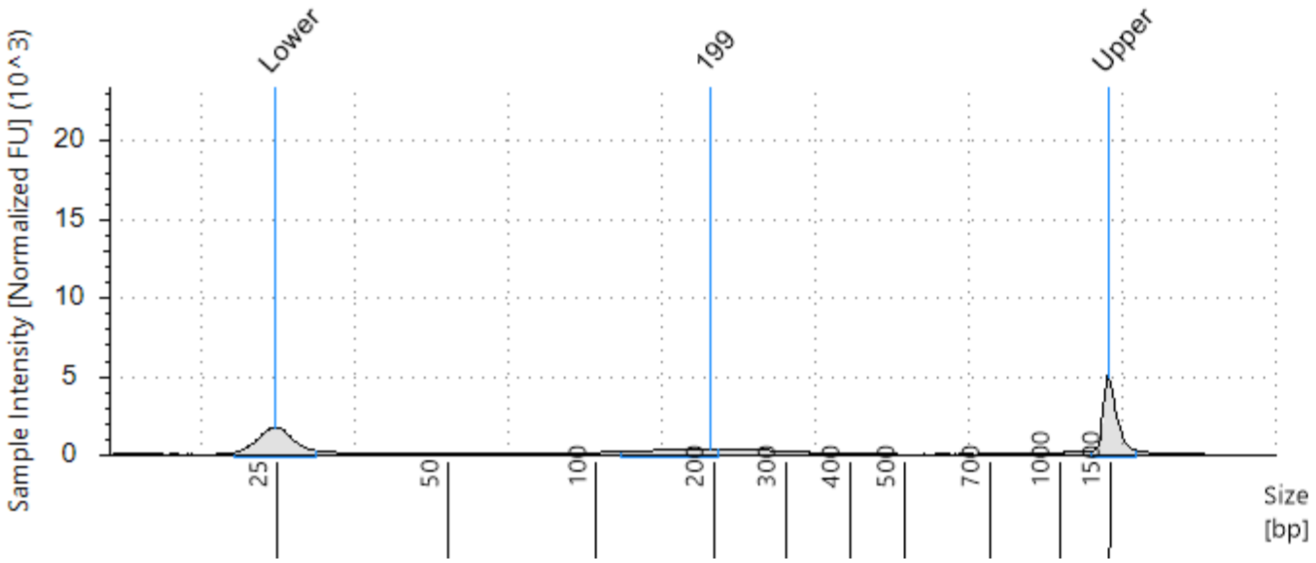

Sample Table

Well	Conc. [ng/µl]	Sample Description	Alert	Observations
F1	2.12	H9 P R3		

Peak Table

Size [bp]	Calibrated Conc. [ng/µl]	Assigned Conc. [ng/µl]	Peak Molarity [nmol/l]	% Integrated Area	Peak Comment	Observations
25	5.80	-	357	-		Lower Marker
199	2.12	-	16.4	100.00		
1500	6.50	6.50	6.67	-		Upper Marker

© Agilent Technologies, Inc. 2017

G1: H11 P R3

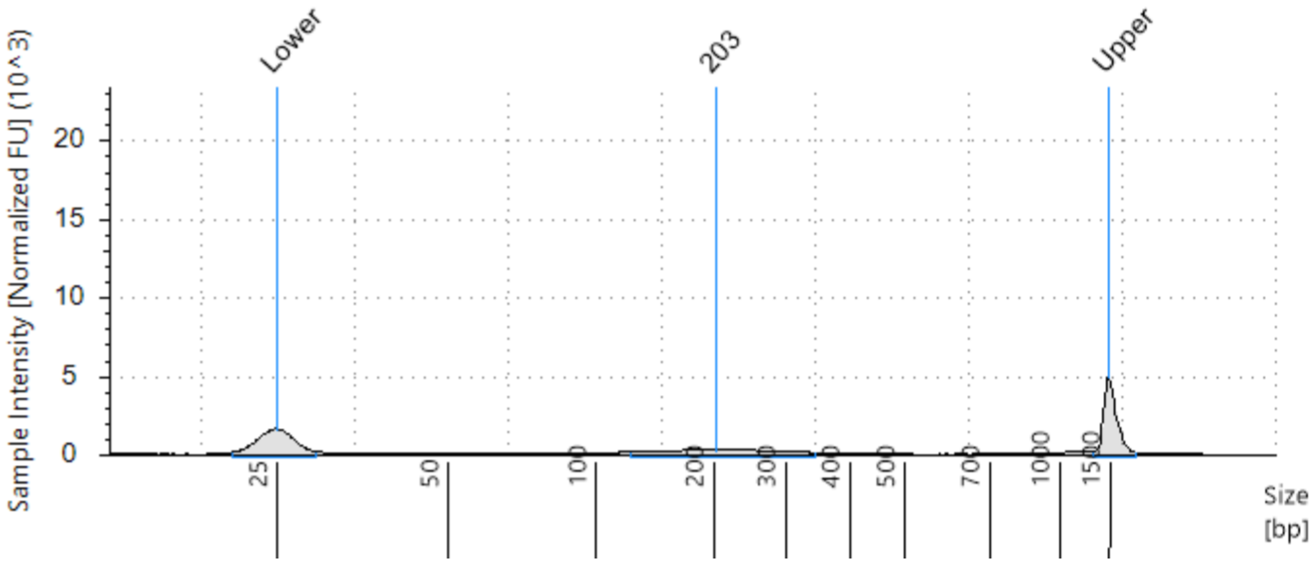

Sample Table

Well	Conc. [ng/µl]	Sample Description	Alert	Observations
G1	3.50	H11 P R3		

Peak Table

Size [bp]	Calibrated Conc. [ng/µl]	Assigned Conc. [ng/µl]	Peak Molarity [nmol/l]	% Integrated Area	Peak Comment	Observations
25	6.11	-	376	-		Lower Marker
203	3.50	-	26.5	100.00		
1500	6.50	6.50	6.67	-		Upper Marker

© Agilent Technologies, Inc. 2017

H1: F9 P R3

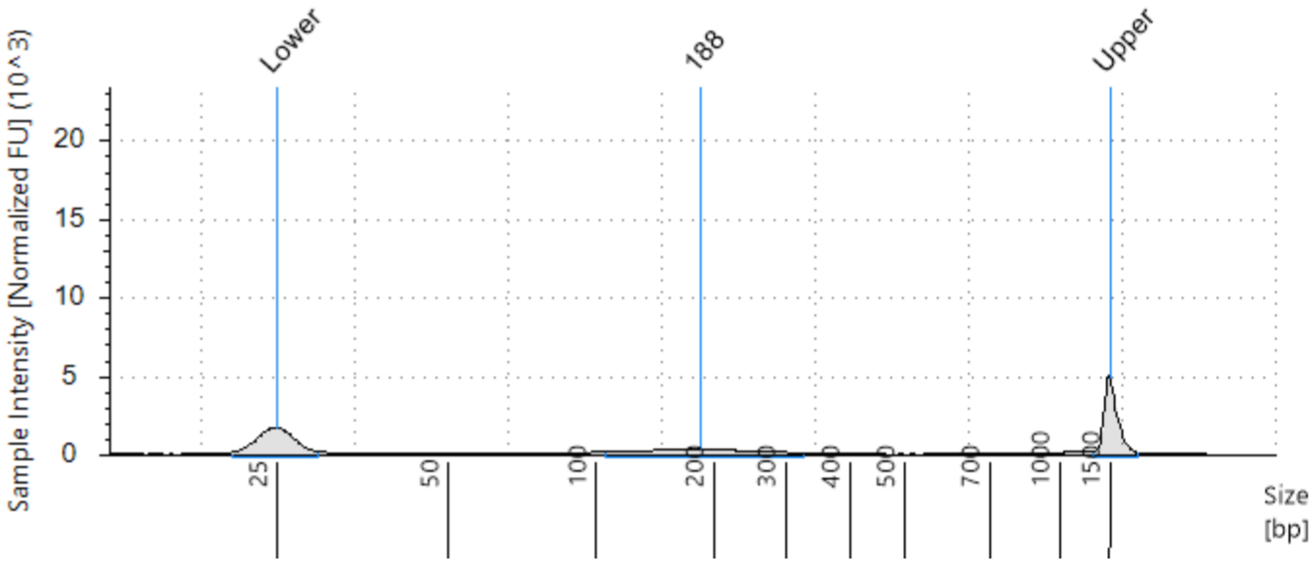

Sample Table

Well	Conc. [ng/µl]	Sample Description	Alert	Observations
H1	3.61	F9 P R3		

Peak Table

Size [bp]	Calibrated Conc. [ng/µl]	Assigned Conc. [ng/µl]	Peak Molarity [nmol/l]	% Integrated Area	Peak Comment	Observations
25	6.34	-	390	-		Lower Marker
188	3.61	-	29.5	100.00		
1500	6.50	6.50	6.67	-		Upper Marker

 © Agilent Technologies, Inc. 2017

A2: H1 M R3

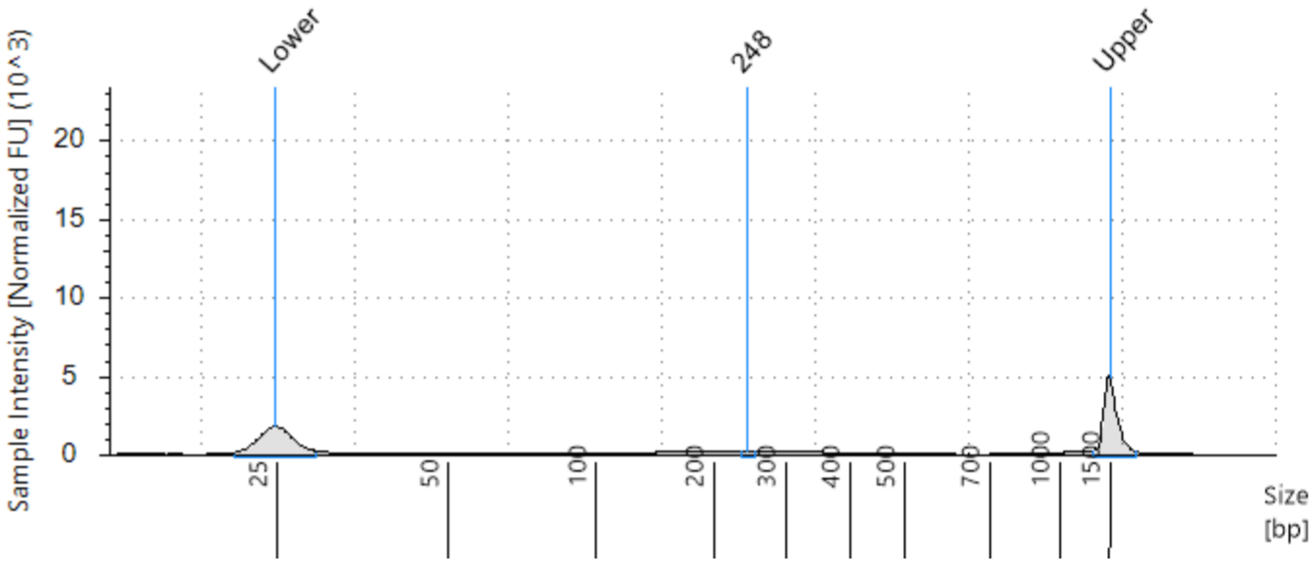

Sample Table

Well	Conc. [ng/µl]	Sample Description	Alert	Observations
A2	0.304	H1 M R3		

Peak Table

Size [bp]	Calibrated Conc. [ng/µl]	Assigned Conc. [ng/µl]	Peak Molarity [nmol/l]	% Integrated Area	Peak Comment	Observations
25	6.03	-	371	-		Lower Marker
248	0.304	-	1.89	100.00		
1500	6.50	6.50	6.67	-		Upper Marker

© Agilent Technologies, Inc. 2017

B2: H3 M R3

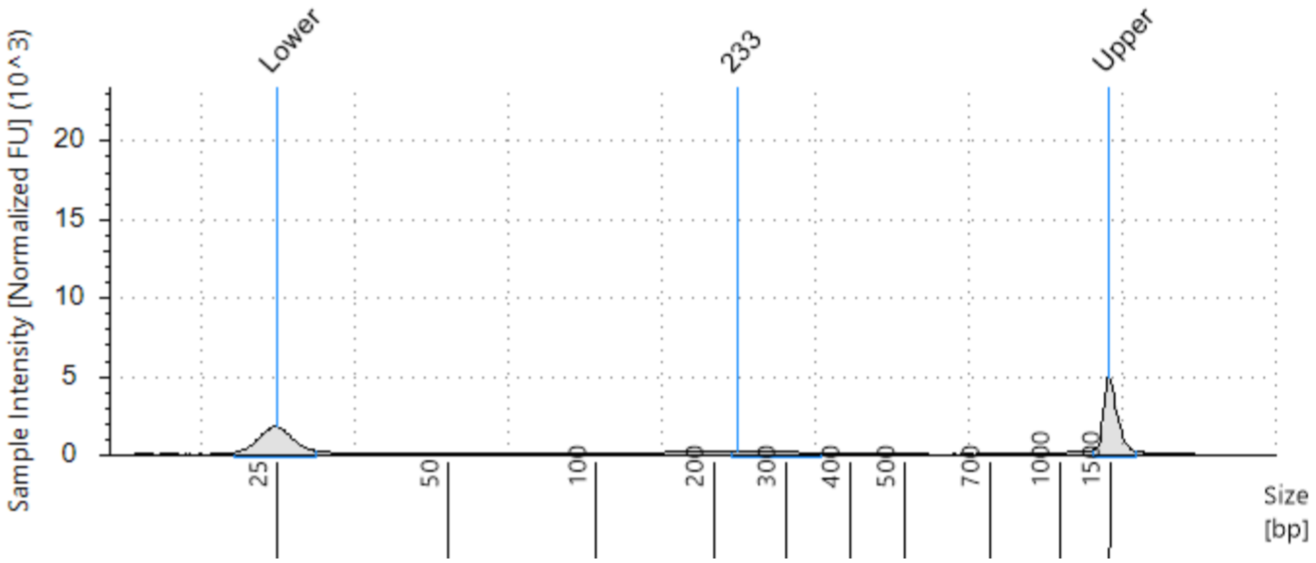

Sample Table

Well	Conc. [ng/µl]	Sample Description	Alert	Observations
B2	1.10	H3 M R3		

Peak Table

Size [bp]	Calibrated Conc. [ng/µl]	Assigned Conc. [ng/µl]	Peak Molarity [nmol/l]	% Integrated Area	Peak Comment	Observations
25	5.75	-	354	-		Lower Marker
233	1.10	-	7.26	100.00		
1500	6.50	6.50	6.67	-		Upper Marker

© Agilent Technologies, Inc. 2017

C2: H5 M R3

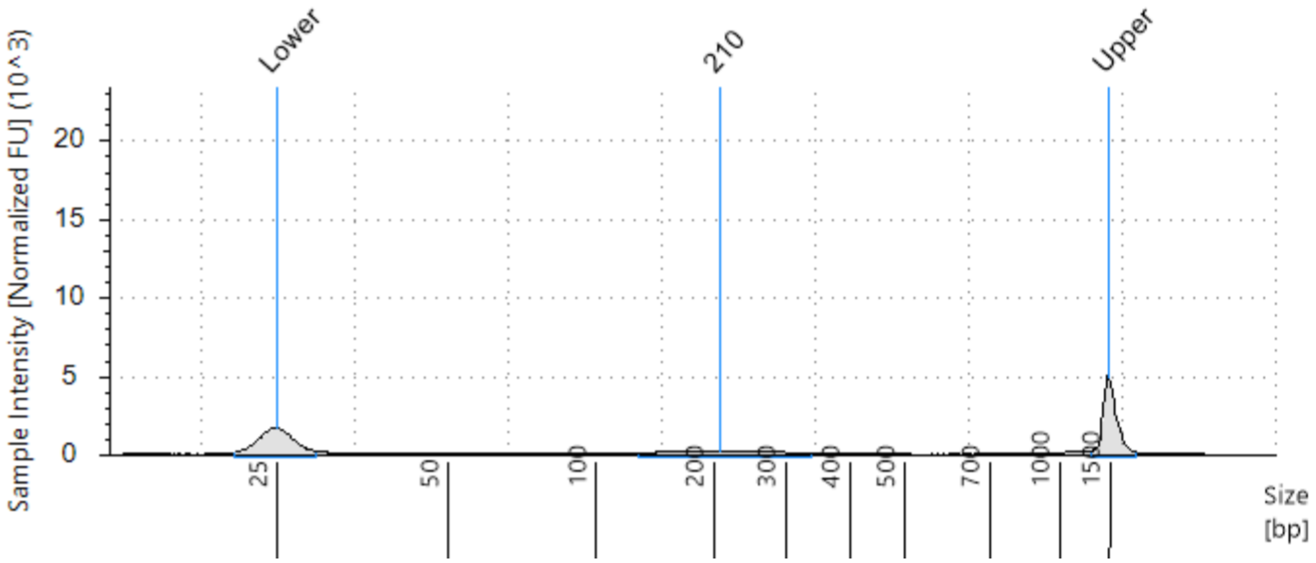

Sample Table

Well	Conc. [ng/µl]	Sample Description	Alert	Observations
C2	2.11	H5 M R3		

Peak Table

Size [bp]	Calibrated Conc. [ng/µl]	Assigned Conc. [ng/µl]	Peak Molarity [nmol/l]	% Integrated Area	Peak Comment	Observations
25	5.82	-	358	-		Lower Marker
210	2.11	-	15.5	100.00		
1500	6.50	6.50	6.67	-		Upper Marker

© Agilent Technologies, Inc. 2017

D2: H7 M R3

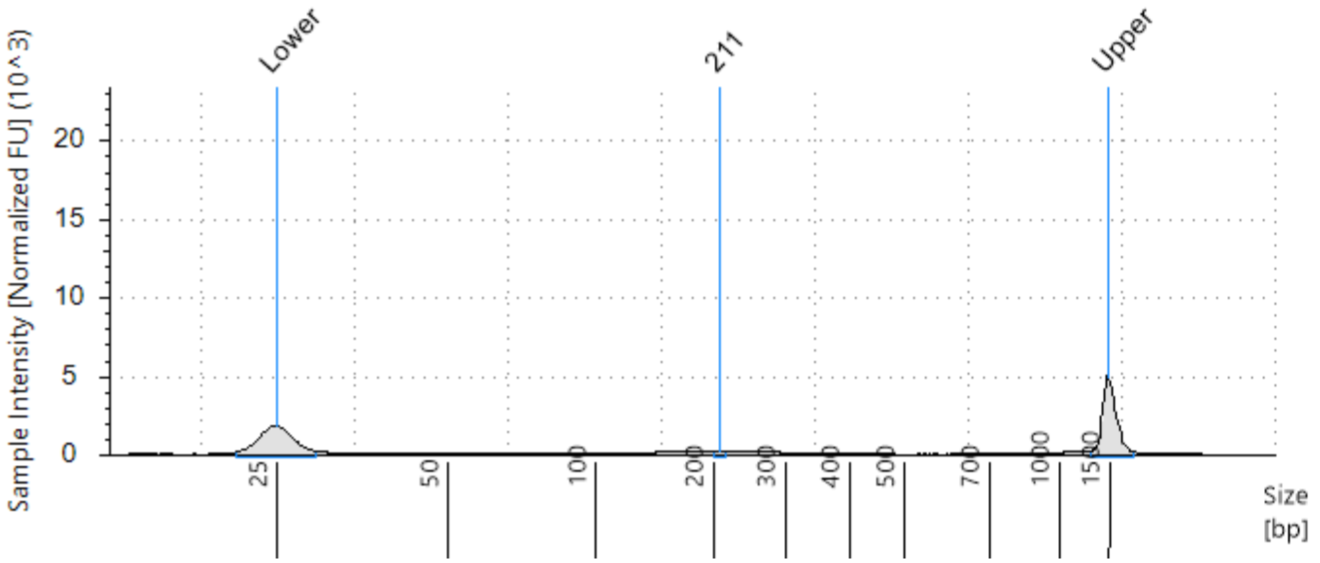

Sample Table

Well	Conc. [ng/µl]	Sample Description	Alert	Observations
D2	0.176	H7 M R3		

Peak Table

Size [bp]	Calibrated Conc. [ng/µl]	Assigned Conc. [ng/µl]	Peak Molarity [nmol/l]	% Integrated Area	Peak Comment	Observations
25	5.96	-	367	-		Lower Marker
211	0.176	-	1.28	100.00		
1500	6.50	6.50	6.67	-		Upper Marker

© Agilent Technologies, Inc. 2017

E2: H9 M R3

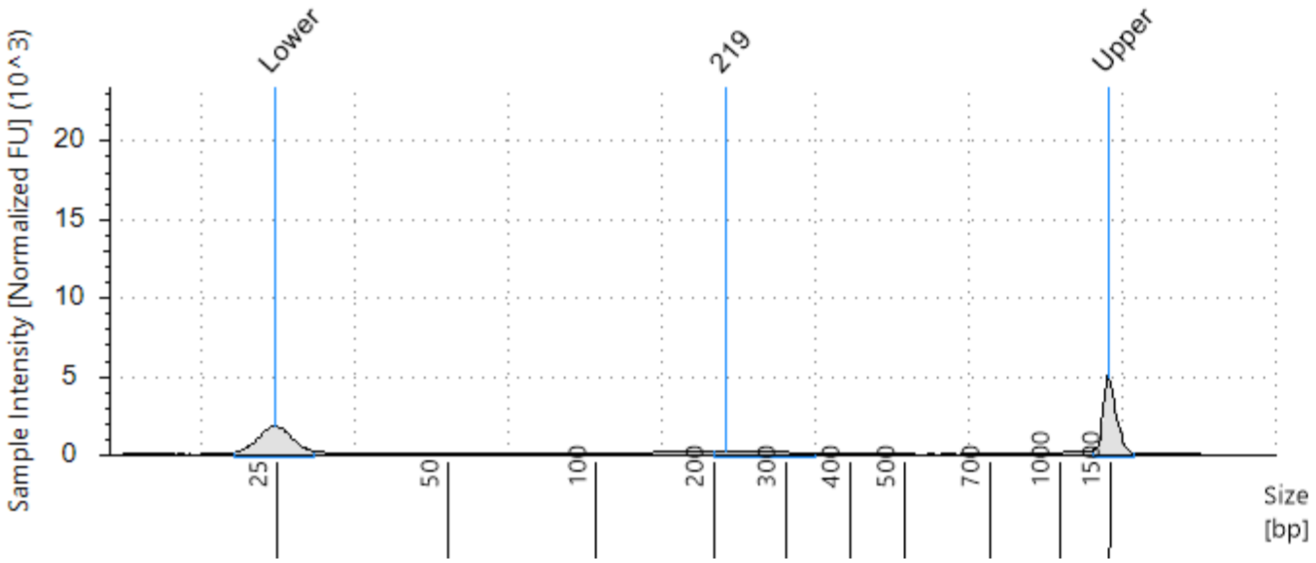

Sample Table

Well	Conc. [ng/µl]	Sample Description	Alert	Observations
E2	1.28	H9 M R3		

Peak Table

Size [bp]	Calibrated Conc. [ng/µl]	Assigned Conc. [ng/µl]	Peak Molarity [nmol/l]	% Integrated Area	Peak Comment	Observations
25	6.12	-	377	-		Lower Marker
219	1.28	-	8.97	100.00		
1500	6.50	6.50	6.67	-		Upper Marker

© Agilent Technologies, Inc. 2017

F2: H11 M R3

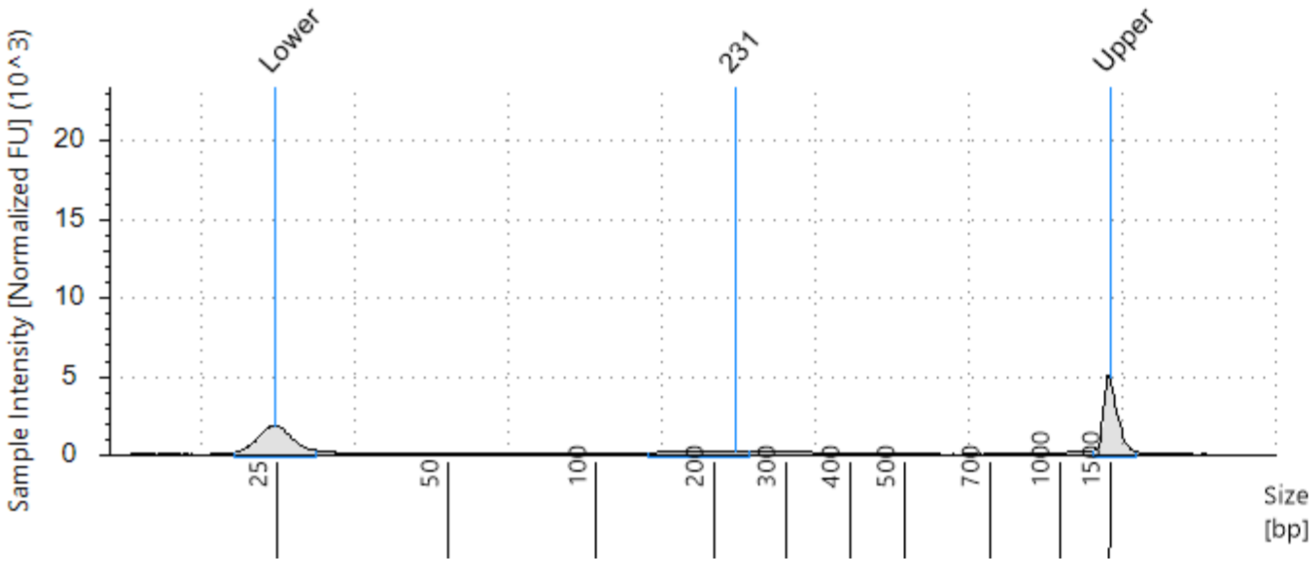

Sample Table

Well	Conc. [ng/µl]	Sample Description	Alert	Observations
F2	1.43	H11 M R3		

Peak Table

Size [bp]	Calibrated Conc. [ng/µl]	Assigned Conc. [ng/µl]	Peak Molarity [nmol/l]	% Integrated Area	Peak Comment	Observations
25	5.93	-	365	-		Lower Marker
231	1.43	-	9.55	100.00		
1500	6.50	6.50	6.67	-		Upper Marker

© Agilent Technologies, Inc. 2017

G2: H4 M R3

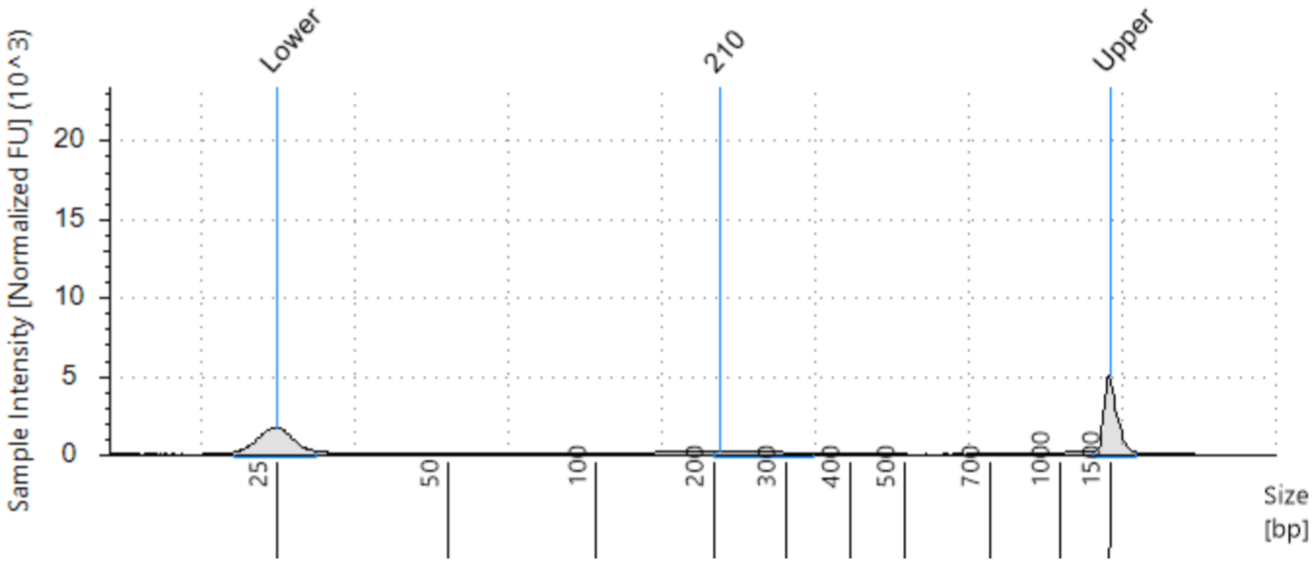

Sample Table

Well	Conc. [ng/µl]	Sample Description	Alert	Observations
G2	1.18	H4 M R3		

Peak Table

Size [bp]	Calibrated Conc. [ng/µl]	Assigned Conc. [ng/µl]	Peak Molarity [nmol/l]	% Integrated Area	Peak Comment	Observations
25	6.03	-	371	-		Lower Marker
210	1.18	-	8.64	100.00		
1500	6.50	6.50	6.67	-		Upper Marker

© Agilent Technologies, Inc. 2017

H2: 700 ng Plus undiluted

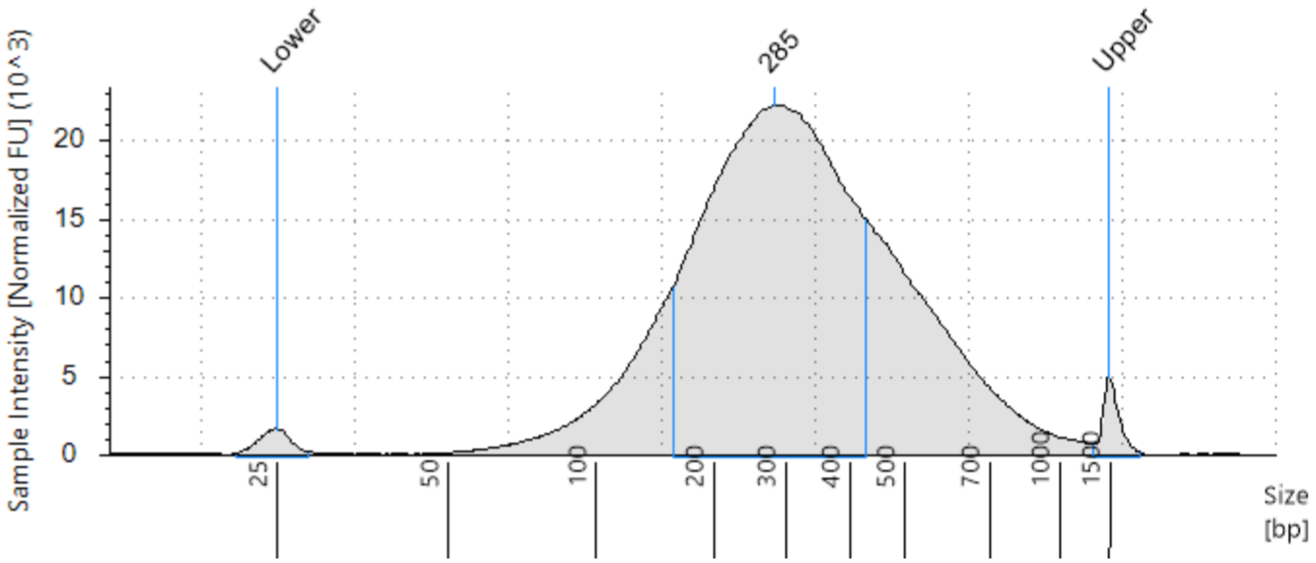

Sample Table

Well	Conc. [ng/µl]	Sample Description	Alert	Observations
H2	246	700 ng Plus undiluted		

Peak Table

Size [bp]	Calibrated Conc. [ng/µl]	Assigned Conc. [ng/µl]	Peak Molarity [nmol/l]	% Integrated Area	Peak Comment	Observations
25	4.17	-	256	-		Lower Marker
285	246	-	1330	100.00		
1500	6.50	6.50	6.67	-		Upper Marker

© Agilent Technologies, Inc. 2017
