# Supplementary material for: 96 sample parallel acoustic fragmentation for high throughput next generation sequencing library preparation
Source: PLoS One. 2026 Feb 17;21(2):e0341139. doi: 10.1371/journal.pone.0341139 (PMC12912608; doi:10.1371/journal.pone.0341139)
Supplement: S5 File — (ZIP) [file pone.0341139.s005.zip › QSonica translator TapeStation raw data/QSonica no cavitation enhancement translator 96-well plate replicate 2.pdf]

Filename: 2020-09-08 DFB MINUS A1- F2,D1000 R2.D1000

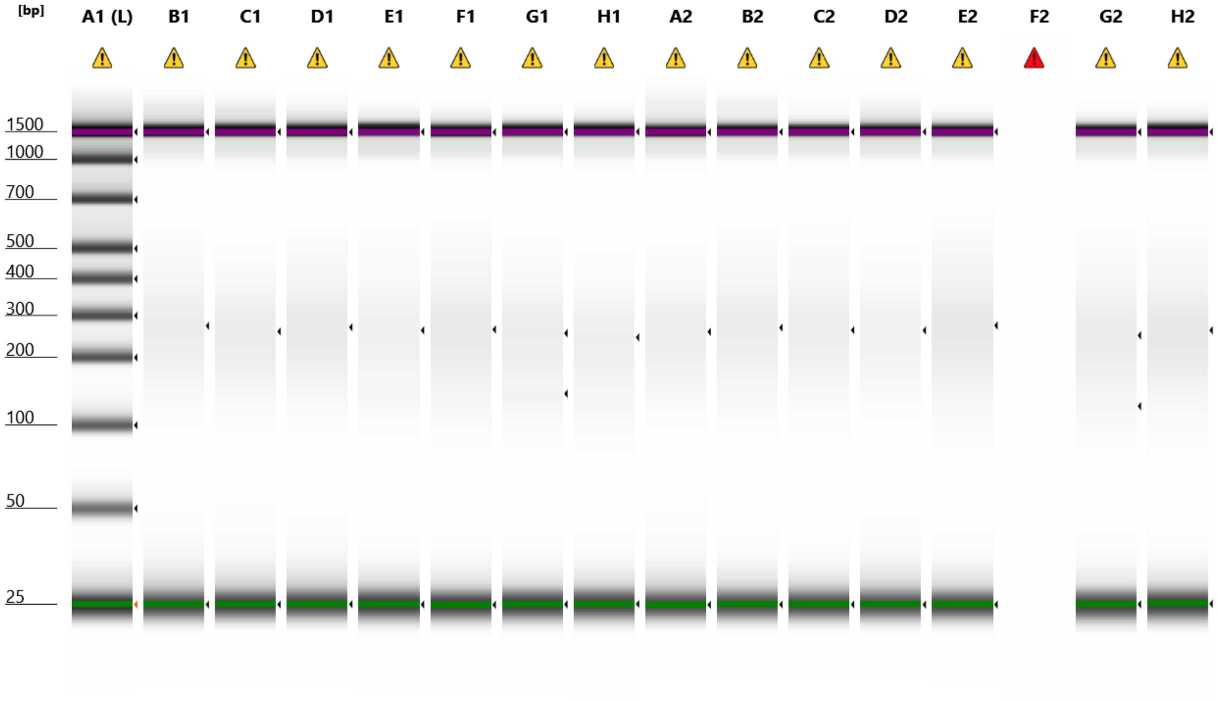

Default image (Contrast 100%)

Sample Info

Well	Conc. [ng/µl]	Sample Description	Alert	Observations
A1	18.7	Ladder	⚠	Caution! Expired ScreenTape device; Ladder
B1	0.157	A1 M R2	⚠	Caution! Expired ScreenTape device
C1	0.206	B1 M R2	⚠	Caution! Expired ScreenTape device
D1	0.172	C1 M R2	⚠	Caution! Expired ScreenTape device
E1	0.0979	D1 M R2	⚠	Caution! Expired ScreenTape device
F1	0.269	E1 M R2	⚠	Caution! Expired ScreenTape device
G1	0.579	F1 M R2	⚠	Caution! Expired ScreenTape device
H1	0.160	G1 M R2	⚠	Caution! Expired ScreenTape device
A2	0.122	H1 M R2	⚠	Caution! Expired ScreenTape device
B2	0.0788	A1 M R2	⚠	Caution! Expired ScreenTape device
C2	0.266	B2 M R2	⚠	Caution! Expired ScreenTape device
D2	0.127	C2 M R2	⚠	Caution! Expired ScreenTape device
E2	0.216	D2 M R2	⚠	Caution! Expired ScreenTape device
F2			⚠	Marker(s) not detected; Caution! Expired ScreenTape device
G2	0.393	E2 M R2	⚠	Caution! Expired ScreenTape device
H2	0.813	F2 M R2	⚠	Caution! Expired ScreenTape device

© Agilent Technologies, Inc. 2017

A1: Ladder

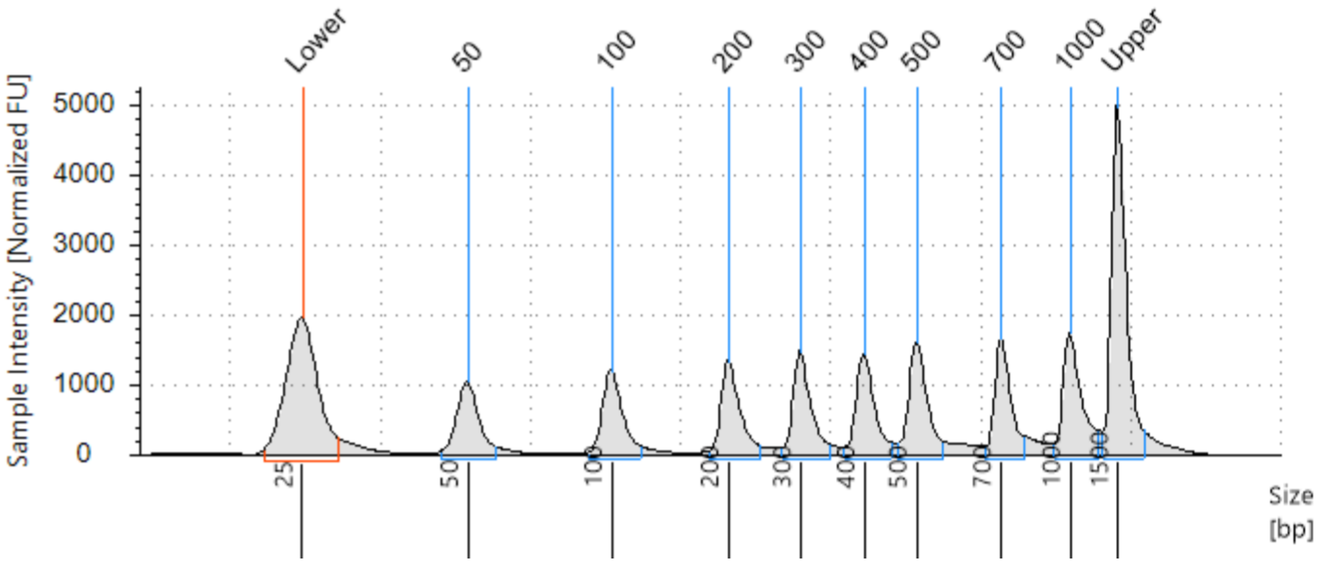

Sample Table

Well	Conc. [ng/µl]	Sample Description	Alert	Observations
A1	18.7	Ladder	⚠	Caution! Expired ScreenTape device; Ladder

Peak Table

Size [bp]	Calibrated Conc. [ng/µl]	Assigned Conc. [ng/µl]	Peak Molarity [nmol/l]	% Integrated Area	Peak Comment	Observations
25	5.41	-	333	-		Lower Marker
50	2.00	-	61.7	10.70		
100	2.13	-	32.7	11.35		
200	2.21	-	17.0	11.82		
300	2.31	-	11.8	12.31		
400	2.34	-	8.99	12.48		
500	2.58	-	7.93	13.76		
700	2.35	-	5.17	12.55		
1000	2.81	-	4.33	15.03		
1500	6.50	6.50	6.67	-		Upper Marker

© Agilent Technologies, Inc. 2017

B1: A1 M R2

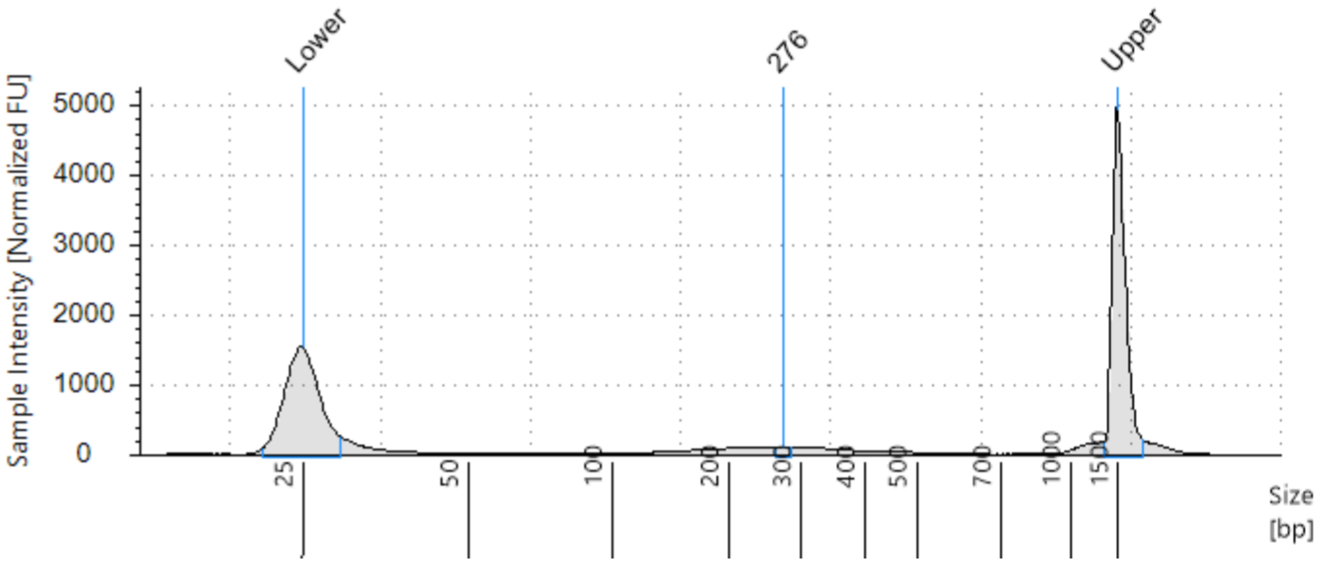

Sample Table

Well	Conc. [ng/µl]	Sample Description	Alert	Observations
B1	0.157	A1 M R2	⚠	Caution! Expired ScreenTape device

Peak Table

Size [bp]	Calibrated Conc. [ng/µl]	Assigned Conc. [ng/µl]	Peak Molarity [nmol/l]	% Integrated Area	Peak Comment	Observations
25	5.41	-	333	-		Lower Marker
276	0.157	-	0.874	100.00		
1500	6.50	6.50	6.67	-		Upper Marker

© Agilent Technologies, Inc. 2017

C1: B1 M R2

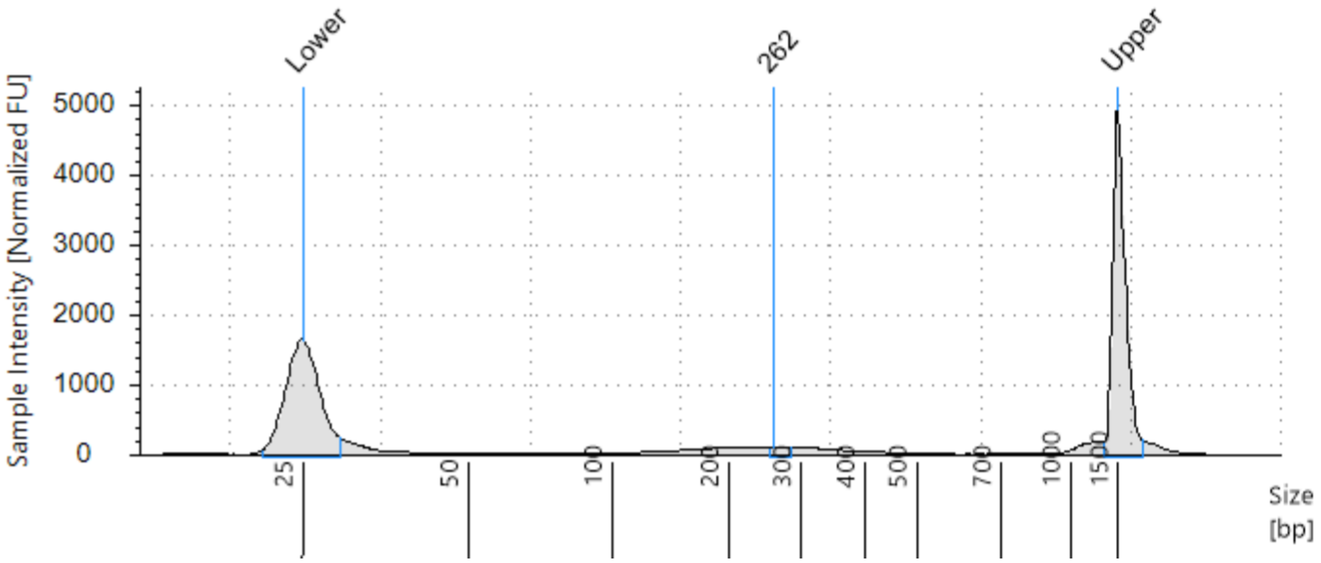

Sample Table

Well	Conc. [ng/µl]	Sample Description	Alert	Observations
C1	0.206	B1 M R2	⚠	Caution! Expired ScreenTape device

Peak Table

Size [bp]	Calibrated Conc. [ng/µl]	Assigned Conc. [ng/µl]	Peak Molarity [nmol/l]	% Integrated Area	Peak Comment	Observations
25	5.76	-	355	-		Lower Marker
262	0.206	-	1.21	100.00		
1500	6.50	6.50	6.67	-		Upper Marker

 © Agilent Technologies, Inc. 2017

D1: C1 M R2

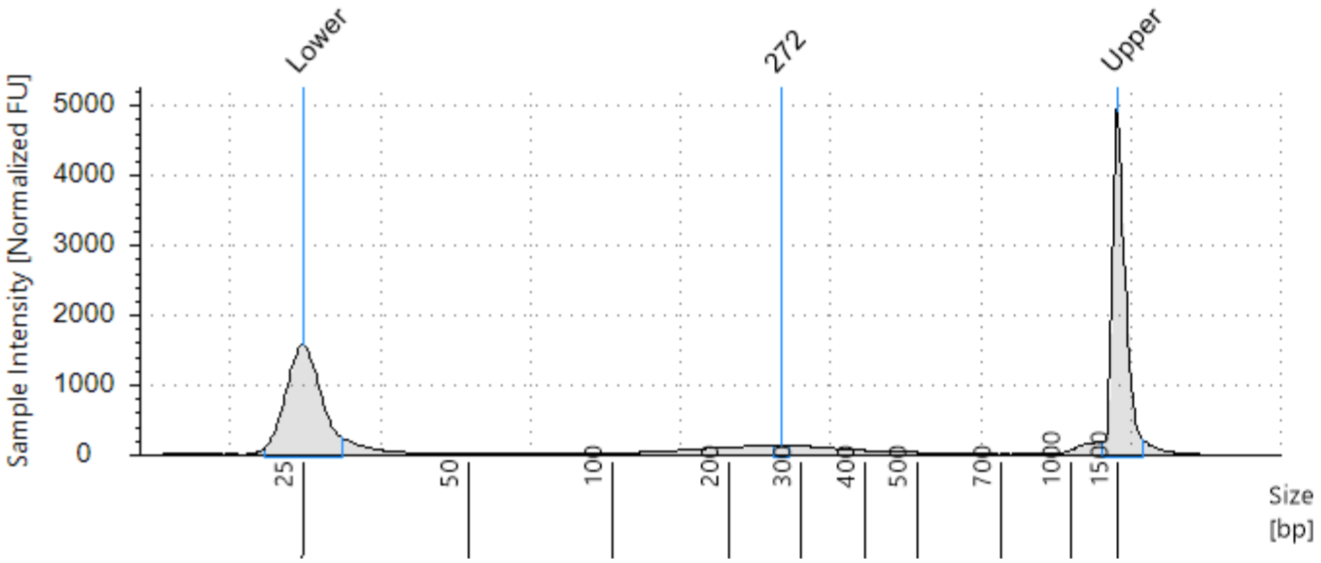

Sample Table

Well	Conc. [ng/µl]	Sample Description	Alert	Observations
D1	0.172	C1 M R2	⚠	Caution! Expired ScreenTape device

Peak Table

Size [bp]	Calibrated Conc. [ng/µl]	Assigned Conc. [ng/µl]	Peak Molarity [nmol/l]	% Integrated Area	Peak Comment	Observations
25	5.60	-	345	-		Lower Marker
272	0.172	-	0.973	100.00		
1500	6.50	6.50	6.67	-		Upper Marker

© Agilent Technologies, Inc. 2017

E1: D1 M R2

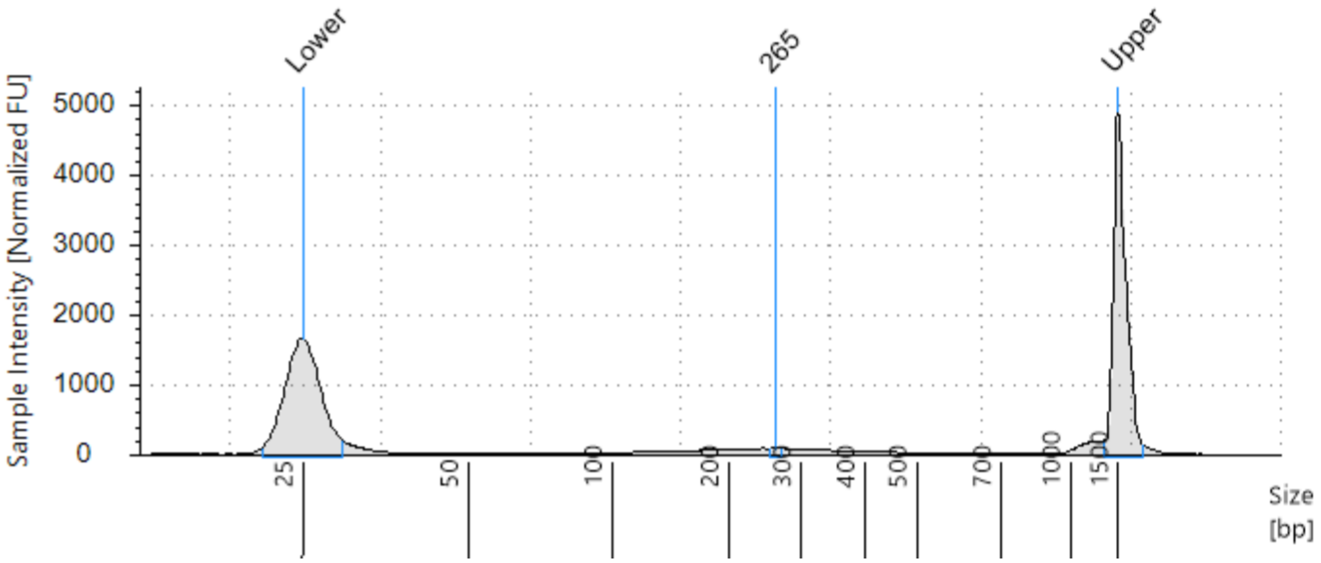

Sample Table

Well	Conc. [ng/µl]	Sample Description	Alert	Observations
E1	0.0979	D1 M R2	⚠	Caution! Expired ScreenTape device

Peak Table

Size [bp]	Calibrated Conc. [ng/µl]	Assigned Conc. [ng/µl]	Peak Molarity [nmol/l]	% Integrated Area	Peak Comment	Observations
25	6.22	-	383	-		Lower Marker
265	0.0979	-	0.569	100.00		
1500	6.50	6.50	6.67	-		Upper Marker

 © Agilent Technologies, Inc. 2017

F1: E1 M R2

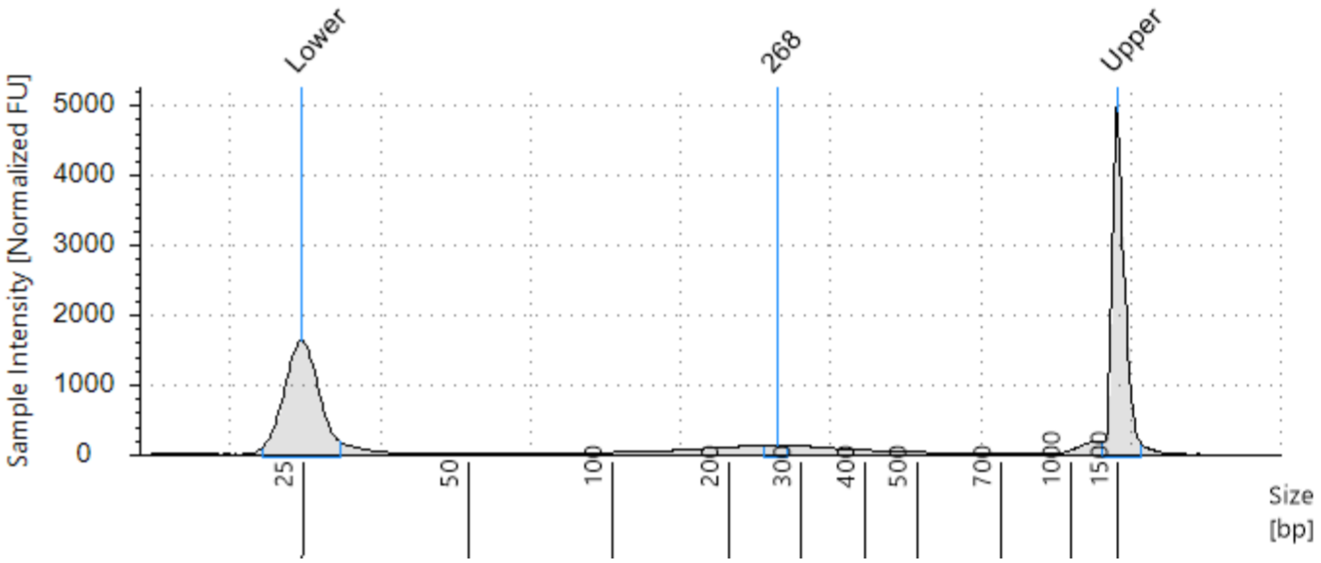

Sample Table

Well	Conc. [ng/µl]	Sample Description	Alert	Observations
F1	0.269	E1 M R2	⚠	Caution! Expired ScreenTape device

Peak Table

Size [bp]	Calibrated Conc. [ng/µl]	Assigned Conc. [ng/µl]	Peak Molarity [nmol/l]	% Integrated Area	Peak Comment	Observations
25	5.97	-	368	-		Lower Marker
268	0.269	-	1.55	100.00		
1500	6.50	6.50	6.67	-		Upper Marker

© Agilent Technologies, Inc. 2017

G1: F1 M R2

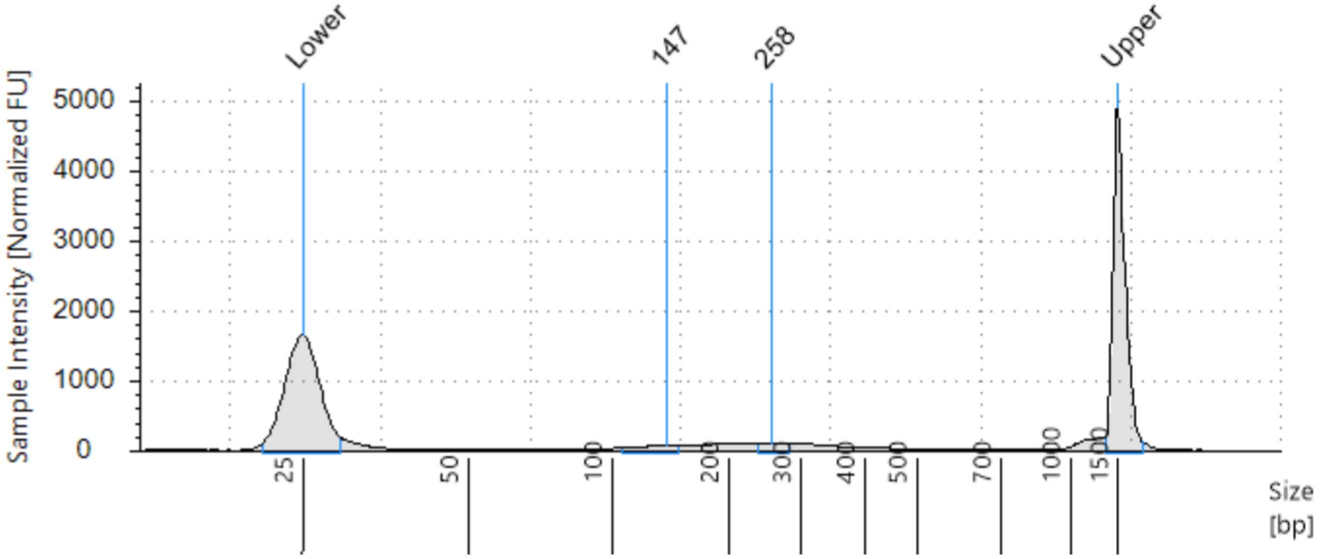

Sample Table

Well	Conc. [ng/µl]	Sample Description	Alert	Observations
G1	0.579	F1 M R2	⚠	Caution! Expired ScreenTape device

Peak Table

Size [bp]	Calibrated Conc. [ng/µl]	Assigned Conc. [ng/µl]	Peak Molarity [nmol/l]	% Integrated Area	Peak Comment	Observations
25	6.26	-	385	-		Lower Marker
147	0.290	-	3.05	50.17		
258	0.289	-	1.72	49.83		
1500	6.50	6.50	6.67	-		Upper Marker

© Agilent Technologies, Inc. 2017

H1: G1 M R2

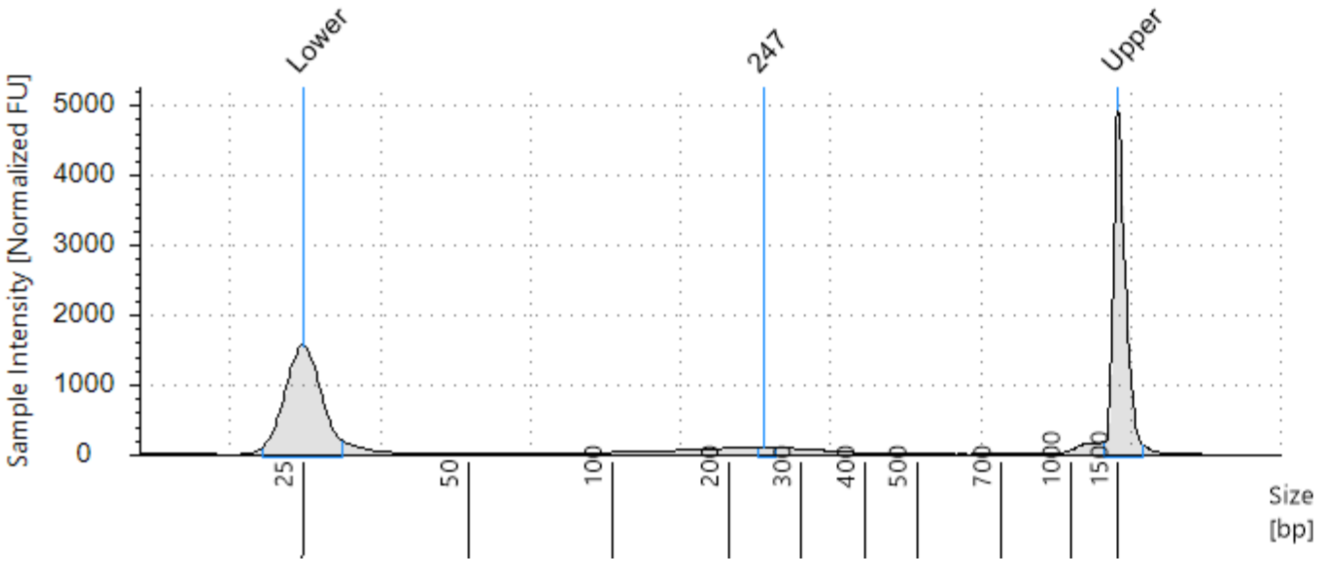

Sample Table

Well	Conc. [ng/µl]	Sample Description	Alert	Observations
H1	0.160	G1 M R2	⚠	Caution! Expired ScreenTape device

Peak Table

Size [bp]	Calibrated Conc. [ng/µl]	Assigned Conc. [ng/µl]	Peak Molarity [nmol/l]	% Integrated Area	Peak Comment	Observations
25	6.06	-	373	-		Lower Marker
247	0.160	-	0.996	100.00		
1500	6.50	6.50	6.67	-		Upper Marker

© Agilent Technologies, Inc. 2017

A2: H1 M R2

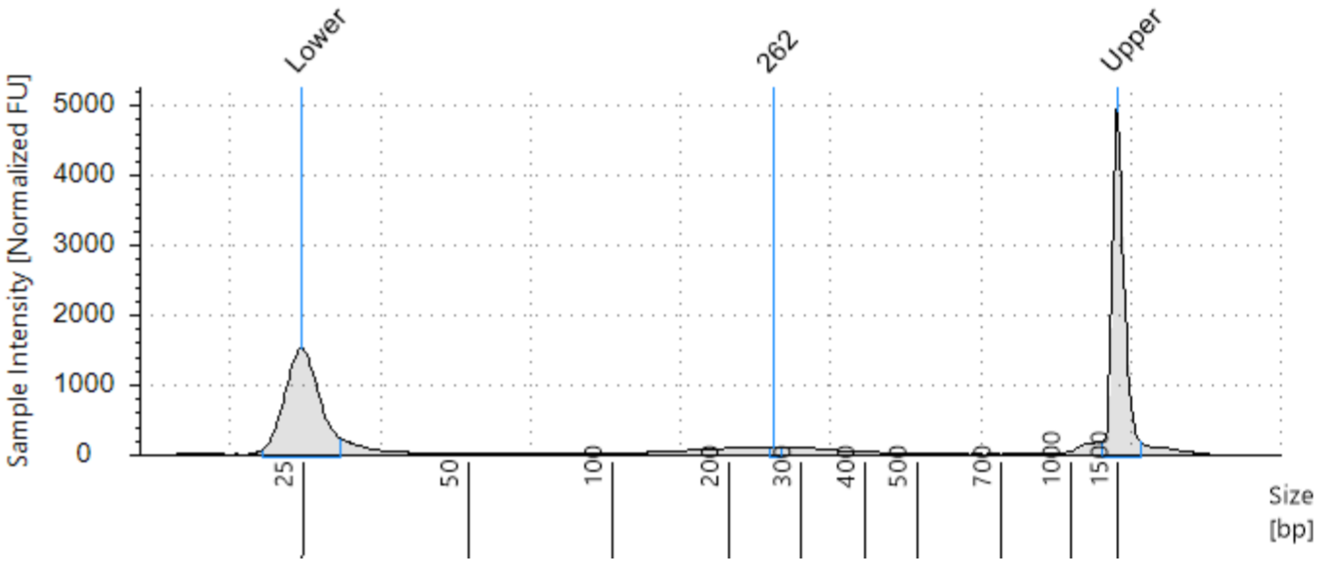

Sample Table

Well	Conc. [ng/µl]	Sample Description	Alert	Observations
A2	0.122	H1 M R2	⚠	Caution! Expired ScreenTape device

Peak Table

Size [bp]	Calibrated Conc. [ng/µl]	Assigned Conc. [ng/µl]	Peak Molarity [nmol/l]	% Integrated Area	Peak Comment	Observations
25	5.72	-	352	-		Lower Marker
262	0.122	-	0.718	100.00		
1500	6.50	6.50	6.67	-		Upper Marker

© Agilent Technologies, Inc. 2017

B2: A1 M R2

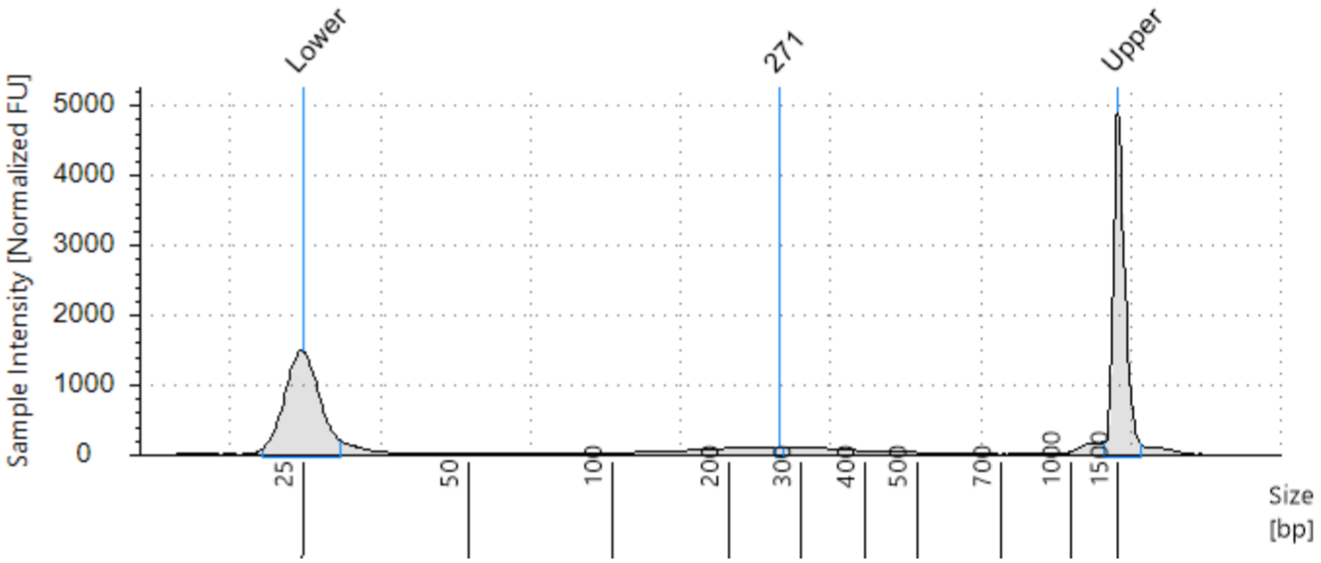

Sample Table

Well	Conc. [ng/µl]	Sample Description	Alert	Observations
B2	0.0788	A1 M R2	⚠	Caution! Expired ScreenTape device

Peak Table

Size [bp]	Calibrated Conc. [ng/µl]	Assigned Conc. [ng/µl]	Peak Molarity [nmol/l]	% Integrated Area	Peak Comment	Observations
25	5.82	-	358	-		Lower Marker
271	0.0788	-	0.446	100.00		
1500	6.50	6.50	6.67	-		Upper Marker

© Agilent Technologies, Inc. 2017

C2: B2 M R2

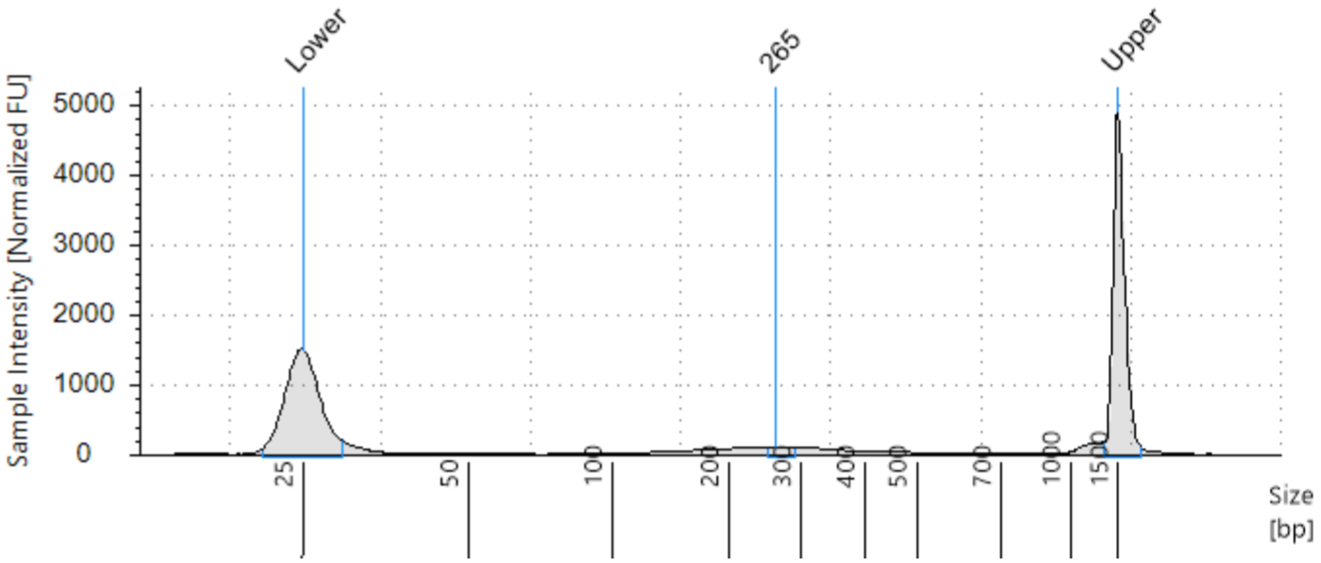

Sample Table

Well	Conc. [ng/µl]	Sample Description	Alert	Observations
C2	0.266	B2 M R2	⚠	Caution! Expired ScreenTape device

Peak Table

Size [bp]	Calibrated Conc. [ng/µl]	Assigned Conc. [ng/µl]	Peak Molarity [nmol/l]	% Integrated Area	Peak Comment	Observations
25	5.96	-	367	-		Lower Marker
265	0.266	-	1.54	100.00		
1500	6.50	6.50	6.67	-		Upper Marker

© Agilent Technologies, Inc. 2017

D2: C2 M R2

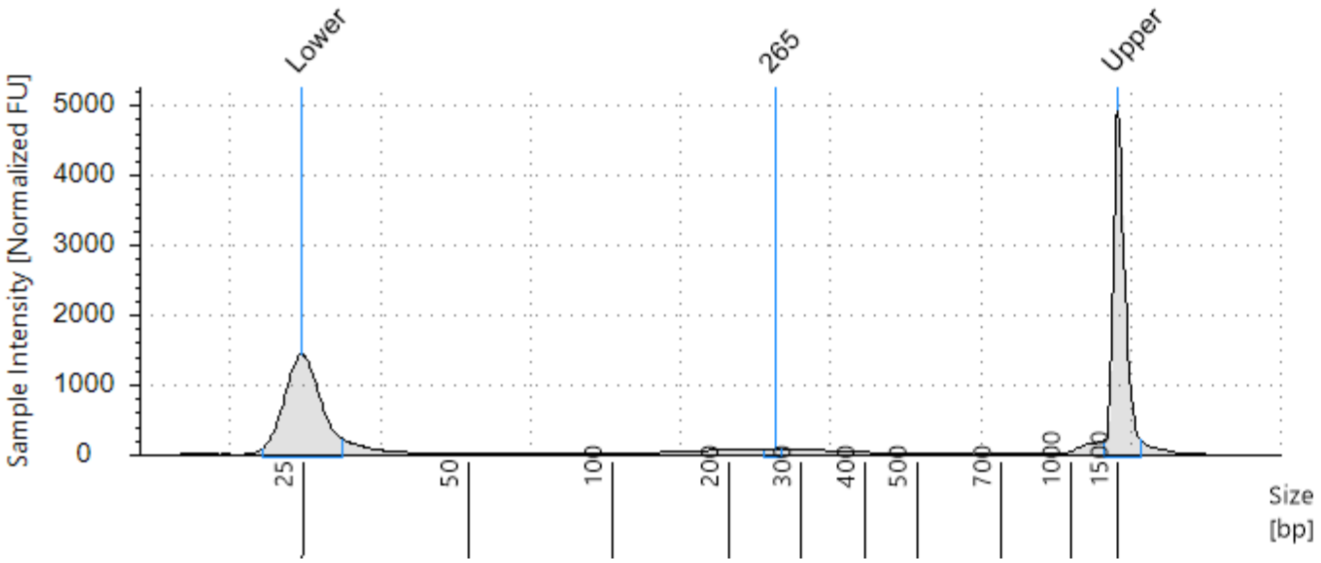

Sample Table

Well	Conc. [ng/µl]	Sample Description	Alert	Observations
D2	0.127	C2 M R2	⚠	Caution! Expired ScreenTape device

Peak Table

Size [bp]	Calibrated Conc. [ng/µl]	Assigned Conc. [ng/µl]	Peak Molarity [nmol/l]	% Integrated Area	Peak Comment	Observations
25	5.71	-	351	-		Lower Marker
265	0.127	-	0.740	100.00		
1500	6.50	6.50	6.67	-		Upper Marker

© Agilent Technologies, Inc. 2017

E2: D2 M R2

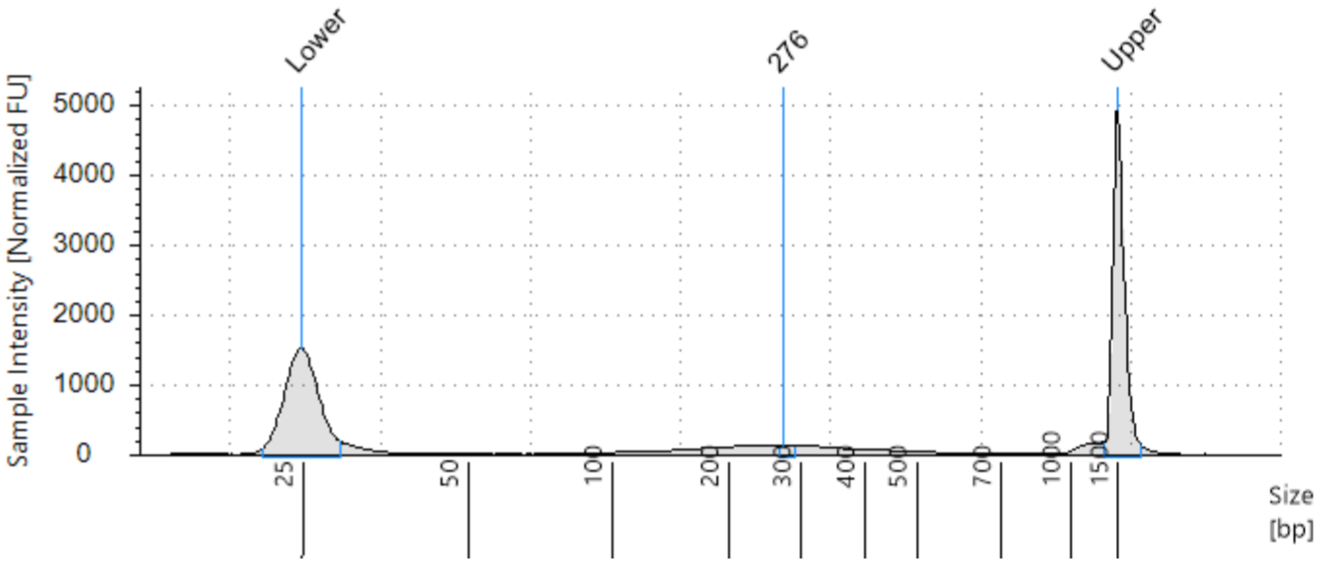

Sample Table

Well	Conc. [ng/µl]	Sample Description	Alert	Observations
E2	0.216	D2 M R2	⚠	Caution! Expired ScreenTape device

Peak Table

Size [bp]	Calibrated Conc. [ng/µl]	Assigned Conc. [ng/µl]	Peak Molarity [nmol/l]	% Integrated Area	Peak Comment	Observations
25	5.89	-	363	-		Lower Marker
276	0.216	-	1.20	100.00		
1500	6.50	6.50	6.67	-		Upper Marker

 © Agilent Technologies, Inc. 2017

F2

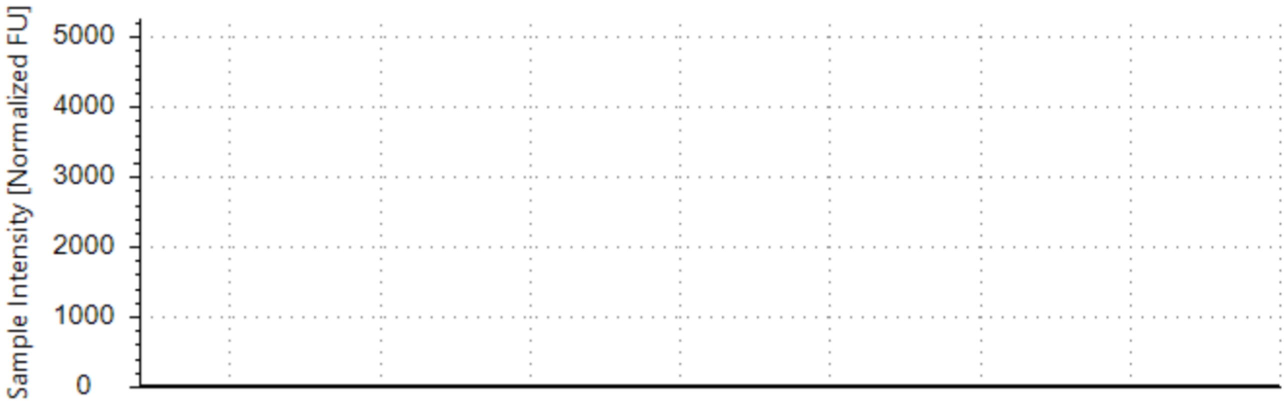

Sample Table

Well	Conc. [ng/µl]	Sample Description	Alert	Observations
F2			▲	Marker(s) not detected; Caution! Expired ScreenTape device

© Agilent Technologies, Inc. 2017

G2: E2 M R2

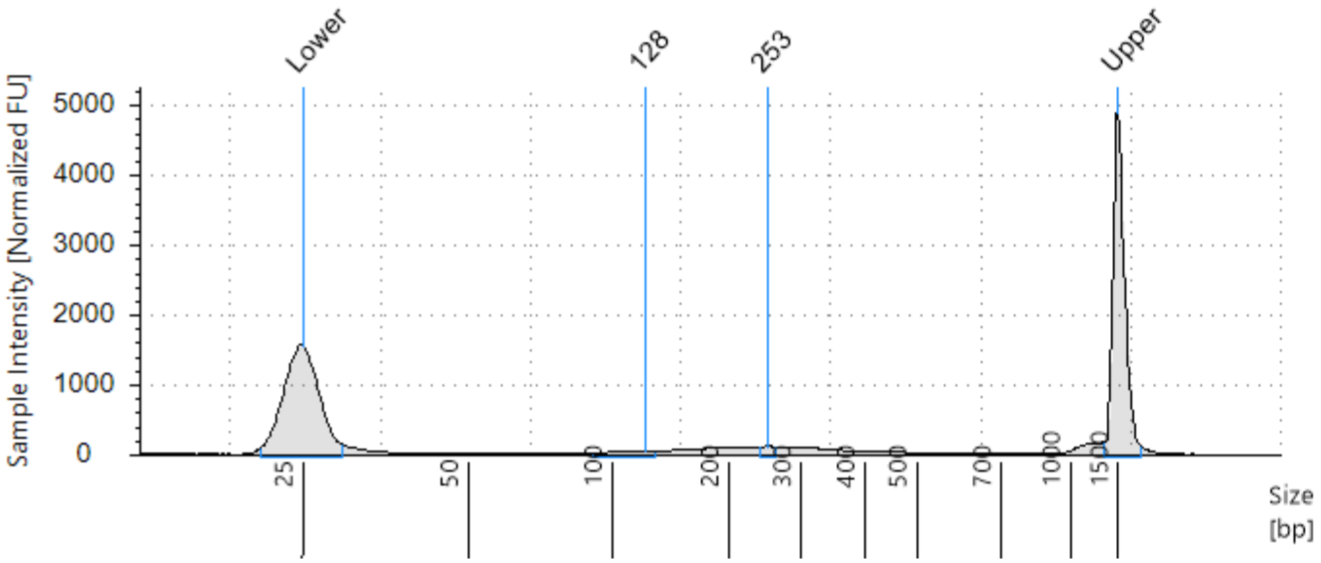

Sample Table

Well	Conc. [ng/µl]	Sample Description	Alert	Observations
G2	0.393	E2 M R2	⚠	Caution! Expired ScreenTape device

Peak Table

Size [bp]	Calibrated Conc. [ng/µl]	Assigned Conc. [ng/µl]	Peak Molarity [nmol/l]	% Integrated Area	Peak Comment	Observations
25	6.24	-	384	-		Lower Marker
128	0.218	-	2.61	55.34		
253	0.176	-	1.07	44.66		
1500	6.50	6.50	6.67	-		Upper Marker

© Agilent Technologies, Inc. 2017

H2: F2 M R2

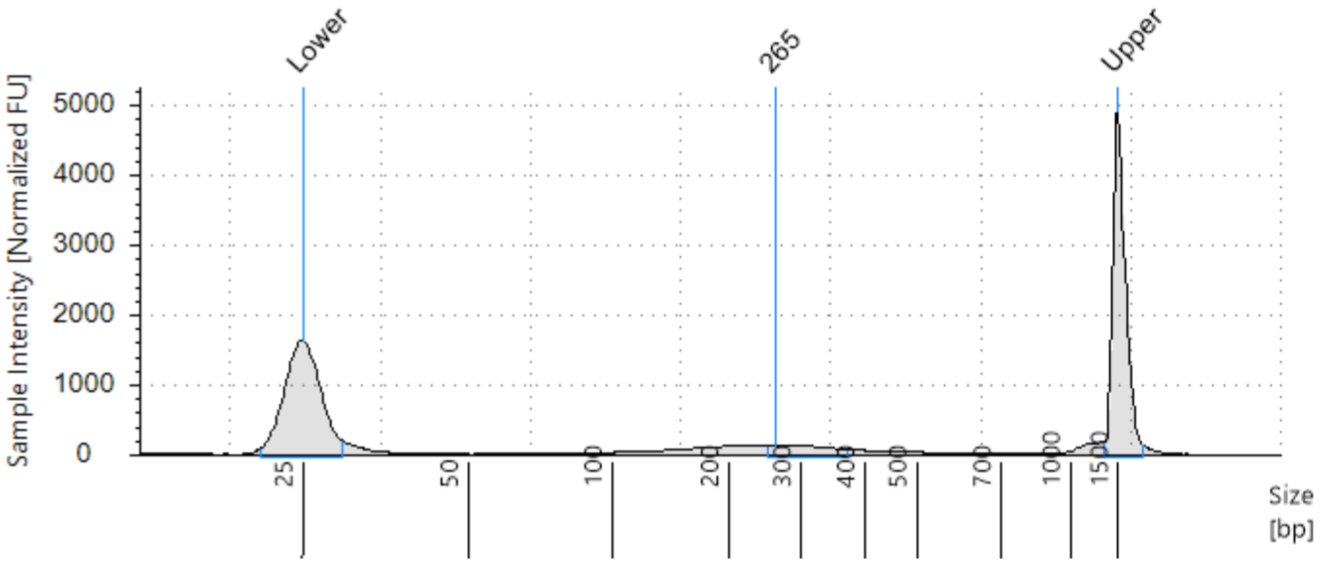

Sample Table

Well	Conc. [ng/µl]	Sample Description	Alert	Observations
H2	0.813	F2 M R2	⚠	Caution! Expired ScreenTape device

Peak Table

Size [bp]	Calibrated Conc. [ng/µl]	Assigned Conc. [ng/µl]	Peak Molarity [nmol/l]	% Integrated Area	Peak Comment	Observations
25	6.12	-	376	-		Lower Marker
265	0.813	-	4.73	100.00		
1500	6.50	6.50	6.67	-		Upper Marker

© Agilent Technologies, Inc. 2017

Filename: 2020-09-08 DFB PLUS G2-E3 Minus G2-D3 D1000 R2.D1000

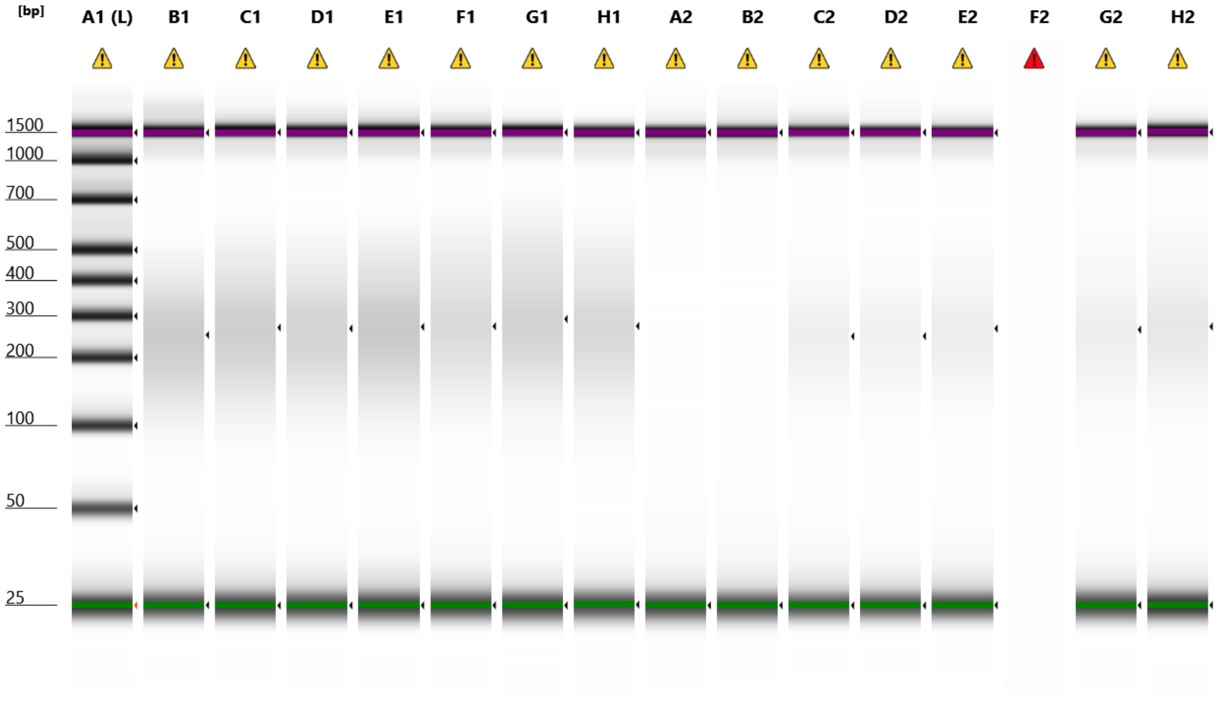

Default image (Contrast 100%)

Sample Info

Well	Conc. [ng/µl]	Sample Description	Alert	Observations
A1	25.9	Ladder	⚠	Caution! Expired ScreenTape device; Ladder
B1	4.11	G2 P R2	⚠	Caution! Expired ScreenTape device
C1	1.75	H2 P R2	⚠	Caution! Expired ScreenTape device
D1	1.75	A3 P R2	⚠	Caution! Expired ScreenTape device
E1	2.14	B3 P R2	⚠	Caution! Expired ScreenTape device
F1	2.79	C3 P R2	⚠	Caution! Expired ScreenTape device
G1	2.67	D3 P R2	⚠	Caution! Expired ScreenTape device
H1	1.49	E3 P R2	⚠	Caution! Expired ScreenTape device
A2		E1 M R1	⚠	Caution! Expired ScreenTape device
B2		E6 M R1	⚠	Caution! Expired ScreenTape device
C2	0.126	G2 M R2	⚠	Caution! Expired ScreenTape device
D2	0.566	H2 M R2	⚠	Caution! Expired ScreenTape device
E2	0.388	A3 M R2	⚠	Caution! Expired ScreenTape device
F2		B3 M R2	⚠	Marker(s) not detected; Caution! Expired ScreenTape device
G2	0.309	C3 M R2	⚠	Caution! Expired ScreenTape device
H2	0.185	D3 M R2	⚠	Caution! Expired ScreenTape device

© Agilent Technologies, Inc. 2017

A1: Ladder

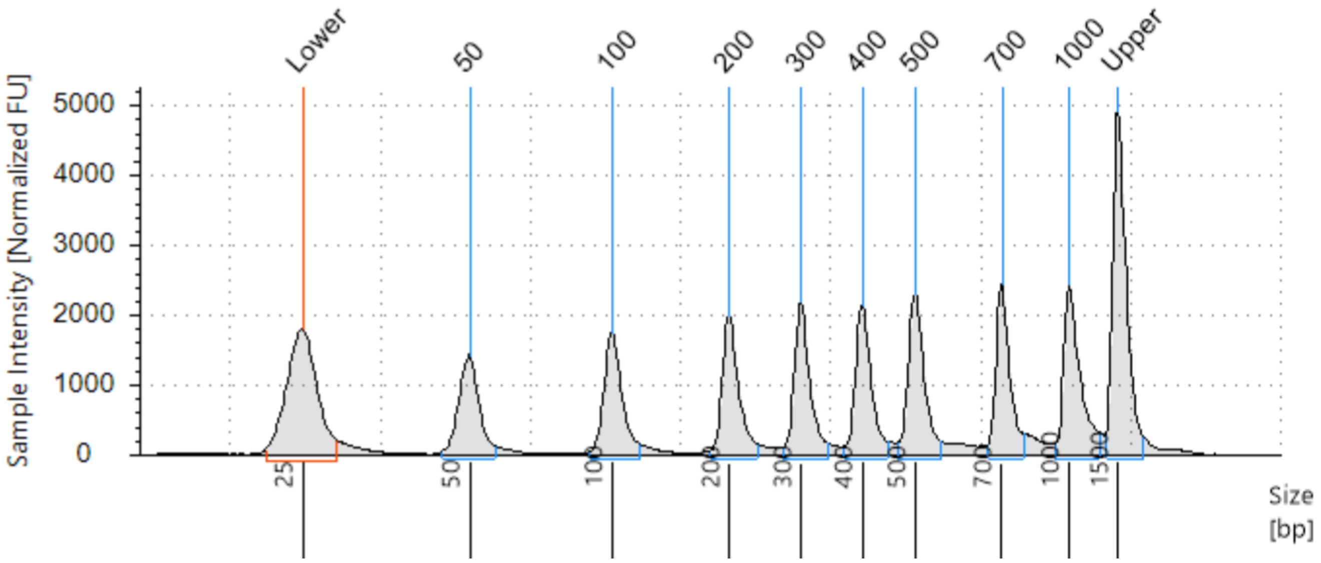

Sample Table

Well	Conc. [ng/µl]	Sample Description	Alert	Observations
A1	25.9	Ladder	⚠	Caution! Expired ScreenTape device; Ladder

Peak Table

Size [bp]	Calibrated Conc. [ng/µl]	Assigned Conc. [ng/µl]	Peak Molarity [nmol/l]	% Integrated Area	Peak Comment	Observations
25	5.21	-	321	-		Lower Marker
50	2.72	-	83.6	10.49		
100	3.01	-	46.3	11.60		
200	3.12	-	24.0	12.04		
300	3.24	-	16.6	12.50		
400	3.25	-	12.5	12.53		
500	3.53	-	10.9	13.62		
700	3.26	-	7.16	12.57		
1000	3.79	-	5.84	14.64		
1500	6.50	6.50	6.67	-		Upper Marker

 © Agilent Technologies, Inc. 2017

B1: G2 P R2

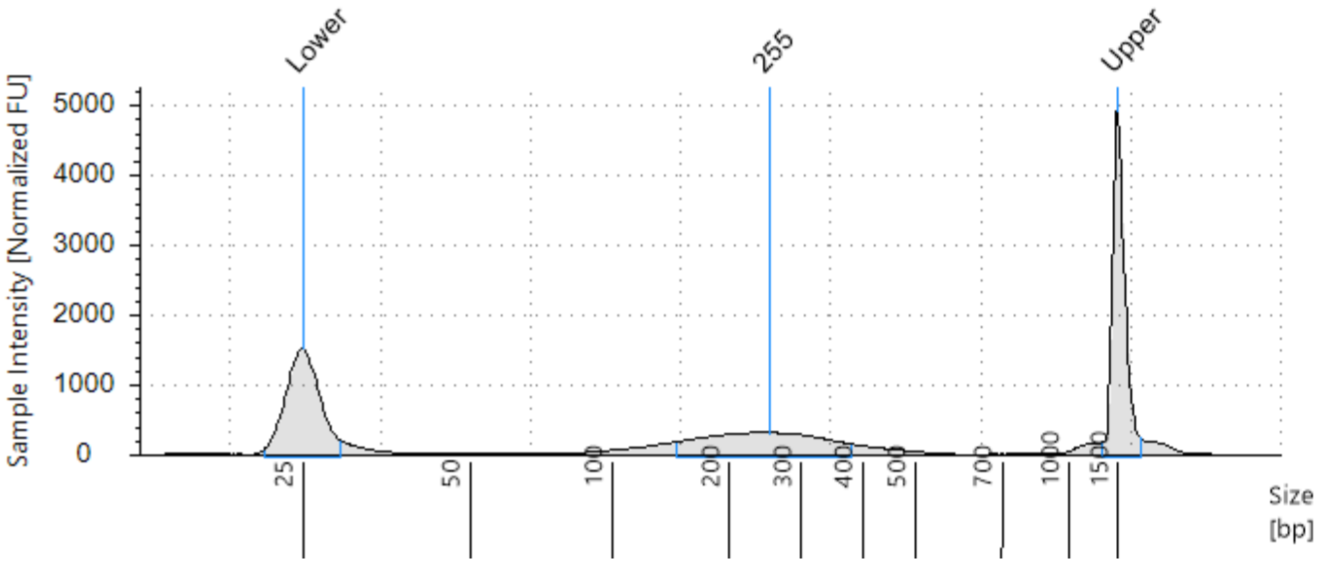

Sample Table

Well	Conc. [ng/µl]	Sample Description	Alert	Observations
B1	4.11	G2 P R2	⚠	Caution! Expired ScreenTape device

Peak Table

Size [bp]	Calibrated Conc. [ng/µl]	Assigned Conc. [ng/µl]	Peak Molarity [nmol/l]	% Integrated Area	Peak Comment	Observations
25	5.53	-	340	-		Lower Marker
255	4.11	-	24.8	100.00		
1500	6.50	6.50	6.67	-		Upper Marker

© Agilent Technologies, Inc. 2017

C1: H2 P R2

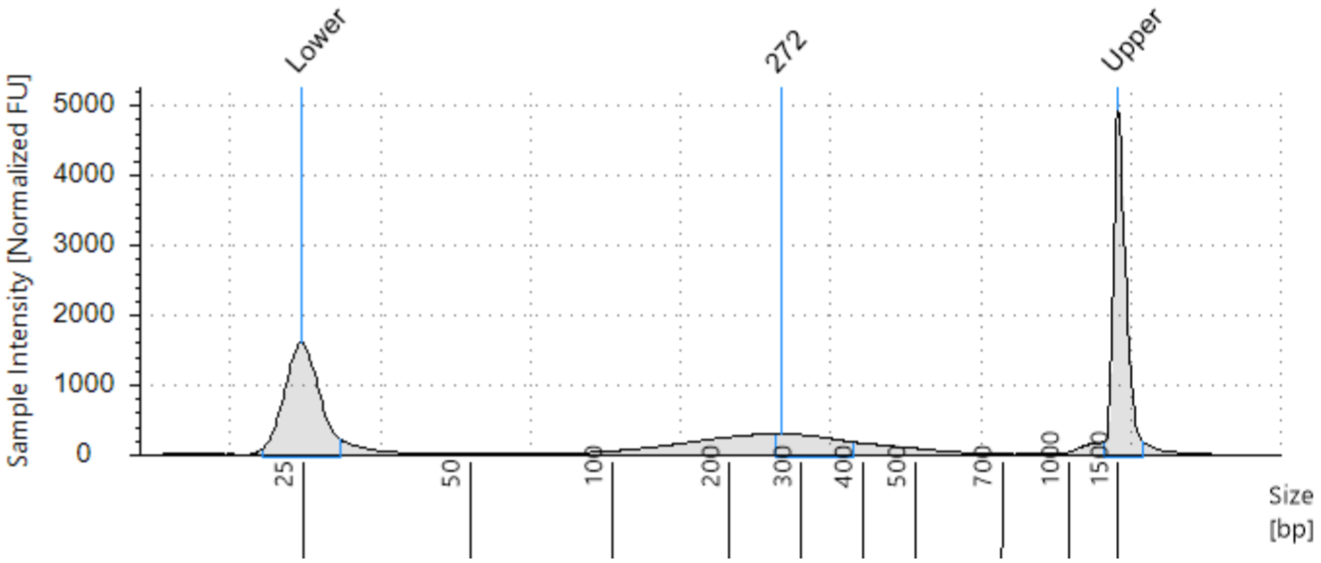

Sample Table

Well	Conc. [ng/µl]	Sample Description	Alert	Observations
C1	1.75	H2 P R2	⚠	Caution! Expired ScreenTape device

Peak Table

Size [bp]	Calibrated Conc. [ng/µl]	Assigned Conc. [ng/µl]	Peak Molarity [nmol/l]	% Integrated Area	Peak Comment	Observations
25	5.74	-	353	-		Lower Marker
272	1.75	-	9.90	100.00		
1500	6.50	6.50	6.67	-		Upper Marker

© Agilent Technologies, Inc. 2017

D1: A3 P R2

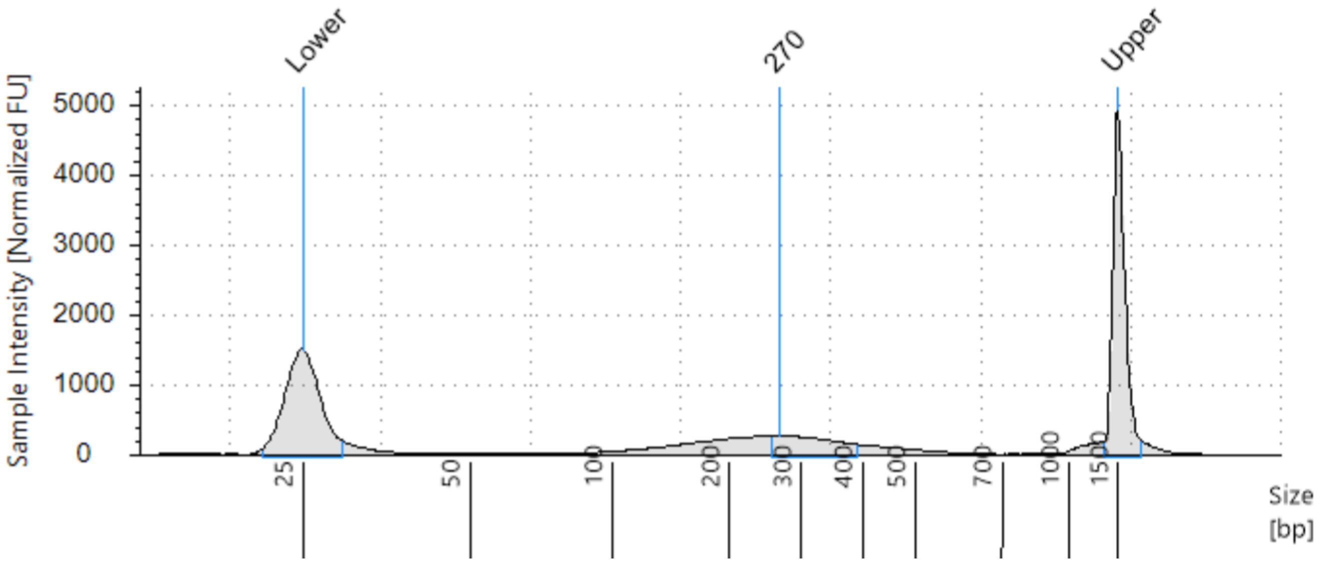

Sample Table

Well	Conc. [ng/µl]	Sample Description	Alert	Observations
D1	1.75	A3 P R2	⚠	Caution! Expired ScreenTape device

Peak Table

Size [bp]	Calibrated Conc. [ng/µl]	Assigned Conc. [ng/µl]	Peak Molarity [nmol/l]	% Integrated Area	Peak Comment	Observations
25	5.73	-	352	-		Lower Marker
270	1.75	-	9.96	100.00		
1500	6.50	6.50	6.67	-		Upper Marker

© Agilent Technologies, Inc. 2017

E1: B3 P R2

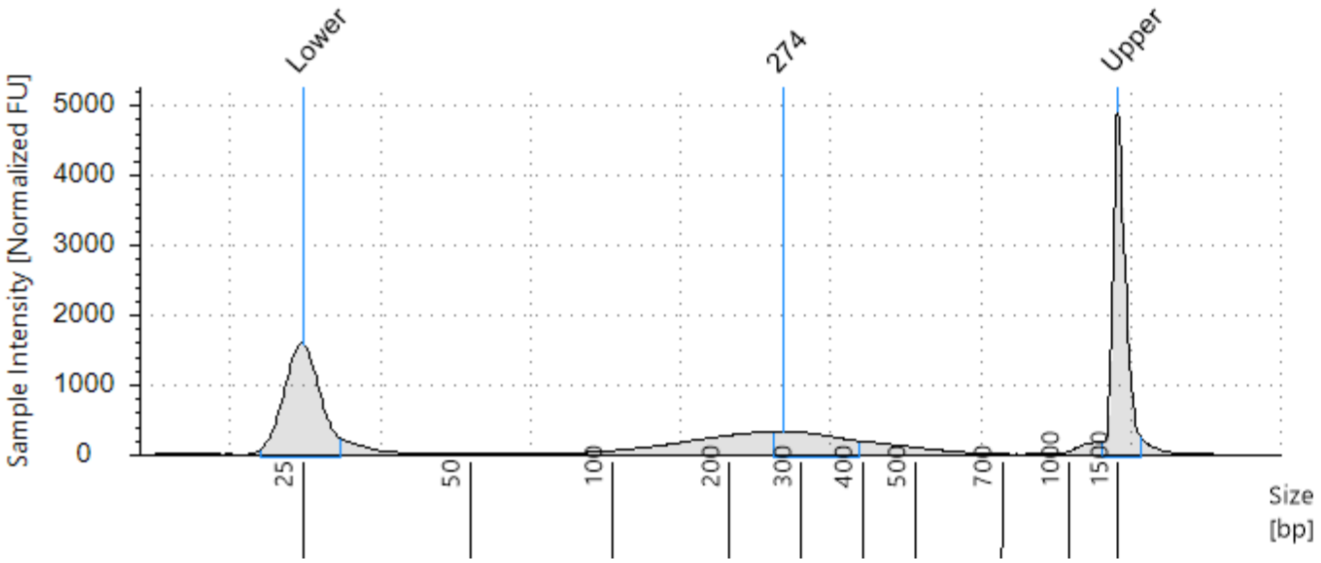

Sample Table

Well	Conc. [ng/µl]	Sample Description	Alert	Observations
E1	2.14	B3 P R2	⚠	Caution! Expired ScreenTape device

Peak Table

Size [bp]	Calibrated Conc. [ng/µl]	Assigned Conc. [ng/µl]	Peak Molarity [nmol/l]	% Integrated Area	Peak Comment	Observations
25	5.92	-	364	-		Lower Marker
274	2.14	-	12.0	100.00		
1500	6.50	6.50	6.67	-		Upper Marker

© Agilent Technologies, Inc. 2017

F1: C3 P R2

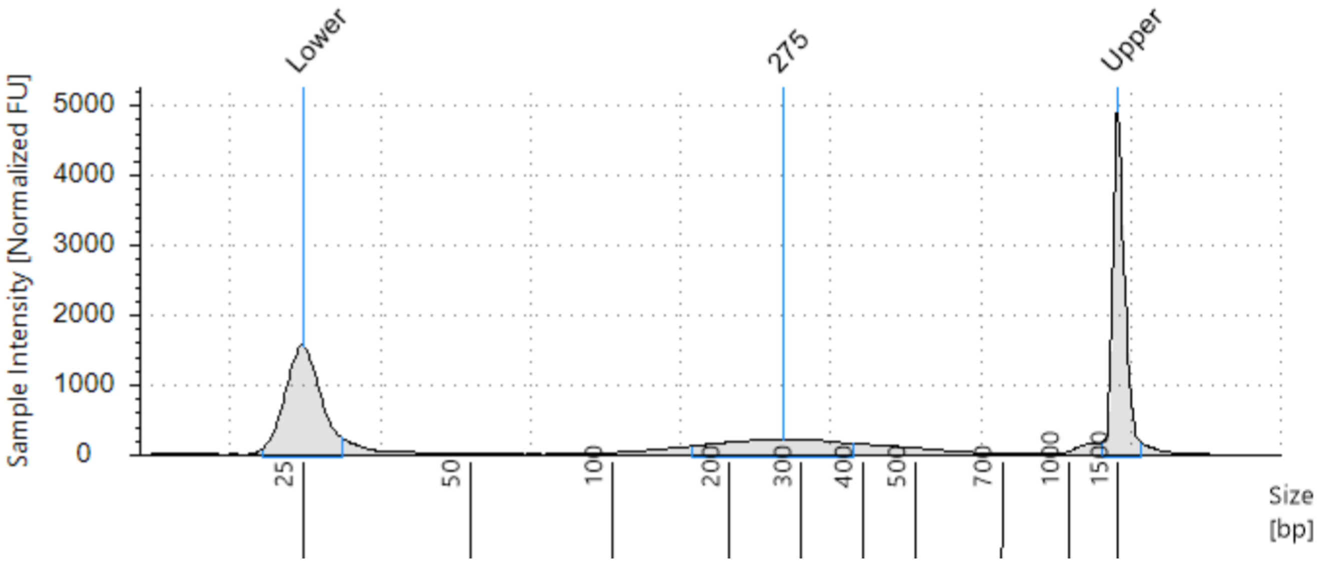

Sample Table

Well	Conc. [ng/µl]	Sample Description	Alert	Observations
F1	2.79	C3 P R2	⚠	Caution! Expired ScreenTape device

Peak Table

Size [bp]	Calibrated Conc. [ng/µl]	Assigned Conc. [ng/µl]	Peak Molarity [nmol/l]	% Integrated Area	Peak Comment	Observations
25	6.01	-	370	-		Lower Marker
275	2.79	-	15.6	100.00		
1500	6.50	6.50	6.67	-		Upper Marker

© Agilent Technologies, Inc. 2017

G1: D3 P R2

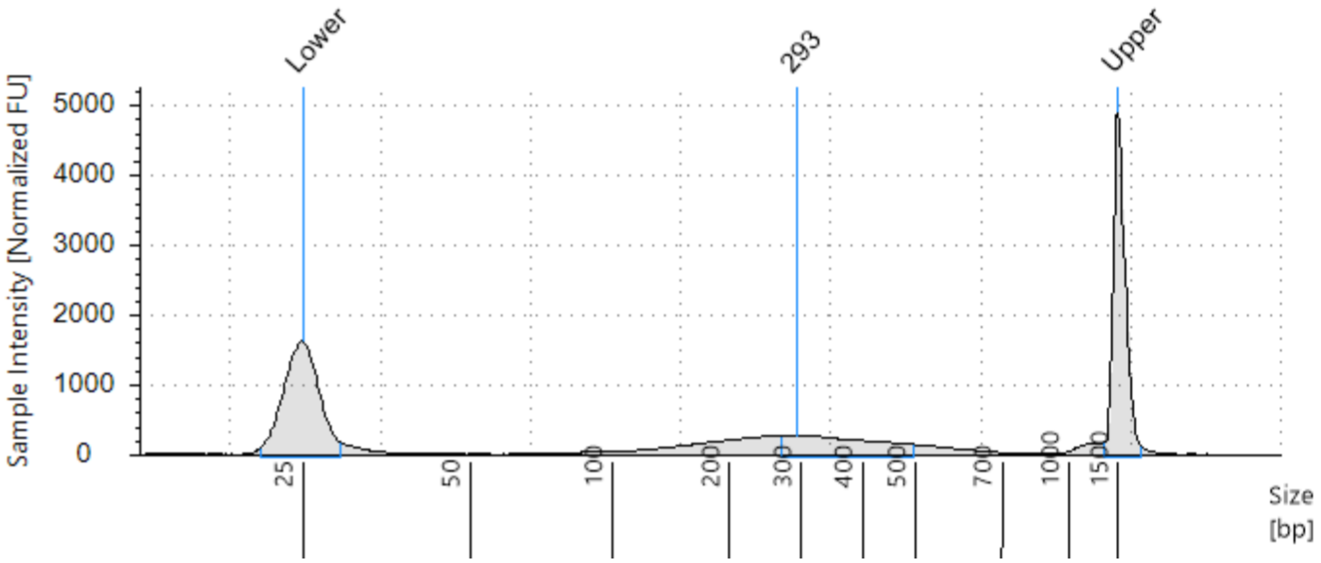

Sample Table

Well	Conc. [ng/µl]	Sample Description	Alert	Observations
G1	2.67	D3 P R2	⚠	Caution! Expired ScreenTape device

Peak Table

Size [bp]	Calibrated Conc. [ng/µl]	Assigned Conc. [ng/µl]	Peak Molarity [nmol/l]	% Integrated Area	Peak Comment	Observations
25	6.28	-	387	-		Lower Marker
293	2.67	-	14.0	100.00		
1500	6.50	6.50	6.67	-		Upper Marker

© Agilent Technologies, Inc. 2017

H1: E3 P R2

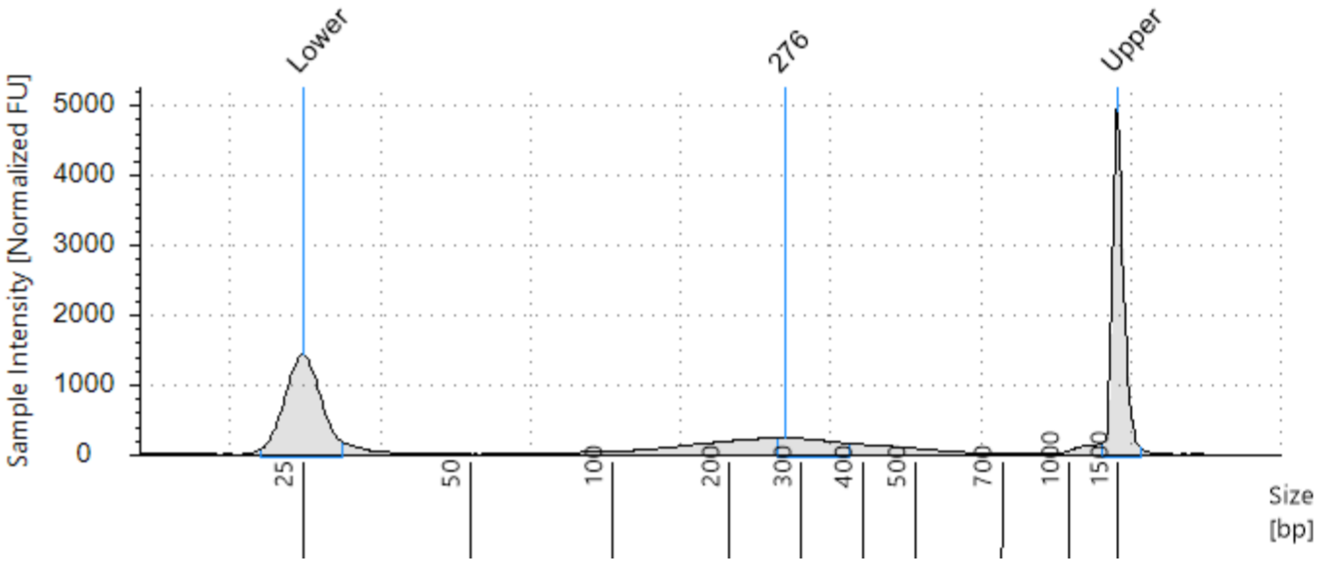

Sample Table

Well	Conc. [ng/µl]	Sample Description	Alert	Observations
H1	1.49	E3 P R2	⚠	Caution! Expired ScreenTape device

Peak Table

Size [bp]	Calibrated Conc. [ng/µl]	Assigned Conc. [ng/µl]	Peak Molarity [nmol/l]	% Integrated Area	Peak Comment	Observations
25	5.98	-	368	-		Lower Marker
276	1.49	-	8.32	100.00		
1500	6.50	6.50	6.67	-		Upper Marker

© Agilent Technologies, Inc. 2017

A2: E1 M R1

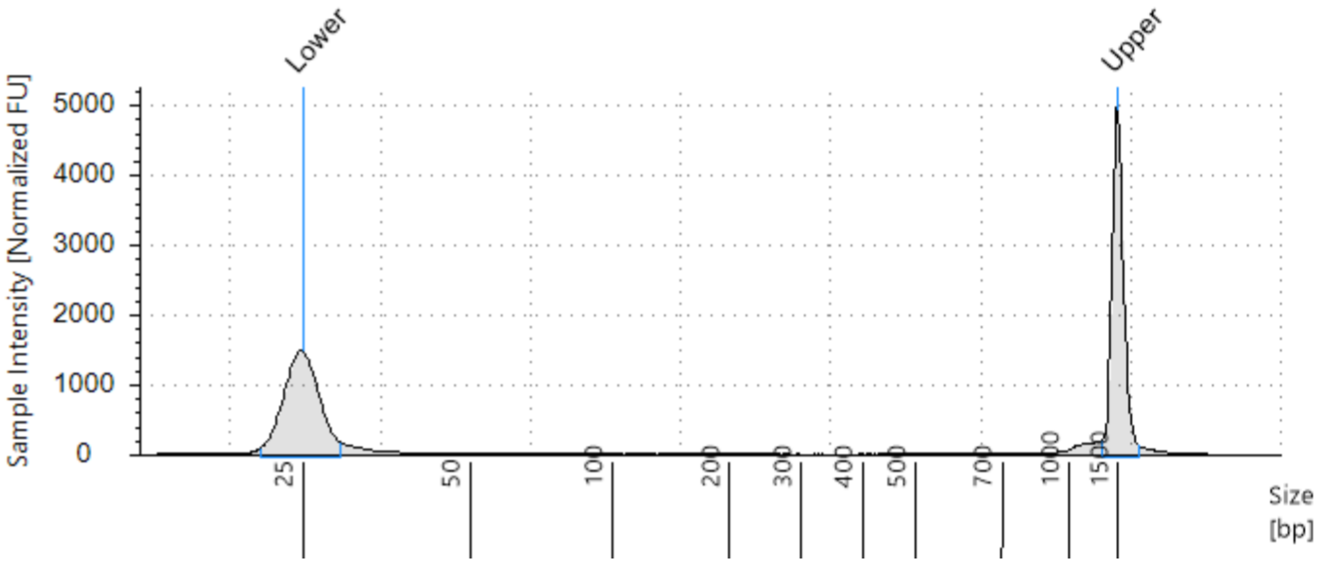

Sample Table

Well	Conc. [ng/µl]	Sample Description	Alert	Observations
A2		E1 M R1	⚠	Caution! Expired ScreenTape device

Peak Table

Size [bp]	Calibrated Conc. [ng/µl]	Assigned Conc. [ng/µl]	Peak Molarity [nmol/l]	% Integrated Area	Peak Comment	Observations
25	5.92	-	364	-		Lower Marker
1500	6.50	6.50	6.67	-		Upper Marker

 © Agilent Technologies, Inc. 2017

B2: E6 M R1

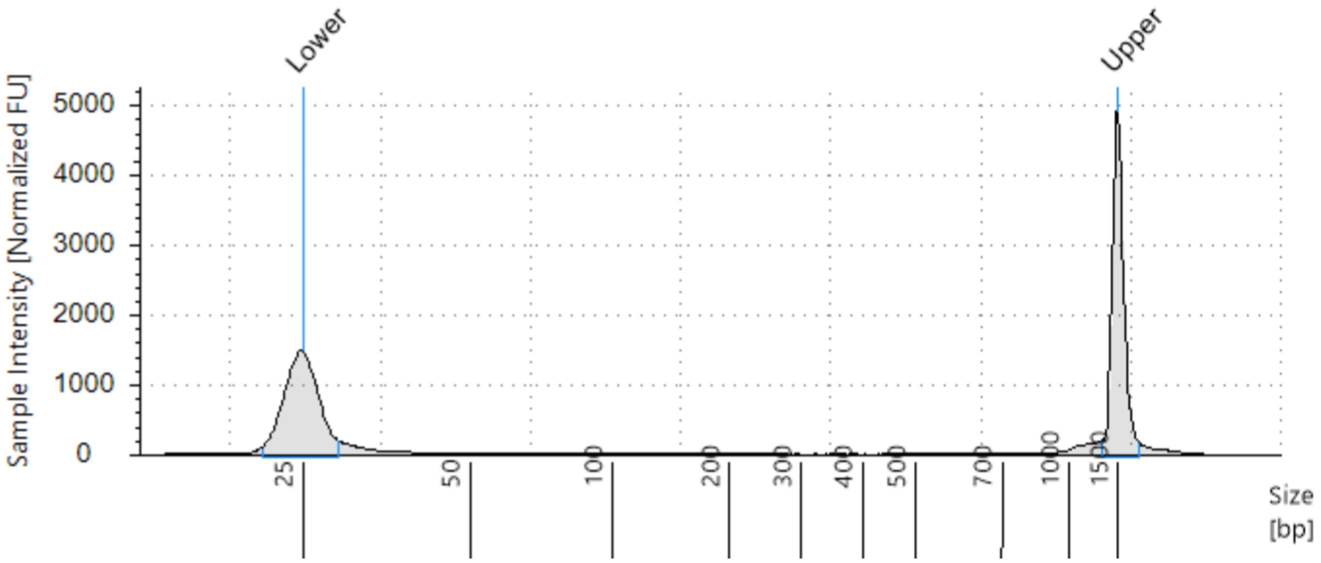

Sample Table

Well	Conc. [ng/µl]	Sample Description	Alert	Observations
B2		E6 M R1	⚠	Caution! Expired ScreenTape device

Peak Table

Size [bp]	Calibrated Conc. [ng/µl]	Assigned Conc. [ng/µl]	Peak Molarity [nmol/l]	% Integrated Area	Peak Comment	Observations
25	5.86	-	360	-		Lower Marker
1500	6.50	6.50	6.67	-		Upper Marker

© Agilent Technologies, Inc. 2017

C2: G2 M R2

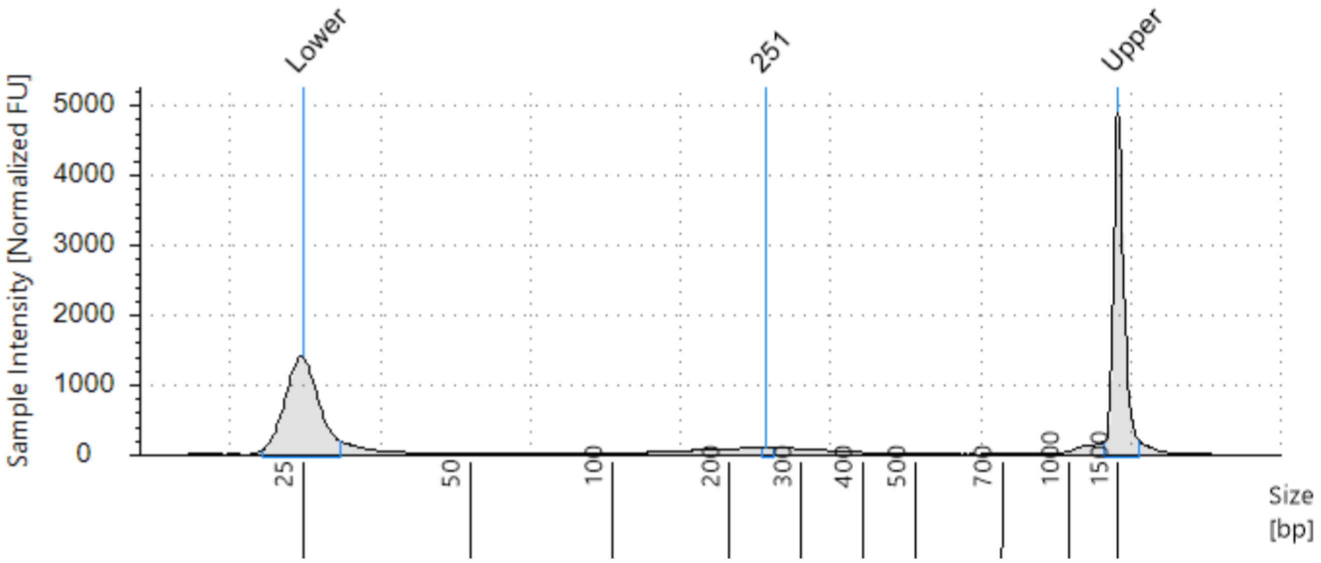

Sample Table

Well	Conc. [ng/µl]	Sample Description	Alert	Observations
C2	0.126	G2 M R2	⚠	Caution! Expired ScreenTape device

Peak Table

Size [bp]	Calibrated Conc. [ng/µl]	Assigned Conc. [ng/µl]	Peak Molarity [nmol/l]	% Integrated Area	Peak Comment	Observations
25	5.75	-	354	-		Lower Marker
251	0.126	-	0.774	100.00		
1500	6.50	6.50	6.67	-		Upper Marker

© Agilent Technologies, Inc. 2017

D2: H2 M R2

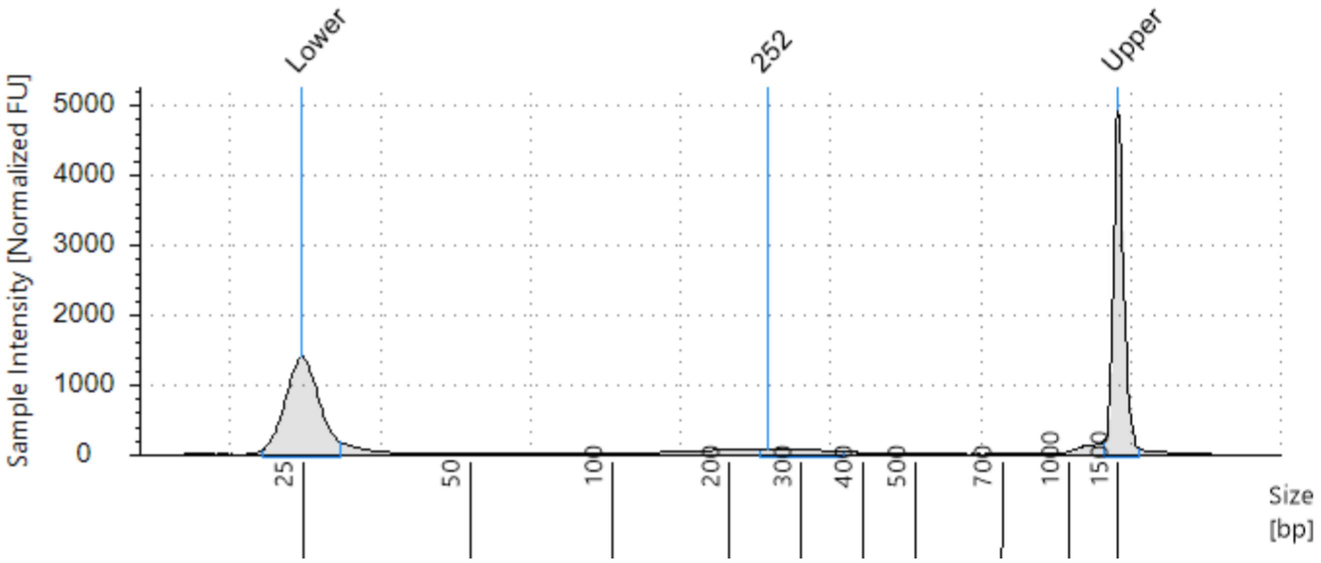

Sample Table

Well	Conc. [ng/µl]	Sample Description	Alert	Observations
D2	0.566	H2 M R2	⚠	Caution! Expired ScreenTape device

Peak Table

Size [bp]	Calibrated Conc. [ng/µl]	Assigned Conc. [ng/µl]	Peak Molarity [nmol/l]	% Integrated Area	Peak Comment	Observations
25	5.79	-	357	-		Lower Marker
252	0.566	-	3.46	100.00		
1500	6.50	6.50	6.67	-		Upper Marker

 © Agilent Technologies, Inc. 2017

E2: A3 M R2

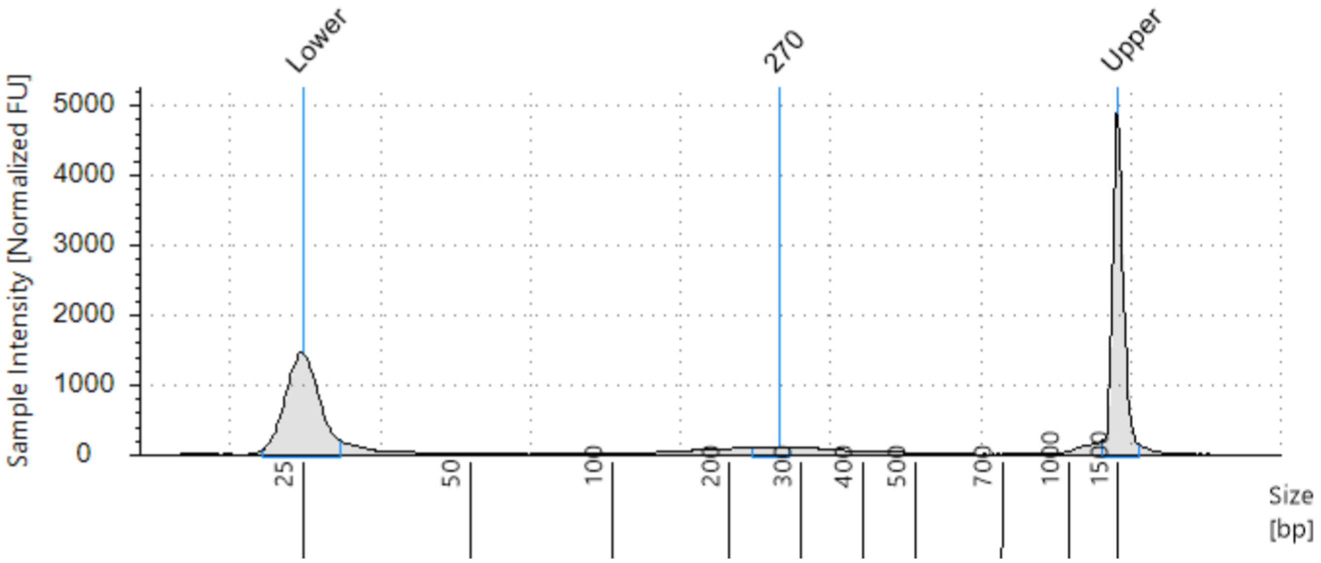

Sample Table

Well	Conc. [ng/µl]	Sample Description	Alert	Observations
E2	0.388	A3 M R2	⚠	Caution! Expired ScreenTape device

Peak Table

Size [bp]	Calibrated Conc. [ng/µl]	Assigned Conc. [ng/µl]	Peak Molarity [nmol/l]	% Integrated Area	Peak Comment	Observations
25	5.98	-	368	-		Lower Marker
270	0.388	-	2.21	100.00		
1500	6.50	6.50	6.67	-		Upper Marker

© Agilent Technologies, Inc. 2017

F2: B3 M R2

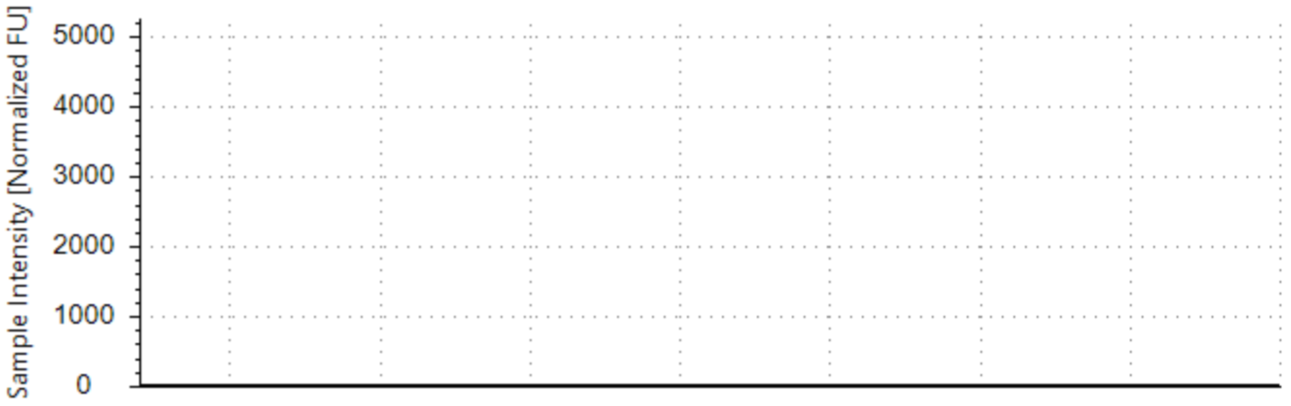

Sample Table

Well	Conc. [ng/µl]	Sample Description	Alert	Observations
F2		B3 M R2	▲	Marker(s) not detected; Caution! Expired ScreenTape device

© Agilent Technologies, Inc. 2017

G2: C3 M R2

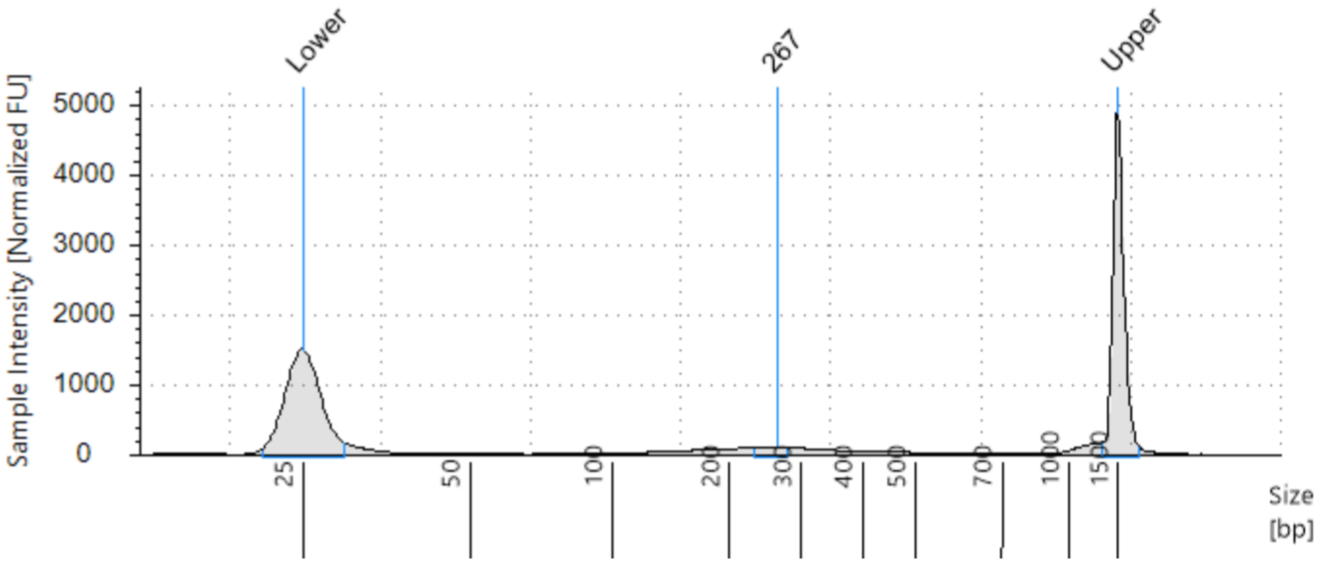

Sample Table

Well	Conc. [ng/µl]	Sample Description	Alert	Observations
G2	0.309	C3 M R2	⚠	Caution! Expired ScreenTape device

Peak Table

Size [bp]	Calibrated Conc. [ng/µl]	Assigned Conc. [ng/µl]	Peak Molarity [nmol/l]	% Integrated Area	Peak Comment	Observations
25	6.34	-	390	-		Lower Marker
267	0.309	-	1.78	100.00		
1500	6.50	6.50	6.67	-		Upper Marker

H2: D3 M R2

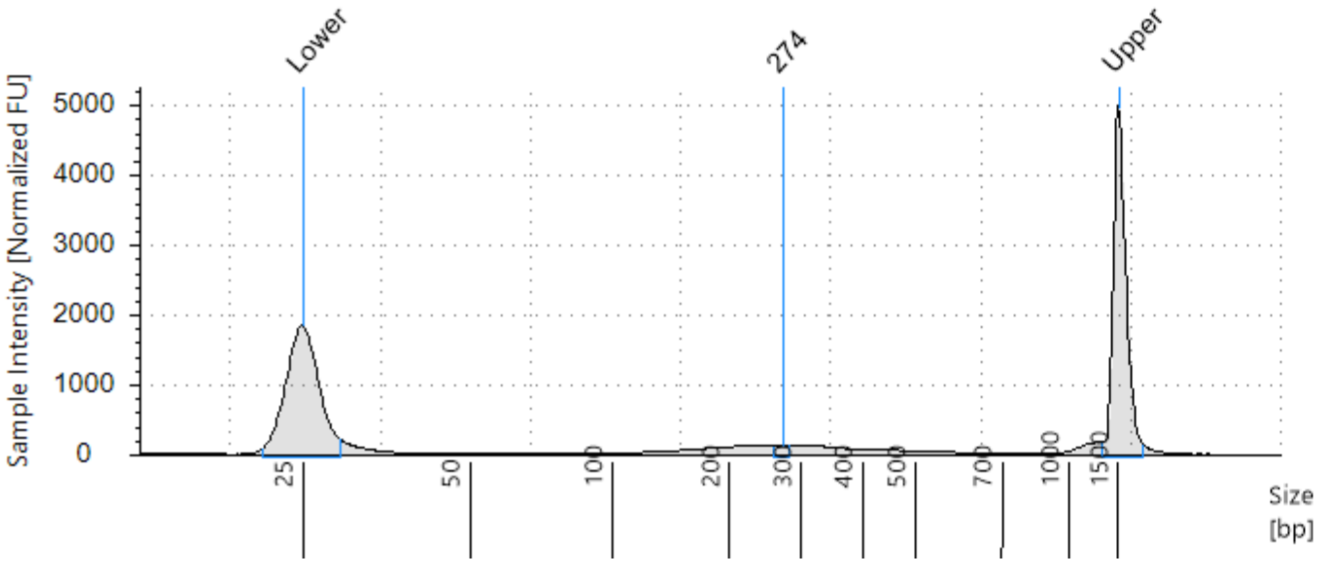

Sample Table

Well	Conc. [ng/µl]	Sample Description	Alert	Observations
H2	0.185	D3 M R2	⚠	Caution! Expired ScreenTape device

Peak Table

Size [bp]	Calibrated Conc. [ng/µl]	Assigned Conc. [ng/µl]	Peak Molarity [nmol/l]	% Integrated Area	Peak Comment	Observations
25	6.26	-	385	-		Lower Marker
274	0.185	-	1.04	100.00		
1500	6.50	6.50	6.67	-		Upper Marker

© Agilent Technologies, Inc. 2017

Filename: 2020-09-11-04- Q-S, MINUS, E3-B5, D1000 R2.D1000

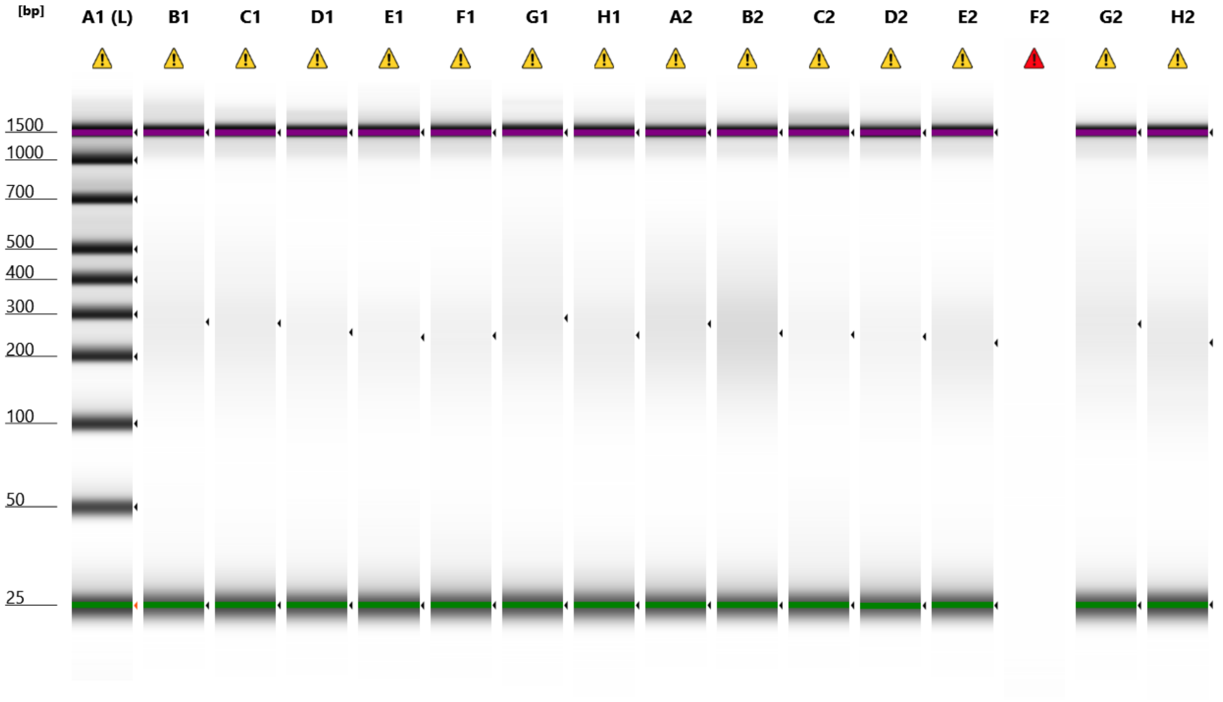

Default image (Contrast 100%)

Sample Info

Well	Conc. [ng/µl]	Sample Description	Alert	Observations
A1	29.4	Ladder	⚠	Caution! Expired ScreenTape device; Ladder
B1	0.101	E3 M R2	⚠	Caution! Expired ScreenTape device
C1	0.171	F3 M R2	⚠	Caution! Expired ScreenTape device
D1	0.0750	G3 M R2	⚠	Caution! Expired ScreenTape device
E1	0.295	H3 M R2	⚠	Caution! Expired ScreenTape device
F1	0.605	A4 M R2	⚠	Caution! Expired ScreenTape device
G1	1.17	B4 M R2	⚠	Caution! Expired ScreenTape device
H1	0.223	C4 M R2	⚠	Caution! Expired ScreenTape device
A2	0.336	D4 M R2	⚠	Caution! Expired ScreenTape device
B2	1.58	E4 M R2	⚠	Caution! Expired ScreenTape device
C2	0.0650	F4 M R2	⚠	Caution! Expired ScreenTape device
D2	0.0677	G4 M R2	⚠	Caution! Expired ScreenTape device
E2	0.126	H4 M R2	⚠	Caution! Expired ScreenTape device
F2			⚠	Marker(s) not detected; Caution! Expired ScreenTape device
G2	0.205	A5 M R2	⚠	Caution! Expired ScreenTape device
H2	0.182	B5 M R2	⚠	Caution! Expired ScreenTape device

 © Agilent Technologies, Inc. 2017

A1: Ladder

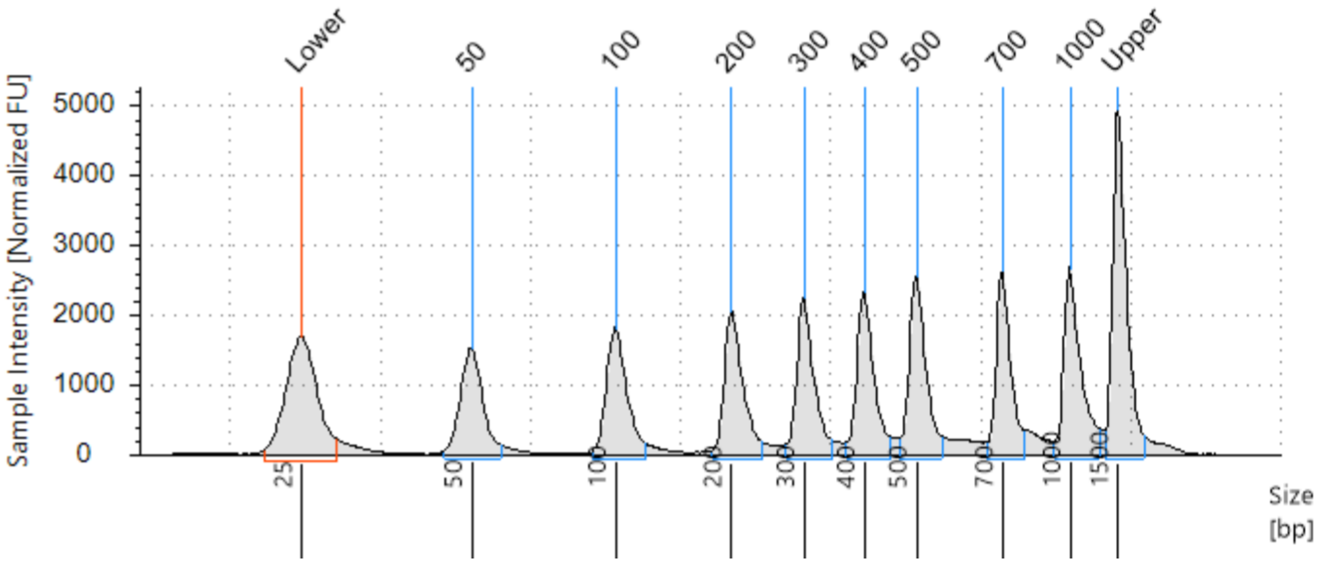

Sample Table

Well	Conc. [ng/µl]	Sample Description	Alert	Observations
A1	29.4	Ladder	⚠	Caution! Expired ScreenTape device; Ladder

Peak Table

Size [bp]	Calibrated Conc. [ng/µl]	Assigned Conc. [ng/µl]	Peak Molarity [nmol/l]	% Integrated Area	Peak Comment	Observations
25	4.94	-	304	-		Lower Marker
50	3.18	-	97.8	10.81		
100	3.48	-	53.5	11.85		
200	3.56	-	27.4	12.11		
300	3.62	-	18.6	12.32		
400	3.72	-	14.3	12.66		
500	3.97	-	12.2	13.51		
700	3.62	-	7.95	12.32		
1000	4.24	-	6.52	14.43		
1500	6.50	6.50	6.67	-		Upper Marker

© Agilent Technologies, Inc. 2017

B1: E3 M R2

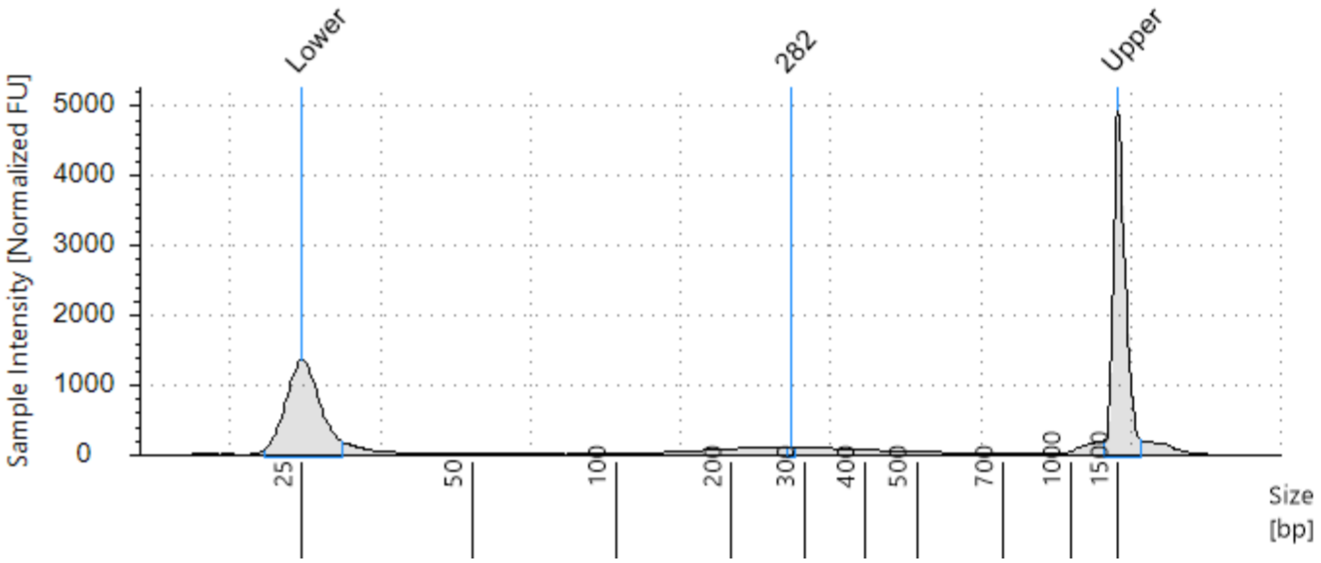

Sample Table

Well	Conc. [ng/µl]	Sample Description	Alert	Observations
B1	0.101	E3 M R2	⚠	Caution! Expired ScreenTape device

Peak Table

Size [bp]	Calibrated Conc. [ng/µl]	Assigned Conc. [ng/µl]	Peak Molarity [nmol/l]	% Integrated Area	Peak Comment	Observations
25	4.99	-	307	-		Lower Marker
282	0.101	-	0.551	100.00		
1500	6.50	6.50	6.67	-		Upper Marker

© Agilent Technologies, Inc. 2017

C1: F3 M R2

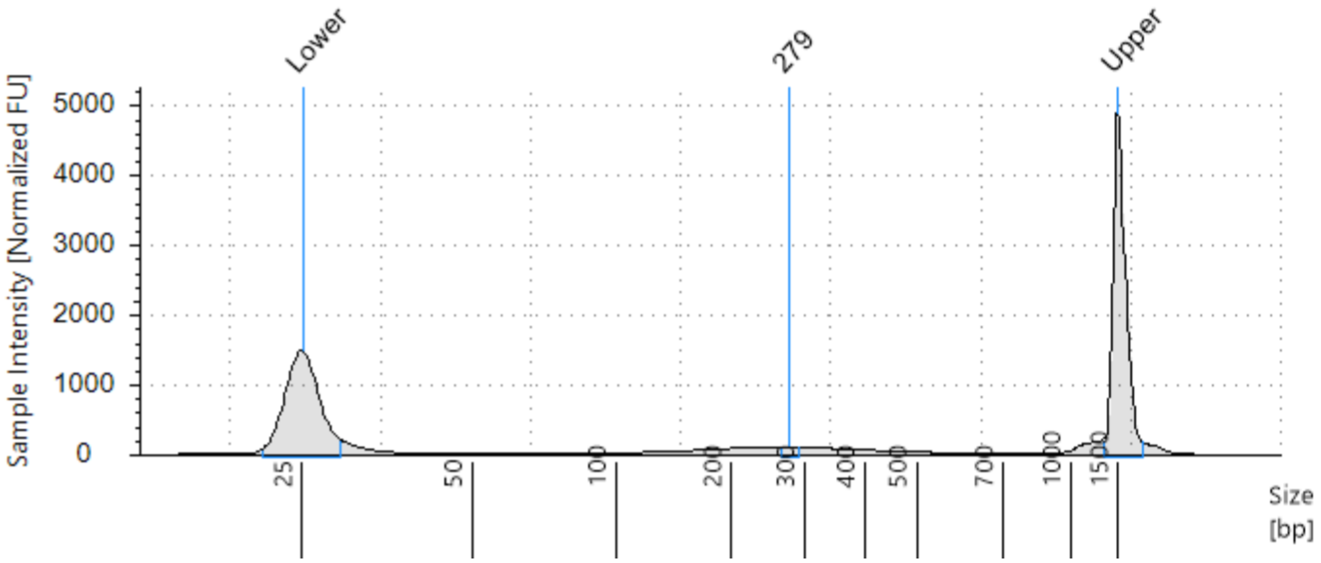

Sample Table

Well	Conc. [ng/µl]	Sample Description	Alert	Observations
C1	0.171	F3 M R2	⚠	Caution! Expired ScreenTape device

Peak Table

Size [bp]	Calibrated Conc. [ng/µl]	Assigned Conc. [ng/µl]	Peak Molarity [nmol/l]	% Integrated Area	Peak Comment	Observations
25	5.30	-	326	-		Lower Marker
279	0.171	-	0.943	100.00		
1500	6.50	6.50	6.67	-		Upper Marker

D1: G3 M R2

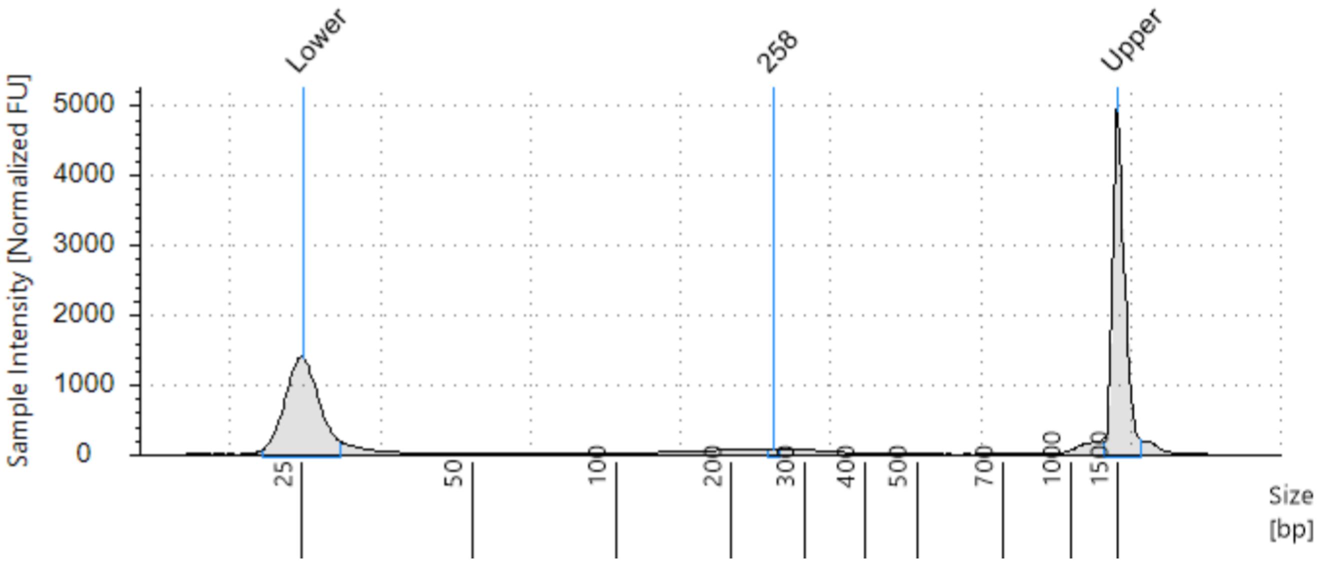

Sample Table

Well	Conc. [ng/µl]	Sample Description	Alert	Observations
D1	0.0750	G3 M R2	⚠	Caution! Expired ScreenTape device

Peak Table

Size [bp]	Calibrated Conc. [ng/µl]	Assigned Conc. [ng/µl]	Peak Molarity [nmol/l]	% Integrated Area	Peak Comment	Observations
25	5.12	-	315	-		Lower Marker
258	0.0750	-	0.448	100.00		
1500	6.50	6.50	6.67	-		Upper Marker

© Agilent Technologies, Inc. 2017

E1: H3 M R2

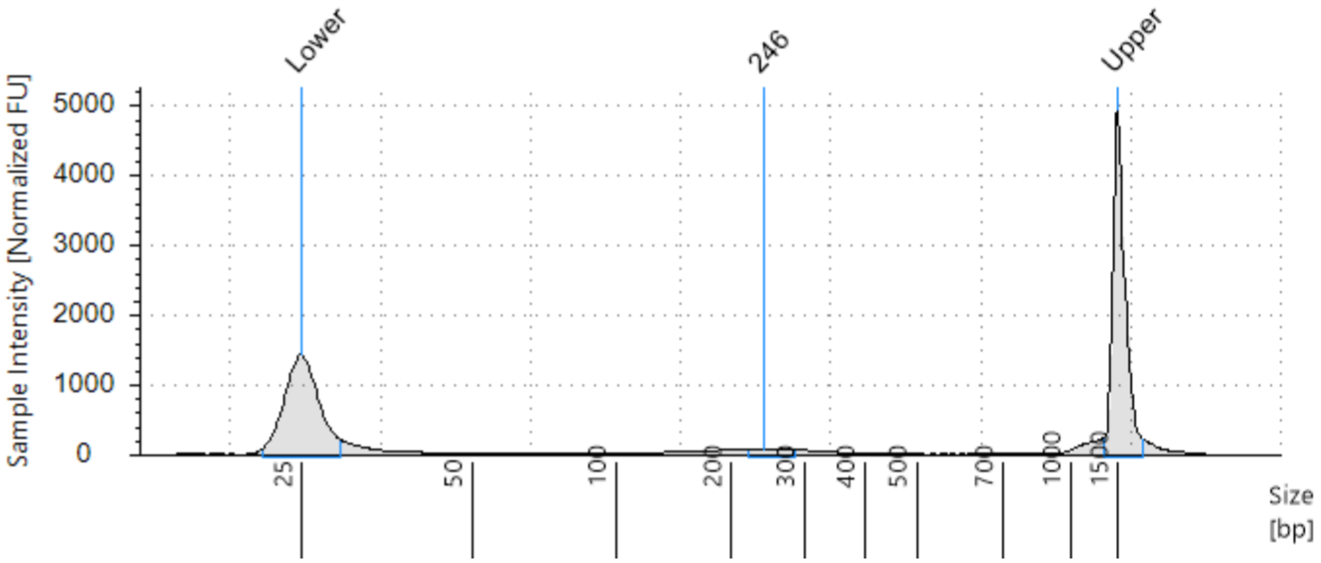

Sample Table

Well	Conc. [ng/µl]	Sample Description	Alert	Observations
E1	0.295	H3 M R2	⚠	Caution! Expired ScreenTape device

Peak Table

Size [bp]	Calibrated Conc. [ng/µl]	Assigned Conc. [ng/µl]	Peak Molarity [nmol/l]	% Integrated Area	Peak Comment	Observations
25	5.25	-	323	-		Lower Marker
246	0.295	-	1.85	100.00		
1500	6.50	6.50	6.67	-		Upper Marker

© Agilent Technologies, Inc. 2017

F1: A4 M R2

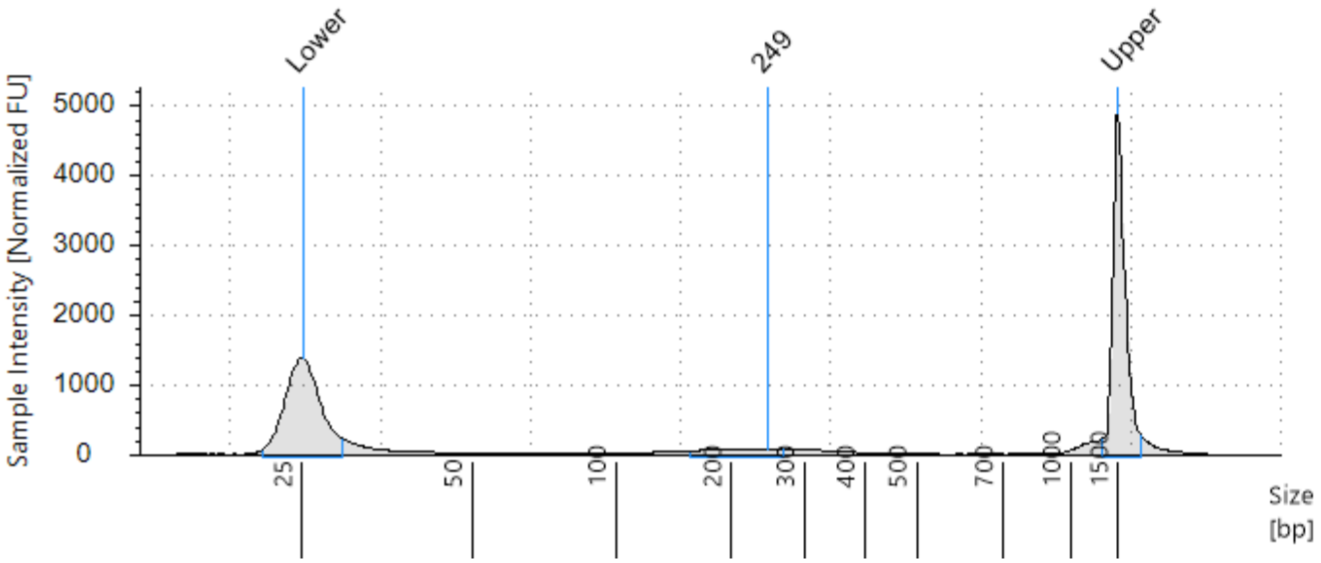

Sample Table

Well	Conc. [ng/µl]	Sample Description	Alert	Observations
F1	0.605	A4 M R2	⚠	Caution! Expired ScreenTape device

Peak Table

Size [bp]	Calibrated Conc. [ng/µl]	Assigned Conc. [ng/µl]	Peak Molarity [nmol/l]	% Integrated Area	Peak Comment	Observations
25	5.21	-	320	-		Lower Marker
249	0.605	-	3.73	100.00		
1500	6.50	6.50	6.67	-		Upper Marker

© Agilent Technologies, Inc. 2017

G1: B4 M R2

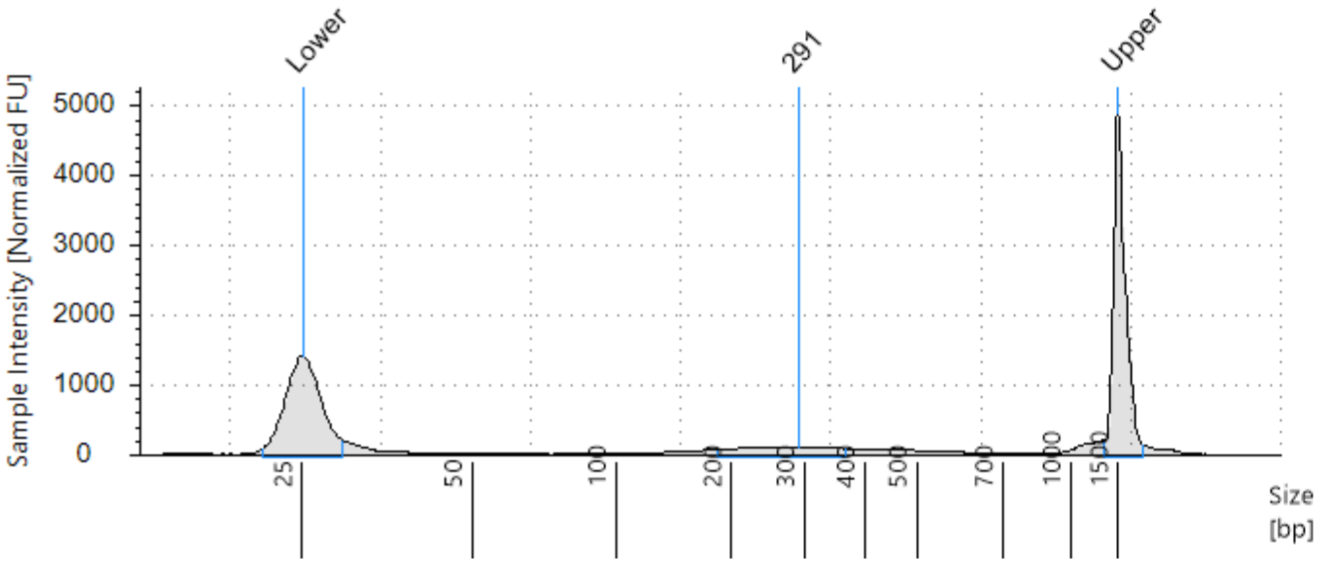

Sample Table

Well	Conc. [ng/µl]	Sample Description	Alert	Observations
G1	1.17	B4 M R2	⚠	Caution! Expired ScreenTape device

Peak Table

Size [bp]	Calibrated Conc. [ng/µl]	Assigned Conc. [ng/µl]	Peak Molarity [nmol/l]	% Integrated Area	Peak Comment	Observations
25	5.54	-	341	-		Lower Marker
291	1.17	-	6.16	100.00		
1500	6.50	6.50	6.67	-		Upper Marker

 © Agilent Technologies, Inc. 2017

H1: C4 M R2

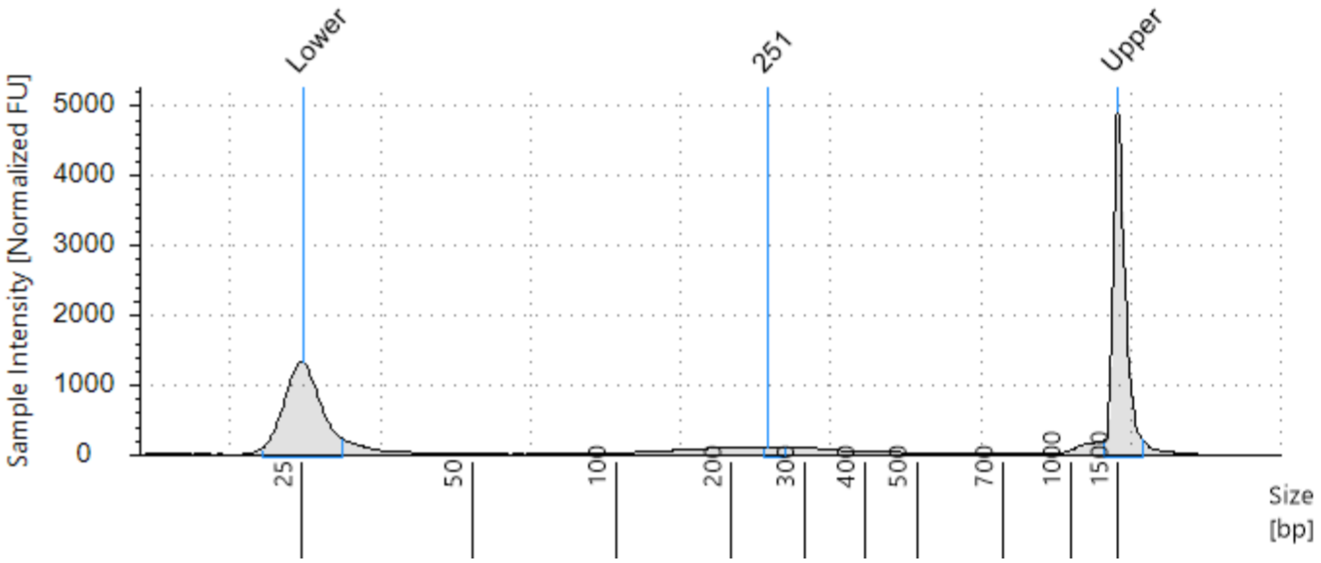

Sample Table

Well	Conc. [ng/µl]	Sample Description	Alert	Observations
H1	0.223	C4 M R2	⚠	Caution! Expired ScreenTape device

Peak Table

Size [bp]	Calibrated Conc. [ng/µl]	Assigned Conc. [ng/µl]	Peak Molarity [nmol/l]	% Integrated Area	Peak Comment	Observations
25	5.24	-	322	-		Lower Marker
251	0.223	-	1.37	100.00		
1500	6.50	6.50	6.67	-		Upper Marker

© Agilent Technologies, Inc. 2017

A2: D4 M R2

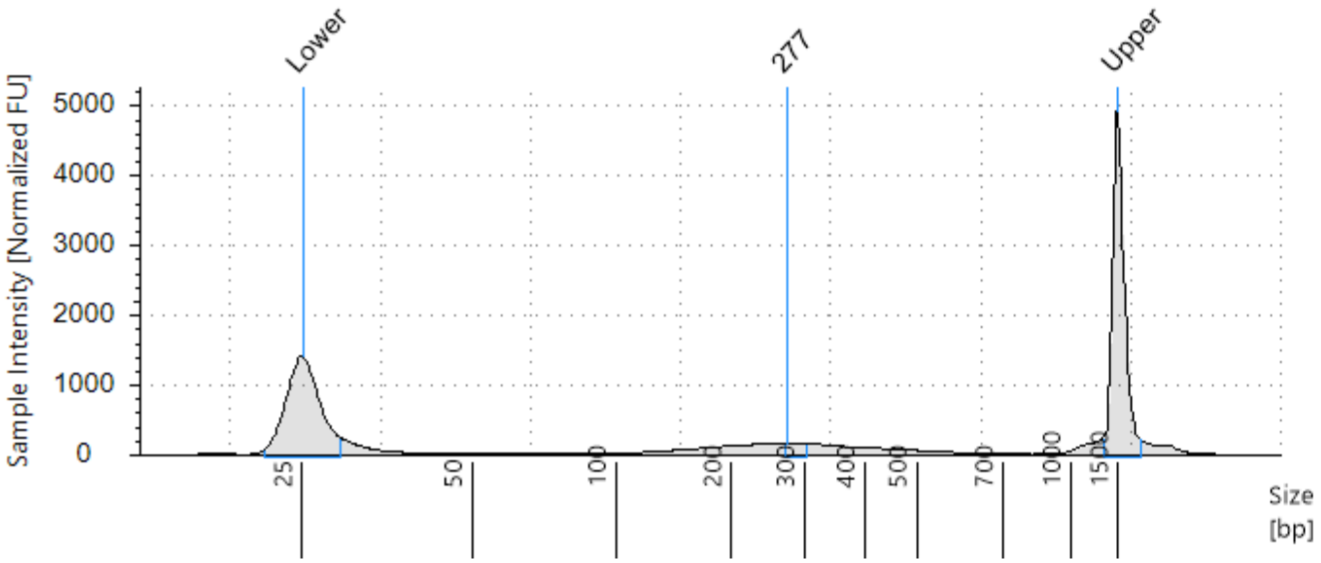

Sample Table

Well	Conc. [ng/µl]	Sample Description	Alert	Observations
A2	0.336	D4 M R2	⚠	Caution! Expired ScreenTape device

Peak Table

Size [bp]	Calibrated Conc. [ng/µl]	Assigned Conc. [ng/µl]	Peak Molarity [nmol/l]	% Integrated Area	Peak Comment	Observations
25	5.26	-	324	-		Lower Marker
277	0.336	-	1.86	100.00		
1500	6.50	6.50	6.67	-		Upper Marker

© Agilent Technologies, Inc. 2017

B2: E4 M R2

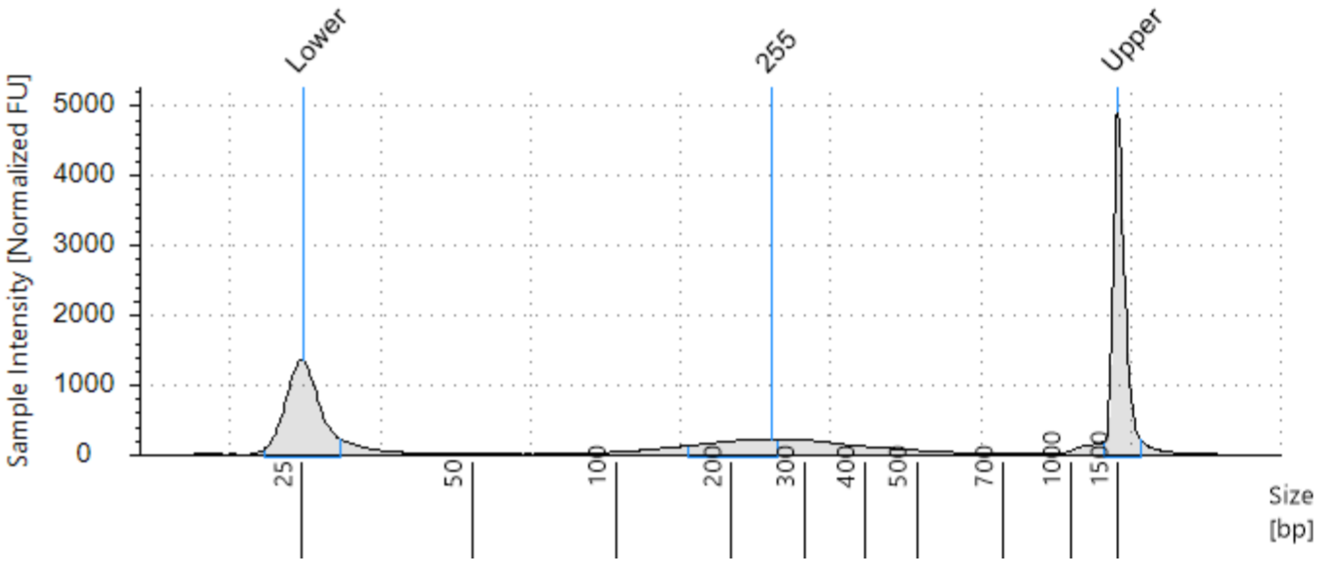

Sample Table

Well	Conc. [ng/µl]	Sample Description	Alert	Observations
B2	1.58	E4 M R2	⚠	Caution! Expired ScreenTape device

Peak Table

Size [bp]	Calibrated Conc. [ng/µl]	Assigned Conc. [ng/µl]	Peak Molarity [nmol/l]	% Integrated Area	Peak Comment	Observations
25	5.04	-	310	-		Lower Marker
255	1.58	-	9.51	100.00		
1500	6.50	6.50	6.67	-		Upper Marker

© Agilent Technologies, Inc. 2017

C2: F4 M R2

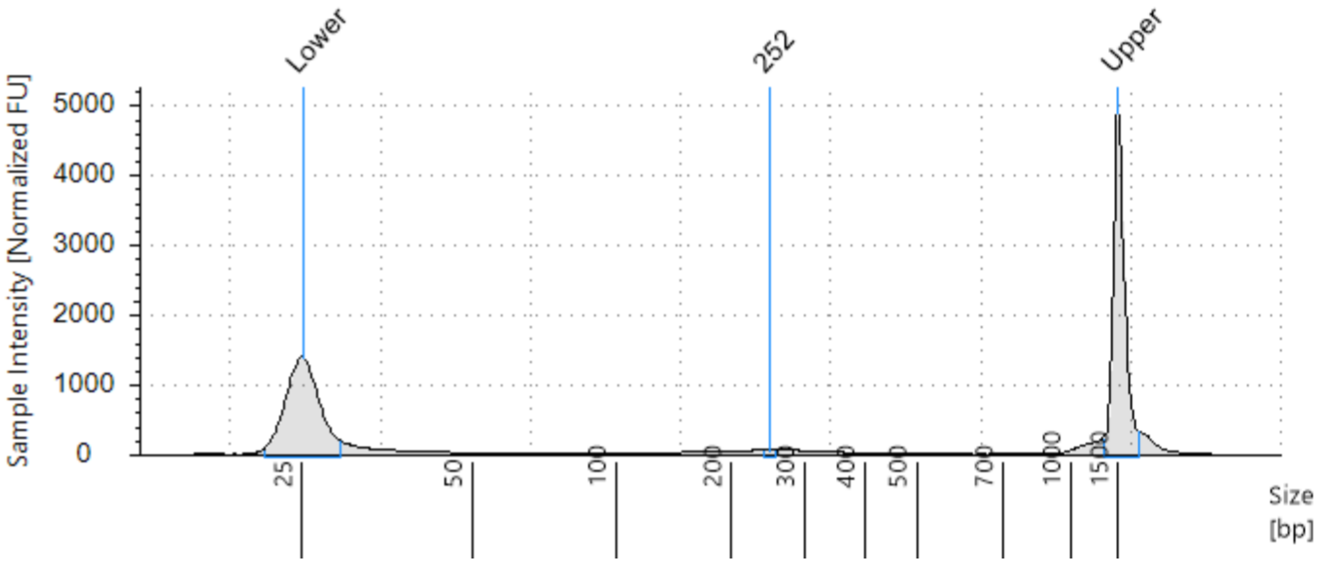

Sample Table

Well	Conc. [ng/µl]	Sample Description	Alert	Observations
C2	0.0650	F4 M R2	⚠	Caution! Expired ScreenTape device

Peak Table

Size [bp]	Calibrated Conc. [ng/µl]	Assigned Conc. [ng/µl]	Peak Molarity [nmol/l]	% Integrated Area	Peak Comment	Observations
25	5.38	-	331	-		Lower Marker
252	0.0650	-	0.397	100.00		
1500	6.50	6.50	6.67	-		Upper Marker

© Agilent Technologies, Inc. 2017

D2: G4 M R2

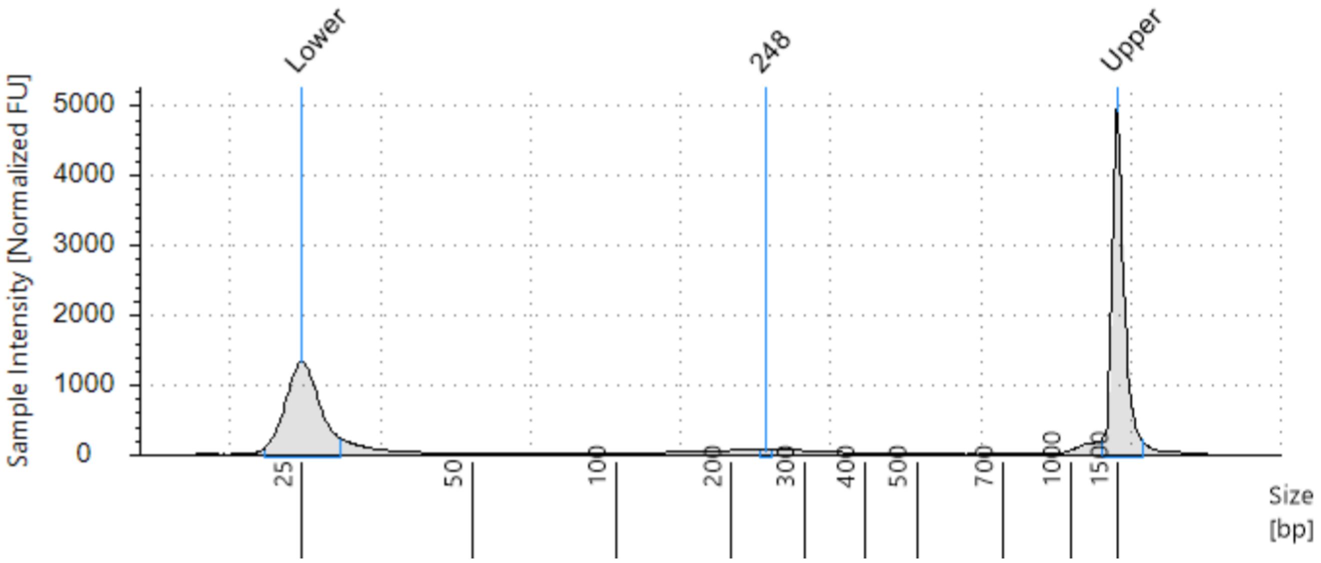

Sample Table

Well	Conc. [ng/µl]	Sample Description	Alert	Observations
D2	0.0677	G4 M R2	⚠	Caution! Expired ScreenTape device

Peak Table

Size [bp]	Calibrated Conc. [ng/µl]	Assigned Conc. [ng/µl]	Peak Molarity [nmol/l]	% Integrated Area	Peak Comment	Observations
25	4.95	-	305	-		Lower Marker
248	0.0677	-	0.420	100.00		
1500	6.50	6.50	6.67	-		Upper Marker

© Agilent Technologies, Inc. 2017

E2: H4 M R2

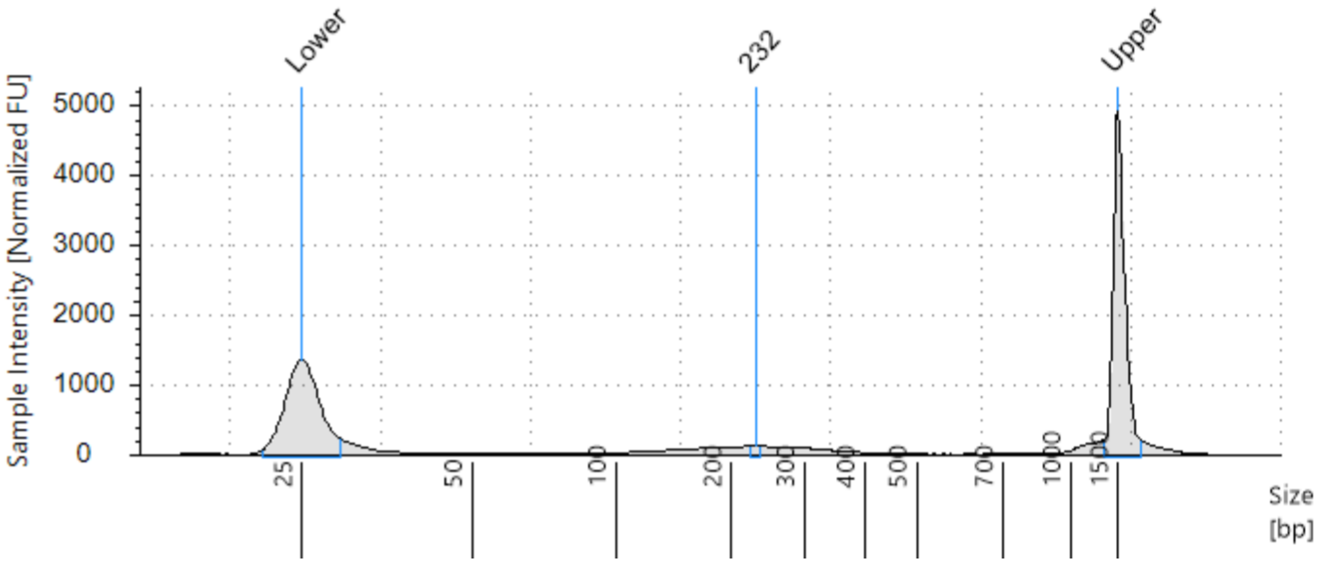

Sample Table

Well	Conc. [ng/µl]	Sample Description	Alert	Observations
E2	0.126	H4 M R2	⚠	Caution! Expired ScreenTape device

Peak Table

Size [bp]	Calibrated Conc. [ng/µl]	Assigned Conc. [ng/µl]	Peak Molarity [nmol/l]	% Integrated Area	Peak Comment	Observations
25	5.29	-	325	-		Lower Marker
232	0.126	-	0.836	100.00		
1500	6.50	6.50	6.67	-		Upper Marker

© Agilent Technologies, Inc. 2017

F2

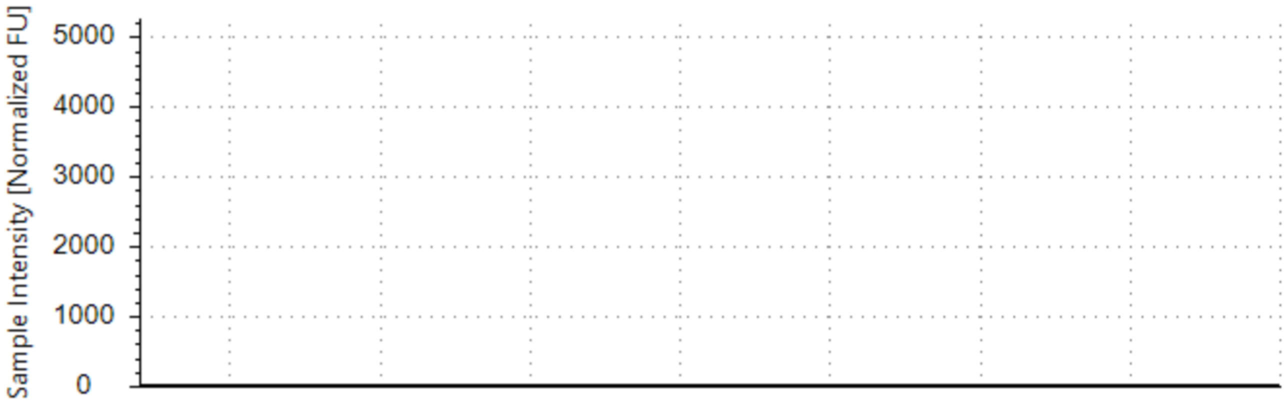

Sample Table

Well	Conc. [ng/µl]	Sample Description	Alert	Observations
F2			▲	Marker(s) not detected; Caution! Expired ScreenTape device

 © Agilent Technologies, Inc. 2017

G2: A5 M R2

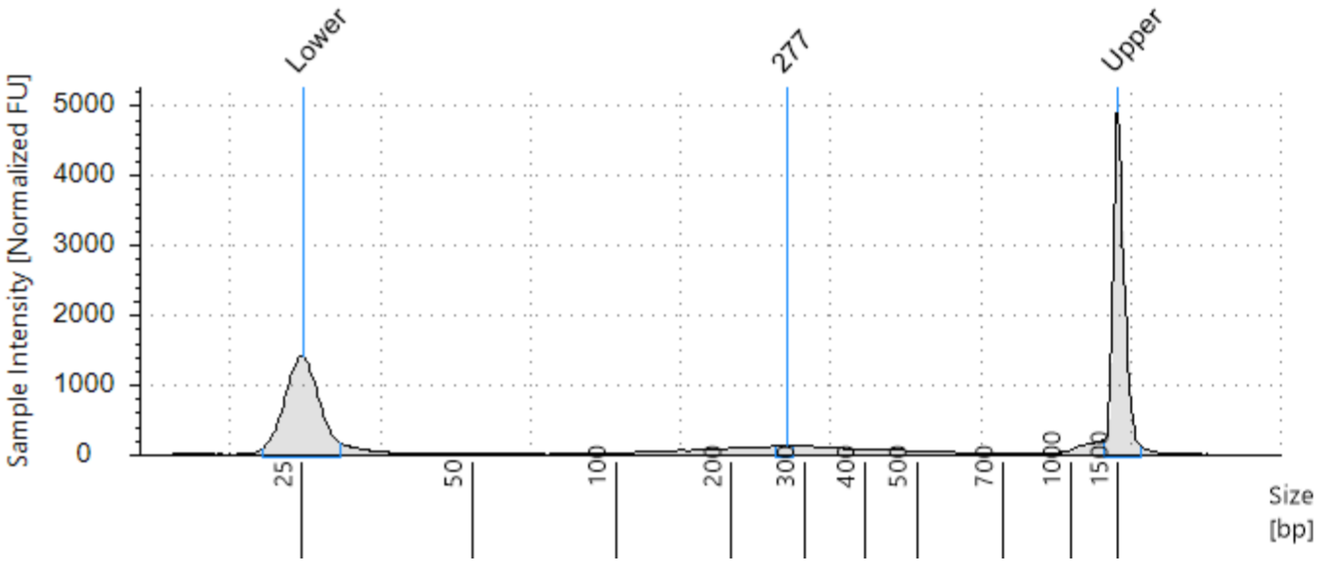

Sample Table

Well	Conc. [ng/µl]	Sample Description	Alert	Observations
G2	0.205	A5 M R2	⚠	Caution! Expired ScreenTape device

Peak Table

Size [bp]	Calibrated Conc. [ng/µl]	Assigned Conc. [ng/µl]	Peak Molarity [nmol/l]	% Integrated Area	Peak Comment	Observations
25	5.55	-	342	-		Lower Marker
277	0.205	-	1.14	100.00		
1500	6.50	6.50	6.67	-		Upper Marker

© Agilent Technologies, Inc. 2017

H2: B5 M R2

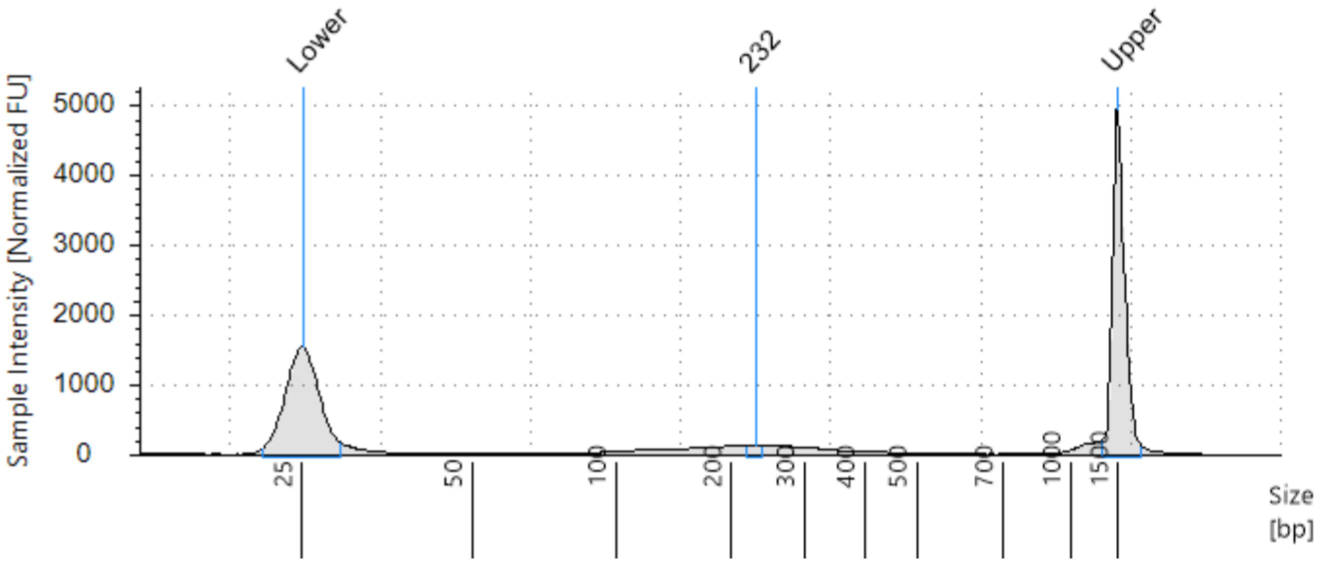

Sample Table

Well	Conc. [ng/µl]	Sample Description	Alert	Observations
H2	0.182	B5 M R2	⚠	Caution! Expired ScreenTape device

Peak Table

Size [bp]	Calibrated Conc. [ng/µl]	Assigned Conc. [ng/µl]	Peak Molarity [nmol/l]	% Integrated Area	Peak Comment	Observations
25	5.62	-	346	-		Lower Marker
232	0.182	-	1.21	100.00		
1500	6.50	6.50	6.67	-		Upper Marker

© Agilent Technologies, Inc. 2017

Filename: 2020-09-11-03- Q-S, MINUS, D5-B7, D1000 R2.D1000

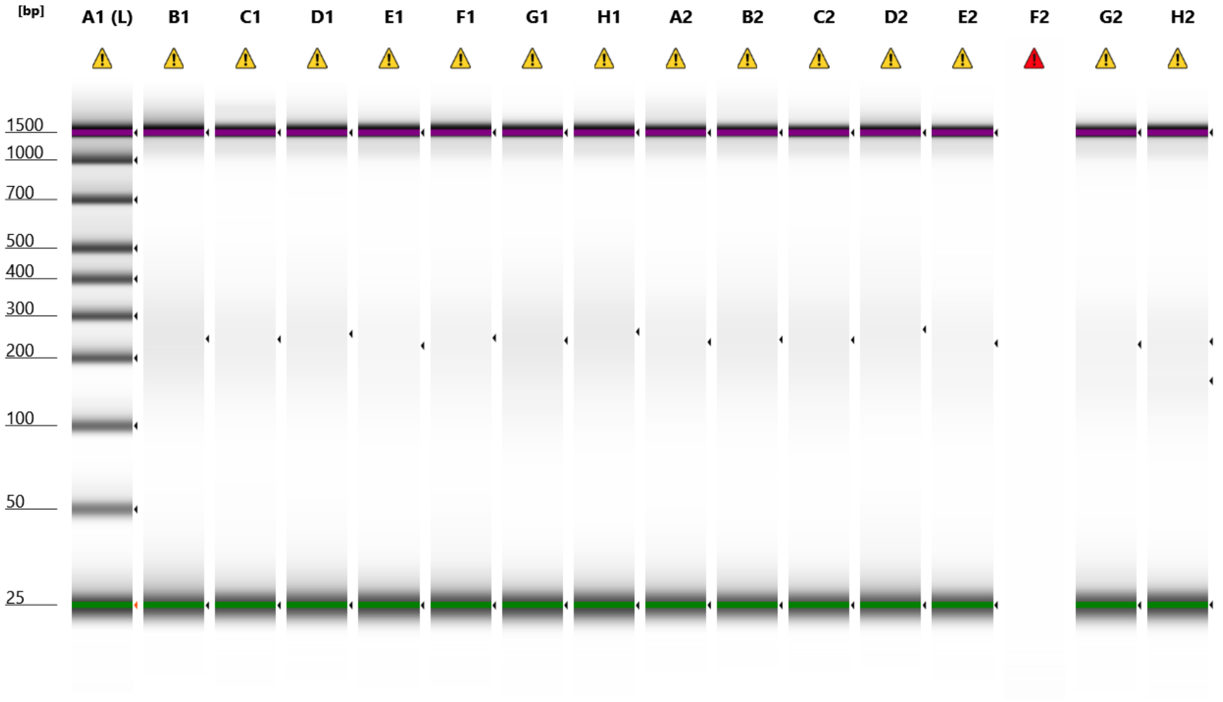

Default image (Contrast 100%)

Sample Info

Well	Conc. [ng/µl]	Sample Description	Alert	Observations
A1	17.1	Ladder	⚠	Caution! Expired ScreenTape device; Ladder
B1	0.195	D5 M R2	⚠	Caution! Expired ScreenTape device
C1	0.261	C5 M R2	⚠	Caution! Expired ScreenTape device
D1	0.111	E5 M R2	⚠	Caution! Expired ScreenTape device
E1	0.0964	F5 M R2	⚠	Caution! Expired ScreenTape device
F1	0.0759	A6 M R2	⚠	Caution! Expired ScreenTape device
G1	0.196	B6 M R2	⚠	Caution! Expired ScreenTape device
H1	0.266	C6 M R2	⚠	Caution! Expired ScreenTape device
A2	0.109	D6 M R2	⚠	Caution! Expired ScreenTape device
B2	0.0807	E6 M R2	⚠	Caution! Expired ScreenTape device
C2	0.111	F6 M R2	⚠	Caution! Expired ScreenTape device
D2	0.0934	G6 M R2	⚠	Caution! Expired ScreenTape device
E2	0.0792	H6 M R2	⚠	Caution! Expired ScreenTape device
F2			⚠	Marker(s) not detected; Caution! Expired ScreenTape device
G2	0.210	A7 M R2	⚠	Caution! Expired ScreenTape device
H2	0.712	B7 M R2	⚠	Caution! Expired ScreenTape device

 © Agilent Technologies, Inc. 2017

A1: Ladder

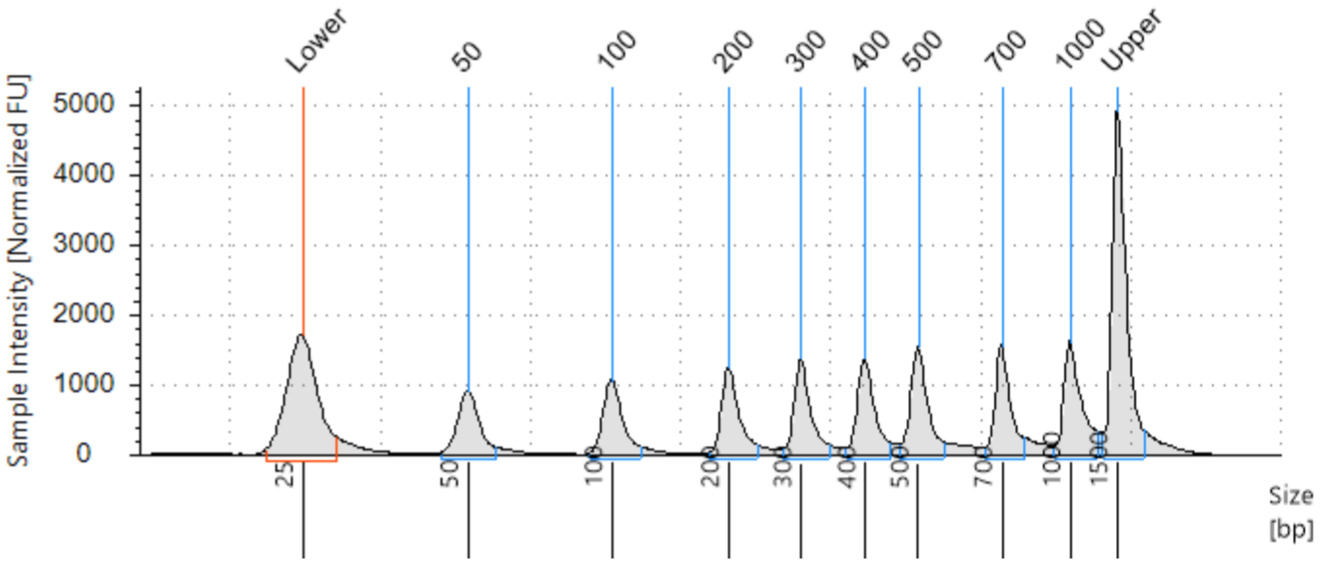

Sample Table

Well	Conc. [ng/µl]	Sample Description	Alert	Observations
A1	17.1	Ladder	⚠	Caution! Expired ScreenTape device; Ladder

Peak Table

Size [bp]	Calibrated Conc. [ng/µl]	Assigned Conc. [ng/µl]	Peak Molarity [nmol/l]	% Integrated Area	Peak Comment	Observations
25	4.86	-	299	-		Lower Marker
50	1.79	-	55.1	10.45		
100	1.91	-	29.3	11.14		
200	2.01	-	15.4	11.73		
300	2.08	-	10.7	12.17		
400	2.19	-	8.40	12.76		
500	2.38	-	7.34	13.93		
700	2.16	-	4.75	12.63		
1000	2.60	-	4.00	15.19		
1500	6.50	6.50	6.67	-		Upper Marker

 © Agilent Technologies, Inc. 2017

B1: D5 M R2

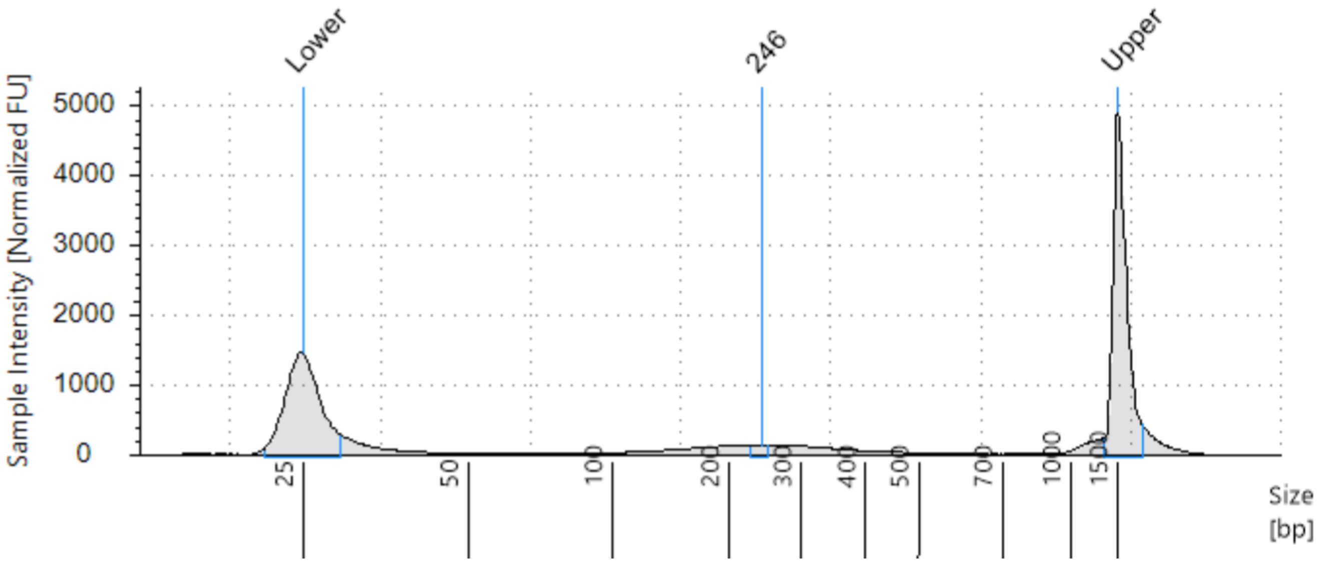

Sample Table

Well	Conc. [ng/µl]	Sample Description	Alert	Observations
B1	0.195	D5 M R2	⚠	Caution! Expired ScreenTape device

Peak Table

Size [bp]	Calibrated Conc. [ng/µl]	Assigned Conc. [ng/µl]	Peak Molarity [nmol/l]	% Integrated Area	Peak Comment	Observations
25	4.90	-	302	-		Lower Marker
246	0.195	-	1.22	100.00		
1500	6.50	6.50	6.67	-		Upper Marker

© Agilent Technologies, Inc. 2017

C1: C5 M R2

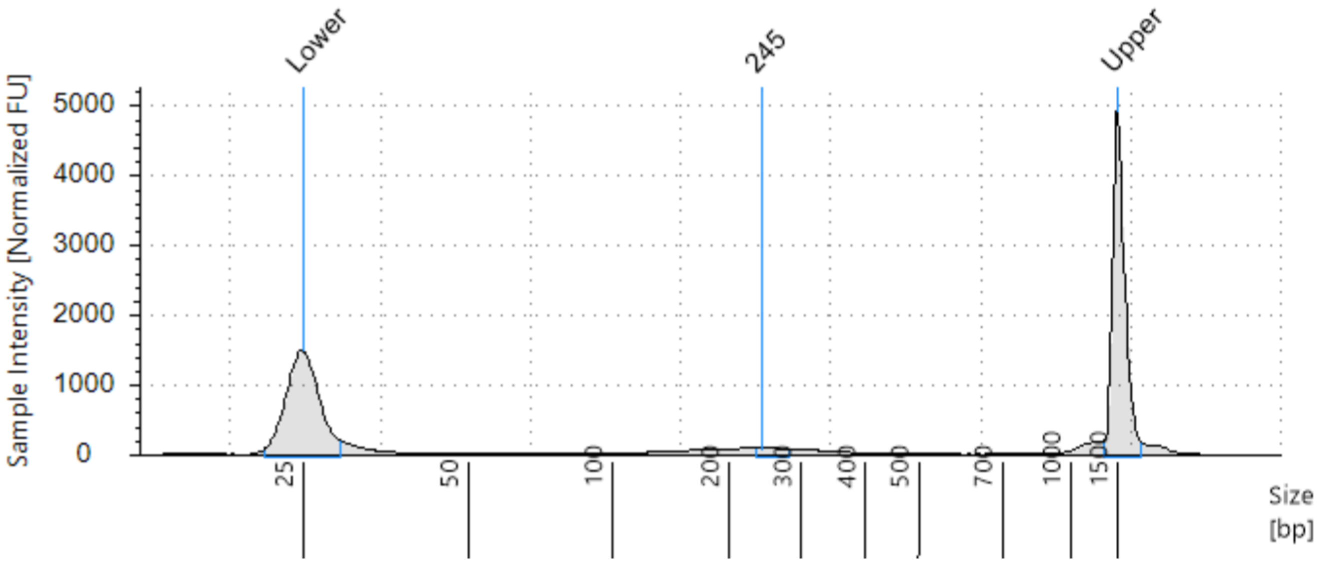

Sample Table

Well	Conc. [ng/µl]	Sample Description	Alert	Observations
C1	0.261	C5 M R2	⚠	Caution! Expired ScreenTape device

Peak Table

Size [bp]	Calibrated Conc. [ng/µl]	Assigned Conc. [ng/µl]	Peak Molarity [nmol/l]	% Integrated Area	Peak Comment	Observations
25	5.28	-	325	-		Lower Marker
245	0.261	-	1.64	100.00		
1500	6.50	6.50	6.67	-		Upper Marker

© Agilent Technologies, Inc. 2017

D1: E5 M R2

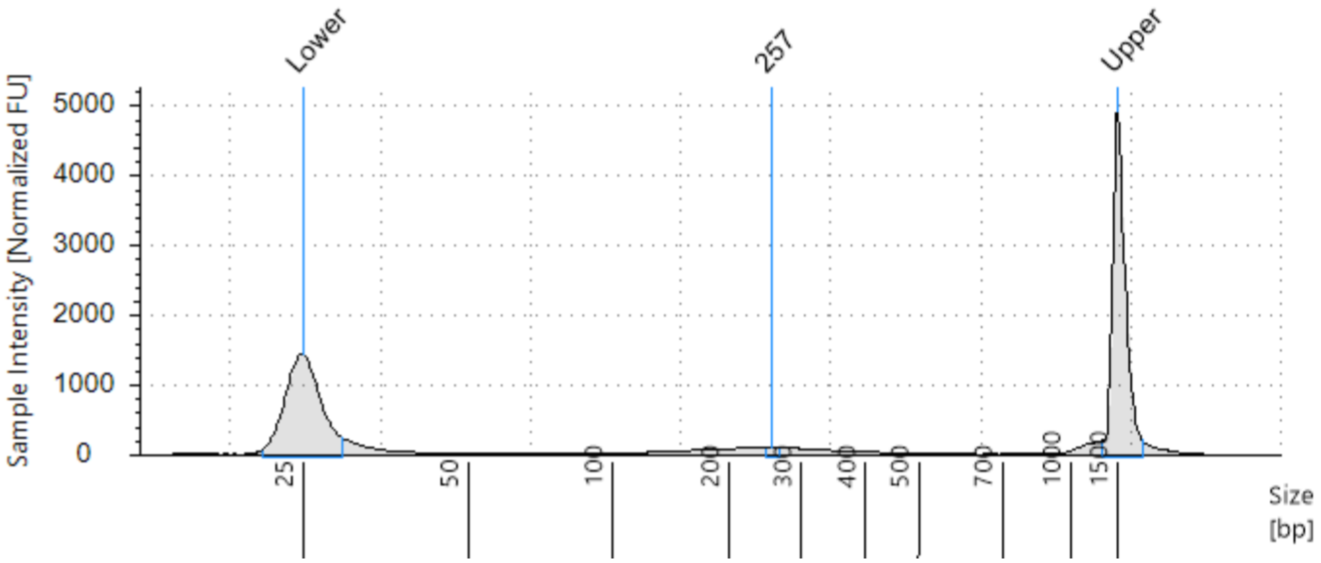

Sample Table

Well	Conc. [ng/µl]	Sample Description	Alert	Observations
D1	0.111	E5 M R2	⚠	Caution! Expired ScreenTape device

Peak Table

Size [bp]	Calibrated Conc. [ng/µl]	Assigned Conc. [ng/µl]	Peak Molarity [nmol/l]	% Integrated Area	Peak Comment	Observations
25	5.28	-	325	-		Lower Marker
257	0.111	-	0.661	100.00		
1500	6.50	6.50	6.67	-		Upper Marker

© Agilent Technologies, Inc. 2017

E1: F5 M R2

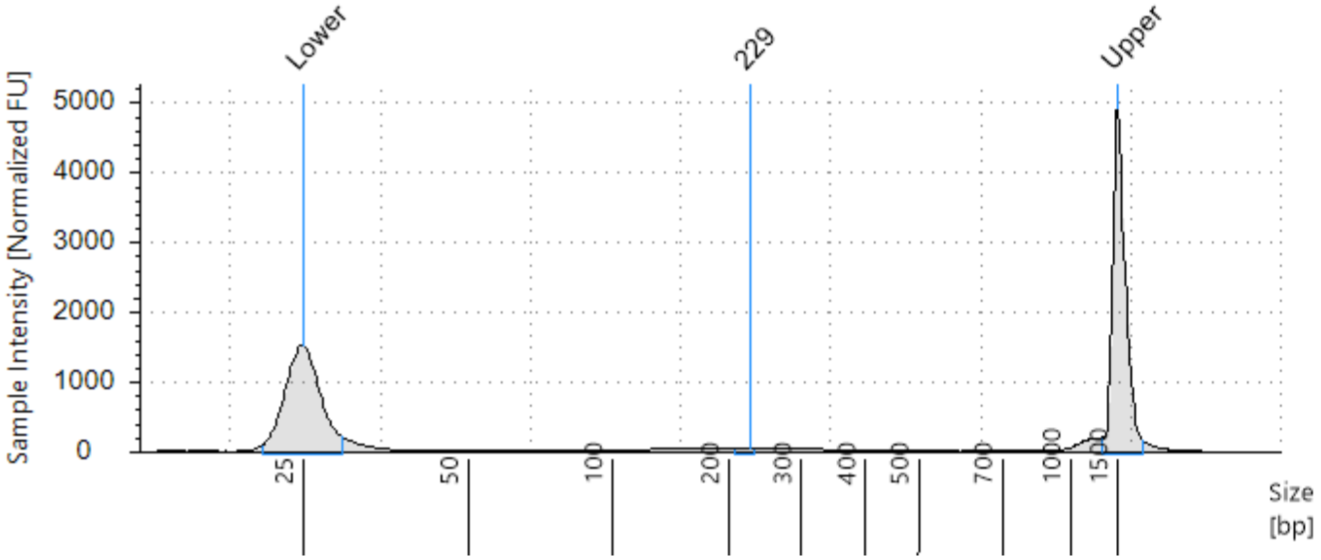

Sample Table

Well	Conc. [ng/µl]	Sample Description	Alert	Observations
E1	0.0964	F5 M R2	⚠	Caution! Expired ScreenTape device

Peak Table

Size [bp]	Calibrated Conc. [ng/µl]	Assigned Conc. [ng/µl]	Peak Molarity [nmol/l]	% Integrated Area	Peak Comment	Observations
25	5.53	-	341	-		Lower Marker
229	0.0964	-	0.646	100.00		
1500	6.50	6.50	6.67	-		Upper Marker

© Agilent Technologies, Inc. 2017

F1: A6 M R2

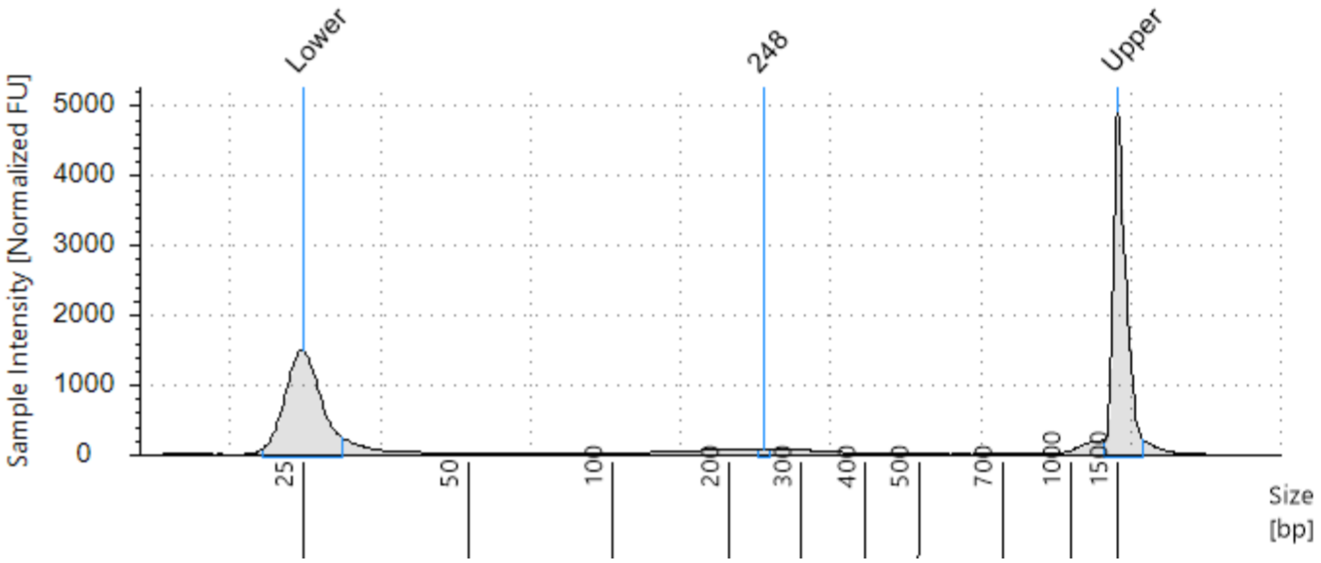

Sample Table

Well	Conc. [ng/µl]	Sample Description	Alert	Observations
F1	0.0759	A6 M R2	⚠	Caution! Expired ScreenTape device

Peak Table

Size [bp]	Calibrated Conc. [ng/µl]	Assigned Conc. [ng/µl]	Peak Molarity [nmol/l]	% Integrated Area	Peak Comment	Observations
25	5.40	-	332	-		Lower Marker
248	0.0759	-	0.471	100.00		
1500	6.50	6.50	6.67	-		Upper Marker

© Agilent Technologies, Inc. 2017

G1: B6 M R2

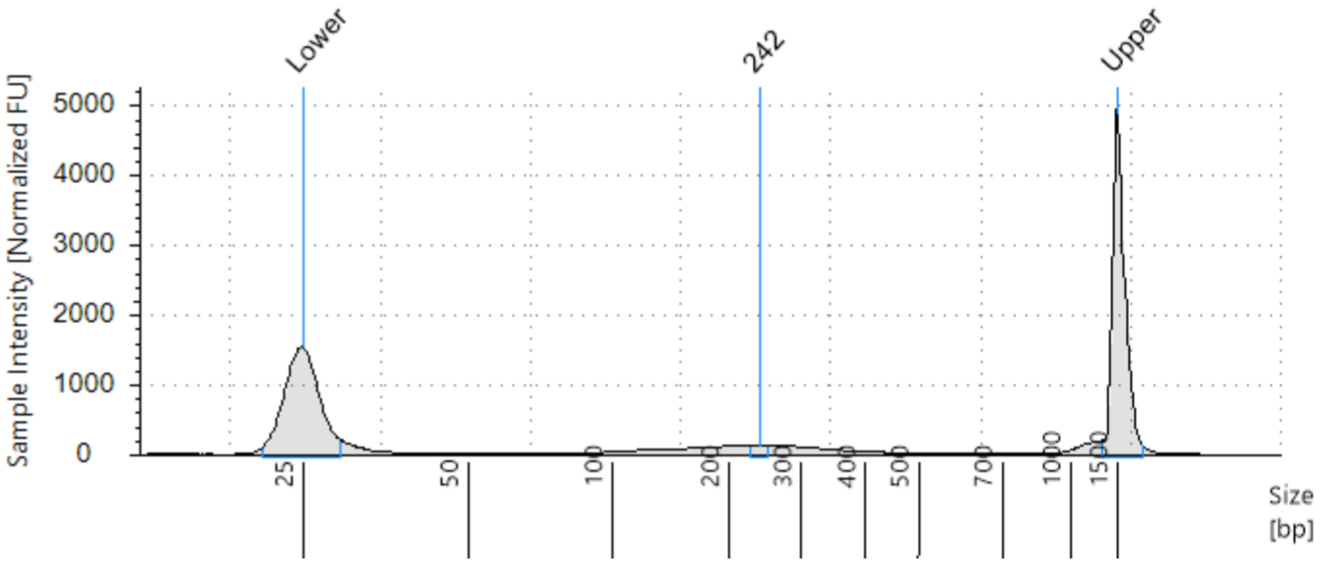

Sample Table

Well	Conc. [ng/µl]	Sample Description	Alert	Observations
G1	0.196	B6 M R2	⚠	Caution! Expired ScreenTape device

Peak Table

Size [bp]	Calibrated Conc. [ng/µl]	Assigned Conc. [ng/µl]	Peak Molarity [nmol/l]	% Integrated Area	Peak Comment	Observations
25	5.72	-	352	-		Lower Marker
242	0.196	-	1.24	100.00		
1500	6.50	6.50	6.67	-		Upper Marker

 © Agilent Technologies, Inc. 2017

H1: C6 M R2

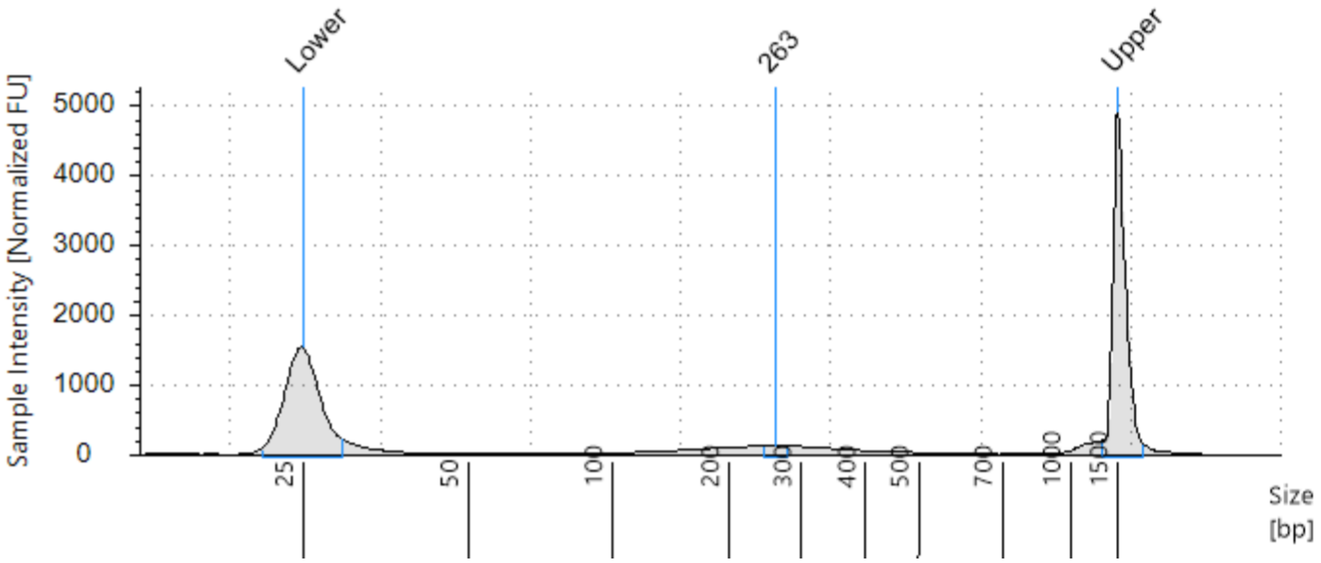

Sample Table

Well	Conc. [ng/µl]	Sample Description	Alert	Observations
H1	0.266	C6 M R2	⚠	Caution! Expired ScreenTape device

Peak Table

Size [bp]	Calibrated Conc. [ng/µl]	Assigned Conc. [ng/µl]	Peak Molarity [nmol/l]	% Integrated Area	Peak Comment	Observations
25	5.69	-	350	-		Lower Marker
263	0.266	-	1.56	100.00		
1500	6.50	6.50	6.67	-		Upper Marker

© Agilent Technologies, Inc. 2017

A2: D6 M R2

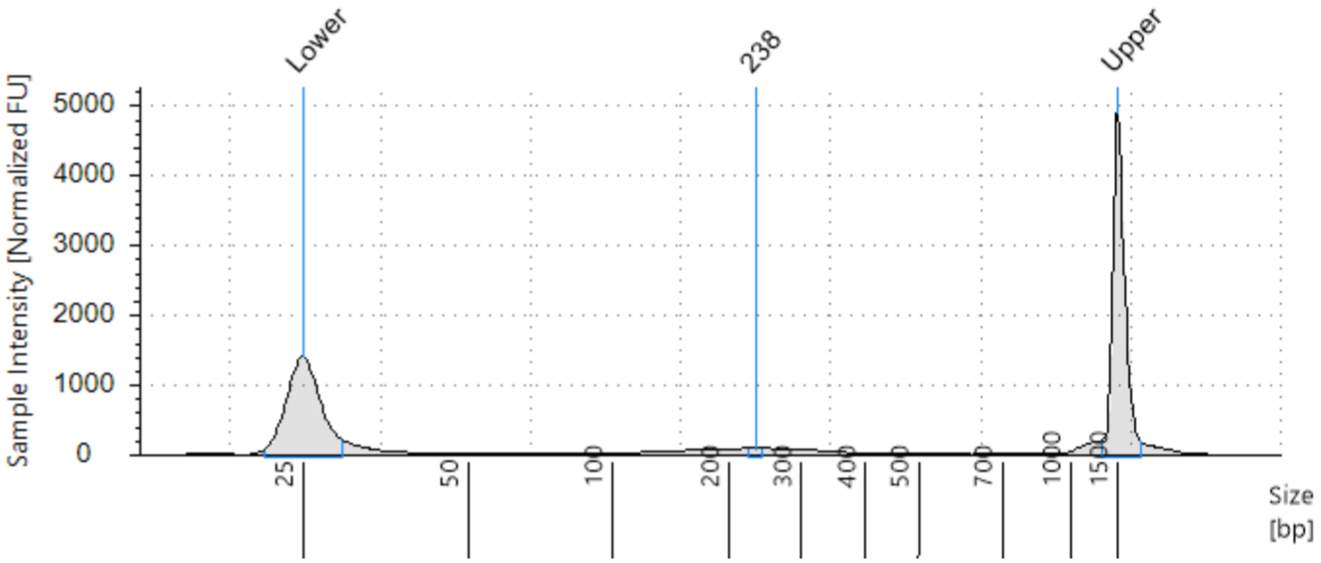

Sample Table

Well	Conc. [ng/µl]	Sample Description	Alert	Observations
A2	0.109	D6 M R2	⚠	Caution! Expired ScreenTape device

Peak Table

Size [bp]	Calibrated Conc. [ng/µl]	Assigned Conc. [ng/µl]	Peak Molarity [nmol/l]	% Integrated Area	Peak Comment	Observations
25	5.20	-	320	-		Lower Marker
238	0.109	-	0.705	100.00		
1500	6.50	6.50	6.67	-		Upper Marker

© Agilent Technologies, Inc. 2017

B2: E6 M R2

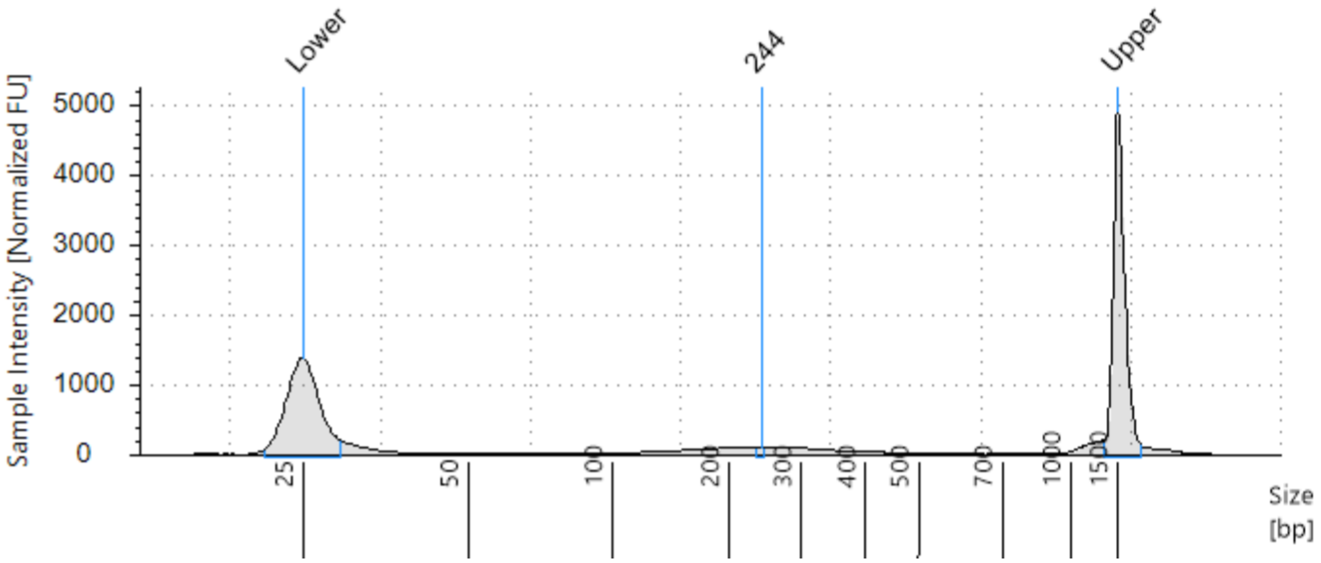

Sample Table

Well	Conc. [ng/µl]	Sample Description	Alert	Observations
B2	0.0807	E6 M R2	⚠	Caution! Expired ScreenTape device

Peak Table

Size [bp]	Calibrated Conc. [ng/µl]	Assigned Conc. [ng/µl]	Peak Molarity [nmol/l]	% Integrated Area	Peak Comment	Observations
25	5.18	-	319	-		Lower Marker
244	0.0807	-	0.509	100.00		
1500	6.50	6.50	6.67	-		Upper Marker

 © Agilent Technologies, Inc. 2017

C2: F6 M R2

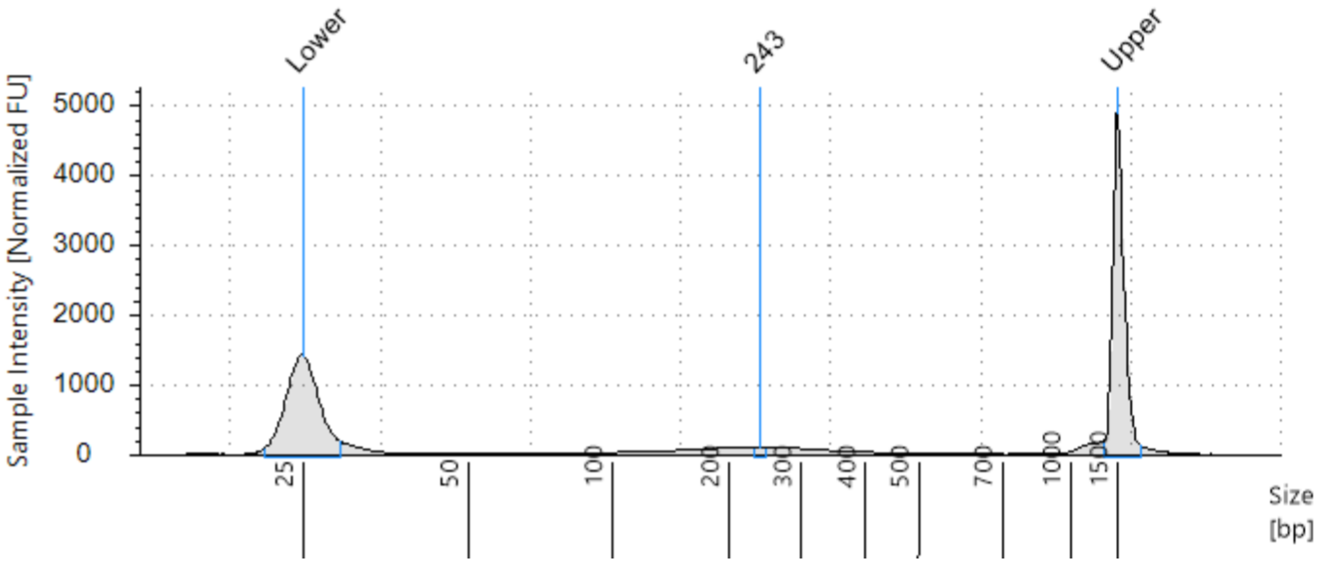

Sample Table

Well	Conc. [ng/µl]	Sample Description	Alert	Observations
C2	0.111	F6 M R2	⚠	Caution! Expired ScreenTape device

Peak Table

Size [bp]	Calibrated Conc. [ng/µl]	Assigned Conc. [ng/µl]	Peak Molarity [nmol/l]	% Integrated Area	Peak Comment	Observations
25	5.40	-	333	-		Lower Marker
243	0.111	-	0.703	100.00		
1500	6.50	6.50	6.67	-		Upper Marker

© Agilent Technologies, Inc. 2017

D2: G6 M R2

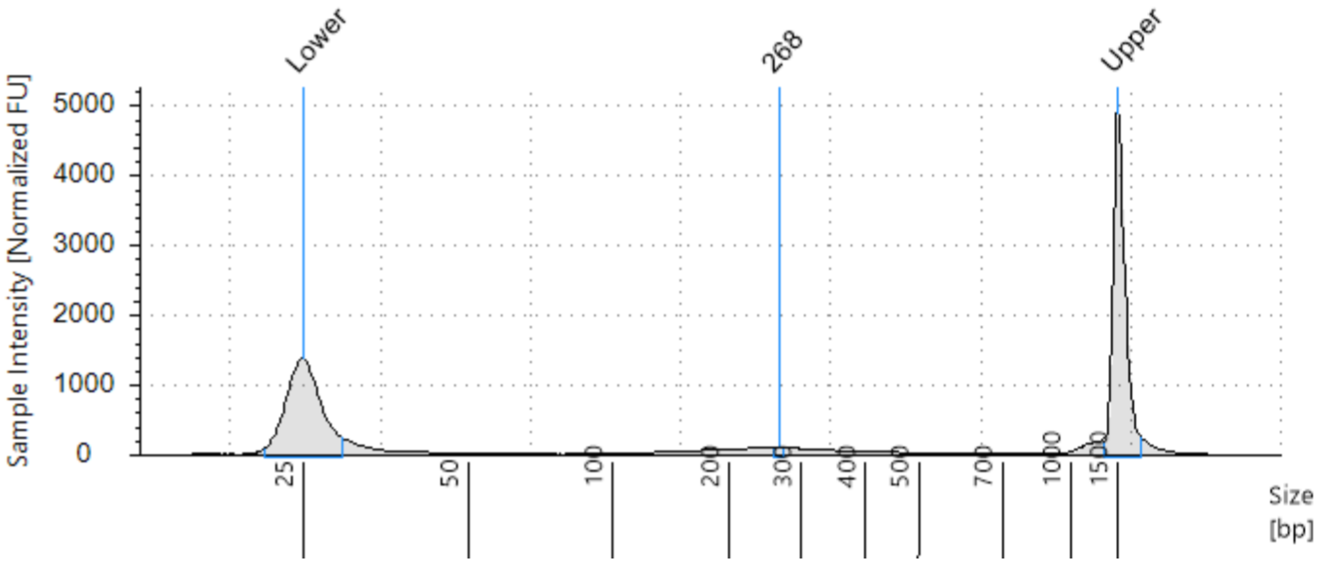

Sample Table

Well	Conc. [ng/µl]	Sample Description	Alert	Observations
D2	0.0934	G6 M R2	⚠	Caution! Expired ScreenTape device

Peak Table

Size [bp]	Calibrated Conc. [ng/µl]	Assigned Conc. [ng/µl]	Peak Molarity [nmol/l]	% Integrated Area	Peak Comment	Observations
25	5.14	-	316	-		Lower Marker
268	0.0934	-	0.536	100.00		
1500	6.50	6.50	6.67	-		Upper Marker

© Agilent Technologies, Inc. 2017

E2: H6 M R2

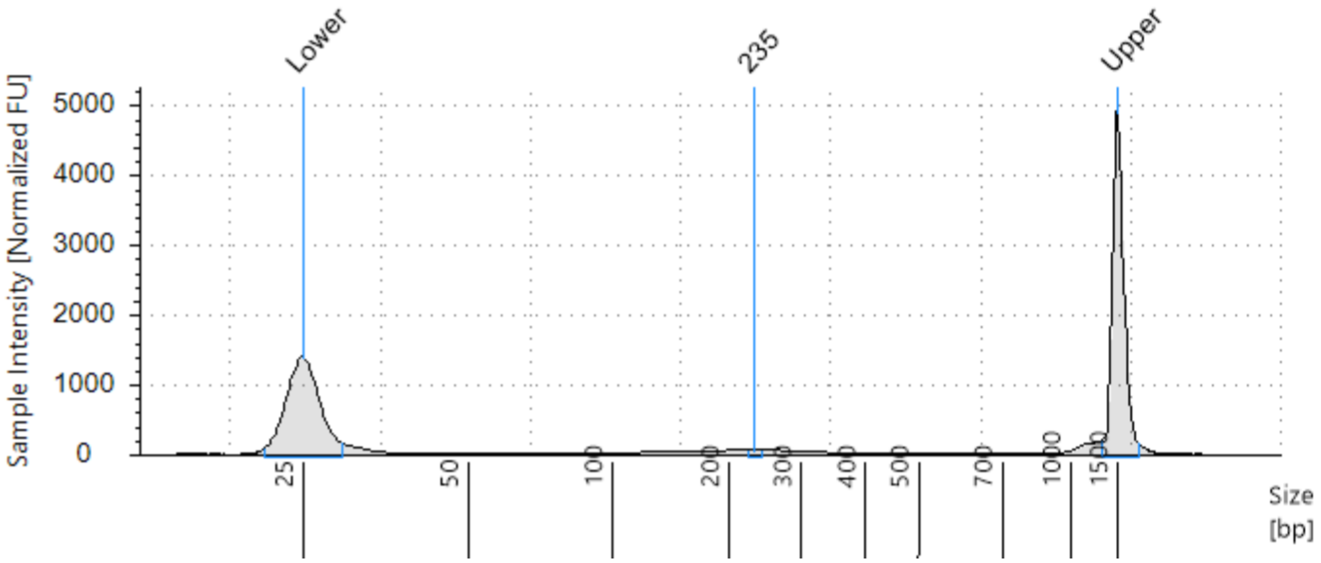

Sample Table

Well	Conc. [ng/µl]	Sample Description	Alert	Observations
E2	0.0792	H6 M R2	⚠	Caution! Expired ScreenTape device

Peak Table

Size [bp]	Calibrated Conc. [ng/µl]	Assigned Conc. [ng/µl]	Peak Molarity [nmol/l]	% Integrated Area	Peak Comment	Observations
25	5.38	-	331	-		Lower Marker
235	0.0792	-	0.518	100.00		
1500	6.50	6.50	6.67	-		Upper Marker

© Agilent Technologies, Inc. 2017

F2

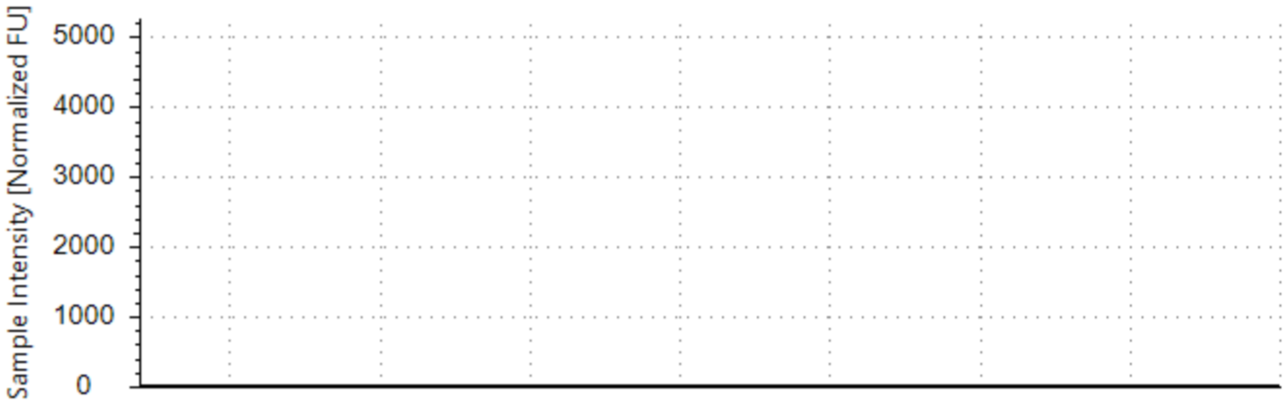

Sample Table

Well	Conc. [ng/µl]	Sample Description	Alert	Observations
F2			▲	Marker(s) not detected; Caution! Expired ScreenTape device

 © Agilent Technologies, Inc. 2017

G2: A7 M R2

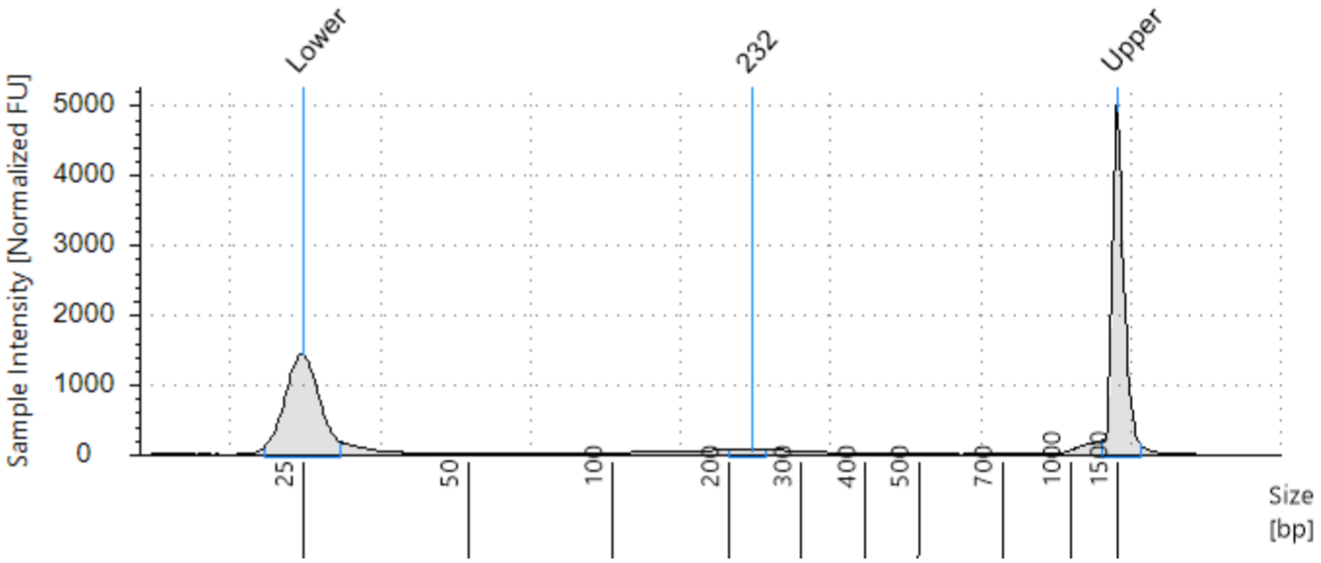

Sample Table

Well	Conc. [ng/µl]	Sample Description	Alert	Observations
G2	0.210	A7 M R2	⚠	Caution! Expired ScreenTape device

Peak Table

Size [bp]	Calibrated Conc. [ng/µl]	Assigned Conc. [ng/µl]	Peak Molarity [nmol/l]	% Integrated Area	Peak Comment	Observations
25	5.46	-	336	-		Lower Marker
232	0.210	-	1.39	100.00		
1500	6.50	6.50	6.67	-		Upper Marker

© Agilent Technologies, Inc. 2017

H2: B7 M R2

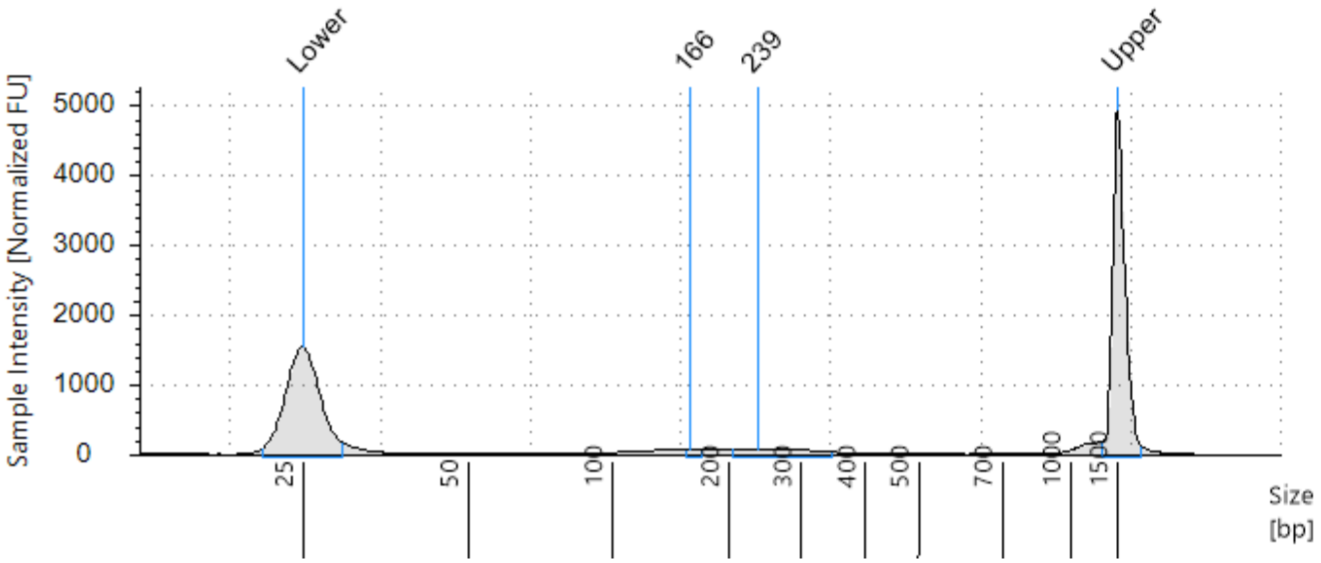

Sample Table

Well	Conc. [ng/µl]	Sample Description	Alert	Observations
H2	0.712	B7 M R2	⚠	Caution! Expired ScreenTape device

Peak Table

Size [bp]	Calibrated Conc. [ng/µl]	Assigned Conc. [ng/µl]	Peak Molarity [nmol/l]	% Integrated Area	Peak Comment	Observations
25	5.66	-	348	-		Lower Marker
166	0.107	-	0.993	15.03		
239	0.605	-	3.90	84.97		
1500	6.50	6.50	6.67	-		Upper Marker

© Agilent Technologies, Inc. 2017

Filename: 2020-09-28-01 Q-S MINUS A7-G8, D5000, R2.D5000

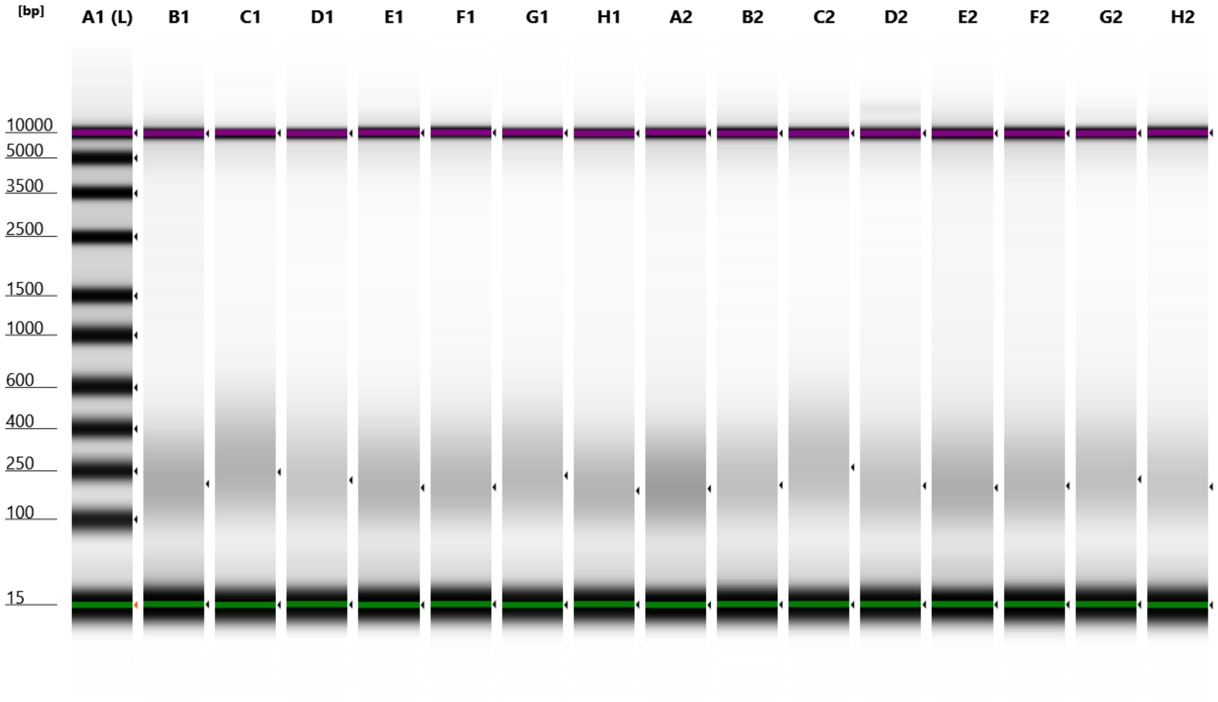

Default image (Contrast 100%)

Sample Info

Well	Conc. [ng/µl]	Sample Description	Alert	Observations
A1	33.4	Ladder		Ladder
B1	0.474	A7 M R2		
C1	1.83	B7 M R2		
D1	2.35	C7 M R2		
E1	1.52	D7 M R2		
F1	2.83	E7 M R2		
G1	3.14	F7 M R2		
H1	3.01	G7 M R2		
A2	4.19	H7 M R2		
B2	0.335	A8 M R2		
C2	1.85	B8 M R2		
D2	1.35	C8 M R2		
E2	1.66	D8 M R2		
F2	1.35	E8 M R2		
G2	1.24	F8 M R2		
H2	2.01	G8 M R2		

© Agilent Technologies, Inc. 2017

A1: Ladder

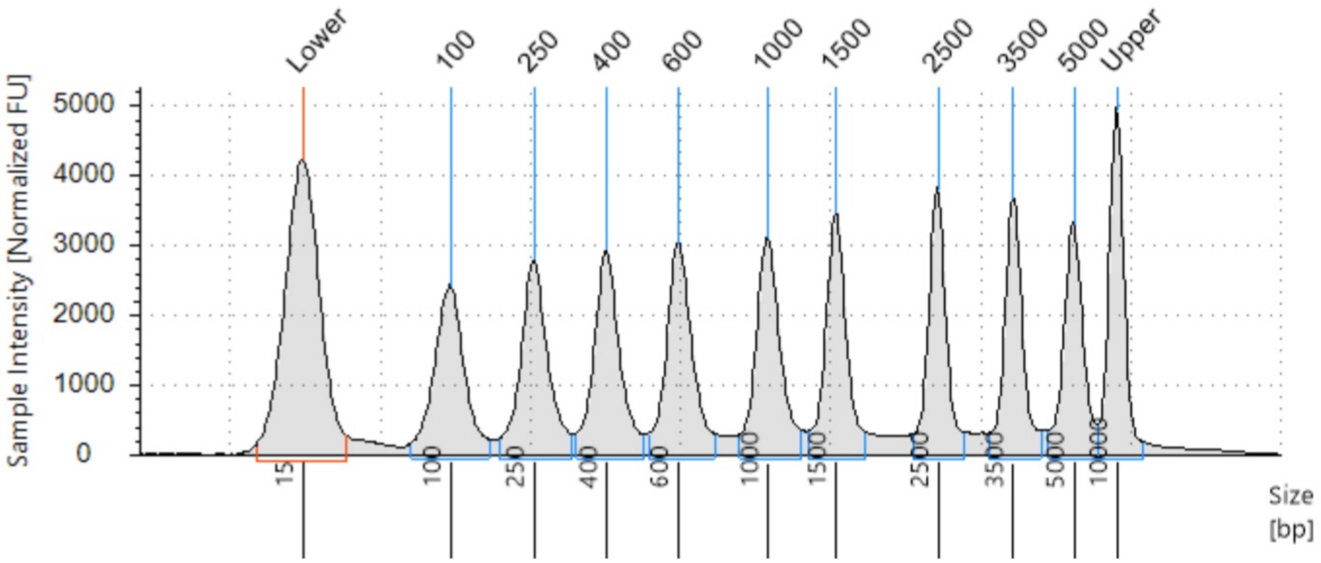

Sample Table

Well	Conc. [ng/µl]	Sample Description	Alert	Observations
A1	33.4	Ladder		Ladder

Peak Table

Size [bp]	Calibrated Conc. [ng/µl]	Assigned Conc. [ng/µl]	Peak Molarity [nmol/l]	% Integrated Area	Peak Comment	Observations
15	6.76	-	693	-		Lower Marker
100	3.75	-	57.7	11.23		
250	3.88	-	23.9	11.60		
400	3.86	-	14.8	11.54		
600	4.00	-	10.3	11.97		
1000	3.89	-	5.99	11.65		
1500	3.73	-	3.82	11.16		
2500	3.53	-	2.17	10.57		
3500	3.43	-	1.51	10.27		
5000	3.35	-	1.03	10.02		
10000	3.25	3.25	0.500	-		Upper Marker

 © Agilent Technologies, Inc. 2017

B1: A7 M R2

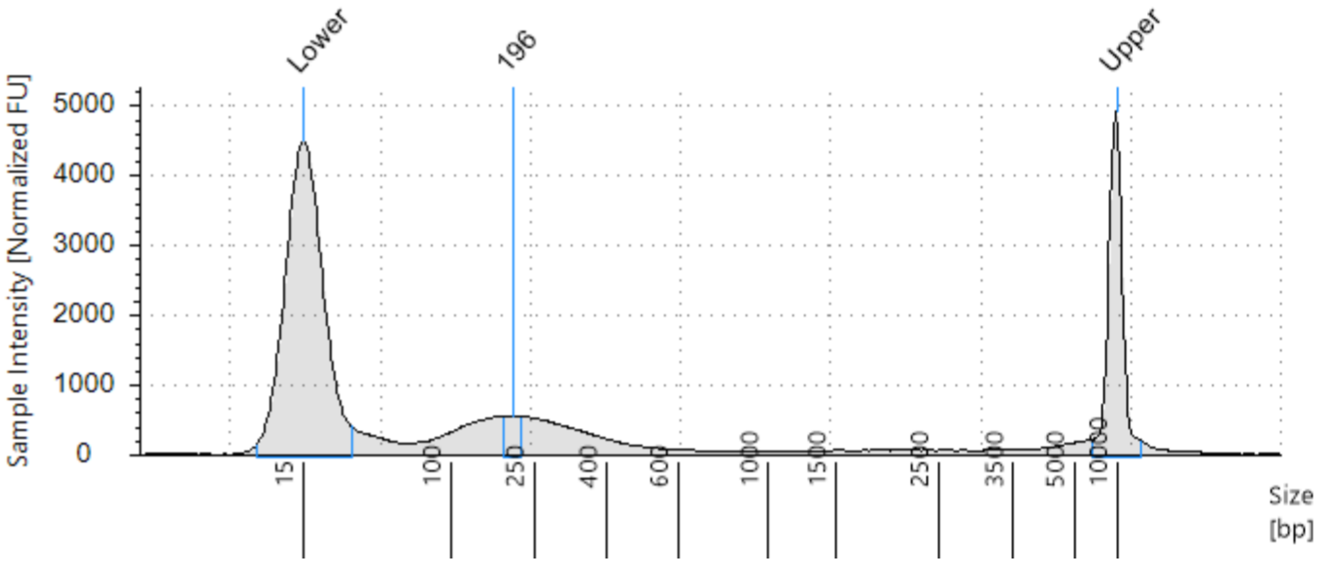

Sample Table

Well	Conc. [ng/µl]	Sample Description	Alert	Observations
B1	0.474	A7 M R2		

Peak Table

Size [bp]	Calibrated Conc. [ng/µl]	Assigned Conc. [ng/µl]	Peak Molarity [nmol/l]	% Integrated Area	Peak Comment	Observations
15	8.13	-	833	-		Lower Marker
196	0.474	-	3.73	100.00		
10000	3.25	3.25	0.500	-		Upper Marker

© Agilent Technologies, Inc. 2017

C1: B7 M R2

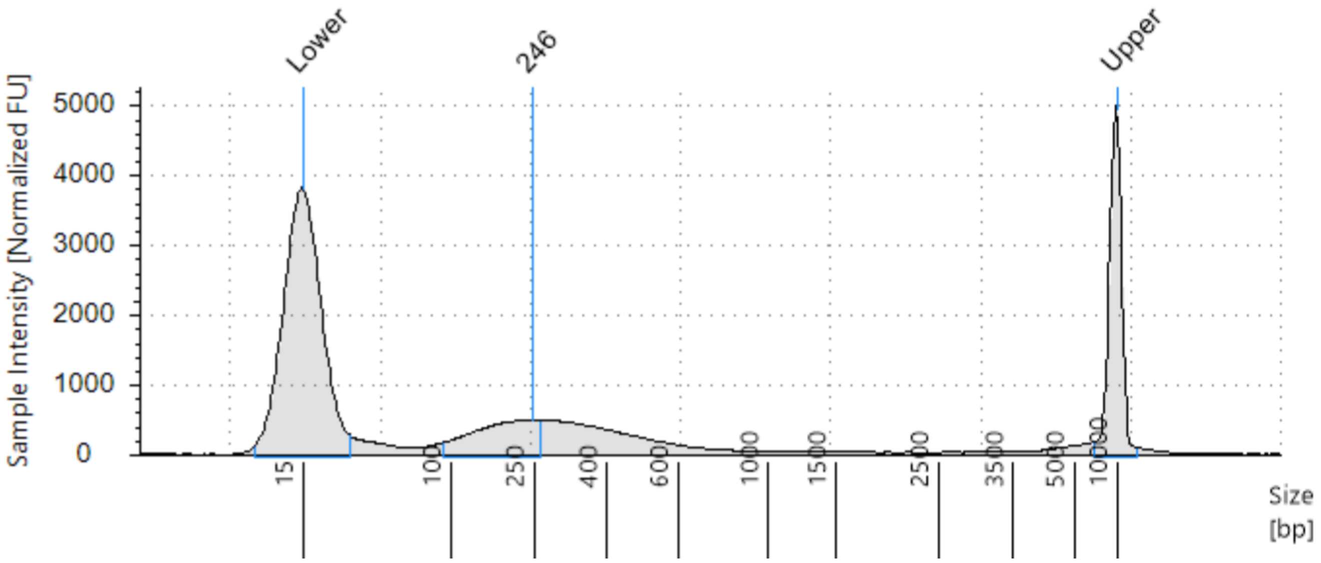

Sample Table

Well	Conc. [ng/µl]	Sample Description	Alert	Observations
C1	1.83	B7 M R2		

Peak Table

Size [bp]	Calibrated Conc. [ng/µl]	Assigned Conc. [ng/µl]	Peak Molarity [nmol/l]	% Integrated Area	Peak Comment	Observations
15	7.37	-	756	-		Lower Marker
246	1.83	-	11.4	100.00		
10000	3.25	3.25	0.500	-		Upper Marker

© Agilent Technologies, Inc. 2017

D1: C7 M R2

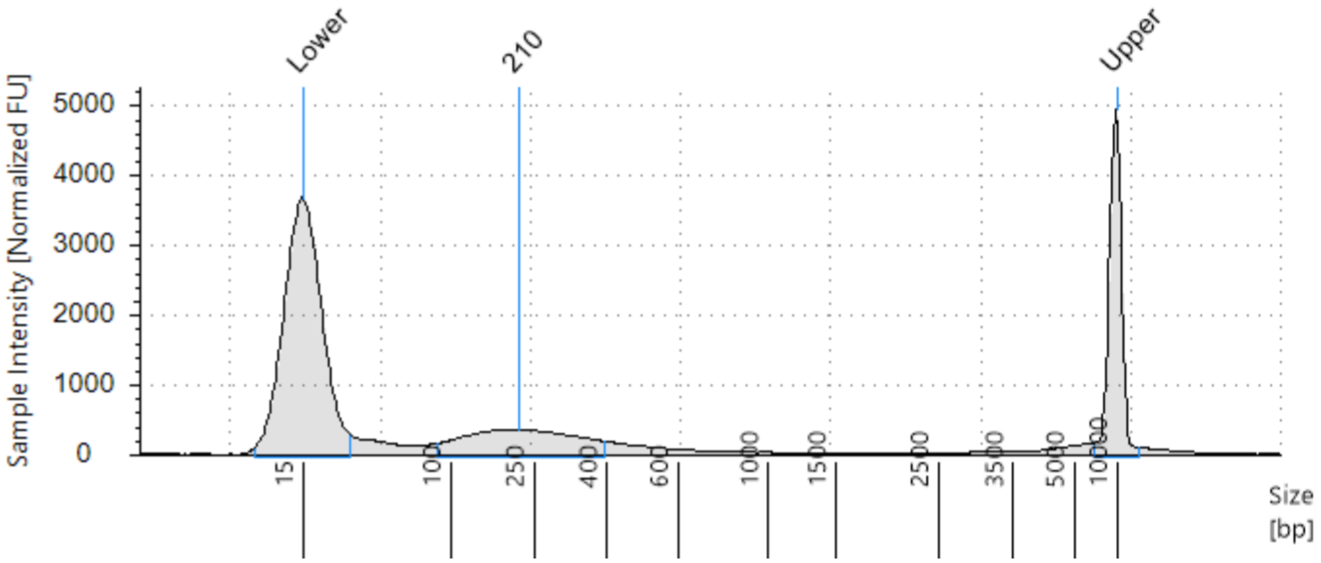

Sample Table

Well	Conc. [ng/µl]	Sample Description	Alert	Observations
D1	2.35	C7 M R2		

Peak Table

Size [bp]	Calibrated Conc. [ng/µl]	Assigned Conc. [ng/µl]	Peak Molarity [nmol/l]	% Integrated Area	Peak Comment	Observations
15	7.14	-	732	-		Lower Marker
210	2.35	-	17.2	100.00		
10000	3.25	3.25	0.500	-		Upper Marker

 © Agilent Technologies, Inc. 2017

E1: D7 M R2

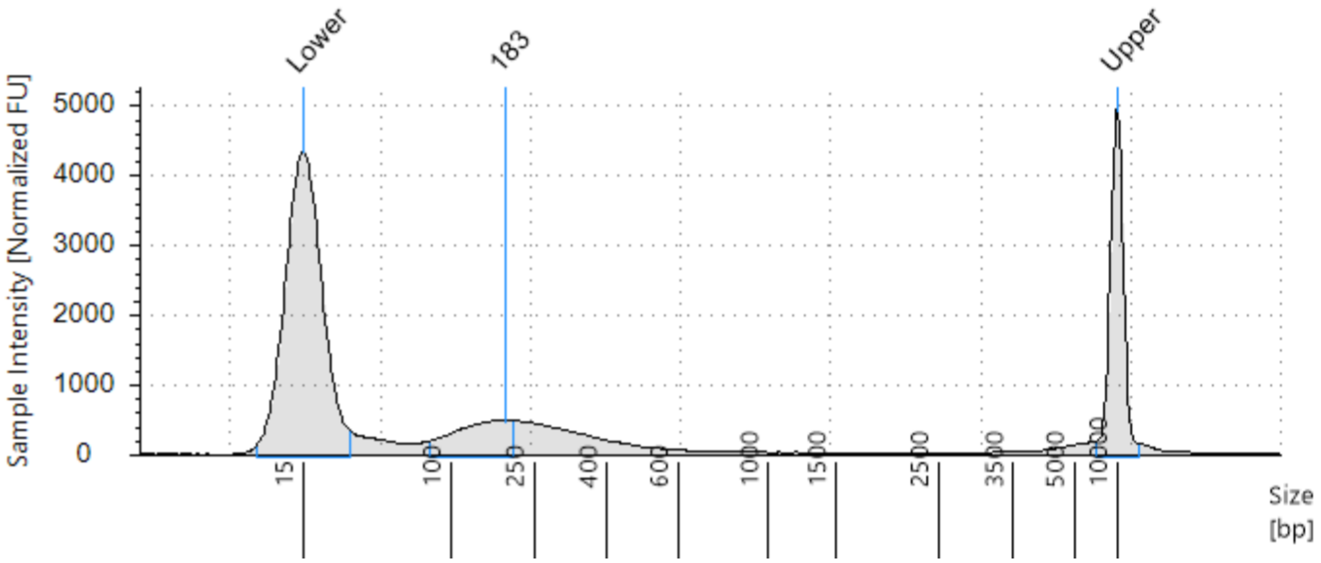

Sample Table

Well	Conc. [ng/µl]	Sample Description	Alert	Observations
E1	1.52	D7 M R2		

Peak Table

Size [bp]	Calibrated Conc. [ng/µl]	Assigned Conc. [ng/µl]	Peak Molarity [nmol/l]	% Integrated Area	Peak Comment	Observations
15	7.65	-	784	-		Lower Marker
183	1.52	-	12.8	100.00		
10000	3.25	3.25	0.500	-		Upper Marker

© Agilent Technologies, Inc. 2017

F1: E7 M R2

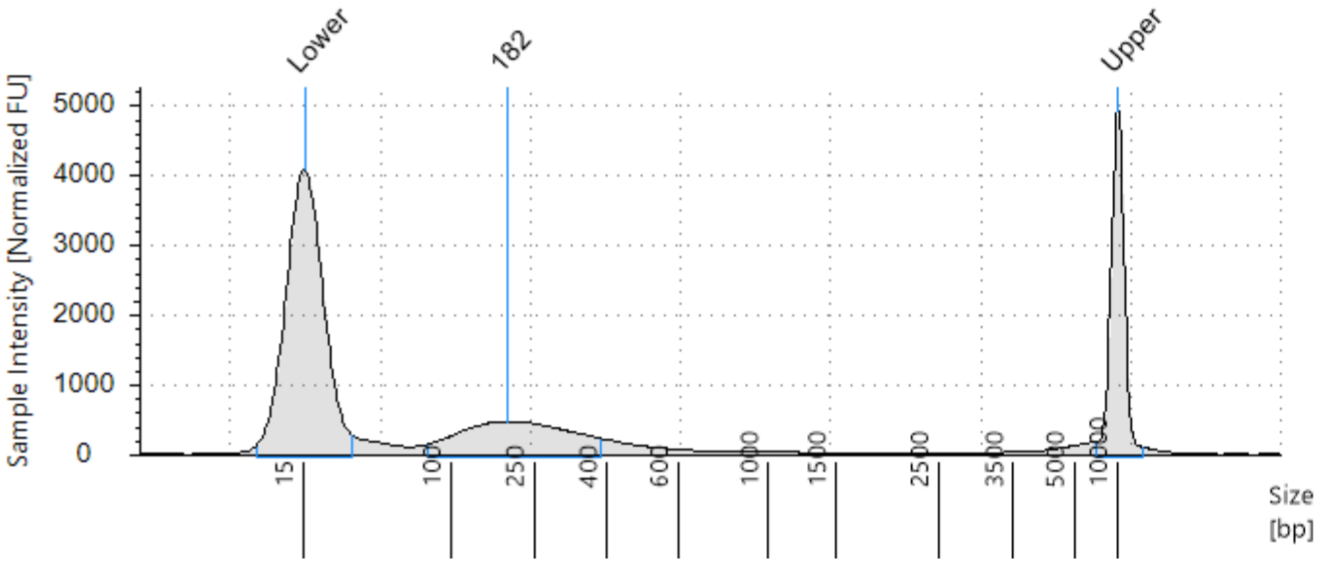

Sample Table

Well	Conc. [ng/µl]	Sample Description	Alert	Observations
F1	2.83	E7 M R2		

Peak Table

Size [bp]	Calibrated Conc. [ng/µl]	Assigned Conc. [ng/µl]	Peak Molarity [nmol/l]	% Integrated Area	Peak Comment	Observations
15	7.30	-	749	-		Lower Marker
182	2.83	-	23.8	100.00		
10000	3.25	3.25	0.500	-		Upper Marker

© Agilent Technologies, Inc. 2017

G1: F7 M R2

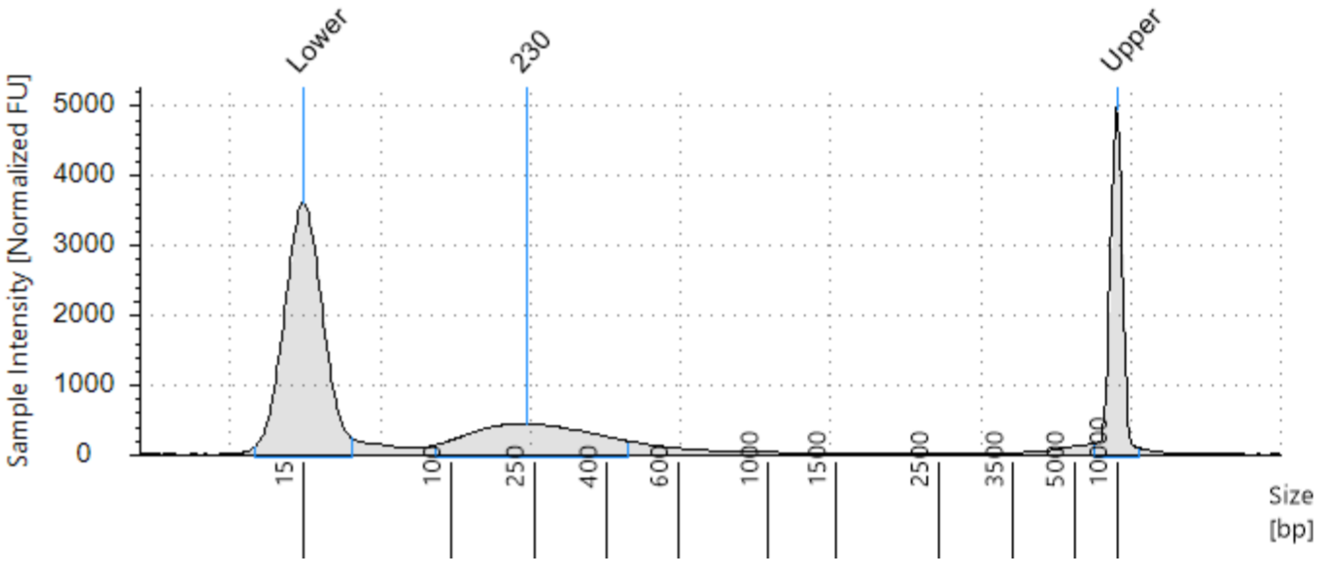

Sample Table

Well	Conc. [ng/µl]	Sample Description	Alert	Observations
Gl	3.14	F7 M R2		

Peak Table

Size [bp]	Calibrated Conc. [ng/µl]	Assigned Conc. [ng/µl]	Peak Molarity [nmol/l]	% Integrated Area	Peak Comment	Observations
15	7.01	-	719	-		Lower Marker
230	3.14	-	21.0	100.00		
10000	3.25	3.25	0.500	-		Upper Marker

 © Agilent Technologies, Inc. 2017

H1: G7 M R2

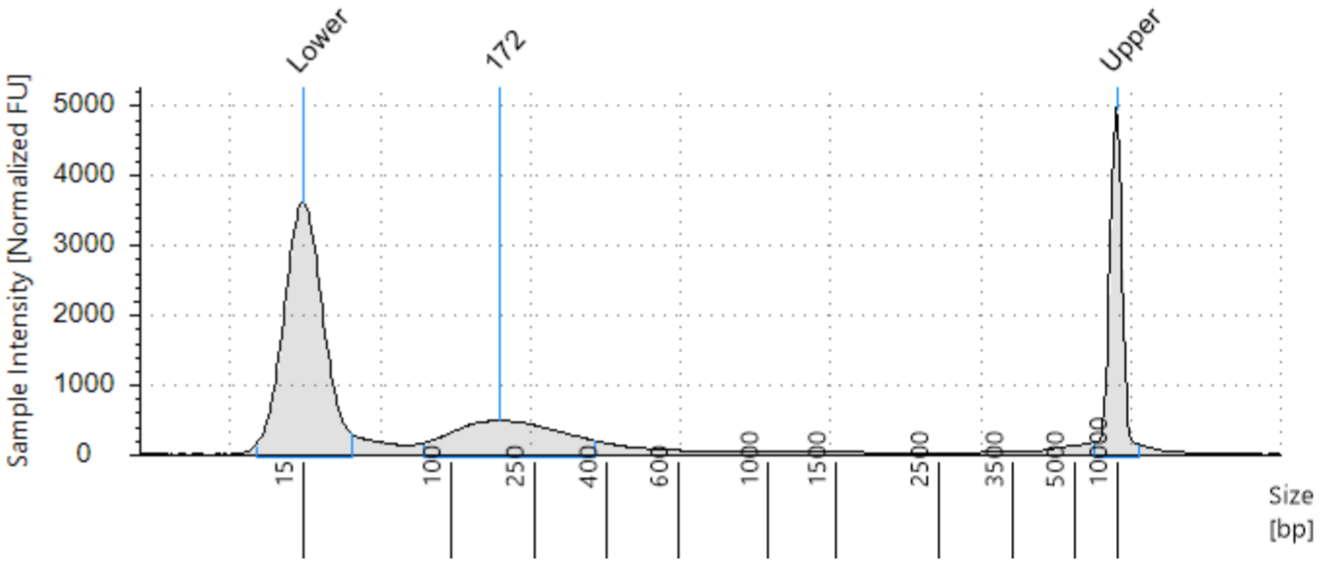

Sample Table

Well	Conc. [ng/µl]	Sample Description	Alert	Observations
H1	3.01	G7 M R2		

Peak Table

Size [bp]	Calibrated Conc. [ng/µl]	Assigned Conc. [ng/µl]	Peak Molarity [nmol/l]	% Integrated Area	Peak Comment	Observations
15	7.02	-	720	-		Lower Marker
172	3.01	-	27.0	100.00		
10000	3.25	3.25	0.500	-		Upper Marker

© Agilent Technologies, Inc. 2017

A2: H7 M R2

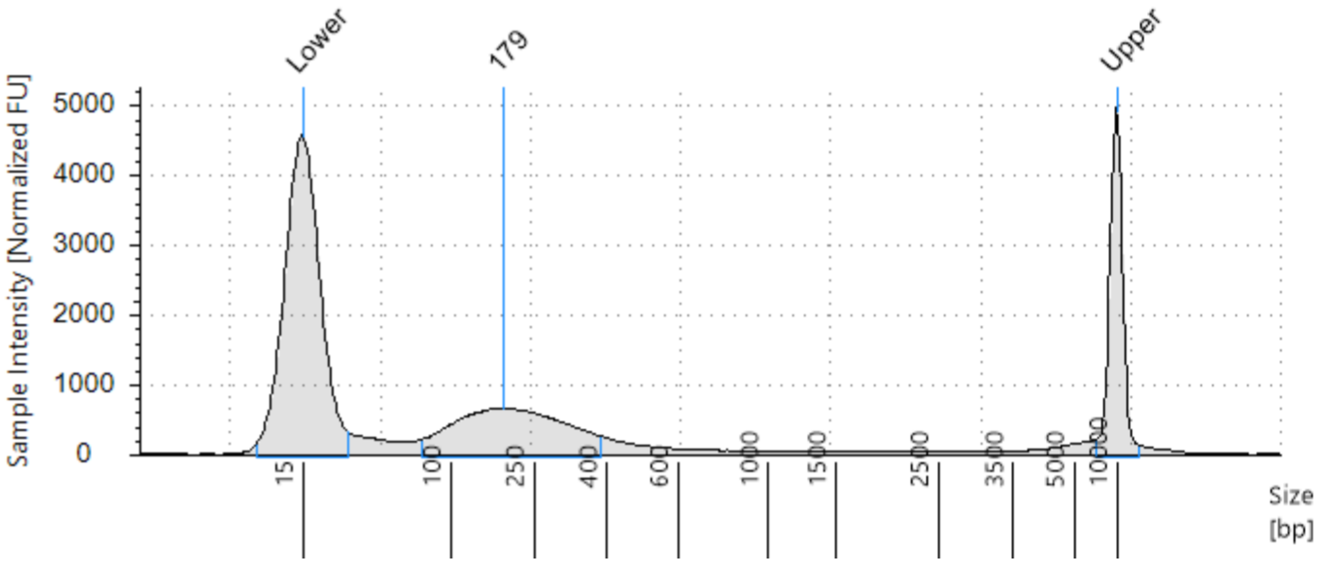

Sample Table

Well	Conc. [ng/µl]	Sample Description	Alert	Observations
A2	4.19	H7 M R2		

Peak Table

Size [bp]	Calibrated Conc. [ng/µl]	Assigned Conc. [ng/µl]	Peak Molarity [nmol/l]	% Integrated Area	Peak Comment	Observations
15	7.97	-	818	-		Lower Marker
179	4.19	-	36.0	100.00		
10000	3.25	3.25	0.500	-		Upper Marker

© Agilent Technologies, Inc. 2017

B2: A8 M R2

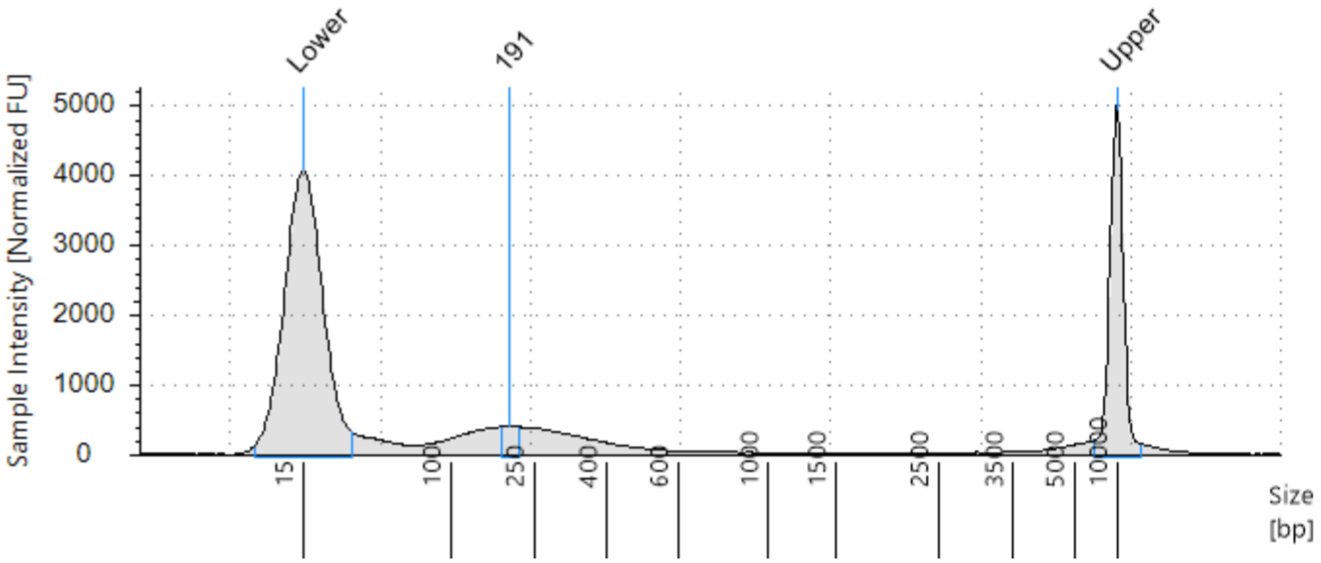

Sample Table

Well	Conc. [ng/µl]	Sample Description	Alert	Observations
B2	0.335	A8 M R2		

Peak Table

Size [bp]	Calibrated Conc. [ng/µl]	Assigned Conc. [ng/µl]	Peak Molarity [nmol/l]	% Integrated Area	Peak Comment	Observations
15	7.31	-	750	-		Lower Marker
191	0.335	-	2.69	100.00		
10000	3.25	3.25	0.500	-		Upper Marker

© Agilent Technologies, Inc. 2017

C2: B8 M R2

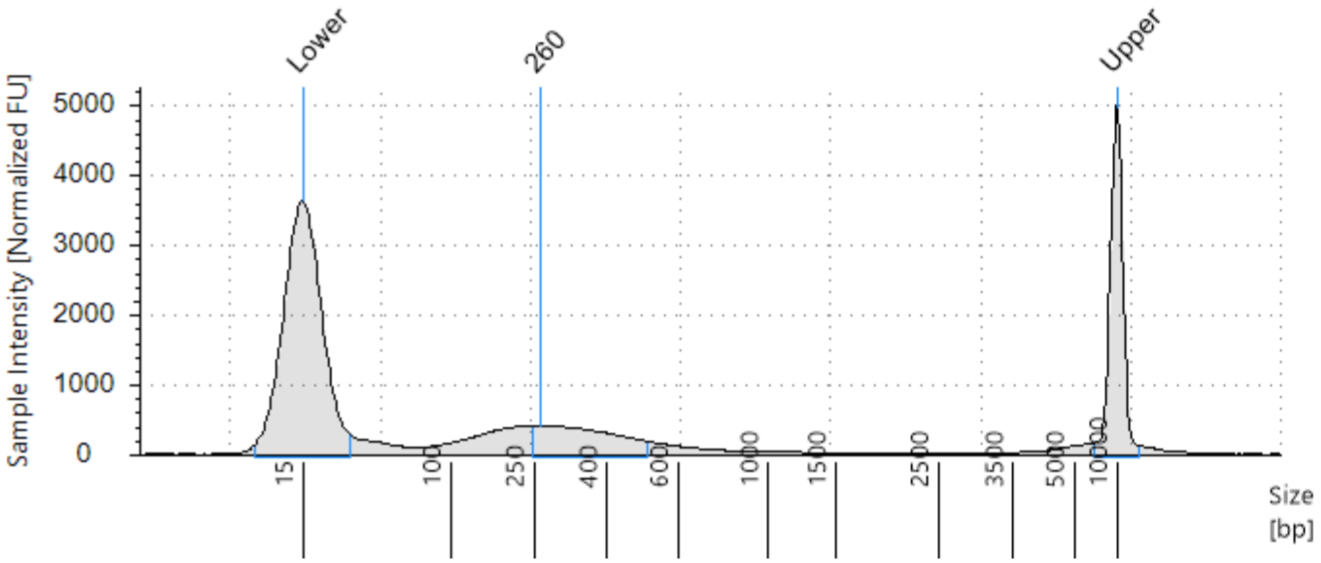

Sample Table

Well	Conc. [ng/µl]	Sample Description	Alert	Observations
C2	1.85	B8 M R2		

Peak Table

Size [bp]	Calibrated Conc. [ng/µl]	Assigned Conc. [ng/µl]	Peak Molarity [nmol/l]	% Integrated Area	Peak Comment	Observations
15	6.83	-	700	-		Lower Marker
260	1.85	-	10.9	100.00		
10000	3.25	3.25	0.500	-		Upper Marker

© Agilent Technologies, Inc. 2017

D2: C8 M R2

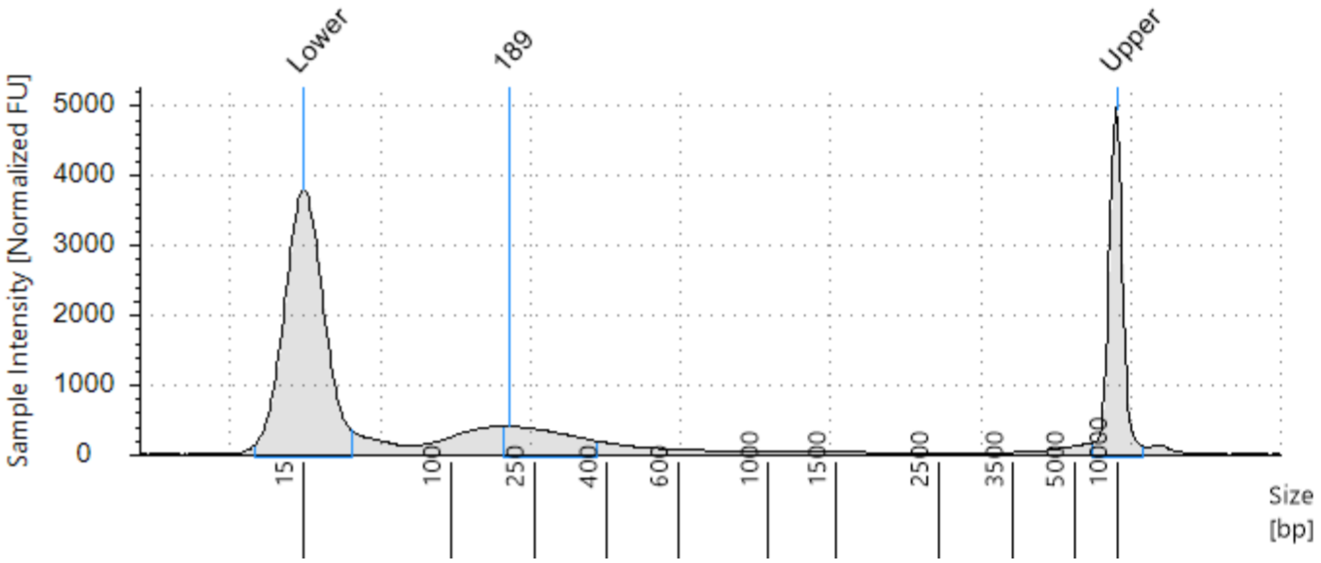

Sample Table

Well	Conc. [ng/µl]	Sample Description	Alert	Observations
D2	1.35	C8 M R2		

Peak Table

Size [bp]	Calibrated Conc. [ng/µl]	Assigned Conc. [ng/µl]	Peak Molarity [nmol/l]	% Integrated Area	Peak Comment	Observations
15	6.73	-	690	-		Lower Marker
189	1.35	-	11.0	100.00		
10000	3.25	3.25	0.500	-		Upper Marker

© Agilent Technologies, Inc. 2017

E2: D8 M R2

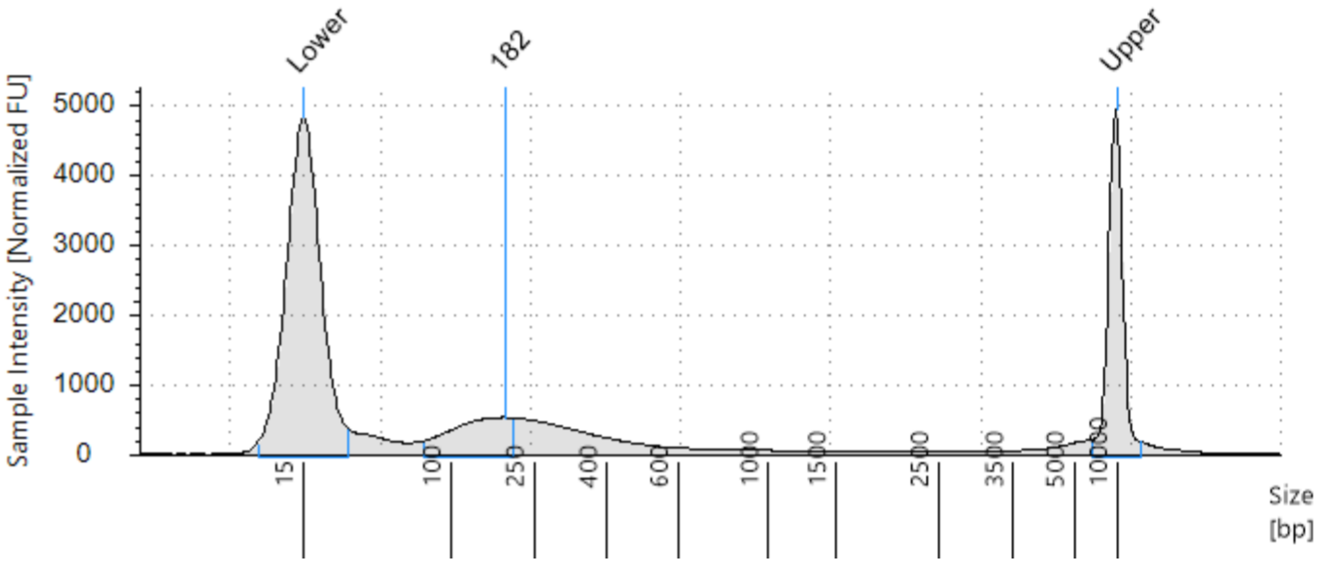

Sample Table

Well	Conc. [ng/µl]	Sample Description	Alert	Observations
E2	1.66	D8 M R2		

Peak Table

Size [bp]	Calibrated Conc. [ng/µl]	Assigned Conc. [ng/µl]	Peak Molarity [nmol/l]	% Integrated Area	Peak Comment	Observations
15	7.52	-	771	-		Lower Marker
182	1.66	-	14.1	100.00		
10000	3.25	3.25	0.500	-		Upper Marker

 © Agilent Technologies, Inc. 2017

F2: E8 M R2

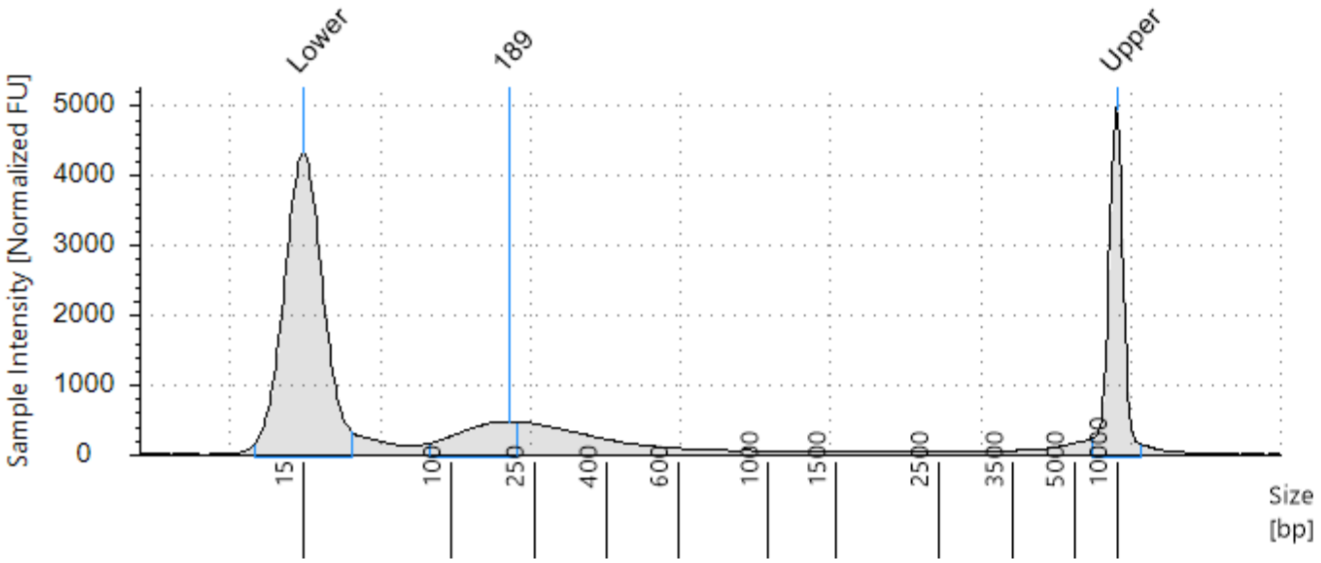

Sample Table

Well	Conc. [ng/µl]	Sample Description	Alert	Observations
F2	1.35	E8 M R2		

Peak Table

Size [bp]	Calibrated Conc. [ng/µl]	Assigned Conc. [ng/µl]	Peak Molarity [nmol/l]	% Integrated Area	Peak Comment	Observations
15	7.42	-	761	-		Lower Marker
189	1.35	-	11.0	100.00		
10000	3.25	3.25	0.500	-		Upper Marker

© Agilent Technologies, Inc. 2017

G2: F8 M R2

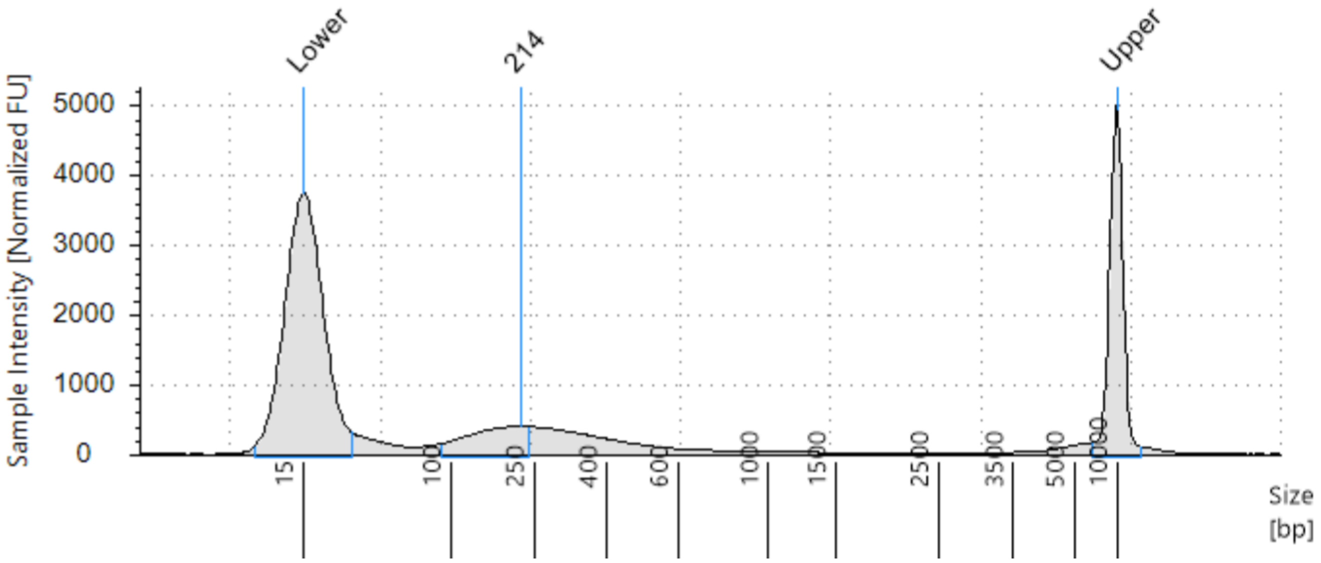

Sample Table

Well	Conc. [ng/µl]	Sample Description	Alert	Observations
G2	1.24	F8 M R2		

Peak Table

Size [bp]	Calibrated Conc. [ng/µl]	Assigned Conc. [ng/µl]	Peak Molarity [nmol/l]	% Integrated Area	Peak Comment	Observations
15	6.61	-	678	-		Lower Marker
214	1.24	-	8.92	100.00		
10000	3.25	3.25	0.500	-		Upper Marker

© Agilent Technologies, Inc. 2017

H2: G8 M R2

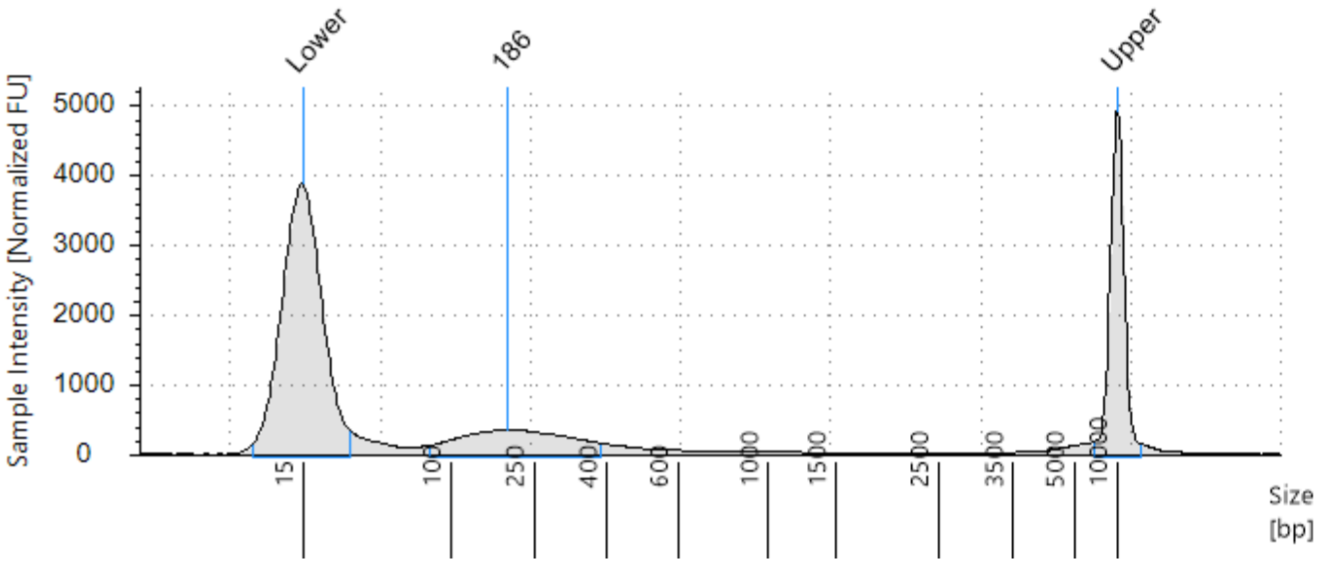

Sample Table

Well	Conc. [ng/µl]	Sample Description	Alert	Observations
H2	2.01	G8 M R2		

Peak Table

Size [bp]	Calibrated Conc. [ng/µl]	Assigned Conc. [ng/µl]	Peak Molarity [nmol/l]	% Integrated Area	Peak Comment	Observations
15	6.95	-	713	-		Lower Marker
186	2.01	-	16.6	100.00		
10000	3.25	3.25	0.500	-		Upper Marker

 © Agilent Technologies, Inc. 2017

Filename: 2020-09-22-01 Q-S,. MINUS , B7-G8, D1000 R2.D1000

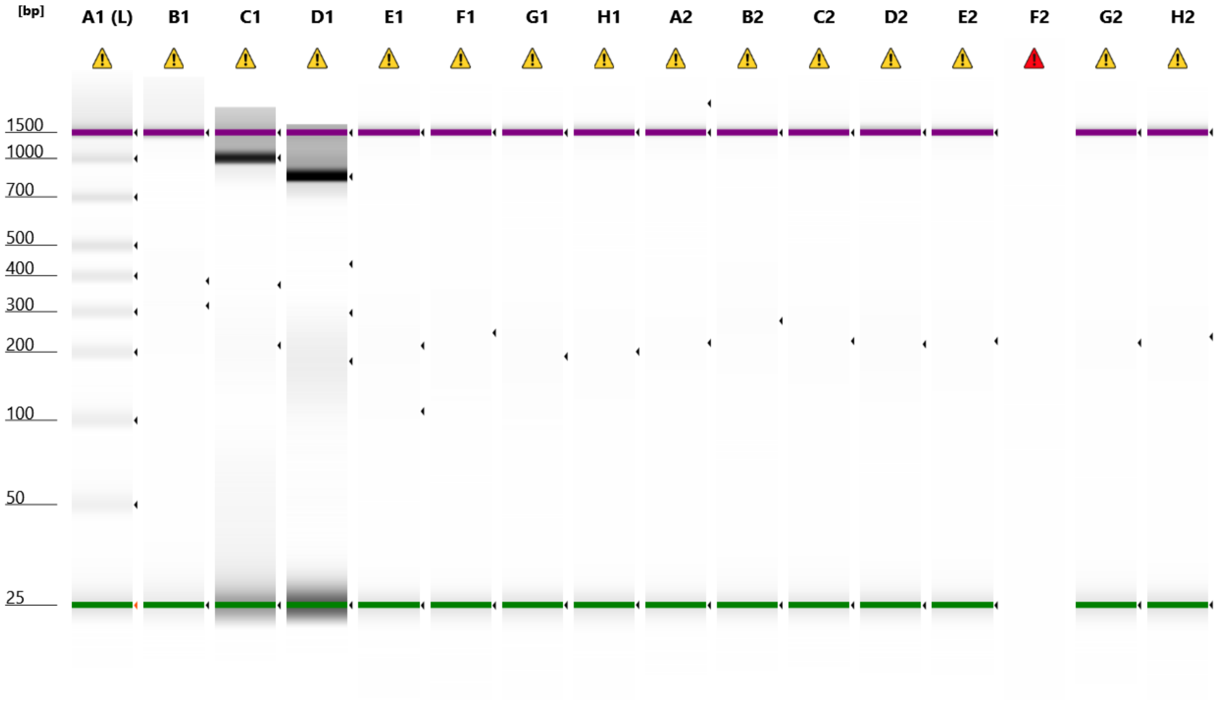

Default image (Contrast 100%)

Sample Info

Well	Conc. [ng/µl]	Sample Description	Alert	Observations
A1	13.1	Ladder	⚠	Caution! Expired ScreenTape device; Ladder
B1	0.476	B7 M R2	⚠	Caution! Expired ScreenTape device
C1	11.4	C7 M R2	⚠	Caution! Expired ScreenTape device
D1	24.0	D7 M R2	⚠	Caution! Expired ScreenTape device
E1	0.443	E7 M R2	⚠	Caution! Expired ScreenTape device
F1	0.714	F7 M R2	⚠	Caution! Expired ScreenTape device
G1	0.294	G7 M R2	⚠	Caution! Expired ScreenTape device
H1	0.127	H7 M R2	⚠	Caution! Expired ScreenTape device
A2	0.228	A8 M R2	⚠	Caution! Expired ScreenTape device
B2	0.250	B8 M R2	⚠	Caution! Expired ScreenTape device
C2	0.198	C8 M R2	⚠	Caution! Expired ScreenTape device
D2	0.913	D8 M R2	⚠	Caution! Expired ScreenTape device
E2	0.142	E8 M R2	⚠	Caution! Expired ScreenTape device
F2		F8 M R2	⚠	Marker(s) not detected; Caution! Expired ScreenTape device
G2	0.0717	G8 M R2	⚠	Caution! Expired ScreenTape device
H2	0.581	H8 M R2	⚠	Caution! Expired ScreenTape device

 © Agilent Technologies, Inc. 2017

A1: Ladder

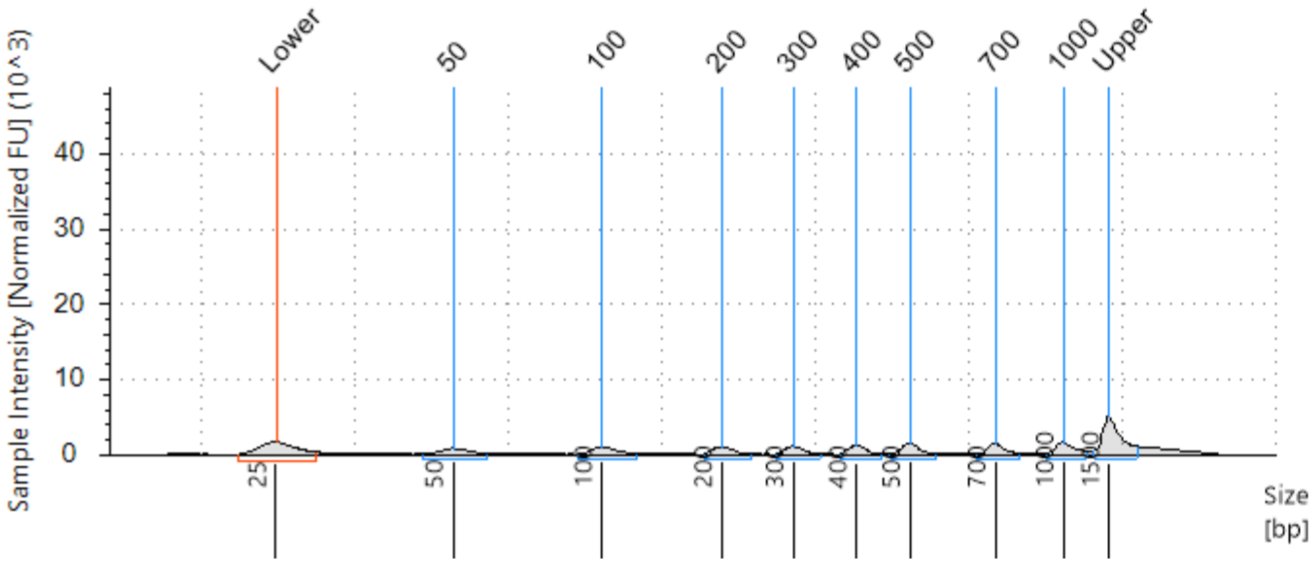

Sample Table

Well	Conc. [ng/µl]	Sample Description	Alert	Observations
A1	13.1	Ladder	⚠	Caution! Expired ScreenTape device; Ladder

Peak Table

Size [bp]	Calibrated Conc. [ng/µl]	Assigned Conc. [ng/µl]	Peak Molarity [nmol/l]	% Integrated Area	Peak Comment	Observations
25	4.10	-	252	-		Lower Marker
50	1.61	-	49.4	12.26		
100	1.65	-	25.4	12.62		
200	1.44	-	11.1	11.02		
300	1.40	-	7.19	10.71		
400	1.46	-	5.60	11.12		
500	1.80	-	5.53	13.72		
700	1.70	-	3.73	12.96		
1000	2.04	-	3.14	15.59		
1500	6.50	6.50	6.67	-		Upper Marker

© Agilent Technologies, Inc. 2017

B1: B7 M R2

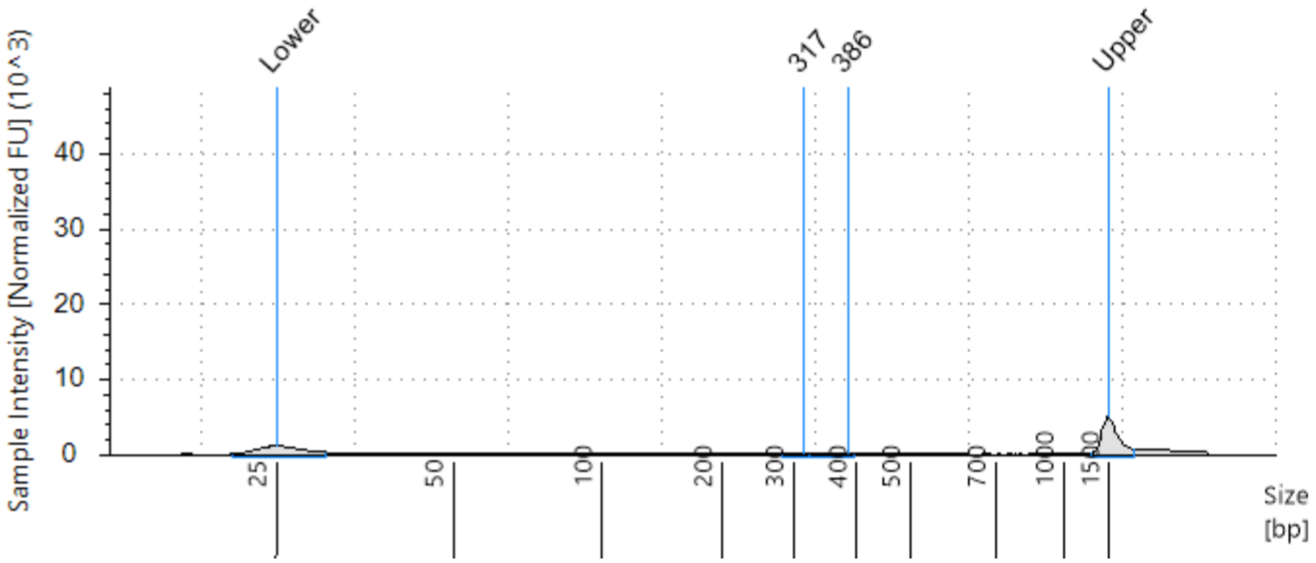

Sample Table

Well	Conc. [ng/µl]	Sample Description	Alert	Observations
B1	0.476	B7 M R2	⚠	Caution! Expired ScreenTape device

Peak Table

Size [bp]	Calibrated Conc. [ng/µl]	Assigned Conc. [ng/µl]	Peak Molarity [nmol/l]	% Integrated Area	Peak Comment	Observations
25	3.86	-	238	-		Lower Marker
317	0.190	-	0.920	39.85		
386	0.286	-	1.14	60.15		
1500	6.50	6.50	6.67	-		Upper Marker

© Agilent Technologies, Inc. 2017

C1: C7 M R2

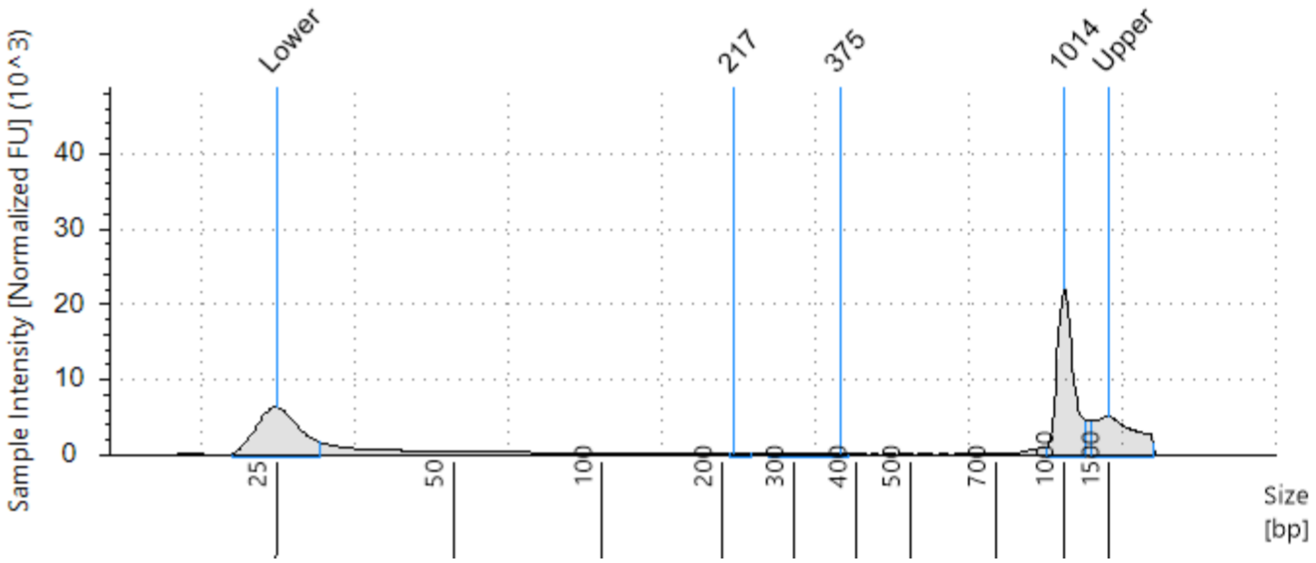

Sample Table

Well	Conc. [ng/µl]	Sample Description	Alert	Observations
C1	11.4	C7 M R2	⚠	Caution! Expired ScreenTape device

Peak Table

Size [bp]	Calibrated Conc. [ng/µl]	Assigned Conc. [ng/µl]	Peak Molarity [nmol/l]	% Integrated Area	Peak Comment	Observations
25	8.18	-	503	-		Lower Marker
217	0.129	-	0.916	1.13		
375	0.187	-	0.767	1.64		
1014	11.1	-	16.8	97.23		
1500	6.50	6.50	6.67	-		Upper Marker

 © Agilent Technologies, Inc. 2017

D1: D7 M R2

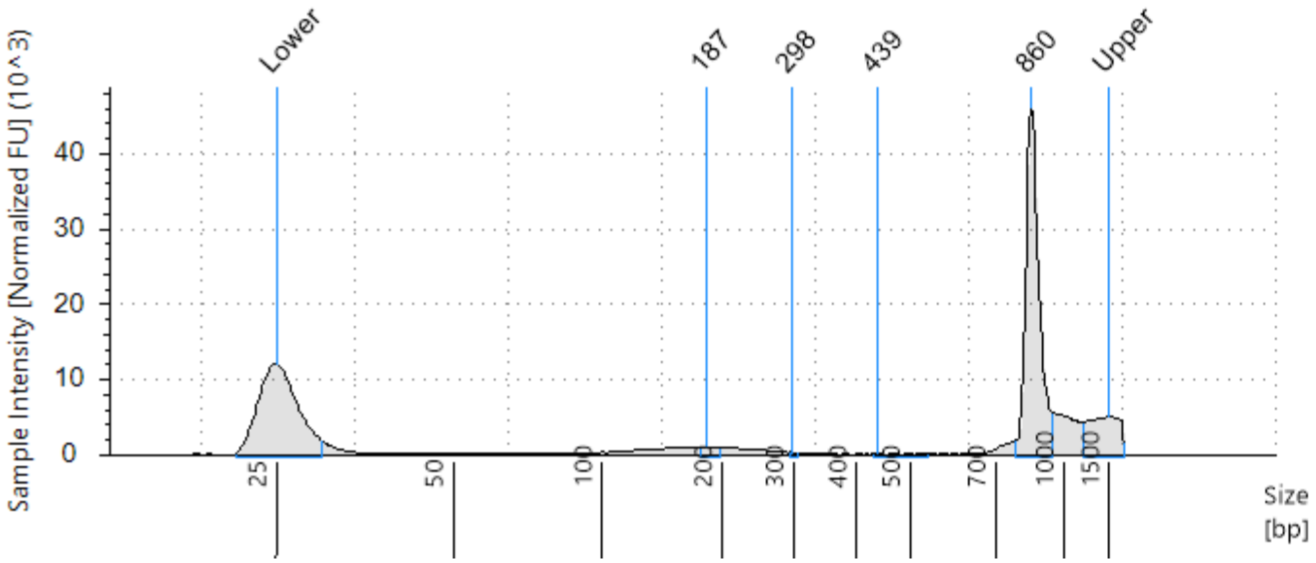

Sample Table

Well	Conc. [ng/µl]	Sample Description	Alert	Observations
D1	24.0	D7 M R2	⚠	Caution! Expired ScreenTape device

Peak Table

Size [bp]	Calibrated Conc. [ng/µl]	Assigned Conc. [ng/µl]	Peak Molarity [nmol/l]	% Integrated Area	Peak Comment	Observations
25	18.1	-	1110	-		Lower Marker
187	0.573	-	4.72	2.38		
298	0.0622	-	0.322	0.26		
439	0.00717	-	0.0251	0.03		
860	23.4	-	41.8	97.33		
1500	6.50	6.50	6.67	-		Upper Marker

© Agilent Technologies, Inc. 2017

E1: E7 M R2

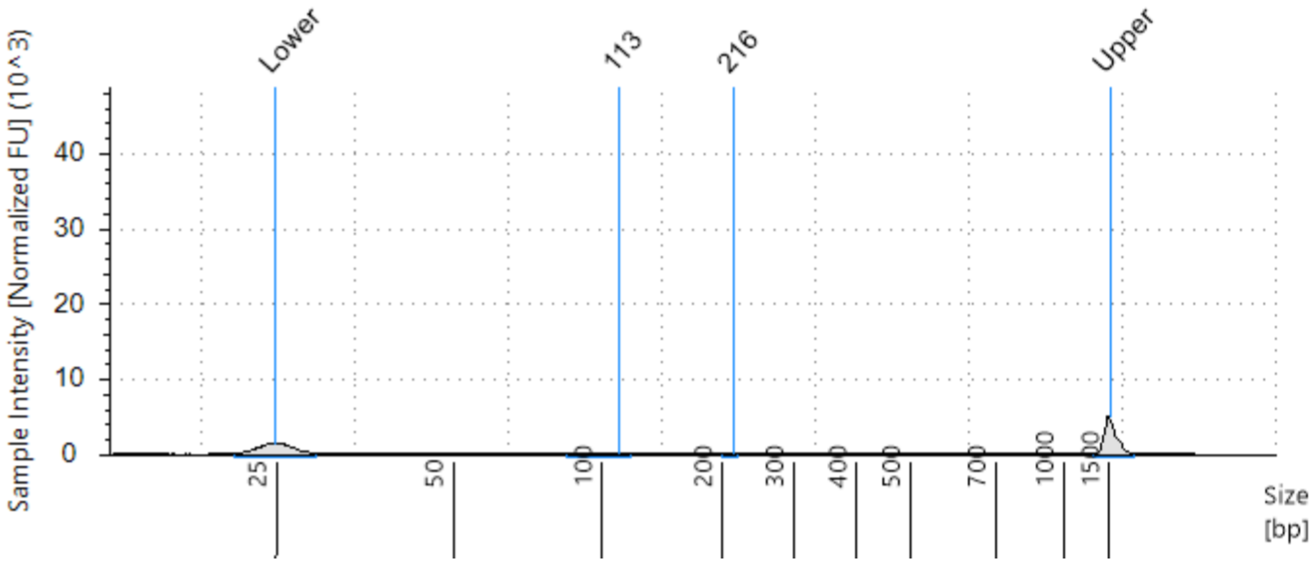

Sample Table

Well	Conc. [ng/µl]	Sample Description	Alert	Observations
E1	0.443	E7 M R2	⚠	Caution! Expired ScreenTape device

Peak Table

Size [bp]	Calibrated Conc. [ng/µl]	Assigned Conc. [ng/µl]	Peak Molarity [nmol/l]	% Integrated Area	Peak Comment	Observations
25	5.71	-	351	-		Lower Marker
113	0.268	-	3.64	60.57		
216	0.175	-	1.24	39.43		
1500	6.50	6.50	6.67	-		Upper Marker

© Agilent Technologies, Inc. 2017

F1: F7 M R2

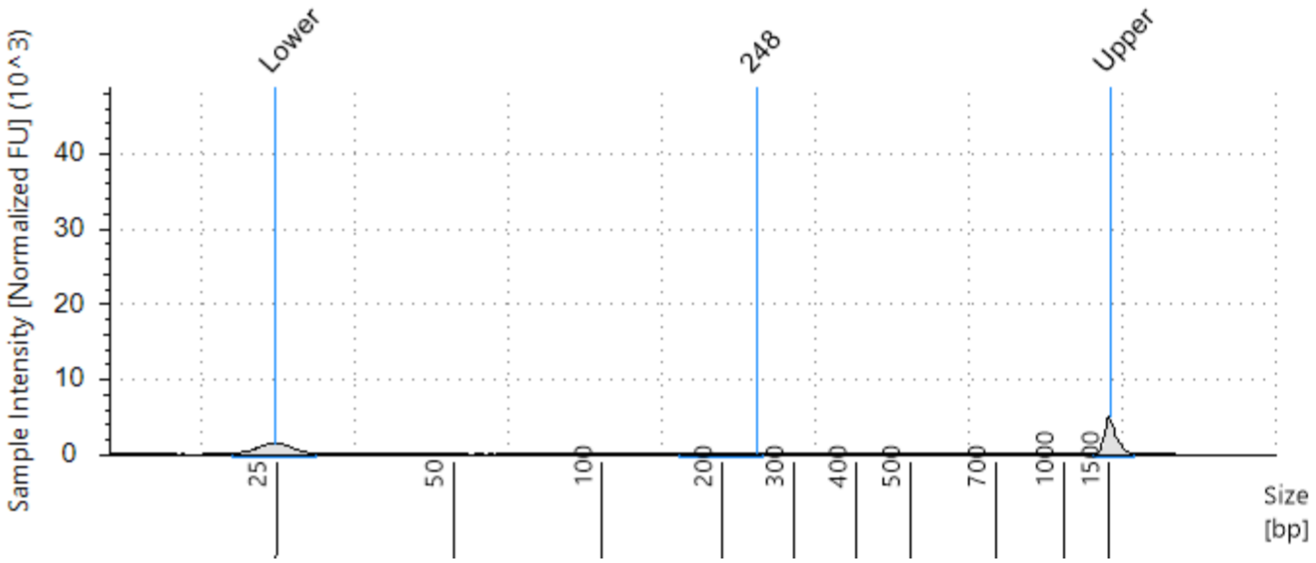

Sample Table

Well	Conc. [ng/µl]	Sample Description	Alert	Observations
F1	0.714	F7 M R2	⚠	Caution! Expired ScreenTape device

Peak Table

Size [bp]	Calibrated Conc. [ng/µl]	Assigned Conc. [ng/µl]	Peak Molarity [nmol/l]	% Integrated Area	Peak Comment	Observations
25	5.77	-	355	-		Lower Marker
248	0.714	-	4.43	100.00		
1500	6.50	6.50	6.67	-		Upper Marker

© Agilent Technologies, Inc. 2017

G1: G7 M R2

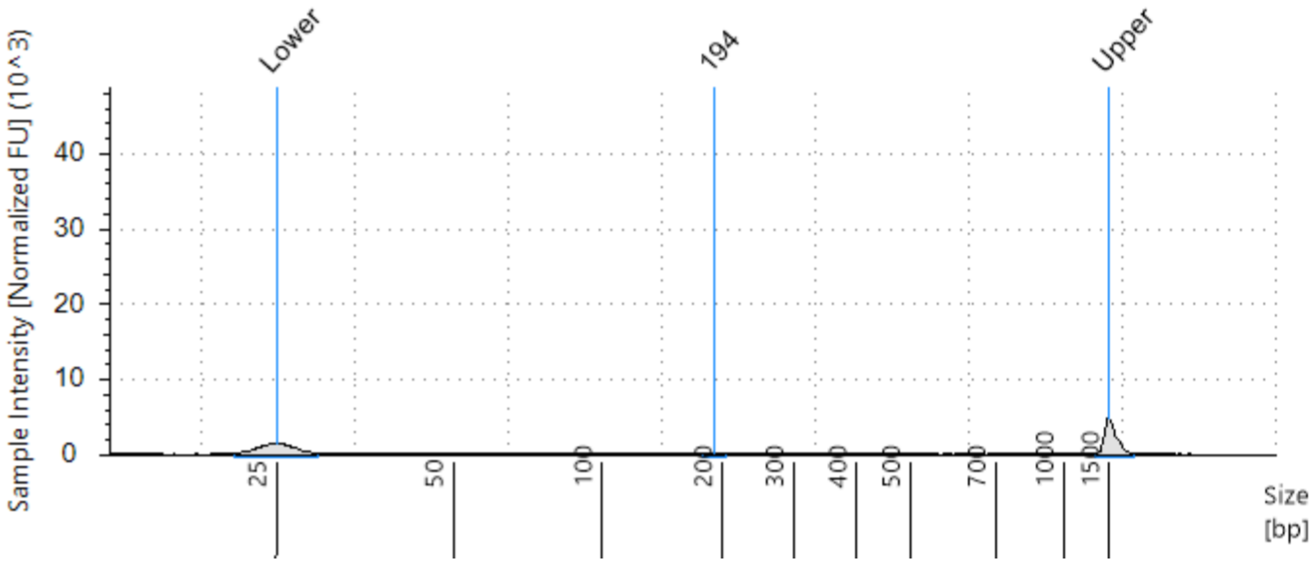

Sample Table

Well	Conc. [ng/µl]	Sample Description	Alert	Observations
G1	0.294	G7 M R2	⚠	Caution! Expired ScreenTape device

Peak Table

Size [bp]	Calibrated Conc. [ng/µl]	Assigned Conc. [ng/µl]	Peak Molarity [nmol/l]	% Integrated Area	Peak Comment	Observations
25	6.04	-	372	-		Lower Marker
194	0.294	-	2.34	100.00		
1500	6.50	6.50	6.67	-		Upper Marker

© Agilent Technologies, Inc. 2017

H1: H7 M R2

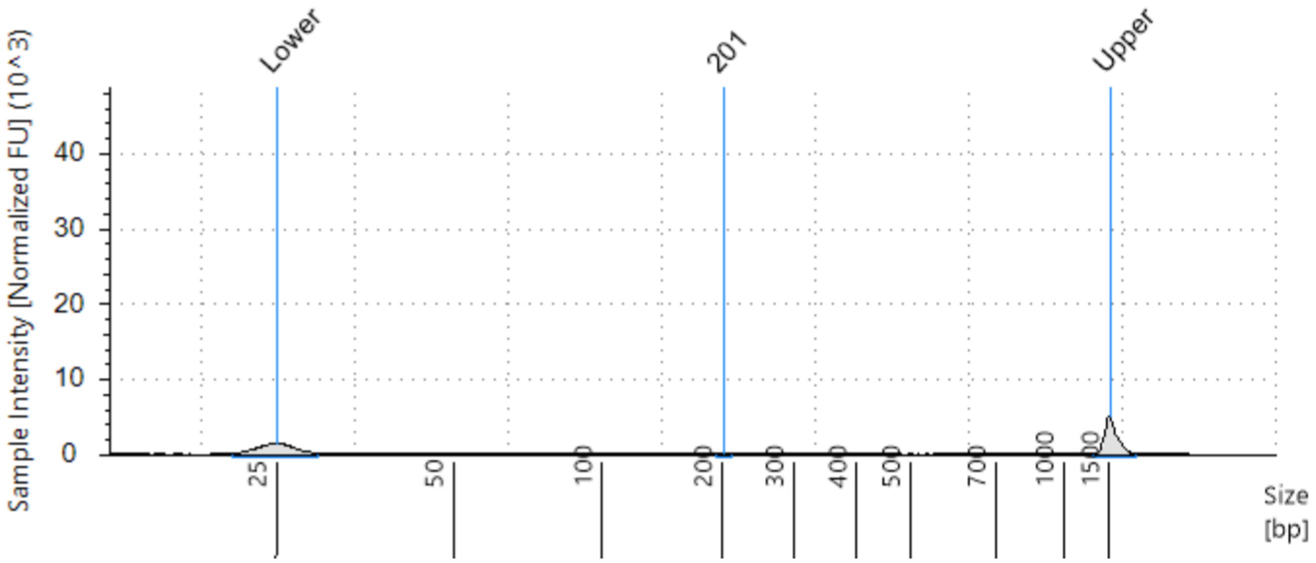

Sample Table

Well	Conc. [ng/µl]	Sample Description	Alert	Observations
H1	0.127	H7 M R2	⚠	Caution! Expired ScreenTape device

Peak Table

Size [bp]	Calibrated Conc. [ng/µl]	Assigned Conc. [ng/µl]	Peak Molarity [nmol/l]	% Integrated Area	Peak Comment	Observations
25	5.76	-	354	-		Lower Marker
201	0.127	-	0.977	100.00		
1500	6.50	6.50	6.67	-		Upper Marker

© Agilent Technologies, Inc. 2017

A2: A8 M R2

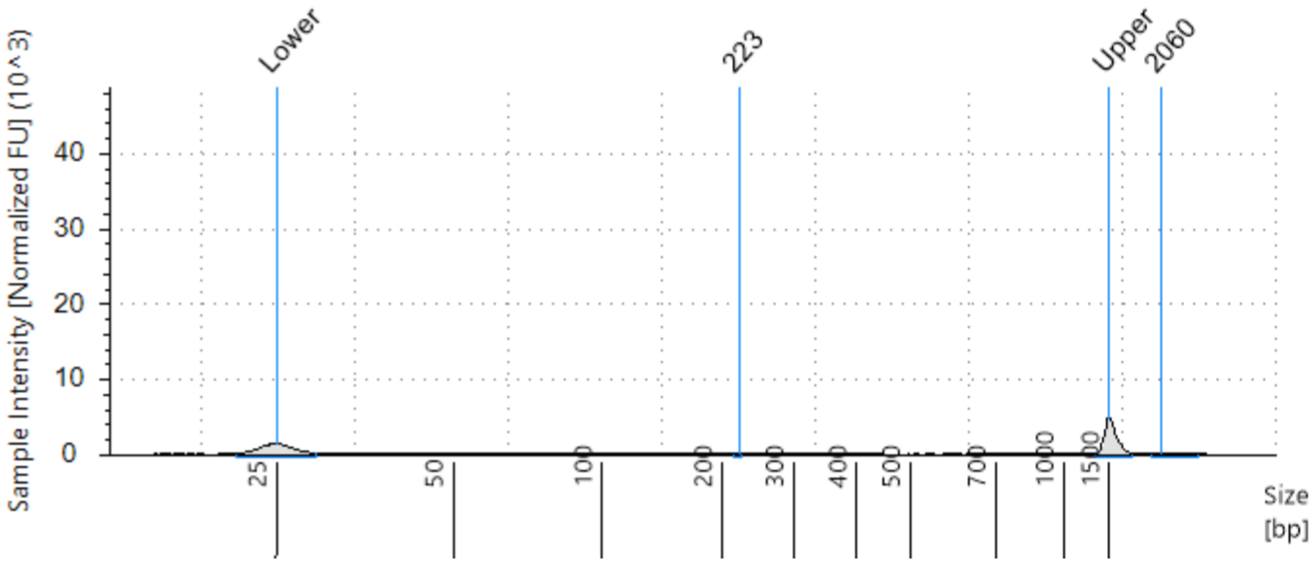

Sample Table

Well	Conc. [ng/µl]	Sample Description	Alert	Observations
A2	0.228	A8 M R2	⚠	Caution! Expired ScreenTape device

Peak Table

Size [bp]	Calibrated Conc. [ng/µl]	Assigned Conc. [ng/µl]	Peak Molarity [nmol/l]	% Integrated Area	Peak Comment	Observations
25	5.37	-	331	-		Lower Marker
223	0.0593	-	0.409	25.96		
1500	6.50	6.50	6.67	-		Upper Marker
2060	0.169	-	0.126	74.04		

© Agilent Technologies, Inc. 2017

B2: B8 M R2

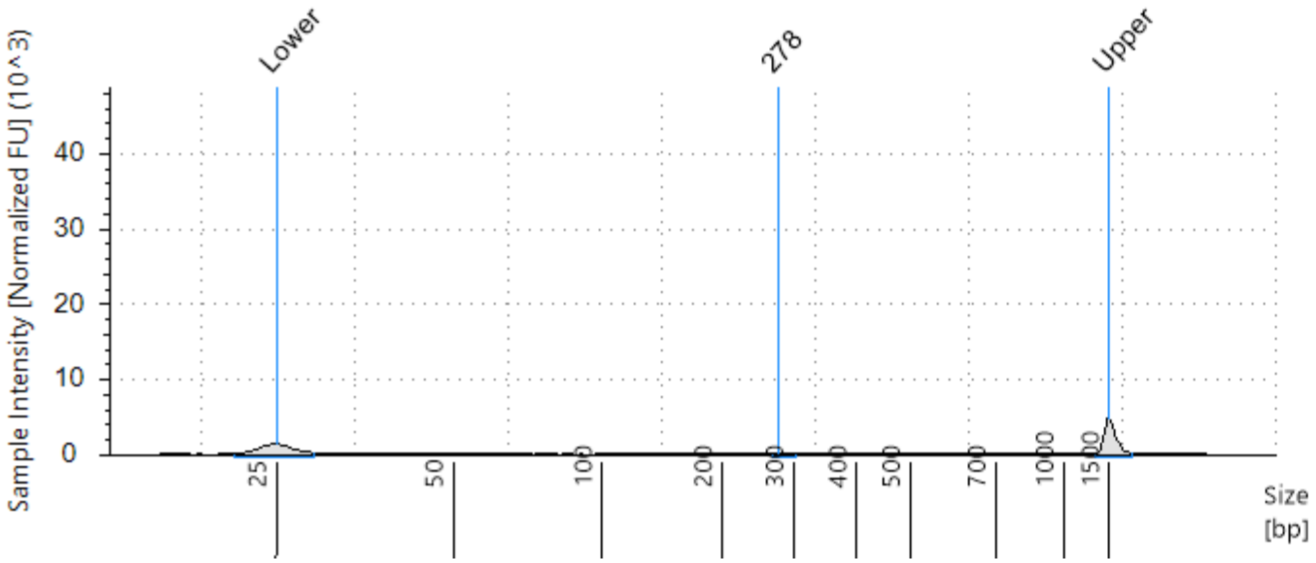

Sample Table

Well	Conc. [ng/µl]	Sample Description	Alert	Observations
B2	0.250	B8 M R2	⚠	Caution! Expired ScreenTape device

Peak Table

Size [bp]	Calibrated Conc. [ng/µl]	Assigned Conc. [ng/µl]	Peak Molarity [nmol/l]	% Integrated Area	Peak Comment	Observations
25	5.37	-	330	-		Lower Marker
278	0.250	-	1.39	100.00		
1500	6.50	6.50	6.67	-		Upper Marker

© Agilent Technologies, Inc. 2017

C2: C8 M R2

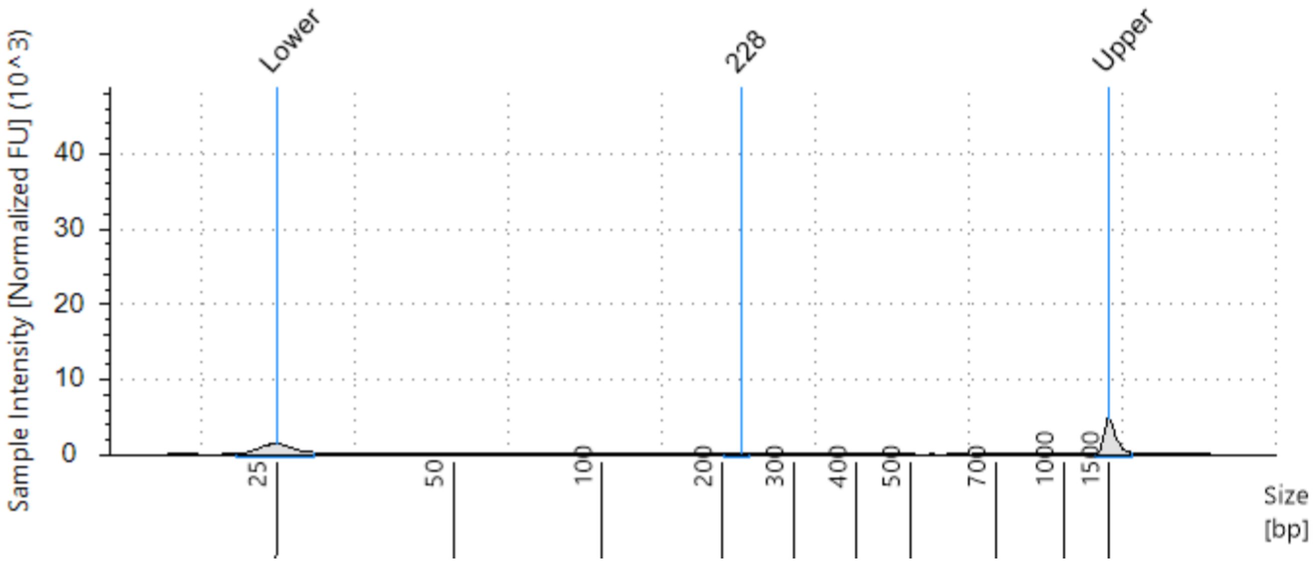

Sample Table

Well	Conc. [ng/µl]	Sample Description	Alert	Observations
C2	0.198	C8 M R2	⚠	Caution! Expired ScreenTape device

Peak Table

Size [bp]	Calibrated Conc. [ng/µl]	Assigned Conc. [ng/µl]	Peak Molarity [nmol/l]	% Integrated Area	Peak Comment	Observations
25	5.39	-	331	-		Lower Marker
228	0.198	-	1.34	100.00		
1500	6.50	6.50	6.67	-		Upper Marker

© Agilent Technologies, Inc. 2017

D2: D8 M R2

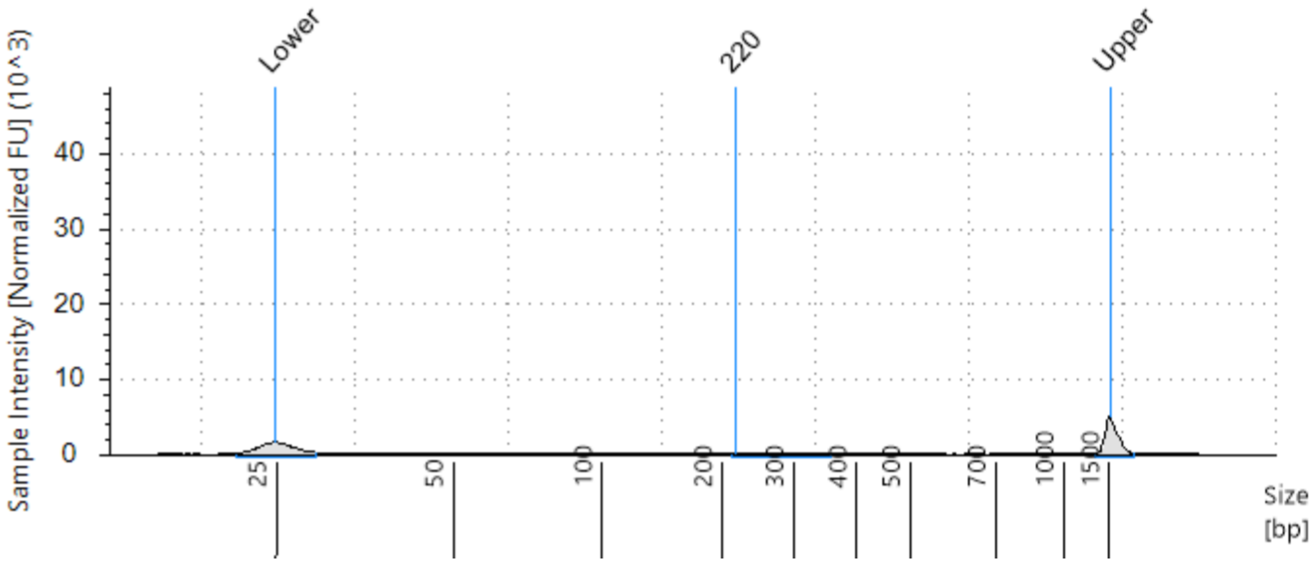

Sample Table

Well	Conc. [ng/µl]	Sample Description	Alert	Observations
D2	0.913	D8 M R2	⚠	Caution! Expired ScreenTape device

Peak Table

Size [bp]	Calibrated Conc. [ng/µl]	Assigned Conc. [ng/µl]	Peak Molarity [nmol/l]	% Integrated Area	Peak Comment	Observations
25	5.78	-	356	-		Lower Marker
220	0.913	-	6.39	100.00		
1500	6.50	6.50	6.67	-		Upper Marker

© Agilent Technologies, Inc. 2017

E2: E8 M R2

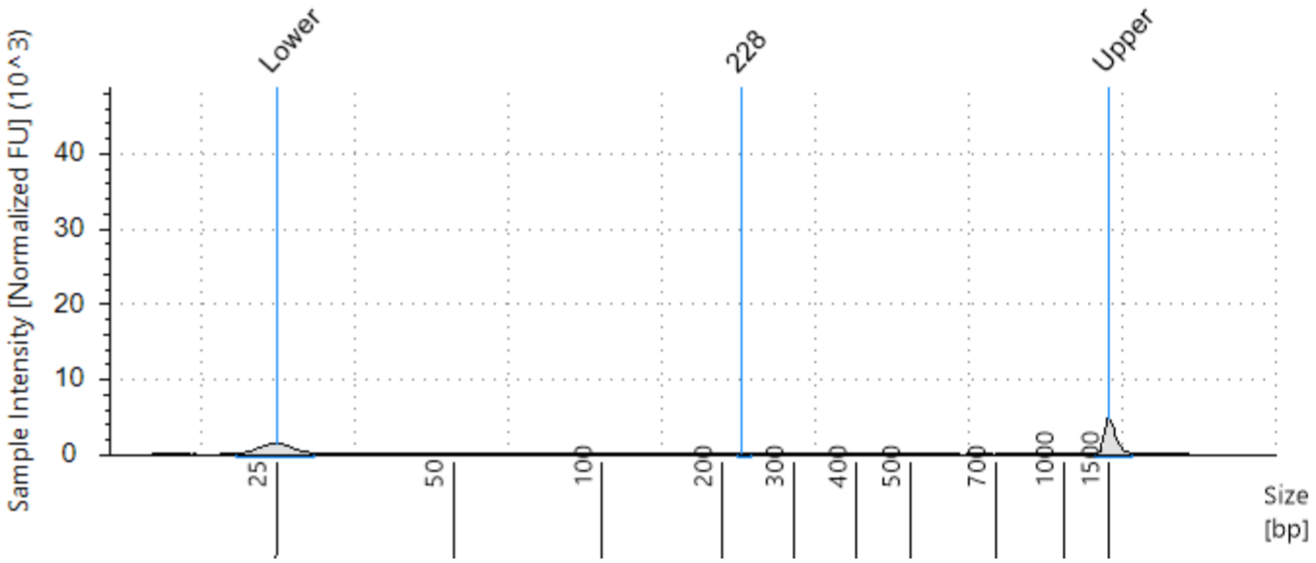

Sample Table

Well	Conc. [ng/µl]	Sample Description	Alert	Observations
E2	0.142	E8 M R2	⚠	Caution! Expired ScreenTape device

Peak Table

Size [bp]	Calibrated Conc. [ng/µl]	Assigned Conc. [ng/µl]	Peak Molarity [nmol/l]	% Integrated Area	Peak Comment	Observations
25	5.64	-	347	-		Lower Marker
228	0.142	-	0.957	100.00		
1500	6.50	6.50	6.67	-		Upper Marker

© Agilent Technologies, Inc. 2017

F2: F8 M R2

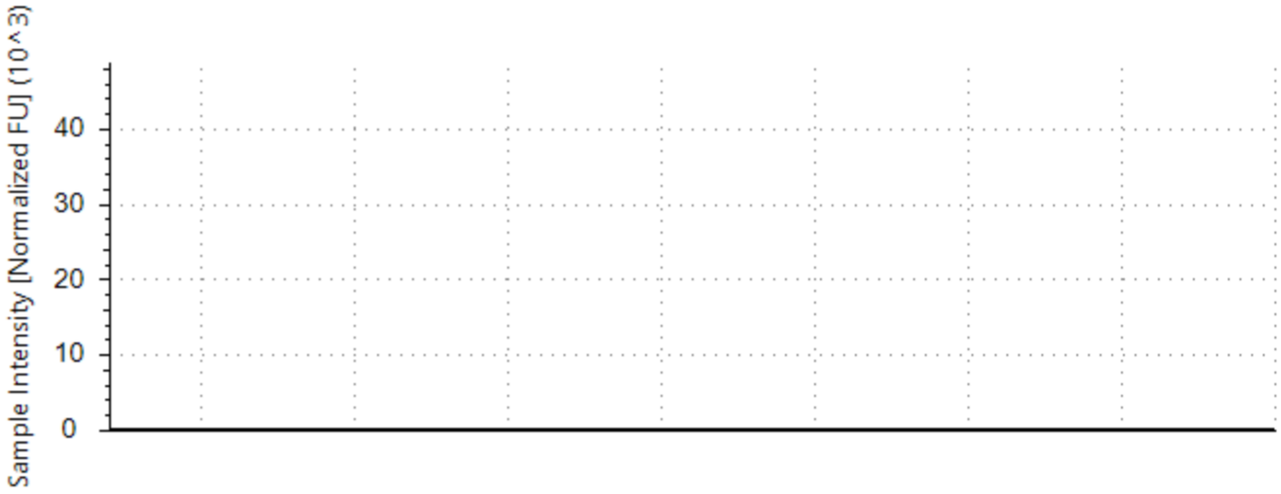

Sample Table

Well	Conc. [ng/µl]	Sample Description	Alert	Observations
F2		F8 M R2	▲	Marker(s) not detected; Caution! Expired ScreenTape device

© Agilent Technologies, Inc. 2017

G2: G8 M R2

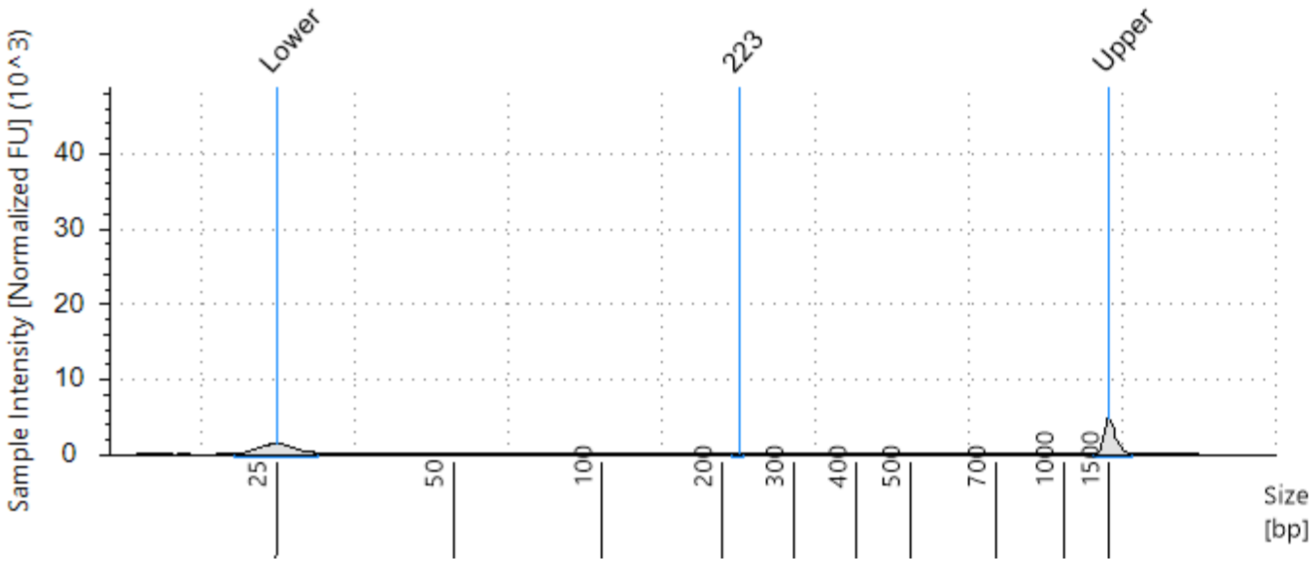

Sample Table

Well	Conc. [ng/µl]	Sample Description	Alert	Observations
G2	0.0717	G8 M R2	⚠	Caution! Expired ScreenTape device

Peak Table

Size [bp]	Calibrated Conc. [ng/µl]	Assigned Conc. [ng/µl]	Peak Molarity [nmol/l]	% Integrated Area	Peak Comment	Observations
25	5.96	-	367	-		Lower Marker
223	0.0717	-	0.494	100.00		
1500	6.50	6.50	6.67	-		Upper Marker

 © Agilent Technologies, Inc. 2017

H2: H8 M R2

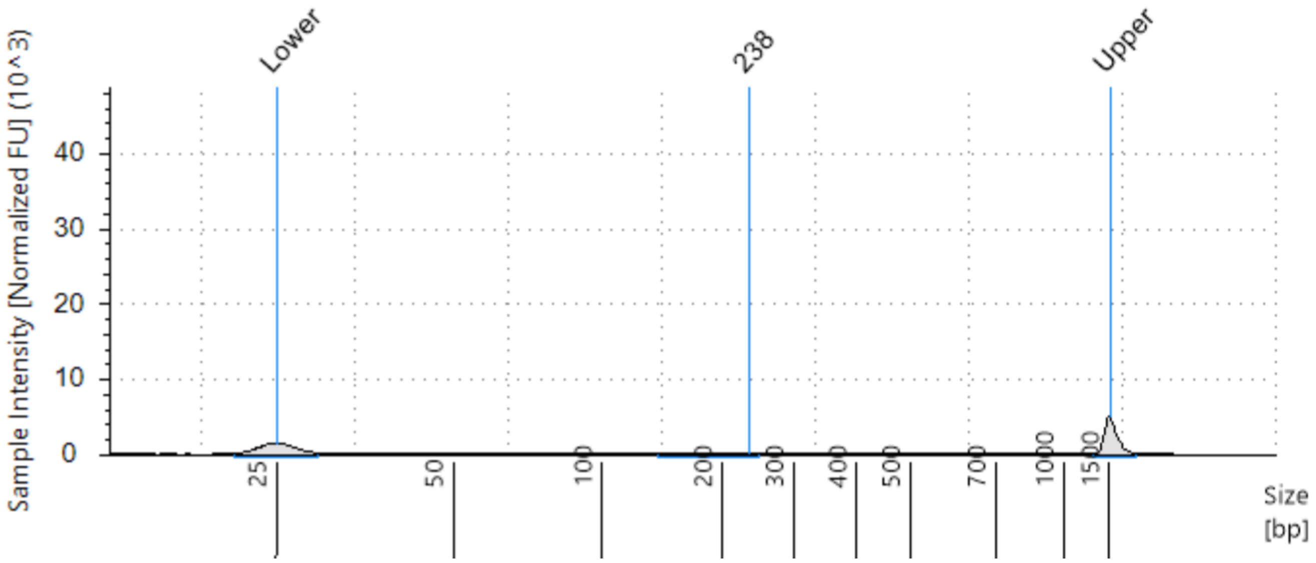

Sample Table

Well	Conc. [ng/µl]	Sample Description	Alert	Observations
H2	0.581	H8 M R2	⚠	Caution! Expired ScreenTape device

Peak Table

Size [bp]	Calibrated Conc. [ng/µl]	Assigned Conc. [ng/µl]	Peak Molarity [nmol/l]	% Integrated Area	Peak Comment	Observations
25	5.76	-	355	-		Lower Marker
238	0.581	-	3.76	100.00		
1500	6.50	6.50	6.67	-		Upper Marker

© Agilent Technologies, Inc. 2017

Filename: 2020-09-22-02 Q-S,. MINUS, A9-G10, D1000 R2.D1000

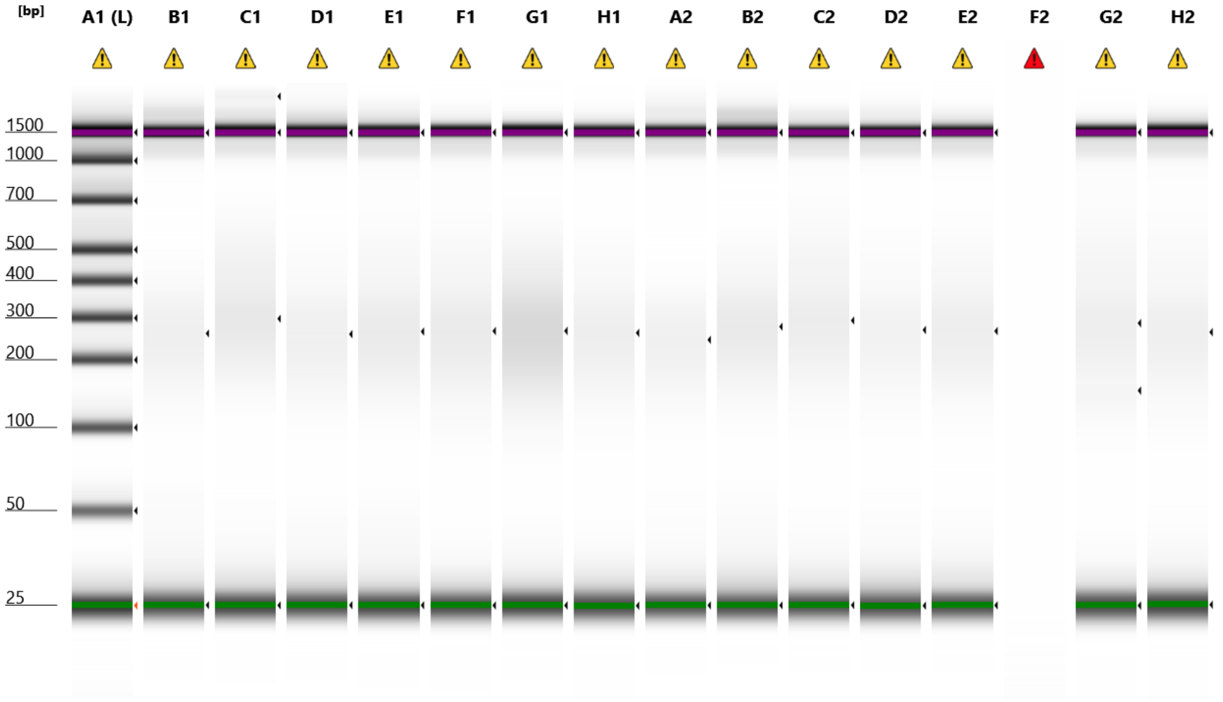

Default image (Contrast 100%)

Sample Info

Well	Conc. [ng/µl]	Sample Description	Alert	Observations
A1	18.6	Ladder	⚠	Caution! Expired ScreenTape device; Ladder
B1	0.537	A9 M R2	⚠	Caution! Expired ScreenTape device
C1	0.825	B9 M R2	⚠	Caution! Expired ScreenTape device
D1	0.0922	C9 M R2	⚠	Caution! Expired ScreenTape device
E1	0.198	D9 M R2	⚠	Caution! Expired ScreenTape device
F1	0.215	E9 M R2	⚠	Caution! Expired ScreenTape device
G1	0.250	F9 M R2	⚠	Caution! Expired ScreenTape device
H1	0.227	G9 M R2	⚠	Caution! Expired ScreenTape device
A2	0.0857	H9 M R2	⚠	Caution! Expired ScreenTape device
B2	0.190	A10 M R2	⚠	Caution! Expired ScreenTape device
C2	0.218	B10 M R2	⚠	Caution! Expired ScreenTape device
D2	0.0791	C10 M R2	⚠	Caution! Expired ScreenTape device
E2	0.113	D10 M R2	⚠	Caution! Expired ScreenTape device
F2		E10 M R2	⚠	Marker(s) not detected; Caution! Expired ScreenTape device
G2	0.810	F10 M R2	⚠	Caution! Expired ScreenTape device
H2	0.177	G10 M R2	⚠	Caution! Expired ScreenTape device

© Agilent Technologies, Inc. 2017

A1: Ladder

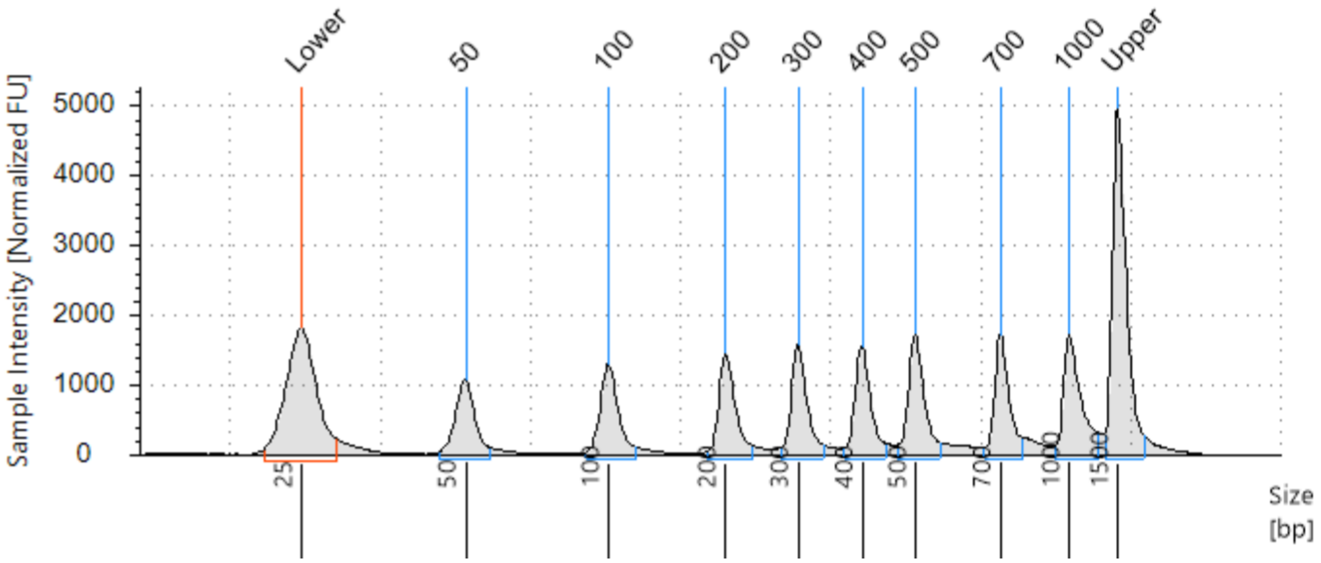

Sample Table

Well	Conc. [ng/µl]	Sample Description	Alert	Observations
A1	18.6	Ladder	⚠	Caution! Expired ScreenTape device; Ladder

Peak Table

Size [bp]	Calibrated Conc. [ng/µl]	Assigned Conc. [ng/µl]	Peak Molarity [nmol/l]	% Integrated Area	Peak Comment	Observations
25	5.13	-	316	-		Lower Marker
50	1.95	-	59.9	10.46		
100	2.16	-	33.2	11.60		
200	2.23	-	17.1	11.98		
300	2.30	-	11.8	12.35		
400	2.30	-	8.85	12.37		
500	2.55	-	7.85	13.71		
700	2.34	-	5.14	12.57		
1000	2.78	-	4.28	14.96		
1500	6.50	6.50	6.67	-		Upper Marker

© Agilent Technologies, Inc. 2017

B1: A9 M R2

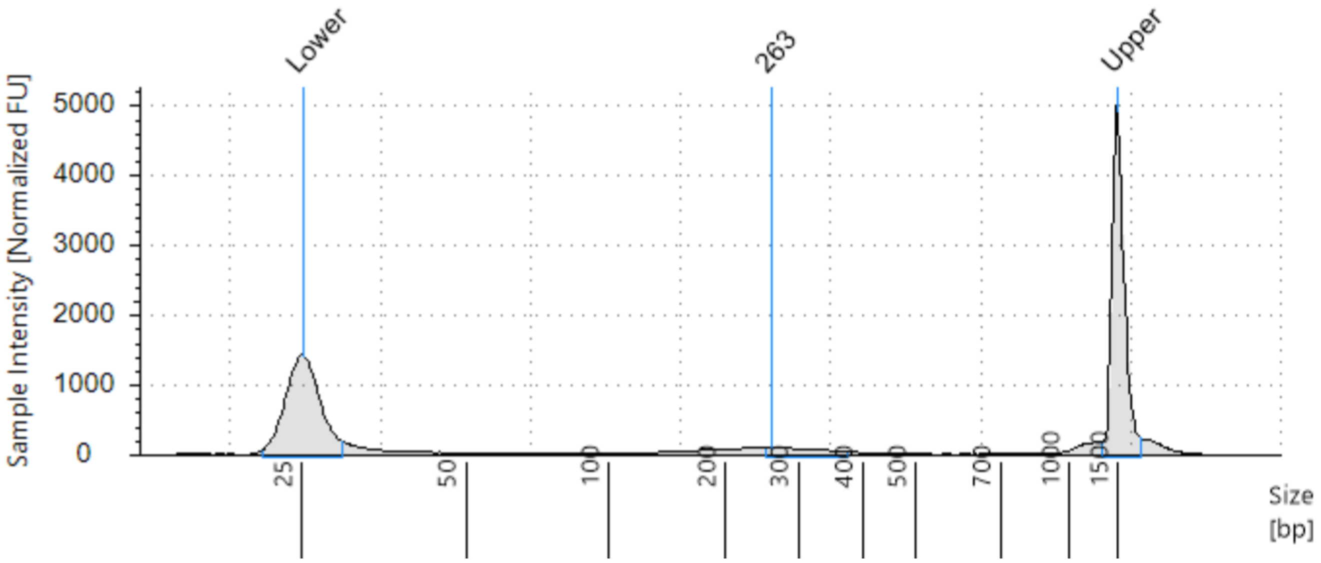

Sample Table

Well	Conc. [ng/µl]	Sample Description	Alert	Observations
B1	0.537	A9 M R2	⚠	Caution! Expired ScreenTape device

Peak Table

Size [bp]	Calibrated Conc. [ng/µl]	Assigned Conc. [ng/µl]	Peak Molarity [nmol/l]	% Integrated Area	Peak Comment	Observations
25	5.21	-	321	-		Lower Marker
263	0.537	-	3.14	100.00		
1500	6.50	6.50	6.67	-		Upper Marker

© Agilent Technologies, Inc. 2017

C1: B9 M R2

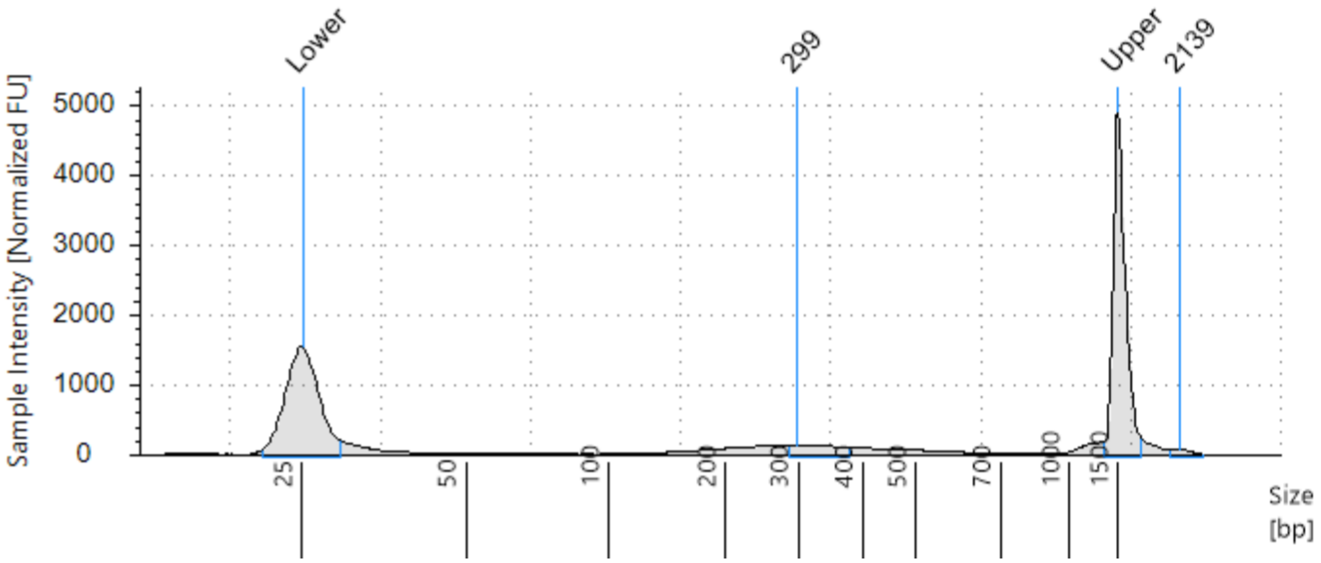

Sample Table

Well	Conc. [ng/µl]	Sample Description	Alert	Observations
C1	0.825	B9 M R2	⚠	Caution! Expired ScreenTape device

Peak Table

Size [bp]	Calibrated Conc. [ng/µl]	Assigned Conc. [ng/µl]	Peak Molarity [nmol/l]	% Integrated Area	Peak Comment	Observations
25	5.57	-	343	-		Lower Marker
299	0.683	-	3.52	82.80		
1500	6.50	6.50	6.67	-		Upper Marker
2139	0.142	-	0.102	17.20		

© Agilent Technologies, Inc. 2017

D1: C9 M R2

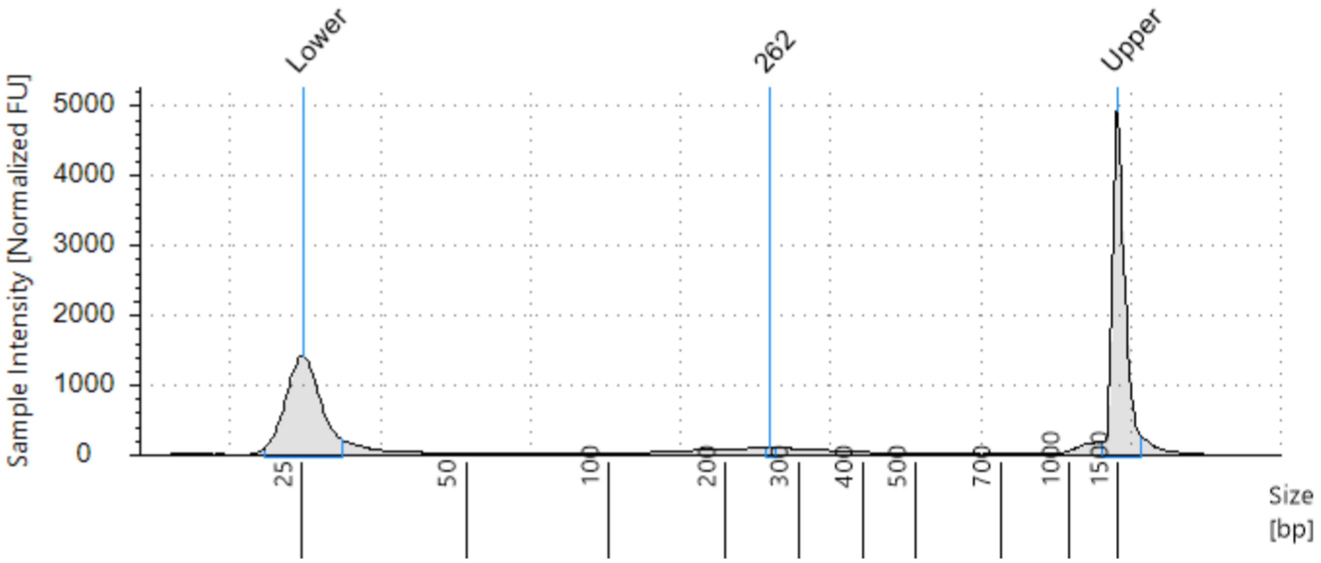

Sample Table

Well	Conc. [ng/µl]	Sample Description	Alert	Observations
D1	0.0922	C9 M R2	⚠	Caution! Expired ScreenTape device

Peak Table

Size [bp]	Calibrated Conc. [ng/µl]	Assigned Conc. [ng/µl]	Peak Molarity [nmol/l]	% Integrated Area	Peak Comment	Observations
25	5.21	-	320	-		Lower Marker
262	0.0922	-	0.542	100.00		
1500	6.50	6.50	6.67	-		Upper Marker

© Agilent Technologies, Inc. 2017

E1: D9 M R2

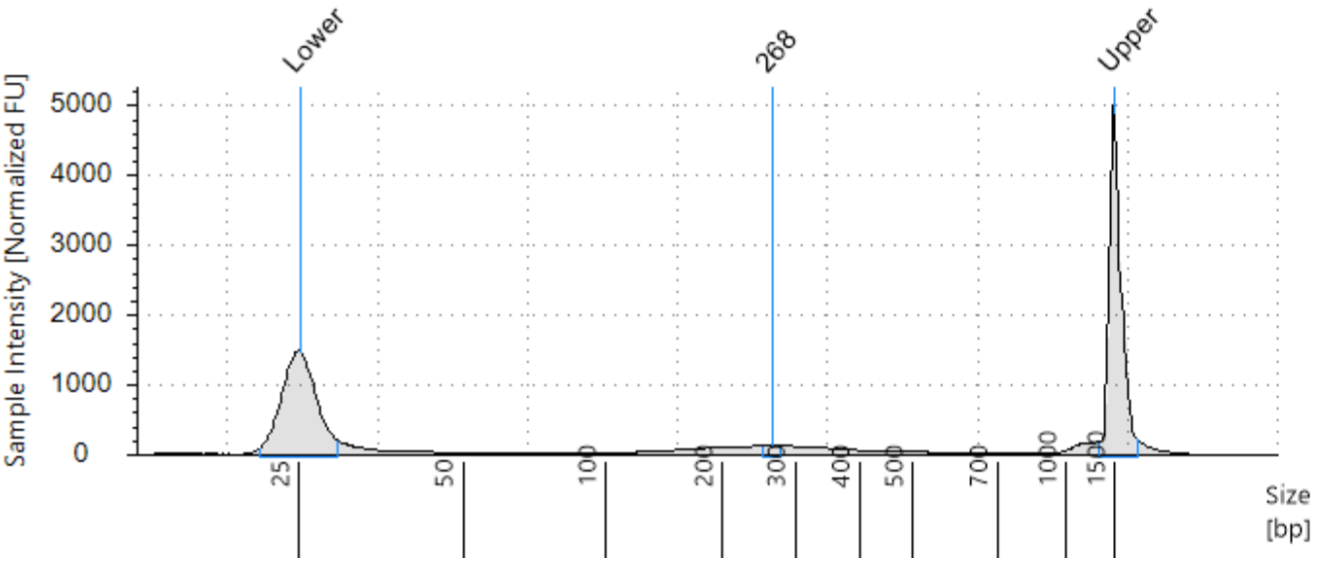

Sample Table

Well	Conc. [ng/µl]	Sample Description	Alert	Observations
E1	0.198	D9 M R2	⚠	Caution! Expired ScreenTape device

Peak Table

Size [bp]	Calibrated Conc. [ng/µl]	Assigned Conc. [ng/µl]	Peak Molarity [nmol/l]	% Integrated Area	Peak Comment	Observations
25	5.66	-	348	-		Lower Marker
268	0.198	-	1.14	100.00		
1500	6.50	6.50	6.67	-		Upper Marker

 © Agilent Technologies, Inc. 2017

F1: E9 M R2

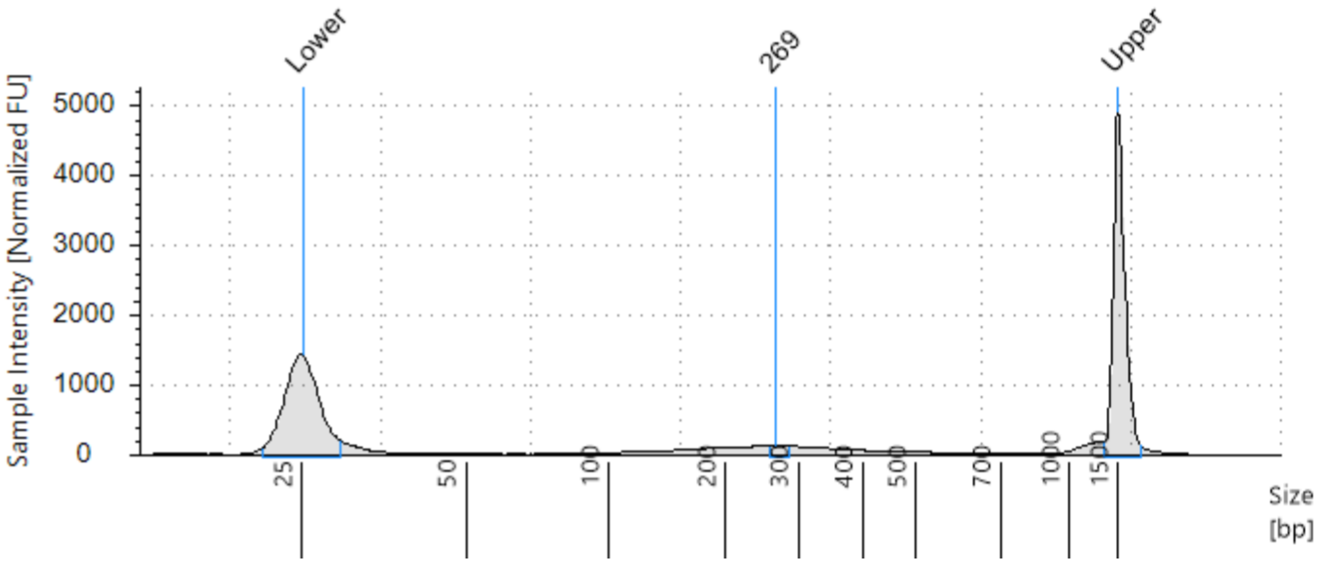

Sample Table

Well	Conc. [ng/µl]	Sample Description	Alert	Observations
F1	0.215	E9 M R2	⚠	Caution! Expired ScreenTape device

Peak Table

Size [bp]	Calibrated Conc. [ng/µl]	Assigned Conc. [ng/µl]	Peak Molarity [nmol/l]	% Integrated Area	Peak Comment	Observations
25	5.62	-	346	-		Lower Marker
269	0.215	-	1.23	100.00		
1500	6.50	6.50	6.67	-		Upper Marker

 © Agilent Technologies, Inc. 2017

G1: F9 M R2

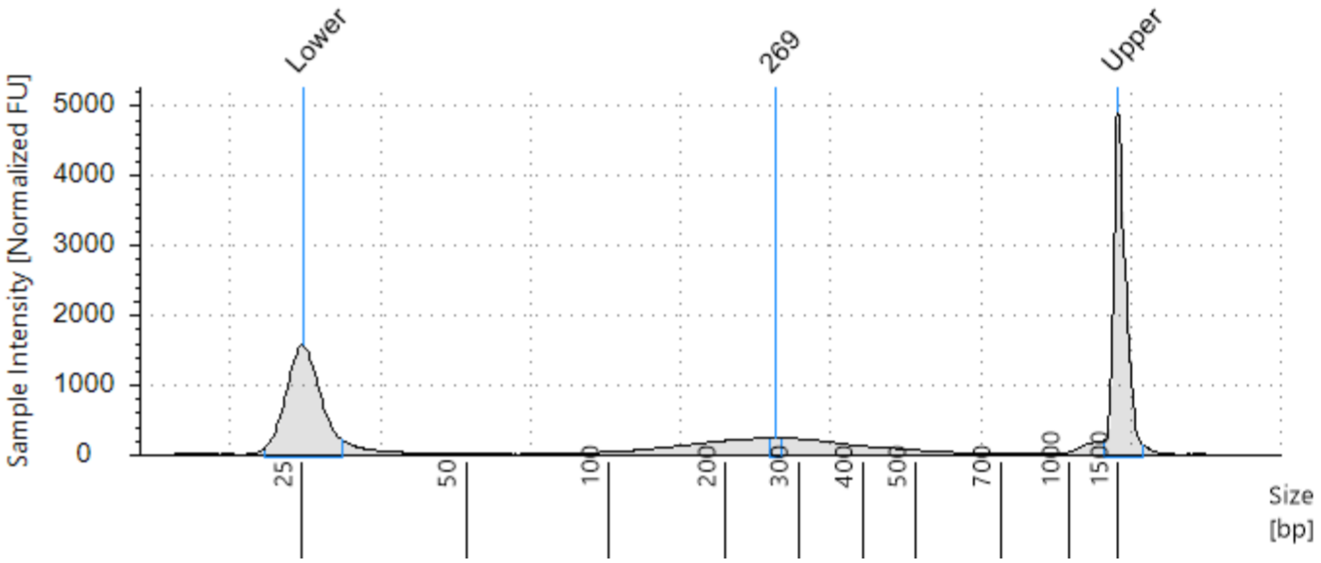

Sample Table

Well	Conc. [ng/µl]	Sample Description	Alert	Observations
G1	0.250	F9 M R2	⚠	Caution! Expired ScreenTape device

Peak Table

Size [bp]	Calibrated Conc. [ng/µl]	Assigned Conc. [ng/µl]	Peak Molarity [nmol/l]	% Integrated Area	Peak Comment	Observations
25	5.80	-	357	-		Lower Marker
269	0.250	-	1.43	100.00		
1500	6.50	6.50	6.67	-		Upper Marker

© Agilent Technologies, Inc. 2017

H1: G9 M R2

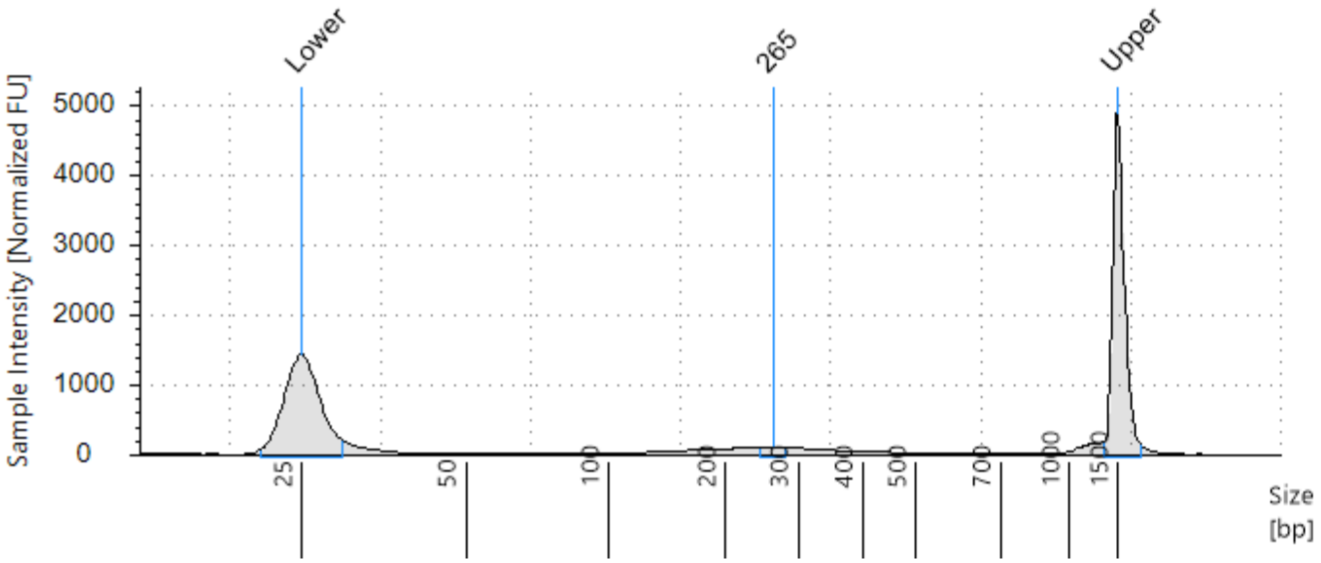

Sample Table

Well	Conc. [ng/µl]	Sample Description	Alert	Observations
H1	0.227	G9 M R2	⚠	Caution! Expired ScreenTape device

Peak Table

Size [bp]	Calibrated Conc. [ng/µl]	Assigned Conc. [ng/µl]	Peak Molarity [nmol/l]	% Integrated Area	Peak Comment	Observations
25	5.79	-	356	-		Lower Marker
265	0.227	-	1.31	100.00		
1500	6.50	6.50	6.67	-		Upper Marker

© Agilent Technologies, Inc. 2017

A2: H9 M R2

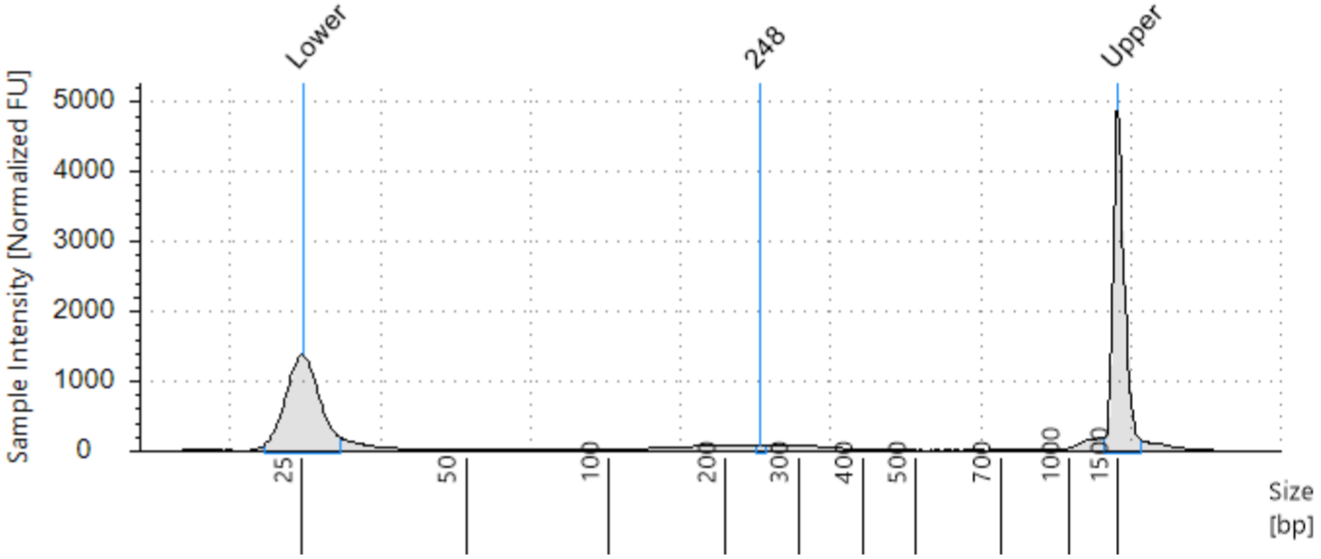

Sample Table

Well	Conc. [ng/µl]	Sample Description	Alert	Observations
A2	0.0857	H9 M R2	⚠	Caution! Expired ScreenTape device

Peak Table

Size [bp]	Calibrated Conc. [ng/µl]	Assigned Conc. [ng/µl]	Peak Molarity [nmol/l]	% Integrated Area	Peak Comment	Observations
25	5.41	-	333	-		Lower Marker
248	0.0857	-	0.531	100.00		
1500	6.50	6.50	6.67	-		Upper Marker

© Agilent Technologies, Inc. 2017

B2: A10 M R2

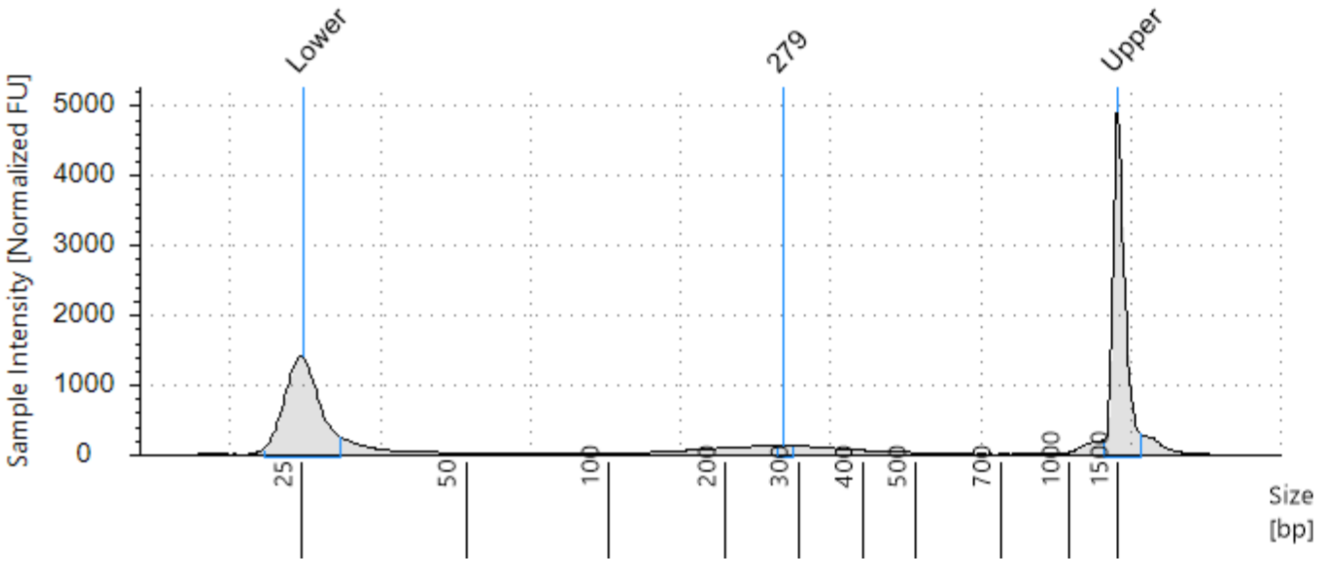

Sample Table

Well	Conc. [ng/µl]	Sample Description	Alert	Observations
B2	0.190	A10 M R2	⚠	Caution! Expired ScreenTape device

Peak Table

Size [bp]	Calibrated Conc. [ng/µl]	Assigned Conc. [ng/µl]	Peak Molarity [nmol/l]	% Integrated Area	Peak Comment	Observations
25	5.40	-	332	-		Lower Marker
279	0.190	-	1.04	100.00		
1500	6.50	6.50	6.67	-		Upper Marker

© Agilent Technologies, Inc. 2017

C2: B10 M R2

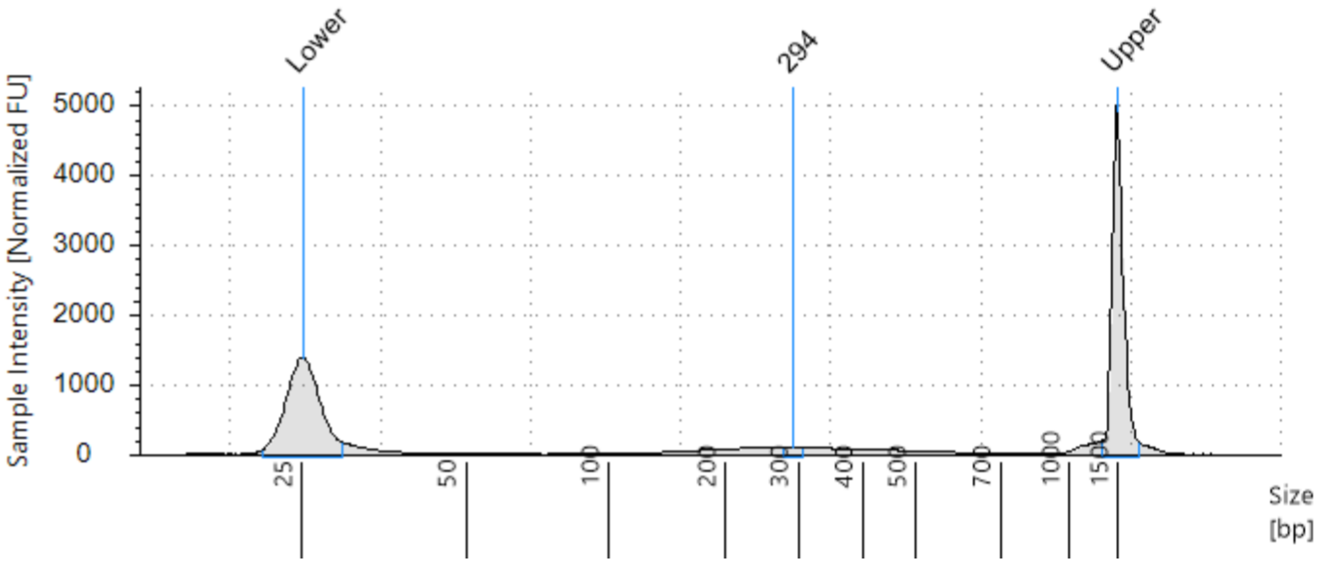

Sample Table

Well	Conc. [ng/µl]	Sample Description	Alert	Observations
C2	0.218	B10 M R2	⚠	Caution! Expired ScreenTape device

Peak Table

Size [bp]	Calibrated Conc. [ng/µl]	Assigned Conc. [ng/µl]	Peak Molarity [nmol/l]	% Integrated Area	Peak Comment	Observations
25	5.44	-	335	-		Lower Marker
294	0.218	-	1.14	100.00		
1500	6.50	6.50	6.67	-		Upper Marker

© Agilent Technologies, Inc. 2017

D2: C10 M R2

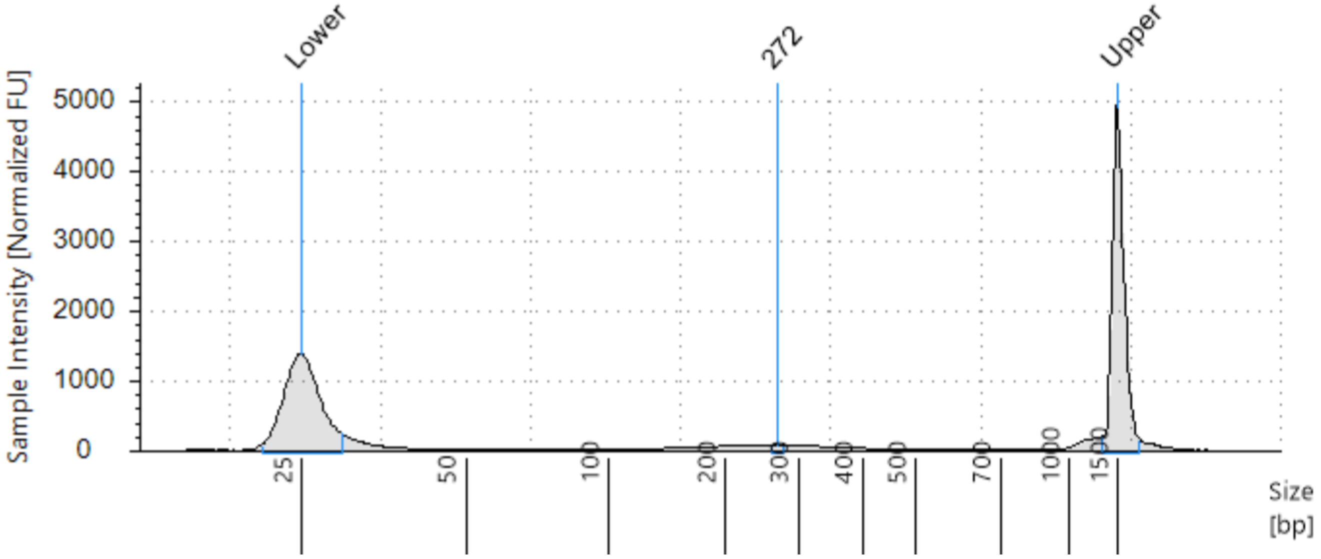

Sample Table

Well	Conc. [ng/µl]	Sample Description	Alert	Observations
D2	0.0791	C10 M R2	⚠	Caution! Expired ScreenTape device

Peak Table

Size [bp]	Calibrated Conc. [ng/µl]	Assigned Conc. [ng/µl]	Peak Molarity [nmol/l]	% Integrated Area	Peak Comment	Observations
25	5.62	-	346	-		Lower Marker
272	0.0791	-	0.447	100.00		
1500	6.50	6.50	6.67	-		Upper Marker

© Agilent Technologies, Inc. 2017

E2: D10 M R2

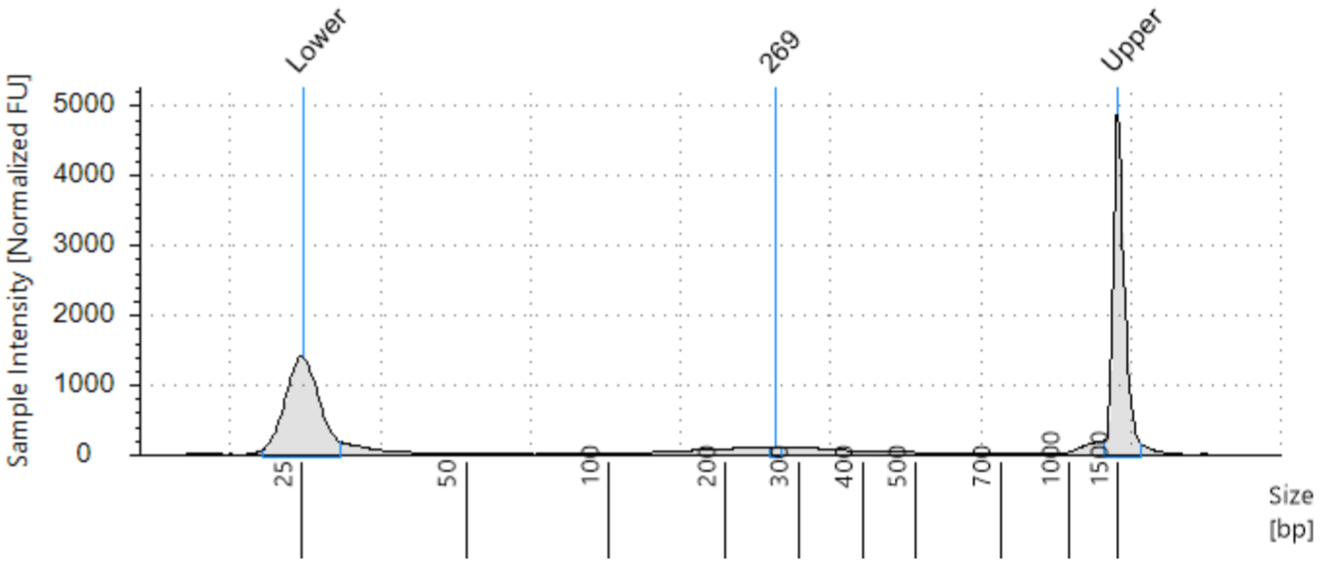

Sample Table

Well	Conc. [ng/µl]	Sample Description	Alert	Observations
E2	0.113	D10 M R2	⚠	Caution! Expired ScreenTape device

Peak Table

Size [bp]	Calibrated Conc. [ng/µl]	Assigned Conc. [ng/µl]	Peak Molarity [nmol/l]	% Integrated Area	Peak Comment	Observations
25	5.51	-	339	-		Lower Marker
269	0.113	-	0.647	100.00		
1500	6.50	6.50	6.67	-		Upper Marker

© Agilent Technologies, Inc. 2017

F2: E10 M R2

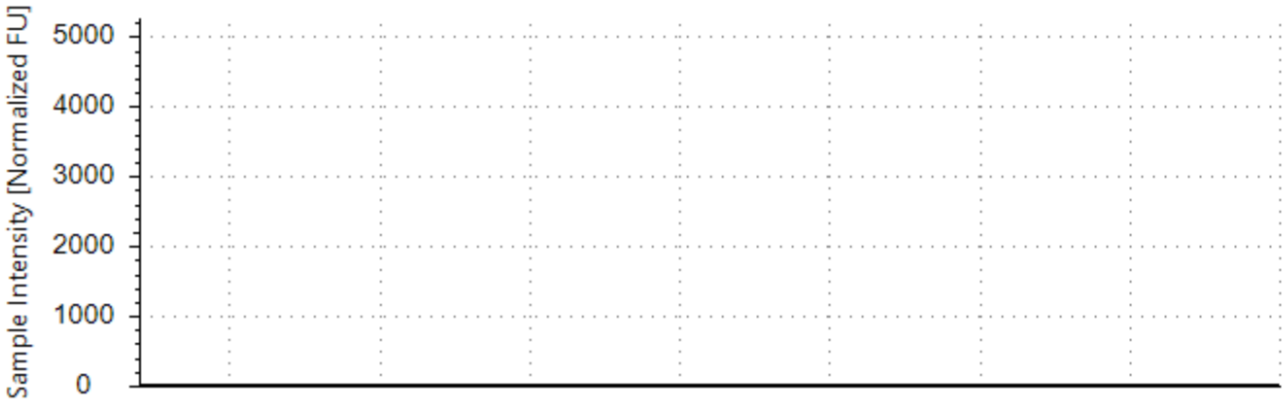

Sample Table

Well	Conc. [ng/µl]	Sample Description	Alert	Observations
F2		E10 M R2	▲	Marker(s) not detected; Caution! Expired ScreenTape device

© Agilent Technologies, Inc. 2017

G2: F10 M R2

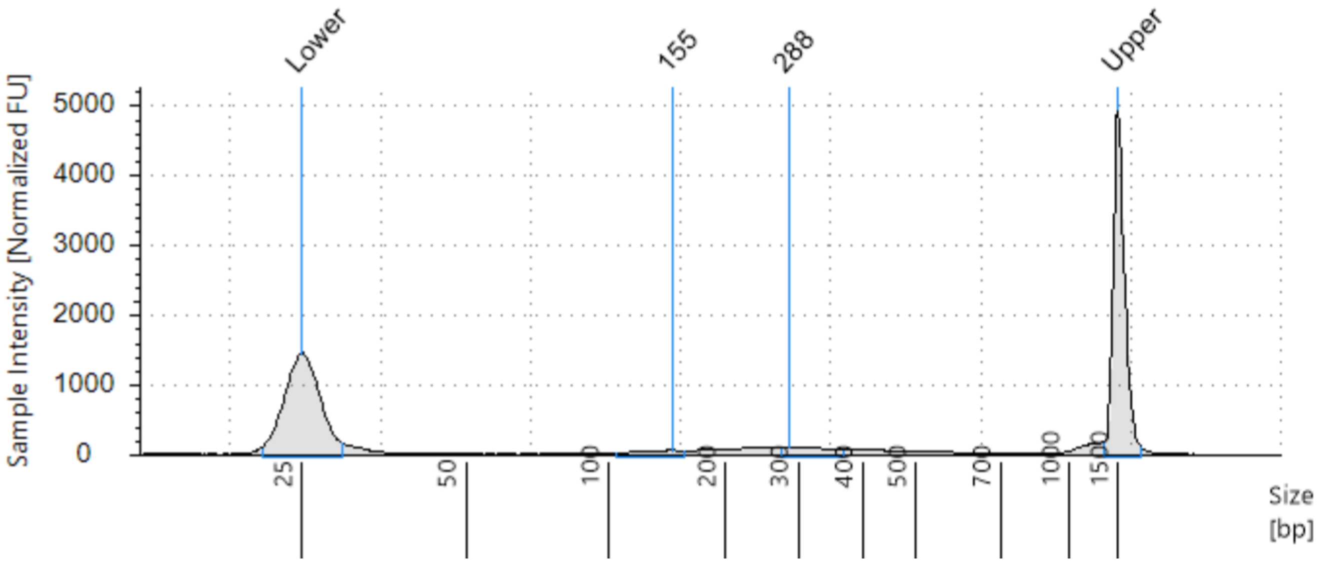

Sample Table

Well	Conc. [ng/µl]	Sample Description	Alert	Observations
G2	0.810	F10 M R2	⚠	Caution! Expired ScreenTape device

Peak Table

Size [bp]	Calibrated Conc. [ng/µl]	Assigned Conc. [ng/µl]	Peak Molarity [nmol/l]	% Integrated Area	Peak Comment	Observations
25	5.87	-	361	-		Lower Marker
155	0.271	-	2.70	33.50		
288	0.539	-	2.88	66.50		
1500	6.50	6.50	6.67	-		Upper Marker

 © Agilent Technologies, Inc. 2017

H2: G10 M R2

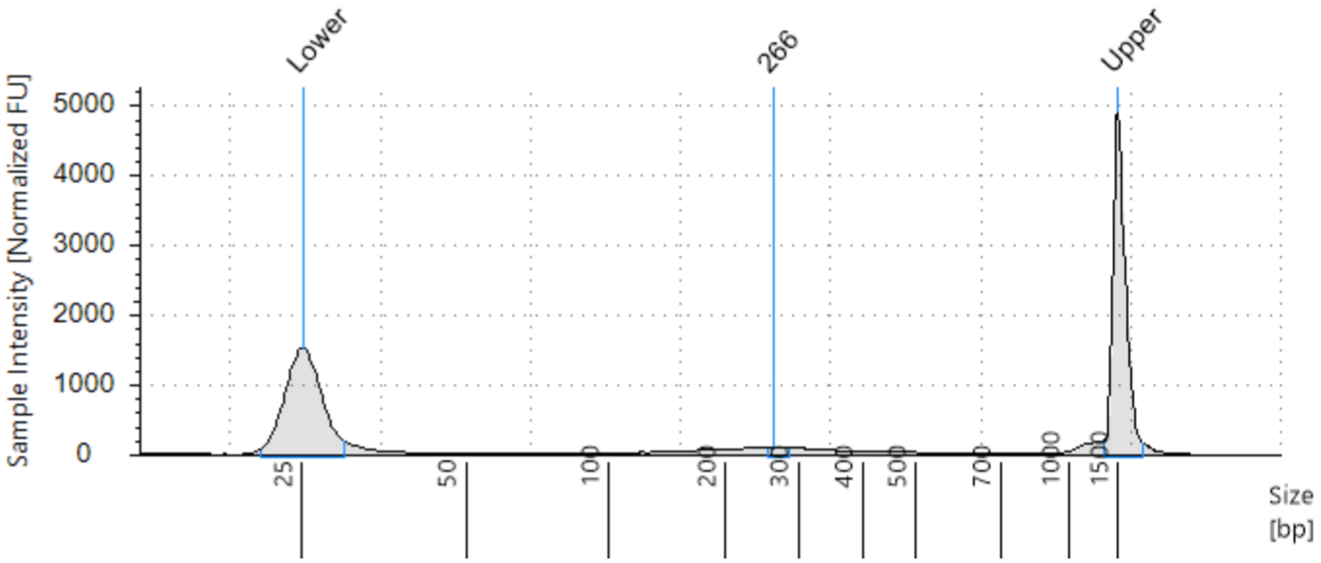

Sample Table

Well	Conc. [ng/µl]	Sample Description	Alert	Observations
H2	0.177	G10 M R2	⚠	Caution! Expired ScreenTape device

Peak Table

Size [bp]	Calibrated Conc. [ng/µl]	Assigned Conc. [ng/µl]	Peak Molarity [nmol/l]	% Integrated Area	Peak Comment	Observations
25	5.84	-	360	-		Lower Marker
266	0.177	-	1.02	100.00		
1500	6.50	6.50	6.67	-		Upper Marker

© Agilent Technologies, Inc. 2017

Filename: 2020-09-30-02, Q-S MINUS A12-G12 R2.D1000

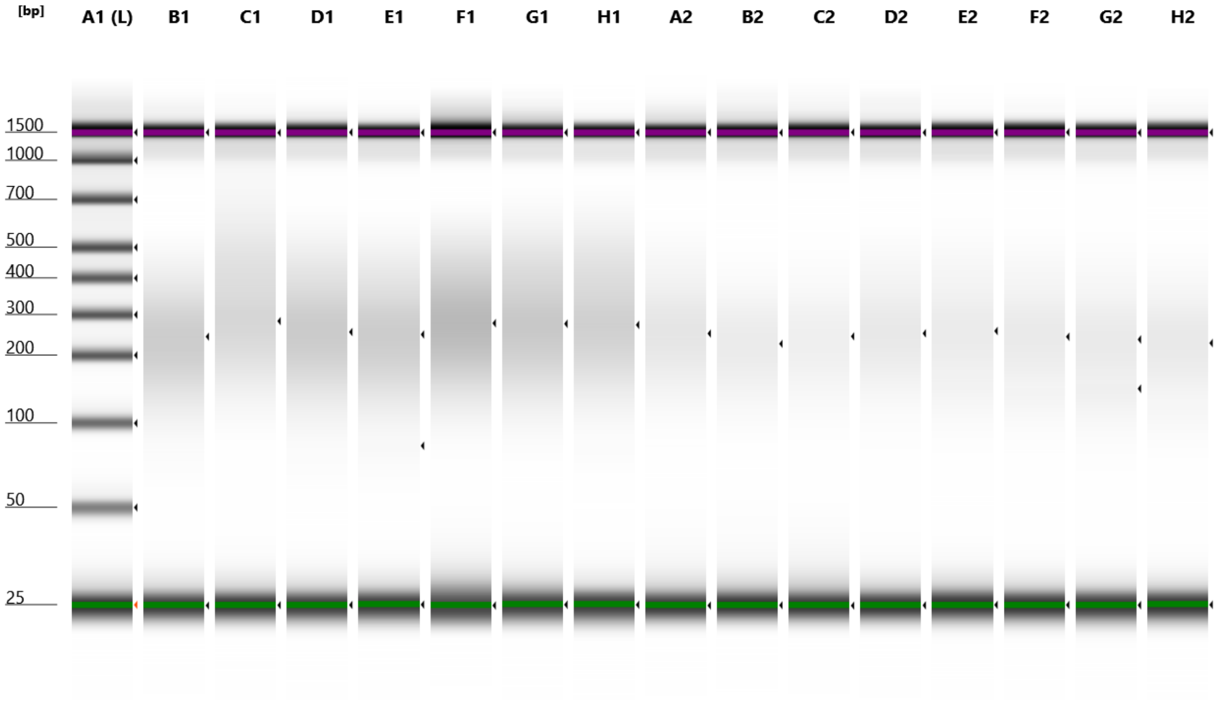

Default image (Contrast 100%)

Sample Info

Well	Conc. [ng/µl]	Sample Description	Alert	Observations
A1	15.7	Ladder		Ladder
B1	0.369	G2 P R2		
C1	1.56	D2 P R2		
D1	3.89	H2 P R2		
E1	3.86	A3 P R2		
F1	0.703	B3 P R2		
G1	3.99	C3 P R2		
H1	3.23	D3 P R2		
A2	0.861	E3 P R2		
B2	0.165	A12 M R2		
C2	0.451	B12 M R2		
D2	1.31	C12 M R2		
E2	0.626	D12 M R2		
F2	0.605	E12 M R2		
G2	0.572	F12 M R2		
H2	0.734	G12 M R2		

© Agilent Technologies, Inc. 2017

A1: Ladder

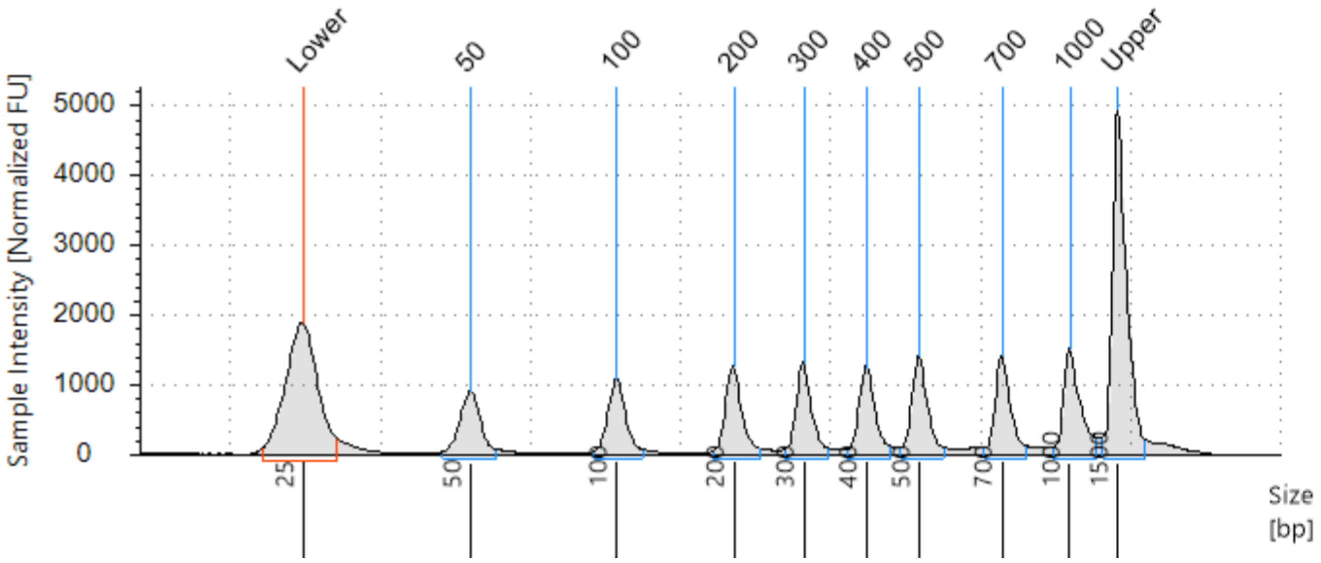

Sample Table

Well	Conc. [ng/µl]	Sample Description	Alert	Observations
A1	15.7	Ladder		Ladder

Peak Table

Size [bp]	Calibrated Conc. [ng/µl]	Assigned Conc. [ng/µl]	Peak Molarity [nmol/l]	% Integrated Area	Peak Comment	Observations
25	5.60	-	345	-		Lower Marker
50	1.72	-	52.9	10.98		
100	1.81	-	27.8	11.53		
200	1.93	-	14.9	12.33		
300	1.88	-	9.65	12.01		
400	1.91	-	7.36	12.21		
500	2.05	-	6.31	13.10		
700	2.03	-	4.46	12.96		
1000	2.33	-	3.59	14.88		
1500	6.50	6.50	6.67	-		Upper Marker

© Agilent Technologies, Inc. 2017

B1: G2 P R2

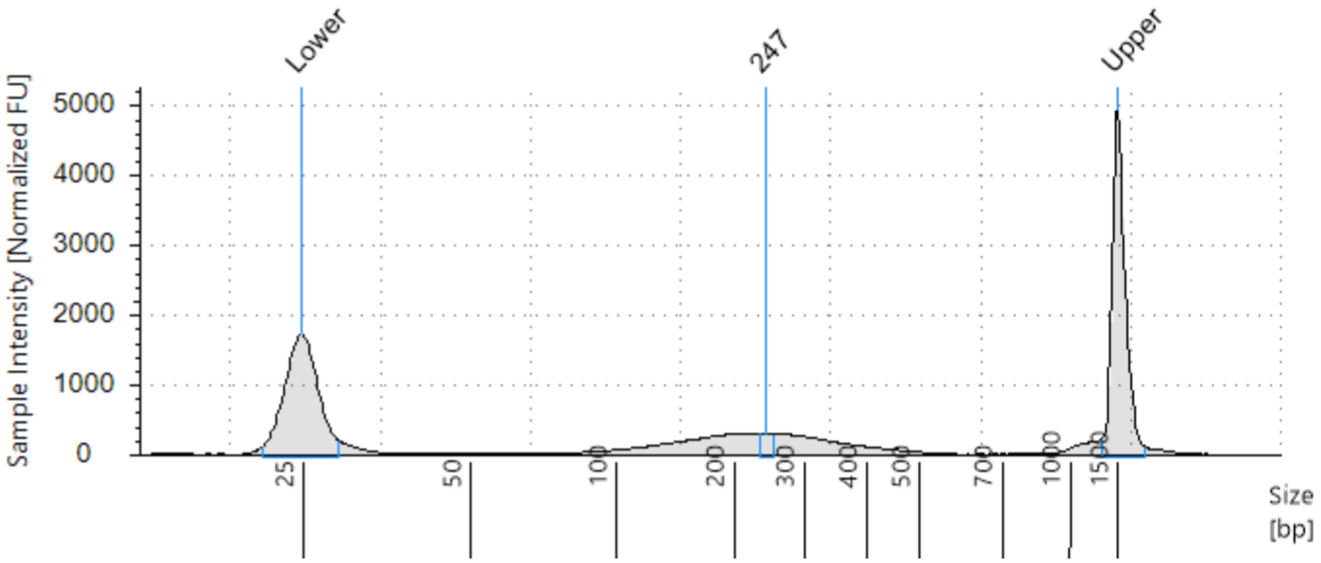

Sample Table

Well	Conc. [ng/µl]	Sample Description	Alert	Observations
B1	0.369	G2 P R2		

Peak Table

Size [bp]	Calibrated Conc. [ng/µl]	Assigned Conc. [ng/µl]	Peak Molarity [nmol/l]	% Integrated Area	Peak Comment	Observations
25	5.78	-	355	-		Lower Marker
247	0.369	-	2.30	100.00		
1500	6.50	6.50	6.67	-		Upper Marker

 © Agilent Technologies, Inc. 2017

C1: D2 P R2

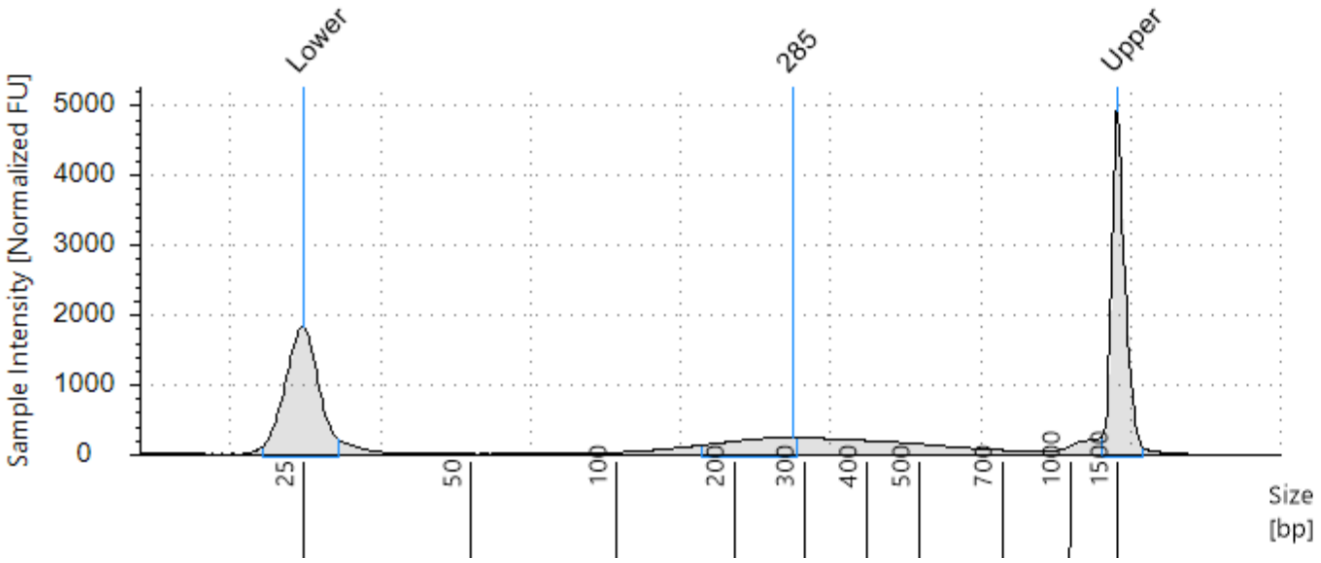

Sample Table

Well	Conc. [ng/µl]	Sample Description	Alert	Observations
C1	1.56	D2 P R2		

Peak Table

Size [bp]	Calibrated Conc. [ng/µl]	Assigned Conc. [ng/µl]	Peak Molarity [nmol/l]	% Integrated Area	Peak Comment	Observations
25	5.95	-	366	-		Lower Marker
285	1.56	-	8.43	100.00		
1500	6.50	6.50	6.67	-		Upper Marker

 © Agilent Technologies, Inc. 2017

D1: H2 P R2

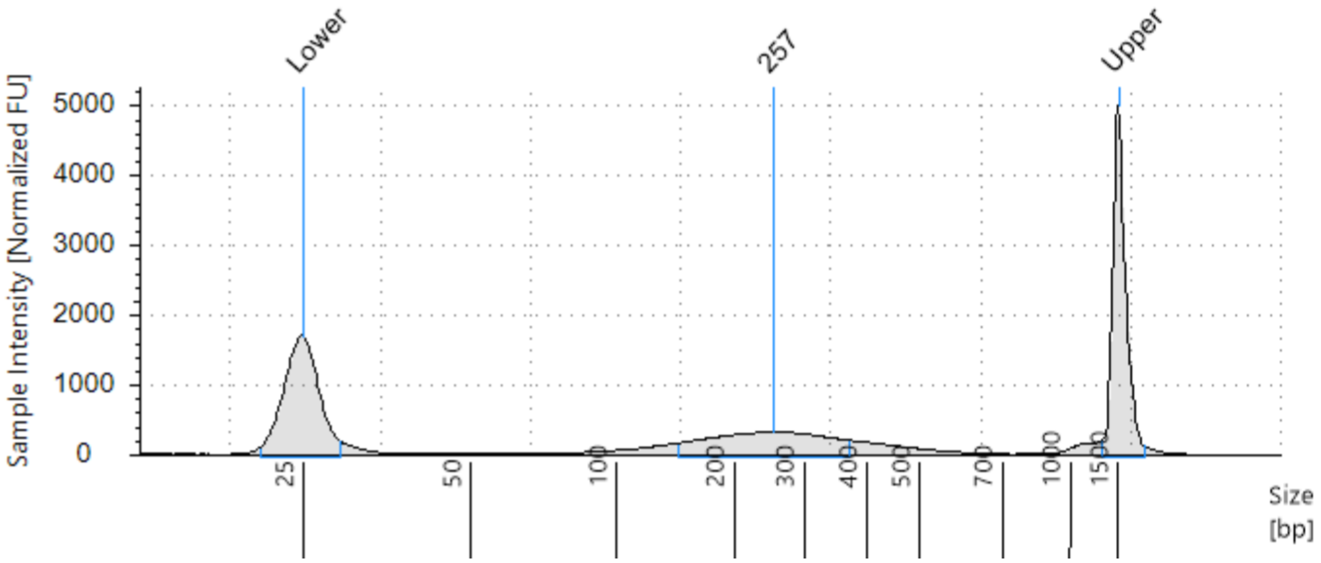

Sample Table

Well	Conc. [ng/µl]	Sample Description	Alert	Observations
D1	3.89	H2 P R2		

Peak Table

Size [bp]	Calibrated Conc. [ng/µl]	Assigned Conc. [ng/µl]	Peak Molarity [nmol/l]	% Integrated Area	Peak Comment	Observations
25	5.96	-	367	-		Lower Marker
257	3.89	-	23.2	100.00		
1500	6.50	6.50	6.67	-		Upper Marker

© Agilent Technologies, Inc. 2017

E1: A3 P R2

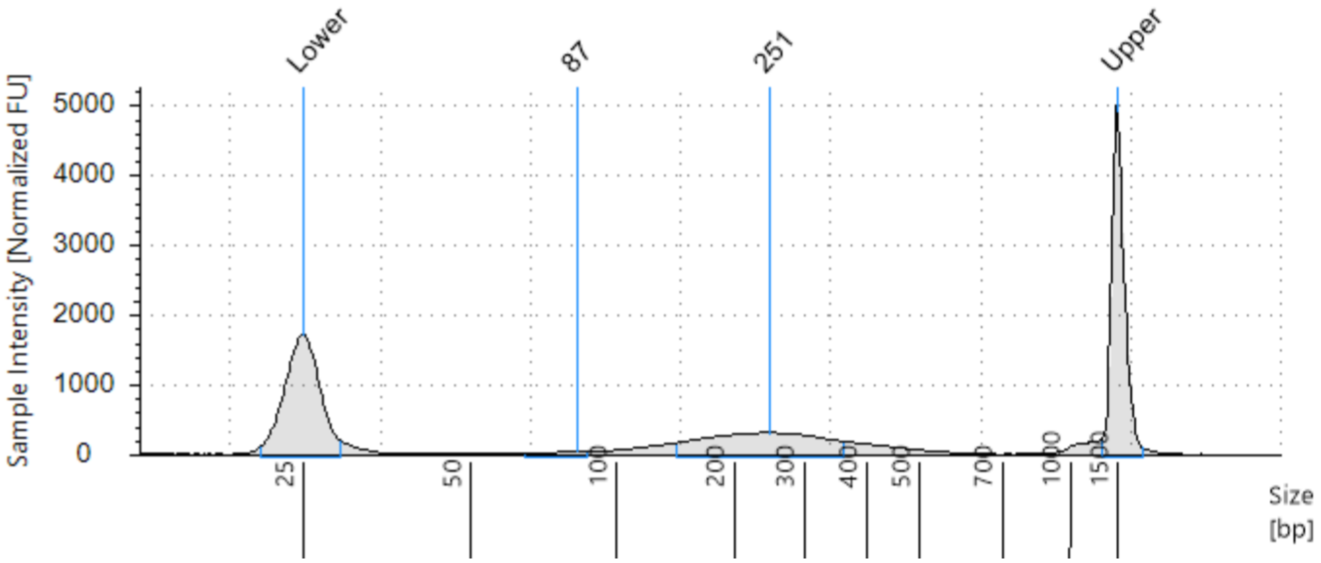

Sample Table

Well	Conc. [ng/µl]	Sample Description	Alert	Observations
E1	3.86	A3 P R2		

Peak Table

Size [bp]	Calibrated Conc. [ng/µl]	Assigned Conc. [ng/µl]	Peak Molarity [nmol/l]	% Integrated Area	Peak Comment	Observations
25	6.12	-	377	-		Lower Marker
87	0.139	-	2.47	3.60		
251	3.72	-	22.8	96.40		
1500	6.50	6.50	6.67	-		Upper Marker

 © Agilent Technologies, Inc. 2017

F1: B3 P R2

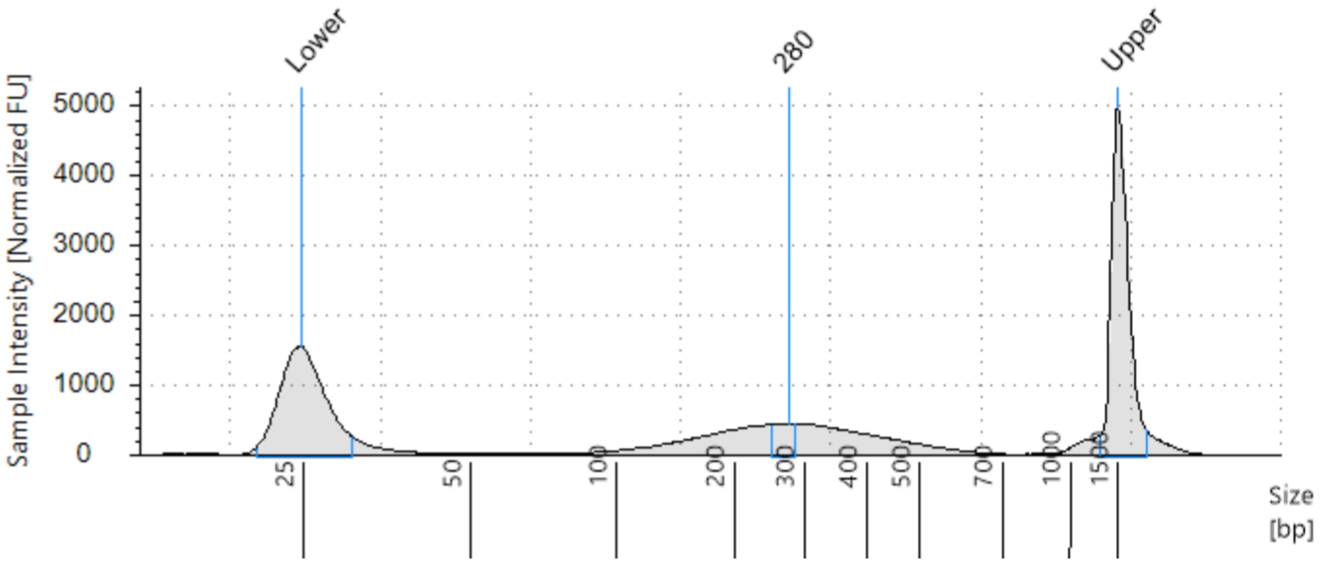

Sample Table

Well	Conc. [ng/µl]	Sample Description	Alert	Observations
F1	0.703	B3 P R2		

Peak Table

Size [bp]	Calibrated Conc. [ng/µl]	Assigned Conc. [ng/µl]	Peak Molarity [nmol/l]	% Integrated Area	Peak Comment	Observations
25	5.32	-	327	-		Lower Marker
280	0.703	-	3.86	100.00		
1500	6.50	6.50	6.67	-		Upper Marker

 © Agilent Technologies, Inc. 2017

GI: C3 P R2

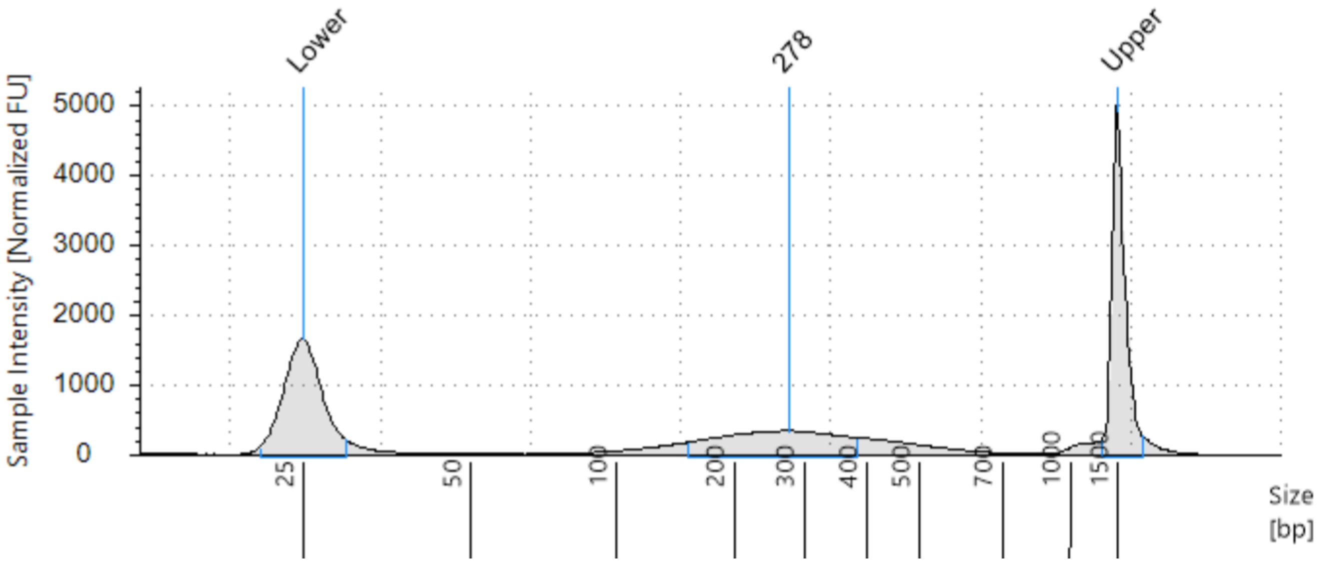

Sample Table

Well	Conc. [ng/µl]	Sample Description	Alert	Observations
GI	3.99	C3 P R2		

Peak Table

Size [bp]	Calibrated Conc. [ng/µl]	Assigned Conc. [ng/µl]	Peak Molarity [nmol/l]	% Integrated Area	Peak Comment	Observations
25	6.20	-	382	-		Lower Marker
278	3.99	-	22.1	100.00		
1500	6.50	6.50	6.67	-		Upper Marker

© Agilent Technologies, Inc. 2017

H1: D3 P R2

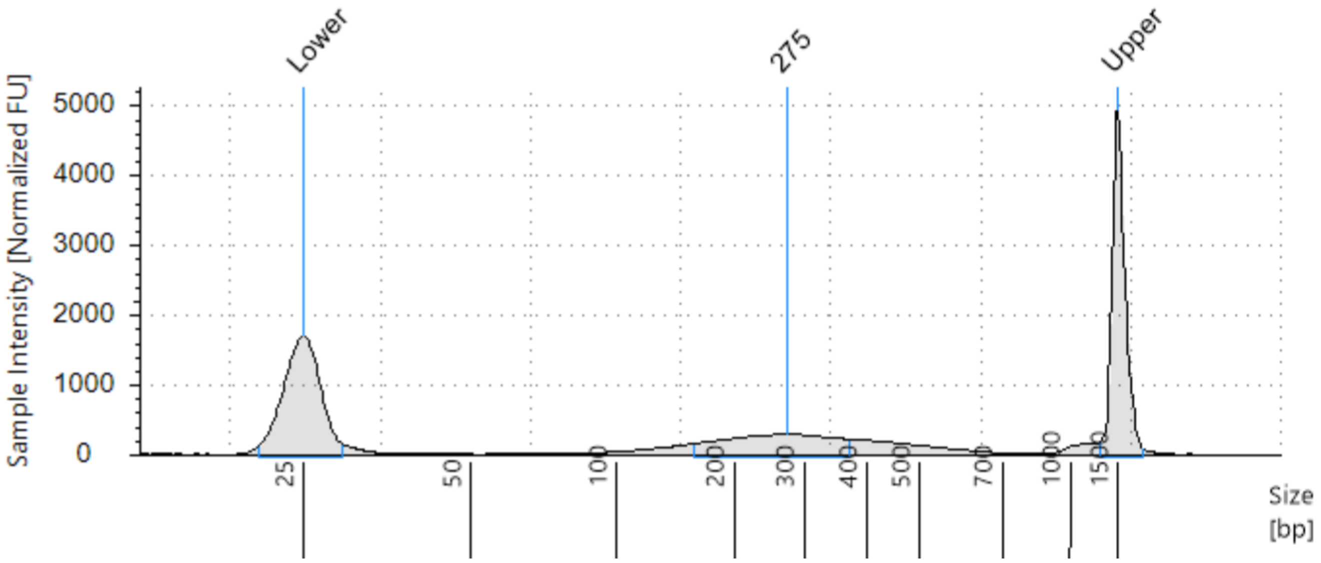

Sample Table

Well	Conc. [ng/µl]	Sample Description	Alert	Observations
H1	3.23	D3 P R2		

Peak Table

Size [bp]	Calibrated Conc. [ng/µl]	Assigned Conc. [ng/µl]	Peak Molarity [nmol/l]	% Integrated Area	Peak Comment	Observations
25	6.34	-	390	-		Lower Marker
275	3.23	-	18.1	100.00		
1500	6.50	6.50	6.67	-		Upper Marker

 © Agilent Technologies, Inc. 2017

A2: E3 P R2

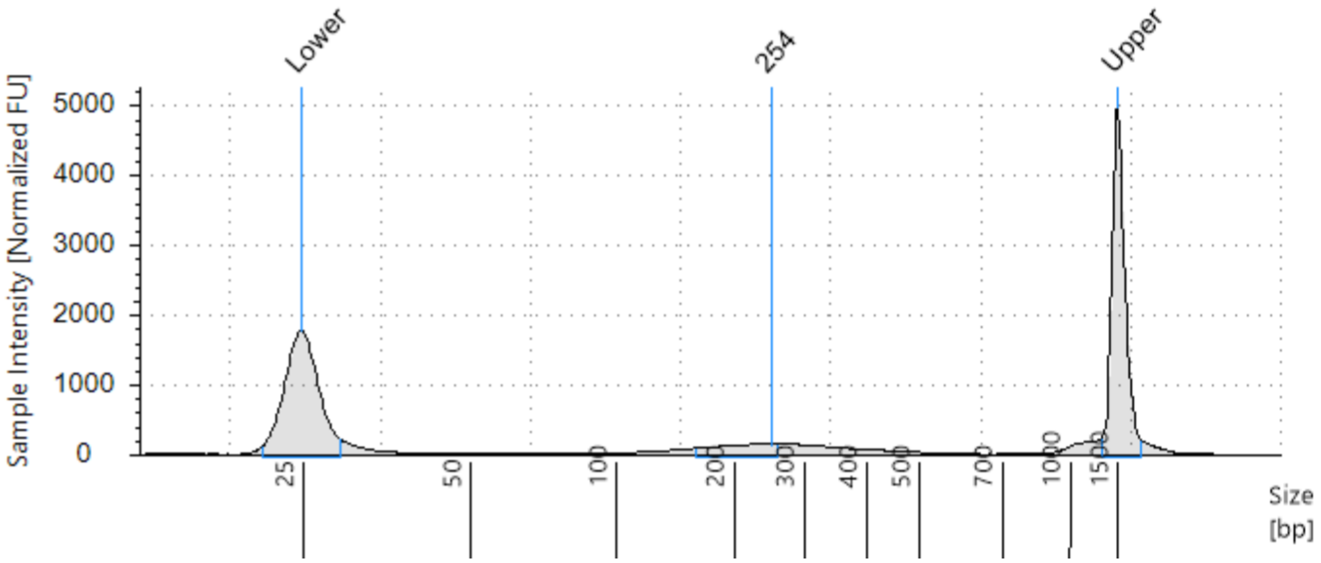

Sample Table

Well	Conc. [ng/µl]	Sample Description	Alert	Observations
A2	0.861	E3 P R2		

Peak Table

Size [bp]	Calibrated Conc. [ng/µl]	Assigned Conc. [ng/µl]	Peak Molarity [nmol/l]	% Integrated Area	Peak Comment	Observations
25	6.00	-	369	-		Lower Marker
254	0.861	-	5.21	100.00		
1500	6.50	6.50	6.67	-		Upper Marker

 © Agilent Technologies, Inc. 2017

B2: A12 M R2

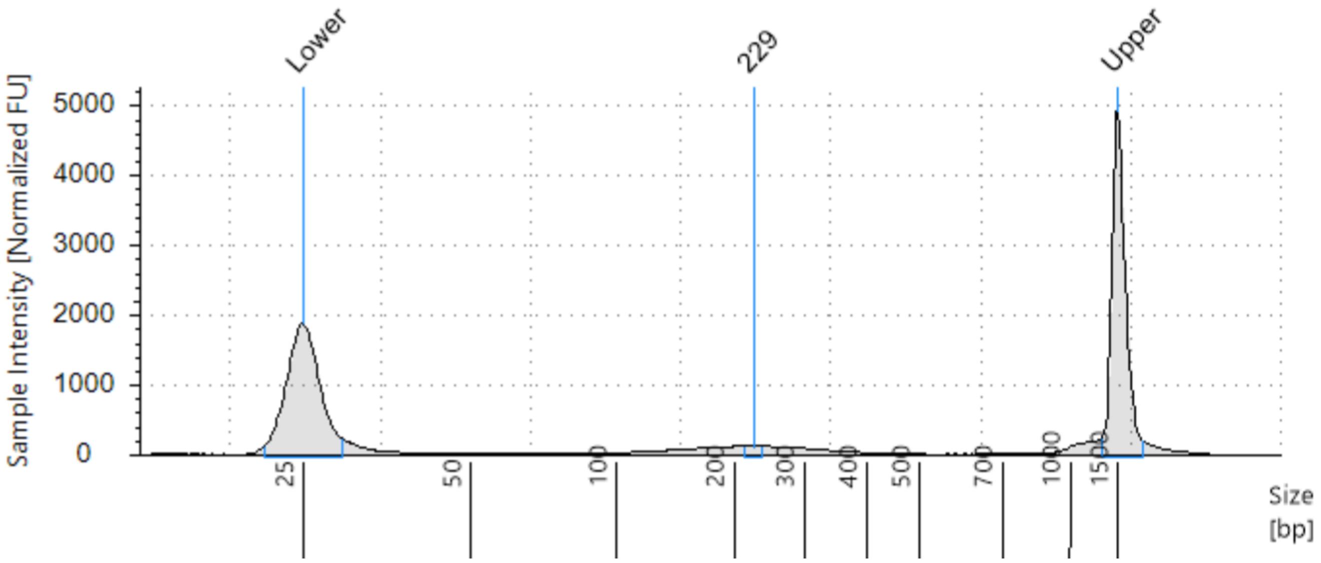

Sample Table

Well	Conc. [ng/µl]	Sample Description	Alert	Observations
B2	0.165	A12 M R2		

Peak Table

Size [bp]	Calibrated Conc. [ng/µl]	Assigned Conc. [ng/µl]	Peak Molarity [nmol/l]	% Integrated Area	Peak Comment	Observations
25	6.07	-	373	-		Lower Marker
229	0.165	-	1.11	100.00		
1500	6.50	6.50	6.67	-		Upper Marker

© Agilent Technologies, Inc. 2017

C2: B12 M R2

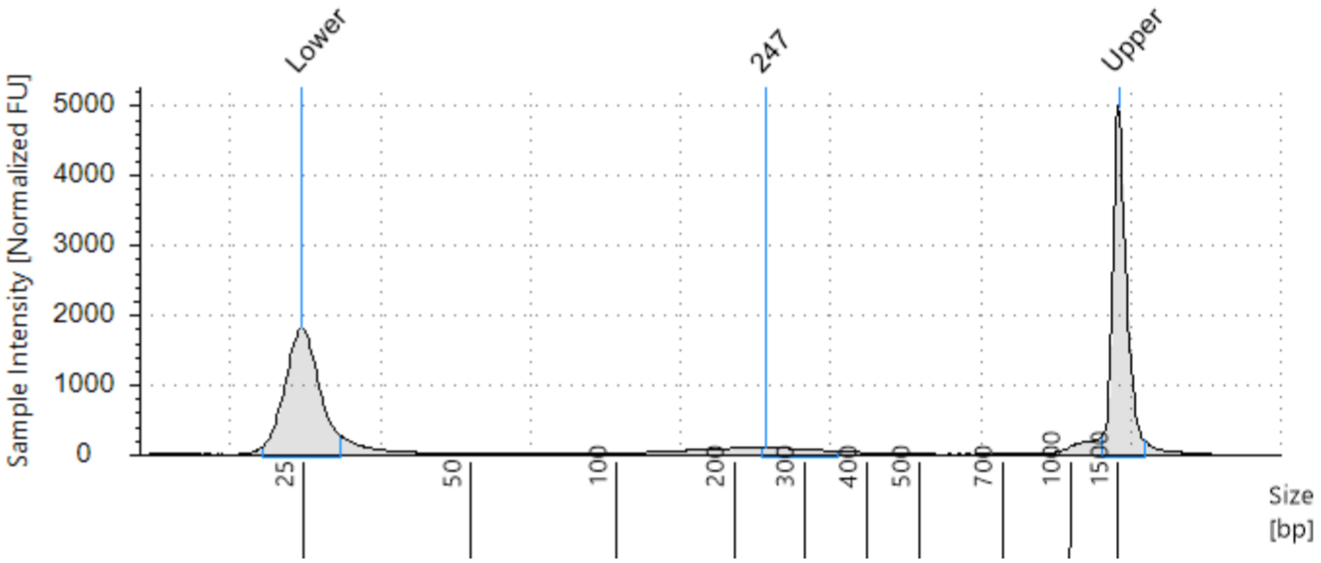

Sample Table

Well	Conc. [ng/µl]	Sample Description	Alert	Observations
C2	0.451	B12 M R2		

Peak Table

Size [bp]	Calibrated Conc. [ng/µl]	Assigned Conc. [ng/µl]	Peak Molarity [nmol/l]	% Integrated Area	Peak Comment	Observations
25	5.90	-	363	-		Lower Marker
247	0.451	-	2.81	100.00		
1500	6.50	6.50	6.67	-		Upper Marker

© Agilent Technologies, Inc. 2017

D2: C12 M R2

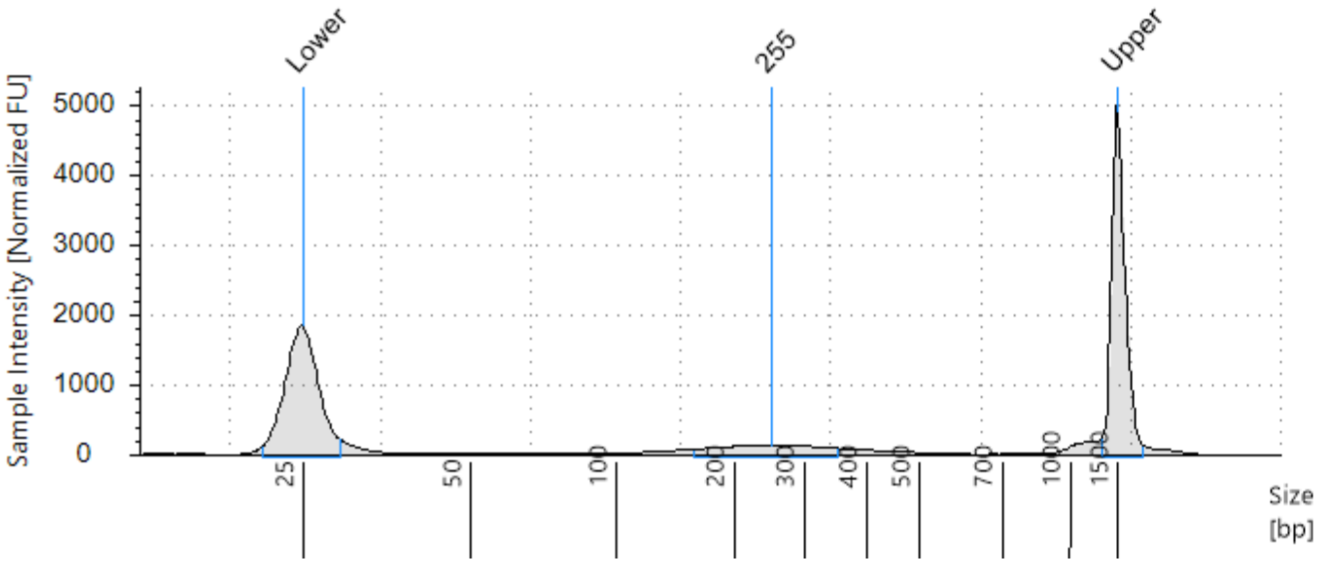

Sample Table

Well	Conc. [ng/µl]	Sample Description	Alert	Observations
D2	1.31	C12 M R2		

Peak Table

Size [bp]	Calibrated Conc. [ng/µl]	Assigned Conc. [ng/µl]	Peak Molarity [nmol/l]	% Integrated Area	Peak Comment	Observations
25	5.95	-	366	-		Lower Marker
255	1.31	-	7.91	100.00		
1500	6.50	6.50	6.67	-		Upper Marker

© Agilent Technologies, Inc. 2017

E2: D12 M R2

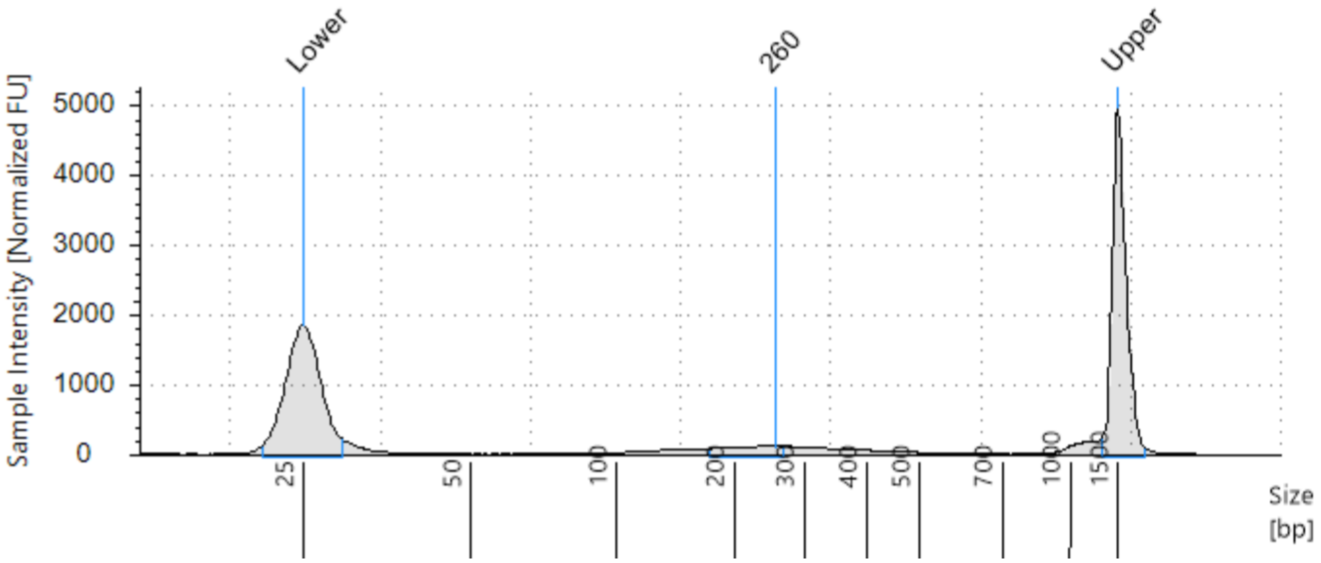

Sample Table

Well	Conc. [ng/µl]	Sample Description	Alert	Observations
E2	0.626	D12 M R2		

Peak Table

Size [bp]	Calibrated Conc. [ng/µl]	Assigned Conc. [ng/µl]	Peak Molarity [nmol/l]	% Integrated Area	Peak Comment	Observations
25	6.17	-	380	-		Lower Marker
260	0.626	-	3.71	100.00		
1500	6.50	6.50	6.67	-		Upper Marker

© Agilent Technologies, Inc. 2017

F2: E12 M R2

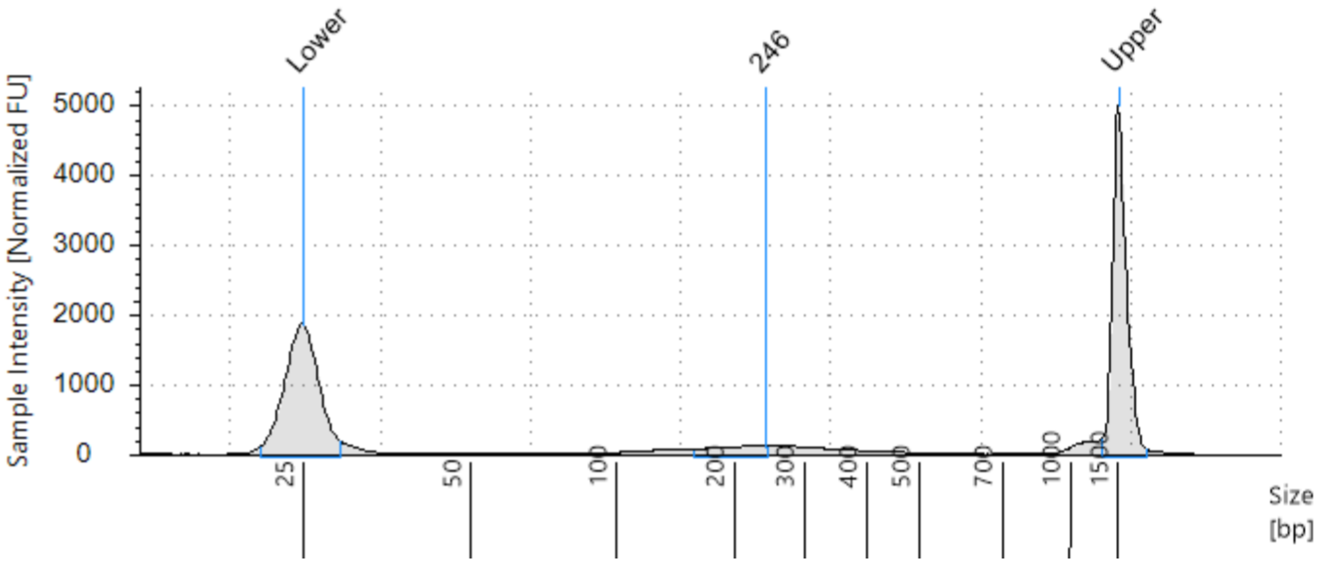

Sample Table

Well	Conc. [ng/µl]	Sample Description	Alert	Observations
F2	0.605	E12 M R2		

Peak Table

Size [bp]	Calibrated Conc. [ng/µl]	Assigned Conc. [ng/µl]	Peak Molarity [nmol/l]	% Integrated Area	Peak Comment	Observations
25	5.98	-	368	-		Lower Marker
246	0.605	-	3.79	100.00		
1500	6.50	6.50	6.67	-		Upper Marker

G2: F12 M R2

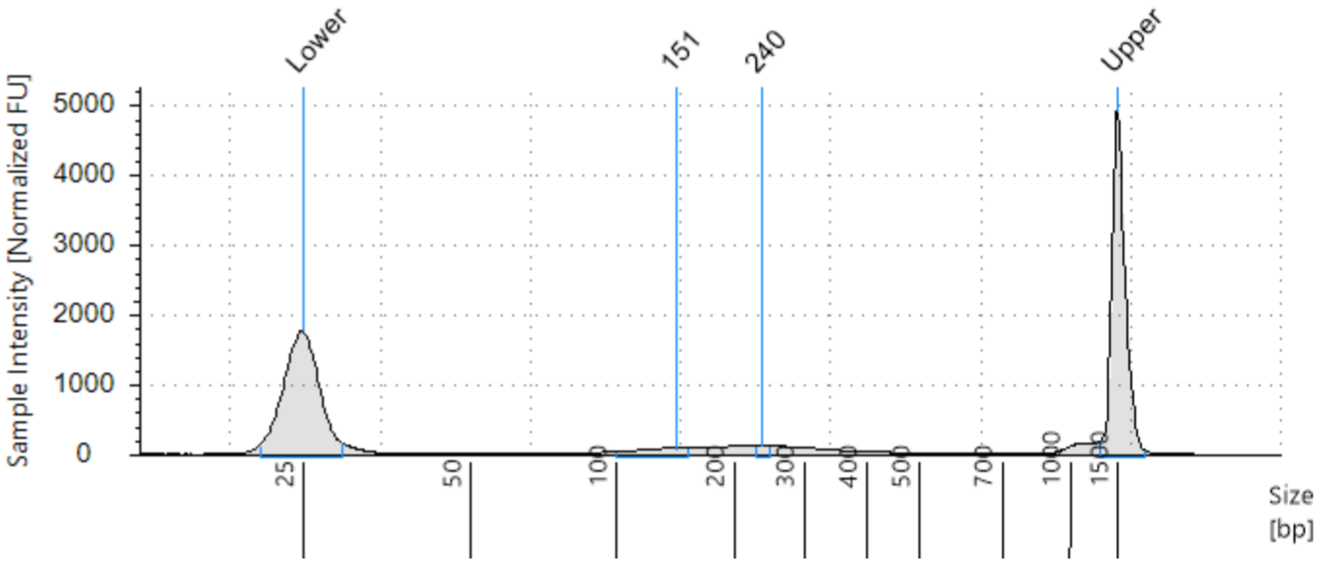

Sample Table

Well	Conc. [ng/µl]	Sample Description	Alert	Observations
G2	0.572	F12 M R2		

Peak Table

Size [bp]	Calibrated Conc. [ng/µl]	Assigned Conc. [ng/µl]	Peak Molarity [nmol/l]	% Integrated Area	Peak Comment	Observations
25	6.22	-	383	-		Lower Marker
151	0.425	-	4.32	74.35		
240	0.147	-	0.941	25.65		
1500	6.50	6.50	6.67	-		Upper Marker

 © Agilent Technologies, Inc. 2017

H2: G12 M R2

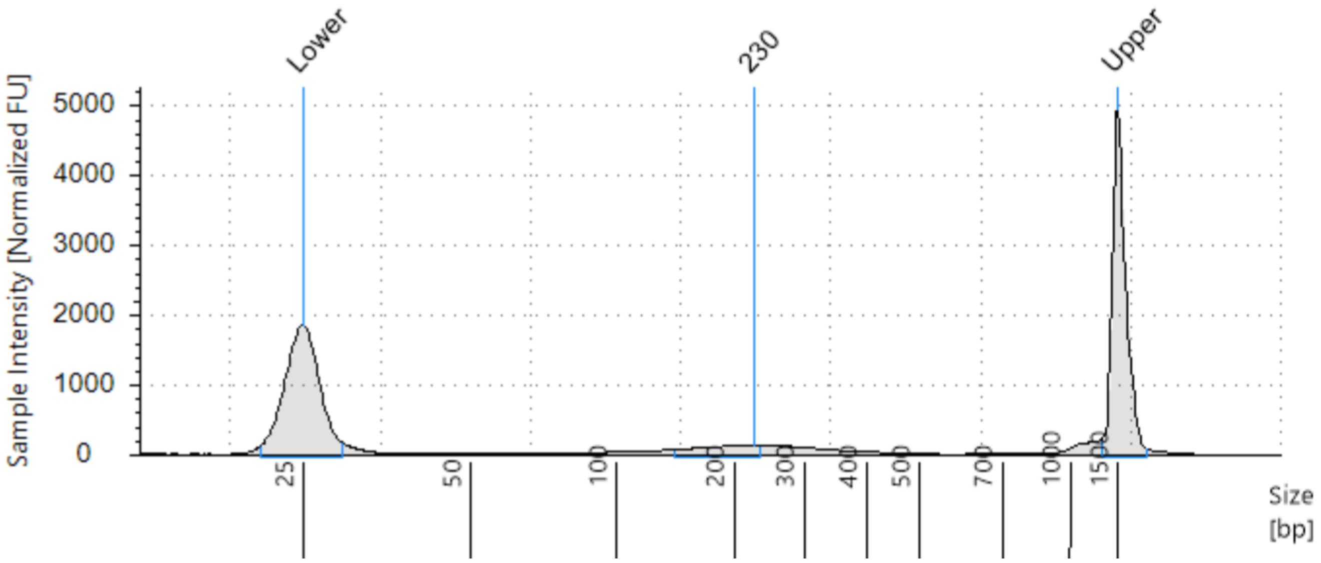

Sample Table

Well	Conc. [ng/µl]	Sample Description	Alert	Observations
H2	0.734	G12 M R2		

Peak Table

Size [bp]	Calibrated Conc. [ng/µl]	Assigned Conc. [ng/µl]	Peak Molarity [nmol/l]	% Integrated Area	Peak Comment	Observations
25	6.26	-	385	-		Lower Marker
230	0.734	-	4.91	100.00		
1500	6.50	6.50	6.67	-		Upper Marker

 © Agilent Technologies, Inc. 2017

Filename: 2020-08-21-03- Q-S MINUS B12-H12 D1000 R2.D1000

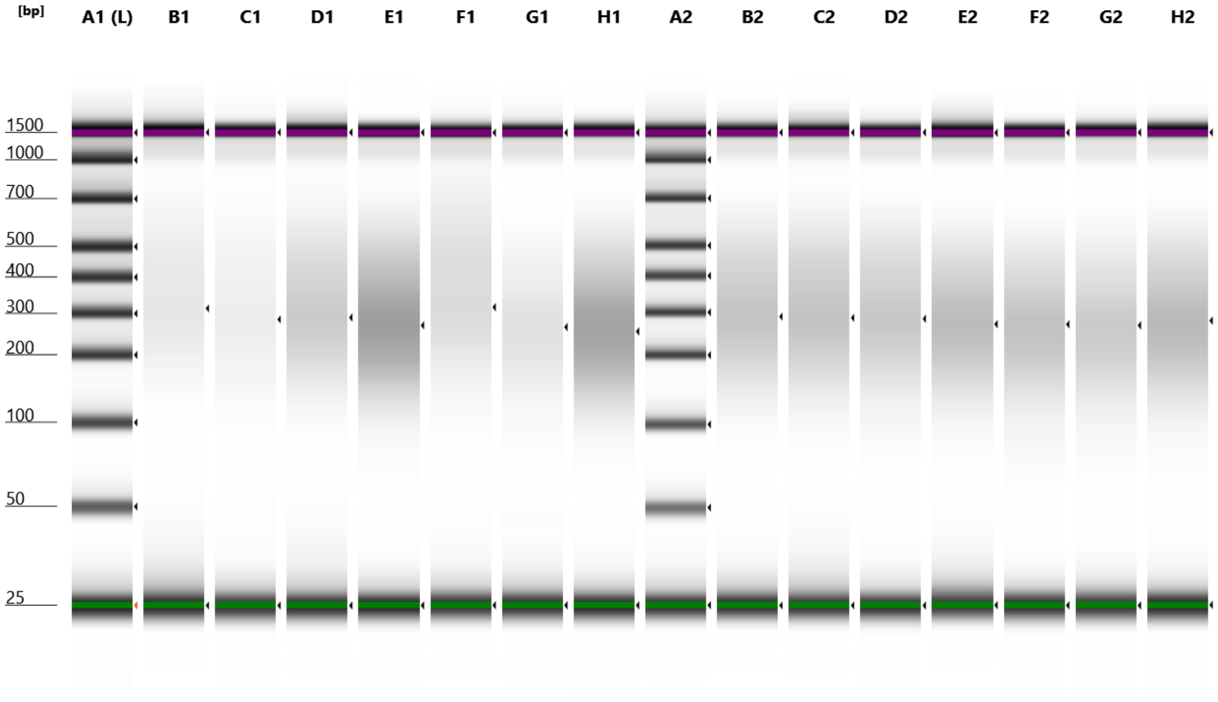

Default image (Contrast 100%)

Sample Info

Well	Conc. [ng/µl]	Sample Description	Alert	Observations
A1	22.4	Ladder		Ladder
B1	0.871	B12 MINUS-QSONICA 96 WELL		
C1	0.662	M-C12		
D1	2.22	M-D12		
E1	8.44	M-E12		
F1	0.585	M-F12		
G1	1.14	M-G12		
H1	4.54	M-H12		
A2	19.8	LADDER		
B2	0.507	PLUS B12		
C2	2.67	P-C12		
D2	0.555	P-D12		
E2	3.03	P-E12		
F2	2.60	P-F12		
G2	1.91	P-G12		
H2	3.29	P-H12		

© Agilent Technologies, Inc. 2017

A1: Ladder

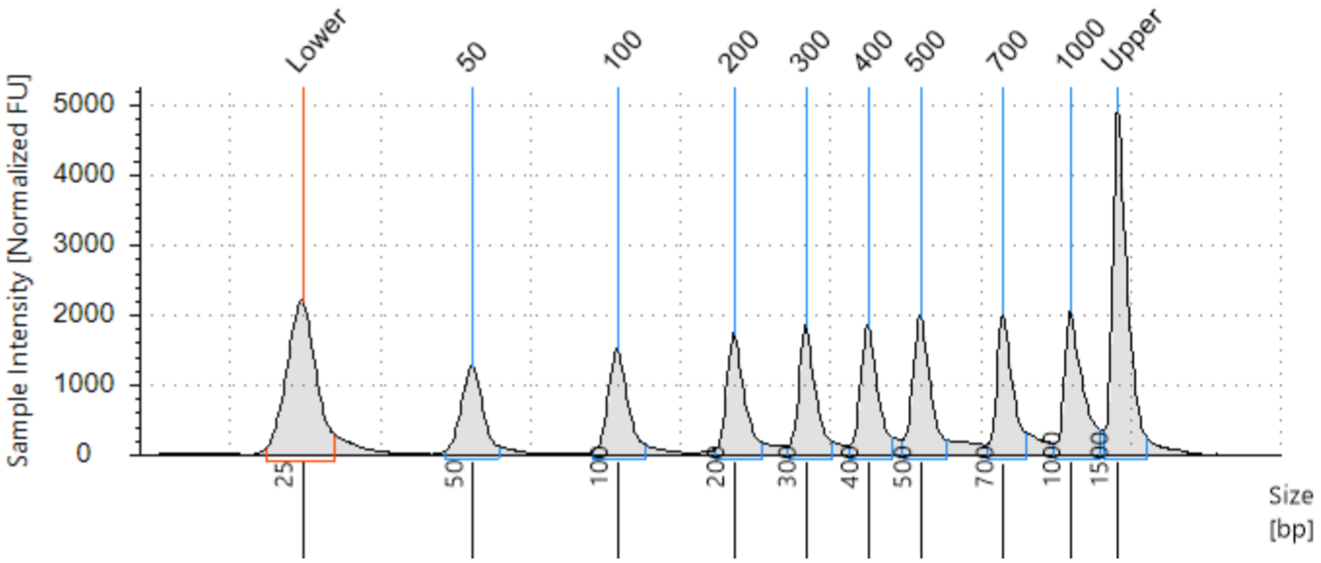

Sample Table

Well	Conc. [ng/µl]	Sample Description	Alert	Observations
A1	22.4	Ladder		Ladder

Peak Table

Size [bp]	Calibrated Conc. [ng/µl]	Assigned Conc. [ng/µl]	Peak Molarity [nmol/l]	% Integrated Area	Peak Comment	Observations
25	5.67	-	349	-		Lower Marker
50	2.41	-	74.2	10.79		
100	2.62	-	40.3	11.71		
200	2.68	-	20.6	11.99		
300	2.71	-	13.9	12.11		
400	2.83	-	10.9	12.66		
500	3.03	-	9.32	13.55		
700	2.80	-	6.16	12.55		
1000	3.27	-	5.04	14.64		
1500	6.50	6.50	6.67	-		Upper Marker

© Agilent Technologies, Inc. 2017

B1: B12 MINUS -QSONICA 96 WELL

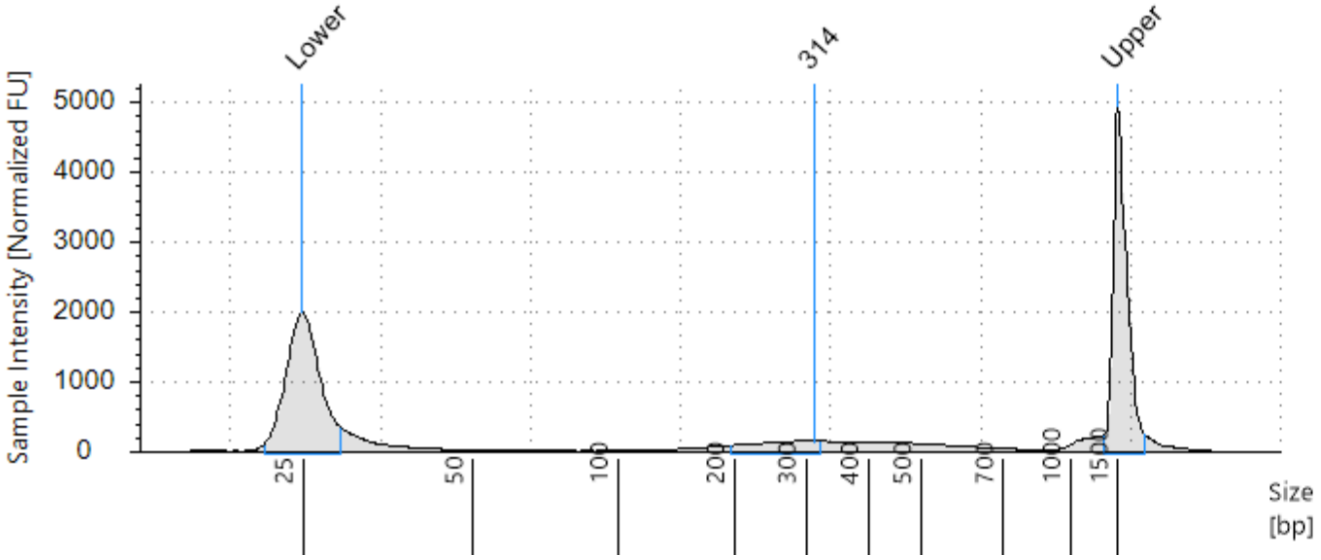

Sample Table

Well	Conc. [ng/µl]	Sample Description	Alert	Observations
B1	0.871	B12 MINUS-QSONICA 96 WELL		

Peak Table

Size [bp]	Calibrated Conc. [ng/µl]	Assigned Conc. [ng/µl]	Peak Molarity [nmol/l]	% Integrated Area	Peak Comment	Observations
25	6.40	-	394	-		Lower Marker
314	0.871	-	4.26	100.00		
1500	6.50	6.50	6.67	-		Upper Marker

© Agilent Technologies, Inc. 2017

C1: M-C12

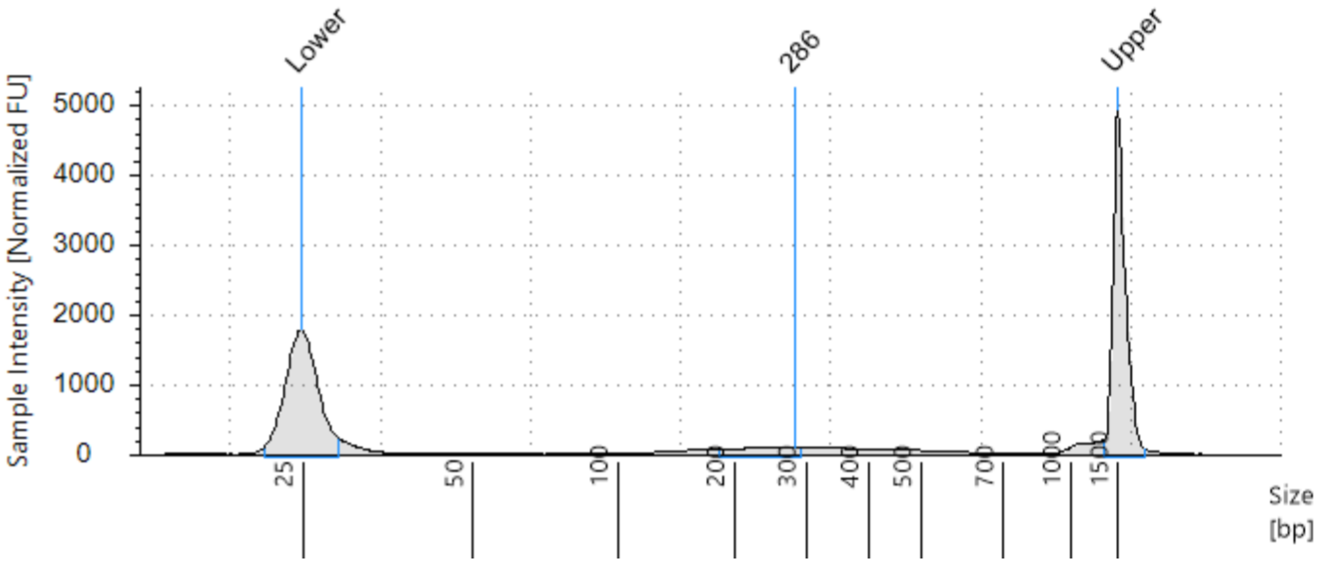

Sample Table

Well	Conc. [ng/µl]	Sample Description	Alert	Observations
C1	0.662	M-C12		

Peak Table

Size [bp]	Calibrated Conc. [ng/µl]	Assigned Conc. [ng/µl]	Peak Molarity [nmol/l]	% Integrated Area	Peak Comment	Observations
25	6.19	-	381	-		Lower Marker
286	0.662	-	3.57	100.00		
1500	6.50	6.50	6.67	-		Upper Marker

© Agilent Technologies, Inc. 2017

D1: M-D12

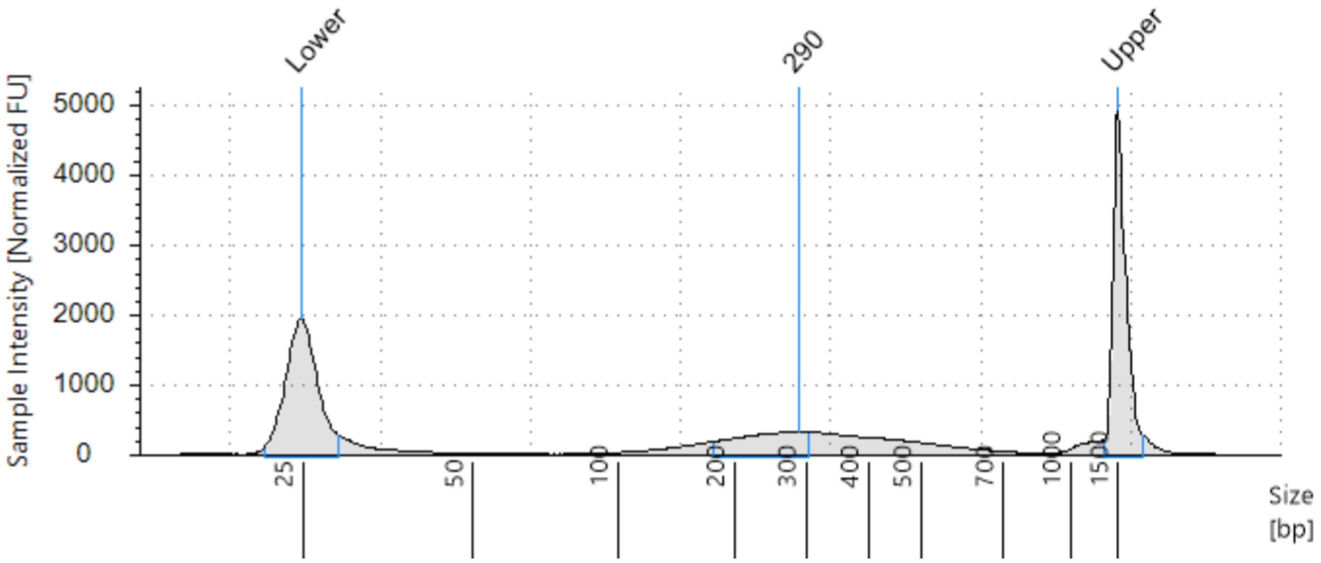

Sample Table

Well	Conc. [ng/µl]	Sample Description	Alert	Observations
D1	2.22	M-D12		

Peak Table

Size [bp]	Calibrated Conc. [ng/µl]	Assigned Conc. [ng/µl]	Peak Molarity [nmol/l]	% Integrated Area	Peak Comment	Observations
25	6.21	-	382	-		Lower Marker
290	2.22	-	11.8	100.00		
1500	6.50	6.50	6.67	-		Upper Marker

© Agilent Technologies, Inc. 2017

E1: M-E12

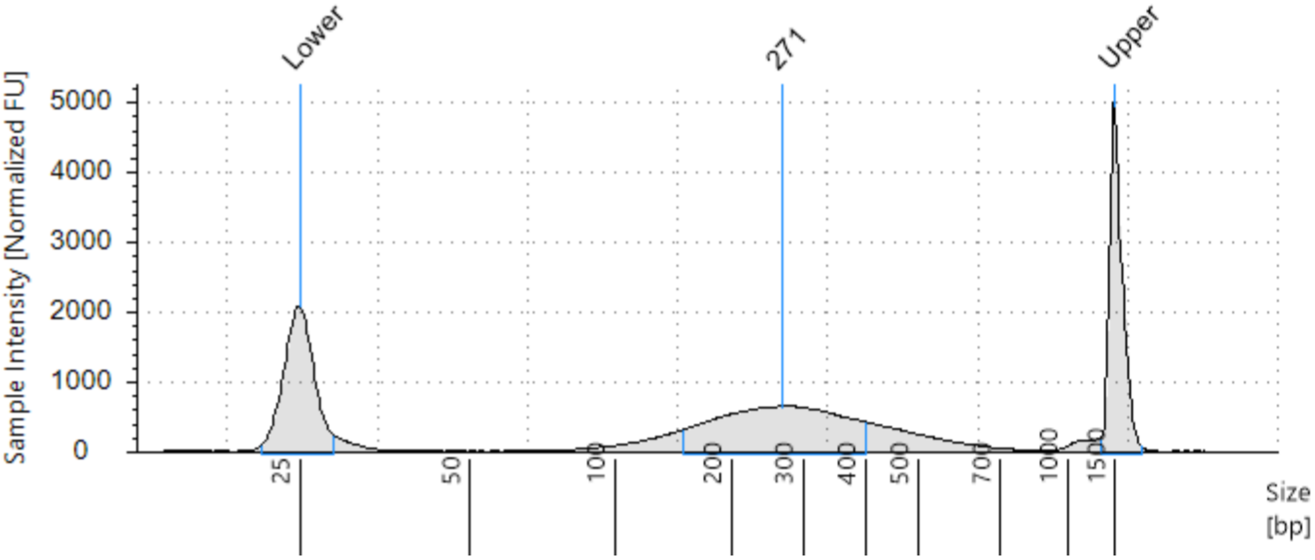

Sample Table

Well	Conc. [ng/µl]	Sample Description	Alert	Observations
E1	8.44	M-E12		

Peak Table

Size [bp]	Calibrated Conc. [ng/µl]	Assigned Conc. [ng/µl]	Peak Molarity [nmol/l]	% Integrated Area	Peak Comment	Observations
25	6.37	-	392	-		Lower Marker
271	8.44	-	47.9	100.00		
1500	6.50	6.50	6.67	-		Upper Marker

© Agilent Technologies, Inc. 2017

F1: M-F12

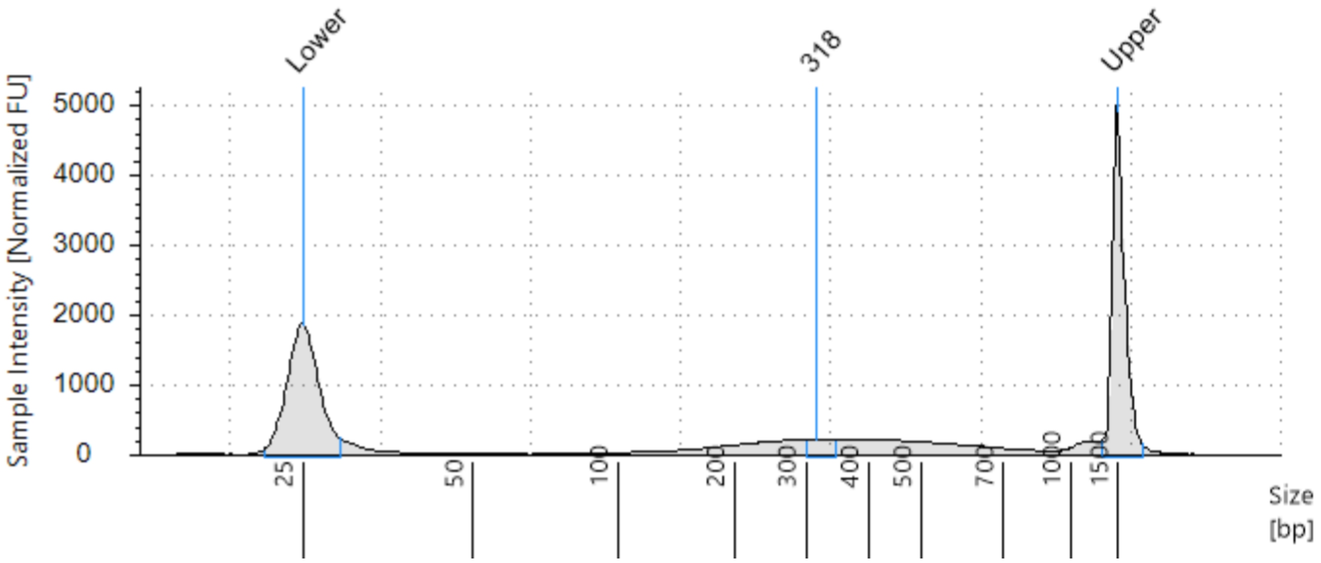

Sample Table

Well	Conc. [ng/µl]	Sample Description	Alert	Observations
F1	0.585	M-F12		

Peak Table

Size [bp]	Calibrated Conc. [ng/µl]	Assigned Conc. [ng/µl]	Peak Molarity [nmol/l]	% Integrated Area	Peak Comment	Observations
25	6.26	-	385	-		Lower Marker
318	0.585	-	2.83	100.00		
1500	6.50	6.50	6.67	-		Upper Marker

© Agilent Technologies, Inc. 2017

Gl: M-Gl2

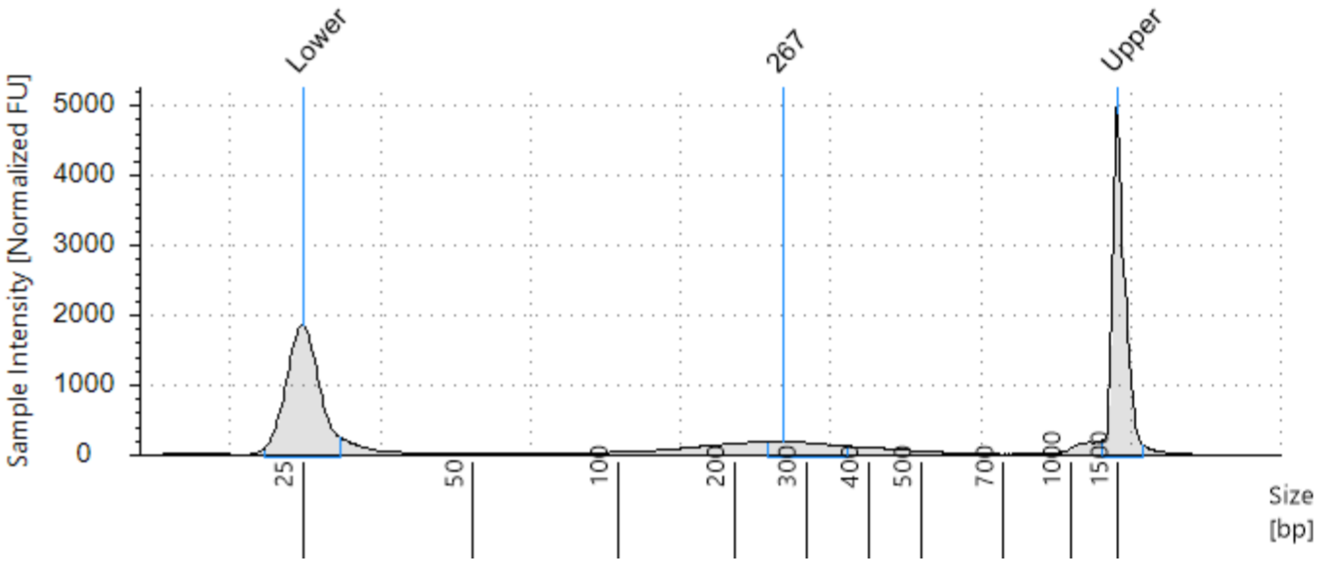

Sample Table

Well	Conc. [ng/µl]	Sample Description	Alert	Observations
Gl	1.14	M-Gl2		

Peak Table

Size [bp]	Calibrated Conc. [ng/µl]	Assigned Conc. [ng/µl]	Peak Molarity [nmol/l]	% Integrated Area	Peak Comment	Observations
25	6.45	-	397	-		Lower Marker
267	1.14	-	6.56	100.00		
1500	6.50	6.50	6.67	-		Upper Marker

 © Agilent Technologies, Inc. 2017

H1: M-H12

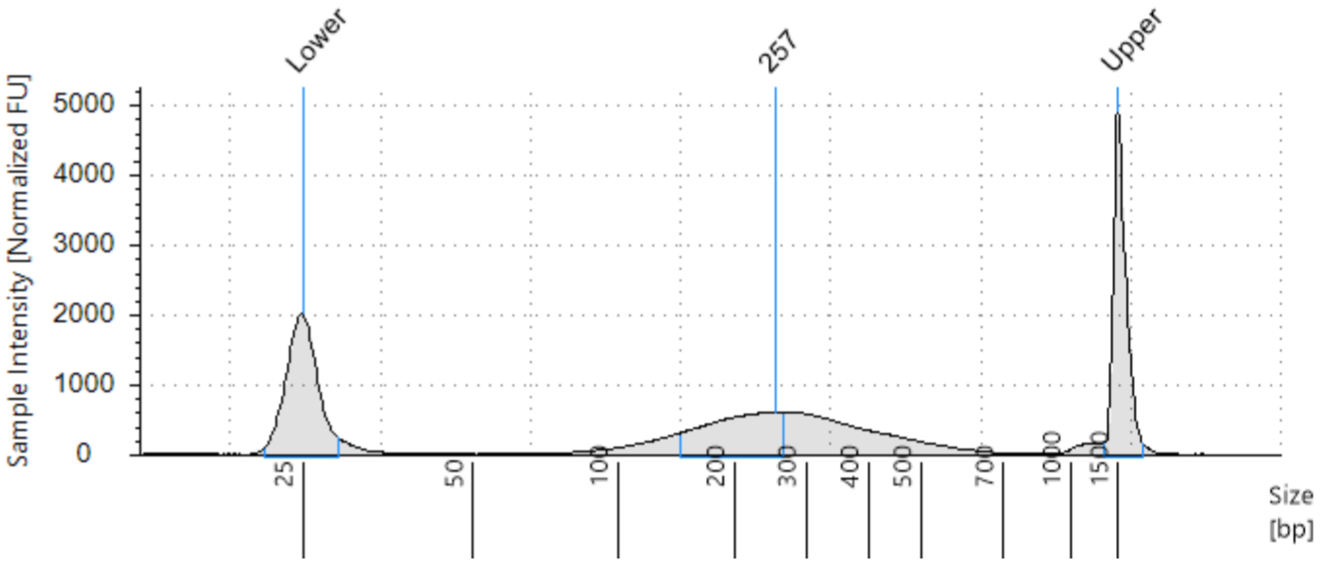

Sample Table

Well	Conc. [ng/µl]	Sample Description	Alert	Observations
H1	4.54	M-H12		

Peak Table

Size [bp]	Calibrated Conc. [ng/µl]	Assigned Conc. [ng/µl]	Peak Molarity [nmol/l]	% Integrated Area	Peak Comment	Observations
25	6.49	-	399	-		Lower Marker
257	4.54	-	27.2	100.00		
1500	6.50	6.50	6.67	-		Upper Marker

© Agilent Technologies, Inc. 2017

A2: LADDER

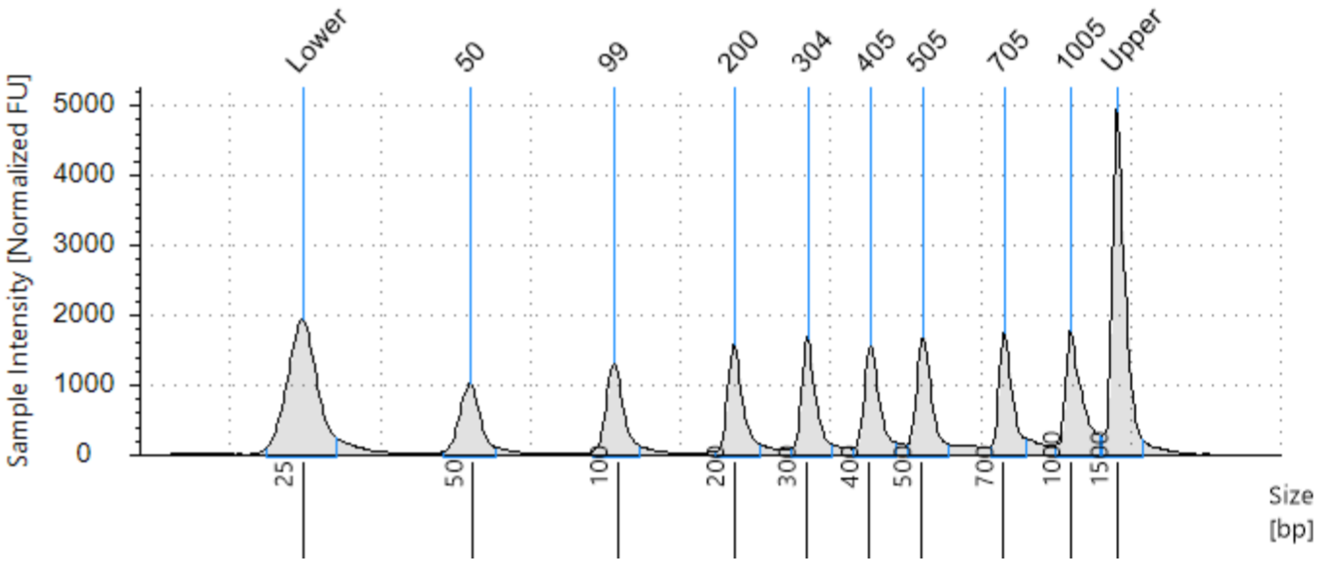

Sample Table

Well	Conc. [ng/µl]	Sample Description	Alert	Observations
A2	19.8	LADDER		

Peak Table

Size [bp]	Calibrated Conc. [ng/µl]	Assigned Conc. [ng/µl]	Peak Molarity [nmol/l]	% Integrated Area	Peak Comment	Observations
25	5.86	-	361	-		Lower Marker
50	2.14	-	66.2	10.78		
99	2.33	-	36.3	11.75		
200	2.45	-	18.9	12.34		
304	2.43	-	12.3	12.24		
405	2.48	-	9.42	12.50		
505	2.63	-	8.00	13.23		
705	2.44	-	5.33	12.32		
1005	2.94	-	4.50	14.83		
1500	6.50	6.50	6.67	-		Upper Marker

© Agilent Technologies, Inc. 2017

B2: PLUS B12

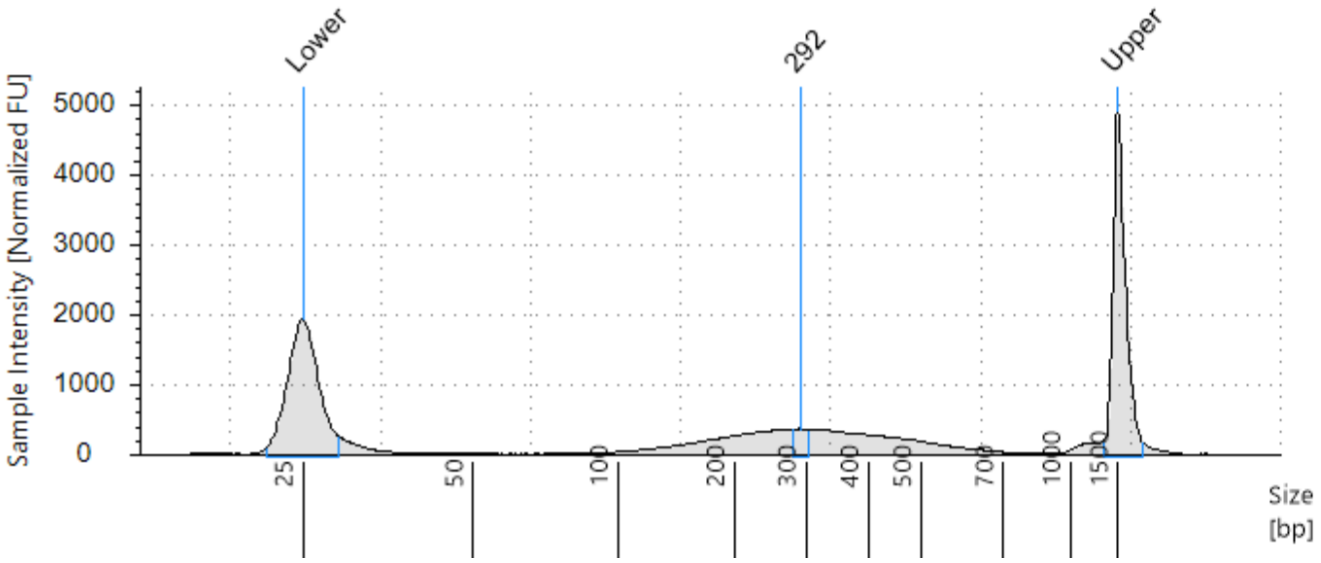

Sample Table

Well	Conc. [ng/µl]	Sample Description	Alert	Observations
B2	0.507	PLUS B12		

Peak Table

Size [bp]	Calibrated Conc. [ng/µl]	Assigned Conc. [ng/µl]	Peak Molarity [nmol/l]	% Integrated Area	Peak Comment	Observations
25	6.27	-	386	-		Lower Marker
292	0.507	-	2.67	100.00		
1500	6.50	6.50	6.67	-		Upper Marker

© Agilent Technologies, Inc. 2017

C2: P-C12

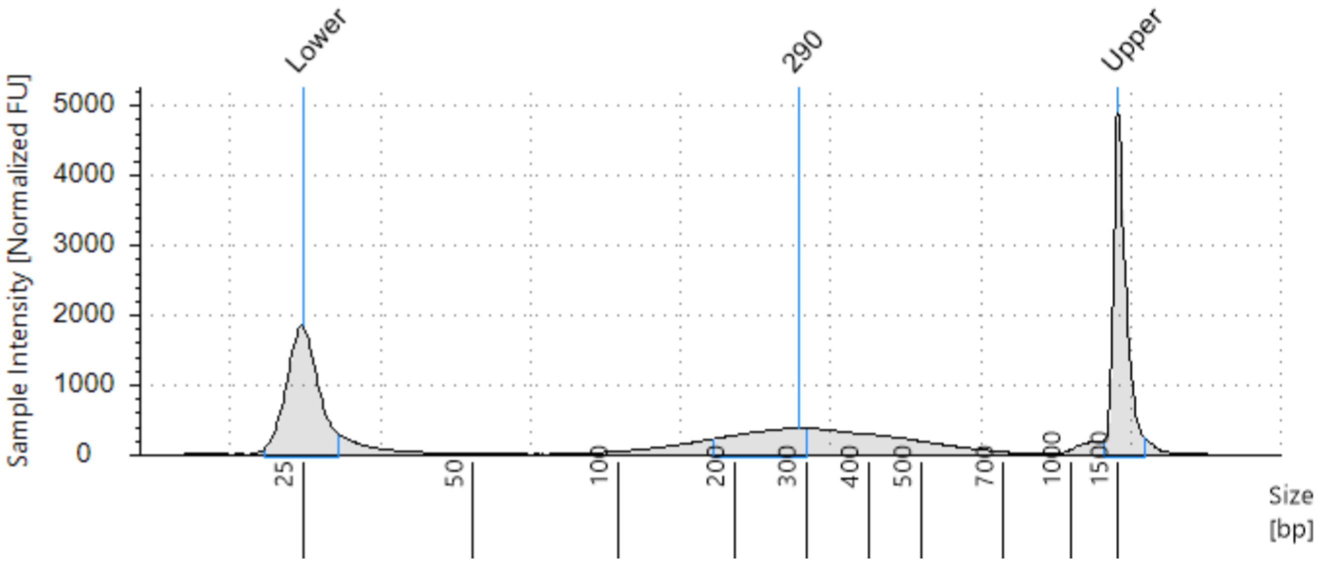

Sample Table

Well	Conc. [ng/µl]	Sample Description	Alert	Observations
C2	2.67	P-C12		

Peak Table

Size [bp]	Calibrated Conc. [ng/µl]	Assigned Conc. [ng/µl]	Peak Molarity [nmol/l]	% Integrated Area	Peak Comment	Observations
25	6.12	-	377	-		Lower Marker
290	2.67	-	14.2	100.00		
1500	6.50	6.50	6.67	-		Upper Marker

 © Agilent Technologies, Inc. 2017

D2: P-D12

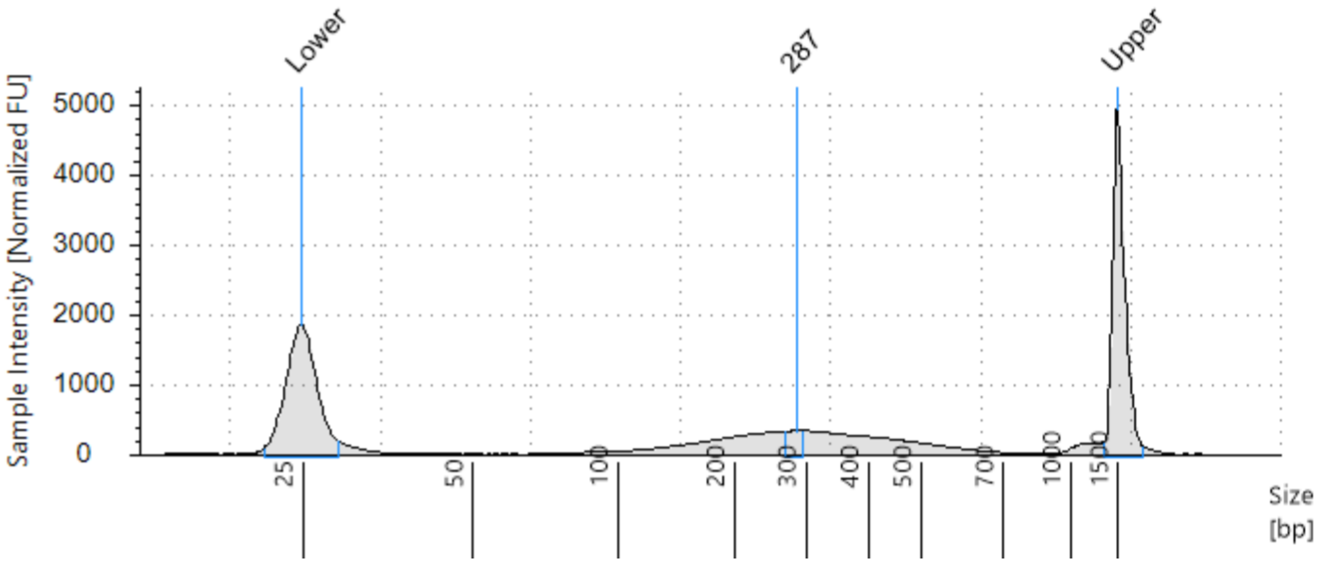

Sample Table

Well	Conc. [ng/µl]	Sample Description	Alert	Observations
D2	0.555	P-D12		

Peak Table

Size [bp]	Calibrated Conc. [ng/µl]	Assigned Conc. [ng/µl]	Peak Molarity [nmol/l]	% Integrated Area	Peak Comment	Observations
25	6.15	-	378	-		Lower Marker
287	0.555	-	2.98	100.00		
1500	6.50	6.50	6.67	-		Upper Marker

 © Agilent Technologies, Inc. 2017

E2: P-E12

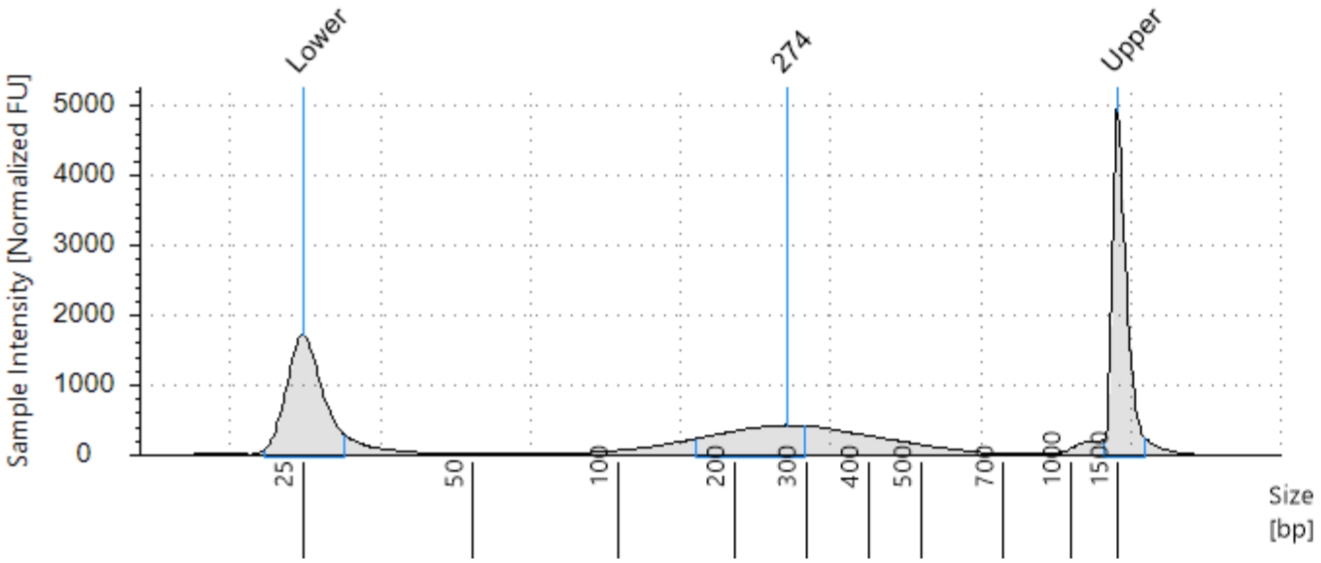

Sample Table

Well	Conc. [ng/µl]	Sample Description	Alert	Observations
E2	3.03	P-E12		

Peak Table

Size [bp]	Calibrated Conc. [ng/µl]	Assigned Conc. [ng/µl]	Peak Molarity [nmol/l]	% Integrated Area	Peak Comment	Observations
25	5.69	-	350	-		Lower Marker
274	3.03	-	17.0	100.00		
1500	6.50	6.50	6.67	-		Upper Marker

© Agilent Technologies, Inc. 2017

F2: P-F12

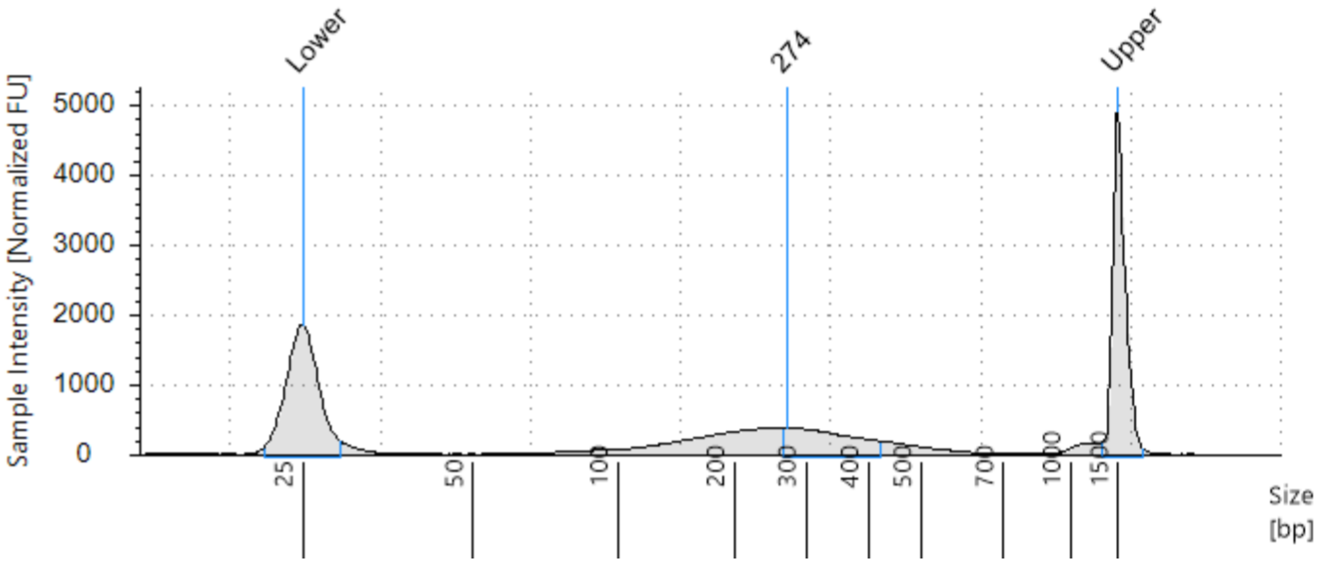

Sample Table

Well	Conc. [ng/µl]	Sample Description	Alert	Observations
F2	2.60	P-F12		

Peak Table

Size [bp]	Calibrated Conc. [ng/µl]	Assigned Conc. [ng/µl]	Peak Molarity [nmol/l]	% Integrated Area	Peak Comment	Observations
25	6.26	-	385	-		Lower Marker
274	2.60	-	14.6	100.00		
1500	6.50	6.50	6.67	-		Upper Marker

© Agilent Technologies, Inc. 2017

G2: P-G12

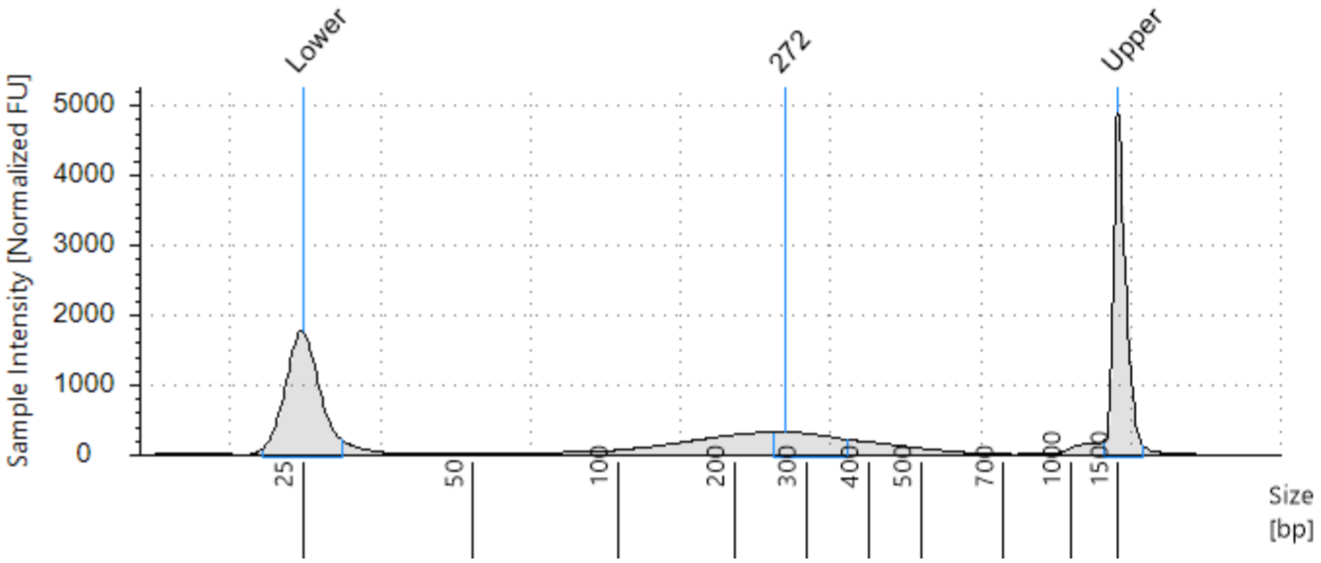

Sample Table

Well	Conc. [ng/µl]	Sample Description	Alert	Observations
G2	1.91	P-G12		

Peak Table

Size [bp]	Calibrated Conc. [ng/µl]	Assigned Conc. [ng/µl]	Peak Molarity [nmol/l]	% Integrated Area	Peak Comment	Observations
25	6.33	-	390	-		Lower Marker
272	1.91	-	10.8	100.00		
1500	6.50	6.50	6.67	-		Upper Marker

 © Agilent Technologies, Inc. 2017

H2: P-H12

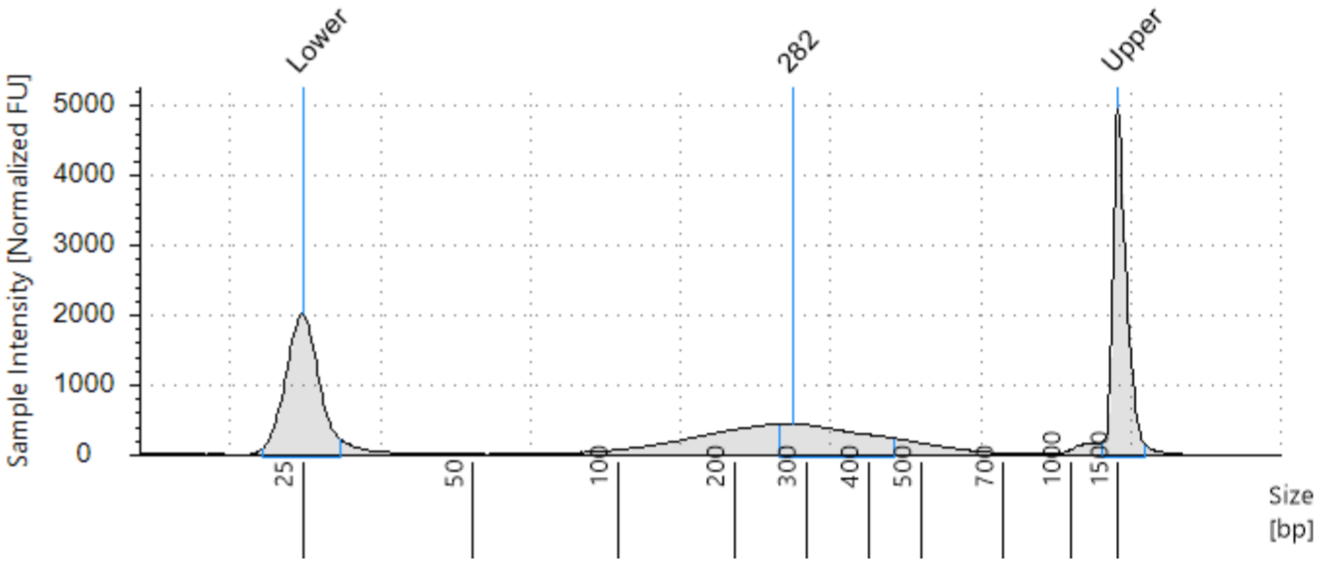

Sample Table

Well	Conc. [ng/µl]	Sample Description	Alert	Observations
H2	3.29	P-H12		

Peak Table

Size [bp]	Calibrated Conc. [ng/µl]	Assigned Conc. [ng/µl]	Peak Molarity [nmol/l]	% Integrated Area	Peak Comment	Observations
25	6.42	-	395	-		Lower Marker
282	3.29	-	18.0	100.00		
1500	6.50	6.50	6.67	-		Upper Marker

© Agilent Technologies, Inc. 2017

Filename: 2020-09-30-02, Q-S G2, D2P ,.... A12,B12, ...M R2.D1000

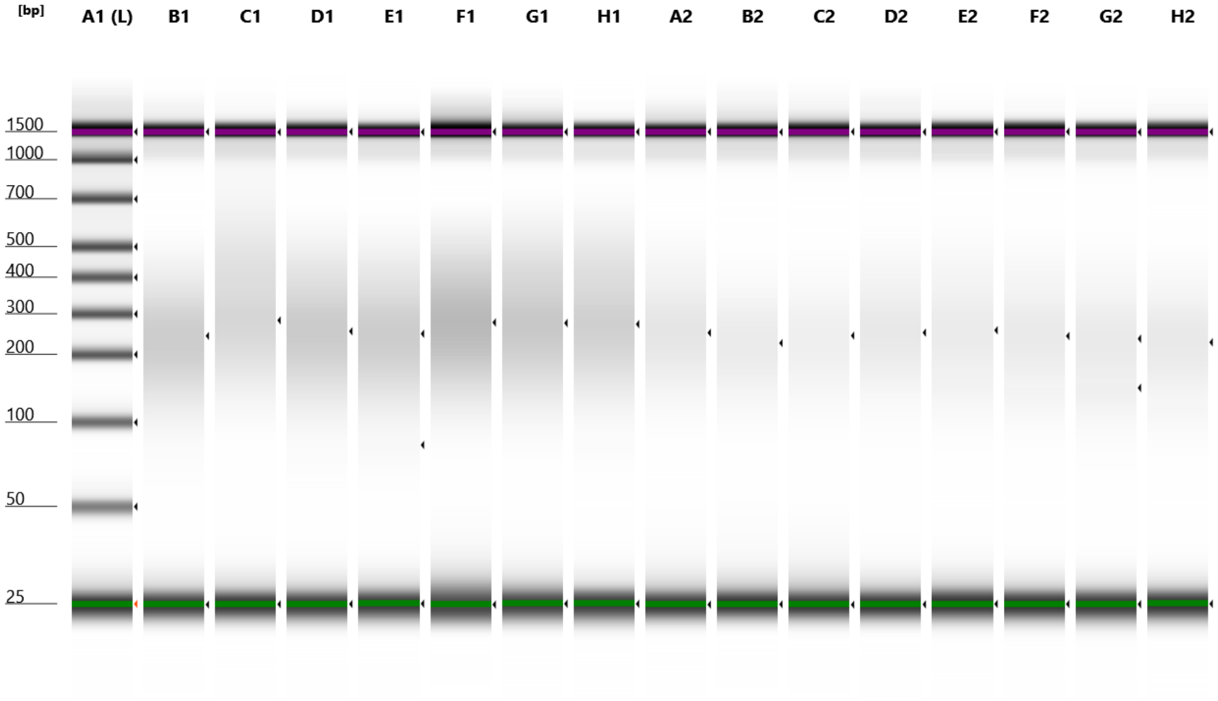

Default image (Contrast 100%)

Sample Info

Well	Conc. [ng/µl]	Sample Description	Alert	Observations
A1	15.7	Ladder		Ladder
B1	0.369	G2 P R2		
C1	1.56	D2 P R2		
D1	3.89	H2 P R2		
E1	3.86	A3 P R2		
F1	0.703	B3 P R2		
G1	3.99	C3 P R2		
H1	3.23	D3 P R2		
A2	0.861	E3 P R2		
B2	0.165	A12 M R2		
C2	0.451	B12 M R2		
D2	1.31	C12 M R2		
E2	0.626	D12 M R2		
F2	0.605	E12 M R2		
G2	0.572	F12 M R2		
H2	0.734	G12 M R2		

© Agilent Technologies, Inc. 2017

A1: Ladder

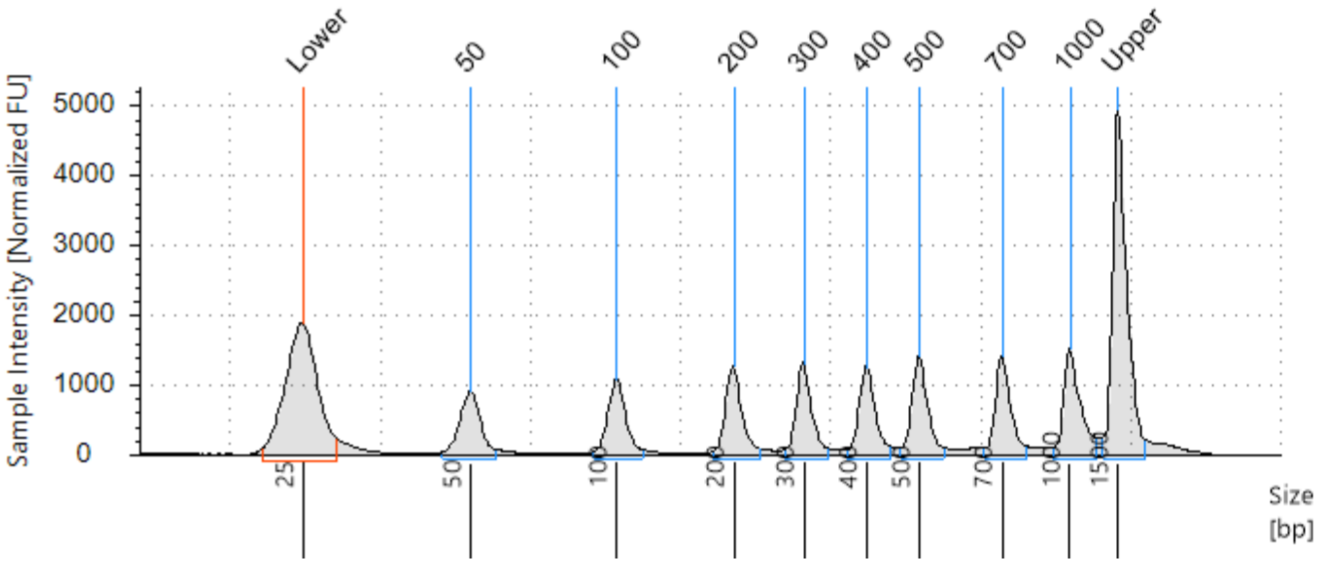

Sample Table

Well	Conc. [ng/µl]	Sample Description	Alert	Observations
A1	15.7	Ladder		Ladder

Peak Table

Size [bp]	Calibrated Conc. [ng/µl]	Assigned Conc. [ng/µl]	Peak Molarity [nmol/l]	% Integrated Area	Peak Comment	Observations
25	5.60	-	345	-		Lower Marker
50	1.72	-	52.9	10.98		
100	1.81	-	27.8	11.53		
200	1.93	-	14.9	12.33		
300	1.88	-	9.65	12.01		
400	1.91	-	7.36	12.21		
500	2.05	-	6.31	13.10		
700	2.03	-	4.46	12.96		
1000	2.33	-	3.59	14.88		
1500	6.50	6.50	6.67	-		Upper Marker

© Agilent Technologies, Inc. 2017

B1: G2 P R2

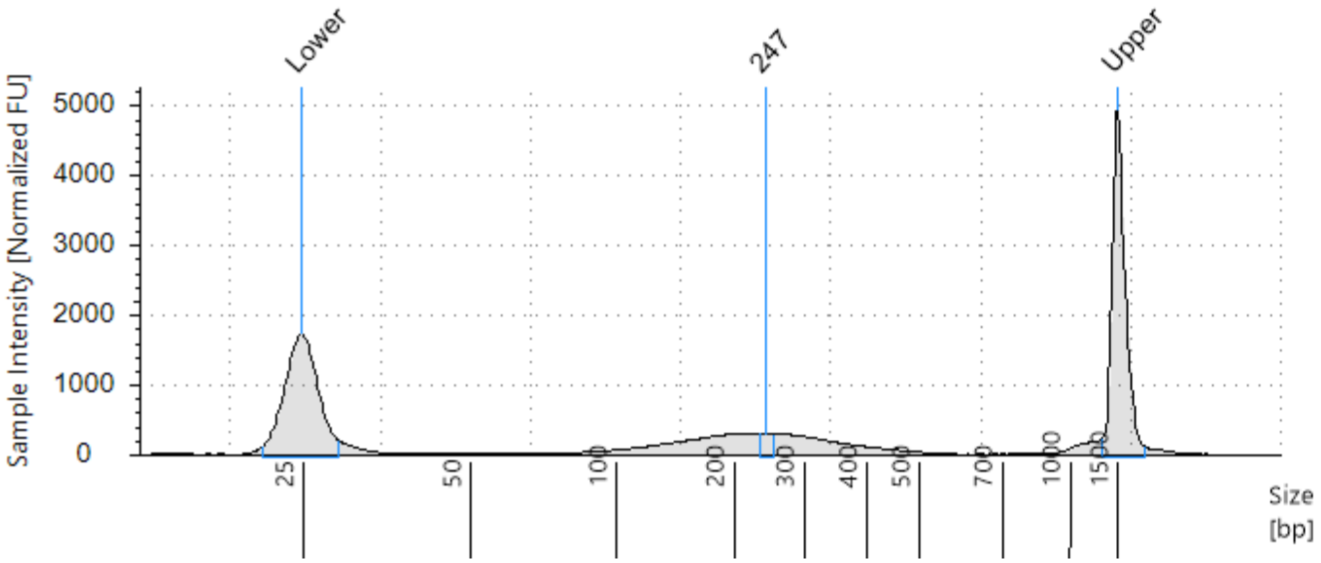

Sample Table

Well	Conc. [ng/µl]	Sample Description	Alert	Observations
B1	0.369	G2 P R2		

Peak Table

Size [bp]	Calibrated Conc. [ng/µl]	Assigned Conc. [ng/µl]	Peak Molarity [nmol/l]	% Integrated Area	Peak Comment	Observations
25	5.78	-	355	-		Lower Marker
247	0.369	-	2.30	100.00		
1500	6.50	6.50	6.67	-		Upper Marker

 © Agilent Technologies, Inc. 2017

C1: D2 P R2

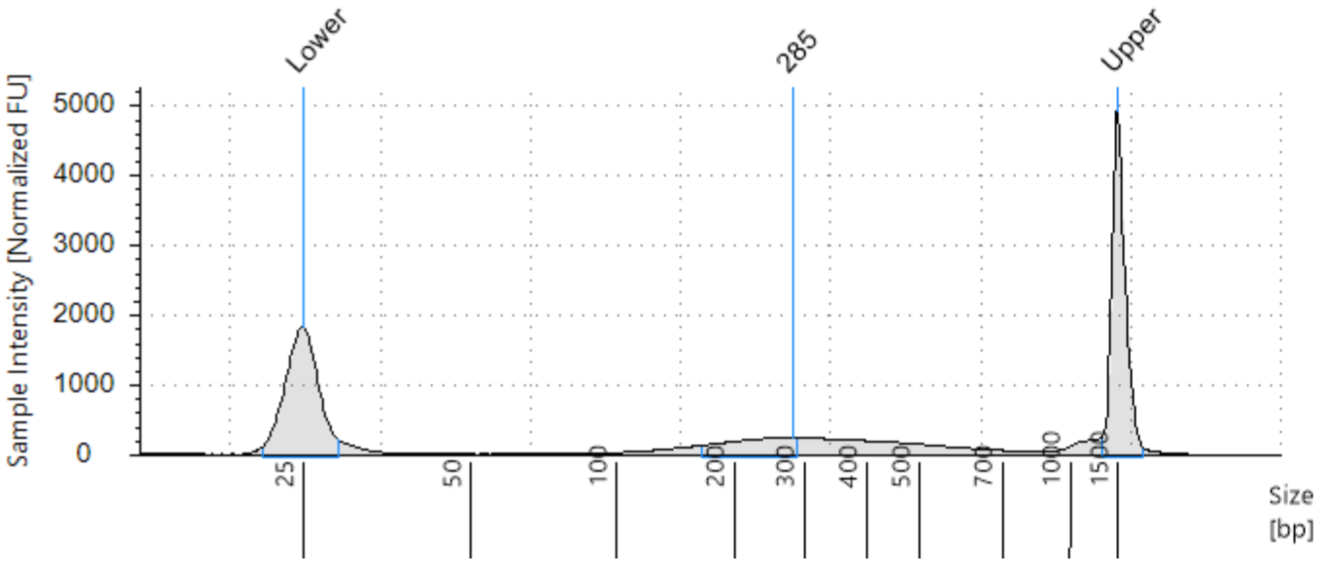

Sample Table

Well	Conc. [ng/µl]	Sample Description	Alert	Observations
C1	1.56	D2 P R2		

Peak Table

Size [bp]	Calibrated Conc. [ng/µl]	Assigned Conc. [ng/µl]	Peak Molarity [nmol/l]	% Integrated Area	Peak Comment	Observations
25	5.95	-	366	-		Lower Marker
285	1.56	-	8.43	100.00		
1500	6.50	6.50	6.67	-		Upper Marker

© Agilent Technologies, Inc. 2017

D1: H2 P R2

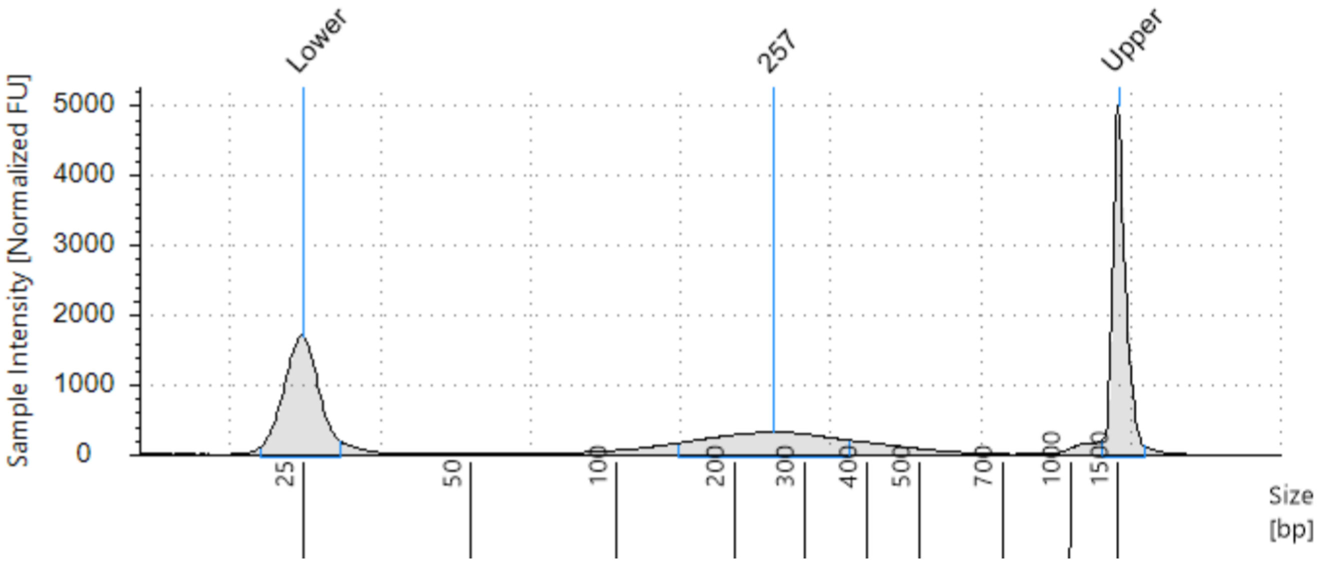

Sample Table

Well	Conc. [ng/µl]	Sample Description	Alert	Observations
D1	3.89	H2 P R2		

Peak Table

Size [bp]	Calibrated Conc. [ng/µl]	Assigned Conc. [ng/µl]	Peak Molarity [nmol/l]	% Integrated Area	Peak Comment	Observations
25	5.96	-	367	-		Lower Marker
257	3.89	-	23.2	100.00		
1500	6.50	6.50	6.67	-		Upper Marker

 © Agilent Technologies, Inc. 2017

E1: A3 P R2

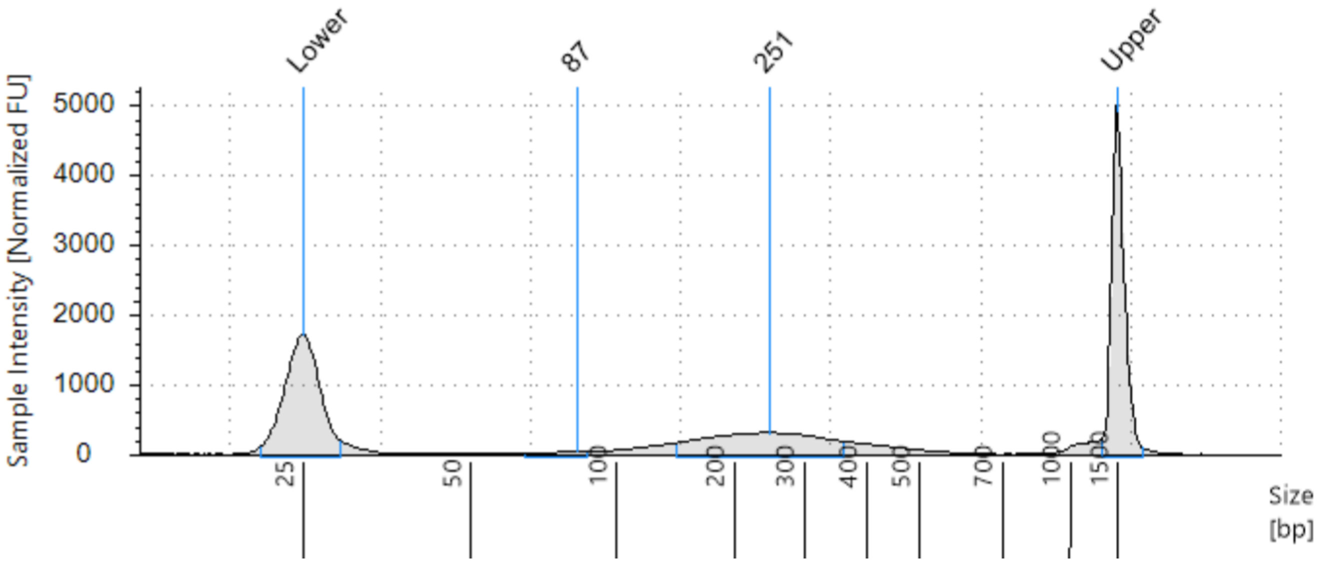

Sample Table

Well	Conc. [ng/µl]	Sample Description	Alert	Observations
E1	3.86	A3 P R2		

Peak Table

Size [bp]	Calibrated Conc. [ng/µl]	Assigned Conc. [ng/µl]	Peak Molarity [nmol/l]	% Integrated Area	Peak Comment	Observations
25	6.12	-	377	-		Lower Marker
87	0.139	-	2.47	3.60		
251	3.72	-	22.8	96.40		
1500	6.50	6.50	6.67	-		Upper Marker

© Agilent Technologies, Inc. 2017

F1: B3 P R2

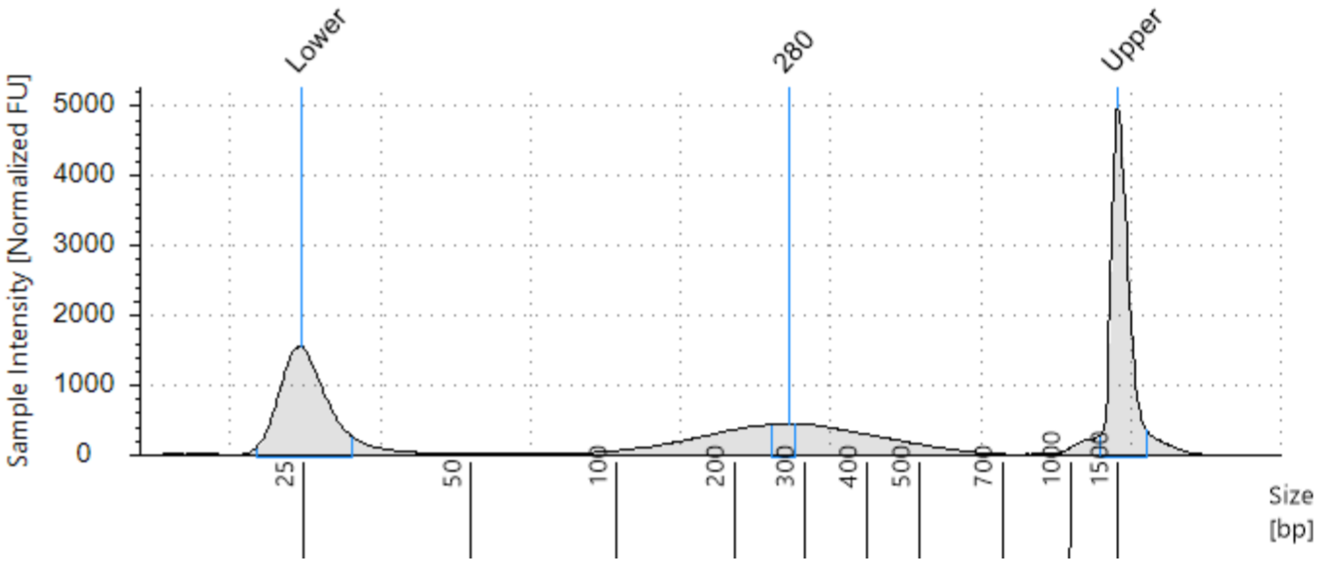

Sample Table

Well	Conc. [ng/µl]	Sample Description	Alert	Observations
F1	0.703	B3 P R2		

Peak Table

Size [bp]	Calibrated Conc. [ng/µl]	Assigned Conc. [ng/µl]	Peak Molarity [nmol/l]	% Integrated Area	Peak Comment	Observations
25	5.32	-	327	-		Lower Marker
280	0.703	-	3.86	100.00		
1500	6.50	6.50	6.67	-		Upper Marker

 © Agilent Technologies, Inc. 2017

G1: C3 P R2

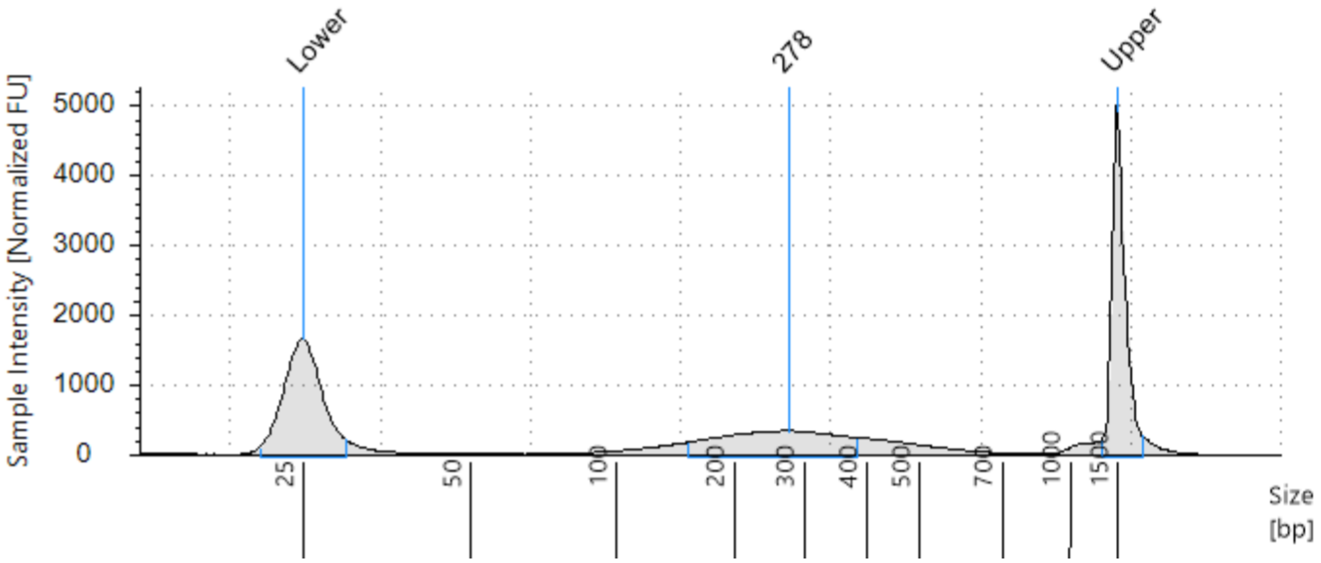

Sample Table

Well	Conc. [ng/µl]	Sample Description	Alert	Observations
G1	3.99	C3 P R2		

Peak Table

Size [bp]	Calibrated Conc. [ng/µl]	Assigned Conc. [ng/µl]	Peak Molarity [nmol/l]	% Integrated Area	Peak Comment	Observations
25	6.20	-	382	-		Lower Marker
278	3.99	-	22.1	100.00		
1500	6.50	6.50	6.67	-		Upper Marker

 © Agilent Technologies, Inc. 2017

H1: D3 P R2

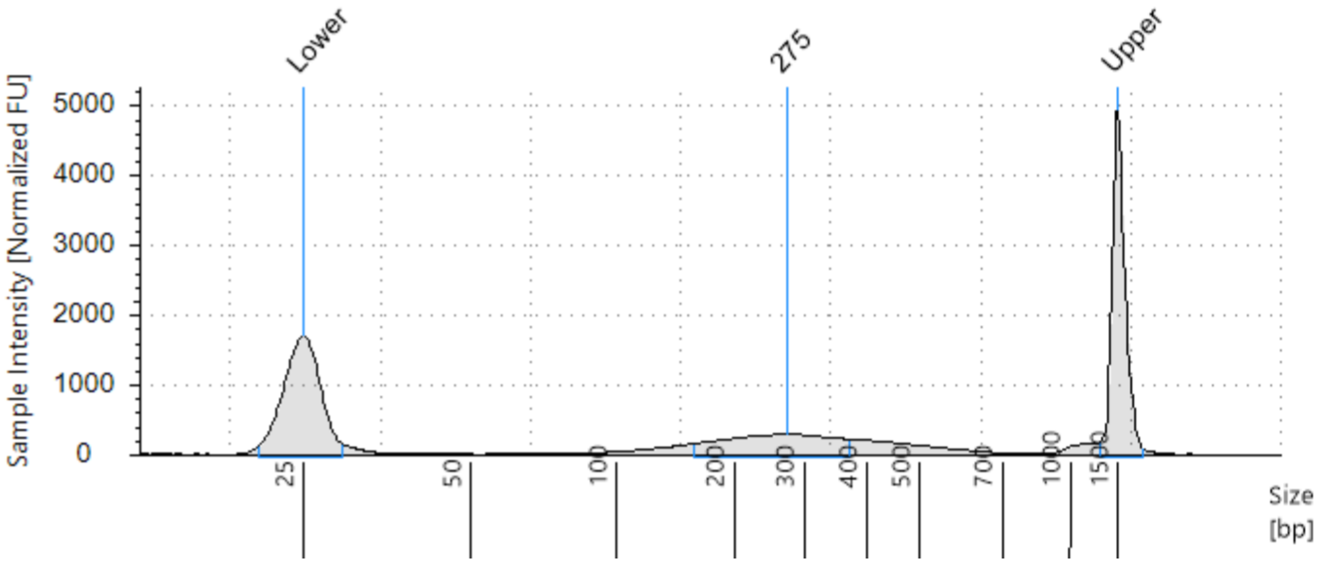

Sample Table

Well	Conc. [ng/µl]	Sample Description	Alert	Observations
H1	3.23	D3 P R2		

Peak Table

Size [bp]	Calibrated Conc. [ng/µl]	Assigned Conc. [ng/µl]	Peak Molarity [nmol/l]	% Integrated Area	Peak Comment	Observations
25	6.34	-	390	-		Lower Marker
275	3.23	-	18.1	100.00		
1500	6.50	6.50	6.67	-		Upper Marker

© Agilent Technologies, Inc. 2017

A2: E3 P R2

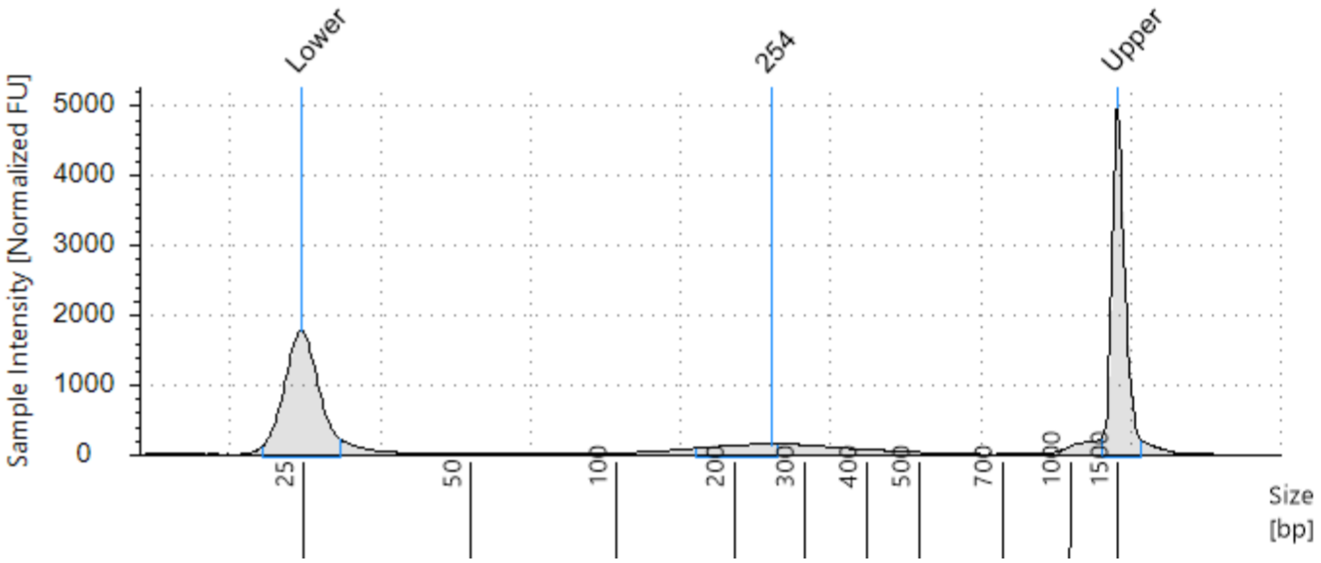

Sample Table

Well	Conc. [ng/µl]	Sample Description	Alert	Observations
A2	0.861	E3 P R2		

Peak Table

Size [bp]	Calibrated Conc. [ng/µl]	Assigned Conc. [ng/µl]	Peak Molarity [nmol/l]	% Integrated Area	Peak Comment	Observations
25	6.00	-	369	-		Lower Marker
254	0.861	-	5.21	100.00		
1500	6.50	6.50	6.67	-		Upper Marker

© Agilent Technologies, Inc. 2017

B2: A12 M R2

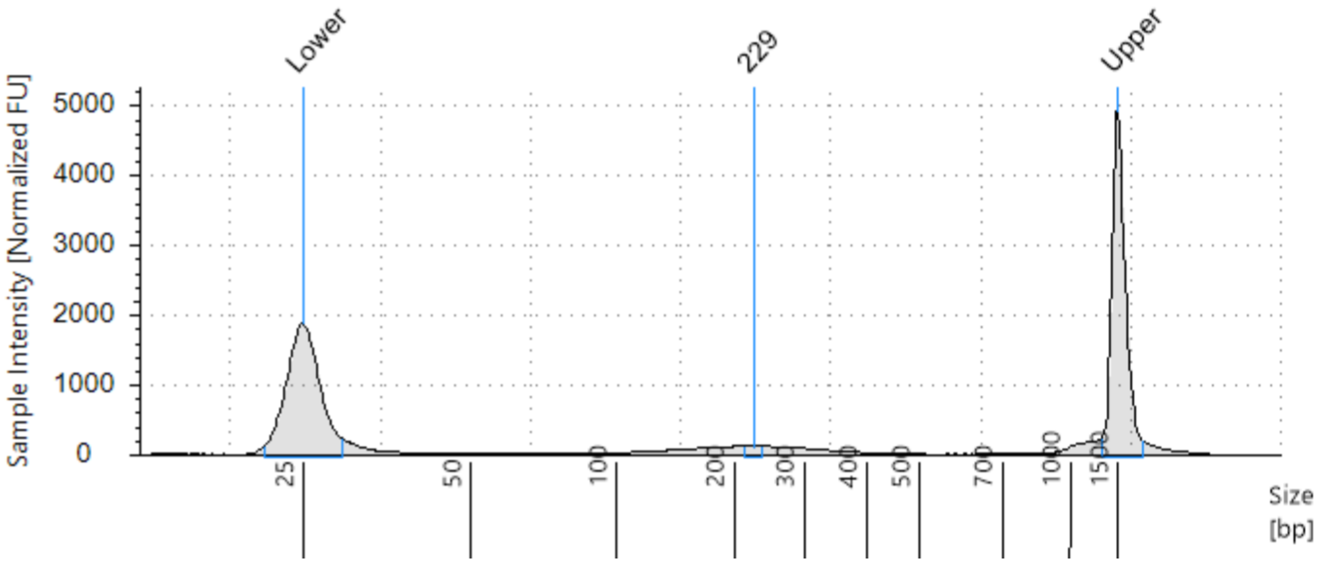

Sample Table

Well	Conc. [ng/µl]	Sample Description	Alert	Observations
B2	0.165	A12 M R2		

Peak Table

Size [bp]	Calibrated Conc. [ng/µl]	Assigned Conc. [ng/µl]	Peak Molarity [nmol/l]	% Integrated Area	Peak Comment	Observations
25	6.07	-	373	-		Lower Marker
229	0.165	-	1.11	100.00		
1500	6.50	6.50	6.67	-		Upper Marker

© Agilent Technologies, Inc. 2017

C2: B12 M R2

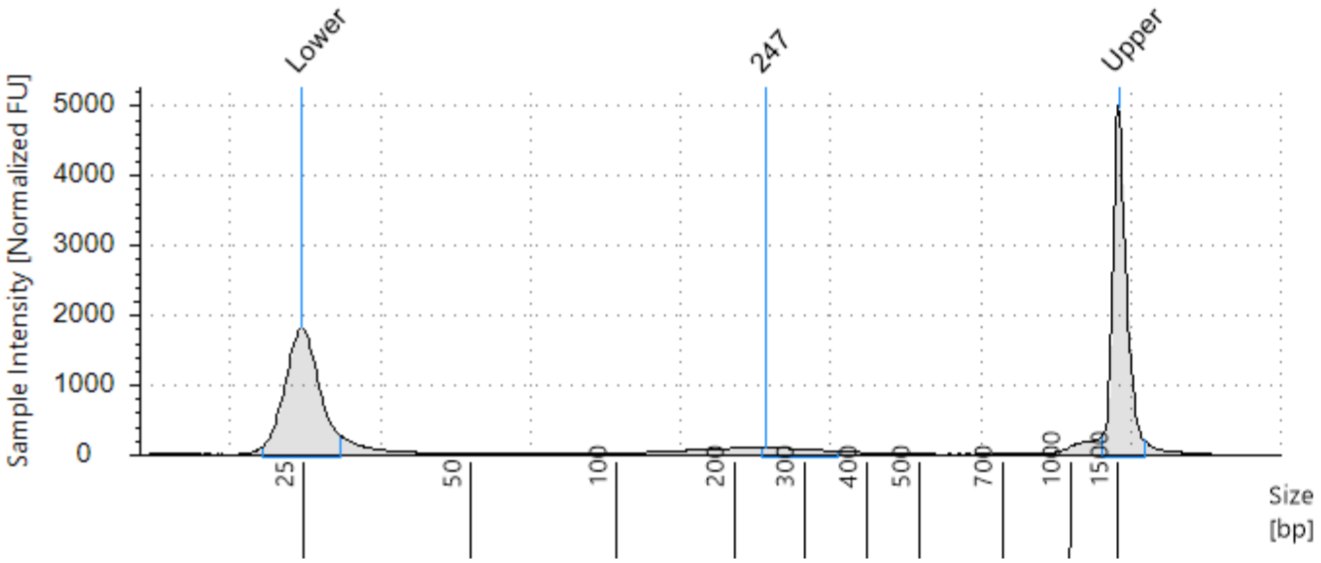

Sample Table

Well	Conc. [ng/µl]	Sample Description	Alert	Observations
C2	0.451	B12 M R2		

Peak Table

Size [bp]	Calibrated Conc. [ng/µl]	Assigned Conc. [ng/µl]	Peak Molarity [nmol/l]	% Integrated Area	Peak Comment	Observations
25	5.90	-	363	-		Lower Marker
247	0.451	-	2.81	100.00		
1500	6.50	6.50	6.67	-		Upper Marker

D2: C12 M R2

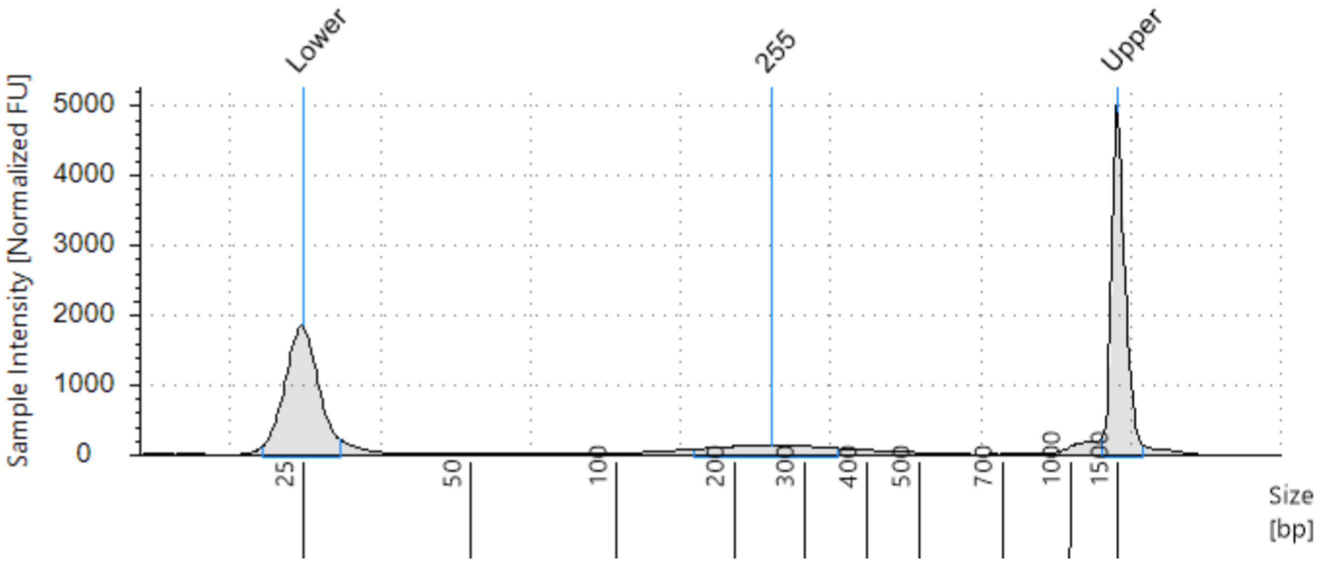

Sample Table

Well	Conc. [ng/µl]	Sample Description	Alert	Observations
D2	1.31	C12 M R2		

Peak Table

Size [bp]	Calibrated Conc. [ng/µl]	Assigned Conc. [ng/µl]	Peak Molarity [nmol/l]	% Integrated Area	Peak Comment	Observations
25	5.95	-	366	-		Lower Marker
255	1.31	-	7.91	100.00		
1500	6.50	6.50	6.67	-		Upper Marker

© Agilent Technologies, Inc. 2017

E2: D12 M R2

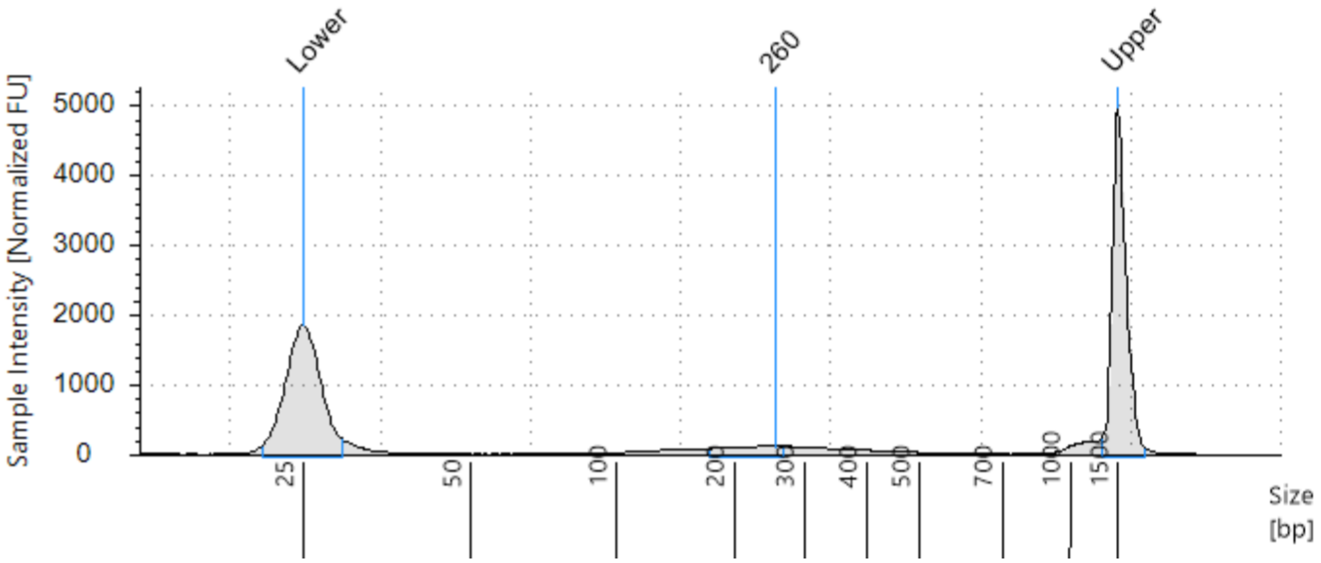

Sample Table

Well	Conc. [ng/µl]	Sample Description	Alert	Observations
E2	0.626	D12 M R2		

Peak Table

Size [bp]	Calibrated Conc. [ng/µl]	Assigned Conc. [ng/µl]	Peak Molarity [nmol/l]	% Integrated Area	Peak Comment	Observations
25	6.17	-	380	-		Lower Marker
260	0.626	-	3.71	100.00		
1500	6.50	6.50	6.67	-		Upper Marker

© Agilent Technologies, Inc. 2017

F2: E12 M R2

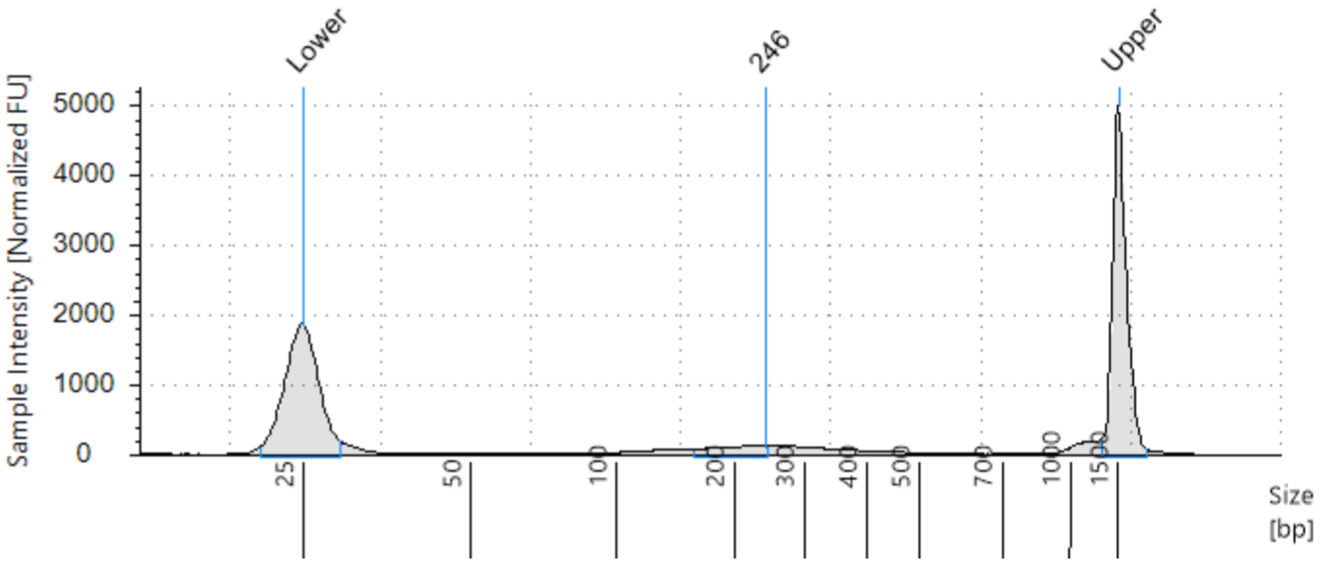

Sample Table

Well	Conc. [ng/µl]	Sample Description	Alert	Observations
F2	0.605	E12 M R2		

Peak Table

Size [bp]	Calibrated Conc. [ng/µl]	Assigned Conc. [ng/µl]	Peak Molarity [nmol/l]	% Integrated Area	Peak Comment	Observations
25	5.98	-	368	-		Lower Marker
246	0.605	-	3.79	100.00		
1500	6.50	6.50	6.67	-		Upper Marker

 © Agilent Technologies, Inc. 2017

G2: F12 M R2

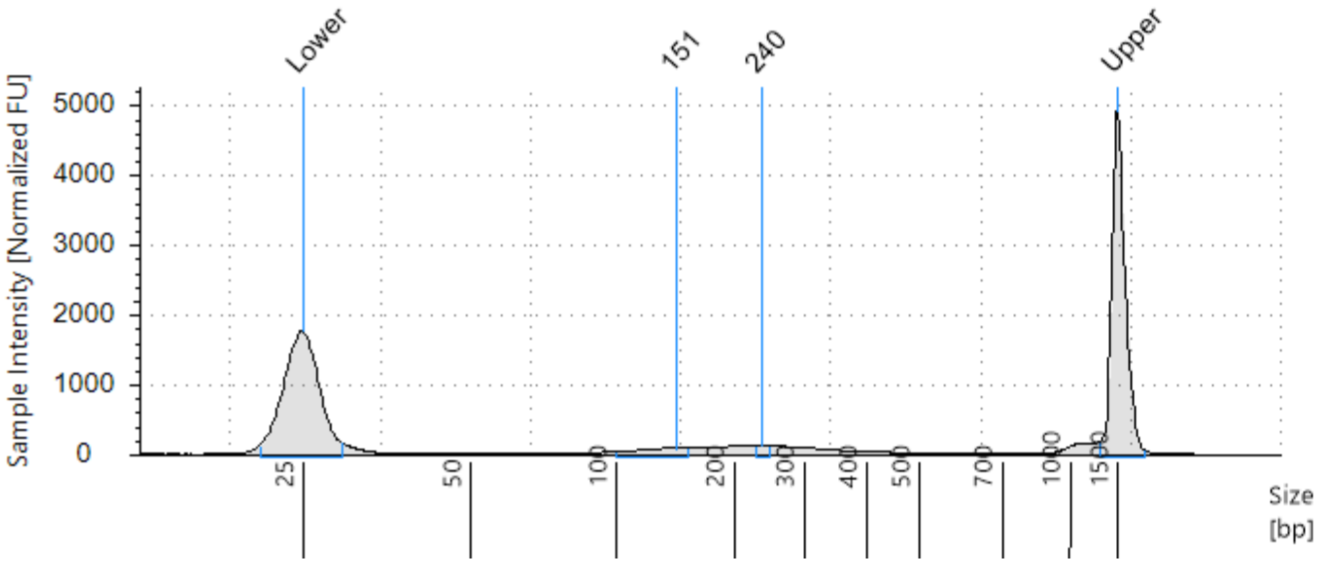

Sample Table

Well	Conc. [ng/µl]	Sample Description	Alert	Observations
G2	0.572	F12 M R2		

Peak Table

Size [bp]	Calibrated Conc. [ng/µl]	Assigned Conc. [ng/µl]	Peak Molarity [nmol/l]	% Integrated Area	Peak Comment	Observations
25	6.22	-	383	-		Lower Marker
151	0.425	-	4.32	74.35		
240	0.147	-	0.941	25.65		
1500	6.50	6.50	6.67	-		Upper Marker

© Agilent Technologies, Inc. 2017

H2: G12 M R2

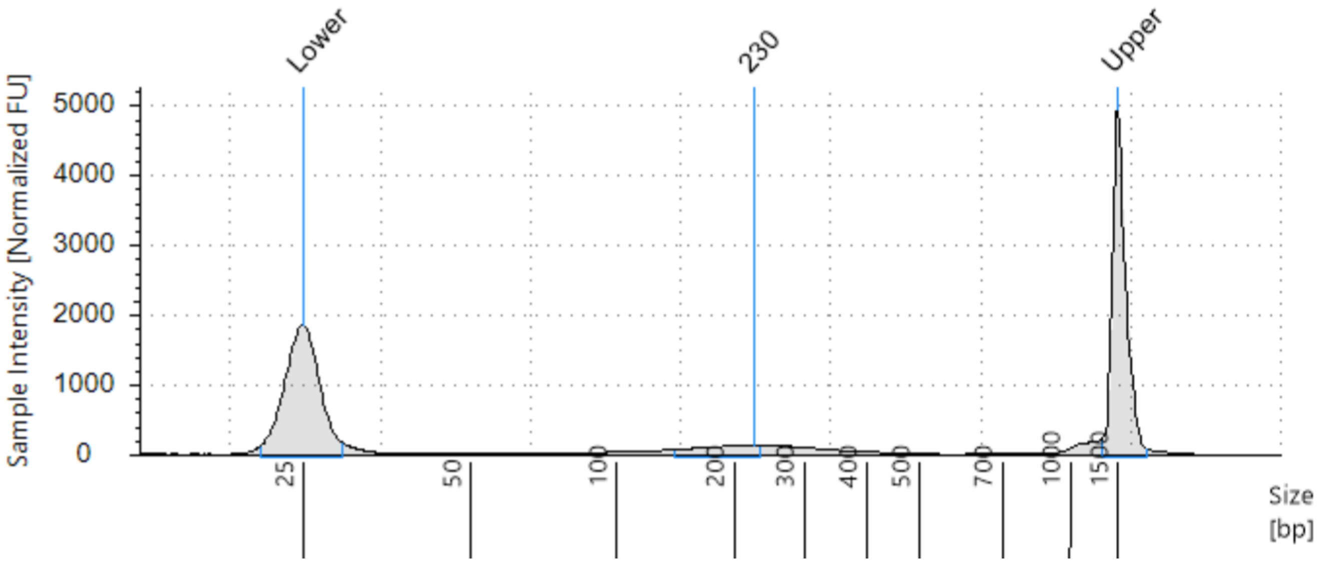

Sample Table

Well	Conc. [ng/µl]	Sample Description	Alert	Observations
H2	0.734	G12 M R2		

Peak Table

Size [bp]	Calibrated Conc. [ng/µl]	Assigned Conc. [ng/µl]	Peak Molarity [nmol/l]	% Integrated Area	Peak Comment	Observations
25	6.26	-	385	-		Lower Marker
230	0.734	-	4.91	100.00		
1500	6.50	6.50	6.67	-		Upper Marker

© Agilent Technologies, Inc. 2017

Filename: 2020-08-31-01- Q-S MINUS F1,G3,C6,A10 D1000 R2.D1000

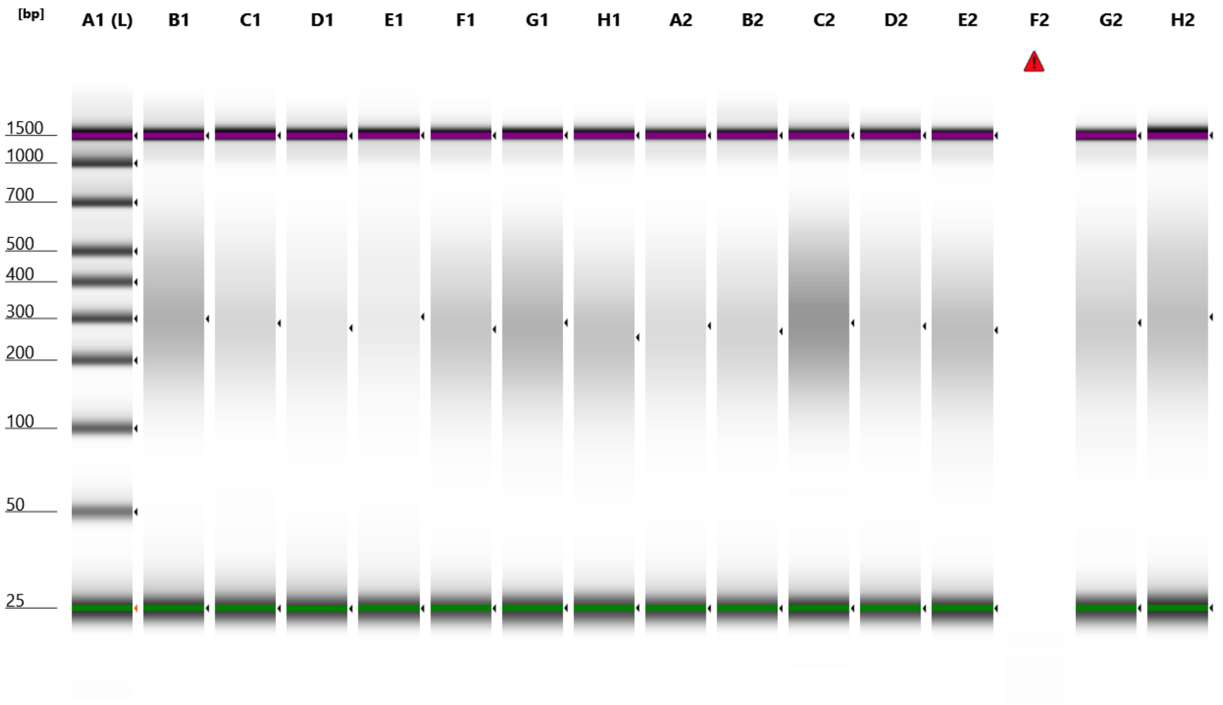

Default image (Contrast 100%)

Sample Info

Well	Conc. [ng/µl]	Sample Description	Alert	Observations
A1	18.5	Ladder		Ladder
B1	3.81	F1 M		
C1	1.22	G3 M		
D1	0.286	C6 M		
E1	0.492	A10 M		
F1	2.83	C6 p		
G1	3.86	A8 P		
H1	3.08	H9 P		
A2	1.41	A11 P		
B2	0.411	B11 P		
C2	1.81	C11 P		
D2	2.53	D11 P		
E2	5.61	E11 P		
F2		F11 P	▲	Marker(s) not detected
G2	1.85	G11 P		
H2	3.40	H11 P		

© Agilent Technologies, Inc. 2017

A1: Ladder

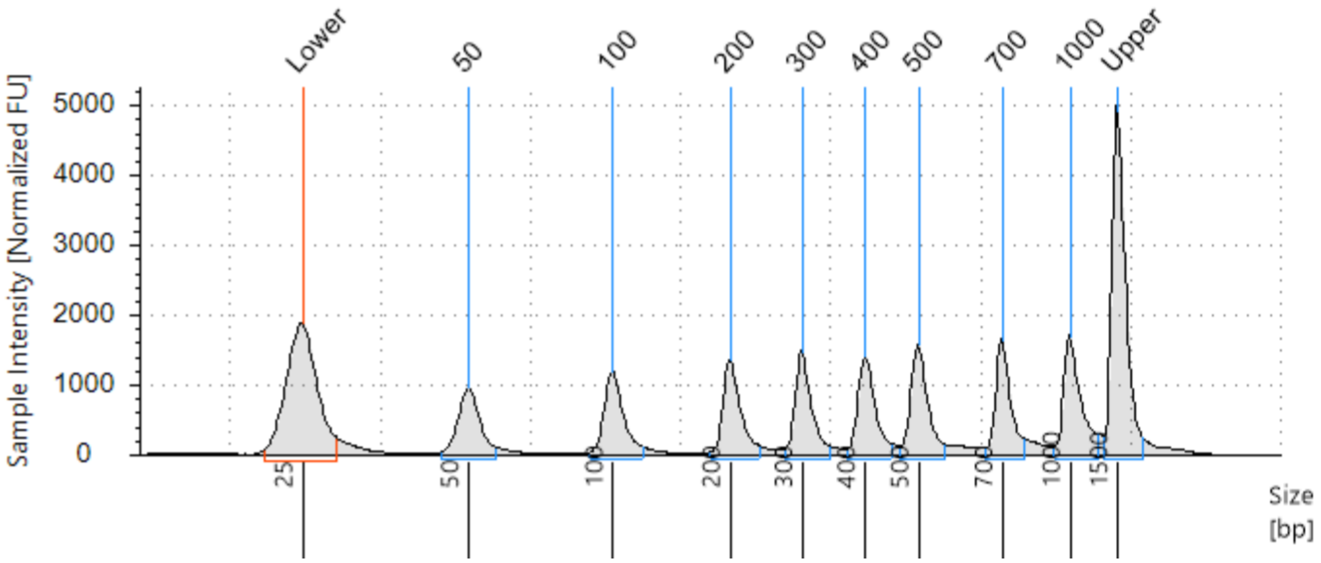

Sample Table

Well	Conc. [ng/µl]	Sample Description	Alert	Observations
A1	18.5	Ladder		Ladder

Peak Table

Size [bp]	Calibrated Conc. [ng/µl]	Assigned Conc. [ng/µl]	Peak Molarity [nmol/l]	% Integrated Area	Peak Comment	Observations
25	5.50	-	338	-		Lower Marker
50	1.95	-	60.0	10.55		
100	2.15	-	33.0	11.61		
200	2.22	-	17.1	12.01		
300	2.27	-	11.7	12.30		
400	2.31	-	8.90	12.52		
500	2.52	-	7.77	13.66		
700	2.32	-	5.10	12.55		
1000	2.73	-	4.21	14.79		
1500	6.50	6.50	6.67	-		Upper Marker

© Agilent Technologies, Inc. 2017

B1: F1 M

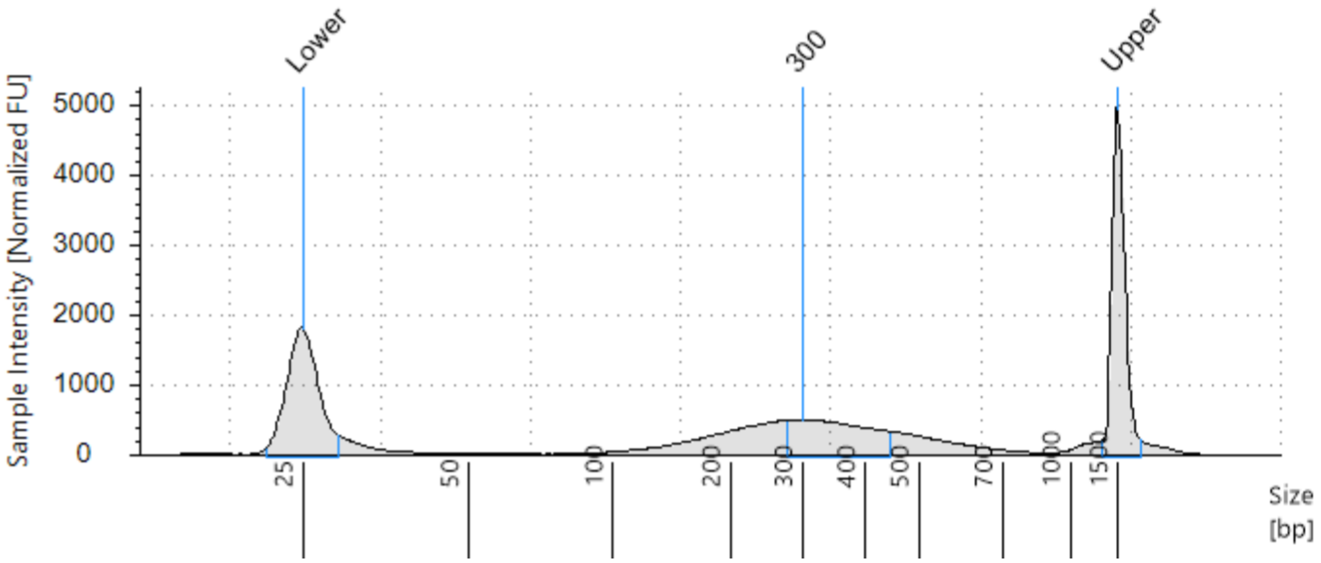

Sample Table

Well	Conc. [ng/µl]	Sample Description	Alert	Observations
B1	3.81	F1 M		

Peak Table

Size [bp]	Calibrated Conc. [ng/µl]	Assigned Conc. [ng/µl]	Peak Molarity [nmol/l]	% Integrated Area	Peak Comment	Observations
25	5.73	-	353	-		Lower Marker
300	3.81	-	19.6	100.00		
1500	6.50	6.50	6.67	-		Upper Marker

© Agilent Technologies, Inc. 2017

C1: G3 M

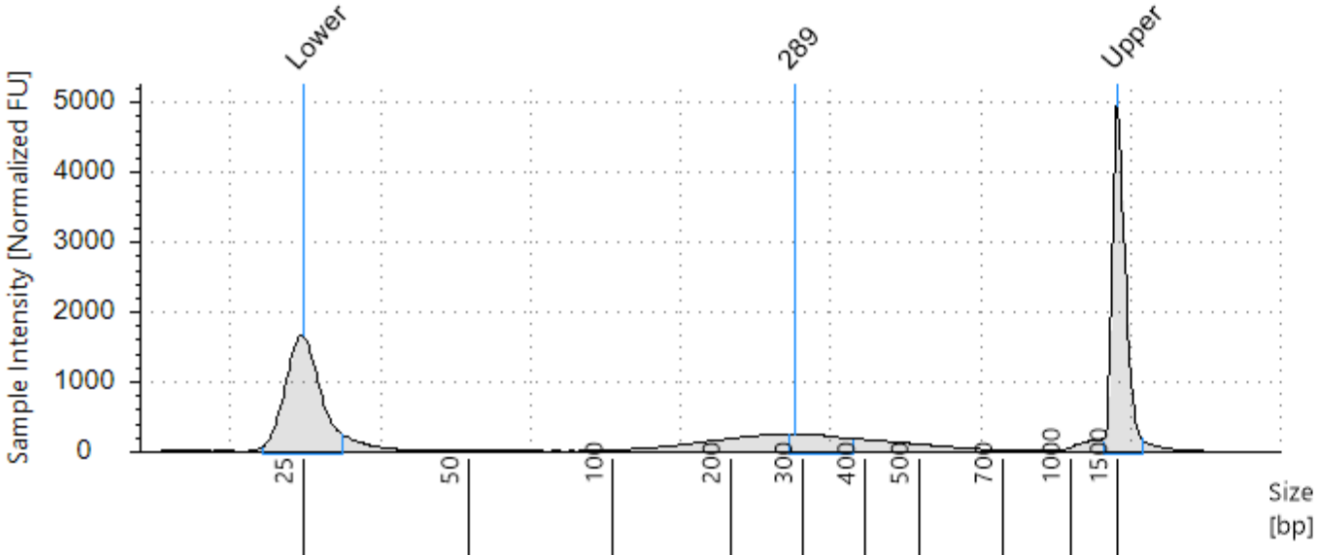

Sample Table

Well	Conc. [ng/µl]	Sample Description	Alert	Observations
C1	1.22	G3 M		

Peak Table

Size [bp]	Calibrated Conc. [ng/µl]	Assigned Conc. [ng/µl]	Peak Molarity [nmol/l]	% Integrated Area	Peak Comment	Observations
25	5.71	-	351	-		Lower Marker
289	1.22	-	6.51	100.00		
1500	6.50	6.50	6.67	-		Upper Marker

© Agilent Technologies, Inc. 2017

D1: C6 M

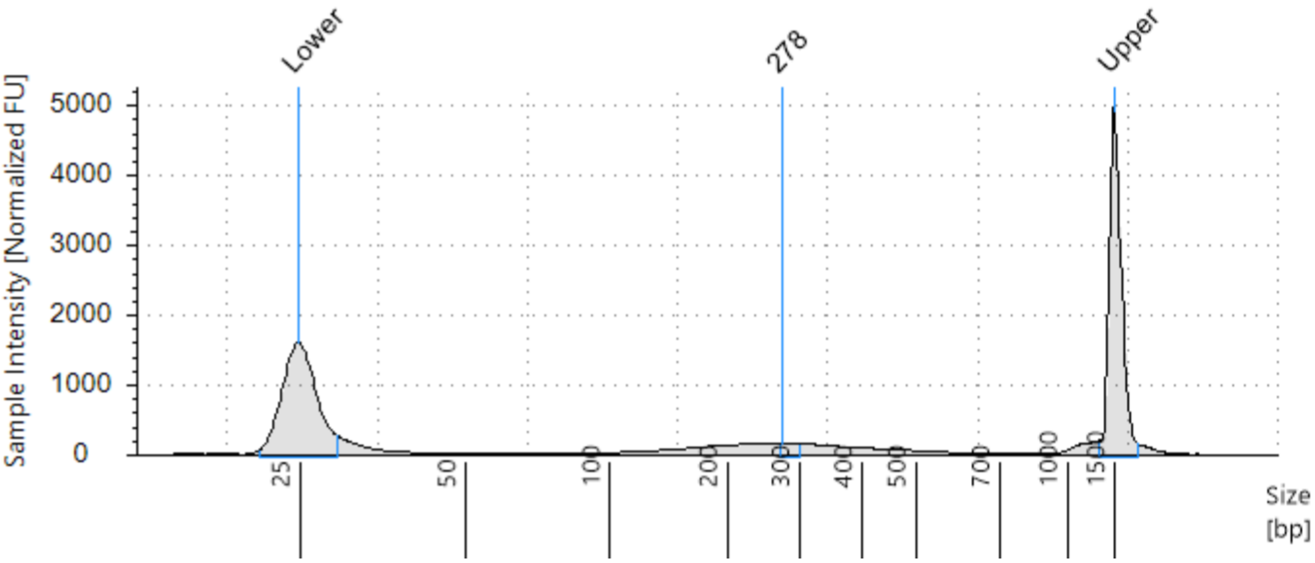

Sample Table

Well	Conc. [ng/µl]	Sample Description	Alert	Observations
D1	0.286	C6 M		

Peak Table

Size [bp]	Calibrated Conc. [ng/µl]	Assigned Conc. [ng/µl]	Peak Molarity [nmol/l]	% Integrated Area	Peak Comment	Observations
25	5.88	-	362	-		Lower Marker
278	0.286	-	1.58	100.00		
1500	6.50	6.50	6.67	-		Upper Marker

© Agilent Technologies, Inc. 2017

E1: A10 M

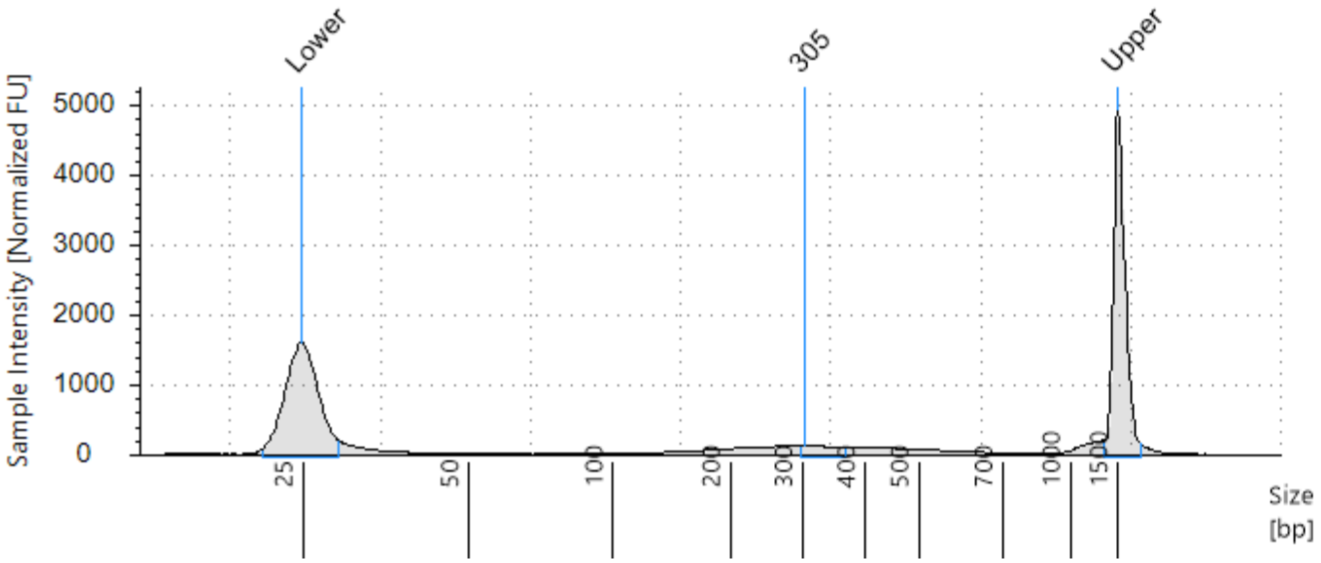

Sample Table

Well	Conc. [ng/µl]	Sample Description	Alert	Observations
E1	0.492	A10 M		

Peak Table

Size [bp]	Calibrated Conc. [ng/µl]	Assigned Conc. [ng/µl]	Peak Molarity [nmol/l]	% Integrated Area	Peak Comment	Observations
25	5.98	-	368	-		Lower Marker
305	0.492	-	2.48	100.00		
1500	6.50	6.50	6.67	-		Upper Marker

© Agilent Technologies, Inc. 2017

F1: C6 p

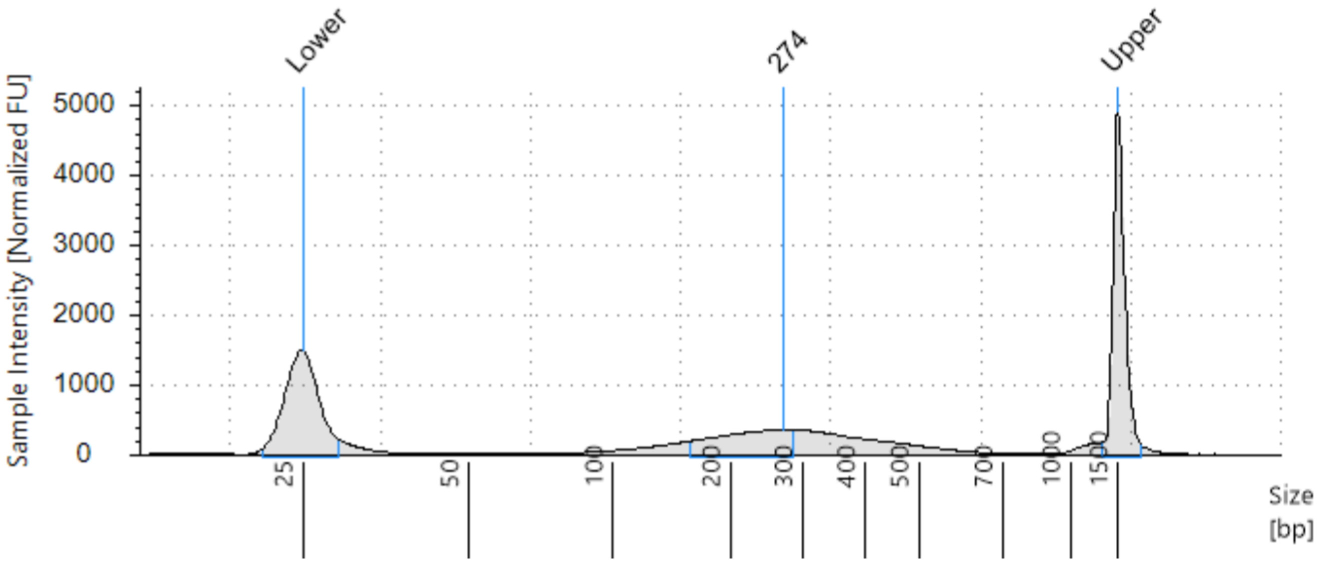

Sample Table

Well	Conc. [ng/µl]	Sample Description	Alert	Observations
F1	2.83	C6 p		

Peak Table

Size [bp]	Calibrated Conc. [ng/µl]	Assigned Conc. [ng/µl]	Peak Molarity [nmol/l]	% Integrated Area	Peak Comment	Observations
25	5.66	-	348	-		Lower Marker
274	2.83	-	15.9	100.00		
1500	6.50	6.50	6.67	-		Upper Marker

 © Agilent Technologies, Inc. 2017

Gl: A8 P

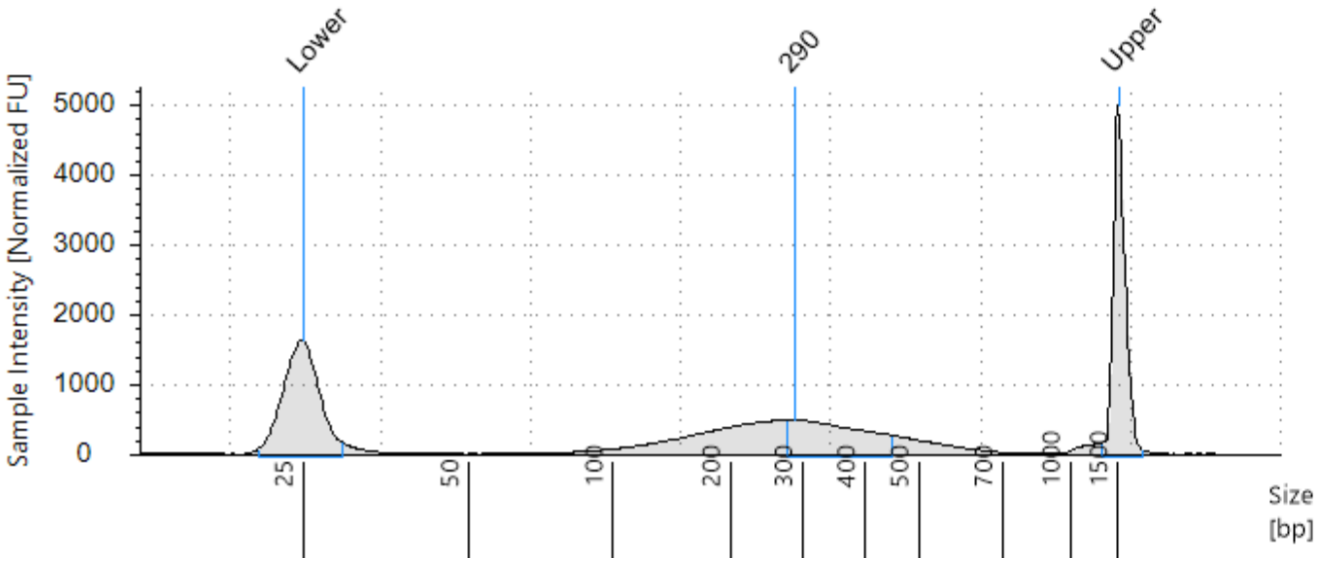

Sample Table

Well	Conc. [ng/µl]	Sample Description	Alert	Observations
Gl	3.86	A8 P		

Peak Table

Size [bp]	Calibrated Conc. [ng/µl]	Assigned Conc. [ng/µl]	Peak Molarity [nmol/l]	% Integrated Area	Peak Comment	Observations
25	6.39	-	393	-		Lower Marker
290	3.86	-	20.5	100.00		
1500	6.50	6.50	6.67	-		Upper Marker

© Agilent Technologies, Inc. 2017

H1: H9 P

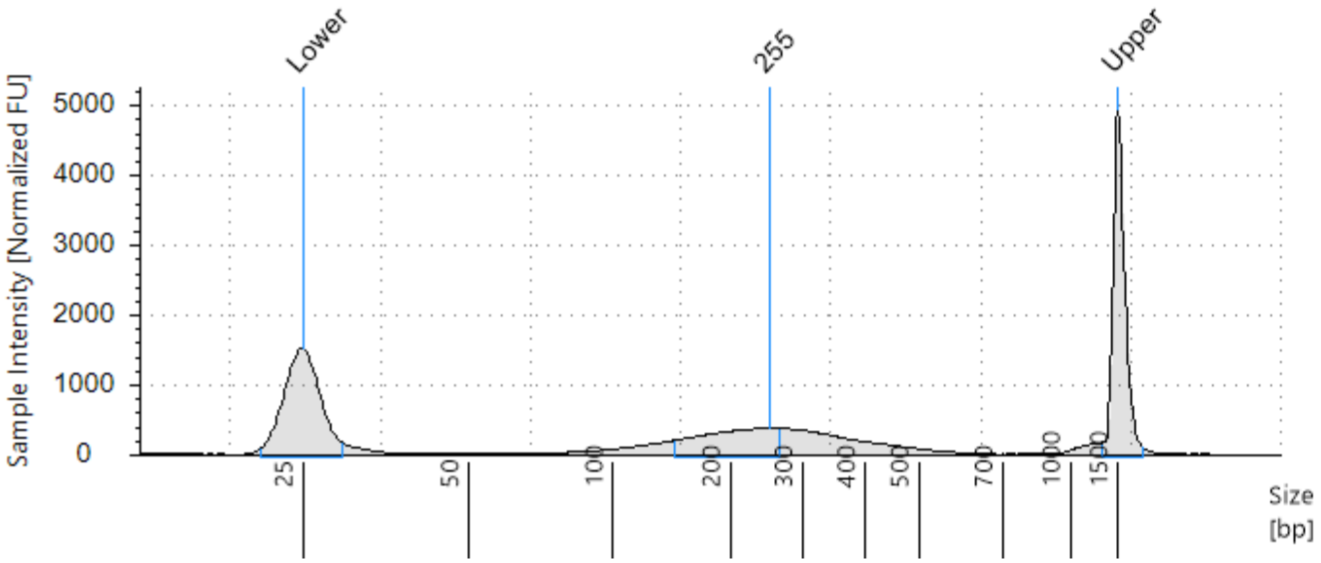

Sample Table

Well	Conc. [ng/µl]	Sample Description	Alert	Observations
H1	3.08	H9 P		

Peak Table

Size [bp]	Calibrated Conc. [ng/µl]	Assigned Conc. [ng/µl]	Peak Molarity [nmol/l]	% Integrated Area	Peak Comment	Observations
25	6.15	-	378	-		Lower Marker
255	3.08	-	18.6	100.00		
1500	6.50	6.50	6.67	-		Upper Marker

 © Agilent Technologies, Inc. 2017

A2: A11 P

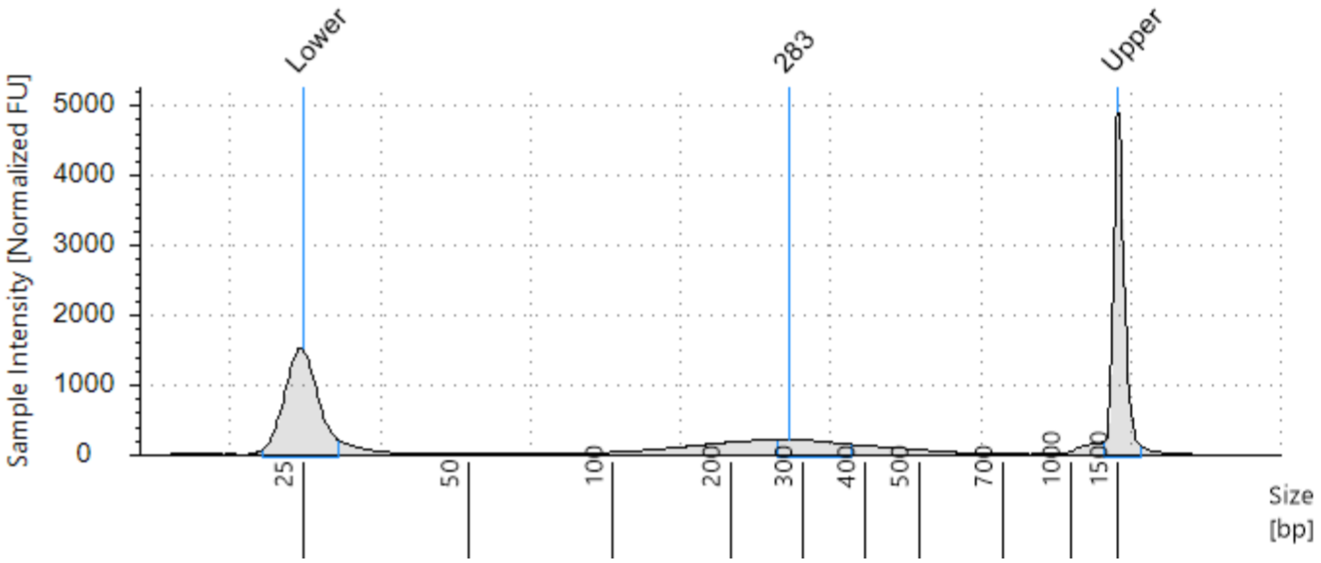

Sample Table

Well	Conc. [ng/µl]	Sample Description	Alert	Observations
A2	1.41	A11 P		

Peak Table

Size [bp]	Calibrated Conc. [ng/µl]	Assigned Conc. [ng/µl]	Peak Molarity [nmol/l]	% Integrated Area	Peak Comment	Observations
25	6.00	-	369	-		Lower Marker
283	1.41	-	7.67	100.00		
1500	6.50	6.50	6.67	-		Upper Marker

© Agilent Technologies, Inc. 2017

B2: B11 P

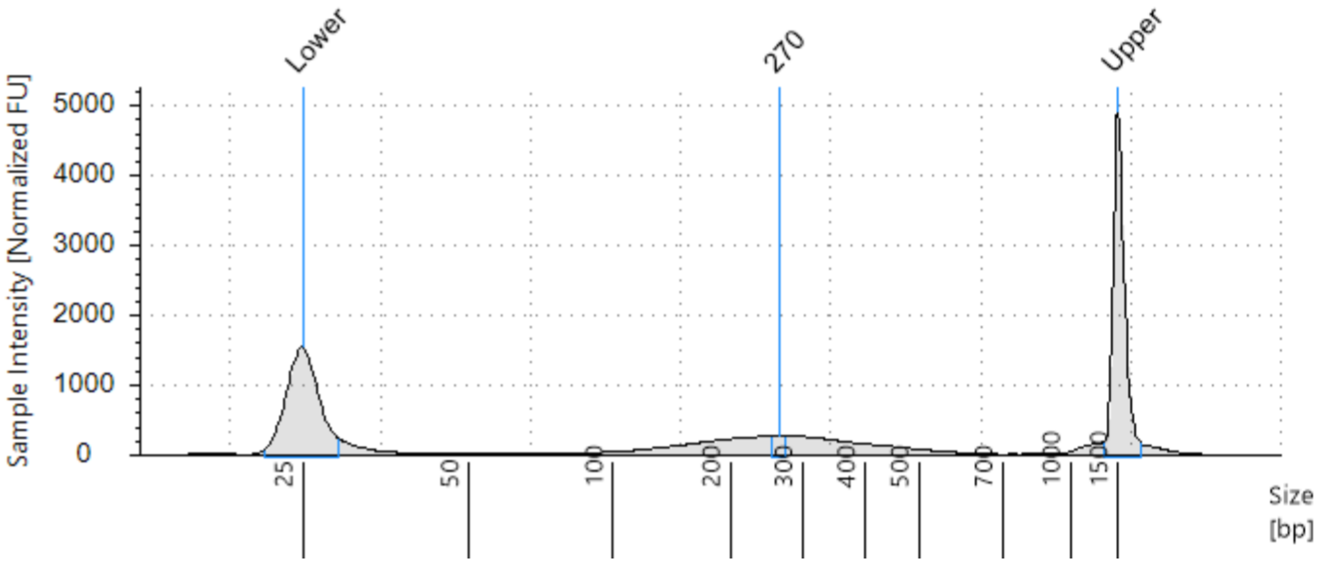

Sample Table

Well	Conc. [ng/µl]	Sample Description	Alert	Observations
B2	0.411	B11 P		

Peak Table

Size [bp]	Calibrated Conc. [ng/µl]	Assigned Conc. [ng/µl]	Peak Molarity [nmol/l]	% Integrated Area	Peak Comment	Observations
25	5.74	-	353	-		Lower Marker
270	0.411	-	2.35	100.00		
1500	6.50	6.50	6.67	-		Upper Marker

© Agilent Technologies, Inc. 2017

C2: C11 P

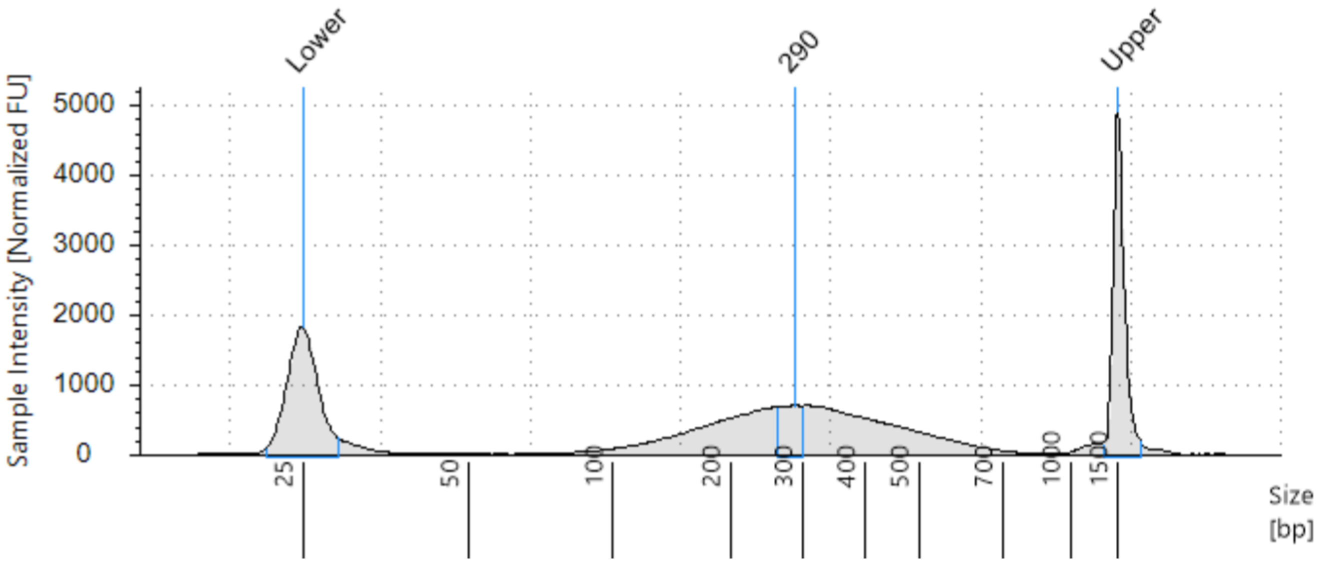

Sample Table

Well	Conc. [ng/µl]	Sample Description	Alert	Observations
C2	1.81	C11 P		

Peak Table

Size [bp]	Calibrated Conc. [ng/µl]	Assigned Conc. [ng/µl]	Peak Molarity [nmol/l]	% Integrated Area	Peak Comment	Observations
25	6.56	-	404	-		Lower Marker
290	1.81	-	9.60	100.00		
1500	6.50	6.50	6.67	-		Upper Marker

© Agilent Technologies, Inc. 2017

D2: D11 P

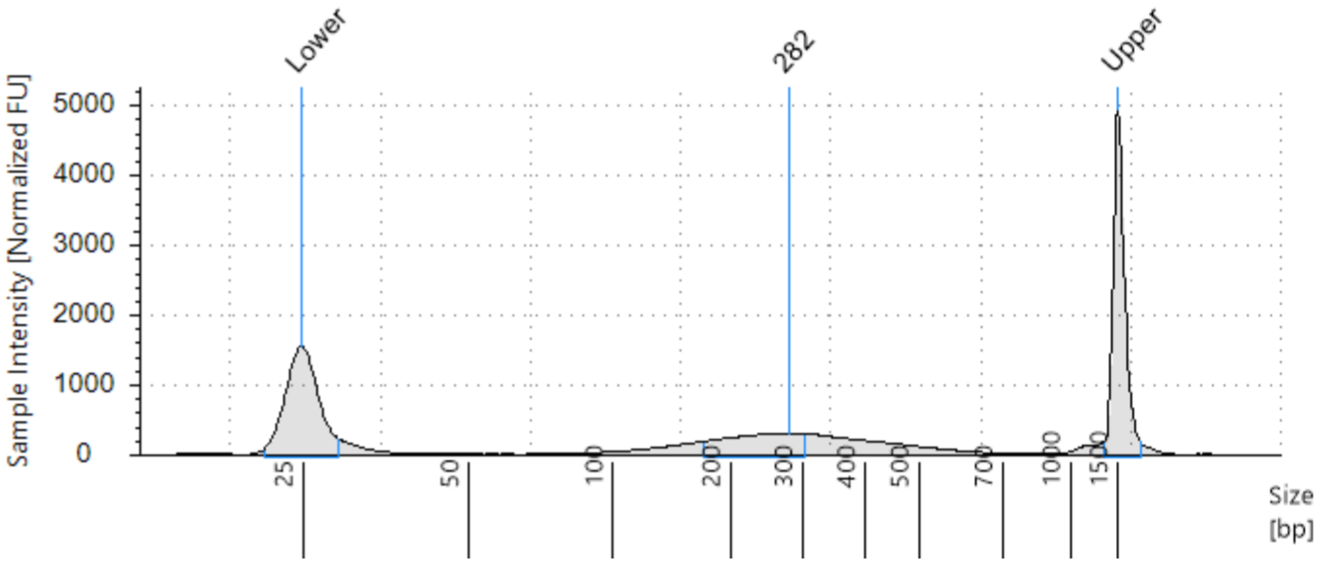

Sample Table

Well	Conc. [ng/µl]	Sample Description	Alert	Observations
D2	2.53	D11 P		

Peak Table

Size [bp]	Calibrated Conc. [ng/µl]	Assigned Conc. [ng/µl]	Peak Molarity [nmol/l]	% Integrated Area	Peak Comment	Observations
25	5.68	-	350	-		Lower Marker
282	2.53	-	13.8	100.00		
1500	6.50	6.50	6.67	-		Upper Marker

© Agilent Technologies, Inc. 2017

E2: E11 P

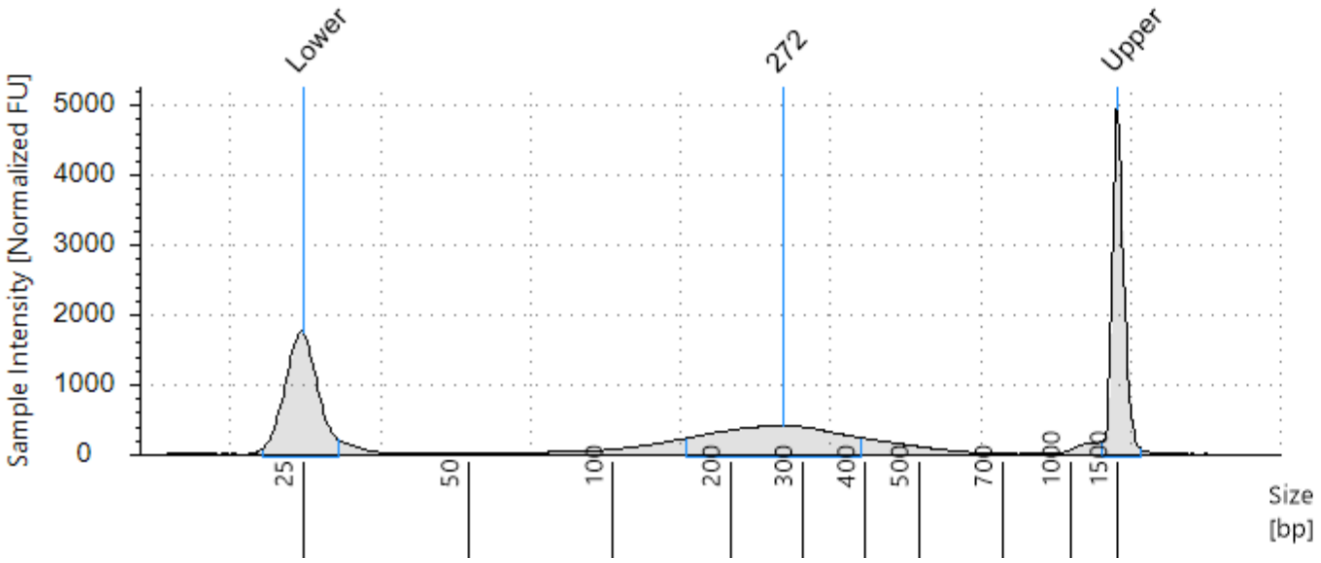

Sample Table

Well	Conc. [ng/µl]	Sample Description	Alert	Observations
E2	5.61	E11 P		

Peak Table

Size [bp]	Calibrated Conc. [ng/µl]	Assigned Conc. [ng/µl]	Peak Molarity [nmol/l]	% Integrated Area	Peak Comment	Observations
25	6.36	-	392	-		Lower Marker
272	5.61	-	31.7	100.00		
1500	6.50	6.50	6.67	-		Upper Marker

 © Agilent Technologies, Inc. 2017

F2: F11 P

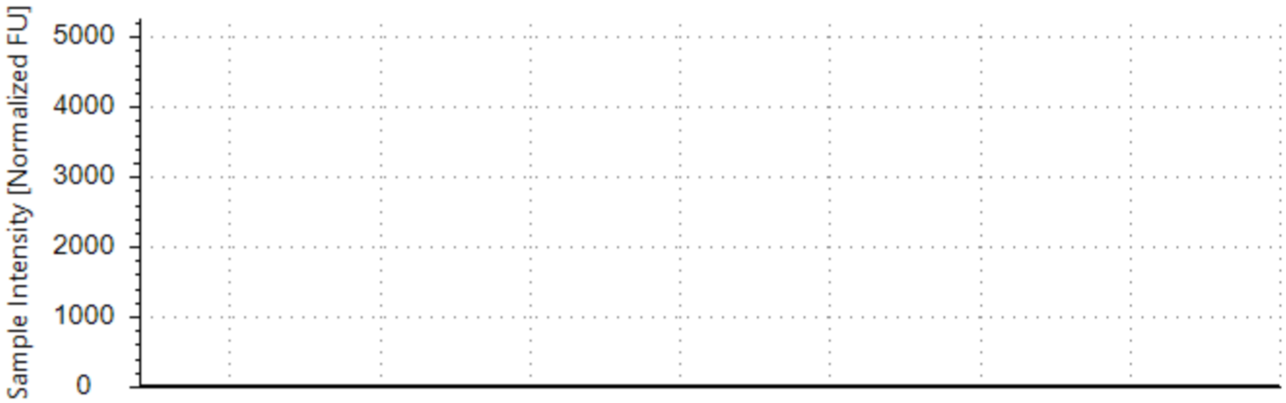

Sample Table

Well	Conc. [ng/µl]	Sample Description	Alert	Observations
F2		F11 P	▲	Marker(s) not detected

 © Agilent Technologies, Inc. 2017

G2: G11 P

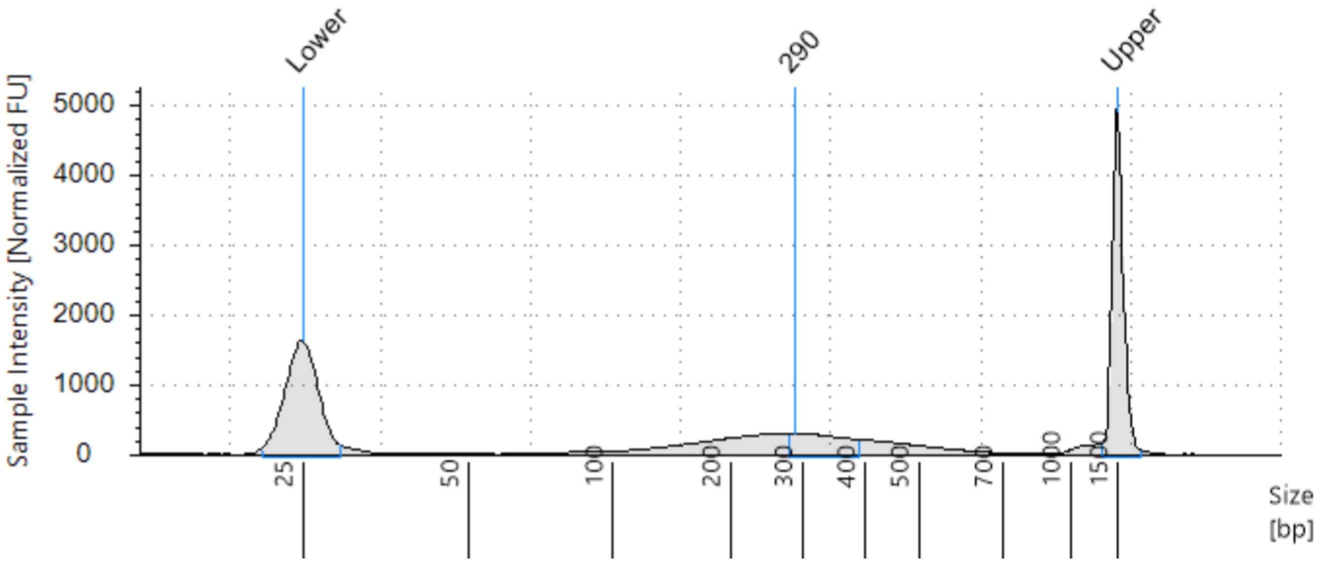

Sample Table

Well	Conc. [ng/µl]	Sample Description	Alert	Observations
G2	1.85	G11 P		

Peak Table

Size [bp]	Calibrated Conc. [ng/µl]	Assigned Conc. [ng/µl]	Peak Molarity [nmol/l]	% Integrated Area	Peak Comment	Observations
25	6.37	-	392	-		Lower Marker
290	1.85	-	9.79	100.00		
1500	6.50	6.50	6.67	-		Upper Marker

 © Agilent Technologies, Inc. 2017

H2: H11 P

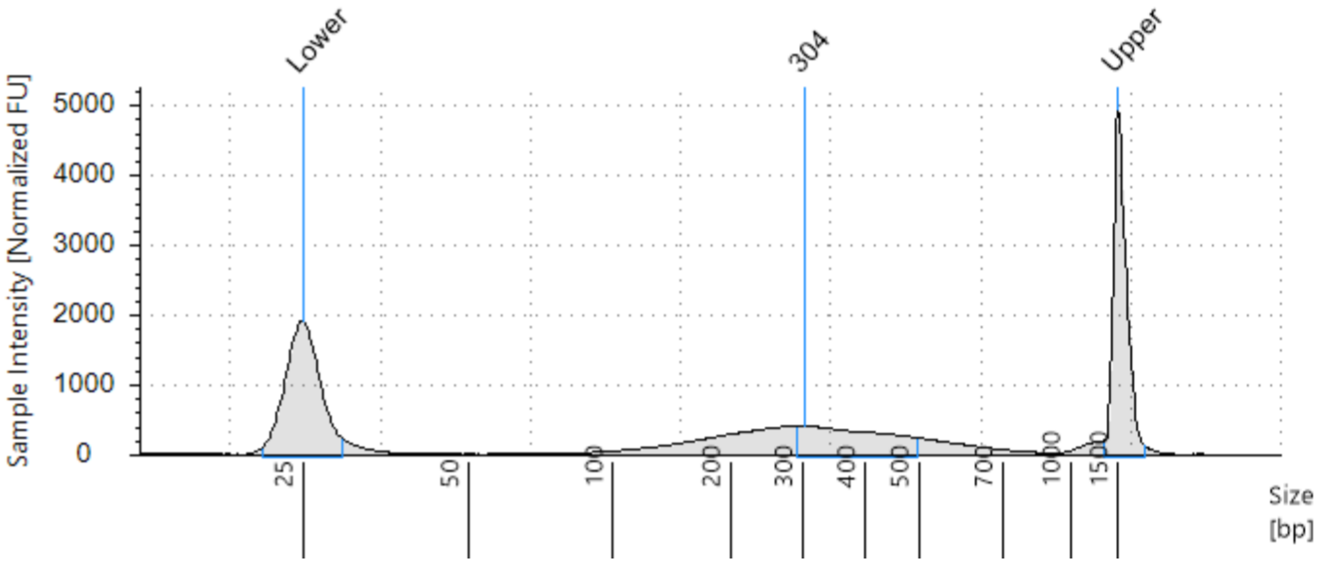

Sample Table

Well	Conc. [ng/µl]	Sample Description	Alert	Observations
H2	3.40	H11 P		

Peak Table

Size [bp]	Calibrated Conc. [ng/µl]	Assigned Conc. [ng/µl]	Peak Molarity [nmol/l]	% Integrated Area	Peak Comment	Observations
25	6.67	-	410	-		Lower Marker
304	3.40	-	17.2	100.00		
1500	6.50	6.50	6.67	-		Upper Marker

© Agilent Technologies, Inc. 2017

Filename: 2020-09-15-04 Q-S MINUS G12, H12, G3,H3, D5,H5,D7, D1000 R2.D1000

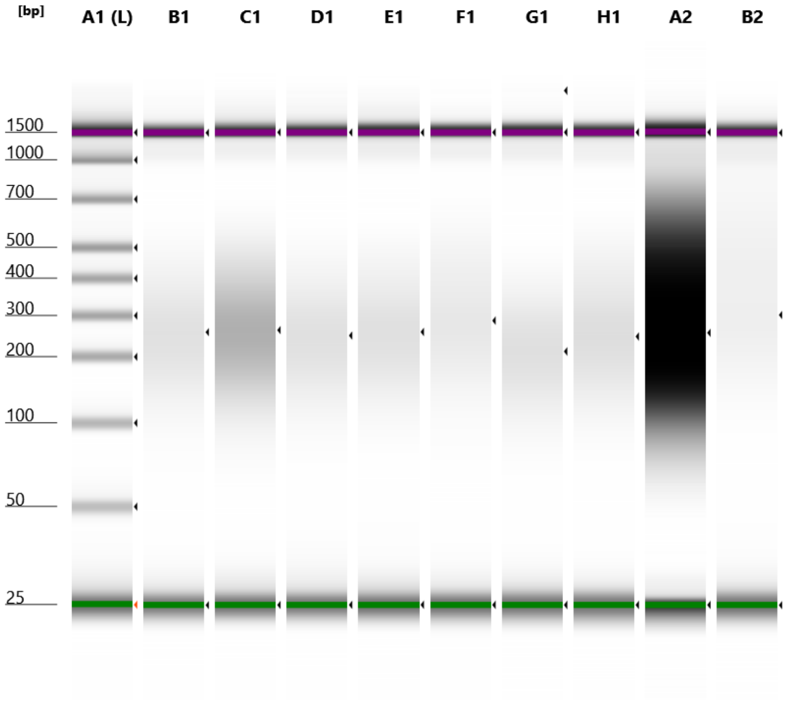

Default image (Contrast 100%)

Sample Info

Well	Conc. [ng/µl]	Sample Description	Alert	Observations
A1	11.5	Ladder		Ladder
B1	3.85	G12 P R2		
C1	10.6	H12 P R2		
D1	2.10	G3 P R2		
E1	1.82	H3 P R2		
F1	3.20	D5 P R2		
G1	3.85	H5 P R2		
H1	4.27	D7 P R2		
A2	47.6	E1 M R1		
B2	0.518	E6 M R1		

 © Agilent Technologies, Inc. 2017

A1: Ladder

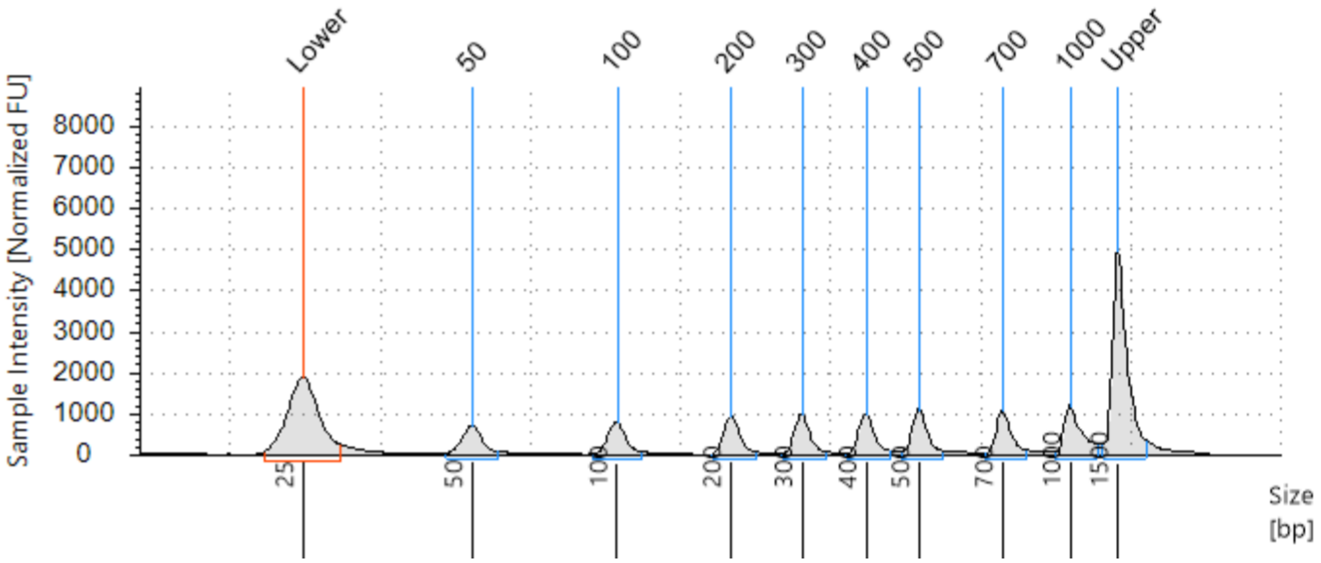

Sample Table

Well	Conc. [ng/µl]	Sample Description	Alert	Observations
A1	11.5	Ladder		Ladder

Peak Table

Size [bp]	Calibrated Conc. [ng/µl]	Assigned Conc. [ng/µl]	Peak Molarity [nmol/l]	% Integrated Area	Peak Comment	Observations
25	5.30	-	326	-		Lower Marker
50	1.27	-	39.0	11.02		
100	1.29	-	19.9	11.22		
200	1.35	-	10.4	11.77		
300	1.39	-	7.12	12.07		
400	1.41	-	5.43	12.28		
500	1.51	-	4.65	13.15		
700	1.47	-	3.23	12.77		
1000	1.81	-	2.78	15.71		
1500	6.50	6.50	6.67	-		Upper Marker

© Agilent Technologies, Inc. 2017

B1: G12 P R2

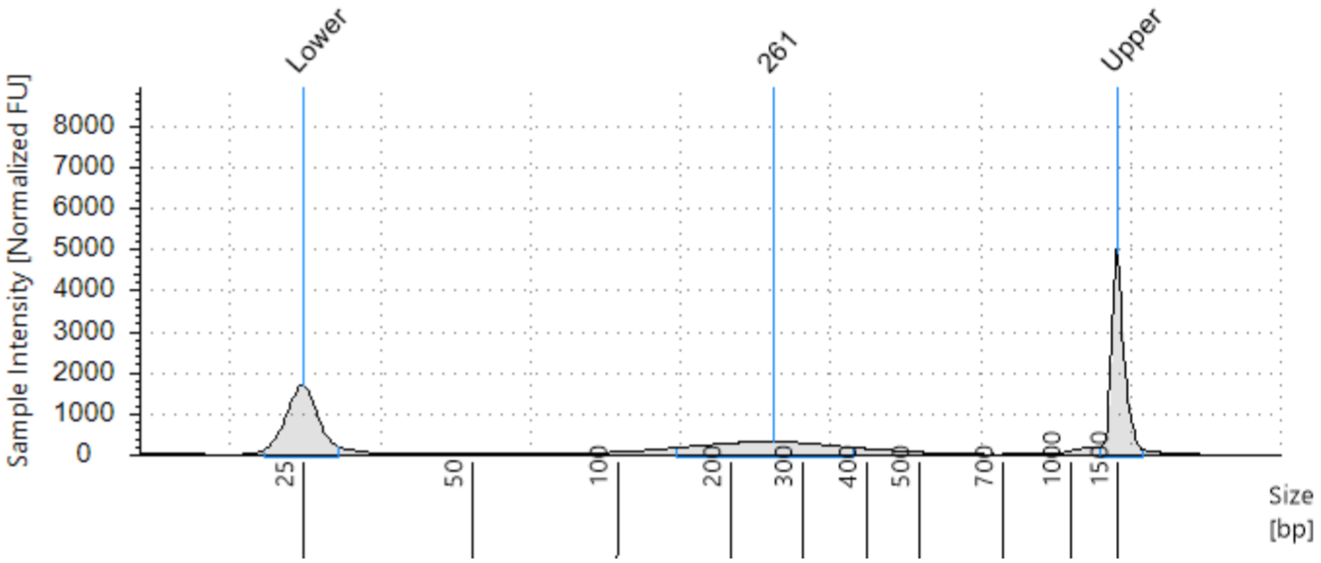

Sample Table

Well	Conc. [ng/µl]	Sample Description	Alert	Observations
B1	3.85	G12 P R2		

Peak Table

Size [bp]	Calibrated Conc. [ng/µl]	Assigned Conc. [ng/µl]	Peak Molarity [nmol/l]	% Integrated Area	Peak Comment	Observations
25	5.58	-	344	-		Lower Marker
261	3.85	-	22.7	100.00		
1500	6.50	6.50	6.67	-		Upper Marker

 © Agilent Technologies, Inc. 2017

C1: H12 P R2

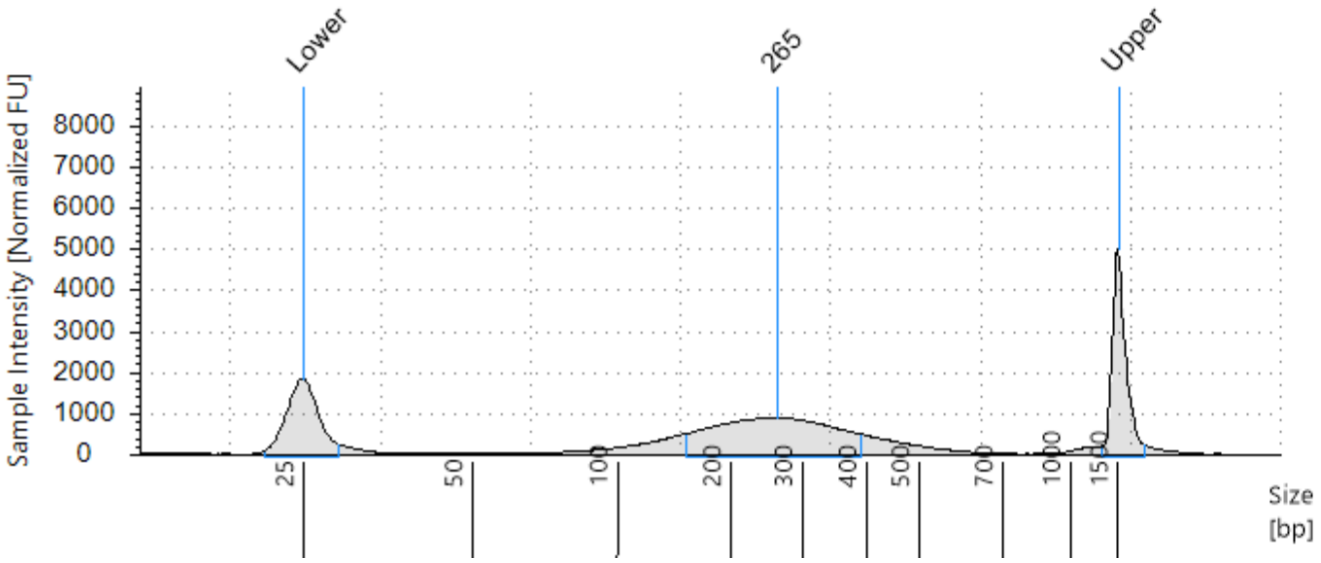

Sample Table

Well	Conc. [ng/µl]	Sample Description	Alert	Observations
C1	10.6	H12 P R2		

Peak Table

Size [bp]	Calibrated Conc. [ng/µl]	Assigned Conc. [ng/µl]	Peak Molarity [nmol/l]	% Integrated Area	Peak Comment	Observations
25	5.64	-	347	-		Lower Marker
265	10.6	-	61.4	100.00		
1500	6.50	6.50	6.67	-		Upper Marker

© Agilent Technologies, Inc. 2017

D1: G3 P R2

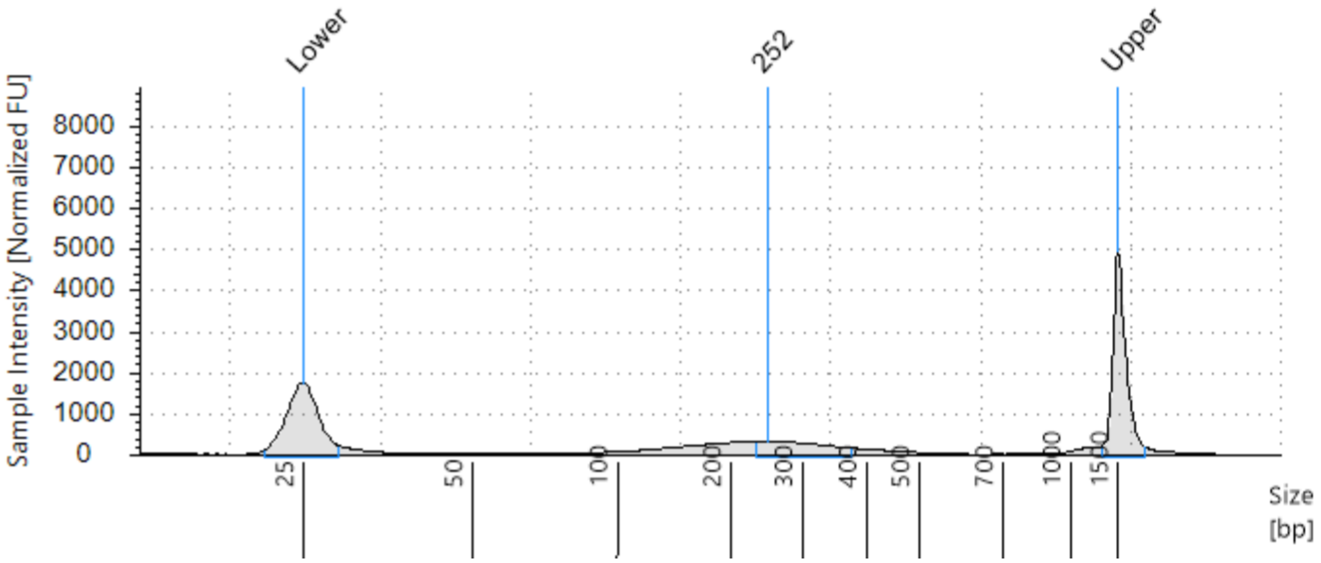

Sample Table

Well	Conc. [ng/µl]	Sample Description	Alert	Observations
D1	2.10	G3 P R2		

Peak Table

Size [bp]	Calibrated Conc. [ng/µl]	Assigned Conc. [ng/µl]	Peak Molarity [nmol/l]	% Integrated Area	Peak Comment	Observations
25	5.65	-	348	-		Lower Marker
252	2.10	-	12.8	100.00		
1500	6.50	6.50	6.67	-		Upper Marker

© Agilent Technologies, Inc. 2017

E1: H3 P R2

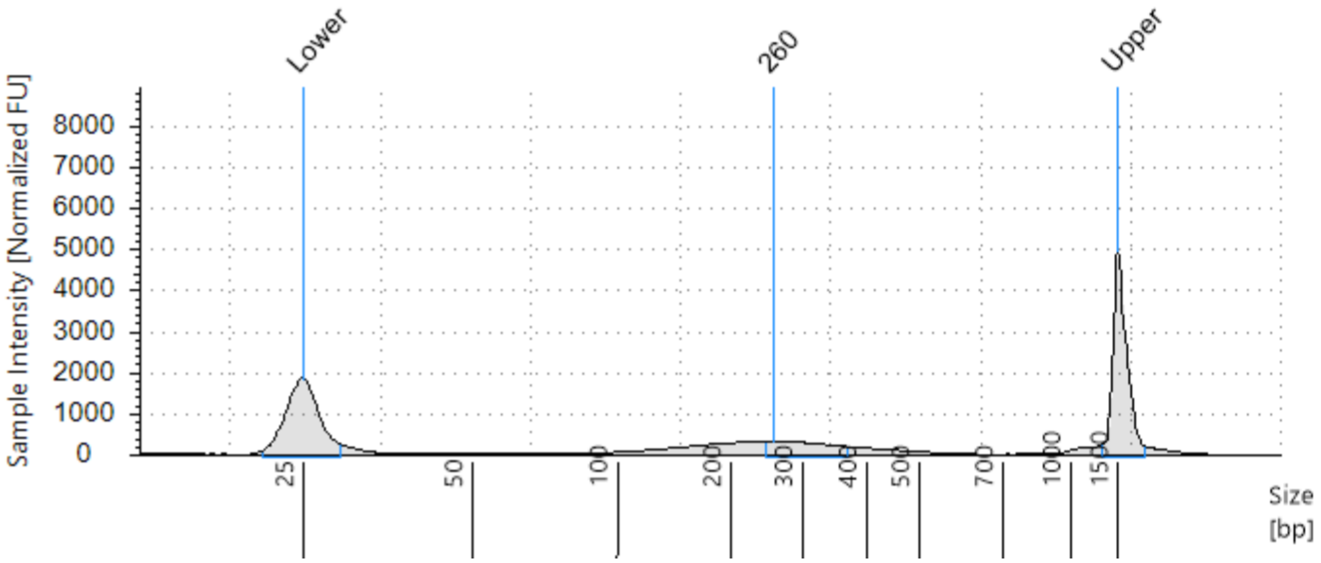

Sample Table

Well	Conc. [ng/µl]	Sample Description	Alert	Observations
E1	1.82	H3 P R2		

Peak Table

Size [bp]	Calibrated Conc. [ng/µl]	Assigned Conc. [ng/µl]	Peak Molarity [nmol/l]	% Integrated Area	Peak Comment	Observations
25	5.85	-	360	-		Lower Marker
260	1.82	-	10.8	100.00		
1500	6.50	6.50	6.67	-		Upper Marker

© Agilent Technologies, Inc. 2017

F1: D5 P R2

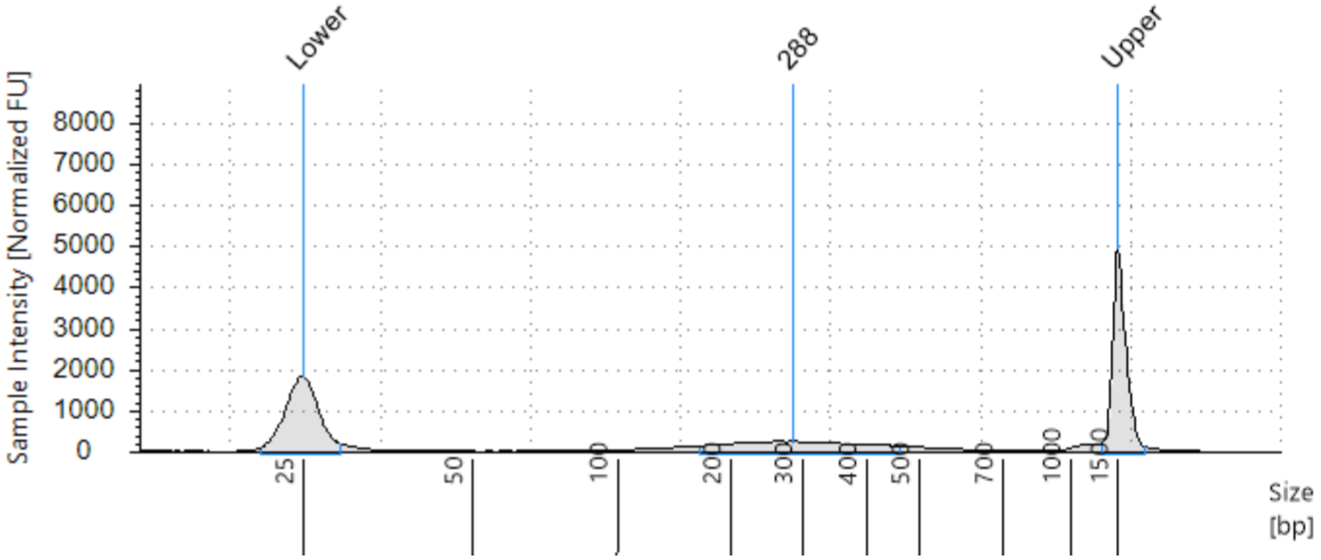

Sample Table

Well	Conc. [ng/µl]	Sample Description	Alert	Observations
F1	3.20	D5 P R2		

Peak Table

Size [bp]	Calibrated Conc. [ng/µl]	Assigned Conc. [ng/µl]	Peak Molarity [nmol/l]	% Integrated Area	Peak Comment	Observations
25	5.98	-	368	-		Lower Marker
288	3.20	-	17.1	100.00		
1500	6.50	6.50	6.67	-		Upper Marker

 © Agilent Technologies, Inc. 2017

G1: H5 P R2

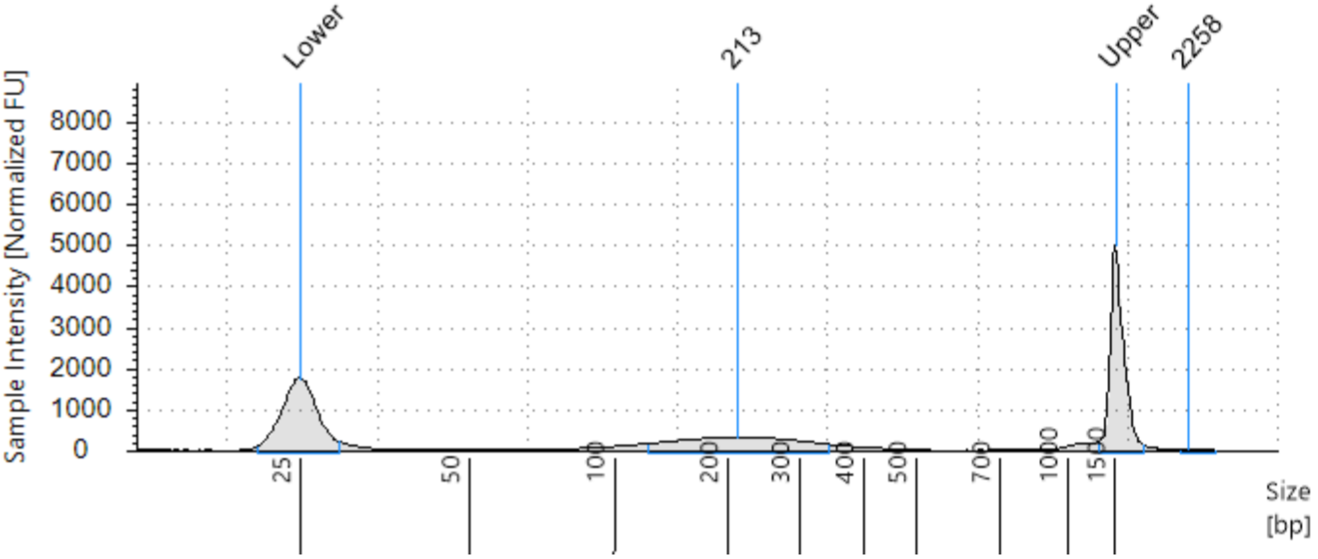

Sample Table

Well	Conc. [ng/µl]	Sample Description	Alert	Observations
G1	3.85	H5 P R2		

Peak Table

Size [bp]	Calibrated Conc. [ng/µl]	Assigned Conc. [ng/µl]	Peak Molarity [nmol/l]	% Integrated Area	Peak Comment	Observations
25	6.04	-	372	-		Lower Marker
213	3.82	-	27.6	99.14		
1500	6.50	6.50	6.67	-		Upper Marker
2258	0.0332	-	0.0226	0.86		

© Agilent Technologies, Inc. 2017

H1: D7 P R2

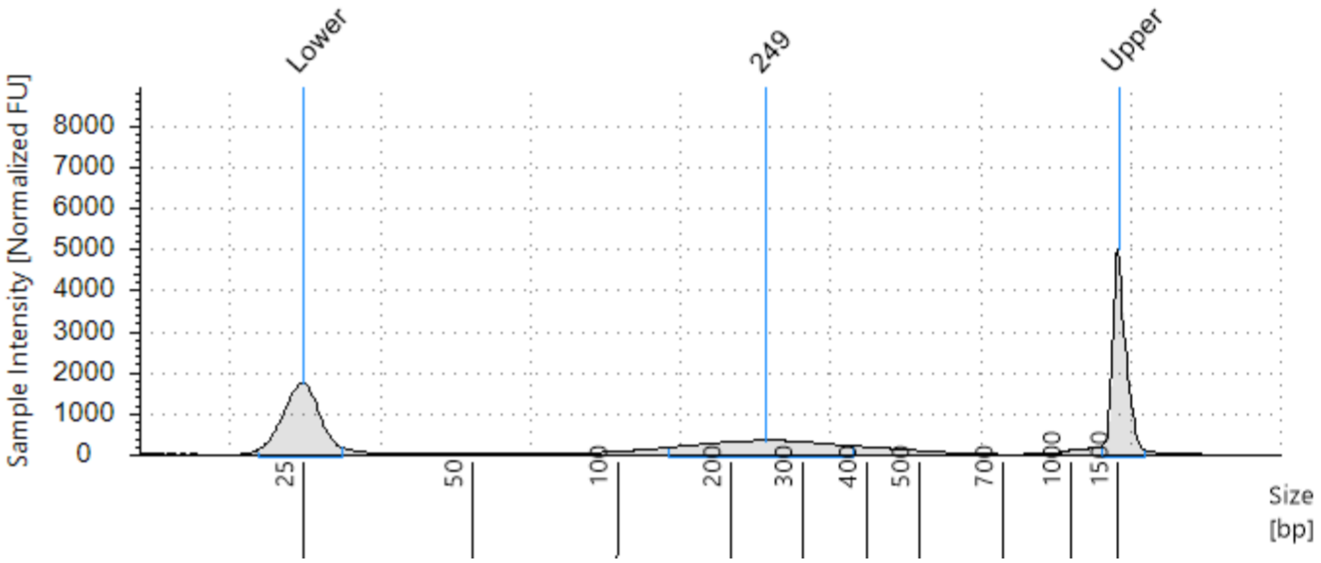

Sample Table

Well	Conc. [ng/µl]	Sample Description	Alert	Observations
H1	4.27	D7 P R2		

Peak Table

Size [bp]	Calibrated Conc. [ng/µl]	Assigned Conc. [ng/µl]	Peak Molarity [nmol/l]	% Integrated Area	Peak Comment	Observations
25	6.26	-	385	-		Lower Marker
249	4.27	-	26.3	100.00		
1500	6.50	6.50	6.67	-		Upper Marker

© Agilent Technologies, Inc. 2017

A2: E1 M R1

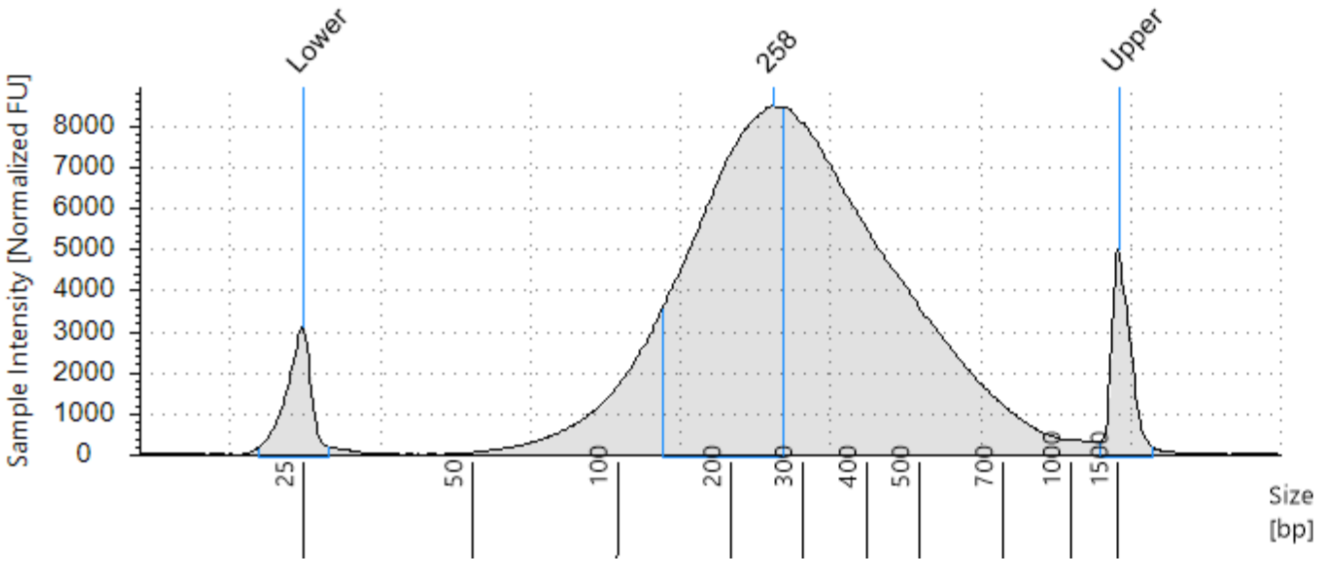

Sample Table

Well	Conc. [ng/µl]	Sample Description	Alert	Observations
A2	47.6	E1 M R1		

Peak Table

Size [bp]	Calibrated Conc. [ng/µl]	Assigned Conc. [ng/µl]	Peak Molarity [nmol/l]	% Integrated Area	Peak Comment	Observations
25	5.34	-	329	-		Lower Marker
258	47.6	-	283	100.00		
1500	6.50	6.50	6.67	-		Upper Marker

© Agilent Technologies, Inc. 2017

B2: E6 M R1

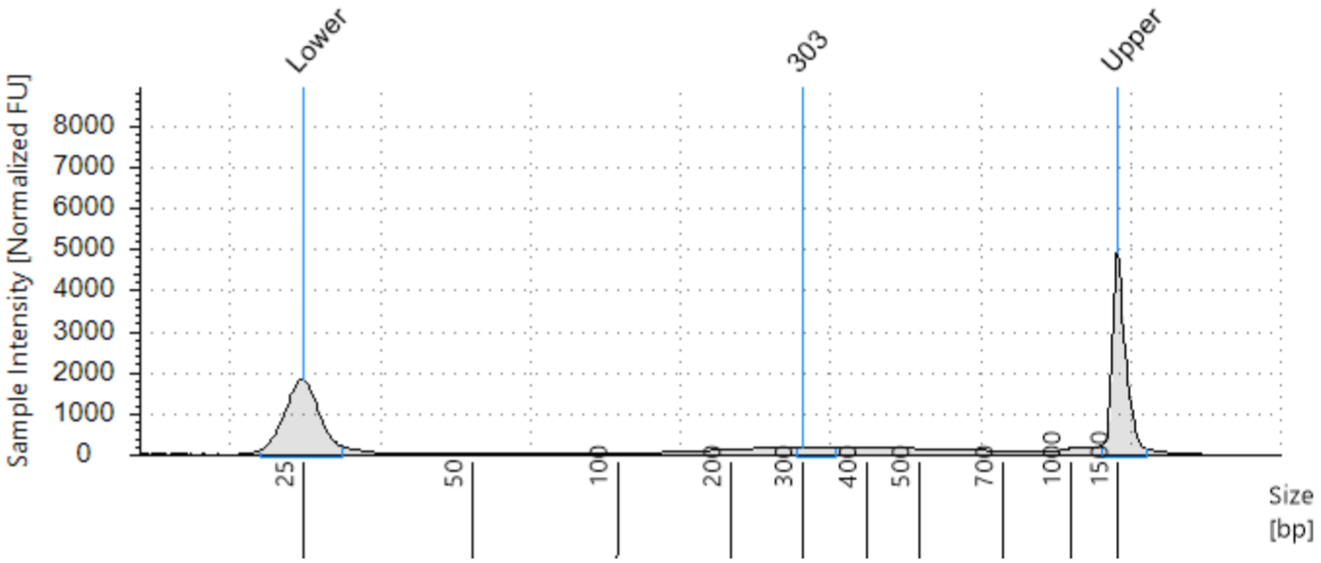

Sample Table

Well	Conc. [ng/µl]	Sample Description	Alert	Observations
B2	0.518	E6 M R1		

Peak Table

Size [bp]	Calibrated Conc. [ng/µl]	Assigned Conc. [ng/µl]	Peak Molarity [nmol/l]	% Integrated Area	Peak Comment	Observations
25	6.29	-	387	-		Lower Marker
303	0.518	-	2.63	100.00		
1500	6.50	6.50	6.67	-		Upper Marker

© Agilent Technologies, Inc. 2017

Filename: 2020-09-30-01Q-S MINUS, B3, G5,H5,A6,B6,B7,D7,E7,A8,B9,E2,C8,C10,E10,F10, R2 , used.D1000

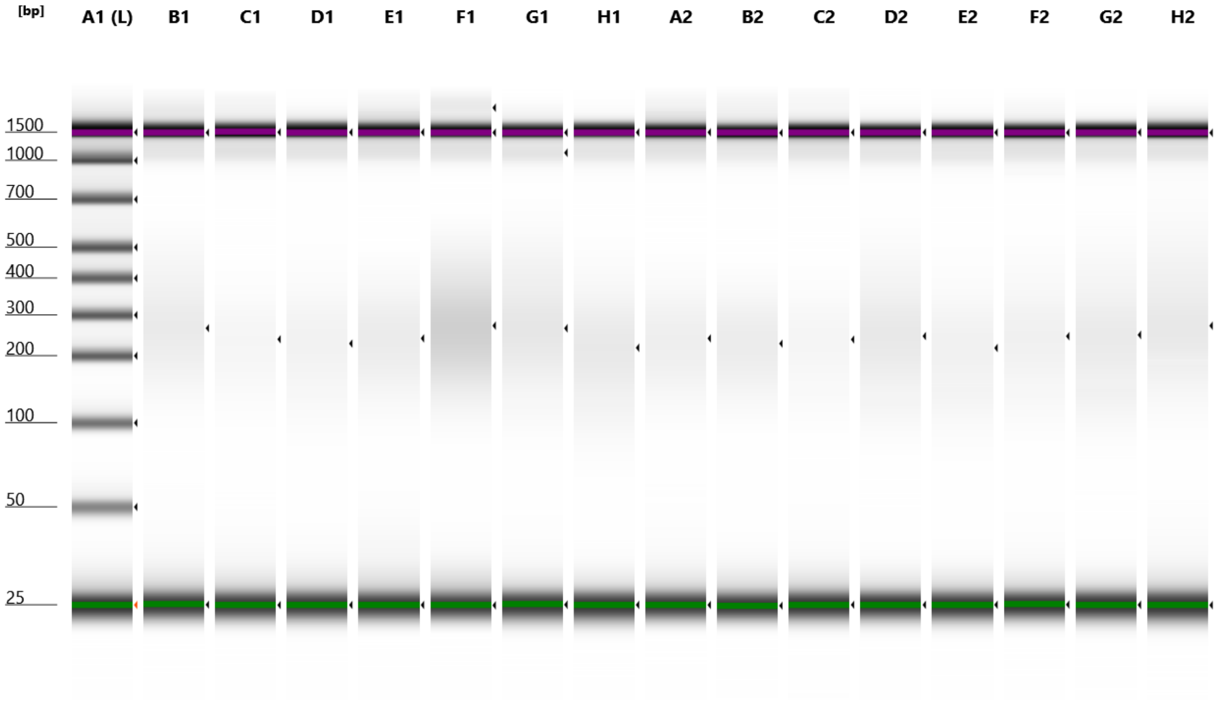

Default image (Contrast 100%)

Sample Info

Well	Conc. [ng/µl]	Sample Description	Alert	Observations
A1	14.4	Ladder		Ladder
B1	0.118	B3 M R2		
C1	0.0523	G5 M R2		
D1	0.0589	H5 M R2		
E1	0.161	A6 M R2		
F1	1.91	B6 M R2		
G1	1.26	B7 M R2		
H1	0.817	D7 M R2		
A2	0.496	E7 M R2		
B2	0.122	A8 M R2		
C2	0.0529	B9 M R2		
D2	0.195	E2 M R2		
E2	0.466	C8 M R2		
F2	0.112	C10 M R2		
G2	0.123	E10 M R2		
H2	1.08	F10 M R2		

© Agilent Technologies, Inc. 2017

A1: Ladder

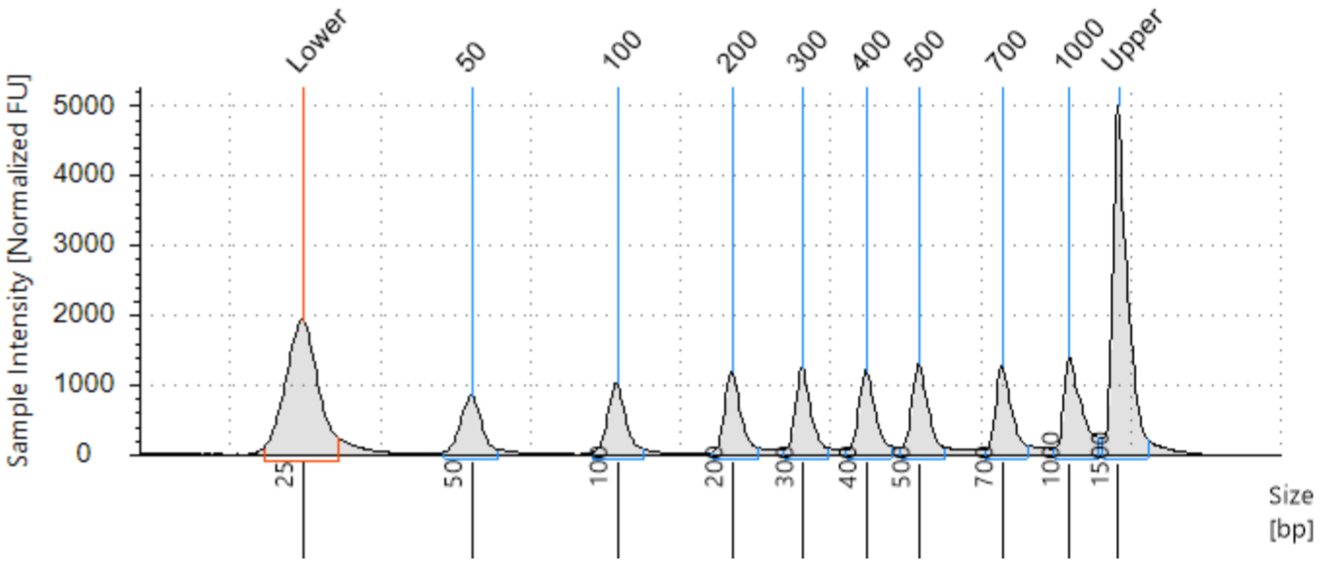

Sample Table

Well	Conc. [ng/µl]	Sample Description	Alert	Observations
A1	14.4	Ladder		Ladder

Peak Table

Size [bp]	Calibrated Conc. [ng/µl]	Assigned Conc. [ng/µl]	Peak Molarity [nmol/l]	% Integrated Area	Peak Comment	Observations
25	5.51	-	339	-		Lower Marker
50	1.58	-	48.6	10.95		
100	1.64	-	25.3	11.37		
200	1.74	-	13.4	12.03		
300	1.76	-	9.02	12.18		
400	1.79	-	6.90	12.42		
500	1.90	-	5.86	13.19		
700	1.85	-	4.06	12.80		
1000	2.17	-	3.34	15.05		
1500	6.50	6.50	6.67	-		Upper Marker

© Agilent Technologies, Inc. 2017

B1: B3 M R2

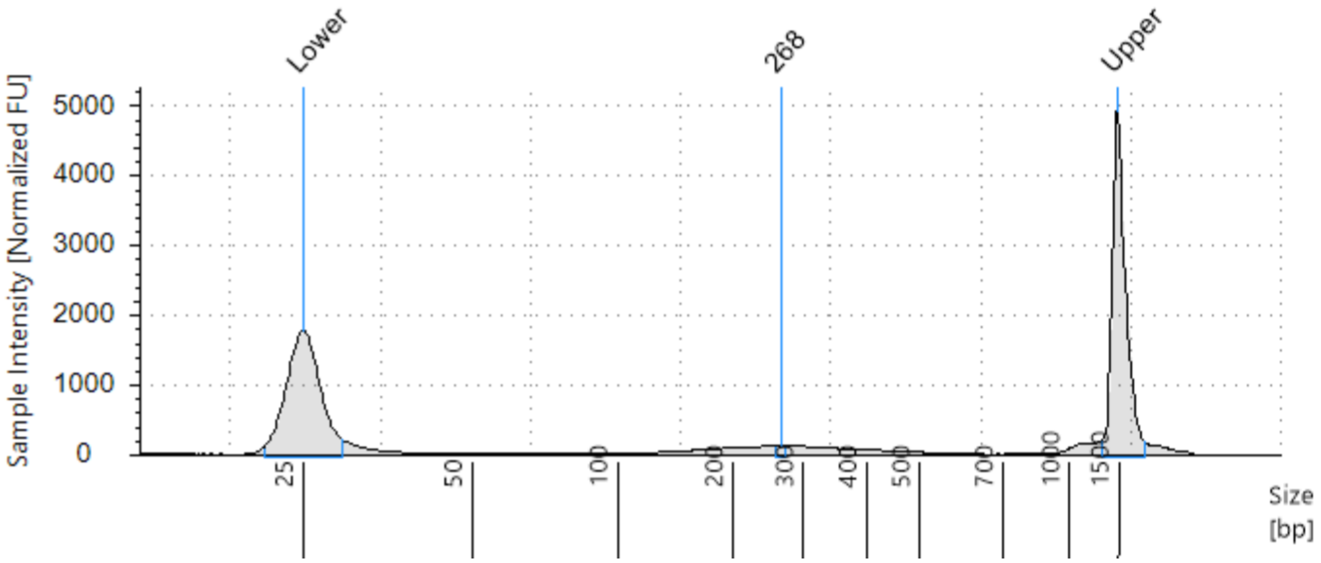

Sample Table

Well	Conc. [ng/µl]	Sample Description	Alert	Observations
B1	0.118	B3 M R2		

Peak Table

Size [bp]	Calibrated Conc. [ng/µl]	Assigned Conc. [ng/µl]	Peak Molarity [nmol/l]	% Integrated Area	Peak Comment	Observations
25	5.91	-	364	-		Lower Marker
268	0.118	-	0.677	100.00		
1500	6.50	6.50	6.67	-		Upper Marker

 © Agilent Technologies, Inc. 2017

C1: G5 M R2

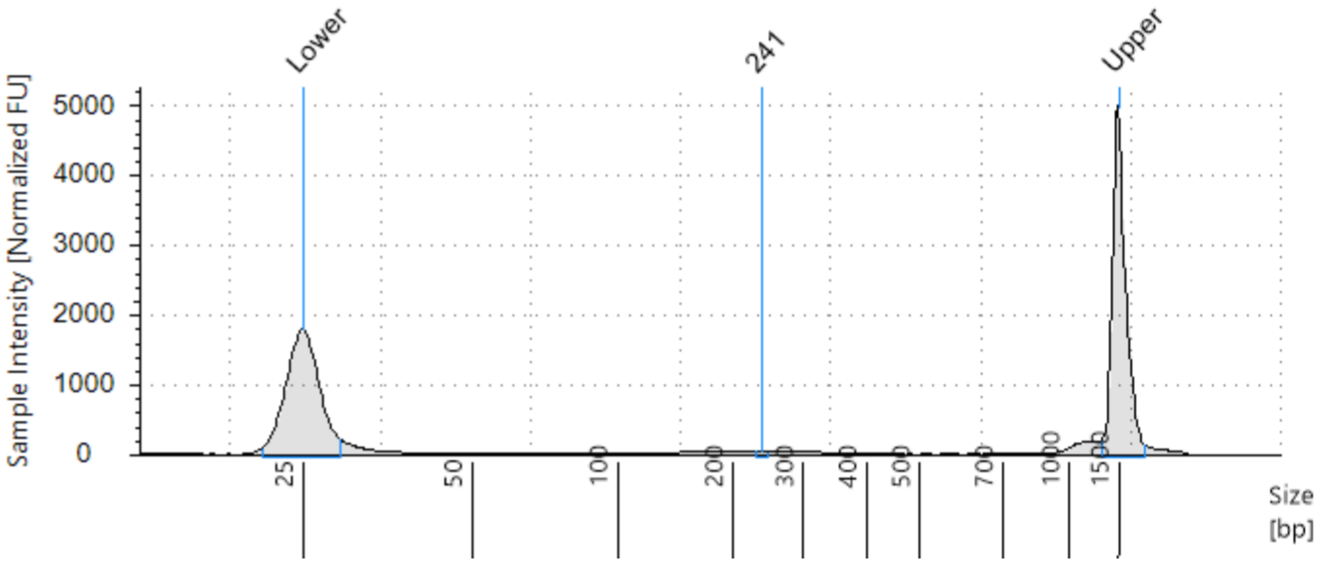

Sample Table

Well	Conc. [ng/µl]	Sample Description	Alert	Observations
C1	0.0523	G5 M R2		

Peak Table

Size [bp]	Calibrated Conc. [ng/µl]	Assigned Conc. [ng/µl]	Peak Molarity [nmol/l]	% Integrated Area	Peak Comment	Observations
25	5.88	-	362	-		Lower Marker
241	0.0523	-	0.335	100.00		
1500	6.50	6.50	6.67	-		Upper Marker

 © Agilent Technologies, Inc. 2017

D1: H5 M R2

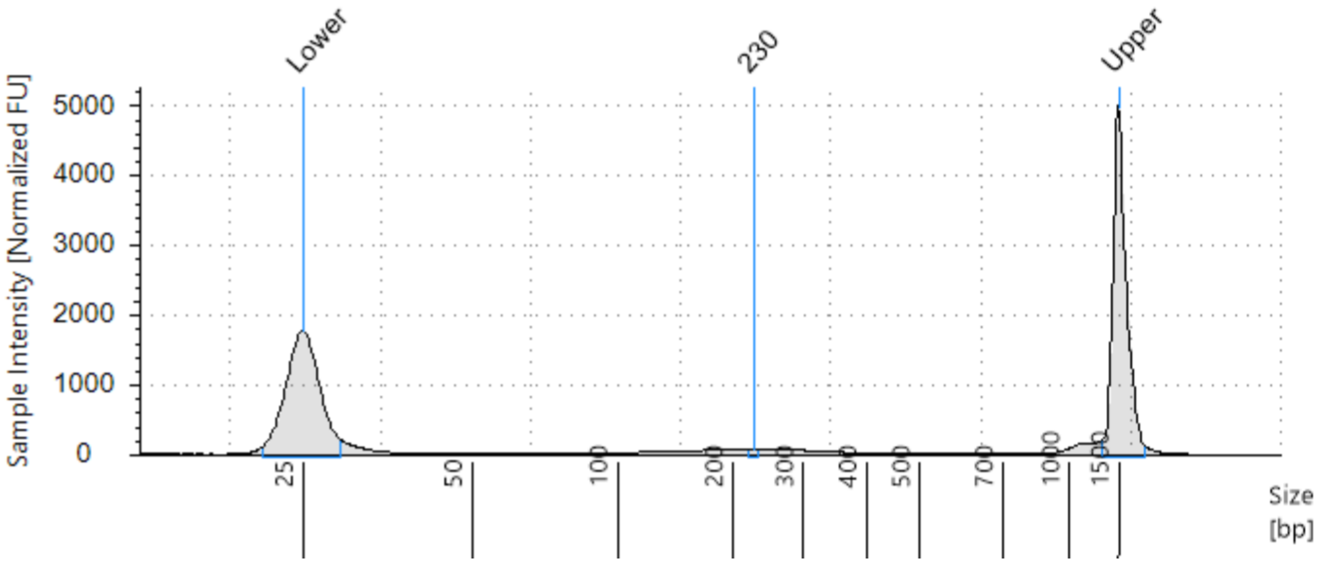

Sample Table

Well	Conc. [ng/µl]	Sample Description	Alert	Observations
D1	0.0589	H5 M R2		

Peak Table

Size [bp]	Calibrated Conc. [ng/µl]	Assigned Conc. [ng/µl]	Peak Molarity [nmol/l]	% Integrated Area	Peak Comment	Observations
25	5.87	-	361	-		Lower Marker
230	0.0589	-	0.394	100.00		
1500	6.50	6.50	6.67	-		Upper Marker

© Agilent Technologies, Inc. 2017

E1: A6 M R2

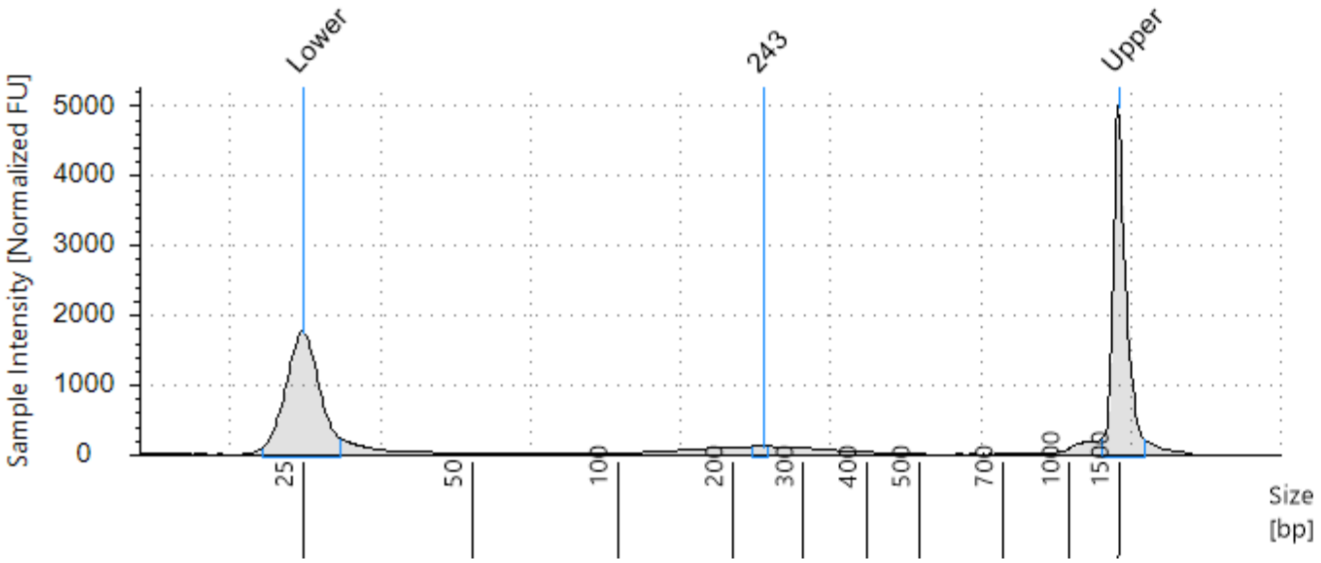

Sample Table

Well	Conc. [ng/µl]	Sample Description	Alert	Observations
E1	0.161	A6 M R2		

Peak Table

Size [bp]	Calibrated Conc. [ng/µl]	Assigned Conc. [ng/µl]	Peak Molarity [nmol/l]	% Integrated Area	Peak Comment	Observations
25	5.99	-	369	-		Lower Marker
243	0.161	-	1.02	100.00		
1500	6.50	6.50	6.67	-		Upper Marker

 © Agilent Technologies, Inc. 2017

F1: B6 M R2

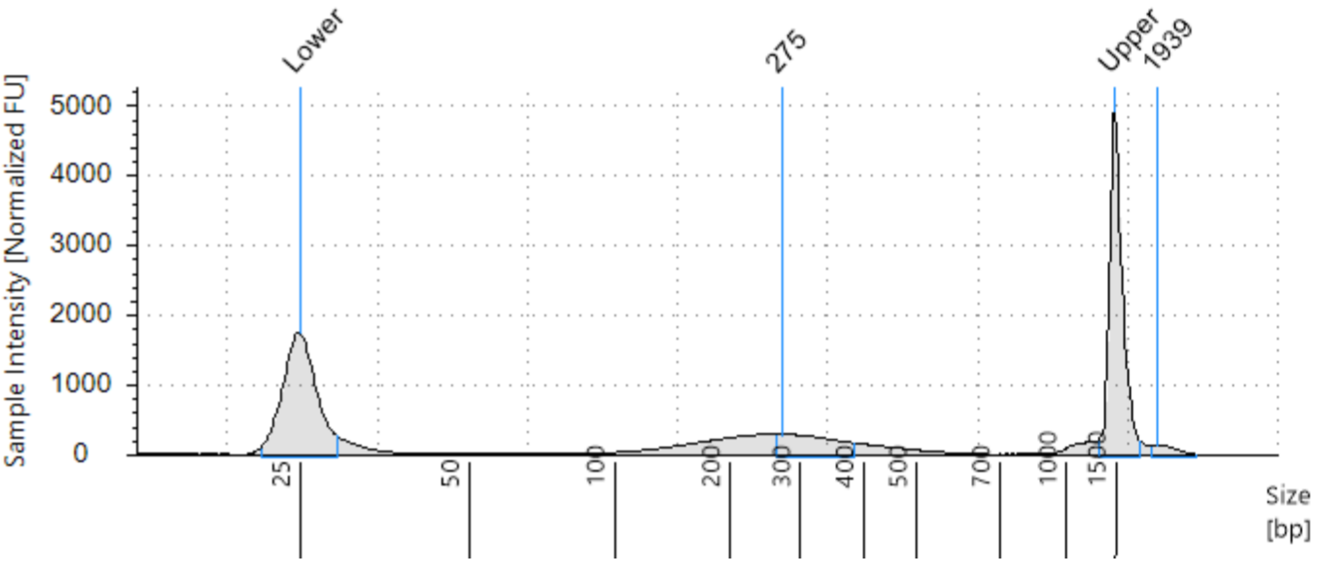

Sample Table

Well	Conc. [ng/µl]	Sample Description	Alert	Observations
F1	1.91	B6 M R2		

Peak Table

Size [bp]	Calibrated Conc. [ng/µl]	Assigned Conc. [ng/µl]	Peak Molarity [nmol/l]	% Integrated Area	Peak Comment	Observations
25	5.91	-	363	-		Lower Marker
275	1.61	-	9.02	84.29		
1500	6.50	6.50	6.67	-		Upper Marker
1939	0.300	-	0.238	15.71		

 © Agilent Technologies, Inc. 2017

G1: B7 M R2

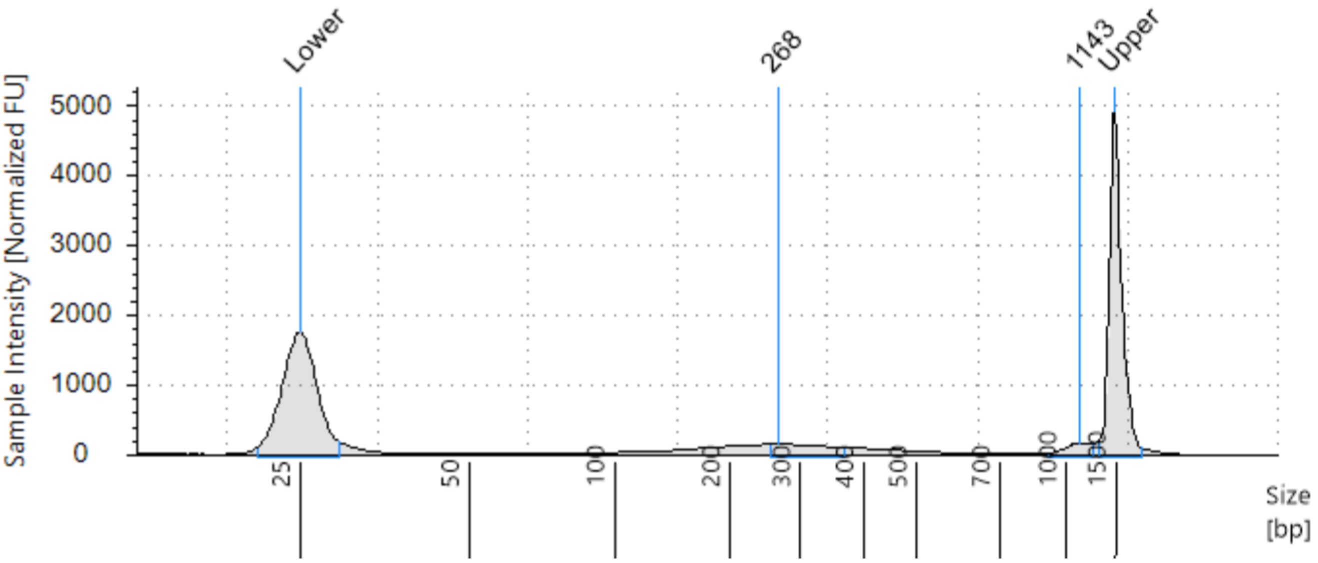

Sample Table

Well	Conc. [ng/µl]	Sample Description	Alert	Observations
G1	1.26	B7 M R2		

Peak Table

Size [bp]	Calibrated Conc. [ng/µl]	Assigned Conc. [ng/µl]	Peak Molarity [nmol/l]	% Integrated Area	Peak Comment	Observations
25	6.43	-	396	-		Lower Marker
268	0.844	-	4.85	66.78		
1143	0.420	-	0.565	33.22		
1500	6.50	6.50	6.67	-		Upper Marker

H1: D7 M R2

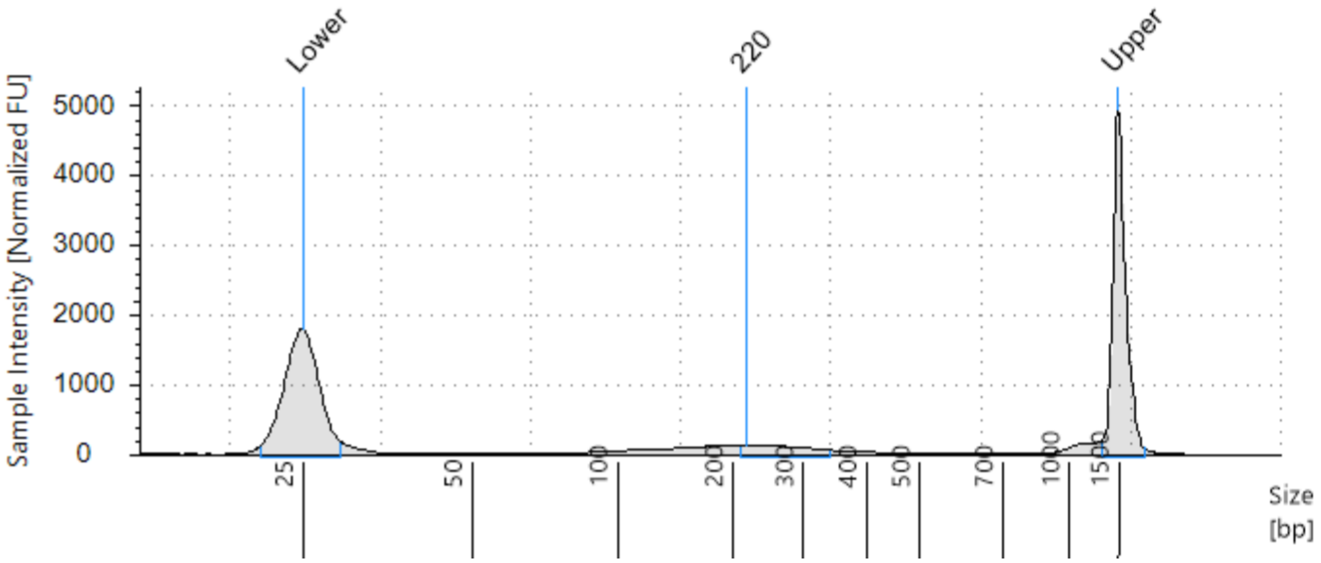

Sample Table

Well	Conc. [ng/µl]	Sample Description	Alert	Observations
H1	0.817	D7 M R2		

Peak Table

Size [bp]	Calibrated Conc. [ng/µl]	Assigned Conc. [ng/µl]	Peak Molarity [nmol/l]	% Integrated Area	Peak Comment	Observations
25	6.46	-	397	-		Lower Marker
220	0.817	-	5.72	100.00		
1500	6.50	6.50	6.67	-		Upper Marker

© Agilent Technologies, Inc. 2017

A2: E7 M R2

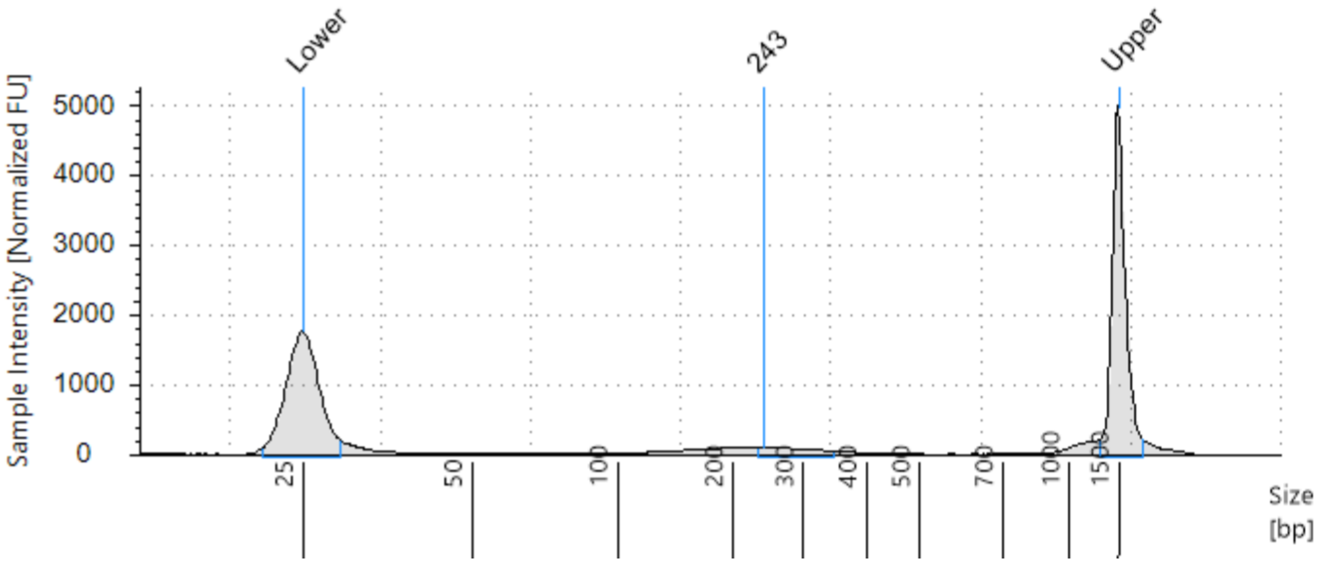

Sample Table

Well	Conc. [ng/µl]	Sample Description	Alert	Observations
A2	0.496	E7 M R2		

Peak Table

Size [bp]	Calibrated Conc. [ng/µl]	Assigned Conc. [ng/µl]	Peak Molarity [nmol/l]	% Integrated Area	Peak Comment	Observations
25	5.83	-	359	-		Lower Marker
243	0.496	-	3.14	100.00		
1500	6.50	6.50	6.67	-		Upper Marker

© Agilent Technologies, Inc. 2017

B2: A8 M R2

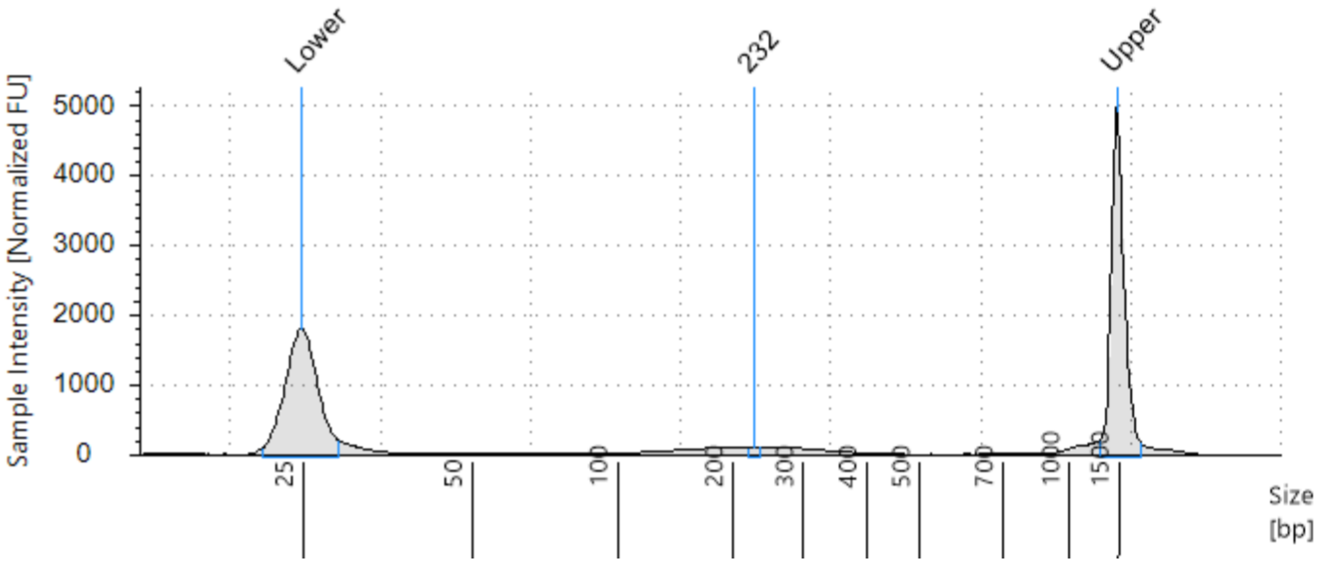

Sample Table

Well	Conc. [ng/µl]	Sample Description	Alert	Observations
B2	0.122	A8 M R2		

Peak Table

Size [bp]	Calibrated Conc. [ng/µl]	Assigned Conc. [ng/µl]	Peak Molarity [nmol/l]	% Integrated Area	Peak Comment	Observations
25	5.91	-	364	-		Lower Marker
232	0.122	-	0.810	100.00		
1500	6.50	6.50	6.67	-		Upper Marker

© Agilent Technologies, Inc. 2017

C2: B9 M R2

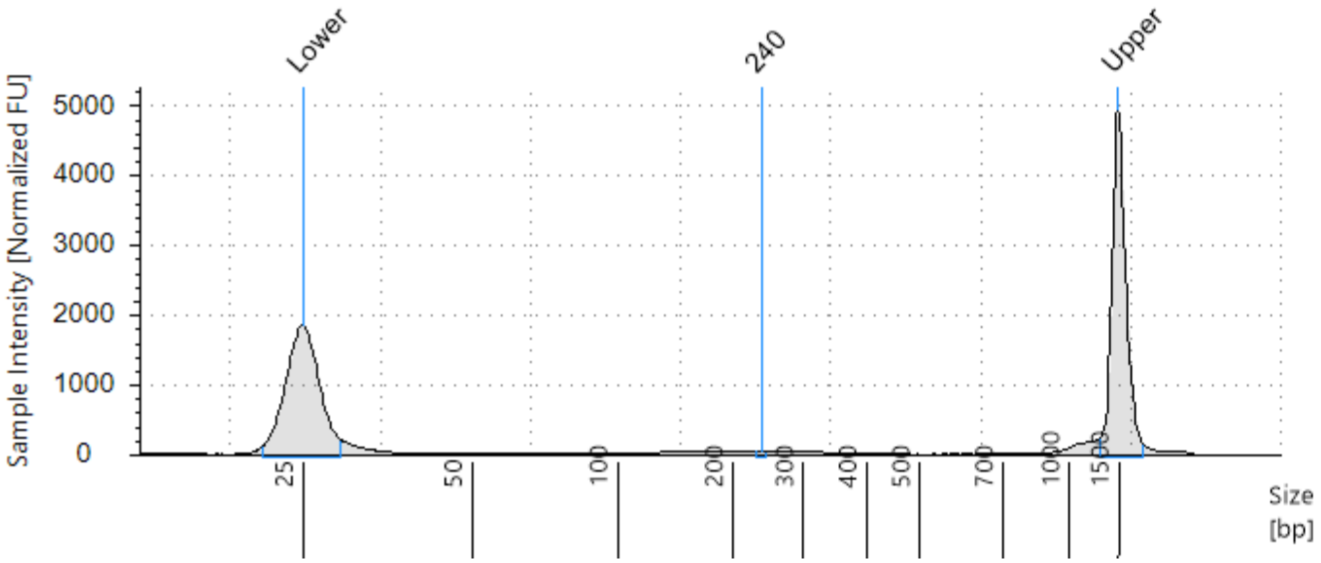

Sample Table

Well	Conc. [ng/µl]	Sample Description	Alert	Observations
C2	0.0529	B9 M R2		

Peak Table

Size [bp]	Calibrated Conc. [ng/µl]	Assigned Conc. [ng/µl]	Peak Molarity [nmol/l]	% Integrated Area	Peak Comment	Observations
25	5.93	-	365	-		Lower Marker
240	0.0529	-	0.339	100.00		
1500	6.50	6.50	6.67	-		Upper Marker

© Agilent Technologies, Inc. 2017

D2: E2 M R2

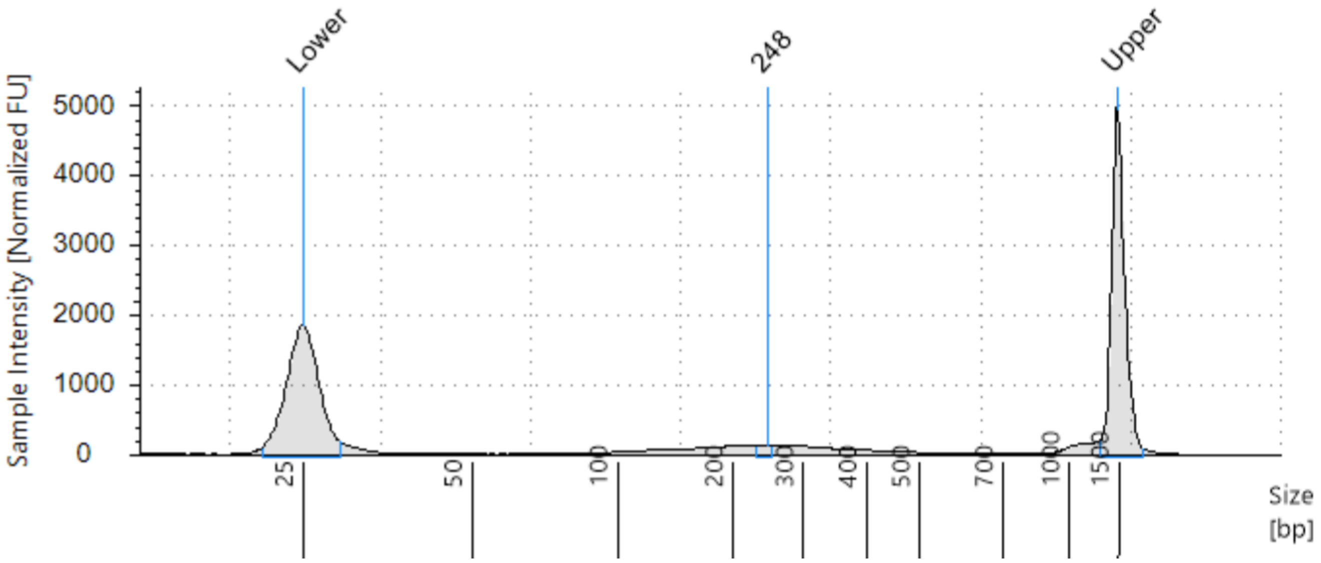

Sample Table

Well	Conc. [ng/µl]	Sample Description	Alert	Observations
D2	0.195	E2 M R2		

Peak Table

Size [bp]	Calibrated Conc. [ng/µl]	Assigned Conc. [ng/µl]	Peak Molarity [nmol/l]	% Integrated Area	Peak Comment	Observations
25	6.02	-	371	-		Lower Marker
248	0.195	-	1.21	100.00		
1500	6.50	6.50	6.67	-		Upper Marker

© Agilent Technologies, Inc. 2017

E2: CB M R2

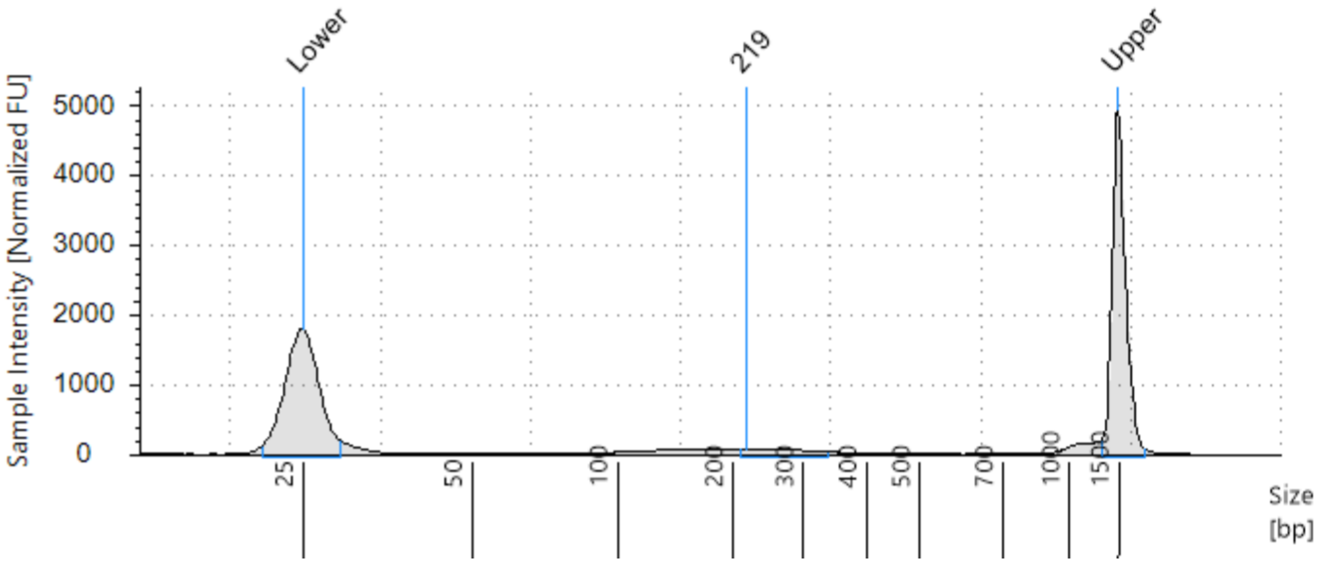

Sample Table

Well	Conc. [ng/µl]	Sample Description	Alert	Observations
E2	0.466	CB M R2		

Peak Table

Size [bp]	Calibrated Conc. [ng/µl]	Assigned Conc. [ng/µl]	Peak Molarity [nmol/l]	% Integrated Area	Peak Comment	Observations
25	6.01	-	370	-		Lower Marker
219	0.466	-	3.27	100.00		
1500	6.50	6.50	6.67	-		Upper Marker

 © Agilent Technologies, Inc. 2017

F2: C10 M R2

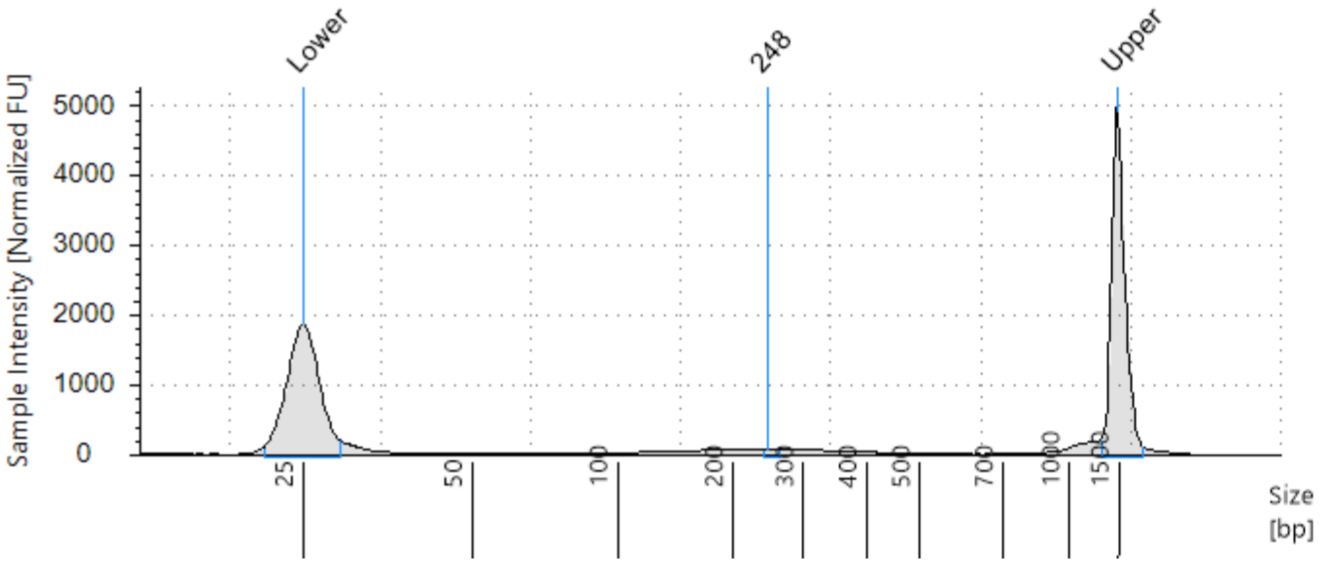

Sample Table

Well	Conc. [ng/µl]	Sample Description	Alert	Observations
F2	0.112	C10 M R2		

Peak Table

Size [bp]	Calibrated Conc. [ng/µl]	Assigned Conc. [ng/µl]	Peak Molarity [nmol/l]	% Integrated Area	Peak Comment	Observations
25	6.01	-	370	-		Lower Marker
248	0.112	-	0.693	100.00		
1500	6.50	6.50	6.67	-		Upper Marker

© Agilent Technologies, Inc. 2017

G2: E10 M R2

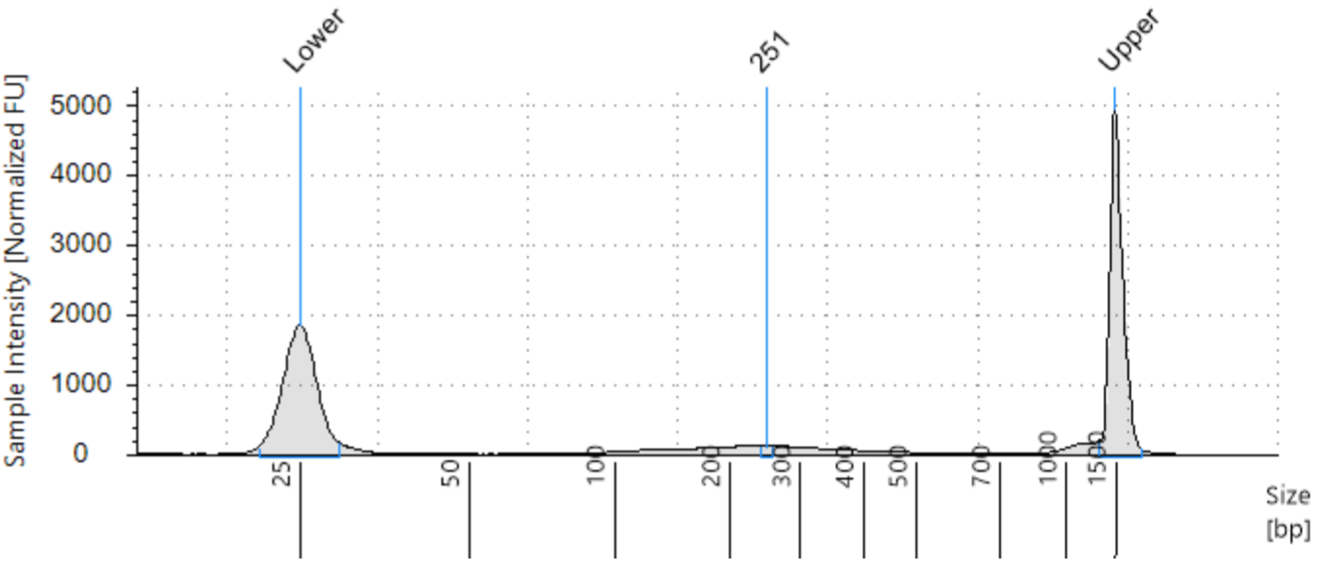

Sample Table

Well	Conc. [ng/µl]	Sample Description	Alert	Observations
G2	0.123	E10 M R2		

Peak Table

Size [bp]	Calibrated Conc. [ng/µl]	Assigned Conc. [ng/µl]	Peak Molarity [nmol/l]	% Integrated Area	Peak Comment	Observations
25	6.32	-	389	-		Lower Marker
251	0.123	-	0.754	100.00		
1500	6.50	6.50	6.67	-		Upper Marker

© Agilent Technologies, Inc. 2017

H2: F10 M R2

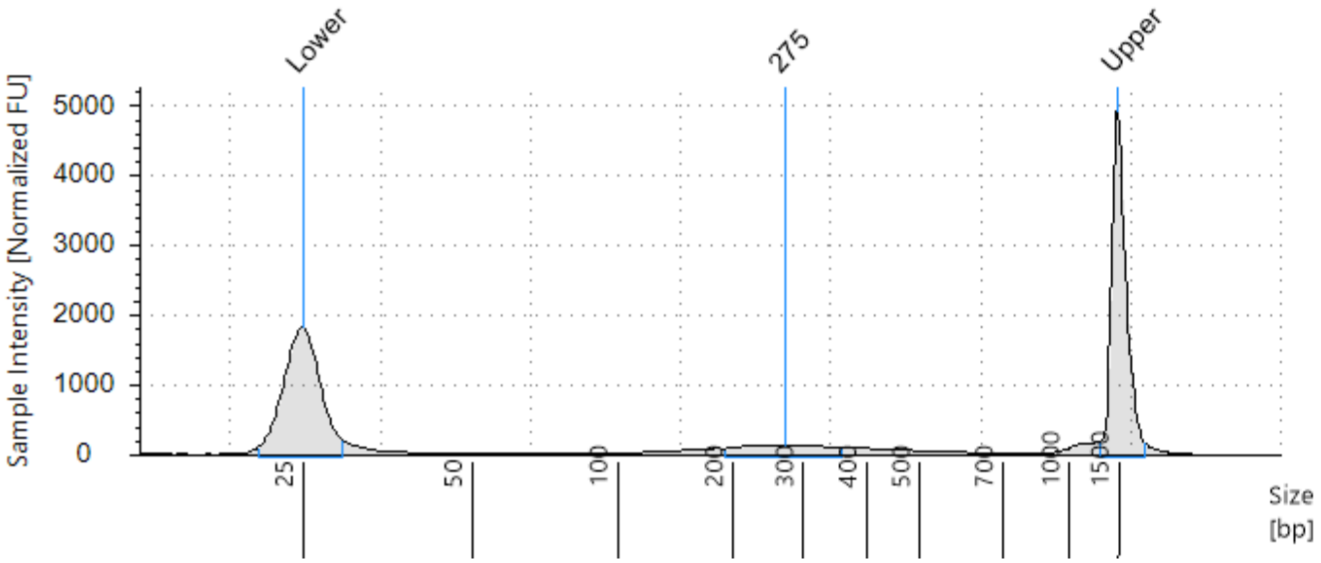

Sample Table

Well	Conc. [ng/µl]	Sample Description	Alert	Observations
H2	1.08	F10 M R2		

Peak Table

Size [bp]	Calibrated Conc. [ng/µl]	Assigned Conc. [ng/µl]	Peak Molarity [nmol/l]	% Integrated Area	Peak Comment	Observations
25	6.08	-	374	-		Lower Marker
275	1.08	-	6.05	100.00		
1500	6.50	6.50	6.67	-		Upper Marker

© Agilent Technologies, Inc. 2017

Filename: 2020-09-28-02 Q-S MINUS A9-G10, D5000, R2.D5000

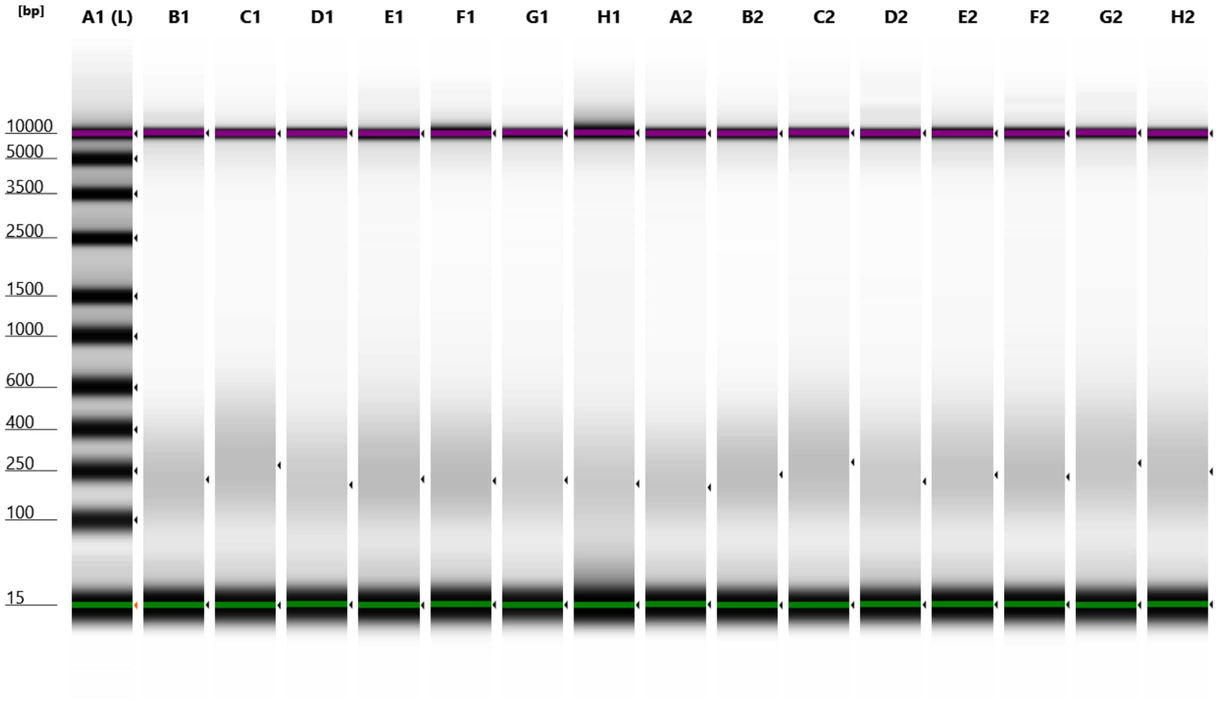

Default image (Contrast 100%)

Sample Info

Well	Conc. [ng/µl]	Sample Description	Alert	Observations
A1	42.5	Ladder		Ladder
B1	1.50	A9 M R2		
C1	1.82	B9 M R2		
D1	0.317	C9 M R2		
E1	1.56	D9 M R2		
F1	1.28	E9 M R2		
G1	1.16	F9 M R2		
H1	0.467	G9 M R2		
A2	1.16	H9 M R2		
B2	0.526	A10 M R2		
C2	1.87	B10 M R2		
D2	0.304	C10 M R2		
E2	1.41	D10 M R2		
F2	1.33	E10 M R2		
G2	0.304	F10 M R2		
H2	1.68	G10 M R2		

© Agilent Technologies, Inc. 2017

A1: Ladder

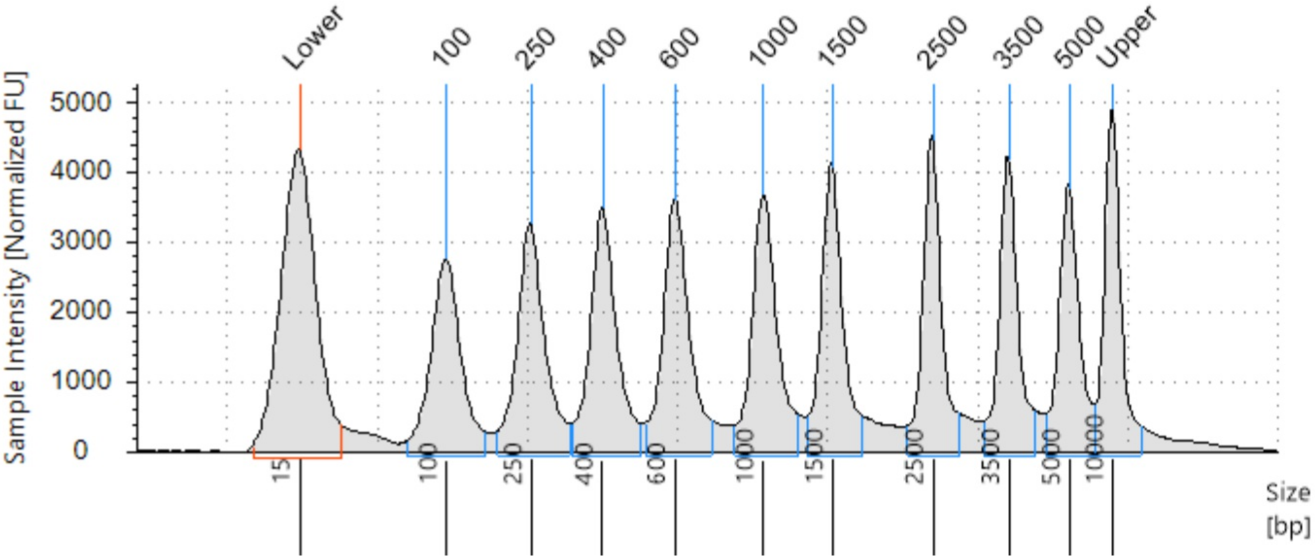

Sample Table

Well	Conc. [ng/µl]	Sample Description	Alert	Observations
A1	42.5	Ladder		Ladder

Peak Table

Size [bp]	Calibrated Conc. [ng/µl]	Assigned Conc. [ng/µl]	Peak Molarity [nmol/l]	% Integrated Area	Peak Comment	Observations
15	7.20	-	738	-		Lower Marker
100	4.49	-	69.1	10.58		
250	4.89	-	30.1	11.50		
400	5.00	-	19.2	11.78		
600	5.14	-	13.2	12.09		
1000	5.02	-	7.73	11.83		
1500	4.82	-	4.94	11.34		
2500	4.55	-	2.80	10.71		
3500	4.37	-	1.92	10.28		
5000	4.21	-	1.29	9.90		
10000	3.25	3.25	0.500	-		Upper Marker

© Agilent Technologies, Inc. 2017

B1: A9 M R2

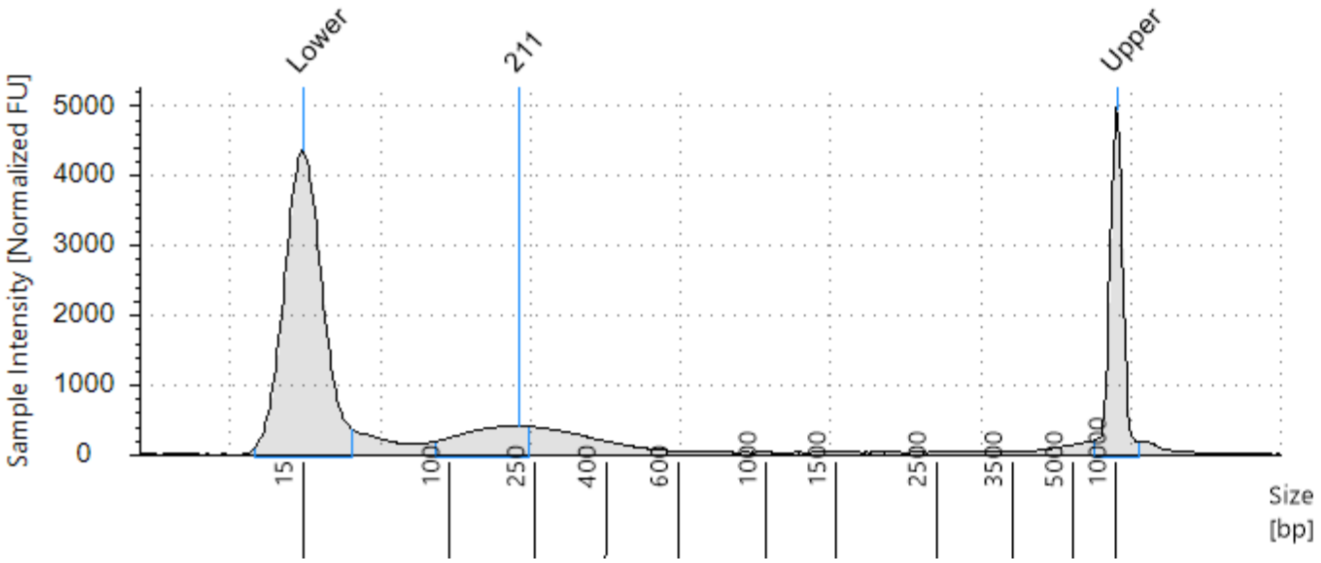

Sample Table

Well	Conc. [ng/µl]	Sample Description	Alert	Observations
B1	1.50	A9 M R2		

Peak Table

Size [bp]	Calibrated Conc. [ng/µl]	Assigned Conc. [ng/µl]	Peak Molarity [nmol/l]	% Integrated Area	Peak Comment	Observations
15	8.19	-	840	-		Lower Marker
211	1.50	-	11.0	100.00		
10000	3.25	3.25	0.500	-		Upper Marker

© Agilent Technologies, Inc. 2017

C1: B9 M R2

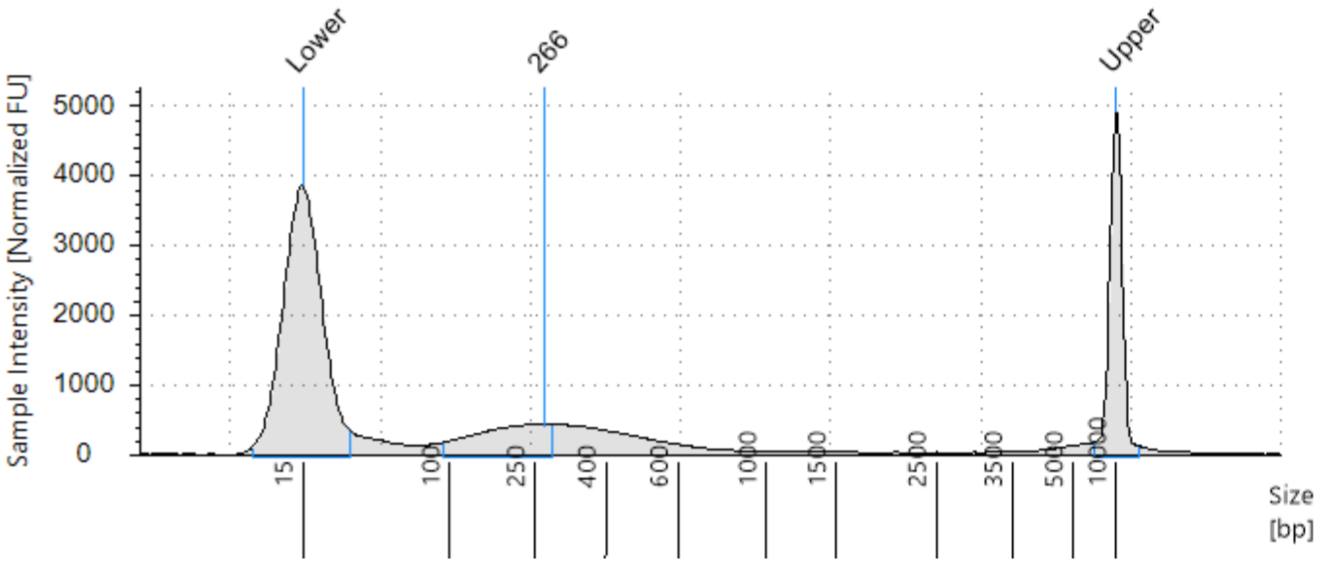

Sample Table

Well	Conc. [ng/µl]	Sample Description	Alert	Observations
C1	1.82	B9 M R2		

Peak Table

Size [bp]	Calibrated Conc. [ng/µl]	Assigned Conc. [ng/µl]	Peak Molarity [nmol/l]	% Integrated Area	Peak Comment	Observations
15	7.50	-	769	-		Lower Marker
266	1.82	-	10.5	100.00		
10000	3.25	3.25	0.500	-		Upper Marker

© Agilent Technologies, Inc. 2017

D1: C9 M R2

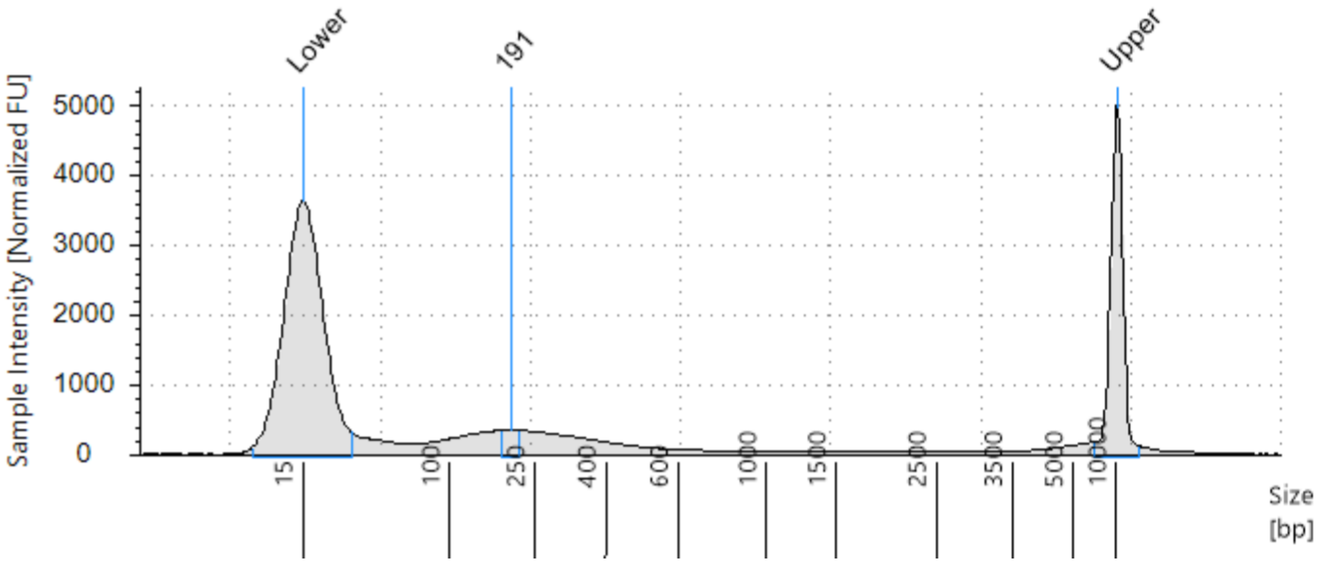

Sample Table

Well	Conc. [ng/µl]	Sample Description	Alert	Observations
D1	0.317	C9 M R2		

Peak Table

Size [bp]	Calibrated Conc. [ng/µl]	Assigned Conc. [ng/µl]	Peak Molarity [nmol/l]	% Integrated Area	Peak Comment	Observations
15	7.19	-	738	-		Lower Marker
191	0.317	-	2.56	100.00		
10000	3.25	3.25	0.500	-		Upper Marker

 © Agilent Technologies, Inc. 2017

E1: D9 M R2

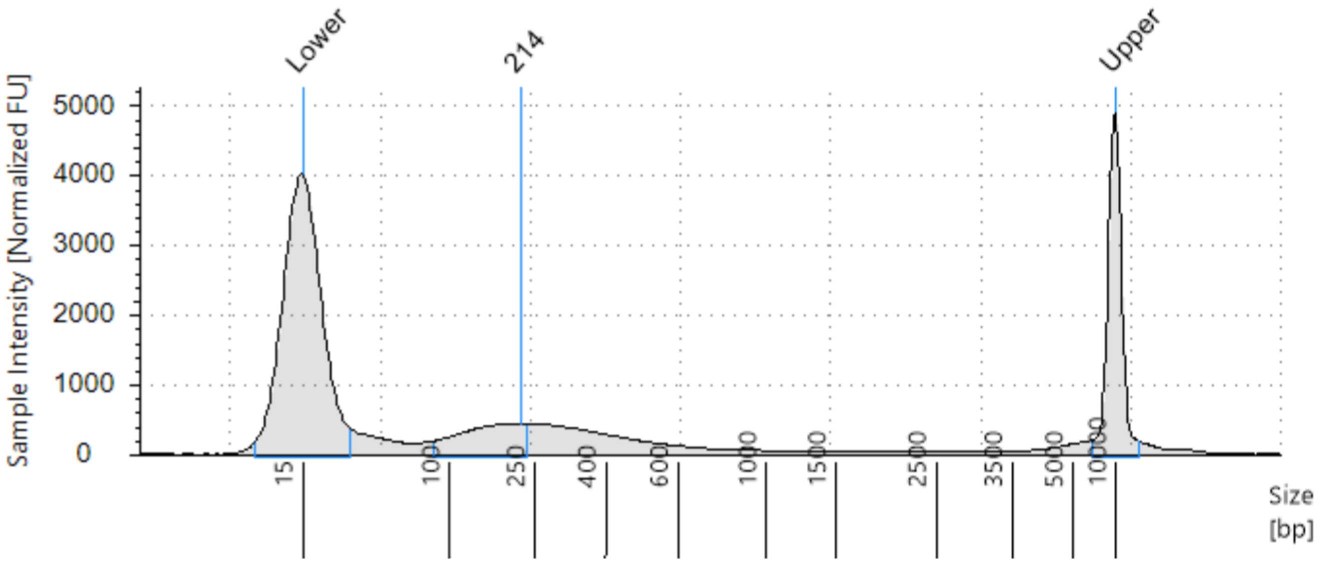

Sample Table

Well	Conc. [ng/µl]	Sample Description	Alert	Observations
E1	1.56	D9 M R2		

Peak Table

Size [bp]	Calibrated Conc. [ng/µl]	Assigned Conc. [ng/µl]	Peak Molarity [nmol/l]	% Integrated Area	Peak Comment	Observations
15	7.27	-	746	-		Lower Marker
214	1.56	-	11.2	100.00		
10000	3.25	3.25	0.500	-		Upper Marker

© Agilent Technologies, Inc. 2017

F1: E9 M R2

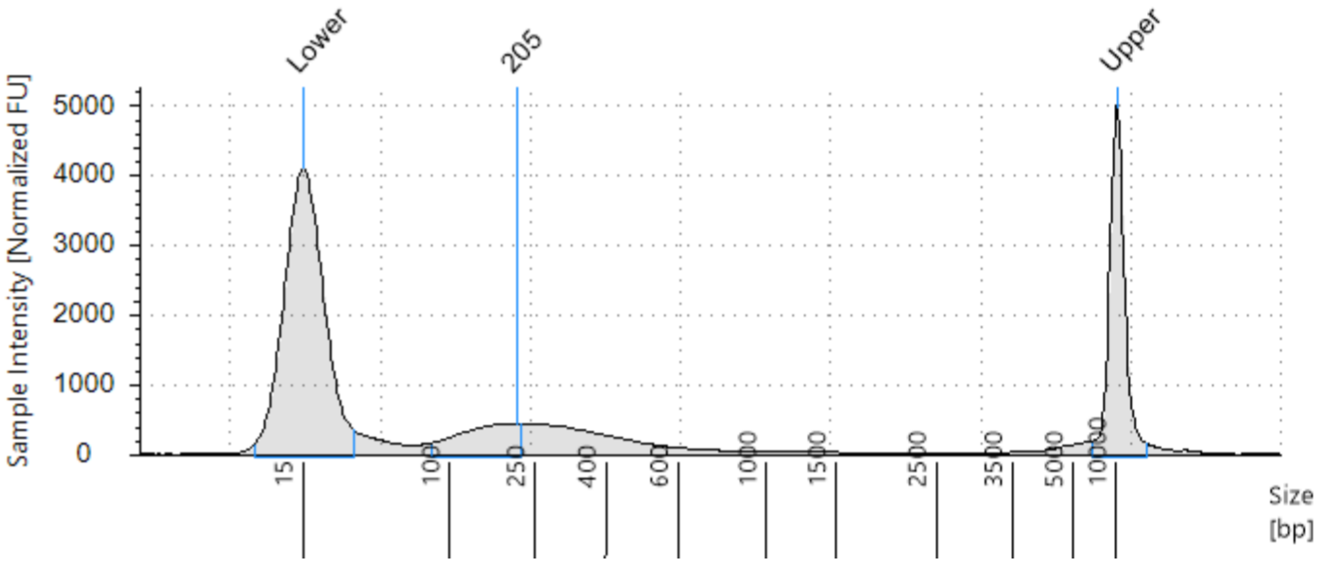

Sample Table

Well	Conc. [ng/µl]	Sample Description	Alert	Observations
F1	1.28	E9 M R2		

Peak Table

Size [bp]	Calibrated Conc. [ng/µl]	Assigned Conc. [ng/µl]	Peak Molarity [nmol/l]	% Integrated Area	Peak Comment	Observations
15	6.86	-	704	-		Lower Marker
205	1.28	-	9.56	100.00		
10000	3.25	3.25	0.500	-		Upper Marker

© Agilent Technologies, Inc. 2017

G1: F9 M R2

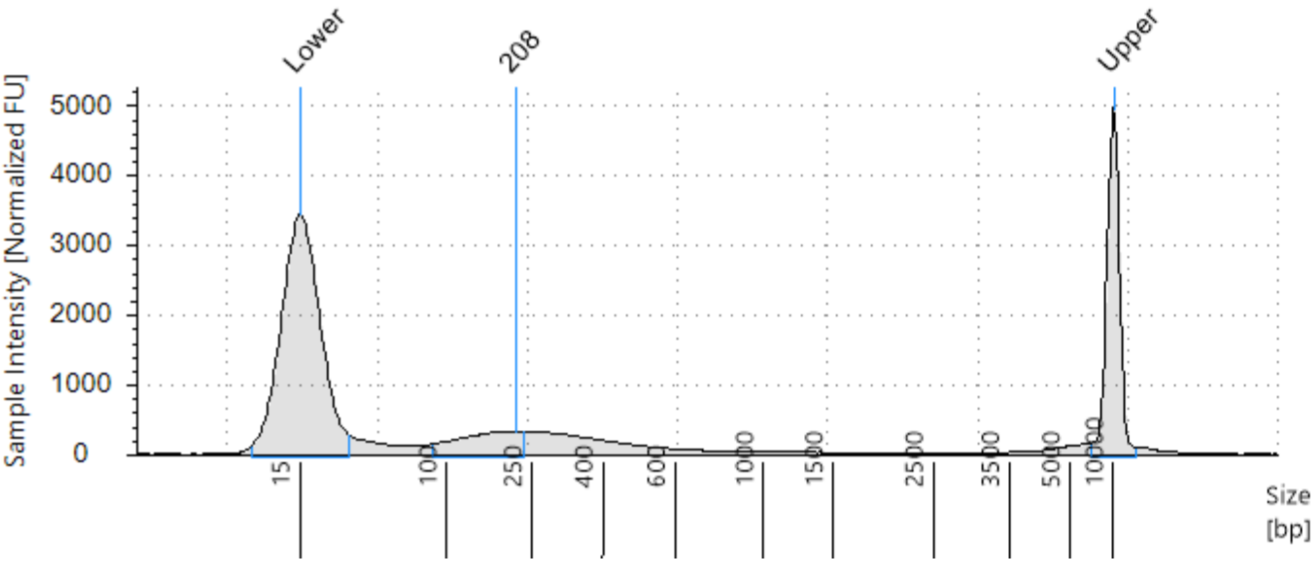

Sample Table

Well	Conc. [ng/µl]	Sample Description	Alert	Observations
Gl	1.16	F9 M R2		

Peak Table

Size [bp]	Calibrated Conc. [ng/µl]	Assigned Conc. [ng/µl]	Peak Molarity [nmol/l]	% Integrated Area	Peak Comment	Observations
15	6.51	-	667	-		Lower Marker
208	1.16	-	8.56	100.00		
10000	3.25	3.25	0.500	-		Upper Marker

 © Agilent Technologies, Inc. 2017

H1: G9 M R2

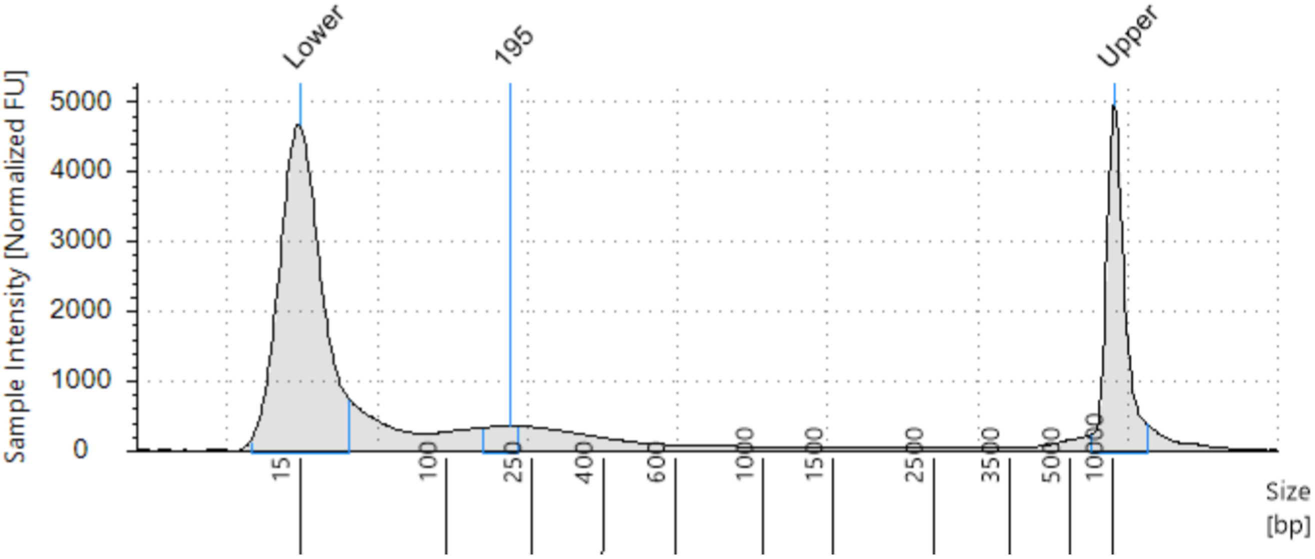

Sample Table

Well	Conc. [ng/µl]	Sample Description	Alert	Observations
H1	0.467	G9 M R2		

Peak Table

Size [bp]	Calibrated Conc. [ng/µl]	Assigned Conc. [ng/µl]	Peak Molarity [nmol/l]	% Integrated Area	Peak Comment	Observations
15	6.79	-	696	-		Lower Marker
195	0.467	-	3.69	100.00		
10000	3.25	3.25	0.500	-		Upper Marker

© Agilent Technologies, Inc. 2017

A2: H9 M R2

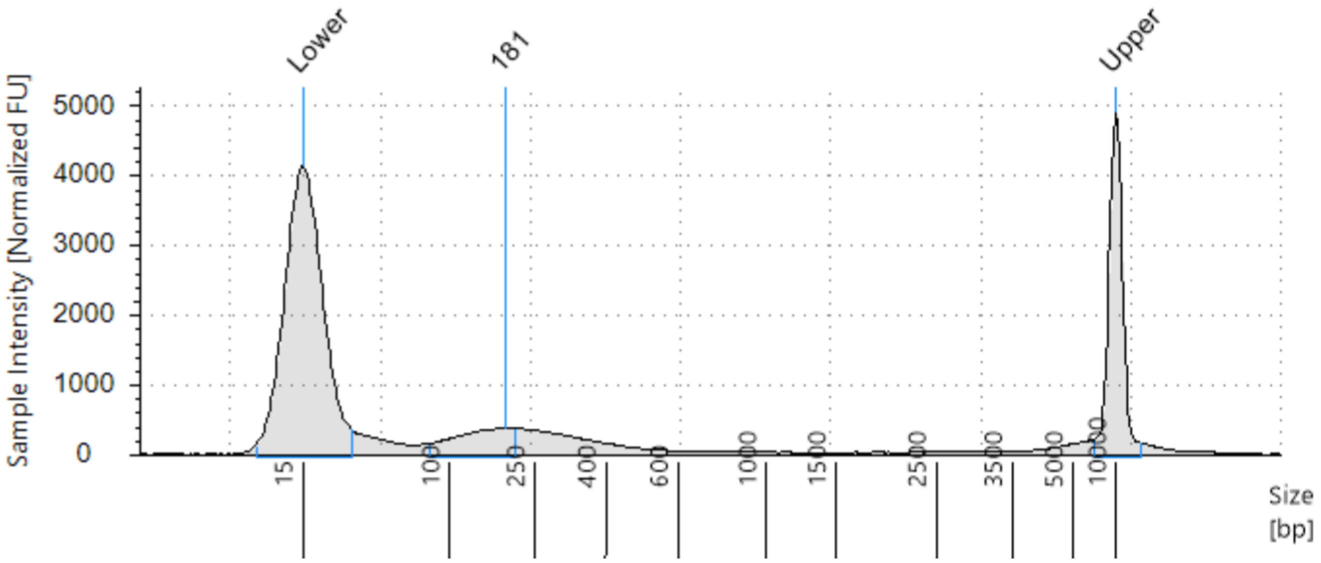

Sample Table

Well	Conc. [ng/µl]	Sample Description	Alert	Observations
A2	1.16	H9 M R2		

Peak Table

Size [bp]	Calibrated Conc. [ng/µl]	Assigned Conc. [ng/µl]	Peak Molarity [nmol/l]	% Integrated Area	Peak Comment	Observations
15	7.42	-	761	-		Lower Marker
181	1.16	-	9.81	100.00		
10000	3.25	3.25	0.500	-		Upper Marker

© Agilent Technologies, Inc. 2017

B2: A10 M R2

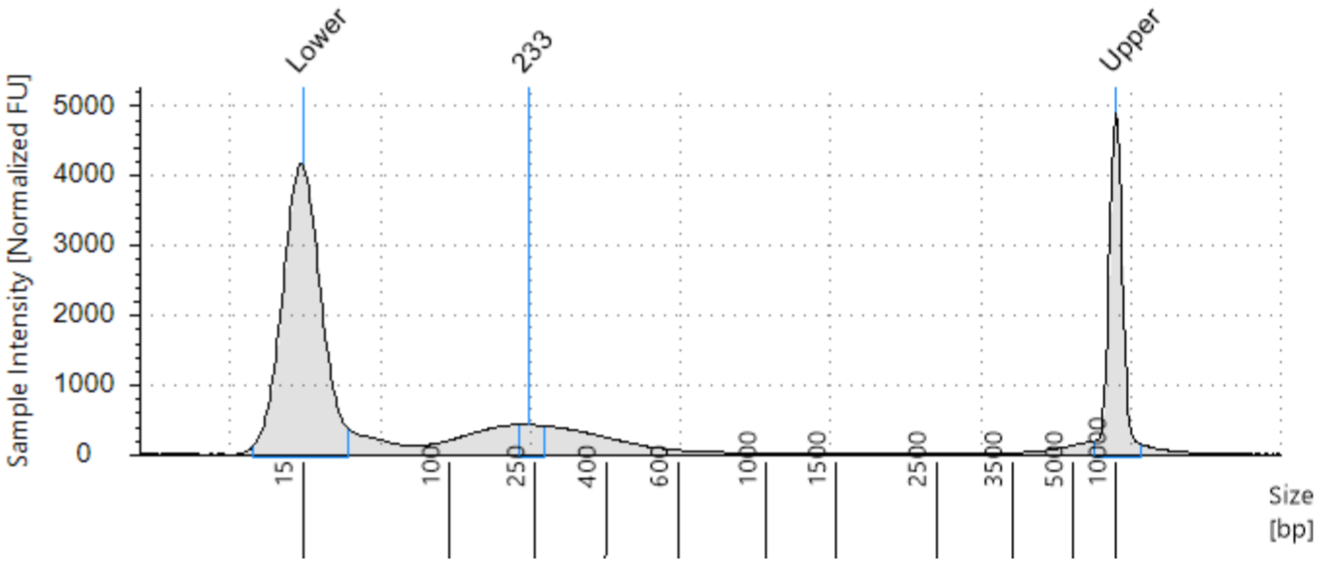

Sample Table

Well	Conc. [ng/µl]	Sample Description	Alert	Observations
B2	0.526	A10 M R2		

Peak Table

Size [bp]	Calibrated Conc. [ng/µl]	Assigned Conc. [ng/µl]	Peak Molarity [nmol/l]	% Integrated Area	Peak Comment	Observations
15	7.50	-	770	-		Lower Marker
233	0.526	-	3.47	100.00		
10000	3.25	3.25	0.500	-		Upper Marker

© Agilent Technologies, Inc. 2017

C2: B10 M R2

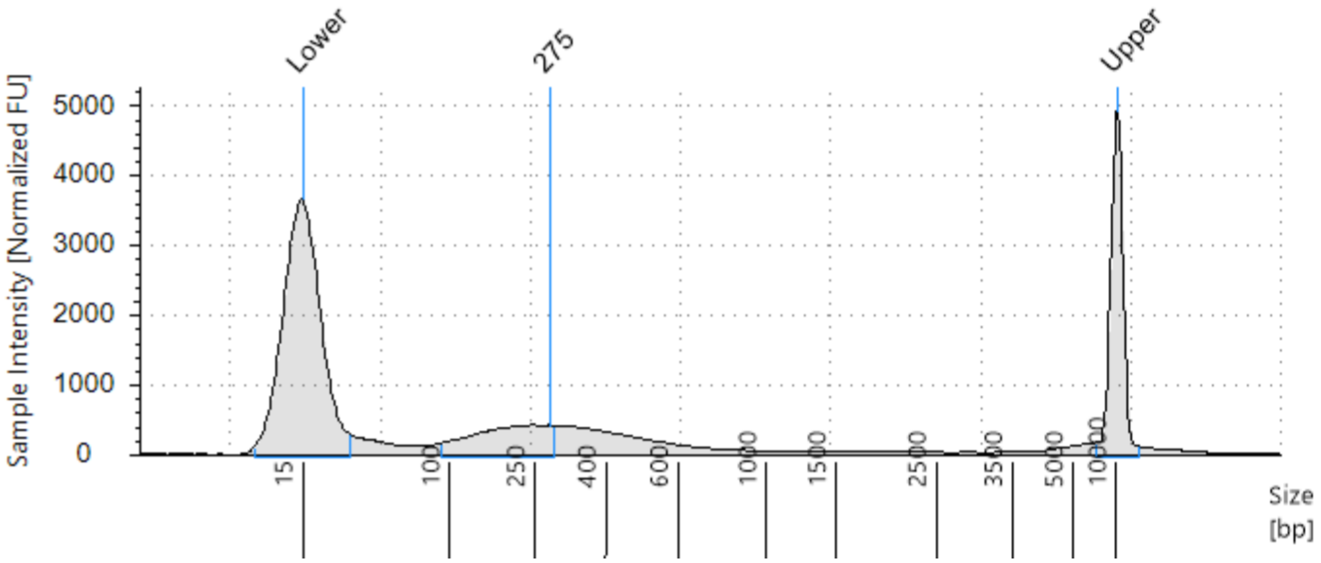

Sample Table

Well	Conc. [ng/µl]	Sample Description	Alert	Observations
C2	1.87	B10 M R2		

Peak Table

Size [bp]	Calibrated Conc. [ng/µl]	Assigned Conc. [ng/µl]	Peak Molarity [nmol/l]	% Integrated Area	Peak Comment	Observations
15	6.91	-	709	-		Lower Marker
275	1.87	-	10.5	100.00		
10000	3.25	3.25	0.500	-		Upper Marker

© Agilent Technologies, Inc. 2017

D2: C10 M R2

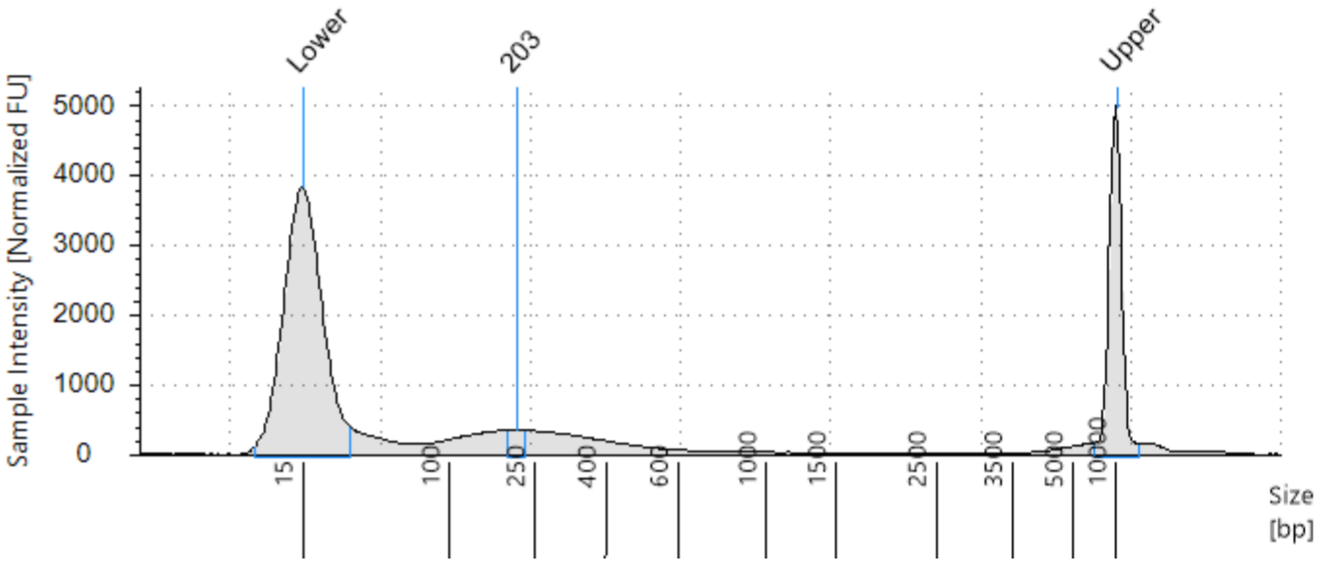

Sample Table

Well	Conc. [ng/µl]	Sample Description	Alert	Observations
D2	0.304	C10 M R2		

Peak Table

Size [bp]	Calibrated Conc. [ng/µl]	Assigned Conc. [ng/µl]	Peak Molarity [nmol/l]	% Integrated Area	Peak Comment	Observations
15	7.20	-	738	-		Lower Marker
203	0.304	-	2.30	100.00		
10000	3.25	3.25	0.500	-		Upper Marker

© Agilent Technologies, Inc. 2017

E2: D10 M R2

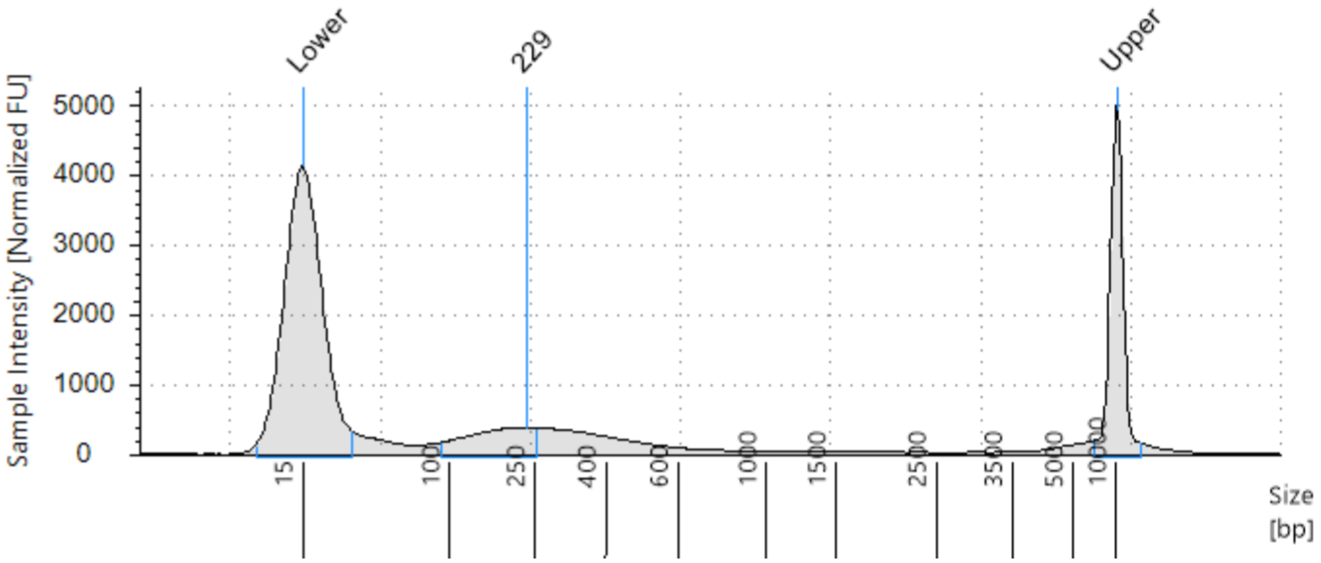

Sample Table

Well	Conc. [ng/µl]	Sample Description	Alert	Observations
E2	1.41	D10 M R2		

Peak Table

Size [bp]	Calibrated Conc. [ng/µl]	Assigned Conc. [ng/µl]	Peak Molarity [nmol/l]	% Integrated Area	Peak Comment	Observations
15	7.69	-	788	-		Lower Marker
229	1.41	-	9.44	100.00		
10000	3.25	3.25	0.500	-		Upper Marker

© Agilent Technologies, Inc. 2017

F2: E10 M R2

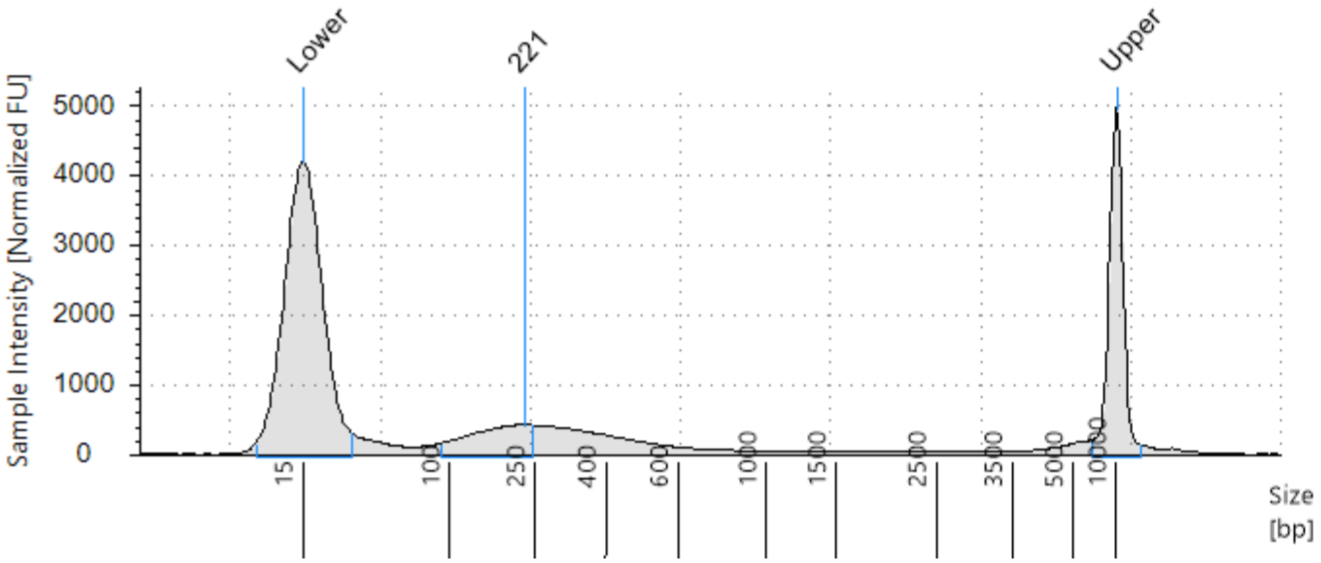

Sample Table

Well	Conc. [ng/µl]	Sample Description	Alert	Observations
F2	1.33	E10 M R2		

Peak Table

Size [bp]	Calibrated Conc. [ng/µl]	Assigned Conc. [ng/µl]	Peak Molarity [nmol/l]	% Integrated Area	Peak Comment	Observations
15	7.38	-	757	-		Lower Marker
221	1.33	-	9.28	100.00		
10000	3.25	3.25	0.500	-		Upper Marker

© Agilent Technologies, Inc. 2017

G2: F10 M R2

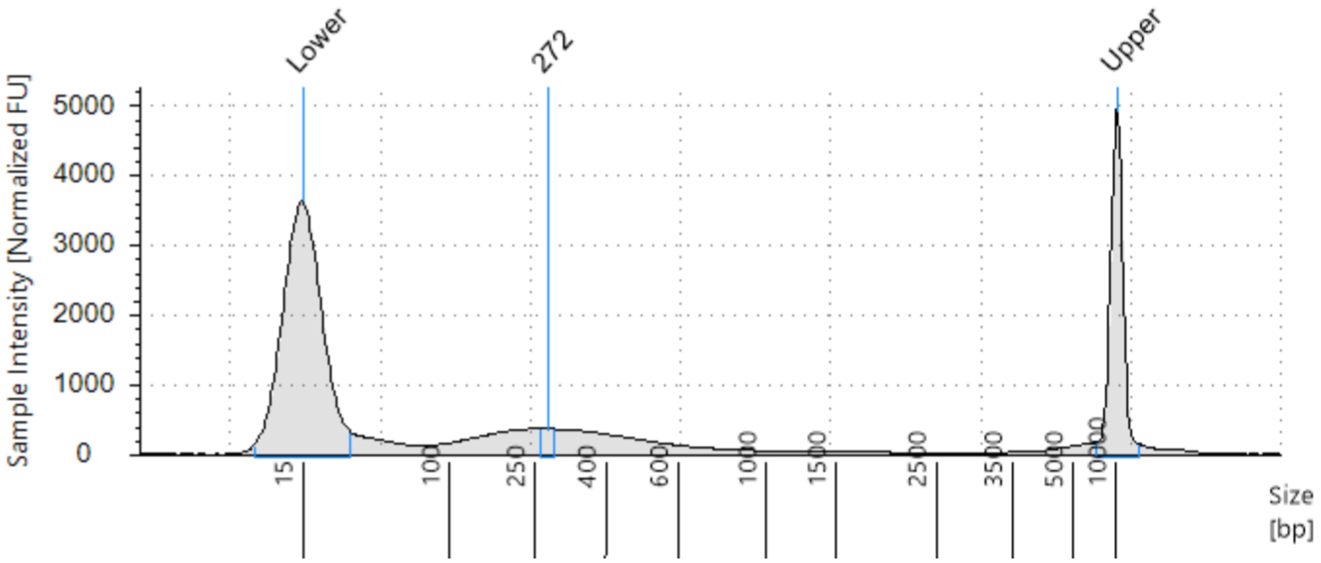

Sample Table

Well	Conc. [ng/µl]	Sample Description	Alert	Observations
G2	0.304	F10 M R2		

Peak Table

Size [bp]	Calibrated Conc. [ng/µl]	Assigned Conc. [ng/µl]	Peak Molarity [nmol/l]	% Integrated Area	Peak Comment	Observations
15	7.10	-	729	-		Lower Marker
272	0.304	-	1.72	100.00		
10000	3.25	3.25	0.500	-		Upper Marker

© Agilent Technologies, Inc. 2017

H2: G10 M R2

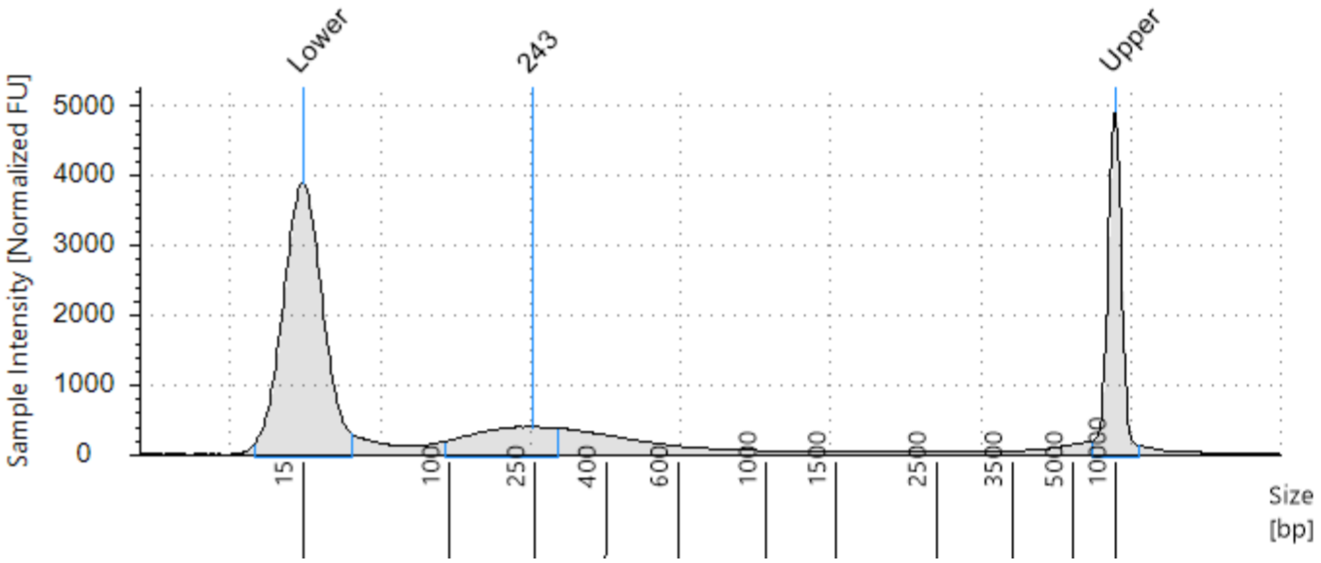

Sample Table

Well	Conc. [ng/µl]	Sample Description	Alert	Observations
H2	1.68	G10 M R2		

Peak Table

Size [bp]	Calibrated Conc. [ng/µl]	Assigned Conc. [ng/µl]	Peak Molarity [nmol/l]	% Integrated Area	Peak Comment	Observations
15	7.02	-	720	-		Lower Marker
243	1.68	-	10.6	100.00		
10000	3.25	3.25	0.500	-		Upper Marker

 © Agilent Technologies, Inc. 2017

Filename: 2020-09-28-03 Q-S MINUS A11-G11,D5000, R2.D5000

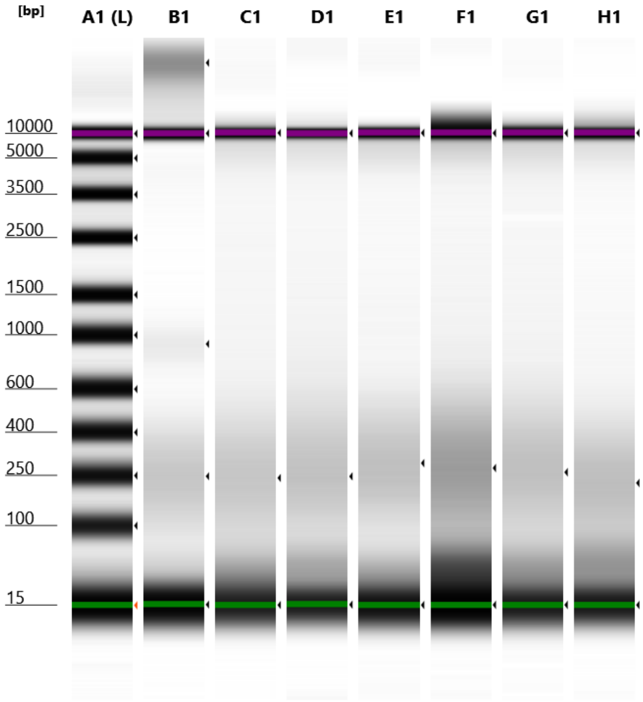

Default image (Contrast 100%)

Sample Info

Well	Conc. [ng/µl]	Sample Description	Alert	Observations
A1	33.3	Ladder		Ladder
B1	2.60	A11 M R2		
C1	0.290	B11 M R2		
D1	0.630	C11 M R2		
E1	1.50	D11 M R2		
F1	0.417	E11 M R2		
G1	0.445	F11 M R2		
H1	0.440	G11 M R2		

 © Agilent Technologies, Inc. 2017

A1: Ladder

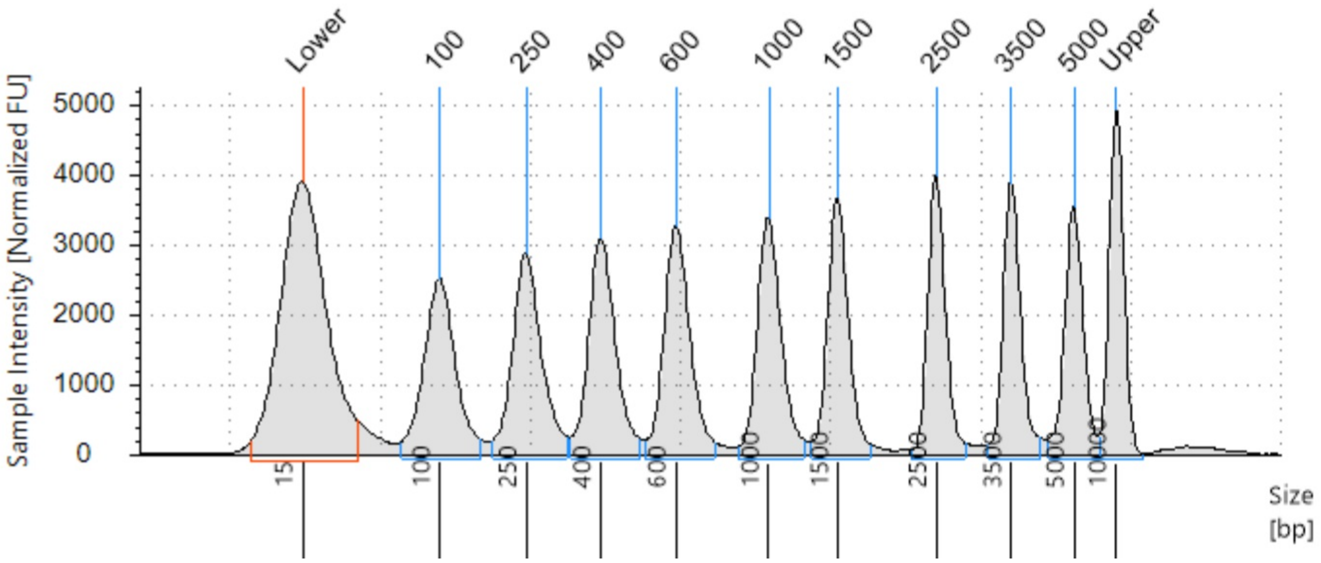

Sample Table

Well	Conc. [ng/µl]	Sample Description	Alert	Observations
A1	33.3	Ladder		Ladder

Peak Table

Size [bp]	Calibrated Conc. [ng/µl]	Assigned Conc. [ng/µl]	Peak Molarity [nmol/l]	% Integrated Area	Peak Comment	Observations
15	6.92	-	710	-		Lower Marker
100	3.79	-	58.3	11.39		
250	3.99	-	24.5	11.99		
400	3.98	-	15.3	11.98		
600	4.07	-	10.4	12.25		
1000	3.88	-	5.97	11.66		
1500	3.68	-	3.77	11.05		
2500	3.41	-	2.10	10.25		
3500	3.30	-	1.45	9.92		
5000	3.16	-	0.974	9.51		
10000	3.25	3.25	0.500	-		Upper Marker

© Agilent Technologies, Inc. 2017

B1: A11 M R2

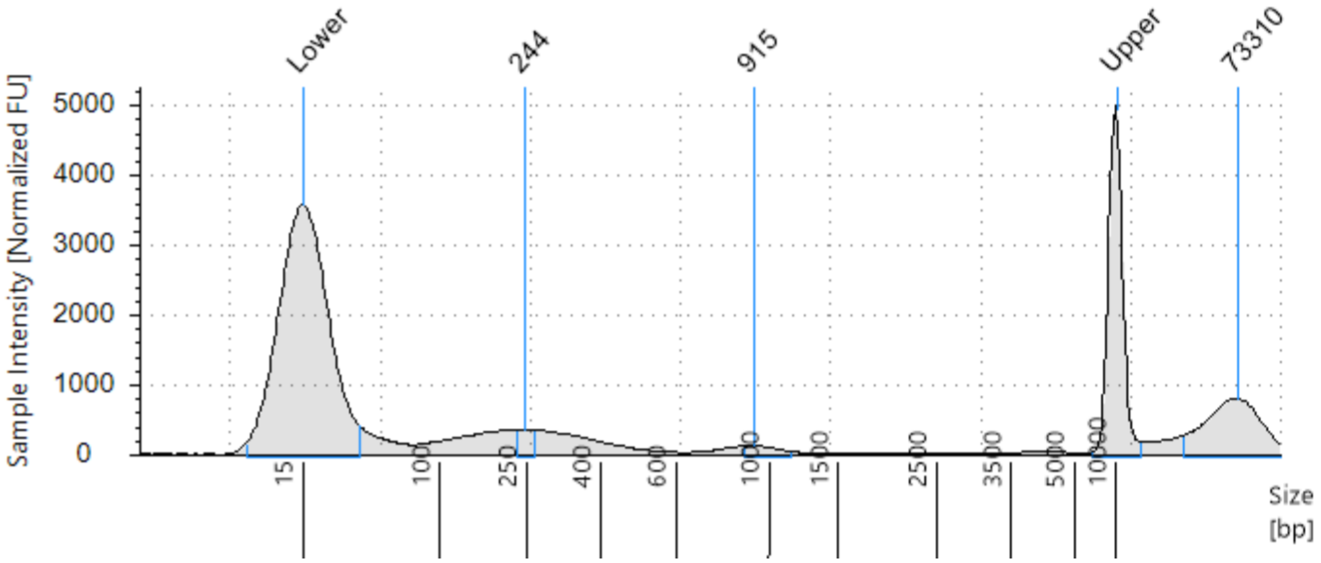

Sample Table

Well	Conc. [ng/µl]	Sample Description	Alert	Observations
B1	2.60	A11 M R2		

Peak Table

Size [bp]	Calibrated Conc. [ng/µl]	Assigned Conc. [ng/µl]	Peak Molarity [nmol/l]	% Integrated Area	Peak Comment	Observations
15	7.20	-	738	-		Lower Marker
244	0.271	-	1.70	10.41		
915	0.180	-	0.302	6.91		
10000	3.25	3.25	0.500	-		Upper Marker
73310	2.15	-	0.0451	82.68		

© Agilent Technologies, Inc. 2017

C1: B11 M R2

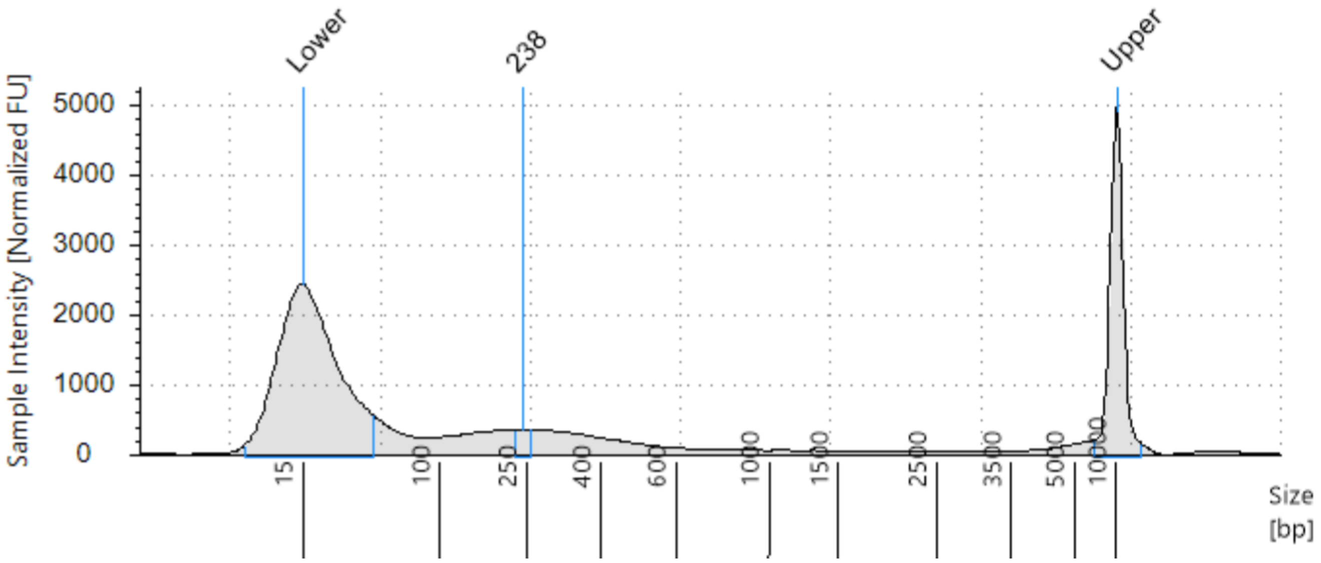

Sample Table

Well	Conc. [ng/µl]	Sample Description	Alert	Observations
C1	0.290	B11 M R2		

Peak Table

Size [bp]	Calibrated Conc. [ng/µl]	Assigned Conc. [ng/µl]	Peak Molarity [nmol/l]	% Integrated Area	Peak Comment	Observations
15	5.79	-	594	-		Lower Marker
238	0.290	-	1.87	100.00		
10000	3.25	3.25	0.500	-		Upper Marker

© Agilent Technologies, Inc. 2017

D1: C11 M R2

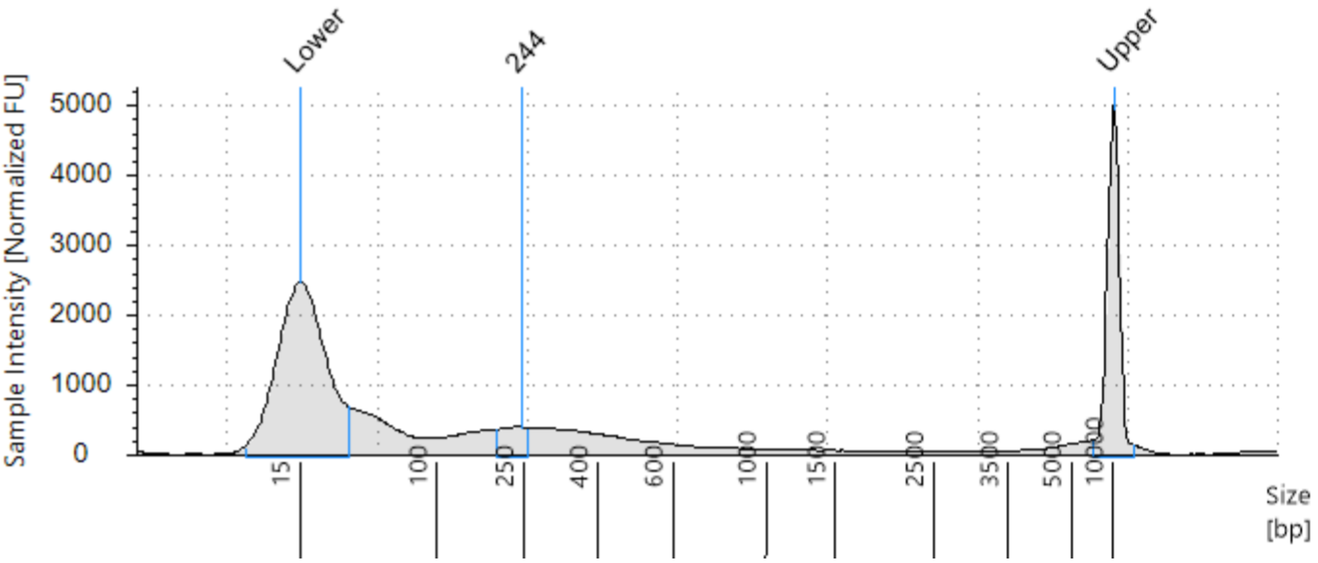

Sample Table

Well	Conc. [ng/µl]	Sample Description	Alert	Observations
D1	0.630	C11 M R2		

Peak Table

Size [bp]	Calibrated Conc. [ng/µl]	Assigned Conc. [ng/µl]	Peak Molarity [nmol/l]	% Integrated Area	Peak Comment	Observations
15	4.95	-	508	-		Lower Marker
244	0.630	-	3.98	100.00		
10000	3.25	3.25	0.500	-		Upper Marker

© Agilent Technologies, Inc. 2017

E1: D11 M R2

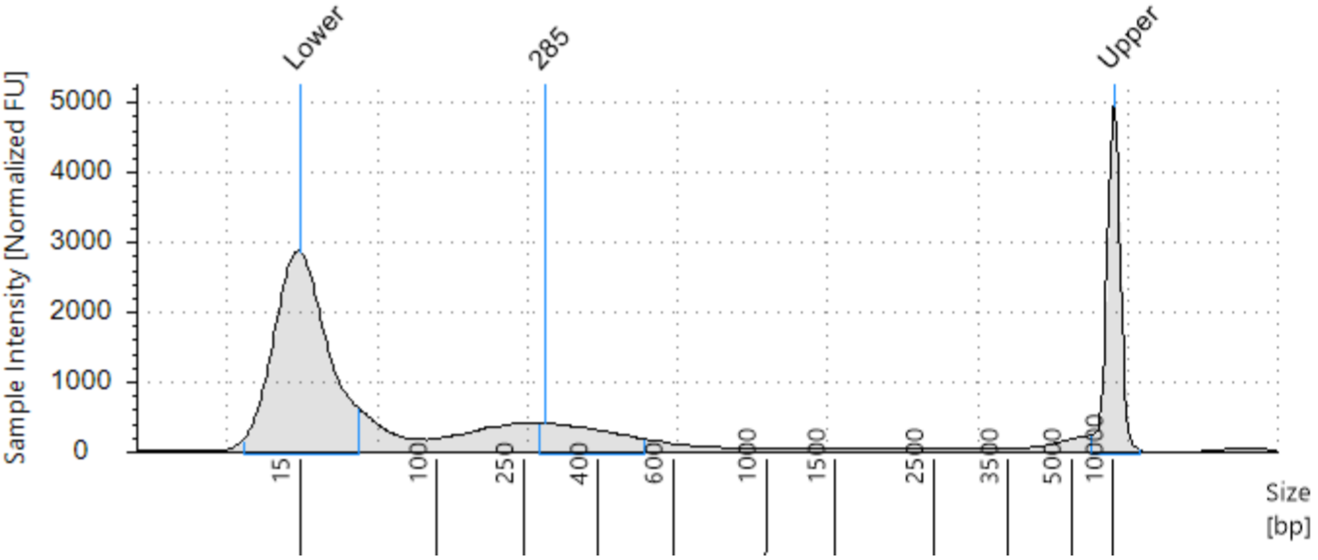

Sample Table

Well	Conc. [ng/µl]	Sample Description	Alert	Observations
E1	1.50	D11 M R2		

Peak Table

Size [bp]	Calibrated Conc. [ng/µl]	Assigned Conc. [ng/µl]	Peak Molarity [nmol/l]	% Integrated Area	Peak Comment	Observations
15	5.97	-	612	-		Lower Marker
285	1.50	-	8.12	100.00		
10000	3.25	3.25	0.500	-		Upper Marker

© Agilent Technologies, Inc. 2017

F1: E11 M R2

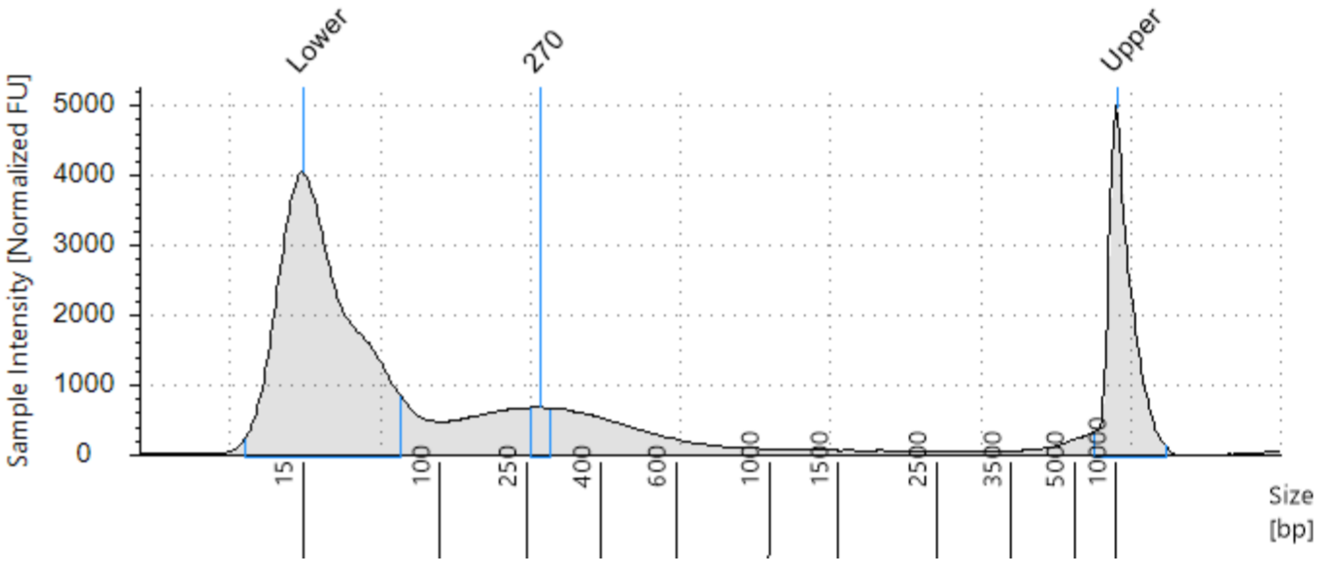

Sample Table

Well	Conc. [ng/µl]	Sample Description	Alert	Observations
F1	0.417	E11 M R2		

Peak Table

Size [bp]	Calibrated Conc. [ng/µl]	Assigned Conc. [ng/µl]	Peak Molarity [nmol/l]	% Integrated Area	Peak Comment	Observations
15	7.11	-	730	-		Lower Marker
270	0.417	-	2.38	100.00		
10000	3.25	3.25	0.500	-		Upper Marker

© Agilent Technologies, Inc. 2017

G1: F11 M R2

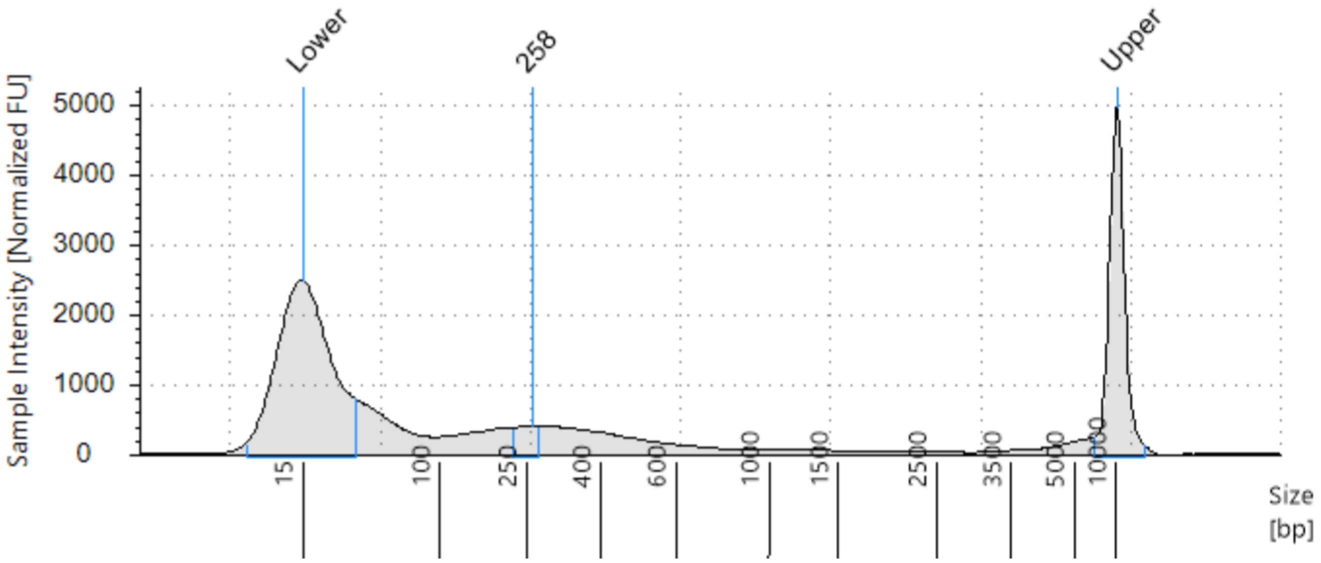

Sample Table

Well	Conc. [ng/µl]	Sample Description	Alert	Observations
G1	0.445	F11 M R2		

Peak Table

Size [bp]	Calibrated Conc. [ng/µl]	Assigned Conc. [ng/µl]	Peak Molarity [nmol/l]	% Integrated Area	Peak Comment	Observations
15	4.24	-	435	-		Lower Marker
258	0.445	-	2.65	100.00		
10000	3.25	3.25	0.500	-		Upper Marker

© Agilent Technologies, Inc. 2017

H1: G11 M R2

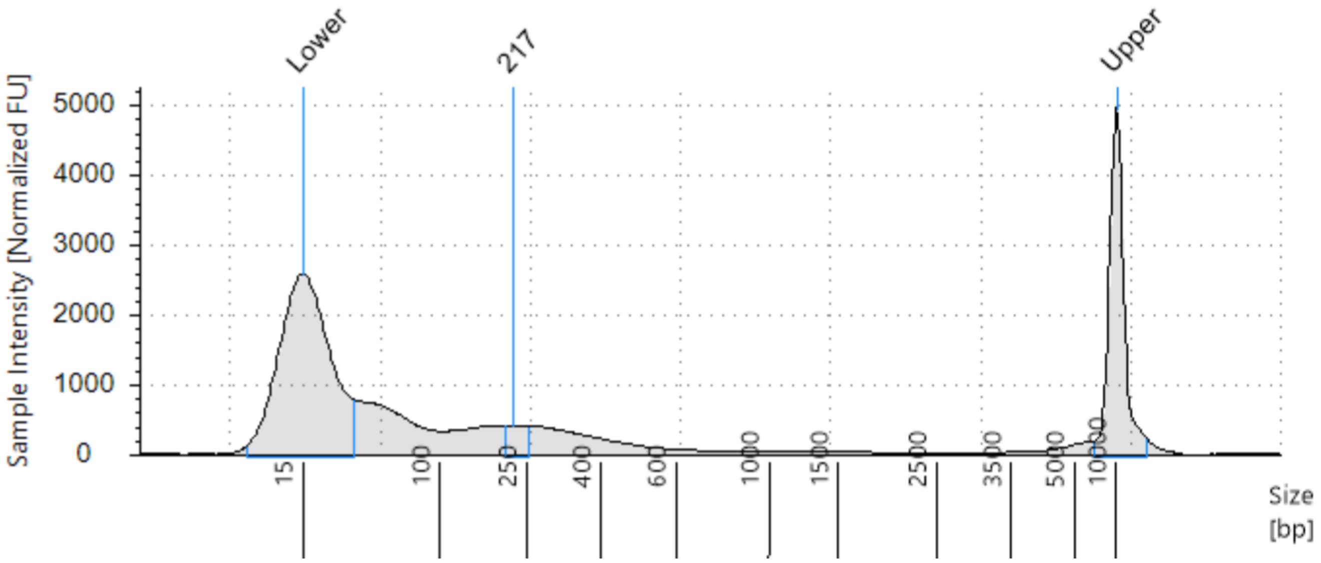

Sample Table

Well	Conc. [ng/µl]	Sample Description	Alert	Observations
H1	0.440	G11 M R2		

Peak Table

Size [bp]	Calibrated Conc. [ng/µl]	Assigned Conc. [ng/µl]	Peak Molarity [nmol/l]	% Integrated Area	Peak Comment	Observations
15	4.61	-	473	-		Lower Marker
217	0.440	-	3.12	100.00		
10000	3.25	3.25	0.500	-		Upper Marker

© Agilent Technologies, Inc. 2017

Filename: 2020-11-10-01- A1B1A2B3A8E9H9 P R1 H9 M R1 A11-F11 M R2F12 M R2.D1000

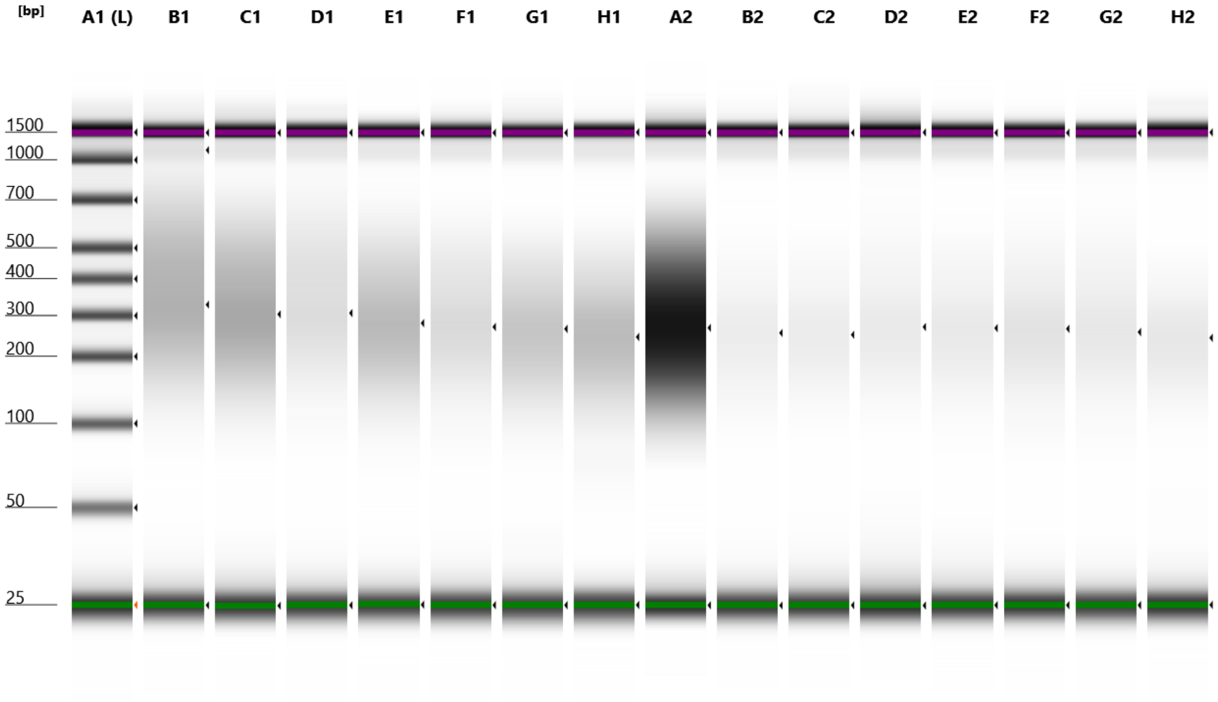

Default image (Contrast 100%)

Sample Info

Well	Conc. [ng/µl]	Sample Description	Alert	Observations
A1	17.3	Ladder		Ladder
B1	1.89	A1 P R1		
C1	7.96	B1 P R1		
D1	1.87	A2 P R1		
E1	2.87	B3 P R1		
F1	2.53	A8 P R1		
G1	4.80	E9 P R1		
H1	5.53	H9 P R1		
A2	17.3	H9 M R1		
B2	0.579	A11 M R2		
C2	0.0893	B11 M R2		
D2	0.109	C11 M R2		
E2	0.603	D11 M R2		
F2	0.193	E11 M R2		
G2	0.835	F11 M R2		
H2	0.928	F12 M R2		

 © Agilent Technologies, Inc. 2017

A1: Ladder

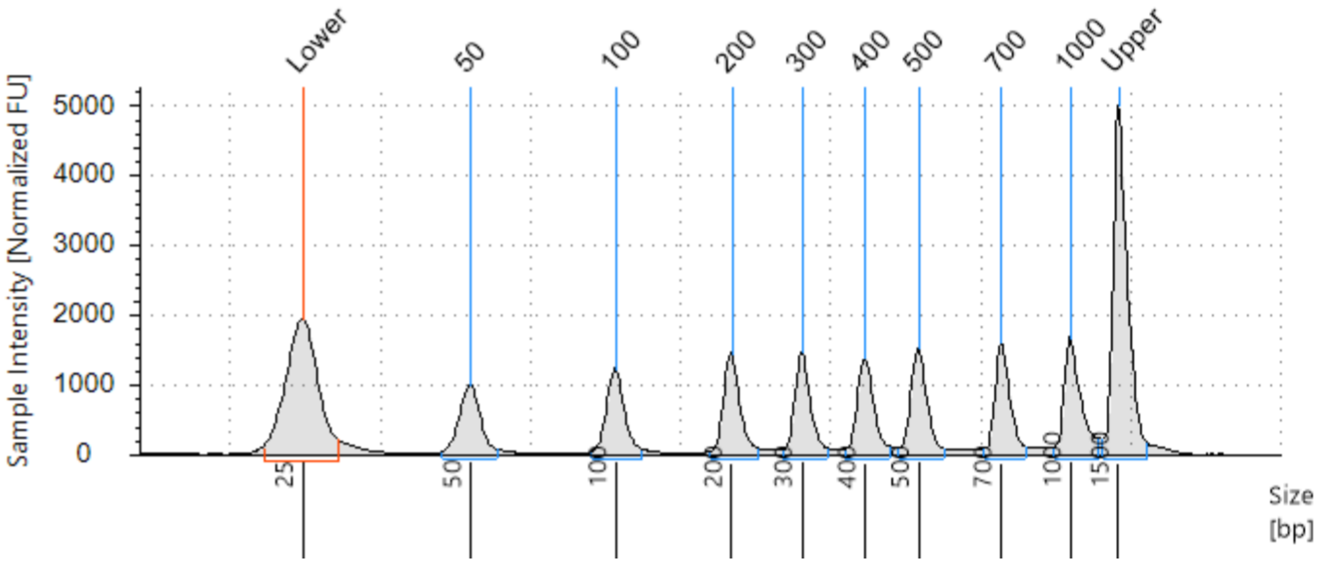

Sample Table

Well	Conc. [ng/µl]	Sample Description	Alert	Observations
A1	17.3	Ladder		Ladder

Peak Table

Size [bp]	Calibrated Conc. [ng/µl]	Assigned Conc. [ng/µl]	Peak Molarity [nmol/l]	% Integrated Area	Peak Comment	Observations
25	5.70	-	351	-		Lower Marker
50	1.93	-	59.3	11.13		
100	2.02	-	31.1	11.69		
200	2.18	-	16.7	12.58		
300	2.11	-	10.8	12.19		
400	2.11	-	8.11	12.19		
500	2.26	-	6.95	13.06		
700	2.21	-	4.85	12.76		
1000	2.49	-	3.84	14.41		
1500	6.50	6.50	6.67	-		Upper Marker

© Agilent Technologies, Inc. 2017

B1: A1 P R1

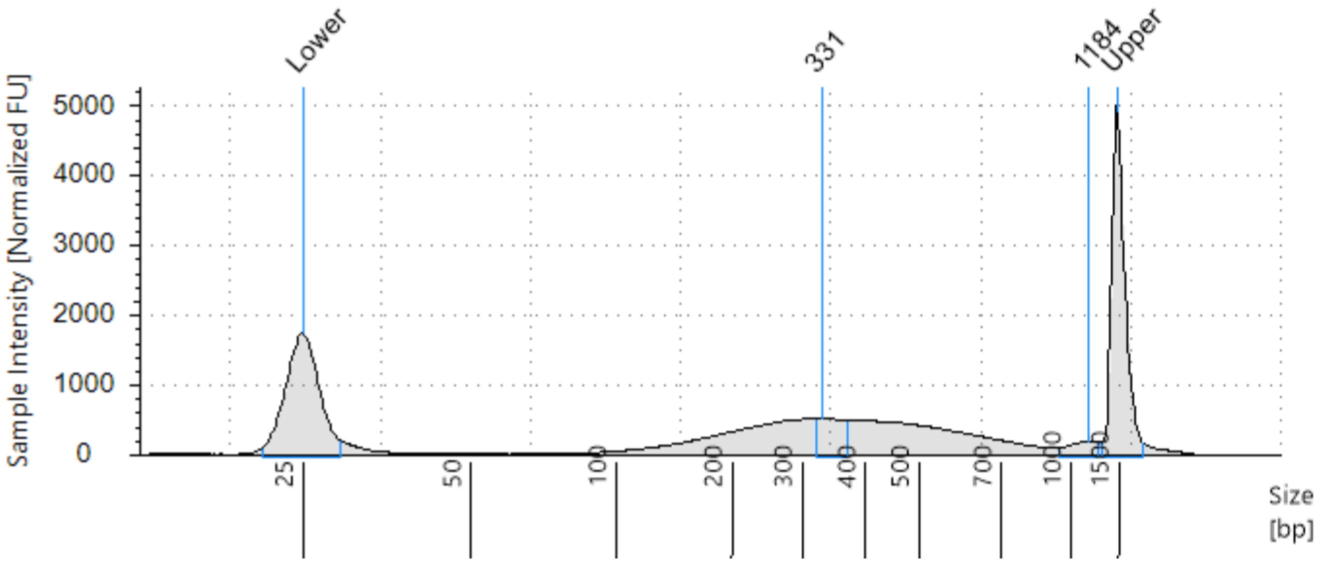

Sample Table

Well	Conc. [ng/µl]	Sample Description	Alert	Observations
B1	1.89	A1 P R1		

Peak Table

Size [bp]	Calibrated Conc. [ng/µl]	Assigned Conc. [ng/µl]	Peak Molarity [nmol/l]	% Integrated Area	Peak Comment	Observations
25	5.74	-	353	-		Lower Marker
331	1.41	-	6.53	74.16		
1184	0.490	-	0.636	25.84		
1500	6.50	6.50	6.67	-		Upper Marker

© Agilent Technologies, Inc. 2017

C1: B1 P R1

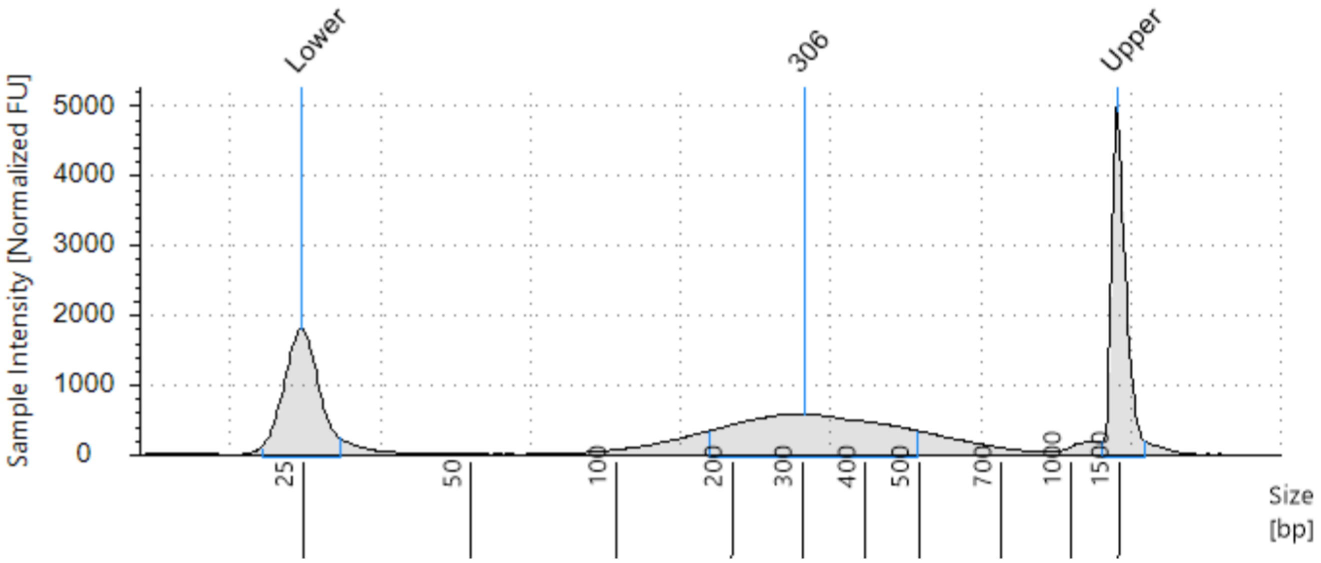

Sample Table

Well	Conc. [ng/µl]	Sample Description	Alert	Observations
C1	7.96	B1 P R1		

Peak Table

Size [bp]	Calibrated Conc. [ng/µl]	Assigned Conc. [ng/µl]	Peak Molarity [nmol/l]	% Integrated Area	Peak Comment	Observations
25	5.81	-	358	-		Lower Marker
306	7.96	-	40.1	100.00		
1500	6.50	6.50	6.67	-		Upper Marker

© Agilent Technologies, Inc. 2017

D1: A2 P R1

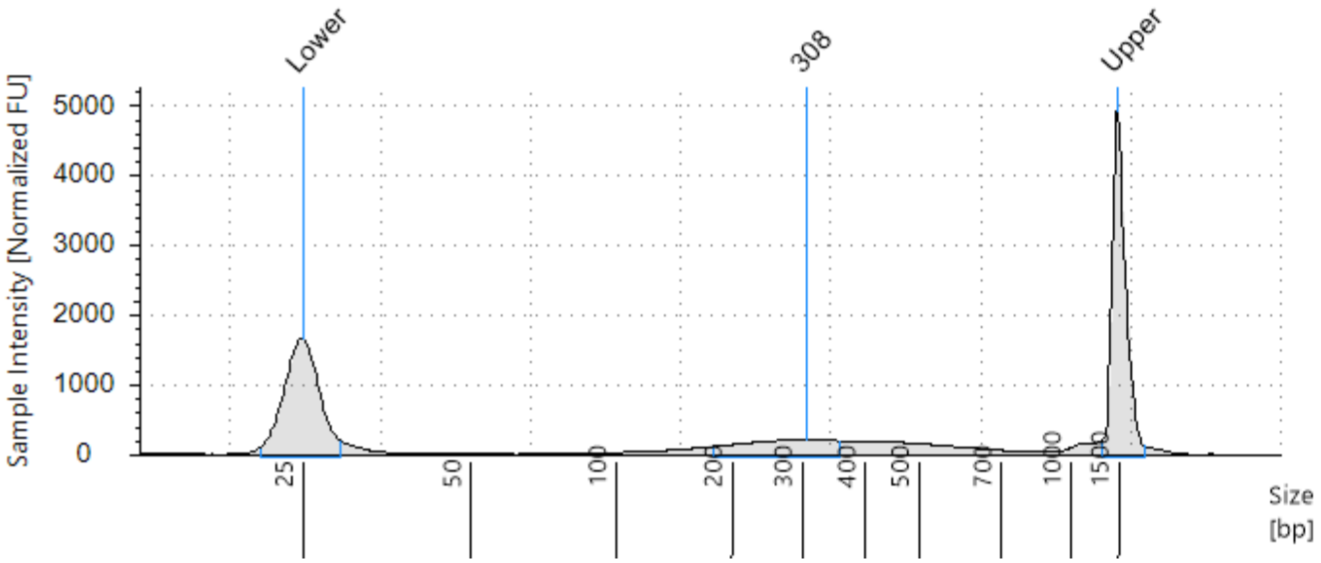

Sample Table

Well	Conc. [ng/µl]	Sample Description	Alert	Observations
D1	1.87	A2 P R1		

Peak Table

Size [bp]	Calibrated Conc. [ng/µl]	Assigned Conc. [ng/µl]	Peak Molarity [nmol/l]	% Integrated Area	Peak Comment	Observations
25	5.67	-	349	-		Lower Marker
308	1.87	-	9.35	100.00		
1500	6.50	6.50	6.67	-		Upper Marker

© Agilent Technologies, Inc. 2017

E1: B3 P R1

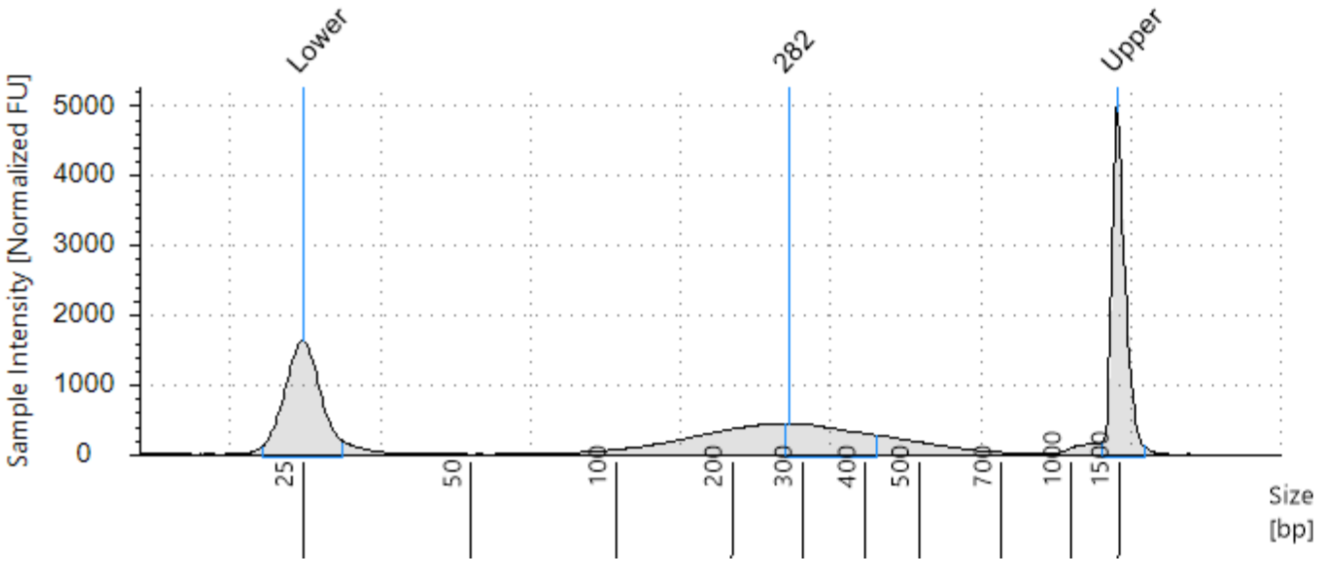

Sample Table

Well	Conc. [ng/µl]	Sample Description	Alert	Observations
E1	2.87	B3 P R1		

Peak Table

Size [bp]	Calibrated Conc. [ng/µl]	Assigned Conc. [ng/µl]	Peak Molarity [nmol/l]	% Integrated Area	Peak Comment	Observations
25	5.69	-	350	-		Lower Marker
282	2.87	-	15.7	100.00		
1500	6.50	6.50	6.67	-		Upper Marker

© Agilent Technologies, Inc. 2017

F1: A8 P R1

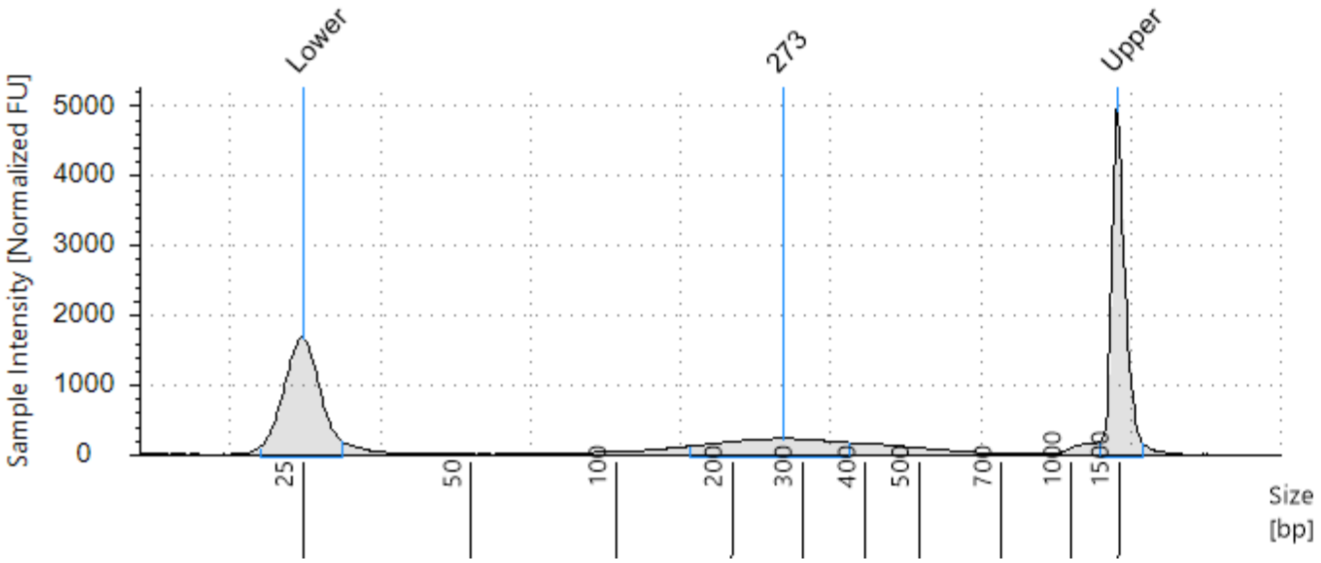

Sample Table

Well	Conc. [ng/µl]	Sample Description	Alert	Observations
F1	2.53	A8 P R1		

Peak Table

Size [bp]	Calibrated Conc. [ng/µl]	Assigned Conc. [ng/µl]	Peak Molarity [nmol/l]	% Integrated Area	Peak Comment	Observations
25	5.89	-	362	-		Lower Marker
273	2.53	-	14.2	100.00		
1500	6.50	6.50	6.67	-		Upper Marker

© Agilent Technologies, Inc. 2017

G1: E9 P R1

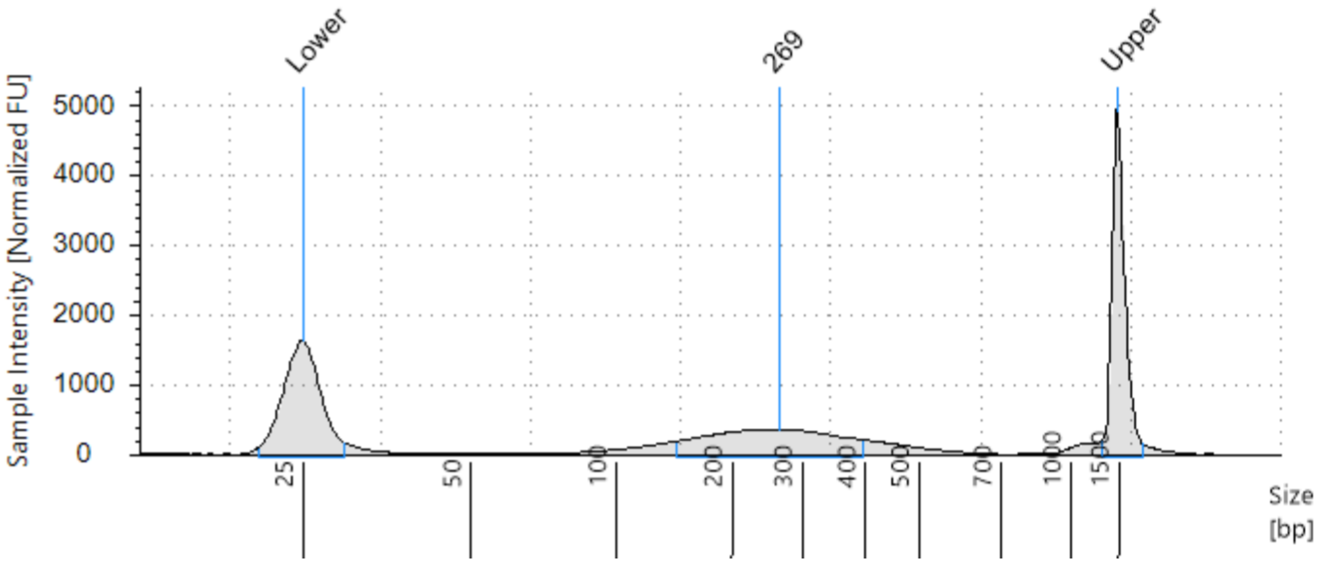

Sample Table

Well	Conc. [ng/µl]	Sample Description	Alert	Observations
G1	4.80	E9 P R1		

Peak Table

Size [bp]	Calibrated Conc. [ng/µl]	Assigned Conc. [ng/µl]	Peak Molarity [nmol/l]	% Integrated Area	Peak Comment	Observations
25	6.15	-	379	-		Lower Marker
269	4.80	-	27.5	100.00		
1500	6.50	6.50	6.67	-		Upper Marker

© Agilent Technologies, Inc. 2017

H1: H9 P R1

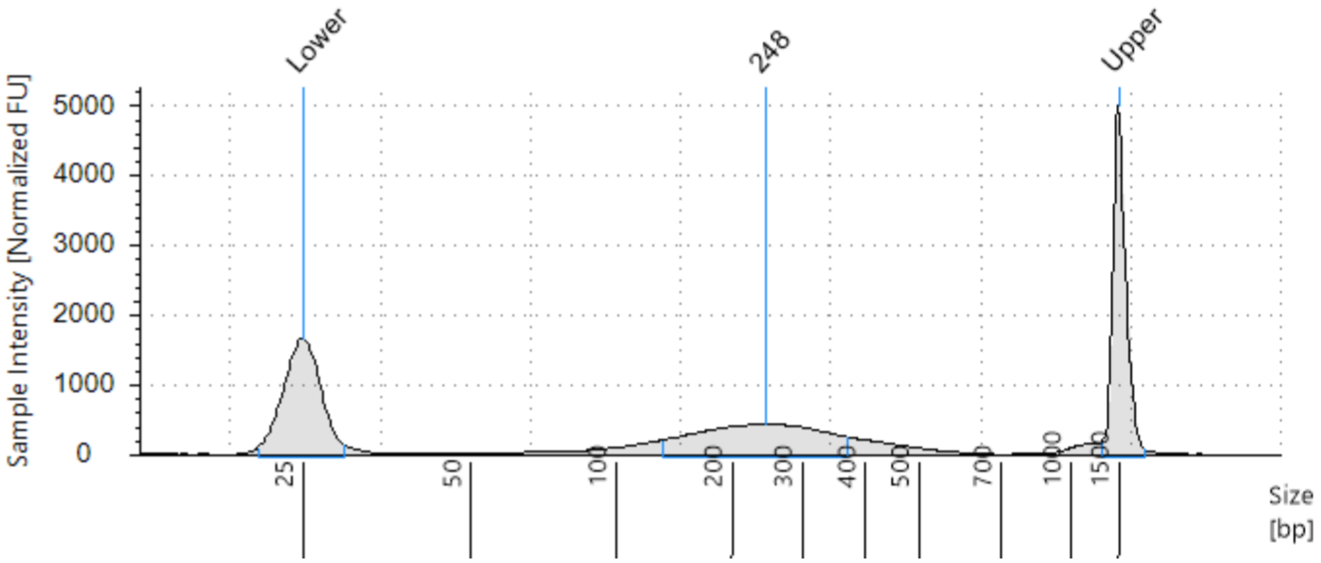

Sample Table

Well	Conc. [ng/µl]	Sample Description	Alert	Observations
H1	5.53	H9 P R1		

Peak Table

Size [bp]	Calibrated Conc. [ng/µl]	Assigned Conc. [ng/µl]	Peak Molarity [nmol/l]	% Integrated Area	Peak Comment	Observations
25	6.24	-	384	-		Lower Marker
248	5.53	-	34.3	100.00		
1500	6.50	6.50	6.67	-		Upper Marker

© Agilent Technologies, Inc. 2017

A2: H9 M R1

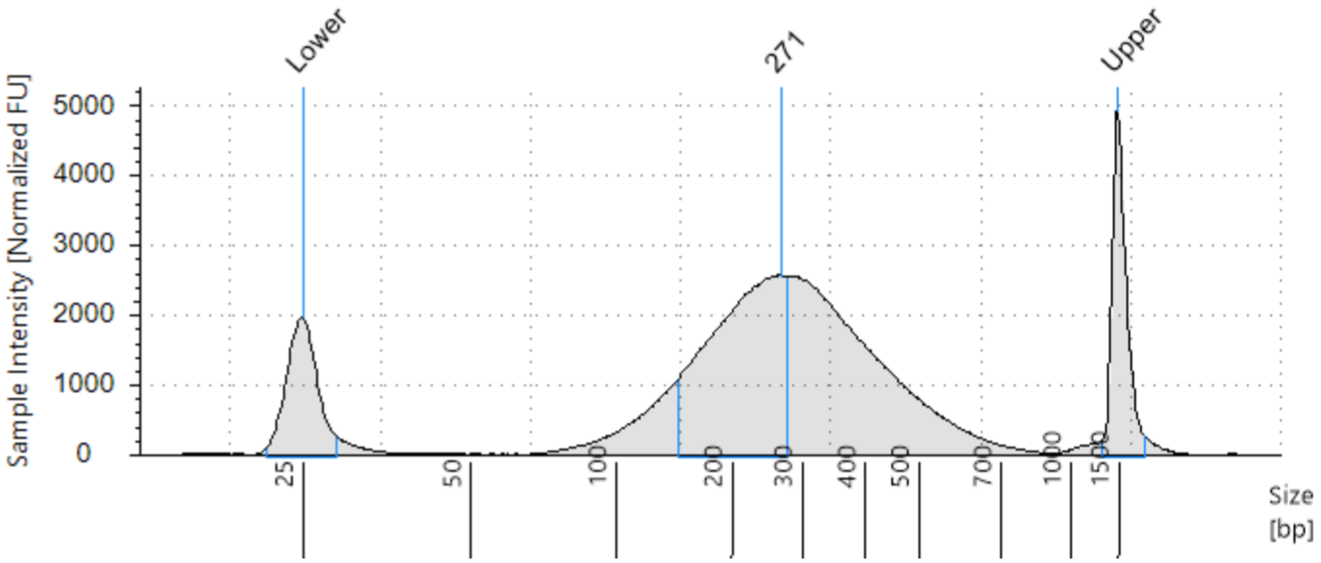

Sample Table

Well	Conc. [ng/µl]	Sample Description	Alert	Observations
A2	17.3	H9 M R1		

Peak Table

Size [bp]	Calibrated Conc. [ng/µl]	Assigned Conc. [ng/µl]	Peak Molarity [nmol/l]	% Integrated Area	Peak Comment	Observations
25	5.55	-	342	-		Lower Marker
271	17.3	-	98.2	100.00		
1500	6.50	6.50	6.67	-		Upper Marker

© Agilent Technologies, Inc. 2017

B2: A11 M R2

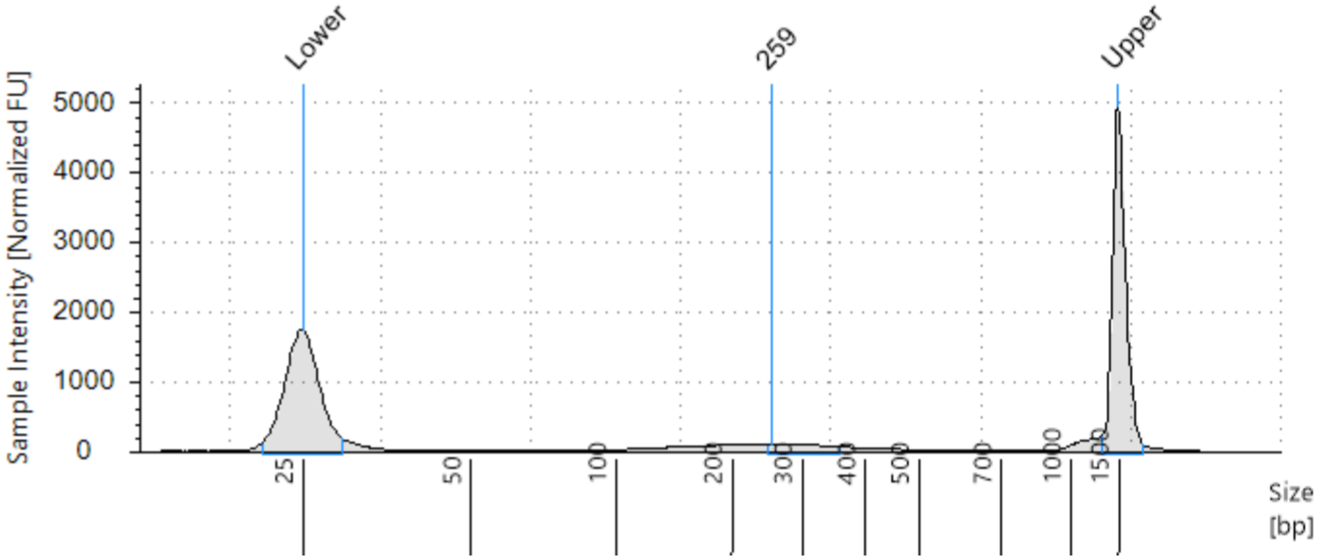

Sample Table

Well	Conc. [ng/µl]	Sample Description	Alert	Observations
B2	0.579	A11 M R2		

Peak Table

Size [bp]	Calibrated Conc. [ng/µl]	Assigned Conc. [ng/µl]	Peak Molarity [nmol/l]	% Integrated Area	Peak Comment	Observations
25	6.00	-	369	-		Lower Marker
259	0.579	-	3.45	100.00		
1500	6.50	6.50	6.67	-		Upper Marker

© Agilent Technologies, Inc. 2017

C2: B11 M R2

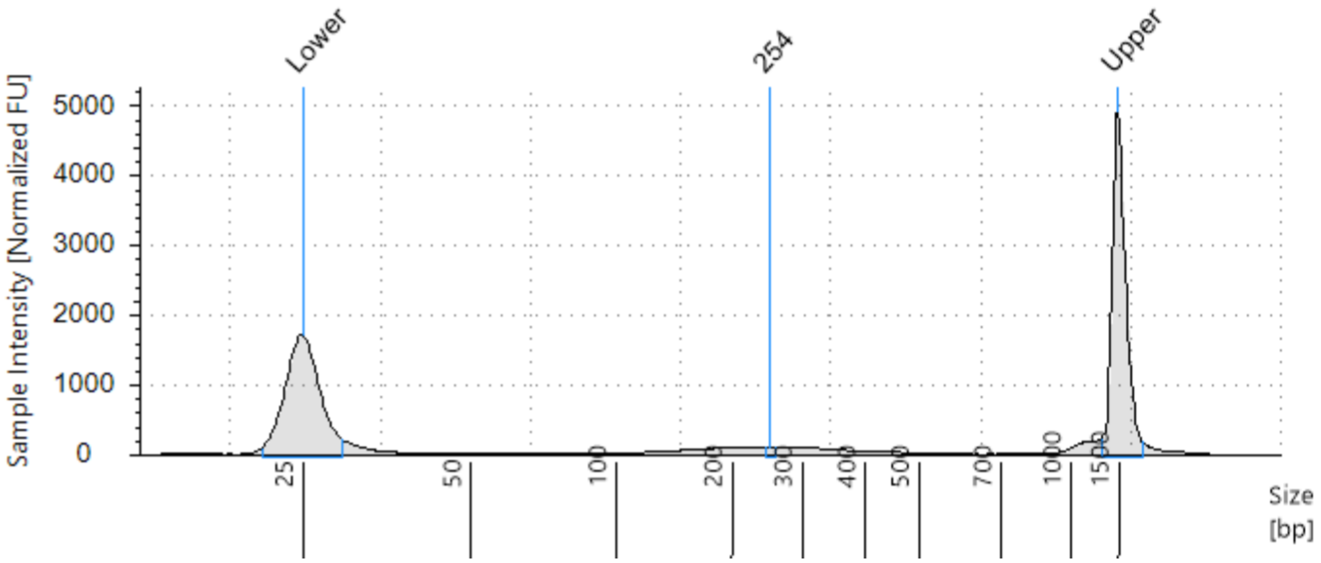

Sample Table

Well	Conc. [ng/µl]	Sample Description	Alert	Observations
C2	0.0893	B11 M R2		

Peak Table

Size [bp]	Calibrated Conc. [ng/µl]	Assigned Conc. [ng/µl]	Peak Molarity [nmol/l]	% Integrated Area	Peak Comment	Observations
25	5.75	-	354	-		Lower Marker
254	0.0893	-	0.541	100.00		
1500	6.50	6.50	6.67	-		Upper Marker

D2: C11 M R2

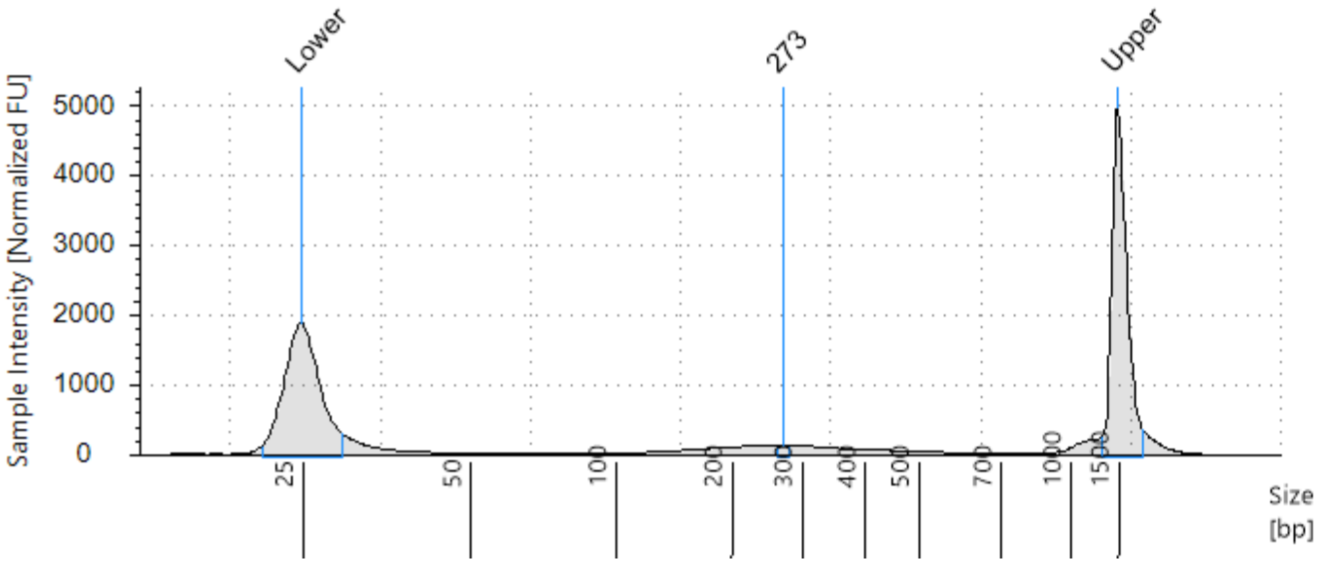

Sample Table

Well	Conc. [ng/µl]	Sample Description	Alert	Observations
D2	0.109	C11 M R2		

Peak Table

Size [bp]	Calibrated Conc. [ng/µl]	Assigned Conc. [ng/µl]	Peak Molarity [nmol/l]	% Integrated Area	Peak Comment	Observations
25	5.81	-	358	-		Lower Marker
273	0.109	-	0.612	100.00		
1500	6.50	6.50	6.67	-		Upper Marker

© Agilent Technologies, Inc. 2017

E2: D11 M R2

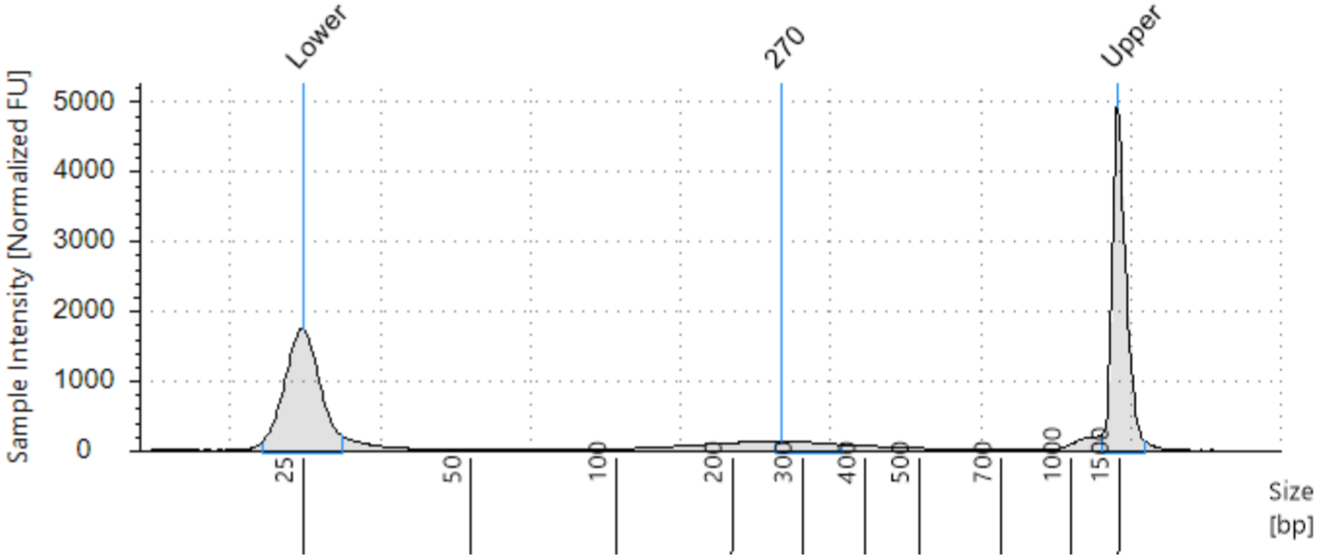

Sample Table

Well	Conc. [ng/µl]	Sample Description	Alert	Observations
E2	0.603	D11 M R2		

Peak Table

Size [bp]	Calibrated Conc. [ng/µl]	Assigned Conc. [ng/µl]	Peak Molarity [nmol/l]	% Integrated Area	Peak Comment	Observations
25	5.82	-	358	-		Lower Marker
270	0.603	-	3.43	100.00		
1500	6.50	6.50	6.67	-		Upper Marker

F2: E11 M R2

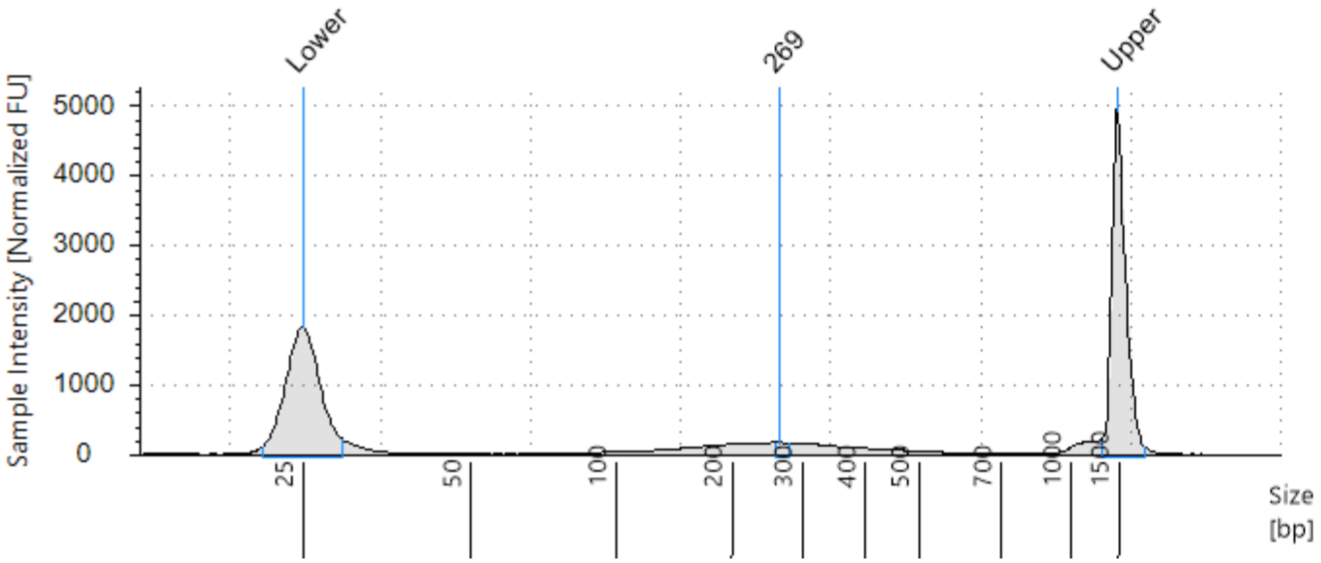

Sample Table

Well	Conc. [ng/µl]	Sample Description	Alert	Observations
F2	0.193	E11 M R2		

Peak Table

Size [bp]	Calibrated Conc. [ng/µl]	Assigned Conc. [ng/µl]	Peak Molarity [nmol/l]	% Integrated Area	Peak Comment	Observations
25	5.97	-	367	-		Lower Marker
269	0.193	-	1.10	100.00		
1500	6.50	6.50	6.67	-		Upper Marker

© Agilent Technologies, Inc. 2017

G2: F11 M R2

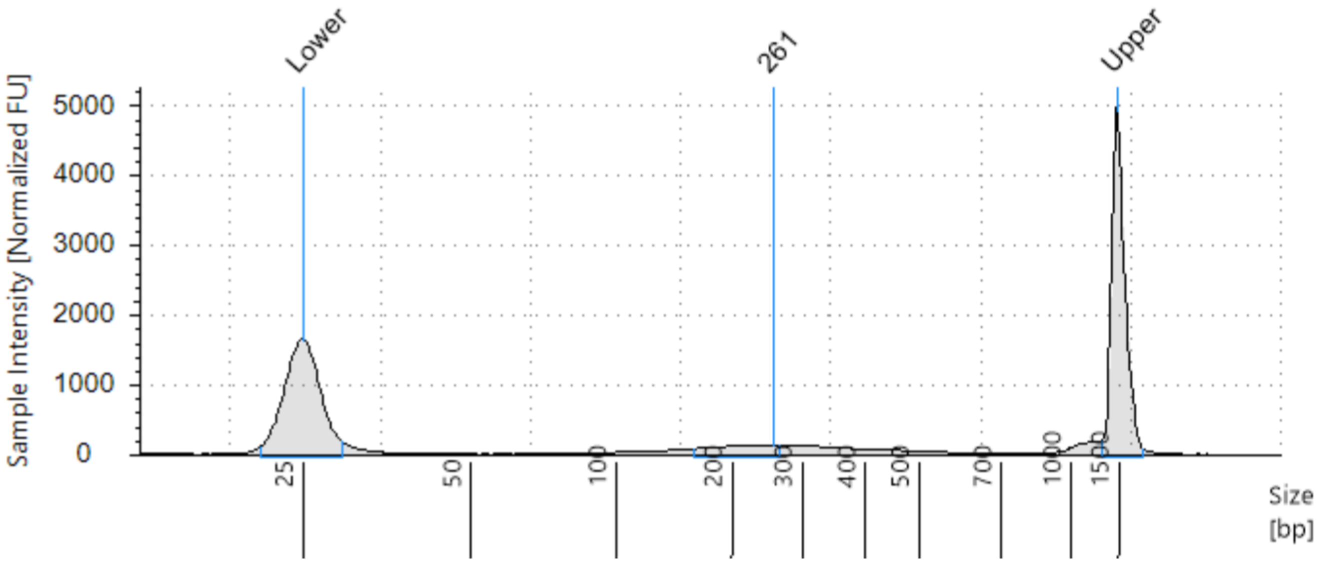

Sample Table

Well	Conc. [ng/µl]	Sample Description	Alert	Observations
G2	0.835	F11 M R2		

Peak Table

Size [bp]	Calibrated Conc. [ng/µl]	Assigned Conc. [ng/µl]	Peak Molarity [nmol/l]	% Integrated Area	Peak Comment	Observations
25	5.86	-	361	-		Lower Marker
261	0.835	-	4.93	100.00		
1500	6.50	6.50	6.67	-		Upper Marker

© Agilent Technologies, Inc. 2017

H2: F12 M R2

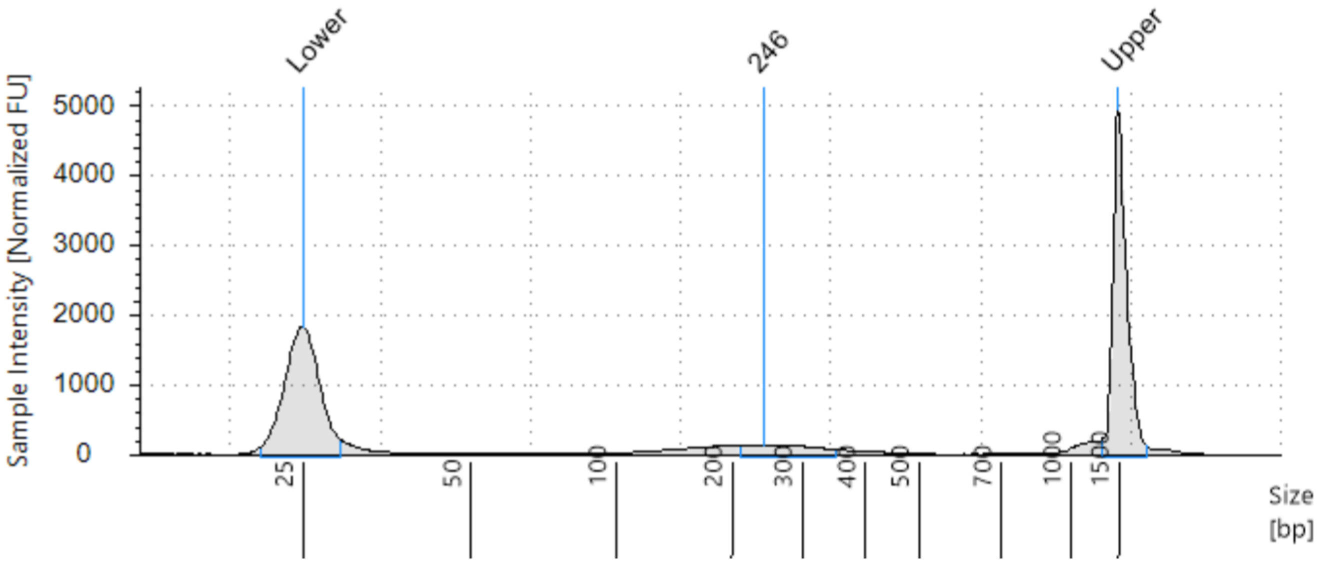

Sample Table

Well	Conc. [ng/µl]	Sample Description	Alert	Observations
H2	0.928	F12 M R2		

Peak Table

Size [bp]	Calibrated Conc. [ng/µl]	Assigned Conc. [ng/µl]	Peak Molarity [nmol/l]	% Integrated Area	Peak Comment	Observations
25	6.13	-	377	-		Lower Marker
246	0.928	-	5.81	100.00		
1500	6.50	6.50	6.67	-		Upper Marker

 © Agilent Technologies, Inc. 2017
