# Supplementary material for: 96 sample parallel acoustic fragmentation for high throughput next generation sequencing library preparation
Source: PLoS One. 2026 Feb 17;21(2):e0341139. doi: 10.1371/journal.pone.0341139 (PMC12912608; doi:10.1371/journal.pone.0341139)
Supplement: S5 File — (ZIP) [file pone.0341139.s005.zip › QSonica translator TapeStation raw data/QSonica no cavitation enhancement translator 96-well plate replicate 1.pdf]

Filename: 2020-03-06-03-.D5000.Q-S, DFB minus from 3.8.20 A1-G2 R1.D5000

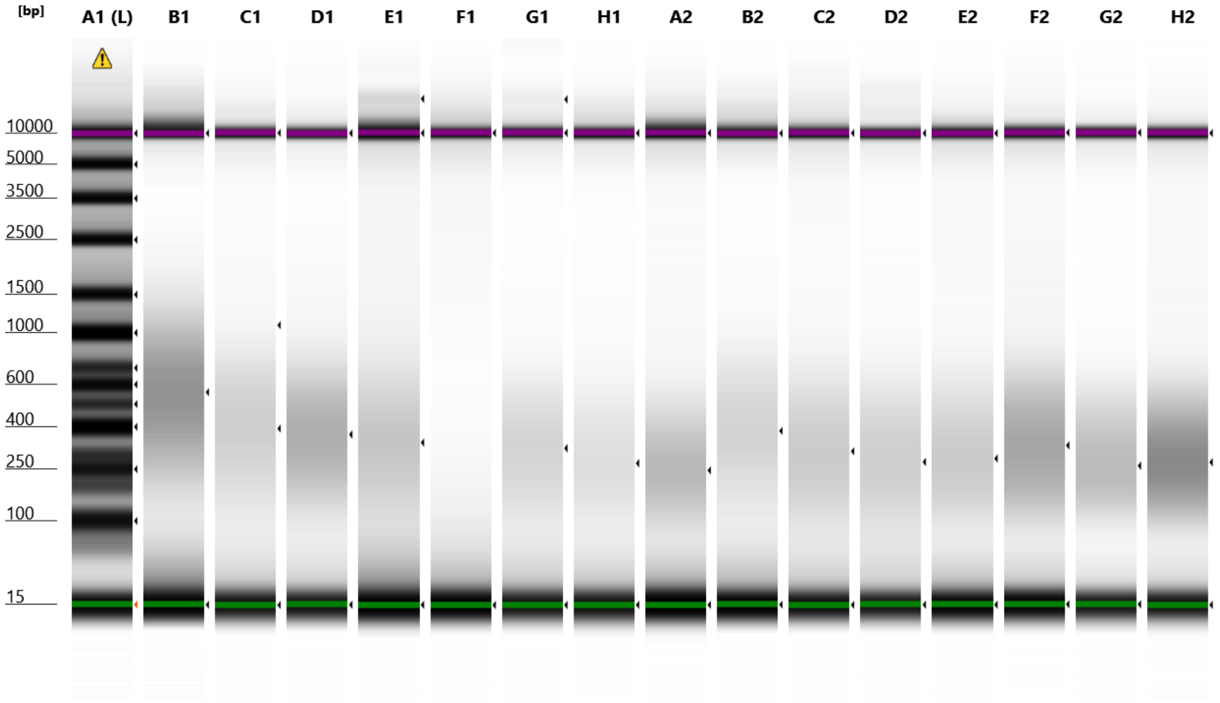

Default image (Contrast 100%)

Sample Info

Well	Conc. [ng/µl]	Sample Description	Alert	Observations
A1	62.9	Ladder	⚠	Issue with ladder peak detection (too many peaks detected); Ladder
B1	0.606	A1 minus R1		
C1	0.225	B1 minus R1		
D1	0.600	C1 minus R1		
E1	1.01	D1 minus R1		
F1		E1 minus R1		
G1	0.500	F1 minus R1		
H1	0.205	G1 minus R1		
A2	0.411	H1 minus R1		
B2	0.252	A2 minus R1		
C2	0.327	B2 minus R1		
D2	0.333	C2 minus R1		
E2	0.360	D2 minus R1		
F2	0.571	E2 minus R1		
G2	0.434	F2 minus R1		
H2	4.57	G2 minus R1		

 © Agilent Technologies, Inc. 2017

A1: Ladder

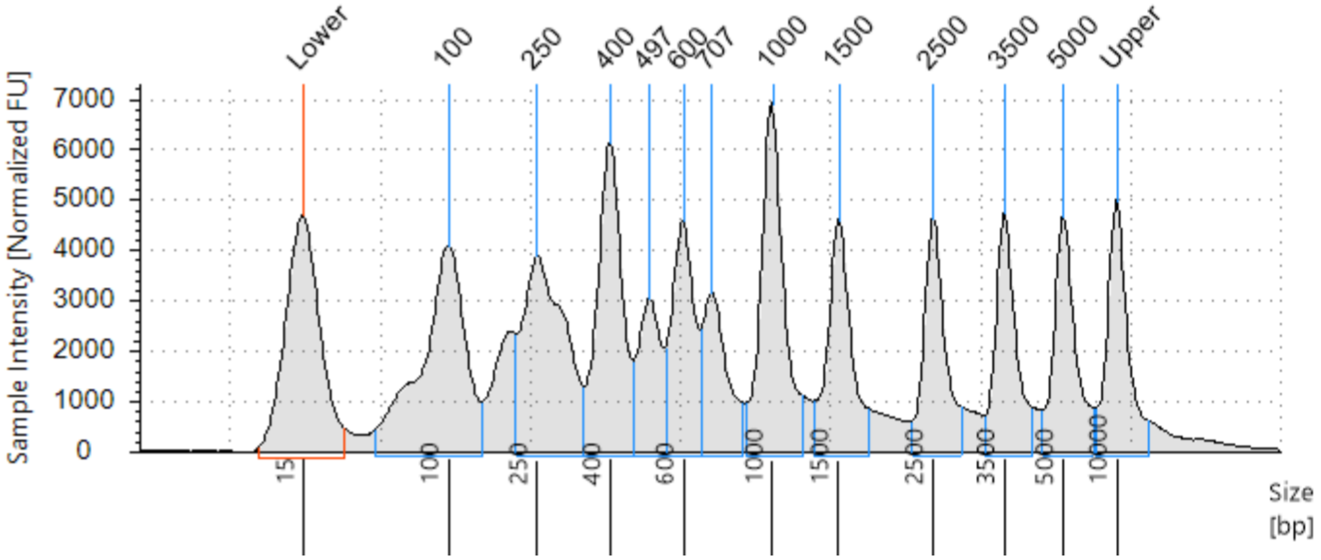

Sample Table

Well	Conc. [ng/µl]	Sample Description	Alert	Observations
A1	62.9	Ladder	⚠	Issue with ladder peak detection (too many peaks detected); Ladder

Peak Table

Size [bp]	Calibrated Conc. [ng/µl]	Assigned Conc. [ng/µl]	Peak Molarity [nmol/l]	% Integrated Area	Peak Comment	Observations
15	6.69	-	686	-		Lower Marker
100	8.53	-	131	13.55		
250	7.93	-	48.8	12.60		
400	7.36	-	28.3	11.69		
497	3.40	-	10.5	5.40		
600	5.10	-	13.1	8.09		
707	3.70	-	8.05	5.87		
1000	7.58	-	11.7	12.05		
1500	5.20	-	5.34	8.27		
2500	4.66	-	2.87	7.41		
3500	4.66	-	2.05	7.41		
5000	4.82	-	1.48	7.66		
10000	3.25	3.25	0.500	-		Upper Marker

© Agilent Technologies, Inc. 2017

B1: A1 minus R1

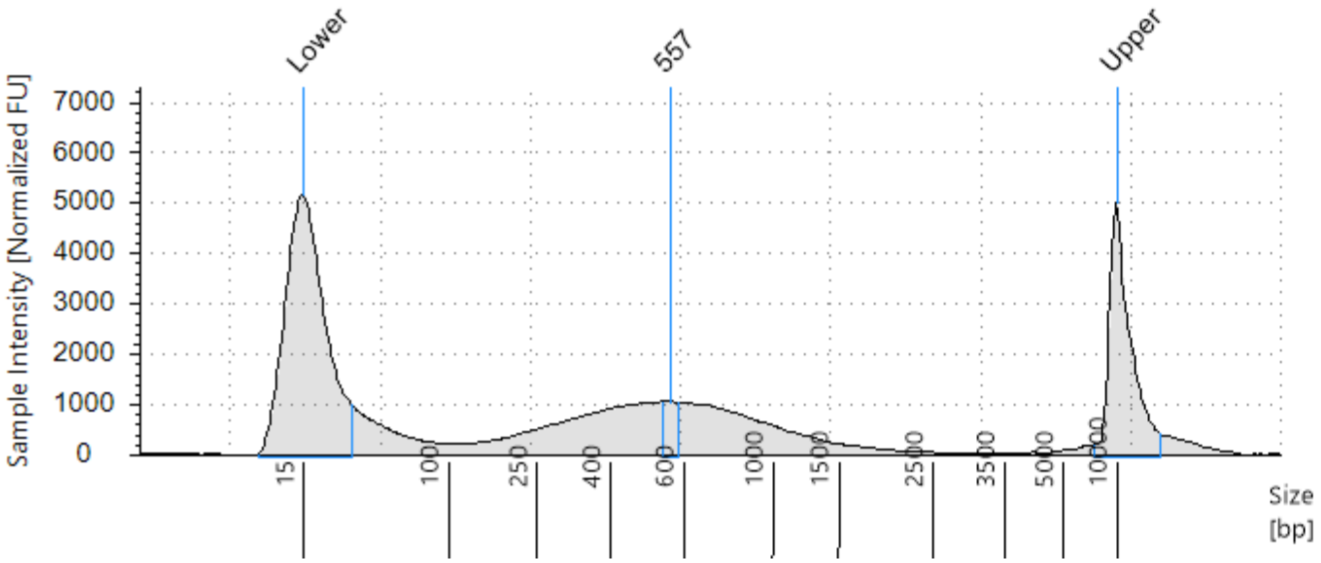

Sample Table

Well	Conc. [ng/µl]	Sample Description	Alert	Observations
B1	0.606	A1 minus R1		

Peak Table

Size [bp]	Calibrated Conc. [ng/µl]	Assigned Conc. [ng/µl]	Peak Molarity [nmol/l]	% Integrated Area	Peak Comment	Observations
15	6.18	-	633	-		Lower Marker
557	0.606	-	1.67	100.00		
10000	3.25	3.25	0.500	-		Upper Marker

© Agilent Technologies, Inc. 2017

C1: B1 minus R1

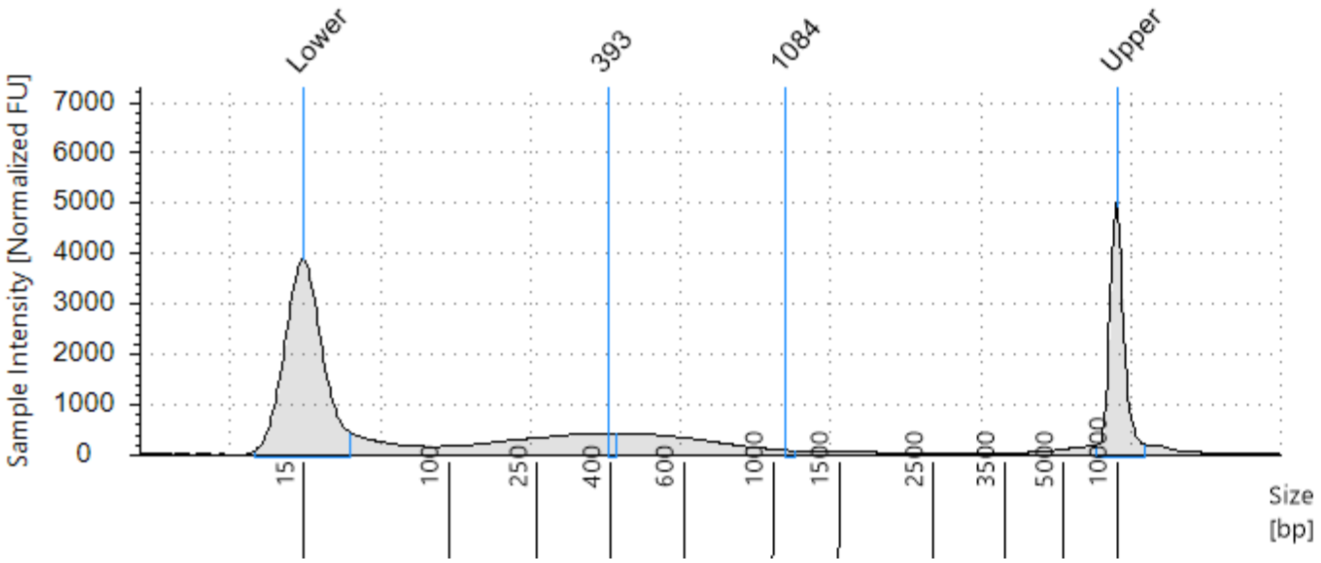

Sample Table

Well	Conc. [ng/µl]	Sample Description	Alert	Observations
C1	0.225	B1 minus R1		

Peak Table

Size [bp]	Calibrated Conc. [ng/µl]	Assigned Conc. [ng/µl]	Peak Molarity [nmol/l]	% Integrated Area	Peak Comment	Observations
15	6.42	-	658	-		Lower Marker
393	0.186	-	0.730	82.64		
1084	0.0391	-	0.0555	17.36		
10000	3.25	3.25	0.500	-		Upper Marker

© Agilent Technologies, Inc. 2017

D1: C1 minus R1

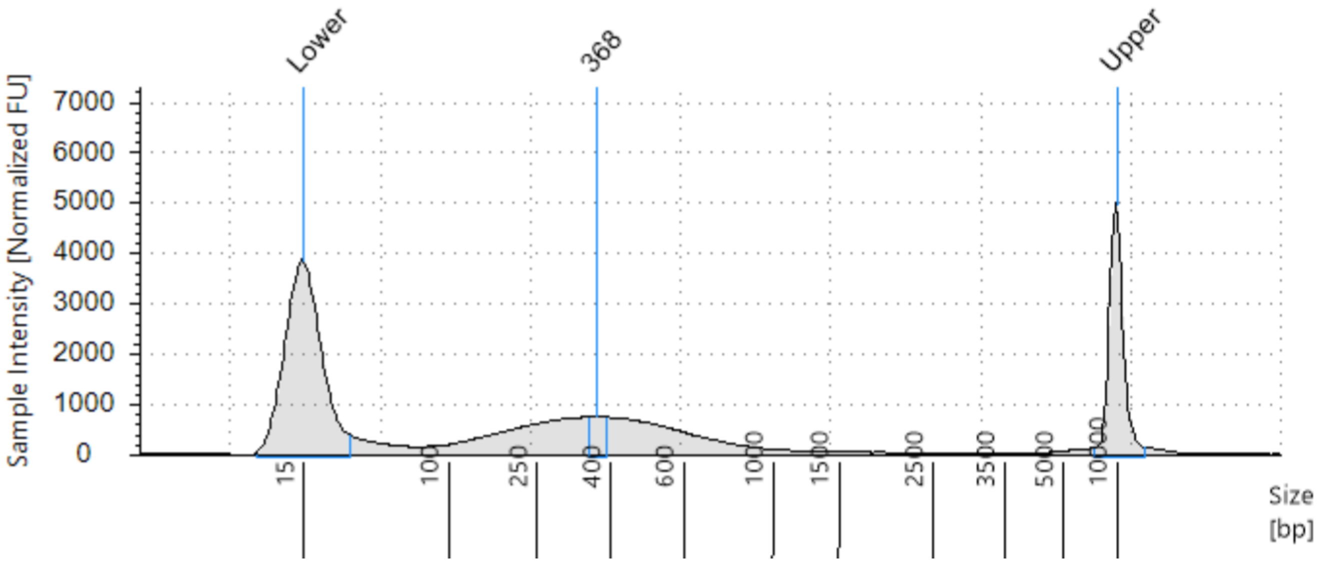

Sample Table

Well	Conc. [ng/µl]	Sample Description	Alert	Observations
D1	0.600	C1 minus R1		

Peak Table

Size [bp]	Calibrated Conc. [ng/µl]	Assigned Conc. [ng/µl]	Peak Molarity [nmol/l]	% Integrated Area	Peak Comment	Observations
15	6.32	-	649	-		Lower Marker
368	0.600	-	2.51	100.00		
10000	3.25	3.25	0.500	-		Upper Marker

© Agilent Technologies, Inc. 2017

E1: D1 minus R1

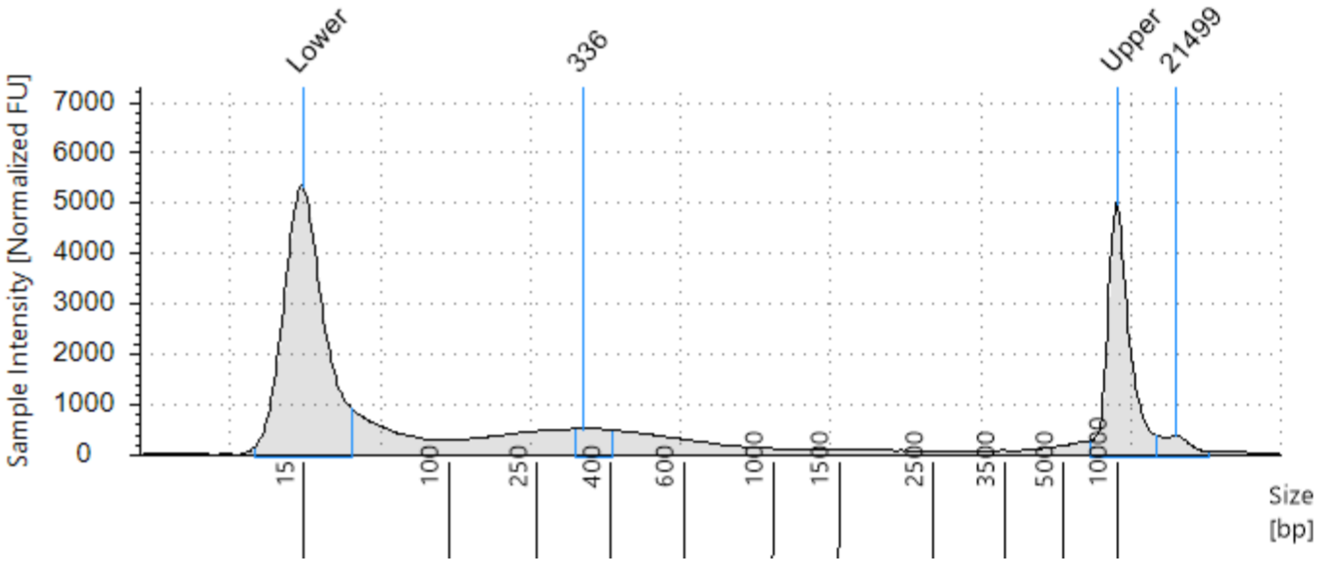

Sample Table

Well	Conc. [ng/µl]	Sample Description	Alert	Observations
E1	1.01	D1 minus R1		

Peak Table

Size [bp]	Calibrated Conc. [ng/µl]	Assigned Conc. [ng/µl]	Peak Molarity [nmol/l]	% Integrated Area	Peak Comment	Observations
15	6.16	-	632	-		Lower Marker
336	0.605	-	2.77	60.07		
10000	3.25	3.25	0.500	-		Upper Marker
21499	0.402	-	0.0288	39.93		

© Agilent Technologies, Inc. 2017

F1: E1 minus R1

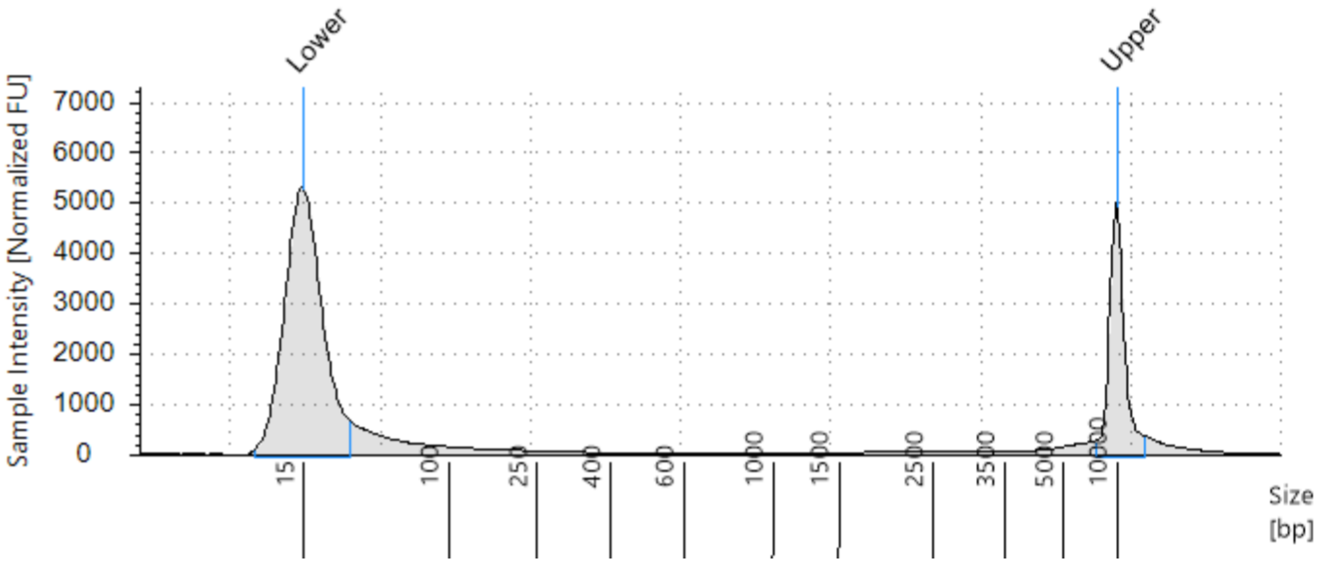

Sample Table

Well	Conc. [ng/µl]	Sample Description	Alert	Observations
F1		E1 minus R1		

Peak Table

Size [bp]	Calibrated Conc. [ng/µl]	Assigned Conc. [ng/µl]	Peak Molarity [nmol/l]	% Integrated Area	Peak Comment	Observations
15	8.99	-	922	-		Lower Marker
10000	3.25	3.25	0.500	-		Upper Marker

© Agilent Technologies, Inc. 2017

G1: F1 minus R1

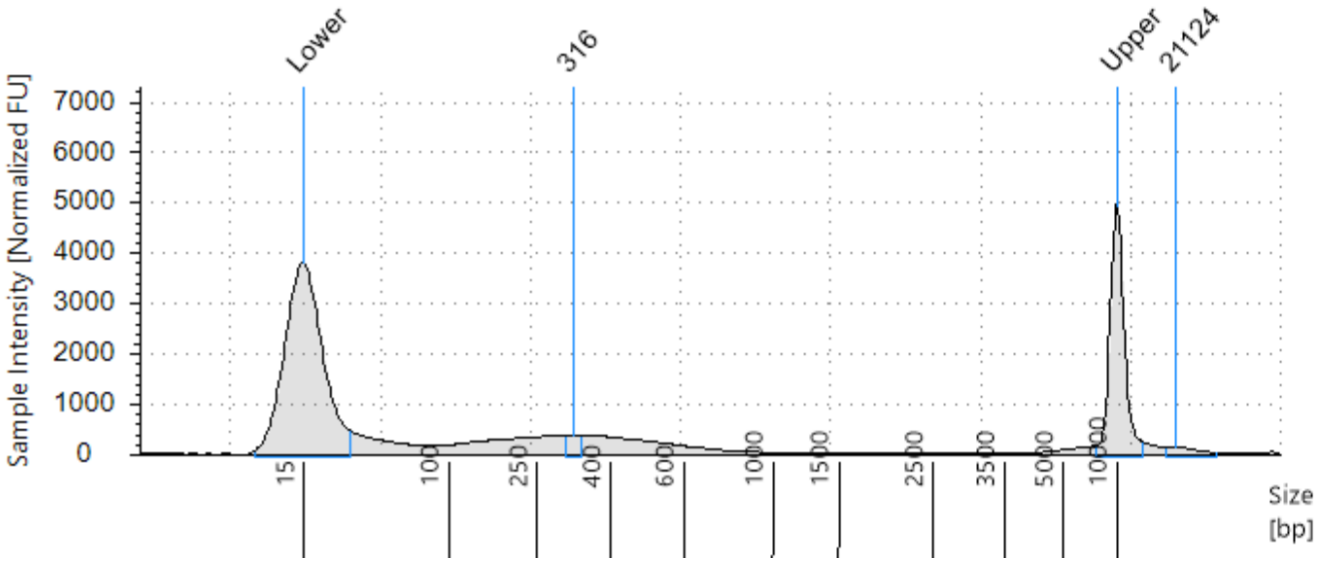

Sample Table

Well	Conc. [ng/µl]	Sample Description	Alert	Observations
G1	0.500	F1 minus R1		

Peak Table

Size [bp]	Calibrated Conc. [ng/µl]	Assigned Conc. [ng/µl]	Peak Molarity [nmol/l]	% Integrated Area	Peak Comment	Observations
15	6.40	-	656	-		Lower Marker
316	0.276	-	1.35	55.30		
10000	3.25	3.25	0.500	-		Upper Marker
21124	0.223	-	0.0163	44.70		

© Agilent Technologies, Inc. 2017

H1: G1 minus R1

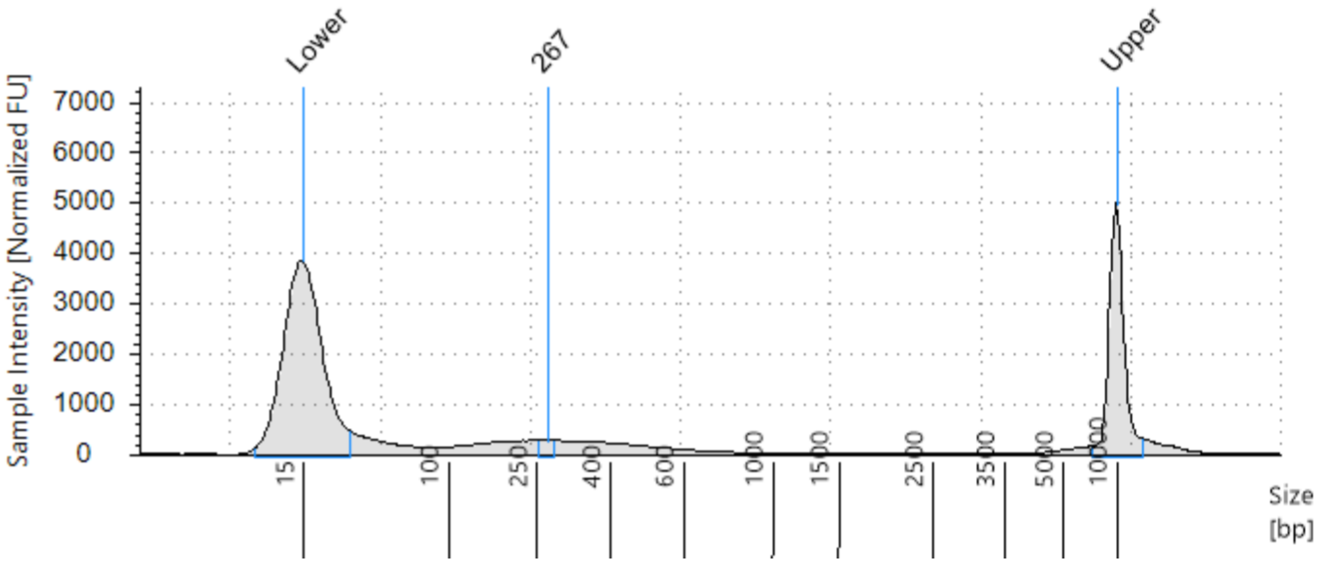

Sample Table

Well	Conc. [ng/µl]	Sample Description	Alert	Observations
H1	0.205	G1 minus R1		

Peak Table

Size [bp]	Calibrated Conc. [ng/µl]	Assigned Conc. [ng/µl]	Peak Molarity [nmol/l]	% Integrated Area	Peak Comment	Observations
15	6.49	-	666	-		Lower Marker
267	0.205	-	1.18	100.00		
10000	3.25	3.25	0.500	-		Upper Marker

© Agilent Technologies, Inc. 2017

A2: H1 minus R1

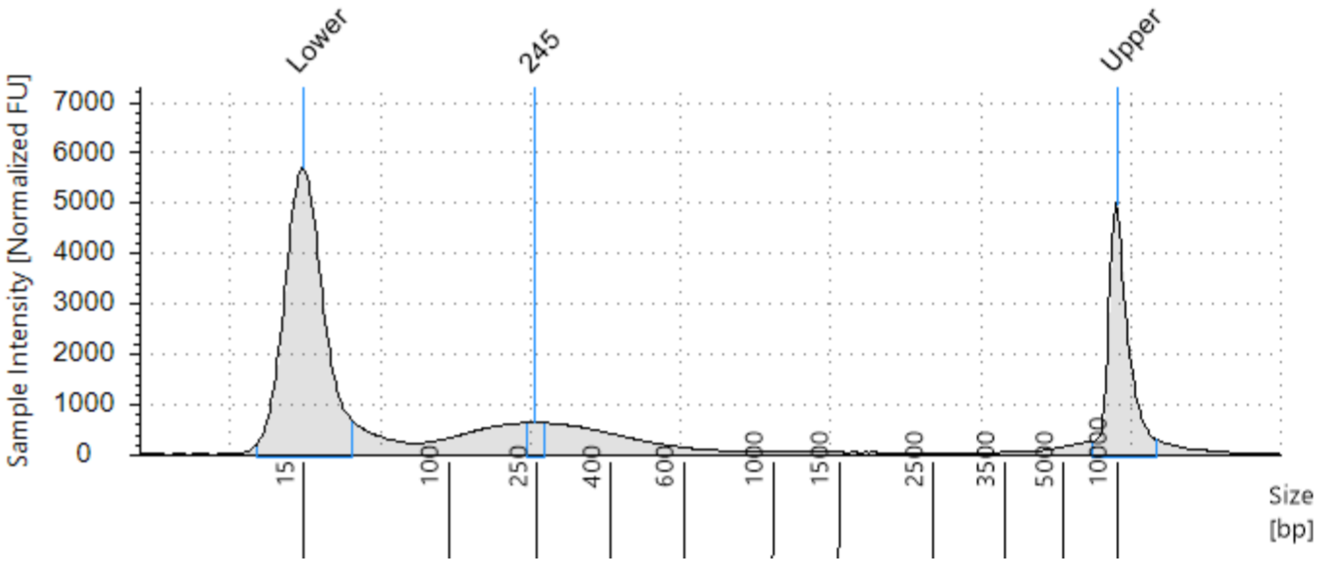

Sample Table

Well	Conc. [ng/µl]	Sample Description	Alert	Observations
A2	0.411	H1 minus R1		

Peak Table

Size [bp]	Calibrated Conc. [ng/µl]	Assigned Conc. [ng/µl]	Peak Molarity [nmol/l]	% Integrated Area	Peak Comment	Observations
15	7.33	-	752	-		Lower Marker
245	0.411	-	2.58	100.00		
10000	3.25	3.25	0.500	-		Upper Marker

 © Agilent Technologies, Inc. 2017

B2: A2 minus R1

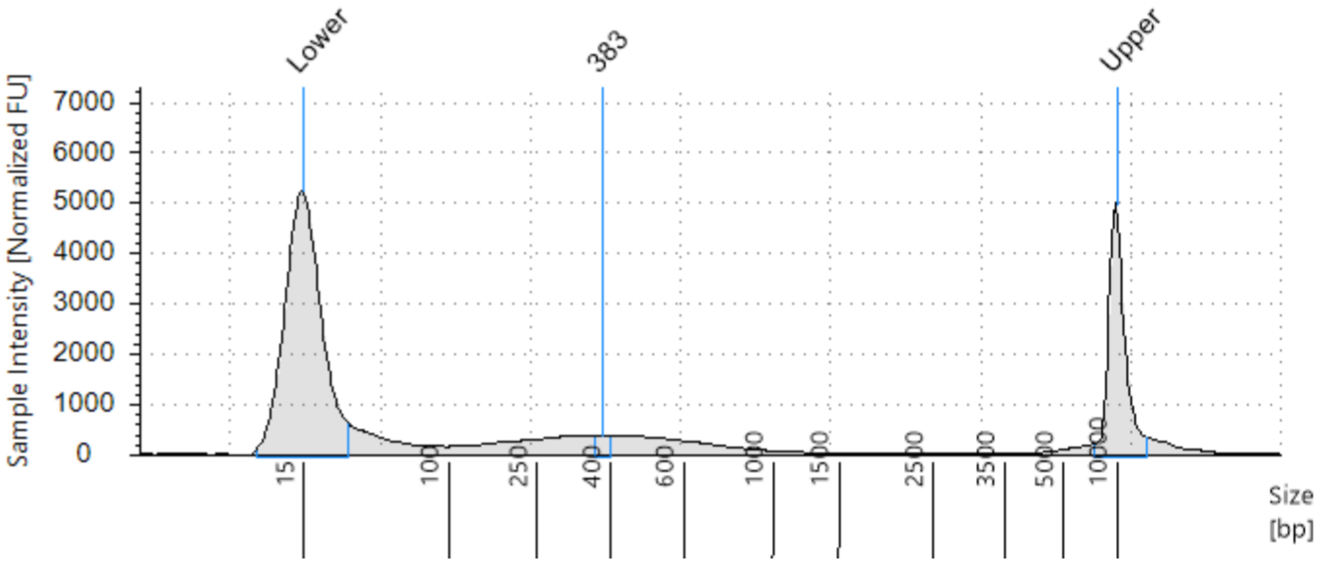

Sample Table

Well	Conc. [ng/µl]	Sample Description	Alert	Observations
B2	0.252	A2 minus R1		

Peak Table

Size [bp]	Calibrated Conc. [ng/µl]	Assigned Conc. [ng/µl]	Peak Molarity [nmol/l]	% Integrated Area	Peak Comment	Observations
15	7.76	-	796	-		Lower Marker
383	0.252	-	1.01	100.00		
10000	3.25	3.25	0.500	-		Upper Marker

 © Agilent Technologies, Inc. 2017

C2: B2 minus R1

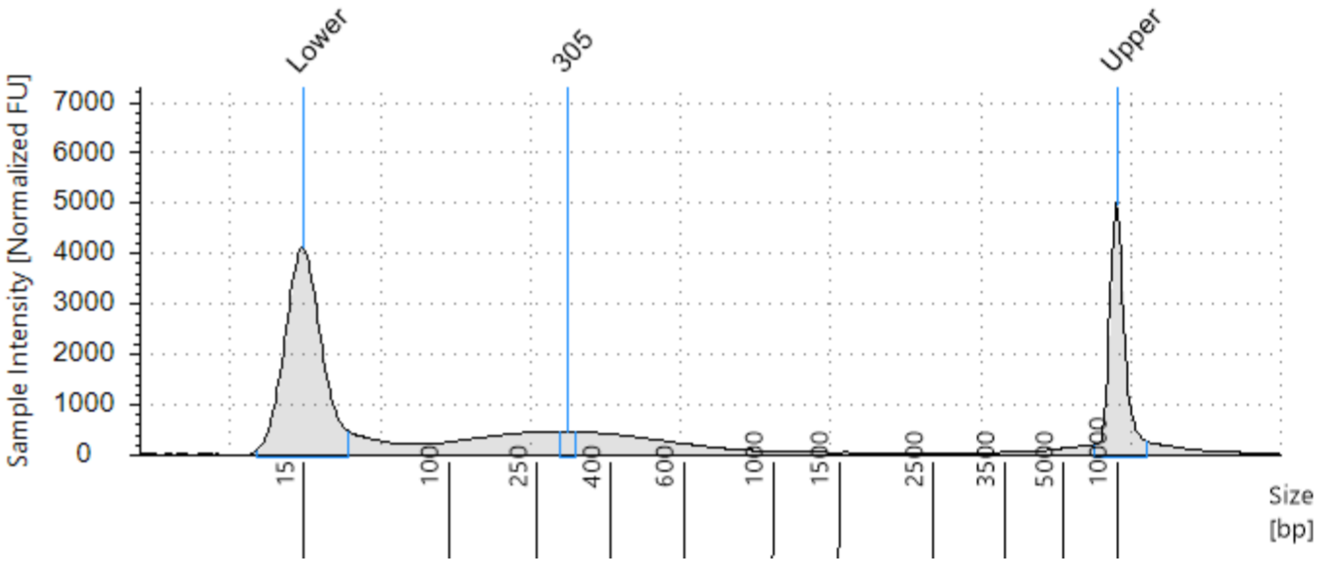

Sample Table

Well	Conc. [ng/µl]	Sample Description	Alert	Observations
C2	0.327	B2 minus R1		

Peak Table

Size [bp]	Calibrated Conc. [ng/µl]	Assigned Conc. [ng/µl]	Peak Molarity [nmol/l]	% Integrated Area	Peak Comment	Observations
15	6.36	-	653	-		Lower Marker
305	0.327	-	1.64	100.00		
10000	3.25	3.25	0.500	-		Upper Marker

© Agilent Technologies, Inc. 2017

D2: C2 minus R1

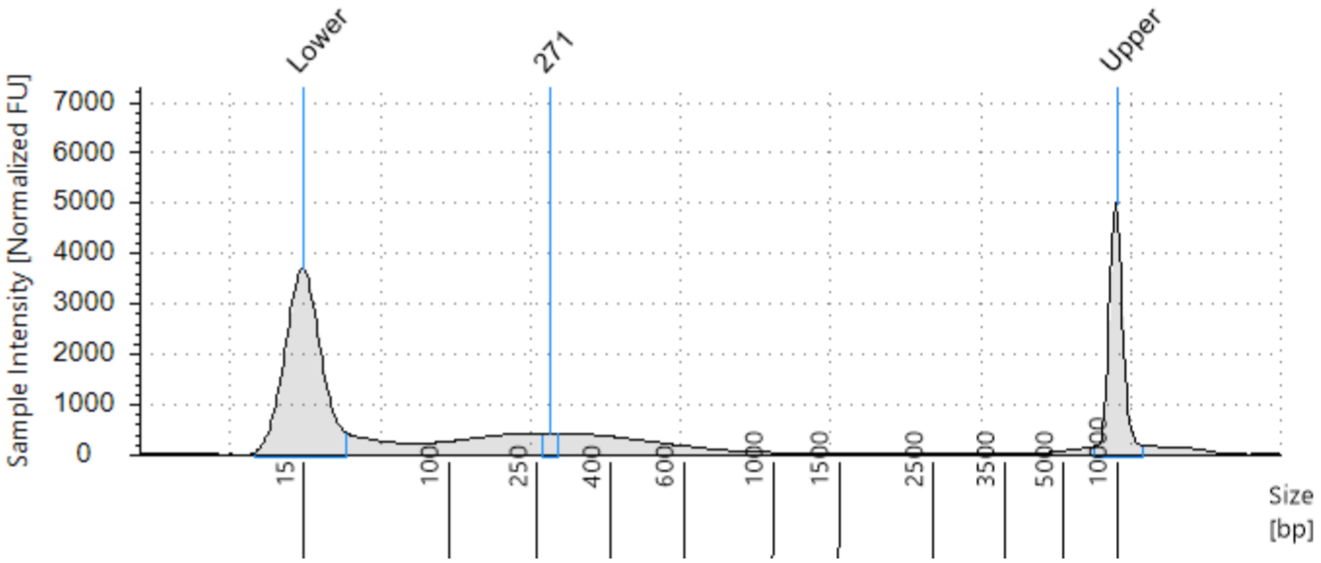

Sample Table

Well	Conc. [ng/µl]	Sample Description	Alert	Observations
D2	0.333	C2 minus R1		

Peak Table

Size [bp]	Calibrated Conc. [ng/µl]	Assigned Conc. [ng/µl]	Peak Molarity [nmol/l]	% Integrated Area	Peak Comment	Observations
15	5.95	-	611	-		Lower Marker
271	0.333	-	1.89	100.00		
10000	3.25	3.25	0.500	-		Upper Marker

© Agilent Technologies, Inc. 2017

E2: D2 minus R1

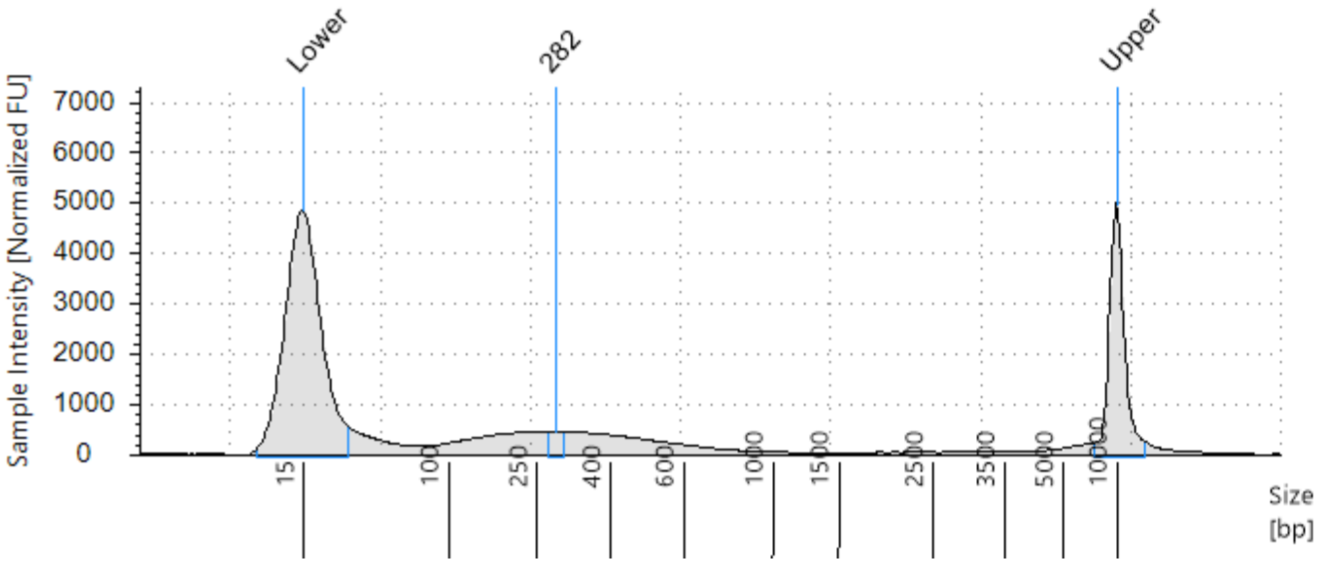

Sample Table

Well	Conc. [ng/µl]	Sample Description	Alert	Observations
E2	0.360	D2 minus R1		

Peak Table

Size [bp]	Calibrated Conc. [ng/µl]	Assigned Conc. [ng/µl]	Peak Molarity [nmol/l]	% Integrated Area	Peak Comment	Observations
15	7.57	-	777	-		Lower Marker
282	0.360	-	1.97	100.00		
10000	3.25	3.25	0.500	-		Upper Marker

© Agilent Technologies, Inc. 2017

F2: E2 minus R1

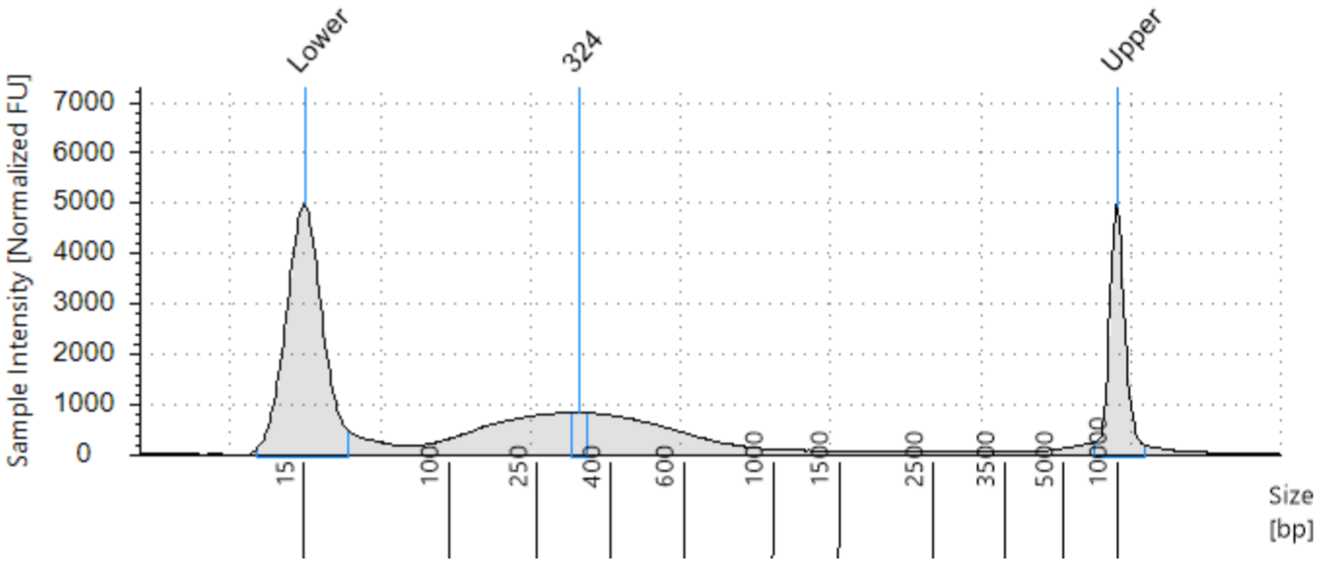

Sample Table

Well	Conc. [ng/µl]	Sample Description	Alert	Observations
F2	0.571	E2 minus R1		

Peak Table

Size [bp]	Calibrated Conc. [ng/µl]	Assigned Conc. [ng/µl]	Peak Molarity [nmol/l]	% Integrated Area	Peak Comment	Observations
15	7.41	-	760	-		Lower Marker
324	0.571	-	2.71	100.00		
10000	3.25	3.25	0.500	-		Upper Marker

 © Agilent Technologies, Inc. 2017

G2: F2 minus R1

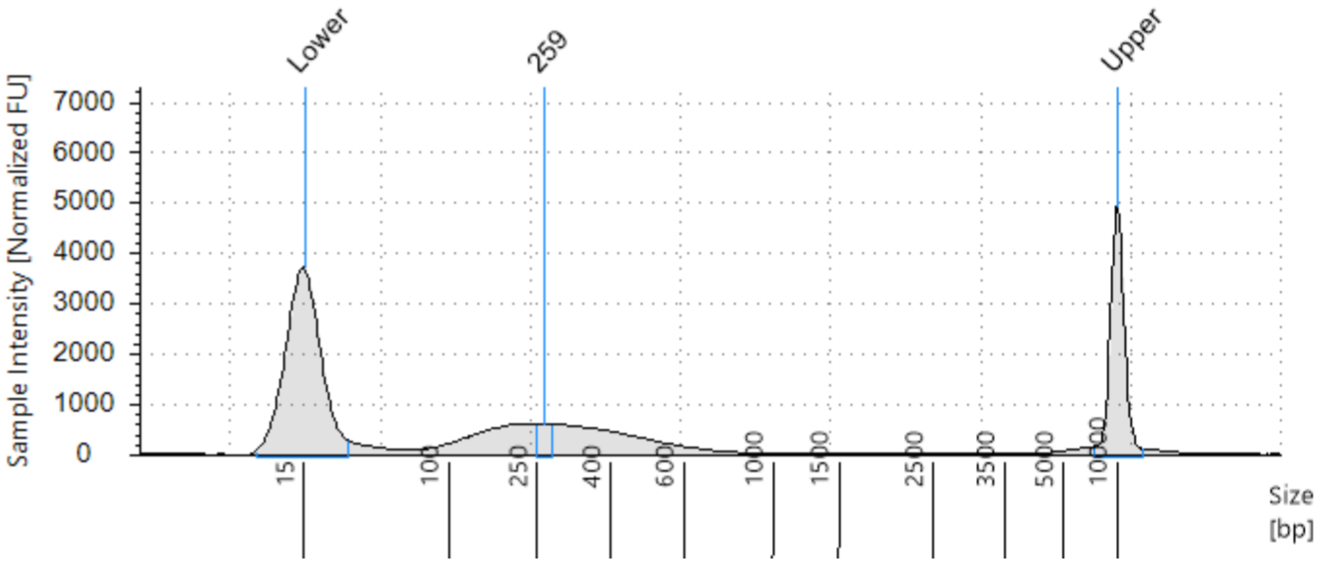

Sample Table

Well	Conc. [ng/µl]	Sample Description	Alert	Observations
G2	0.434	F2 minus R1		

Peak Table

Size [bp]	Calibrated Conc. [ng/µl]	Assigned Conc. [ng/µl]	Peak Molarity [nmol/l]	% Integrated Area	Peak Comment	Observations
15	5.95	-	610	-		Lower Marker
259	0.434	-	2.58	100.00		
10000	3.25	3.25	0.500	-		Upper Marker

© Agilent Technologies, Inc. 2017

H2: G2 minus R1

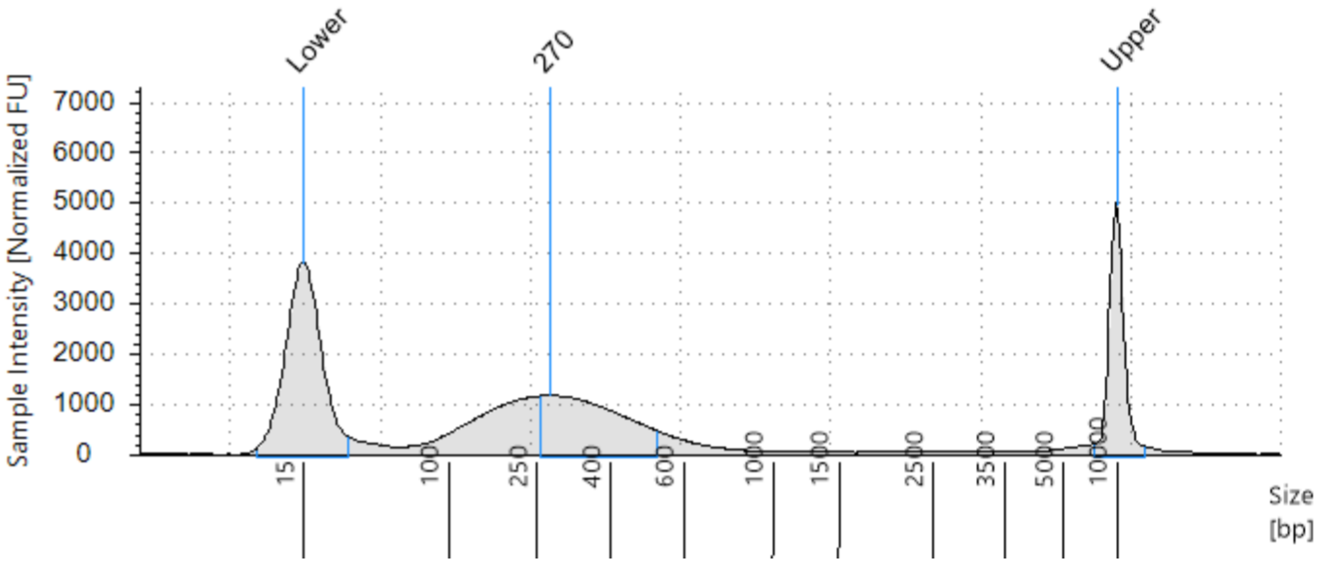

Sample Table

Well	Conc. [ng/µl]	Sample Description	Alert	Observations
H2	4.57	G2 minus R1		

Peak Table

Size [bp]	Calibrated Conc. [ng/µl]	Assigned Conc. [ng/µl]	Peak Molarity [nmol/l]	% Integrated Area	Peak Comment	Observations
15	5.89	-	604	-		Lower Marker
270	4.57	-	26.0	100.00		
10000	3.25	3.25	0.500	-		Upper Marker

© Agilent Technologies, Inc. 2017

Filename: 2020-03-06-04- D5000.Q-S,DFB minus from 3.8.20 H2-F4 R1.D5000

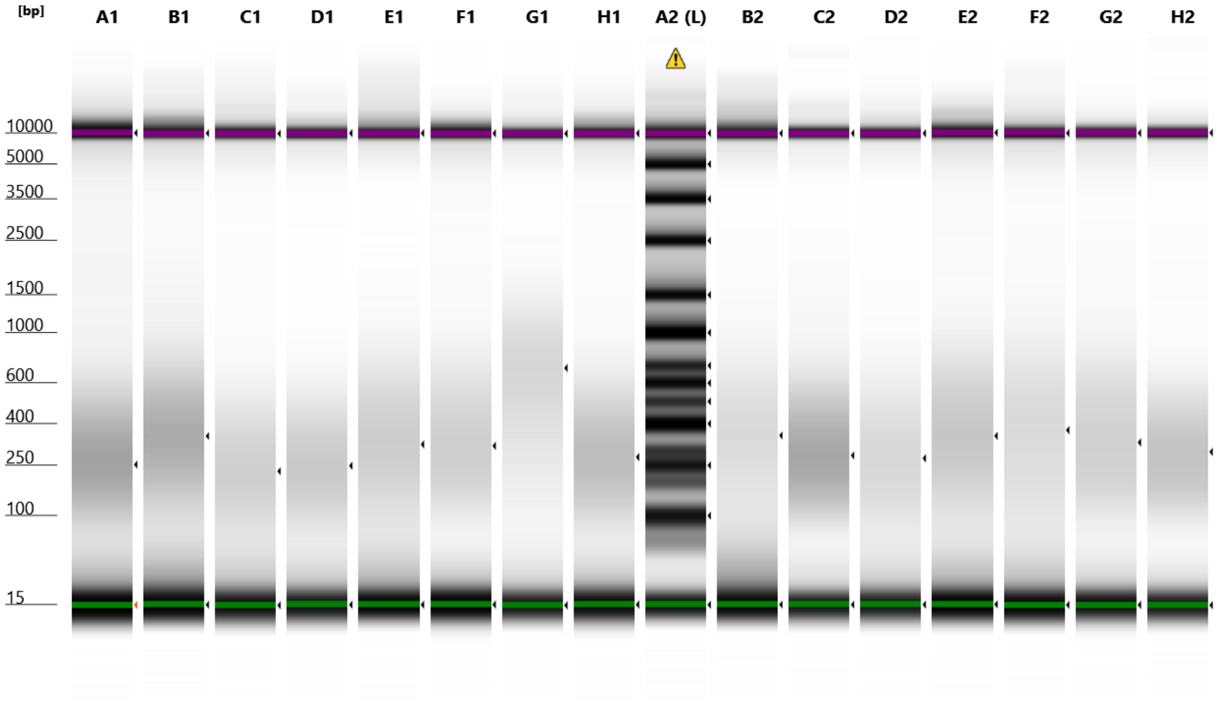

Default image (Contrast 100%)

Sample Info

Well	Conc. [ng/µl]	Sample Description	Alert	Observations
A1	2.28	Ladder		
B1	0.496	H2 minus R1		
C1	0.323	A3 minus R1		
D1	0.371	B3 minus R1		
E1	0.295	C3 minus R1		
F1	0.280	D3 minus R1		
G1	0.159	E3 minus R1		
H1	0.436	F3 minus R1		
A2	55.1	G3 minus R1	⚠	Issue with ladder peak detection (too many peaks detected); Ladder
B2	0.199	H3 minus R1		
C2	1.12	A4 minus R1		
D2	0.225	B4 minus R1		
E2	0.364	C4 minus R1		
F2	0.260	D4 minus R1		
G2	0.333	E4 minus R1		
H2	2.53	F4 minus R1		

 © Agilent Technologies, Inc. 2017

A1: Ladder

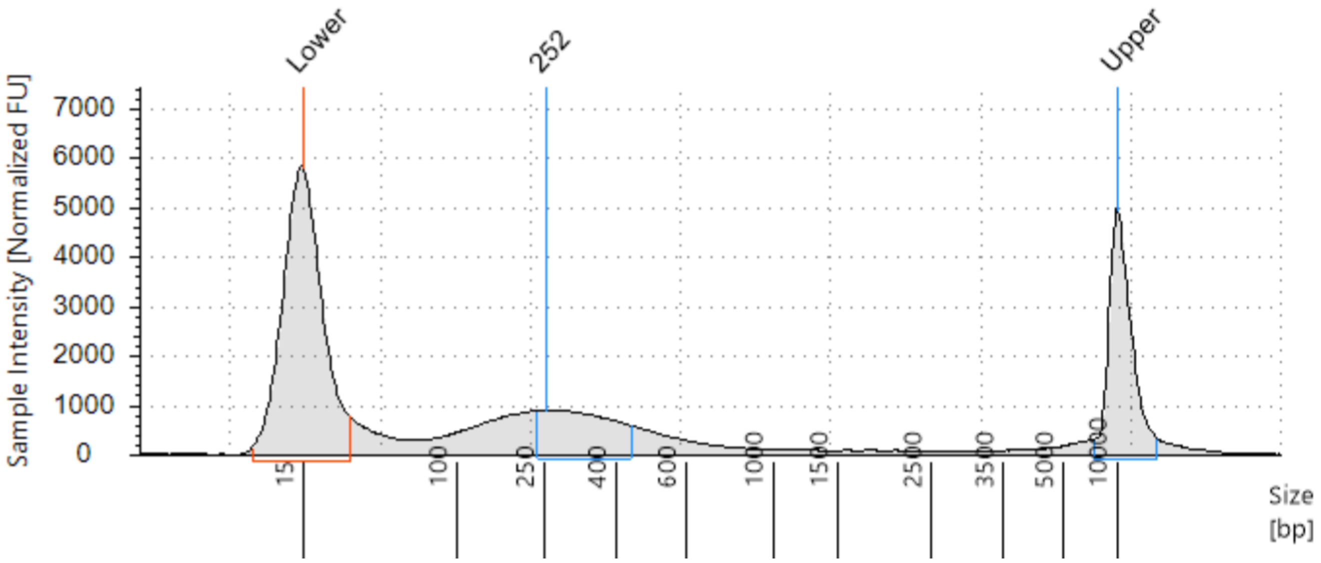

Sample Table

Well	Conc. [ng/µl]	Sample Description	Alert	Observations
A1	2.28	Ladder		

Peak Table

Size [bp]	Calibrated Conc. [ng/µl]	Assigned Conc. [ng/µl]	Peak Molarity [nmol/l]	% Integrated Area	Peak Comment	Observations
15	6.58	-	675	-		Lower Marker
252	2.28	-	13.9	100.00		
10000	3.25	3.25	0.500	-		Upper Marker

© Agilent Technologies, Inc. 2017

B1: H2 minus R1

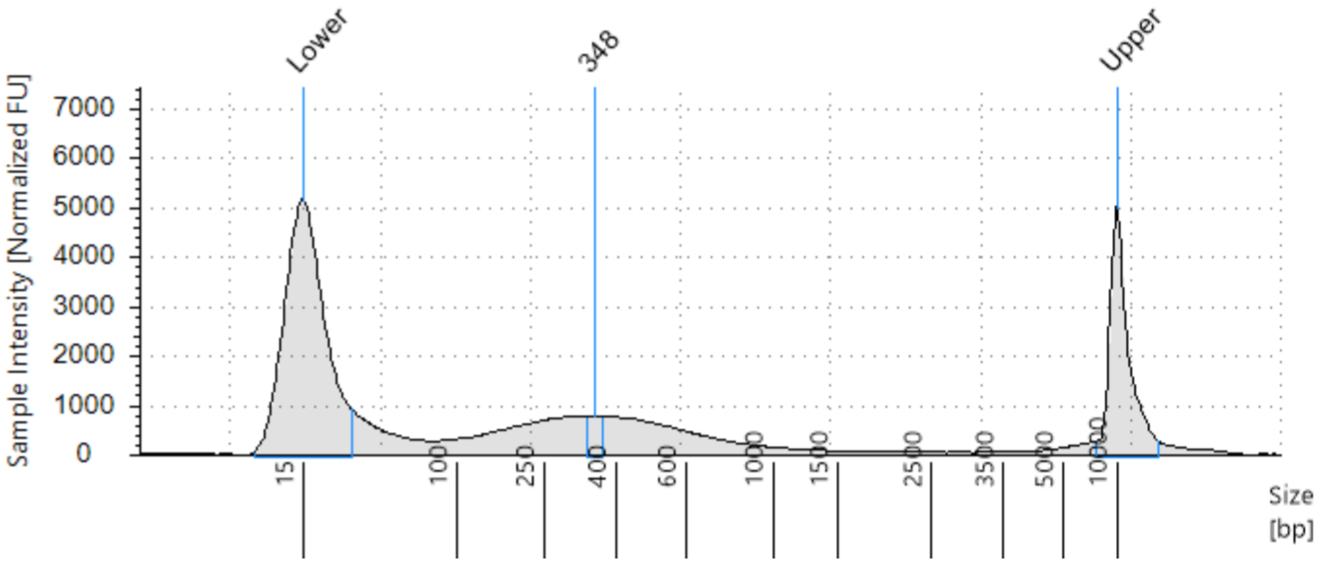

Sample Table

Well	Conc. [ng/µl]	Sample Description	Alert	Observations
B1	0.496	H2 minus R1		

Peak Table

Size [bp]	Calibrated Conc. [ng/µl]	Assigned Conc. [ng/µl]	Peak Molarity [nmol/l]	% Integrated Area	Peak Comment	Observations
15	6.88	-	705	-		Lower Marker
348	0.496	-	2.20	100.00		
10000	3.25	3.25	0.500	-		Upper Marker

© Agilent Technologies, Inc. 2017

C1: A3 minus R1

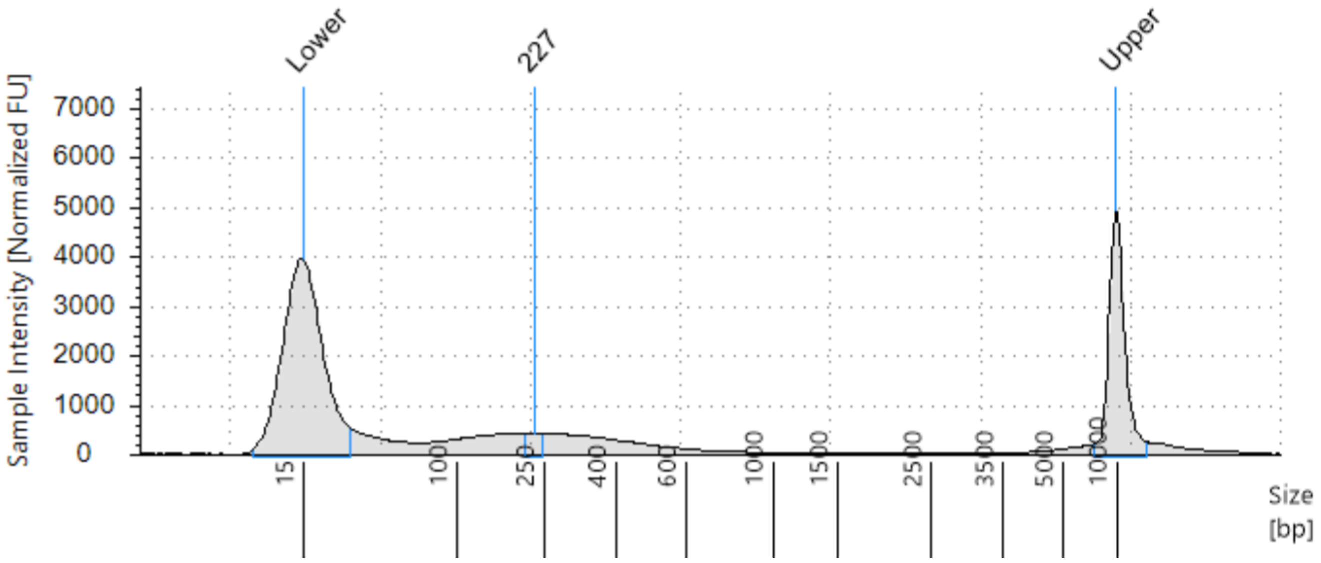

Sample Table

Well	Conc. [ng/µl]	Sample Description	Alert	Observations
C1	0.323	A3 minus R1		

Peak Table

Size [bp]	Calibrated Conc. [ng/µl]	Assigned Conc. [ng/µl]	Peak Molarity [nmol/l]	% Integrated Area	Peak Comment	Observations
15	6.32	-	649	-		Lower Marker
227	0.323	-	2.19	100.00		
10000	3.25	3.25	0.500	-		Upper Marker

© Agilent Technologies, Inc. 2017

D1: B3 minus R1

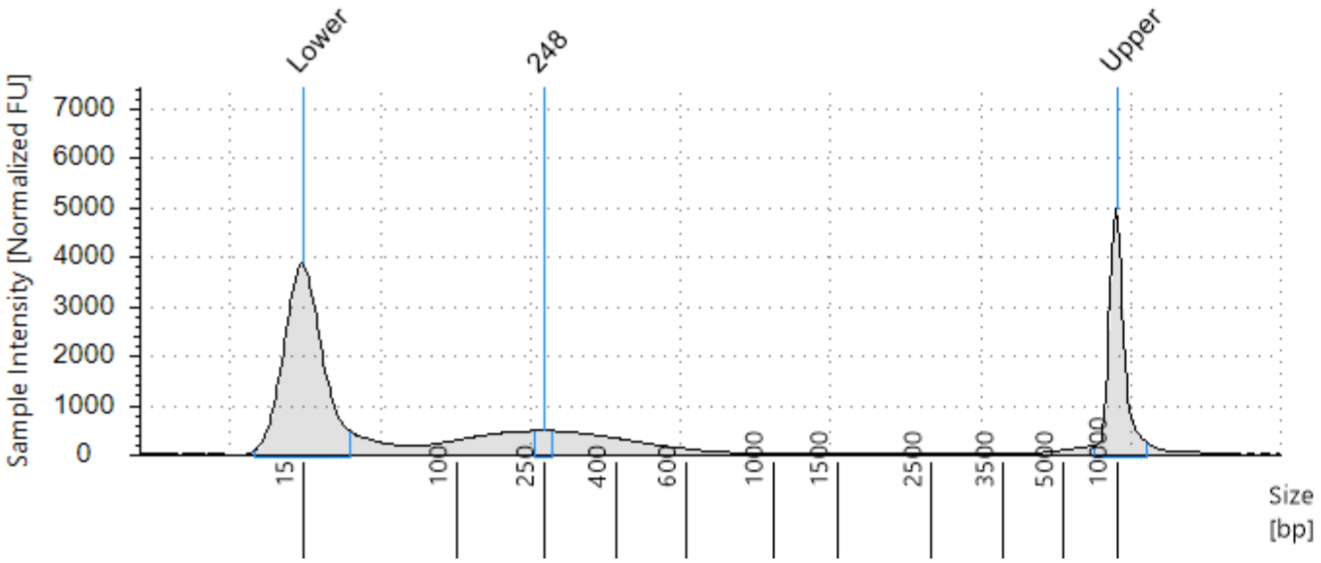

Sample Table

Well	Conc. [ng/µl]	Sample Description	Alert	Observations
D1	0.371	B3 minus R1		

Peak Table

Size [bp]	Calibrated Conc. [ng/µl]	Assigned Conc. [ng/µl]	Peak Molarity [nmol/l]	% Integrated Area	Peak Comment	Observations
15	6.26	-	642	-		Lower Marker
248	0.371	-	2.30	100.00		
10000	3.25	3.25	0.500	-		Upper Marker

© Agilent Technologies, Inc. 2017

E1: C3 minus R1

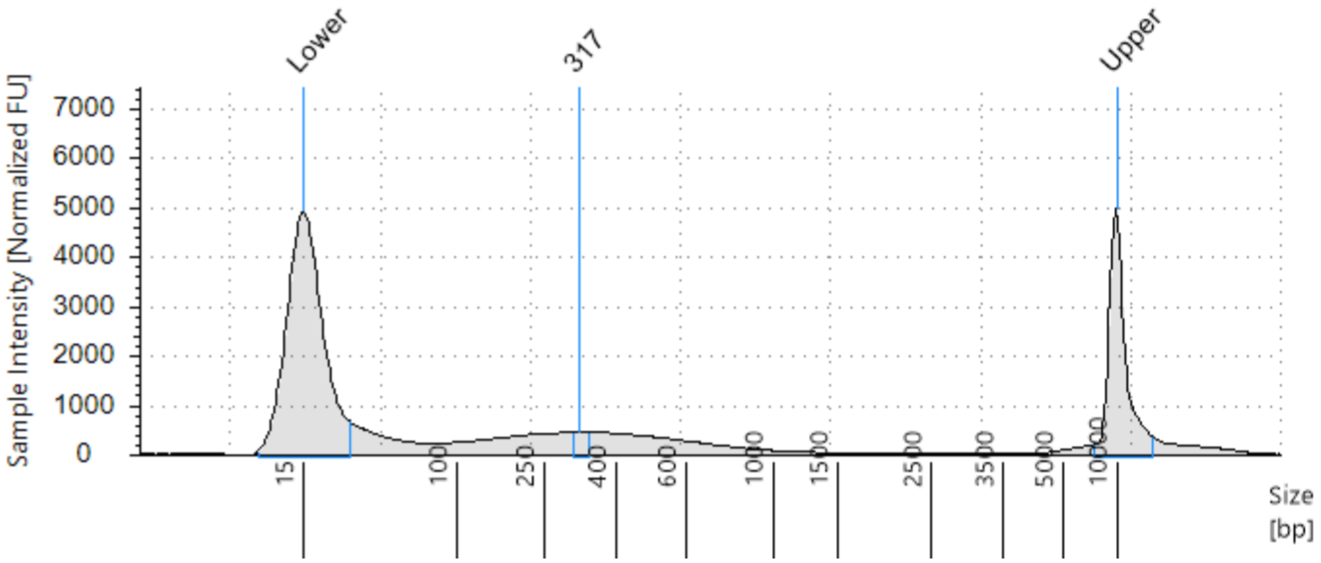

Sample Table

Well	Conc. [ng/µl]	Sample Description	Alert	Observations
E1	0.295	C3 minus R1		

Peak Table

Size [bp]	Calibrated Conc. [ng/µl]	Assigned Conc. [ng/µl]	Peak Molarity [nmol/l]	% Integrated Area	Peak Comment	Observations
15	7.07	-	725	-		Lower Marker
317	0.295	-	1.44	100.00		
10000	3.25	3.25	0.500	-		Upper Marker

© Agilent Technologies, Inc. 2017

F1: D3 minus R1

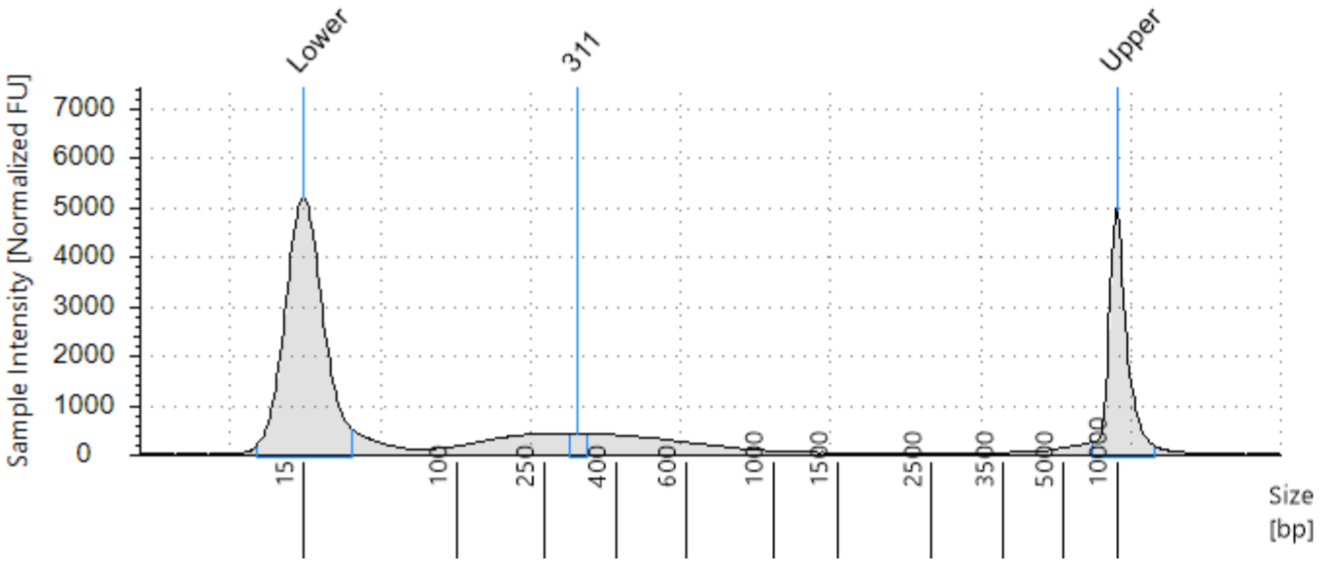

Sample Table

Well	Conc. [ng/µl]	Sample Description	Alert	Observations
F1	0.280	D3 minus R1		

Peak Table

Size [bp]	Calibrated Conc. [ng/µl]	Assigned Conc. [ng/µl]	Peak Molarity [nmol/l]	% Integrated Area	Peak Comment	Observations
15	7.18	-	736	-		Lower Marker
311	0.280	-	1.39	100.00		
10000	3.25	3.25	0.500	-		Upper Marker

 © Agilent Technologies, Inc. 2017

G1: E3 minus R1

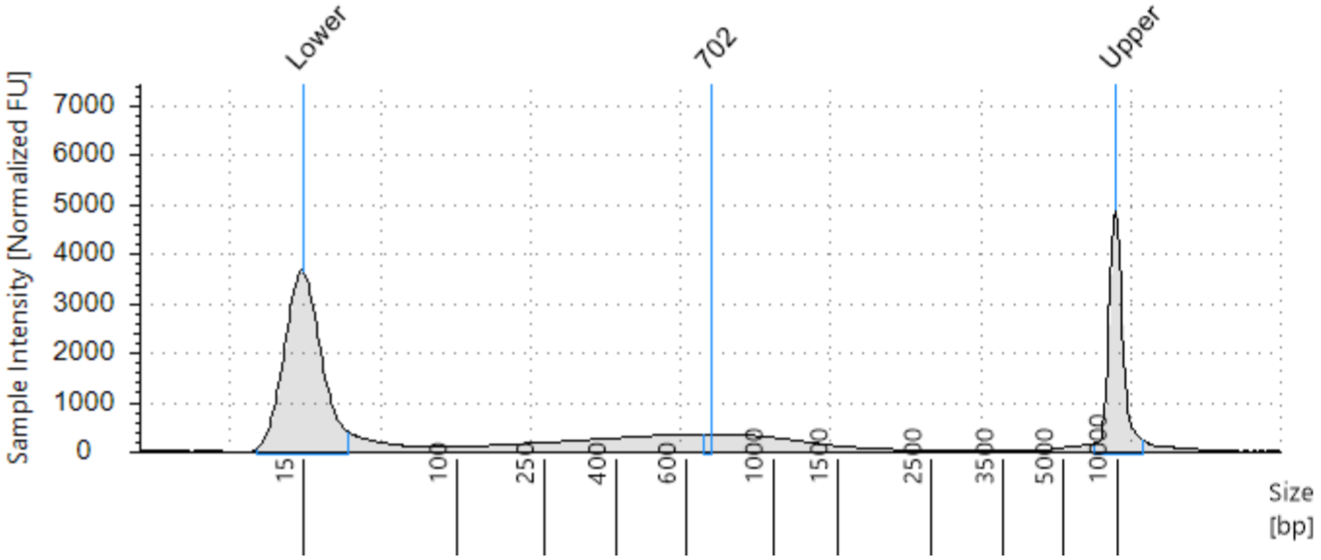

Sample Table

Well	Conc. [ng/µl]	Sample Description	Alert	Observations
G1	0.159	E3 minus R1		

Peak Table

Size [bp]	Calibrated Conc. [ng/µl]	Assigned Conc. [ng/µl]	Peak Molarity [nmol/l]	% Integrated Area	Peak Comment	Observations
15	6.32	-	648	-		Lower Marker
702	0.159	-	0.348	100.00		
10000	3.25	3.25	0.500	-		Upper Marker

© Agilent Technologies, Inc. 2017

H1: F3 minus R1

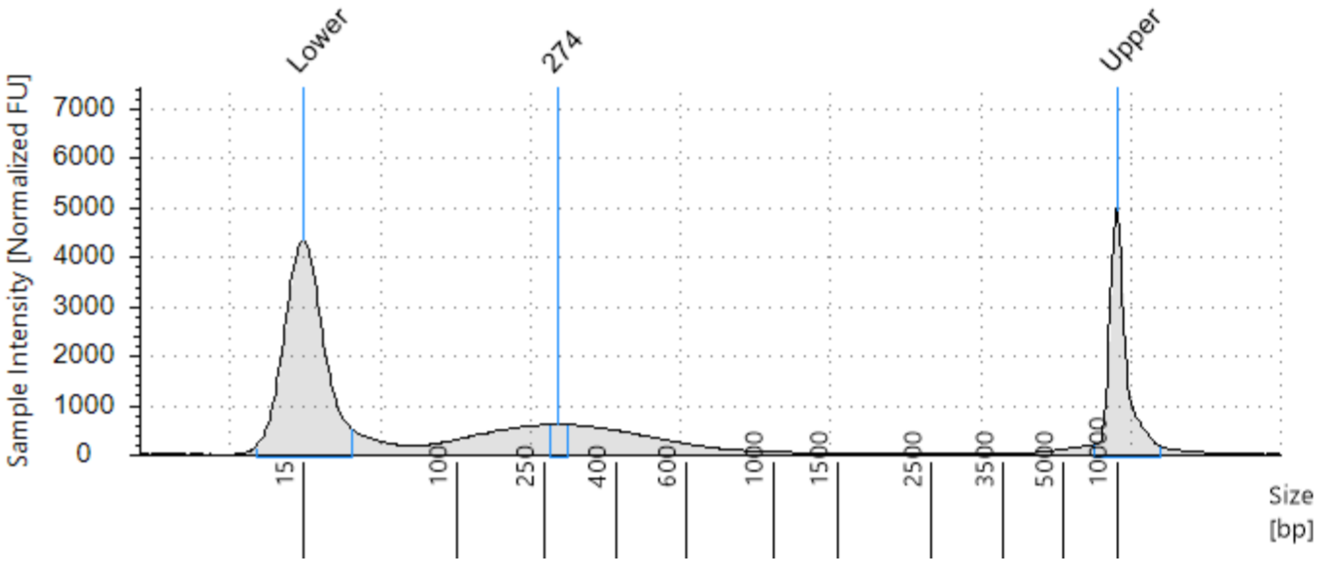

Sample Table

Well	Conc. [ng/µl]	Sample Description	Alert	Observations
H1	0.436	F3 minus R1		

Peak Table

Size [bp]	Calibrated Conc. [ng/µl]	Assigned Conc. [ng/µl]	Peak Molarity [nmol/l]	% Integrated Area	Peak Comment	Observations
15	6.45	-	662	-		Lower Marker
274	0.436	-	2.45	100.00		
10000	3.25	3.25	0.500	-		Upper Marker

 © Agilent Technologies, Inc. 2017

A2: G3 minus R1

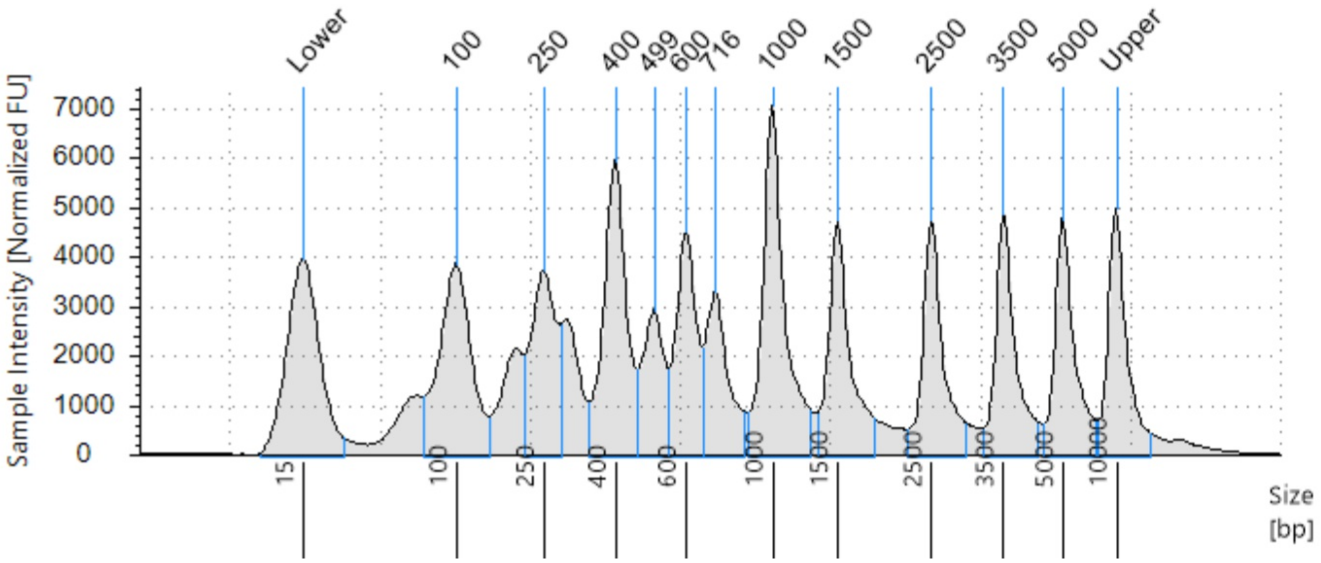

Sample Table

Well	Conc. [ng/µl]	Sample Description	Alert	Observations
A2	55.1	G3 minus R1	⚠	Issue with ladder peak detection (too many peaks detected); Ladder

Peak Table

Size [bp]	Calibrated Conc. [ng/µl]	Assigned Conc. [ng/µl]	Peak Molarity [nmol/l]	% Integrated Area	Peak Comment	Observations
15	5.60	-	575	-		Lower Marker
100	6.06	-	93.3	11.00		
250	4.65	-	28.6	8.43		
400	6.60	-	25.4	11.97		
499	3.14	-	9.69	5.70		
600	4.52	-	11.6	8.20		
716	3.68	-	7.90	6.67		
1000	7.55	-	11.6	13.69		
1500	4.98	-	5.11	9.04		
2500	4.67	-	2.87	8.47		
3500	4.69	-	2.06	8.50		
5000	4.60	-	1.41	8.34		
10000	3.25	3.25	0.500	-		Upper Marker

© Agilent Technologies, Inc. 2017

B2: H3 minus R1

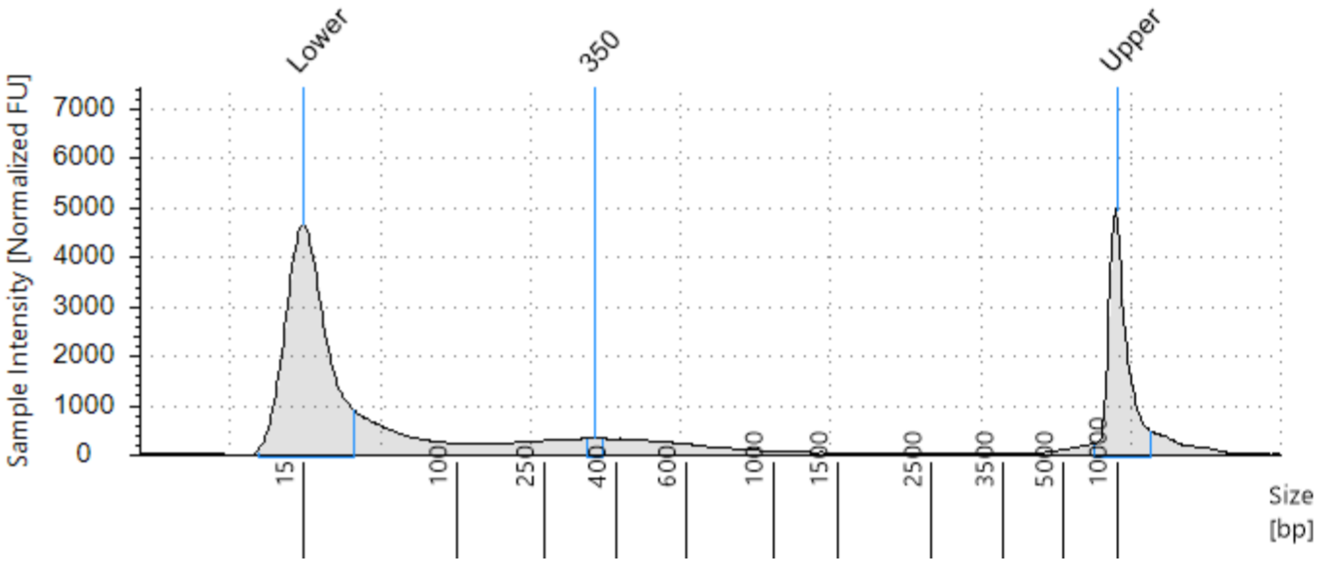

Sample Table

Well	Conc. [ng/µl]	Sample Description	Alert	Observations
B2	0.199	H3 minus R1		

Peak Table

Size [bp]	Calibrated Conc. [ng/µl]	Assigned Conc. [ng/µl]	Peak Molarity [nmol/l]	% Integrated Area	Peak Comment	Observations
15	6.59	-	676	-		Lower Marker
350	0.199	-	0.875	100.00		
10000	3.25	3.25	0.500	-		Upper Marker

 © Agilent Technologies, Inc. 2017

C2: A4 minus R1

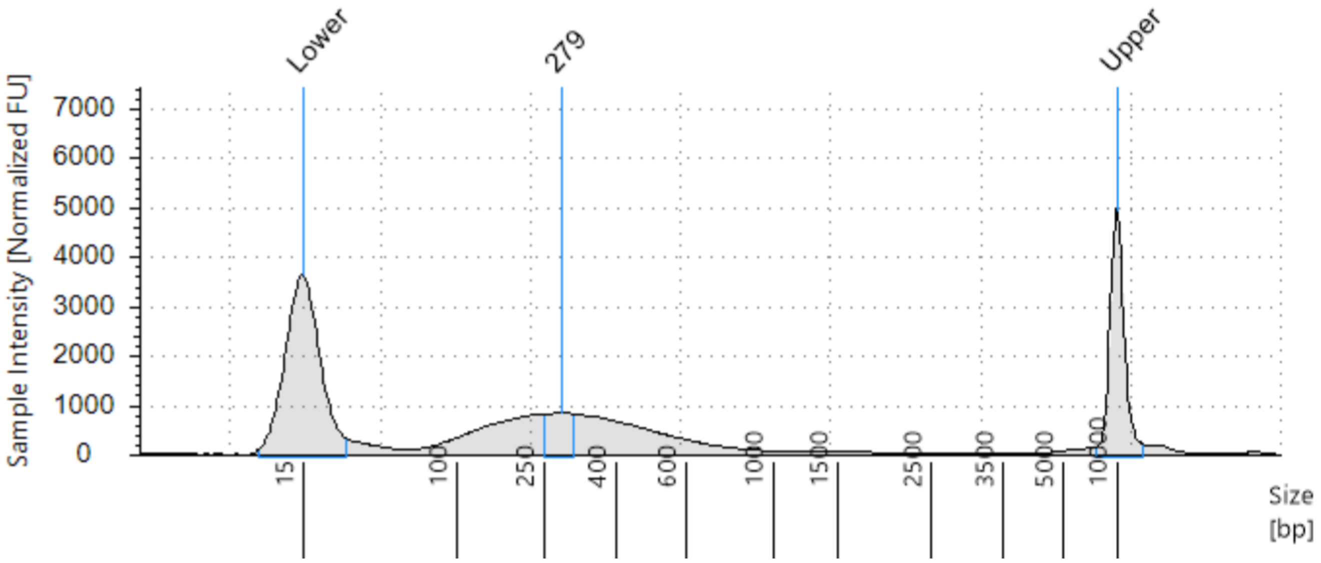

Sample Table

Well	Conc. [ng/µl]	Sample Description	Alert	Observations
C2	1.12	A4 minus R1		

Peak Table

Size [bp]	Calibrated Conc. [ng/µl]	Assigned Conc. [ng/µl]	Peak Molarity [nmol/l]	% Integrated Area	Peak Comment	Observations
15	5.65	-	579	-		Lower Marker
279	1.12	-	6.19	100.00		
10000	3.25	3.25	0.500	-		Upper Marker

© Agilent Technologies, Inc. 2017

D2: B4 minus R1

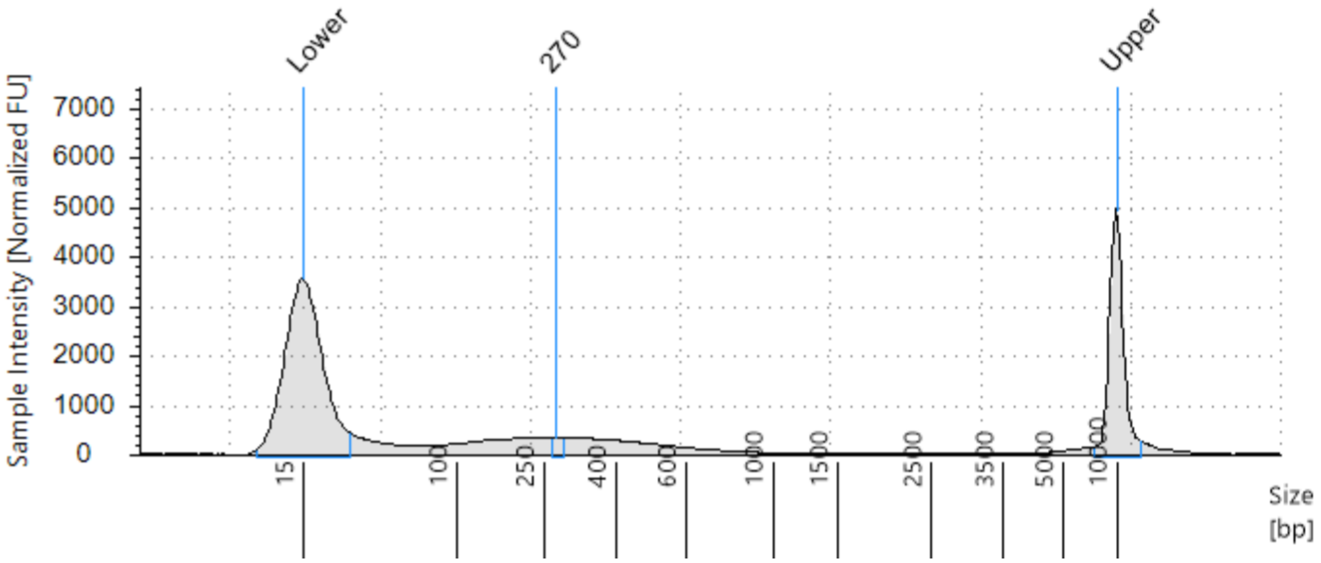

Sample Table

Well	Conc. [ng/µl]	Sample Description	Alert	Observations
D2	0.225	B4 minus R1		

Peak Table

Size [bp]	Calibrated Conc. [ng/µl]	Assigned Conc. [ng/µl]	Peak Molarity [nmol/l]	% Integrated Area	Peak Comment	Observations
15	6.09	-	625	-		Lower Marker
270	0.225	-	1.28	100.00		
10000	3.25	3.25	0.500	-		Upper Marker

© Agilent Technologies, Inc. 2017

E2: C4 minus R1

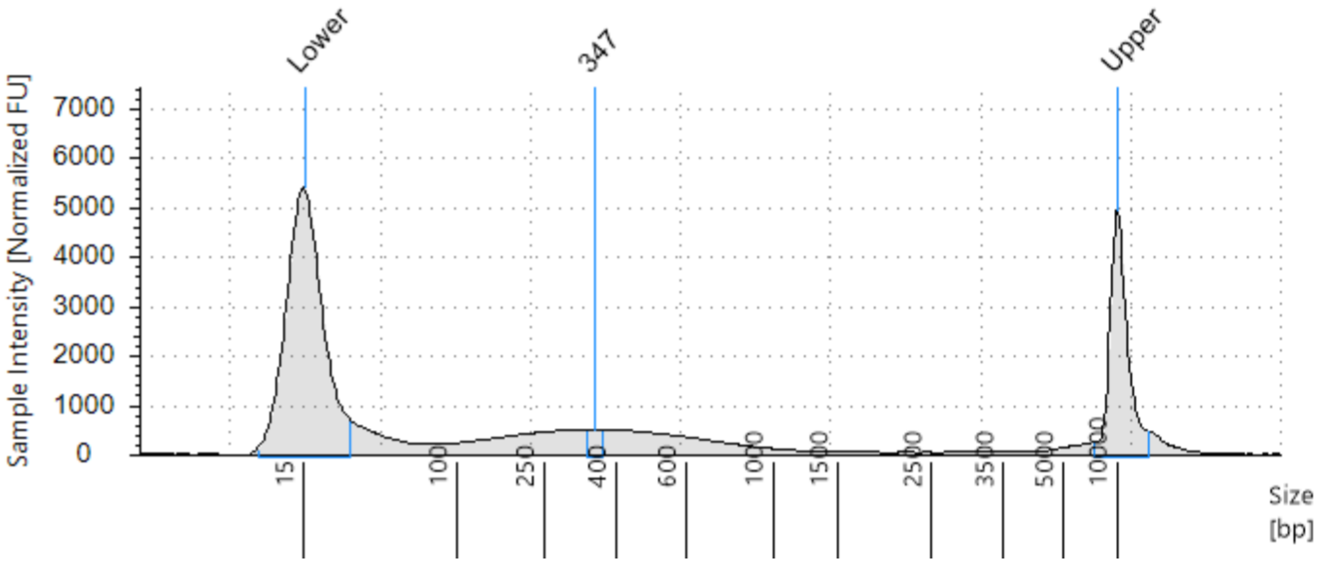

Sample Table

Well	Conc. [ng/µl]	Sample Description	Alert	Observations
E2	0.364	C4 minus R1		

Peak Table

Size [bp]	Calibrated Conc. [ng/µl]	Assigned Conc. [ng/µl]	Peak Molarity [nmol/l]	% Integrated Area	Peak Comment	Observations
15	7.68	-	788	-		Lower Marker
347	0.364	-	1.61	100.00		
10000	3.25	3.25	0.500	-		Upper Marker

 © Agilent Technologies, Inc. 2017

F2: D4 minus R1

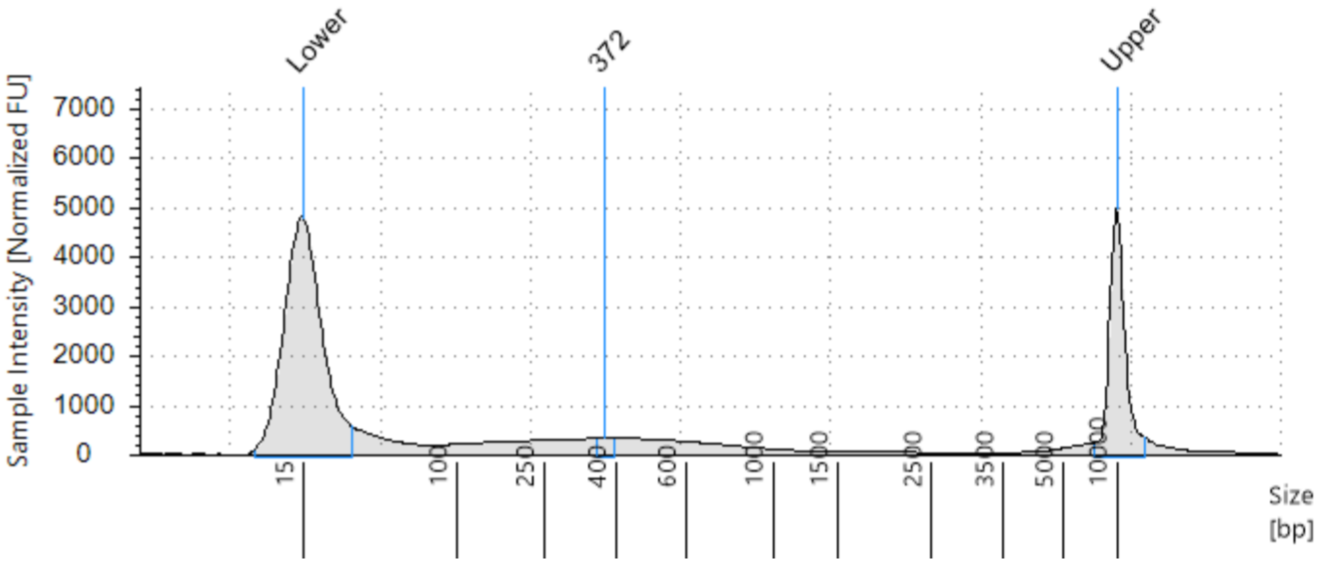

Sample Table

Well	Conc. [ng/µl]	Sample Description	Alert	Observations
F2	0.260	D4 minus R1		

Peak Table

Size [bp]	Calibrated Conc. [ng/µl]	Assigned Conc. [ng/µl]	Peak Molarity [nmol/l]	% Integrated Area	Peak Comment	Observations
15	7.74	-	794	-		Lower Marker
372	0.260	-	1.08	100.00		
10000	3.25	3.25	0.500	-		Upper Marker

G2: E4 minus R1

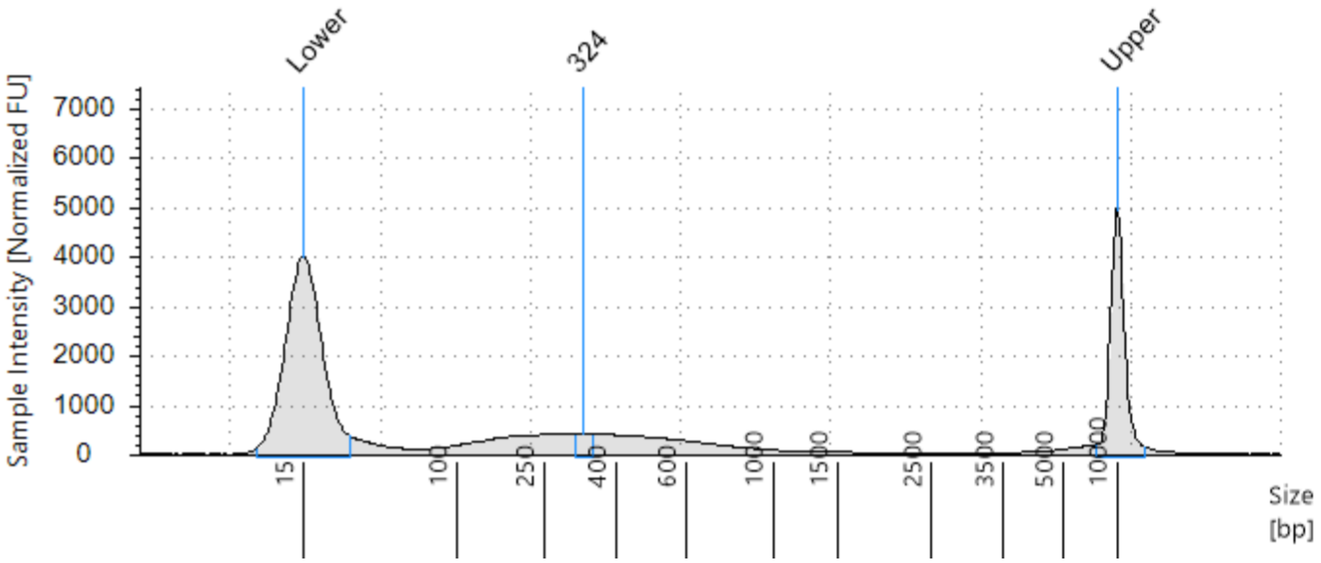

Sample Table

Well	Conc. [ng/µl]	Sample Description	Alert	Observations
G2	0.333	E4 minus R1		

Peak Table

Size [bp]	Calibrated Conc. [ng/µl]	Assigned Conc. [ng/µl]	Peak Molarity [nmol/l]	% Integrated Area	Peak Comment	Observations
15	6.63	-	680	-		Lower Marker
324	0.333	-	1.58	100.00		
10000	3.25	3.25	0.500	-		Upper Marker

© Agilent Technologies, Inc. 2017

H2: F4 minus R1

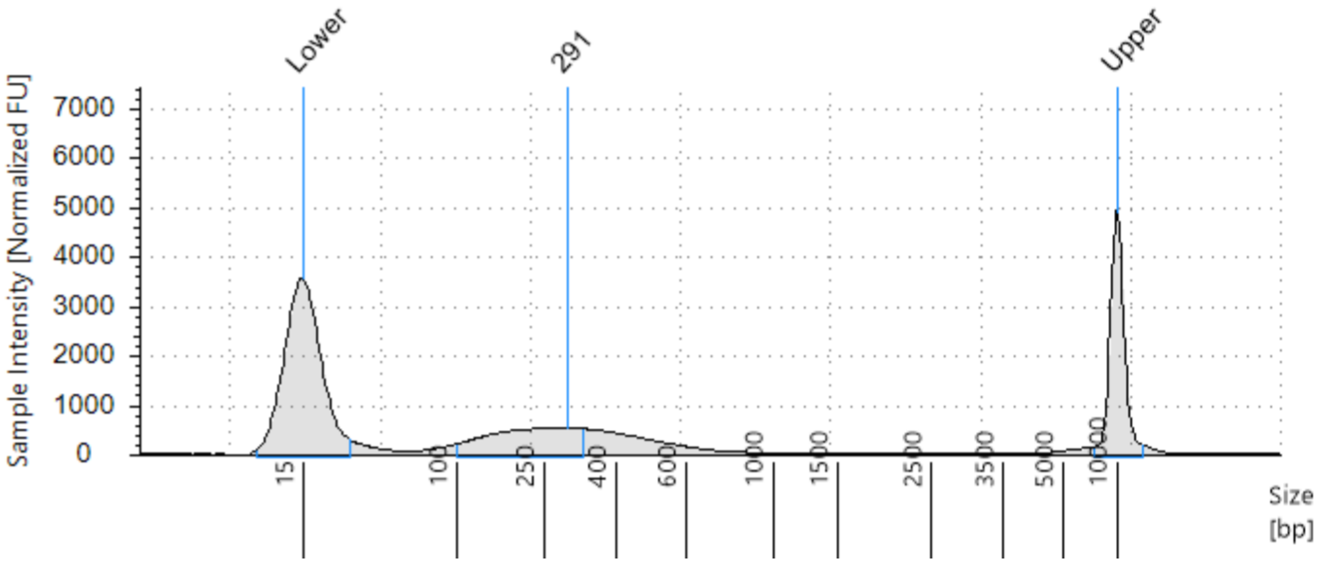

Sample Table

Well	Conc. [ng/µl]	Sample Description	Alert	Observations
H2	2.53	F4 minus R1		

Peak Table

Size [bp]	Calibrated Conc. [ng/µl]	Assigned Conc. [ng/µl]	Peak Molarity [nmol/l]	% Integrated Area	Peak Comment	Observations
15	6.05	-	621	-		Lower Marker
291	2.53	-	13.4	100.00		
10000	3.25	3.25	0.500	-		Upper Marker

© Agilent Technologies, Inc. 2017

Filename: 2020-08-03-02.D1000.Q-S DFB minus, from 3.8.20, H3-E5 R1.D1000

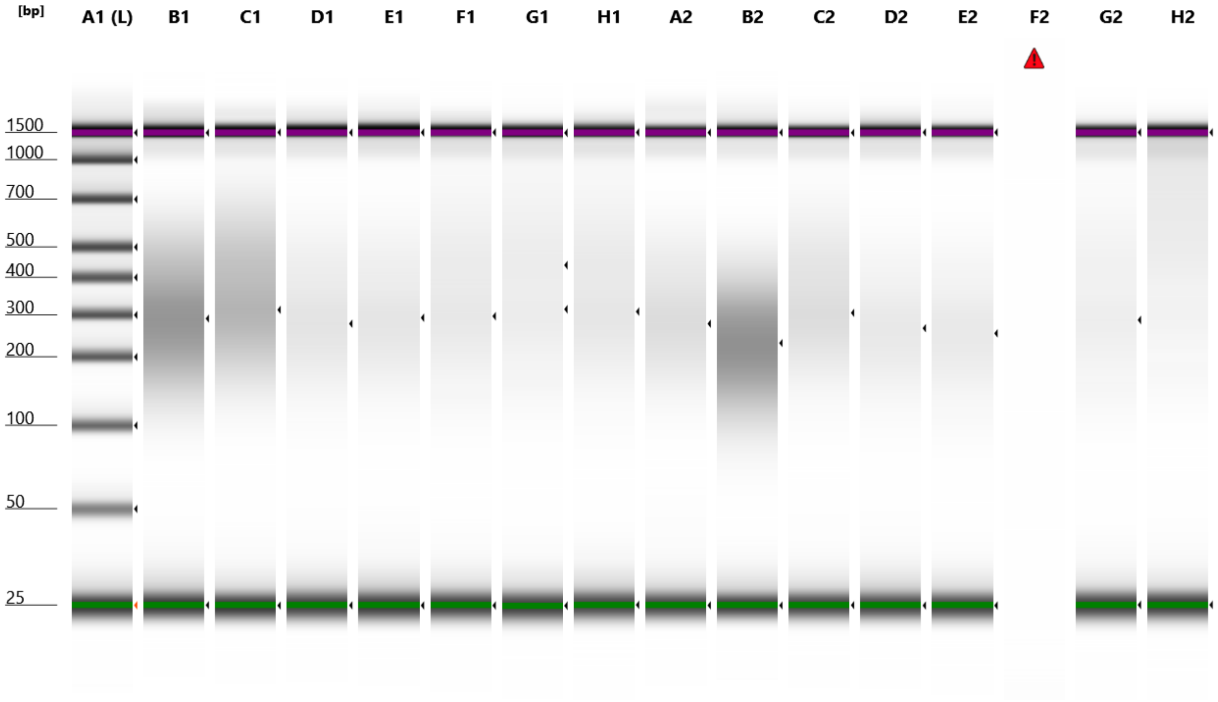

Default image (Contrast 100%)

Sample Info

Well	Conc. [ng/µl]	Sample Description	Alert	Observations
A1	16.8	Ladder		Ladder
B1	8.58	H3 DFB minus from 3.8.20		
C1	7.15	A4		
D1	0.238	B4		
E1	0.864	C4		
F1	0.255	D4		
G1	0.402	E4		
H1	0.530	F4		
A2	1.60	G4		
B2	5.71	H4		
C2	2.28	A5		
D2	0.267	B5		
E2	0.251	C5		
F2			▲	Marker(s) not detected
G2	0.134	D5		
H2		E5		

© Agilent Technologies, Inc. 2017

A1: Ladder

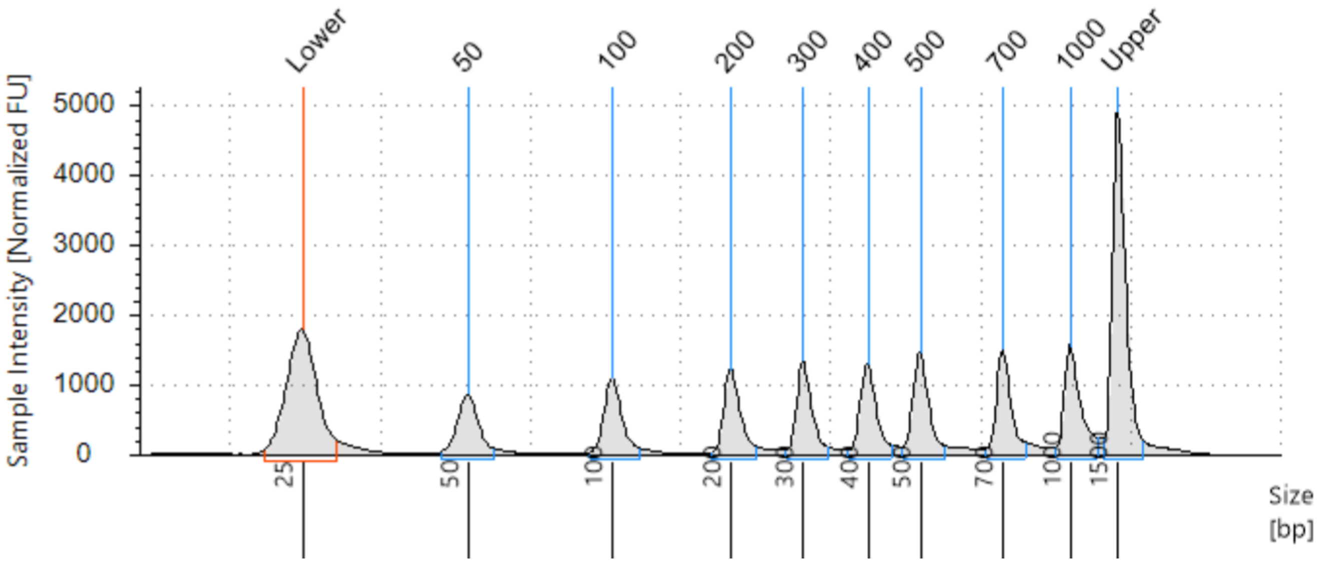

Sample Table

Well	Conc. [ng/µl]	Sample Description	Alert	Observations
A1	16.8	Ladder		Ladder

Peak Table

Size [bp]	Calibrated Conc. [ng/µl]	Assigned Conc. [ng/µl]	Peak Molarity [nmol/l]	% Integrated Area	Peak Comment	Observations
25	5.36	-	330	-		Lower Marker
50	1.79	-	55.1	10.68		
100	1.94	-	29.8	11.56		
200	2.00	-	15.4	11.91		
300	2.02	-	10.4	12.07		
400	2.09	-	8.05	12.48		
500	2.28	-	7.01	13.59		
700	2.17	-	4.77	12.94		
1000	2.48	-	3.81	14.78		
1500	6.50	6.50	6.67	-		Upper Marker

© Agilent Technologies, Inc. 2017

B1: H3 DFB minus from 3.8.20

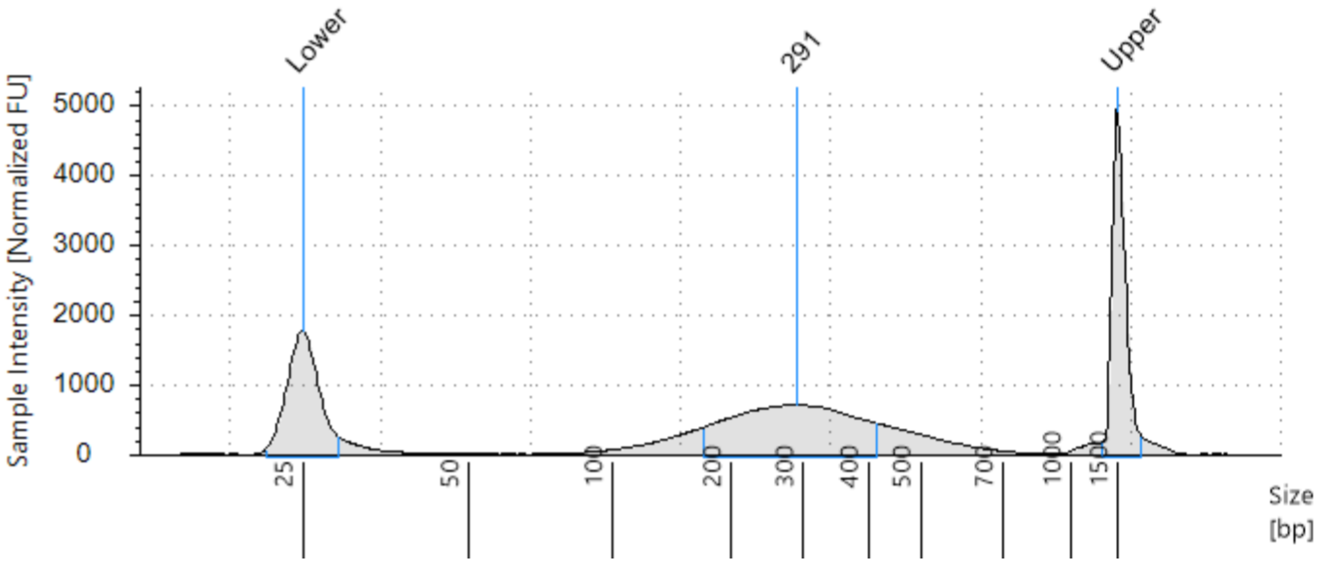

Sample Table

Well	Conc. [ng/µl]	Sample Description	Alert	Observations
B1	8.58	H3 DFB minus from 3.8.20		

Peak Table

Size [bp]	Calibrated Conc. [ng/µl]	Assigned Conc. [ng/µl]	Peak Molarity [nmol/l]	% Integrated Area	Peak Comment	Observations
25	5.48	-	337	-		Lower Marker
291	8.58	-	45.3	100.00		
1500	6.50	6.50	6.67	-		Upper Marker

© Agilent Technologies, Inc. 2017

C1: A4

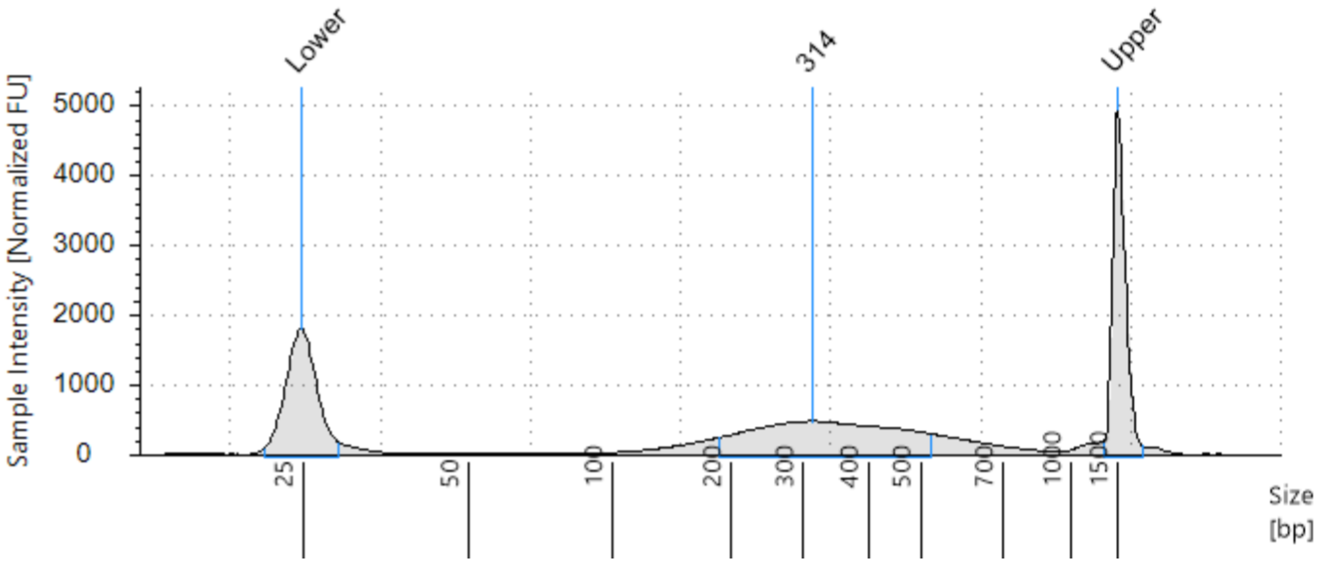

Sample Table

Well	Conc. [ng/µl]	Sample Description	Alert	Observations
C1	7.15	A4		

Peak Table

Size [bp]	Calibrated Conc. [ng/µl]	Assigned Conc. [ng/µl]	Peak Molarity [nmol/l]	% Integrated Area	Peak Comment	Observations
25	5.68	-	349	-		Lower Marker
314	7.15	-	35.1	100.00		
1500	6.50	6.50	6.67	-		Upper Marker

© Agilent Technologies, Inc. 2017

D1: B4

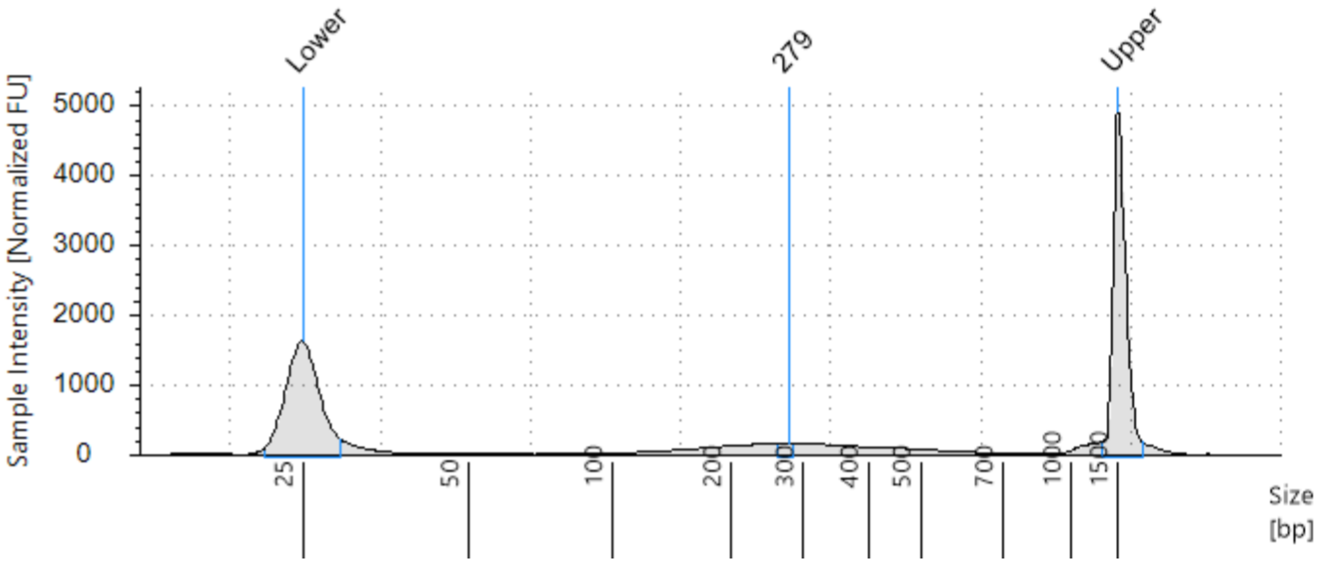

Sample Table

Well	Conc. [ng/µl]	Sample Description	Alert	Observations
D1	0.238	B4		

Peak Table

Size [bp]	Calibrated Conc. [ng/µl]	Assigned Conc. [ng/µl]	Peak Molarity [nmol/l]	% Integrated Area	Peak Comment	Observations
25	5.67	-	349	-		Lower Marker
279	0.238	-	1.31	100.00		
1500	6.50	6.50	6.67	-		Upper Marker

© Agilent Technologies, Inc. 2017

E1: C4

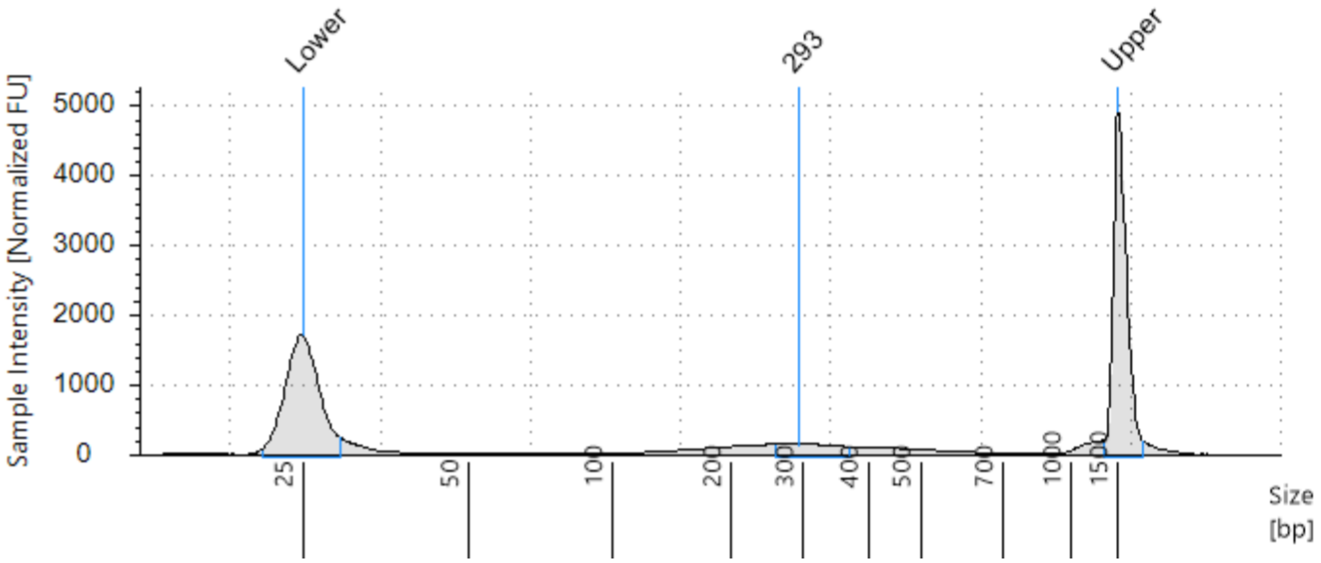

Sample Table

Well	Conc. [ng/µl]	Sample Description	Alert	Observations
E1	0.864	C4		

Peak Table

Size [bp]	Calibrated Conc. [ng/µl]	Assigned Conc. [ng/µl]	Peak Molarity [nmol/l]	% Integrated Area	Peak Comment	Observations
25	5.76	-	354	-		Lower Marker
293	0.864	-	4.53	100.00		
1500	6.50	6.50	6.67	-		Upper Marker

 © Agilent Technologies, Inc. 2017

F1: D4

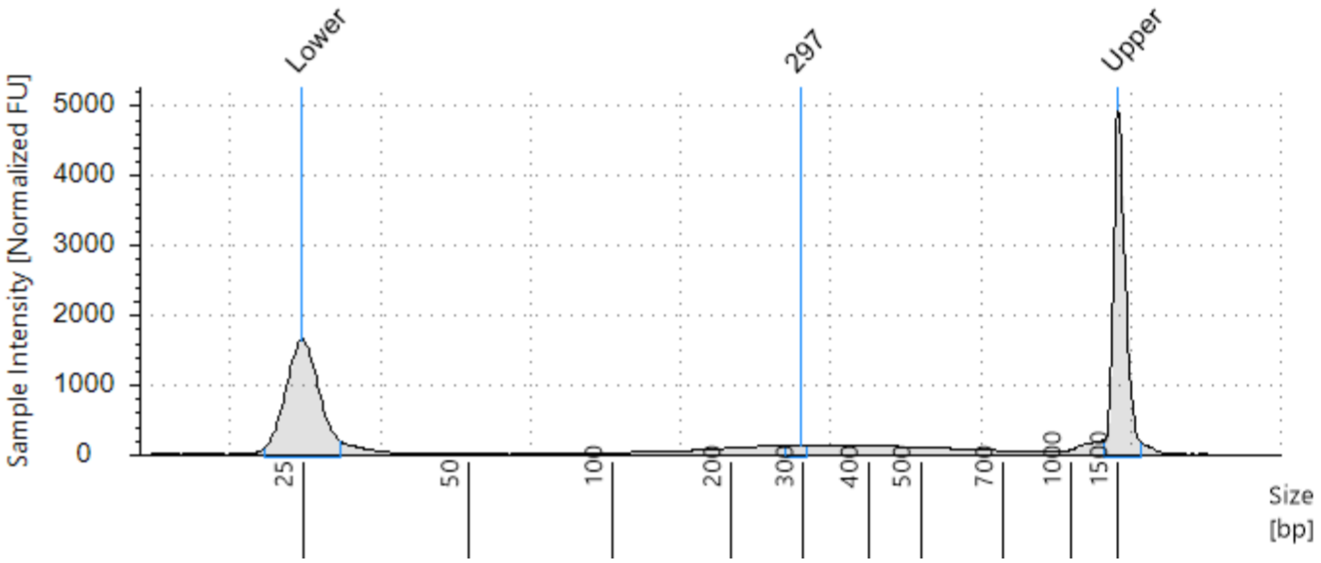

Sample Table

Well	Conc. [ng/µl]	Sample Description	Alert	Observations
F1	0.255	D4		

Peak Table

Size [bp]	Calibrated Conc. [ng/µl]	Assigned Conc. [ng/µl]	Peak Molarity [nmol/l]	% Integrated Area	Peak Comment	Observations
25	5.96	-	367	-		Lower Marker
297	0.255	-	1.32	100.00		
1500	6.50	6.50	6.67	-		Upper Marker

© Agilent Technologies, Inc. 2017

G1: E4

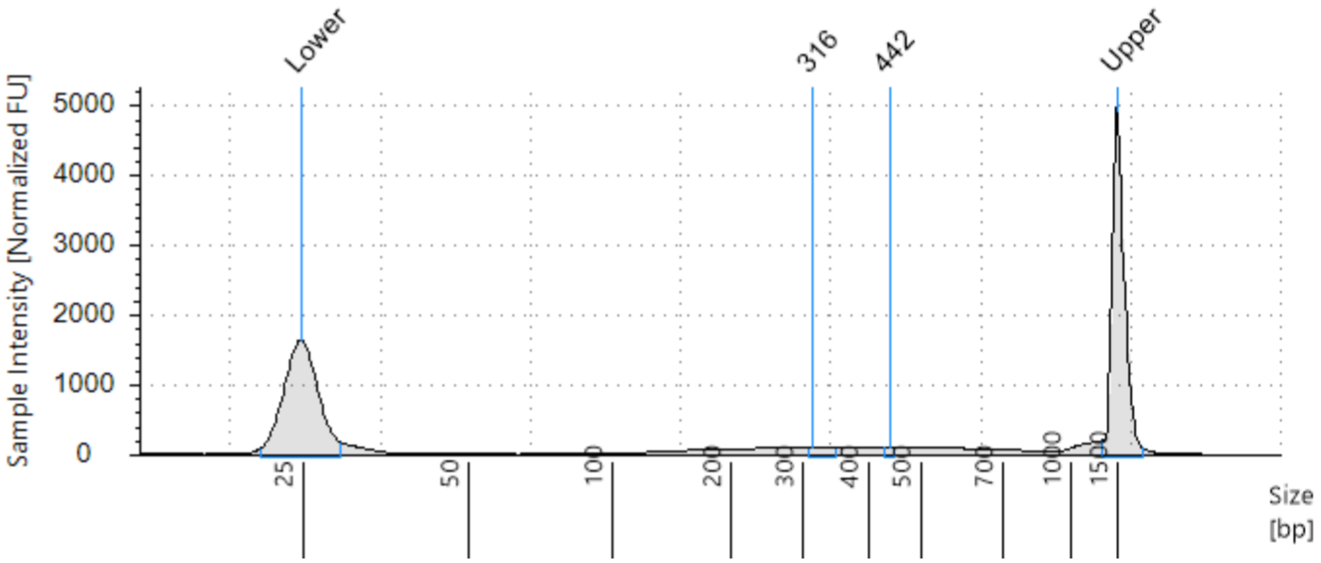

Sample Table

Well	Conc. [ng/µl]	Sample Description	Alert	Observations
G1	0.402	E4		

Peak Table

Size [bp]	Calibrated Conc. [ng/µl]	Assigned Conc. [ng/µl]	Peak Molarity [nmol/l]	% Integrated Area	Peak Comment	Observations
25	5.99	-	368	-		Lower Marker
316	0.284	-	1.38	70.60		
442	0.118	-	0.412	29.40		
1500	6.50	6.50	6.67	-		Upper Marker

 © Agilent Technologies, Inc. 2017

H1: F4

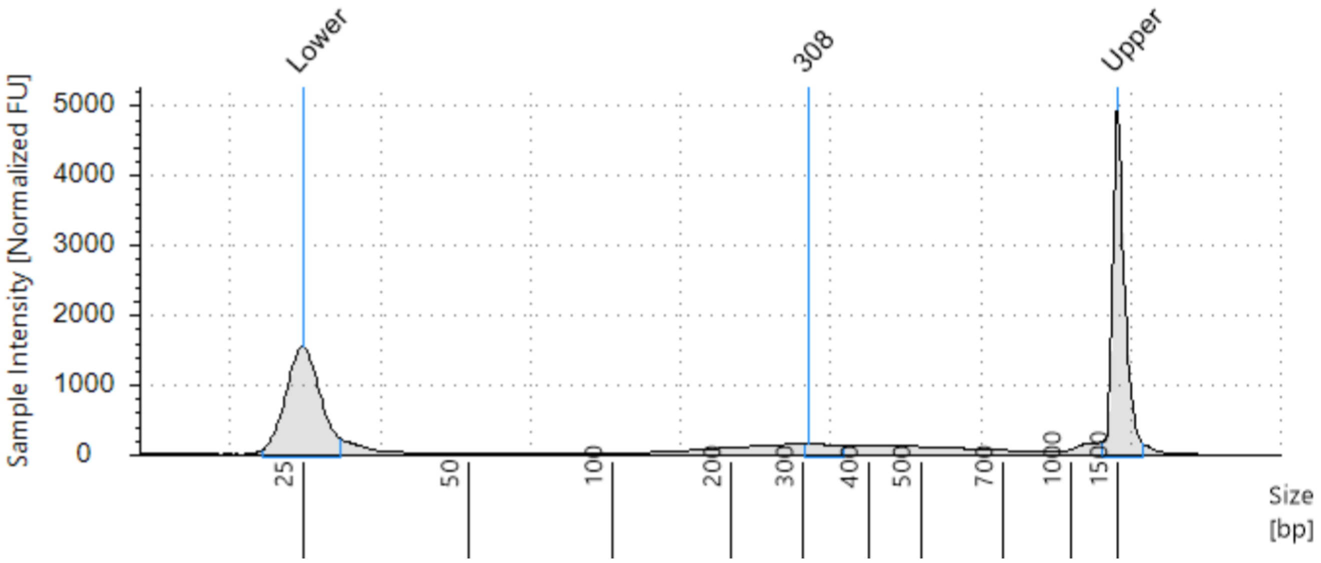

Sample Table

Well	Conc. [ng/µl]	Sample Description	Alert	Observations
H1	0.530	F4		

Peak Table

Size [bp]	Calibrated Conc. [ng/µl]	Assigned Conc. [ng/µl]	Peak Molarity [nmol/l]	% Integrated Area	Peak Comment	Observations
25	5.78	-	356	-		Lower Marker
308	0.530	-	2.64	100.00		
1500	6.50	6.50	6.67	-		Upper Marker

© Agilent Technologies, Inc. 2017

A2: G4

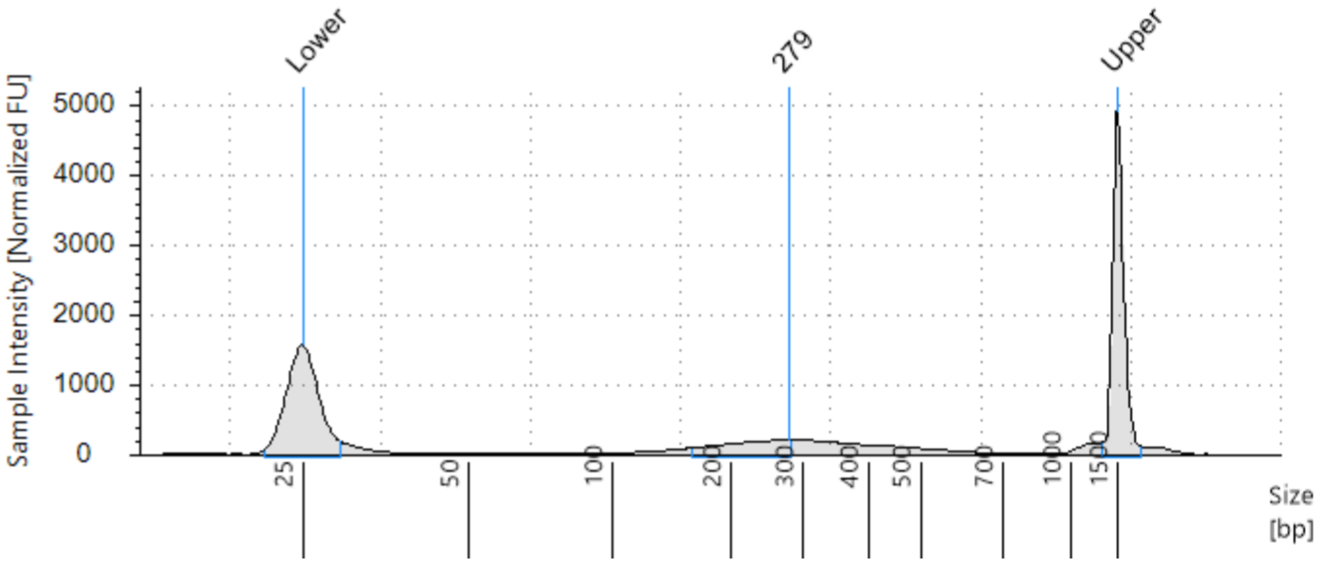

Sample Table

Well	Conc. [ng/µl]	Sample Description	Alert	Observations
A2	1.60	G4		

Peak Table

Size [bp]	Calibrated Conc. [ng/µl]	Assigned Conc. [ng/µl]	Peak Molarity [nmol/l]	% Integrated Area	Peak Comment	Observations
25	5.95	-	366	-		Lower Marker
279	1.60	-	8.81	100.00		
1500	6.50	6.50	6.67	-		Upper Marker

© Agilent Technologies, Inc. 2017

B2: H4

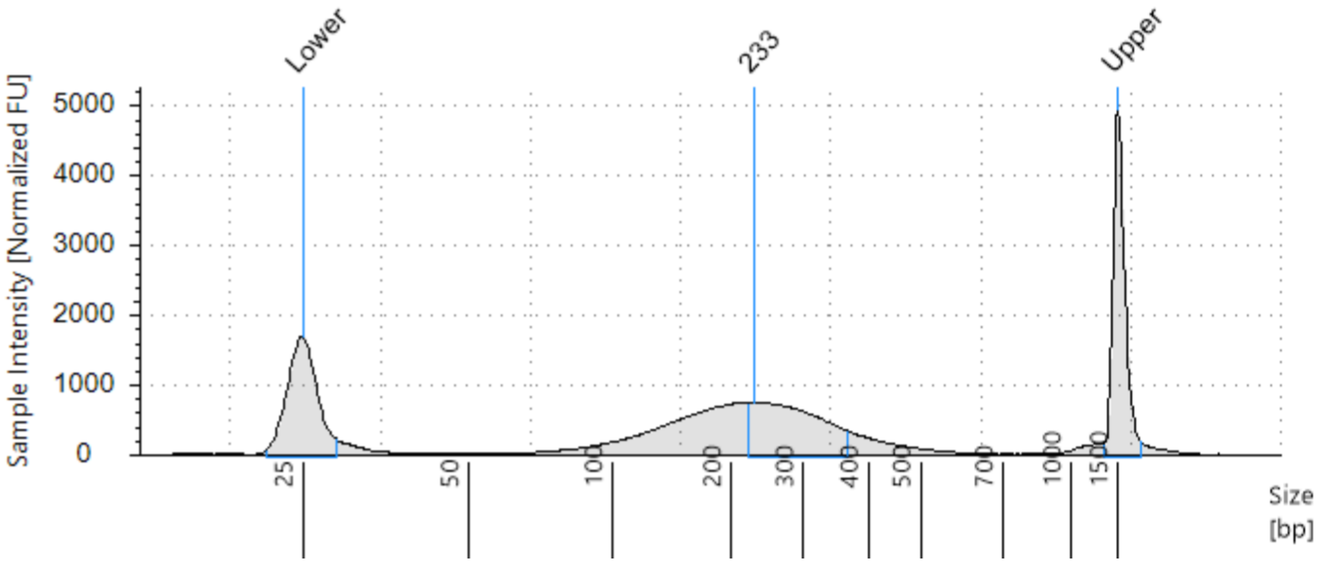

Sample Table

Well	Conc. [ng/µl]	Sample Description	Alert	Observations
B2	5.71	H4		

Peak Table

Size [bp]	Calibrated Conc. [ng/µl]	Assigned Conc. [ng/µl]	Peak Molarity [nmol/l]	% Integrated Area	Peak Comment	Observations
25	5.55	-	341	-		Lower Marker
233	5.71	-	37.7	100.00		
1500	6.50	6.50	6.67	-		Upper Marker

 © Agilent Technologies, Inc. 2017

C2: A5

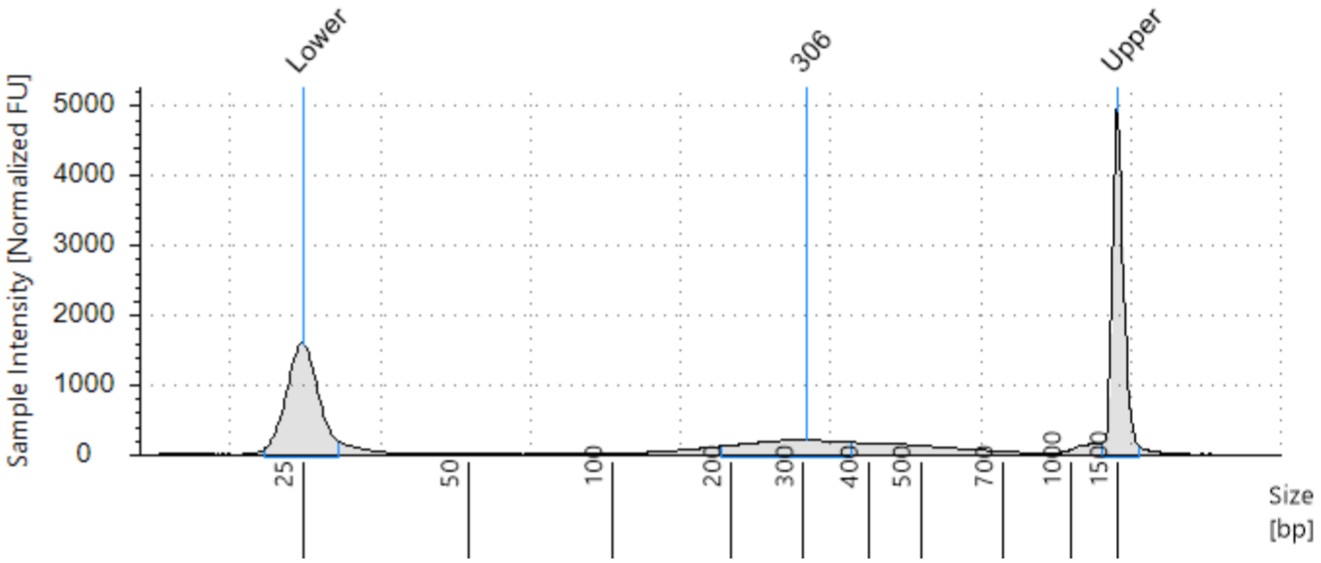

Sample Table

Well	Conc. [ng/µl]	Sample Description	Alert	Observations
C2	2.28	A5		

Peak Table

Size [bp]	Calibrated Conc. [ng/µl]	Assigned Conc. [ng/µl]	Peak Molarity [nmol/l]	% Integrated Area	Peak Comment	Observations
25	5.88	-	362	-		Lower Marker
306	2.28	-	11.5	100.00		
1500	6.50	6.50	6.67	-		Upper Marker

© Agilent Technologies, Inc. 2017

D2: B5

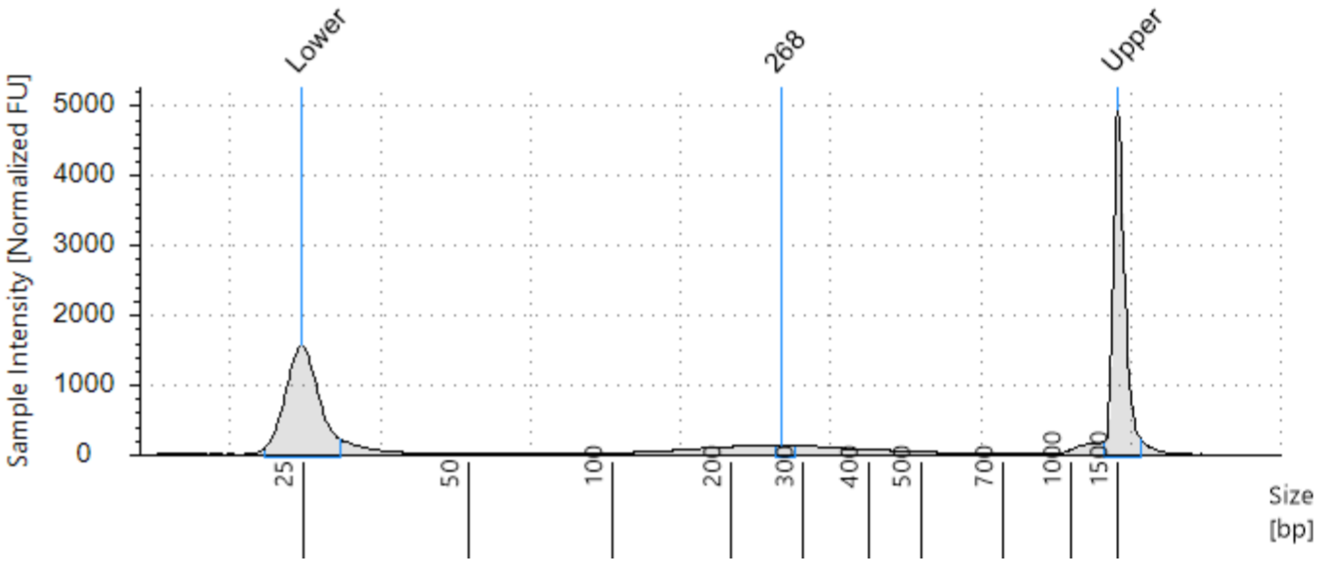

Sample Table

Well	Conc. [ng/µl]	Sample Description	Alert	Observations
D2	0.267	B5		

Peak Table

Size [bp]	Calibrated Conc. [ng/µl]	Assigned Conc. [ng/µl]	Peak Molarity [nmol/l]	% Integrated Area	Peak Comment	Observations
25	5.72	-	352	-		Lower Marker
268	0.267	-	1.53	100.00		
1500	6.50	6.50	6.67	-		Upper Marker

© Agilent Technologies, Inc. 2017

E2: C5

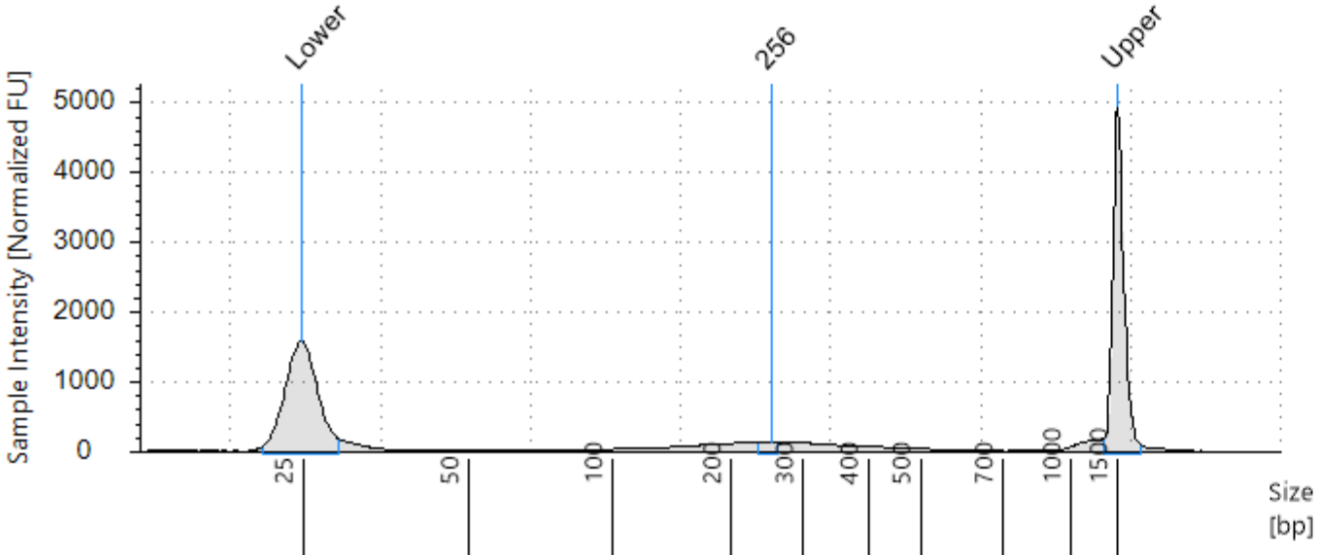

Sample Table

Well	Conc. [ng/µl]	Sample Description	Alert	Observations
E2	0.251	C5		

Peak Table

Size [bp]	Calibrated Conc. [ng/µl]	Assigned Conc. [ng/µl]	Peak Molarity [nmol/l]	% Integrated Area	Peak Comment	Observations
25	6.08	-	374	-		Lower Marker
256	0.251	-	1.51	100.00		
1500	6.50	6.50	6.67	-		Upper Marker

© Agilent Technologies, Inc. 2017

F2

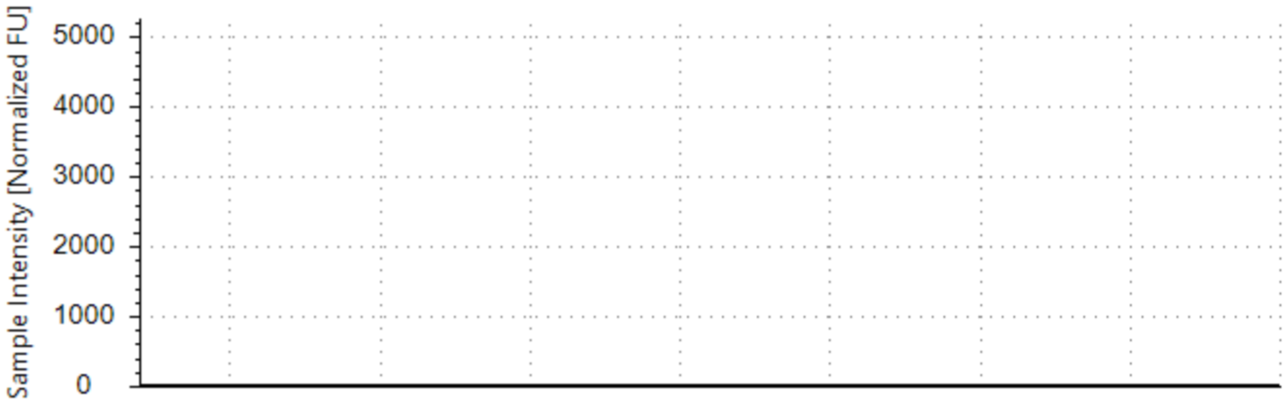

Sample Table

Well	Conc. [ng/µl]	Sample Description	Alert	Observations
F2			▲	Marker(s) not detected

© Agilent Technologies, Inc. 2017

G2: D5

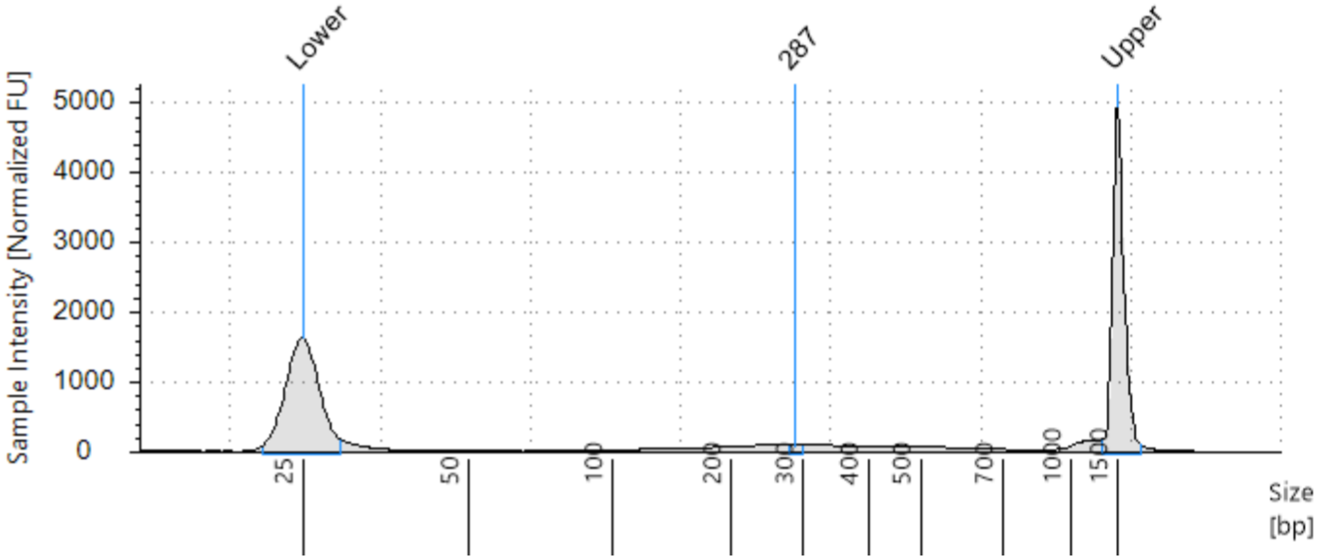

Sample Table

Well	Conc. [ng/µl]	Sample Description	Alert	Observations
G2	0.134	D5		

Peak Table

Size [bp]	Calibrated Conc. [ng/µl]	Assigned Conc. [ng/µl]	Peak Molarity [nmol/l]	% Integrated Area	Peak Comment	Observations
25	6.27	-	386	-		Lower Marker
287	0.134	-	0.720	100.00		
1500	6.50	6.50	6.67	-		Upper Marker

© Agilent Technologies, Inc. 2017

H2: E5

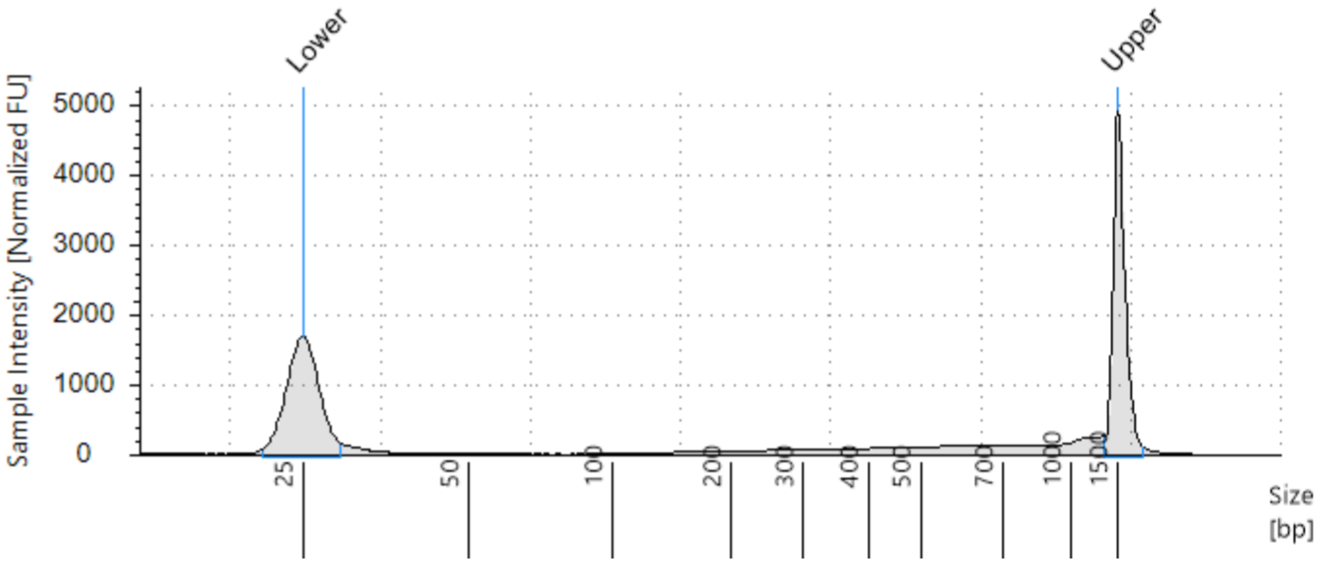

Sample Table

Well	Conc. [ng/µl]	Sample Description	Alert	Observations
H2		E5		

Peak Table

Size [bp]	Calibrated Conc. [ng/µl]	Assigned Conc. [ng/µl]	Peak Molarity [nmol/l]	% Integrated Area	Peak Comment	Observations
25	6.10	-	375	-		Lower Marker
1500	6.50	6.50	6.67	-		Upper Marker

 © Agilent Technologies, Inc. 2017

Filename: 2020-08-10-01.D1000.Q-S DFB minus from 3.8.20, G4-B5 R1.D1000

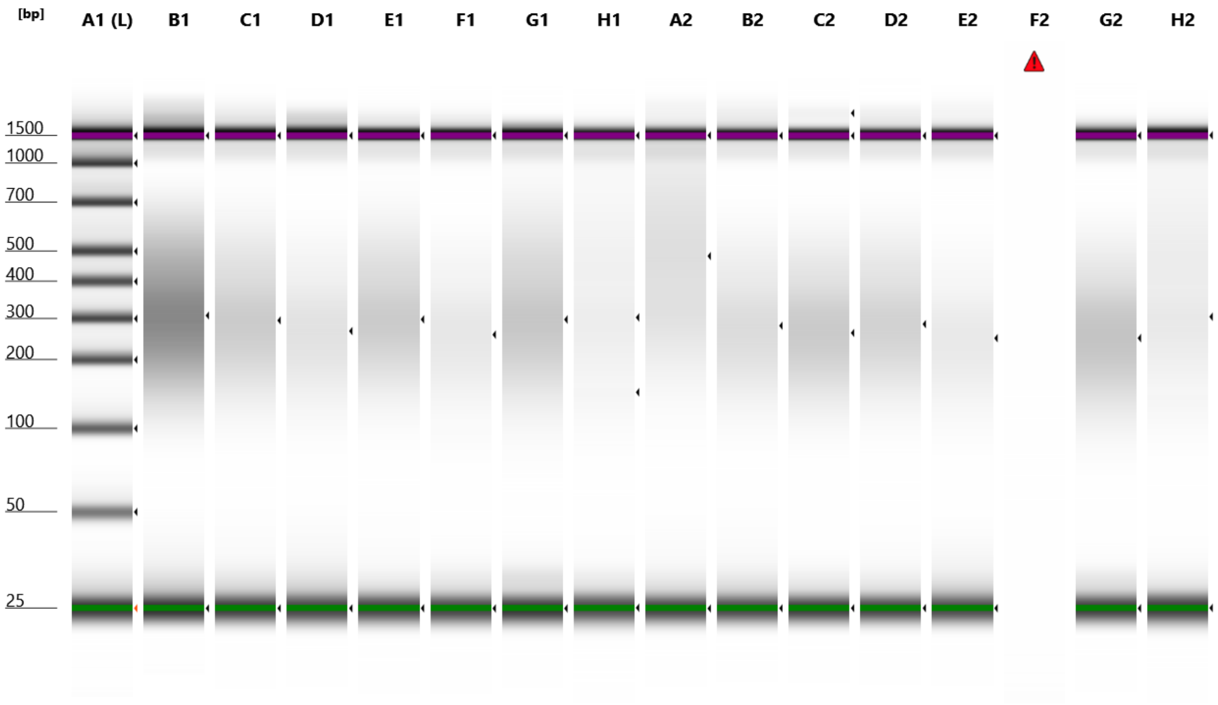

Default image (Contrast 100%)

Sample Info

Well	Conc. [ng/µl]	Sample Description	Alert	Observations
A1	17.8	Ladder		Ladder
B1	1.62	G4		
C1	1.74	E4		
D1	0.335	F4		
E1	2.09	G4		
F1	0.342	H4		
G1	1.85	A5		
H1	1.42	B5		
A2	1.15	C5		
B2	1.35	D5		
C2	4.56	E5		
D2	1.78	F5		
E2	0.383	G5		
F2		H5	▲	Marker(s) not detected
G2	4.99	A5		
H2	0.223	B5		

© Agilent Technologies, Inc. 2017

A1: Ladder

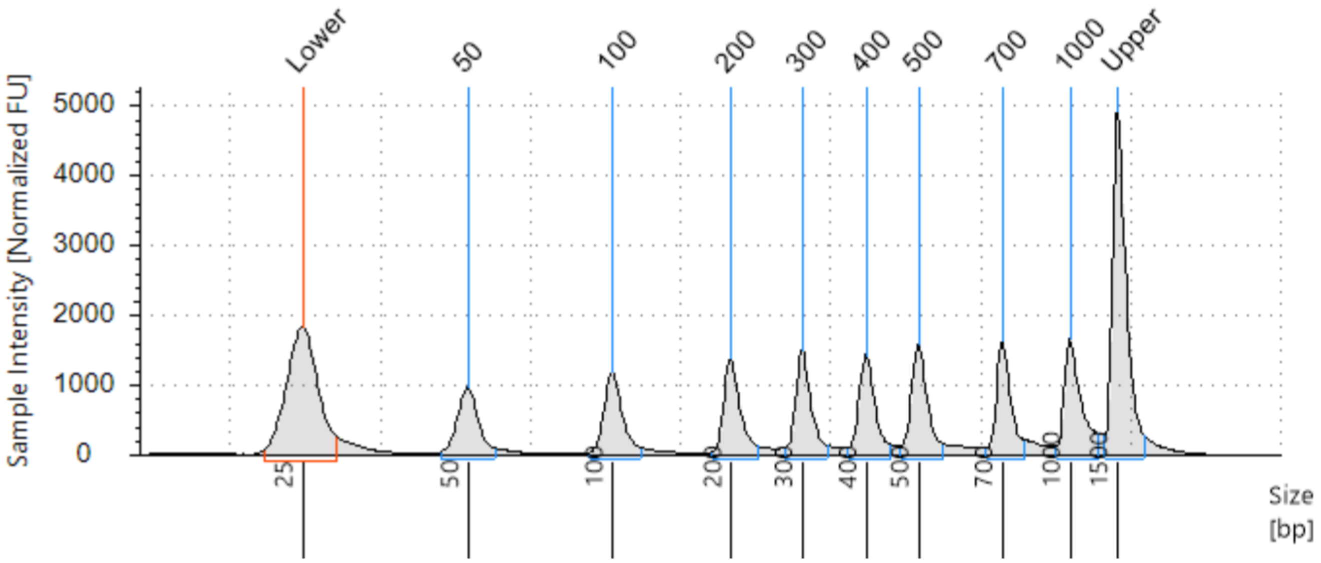

Sample Table

Well	Conc. [ng/µl]	Sample Description	Alert	Observations
A1	17.8	Ladder		Ladder

Peak Table

Size [bp]	Calibrated Conc. [ng/µl]	Assigned Conc. [ng/µl]	Peak Molarity [nmol/l]	% Integrated Area	Peak Comment	Observations
25	5.49	-	338	-		Lower Marker
50	1.91	-	58.7	10.71		
100	2.06	-	31.7	11.56		
200	2.15	-	16.5	12.07		
300	2.21	-	11.3	12.41		
400	2.21	-	8.49	12.39		
500	2.41	-	7.40	13.50		
700	2.24	-	4.93	12.59		
1000	2.63	-	4.05	14.76		
1500	6.50	6.50	6.67	-		Upper Marker

© Agilent Technologies, Inc. 2017

B1: G4

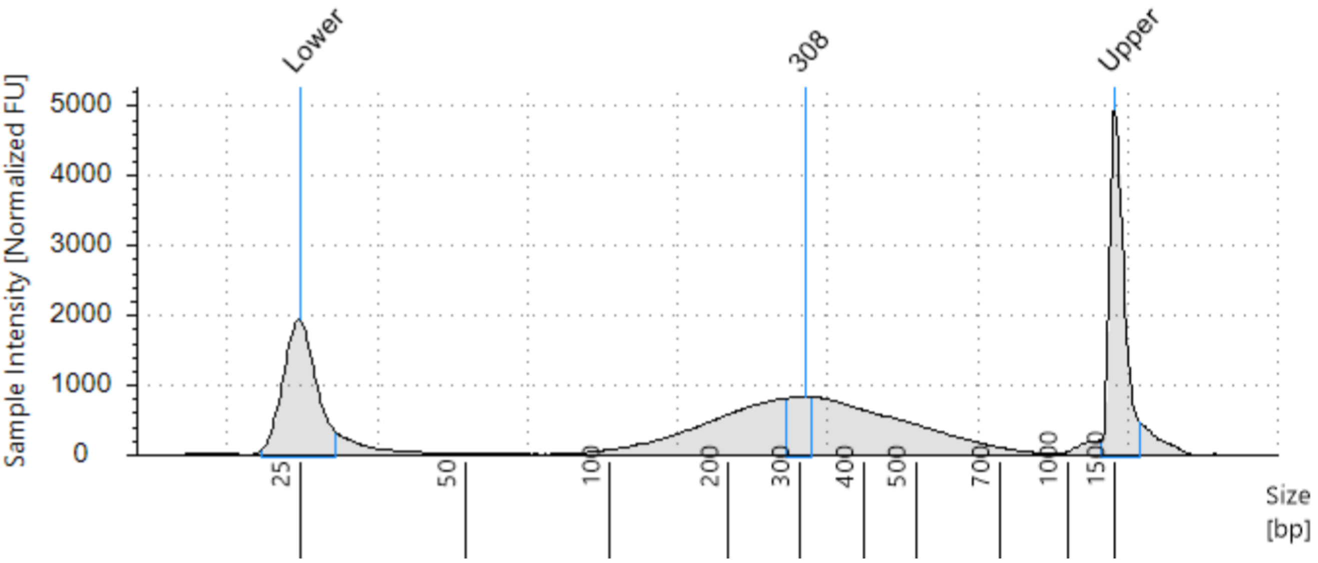

Sample Table

Well	Conc. [ng/µl]	Sample Description	Alert	Observations
B1	1.62	G4		

Peak Table

Size [bp]	Calibrated Conc. [ng/µl]	Assigned Conc. [ng/µl]	Peak Molarity [nmol/l]	% Integrated Area	Peak Comment	Observations
25	5.71	-	351	-		Lower Marker
308	1.62	-	8.08	100.00		
1500	6.50	6.50	6.67	-		Upper Marker

© Agilent Technologies, Inc. 2017

C1: E4

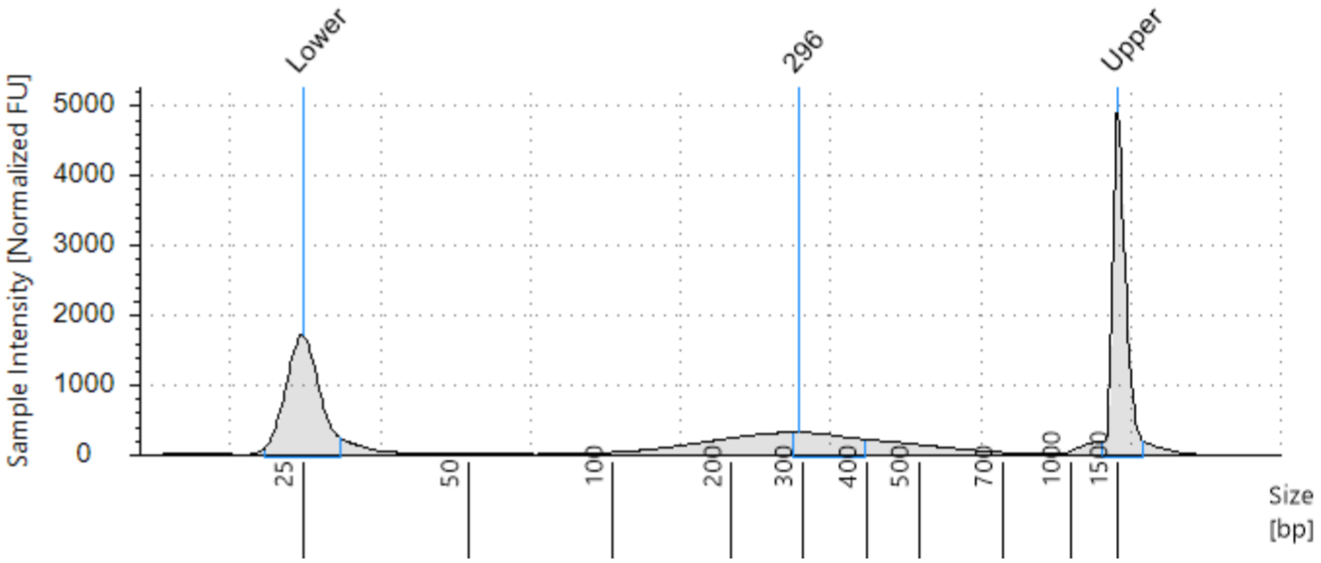

Sample Table

Well	Conc. [ng/µl]	Sample Description	Alert	Observations
C1	1.74	E4		

Peak Table

Size [bp]	Calibrated Conc. [ng/µl]	Assigned Conc. [ng/µl]	Peak Molarity [nmol/l]	% Integrated Area	Peak Comment	Observations
25	5.77	-	355	-		Lower Marker
296	1.74	-	9.06	100.00		
1500	6.50	6.50	6.67	-		Upper Marker

© Agilent Technologies, Inc. 2017

D1: F4

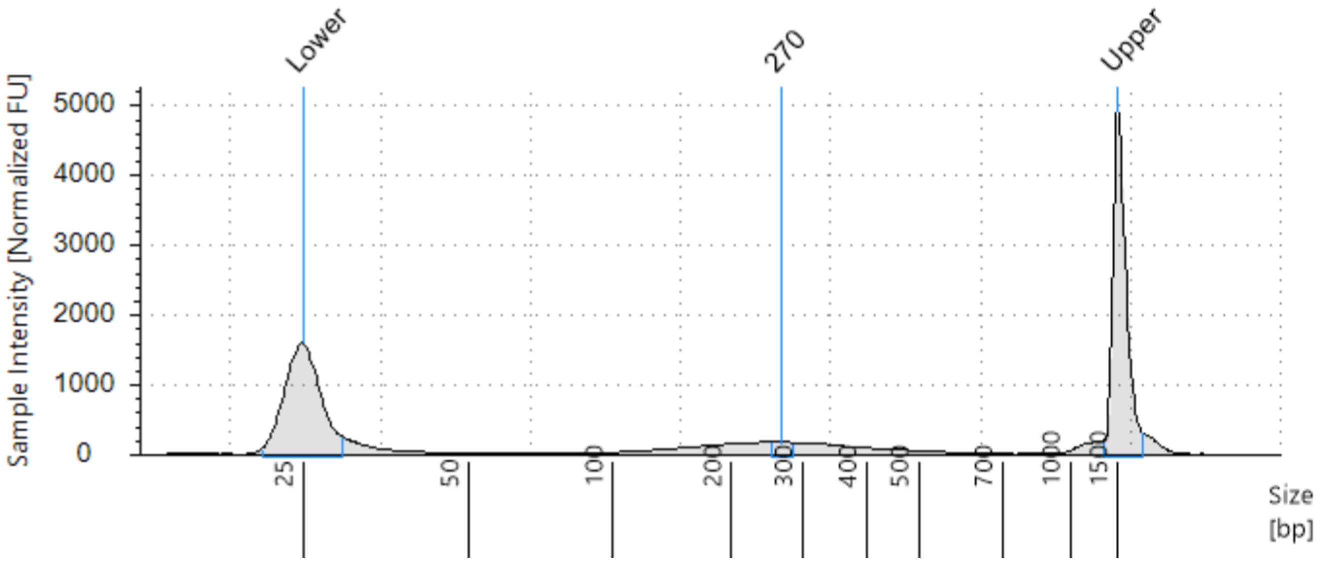

Sample Table

Well	Conc. [ng/µl]	Sample Description	Alert	Observations
D1	0.335	F4		

Peak Table

Size [bp]	Calibrated Conc. [ng/µl]	Assigned Conc. [ng/µl]	Peak Molarity [nmol/l]	% Integrated Area	Peak Comment	Observations
25	5.60	-	345	-		Lower Marker
270	0.335	-	1.91	100.00		
1500	6.50	6.50	6.67	-		Upper Marker

© Agilent Technologies, Inc. 2017

E1: G4

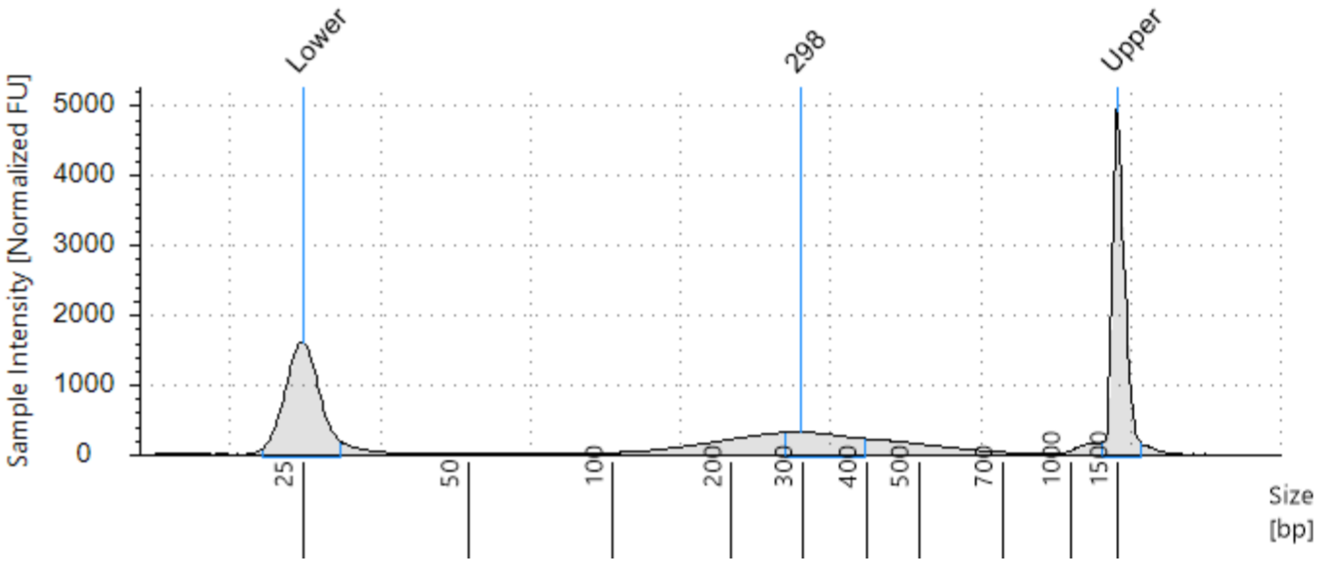

Sample Table

Well	Conc. [ng/µl]	Sample Description	Alert	Observations
E1	2.09	G4		

Peak Table

Size [bp]	Calibrated Conc. [ng/µl]	Assigned Conc. [ng/µl]	Peak Molarity [nmol/l]	% Integrated Area	Peak Comment	Observations
25	5.86	-	360	-		Lower Marker
298	2.09	-	10.8	100.00		
1500	6.50	6.50	6.67	-		Upper Marker

© Agilent Technologies, Inc. 2017

F1: H4

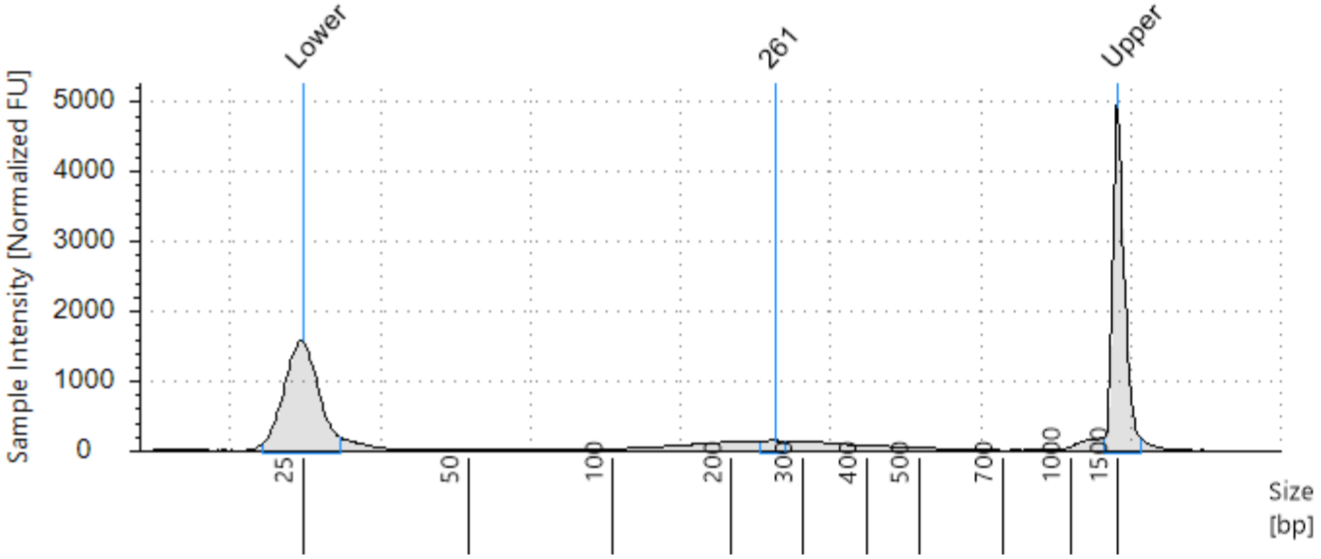

Sample Table

Well	Conc. [ng/µl]	Sample Description	Alert	Observations
F1	0.342	H4		

Peak Table

Size [bp]	Calibrated Conc. [ng/µl]	Assigned Conc. [ng/µl]	Peak Molarity [nmol/l]	% Integrated Area	Peak Comment	Observations
25	5.99	-	368	-		Lower Marker
261	0.342	-	2.02	100.00		
1500	6.50	6.50	6.67	-		Upper Marker

© Agilent Technologies, Inc. 2017

G1: A5

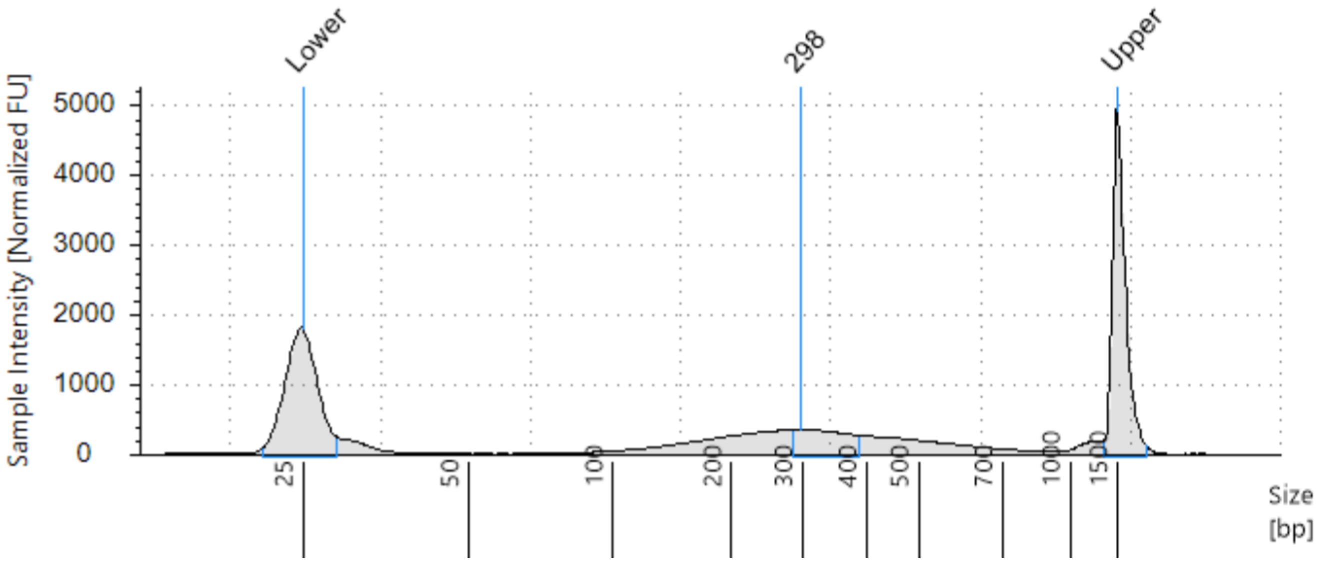

Sample Table

Well	Conc. [ng/µl]	Sample Description	Alert	Observations
G1	1.85	A5		

Peak Table

Size [bp]	Calibrated Conc. [ng/µl]	Assigned Conc. [ng/µl]	Peak Molarity [nmol/l]	% Integrated Area	Peak Comment	Observations
25	5.95	-	366	-		Lower Marker
298	1.85	-	9.56	100.00		
1500	6.50	6.50	6.67	-		Upper Marker

© Agilent Technologies, Inc. 2017

H1: B5

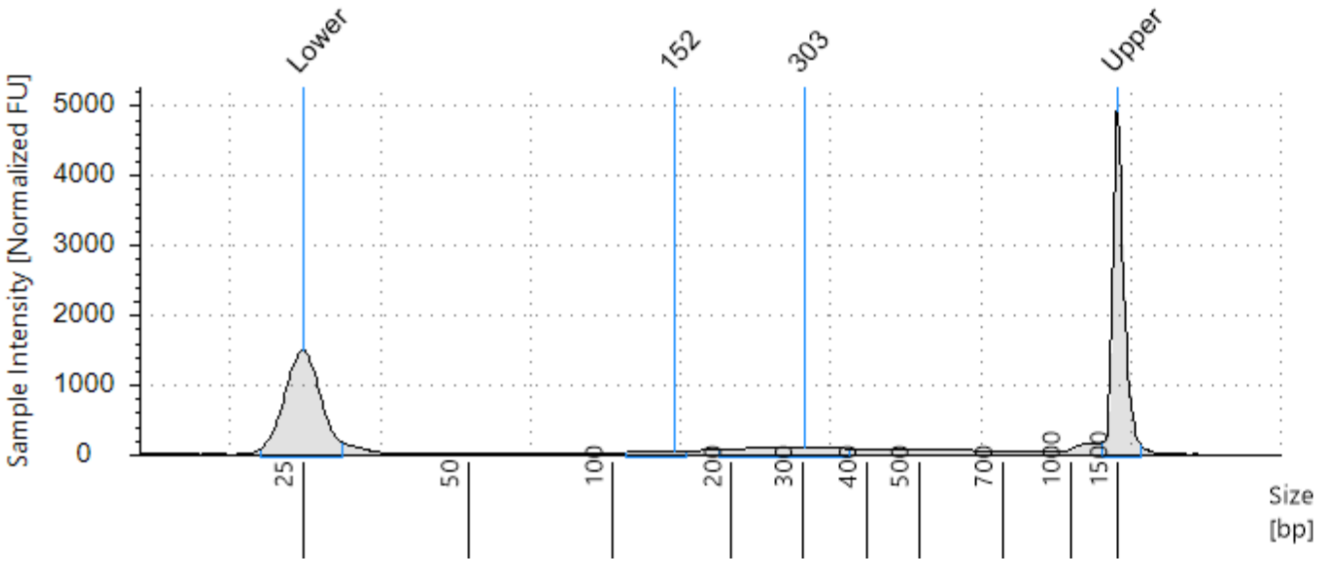

Sample Table

Well	Conc. [ng/µl]	Sample Description	Alert	Observations
H1	1.42	B5		

Peak Table

Size [bp]	Calibrated Conc. [ng/µl]	Assigned Conc. [ng/µl]	Peak Molarity [nmol/l]	% Integrated Area	Peak Comment	Observations
25	6.12	-	376	-		Lower Marker
152	0.254	-	2.57	17.92		
303	1.16	-	5.91	82.08		
1500	6.50	6.50	6.67	-		Upper Marker

© Agilent Technologies, Inc. 2017

A2: C5

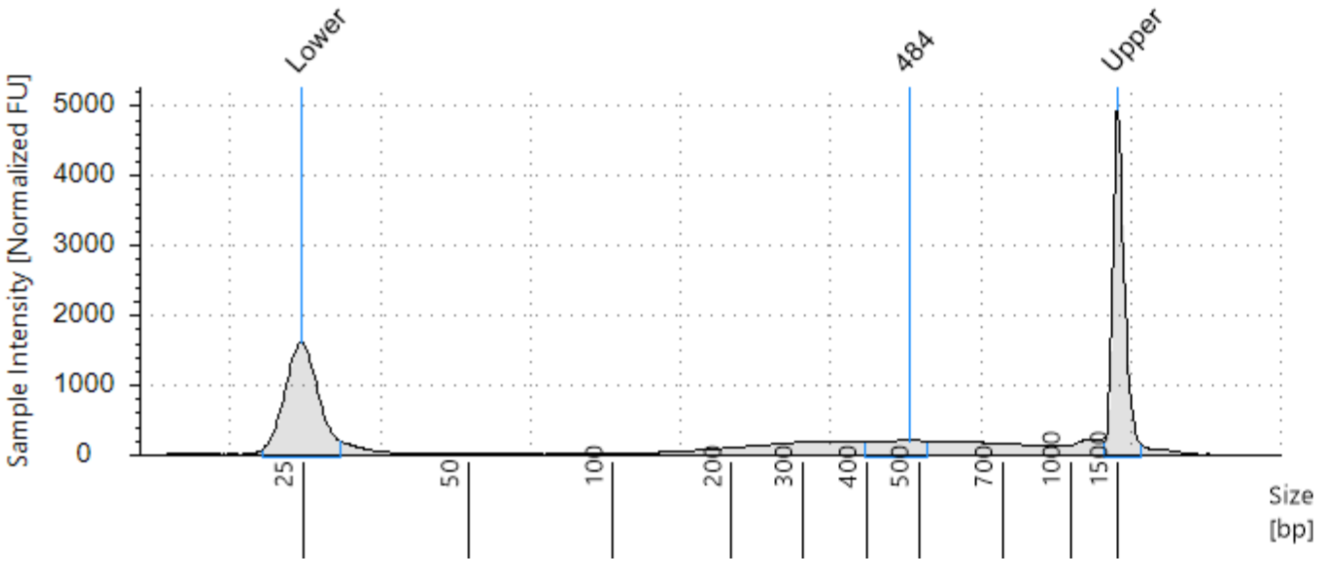

Sample Table

Well	Conc. [ng/µl]	Sample Description	Alert	Observations
A2	1.15	C5		

Peak Table

Size [bp]	Calibrated Conc. [ng/µl]	Assigned Conc. [ng/µl]	Peak Molarity [nmol/l]	% Integrated Area	Peak Comment	Observations
25	5.91	-	364	-		Lower Marker
484	1.15	-	3.67	100.00		
1500	6.50	6.50	6.67	-		Upper Marker

 © Agilent Technologies, Inc. 2017

B2: D5

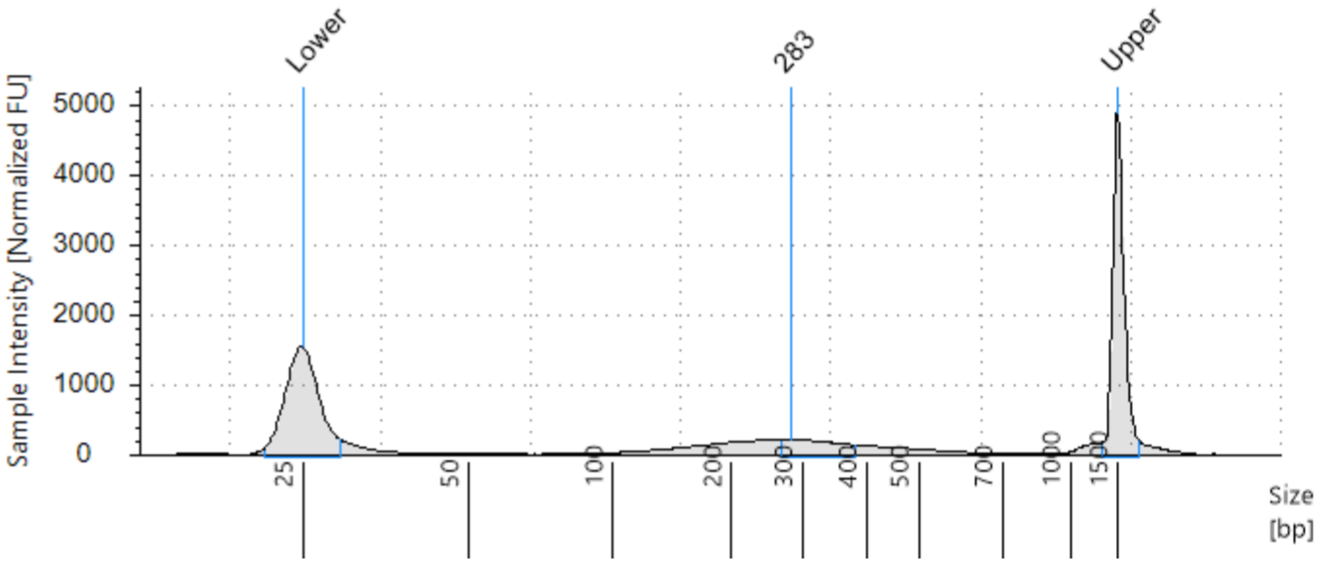

Sample Table

Well	Conc. [ng/µl]	Sample Description	Alert	Observations
B2	1.35	D5		

Peak Table

Size [bp]	Calibrated Conc. [ng/µl]	Assigned Conc. [ng/µl]	Peak Molarity [nmol/l]	% Integrated Area	Peak Comment	Observations
25	5.96	-	367	-		Lower Marker
283	1.35	-	7.34	100.00		
1500	6.50	6.50	6.67	-		Upper Marker

© Agilent Technologies, Inc. 2017

C2: E5

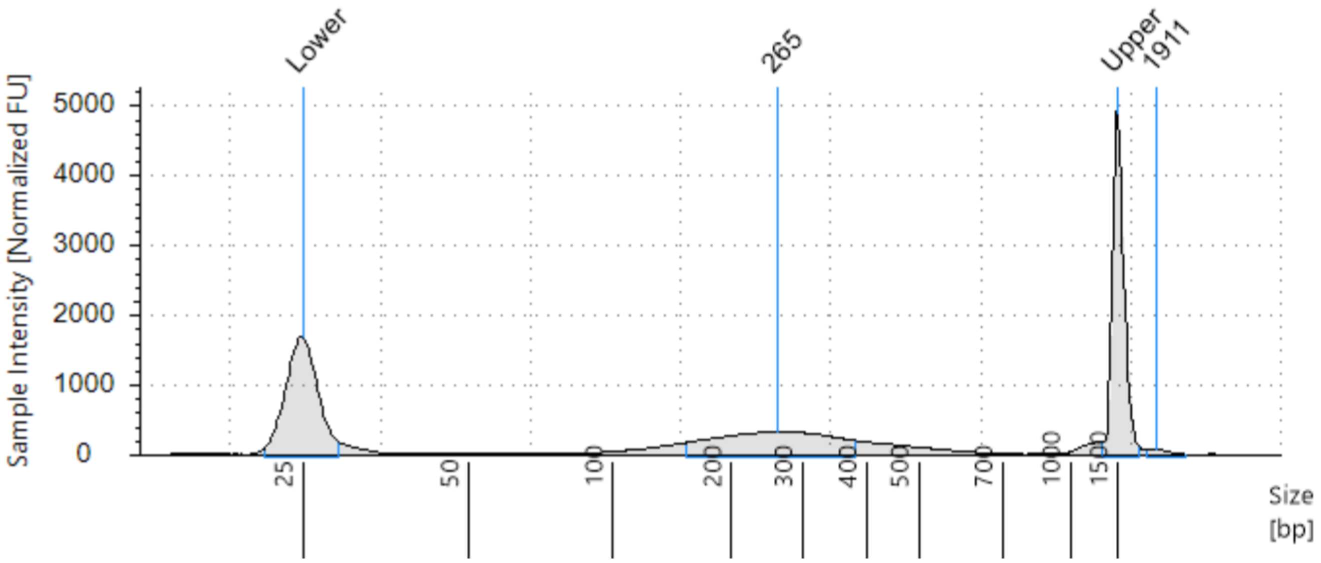

Sample Table

Well	Conc. [ng/µl]	Sample Description	Alert	Observations
C2	4.56	E5		

Peak Table

Size [bp]	Calibrated Conc. [ng/µl]	Assigned Conc. [ng/µl]	Peak Molarity [nmol/l]	% Integrated Area	Peak Comment	Observations
25	6.14	-	378	-		Lower Marker
265	4.39	-	25.5	96.37		
1500	6.50	6.50	6.67	-		Upper Marker
1911	0.166	-	0.133	3.63		

 © Agilent Technologies, Inc. 2017

D2: F5

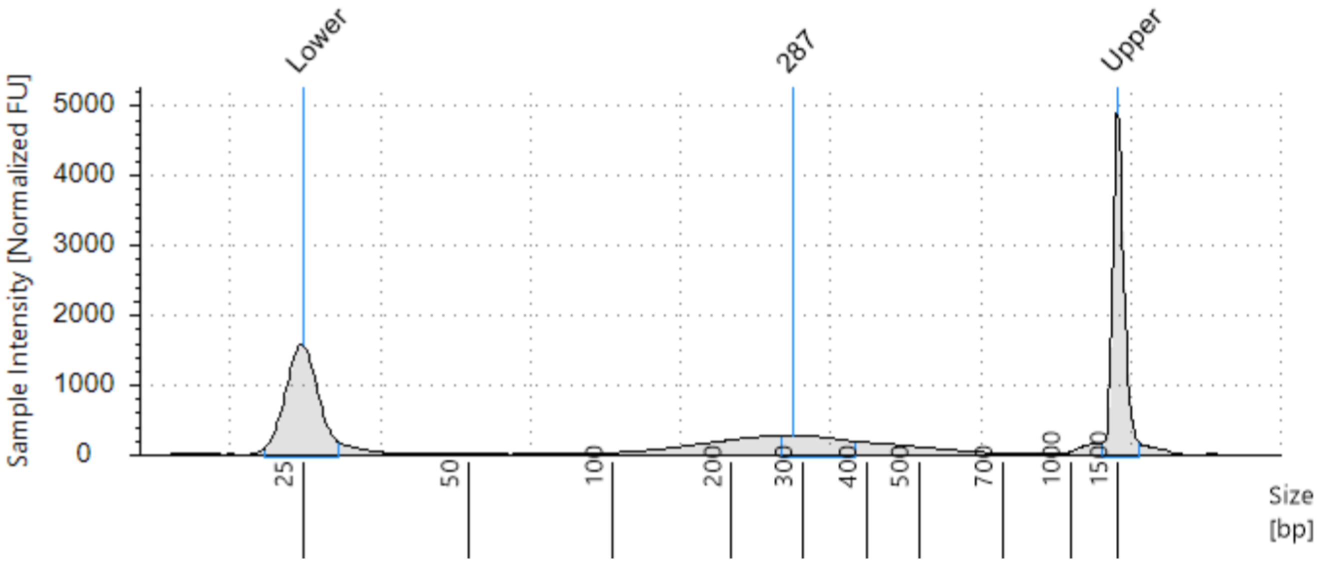

Sample Table

Well	Conc. [ng/µl]	Sample Description	Alert	Observations
D2	1.78	F5		

Peak Table

Size [bp]	Calibrated Conc. [ng/µl]	Assigned Conc. [ng/µl]	Peak Molarity [nmol/l]	% Integrated Area	Peak Comment	Observations
25	5.89	-	363	-		Lower Marker
287	1.78	-	9.54	100.00		
1500	6.50	6.50	6.67	-		Upper Marker

© Agilent Technologies, Inc. 2017

E2: G5

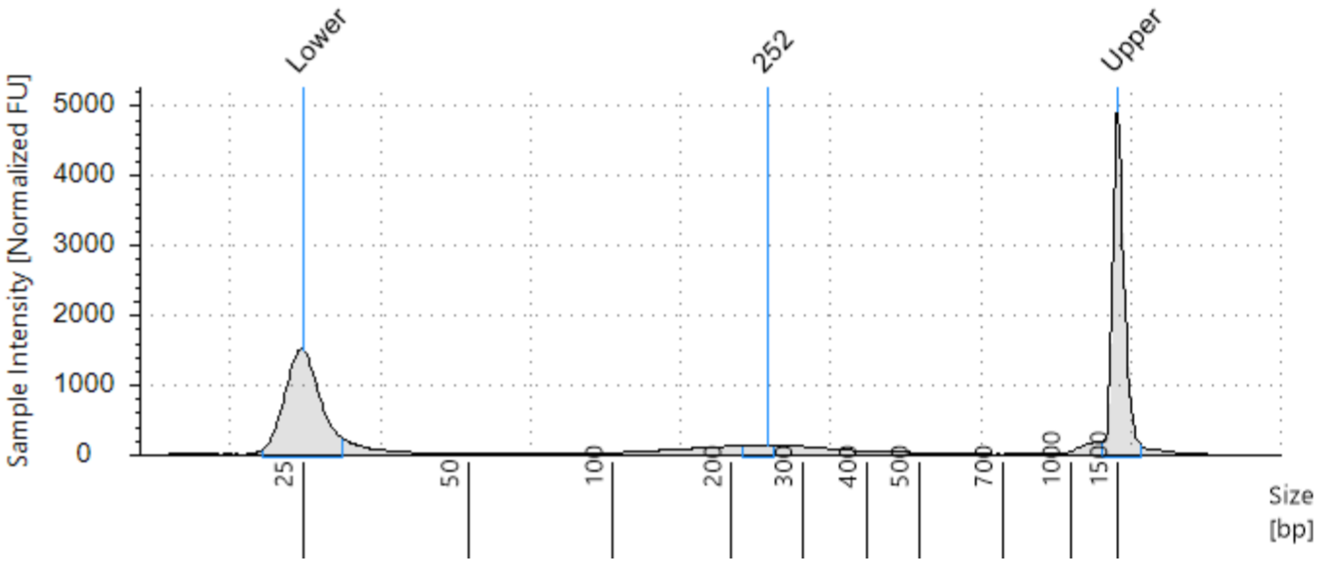

Sample Table

Well	Conc. [ng/µl]	Sample Description	Alert	Observations
E2	0.383	G5		

Peak Table

Size [bp]	Calibrated Conc. [ng/µl]	Assigned Conc. [ng/µl]	Peak Molarity [nmol/l]	% Integrated Area	Peak Comment	Observations
25	5.98	-	368	-		Lower Marker
252	0.383	-	2.33	100.00		
1500	6.50	6.50	6.67	-		Upper Marker

 © Agilent Technologies, Inc. 2017

F2: H5

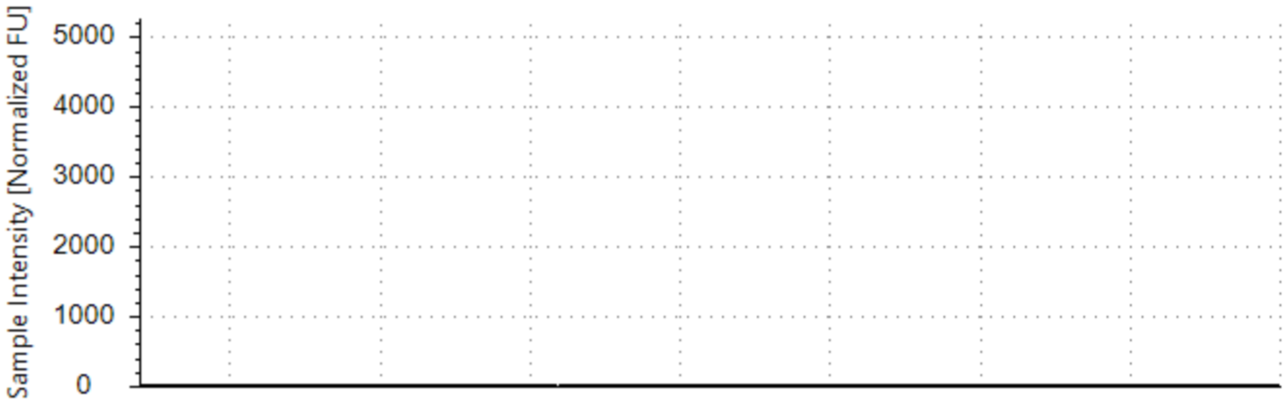

Sample Table

Well	Conc. [ng/µl]	Sample Description	Alert	Observations
F2		H5	▲	Marker(s) not detected

© Agilent Technologies, Inc. 2017

G2: A5

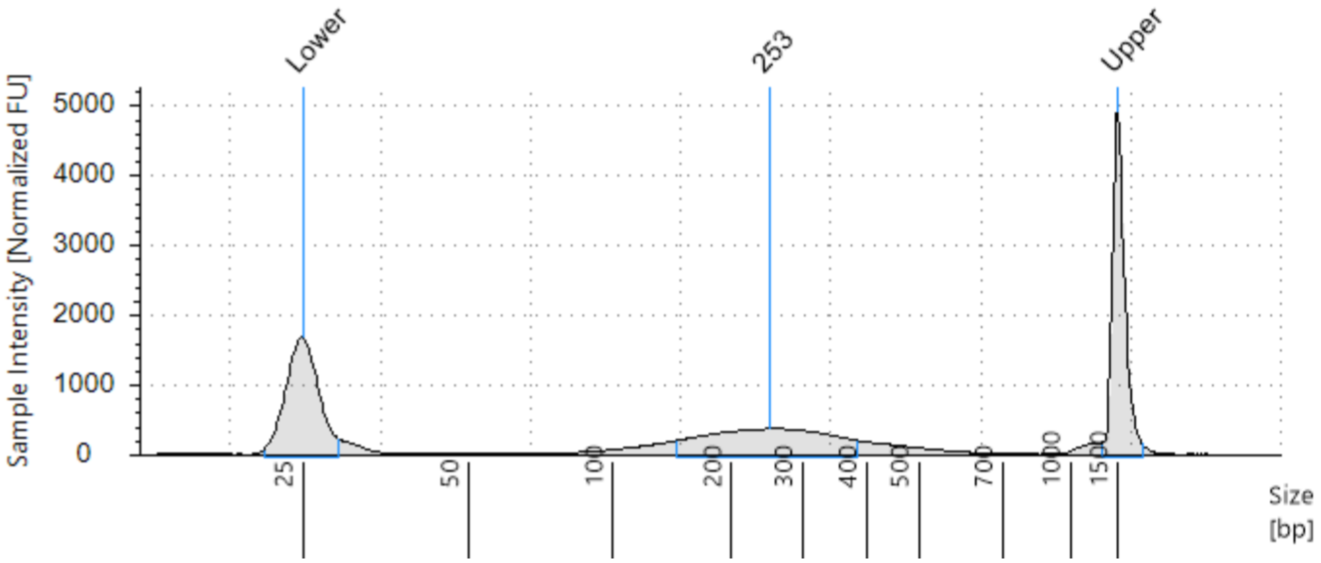

Sample Table

Well	Conc. [ng/µl]	Sample Description	Alert	Observations
G2	4.99	A5		

Peak Table

Size [bp]	Calibrated Conc. [ng/µl]	Assigned Conc. [ng/µl]	Peak Molarity [nmol/l]	% Integrated Area	Peak Comment	Observations
25	5.84	-	360	-		Lower Marker
253	4.99	-	30.4	100.00		
1500	6.50	6.50	6.67	-		Upper Marker

© Agilent Technologies, Inc. 2017

H2: B5

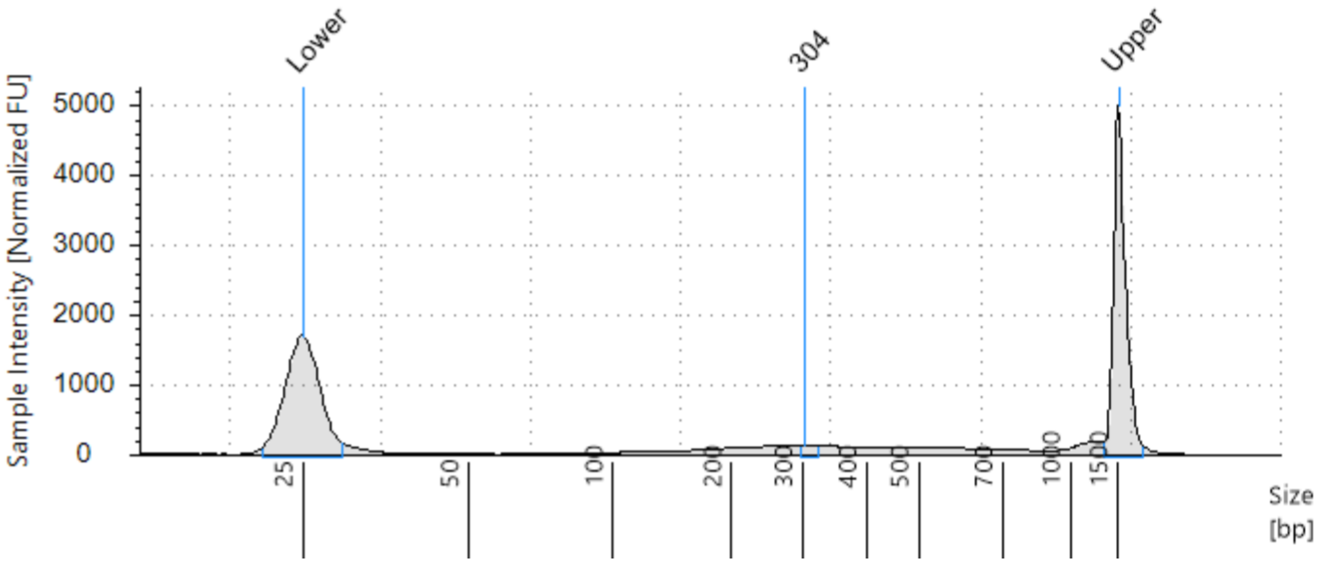

Sample Table

Well	Conc. [ng/µl]	Sample Description	Alert	Observations
H2	0.223	B5		

Peak Table

Size [bp]	Calibrated Conc. [ng/µl]	Assigned Conc. [ng/µl]	Peak Molarity [nmol/l]	% Integrated Area	Peak Comment	Observations
25	6.36	-	391	-		Lower Marker
304	0.223	-	1.13	100.00		
1500	6.50	6.50	6.67	-		Upper Marker

© Agilent Technologies, Inc. 2017

Filename: 2020-08-10-02.D1000,Q-S DFB minus from 3.8.20, F6-E8 R1.D1000

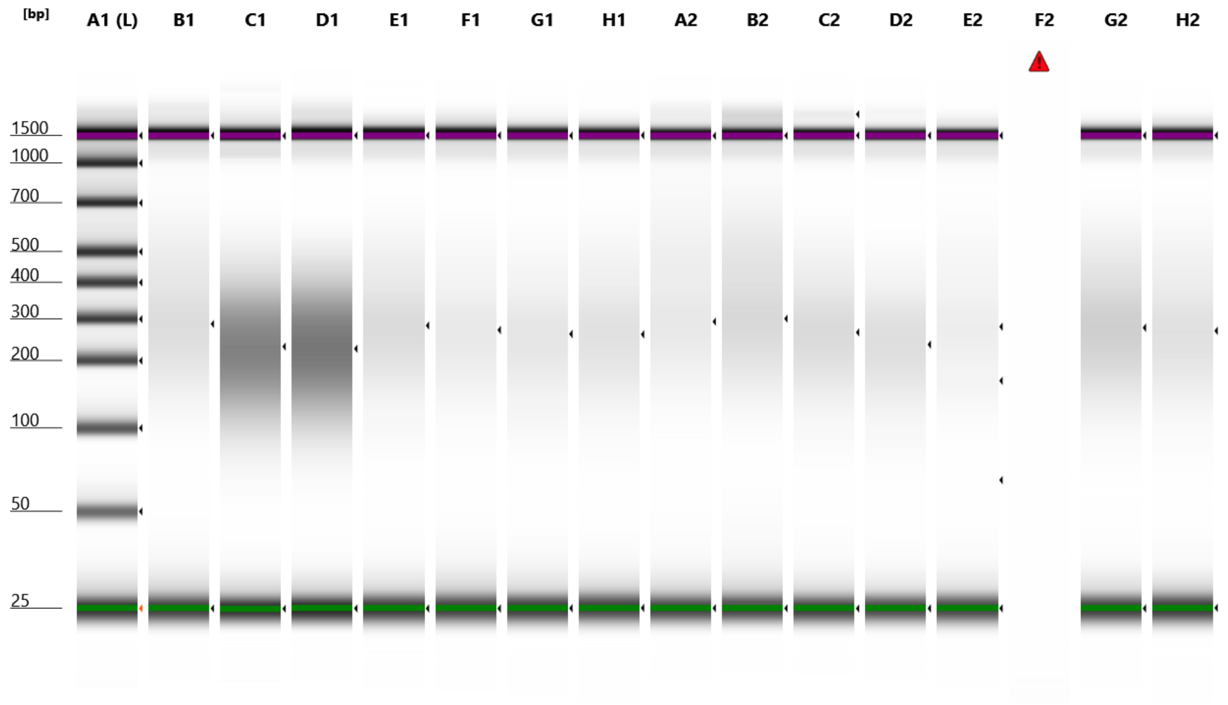

Default image (Contrast 100%)

Sample Info

Well	Conc. [ng/µl]	Sample Description	Alert	Observations
A1	21.1	Ladder		Ladder
B1	1.45	F6		
C1	5.82	G6		
D1	6.52	H6		
E1	1.26	A7		
F1	0.197	B7		
G1	0.850	C7		
H1	0.386	D7		
A2	0.174	E7		
B2	0.484	F7		
C2	0.462	G7		
D2	1.74	H7		
E2	1.11	A8		
F2		B8	▲	Marker(s) not detected
G2	0.753	C8		
H2	0.266	D8		

© Agilent Technologies, Inc. 2017

A1: Ladder

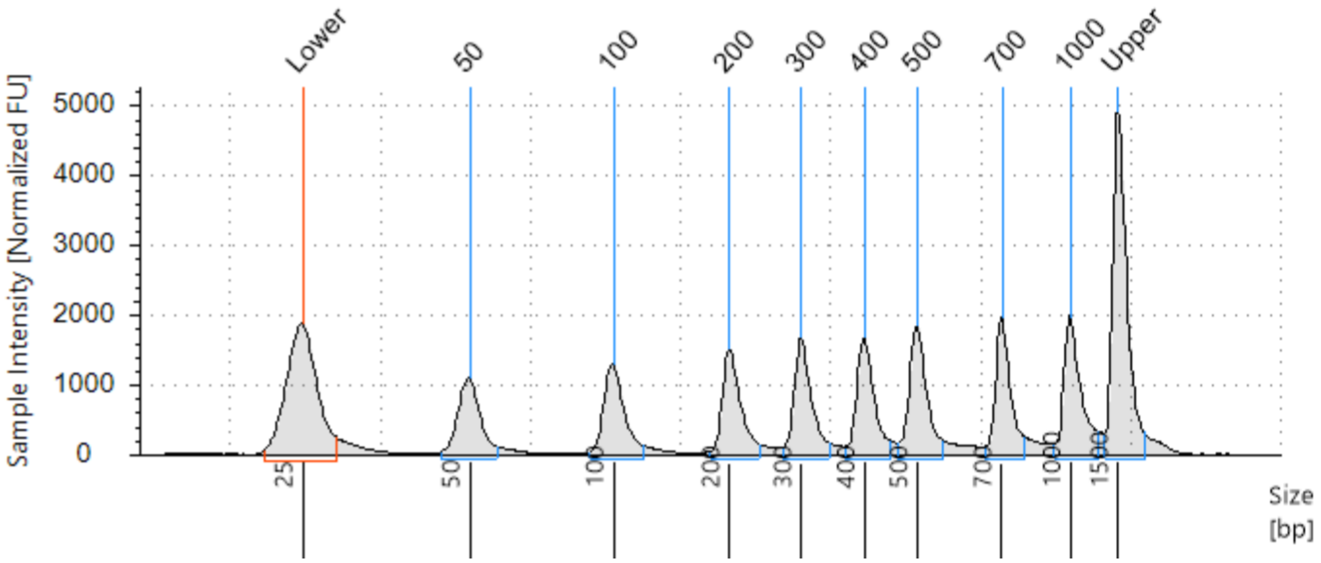

Sample Table

Well	Conc. [ng/µl]	Sample Description	Alert	Observations
A1	21.1	Ladder		Ladder

Peak Table

Size [bp]	Calibrated Conc. [ng/µl]	Assigned Conc. [ng/µl]	Peak Molarity [nmol/l]	% Integrated Area	Peak Comment	Observations
25	5.26	-	324	-		Lower Marker
50	2.21	-	68.0	10.49		
100	2.41	-	37.1	11.45		
200	2.52	-	19.4	11.95		
300	2.63	-	13.5	12.46		
400	2.67	-	10.3	12.66		
500	2.92	-	8.99	13.87		
700	2.66	-	5.85	12.62		
1000	3.05	-	4.70	14.49		
1500	6.50	6.50	6.67	-		Upper Marker

© Agilent Technologies, Inc. 2017

B1: F6

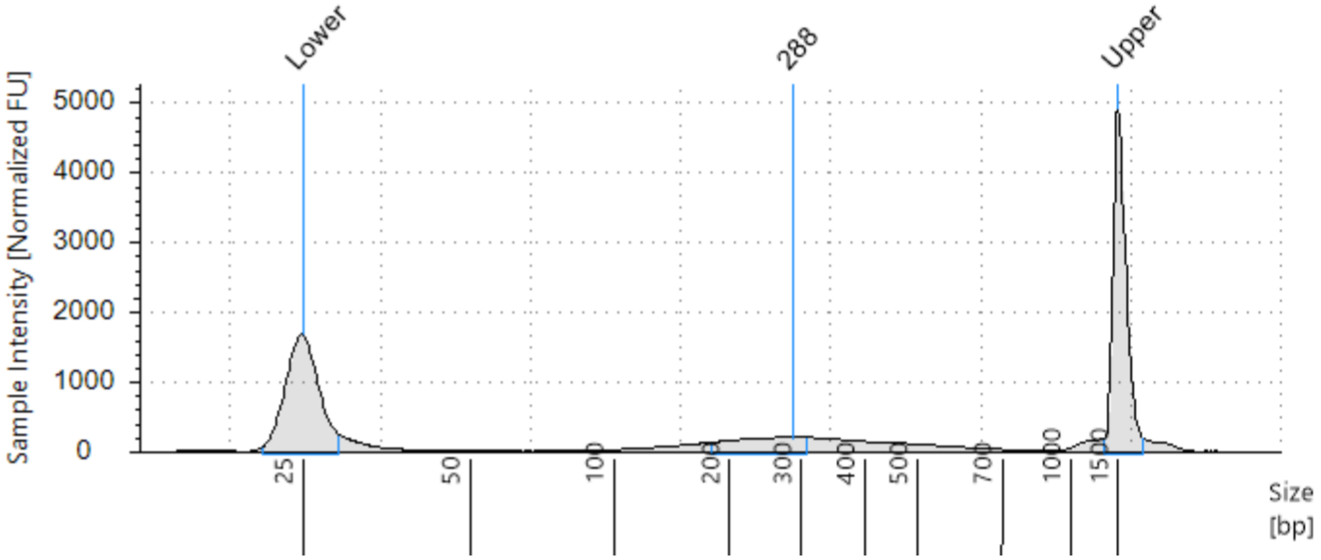

Sample Table

Well	Conc. [ng/µl]	Sample Description	Alert	Observations
B1	1.45	F6		

Peak Table

Size [bp]	Calibrated Conc. [ng/µl]	Assigned Conc. [ng/µl]	Peak Molarity [nmol/l]	% Integrated Area	Peak Comment	Observations
25	5.59	-	344	-		Lower Marker
288	1.45	-	7.75	100.00		
1500	6.50	6.50	6.67	-		Upper Marker

© Agilent Technologies, Inc. 2017

C1: G6

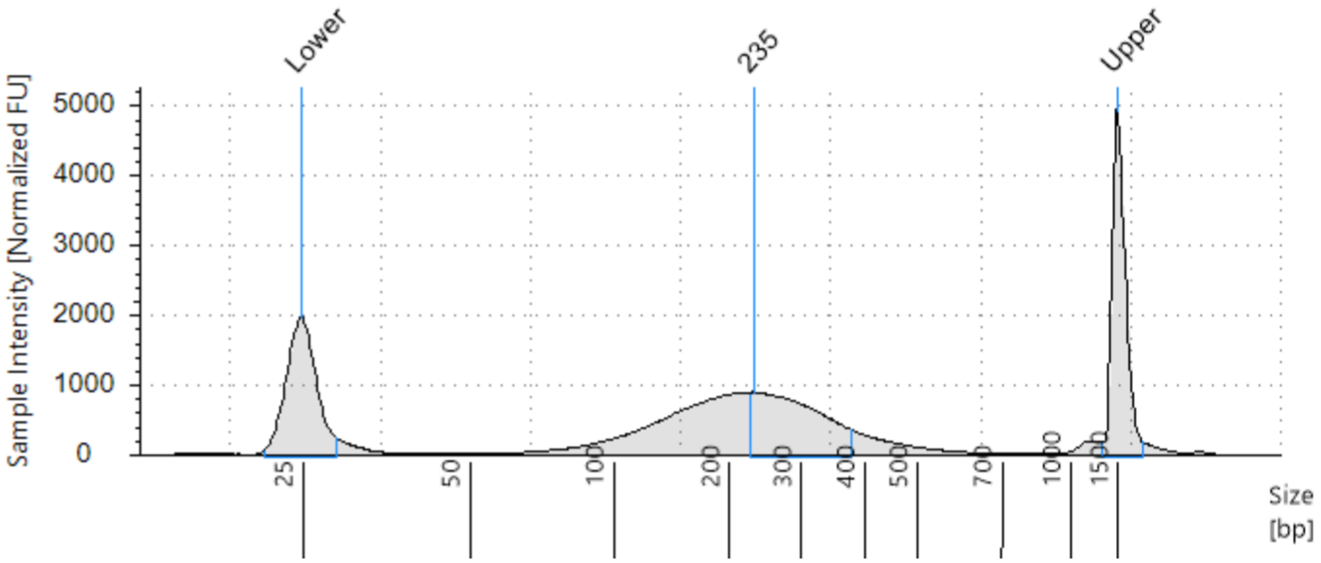

Sample Table

Well	Conc. [ng/µl]	Sample Description	Alert	Observations
C1	5.82	G6		

Peak Table

Size [bp]	Calibrated Conc. [ng/µl]	Assigned Conc. [ng/µl]	Peak Molarity [nmol/l]	% Integrated Area	Peak Comment	Observations
25	5.72	-	352	-		Lower Marker
235	5.82	-	38.2	100.00		
1500	6.50	6.50	6.67	-		Upper Marker

 © Agilent Technologies, Inc. 2017

D1: H6

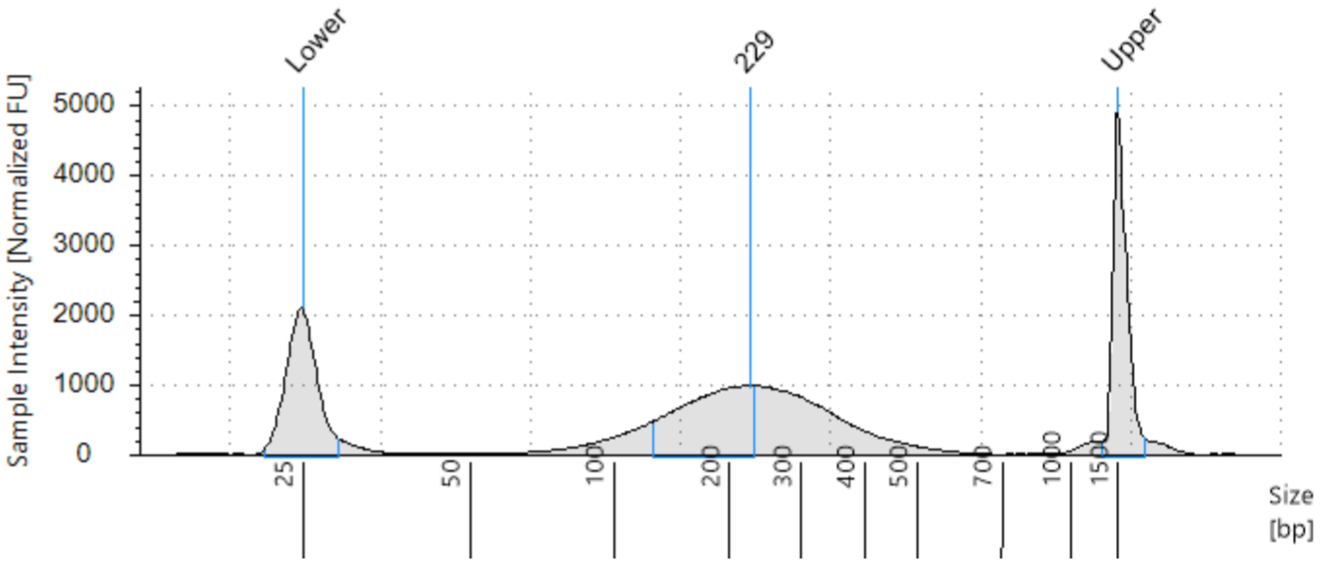

Sample Table

Well	Conc. [ng/µl]	Sample Description	Alert	Observations
D1	6.52	H6		

Peak Table

Size [bp]	Calibrated Conc. [ng/µl]	Assigned Conc. [ng/µl]	Peak Molarity [nmol/l]	% Integrated Area	Peak Comment	Observations
25	6.04	-	372	-		Lower Marker
229	6.52	-	43.8	100.00		
1500	6.50	6.50	6.67	-		Upper Marker

© Agilent Technologies, Inc. 2017

E1: A7

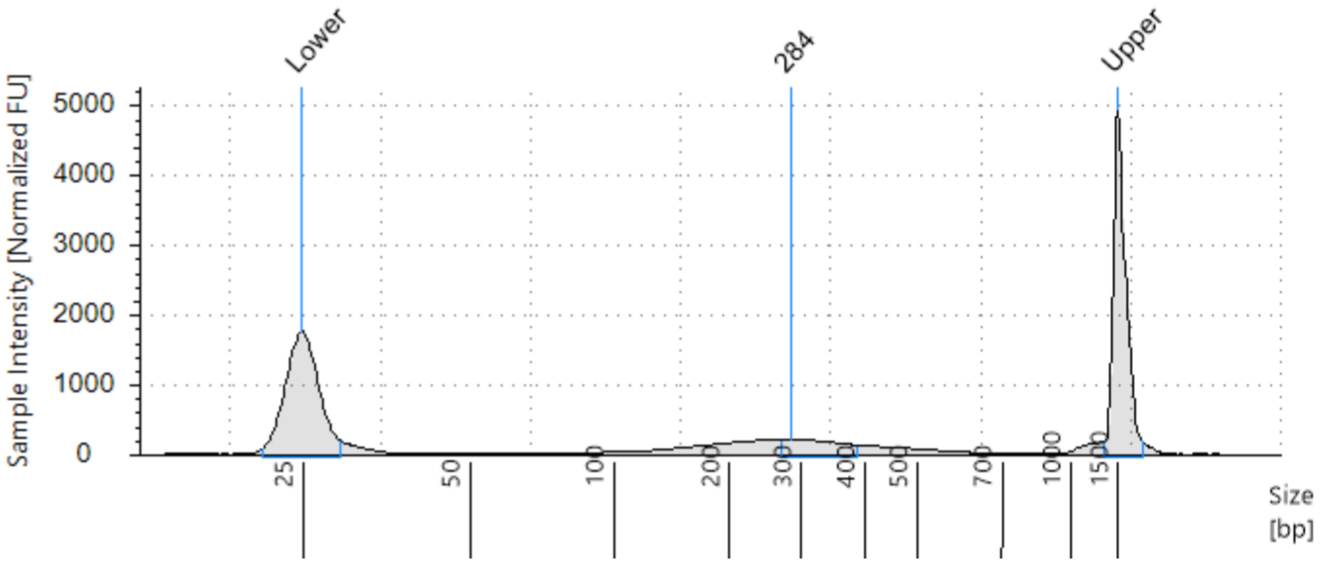

Sample Table

Well	Conc. [ng/µl]	Sample Description	Alert	Observations
E1	1.26	A7		

Peak Table

Size [bp]	Calibrated Conc. [ng/µl]	Assigned Conc. [ng/µl]	Peak Molarity [nmol/l]	% Integrated Area	Peak Comment	Observations
25	6.18	-	380	-		Lower Marker
284	1.26	-	6.80	100.00		
1500	6.50	6.50	6.67	-		Upper Marker

© Agilent Technologies, Inc. 2017

F1: B7

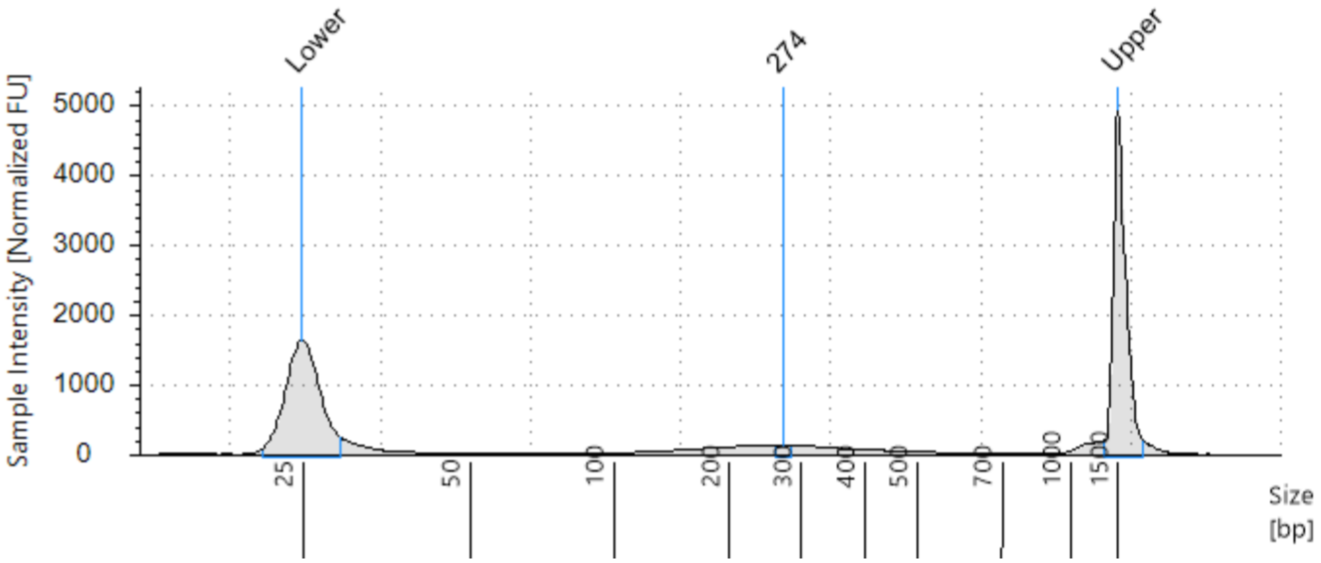

Sample Table

Well	Conc. [ng/µl]	Sample Description	Alert	Observations
F1	0.197	B7		

Peak Table

Size [bp]	Calibrated Conc. [ng/µl]	Assigned Conc. [ng/µl]	Peak Molarity [nmol/l]	% Integrated Area	Peak Comment	Observations
25	5.89	-	362	-		Lower Marker
274	0.197	-	1.11	100.00		
1500	6.50	6.50	6.67	-		Upper Marker

Gl: C7

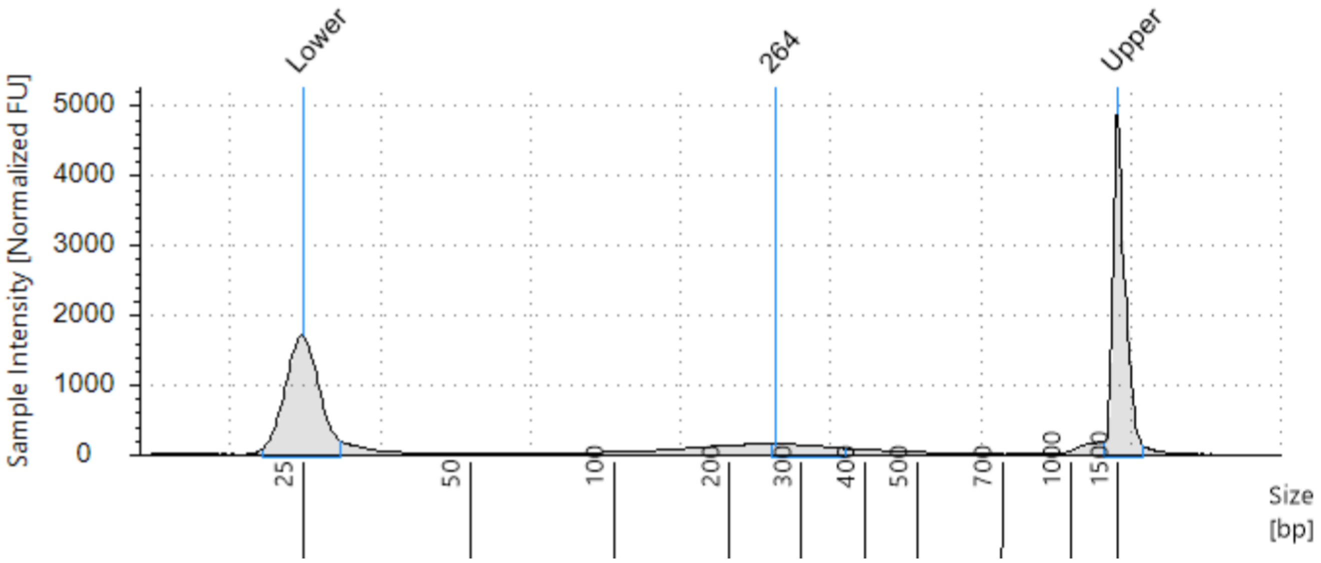

Sample Table

Well	Conc. [ng/µl]	Sample Description	Alert	Observations
Gl	0.850	C7		

Peak Table

Size [bp]	Calibrated Conc. [ng/µl]	Assigned Conc. [ng/µl]	Peak Molarity [nmol/l]	% Integrated Area	Peak Comment	Observations
25	6.21	-	382	-		Lower Marker
264	0.850	-	4.95	100.00		
1500	6.50	6.50	6.67	-		Upper Marker

 © Agilent Technologies, Inc. 2017

H1: D7

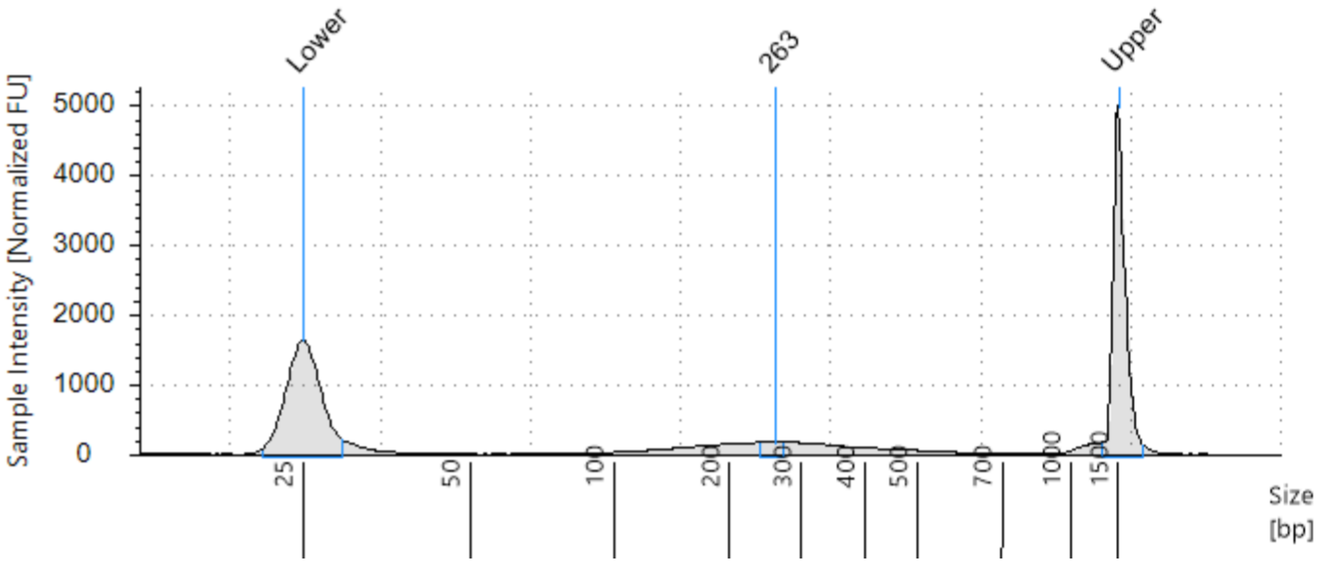

Sample Table

Well	Conc. [ng/µl]	Sample Description	Alert	Observations
H1	0.386	D7		

Peak Table

Size [bp]	Calibrated Conc. [ng/µl]	Assigned Conc. [ng/µl]	Peak Molarity [nmol/l]	% Integrated Area	Peak Comment	Observations
25	6.15	-	378	-		Lower Marker
263	0.386	-	2.26	100.00		
1500	6.50	6.50	6.67	-		Upper Marker

© Agilent Technologies, Inc. 2017

A2: E7

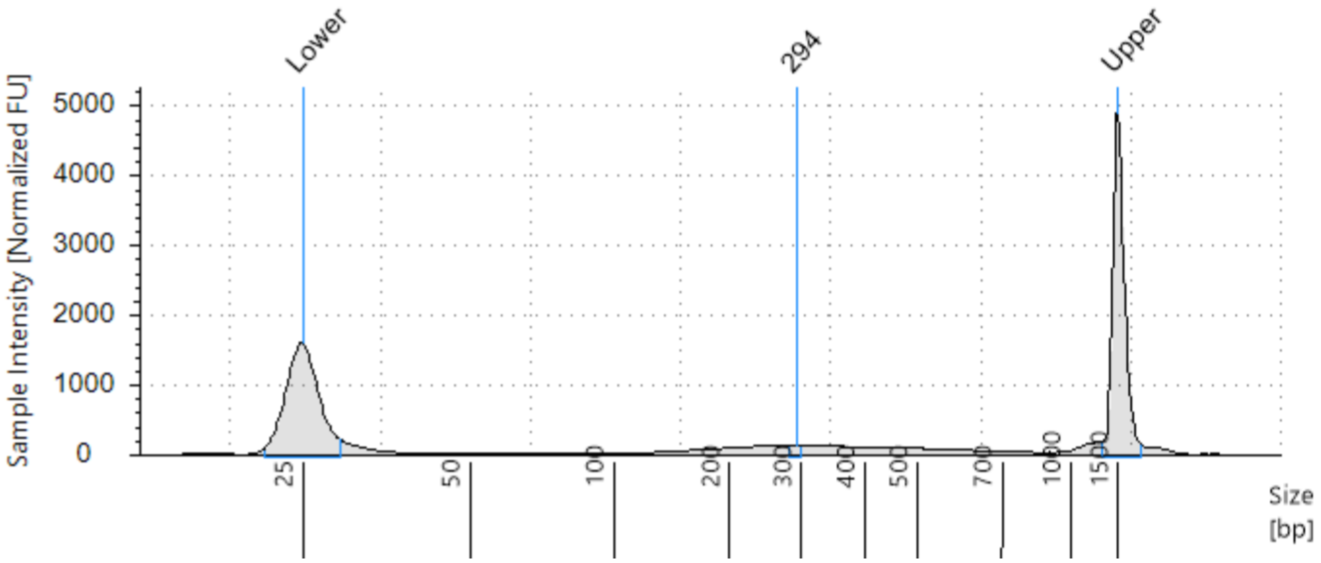

Sample Table

Well	Conc. [ng/µl]	Sample Description	Alert	Observations
A2	0.174	E7		

Peak Table

Size [bp]	Calibrated Conc. [ng/µl]	Assigned Conc. [ng/µl]	Peak Molarity [nmol/l]	% Integrated Area	Peak Comment	Observations
25	5.89	-	363	-		Lower Marker
294	0.174	-	0.911	100.00		
1500	6.50	6.50	6.67	-		Upper Marker

B2: F7

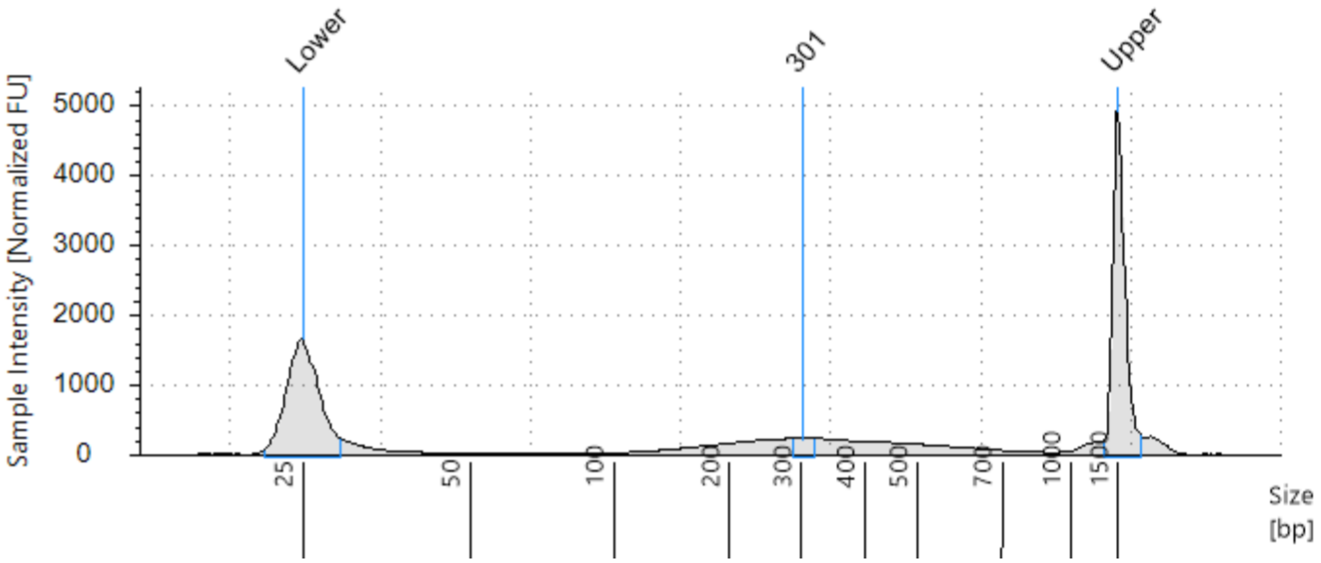

Sample Table

Well	Conc. [ng/µl]	Sample Description	Alert	Observations
B2	0.484	F7		

Peak Table

Size [bp]	Calibrated Conc. [ng/µl]	Assigned Conc. [ng/µl]	Peak Molarity [nmol/l]	% Integrated Area	Peak Comment	Observations
25	5.73	-	353	-		Lower Marker
301	0.484	-	2.47	100.00		
1500	6.50	6.50	6.67	-		Upper Marker

© Agilent Technologies, Inc. 2017

C2: G7

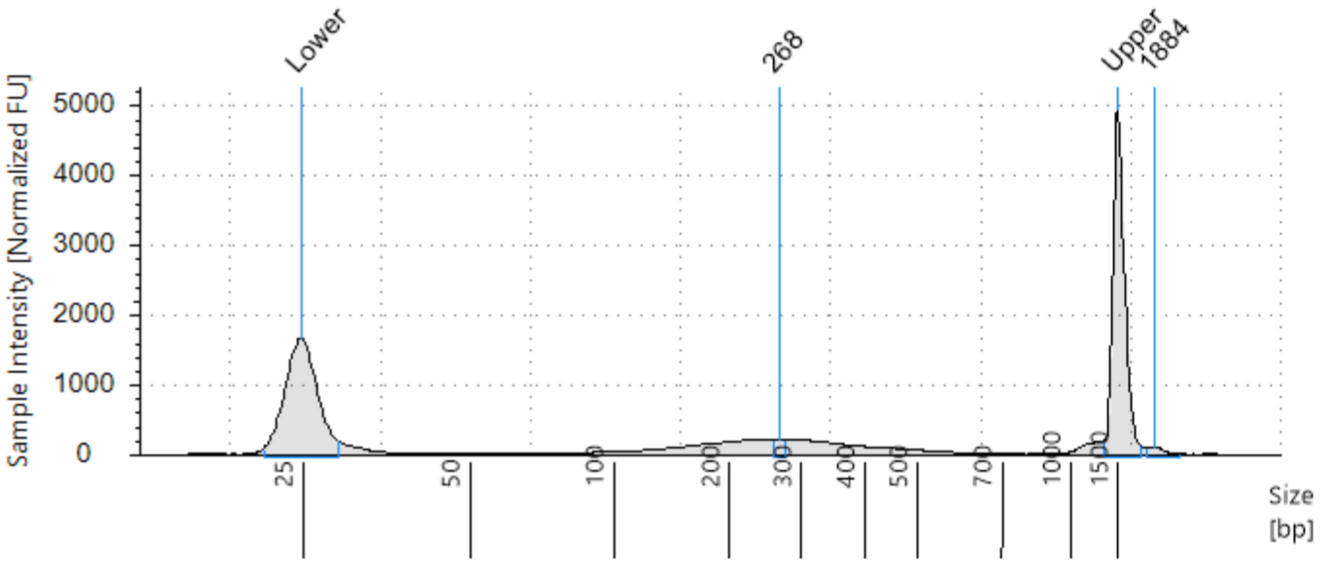

Sample Table

Well	Conc. [ng/µl]	Sample Description	Alert	Observations
C2	0.462	G7		

Peak Table

Size [bp]	Calibrated Conc. [ng/µl]	Assigned Conc. [ng/µl]	Peak Molarity [nmol/l]	% Integrated Area	Peak Comment	Observations
25	6.04	-	372	-		Lower Marker
268	0.264	-	1.51	57.07		
1500	6.50	6.50	6.67	-		Upper Marker
1884	0.198	-	0.162	42.93		

 © Agilent Technologies, Inc. 2017

D2: H7

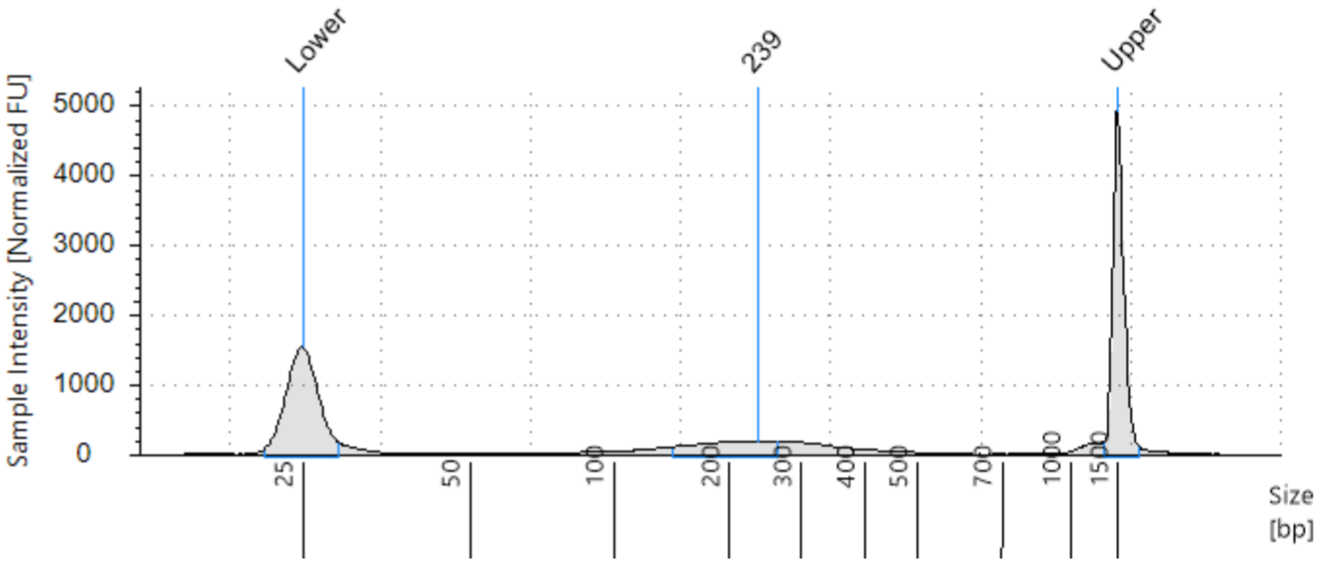

Sample Table

Well	Conc. [ng/µl]	Sample Description	Alert	Observations
D2	1.74	H7		

Peak Table

Size [bp]	Calibrated Conc. [ng/µl]	Assigned Conc. [ng/µl]	Peak Molarity [nmol/l]	% Integrated Area	Peak Comment	Observations
25	5.84	-	360	-		Lower Marker
239	1.74	-	11.2	100.00		
1500	6.50	6.50	6.67	-		Upper Marker

© Agilent Technologies, Inc. 2017

E2: A8

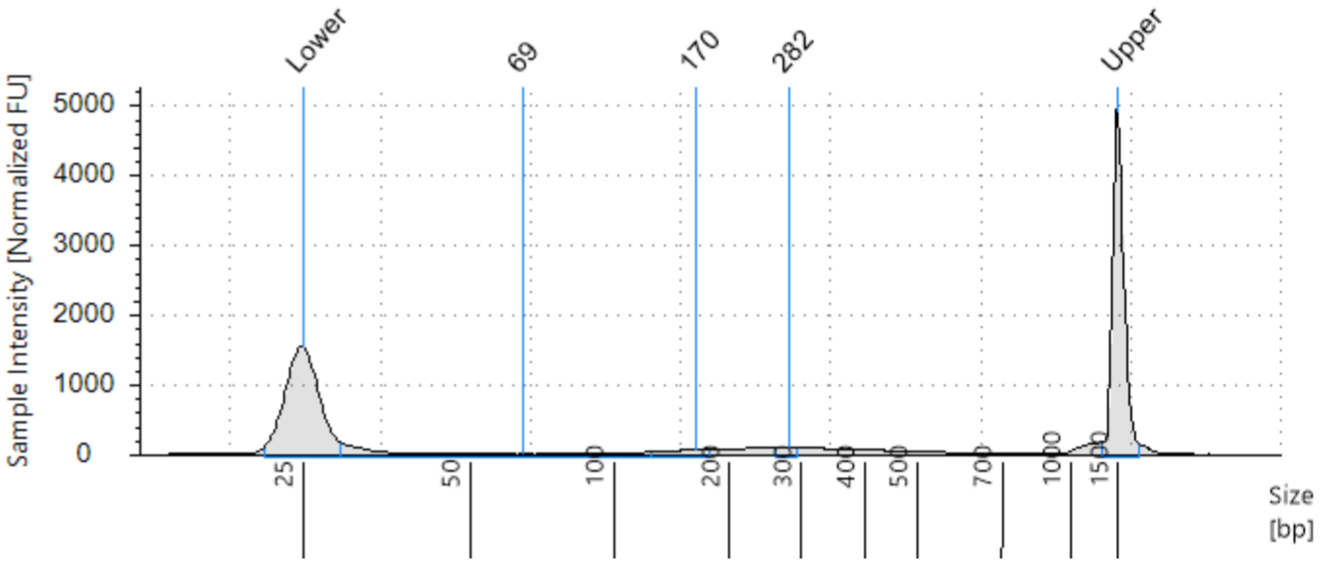

Sample Table

Well	Conc. [ng/µl]	Sample Description	Alert	Observations
E2	1.11	A8		

Peak Table

Size [bp]	Calibrated Conc. [ng/µl]	Assigned Conc. [ng/µl]	Peak Molarity [nmol/l]	% Integrated Area	Peak Comment	Observations
25	6.01	-	370	-		Lower Marker
69	0.599	-	13.4	54.10		
170	0.302	-	2.73	27.27		
282	0.206	-	1.13	18.63		
1500	6.50	6.50	6.67	-		Upper Marker

© Agilent Technologies, Inc. 2017

F2: B8

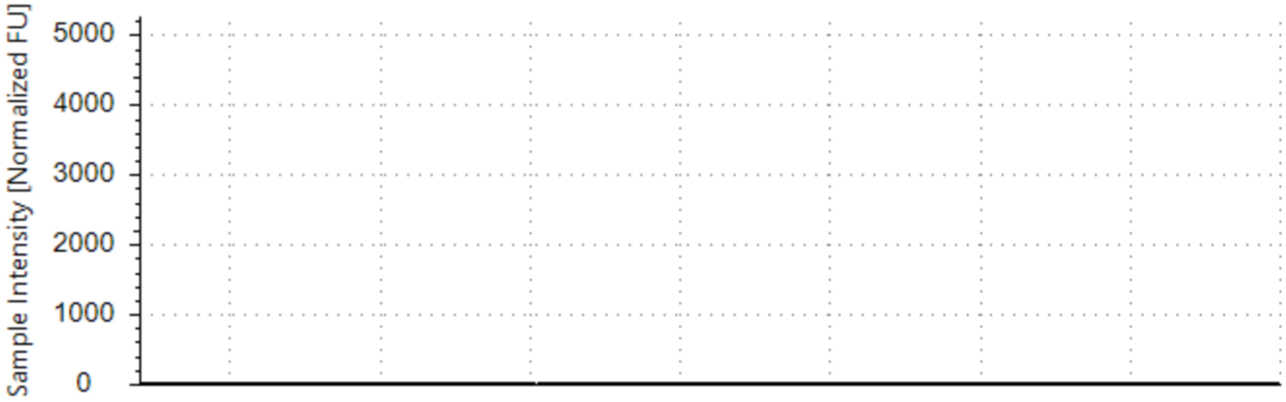

Sample Table

Well	Conc. [ng/µl]	Sample Description	Alert	Observations
F2		B8	▲	Marker(s) not detected

© Agilent Technologies, Inc. 2017

G2: C8

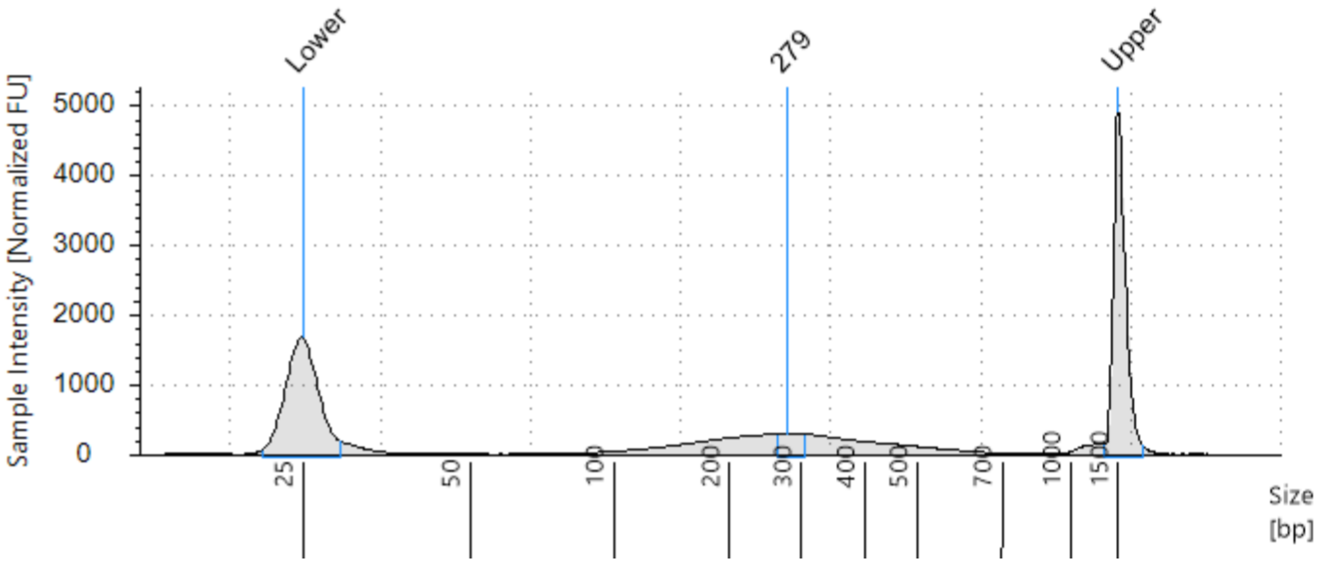

Sample Table

Well	Conc. [ng/µl]	Sample Description	Alert	Observations
G2	0.753	C8		

Peak Table

Size [bp]	Calibrated Conc. [ng/µl]	Assigned Conc. [ng/µl]	Peak Molarity [nmol/l]	% Integrated Area	Peak Comment	Observations
25	5.97	-	367	-		Lower Marker
279	0.753	-	4.15	100.00		
1500	6.50	6.50	6.67	-		Upper Marker

© Agilent Technologies, Inc. 2017

H2: D8

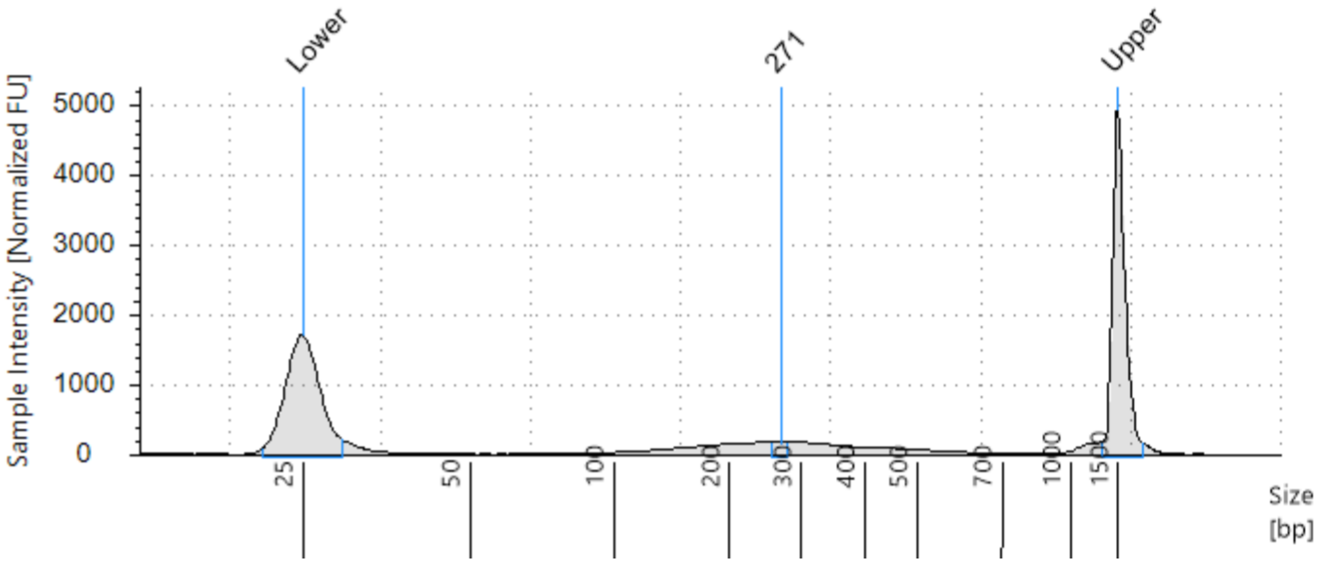

Sample Table

Well	Conc. [ng/µl]	Sample Description	Alert	Observations
H2	0.266	D8		

Peak Table

Size [bp]	Calibrated Conc. [ng/µl]	Assigned Conc. [ng/µl]	Peak Molarity [nmol/l]	% Integrated Area	Peak Comment	Observations
25	6.22	-	383	-		Lower Marker
271	0.266	-	1.51	100.00		
1500	6.50	6.50	6.67	-		Upper Marker

© Agilent Technologies, Inc. 2017

Filename: 2020-08-10-03.D1000,Q-S DFB minus from 3.8.20, E8-C10 R1.D1000

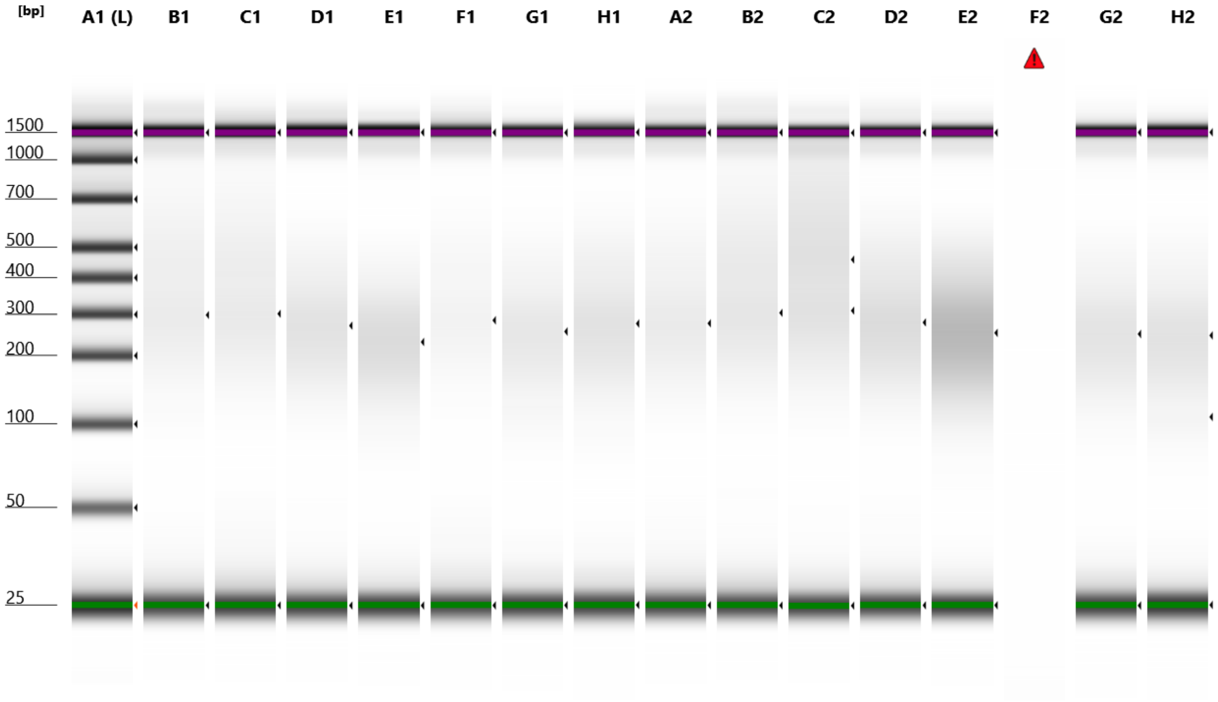

Default image (Contrast 100%)

Sample Info

Well	Conc. [ng/µl]	Sample Description	Alert	Observations
A1	20.2	Ladder		Ladder
B1	0.152	E8		
C1	0.434	F8		
D1	0.360	G8		
E1	1.60	H8		
F1	0.0960	A9		
G1	0.355	B9		
H1	1.07	C9		
A2	0.280	D9		
B2	0.679	E9		
C2	1.69	F9		
D2	2.72	G9		
E2	3.71	H9		
F2		A10	▲	Marker(s) not detected
G2	0.286	B10		
H2	0.464	C10		

© Agilent Technologies, Inc. 2017

A1: Ladder

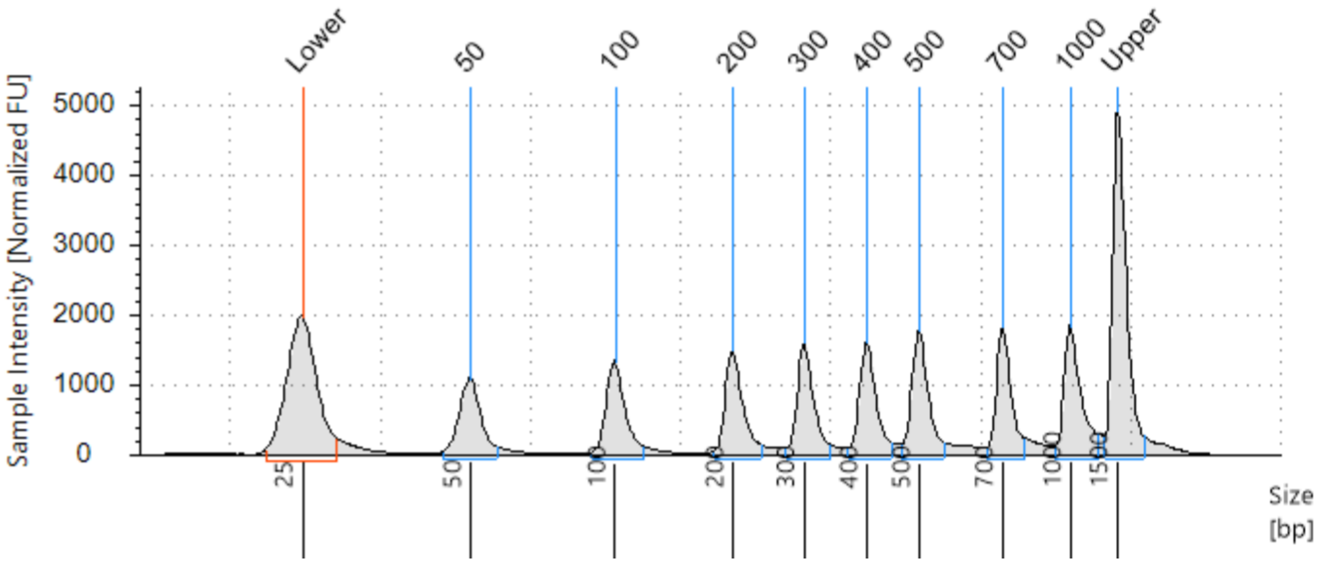

Sample Table

Well	Conc. [ng/µl]	Sample Description	Alert	Observations
A1	20.2	Ladder		Ladder

Peak Table

Size [bp]	Calibrated Conc. [ng/µl]	Assigned Conc. [ng/µl]	Peak Molarity [nmol/l]	% Integrated Area	Peak Comment	Observations
25	5.56	-	342	-		Lower Marker
50	2.19	-	67.5	10.88		
100	2.37	-	36.5	11.77		
200	2.45	-	18.9	12.17		
300	2.46	-	12.6	12.23		
400	2.51	-	9.65	12.44		
500	2.72	-	8.38	13.51		
700	2.53	-	5.56	12.54		
1000	2.92	-	4.48	14.46		
1500	6.50	6.50	6.67	-		Upper Marker

© Agilent Technologies, Inc. 2017

B1: E8

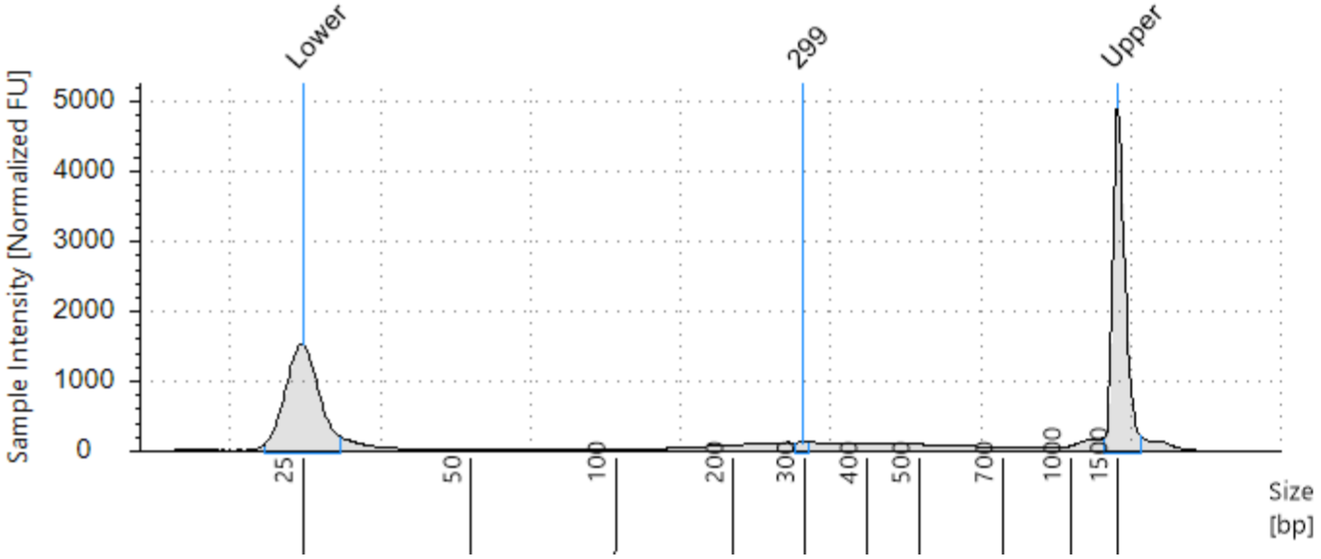

Sample Table

Well	Conc. [ng/µl]	Sample Description	Alert	Observations
B1	0.152	E8		

Peak Table

Size [bp]	Calibrated Conc. [ng/µl]	Assigned Conc. [ng/µl]	Peak Molarity [nmol/l]	% Integrated Area	Peak Comment	Observations
25	5.40	-	332	-		Lower Marker
299	0.152	-	0.782	100.00		
1500	6.50	6.50	6.67	-		Upper Marker

© Agilent Technologies, Inc. 2017

C1: F8

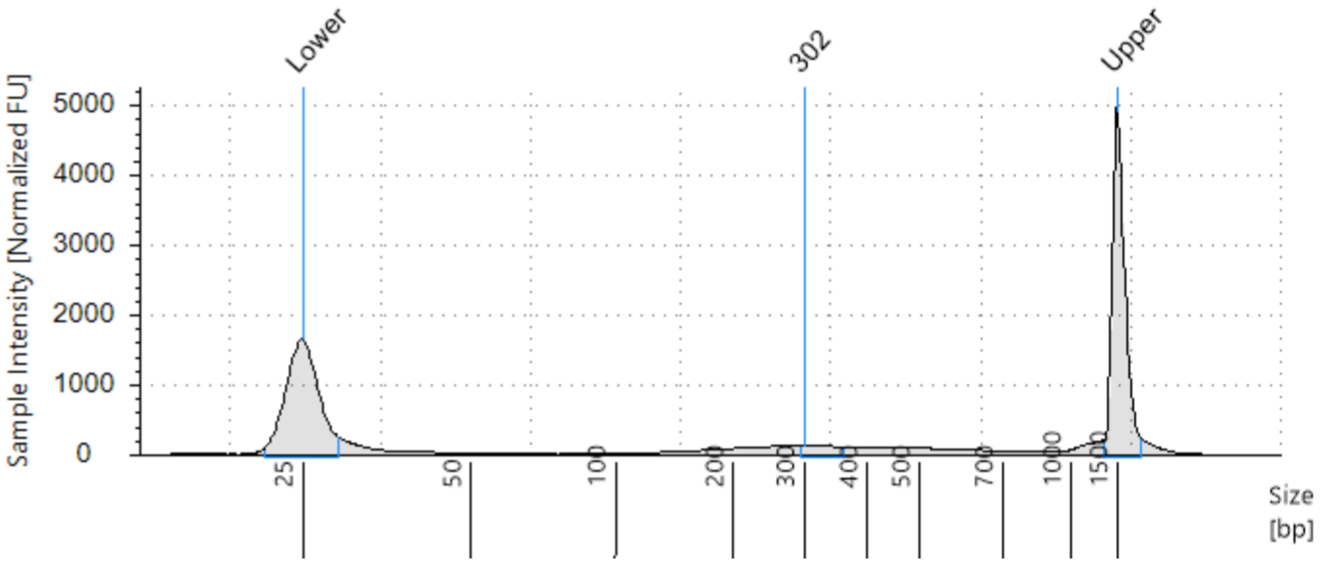

Sample Table

Well	Conc. [ng/µl]	Sample Description	Alert	Observations
C1	0.434	F8		

Peak Table

Size [bp]	Calibrated Conc. [ng/µl]	Assigned Conc. [ng/µl]	Peak Molarity [nmol/l]	% Integrated Area	Peak Comment	Observations
25	5.59	-	344	-		Lower Marker
302	0.434	-	2.21	100.00		
1500	6.50	6.50	6.67	-		Upper Marker

© Agilent Technologies, Inc. 2017

D1: G8

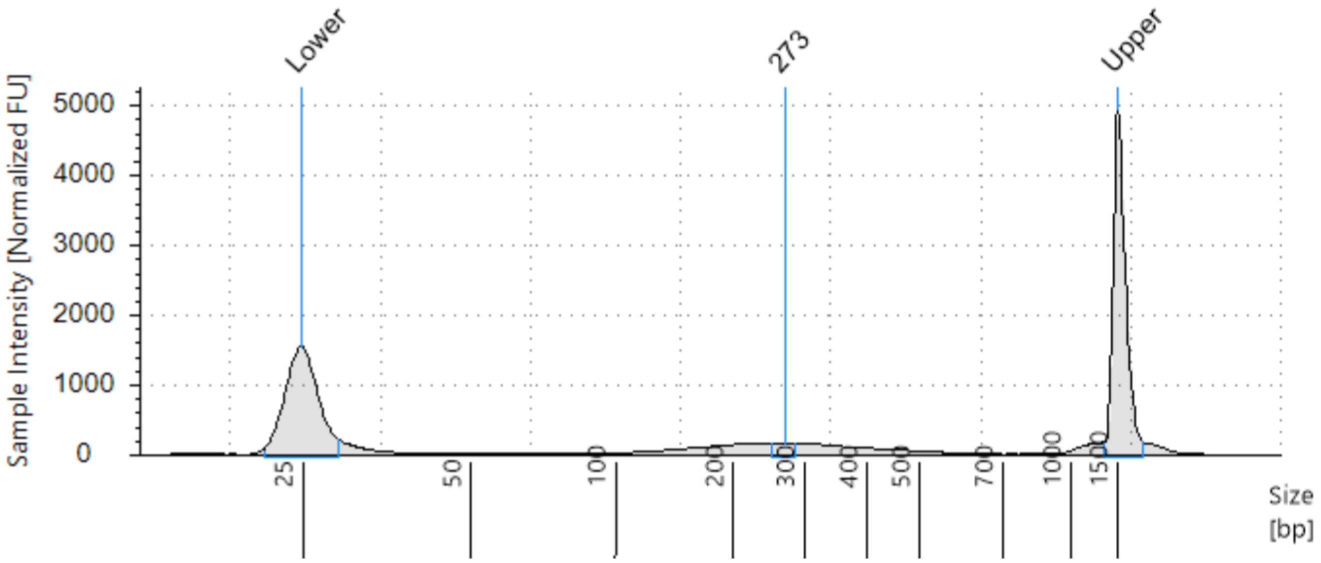

Sample Table

Well	Conc. [ng/µl]	Sample Description	Alert	Observations
D1	0.360	G8		

Peak Table

Size [bp]	Calibrated Conc. [ng/µl]	Assigned Conc. [ng/µl]	Peak Molarity [nmol/l]	% Integrated Area	Peak Comment	Observations
25	5.38	-	331	-		Lower Marker
273	0.360	-	2.03	100.00		
1500	6.50	6.50	6.67	-		Upper Marker

© Agilent Technologies, Inc. 2017

E1: H8

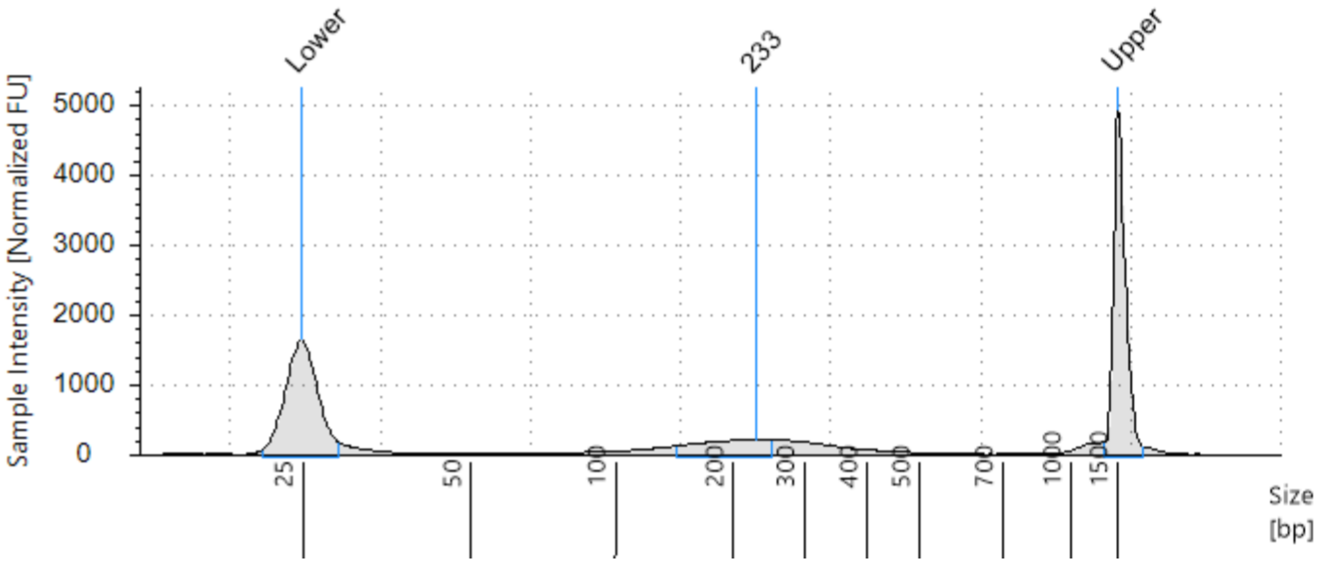

Sample Table

Well	Conc. [ng/µl]	Sample Description	Alert	Observations
E1	1.60	H8		

Peak Table

Size [bp]	Calibrated Conc. [ng/µl]	Assigned Conc. [ng/µl]	Peak Molarity [nmol/l]	% Integrated Area	Peak Comment	Observations
25	5.81	-	357	-		Lower Marker
233	1.60	-	10.6	100.00		
1500	6.50	6.50	6.67	-		Upper Marker

 © Agilent Technologies, Inc. 2017

F1: A9

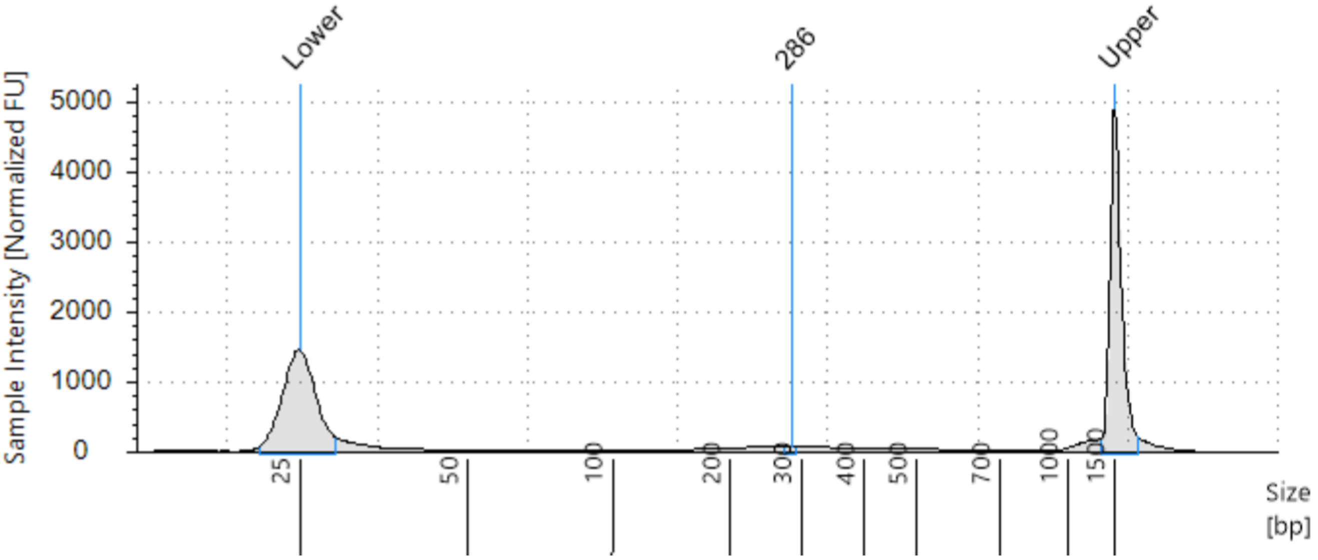

Sample Table

Well	Conc. [ng/µl]	Sample Description	Alert	Observations
F1	0.0960	A9		

Peak Table

Size [bp]	Calibrated Conc. [ng/µl]	Assigned Conc. [ng/µl]	Peak Molarity [nmol/l]	% Integrated Area	Peak Comment	Observations
25	5.54	-	341	-		Lower Marker
286	0.0960	-	0.517	100.00		
1500	6.50	6.50	6.67	-		Upper Marker

© Agilent Technologies, Inc. 2017

G1: B9

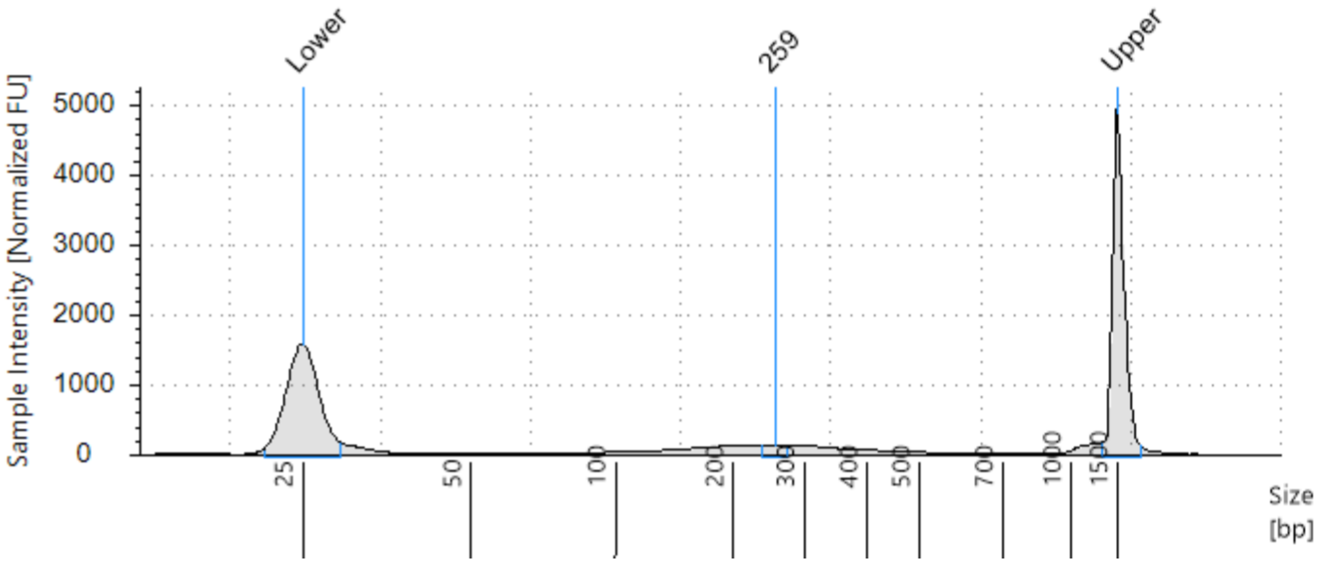

Sample Table

Well	Conc. [ng/µl]	Sample Description	Alert	Observations
G1	0.355	B9		

Peak Table

Size [bp]	Calibrated Conc. [ng/µl]	Assigned Conc. [ng/µl]	Peak Molarity [nmol/l]	% Integrated Area	Peak Comment	Observations
25	5.90	-	363	-		Lower Marker
259	0.355	-	2.11	100.00		
1500	6.50	6.50	6.67	-		Upper Marker

© Agilent Technologies, Inc. 2017

H1: C9

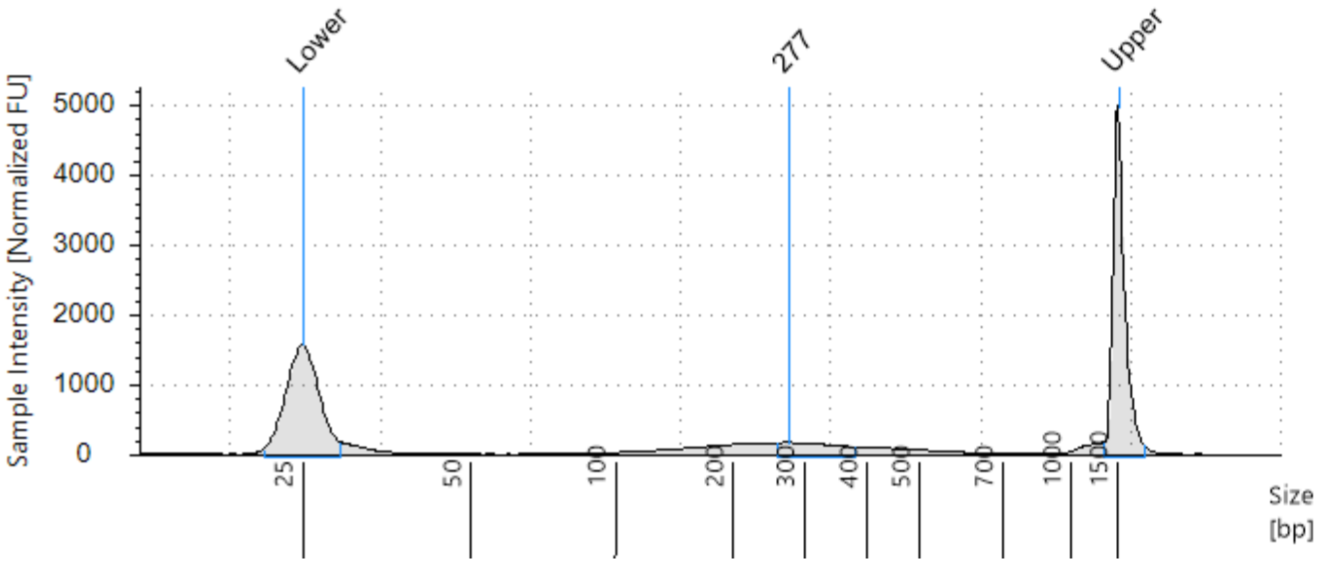

Sample Table

Well	Conc. [ng/µl]	Sample Description	Alert	Observations
H1	1.07	C9		

Peak Table

Size [bp]	Calibrated Conc. [ng/µl]	Assigned Conc. [ng/µl]	Peak Molarity [nmol/l]	% Integrated Area	Peak Comment	Observations
25	5.83	-	359	-		Lower Marker
277	1.07	-	5.93	100.00		
1500	6.50	6.50	6.67	-		Upper Marker

© Agilent Technologies, Inc. 2017

A2: D9

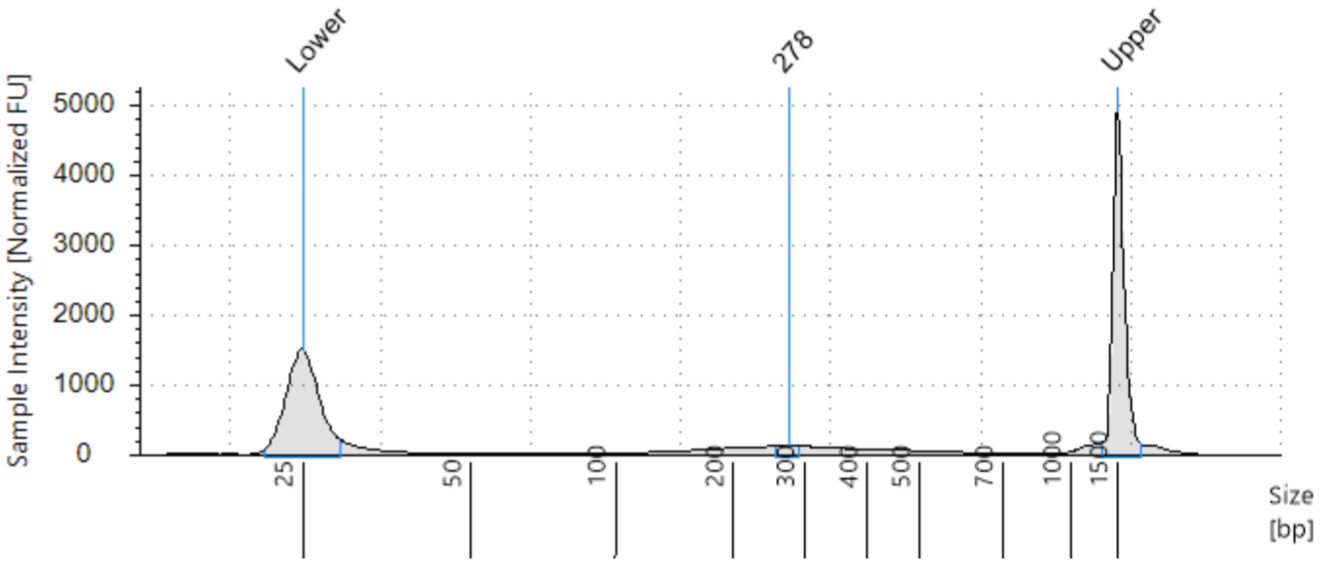

Sample Table

Well	Conc. [ng/µl]	Sample Description	Alert	Observations
A2	0.280	D9		

Peak Table

Size [bp]	Calibrated Conc. [ng/µl]	Assigned Conc. [ng/µl]	Peak Molarity [nmol/l]	% Integrated Area	Peak Comment	Observations
25	5.68	-	350	-		Lower Marker
278	0.280	-	1.55	100.00		
1500	6.50	6.50	6.67	-		Upper Marker

 © Agilent Technologies, Inc. 2017

B2: E9

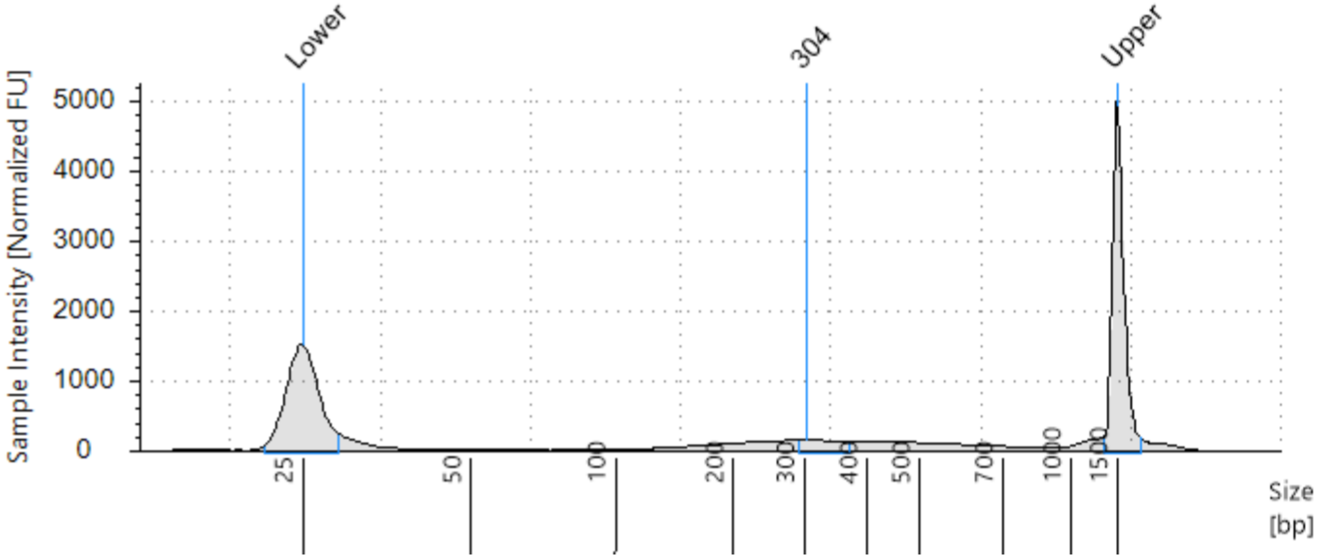

Sample Table

Well	Conc. [ng/µl]	Sample Description	Alert	Observations
B2	0.679	E9		

Peak Table

Size [bp]	Calibrated Conc. [ng/µl]	Assigned Conc. [ng/µl]	Peak Molarity [nmol/l]	% Integrated Area	Peak Comment	Observations
25	5.61	-	345	-		Lower Marker
304	0.679	-	3.43	100.00		
1500	6.50	6.50	6.67	-		Upper Marker

© Agilent Technologies, Inc. 2017

C2: F9

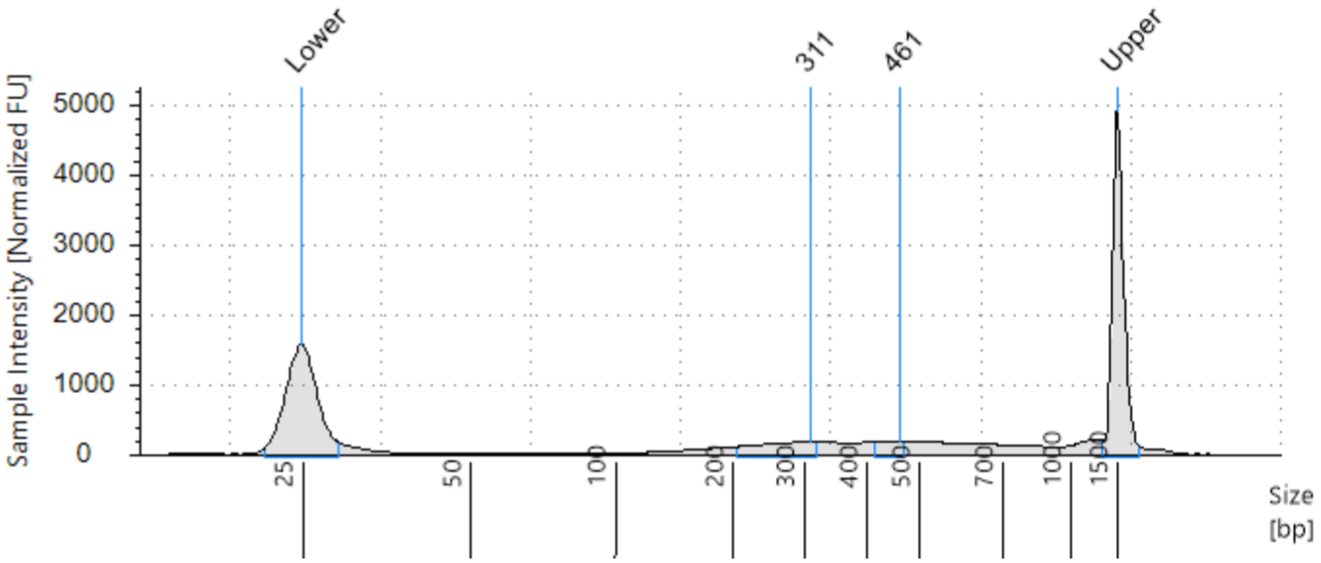

Sample Table

Well	Conc. [ng/µl]	Sample Description	Alert	Observations
C2	1.69	F9		

Peak Table

Size [bp]	Calibrated Conc. [ng/µl]	Assigned Conc. [ng/µl]	Peak Molarity [nmol/l]	% Integrated Area	Peak Comment	Observations
25	5.74	-	353	-		Lower Marker
311	1.18	-	5.83	69.93		
461	0.507	-	1.69	30.07		
1500	6.50	6.50	6.67	-		Upper Marker

© Agilent Technologies, Inc. 2017

D2: G9

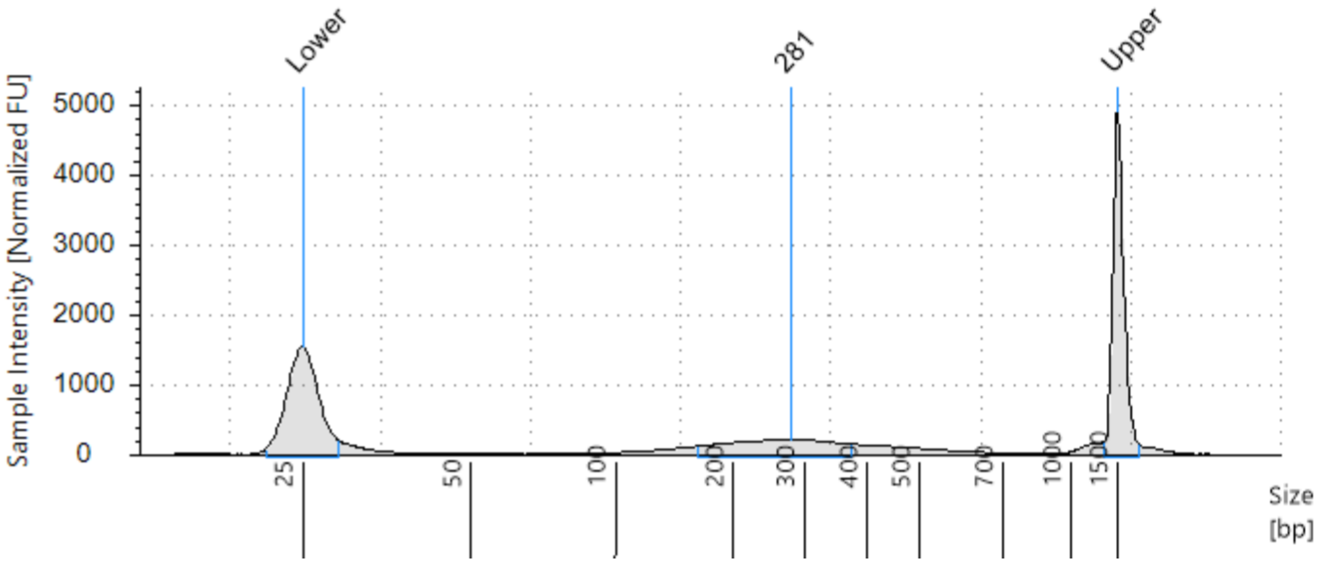

Sample Table

Well	Conc. [ng/µl]	Sample Description	Alert	Observations
D2	2.72	G9		

Peak Table

Size [bp]	Calibrated Conc. [ng/µl]	Assigned Conc. [ng/µl]	Peak Molarity [nmol/l]	% Integrated Area	Peak Comment	Observations
25	5.77	-	355	-		Lower Marker
281	2.72	-	14.9	100.00		
1500	6.50	6.50	6.67	-		Upper Marker

 © Agilent Technologies, Inc. 2017

E2: H9

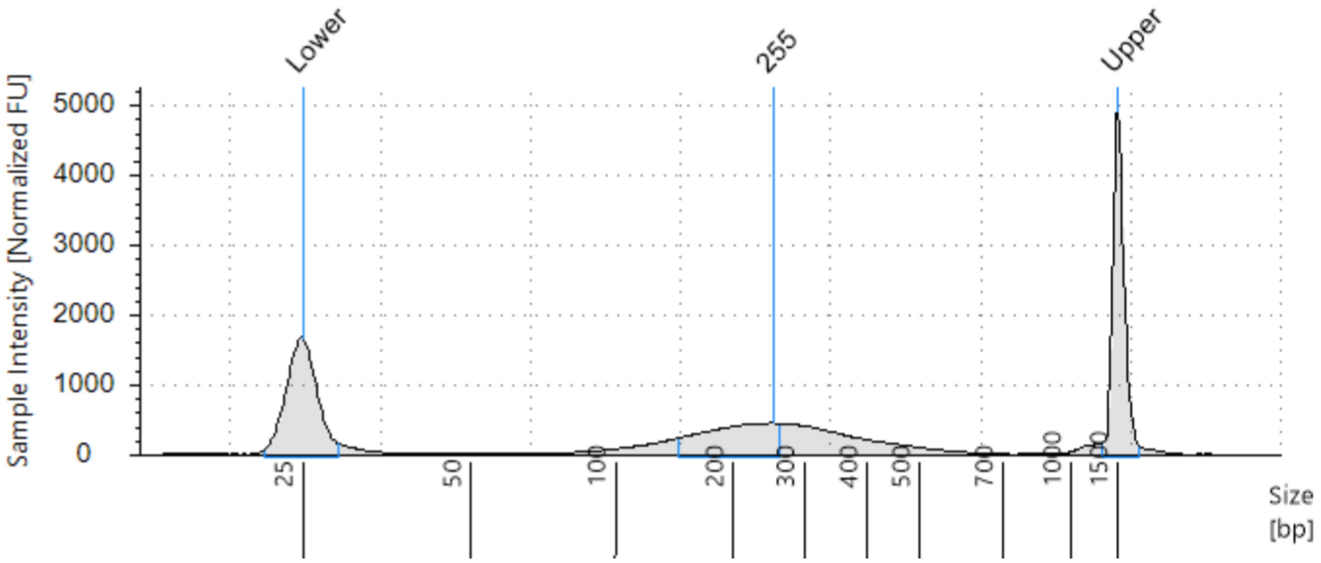

Sample Table

Well	Conc. [ng/µl]	Sample Description	Alert	Observations
E2	3.71	H9		

Peak Table

Size [bp]	Calibrated Conc. [ng/µl]	Assigned Conc. [ng/µl]	Peak Molarity [nmol/l]	% Integrated Area	Peak Comment	Observations
25	5.83	-	359	-		Lower Marker
255	3.71	-	22.3	100.00		
1500	6.50	6.50	6.67	-		Upper Marker

© Agilent Technologies, Inc. 2017

F2: A10

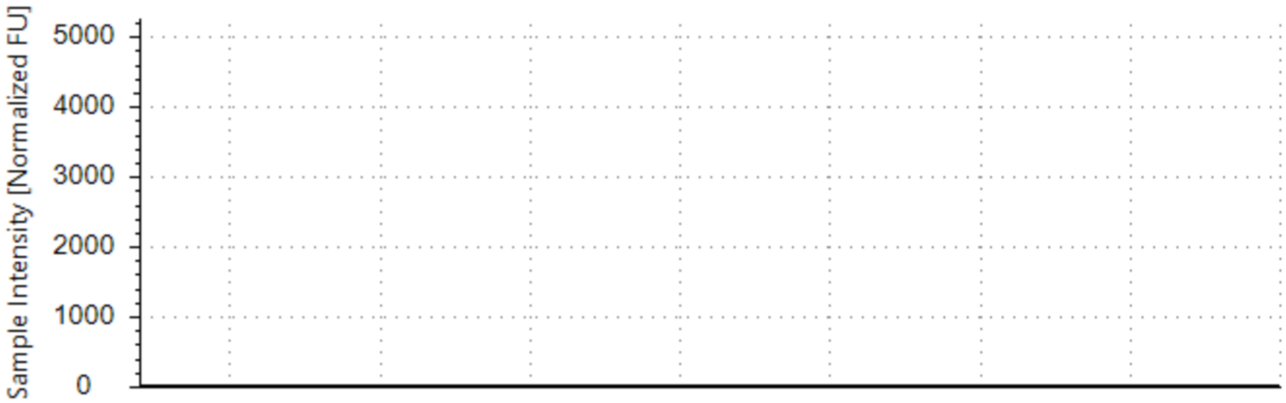

Sample Table

Well	Conc. [ng/µl]	Sample Description	Alert	Observations
F2		A10	▲	Marker(s) not detected

© Agilent Technologies, Inc. 2017

G2: B10

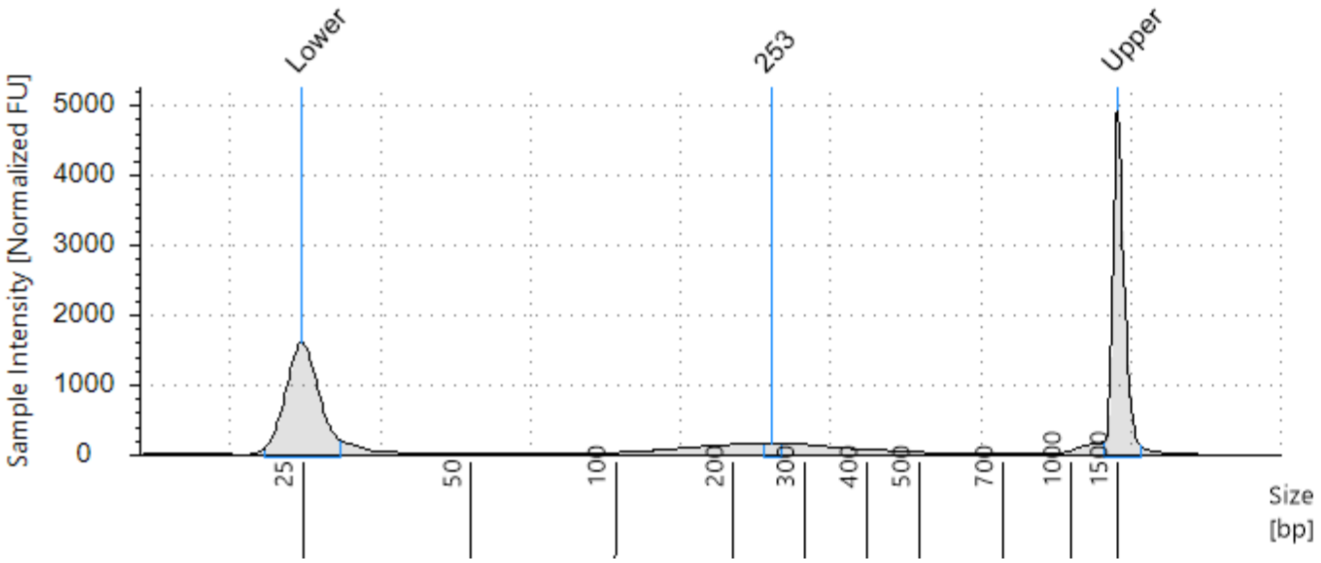

Sample Table

Well	Conc. [ng/µl]	Sample Description	Alert	Observations
G2	0.286	B10		

Peak Table

Size [bp]	Calibrated Conc. [ng/µl]	Assigned Conc. [ng/µl]	Peak Molarity [nmol/l]	% Integrated Area	Peak Comment	Observations
25	6.11	-	376	-		Lower Marker
253	0.286	-	1.74	100.00		
1500	6.50	6.50	6.67	-		Upper Marker

H2: C10

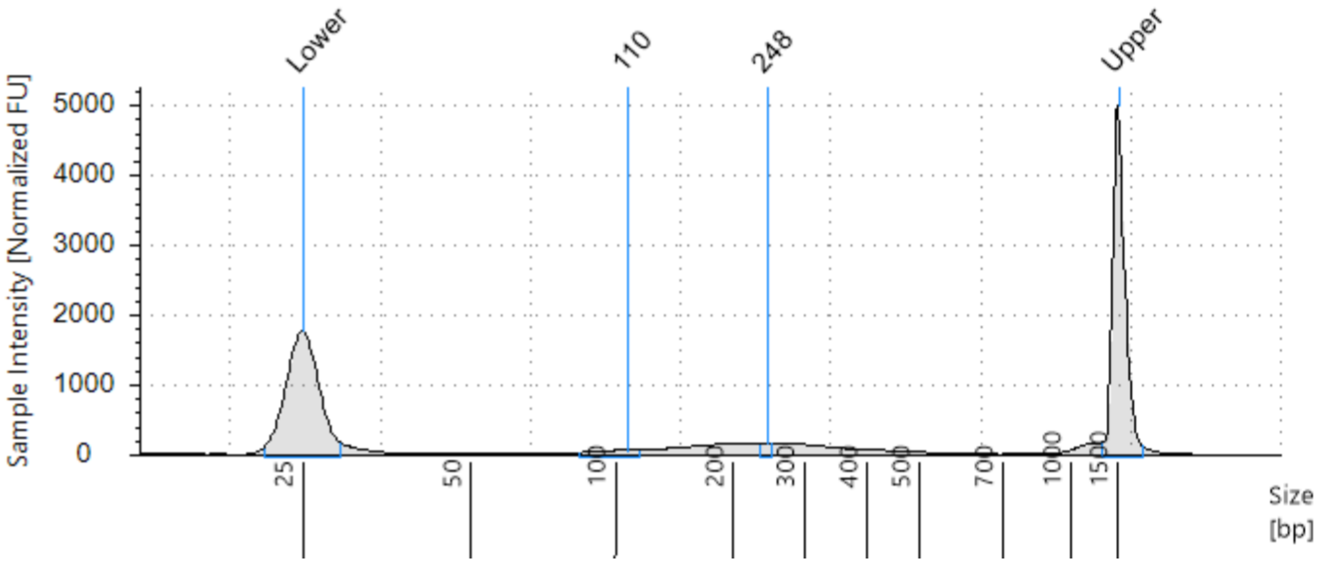

Sample Table

Well	Conc. [ng/µl]	Sample Description	Alert	Observations
H2	0.464	C10		

Peak Table

Size [bp]	Calibrated Conc. [ng/µl]	Assigned Conc. [ng/µl]	Peak Molarity [nmol/l]	% Integrated Area	Peak Comment	Observations
25	6.21	-	382	-		Lower Marker
110	0.262	-	3.67	56.51		
248	0.202	-	1.25	43.49		
1500	6.50	6.50	6.67	-		Upper Marker

 © Agilent Technologies, Inc. 2017

Filename: 2020-08-21-01.D1000,Q-S, DFB minus from3.8.20 C10-A12 R1.D1000

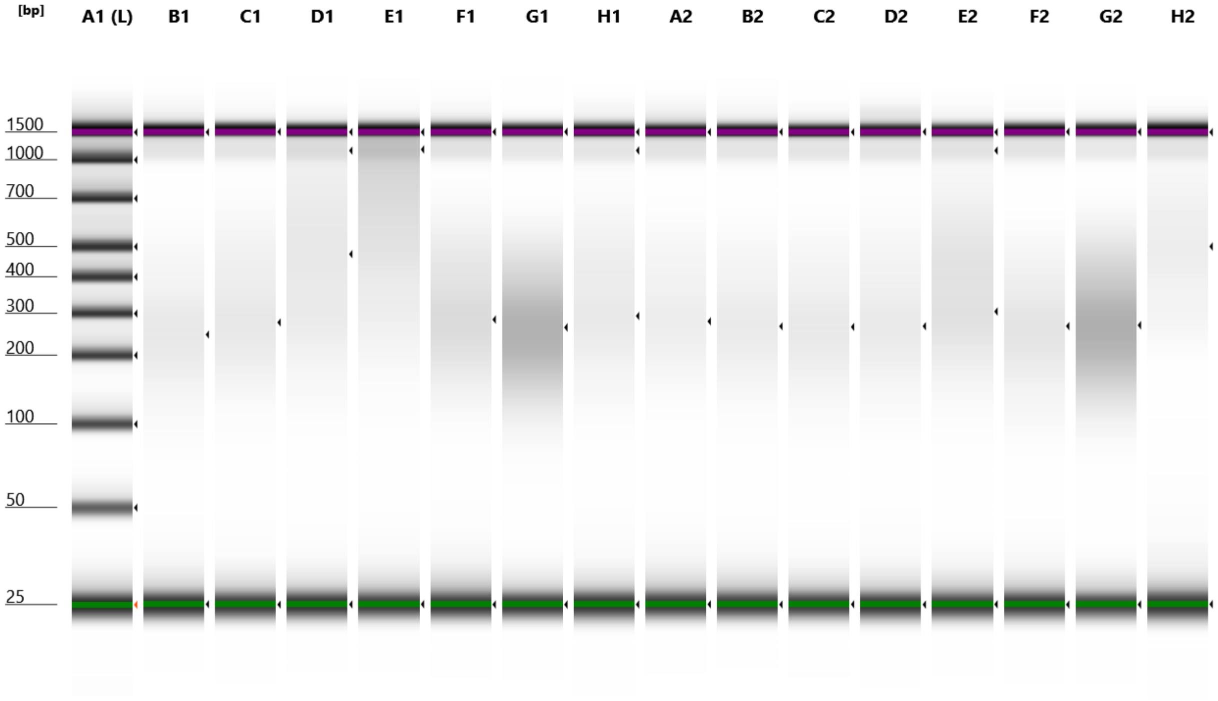

Default image (Contrast 100%)

Sample Info

Well	Conc. [ng/µl]	Sample Description	Alert	Observations
A1	21.5	Ladder		Ladder
B1	0.252	Minus C10 -Q-SONICA 96 WELL		
C1	1.13	M-D10		
D1	0.705	M-E10		
E1	1.26	M-f10		
F1	1.68	M-G10		
G1	3.76	M-H10		
H1	0.667	M-A11		
A2	0.158	M-B11		
B2	0.149	M-C11		
C2	0.773	M-D11		
D2	0.821	M-E11		
E2	0.808	M-F11		
F2	0.198	M-G11		
G2	6.95	M-H11		
H2	0.0995	M-A12		

© Agilent Technologies, Inc. 2017

A1: Ladder

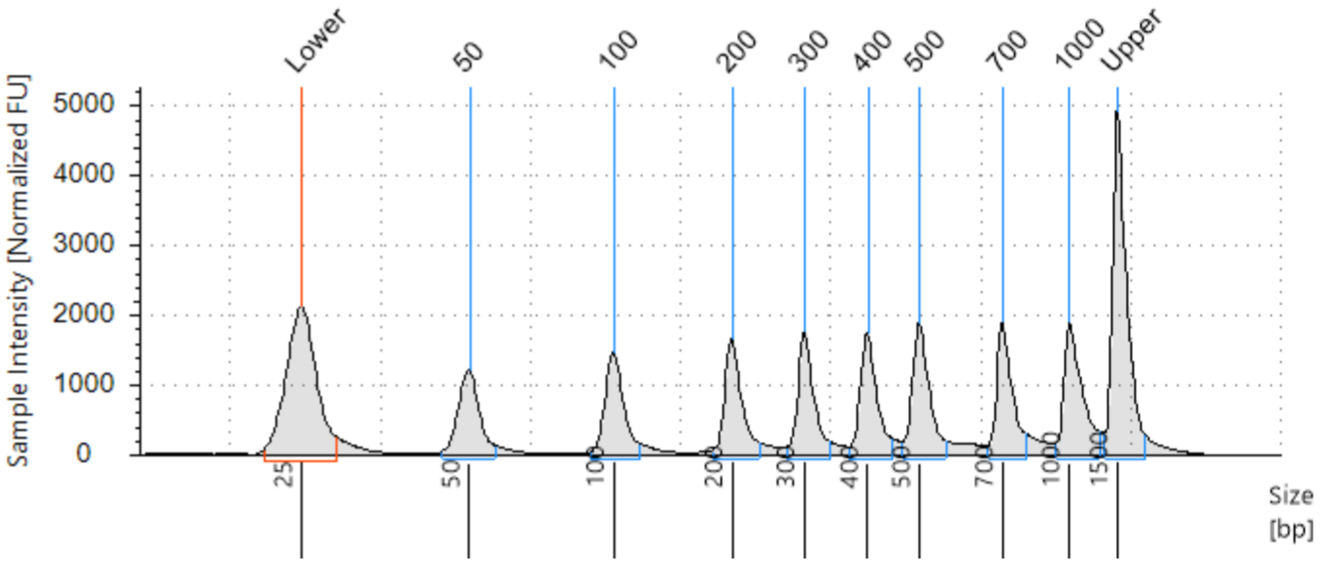

Sample Table

Well	Conc. [ng/µl]	Sample Description	Alert	Observations
A1	21.5	Ladder		Ladder

Peak Table

Size [bp]	Calibrated Conc. [ng/µl]	Assigned Conc. [ng/µl]	Peak Molarity [nmol/l]	% Integrated Area	Peak Comment	Observations
25	5.91	-	363	-		Lower Marker
50	2.35	-	72.2	10.90		
100	2.53	-	38.9	11.74		
200	2.58	-	19.8	11.98		
300	2.58	-	13.2	11.98		
400	2.70	-	10.4	12.55		
500	2.93	-	9.02	13.62		
700	2.71	-	5.95	12.57		
1000	3.16	-	4.86	14.67		
1500	6.50	6.50	6.67	-		Upper Marker

© Agilent Technologies, Inc. 2017

B1: Minus C10 -Q-SONICA 96 WELL

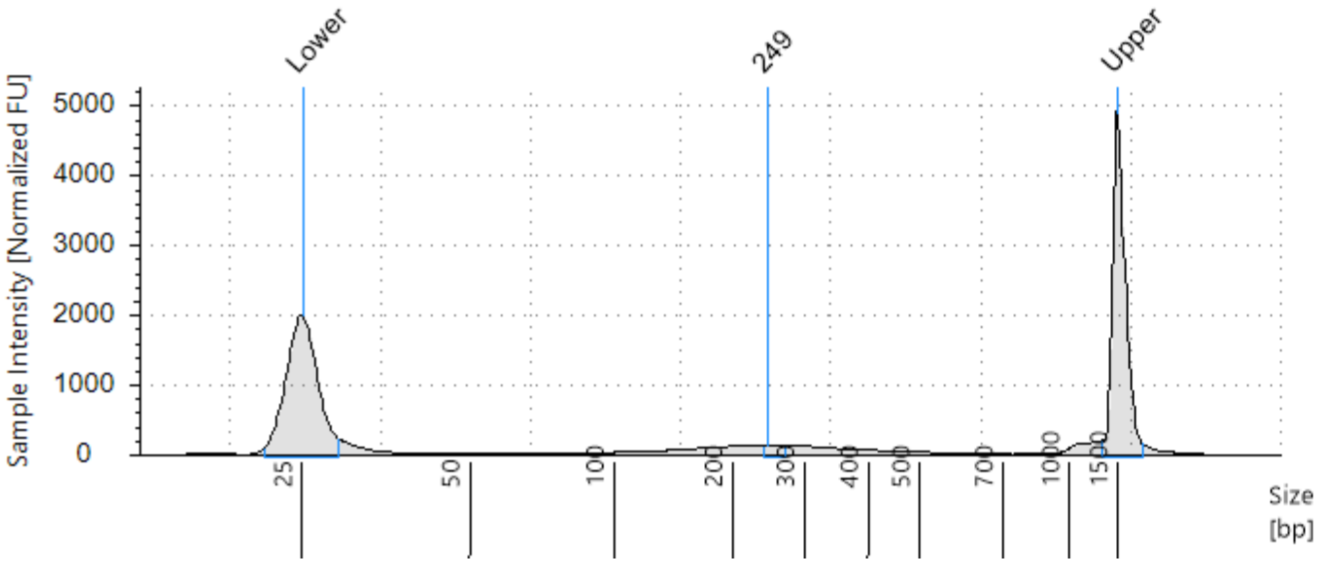

Sample Table

Well	Conc. [ng/µl]	Sample Description	Alert	Observations
B1	0.252	Minus C10 -Q-SONICA 96 WELL		

Peak Table

Size [bp]	Calibrated Conc. [ng/µl]	Assigned Conc. [ng/µl]	Peak Molarity [nmol/l]	% Integrated Area	Peak Comment	Observations
25	6.52	-	401	-		Lower Marker
249	0.252	-	1.55	100.00		
1500	6.50	6.50	6.67	-		Upper Marker

© Agilent Technologies, Inc. 2017

C1: M-D10

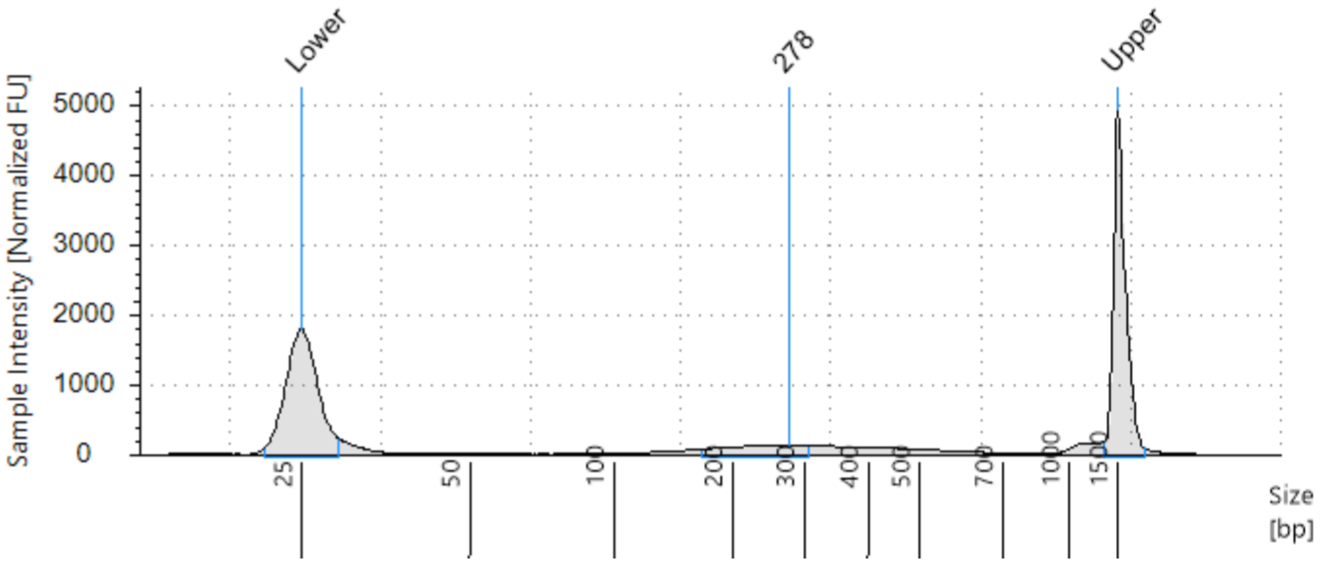

Sample Table

Well	Conc. [ng/µl]	Sample Description	Alert	Observations
C1	1.13	M-D10		

Peak Table

Size [bp]	Calibrated Conc. [ng/µl]	Assigned Conc. [ng/µl]	Peak Molarity [nmol/l]	% Integrated Area	Peak Comment	Observations
25	6.25	-	385	-		Lower Marker
278	1.13	-	6.24	100.00		
1500	6.50	6.50	6.67	-		Upper Marker

D1: M-E10

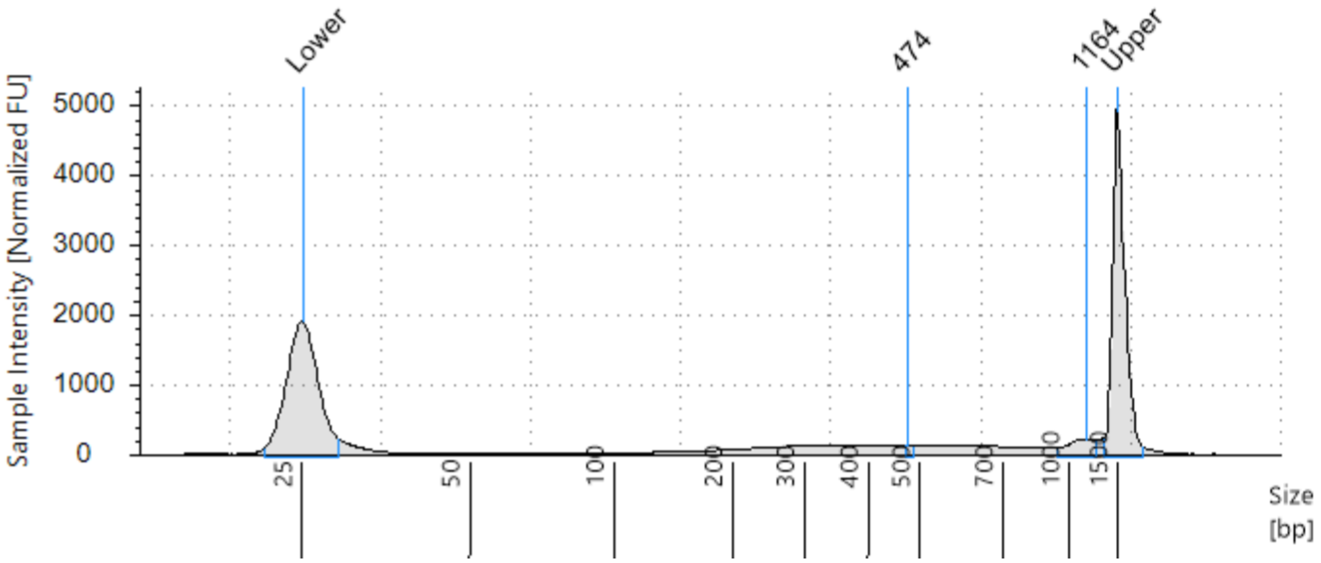

Sample Table

Well	Conc. [ng/µl]	Sample Description	Alert	Observations
D1	0.705	M-E10		

Peak Table

Size [bp]	Calibrated Conc. [ng/µl]	Assigned Conc. [ng/µl]	Peak Molarity [nmol/l]	% Integrated Area	Peak Comment	Observations
25	6.49	-	399	-		Lower Marker
474	0.107	-	0.347	15.20		
1164	0.597	-	0.790	84.80		
1500	6.50	6.50	6.67	-		Upper Marker

© Agilent Technologies, Inc. 2017

E1: M-f10

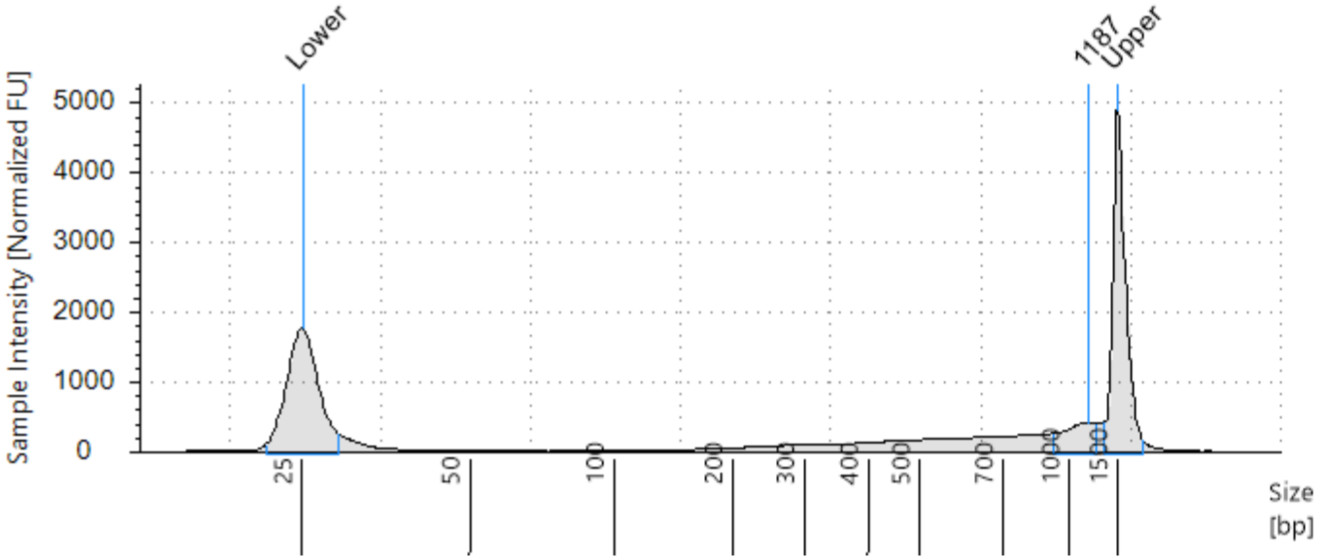

Sample Table

Well	Conc. [ng/µl]	Sample Description	Alert	Observations
E1	1.26	M-f10		

Peak Table

Size [bp]	Calibrated Conc. [ng/µl]	Assigned Conc. [ng/µl]	Peak Molarity [nmol/l]	% Integrated Area	Peak Comment	Observations
25	5.80	-	357	-		Lower Marker
1187	1.26	-	1.64	100.00		
1500	6.50	6.50	6.67	-		Upper Marker

 © Agilent Technologies, Inc. 2017

F1: M-G10

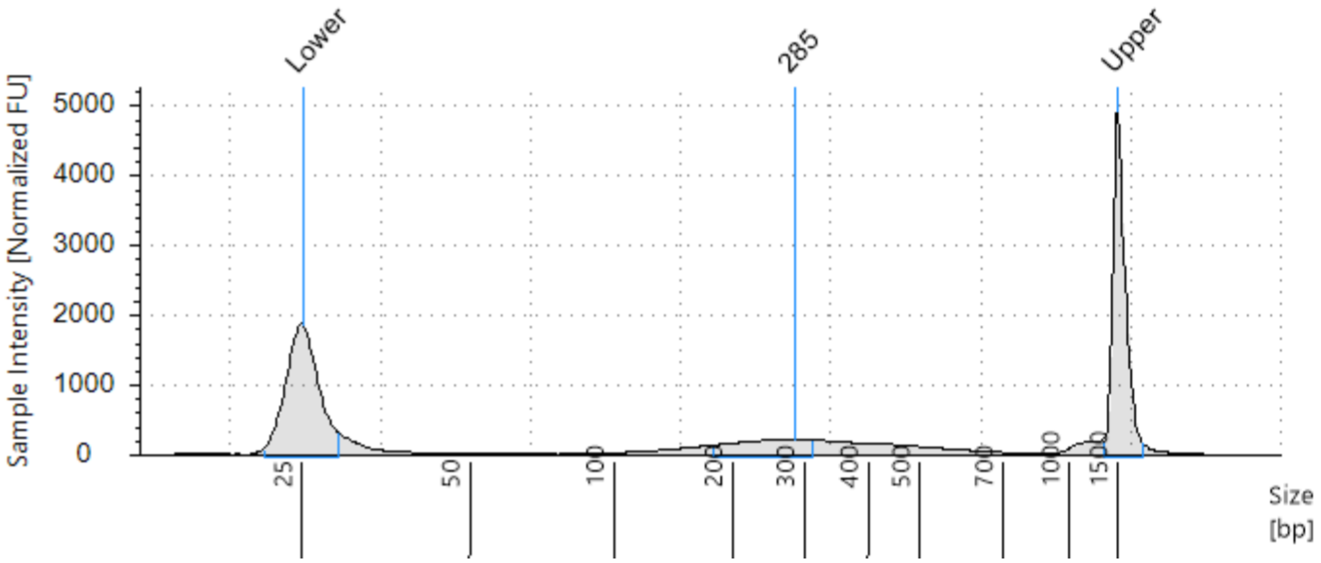

Sample Table

Well	Conc. [ng/µl]	Sample Description	Alert	Observations
F1	1.68	M-G10		

Peak Table

Size [bp]	Calibrated Conc. [ng/µl]	Assigned Conc. [ng/µl]	Peak Molarity [nmol/l]	% Integrated Area	Peak Comment	Observations
25	6.41	-	395	-		Lower Marker
285	1.68	-	9.07	100.00		
1500	6.50	6.50	6.67	-		Upper Marker

© Agilent Technologies, Inc. 2017

G1: M-H10

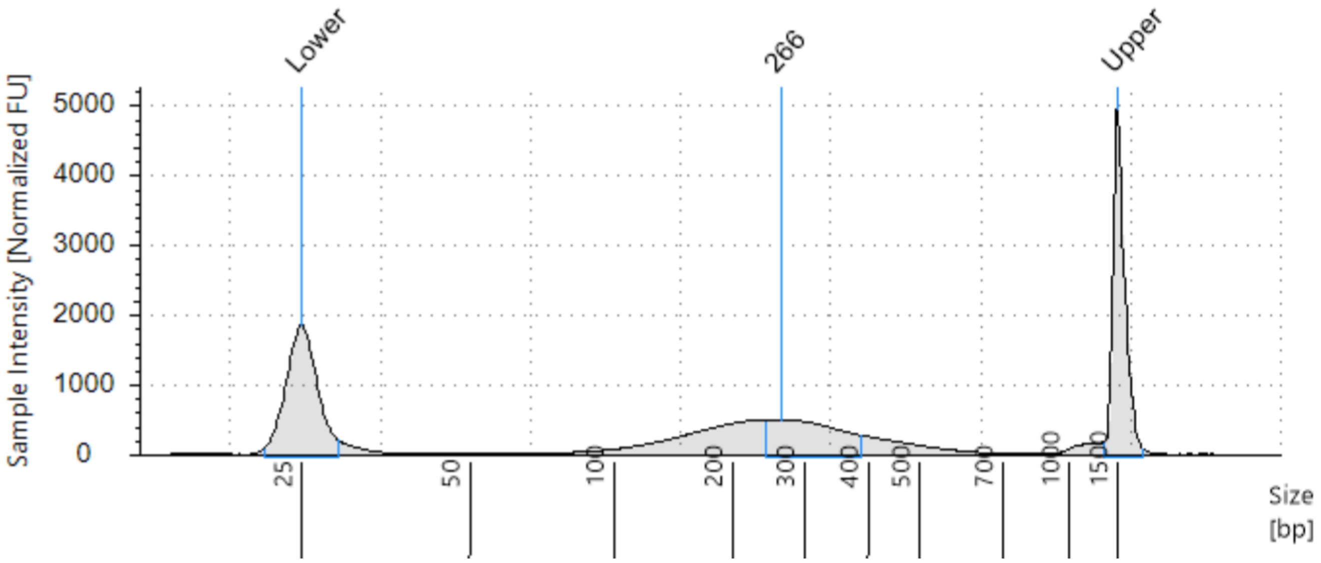

Sample Table

Well	Conc. [ng/µl]	Sample Description	Alert	Observations
G1	3.76	M-H10		

Peak Table

Size [bp]	Calibrated Conc. [ng/µl]	Assigned Conc. [ng/µl]	Peak Molarity [nmol/l]	% Integrated Area	Peak Comment	Observations
25	6.34	-	390	-		Lower Marker
266	3.76	-	21.7	100.00		
1500	6.50	6.50	6.67	-		Upper Marker

© Agilent Technologies, Inc. 2017

H1: M-A11

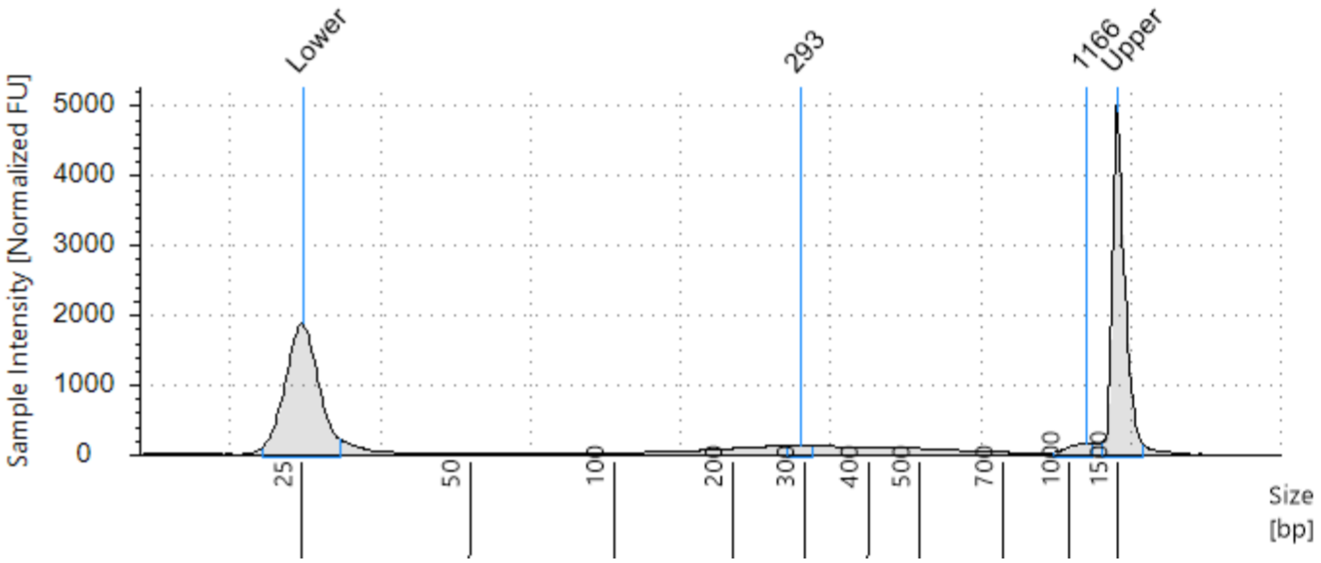

Sample Table

Well	Conc. [ng/µl]	Sample Description	Alert	Observations
H1	0.667	M-A11		

Peak Table

Size [bp]	Calibrated Conc. [ng/µl]	Assigned Conc. [ng/µl]	Peak Molarity [nmol/l]	% Integrated Area	Peak Comment	Observations
25	6.51	-	400	-		Lower Marker
293	0.297	-	1.56	44.52		
1166	0.370	-	0.489	55.48		
1500	6.50	6.50	6.67	-		Upper Marker

© Agilent Technologies, Inc. 2017

A2: M-B11

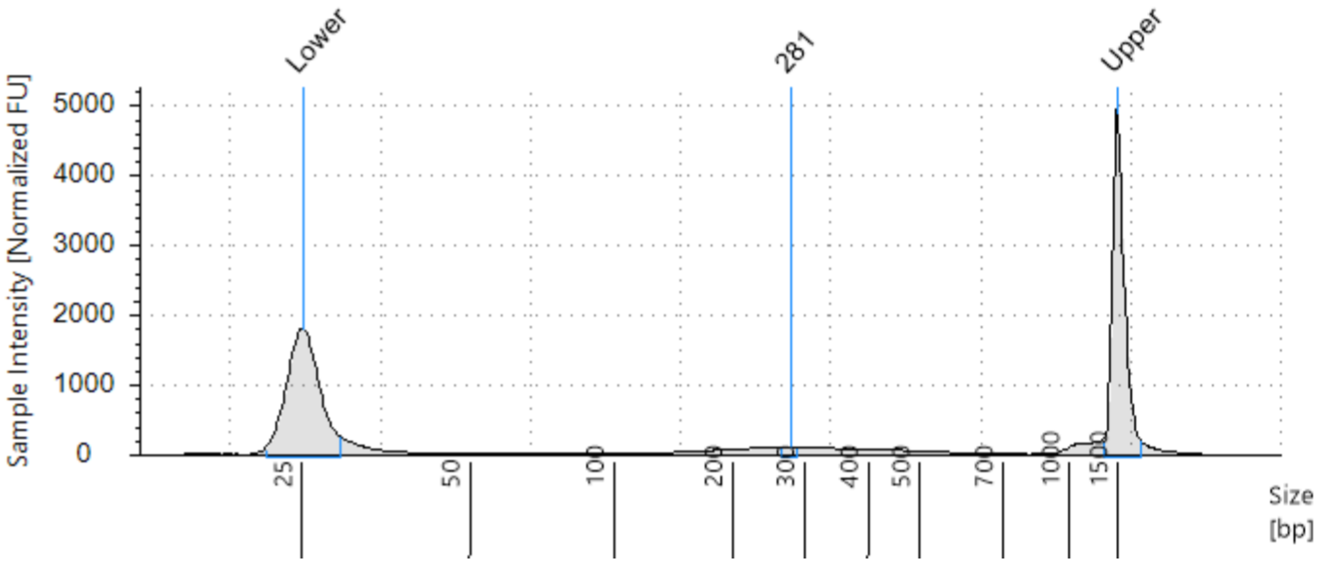

Sample Table

Well	Conc. [ng/µl]	Sample Description	Alert	Observations
A2	0.158	M-B11		

Peak Table

Size [bp]	Calibrated Conc. [ng/µl]	Assigned Conc. [ng/µl]	Peak Molarity [nmol/l]	% Integrated Area	Peak Comment	Observations
25	6.43	-	396	-		Lower Marker
281	0.158	-	0.868	100.00		
1500	6.50	6.50	6.67	-		Upper Marker

B2: M-C11

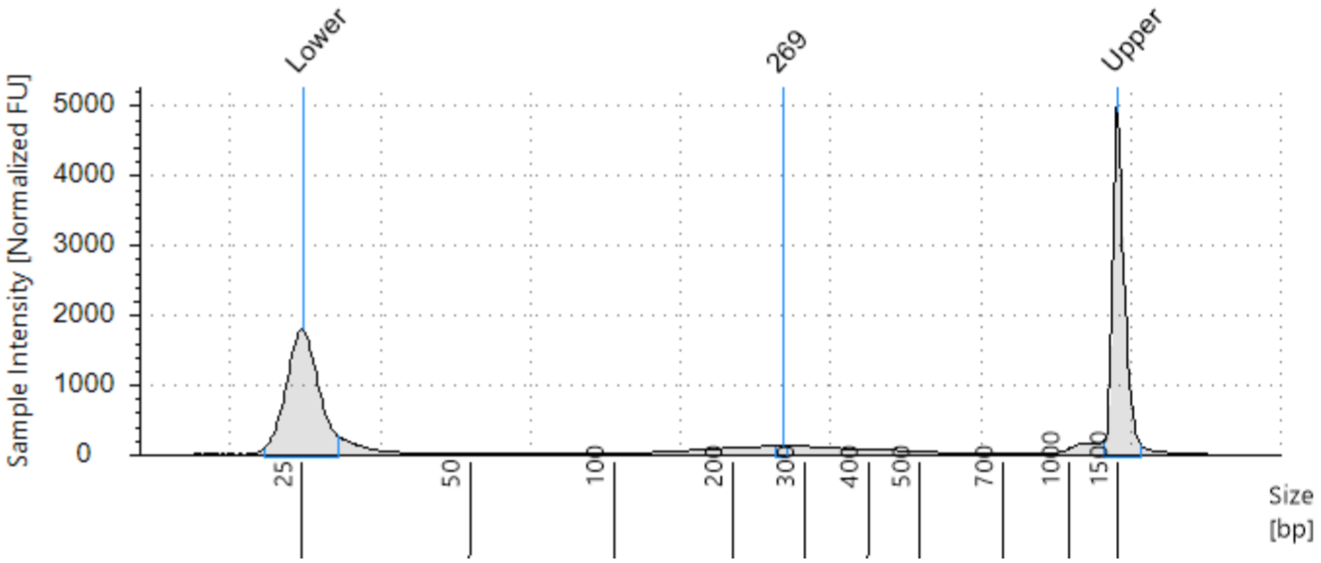

Sample Table

Well	Conc. [ng/µl]	Sample Description	Alert	Observations
B2	0.149	M-C11		

Peak Table

Size [bp]	Calibrated Conc. [ng/µl]	Assigned Conc. [ng/µl]	Peak Molarity [nmol/l]	% Integrated Area	Peak Comment	Observations
25	6.38	-	393	-		Lower Marker
269	0.149	-	0.854	100.00		
1500	6.50	6.50	6.67	-		Upper Marker

 © Agilent Technologies, Inc. 2017

C2: M-D11

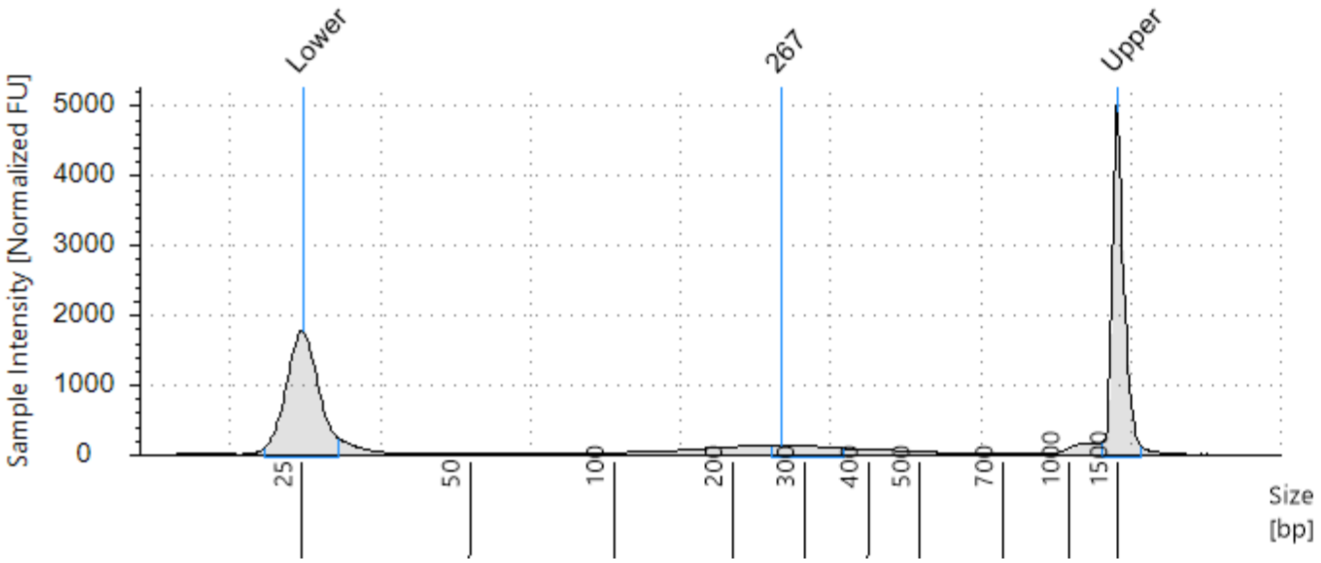

Sample Table

Well	Conc. [ng/µl]	Sample Description	Alert	Observations
C2	0.773	M-D11		

Peak Table

Size [bp]	Calibrated Conc. [ng/µl]	Assigned Conc. [ng/µl]	Peak Molarity [nmol/l]	% Integrated Area	Peak Comment	Observations
25	6.34	-	390	-		Lower Marker
267	0.773	-	4.45	100.00		
1500	6.50	6.50	6.67	-		Upper Marker

 © Agilent Technologies, Inc. 2017

D2: M-E11

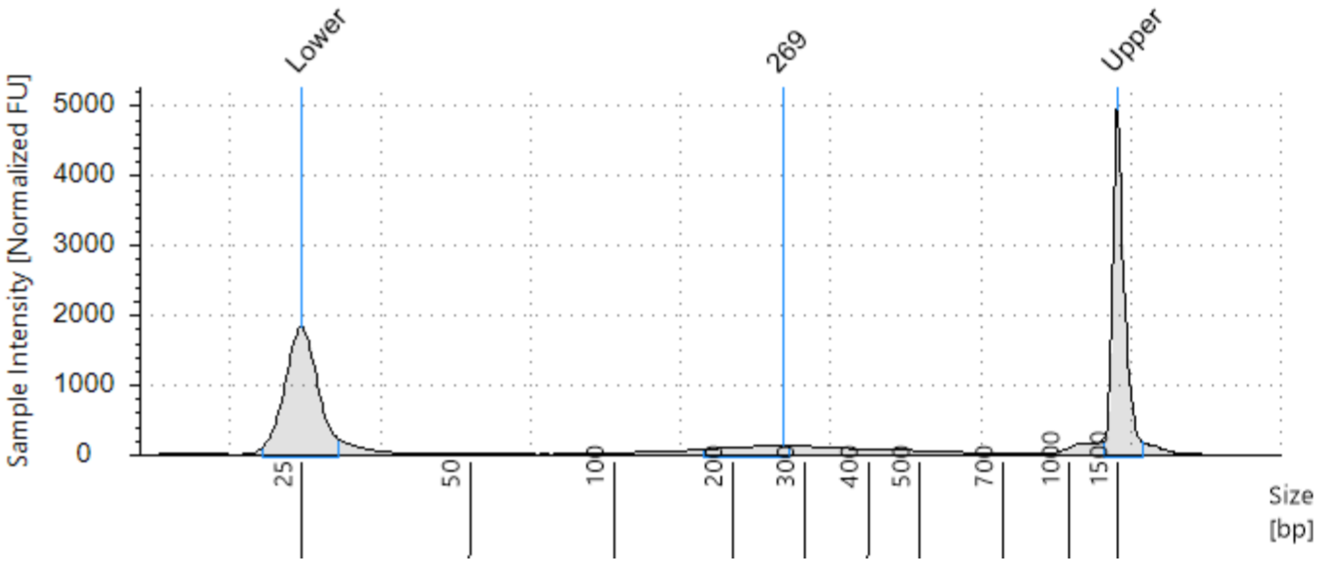

Sample Table

Well	Conc. [ng/µl]	Sample Description	Alert	Observations
D2	0.821	M-E11		

Peak Table

Size [bp]	Calibrated Conc. [ng/µl]	Assigned Conc. [ng/µl]	Peak Molarity [nmol/l]	% Integrated Area	Peak Comment	Observations
25	6.64	-	409	-		Lower Marker
269	0.821	-	4.70	100.00		
1500	6.50	6.50	6.67	-		Upper Marker

© Agilent Technologies, Inc. 2017

E2: M-F11

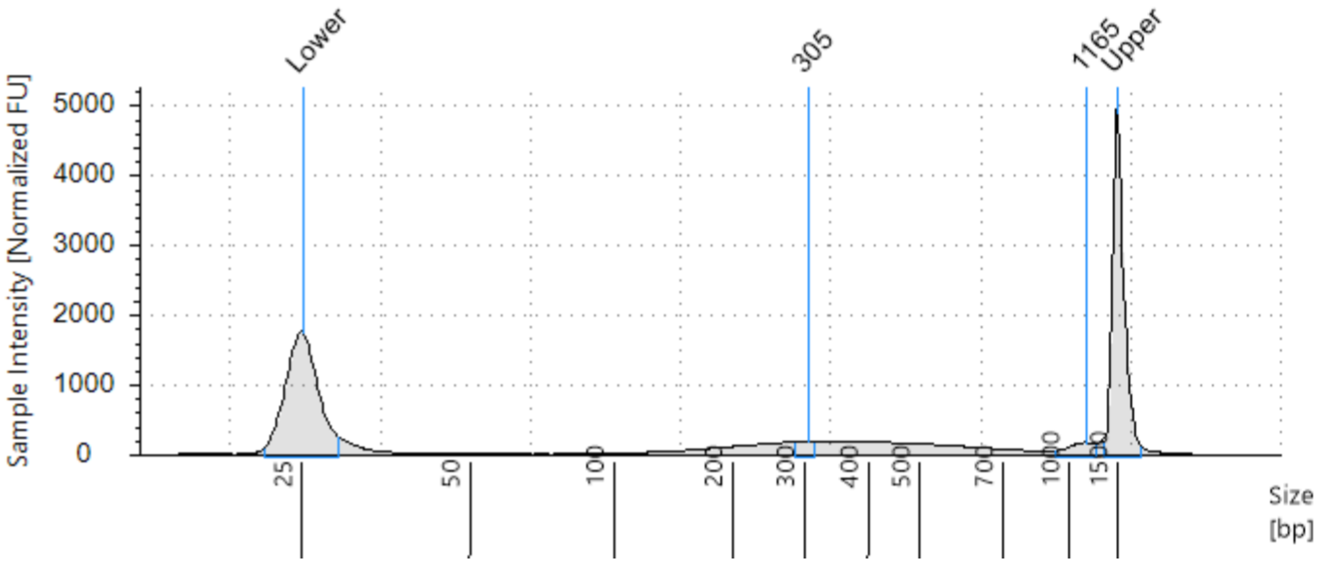

Sample Table

Well	Conc. [ng/µl]	Sample Description	Alert	Observations
E2	0.808	M-F11		

Peak Table

Size [bp]	Calibrated Conc. [ng/µl]	Assigned Conc. [ng/µl]	Peak Molarity [nmol/l]	% Integrated Area	Peak Comment	Observations
25	6.51	-	401	-		Lower Marker
305	0.328	-	1.65	40.56		
1165	0.480	-	0.634	59.44		
1500	6.50	6.50	6.67	-		Upper Marker

© Agilent Technologies, Inc. 2017

F2: M-Gl1

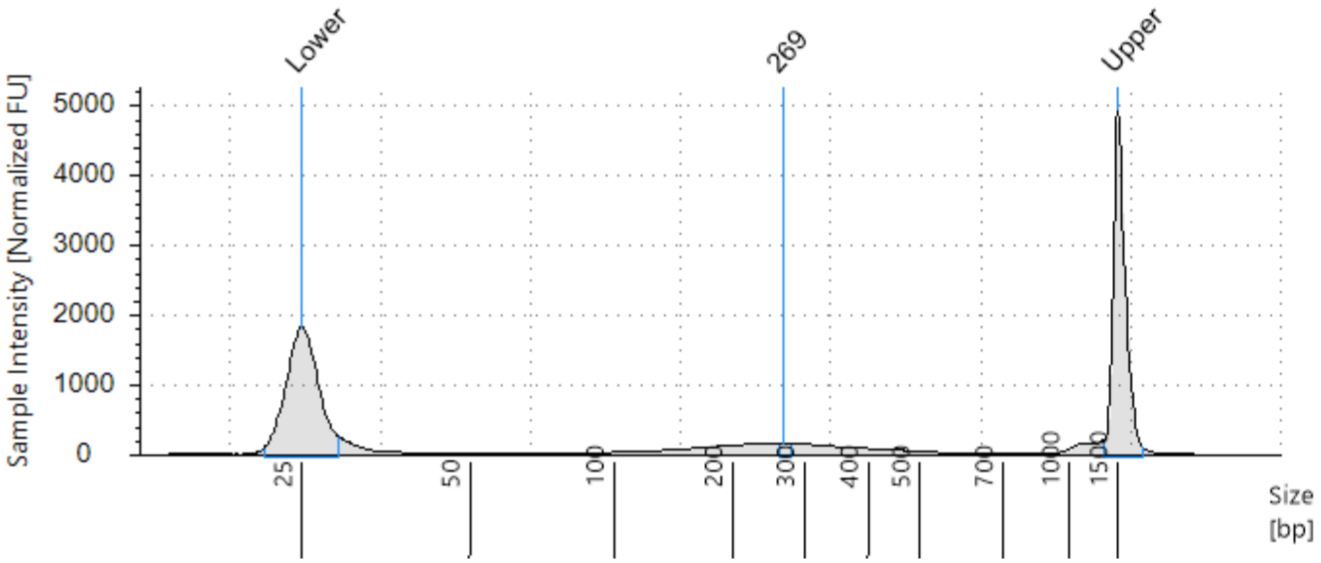

Sample Table

Well	Conc. [ng/µl]	Sample Description	Alert	Observations
F2	0.198	M-Gl1		

Peak Table

Size [bp]	Calibrated Conc. [ng/µl]	Assigned Conc. [ng/µl]	Peak Molarity [nmol/l]	% Integrated Area	Peak Comment	Observations
25	6.47	-	398	-		Lower Marker
269	0.198	-	1.13	100.00		
1500	6.50	6.50	6.67	-		Upper Marker

© Agilent Technologies, Inc. 2017

G2: M-H11

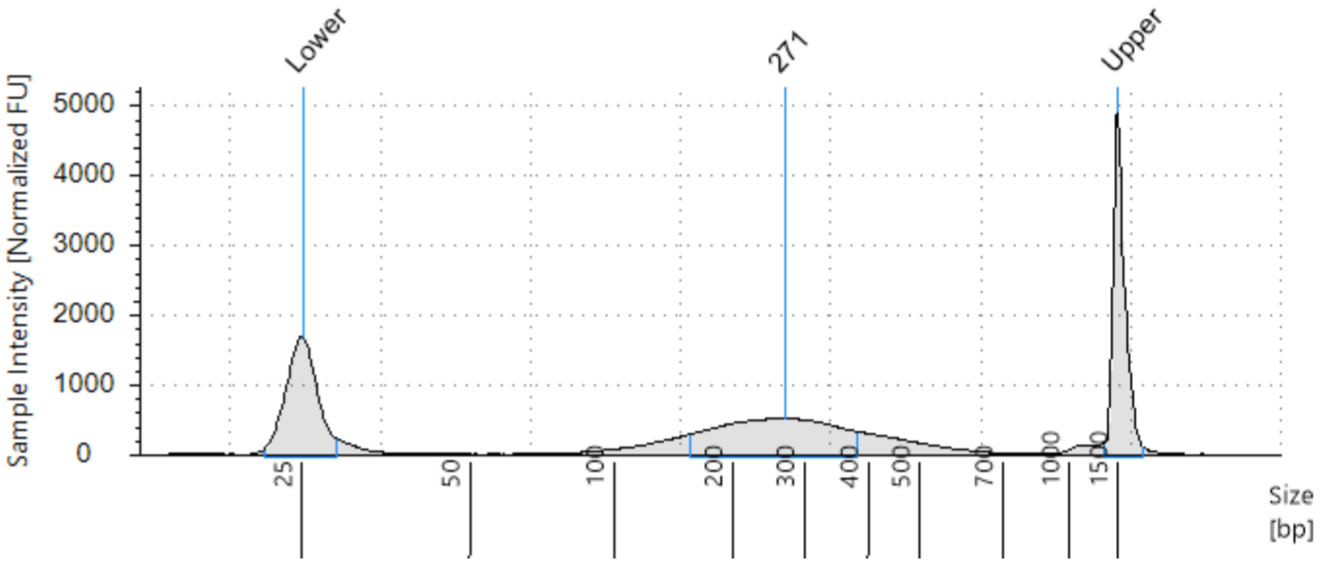

Sample Table

Well	Conc. [ng/µl]	Sample Description	Alert	Observations
G2	6.95	M-H11		

Peak Table

Size [bp]	Calibrated Conc. [ng/µl]	Assigned Conc. [ng/µl]	Peak Molarity [nmol/l]	% Integrated Area	Peak Comment	Observations
25	5.87	-	361	-		Lower Marker
271	6.95	-	39.4	100.00		
1500	6.50	6.50	6.67	-		Upper Marker

© Agilent Technologies, Inc. 2017

H2: M-A12

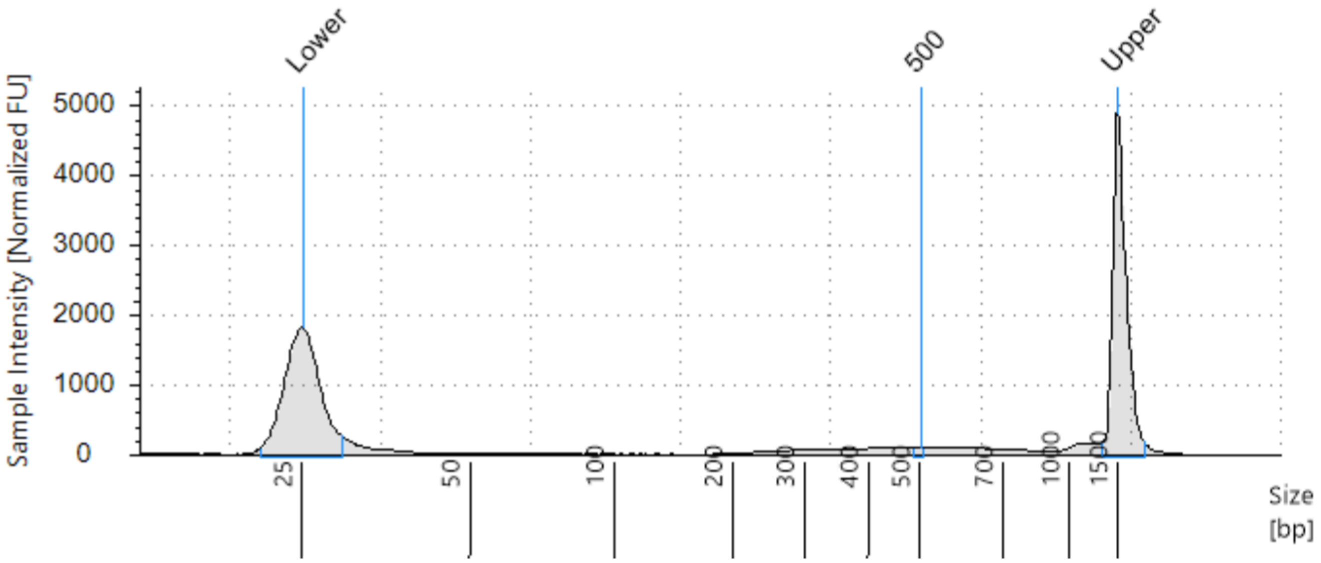

Sample Table

Well	Conc. [ng/µl]	Sample Description	Alert	Observations
H2	0.0995	M-A12		

Peak Table

Size [bp]	Calibrated Conc. [ng/µl]	Assigned Conc. [ng/µl]	Peak Molarity [nmol/l]	% Integrated Area	Peak Comment	Observations
25	6.31	-	389	-		Lower Marker
500	0.0995	-	0.306	100.00		
1500	6.50	6.50	6.67	-		Upper Marker

Filename: 2020-09-15-04 Q-S MINUS E1,E6 D1000 R1.D1000

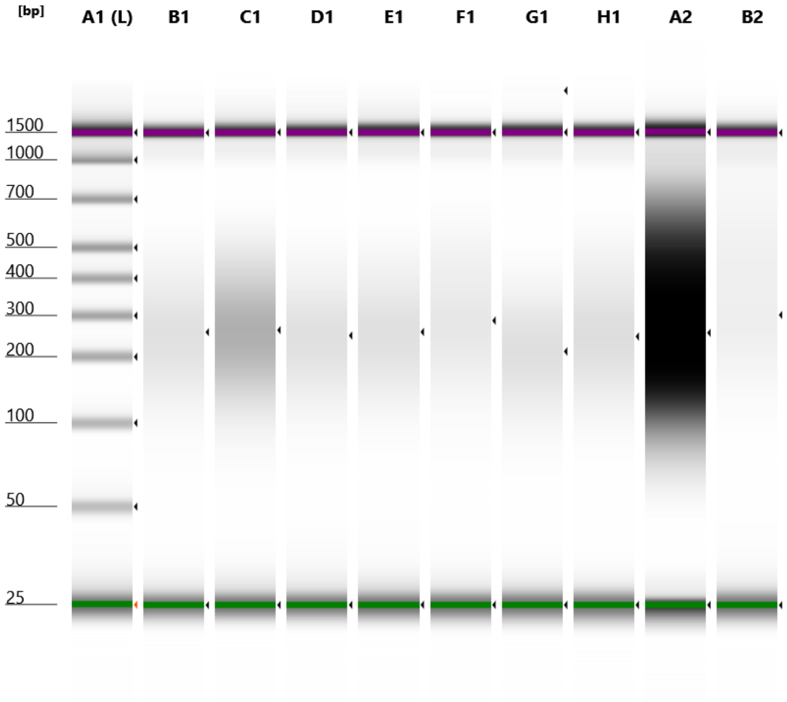

Default image (Contrast 100%)

Sample Info

Well	Conc. [ng/µl]	Sample Description	Alert	Observations
A1	11.5	Ladder		Ladder
B1	3.85	G12 P R2		
C1	10.6	H12 P R2		
D1	2.10	G3 P R2		
E1	1.82	H3 P R2		
F1	3.20	D5 P R2		
G1	3.85	H5 P R2		
H1	4.27	D7 P R2		
A2	47.6	E1 M R1		
B2	0.518	E6 M R1		

 © Agilent Technologies, Inc. 2017

A1: Ladder

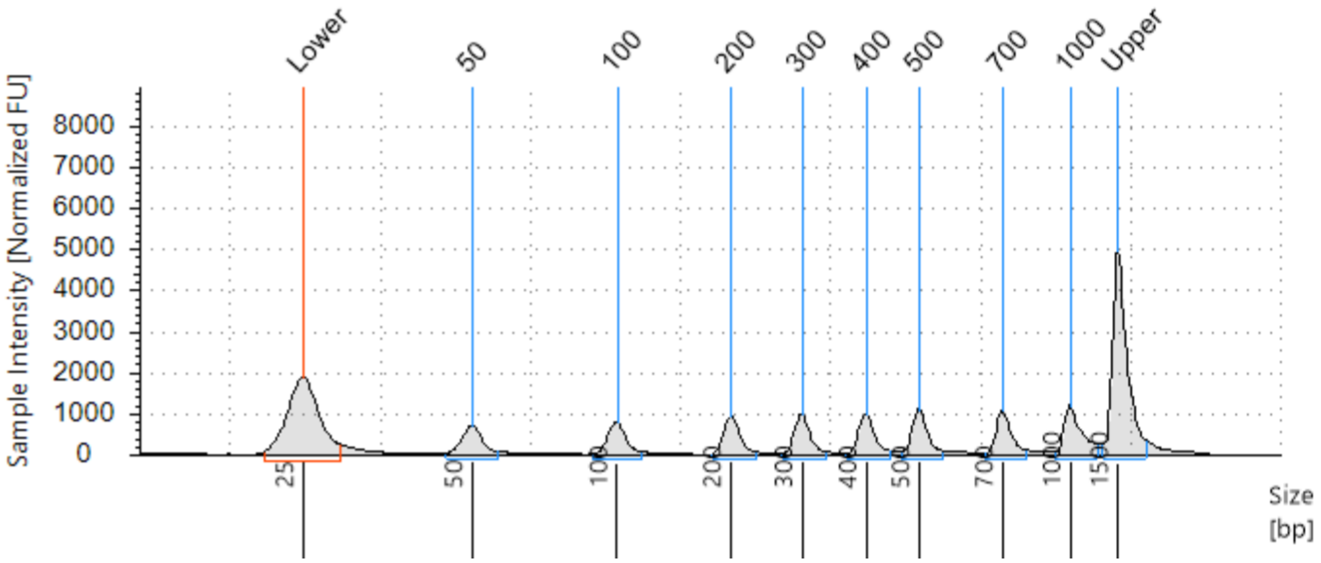

Sample Table

Well	Conc. [ng/µl]	Sample Description	Alert	Observations
A1	11.5	Ladder		Ladder

Peak Table

Size [bp]	Calibrated Conc. [ng/µl]	Assigned Conc. [ng/µl]	Peak Molarity [nmol/l]	% Integrated Area	Peak Comment	Observations
25	5.30	-	326	-		Lower Marker
50	1.27	-	39.0	11.02		
100	1.29	-	19.9	11.22		
200	1.35	-	10.4	11.77		
300	1.39	-	7.12	12.07		
400	1.41	-	5.43	12.28		
500	1.51	-	4.65	13.15		
700	1.47	-	3.23	12.77		
1000	1.81	-	2.78	15.71		
1500	6.50	6.50	6.67	-		Upper Marker

© Agilent Technologies, Inc. 2017

A2: E1 M R1

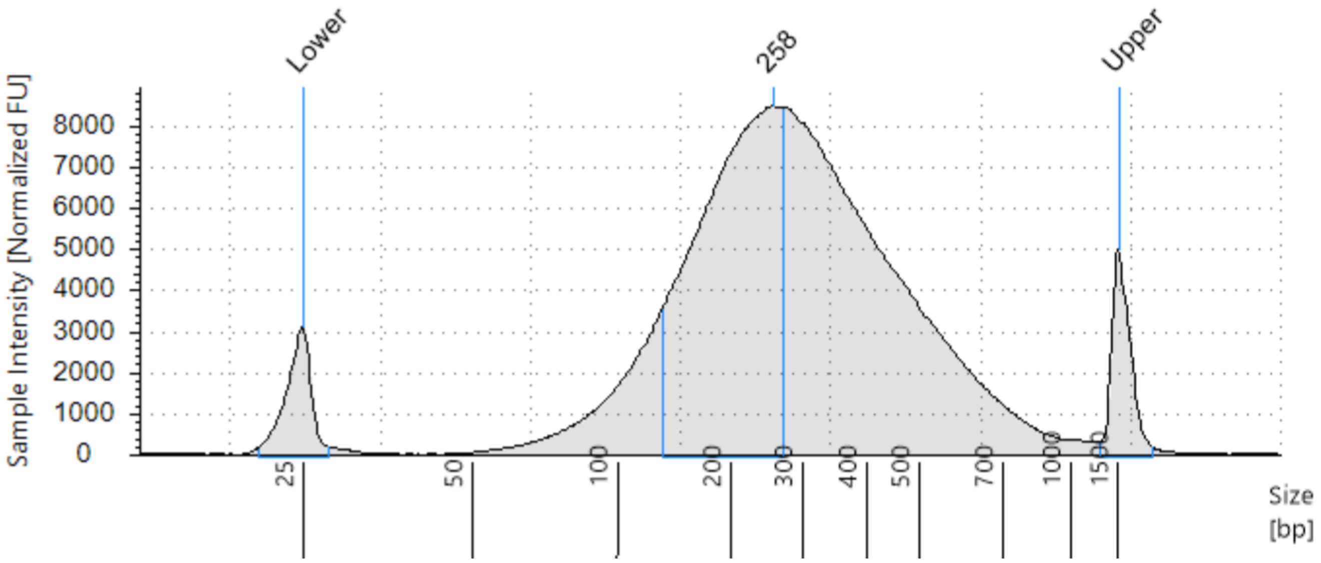

Sample Table

Well	Conc. [ng/µl]	Sample Description	Alert	Observations
A2	47.6	E1 M R1		

Peak Table

Size [bp]	Calibrated Conc. [ng/µl]	Assigned Conc. [ng/µl]	Peak Molarity [nmol/l]	% Integrated Area	Peak Comment	Observations
25	5.34	-	329	-		Lower Marker
258	47.6	-	283	100.00		
1500	6.50	6.50	6.67	-		Upper Marker

© Agilent Technologies, Inc. 2017

B2: E6 M R1

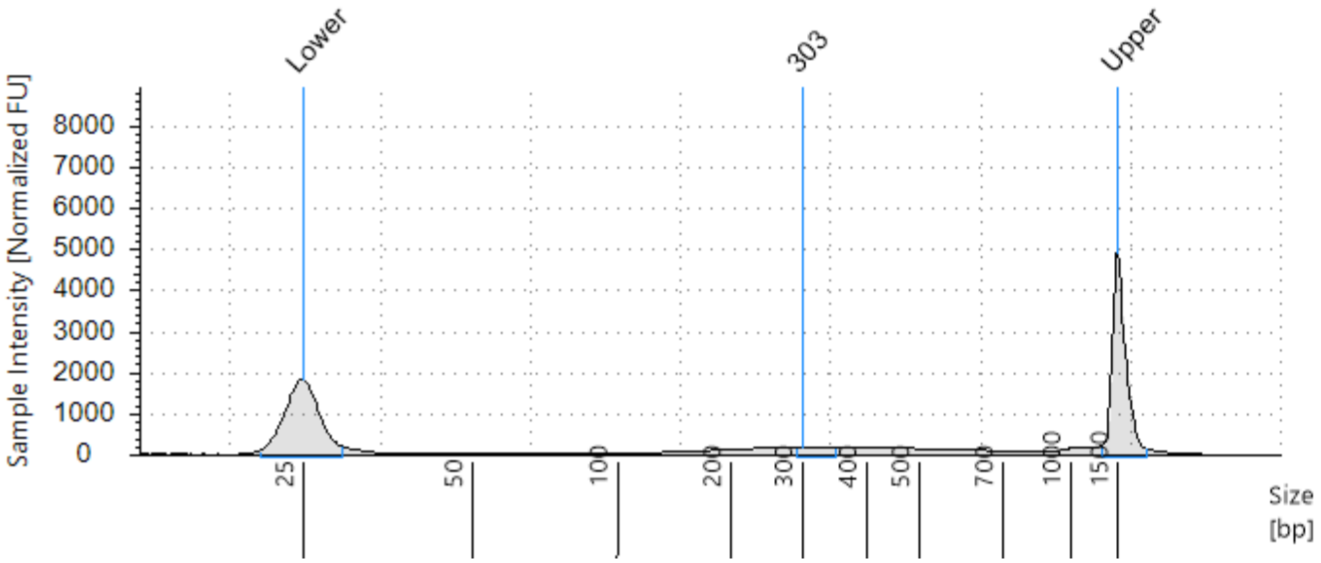

Sample Table

Well	Conc. [ng/µl]	Sample Description	Alert	Observations
B2	0.518	E6 M R1		

Peak Table

Size [bp]	Calibrated Conc. [ng/µl]	Assigned Conc. [ng/µl]	Peak Molarity [nmol/l]	% Integrated Area	Peak Comment	Observations
25	6.29	-	387	-		Lower Marker
303	0.518	-	2.63	100.00		
1500	6.50	6.50	6.67	-		Upper Marker

© Agilent Technologies, Inc. 2017

Filename: D1000, Q-S, MINUS from 3.8.20, A4-B5 R1.cD1000

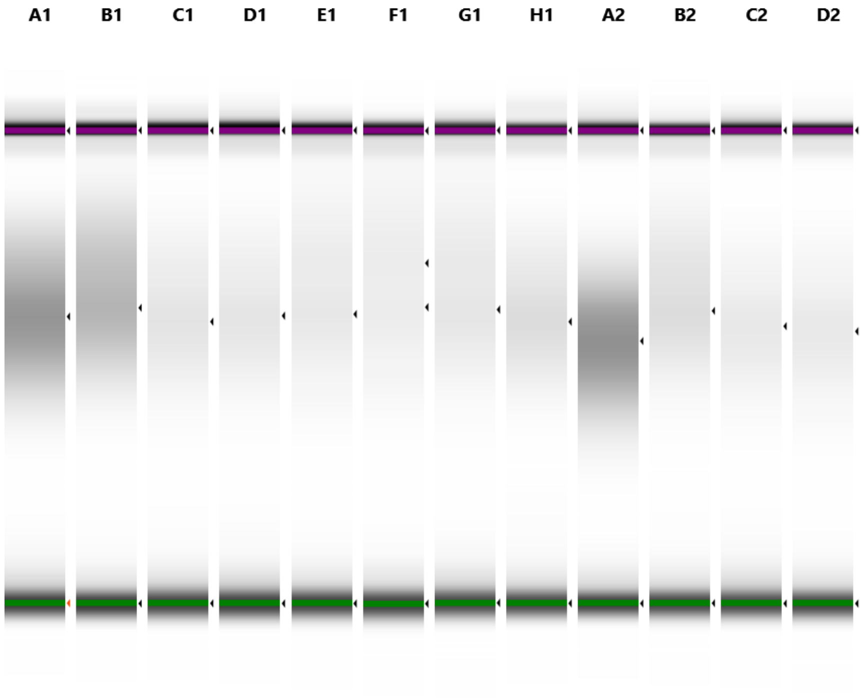

Default image (Contrast 100%)

Sample Info

Well	Conc. [ng/µl]	Sample Description	Alert	Observations
A1	8.58	H3 DFB minus from 3.8.20		
B1	7.15	A4		
C1	0.238	B4		
D1	0.864	C4		
E1	0.255	D4		
F1	0.402	E4		
G1	0.530	F4		
H1	1.60	G4		
A2	5.71	H4		
B2	2.28	A5		
C2	0.267	B5		
D2	0.251			

© Agilent Technologies, Inc. 2017

A1: H3 DFB minus from 3.8.20

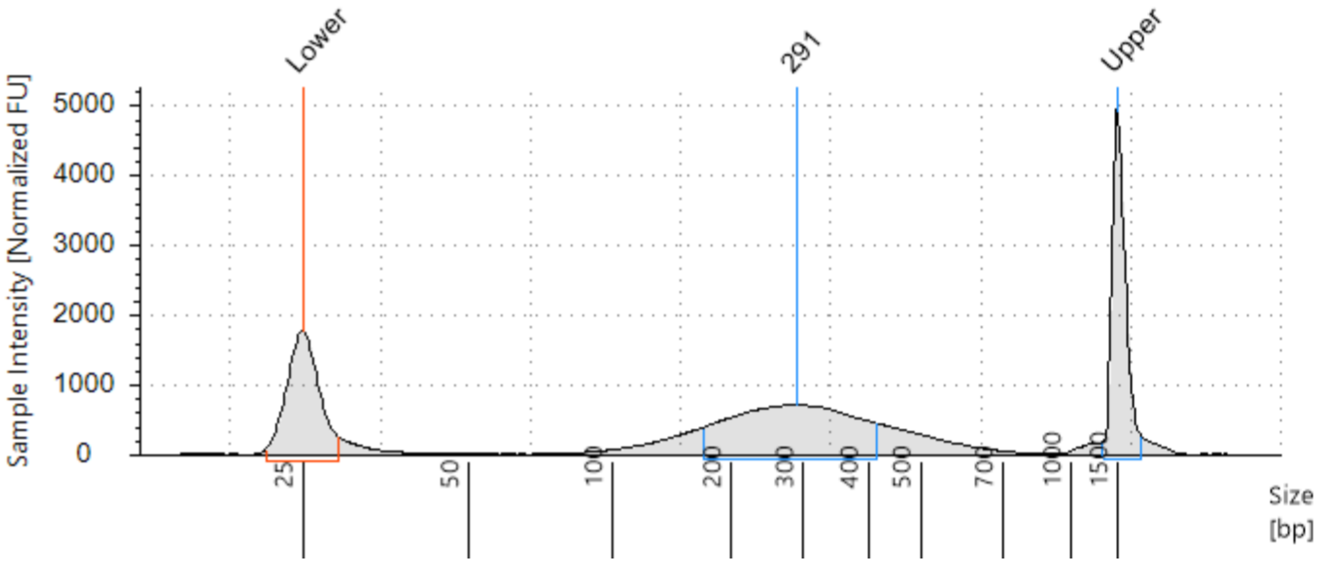

Sample Table

Well	Conc. [ng/µl]	Sample Description	Alert	Observations
A1	8.58	H3 DFB minus from 3.8.20		

Peak Table

Size [bp]	Calibrated Conc. [ng/µl]	Assigned Conc. [ng/µl]	Peak Molarity [nmol/l]	% Integrated Area	Peak Comment	Observations
25	5.48	-	337	-		Lower Marker
291	8.58	-	45.3	100.00		
1500	6.50	6.50	6.67	-		Upper Marker

© Agilent Technologies, Inc. 2017

B1: A4

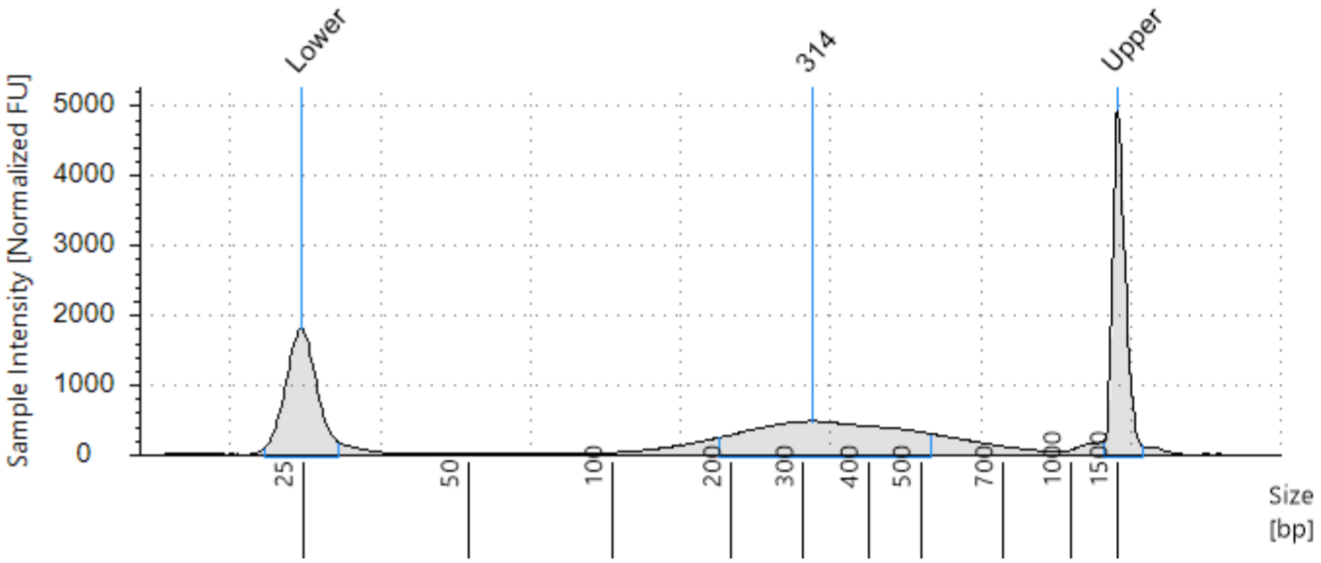

Sample Table

Well	Conc. [ng/µl]	Sample Description	Alert	Observations
B1	7.15	A4		

Peak Table

Size [bp]	Calibrated Conc. [ng/µl]	Assigned Conc. [ng/µl]	Peak Molarity [nmol/l]	% Integrated Area	Peak Comment	Observations
25	5.68	-	349	-		Lower Marker
314	7.15	-	35.1	100.00		
1500	6.50	6.50	6.67	-		Upper Marker

 © Agilent Technologies, Inc. 2017

C1: B4

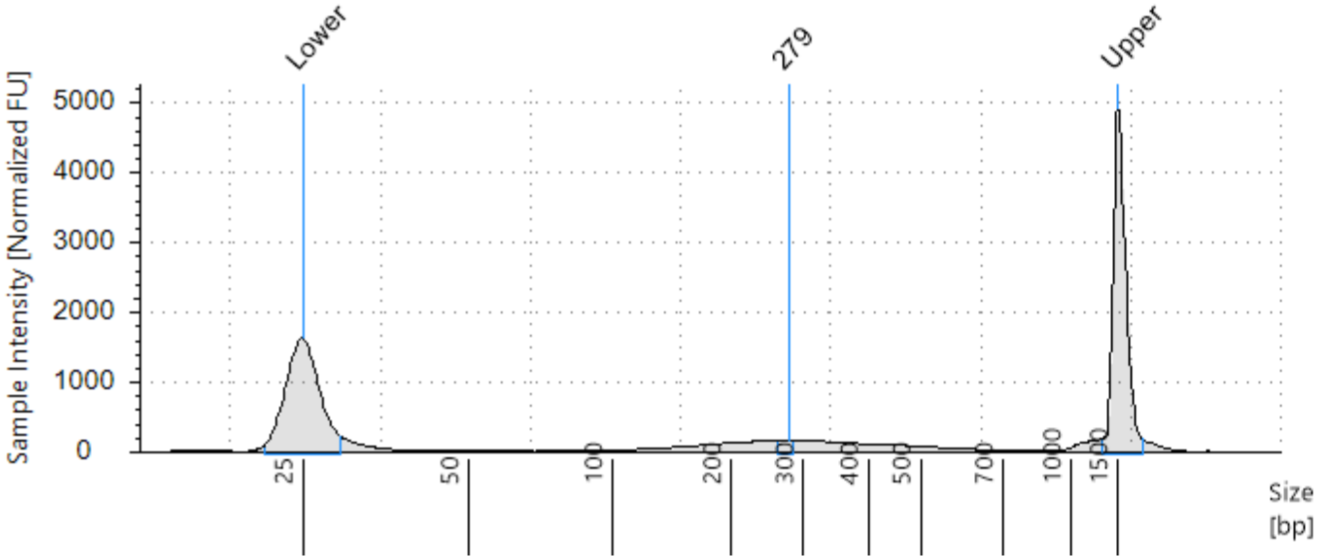

Sample Table

Well	Conc. [ng/µl]	Sample Description	Alert	Observations
C1	0.238	B4		

Peak Table

Size [bp]	Calibrated Conc. [ng/µl]	Assigned Conc. [ng/µl]	Peak Molarity [nmol/l]	% Integrated Area	Peak Comment	Observations
25	5.67	-	349	-		Lower Marker
279	0.238	-	1.31	100.00		
1500	6.50	6.50	6.67	-		Upper Marker

© Agilent Technologies, Inc. 2017

D1: C4

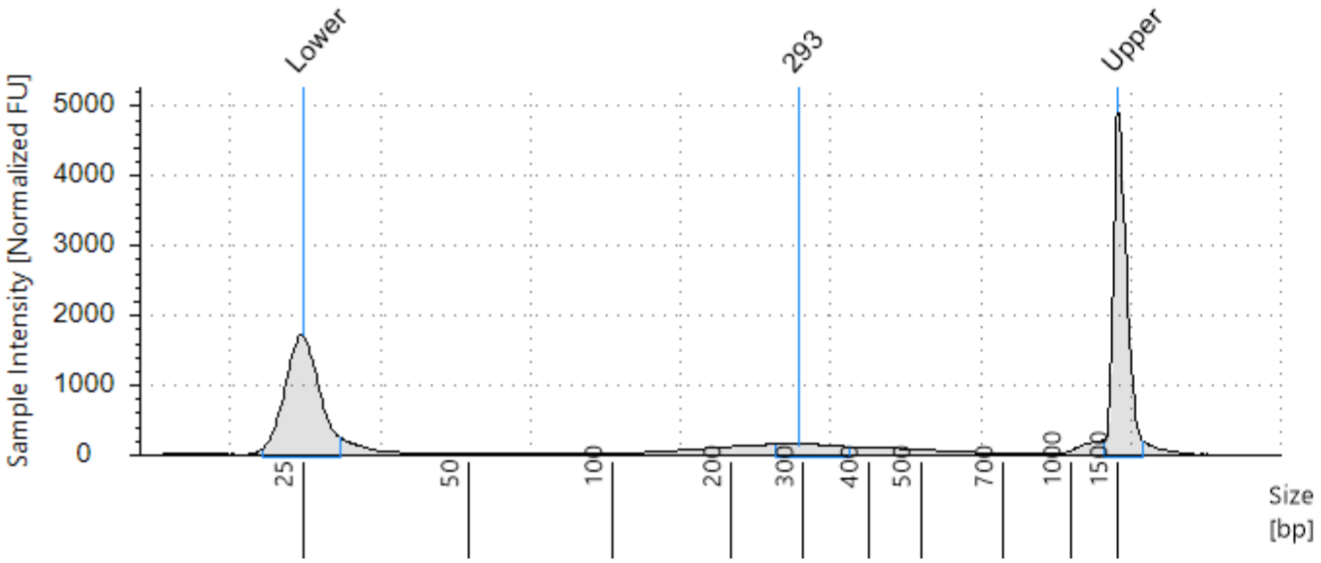

Sample Table

Well	Conc. [ng/µl]	Sample Description	Alert	Observations
D1	0.864	C4		

Peak Table

Size [bp]	Calibrated Conc. [ng/µl]	Assigned Conc. [ng/µl]	Peak Molarity [nmol/l]	% Integrated Area	Peak Comment	Observations
25	5.76	-	354	-		Lower Marker
293	0.864	-	4.53	100.00		
1500	6.50	6.50	6.67	-		Upper Marker

© Agilent Technologies, Inc. 2017

E1: D4

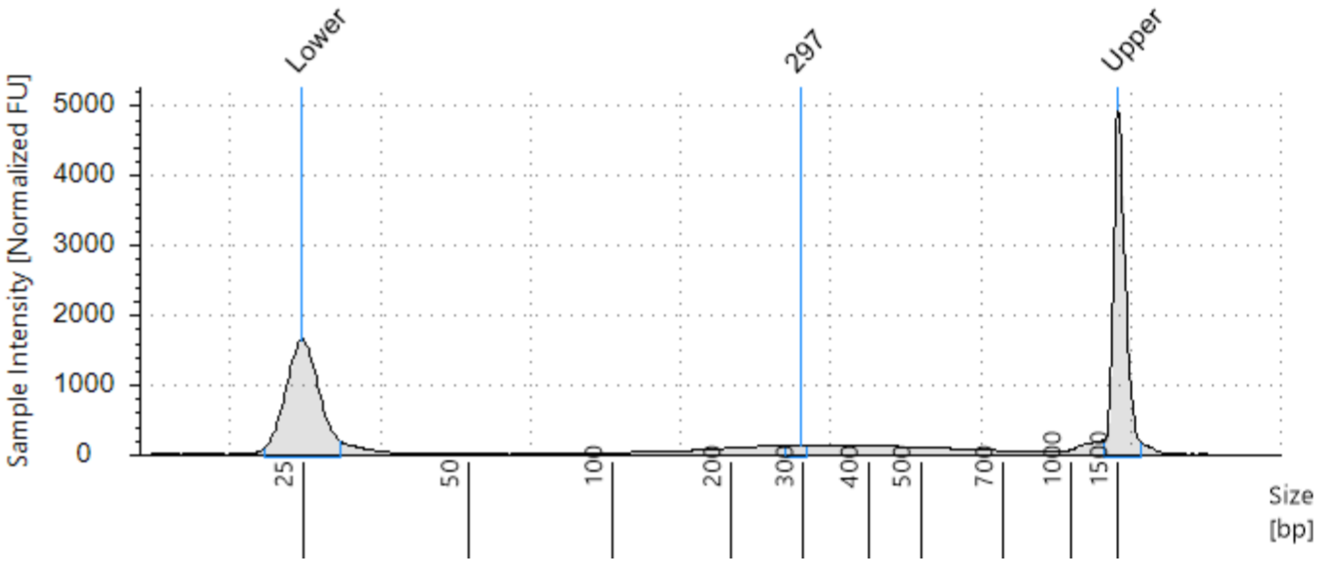

Sample Table

Well	Conc. [ng/µl]	Sample Description	Alert	Observations
E1	0.255	D4		

Peak Table

Size [bp]	Calibrated Conc. [ng/µl]	Assigned Conc. [ng/µl]	Peak Molarity [nmol/l]	% Integrated Area	Peak Comment	Observations
25	5.96	-	367	-		Lower Marker
297	0.255	-	1.32	100.00		
1500	6.50	6.50	6.67	-		Upper Marker

© Agilent Technologies, Inc. 2017

F1: E4

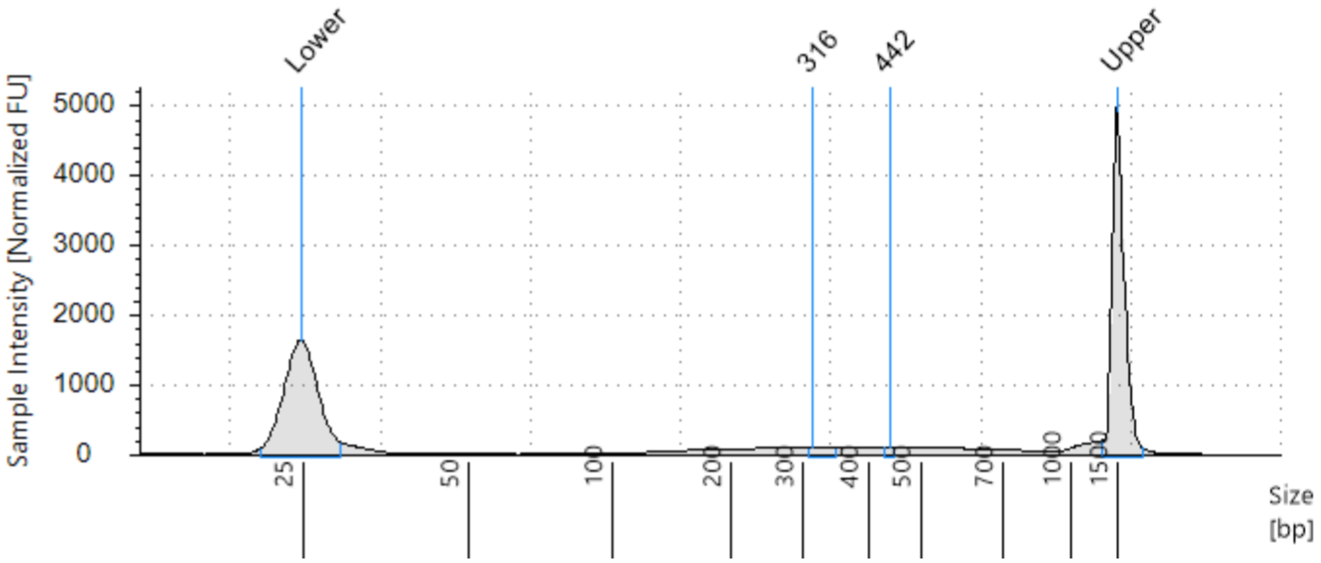

Sample Table

Well	Conc. [ng/µl]	Sample Description	Alert	Observations
F1	0.402	E4		

Peak Table

Size [bp]	Calibrated Conc. [ng/µl]	Assigned Conc. [ng/µl]	Peak Molarity [nmol/l]	% Integrated Area	Peak Comment	Observations
25	5.99	-	368	-		Lower Marker
316	0.284	-	1.38	70.60		
442	0.118	-	0.412	29.40		
1500	6.50	6.50	6.67	-		Upper Marker

 © Agilent Technologies, Inc. 2017

G1: F4

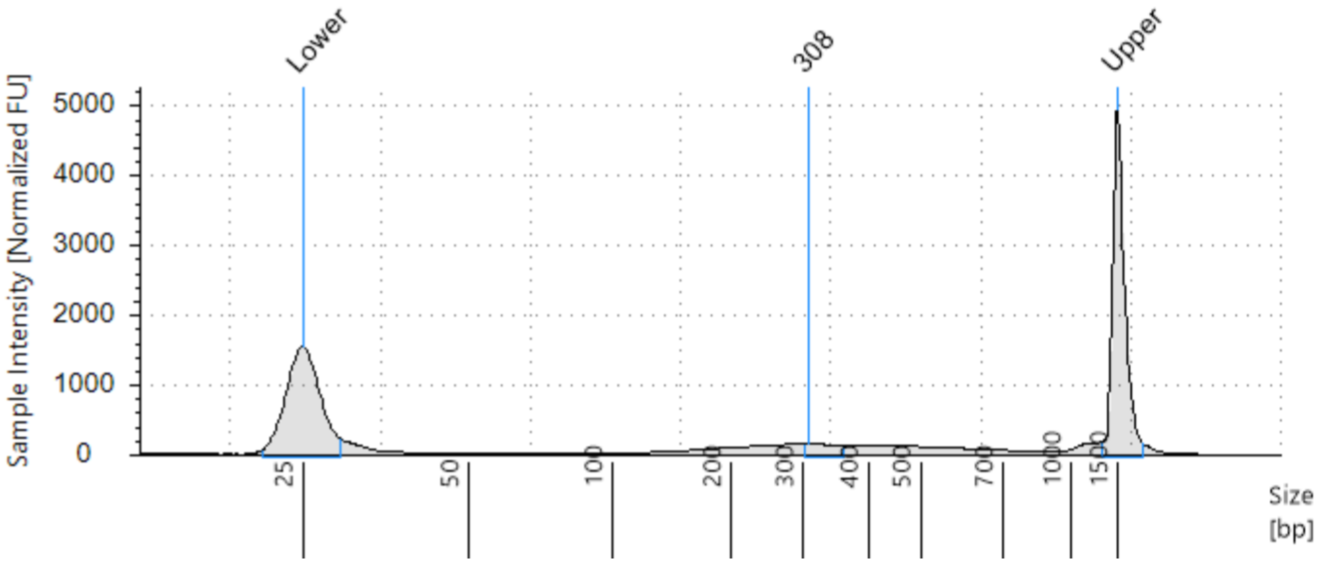

Sample Table

Well	Conc. [ng/µl]	Sample Description	Alert	Observations
G1	0.530	F4		

Peak Table

Size [bp]	Calibrated Conc. [ng/µl]	Assigned Conc. [ng/µl]	Peak Molarity [nmol/l]	% Integrated Area	Peak Comment	Observations
25	5.78	-	356	-		Lower Marker
308	0.530	-	2.64	100.00		
1500	6.50	6.50	6.67	-		Upper Marker

 © Agilent Technologies, Inc. 2017

H1: G4

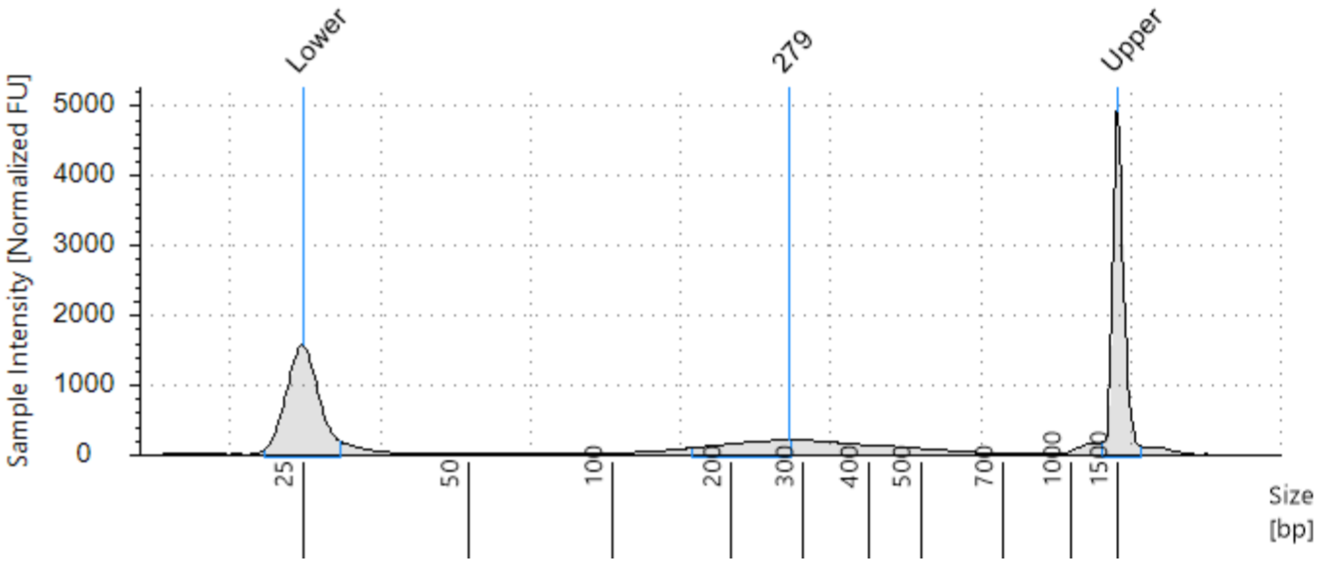

Sample Table

Well	Conc. [ng/µl]	Sample Description	Alert	Observations
H1	1.60	G4		

Peak Table

Size [bp]	Calibrated Conc. [ng/µl]	Assigned Conc. [ng/µl]	Peak Molarity [nmol/l]	% Integrated Area	Peak Comment	Observations
25	5.95	-	366	-		Lower Marker
279	1.60	-	8.81	100.00		
1500	6.50	6.50	6.67	-		Upper Marker

© Agilent Technologies, Inc. 2017

A2: H4

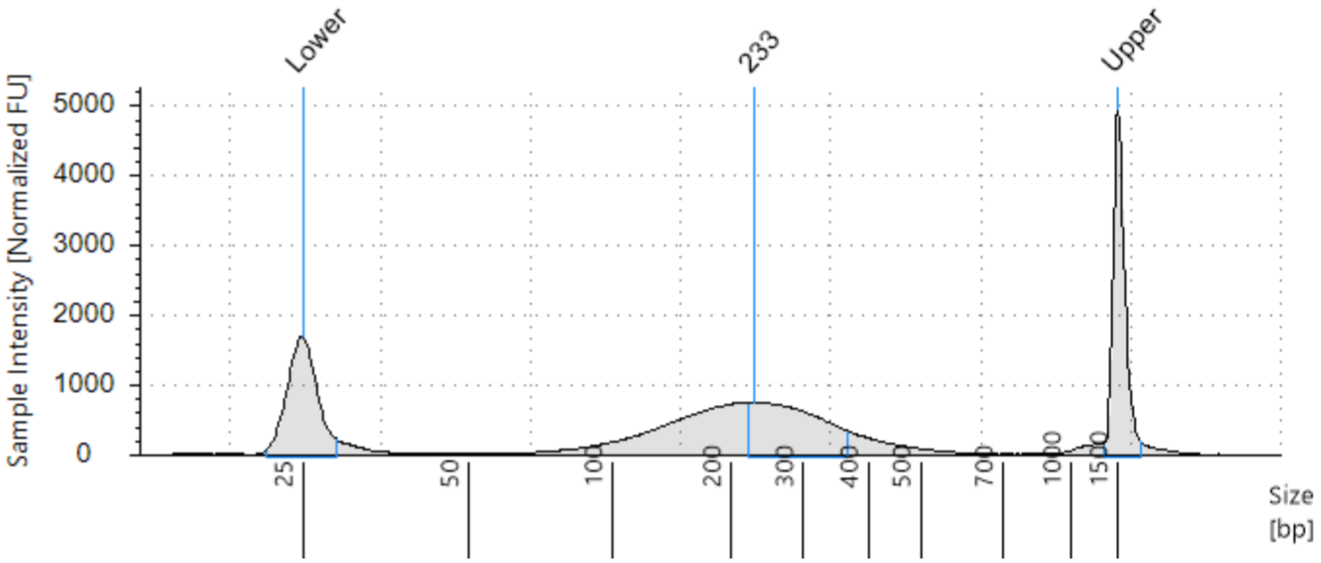

Sample Table

Well	Conc. [ng/µl]	Sample Description	Alert	Observations
A2	5.71	H4		

Peak Table

Size [bp]	Calibrated Conc. [ng/µl]	Assigned Conc. [ng/µl]	Peak Molarity [nmol/l]	% Integrated Area	Peak Comment	Observations
25	5.55	-	341	-		Lower Marker
233	5.71	-	37.7	100.00		
1500	6.50	6.50	6.67	-		Upper Marker

 © Agilent Technologies, Inc. 2017

B2: A5

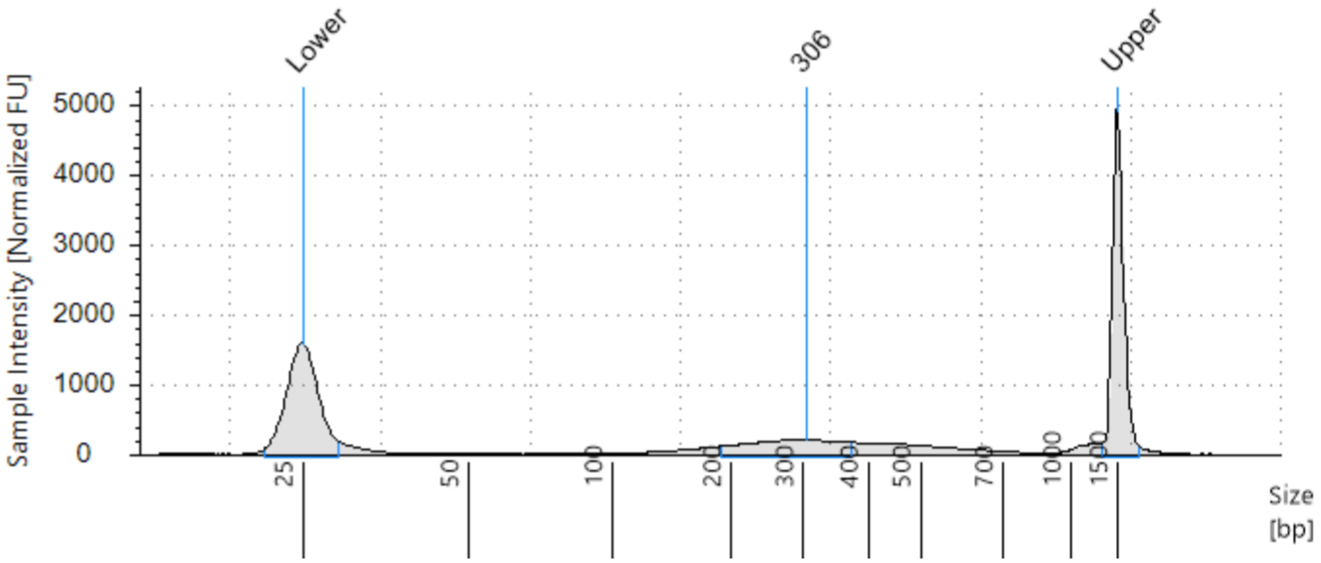

Sample Table

Well	Conc. [ng/µl]	Sample Description	Alert	Observations
B2	2.28	A5		

Peak Table

Size [bp]	Calibrated Conc. [ng/µl]	Assigned Conc. [ng/µl]	Peak Molarity [nmol/l]	% Integrated Area	Peak Comment	Observations
25	5.88	-	362	-		Lower Marker
306	2.28	-	11.5	100.00		
1500	6.50	6.50	6.67	-		Upper Marker

© Agilent Technologies, Inc. 2017

C2: B5

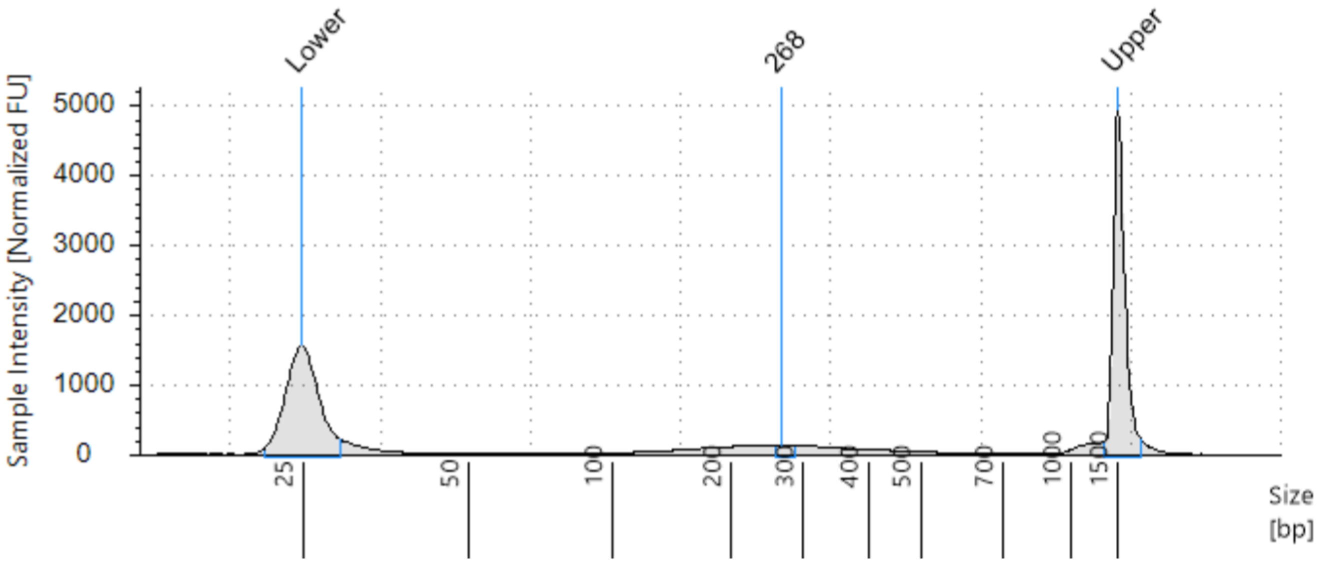

Sample Table

Well	Conc. [ng/µl]	Sample Description	Alert	Observations
C2	0.267	B5		

Peak Table

Size [bp]	Calibrated Conc. [ng/µl]	Assigned Conc. [ng/µl]	Peak Molarity [nmol/l]	% Integrated Area	Peak Comment	Observations
25	5.72	-	352	-		Lower Marker
268	0.267	-	1.53	100.00		
1500	6.50	6.50	6.67	-		Upper Marker

© Agilent Technologies, Inc. 2017

D2

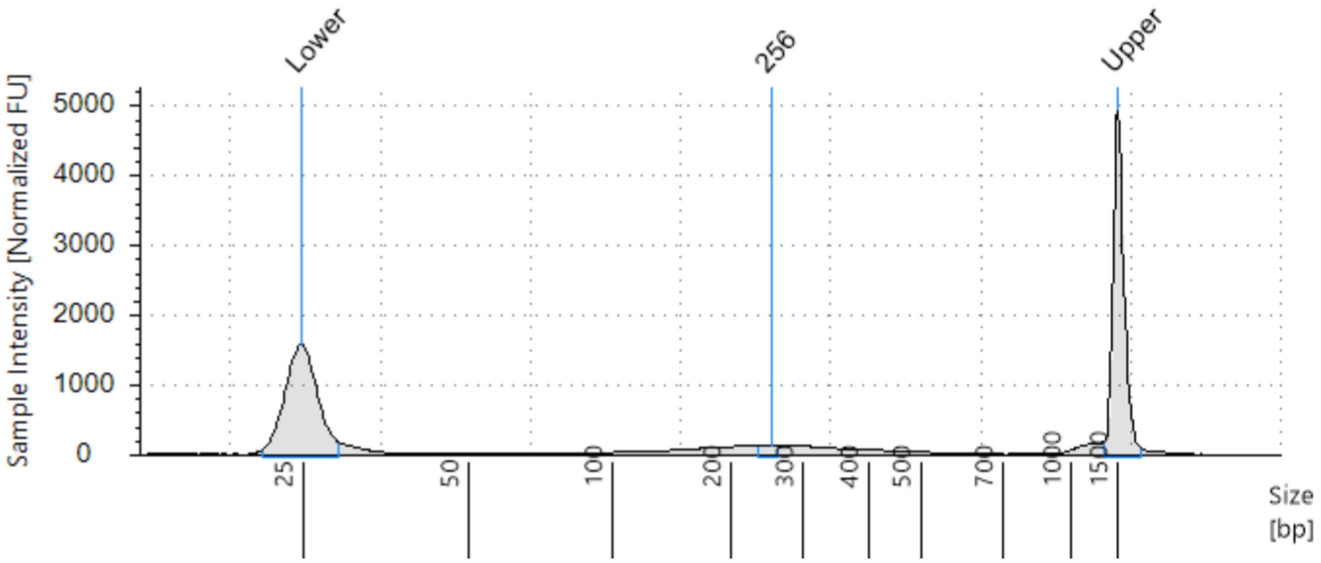

Sample Table

Well	Conc. [ng/µl]	Sample Description	Alert	Observations
D2	0.251			

Peak Table

Size [bp]	Calibrated Conc. [ng/µl]	Assigned Conc. [ng/µl]	Peak Molarity [nmol/l]	% Integrated Area	Peak Comment	Observations
25	6.08	-	374	-		Lower Marker
256	0.251	-	1.51	100.00		
1500	6.50	6.50	6.67	-		Upper Marker

© Agilent Technologies, Inc. 2017
