# Supplementary material for: 96 sample parallel acoustic fragmentation for high throughput next generation sequencing library preparation
Source: PLoS One. 2026 Feb 17;21(2):e0341139. doi: 10.1371/journal.pone.0341139 (PMC12912608; doi:10.1371/journal.pone.0341139)
Supplement: S5 File — (ZIP) [file pone.0341139.s005.zip › QSonica translator TapeStation raw data/QSonica nanodroplets translator 96-well plate replicate 1.pdf]

Filename: 2020-03-06-01- Q_S DFB plus A1-G2 R1.D1000

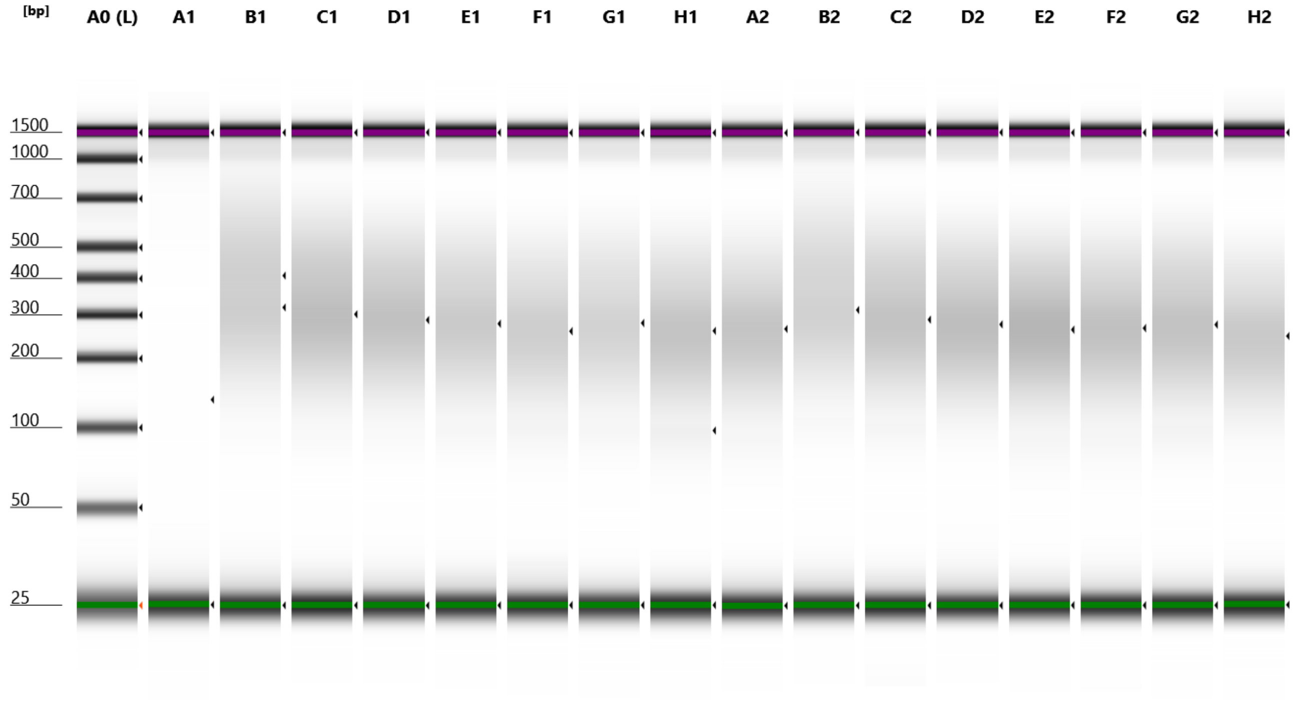

Default image (Contrast 100%)

Sample Info

Well	Conc. [ng/µl]	Sample Description	Alert	Observations
A0	20.3	Electronic Ladder		Ladder
A1	0.407	Ladder		
B1	0.792	A1, plus , R1		
C1	0.398	B1		
D1	2.72	C1		
E1	2.25	D1		
F1	2.06	E1		
G1	1.40	F1		
H1	2.48	G1		
A2	4.38	H1		
B2	2.60	A2		
C2	2.59	B2		
D2	4.94	C2		
E2	3.62	D2		
F2	4.86	E2		
G2	2.07	F2		
H2	0.400	G2		

© Agilent Technologies, Inc. 2017

A0: Electronic Ladder

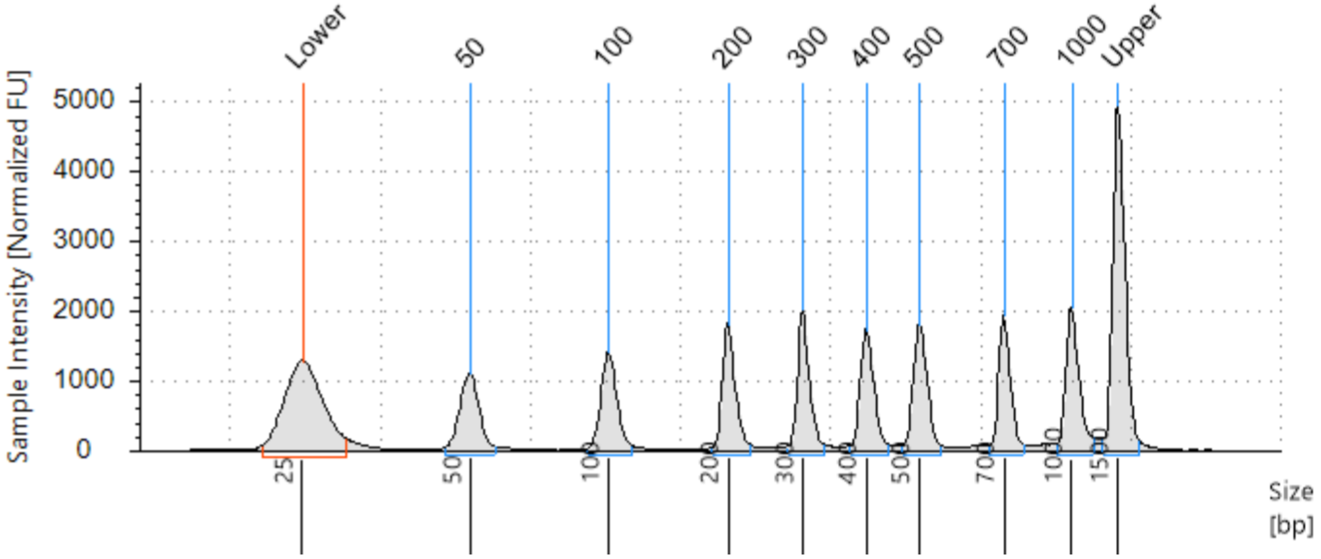

Sample Table

Well	Conc. [ng/µl]	Sample Description	Alert	Observations
A0	20.3	Electronic Ladder		Ladder

Peak Table

Size [bp]	Calibrated Conc. [ng/µl]	Assigned Conc. [ng/µl]	Peak Molarity [nmol/l]	% Integrated Area	Peak Comment	Observations
25	5.22	-	321	-		Lower Marker
50	2.25	-	69.3	11.11		
100	2.37	-	36.5	11.71		
200	2.47	-	19.0	12.20		
300	2.55	-	13.1	12.56		
400	2.57	-	9.87	12.66		
500	2.71	-	8.33	13.36		
700	2.46	-	5.41	12.15		
1000	2.89	-	4.44	14.25		
1500	6.50	6.50	6.67	-		Upper Marker

 © Agilent Technologies, Inc. 2017

A1: Ladder

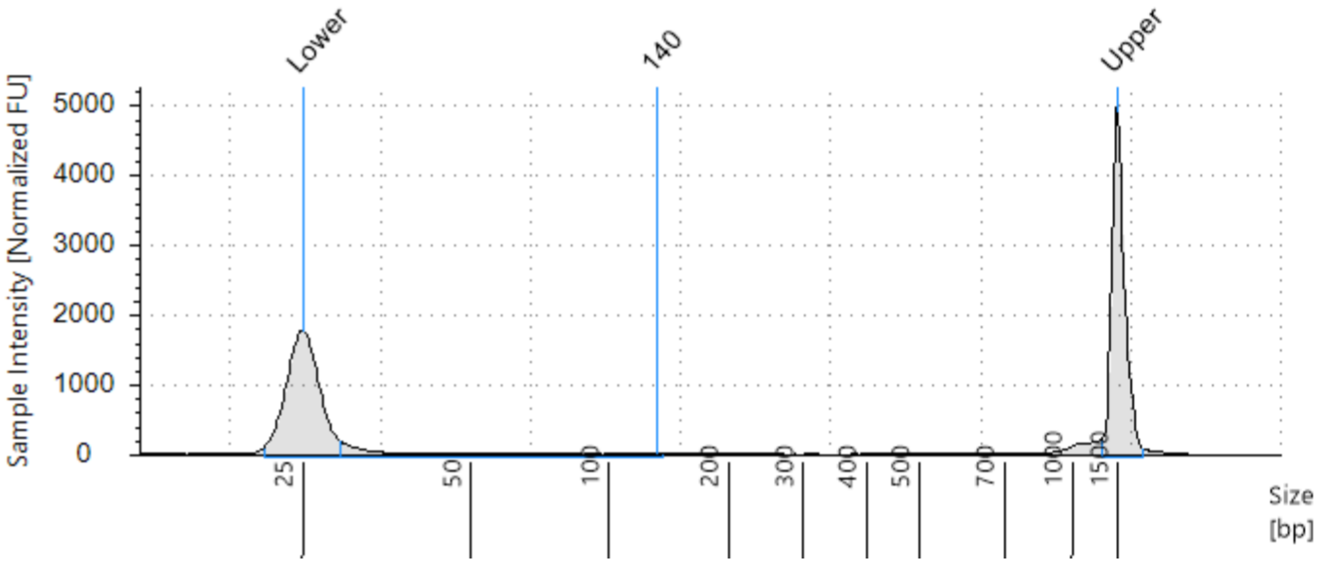

Sample Table

Well	Conc. [ng/µl]	Sample Description	Alert	Observations
A1	0.407	Ladder		

Peak Table

Size [bp]	Calibrated Conc. [ng/µl]	Assigned Conc. [ng/µl]	Peak Molarity [nmol/l]	% Integrated Area	Peak Comment	Observations
25	6.01	-	370	-		Lower Marker
140	0.407	-	4.47	100.00		
1500	6.50	6.50	6.67	-		Upper Marker

© Agilent Technologies, Inc. 2017

B1: A1, plus , R1

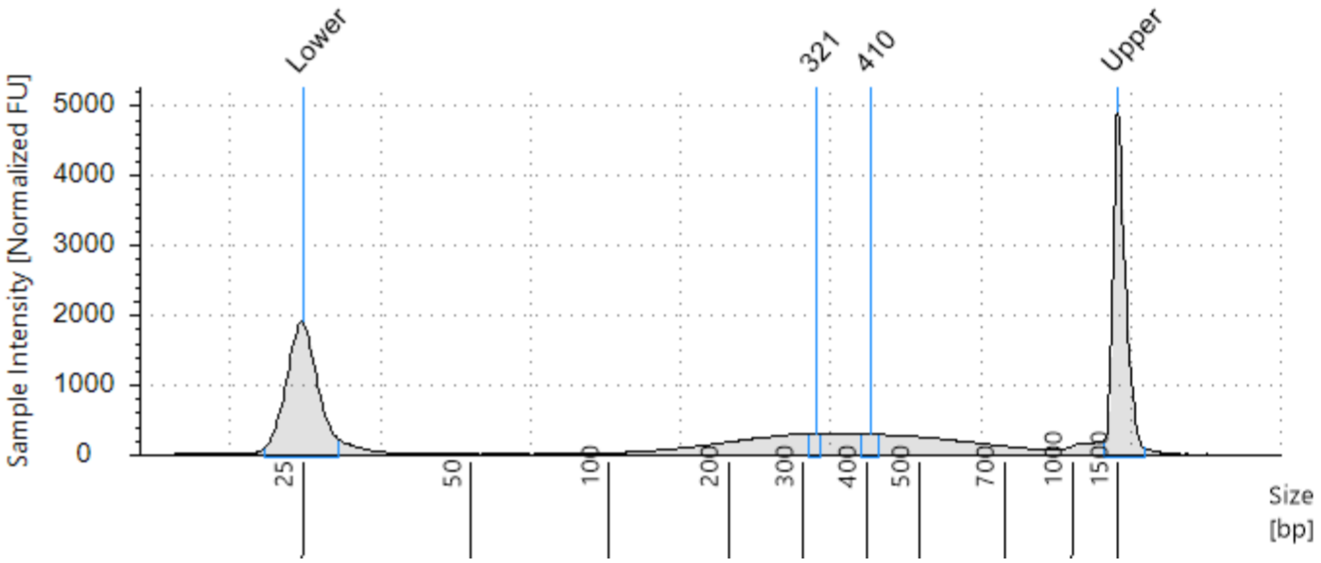

Sample Table

Well	Conc. [ng/µl]	Sample Description	Alert	Observations
B1	0.792	A1, plus , R1		

Peak Table

Size [bp]	Calibrated Conc. [ng/µl]	Assigned Conc. [ng/µl]	Peak Molarity [nmol/l]	% Integrated Area	Peak Comment	Observations
25	6.15	-	378	-		Lower Marker
321	0.335	-	1.61	42.26		
410	0.458	-	1.72	57.74		
1500	6.50	6.50	6.67	-		Upper Marker

© Agilent Technologies, Inc. 2017

C1: B1

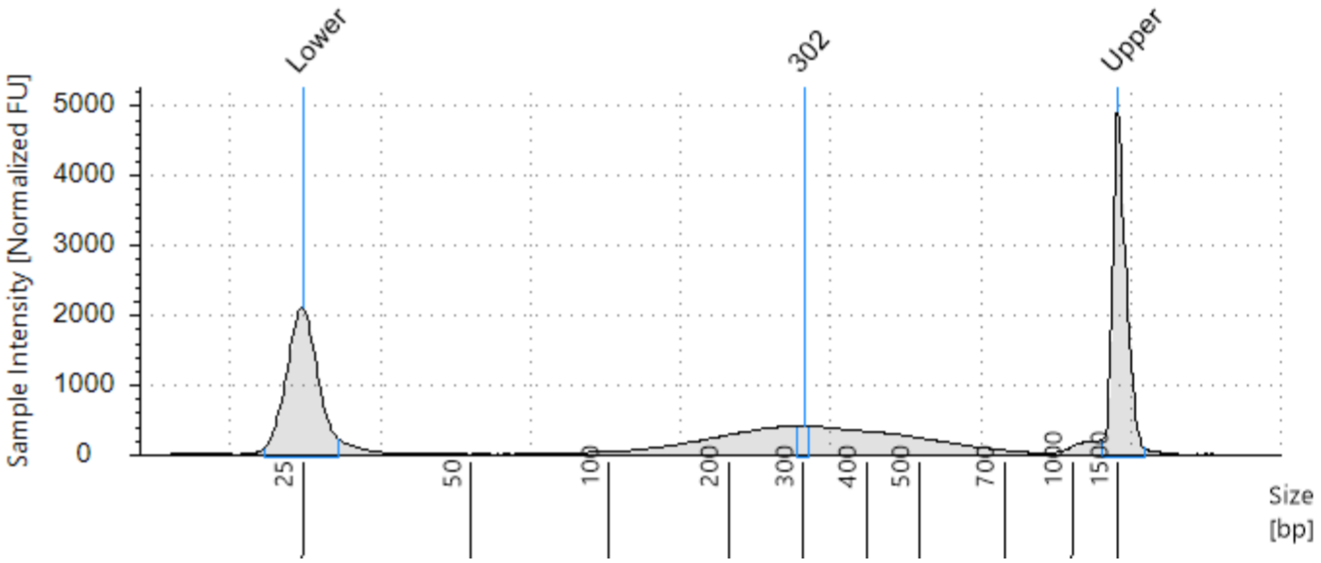

Sample Table

Well	Conc. [ng/µl]	Sample Description	Alert	Observations
C1	0.398	B1		

Peak Table

Size [bp]	Calibrated Conc. [ng/µl]	Assigned Conc. [ng/µl]	Peak Molarity [nmol/l]	% Integrated Area	Peak Comment	Observations
25	6.32	-	389	-		Lower Marker
302	0.398	-	2.03	100.00		
1500	6.50	6.50	6.67	-		Upper Marker

 © Agilent Technologies, Inc. 2017

D1: C1

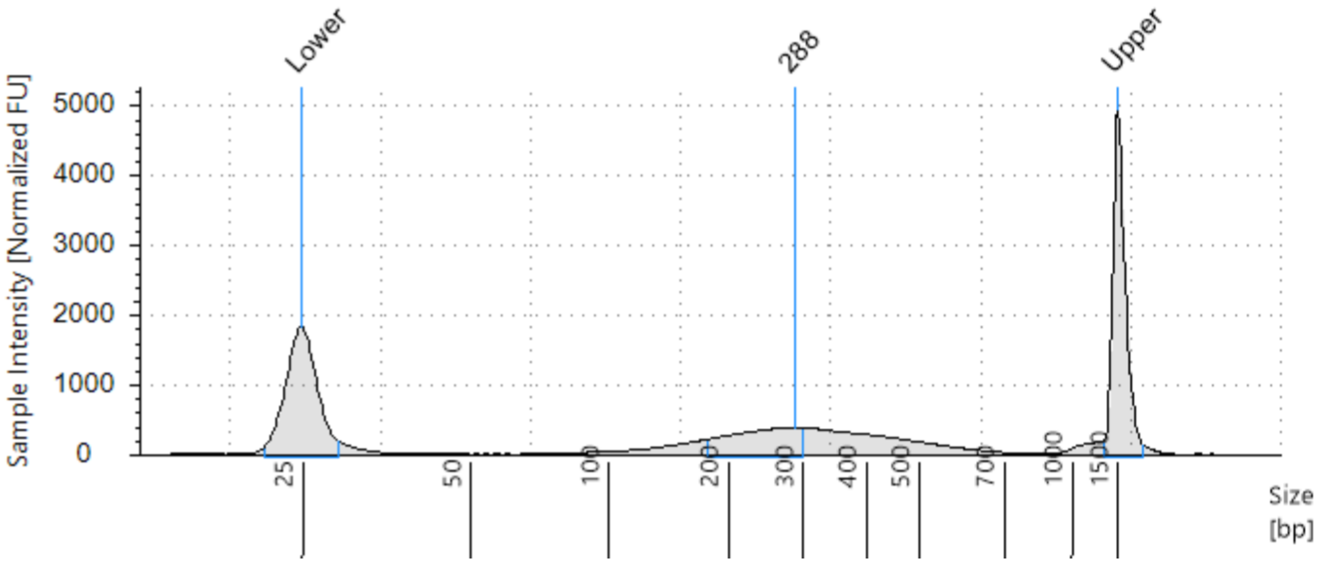

Sample Table

Well	Conc. [ng/µl]	Sample Description	Alert	Observations
D1	2.72	C1		

Peak Table

Size [bp]	Calibrated Conc. [ng/µl]	Assigned Conc. [ng/µl]	Peak Molarity [nmol/l]	% Integrated Area	Peak Comment	Observations
25	6.00	-	369	-		Lower Marker
288	2.72	-	14.5	100.00		
1500	6.50	6.50	6.67	-		Upper Marker

© Agilent Technologies, Inc. 2017

E1: D1

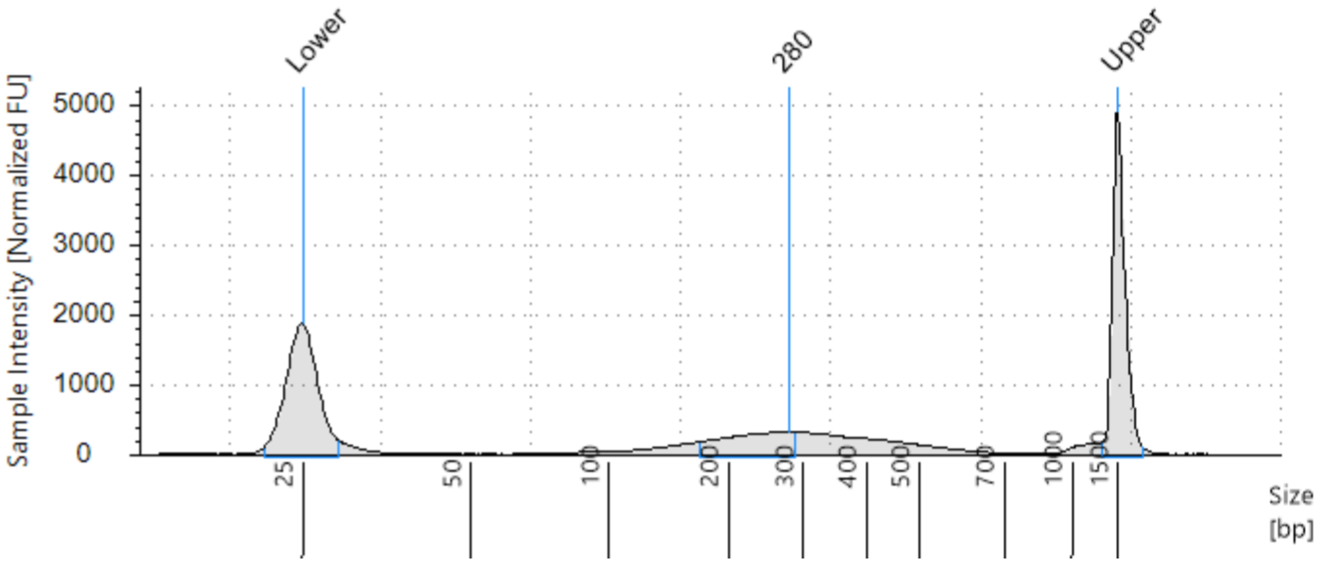

Sample Table

Well	Conc. [ng/µl]	Sample Description	Alert	Observations
E1	2.25	D1		

Peak Table

Size [bp]	Calibrated Conc. [ng/µl]	Assigned Conc. [ng/µl]	Peak Molarity [nmol/l]	% Integrated Area	Peak Comment	Observations
25	6.17	-	380	-		Lower Marker
280	2.25	-	12.4	100.00		
1500	6.50	6.50	6.67	-		Upper Marker

© Agilent Technologies, Inc. 2017

F1: E1

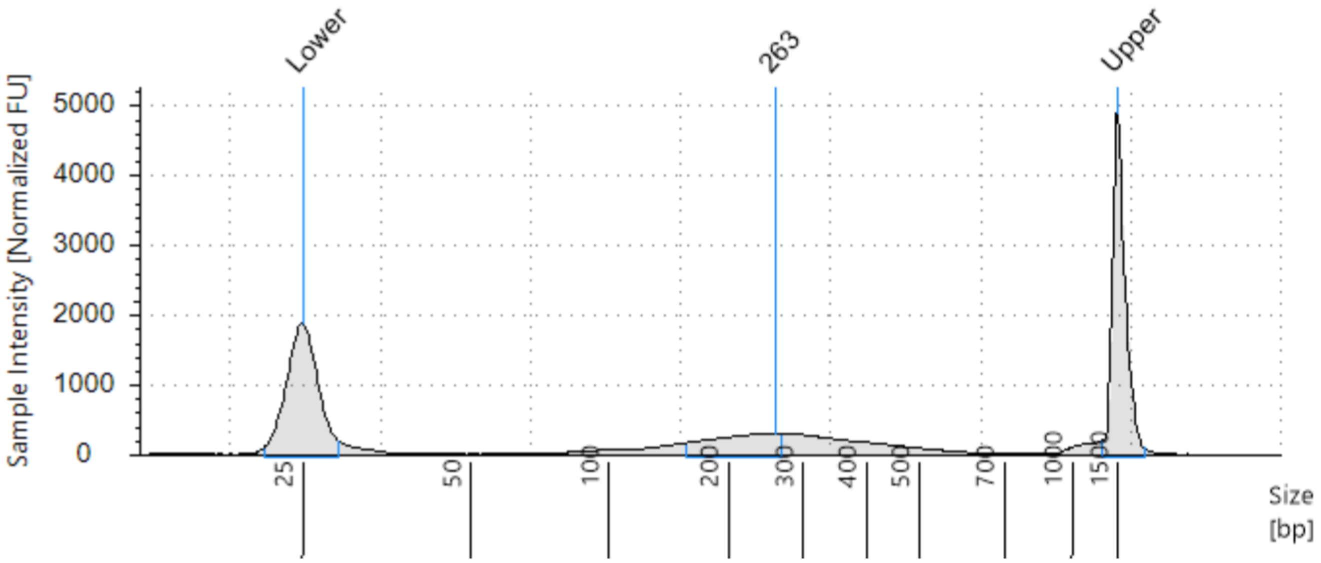

Sample Table

Well	Conc. [ng/µl]	Sample Description	Alert	Observations
F1	2.06	E1		

Peak Table

Size [bp]	Calibrated Conc. [ng/µl]	Assigned Conc. [ng/µl]	Peak Molarity [nmol/l]	% Integrated Area	Peak Comment	Observations
25	6.16	-	379	-		Lower Marker
263	2.06	-	12.1	100.00		
1500	6.50	6.50	6.67	-		Upper Marker

© Agilent Technologies, Inc. 2017

G1: F1

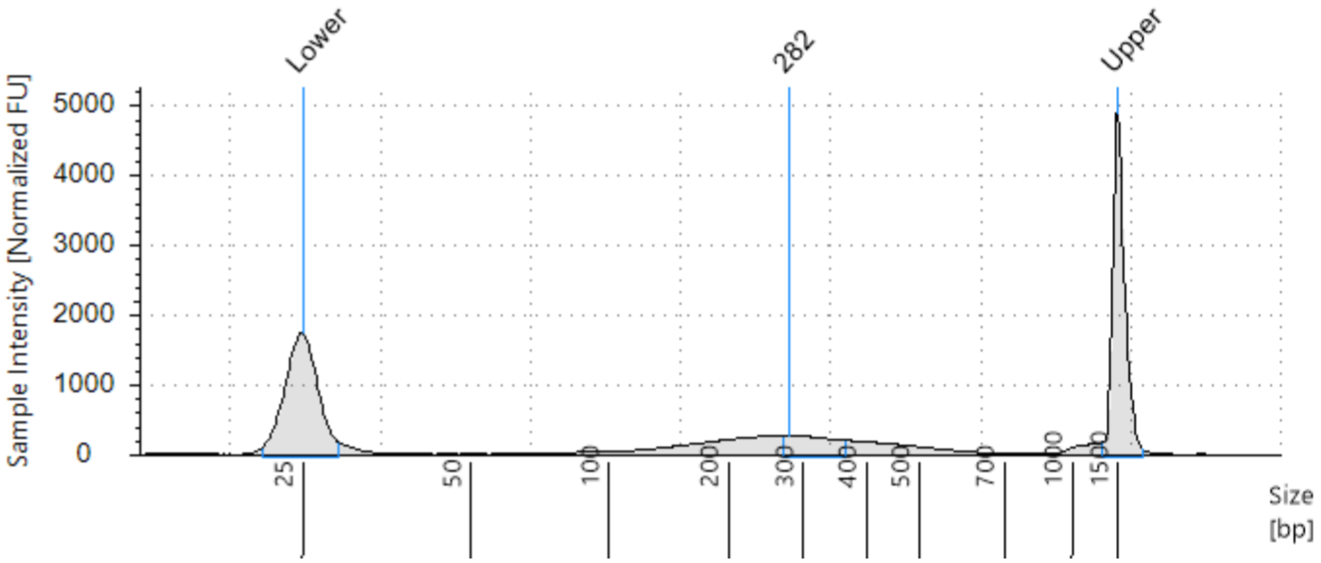

Sample Table

Well	Conc. [ng/µl]	Sample Description	Alert	Observations
G1	1.40	F1		

Peak Table

Size [bp]	Calibrated Conc. [ng/µl]	Assigned Conc. [ng/µl]	Peak Molarity [nmol/l]	% Integrated Area	Peak Comment	Observations
25	6.25	-	385	-		Lower Marker
282	1.40	-	7.62	100.00		
1500	6.50	6.50	6.67	-		Upper Marker

© Agilent Technologies, Inc. 2017

H1: G1

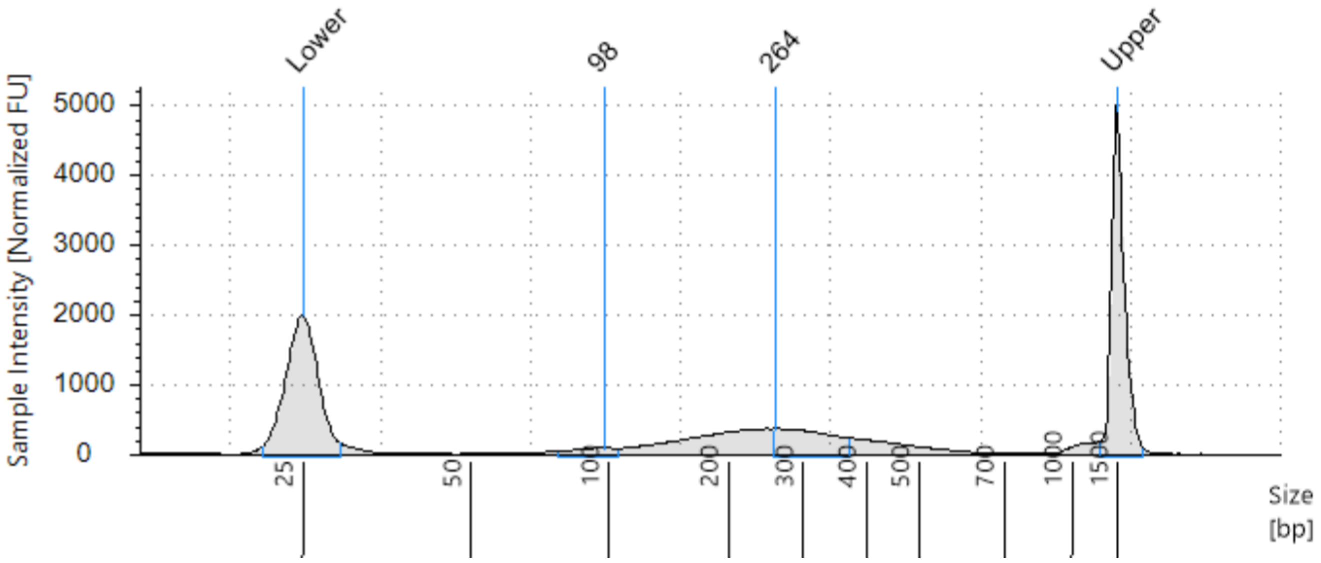

Sample Table

Well	Conc. [ng/µl]	Sample Description	Alert	Observations
H1	2.48	G1		

Peak Table

Size [bp]	Calibrated Conc. [ng/µl]	Assigned Conc. [ng/µl]	Peak Molarity [nmol/l]	% Integrated Area	Peak Comment	Observations
25	6.57	-	404	-		Lower Marker
98	0.369	-	5.78	14.86		
264	2.11	-	12.3	85.14		
1500	6.50	6.50	6.67	-		Upper Marker

 © Agilent Technologies, Inc. 2017

A2: H1

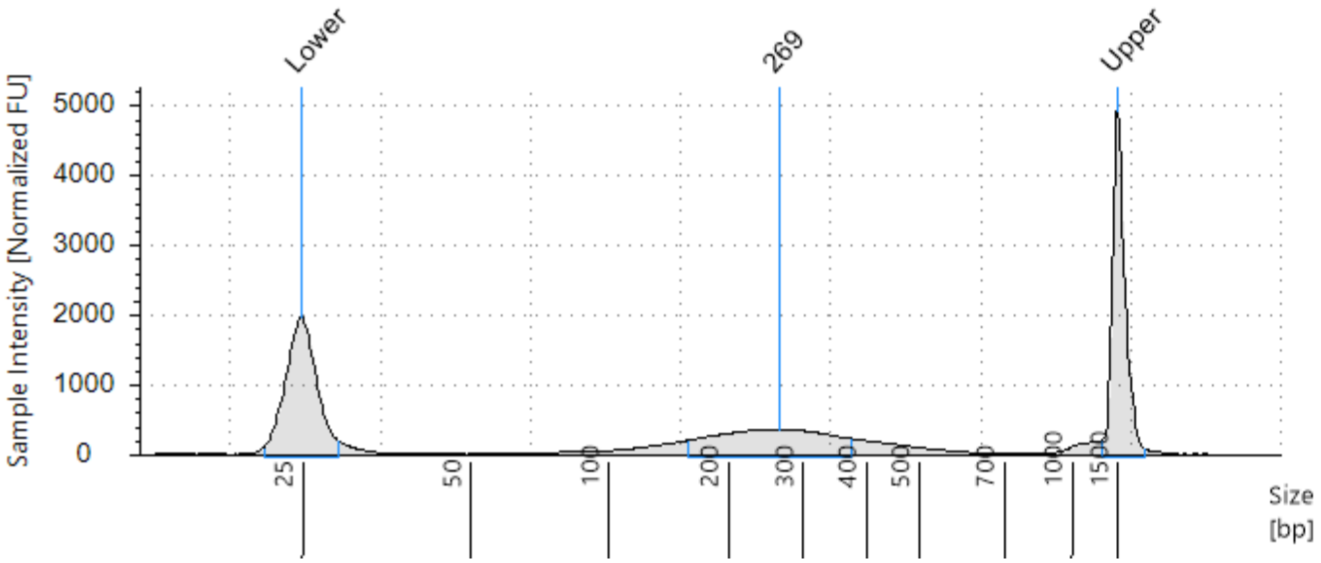

Sample Table

Well	Conc. [ng/µl]	Sample Description	Alert	Observations
A2	4.38	H1		

Peak Table

Size [bp]	Calibrated Conc. [ng/µl]	Assigned Conc. [ng/µl]	Peak Molarity [nmol/l]	% Integrated Area	Peak Comment	Observations
25	6.34	-	390	-		Lower Marker
269	4.38	-	25.1	100.00		
1500	6.50	6.50	6.67	-		Upper Marker

© Agilent Technologies, Inc. 2017

B2: A2

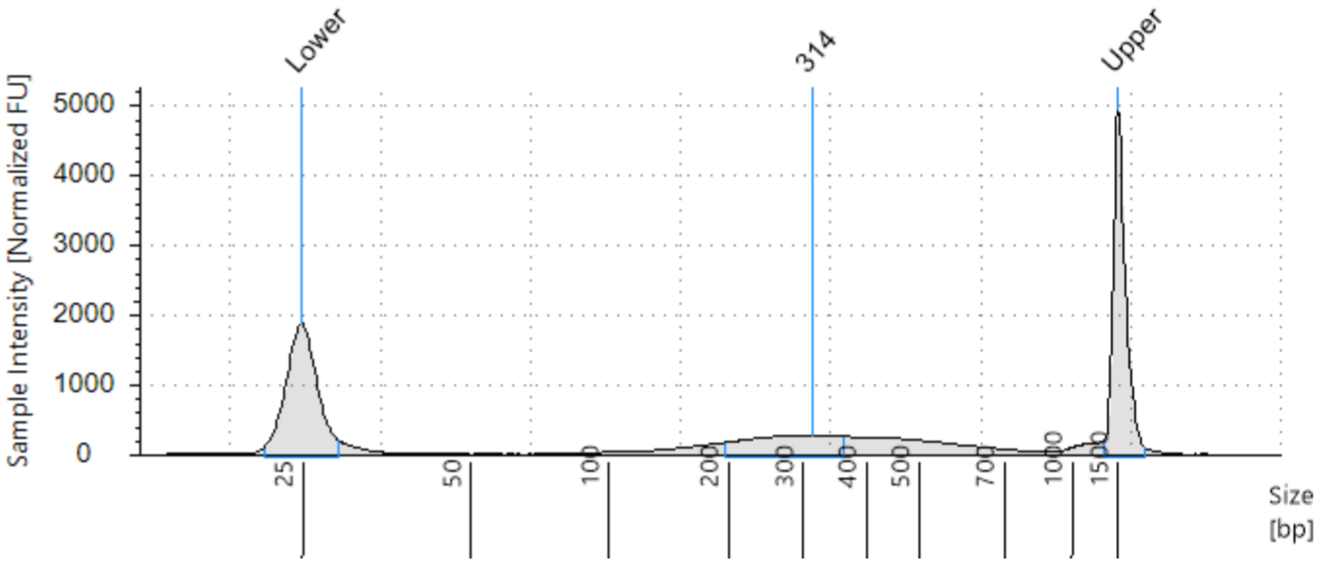

Sample Table

Well	Conc. [ng/µl]	Sample Description	Alert	Observations
B2	2.60	A2		

Peak Table

Size [bp]	Calibrated Conc. [ng/µl]	Assigned Conc. [ng/µl]	Peak Molarity [nmol/l]	% Integrated Area	Peak Comment	Observations
25	6.12	-	376	-		Lower Marker
314	2.60	-	12.7	100.00		
1500	6.50	6.50	6.67	-		Upper Marker

© Agilent Technologies, Inc. 2017

C2: B2

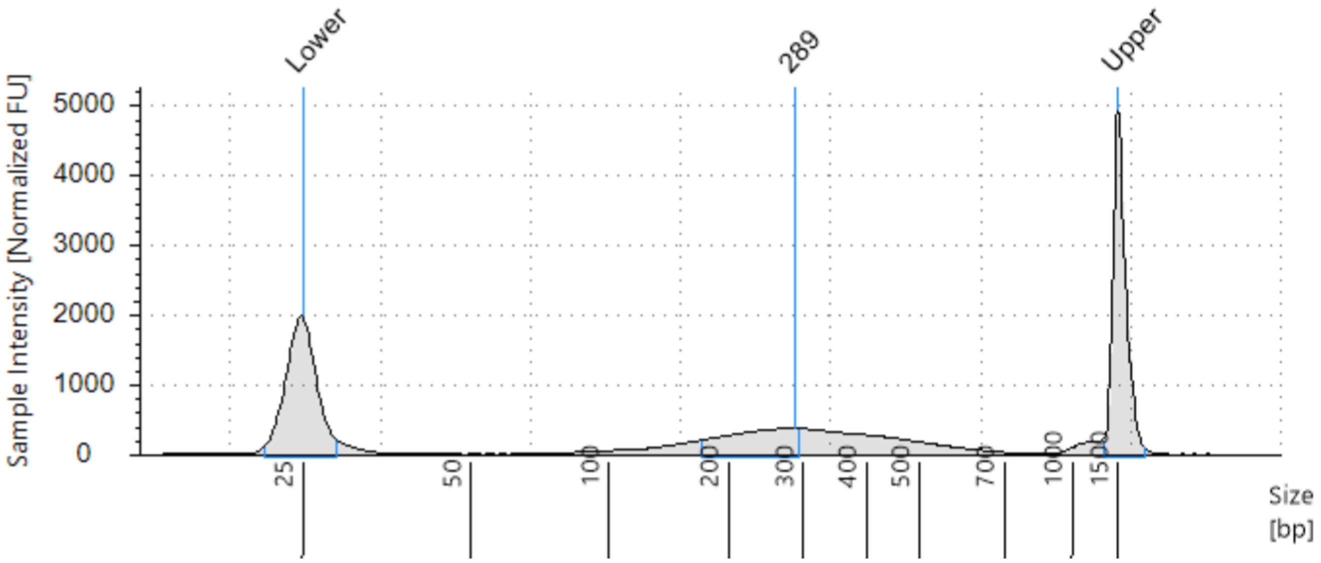

Sample Table

Well	Conc. [ng/µl]	Sample Description	Alert	Observations
C2	2.59	B2		

Peak Table

Size [bp]	Calibrated Conc. [ng/µl]	Assigned Conc. [ng/µl]	Peak Molarity [nmol/l]	% Integrated Area	Peak Comment	Observations
25	6.16	-	379	-		Lower Marker
289	2.59	-	13.8	100.00		
1500	6.50	6.50	6.67	-		Upper Marker

© Agilent Technologies, Inc. 2017

D2: C2

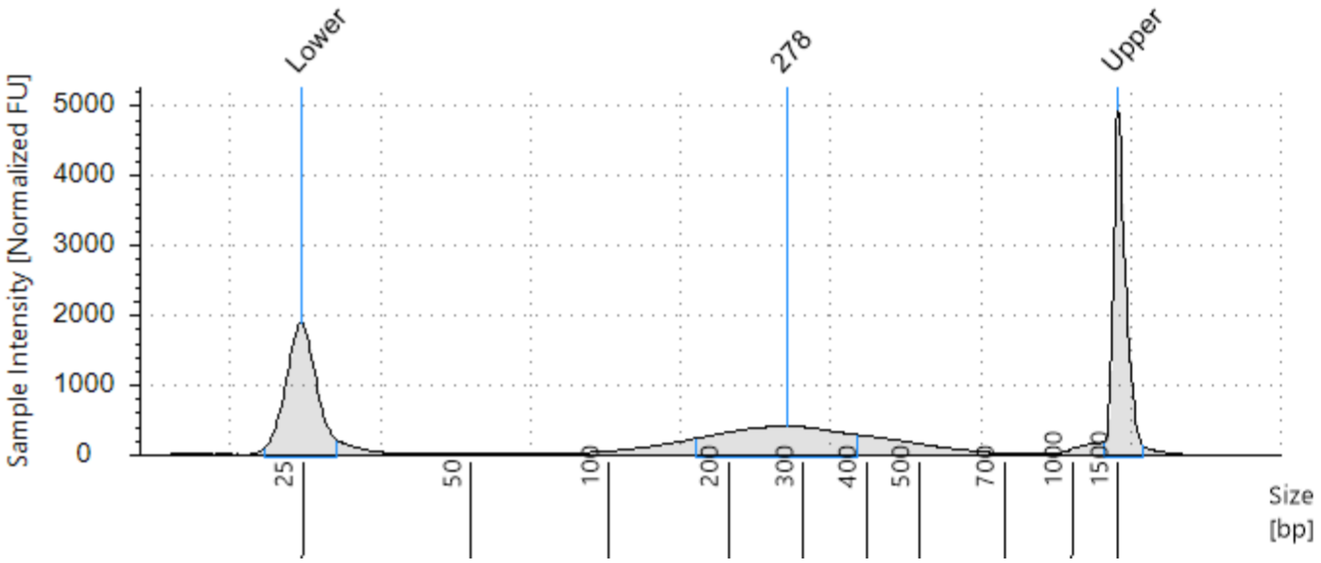

Sample Table

Well	Conc. [ng/µl]	Sample Description	Alert	Observations
D2	4.94	C2		

Peak Table

Size [bp]	Calibrated Conc. [ng/µl]	Assigned Conc. [ng/µl]	Peak Molarity [nmol/l]	% Integrated Area	Peak Comment	Observations
25	6.01	-	370	-		Lower Marker
278	4.94	-	27.3	100.00		
1500	6.50	6.50	6.67	-		Upper Marker

© Agilent Technologies, Inc. 2017

E2: D2

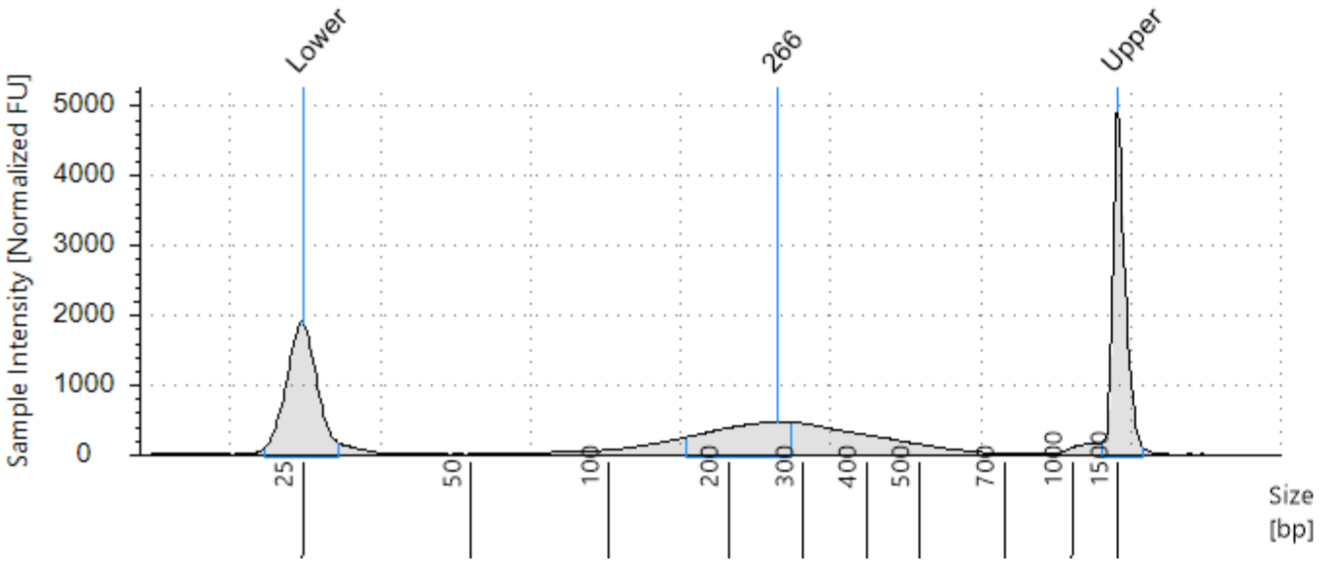

Sample Table

Well	Conc. [ng/µl]	Sample Description	Alert	Observations
E2	3.62	D2		

Peak Table

Size [bp]	Calibrated Conc. [ng/µl]	Assigned Conc. [ng/µl]	Peak Molarity [nmol/l]	% Integrated Area	Peak Comment	Observations
25	6.07	-	374	-		Lower Marker
266	3.62	-	20.9	100.00		
1500	6.50	6.50	6.67	-		Upper Marker

© Agilent Technologies, Inc. 2017

F2: E2

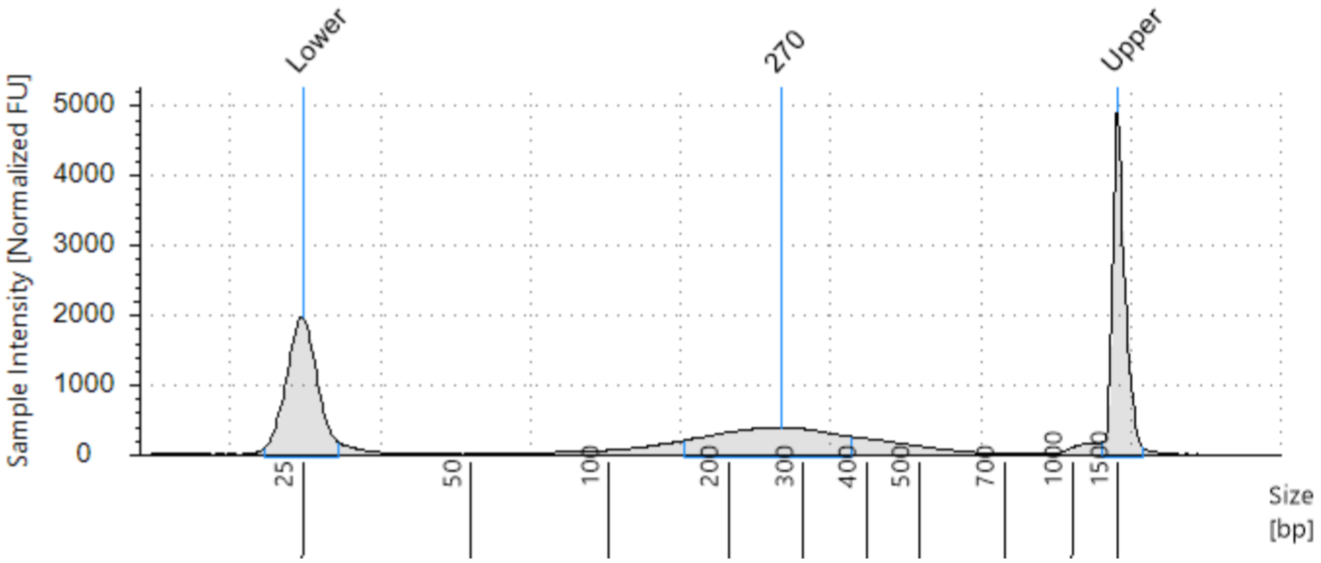

Sample Table

Well	Conc. [ng/µl]	Sample Description	Alert	Observations
F2	4.86	E2		

Peak Table

Size [bp]	Calibrated Conc. [ng/µl]	Assigned Conc. [ng/µl]	Peak Molarity [nmol/l]	% Integrated Area	Peak Comment	Observations
25	6.41	-	395	-		Lower Marker
270	4.86	-	27.7	100.00		
1500	6.50	6.50	6.67	-		Upper Marker

© Agilent Technologies, Inc. 2017

G2: F2

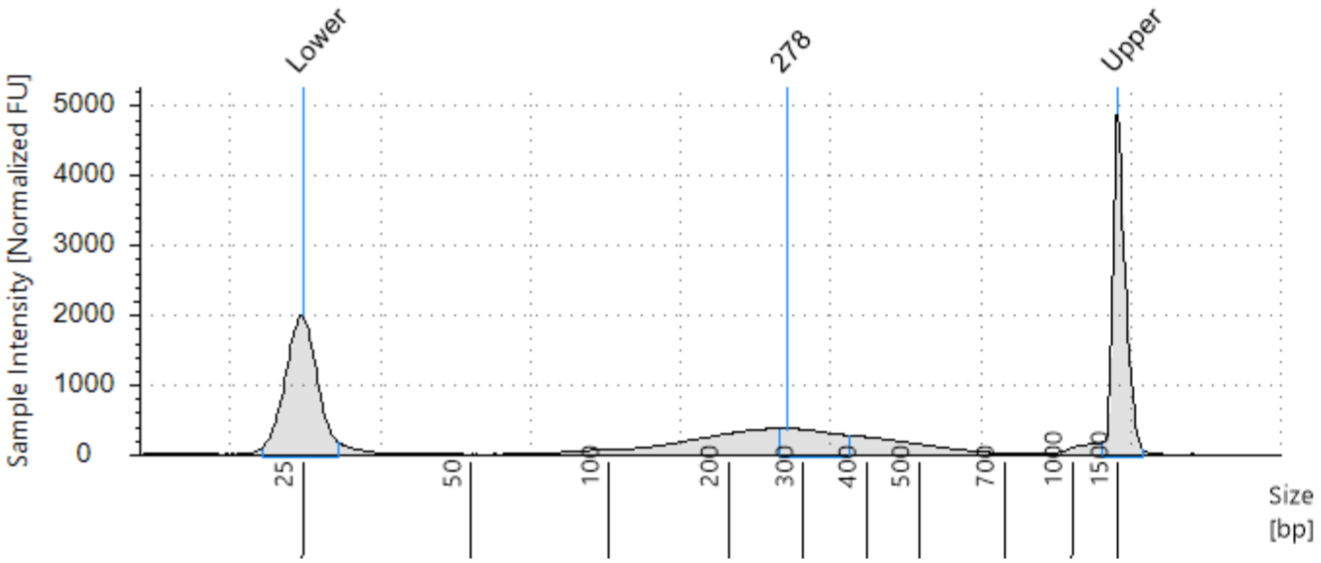

Sample Table

Well	Conc. [ng/µl]	Sample Description	Alert	Observations
G2	2.07	F2		

Peak Table

Size [bp]	Calibrated Conc. [ng/µl]	Assigned Conc. [ng/µl]	Peak Molarity [nmol/l]	% Integrated Area	Peak Comment	Observations
25	6.64	-	408	-		Lower Marker
278	2.07	-	11.5	100.00		
1500	6.50	6.50	6.67	-		Upper Marker

© Agilent Technologies, Inc. 2017

H2: G2

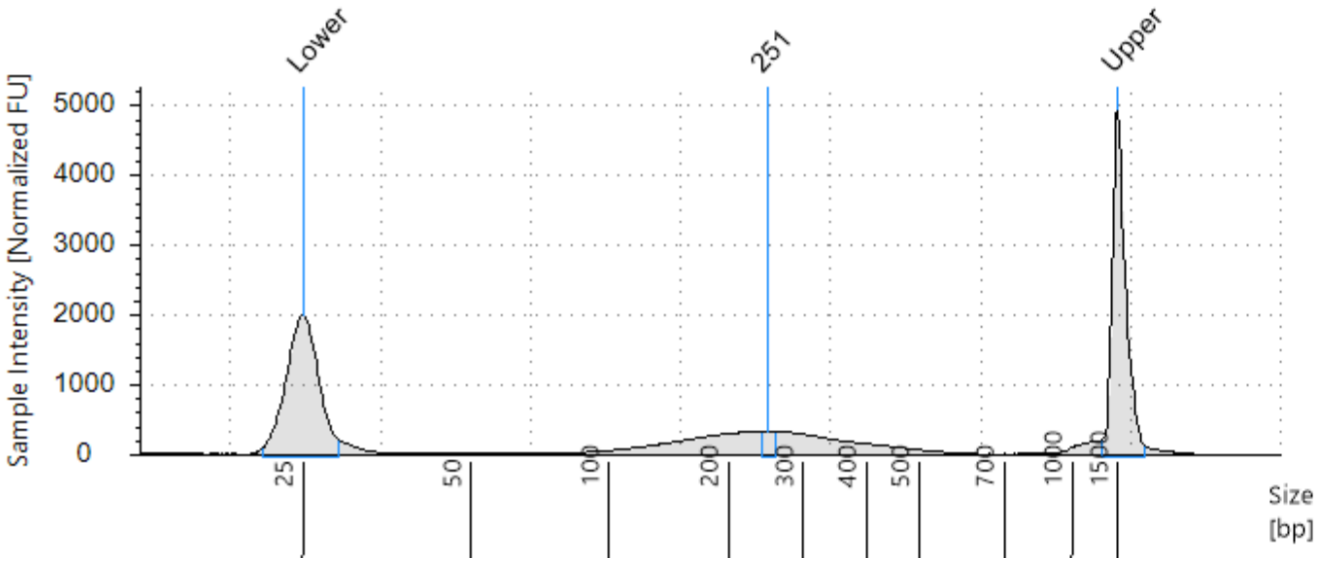

Sample Table

Well	Conc. [ng/µl]	Sample Description	Alert	Observations
H2	0.400	G2		

Peak Table

Size [bp]	Calibrated Conc. [ng/µl]	Assigned Conc. [ng/µl]	Peak Molarity [nmol/l]	% Integrated Area	Peak Comment	Observations
25	6.31	-	388	-		Lower Marker
251	0.400	-	2.45	100.00		
1500	6.50	6.50	6.67	-		Upper Marker

© Agilent Technologies, Inc. 2017

Filename: 2020-03-06-01-Q-S DFB plus H2-F4 R1.D1000

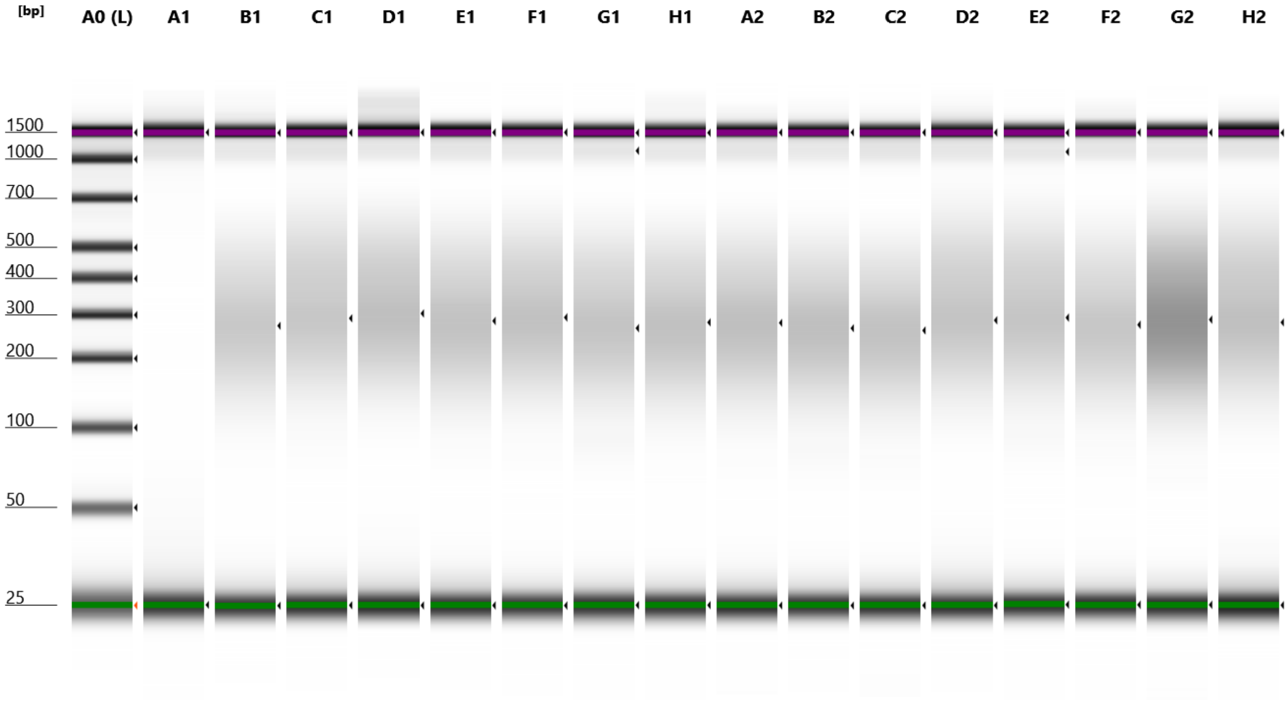

Default image (Contrast 100%)

Sample Info

Well	Conc. [ng/µl]	Sample Description	Alert	Observations
A0	20.3	Electronic Ladder		Ladder
A1		Ladder		
B1	4.57	H2 Plus R1		
C1	2.33	A3 plus R1		
D1	1.96	B3 Plus R1		
E1	4.77	c3 Plus R1		
F1	3.01	D3 Plus R1		
G1	3.06	E3 Plus R1		
H1	5.40	F3 Plus R1		
A2	5.11	G3 Plus R1		
B2	0.548	H3 Plus R1		
C2	0.487	A4 Plus R1		
D2	5.02	B4 Plus R1		
E2	5.90	C4 Plus R1		
F2	1.92	D4 Plus R1		
G2	6.96	E4 Plus R1		
H2	5.19	F4 Plus R1		

© Agilent Technologies, Inc. 2017

A0: Electronic Ladder

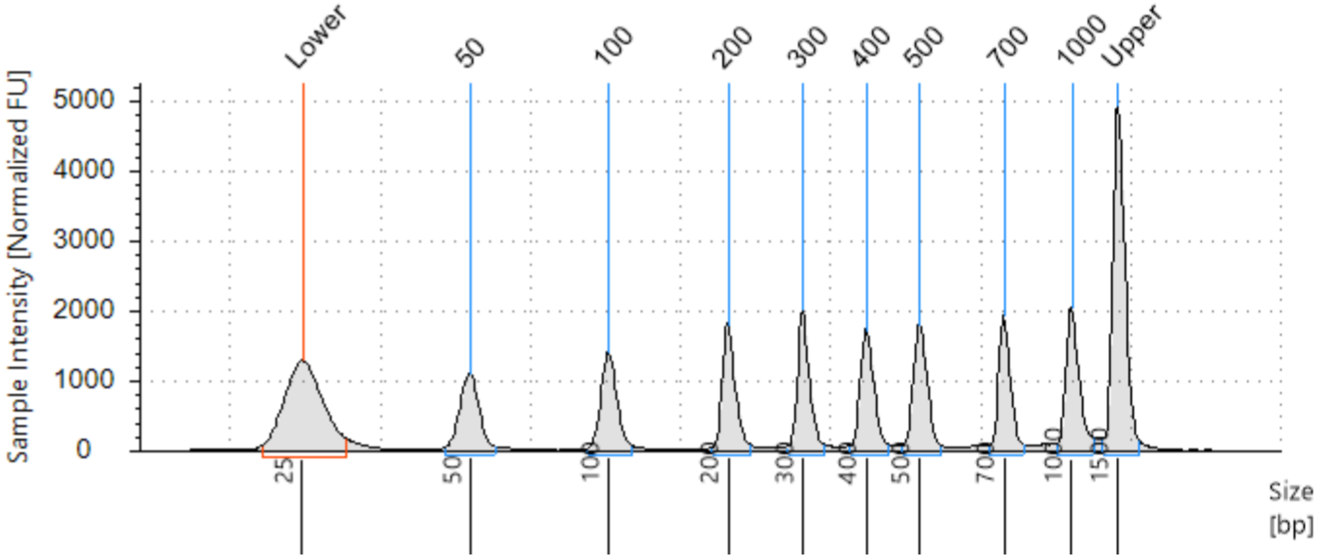

Sample Table

Well	Conc. [ng/µl]	Sample Description	Alert	Observations
A0	20.3	Electronic Ladder		Ladder

Peak Table

Size [bp]	Calibrated Conc. [ng/µl]	Assigned Conc. [ng/µl]	Peak Molarity [nmol/l]	% Integrated Area	Peak Comment	Observations
25	5.22	-	321	-		Lower Marker
50	2.25	-	69.3	11.11		
100	2.37	-	36.5	11.71		
200	2.47	-	19.0	12.20		
300	2.55	-	13.1	12.56		
400	2.57	-	9.87	12.66		
500	2.71	-	8.33	13.36		
700	2.46	-	5.41	12.15		
1000	2.89	-	4.44	14.25		
1500	6.50	6.50	6.67	-		Upper Marker

© Agilent Technologies, Inc. 2017

A1: Ladder

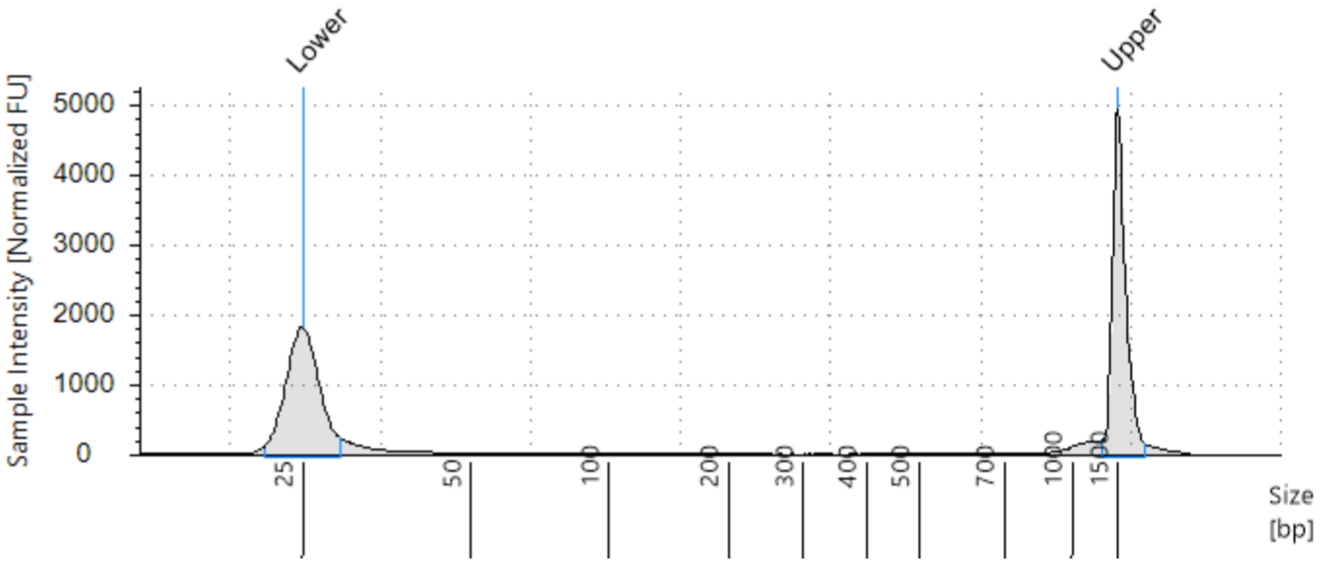

Sample Table

Well	Conc. [ng/µl]	Sample Description	Alert	Observations
A1		Ladder		

Peak Table

Size [bp]	Calibrated Conc. [ng/µl]	Assigned Conc. [ng/µl]	Peak Molarity [nmol/l]	% Integrated Area	Peak Comment	Observations
25	6.02	-	370	-		Lower Marker
1500	6.50	6.50	6.67	-		Upper Marker

© Agilent Technologies, Inc. 2017

B1: H2 Plus R1

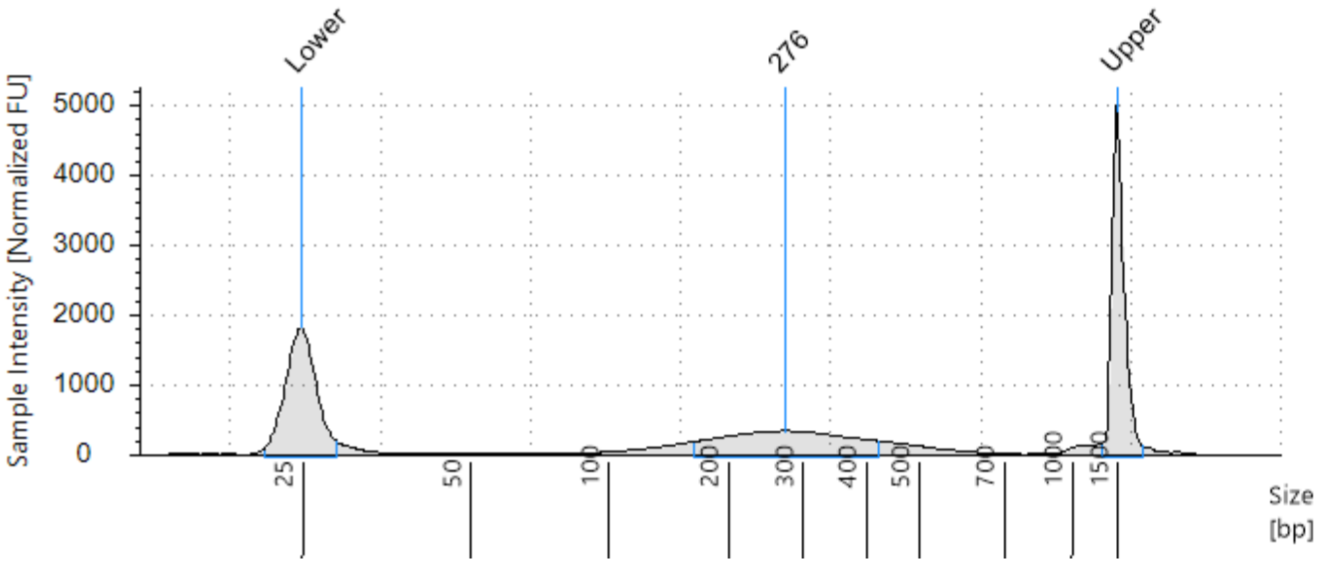

Sample Table

Well	Conc. [ng/µl]	Sample Description	Alert	Observations
B1	4.57	H2 Plus R1		

Peak Table

Size [bp]	Calibrated Conc. [ng/µl]	Assigned Conc. [ng/µl]	Peak Molarity [nmol/l]	% Integrated Area	Peak Comment	Observations
25	5.93	-	365	-		Lower Marker
276	4.57	-	25.5	100.00		
1500	6.50	6.50	6.67	-		Upper Marker

 © Agilent Technologies, Inc. 2017

C1: A3 plus R1

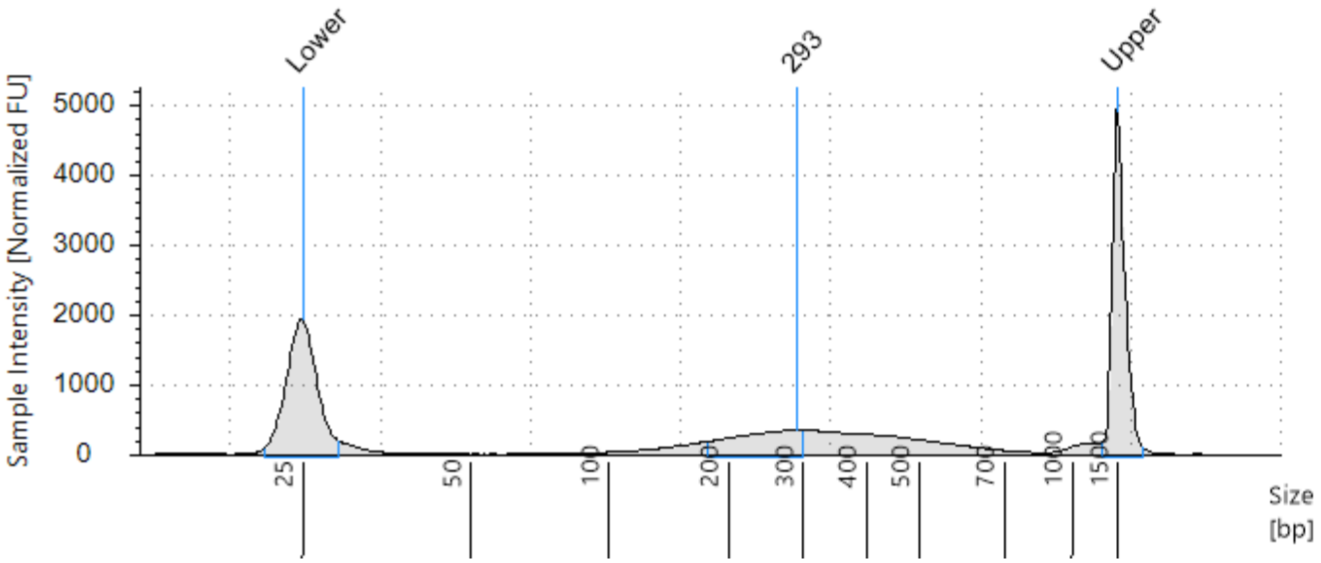

Sample Table

Well	Conc. [ng/µl]	Sample Description	Alert	Observations
C1	2.33	A3 plus R1		

Peak Table

Size [bp]	Calibrated Conc. [ng/µl]	Assigned Conc. [ng/µl]	Peak Molarity [nmol/l]	% Integrated Area	Peak Comment	Observations
25	6.07	-	373	-		Lower Marker
293	2.33	-	12.2	100.00		
1500	6.50	6.50	6.67	-		Upper Marker

© Agilent Technologies, Inc. 2017

D1: B3 Plus R1

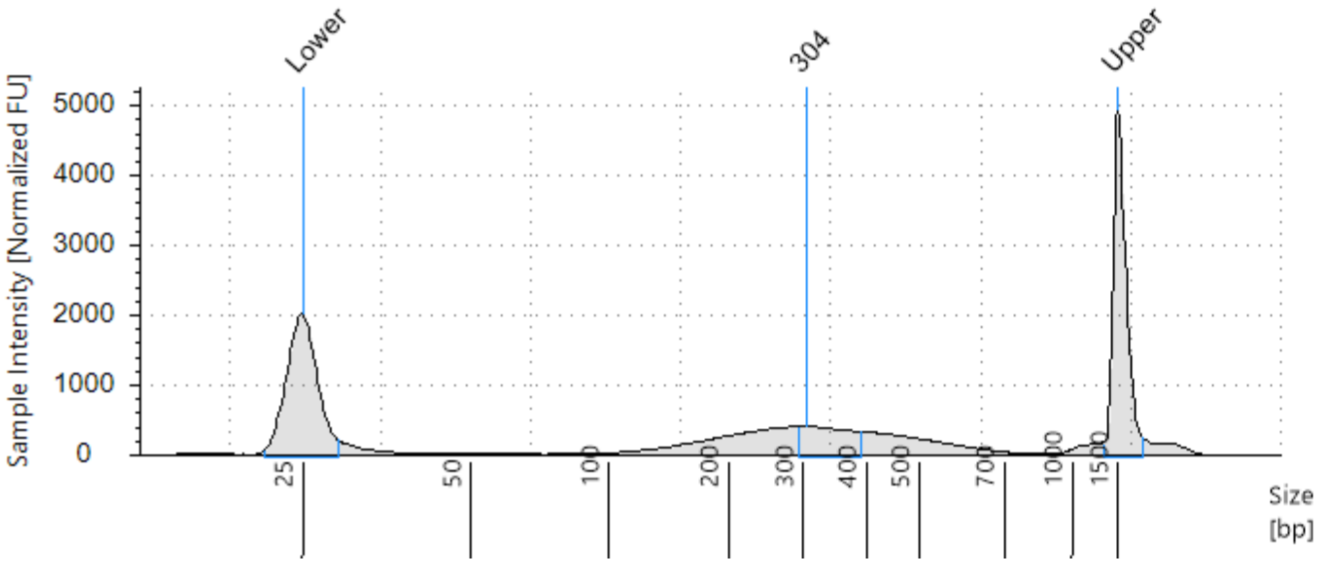

Sample Table

Well	Conc. [ng/µl]	Sample Description	Alert	Observations
D1	1.96	B3 Plus R1		

Peak Table

Size [bp]	Calibrated Conc. [ng/µl]	Assigned Conc. [ng/µl]	Peak Molarity [nmol/l]	% Integrated Area	Peak Comment	Observations
25	6.24	-	384	-		Lower Marker
304	1.96	-	9.90	100.00		
1500	6.50	6.50	6.67	-		Upper Marker

© Agilent Technologies, Inc. 2017

E1: c3 Plus R1

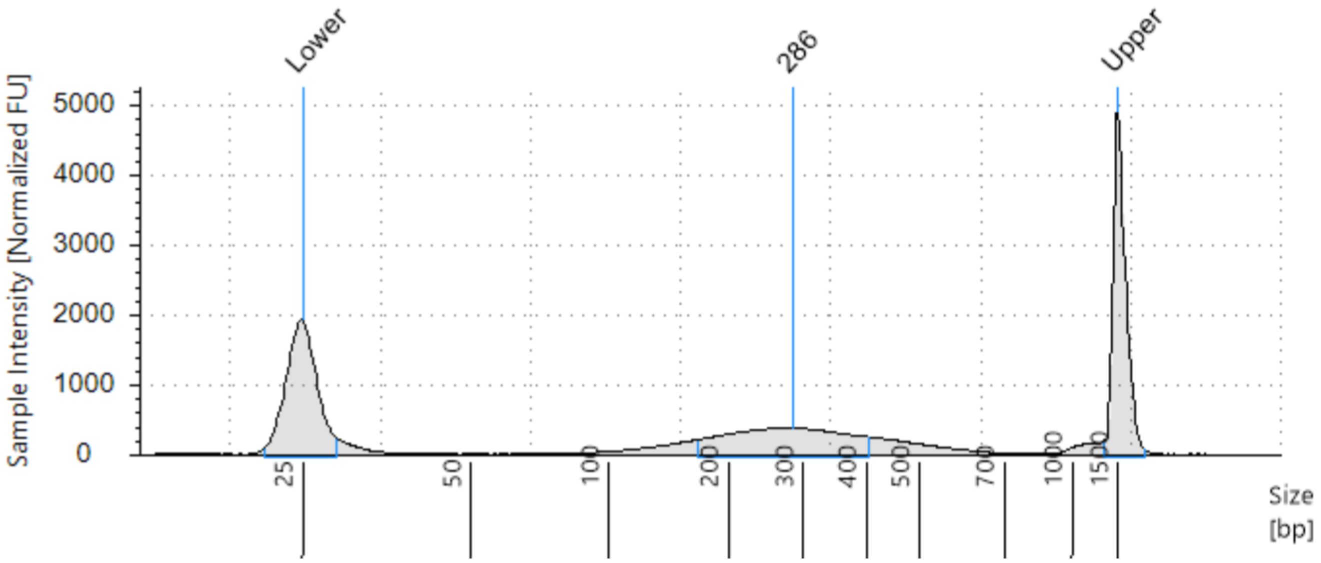

Sample Table

Well	Conc. [ng/µl]	Sample Description	Alert	Observations
E1	4.77	c3 Plus R1		

Peak Table

Size [bp]	Calibrated Conc. [ng/µl]	Assigned Conc. [ng/µl]	Peak Molarity [nmol/l]	% Integrated Area	Peak Comment	Observations
25	6.16	-	379	-		Lower Marker
286	4.77	-	25.6	100.00		
1500	6.50	6.50	6.67	-		Upper Marker

 © Agilent Technologies, Inc. 2017

F1: D3 Plus R1

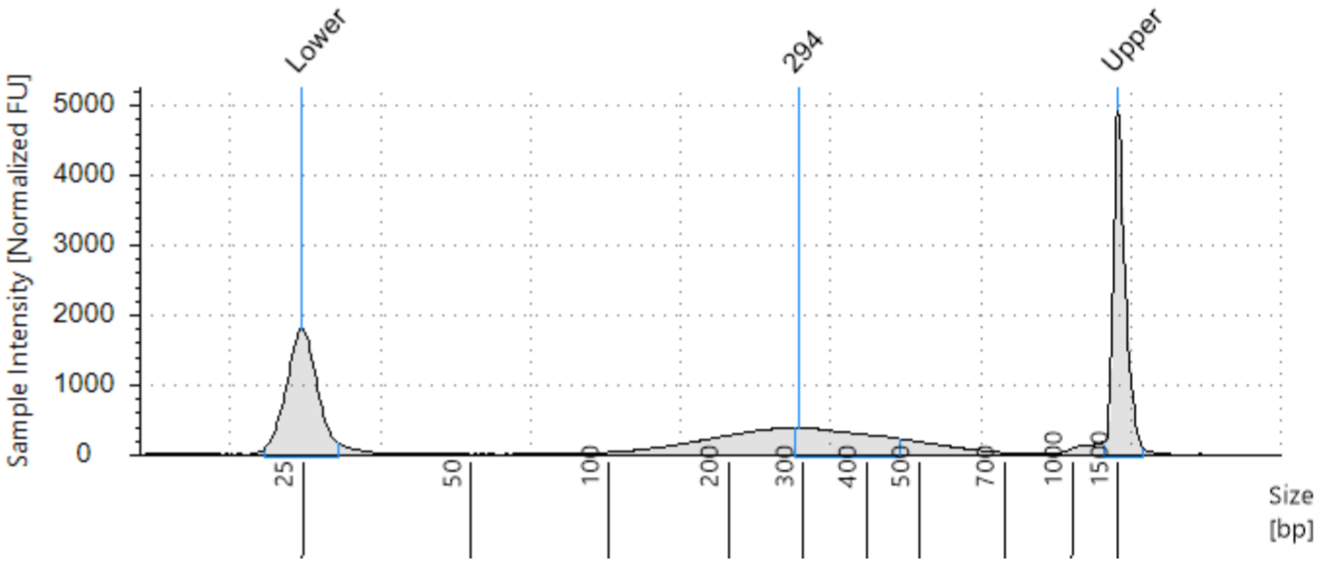

Sample Table

Well	Conc. [ng/µl]	Sample Description	Alert	Observations
F1	3.01	D3 Plus R1		

Peak Table

Size [bp]	Calibrated Conc. [ng/µl]	Assigned Conc. [ng/µl]	Peak Molarity [nmol/l]	% Integrated Area	Peak Comment	Observations
25	5.93	-	365	-		Lower Marker
294	3.01	-	15.7	100.00		
1500	6.50	6.50	6.67	-		Upper Marker

 © Agilent Technologies, Inc. 2017

G1: E3 Plus R1

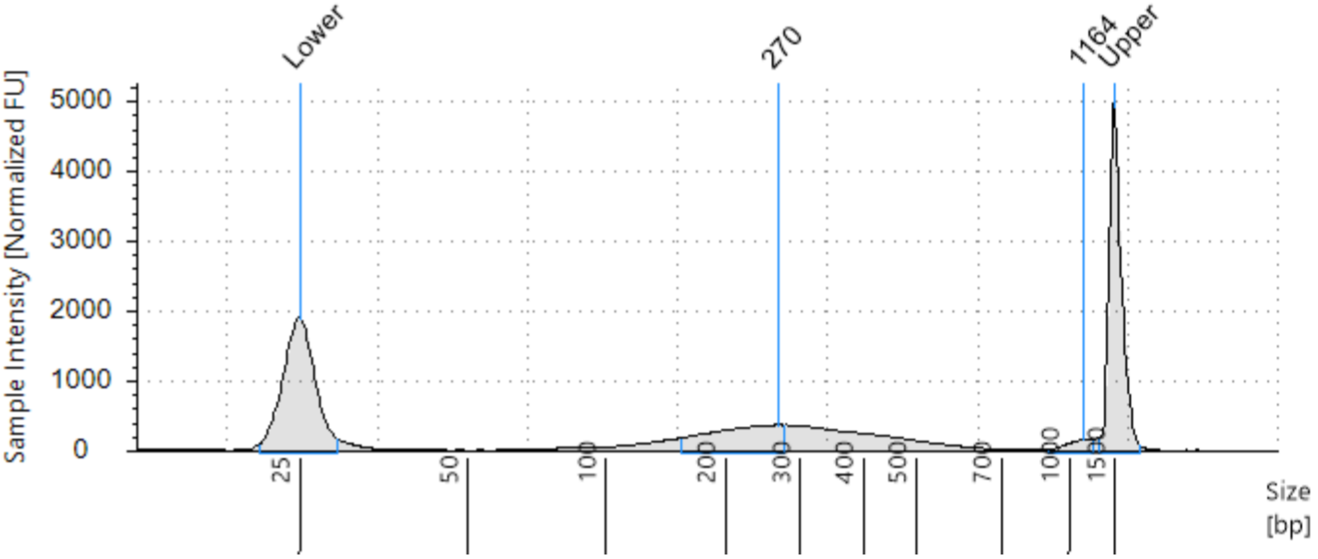

Sample Table

Well	Conc. [ng/µl]	Sample Description	Alert	Observations
G1	3.06	E3 Plus R1		

Peak Table

Size [bp]	Calibrated Conc. [ng/µl]	Assigned Conc. [ng/µl]	Peak Molarity [nmol/l]	% Integrated Area	Peak Comment	Observations
25	6.34	-	390	-		Lower Marker
270	2.69	-	15.4	88.03		
1164	0.366	-	0.484	11.97		
1500	6.50	6.50	6.67	-		Upper Marker

© Agilent Technologies, Inc. 2017

H1: F3 Plus R1

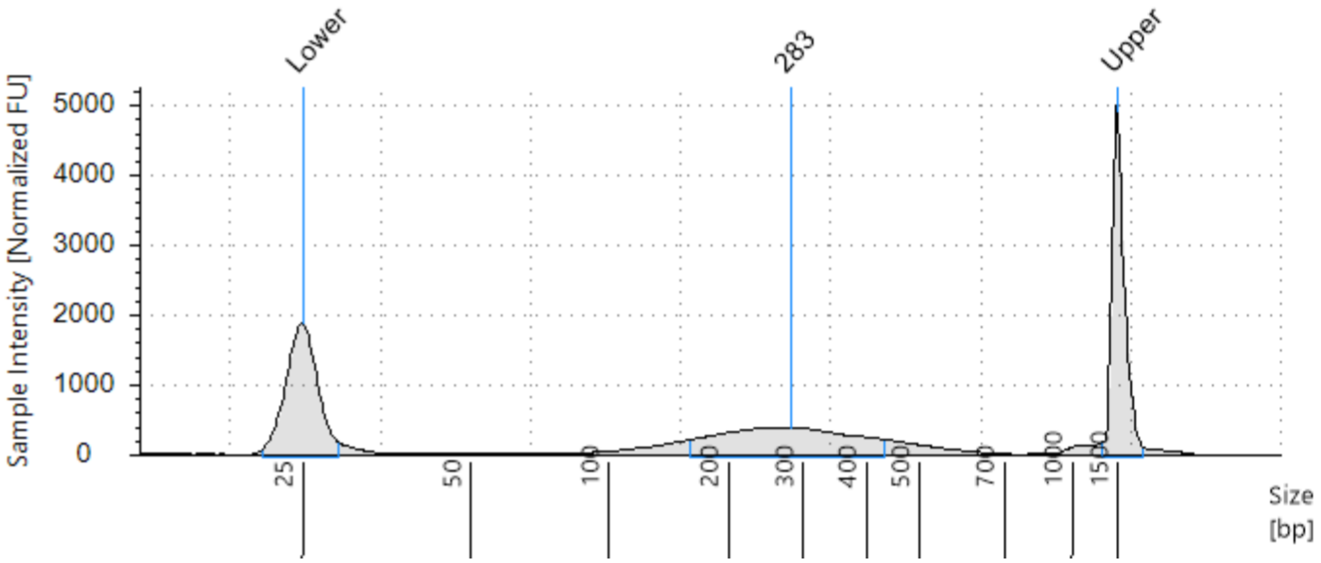

Sample Table

Well	Conc. [ng/µl]	Sample Description	Alert	Observations
H1	5.40	F3 Plus R1		

Peak Table

Size [bp]	Calibrated Conc. [ng/µl]	Assigned Conc. [ng/µl]	Peak Molarity [nmol/l]	% Integrated Area	Peak Comment	Observations
25	6.22	-	383	-		Lower Marker
283	5.40	-	29.3	100.00		
1500	6.50	6.50	6.67	-		Upper Marker

 © Agilent Technologies, Inc. 2017

A2: G3 Plus R1

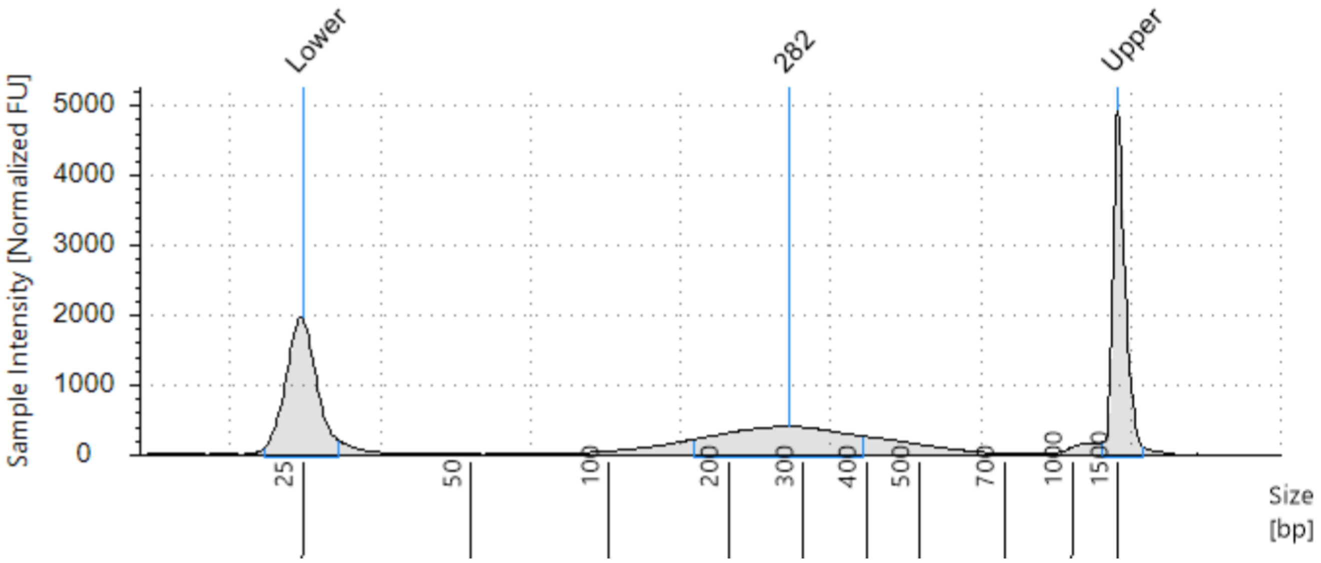

Sample Table

Well	Conc. [ng/µl]	Sample Description	Alert	Observations
A2	5.11	G3 Plus R1		

Peak Table

Size [bp]	Calibrated Conc. [ng/µl]	Assigned Conc. [ng/µl]	Peak Molarity [nmol/l]	% Integrated Area	Peak Comment	Observations
25	6.38	-	392	-		Lower Marker
282	5.11	-	27.9	100.00		
1500	6.50	6.50	6.67	-		Upper Marker

© Agilent Technologies, Inc. 2017

B2: H3 Plus R1

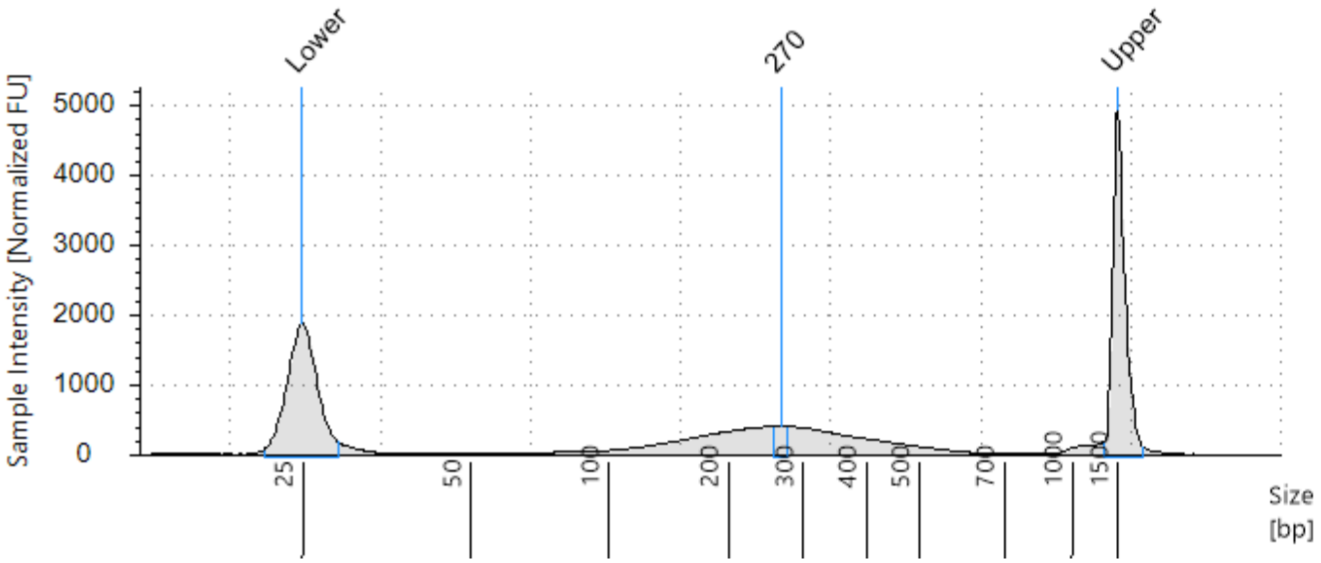

Sample Table

Well	Conc. [ng/µl]	Sample Description	Alert	Observations
B2	0.548	H3 Plus R1		

Peak Table

Size [bp]	Calibrated Conc. [ng/µl]	Assigned Conc. [ng/µl]	Peak Molarity [nmol/l]	% Integrated Area	Peak Comment	Observations
25	6.03	-	371	-		Lower Marker
270	0.548	-	3.13	100.00		
1500	6.50	6.50	6.67	-		Upper Marker

© Agilent Technologies, Inc. 2017

C2: A4 Plus R1

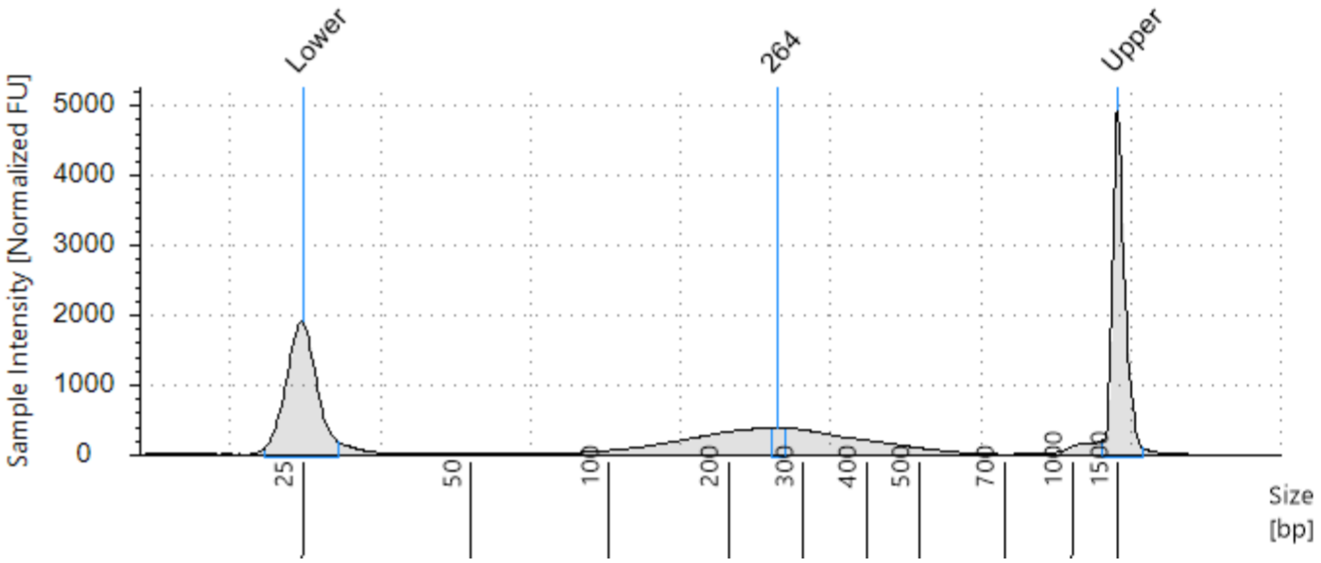

Sample Table

Well	Conc. [ng/µl]	Sample Description	Alert	Observations
C2	0.487	A4 Plus R1		

Peak Table

Size [bp]	Calibrated Conc. [ng/µl]	Assigned Conc. [ng/µl]	Peak Molarity [nmol/l]	% Integrated Area	Peak Comment	Observations
25	6.20	-	381	-		Lower Marker
264	0.487	-	2.83	100.00		
1500	6.50	6.50	6.67	-		Upper Marker

© Agilent Technologies, Inc. 2017

D2: B4 Plus R1

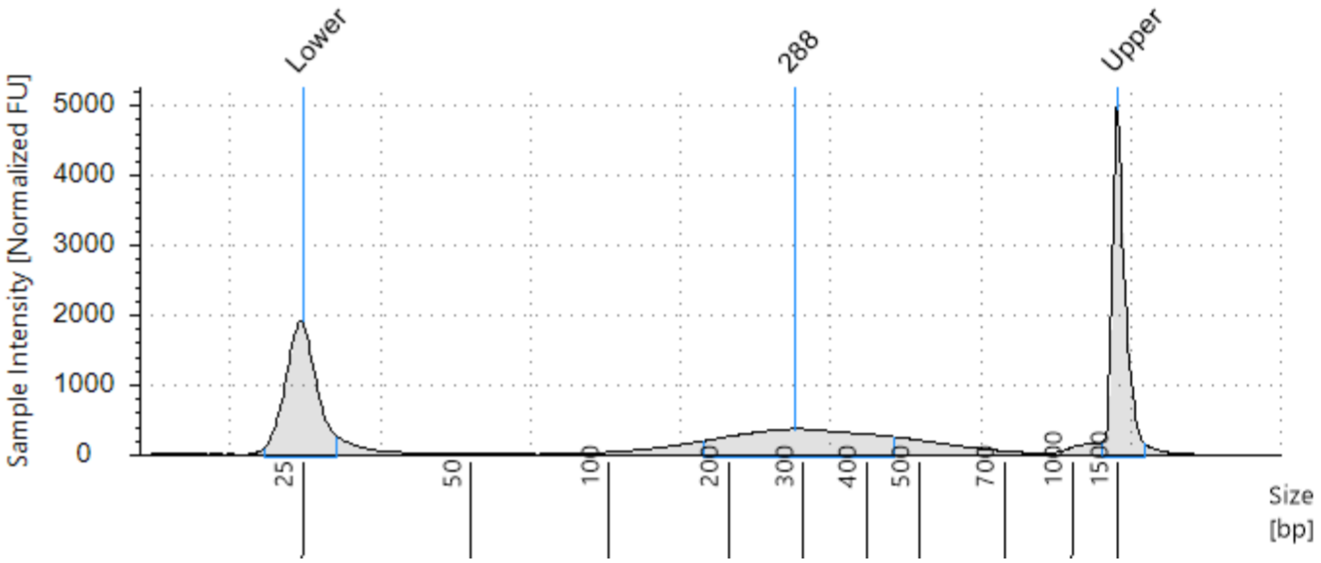

Sample Table

Well	Conc. [ng/µl]	Sample Description	Alert	Observations
D2	5.02	B4 Plus R1		

Peak Table

Size [bp]	Calibrated Conc. [ng/µl]	Assigned Conc. [ng/µl]	Peak Molarity [nmol/l]	% Integrated Area	Peak Comment	Observations
25	6.02	-	370	-		Lower Marker
288	5.02	-	26.8	100.00		
1500	6.50	6.50	6.67	-		Upper Marker

© Agilent Technologies, Inc. 2017

E2: C4 Plus R1

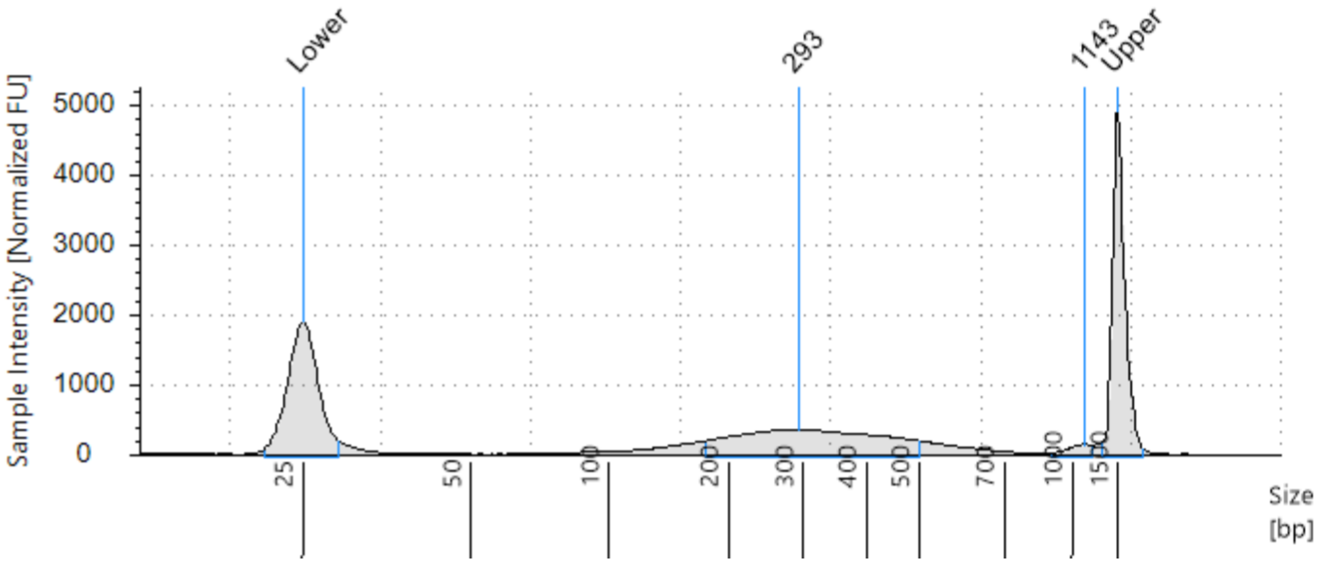

Sample Table

Well	Conc. [ng/µl]	Sample Description	Alert	Observations
E2	5.90	C4 Plus R1		

Peak Table

Size [bp]	Calibrated Conc. [ng/µl]	Assigned Conc. [ng/µl]	Peak Molarity [nmol/l]	% Integrated Area	Peak Comment	Observations
25	6.18	-	381	-		Lower Marker
293	5.57	-	29.2	94.37		
1143	0.333	-	0.448	5.63		
1500	6.50	6.50	6.67	-		Upper Marker

F2: D4 Plus R1

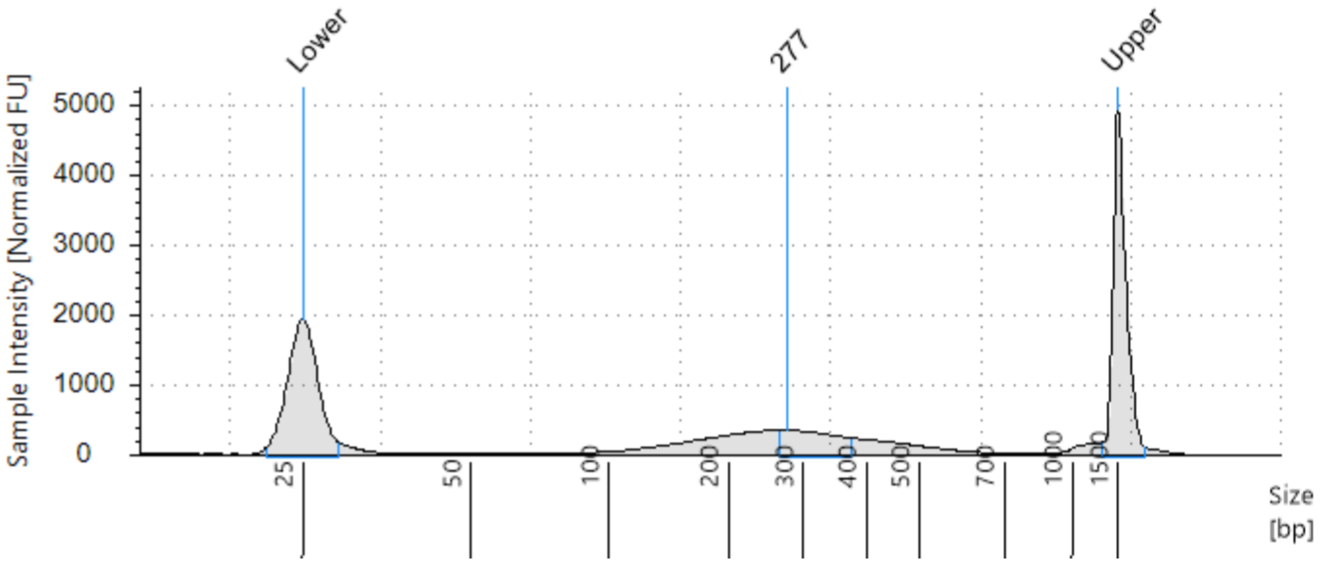

Sample Table

Well	Conc. [ng/µl]	Sample Description	Alert	Observations
F2	1.92	D4 Plus R1		

Peak Table

Size [bp]	Calibrated Conc. [ng/µl]	Assigned Conc. [ng/µl]	Peak Molarity [nmol/l]	% Integrated Area	Peak Comment	Observations
25	6.00	-	369	-		Lower Marker
277	1.92	-	10.7	100.00		
1500	6.50	6.50	6.67	-		Upper Marker

© Agilent Technologies, Inc. 2017

G2: E4 Plus R1

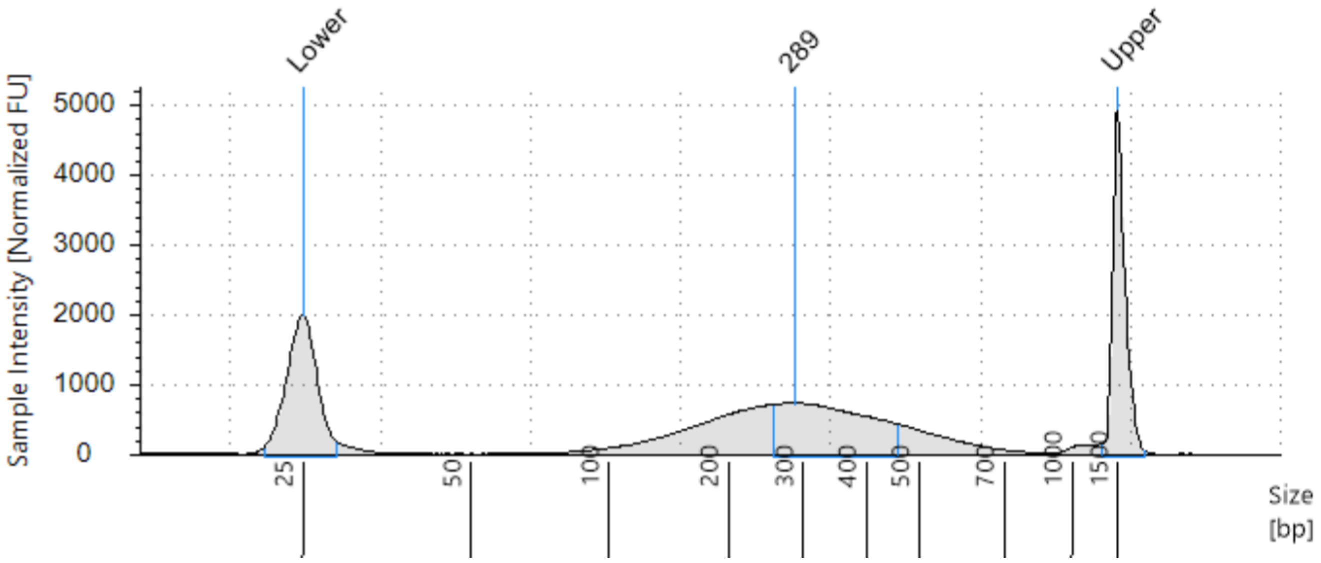

Sample Table

Well	Conc. [ng/µl]	Sample Description	Alert	Observations
G2	6.96	E4 Plus R1		

Peak Table

Size [bp]	Calibrated Conc. [ng/µl]	Assigned Conc. [ng/µl]	Peak Molarity [nmol/l]	% Integrated Area	Peak Comment	Observations
25	6.42	-	395	-		Lower Marker
289	6.96	-	37.1	100.00		
1500	6.50	6.50	6.67	-		Upper Marker

 © Agilent Technologies, Inc. 2017

H2: F4 Plus R1

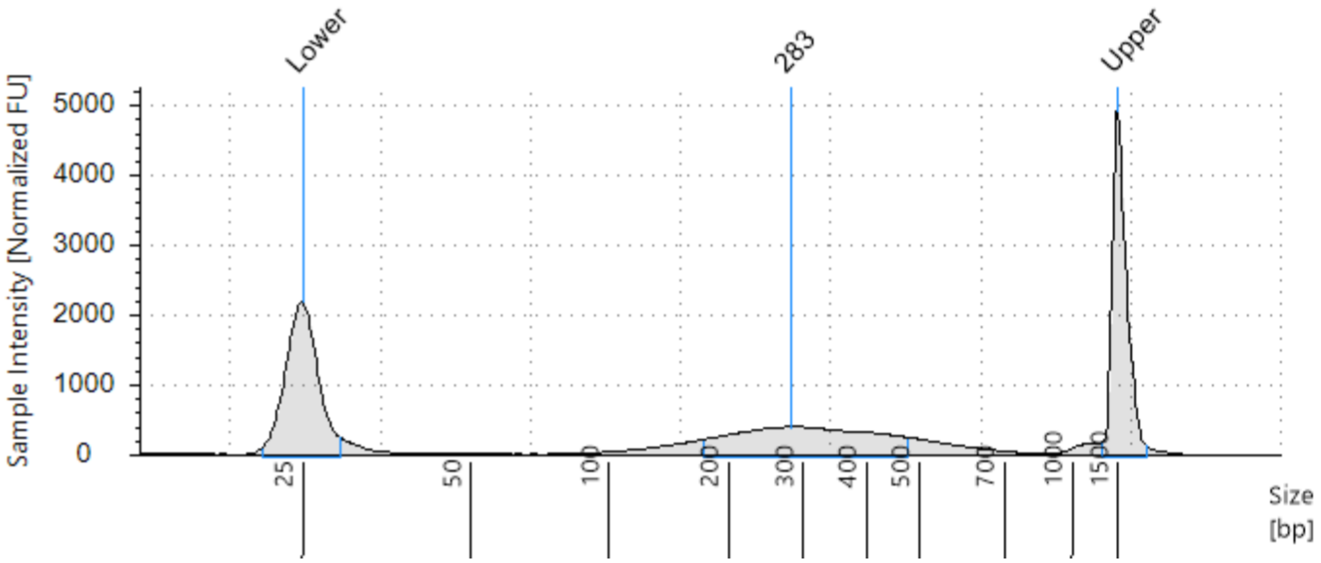

Sample Table

Well	Conc. [ng/µl]	Sample Description	Alert	Observations
H2	5.19	F4 Plus R1		

Peak Table

Size [bp]	Calibrated Conc. [ng/µl]	Assigned Conc. [ng/µl]	Peak Molarity [nmol/l]	% Integrated Area	Peak Comment	Observations
25	6.45	-	397	-		Lower Marker
283	5.19	-	28.2	100.00		
1500	6.50	6.50	6.67	-		Upper Marker

 © Agilent Technologies, Inc. 2017

Filename: 2020-03-06-02-Q-S DFB plus G4-F6 R1.D1000

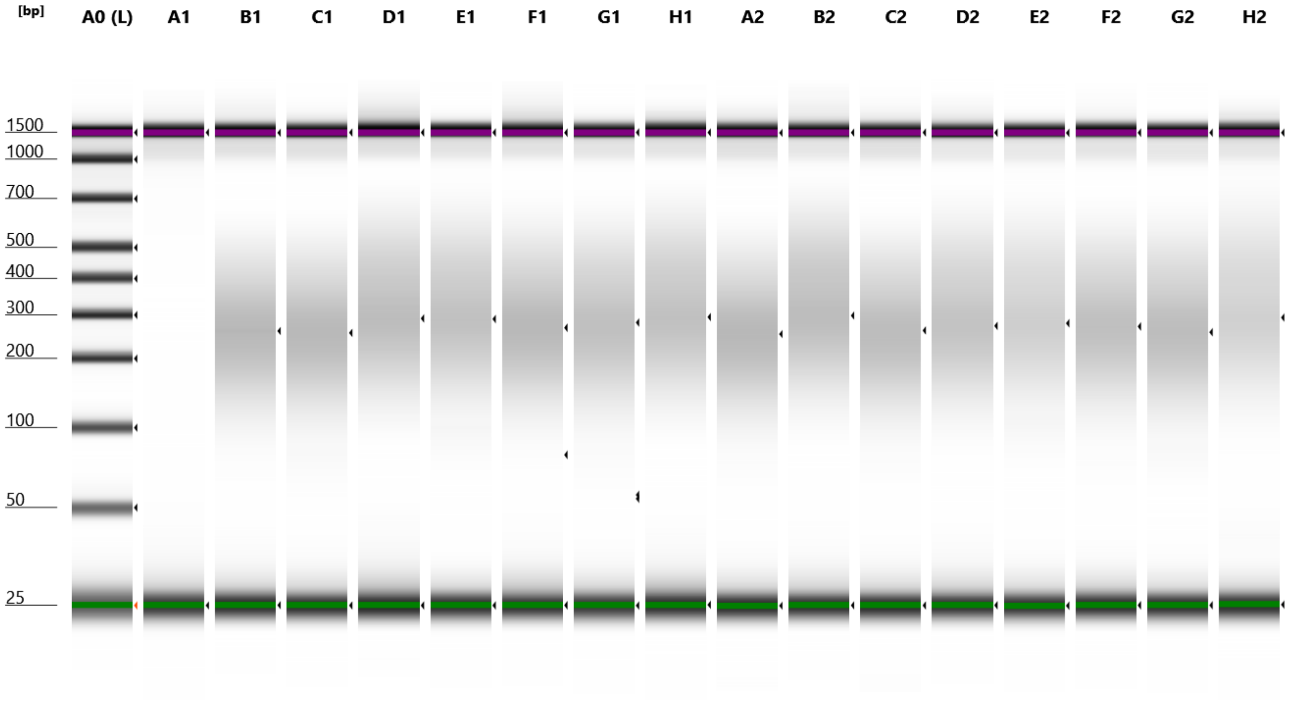

Default image (Contrast 100%)

Sample Info

Well	Conc. [ng/µl]	Sample Description	Alert	Observations
A0	20.3	Electronic Ladder		Ladder
A1		Ladder		
B1	3.15	G4 plus R1		
C1	3.09	H4 plus R1		
D1	0.652	A5 plus R1		
E1	5.23	B5 plus R1		
F1	6.60	C5 plus R1		
G1	6.76	D5 plus R1		
H1	5.28	E5 plus R1		
A2	5.49	F5 plus R1		
B2	3.02	G5 plus R1		
C2	5.23	H5 plus R1		
D2	4.43	A6 plus R1		
E2	4.23	B6 plus R1		
F2	2.90	D6 plus R1		
G2	4.99	E6 plus R1		
H2	2.83	F6 plus R1		

 © Agilent Technologies, Inc. 2017

A0: Electronic Ladder

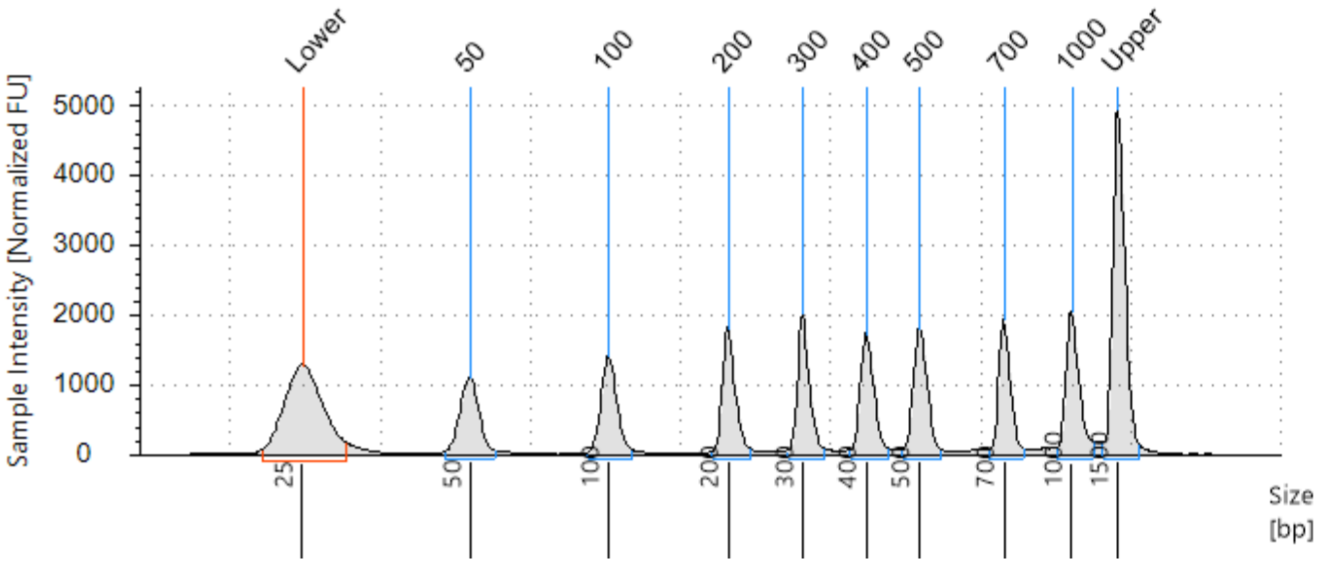

Sample Table

Well	Conc. [ng/µl]	Sample Description	Alert	Observations
A0	20.3	Electronic Ladder		Ladder

Peak Table

Size [bp]	Calibrated Conc. [ng/µl]	Assigned Conc. [ng/µl]	Peak Molarity [nmol/l]	% Integrated Area	Peak Comment	Observations
25	5.22	-	321	-		Lower Marker
50	2.25	-	69.3	11.11		
100	2.37	-	36.5	11.71		
200	2.47	-	19.0	12.20		
300	2.55	-	13.1	12.56		
400	2.57	-	9.87	12.66		
500	2.71	-	8.33	13.36		
700	2.46	-	5.41	12.15		
1000	2.89	-	4.44	14.25		
1500	6.50	6.50	6.67	-		Upper Marker

© Agilent Technologies, Inc. 2017

A1: Ladder

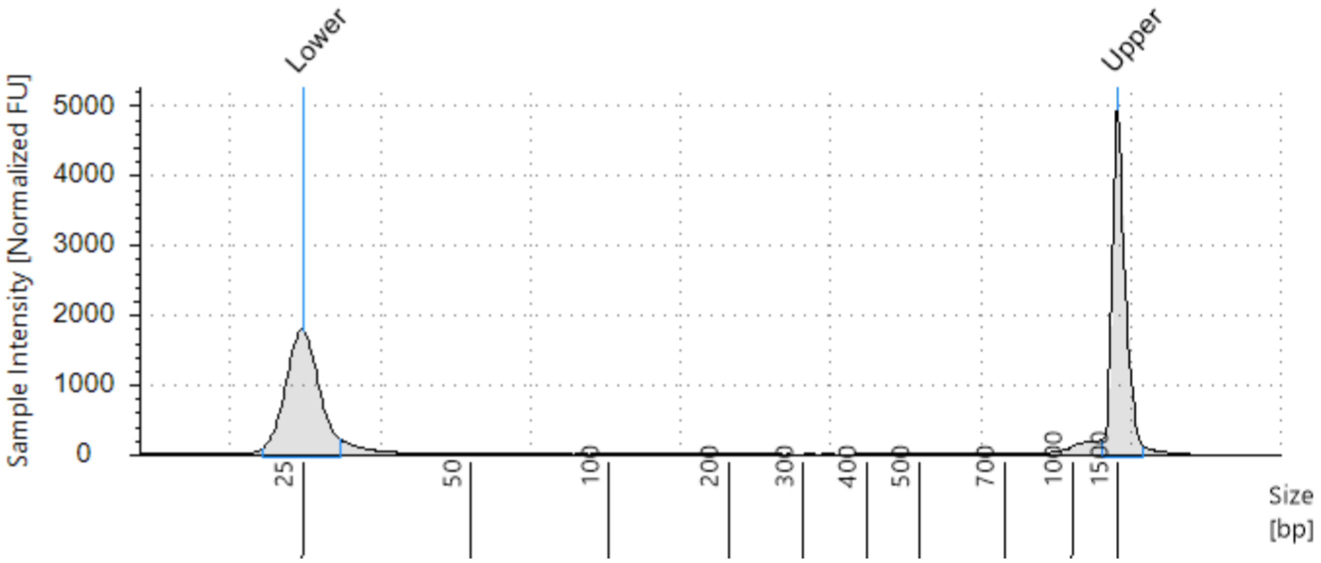

Sample Table

Well	Conc. [ng/µl]	Sample Description	Alert	Observations
A1		Ladder		

Peak Table

Size [bp]	Calibrated Conc. [ng/µl]	Assigned Conc. [ng/µl]	Peak Molarity [nmol/l]	% Integrated Area	Peak Comment	Observations
25	5.95	-	366	-		Lower Marker
1500	6.50	6.50	6.67	-		Upper Marker

 © Agilent Technologies, Inc. 2017

B1: G4 plus R1

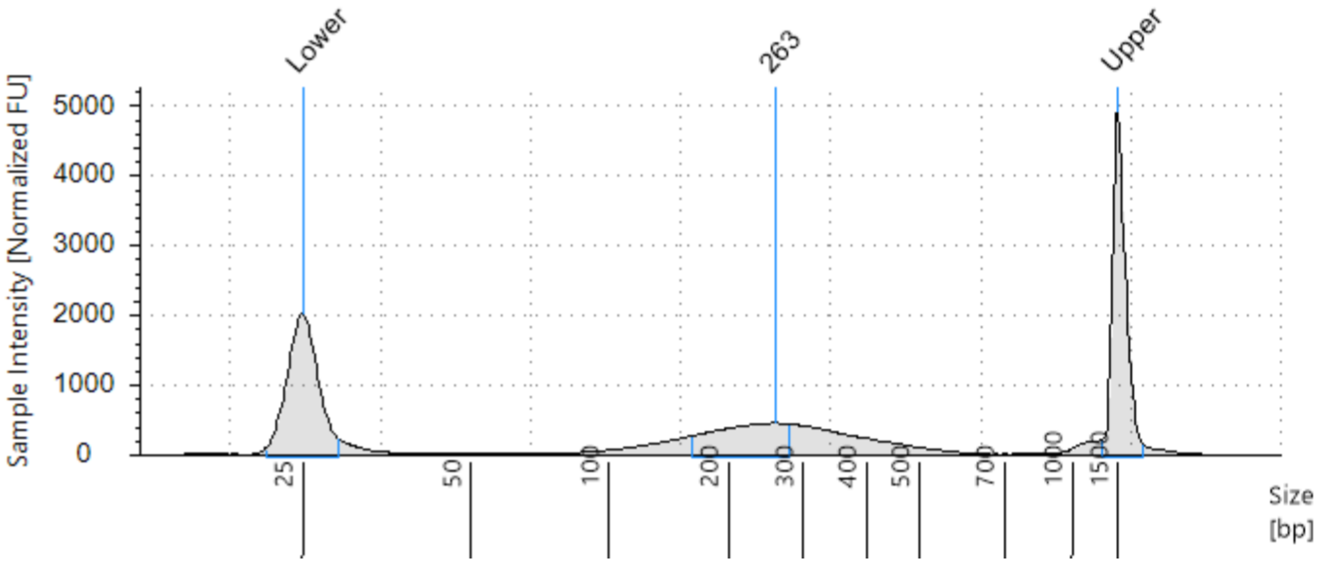

Sample Table

Well	Conc. [ng/µl]	Sample Description	Alert	Observations
B1	3.15	G4 plus R1		

Peak Table

Size [bp]	Calibrated Conc. [ng/µl]	Assigned Conc. [ng/µl]	Peak Molarity [nmol/l]	% Integrated Area	Peak Comment	Observations
25	6.21	-	382	-		Lower Marker
263	3.15	-	18.4	100.00		
1500	6.50	6.50	6.67	-		Upper Marker

 © Agilent Technologies, Inc. 2017

C1: H4 plus R1

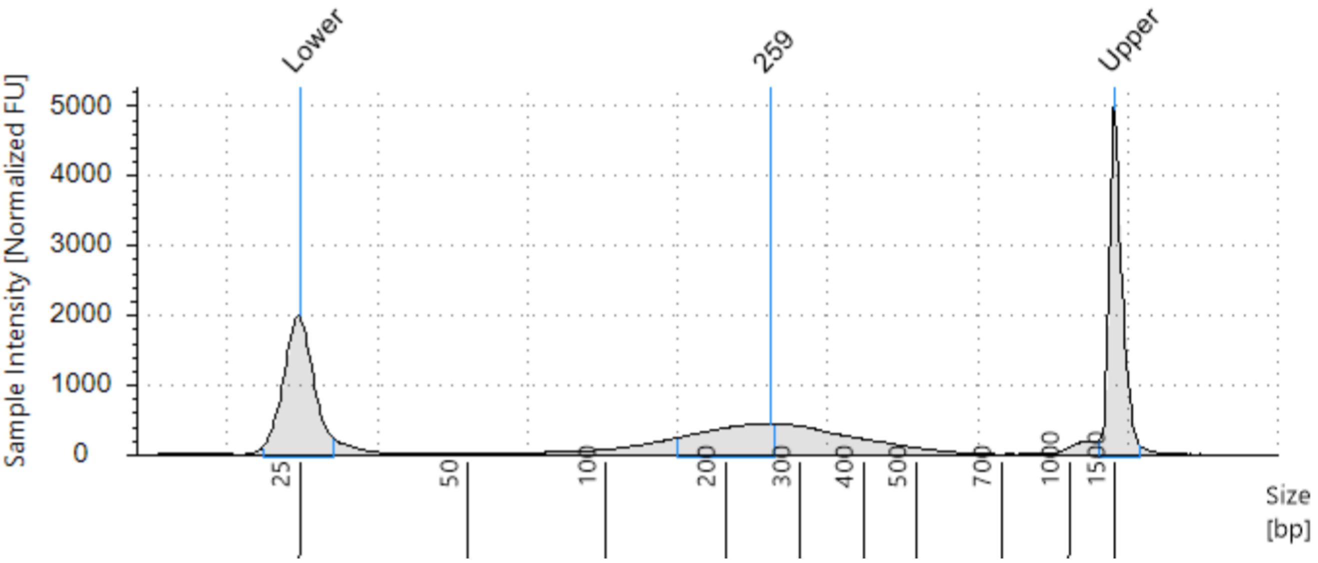

Sample Table

Well	Conc. [ng/µl]	Sample Description	Alert	Observations
C1	3.09	H4 plus R1		

Peak Table

Size [bp]	Calibrated Conc. [ng/µl]	Assigned Conc. [ng/µl]	Peak Molarity [nmol/l]	% Integrated Area	Peak Comment	Observations
25	6.04	-	372	-		Lower Marker
259	3.09	-	18.4	100.00		
1500	6.50	6.50	6.67	-		Upper Marker

 © Agilent Technologies, Inc. 2017

D1: A5 plus R1

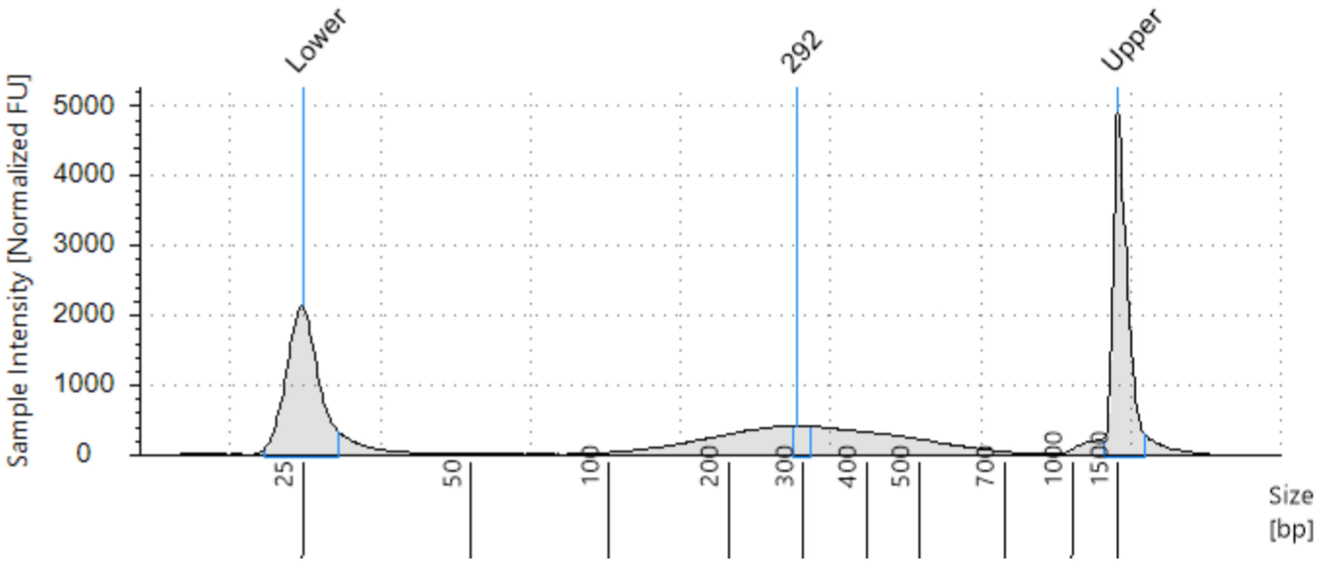

Sample Table

Well	Conc. [ng/µl]	Sample Description	Alert	Observations
D1	0.652	A5 plus R1		

Peak Table

Size [bp]	Calibrated Conc. [ng/µl]	Assigned Conc. [ng/µl]	Peak Molarity [nmol/l]	% Integrated Area	Peak Comment	Observations
25	6.21	-	382	-		Lower Marker
292	0.652	-	3.43	100.00		
1500	6.50	6.50	6.67	-		Upper Marker

© Agilent Technologies, Inc. 2017

E1: B5 plus R1

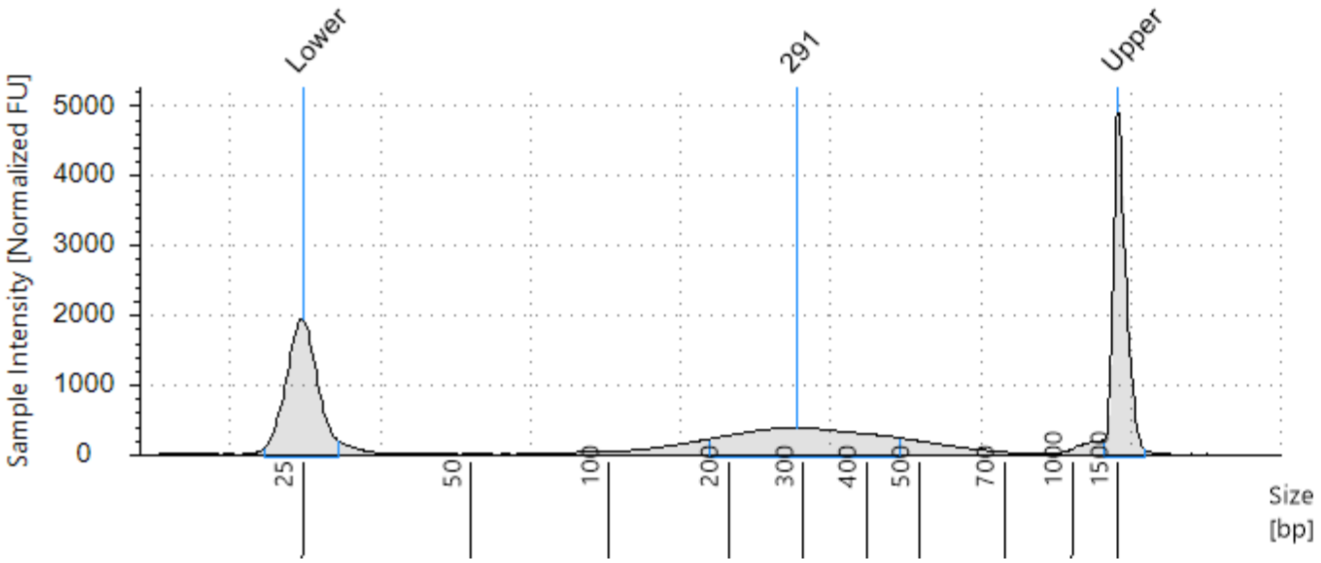

Sample Table

Well	Conc. [ng/µl]	Sample Description	Alert	Observations
E1	5.23	B5 plus R1		

Peak Table

Size [bp]	Calibrated Conc. [ng/µl]	Assigned Conc. [ng/µl]	Peak Molarity [nmol/l]	% Integrated Area	Peak Comment	Observations
25	6.12	-	376	-		Lower Marker
291	5.23	-	27.7	100.00		
1500	6.50	6.50	6.67	-		Upper Marker

© Agilent Technologies, Inc. 2017

F1: C5 plus R1

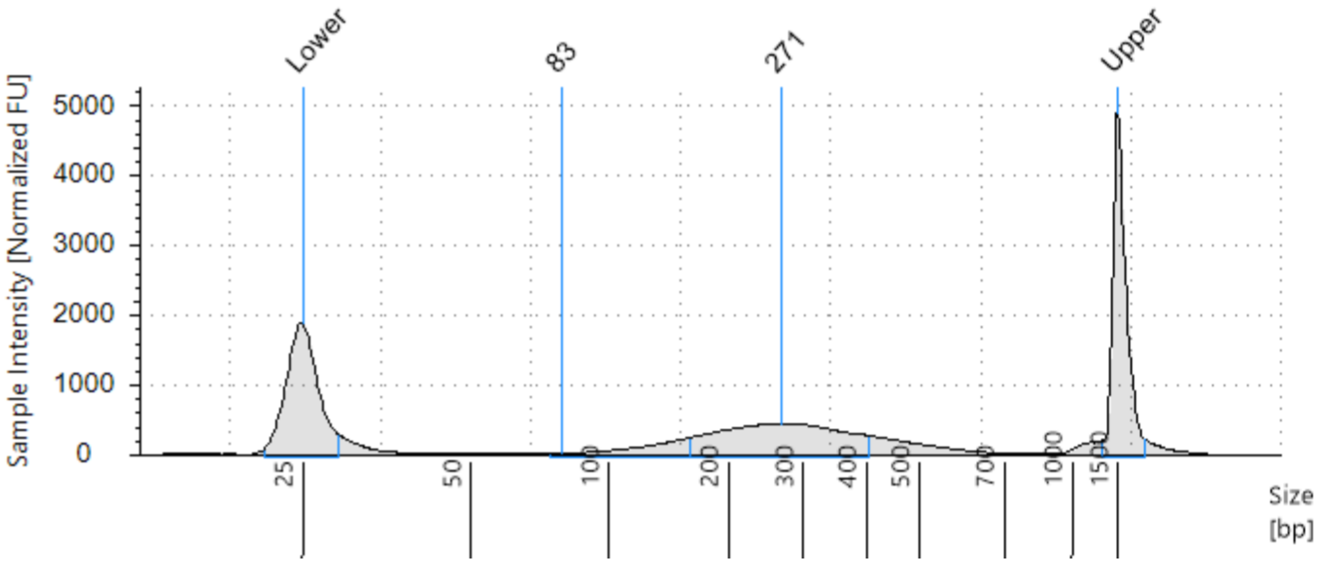

Sample Table

Well	Conc. [ng/µl]	Sample Description	Alert	Observations
F1	6.60	C5 plus R1		

Peak Table

Size [bp]	Calibrated Conc. [ng/µl]	Assigned Conc. [ng/µl]	Peak Molarity [nmol/l]	% Integrated Area	Peak Comment	Observations
25	5.96	-	367	-		Lower Marker
83	1.01	-	18.7	15.29		
271	5.59	-	31.7	84.71		
1500	6.50	6.50	6.67	-		Upper Marker

© Agilent Technologies, Inc. 2017

G1: D5 plus R1

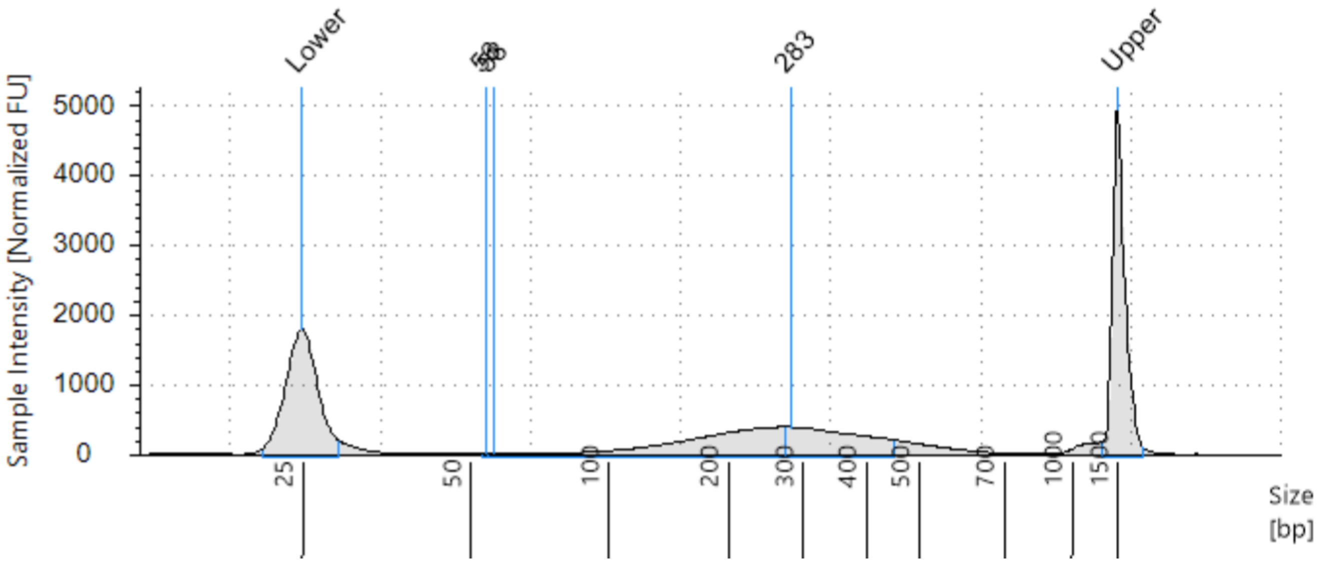

Sample Table

Well	Conc. [ng/µl]	Sample Description	Alert	Observations
G1	6.76	D5 plus R1		

Peak Table

Size [bp]	Calibrated Conc. [ng/µl]	Assigned Conc. [ng/µl]	Peak Molarity [nmol/l]	% Integrated Area	Peak Comment	Observations
25	5.99	-	369	-		Lower Marker
56	0.000974	-	0.0269	0.01		
58	3.75	-	99.5	55.46		
283	3.01	-	16.4	44.53		
1500	6.50	6.50	6.67	-		Upper Marker

© Agilent Technologies, Inc. 2017

H1: E5 plus R1

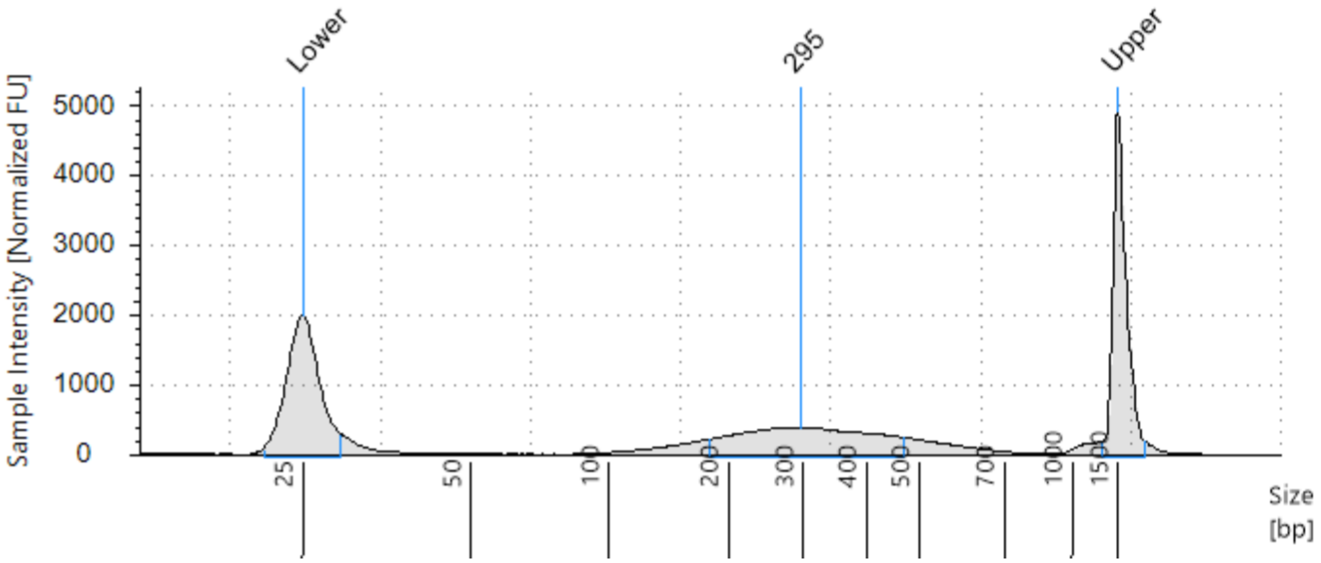

Sample Table

Well	Conc. [ng/µl]	Sample Description	Alert	Observations
H1	5.28	E5 plus R1		

Peak Table

Size [bp]	Calibrated Conc. [ng/µl]	Assigned Conc. [ng/µl]	Peak Molarity [nmol/l]	% Integrated Area	Peak Comment	Observations
25	6.52	-	401	-		Lower Marker
295	5.28	-	27.6	100.00		
1500	6.50	6.50	6.67	-		Upper Marker

A2: F5 plus R1

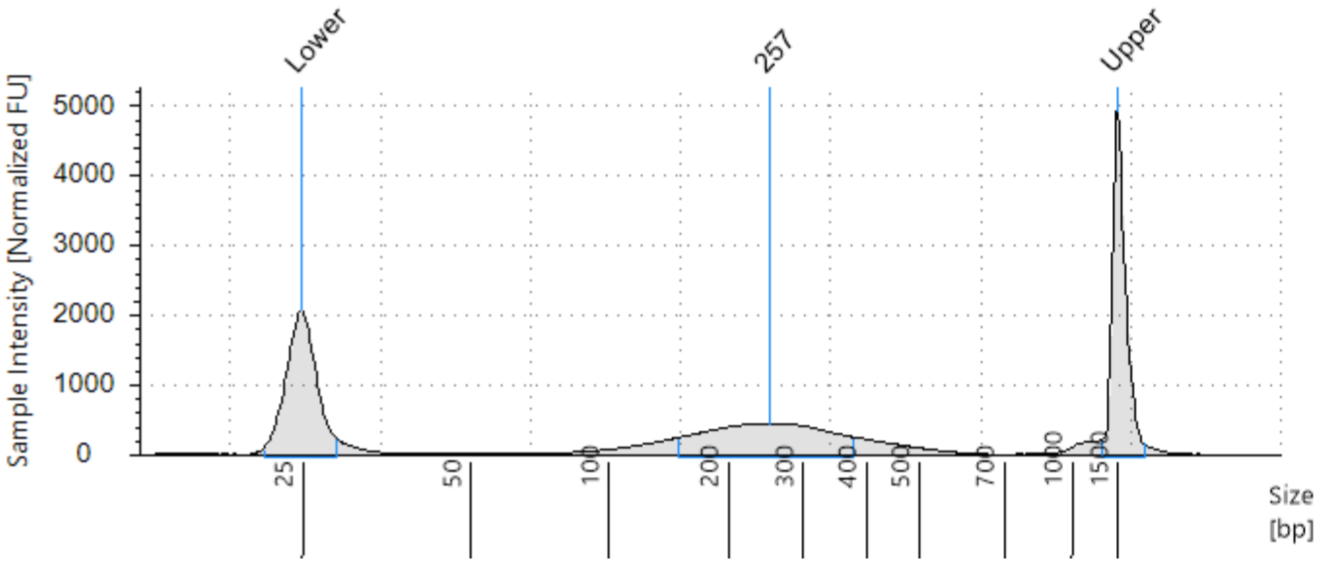

Sample Table

Well	Conc. [ng/µl]	Sample Description	Alert	Observations
A2	5.49	F5 plus R1		

Peak Table

Size [bp]	Calibrated Conc. [ng/µl]	Assigned Conc. [ng/µl]	Peak Molarity [nmol/l]	% Integrated Area	Peak Comment	Observations
25	6.23	-	383	-		Lower Marker
257	5.49	-	32.9	100.00		
1500	6.50	6.50	6.67	-		Upper Marker

 © Agilent Technologies, Inc. 2017

B2: G5 plus R1

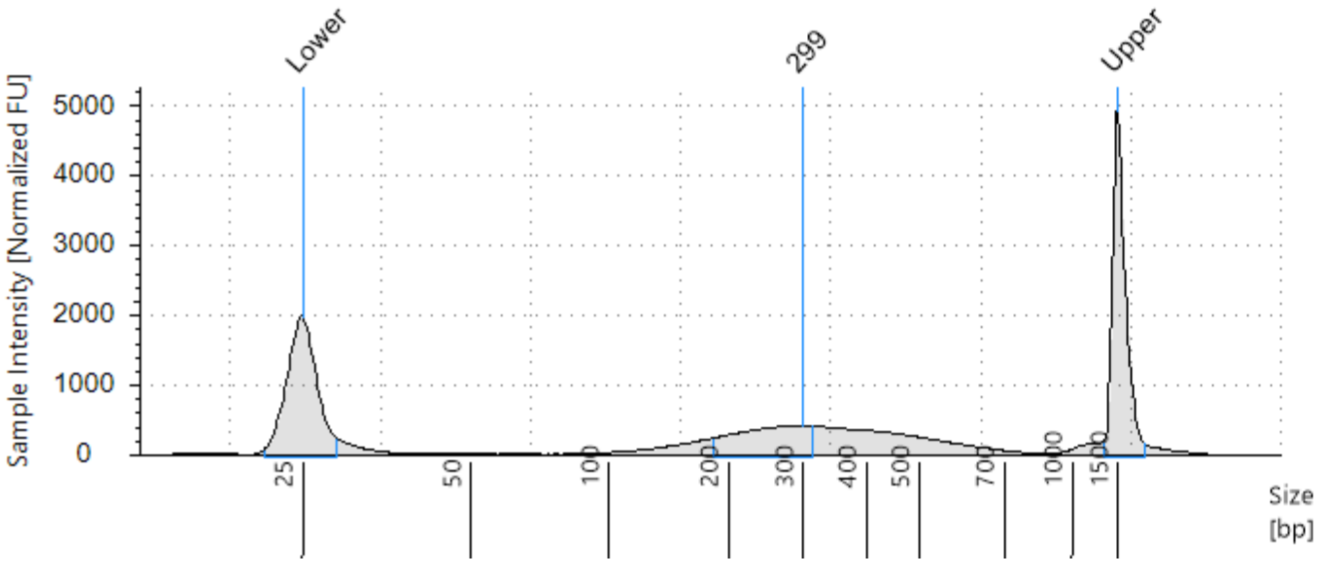

Sample Table

Well	Conc. [ng/µl]	Sample Description	Alert	Observations
B2	3.02	G5 plus R1		

Peak Table

Size [bp]	Calibrated Conc. [ng/µl]	Assigned Conc. [ng/µl]	Peak Molarity [nmol/l]	% Integrated Area	Peak Comment	Observations
25	6.01	-	370	-		Lower Marker
299	3.02	-	15.5	100.00		
1500	6.50	6.50	6.67	-		Upper Marker

 © Agilent Technologies, Inc. 2017

C2: H5 plus R1

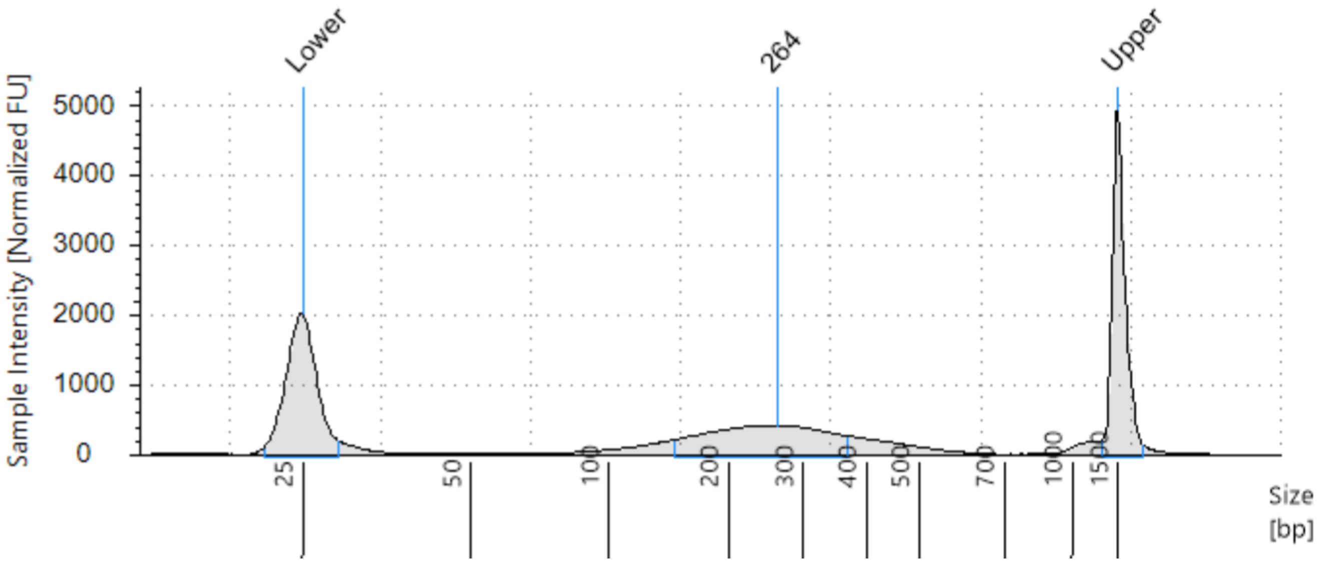

Sample Table

Well	Conc. [ng/µl]	Sample Description	Alert	Observations
C2	5.23	H5 plus R1		

Peak Table

Size [bp]	Calibrated Conc. [ng/µl]	Assigned Conc. [ng/µl]	Peak Molarity [nmol/l]	% Integrated Area	Peak Comment	Observations
25	6.31	-	388	-		Lower Marker
264	5.23	-	30.4	100.00		
1500	6.50	6.50	6.67	-		Upper Marker

© Agilent Technologies, Inc. 2017

D2: A6 plus R1

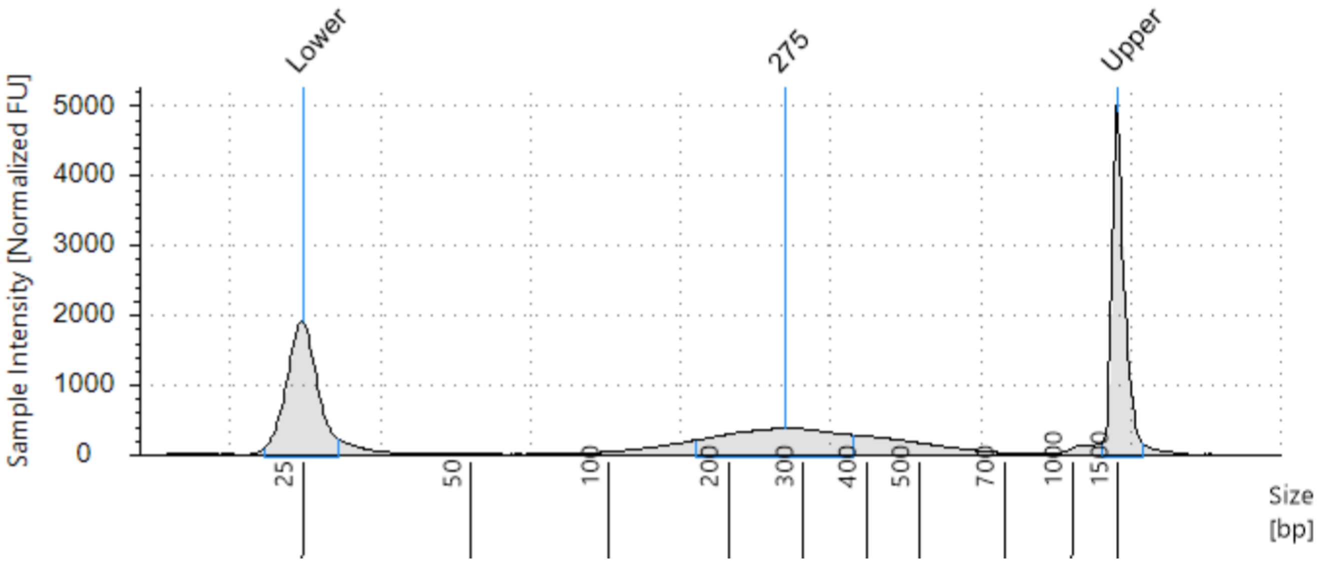

Sample Table

Well	Conc. [ng/µl]	Sample Description	Alert	Observations
D2	4.43	A6 plus R1		

Peak Table

Size [bp]	Calibrated Conc. [ng/µl]	Assigned Conc. [ng/µl]	Peak Molarity [nmol/l]	% Integrated Area	Peak Comment	Observations
25	6.06	-	373	-		Lower Marker
275	4.43	-	24.8	100.00		
1500	6.50	6.50	6.67	-		Upper Marker

© Agilent Technologies, Inc. 2017

E2: B6 plus R1

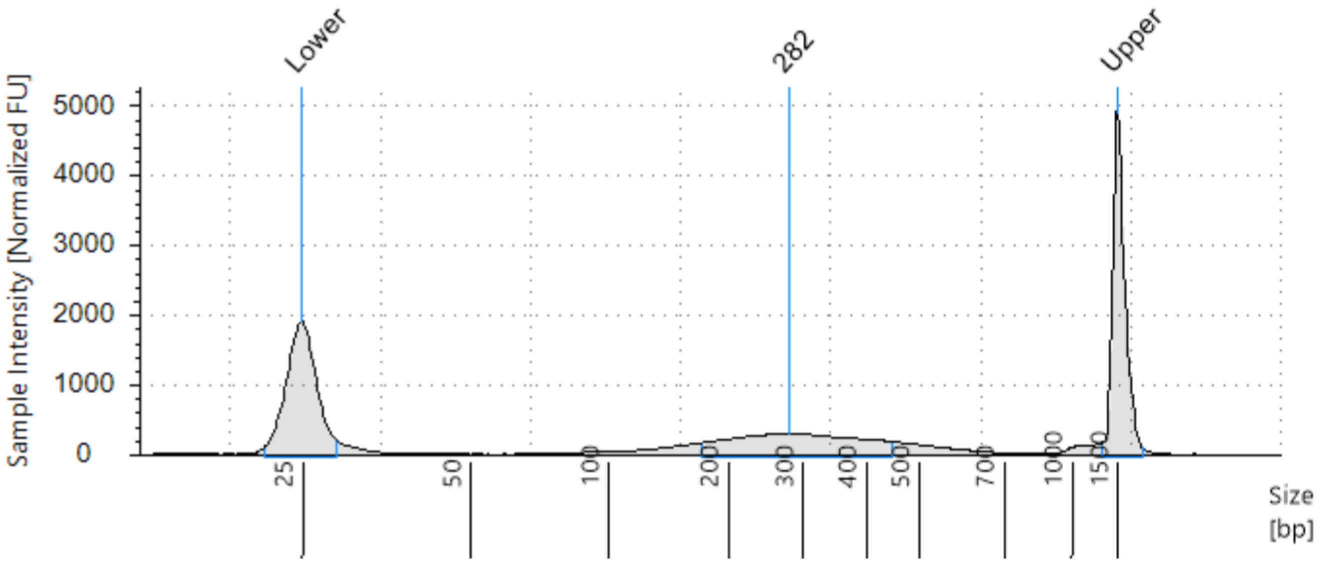

Sample Table

Well	Conc. [ng/µl]	Sample Description	Alert	Observations
E2	4.23	B6 plus R1		

Peak Table

Size [bp]	Calibrated Conc. [ng/µl]	Assigned Conc. [ng/µl]	Peak Molarity [nmol/l]	% Integrated Area	Peak Comment	Observations
25	6.19	-	381	-		Lower Marker
282	4.23	-	23.1	100.00		
1500	6.50	6.50	6.67	-		Upper Marker

© Agilent Technologies, Inc. 2017

F2: D6 plus R1

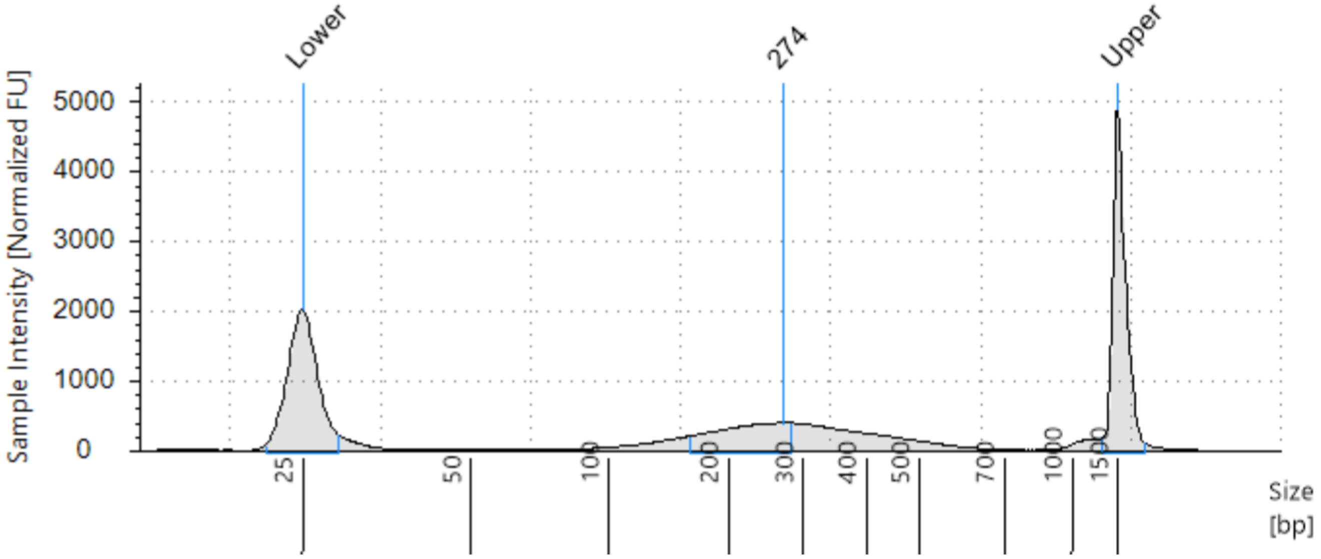

Sample Table

Well	Conc. [ng/µl]	Sample Description	Alert	Observations
F2	2.90	D6 plus R1		

Peak Table

Size [bp]	Calibrated Conc. [ng/µl]	Assigned Conc. [ng/µl]	Peak Molarity [nmol/l]	% Integrated Area	Peak Comment	Observations
25	6.29	-	387	-		Lower Marker
274	2.90	-	16.3	100.00		
1500	6.50	6.50	6.67	-		Upper Marker

© Agilent Technologies, Inc. 2017

G2: E6 plus R1

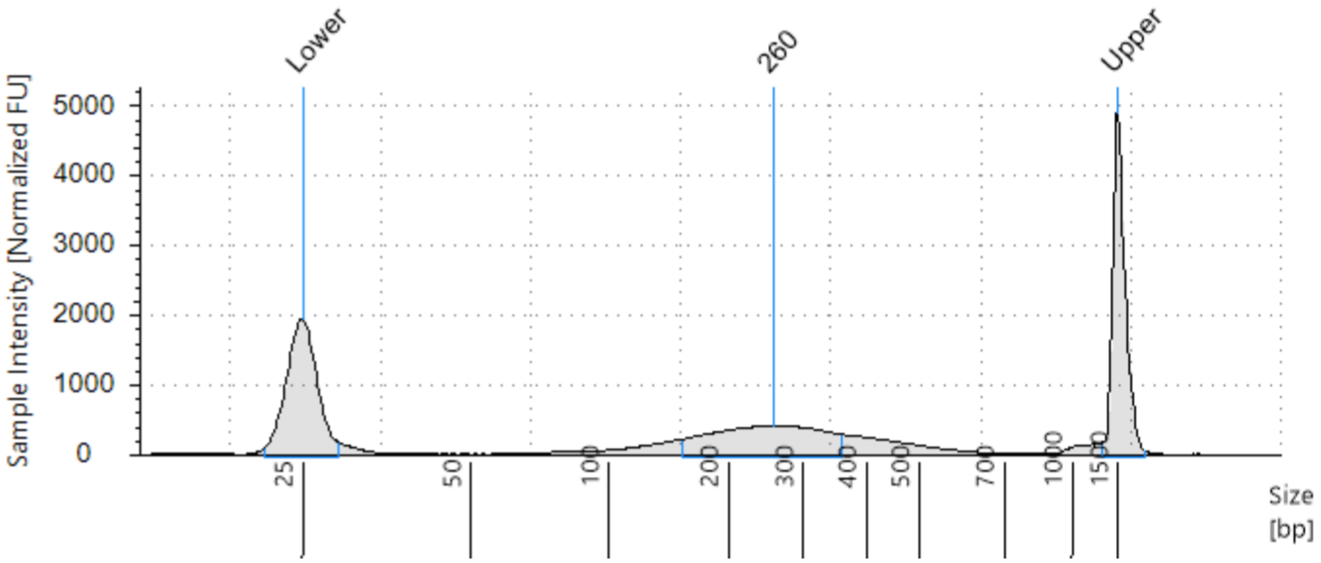

Sample Table

Well	Conc. [ng/µl]	Sample Description	Alert	Observations
G2	4.99	E6 plus R1		

Peak Table

Size [bp]	Calibrated Conc. [ng/µl]	Assigned Conc. [ng/µl]	Peak Molarity [nmol/l]	% Integrated Area	Peak Comment	Observations
25	6.31	-	389	-		Lower Marker
260	4.99	-	29.5	100.00		
1500	6.50	6.50	6.67	-		Upper Marker

© Agilent Technologies, Inc. 2017

H2: F6 plus R1

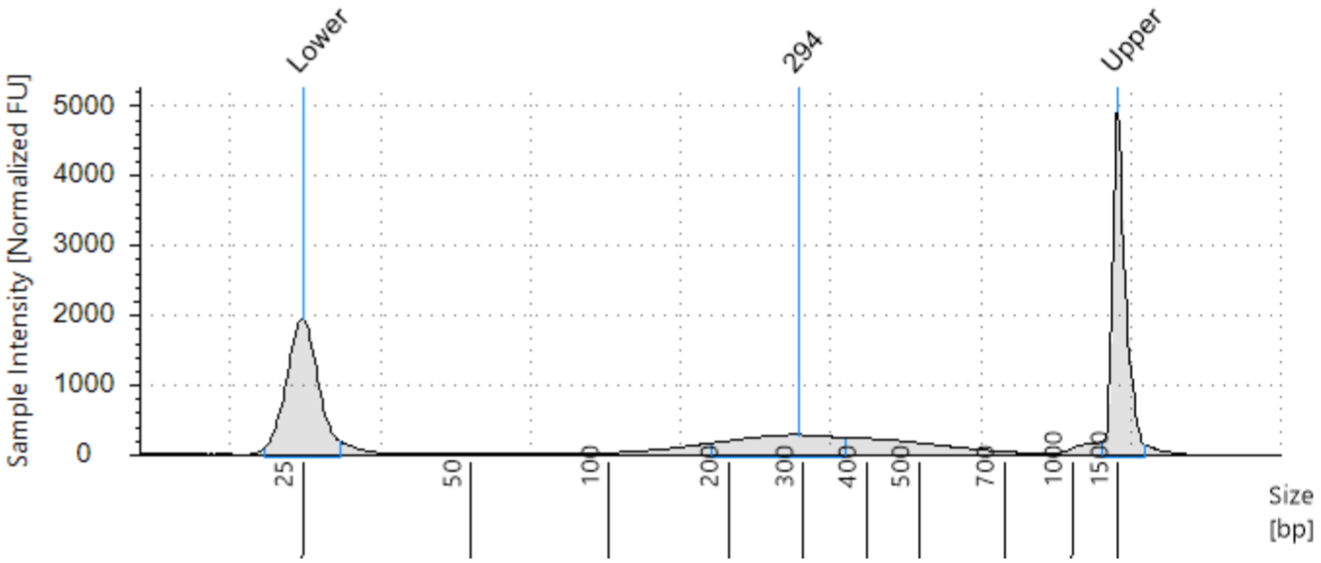

Sample Table

Well	Conc. [ng/µl]	Sample Description	Alert	Observations
H2	2.83	F6 plus R1		

Peak Table

Size [bp]	Calibrated Conc. [ng/µl]	Assigned Conc. [ng/µl]	Peak Molarity [nmol/l]	% Integrated Area	Peak Comment	Observations
25	6.19	-	381	-		Lower Marker
294	2.83	-	14.8	100.00		
1500	6.50	6.50	6.67	-		Upper Marker

© Agilent Technologies, Inc. 2017

Filename: 2020-07-20-01-Q-S DFB Plus E6-C8 R1.D1000

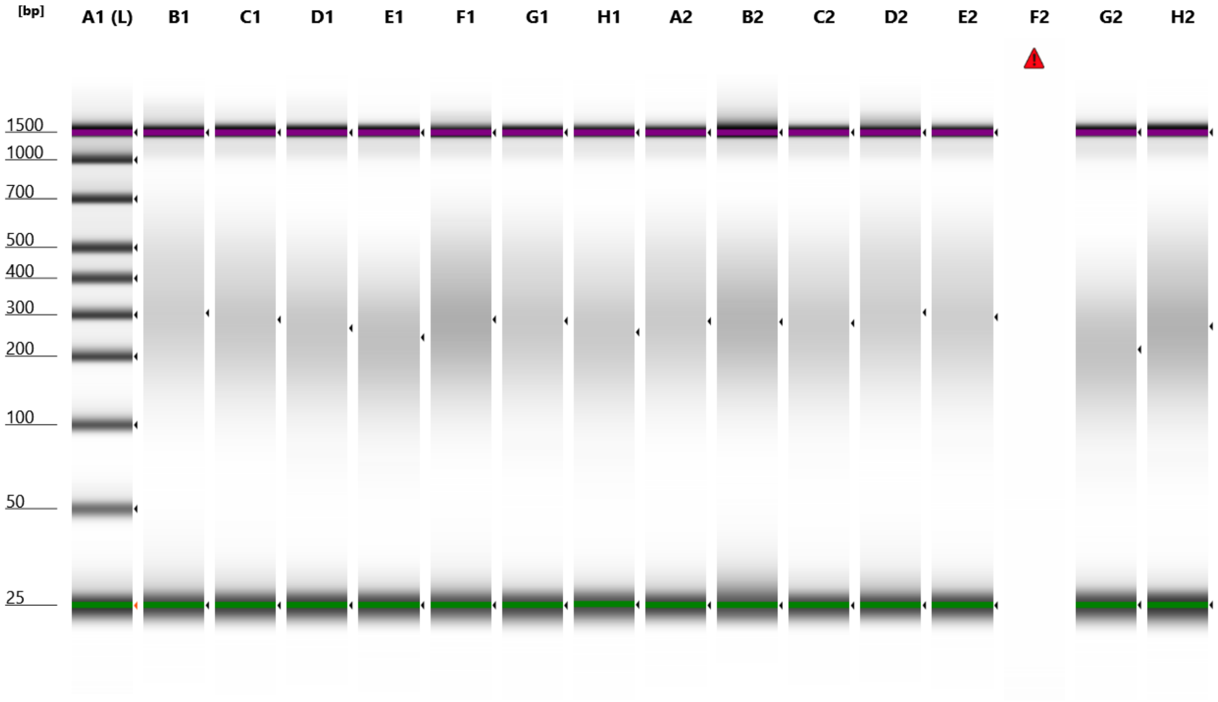

Default image (Contrast 100%)

Sample Info

Well	Conc. [ng/µl]	Sample Description	Alert	Observations
A1	20.1	Ladder		Ladder
B1	4.40	6E		
C1	2.79	6F		
D1	2.62	6G		
E1	2.75	6H		
F1	3.89	7A		
G1	2.34	7B		
H1	2.81	7C		
A2	2.45	7D		
B2	0.849	7E		
C2	2.16	7F		
D2	2.76	7G		
E2	1.87	7H		
F2		8A	▲	Marker(s) not detected
G2	3.07	8B		
H2	2.97	8C		

© Agilent Technologies, Inc. 2017

A1: Ladder

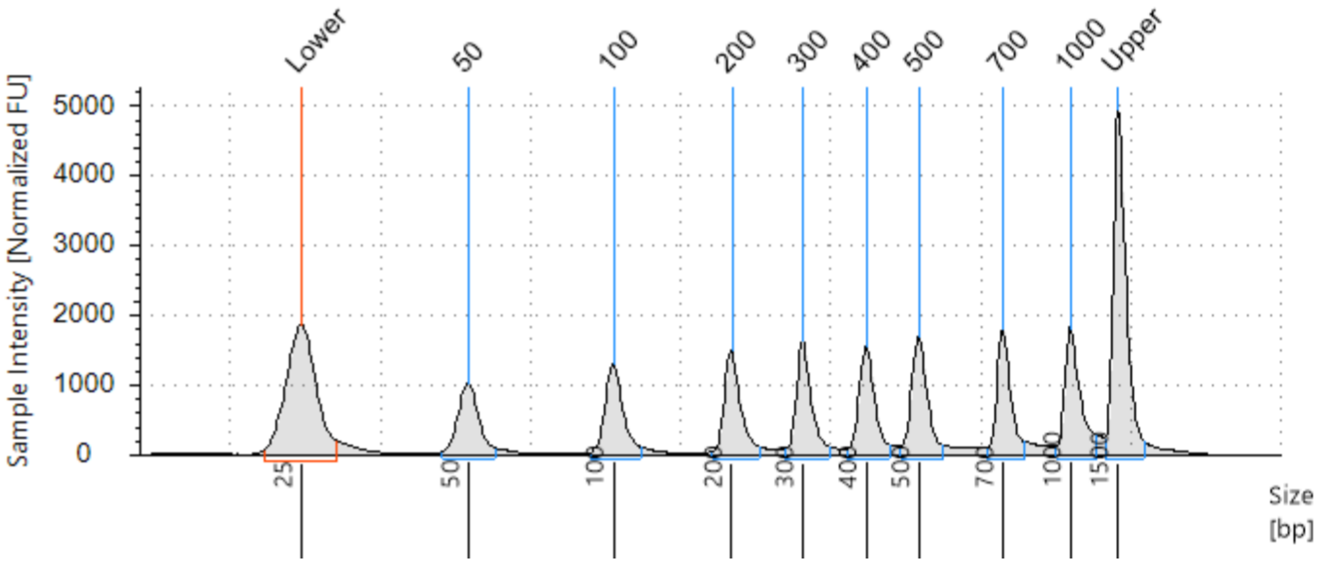

Sample Table

Well	Conc. [ng/µl]	Sample Description	Alert	Observations
A1	20.1	Ladder		Ladder

Peak Table

Size [bp]	Calibrated Conc. [ng/µl]	Assigned Conc. [ng/µl]	Peak Molarity [nmol/l]	% Integrated Area	Peak Comment	Observations
25	5.66	-	348	-		Lower Marker
50	2.17	-	66.6	10.79		
100	2.37	-	36.5	11.82		
200	2.46	-	19.0	12.28		
300	2.50	-	12.8	12.44		
400	2.50	-	9.62	12.46		
500	2.71	-	8.34	13.51		
700	2.54	-	5.57	12.63		
1000	2.82	-	4.34	14.06		
1500	6.50	6.50	6.67	-		Upper Marker

© Agilent Technologies, Inc. 2017

B1: 6E

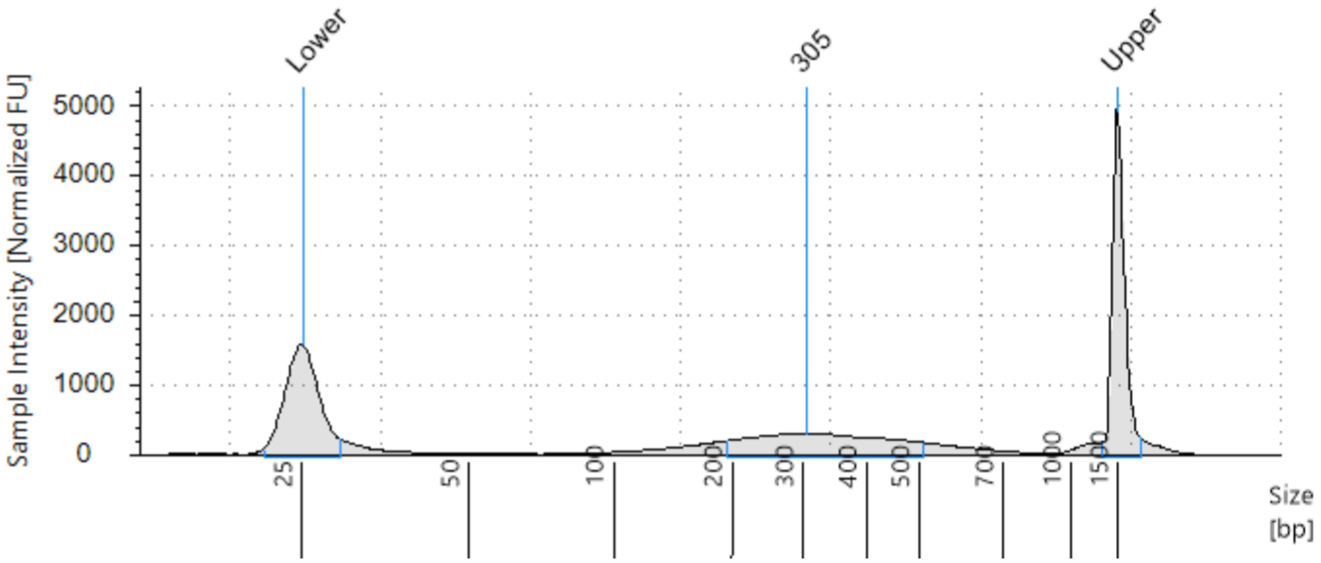

Sample Table

Well	Conc. [ng/µl]	Sample Description	Alert	Observations
B1	4.40	6E		

Peak Table

Size [bp]	Calibrated Conc. [ng/µl]	Assigned Conc. [ng/µl]	Peak Molarity [nmol/l]	% Integrated Area	Peak Comment	Observations
25	5.67	-	349	-		Lower Marker
305	4.40	-	22.2	100.00		
1500	6.50	6.50	6.67	-		Upper Marker

© Agilent Technologies, Inc. 2017

C1: 6F

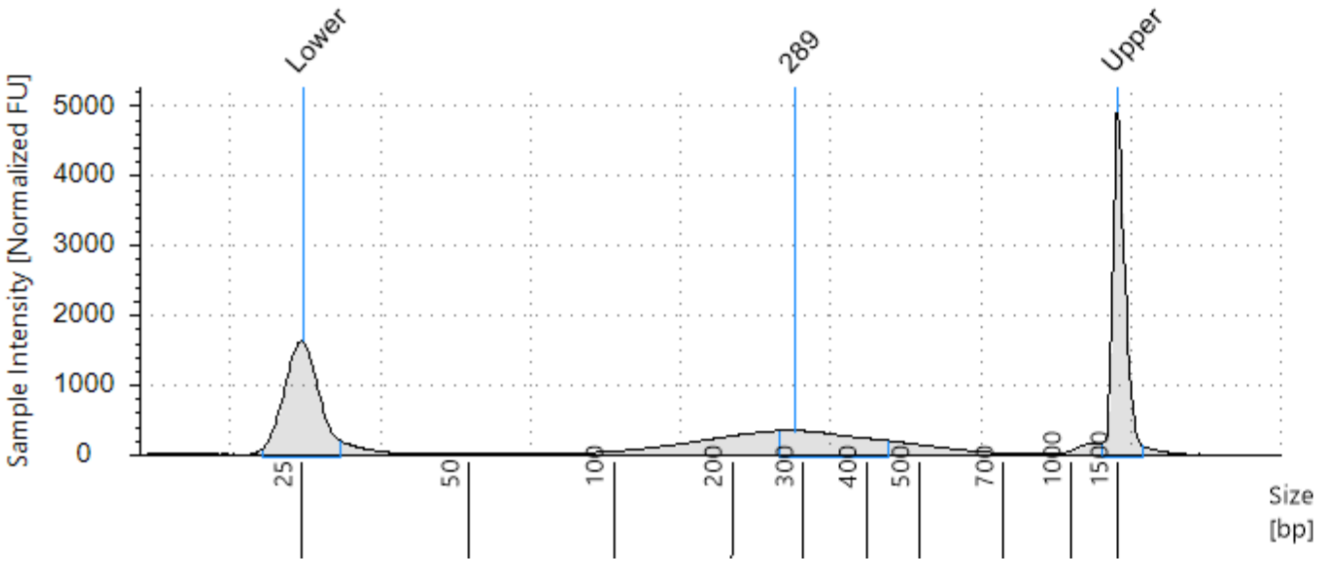

Sample Table

Well	Conc. [ng/µl]	Sample Description	Alert	Observations
C1	2.79	6F		

Peak Table

Size [bp]	Calibrated Conc. [ng/µl]	Assigned Conc. [ng/µl]	Peak Molarity [nmol/l]	% Integrated Area	Peak Comment	Observations
25	5.94	-	366	-		Lower Marker
289	2.79	-	14.9	100.00		
1500	6.50	6.50	6.67	-		Upper Marker

© Agilent Technologies, Inc. 2017

D1: 6G

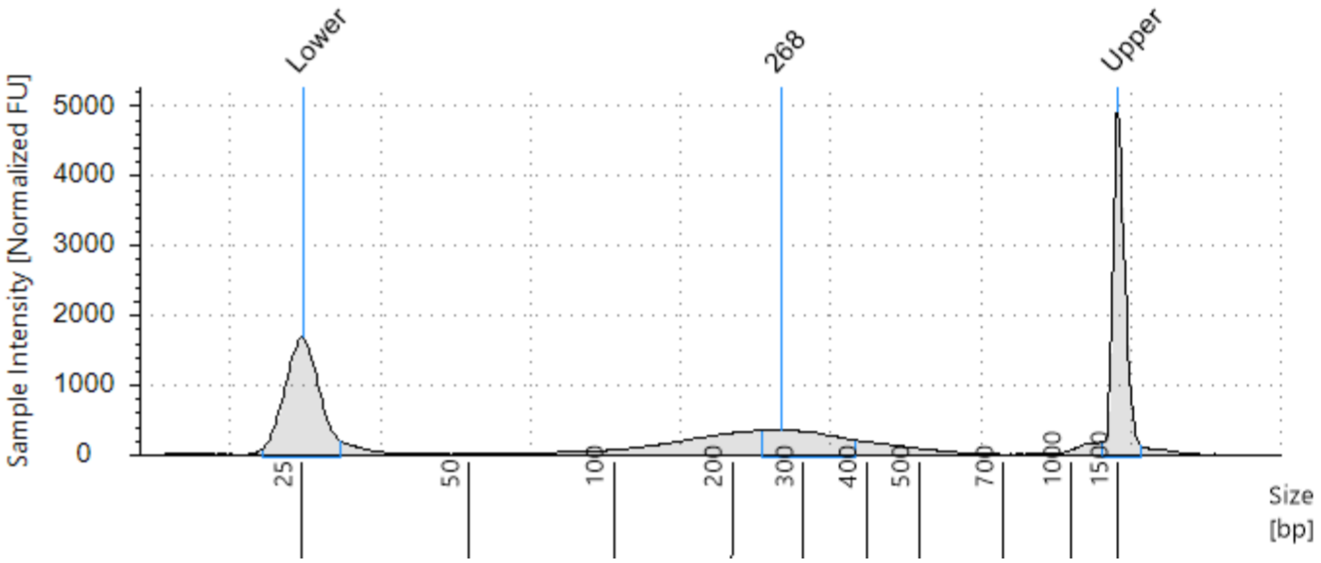

Sample Table

Well	Conc. [ng/µl]	Sample Description	Alert	Observations
D1	2.62	6G		

Peak Table

Size [bp]	Calibrated Conc. [ng/µl]	Assigned Conc. [ng/µl]	Peak Molarity [nmol/l]	% Integrated Area	Peak Comment	Observations
25	6.15	-	379	-		Lower Marker
268	2.62	-	15.1	100.00		
1500	6.50	6.50	6.67	-		Upper Marker

© Agilent Technologies, Inc. 2017

E1: 6H

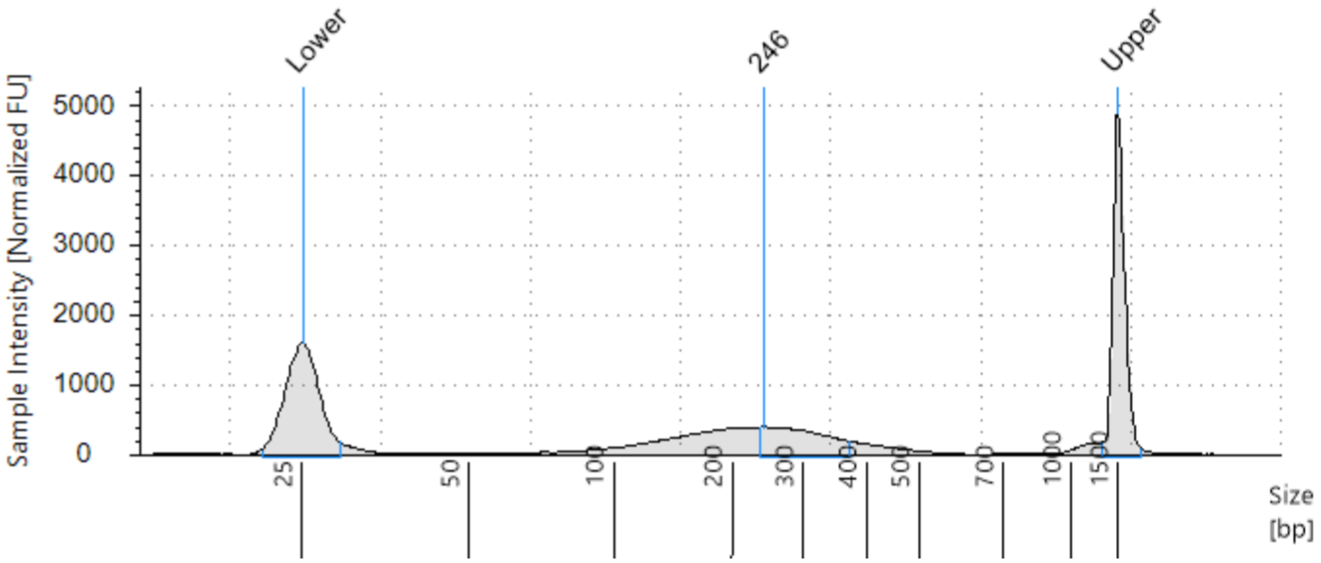

Sample Table

Well	Conc. [ng/µl]	Sample Description	Alert	Observations
E1	2.75	6H		

Peak Table

Size [bp]	Calibrated Conc. [ng/µl]	Assigned Conc. [ng/µl]	Peak Molarity [nmol/l]	% Integrated Area	Peak Comment	Observations
25	6.11	-	376	-		Lower Marker
246	2.75	-	17.2	100.00		
1500	6.50	6.50	6.67	-		Upper Marker

F1: 7A

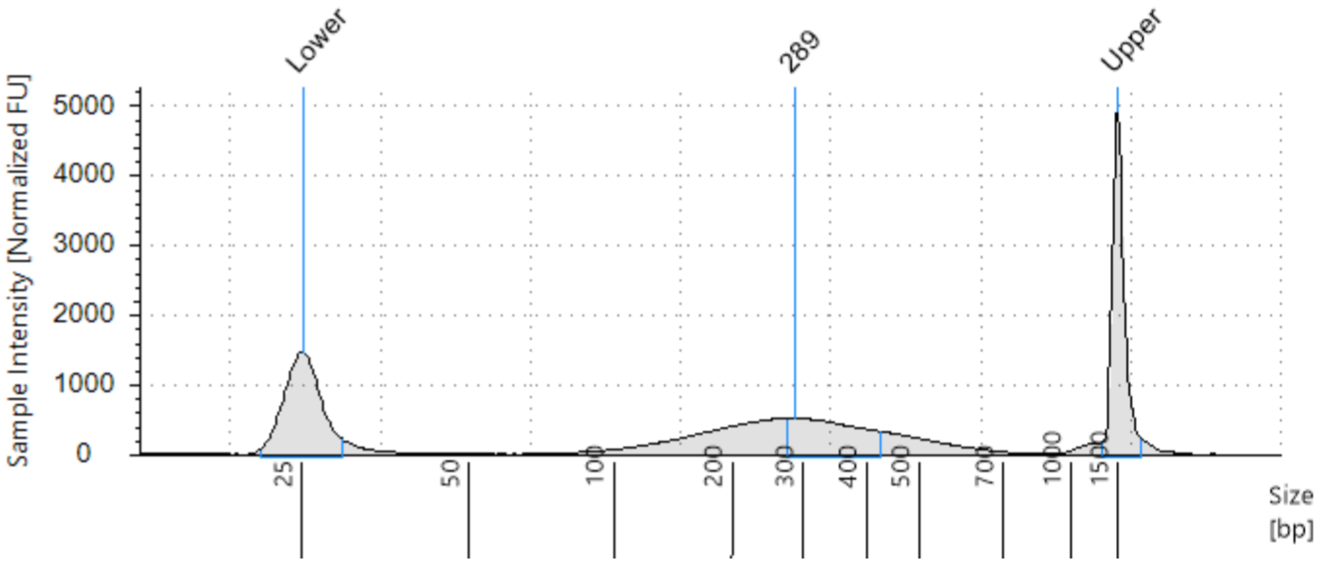

Sample Table

Well	Conc. [ng/µl]	Sample Description	Alert	Observations
F1	3.89	7A		

Peak Table

Size [bp]	Calibrated Conc. [ng/µl]	Assigned Conc. [ng/µl]	Peak Molarity [nmol/l]	% Integrated Area	Peak Comment	Observations
25	5.90	-	363	-		Lower Marker
289	3.89	-	20.7	100.00		
1500	6.50	6.50	6.67	-		Upper Marker

© Agilent Technologies, Inc. 2017

G1: 7B

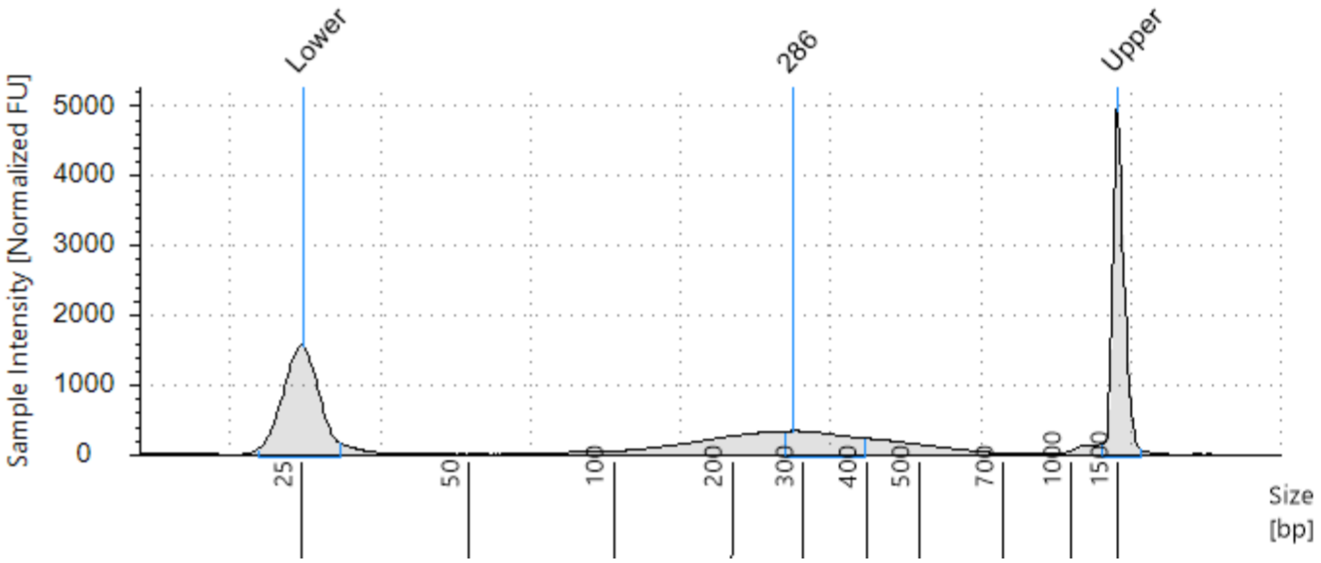

Sample Table

Well	Conc. [ng/µl]	Sample Description	Alert	Observations
G1	2.34	7B		

Peak Table

Size [bp]	Calibrated Conc. [ng/µl]	Assigned Conc. [ng/µl]	Peak Molarity [nmol/l]	% Integrated Area	Peak Comment	Observations
25	6.39	-	393	-		Lower Marker
286	2.34	-	12.6	100.00		
1500	6.50	6.50	6.67	-		Upper Marker

© Agilent Technologies, Inc. 2017

H1: 7C

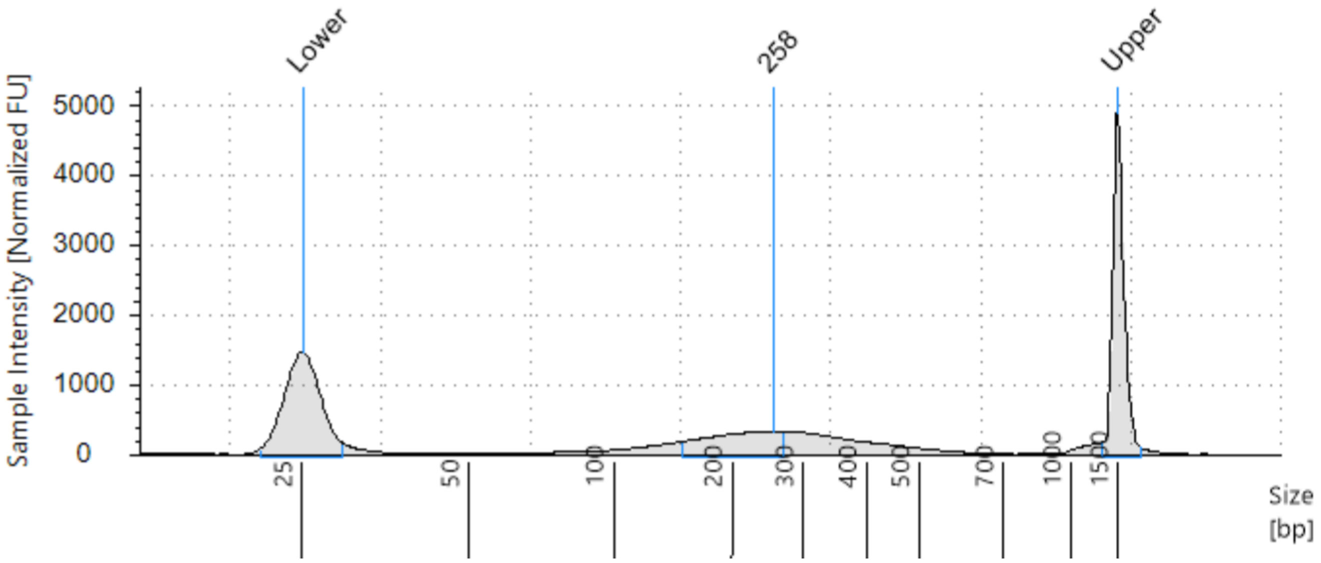

Sample Table

Well	Conc. [ng/µl]	Sample Description	Alert	Observations
H1	2.81	7C		

Peak Table

Size [bp]	Calibrated Conc. [ng/µl]	Assigned Conc. [ng/µl]	Peak Molarity [nmol/l]	% Integrated Area	Peak Comment	Observations
25	6.29	-	387	-		Lower Marker
258	2.81	-	16.8	100.00		
1500	6.50	6.50	6.67	-		Upper Marker

© Agilent Technologies, Inc. 2017

A2: 7D

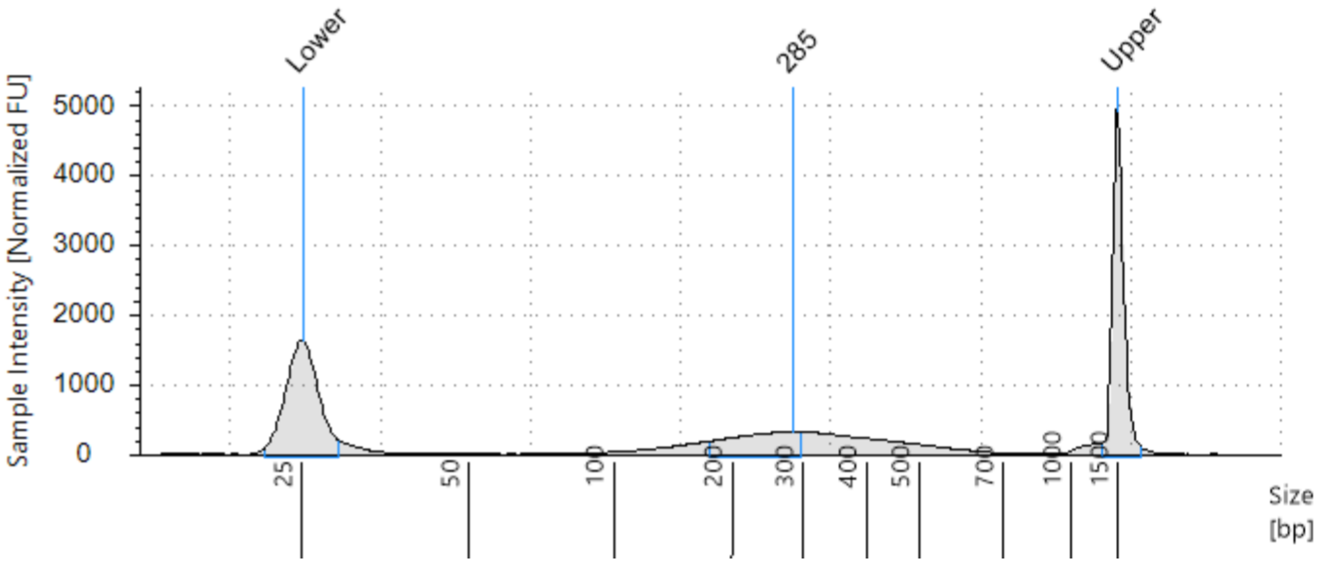

Sample Table

Well	Conc. [ng/µl]	Sample Description	Alert	Observations
A2	2.45	7D		

Peak Table

Size [bp]	Calibrated Conc. [ng/µl]	Assigned Conc. [ng/µl]	Peak Molarity [nmol/l]	% Integrated Area	Peak Comment	Observations
25	6.25	-	384	-		Lower Marker
285	2.45	-	13.2	100.00		
1500	6.50	6.50	6.67	-		Upper Marker

 © Agilent Technologies, Inc. 2017

B2: 7E

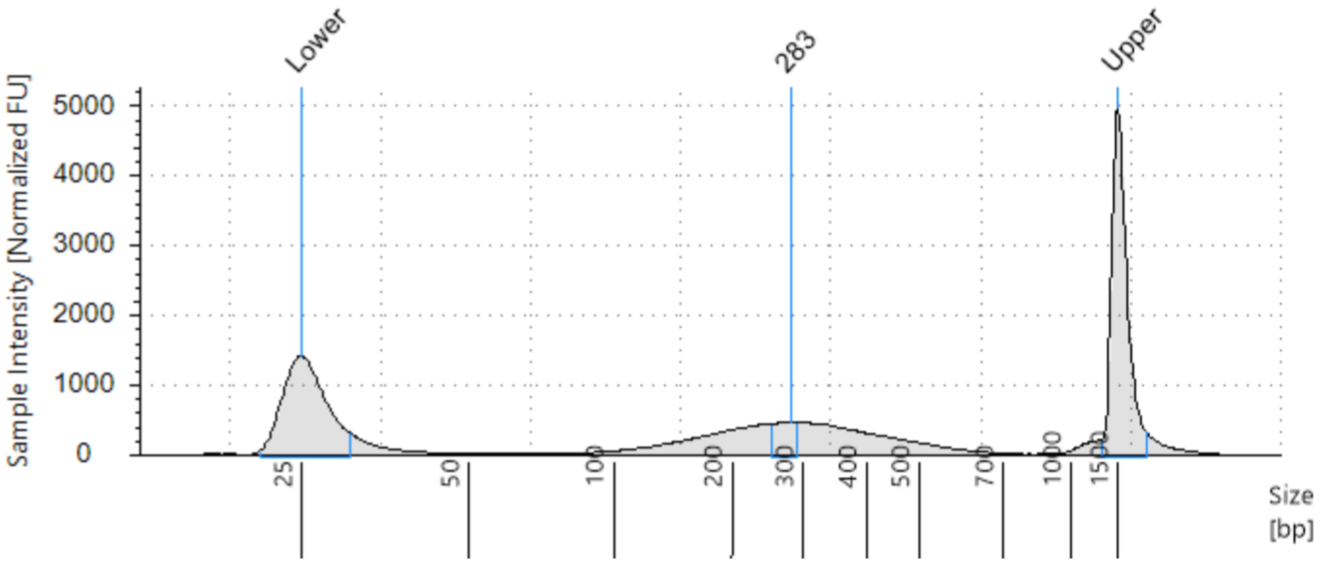

Sample Table

Well	Conc. [ng/µl]	Sample Description	Alert	Observations
B2	0.849	7E		

Peak Table

Size [bp]	Calibrated Conc. [ng/µl]	Assigned Conc. [ng/µl]	Peak Molarity [nmol/l]	% Integrated Area	Peak Comment	Observations
25	5.10	-	314	-		Lower Marker
283	0.849	-	4.62	100.00		
1500	6.50	6.50	6.67	-		Upper Marker

 © Agilent Technologies, Inc. 2017

C2: 7F

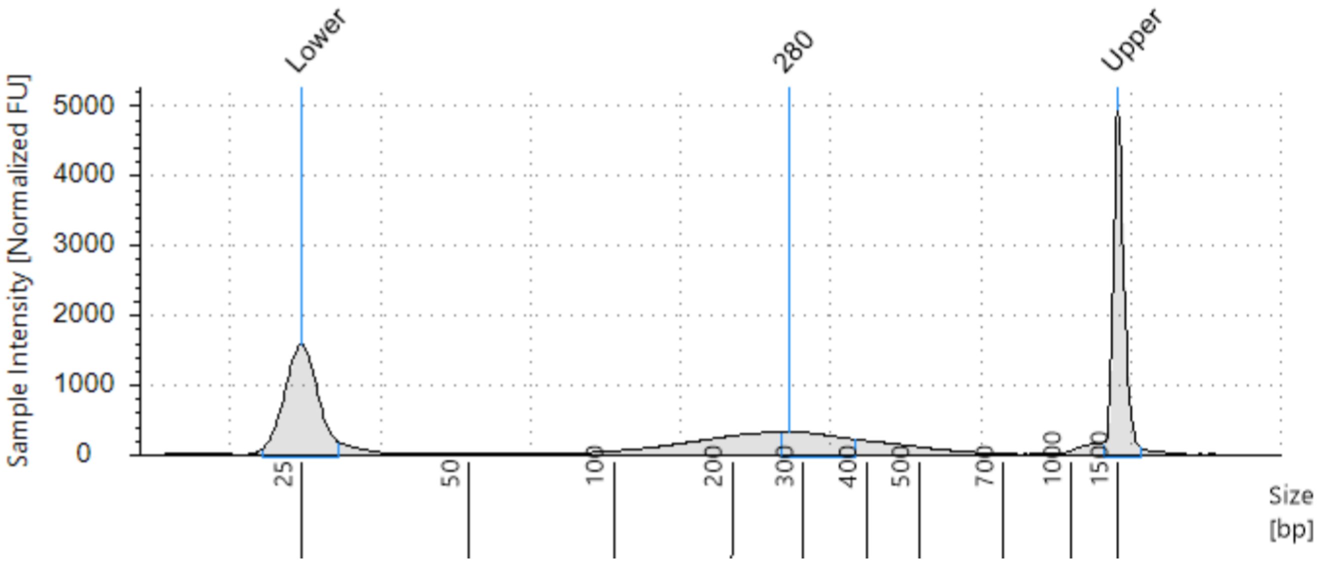

Sample Table

Well	Conc. [ng/µl]	Sample Description	Alert	Observations
C2	2.16	7F		

Peak Table

Size [bp]	Calibrated Conc. [ng/µl]	Assigned Conc. [ng/µl]	Peak Molarity [nmol/l]	% Integrated Area	Peak Comment	Observations
25	6.21	-	382	-		Lower Marker
280	2.16	-	11.9	100.00		
1500	6.50	6.50	6.67	-		Upper Marker

© Agilent Technologies, Inc. 2017

D2: 7G

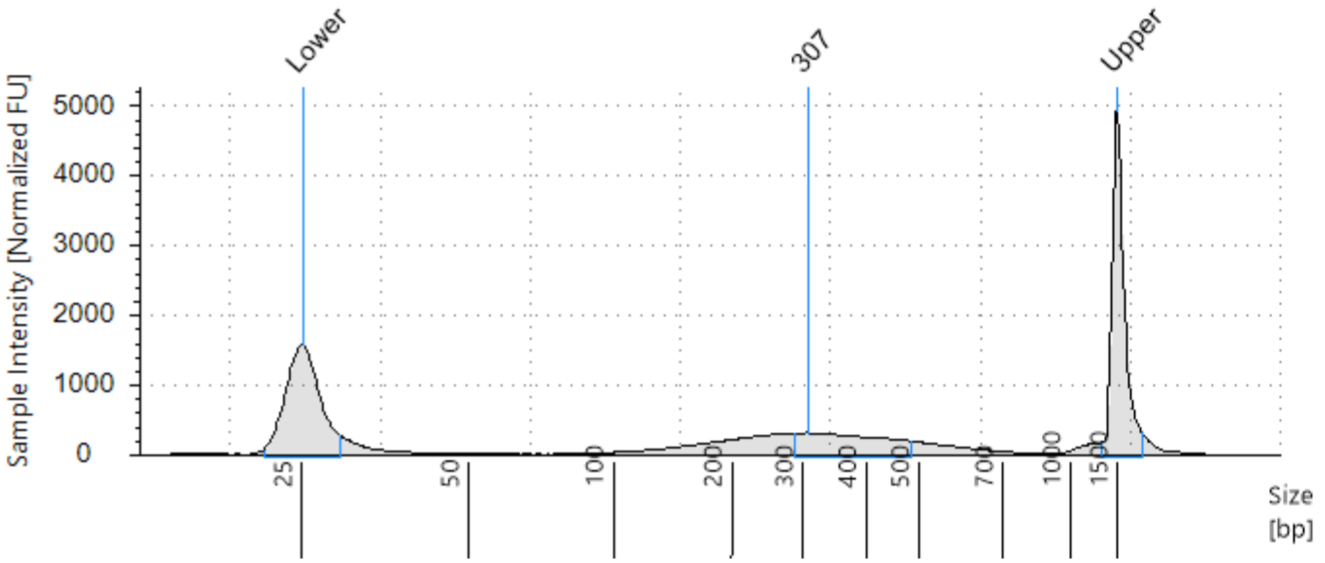

Sample Table

Well	Conc. [ng/µl]	Sample Description	Alert	Observations
D2	2.76	7G		

Peak Table

Size [bp]	Calibrated Conc. [ng/µl]	Assigned Conc. [ng/µl]	Peak Molarity [nmol/l]	% Integrated Area	Peak Comment	Observations
25	5.76	-	354	-		Lower Marker
307	2.76	-	13.9	100.00		
1500	6.50	6.50	6.67	-		Upper Marker

© Agilent Technologies, Inc. 2017

E2: 7H

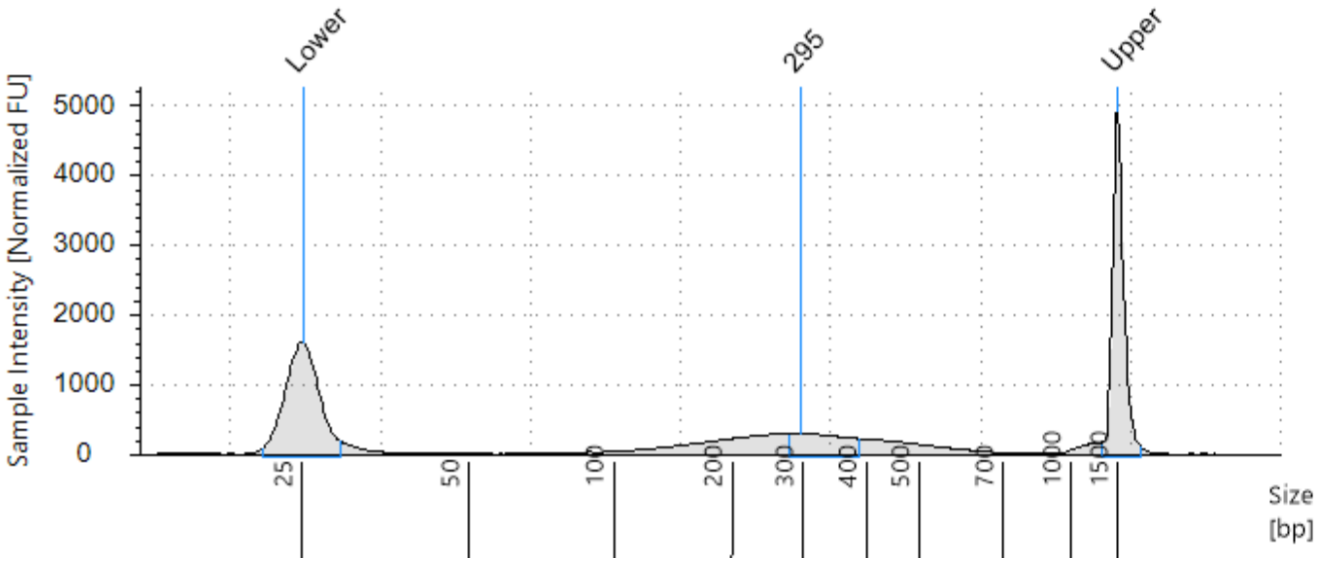

Sample Table

Well	Conc. [ng/µl]	Sample Description	Alert	Observations
E2	1.87	7H		

Peak Table

Size [bp]	Calibrated Conc. [ng/µl]	Assigned Conc. [ng/µl]	Peak Molarity [nmol/l]	% Integrated Area	Peak Comment	Observations
25	6.36	-	392	-		Lower Marker
295	1.87	-	9.77	100.00		
1500	6.50	6.50	6.67	-		Upper Marker

 © Agilent Technologies, Inc. 2017

F2: 8A

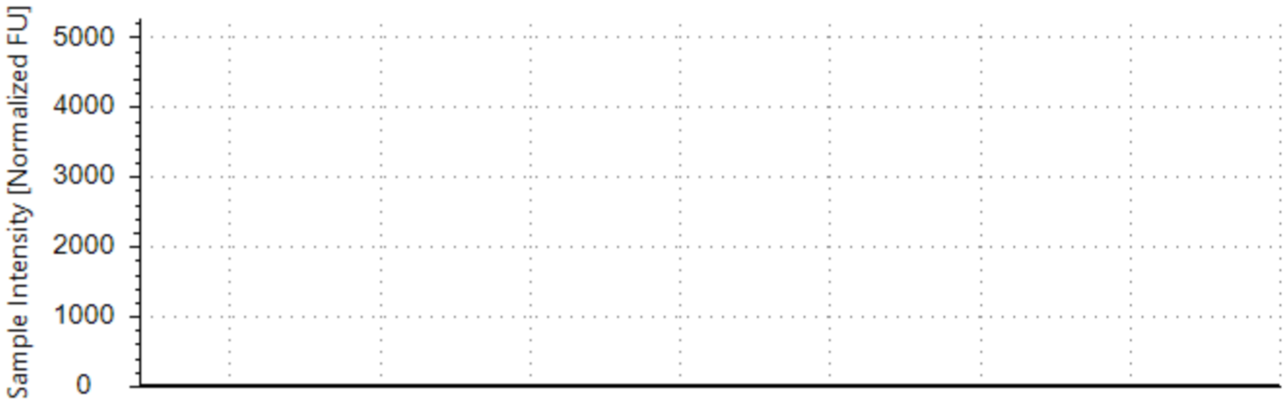

Sample Table

Well	Conc. [ng/µl]	Sample Description	Alert	Observations
F2		8A	▲	Marker(s) not detected

© Agilent Technologies, Inc. 2017

G2: 8B

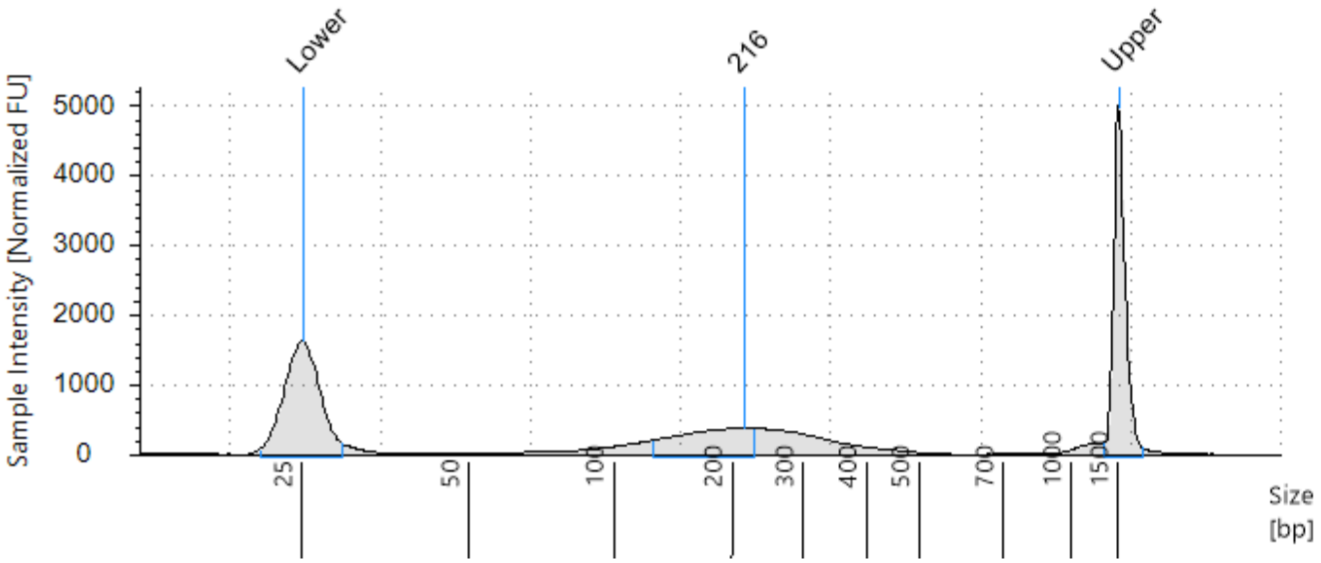

Sample Table

Well	Conc. [ng/µl]	Sample Description	Alert	Observations
G2	3.07	8B		

Peak Table

Size [bp]	Calibrated Conc. [ng/µl]	Assigned Conc. [ng/µl]	Peak Molarity [nmol/l]	% Integrated Area	Peak Comment	Observations
25	6.51	-	401	-		Lower Marker
216	3.07	-	21.8	100.00		
1500	6.50	6.50	6.67	-		Upper Marker

© Agilent Technologies, Inc. 2017

H2: 8C

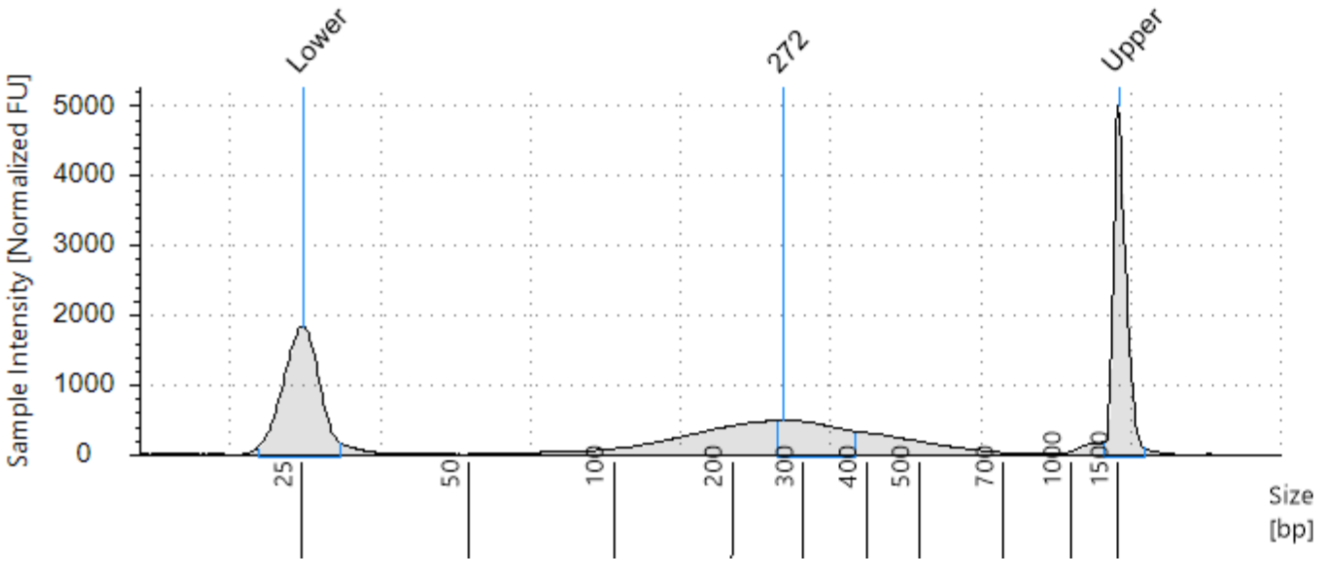

Sample Table

Well	Conc. [ng/µl]	Sample Description	Alert	Observations
H2	2.97	8C		

Peak Table

Size [bp]	Calibrated Conc. [ng/µl]	Assigned Conc. [ng/µl]	Peak Molarity [nmol/l]	% Integrated Area	Peak Comment	Observations
25	6.82	-	420	-		Lower Marker
272	2.97	-	16.8	100.00		
1500	6.50	6.50	6.67	-		Upper Marker

© Agilent Technologies, Inc. 2017

Filename: 2020-07-20-02.Q-S DFB Plus D8-B10 R1.D1000

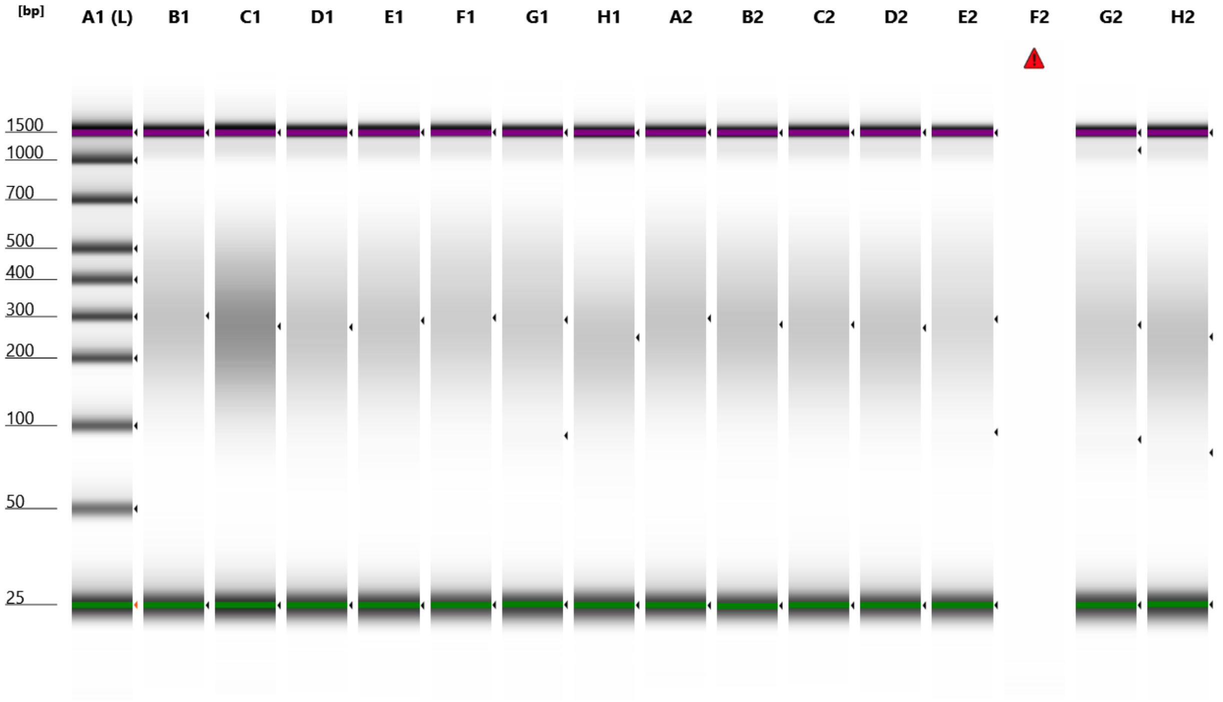

Default image (Contrast 100%)

Sample Info

Well	Conc. [ng/µl]	Sample Description	Alert	Observations
A1	18.4	Ladder		Ladder
B1	5.02	PLus 8D		
C1	4.49	8E		
D1	2.16	8F		
E1	4.15	8G		
F1	3.87	8H		
G1	5.24	9A		
H1	0.598	9B		
A2	3.07	9C		
B2	3.06	9D		
C2	2.88	9E		
D2	4.61	9F		
E2	1.51	9G		
F2		9H	▲	Marker(s) not detected
G2	2.50	10A		
H2	5.03	10 B		

© Agilent Technologies, Inc. 2017

A1: Ladder

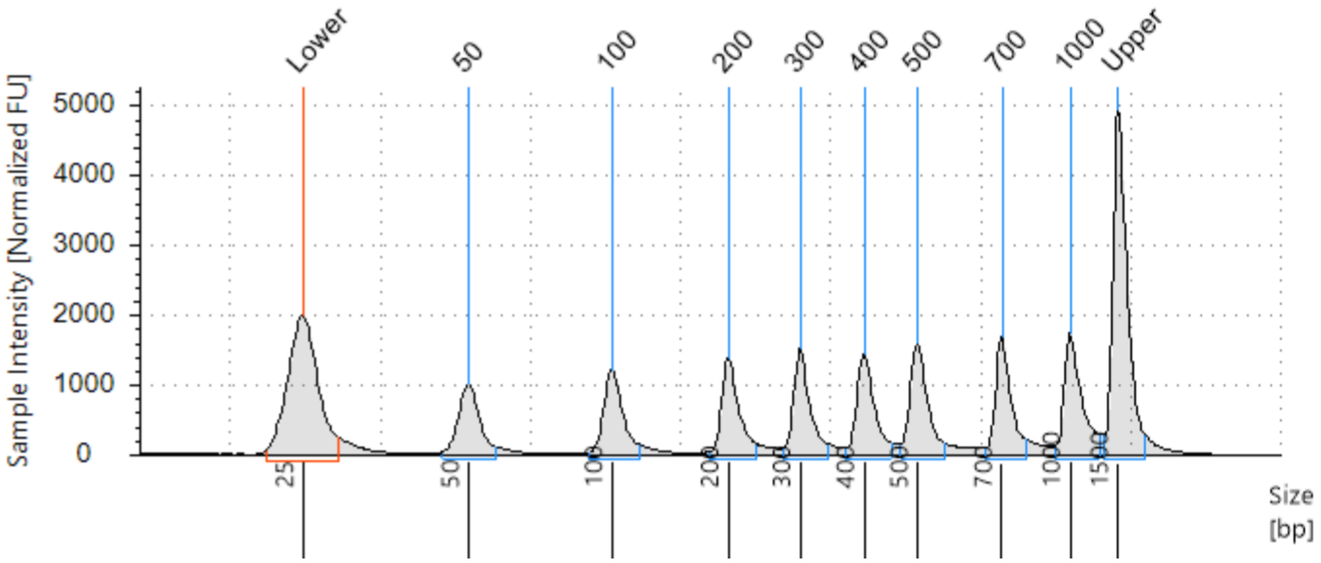

Sample Table

Well	Conc. [ng/µl]	Sample Description	Alert	Observations
A1	18.4	Ladder		Ladder

Peak Table

Size [bp]	Calibrated Conc. [ng/µl]	Assigned Conc. [ng/µl]	Peak Molarity [nmol/l]	% Integrated Area	Peak Comment	Observations
25	5.45	-	335	-		Lower Marker
50	1.93	-	59.3	10.48		
100	2.11	-	32.4	11.47		
200	2.17	-	16.7	11.79		
300	2.24	-	11.5	12.21		
400	2.31	-	8.88	12.56		
500	2.53	-	7.79	13.78		
700	2.36	-	5.19	12.85		
1000	2.73	-	4.20	14.85		
1500	6.50	6.50	6.67	-		Upper Marker

© Agilent Technologies, Inc. 2017

B1: PLus 8D

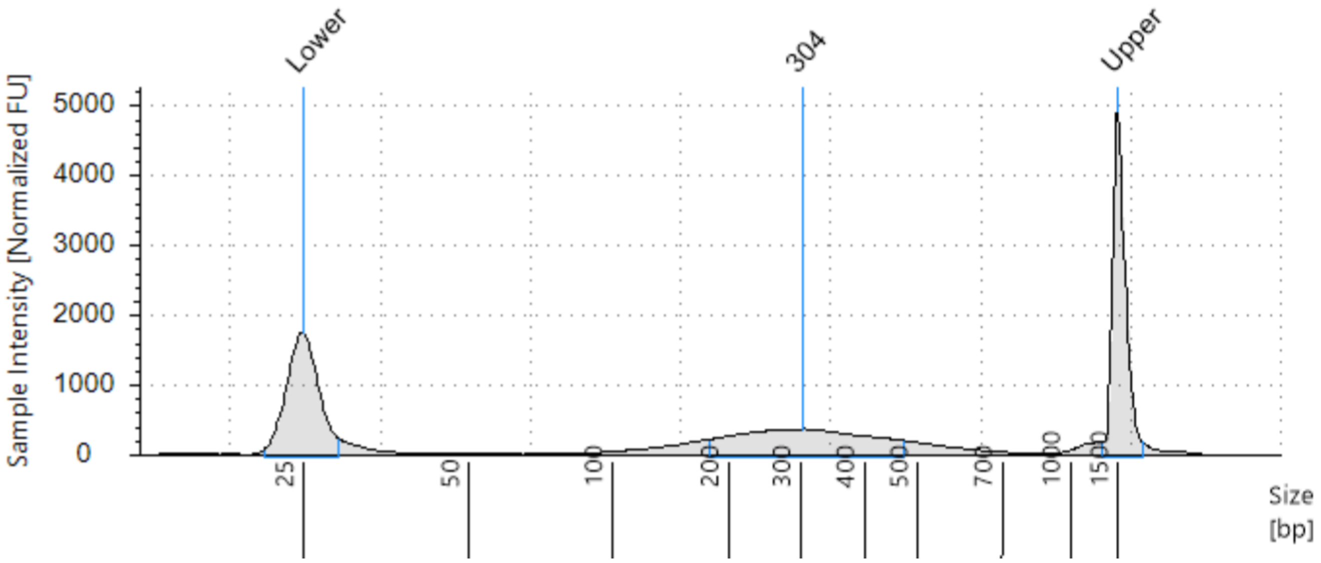

Sample Table

Well	Conc. [ng/µl]	Sample Description	Alert	Observations
B1	5.02	PLus 8D		

Peak Table

Size [bp]	Calibrated Conc. [ng/µl]	Assigned Conc. [ng/µl]	Peak Molarity [nmol/l]	% Integrated Area	Peak Comment	Observations
25	5.77	-	355	-		Lower Marker
304	5.02	-	25.4	100.00		
1500	6.50	6.50	6.67	-		Upper Marker

 © Agilent Technologies, Inc. 2017

C1: 8E

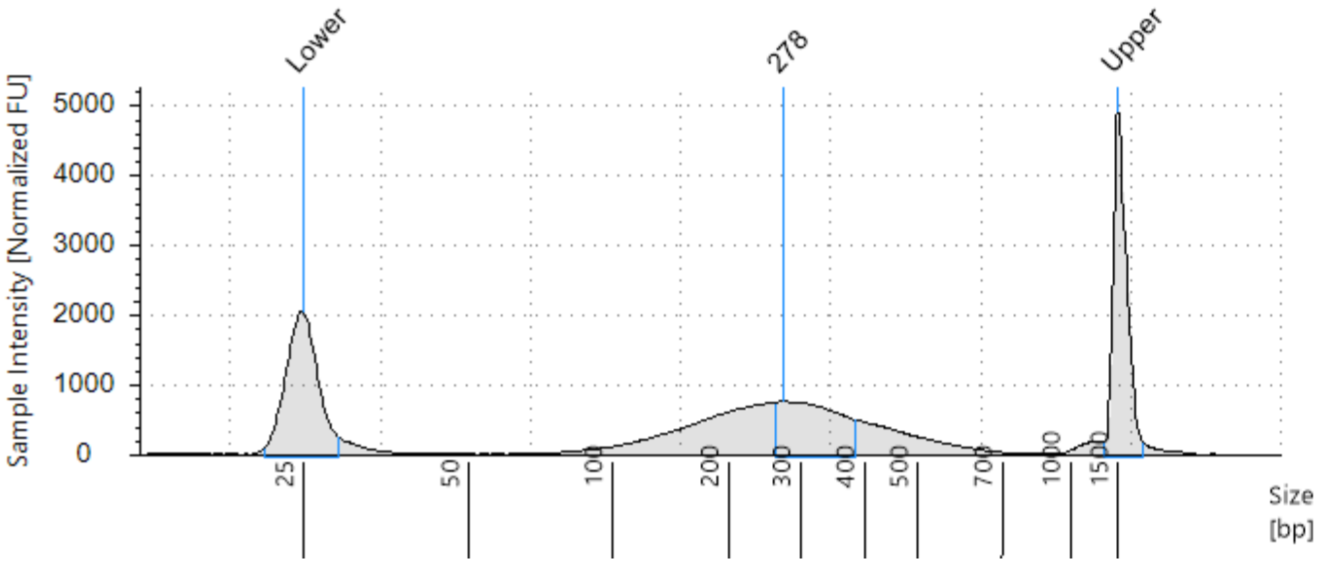

Sample Table

Well	Conc. [ng/µl]	Sample Description	Alert	Observations
C1	4.49	8E		

Peak Table

Size [bp]	Calibrated Conc. [ng/µl]	Assigned Conc. [ng/µl]	Peak Molarity [nmol/l]	% Integrated Area	Peak Comment	Observations
25	6.32	-	389	-		Lower Marker
278	4.49	-	24.9	100.00		
1500	6.50	6.50	6.67	-		Upper Marker

© Agilent Technologies, Inc. 2017

D1: 8F

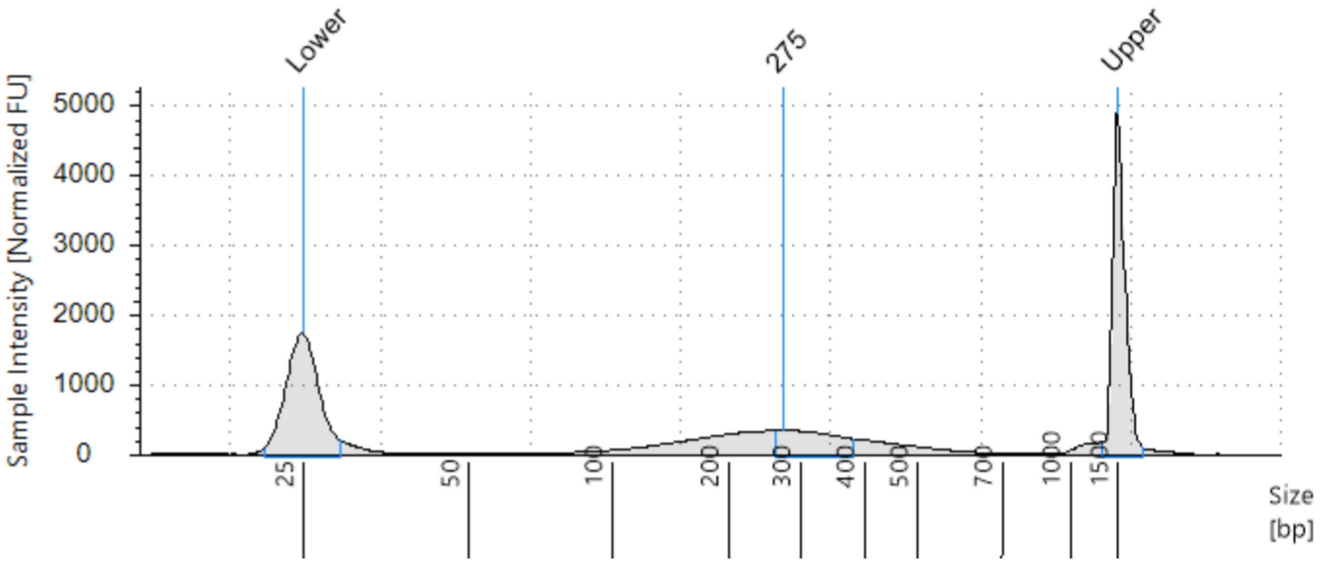

Sample Table

Well	Conc. [ng/µl]	Sample Description	Alert	Observations
D1	2.16	8F		

Peak Table

Size [bp]	Calibrated Conc. [ng/µl]	Assigned Conc. [ng/µl]	Peak Molarity [nmol/l]	% Integrated Area	Peak Comment	Observations
25	6.05	-	372	-		Lower Marker
275	2.16	-	12.1	100.00		
1500	6.50	6.50	6.67	-		Upper Marker

© Agilent Technologies, Inc. 2017

E1: 8G

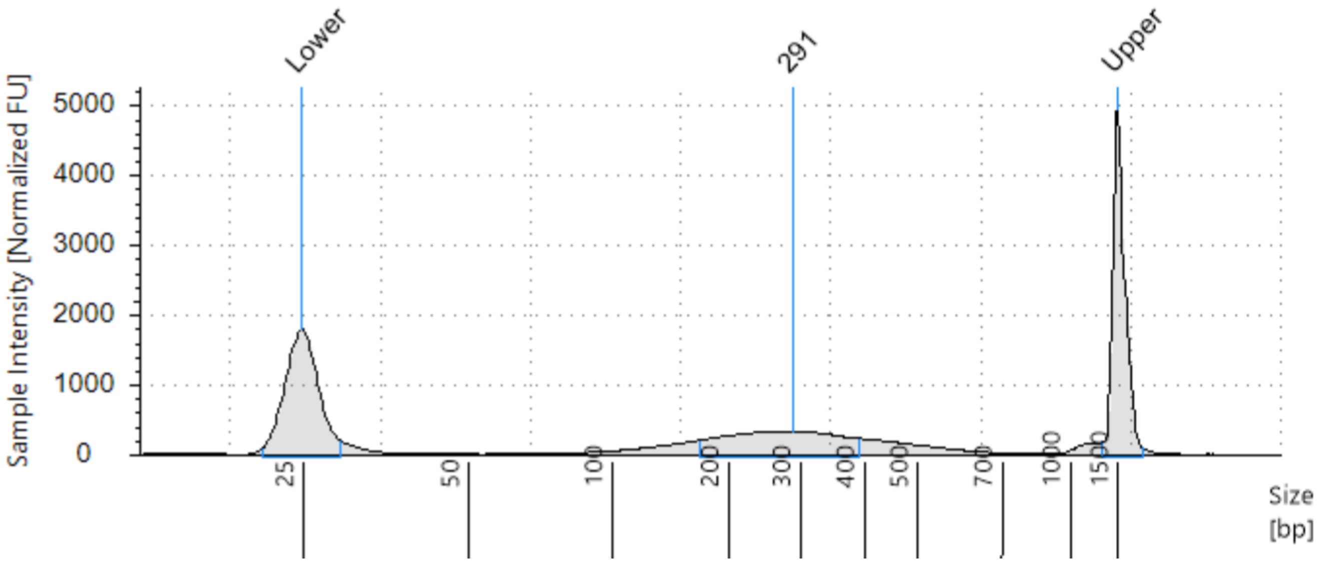

Sample Table

Well	Conc. [ng/µl]	Sample Description	Alert	Observations
E1	4.15	8G		

Peak Table

Size [bp]	Calibrated Conc. [ng/µl]	Assigned Conc. [ng/µl]	Peak Molarity [nmol/l]	% Integrated Area	Peak Comment	Observations
25	6.44	-	397	-		Lower Marker
291	4.15	-	21.9	100.00		
1500	6.50	6.50	6.67	-		Upper Marker

 © Agilent Technologies, Inc. 2017

F1: 8H

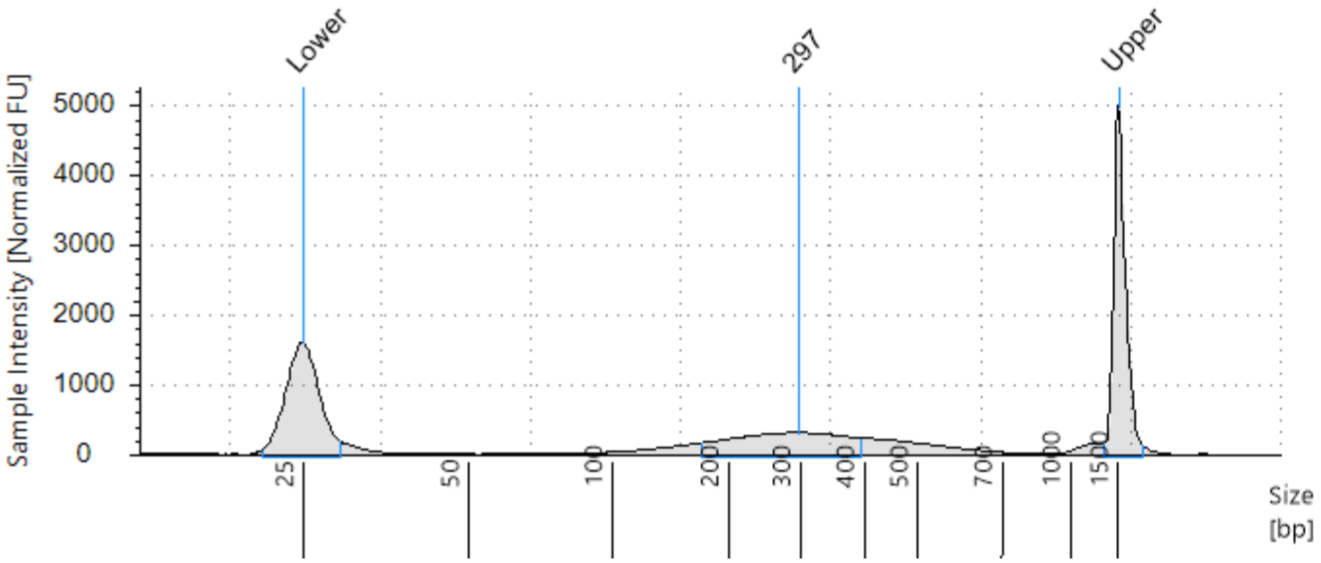

Sample Table

Well	Conc. [ng/µl]	Sample Description	Alert	Observations
F1	3.87	8H		

Peak Table

Size [bp]	Calibrated Conc. [ng/µl]	Assigned Conc. [ng/µl]	Peak Molarity [nmol/l]	% Integrated Area	Peak Comment	Observations
25	5.85	-	360	-		Lower Marker
297	3.87	-	20.0	100.00		
1500	6.50	6.50	6.67	-		Upper Marker

© Agilent Technologies, Inc. 2017

Gl: 9A

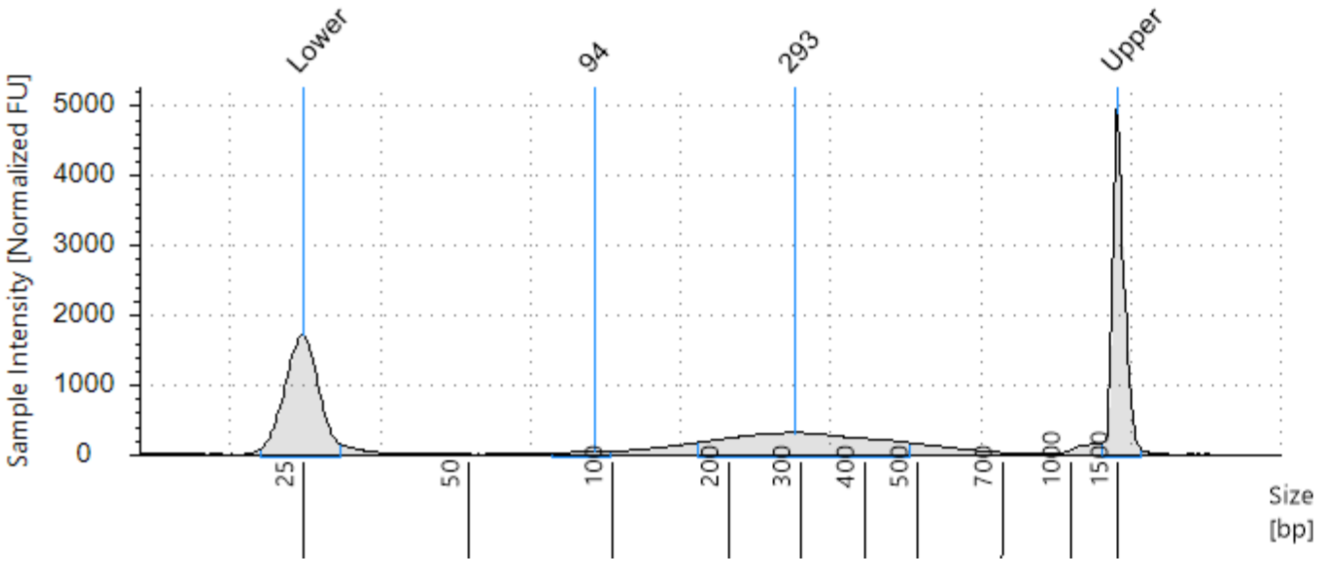

Sample Table

Well	Conc. [ng/µl]	Sample Description	Alert	Observations
Gl	5.24	9A		

Peak Table

Size [bp]	Calibrated Conc. [ng/µl]	Assigned Conc. [ng/µl]	Peak Molarity [nmol/l]	% Integrated Area	Peak Comment	Observations
25	6.51	-	401	-		Lower Marker
94	0.183	-	2.99	3.49		
293	5.06	-	26.6	96.51		
1500	6.50	6.50	6.67	-		Upper Marker

 © Agilent Technologies, Inc. 2017

H1: 9B

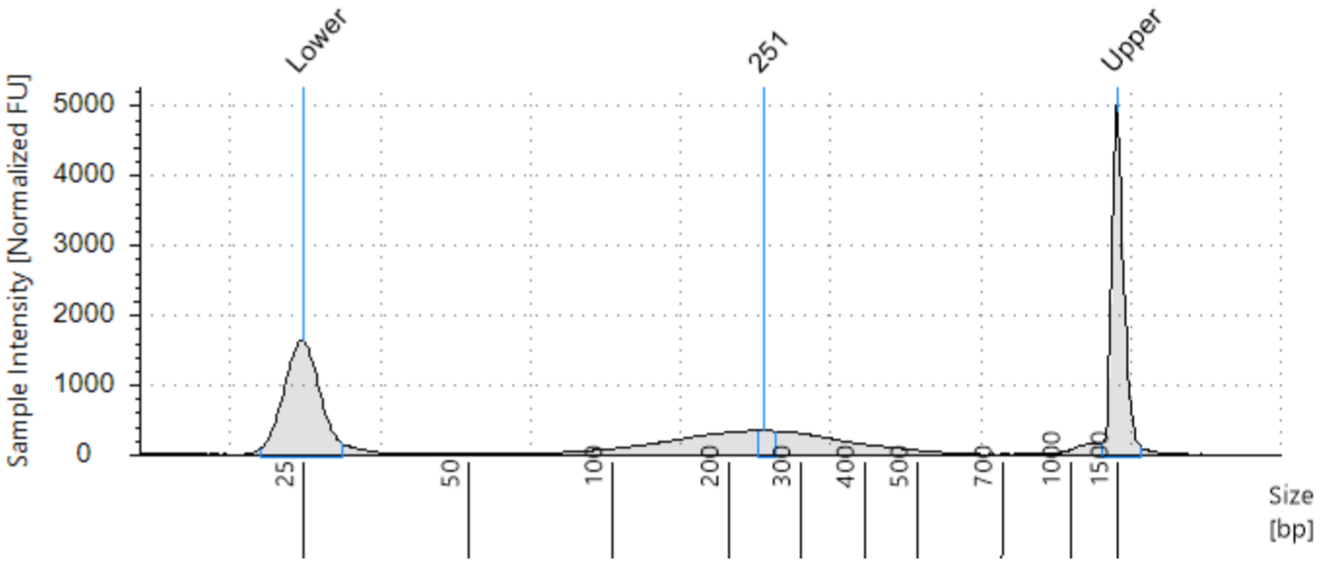

Sample Table

Well	Conc. [ng/µl]	Sample Description	Alert	Observations
H1	0.598	9B		

Peak Table

Size [bp]	Calibrated Conc. [ng/µl]	Assigned Conc. [ng/µl]	Peak Molarity [nmol/l]	% Integrated Area	Peak Comment	Observations
25	6.29	-	387	-		Lower Marker
251	0.598	-	3.67	100.00		
1500	6.50	6.50	6.67	-		Upper Marker

 © Agilent Technologies, Inc. 2017

A2: 9C

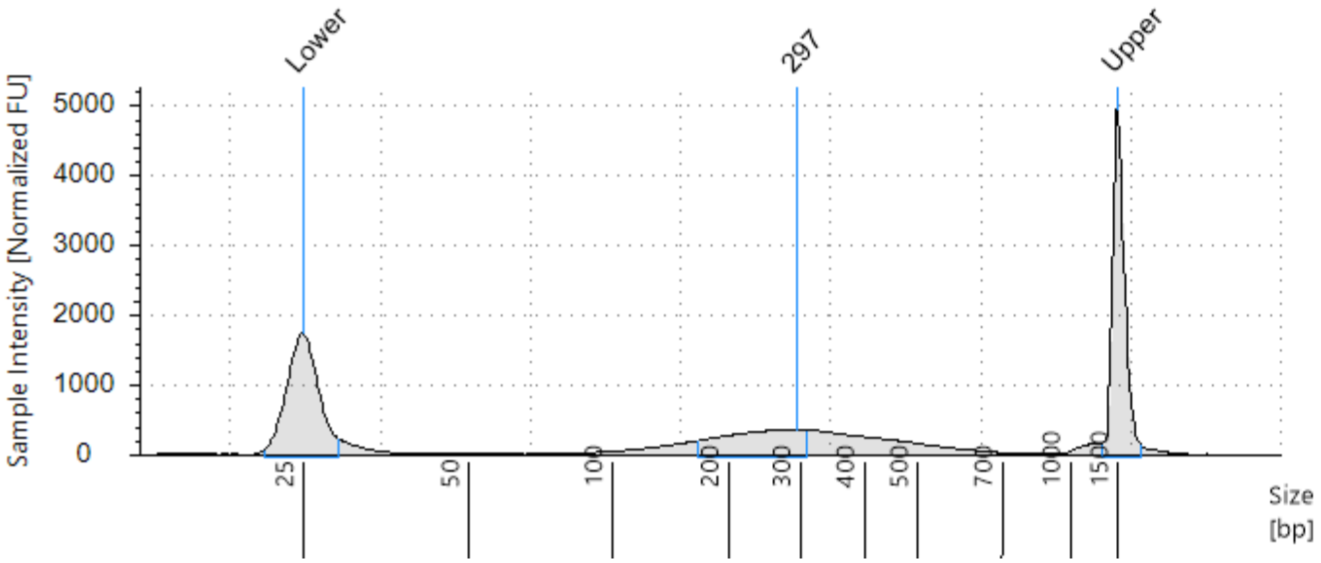

Sample Table

Well	Conc. [ng/µl]	Sample Description	Alert	Observations
A2	3.07	9C		

Peak Table

Size [bp]	Calibrated Conc. [ng/µl]	Assigned Conc. [ng/µl]	Peak Molarity [nmol/l]	% Integrated Area	Peak Comment	Observations
25	5.99	-	369	-		Lower Marker
297	3.07	-	15.9	100.00		
1500	6.50	6.50	6.67	-		Upper Marker

 © Agilent Technologies, Inc. 2017

B2: 9D

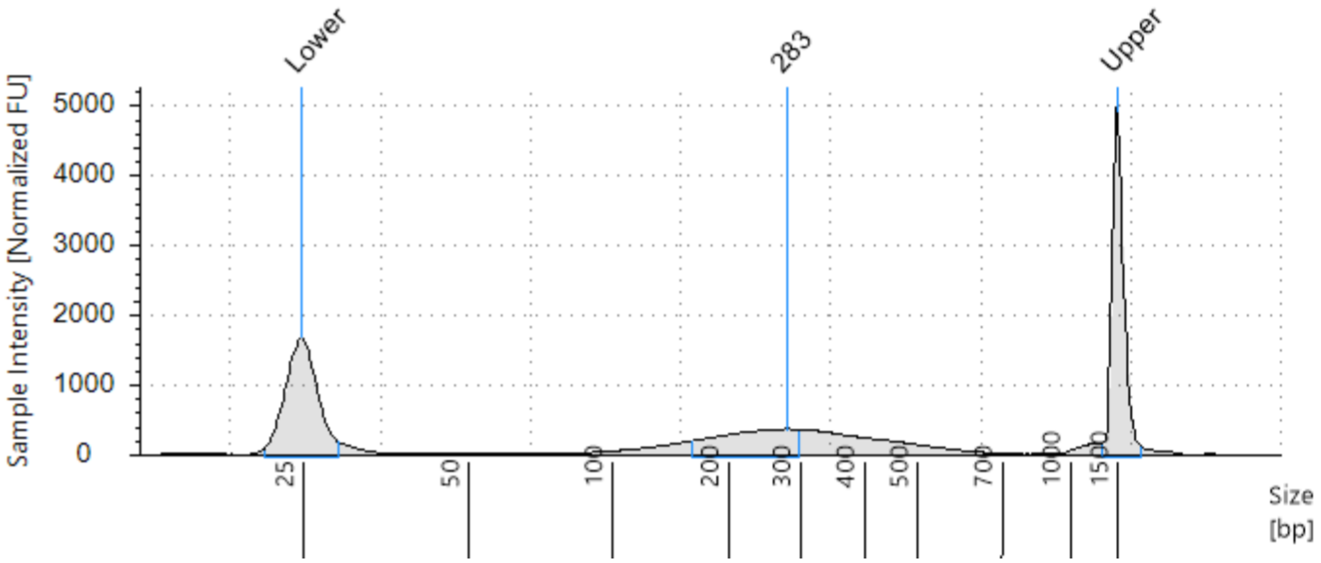

Sample Table

Well	Conc. [ng/µl]	Sample Description	Alert	Observations
B2	3.06	9D		

Peak Table

Size [bp]	Calibrated Conc. [ng/µl]	Assigned Conc. [ng/µl]	Peak Molarity [nmol/l]	% Integrated Area	Peak Comment	Observations
25	5.98	-	368	-		Lower Marker
283	3.06	-	16.7	100.00		
1500	6.50	6.50	6.67	-		Upper Marker

© Agilent Technologies, Inc. 2017

C2: 9E

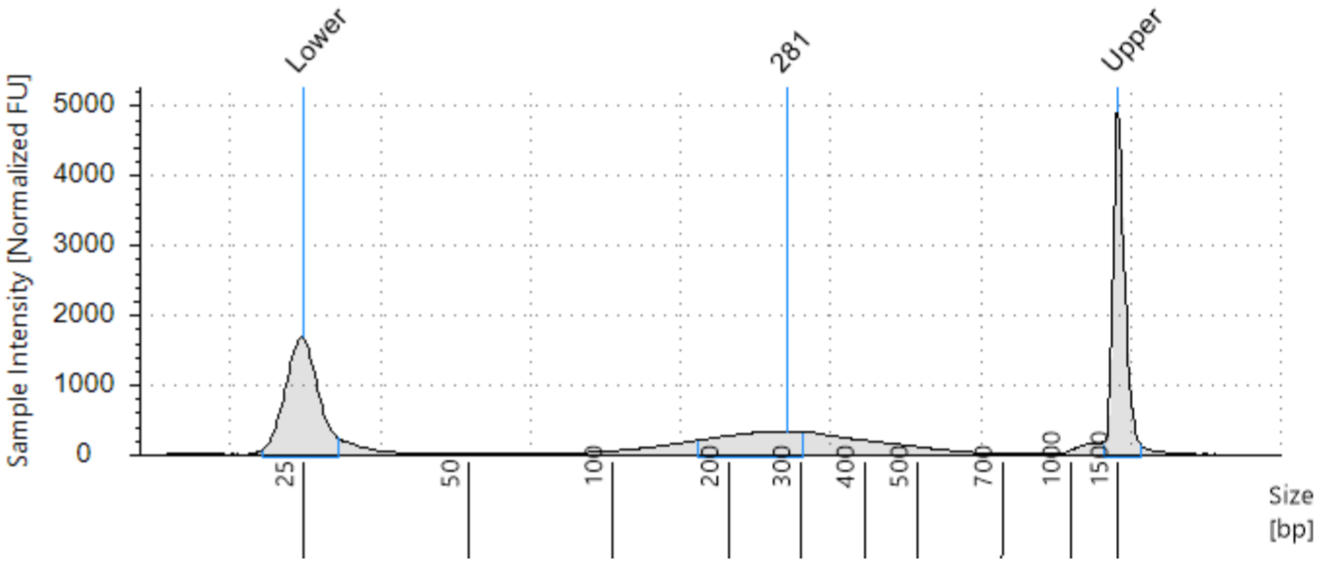

Sample Table

Well	Conc. [ng/µl]	Sample Description	Alert	Observations
C2	2.88	9E		

Peak Table

Size [bp]	Calibrated Conc. [ng/µl]	Assigned Conc. [ng/µl]	Peak Molarity [nmol/l]	% Integrated Area	Peak Comment	Observations
25	6.09	-	375	-		Lower Marker
281	2.88	-	15.8	100.00		
1500	6.50	6.50	6.67	-		Upper Marker

 © Agilent Technologies, Inc. 2017

D2: 9F

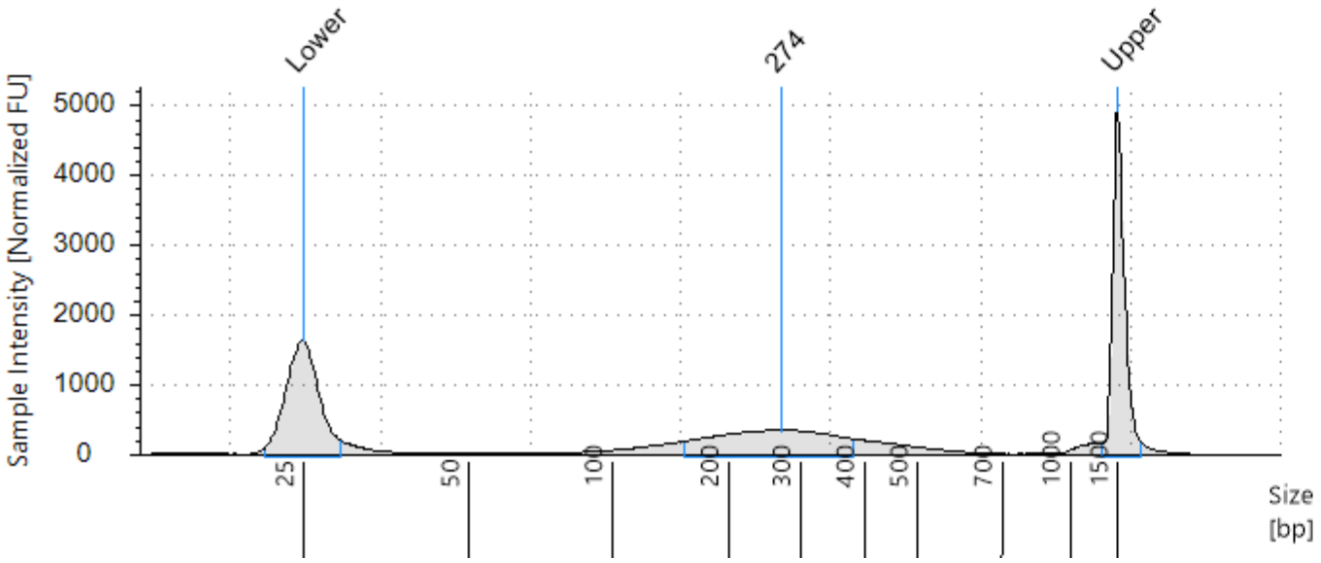

Sample Table

Well	Conc. [ng/µl]	Sample Description	Alert	Observations
D2	4.61	9F		

Peak Table

Size [bp]	Calibrated Conc. [ng/µl]	Assigned Conc. [ng/µl]	Peak Molarity [nmol/l]	% Integrated Area	Peak Comment	Observations
25	5.92	-	365	-		Lower Marker
274	4.61	-	25.9	100.00		
1500	6.50	6.50	6.67	-		Upper Marker

© Agilent Technologies, Inc. 2017

E2: 9G

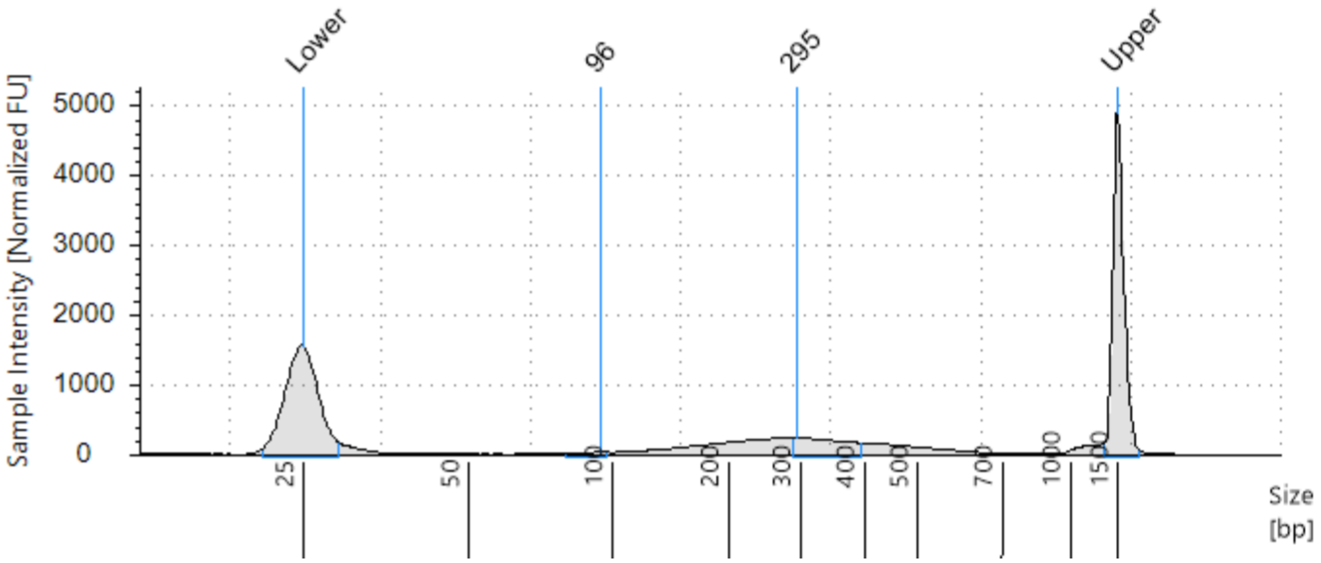

Sample Table

Well	Conc. [ng/µl]	Sample Description	Alert	Observations
E2	1.51	9G		

Peak Table

Size [bp]	Calibrated Conc. [ng/µl]	Assigned Conc. [ng/µl]	Peak Molarity [nmol/l]	% Integrated Area	Peak Comment	Observations
25	6.18	-	380	-		Lower Marker
96	0.0936	-	1.49	6.21		
295	1.41	-	7.37	93.79		
1500	6.50	6.50	6.67	-		Upper Marker

© Agilent Technologies, Inc. 2017

F2: 9H

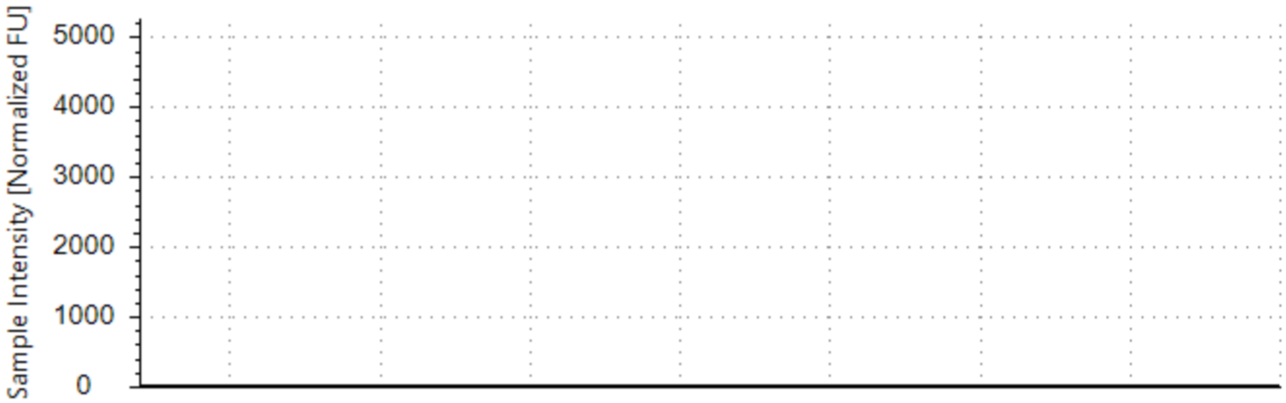

Sample Table

Well	Conc. [ng/µl]	Sample Description	Alert	Observations
F2		9H	▲	Marker(s) not detected

 © Agilent Technologies, Inc. 2017

G2: 10A

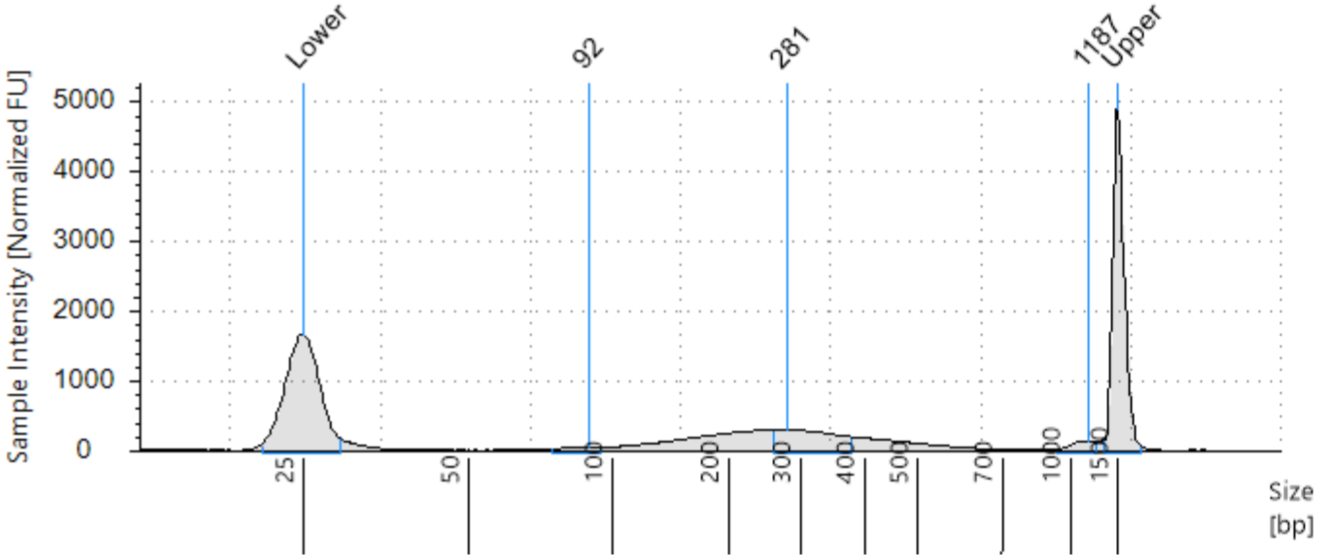

Sample Table

Well	Conc. [ng/µl]	Sample Description	Alert	Observations
G2	2.50	10A		

Peak Table

Size [bp]	Calibrated Conc. [ng/µl]	Assigned Conc. [ng/µl]	Peak Molarity [nmol/l]	% Integrated Area	Peak Comment	Observations
25	6.60	-	406	-		Lower Marker
92	0.165	-	2.76	6.59		
281	1.98	-	10.9	79.41		
1187	0.350	-	0.454	14.00		
1500	6.50	6.50	6.67	-		Upper Marker

© Agilent Technologies, Inc. 2017

H2: 10 B

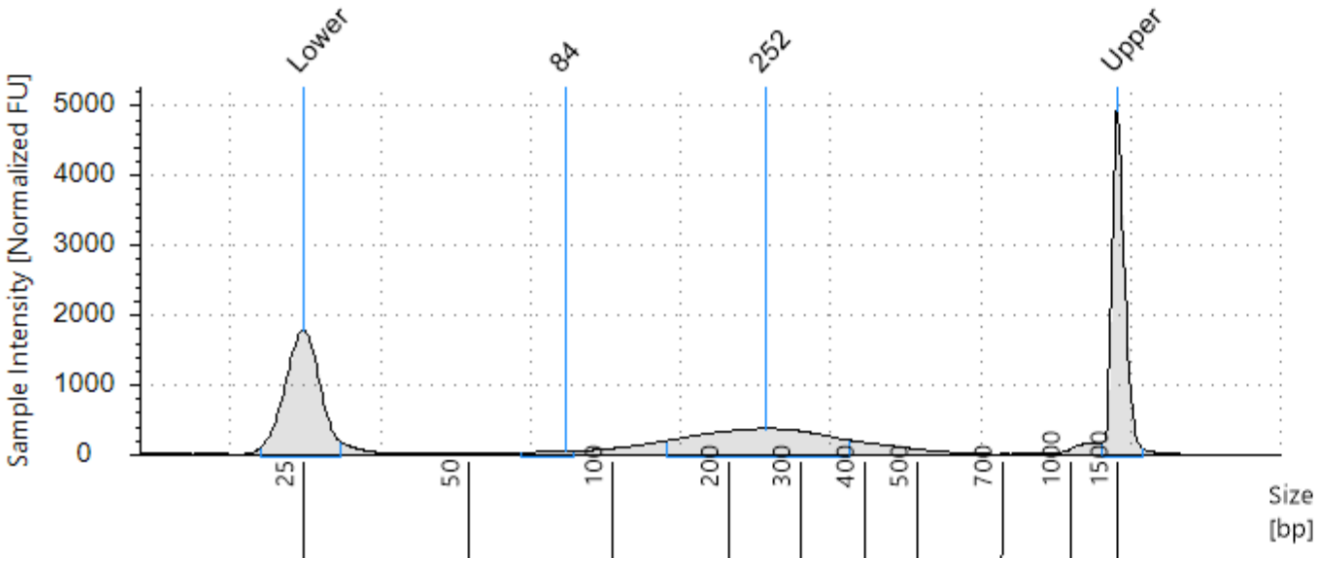

Sample Table

Well	Conc. [ng/µl]	Sample Description	Alert	Observations
H2	5.03	10 B		

Peak Table

Size [bp]	Calibrated Conc. [ng/µl]	Assigned Conc. [ng/µl]	Peak Molarity [nmol/l]	% Integrated Area	Peak Comment	Observations
25	6.42	-	395	-		Lower Marker
84	0.159	-	2.92	3.16		
252	4.87	-	29.8	96.84		
1500	6.50	6.50	6.67	-		Upper Marker

© Agilent Technologies, Inc. 2017

Filename: 2020-08-21-02- Q-S DFB plus C10- A12 R1.D1000

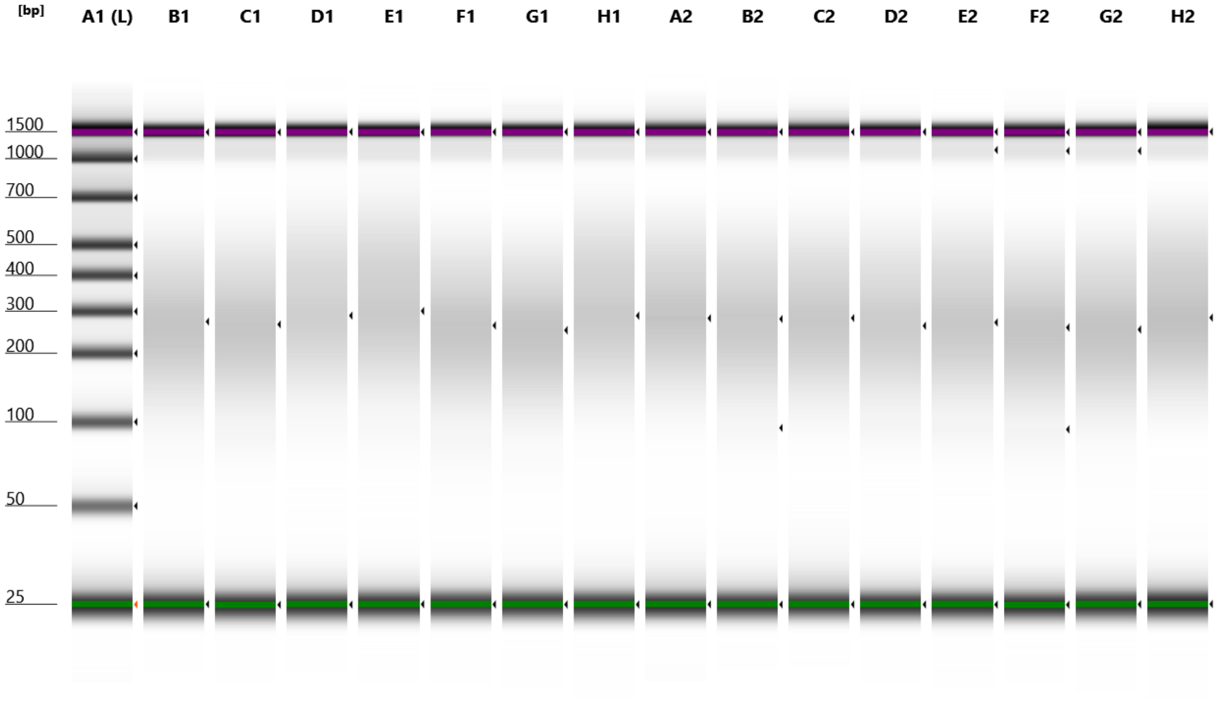

Default image (Contrast 100%)

Sample Info

Well	Conc. [ng/µl]	Sample Description	Alert	Observations
A1	19.6	Ladder		Ladder
B1	2.02	PLUS C10 -Q-SONICA 96 WELL PLATE		
C1	2.48	P-D10		
D1	2.09	P-E10		
E1	1.41	P-F10		
F1	2.19	P-G10		
G1	4.99	P-H10		
H1	5.17	P-A11		
A2	2.42	P-B11		
B2	2.03	P-C11		
C2	4.73	P-D11		
D2	2.22	P-E11		
E2	0.940	P-F11		
F2	1.05	P-G11		
G2	0.867	P-H11		
H2	3.28	P-A12		

© Agilent Technologies, Inc. 2017

A1: Ladder

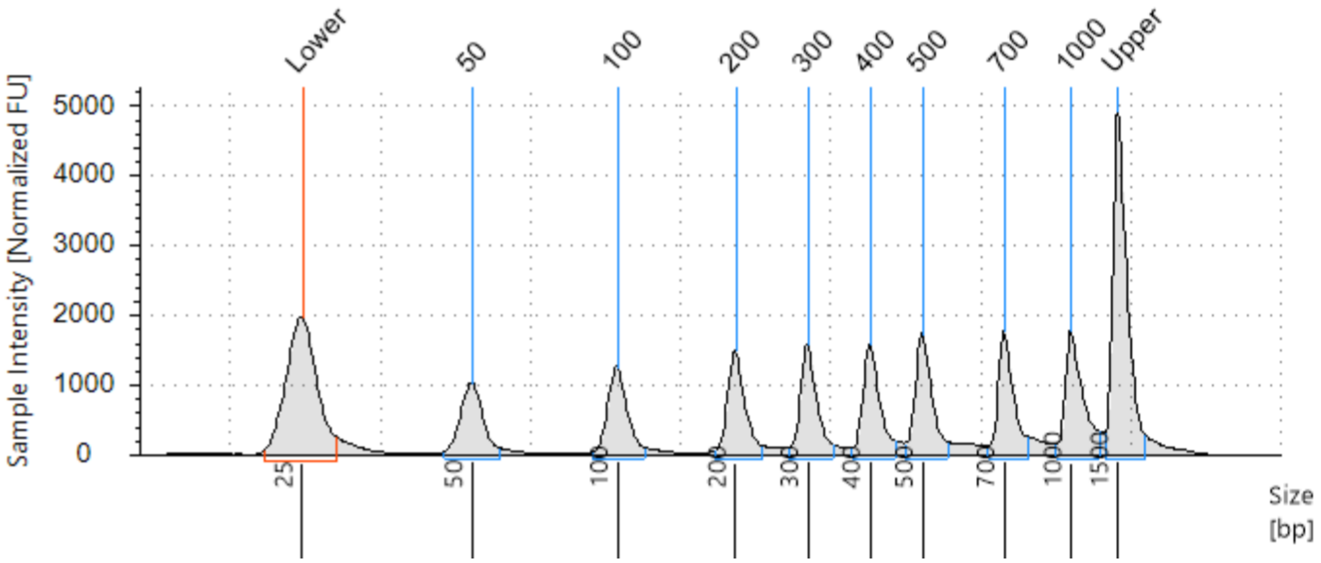

Sample Table

Well	Conc. [ng/µl]	Sample Description	Alert	Observations
A1	19.6	Ladder		Ladder

Peak Table

Size [bp]	Calibrated Conc. [ng/µl]	Assigned Conc. [ng/µl]	Peak Molarity [nmol/l]	% Integrated Area	Peak Comment	Observations
25	5.55	-	342	-		Lower Marker
50	2.11	-	64.8	10.75		
100	2.27	-	34.9	11.59		
200	2.37	-	18.2	12.08		
300	2.39	-	12.2	12.18		
400	2.45	-	9.41	12.50		
500	2.65	-	8.15	13.53		
700	2.50	-	5.49	12.76		
1000	2.86	-	4.40	14.61		
1500	6.50	6.50	6.67	-		Upper Marker

© Agilent Technologies, Inc. 2017

B1: PLUS C10 -Q-SONICA 96 WELL PLATE

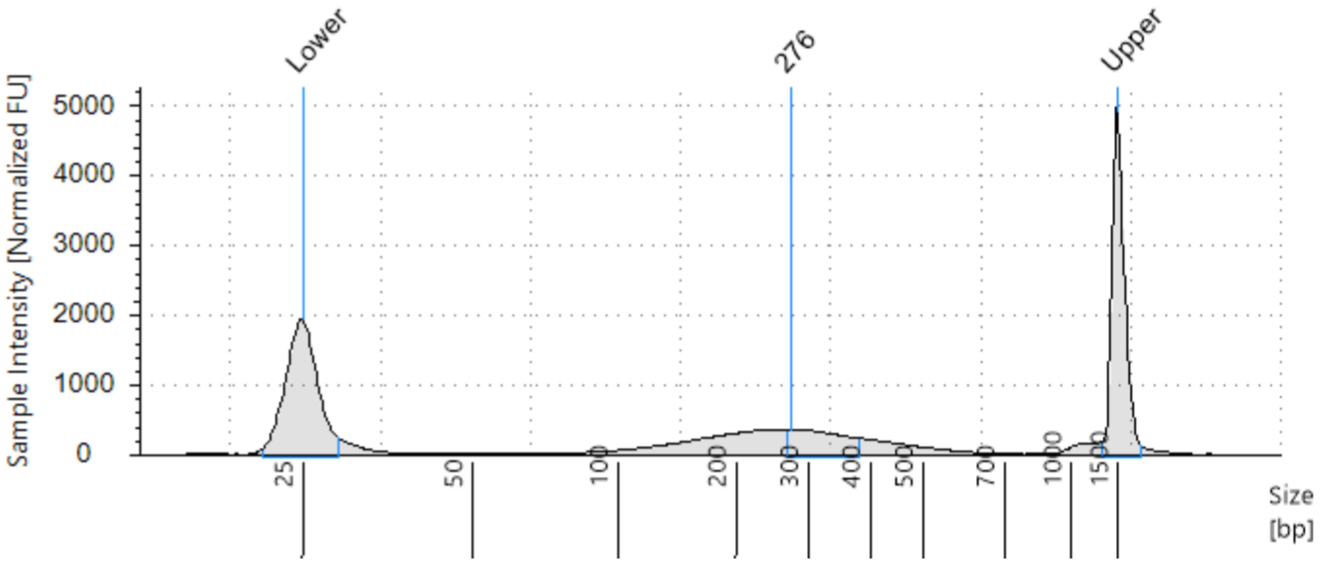

Sample Table

Well	Conc. [ng/µl]	Sample Description	Alert	Observations
B1	2.02	PLUS C10 -Q-SONICA 96 WELL PLATE		

Peak Table

Size [bp]	Calibrated Conc. [ng/µl]	Assigned Conc. [ng/µl]	Peak Molarity [nmol/l]	% Integrated Area	Peak Comment	Observations
25	6.40	-	394	-		Lower Marker
276	2.02	-	11.2	100.00		
1500	6.50	6.50	6.67	-		Upper Marker

© Agilent Technologies, Inc. 2017

C1: P-D10

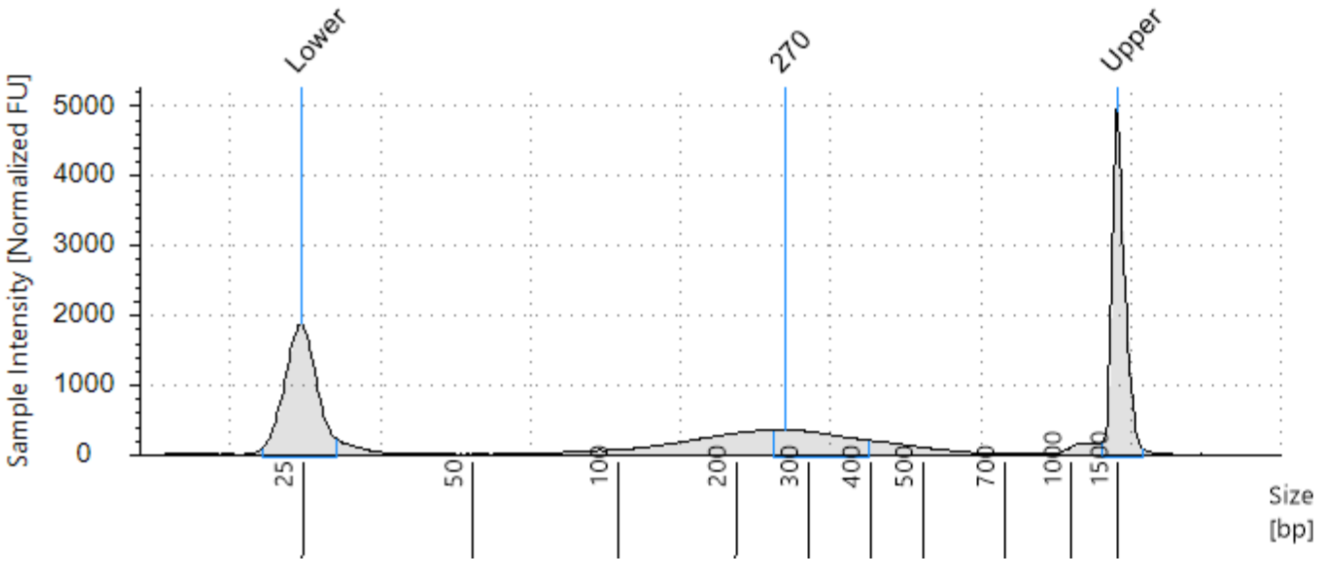

Sample Table

Well	Conc. [ng/µl]	Sample Description	Alert	Observations
C1	2.48	P-D10		

Peak Table

Size [bp]	Calibrated Conc. [ng/µl]	Assigned Conc. [ng/µl]	Peak Molarity [nmol/l]	% Integrated Area	Peak Comment	Observations
25	6.15	-	378	-		Lower Marker
270	2.48	-	14.2	100.00		
1500	6.50	6.50	6.67	-		Upper Marker

 © Agilent Technologies, Inc. 2017

D1: P-E10

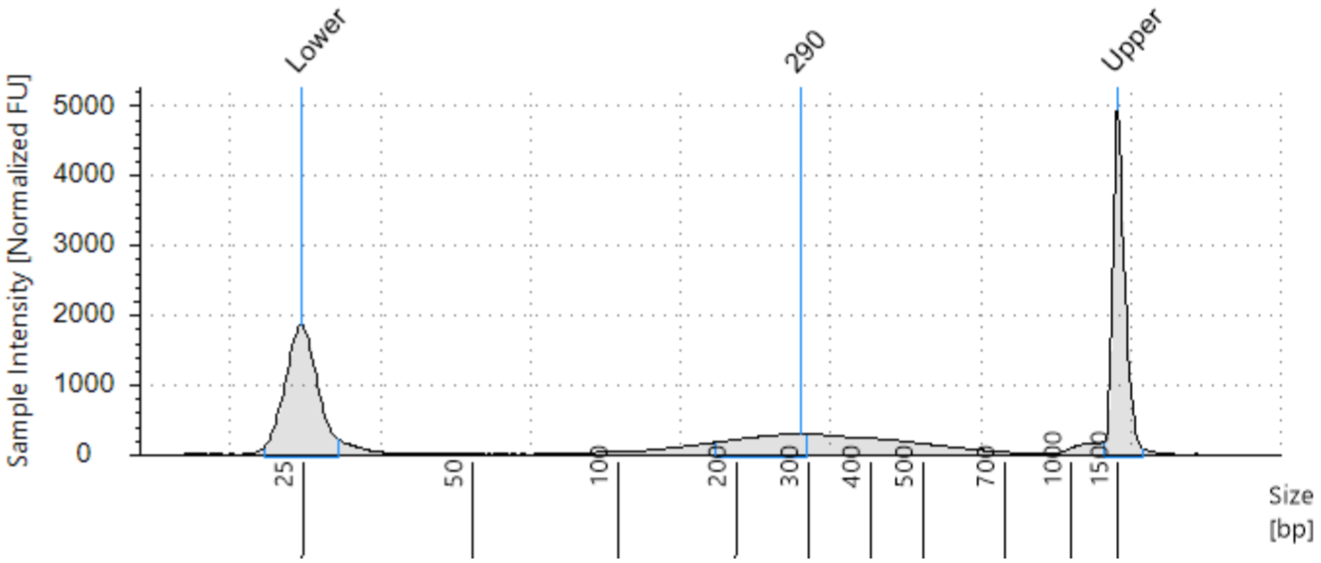

Sample Table

Well	Conc. [ng/µl]	Sample Description	Alert	Observations
D1	2.09	P-E10		

Peak Table

Size [bp]	Calibrated Conc. [ng/µl]	Assigned Conc. [ng/µl]	Peak Molarity [nmol/l]	% Integrated Area	Peak Comment	Observations
25	6.48	-	399	-		Lower Marker
290	2.09	-	11.1	100.00		
1500	6.50	6.50	6.67	-		Upper Marker

© Agilent Technologies, Inc. 2017

E1: P-F10

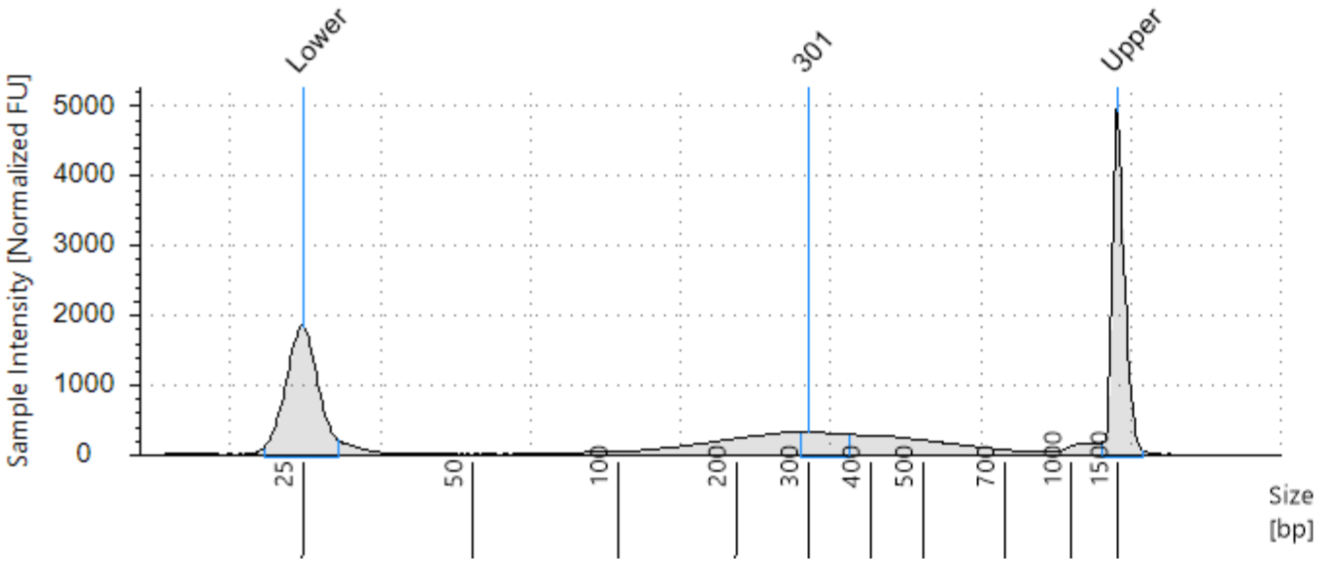

Sample Table

Well	Conc. [ng/µl]	Sample Description	Alert	Observations
E1	1.41	P-F10		

Peak Table

Size [bp]	Calibrated Conc. [ng/µl]	Assigned Conc. [ng/µl]	Peak Molarity [nmol/l]	% Integrated Area	Peak Comment	Observations
25	6.30	-	388	-		Lower Marker
301	1.41	-	7.18	100.00		
1500	6.50	6.50	6.67	-		Upper Marker

F1: P-G10

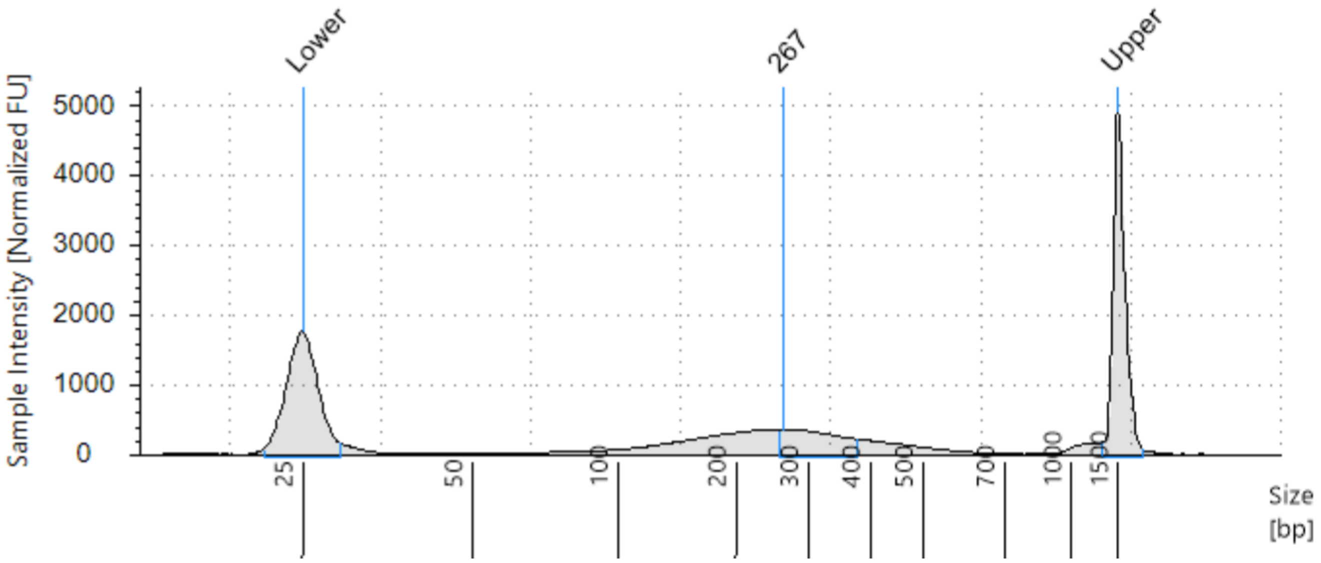

Sample Table

Well	Conc. [ng/µl]	Sample Description	Alert	Observations
F1	2.19	P-G10		

Peak Table

Size [bp]	Calibrated Conc. [ng/µl]	Assigned Conc. [ng/µl]	Peak Molarity [nmol/l]	% Integrated Area	Peak Comment	Observations
25	6.17	-	380	-		Lower Marker
267	2.19	-	12.6	100.00		
1500	6.50	6.50	6.67	-		Upper Marker

© Agilent Technologies, Inc. 2017

G1: P-H10

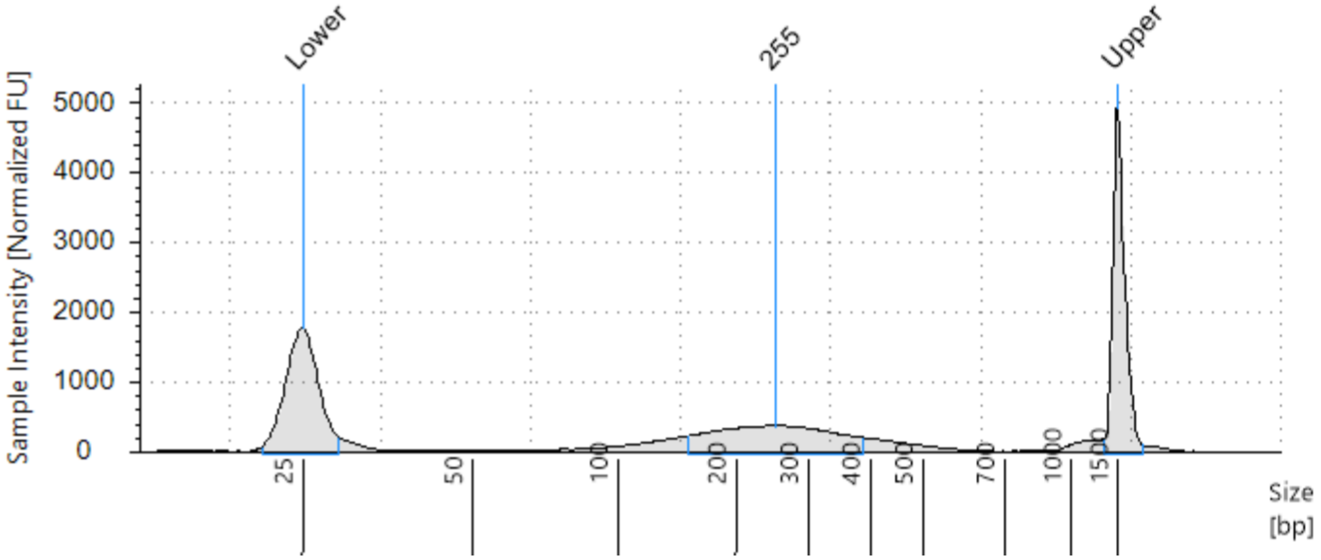

Sample Table

Well	Conc. [ng/µl]	Sample Description	Alert	Observations
G1	4.99	P-H10		

Peak Table

Size [bp]	Calibrated Conc. [ng/µl]	Assigned Conc. [ng/µl]	Peak Molarity [nmol/l]	% Integrated Area	Peak Comment	Observations
25	6.37	-	392	-		Lower Marker
255	4.99	-	30.1	100.00		
1500	6.50	6.50	6.67	-		Upper Marker

© Agilent Technologies, Inc. 2017

H1: P-A11

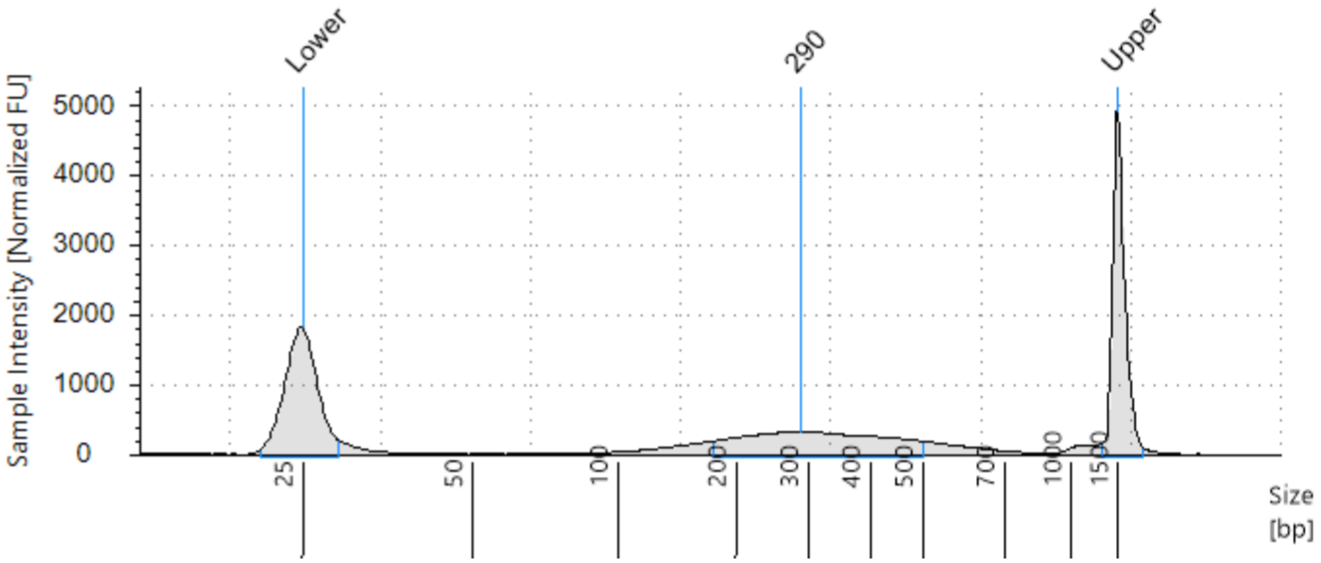

Sample Table

Well	Conc. [ng/µl]	Sample Description	Alert	Observations
H1	5.17	P-A11		

Peak Table

Size [bp]	Calibrated Conc. [ng/µl]	Assigned Conc. [ng/µl]	Peak Molarity [nmol/l]	% Integrated Area	Peak Comment	Observations
25	6.45	-	397	-		Lower Marker
290	5.17	-	27.5	100.00		
1500	6.50	6.50	6.67	-		Upper Marker

© Agilent Technologies, Inc. 2017

A2: P-B11

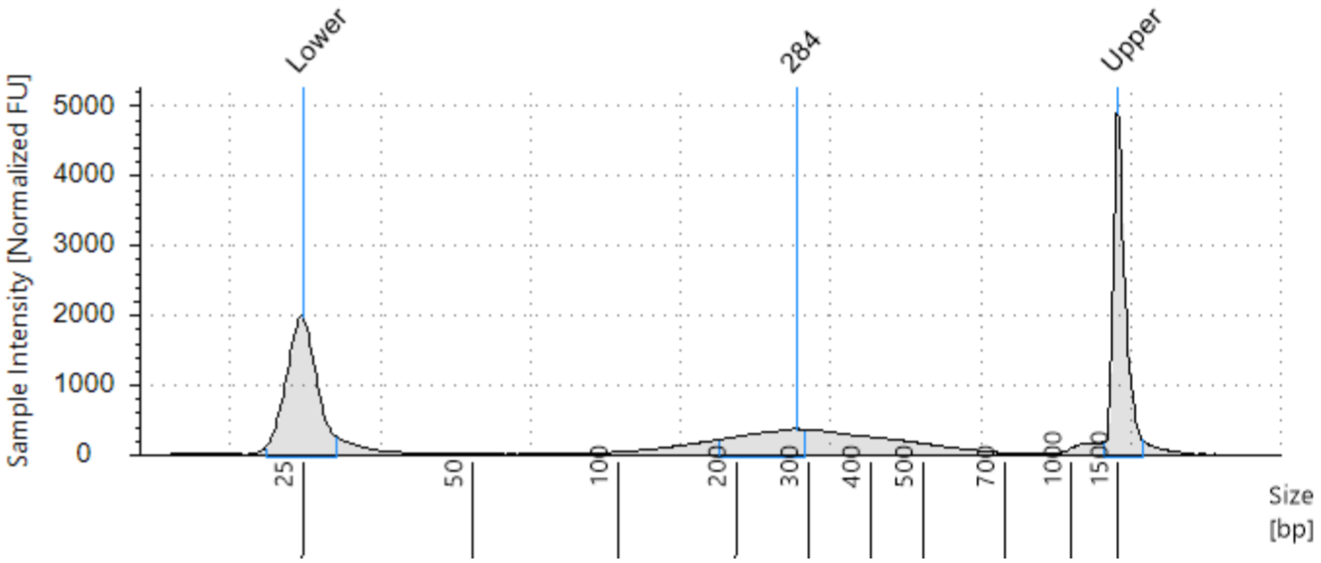

Sample Table

Well	Conc. [ng/µl]	Sample Description	Alert	Observations
A2	2.42	P-B11		

Peak Table

Size [bp]	Calibrated Conc. [ng/µl]	Assigned Conc. [ng/µl]	Peak Molarity [nmol/l]	% Integrated Area	Peak Comment	Observations
25	6.53	-	402	-		Lower Marker
284	2.42	-	13.1	100.00		
1500	6.50	6.50	6.67	-		Upper Marker

© Agilent Technologies, Inc. 2017

B2: P-C11

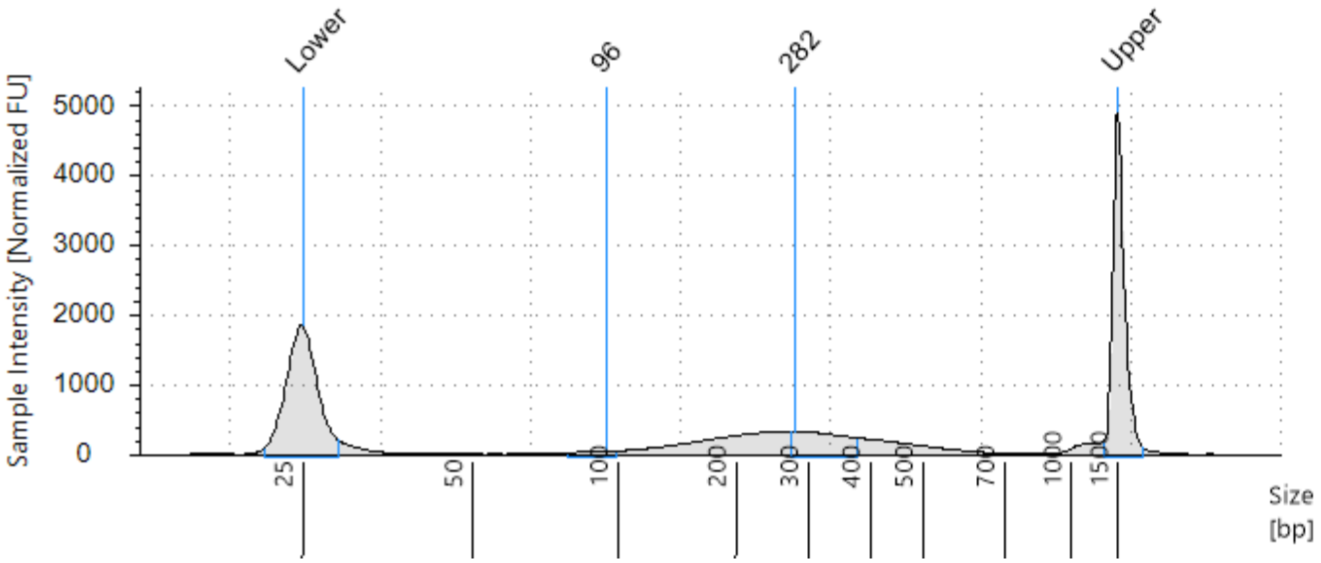

Sample Table

Well	Conc. [ng/µl]	Sample Description	Alert	Observations
B2	2.03	P-C11		

Peak Table

Size [bp]	Calibrated Conc. [ng/µl]	Assigned Conc. [ng/µl]	Peak Molarity [nmol/l]	% Integrated Area	Peak Comment	Observations
25	6.40	-	394	-		Lower Marker
96	0.175	-	2.79	8.63		
282	1.85	-	10.1	91.37		
1500	6.50	6.50	6.67	-		Upper Marker

© Agilent Technologies, Inc. 2017

C2: P-D11

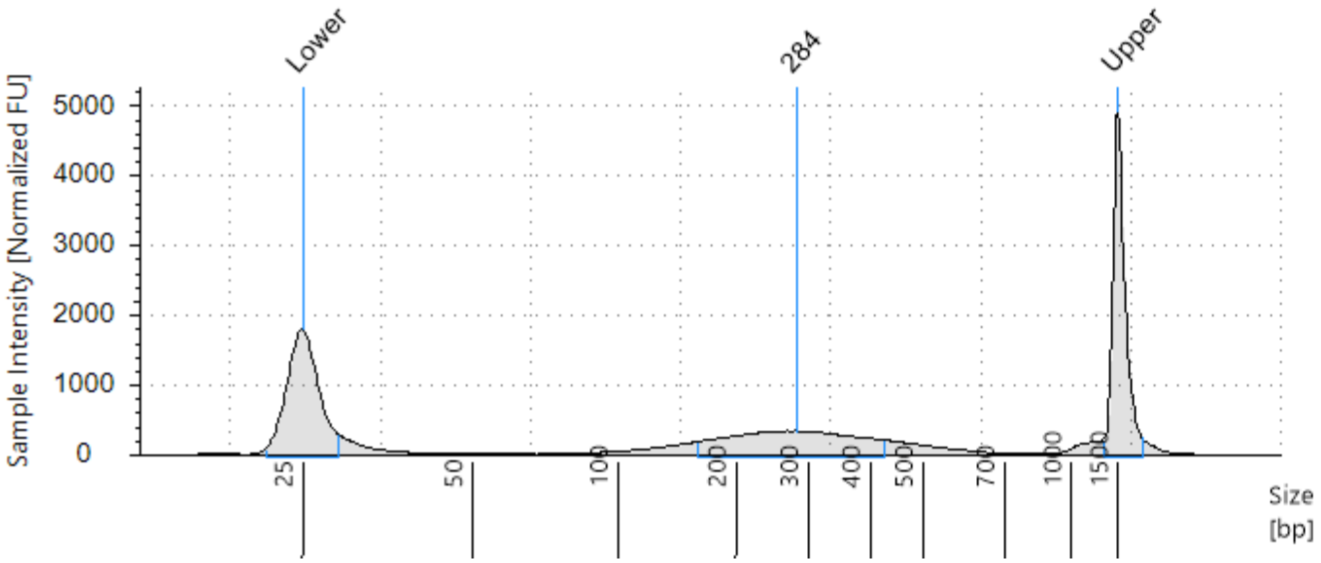

Sample Table

Well	Conc. [ng/µl]	Sample Description	Alert	Observations
C2	4.73	P-D11		

Peak Table

Size [bp]	Calibrated Conc. [ng/µl]	Assigned Conc. [ng/µl]	Peak Molarity [nmol/l]	% Integrated Area	Peak Comment	Observations
25	6.14	-	378	-		Lower Marker
284	4.73	-	25.6	100.00		
1500	6.50	6.50	6.67	-		Upper Marker

 © Agilent Technologies, Inc. 2017

D2: P-E11

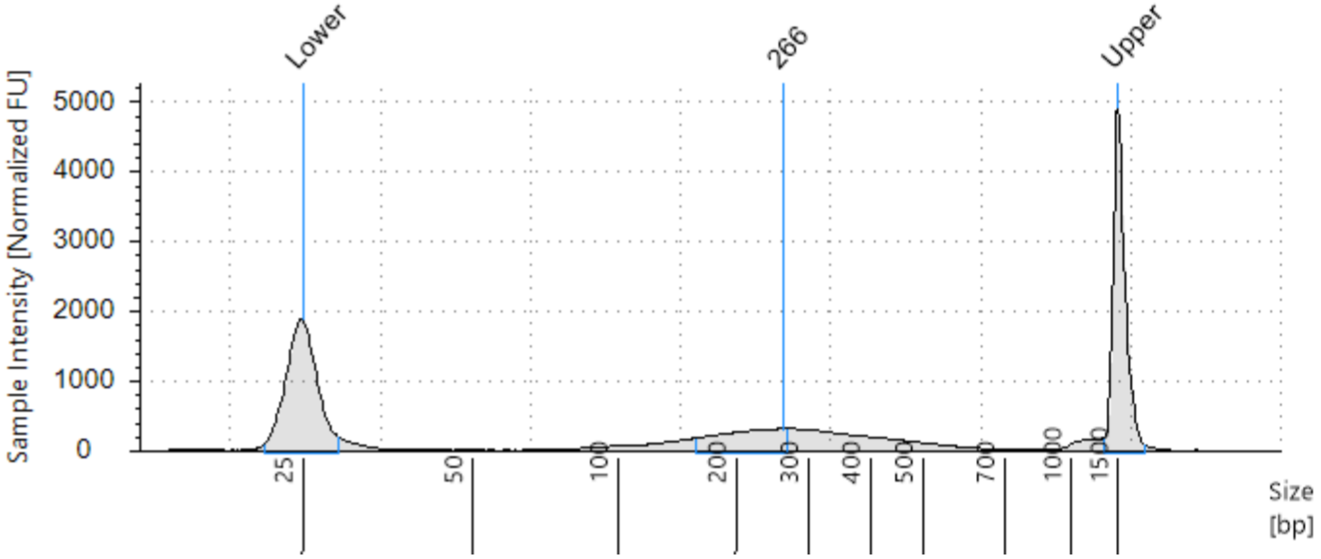

Sample Table

Well	Conc. [ng/µl]	Sample Description	Alert	Observations
D2	2.22	P-E11		

Peak Table

Size [bp]	Calibrated Conc. [ng/µl]	Assigned Conc. [ng/µl]	Peak Molarity [nmol/l]	% Integrated Area	Peak Comment	Observations
25	6.36	-	392	-		Lower Marker
266	2.22	-	12.8	100.00		
1500	6.50	6.50	6.67	-		Upper Marker

© Agilent Technologies, Inc. 2017

E2: P-F11

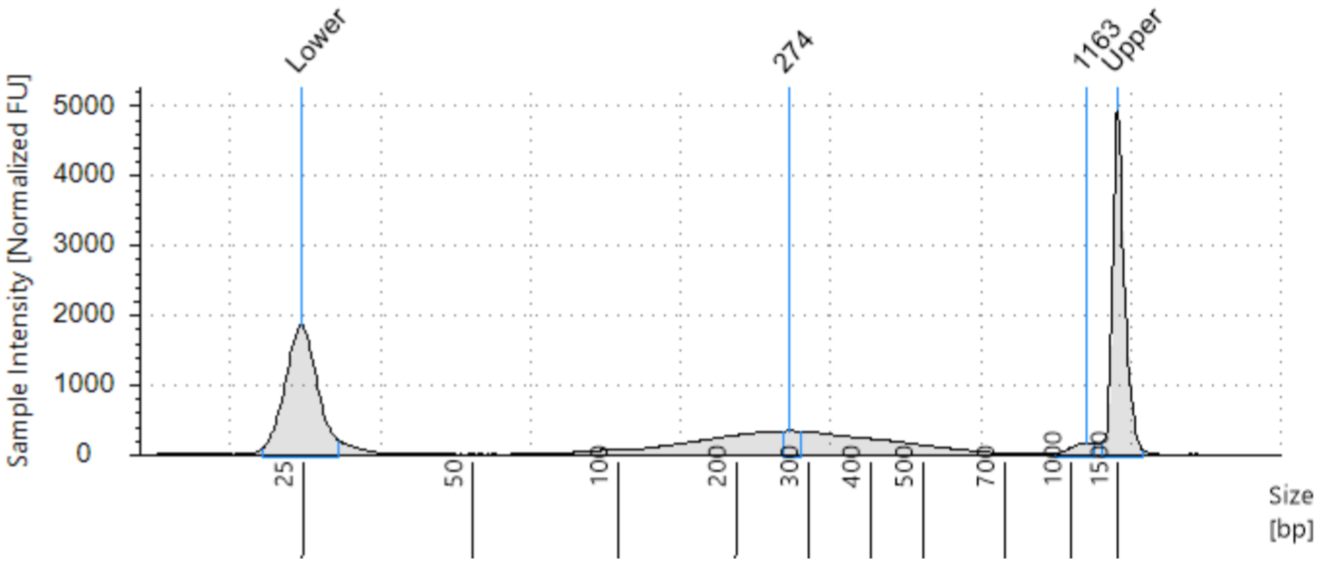

Sample Table

Well	Conc. [ng/µl]	Sample Description	Alert	Observations
E2	0.940	P-F11		

Peak Table

Size [bp]	Calibrated Conc. [ng/µl]	Assigned Conc. [ng/µl]	Peak Molarity [nmol/l]	% Integrated Area	Peak Comment	Observations
25	6.45	-	397	-		Lower Marker
274	0.571	-	3.21	60.77		
1163	0.369	-	0.488	39.23		
1500	6.50	6.50	6.67	-		Upper Marker

© Agilent Technologies, Inc. 2017

F2: P-G11

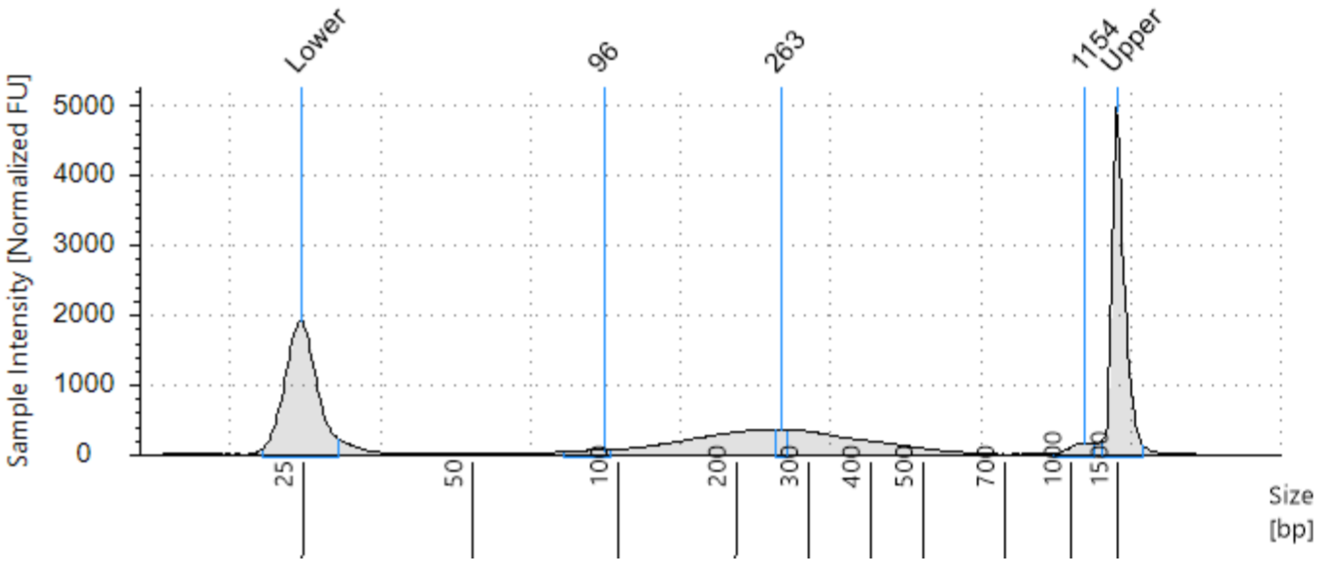

Sample Table

Well	Conc. [ng/µl]	Sample Description	Alert	Observations
F2	1.05	P-G11		

Peak Table

Size [bp]	Calibrated Conc. [ng/µl]	Assigned Conc. [ng/µl]	Peak Molarity [nmol/l]	% Integrated Area	Peak Comment	Observations
25	6.30	-	388	-		Lower Marker
96	0.216	-	3.48	20.63		
263	0.442	-	2.59	42.21		
1154	0.389	-	0.519	37.16		
1500	6.50	6.50	6.67	-		Upper Marker

© Agilent Technologies, Inc. 2017

G2: P-H11

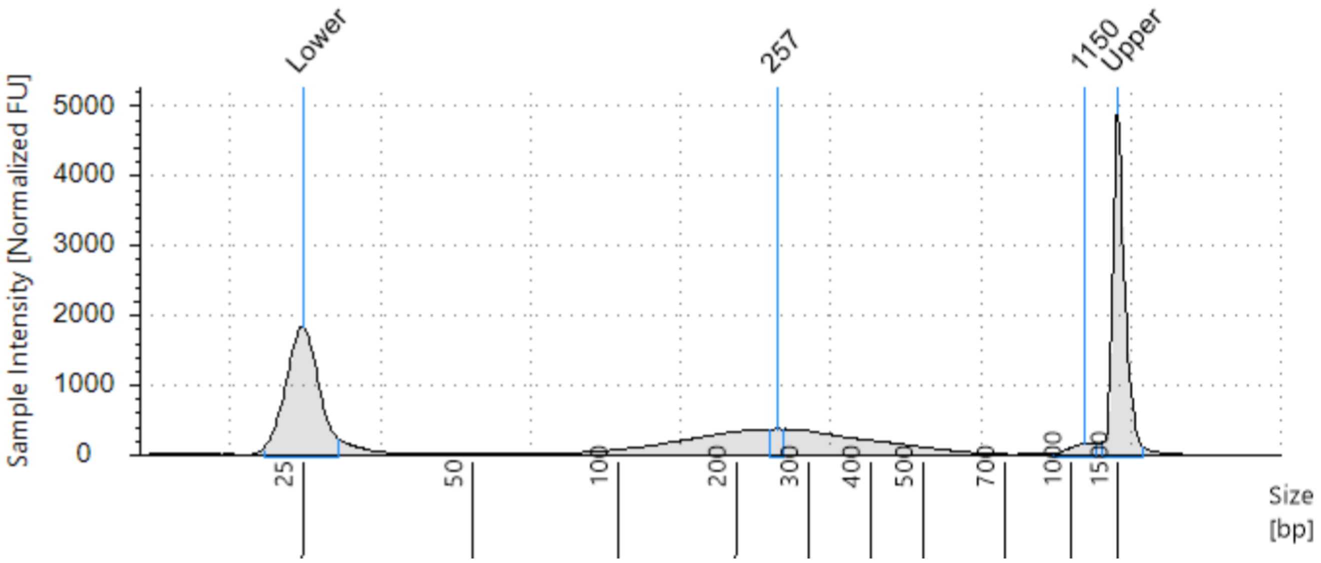

Sample Table

Well	Conc. [ng/µl]	Sample Description	Alert	Observations
G2	0.867	P-H11		

Peak Table

Size [bp]	Calibrated Conc. [ng/µl]	Assigned Conc. [ng/µl]	Peak Molarity [nmol/l]	% Integrated Area	Peak Comment	Observations
25	6.54	-	403	-		Lower Marker
257	0.473	-	2.83	54.60		
1150	0.394	-	0.526	45.40		
1500	6.50	6.50	6.67	-		Upper Marker

© Agilent Technologies, Inc. 2017

H2: P-A12

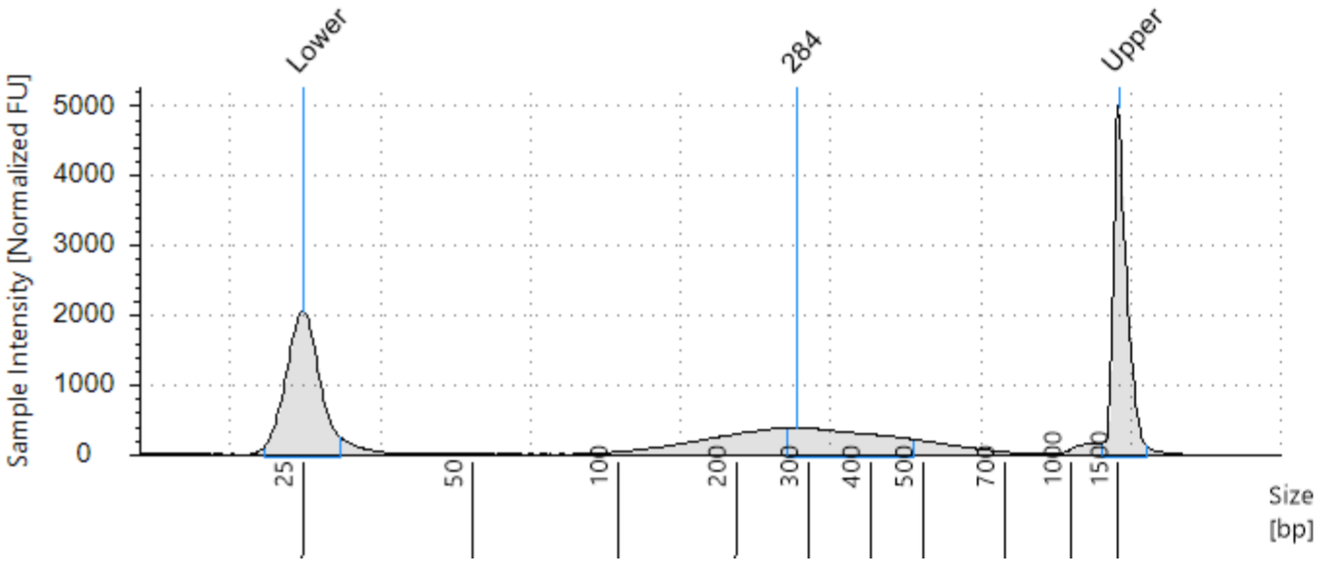

Sample Table

Well	Conc. [ng/µl]	Sample Description	Alert	Observations
H2	3.28	P-A12		

Peak Table

Size [bp]	Calibrated Conc. [ng/µl]	Assigned Conc. [ng/µl]	Peak Molarity [nmol/l]	% Integrated Area	Peak Comment	Observations
25	6.59	-	406	-		Lower Marker
284	3.28	-	17.7	100.00		
1500	6.50	6.50	6.67	-		Upper Marker

 © Agilent Technologies, Inc. 2017

Filename: 2020-08-21-03- Q-S DFB plus B12-H12 R1.D1000

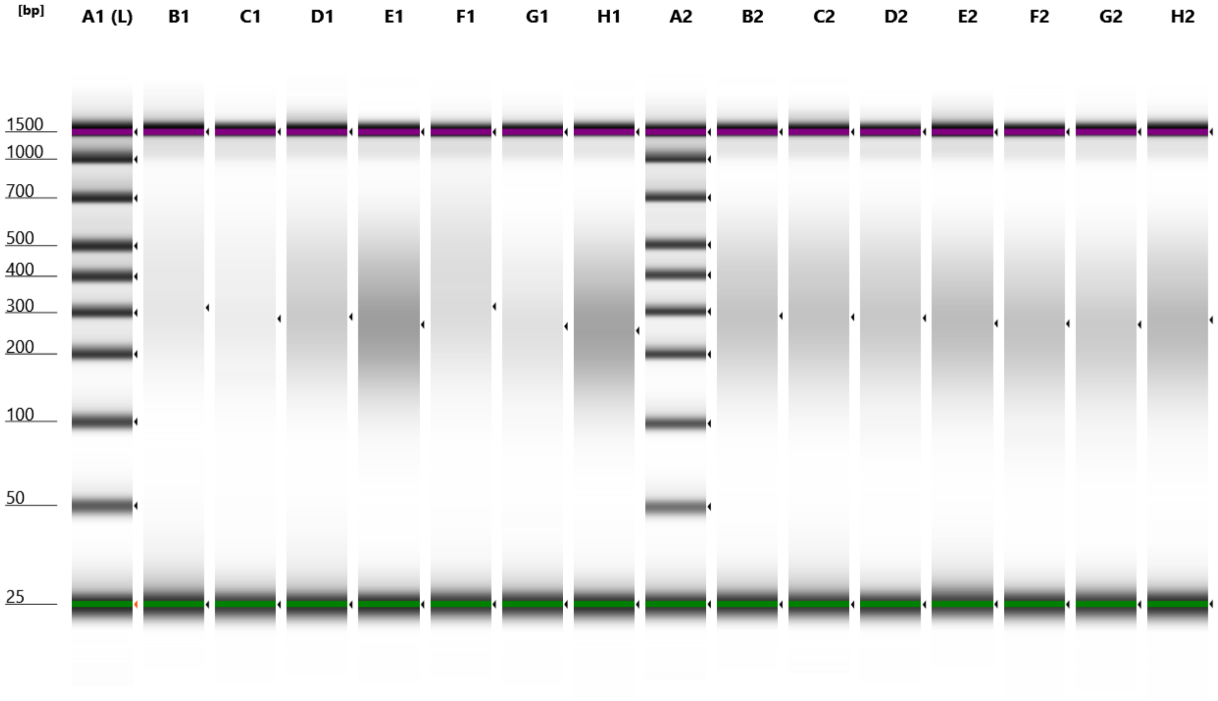

Default image (Contrast 100%)

Sample Info

Well	Conc. [ng/µl]	Sample Description	Alert	Observations
A1	22.4	Ladder		Ladder
B1	0.871	B12 MINUS-QSONICA 96 WELL		
C1	0.662	M-C12		
D1	2.22	M-D12		
E1	8.44	M-E12		
F1	0.585	M-F12		
G1	1.14	M-G12		
H1	4.54	M-H12		
A2	19.8	LADDER		
B2	0.507	PLUS B12		
C2	2.67	P-C12		
D2	0.555	P-D12		
E2	3.03	P-E12		
F2	2.60	P-F12		
G2	1.91	P-G12		
H2	3.29	P-H12		

© Agilent Technologies, Inc. 2017

A1: Ladder

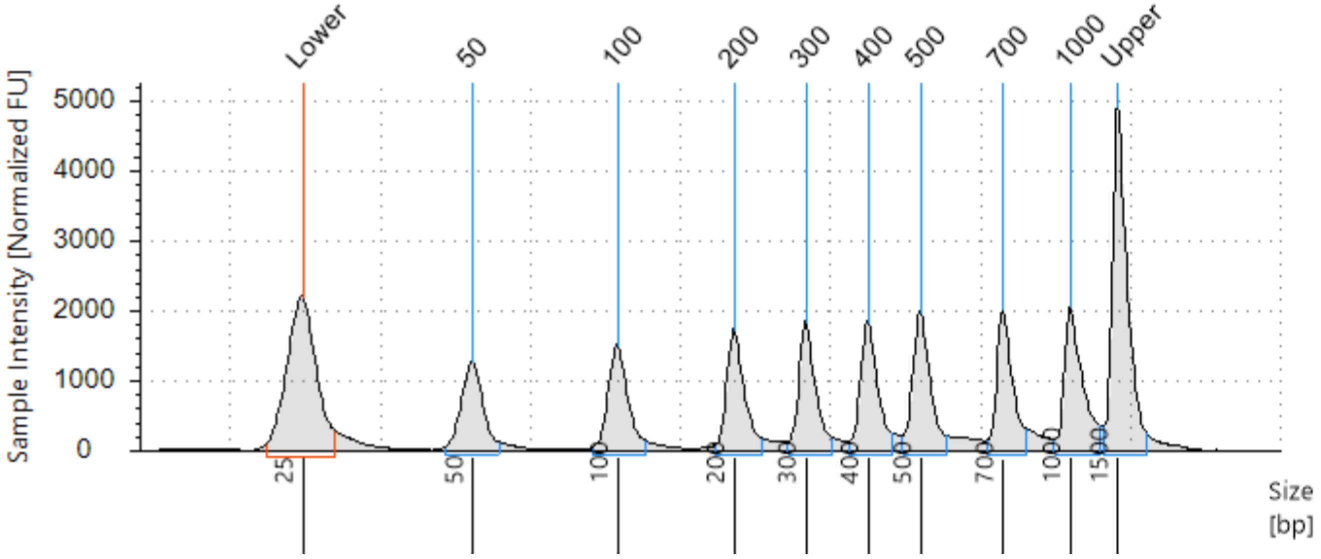

Sample Table

Well	Conc. [ng/µl]	Sample Description	Alert	Observations
A1	22.4	Ladder		Ladder

Peak Table

Size [bp]	Calibrated Conc. [ng/µl]	Assigned Conc. [ng/µl]	Peak Molarity [nmol/l]	% Integrated Area	Peak Comment	Observations
25	5.67	-	349	-		Lower Marker
50	2.41	-	74.2	10.79		
100	2.62	-	40.3	11.71		
200	2.68	-	20.6	11.99		
300	2.71	-	13.9	12.11		
400	2.83	-	10.9	12.66		
500	3.03	-	9.32	13.55		
700	2.80	-	6.16	12.55		
1000	3.27	-	5.04	14.64		
1500	6.50	6.50	6.67	-		Upper Marker

© Agilent Technologies, Inc. 2017

B1: B12 MINUS -QSONICA 96 WELL

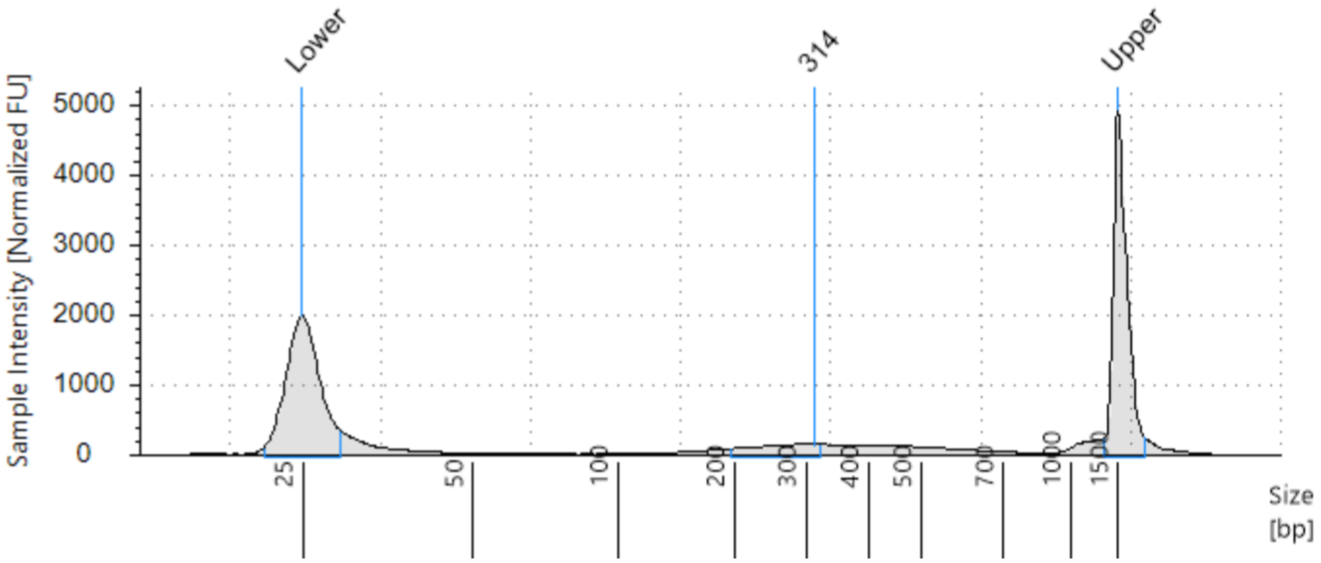

Sample Table

Well	Conc. [ng/µl]	Sample Description	Alert	Observations
B1	0.871	B12 MINUS-QSONICA 96 WELL		

Peak Table

Size [bp]	Calibrated Conc. [ng/µl]	Assigned Conc. [ng/µl]	Peak Molarity [nmol/l]	% Integrated Area	Peak Comment	Observations
25	6.40	-	394	-		Lower Marker
314	0.871	-	4.26	100.00		
1500	6.50	6.50	6.67	-		Upper Marker

 © Agilent Technologies, Inc. 2017

C1: M-C12

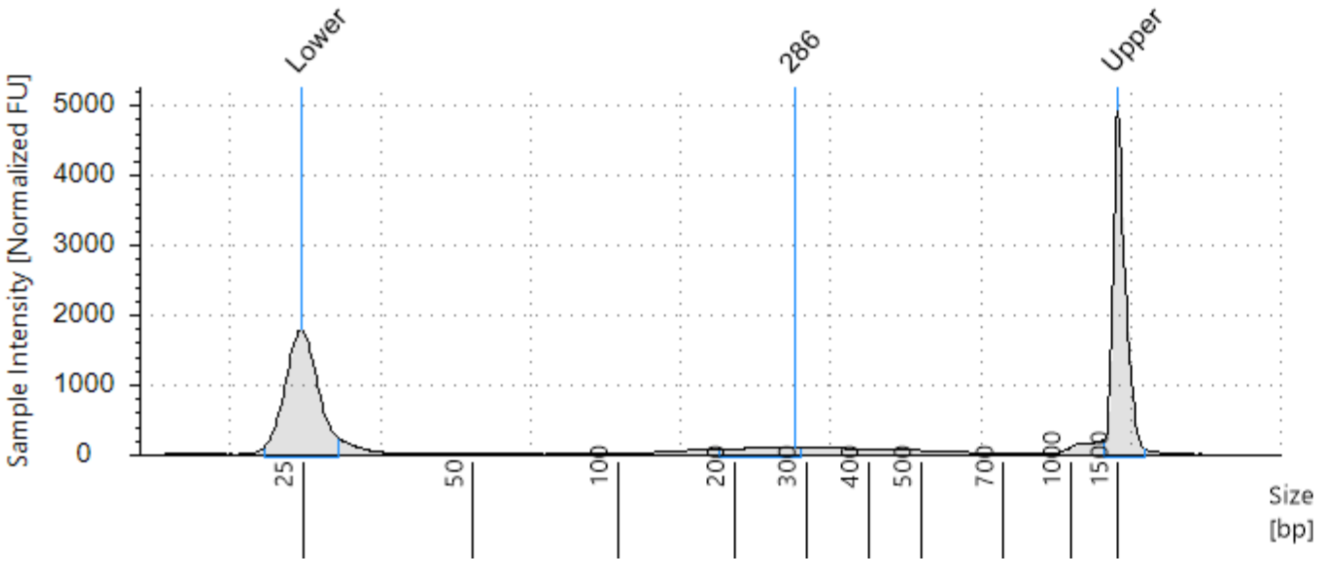

Sample Table

Well	Conc. [ng/µl]	Sample Description	Alert	Observations
C1	0.662	M-C12		

Peak Table

Size [bp]	Calibrated Conc. [ng/µl]	Assigned Conc. [ng/µl]	Peak Molarity [nmol/l]	% Integrated Area	Peak Comment	Observations
25	6.19	-	381	-		Lower Marker
286	0.662	-	3.57	100.00		
1500	6.50	6.50	6.67	-		Upper Marker

© Agilent Technologies, Inc. 2017

D1: M-D12

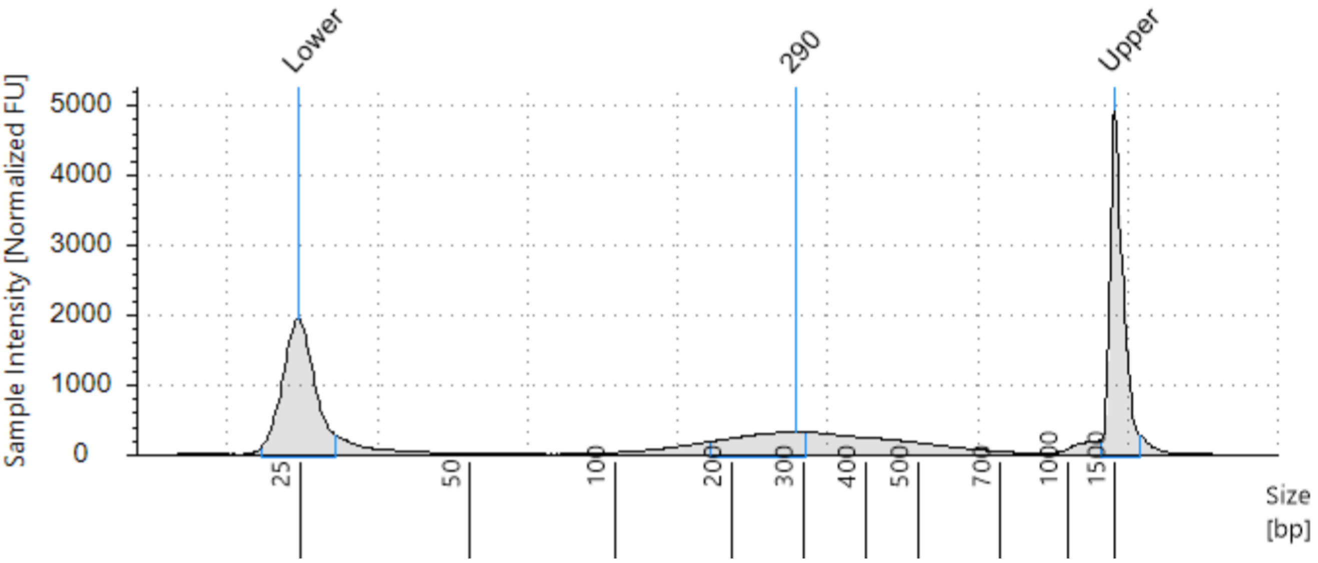

Sample Table

Well	Conc. [ng/µl]	Sample Description	Alert	Observations
D1	2.22	M-D12		

Peak Table

Size [bp]	Calibrated Conc. [ng/µl]	Assigned Conc. [ng/µl]	Peak Molarity [nmol/l]	% Integrated Area	Peak Comment	Observations
25	6.21	-	382	-		Lower Marker
290	2.22	-	11.8	100.00		
1500	6.50	6.50	6.67	-		Upper Marker

© Agilent Technologies, Inc. 2017

E1: M-E12

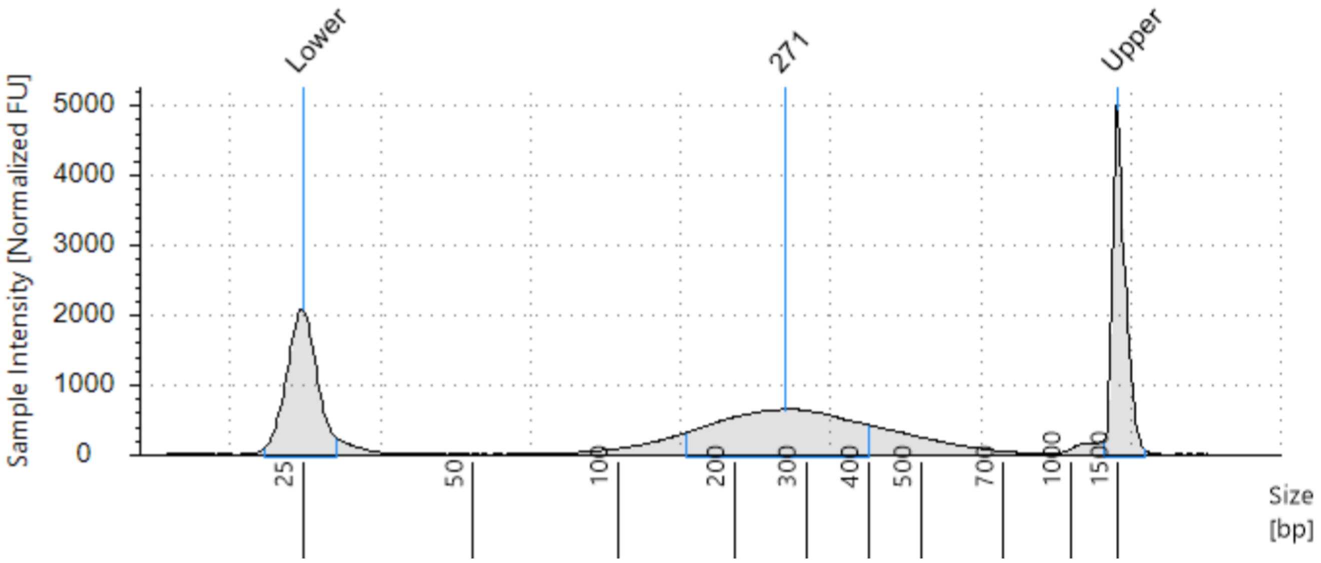

Sample Table

Well	Conc. [ng/µl]	Sample Description	Alert	Observations
E1	8.44	M-E12		

Peak Table

Size [bp]	Calibrated Conc. [ng/µl]	Assigned Conc. [ng/µl]	Peak Molarity [nmol/l]	% Integrated Area	Peak Comment	Observations
25	6.37	-	392	-		Lower Marker
271	8.44	-	47.9	100.00		
1500	6.50	6.50	6.67	-		Upper Marker

© Agilent Technologies, Inc. 2017

F1: M-F12

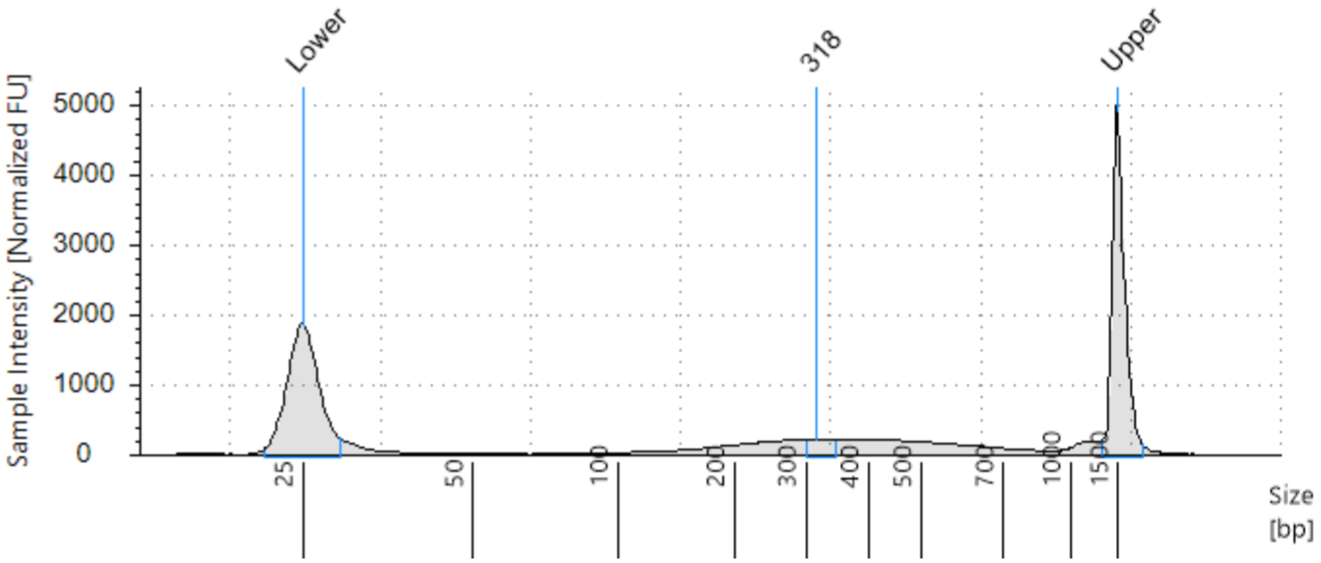

Sample Table

Well	Conc. [ng/µl]	Sample Description	Alert	Observations
F1	0.585	M-F12		

Peak Table

Size [bp]	Calibrated Conc. [ng/µl]	Assigned Conc. [ng/µl]	Peak Molarity [nmol/l]	% Integrated Area	Peak Comment	Observations
25	6.26	-	385	-		Lower Marker
318	0.585	-	2.83	100.00		
1500	6.50	6.50	6.67	-		Upper Marker

© Agilent Technologies, Inc. 2017

G1: M-G12

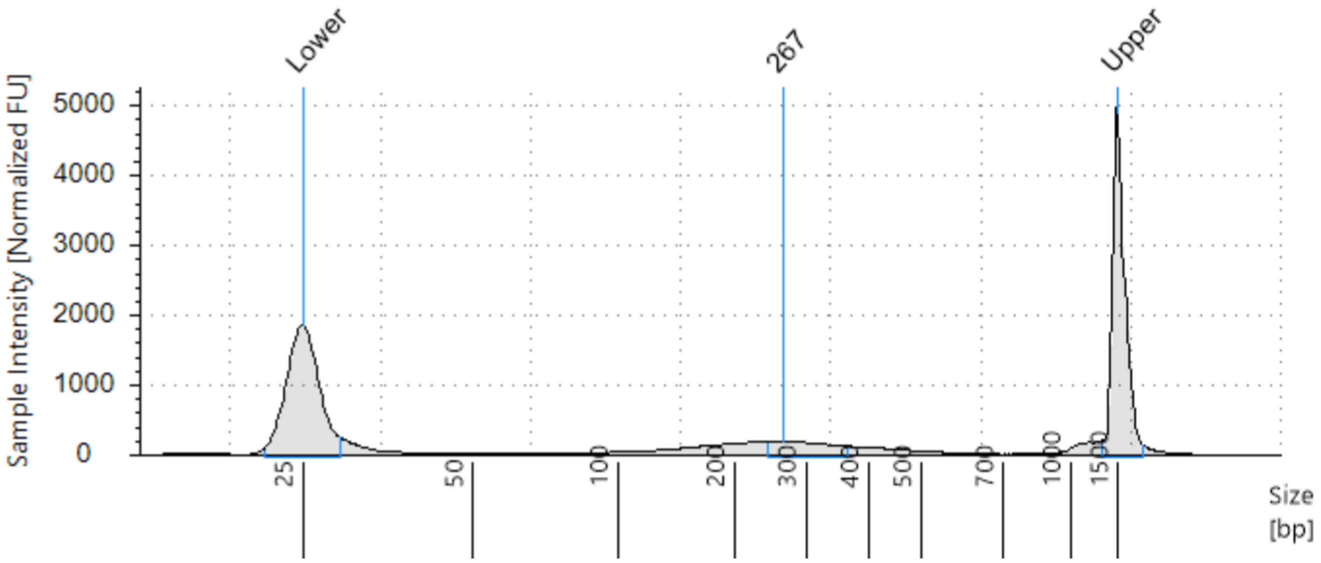

Sample Table

Well	Conc. [ng/µl]	Sample Description	Alert	Observations
G1	1.14	M-G12		

Peak Table

Size [bp]	Calibrated Conc. [ng/µl]	Assigned Conc. [ng/µl]	Peak Molarity [nmol/l]	% Integrated Area	Peak Comment	Observations
25	6.45	-	397	-		Lower Marker
267	1.14	-	6.56	100.00		
1500	6.50	6.50	6.67	-		Upper Marker

© Agilent Technologies, Inc. 2017

H1: M-H12

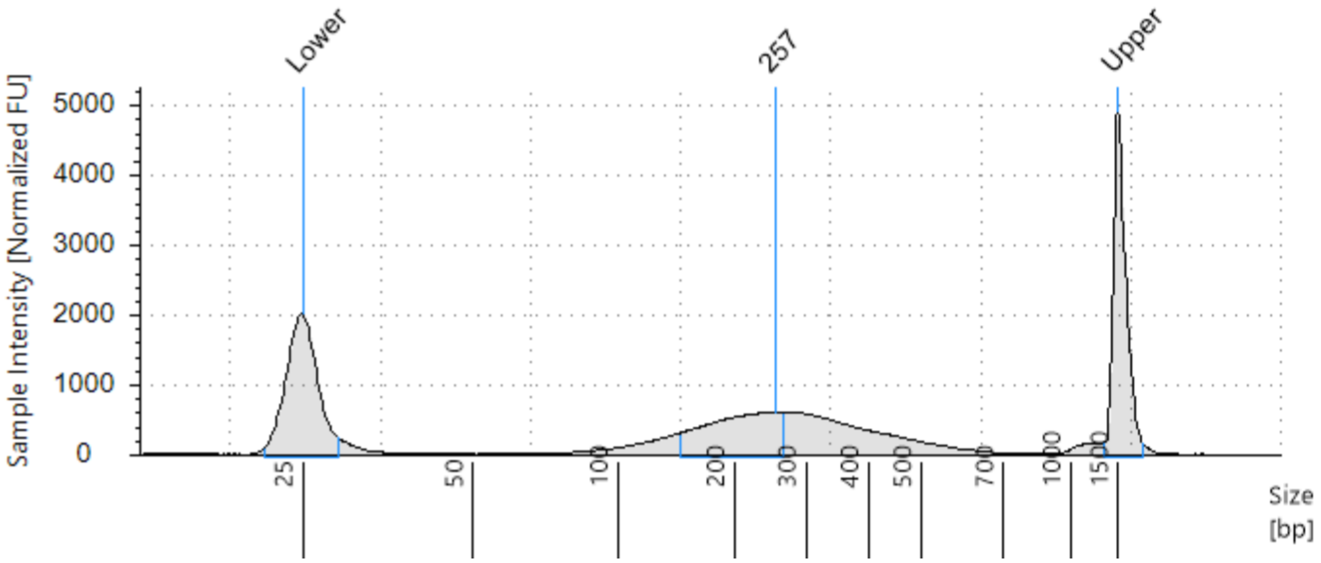

Sample Table

Well	Conc. [ng/µl]	Sample Description	Alert	Observations
H1	4.54	M-H12		

Peak Table

Size [bp]	Calibrated Conc. [ng/µl]	Assigned Conc. [ng/µl]	Peak Molarity [nmol/l]	% Integrated Area	Peak Comment	Observations
25	6.49	-	399	-		Lower Marker
257	4.54	-	27.2	100.00		
1500	6.50	6.50	6.67	-		Upper Marker

© Agilent Technologies, Inc. 2017

A2: LADDER

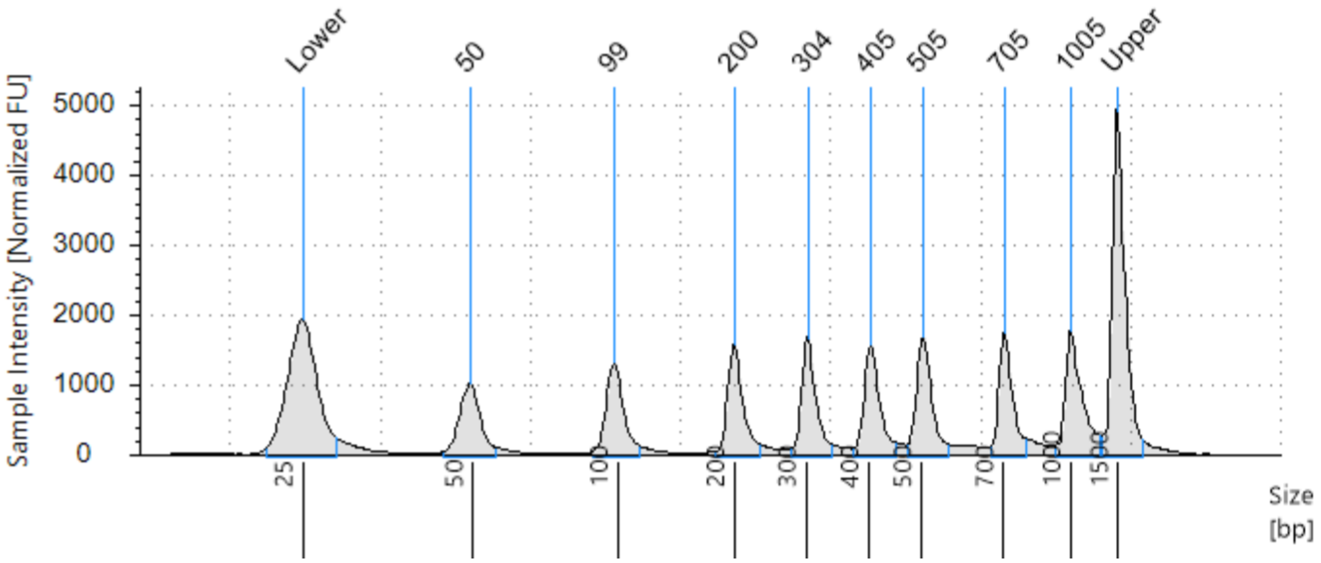

Sample Table

Well	Conc. [ng/µl]	Sample Description	Alert	Observations
A2	19.8	LADDER		

Peak Table

Size [bp]	Calibrated Conc. [ng/µl]	Assigned Conc. [ng/µl]	Peak Molarity [nmol/l]	% Integrated Area	Peak Comment	Observations
25	5.86	-	361	-		Lower Marker
50	2.14	-	66.2	10.78		
99	2.33	-	36.3	11.75		
200	2.45	-	18.9	12.34		
304	2.43	-	12.3	12.24		
405	2.48	-	9.42	12.50		
505	2.63	-	8.00	13.23		
705	2.44	-	5.33	12.32		
1005	2.94	-	4.50	14.83		
1500	6.50	6.50	6.67	-		Upper Marker

© Agilent Technologies, Inc. 2017

B2: PLUS B12

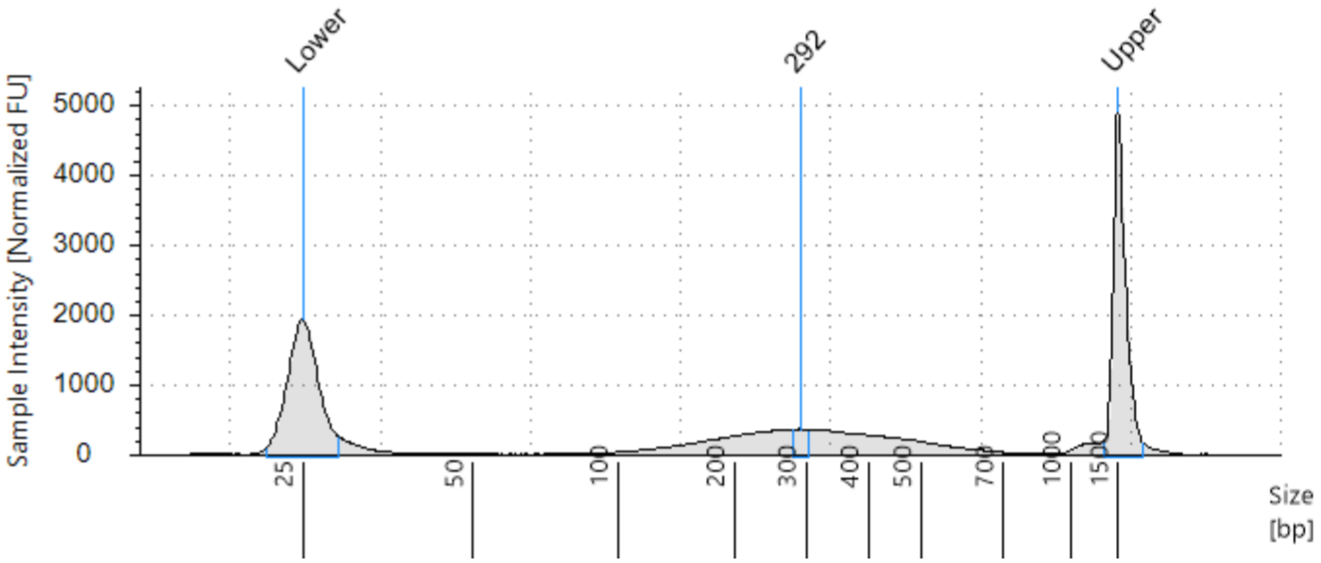

Sample Table

Well	Conc. [ng/µl]	Sample Description	Alert	Observations
B2	0.507	PLUS B12		

Peak Table

Size [bp]	Calibrated Conc. [ng/µl]	Assigned Conc. [ng/µl]	Peak Molarity [nmol/l]	% Integrated Area	Peak Comment	Observations
25	6.27	-	386	-		Lower Marker
292	0.507	-	2.67	100.00		
1500	6.50	6.50	6.67	-		Upper Marker

© Agilent Technologies, Inc. 2017

C2: P-C12

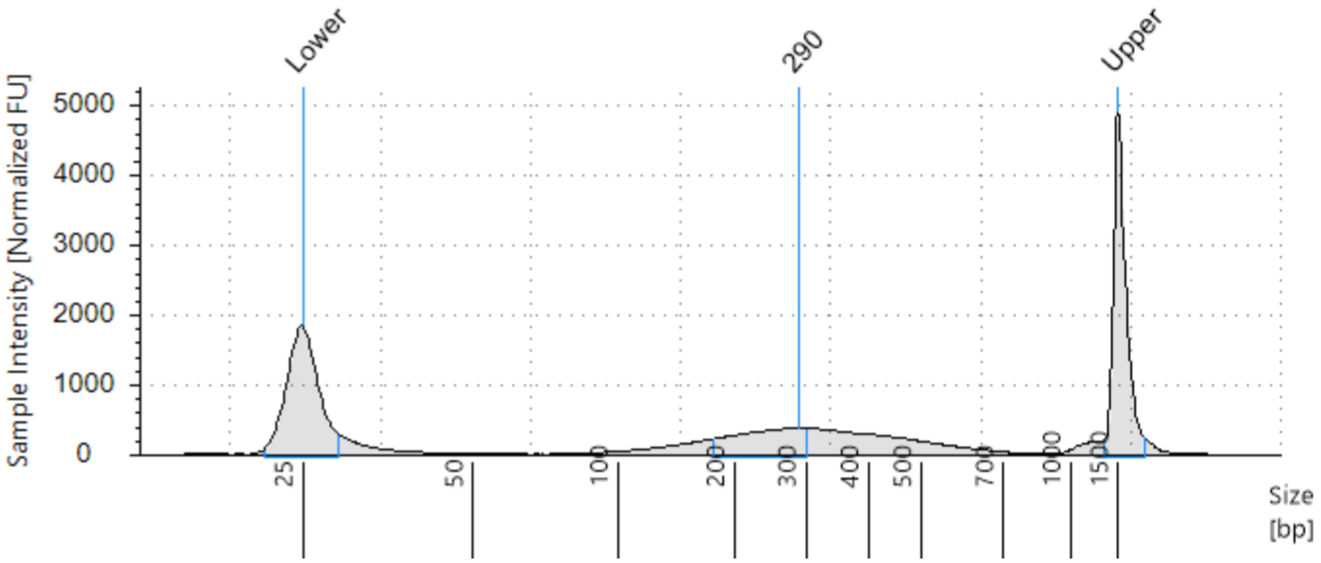

Sample Table

Well	Conc. [ng/µl]	Sample Description	Alert	Observations
C2	2.67	P-C12		

Peak Table

Size [bp]	Calibrated Conc. [ng/µl]	Assigned Conc. [ng/µl]	Peak Molarity [nmol/l]	% Integrated Area	Peak Comment	Observations
25	6.12	-	377	-		Lower Marker
290	2.67	-	14.2	100.00		
1500	6.50	6.50	6.67	-		Upper Marker

D2: P-D12

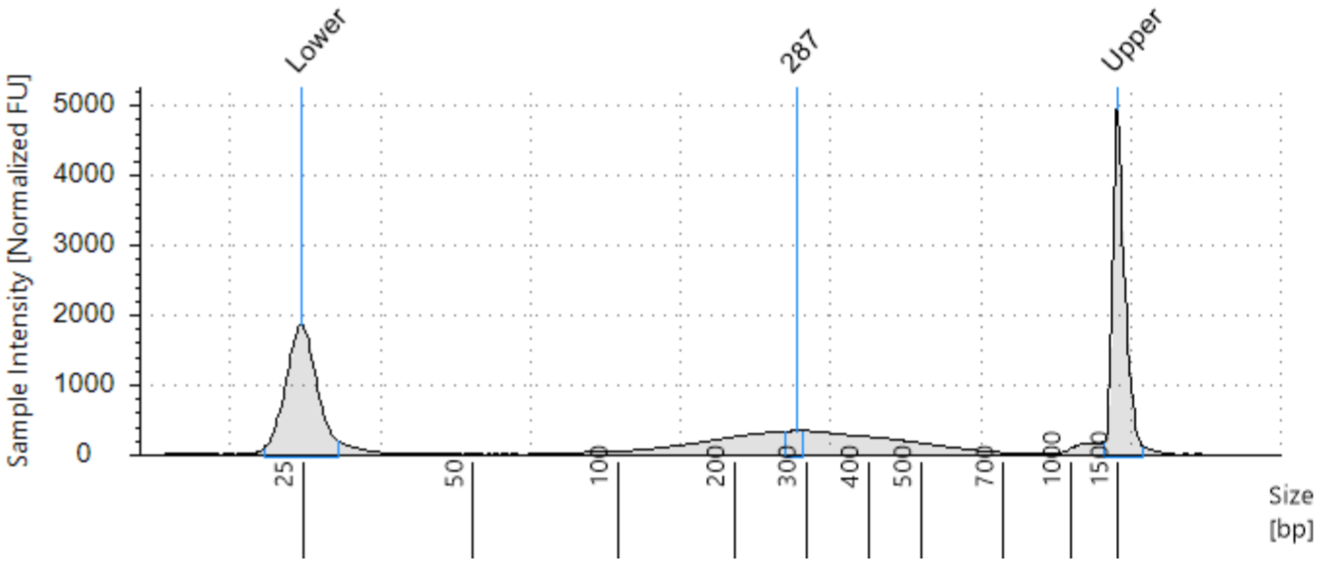

Sample Table

Well	Conc. [ng/µl]	Sample Description	Alert	Observations
D2	0.555	P-D12		

Peak Table

Size [bp]	Calibrated Conc. [ng/µl]	Assigned Conc. [ng/µl]	Peak Molarity [nmol/l]	% Integrated Area	Peak Comment	Observations
25	6.15	-	378	-		Lower Marker
287	0.555	-	2.98	100.00		
1500	6.50	6.50	6.67	-		Upper Marker

© Agilent Technologies, Inc. 2017

E2: P-E12

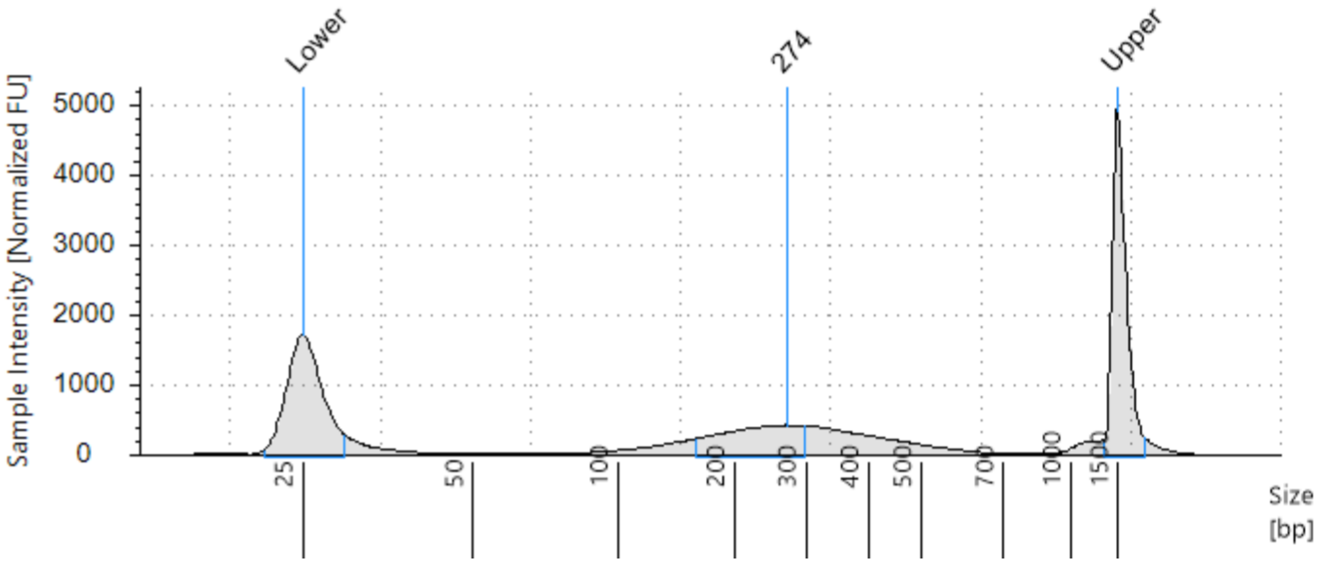

Sample Table

Well	Conc. [ng/µl]	Sample Description	Alert	Observations
E2	3.03	P-E12		

Peak Table

Size [bp]	Calibrated Conc. [ng/µl]	Assigned Conc. [ng/µl]	Peak Molarity [nmol/l]	% Integrated Area	Peak Comment	Observations
25	5.69	-	350	-		Lower Marker
274	3.03	-	17.0	100.00		
1500	6.50	6.50	6.67	-		Upper Marker

© Agilent Technologies, Inc. 2017

F2: P-F12

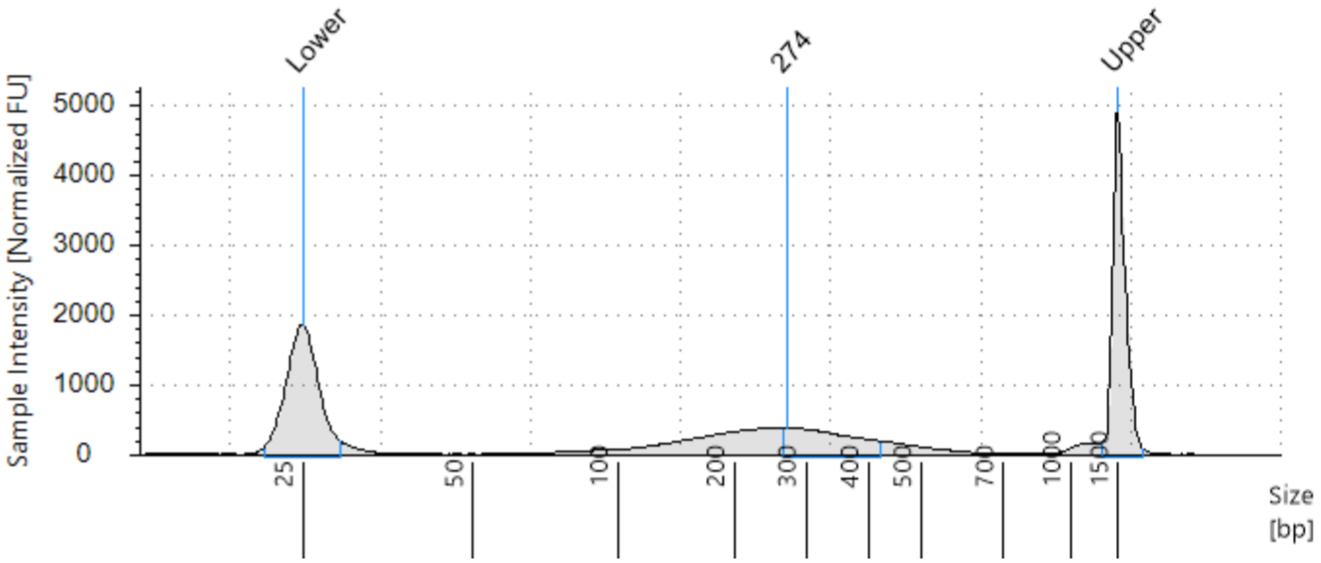

Sample Table

Well	Conc. [ng/µl]	Sample Description	Alert	Observations
F2	2.60	P-F12		

Peak Table

Size [bp]	Calibrated Conc. [ng/µl]	Assigned Conc. [ng/µl]	Peak Molarity [nmol/l]	% Integrated Area	Peak Comment	Observations
25	6.26	-	385	-		Lower Marker
274	2.60	-	14.6	100.00		
1500	6.50	6.50	6.67	-		Upper Marker

G2: P-G12

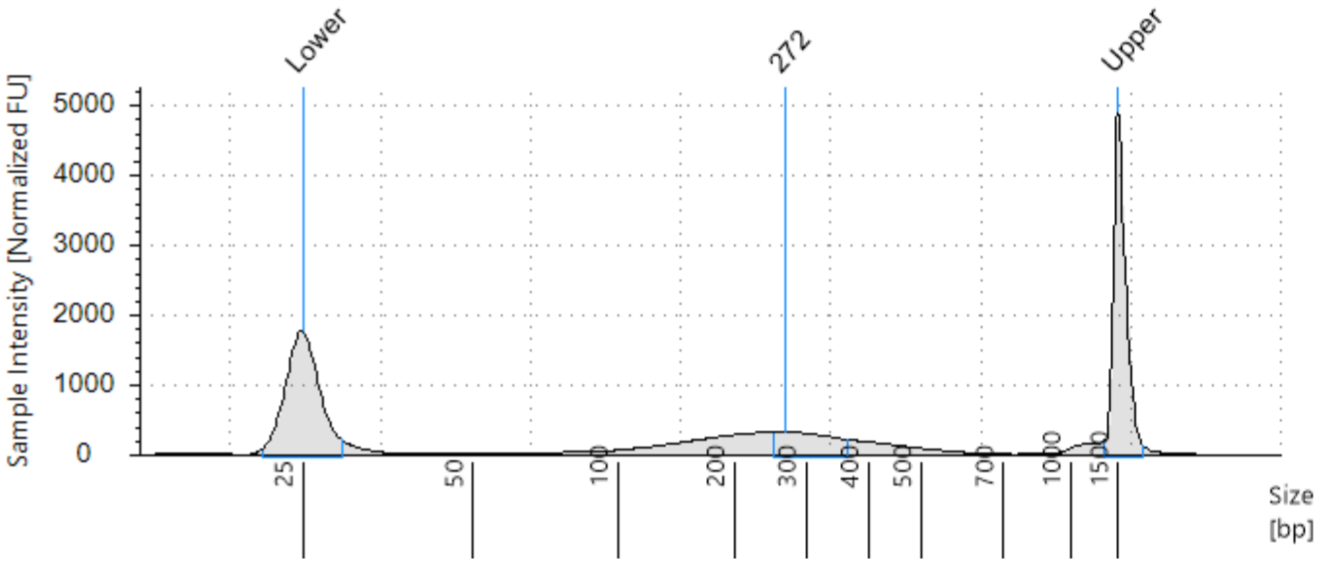

Sample Table

Well	Conc. [ng/µl]	Sample Description	Alert	Observations
G2	1.91	P-G12		

Peak Table

Size [bp]	Calibrated Conc. [ng/µl]	Assigned Conc. [ng/µl]	Peak Molarity [nmol/l]	% Integrated Area	Peak Comment	Observations
25	6.33	-	390	-		Lower Marker
272	1.91	-	10.8	100.00		
1500	6.50	6.50	6.67	-		Upper Marker

H2: P-H12

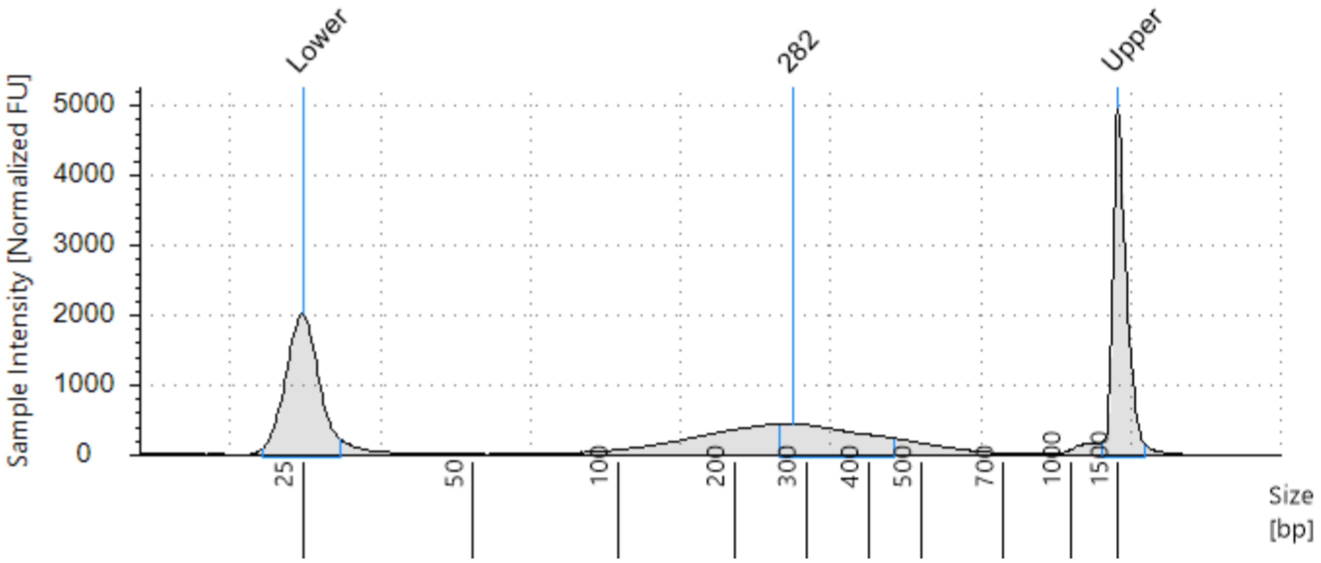

Sample Table

Well	Conc. [ng/µl]	Sample Description	Alert	Observations
H2	3.29	P-H12		

Peak Table

Size [bp]	Calibrated Conc. [ng/µl]	Assigned Conc. [ng/µl]	Peak Molarity [nmol/l]	% Integrated Area	Peak Comment	Observations
25	6.42	-	395	-		Lower Marker
282	3.29	-	18.0	100.00		
1500	6.50	6.50	6.67	-		Upper Marker

© Agilent Technologies, Inc. 2017

Filename: 2020-08-31-01 Q-S PLUS C6,A8,H9,A11,H11 R1.D1000

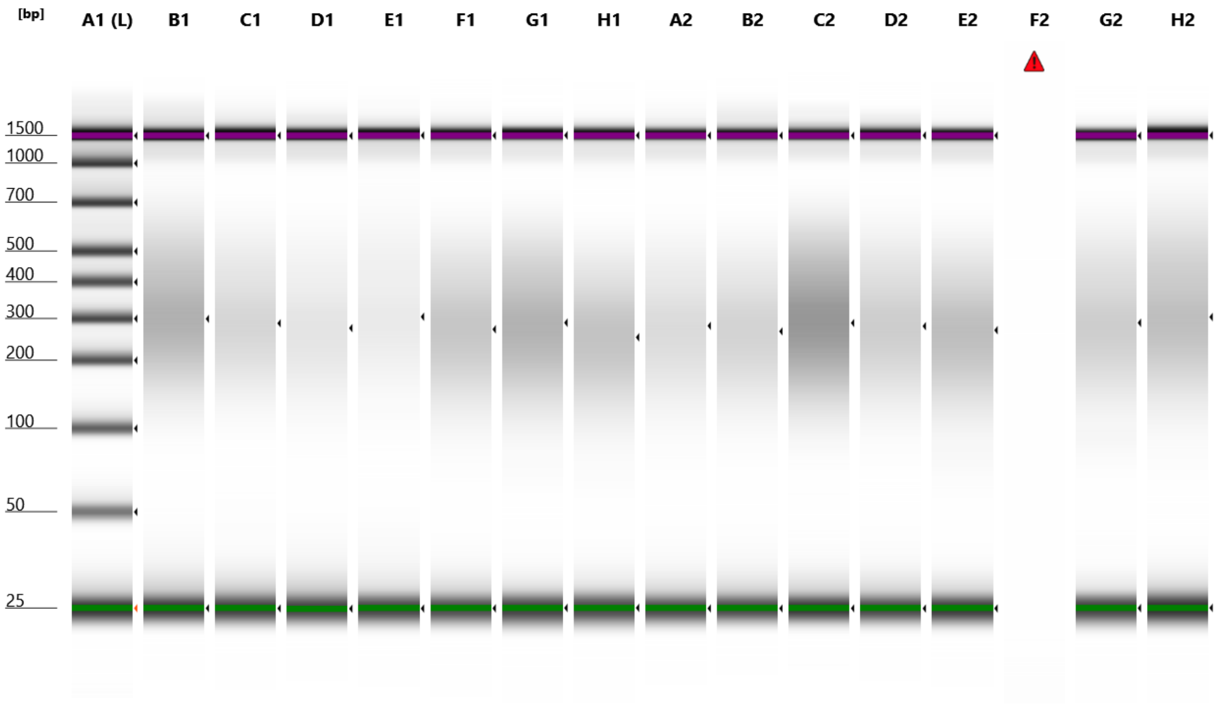

Default image (Contrast 100%)

Sample Info

Well	Conc. [ng/µl]	Sample Description	Alert	Observations
A1	18.5	Ladder		Ladder
B1	3.81	F1 M		
C1	1.22	G3 M		
D1	0.286	C6 M		
E1	0.492	A10 M		
F1	2.83	C6 p		
G1	3.86	A8 P		
H1	3.08	H9 P		
A2	1.41	A11 P		
B2	0.411	B11 P		
C2	1.81	C11 P		
D2	2.53	D11 P		
E2	5.61	E11 P		
F2		F11 P	▲	Marker(s) not detected
G2	1.85	G11 P		
H2	3.40	H11 P		

 © Agilent Technologies, Inc. 2017

A1: Ladder

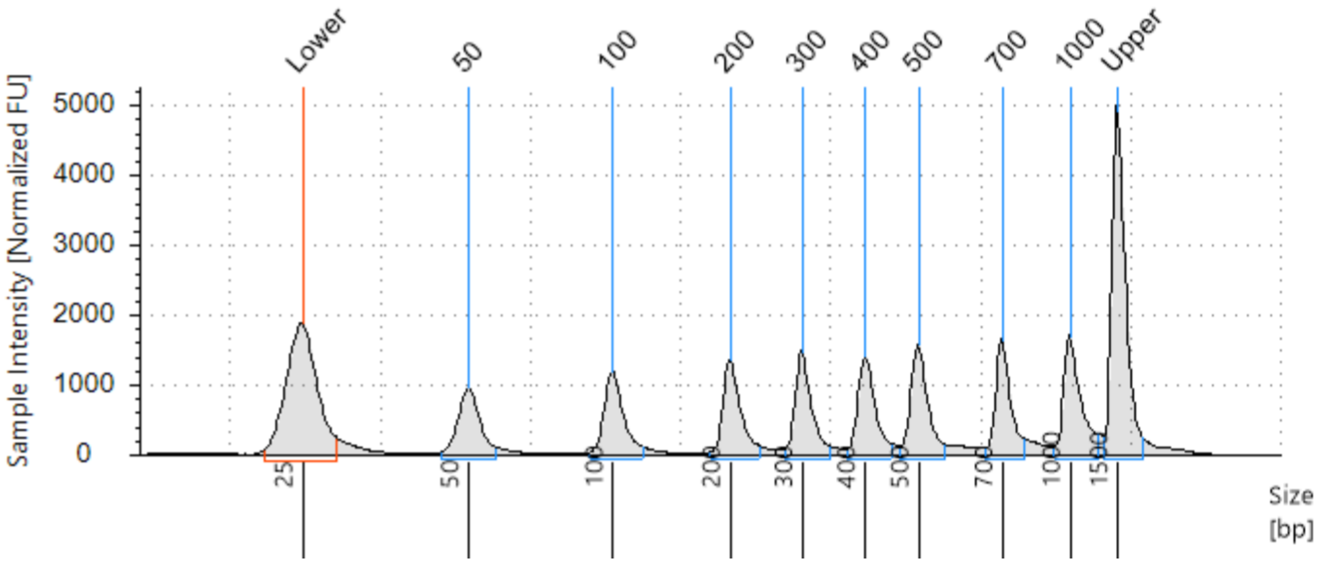

Sample Table

Well	Conc. [ng/µl]	Sample Description	Alert	Observations
A1	18.5	Ladder		Ladder

Peak Table

Size [bp]	Calibrated Conc. [ng/µl]	Assigned Conc. [ng/µl]	Peak Molarity [nmol/l]	% Integrated Area	Peak Comment	Observations
25	5.50	-	338	-		Lower Marker
50	1.95	-	60.0	10.55		
100	2.15	-	33.0	11.61		
200	2.22	-	17.1	12.01		
300	2.27	-	11.7	12.30		
400	2.31	-	8.90	12.52		
500	2.52	-	7.77	13.66		
700	2.32	-	5.10	12.55		
1000	2.73	-	4.21	14.79		
1500	6.50	6.50	6.67	-		Upper Marker

© Agilent Technologies, Inc. 2017

B1: F1 M

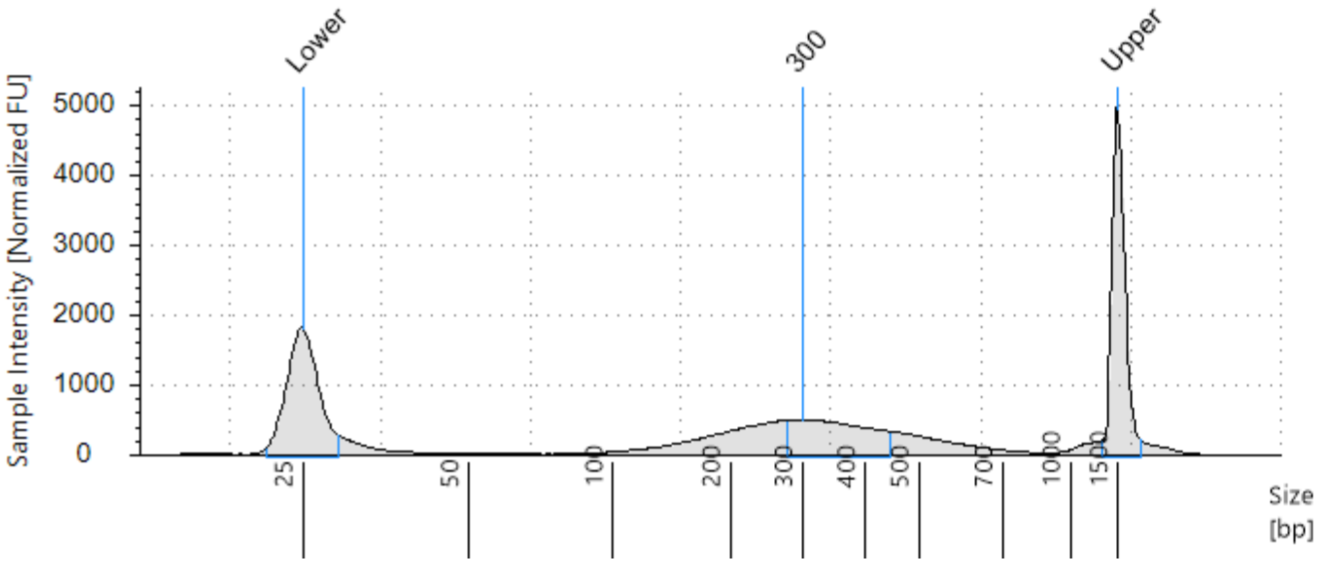

Sample Table

Well	Conc. [ng/µl]	Sample Description	Alert	Observations
B1	3.81	F1 M		

Peak Table

Size [bp]	Calibrated Conc. [ng/µl]	Assigned Conc. [ng/µl]	Peak Molarity [nmol/l]	% Integrated Area	Peak Comment	Observations
25	5.73	-	353	-		Lower Marker
300	3.81	-	19.6	100.00		
1500	6.50	6.50	6.67	-		Upper Marker

© Agilent Technologies, Inc. 2017

C1: G3 M

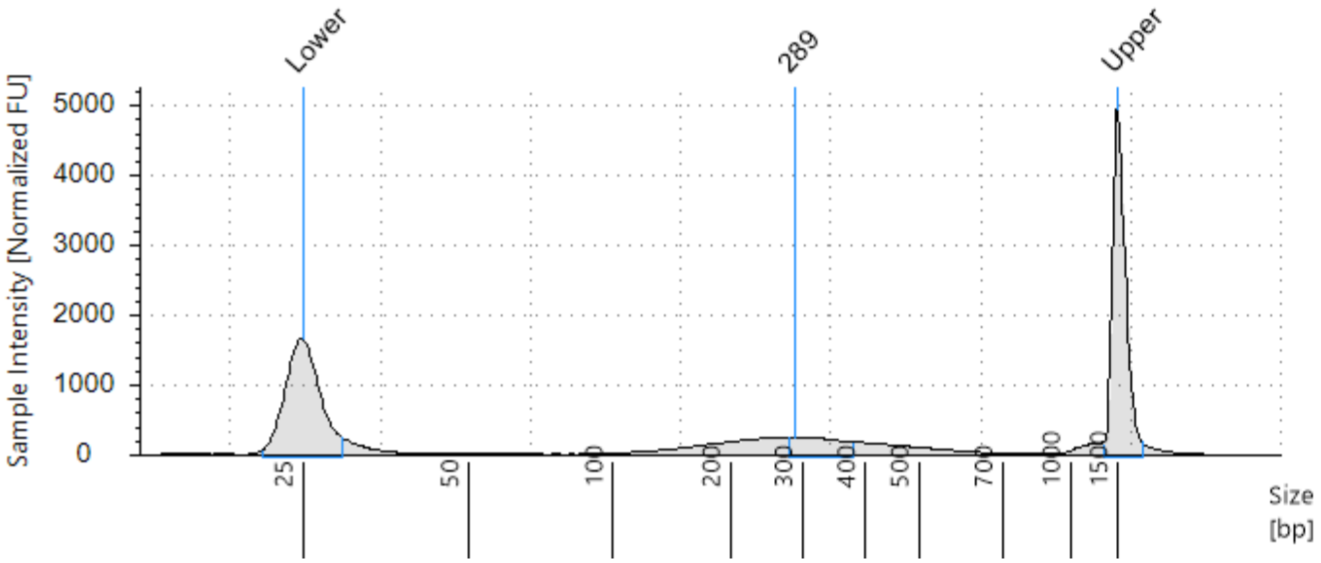

Sample Table

Well	Conc. [ng/µl]	Sample Description	Alert	Observations
C1	1.22	G3 M		

Peak Table

Size [bp]	Calibrated Conc. [ng/µl]	Assigned Conc. [ng/µl]	Peak Molarity [nmol/l]	% Integrated Area	Peak Comment	Observations
25	5.71	-	351	-		Lower Marker
289	1.22	-	6.51	100.00		
1500	6.50	6.50	6.67	-		Upper Marker

© Agilent Technologies, Inc. 2017

D1: C6 M

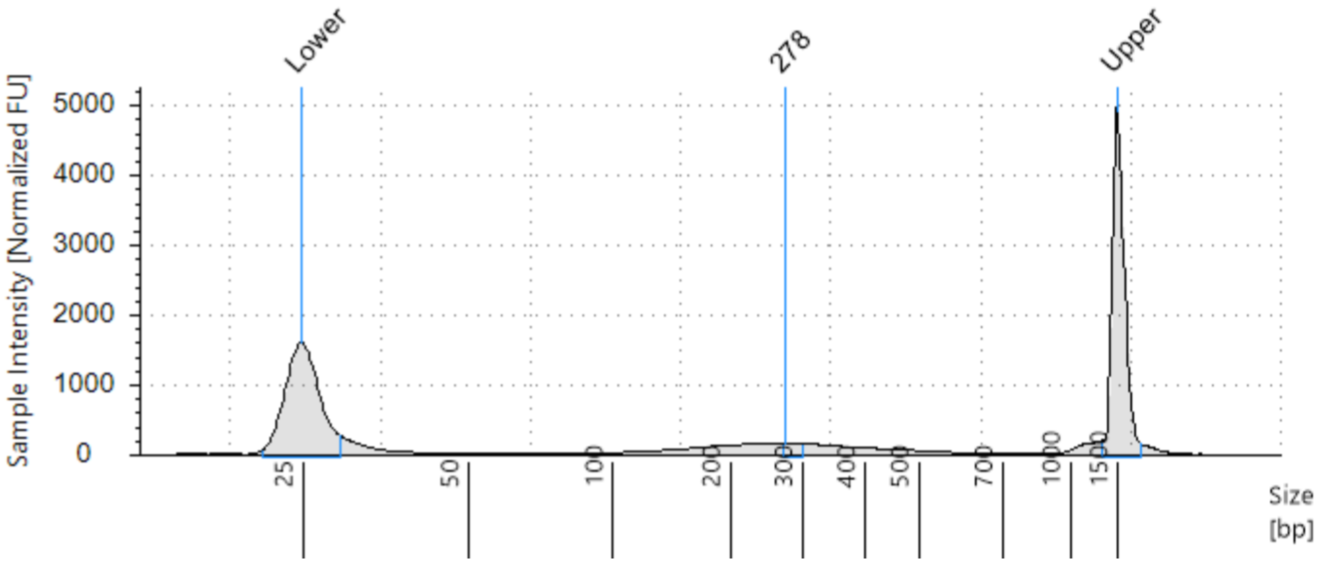

Sample Table

Well	Conc. [ng/µl]	Sample Description	Alert	Observations
D1	0.286	C6 M		

Peak Table

Size [bp]	Calibrated Conc. [ng/µl]	Assigned Conc. [ng/µl]	Peak Molarity [nmol/l]	% Integrated Area	Peak Comment	Observations
25	5.88	-	362	-		Lower Marker
278	0.286	-	1.58	100.00		
1500	6.50	6.50	6.67	-		Upper Marker

E1: A10 M

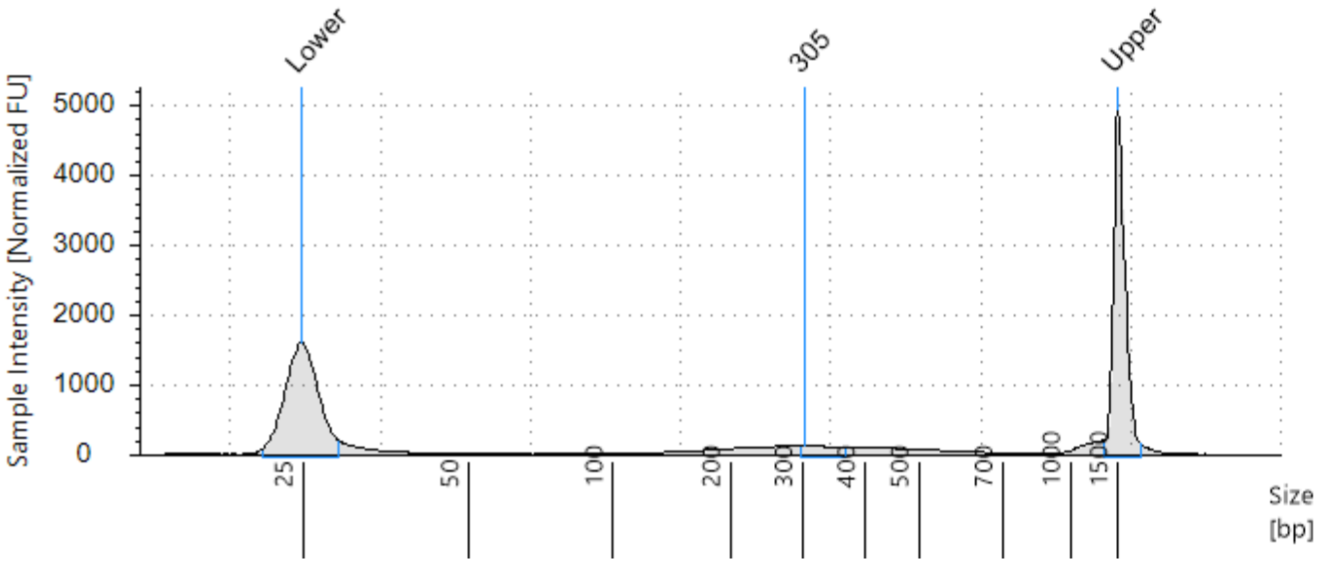

Sample Table

Well	Conc. [ng/µl]	Sample Description	Alert	Observations
E1	0.492	A10 M		

Peak Table

Size [bp]	Calibrated Conc. [ng/µl]	Assigned Conc. [ng/µl]	Peak Molarity [nmol/l]	% Integrated Area	Peak Comment	Observations
25	5.98	-	368	-		Lower Marker
305	0.492	-	2.48	100.00		
1500	6.50	6.50	6.67	-		Upper Marker

 © Agilent Technologies, Inc. 2017

F1: C6 p

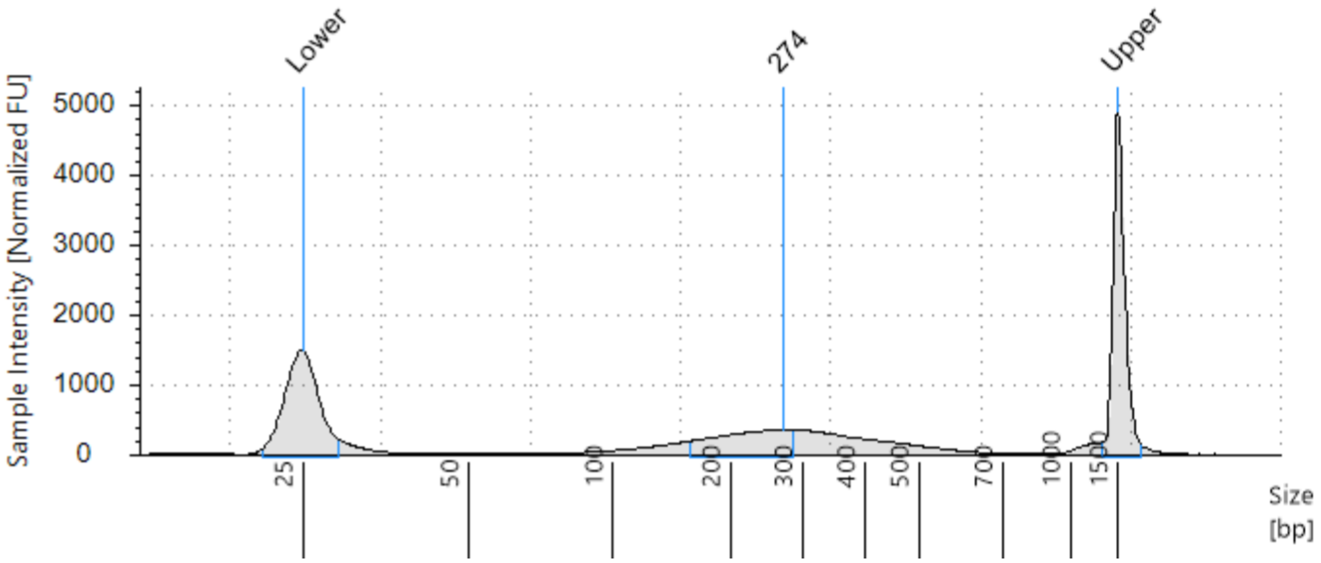

Sample Table

Well	Conc. [ng/µl]	Sample Description	Alert	Observations
F1	2.83	C6 p		

Peak Table

Size [bp]	Calibrated Conc. [ng/µl]	Assigned Conc. [ng/µl]	Peak Molarity [nmol/l]	% Integrated Area	Peak Comment	Observations
25	5.66	-	348	-		Lower Marker
274	2.83	-	15.9	100.00		
1500	6.50	6.50	6.67	-		Upper Marker

© Agilent Technologies, Inc. 2017

G1: A8 P

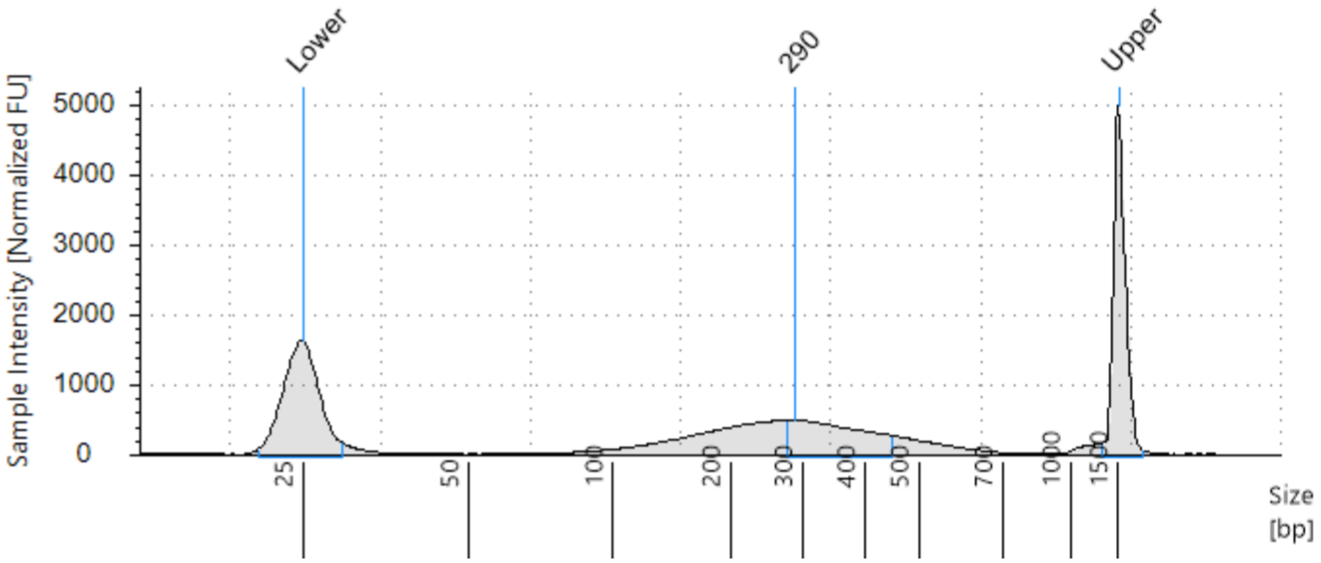

Sample Table

Well	Conc. [ng/µl]	Sample Description	Alert	Observations
G1	3.86	A8 P		

Peak Table

Size [bp]	Calibrated Conc. [ng/µl]	Assigned Conc. [ng/µl]	Peak Molarity [nmol/l]	% Integrated Area	Peak Comment	Observations
25	6.39	-	393	-		Lower Marker
290	3.86	-	20.5	100.00		
1500	6.50	6.50	6.67	-		Upper Marker

© Agilent Technologies, Inc. 2017

H1: H9 P

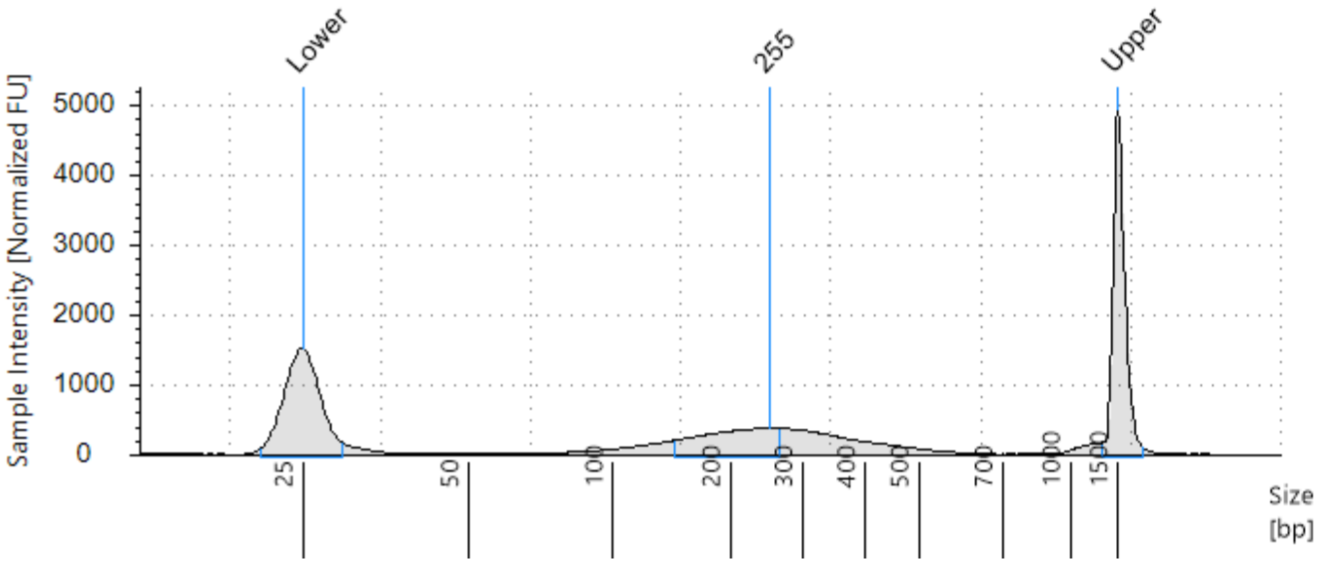

Sample Table

Well	Conc. [ng/µl]	Sample Description	Alert	Observations
H1	3.08	H9 P		

Peak Table

Size [bp]	Calibrated Conc. [ng/µl]	Assigned Conc. [ng/µl]	Peak Molarity [nmol/l]	% Integrated Area	Peak Comment	Observations
25	6.15	-	378	-		Lower Marker
255	3.08	-	18.6	100.00		
1500	6.50	6.50	6.67	-		Upper Marker

© Agilent Technologies, Inc. 2017

A2: A11 P

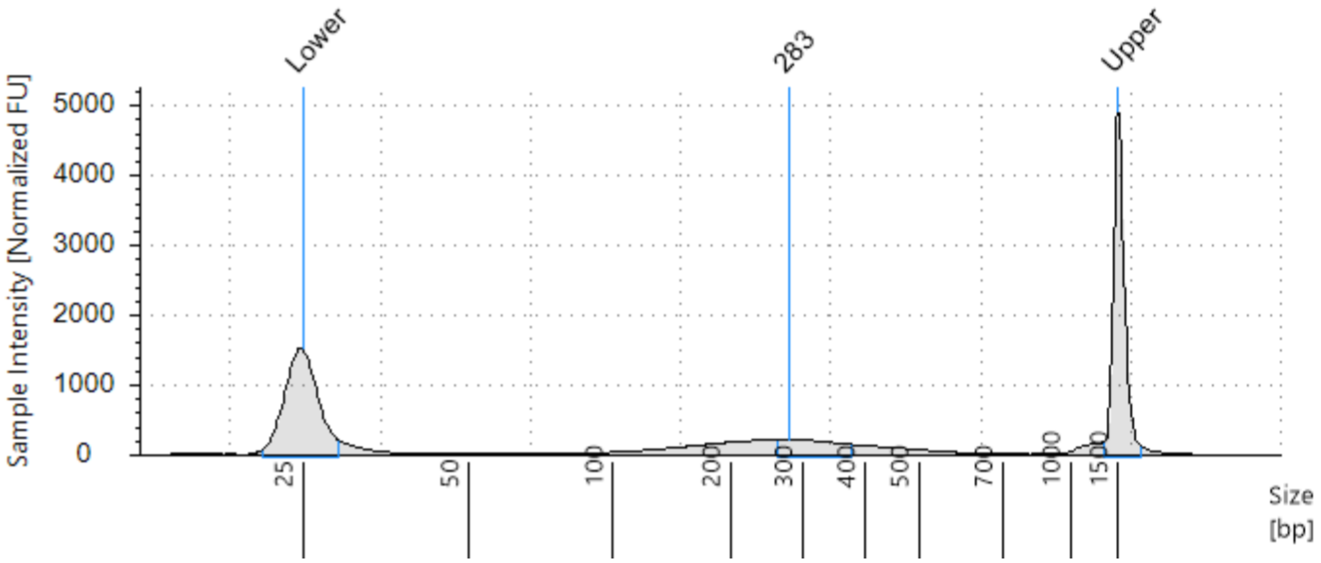

Sample Table

Well	Conc. [ng/µl]	Sample Description	Alert	Observations
A2	1.41	A11 P		

Peak Table

Size [bp]	Calibrated Conc. [ng/µl]	Assigned Conc. [ng/µl]	Peak Molarity [nmol/l]	% Integrated Area	Peak Comment	Observations
25	6.00	-	369	-		Lower Marker
283	1.41	-	7.67	100.00		
1500	6.50	6.50	6.67	-		Upper Marker

© Agilent Technologies, Inc. 2017

B2: B11 P

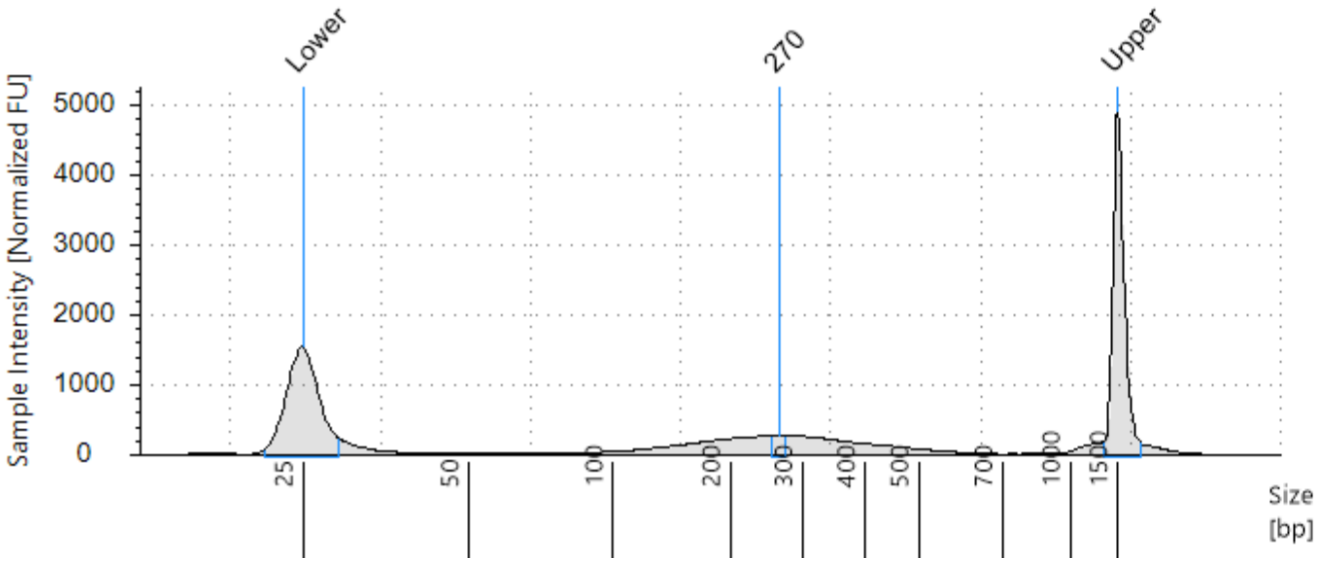

Sample Table

Well	Conc. [ng/µl]	Sample Description	Alert	Observations
B2	0.411	B11 P		

Peak Table

Size [bp]	Calibrated Conc. [ng/µl]	Assigned Conc. [ng/µl]	Peak Molarity [nmol/l]	% Integrated Area	Peak Comment	Observations
25	5.74	-	353	-		Lower Marker
270	0.411	-	2.35	100.00		
1500	6.50	6.50	6.67	-		Upper Marker

© Agilent Technologies, Inc. 2017

C2: C11 P

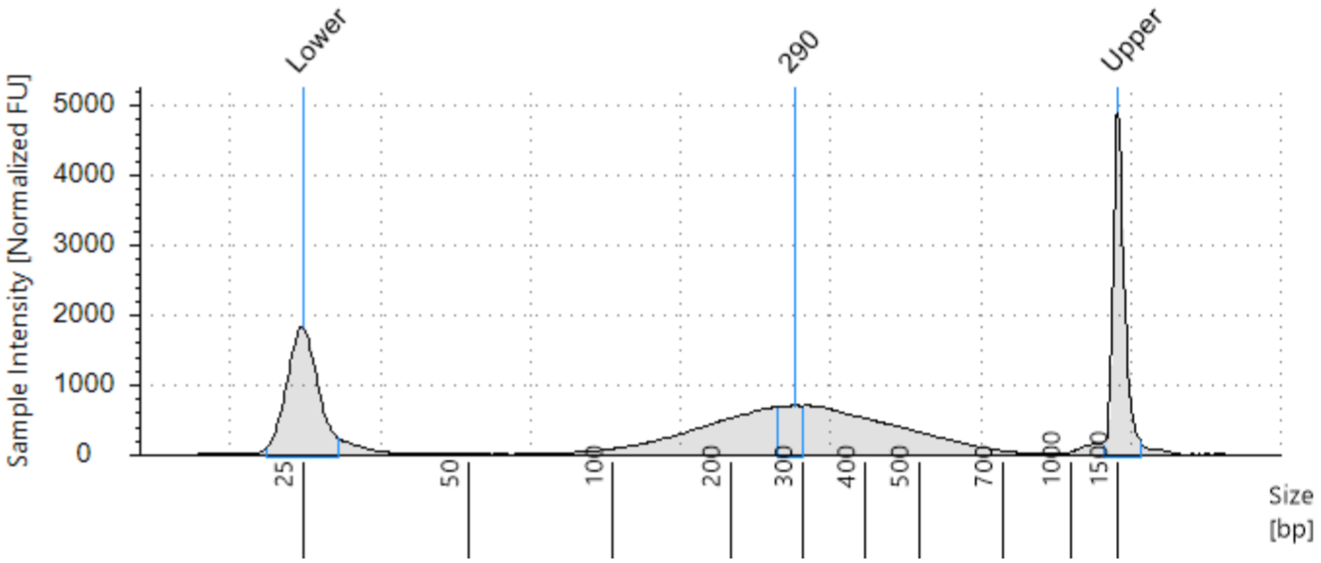

Sample Table

Well	Conc. [ng/µl]	Sample Description	Alert	Observations
C2	1.81	C11 P		

Peak Table

Size [bp]	Calibrated Conc. [ng/µl]	Assigned Conc. [ng/µl]	Peak Molarity [nmol/l]	% Integrated Area	Peak Comment	Observations
25	6.56	-	404	-		Lower Marker
290	1.81	-	9.60	100.00		
1500	6.50	6.50	6.67	-		Upper Marker

© Agilent Technologies, Inc. 2017

D2: D11 P

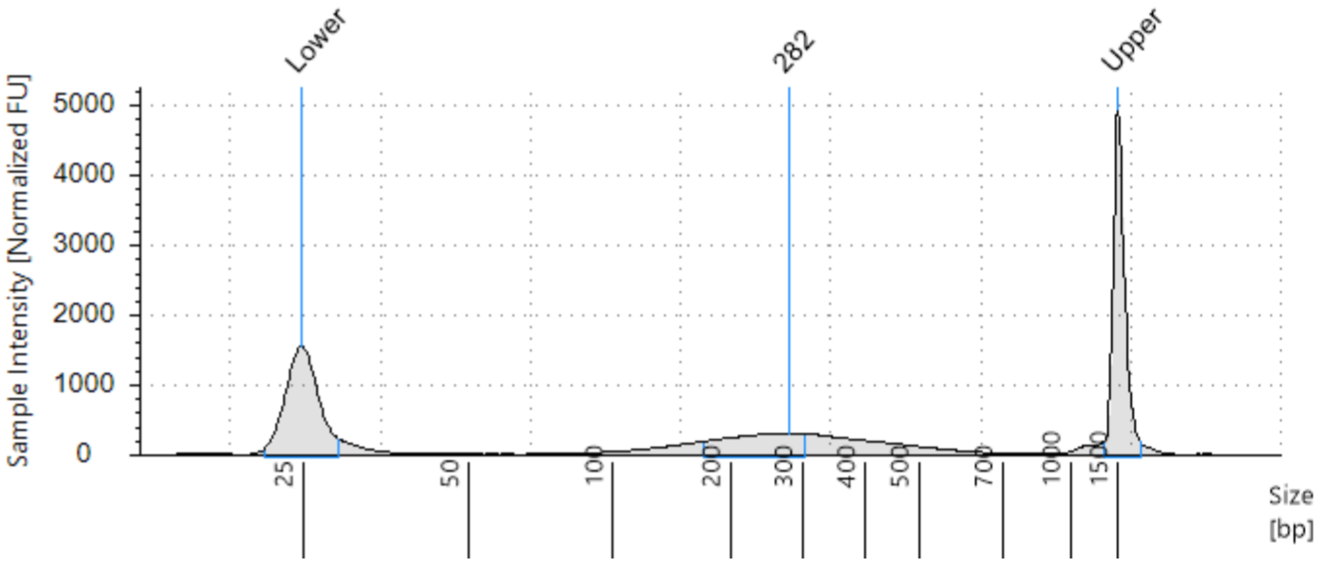

Sample Table

Well	Conc. [ng/µl]	Sample Description	Alert	Observations
D2	2.53	D11 P		

Peak Table

Size [bp]	Calibrated Conc. [ng/µl]	Assigned Conc. [ng/µl]	Peak Molarity [nmol/l]	% Integrated Area	Peak Comment	Observations
25	5.68	-	350	-		Lower Marker
282	2.53	-	13.8	100.00		
1500	6.50	6.50	6.67	-		Upper Marker

 © Agilent Technologies, Inc. 2017

E2: E11 P

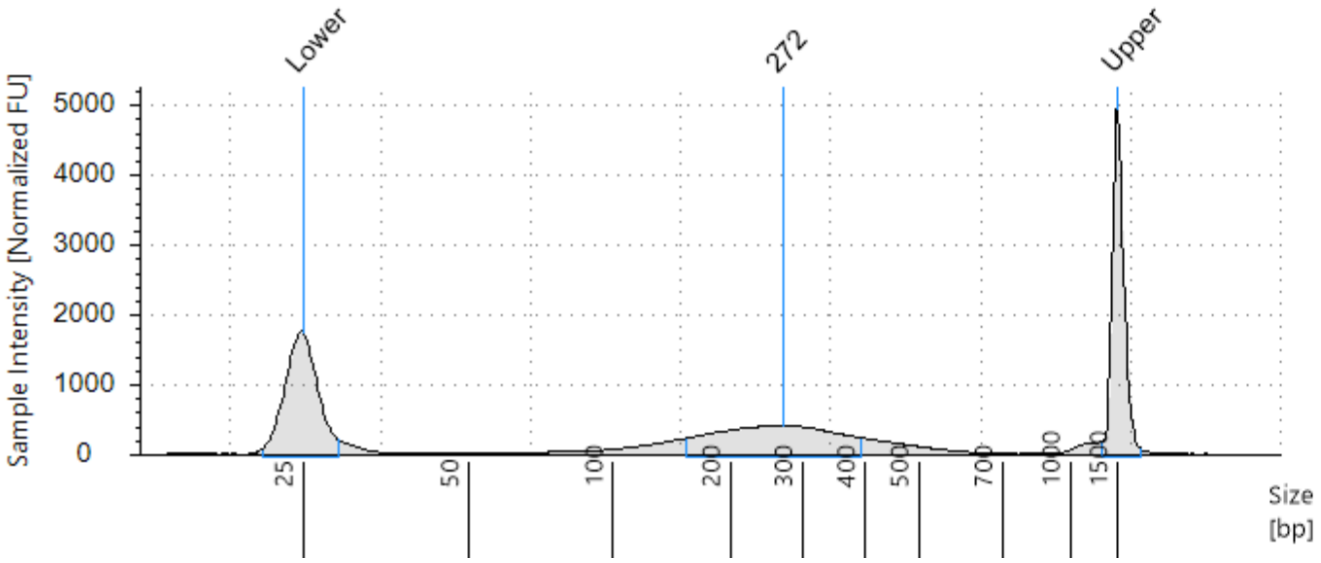

Sample Table

Well	Conc. [ng/µl]	Sample Description	Alert	Observations
E2	5.61	E11 P		

Peak Table

Size [bp]	Calibrated Conc. [ng/µl]	Assigned Conc. [ng/µl]	Peak Molarity [nmol/l]	% Integrated Area	Peak Comment	Observations
25	6.36	-	392	-		Lower Marker
272	5.61	-	31.7	100.00		
1500	6.50	6.50	6.67	-		Upper Marker

© Agilent Technologies, Inc. 2017

F2: F11 P

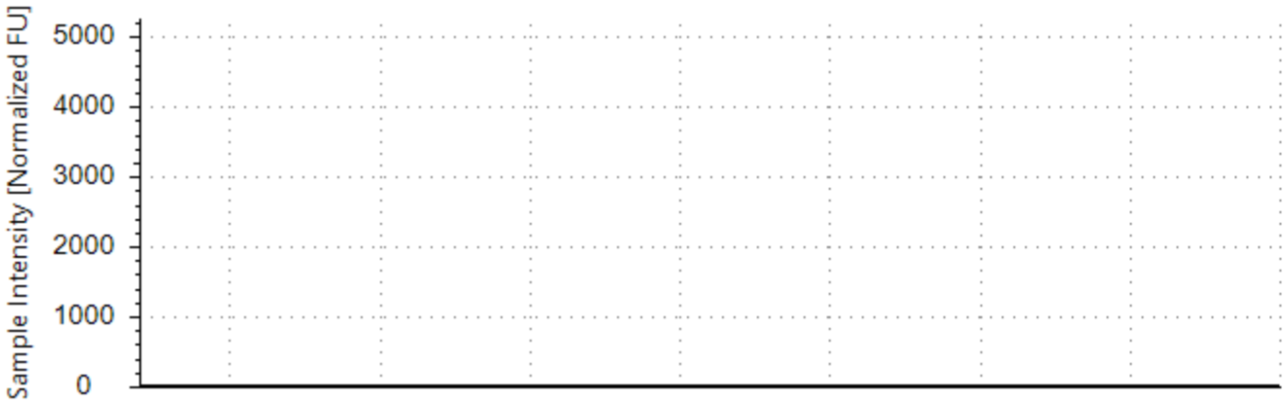

Sample Table

Well	Conc. [ng/µl]	Sample Description	Alert	Observations
F2		F11 P	▲	Marker(s) not detected

 © Agilent Technologies, Inc. 2017

G2: G11 P

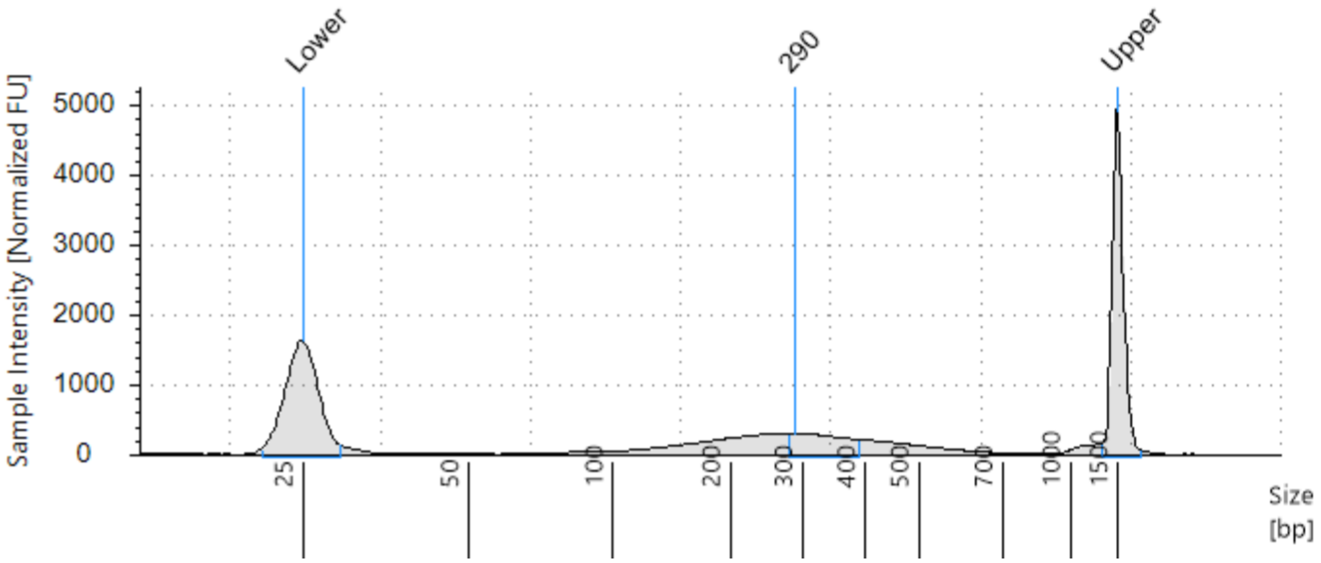

Sample Table

Well	Conc. [ng/µl]	Sample Description	Alert	Observations
G2	1.85	G11 P		

Peak Table

Size [bp]	Calibrated Conc. [ng/µl]	Assigned Conc. [ng/µl]	Peak Molarity [nmol/l]	% Integrated Area	Peak Comment	Observations
25	6.37	-	392	-		Lower Marker
290	1.85	-	9.79	100.00		
1500	6.50	6.50	6.67	-		Upper Marker

 © Agilent Technologies, Inc. 2017

H2: H11 P

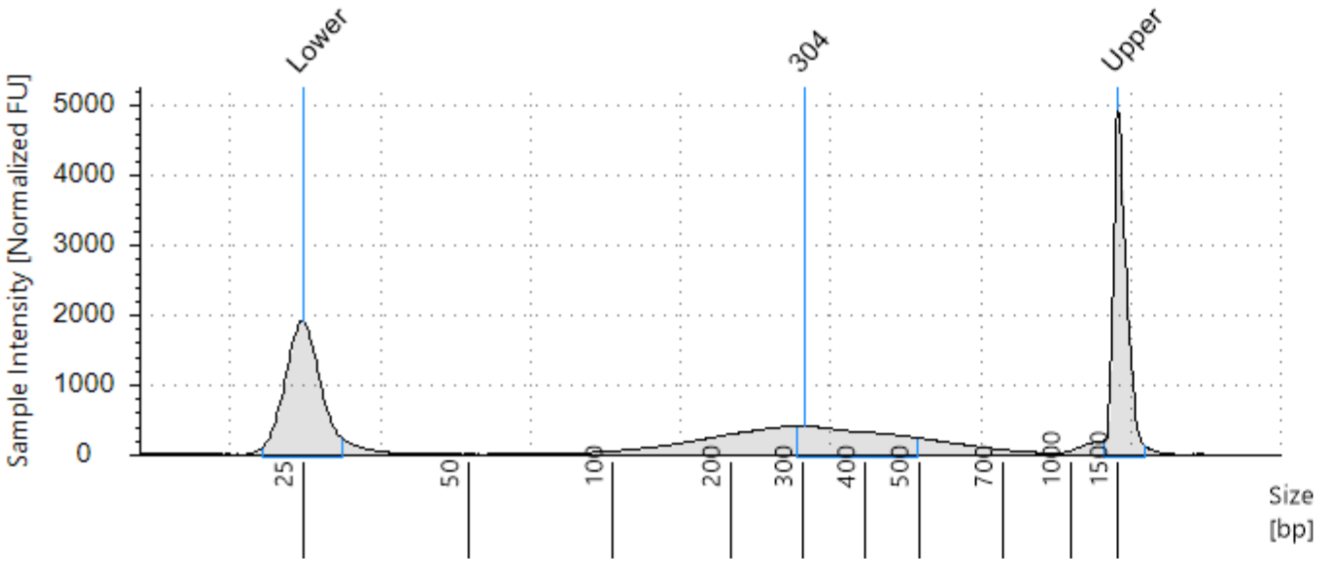

Sample Table

Well	Conc. [ng/µl]	Sample Description	Alert	Observations
H2	3.40	H11 P		

Peak Table

Size [bp]	Calibrated Conc. [ng/µl]	Assigned Conc. [ng/µl]	Peak Molarity [nmol/l]	% Integrated Area	Peak Comment	Observations
25	6.67	-	410	-		Lower Marker
304	3.40	-	17.2	100.00		
1500	6.50	6.50	6.67	-		Upper Marker

© Agilent Technologies, Inc. 2017

Filename: 2020-09-15-04 Q-S plus G12,H12 R2 G3,H3,D5,H5,D7, R2 E1,E6 M R1.D1000

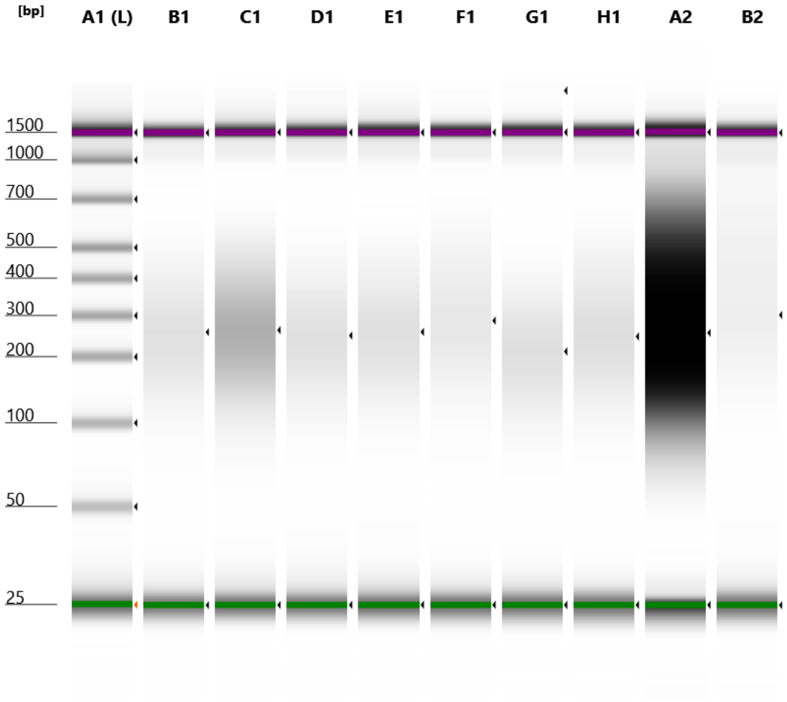

Default image (Contrast 100%)

Sample Info

Well	Conc. [ng/µl]	Sample Description	Alert	Observations
A1	11.5	Ladder		Ladder
B1	3.85	G12 P R2		
C1	10.6	H12 P R2		
D1	2.10	G3 P R2		
E1	1.82	H3 P R2		
F1	3.20	D5 P R2		
G1	3.85	H5 P R2		
H1	4.27	D7 P R2		
A2	47.6	E1 M R1		
B2	0.518	E6 M R1		

© Agilent Technologies, Inc. 2017

A1: Ladder

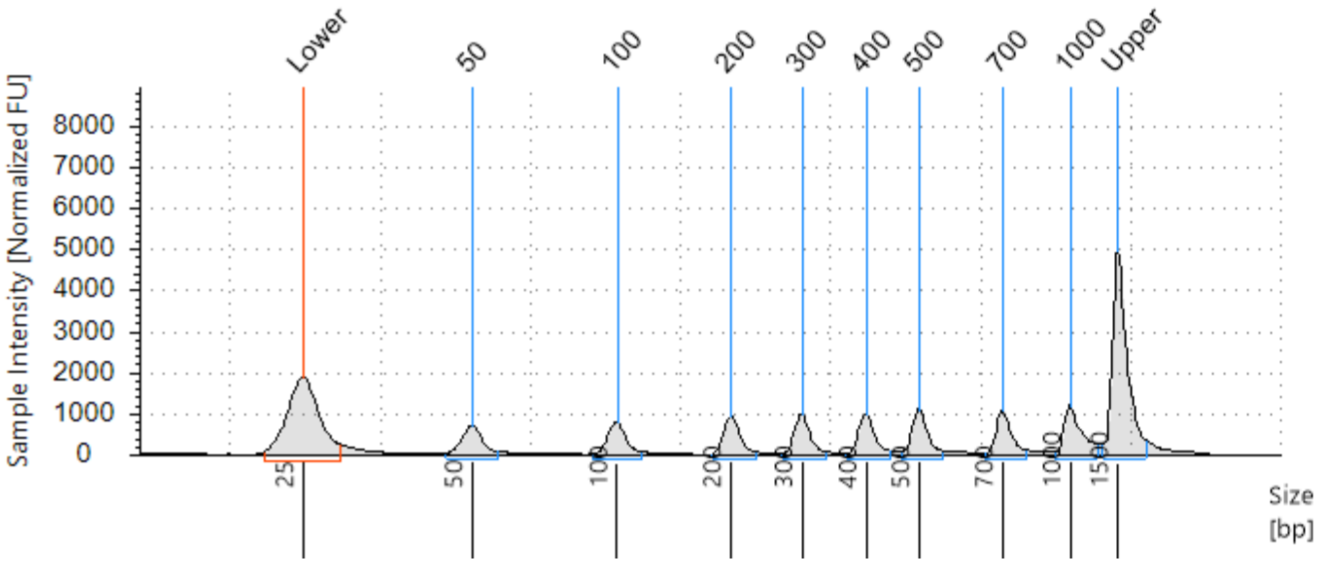

Sample Table

Well	Conc. [ng/µl]	Sample Description	Alert	Observations
A1	11.5	Ladder		Ladder

Peak Table

Size [bp]	Calibrated Conc. [ng/µl]	Assigned Conc. [ng/µl]	Peak Molarity [nmol/l]	% Integrated Area	Peak Comment	Observations
25	5.30	-	326	-		Lower Marker
50	1.27	-	39.0	11.02		
100	1.29	-	19.9	11.22		
200	1.35	-	10.4	11.77		
300	1.39	-	7.12	12.07		
400	1.41	-	5.43	12.28		
500	1.51	-	4.65	13.15		
700	1.47	-	3.23	12.77		
1000	1.81	-	2.78	15.71		
1500	6.50	6.50	6.67	-		Upper Marker

 © Agilent Technologies, Inc. 2017

B1: Gl2 P R2

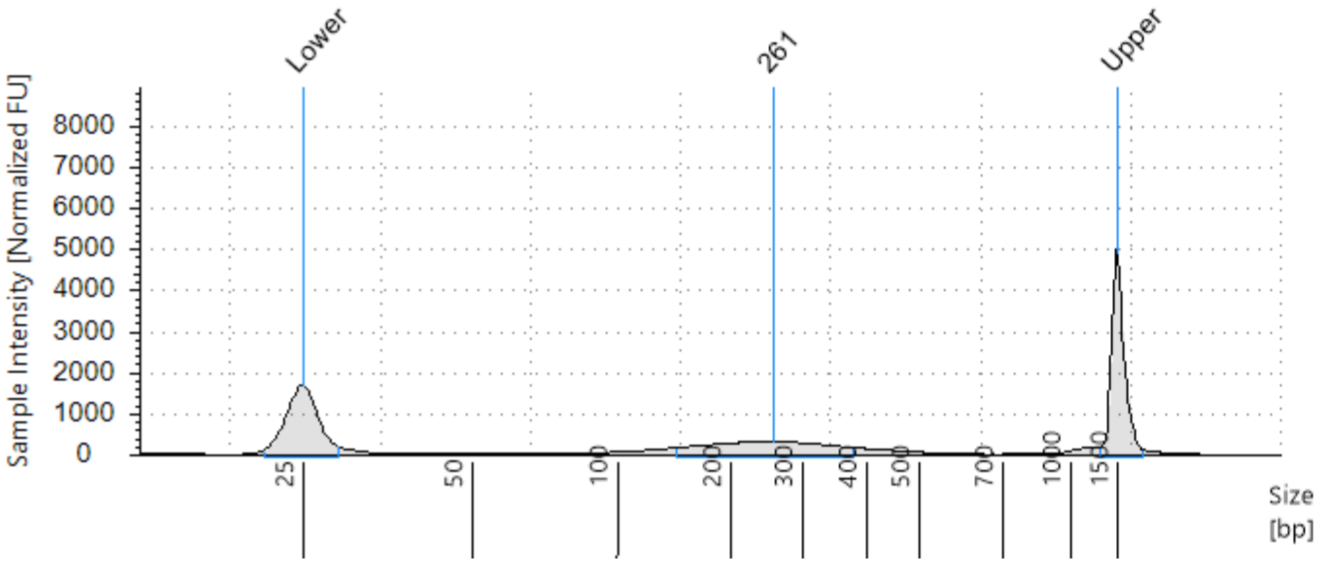

Sample Table

Well	Conc. [ng/µl]	Sample Description	Alert	Observations
B1	3.85	Gl2 P R2		

Peak Table

Size [bp]	Calibrated Conc. [ng/µl]	Assigned Conc. [ng/µl]	Peak Molarity [nmol/l]	% Integrated Area	Peak Comment	Observations
25	5.58	-	344	-		Lower Marker
261	3.85	-	22.7	100.00		
1500	6.50	6.50	6.67	-		Upper Marker

© Agilent Technologies, Inc. 2017

C1: H12 P R2

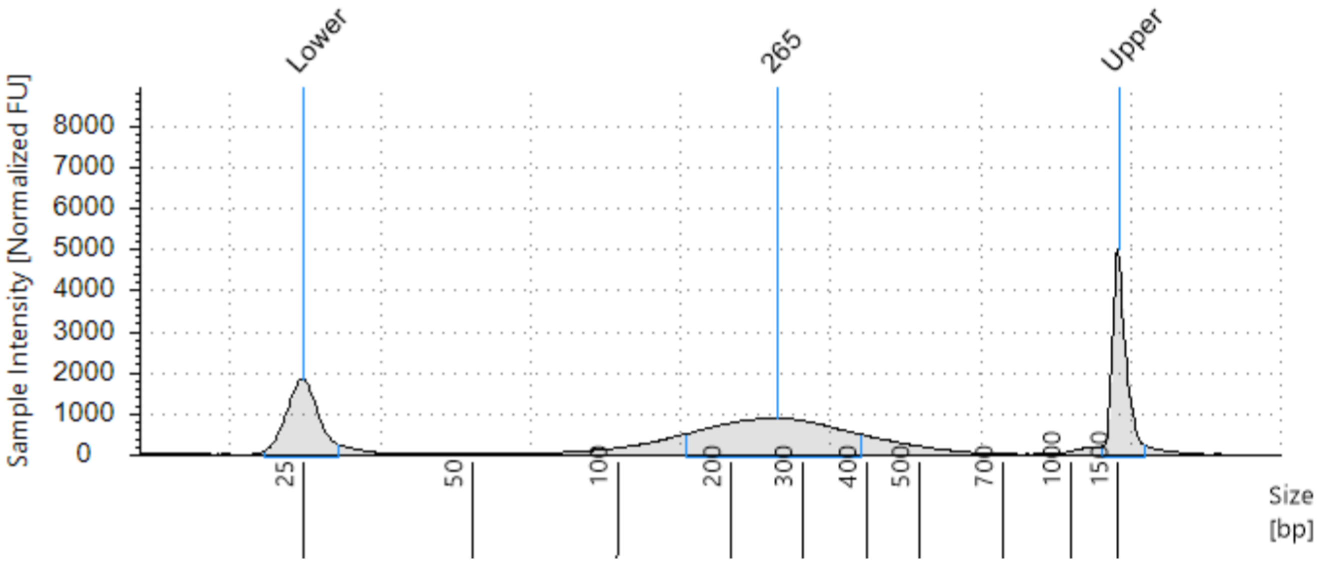

Sample Table

Well	Conc. [ng/µl]	Sample Description	Alert	Observations
C1	10.6	H12 P R2		

Peak Table

Size [bp]	Calibrated Conc. [ng/µl]	Assigned Conc. [ng/µl]	Peak Molarity [nmol/l]	% Integrated Area	Peak Comment	Observations
25	5.64	-	347	-		Lower Marker
265	10.6	-	61.4	100.00		
1500	6.50	6.50	6.67	-		Upper Marker

© Agilent Technologies, Inc. 2017

D1: G3 P R2

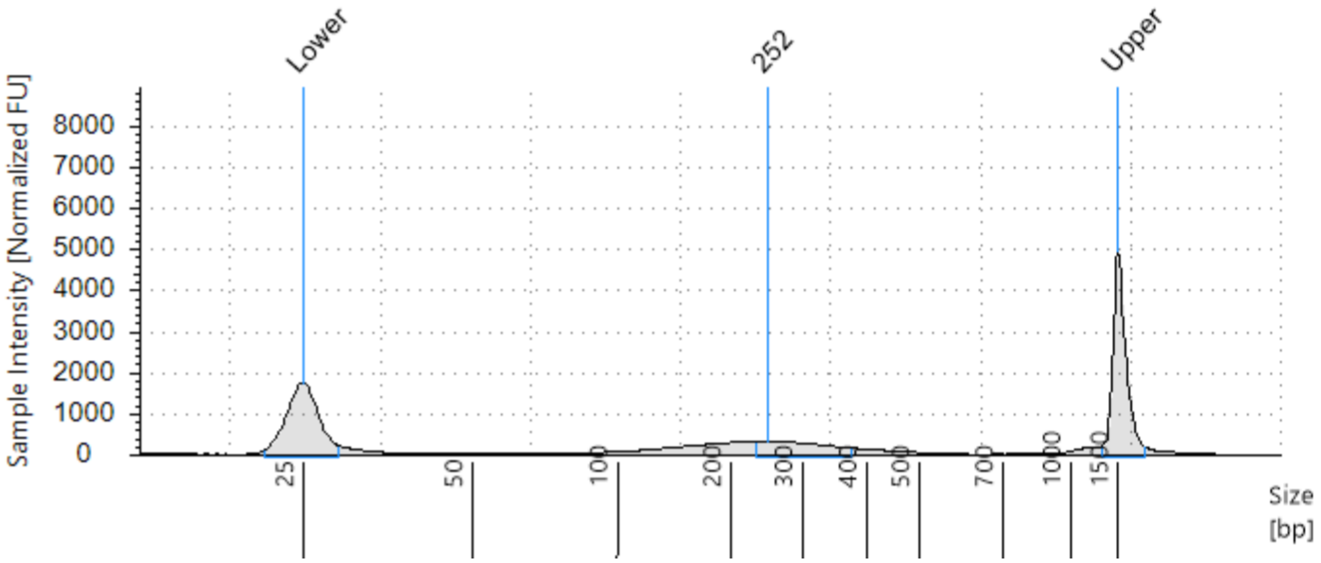

Sample Table

Well	Conc. [ng/µl]	Sample Description	Alert	Observations
D1	2.10	G3 P R2		

Peak Table

Size [bp]	Calibrated Conc. [ng/µl]	Assigned Conc. [ng/µl]	Peak Molarity [nmol/l]	% Integrated Area	Peak Comment	Observations
25	5.65	-	348	-		Lower Marker
252	2.10	-	12.8	100.00		
1500	6.50	6.50	6.67	-		Upper Marker

© Agilent Technologies, Inc. 2017

E1: H3 P R2

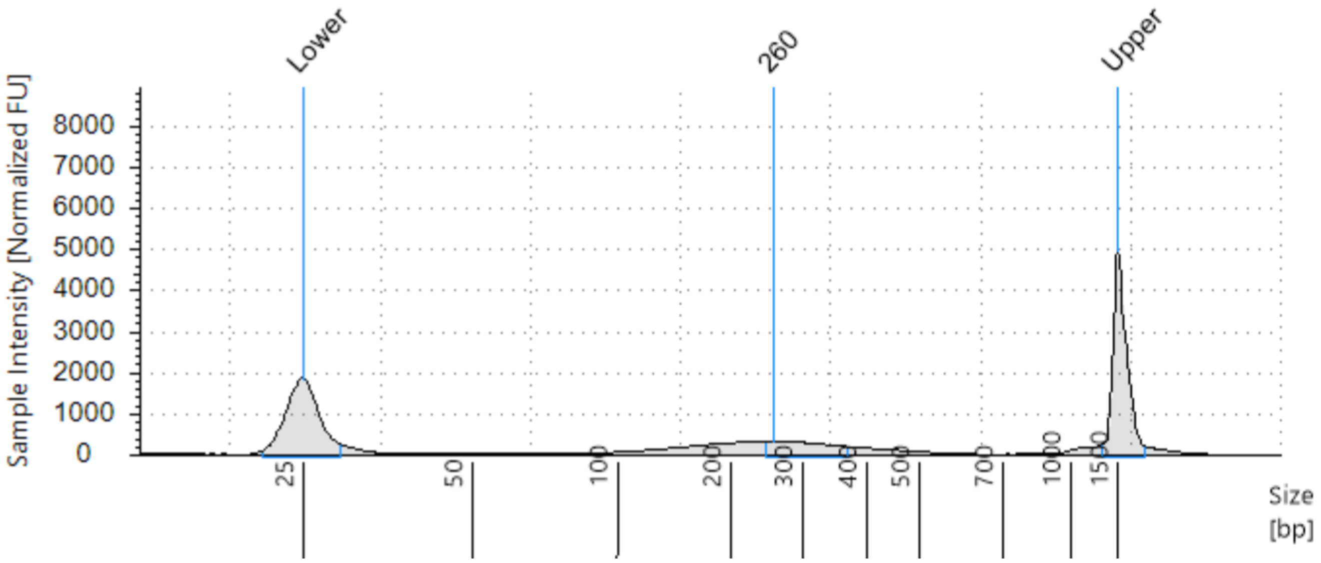

Sample Table

Well	Conc. [ng/µl]	Sample Description	Alert	Observations
E1	1.82	H3 P R2		

Peak Table

Size [bp]	Calibrated Conc. [ng/µl]	Assigned Conc. [ng/µl]	Peak Molarity [nmol/l]	% Integrated Area	Peak Comment	Observations
25	5.85	-	360	-		Lower Marker
260	1.82	-	10.8	100.00		
1500	6.50	6.50	6.67	-		Upper Marker

© Agilent Technologies, Inc. 2017

F1: D5 P R2

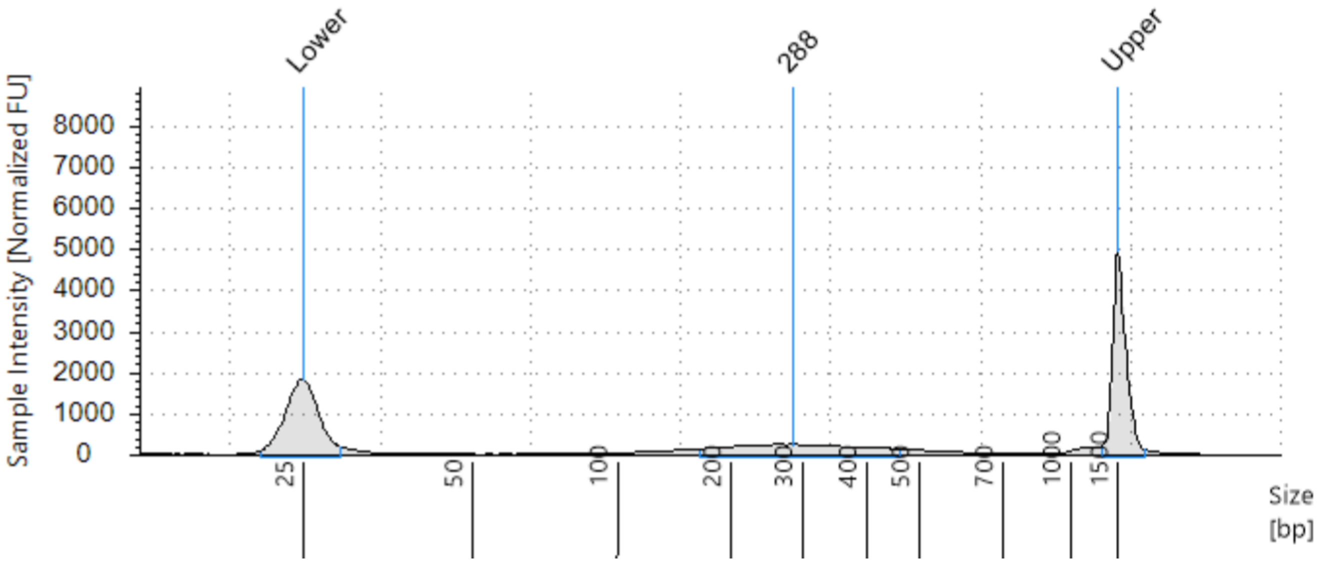

Sample Table

Well	Conc. [ng/µl]	Sample Description	Alert	Observations
F1	3.20	D5 P R2		

Peak Table

Size [bp]	Calibrated Conc. [ng/µl]	Assigned Conc. [ng/µl]	Peak Molarity [nmol/l]	% Integrated Area	Peak Comment	Observations
25	5.98	-	368	-		Lower Marker
288	3.20	-	17.1	100.00		
1500	6.50	6.50	6.67	-		Upper Marker

 © Agilent Technologies, Inc. 2017

G1: H5 P R2

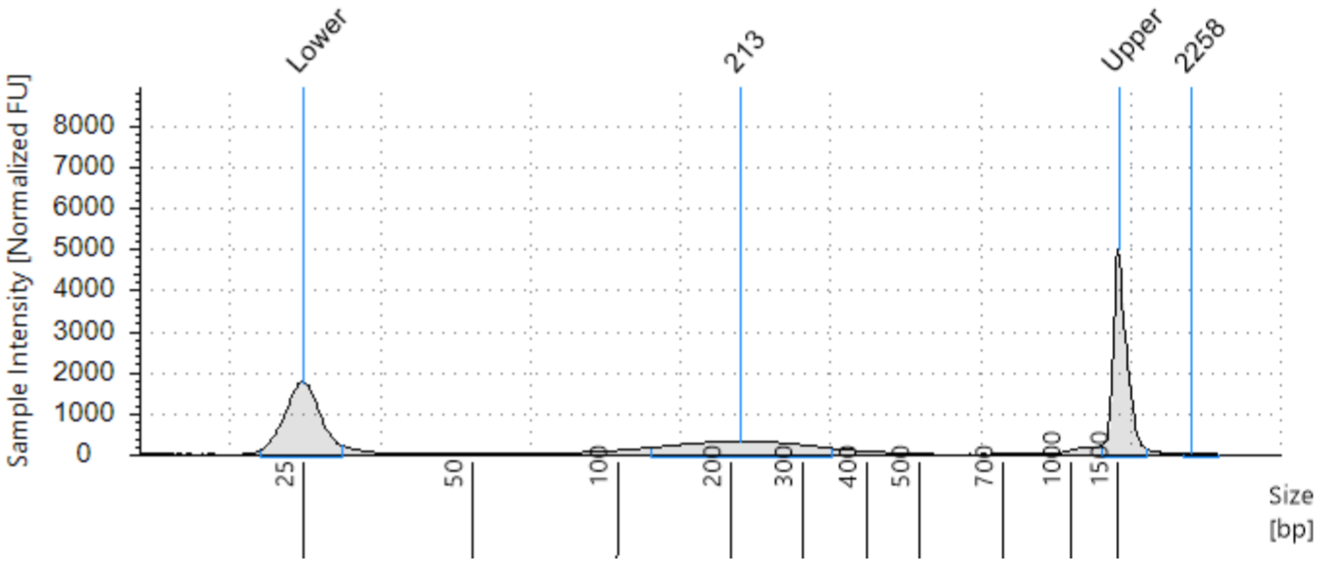

Sample Table

Well	Conc. [ng/µl]	Sample Description	Alert	Observations
G1	3.85	H5 P R2		

Peak Table

Size [bp]	Calibrated Conc. [ng/µl]	Assigned Conc. [ng/µl]	Peak Molarity [nmol/l]	% Integrated Area	Peak Comment	Observations
25	6.04	-	372	-		Lower Marker
213	3.82	-	27.6	99.14		
1500	6.50	6.50	6.67	-		Upper Marker
2258	0.0332	-	0.0226	0.86		

© Agilent Technologies, Inc. 2017

H1: D7 P R2

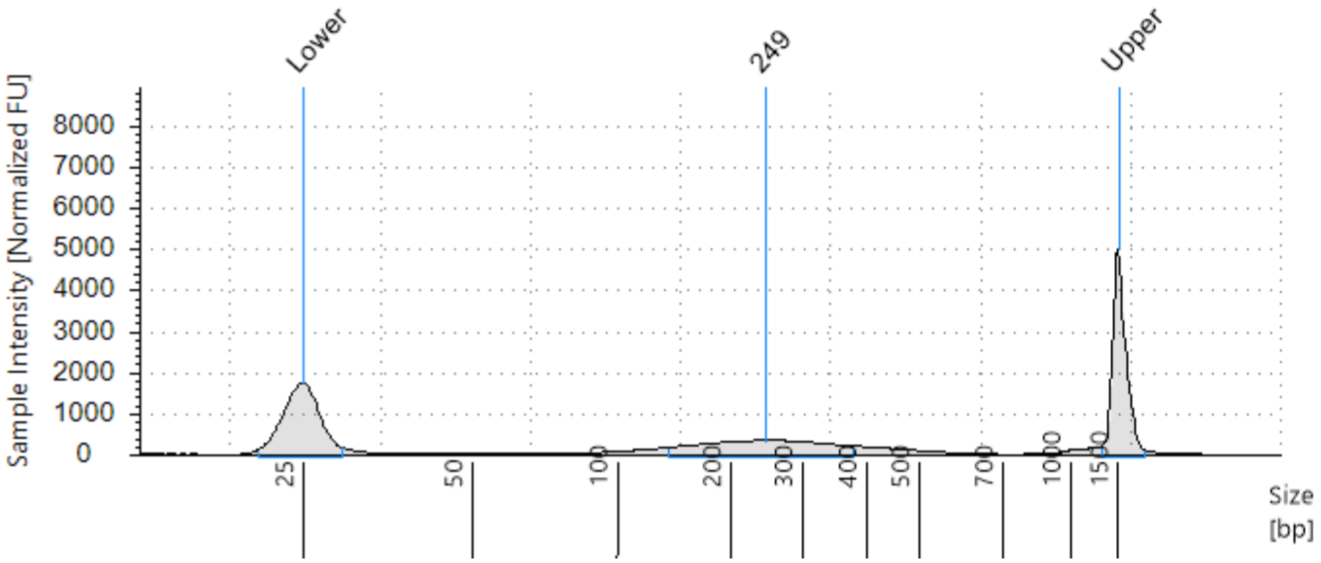

Sample Table

Well	Conc. [ng/µl]	Sample Description	Alert	Observations
H1	4.27	D7 P R2		

Peak Table

Size [bp]	Calibrated Conc. [ng/µl]	Assigned Conc. [ng/µl]	Peak Molarity [nmol/l]	% Integrated Area	Peak Comment	Observations
25	6.26	-	385	-		Lower Marker
249	4.27	-	26.3	100.00		
1500	6.50	6.50	6.67	-		Upper Marker

© Agilent Technologies, Inc. 2017

A2: E1 M R1

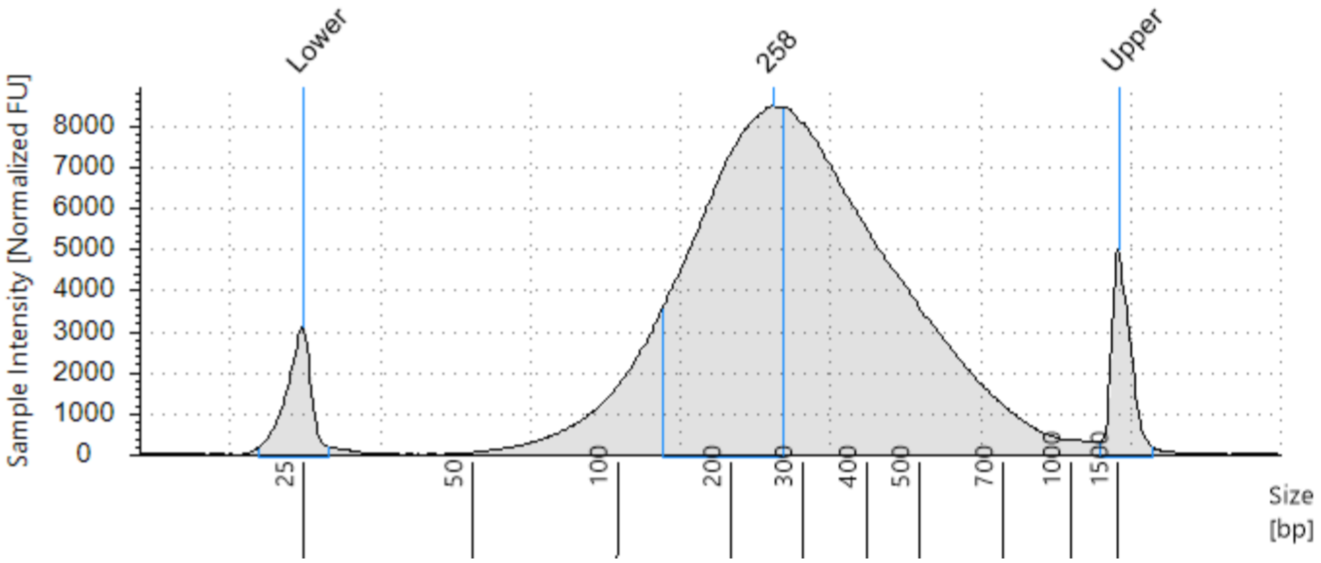

Sample Table

Well	Conc. [ng/µl]	Sample Description	Alert	Observations
A2	47.6	E1 M R1		

Peak Table

Size [bp]	Calibrated Conc. [ng/µl]	Assigned Conc. [ng/µl]	Peak Molarity [nmol/l]	% Integrated Area	Peak Comment	Observations
25	5.34	-	329	-		Lower Marker
258	47.6	-	283	100.00		
1500	6.50	6.50	6.67	-		Upper Marker

© Agilent Technologies, Inc. 2017

B2: E6 M R1

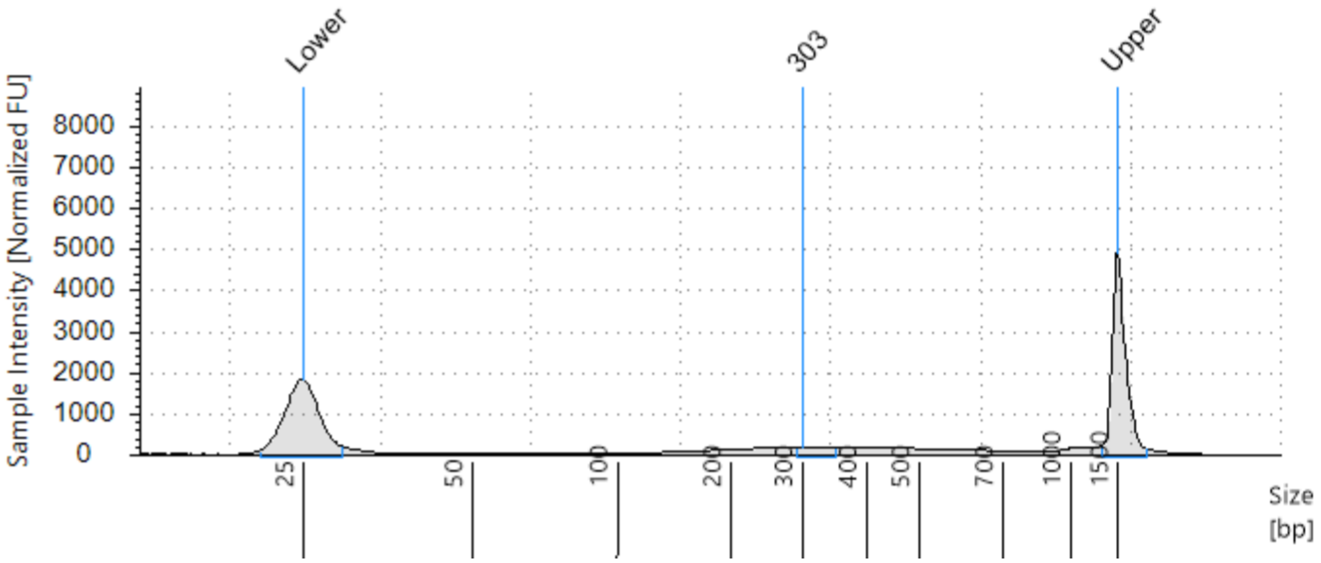

Sample Table

Well	Conc. [ng/µl]	Sample Description	Alert	Observations
B2	0.518	E6 M R1		

Peak Table

Size [bp]	Calibrated Conc. [ng/µl]	Assigned Conc. [ng/µl]	Peak Molarity [nmol/l]	% Integrated Area	Peak Comment	Observations
25	6.29	-	387	-		Lower Marker
303	0.518	-	2.63	100.00		
1500	6.50	6.50	6.67	-		Upper Marker

© Agilent Technologies, Inc. 2017
