# Supplementary material for: 96 sample parallel acoustic fragmentation for high throughput next generation sequencing library preparation
Source: PLoS One. 2026 Feb 17;21(2):e0341139. doi: 10.1371/journal.pone.0341139 (PMC12912608; doi:10.1371/journal.pone.0341139)
Supplement: S5 File — (ZIP) [file pone.0341139.s005.zip › QSonica translator TapeStation raw data/QSonica nanodroplets translator 96-well plate replicate 3.pdf]

Filename: 2020-10-06-01 Q-S plus A1-H2 EXP H1 R3.D1000

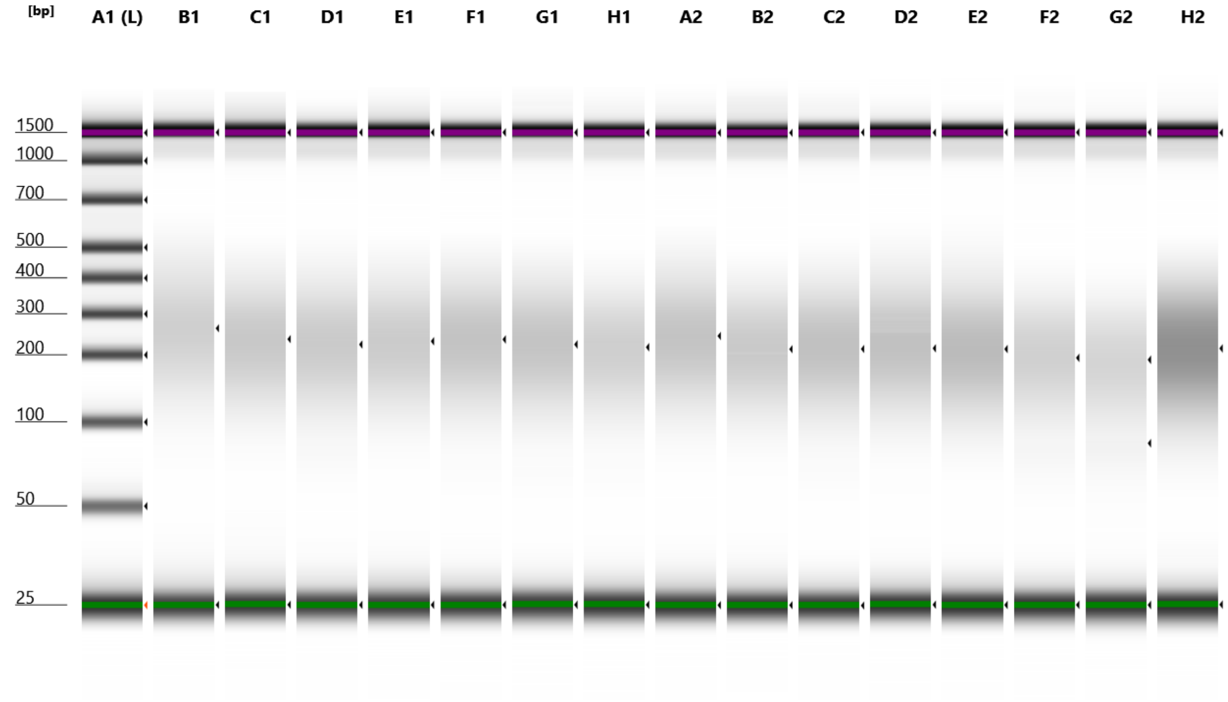

Default image (Contrast 100%)

Sample Info

Well	Conc. [ng/µl]	Sample Description	Alert	Observations
A1	17.5	Ladder		Ladder
B1	2.08	A1 P R3		
C1	2.08	B1 P R3		
D1	2.22	C1 P R3		
E1	2.16	D1 P R3		
F1	4.51	E1 P R3		
G1	4.48	F1 P R3		
H1	0.219	G1 P R3		
A2	2.30	A2 P R3		
B2	2.15	B2 P R3		
C2	2.52	C2 P R3		
D2	3.07	D2 P R3		
E2	2.63	E2 P R3		
F2	1.63	F2 P R3		
G2	1.68	G2 P R3		
H2	5.03	H2 P R3		

© Agilent Technologies, Inc. 2017

A1: Ladder

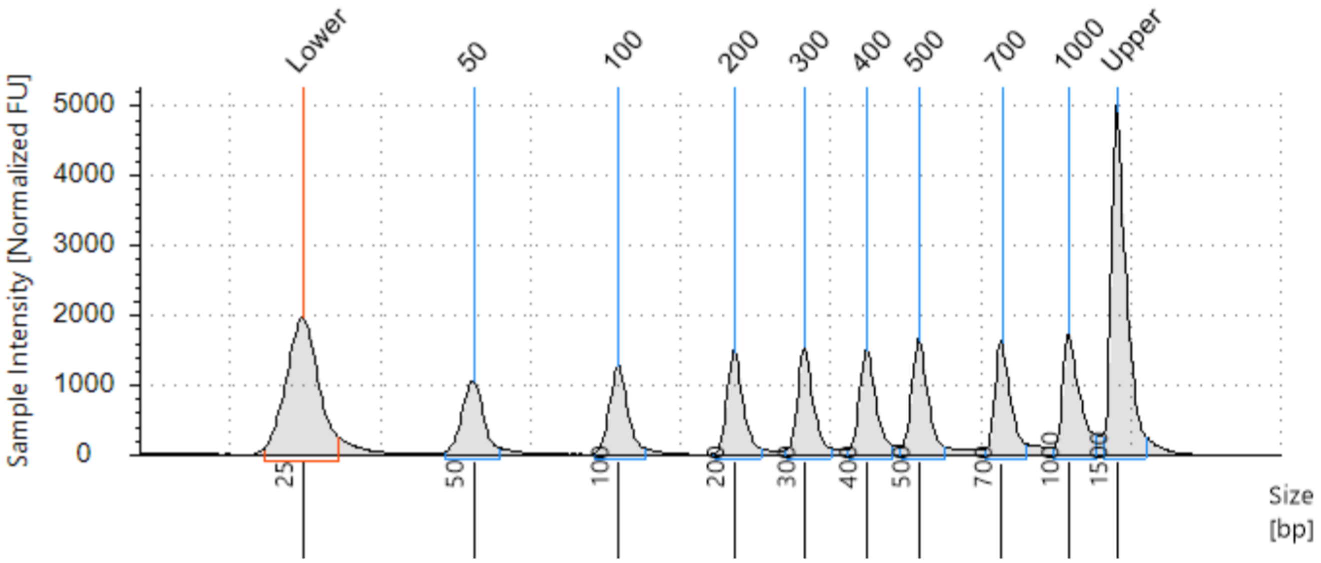

Sample Table

Well	Conc. [ng/µl]	Sample Description	Alert	Observations
A1	17.5	Ladder		Ladder

Peak Table

Size [bp]	Calibrated Conc. [ng/µl]	Assigned Conc. [ng/µl]	Peak Molarity [nmol/l]	% Integrated Area	Peak Comment	Observations
25	5.52	-	339	-		Lower Marker
50	1.97	-	60.8	11.27		
100	2.02	-	31.0	11.50		
200	2.10	-	16.1	11.96		
300	2.12	-	10.9	12.09		
400	2.20	-	8.47	12.56		
500	2.34	-	7.19	13.34		
700	2.21	-	4.86	12.63		
1000	2.57	-	3.95	14.65		
1500	6.50	6.50	6.67	-		Upper Marker

© Agilent Technologies, Inc. 2017

B1: A1 P R3

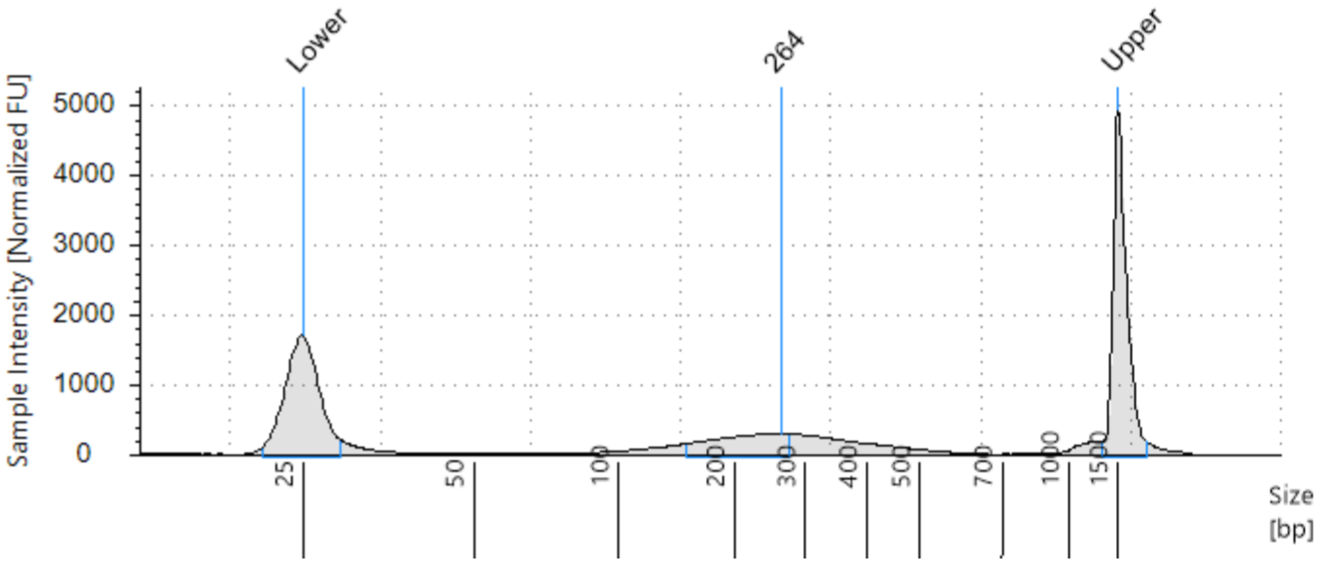

Sample Table

Well	Conc. [ng/µl]	Sample Description	Alert	Observations
B1	2.08	A1 P R3		

Peak Table

Size [bp]	Calibrated Conc. [ng/µl]	Assigned Conc. [ng/µl]	Peak Molarity [nmol/l]	% Integrated Area	Peak Comment	Observations
25	5.65	-	348	-		Lower Marker
264	2.08	-	12.1	100.00		
1500	6.50	6.50	6.67	-		Upper Marker

© Agilent Technologies, Inc. 2017

C1: B1 P R3

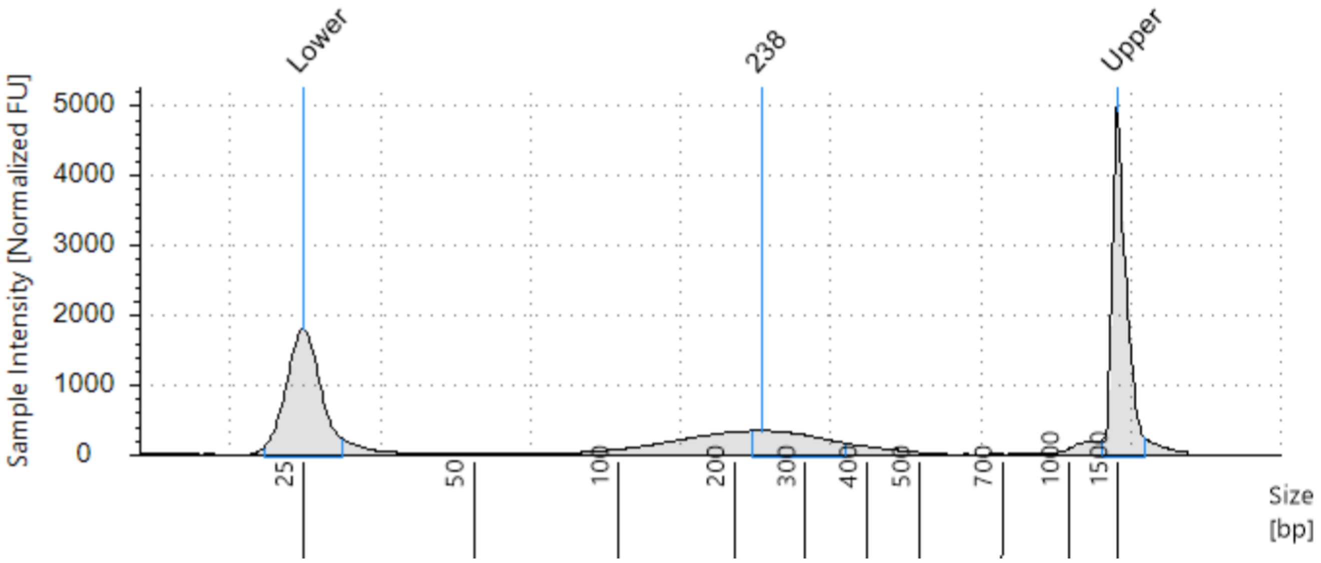

Sample Table

Well	Conc. [ng/µl]	Sample Description	Alert	Observations
C1	2.08	B1 P R3		

Peak Table

Size [bp]	Calibrated Conc. [ng/µl]	Assigned Conc. [ng/µl]	Peak Molarity [nmol/l]	% Integrated Area	Peak Comment	Observations
25	5.73	-	352	-		Lower Marker
238	2.08	-	13.5	100.00		
1500	6.50	6.50	6.67	-		Upper Marker

© Agilent Technologies, Inc. 2017

D1: C1 P R3

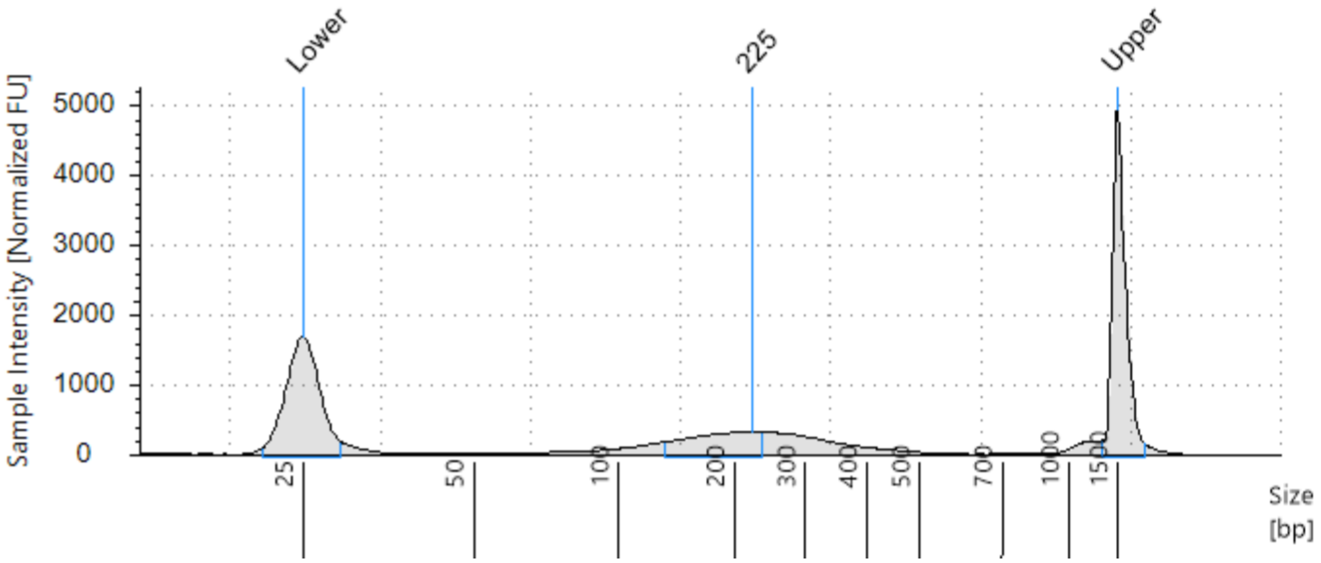

Sample Table

Well	Conc. [ng/µl]	Sample Description	Alert	Observations
D1	2.22	C1 P R3		

Peak Table

Size [bp]	Calibrated Conc. [ng/µl]	Assigned Conc. [ng/µl]	Peak Molarity [nmol/l]	% Integrated Area	Peak Comment	Observations
25	5.68	-	349	-		Lower Marker
225	2.22	-	15.2	100.00		
1500	6.50	6.50	6.67	-		Upper Marker

© Agilent Technologies, Inc. 2017

E1: D1 P R3

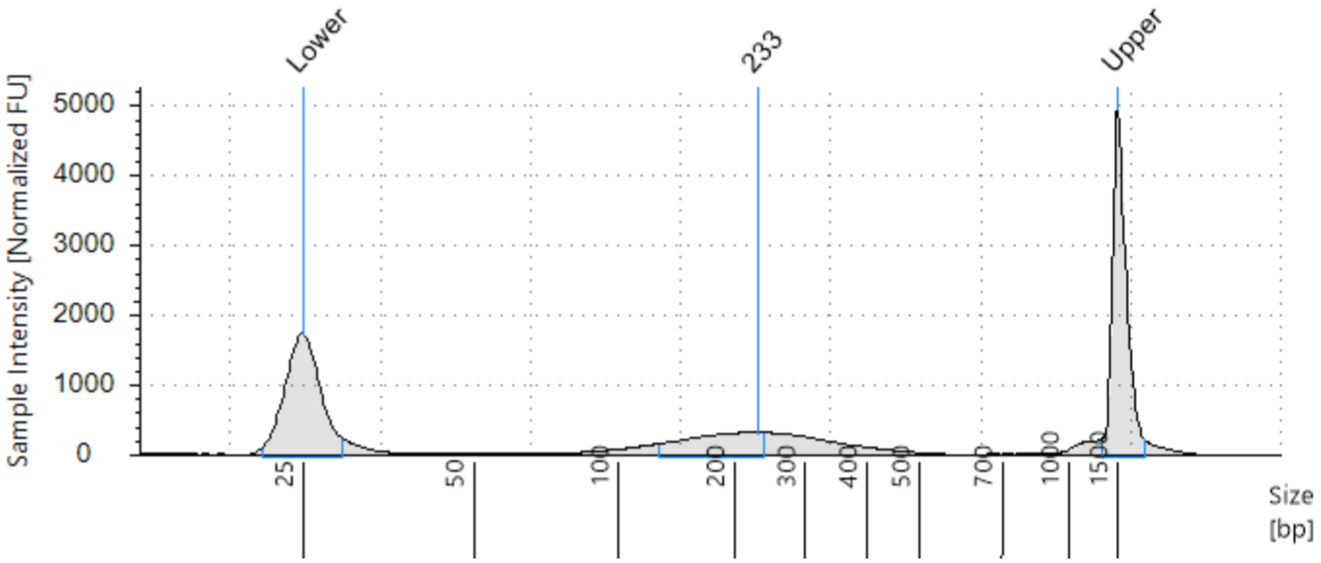

Sample Table

Well	Conc. [ng/µl]	Sample Description	Alert	Observations
E1	2.16	D1 P R3		

Peak Table

Size [bp]	Calibrated Conc. [ng/µl]	Assigned Conc. [ng/µl]	Peak Molarity [nmol/l]	% Integrated Area	Peak Comment	Observations
25	5.73	-	353	-		Lower Marker
233	2.16	-	14.3	100.00		
1500	6.50	6.50	6.67	-		Upper Marker

© Agilent Technologies, Inc. 2017

F1: E1 P R3

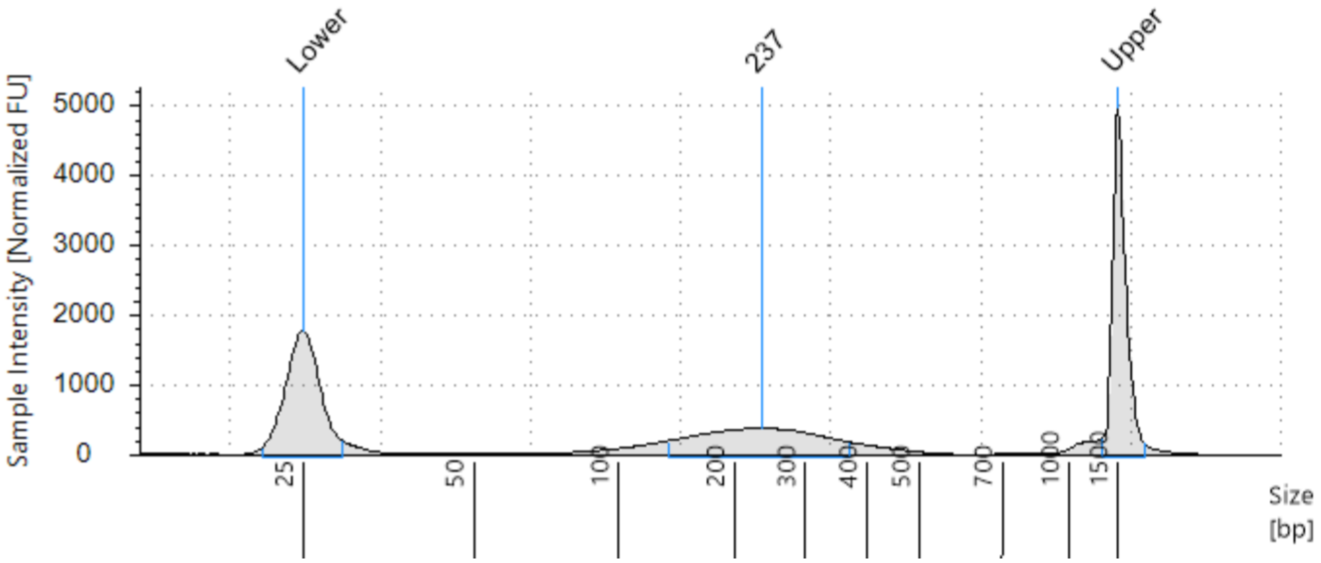

Sample Table

Well	Conc. [ng/µl]	Sample Description	Alert	Observations
F1	4.51	E1 P R3		

Peak Table

Size [bp]	Calibrated Conc. [ng/µl]	Assigned Conc. [ng/µl]	Peak Molarity [nmol/l]	% Integrated Area	Peak Comment	Observations
25	5.92	-	364	-		Lower Marker
237	4.51	-	29.3	100.00		
1500	6.50	6.50	6.67	-		Upper Marker

© Agilent Technologies, Inc. 2017

G1: F1 P R3

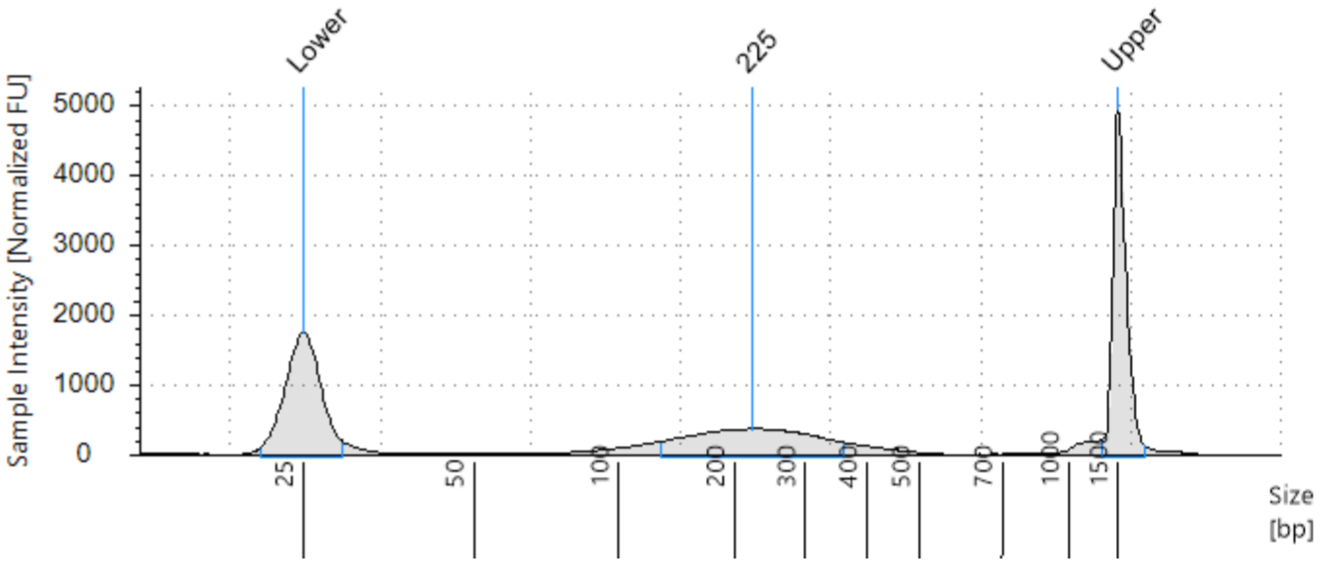

Sample Table

Well	Conc. [ng/µl]	Sample Description	Alert	Observations
G1	4.48	F1 P R3		

Peak Table

Size [bp]	Calibrated Conc. [ng/µl]	Assigned Conc. [ng/µl]	Peak Molarity [nmol/l]	% Integrated Area	Peak Comment	Observations
25	6.10	-	376	-		Lower Marker
225	4.48	-	30.7	100.00		
1500	6.50	6.50	6.67	-		Upper Marker

© Agilent Technologies, Inc. 2017

H1: G1 P R3

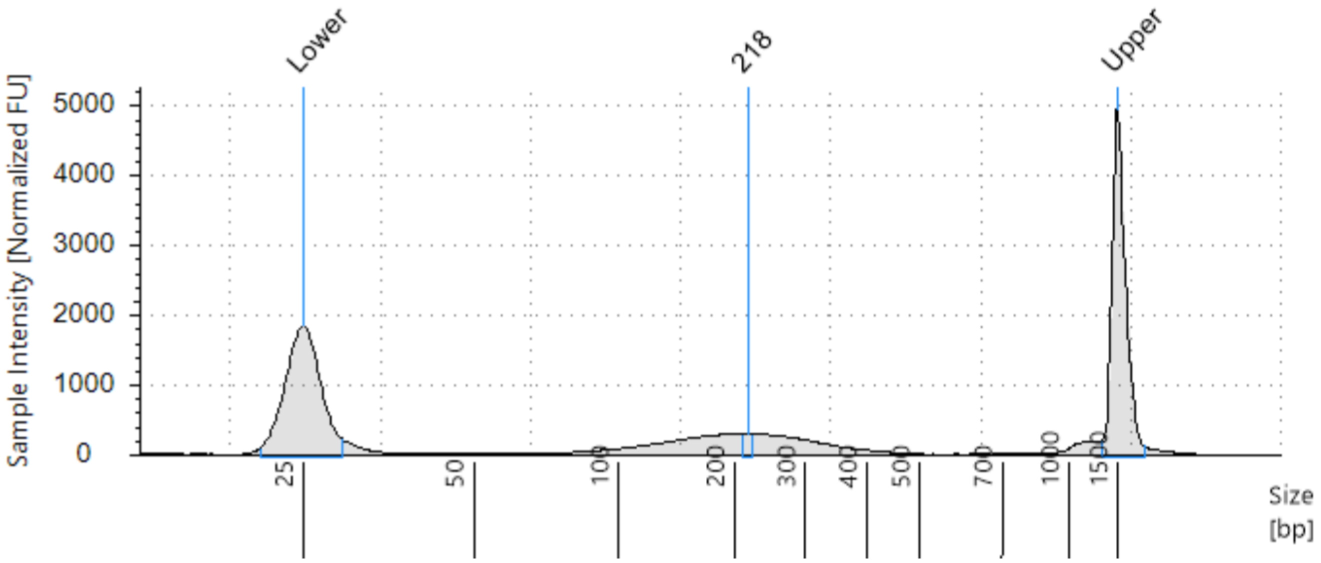

Sample Table

Well	Conc. [ng/µl]	Sample Description	Alert	Observations
H1	0.219	G1 P R3		

Peak Table

Size [bp]	Calibrated Conc. [ng/µl]	Assigned Conc. [ng/µl]	Peak Molarity [nmol/l]	% Integrated Area	Peak Comment	Observations
25	6.31	-	388	-		Lower Marker
218	0.219	-	1.54	100.00		
1500	6.50	6.50	6.67	-		Upper Marker

© Agilent Technologies, Inc. 2017

A2: A2 P R3

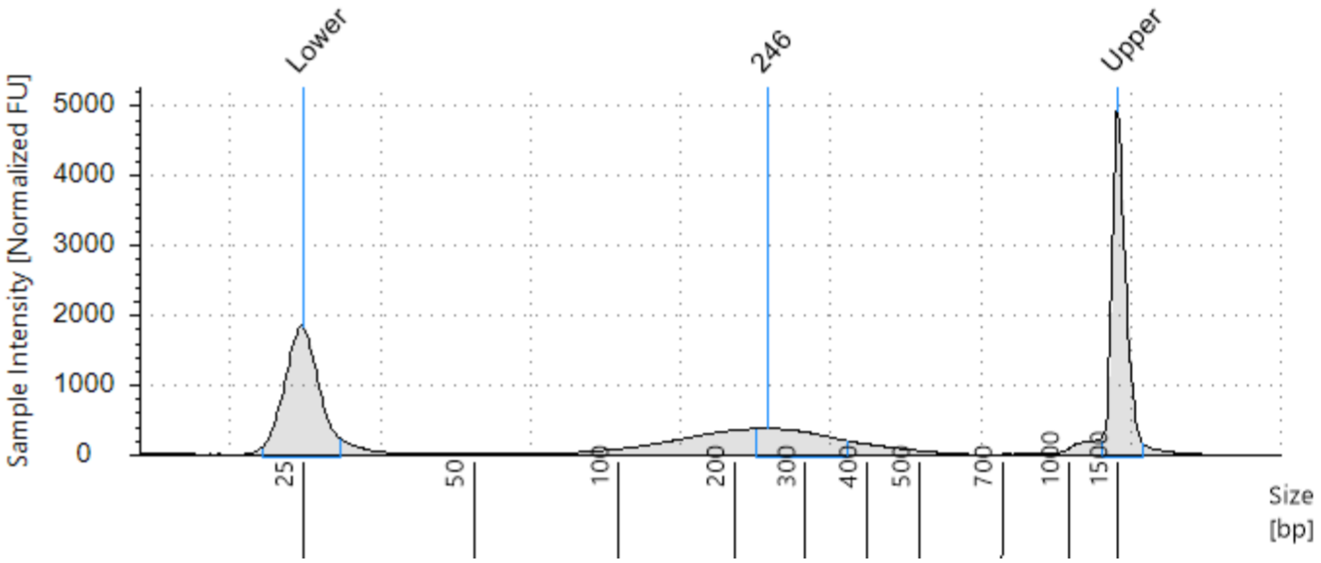

Sample Table

Well	Conc. [ng/µl]	Sample Description	Alert	Observations
A2	2.30	A2 P R3		

Peak Table

Size [bp]	Calibrated Conc. [ng/µl]	Assigned Conc. [ng/µl]	Peak Molarity [nmol/l]	% Integrated Area	Peak Comment	Observations
25	5.89	-	363	-		Lower Marker
246	2.30	-	14.4	100.00		
1500	6.50	6.50	6.67	-		Upper Marker

© Agilent Technologies, Inc. 2017

B2: B2 P R3

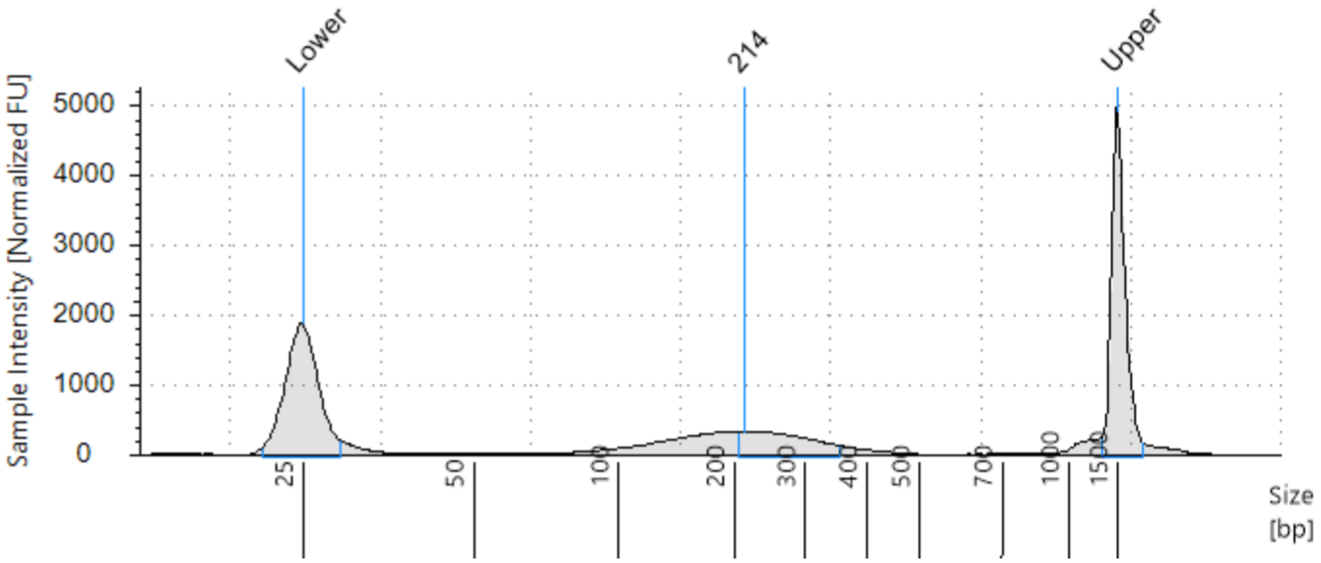

Sample Table

Well	Conc. [ng/µl]	Sample Description	Alert	Observations
B2	2.15	B2 P R3		

Peak Table

Size [bp]	Calibrated Conc. [ng/µl]	Assigned Conc. [ng/µl]	Peak Molarity [nmol/l]	% Integrated Area	Peak Comment	Observations
25	5.91	-	364	-		Lower Marker
214	2.15	-	15.5	100.00		
1500	6.50	6.50	6.67	-		Upper Marker

© Agilent Technologies, Inc. 2017

C2: C2 P R3

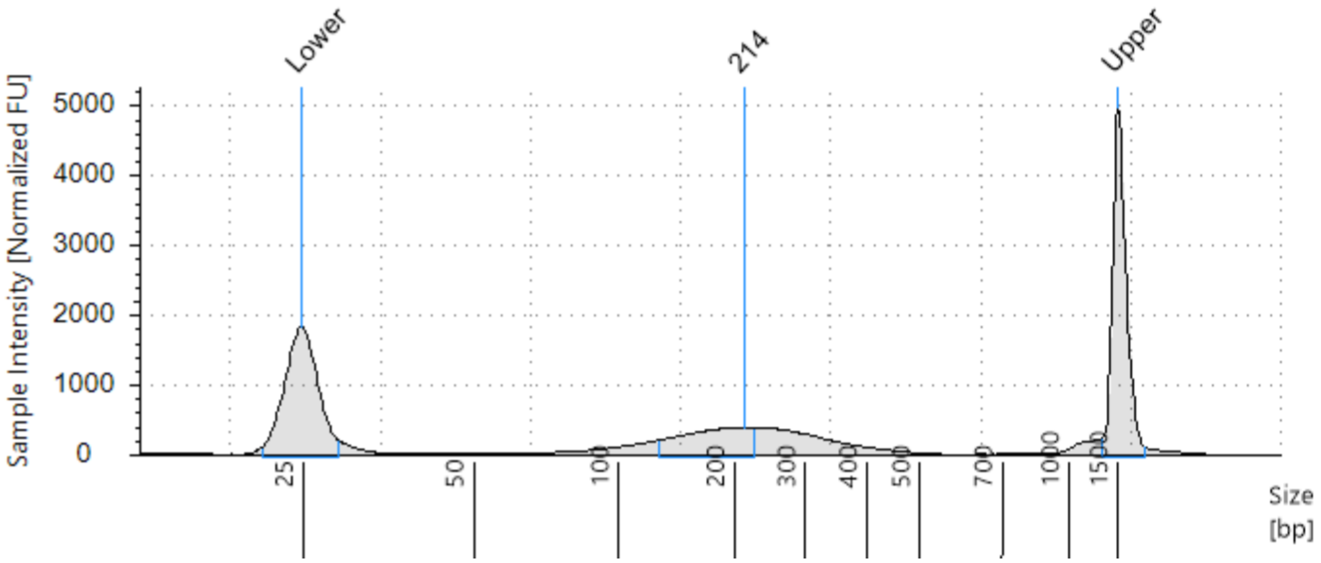

Sample Table

Well	Conc. [ng/µl]	Sample Description	Alert	Observations
C2	2.52	C2 P R3		

Peak Table

Size [bp]	Calibrated Conc. [ng/µl]	Assigned Conc. [ng/µl]	Peak Molarity [nmol/l]	% Integrated Area	Peak Comment	Observations
25	5.85	-	360	-		Lower Marker
214	2.52	-	18.1	100.00		
1500	6.50	6.50	6.67	-		Upper Marker

© Agilent Technologies, Inc. 2017

D2: D2 P R3

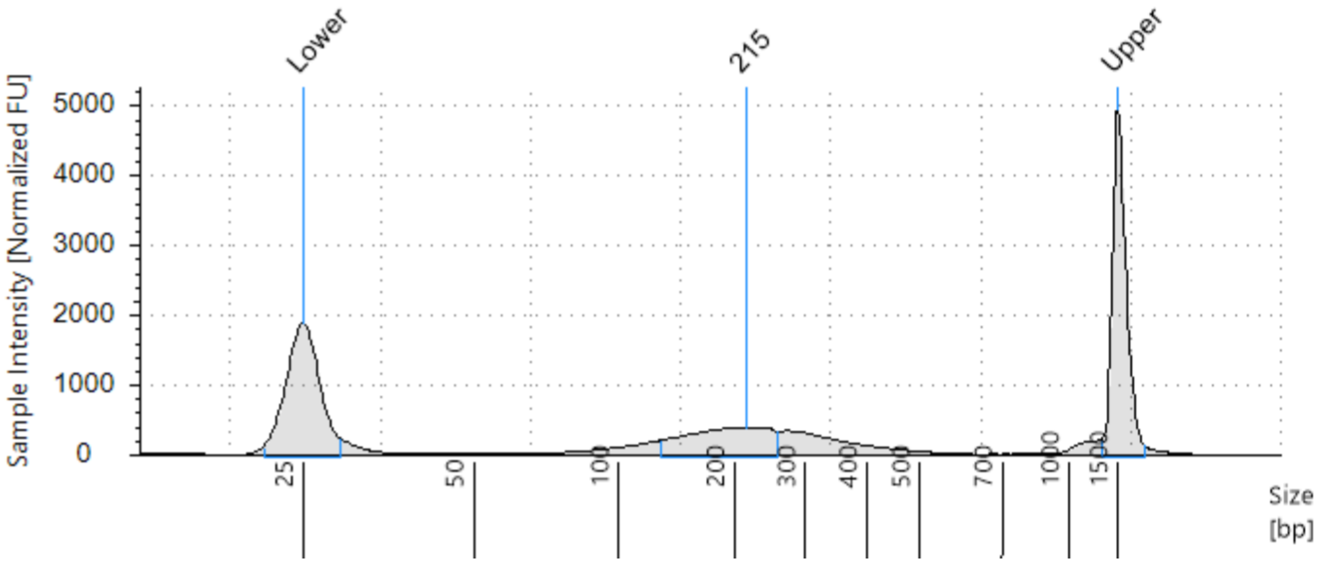

Sample Table

Well	Conc. [ng/µl]	Sample Description	Alert	Observations
D2	3.07	D2 P R3		

Peak Table

Size [bp]	Calibrated Conc. [ng/µl]	Assigned Conc. [ng/µl]	Peak Molarity [nmol/l]	% Integrated Area	Peak Comment	Observations
25	5.93	-	365	-		Lower Marker
215	3.07	-	21.9	100.00		
1500	6.50	6.50	6.67	-		Upper Marker

© Agilent Technologies, Inc. 2017

E2: E2 P R3

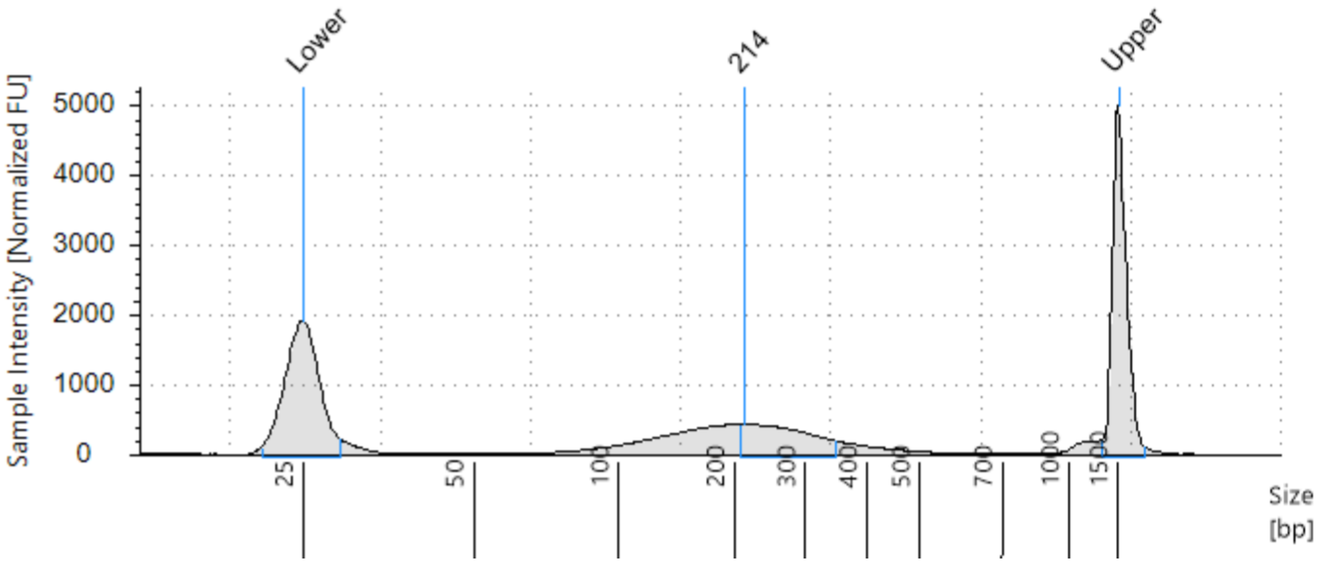

Sample Table

Well	Conc. [ng/µl]	Sample Description	Alert	Observations
E2	2.63	E2 P R3		

Peak Table

Size [bp]	Calibrated Conc. [ng/µl]	Assigned Conc. [ng/µl]	Peak Molarity [nmol/l]	% Integrated Area	Peak Comment	Observations
25	6.13	-	377	-		Lower Marker
214	2.63	-	19.0	100.00		
1500	6.50	6.50	6.67	-		Upper Marker

© Agilent Technologies, Inc. 2017

F2: F2 P R3

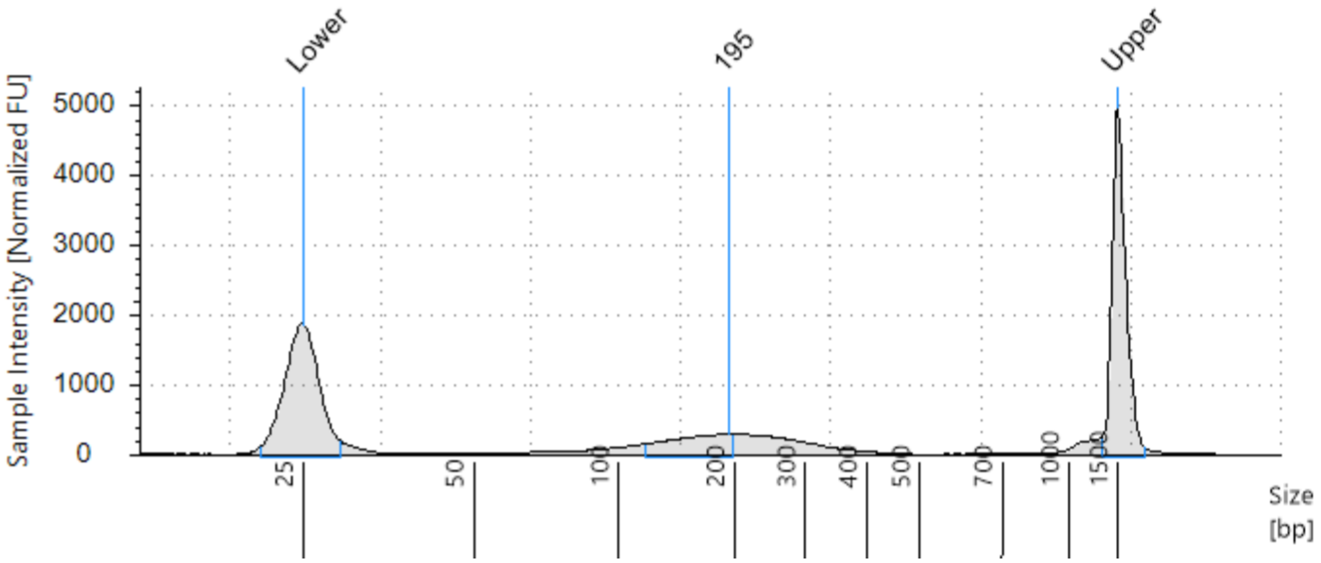

Sample Table

Well	Conc. [ng/µl]	Sample Description	Alert	Observations
F2	1.63	F2 P R3		

Peak Table

Size [bp]	Calibrated Conc. [ng/µl]	Assigned Conc. [ng/µl]	Peak Molarity [nmol/l]	% Integrated Area	Peak Comment	Observations
25	6.10	-	376	-		Lower Marker
195	1.63	-	12.8	100.00		
1500	6.50	6.50	6.67	-		Upper Marker

© Agilent Technologies, Inc. 2017

G2: G2 P R3

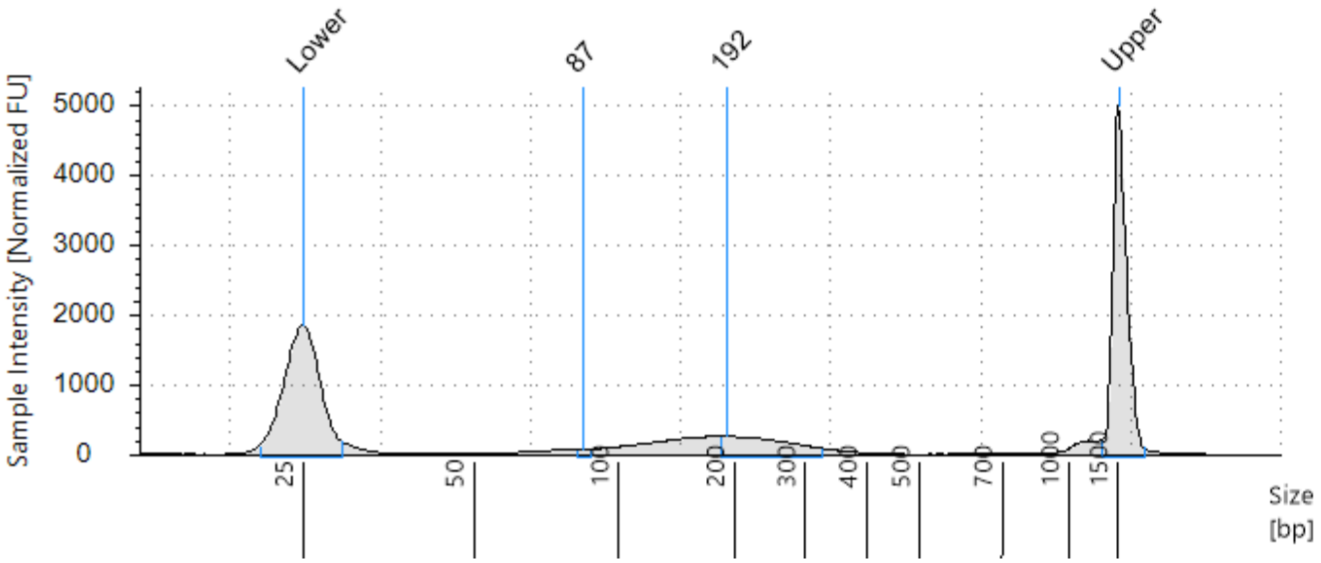

Sample Table

Well	Conc. [ng/µl]	Sample Description	Alert	Observations
G2	1.68	G2 P R3		

Peak Table

Size [bp]	Calibrated Conc. [ng/µl]	Assigned Conc. [ng/µl]	Peak Molarity [nmol/l]	% Integrated Area	Peak Comment	Observations
25	6.29	-	387	-		Lower Marker
87	0.0878	-	1.55	5.23		
192	1.59	-	12.7	94.77		
1500	6.50	6.50	6.67	-		Upper Marker

 © Agilent Technologies, Inc. 2017

H2: H2 P R3

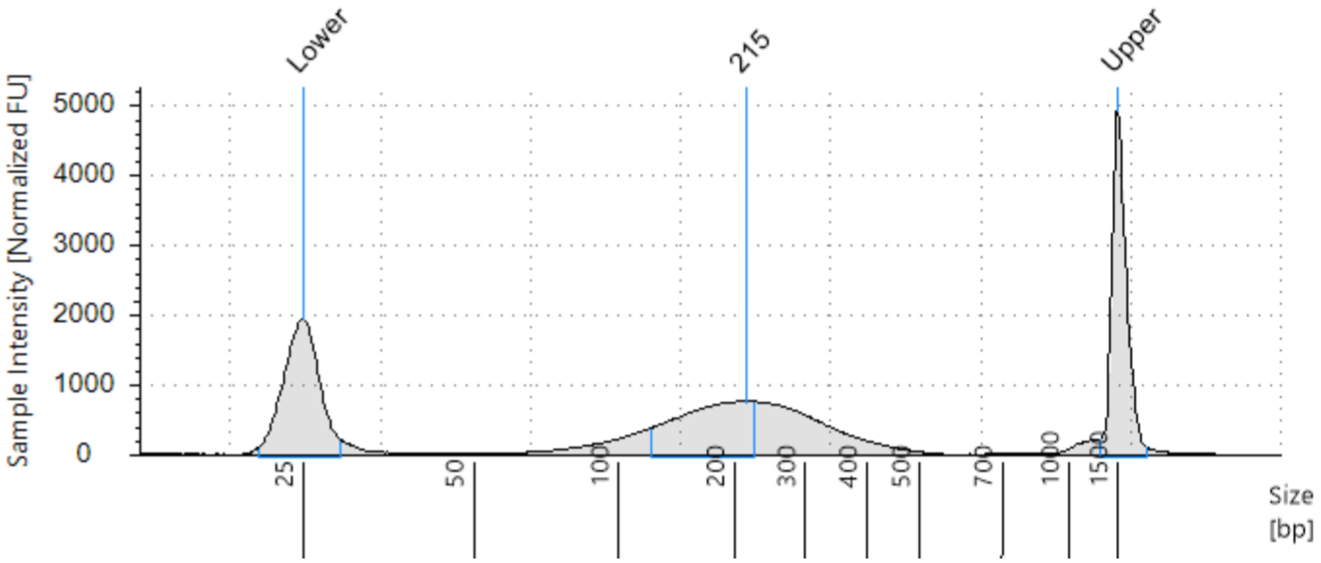

Sample Table

Well	Conc. [ng/µl]	Sample Description	Alert	Observations
H2	5.03	H2 P R3		

Peak Table

Size [bp]	Calibrated Conc. [ng/µl]	Assigned Conc. [ng/µl]	Peak Molarity [nmol/l]	% Integrated Area	Peak Comment	Observations
25	6.22	-	383	-		Lower Marker
215	5.03	-	35.9	100.00		
1500	6.50	6.50	6.67	-		Upper Marker

© Agilent Technologies, Inc. 2017

Filename: 2020-10-06-02 Q-S plus A3-H4 EXP H3 R3.D1000

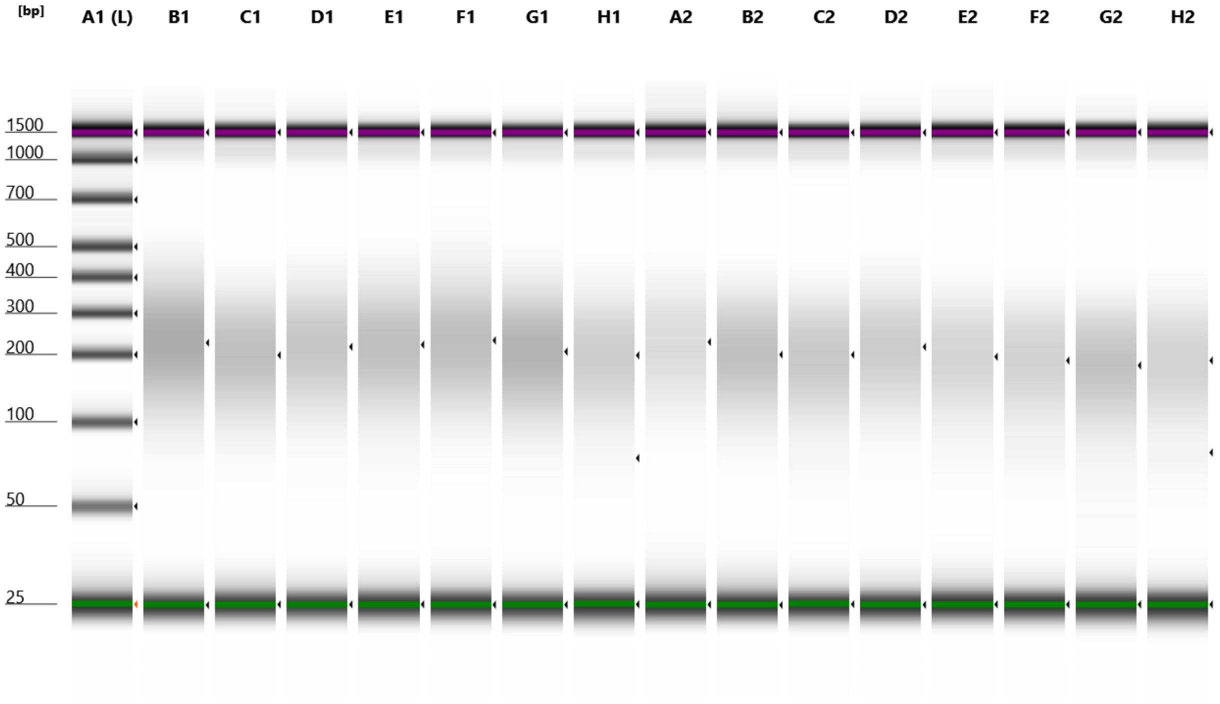

Default image (Contrast 100%)

Sample Info

Well	Conc. [ng/µl]	Sample Description	Alert	Observations
A1	16.5	Ladder		Ladder
B1	0.505	A3 P R3		
C1	2.64	B3 P R3		
D1	4.44	C3 P R3		
E1	5.13	D3 P R3		
F1	4.97	E3 P R3		
G1	3.44	F3 P R3		
H1	3.98	G3 P R3		
A2	0.176	A4 P R3		
B2	2.37	B4 P R3		
C2	2.16	C4 P R3		
D2	1.91	D4 P R3		
E2	0.244	E4 P R3		
F2	1.68	F4 P R3		
G2	4.49	G4 P R3		
H2	2.08	H4 P R3		

 © Agilent Technologies, Inc. 2017

A1: Ladder

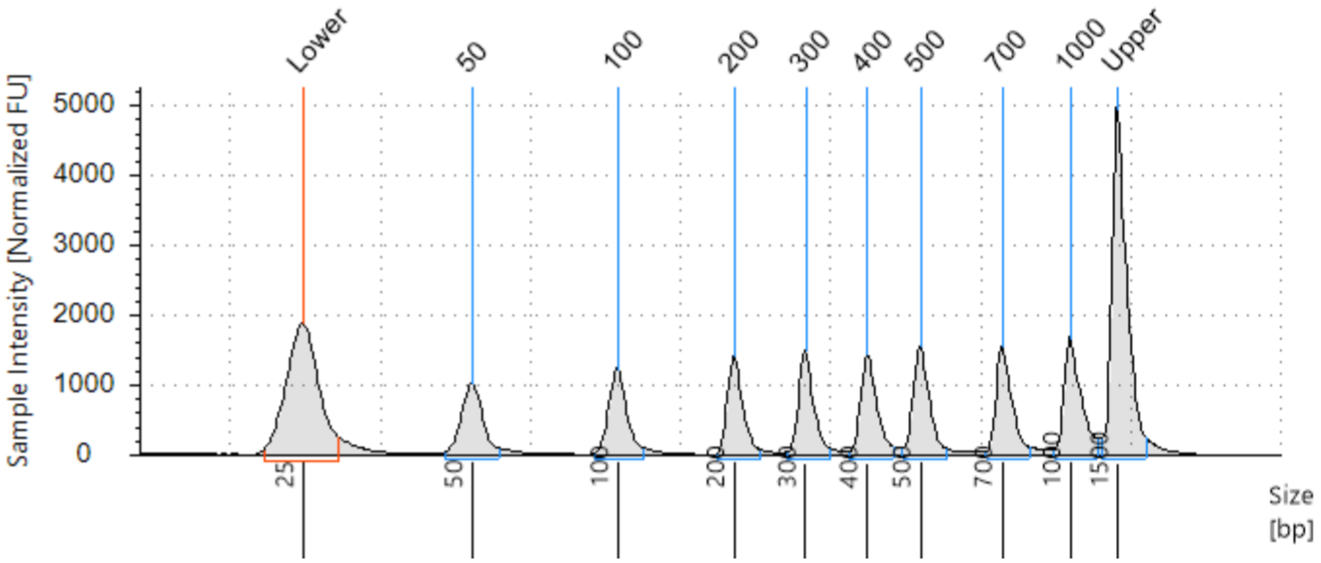

Sample Table

Well	Conc. [ng/µl]	Sample Description	Alert	Observations
A1	16.5	Ladder		Ladder

Peak Table

Size [bp]	Calibrated Conc. [ng/µl]	Assigned Conc. [ng/µl]	Peak Molarity [nmol/l]	% Integrated Area	Peak Comment	Observations
25	5.31	-	327	-		Lower Marker
50	1.88	-	57.7	11.36		
100	1.94	-	29.9	11.75		
200	1.91	-	14.7	11.57		
300	1.97	-	10.1	11.93		
400	2.05	-	7.87	12.39		
500	2.19	-	6.74	13.26		
700	2.13	-	4.69	12.92		
1000	2.45	-	3.77	14.83		
1500	6.50	6.50	6.67	-		Upper Marker

© Agilent Technologies, Inc. 2017

B1: A3 P R3

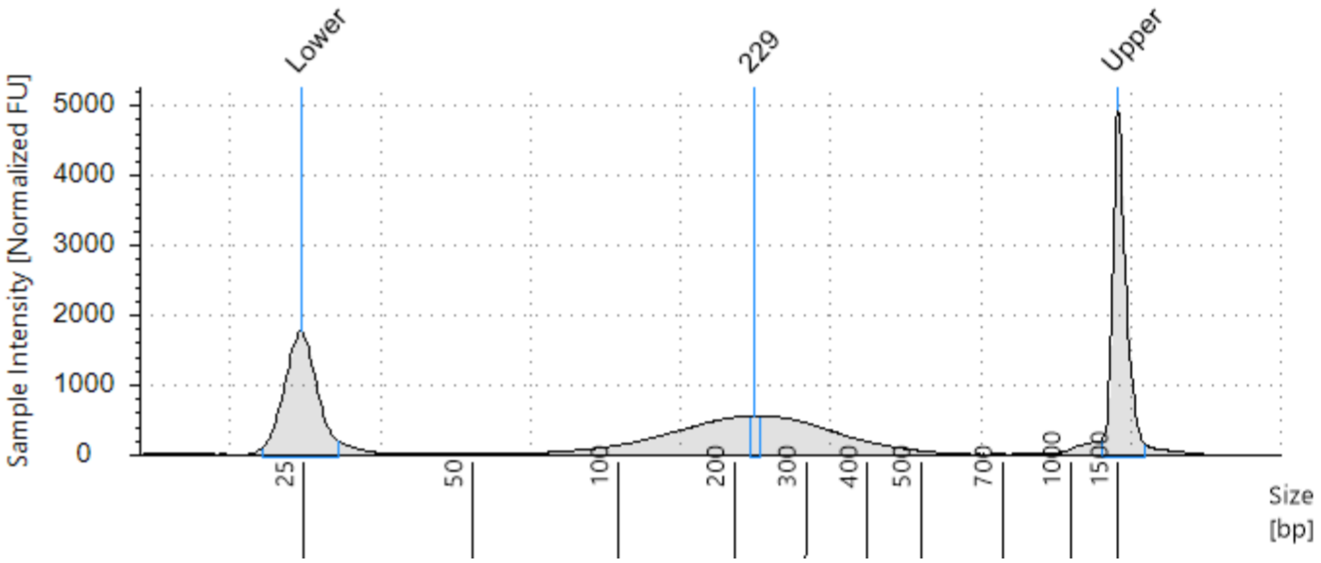

Sample Table

Well	Conc. [ng/µl]	Sample Description	Alert	Observations
B1	0.505	A3 P R3		

Peak Table

Size [bp]	Calibrated Conc. [ng/µl]	Assigned Conc. [ng/µl]	Peak Molarity [nmol/l]	% Integrated Area	Peak Comment	Observations
25	5.60	-	344	-		Lower Marker
229	0.505	-	3.38	100.00		
1500	6.50	6.50	6.67	-		Upper Marker

© Agilent Technologies, Inc. 2017

C1: B3 P R3

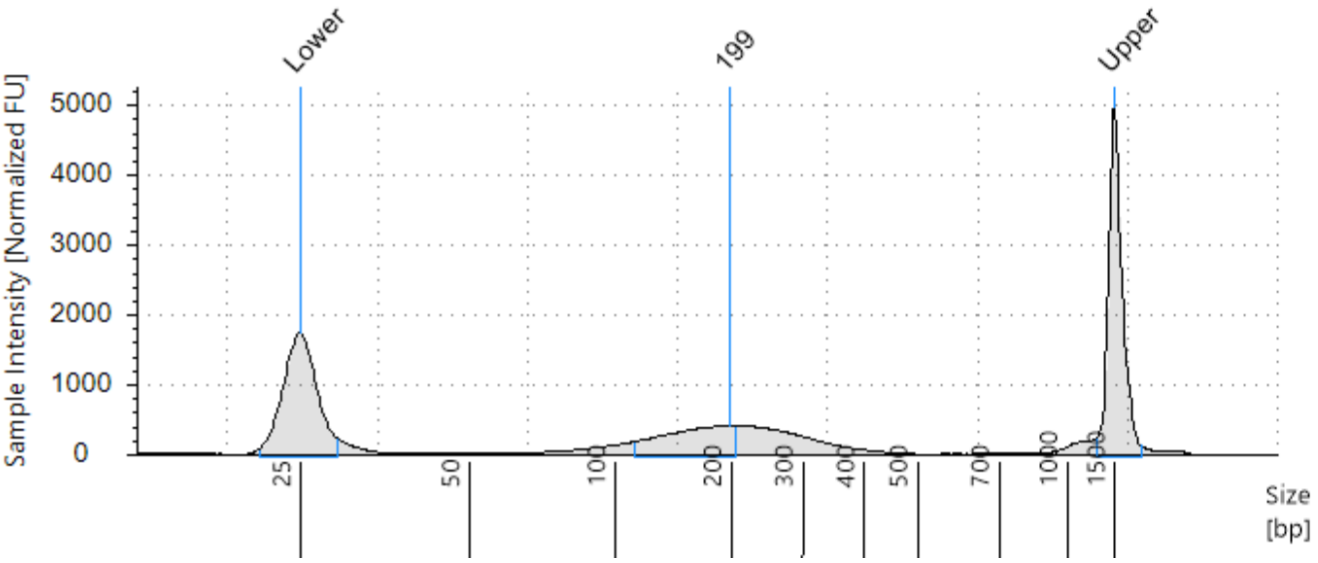

Sample Table

Well	Conc. [ng/µl]	Sample Description	Alert	Observations
C1	2.64	B3 P R3		

Peak Table

Size [bp]	Calibrated Conc. [ng/µl]	Assigned Conc. [ng/µl]	Peak Molarity [nmol/l]	% Integrated Area	Peak Comment	Observations
25	5.59	-	344	-		Lower Marker
199	2.64	-	20.4	100.00		
1500	6.50	6.50	6.67	-		Upper Marker

 © Agilent Technologies, Inc. 2017

D1: C3 P R3

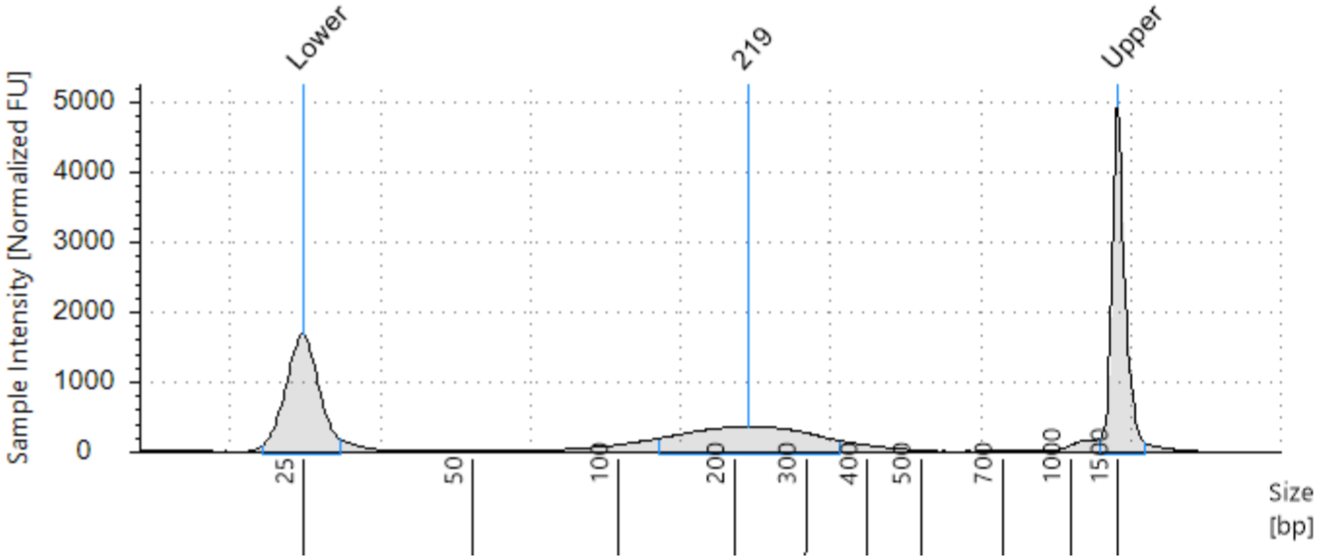

Sample Table

Well	Conc. [ng/µl]	Sample Description	Alert	Observations
D1	4.44	C3 P R3		

Peak Table

Size [bp]	Calibrated Conc. [ng/µl]	Assigned Conc. [ng/µl]	Peak Molarity [nmol/l]	% Integrated Area	Peak Comment	Observations
25	5.59	-	344	-		Lower Marker
219	4.44	-	31.2	100.00		
1500	6.50	6.50	6.67	-		Upper Marker

© Agilent Technologies, Inc. 2017

E1: D3 P R3

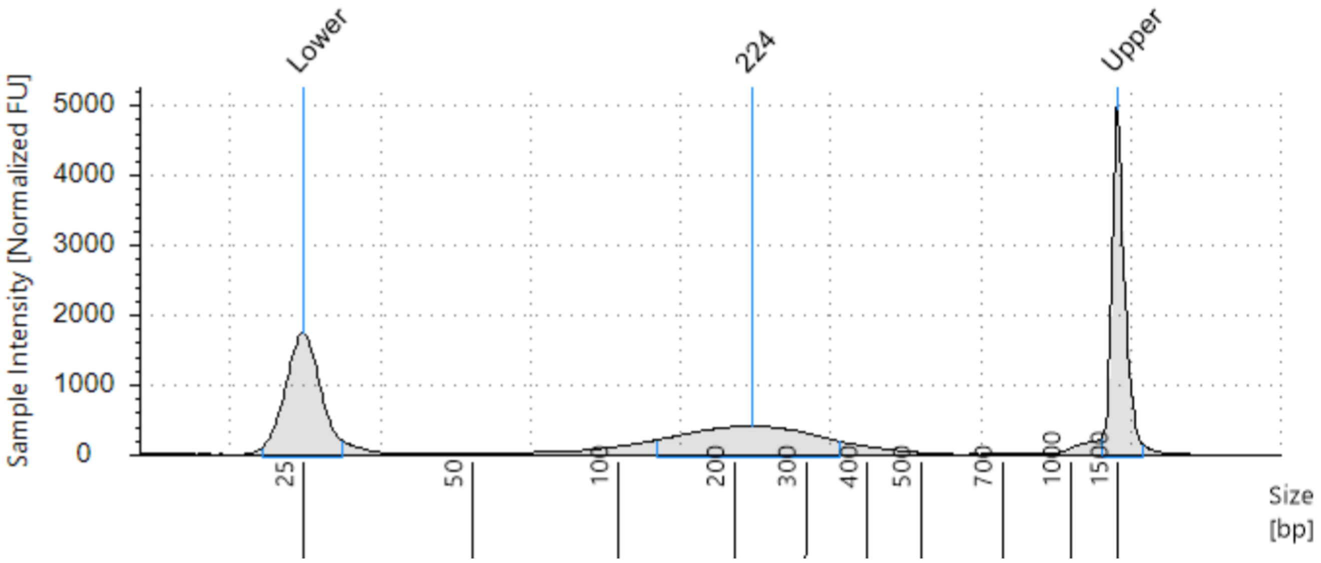

Sample Table

Well	Conc. [ng/µl]	Sample Description	Alert	Observations
E1	5.13	D3 P R3		

Peak Table

Size [bp]	Calibrated Conc. [ng/µl]	Assigned Conc. [ng/µl]	Peak Molarity [nmol/l]	% Integrated Area	Peak Comment	Observations
25	6.11	-	376	-		Lower Marker
224	5.13	-	35.2	100.00		
1500	6.50	6.50	6.67	-		Upper Marker

© Agilent Technologies, Inc. 2017

F1: E3 P R3

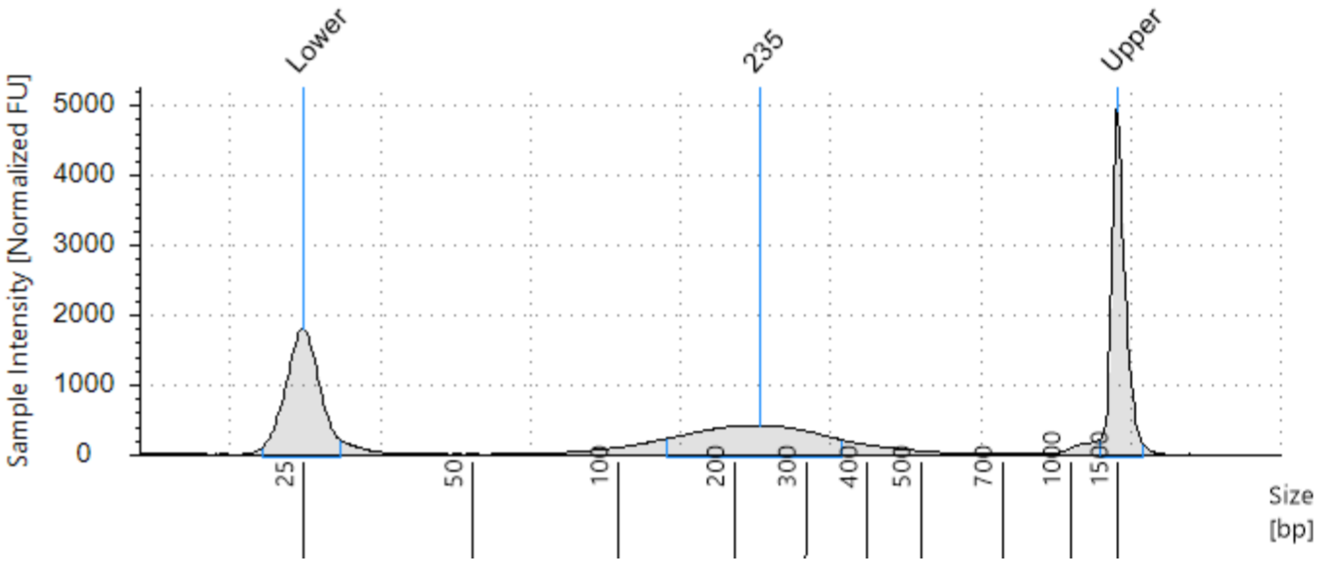

Sample Table

Well	Conc. [ng/µl]	Sample Description	Alert	Observations
F1	4.97	E3 P R3		

Peak Table

Size [bp]	Calibrated Conc. [ng/µl]	Assigned Conc. [ng/µl]	Peak Molarity [nmol/l]	% Integrated Area	Peak Comment	Observations
25	5.93	-	365	-		Lower Marker
235	4.97	-	32.5	100.00		
1500	6.50	6.50	6.67	-		Upper Marker

© Agilent Technologies, Inc. 2017

G1: F3 P R3

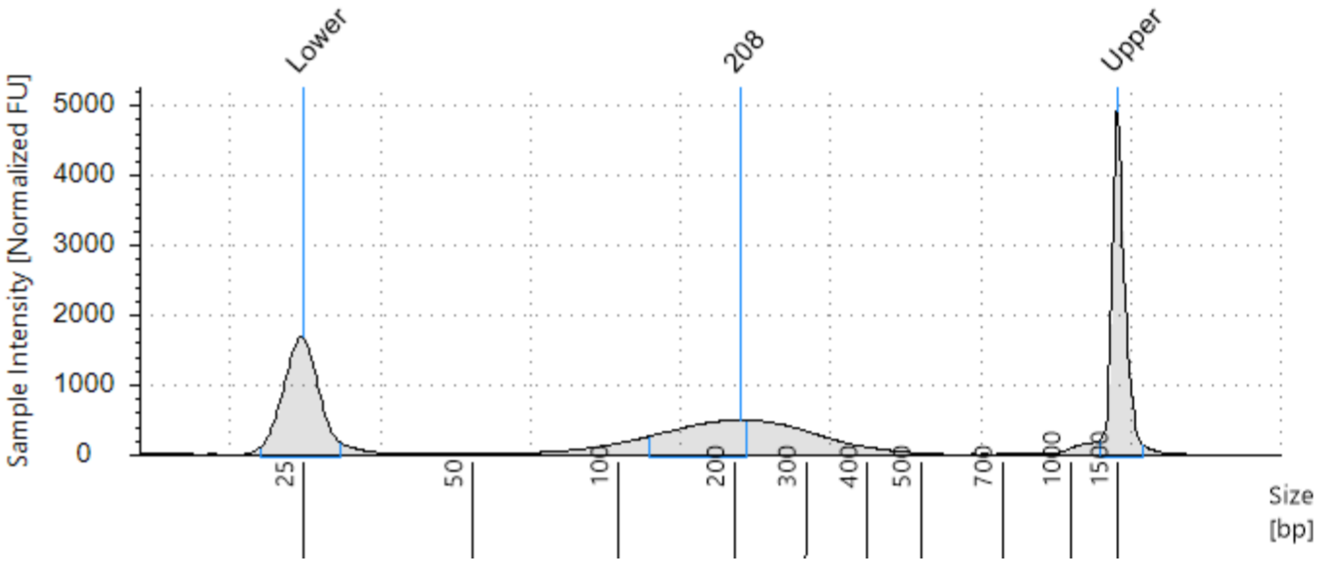

Sample Table

Well	Conc. [ng/µl]	Sample Description	Alert	Observations
G1	3.44	F3 P R3		

Peak Table

Size [bp]	Calibrated Conc. [ng/µl]	Assigned Conc. [ng/µl]	Peak Molarity [nmol/l]	% Integrated Area	Peak Comment	Observations
25	6.09	-	375	-		Lower Marker
208	3.44	-	25.5	100.00		
1500	6.50	6.50	6.67	-		Upper Marker

© Agilent Technologies, Inc. 2017

H1: G3 P R3

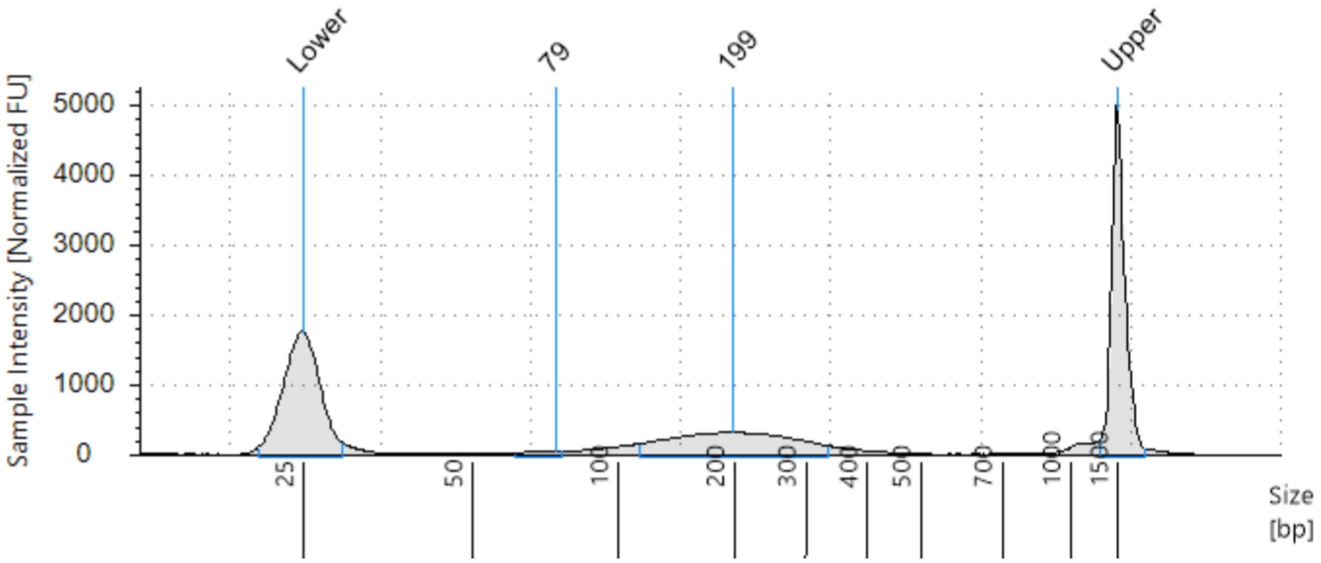

Sample Table

Well	Conc. [ng/µl]	Sample Description	Alert	Observations
H1	3.98	G3 P R3		

Peak Table

Size [bp]	Calibrated Conc. [ng/µl]	Assigned Conc. [ng/µl]	Peak Molarity [nmol/l]	% Integrated Area	Peak Comment	Observations
25	6.28	-	387	-		Lower Marker
79	0.139	-	2.72	3.49		
199	3.84	-	29.7	96.51		
1500	6.50	6.50	6.67	-		Upper Marker

© Agilent Technologies, Inc. 2017

A2: A4 P R3

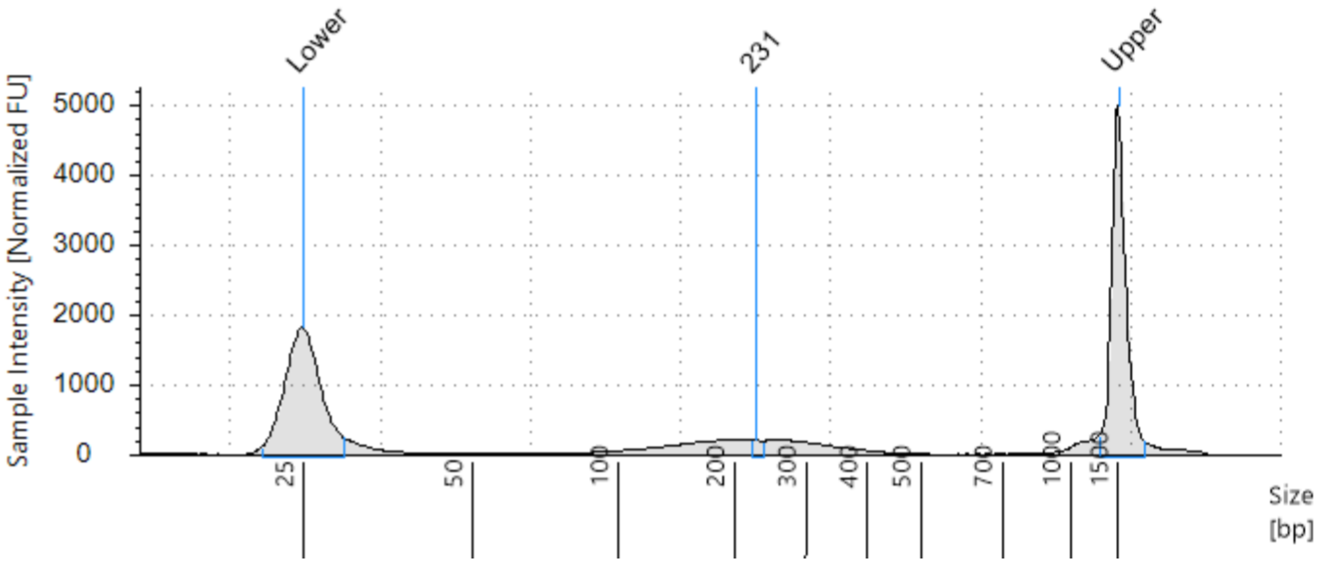

Sample Table

Well	Conc. [ng/µl]	Sample Description	Alert	Observations
A2	0.176	A4 P R3		

Peak Table

Size [bp]	Calibrated Conc. [ng/µl]	Assigned Conc. [ng/µl]	Peak Molarity [nmol/l]	% Integrated Area	Peak Comment	Observations
25	5.92	-	364	-		Lower Marker
231	0.176	-	1.17	100.00		
1500	6.50	6.50	6.67	-		Upper Marker

© Agilent Technologies, Inc. 2017

B2: B4 P R3

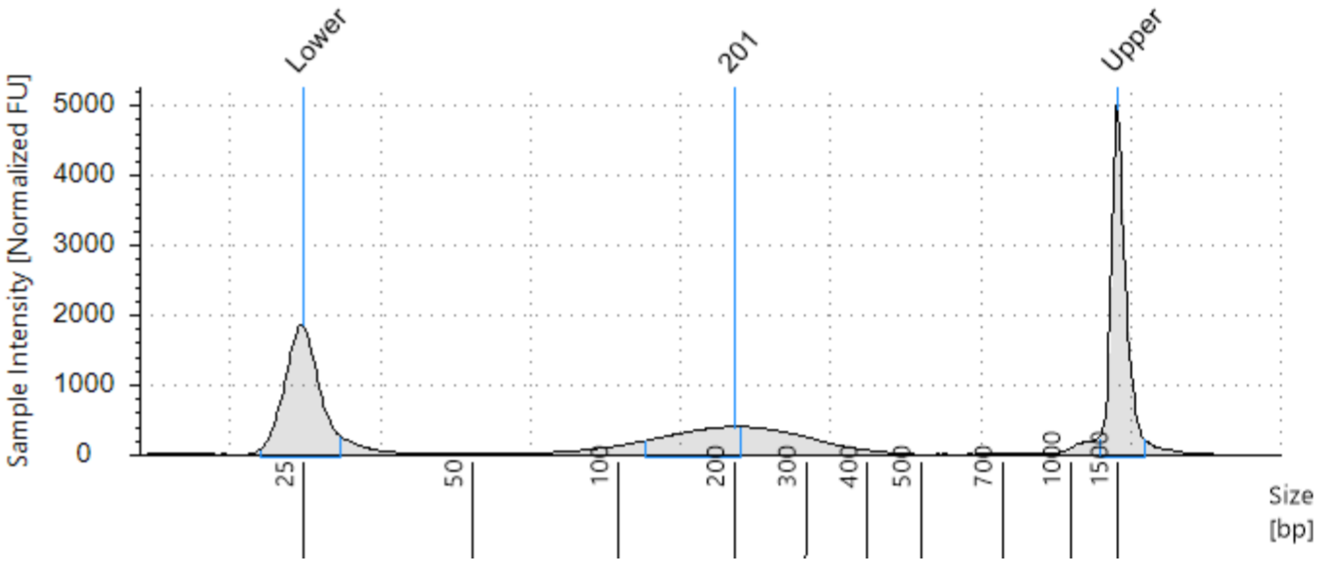

Sample Table

Well	Conc. [ng/µl]	Sample Description	Alert	Observations
B2	2.37	B4 P R3		

Peak Table

Size [bp]	Calibrated Conc. [ng/µl]	Assigned Conc. [ng/µl]	Peak Molarity [nmol/l]	% Integrated Area	Peak Comment	Observations
25	5.70	-	351	-		Lower Marker
201	2.37	-	18.1	100.00		
1500	6.50	6.50	6.67	-		Upper Marker

© Agilent Technologies, Inc. 2017

C2: C4 P R3

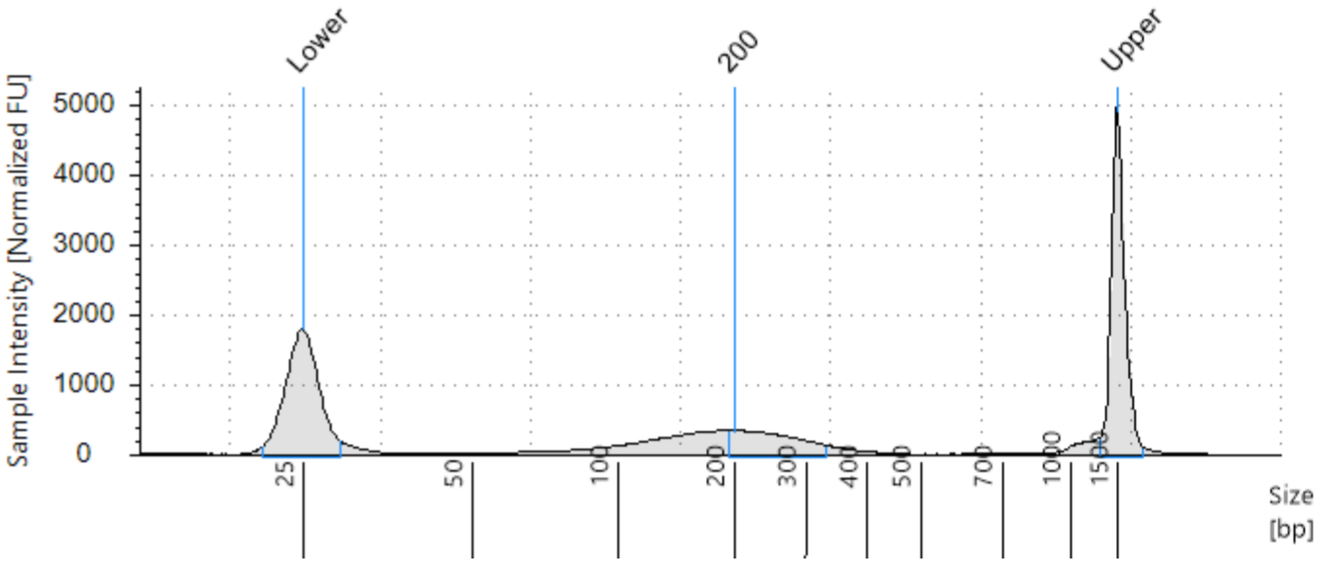

Sample Table

Well	Conc. [ng/µl]	Sample Description	Alert	Observations
C2	2.16	C4 P R3		

Peak Table

Size [bp]	Calibrated Conc. [ng/µl]	Assigned Conc. [ng/µl]	Peak Molarity [nmol/l]	% Integrated Area	Peak Comment	Observations
25	5.82	-	358	-		Lower Marker
200	2.16	-	16.6	100.00		
1500	6.50	6.50	6.67	-		Upper Marker

© Agilent Technologies, Inc. 2017

D2: D4 P R3

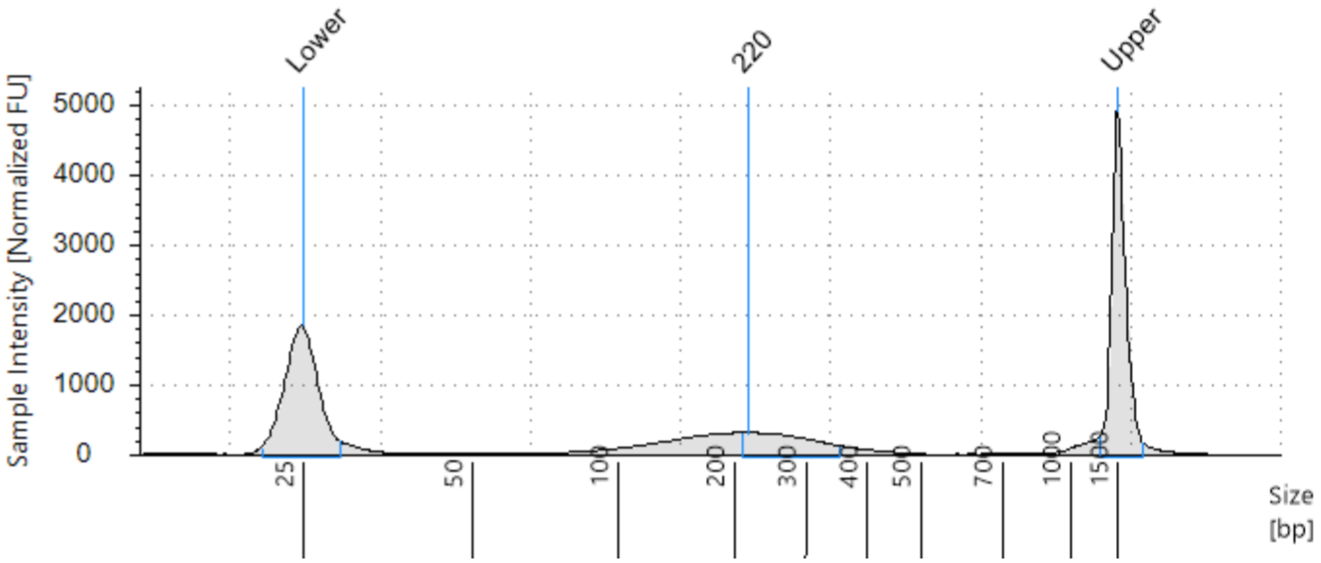

Sample Table

Well	Conc. [ng/µl]	Sample Description	Alert	Observations
D2	1.91	D4 P R3		

Peak Table

Size [bp]	Calibrated Conc. [ng/µl]	Assigned Conc. [ng/µl]	Peak Molarity [nmol/l]	% Integrated Area	Peak Comment	Observations
25	5.79	-	356	-		Lower Marker
220	1.91	-	13.4	100.00		
1500	6.50	6.50	6.67	-		Upper Marker

© Agilent Technologies, Inc. 2017

E2: E4 P R3

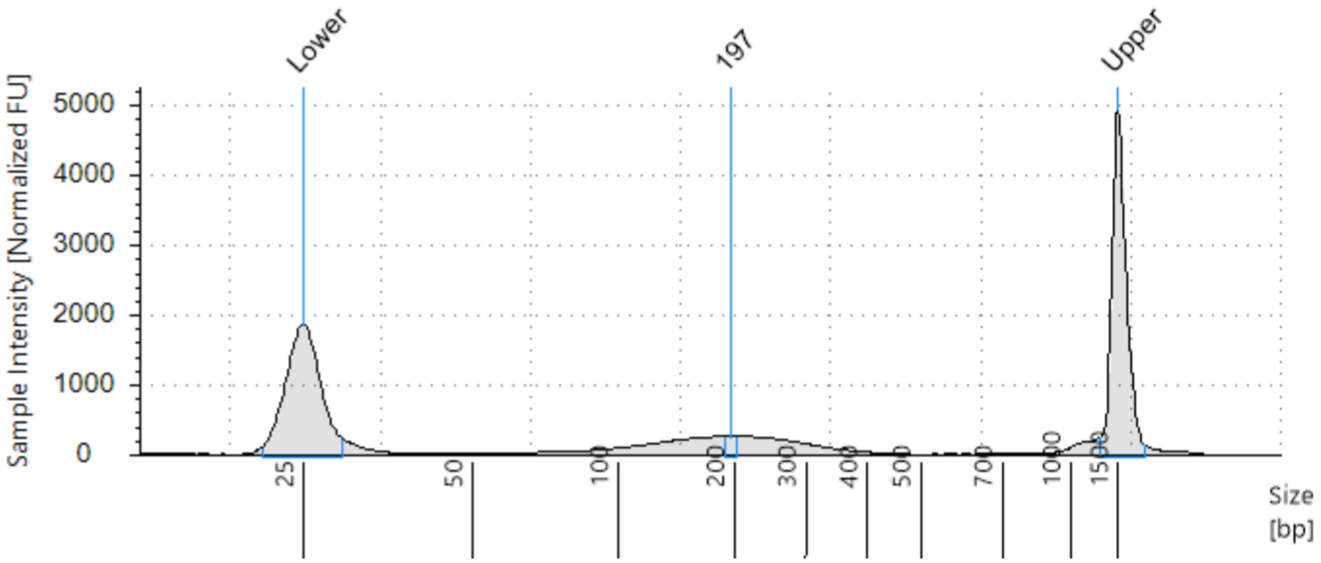

Sample Table

Well	Conc. [ng/µl]	Sample Description	Alert	Observations
E2	0.244	E4 P R3		

Peak Table

Size [bp]	Calibrated Conc. [ng/µl]	Assigned Conc. [ng/µl]	Peak Molarity [nmol/l]	% Integrated Area	Peak Comment	Observations
25	5.90	-	363	-		Lower Marker
197	0.244	-	1.90	100.00		
1500	6.50	6.50	6.67	-		Upper Marker

© Agilent Technologies, Inc. 2017

F2: F4 P R3

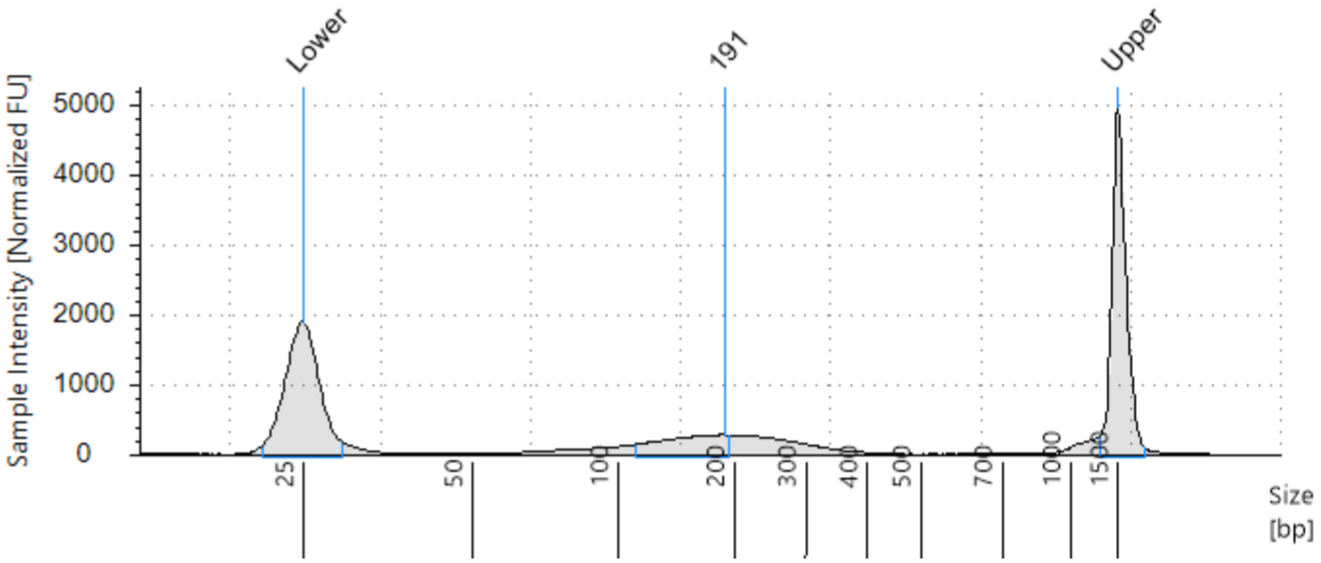

Sample Table

Well	Conc. [ng/µl]	Sample Description	Alert	Observations
F2	1.68	F4 P R3		

Peak Table

Size [bp]	Calibrated Conc. [ng/µl]	Assigned Conc. [ng/µl]	Peak Molarity [nmol/l]	% Integrated Area	Peak Comment	Observations
25	6.00	-	369	-		Lower Marker
191	1.68	-	13.5	100.00		
1500	6.50	6.50	6.67	-		Upper Marker

© Agilent Technologies, Inc. 2017

G2: G4 P R3

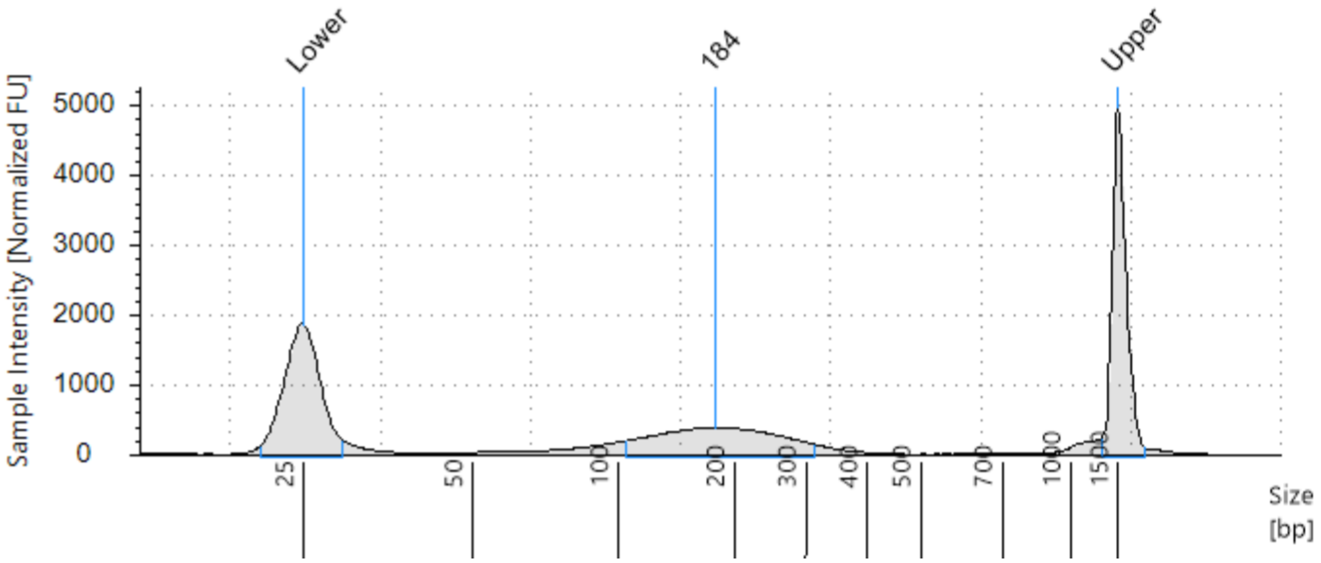

Sample Table

Well	Conc. [ng/µl]	Sample Description	Alert	Observations
G2	4.49	G4 P R3		

Peak Table

Size [bp]	Calibrated Conc. [ng/µl]	Assigned Conc. [ng/µl]	Peak Molarity [nmol/l]	% Integrated Area	Peak Comment	Observations
25	6.31	-	389	-		Lower Marker
184	4.49	-	37.5	100.00		
1500	6.50	6.50	6.67	-		Upper Marker

© Agilent Technologies, Inc. 2017

H2: H4 P R3

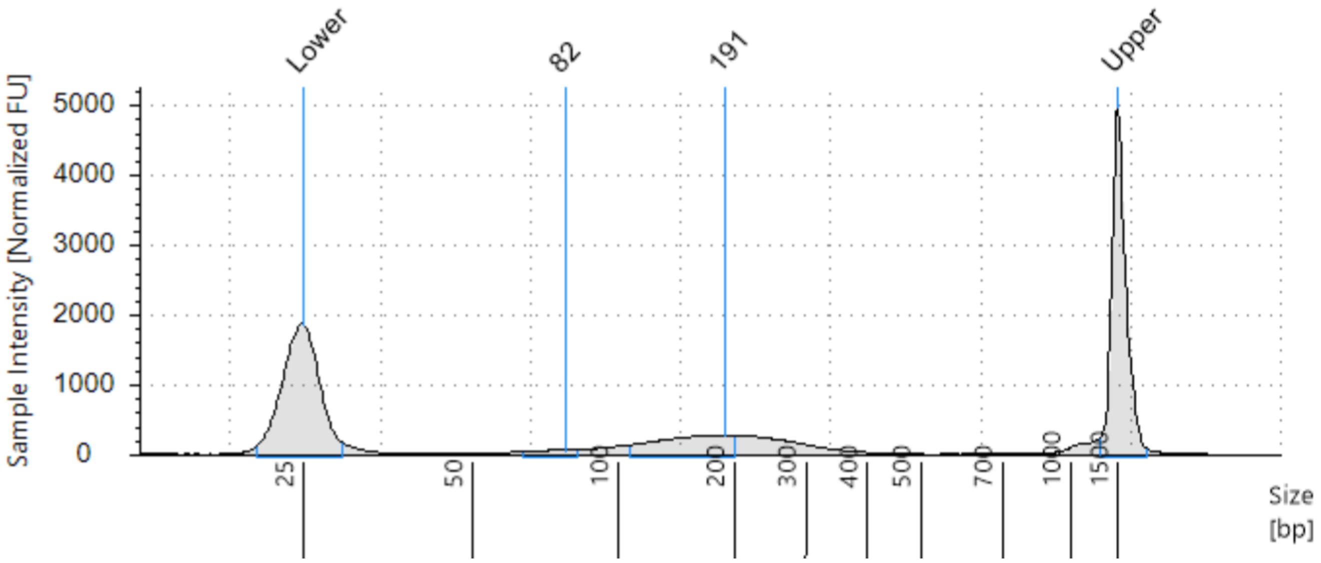

Sample Table

Well	Conc. [ng/µl]	Sample Description	Alert	Observations
H2	2.08	H4 P R3		

Peak Table

Size [bp]	Calibrated Conc. [ng/µl]	Assigned Conc. [ng/µl]	Peak Molarity [nmol/l]	% Integrated Area	Peak Comment	Observations
25	6.39	-	393	-		Lower Marker
82	0.239	-	4.49	11.46		
191	1.84	-	14.8	88.54		
1500	6.50	6.50	6.67	-		Upper Marker

© Agilent Technologies, Inc. 2017

Filename: 2020-10-06-03 Q-S plus A5-H6 EXP H5 R3.D1000

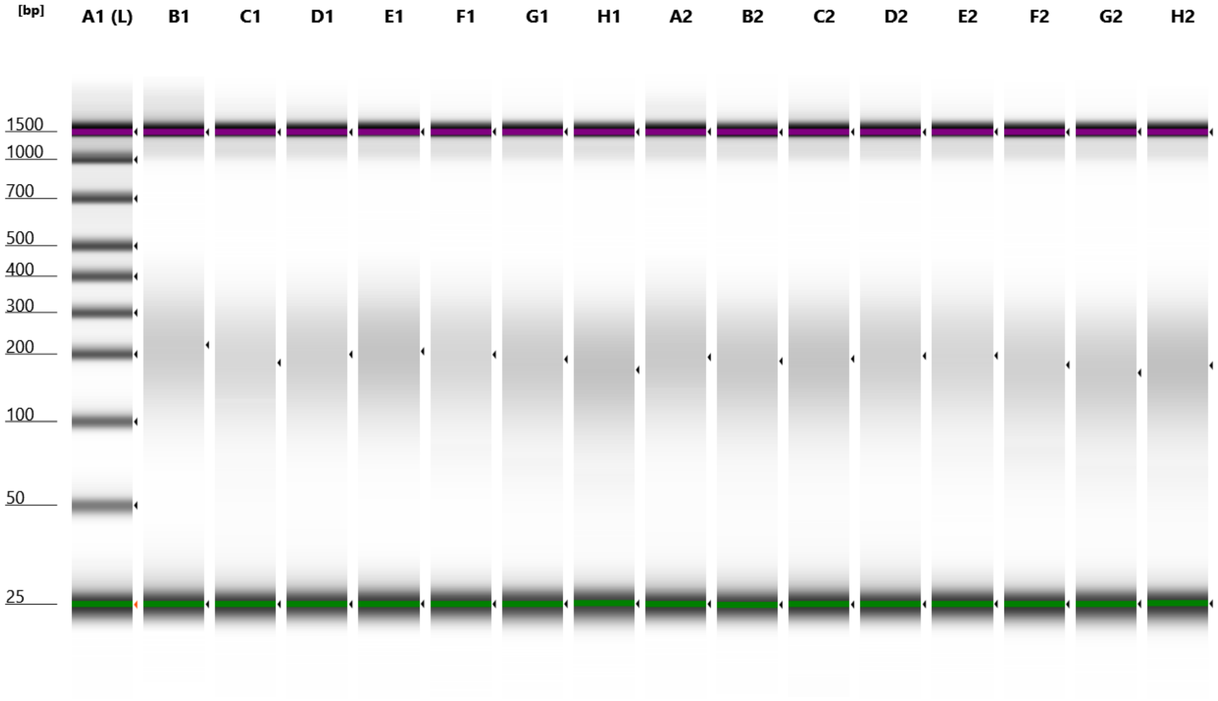

Default image (Contrast 100%)

Sample Info

Well	Conc. [ng/µl]	Sample Description	Alert	Observations
A1	16.2	Ladder		Ladder
B1	1.78	A5 P R3		
C1	1.35	B5 P R3		
D1	1.76	C5 P R3		
E1	2.48	D5 P R3		
F1	1.65	E5 P R3		
G1	4.01	F5 P R3		
H1	2.74	G5 P R3		
A2	2.06	A6 P R3		
B2	3.85	B6 P R3		
C2	4.08	C6 P R3		
D2	3.48	D6 P R3		
E2	1.62	E6 P R3		
F2	1.74	F6 P R3		
G2	3.82	G6 P R3		
H2	4.66	H6 P R3		

© Agilent Technologies, Inc. 2017

A1: Ladder

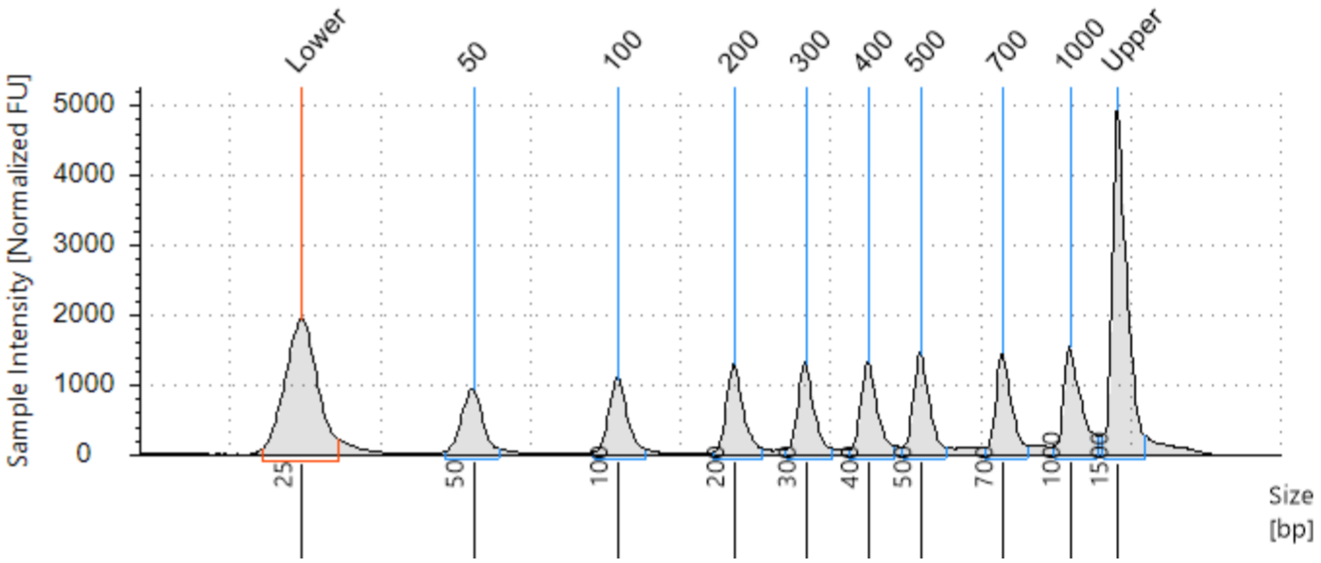

Sample Table

Well	Conc. [ng/µl]	Sample Description	Alert	Observations
A1	16.2	Ladder		Ladder

Peak Table

Size [bp]	Calibrated Conc. [ng/µl]	Assigned Conc. [ng/µl]	Peak Molarity [nmol/l]	% Integrated Area	Peak Comment	Observations
25	5.64	-	347	-		Lower Marker
50	1.79	-	55.2	11.08		
100	1.87	-	28.7	11.55		
200	1.99	-	15.3	12.29		
300	1.94	-	9.93	11.97		
400	2.01	-	7.73	12.42		
500	2.12	-	6.52	13.10		
700	2.08	-	4.58	12.87		
1000	2.38	-	3.66	14.71		
1500	6.50	6.50	6.67	-		Upper Marker

© Agilent Technologies, Inc. 2017

B1: A5 P R3

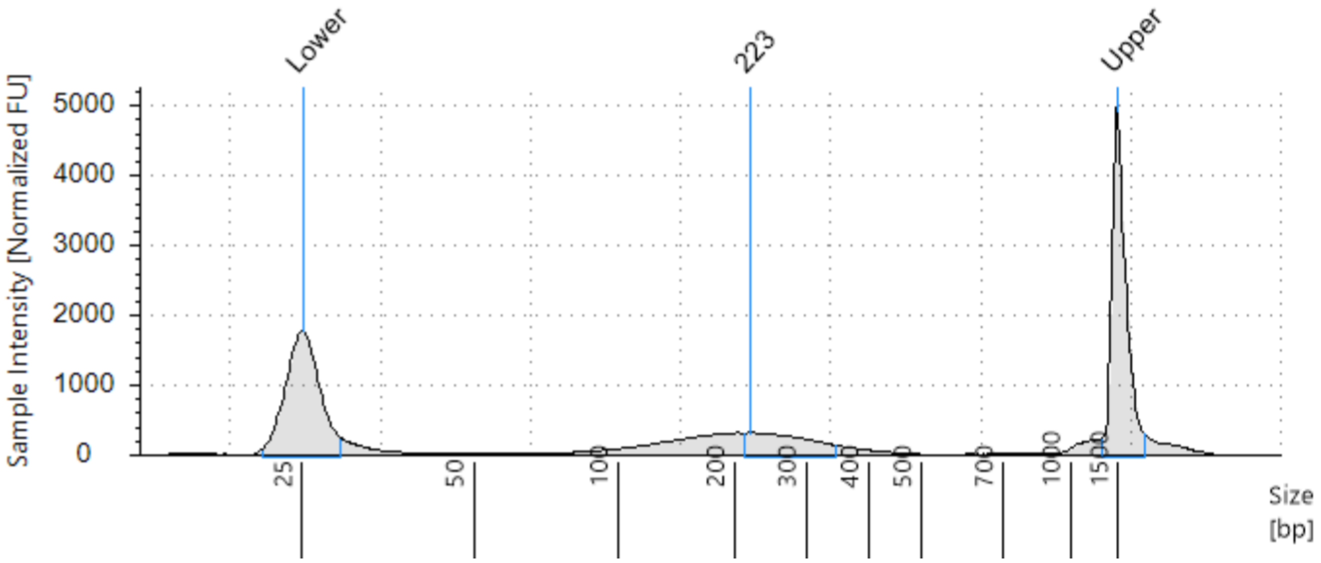

Sample Table

Well	Conc. [ng/µl]	Sample Description	Alert	Observations
B1	1.78	A5 P R3		

Peak Table

Size [bp]	Calibrated Conc. [ng/µl]	Assigned Conc. [ng/µl]	Peak Molarity [nmol/l]	% Integrated Area	Peak Comment	Observations
25	5.67	-	349	-		Lower Marker
223	1.78	-	12.3	100.00		
1500	6.50	6.50	6.67	-		Upper Marker

© Agilent Technologies, Inc. 2017

C1: B5 P R3

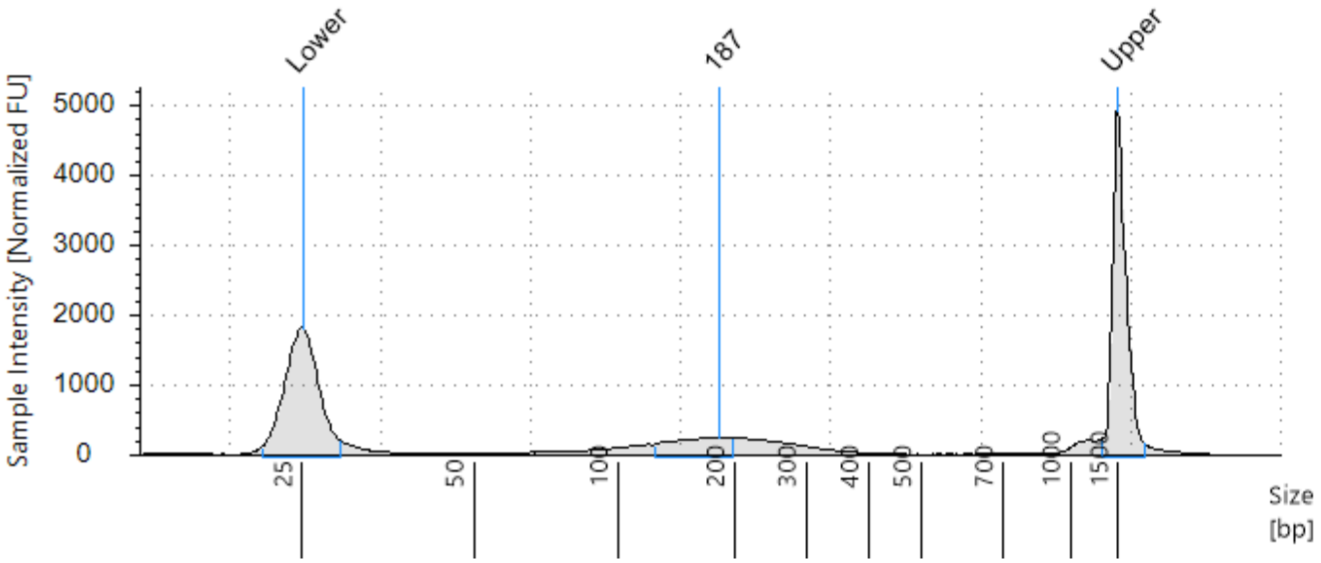

Sample Table

Well	Conc. [ng/µl]	Sample Description	Alert	Observations
C1	1.35	B5 P R3		

Peak Table

Size [bp]	Calibrated Conc. [ng/µl]	Assigned Conc. [ng/µl]	Peak Molarity [nmol/l]	% Integrated Area	Peak Comment	Observations
25	5.99	-	368	-		Lower Marker
187	1.35	-	11.1	100.00		
1500	6.50	6.50	6.67	-		Upper Marker

© Agilent Technologies, Inc. 2017

D1: C5 P R3

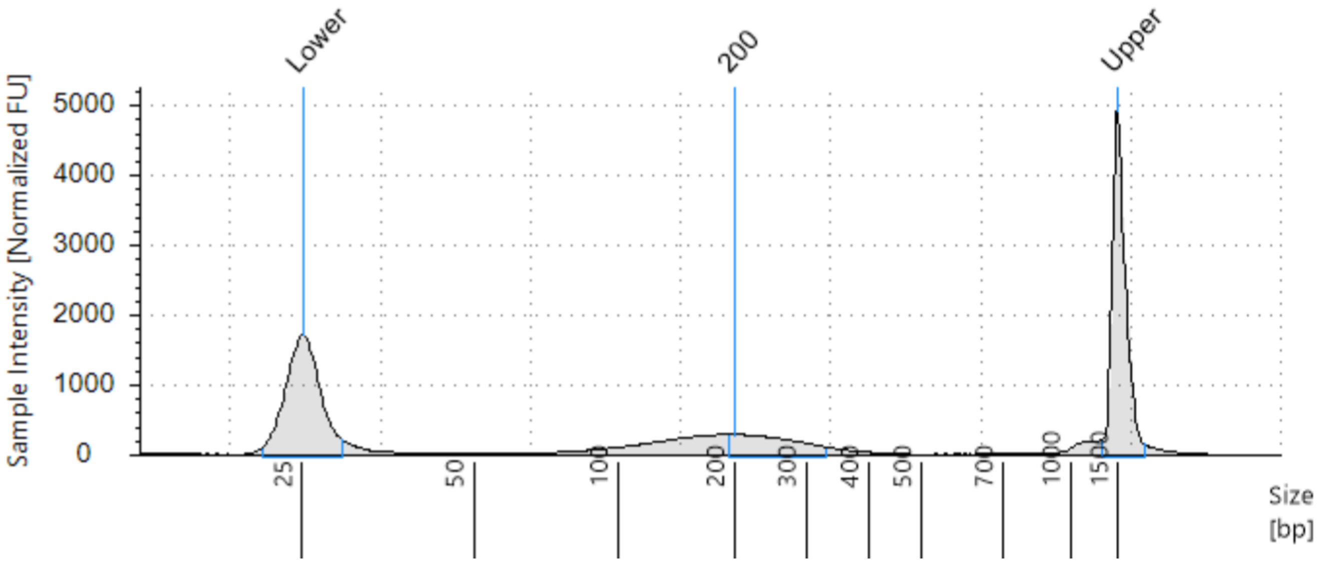

Sample Table

Well	Conc. [ng/µl]	Sample Description	Alert	Observations
D1	1.76	C5 P R3		

Peak Table

Size [bp]	Calibrated Conc. [ng/µl]	Assigned Conc. [ng/µl]	Peak Molarity [nmol/l]	% Integrated Area	Peak Comment	Observations
25	5.79	-	356	-		Lower Marker
200	1.76	-	13.6	100.00		
1500	6.50	6.50	6.67	-		Upper Marker

 © Agilent Technologies, Inc. 2017

E1: D5 P R3

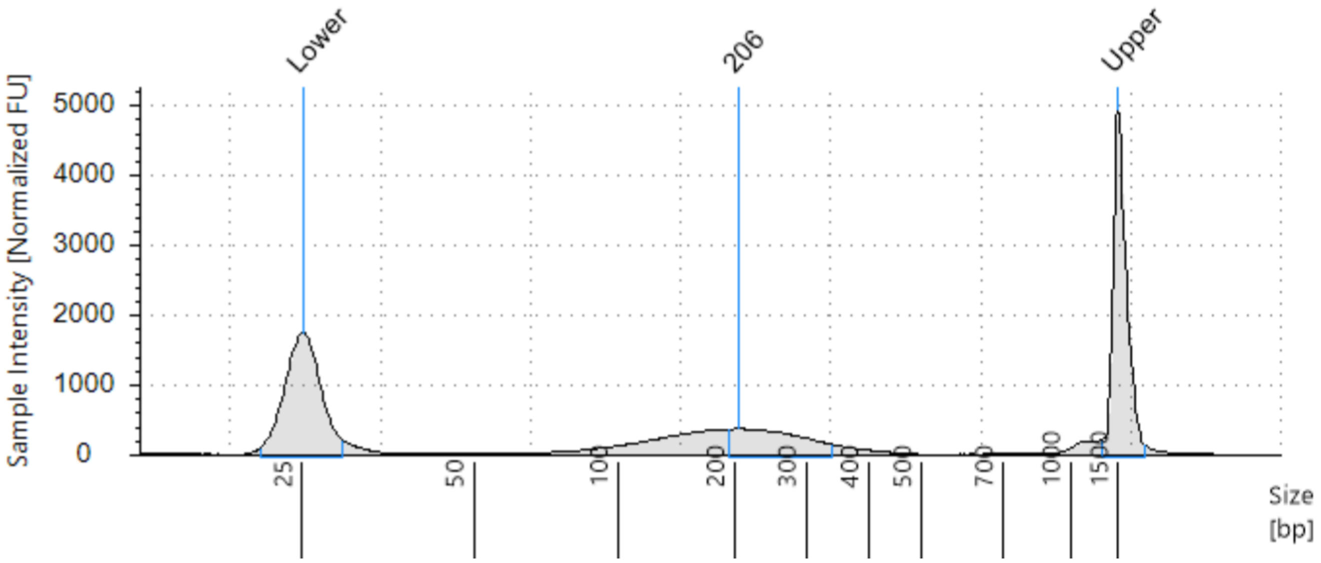

Sample Table

Well	Conc. [ng/µl]	Sample Description	Alert	Observations
E1	2.48	D5 P R3		

Peak Table

Size [bp]	Calibrated Conc. [ng/µl]	Assigned Conc. [ng/µl]	Peak Molarity [nmol/l]	% Integrated Area	Peak Comment	Observations
25	6.10	-	375	-		Lower Marker
206	2.48	-	18.5	100.00		
1500	6.50	6.50	6.67	-		Upper Marker

© Agilent Technologies, Inc. 2017

F1: E5 P R3

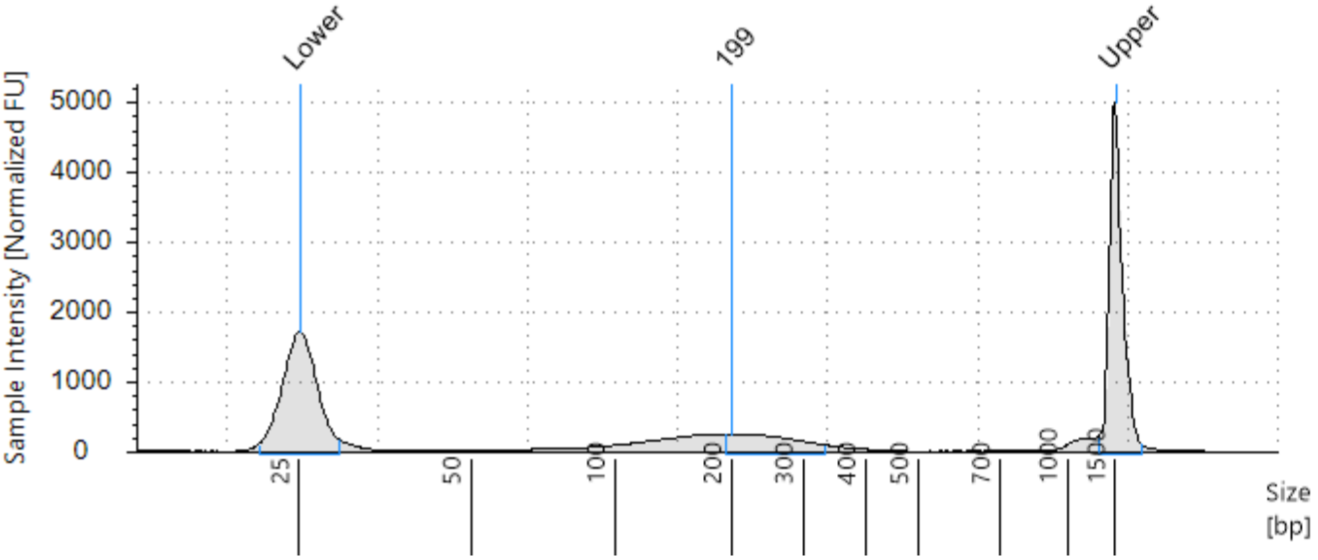

Sample Table

Well	Conc. [ng/µl]	Sample Description	Alert	Observations
F1	1.65	E5 P R3		

Peak Table

Size [bp]	Calibrated Conc. [ng/µl]	Assigned Conc. [ng/µl]	Peak Molarity [nmol/l]	% Integrated Area	Peak Comment	Observations
25	5.89	-	363	-		Lower Marker
199	1.65	-	12.7	100.00		
1500	6.50	6.50	6.67	-		Upper Marker

© Agilent Technologies, Inc. 2017

G1: F5 P R3

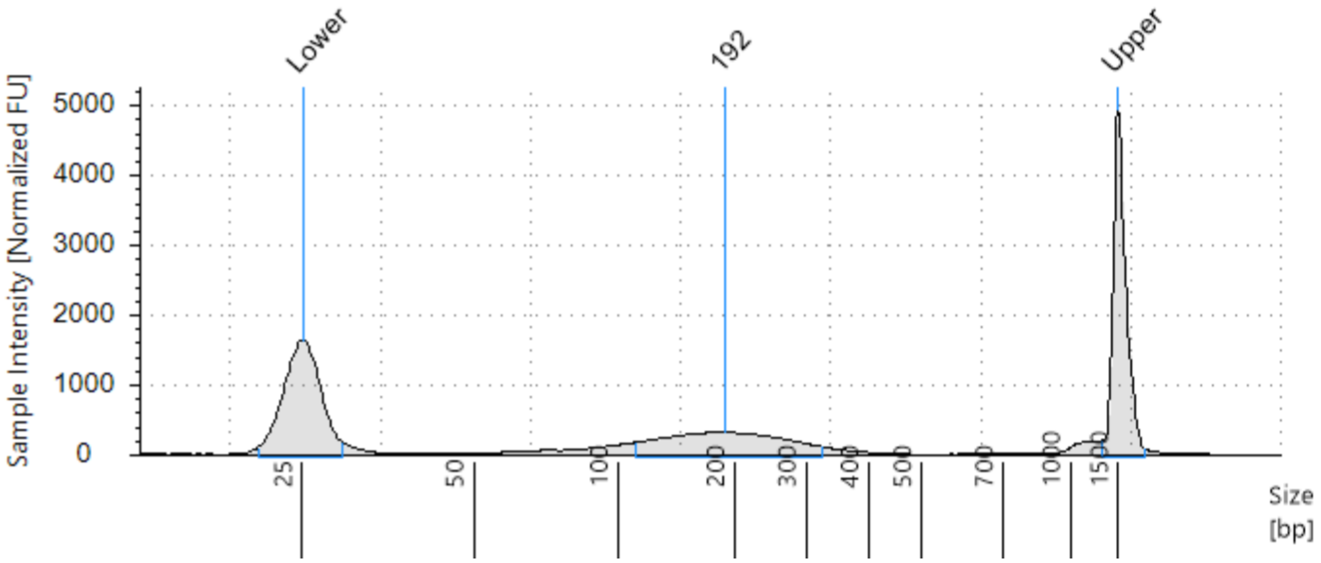

Sample Table

Well	Conc. [ng/µl]	Sample Description	Alert	Observations
G1	4.01	F5 P R3		

Peak Table

Size [bp]	Calibrated Conc. [ng/µl]	Assigned Conc. [ng/µl]	Peak Molarity [nmol/l]	% Integrated Area	Peak Comment	Observations
25	6.28	-	387	-		Lower Marker
192	4.01	-	32.2	100.00		
1500	6.50	6.50	6.67	-		Upper Marker

© Agilent Technologies, Inc. 2017

H1: G5 P R3

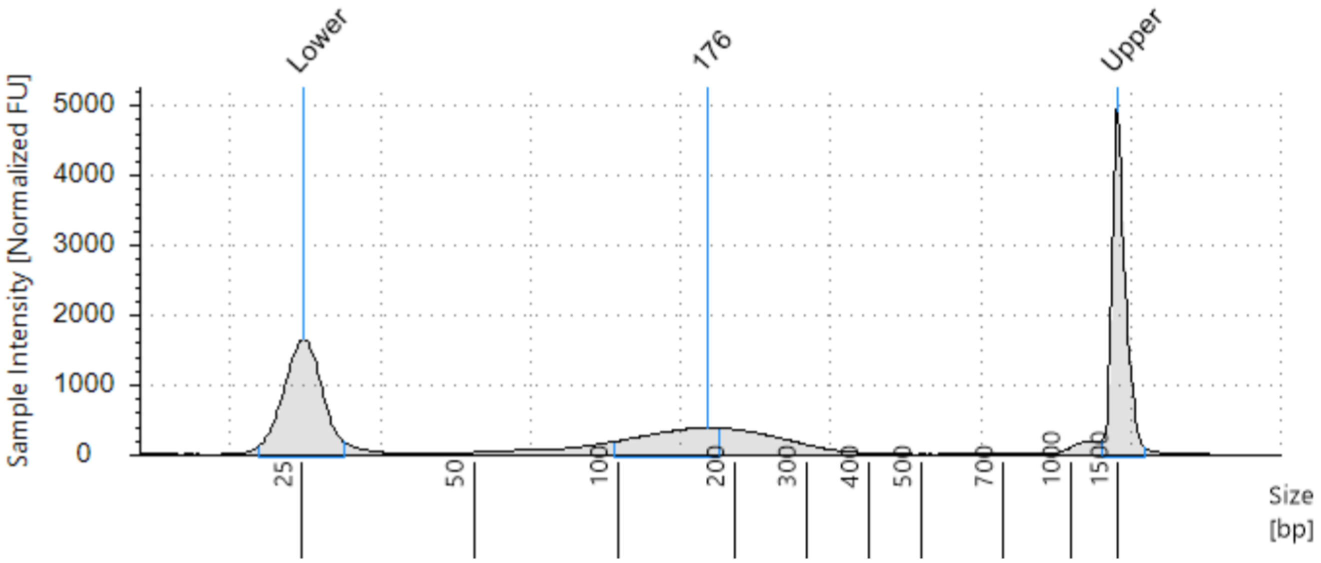

Sample Table

Well	Conc. [ng/µl]	Sample Description	Alert	Observations
H1	2.74	G5 P R3		

Peak Table

Size [bp]	Calibrated Conc. [ng/µl]	Assigned Conc. [ng/µl]	Peak Molarity [nmol/l]	% Integrated Area	Peak Comment	Observations
25	6.25	-	384	-		Lower Marker
176	2.74	-	23.9	100.00		
1500	6.50	6.50	6.67	-		Upper Marker

 © Agilent Technologies, Inc. 2017

A2: A6 P R3

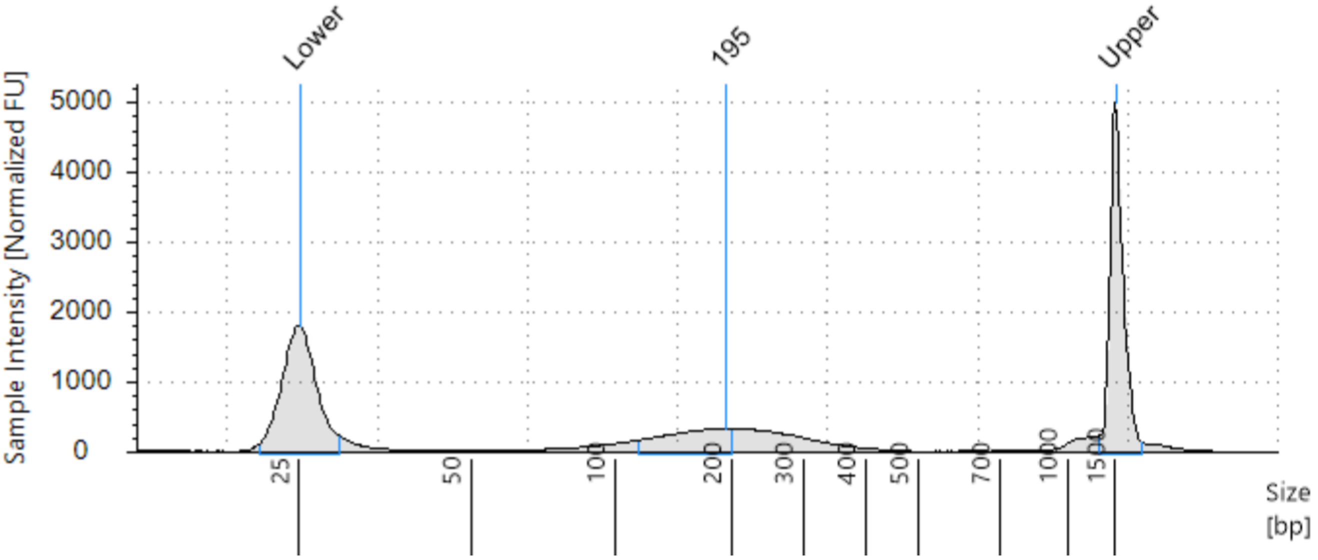

Sample Table

Well	Conc. [ng/µl]	Sample Description	Alert	Observations
A2	2.06	A6 P R3		

Peak Table

Size [bp]	Calibrated Conc. [ng/µl]	Assigned Conc. [ng/µl]	Peak Molarity [nmol/l]	% Integrated Area	Peak Comment	Observations
25	6.10	-	375	-		Lower Marker
195	2.06	-	16.2	100.00		
1500	6.50	6.50	6.67	-		Upper Marker

© Agilent Technologies, Inc. 2017

B2: B6 P R3

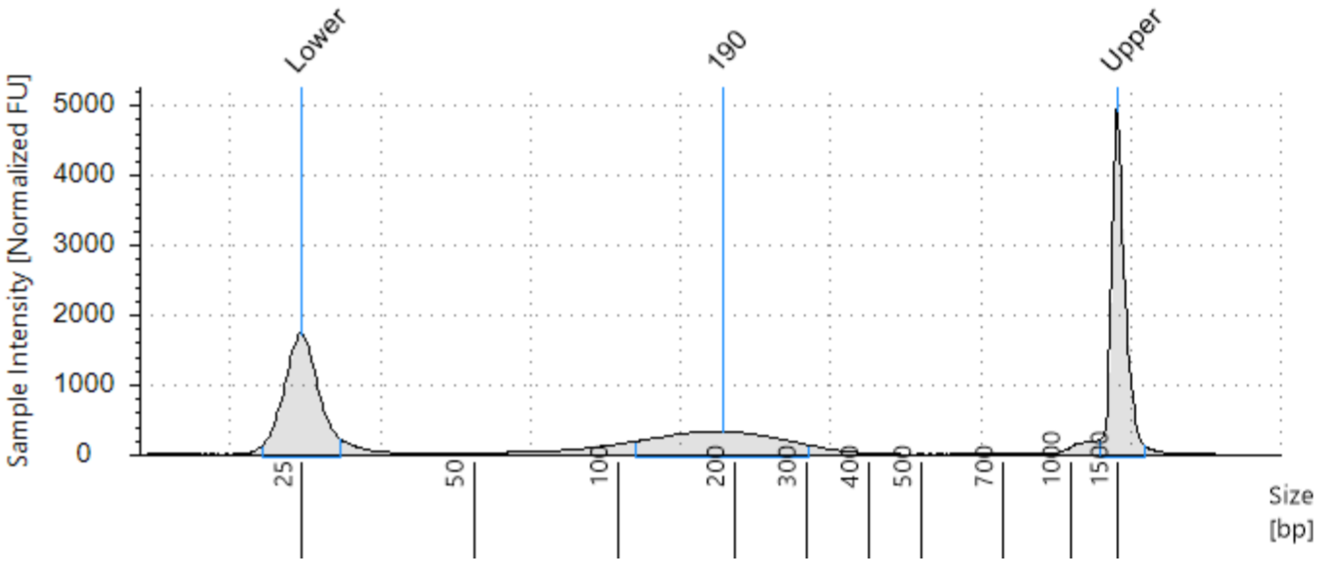

Sample Table

Well	Conc. [ng/µl]	Sample Description	Alert	Observations
B2	3.85	B6 P R3		

Peak Table

Size [bp]	Calibrated Conc. [ng/µl]	Assigned Conc. [ng/µl]	Peak Molarity [nmol/l]	% Integrated Area	Peak Comment	Observations
25	5.84	-	359	-		Lower Marker
190	3.85	-	31.1	100.00		
1500	6.50	6.50	6.67	-		Upper Marker

 © Agilent Technologies, Inc. 2017

C2: C6 P R3

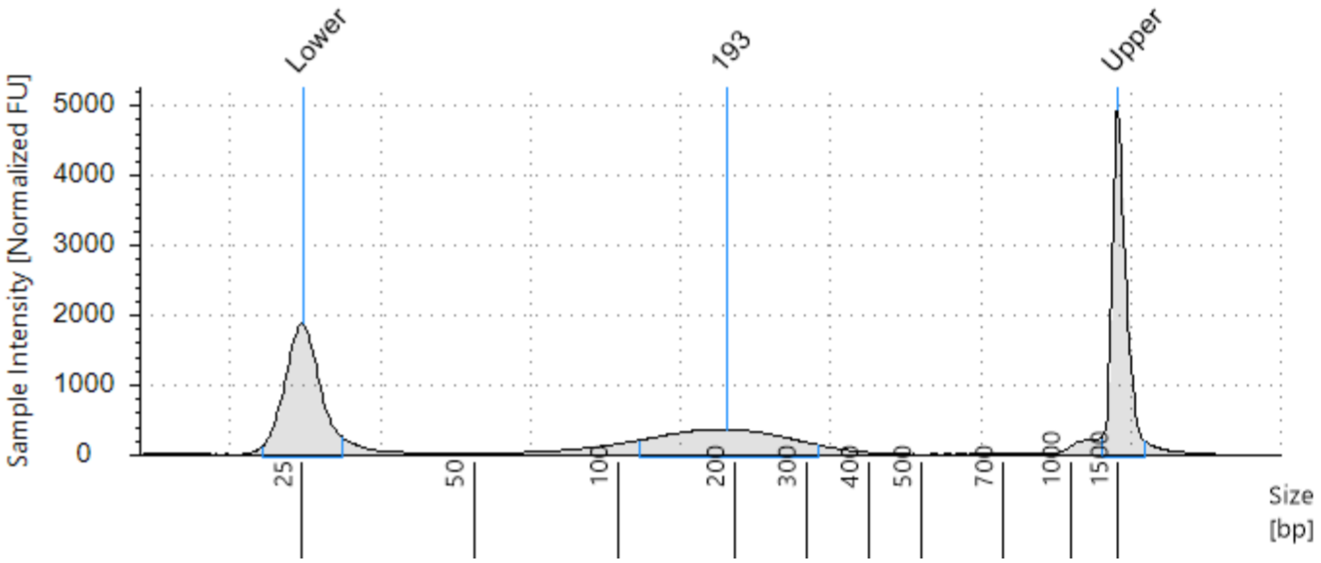

Sample Table

Well	Conc. [ng/µl]	Sample Description	Alert	Observations
C2	4.08	C6 P R3		

Peak Table

Size [bp]	Calibrated Conc. [ng/µl]	Assigned Conc. [ng/µl]	Peak Molarity [nmol/l]	% Integrated Area	Peak Comment	Observations
25	6.09	-	375	-		Lower Marker
193	4.08	-	32.5	100.00		
1500	6.50	6.50	6.67	-		Upper Marker

© Agilent Technologies, Inc. 2017

D2: D6 P R3

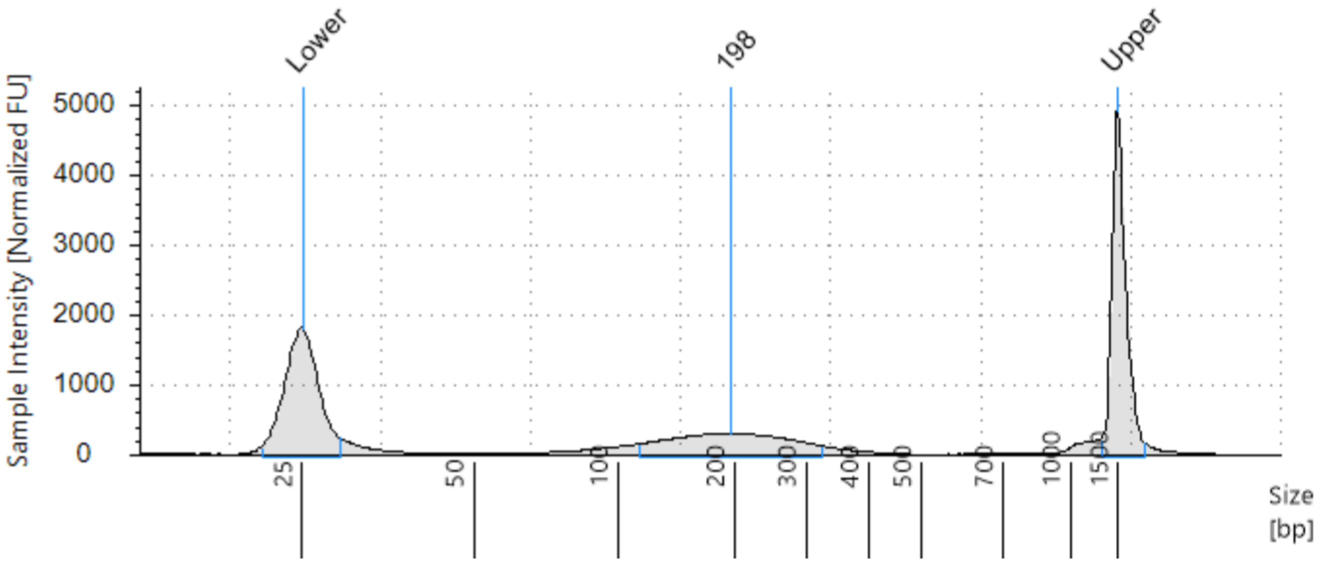

Sample Table

Well	Conc. [ng/µl]	Sample Description	Alert	Observations
D2	3.48	D6 P R3		

Peak Table

Size [bp]	Calibrated Conc. [ng/µl]	Assigned Conc. [ng/µl]	Peak Molarity [nmol/l]	% Integrated Area	Peak Comment	Observations
25	5.83	-	358	-		Lower Marker
198	3.48	-	27.1	100.00		
1500	6.50	6.50	6.67	-		Upper Marker

© Agilent Technologies, Inc. 2017

E2: E6 P R3

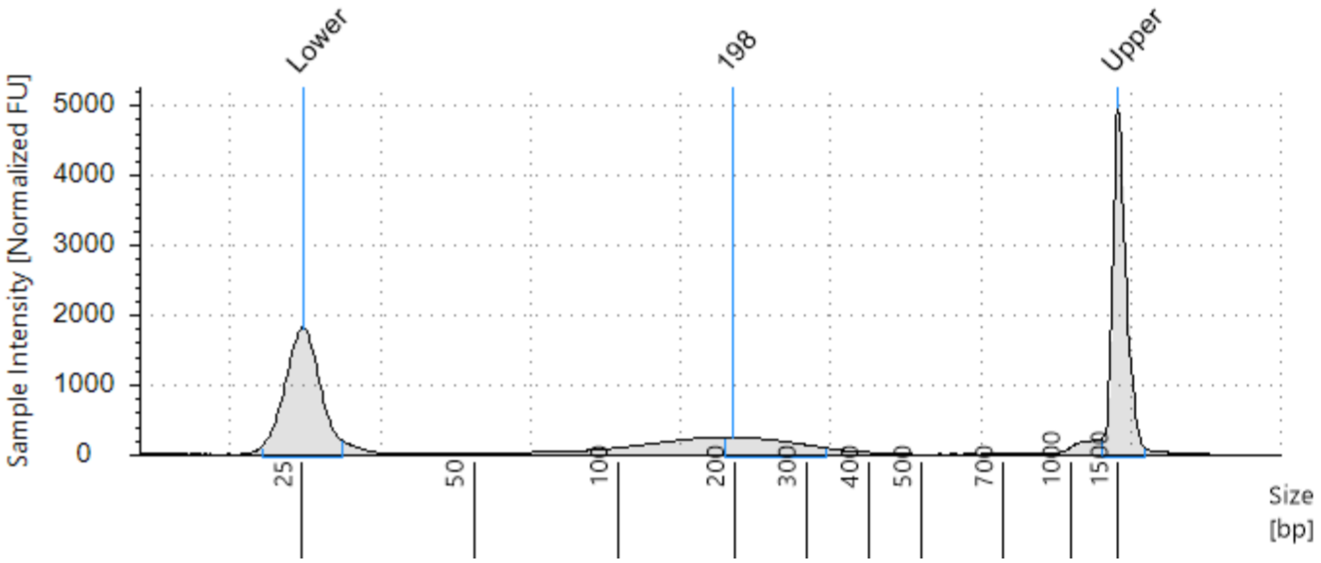

Sample Table

Well	Conc. [ng/µl]	Sample Description	Alert	Observations
E2	1.62	E6 P R3		

Peak Table

Size [bp]	Calibrated Conc. [ng/µl]	Assigned Conc. [ng/µl]	Peak Molarity [nmol/l]	% Integrated Area	Peak Comment	Observations
25	6.11	-	376	-		Lower Marker
198	1.62	-	12.6	100.00		
1500	6.50	6.50	6.67	-		Upper Marker

 © Agilent Technologies, Inc. 2017

F2: F6 P R3

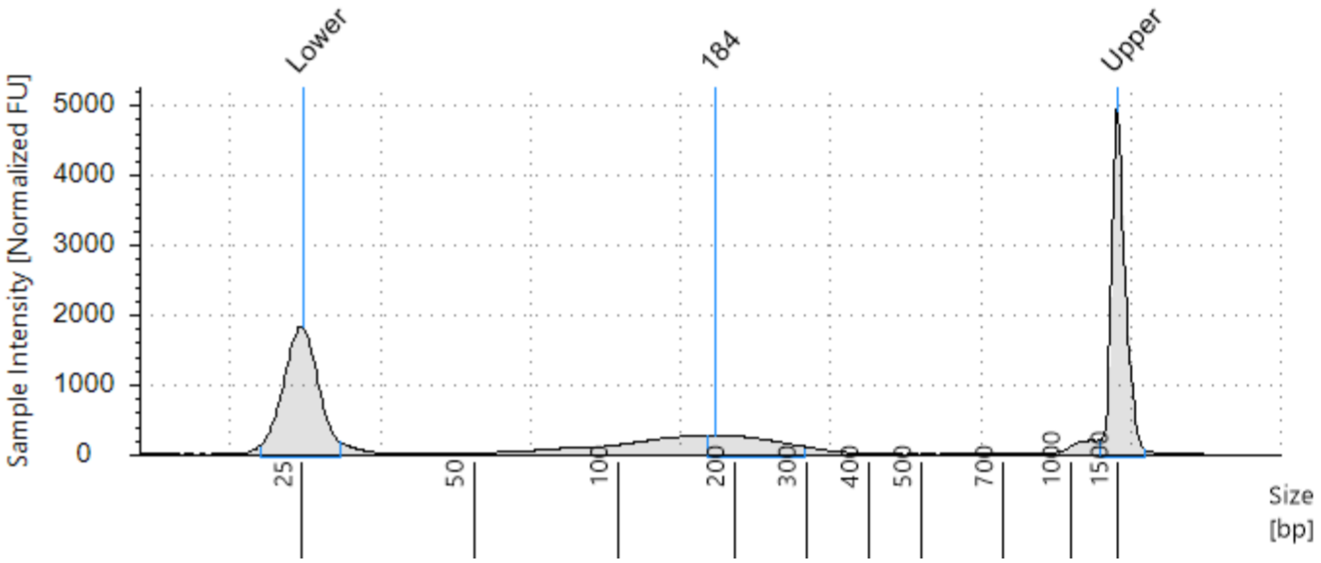

Sample Table

Well	Conc. [ng/µl]	Sample Description	Alert	Observations
F2	1.74	F6 P R3		

Peak Table

Size [bp]	Calibrated Conc. [ng/µl]	Assigned Conc. [ng/µl]	Peak Molarity [nmol/l]	% Integrated Area	Peak Comment	Observations
25	6.12	-	376	-		Lower Marker
184	1.74	-	14.5	100.00		
1500	6.50	6.50	6.67	-		Upper Marker

© Agilent Technologies, Inc. 2017

G2: G6 P R3

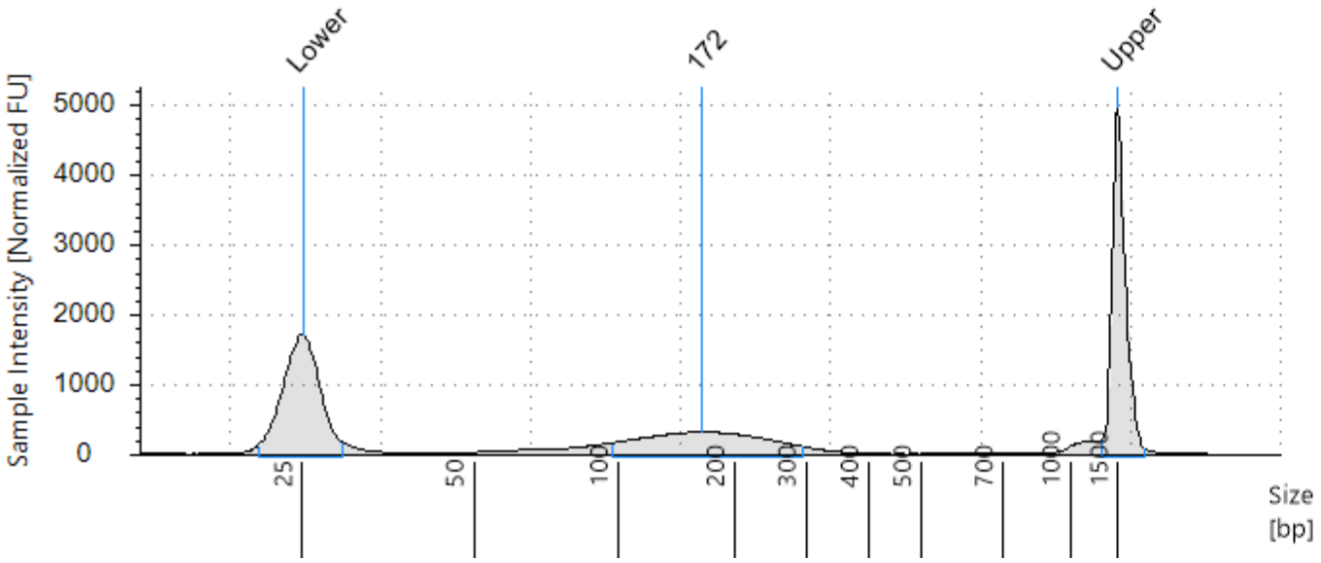

Sample Table

Well	Conc. [ng/µl]	Sample Description	Alert	Observations
G2	3.82	G6 P R3		

Peak Table

Size [bp]	Calibrated Conc. [ng/µl]	Assigned Conc. [ng/µl]	Peak Molarity [nmol/l]	% Integrated Area	Peak Comment	Observations
25	6.16	-	379	-		Lower Marker
172	3.82	-	34.2	100.00		
1500	6.50	6.50	6.67	-		Upper Marker

 © Agilent Technologies, Inc. 2017

H2: H6 P R3

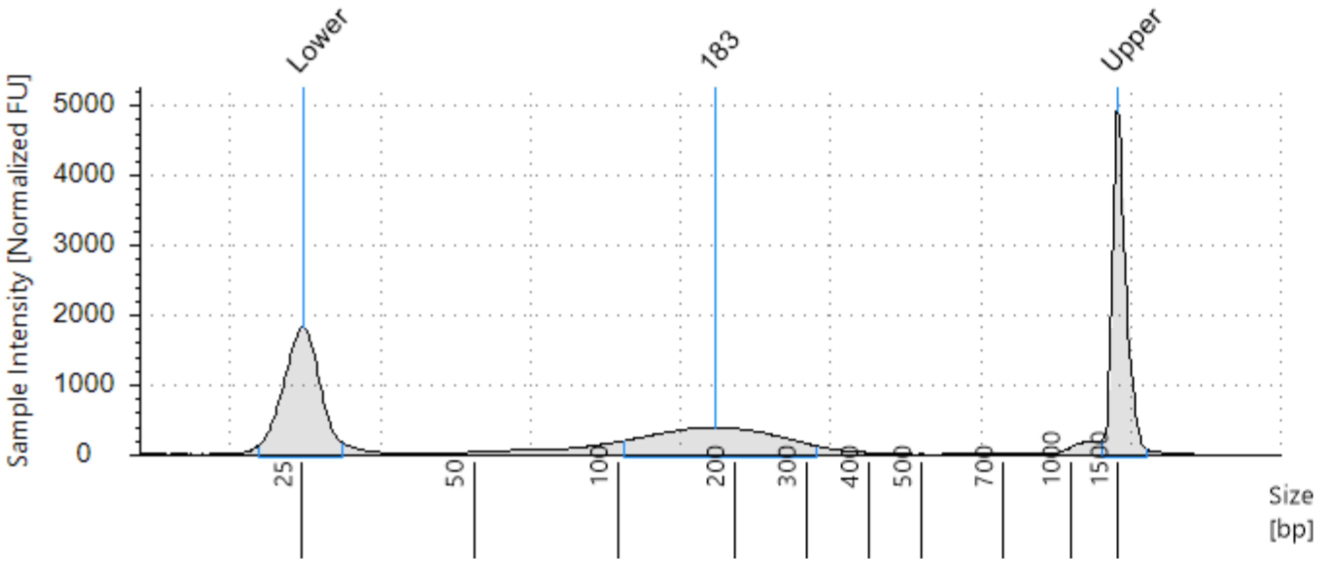

Sample Table

Well	Conc. [ng/µl]	Sample Description	Alert	Observations
H2	4.66	H6 P R3		

Peak Table

Size [bp]	Calibrated Conc. [ng/µl]	Assigned Conc. [ng/µl]	Peak Molarity [nmol/l]	% Integrated Area	Peak Comment	Observations
25	6.30	-	388	-		Lower Marker
183	4.66	-	39.2	100.00		
1500	6.50	6.50	6.67	-		Upper Marker

© Agilent Technologies, Inc. 2017

Filename: 2020-10-12-01 Q_S plus A7-H8 EXP H7 R3.D1000

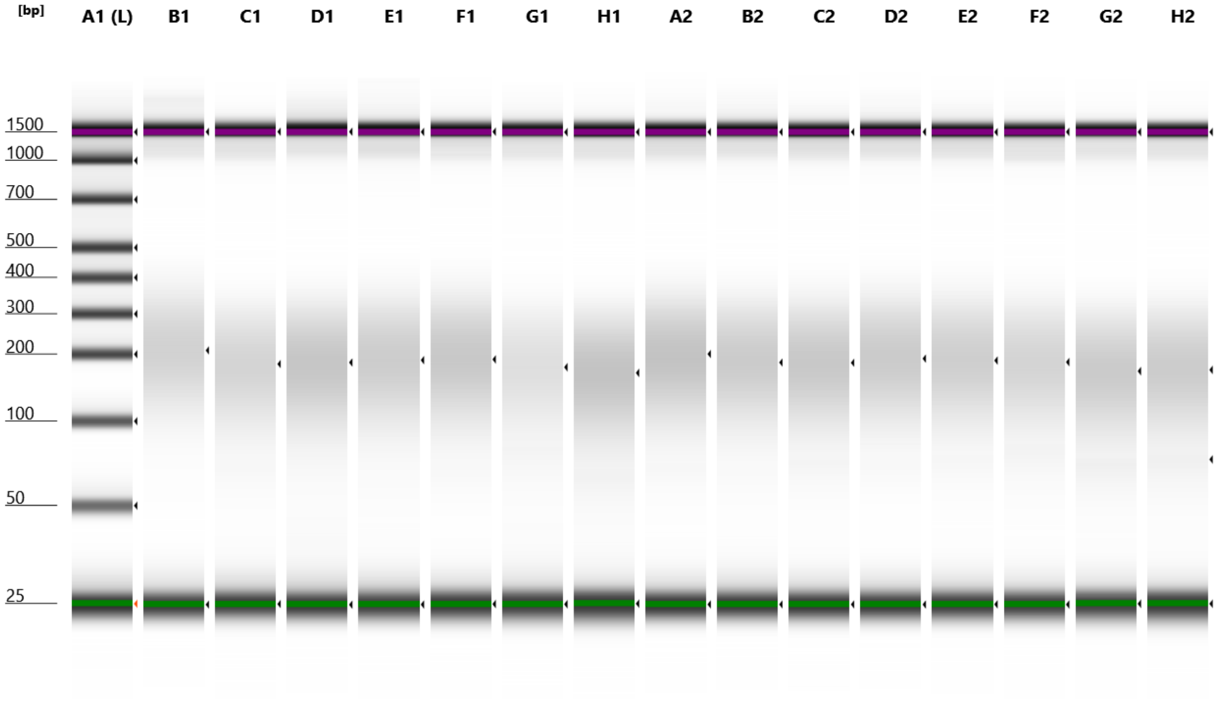

Default image (Contrast 100%)

Sample Info

Well	Conc. [ng/µl]	Sample Description	Alert	Observations
A1	19.2	Ladder		Ladder
B1	1.82	A7 P R3		
C1	1.72	B7 P R3		
D1	2.12	C7 P R3		
E1	2.13	D7 P R3		
F1	4.26	E7 P R3		
G1	1.35	F7 P R3		
H1	4.58	G7 P R3		
A2	4.43	A8 P R3		
B2	1.89	B8 P R3		
C2	3.98	C8 P R3		
D2	2.00	D8 P R3		
E2	3.59	E8 P R3		
F2	3.16	F8 P R3		
G2	2.32	G8 P R3		
H2	2.34	H8 P R3		

 © Agilent Technologies, Inc. 2017

A1: Ladder

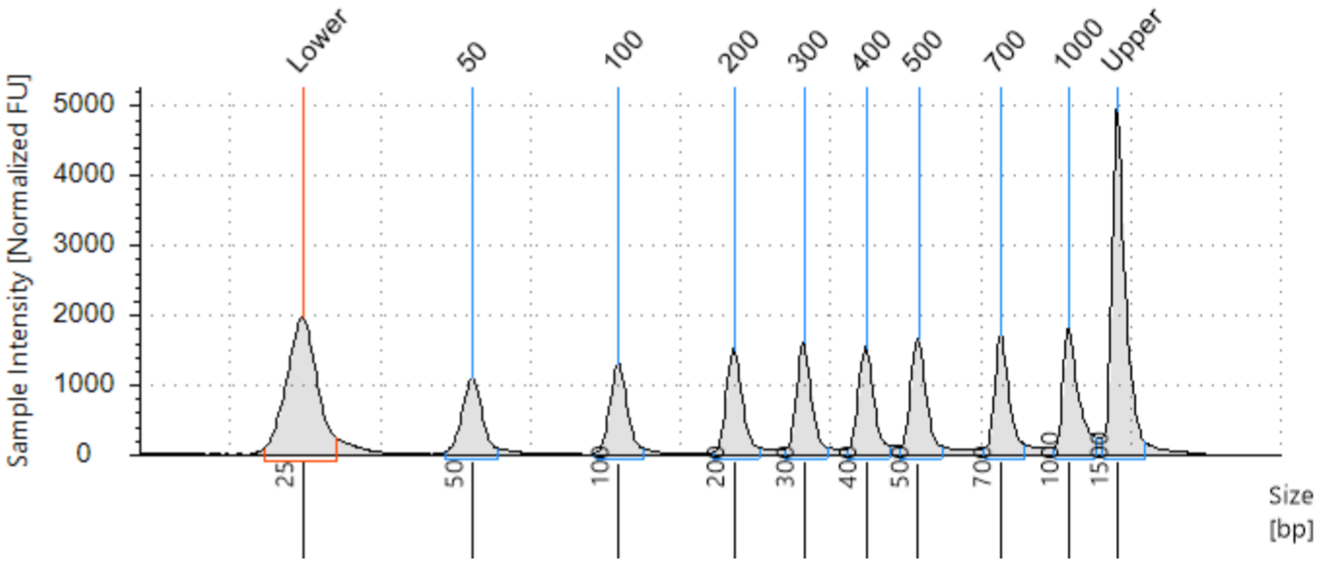

Sample Table

Well	Conc. [ng/µl]	Sample Description	Alert	Observations
A1	19.2	Ladder		Ladder

Peak Table

Size [bp]	Calibrated Conc. [ng/µl]	Assigned Conc. [ng/µl]	Peak Molarity [nmol/l]	% Integrated Area	Peak Comment	Observations
25	5.79	-	356	-		Lower Marker
50	2.12	-	65.1	11.04		
100	2.19	-	33.6	11.41		
200	2.37	-	18.3	12.39		
300	2.38	-	12.2	12.40		
400	2.37	-	9.13	12.38		
500	2.55	-	7.83	13.29		
700	2.45	-	5.38	12.77		
1000	2.74	-	4.22	14.32		
1500	6.50	6.50	6.67	-		Upper Marker

© Agilent Technologies, Inc. 2017

B1: A7 P R3

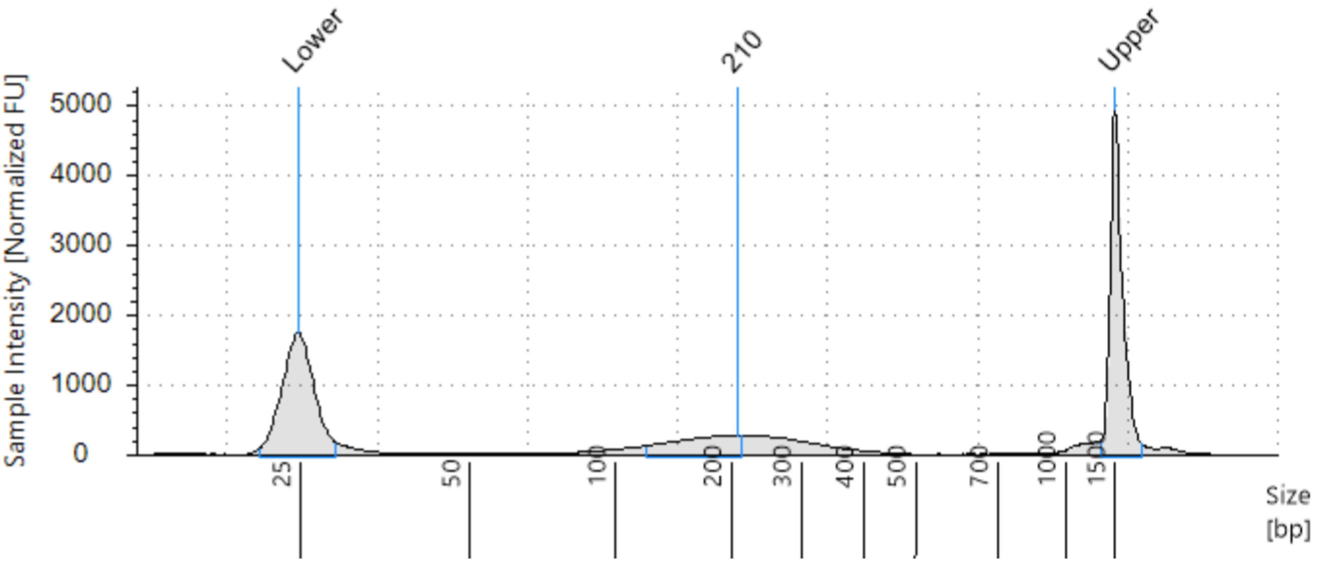

Sample Table

Well	Conc. [ng/µl]	Sample Description	Alert	Observations
B1	1.82	A7 P R3		

Peak Table

Size [bp]	Calibrated Conc. [ng/µl]	Assigned Conc. [ng/µl]	Peak Molarity [nmol/l]	% Integrated Area	Peak Comment	Observations
25	5.84	-	360	-		Lower Marker
210	1.82	-	13.3	100.00		
1500	6.50	6.50	6.67	-		Upper Marker

© Agilent Technologies, Inc. 2017

C1: B7 P R3

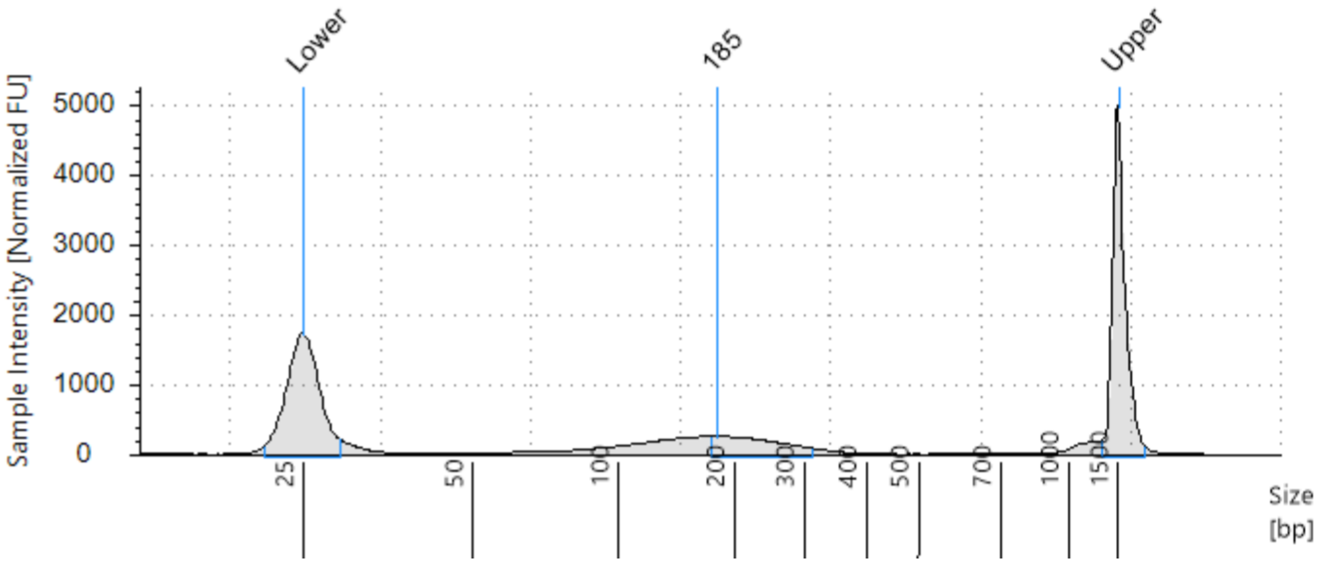

Sample Table

Well	Conc. [ng/µl]	Sample Description	Alert	Observations
C1	1.72	B7 P R3		

Peak Table

Size [bp]	Calibrated Conc. [ng/µl]	Assigned Conc. [ng/µl]	Peak Molarity [nmol/l]	% Integrated Area	Peak Comment	Observations
25	5.88	-	362	-		Lower Marker
185	1.72	-	14.3	100.00		
1500	6.50	6.50	6.67	-		Upper Marker

© Agilent Technologies, Inc. 2017

D1: C7 P R3

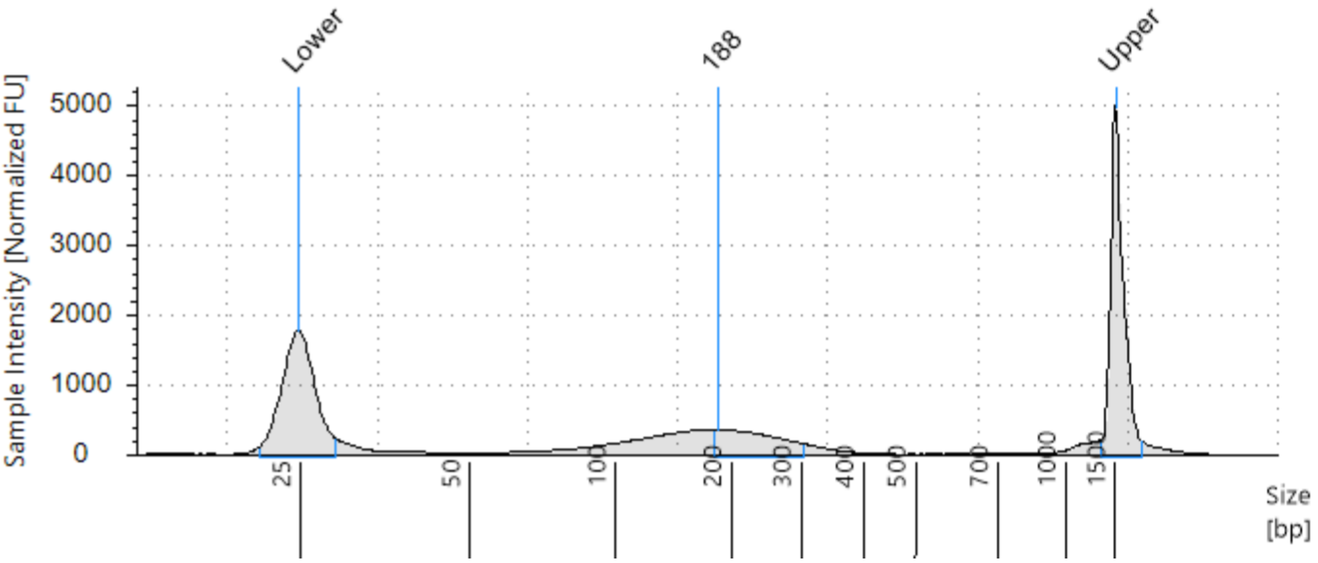

Sample Table

Well	Conc. [ng/µl]	Sample Description	Alert	Observations
D1	2.12	C7 P R3		

Peak Table

Size [bp]	Calibrated Conc. [ng/µl]	Assigned Conc. [ng/µl]	Peak Molarity [nmol/l]	% Integrated Area	Peak Comment	Observations
25	5.93	-	365	-		Lower Marker
188	2.12	-	17.3	100.00		
1500	6.50	6.50	6.67	-		Upper Marker

© Agilent Technologies, Inc. 2017

E1: D7 P R3

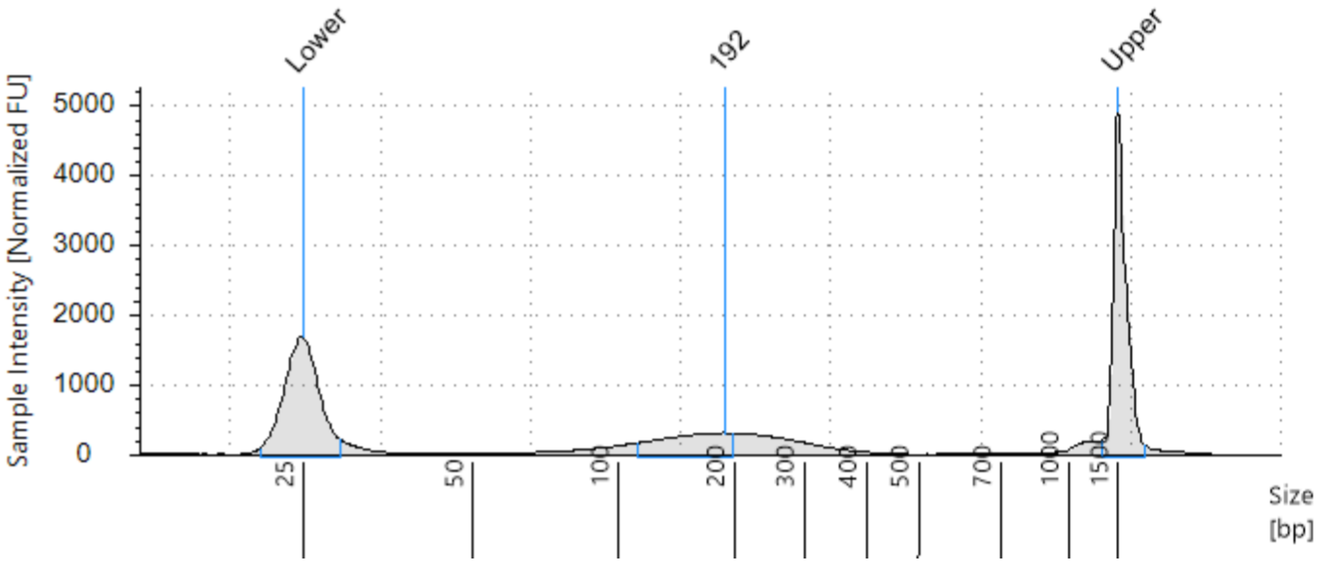

Sample Table

Well	Conc. [ng/µl]	Sample Description	Alert	Observations
E1	2.13	D7 P R3		

Peak Table

Size [bp]	Calibrated Conc. [ng/µl]	Assigned Conc. [ng/µl]	Peak Molarity [nmol/l]	% Integrated Area	Peak Comment	Observations
25	5.99	-	368	-		Lower Marker
192	2.13	-	17.1	100.00		
1500	6.50	6.50	6.67	-		Upper Marker

© Agilent Technologies, Inc. 2017

F1: E7 P R3

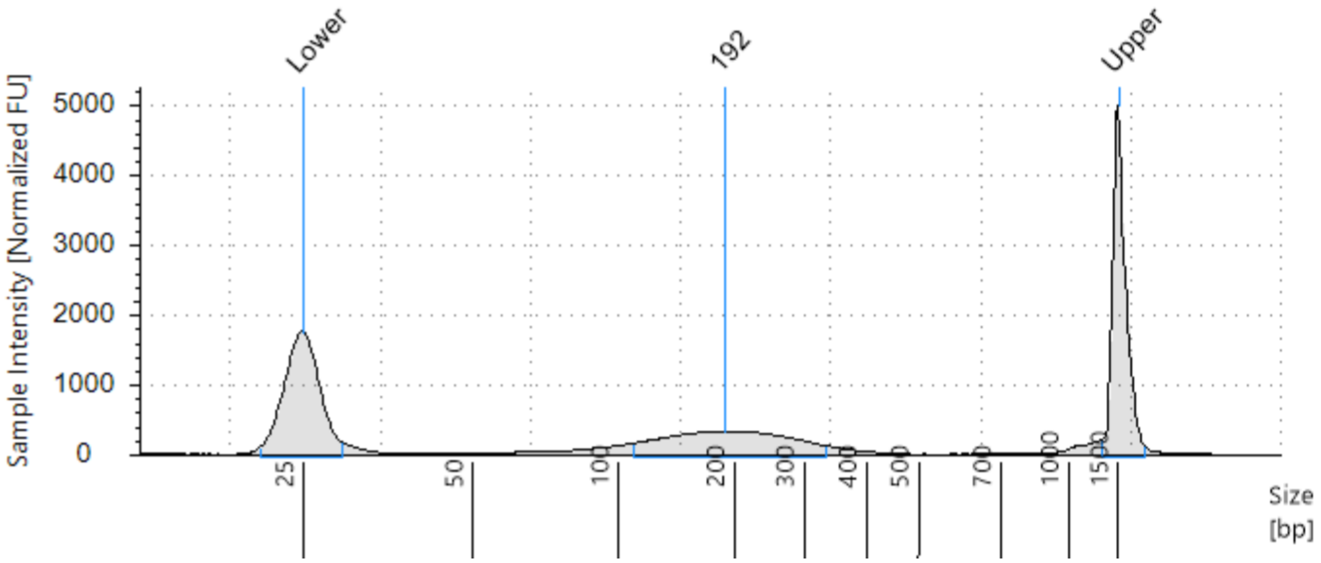

Sample Table

Well	Conc. [ng/µl]	Sample Description	Alert	Observations
F1	4.26	E7 P R3		

Peak Table

Size [bp]	Calibrated Conc. [ng/µl]	Assigned Conc. [ng/µl]	Peak Molarity [nmol/l]	% Integrated Area	Peak Comment	Observations
25	6.22	-	383	-		Lower Marker
192	4.26	-	34.1	100.00		
1500	6.50	6.50	6.67	-		Upper Marker

 © Agilent Technologies, Inc. 2017

G1: F7 P R3

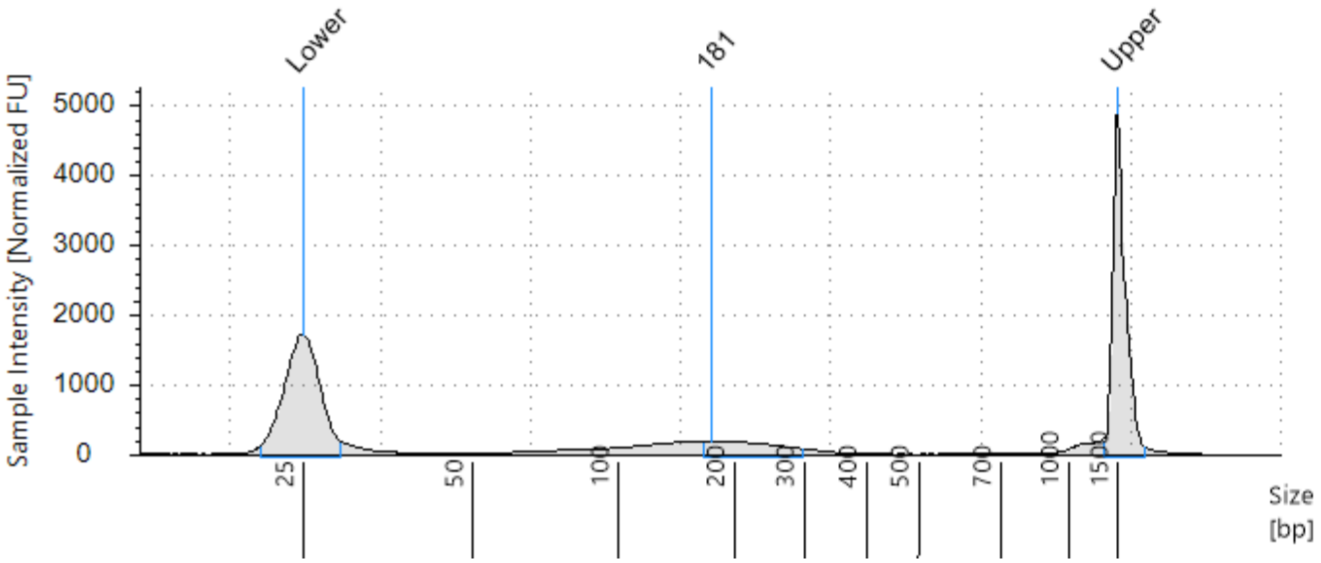

Sample Table

Well	Conc. [ng/µl]	Sample Description	Alert	Observations
G1	1.35	F7 P R3		

Peak Table

Size [bp]	Calibrated Conc. [ng/µl]	Assigned Conc. [ng/µl]	Peak Molarity [nmol/l]	% Integrated Area	Peak Comment	Observations
25	6.49	-	400	-		Lower Marker
181	1.35	-	11.5	100.00		
1500	6.50	6.50	6.67	-		Upper Marker

 © Agilent Technologies, Inc. 2017

H1: G7 P R3

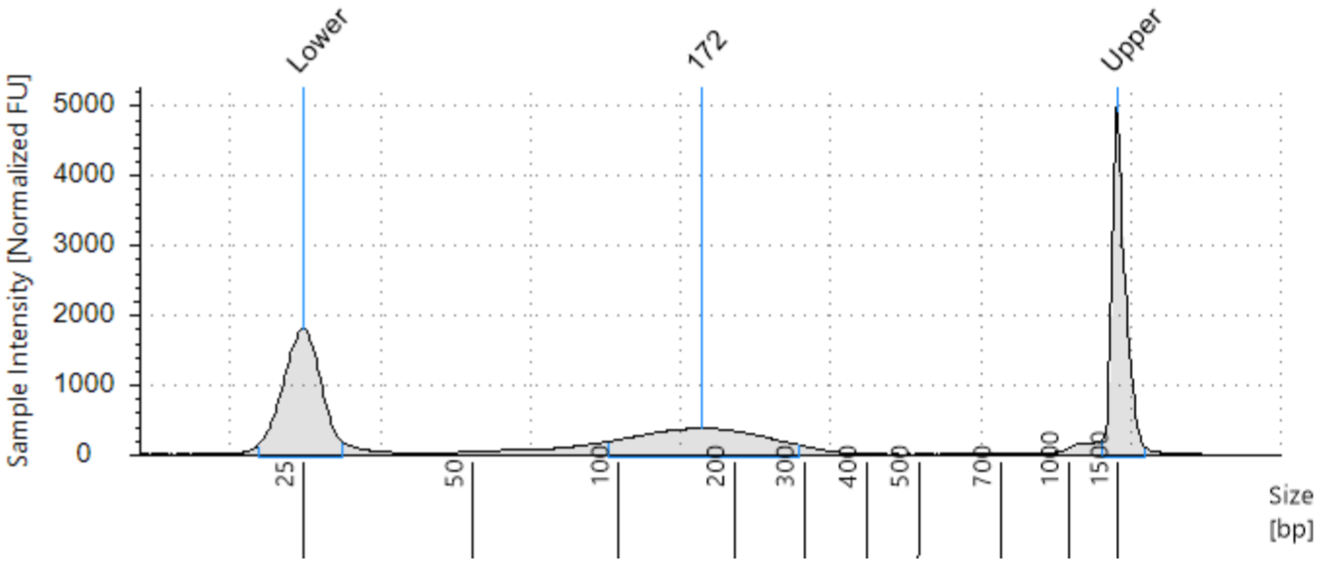

Sample Table

Well	Conc. [ng/µl]	Sample Description	Alert	Observations
H1	4.58	G7 P R3		

Peak Table

Size [bp]	Calibrated Conc. [ng/µl]	Assigned Conc. [ng/µl]	Peak Molarity [nmol/l]	% Integrated Area	Peak Comment	Observations
25	6.70	-	412	-		Lower Marker
172	4.58	-	41.0	100.00		
1500	6.50	6.50	6.67	-		Upper Marker

© Agilent Technologies, Inc. 2017

A2: A8 P R3

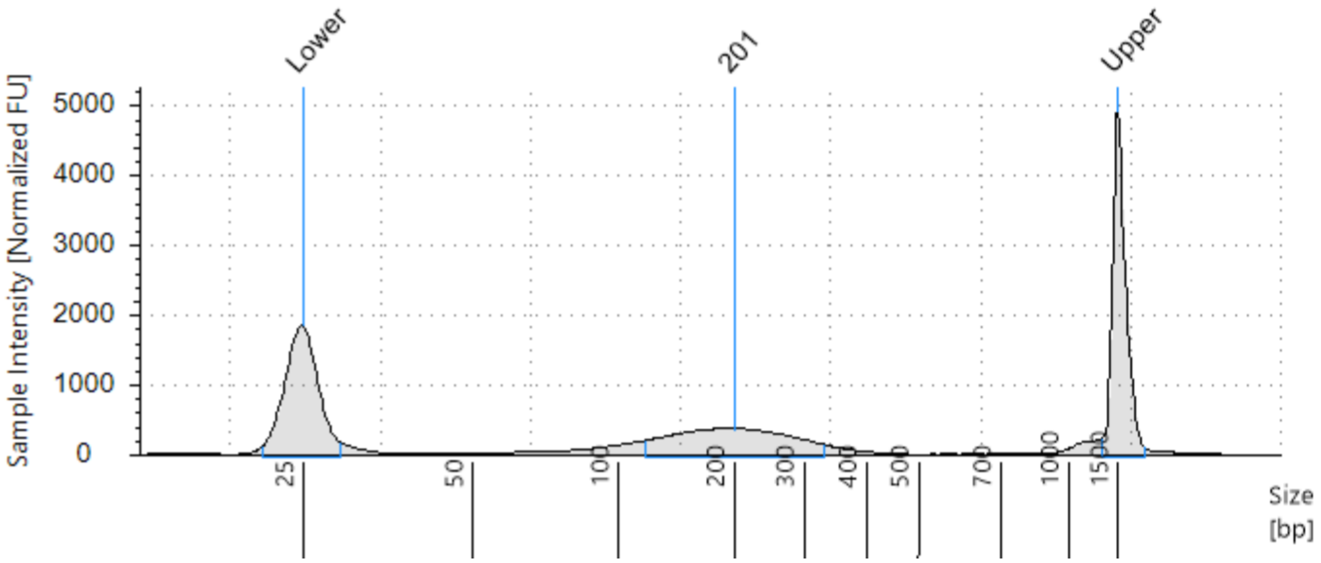

Sample Table

Well	Conc. [ng/µl]	Sample Description	Alert	Observations
A2	4.43	A8 P R3		

Peak Table

Size [bp]	Calibrated Conc. [ng/µl]	Assigned Conc. [ng/µl]	Peak Molarity [nmol/l]	% Integrated Area	Peak Comment	Observations
25	6.10	-	375	-		Lower Marker
201	4.43	-	33.8	100.00		
1500	6.50	6.50	6.67	-		Upper Marker

© Agilent Technologies, Inc. 2017

B2: B8 P R3

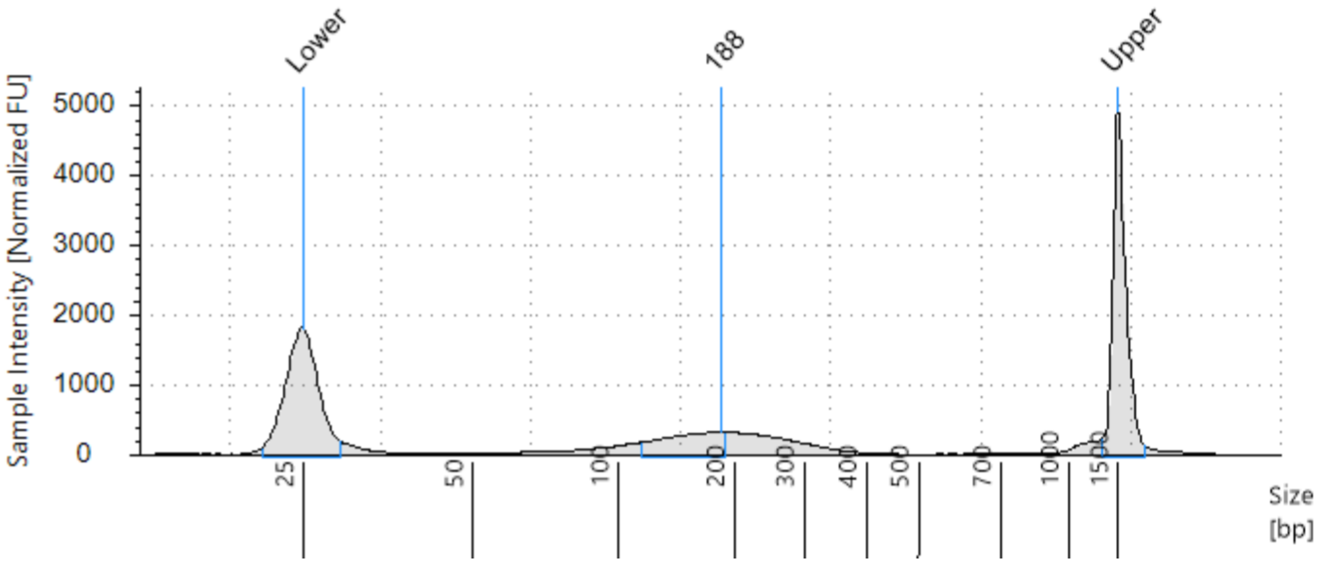

Sample Table

Well	Conc. [ng/µl]	Sample Description	Alert	Observations
B2	1.89	B8 P R3		

Peak Table

Size [bp]	Calibrated Conc. [ng/µl]	Assigned Conc. [ng/µl]	Peak Molarity [nmol/l]	% Integrated Area	Peak Comment	Observations
25	5.97	-	367	-		Lower Marker
188	1.89	-	15.4	100.00		
1500	6.50	6.50	6.67	-		Upper Marker

© Agilent Technologies, Inc. 2017

C2: C8 P R3

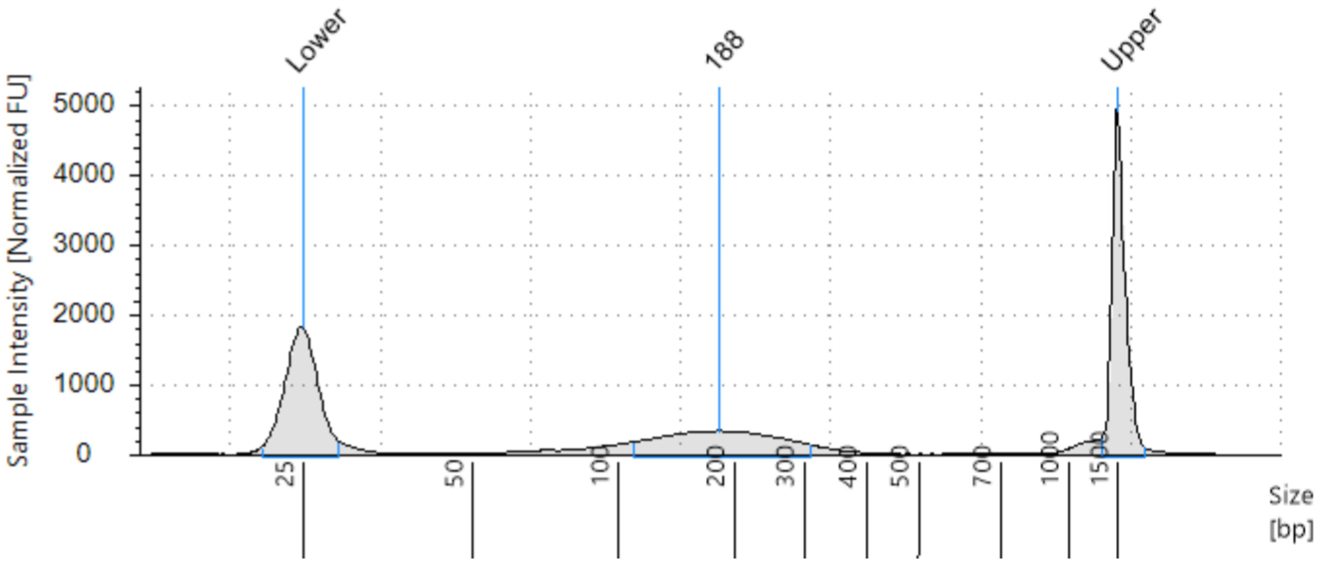

Sample Table

Well	Conc. [ng/µl]	Sample Description	Alert	Observations
C2	3.98	C8 P R3		

Peak Table

Size [bp]	Calibrated Conc. [ng/µl]	Assigned Conc. [ng/µl]	Peak Molarity [nmol/l]	% Integrated Area	Peak Comment	Observations
25	6.01	-	370	-		Lower Marker
188	3.98	-	32.6	100.00		
1500	6.50	6.50	6.67	-		Upper Marker

D2: D8 P R3

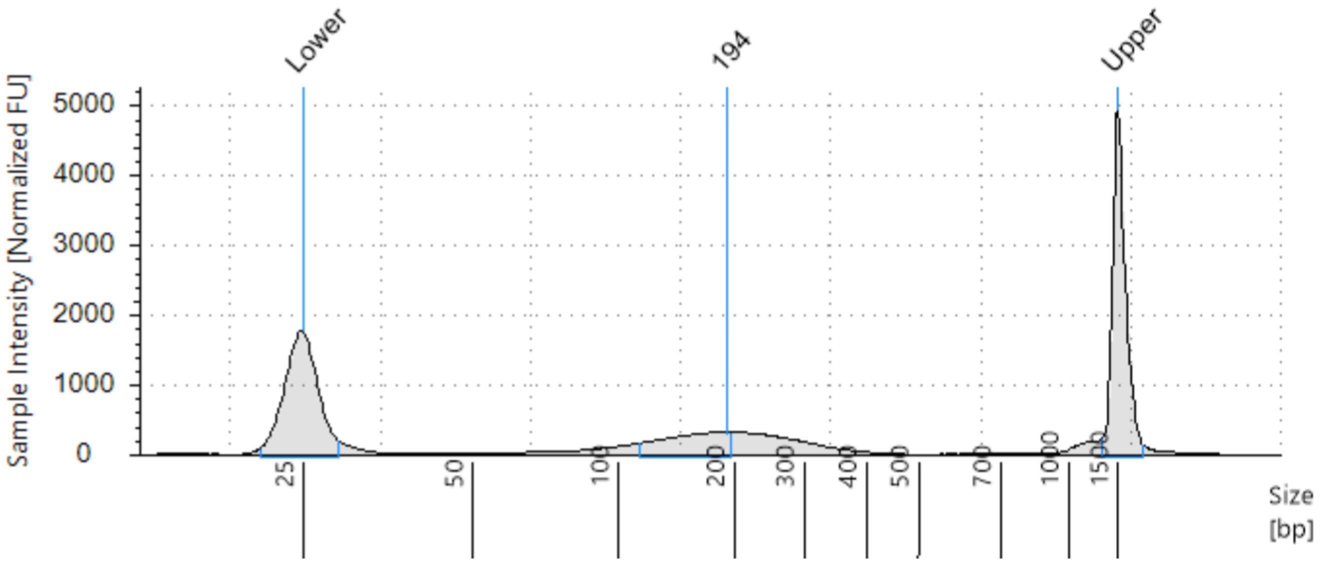

Sample Table

Well	Conc. [ng/µl]	Sample Description	Alert	Observations
D2	2.00	D8 P R3		

Peak Table

Size [bp]	Calibrated Conc. [ng/µl]	Assigned Conc. [ng/µl]	Peak Molarity [nmol/l]	% Integrated Area	Peak Comment	Observations
25	5.92	-	364	-		Lower Marker
194	2.00	-	15.8	100.00		
1500	6.50	6.50	6.67	-		Upper Marker

© Agilent Technologies, Inc. 2017

E2: E8 P R3

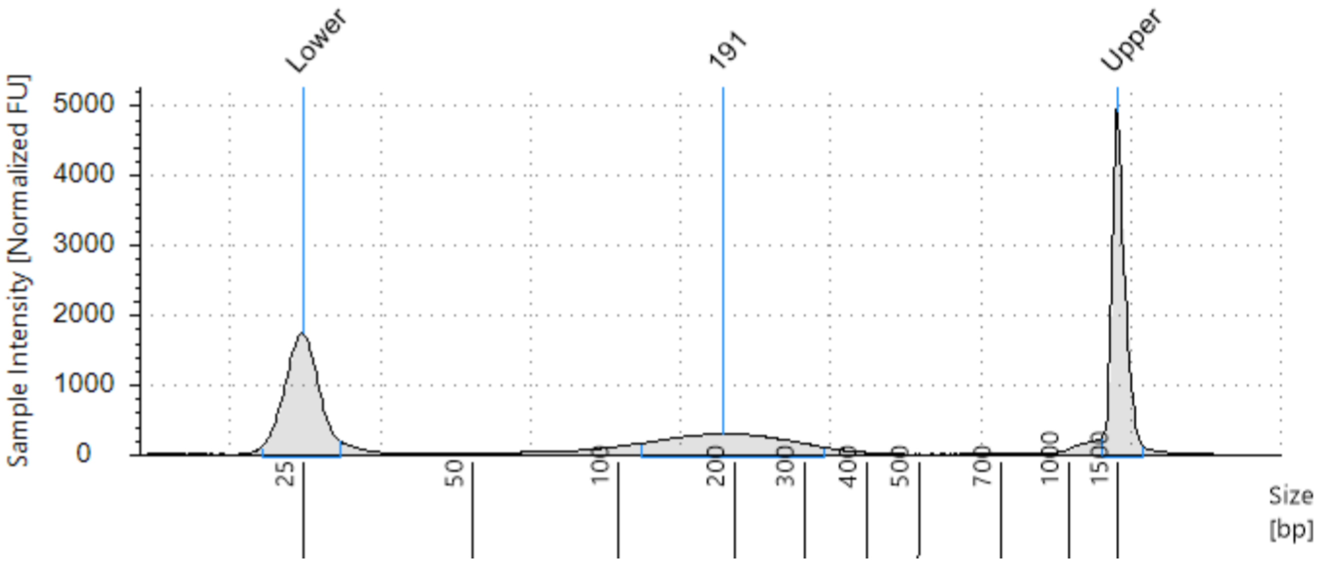

Sample Table

Well	Conc. [ng/µl]	Sample Description	Alert	Observations
E2	3.59	E8 P R3		

Peak Table

Size [bp]	Calibrated Conc. [ng/µl]	Assigned Conc. [ng/µl]	Peak Molarity [nmol/l]	% Integrated Area	Peak Comment	Observations
25	5.96	-	366	-		Lower Marker
191	3.59	-	28.9	100.00		
1500	6.50	6.50	6.67	-		Upper Marker

 © Agilent Technologies, Inc. 2017

F2: F8 P R3

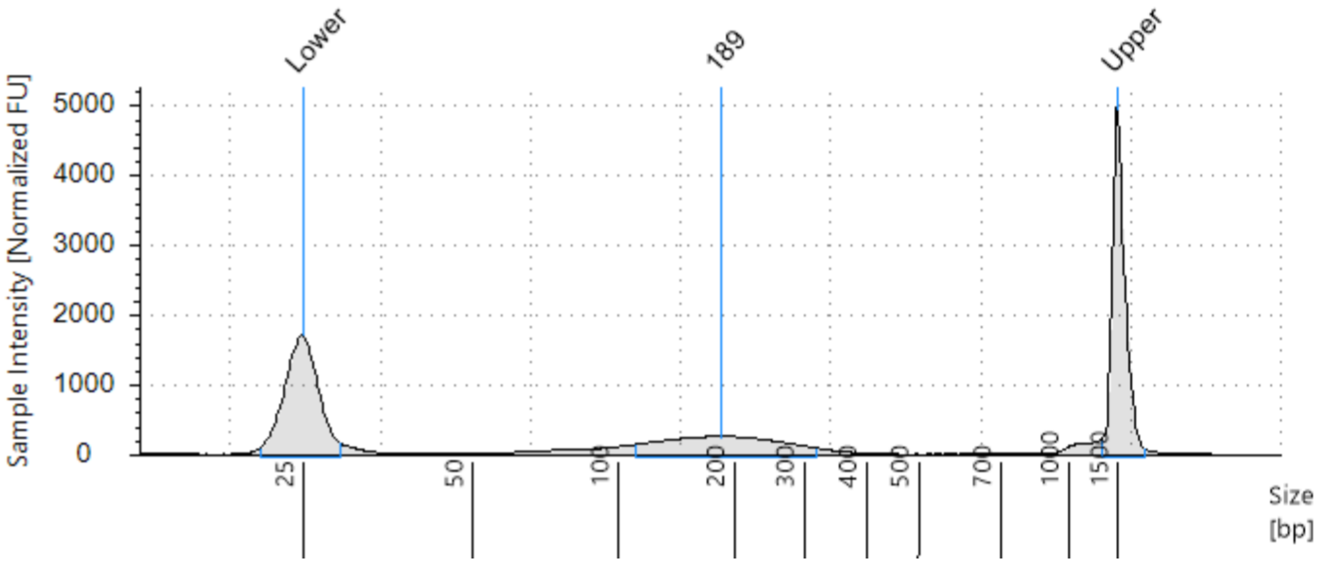

Sample Table

Well	Conc. [ng/µl]	Sample Description	Alert	Observations
F2	3.16	F8 P R3		

Peak Table

Size [bp]	Calibrated Conc. [ng/µl]	Assigned Conc. [ng/µl]	Peak Molarity [nmol/l]	% Integrated Area	Peak Comment	Observations
25	5.91	-	364	-		Lower Marker
189	3.16	-	25.8	100.00		
1500	6.50	6.50	6.67	-		Upper Marker

© Agilent Technologies, Inc. 2017

G2: G8 P R3

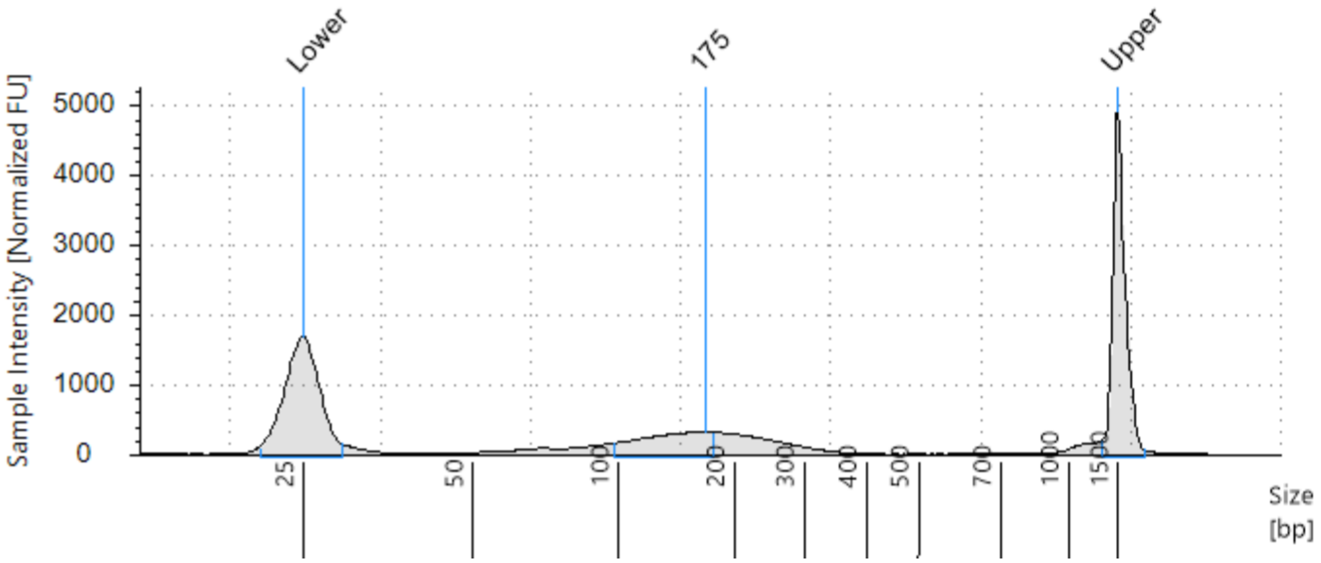

Sample Table

Well	Conc. [ng/µl]	Sample Description	Alert	Observations
G2	2.32	G8 P R3		

Peak Table

Size [bp]	Calibrated Conc. [ng/µl]	Assigned Conc. [ng/µl]	Peak Molarity [nmol/l]	% Integrated Area	Peak Comment	Observations
25	6.26	-	385	-		Lower Marker
175	2.32	-	20.4	100.00		
1500	6.50	6.50	6.67	-		Upper Marker

© Agilent Technologies, Inc. 2017

H2: H8 P R3

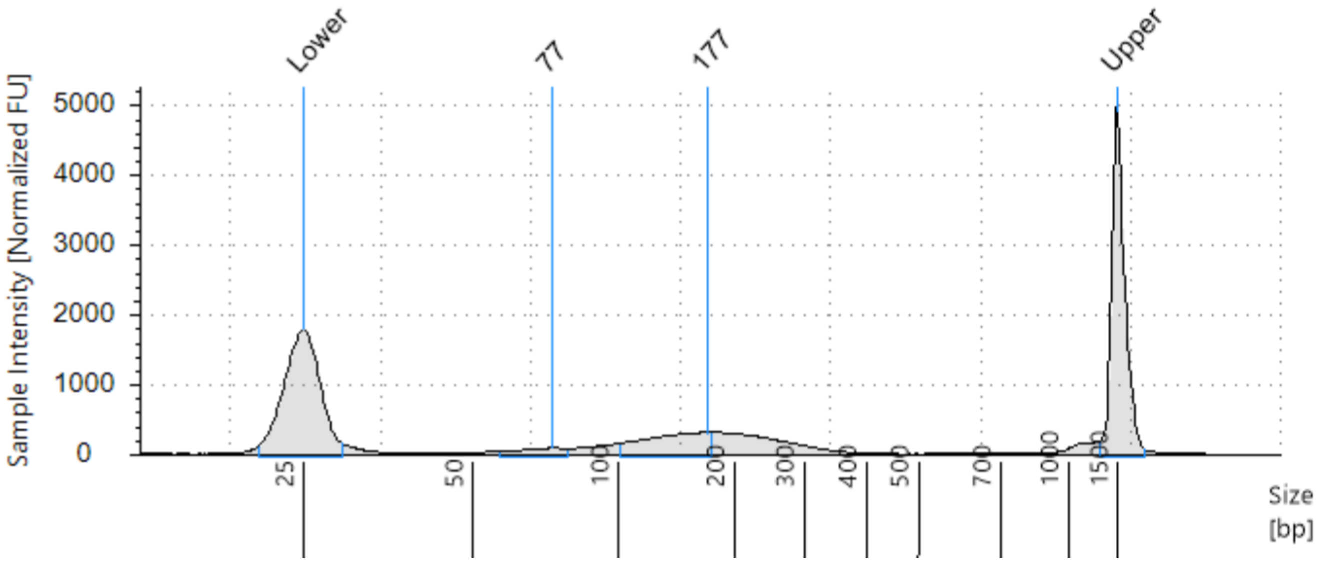

Sample Table

Well	Conc. [ng/µl]	Sample Description	Alert	Observations
H2	2.34	H8 P R3		

Peak Table

Size [bp]	Calibrated Conc. [ng/µl]	Assigned Conc. [ng/µl]	Peak Molarity [nmol/l]	% Integrated Area	Peak Comment	Observations
25	6.44	-	396	-		Lower Marker
77	0.372	-	7.40	15.93		
177	1.96	-	17.1	84.07		
1500	6.50	6.50	6.67	-		Upper Marker

© Agilent Technologies, Inc. 2017

Filename: 2020-10-12-02 Q_S plus A9-H10 EXP H9 R3.D1000

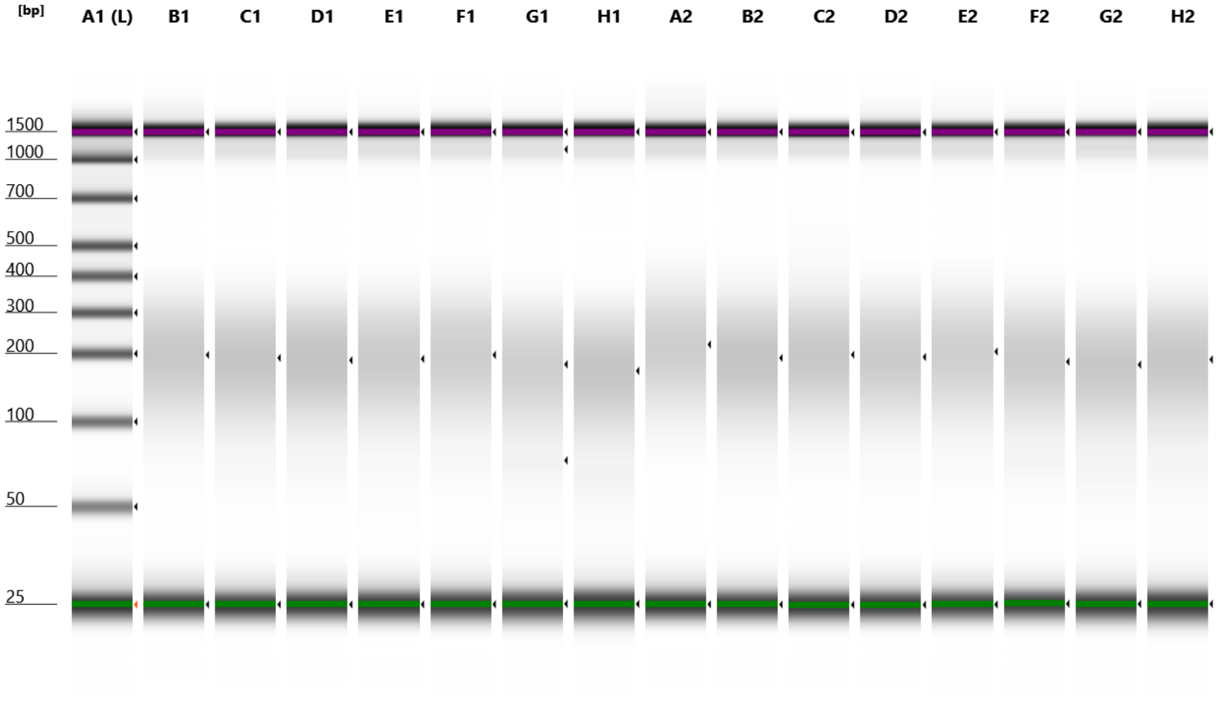

Default image (Contrast 100%)

Sample Info

Well	Conc. [ng/µl]	Sample Description	Alert	Observations
A1	15.2	Ladder		Ladder
B1	4.18	A9 P R3		
C1	2.40	B9 P R3		
D1	2.40	C9 P R3		
E1	4.02	D9 P R3		
F1	2.09	E9 P R3		
G1	2.70	F9 P R3		
H1	4.34	G9 P R3		
A2	1.87	A10 P R3		
B2	4.47	B10 P R3		
C2	4.07	C10 P R3		
D2	3.82	D10 PR3		
E2	3.73	E10 P R3		
F2	2.05	F10 P R3		
G2	2.26	G10 P R3		
H2	2.21	H10 P R3		

© Agilent Technologies, Inc. 2017

A1: Ladder

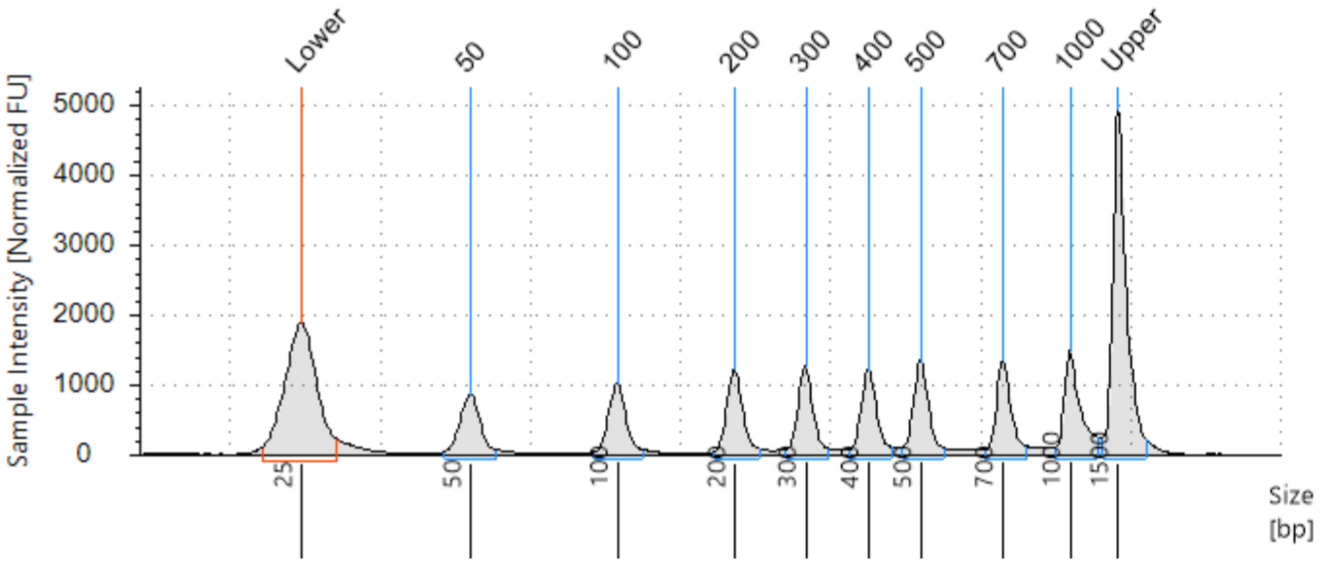

Sample Table

Well	Conc. [ng/µl]	Sample Description	Alert	Observations
A1	15.2	Ladder		Ladder

Peak Table

Size [bp]	Calibrated Conc. [ng/µl]	Assigned Conc. [ng/µl]	Peak Molarity [nmol/l]	% Integrated Area	Peak Comment	Observations
25	5.74	-	353	-		Lower Marker
50	1.67	-	51.4	10.99		
100	1.75	-	26.9	11.51		
200	1.90	-	14.6	12.49		
300	1.88	-	9.63	12.35		
400	1.86	-	7.15	12.23		
500	1.98	-	6.09	13.02		
700	1.92	-	4.21	12.60		
1000	2.25	-	3.47	14.81		
1500	6.50	6.50	6.67	-		Upper Marker

© Agilent Technologies, Inc. 2017

B1: A9 P R3

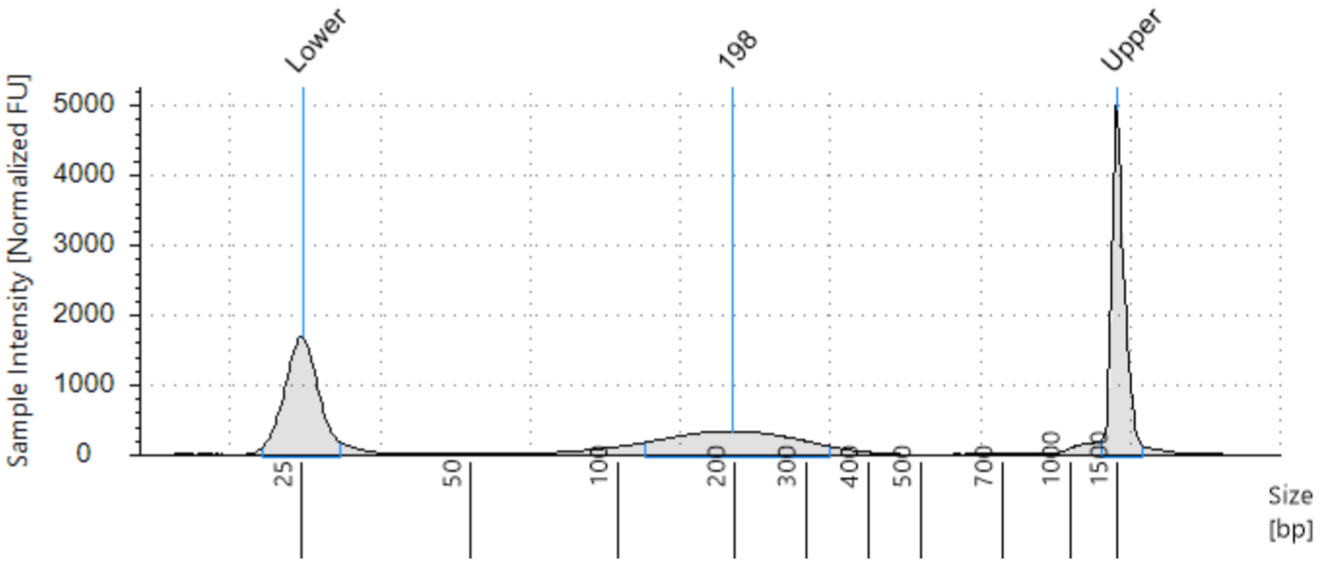

Sample Table

Well	Conc. [ng/µl]	Sample Description	Alert	Observations
B1	4.18	A9 P R3		

Peak Table

Size [bp]	Calibrated Conc. [ng/µl]	Assigned Conc. [ng/µl]	Peak Molarity [nmol/l]	% Integrated Area	Peak Comment	Observations
25	5.77	-	355	-		Lower Marker
198	4.18	-	32.4	100.00		
1500	6.50	6.50	6.67	-		Upper Marker

© Agilent Technologies, Inc. 2017

C1: B9 P R3

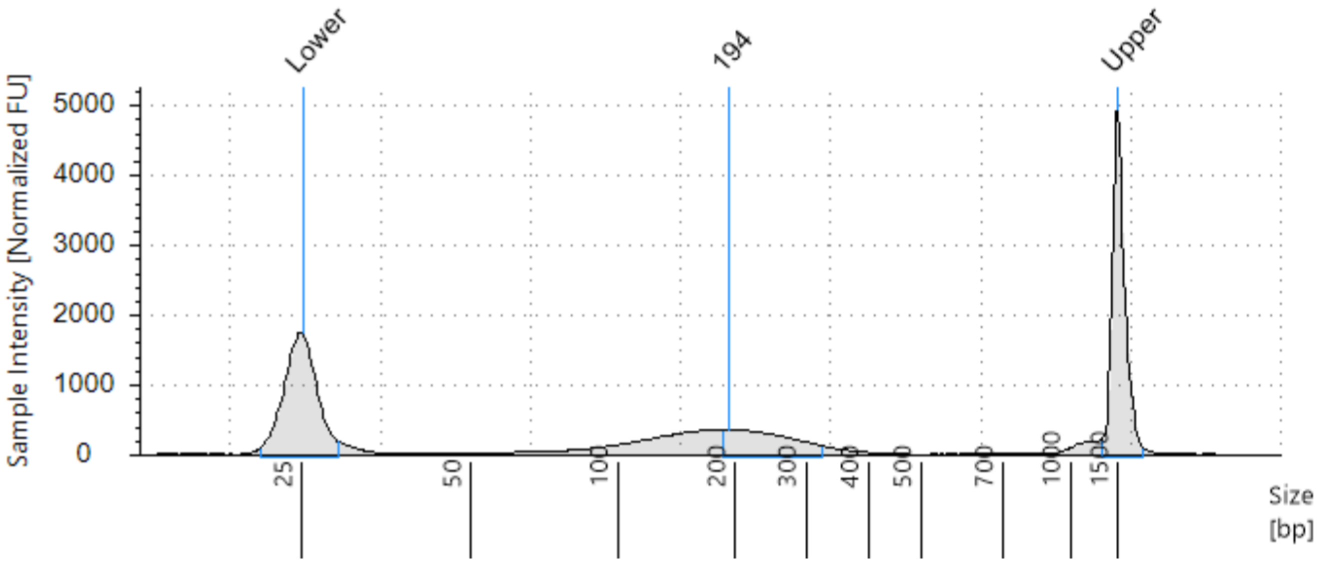

Sample Table

Well	Conc. [ng/µl]	Sample Description	Alert	Observations
C1	2.40	B9 P R3		

Peak Table

Size [bp]	Calibrated Conc. [ng/µl]	Assigned Conc. [ng/µl]	Peak Molarity [nmol/l]	% Integrated Area	Peak Comment	Observations
25	6.01	-	370	-		Lower Marker
194	2.40	-	19.1	100.00		
1500	6.50	6.50	6.67	-		Upper Marker

© Agilent Technologies, Inc. 2017

D1: C9 P R3

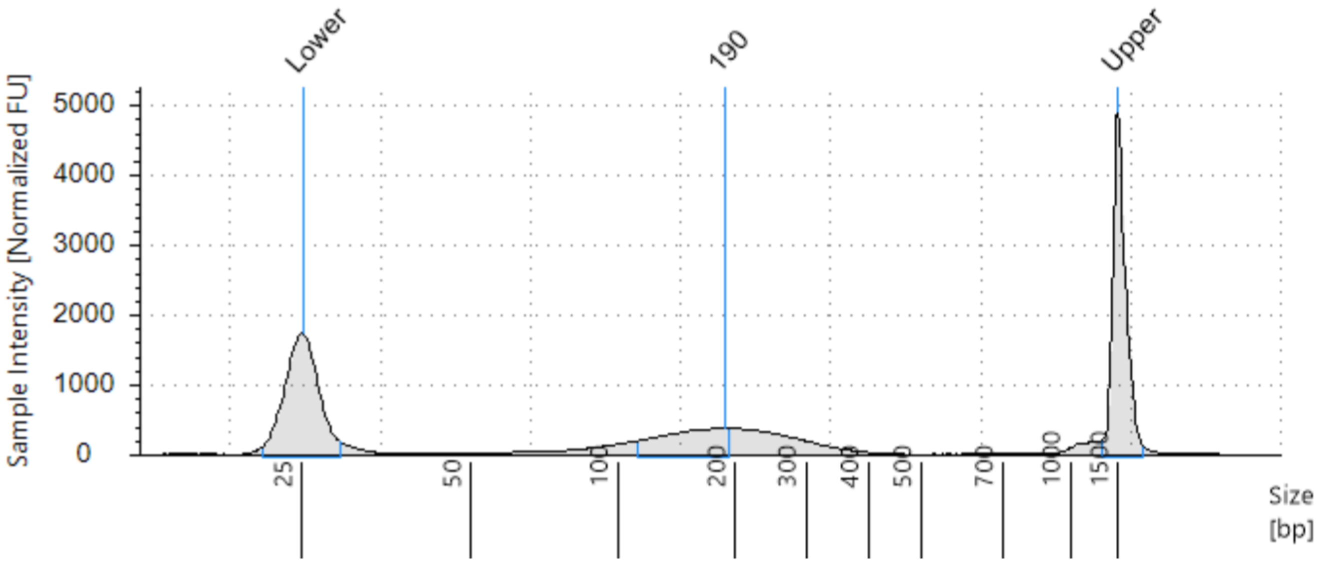

Sample Table

Well	Conc. [ng/µl]	Sample Description	Alert	Observations
D1	2.40	C9 P R3		

Peak Table

Size [bp]	Calibrated Conc. [ng/µl]	Assigned Conc. [ng/µl]	Peak Molarity [nmol/l]	% Integrated Area	Peak Comment	Observations
25	6.00	-	369	-		Lower Marker
190	2.40	-	19.4	100.00		
1500	6.50	6.50	6.67	-		Upper Marker

© Agilent Technologies, Inc. 2017

E1: D9 P R3

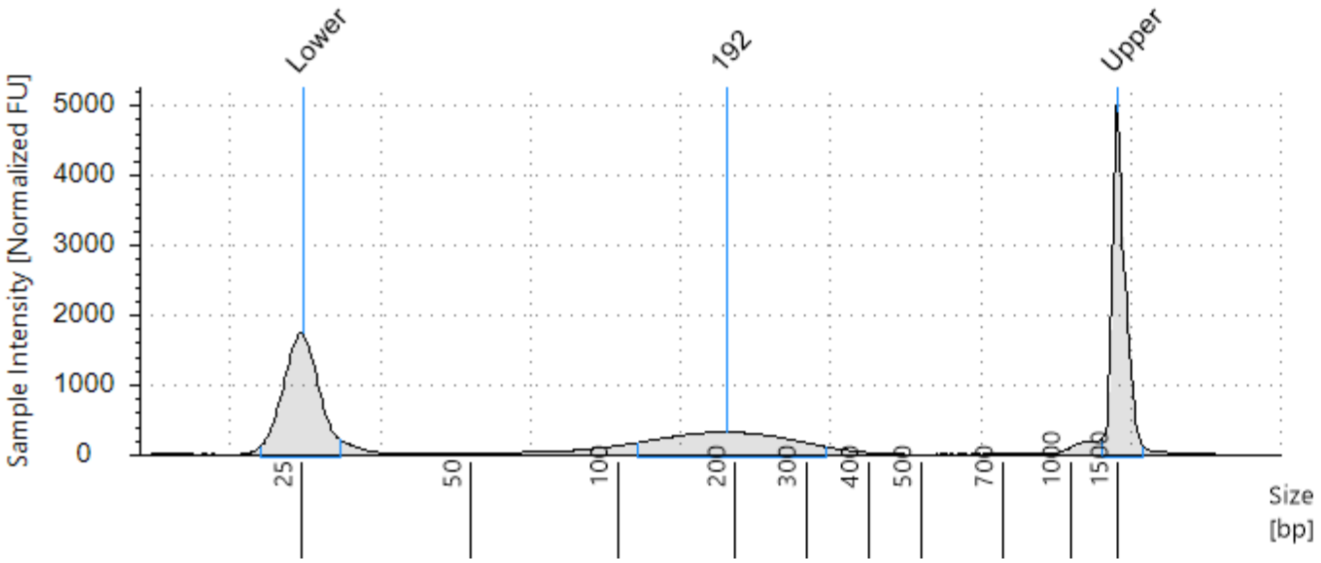

Sample Table

Well	Conc. [ng/µl]	Sample Description	Alert	Observations
E1	4.02	D9 P R3		

Peak Table

Size [bp]	Calibrated Conc. [ng/µl]	Assigned Conc. [ng/µl]	Peak Molarity [nmol/l]	% Integrated Area	Peak Comment	Observations
25	6.19	-	381	-		Lower Marker
192	4.02	-	32.1	100.00		
1500	6.50	6.50	6.67	-		Upper Marker

© Agilent Technologies, Inc. 2017

F1: E9 P R3

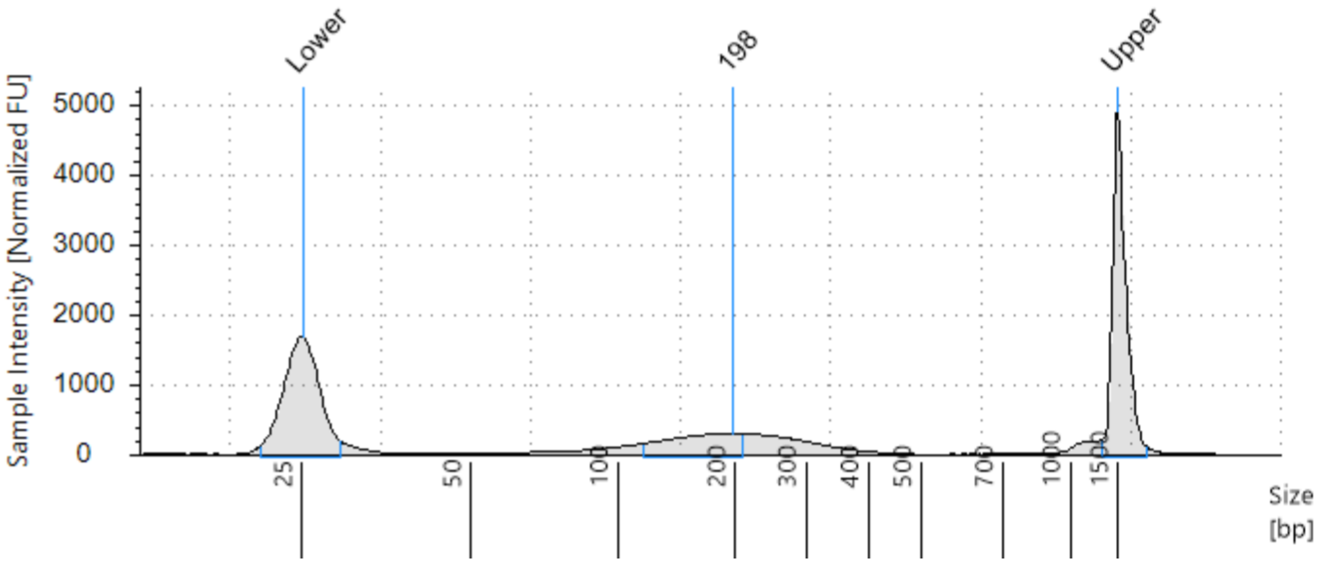

Sample Table

Well	Conc. [ng/µl]	Sample Description	Alert	Observations
F1	2.09	E9 P R3		

Peak Table

Size [bp]	Calibrated Conc. [ng/µl]	Assigned Conc. [ng/µl]	Peak Molarity [nmol/l]	% Integrated Area	Peak Comment	Observations
25	5.97	-	367	-		Lower Marker
198	2.09	-	16.2	100.00		
1500	6.50	6.50	6.67	-		Upper Marker

© Agilent Technologies, Inc. 2017

G1: F9 P R3

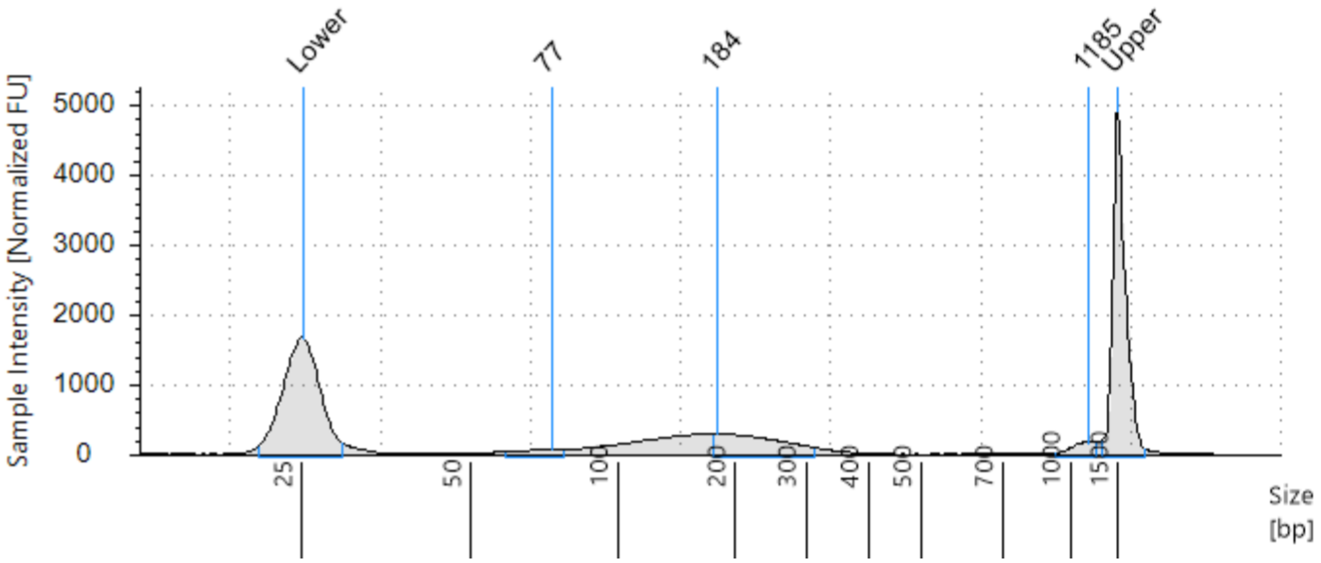

Sample Table

Well	Conc. [ng/µl]	Sample Description	Alert	Observations
G1	2.70	F9 P R3		

Peak Table

Size [bp]	Calibrated Conc. [ng/µl]	Assigned Conc. [ng/µl]	Peak Molarity [nmol/l]	% Integrated Area	Peak Comment	Observations
25	6.46	-	397	-		Lower Marker
77	0.301	-	6.01	11.15		
184	1.99	-	16.7	73.98		
1185	0.401	-	0.520	14.87		
1500	6.50	6.50	6.67	-		Upper Marker

 © Agilent Technologies, Inc. 2017

H1: G9 P R3

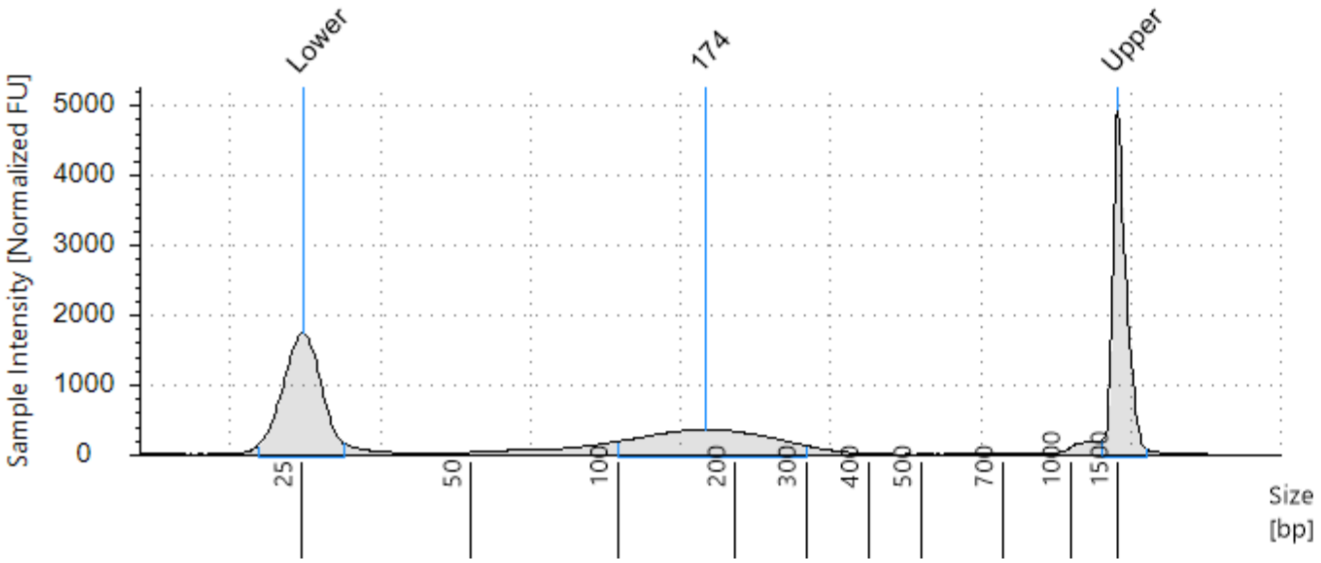

Sample Table

Well	Conc. [ng/µl]	Sample Description	Alert	Observations
H1	4.34	G9 P R3		

Peak Table

Size [bp]	Calibrated Conc. [ng/µl]	Assigned Conc. [ng/µl]	Peak Molarity [nmol/l]	% Integrated Area	Peak Comment	Observations
25	6.53	-	402	-		Lower Marker
174	4.34	-	38.3	100.00		
1500	6.50	6.50	6.67	-		Upper Marker

© Agilent Technologies, Inc. 2017

A2: A10 P R3

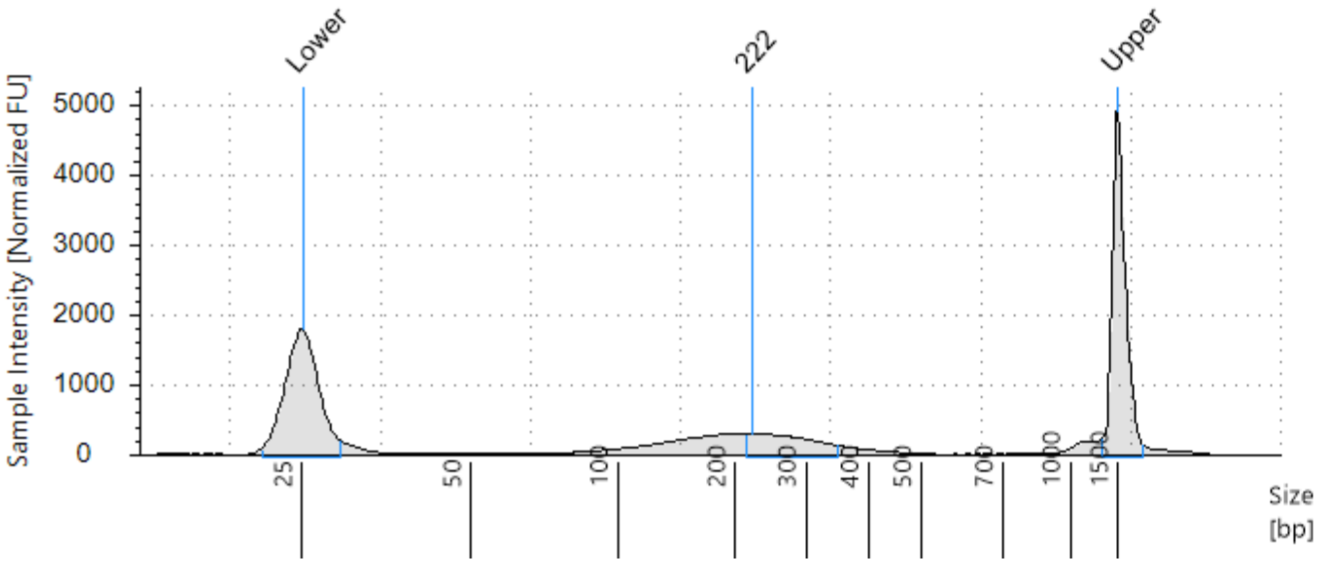

Sample Table

Well	Conc. [ng/µl]	Sample Description	Alert	Observations
A2	1.87	A10 P R3		

Peak Table

Size [bp]	Calibrated Conc. [ng/µl]	Assigned Conc. [ng/µl]	Peak Molarity [nmol/l]	% Integrated Area	Peak Comment	Observations
25	6.04	-	372	-		Lower Marker
222	1.87	-	12.9	100.00		
1500	6.50	6.50	6.67	-		Upper Marker

 © Agilent Technologies, Inc. 2017

B2: B10 P R3

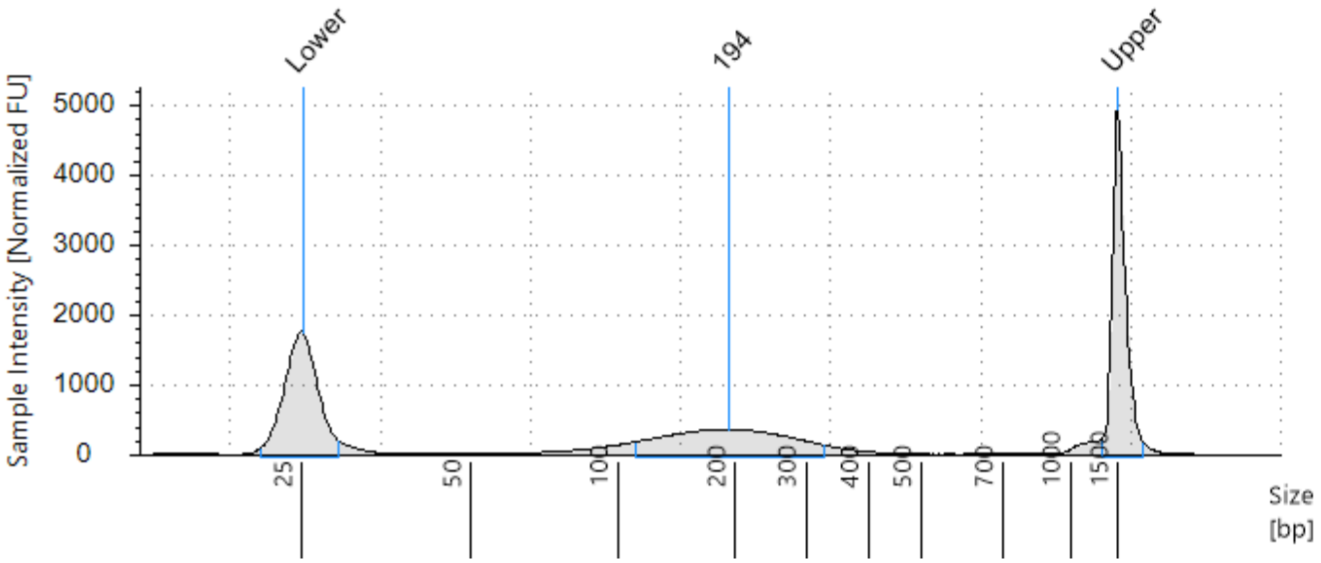

Sample Table

Well	Conc. [ng/µl]	Sample Description	Alert	Observations
B2	4.47	B10 P R3		

Peak Table

Size [bp]	Calibrated Conc. [ng/µl]	Assigned Conc. [ng/µl]	Peak Molarity [nmol/l]	% Integrated Area	Peak Comment	Observations
25	5.82	-	358	-		Lower Marker
194	4.47	-	35.5	100.00		
1500	6.50	6.50	6.67	-		Upper Marker

 © Agilent Technologies, Inc. 2017

C2: C10 P R3

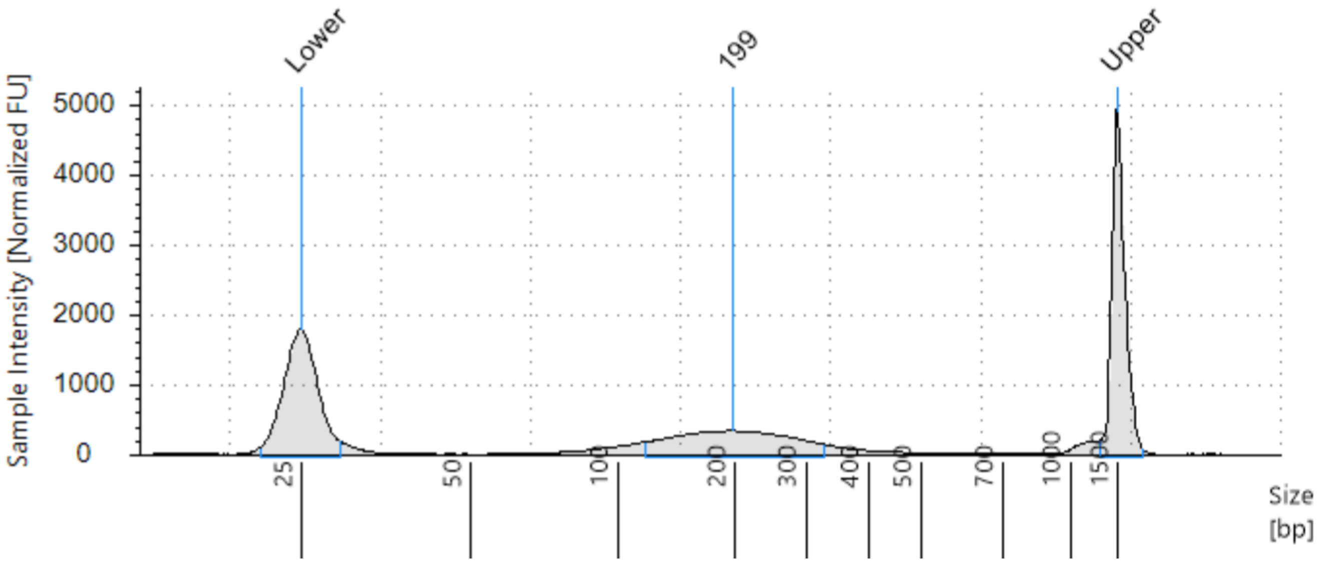

Sample Table

Well	Conc. [ng/µl]	Sample Description	Alert	Observations
C2	4.07	C10 P R3		

Peak Table

Size [bp]	Calibrated Conc. [ng/µl]	Assigned Conc. [ng/µl]	Peak Molarity [nmol/l]	% Integrated Area	Peak Comment	Observations
25	6.10	-	376	-		Lower Marker
199	4.07	-	31.5	100.00		
1500	6.50	6.50	6.67	-		Upper Marker

© Agilent Technologies, Inc. 2017

D2: D10 PR3

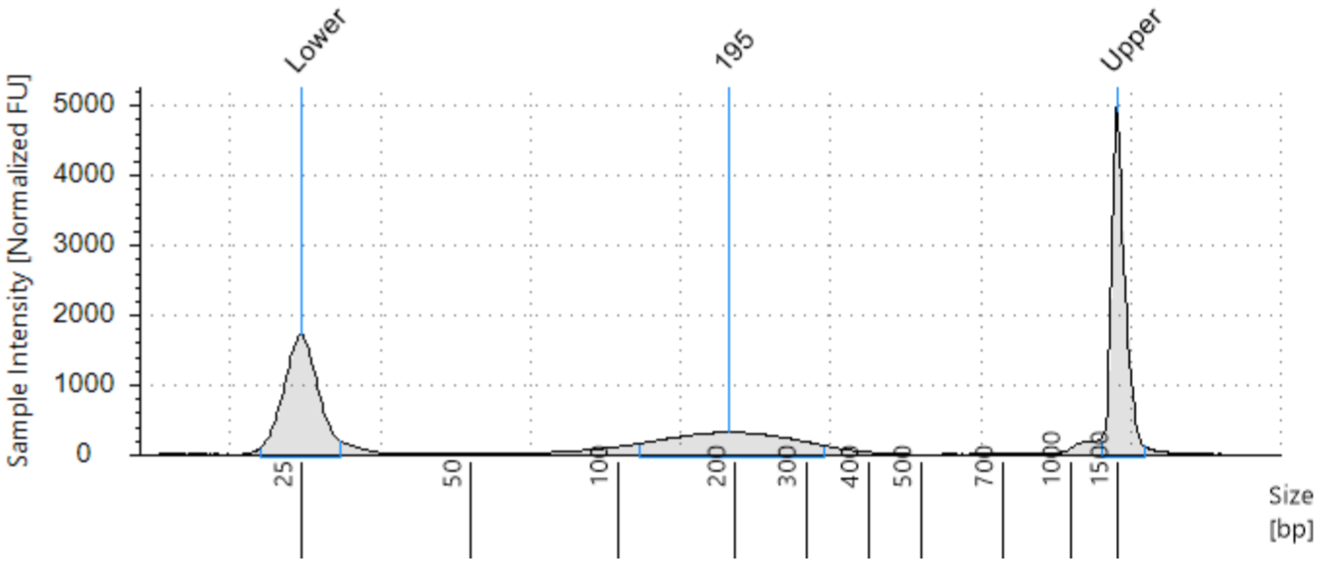

Sample Table

Well	Conc. [ng/µl]	Sample Description	Alert	Observations
D2	3.82	D10 PR3		

Peak Table

Size [bp]	Calibrated Conc. [ng/µl]	Assigned Conc. [ng/µl]	Peak Molarity [nmol/l]	% Integrated Area	Peak Comment	Observations
25	5.70	-	351	-		Lower Marker
195	3.82	-	30.0	100.00		
1500	6.50	6.50	6.67	-		Upper Marker

© Agilent Technologies, Inc. 2017

E2: E10 P R3

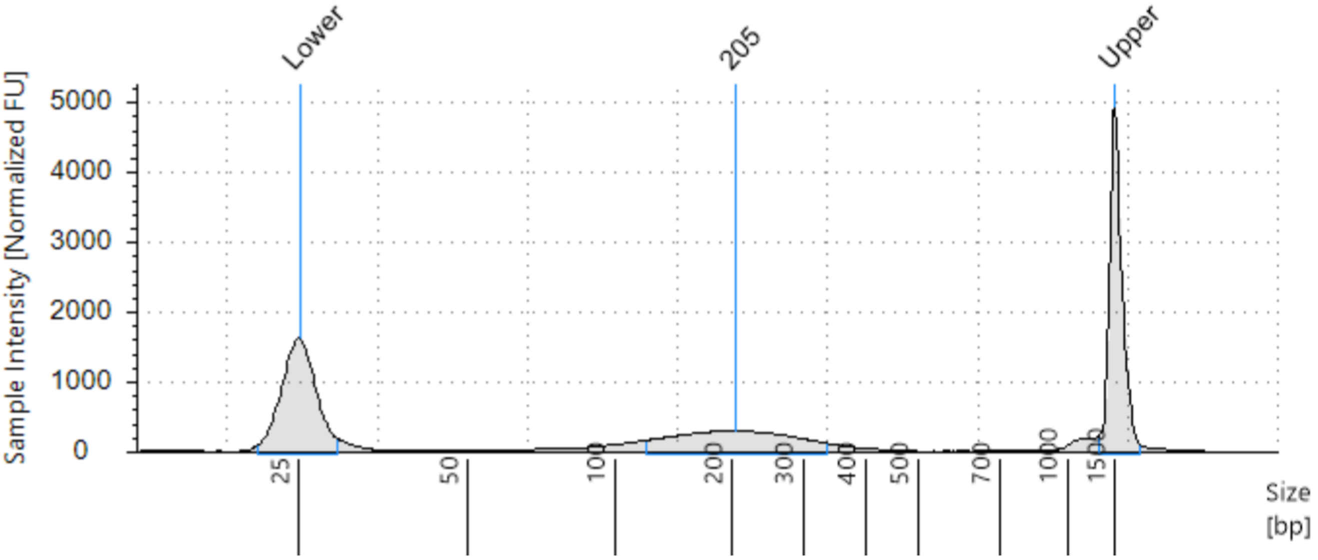

Sample Table

Well	Conc. [ng/µl]	Sample Description	Alert	Observations
E2	3.73	E10 P R3		

Peak Table

Size [bp]	Calibrated Conc. [ng/µl]	Assigned Conc. [ng/µl]	Peak Molarity [nmol/l]	% Integrated Area	Peak Comment	Observations
25	5.81	-	358	-		Lower Marker
205	3.73	-	28.0	100.00		
1500	6.50	6.50	6.67	-		Upper Marker

© Agilent Technologies, Inc. 2017

F2: F10 P R3

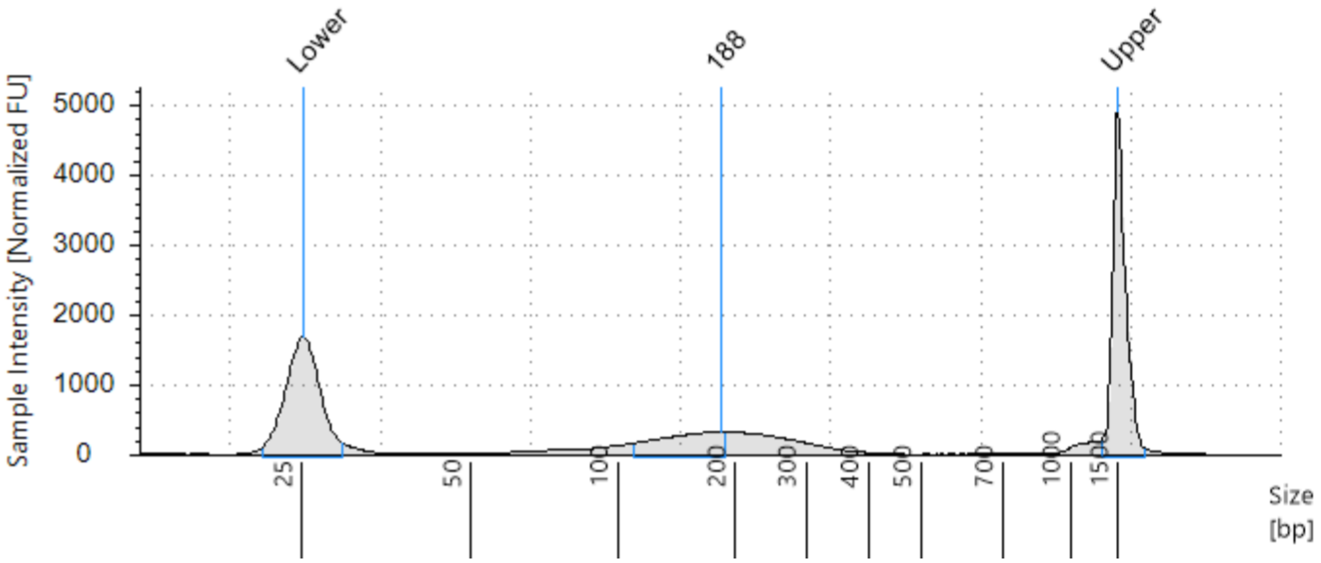

Sample Table

Well	Conc. [ng/µl]	Sample Description	Alert	Observations
F2	2.05	F10 P R3		

Peak Table

Size [bp]	Calibrated Conc. [ng/µl]	Assigned Conc. [ng/µl]	Peak Molarity [nmol/l]	% Integrated Area	Peak Comment	Observations
25	5.86	-	360	-		Lower Marker
188	2.05	-	16.8	100.00		
1500	6.50	6.50	6.67	-		Upper Marker

© Agilent Technologies, Inc. 2017

G2: G10 P R3

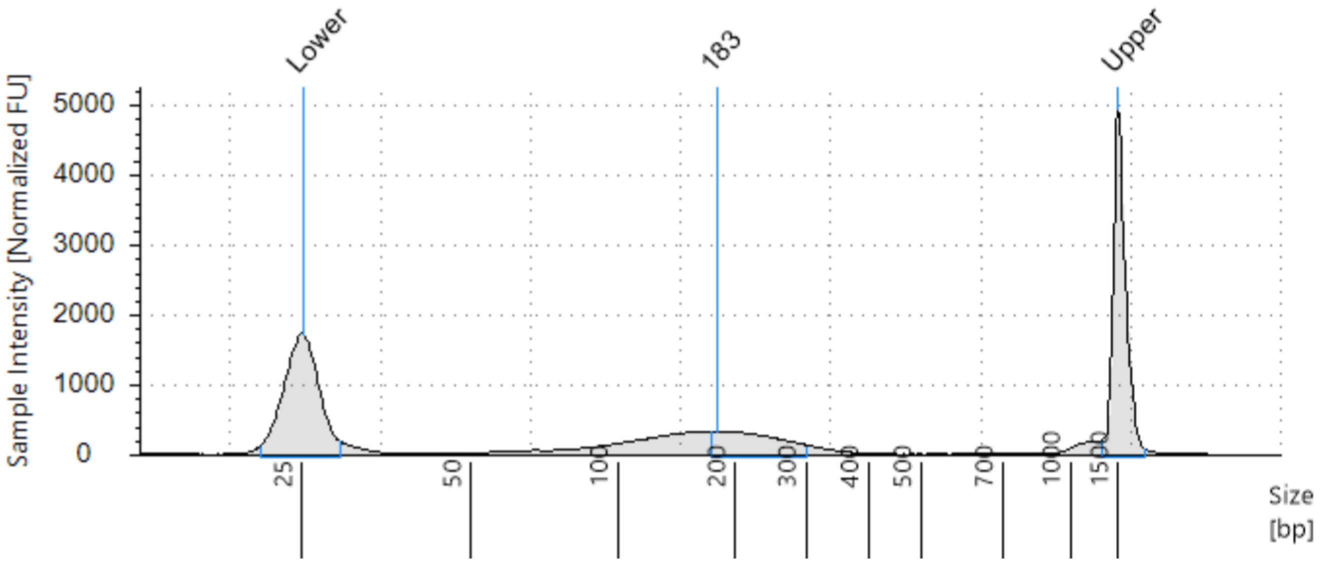

Sample Table

Well	Conc. [ng/µl]	Sample Description	Alert	Observations
G2	2.26	G10 P R3		

Peak Table

Size [bp]	Calibrated Conc. [ng/µl]	Assigned Conc. [ng/µl]	Peak Molarity [nmol/l]	% Integrated Area	Peak Comment	Observations
25	6.39	-	393	-		Lower Marker
183	2.26	-	19.0	100.00		
1500	6.50	6.50	6.67	-		Upper Marker

© Agilent Technologies, Inc. 2017

H2: H10 P R3

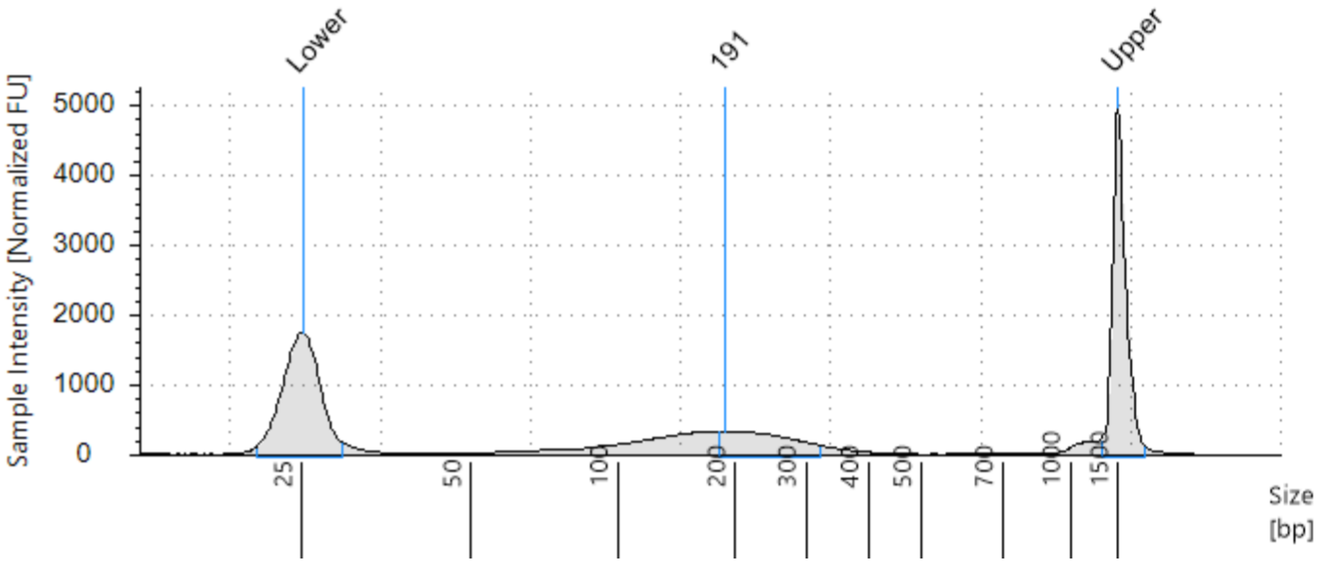

Sample Table

Well	Conc. [ng/µl]	Sample Description	Alert	Observations
H2	2.21	H10 P R3		

Peak Table

Size [bp]	Calibrated Conc. [ng/µl]	Assigned Conc. [ng/µl]	Peak Molarity [nmol/l]	% Integrated Area	Peak Comment	Observations
25	6.40	-	394	-		Lower Marker
191	2.21	-	17.8	100.00		
1500	6.50	6.50	6.67	-		Upper Marker

© Agilent Technologies, Inc. 2017

Filename: 2020-10-27-01 Q-S plus A11-H12 Exp H11 R3.D1000

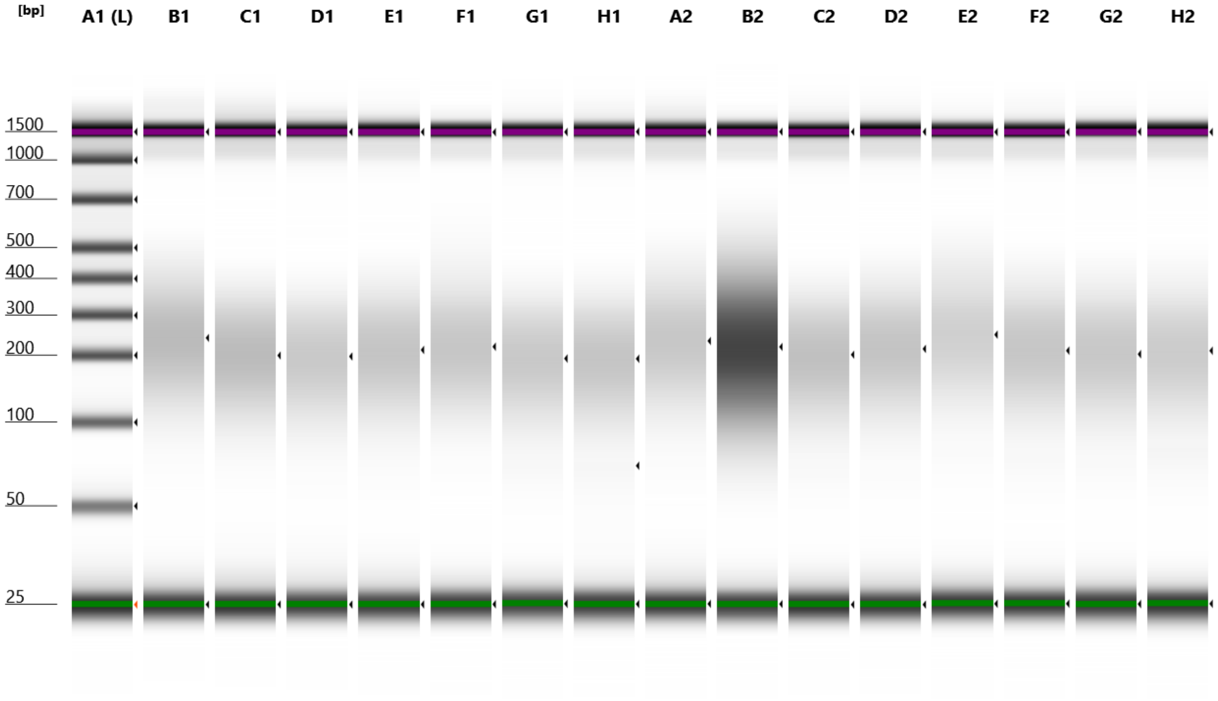

Default image (Contrast 100%)

Sample Info

Well	Conc. [ng/µl]	Sample Description	Alert	Observations
A1	16.1	Ladder		Ladder
B1	3.03	A11 P R3		
C1	4.94	B11 P R3		
D1	2.23	C11 P R3		
E1	4.42	D11 P R3		
F1	2.35	E11 P R3		
G1	4.36	F11 P R3		
H1	4.68	G11 P R3		
A2	2.28	A12 P R3		
B2	21.1	B12 P R3		
C2	2.44	C12 P R3		
D2	2.41	D12 P R3		
E2	3.35	E12 P R3		
F2	2.25	F12 P R3		
G2	4.13	G12 P R3		
H2	0.524	H12 P R3		

 © Agilent Technologies, Inc. 2017

A1: Ladder

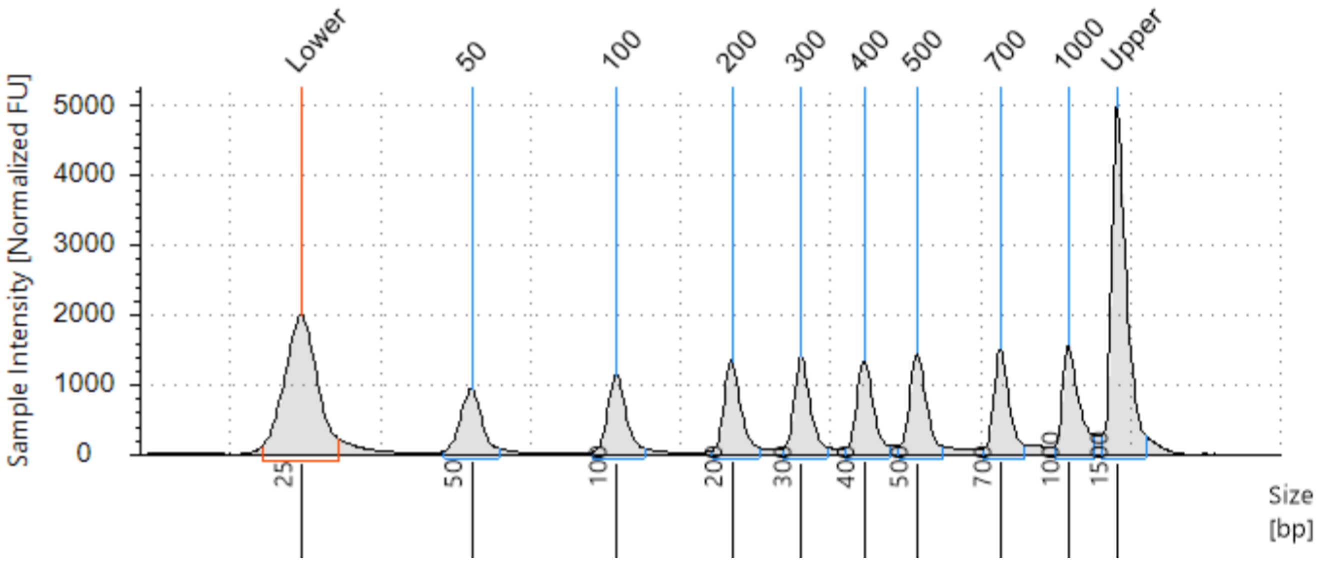

Sample Table

Well	Conc. [ng/µl]	Sample Description	Alert	Observations
A1	16.1	Ladder		Ladder

Peak Table

Size [bp]	Calibrated Conc. [ng/µl]	Assigned Conc. [ng/µl]	Peak Molarity [nmol/l]	% Integrated Area	Peak Comment	Observations
25	5.65	-	348	-		Lower Marker
50	1.76	-	54.0	10.94		
100	1.85	-	28.5	11.55		
200	2.00	-	15.3	12.43		
300	2.00	-	10.3	12.46		
400	2.01	-	7.72	12.51		
500	2.14	-	6.57	13.31		
700	2.05	-	4.51	12.77		
1000	2.25	-	3.46	14.03		
1500	6.50	6.50	6.67	-		Upper Marker

© Agilent Technologies, Inc. 2017

B1: A11 P R3

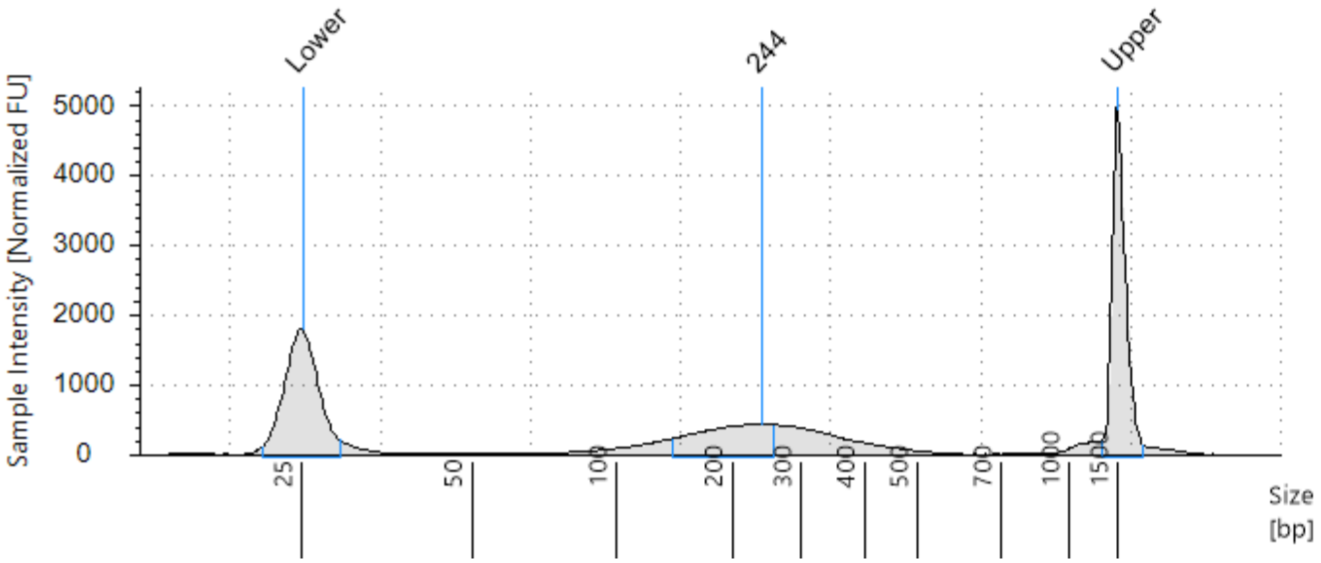

Sample Table

Well	Conc. [ng/µl]	Sample Description	Alert	Observations
B1	3.03	A11 P R3		

Peak Table

Size [bp]	Calibrated Conc. [ng/µl]	Assigned Conc. [ng/µl]	Peak Molarity [nmol/l]	% Integrated Area	Peak Comment	Observations
25	5.89	-	362	-		Lower Marker
244	3.03	-	19.1	100.00		
1500	6.50	6.50	6.67	-		Upper Marker

© Agilent Technologies, Inc. 2017

C1: B11 P R3

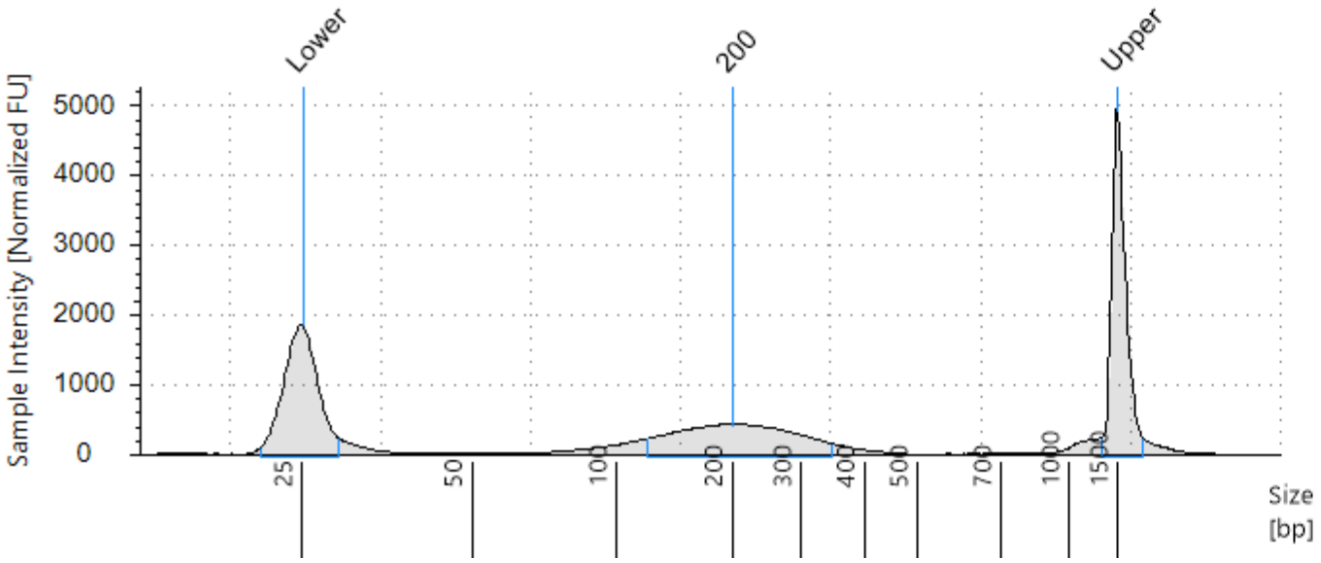

Sample Table

Well	Conc. [ng/µl]	Sample Description	Alert	Observations
C1	4.94	B11 P R3		

Peak Table

Size [bp]	Calibrated Conc. [ng/µl]	Assigned Conc. [ng/µl]	Peak Molarity [nmol/l]	% Integrated Area	Peak Comment	Observations
25	5.97	-	368	-		Lower Marker
200	4.94	-	38.0	100.00		
1500	6.50	6.50	6.67	-		Upper Marker

© Agilent Technologies, Inc. 2017

D1: C11 P R3

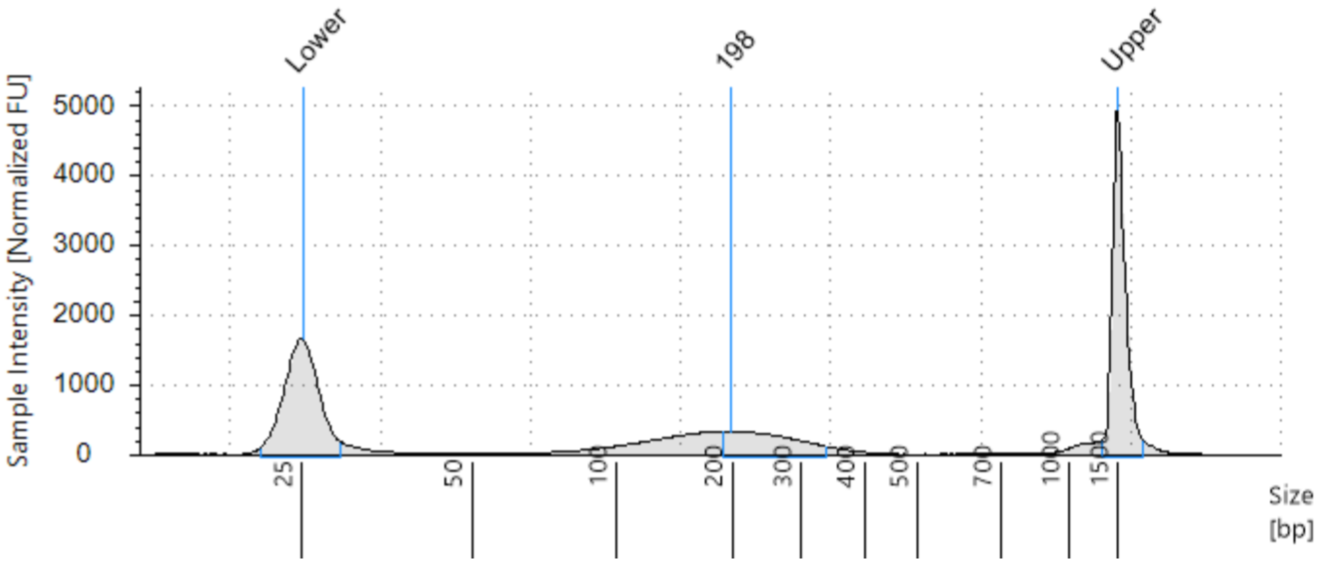

Sample Table

Well	Conc. [ng/µl]	Sample Description	Alert	Observations
D1	2.23	C11 P R3		

Peak Table

Size [bp]	Calibrated Conc. [ng/µl]	Assigned Conc. [ng/µl]	Peak Molarity [nmol/l]	% Integrated Area	Peak Comment	Observations
25	5.72	-	352	-		Lower Marker
198	2.23	-	17.3	100.00		
1500	6.50	6.50	6.67	-		Upper Marker

© Agilent Technologies, Inc. 2017

E1: D11 P R3

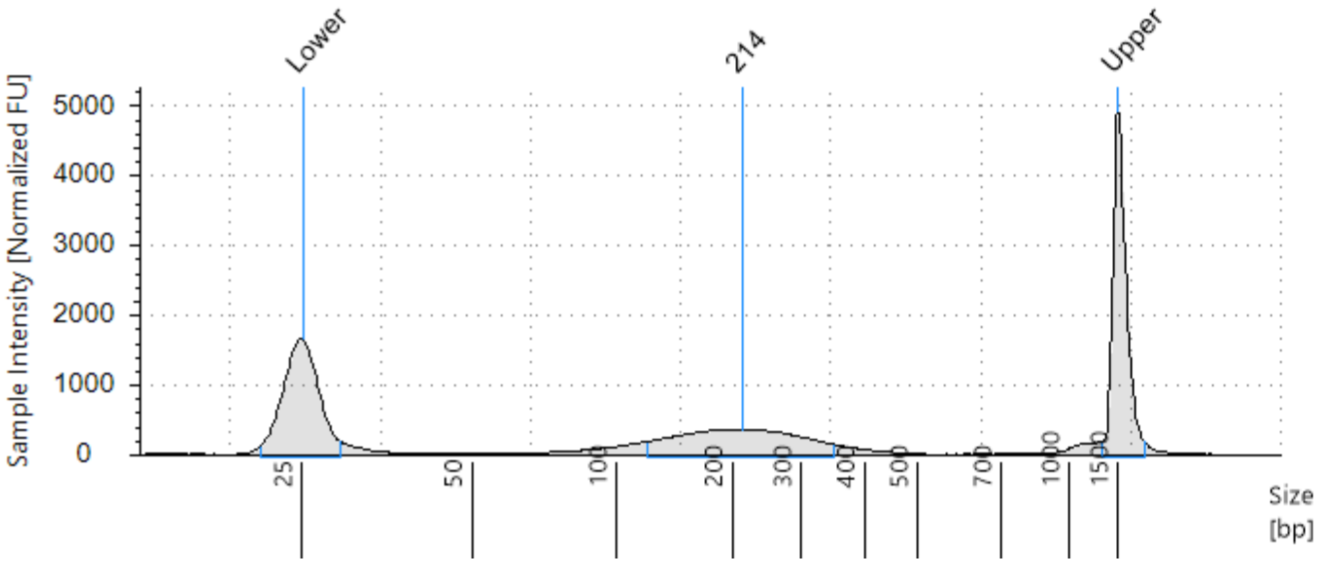

Sample Table

Well	Conc. [ng/µl]	Sample Description	Alert	Observations
E1	4.42	D11 P R3		

Peak Table

Size [bp]	Calibrated Conc. [ng/µl]	Assigned Conc. [ng/µl]	Peak Molarity [nmol/l]	% Integrated Area	Peak Comment	Observations
25	5.76	-	354	-		Lower Marker
214	4.42	-	31.9	100.00		
1500	6.50	6.50	6.67	-		Upper Marker

© Agilent Technologies, Inc. 2017

F1: E11 P R3

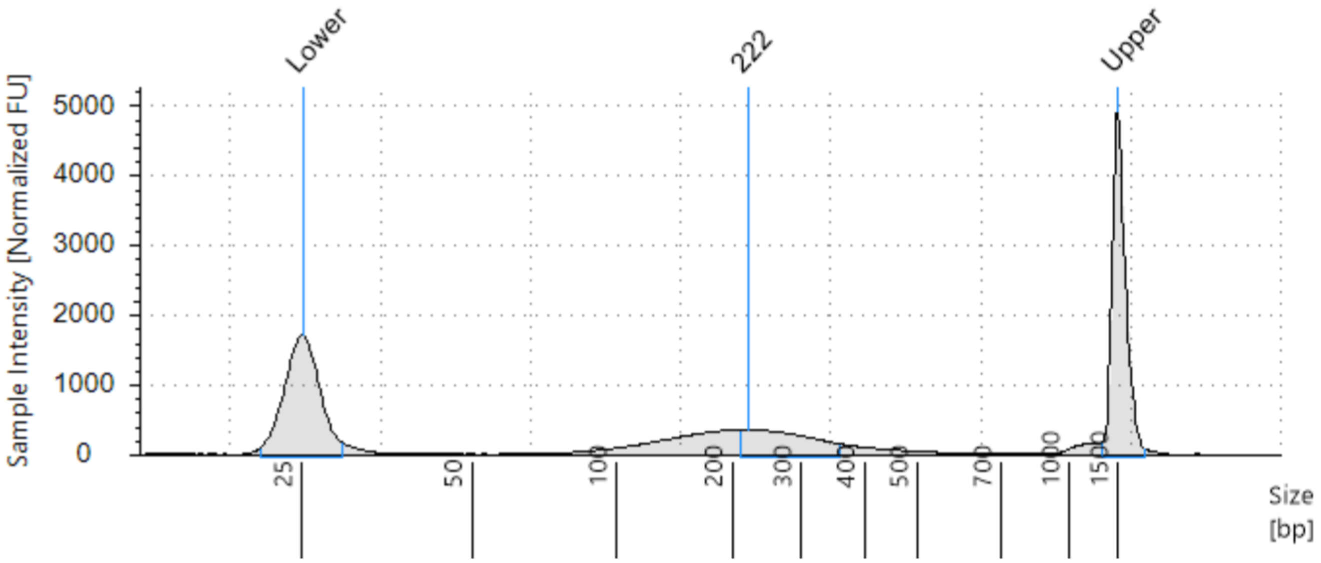

Sample Table

Well	Conc. [ng/µl]	Sample Description	Alert	Observations
F1	2.35	E11 P R3		

Peak Table

Size [bp]	Calibrated Conc. [ng/µl]	Assigned Conc. [ng/µl]	Peak Molarity [nmol/l]	% Integrated Area	Peak Comment	Observations
25	5.89	-	362	-		Lower Marker
222	2.35	-	16.3	100.00		
1500	6.50	6.50	6.67	-		Upper Marker

© Agilent Technologies, Inc. 2017

G1: F11 P R3

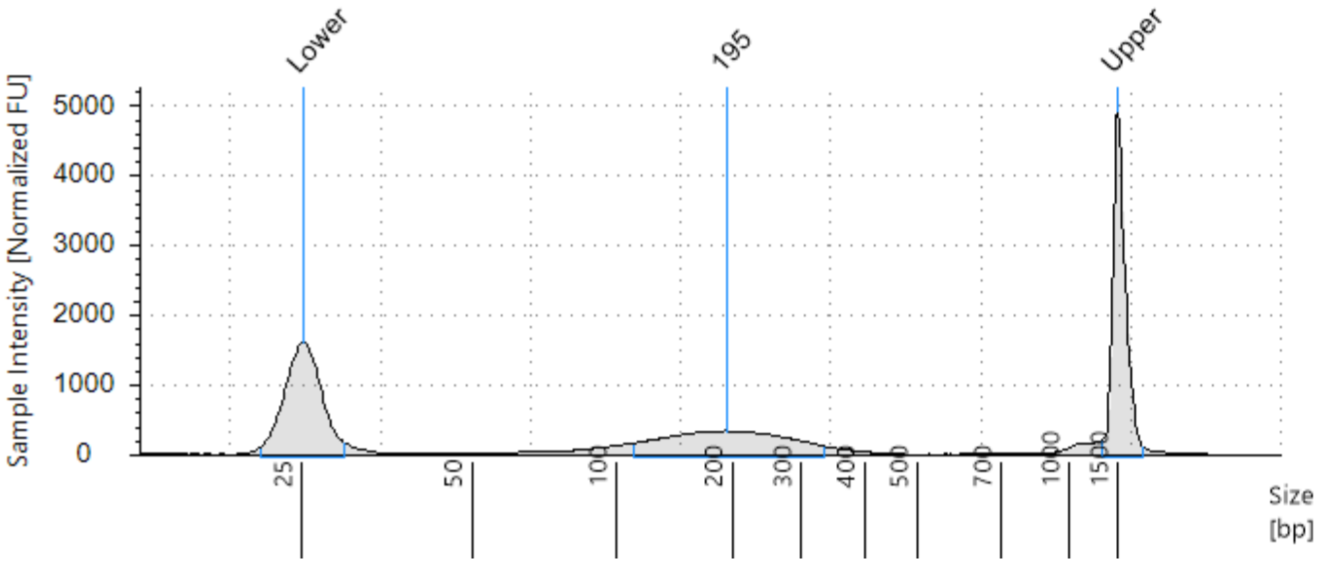

Sample Table

Well	Conc. [ng/µl]	Sample Description	Alert	Observations
G1	4.36	F11 P R3		

Peak Table

Size [bp]	Calibrated Conc. [ng/µl]	Assigned Conc. [ng/µl]	Peak Molarity [nmol/l]	% Integrated Area	Peak Comment	Observations
25	6.15	-	379	-		Lower Marker
195	4.36	-	34.5	100.00		
1500	6.50	6.50	6.67	-		Upper Marker

© Agilent Technologies, Inc. 2017

H1: Gl1 P R3

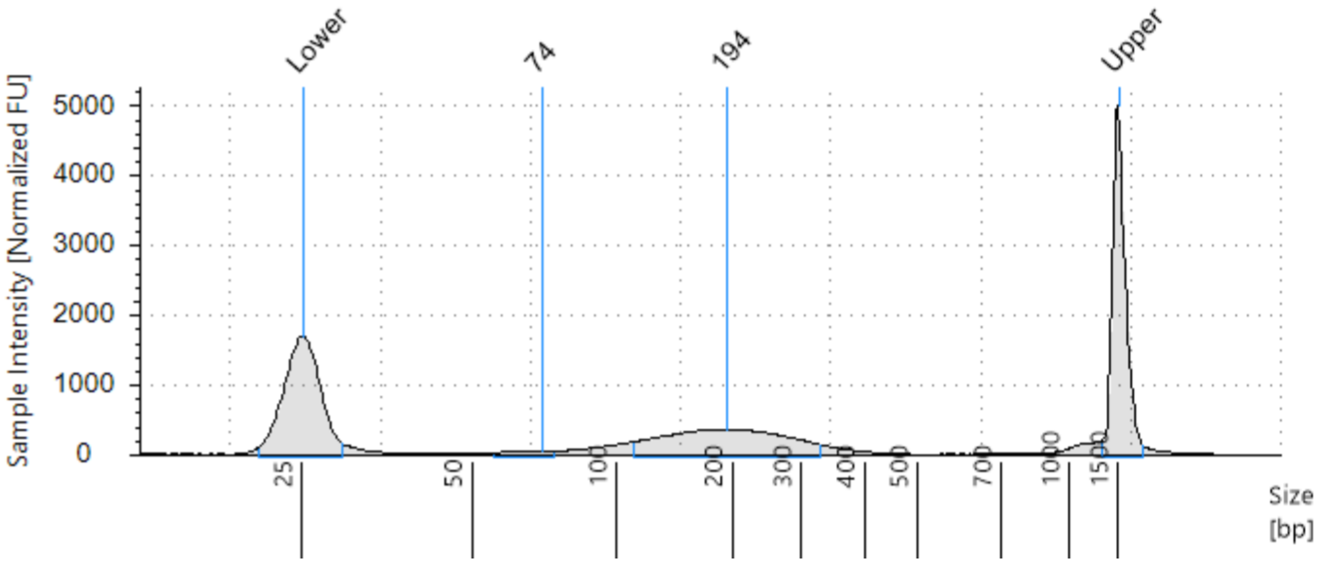

Sample Table

Well	Conc. [ng/µl]	Sample Description	Alert	Observations
H1	4.68	Gl1 P R3		

Peak Table

Size [bp]	Calibrated Conc. [ng/µl]	Assigned Conc. [ng/µl]	Peak Molarity [nmol/l]	% Integrated Area	Peak Comment	Observations
25	6.19	-	381	-		Lower Marker
74	0.194	-	4.05	4.15		
194	4.48	-	35.5	95.85		
1500	6.50	6.50	6.67	-		Upper Marker

 © Agilent Technologies, Inc. 2017

A2: A12 P R3

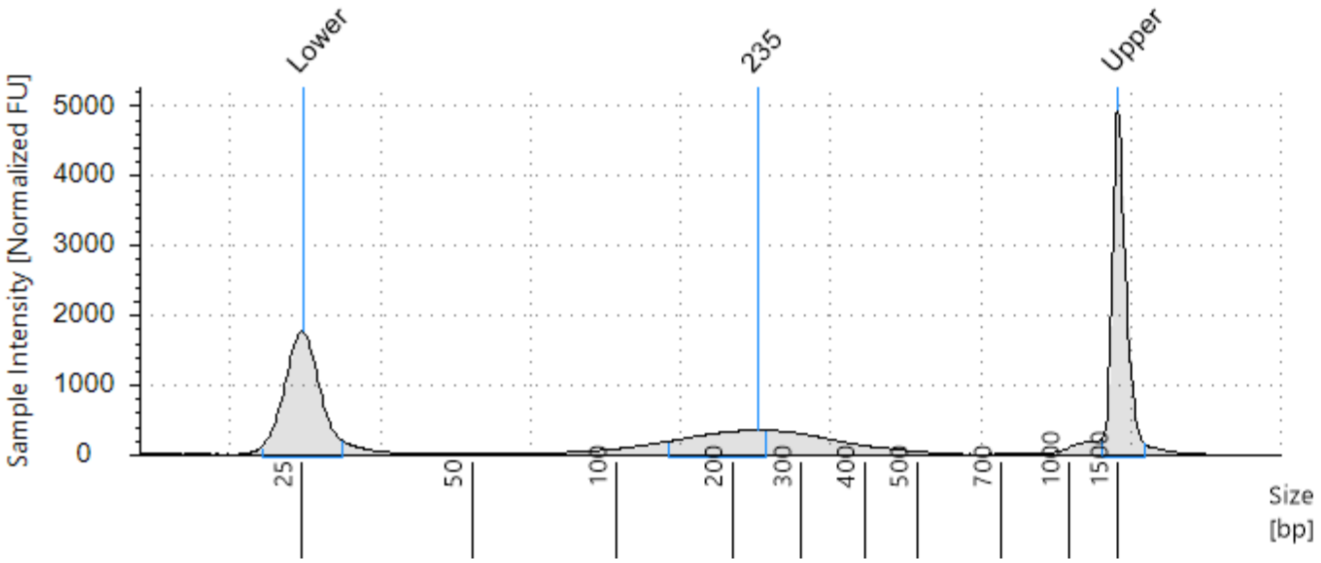

Sample Table

Well	Conc. [ng/µl]	Sample Description	Alert	Observations
A2	2.28	A12 P R3		

Peak Table

Size [bp]	Calibrated Conc. [ng/µl]	Assigned Conc. [ng/µl]	Peak Molarity [nmol/l]	% Integrated Area	Peak Comment	Observations
25	5.87	-	361	-		Lower Marker
235	2.28	-	14.9	100.00		
1500	6.50	6.50	6.67	-		Upper Marker

 © Agilent Technologies, Inc. 2017

B2: B12 P R3

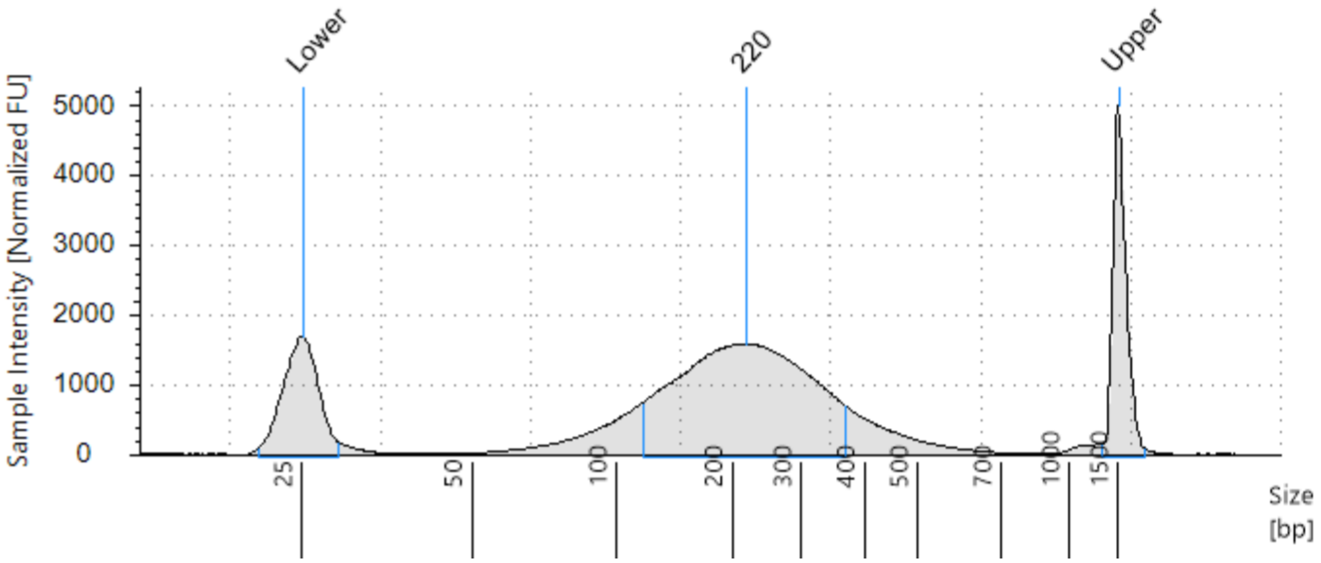

Sample Table

Well	Conc. [ng/µl]	Sample Description	Alert	Observations
B2	21.1	B12 P R3		

Peak Table

Size [bp]	Calibrated Conc. [ng/µl]	Assigned Conc. [ng/µl]	Peak Molarity [nmol/l]	% Integrated Area	Peak Comment	Observations
25	5.89	-	362	-		Lower Marker
220	21.1	-	147	100.00		
1500	6.50	6.50	6.67	-		Upper Marker

© Agilent Technologies, Inc. 2017

C2: C12 P R3

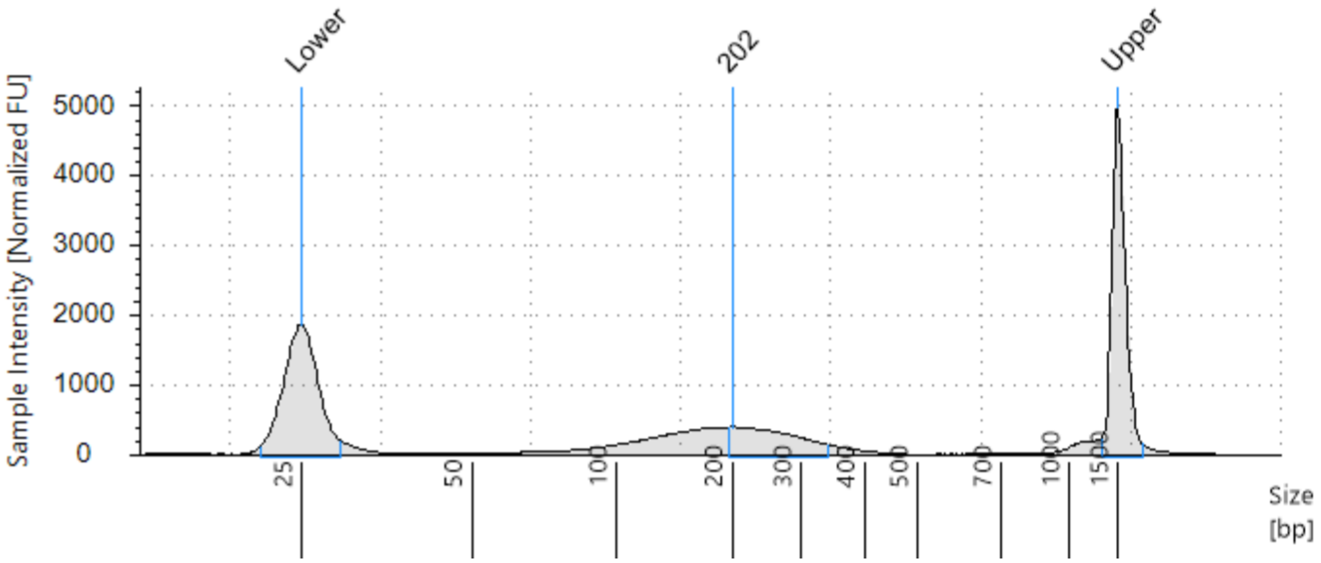

Sample Table

Well	Conc. [ng/µl]	Sample Description	Alert	Observations
C2	2.44	C12 P R3		

Peak Table

Size [bp]	Calibrated Conc. [ng/µl]	Assigned Conc. [ng/µl]	Peak Molarity [nmol/l]	% Integrated Area	Peak Comment	Observations
25	6.15	-	379	-		Lower Marker
202	2.44	-	18.6	100.00		
1500	6.50	6.50	6.67	-		Upper Marker

© Agilent Technologies, Inc. 2017

D2: D12 P R3

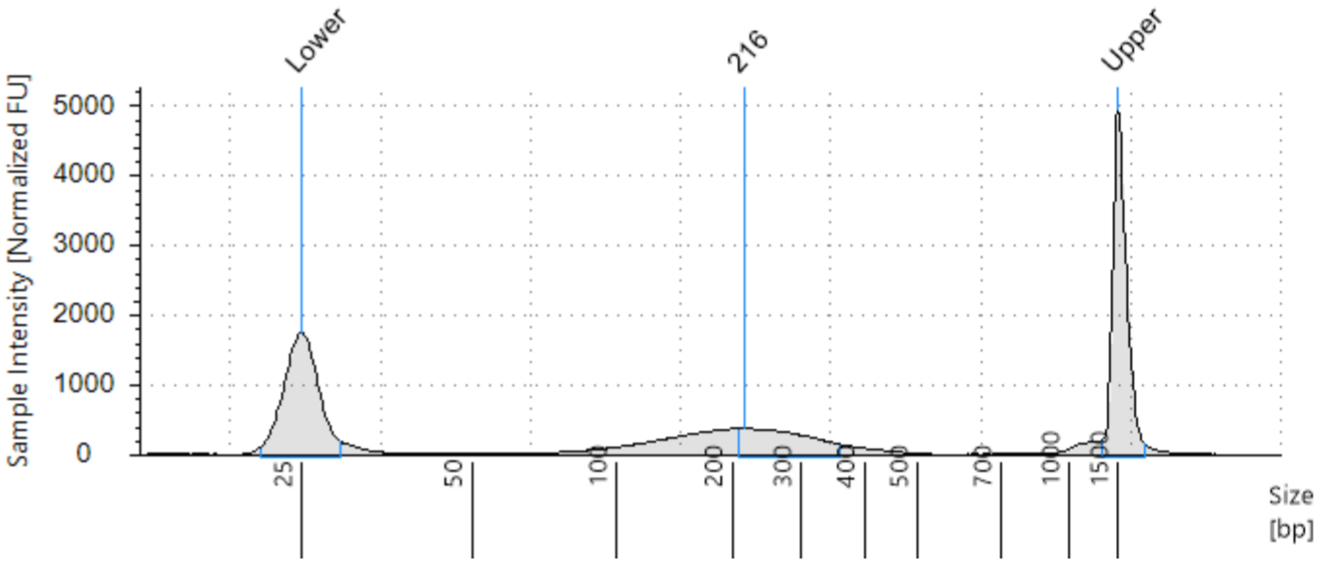

Sample Table

Well	Conc. [ng/µl]	Sample Description	Alert	Observations
D2	2.41	D12 P R3		

Peak Table

Size [bp]	Calibrated Conc. [ng/µl]	Assigned Conc. [ng/µl]	Peak Molarity [nmol/l]	% Integrated Area	Peak Comment	Observations
25	5.75	-	354	-		Lower Marker
216	2.41	-	17.1	100.00		
1500	6.50	6.50	6.67	-		Upper Marker

© Agilent Technologies, Inc. 2017

E2: E12 P R3

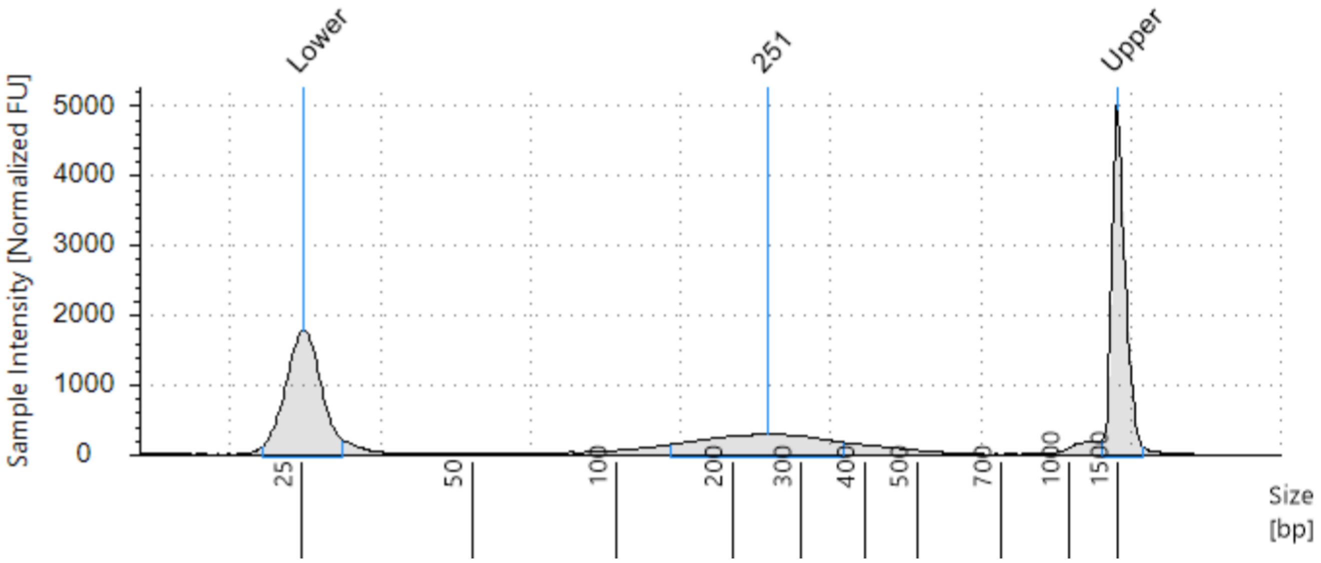

Sample Table

Well	Conc. [ng/µl]	Sample Description	Alert	Observations
E2	3.35	E12 P R3		

Peak Table

Size [bp]	Calibrated Conc. [ng/µl]	Assigned Conc. [ng/µl]	Peak Molarity [nmol/l]	% Integrated Area	Peak Comment	Observations
25	6.07	-	374	-		Lower Marker
251	3.35	-	20.5	100.00		
1500	6.50	6.50	6.67	-		Upper Marker

 © Agilent Technologies, Inc. 2017

F2: F12 P R3

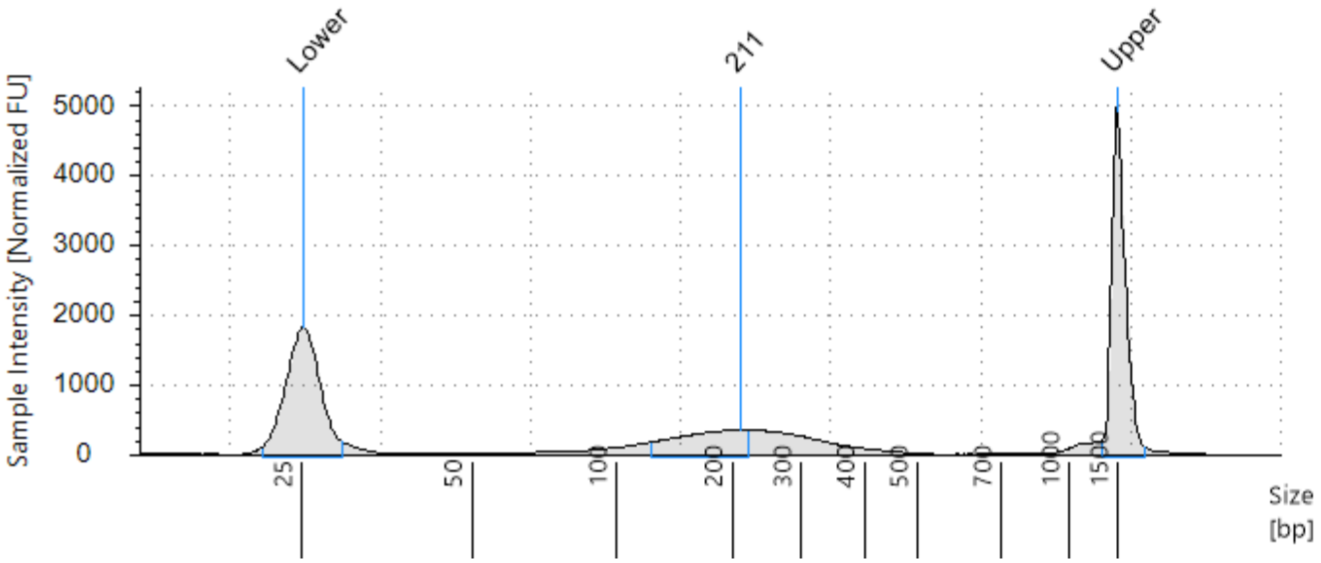

Sample Table

Well	Conc. [ng/µl]	Sample Description	Alert	Observations
F2	2.25	F12 P R3		

Peak Table

Size [bp]	Calibrated Conc. [ng/µl]	Assigned Conc. [ng/µl]	Peak Molarity [nmol/l]	% Integrated Area	Peak Comment	Observations
25	5.94	-	365	-		Lower Marker
211	2.25	-	16.4	100.00		
1500	6.50	6.50	6.67	-		Upper Marker

© Agilent Technologies, Inc. 2017

G2: G12 P R3

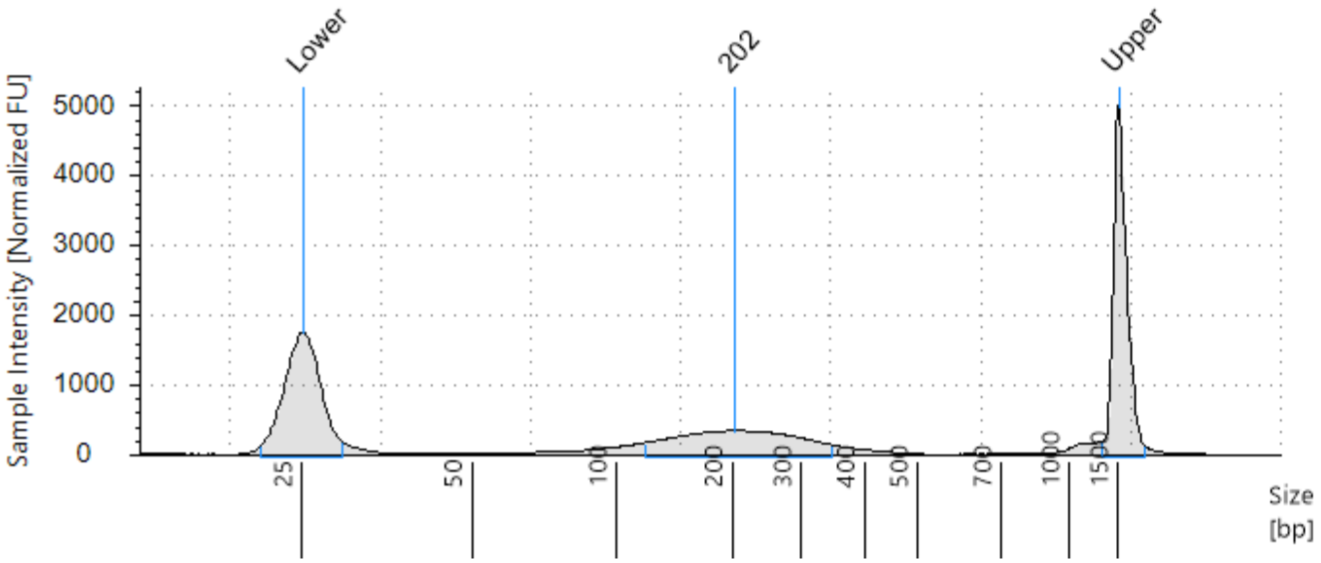

Sample Table

Well	Conc. [ng/µl]	Sample Description	Alert	Observations
G2	4.13	G12 P R3		

Peak Table

Size [bp]	Calibrated Conc. [ng/µl]	Assigned Conc. [ng/µl]	Peak Molarity [nmol/l]	% Integrated Area	Peak Comment	Observations
25	6.12	-	376	-		Lower Marker
202	4.13	-	31.4	100.00		
1500	6.50	6.50	6.67	-		Upper Marker

 © Agilent Technologies, Inc. 2017

H2: H12 P R3

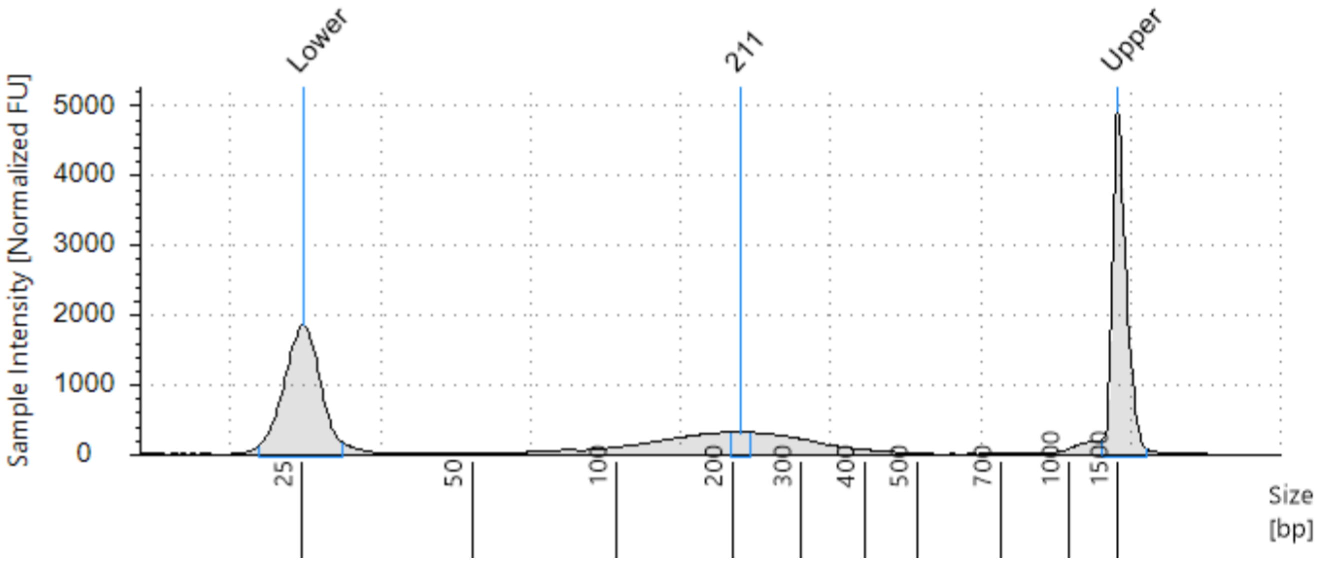

Sample Table

Well	Conc. [ng/µl]	Sample Description	Alert	Observations
H2	0.524	H12 P R3		

Peak Table

Size [bp]	Calibrated Conc. [ng/µl]	Assigned Conc. [ng/µl]	Peak Molarity [nmol/l]	% Integrated Area	Peak Comment	Observations
25	6.28	-	387	-		Lower Marker
211	0.524	-	3.83	100.00		
1500	6.50	6.50	6.67	-		Upper Marker

© Agilent Technologies, Inc. 2017

Filename: 2020-10-27-02 H1,3,5,5,9,11, F9 plus H1,2,3,5,9,11, H4 M R3.D1000

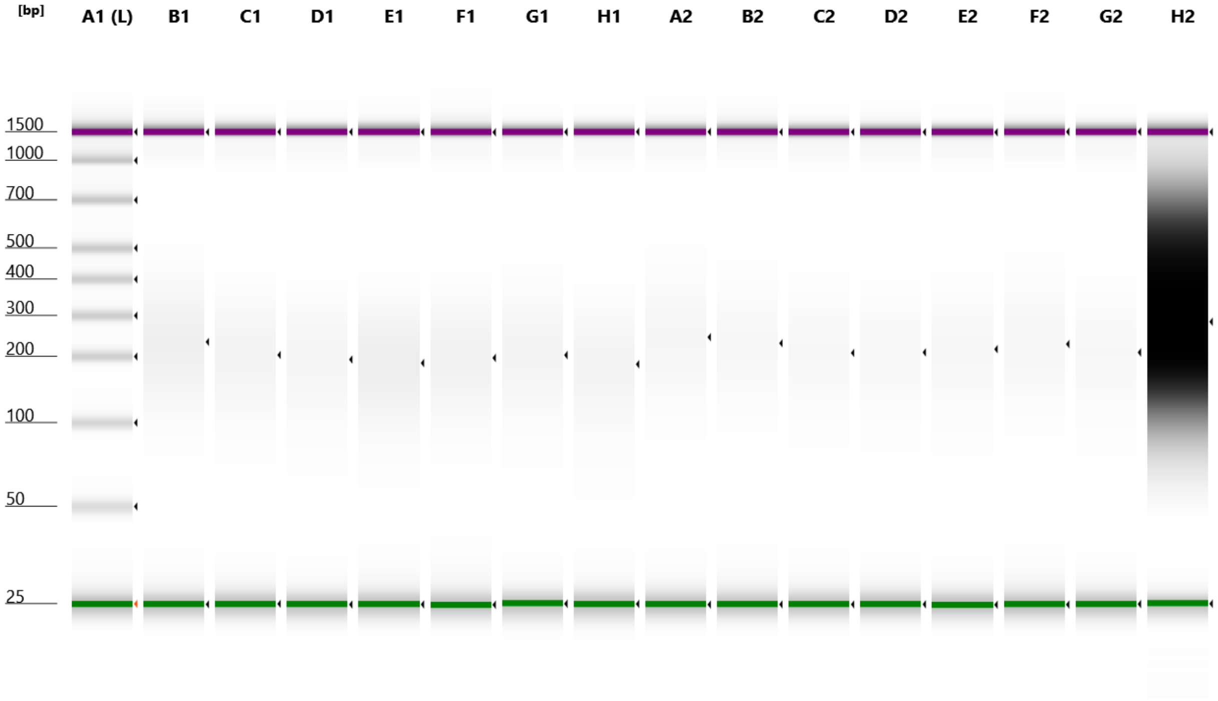

Default image (Contrast 100%)

Sample Info

Well	Conc. [ng/µl]	Sample Description	Alert	Observations
A1	15.4	Ladder		Ladder
B1	2.50	H1 P R3		
C1	3.32	H3 P R3		
D1	1.70	H5 P R3		
E1	5.13	H7 P R3		
F1	2.12	H9 P R3		
G1	3.50	H11 P R3		
H1	3.61	F9 P R3		
A2	0.304	H1 M R3		
B2	1.10	H3 M R3		
C2	2.11	H5 M R3		
D2	0.176	H7 M R3		
E2	1.28	H9 M R3		
F2	1.43	H11 M R3		
G2	1.18	H4 M R3		
H2	246	700 ng Plus undiluted		

© Agilent Technologies, Inc. 2017

A1: Ladder

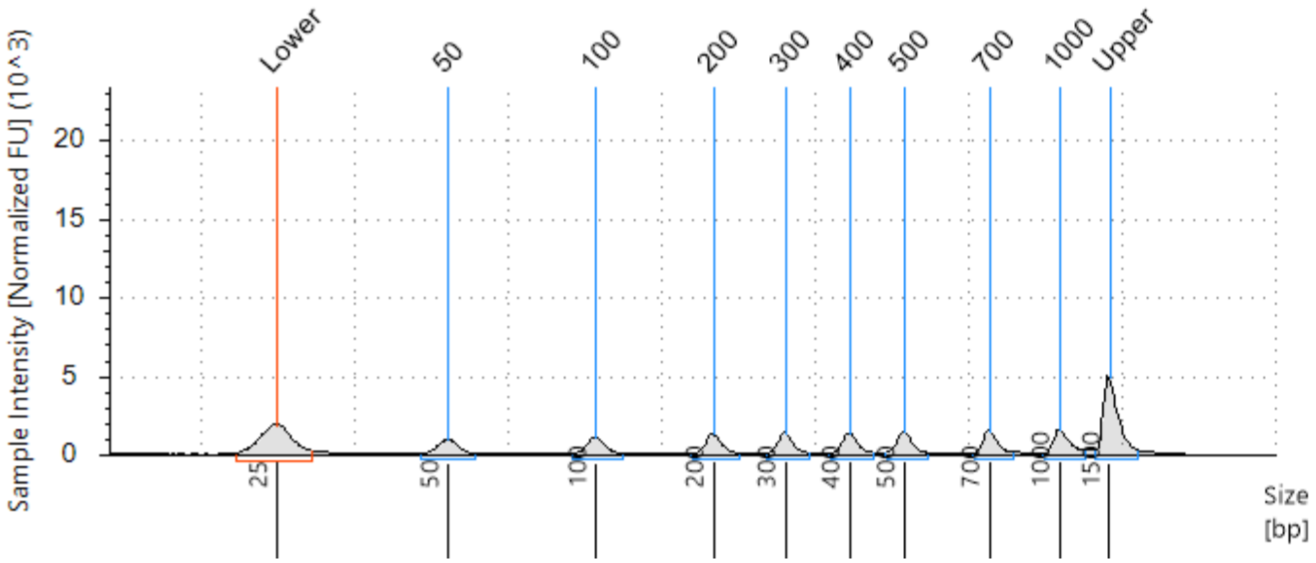

Sample Table

Well	Conc. [ng/µl]	Sample Description	Alert	Observations
A1	15.4	Ladder		Ladder

Peak Table

Size [bp]	Calibrated Conc. [ng/µl]	Assigned Conc. [ng/µl]	Peak Molarity [nmol/l]	% Integrated Area	Peak Comment	Observations
25	5.33	-	328	-		Lower Marker
50	1.67	-	51.5	10.88		
100	1.74	-	26.8	11.31		
200	1.87	-	14.4	12.18		
300	1.89	-	9.67	12.26		
400	1.92	-	7.38	12.47		
500	2.05	-	6.30	13.32		
700	1.98	-	4.36	12.90		
1000	2.26	-	3.47	14.68		
1500	6.50	6.50	6.67	-		Upper Marker

© Agilent Technologies, Inc. 2017

B1: H1 P R3

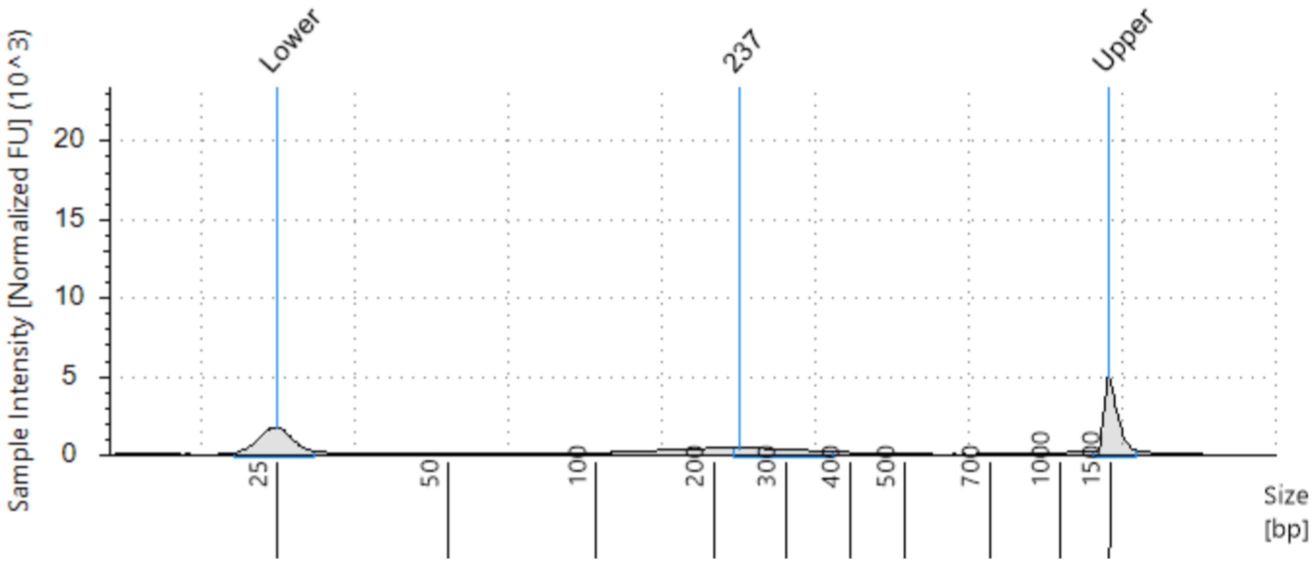

Sample Table

Well	Conc. [ng/µl]	Sample Description	Alert	Observations
B1	2.50	H1 P R3		

Peak Table

Size [bp]	Calibrated Conc. [ng/µl]	Assigned Conc. [ng/µl]	Peak Molarity [nmol/l]	% Integrated Area	Peak Comment	Observations
25	5.65	-	348	-		Lower Marker
237	2.50	-	16.3	100.00		
1500	6.50	6.50	6.67	-		Upper Marker

© Agilent Technologies, Inc. 2017

C1: H3 P R3

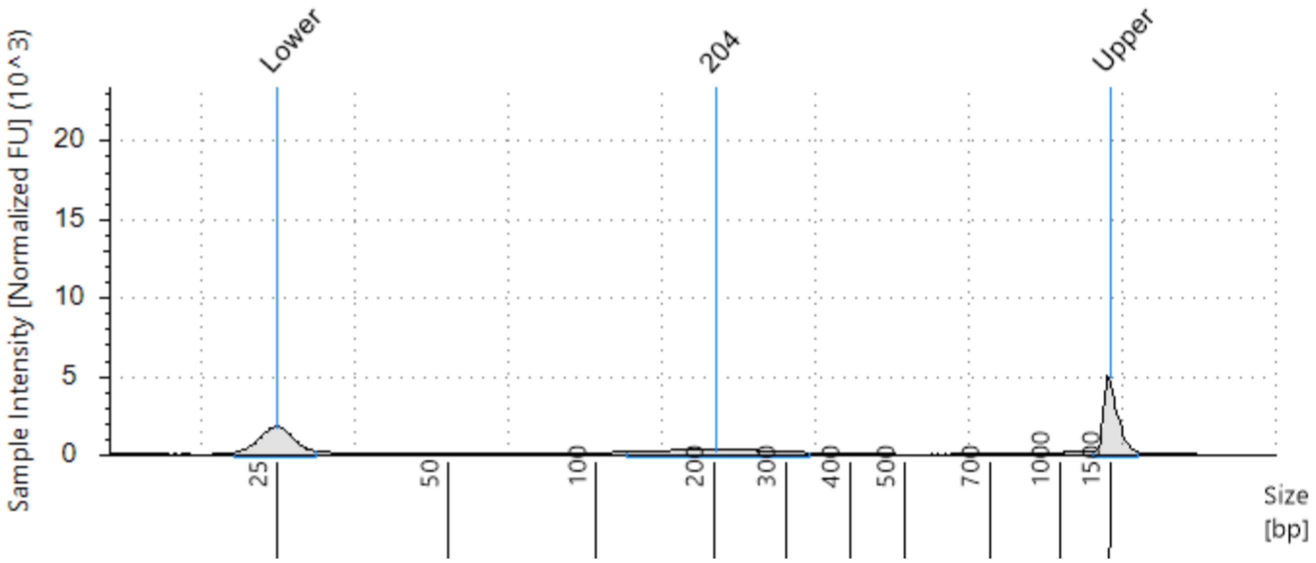

Sample Table

Well	Conc. [ng/µl]	Sample Description	Alert	Observations
C1	3.32	H3 P R3		

Peak Table

Size [bp]	Calibrated Conc. [ng/µl]	Assigned Conc. [ng/µl]	Peak Molarity [nmol/l]	% Integrated Area	Peak Comment	Observations
25	5.69	-	350	-		Lower Marker
204	3.32	-	25.0	100.00		
1500	6.50	6.50	6.67	-		Upper Marker

© Agilent Technologies, Inc. 2017

D1: H5 P R3

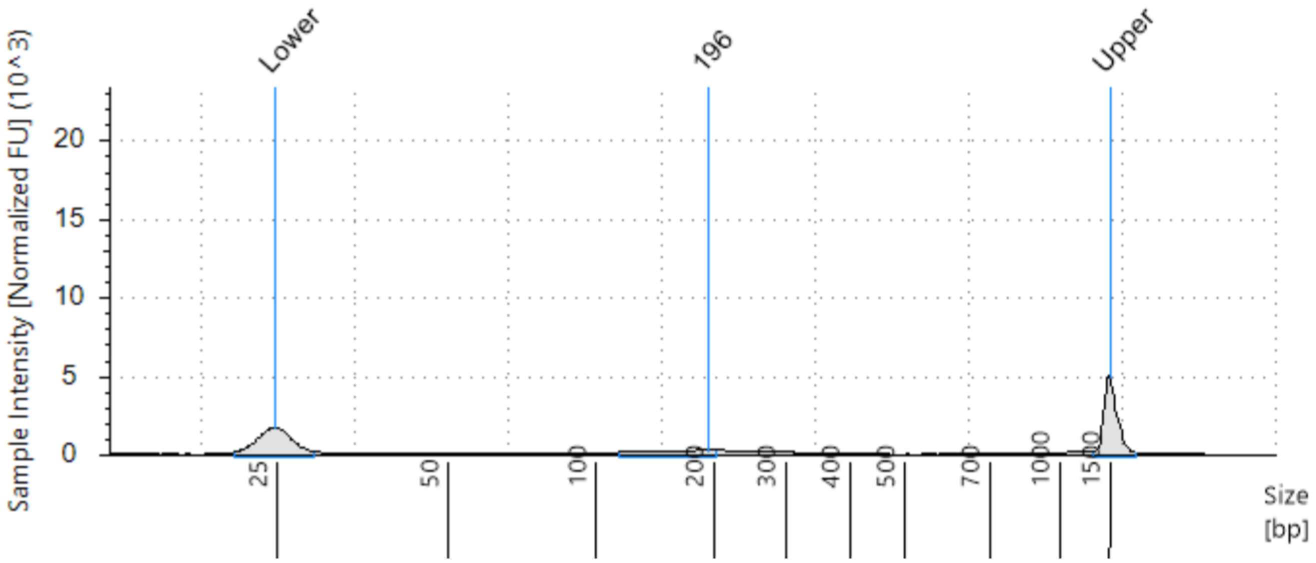

Sample Table

Well	Conc. [ng/µl]	Sample Description	Alert	Observations
D1	1.70	H5 P R3		

Peak Table

Size [bp]	Calibrated Conc. [ng/µl]	Assigned Conc. [ng/µl]	Peak Molarity [nmol/l]	% Integrated Area	Peak Comment	Observations
25	5.91	-	363	-		Lower Marker
196	1.70	-	13.3	100.00		
1500	6.50	6.50	6.67	-		Upper Marker

 © Agilent Technologies, Inc. 2017

E1: H7 P R3

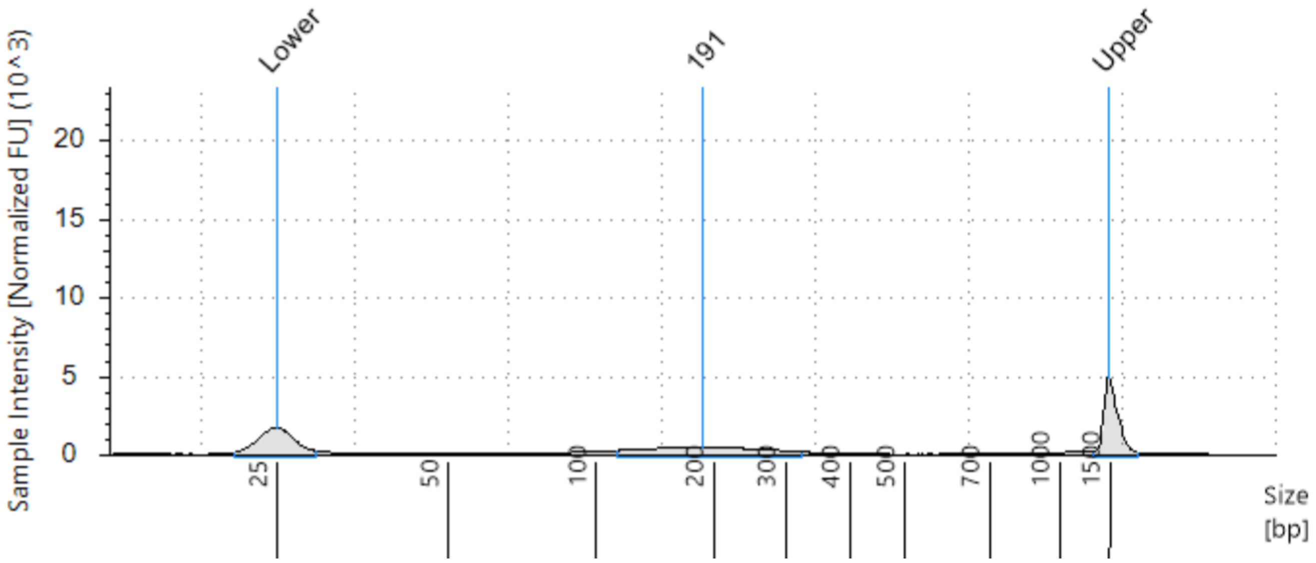

Sample Table

Well	Conc. [ng/µl]	Sample Description	Alert	Observations
E1	5.13	H7 P R3		

Peak Table

Size [bp]	Calibrated Conc. [ng/µl]	Assigned Conc. [ng/µl]	Peak Molarity [nmol/l]	% Integrated Area	Peak Comment	Observations
25	5.83	-	359	-		Lower Marker
191	5.13	-	41.4	100.00		
1500	6.50	6.50	6.67	-		Upper Marker

 © Agilent Technologies, Inc. 2017

F1: H9 P R3

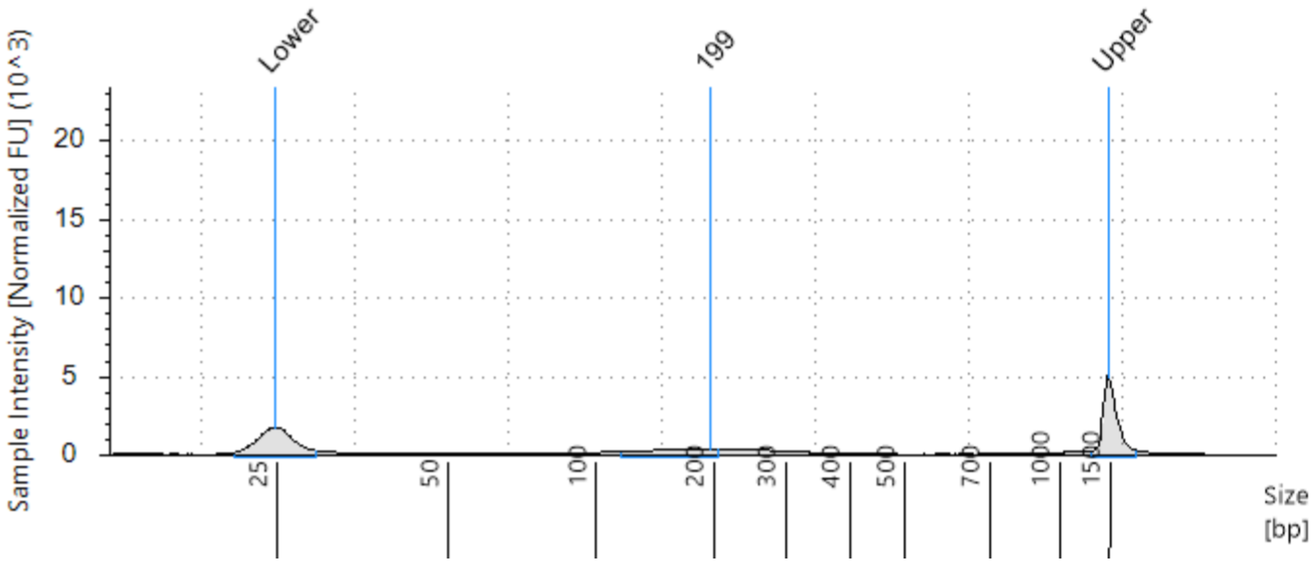

Sample Table

Well	Conc. [ng/µl]	Sample Description	Alert	Observations
F1	2.12	H9 P R3		

Peak Table

Size [bp]	Calibrated Conc. [ng/µl]	Assigned Conc. [ng/µl]	Peak Molarity [nmol/l]	% Integrated Area	Peak Comment	Observations
25	5.80	-	357	-		Lower Marker
199	2.12	-	16.4	100.00		
1500	6.50	6.50	6.67	-		Upper Marker

© Agilent Technologies, Inc. 2017

G1: H11 P R3

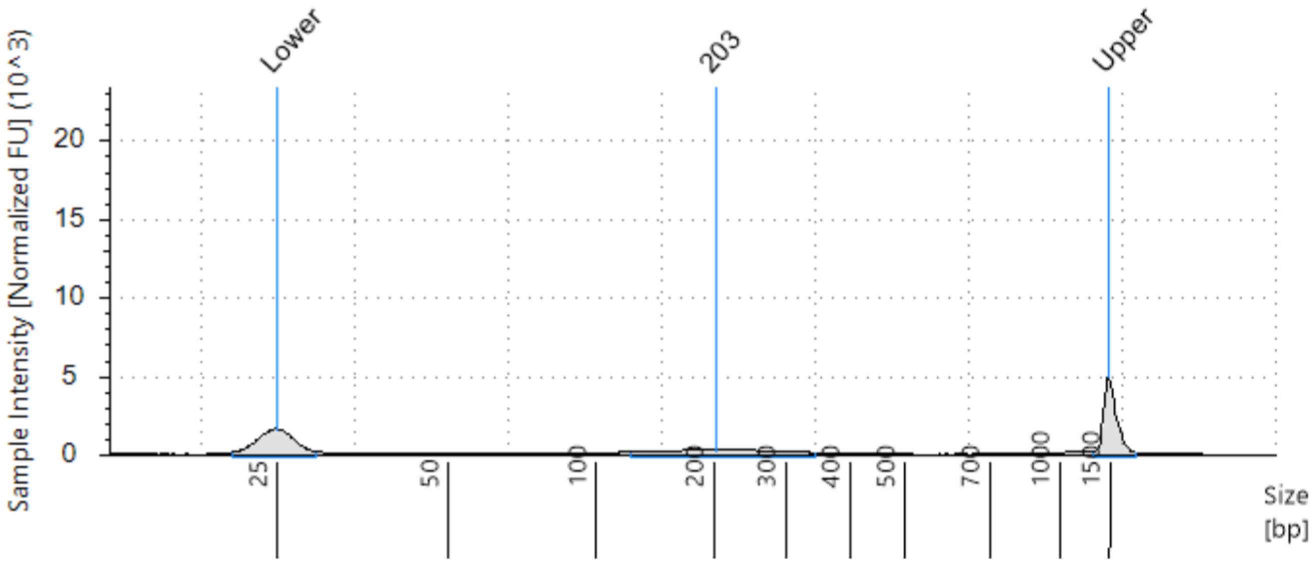

Sample Table

Well	Conc. [ng/µl]	Sample Description	Alert	Observations
G1	3.50	H11 P R3		

Peak Table

Size [bp]	Calibrated Conc. [ng/µl]	Assigned Conc. [ng/µl]	Peak Molarity [nmol/l]	% Integrated Area	Peak Comment	Observations
25	6.11	-	376	-		Lower Marker
203	3.50	-	26.5	100.00		
1500	6.50	6.50	6.67	-		Upper Marker

© Agilent Technologies, Inc. 2017

H1: F9 P R3

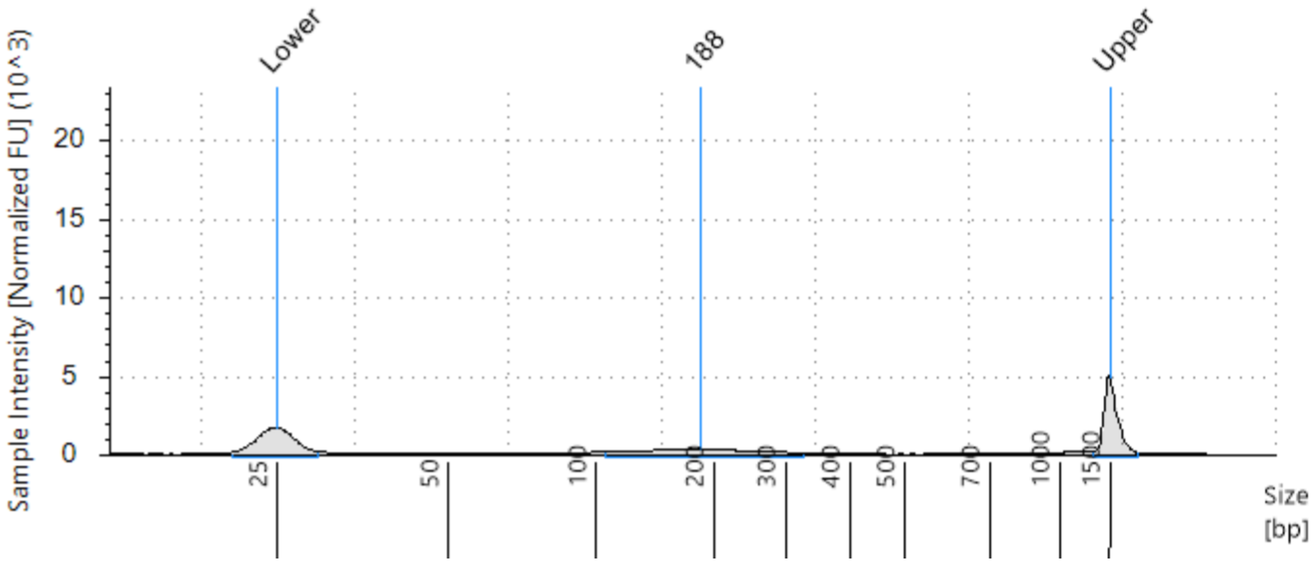

Sample Table

Well	Conc. [ng/µl]	Sample Description	Alert	Observations
H1	3.61	F9 P R3		

Peak Table

Size [bp]	Calibrated Conc. [ng/µl]	Assigned Conc. [ng/µl]	Peak Molarity [nmol/l]	% Integrated Area	Peak Comment	Observations
25	6.34	-	390	-		Lower Marker
188	3.61	-	29.5	100.00		
1500	6.50	6.50	6.67	-		Upper Marker

 © Agilent Technologies, Inc. 2017

A2: H1 M R3

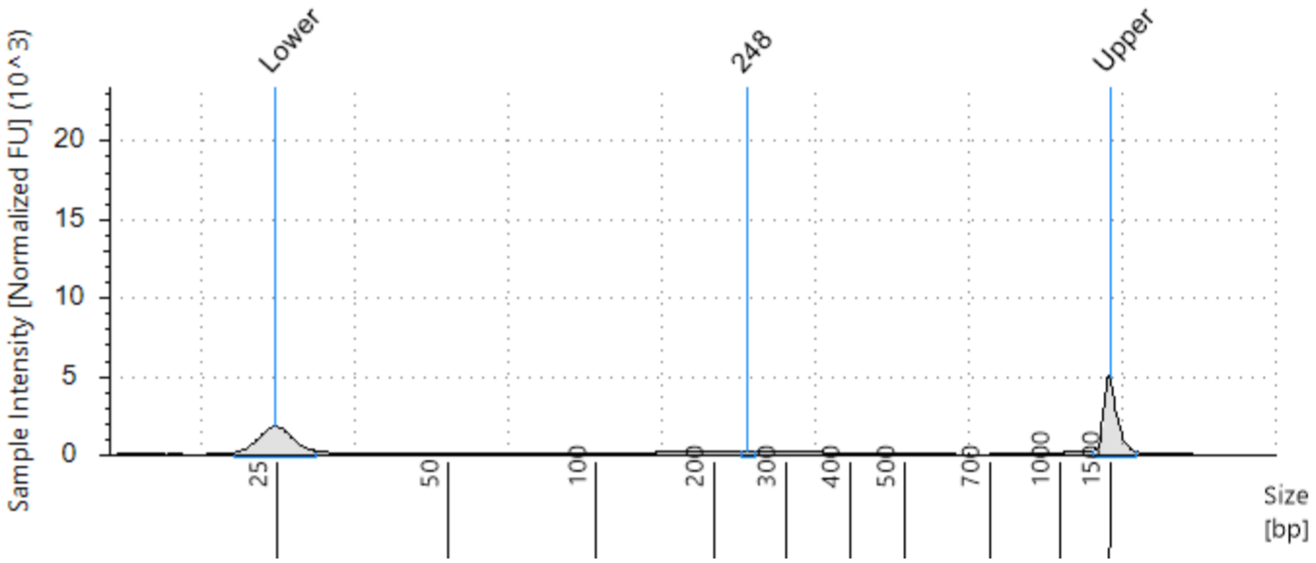

Sample Table

Well	Conc. [ng/µl]	Sample Description	Alert	Observations
A2	0.304	H1 M R3		

Peak Table

Size [bp]	Calibrated Conc. [ng/µl]	Assigned Conc. [ng/µl]	Peak Molarity [nmol/l]	% Integrated Area	Peak Comment	Observations
25	6.03	-	371	-		Lower Marker
248	0.304	-	1.89	100.00		
1500	6.50	6.50	6.67	-		Upper Marker

 © Agilent Technologies, Inc. 2017

B2: H3 M R3

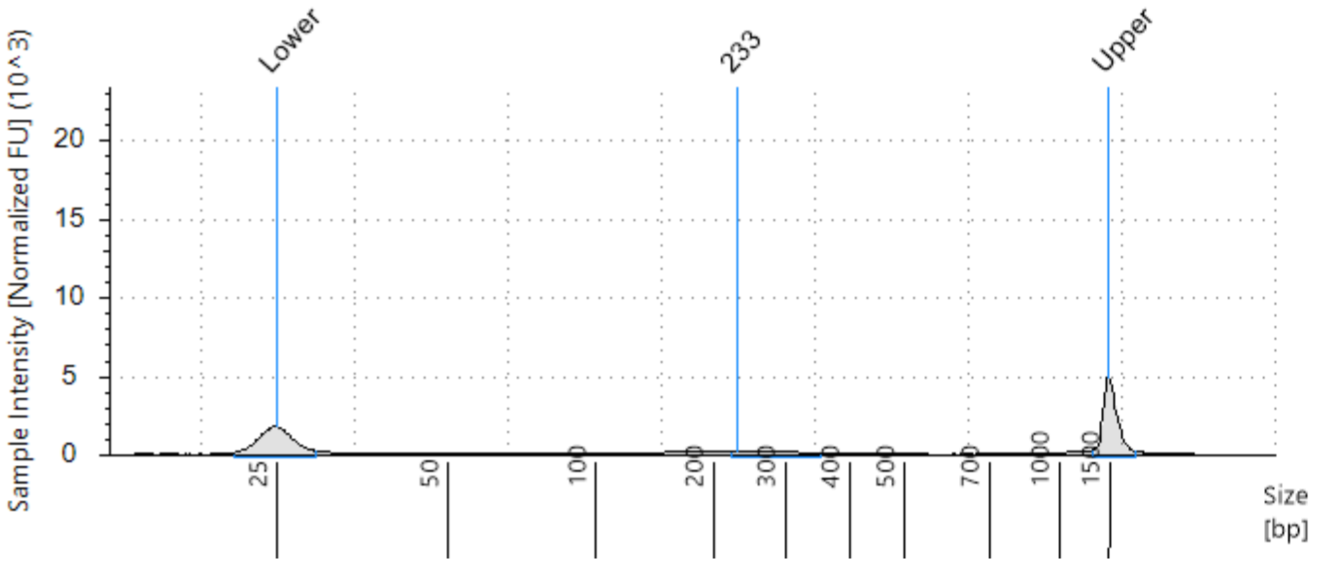

Sample Table

Well	Conc. [ng/µl]	Sample Description	Alert	Observations
B2	1.10	H3 M R3		

Peak Table

Size [bp]	Calibrated Conc. [ng/µl]	Assigned Conc. [ng/µl]	Peak Molarity [nmol/l]	% Integrated Area	Peak Comment	Observations
25	5.75	-	354	-		Lower Marker
233	1.10	-	7.26	100.00		
1500	6.50	6.50	6.67	-		Upper Marker

© Agilent Technologies, Inc. 2017

C2: H5 M R3

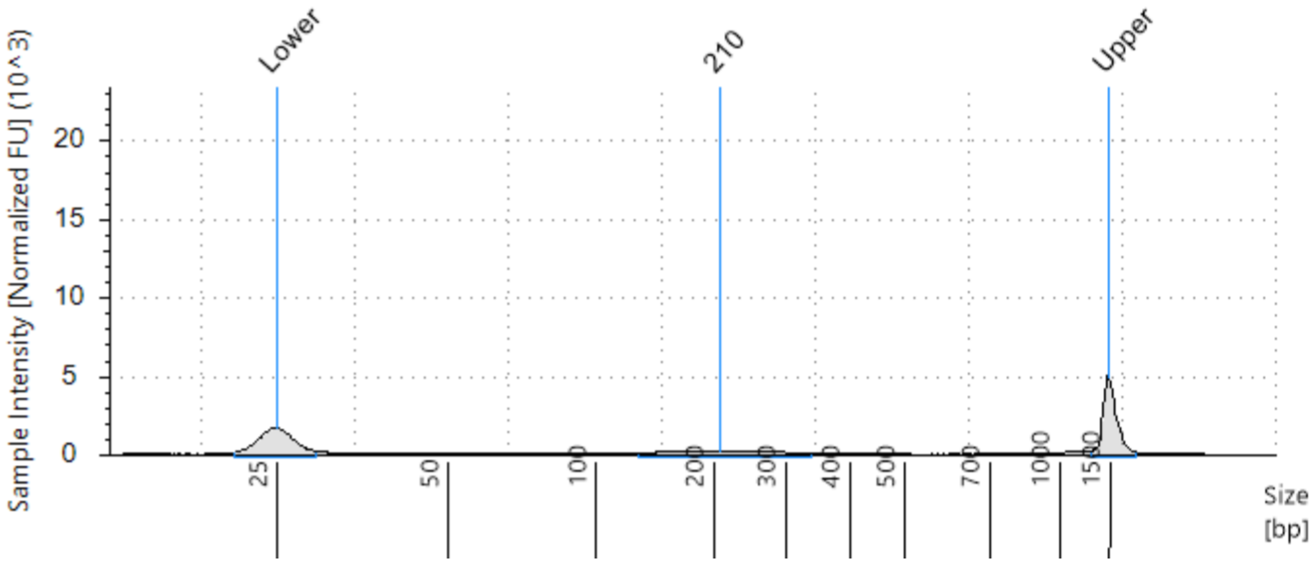

Sample Table

Well	Conc. [ng/µl]	Sample Description	Alert	Observations
C2	2.11	H5 M R3		

Peak Table

Size [bp]	Calibrated Conc. [ng/µl]	Assigned Conc. [ng/µl]	Peak Molarity [nmol/l]	% Integrated Area	Peak Comment	Observations
25	5.82	-	358	-		Lower Marker
210	2.11	-	15.5	100.00		
1500	6.50	6.50	6.67	-		Upper Marker

 © Agilent Technologies, Inc. 2017

D2: H7 M R3

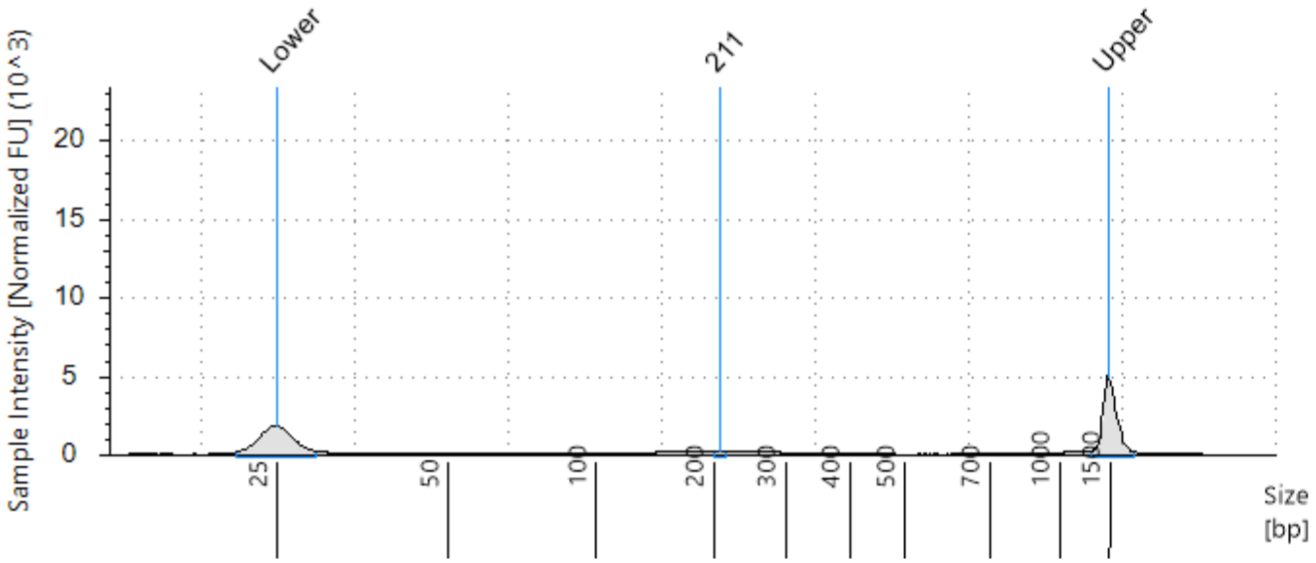

Sample Table

Well	Conc. [ng/µl]	Sample Description	Alert	Observations
D2	0.176	H7 M R3		

Peak Table

Size [bp]	Calibrated Conc. [ng/µl]	Assigned Conc. [ng/µl]	Peak Molarity [nmol/l]	% Integrated Area	Peak Comment	Observations
25	5.96	-	367	-		Lower Marker
211	0.176	-	1.28	100.00		
1500	6.50	6.50	6.67	-		Upper Marker

© Agilent Technologies, Inc. 2017

E2: H9 M R3

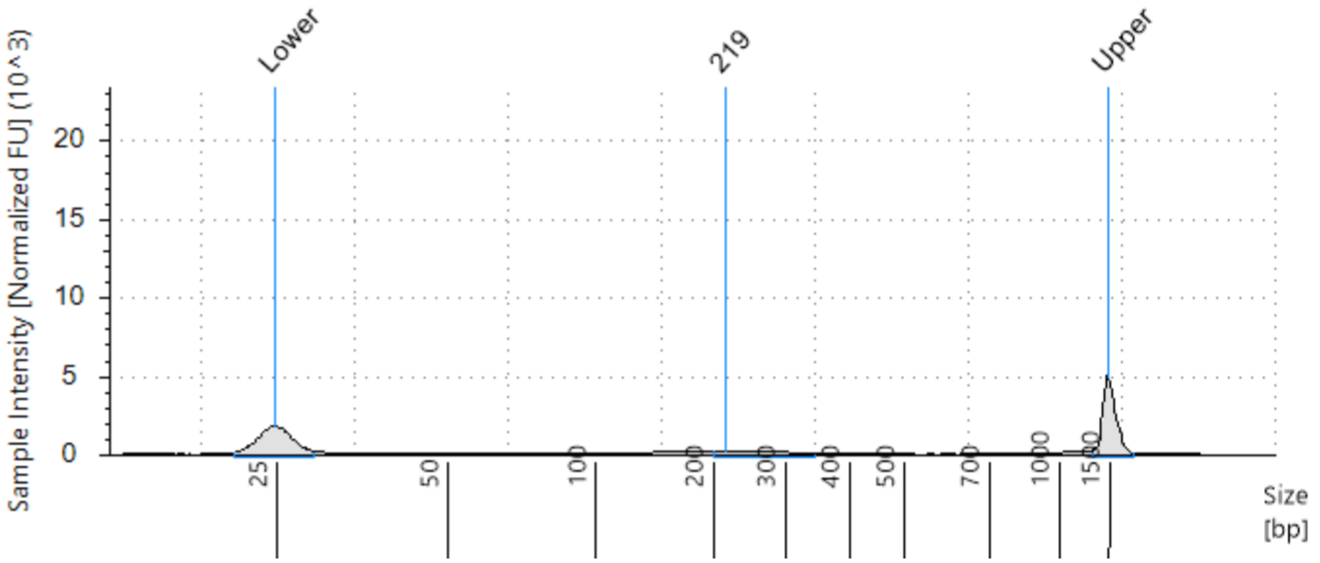

Sample Table

Well	Conc. [ng/µl]	Sample Description	Alert	Observations
E2	1.28	H9 M R3		

Peak Table

Size [bp]	Calibrated Conc. [ng/µl]	Assigned Conc. [ng/µl]	Peak Molarity [nmol/l]	% Integrated Area	Peak Comment	Observations
25	6.12	-	377	-		Lower Marker
219	1.28	-	8.97	100.00		
1500	6.50	6.50	6.67	-		Upper Marker

© Agilent Technologies, Inc. 2017

F2: H11 M R3

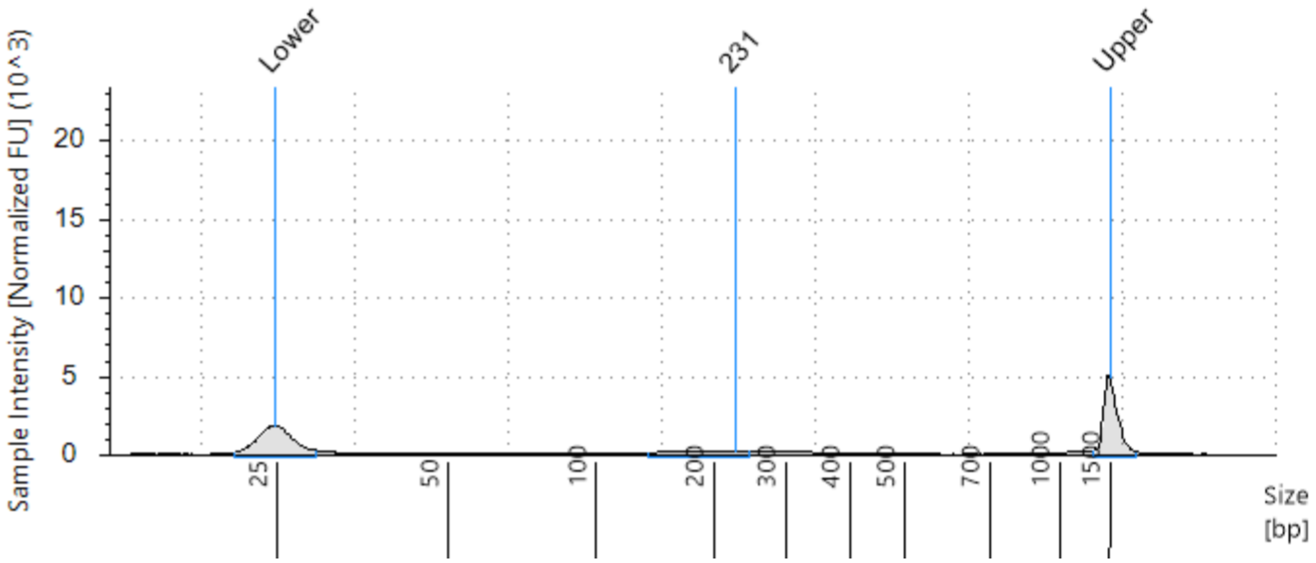

Sample Table

Well	Conc. [ng/µl]	Sample Description	Alert	Observations
F2	1.43	H11 M R3		

Peak Table

Size [bp]	Calibrated Conc. [ng/µl]	Assigned Conc. [ng/µl]	Peak Molarity [nmol/l]	% Integrated Area	Peak Comment	Observations
25	5.93	-	365	-		Lower Marker
231	1.43	-	9.55	100.00		
1500	6.50	6.50	6.67	-		Upper Marker

 © Agilent Technologies, Inc. 2017

G2: H4 M R3

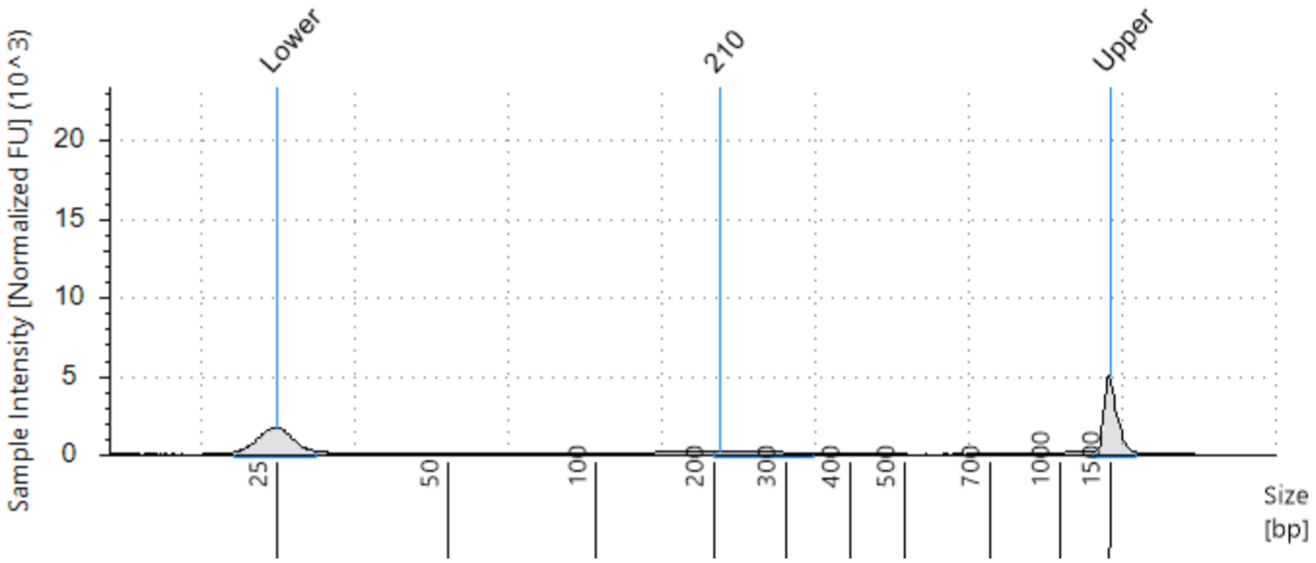

Sample Table

Well	Conc. [ng/µl]	Sample Description	Alert	Observations
G2	1.18	H4 M R3		

Peak Table

Size [bp]	Calibrated Conc. [ng/µl]	Assigned Conc. [ng/µl]	Peak Molarity [nmol/l]	% Integrated Area	Peak Comment	Observations
25	6.03	-	371	-		Lower Marker
210	1.18	-	8.64	100.00		
1500	6.50	6.50	6.67	-		Upper Marker

© Agilent Technologies, Inc. 2017

H2: 700 ng Plus undiluted

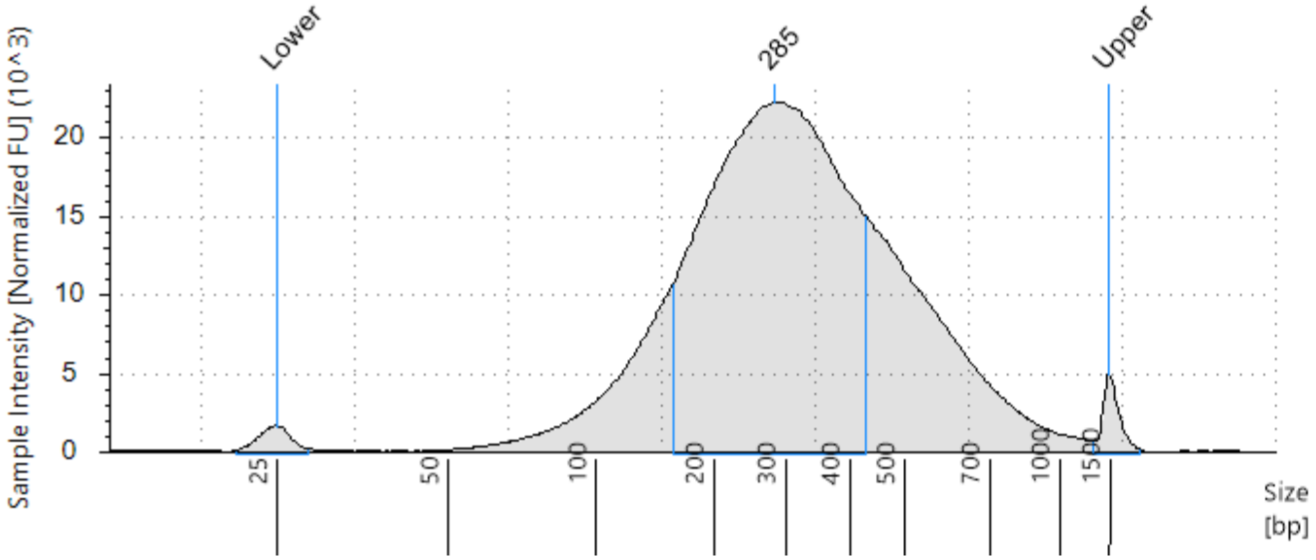

Sample Table

Well	Conc. [ng/µl]	Sample Description	Alert	Observations
H2	246	700 ng Plus undiluted		

Peak Table

Size [bp]	Calibrated Conc. [ng/µl]	Assigned Conc. [ng/µl]	Peak Molarity [nmol/l]	% Integrated Area	Peak Comment	Observations
25	4.17	-	256	-		Lower Marker
285	246	-	1330	100.00		
1500	6.50	6.50	6.67	-		Upper Marker

 © Agilent Technologies, Inc. 2017
