# Supplementary material for: 96 sample parallel acoustic fragmentation for high throughput next generation sequencing library preparation
Source: PLoS One. 2026 Feb 17;21(2):e0341139. doi: 10.1371/journal.pone.0341139 (PMC12912608; doi:10.1371/journal.pone.0341139)
Supplement: S5 File — (ZIP) [file pone.0341139.s005.zip › QSonica translator TapeStation raw data/QSonica nanodroplets translator 96-well plate replicate 2.pdf]

Filename: 2020-09-08 Q-S DFB plus A1- F2 R2.D1000

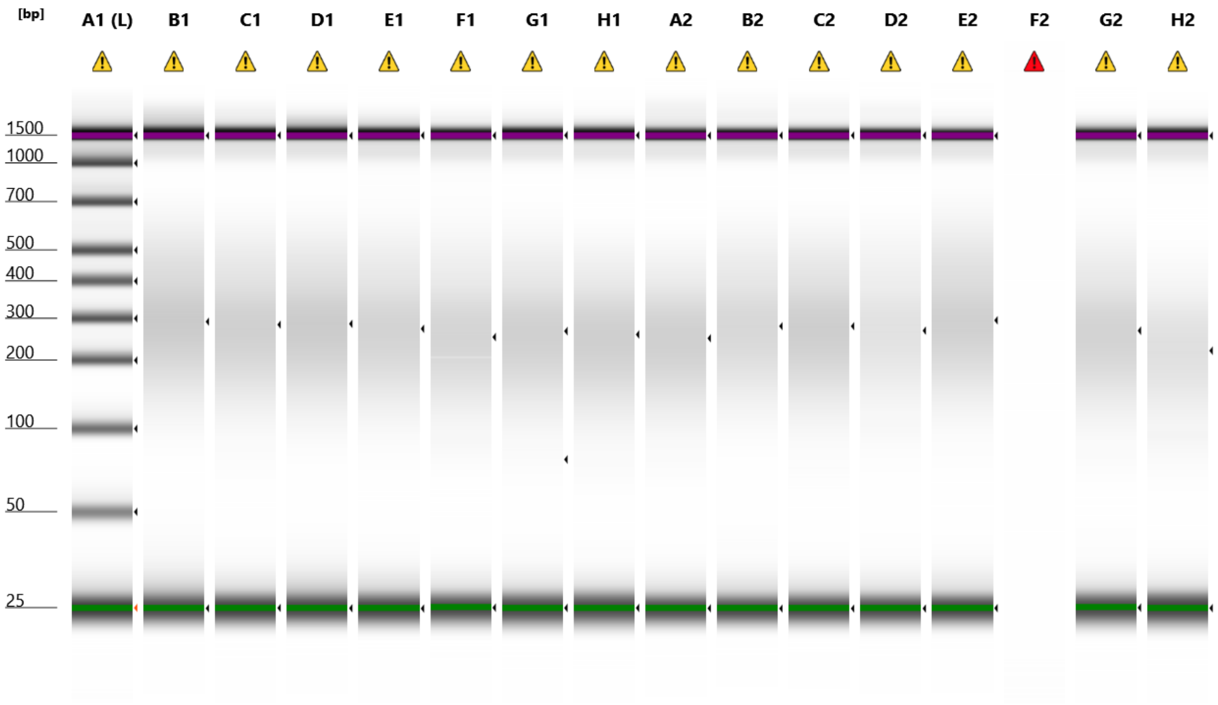

Default image (Contrast 100%)

Sample Info

Well	Conc. [ng/µl]	Sample Description	Alert	Observations
A1	15.7	Ladder	⚠	Caution! Expired ScreenTape device; Ladder
B1	2.25	A1 p R2	⚠	Caution! Expired ScreenTape device
C1	1.47	B1 p R2	⚠	Caution! Expired ScreenTape device
D1	1.84	C1 P R2	⚠	Caution! Expired ScreenTape device
E1	1.66	D1 P R2	⚠	Caution! Expired ScreenTape device
F1	0.842	E1 p R2	⚠	Caution! Expired ScreenTape device
G1	4.58	F1 p R2	⚠	Caution! Expired ScreenTape device
H1	4.03	F1 p R2	⚠	Caution! Expired ScreenTape device
A2	0.510	G1 p R2	⚠	Caution! Expired ScreenTape device
B2	0.577	H1 p R2	⚠	Caution! Expired ScreenTape device
C2	1.95	A2 p R2	⚠	Caution! Expired ScreenTape device
D2	1.35	B2 p R2	⚠	Caution! Expired ScreenTape device
E2	1.86	C2 p R2	⚠	Caution! Expired ScreenTape device
F2		D2 p R2	⚠	Marker(s) not detected; Caution! Expired ScreenTape device
G2	3.70	E2 p R2	⚠	Caution! Expired ScreenTape device
H2	1.38	F2 p R2	⚠	Caution! Expired ScreenTape device

© Agilent Technologies, Inc. 2017

A1: Ladder

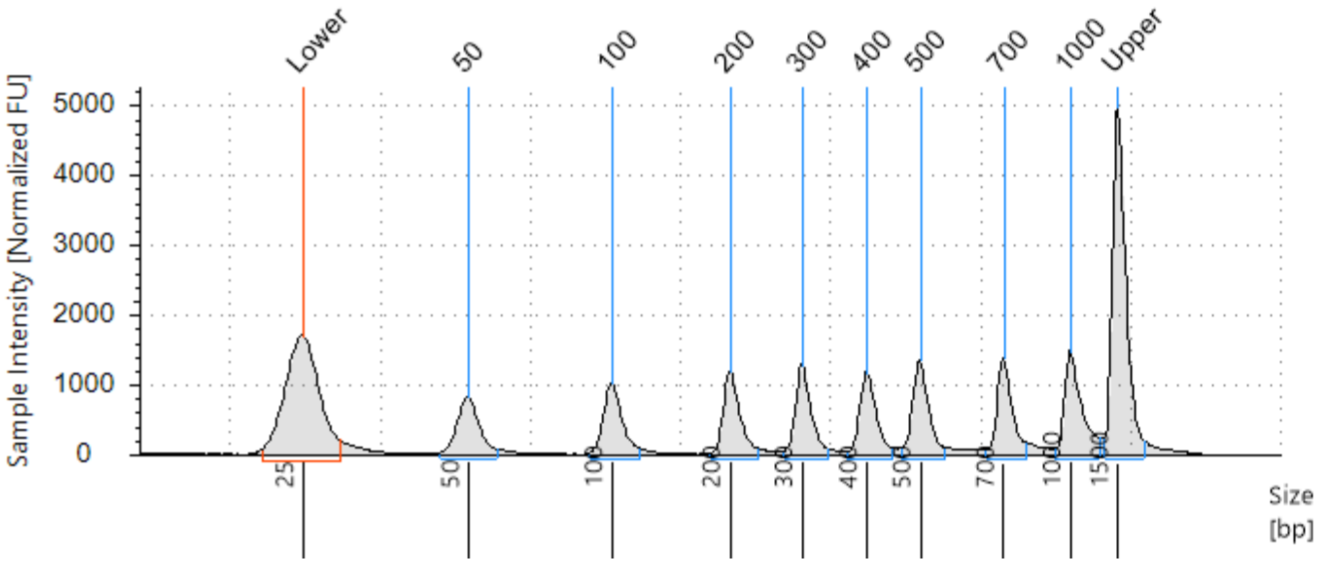

Sample Table

Well	Conc. [ng/µl]	Sample Description	Alert	Observations
A1	15.7	Ladder	⚠	Caution! Expired ScreenTape device; Ladder

Peak Table

Size [bp]	Calibrated Conc. [ng/µl]	Assigned Conc. [ng/µl]	Peak Molarity [nmol/l]	% Integrated Area	Peak Comment	Observations
25	5.56	-	342	-		Lower Marker
50	1.74	-	53.6	11.08		
100	1.83	-	28.1	11.64		
200	1.89	-	14.5	12.02		
300	1.92	-	9.83	12.20		
400	1.89	-	7.28	12.04		
500	2.07	-	6.38	13.18		
700	1.98	-	4.35	12.60		
1000	2.40	-	3.69	15.24		
1500	6.50	6.50	6.67	-		Upper Marker

© Agilent Technologies, Inc. 2017

B1: A1 p R2

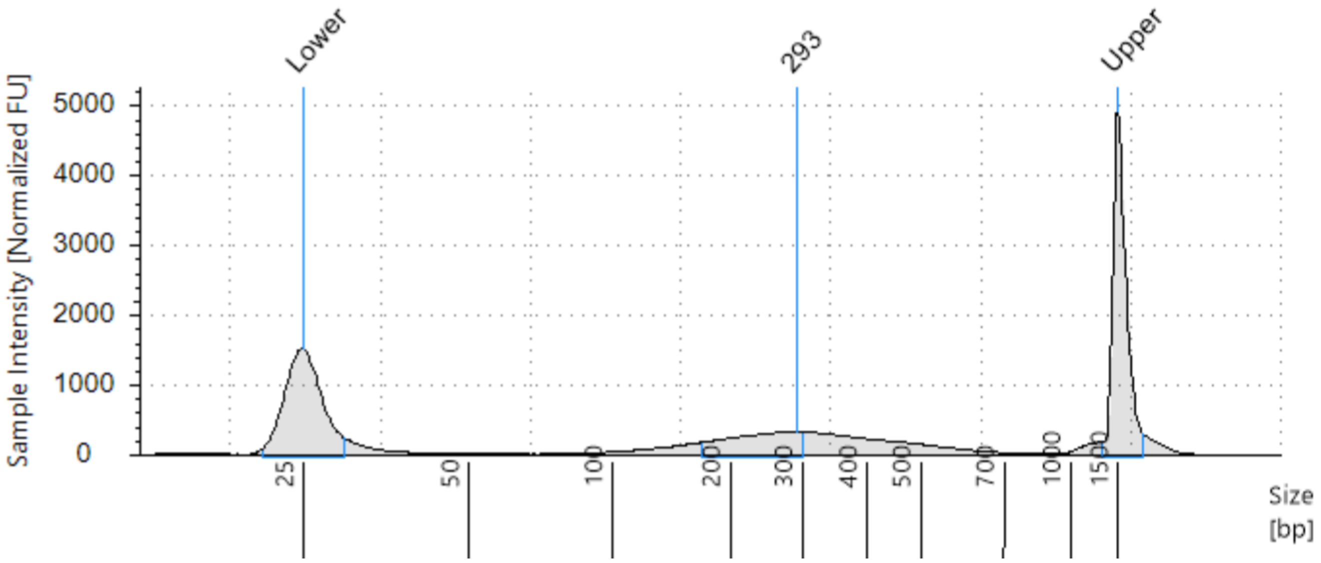

Sample Table

Well	Conc. [ng/µl]	Sample Description	Alert	Observations
B1	2.25	A1 p R2	⚠	Caution! Expired ScreenTape device

Peak Table

Size [bp]	Calibrated Conc. [ng/µl]	Assigned Conc. [ng/µl]	Peak Molarity [nmol/l]	% Integrated Area	Peak Comment	Observations
25	5.55	-	341	-		Lower Marker
293	2.25	-	11.8	100.00		
1500	6.50	6.50	6.67	-		Upper Marker

© Agilent Technologies, Inc. 2017

C1: B1 p R2

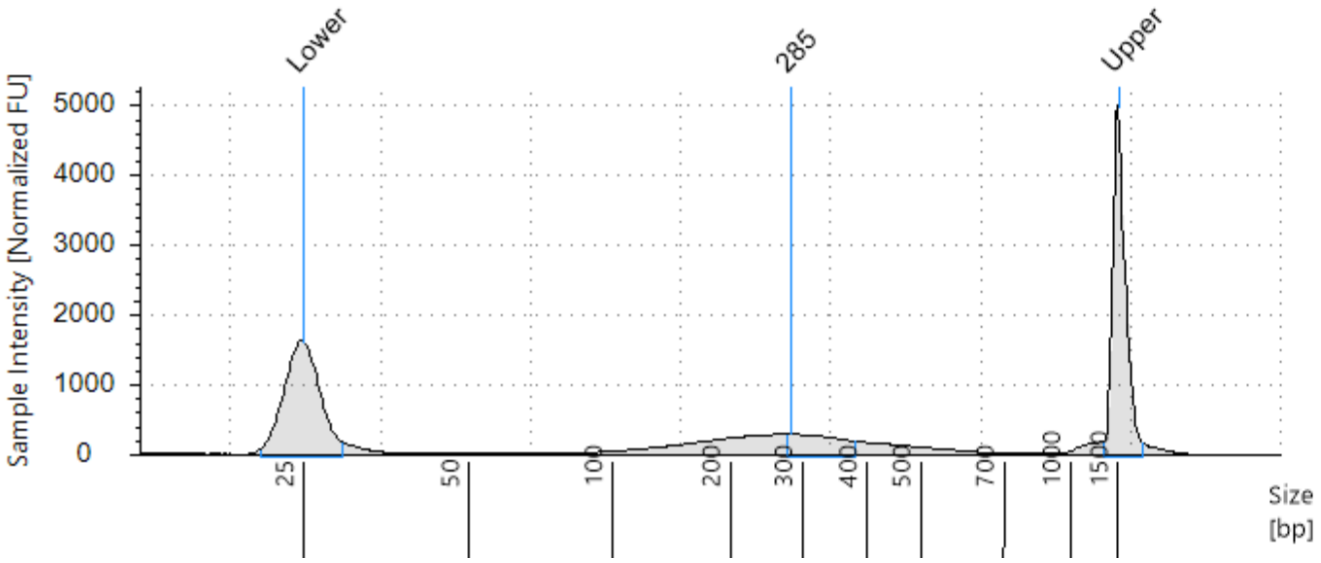

Sample Table

Well	Conc. [ng/µl]	Sample Description	Alert	Observations
C1	1.47	B1 p R2	⚠	Caution! Expired ScreenTape device

Peak Table

Size [bp]	Calibrated Conc. [ng/µl]	Assigned Conc. [ng/µl]	Peak Molarity [nmol/l]	% Integrated Area	Peak Comment	Observations
25	5.94	-	365	-		Lower Marker
285	1.47	-	7.95	100.00		
1500	6.50	6.50	6.67	-		Upper Marker

© Agilent Technologies, Inc. 2017

D1: C1 P R2

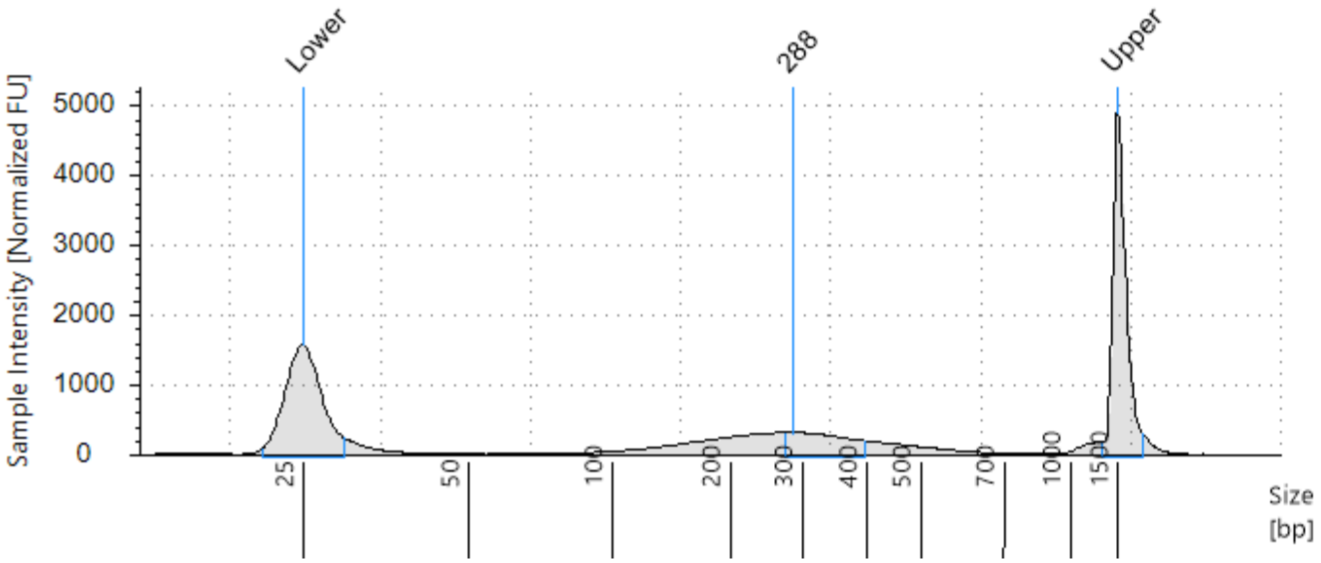

Sample Table

Well	Conc. [ng/µl]	Sample Description	Alert	Observations
D1	1.84	C1 P R2	⚠	Caution! Expired ScreenTape device

Peak Table

Size [bp]	Calibrated Conc. [ng/µl]	Assigned Conc. [ng/µl]	Peak Molarity [nmol/l]	% Integrated Area	Peak Comment	Observations
25	5.72	-	352	-		Lower Marker
288	1.84	-	9.85	100.00		
1500	6.50	6.50	6.67	-		Upper Marker

© Agilent Technologies, Inc. 2017

E1: D1 P R2

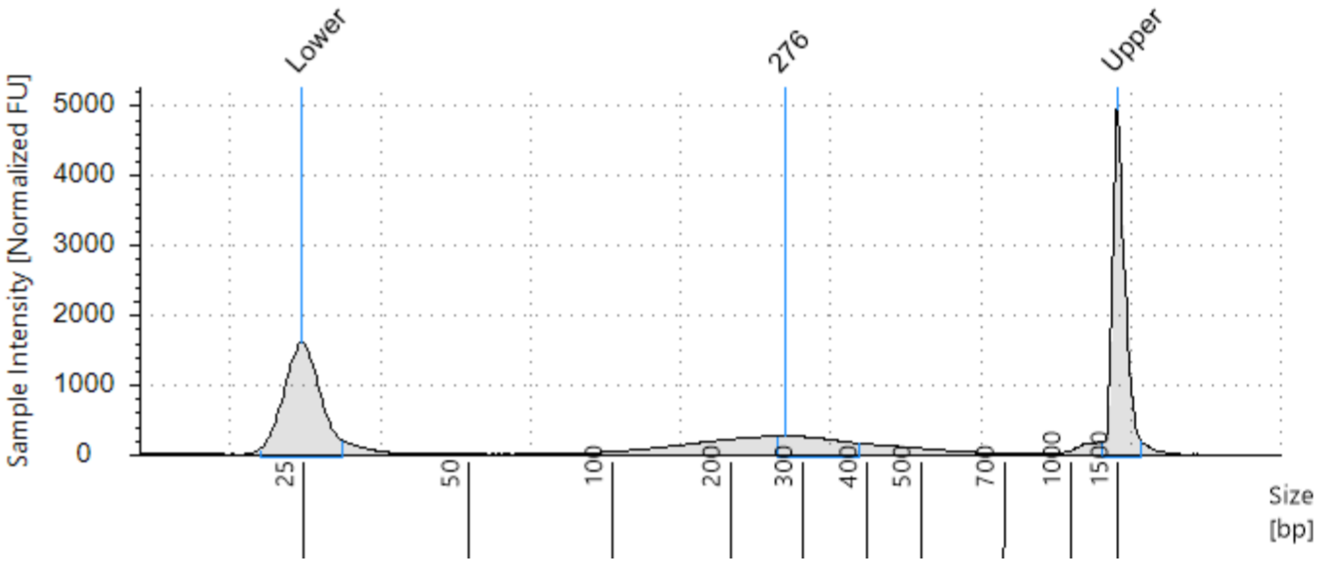

Sample Table

Well	Conc. [ng/µl]	Sample Description	Alert	Observations
E1	1.66	D1 P R2	⚠	Caution! Expired ScreenTape device

Peak Table

Size [bp]	Calibrated Conc. [ng/µl]	Assigned Conc. [ng/µl]	Peak Molarity [nmol/l]	% Integrated Area	Peak Comment	Observations
25	6.05	-	373	-		Lower Marker
276	1.66	-	9.26	100.00		
1500	6.50	6.50	6.67	-		Upper Marker

© Agilent Technologies, Inc. 2017

F1: E1 p R2

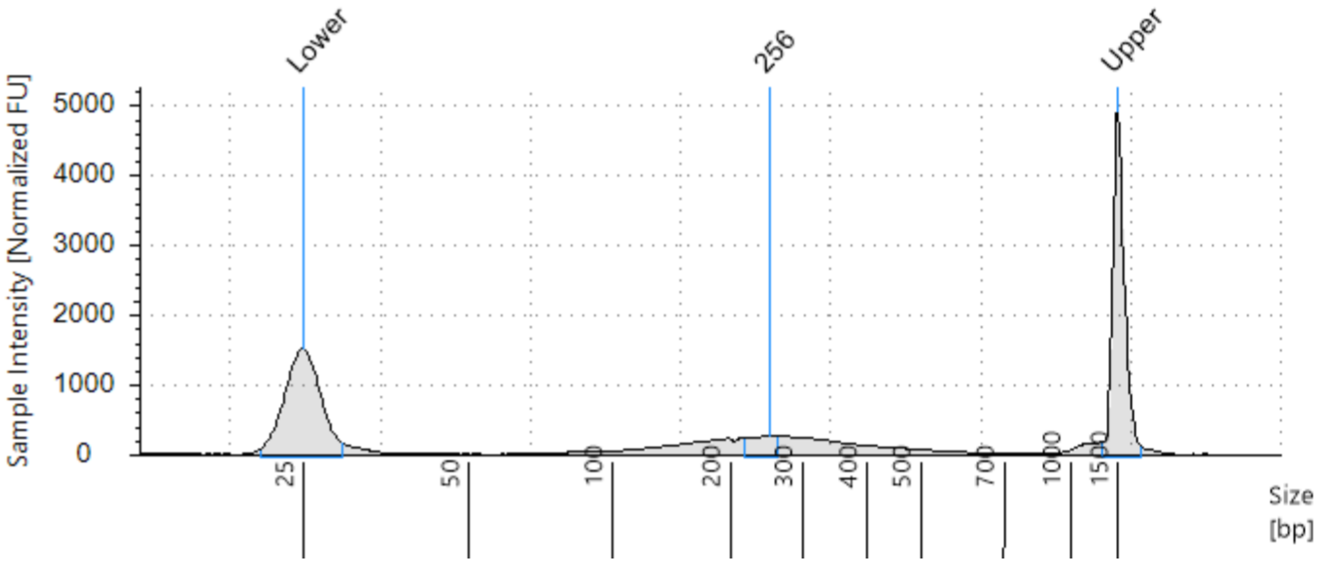

Sample Table

Well	Conc. [ng/µl]	Sample Description	Alert	Observations
F1	0.842	E1 p R2	⚠	Caution! Expired ScreenTape device

Peak Table

Size [bp]	Calibrated Conc. [ng/µl]	Assigned Conc. [ng/µl]	Peak Molarity [nmol/l]	% Integrated Area	Peak Comment	Observations
25	6.06	-	373	-		Lower Marker
256	0.842	-	5.07	100.00		
1500	6.50	6.50	6.67	-		Upper Marker

© Agilent Technologies, Inc. 2017

G1: F1 p R2

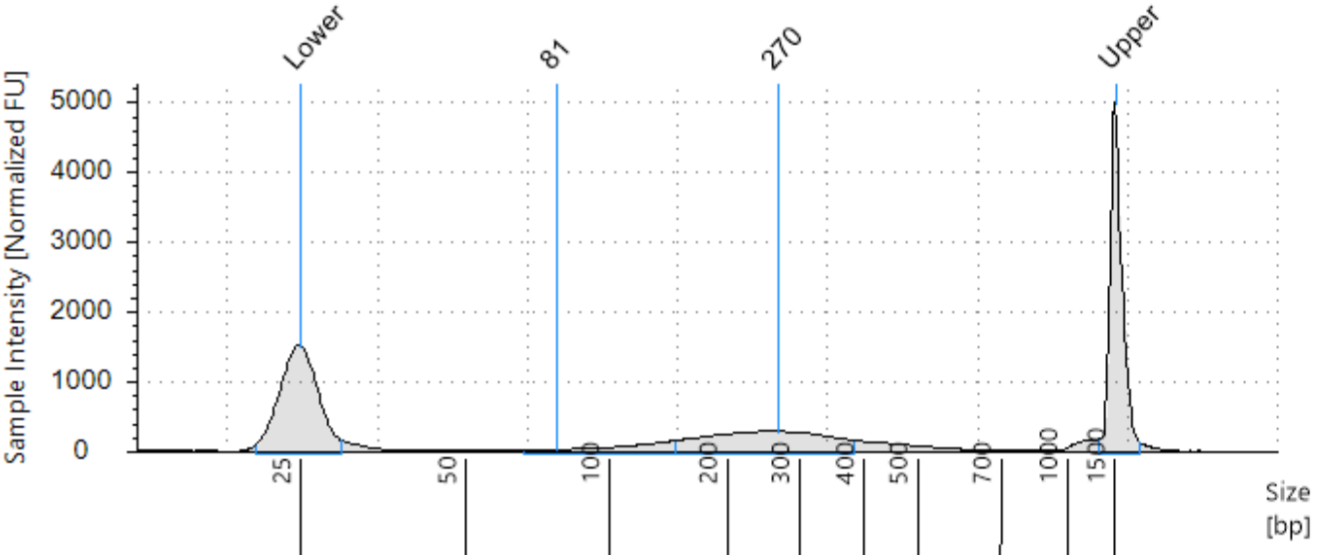

Sample Table

Well	Conc. [ng/µl]	Sample Description	Alert	Observations
G1	4.58	F1 p R2	⚠	Caution! Expired ScreenTape device

Peak Table

Size [bp]	Calibrated Conc. [ng/µl]	Assigned Conc. [ng/µl]	Peak Molarity [nmol/l]	% Integrated Area	Peak Comment	Observations
25	6.24	-	384	-		Lower Marker
81	0.781	-	14.7	17.04		
270	3.80	-	21.7	82.96		
1500	6.50	6.50	6.67	-		Upper Marker

© Agilent Technologies, Inc. 2017

H1: F1 p R2

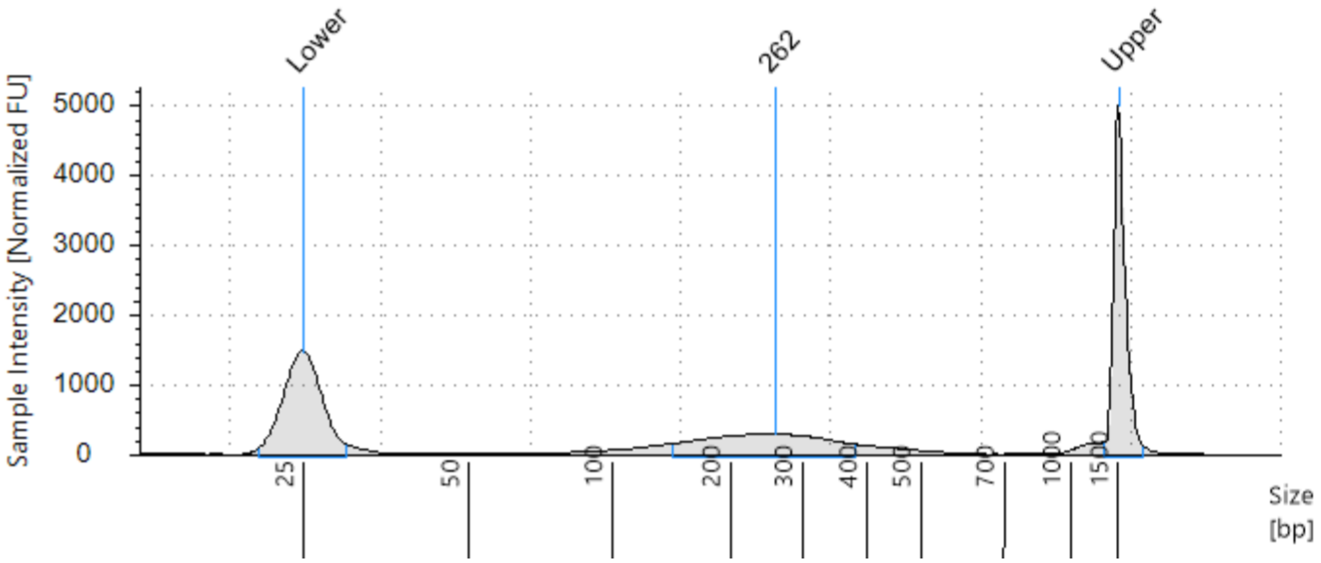

Sample Table

Well	Conc. [ng/µl]	Sample Description	Alert	Observations
H1	4.03	F1 p R2	⚠	Caution! Expired ScreenTape device

Peak Table

Size [bp]	Calibrated Conc. [ng/µl]	Assigned Conc. [ng/µl]	Peak Molarity [nmol/l]	% Integrated Area	Peak Comment	Observations
25	6.26	-	385	-		Lower Marker
262	4.03	-	23.7	100.00		
1500	6.50	6.50	6.67	-		Upper Marker

© Agilent Technologies, Inc. 2017

A2: Gl p R2

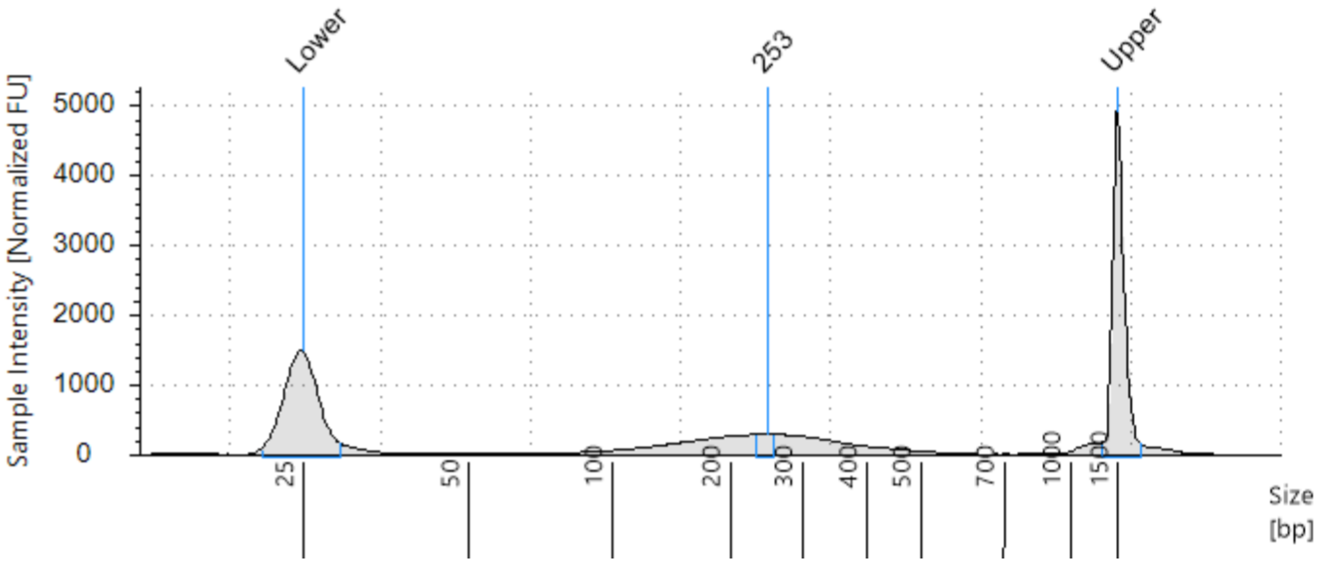

Sample Table

Well	Conc. [ng/µl]	Sample Description	Alert	Observations
A2	0.510	Gl p R2	⚠	Caution! Expired ScreenTape device

Peak Table

Size [bp]	Calibrated Conc. [ng/µl]	Assigned Conc. [ng/µl]	Peak Molarity [nmol/l]	% Integrated Area	Peak Comment	Observations
25	5.82	-	358	-		Lower Marker
253	0.510	-	3.09	100.00		
1500	6.50	6.50	6.67	-		Upper Marker

© Agilent Technologies, Inc. 2017

B2: H1 p R2

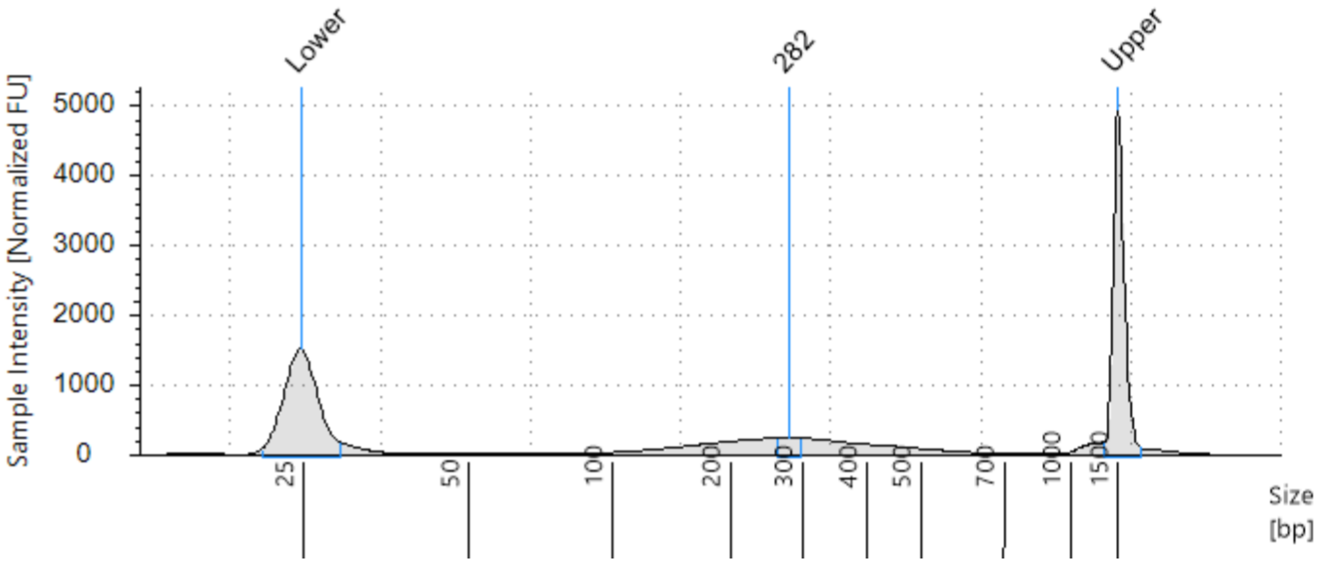

Sample Table

Well	Conc. [ng/µl]	Sample Description	Alert	Observations
B2	0.577	H1 p R2	⚠	Caution! Expired ScreenTape device

Peak Table

Size [bp]	Calibrated Conc. [ng/µl]	Assigned Conc. [ng/µl]	Peak Molarity [nmol/l]	% Integrated Area	Peak Comment	Observations
25	5.98	-	368	-		Lower Marker
282	0.577	-	3.14	100.00		
1500	6.50	6.50	6.67	-		Upper Marker

© Agilent Technologies, Inc. 2017

C2: A2 p R2

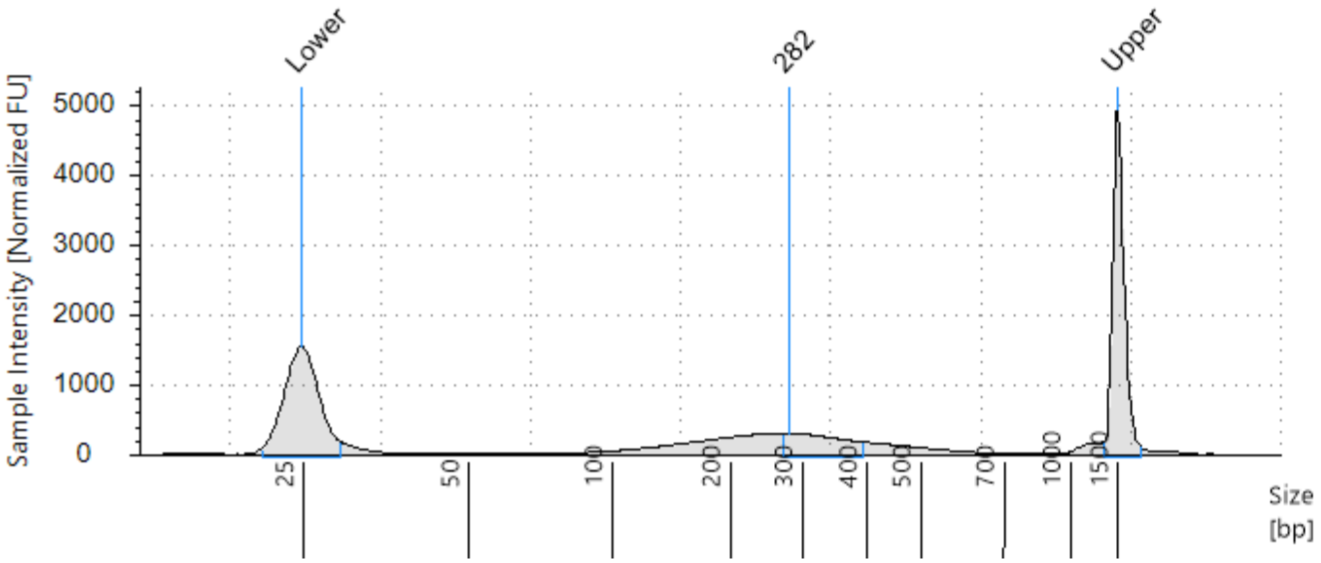

Sample Table

Well	Conc. [ng/µl]	Sample Description	Alert	Observations
C2	1.95	A2 p R2	⚠	Caution! Expired ScreenTape device

Peak Table

Size [bp]	Calibrated Conc. [ng/µl]	Assigned Conc. [ng/µl]	Peak Molarity [nmol/l]	% Integrated Area	Peak Comment	Observations
25	6.09	-	374	-		Lower Marker
282	1.95	-	10.6	100.00		
1500	6.50	6.50	6.67	-		Upper Marker

© Agilent Technologies, Inc. 2017

D2: B2 p R2

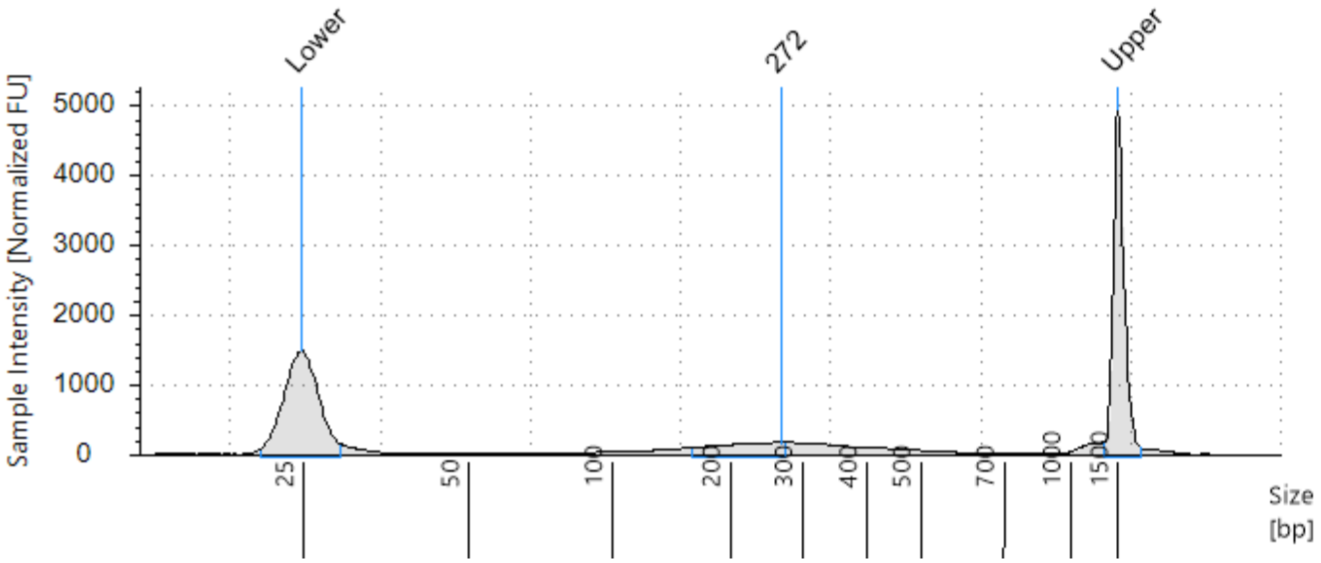

Sample Table

Well	Conc. [ng/µl]	Sample Description	Alert	Observations
D2	1.35	B2 p R2	⚠	Caution! Expired ScreenTape device

Peak Table

Size [bp]	Calibrated Conc. [ng/µl]	Assigned Conc. [ng/µl]	Peak Molarity [nmol/l]	% Integrated Area	Peak Comment	Observations
25	6.13	-	377	-		Lower Marker
272	1.35	-	7.66	100.00		
1500	6.50	6.50	6.67	-		Upper Marker

© Agilent Technologies, Inc. 2017

E2: C2 p R2

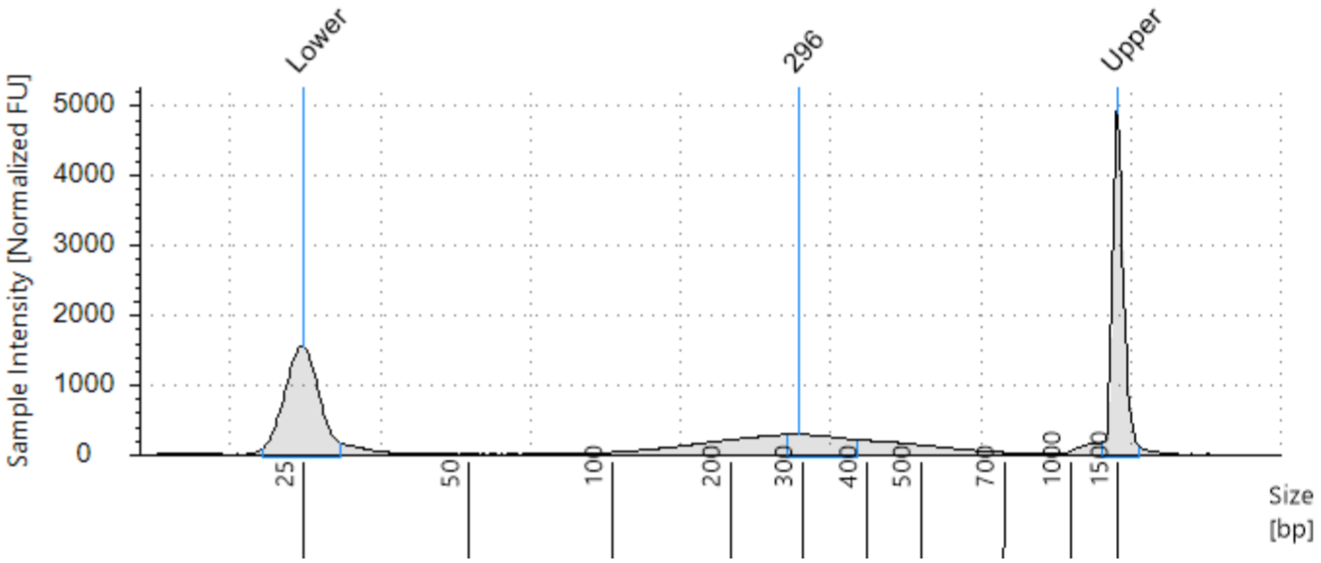

Sample Table

Well	Conc. [ng/µl]	Sample Description	Alert	Observations
E2	1.86	C2 p R2	⚠	Caution! Expired ScreenTape device

Peak Table

Size [bp]	Calibrated Conc. [ng/µl]	Assigned Conc. [ng/µl]	Peak Molarity [nmol/l]	% Integrated Area	Peak Comment	Observations
25	6.27	-	386	-		Lower Marker
296	1.86	-	9.64	100.00		
1500	6.50	6.50	6.67	-		Upper Marker

© Agilent Technologies, Inc. 2017

F2: D2 p R2

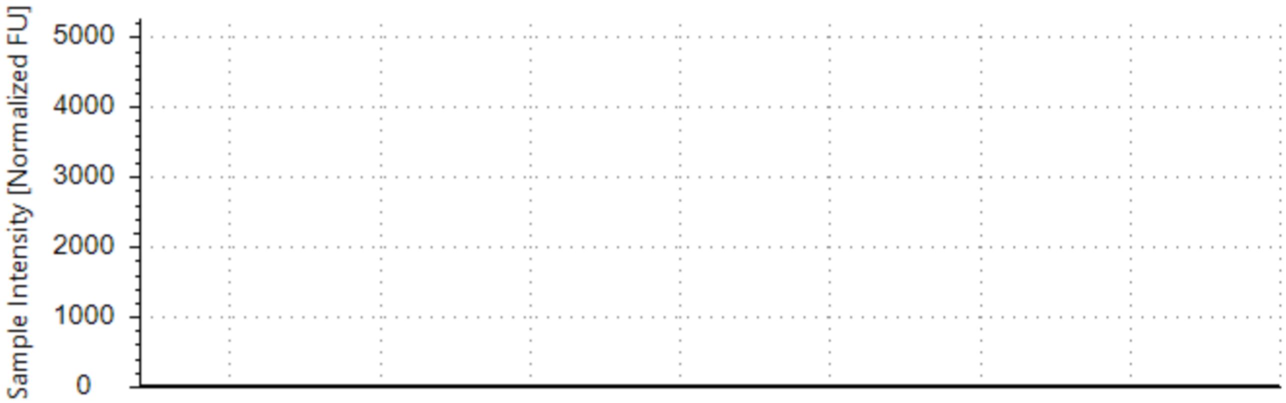

Sample Table

Well	Conc. [ng/µl]	Sample Description	Alert	Observations
F2		D2 p R2	▲	Marker(s) not detected; Caution! Expired ScreenTape device

 © Agilent Technologies, Inc. 2017

G2: E2 p R2

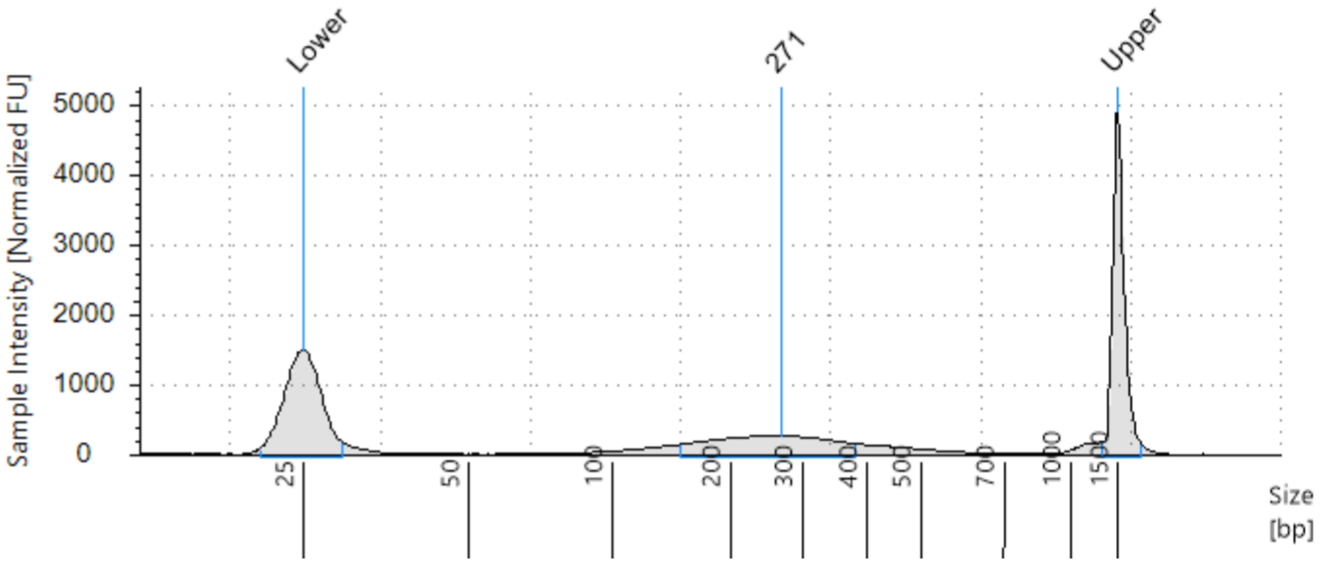

Sample Table

Well	Conc. [ng/µl]	Sample Description	Alert	Observations
G2	3.70	E2 p R2	⚠	Caution! Expired ScreenTape device

Peak Table

Size [bp]	Calibrated Conc. [ng/µl]	Assigned Conc. [ng/µl]	Peak Molarity [nmol/l]	% Integrated Area	Peak Comment	Observations
25	6.29	-	387	-		Lower Marker
271	3.70	-	21.0	100.00		
1500	6.50	6.50	6.67	-		Upper Marker

© Agilent Technologies, Inc. 2017

H2: F2 p R2

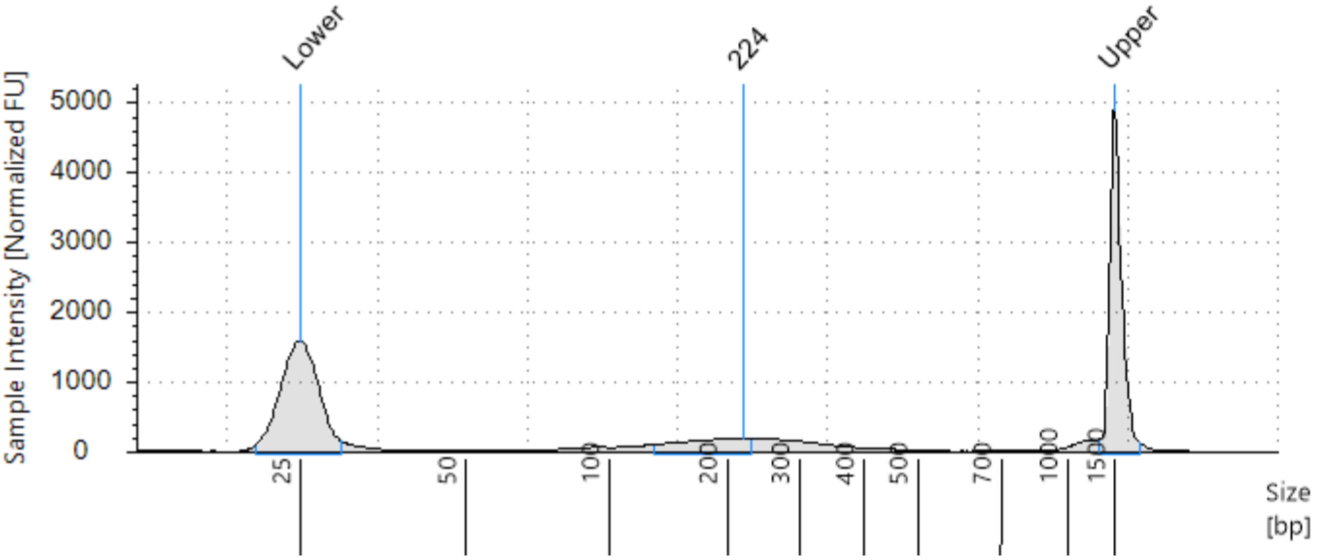

Sample Table

Well	Conc. [ng/µl]	Sample Description	Alert	Observations
H2	1.38	F2 p R2	⚠	Caution! Expired ScreenTape device

Peak Table

Size [bp]	Calibrated Conc. [ng/µl]	Assigned Conc. [ng/µl]	Peak Molarity [nmol/l]	% Integrated Area	Peak Comment	Observations
25	6.65	-	409	-		Lower Marker
224	1.38	-	9.48	100.00		
1500	6.50	6.50	6.67	-		Upper Marker

© Agilent Technologies, Inc. 2017

Filename: 2020-09-08 Q-S DFB PLUS G2-E3 Minus G2-D3 R2.D1000

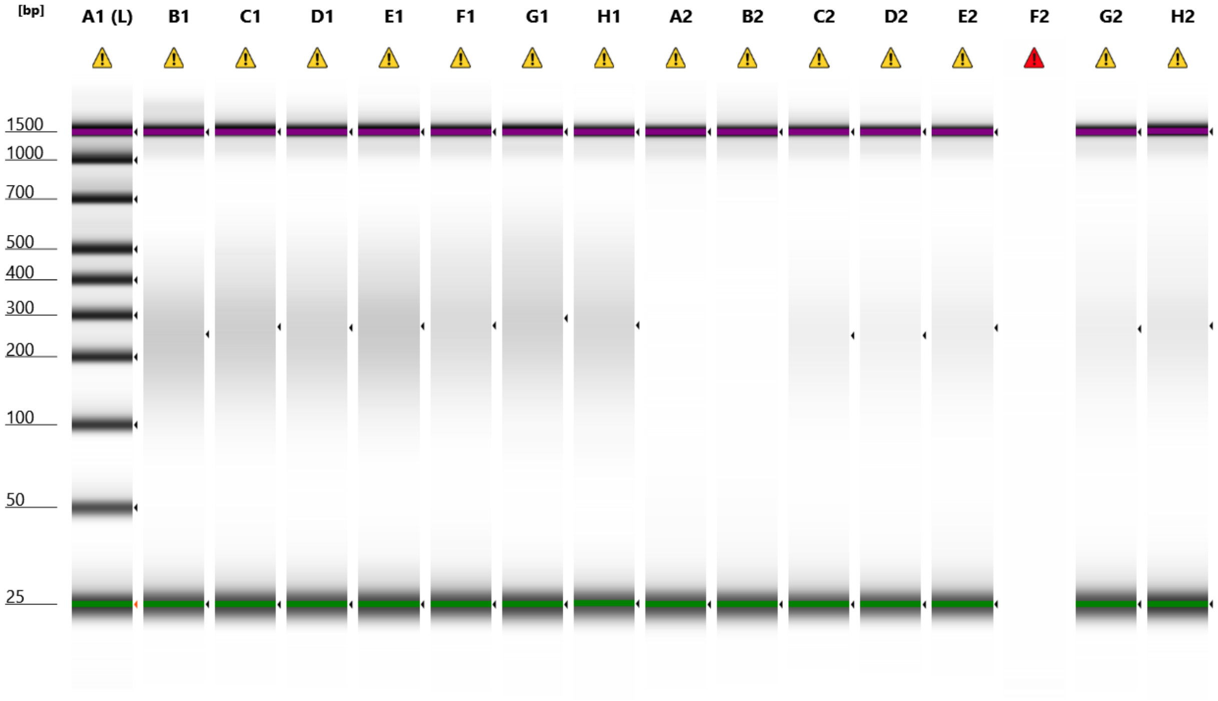

Default image (Contrast 100%)

Sample Info

Well	Conc. [ng/µl]	Sample Description	Alert	Observations
A1	25.9	Ladder	⚠	Caution! Expired ScreenTape device; Ladder
B1	4.11	G2 P R2	⚠	Caution! Expired ScreenTape device
C1	1.75	H2 P R2	⚠	Caution! Expired ScreenTape device
D1	1.75	A3 P R2	⚠	Caution! Expired ScreenTape device
E1	2.14	B3 P R2	⚠	Caution! Expired ScreenTape device
F1	2.79	C3 P R2	⚠	Caution! Expired ScreenTape device
G1	2.67	D3 P R2	⚠	Caution! Expired ScreenTape device
H1	1.49	E3 P R2	⚠	Caution! Expired ScreenTape device
A2		E1 M R1	⚠	Caution! Expired ScreenTape device
B2		E6 M R1	⚠	Caution! Expired ScreenTape device
C2	0.126	G2 M R2	⚠	Caution! Expired ScreenTape device
D2	0.566	H2 M R2	⚠	Caution! Expired ScreenTape device
E2	0.388	A3 M R2	⚠	Caution! Expired ScreenTape device
F2		B3 M R2	⚠	Marker(s) not detected; Caution! Expired ScreenTape device
G2	0.309	C3 M R2	⚠	Caution! Expired ScreenTape device
H2	0.185	D3 M R2	⚠	Caution! Expired ScreenTape device

© Agilent Technologies, Inc. 2017

A1: Ladder

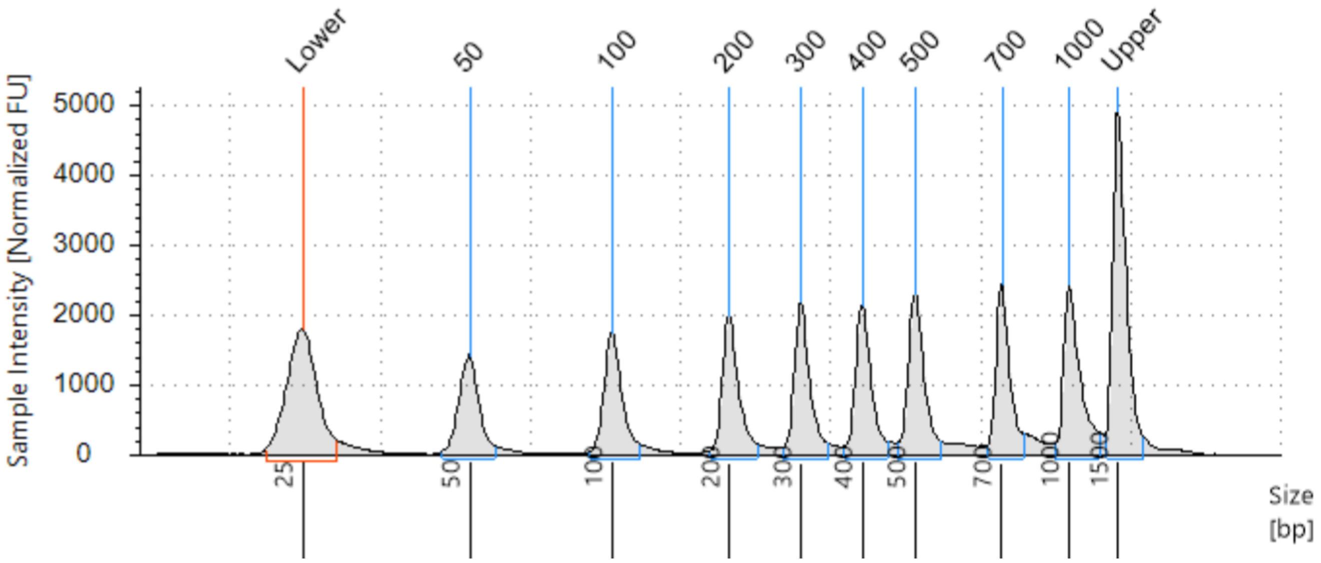

Sample Table

Well	Conc. [ng/µl]	Sample Description	Alert	Observations
A1	25.9	Ladder	⚠	Caution! Expired ScreenTape device; Ladder

Peak Table

Size [bp]	Calibrated Conc. [ng/µl]	Assigned Conc. [ng/µl]	Peak Molarity [nmol/l]	% Integrated Area	Peak Comment	Observations
25	5.21	-	321	-		Lower Marker
50	2.72	-	83.6	10.49		
100	3.01	-	46.3	11.60		
200	3.12	-	24.0	12.04		
300	3.24	-	16.6	12.50		
400	3.25	-	12.5	12.53		
500	3.53	-	10.9	13.62		
700	3.26	-	7.16	12.57		
1000	3.79	-	5.84	14.64		
1500	6.50	6.50	6.67	-		Upper Marker

© Agilent Technologies, Inc. 2017

B1: G2 P R2

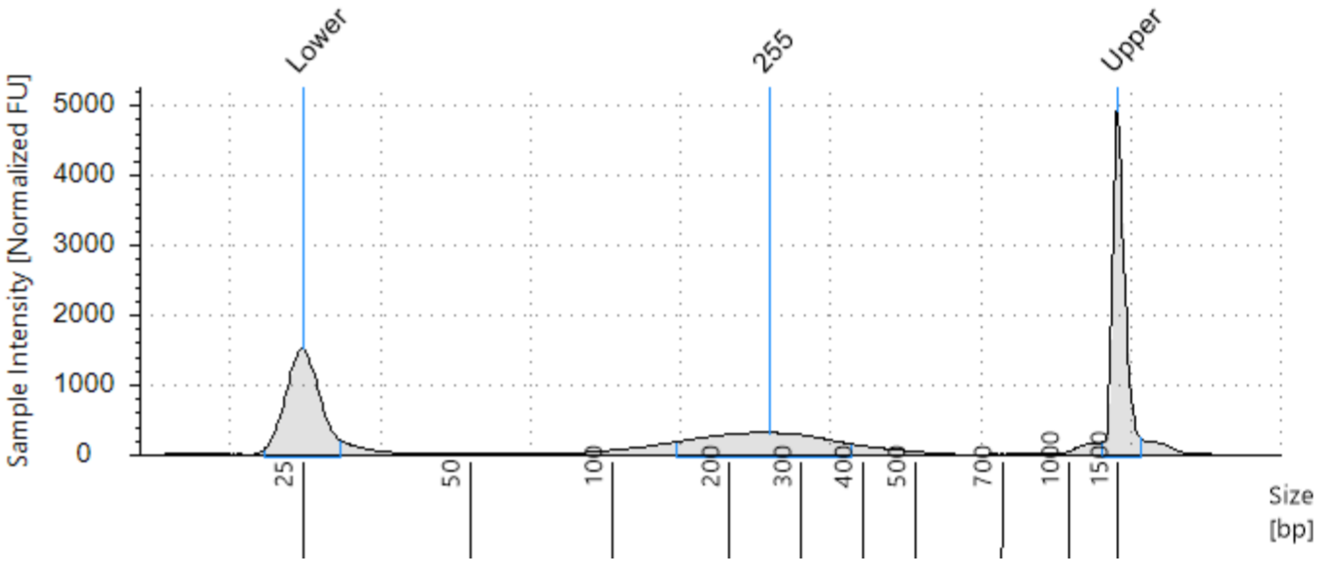

Sample Table

Well	Conc. [ng/µl]	Sample Description	Alert	Observations
B1	4.11	G2 P R2	⚠	Caution! Expired ScreenTape device

Peak Table

Size [bp]	Calibrated Conc. [ng/µl]	Assigned Conc. [ng/µl]	Peak Molarity [nmol/l]	% Integrated Area	Peak Comment	Observations
25	5.53	-	340	-		Lower Marker
255	4.11	-	24.8	100.00		
1500	6.50	6.50	6.67	-		Upper Marker

© Agilent Technologies, Inc. 2017

C1: H2 P R2

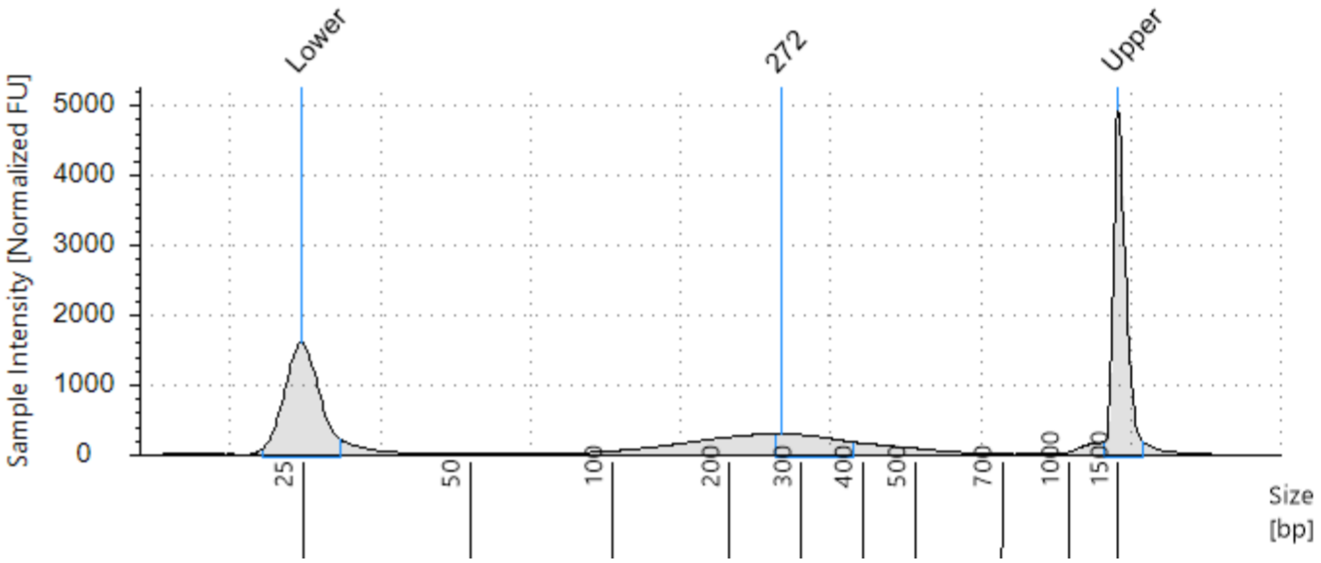

Sample Table

Well	Conc. [ng/µl]	Sample Description	Alert	Observations
C1	1.75	H2 P R2	⚠	Caution! Expired ScreenTape device

Peak Table

Size [bp]	Calibrated Conc. [ng/µl]	Assigned Conc. [ng/µl]	Peak Molarity [nmol/l]	% Integrated Area	Peak Comment	Observations
25	5.74	-	353	-		Lower Marker
272	1.75	-	9.90	100.00		
1500	6.50	6.50	6.67	-		Upper Marker

 © Agilent Technologies, Inc. 2017

D1: A3 P R2

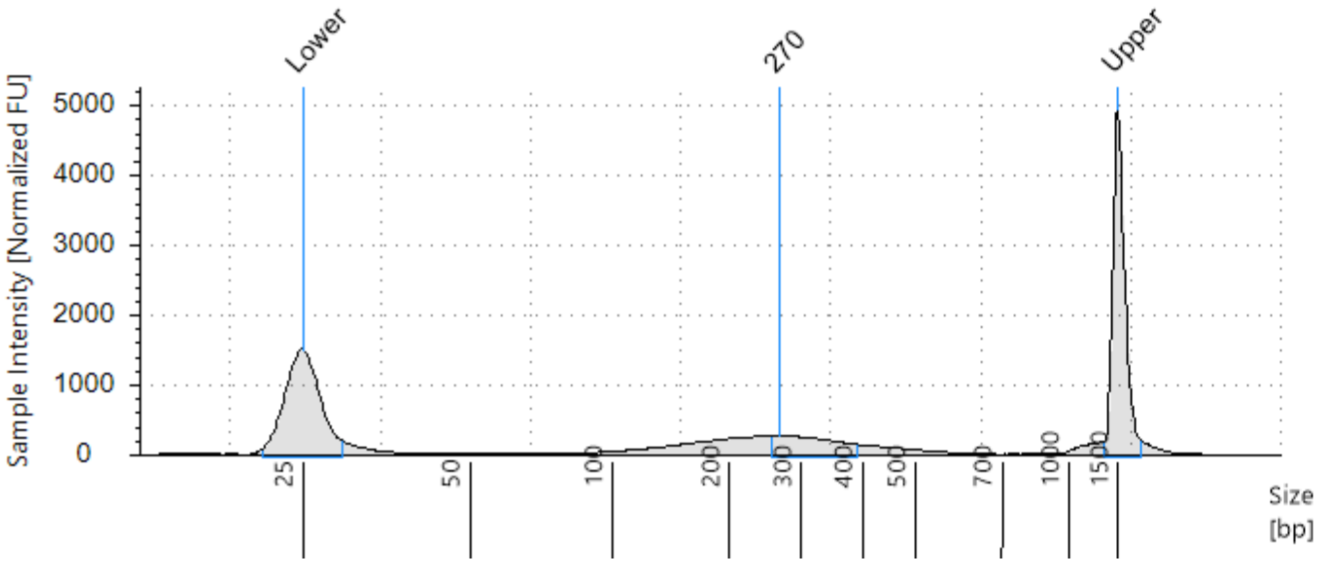

Sample Table

Well	Conc. [ng/µl]	Sample Description	Alert	Observations
D1	1.75	A3 P R2	⚠	Caution! Expired ScreenTape device

Peak Table

Size [bp]	Calibrated Conc. [ng/µl]	Assigned Conc. [ng/µl]	Peak Molarity [nmol/l]	% Integrated Area	Peak Comment	Observations
25	5.73	-	352	-		Lower Marker
270	1.75	-	9.96	100.00		
1500	6.50	6.50	6.67	-		Upper Marker

© Agilent Technologies, Inc. 2017

E1: B3 P R2

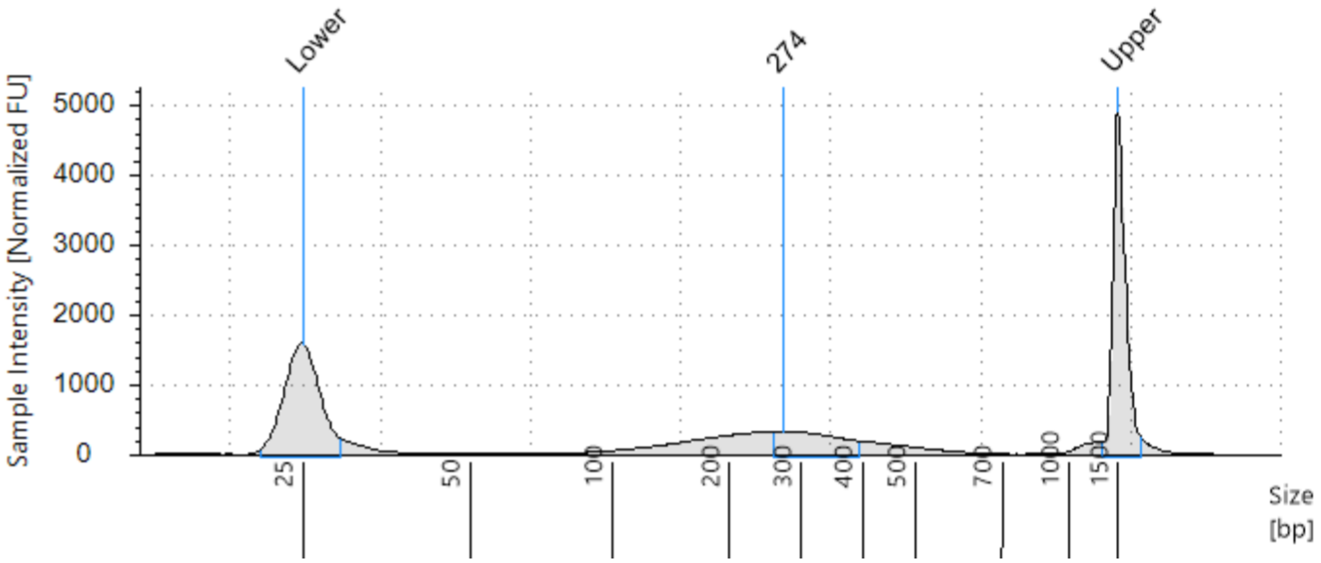

Sample Table

Well	Conc. [ng/µl]	Sample Description	Alert	Observations
E1	2.14	B3 P R2	⚠	Caution! Expired ScreenTape device

Peak Table

Size [bp]	Calibrated Conc. [ng/µl]	Assigned Conc. [ng/µl]	Peak Molarity [nmol/l]	% Integrated Area	Peak Comment	Observations
25	5.92	-	364	-		Lower Marker
274	2.14	-	12.0	100.00		
1500	6.50	6.50	6.67	-		Upper Marker

 © Agilent Technologies, Inc. 2017

F1: C3 P R2

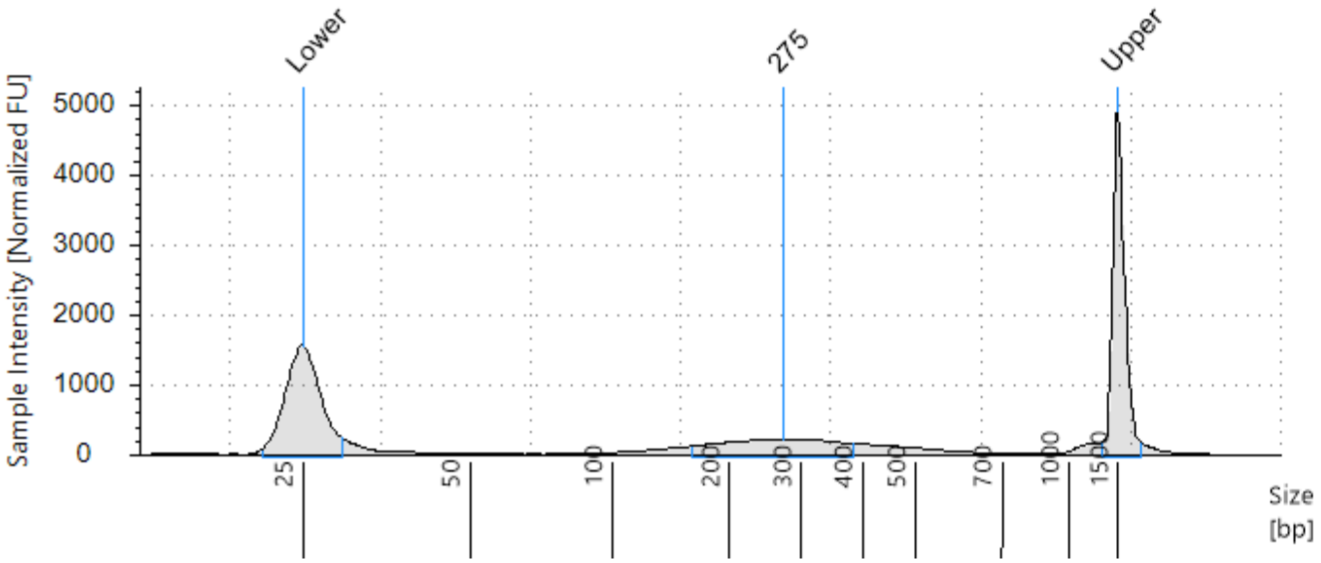

Sample Table

Well	Conc. [ng/µl]	Sample Description	Alert	Observations
F1	2.79	C3 P R2	⚠	Caution! Expired ScreenTape device

Peak Table

Size [bp]	Calibrated Conc. [ng/µl]	Assigned Conc. [ng/µl]	Peak Molarity [nmol/l]	% Integrated Area	Peak Comment	Observations
25	6.01	-	370	-		Lower Marker
275	2.79	-	15.6	100.00		
1500	6.50	6.50	6.67	-		Upper Marker

© Agilent Technologies, Inc. 2017

G1: D3 P R2

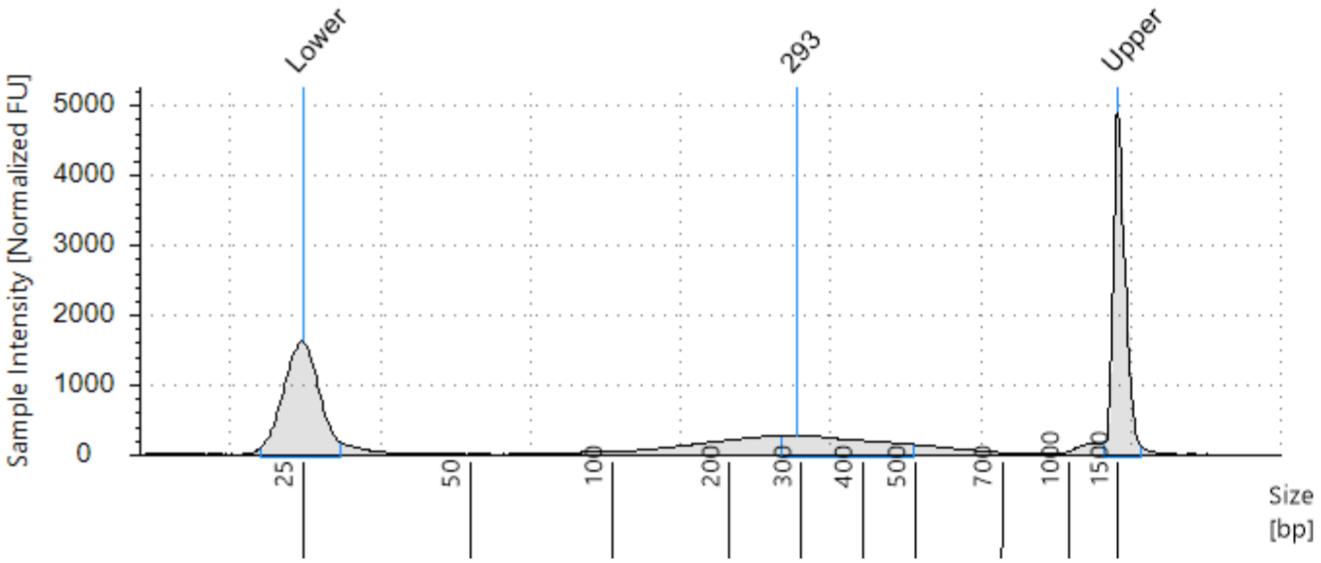

Sample Table

Well	Conc. [ng/µl]	Sample Description	Alert	Observations
G1	2.67	D3 P R2	⚠	Caution! Expired ScreenTape device

Peak Table

Size [bp]	Calibrated Conc. [ng/µl]	Assigned Conc. [ng/µl]	Peak Molarity [nmol/l]	% Integrated Area	Peak Comment	Observations
25	6.28	-	387	-		Lower Marker
293	2.67	-	14.0	100.00		
1500	6.50	6.50	6.67	-		Upper Marker

 © Agilent Technologies, Inc. 2017

H1: E3 P R2

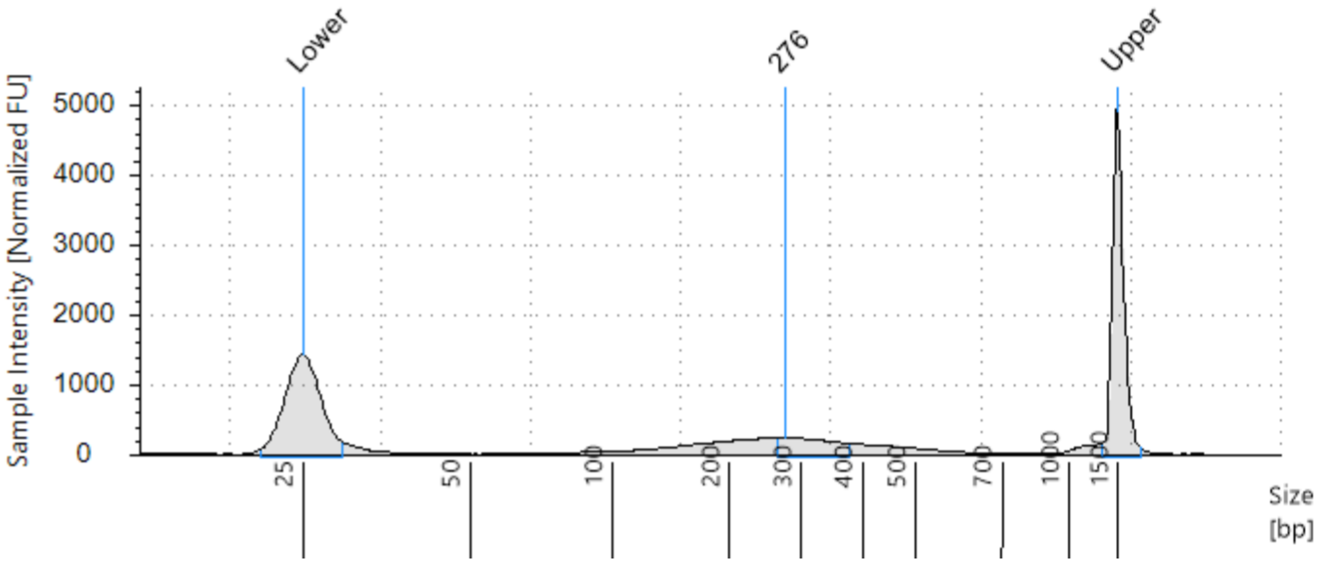

Sample Table

Well	Conc. [ng/µl]	Sample Description	Alert	Observations
H1	1.49	E3 P R2	⚠	Caution! Expired ScreenTape device

Peak Table

Size [bp]	Calibrated Conc. [ng/µl]	Assigned Conc. [ng/µl]	Peak Molarity [nmol/l]	% Integrated Area	Peak Comment	Observations
25	5.98	-	368	-		Lower Marker
276	1.49	-	8.32	100.00		
1500	6.50	6.50	6.67	-		Upper Marker

 © Agilent Technologies, Inc. 2017

A2: E1 M R1

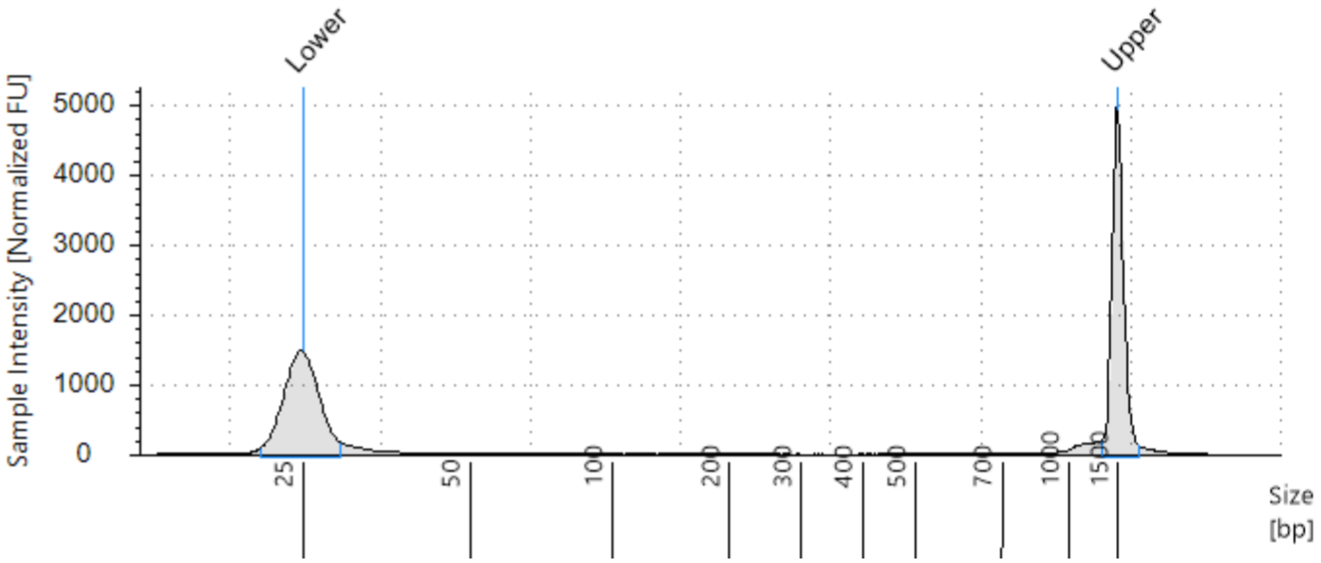

Sample Table

Well	Conc. [ng/µl]	Sample Description	Alert	Observations
A2		E1 M R1	⚠	Caution! Expired ScreenTape device

Peak Table

Size [bp]	Calibrated Conc. [ng/µl]	Assigned Conc. [ng/µl]	Peak Molarity [nmol/l]	% Integrated Area	Peak Comment	Observations
25	5.92	-	364	-		Lower Marker
1500	6.50	6.50	6.67	-		Upper Marker

B2: E6 M R1

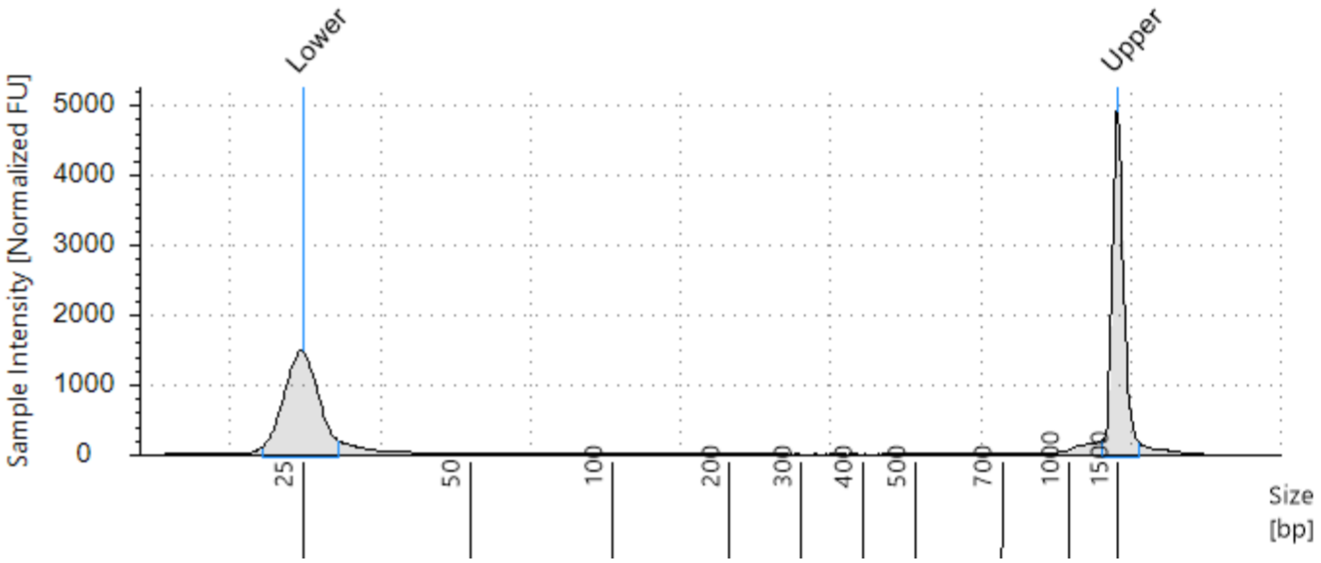

Sample Table

Well	Conc. [ng/µl]	Sample Description	Alert	Observations
B2		E6 M R1	⚠	Caution! Expired ScreenTape device

Peak Table

Size [bp]	Calibrated Conc. [ng/µl]	Assigned Conc. [ng/µl]	Peak Molarity [nmol/l]	% Integrated Area	Peak Comment	Observations
25	5.86	-	360	-		Lower Marker
1500	6.50	6.50	6.67	-		Upper Marker

© Agilent Technologies, Inc. 2017

C2: G2 M R2

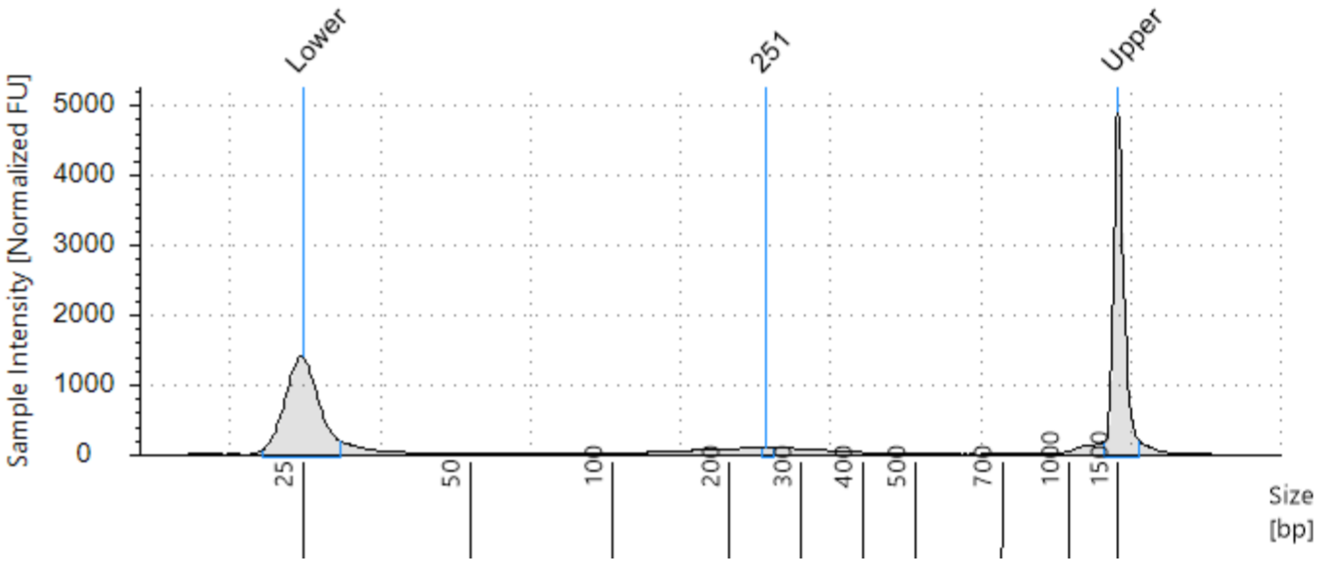

Sample Table

Well	Conc. [ng/µl]	Sample Description	Alert	Observations
C2	0.126	G2 M R2	⚠	Caution! Expired ScreenTape device

Peak Table

Size [bp]	Calibrated Conc. [ng/µl]	Assigned Conc. [ng/µl]	Peak Molarity [nmol/l]	% Integrated Area	Peak Comment	Observations
25	5.75	-	354	-		Lower Marker
251	0.126	-	0.774	100.00		
1500	6.50	6.50	6.67	-		Upper Marker

© Agilent Technologies, Inc. 2017

D2: H2 M R2

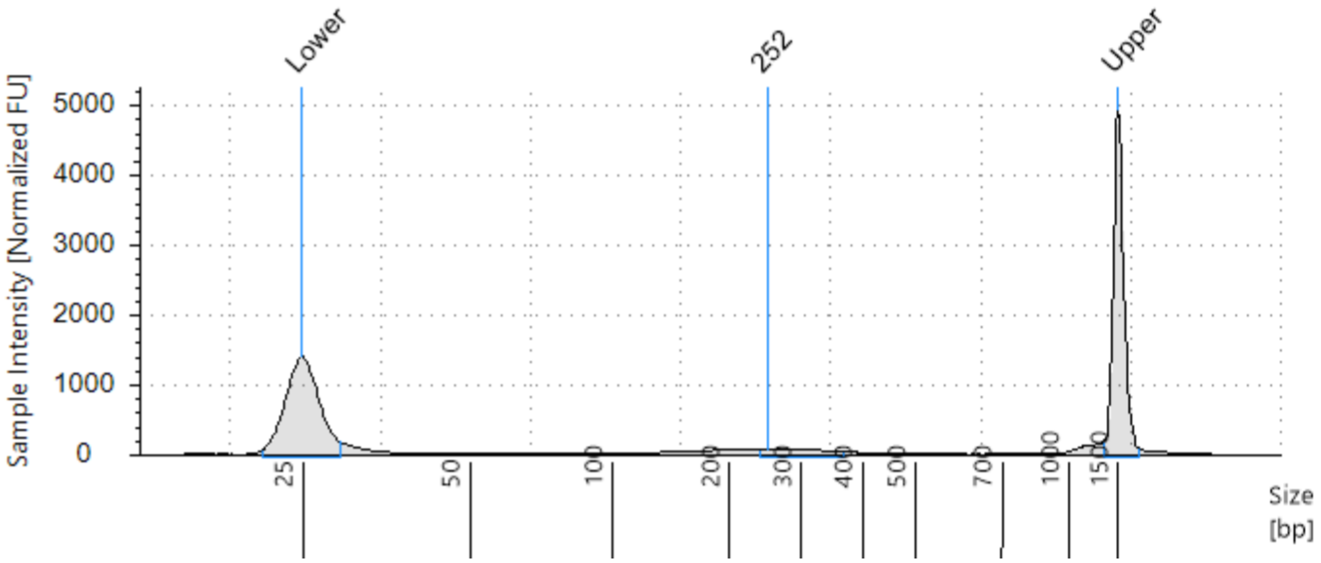

Sample Table

Well	Conc. [ng/µl]	Sample Description	Alert	Observations
D2	0.566	H2 M R2	⚠	Caution! Expired ScreenTape device

Peak Table

Size [bp]	Calibrated Conc. [ng/µl]	Assigned Conc. [ng/µl]	Peak Molarity [nmol/l]	% Integrated Area	Peak Comment	Observations
25	5.79	-	357	-		Lower Marker
252	0.566	-	3.46	100.00		
1500	6.50	6.50	6.67	-		Upper Marker

© Agilent Technologies, Inc. 2017

E2: A3 M R2

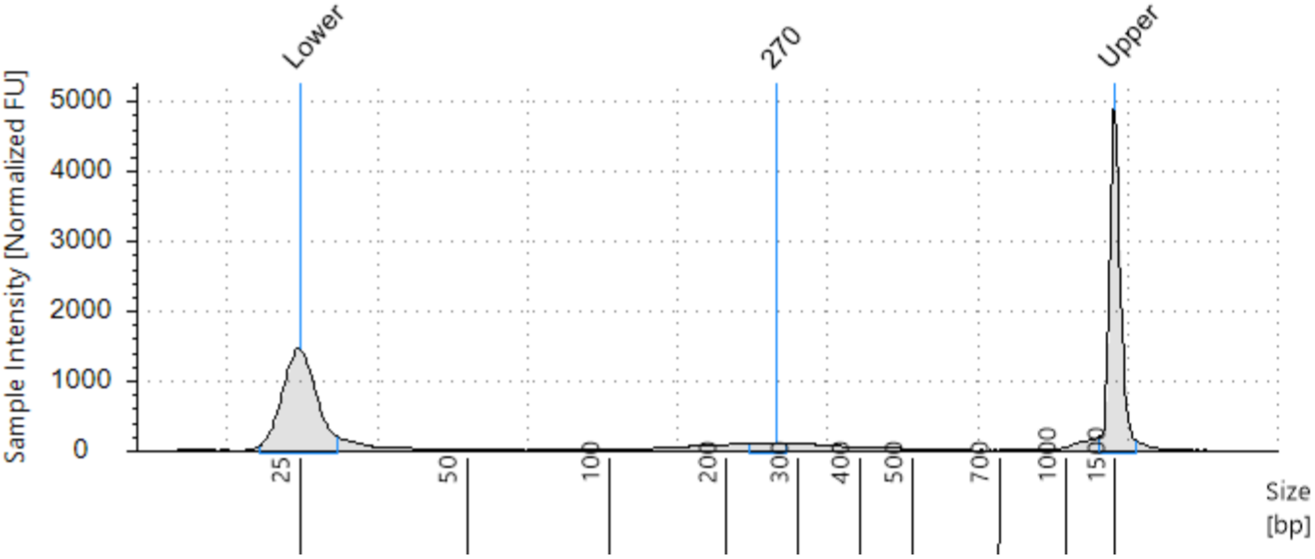

Sample Table

Well	Conc. [ng/µl]	Sample Description	Alert	Observations
E2	0.388	A3 M R2	⚠	Caution! Expired ScreenTape device

Peak Table

Size [bp]	Calibrated Conc. [ng/µl]	Assigned Conc. [ng/µl]	Peak Molarity [nmol/l]	% Integrated Area	Peak Comment	Observations
25	5.98	-	368	-		Lower Marker
270	0.388	-	2.21	100.00		
1500	6.50	6.50	6.67	-		Upper Marker

© Agilent Technologies, Inc. 2017

F2: B3 M R2

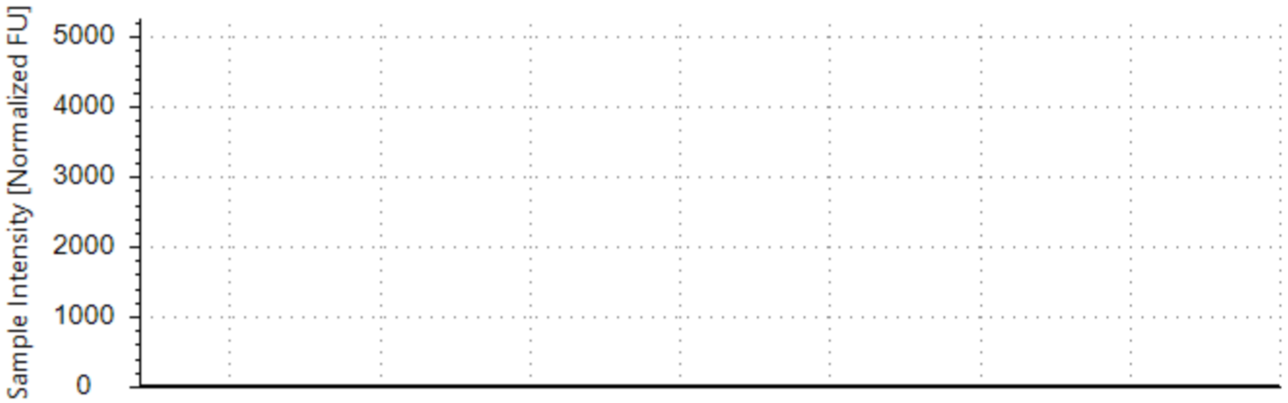

Sample Table

Well	Conc. [ng/µl]	Sample Description	Alert	Observations
F2		B3 M R2	▲	Marker(s) not detected; Caution! Expired ScreenTape device

 © Agilent Technologies, Inc. 2017

G2: C3 M R2

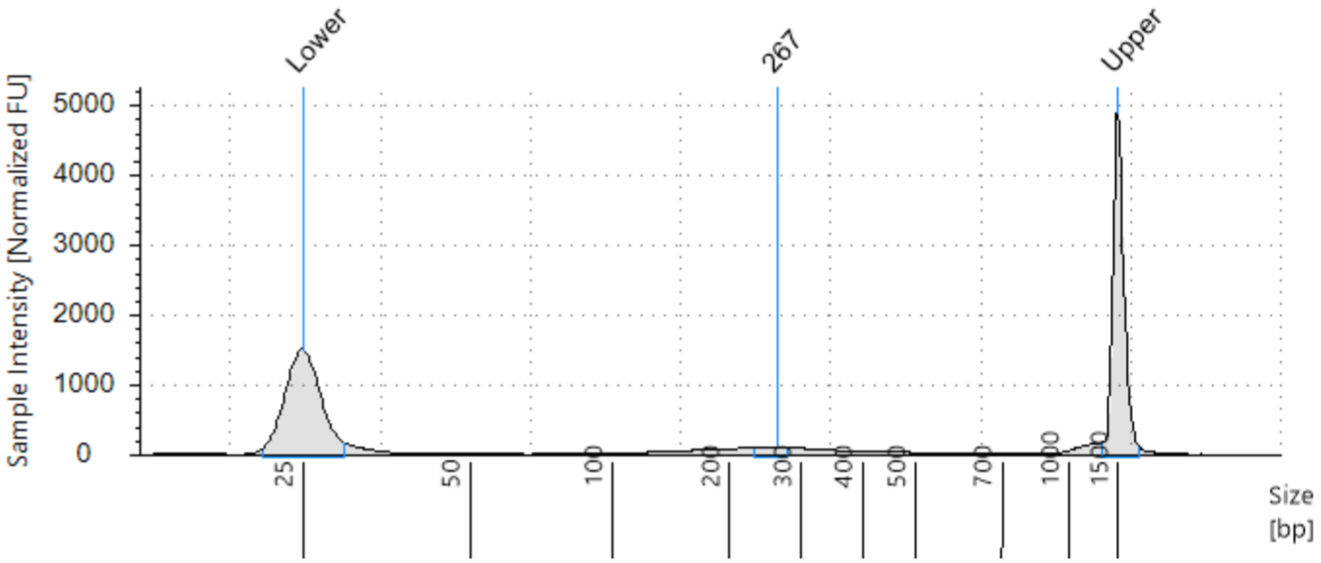

Sample Table

Well	Conc. [ng/µl]	Sample Description	Alert	Observations
G2	0.309	C3 M R2	⚠	Caution! Expired ScreenTape device

Peak Table

Size [bp]	Calibrated Conc. [ng/µl]	Assigned Conc. [ng/µl]	Peak Molarity [nmol/l]	% Integrated Area	Peak Comment	Observations
25	6.34	-	390	-		Lower Marker
267	0.309	-	1.78	100.00		
1500	6.50	6.50	6.67	-		Upper Marker

© Agilent Technologies, Inc. 2017

H2: D3 M R2

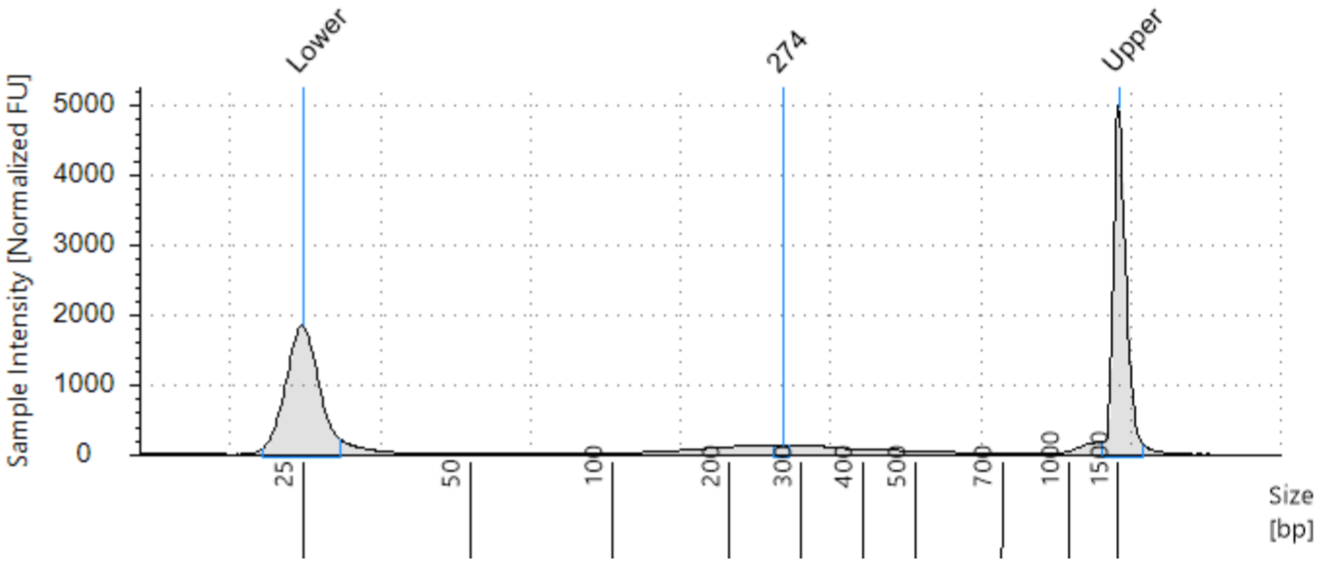

Sample Table

Well	Conc. [ng/µl]	Sample Description	Alert	Observations
H2	0.185	D3 M R2	⚠	Caution! Expired ScreenTape device

Peak Table

Size [bp]	Calibrated Conc. [ng/µl]	Assigned Conc. [ng/µl]	Peak Molarity [nmol/l]	% Integrated Area	Peak Comment	Observations
25	6.26	-	385	-		Lower Marker
274	0.185	-	1.04	100.00		
1500	6.50	6.50	6.67	-		Upper Marker

 © Agilent Technologies, Inc. 2017

Filename: 2020-09-11-02- Q-S, plus, D5-D7, R2.D1000

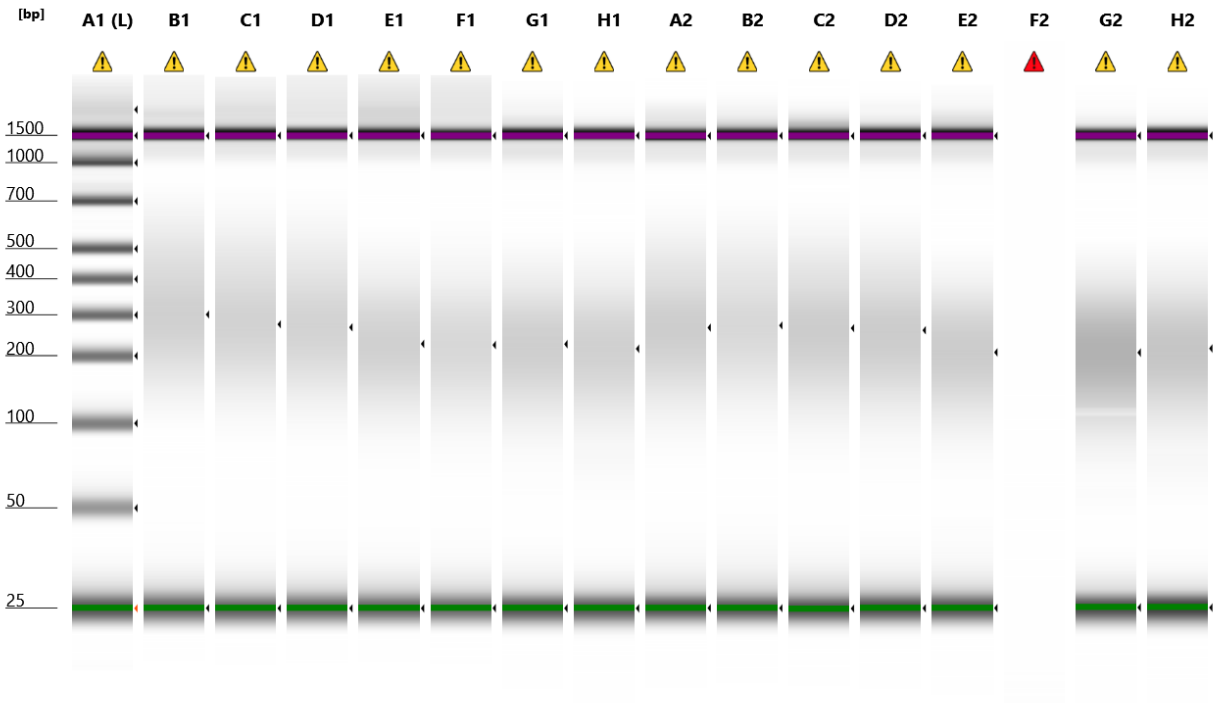

Default image (Contrast 100%)

Sample Info

Well	Conc. [ng/µl]	Sample Description	Alert	Observations
A1	16.9	Ladder	⚠	Caution! Expired ScreenTape device; Issue with ladder peak detection (too many peaks detected); Ladder
B1	0.643	D5 P R2	⚠	Caution! Expired ScreenTape device
C1	0.535	E5 P R2	⚠	Caution! Expired ScreenTape device
D1	1.99	F5 P R2	⚠	Caution! Expired ScreenTape device
E1	0.322	G5 P R2	⚠	Caution! Expired ScreenTape device
F1	0.466	H5 P R2	⚠	Caution! Expired ScreenTape device
G1	0.660	A6 P R2	⚠	Caution! Expired ScreenTape device
H1	2.37	B6 P R2	⚠	Caution! Expired ScreenTape device
A2	0.648	C6 P R2	⚠	Caution! Expired ScreenTape device
B2	0.541	D6 P R2	⚠	Caution! Expired ScreenTape device
C2	4.24	E6 P R2	⚠	Caution! Expired ScreenTape device
D2	4.17	F6 P R2	⚠	Caution! Expired ScreenTape device
E2	2.46	G6 P R2	⚠	Caution! Expired ScreenTape device
F2			⚠	Marker(s) not detected; Caution! Expired ScreenTape device
G2	4.25	H6 P R2	⚠	Caution! Expired ScreenTape device
H2	2.72	A7 P R2	⚠	Caution! Expired ScreenTape device

© Agilent Technologies, Inc. 2017

A1: Ladder

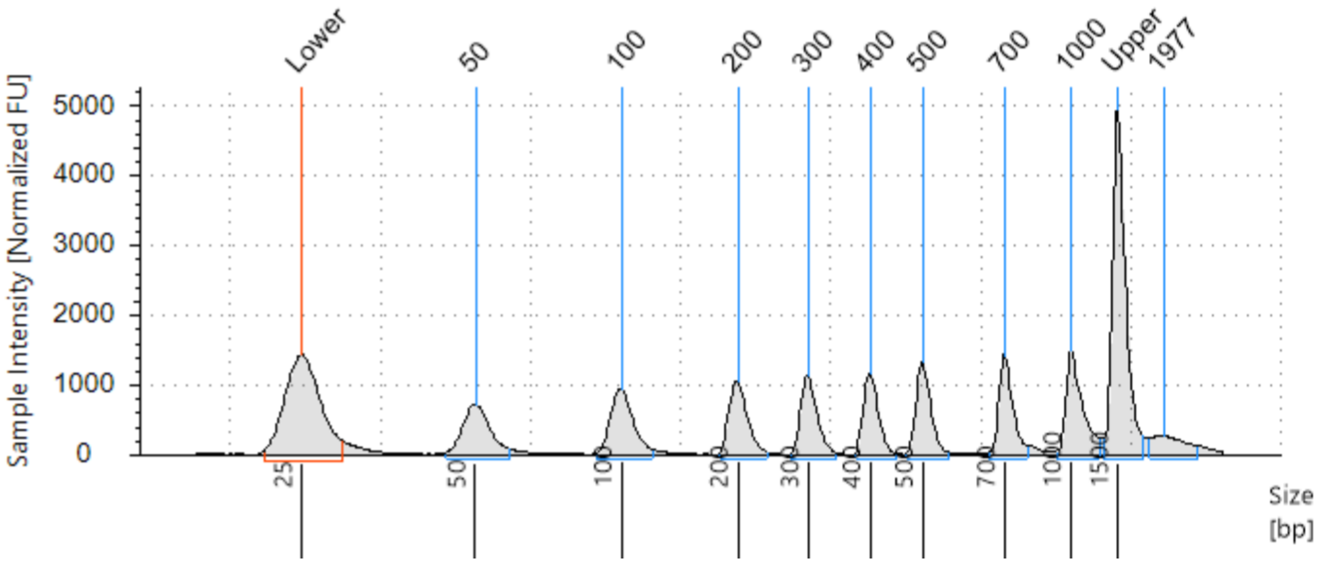

Sample Table

Well	Conc. [ng/µl]	Sample Description	Alert	Observations
A1	16.9	Ladder	⚠	Caution! Expired ScreenTape device; Issue with ladder peak detection (too many peaks detected); Ladder

Peak Table

Size [bp]	Calibrated Conc. [ng/µl]	Assigned Conc. [ng/µl]	Peak Molarity [nmol/l]	% Integrated Area	Peak Comment	Observations
25	5.01	-	308	-		Lower Marker
50	1.93	-	59.5	11.48		
100	2.13	-	32.7	12.62		
200	1.95	-	15.0	11.54		
300	1.88	-	9.62	11.12		
400	1.77	-	6.81	10.50		
500	1.94	-	5.98	11.53		
700	1.98	-	4.34	11.72		
1000	2.40	-	3.69	14.24		
1500	6.50	6.50	6.67	-		Upper Marker
1977	0.886	-	0.689	5.25		

© Agilent Technologies, Inc. 2017

B1: D5 P R2

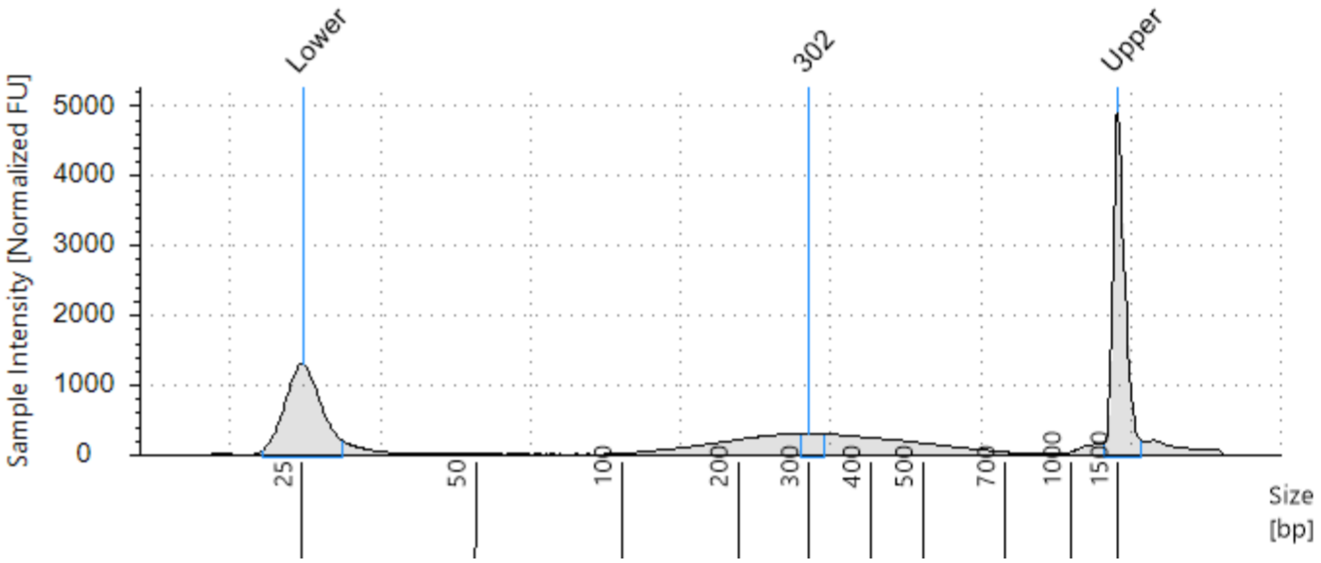

Sample Table

Well	Conc. [ng/µl]	Sample Description	Alert	Observations
B1	0.643	D5 P R2	⚠	Caution! Expired ScreenTape device

Peak Table

Size [bp]	Calibrated Conc. [ng/µl]	Assigned Conc. [ng/µl]	Peak Molarity [nmol/l]	% Integrated Area	Peak Comment	Observations
25	5.13	-	316	-		Lower Marker
302	0.643	-	3.28	100.00		
1500	6.50	6.50	6.67	-		Upper Marker

© Agilent Technologies, Inc. 2017

C1: E5 P R2

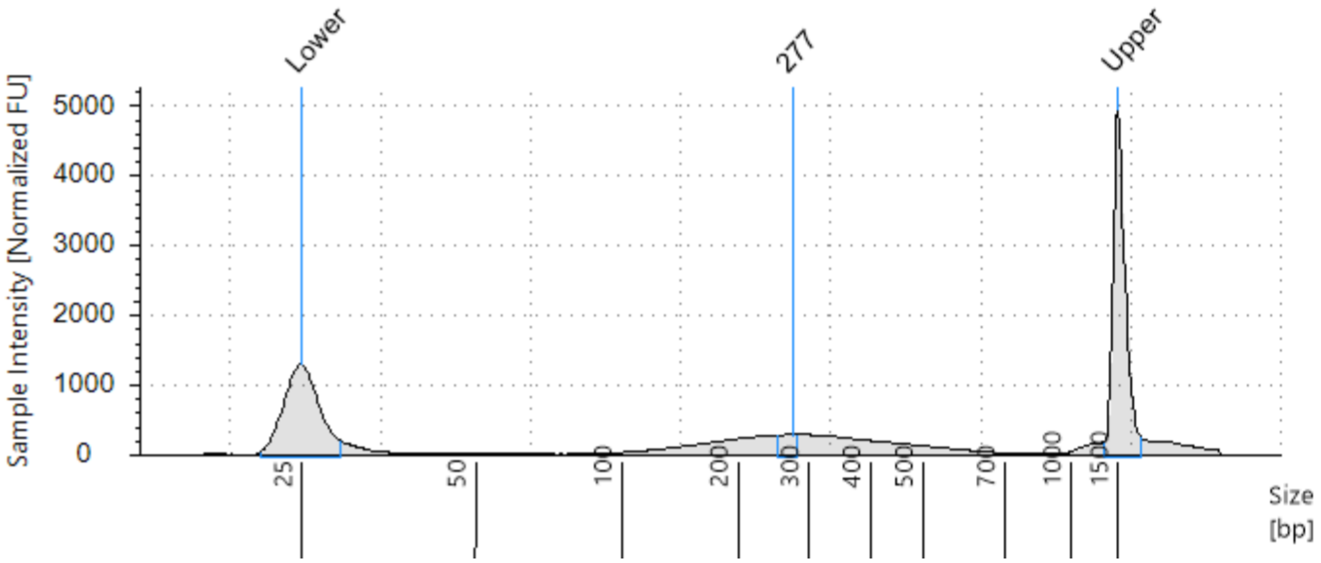

Sample Table

Well	Conc. [ng/µl]	Sample Description	Alert	Observations
C1	0.535	E5 P R2	⚠	Caution! Expired ScreenTape device

Peak Table

Size [bp]	Calibrated Conc. [ng/µl]	Assigned Conc. [ng/µl]	Peak Molarity [nmol/l]	% Integrated Area	Peak Comment	Observations
25	4.86	-	299	-		Lower Marker
277	0.535	-	2.97	100.00		
1500	6.50	6.50	6.67	-		Upper Marker

© Agilent Technologies, Inc. 2017

D1: F5 P R2

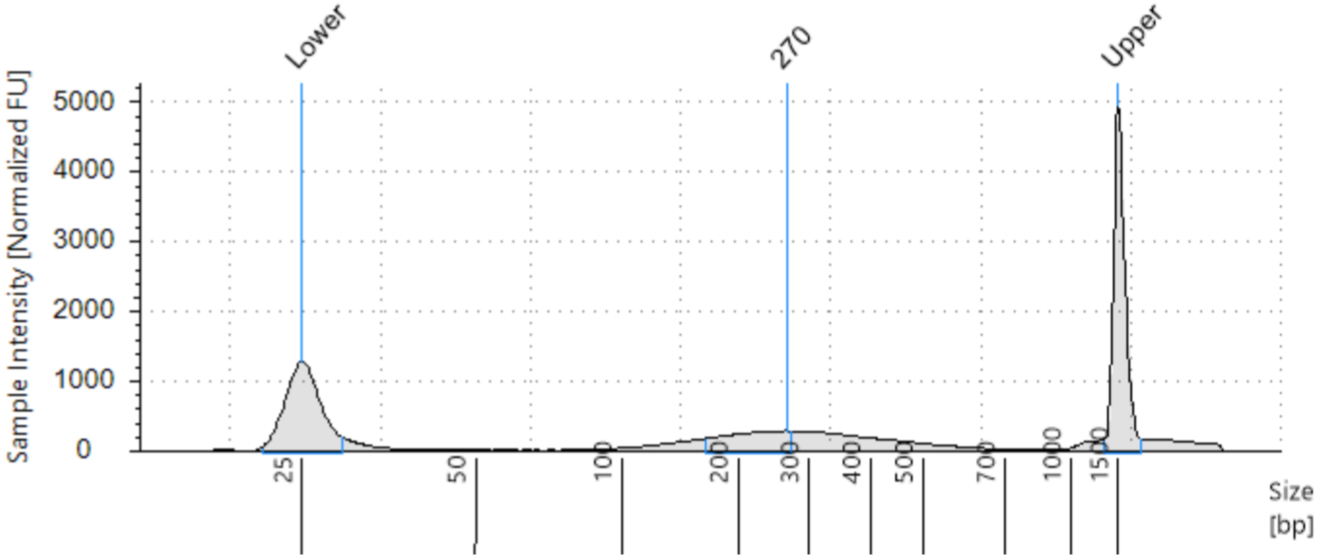

Sample Table

Well	Conc. [ng/µl]	Sample Description	Alert	Observations
D1	1.99	F5 P R2	⚠	Caution! Expired ScreenTape device

Peak Table

Size [bp]	Calibrated Conc. [ng/µl]	Assigned Conc. [ng/µl]	Peak Molarity [nmol/l]	% Integrated Area	Peak Comment	Observations
25	5.03	-	310	-		Lower Marker
270	1.99	-	11.3	100.00		
1500	6.50	6.50	6.67	-		Upper Marker

© Agilent Technologies, Inc. 2017

E1: G5 P R2

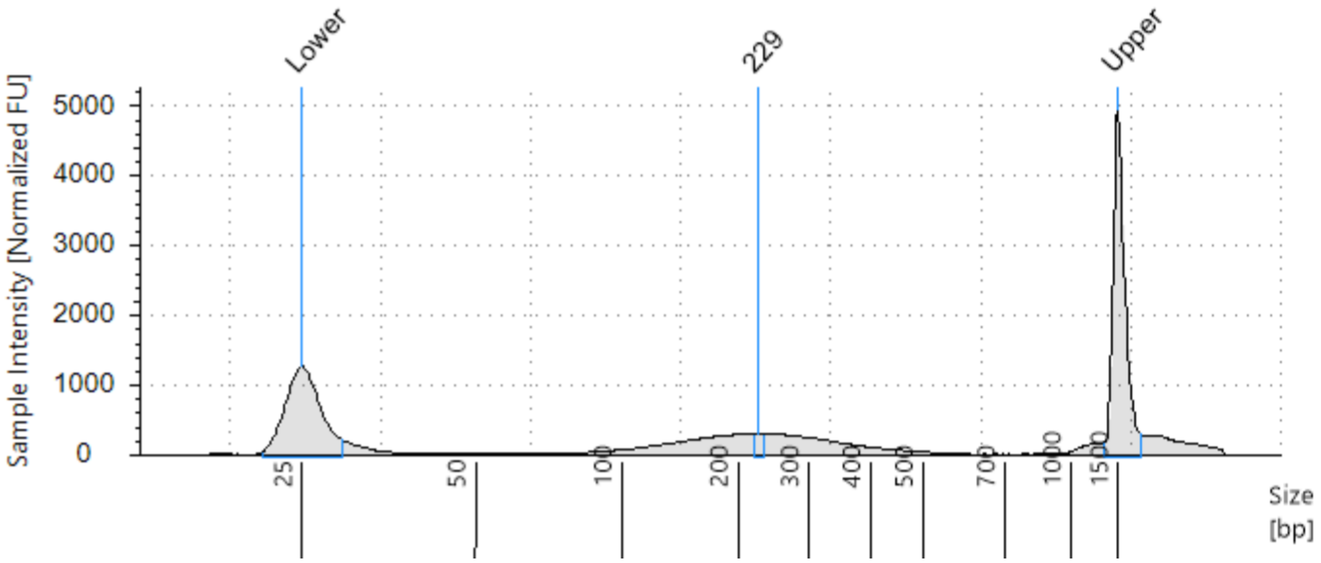

Sample Table

Well	Conc. [ng/µl]	Sample Description	Alert	Observations
E1	0.322	G5 P R2	⚠	Caution! Expired ScreenTape device

Peak Table

Size [bp]	Calibrated Conc. [ng/µl]	Assigned Conc. [ng/µl]	Peak Molarity [nmol/l]	% Integrated Area	Peak Comment	Observations
25	4.87	-	300	-		Lower Marker
229	0.322	-	2.16	100.00		
1500	6.50	6.50	6.67	-		Upper Marker

© Agilent Technologies, Inc. 2017

F1: H5 P R2

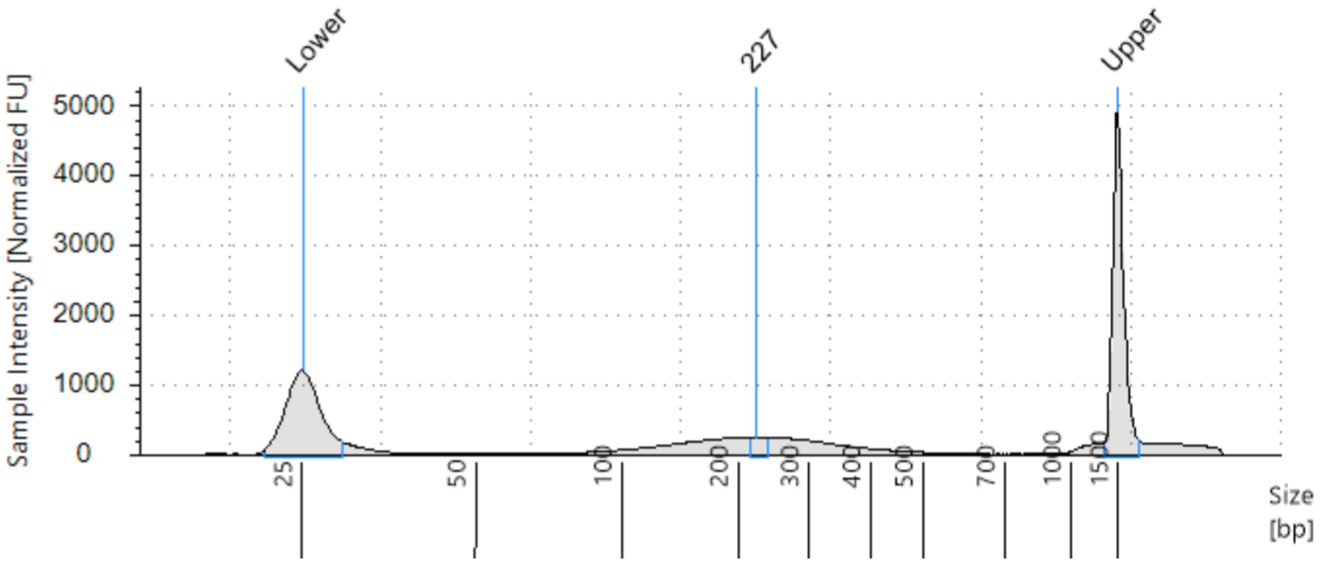

Sample Table

Well	Conc. [ng/µl]	Sample Description	Alert	Observations
F1	0.466	H5 P R2	⚠	Caution! Expired ScreenTape device

Peak Table

Size [bp]	Calibrated Conc. [ng/µl]	Assigned Conc. [ng/µl]	Peak Molarity [nmol/l]	% Integrated Area	Peak Comment	Observations
25	4.96	-	305	-		Lower Marker
227	0.466	-	3.17	100.00		
1500	6.50	6.50	6.67	-		Upper Marker

 © Agilent Technologies, Inc. 2017

G1: A6 P R2

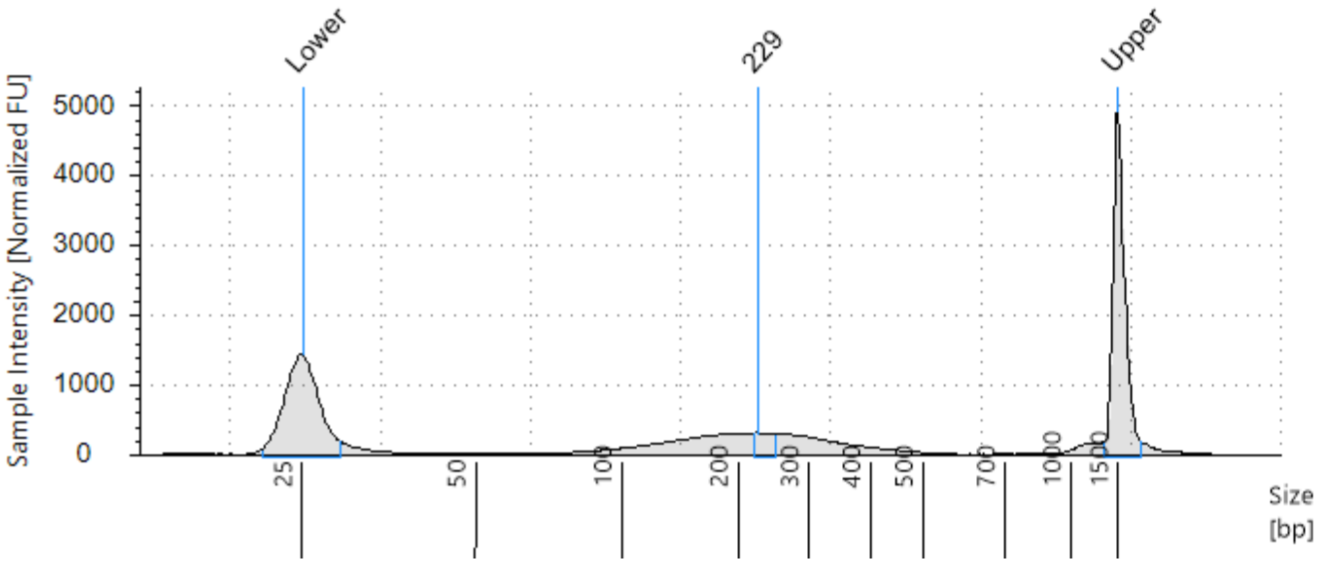

Sample Table

Well	Conc. [ng/µl]	Sample Description	Alert	Observations
G1	0.660	A6 P R2	⚠	Caution! Expired ScreenTape device

Peak Table

Size [bp]	Calibrated Conc. [ng/µl]	Assigned Conc. [ng/µl]	Peak Molarity [nmol/l]	% Integrated Area	Peak Comment	Observations
25	5.50	-	338	-		Lower Marker
229	0.660	-	4.44	100.00		
1500	6.50	6.50	6.67	-		Upper Marker

© Agilent Technologies, Inc. 2017

H1: B6 P R2

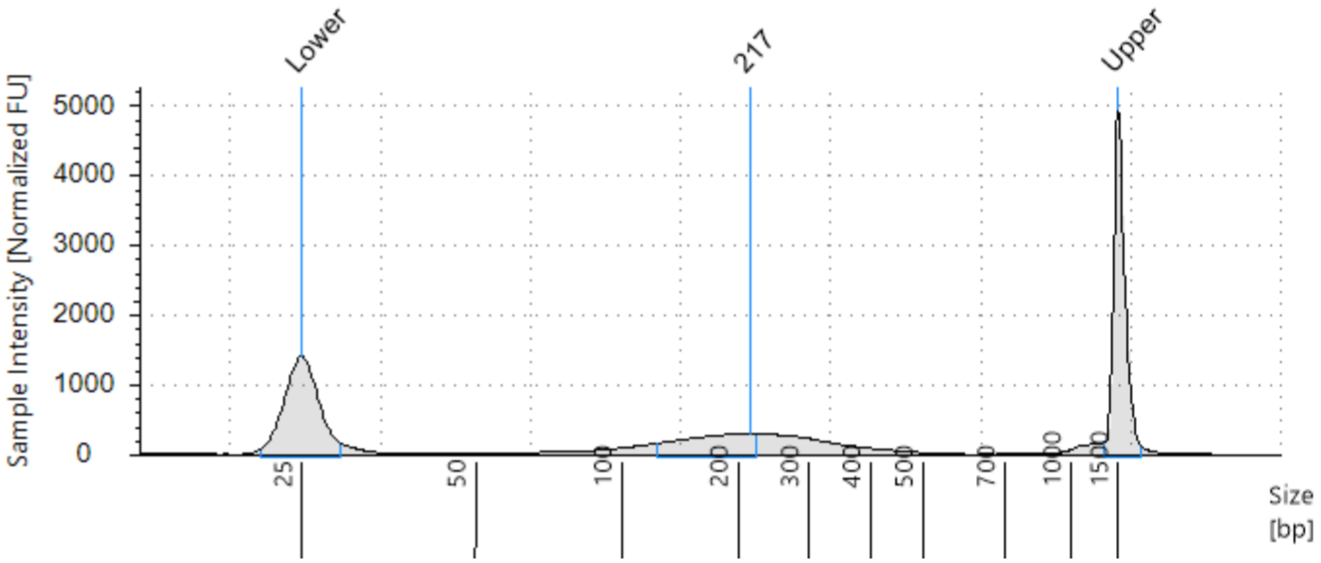

Sample Table

Well	Conc. [ng/µl]	Sample Description	Alert	Observations
H1	2.37	B6 P R2	⚠	Caution! Expired ScreenTape device

Peak Table

Size [bp]	Calibrated Conc. [ng/µl]	Assigned Conc. [ng/µl]	Peak Molarity [nmol/l]	% Integrated Area	Peak Comment	Observations
25	5.59	-	344	-		Lower Marker
217	2.37	-	16.8	100.00		
1500	6.50	6.50	6.67	-		Upper Marker

© Agilent Technologies, Inc. 2017

A2: C6 P R2

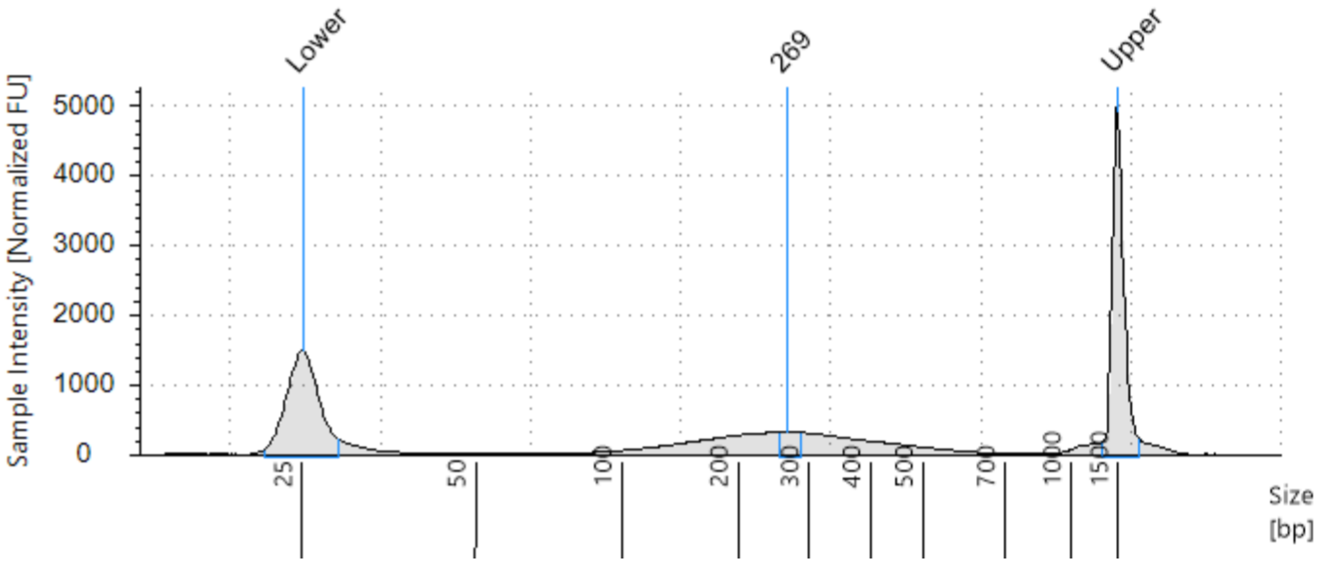

Sample Table

Well	Conc. [ng/µl]	Sample Description	Alert	Observations
A2	0.648	C6 P R2	⚠	Caution! Expired ScreenTape device

Peak Table

Size [bp]	Calibrated Conc. [ng/µl]	Assigned Conc. [ng/µl]	Peak Molarity [nmol/l]	% Integrated Area	Peak Comment	Observations
25	5.34	-	329	-		Lower Marker
269	0.648	-	3.70	100.00		
1500	6.50	6.50	6.67	-		Upper Marker

© Agilent Technologies, Inc. 2017

B2: D6 P R2

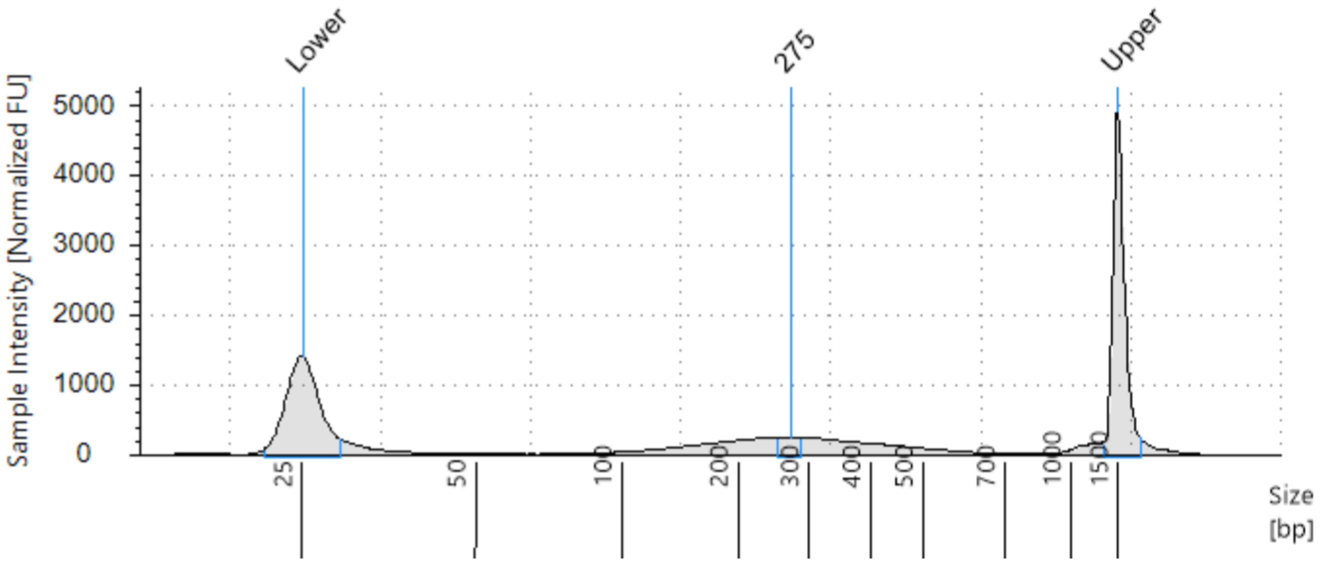

Sample Table

Well	Conc. [ng/µl]	Sample Description	Alert	Observations
B2	0.541	D6 P R2	⚠	Caution! Expired ScreenTape device

Peak Table

Size [bp]	Calibrated Conc. [ng/µl]	Assigned Conc. [ng/µl]	Peak Molarity [nmol/l]	% Integrated Area	Peak Comment	Observations
25	5.27	-	325	-		Lower Marker
275	0.541	-	3.03	100.00		
1500	6.50	6.50	6.67	-		Upper Marker

© Agilent Technologies, Inc. 2017

C2: E6 P R2

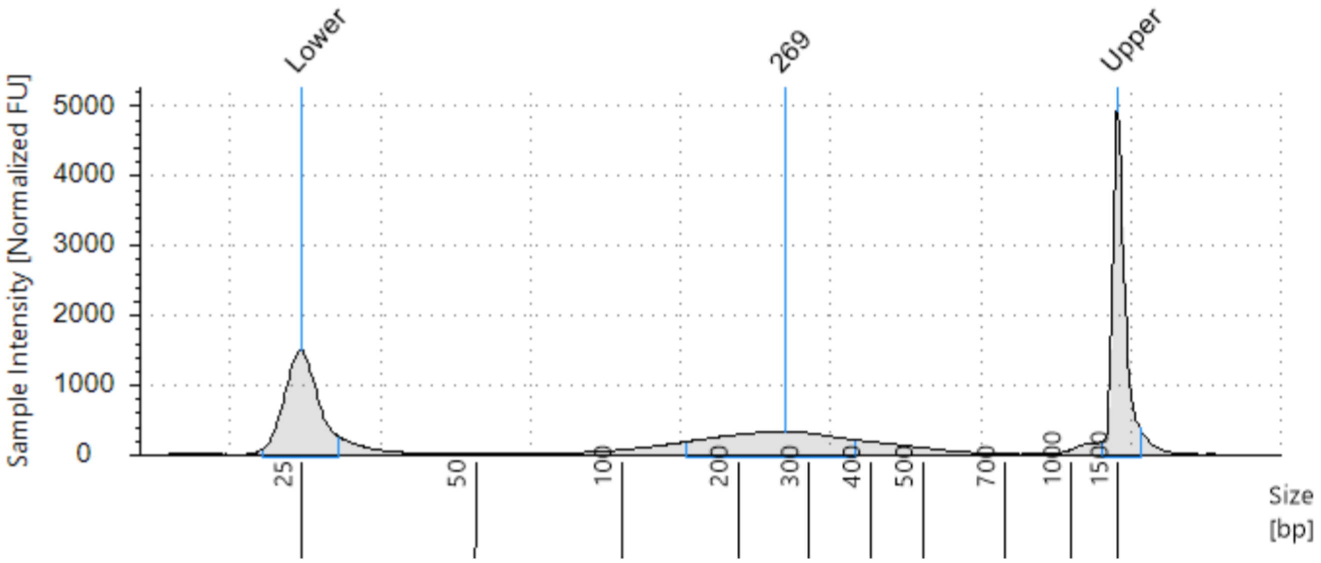

Sample Table

Well	Conc. [ng/µl]	Sample Description	Alert	Observations
C2	4.24	E6 P R2	⚠	Caution! Expired ScreenTape device

Peak Table

Size [bp]	Calibrated Conc. [ng/µl]	Assigned Conc. [ng/µl]	Peak Molarity [nmol/l]	% Integrated Area	Peak Comment	Observations
25	5.39	-	332	-		Lower Marker
269	4.24	-	24.3	100.00		
1500	6.50	6.50	6.67	-		Upper Marker

© Agilent Technologies, Inc. 2017

D2: F6 P R2

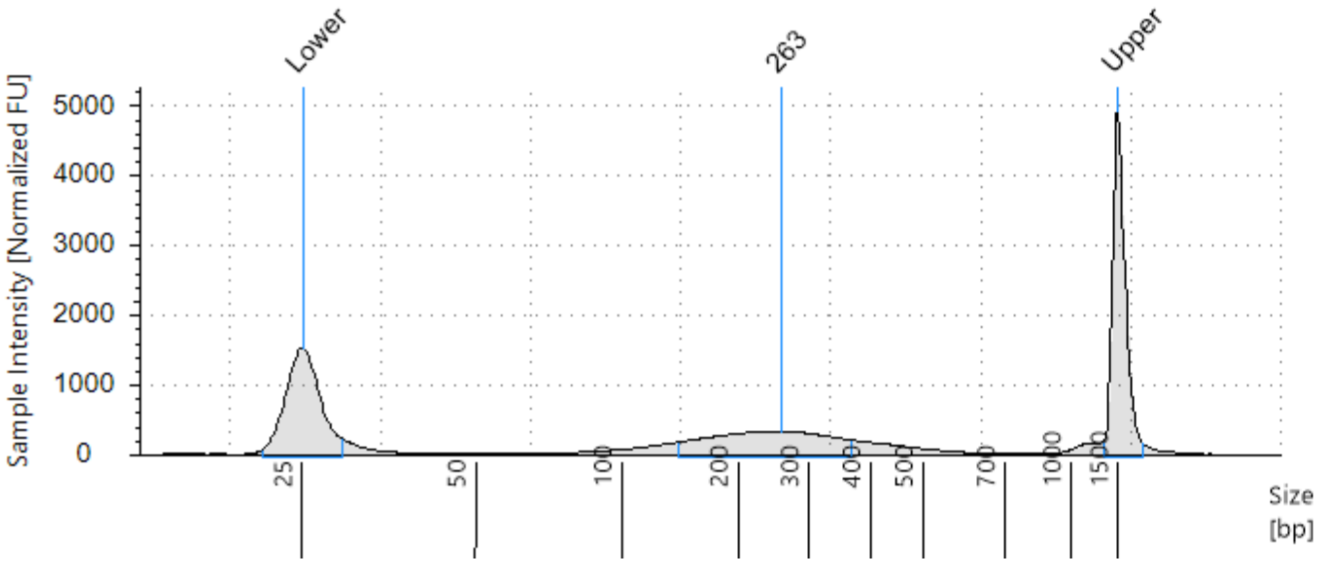

Sample Table

Well	Conc. [ng/µl]	Sample Description	Alert	Observations
D2	4.17	F6 P R2	⚠	Caution! Expired ScreenTape device

Peak Table

Size [bp]	Calibrated Conc. [ng/µl]	Assigned Conc. [ng/µl]	Peak Molarity [nmol/l]	% Integrated Area	Peak Comment	Observations
25	5.39	-	332	-		Lower Marker
263	4.17	-	24.4	100.00		
1500	6.50	6.50	6.67	-		Upper Marker

© Agilent Technologies, Inc. 2017

E2: G6 P R2

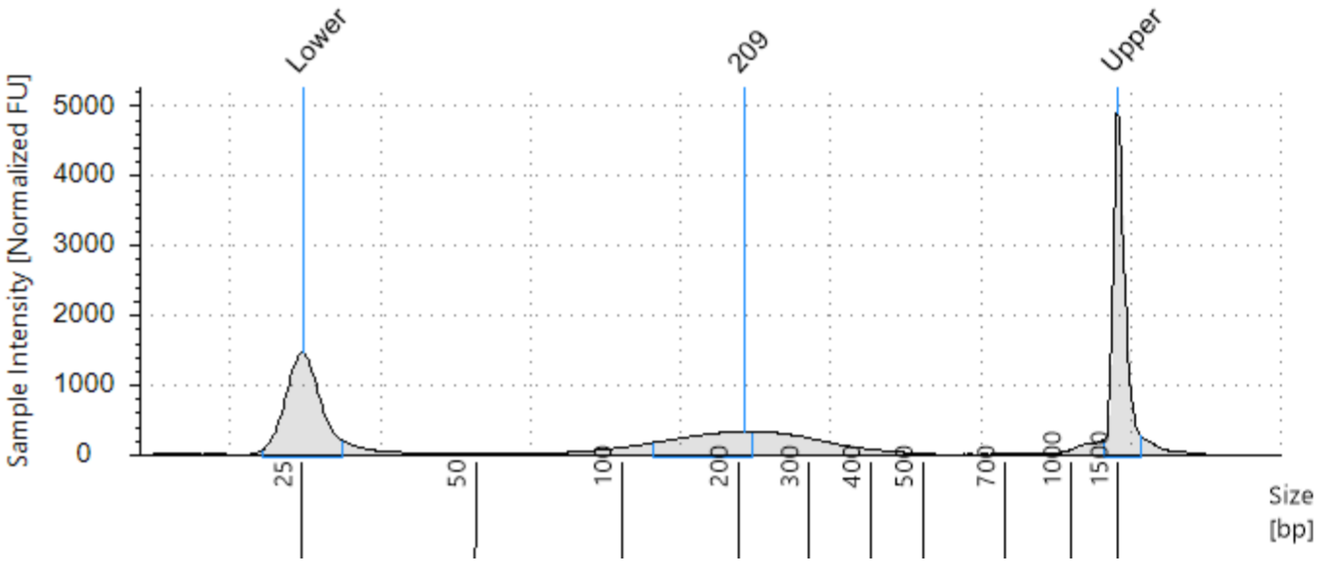

Sample Table

Well	Conc. [ng/µl]	Sample Description	Alert	Observations
E2	2.46	G6 P R2	⚠	Caution! Expired ScreenTape device

Peak Table

Size [bp]	Calibrated Conc. [ng/µl]	Assigned Conc. [ng/µl]	Peak Molarity [nmol/l]	% Integrated Area	Peak Comment	Observations
25	5.48	-	337	-		Lower Marker
209	2.46	-	18.1	100.00		
1500	6.50	6.50	6.67	-		Upper Marker

© Agilent Technologies, Inc. 2017

F2

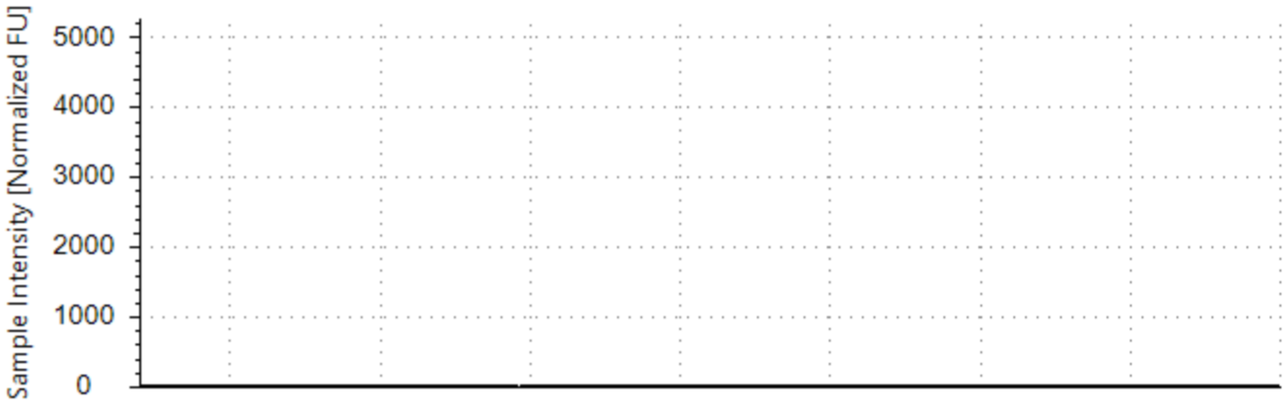

Sample Table

Well	Conc. [ng/µl]	Sample Description	Alert	Observations
F2			▲	Marker(s) not detected; Caution! Expired ScreenTape device

 © Agilent Technologies, Inc. 2017

G2: H6 P R2

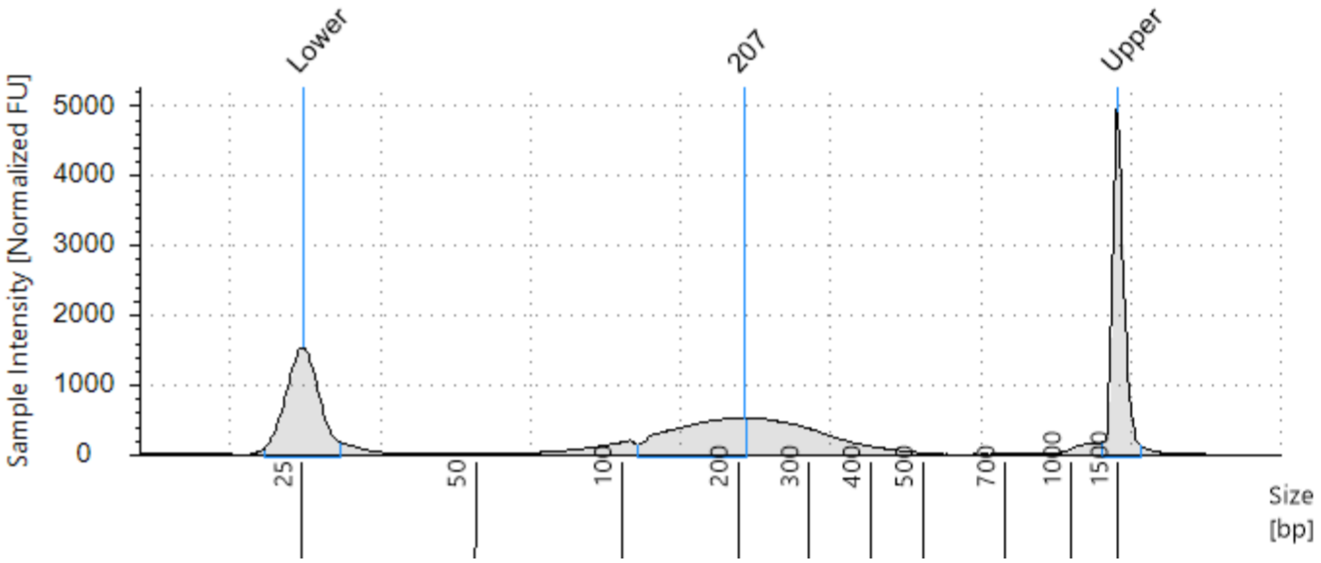

Sample Table

Well	Conc. [ng/µl]	Sample Description	Alert	Observations
G2	4.25	H6 P R2	⚠	Caution! Expired ScreenTape device

Peak Table

Size [bp]	Calibrated Conc. [ng/µl]	Assigned Conc. [ng/µl]	Peak Molarity [nmol/l]	% Integrated Area	Peak Comment	Observations
25	5.79	-	356	-		Lower Marker
207	4.25	-	31.5	100.00		
1500	6.50	6.50	6.67	-		Upper Marker

H2: A7 P R2

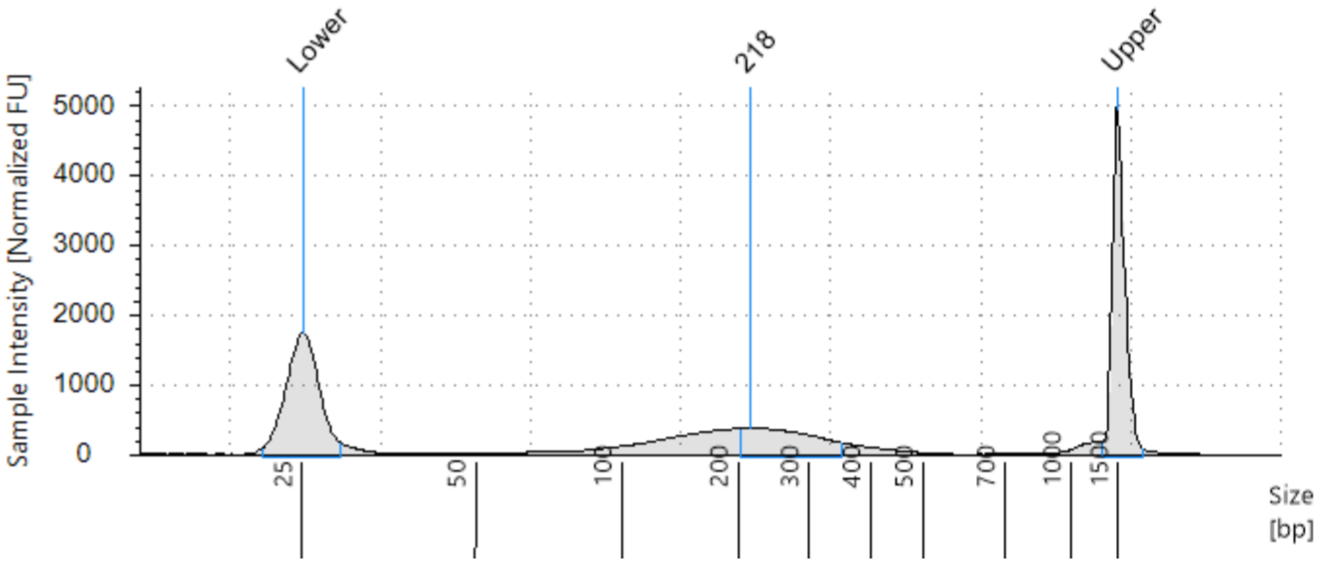

Sample Table

Well	Conc. [ng/µl]	Sample Description	Alert	Observations
H2	2.72	A7 P R2	⚠	Caution! Expired ScreenTape device

Peak Table

Size [bp]	Calibrated Conc. [ng/µl]	Assigned Conc. [ng/µl]	Peak Molarity [nmol/l]	% Integrated Area	Peak Comment	Observations
25	5.97	-	368	-		Lower Marker
218	2.72	-	19.3	100.00		
1500	6.50	6.50	6.67	-		Upper Marker

© Agilent Technologies, Inc. 2017

Filename: 2020-09-11-02- Q-S, plus, F3-C5, R2.D1000

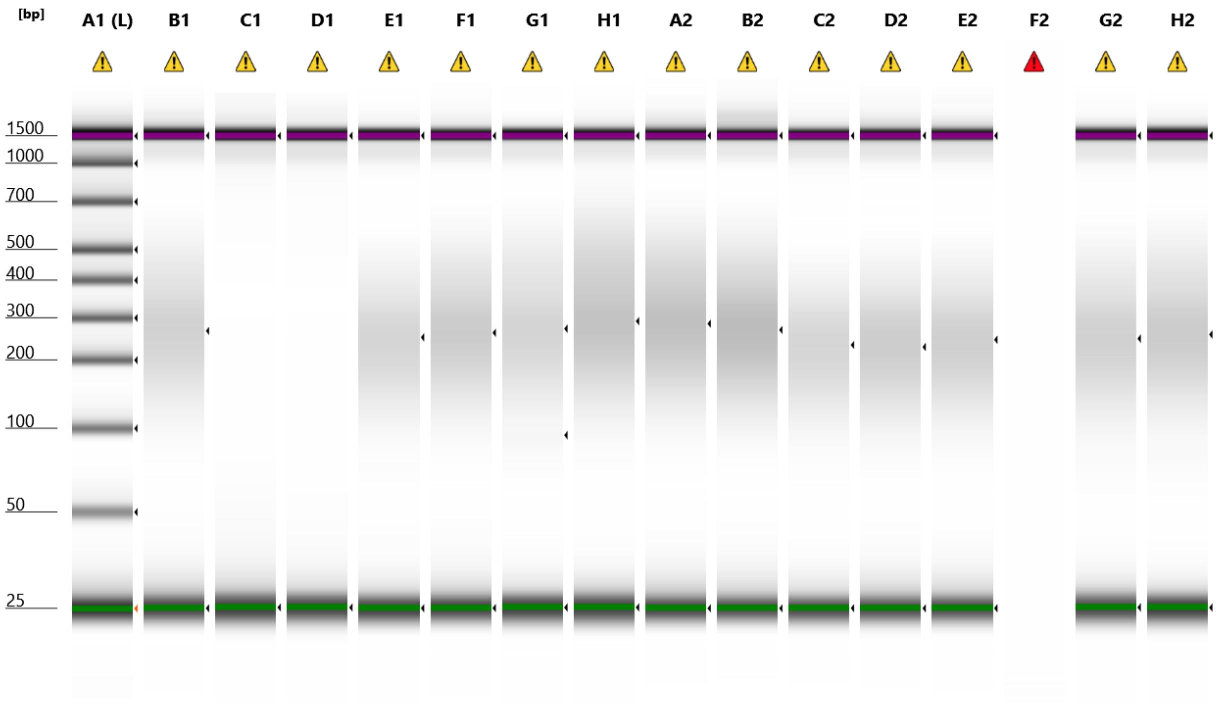

Default image (Contrast 100%)

Sample Info

Well	Conc. [ng/µl]	Sample Description	Alert	Observations
A1	13.9	Ladder	⚠	Caution! Expired ScreenTape device; Ladder
B1	2.06	F3 P R2	⚠	Caution! Expired ScreenTape device
C1		G3 P R2	⚠	Caution! Expired ScreenTape device
D1		H3 P R2	⚠	Caution! Expired ScreenTape device
E1	1.64	A4 P R2	⚠	Caution! Expired ScreenTape device
F1	0.727	B4 P R2	⚠	Caution! Expired ScreenTape device
G1	3.63	C4 P R2	⚠	Caution! Expired ScreenTape device
H1	0.527	D4 P R2	⚠	Caution! Expired ScreenTape device
A2	0.871	E4 P R2	⚠	Caution! Expired ScreenTape device
B2	3.40	F4 P R2	⚠	Caution! Expired ScreenTape device
C2	1.92	G4 P R2	⚠	Caution! Expired ScreenTape device
D2	2.35	H4 P R2	⚠	Caution! Expired ScreenTape device
E2	2.03	A5 P R2	⚠	Caution! Expired ScreenTape device
F2			⚠	Marker(s) not detected; Caution! Expired ScreenTape device
G2	0.547	B5 P R2	⚠	Caution! Expired ScreenTape device
H2	2.60	C5 P R2	⚠	Caution! Expired ScreenTape device

© Agilent Technologies, Inc. 2017

A1: Ladder

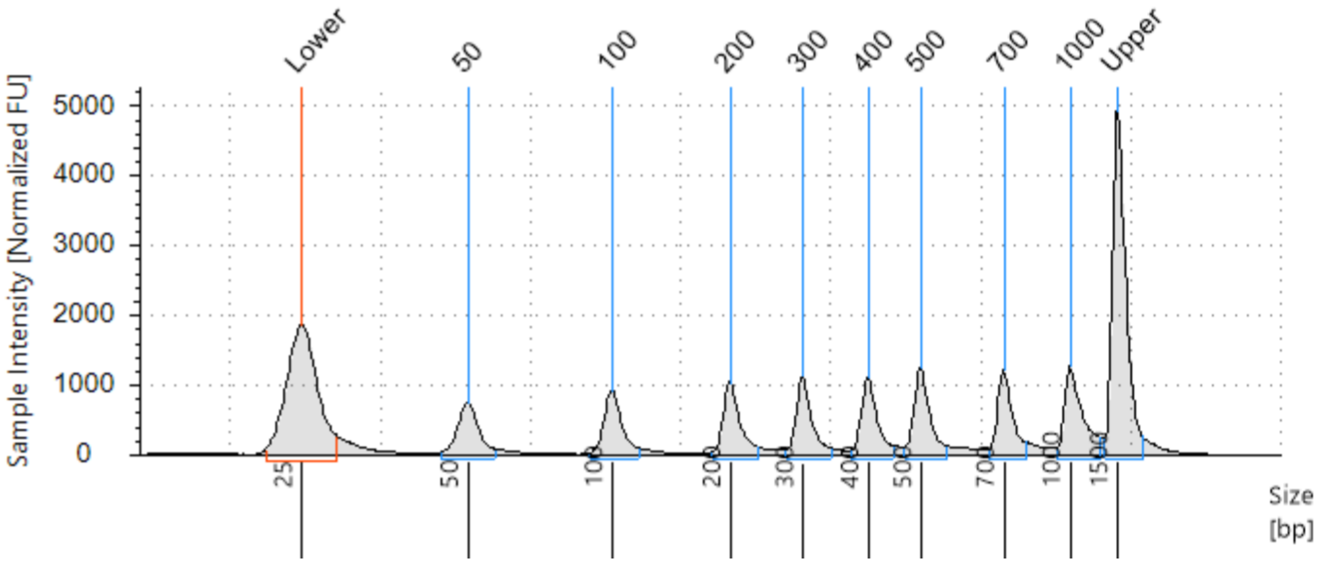

Sample Table

Well	Conc. [ng/µl]	Sample Description	Alert	Observations
A1	13.9	Ladder	⚠	Caution! Expired ScreenTape device; Ladder

Peak Table

Size [bp]	Calibrated Conc. [ng/µl]	Assigned Conc. [ng/µl]	Peak Molarity [nmol/l]	% Integrated Area	Peak Comment	Observations
25	5.58	-	343	-		Lower Marker
50	1.50	-	46.1	10.80		
100	1.58	-	24.3	11.40		
200	1.65	-	12.7	11.88		
300	1.68	-	8.59	12.07		
400	1.71	-	6.58	12.33		
500	1.88	-	5.78	13.55		
700	1.74	-	3.83	12.55		
1000	2.14	-	3.29	15.42		
1500	6.50	6.50	6.67	-		Upper Marker

© Agilent Technologies, Inc. 2017

B1: F3 P R2

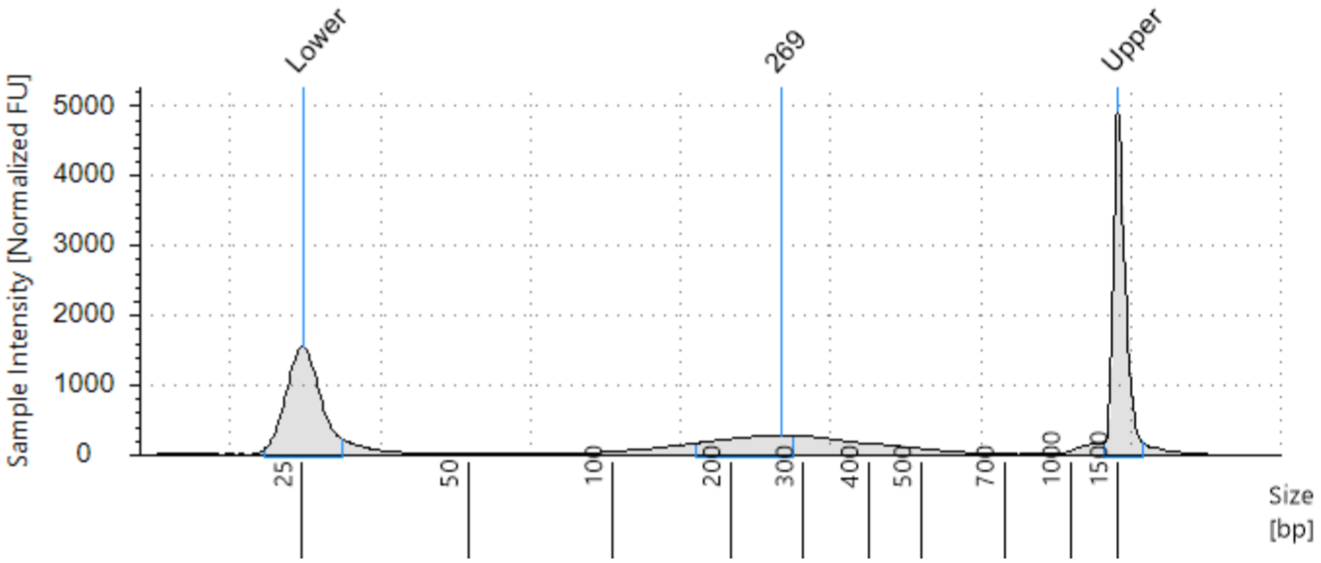

Sample Table

Well	Conc. [ng/µl]	Sample Description	Alert	Observations
B1	2.06	F3 P R2	⚠	Caution! Expired ScreenTape device

Peak Table

Size [bp]	Calibrated Conc. [ng/µl]	Assigned Conc. [ng/µl]	Peak Molarity [nmol/l]	% Integrated Area	Peak Comment	Observations
25	5.66	-	349	-		Lower Marker
269	2.06	-	11.8	100.00		
1500	6.50	6.50	6.67	-		Upper Marker

© Agilent Technologies, Inc. 2017

C1: G3 P R2

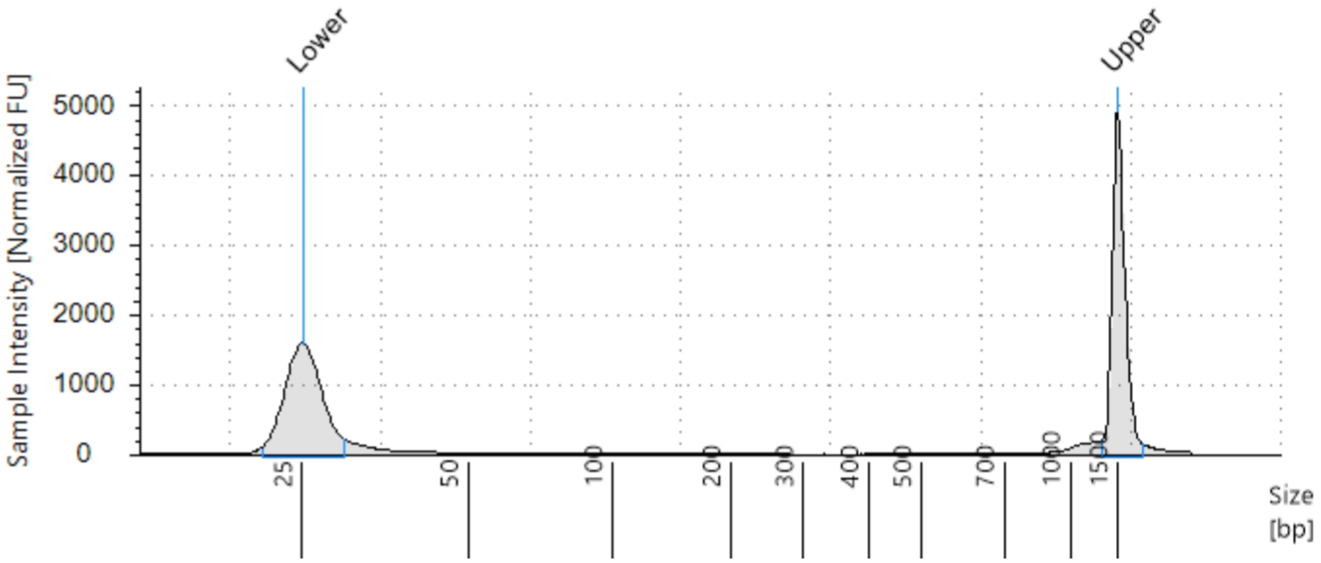

Sample Table

Well	Conc. [ng/µl]	Sample Description	Alert	Observations
C1		G3 P R2	⚠	Caution! Expired ScreenTape device

Peak Table

Size [bp]	Calibrated Conc. [ng/µl]	Assigned Conc. [ng/µl]	Peak Molarity [nmol/l]	% Integrated Area	Peak Comment	Observations
25	6.09	-	375	-		Lower Marker
1500	6.50	6.50	6.67	-		Upper Marker

 © Agilent Technologies, Inc. 2017

D1: H3 P R2

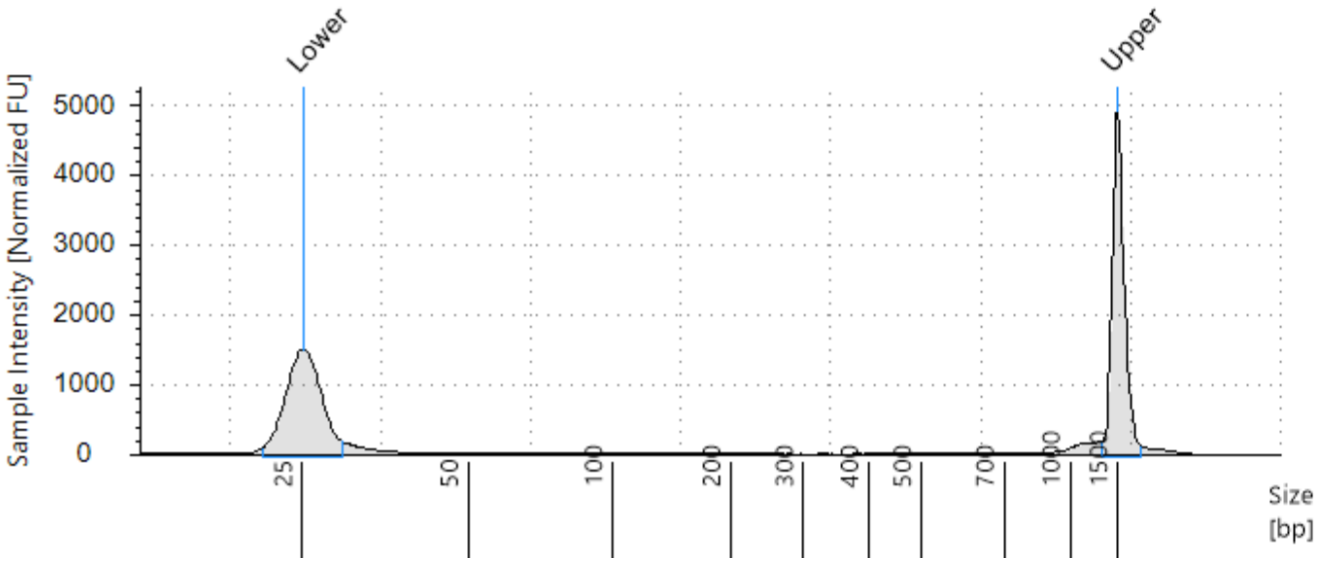

Sample Table

Well	Conc. [ng/µl]	Sample Description	Alert	Observations
D1		H3 P R2	⚠	Caution! Expired ScreenTape device

Peak Table

Size [bp]	Calibrated Conc. [ng/µl]	Assigned Conc. [ng/µl]	Peak Molarity [nmol/l]	% Integrated Area	Peak Comment	Observations
25	5.84	-	359	-		Lower Marker
1500	6.50	6.50	6.67	-		Upper Marker

© Agilent Technologies, Inc. 2017

E1: A4 P R2

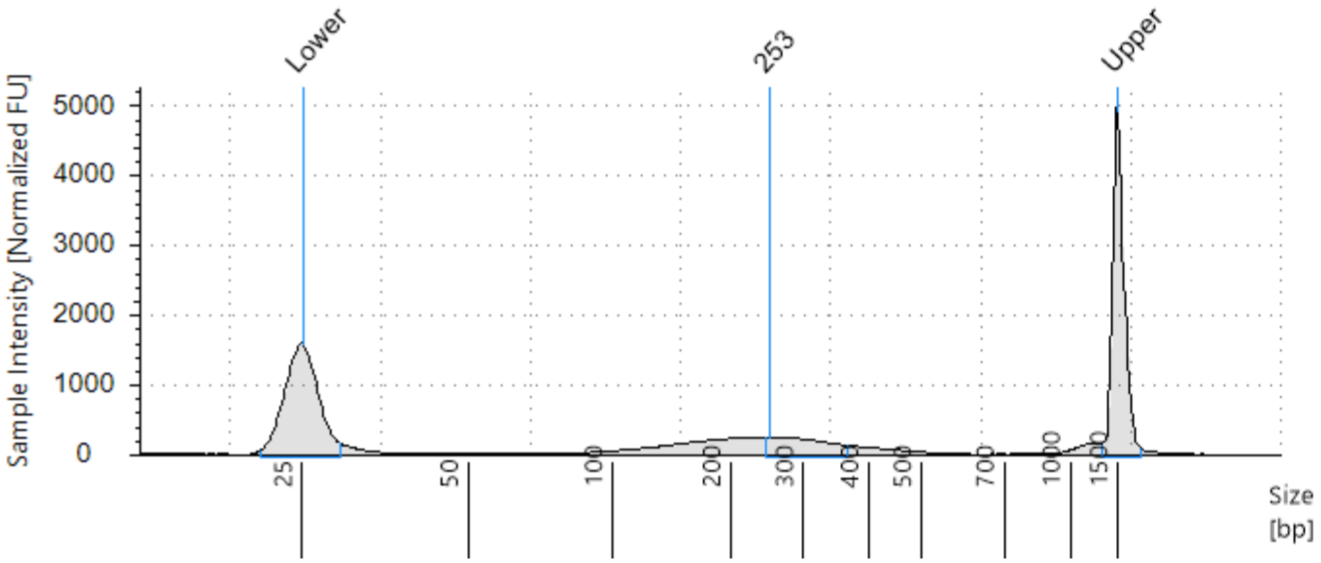

Sample Table

Well	Conc. [ng/µl]	Sample Description	Alert	Observations
E1	1.64	A4 P R2	⚠	Caution! Expired ScreenTape device

Peak Table

Size [bp]	Calibrated Conc. [ng/µl]	Assigned Conc. [ng/µl]	Peak Molarity [nmol/l]	% Integrated Area	Peak Comment	Observations
25	6.08	-	374	-		Lower Marker
253	1.64	-	9.98	100.00		
1500	6.50	6.50	6.67	-		Upper Marker

© Agilent Technologies, Inc. 2017

F1: B4 P R2

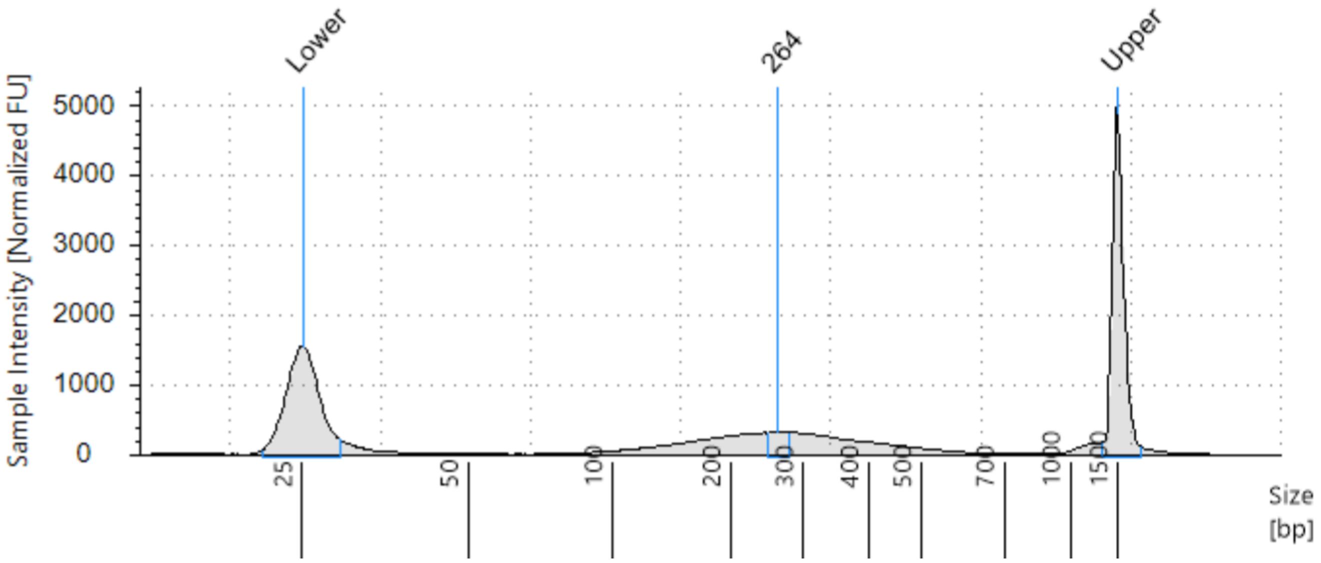

Sample Table

Well	Conc. [ng/µl]	Sample Description	Alert	Observations
F1	0.727	B4 P R2	⚠	Caution! Expired ScreenTape device

Peak Table

Size [bp]	Calibrated Conc. [ng/µl]	Assigned Conc. [ng/µl]	Peak Molarity [nmol/l]	% Integrated Area	Peak Comment	Observations
25	5.88	-	362	-		Lower Marker
264	0.727	-	4.23	100.00		
1500	6.50	6.50	6.67	-		Upper Marker

© Agilent Technologies, Inc. 2017

G1: C4 P R2

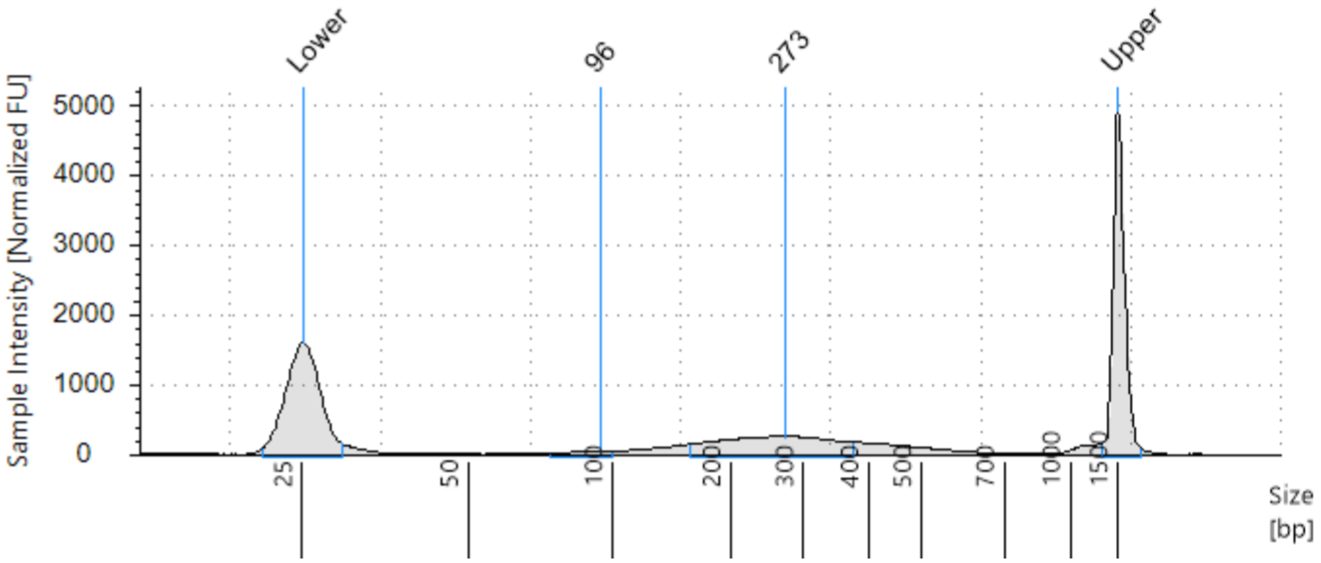

Sample Table

Well	Conc. [ng/µl]	Sample Description	Alert	Observations
G1	3.63	C4 P R2	⚠	Caution! Expired ScreenTape device

Peak Table

Size [bp]	Calibrated Conc. [ng/µl]	Assigned Conc. [ng/µl]	Peak Molarity [nmol/l]	% Integrated Area	Peak Comment	Observations
25	6.29	-	387	-		Lower Marker
96	0.202	-	3.25	5.56		
273	3.43	-	19.3	94.44		
1500	6.50	6.50	6.67	-		Upper Marker

 © Agilent Technologies, Inc. 2017

H1: D4 P R2

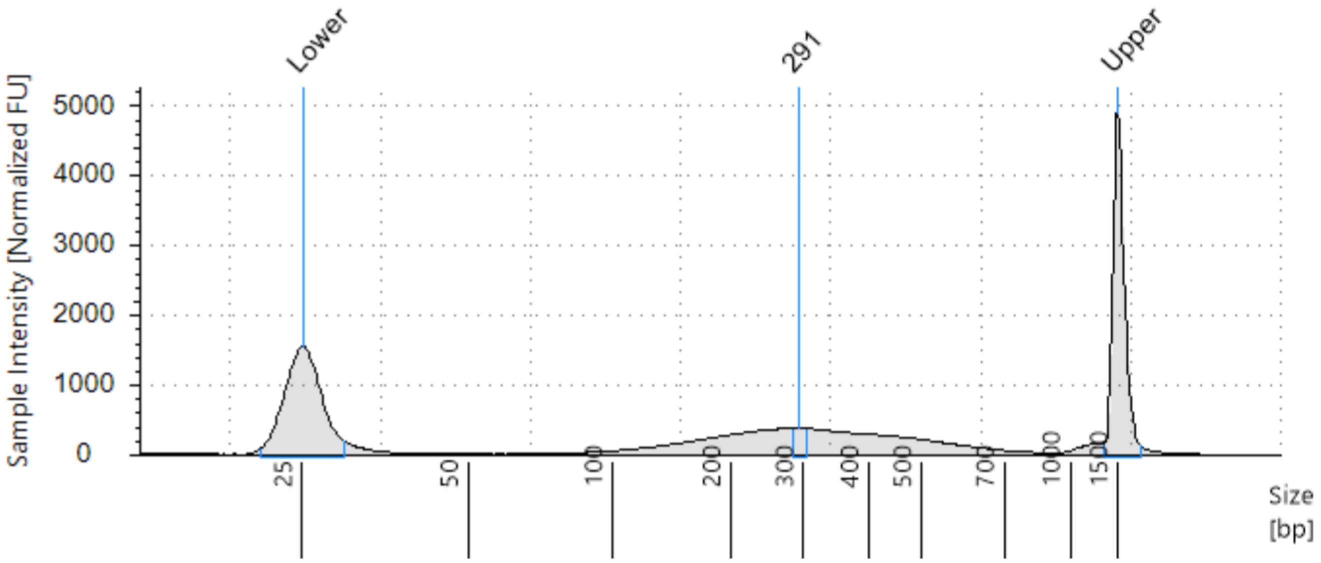

Sample Table

Well	Conc. [ng/µl]	Sample Description	Alert	Observations
H1	0.527	D4 P R2	⚠	Caution! Expired ScreenTape device

Peak Table

Size [bp]	Calibrated Conc. [ng/µl]	Assigned Conc. [ng/µl]	Peak Molarity [nmol/l]	% Integrated Area	Peak Comment	Observations
25	6.43	-	396	-		Lower Marker
291	0.527	-	2.78	100.00		
1500	6.50	6.50	6.67	-		Upper Marker

 © Agilent Technologies, Inc. 2017

A2: E4 P R2

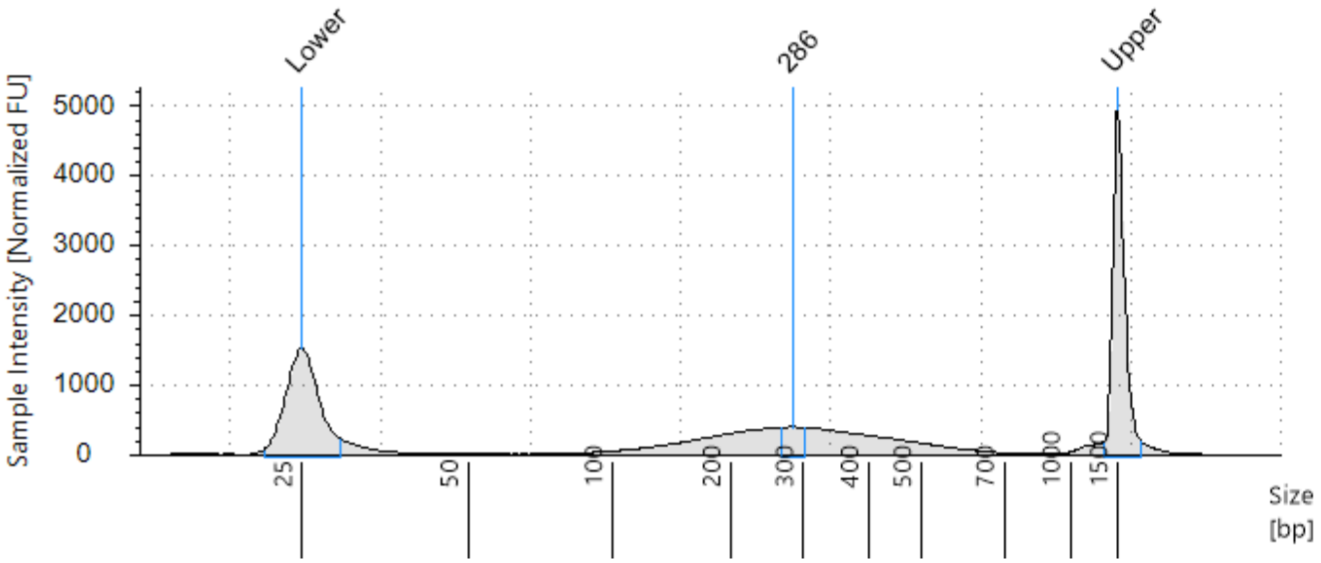

Sample Table

Well	Conc. [ng/µl]	Sample Description	Alert	Observations
A2	0.871	E4 P R2	⚠	Caution! Expired ScreenTape device

Peak Table

Size [bp]	Calibrated Conc. [ng/µl]	Assigned Conc. [ng/µl]	Peak Molarity [nmol/l]	% Integrated Area	Peak Comment	Observations
25	5.50	-	338	-		Lower Marker
286	0.871	-	4.69	100.00		
1500	6.50	6.50	6.67	-		Upper Marker

© Agilent Technologies, Inc. 2017

B2: F4 P R2

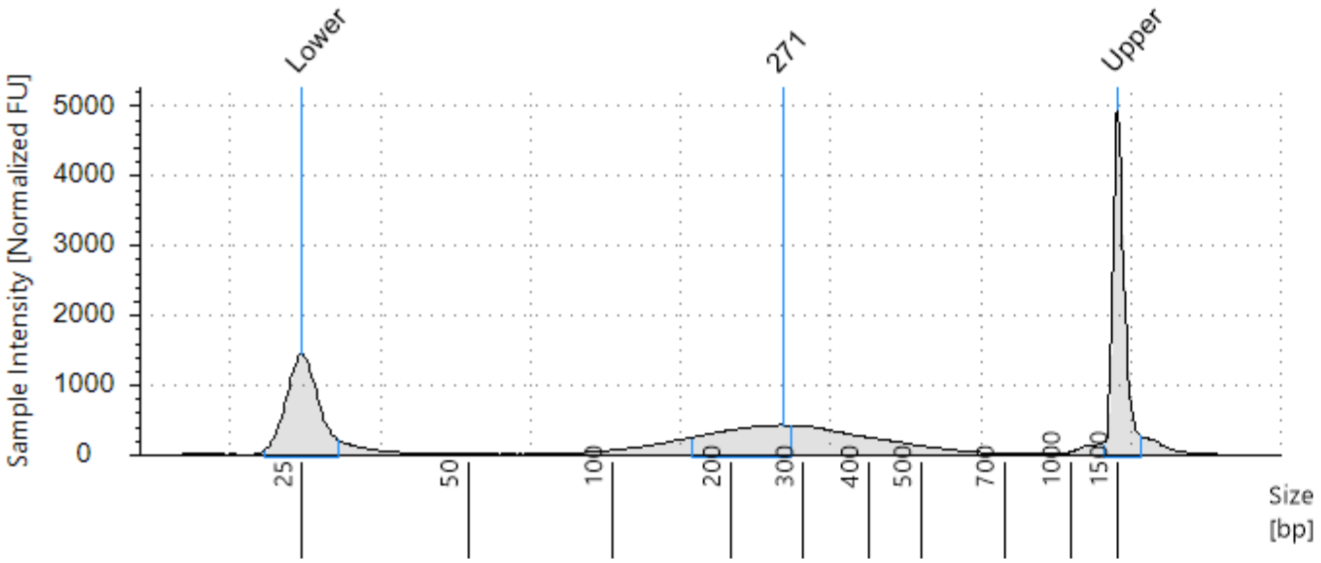

Sample Table

Well	Conc. [ng/µl]	Sample Description	Alert	Observations
B2	3.40	F4 P R2	⚠	Caution! Expired ScreenTape device

Peak Table

Size [bp]	Calibrated Conc. [ng/µl]	Assigned Conc. [ng/µl]	Peak Molarity [nmol/l]	% Integrated Area	Peak Comment	Observations
25	5.21	-	321	-		Lower Marker
271	3.40	-	19.3	100.00		
1500	6.50	6.50	6.67	-		Upper Marker

© Agilent Technologies, Inc. 2017

C2: G4 P R2

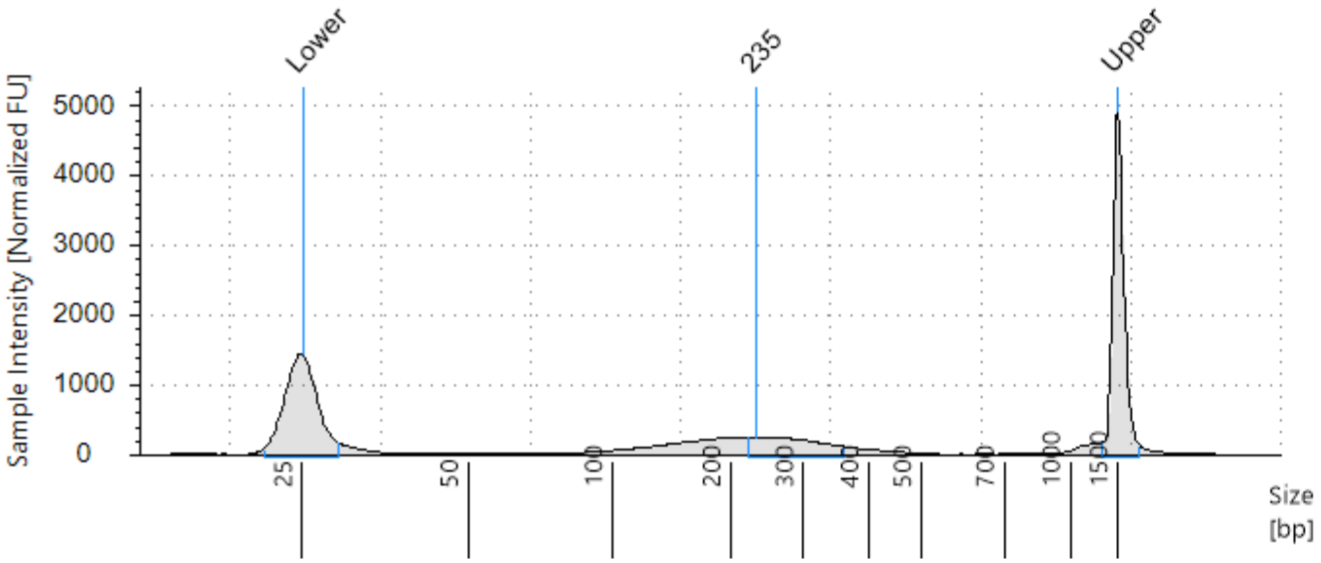

Sample Table

Well	Conc. [ng/µl]	Sample Description	Alert	Observations
C2	1.92	G4 P R2	⚠	Caution! Expired ScreenTape device

Peak Table

Size [bp]	Calibrated Conc. [ng/µl]	Assigned Conc. [ng/µl]	Peak Molarity [nmol/l]	% Integrated Area	Peak Comment	Observations
25	5.51	-	339	-		Lower Marker
235	1.92	-	12.6	100.00		
1500	6.50	6.50	6.67	-		Upper Marker

© Agilent Technologies, Inc. 2017

D2: H4 P R2

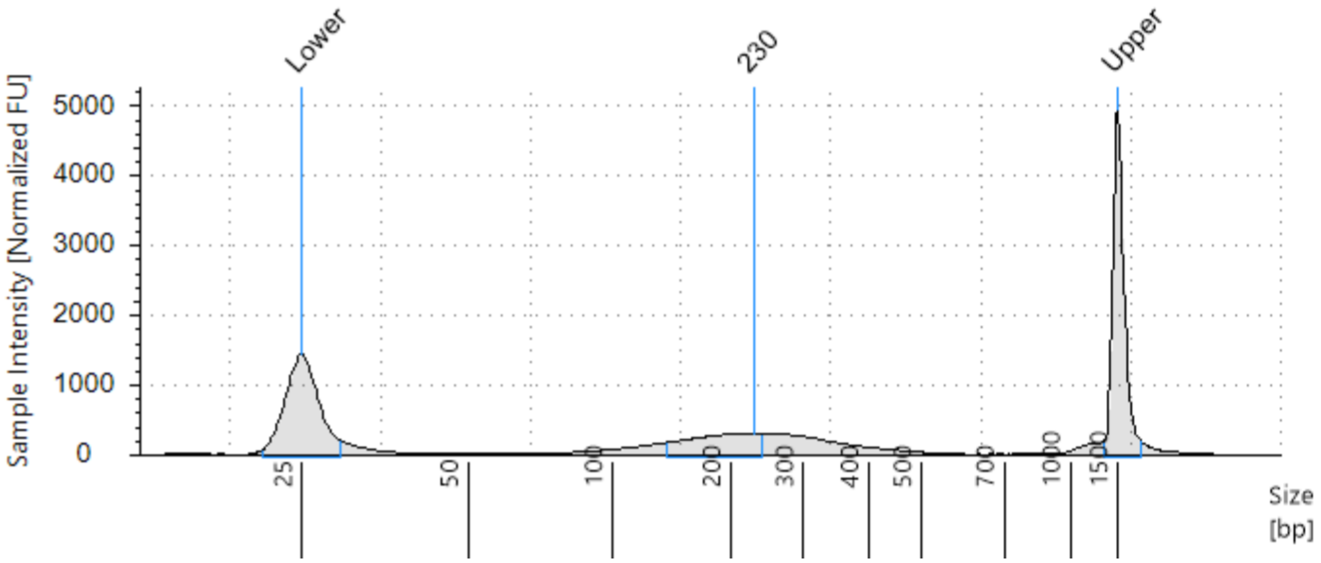

Sample Table

Well	Conc. [ng/µl]	Sample Description	Alert	Observations
D2	2.35	H4 P R2	⚠	Caution! Expired ScreenTape device

Peak Table

Size [bp]	Calibrated Conc. [ng/µl]	Assigned Conc. [ng/µl]	Peak Molarity [nmol/l]	% Integrated Area	Peak Comment	Observations
25	5.47	-	337	-		Lower Marker
230	2.35	-	15.7	100.00		
1500	6.50	6.50	6.67	-		Upper Marker

© Agilent Technologies, Inc. 2017

E2: A5 P R2

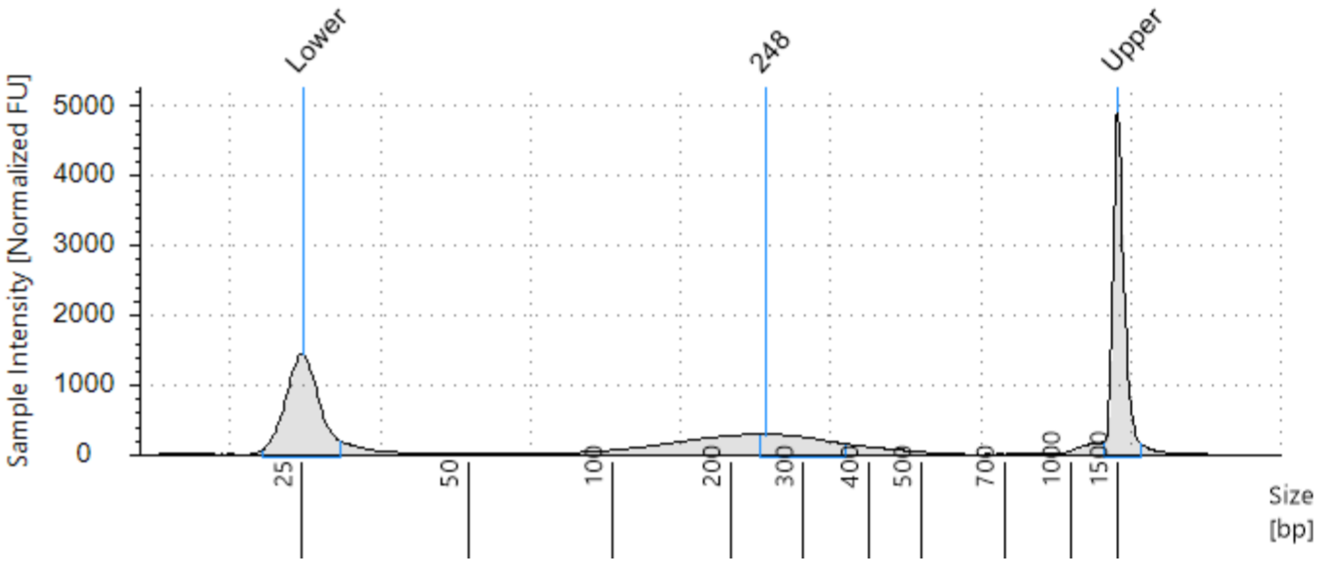

Sample Table

Well	Conc. [ng/µl]	Sample Description	Alert	Observations
E2	2.03	A5 P R2	⚠	Caution! Expired ScreenTape device

Peak Table

Size [bp]	Calibrated Conc. [ng/µl]	Assigned Conc. [ng/µl]	Peak Molarity [nmol/l]	% Integrated Area	Peak Comment	Observations
25	5.54	-	341	-		Lower Marker
248	2.03	-	12.6	100.00		
1500	6.50	6.50	6.67	-		Upper Marker

© Agilent Technologies, Inc. 2017

F2

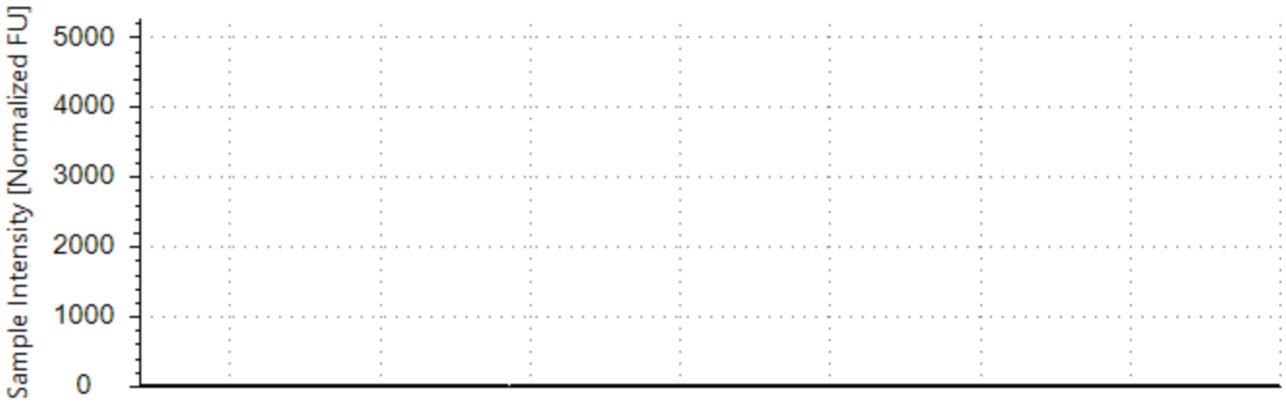

Sample Table

Well	Conc. [ng/µl]	Sample Description	Alert	Observations
F2			▲	Marker(s) not detected; Caution! Expired ScreenTape device

© Agilent Technologies, Inc. 2017

G2: B5 P R2

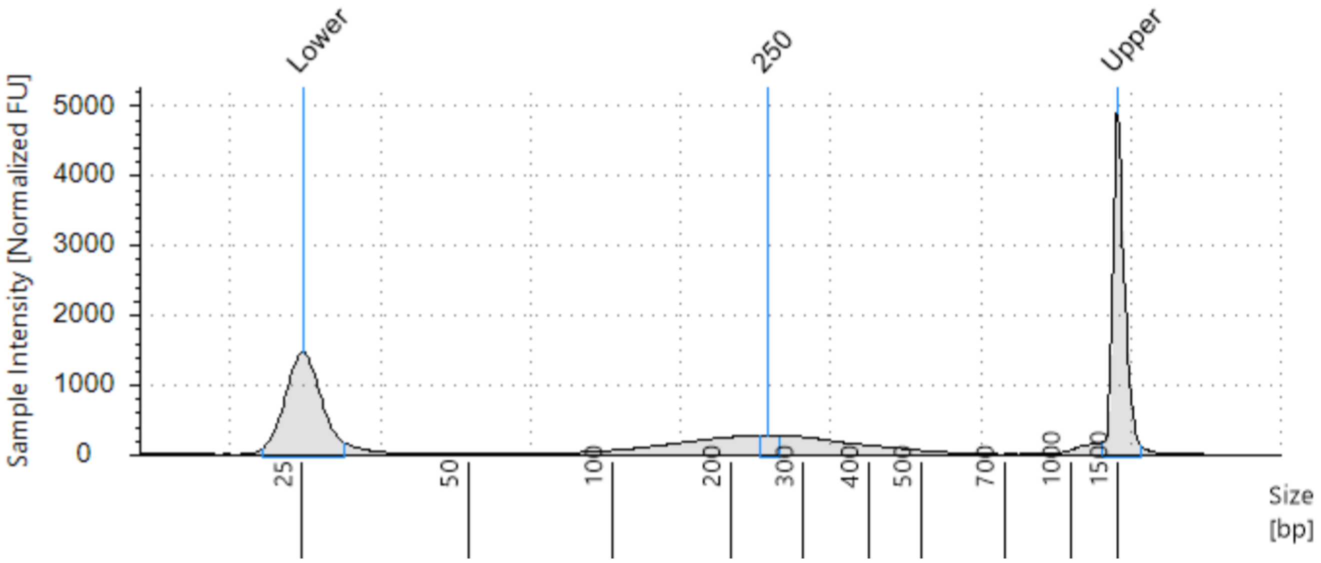

Sample Table

Well	Conc. [ng/µl]	Sample Description	Alert	Observations
G2	0.547	B5 P R2	⚠	Caution! Expired ScreenTape device

Peak Table

Size [bp]	Calibrated Conc. [ng/µl]	Assigned Conc. [ng/µl]	Peak Molarity [nmol/l]	% Integrated Area	Peak Comment	Observations
25	5.85	-	360	-		Lower Marker
250	0.547	-	3.37	100.00		
1500	6.50	6.50	6.67	-		Upper Marker

© Agilent Technologies, Inc. 2017

H2: C5 P R2

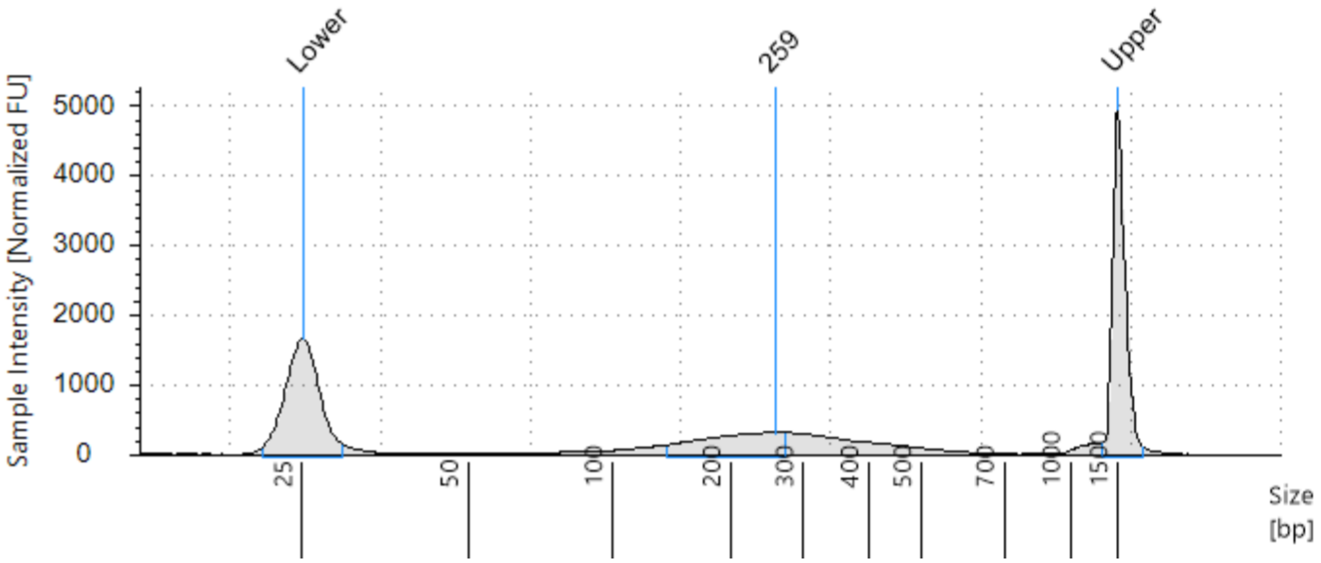

Sample Table

Well	Conc. [ng/µl]	Sample Description	Alert	Observations
H2	2.60	C5 P R2	⚠	Caution! Expired ScreenTape device

Peak Table

Size [bp]	Calibrated Conc. [ng/µl]	Assigned Conc. [ng/µl]	Peak Molarity [nmol/l]	% Integrated Area	Peak Comment	Observations
25	5.85	-	360	-		Lower Marker
259	2.60	-	15.4	100.00		
1500	6.50	6.50	6.67	-		Upper Marker

© Agilent Technologies, Inc. 2017

Filename: 2020-09-15-01 Q-S plus B7-H8 R2.D1000

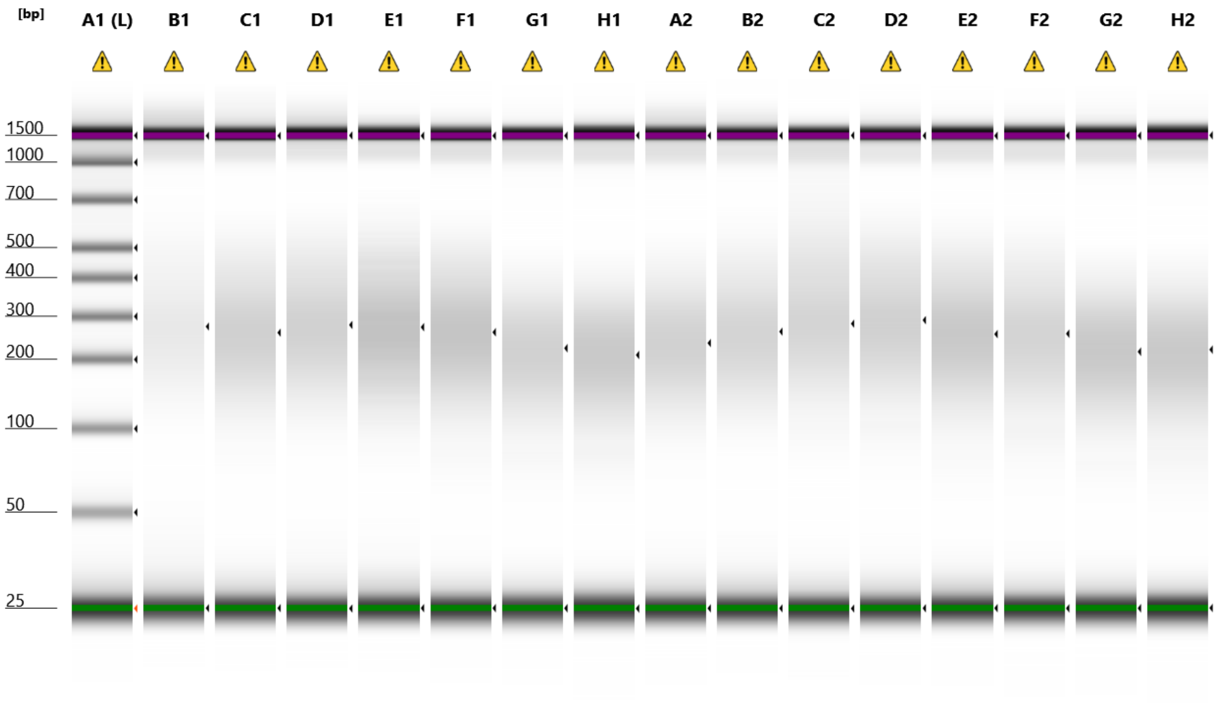

Default image (Contrast 100%)

Sample Info

Well	Conc. [ng/µl]	Sample Description	Alert	Observations
A1	10.6	Ladder	⚠	Caution! Expired ScreenTape device; Ladder
B1	0.621	B7 P R2	⚠	Caution! Expired ScreenTape device
C1	1.97	C7 P R2	⚠	Caution! Expired ScreenTape device
D1	1.60	D7 P R2	⚠	Caution! Expired ScreenTape device
E1	3.28	E7 P R2	⚠	Caution! Expired ScreenTape device
F1	2.46	F7 P R2	⚠	Caution! Expired ScreenTape device
G1	1.91	G7 P R2	⚠	Caution! Expired ScreenTape device
H1	0.532	H7 P R2	⚠	Caution! Expired ScreenTape device
A2	0.501	A8 P R2	⚠	Caution! Expired ScreenTape device
B2	1.49	B8 P R2	⚠	Caution! Expired ScreenTape device
C2	1.94	C8 P R2	⚠	Caution! Expired ScreenTape device
D2	3.43	D8 P R2	⚠	Caution! Expired ScreenTape device
E2	2.35	E8 P R2	⚠	Caution! Expired ScreenTape device
F2	1.59	F8 P R2	⚠	Caution! Expired ScreenTape device
G2	2.28	G8 P R2	⚠	Caution! Expired ScreenTape device
H2	2.12	H8 P R2	⚠	Caution! Expired ScreenTape device

© Agilent Technologies, Inc. 2017

A1: Ladder

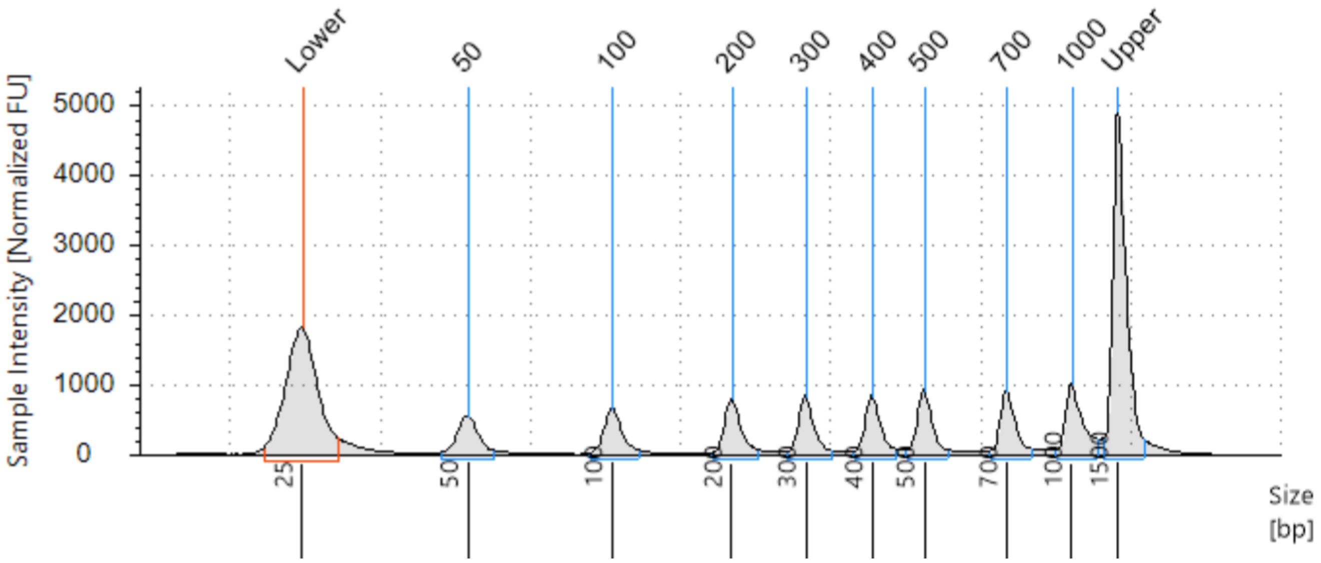

Sample Table

Well	Conc. [ng/µl]	Sample Description	Alert	Observations
A1	10.6	Ladder	⚠	Caution! Expired ScreenTape device; Ladder

Peak Table

Size [bp]	Calibrated Conc. [ng/µl]	Assigned Conc. [ng/µl]	Peak Molarity [nmol/l]	% Integrated Area	Peak Comment	Observations
25	5.69	-	350	-		Lower Marker
50	1.15	-	35.5	10.90		
100	1.17	-	18.1	11.11		
200	1.29	-	9.89	12.16		
300	1.28	-	6.57	12.11		
400	1.30	-	4.99	12.27		
500	1.36	-	4.19	12.87		
700	1.35	-	2.98	12.81		
1000	1.67	-	2.56	15.76		
1500	6.50	6.50	6.67	-		Upper Marker

 © Agilent Technologies, Inc. 2017

B1: B7 P R2

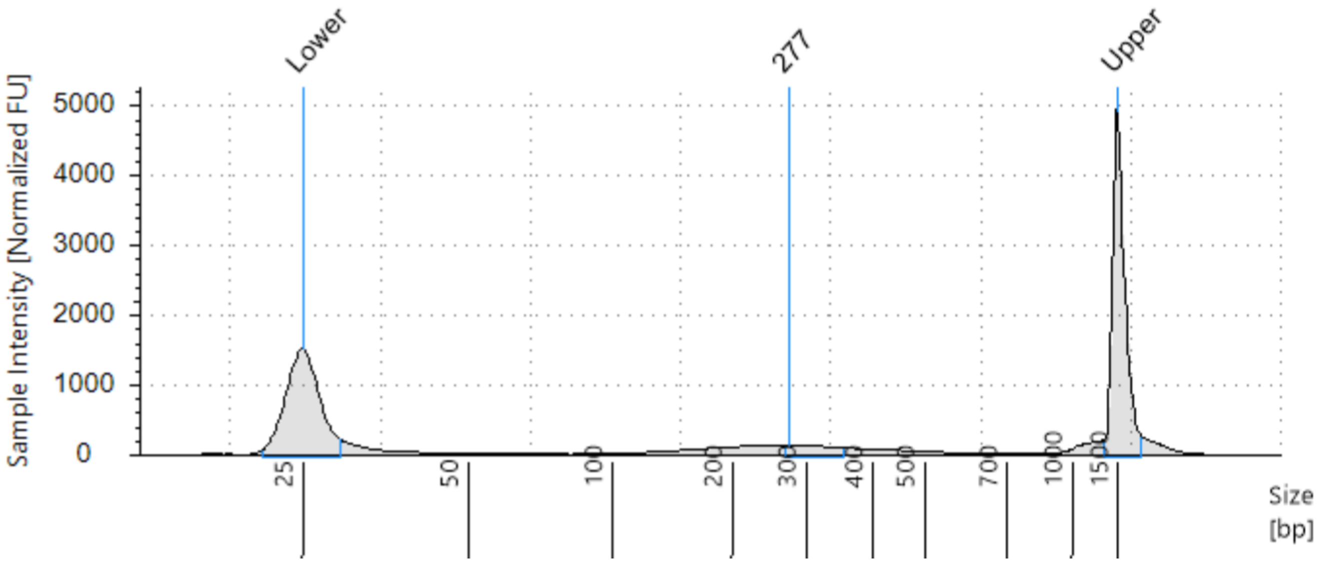

Sample Table

Well	Conc. [ng/µl]	Sample Description	Alert	Observations
B1	0.621	B7 P R2	⚠	Caution! Expired ScreenTape device

Peak Table

Size [bp]	Calibrated Conc. [ng/µl]	Assigned Conc. [ng/µl]	Peak Molarity [nmol/l]	% Integrated Area	Peak Comment	Observations
25	5.43	-	334	-		Lower Marker
277	0.621	-	3.45	100.00		
1500	6.50	6.50	6.67	-		Upper Marker

© Agilent Technologies, Inc. 2017

C1: C7 P R2

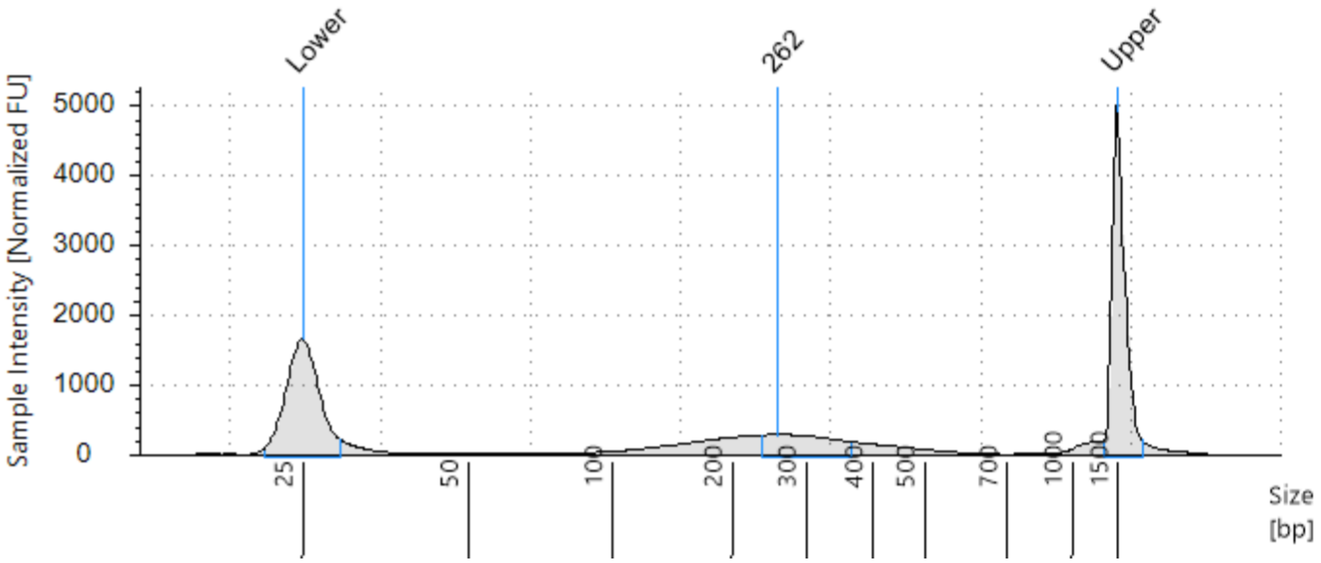

Sample Table

Well	Conc. [ng/µl]	Sample Description	Alert	Observations
C1	1.97	C7 P R2	⚠	Caution! Expired ScreenTape device

Peak Table

Size [bp]	Calibrated Conc. [ng/µl]	Assigned Conc. [ng/µl]	Peak Molarity [nmol/l]	% Integrated Area	Peak Comment	Observations
25	5.55	-	342	-		Lower Marker
262	1.97	-	11.6	100.00		
1500	6.50	6.50	6.67	-		Upper Marker

© Agilent Technologies, Inc. 2017

D1: D7 P R2

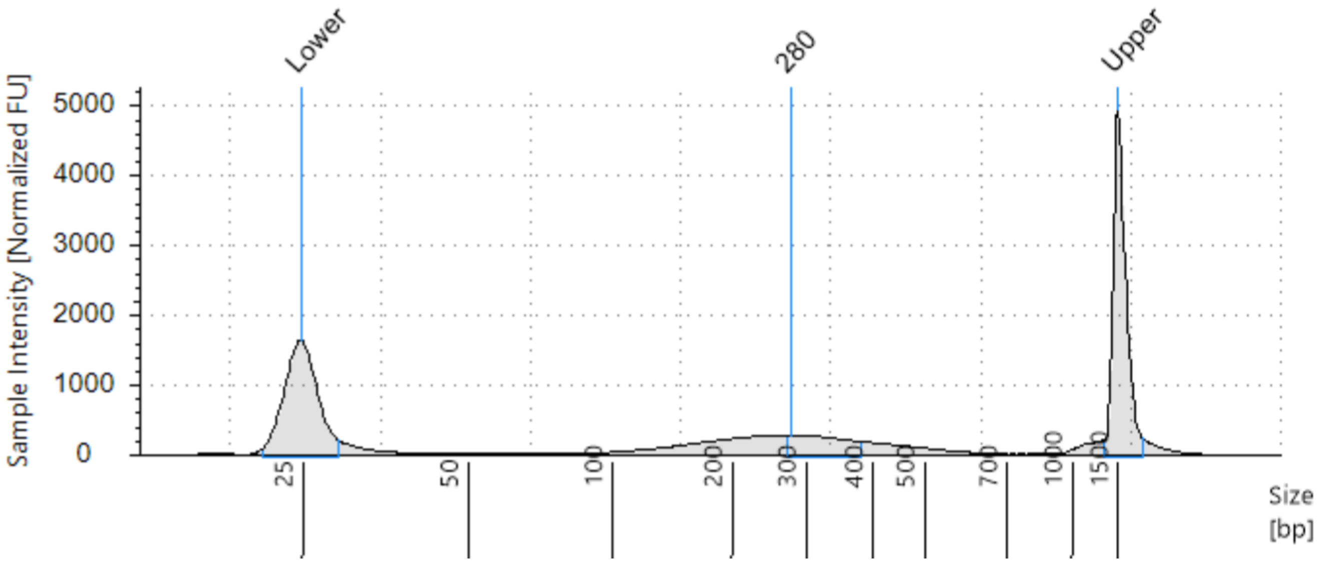

Sample Table

Well	Conc. [ng/µl]	Sample Description	Alert	Observations
D1	1.60	D7 P R2	⚠	Caution! Expired ScreenTape device

Peak Table

Size [bp]	Calibrated Conc. [ng/µl]	Assigned Conc. [ng/µl]	Peak Molarity [nmol/l]	% Integrated Area	Peak Comment	Observations
25	5.59	-	344	-		Lower Marker
280	1.60	-	8.79	100.00		
1500	6.50	6.50	6.67	-		Upper Marker

 © Agilent Technologies, Inc. 2017

E1: E7 P R2

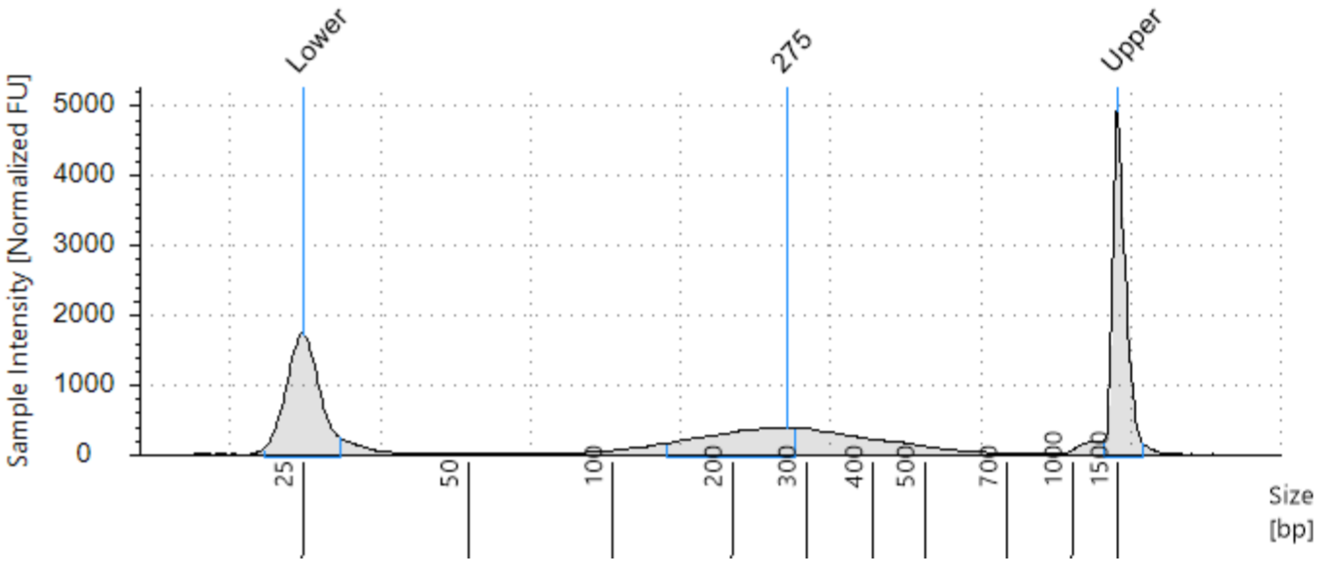

Sample Table

Well	Conc. [ng/µl]	Sample Description	Alert	Observations
E1	3.28	E7 P R2	⚠	Caution! Expired ScreenTape device

Peak Table

Size [bp]	Calibrated Conc. [ng/µl]	Assigned Conc. [ng/µl]	Peak Molarity [nmol/l]	% Integrated Area	Peak Comment	Observations
25	5.79	-	356	-		Lower Marker
275	3.28	-	18.3	100.00		
1500	6.50	6.50	6.67	-		Upper Marker

 © Agilent Technologies, Inc. 2017

F1: F7 P R2

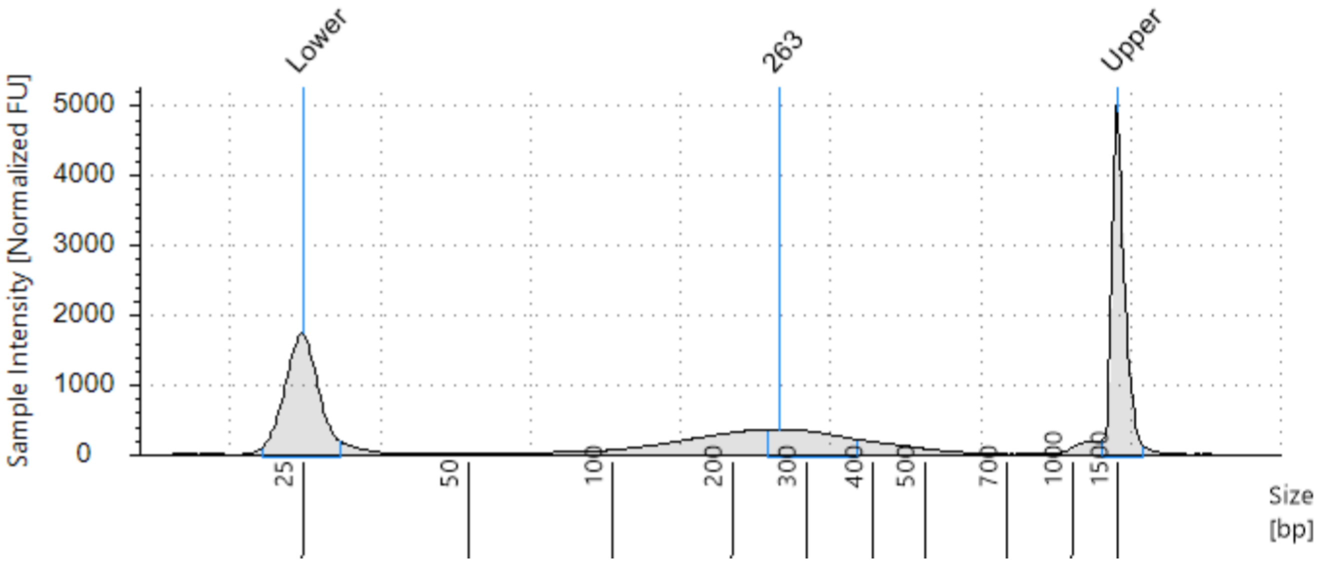

Sample Table

Well	Conc. [ng/µl]	Sample Description	Alert	Observations
F1	2.46	F7 P R2	⚠	Caution! Expired ScreenTape device

Peak Table

Size [bp]	Calibrated Conc. [ng/µl]	Assigned Conc. [ng/µl]	Peak Molarity [nmol/l]	% Integrated Area	Peak Comment	Observations
25	5.91	-	364	-		Lower Marker
263	2.46	-	14.4	100.00		
1500	6.50	6.50	6.67	-		Upper Marker

© Agilent Technologies, Inc. 2017

G1: G7 P R2

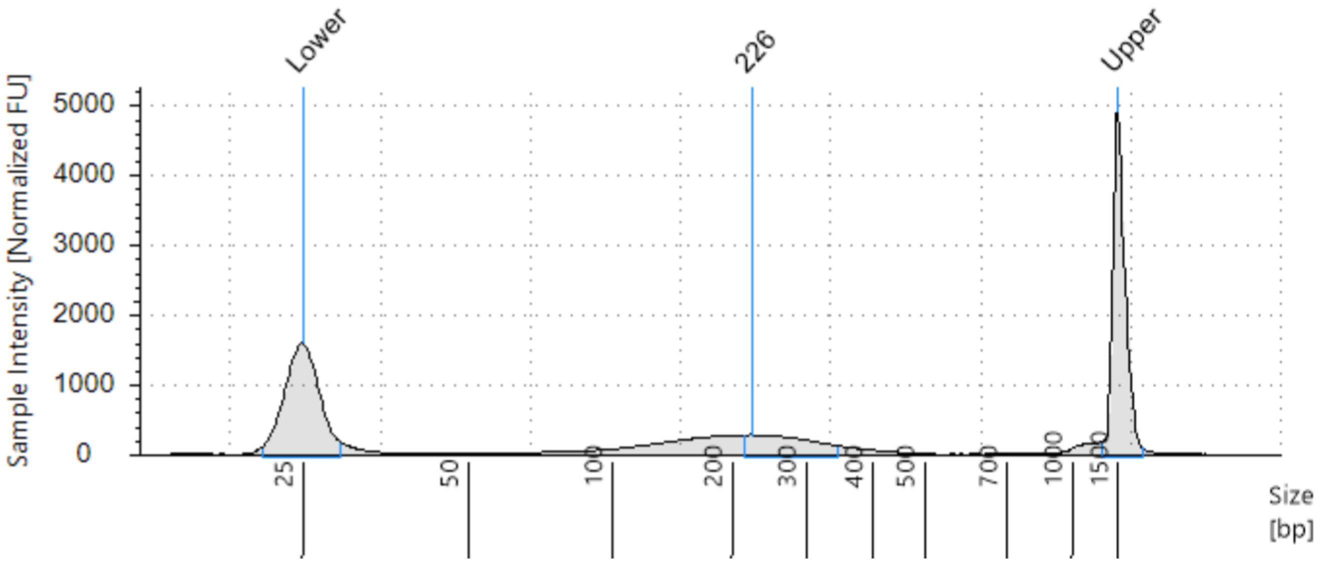

Sample Table

Well	Conc. [ng/µl]	Sample Description	Alert	Observations
G1	1.91	G7 P R2	⚠	Caution! Expired ScreenTape device

Peak Table

Size [bp]	Calibrated Conc. [ng/µl]	Assigned Conc. [ng/µl]	Peak Molarity [nmol/l]	% Integrated Area	Peak Comment	Observations
25	5.89	-	362	-		Lower Marker
226	1.91	-	13.0	100.00		
1500	6.50	6.50	6.67	-		Upper Marker

© Agilent Technologies, Inc. 2017

H1: H7 P R2

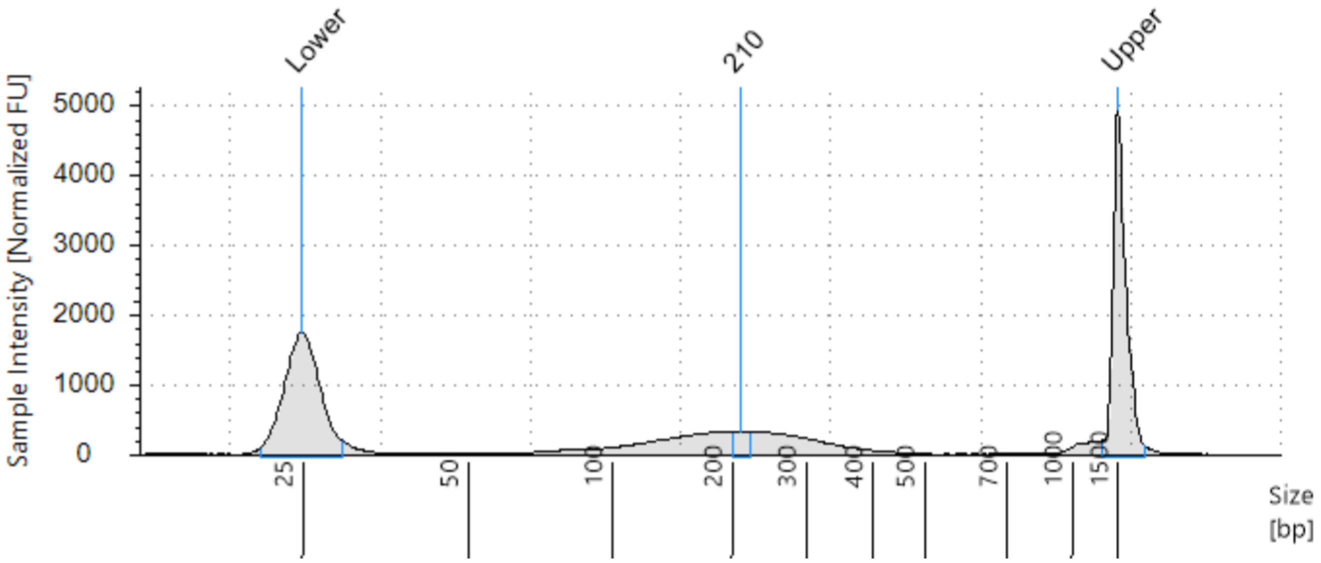

Sample Table

Well	Conc. [ng/µl]	Sample Description	Alert	Observations
H1	0.532	H7 P R2	⚠	Caution! Expired ScreenTape device

Peak Table

Size [bp]	Calibrated Conc. [ng/µl]	Assigned Conc. [ng/µl]	Peak Molarity [nmol/l]	% Integrated Area	Peak Comment	Observations
25	6.31	-	388	-		Lower Marker
210	0.532	-	3.89	100.00		
1500	6.50	6.50	6.67	-		Upper Marker

A2: A8 P R2

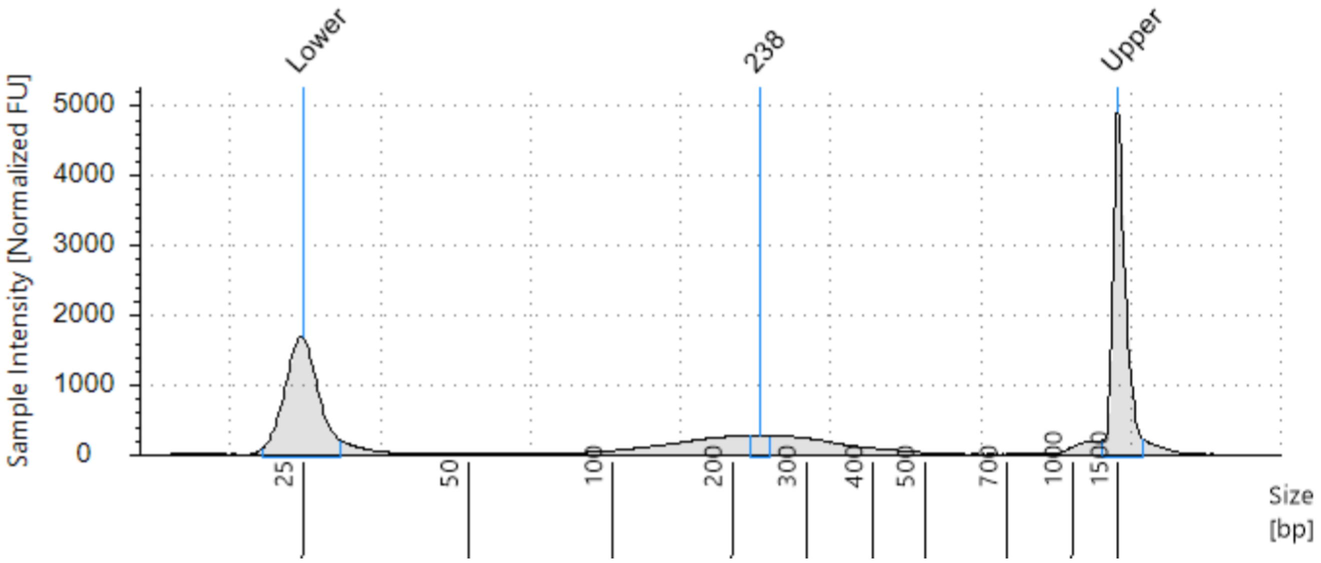

Sample Table

Well	Conc. [ng/µl]	Sample Description	Alert	Observations
A2	0.501	A8 P R2	⚠	Caution! Expired ScreenTape device

Peak Table

Size [bp]	Calibrated Conc. [ng/µl]	Assigned Conc. [ng/µl]	Peak Molarity [nmol/l]	% Integrated Area	Peak Comment	Observations
25	5.86	-	361	-		Lower Marker
238	0.501	-	3.24	100.00		
1500	6.50	6.50	6.67	-		Upper Marker

© Agilent Technologies, Inc. 2017

B2: B8 P R2

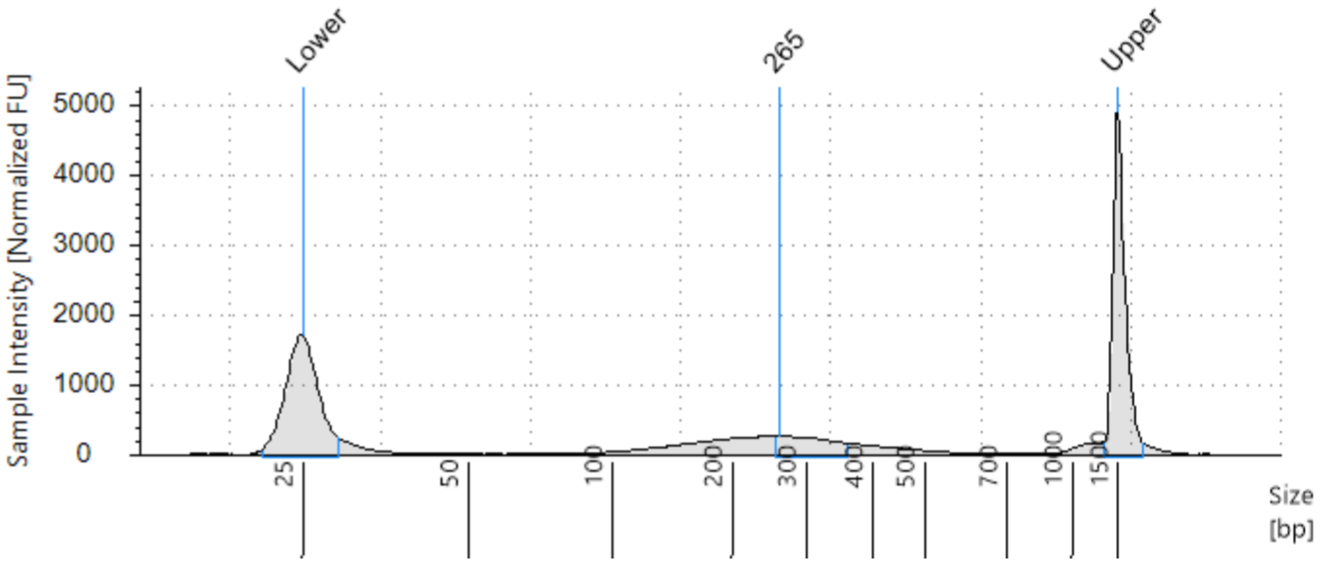

Sample Table

Well	Conc. [ng/µl]	Sample Description	Alert	Observations
B2	1.49	B8 P R2	⚠	Caution! Expired ScreenTape device

Peak Table

Size [bp]	Calibrated Conc. [ng/µl]	Assigned Conc. [ng/µl]	Peak Molarity [nmol/l]	% Integrated Area	Peak Comment	Observations
25	5.96	-	367	-		Lower Marker
265	1.49	-	8.64	100.00		
1500	6.50	6.50	6.67	-		Upper Marker

© Agilent Technologies, Inc. 2017

C2: C8 P R2

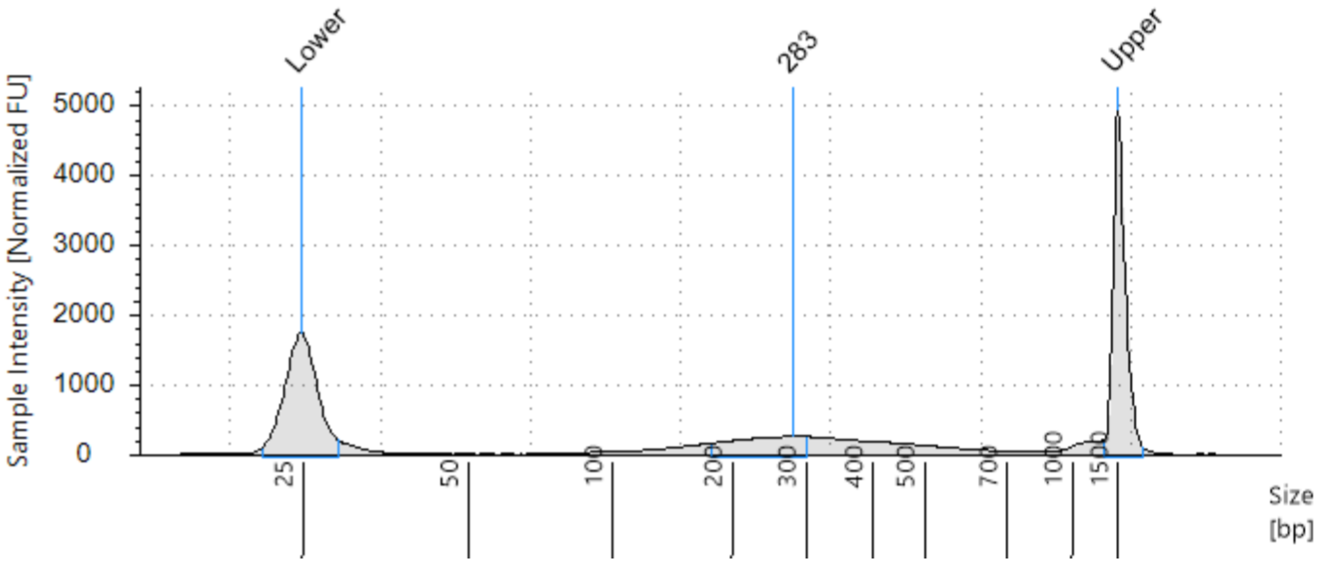

Sample Table

Well	Conc. [ng/µl]	Sample Description	Alert	Observations
C2	1.94	C8 P R2	⚠	Caution! Expired ScreenTape device

Peak Table

Size [bp]	Calibrated Conc. [ng/µl]	Assigned Conc. [ng/µl]	Peak Molarity [nmol/l]	% Integrated Area	Peak Comment	Observations
25	6.04	-	371	-		Lower Marker
283	1.94	-	10.5	100.00		
1500	6.50	6.50	6.67	-		Upper Marker

 © Agilent Technologies, Inc. 2017

D2: D8 P R2

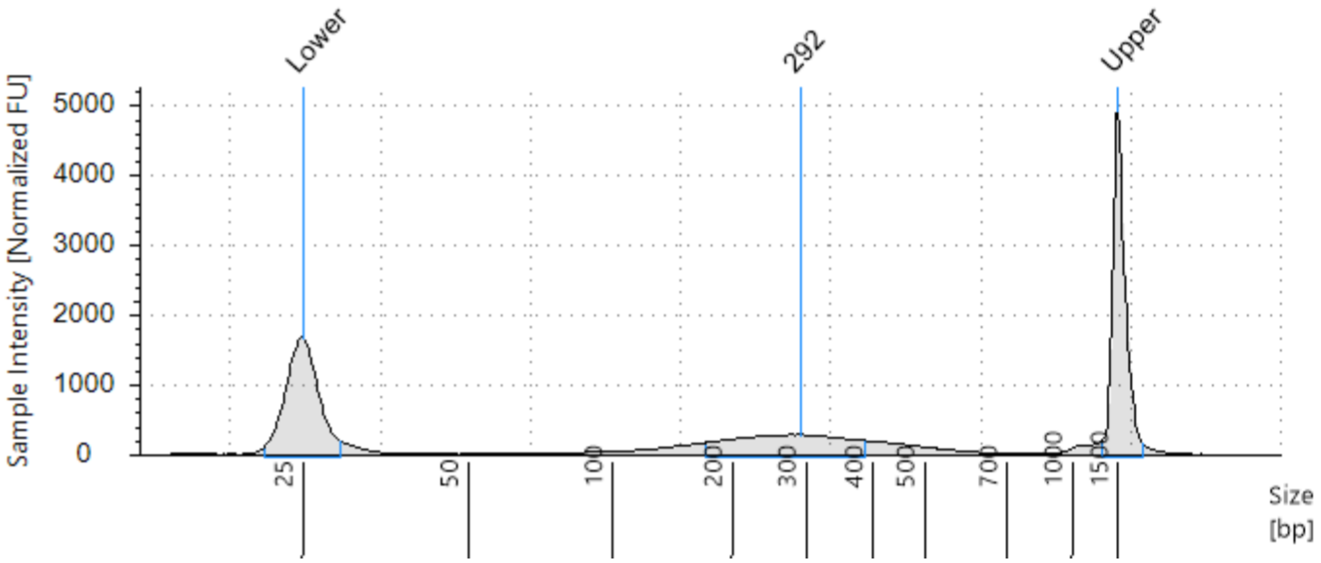

Sample Table

Well	Conc. [ng/µl]	Sample Description	Alert	Observations
D2	3.43	D8 P R2	⚠	Caution! Expired ScreenTape device

Peak Table

Size [bp]	Calibrated Conc. [ng/µl]	Assigned Conc. [ng/µl]	Peak Molarity [nmol/l]	% Integrated Area	Peak Comment	Observations
25	5.68	-	350	-		Lower Marker
292	3.43	-	18.1	100.00		
1500	6.50	6.50	6.67	-		Upper Marker

© Agilent Technologies, Inc. 2017

E2: E8 P R2

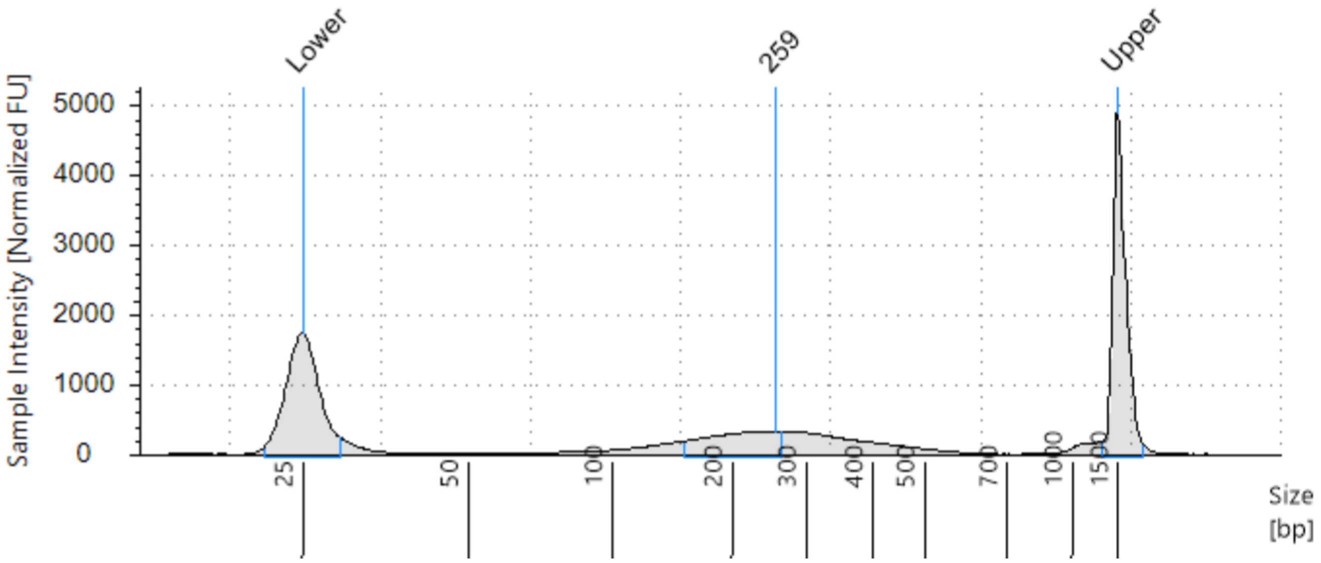

Sample Table

Well	Conc. [ng/µl]	Sample Description	Alert	Observations
E2	2.35	E8 P R2	⚠	Caution! Expired ScreenTape device

Peak Table

Size [bp]	Calibrated Conc. [ng/µl]	Assigned Conc. [ng/µl]	Peak Molarity [nmol/l]	% Integrated Area	Peak Comment	Observations
25	5.87	-	361	-		Lower Marker
259	2.35	-	14.0	100.00		
1500	6.50	6.50	6.67	-		Upper Marker

© Agilent Technologies, Inc. 2017

F2: F8 P R2

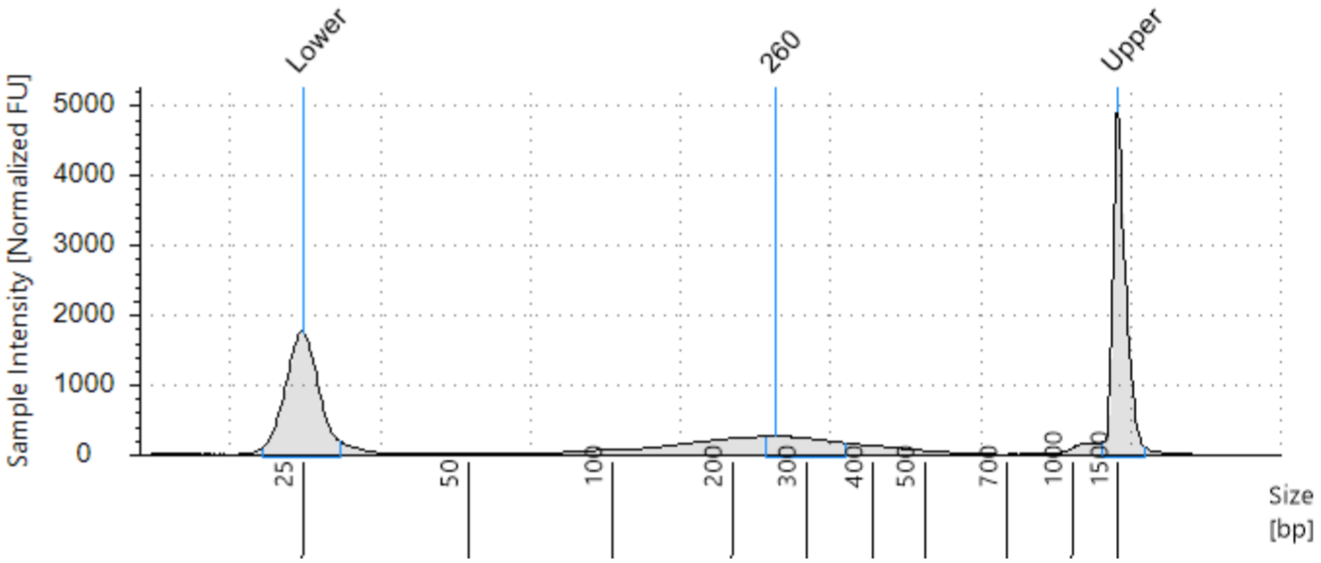

Sample Table

Well	Conc. [ng/µl]	Sample Description	Alert	Observations
F2	1.59	F8 P R2	⚠	Caution! Expired ScreenTape device

Peak Table

Size [bp]	Calibrated Conc. [ng/µl]	Assigned Conc. [ng/µl]	Peak Molarity [nmol/l]	% Integrated Area	Peak Comment	Observations
25	5.94	-	365	-		Lower Marker
260	1.59	-	9.44	100.00		
1500	6.50	6.50	6.67	-		Upper Marker

© Agilent Technologies, Inc. 2017

G2: G8 P R2

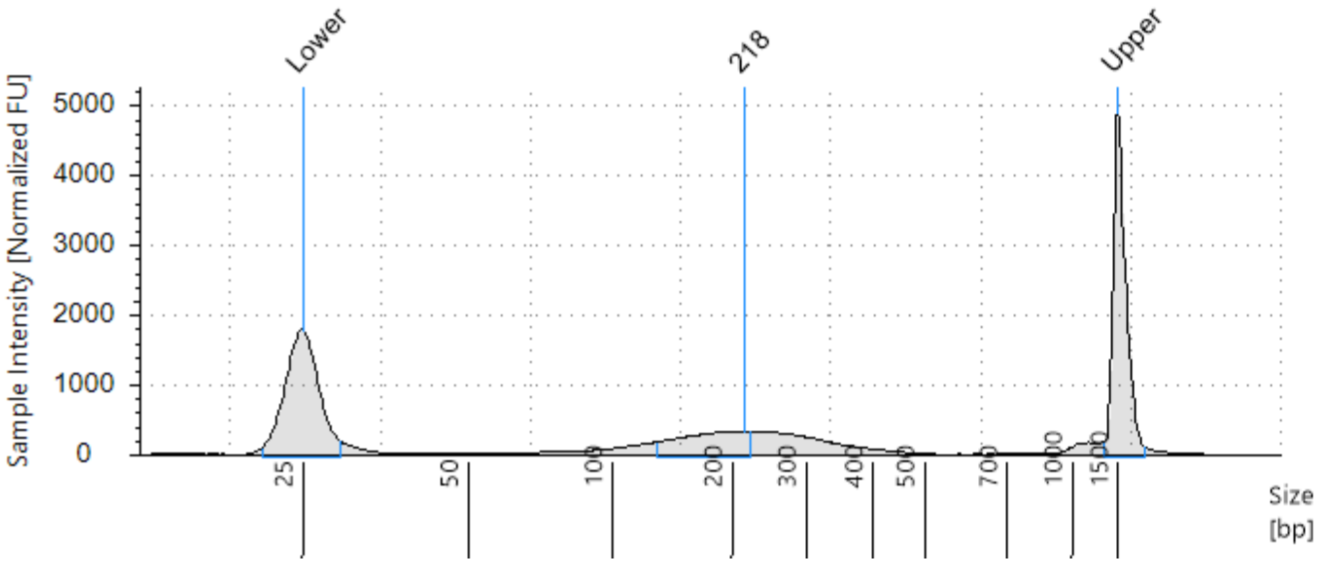

Sample Table

Well	Conc. [ng/µl]	Sample Description	Alert	Observations
G2	2.28	G8 P R2	⚠	Caution! Expired ScreenTape device

Peak Table

Size [bp]	Calibrated Conc. [ng/µl]	Assigned Conc. [ng/µl]	Peak Molarity [nmol/l]	% Integrated Area	Peak Comment	Observations
25	6.20	-	382	-		Lower Marker
218	2.28	-	16.1	100.00		
1500	6.50	6.50	6.67	-		Upper Marker

 © Agilent Technologies, Inc. 2017

H2: H8 P R2

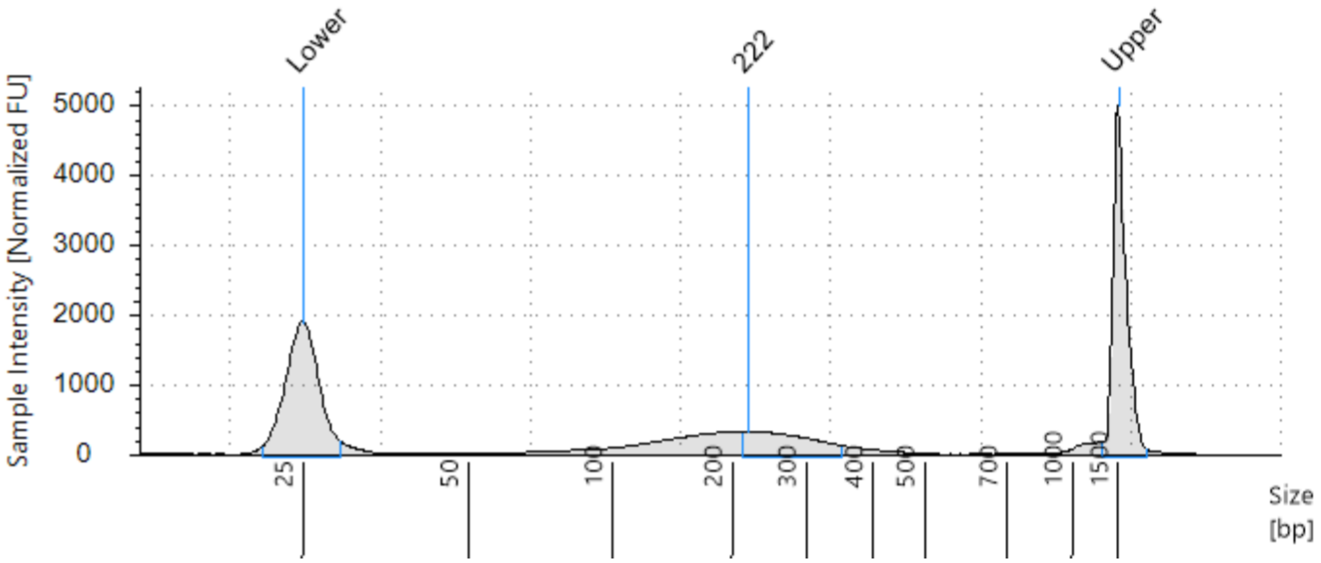

Sample Table

Well	Conc. [ng/µl]	Sample Description	Alert	Observations
H2	2.12	H8 P R2	⚠	Caution! Expired ScreenTape device

Peak Table

Size [bp]	Calibrated Conc. [ng/µl]	Assigned Conc. [ng/µl]	Peak Molarity [nmol/l]	% Integrated Area	Peak Comment	Observations
25	6.28	-	386	-		Lower Marker
222	2.12	-	14.6	100.00		
1500	6.50	6.50	6.67	-		Upper Marker

© Agilent Technologies, Inc. 2017

Filename: 2020-09-15-02 Q-S plus A9-F10 R2.D1000

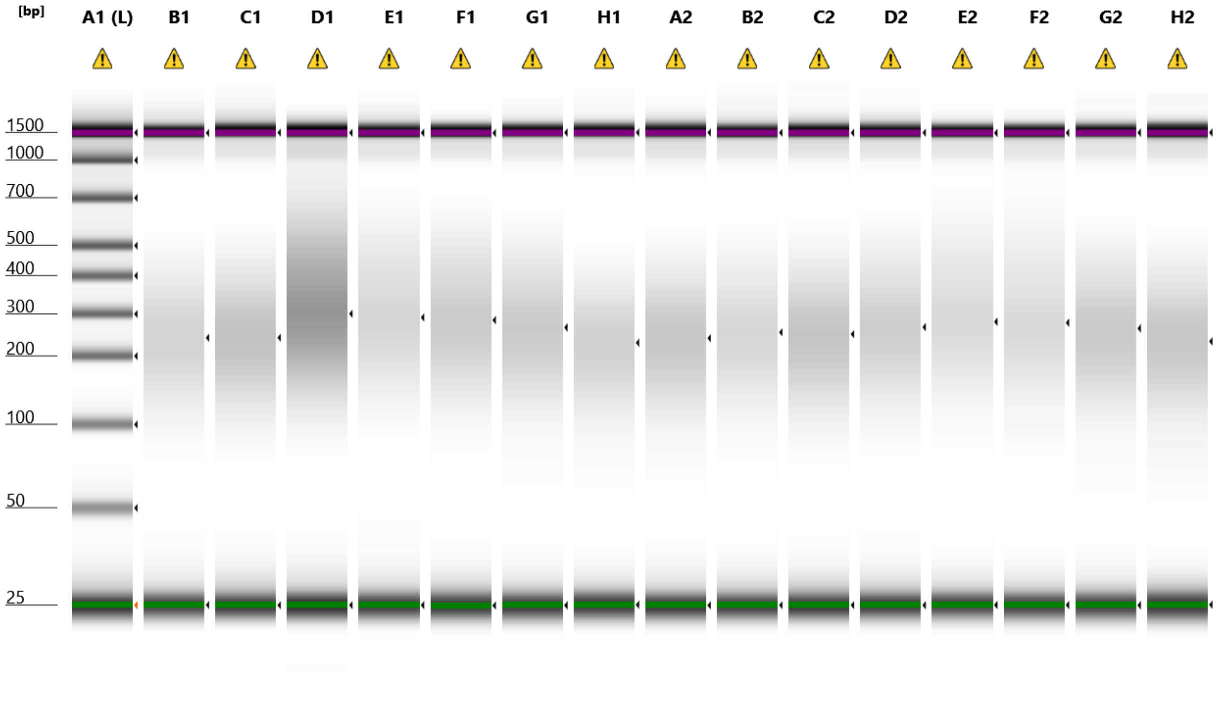

Default image (Contrast 100%)

Sample Info

Well	Conc. [ng/µl]	Sample Description	Alert	Observations
A1	13.5	Ladder	⚠	Caution! Expired ScreenTape device; Ladder
B1	1.81	A9 P R2	⚠	Caution! Expired ScreenTape device
C1	2.62	B9 P R2	⚠	Caution! Expired ScreenTape device
D1	4.19	C9 P R2	⚠	Caution! Expired ScreenTape device
E1	0.360	D9 P R2	⚠	Caution! Expired ScreenTape device
F1	0.510	E9 P R2	⚠	Caution! Expired ScreenTape device
G1	4.13	F9 P R2	⚠	Caution! Expired ScreenTape device
H1	2.14	G9 P R2	⚠	Caution! Expired ScreenTape device
A2	4.36	H9 P R2	⚠	Caution! Expired ScreenTape device
B2	1.57	A10 P R2	⚠	Caution! Expired ScreenTape device
C2	2.26	B10 P R2	⚠	Caution! Expired ScreenTape device
D2	1.88	C10 P R2	⚠	Caution! Expired ScreenTape device
E2	1.66	D10 P R2	⚠	Caution! Expired ScreenTape device
F2	1.73	E10 P R2	⚠	Caution! Expired ScreenTape device
G2	1.93	F10 P R2	⚠	Caution! Expired ScreenTape device
H2	2.24	G10 P R2	⚠	Caution! Expired ScreenTape device

© Agilent Technologies, Inc. 2017

A1: Ladder

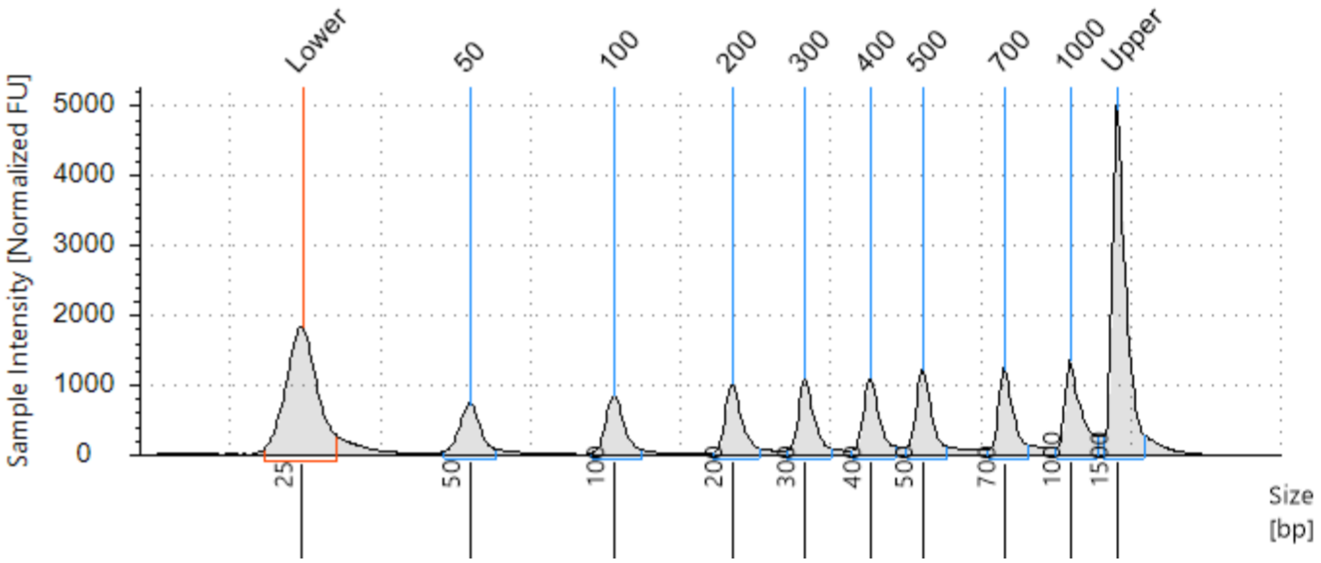

Sample Table

Well	Conc. [ng/µl]	Sample Description	Alert	Observations
A1	13.5	Ladder	⚠	Caution! Expired ScreenTape device; Ladder

Peak Table

Size [bp]	Calibrated Conc. [ng/µl]	Assigned Conc. [ng/µl]	Peak Molarity [nmol/l]	% Integrated Area	Peak Comment	Observations
25	5.44	-	335	-		Lower Marker
50	1.46	-	45.0	10.81		
100	1.49	-	22.9	11.00		
200	1.62	-	12.4	11.96		
300	1.65	-	8.45	12.17		
400	1.69	-	6.48	12.46		
500	1.80	-	5.53	13.30		
700	1.75	-	3.85	12.94		
1000	2.08	-	3.20	15.36		
1500	6.50	6.50	6.67	-		Upper Marker

© Agilent Technologies, Inc. 2017

B1: A9 P R2

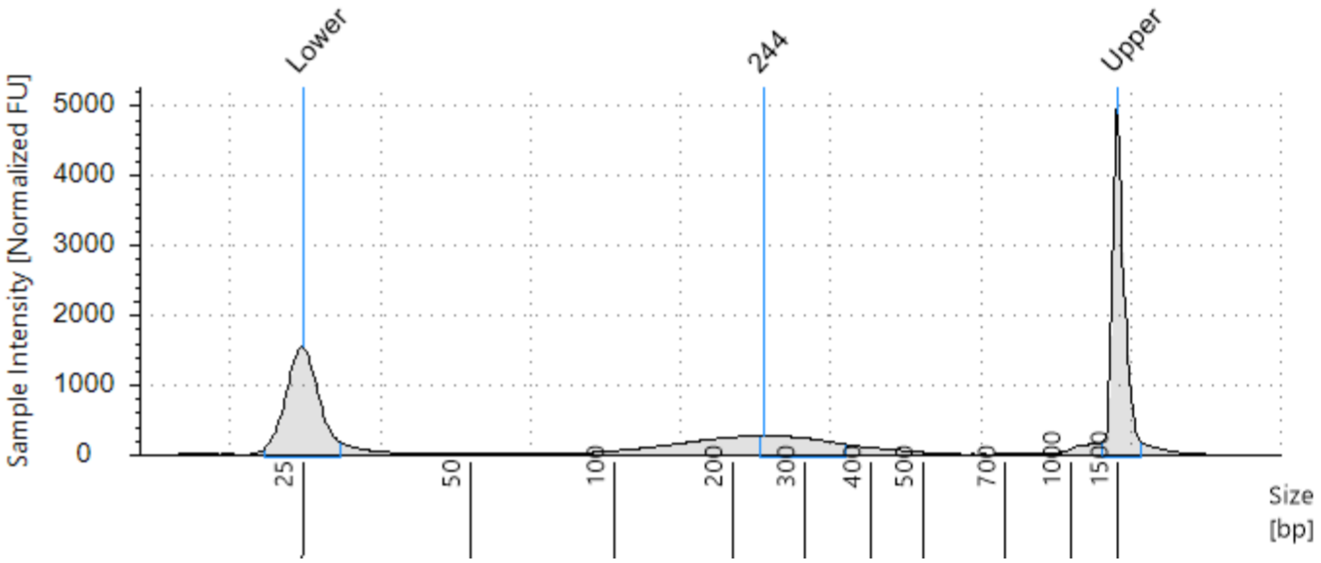

Sample Table

Well	Conc. [ng/µl]	Sample Description	Alert	Observations
B1	1.81	A9 P R2	⚠	Caution! Expired ScreenTape device

Peak Table

Size [bp]	Calibrated Conc. [ng/µl]	Assigned Conc. [ng/µl]	Peak Molarity [nmol/l]	% Integrated Area	Peak Comment	Observations
25	5.55	-	342	-		Lower Marker
244	1.81	-	11.5	100.00		
1500	6.50	6.50	6.67	-		Upper Marker

© Agilent Technologies, Inc. 2017

C1: B9 P R2

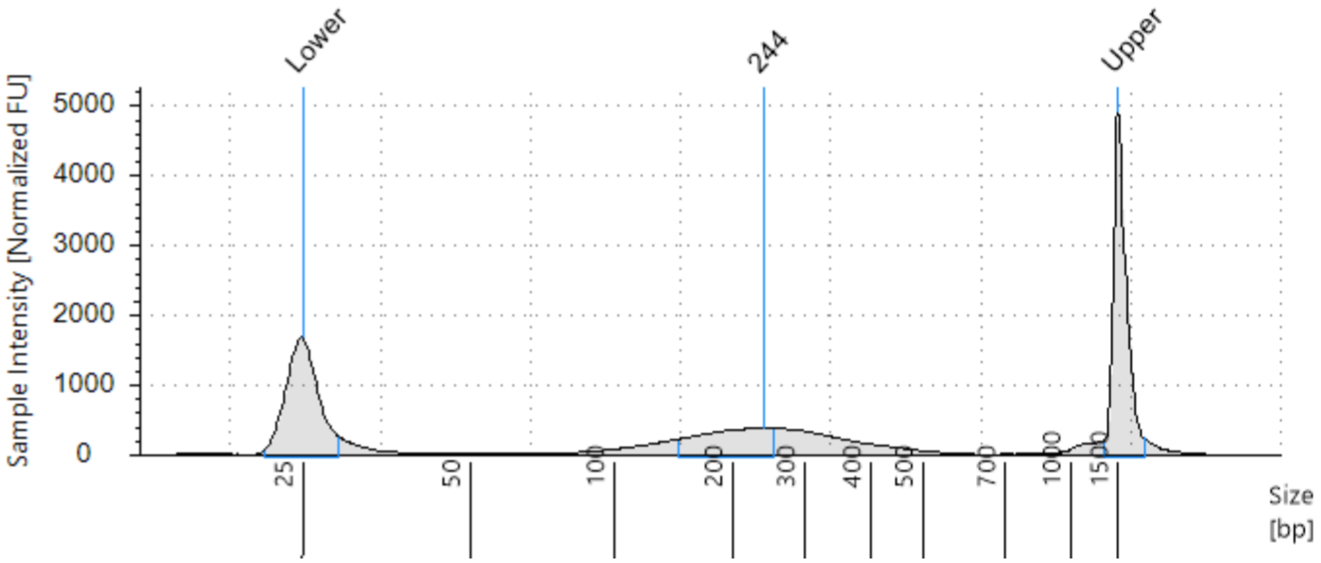

Sample Table

Well	Conc. [ng/µl]	Sample Description	Alert	Observations
C1	2.62	B9 P R2	⚠	Caution! Expired ScreenTape device

Peak Table

Size [bp]	Calibrated Conc. [ng/µl]	Assigned Conc. [ng/µl]	Peak Molarity [nmol/l]	% Integrated Area	Peak Comment	Observations
25	5.46	-	336	-		Lower Marker
244	2.62	-	16.6	100.00		
1500	6.50	6.50	6.67	-		Upper Marker

© Agilent Technologies, Inc. 2017

D1: C9 P R2

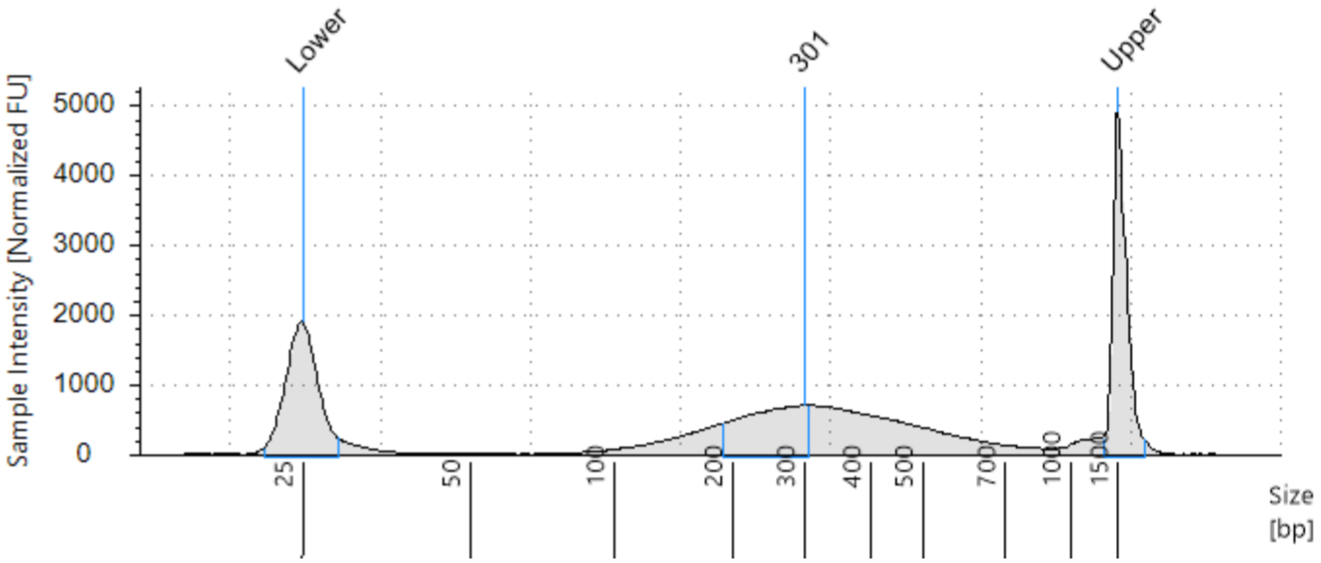

Sample Table

Well	Conc. [ng/µl]	Sample Description	Alert	Observations
D1	4.19	C9 P R2	⚠	Caution! Expired ScreenTape device

Peak Table

Size [bp]	Calibrated Conc. [ng/µl]	Assigned Conc. [ng/µl]	Peak Molarity [nmol/l]	% Integrated Area	Peak Comment	Observations
25	5.89	-	362	-		Lower Marker
301	4.19	-	21.5	100.00		
1500	6.50	6.50	6.67	-		Upper Marker

© Agilent Technologies, Inc. 2017

E1: D9 P R2

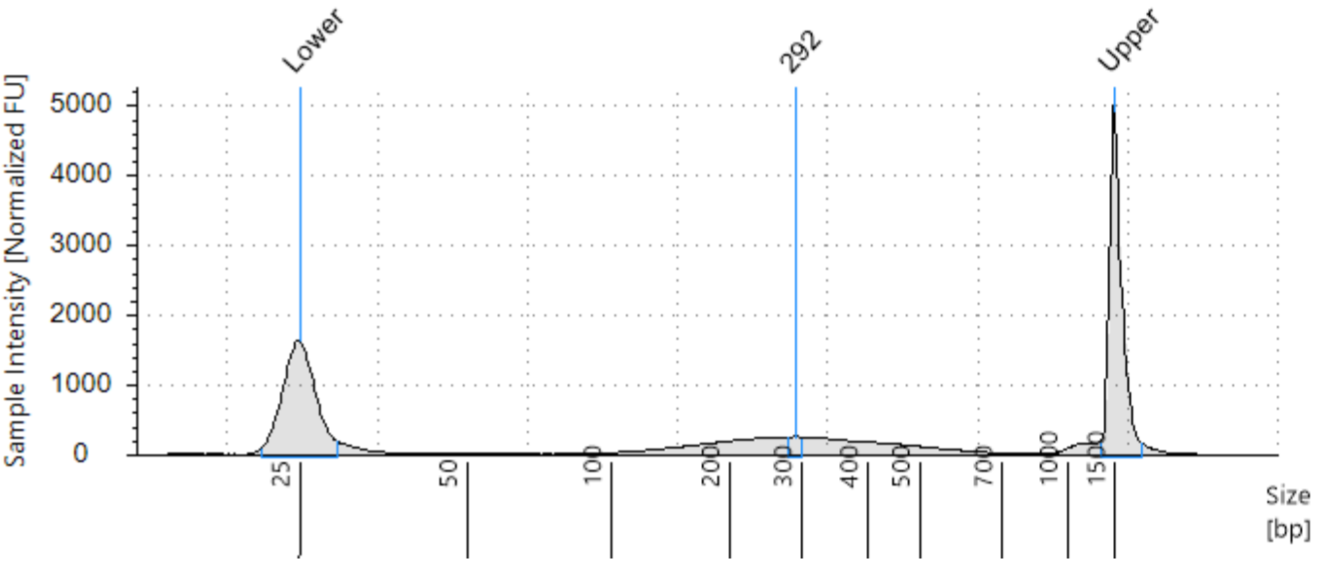

Sample Table

Well	Conc. [ng/µl]	Sample Description	Alert	Observations
E1	0.360	D9 P R2	⚠	Caution! Expired ScreenTape device

Peak Table

Size [bp]	Calibrated Conc. [ng/µl]	Assigned Conc. [ng/µl]	Peak Molarity [nmol/l]	% Integrated Area	Peak Comment	Observations
25	5.66	-	348	-		Lower Marker
292	0.360	-	1.90	100.00		
1500	6.50	6.50	6.67	-		Upper Marker

 © Agilent Technologies, Inc. 2017

F1: E9 P R2

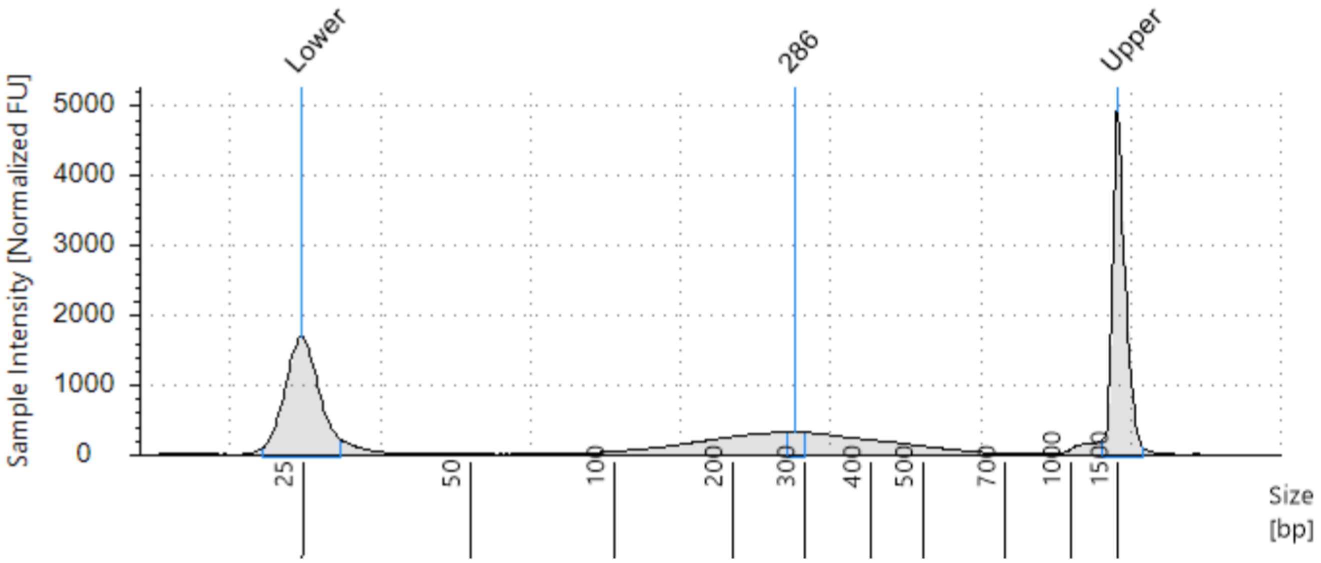

Sample Table

Well	Conc. [ng/µl]	Sample Description	Alert	Observations
F1	0.510	E9 P R2	⚠	Caution! Expired ScreenTape device

Peak Table

Size [bp]	Calibrated Conc. [ng/µl]	Assigned Conc. [ng/µl]	Peak Molarity [nmol/l]	% Integrated Area	Peak Comment	Observations
25	5.88	-	362	-		Lower Marker
286	0.510	-	2.74	100.00		
1500	6.50	6.50	6.67	-		Upper Marker

 © Agilent Technologies, Inc. 2017

G1: F9 P R2

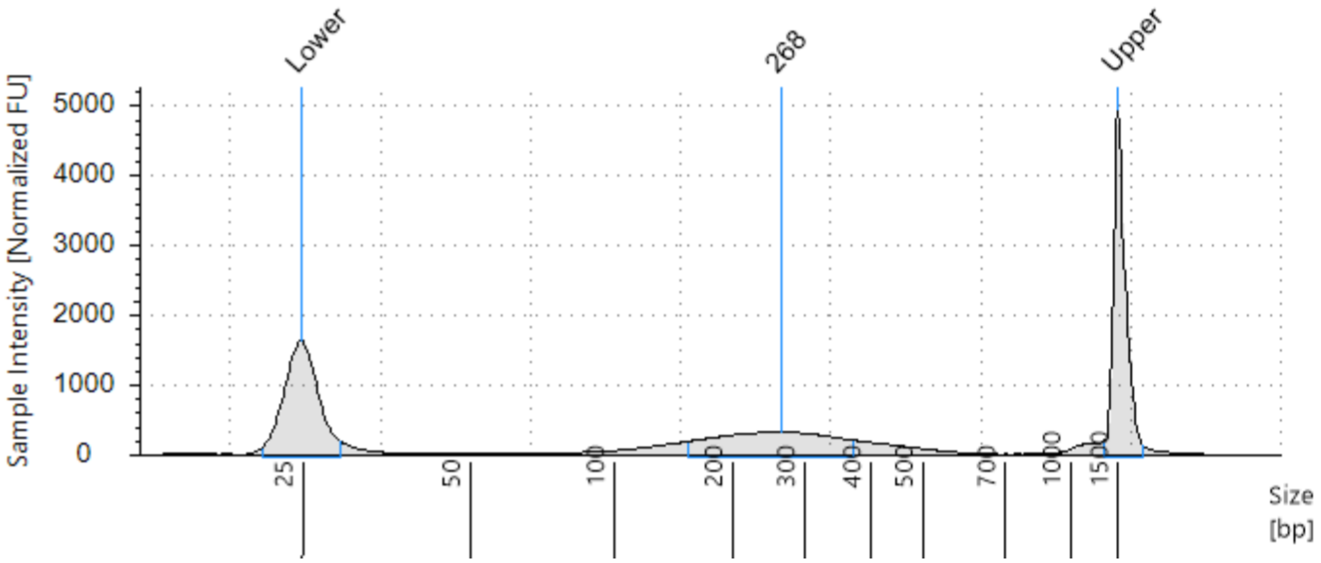

Sample Table

Well	Conc. [ng/µl]	Sample Description	Alert	Observations
G1	4.13	F9 P R2	⚠	Caution! Expired ScreenTape device

Peak Table

Size [bp]	Calibrated Conc. [ng/µl]	Assigned Conc. [ng/µl]	Peak Molarity [nmol/l]	% Integrated Area	Peak Comment	Observations
25	5.97	-	367	-		Lower Marker
268	4.13	-	23.7	100.00		
1500	6.50	6.50	6.67	-		Upper Marker

H1: G9 P R2

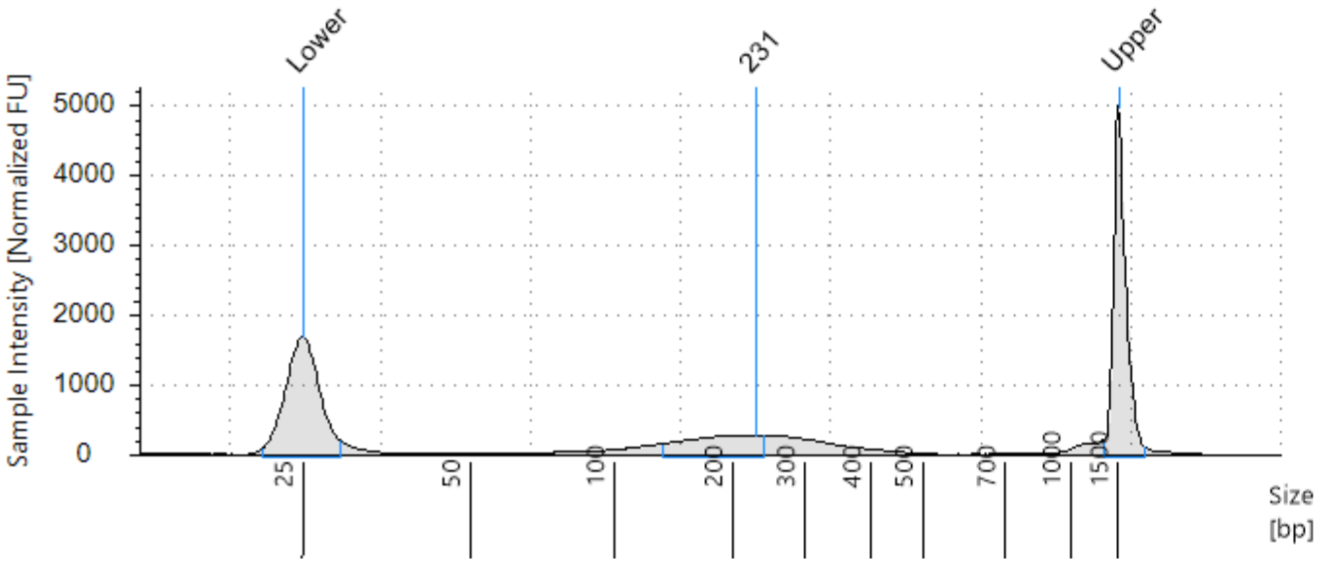

Sample Table

Well	Conc. [ng/µl]	Sample Description	Alert	Observations
H1	2.14	G9 P R2	⚠	Caution! Expired ScreenTape device

Peak Table

Size [bp]	Calibrated Conc. [ng/µl]	Assigned Conc. [ng/µl]	Peak Molarity [nmol/l]	% Integrated Area	Peak Comment	Observations
25	6.04	-	372	-		Lower Marker
231	2.14	-	14.2	100.00		
1500	6.50	6.50	6.67	-		Upper Marker

© Agilent Technologies, Inc. 2017

A2: H9 P R2

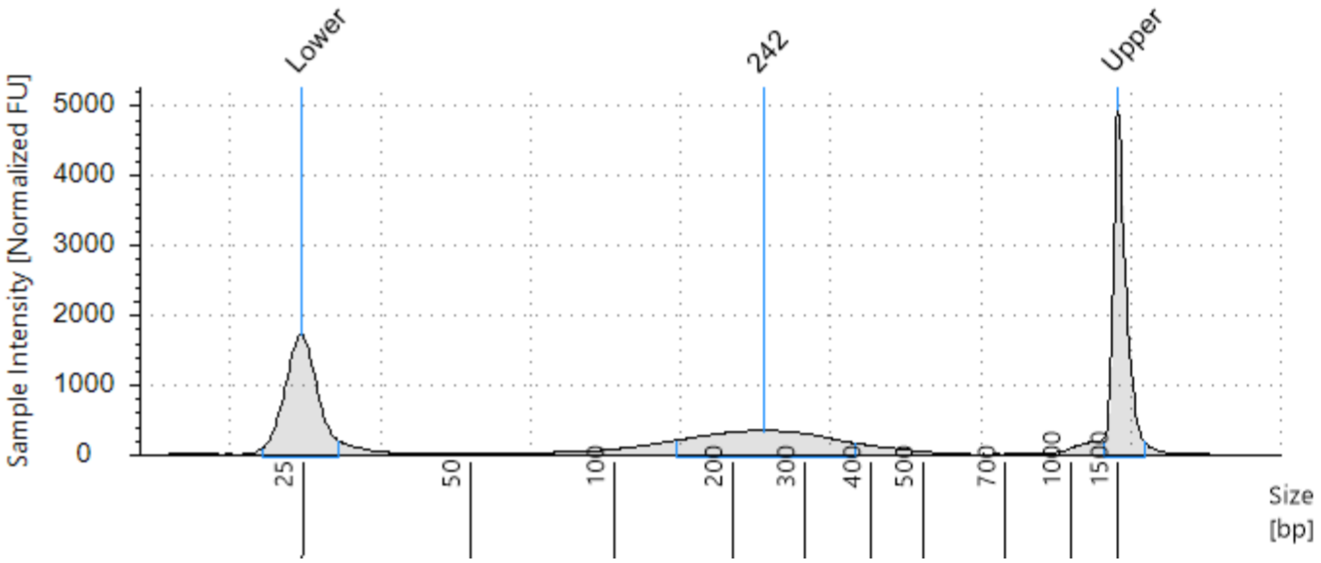

Sample Table

Well	Conc. [ng/µl]	Sample Description	Alert	Observations
A2	4.36	H9 P R2	⚠	Caution! Expired ScreenTape device

Peak Table

Size [bp]	Calibrated Conc. [ng/µl]	Assigned Conc. [ng/µl]	Peak Molarity [nmol/l]	% Integrated Area	Peak Comment	Observations
25	5.62	-	346	-		Lower Marker
242	4.36	-	27.7	100.00		
1500	6.50	6.50	6.67	-		Upper Marker

© Agilent Technologies, Inc. 2017

B2: A10 P R2

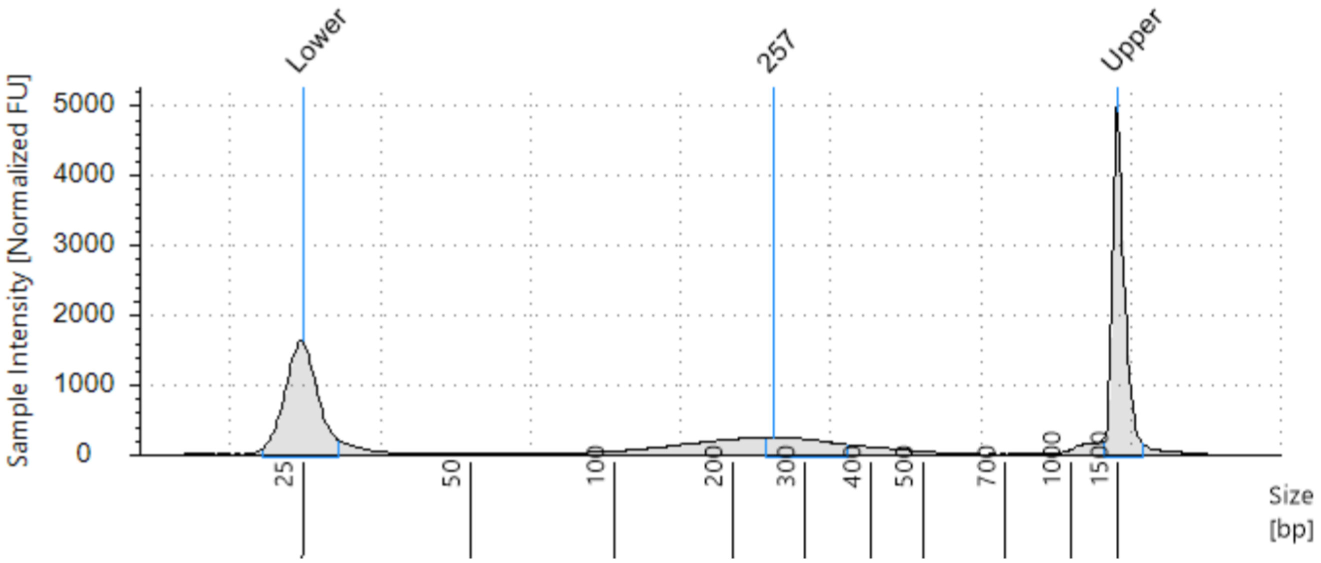

Sample Table

Well	Conc. [ng/µl]	Sample Description	Alert	Observations
B2	1.57	A10 P R2	⚠	Caution! Expired ScreenTape device

Peak Table

Size [bp]	Calibrated Conc. [ng/µl]	Assigned Conc. [ng/µl]	Peak Molarity [nmol/l]	% Integrated Area	Peak Comment	Observations
25	5.71	-	351	-		Lower Marker
257	1.57	-	9.42	100.00		
1500	6.50	6.50	6.67	-		Upper Marker

© Agilent Technologies, Inc. 2017

C2: B10 P R2

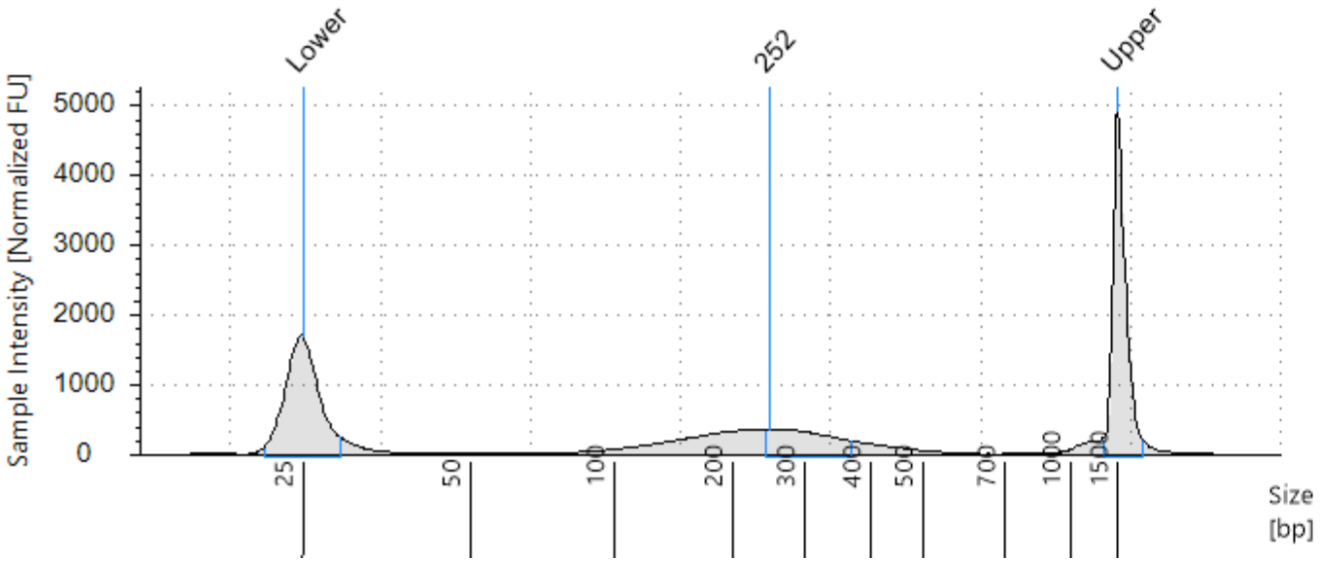

Sample Table

Well	Conc. [ng/µl]	Sample Description	Alert	Observations
C2	2.26	B10 P R2	⚠	Caution! Expired ScreenTape device

Peak Table

Size [bp]	Calibrated Conc. [ng/µl]	Assigned Conc. [ng/µl]	Peak Molarity [nmol/l]	% Integrated Area	Peak Comment	Observations
25	5.76	-	355	-		Lower Marker
252	2.26	-	13.8	100.00		
1500	6.50	6.50	6.67	-		Upper Marker

 © Agilent Technologies, Inc. 2017

D2: C10 P R2

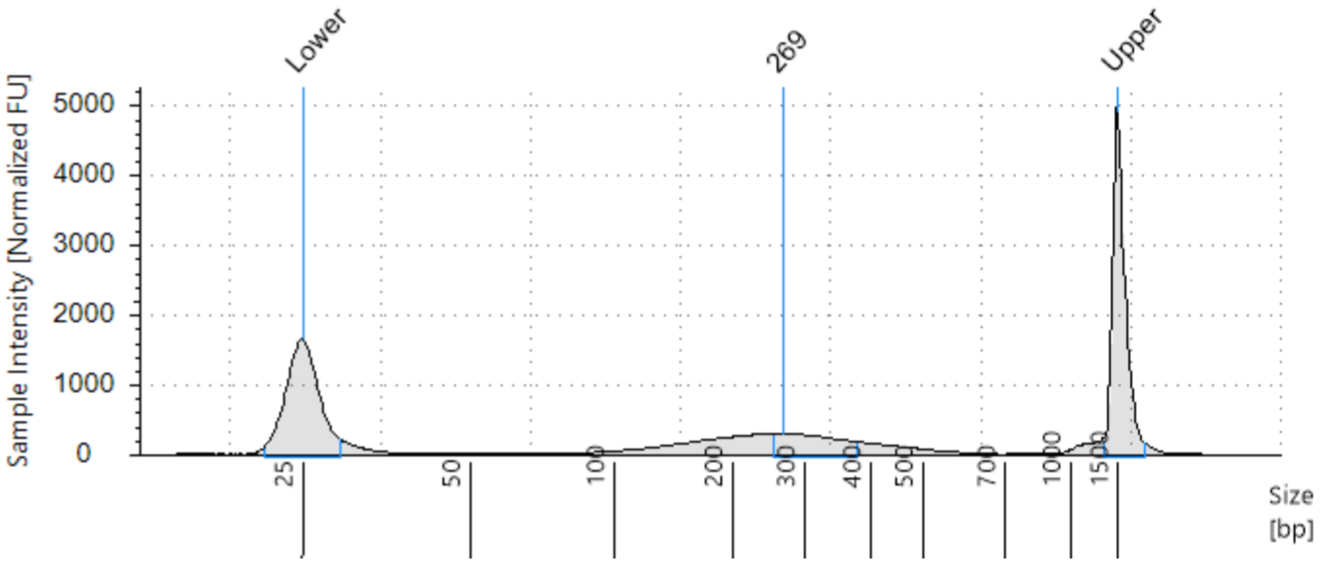

Sample Table

Well	Conc. [ng/µl]	Sample Description	Alert	Observations
D2	1.88	C10 P R2	⚠	Caution! Expired ScreenTape device

Peak Table

Size [bp]	Calibrated Conc. [ng/µl]	Assigned Conc. [ng/µl]	Peak Molarity [nmol/l]	% Integrated Area	Peak Comment	Observations
25	5.64	-	347	-		Lower Marker
269	1.88	-	10.8	100.00		
1500	6.50	6.50	6.67	-		Upper Marker

© Agilent Technologies, Inc. 2017

E2: D10 P R2

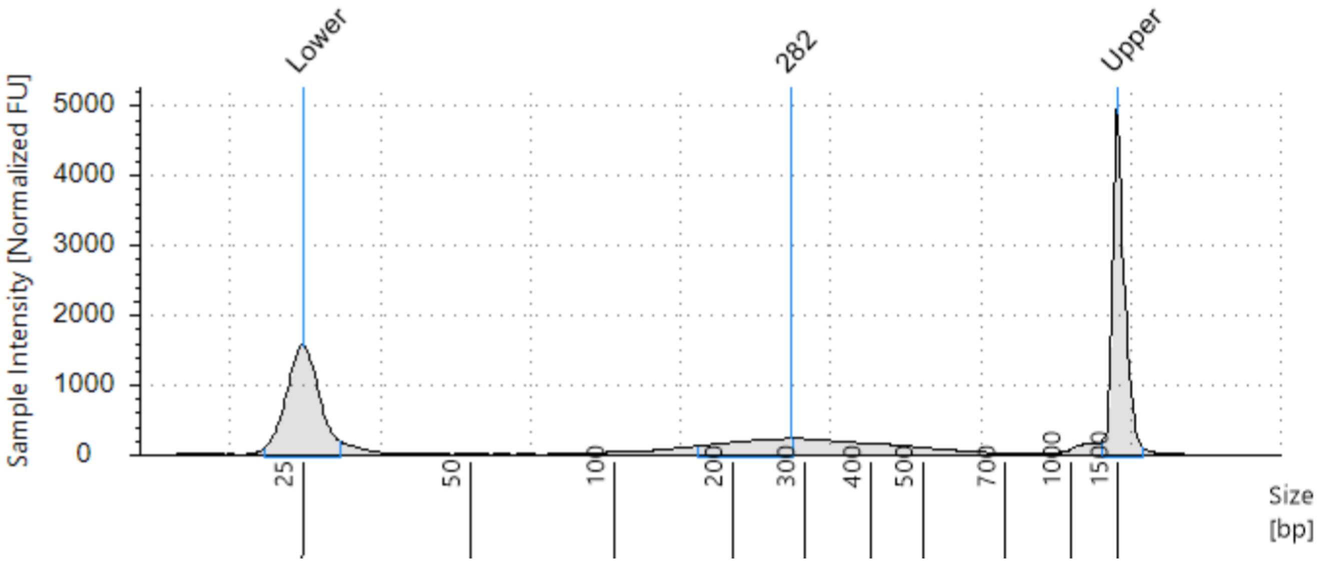

Sample Table

Well	Conc. [ng/µl]	Sample Description	Alert	Observations
E2	1.66	D10 P R2	⚠	Caution! Expired ScreenTape device

Peak Table

Size [bp]	Calibrated Conc. [ng/µl]	Assigned Conc. [ng/µl]	Peak Molarity [nmol/l]	% Integrated Area	Peak Comment	Observations
25	5.62	-	346	-		Lower Marker
282	1.66	-	9.06	100.00		
1500	6.50	6.50	6.67	-		Upper Marker

 © Agilent Technologies, Inc. 2017

F2: E10 P R2

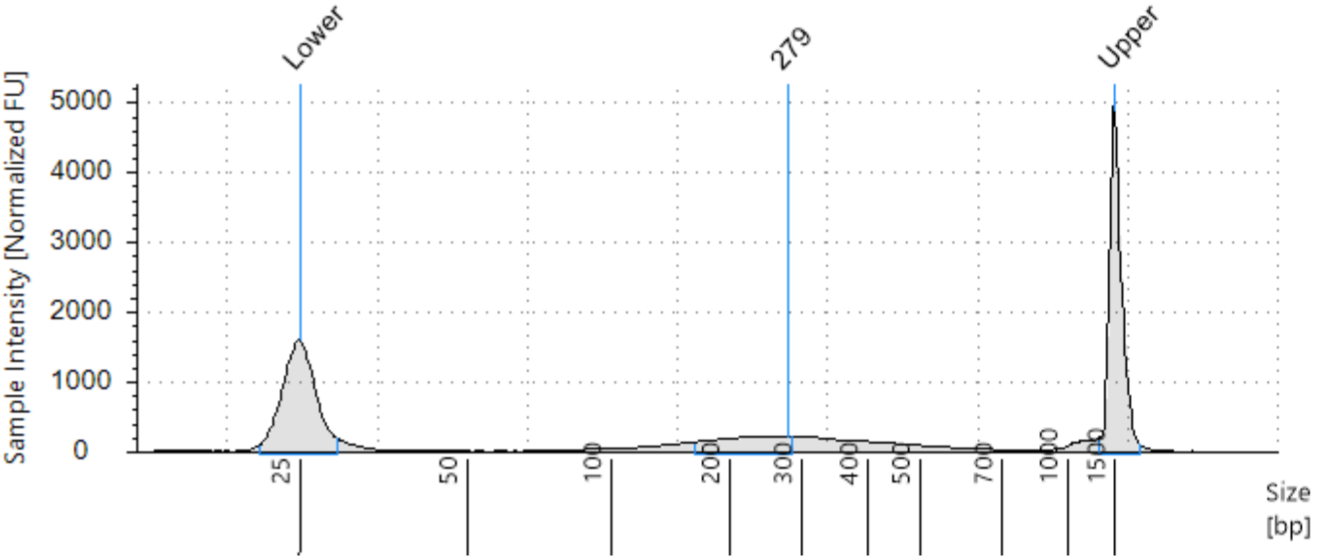

Sample Table

Well	Conc. [ng/µl]	Sample Description	Alert	Observations
F2	1.73	E10 P R2	⚠	Caution! Expired ScreenTape device

Peak Table

Size [bp]	Calibrated Conc. [ng/µl]	Assigned Conc. [ng/µl]	Peak Molarity [nmol/l]	% Integrated Area	Peak Comment	Observations
25	5.67	-	349	-		Lower Marker
279	1.73	-	9.53	100.00		
1500	6.50	6.50	6.67	-		Upper Marker

© Agilent Technologies, Inc. 2017

G2: F10 P R2

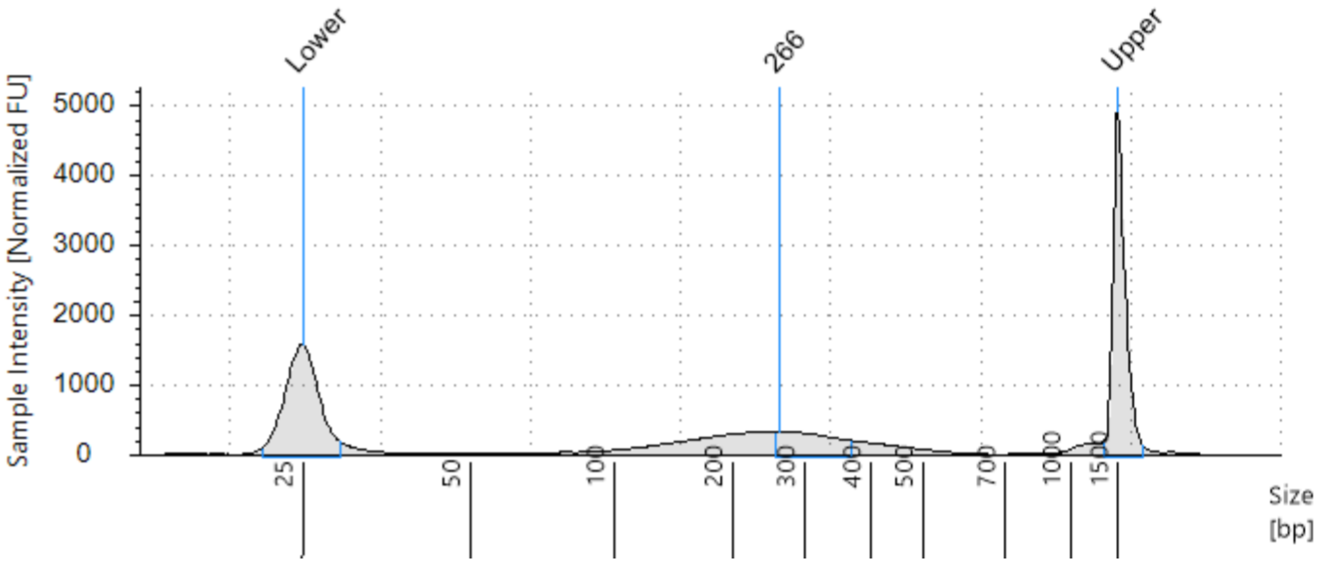

Sample Table

Well	Conc. [ng/µl]	Sample Description	Alert	Observations
G2	1.93	F10 P R2	⚠	Caution! Expired ScreenTape device

Peak Table

Size [bp]	Calibrated Conc. [ng/µl]	Assigned Conc. [ng/µl]	Peak Molarity [nmol/l]	% Integrated Area	Peak Comment	Observations
25	5.71	-	351	-		Lower Marker
266	1.93	-	11.1	100.00		
1500	6.50	6.50	6.67	-		Upper Marker

© Agilent Technologies, Inc. 2017

H2: G10 P R2

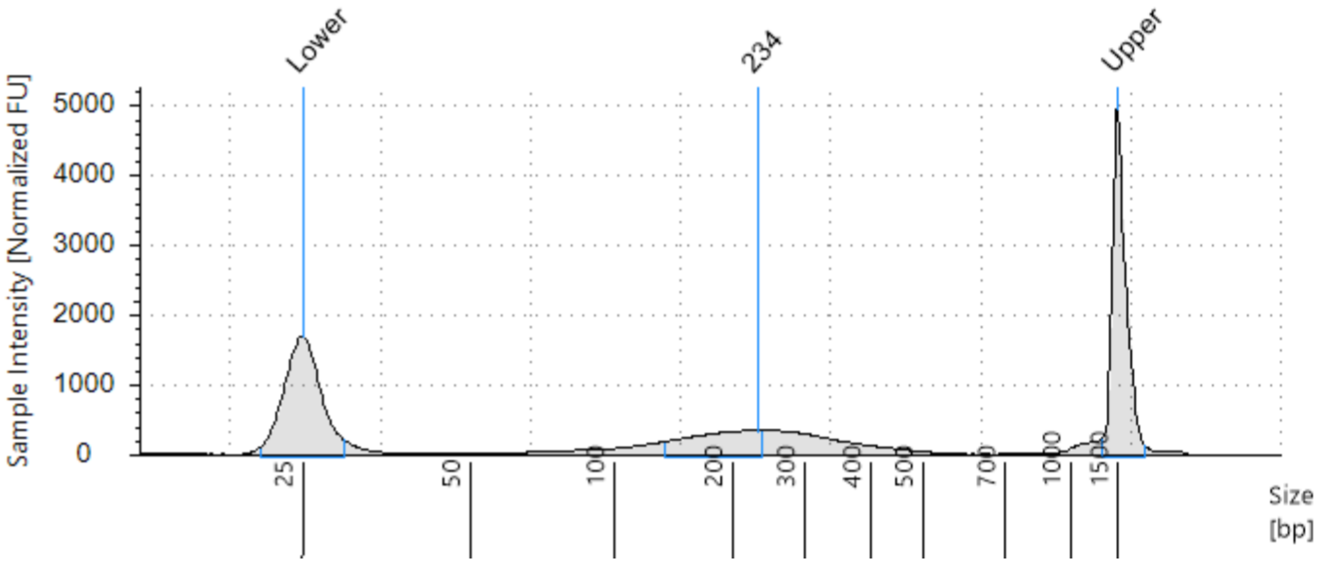

Sample Table

Well	Conc. [ng/µl]	Sample Description	Alert	Observations
H2	2.24	G10 P R2	⚠	Caution! Expired ScreenTape device

Peak Table

Size [bp]	Calibrated Conc. [ng/µl]	Assigned Conc. [ng/µl]	Peak Molarity [nmol/l]	% Integrated Area	Peak Comment	Observations
25	5.88	-	362	-		Lower Marker
234	2.24	-	14.7	100.00		
1500	6.50	6.50	6.67	-		Upper Marker

© Agilent Technologies, Inc. 2017

Filename: 2020-09-15-03 Q-S plus H10-F12 R2.D1000

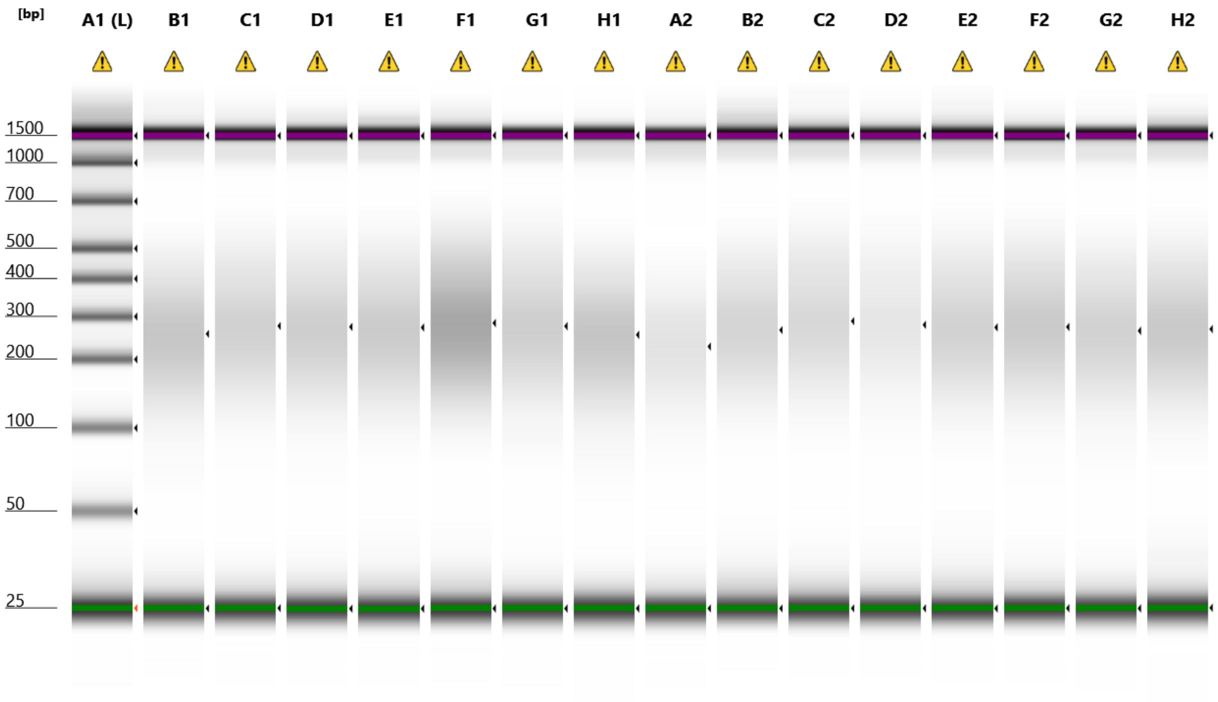

Default image (Contrast 100%)

Sample Info

Well	Conc. [ng/µl]	Sample Description	Alert	Observations
A1	13.2	Ladder	⚠	Caution! Expired ScreenTape device; Ladder
B1	0.558	H10 P R2	⚠	Caution! Expired ScreenTape device
C1	2.07	A11 P R2	⚠	Caution! Expired ScreenTape device
D1	1.59	B11 P R2	⚠	Caution! Expired ScreenTape device
E1	1.86	C11 P R2	⚠	Caution! Expired ScreenTape device
F1	0.774	D11 P R2	⚠	Caution! Expired ScreenTape device
G1	0.446	E11 P R2	⚠	Caution! Expired ScreenTape device
H1	2.55	F11 P R2	⚠	Caution! Expired ScreenTape device
A2	1.17	G11 P R2	⚠	Caution! Expired ScreenTape device
B2	1.68	H11 P R2	⚠	Caution! Expired ScreenTape device
C2	1.77	A12 P R2	⚠	Caution! Expired ScreenTape device
D2	1.50	B12 P R2	⚠	Caution! Expired ScreenTape device
E2	2.03	C12 P R2	⚠	Caution! Expired ScreenTape device
F2	2.15	D12 P R2	⚠	Caution! Expired ScreenTape device
G2	2.00	E12 P R2	⚠	Caution! Expired ScreenTape device
H2	3.80	F12 P R2	⚠	Caution! Expired ScreenTape device

 © Agilent Technologies, Inc. 2017

A1: Ladder

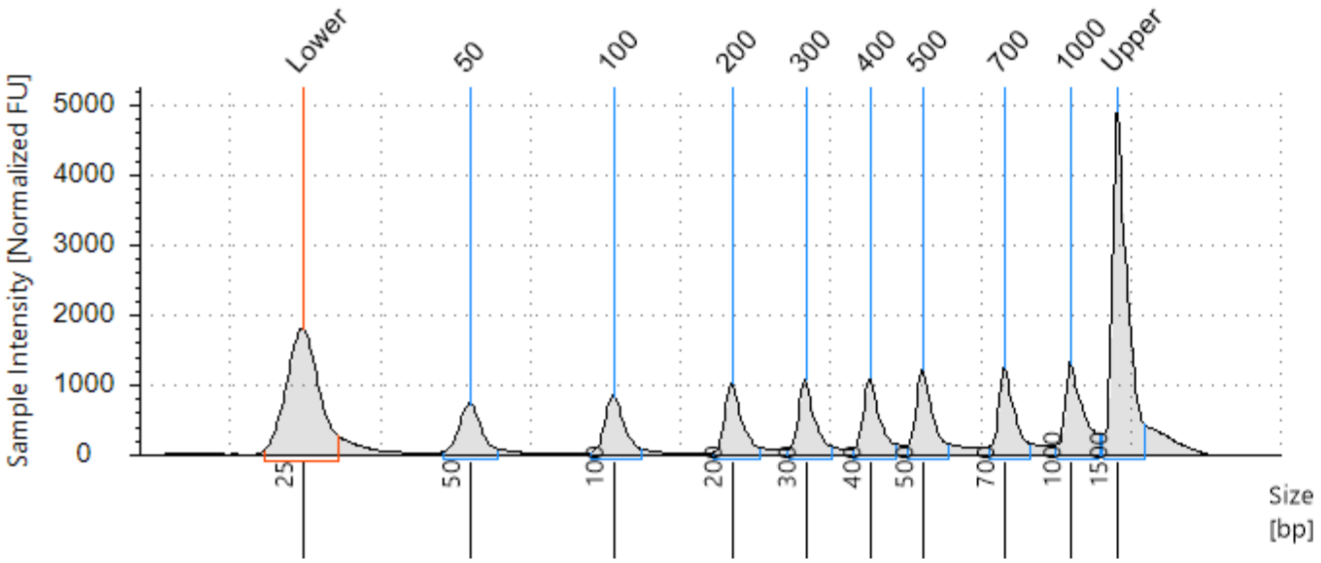

Sample Table

Well	Conc. [ng/µl]	Sample Description	Alert	Observations
A1	13.2	Ladder	⚠	Caution! Expired ScreenTape device; Ladder

Peak Table

Size [bp]	Calibrated Conc. [ng/µl]	Assigned Conc. [ng/µl]	Peak Molarity [nmol/l]	% Integrated Area	Peak Comment	Observations
25	5.18	-	319	-		Lower Marker
50	1.44	-	44.3	10.88		
100	1.45	-	22.4	10.98		
200	1.55	-	11.9	11.69		
300	1.57	-	8.03	11.83		
400	1.62	-	6.23	12.23		
500	1.77	-	5.44	13.36		
700	1.77	-	3.89	13.36		
1000	2.07	-	3.19	15.67		
1500	6.50	6.50	6.67	-		Upper Marker

© Agilent Technologies, Inc. 2017

B1: H10 P R2

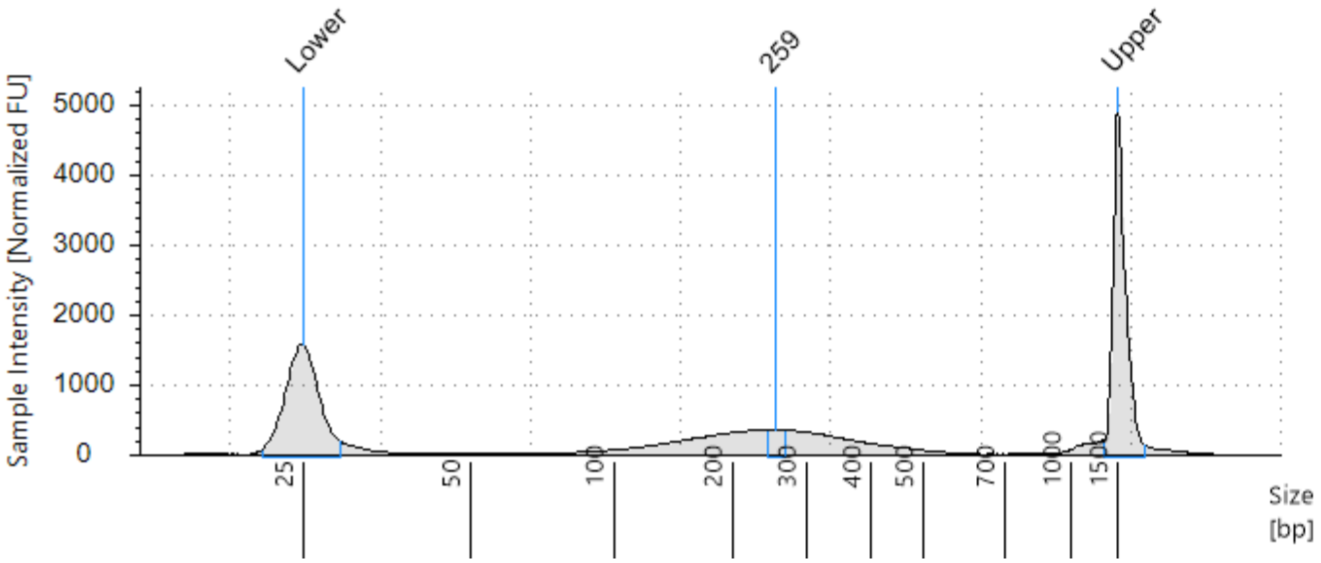

Sample Table

Well	Conc. [ng/µl]	Sample Description	Alert	Observations
B1	0.558	H10 P R2	⚠	Caution! Expired ScreenTape device

Peak Table

Size [bp]	Calibrated Conc. [ng/µl]	Assigned Conc. [ng/µl]	Peak Molarity [nmol/l]	% Integrated Area	Peak Comment	Observations
25	5.52	-	340	-		Lower Marker
259	0.558	-	3.31	100.00		
1500	6.50	6.50	6.67	-		Upper Marker

© Agilent Technologies, Inc. 2017

C1: A11 P R2

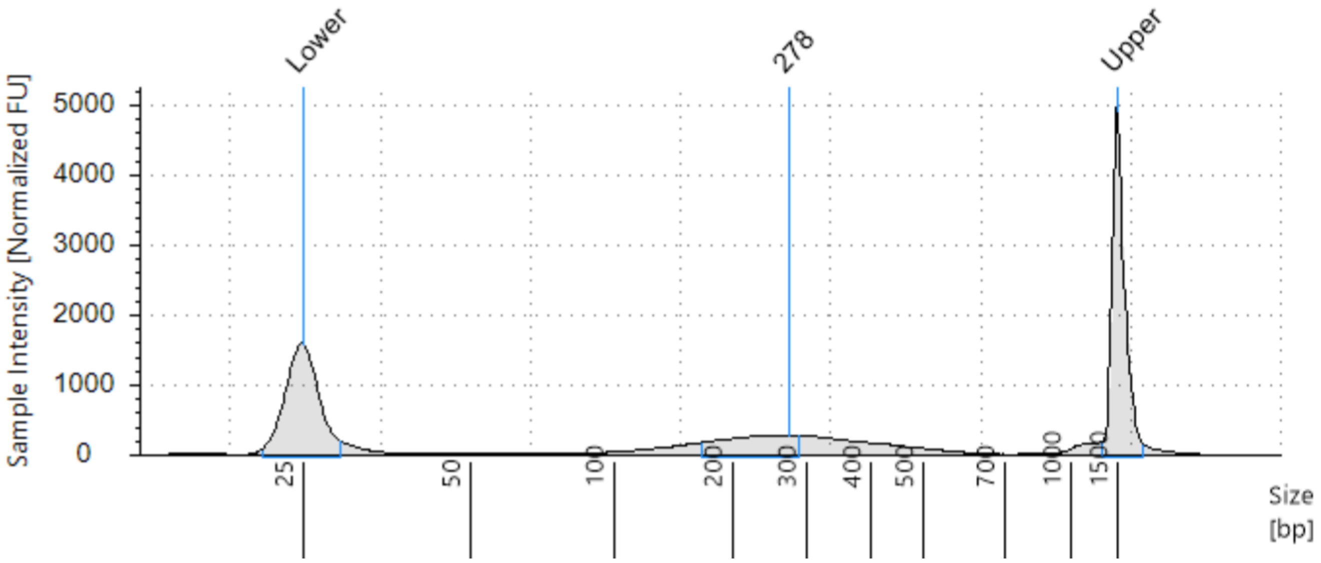

Sample Table

Well	Conc. [ng/µl]	Sample Description	Alert	Observations
C1	2.07	A11 P R2	⚠	Caution! Expired ScreenTape device

Peak Table

Size [bp]	Calibrated Conc. [ng/µl]	Assigned Conc. [ng/µl]	Peak Molarity [nmol/l]	% Integrated Area	Peak Comment	Observations
25	5.48	-	337	-		Lower Marker
278	2.07	-	11.5	100.00		
1500	6.50	6.50	6.67	-		Upper Marker

 © Agilent Technologies, Inc. 2017

D1: B11 P R2

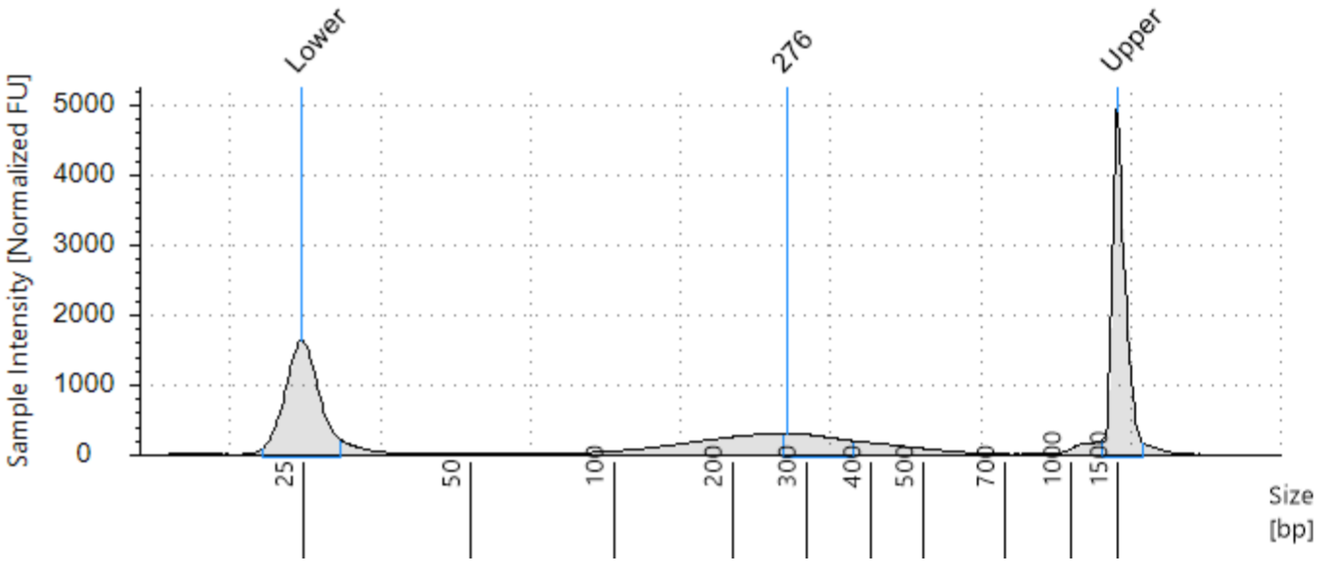

Sample Table

Well	Conc. [ng/µl]	Sample Description	Alert	Observations
D1	1.59	B11 P R2	⚠	Caution! Expired ScreenTape device

Peak Table

Size [bp]	Calibrated Conc. [ng/µl]	Assigned Conc. [ng/µl]	Peak Molarity [nmol/l]	% Integrated Area	Peak Comment	Observations
25	5.55	-	341	-		Lower Marker
276	1.59	-	8.87	100.00		
1500	6.50	6.50	6.67	-		Upper Marker

© Agilent Technologies, Inc. 2017

E1: C11 P R2

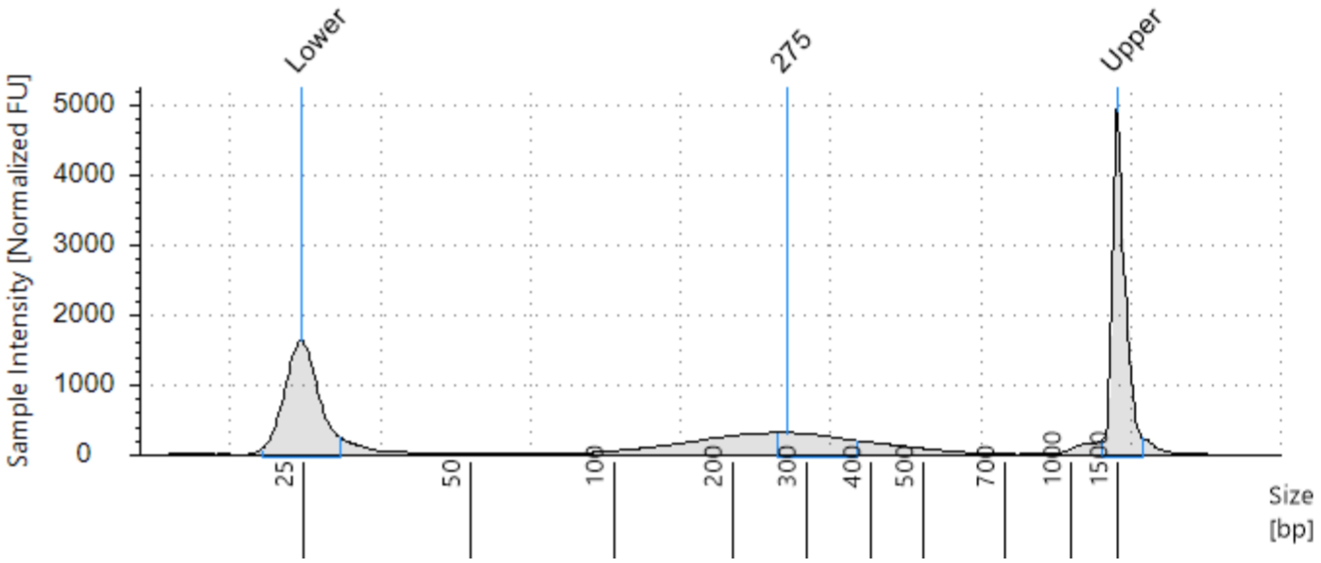

Sample Table

Well	Conc. [ng/µl]	Sample Description	Alert	Observations
E1	1.86	C11 P R2	⚠	Caution! Expired ScreenTape device

Peak Table

Size [bp]	Calibrated Conc. [ng/µl]	Assigned Conc. [ng/µl]	Peak Molarity [nmol/l]	% Integrated Area	Peak Comment	Observations
25	5.65	-	348	-		Lower Marker
275	1.86	-	10.4	100.00		
1500	6.50	6.50	6.67	-		Upper Marker

© Agilent Technologies, Inc. 2017

F1: D11 P R2

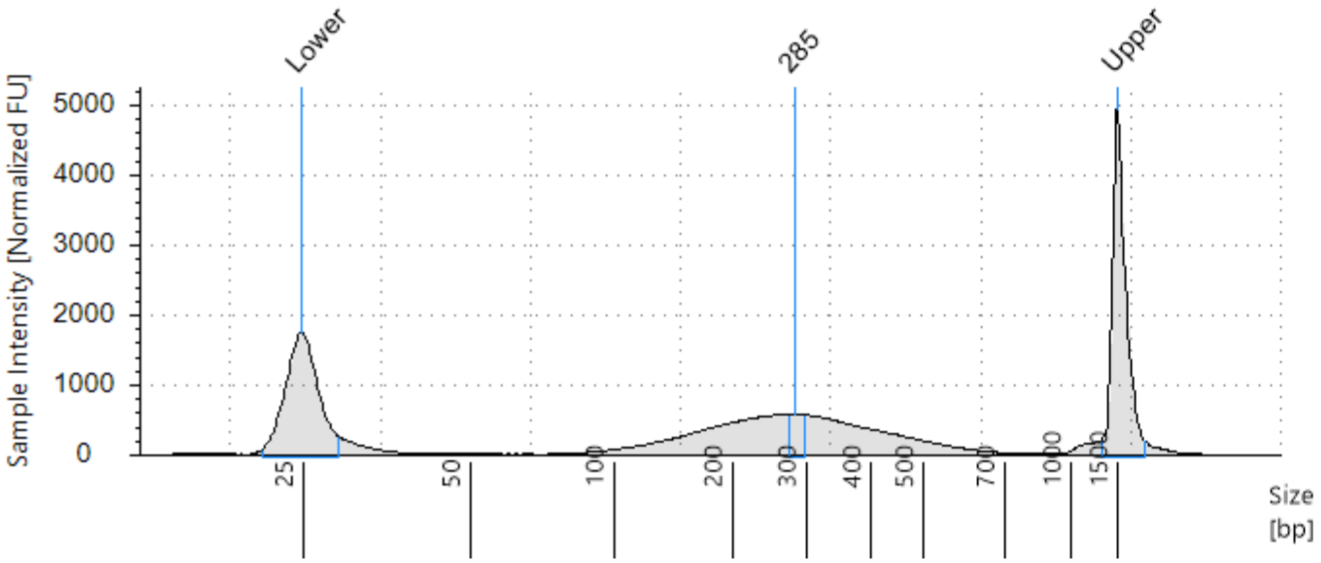

Sample Table

Well	Conc. [ng/µl]	Sample Description	Alert	Observations
F1	0.774	D11 P R2	⚠	Caution! Expired ScreenTape device

Peak Table

Size [bp]	Calibrated Conc. [ng/µl]	Assigned Conc. [ng/µl]	Peak Molarity [nmol/l]	% Integrated Area	Peak Comment	Observations
25	5.62	-	346	-		Lower Marker
285	0.774	-	4.18	100.00		
1500	6.50	6.50	6.67	-		Upper Marker

© Agilent Technologies, Inc. 2017

G1: E11 P R2

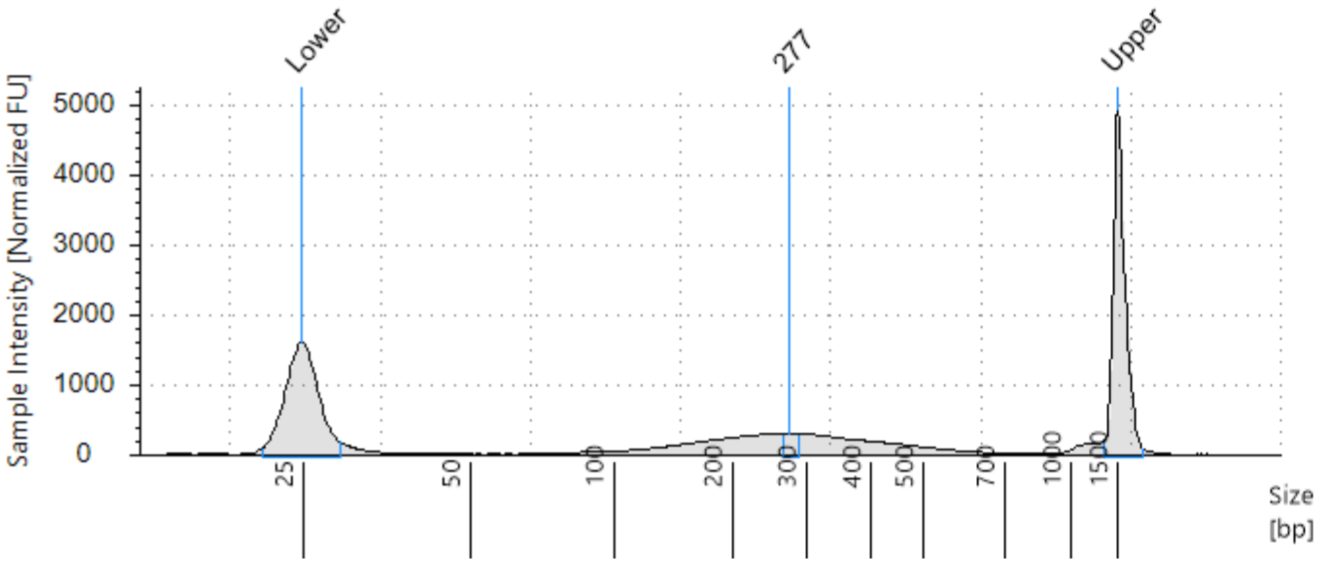

Sample Table

Well	Conc. [ng/µl]	Sample Description	Alert	Observations
G1	0.446	E11 P R2	⚠	Caution! Expired ScreenTape device

Peak Table

Size [bp]	Calibrated Conc. [ng/µl]	Assigned Conc. [ng/µl]	Peak Molarity [nmol/l]	% Integrated Area	Peak Comment	Observations
25	5.87	-	361	-		Lower Marker
277	0.446	-	2.47	100.00		
1500	6.50	6.50	6.67	-		Upper Marker

 © Agilent Technologies, Inc. 2017

H1: F11 P R2

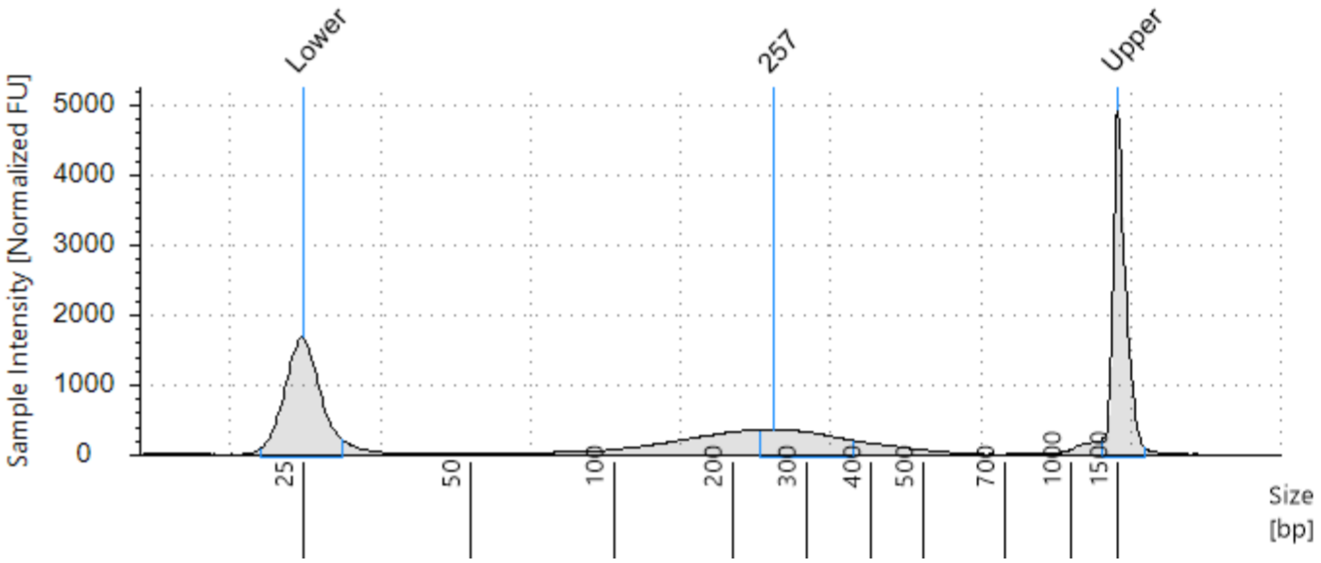

Sample Table

Well	Conc. [ng/µl]	Sample Description	Alert	Observations
H1	2.55	F11 P R2	⚠	Caution! Expired ScreenTape device

Peak Table

Size [bp]	Calibrated Conc. [ng/µl]	Assigned Conc. [ng/µl]	Peak Molarity [nmol/l]	% Integrated Area	Peak Comment	Observations
25	6.08	-	374	-		Lower Marker
257	2.55	-	15.2	100.00		
1500	6.50	6.50	6.67	-		Upper Marker

© Agilent Technologies, Inc. 2017

A2: G11 P R2

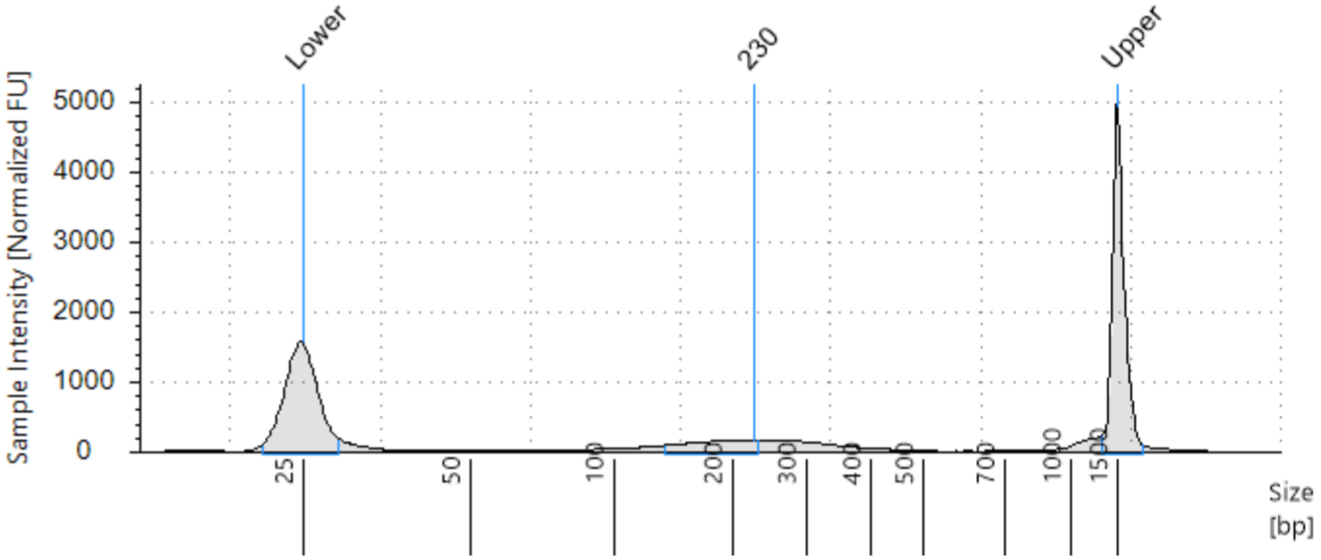

Sample Table

Well	Conc. [ng/µl]	Sample Description	Alert	Observations
A2	1.17	G11 P R2	⚠	Caution! Expired ScreenTape device

Peak Table

Size [bp]	Calibrated Conc. [ng/µl]	Assigned Conc. [ng/µl]	Peak Molarity [nmol/l]	% Integrated Area	Peak Comment	Observations
25	5.72	-	352	-		Lower Marker
230	1.17	-	7.81	100.00		
1500	6.50	6.50	6.67	-		Upper Marker

© Agilent Technologies, Inc. 2017

B2: H11 P R2

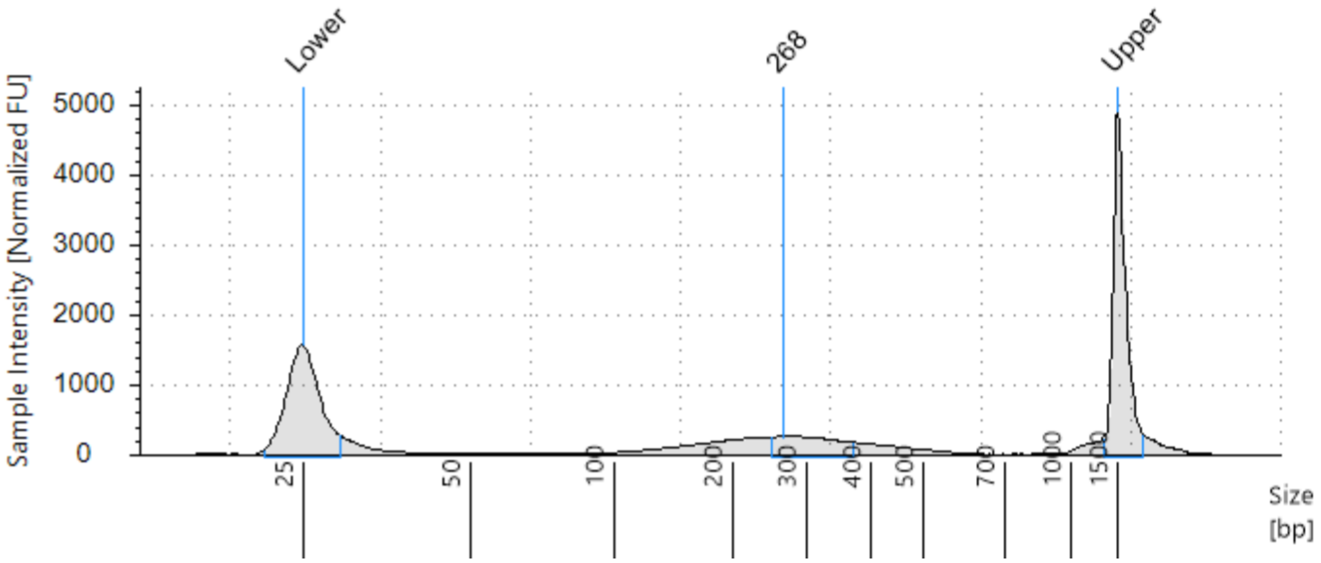

Sample Table

Well	Conc. [ng/µl]	Sample Description	Alert	Observations
B2	1.68	H11 P R2	⚠	Caution! Expired ScreenTape device

Peak Table

Size [bp]	Calibrated Conc. [ng/µl]	Assigned Conc. [ng/µl]	Peak Molarity [nmol/l]	% Integrated Area	Peak Comment	Observations
25	5.38	-	331	-		Lower Marker
268	1.68	-	9.63	100.00		
1500	6.50	6.50	6.67	-		Upper Marker

© Agilent Technologies, Inc. 2017

C2: A12 P R2

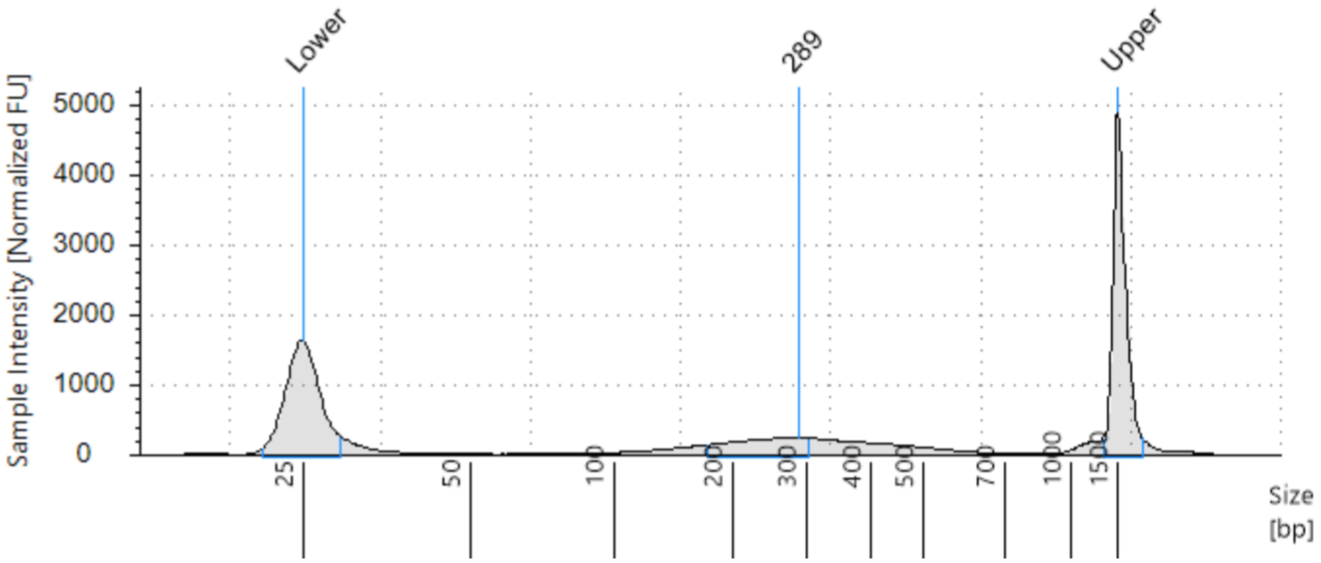

Sample Table

Well	Conc. [ng/µl]	Sample Description	Alert	Observations
C2	1.77	A12 P R2	⚠	Caution! Expired ScreenTape device

Peak Table

Size [bp]	Calibrated Conc. [ng/µl]	Assigned Conc. [ng/µl]	Peak Molarity [nmol/l]	% Integrated Area	Peak Comment	Observations
25	5.81	-	357	-		Lower Marker
289	1.77	-	9.41	100.00		
1500	6.50	6.50	6.67	-		Upper Marker

© Agilent Technologies, Inc. 2017

D2: B12 P R2

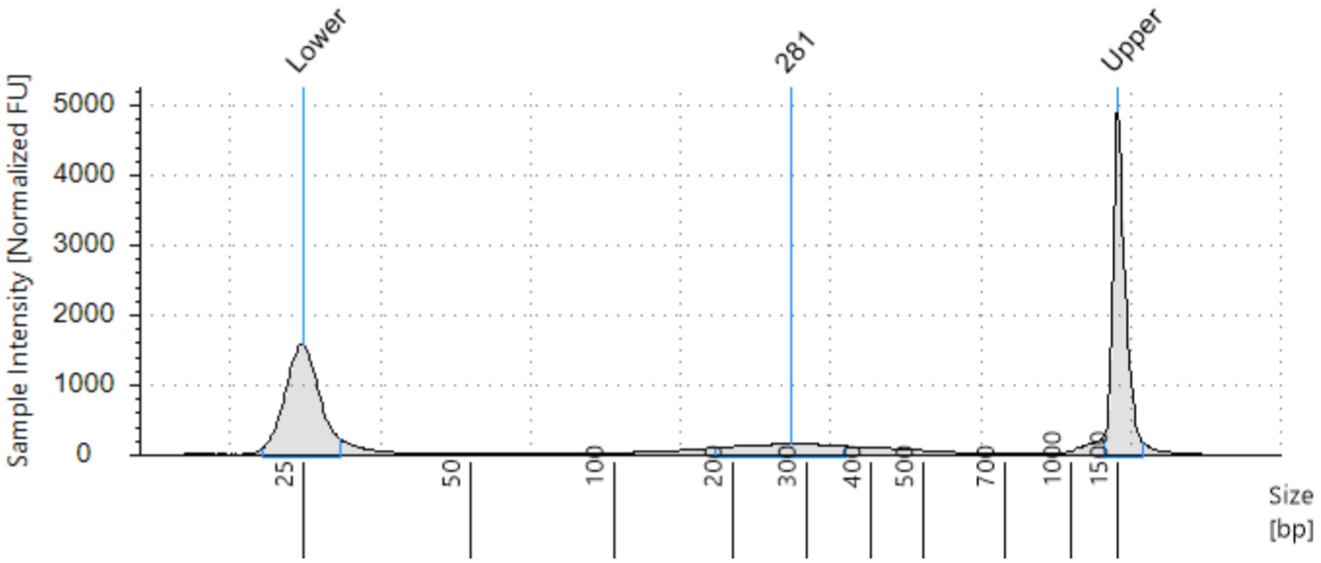

Sample Table

Well	Conc. [ng/µl]	Sample Description	Alert	Observations
D2	1.50	B12 P R2	⚠	Caution! Expired ScreenTape device

Peak Table

Size [bp]	Calibrated Conc. [ng/µl]	Assigned Conc. [ng/µl]	Peak Molarity [nmol/l]	% Integrated Area	Peak Comment	Observations
25	5.65	-	348	-		Lower Marker
281	1.50	-	8.25	100.00		
1500	6.50	6.50	6.67	-		Upper Marker

 © Agilent Technologies, Inc. 2017

E2: C12 P R2

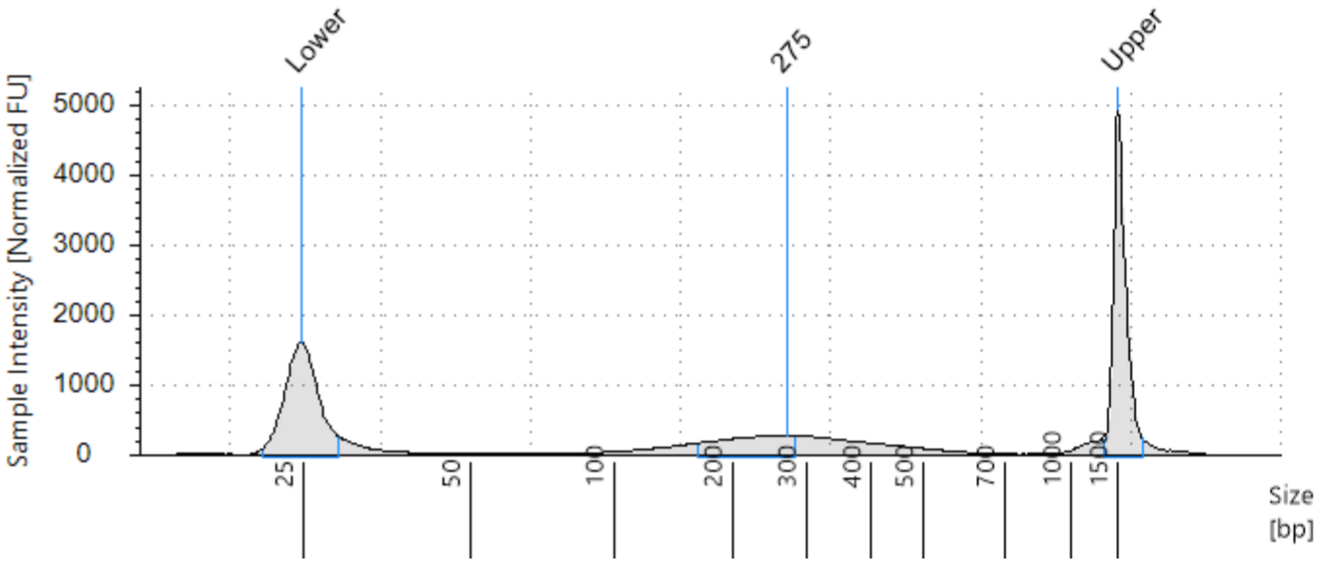

Sample Table

Well	Conc. [ng/µl]	Sample Description	Alert	Observations
E2	2.03	C12 P R2	⚠	Caution! Expired ScreenTape device

Peak Table

Size [bp]	Calibrated Conc. [ng/µl]	Assigned Conc. [ng/µl]	Peak Molarity [nmol/l]	% Integrated Area	Peak Comment	Observations
25	5.58	-	344	-		Lower Marker
275	2.03	-	11.4	100.00		
1500	6.50	6.50	6.67	-		Upper Marker

© Agilent Technologies, Inc. 2017

F2: D12 P R2

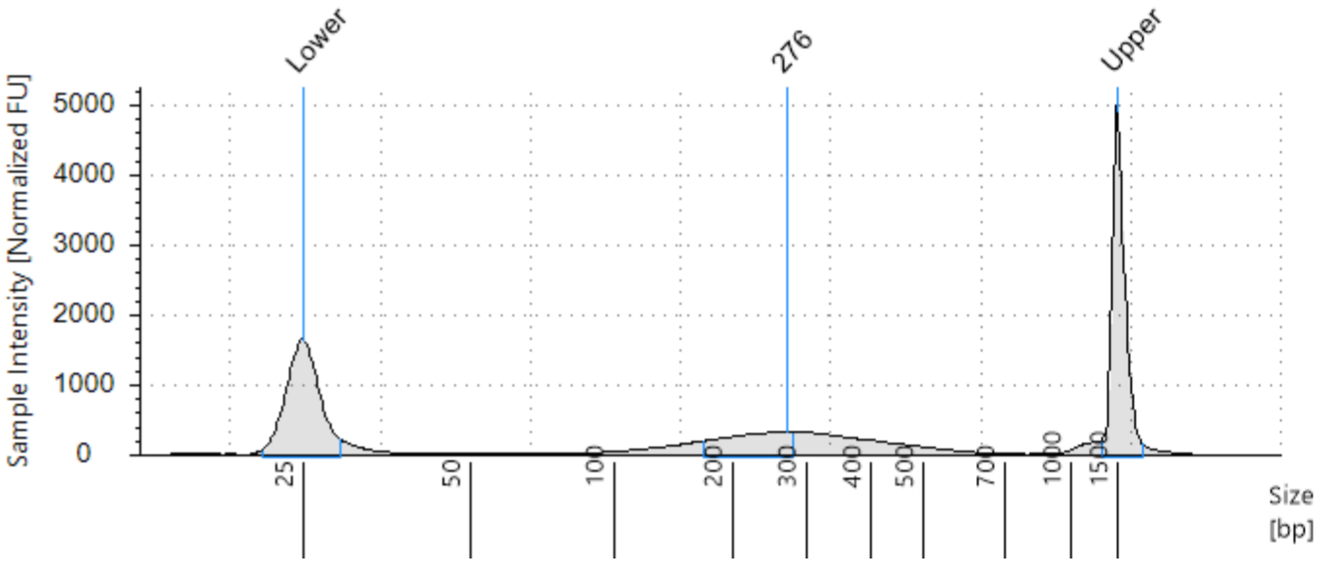

Sample Table

Well	Conc. [ng/µl]	Sample Description	Alert	Observations
F2	2.15	D12 P R2	⚠	Caution! Expired ScreenTape device

Peak Table

Size [bp]	Calibrated Conc. [ng/µl]	Assigned Conc. [ng/µl]	Peak Molarity [nmol/l]	% Integrated Area	Peak Comment	Observations
25	5.60	-	345	-		Lower Marker
276	2.15	-	12.0	100.00		
1500	6.50	6.50	6.67	-		Upper Marker

© Agilent Technologies, Inc. 2017

G2: E12 P R2

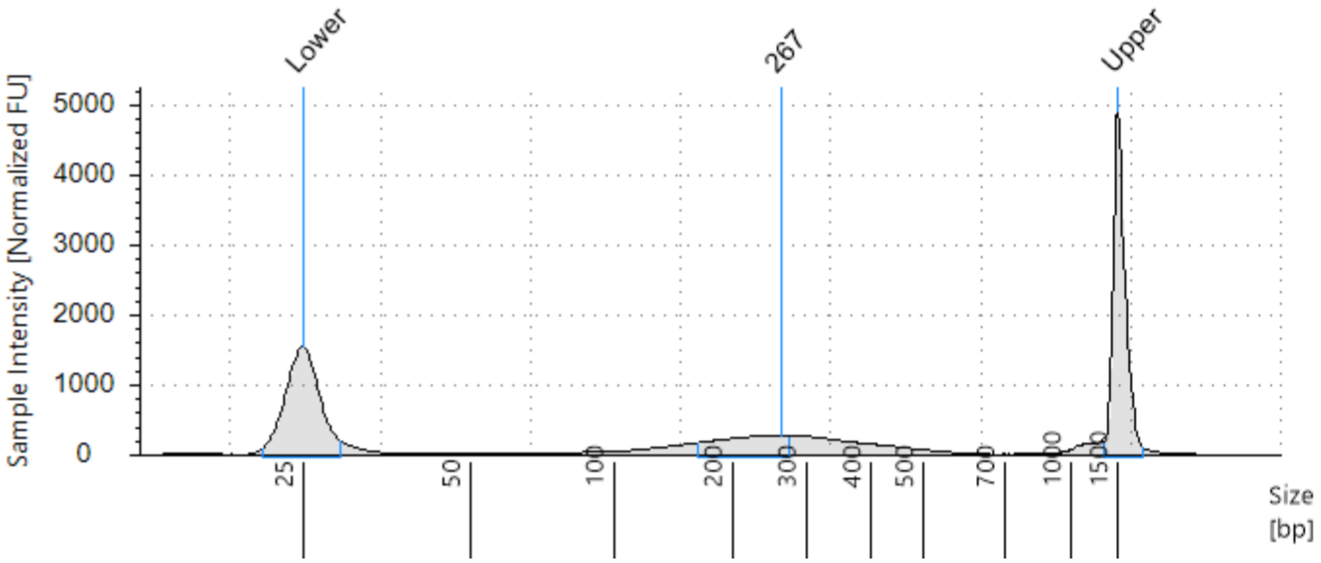

Sample Table

Well	Conc. [ng/µl]	Sample Description	Alert	Observations
G2	2.00	E12 P R2	⚠	Caution! Expired ScreenTape device

Peak Table

Size [bp]	Calibrated Conc. [ng/µl]	Assigned Conc. [ng/µl]	Peak Molarity [nmol/l]	% Integrated Area	Peak Comment	Observations
25	5.68	-	349	-		Lower Marker
267	2.00	-	11.5	100.00		
1500	6.50	6.50	6.67	-		Upper Marker

© Agilent Technologies, Inc. 2017

H2: F12 P R2

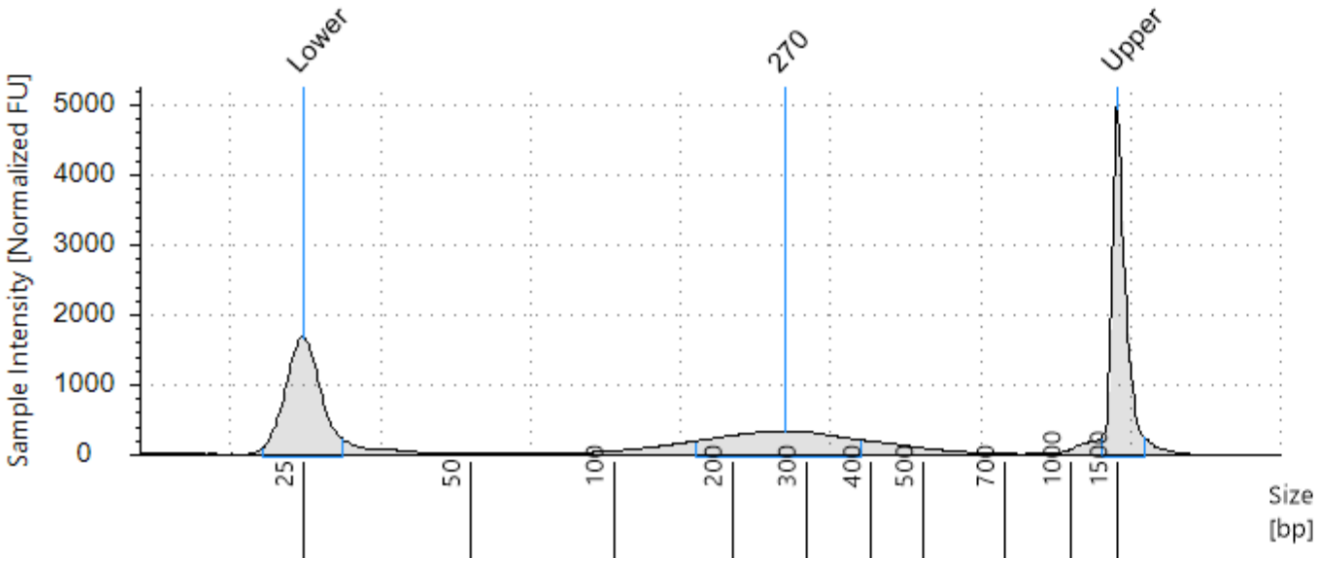

Sample Table

Well	Conc. [ng/µl]	Sample Description	Alert	Observations
H2	3.80	F12 P R2	⚠	Caution! Expired ScreenTape device

Peak Table

Size [bp]	Calibrated Conc. [ng/µl]	Assigned Conc. [ng/µl]	Peak Molarity [nmol/l]	% Integrated Area	Peak Comment	Observations
25	5.75	-	354	-		Lower Marker
270	3.80	-	21.6	100.00		
1500	6.50	6.50	6.67	-		Upper Marker

© Agilent Technologies, Inc. 2017

Filename: 2020-09-28-01 Q-S MINUS A7-G8, D5000, R2.D5000

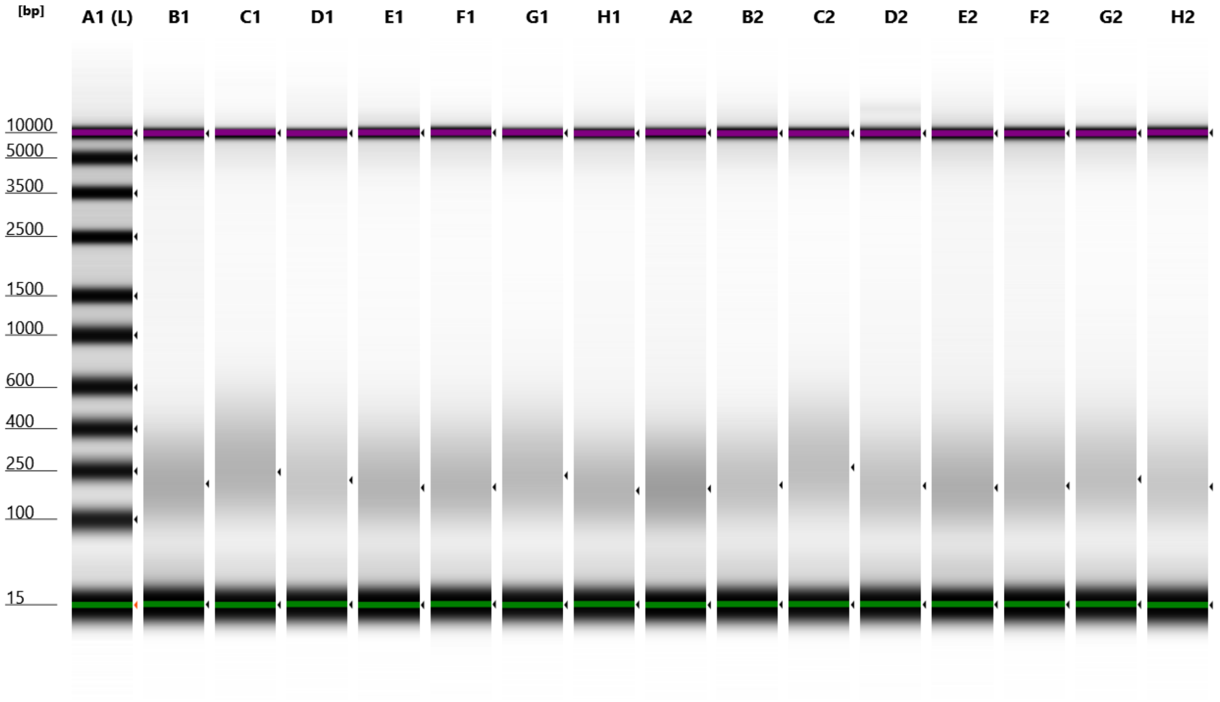

Default image (Contrast 100%)

Sample Info

Well	Conc. [ng/µl]	Sample Description	Alert	Observations
A1	33.4	Ladder		Ladder
B1	0.474	A7 M R2		
C1	1.83	B7 M R2		
D1	2.35	C7 M R2		
E1	1.52	D7 M R2		
F1	2.83	E7 M R2		
G1	3.14	F7 M R2		
H1	3.01	G7 M R2		
A2	4.19	H7 M R2		
B2	0.335	A8 M R2		
C2	1.85	B8 M R2		
D2	1.35	C8 M R2		
E2	1.66	D8 M R2		
F2	1.35	E8 M R2		
G2	1.24	F8 M R2		
H2	2.01	G8 M R2		

© Agilent Technologies, Inc. 2017

A1: Ladder

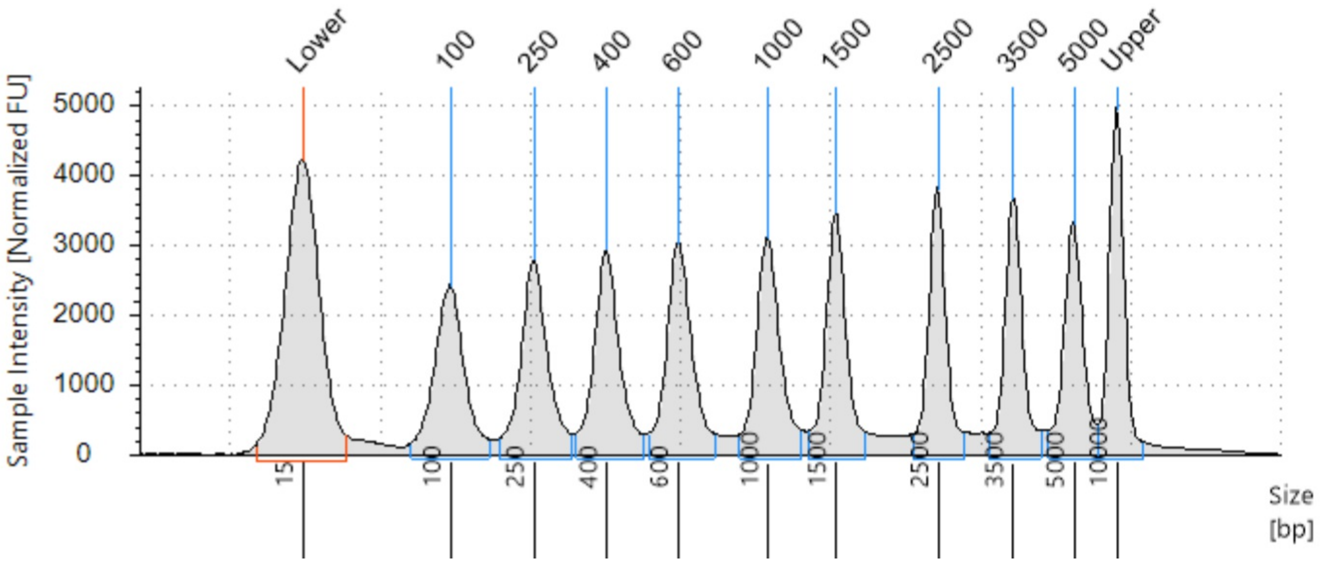

Sample Table

Well	Conc. [ng/µl]	Sample Description	Alert	Observations
A1	33.4	Ladder		Ladder

Peak Table

Size [bp]	Calibrated Conc. [ng/µl]	Assigned Conc. [ng/µl]	Peak Molarity [nmol/l]	% Integrated Area	Peak Comment	Observations
15	6.76	-	693	-		Lower Marker
100	3.75	-	57.7	11.23		
250	3.88	-	23.9	11.60		
400	3.86	-	14.8	11.54		
600	4.00	-	10.3	11.97		
1000	3.89	-	5.99	11.65		
1500	3.73	-	3.82	11.16		
2500	3.53	-	2.17	10.57		
3500	3.43	-	1.51	10.27		
5000	3.35	-	1.03	10.02		
10000	3.25	3.25	0.500	-		Upper Marker

© Agilent Technologies, Inc. 2017

B1: A7 M R2

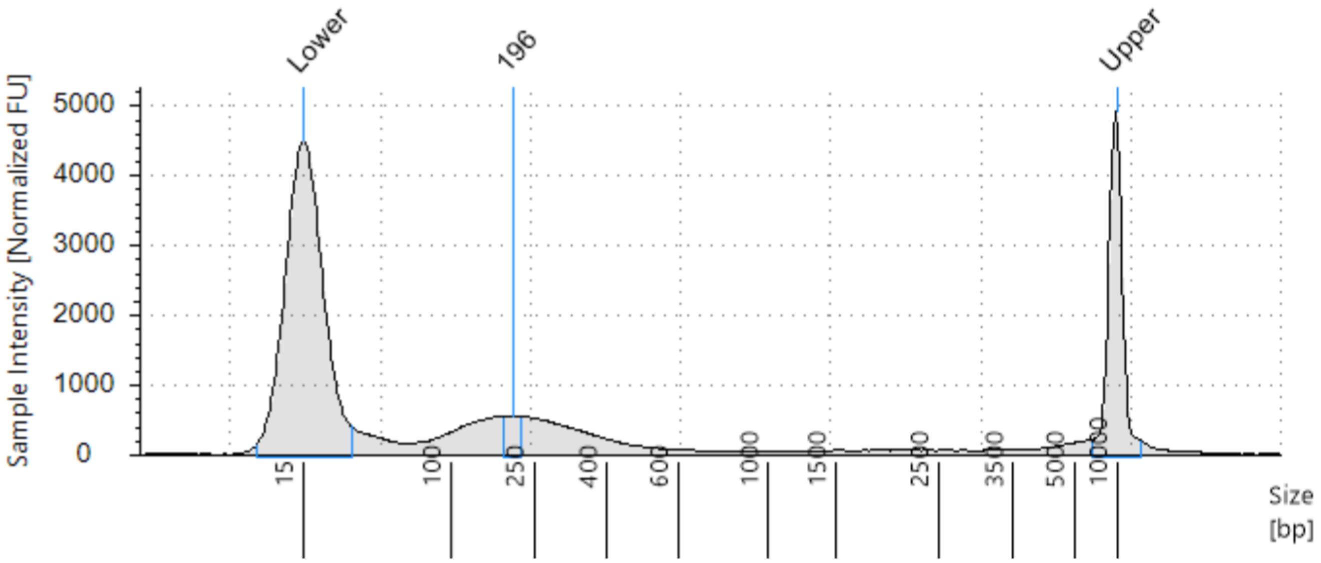

Sample Table

Well	Conc. [ng/µl]	Sample Description	Alert	Observations
B1	0.474	A7 M R2		

Peak Table

Size [bp]	Calibrated Conc. [ng/µl]	Assigned Conc. [ng/µl]	Peak Molarity [nmol/l]	% Integrated Area	Peak Comment	Observations
15	8.13	-	833	-		Lower Marker
196	0.474	-	3.73	100.00		
10000	3.25	3.25	0.500	-		Upper Marker

© Agilent Technologies, Inc. 2017

C1: B7 M R2

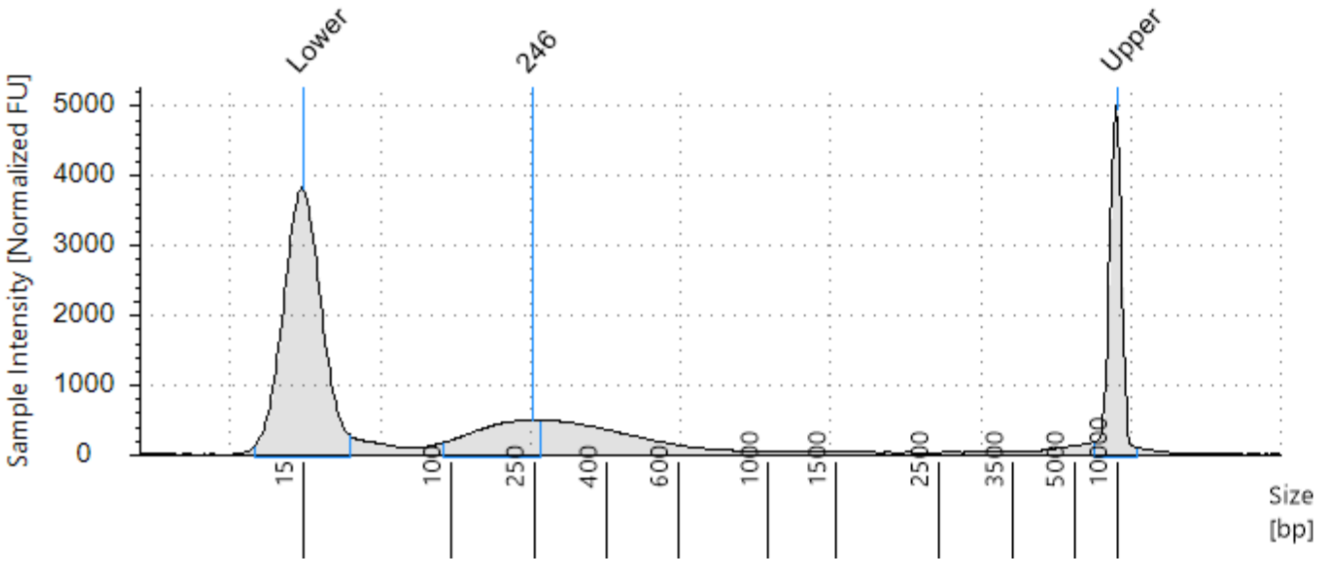

Sample Table

Well	Conc. [ng/µl]	Sample Description	Alert	Observations
C1	1.83	B7 M R2		

Peak Table

Size [bp]	Calibrated Conc. [ng/µl]	Assigned Conc. [ng/µl]	Peak Molarity [nmol/l]	% Integrated Area	Peak Comment	Observations
15	7.37	-	756	-		Lower Marker
246	1.83	-	11.4	100.00		
10000	3.25	3.25	0.500	-		Upper Marker

 © Agilent Technologies, Inc. 2017

D1: C7 M R2

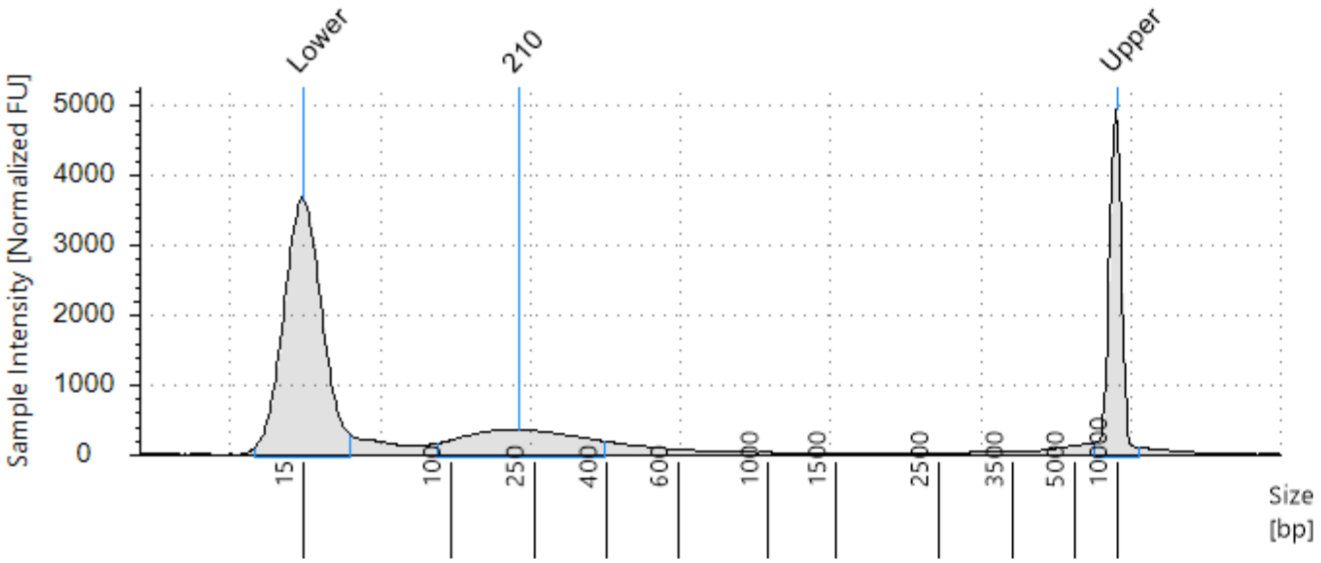

Sample Table

Well	Conc. [ng/µl]	Sample Description	Alert	Observations
D1	2.35	C7 M R2		

Peak Table

Size [bp]	Calibrated Conc. [ng/µl]	Assigned Conc. [ng/µl]	Peak Molarity [nmol/l]	% Integrated Area	Peak Comment	Observations
15	7.14	-	732	-		Lower Marker
210	2.35	-	17.2	100.00		
10000	3.25	3.25	0.500	-		Upper Marker

© Agilent Technologies, Inc. 2017

E1: D7 M R2

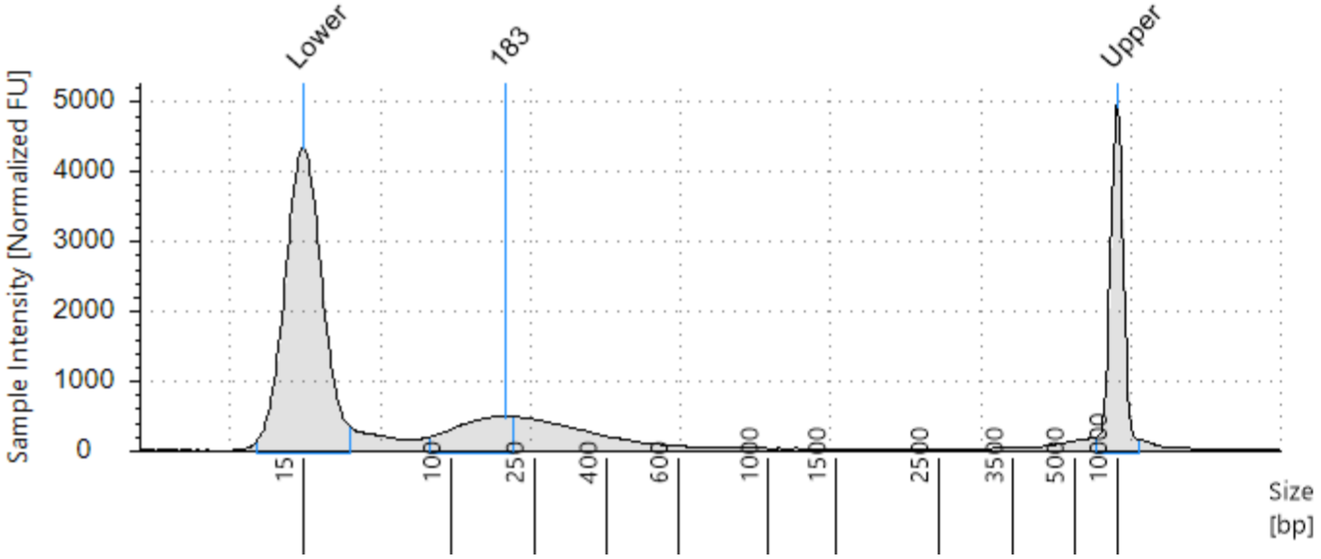

Sample Table

Well	Conc. [ng/µl]	Sample Description	Alert	Observations
E1	1.52	D7 M R2		

Peak Table

Size [bp]	Calibrated Conc. [ng/µl]	Assigned Conc. [ng/µl]	Peak Molarity [nmol/l]	% Integrated Area	Peak Comment	Observations
15	7.65	-	784	-		Lower Marker
183	1.52	-	12.8	100.00		
10000	3.25	3.25	0.500	-		Upper Marker

© Agilent Technologies, Inc. 2017

F1: E7 M R2

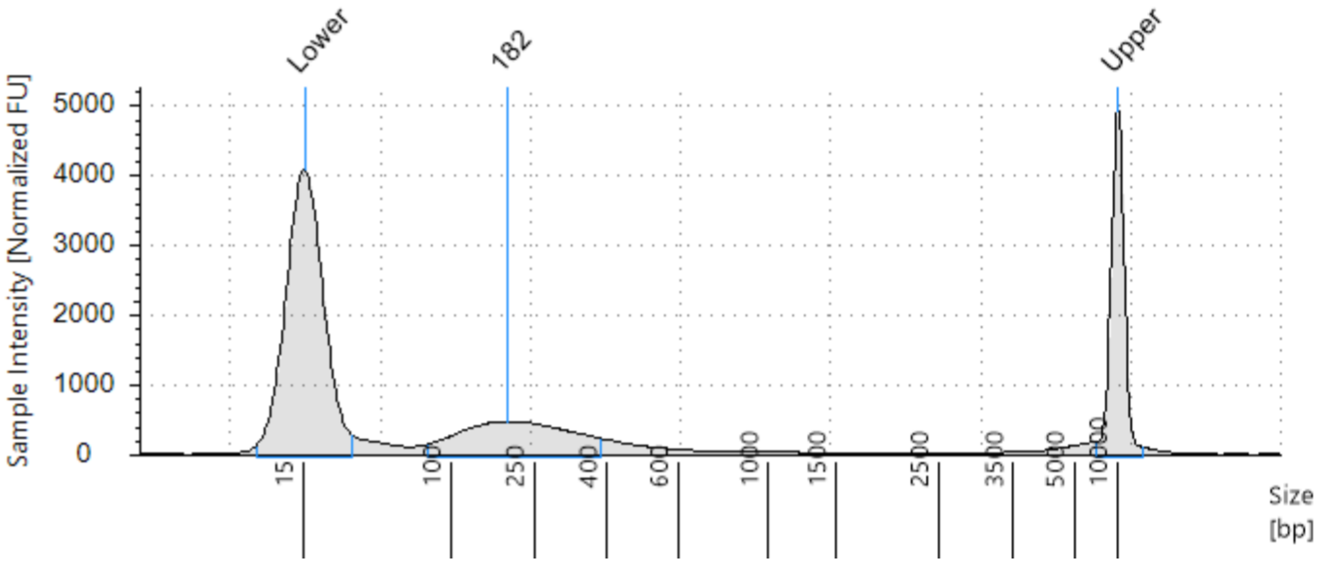

Sample Table

Well	Conc. [ng/µl]	Sample Description	Alert	Observations
F1	2.83	E7 M R2		

Peak Table

Size [bp]	Calibrated Conc. [ng/µl]	Assigned Conc. [ng/µl]	Peak Molarity [nmol/l]	% Integrated Area	Peak Comment	Observations
15	7.30	-	749	-		Lower Marker
182	2.83	-	23.8	100.00		
10000	3.25	3.25	0.500	-		Upper Marker

 © Agilent Technologies, Inc. 2017

G1: F7 M R2

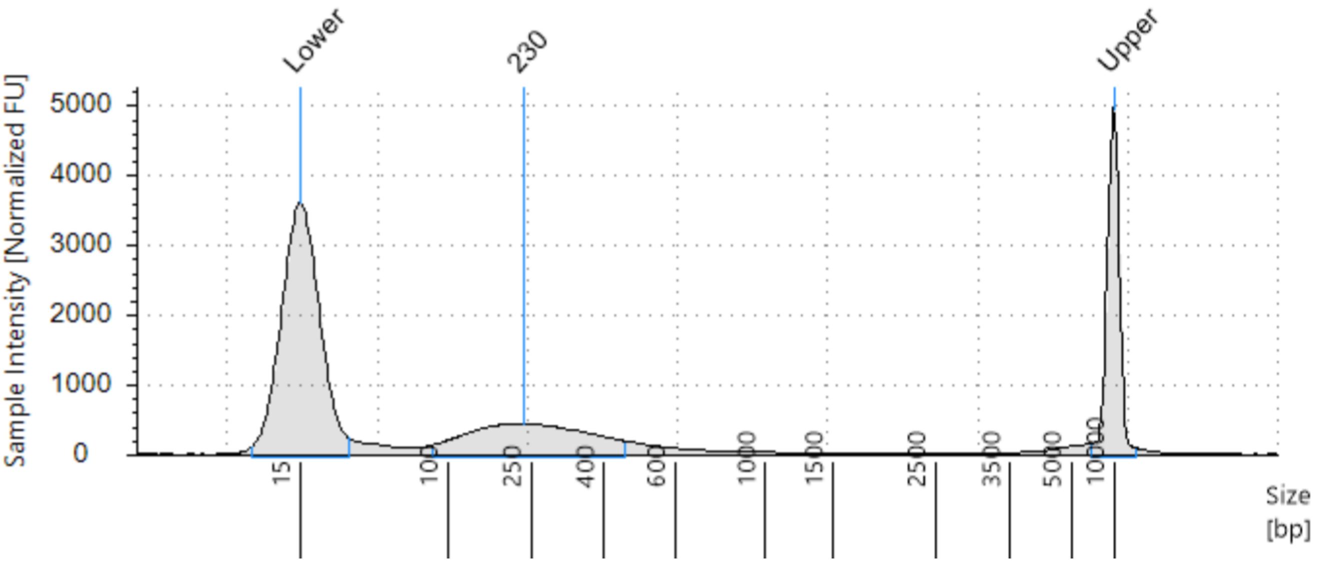

Sample Table

Well	Conc. [ng/µl]	Sample Description	Alert	Observations
G1	3.14	F7 M R2		

Peak Table

Size [bp]	Calibrated Conc. [ng/µl]	Assigned Conc. [ng/µl]	Peak Molarity [nmol/l]	% Integrated Area	Peak Comment	Observations
15	7.01	-	719	-		Lower Marker
230	3.14	-	21.0	100.00		
10000	3.25	3.25	0.500	-		Upper Marker

© Agilent Technologies, Inc. 2017

H1: G7 M R2

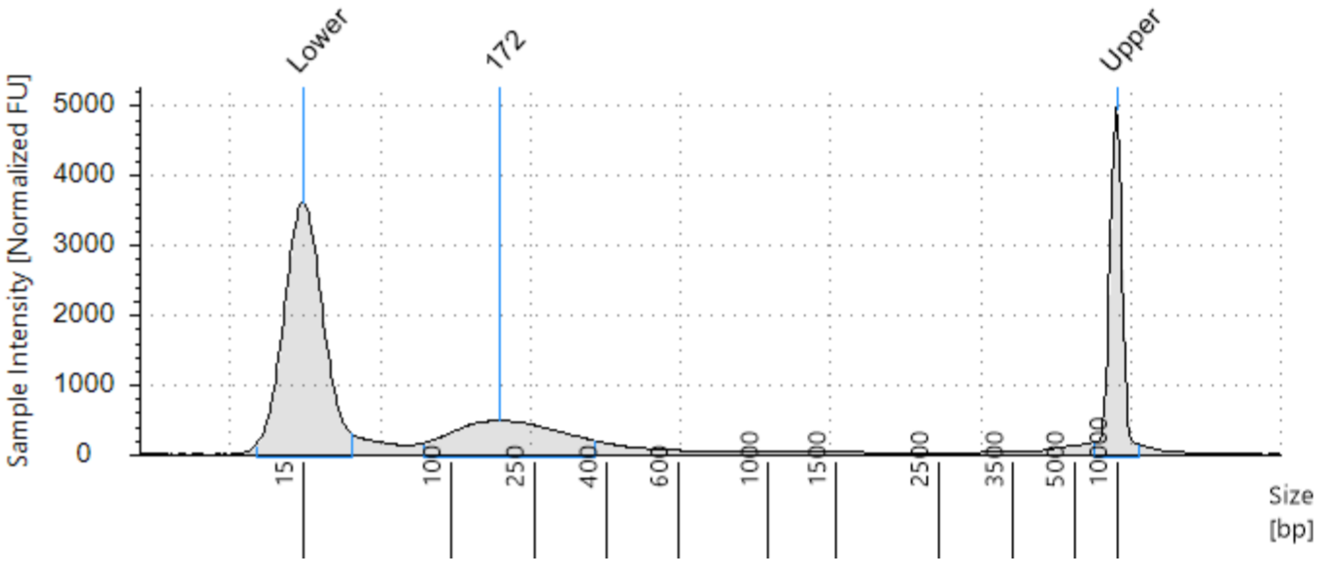

Sample Table

Well	Conc. [ng/µl]	Sample Description	Alert	Observations
H1	3.01	G7 M R2		

Peak Table

Size [bp]	Calibrated Conc. [ng/µl]	Assigned Conc. [ng/µl]	Peak Molarity [nmol/l]	% Integrated Area	Peak Comment	Observations
15	7.02	-	720	-		Lower Marker
172	3.01	-	27.0	100.00		
10000	3.25	3.25	0.500	-		Upper Marker

© Agilent Technologies, Inc. 2017

A2: H7 M R2

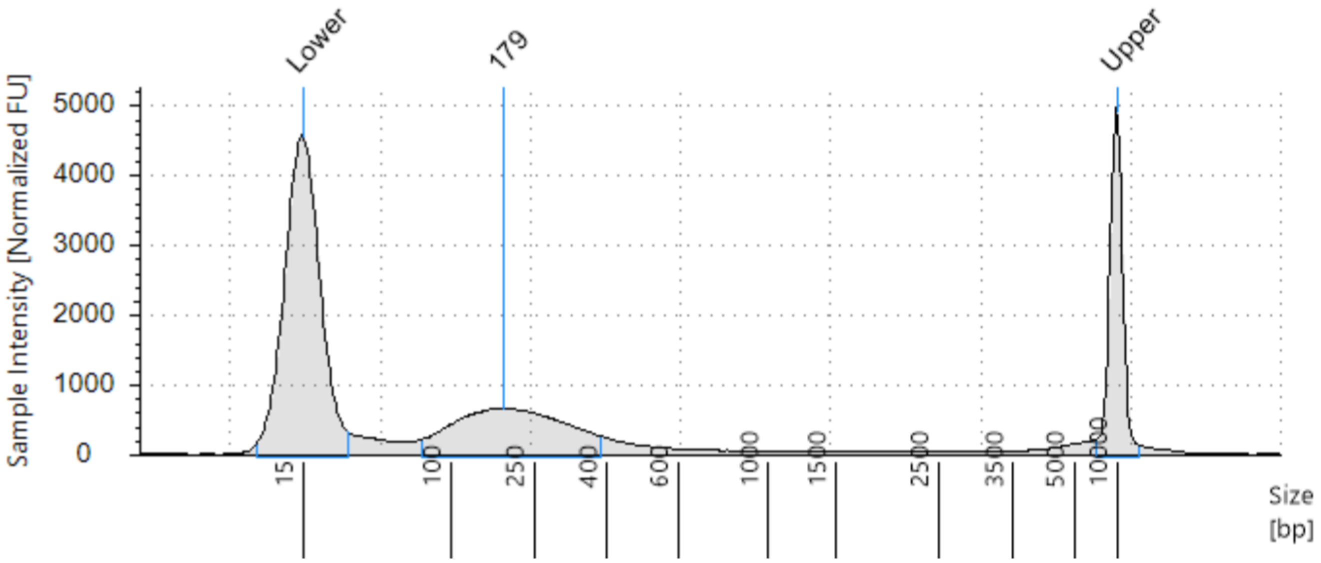

Sample Table

Well	Conc. [ng/µl]	Sample Description	Alert	Observations
A2	4.19	H7 M R2		

Peak Table

Size [bp]	Calibrated Conc. [ng/µl]	Assigned Conc. [ng/µl]	Peak Molarity [nmol/l]	% Integrated Area	Peak Comment	Observations
15	7.97	-	818	-		Lower Marker
179	4.19	-	36.0	100.00		
10000	3.25	3.25	0.500	-		Upper Marker

© Agilent Technologies, Inc. 2017

B2: A8 M R2

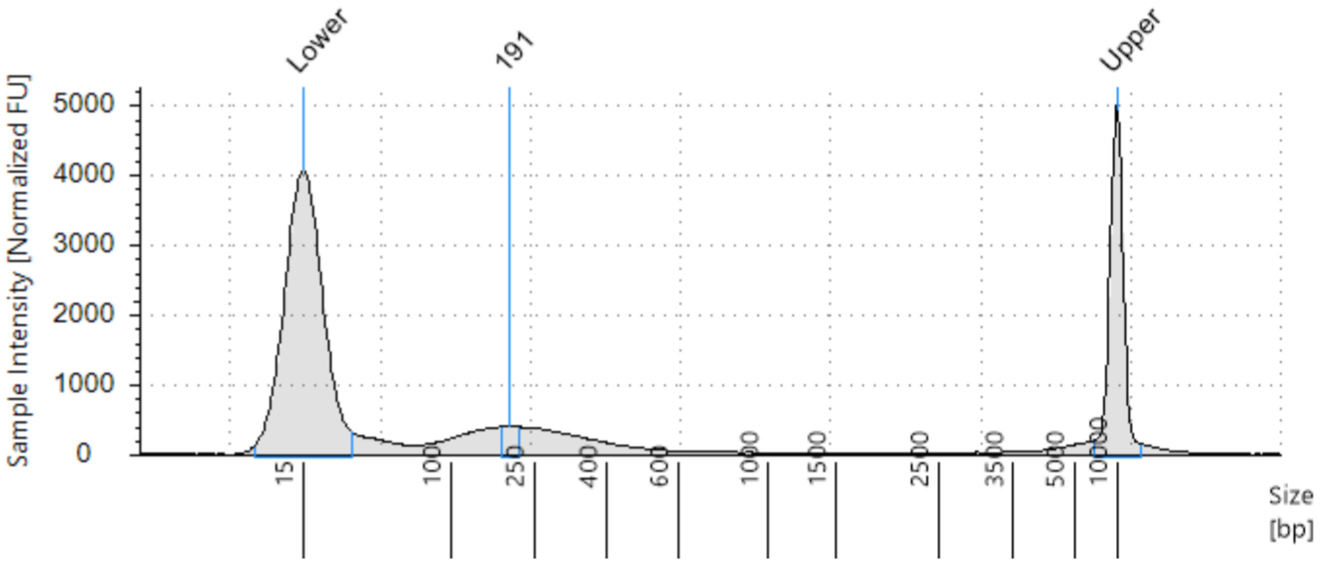

Sample Table

Well	Conc. [ng/µl]	Sample Description	Alert	Observations
B2	0.335	A8 M R2		

Peak Table

Size [bp]	Calibrated Conc. [ng/µl]	Assigned Conc. [ng/µl]	Peak Molarity [nmol/l]	% Integrated Area	Peak Comment	Observations
15	7.31	-	750	-		Lower Marker
191	0.335	-	2.69	100.00		
10000	3.25	3.25	0.500	-		Upper Marker

© Agilent Technologies, Inc. 2017

C2: B8 M R2

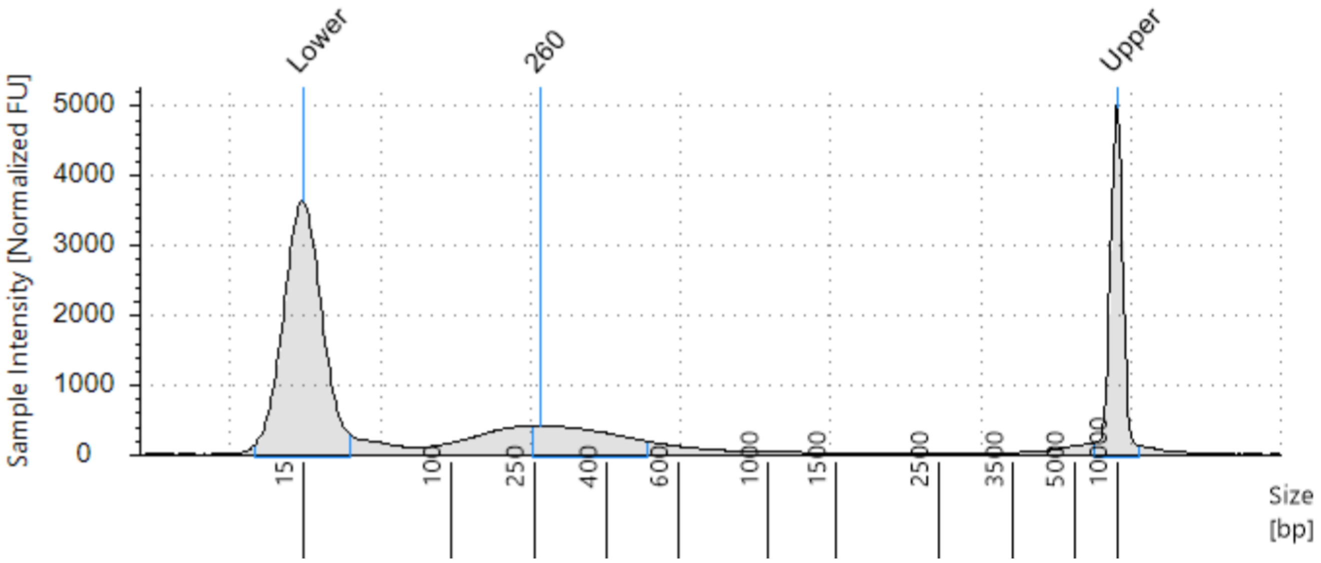

Sample Table

Well	Conc. [ng/µl]	Sample Description	Alert	Observations
C2	1.85	B8 M R2		

Peak Table

Size [bp]	Calibrated Conc. [ng/µl]	Assigned Conc. [ng/µl]	Peak Molarity [nmol/l]	% Integrated Area	Peak Comment	Observations
15	6.83	-	700	-		Lower Marker
260	1.85	-	10.9	100.00		
10000	3.25	3.25	0.500	-		Upper Marker

© Agilent Technologies, Inc. 2017

D2: C8 M R2

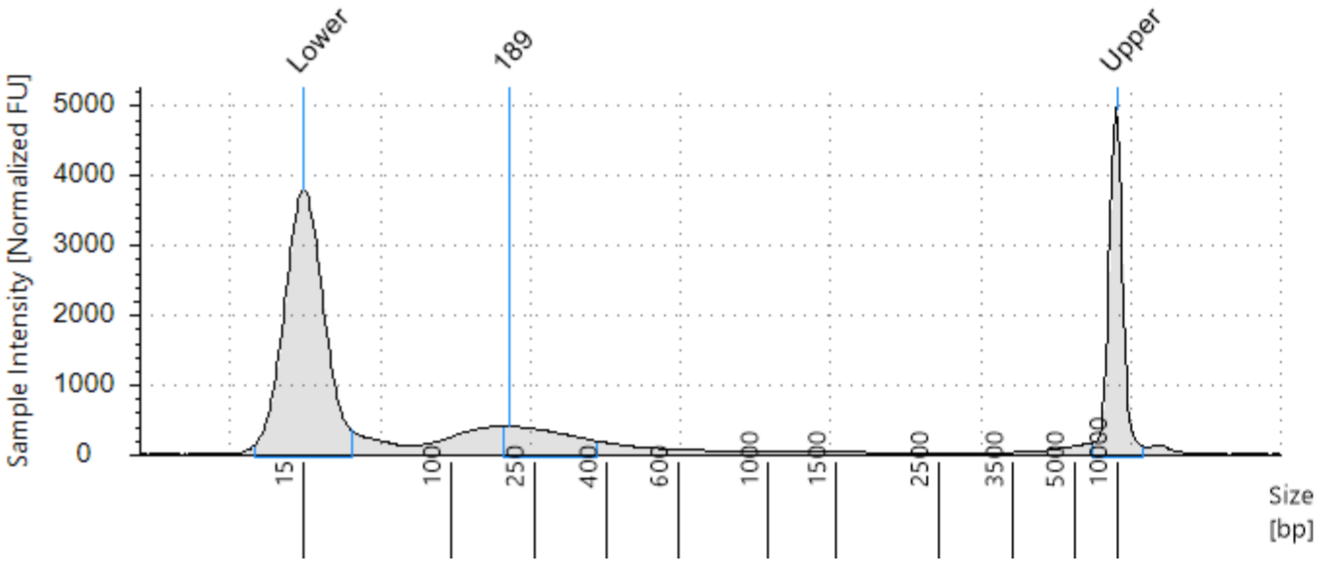

Sample Table

Well	Conc. [ng/µl]	Sample Description	Alert	Observations
D2	1.35	C8 M R2		

Peak Table

Size [bp]	Calibrated Conc. [ng/µl]	Assigned Conc. [ng/µl]	Peak Molarity [nmol/l]	% Integrated Area	Peak Comment	Observations
15	6.73	-	690	-		Lower Marker
189	1.35	-	11.0	100.00		
10000	3.25	3.25	0.500	-		Upper Marker

© Agilent Technologies, Inc. 2017

E2: D8 M R2

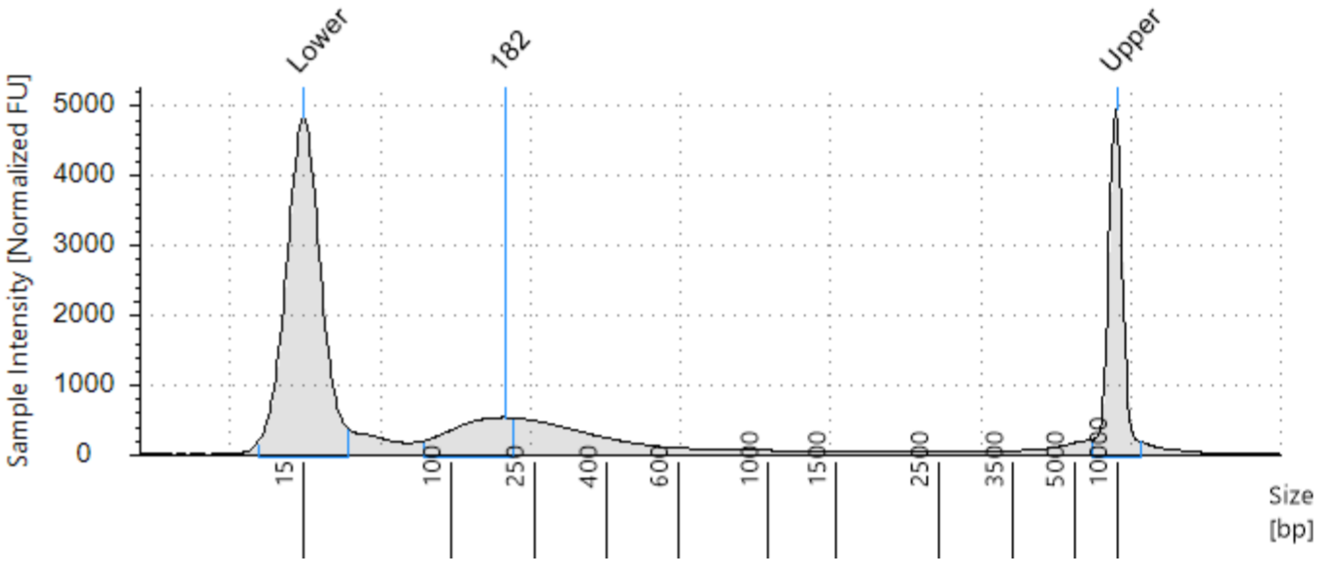

Sample Table

Well	Conc. [ng/µl]	Sample Description	Alert	Observations
E2	1.66	D8 M R2		

Peak Table

Size [bp]	Calibrated Conc. [ng/µl]	Assigned Conc. [ng/µl]	Peak Molarity [nmol/l]	% Integrated Area	Peak Comment	Observations
15	7.52	-	771	-		Lower Marker
182	1.66	-	14.1	100.00		
10000	3.25	3.25	0.500	-		Upper Marker

© Agilent Technologies, Inc. 2017

F2: E8 M R2

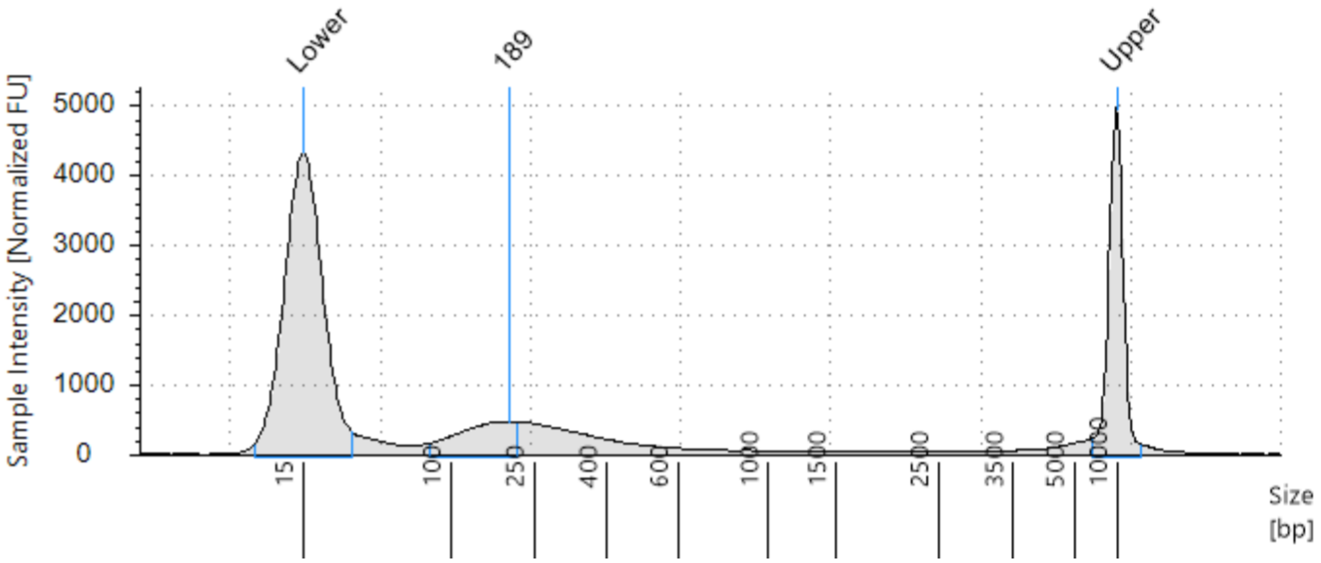

Sample Table

Well	Conc. [ng/µl]	Sample Description	Alert	Observations
F2	1.35	E8 M R2		

Peak Table

Size [bp]	Calibrated Conc. [ng/µl]	Assigned Conc. [ng/µl]	Peak Molarity [nmol/l]	% Integrated Area	Peak Comment	Observations
15	7.42	-	761	-		Lower Marker
189	1.35	-	11.0	100.00		
10000	3.25	3.25	0.500	-		Upper Marker

G2: F8 M R2

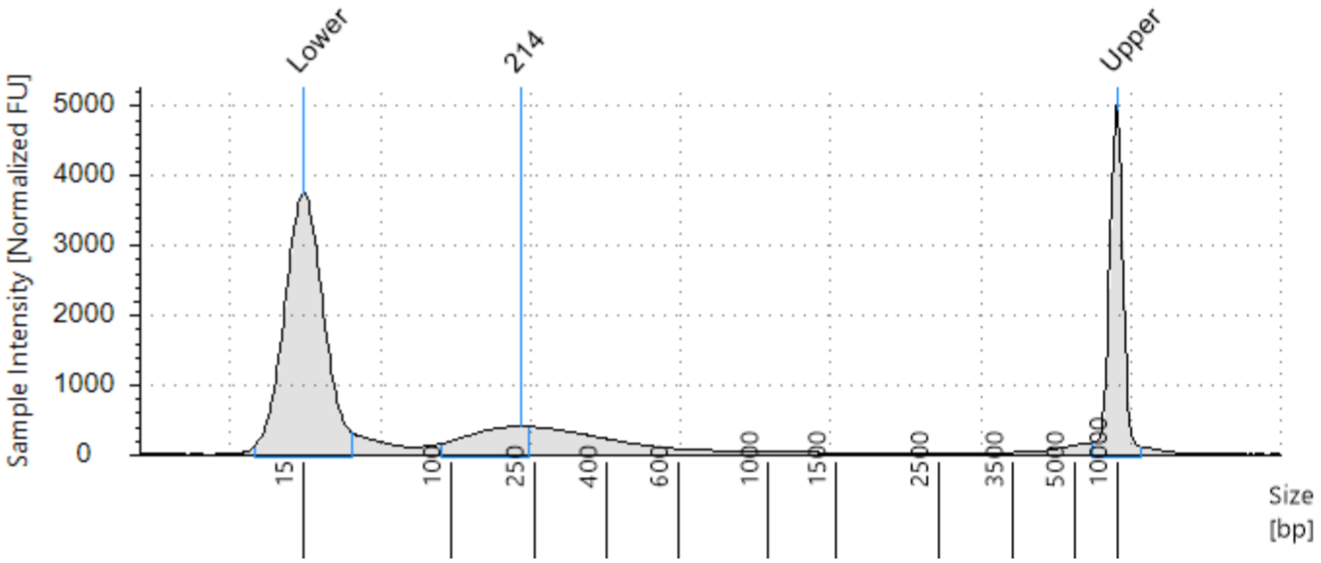

Sample Table

Well	Conc. [ng/µl]	Sample Description	Alert	Observations
G2	1.24	F8 M R2		

Peak Table

Size [bp]	Calibrated Conc. [ng/µl]	Assigned Conc. [ng/µl]	Peak Molarity [nmol/l]	% Integrated Area	Peak Comment	Observations
15	6.61	-	678	-		Lower Marker
214	1.24	-	8.92	100.00		
10000	3.25	3.25	0.500	-		Upper Marker

© Agilent Technologies, Inc. 2017

H2: G8 M R2

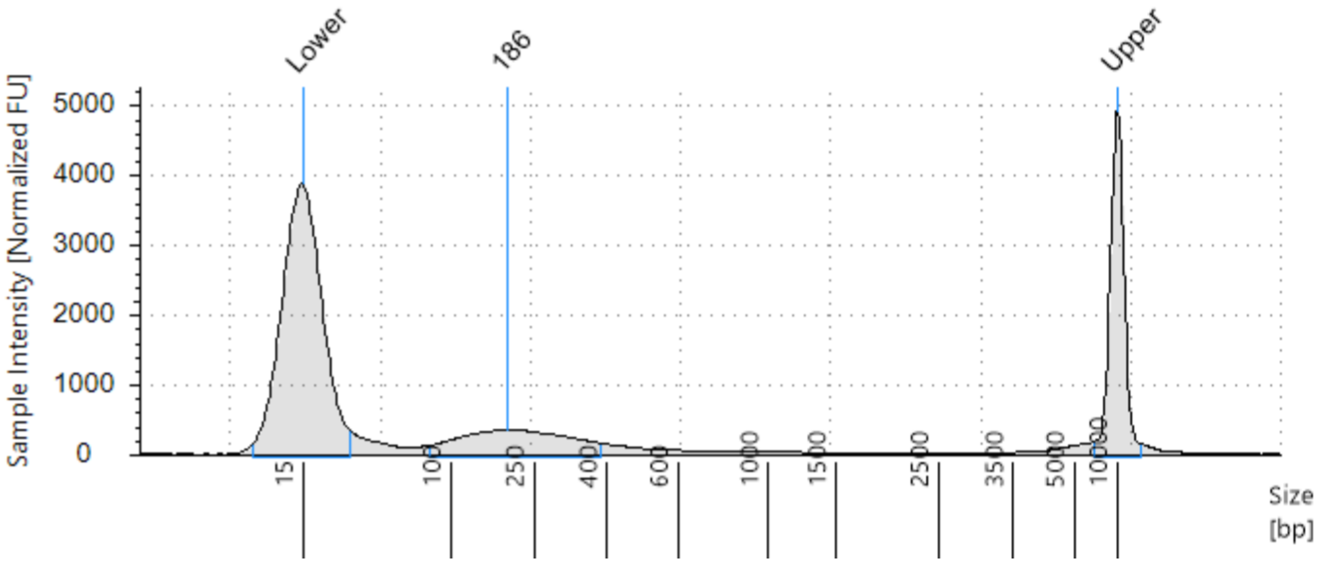

Sample Table

Well	Conc. [ng/µl]	Sample Description	Alert	Observations
H2	2.01	G8 M R2		

Peak Table

Size [bp]	Calibrated Conc. [ng/µl]	Assigned Conc. [ng/µl]	Peak Molarity [nmol/l]	% Integrated Area	Peak Comment	Observations
15	6.95	-	713	-		Lower Marker
186	2.01	-	16.6	100.00		
10000	3.25	3.25	0.500	-		Upper Marker

© Agilent Technologies, Inc. 2017

Filename: 2020-09-30-02, Q-S Plus G2,D2,H2,A3,B3,C3,D3,E3 R2.D1000

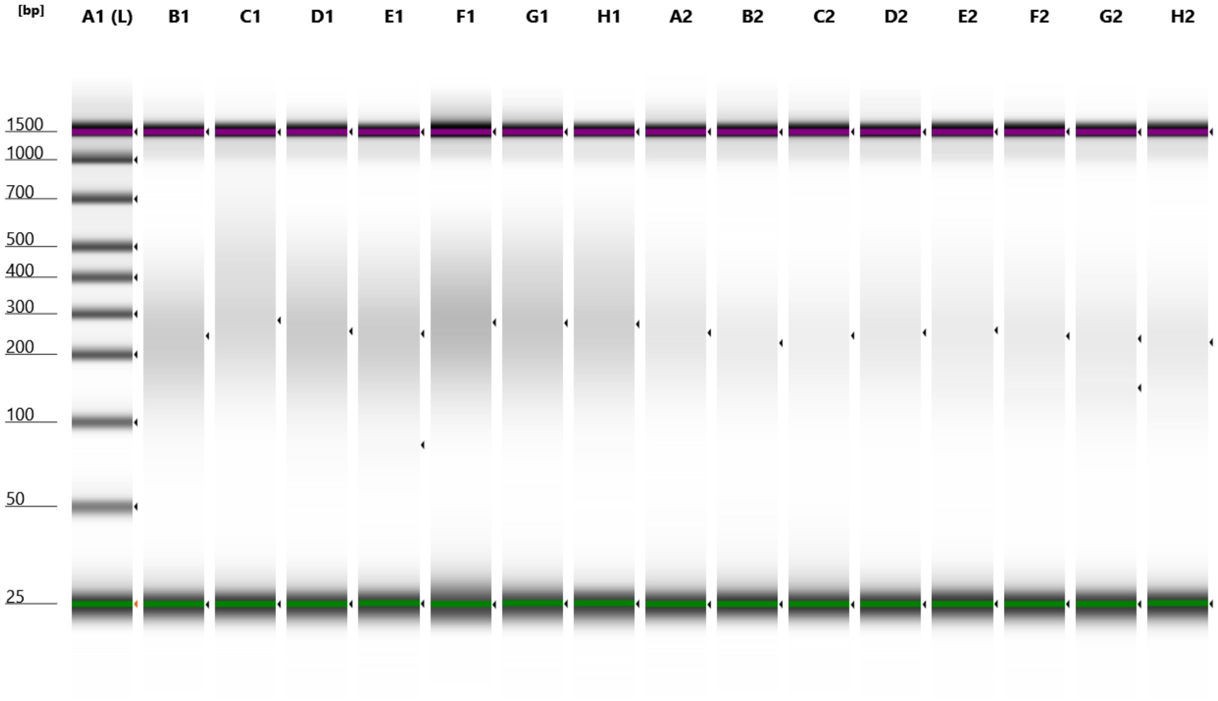

Default image (Contrast 100%)

Sample Info

Well	Conc. [ng/µl]	Sample Description	Alert	Observations
A1	15.7	Ladder		Ladder
B1	0.369	G2 P R2		
C1	1.56	D2 P R2		
D1	3.89	H2 P R2		
E1	3.86	A3 P R2		
F1	0.703	B3 P R2		
G1	3.99	C3 P R2		
H1	3.23	D3 P R2		
A2	0.861	E3 P R2		
B2	0.165	A12 M R2		
C2	0.451	B12 M R2		
D2	1.31	C12 M R2		
E2	0.626	D12 M R2		
F2	0.605	E12 M R2		
G2	0.572	F12 M R2		
H2	0.734	G12 M R2		

© Agilent Technologies, Inc. 2017

A1: Ladder

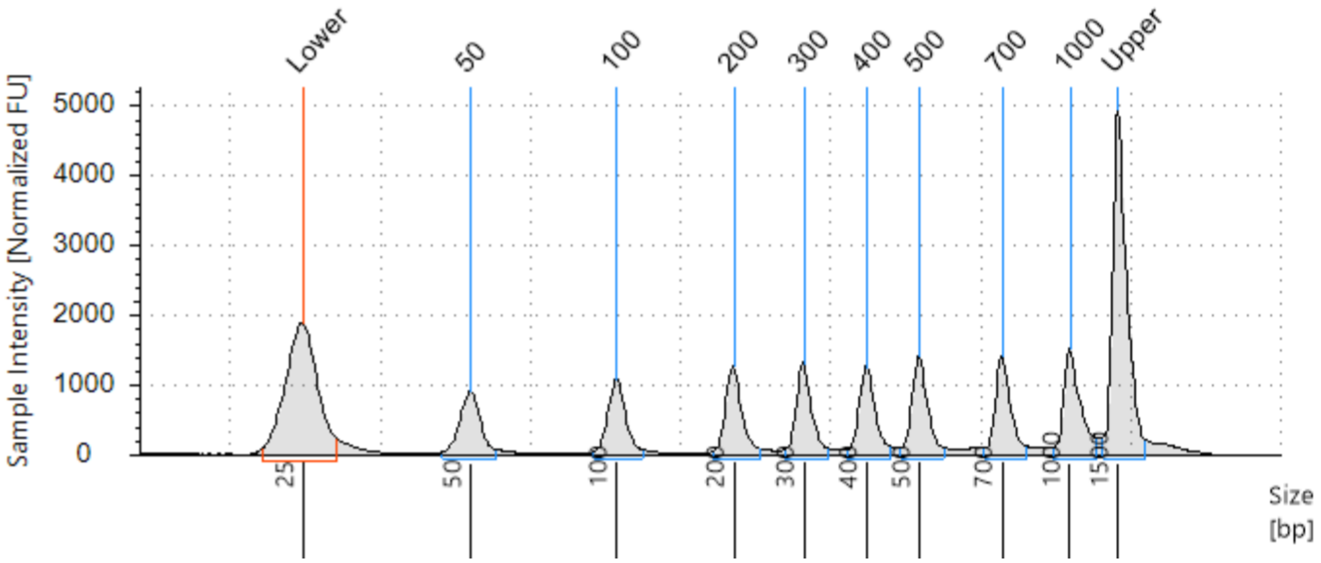

Sample Table

Well	Conc. [ng/µl]	Sample Description	Alert	Observations
A1	15.7	Ladder		Ladder

Peak Table

Size [bp]	Calibrated Conc. [ng/µl]	Assigned Conc. [ng/µl]	Peak Molarity [nmol/l]	% Integrated Area	Peak Comment	Observations
25	5.60	-	345	-		Lower Marker
50	1.72	-	52.9	10.98		
100	1.81	-	27.8	11.53		
200	1.93	-	14.9	12.33		
300	1.88	-	9.65	12.01		
400	1.91	-	7.36	12.21		
500	2.05	-	6.31	13.10		
700	2.03	-	4.46	12.96		
1000	2.33	-	3.59	14.88		
1500	6.50	6.50	6.67	-		Upper Marker

© Agilent Technologies, Inc. 2017

B1: G2 P R2

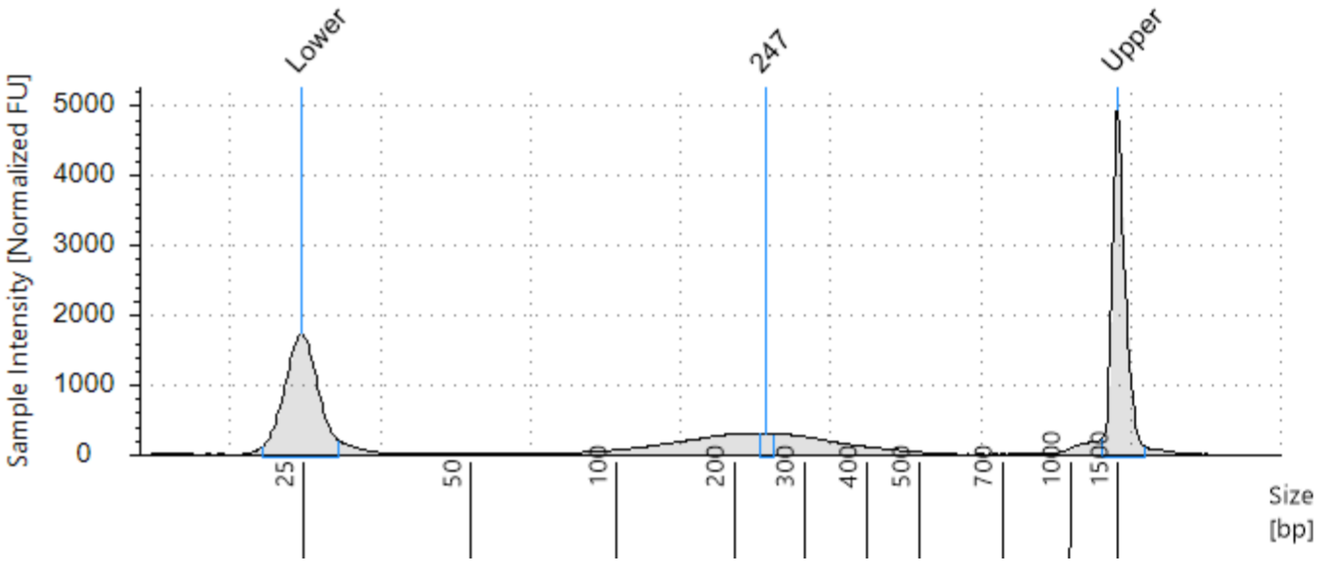

Sample Table

Well	Conc. [ng/µl]	Sample Description	Alert	Observations
B1	0.369	G2 P R2		

Peak Table

Size [bp]	Calibrated Conc. [ng/µl]	Assigned Conc. [ng/µl]	Peak Molarity [nmol/l]	% Integrated Area	Peak Comment	Observations
25	5.78	-	355	-		Lower Marker
247	0.369	-	2.30	100.00		
1500	6.50	6.50	6.67	-		Upper Marker

 © Agilent Technologies, Inc. 2017

C1: D2 P R2

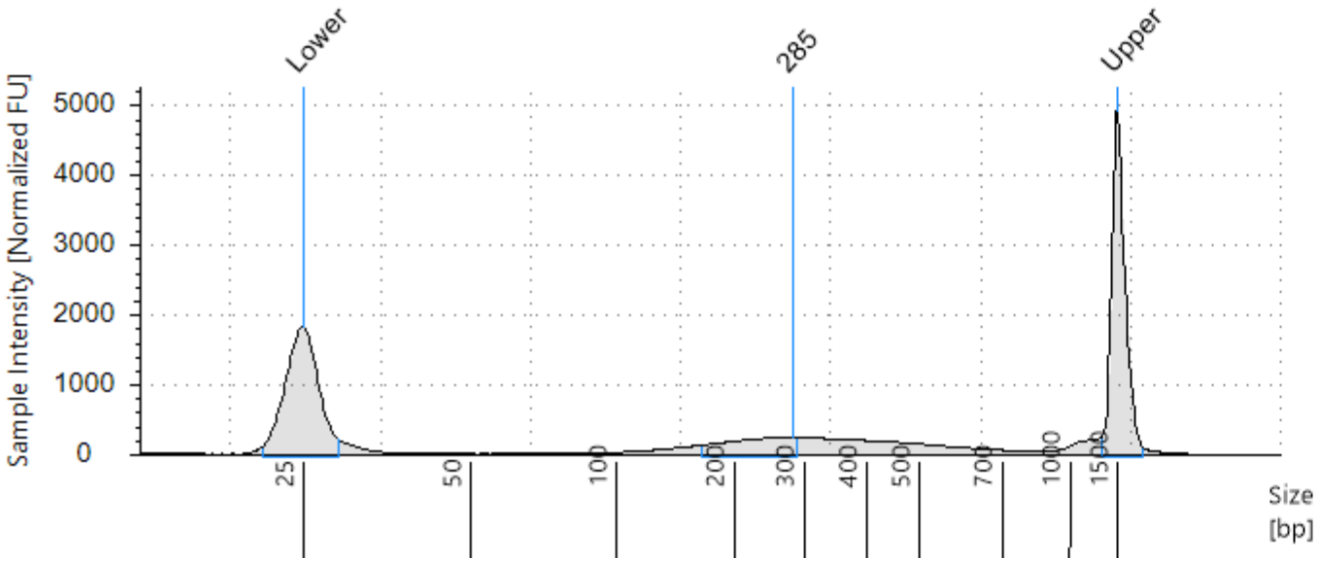

Sample Table

Well	Conc. [ng/µl]	Sample Description	Alert	Observations
C1	1.56	D2 P R2		

Peak Table

Size [bp]	Calibrated Conc. [ng/µl]	Assigned Conc. [ng/µl]	Peak Molarity [nmol/l]	% Integrated Area	Peak Comment	Observations
25	5.95	-	366	-		Lower Marker
285	1.56	-	8.43	100.00		
1500	6.50	6.50	6.67	-		Upper Marker

© Agilent Technologies, Inc. 2017

D1: H2 P R2

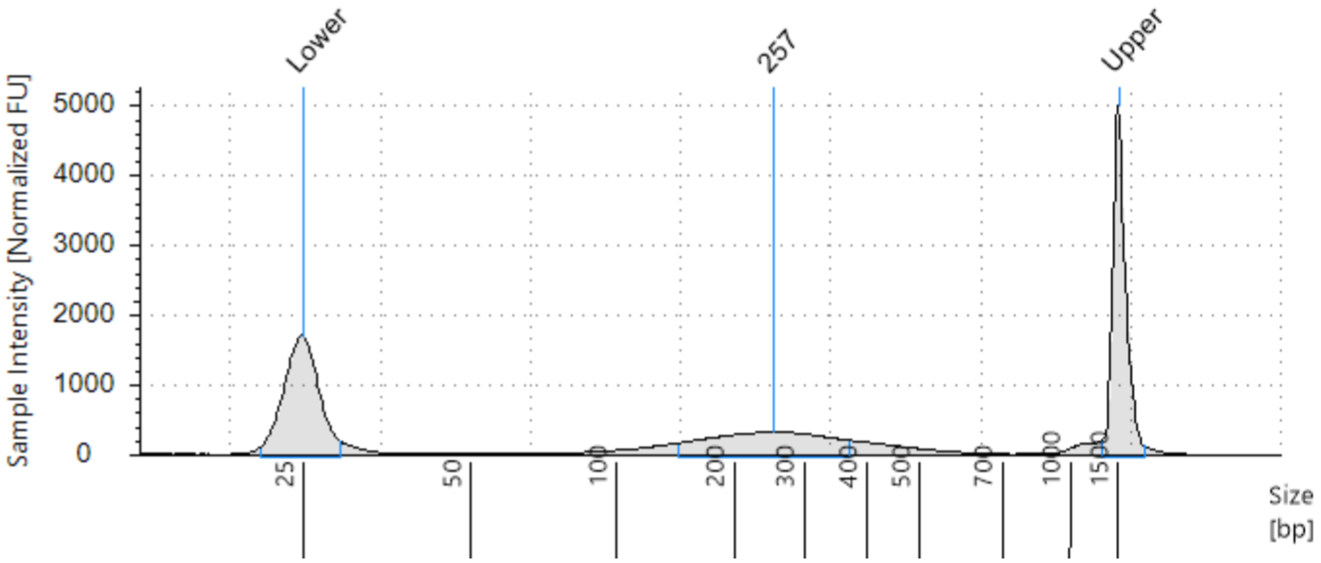

Sample Table

Well	Conc. [ng/µl]	Sample Description	Alert	Observations
D1	3.89	H2 P R2		

Peak Table

Size [bp]	Calibrated Conc. [ng/µl]	Assigned Conc. [ng/µl]	Peak Molarity [nmol/l]	% Integrated Area	Peak Comment	Observations
25	5.96	-	367	-		Lower Marker
257	3.89	-	23.2	100.00		
1500	6.50	6.50	6.67	-		Upper Marker

© Agilent Technologies, Inc. 2017

E1: A3 P R2

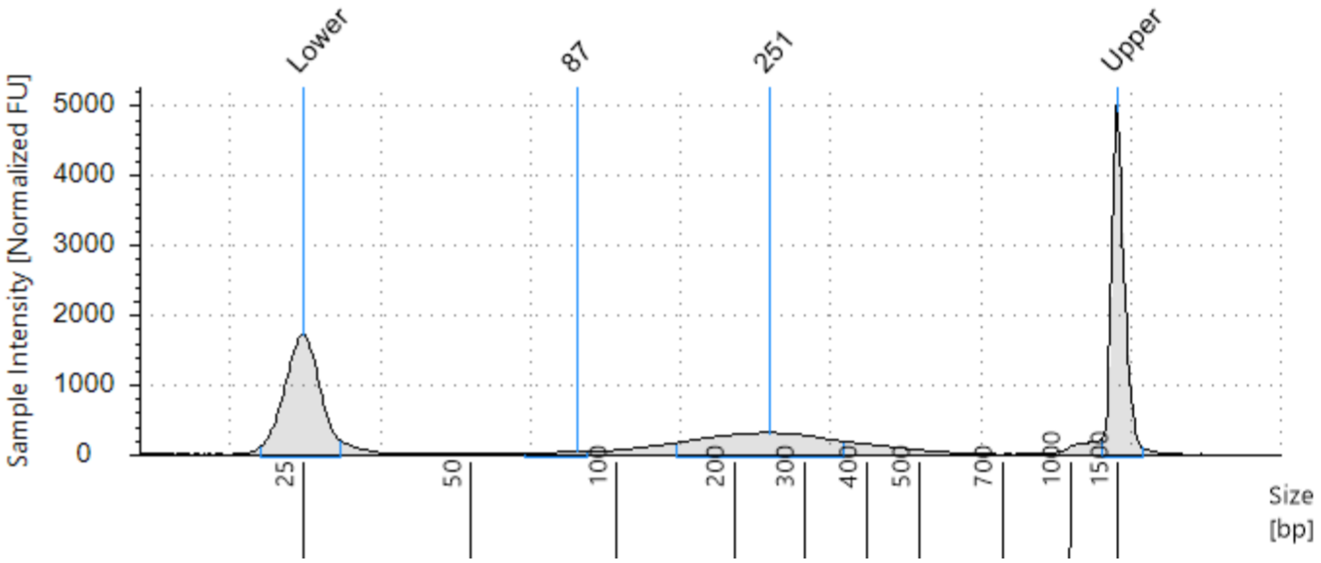

Sample Table

Well	Conc. [ng/µl]	Sample Description	Alert	Observations
E1	3.86	A3 P R2		

Peak Table

Size [bp]	Calibrated Conc. [ng/µl]	Assigned Conc. [ng/µl]	Peak Molarity [nmol/l]	% Integrated Area	Peak Comment	Observations
25	6.12	-	377	-		Lower Marker
87	0.139	-	2.47	3.60		
251	3.72	-	22.8	96.40		
1500	6.50	6.50	6.67	-		Upper Marker

© Agilent Technologies, Inc. 2017

F1: B3 P R2

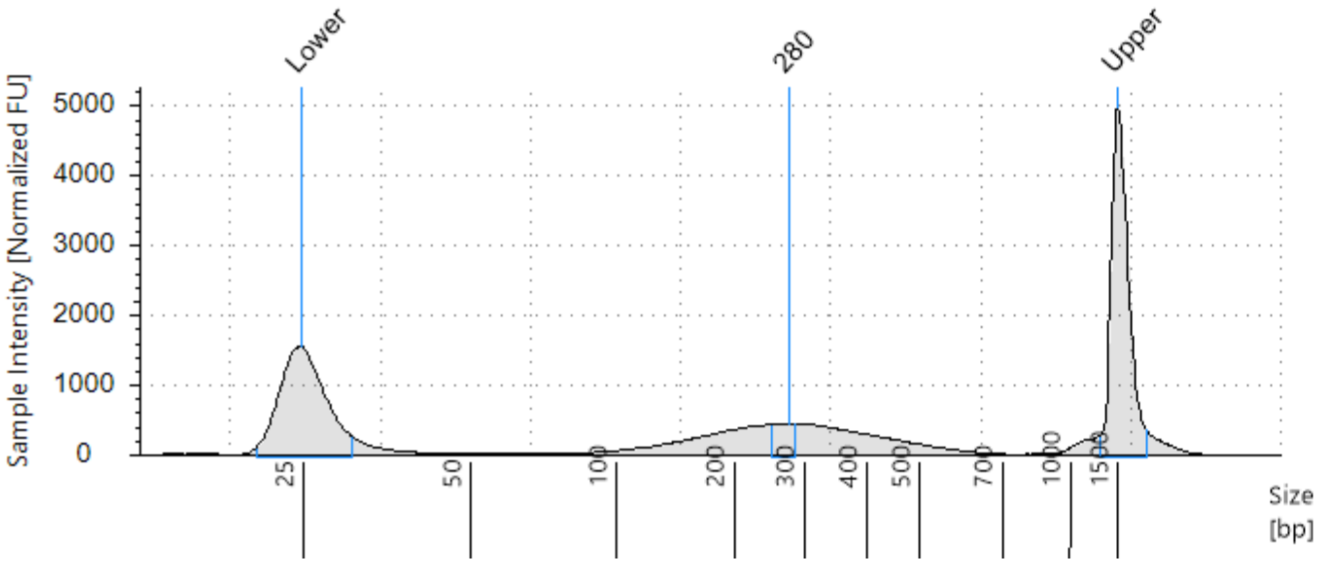

Sample Table

Well	Conc. [ng/µl]	Sample Description	Alert	Observations
F1	0.703	B3 P R2		

Peak Table

Size [bp]	Calibrated Conc. [ng/µl]	Assigned Conc. [ng/µl]	Peak Molarity [nmol/l]	% Integrated Area	Peak Comment	Observations
25	5.32	-	327	-		Lower Marker
280	0.703	-	3.86	100.00		
1500	6.50	6.50	6.67	-		Upper Marker

G1: C3 P R2

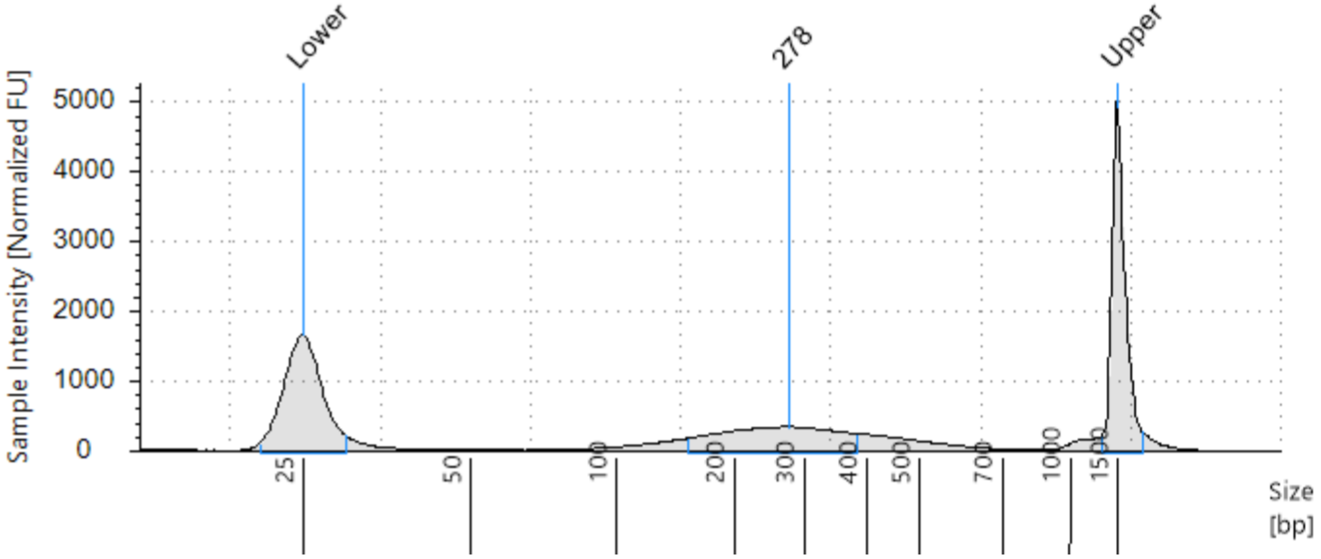

Sample Table

Well	Conc. [ng/µl]	Sample Description	Alert	Observations
G1	3.99	C3 P R2		

Peak Table

Size [bp]	Calibrated Conc. [ng/µl]	Assigned Conc. [ng/µl]	Peak Molarity [nmol/l]	% Integrated Area	Peak Comment	Observations
25	6.20	-	382	-		Lower Marker
278	3.99	-	22.1	100.00		
1500	6.50	6.50	6.67	-		Upper Marker

 © Agilent Technologies, Inc. 2017

H1: D3 P R2

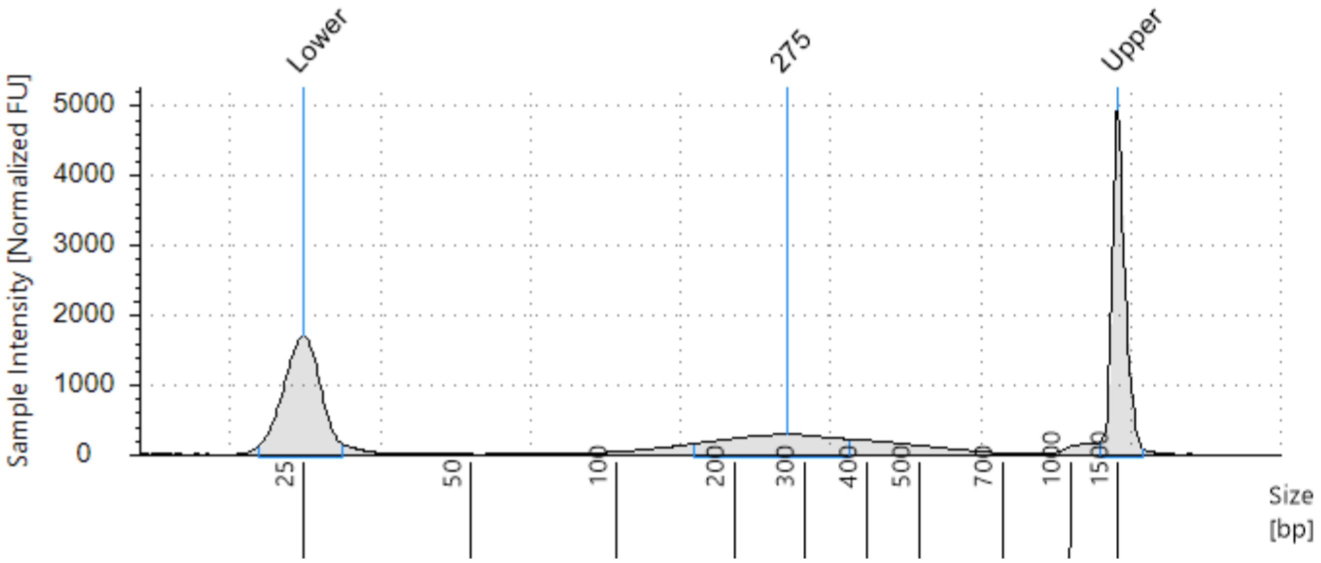

Sample Table

Well	Conc. [ng/µl]	Sample Description	Alert	Observations
H1	3.23	D3 P R2		

Peak Table

Size [bp]	Calibrated Conc. [ng/µl]	Assigned Conc. [ng/µl]	Peak Molarity [nmol/l]	% Integrated Area	Peak Comment	Observations
25	6.34	-	390	-		Lower Marker
275	3.23	-	18.1	100.00		
1500	6.50	6.50	6.67	-		Upper Marker

© Agilent Technologies, Inc. 2017

A2: E3 P R2

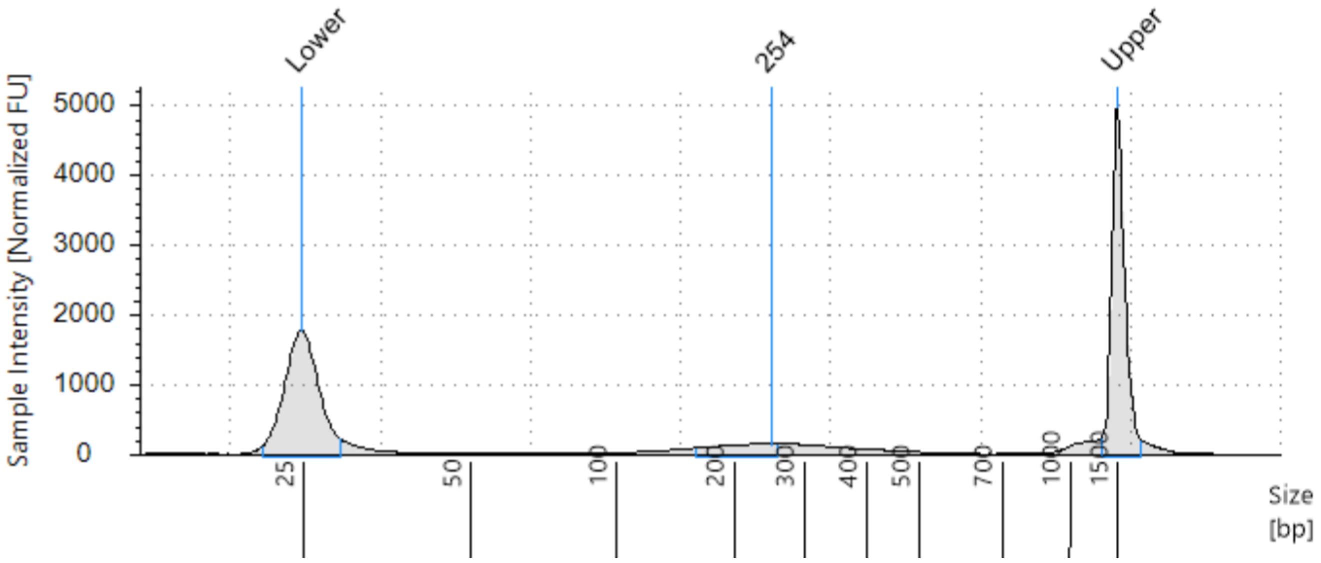

Sample Table

Well	Conc. [ng/µl]	Sample Description	Alert	Observations
A2	0.861	E3 P R2		

Peak Table

Size [bp]	Calibrated Conc. [ng/µl]	Assigned Conc. [ng/µl]	Peak Molarity [nmol/l]	% Integrated Area	Peak Comment	Observations
25	6.00	-	369	-		Lower Marker
254	0.861	-	5.21	100.00		
1500	6.50	6.50	6.67	-		Upper Marker

 © Agilent Technologies, Inc. 2017

B2: A12 M R2

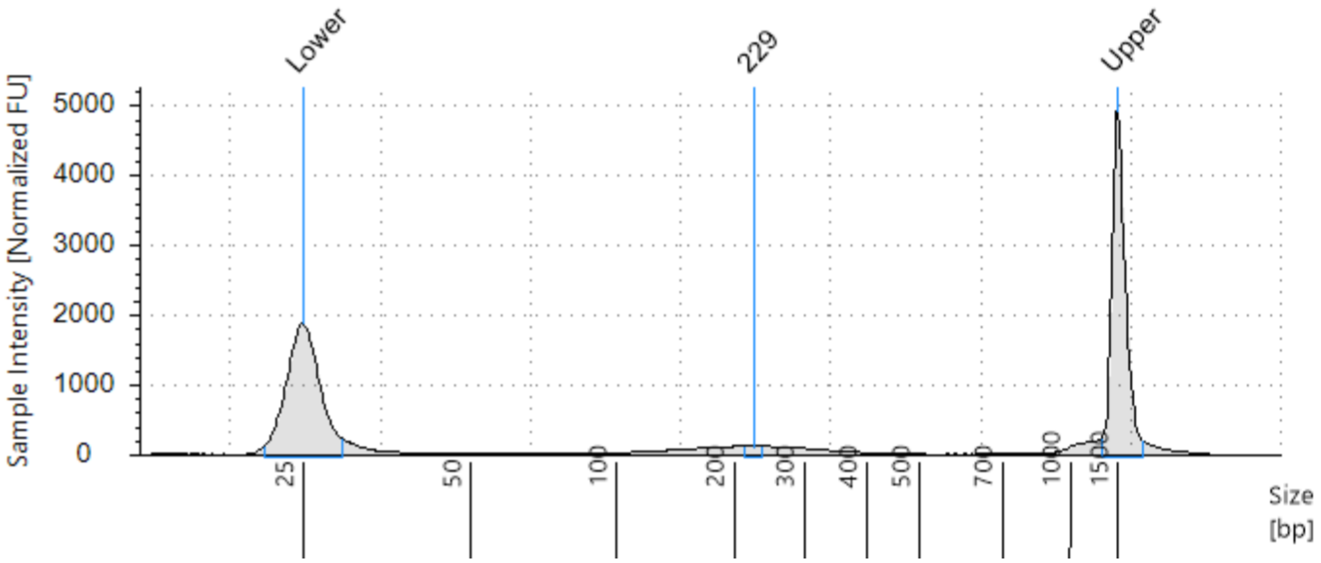

Sample Table

Well	Conc. [ng/µl]	Sample Description	Alert	Observations
B2	0.165	A12 M R2		

Peak Table

Size [bp]	Calibrated Conc. [ng/µl]	Assigned Conc. [ng/µl]	Peak Molarity [nmol/l]	% Integrated Area	Peak Comment	Observations
25	6.07	-	373	-		Lower Marker
229	0.165	-	1.11	100.00		
1500	6.50	6.50	6.67	-		Upper Marker

© Agilent Technologies, Inc. 2017

C2: B12 M R2

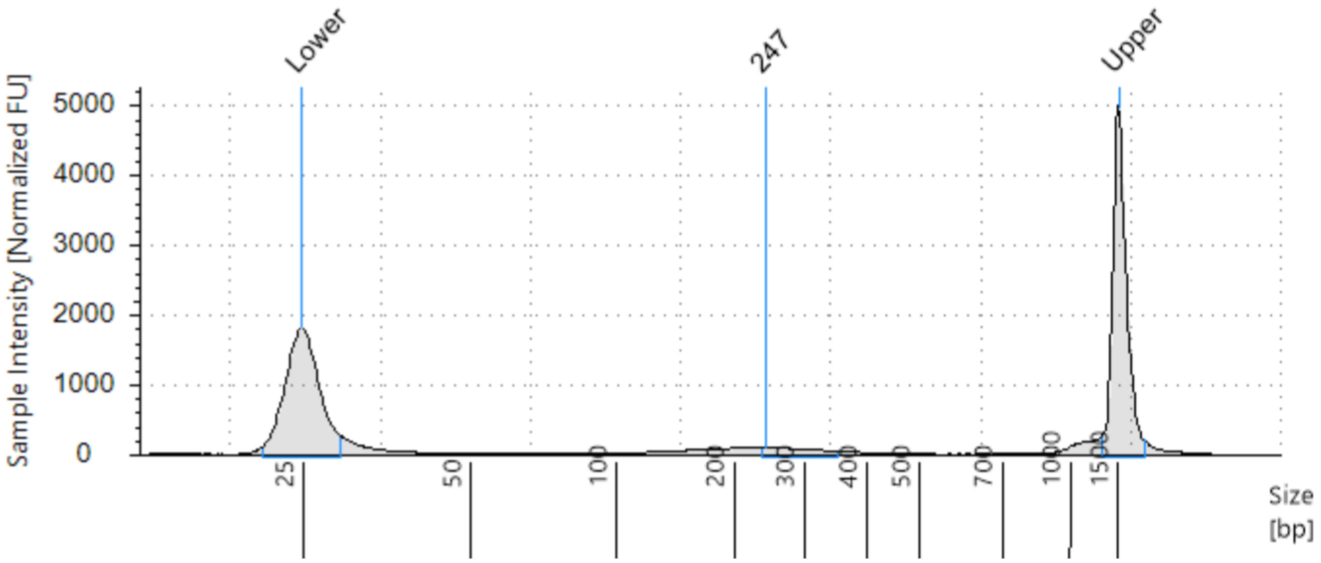

Sample Table

Well	Conc. [ng/µl]	Sample Description	Alert	Observations
C2	0.451	B12 M R2		

Peak Table

Size [bp]	Calibrated Conc. [ng/µl]	Assigned Conc. [ng/µl]	Peak Molarity [nmol/l]	% Integrated Area	Peak Comment	Observations
25	5.90	-	363	-		Lower Marker
247	0.451	-	2.81	100.00		
1500	6.50	6.50	6.67	-		Upper Marker

© Agilent Technologies, Inc. 2017

D2: C12 M R2

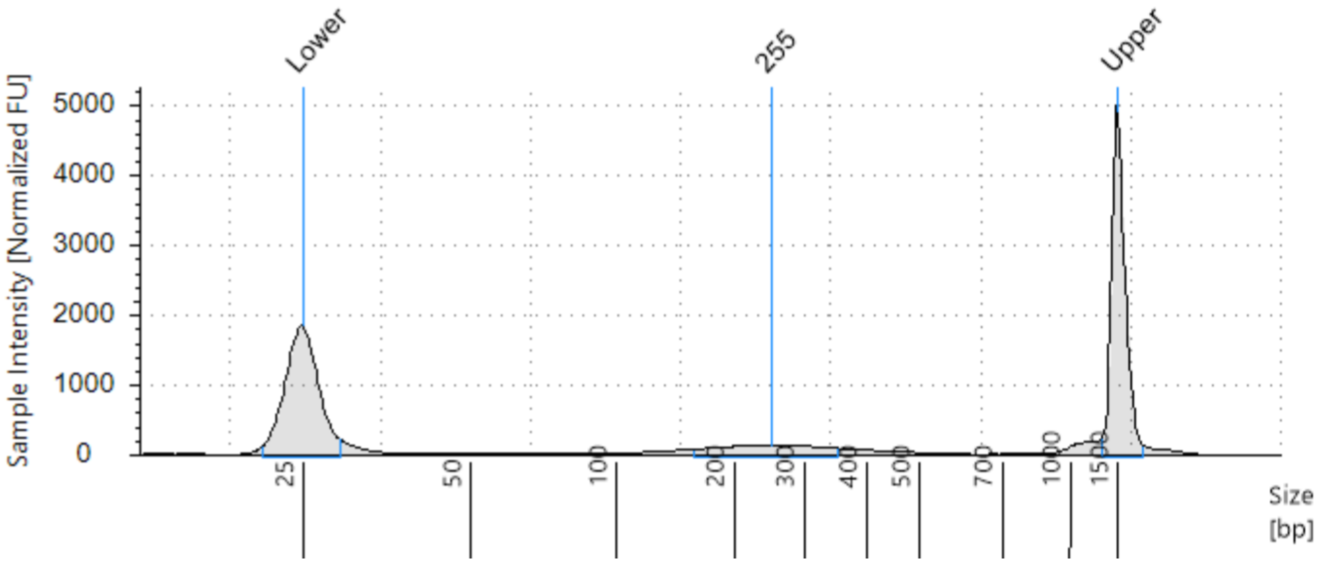

Sample Table

Well	Conc. [ng/µl]	Sample Description	Alert	Observations
D2	1.31	C12 M R2		

Peak Table

Size [bp]	Calibrated Conc. [ng/µl]	Assigned Conc. [ng/µl]	Peak Molarity [nmol/l]	% Integrated Area	Peak Comment	Observations
25	5.95	-	366	-		Lower Marker
255	1.31	-	7.91	100.00		
1500	6.50	6.50	6.67	-		Upper Marker

E2: D12 M R2

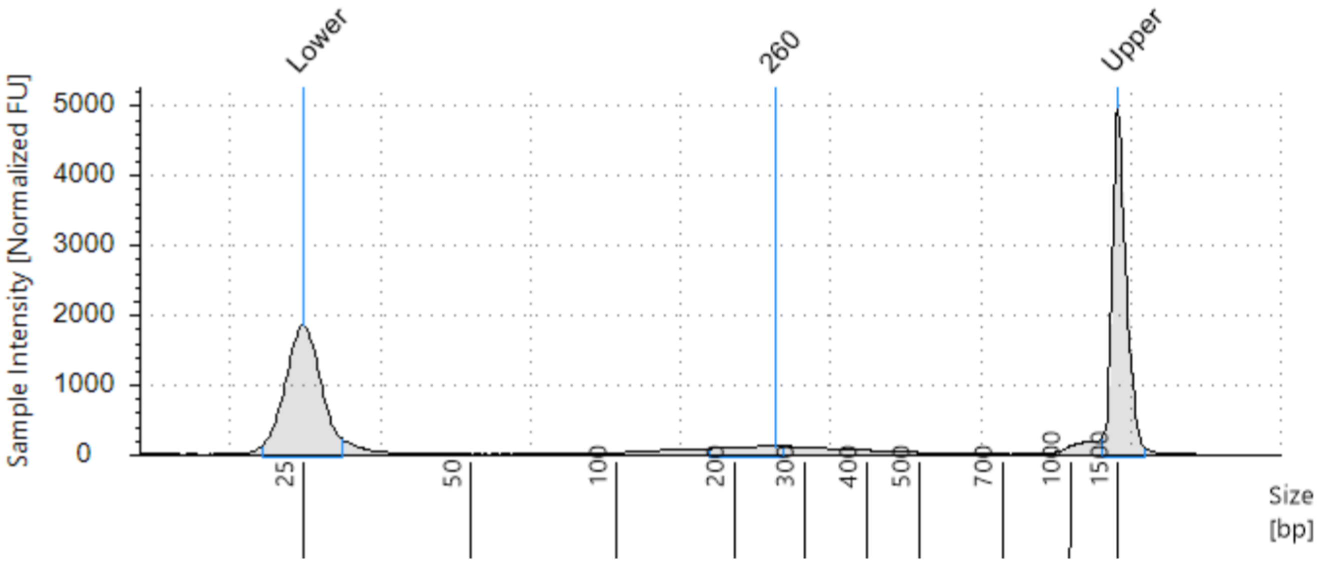

Sample Table

Well	Conc. [ng/µl]	Sample Description	Alert	Observations
E2	0.626	D12 M R2		

Peak Table

Size [bp]	Calibrated Conc. [ng/µl]	Assigned Conc. [ng/µl]	Peak Molarity [nmol/l]	% Integrated Area	Peak Comment	Observations
25	6.17	-	380	-		Lower Marker
260	0.626	-	3.71	100.00		
1500	6.50	6.50	6.67	-		Upper Marker

© Agilent Technologies, Inc. 2017

F2: E12 M R2

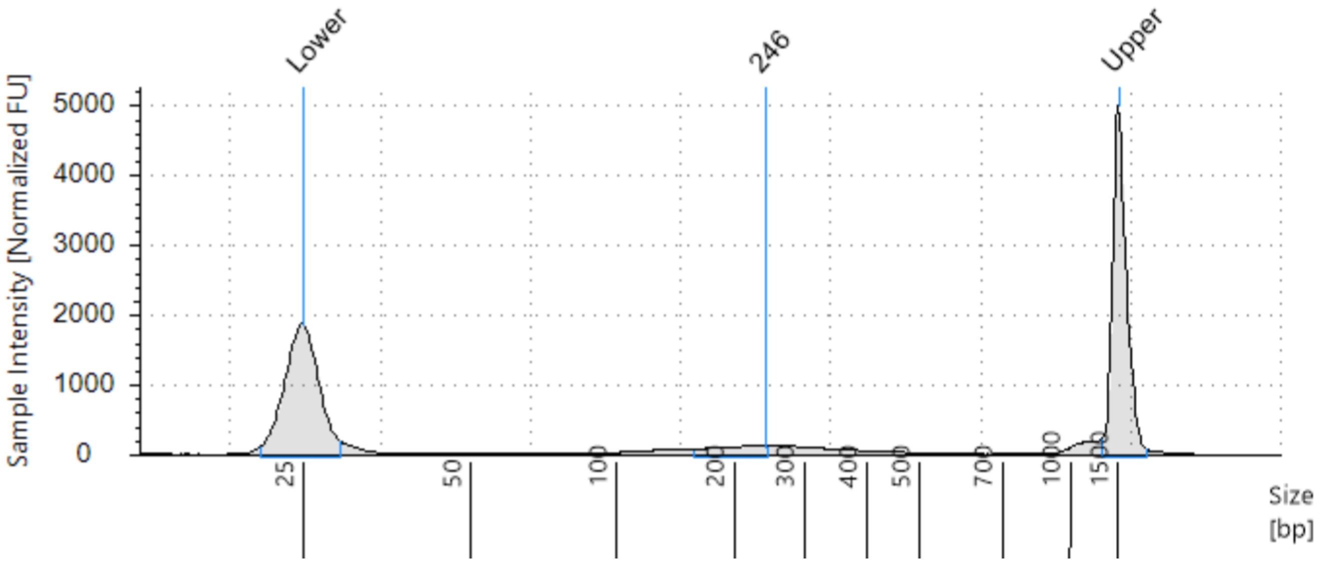

Sample Table

Well	Conc. [ng/µl]	Sample Description	Alert	Observations
F2	0.605	E12 M R2		

Peak Table

Size [bp]	Calibrated Conc. [ng/µl]	Assigned Conc. [ng/µl]	Peak Molarity [nmol/l]	% Integrated Area	Peak Comment	Observations
25	5.98	-	368	-		Lower Marker
246	0.605	-	3.79	100.00		
1500	6.50	6.50	6.67	-		Upper Marker

© Agilent Technologies, Inc. 2017

G2: F12 M R2

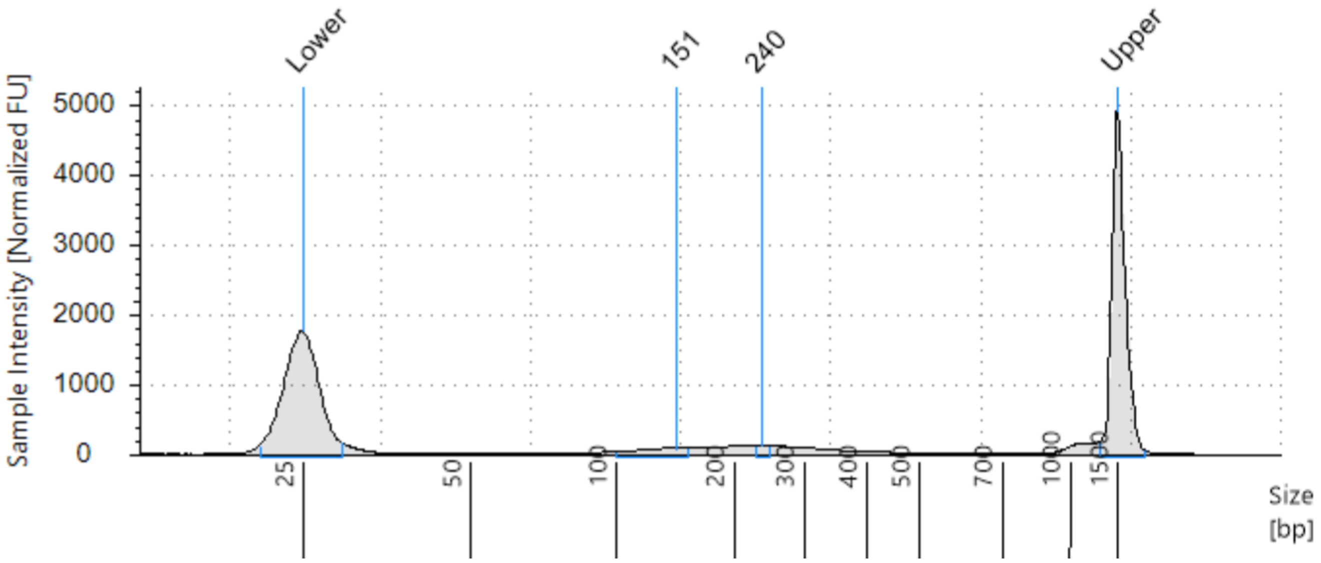

Sample Table

Well	Conc. [ng/µl]	Sample Description	Alert	Observations
G2	0.572	F12 M R2		

Peak Table

Size [bp]	Calibrated Conc. [ng/µl]	Assigned Conc. [ng/µl]	Peak Molarity [nmol/l]	% Integrated Area	Peak Comment	Observations
25	6.22	-	383	-		Lower Marker
151	0.425	-	4.32	74.35		
240	0.147	-	0.941	25.65		
1500	6.50	6.50	6.67	-		Upper Marker

© Agilent Technologies, Inc. 2017

H2: G12 M R2

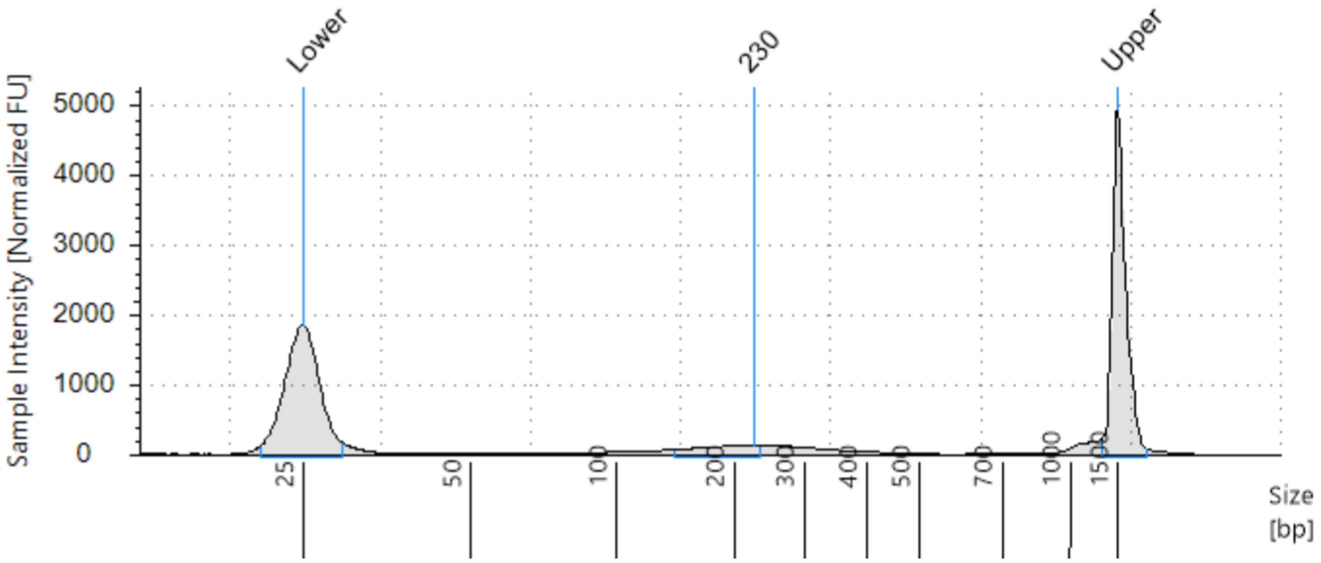

Sample Table

Well	Conc. [ng/µl]	Sample Description	Alert	Observations
H2	0.734	G12 M R2		

Peak Table

Size [bp]	Calibrated Conc. [ng/µl]	Assigned Conc. [ng/µl]	Peak Molarity [nmol/l]	% Integrated Area	Peak Comment	Observations
25	6.26	-	385	-		Lower Marker
230	0.734	-	4.91	100.00		
1500	6.50	6.50	6.67	-		Upper Marker

 © Agilent Technologies, Inc. 2017

Filename: 2020-11-10-01- A1B1A2B3A8E9H9 P R1 H9 M R1 A11-F11 M R2F12 M R2.D1000

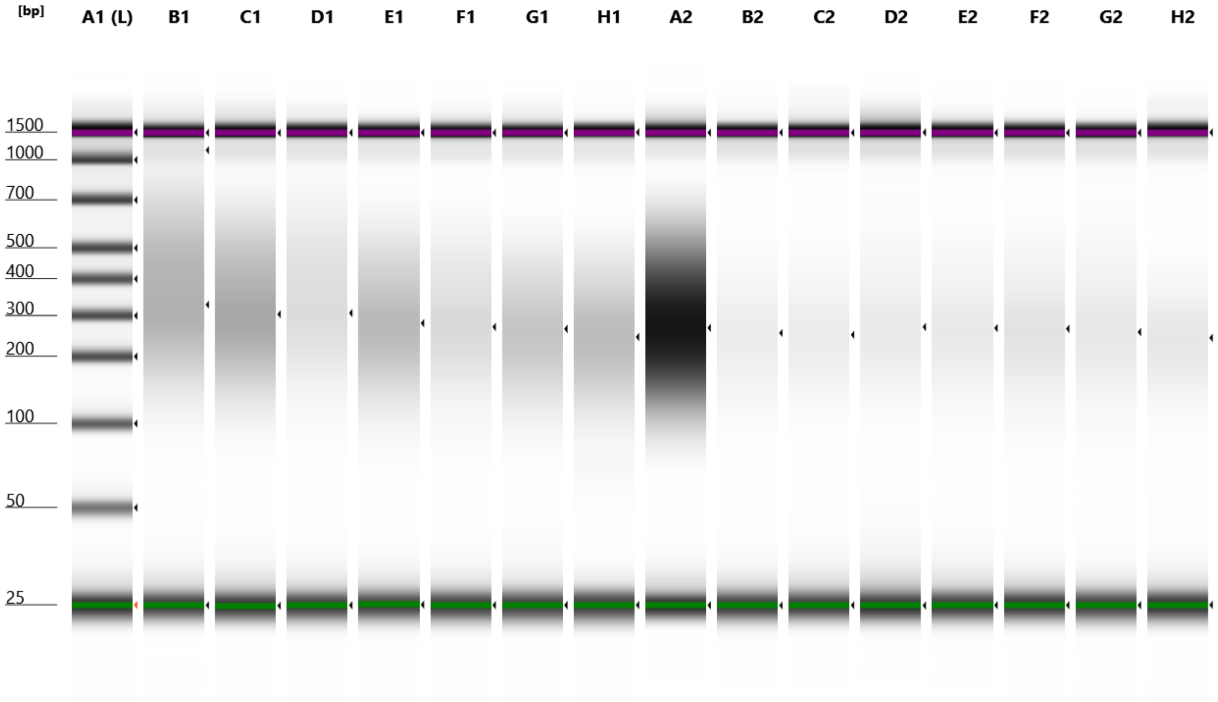

Default image (Contrast 100%)

Sample Info

Well	Conc. [ng/µl]	Sample Description	Alert	Observations
A1	17.3	Ladder		Ladder
B1	1.89	A1 P R1		
C1	7.96	B1 P R1		
D1	1.87	A2 P R1		
E1	2.87	B3 P R1		
F1	2.53	A8 P R1		
G1	4.80	E9 P R1		
H1	5.53	H9 P R1		
A2	17.3	H9 M R1		
B2	0.579	A11 M R2		
C2	0.0893	B11 M R2		
D2	0.109	C11 M R2		
E2	0.603	D11 M R2		
F2	0.193	E11 M R2		
G2	0.835	F11 M R2		
H2	0.928	F12 M R2		

 © Agilent Technologies, Inc. 2017

A1: Ladder

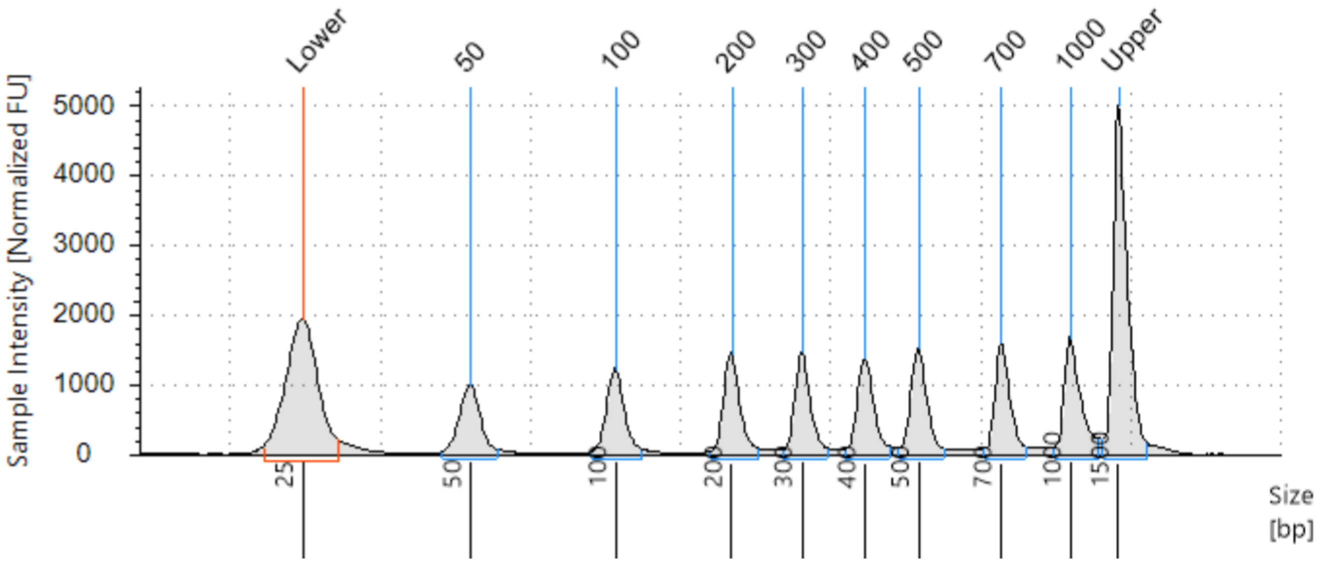

Sample Table

Well	Conc. [ng/µl]	Sample Description	Alert	Observations
A1	17.3	Ladder		Ladder

Peak Table

Size [bp]	Calibrated Conc. [ng/µl]	Assigned Conc. [ng/µl]	Peak Molarity [nmol/l]	% Integrated Area	Peak Comment	Observations
25	5.70	-	351	-		Lower Marker
50	1.93	-	59.3	11.13		
100	2.02	-	31.1	11.69		
200	2.18	-	16.7	12.58		
300	2.11	-	10.8	12.19		
400	2.11	-	8.11	12.19		
500	2.26	-	6.95	13.06		
700	2.21	-	4.85	12.76		
1000	2.49	-	3.84	14.41		
1500	6.50	6.50	6.67	-		Upper Marker

 © Agilent Technologies, Inc. 2017

B1: A1 P R1

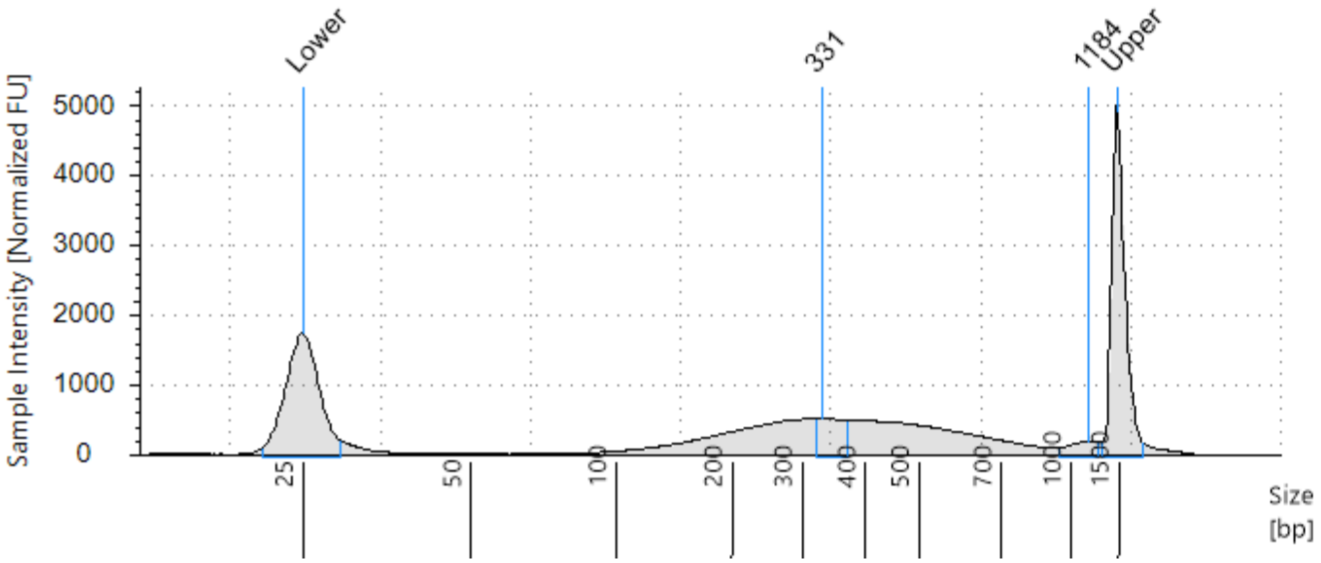

Sample Table

Well	Conc. [ng/µl]	Sample Description	Alert	Observations
B1	1.89	A1 P R1		

Peak Table

Size [bp]	Calibrated Conc. [ng/µl]	Assigned Conc. [ng/µl]	Peak Molarity [nmol/l]	% Integrated Area	Peak Comment	Observations
25	5.74	-	353	-		Lower Marker
331	1.41	-	6.53	74.16		
1184	0.490	-	0.636	25.84		
1500	6.50	6.50	6.67	-		Upper Marker

 © Agilent Technologies, Inc. 2017

C1: B1 P R1

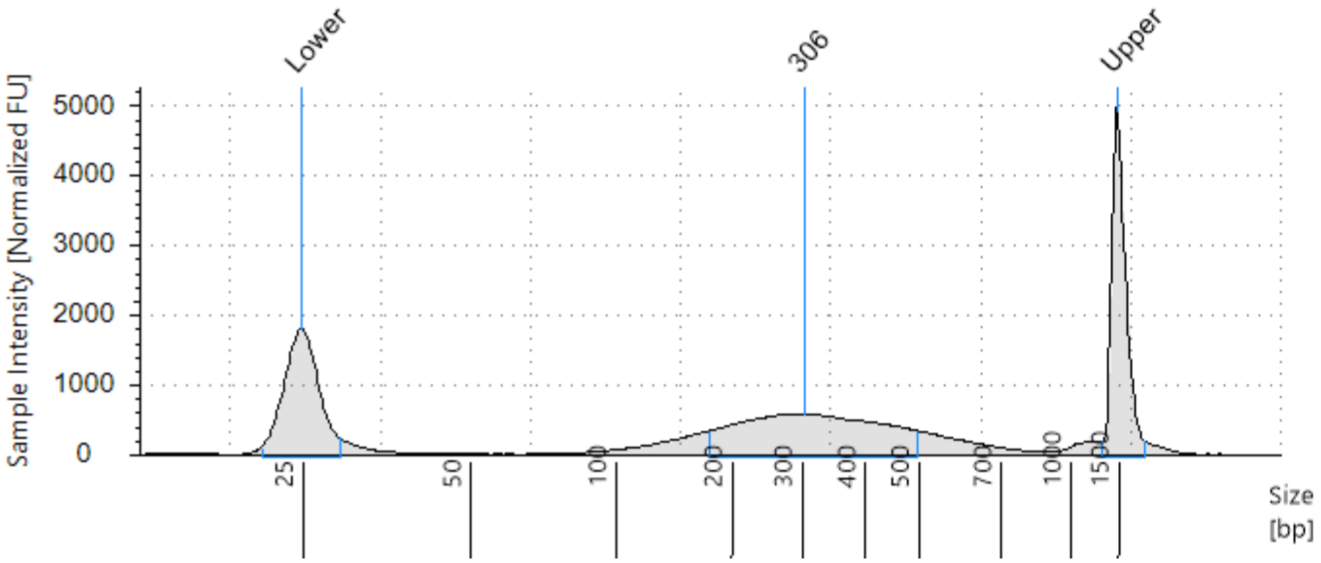

Sample Table

Well	Conc. [ng/µl]	Sample Description	Alert	Observations
C1	7.96	B1 P R1		

Peak Table

Size [bp]	Calibrated Conc. [ng/µl]	Assigned Conc. [ng/µl]	Peak Molarity [nmol/l]	% Integrated Area	Peak Comment	Observations
25	5.81	-	358	-		Lower Marker
306	7.96	-	40.1	100.00		
1500	6.50	6.50	6.67	-		Upper Marker

© Agilent Technologies, Inc. 2017

D1: A2 P R1

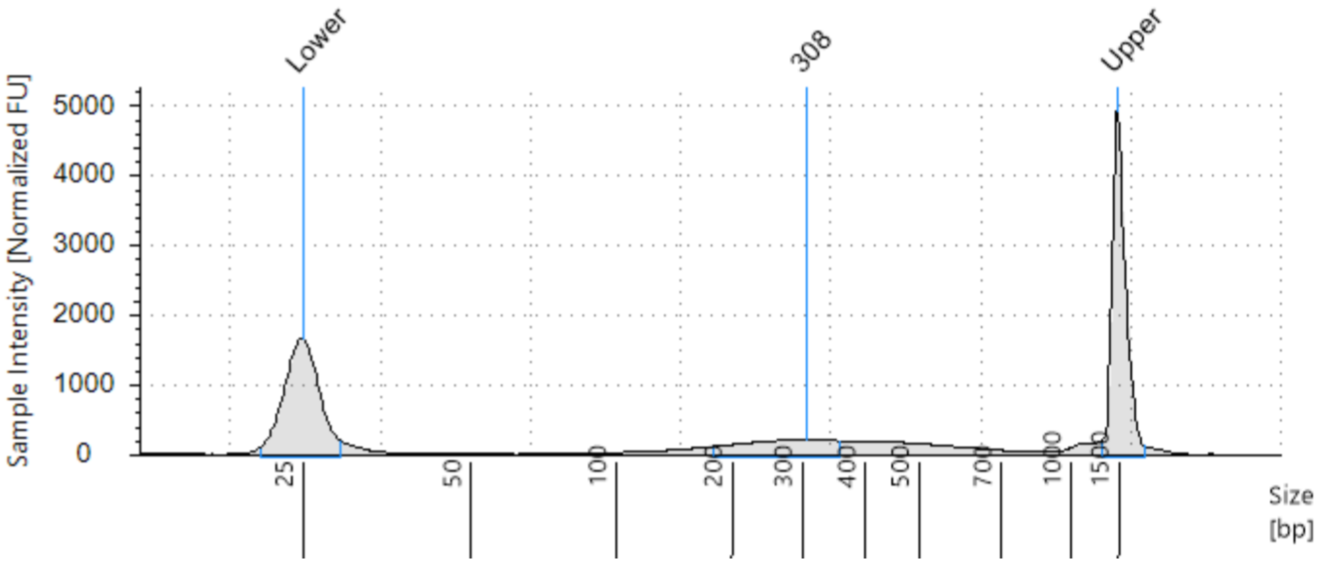

Sample Table

Well	Conc. [ng/µl]	Sample Description	Alert	Observations
D1	1.87	A2 P R1		

Peak Table

Size [bp]	Calibrated Conc. [ng/µl]	Assigned Conc. [ng/µl]	Peak Molarity [nmol/l]	% Integrated Area	Peak Comment	Observations
25	5.67	-	349	-		Lower Marker
308	1.87	-	9.35	100.00		
1500	6.50	6.50	6.67	-		Upper Marker

© Agilent Technologies, Inc. 2017

E1: B3 P R1

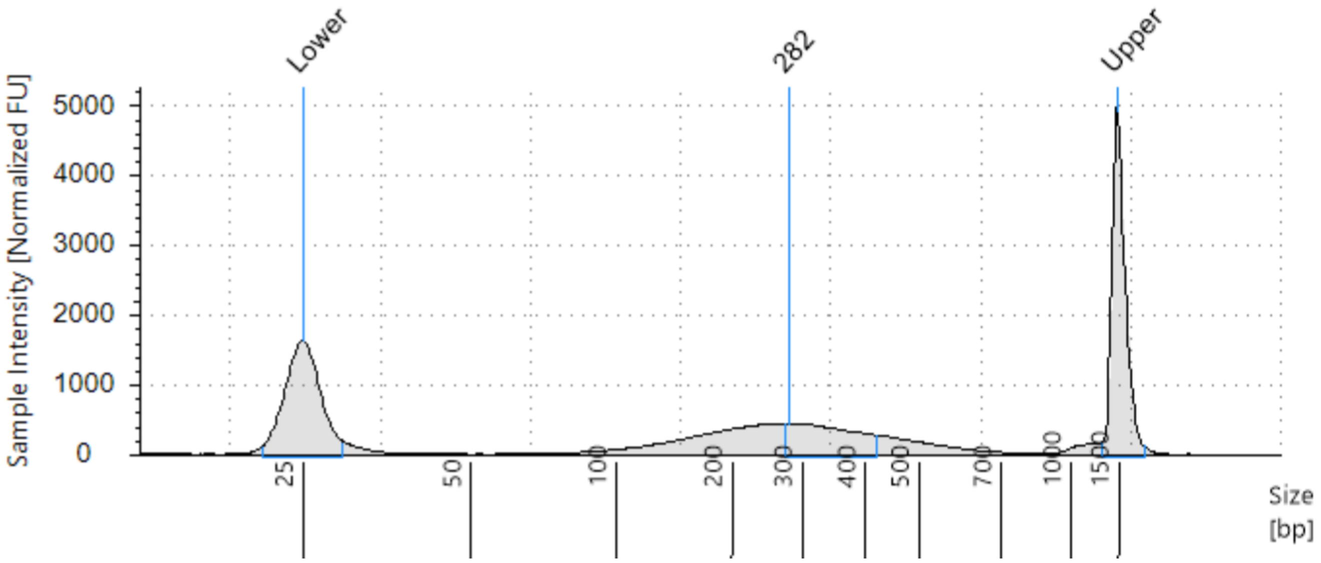

Sample Table

Well	Conc. [ng/µl]	Sample Description	Alert	Observations
E1	2.87	B3 P R1		

Peak Table

Size [bp]	Calibrated Conc. [ng/µl]	Assigned Conc. [ng/µl]	Peak Molarity [nmol/l]	% Integrated Area	Peak Comment	Observations
25	5.69	-	350	-		Lower Marker
282	2.87	-	15.7	100.00		
1500	6.50	6.50	6.67	-		Upper Marker

© Agilent Technologies, Inc. 2017

F1: A8 P R1

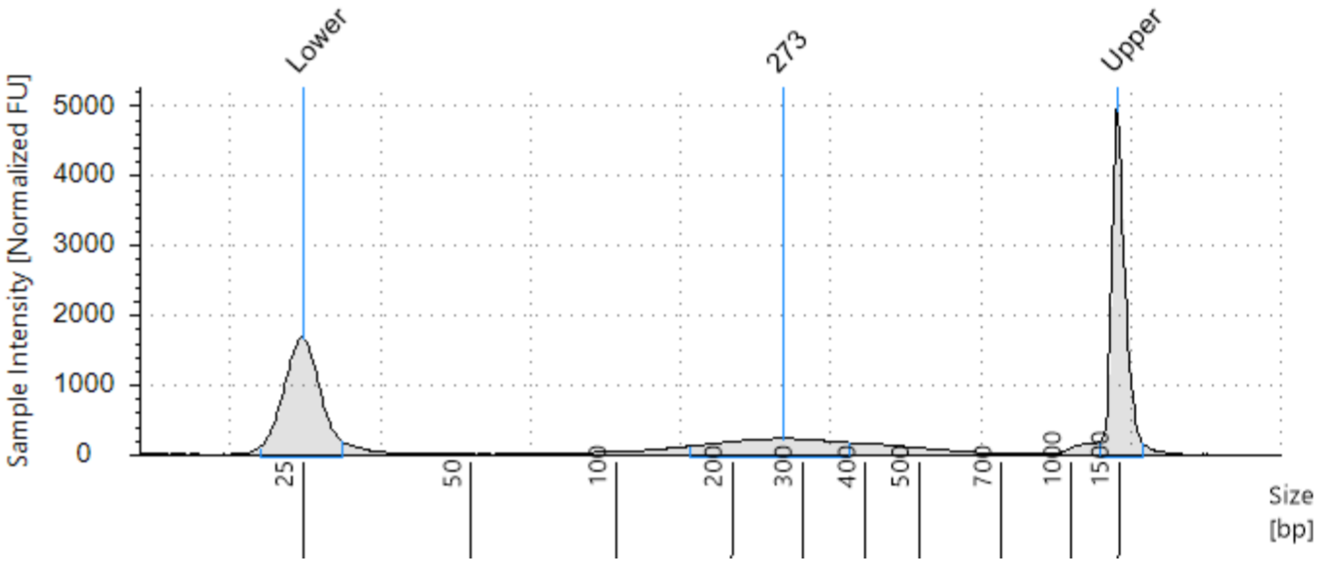

Sample Table

Well	Conc. [ng/µl]	Sample Description	Alert	Observations
F1	2.53	A8 P R1		

Peak Table

Size [bp]	Calibrated Conc. [ng/µl]	Assigned Conc. [ng/µl]	Peak Molarity [nmol/l]	% Integrated Area	Peak Comment	Observations
25	5.89	-	362	-		Lower Marker
273	2.53	-	14.2	100.00		
1500	6.50	6.50	6.67	-		Upper Marker

© Agilent Technologies, Inc. 2017

G1: E9 P R1

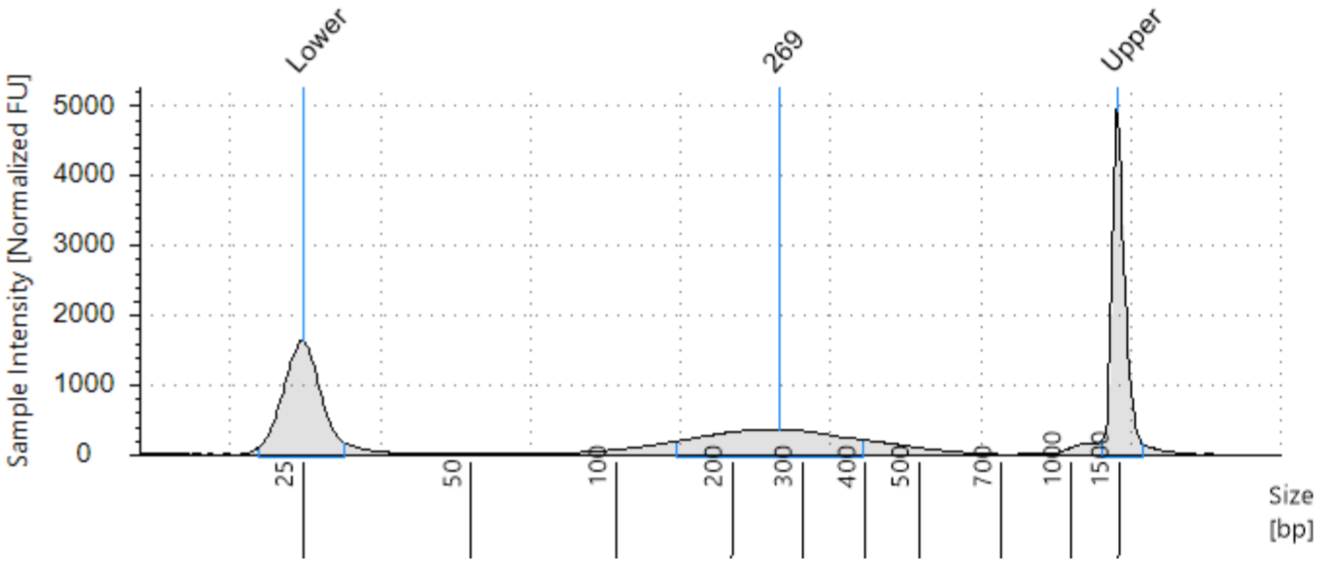

Sample Table

Well	Conc. [ng/µl]	Sample Description	Alert	Observations
G1	4.80	E9 P R1		

Peak Table

Size [bp]	Calibrated Conc. [ng/µl]	Assigned Conc. [ng/µl]	Peak Molarity [nmol/l]	% Integrated Area	Peak Comment	Observations
25	6.15	-	379	-		Lower Marker
269	4.80	-	27.5	100.00		
1500	6.50	6.50	6.67	-		Upper Marker

 © Agilent Technologies, Inc. 2017

H1: H9 P R1

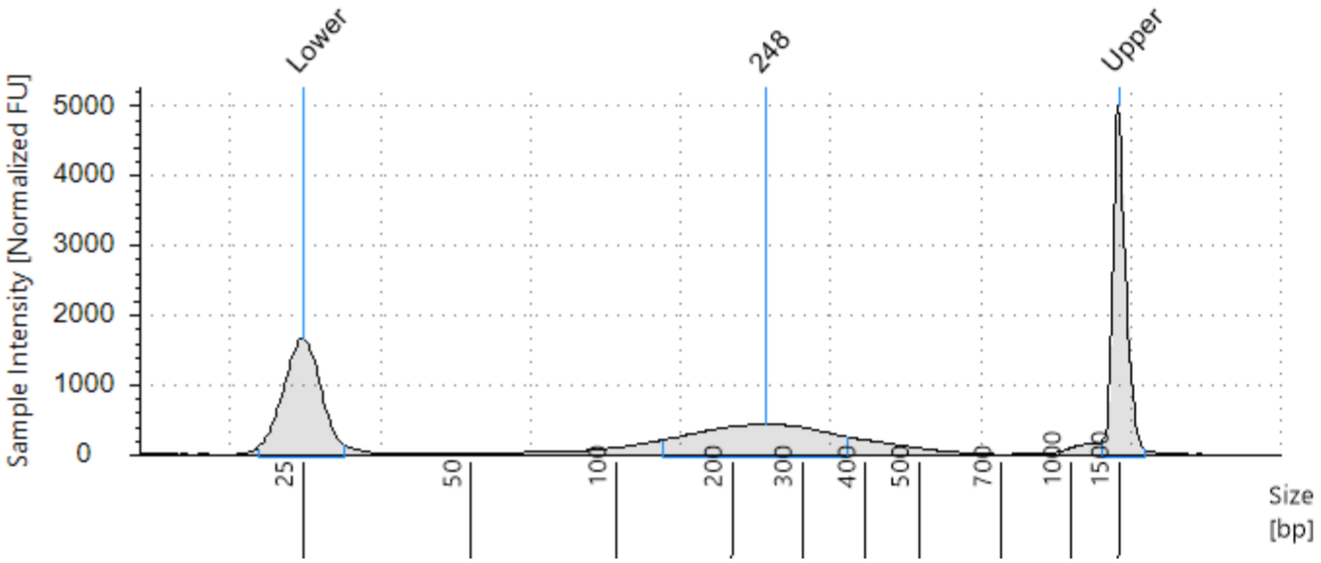

Sample Table

Well	Conc. [ng/µl]	Sample Description	Alert	Observations
H1	5.53	H9 P R1		

Peak Table

Size [bp]	Calibrated Conc. [ng/µl]	Assigned Conc. [ng/µl]	Peak Molarity [nmol/l]	% Integrated Area	Peak Comment	Observations
25	6.24	-	384	-		Lower Marker
248	5.53	-	34.3	100.00		
1500	6.50	6.50	6.67	-		Upper Marker

 © Agilent Technologies, Inc. 2017

A2: H9 M R1

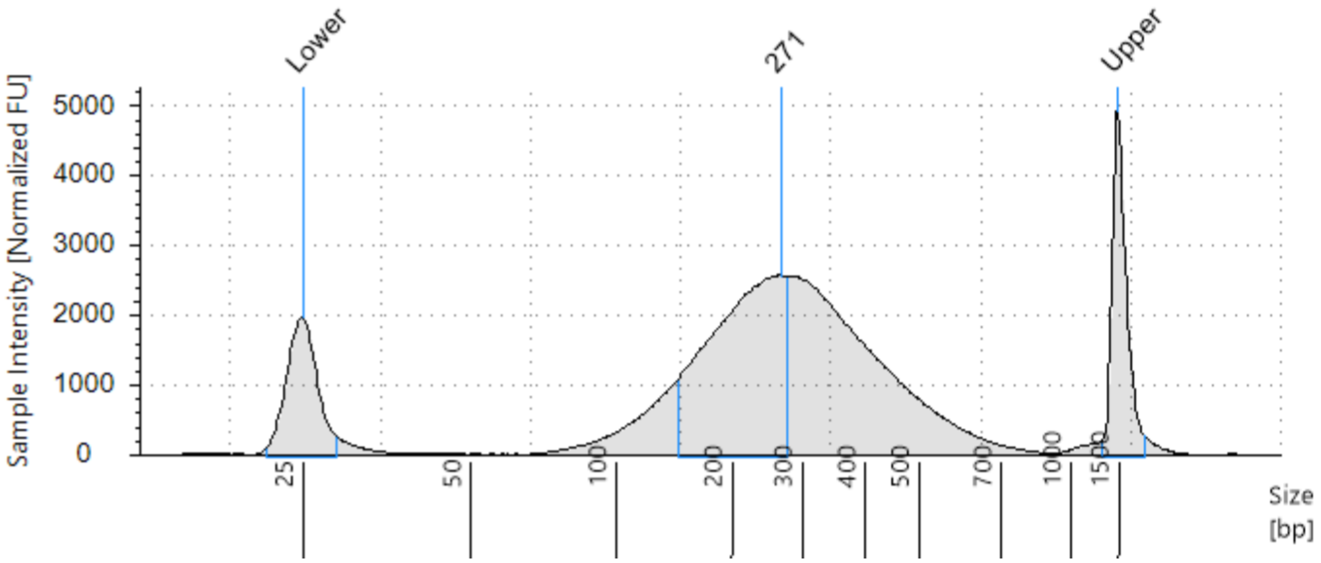

Sample Table

Well	Conc. [ng/µl]	Sample Description	Alert	Observations
A2	17.3	H9 M R1		

Peak Table

Size [bp]	Calibrated Conc. [ng/µl]	Assigned Conc. [ng/µl]	Peak Molarity [nmol/l]	% Integrated Area	Peak Comment	Observations
25	5.55	-	342	-		Lower Marker
271	17.3	-	98.2	100.00		
1500	6.50	6.50	6.67	-		Upper Marker

 © Agilent Technologies, Inc. 2017

B2: A11 M R2

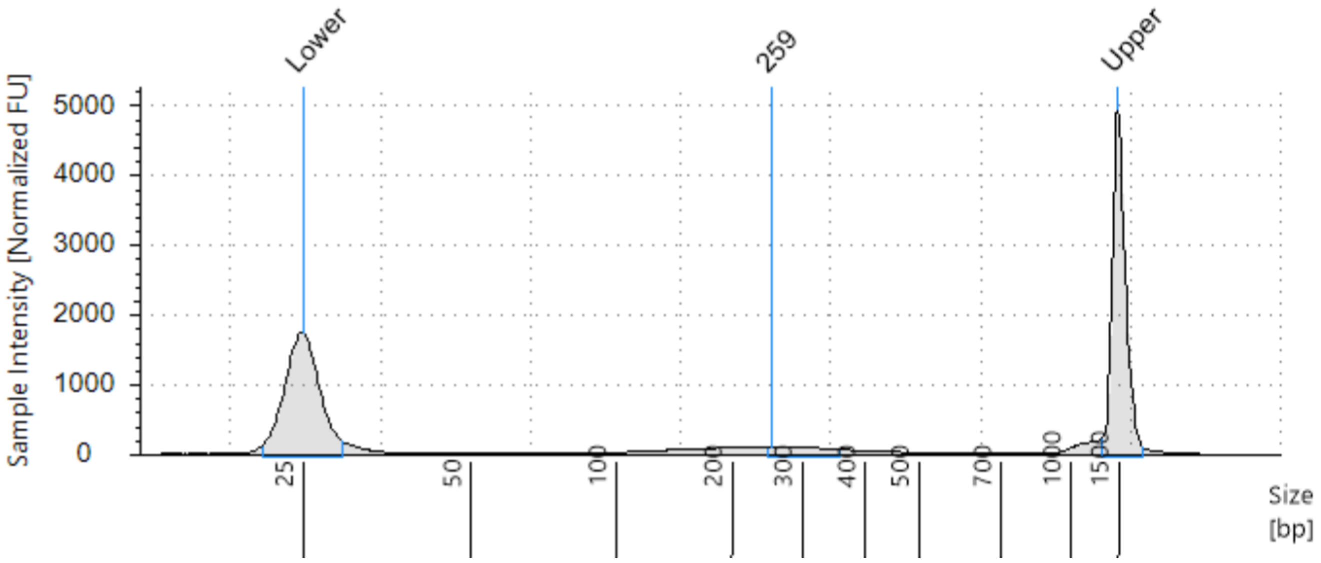

Sample Table

Well	Conc. [ng/µl]	Sample Description	Alert	Observations
B2	0.579	A11 M R2		

Peak Table

Size [bp]	Calibrated Conc. [ng/µl]	Assigned Conc. [ng/µl]	Peak Molarity [nmol/l]	% Integrated Area	Peak Comment	Observations
25	6.00	-	369	-		Lower Marker
259	0.579	-	3.45	100.00		
1500	6.50	6.50	6.67	-		Upper Marker

© Agilent Technologies, Inc. 2017

C2: B11 M R2

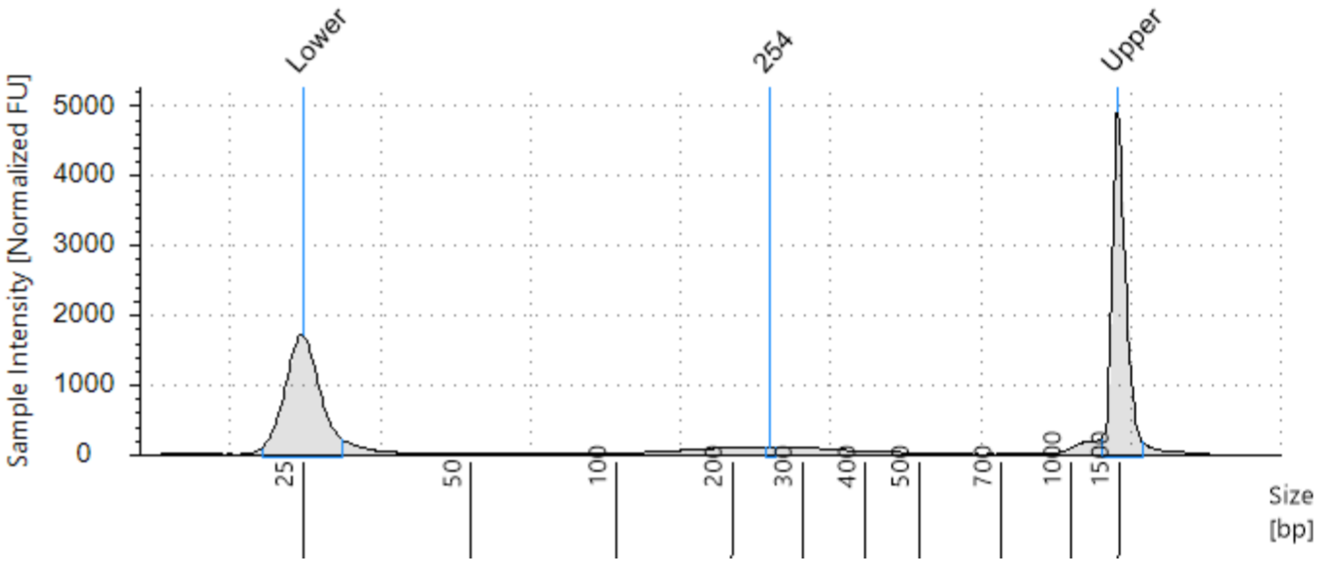

Sample Table

Well	Conc. [ng/µl]	Sample Description	Alert	Observations
C2	0.0893	B11 M R2		

Peak Table

Size [bp]	Calibrated Conc. [ng/µl]	Assigned Conc. [ng/µl]	Peak Molarity [nmol/l]	% Integrated Area	Peak Comment	Observations
25	5.75	-	354	-		Lower Marker
254	0.0893	-	0.541	100.00		
1500	6.50	6.50	6.67	-		Upper Marker

© Agilent Technologies, Inc. 2017

D2: C11 M R2

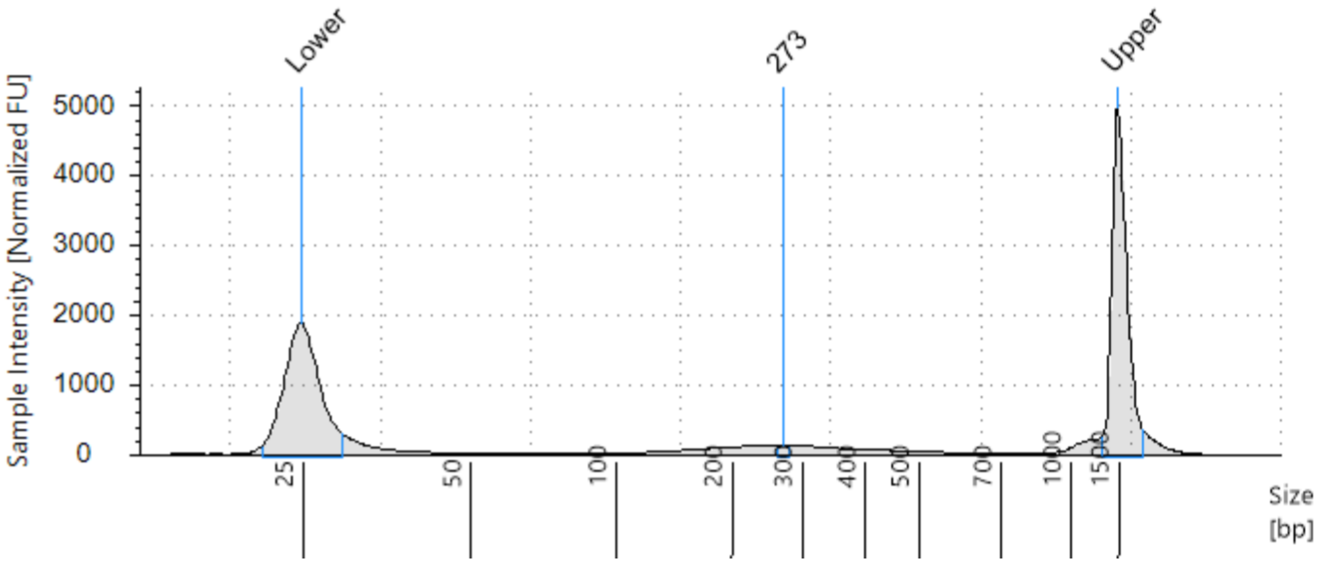

Sample Table

Well	Conc. [ng/µl]	Sample Description	Alert	Observations
D2	0.109	C11 M R2		

Peak Table

Size [bp]	Calibrated Conc. [ng/µl]	Assigned Conc. [ng/µl]	Peak Molarity [nmol/l]	% Integrated Area	Peak Comment	Observations
25	5.81	-	358	-		Lower Marker
273	0.109	-	0.612	100.00		
1500	6.50	6.50	6.67	-		Upper Marker

© Agilent Technologies, Inc. 2017

E2: D11 M R2

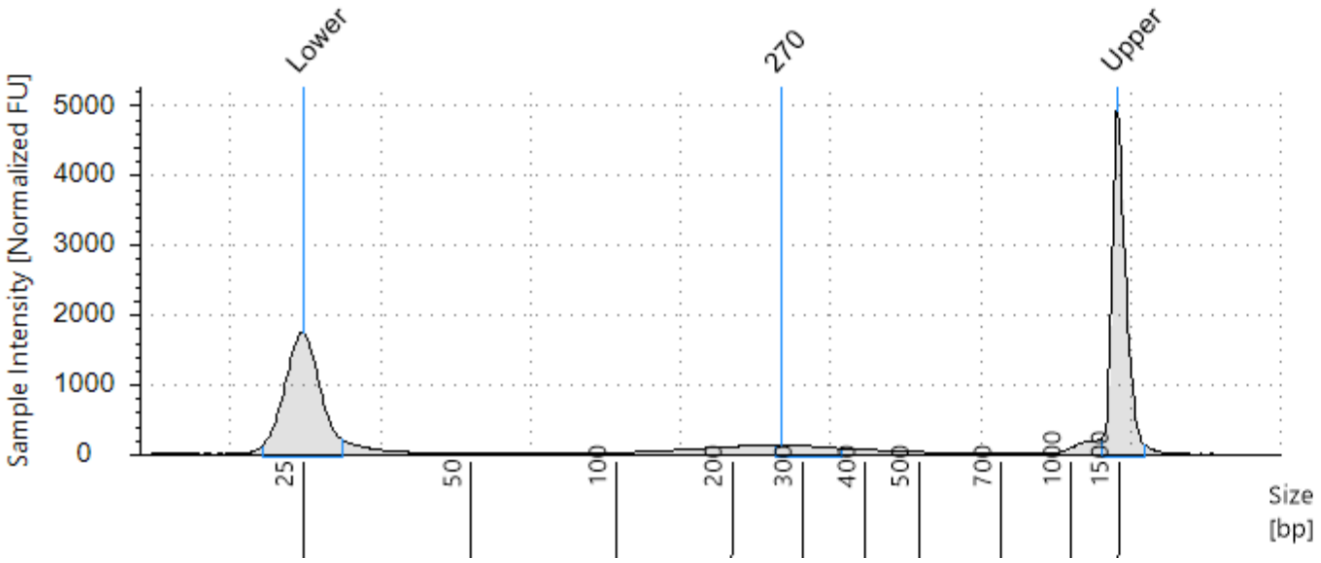

Sample Table

Well	Conc. [ng/µl]	Sample Description	Alert	Observations
E2	0.603	D11 M R2		

Peak Table

Size [bp]	Calibrated Conc. [ng/µl]	Assigned Conc. [ng/µl]	Peak Molarity [nmol/l]	% Integrated Area	Peak Comment	Observations
25	5.82	-	358	-		Lower Marker
270	0.603	-	3.43	100.00		
1500	6.50	6.50	6.67	-		Upper Marker

 © Agilent Technologies, Inc. 2017

F2: E11 M R2

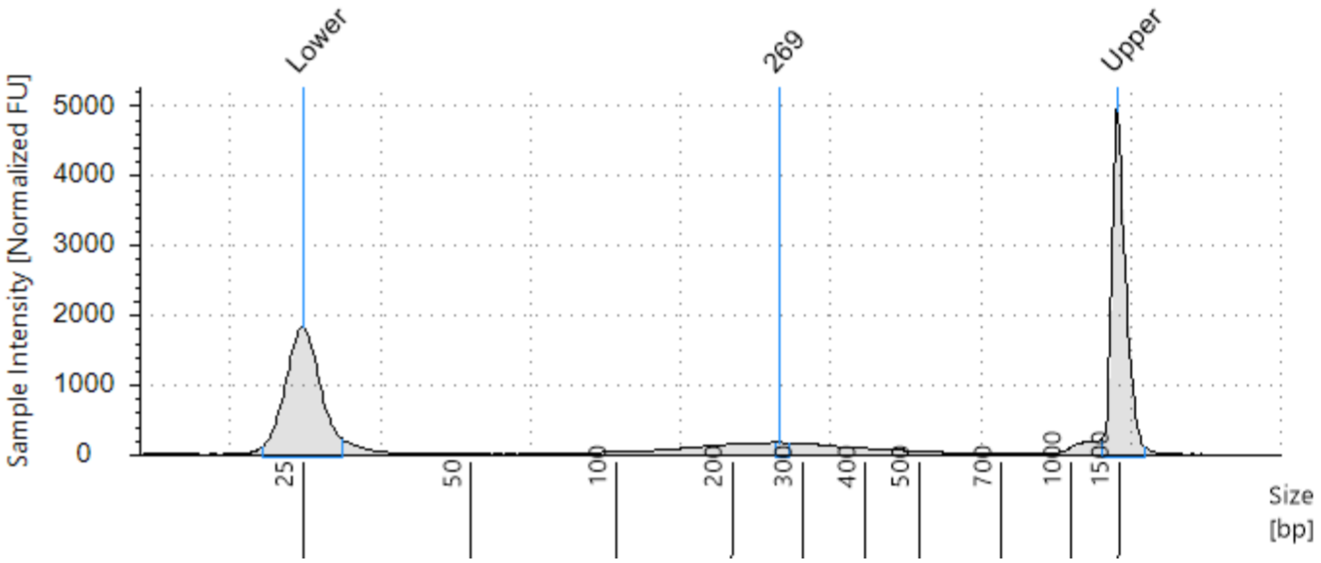

Sample Table

Well	Conc. [ng/µl]	Sample Description	Alert	Observations
F2	0.193	E11 M R2		

Peak Table

Size [bp]	Calibrated Conc. [ng/µl]	Assigned Conc. [ng/µl]	Peak Molarity [nmol/l]	% Integrated Area	Peak Comment	Observations
25	5.97	-	367	-		Lower Marker
269	0.193	-	1.10	100.00		
1500	6.50	6.50	6.67	-		Upper Marker

© Agilent Technologies, Inc. 2017

G2: F11 M R2

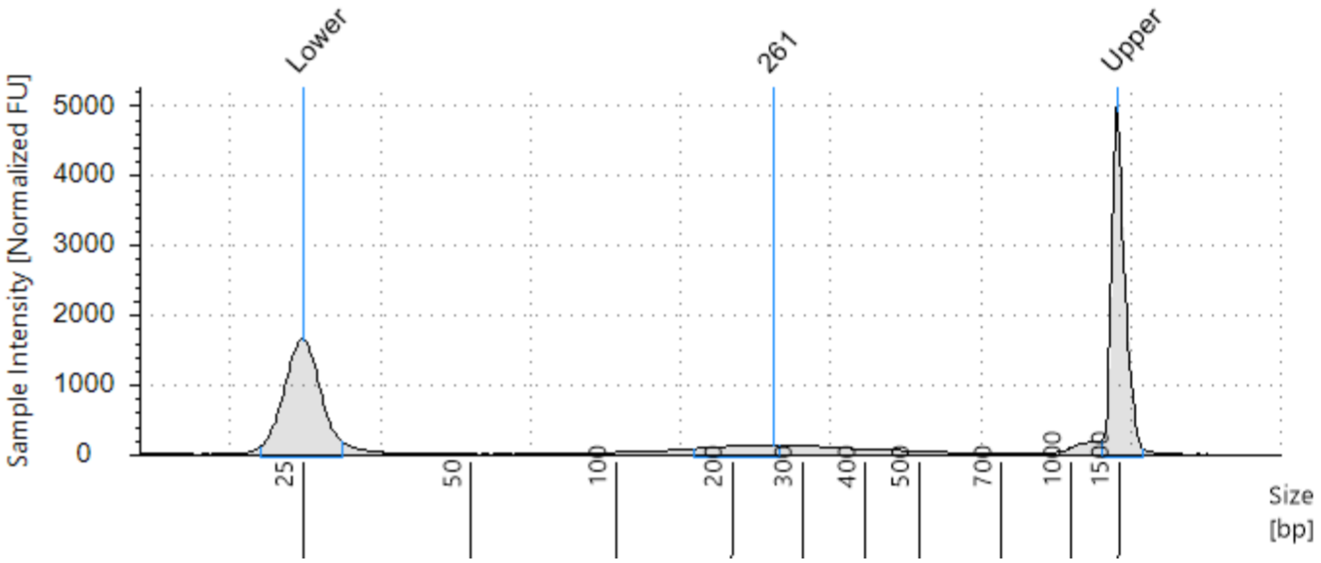

Sample Table

Well	Conc. [ng/µl]	Sample Description	Alert	Observations
G2	0.835	F11 M R2		

Peak Table

Size [bp]	Calibrated Conc. [ng/µl]	Assigned Conc. [ng/µl]	Peak Molarity [nmol/l]	% Integrated Area	Peak Comment	Observations
25	5.86	-	361	-		Lower Marker
261	0.835	-	4.93	100.00		
1500	6.50	6.50	6.67	-		Upper Marker

 © Agilent Technologies, Inc. 2017

H2: F12 M R2

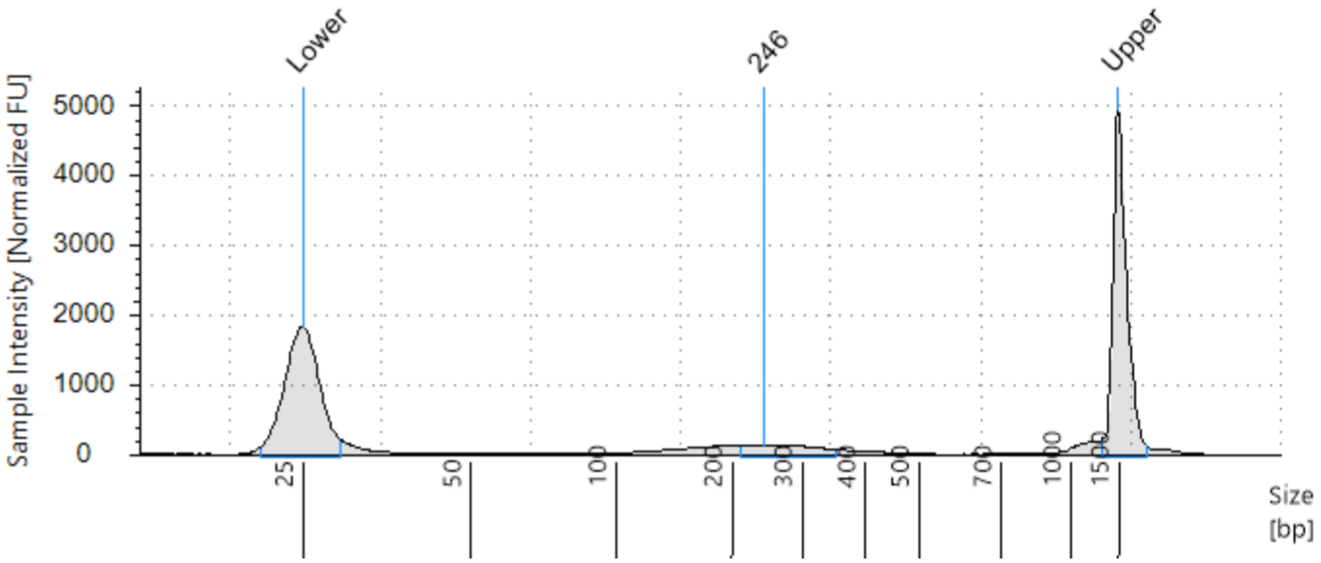

Sample Table

Well	Conc. [ng/µl]	Sample Description	Alert	Observations
H2	0.928	F12 M R2		

Peak Table

Size [bp]	Calibrated Conc. [ng/µl]	Assigned Conc. [ng/µl]	Peak Molarity [nmol/l]	% Integrated Area	Peak Comment	Observations
25	6.13	-	377	-		Lower Marker
246	0.928	-	5.81	100.00		
1500	6.50	6.50	6.67	-		Upper Marker

 © Agilent Technologies, Inc. 2017
